# Supplementary material for: Machine Learning Application for Medicinal Chemistry: Colchicine Case, New Structures, and Anticancer Activity Prediction
Source: Pharmaceuticals (Basel). 2024 Jan 29;17(2):173. doi: 10.3390/ph17020173 (PMC10892630; doi:10.3390/ph17020173)
Supplement: Supplementary file 1 [file Supplementary code.pdf › Supplementary code.pdf]

Notebook

December 19, 2023

[ ]:
```
##########
# FILE 2
##########

from rdkit import Chem
import sys
from pathlib import Path
SELFIES_coder_path = Path("../SELFIES_coder")
sys.path.append(SELFIES_coder_path.as_posix())
import SELFIES_coder as SELFIES_coder
import pandas as pd

def read_and_prepare_data(file, reps):
    #read file
    data = pd.read_excel(file)
    #read SMILES
    #data = data[data['SELFIES_length'] < 55]
    data = data['SMILES']
    #prepare rdkit representations
    mols = [Chem.MolFromSmiles(smi) for smi in data]

    smiles_training = []
    #prepare various representations for each of molecule
    for i in range(reps):
        for smi in mols:
            try:
                new_smi = Chem.MolToSmiles(smi, doRandom=True)
                smiles_training.append(new_smi)
            except:
                print("Error when processing", i, smi)

    selfies_training = [SELFIES_coder.SMILES_to_SELFIES(smi) for smi in␣
 ↪smiles_training]
    drop_duplicates_self_train = list(set(selfies_training))

    to_be_tested__ = [SELFIES_coder.SELFIES_to_SMILES(self) for self in␣
 ↪drop_duplicates_self_train]
```

```
    drop_duplicates_smmi_train = list(set(to_be_tested__))
    print("The number of training data is␣
↪"+str(len(drop_duplicates_smmi_train)))

    training_smiles = pd.DataFrame(data=drop_duplicates_smmi_train,␣
↪columns=['SMILES'])

    return training_smiles.to_parquet('../Data/training_smiles.parquet')


if __name__ == "__main__":

    read_and_prepare_data('../Data/Kolchicyna_prepared_data.xlsx', 1000)
```

Notebook

December 19, 2023

[ ]:
```
##########
# FILE 4
##########

# General Imports
import os
import pandas as pd
import numpy as np
import time
import sys
from pathlib import Path
SELFIES_coder_path = Path("../SELFIES_coder")
sys.path.append(SELFIES_coder_path.as_posix())
import SELFIES_coder as SELFIES_CODER
import selfies as sf
from sklearn.model_selection import train_test_split
from keras.models import Model
from keras.layers import Input
from keras.layers import LSTM
from keras.layers import Dense
from keras.layers import Concatenate
from keras import regularizers
from keras.callbacks import History, ReduceLROnPlateau
from tensorflow.keras.optimizers import RMSprop, Adam
import pickle

SELFIES_CODER.TEST()



def initialize():
    # Know where user actually is
    directory_path = os.getcwd()
    print("My current directory is : " + directory_path)
    folder_name = os.path.basename(directory_path)
    print("My directory name is : " + folder_name)
```

```python
def load_and_prepare_data(parquet_file):

    data = pd.read_parquet(parquet_file)

    data = SELFIES_CODER.get_encoded_SELFIES(data['SMILES'].to_list())

    # characters that are used in given SMILES dataset along with initial and stopping characters
    charset = set("".join(list(data[0]))+"!E")
    char_to_int = dict((c,i) for i,c in enumerate(charset))
    int_to_char = dict((i,c) for i,c in enumerate(charset))
    embed = data[3] + 5 #20
    print("Charset is "+str(charset))

    import json
    json = json.dumps(data[2])
    f = open("SELFIES_to_mol_seq.json","w")
    f.write(json)
    f.close()

    import json
    json = json.dumps(data[1])
    f = open("mol_seq_to_SELFIES.json","w")
    f.write(json)
    f.close()

    import json
    json = json.dumps(char_to_int)
    f = open("mol_seq_to_int.json","w")
    f.write(json)
    f.close()

    import json
    json1 = json.dumps(int_to_char)
    f = open("int_to_mol_seq.json","w")
    f.write(json1)
    f.close()


    mol_seq_train, mol_seq_test = train_test_split(data[0], test_size=0.1, train_size=0.9, random_state=42)
    return data, charset, char_to_int, embed, mol_seq_train, mol_seq_test

#vectorization of molecular sequences
def vectorize(mol_seq, shap, embed, char_to_int, charset):
        one_hot =  np.zeros((shap, embed , len(charset)),dtype=np.int8)
        for i,smile in enumerate(mol_seq):
```

```
            #encode the startchar
            one_hot[i,0,char_to_int["!"]] = 1
            #encode the rest of the chars
            for j,c in enumerate(smile):
                one_hot[i,j+1,char_to_int[c]] = 1
            #Encode endchar
            one_hot[i,len(smile)+1:,char_to_int["E"]] = 1
        #Return two, one for input and the other for output
        return one_hot[:,0:-1,:], one_hot[:,1:,:]

def data_vectorization(mol_seq_train, mol_seq_test, embed, char_to_int,␣
↪charset):

    X_train, Y_train = vectorize(mol_seq_train, len(mol_seq_train), embed,␣
↪char_to_int, charset)
    X_test, Y_test = vectorize(mol_seq_test, len(mol_seq_test), embed,␣
↪char_to_int, charset)

    return X_train, Y_train, X_test, Y_test



def build_model(X_train, Y_train, X_test, Y_test):

    input_shape = X_train.shape[1:]
    output_dim = Y_train.shape[-1]
    latent_dim = 128
    lstm_dim = 128
    #encoder-decoder architecture
    unroll = False
    encoder_inputs = Input(shape=input_shape)
    encoder = LSTM(lstm_dim, return_state=True,
                unroll=unroll)
    encoder_outputs, state_h, state_c = encoder(encoder_inputs)
    states = Concatenate(axis=-1)([state_h, state_c])
    neck = Dense(latent_dim, activation="relu")
    neck_outputs = neck(states)

    decode_h = Dense(lstm_dim, activation="relu")
    decode_c = Dense(lstm_dim, activation="relu")
    state_h_decoded =  decode_h(neck_outputs)
    state_c_decoded =  decode_c(neck_outputs)
    encoder_states = [state_h_decoded, state_c_decoded]
    decoder_inputs = Input(shape=input_shape)
    decoder_lstm = LSTM(lstm_dim,
                        return_sequences=True,
                        unroll=unroll
```

```
                )
decoder_outputs = decoder_lstm(decoder_inputs, initial_state=encoder_states)
decoder_dense = Dense(output_dim, activation='softmax')
decoder_outputs = decoder_dense(decoder_outputs)
#Define the model, that inputs the training vector for two places, and␣
↪predicts one character ahead of the input
model = Model([encoder_inputs, decoder_inputs], decoder_outputs)
print(model.summary())

h = History()
rlr = ReduceLROnPlateau(monitor='val_loss', factor=0.5,patience=10,␣
↪min_lr=0.0000001, verbose=1, min_delta=1e-6) #epsilon=min_delta
opt=Adam(learning_rate=0.005) #Default 0.001
model.compile(optimizer=opt, loss='categorical_crossentropy')

model.fit([X_train,X_train],Y_train, epochs=250, batch_size=128,␣
↪shuffle=True, callbacks=[h, rlr], validation_data=([X_test,X_test],Y_test))␣
↪#100

f = open("Neural_network_history.pickle","wb")
pickle.dump(h.history, f)


smiles_to_latent_model = Model(encoder_inputs, neck_outputs)

smiles_to_latent_model.save("mol_seq2lat.h5")


latent_input = Input(shape=(latent_dim,))
#reuse_layers
state_h_decoded_2 =  decode_h(latent_input)
state_c_decoded_2 =  decode_c(latent_input)
latent_to_states_model = Model(latent_input, [state_h_decoded_2,␣
↪state_c_decoded_2])
latent_to_states_model.save("lat2state.h5")


#Last one is special, we need to change it to stateful, and change the␣
↪input shape
inf_decoder_inputs = Input(batch_shape=(1, 1, input_shape[1]))
inf_decoder_lstm = LSTM(lstm_dim,
                    return_sequences=True,
                    unroll=unroll,
                    stateful=True
                )
inf_decoder_outputs = inf_decoder_lstm(inf_decoder_inputs)
inf_decoder_dense = Dense(output_dim, activation='softmax')
```

```python
    inf_decoder_outputs = inf_decoder_dense(inf_decoder_outputs)
    sample_model = Model(inf_decoder_inputs, inf_decoder_outputs)


    #Transfer Weights
    for i in range(1,3):
        sample_model.layers[i].set_weights(model.layers[i+6].get_weights())
    sample_model.save("samplemodel.h5")

    return print("The model has been successfully build...")


if __name__ == "__main__":

     initialize()

     data, charset, char_to_int, embed, mol_seq_train, mol_seq_test =␣
 ↪load_and_prepare_data('../Data/training_smiles.parquet')

     X_train, Y_train, X_test, Y_test = data_vectorization(mol_seq_train,␣
 ↪mol_seq_test, embed, char_to_int, charset)

     build_model(X_train, Y_train, X_test, Y_test)
```

Notebook

December 19, 2023

1 File 8

[1]:
```
import pandas as pd
import numpy as np
```

[2]:
```
history__ = pd.read_pickle('Neural_network_history.pickle')
history__.head()
```

[2]:

	loss	val_loss
0	0.7097	0.4202
1	0.3570	0.3229
2	0.2981	0.2836
3	0.2714	0.2648
4	0.2560	0.2551

[15]:
```
from matplotlib import pyplot as plt
%matplotlib inline
plt.plot(history__["loss"], label="Training Loss")
plt.plot(history__["val_loss"], label="Validation Loss")
plt.yscale("log", base=10)
plt.ylim(0.02,1.0)
#plt.yticks(np.arange(0.01, 0.01, 1.0))
plt.rc('grid', linestyle="-.", color='black')
plt.grid(True)
plt.text(250, history__['loss'][249], str(history__['loss'][249]),␣
  ↪horizontalalignment='left', fontweight='bold', color='blue')
plt.text(250, history__['val_loss'][249], str(history__['val_loss'][249])+'0',␣
  ↪horizontalalignment='left', fontweight='bold', color='blue')
plt.legend()
plt.xlabel("Epoch number", fontweight='bold')
plt.ylabel("Loss value", fontweight='bold')
plt.savefig('model_loss_plot_log.pdf', bbox_inches='tight')
plt.show()
```

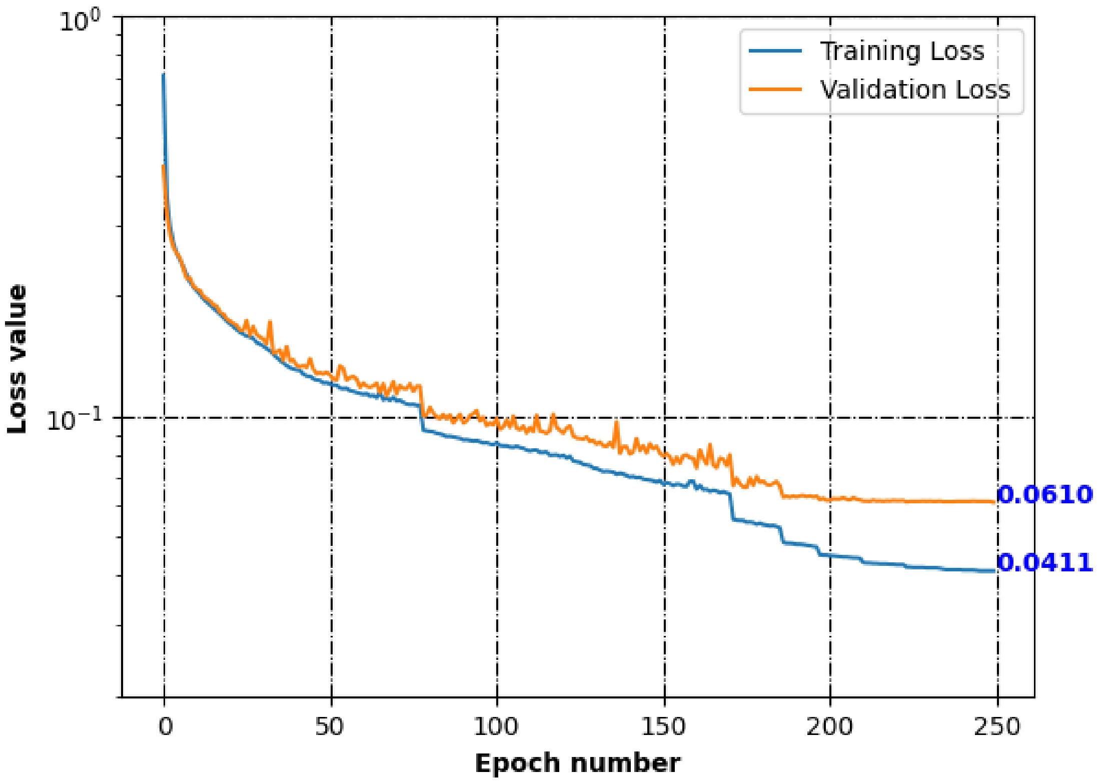

[2]:
```
file1 = open("issue.txt","r")
```

[3]:
```
content = file1.readlines()
```

[4]:
```
content[290]
```

[4]:
```
'831/831 [==============================] - 27s 25ms/step - loss: 0.7097 -
val_loss: 0.4202\n'
```

[5]:
```
content[504]
```

[5]:
```
'831/831 [==============================] - 13s 15ms/step - loss: 0.3570 -
val_loss: 0.3229\n'
```

[6]:
```
step = 504-290
step
```

[6]:
```
214
```

[7]:
```
lk = []
for i in range(250):
    lk.append(content[290+step*i])
```

[8]:
```
lk[0:5]
```

[8]:
```
['831/831 [==============================] - 27s 25ms/step - loss: 0.7097 -
val_loss: 0.4202\n',
 '831/831 [==============================] - 13s 15ms/step - loss: 0.3570 -
val_loss: 0.3229\n',
 '831/831 [==============================] - 13s 15ms/step - loss: 0.2981 -
val_loss: 0.2836\n',
 '653/831 [======================>…] - ETA: 3s - loss: 0.2731\n',
 '377/831 [============>…] - ETA: 10s - loss: 0.2593\n']
```

[9]:
```
lk = []
for i in range(len(content)):
    if "val" in content[i]:
        lk.append(content[i])
    else:
        pass
```

[10]:
```
lk[0:5]
```

[10]:
```
['831/831 [==============================] - 27s 25ms/step - loss: 0.7097 -
val_loss: 0.4202\n',
 '831/831 [==============================] - 13s 15ms/step - loss: 0.3570 -
val_loss: 0.3229\n',
 '831/831 [==============================] - 13s 15ms/step - loss: 0.2981 -
val_loss: 0.2836\n',
 '831/831 [==============================] - 18s 22ms/step - loss: 0.2714 -
val_loss: 0.2648\n',
 '831/831 [==============================] - 21s 25ms/step - loss: 0.2560 -
val_loss: 0.2551\n']
```

[11]:
```
lk[0][65:71]
```

[11]:
```
'0.7097'
```

[12]:
```
float(lk[0][65:71])
```

[12]:
```
0.7097
```

[13]:
```
lk[0][84:-1]
```

[13]:
```
'0.4202'
```

[14]:
```
train_loss = []
test_loss = []
for i in range(len(lk)):
    train_loss.append(float(lk[i][65:71]))
```

```
    test_loss.append(float(lk[i][84:-1]))
```

[15]:
```
train_loss
```

[15]:
```
[0.7097,
 0.357,
 0.2981,
 0.2714,
 0.256,
 0.2461,
 0.236,
 0.2259,
 0.2165,
 0.2104,
 0.2058,
 0.2018,
 0.1965,
 0.1931,
 0.1897,
 0.1865,
 0.183,
 0.1799,
 0.1766,
 0.173,
 0.1701,
 0.1677,
 0.1645,
 0.1623,
 0.1603,
 0.1586,
 0.1579,
 0.1564,
 0.1529,
 0.1512,
 0.1497,
 0.1475,
 0.1456,
 0.1429,
 0.1401,
 0.138,
 0.136,
 0.1339,
 0.1323,
 0.1312,
 0.1306,
 0.1302,
 0.1275,
```

0.126,
0.1259,
0.1241,
0.1231,
0.1229,
0.1211,
0.1214,
0.1203,
0.1196,
0.1196,
0.1176,
0.1172,
0.1175,
0.1158,
0.1154,
0.1146,
0.114,
0.1135,
0.1139,
0.1122,
0.1127,
0.1111,
0.1149,
0.1099,
0.111,
0.1096,
0.1116,
0.1092,
0.1096,
0.1082,
0.1073,
0.1069,
0.1071,
0.1064,
0.1065,
0.0924,
0.0919,
0.0917,
0.0914,
0.0907,
0.0904,
0.0898,
0.089,
0.0891,
0.0887,
0.0884,
0.0881,

0.0873,
0.0874,
0.087,
0.0867,
0.0869,
0.0864,
0.0857,
0.0859,
0.0853,
0.0849,
0.0859,
0.0845,
0.0843,
0.0843,
0.0838,
0.0836,
0.0841,
0.0835,
0.0831,
0.0824,
0.0819,
0.0823,
0.0824,
0.0812,
0.0812,
0.0814,
0.0799,
0.08,
0.0801,
0.0793,
0.0799,
0.0786,
0.0794,
0.0773,
0.077,
0.0767,
0.0758,
0.0755,
0.0753,
0.0742,
0.074,
0.0733,
0.0727,
0.0729,
0.0727,
0.0725,
0.0716,

0.072,
0.0717,
0.0711,
0.0705,
0.0707,
0.0702,
0.0704,
0.0697,
0.0695,
0.0693,
0.069,
0.0685,
0.0691,
0.0675,
0.0682,
0.0679,
0.0671,
0.0677,
0.0676,
0.0669,
0.0668,
0.0688,
0.0688,
0.0657,
0.0672,
0.0655,
0.0657,
0.0649,
0.0654,
0.0651,
0.0644,
0.0648,
0.0643,
0.0639,
0.0553,
0.055,
0.0549,
0.0549,
0.0543,
0.0544,
0.0542,
0.0537,
0.0541,
0.0536,
0.0534,
0.0532,
0.0533,

0.053,
0.0526,
0.0485,
0.0482,
0.0482,
0.0481,
0.0479,
0.0479,
0.0478,
0.0476,
0.0475,
0.0473,
0.0472,
0.0451,
0.045,
0.045,
0.0448,
0.0448,
0.0447,
0.0446,
0.0445,
0.0445,
0.0444,
0.0443,
0.0443,
0.0442,
0.0431,
0.043,
0.043,
0.0429,
0.0429,
0.0428,
0.0428,
0.0428,
0.0427,
0.0427,
0.0426,
0.0426,
0.0426,
0.042,
0.042,
0.0419,
0.0419,
0.0419,
0.0419,
0.0418,
0.0418,

```
 0.0418,
 0.0418,
 0.0417,
 0.0415,
 0.0414,
 0.0414,
 0.0414,
 0.0414,
 0.0414,
 0.0413,
 0.0413,
 0.0413,
 0.0413,
 0.0412,
 0.0411,
 0.0411,
 0.0411,
 0.0411,
 0.0411]
```

[16]:
```
test_loss
```

[16]:
```
[0.4202,
 0.3229,
 0.2836,
 0.2648,
 0.2551,
 0.2486,
 0.2343,
 0.2206,
 0.2218,
 0.2115,
 0.2079,
 0.2061,
 0.1995,
 0.1964,
 0.194,
 0.1897,
 0.1886,
 0.1818,
 0.1802,
 0.1752,
 0.1729,
 0.1707,
 0.1689,
 0.1643,
 0.1639,
```

0.1733,
0.1591,
0.1676,
0.1602,
0.1575,
0.1558,
0.1515,
0.1721,
0.1436,
0.1448,
0.1464,
0.1377,
0.1494,
0.1374,
0.1382,
0.1341,
0.1329,
0.1339,
0.1326,
0.1391,
0.1311,
0.1278,
0.1284,
0.1276,
0.1287,
0.1269,
0.1239,
0.1231,
0.1337,
0.1316,
0.123,
0.125,
0.1258,
0.1237,
0.118,
0.121,
0.1189,
0.1181,
0.1191,
0.1209,
0.1146,
0.1205,
0.1125,
0.1167,
0.1222,
0.1137,
0.1193,

0.1173,
0.1172,
0.1205,
0.1154,
0.1189,
0.1189,
0.1002,
0.1007,
0.1052,
0.1018,
0.0995,
0.0987,
0.1011,
0.0986,
0.1008,
0.0963,
0.0985,
0.1011,
0.0962,
0.0973,
0.1003,
0.1015,
0.1032,
0.0973,
0.0993,
0.0948,
0.0963,
0.0953,
0.0987,
0.0929,
0.094,
0.0978,
0.0938,
0.1,
0.0934,
0.0926,
0.0946,
0.0908,
0.0938,
0.0916,
0.1006,
0.0918,
0.0915,
0.0906,
0.0921,
0.1012,
0.0938,

0.0923,
0.0907,
0.0935,
0.0947,
0.0893,
0.0882,
0.088,
0.0876,
0.0888,
0.0866,
0.087,
0.0852,
0.0862,
0.0844,
0.0841,
0.086,
0.0836,
0.097,
0.0808,
0.0839,
0.0839,
0.0804,
0.0815,
0.088,
0.082,
0.0844,
0.081,
0.0854,
0.0829,
0.0841,
0.0808,
0.0795,
0.0806,
0.0792,
0.0758,
0.0789,
0.0763,
0.0797,
0.0801,
0.0787,
0.0785,
0.0742,
0.0824,
0.0783,
0.076,
0.085,
0.0755,

0.0744,
0.0786,
0.078,
0.0752,
0.0802,
0.0671,
0.0701,
0.0678,
0.0666,
0.0665,
0.0702,
0.0672,
0.0707,
0.0684,
0.0688,
0.0676,
0.0673,
0.0679,
0.0687,
0.0669,
0.0628,
0.0632,
0.0628,
0.0633,
0.0629,
0.0633,
0.0634,
0.063,
0.0633,
0.0629,
0.0632,
0.0619,
0.0624,
0.0616,
0.0617,
0.0622,
0.0621,
0.0621,
0.0626,
0.062,
0.0618,
0.0626,
0.062,
0.0618,
0.0614,
0.0614,
0.0613,

```
 0.0617,
 0.0614,
 0.0615,
 0.0613,
 0.0615,
 0.0614,
 0.0616,
 0.0615,
 0.0615,
 0.0616,
 0.0611,
 0.0613,
 0.0613,
 0.0613,
 0.0613,
 0.0614,
 0.0613,
 0.0614,
 0.0613,
 0.0613,
 0.0614,
 0.0612,
 0.0612,
 0.0612,
 0.0612,
 0.0613,
 0.0613,
 0.0613,
 0.0613,
 0.0613,
 0.0614,
 0.0613,
 0.0613,
 0.0613,
 0.0613,
 0.0613,
 0.061]
```

[17]:

```
dff = pd.DataFrame(data=train_loss, columns=['loss'])
dff['val_loss'] = test_loss
dff.head()
```

[17]:

	loss	val_loss
0	0.7097	0.4202
1	0.3570	0.3229
2	0.2981	0.2836
3	0.2714	0.2648

```
4  0.2560    0.2551
```

[18]:
```
import pickle
```

[21]:
```
f = open("Neural_network_history.pickle","wb")
pickle.dump(dff, f)
```

Notebook

December 19, 2023

1 File 12

[1]:
```
import sys
from pathlib import Path
prediction_mode_path = Path("../module")
sys.path.append(prediction_mode_path.as_posix())
import models_creation as pred_model

import pandas as pd
import numpy as np

from matplotlib import pyplot as plt
```

[2]:
```
pad = pd.read_excel('../Data/Kolchicyna_prepared_data.xlsx')
pad.head()
```

[2]:
```
   Unnamed: 0                              Publikacja DOI  \
0           0  https://doi.org/10.1016/j.bmcl.2021.128382
1           1  https://doi.org/10.1016/j.bmcl.2021.128382
2           2  https://doi.org/10.1016/j.bmcl.2021.128382
3           3  https://doi.org/10.1016/j.bmcl.2021.128382
4           4  https://doi.org/10.1016/j.bmcl.2021.128382

  Numer związku w publikacji  \
0                          1
1                          2
2                          3
3                          4
4                          5

                                              SMILES  Atywność [nM]   A549  \
0  COc2c3C1=CC=C(SC)C(=O)C=C1[C@H](CCc3cc(OC)c2OC…  Atywność [nM]  10,8
1  COc2c3C1=CC=C(SC)C(=O)C=C1[C@H](CCc3cc(OC)c2OC…  Atywność [nM]  11,6
2  COc2c3C1=CC=C(SC)C(=O)C=C1[C@H](CCc3cc(OC)c2OC…  Atywność [nM]  10,9
3  CC(C)CN[C@H]2CCc3cc(OC)c(OC)c(OC)c3C1=CC=C(SC)…  Atywność [nM]  10,5
4  COc2c3C1=CC=C(SC)C(=O)C=C1[C@H](CCc3cc(OC)c2OC…  Atywność [nM]  89,5

   MCF-7   LoVo LoVo/DX BALB/3T3  A549_float  MCF-7_float  LoVo_float  \
```

```
0  10,3    6,5    54,9    10,2          10.8          10.3          6.5
1  12,0    8,5     31.1    14.3         11.6          12.0          8.5
2  12,2    8,8    17,9    11,7          10.9          12.2          8.8
3  11,3    8,5    10,2    11,0          10.5          11.3          8.5
4  92,7   52,8    77,8    99,4          89.5          92.7         52.8

   LoVo/DX_float  BALB/3T3_float
0           54.9            10.2
1           31.1            14.3
2           17.9            11.7
3           10.2            11.0
4           77.8            99.4
```

[3]:
```python
list(pad.columns)[10:]
```

[3]:
```
['A549_float', 'MCF-7_float', 'LoVo_float', 'LoVo/DX_float', 'BALB/3T3_float']
```

[4]:
```python
molecular_descriptors_df = pred_model.prepare_data('../Data/
 ↪Kolchicyna_prepared_data.xlsx')
```

```
100%|
   | 120/120 [00:04<00:00, 29.90it/s]

Data size (rows, columns): (120, 1613)
Data size after first reduction (rows, columns): (120, 1443)
Data size after second reduction (rows, columns): (120, 1211)
```

[5]:
```python
molecular_descriptors_df.head()
```

[5]:
```
      AATS0Z  AATS0are    AATS0d   AATS0dv      AATS0i     AATS0m    AATS0p  \
0  25.090909  6.046518  3.109091  6.662626  160.775045  99.075564  1.555512
1  24.448276  6.008422  3.051724  6.386973  161.021674  96.473315  1.538471
2  23.868852  5.974074  3.000000  6.138434  161.244045  94.127025  1.523105
3  23.868852  5.974074  3.032787  6.171220  161.244045  94.127025  1.523105
4  23.343750  5.942945  2.953125  5.913194  161.445569  92.000700  1.509180

    AATS0pe    AATS0s   AATS0se  …     piPC5     piPC6     piPC7     piPC8  \
0  6.126680  3.435416  7.629859  …  6.098566  6.595759  6.979116  7.337313
1  6.088791  3.330998  7.596891  …  6.103048  6.601209  6.985613  7.349442
2  6.054630  3.236850  7.567166  …  6.105281  6.603923  6.989306  7.353932
3  6.054630  3.257798  7.567166  …  6.107510  6.606629  6.992068  7.361425
4  6.023670  3.151529  7.540228  …  6.107510  6.605277  6.991148  7.356489

      piPC9  A549_float  MCF-7_float  LoVo_float  LoVo/DX_float  \
0  7.681445        10.8         10.3         6.5           54.9
1  7.695531        11.6         12.0         8.5           31.1
2  7.704023        10.9         12.2         8.8           17.9
3  7.709420        10.5         11.3         8.5           10.2
```

```
4  7.707175        89.5         92.7        52.8           77.8

   BALB/3T3_float
0            10.2
1            14.3
2            11.7
3            11.0
4            99.4

[5 rows x 1216 columns]
```

[6]:
```python
temp_li = list(pad.columns)[10:]
```

[7]:
```python
temp_li
```

[7]:
```
['A549_float', 'MCF-7_float', 'LoVo_float', 'LoVo/DX_float', 'BALB/3T3_float']
```

[8]:
```python
for element in temp_li:
    molecular_descriptors_df[str(element+('_transformed'))] = pred_model.
↪transform(molecular_descriptors_df[element])
```

[9]:
```python
molecular_descriptors_df.head()
```

[9]:
```
      AATS0Z  AATS0are    AATS0d   AATS0dv      AATS0i     AATS0m    AATS0p  \
0  25.090909  6.046518  3.109091  6.662626  160.775045  99.075564  1.555512
1  24.448276  6.008422  3.051724  6.386973  161.021674  96.473315  1.538471
2  23.868852  5.974074  3.000000  6.138434  161.244045  94.127025  1.523105
3  23.868852  5.974074  3.032787  6.171220  161.244045  94.127025  1.523105
4  23.343750  5.942945  2.953125  5.913194  161.445569  92.000700  1.509180

    AATS0pe    AATS0s   AATS0se  …  A549_float  MCF-7_float  LoVo_float  \
0  6.126680  3.435416  7.629859  …        10.8         10.3         6.5
1  6.088791  3.330998  7.596891  …        11.6         12.0         8.5
2  6.054630  3.236850  7.567166  …        10.9         12.2         8.8
3  6.054630  3.257798  7.567166  …        10.5         11.3         8.5
4  6.023670  3.151529  7.540228  …        89.5         92.7        52.8

   LoVo/DX_float  BALB/3T3_float  A549_float_transformed  \
0           54.9            10.2                7.966576
1           31.1            14.3                7.935542
2           17.9            11.7                7.962574
3           10.2            11.0                7.978811
4           77.8            99.4                7.048177

   MCF-7_float_transformed  LoVo_float_transformed  LoVo/DX_float_transformed  \
0                 7.987163                8.187087                   7.260428
1                 7.920819                8.070581                   7.507240
```

```
2                 7.913640             8.055517              7.747147
3                 7.946922             8.070581              7.991400
4                 7.032920             7.277366              7.109020

   BALB/3T3_float_transformed
0                    7.991400
1                    7.844664
2                    7.931814
3                    7.958607
4                    7.002614

[5 rows x 1221 columns]
```

[10]:
```
molecular_descriptors_df.to_excel('../Data/Kolchicyna_machine_learning.xlsx')
```

[11]:
```
for hist_ in temp_li:
    fig, axes = plt.subplots(1, 2, layout="constrained")

    print(molecular_descriptors_df[hist_].hist(ax=axes[0]))
    print(molecular_descriptors_df[hist_+'_transformed'].hist(ax=axes[1]))
    print(fig.suptitle('Before (left) and after (right) transformation:␣
 ↪'+(hist_)))
```

```
AxesSubplot(0.125,0.11;0.352273x0.77)
AxesSubplot(0.547727,0.11;0.352273x0.77)
Text(0.5, 0.98, 'Before (left) and after (right) transformation: A549_float')
AxesSubplot(0.125,0.11;0.352273x0.77)
AxesSubplot(0.547727,0.11;0.352273x0.77)
Text(0.5, 0.98, 'Before (left) and after (right) transformation: MCF-7_float')
AxesSubplot(0.125,0.11;0.352273x0.77)
AxesSubplot(0.547727,0.11;0.352273x0.77)
Text(0.5, 0.98, 'Before (left) and after (right) transformation: LoVo_float')
AxesSubplot(0.125,0.11;0.352273x0.77)
AxesSubplot(0.547727,0.11;0.352273x0.77)
Text(0.5, 0.98, 'Before (left) and after (right) transformation: LoVo/DX_float')
AxesSubplot(0.125,0.11;0.352273x0.77)
AxesSubplot(0.547727,0.11;0.352273x0.77)
Text(0.5, 0.98, 'Before (left) and after (right) transformation:
BALB/3T3_float')
```

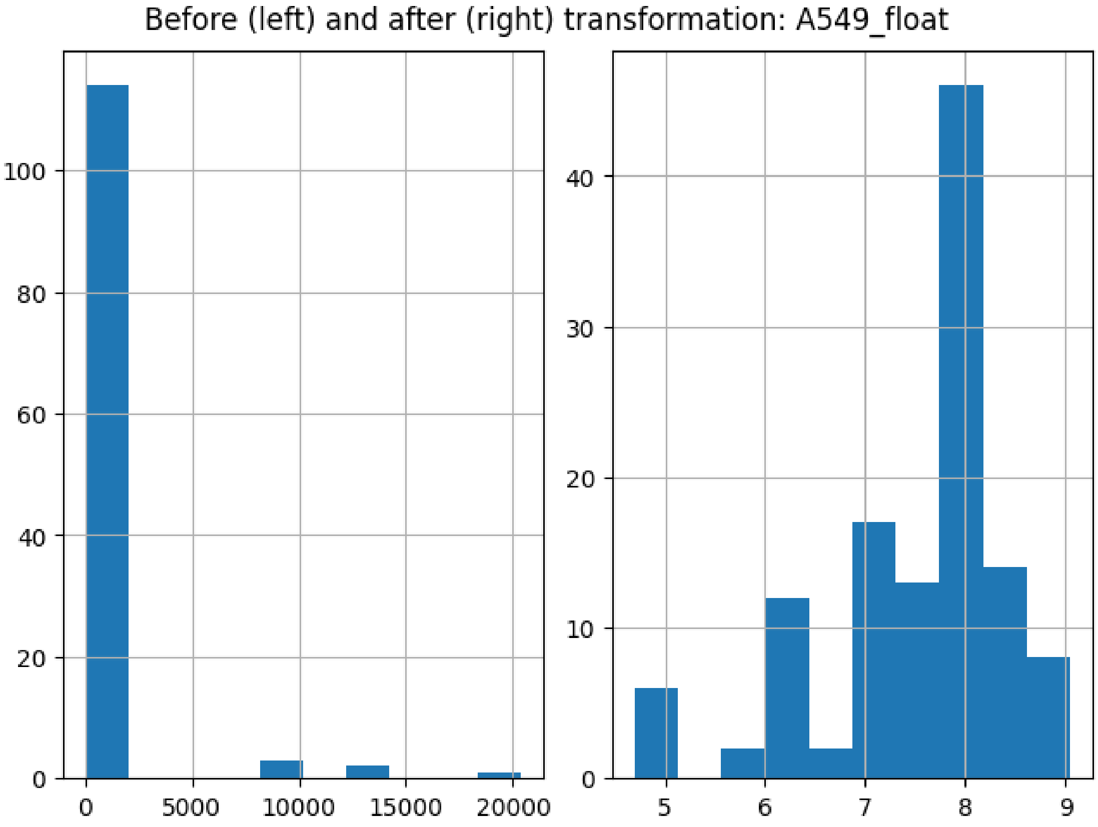
Before (left) and after (right) transformation: A549_float
0
20
40
60
80
100
0
5000
10000
15000
20000
0
10
20
30
40
5
6
7
8
9

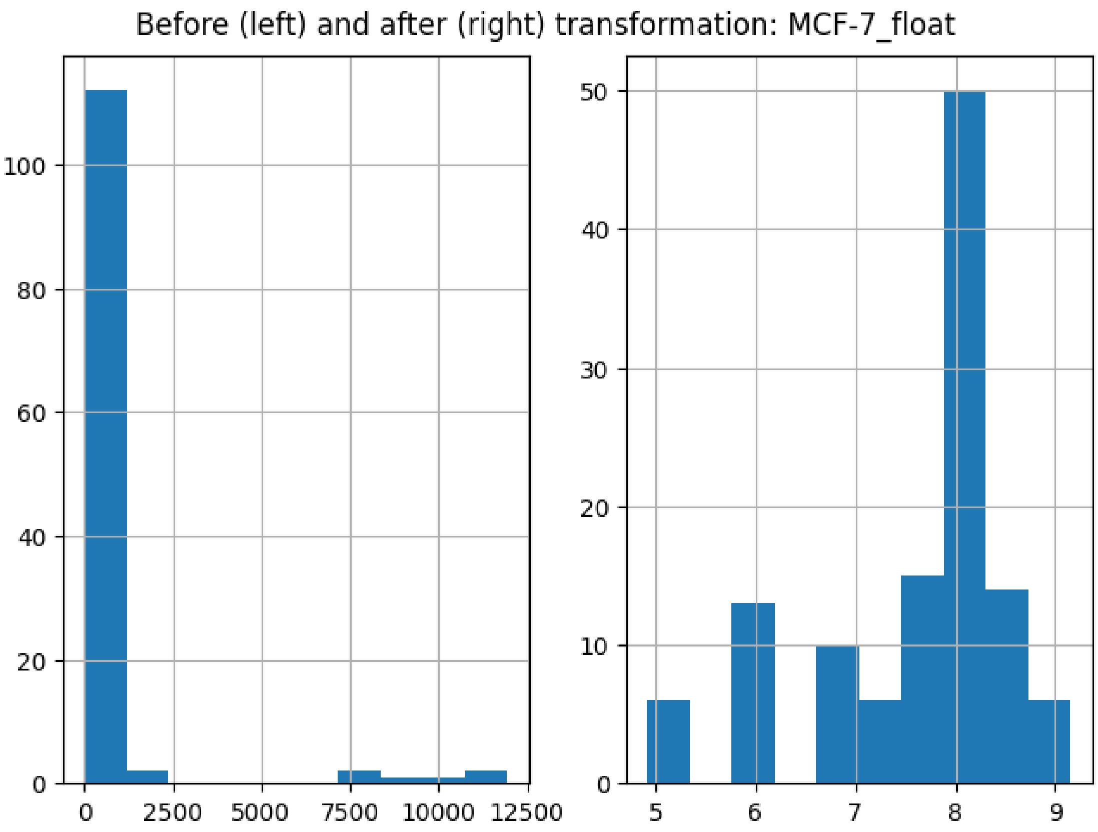

Before (left) and after (right) transformation: MCF-7_float
0
20
40
60
80
100
0
2500
5000
7500
10000
12500
0
10
20
30
40
50
5
6
7
8
9

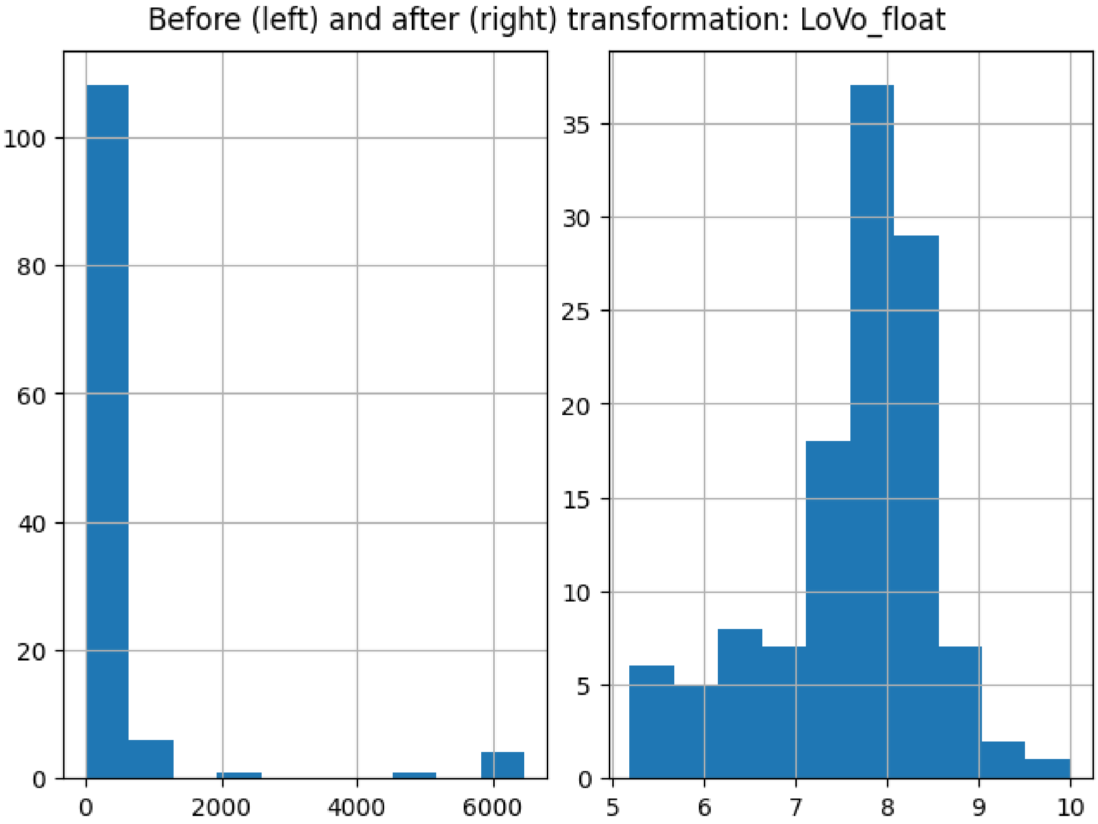
Before (left) and after (right) transformation: LoVo_float
0
20
40
60
80
100
0
2000
4000
6000
0
5
10
15
20
25
30
35
5
6
7
8
9
10

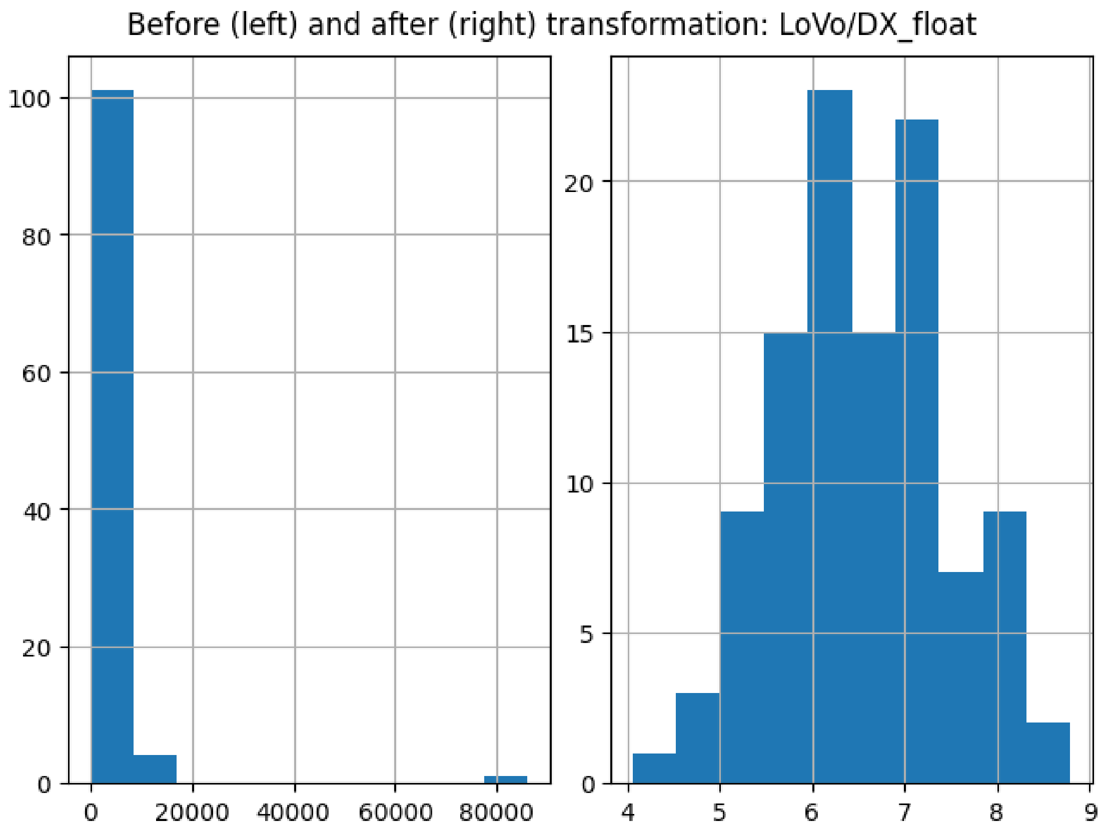
Before (left) and after (right) transformation: LoVo/DX_float
0
20
40
60
80
100
0
20000
40000
60000
80000
0
5
10
15
20
4
5
6
7
8
9

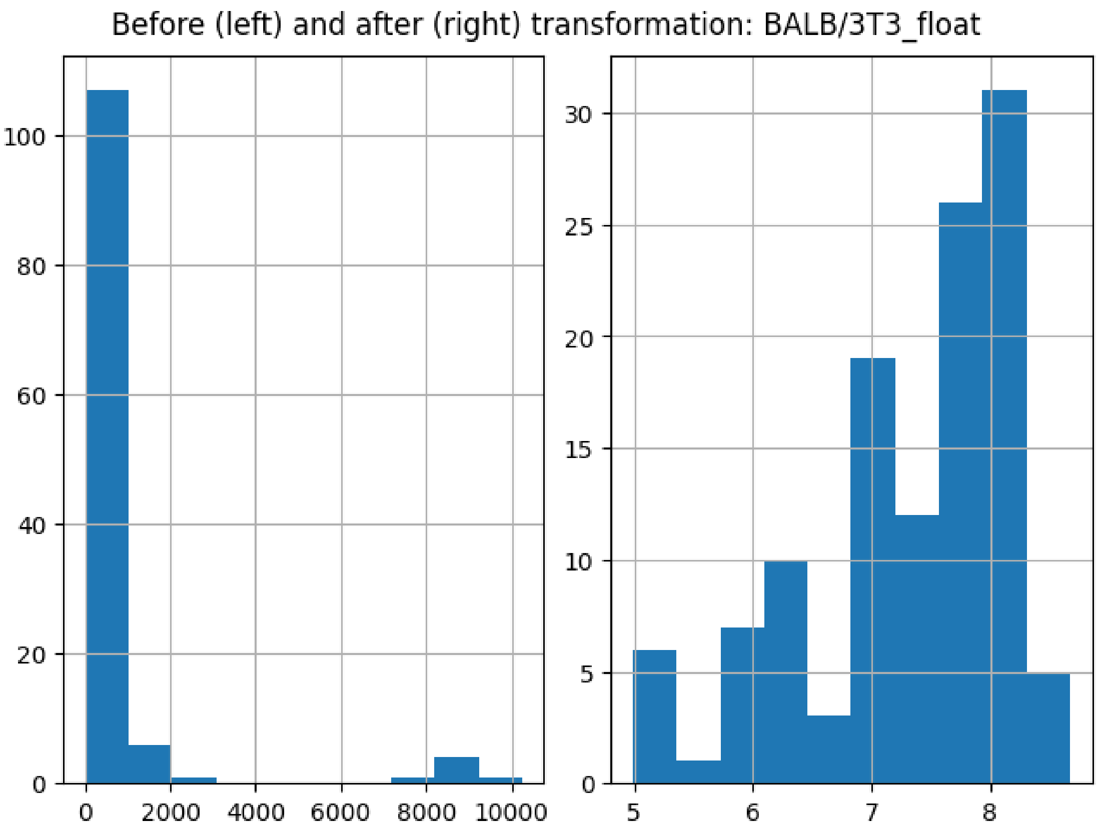

Before (left) and after (right) transformation: BALB/3T3_float
0
20
40
60
80
100
0
2000
4000
6000
8000
10000
0
5
10
15
20
25
30
5
6
7
8

Notebook

December 19, 2023

1 File 14

[1]:
```
import sys
from pathlib import Path
prediction_mode_path = Path("../module")
sys.path.append(prediction_mode_path.as_posix())
import models_creation as pred_model

import pandas as pd
import numpy as np

from sklearn.metrics import r2_score

import matplotlib.pyplot as plt
from sklearn.tree import plot_tree

from matplotlib import pyplot as plt

import joblib

import warnings
warnings.filterwarnings('ignore')
```

[2]:
```
load_prepared_data = pd.read_excel('../Data/Kolchicyna_machine_learning.xlsx')
load_prepared_data = load_prepared_data.drop(columns=['Unnamed: 0'])
```

[3]:
```
load_prepared_data.head()
```

[3]:
```
      AATS0Z  AATS0are   AATS0d   AATS0dv      AATS0i     AATS0m    AATS0p  \
0  25.090909  6.046518  3.109091  6.662626  160.775045  99.075564  1.555512
1  24.448276  6.008422  3.051724  6.386973  161.021674  96.473315  1.538471
2  23.868852  5.974074  3.000000  6.138434  161.244045  94.127025  1.523105
3  23.868852  5.974074  3.032787  6.171220  161.244045  94.127025  1.523105
4  23.343750  5.942945  2.953125  5.913194  161.445569  92.000700  1.509180

    AATS0pe    AATS0s   AATS0se  …  A549_float  MCF-7_float  LoVo_float  \
0  6.126680  3.435416  7.629859  …        10.8         10.3         6.5
1  6.088791  3.330998  7.596891  …        11.6         12.0         8.5
```

```
2  6.054630  3.236850  7.567166  …        10.9         12.2          8.8
3  6.054630  3.257798  7.567166  …        10.5         11.3          8.5
4  6.023670  3.151529  7.540228  …        89.5         92.7         52.8

   LoVo/DX_float  BALB/3T3_float  A549_float_transformed  \
0           54.9            10.2                7.966576
1           31.1            14.3                7.935542
2           17.9            11.7                7.962574
3           10.2            11.0                7.978811
4           77.8            99.4                7.048177

   MCF-7_float_transformed  LoVo_float_transformed  LoVo/DX_float_transformed  \
0                 7.987163                8.187087                   7.260428
1                 7.920819                8.070581                   7.507240
2                 7.913640                8.055517                   7.747147
3                 7.946922                8.070581                   7.991400
4                 7.032920                7.277366                   7.109020

   BALB/3T3_float_transformed
0                    7.991400
1                    7.844664
2                    7.931814
3                    7.958607
4                    7.002614

[5 rows x 1221 columns]
```

[4]:
```
list(load_prepared_data.columns)[-5:]
```

[4]:
```
['A549_float_transformed',
 'MCF-7_float_transformed',
 'LoVo_float_transformed',
 'LoVo/DX_float_transformed',
 'BALB/3T3_float_transformed']
```

[5]:
```
list(load_prepared_data.columns)[0:-5][-6] #last molecular descriptor
```

[5]:
```
'piPC9'
```

[6]:
```
list(load_prepared_data.columns)[0:-10][-1]
```

[6]:
```
'piPC9'
```

[7]:
```
len(list(load_prepared_data.columns)[0:-10])
```

[7]:
```
1211
```

1.1 A549

[8]:
```
data = load_prepared_data[list(load_prepared_data.columns)[0:-10]]
```

[9]:
```
data['A549'] = load_prepared_data[load_prepared_data.columns[-5]]
```

[10]:
```
data.head()
```

[10]:
```
     AATS0Z  AATS0are   AATS0d   AATS0dv      AATS0i     AATS0m    AATS0p  \
0  25.090909  6.046518  3.109091  6.662626  160.775045  99.075564  1.555512
1  24.448276  6.008422  3.051724  6.386973  161.021674  96.473315  1.538471
2  23.868852  5.974074  3.000000  6.138434  161.244045  94.127025  1.523105
3  23.868852  5.974074  3.032787  6.171220  161.244045  94.127025  1.523105
4  23.343750  5.942945  2.953125  5.913194  161.445569  92.000700  1.509180

    AATS0pe   AATS0s  AATS0se  …   piPC10    piPC2    piPC3    piPC4  \
0  6.126680  3.435416  7.629859  …  7.999655  4.266195  4.915408  5.516700
1  6.088791  3.330998  7.596891  …  8.018726  4.280132  4.922714  5.520711
2  6.054630  3.236850  7.567166  …  8.028799  4.293878  4.929967  5.524706
3  6.054630  3.257798  7.567166  …  8.037440  4.307438  4.929967  5.524706
4  6.023670  3.151529  7.540228  …  8.034892  4.307438  4.937168  5.528685

     piPC5    piPC6    piPC7    piPC8    piPC9     A549
0  6.098566  6.595759  6.979116  7.337313  7.681445  7.966576
1  6.103048  6.601209  6.985613  7.349442  7.695531  7.935542
2  6.105281  6.603923  6.989306  7.353932  7.704023  7.962574
3  6.107510  6.606629  6.992068  7.361425  7.709420  7.978811
4  6.107510  6.605277  6.991148  7.356489  7.707175  7.048177

[5 rows x 1212 columns]
```

[11]:
```
pred_model.correlation_dataframe(data, 0.3, 'A549').shape
```

[11]: (127, 3)

[12]:
```
pred_model.correlation_dataframe(data, 0.5, 'A549')
```

[12]:
```
     molecular descriptor name  corr_value  absolute correlation value
226                     AMID_O   -0.557717                    0.557717
505                 EState_VSA5   -0.534748                    0.534748
791                    MDEO-12   -0.555724                    0.555724
921                      SaasC    0.513071                    0.513071
1091               VSA_EState5    0.526082                    0.526082
```

[13]:
```
pred_model.correlation_dataframe(data, 0.54, 'A549') # => these descriptors␣
↪will be used to build models
```

[13]:
```
     molecular descriptor name  corr_value  absolute correlation value
226                     AMID_O   -0.557717                    0.557717
791                   MDEO-12   -0.555724                    0.555724
```

1.2 MCF-7

[14]:
```python
data = load_prepared_data[list(load_prepared_data.columns)[0:-10]]
data['MCF-7'] = load_prepared_data[load_prepared_data.columns[-4]]
data.head()
```

[14]:
```
     AATS0Z  AATS0are    AATS0d   AATS0dv      AATS0i     AATS0m    AATS0p  \
0  25.090909  6.046518  3.109091  6.662626  160.775045  99.075564  1.555512
1  24.448276  6.008422  3.051724  6.386973  161.021674  96.473315  1.538471
2  23.868852  5.974074  3.000000  6.138434  161.244045  94.127025  1.523105
3  23.868852  5.974074  3.032787  6.171220  161.244045  94.127025  1.523105
4  23.343750  5.942945  2.953125  5.913194  161.445569  92.000700  1.509180

   AATS0pe   AATS0s   AATS0se  …    piPC10     piPC2     piPC3     piPC4  \
0  6.126680  3.435416  7.629859  …  7.999655  4.266195  4.915408  5.516700
1  6.088791  3.330998  7.596891  …  8.018726  4.280132  4.922714  5.520711
2  6.054630  3.236850  7.567166  …  8.028799  4.293878  4.929967  5.524706
3  6.054630  3.257798  7.567166  …  8.037440  4.307438  4.929967  5.524706
4  6.023670  3.151529  7.540228  …  8.034892  4.307438  4.937168  5.528685

     piPC5     piPC6     piPC7     piPC8     piPC9     MCF-7
0  6.098566  6.595759  6.979116  7.337313  7.681445  7.987163
1  6.103048  6.601209  6.985613  7.349442  7.695531  7.920819
2  6.105281  6.603923  6.989306  7.353932  7.704023  7.913640
3  6.107510  6.606629  6.992068  7.361425  7.709420  7.946922
4  6.107510  6.605277  6.991148  7.356489  7.707175  7.032920

[5 rows x 1212 columns]
```

[15]:
```python
pred_model.correlation_dataframe(data, 0.3, 'MCF-7').shape
```

[15]:
```
(135, 3)
```

[16]:
```python
pred_model.correlation_dataframe(data, 0.5, 'MCF-7')
```

[16]:
```
     molecular descriptor name  corr_value  absolute correlation value
226                     AMID_O   -0.520578                    0.520578
505                EState_VSA5   -0.578904                    0.578904
506                EState_VSA6    0.519794                    0.519794
791                   MDEO-12   -0.525441                    0.525441
```

[17]:
```python
pred_model.correlation_dataframe(data, 0.525, 'MCF-7') # => these descriptors␣
↪will be used to build models
```

[17]:
```
     molecular descriptor name  corr_value  absolute correlation value
505                EState_VSA5   -0.578904                    0.578904
791                   MDEO-12   -0.525441                    0.525441
```

1.3 LoVo

[18]:
```
data = load_prepared_data[list(load_prepared_data.columns)[0:-10]]
data['LoVo'] = load_prepared_data[load_prepared_data.columns[-3]]
data.head()
```

[18]:
```
     AATS0Z  AATS0are   AATS0d   AATS0dv      AATS0i     AATS0m    AATS0p  \
0  25.090909  6.046518  3.109091  6.662626  160.775045  99.075564  1.555512
1  24.448276  6.008422  3.051724  6.386973  161.021674  96.473315  1.538471
2  23.868852  5.974074  3.000000  6.138434  161.244045  94.127025  1.523105
3  23.868852  5.974074  3.032787  6.171220  161.244045  94.127025  1.523105
4  23.343750  5.942945  2.953125  5.913194  161.445569  92.000700  1.509180

    AATS0pe   AATS0s   AATS0se  …    piPC10     piPC2     piPC3     piPC4  \
0  6.126680  3.435416  7.629859  …  7.999655  4.266195  4.915408  5.516700
1  6.088791  3.330998  7.596891  …  8.018726  4.280132  4.922714  5.520711
2  6.054630  3.236850  7.567166  …  8.028799  4.293878  4.929967  5.524706
3  6.054630  3.257798  7.567166  …  8.037440  4.307438  4.929967  5.524706
4  6.023670  3.151529  7.540228  …  8.034892  4.307438  4.937168  5.528685

      piPC5     piPC6     piPC7     piPC8     piPC9      LoVo
0  6.098566  6.595759  6.979116  7.337313  7.681445  8.187087
1  6.103048  6.601209  6.985613  7.349442  7.695531  8.070581
2  6.105281  6.603923  6.989306  7.353932  7.704023  8.055517
3  6.107510  6.606629  6.992068  7.361425  7.709420  8.070581
4  6.107510  6.605277  6.991148  7.356489  7.707175  7.277366

[5 rows x 1212 columns]
```

[19]:
```
pred_model.correlation_dataframe(data, 0.3, 'LoVo').shape
```

[19]: (83, 3)

[20]:
```
pred_model.correlation_dataframe(data, 0.5, 'LoVo')
```

[20]:
```
      molecular descriptor name  corr_value  absolute correlation value
226                      AMID_O   -0.524157                    0.524157
505                 EState_VSA5   -0.579664                    0.579664
791                     MDEO-12   -0.558727                    0.558727
851                       NdssC   -0.506855                    0.506855
1091                VSA_EState5    0.503578                    0.503578
```

[21]:
```
pred_model.correlation_dataframe(data, 0.55, 'LoVo') # => these descriptors␣
↪will be used to build models
```

[21]:
```
     molecular descriptor name  corr_value  absolute correlation value
505                EState_VSA5   -0.579664                    0.579664
791                    MDEO-12   -0.558727                    0.558727
```

1.4 LoVo/DX

[22]:
```python
data = load_prepared_data[list(load_prepared_data.columns)[0:-10]]
data['LoVo/DX'] = load_prepared_data[load_prepared_data.columns[-2]]
data.head()
```

[22]:
```
      AATS0Z  AATS0are    AATS0d    AATS0dv      AATS0i     AATS0m    AATS0p  \
0  25.090909  6.046518  3.109091  6.662626  160.775045  99.075564  1.555512
1  24.448276  6.008422  3.051724  6.386973  161.021674  96.473315  1.538471
2  23.868852  5.974074  3.000000  6.138434  161.244045  94.127025  1.523105
3  23.868852  5.974074  3.032787  6.171220  161.244045  94.127025  1.523105
4  23.343750  5.942945  2.953125  5.913194  161.445569  92.000700  1.509180

    AATS0pe    AATS0s   AATS0se  …    piPC10     piPC2     piPC3     piPC4  \
0  6.126680  3.435416  7.629859  …  7.999655  4.266195  4.915408  5.516700
1  6.088791  3.330998  7.596891  …  8.018726  4.280132  4.922714  5.520711
2  6.054630  3.236850  7.567166  …  8.028799  4.293878  4.929967  5.524706
3  6.054630  3.257798  7.567166  …  8.037440  4.307438  4.929967  5.524706
4  6.023670  3.151529  7.540228  …  8.034892  4.307438  4.937168  5.528685

      piPC5     piPC6     piPC7     piPC8     piPC9   LoVo/DX
0  6.098566  6.595759  6.979116  7.337313  7.681445  7.260428
1  6.103048  6.601209  6.985613  7.349442  7.695531  7.507240
2  6.105281  6.603923  6.989306  7.353932  7.704023  7.747147
3  6.107510  6.606629  6.992068  7.361425  7.709420  7.991400
4  6.107510  6.605277  6.991148  7.356489  7.707175  7.109020

[5 rows x 1212 columns]
```

[23]:
```python
print(data.shape)
data = data.dropna()
```

```
(120, 1212)
```

[24]:
```python
data.shape
```

[24]:
```
(106, 1212)
```

[25]:
```python
pred_model.correlation_dataframe(data, 0.3, 'LoVo/DX').shape
```

[25]:
```
(601, 3)
```

[26]:
```python
pred_model.correlation_dataframe(data, 0.5, 'LoVo/DX').shape
```

[26]: (50, 3)

[27]:
```
pred_model.correlation_dataframe(data, 0.64, 'LoVo/DX') # => these descriptors␣
↪will be used to build models
```

[27]:

	molecular descriptor name	corr_value	absolute correlation value
556	GATS2c	0.652245	0.652245
851	NdssC	-0.647960	0.647960
881	RNCG	0.663041	0.663041

1.5 BALB/3T3

[28]:
```
data = load_prepared_data[list(load_prepared_data.columns)[0:-10]]
data['BALB/3T3'] = load_prepared_data[load_prepared_data.columns[-1]]
data.head()
```

[28]:

	AATS0Z	AATS0are	AATS0d	AATS0dv	AATS0i	AATS0m	AATS0p	\
0	25.090909	6.046518	3.109091	6.662626	160.775045	99.075564	1.555512	
1	24.448276	6.008422	3.051724	6.386973	161.021674	96.473315	1.538471	
2	23.868852	5.974074	3.000000	6.138434	161.244045	94.127025	1.523105	
3	23.868852	5.974074	3.032787	6.171220	161.244045	94.127025	1.523105	
4	23.343750	5.942945	2.953125	5.913194	161.445569	92.000700	1.509180	

	AATS0pe	AATS0s	AATS0se	…	piPC10	piPC2	piPC3	piPC4	\
0	6.126680	3.435416	7.629859	…	7.999655	4.266195	4.915408	5.516700	
1	6.088791	3.330998	7.596891	…	8.018726	4.280132	4.922714	5.520711	
2	6.054630	3.236850	7.567166	…	8.028799	4.293878	4.929967	5.524706	
3	6.054630	3.257798	7.567166	…	8.037440	4.307438	4.929967	5.524706	
4	6.023670	3.151529	7.540228	…	8.034892	4.307438	4.937168	5.528685	

	piPC5	piPC6	piPC7	piPC8	piPC9	BALB/3T3
0	6.098566	6.595759	6.979116	7.337313	7.681445	7.991400
1	6.103048	6.601209	6.985613	7.349442	7.695531	7.844664
2	6.105281	6.603923	6.989306	7.353932	7.704023	7.931814
3	6.107510	6.606629	6.992068	7.361425	7.709420	7.958607
4	6.107510	6.605277	6.991148	7.356489	7.707175	7.002614

[5 rows x 1212 columns]

[29]:
```
pred_model.correlation_dataframe(data, 0.3, 'BALB/3T3').shape
```

[29]: (112, 3)

[30]:
```
pred_model.correlation_dataframe(data, 0.5, 'BALB/3T3')
```

[30]:

	molecular descriptor name	corr_value	absolute correlation value
226	AMID_O	-0.546843	0.546843
505	EState_VSA5	-0.588780	0.588780

```
556                         GATS2c    0.511671                        0.511671
791                        MDEO-12   -0.582747                        0.582747
847                            NdO   -0.500462                        0.500462
851                          NdssC   -0.526783                        0.526783
1091                   VSA_EState5    0.529677                        0.529677
```

[31]:
```python
pred_model.correlation_dataframe(data, 0.58, 'BALB/3T3') # => these descriptors␣
↪will be used to build models
```

[31]:
```
    molecular descriptor name  corr_value  absolute correlation value
505               EState_VSA5   -0.588780                    0.588780
791                   MDEO-12   -0.582747                    0.582747
```

Notebook

January 18, 2024

1 File 15

[1]:
```
import sys
from pathlib import Path
prediction_mode_path = Path("../module")
sys.path.append(prediction_mode_path.as_posix())
import models_creation as pred_model

import pandas as pd
import numpy as np

from sklearn.metrics import r2_score

import matplotlib.pyplot as plt
from sklearn.tree import plot_tree

from matplotlib import pyplot as plt

import joblib

import warnings
warnings.filterwarnings('ignore')
```

[2]:
```
load_prepared_data = pd.read_excel('../Data/Kolchicyna_machine_learning.xlsx')
load_prepared_data = load_prepared_data.drop(columns=['Unnamed: 0'])

target = 'A549'

corr_low = 0.33
corr_high = 0.54

random_state = 15
```

[3]:
```
load_prepared_data.head()
```

[3]:
```
      AATS0Z  AATS0are    AATS0d   AATS0dv      AATS0i     AATS0m    AATS0p  \
0  25.090909  6.046518  3.109091  6.662626  160.775045  99.075564  1.555512
1  24.448276  6.008422  3.051724  6.386973  161.021674  96.473315  1.538471
```

```
2  23.868852  5.974074  3.000000  6.138434  161.244045  94.127025  1.523105
3  23.868852  5.974074  3.032787  6.171220  161.244045  94.127025  1.523105
4  23.343750  5.942945  2.953125  5.913194  161.445569  92.000700  1.509180

    AATS0pe    AATS0s    AATS0se  …  A549_float  MCF-7_float  LoVo_float  \
0  6.126680  3.435416  7.629859  …        10.8         10.3         6.5
1  6.088791  3.330998  7.596891  …        11.6         12.0         8.5
2  6.054630  3.236850  7.567166  …        10.9         12.2         8.8
3  6.054630  3.257798  7.567166  …        10.5         11.3         8.5
4  6.023670  3.151529  7.540228  …        89.5         92.7        52.8

   LoVo/DX_float  BALB/3T3_float  A549_float_transformed  \
0           54.9            10.2                7.966576
1           31.1            14.3                7.935542
2           17.9            11.7                7.962574
3           10.2            11.0                7.978811
4           77.8            99.4                7.048177

   MCF-7_float_transformed  LoVo_float_transformed  LoVo/DX_float_transformed  \
0                 7.987163                8.187087                   7.260428
1                 7.920819                8.070581                   7.507240
2                 7.913640                8.055517                   7.747147
3                 7.946922                8.070581                   7.991400
4                 7.032920                7.277366                   7.109020

   BALB/3T3_float_transformed
0                    7.991400
1                    7.844664
2                    7.931814
3                    7.958607
4                    7.002614

[5 rows x 1221 columns]
```

[4]:
```python
data = load_prepared_data[list(load_prepared_data.columns)[0:-10]]
data[target] = load_prepared_data[load_prepared_data.columns[-5]]
data.head()
```

[4]:
```
      AATS0Z  AATS0are    AATS0d   AATS0dv      AATS0i     AATS0m    AATS0p  \
0  25.090909  6.046518  3.109091  6.662626  160.775045  99.075564  1.555512
1  24.448276  6.008422  3.051724  6.386973  161.021674  96.473315  1.538471
2  23.868852  5.974074  3.000000  6.138434  161.244045  94.127025  1.523105
3  23.868852  5.974074  3.032787  6.171220  161.244045  94.127025  1.523105
4  23.343750  5.942945  2.953125  5.913194  161.445569  92.000700  1.509180

    AATS0pe    AATS0s    AATS0se  …    piPC10     piPC2     piPC3     piPC4  \
0  6.126680  3.435416  7.629859  …  7.999655  4.266195  4.915408  5.516700
```

```
1  6.088791  3.330998  7.596891  …  8.018726  4.280132  4.922714  5.520711
2  6.054630  3.236850  7.567166  …  8.028799  4.293878  4.929967  5.524706
3  6.054630  3.257798  7.567166  …  8.037440  4.307438  4.929967  5.524706
4  6.023670  3.151529  7.540228  …  8.034892  4.307438  4.937168  5.528685

     piPC5     piPC6     piPC7     piPC8     piPC9      A549
0  6.098566  6.595759  6.979116  7.337313  7.681445  7.966576
1  6.103048  6.601209  6.985613  7.349442  7.695531  7.935542
2  6.105281  6.603923  6.989306  7.353932  7.704023  7.962574
3  6.107510  6.606629  6.992068  7.361425  7.709420  7.978811
4  6.107510  6.605277  6.991148  7.356489  7.707175  7.048177

[5 rows x 1212 columns]
```

2 Multiple Linear regression (MLR)

[5]:
```
without_standardization, train_r2_, test_r2_, hist1, hist2, target_column_name,␣
↪training_data_RMSE, test_data_RMSE  = pred_model.
↪prepare_data_and_create_model(molecular_descriptors_df=data,
                                                                               ␣
↪                    correlation_threshold=0.5,
                                                                               ␣
↪                    standardization=False,
                                                                               ␣
↪                    model_type='linear_model',
                                                                               ␣
↪                    target_column_name = str(target),
                                                                               ␣
↪                    random_state=random_state,
                                                                               ␣
↪                    train_test_split_=True,
                                                                               ␣
↪                    verbose=True)
```

```
I am not doing standardization…
  molecular descriptor name
0                    AATS0Z
1                  AATS0are
2                    AATS0d
3                   AATS0dv
4                    AATS0i
  molecular descriptor name  corr_value
0                    AATS0Z   -0.022797
1                  AATS0are   -0.139064
2                    AATS0d    0.027592
3                   AATS0dv   -0.136049
```

```
4                  AATS0i    0.168958
  molecular descriptor name  corr_value  absolute correlation value
0                  AATS0Z   -0.022797                    0.022797
1                AATS0are   -0.139064                    0.139064
2                  AATS0d    0.027592                    0.027592
3                 AATS0dv   -0.136049                    0.136049
4                  AATS0i    0.168958                    0.168958
     molecular descriptor name  corr_value  absolute correlation value
226                     AMID_O   -0.557717                    0.557717
505                EState_VSA5   -0.534748                    0.534748
791                    MDEO-12   -0.555724                    0.555724
921                      SaasC    0.513071                    0.513071
1091               VSA_EState5    0.526082                    0.526082
     molecular descriptor name  corr_value  absolute correlation value
226                     AMID_O   -0.557717                    0.557717
505                EState_VSA5   -0.534748                    0.534748
791                    MDEO-12   -0.555724                    0.555724
921                      SaasC    0.513071                    0.513071
1091               VSA_EState5    0.526082                    0.526082
The model used is: LinearReg…
Return the coefficient of determination of the prediction:
0.607684302836734
R^2 score: 0.45207360740354974
Correlation coefficient: 0.6723641925352284
Test data - unseen during training:
R^2 score: 0.607684302836734
Correlation coefficient: 0.7795410847650905
[7.84879747 6.24299325 7.37407883 8.0208511  7.9100943  8.19522969
 8.19061816 7.98447887 7.96950463 7.58923738 6.86384061 7.61628624
 7.4997649  7.87840731 8.14591404 8.10506343 6.33645764 5.85493895]
102    7.920819
38     6.019997
8      5.996841
109    7.886057
11     7.962574
51     7.886057
88     8.075721
21     7.325139
82     7.978811
98     7.481486
58     7.677781
64     8.537602
74     8.142668
103    8.397940
91     8.958607
43     7.823909
25     4.886525
30     6.009661
```

```
Name: A549, dtype: float64
Training Root Mean Square Error: 0.6910343772412224
Testing Root Mean Square Error: 0.6492799270001024
```

[6]:
```python
print(target_column_name+str('_transformed'))
print(hist1[target_column_name].hist())
```

```
A549_transformed
AxesSubplot(0.125,0.11;0.775x0.77)
```

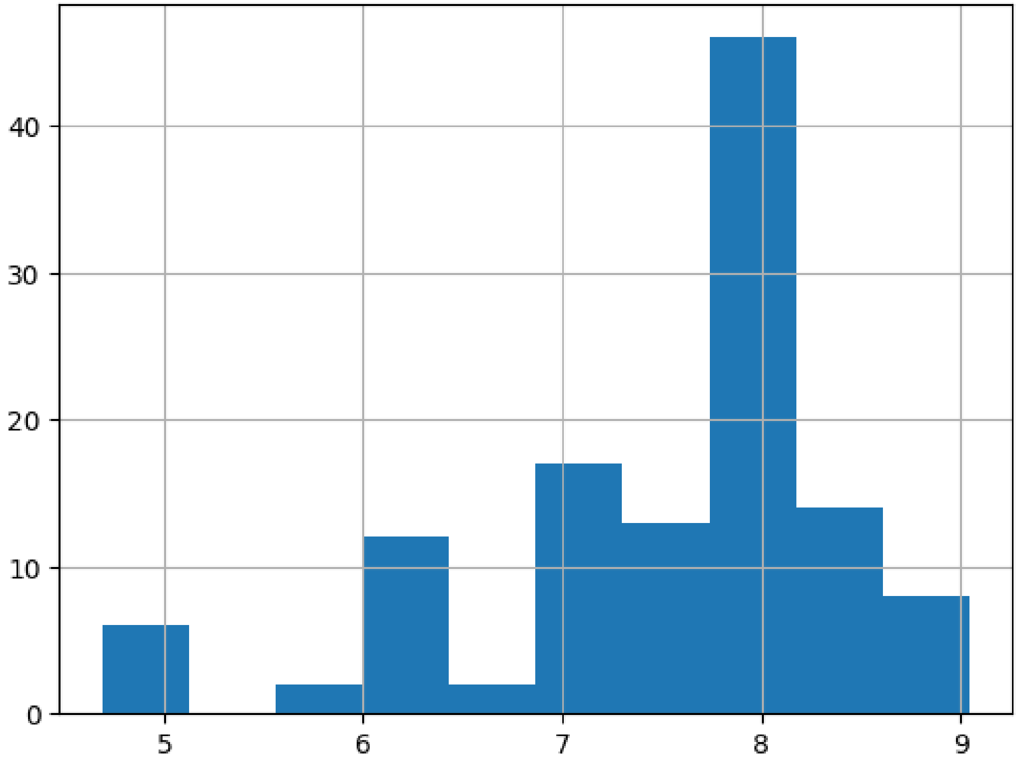

[7]:
```python
with_standardization, train_r2_, test_r2_, hist1, hist2, target_column_name,␣
↪training_data_RMSE, test_data_RMSE  = pred_model.
↪prepare_data_and_create_model(molecular_descriptors_df=data,
                                                                          ␣
↪                       correlation_threshold=0.50,
                                                                          ␣
↪                       standardization=True,
                                                                          ␣
↪                       model_type='linear_model',
                                                                          ␣
↪                       target_column_name = target,
```

```
                    random_state=random_state,
                    train_test_split_=True,
                    verbose=True)
```

```
I am doing standardization…
  molecular descriptor name
0                    AATS0Z
1                  AATS0are
2                    AATS0d
3                   AATS0dv
4                    AATS0i
  molecular descriptor name  corr_value
0                    AATS0Z   -0.022797
1                  AATS0are   -0.139064
2                    AATS0d    0.027592
3                   AATS0dv   -0.136049
4                    AATS0i    0.168958
  molecular descriptor name  corr_value  absolute correlation value
0                    AATS0Z   -0.022797                    0.022797
1                  AATS0are   -0.139064                    0.139064
2                    AATS0d    0.027592                    0.027592
3                   AATS0dv   -0.136049                    0.136049
4                    AATS0i    0.168958                    0.168958
     molecular descriptor name  corr_value  absolute correlation value
226                     AMID_O   -0.557717                    0.557717
505                EState_VSA5   -0.534748                    0.534748
791                    MDEO-12   -0.555724                    0.555724
921                      SaasC    0.513071                    0.513071
1091               VSA_EState5    0.526082                    0.526082
     molecular descriptor name  corr_value  absolute correlation value
226                     AMID_O   -0.557717                    0.557717
505                EState_VSA5   -0.534748                    0.534748
791                    MDEO-12   -0.555724                    0.555724
921                      SaasC    0.513071                    0.513071
1091               VSA_EState5    0.526082                    0.526082
The model used is: LinearReg…
Return the coefficient of determination of the prediction:
0.607684302836734
R^2 score: 0.45207360740354974
Correlation coefficient: 0.6723641925352284
Test data - unseen during training:
R^2 score: 0.607684302836734
Correlation coefficient: 0.7795410847650905
[7.84879747 6.24299325 7.37407883 8.0208511  7.9100943  8.19522969
```

```
 8.19061816 7.98447887 7.96950463 7.58923738 6.86384061 7.61628624
 7.4997649  7.87840731 8.14591404 8.10506343 6.33645764 5.85493895]
102    7.920819
38     6.019997
8      5.996841
109    7.886057
11     7.962574
51     7.886057
88     8.075721
21     7.325139
82     7.978811
98     7.481486
58     7.677781
64     8.537602
74     8.142668
103    8.397940
91     8.958607
43     7.823909
25     4.886525
30     6.009661
Name: A549, dtype: float64
Training Root Mean Square Error: 0.6910343772412224
Testing Root Mean Square Error: 0.6492799270001024
```

2.1 Search inside correlation space

[8]:
```
step = 0.01
initial_step = corr_low
last_step = corr_high
first_list = [x / 100.0 for x in range(int(initial_step*100),␣
 ↪int(last_step*100), int(step*100))]
second_list = []
third_list = []
fourth_l = []
fifth_l = []
f_list = []
for i in first_list:
    without_standardization, train_r2, test_r2, _, h_, target_column_name,␣
 ↪training_data_RMSE, test_data_RMSE = pred_model.
 ↪prepare_data_and_create_model(molecular_descriptors_df = data,
                                                                          ␣
 ↪                      correlation_threshold = i,
                                                                          ␣
 ↪                      standardization = False,
                                                                          ␣
 ↪                      model_type = 'linear_model',
```

```
                        target_column_name = target,
                        random_state=random_state,
                        train_test_split_ = True,
                        verbose = False)
    second_list.append(train_r2)
    third_list.append(test_r2)
    fourth_l.append(training_data_RMSE)
    fifth_l.append(test_data_RMSE)
    f_list.append(len(h_))
```

[9]:
```
df_without_standardization = pd.DataFrame(data=first_list,
 columns=["Correlation threshold"])
df_without_standardization['Training data R^2 score'] = second_list
df_without_standardization['Test data R^2 score'] = third_list
df_without_standardization['Training RMSE'] = fourth_l
df_without_standardization['Test RMSE'] = fifth_l
df_without_standardization['Number of features'] = f_list
```

[10]:
```
df_linear = df_without_standardization.copy()
df_without_standardization
```

[10]:

	Correlation threshold	Training data R^2 score	Test data R^2 score \
0	0.33	0.990987	-3.202429
1	0.34	0.956906	0.343203
2	0.35	0.947797	0.394503
3	0.36	0.930886	0.567737
4	0.37	0.909806	0.499993
5	0.38	0.865903	0.664443
6	0.39	0.771356	0.749644
7	0.40	0.666720	0.577809
8	0.41	0.660500	0.648843
9	0.42	0.603301	0.652730
10	0.43	0.595478	0.638470
11	0.44	0.579065	0.647024
12	0.45	0.579065	0.647024
13	0.46	0.555497	0.636815
14	0.47	0.532003	0.636513
15	0.48	0.503811	0.591147
16	0.49	0.465449	0.603340
17	0.50	0.452074	0.607684
18	0.51	0.452074	0.607684
19	0.52	0.452073	0.607536
20	0.53	0.428089	0.626633

```
    Training RMSE  Test RMSE  Number of features
0        0.088631   2.125026                  94
1        0.193797   0.840098                  79
2        0.213297   0.806622                  73
3        0.245427   0.681535                  68
4        0.280367   0.732997                  57
5        0.341859   0.600478                  47
6        0.446394   0.518673                  39
7        0.538943   0.673548                  31
8        0.543949   0.614278                  27
9        0.587989   0.610869                  22
10       0.593758   0.623284                  18
11       0.605684   0.615866                  16
12       0.605684   0.615866                  16
13       0.622409   0.624709                  13
14       0.638646   0.624969                  11
15       0.657601   0.662823                   8
16       0.682548   0.652865                   7
17       0.691034   0.649280                   5
18       0.691034   0.649280                   5
19       0.691035   0.649403                   4
20       0.705997   0.633406                   3
```

[ ]:

2.2 Plots

[11]:

```
plt.plot(df_without_standardization['Correlation threshold'],␣
 ↪df_without_standardization['Training data R^2 score'], label = "Training␣
 ↪data R^2 score", marker='v')
plt.plot(df_without_standardization['Correlation threshold'],␣
 ↪df_without_standardization['Test data R^2 score'], label = "Test data R^2␣
 ↪score", marker='D')
plt.legend()
plt.xlabel('Correlation threshold')
plt.ylabel('R^2 score')
plt.ylim([0, 1])
plt.show()
```

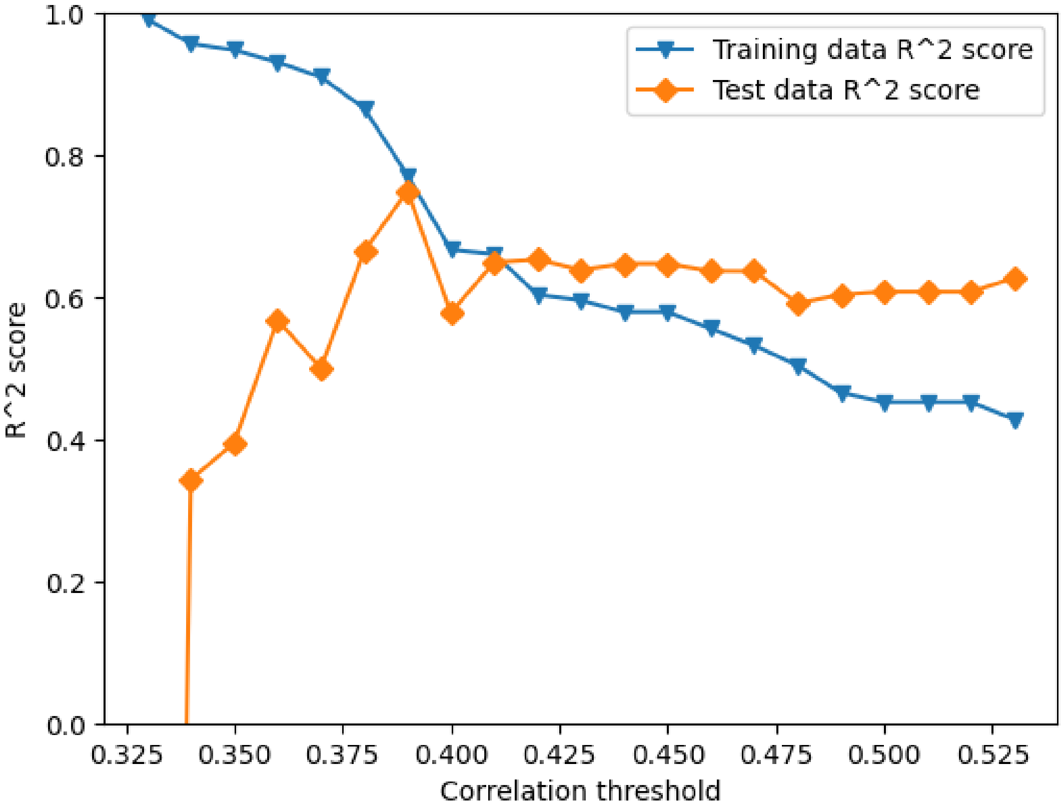

[12]:
```
plt.plot(df_without_standardization['Correlation threshold'],␣
↪df_without_standardization['Number of features'], label = "Number of␣
↪features", marker='.')
plt.legend()
plt.xlabel('Correlation threshold')
plt.ylabel('Number of features')
plt.show()
```

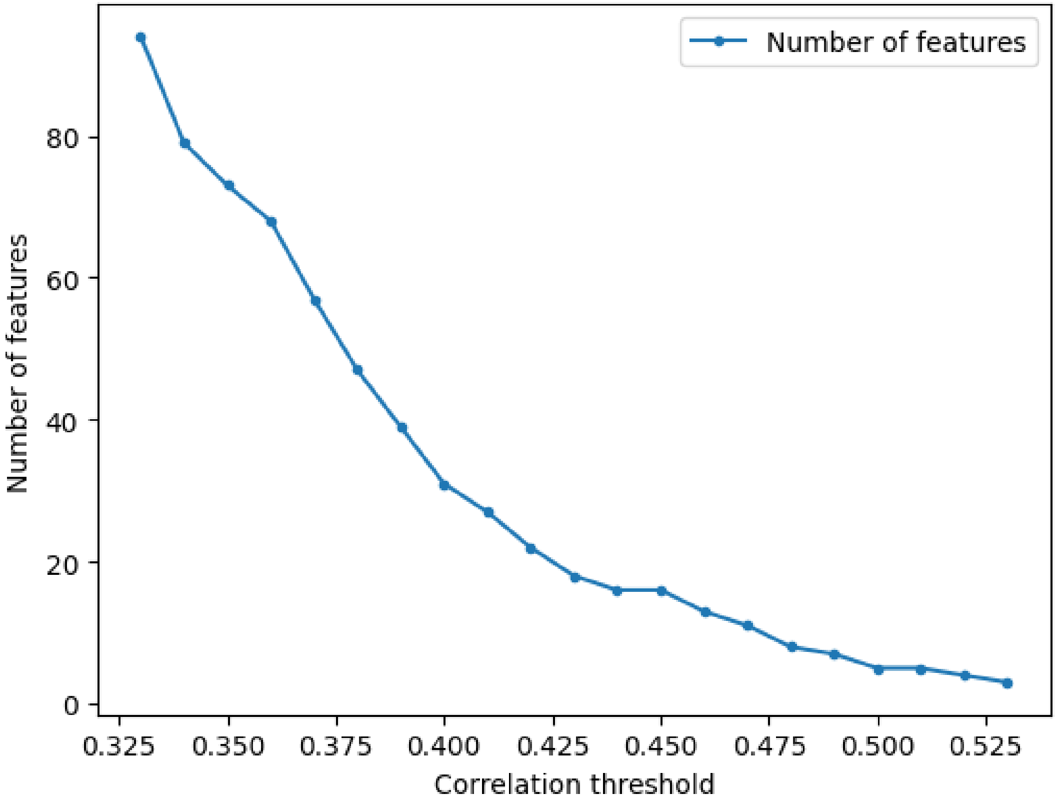

[ ]:

```

```

2.3 Decision Tree

[13]:

```
without_standardization, train_r2_, test_r2_, hist1, hist2, target_column_name,␣
↪training_data_RMSE, test_data_RMSE  = pred_model.
↪prepare_data_and_create_model(molecular_descriptors_df=data,
                                                                                ␣
↪                    correlation_threshold=0.50,
                                                                                ␣
↪                    standardization=False,
                                                                                ␣
↪                    model_type='DecisionTreeRegressor',
                                                                                ␣
↪                    max_depth=5,
                                                                                ␣
↪                    target_column_name = target,
                                                                                ␣
↪                    random_state=random_state,
```

```
                    train_test_split_=True,
                    verbose=True)
```

```
I am not doing standardization…
  molecular descriptor name
0                    AATS0Z
1                  AATS0are
2                    AATS0d
3                   AATS0dv
4                    AATS0i
  molecular descriptor name  corr_value
0                    AATS0Z   -0.022797
1                  AATS0are   -0.139064
2                    AATS0d    0.027592
3                   AATS0dv   -0.136049
4                    AATS0i    0.168958
  molecular descriptor name  corr_value  absolute correlation value
0                    AATS0Z   -0.022797                    0.022797
1                  AATS0are   -0.139064                    0.139064
2                    AATS0d    0.027592                    0.027592
3                   AATS0dv   -0.136049                    0.136049
4                    AATS0i    0.168958                    0.168958
     molecular descriptor name  corr_value  absolute correlation value
226                     AMID_O   -0.557717                    0.557717
505                EState_VSA5   -0.534748                    0.534748
791                    MDEO-12   -0.555724                    0.555724
921                      SaasC    0.513071                    0.513071
1091               VSA_EState5    0.526082                    0.526082
     molecular descriptor name  corr_value  absolute correlation value
226                     AMID_O   -0.557717                    0.557717
505                EState_VSA5   -0.534748                    0.534748
791                    MDEO-12   -0.555724                    0.555724
921                      SaasC    0.513071                    0.513071
1091               VSA_EState5    0.526082                    0.526082
The model used is: DecisionTree…
Return the coefficient of determination of the prediction:
0.4511327118987092
R^2 score: 0.9133233269283261
Correlation coefficient: 0.9556795105726219
Test data - unseen during training:
R^2 score: 0.4511327118987092
Correlation coefficient: 0.6716641362308317
[8.18943869 6.01979892 7.04240039 7.95366066 7.95366066 7.71707609
 7.95366066 7.08704364 7.95366066 8.68324418 8.68324418 8.18943869
 8.18943869 8.18943869 7.08704364 7.95366066 6.31695296 4.93372809]
102    7.920819
```

```
38     6.019997
8      5.996841
109    7.886057
11     7.962574
51     7.886057
88     8.075721
21     7.325139
82     7.978811
98     7.481486
58     7.677781
64     8.537602
74     8.142668
103    8.397940
91     8.958607
43     7.823909
25     4.886525
30     6.009661
Name: A549, dtype: float64
Training Root Mean Square Error: 0.2748461511342915
Testing Root Mean Square Error: 0.7679762825865003
```

[14]:
```python
print(target_column_name+str('_transformed'))
print(hist1[target_column_name].hist())
```

```
A549_transformed
AxesSubplot(0.125,0.11;0.775x0.77)
```

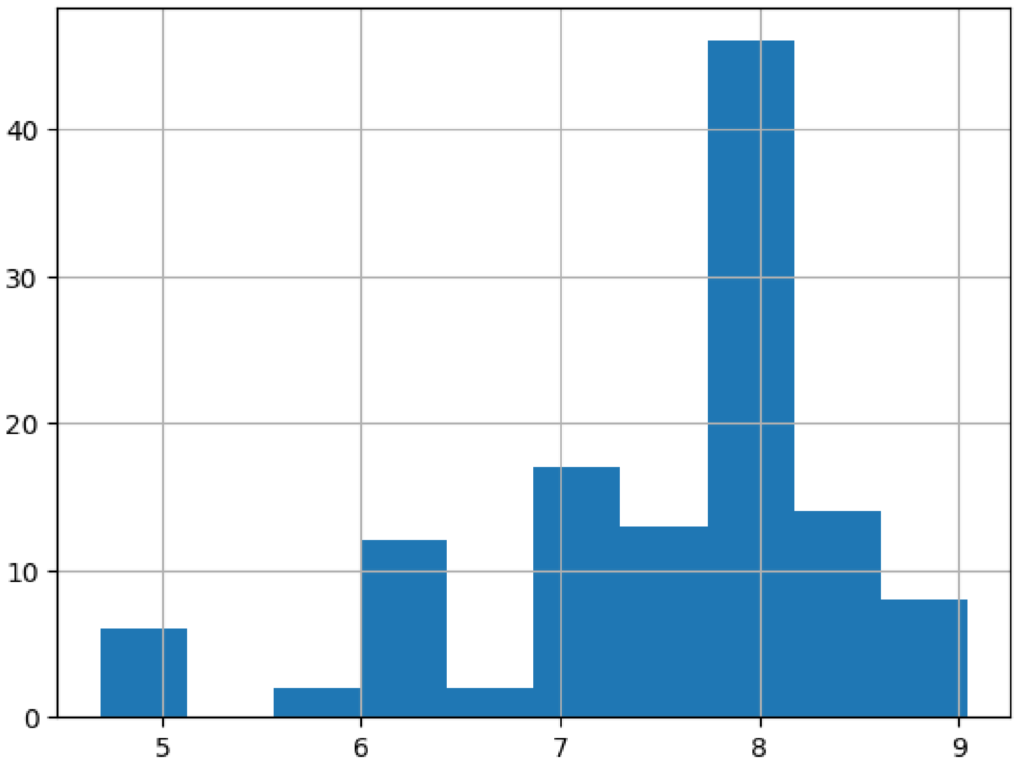

[15]:
```
with_standardization, train_r2_, test_r2_, hist1, hist2, target_column_name,␣
↪training_data_RMSE, test_data_RMSE  = pred_model.
↪prepare_data_and_create_model(molecular_descriptors_df=data,
                                                                         ␣
↪                          correlation_threshold=0.50,
                                                                         ␣
↪                          standardization=True,
                                                                         ␣
↪                          model_type='DecisionTreeRegressor',
                                                                         ␣
↪                          max_depth=5,
                                                                         ␣
↪                          target_column_name = target,
                                                                         ␣
↪                          random_state=random_state,
                                                                         ␣
↪                          train_test_split_=True,
                                                                         ␣
↪                          verbose=True)
```

```
I am doing standardization…
  molecular descriptor name
```

```
0                    AATS0Z
1                  AATS0are
2                    AATS0d
3                   AATS0dv
4                    AATS0i
  molecular descriptor name  corr_value
0                    AATS0Z   -0.022797
1                  AATS0are   -0.139064
2                    AATS0d    0.027592
3                   AATS0dv   -0.136049
4                    AATS0i    0.168958
  molecular descriptor name  corr_value  absolute correlation value
0                    AATS0Z   -0.022797                    0.022797
1                  AATS0are   -0.139064                    0.139064
2                    AATS0d    0.027592                    0.027592
3                   AATS0dv   -0.136049                    0.136049
4                    AATS0i    0.168958                    0.168958
     molecular descriptor name  corr_value  absolute correlation value
226                     AMID_O   -0.557717                    0.557717
505                 EState_VSA5   -0.534748                    0.534748
791                    MDEO-12   -0.555724                    0.555724
921                      SaasC    0.513071                    0.513071
1091                VSA_EState5    0.526082                    0.526082
     molecular descriptor name  corr_value  absolute correlation value
226                     AMID_O   -0.557717                    0.557717
505                 EState_VSA5   -0.534748                    0.534748
791                    MDEO-12   -0.555724                    0.555724
921                      SaasC    0.513071                    0.513071
1091                VSA_EState5    0.526082                    0.526082
The model used is: DecisionTree…
Return the coefficient of determination of the prediction:
0.4511327118987092
R^2 score: 0.9133233269283261
Correlation coefficient: 0.9556795105726219
Test data - unseen during training:
R^2 score: 0.4511327118987092
Correlation coefficient: 0.6716641362308317
[8.18943869 6.01979892 7.04240039 7.95366066 7.95366066 7.71707609
 7.95366066 7.08704364 7.95366066 8.68324418 8.68324418 8.18943869
 8.18943869 8.18943869 7.08704364 7.95366066 6.31695296 4.93372809]
102    7.920819
38     6.019997
8      5.996841
109    7.886057
11     7.962574
51     7.886057
88     8.075721
21     7.325139
```

```
82     7.978811
98     7.481486
58     7.677781
64     8.537602
74     8.142668
103    8.397940
91     8.958607
43     7.823909
25     4.886525
30     6.009661
Name: A549, dtype: float64
Training Root Mean Square Error: 0.2748461511342915
Testing Root Mean Square Error: 0.7679762825865003
```

2.4 Search inside correlation space

[16]:
```
step = 0.01
initial_step = corr_low
last_step = corr_high
first_list = [x / 100.0 for x in range(int(initial_step*100),␣
 ↪int(last_step*100), int(step*100))]
max_depth = [range(2, 30, 1)]
corr_th = []
second_list = []
third_list = []
fourth_l = []
fifth_l = []
f_list = []
fif_list = []
for i in first_list:
    for depth in max_depth[0]:

        without_standardization, train_r2, test_r2, _, h_, target_column_name,␣
 ↪training_data_RMSE, test_data_RMSE = pred_model.
 ↪prepare_data_and_create_model(molecular_descriptors_df=data,
                                                                             ␣
 ↪                     correlation_threshold=i,
                                                                             ␣
 ↪                     standardization=False,
                                                                             ␣
 ↪                     model_type='DecisionTreeRegressor',
                                                                             ␣
 ↪                     max_depth=depth,
                                                                             ␣
 ↪                     target_column_name = target,
                                                                             ␣
 ↪                     random_state=random_state,
```

```
                    train_test_split_=True,
                    verbose=False)
        corr_th.append(i)
        second_list.append(train_r2)
        third_list.append(test_r2)
        fourth_l.append(training_data_RMSE)
        fifth_l.append(test_data_RMSE)
        f_list.append(len(h_))
        fif_list.append(depth)
```

[17]:
```
df_without_standardization = pd.DataFrame(data=corr_th, columns=["Correlation threshold"])
df_without_standardization['Training data R^2 score'] = second_list
df_without_standardization['Test data R^2 score'] = third_list
df_without_standardization['Training RMSE'] = fourth_l
df_without_standardization['Test RMSE'] = fifth_l
df_without_standardization['Number of features'] = f_list
df_without_standardization['Depth number'] = fif_list
```

[18]:
```
df_decision_tree = df_without_standardization.copy()
#df_without_standardization.to_excel('../Data/A549_Decision_tree_rs_'+str(random_state)+'.xlsx')
```

2.5 Plots

[19]:
```
corre_list = [x / 100.0 for x in range(int(initial_step*100),␣
↪int(last_step*100), int(0.01*100))]
for element in corre_list:
    element_ =␣
↪df_without_standardization[df_without_standardization['Correlation␣
↪threshold'] == float(element)]
    plt.plot(element_['Depth number'], element_['Training data R^2 score'],␣
↪label = "Training data R^2 score", marker='v')
    plt.plot(element_['Depth number'], element_['Test data R^2 score'], label =␣
↪"Test data R^2 score", marker='D')
    plt.legend()
    plt.xlabel('Depth number -> correlation threshold: '+str(element)+'. \n␣
↪Number of features: '+str(element_['Number of features'].iloc[0]),␣
↪fontweight='bold')
    plt.xlim(max(element_['Depth number'])+1, min(element_['Depth number'])-1)
    plt.ylabel('R^2 score')
    plt.ylim([0, 1])
    plt.rc('grid', linestyle="-.", color='black')
    plt.grid(True)
    plt.show()
```

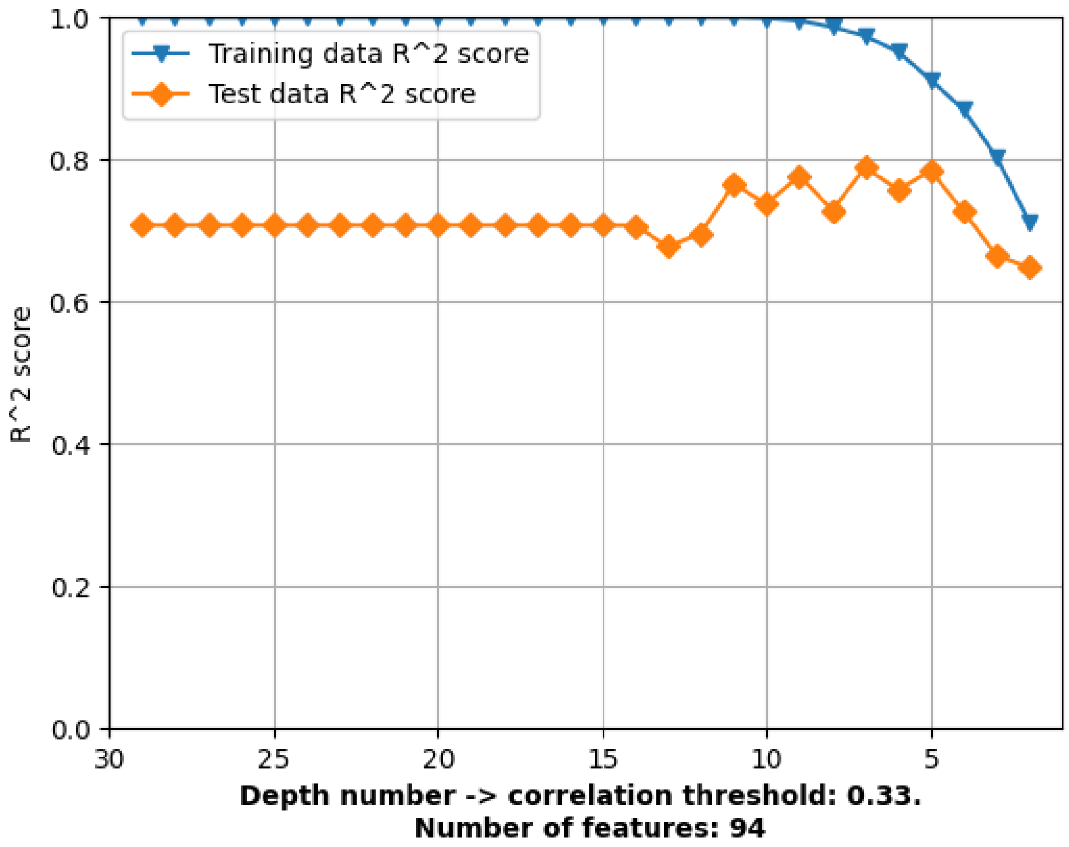

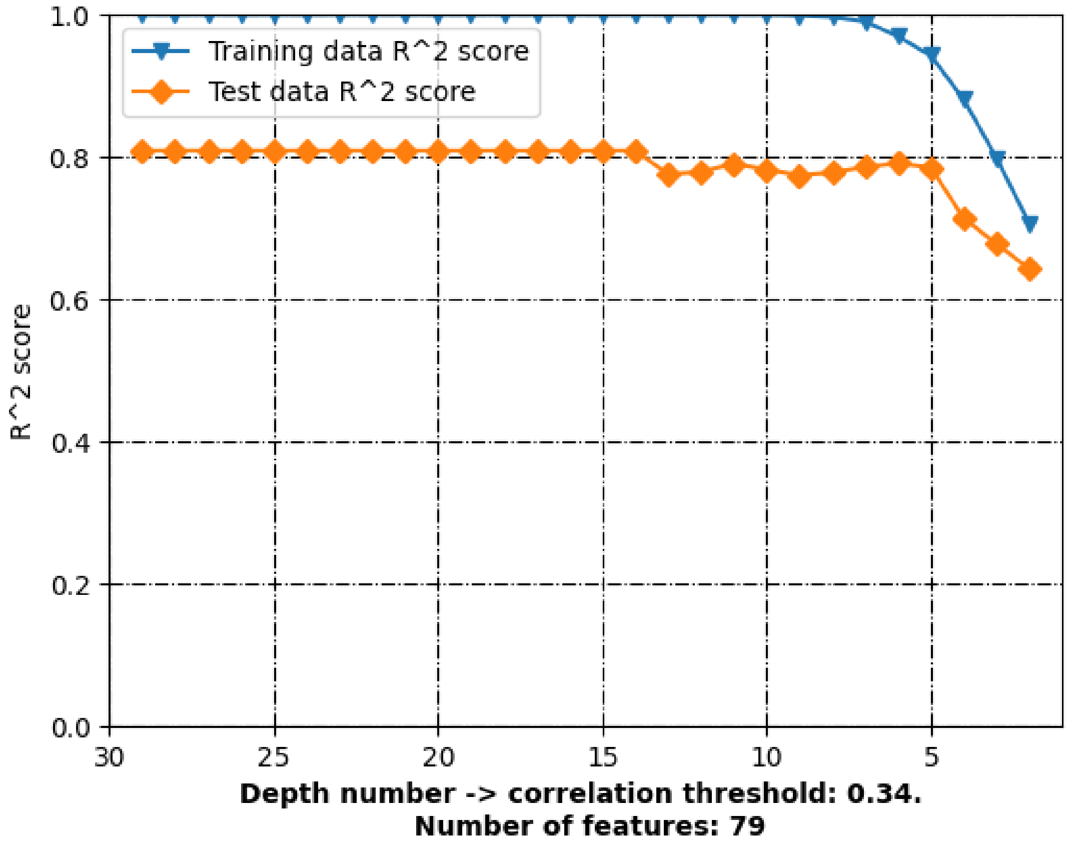

Training data R^2 score
Test data R^2 score
R^2 score
1.0
0.8
0.6
0.4
0.2
0.0
30
25
20
15
10
5
Depth number -> correlation threshold: 0.34.
Number of features: 79

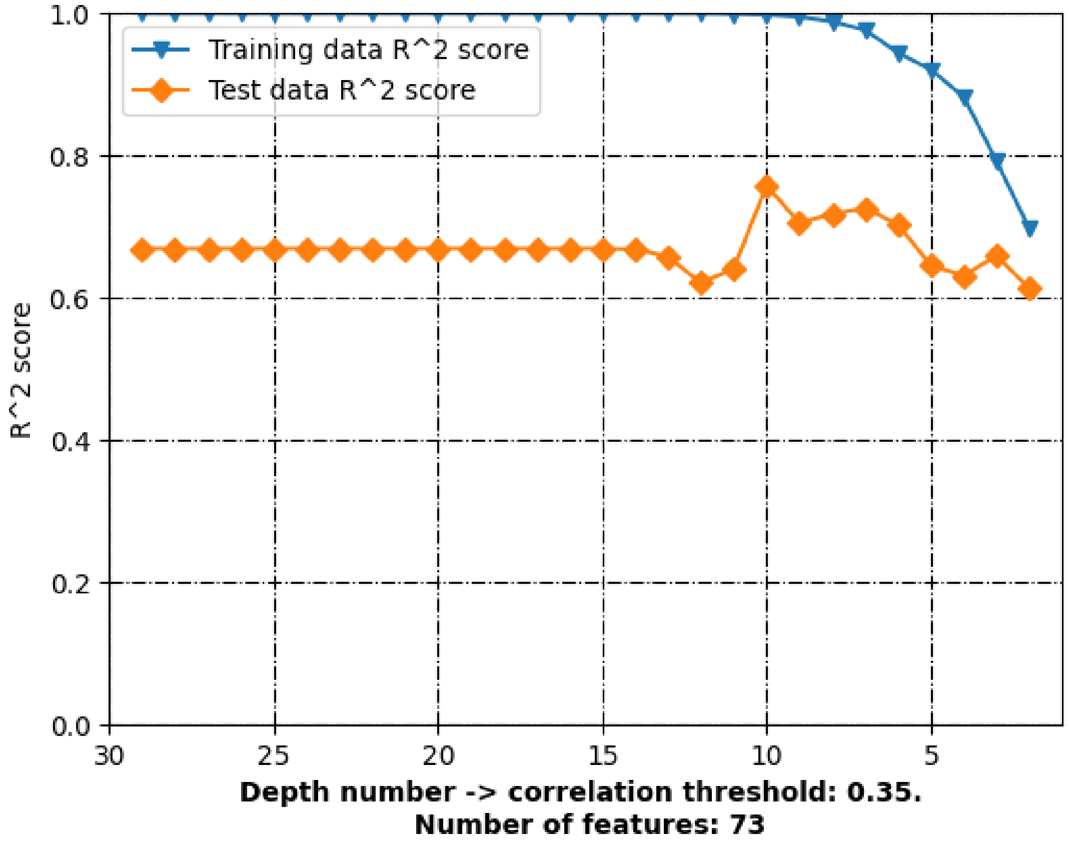

1.0
0.8
0.6
0.4
0.2
0.0
R^2 score
Training data R^2 score
Test data R^2 score
30
25
20
15
10
5
Depth number -> correlation threshold: 0.35.
Number of features: 73

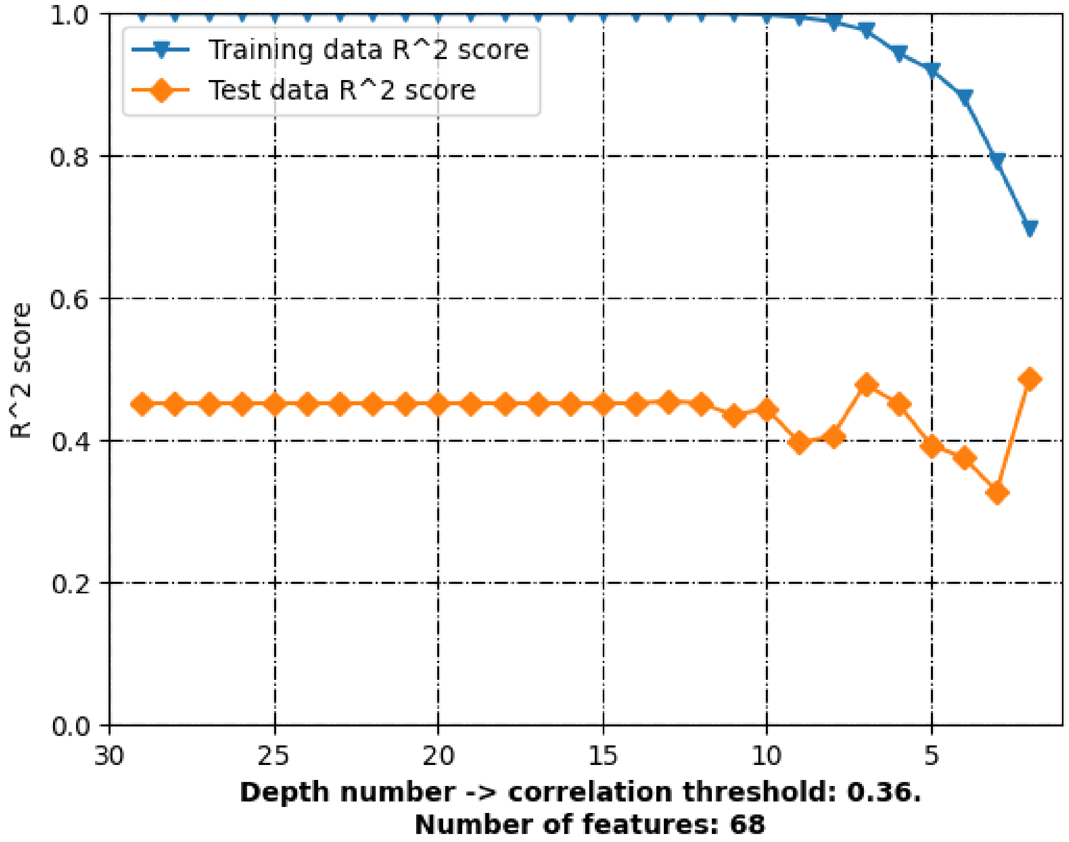

Training data R^2 score
Test data R^2 score
R^2 score
1.0
0.8
0.6
0.4
0.2
0.0
30
25
20
15
10
5
Depth number -> correlation threshold: 0.36.
Number of features: 68

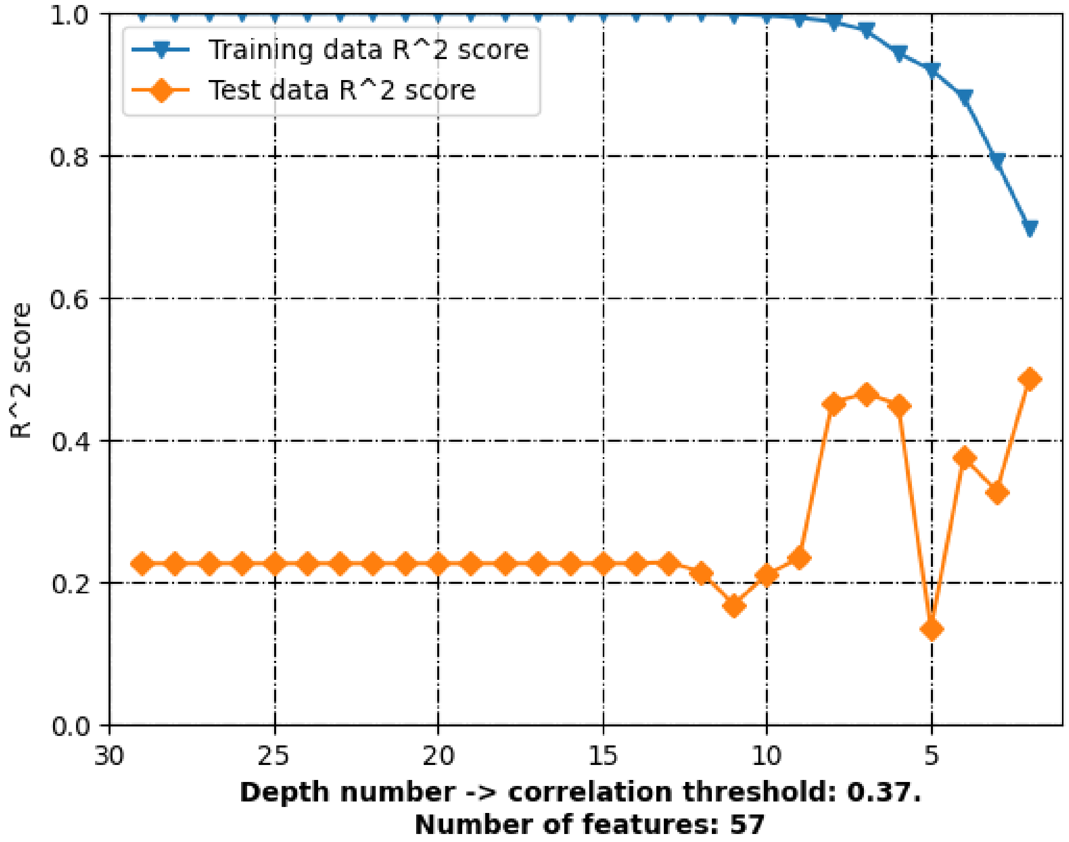

1.0
0.8
0.6
0.4
0.2
0.0
R^2 score
Training data R^2 score
Test data R^2 score
30
25
20
15
10
5
Depth number -> correlation threshold: 0.37.
Number of features: 57

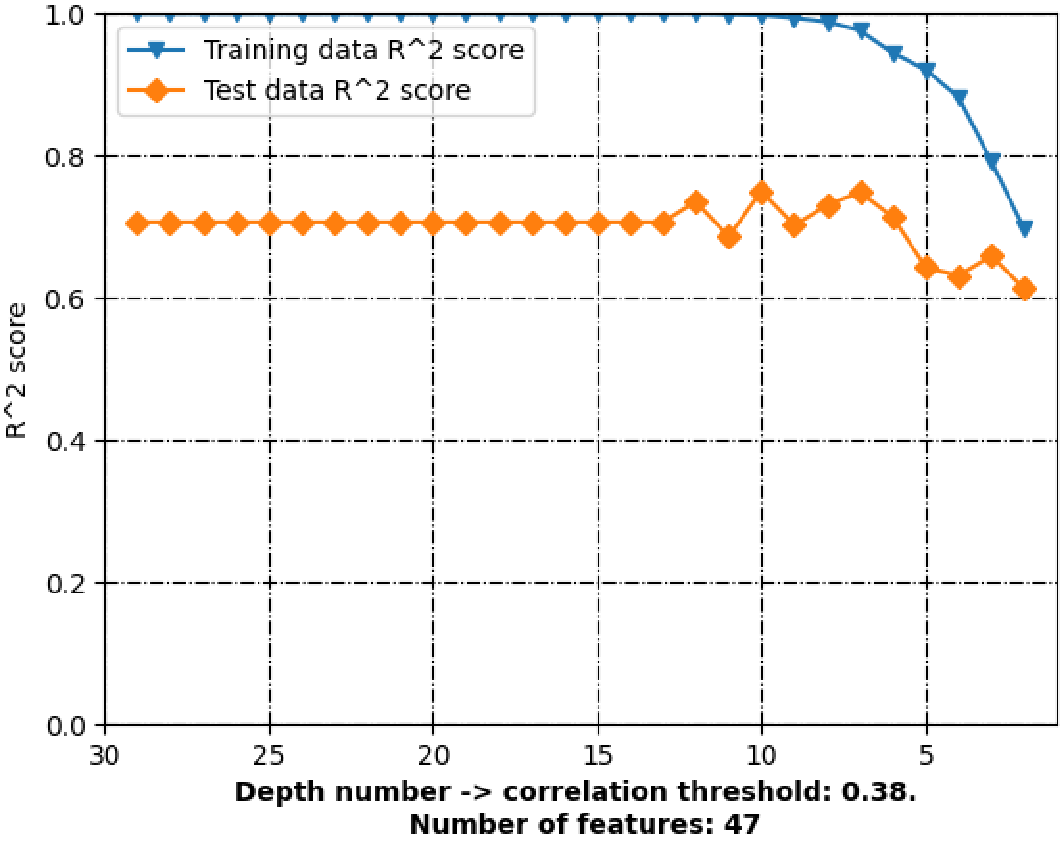
Training data R^2 score
Test data R^2 score
R^2 score
1.0
0.8
0.6
0.4
0.2
0.0
30
25
20
15
10
5
Depth number -> correlation threshold: 0.38.
Number of features: 47

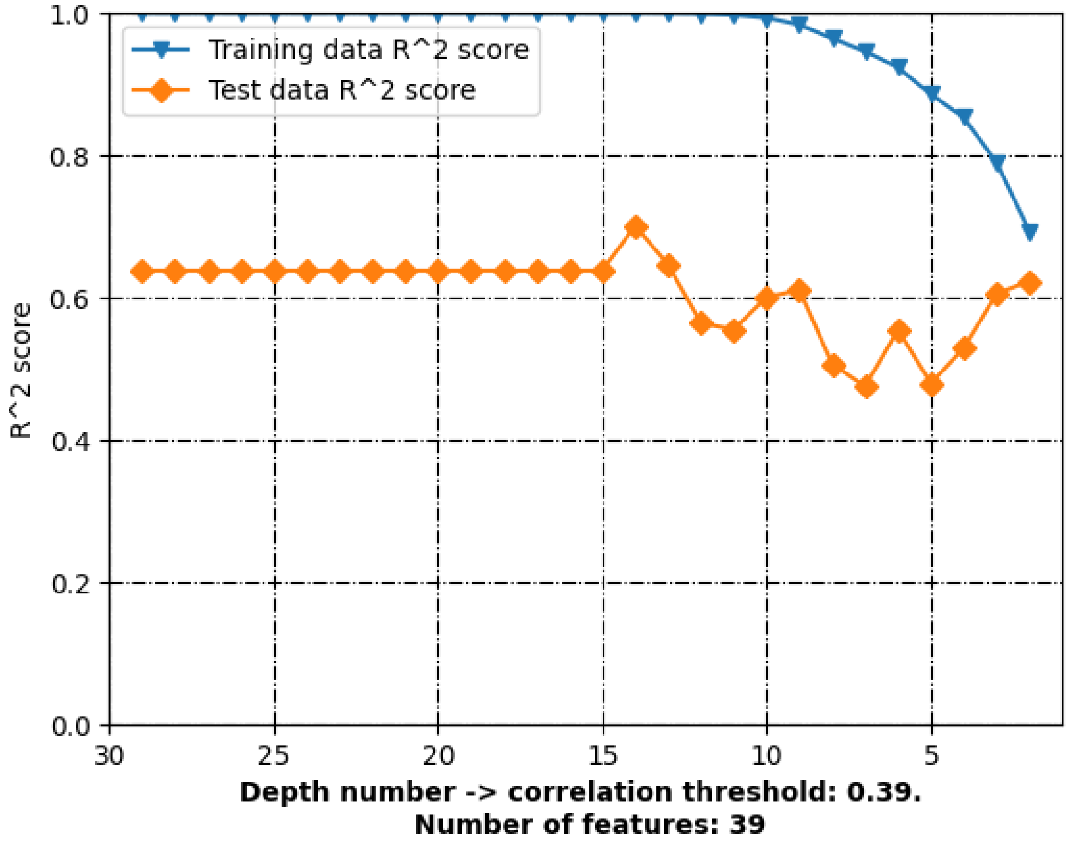
Training data R^2 score
Test data R^2 score
R^2 score
1.0
0.8
0.6
0.4
0.2
0.0
30
25
20
15
10
5
Depth number -> correlation threshold: 0.39.
Number of features: 39

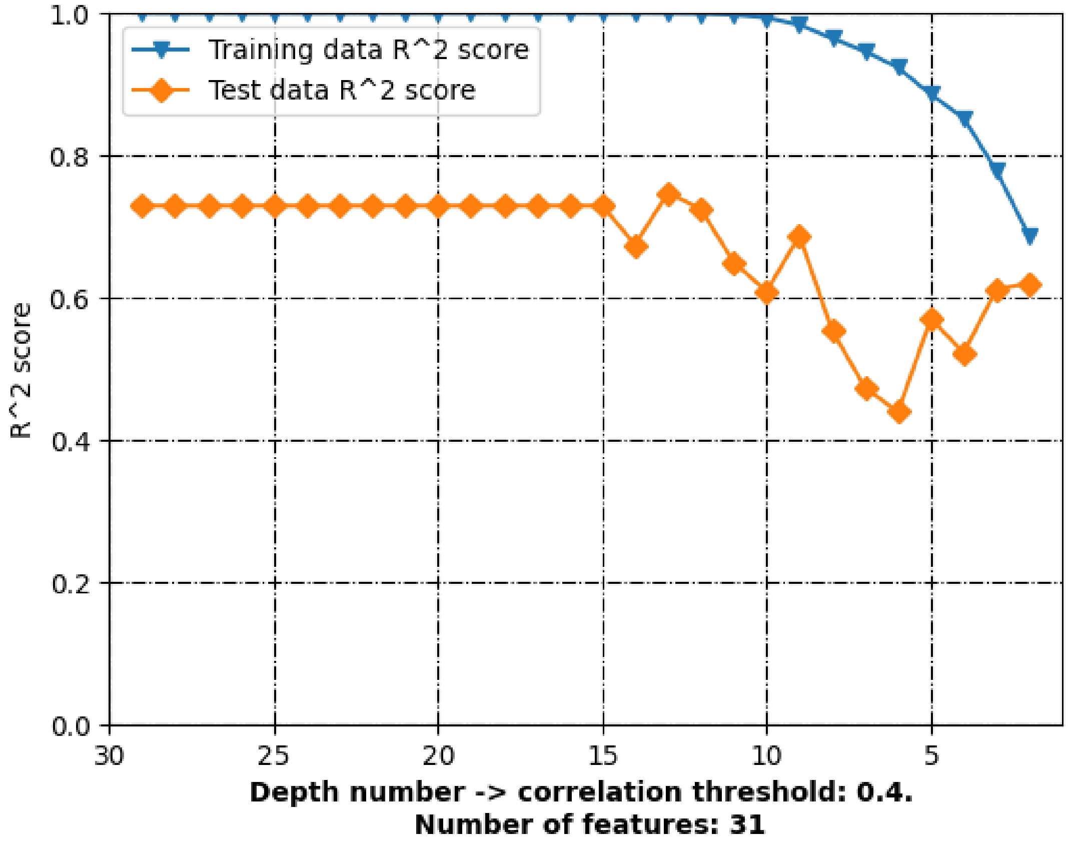

Training data R^2 score
Test data R^2 score
R^2 score
1.0
0.8
0.6
0.4
0.2
0.0
30
25
20
15
10
5
Depth number -> correlation threshold: 0.4.
Number of features: 31

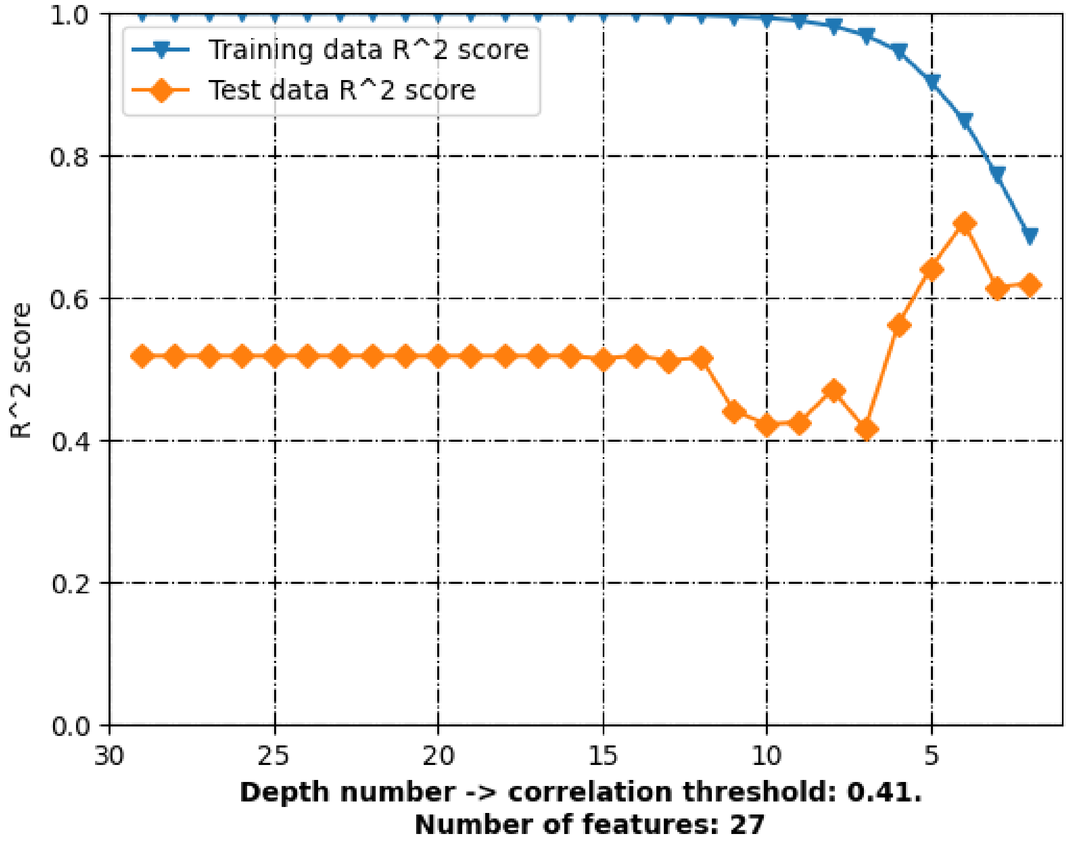
Training data R^2 score
Test data R^2 score
R^2 score
1.0
0.8
0.6
0.4
0.2
0.0
30
25
20
15
10
5
Depth number -> correlation threshold: 0.41.
Number of features: 27

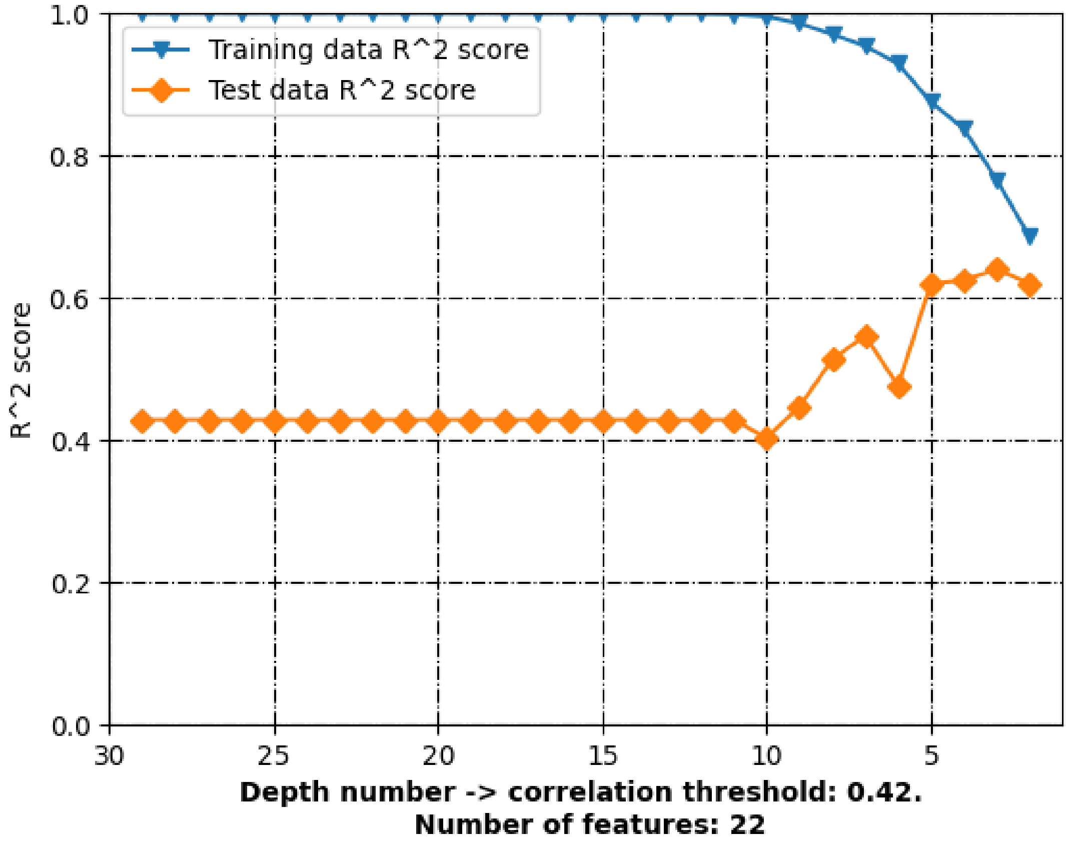

Training data R^2 score
Test data R^2 score
1.0
0.8
0.6
0.4
0.2
0.0
R^2 score
30
25
20
15
10
5
Depth number -> correlation threshold: 0.42.
Number of features: 22

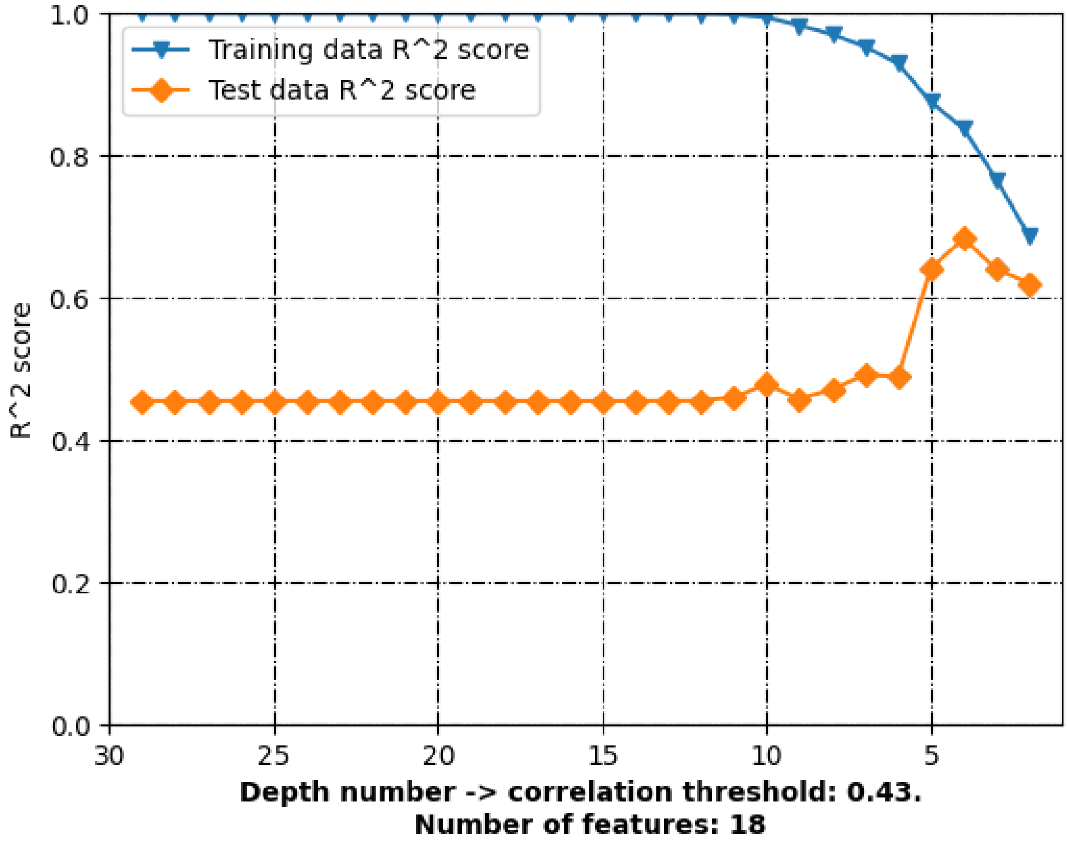

Training data R^2 score
Test data R^2 score
R^2 score
1.0
0.8
0.6
0.4
0.2
0.0
30
25
20
15
10
5
Depth number -> correlation threshold: 0.43.
Number of features: 18

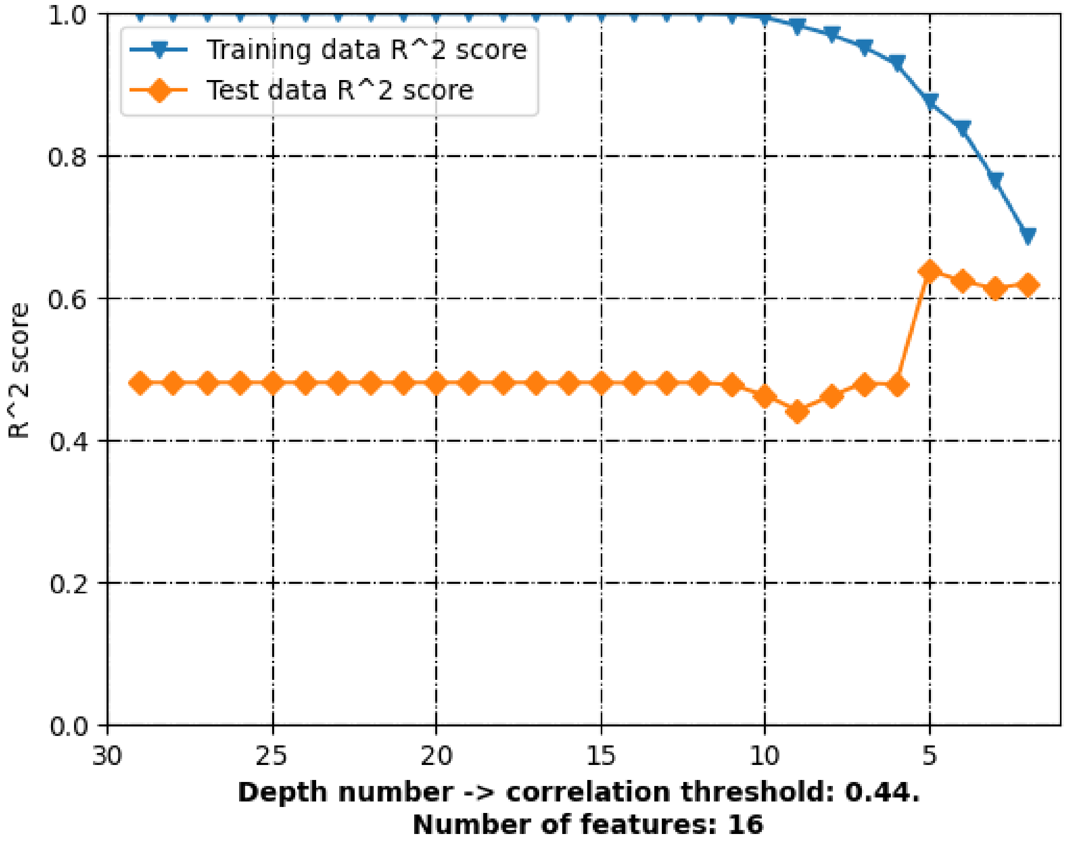

Training data R^2 score
Test data R^2 score
R^2 score
1.0
0.8
0.6
0.4
0.2
0.0
30
25
20
15
10
5
Depth number -> correlation threshold: 0.44.
Number of features: 16

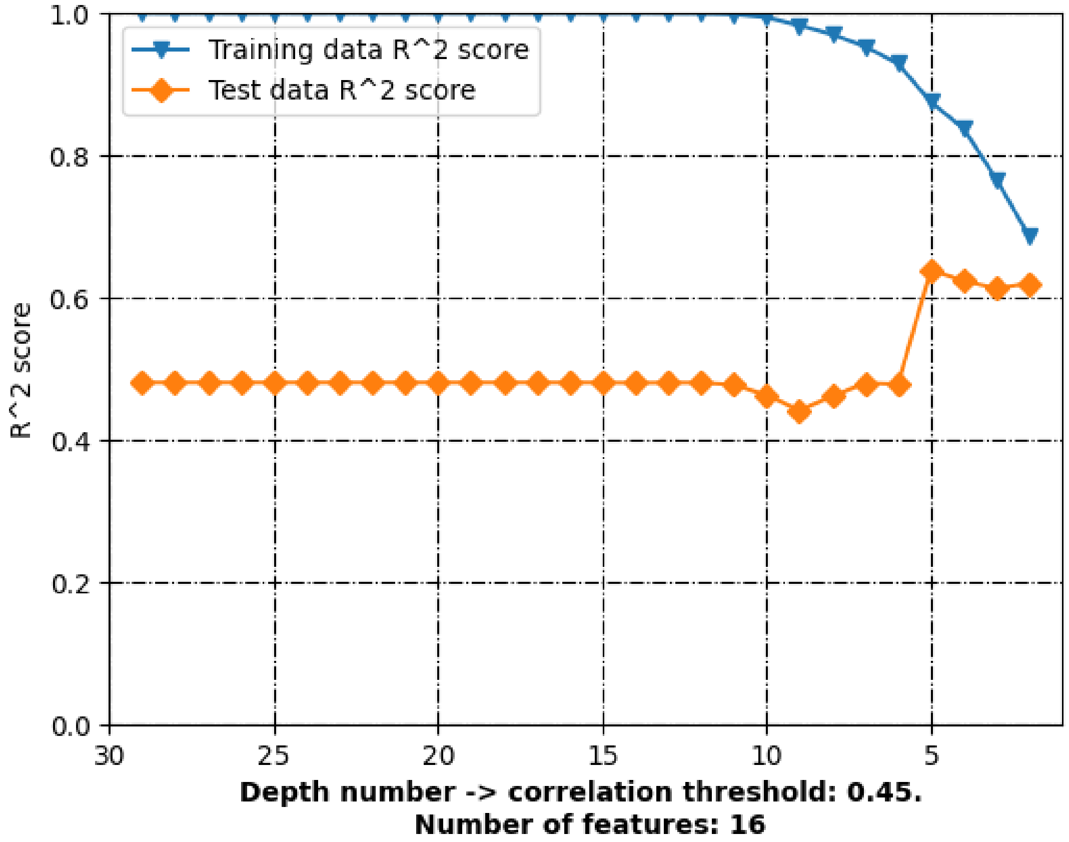

Training data R^2 score
Test data R^2 score
R^2 score
1.0
0.8
0.6
0.4
0.2
0.0
30
25
20
15
10
5
Depth number -> correlation threshold: 0.45.
Number of features: 16

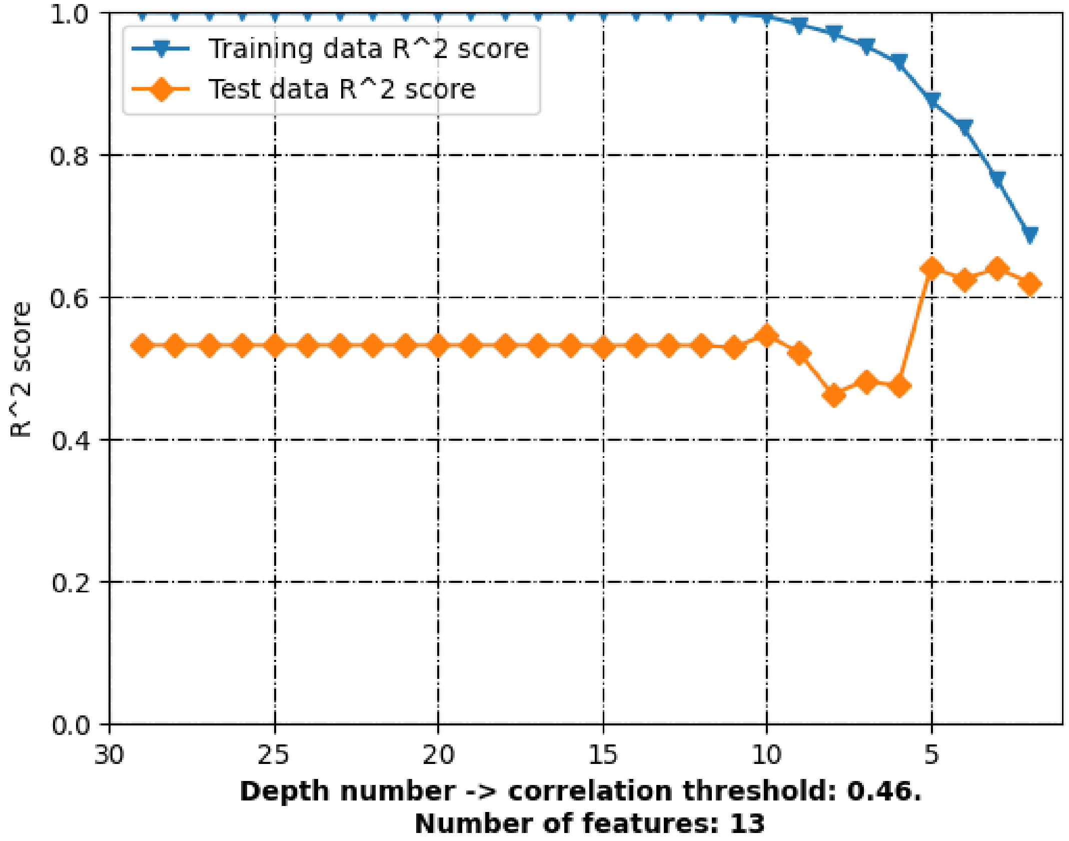

Training data R^2 score
Test data R^2 score
R^2 score
1.0
0.8
0.6
0.4
0.2
0.0
30
25
20
15
10
5
Depth number -> correlation threshold: 0.46.
Number of features: 13

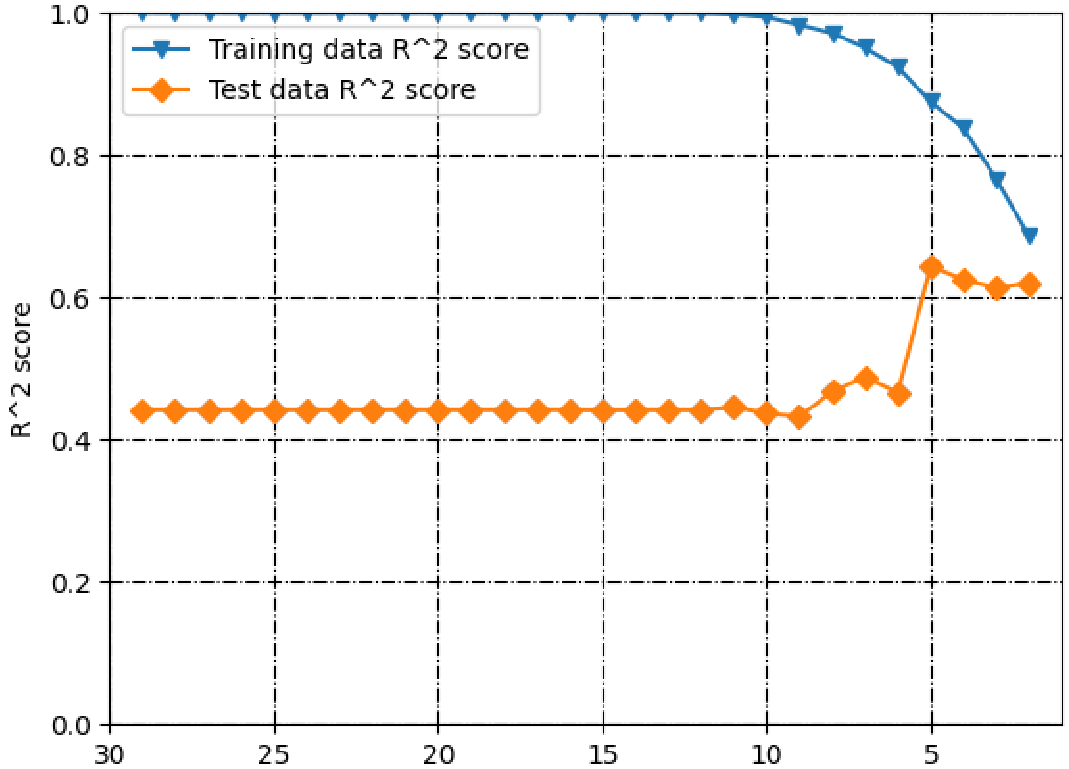

Depth number -> correlation threshold: 0.47.
Number of features: 11

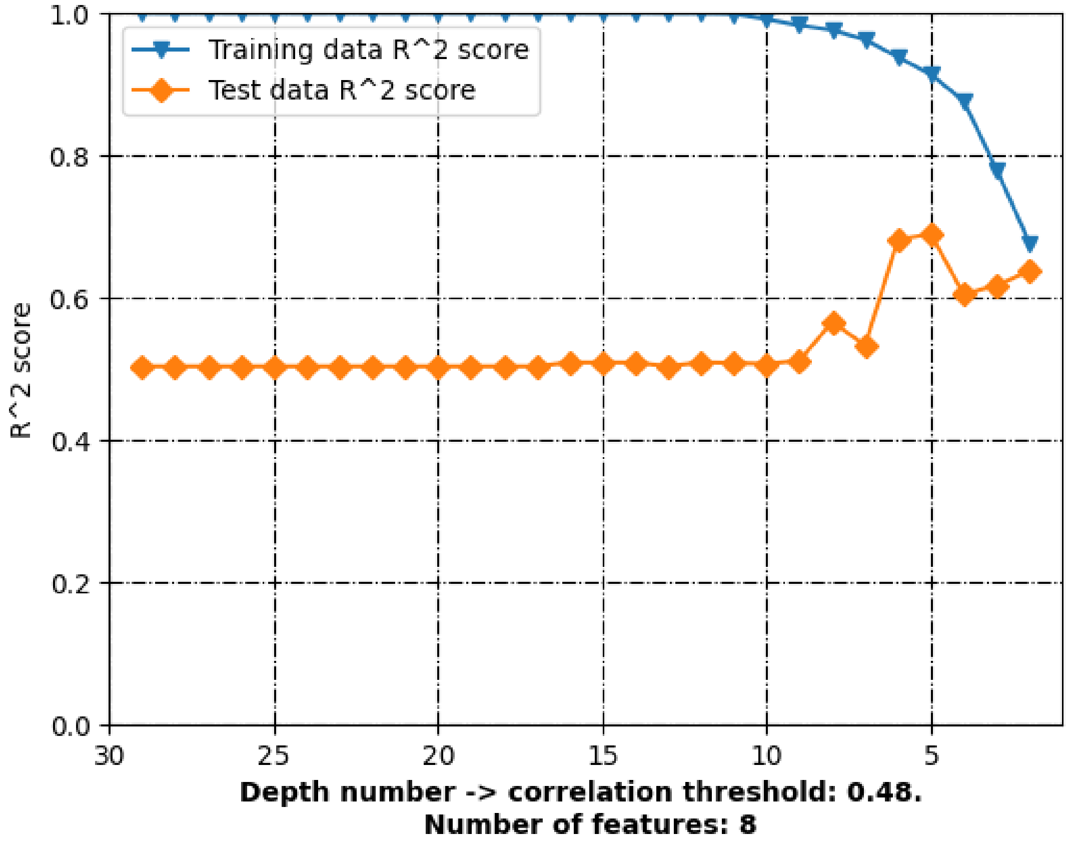

Training data R^2 score
Test data R^2 score
R^2 score
1.0
0.8
0.6
0.4
0.2
0.0
30
25
20
15
10
5
Depth number -> correlation threshold: 0.48.
Number of features: 8

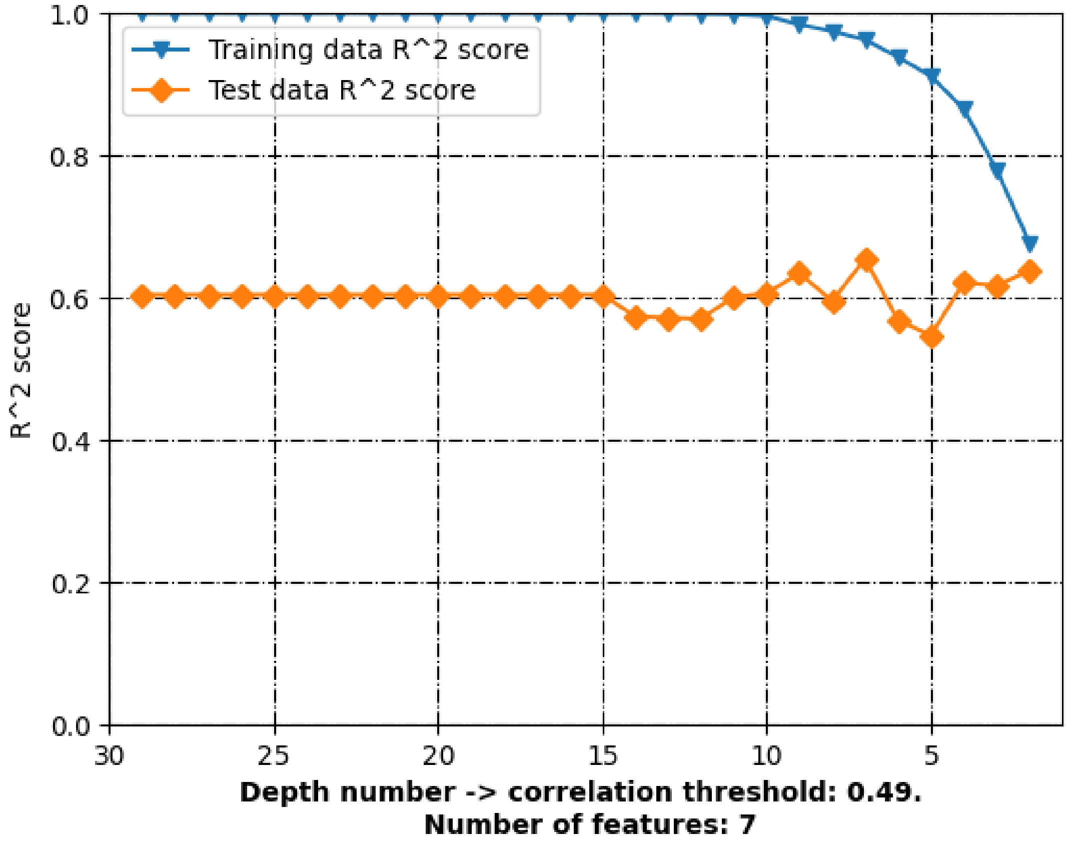

1.0
0.8
0.6
0.4
0.2
0.0
R^2 score
Training data R^2 score
Test data R^2 score
30
25
20
15
10
5
Depth number -> correlation threshold: 0.49.
Number of features: 7

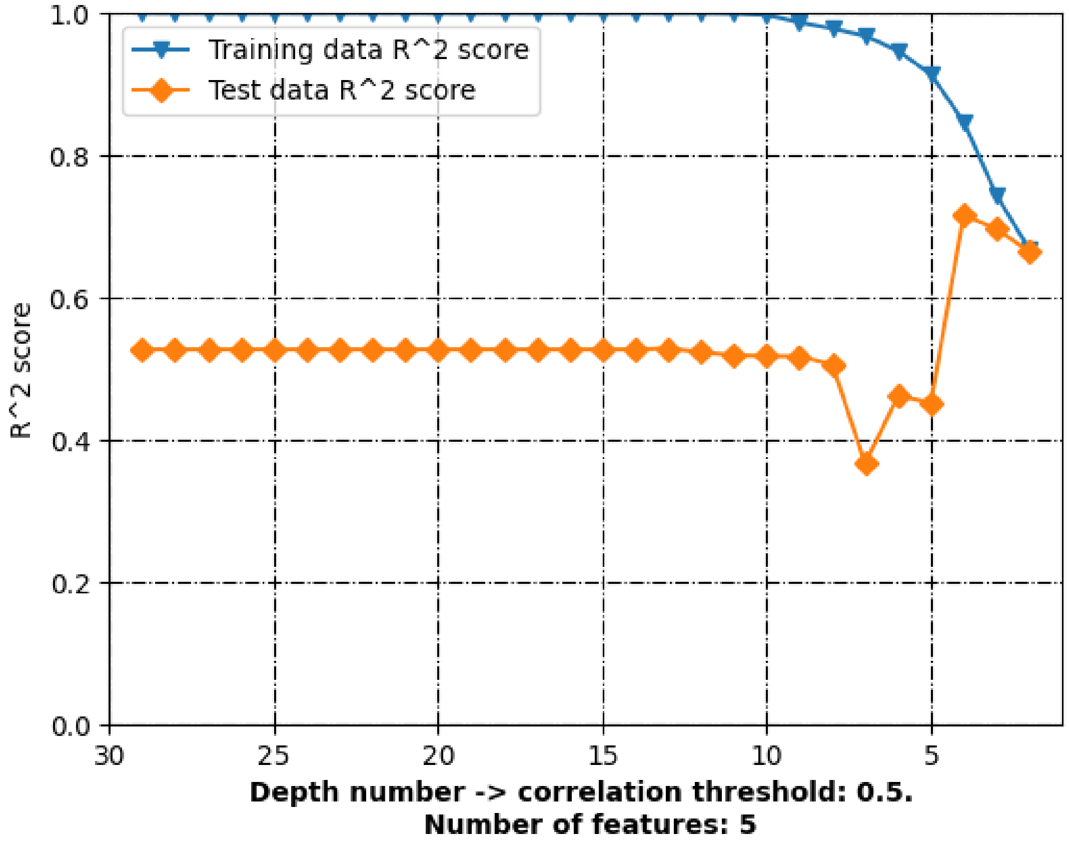
Training data R^2 score
Test data R^2 score
R^2 score
1.0
0.8
0.6
0.4
0.2
0.0
30
25
20
15
10
5
Depth number -> correlation threshold: 0.5.
Number of features: 5

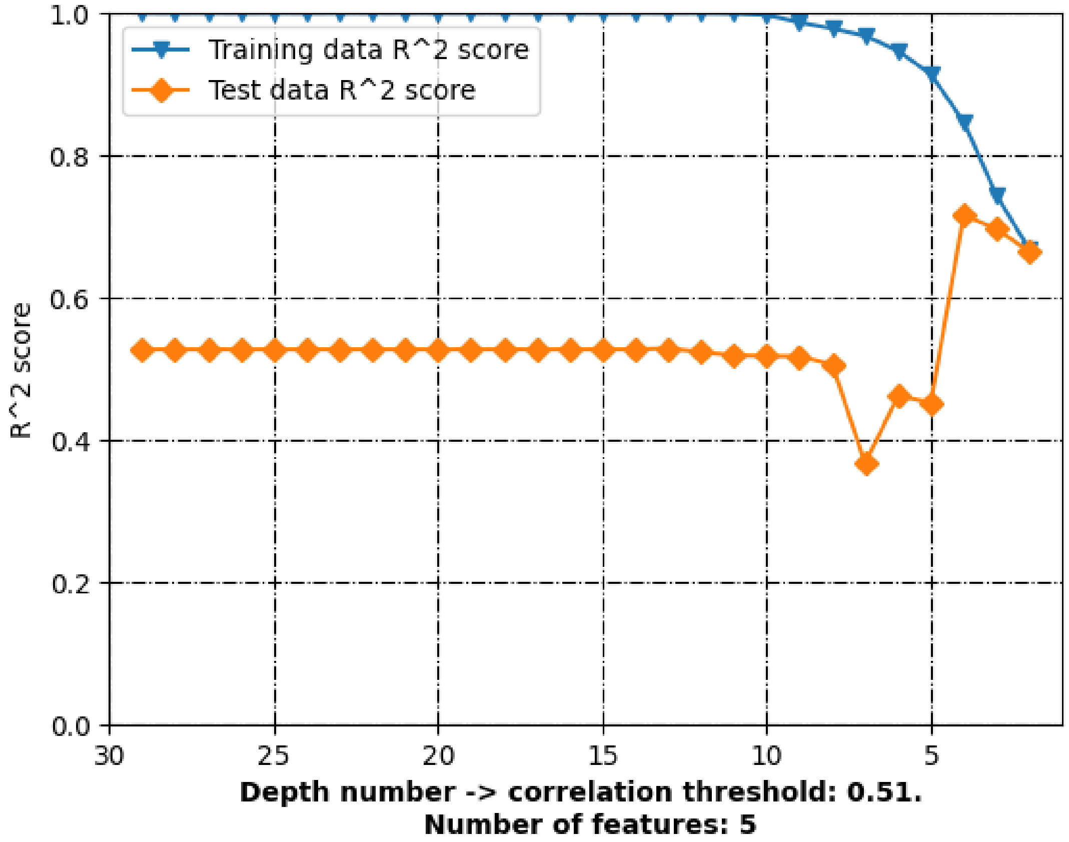
Training data R^2 score
Test data R^2 score
R^2 score
1.0
0.8
0.6
0.4
0.2
0.0
30
25
20
15
10
5
Depth number -> correlation threshold: 0.51.
Number of features: 5

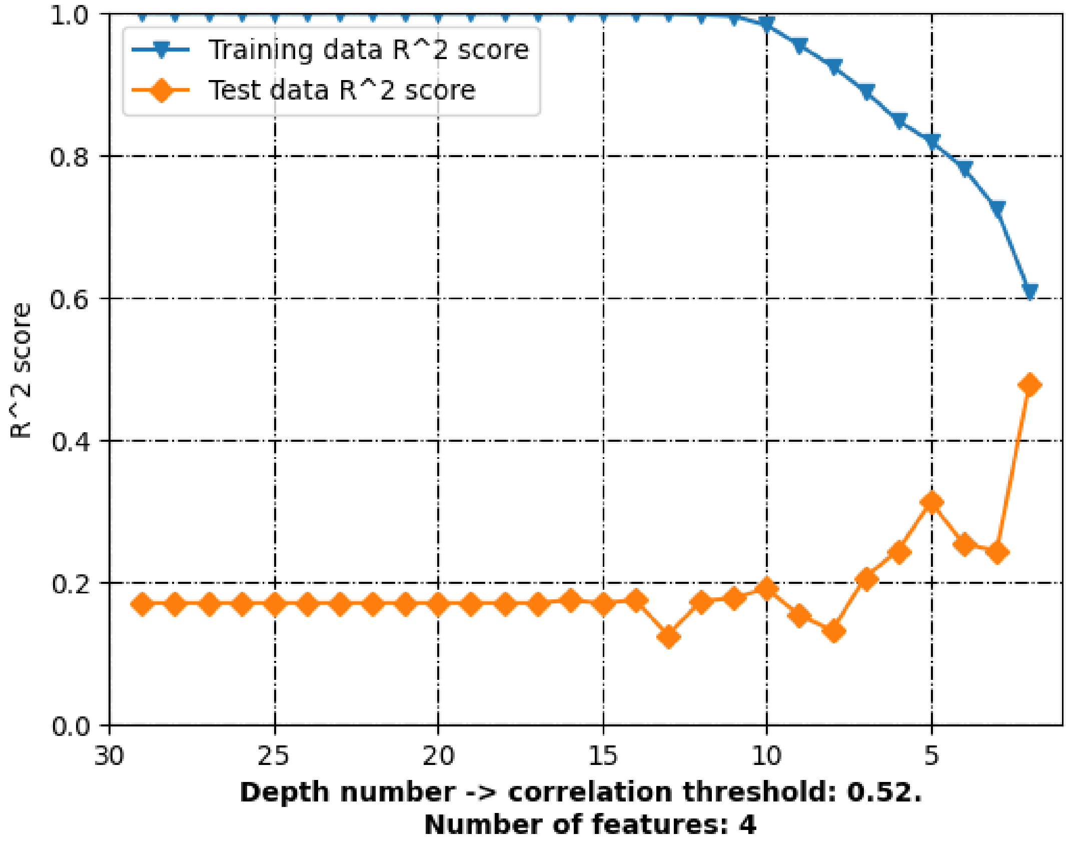

Training data R^2 score
Test data R^2 score
R^2 score
1.0
0.8
0.6
0.4
0.2
0.0
30
25
20
15
10
5
Depth number -> correlation threshold: 0.52.
Number of features: 4

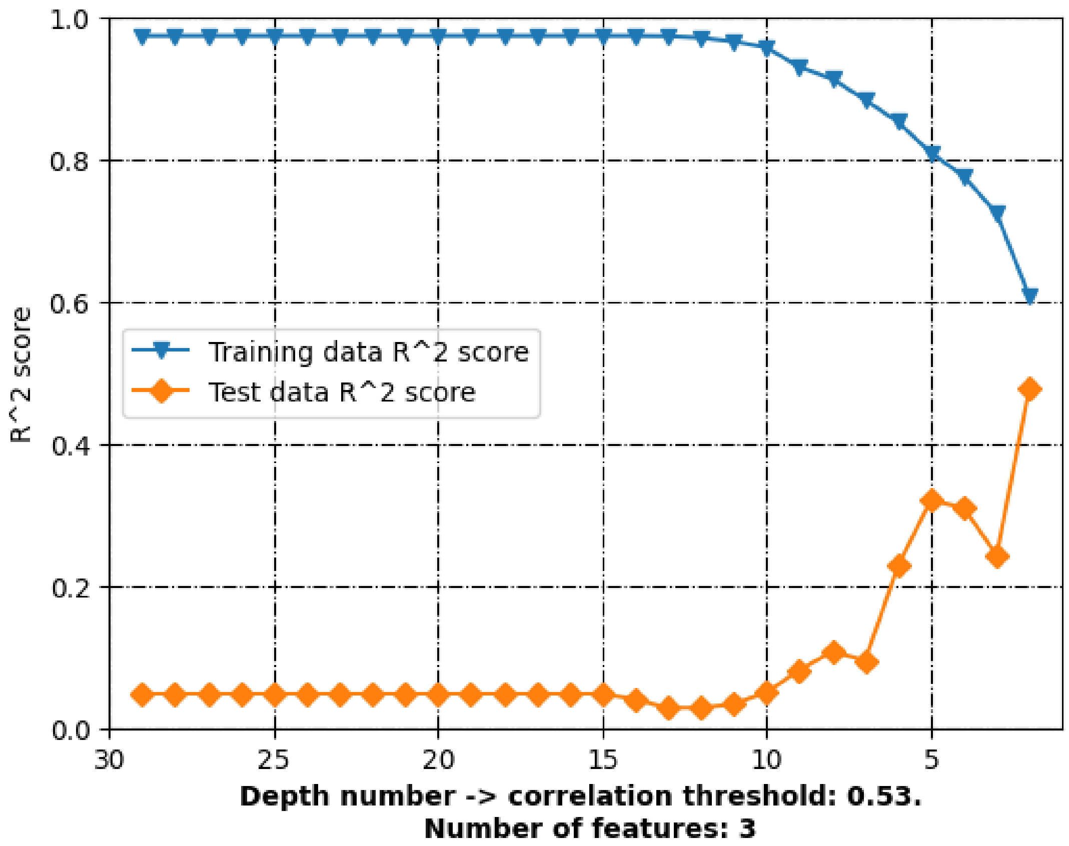

[20]:
```
plt.plot(df_without_standardization['Correlation threshold'],␣
 ↪df_without_standardization['Number of features'], label = "Number of␣
 ↪features", marker='.')
plt.legend()
plt.xlabel('Correlation threshold')
plt.ylabel('Number of features')
plt.show()
```

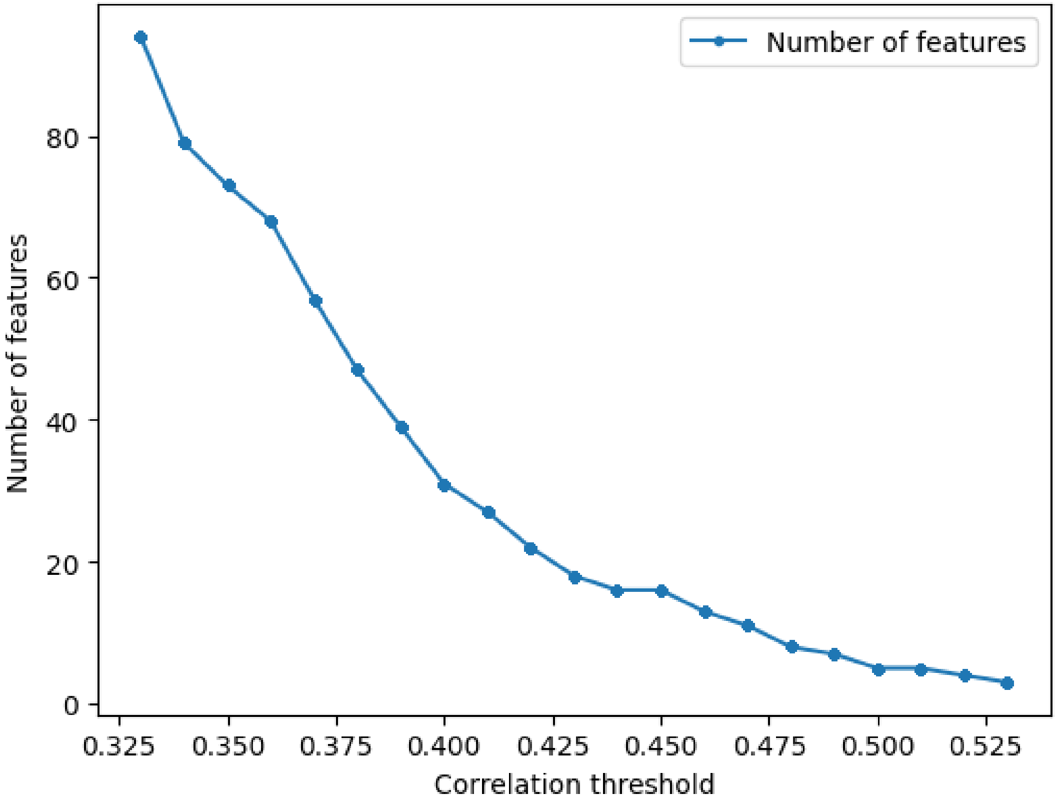

[ ]:

```

```

3 Random Forest

[21]:

```
without_standardization, train_r2_, test_r2_, hist1, hist2, target_column_name,␣
↪training_data_RMSE, test_data_RMSE  = pred_model.
↪prepare_data_and_create_model(molecular_descriptors_df=data,
                                                                                   ␣
↪                         correlation_threshold=0.50,
                                                                                   ␣
↪                         standardization=False,
                                                                                   ␣
↪                         model_type='RandomForestRegressor',
                                                                                   ␣
↪                         n_estimators_=12,
                                                                                   ␣
↪                         target_column_name = target,
                                                                                   ␣
↪                         random_state=random_state,
```

```
↪                         train_test_split_=True,
↪                         verbose=True)
```

```
I am not doing standardization…
  molecular descriptor name
0                    AATS0Z
1                  AATS0are
2                    AATS0d
3                   AATS0dv
4                    AATS0i
  molecular descriptor name  corr_value
0                    AATS0Z   -0.022797
1                  AATS0are   -0.139064
2                    AATS0d    0.027592
3                   AATS0dv   -0.136049
4                    AATS0i    0.168958
  molecular descriptor name  corr_value  absolute correlation value
0                    AATS0Z   -0.022797                    0.022797
1                  AATS0are   -0.139064                    0.139064
2                    AATS0d    0.027592                    0.027592
3                   AATS0dv   -0.136049                    0.136049
4                    AATS0i    0.168958                    0.168958
     molecular descriptor name  corr_value  absolute correlation value
226                     AMID_O   -0.557717                    0.557717
505                 EState_VSA5  -0.534748                    0.534748
791                    MDEO-12   -0.555724                    0.555724
921                      SaasC    0.513071                    0.513071
1091               VSA_EState5    0.526082                    0.526082
     molecular descriptor name  corr_value  absolute correlation value
226                     AMID_O   -0.557717                    0.557717
505                 EState_VSA5  -0.534748                    0.534748
791                    MDEO-12   -0.555724                    0.555724
921                      SaasC    0.513071                    0.513071
1091               VSA_EState5    0.526082                    0.526082
The model used is: RandomForest…
Return the coefficient of determination of the prediction:
0.7074034461598402
R^2 score: 0.9176000098861355
Correlation coefficient: 0.9579144063464833
Test data - unseen during training:
R^2 score: 0.7074034461598402
Correlation coefficient: 0.8410727948042549
[8.0030081  5.74447545 7.05947768 7.97223228 7.99229063 7.63253623
 8.01751878 7.29942536 8.03543867 7.63579976 8.39302261 8.08632736
 8.30093813 7.72989841 7.6806442  8.01313613 5.99467593 5.49942963]
102    7.920819
```

```
38     6.019997
8      5.996841
109    7.886057
11     7.962574
51     7.886057
88     8.075721
21     7.325139
82     7.978811
98     7.481486
58     7.677781
64     8.537602
74     8.142668
103    8.397940
91     8.958607
43     7.823909
25     4.886525
30     6.009661
Name: A549, dtype: float64
Training Root Mean Square Error: 0.2679798395343317
Testing Root Mean Square Error: 0.5607234734609626
```

[22]:

```
print(target_column_name+str('_transformed'))
print(hist1[target_column_name].hist())
```

```
A549_transformed
AxesSubplot(0.125,0.11;0.775x0.77)
```

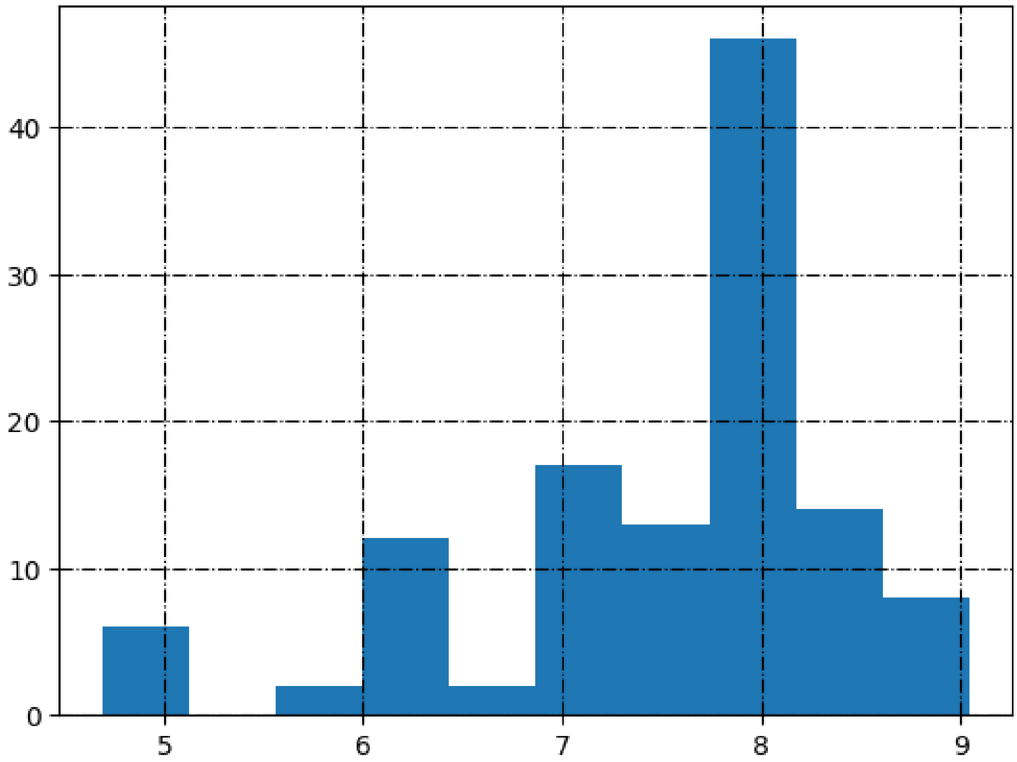

[23]:
```
with_standardization, train_r2_, test_r2_, hist1, hist2, target_column_name,␣
↪training_data_RMSE, test_data_RMSE  = pred_model.
↪prepare_data_and_create_model(molecular_descriptors_df=data,
                                                                              ␣
↪                    correlation_threshold=0.50,
                                                                              ␣
↪                    standardization=True,
                                                                              ␣
↪                    model_type='RandomForestRegressor',
                                                                              ␣
↪                    n_estimators_=12,
                                                                              ␣
↪                    target_column_name = target,
                                                                              ␣
↪                    random_state=random_state,
                                                                              ␣
↪                    train_test_split_=True,
                                                                              ␣
↪                    verbose=True)
```

```
I am doing standardization…
  molecular descriptor name
```

```
0                  AATS0Z
1                AATS0are
2                  AATS0d
3                 AATS0dv
4                  AATS0i
  molecular descriptor name  corr_value
0                  AATS0Z   -0.022797
1                AATS0are   -0.139064
2                  AATS0d    0.027592
3                 AATS0dv   -0.136049
4                  AATS0i    0.168958
  molecular descriptor name  corr_value  absolute correlation value
0                  AATS0Z   -0.022797                    0.022797
1                AATS0are   -0.139064                    0.139064
2                  AATS0d    0.027592                    0.027592
3                 AATS0dv   -0.136049                    0.136049
4                  AATS0i    0.168958                    0.168958
     molecular descriptor name  corr_value  absolute correlation value
226                  AMID_O   -0.557717                    0.557717
505              EState_VSA5   -0.534748                    0.534748
791                  MDEO-12   -0.555724                    0.555724
921                    SaasC    0.513071                    0.513071
1091             VSA_EState5    0.526082                    0.526082
     molecular descriptor name  corr_value  absolute correlation value
226                  AMID_O   -0.557717                    0.557717
505              EState_VSA5   -0.534748                    0.534748
791                  MDEO-12   -0.555724                    0.555724
921                    SaasC    0.513071                    0.513071
1091             VSA_EState5    0.526082                    0.526082
The model used is: RandomForest…
Return the coefficient of determination of the prediction:
0.7074034461598402
R^2 score: 0.9176000098861355
Correlation coefficient: 0.9579144063464833
Test data - unseen during training:
R^2 score: 0.7074034461598402
Correlation coefficient: 0.8410727948042549
[8.0030081  5.74447545 7.05947768 7.97223228 7.99229063 7.63253623
 8.01751878 7.29942536 8.03543867 7.63579976 8.39302261 8.08632736
 8.30093813 7.72989841 7.6806442  8.01313613 5.99467593 5.49942963]
102    7.920819
38     6.019997
8      5.996841
109    7.886057
11     7.962574
51     7.886057
88     8.075721
21     7.325139
```

```
82     7.978811
98     7.481486
58     7.677781
64     8.537602
74     8.142668
103    8.397940
91     8.958607
43     7.823909
25     4.886525
30     6.009661
Name: A549, dtype: float64
Training Root Mean Square Error: 0.2679798395343317
Testing Root Mean Square Error: 0.5607234734609626
```

3.1 Search inside correlation space

[24]:

```
step = 0.01
initial_step = corr_low
last_step = corr_high
first_list = [x / 100.0 for x in range(int(initial_step*100),␣
 ↪int(last_step*100), int(step*100))]
n_estimators = [range(2,21,1)]
corr_th = []
second_list = []
third_list = []
fourth_l = []
fifth_l = []
f_list = []
fif_list = []
for i in first_list:

    for estimator in n_estimators[0]:

        without_standardization, train_r2, test_r2, _, h_, target_column_name,␣
 ↪training_data_RMSE, test_data_RMSE = pred_model.
 ↪prepare_data_and_create_model(molecular_descriptors_df=data,
                                                                               ␣
 ↪                    correlation_threshold=i,
                                                                               ␣
 ↪                    standardization=False,
                                                                               ␣
 ↪                    model_type='RandomForestRegressor',
                                                                               ␣
 ↪                    n_estimators_=estimator,
                                                                               ␣
 ↪                    target_column_name = target,
```

```
                                                                                    ␣
↪                    random_state=random_state,
                                                                                    ␣
↪                    train_test_split_=True,
                                                                                    ␣
↪                    verbose=False)
        corr_th.append(i)
        second_list.append(train_r2)
        third_list.append(test_r2)
        fourth_l.append(training_data_RMSE)
        fifth_l.append(test_data_RMSE)
        f_list.append(len(h_))
        fif_list.append(estimator)
```

[25]:
```
df_without_standardization = pd.DataFrame(data=corr_th, columns=["Correlation␣
↪threshold"])
df_without_standardization['Training data R^2 score'] = second_list
df_without_standardization['Test data R^2 score'] = third_list
df_without_standardization['Training RMSE'] = fourth_l
df_without_standardization['Test RMSE'] = fifth_l
df_without_standardization['Number of features'] = f_list
df_without_standardization['Number of estimators'] = fif_list
```

[26]:
```
df_random_forest = df_without_standardization.copy()
#df_without_standardization.to_excel('../Data/
↪A549_Random_forest_rs_'+str(random_state)+'.xlsx')
```

[27]:
```
corre_list = [x / 100.0 for x in range(int(initial_step*100),␣
↪int(last_step*100), int(0.01*100))]
for element in corre_list:
    element_ =␣
↪df_without_standardization[df_without_standardization['Correlation␣
↪threshold'] == float(element)]
    plt.plot(element_['Number of estimators'], element_['Training data R^2␣
↪score'], label = "Training data R^2 score", marker='v')
    plt.plot(element_['Number of estimators'], element_['Test data R^2 score'],␣
↪label = "Test data R^2 score", marker='D')
    plt.legend()
    plt.xlabel('Number of estimators -> correlation threshold: '+str(element)+'.
↪ \n Number of features: '+str(element_['Number of features'].iloc[0]))
    plt.xlim(max(element_['Number of estimators'])+1, min(element_['Number of␣
↪estimators'])-1)
    plt.ylabel('R^2 score')
    plt.ylim([0, 1])
    plt.rc('grid', linestyle="-.", color='black')
    plt.grid(True)
```

```
plt.show()
```

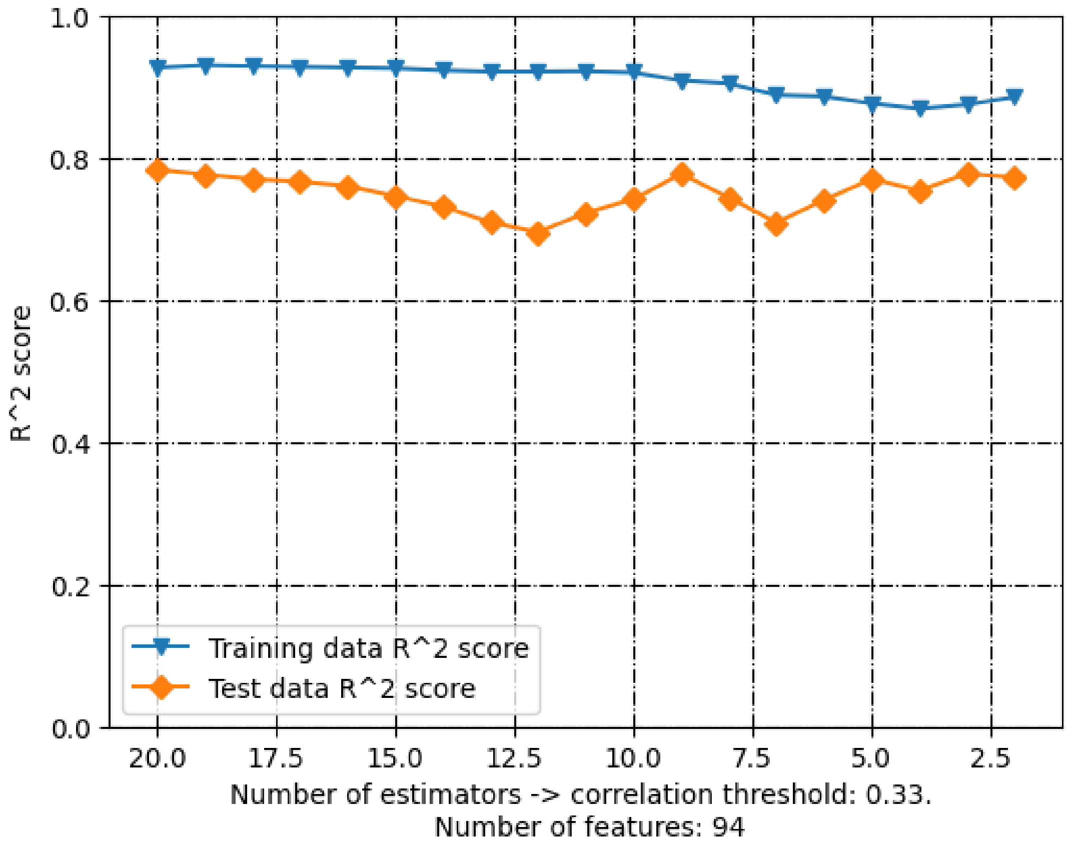

1.0
0.8
0.6
0.4
0.2
0.0
R^2 score
20.0
17.5
15.0
12.5
10.0
7.5
5.0
2.5
Number of estimators -> correlation threshold: 0.33.
Number of features: 94
Training data R^2 score
Test data R^2 score

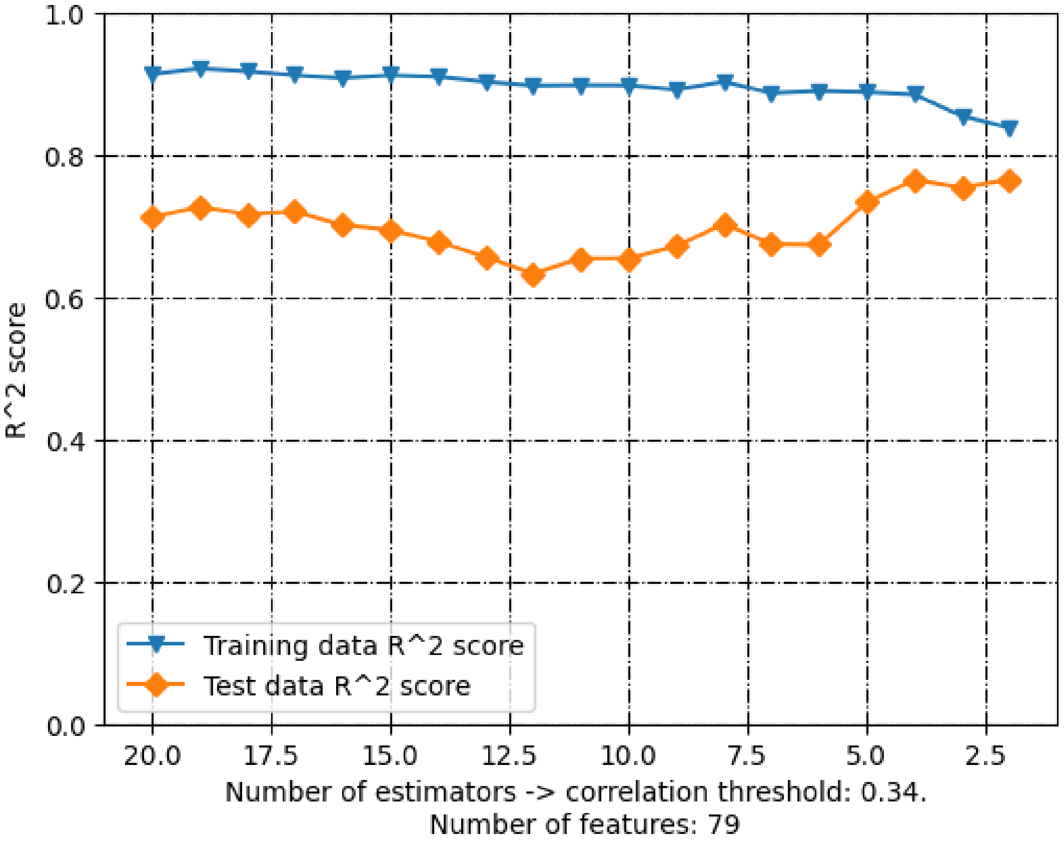
1.0
0.8
0.6
0.4
0.2
0.0
R^2 score
Training data R^2 score
Test data R^2 score
20.0
17.5
15.0
12.5
10.0
7.5
5.0
2.5
Number of estimators -> correlation threshold: 0.34.
Number of features: 79

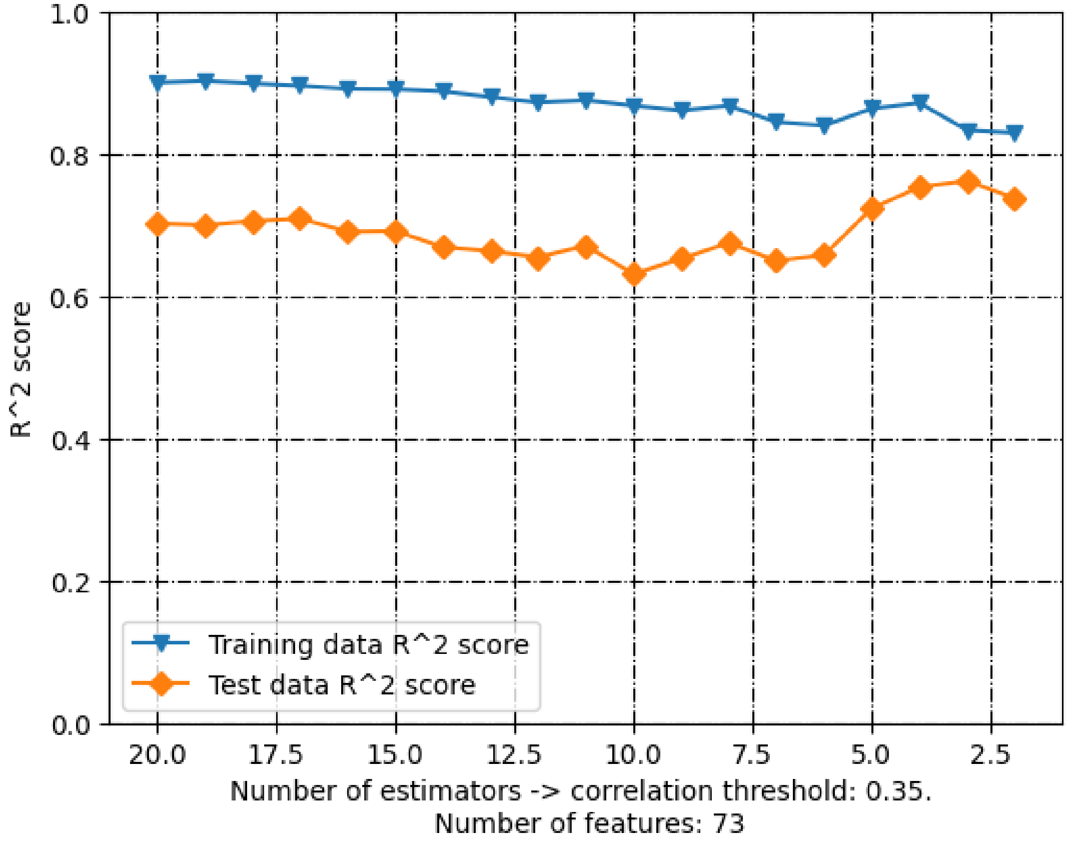

Training data R^2 score
Test data R^2 score
R^2 score
Number of estimators -> correlation threshold: 0.35.
Number of features: 73
1.0
0.8
0.6
0.4
0.2
0.0
20.0
17.5
15.0
12.5
10.0
7.5
5.0
2.5

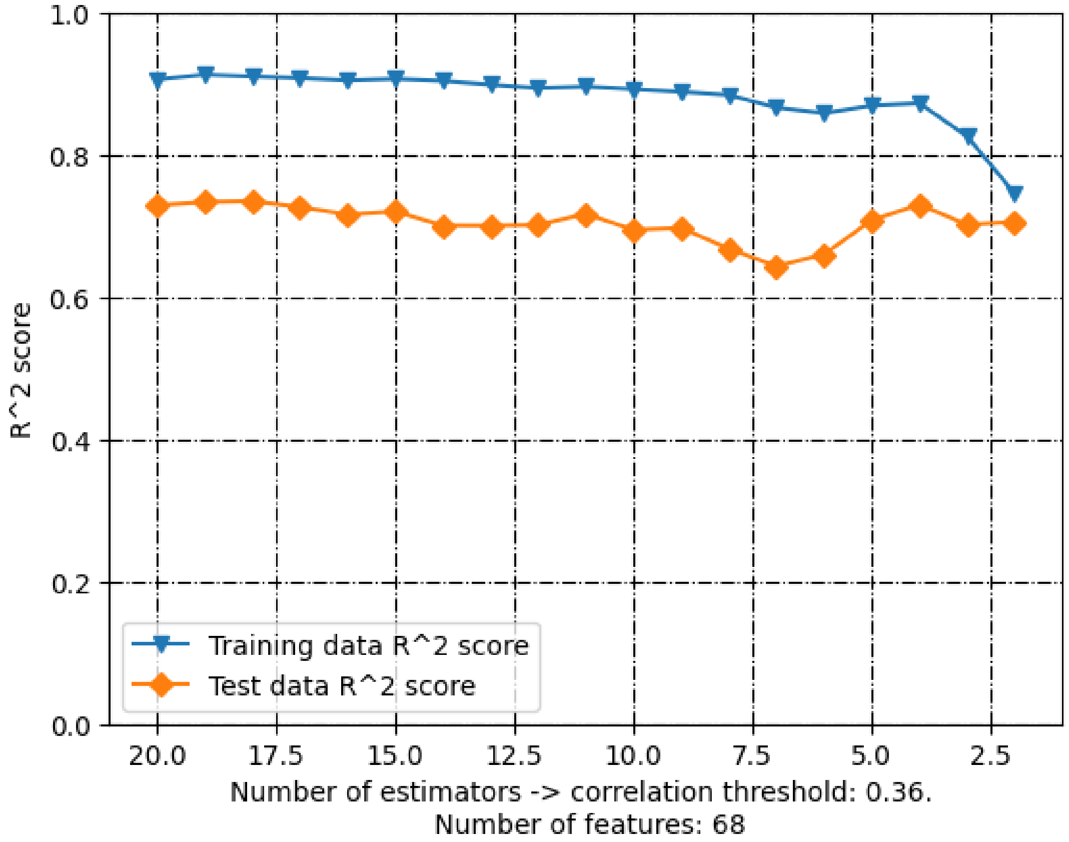

1.0
0.8
0.6
0.4
0.2
0.0
R^2 score
20.0
17.5
15.0
12.5
10.0
7.5
5.0
2.5
Number of estimators -> correlation threshold: 0.36.
Number of features: 68
Training data R^2 score
Test data R^2 score

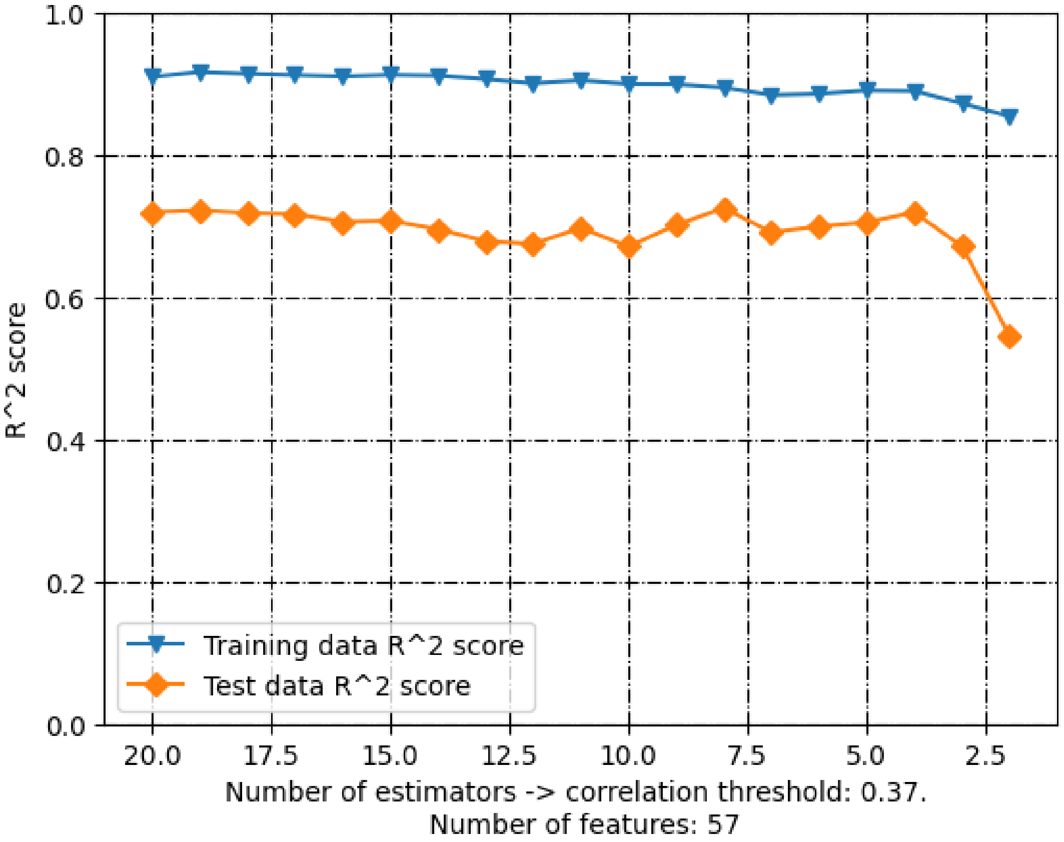

1.0
0.8
0.6
0.4
0.2
0.0
R^2 score
Training data R^2 score
Test data R^2 score
20.0
17.5
15.0
12.5
10.0
7.5
5.0
2.5
Number of estimators -> correlation threshold: 0.37.
Number of features: 57

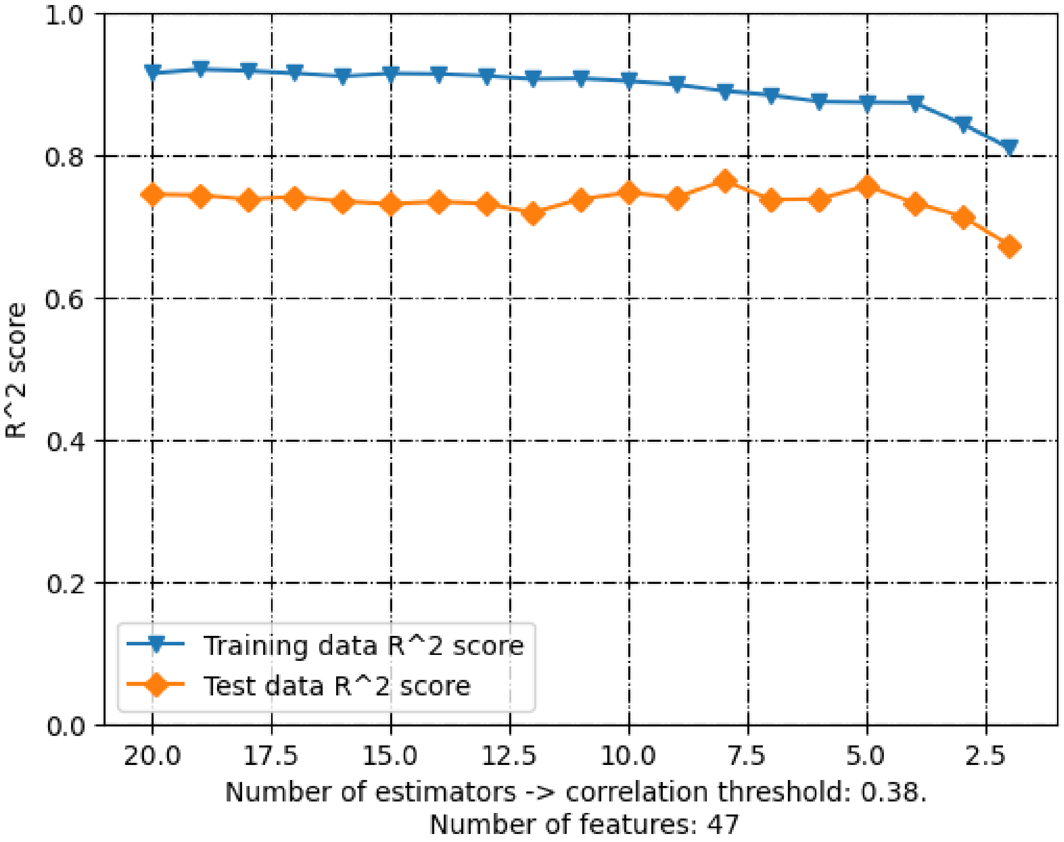
1.0
0.8
0.6
0.4
0.2
0.0
R^2 score
20.0
17.5
15.0
12.5
10.0
7.5
5.0
2.5
Number of estimators -> correlation threshold: 0.38.
Number of features: 47
Training data R^2 score
Test data R^2 score

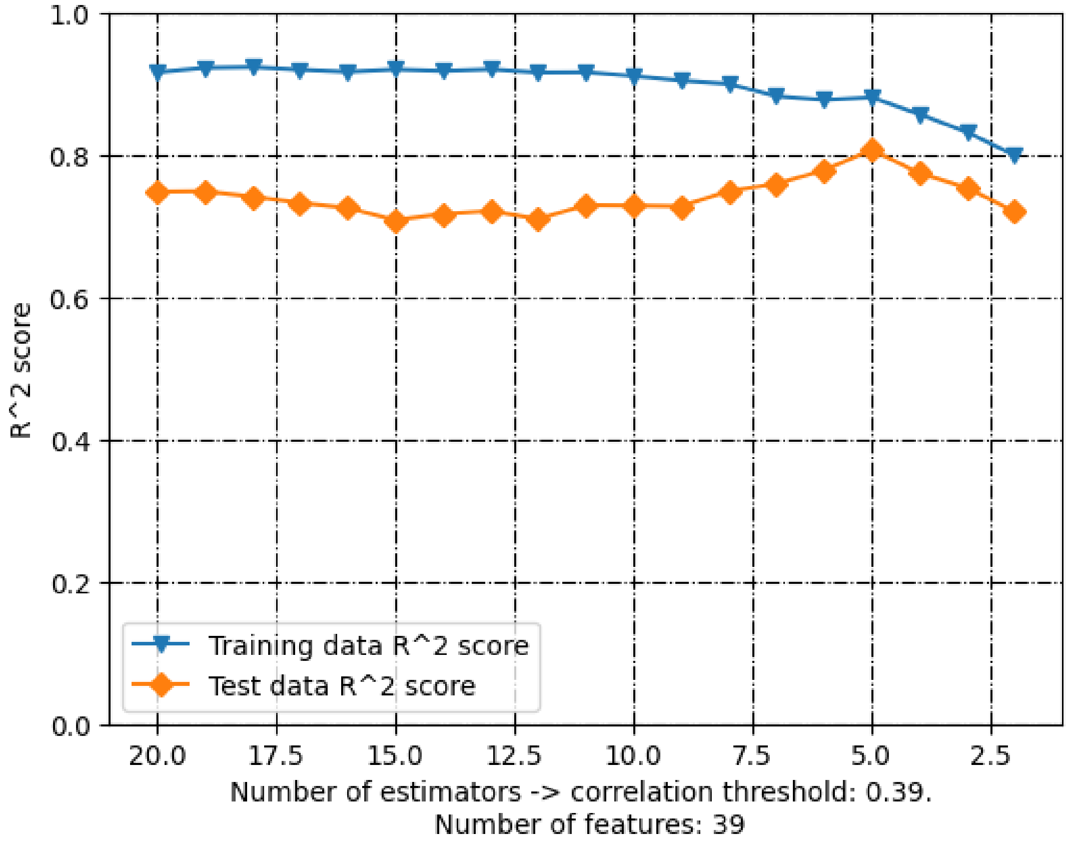
1.0
0.8
0.6
0.4
0.2
0.0
R^2 score
20.0
17.5
15.0
12.5
10.0
7.5
5.0
2.5
Number of estimators -> correlation threshold: 0.39.
Number of features: 39
Training data R^2 score
Test data R^2 score

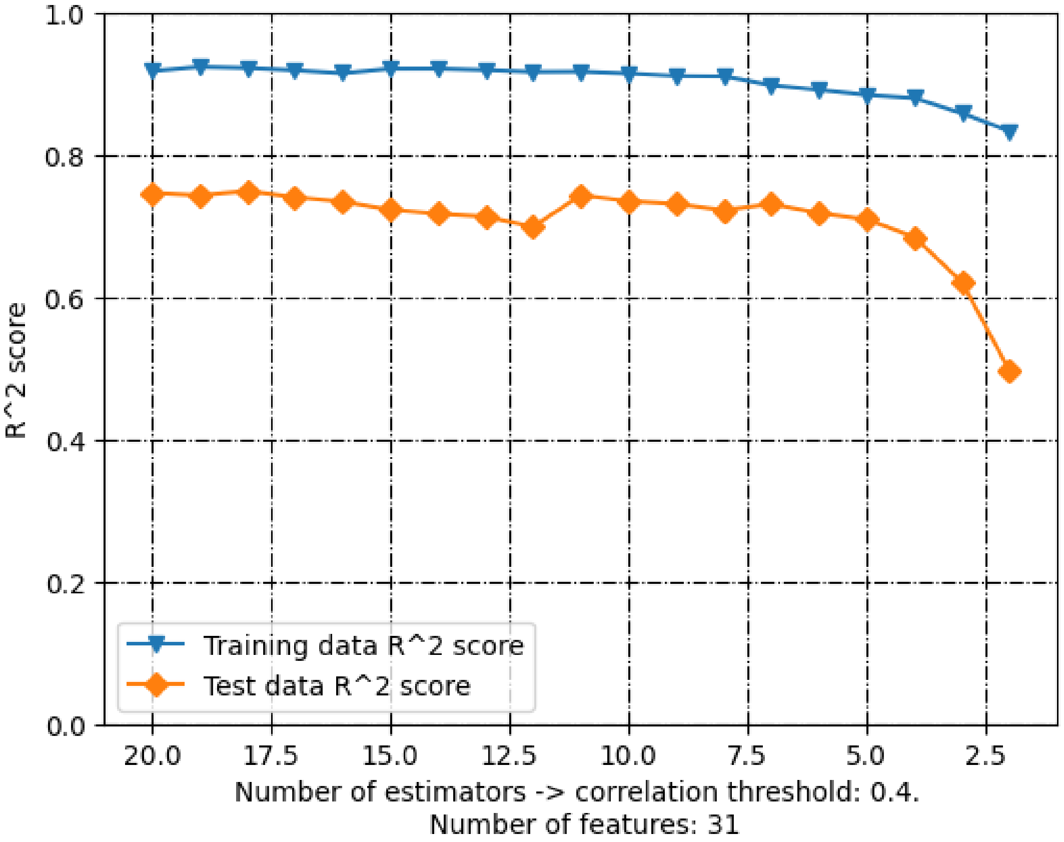
1.0
0.8
0.6
0.4
0.2
0.0
R^2 score
Training data R^2 score
Test data R^2 score
20.0
17.5
15.0
12.5
10.0
7.5
5.0
2.5
Number of estimators -> correlation threshold: 0.4.
Number of features: 31

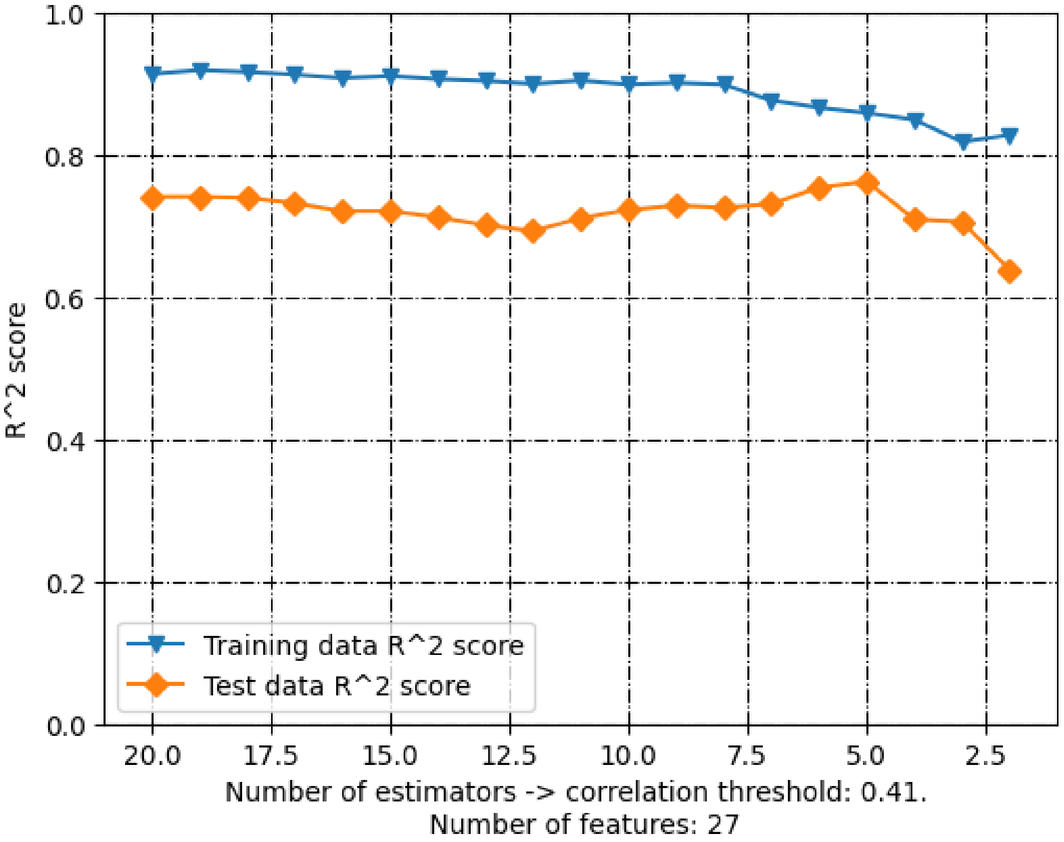

1.0
0.8
0.6
0.4
0.2
0.0
R^2 score
20.0
17.5
15.0
12.5
10.0
7.5
5.0
2.5
Training data R^2 score
Test data R^2 score
Number of estimators -> correlation threshold: 0.41.
Number of features: 27

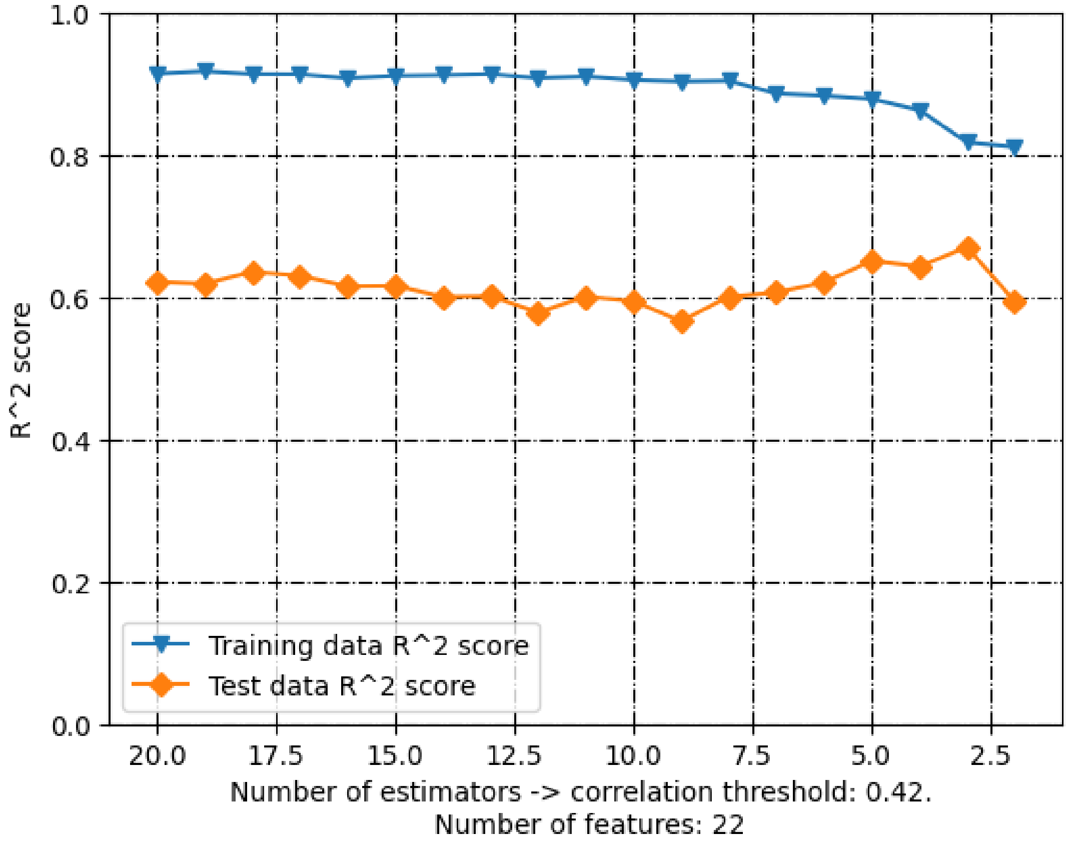
1.0
0.8
0.6
0.4
0.2
0.0
R^2 score
Training data R^2 score
Test data R^2 score
20.0
17.5
15.0
12.5
10.0
7.5
5.0
2.5
Number of estimators -> correlation threshold: 0.42.
Number of features: 22

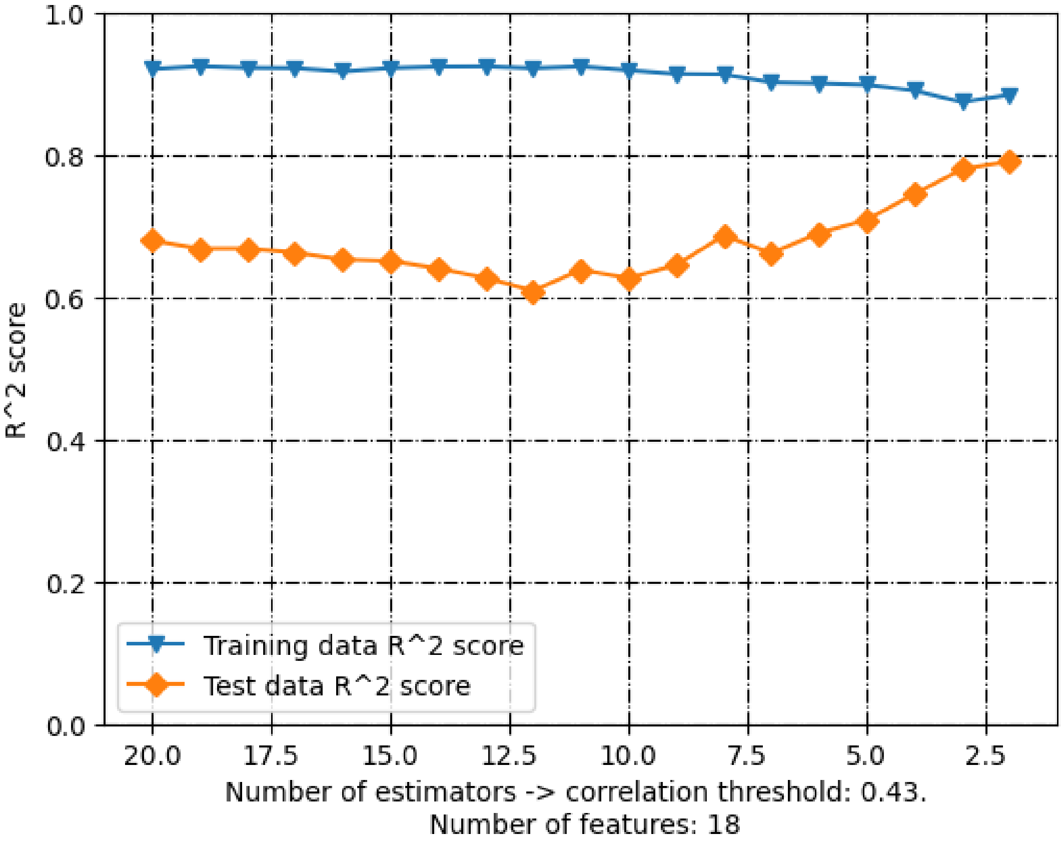

1.0
0.8
0.6
0.4
0.2
0.0
R^2 score
20.0
17.5
15.0
12.5
10.0
7.5
5.0
2.5
Training data R^2 score
Test data R^2 score
Number of estimators -> correlation threshold: 0.43.
Number of features: 18

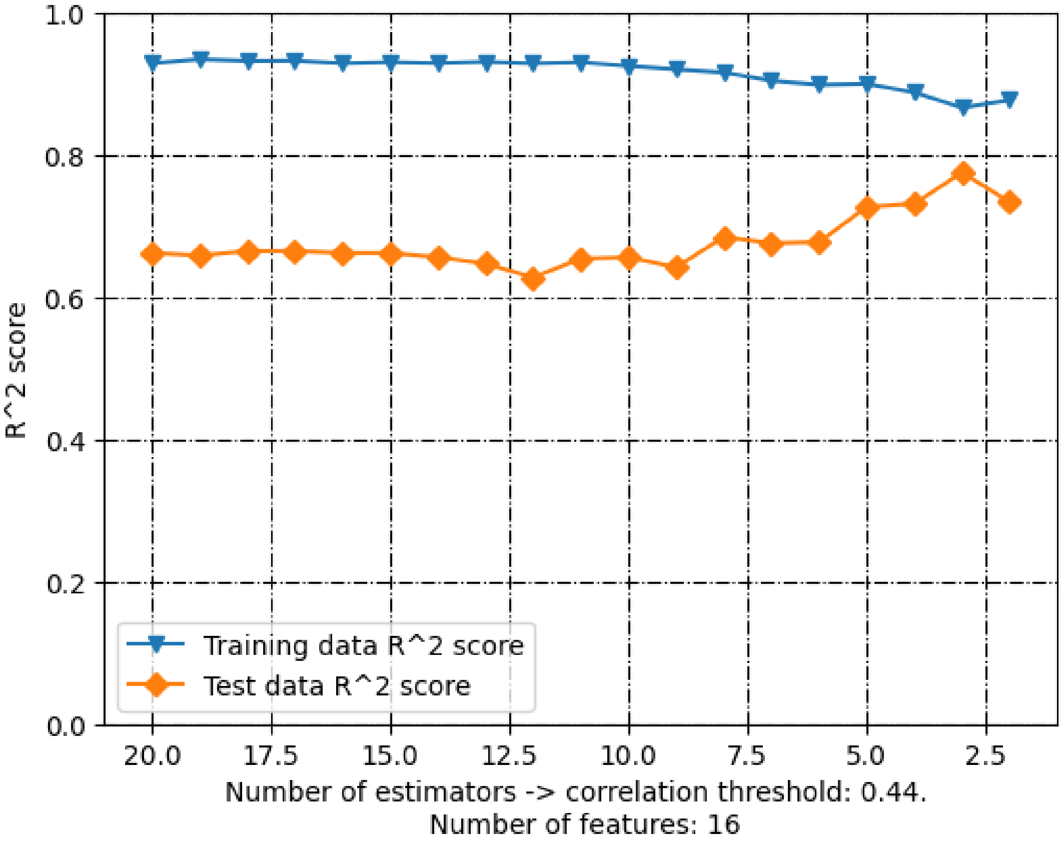
1.0
0.8
0.6
0.4
0.2
0.0
R^2 score
Training data R^2 score
Test data R^2 score
20.0
17.5
15.0
12.5
10.0
7.5
5.0
2.5
Number of estimators -> correlation threshold: 0.44.
Number of features: 16

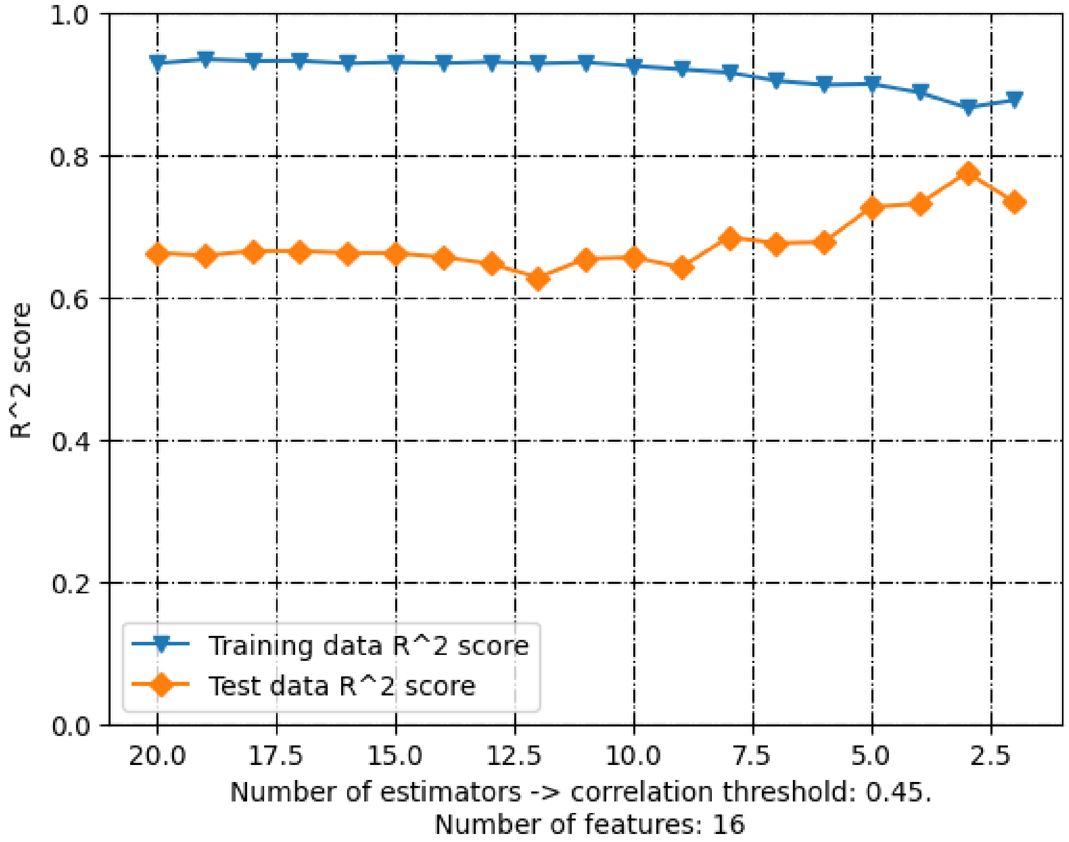
1.0
0.8
0.6
0.4
0.2
0.0
R^2 score
Training data R^2 score
Test data R^2 score
20.0
17.5
15.0
12.5
10.0
7.5
5.0
2.5
Number of estimators -> correlation threshold: 0.45.
Number of features: 16

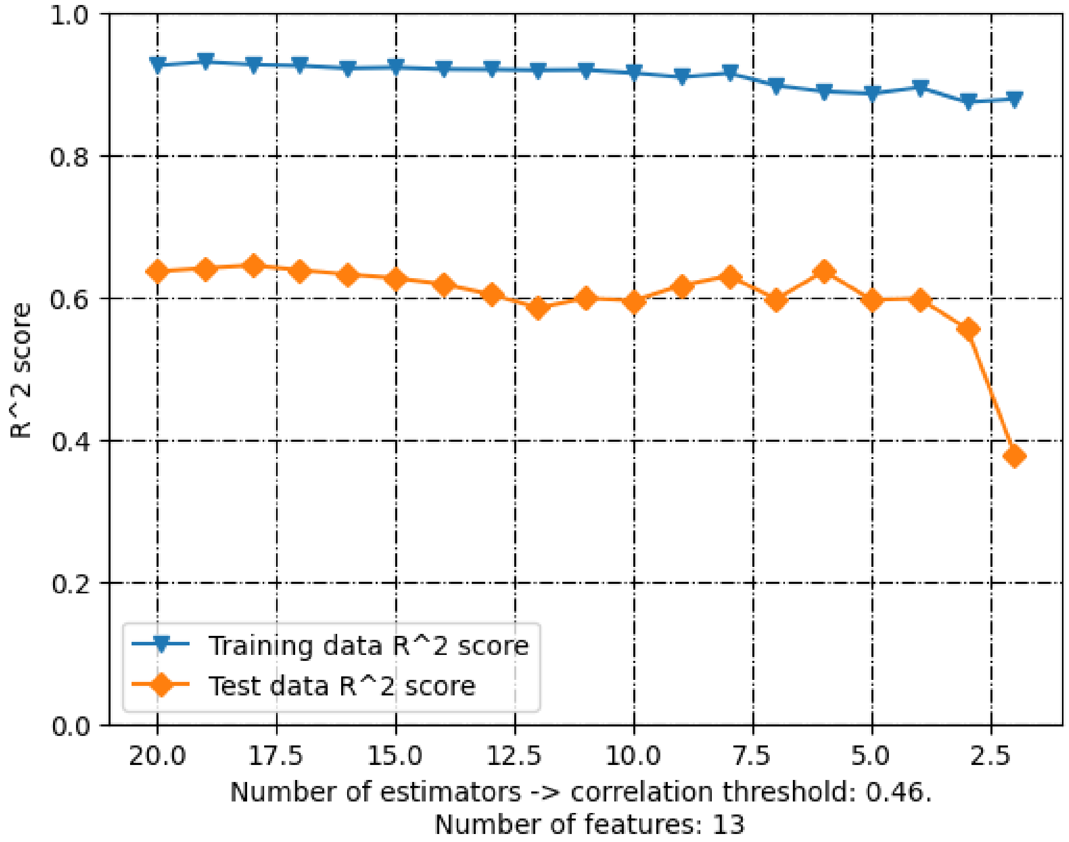

1.0
0.8
0.6
0.4
0.2
0.0
R^2 score
20.0
17.5
15.0
12.5
10.0
7.5
5.0
2.5
Number of estimators -> correlation threshold: 0.46.
Number of features: 13
Training data R^2 score
Test data R^2 score

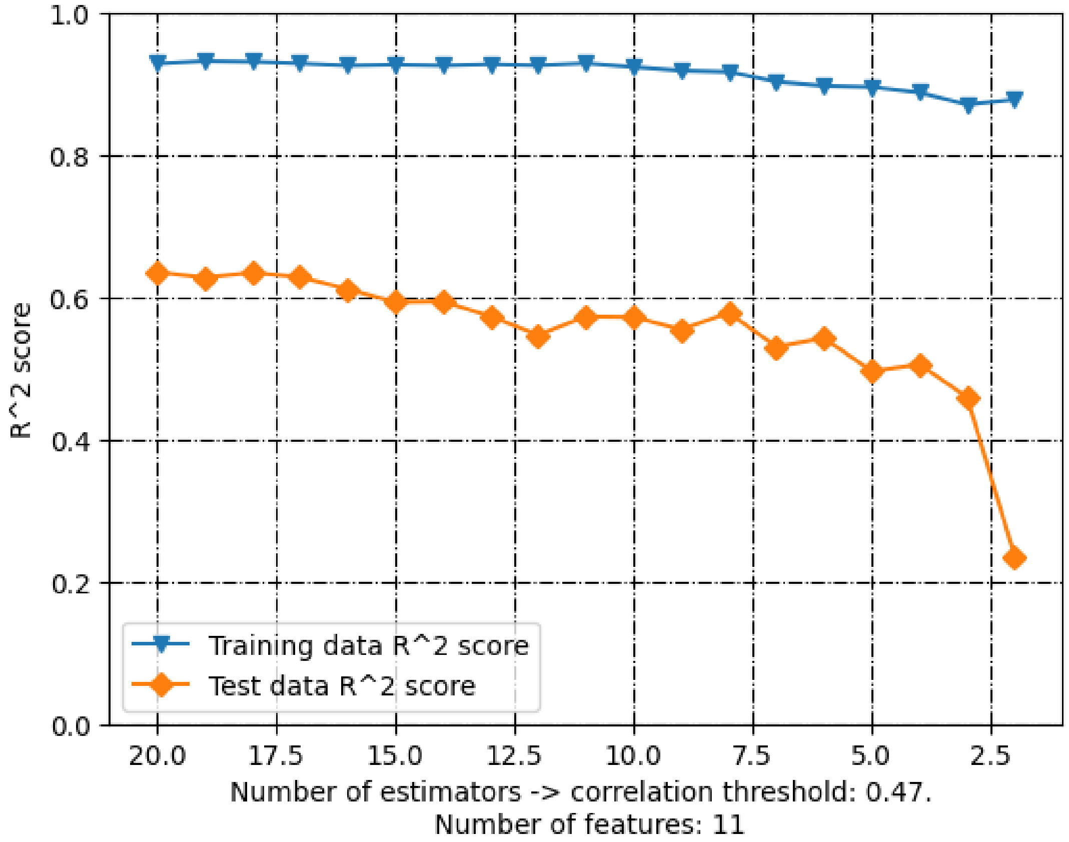

1.0
0.8
0.6
0.4
0.2
0.0
R^2 score
20.0
17.5
15.0
12.5
10.0
7.5
5.0
2.5
Training data R^2 score
Test data R^2 score
Number of estimators -> correlation threshold: 0.47.
Number of features: 11

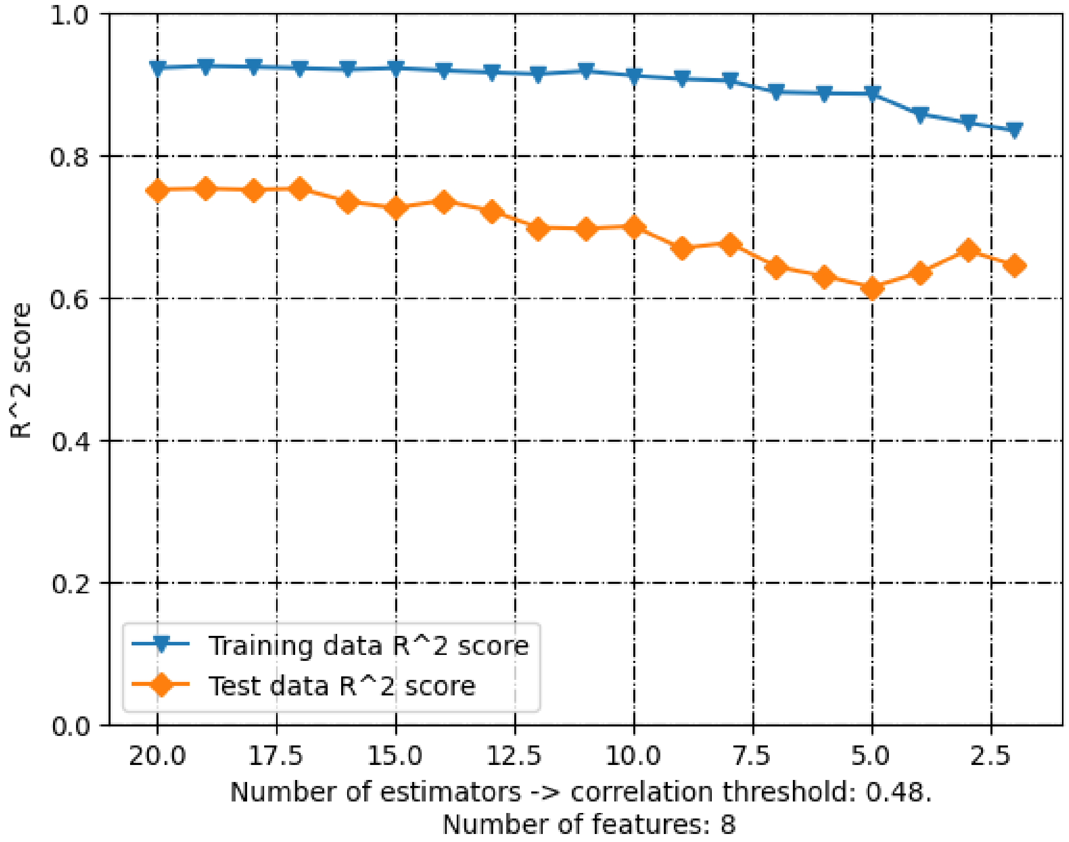
1.0
0.8
0.6
0.4
0.2
0.0
R^2 score
Training data R^2 score
Test data R^2 score
20.0
17.5
15.0
12.5
10.0
7.5
5.0
2.5
Number of estimators -> correlation threshold: 0.48.
Number of features: 8

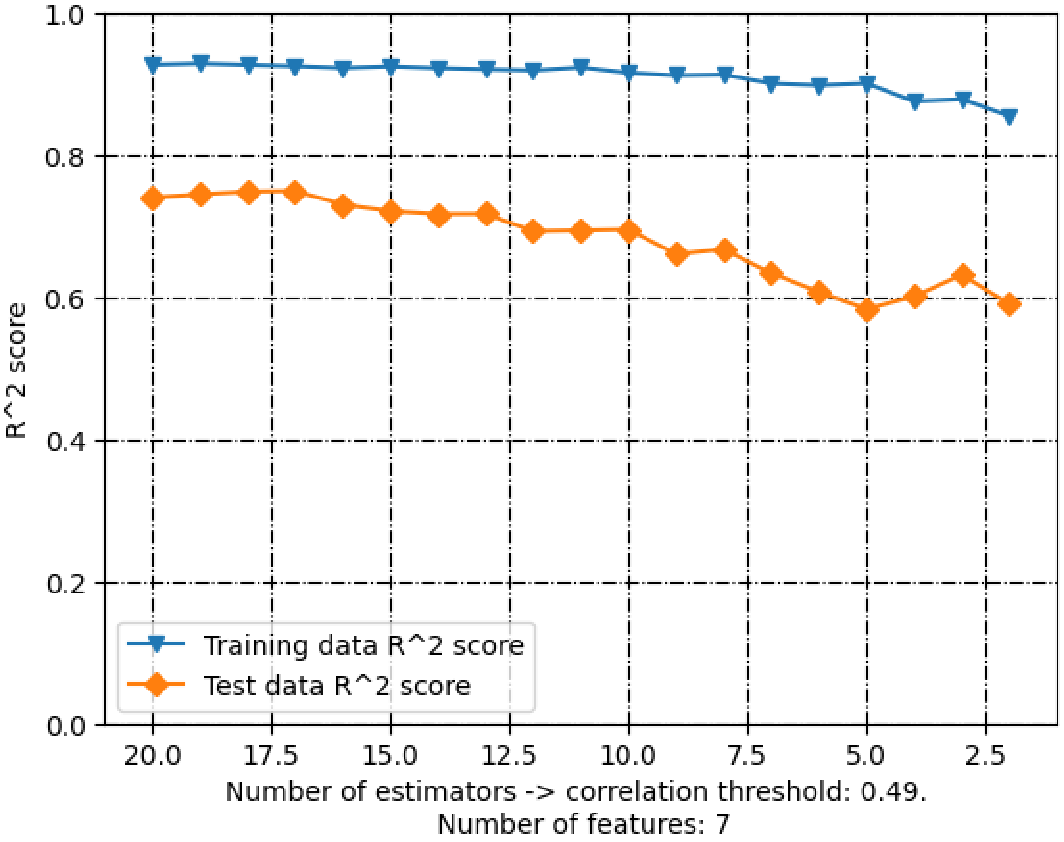

1.0
0.8
0.6
0.4
0.2
0.0
R^2 score
Training data R^2 score
Test data R^2 score
20.0
17.5
15.0
12.5
10.0
7.5
5.0
2.5
Number of estimators -> correlation threshold: 0.49.
Number of features: 7

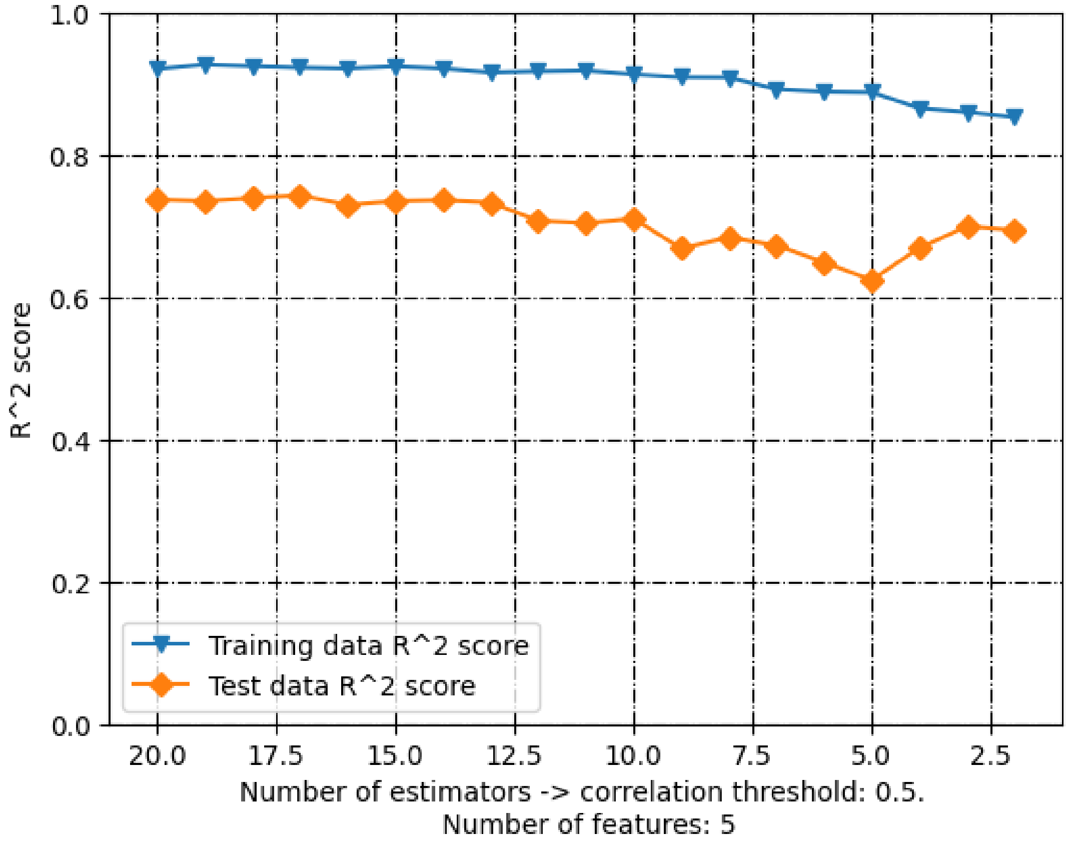

1.0
0.8
0.6
0.4
0.2
0.0
R^2 score
Training data R^2 score
Test data R^2 score
20.0
17.5
15.0
12.5
10.0
7.5
5.0
2.5
Number of estimators -> correlation threshold: 0.5.
Number of features: 5

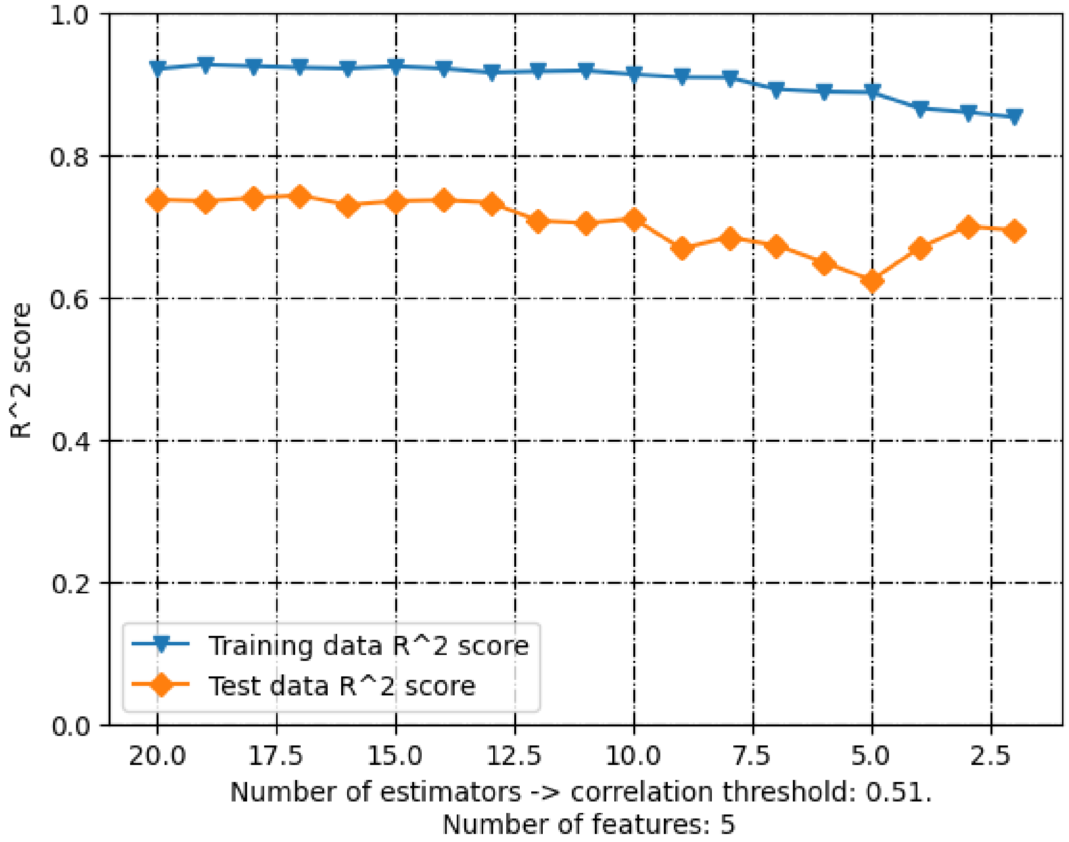
1.0
0.8
0.6
0.4
0.2
0.0
R^2 score
20.0
17.5
15.0
12.5
10.0
7.5
5.0
2.5
Training data R^2 score
Test data R^2 score
Number of estimators -> correlation threshold: 0.51.
Number of features: 5

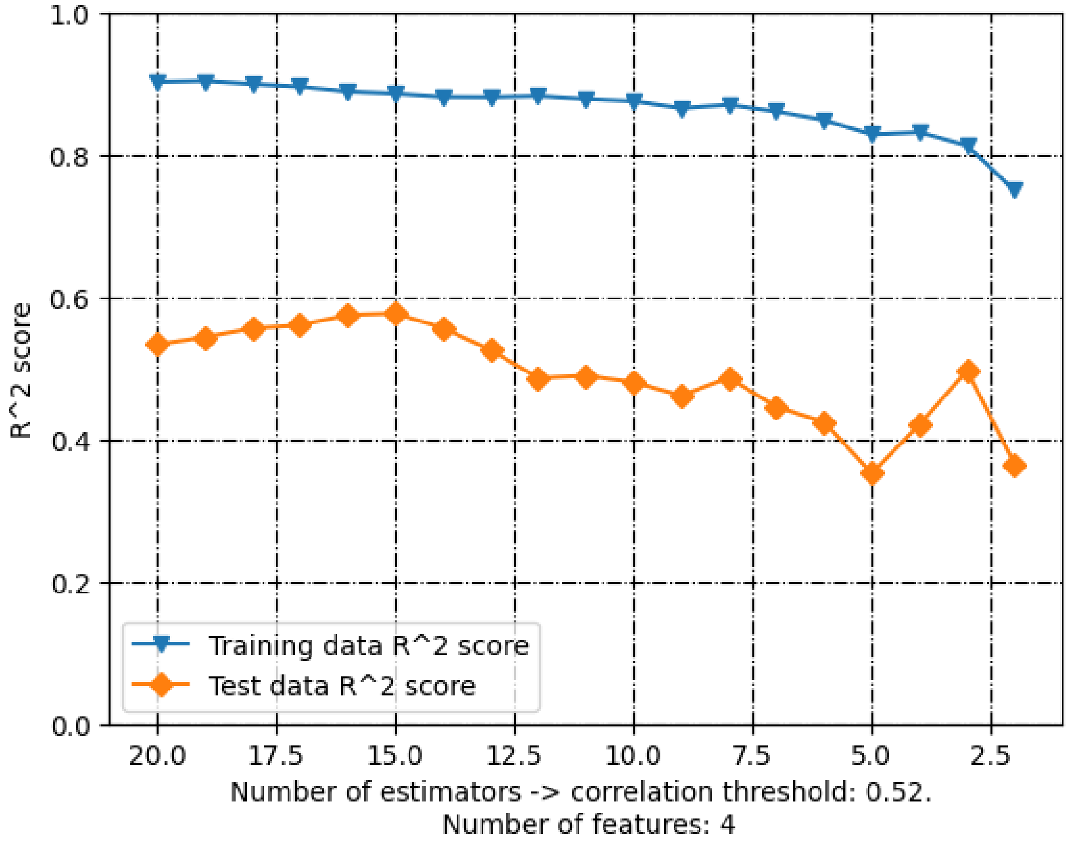
1.0
0.8
0.6
0.4
0.2
0.0
R^2 score
20.0
17.5
15.0
12.5
10.0
7.5
5.0
2.5
Number of estimators -> correlation threshold: 0.52.
Number of features: 4
Training data R^2 score
Test data R^2 score

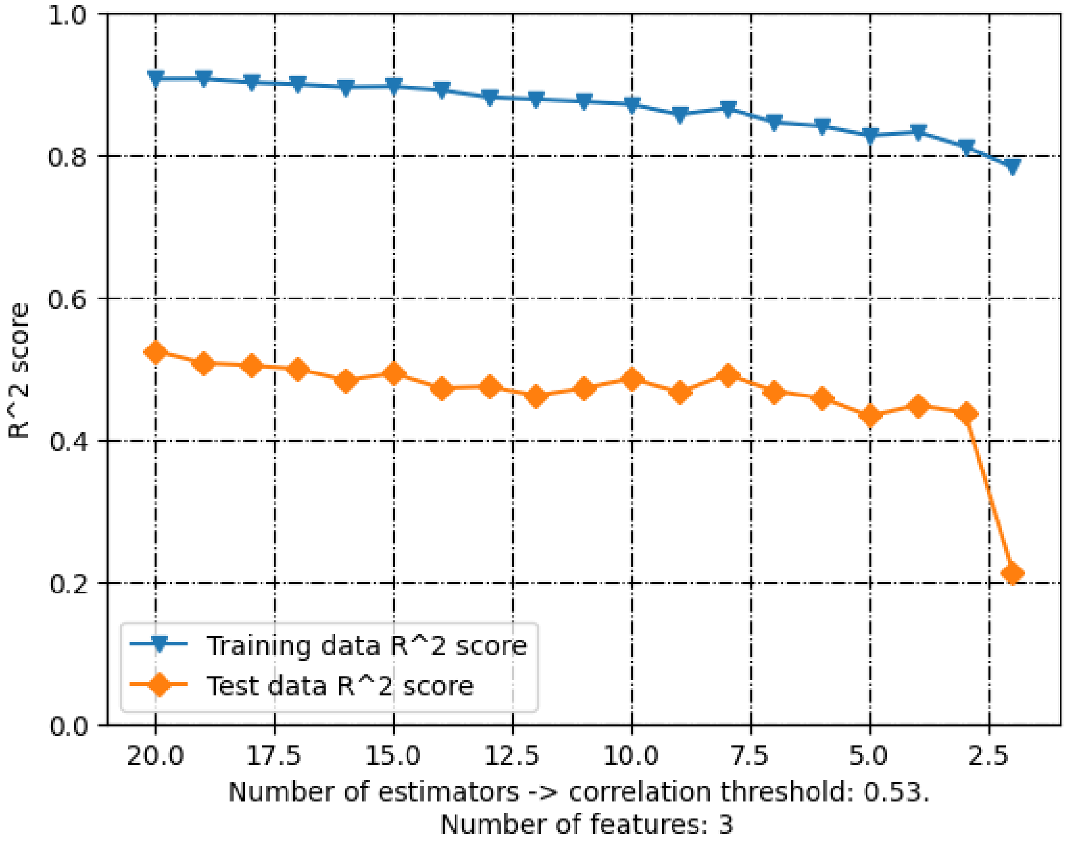

[28]:
```
plt.plot(df_without_standardization['Correlation threshold'],␣
 ↪df_without_standardization['Number of features'], label = "Number of␣
 ↪features", marker='.')
plt.legend()
plt.xlabel('Correlation threshold')
plt.ylabel('Number of features')
plt.show()
```

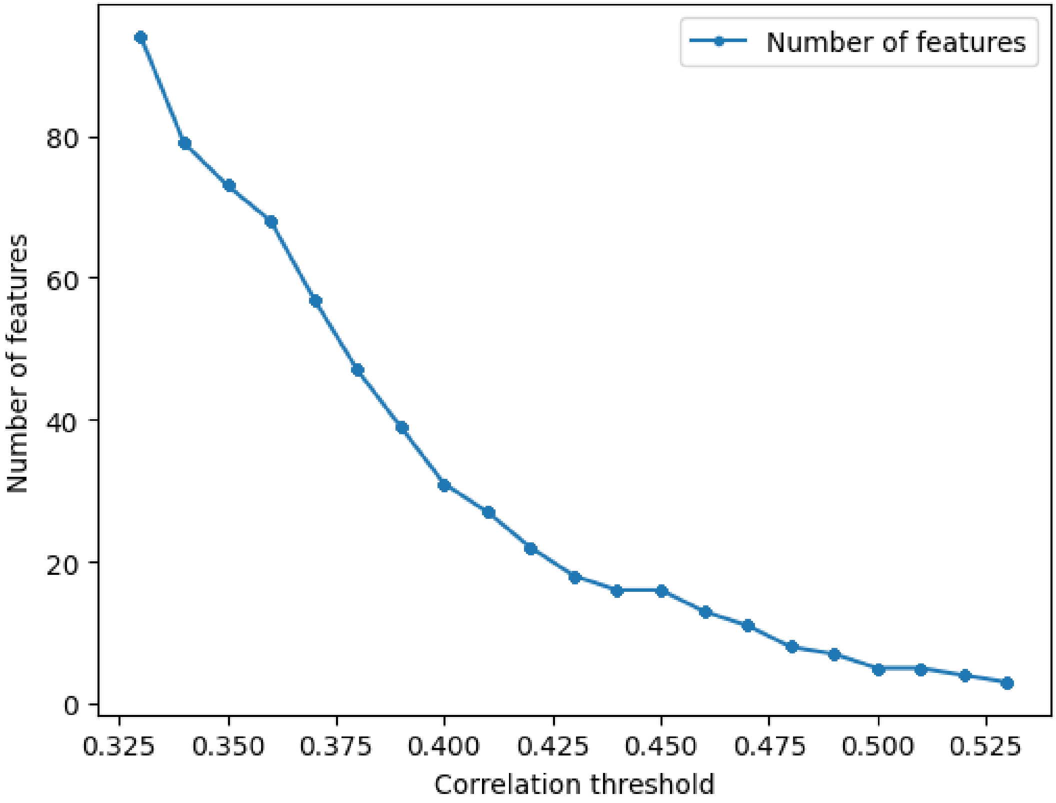

[ ]:

4 KNeighborsRegressor

[29]:
```
without_standardization, train_r2_, test_r2_, hist1, hist2, target_column_name,␣
↪training_data_RMSE, test_data_RMSE  = pred_model.
↪prepare_data_and_create_model(molecular_descriptors_df=data,
                                                                            ␣
↪                    correlation_threshold=0.50,
                                                                            ␣
↪                    standardization=False,
                                                                            ␣
↪                    model_type='KNeighborsRegressor',
                                                                            ␣
↪                    target_column_name = target,
                                                                            ␣
↪                    random_state=random_state,
                                                                            ␣
↪                    train_test_split_=True,
```

```
↪                    verbose=True)
```

```
I am not doing standardization…
  molecular descriptor name
0                    AATS0Z
1                  AATS0are
2                    AATS0d
3                   AATS0dv
4                    AATS0i
  molecular descriptor name  corr_value
0                    AATS0Z   -0.022797
1                  AATS0are   -0.139064
2                    AATS0d    0.027592
3                   AATS0dv   -0.136049
4                    AATS0i    0.168958
  molecular descriptor name  corr_value  absolute correlation value
0                    AATS0Z   -0.022797                    0.022797
1                  AATS0are   -0.139064                    0.139064
2                    AATS0d    0.027592                    0.027592
3                   AATS0dv   -0.136049                    0.136049
4                    AATS0i    0.168958                    0.168958
     molecular descriptor name  corr_value  absolute correlation value
226                     AMID_O   -0.557717                    0.557717
505                 EState_VSA5   -0.534748                    0.534748
791                    MDEO-12   -0.555724                    0.555724
921                      SaasC    0.513071                    0.513071
1091               VSA_EState5    0.526082                    0.526082
     molecular descriptor name  corr_value  absolute correlation value
226                     AMID_O   -0.557717                    0.557717
505                 EState_VSA5   -0.534748                    0.534748
791                    MDEO-12   -0.555724                    0.555724
921                      SaasC    0.513071                    0.513071
1091               VSA_EState5    0.526082                    0.526082
The model used is: KNeighbors…
Return the coefficient of determination of the prediction:
0.7385820073958993
R^2 score: 0.5920842564446827
Correlation coefficient: 0.7694701140685599
Test data - unseen during training:
R^2 score: 0.7385820073958993
Correlation coefficient: 0.8594079400354055
[7.99503884 5.36315619 5.86699104 7.99067194 7.62074397 7.7994894
 8.04916244 7.62074397 7.8354149  8.28987278 6.48251944 7.97780679
 8.28987278 7.67599179 7.91842501 7.75521654 5.36946488 6.326328  ]
102    7.920819
38     6.019997
8      5.996841
```

```
109    7.886057
11     7.962574
51     7.886057
88     8.075721
21     7.325139
82     7.978811
98     7.481486
58     7.677781
64     8.537602
74     8.142668
103    8.397940
91     8.958607
43     7.823909
25     4.886525
30     6.009661
Name: A549, dtype: float64
Training Root Mean Square Error: 0.5962436633769816
Testing Root Mean Square Error: 0.5300073247180529
```

[30]:
```python
print(target_column_name+str('_transformed'))
print(hist1[target_column_name].hist())
```

```
A549_transformed
AxesSubplot(0.125,0.11;0.775x0.77)
```

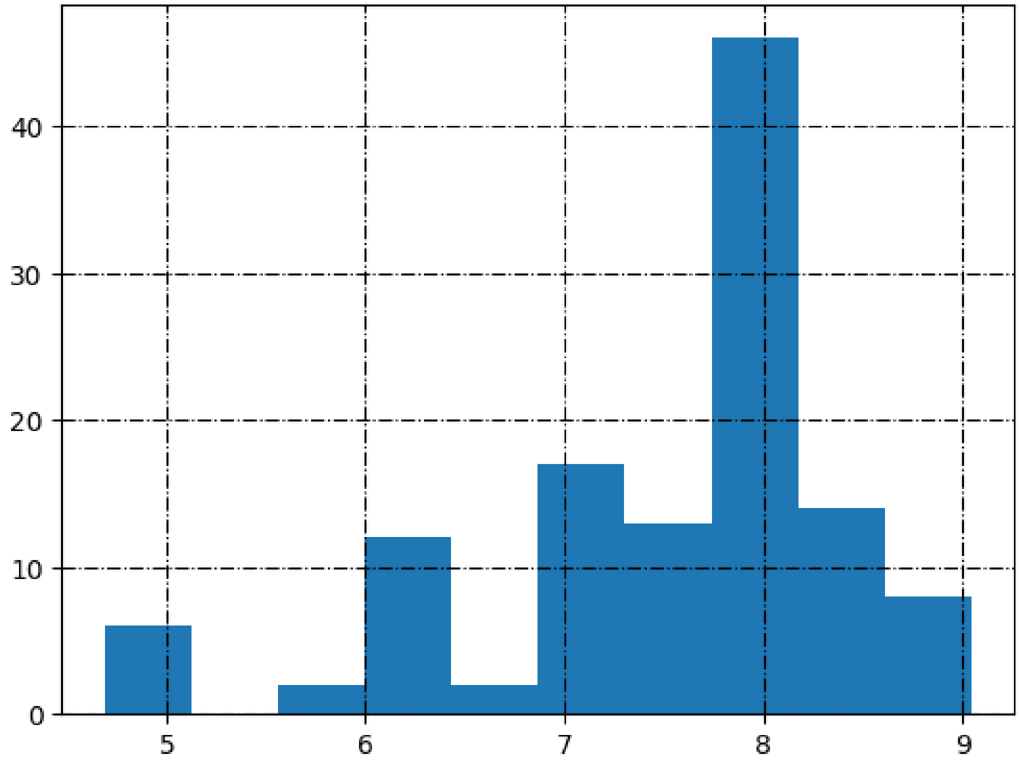

[31]:
```
with_standardization, train_r2_, test_r2_, hist1, hist2, target_column_name,␣
↪training_data_RMSE, test_data_RMSE  = pred_model.
↪prepare_data_and_create_model(molecular_descriptors_df=data,
                                                                                  ␣
↪                          correlation_threshold=0.50,
                                                                                  ␣
↪                          standardization=True,
                                                                                  ␣
↪                          model_type='KNeighborsRegressor',
                                                                                  ␣
↪                          target_column_name = target,
                                                                                  ␣
↪                          random_state=random_state,
                                                                                  ␣
↪                          train_test_split_=True,
                                                                                  ␣
↪                          verbose=True)
```

```
I am doing standardization…
  molecular descriptor name
0                    AATS0Z
1                  AATS0are
2                    AATS0d
3                   AATS0dv
4                    AATS0i
  molecular descriptor name  corr_value
0                    AATS0Z   -0.022797
1                  AATS0are   -0.139064
2                    AATS0d    0.027592
3                   AATS0dv   -0.136049
4                    AATS0i    0.168958
  molecular descriptor name  corr_value  absolute correlation value
0                    AATS0Z   -0.022797                    0.022797
1                  AATS0are   -0.139064                    0.139064
2                    AATS0d    0.027592                    0.027592
3                   AATS0dv   -0.136049                    0.136049
4                    AATS0i    0.168958                    0.168958
     molecular descriptor name  corr_value  absolute correlation value
226                     AMID_O   -0.557717                    0.557717
505                 EState_VSA5   -0.534748                    0.534748
791                    MDEO-12   -0.555724                    0.555724
921                      SaasC    0.513071                    0.513071
1091               VSA_EState5    0.526082                    0.526082
     molecular descriptor name  corr_value  absolute correlation value
226                     AMID_O   -0.557717                    0.557717
505                 EState_VSA5   -0.534748                    0.534748
```

```
791                    MDEO-12   -0.555724                    0.555724
921                      SaasC    0.513071                    0.513071
1091                VSA_EState5    0.526082                    0.526082
The model used is: KNeighbors…
Return the coefficient of determination of the prediction:
0.7385820073958993
R^2 score: 0.5920842564446827
Correlation coefficient: 0.7694701140685599
Test data - unseen during training:
R^2 score: 0.7385820073958993
Correlation coefficient: 0.8594079400354055
[7.99503884 5.36315619 5.86699104 7.99067194 7.62074397 7.7994894
 8.04916244 7.62074397 7.8354149  8.28987278 6.48251944 7.97780679
 8.28987278 7.67599179 7.91842501 7.75521654 5.36946488 6.326328  ]
102    7.920819
38     6.019997
8      5.996841
109    7.886057
11     7.962574
51     7.886057
88     8.075721
21     7.325139
82     7.978811
98     7.481486
58     7.677781
64     8.537602
74     8.142668
103    8.397940
91     8.958607
43     7.823909
25     4.886525
30     6.009661
Name: A549, dtype: float64
Training Root Mean Square Error: 0.5962436633769816
Testing Root Mean Square Error: 0.5300073247180529
```

4.1 Search inside correlation space

[32]:
```
step = 0.01
initial_step = corr_low
last_step = corr_high
first_list = [x / 100.0 for x in range(int(initial_step*100),
 ↪int(last_step*100), int(step*100))]
second_list = []
third_list = []
fourth_l = []
fifth_l = []
```

```
f_list = []
for i in first_list:
    without_standardization, train_r2, test_r2, _, h_, target_column_name,␣
 ↪training_data_RMSE, test_data_RMSE = pred_model.
 ↪prepare_data_and_create_model(molecular_descriptors_df = data,
                                                                            ␣
 ↪                    correlation_threshold = i,
                                                                            ␣
 ↪                    standardization = False,
                                                                            ␣
 ↪                    model_type = 'KNeighborsRegressor',
                                                                            ␣
 ↪                    target_column_name = target,
                                                                            ␣
 ↪                    random_state=random_state,
                                                                            ␣
 ↪                    train_test_split_ = True,
                                                                            ␣
 ↪                    verbose = False)
    second_list.append(train_r2)
    third_list.append(test_r2)
    fourth_l.append(training_data_RMSE)
    fifth_l.append(test_data_RMSE)
    f_list.append(len(h_))
```

```
  File "C:\Users\aleks\anaconda3\envs\cheminf_gpu\lib\site-
packages\joblib\externals\loky\backend\context.py", line 217, in
_count_physical_cores
    raise ValueError(
```

[33]:
```
df_without_standardization = pd.DataFrame(data=first_list,␣
 ↪columns=["Correlation threshold"])
df_without_standardization['Training data R^2 score'] = second_list
df_without_standardization['Test data R^2 score'] = third_list
df_without_standardization['Training RMSE'] = fourth_l
df_without_standardization['Test RMSE'] = fifth_l
df_without_standardization['Number of features'] = f_list
```

[34]:
```
df_k_nearest = df_without_standardization.copy()
df_without_standardization
```

[34]:
```
    Correlation threshold  Training data R^2 score  Test data R^2 score  \
0                    0.33                 0.365578             0.403549
1                    0.34                 0.576594             0.678750
2                    0.35                 0.579578             0.683797
3                    0.36                 0.579087             0.683797
4                    0.37                 0.586911             0.675784
```

5	0.38	0.656357	0.725957
6	0.39	0.670313	0.656381
7	0.40	0.657352	0.400121
8	0.41	0.657352	0.400121
9	0.42	0.643987	0.438148
10	0.43	0.666988	0.371349
11	0.44	0.660508	0.370829
12	0.45	0.660508	0.370829
13	0.46	0.531770	0.798907
14	0.47	0.523515	0.795108
15	0.48	0.581962	0.830782
16	0.49	0.570531	0.740497
17	0.50	0.592084	0.738582
18	0.51	0.592084	0.738582
19	0.52	0.563023	0.648450
20	0.53	0.553936	0.654096

	Training RMSE	Test RMSE	Number of features
0	0.743580	0.800574	94
1	0.607459	0.587537	79
2	0.605315	0.582905	73
3	0.605668	0.582905	68
4	0.600012	0.590244	57
5	0.547258	0.542654	47
6	0.536030	0.607649	39
7	0.546465	0.802871	31
8	0.546465	0.802871	27
9	0.557021	0.777007	22
10	0.538727	0.821900	18
11	0.543943	0.822240	16
12	0.543943	0.822240	16
13	0.638804	0.464850	13
14	0.644411	0.469221	11
15	0.603596	0.426420	8
16	0.611793	0.528062	7
17	0.596244	0.530007	5
18	0.596244	0.530007	5
19	0.617118	0.614621	4
20	0.623501	0.609666	3

4.2 Plots

[35]:
```
plt.plot(df_without_standardization['Correlation threshold'],␣
↪df_without_standardization['Training data R^2 score'], label = "Training␣
↪data R^2 score", marker='v')
```

```
plt.plot(df_without_standardization['Correlation threshold'],␣
    ↪df_without_standardization['Test data R^2 score'], label = "Test data R^2␣
    ↪score", marker='D')
plt.legend()
plt.xlabel('Correlation threshold')
plt.ylabel('R^2 score')
plt.ylim([0, 1])
plt.show()
```

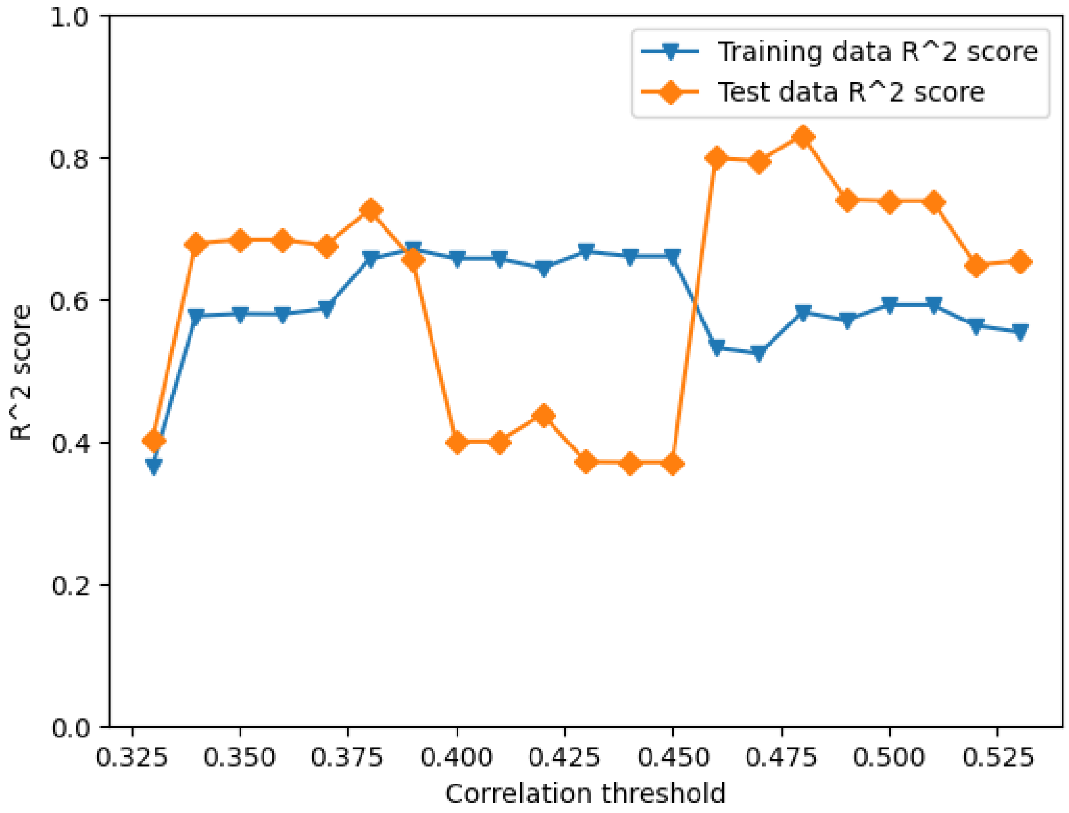

[36]:
```
plt.plot(df_without_standardization['Correlation threshold'],␣
    ↪df_without_standardization['Number of features'], label = "Number of␣
    ↪features", marker='.')
plt.legend()
plt.xlabel('Correlation threshold')
plt.ylabel('Number of features')
plt.show()
```

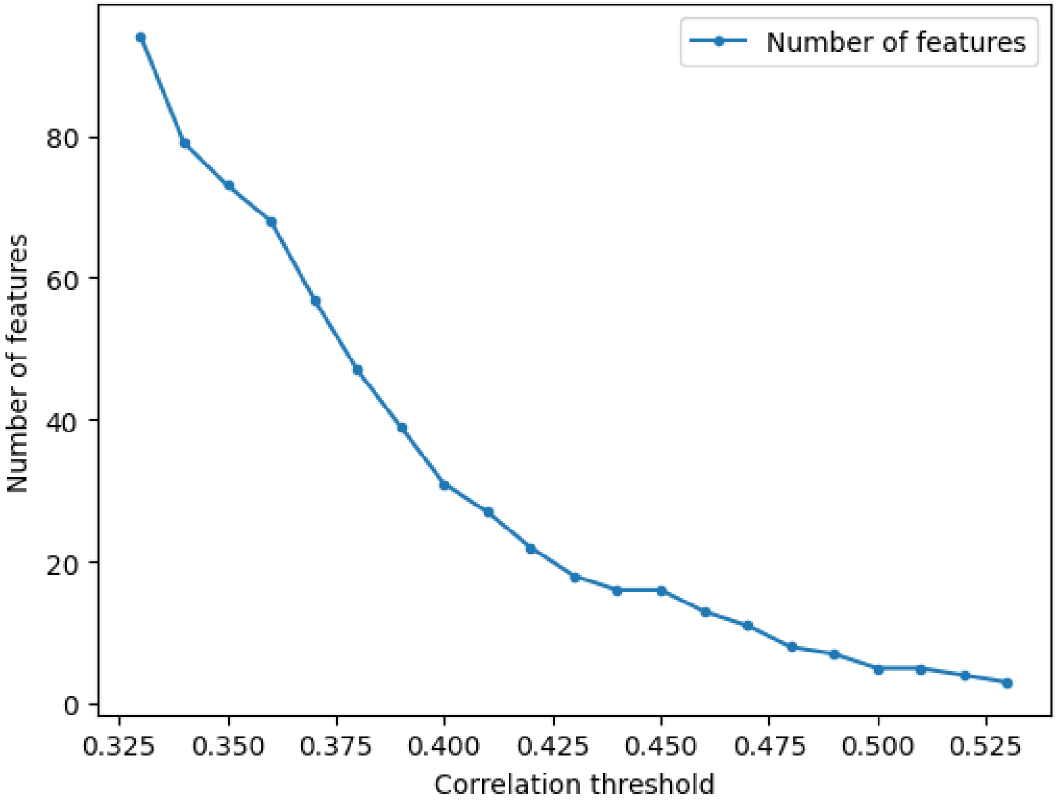


```
[ ]:
```

5 Support Vector Machines (SVM)

```
[37]: without_standardization, train_r2_, test_r2_, hist1, hist2, target_column_name,␣
       ↪training_data_RMSE, test_data_RMSE  = pred_model.
       ↪prepare_data_and_create_model(molecular_descriptors_df=data,
                                                                                    ␣
       ↪                    correlation_threshold=0.50,
                                                                                    ␣
       ↪                    standardization=False,
                                                                                    ␣
       ↪                    model_type='SVR',
                                                                                    ␣
       ↪                    kernel_ = 'linear',
                                                                                    ␣
       ↪                    gamma_ = 'auto',
                                                                                    ␣
       ↪                    target_column_name = target,
```

```
↪                    random_state=random_state,
↪                    train_test_split_=True,
↪                    verbose=True)
```

```
I am not doing standardization…
  molecular descriptor name
0                    AATS0Z
1                  AATS0are
2                    AATS0d
3                   AATS0dv
4                    AATS0i
  molecular descriptor name  corr_value
0                    AATS0Z   -0.022797
1                  AATS0are   -0.139064
2                    AATS0d    0.027592
3                   AATS0dv   -0.136049
4                    AATS0i    0.168958
  molecular descriptor name  corr_value  absolute correlation value
0                    AATS0Z   -0.022797                    0.022797
1                  AATS0are   -0.139064                    0.139064
2                    AATS0d    0.027592                    0.027592
3                   AATS0dv   -0.136049                    0.136049
4                    AATS0i    0.168958                    0.168958
     molecular descriptor name  corr_value  absolute correlation value
226                     AMID_O   -0.557717                    0.557717
505                EState_VSA5   -0.534748                    0.534748
791                    MDEO-12   -0.555724                    0.555724
921                      SaasC    0.513071                    0.513071
1091               VSA_EState5    0.526082                    0.526082
     molecular descriptor name  corr_value  absolute correlation value
226                     AMID_O   -0.557717                    0.557717
505                EState_VSA5   -0.534748                    0.534748
791                    MDEO-12   -0.555724                    0.555724
921                      SaasC    0.513071                    0.513071
1091               VSA_EState5    0.526082                    0.526082
The model used is: SVR…
Return the coefficient of determination of the prediction:
0.5885571370707737
R^2 score: 0.4153462878154097
Correlation coefficient: 0.6444736517619706
Test data - unseen during training:
R^2 score: 0.5885571370707737
Correlation coefficient: 0.7671747760913245
[7.78405904 6.26070156 7.24434095 7.90858847 7.99063574 8.18591758
```

```
 8.16006148 8.00461122 8.02839849 7.60464728 6.66113788 7.62701574
 7.61098367 7.84099337 8.2343655  8.08338059 6.52621018 6.11715821]
102    7.920819
38     6.019997
8      5.996841
109    7.886057
11     7.962574
51     7.886057
88     8.075721
21     7.325139
82     7.978811
98     7.481486
58     7.677781
64     8.537602
74     8.142668
103    8.397940
91     8.958607
43     7.823909
25     4.886525
30     6.009661
Name: A549, dtype: float64
Training Root Mean Square Error: 0.7138186632786595
Testing Root Mean Square Error: 0.6649192410798268
```

[38]:

```python
print(target_column_name+str('_transformed'))
print(hist1[target_column_name].hist())
```

```
A549_transformed
AxesSubplot(0.125,0.11;0.775x0.77)
```

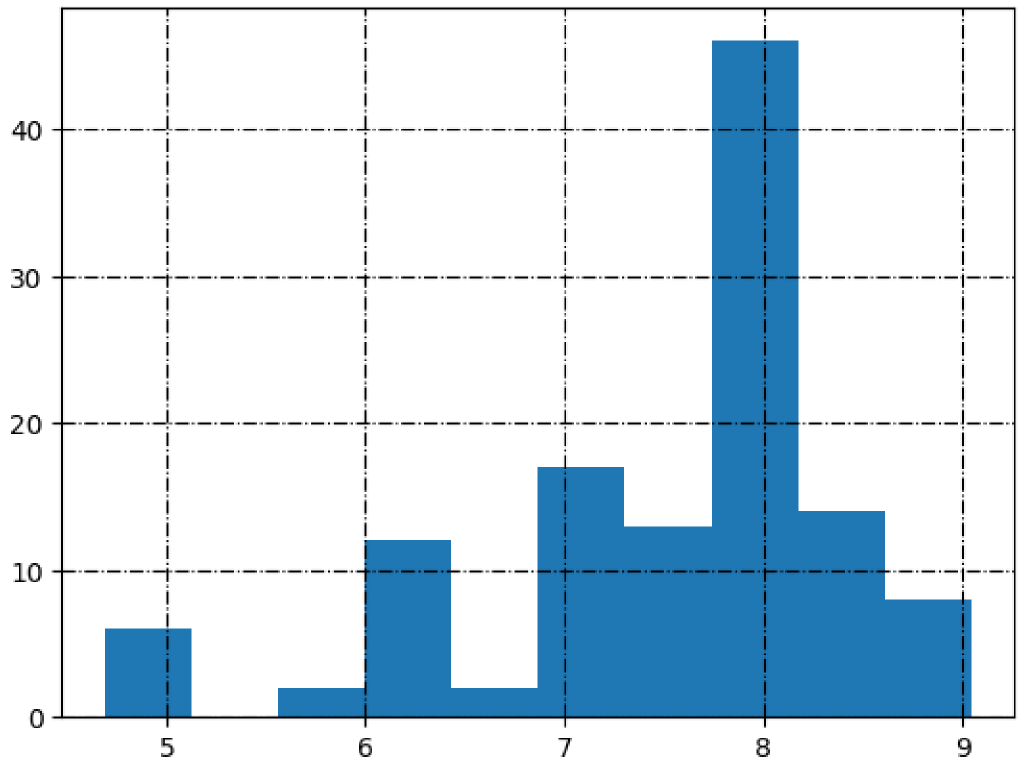

[39]:
```
with_standardization, train_r2_, test_r2_, hist1, hist2, target_column_name,␣
↪training_data_RMSE, test_data_RMSE  = pred_model.
↪prepare_data_and_create_model(molecular_descriptors_df=data,
                                                                                   ␣
↪                         correlation_threshold=0.50,
                                                                                   ␣
↪                         standardization=True,
                                                                                   ␣
↪                         model_type='SVR',
                                                                                   ␣
↪                         kernel_ = 'linear',
                                                                                   ␣
↪                         gamma_ = 'auto',
                                                                                   ␣
↪                         target_column_name = target,
                                                                                   ␣
↪                         random_state=random_state,
                                                                                   ␣
↪                         train_test_split_=True,
                                                                                   ␣
↪                         verbose=True)
```

```
I am doing standardization…
  molecular descriptor name
0                    AATS0Z
1                  AATS0are
2                    AATS0d
3                   AATS0dv
4                    AATS0i
  molecular descriptor name  corr_value
0                    AATS0Z   -0.022797
1                  AATS0are   -0.139064
2                    AATS0d    0.027592
3                   AATS0dv   -0.136049
4                    AATS0i    0.168958
  molecular descriptor name  corr_value  absolute correlation value
0                    AATS0Z   -0.022797                    0.022797
1                  AATS0are   -0.139064                    0.139064
2                    AATS0d    0.027592                    0.027592
3                   AATS0dv   -0.136049                    0.136049
4                    AATS0i    0.168958                    0.168958
     molecular descriptor name  corr_value  absolute correlation value
226                     AMID_O   -0.557717                    0.557717
505                EState_VSA5   -0.534748                    0.534748
791                    MDEO-12   -0.555724                    0.555724
921                      SaasC    0.513071                    0.513071
1091               VSA_EState5    0.526082                    0.526082
     molecular descriptor name  corr_value  absolute correlation value
226                     AMID_O   -0.557717                    0.557717
505                EState_VSA5   -0.534748                    0.534748
791                    MDEO-12   -0.555724                    0.555724
921                      SaasC    0.513071                    0.513071
1091               VSA_EState5    0.526082                    0.526082
The model used is: SVR…
Return the coefficient of determination of the prediction:
0.5885571370707737
R^2 score: 0.4153462878154097
Correlation coefficient: 0.6444736517619706
Test data - unseen during training:
R^2 score: 0.5885571370707737
Correlation coefficient: 0.7671747760913245
[7.78405904 6.26070156 7.24434095 7.90858847 7.99063574 8.18591758
 8.16006148 8.00461122 8.02839849 7.60464728 6.66113788 7.62701574
 7.61098367 7.84099337 8.2343655  8.08338059 6.52621018 6.11715821]
102    7.920819
38     6.019997
8      5.996841
109    7.886057
11     7.962574
51     7.886057
```

```
88     8.075721
21     7.325139
82     7.978811
98     7.481486
58     7.677781
64     8.537602
74     8.142668
103    8.397940
91     8.958607
43     7.823909
25     4.886525
30     6.009661
Name: A549, dtype: float64
Training Root Mean Square Error: 0.7138186632786595
Testing Root Mean Square Error: 0.6649192410798268
```

5.1 Search inside correlation space

[40]:
```
step = 0.01
initial_step = corr_low
last_step = corr_high
first_list = [x / 100.0 for x in range(int(initial_step*100),␣
 ↪int(last_step*100), int(step*100))]
second_list = []
third_list = []
fourth_l = []
fifth_l = []
f_list = []
for i in first_list:
    without_standardization, train_r2, test_r2, _, h_, target_column_name,␣
 ↪training_data_RMSE, test_data_RMSE = pred_model.
 ↪prepare_data_and_create_model(molecular_descriptors_df = data,
                                                                        ␣
 ↪                      correlation_threshold = i,
                                                                        ␣
 ↪                      standardization = False,
                                                                        ␣
 ↪                      model_type = 'SVR',
                                                                        ␣
 ↪                      kernel_ = 'linear',
                                                                        ␣
 ↪                      gamma_ = 'auto',
                                                                        ␣
 ↪                      target_column_name = target,
                                                                        ␣
 ↪                      random_state=random_state,
```

```
↪                         train_test_split_ = True,
                                                                       ␣
↪                         verbose = False)
    second_list.append(train_r2)
    third_list.append(test_r2)
    fourth_l.append(training_data_RMSE)
    fifth_l.append(test_data_RMSE)
    f_list.append(len(h_))
```

[41]:
```
df_without_standardization = pd.DataFrame(data=first_list,␣
 ↪columns=["Correlation threshold"])
df_without_standardization['Training data R^2 score'] = second_list
df_without_standardization['Test data R^2 score'] = third_list
df_without_standardization['Training RMSE'] = fourth_l
df_without_standardization['Test RMSE'] = fifth_l
df_without_standardization['Number of features'] = f_list
```

[42]:
```
df_svm = df_without_standardization.copy()
df_without_standardization
```

[42]:

	Correlation threshold	Training data R^2 score	Test data R^2 score	\
0	0.33	0.757913	0.743592	
1	0.34	0.702054	0.667643	
2	0.35	0.698608	0.647828	
3	0.36	0.650943	0.556002	
4	0.37	0.646026	0.464720	
5	0.38	0.605983	0.607521	
6	0.39	0.582467	0.589989	
7	0.40	0.555130	0.573021	
8	0.41	0.544207	0.560628	
9	0.42	0.533739	0.545440	
10	0.43	0.539155	0.586383	
11	0.44	0.530397	0.596339	
12	0.45	0.530397	0.596339	
13	0.46	0.526020	0.585876	
14	0.47	0.514480	0.587514	
15	0.48	0.477323	0.463741	
16	0.49	0.427488	0.538032	
17	0.50	0.415346	0.588557	
18	0.51	0.415346	0.588557	
19	0.52	0.424873	0.519240	
20	0.53	0.400334	0.538101	

	Training RMSE	Test RMSE	Number of features
0	0.459330	0.524904	94
1	0.509574	0.597608	79

2	0.512512	0.615165	73
3	0.551552	0.690724	68
4	0.555423	0.758411	57
5	0.585998	0.649415	47
6	0.603231	0.663761	39
7	0.622666	0.677356	31
8	0.630264	0.687116	27
9	0.637460	0.698891	22
10	0.633747	0.666674	18
11	0.639741	0.658601	16
12	0.639741	0.658601	16
13	0.642715	0.667082	13
14	0.650492	0.665762	11
15	0.674924	0.759104	8
16	0.706368	0.704564	7
17	0.713819	0.664919	5
18	0.713819	0.664919	5
19	0.707979	0.718750	4
20	0.722925	0.704510	3

5.2 Plots

[43]:
```
plt.plot(df_without_standardization['Correlation threshold'],␣
    ↪df_without_standardization['Training data R^2 score'], label = "Training␣
    ↪data R^2 score", marker='v')
plt.plot(df_without_standardization['Correlation threshold'],␣
    ↪df_without_standardization['Test data R^2 score'], label = "Test data R^2␣
    ↪score", marker='D')
plt.legend()
plt.xlabel('Correlation threshold')
plt.ylabel('R^2 score')
plt.ylim([0, 1])
plt.show()
```

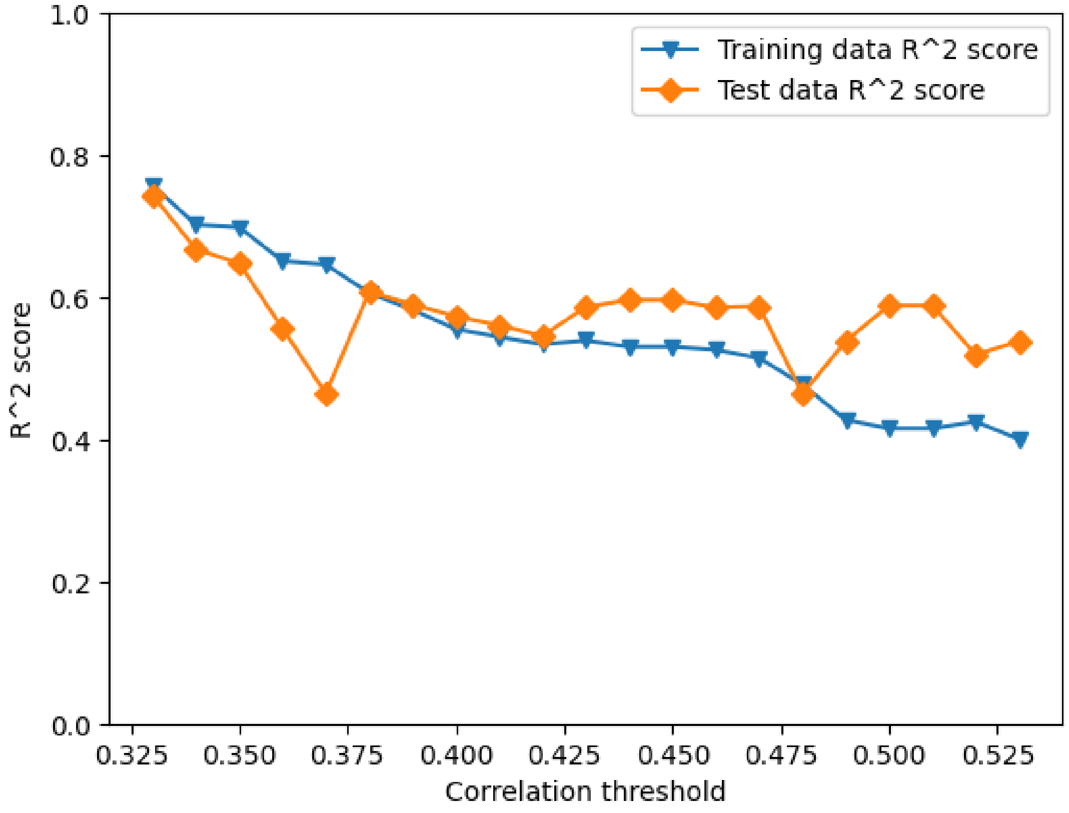

[44]:
```
plt.plot(df_without_standardization['Correlation threshold'],␣
  ↪df_without_standardization['Number of features'], label = "Number of␣
  ↪features", marker='.')
plt.legend()
plt.xlabel('Correlation threshold')
plt.ylabel('Number of features')
plt.show()
```

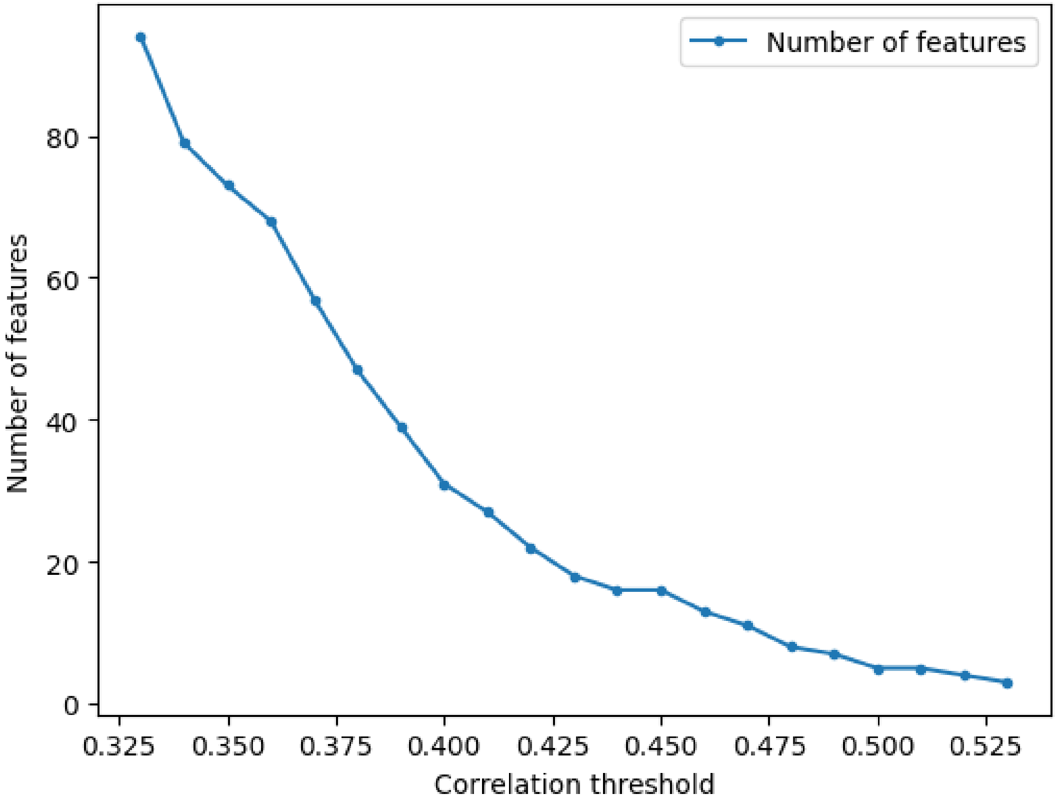

5.3 Store quality measurements

[45]:
```
with pd.ExcelWriter('../Data/Quality_'+str(target)+'_'+str(random_state)+'_.
↪xlsx') as writer:
    df_linear.to_excel(writer, sheet_name='MLR')
    df_decision_tree.to_excel(writer, sheet_name='DT')
    df_random_forest.to_excel(writer, sheet_name='RF')
    df_k_nearest.to_excel(writer, sheet_name='KNN')
    df_svm.to_excel(writer, sheet_name='SVM')
```

Notebook

January 18, 2024

1 File 16

[1]:
```
import sys
from pathlib import Path
prediction_mode_path = Path("../module")
sys.path.append(prediction_mode_path.as_posix())
import models_creation as pred_model

import pandas as pd
import numpy as np

from sklearn.metrics import r2_score

import matplotlib.pyplot as plt
from sklearn.tree import plot_tree

from matplotlib import pyplot as plt

import joblib

import warnings
warnings.filterwarnings('ignore')
```

[2]:
```
load_prepared_data = pd.read_excel('../Data/Kolchicyna_machine_learning.xlsx')
load_prepared_data = load_prepared_data.drop(columns=['Unnamed: 0'])

target = 'A549'

corr_low = 0.33
corr_high = 0.54

random_state = 28
```

[3]:
```
load_prepared_data.head()
```

[3]:
```
      AATS0Z  AATS0are    AATS0d   AATS0dv      AATS0i     AATS0m    AATS0p  \
0  25.090909  6.046518  3.109091  6.662626  160.775045  99.075564  1.555512
1  24.448276  6.008422  3.051724  6.386973  161.021674  96.473315  1.538471
```

```
2  23.868852  5.974074  3.000000  6.138434  161.244045  94.127025  1.523105
3  23.868852  5.974074  3.032787  6.171220  161.244045  94.127025  1.523105
4  23.343750  5.942945  2.953125  5.913194  161.445569  92.000700  1.509180

    AATS0pe    AATS0s    AATS0se  …  A549_float  MCF-7_float  LoVo_float  \
0  6.126680  3.435416  7.629859  …        10.8         10.3         6.5
1  6.088791  3.330998  7.596891  …        11.6         12.0         8.5
2  6.054630  3.236850  7.567166  …        10.9         12.2         8.8
3  6.054630  3.257798  7.567166  …        10.5         11.3         8.5
4  6.023670  3.151529  7.540228  …        89.5         92.7        52.8

   LoVo/DX_float  BALB/3T3_float  A549_float_transformed  \
0           54.9            10.2                7.966576
1           31.1            14.3                7.935542
2           17.9            11.7                7.962574
3           10.2            11.0                7.978811
4           77.8            99.4                7.048177

   MCF-7_float_transformed  LoVo_float_transformed  LoVo/DX_float_transformed  \
0                 7.987163                8.187087                   7.260428
1                 7.920819                8.070581                   7.507240
2                 7.913640                8.055517                   7.747147
3                 7.946922                8.070581                   7.991400
4                 7.032920                7.277366                   7.109020

   BALB/3T3_float_transformed
0                    7.991400
1                    7.844664
2                    7.931814
3                    7.958607
4                    7.002614

[5 rows x 1221 columns]
```

[4]:
```
data = load_prepared_data[list(load_prepared_data.columns)[0:-10]]
data[target] = load_prepared_data[load_prepared_data.columns[-5]]
data.head()
```

[4]:
```
      AATS0Z  AATS0are    AATS0d   AATS0dv      AATS0i     AATS0m    AATS0p  \
0  25.090909  6.046518  3.109091  6.662626  160.775045  99.075564  1.555512
1  24.448276  6.008422  3.051724  6.386973  161.021674  96.473315  1.538471
2  23.868852  5.974074  3.000000  6.138434  161.244045  94.127025  1.523105
3  23.868852  5.974074  3.032787  6.171220  161.244045  94.127025  1.523105
4  23.343750  5.942945  2.953125  5.913194  161.445569  92.000700  1.509180

    AATS0pe    AATS0s    AATS0se  …    piPC10     piPC2     piPC3     piPC4  \
0  6.126680  3.435416  7.629859  …  7.999655  4.266195  4.915408  5.516700
```

```
1  6.088791  3.330998  7.596891  …  8.018726  4.280132  4.922714  5.520711
2  6.054630  3.236850  7.567166  …  8.028799  4.293878  4.929967  5.524706
3  6.054630  3.257798  7.567166  …  8.037440  4.307438  4.929967  5.524706
4  6.023670  3.151529  7.540228  …  8.034892  4.307438  4.937168  5.528685

      piPC5     piPC6     piPC7     piPC8     piPC9      A549
0  6.098566  6.595759  6.979116  7.337313  7.681445  7.966576
1  6.103048  6.601209  6.985613  7.349442  7.695531  7.935542
2  6.105281  6.603923  6.989306  7.353932  7.704023  7.962574
3  6.107510  6.606629  6.992068  7.361425  7.709420  7.978811
4  6.107510  6.605277  6.991148  7.356489  7.707175  7.048177

[5 rows x 1212 columns]
```

2 Multiple Linear regression (MLR)

[5]:
```
without_standardization, train_r2_, test_r2_, hist1, hist2, target_column_name,␣
↪training_data_RMSE, test_data_RMSE  = pred_model.
↪prepare_data_and_create_model(molecular_descriptors_df=data,
                                                                                    ␣
↪                        correlation_threshold=0.5,
                                                                                    ␣
↪                        standardization=False,
                                                                                    ␣
↪                        model_type='linear_model',
                                                                                    ␣
↪                        target_column_name = str(target),
                                                                                    ␣
↪                        random_state=random_state,
                                                                                    ␣
↪                        train_test_split_=True,
                                                                                    ␣
↪                        verbose=True)
```

```
I am not doing standardization…
  molecular descriptor name
0                   AATS0Z
1                 AATS0are
2                   AATS0d
3                  AATS0dv
4                   AATS0i
  molecular descriptor name  corr_value
0                   AATS0Z   -0.022797
1                 AATS0are   -0.139064
2                   AATS0d    0.027592
3                  AATS0dv   -0.136049
```

```
4                  AATS0i    0.168958
  molecular descriptor name  corr_value  absolute correlation value
0                  AATS0Z   -0.022797                    0.022797
1                AATS0are   -0.139064                    0.139064
2                  AATS0d    0.027592                    0.027592
3                 AATS0dv   -0.136049                    0.136049
4                  AATS0i    0.168958                    0.168958
     molecular descriptor name  corr_value  absolute correlation value
226                     AMID_O   -0.557717                    0.557717
505                 EState_VSA5  -0.534748                    0.534748
791                     MDEO-12  -0.555724                    0.555724
921                       SaasC   0.513071                    0.513071
1091                VSA_EState5   0.526082                    0.526082
     molecular descriptor name  corr_value  absolute correlation value
226                     AMID_O   -0.557717                    0.557717
505                 EState_VSA5  -0.534748                    0.534748
791                     MDEO-12  -0.555724                    0.555724
921                       SaasC   0.513071                    0.513071
1091                VSA_EState5   0.526082                    0.526082
The model used is: LinearReg…
Return the coefficient of determination of the prediction:
0.3263597901499842
R^2 score: 0.5008978125943887
Correlation coefficient: 0.7077413458279718
Test data - unseen during training:
R^2 score: 0.3263597901499842
Correlation coefficient: 0.5712790825419606
[8.17050812 6.41656195 7.51260775 6.65848085 7.9580164  7.32723458
 7.55473012 8.29466015 6.92867321 7.59751174 7.65932317 7.99323287
 7.80547787 7.31200666 7.3326104  6.53208066 7.98252888 6.66784957]
113    7.823909
35     6.053057
101    6.721246
36     6.221126
100    7.130768
13     7.966576
0      7.966576
114    7.920819
104    8.309804
96     7.102373
40     7.920819
103    8.397940
48     7.853872
39     8.356547
14     8.148742
117    6.031517
21     7.325139
9      6.818728
```

```
Name: A549, dtype: float64
Training Root Mean Square Error: 0.6900454953161479
Testing Root Mean Square Error: 0.637795296583113
```

[6]:
```
print(target_column_name+str('_transformed'))
print(hist1[target_column_name].hist())
```

```
A549_transformed
AxesSubplot(0.125,0.11;0.775x0.77)
```

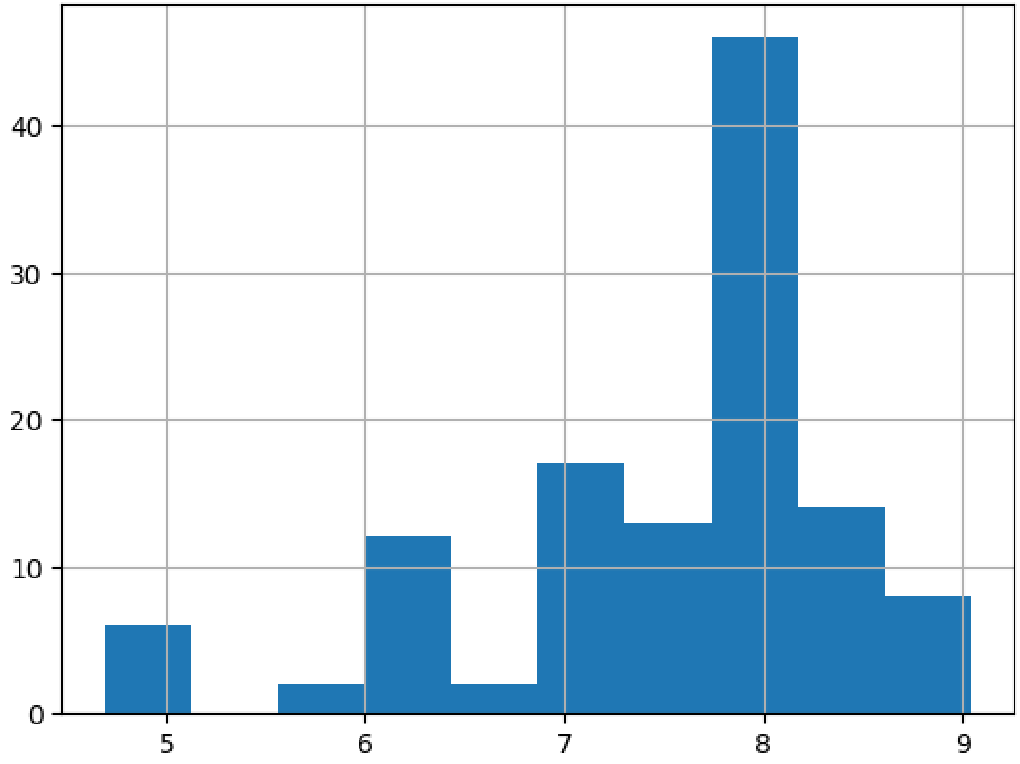

[7]:
```
with_standardization, train_r2_, test_r2_, hist1, hist2, target_column_name,␣
↪training_data_RMSE, test_data_RMSE  = pred_model.
↪prepare_data_and_create_model(molecular_descriptors_df=data,
                                                                            ␣
↪                         correlation_threshold=0.50,
                                                                            ␣
↪                         standardization=True,
                                                                            ␣
↪                         model_type='linear_model',
                                                                            ␣
↪                         target_column_name = target,
```

```
↪                     random_state=random_state,
↪                     train_test_split_=True,
↪                     verbose=True)
```

```
I am doing standardization…
  molecular descriptor name
0                    AATS0Z
1                  AATS0are
2                    AATS0d
3                   AATS0dv
4                    AATS0i
  molecular descriptor name  corr_value
0                    AATS0Z   -0.022797
1                  AATS0are   -0.139064
2                    AATS0d    0.027592
3                   AATS0dv   -0.136049
4                    AATS0i    0.168958
  molecular descriptor name  corr_value  absolute correlation value
0                    AATS0Z   -0.022797                    0.022797
1                  AATS0are   -0.139064                    0.139064
2                    AATS0d    0.027592                    0.027592
3                   AATS0dv   -0.136049                    0.136049
4                    AATS0i    0.168958                    0.168958
     molecular descriptor name  corr_value  absolute correlation value
226                     AMID_O   -0.557717                    0.557717
505                EState_VSA5   -0.534748                    0.534748
791                    MDEO-12   -0.555724                    0.555724
921                      SaasC    0.513071                    0.513071
1091               VSA_EState5    0.526082                    0.526082
     molecular descriptor name  corr_value  absolute correlation value
226                     AMID_O   -0.557717                    0.557717
505                EState_VSA5   -0.534748                    0.534748
791                    MDEO-12   -0.555724                    0.555724
921                      SaasC    0.513071                    0.513071
1091               VSA_EState5    0.526082                    0.526082
The model used is: LinearReg…
Return the coefficient of determination of the prediction:
0.3263597901499842
R^2 score: 0.5008978125943887
Correlation coefficient: 0.7077413458279718
Test data - unseen during training:
R^2 score: 0.3263597901499842
Correlation coefficient: 0.5712790825419606
[8.17050812 6.41656195 7.51260775 6.65848085 7.9580164  7.32723458
```

```
 7.55473012 8.29466015 6.92867321 7.59751174 7.65932317 7.99323287
 7.80547787 7.31200666 7.3326104  6.53208066 7.98252888 6.66784957]
113    7.823909
35     6.053057
101    6.721246
36     6.221126
100    7.130768
13     7.966576
0      7.966576
114    7.920819
104    8.309804
96     7.102373
40     7.920819
103    8.397940
48     7.853872
39     8.356547
14     8.148742
117    6.031517
21     7.325139
9      6.818728
Name: A549, dtype: float64
Training Root Mean Square Error: 0.6900454953161479
Testing Root Mean Square Error: 0.637795296583113
```

2.1 Search inside correlation space

[8]:

```
step = 0.01
initial_step = corr_low
last_step = corr_high
first_list = [x / 100.0 for x in range(int(initial_step*100),␣
 ↪int(last_step*100), int(step*100))]
second_list = []
third_list = []
fourth_l = []
fifth_l = []
f_list = []
for i in first_list:
    without_standardization, train_r2, test_r2, _, h_, target_column_name,␣
 ↪training_data_RMSE, test_data_RMSE = pred_model.
 ↪prepare_data_and_create_model(molecular_descriptors_df = data,
                                                                                ␣
 ↪                      correlation_threshold = i,
                                                                                ␣
 ↪                      standardization = False,
                                                                                ␣
 ↪                      model_type = 'linear_model',
```

```
                    target_column_name = target,
                    random_state=random_state,
                    train_test_split_ = True,
                    verbose = False)
    second_list.append(train_r2)
    third_list.append(test_r2)
    fourth_l.append(training_data_RMSE)
    fifth_l.append(test_data_RMSE)
    f_list.append(len(h_))
```

[9]:
```
df_without_standardization = pd.DataFrame(data=first_list,
 columns=["Correlation threshold"])
df_without_standardization['Training data R^2 score'] = second_list
df_without_standardization['Test data R^2 score'] = third_list
df_without_standardization['Training RMSE'] = fourth_l
df_without_standardization['Test RMSE'] = fifth_l
df_without_standardization['Number of features'] = f_list
```

[10]:
```
df_linear = df_without_standardization.copy()
df_without_standardization
```

[10]:

	Correlation threshold	Training data R^2 score	Test data R^2 score	\
0	0.33	0.988674	-5.539361	
1	0.34	0.971113	-1.646468	
2	0.35	0.959683	-0.619875	
3	0.36	0.933406	-0.488174	
4	0.37	0.914824	-0.459014	
5	0.38	0.886734	-0.098428	
6	0.39	0.816081	0.157274	
7	0.40	0.712155	-0.031857	
8	0.41	0.710437	-0.029820	
9	0.42	0.667863	-0.158184	
10	0.43	0.653445	-0.050300	
11	0.44	0.634097	0.091773	
12	0.45	0.634097	0.091773	
13	0.46	0.607476	0.156489	
14	0.47	0.567309	0.443574	
15	0.48	0.538457	0.426137	
16	0.49	0.511387	0.356796	
17	0.50	0.500898	0.326360	
18	0.51	0.500898	0.326360	
19	0.52	0.500883	0.328066	
20	0.53	0.489652	0.256984	

	Training RMSE	Test RMSE	Number of features
0	0.103949	1.987170	94
1	0.166010	1.264156	79
2	0.196122	0.989027	73
3	0.252059	0.947969	68
4	0.285064	0.938636	57
5	0.328725	0.814429	47
6	0.418886	0.713363	39
7	0.524037	0.789364	31
8	0.525599	0.788584	27
9	0.562914	0.836288	22
10	0.575002	0.796387	18
11	0.590834	0.740567	16
12	0.590834	0.740567	16
13	0.611950	0.713695	13
14	0.642498	0.579657	11
15	0.663573	0.588669	8
16	0.682756	0.623220	7
17	0.690045	0.637795	5
18	0.690045	0.637795	5
19	0.690056	0.636987	4
20	0.697776	0.669833	3

[ ]:

```

```

2.2 Plots

[11]:

```
plt.plot(df_without_standardization['Correlation threshold'],␣
  ↪df_without_standardization['Training data R^2 score'], label = "Training␣
  ↪data R^2 score", marker='v')
plt.plot(df_without_standardization['Correlation threshold'],␣
  ↪df_without_standardization['Test data R^2 score'], label = "Test data R^2␣
  ↪score", marker='D')
plt.legend()
plt.xlabel('Correlation threshold')
plt.ylabel('R^2 score')
plt.ylim([0, 1])
plt.show()
```

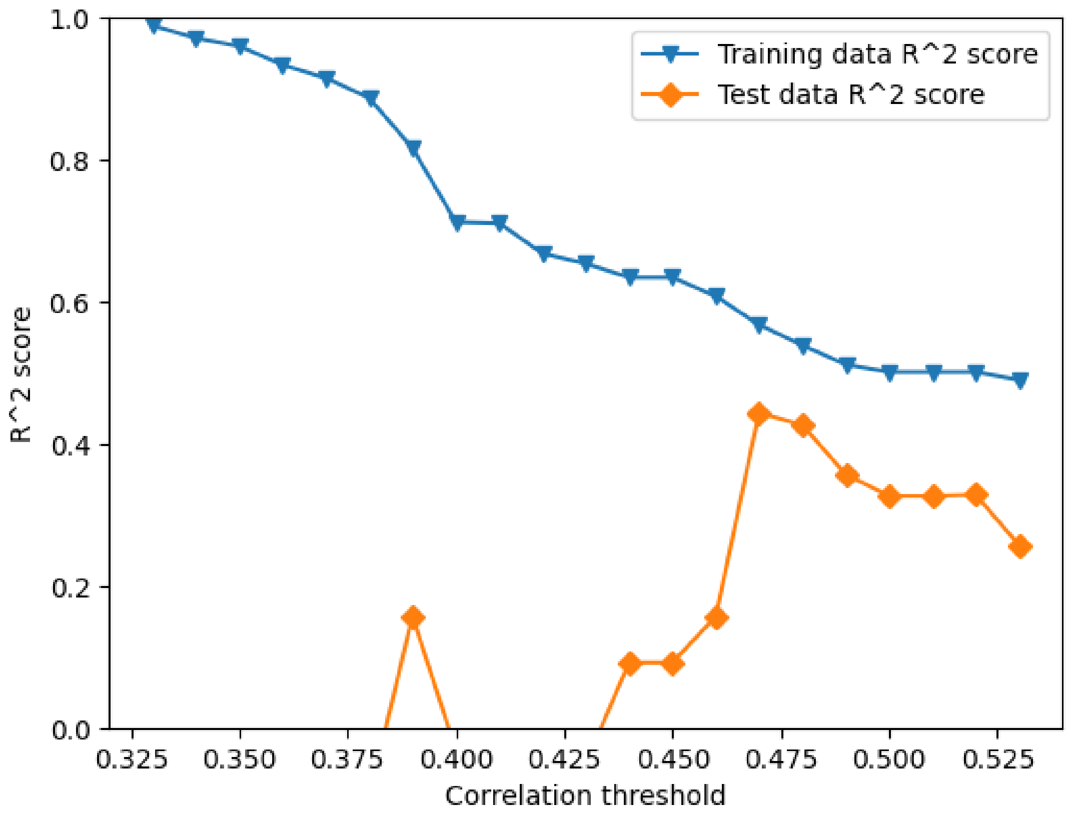

[12]:
```
plt.plot(df_without_standardization['Correlation threshold'],␣
  ↪df_without_standardization['Number of features'], label = "Number of␣
  ↪features", marker='.')
plt.legend()
plt.xlabel('Correlation threshold')
plt.ylabel('Number of features')
plt.show()
```

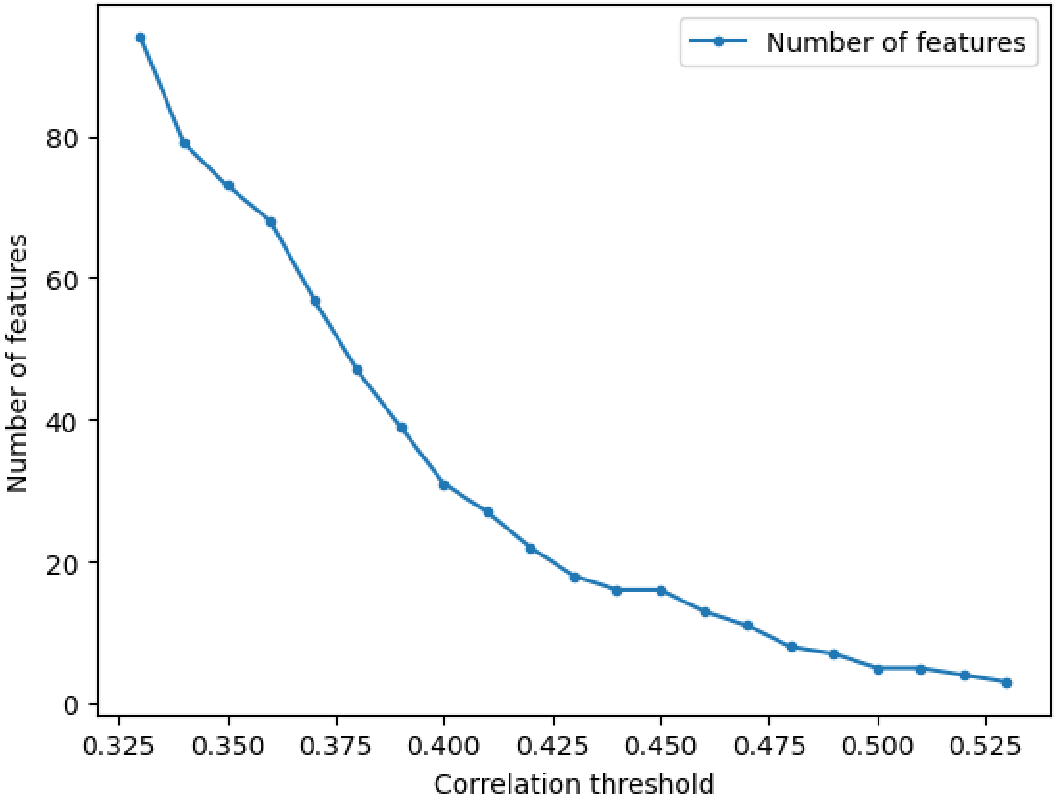

[ ]:

```
```

2.3 Decision Tree

[13]:

```
without_standardization, train_r2_, test_r2_, hist1, hist2, target_column_name,␣
↪training_data_RMSE, test_data_RMSE  = pred_model.
↪prepare_data_and_create_model(molecular_descriptors_df=data,
                                                                              ␣
↪                         correlation_threshold=0.50,
                                                                              ␣
↪                         standardization=False,
                                                                              ␣
↪                         model_type='DecisionTreeRegressor',
                                                                              ␣
↪                         max_depth=5,
                                                                              ␣
↪                         target_column_name = target,
                                                                              ␣
↪                         random_state=random_state,
```

```
↪                          train_test_split_=True,
↪                          verbose=True)
```

```
I am not doing standardization…
  molecular descriptor name
0                    AATS0Z
1                  AATS0are
2                    AATS0d
3                   AATS0dv
4                    AATS0i
  molecular descriptor name  corr_value
0                    AATS0Z   -0.022797
1                  AATS0are   -0.139064
2                    AATS0d    0.027592
3                   AATS0dv   -0.136049
4                    AATS0i    0.168958
  molecular descriptor name  corr_value  absolute correlation value
0                    AATS0Z   -0.022797                    0.022797
1                  AATS0are   -0.139064                    0.139064
2                    AATS0d    0.027592                    0.027592
3                   AATS0dv   -0.136049                    0.136049
4                    AATS0i    0.168958                    0.168958
     molecular descriptor name  corr_value  absolute correlation value
226                     AMID_O   -0.557717                    0.557717
505                EState_VSA5   -0.534748                    0.534748
791                   MDEO-12   -0.555724                    0.555724
921                      SaasC    0.513071                    0.513071
1091               VSA_EState5    0.526082                    0.526082
     molecular descriptor name  corr_value  absolute correlation value
226                     AMID_O   -0.557717                    0.557717
505                EState_VSA5   -0.534748                    0.534748
791                   MDEO-12   -0.555724                    0.555724
921                      SaasC    0.513071                    0.513071
1091               VSA_EState5    0.526082                    0.526082
The model used is: DecisionTree…
Return the coefficient of determination of the prediction:
0.07551134844737695
R^2 score: 0.821196391427611
Correlation coefficient: 0.9061988696900979
Test data - unseen during training:
R^2 score: 0.07551134844737695
Correlation coefficient: 0.2747932831191056
[7.89470296 6.31695296 8.21966244 5.88605665 7.89470296 8.21966244
 7.89470296 7.54176295 7.89470296 7.89470296 7.89470296 7.89470296
 7.89470296 7.89470296 8.21966244 8.21966244 7.03791206 5.99684117]
113    7.823909
```

```
35     6.053057
101    6.721246
36     6.221126
100    7.130768
13     7.966576
0      7.966576
114    7.920819
104    8.309804
96     7.102373
40     7.920819
103    8.397940
48     7.853872
39     8.356547
14     8.148742
117    6.031517
21     7.325139
9      6.818728
Name: A549, dtype: float64
Training Root Mean Square Error: 0.41302004356980293
Testing Root Mean Square Error: 0.7471677242019531
```

[14]:
```
print(target_column_name+str('_transformed'))
print(hist1[target_column_name].hist())
```

```
A549_transformed
AxesSubplot(0.125,0.11;0.775x0.77)
```

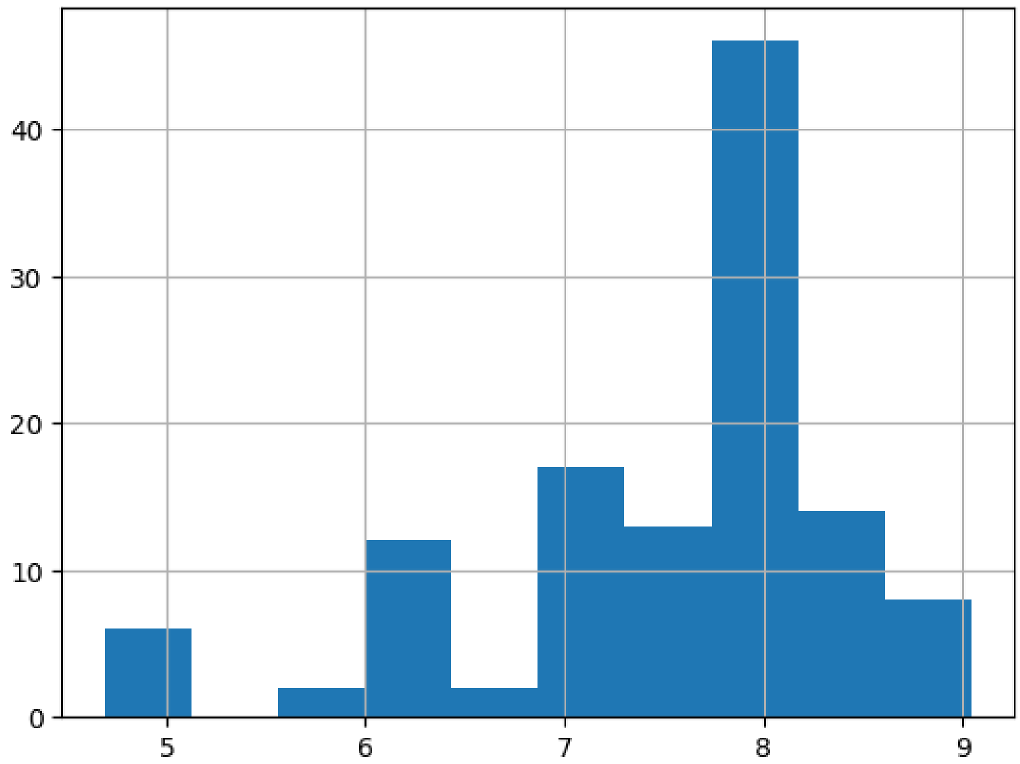

[15]:
```
with_standardization, train_r2_, test_r2_, hist1, hist2, target_column_name,␣
↪training_data_RMSE, test_data_RMSE  = pred_model.
↪prepare_data_and_create_model(molecular_descriptors_df=data,
                                                                                ␣
↪                        correlation_threshold=0.50,
                                                                                ␣
↪                        standardization=True,
                                                                                ␣
↪                        model_type='DecisionTreeRegressor',
                                                                                ␣
↪                        max_depth=5,
                                                                                ␣
↪                        target_column_name = target,
                                                                                ␣
↪                        random_state=random_state,
                                                                                ␣
↪                        train_test_split_=True,
                                                                                ␣
↪                        verbose=True)
```

```
I am doing standardization…
  molecular descriptor name
```

```
0                   AATS0Z
1                 AATS0are
2                   AATS0d
3                  AATS0dv
4                   AATS0i
  molecular descriptor name  corr_value
0                   AATS0Z   -0.022797
1                 AATS0are   -0.139064
2                   AATS0d    0.027592
3                  AATS0dv   -0.136049
4                   AATS0i    0.168958
  molecular descriptor name  corr_value  absolute correlation value
0                   AATS0Z   -0.022797                    0.022797
1                 AATS0are   -0.139064                    0.139064
2                   AATS0d    0.027592                    0.027592
3                  AATS0dv   -0.136049                    0.136049
4                   AATS0i    0.168958                    0.168958
     molecular descriptor name  corr_value  absolute correlation value
226                     AMID_O   -0.557717                    0.557717
505                 EState_VSA5   -0.534748                    0.534748
791                    MDEO-12   -0.555724                    0.555724
921                      SaasC    0.513071                    0.513071
1091                VSA_EState5    0.526082                    0.526082
     molecular descriptor name  corr_value  absolute correlation value
226                     AMID_O   -0.557717                    0.557717
505                 EState_VSA5   -0.534748                    0.534748
791                    MDEO-12   -0.555724                    0.555724
921                      SaasC    0.513071                    0.513071
1091                VSA_EState5    0.526082                    0.526082
The model used is: DecisionTree…
Return the coefficient of determination of the prediction:
0.07551134844737695
R^2 score: 0.821196391427611
Correlation coefficient: 0.9061988696900979
Test data - unseen during training:
R^2 score: 0.07551134844737695
Correlation coefficient: 0.2747932831191056
[7.89470296 6.31695296 8.21966244 5.88605665 7.89470296 8.21966244
 7.89470296 7.54176295 7.89470296 7.89470296 7.89470296 7.89470296
 7.89470296 7.89470296 8.21966244 8.21966244 7.03791206 5.99684117]
113    7.823909
35     6.053057
101    6.721246
36     6.221126
100    7.130768
13     7.966576
0      7.966576
114    7.920819
```

```
104     8.309804
96      7.102373
40      7.920819
103     8.397940
48      7.853872
39      8.356547
14      8.148742
117     6.031517
21      7.325139
9       6.818728
Name: A549, dtype: float64
Training Root Mean Square Error: 0.41302004356980293
Testing Root Mean Square Error: 0.7471677242019531
```

2.4 Search inside correlation space

[16]:
```
step = 0.01
initial_step = corr_low
last_step = corr_high
first_list = [x / 100.0 for x in range(int(initial_step*100),␣
 ↪int(last_step*100), int(step*100))]
max_depth = [range(2, 30, 1)]
corr_th = []
second_list = []
third_list = []
fourth_l = []
fifth_l = []
f_list = []
fif_list = []
for i in first_list:
    for depth in max_depth[0]:

        without_standardization, train_r2, test_r2, _, h_, target_column_name,␣
 ↪training_data_RMSE, test_data_RMSE = pred_model.
 ↪prepare_data_and_create_model(molecular_descriptors_df=data,
                                                                                ␣
 ↪                      correlation_threshold=i,
                                                                                ␣
 ↪                      standardization=False,
                                                                                ␣
 ↪                      model_type='DecisionTreeRegressor',
                                                                                ␣
 ↪                      max_depth=depth,
                                                                                ␣
 ↪                      target_column_name = target,
                                                                                ␣
 ↪                      random_state=random_state,
```

```
↪                       train_test_split_=True,
↪                       verbose=False)
        corr_th.append(i)
        second_list.append(train_r2)
        third_list.append(test_r2)
        fourth_l.append(training_data_RMSE)
        fifth_l.append(test_data_RMSE)
        f_list.append(len(h_))
        fif_list.append(depth)
```

[17]:
```
df_without_standardization = pd.DataFrame(data=corr_th, columns=["Correlation␣
↪threshold"])
df_without_standardization['Training data R^2 score'] = second_list
df_without_standardization['Test data R^2 score'] = third_list
df_without_standardization['Training RMSE'] = fourth_l
df_without_standardization['Test RMSE'] = fifth_l
df_without_standardization['Number of features'] = f_list
df_without_standardization['Depth number'] = fif_list
```

[18]:
```
df_decision_tree = df_without_standardization.copy()
#df_without_standardization.to_excel('../Data/
↪A549_Decision_tree_rs_'+str(random_state)+'.xlsx')
```

2.5 Plots

[19]:

```
corre_list = [x / 100.0 for x in range(int(initial_step*100),␣
↪int(last_step*100), int(0.01*100))]
for element in corre_list:
    element_ =␣
↪df_without_standardization[df_without_standardization['Correlation␣
↪threshold'] == float(element)]
    plt.plot(element_['Depth number'], element_['Training data R^2 score'],␣
↪label = "Training data R^2 score", marker='v')
    plt.plot(element_['Depth number'], element_['Test data R^2 score'], label =␣
↪"Test data R^2 score", marker='D')
    plt.legend()
    plt.xlabel('Depth number -> correlation threshold: '+str(element)+'. \n␣
↪Number of features: '+str(element_['Number of features'].iloc[0]),␣
↪fontweight='bold')
    plt.xlim(max(element_['Depth number'])+1, min(element_['Depth number'])-1)
    plt.ylabel('R^2 score')
    plt.ylim([0, 1])
    plt.rc('grid', linestyle="-.", color='black')
    plt.grid(True)
    plt.show()
```

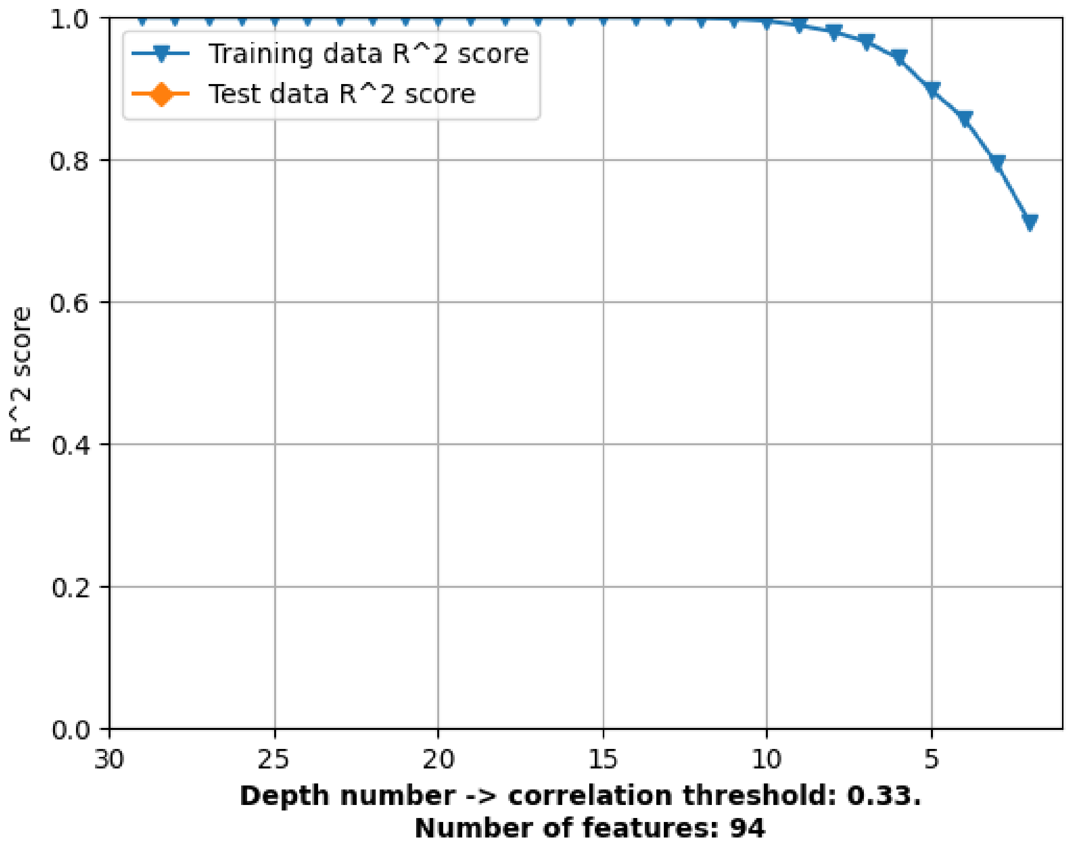

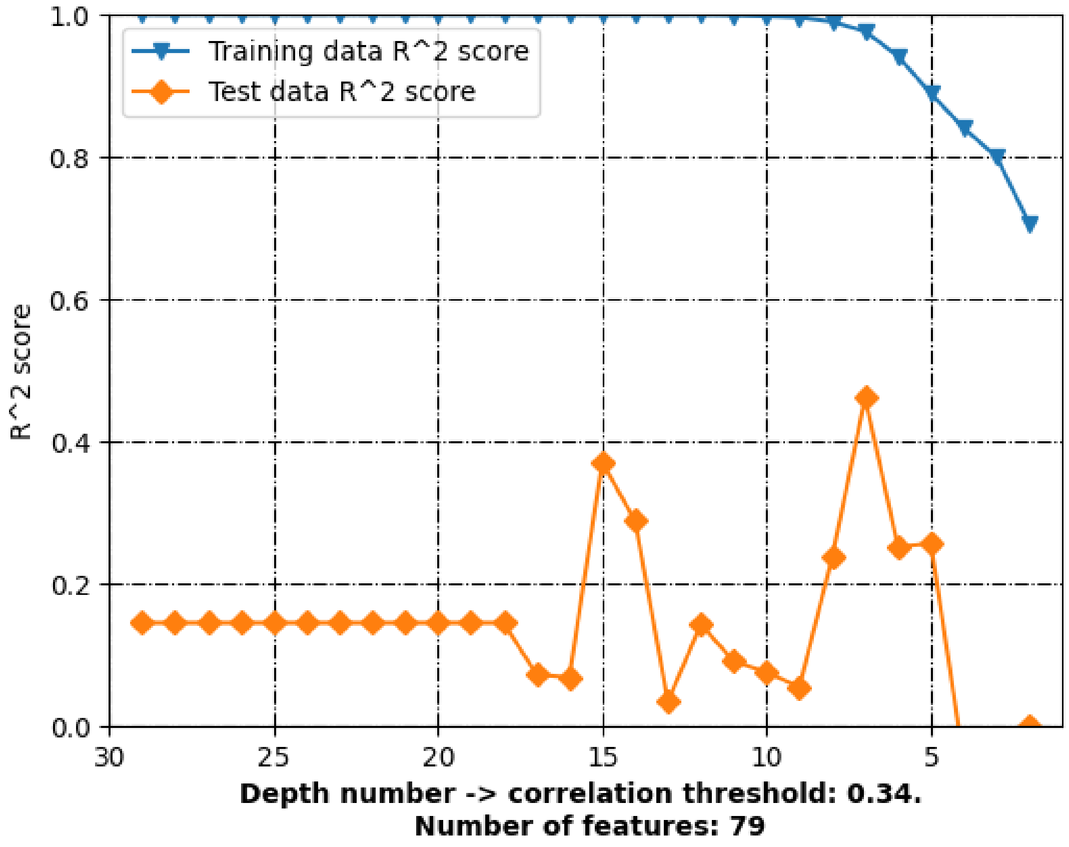
Training data R^2 score
Test data R^2 score
R^2 score
1.0
0.8
0.6
0.4
0.2
0.0
30
25
20
15
10
5
Depth number -> correlation threshold: 0.34.
Number of features: 79

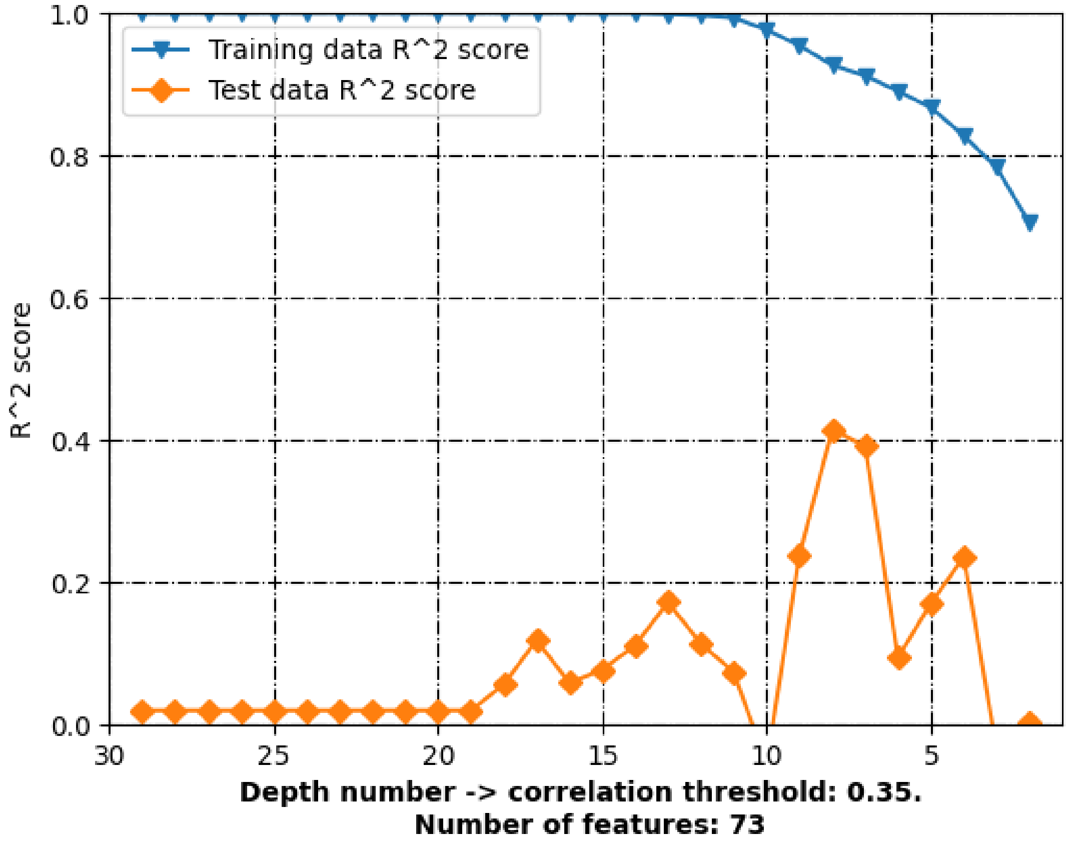
Training data R^2 score
Test data R^2 score
R^2 score
1.0
0.8
0.6
0.4
0.2
0.0
30
25
20
15
10
5
Depth number -> correlation threshold: 0.35.
Number of features: 73

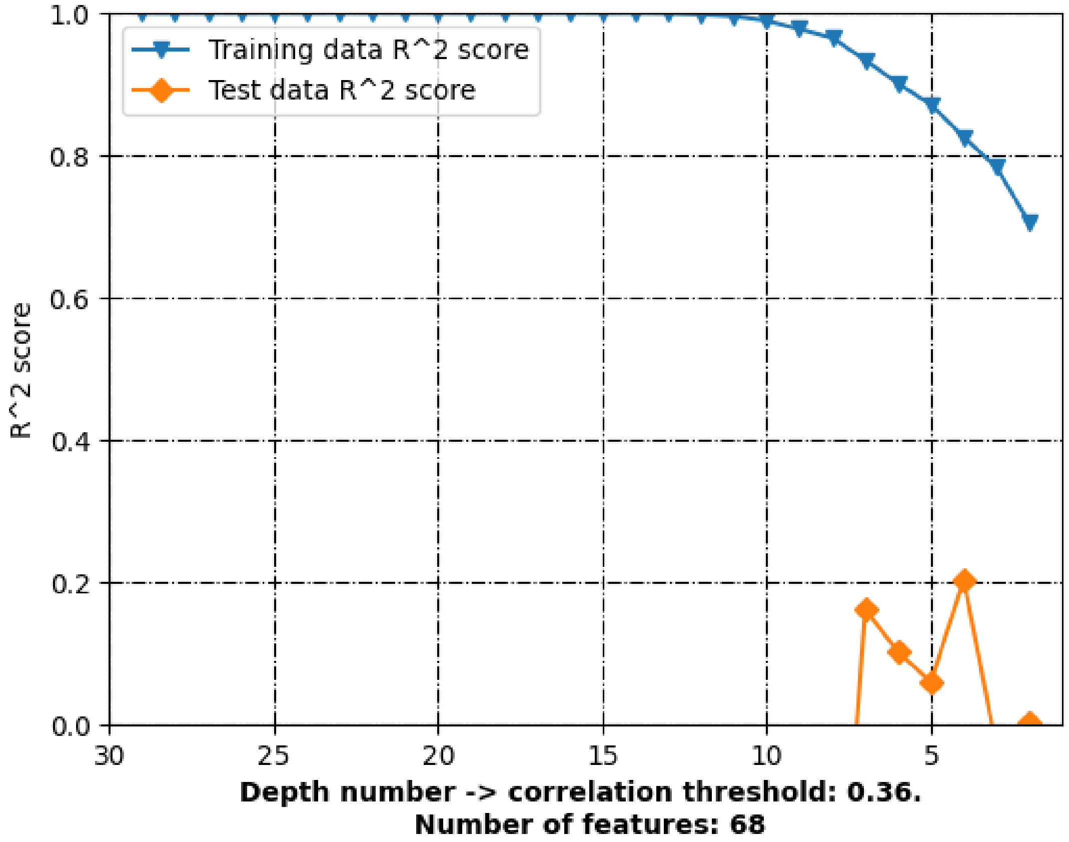
Training data R^2 score
Test data R^2 score
R^2 score
1.0
0.8
0.6
0.4
0.2
0.0
30
25
20
15
10
5
Depth number -> correlation threshold: 0.36.
Number of features: 68

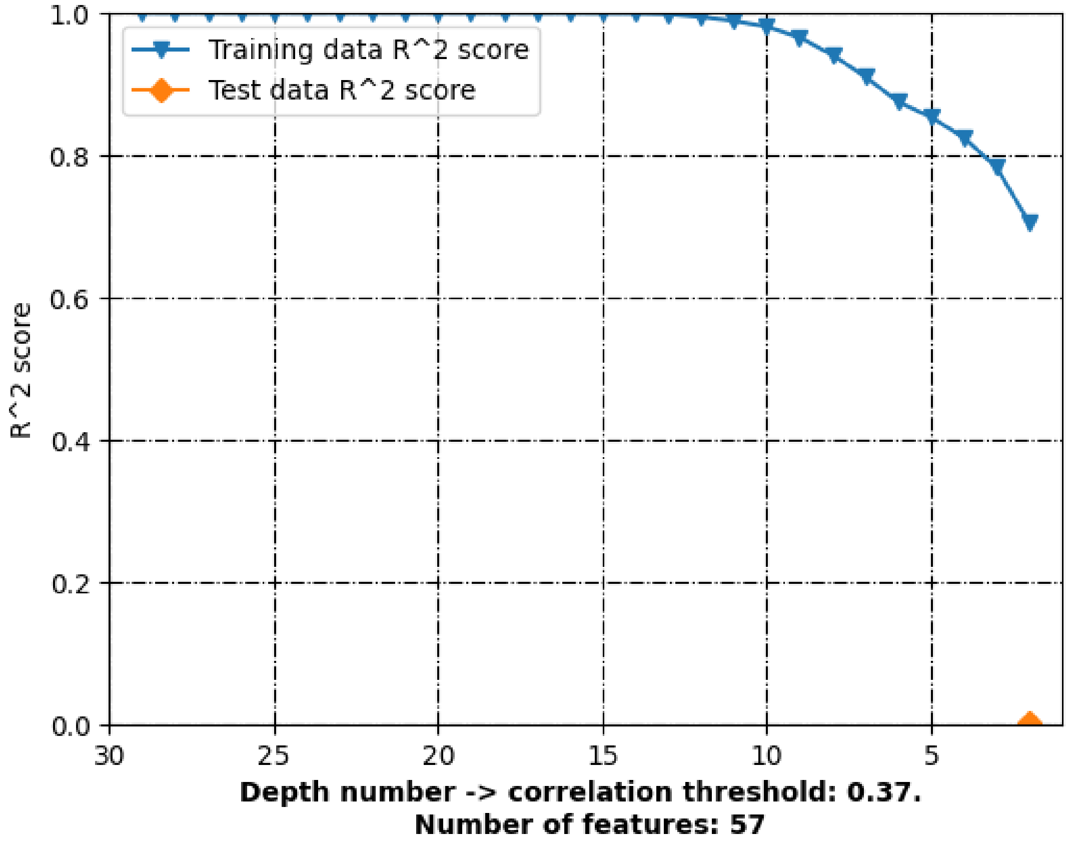

Training data R^2 score
Test data R^2 score
R^2 score
1.0
0.8
0.6
0.4
0.2
0.0
30
25
20
15
10
5
Depth number -> correlation threshold: 0.37.
Number of features: 57

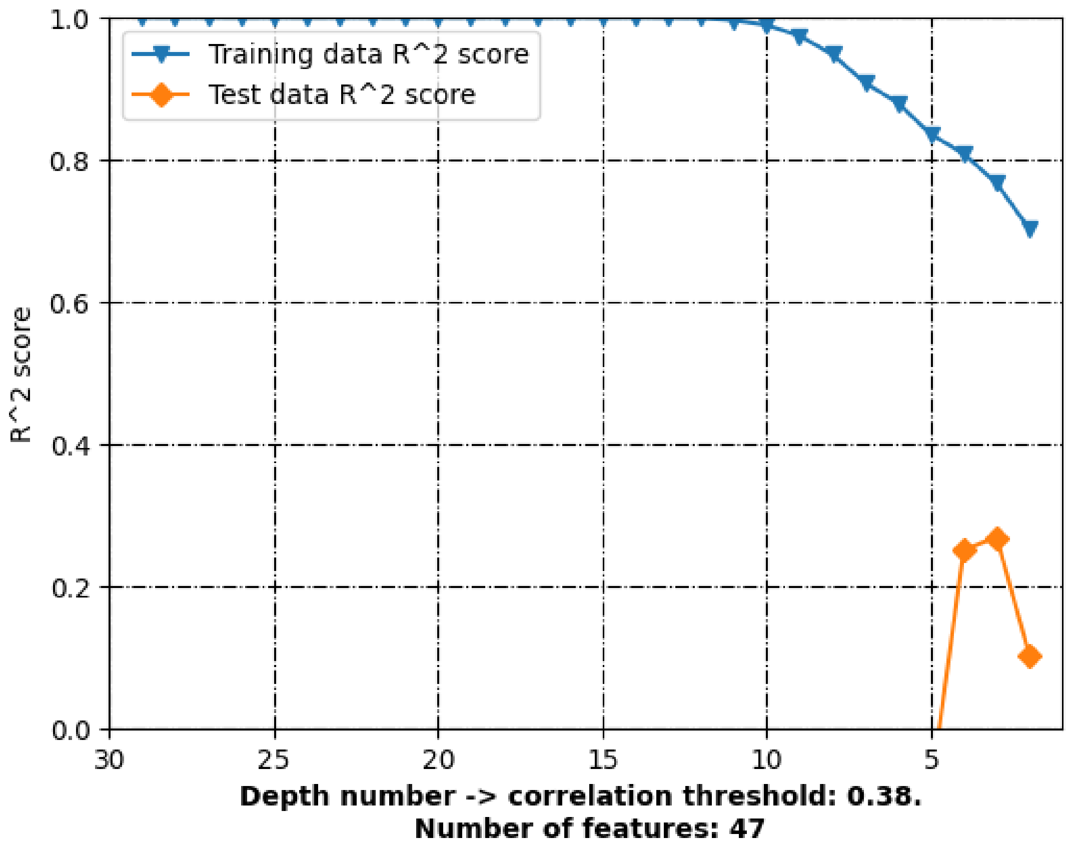

Training data R^2 score
Test data R^2 score
R^2 score
1.0
0.8
0.6
0.4
0.2
0.0
30
25
20
15
10
5
Depth number -> correlation threshold: 0.38.
Number of features: 47

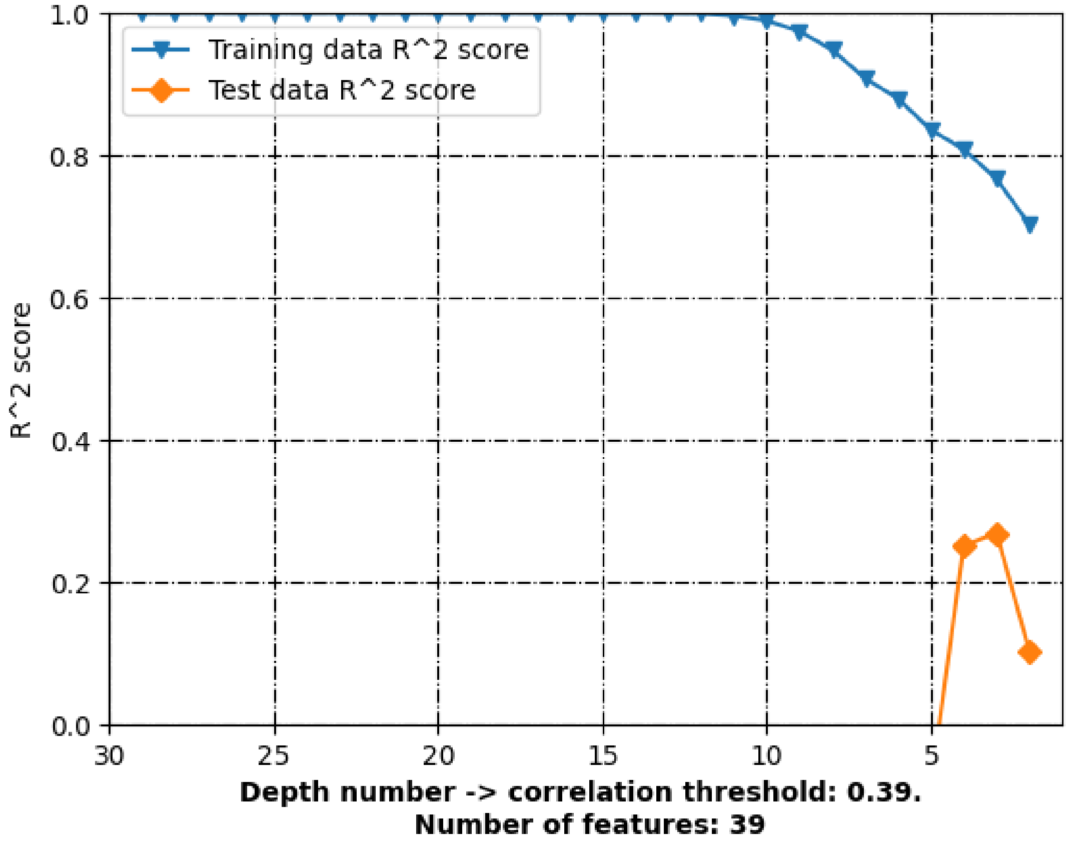
1.0
0.8
0.6
0.4
0.2
0.0
R^2 score
Training data R^2 score
Test data R^2 score
30
25
20
15
10
5
Depth number -> correlation threshold: 0.39.
Number of features: 39

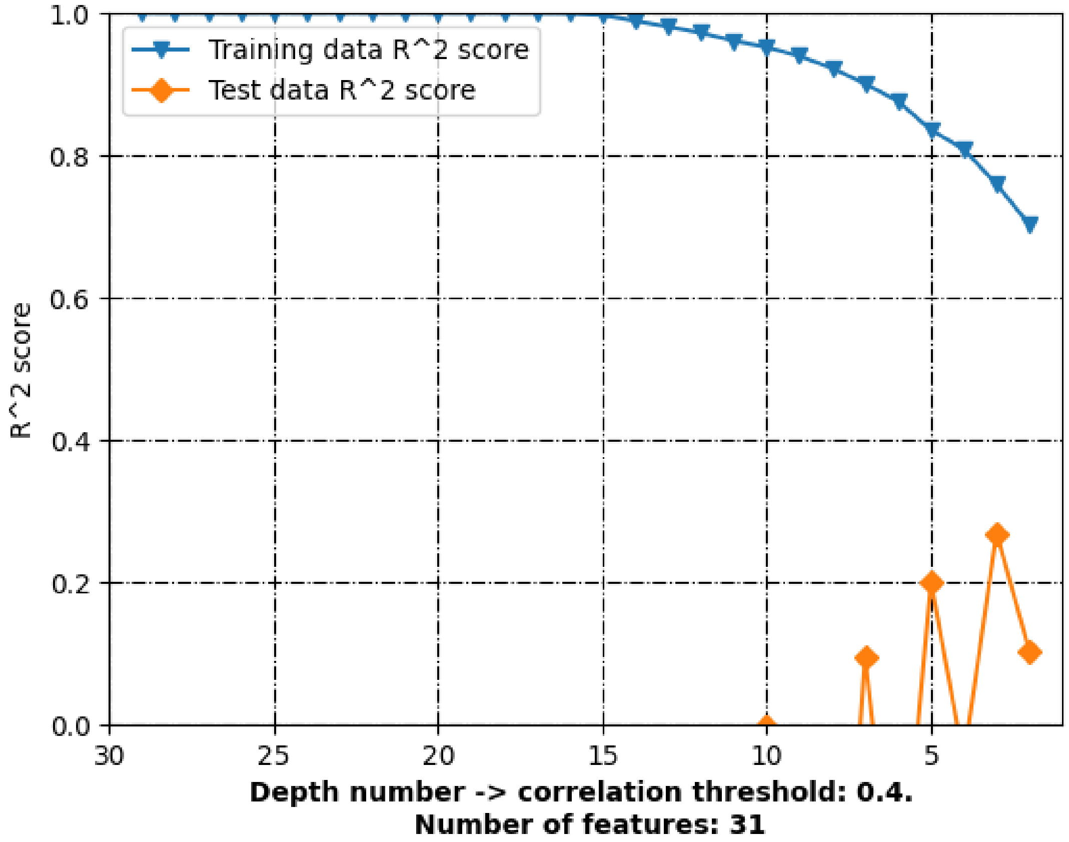

Training data R^2 score
Test data R^2 score
R^2 score
1.0
0.8
0.6
0.4
0.2
0.0
30
25
20
15
10
5
Depth number -> correlation threshold: 0.4.
Number of features: 31

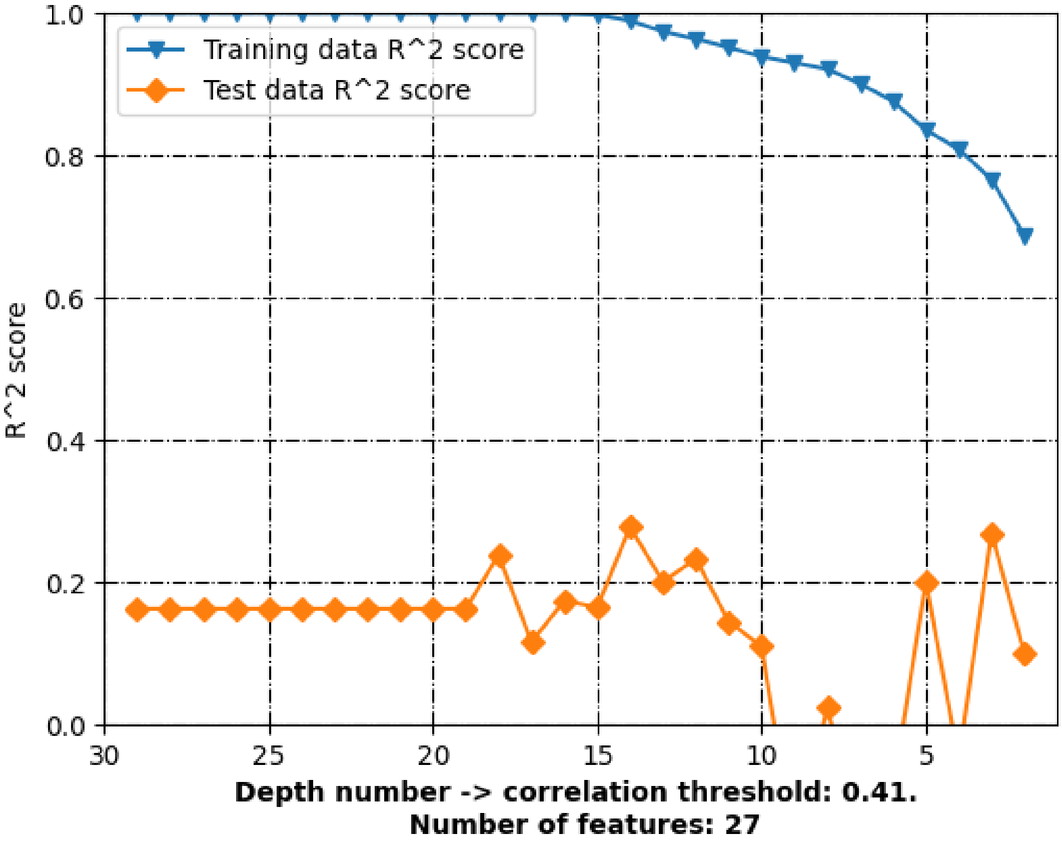
Training data R^2 score
Test data R^2 score
R^2 score
1.0
0.8
0.6
0.4
0.2
0.0
30
25
20
15
10
5
Depth number -> correlation threshold: 0.41.
Number of features: 27

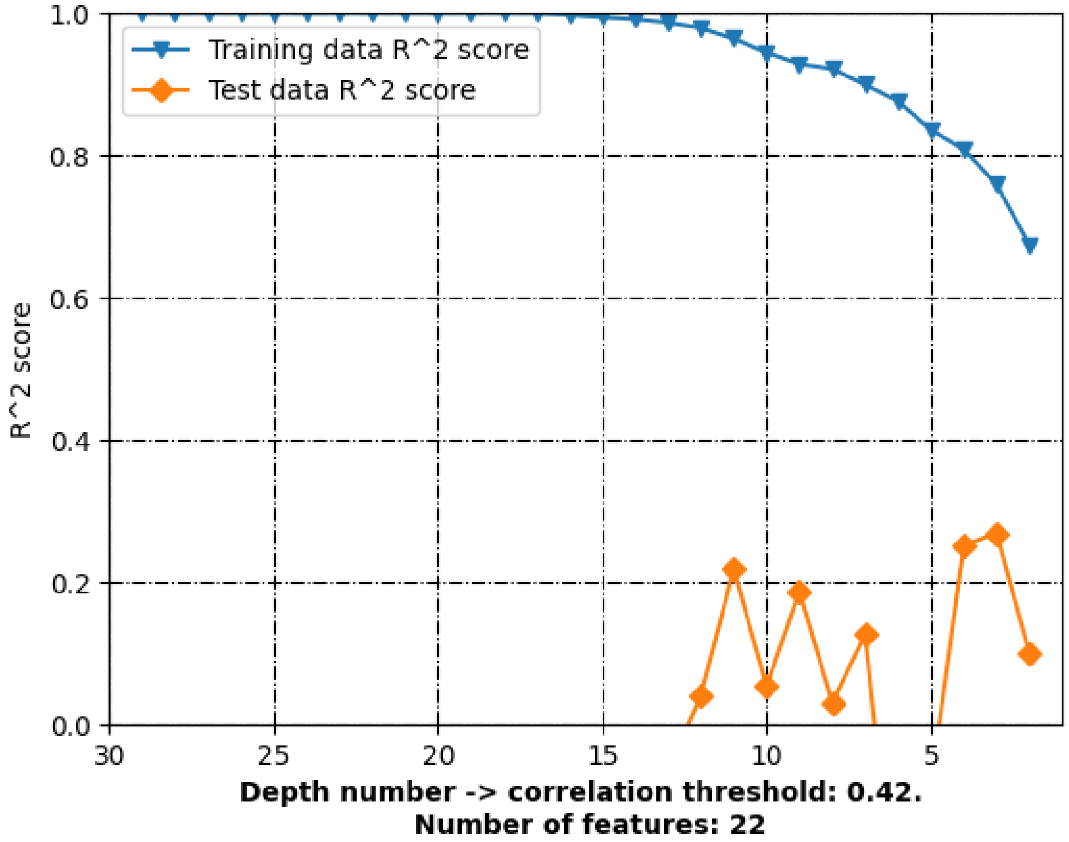

Training data R^2 score
Test data R^2 score
R^2 score
1.0
0.8
0.6
0.4
0.2
0.0
30
25
20
15
10
5
Depth number -> correlation threshold: 0.42.
Number of features: 22

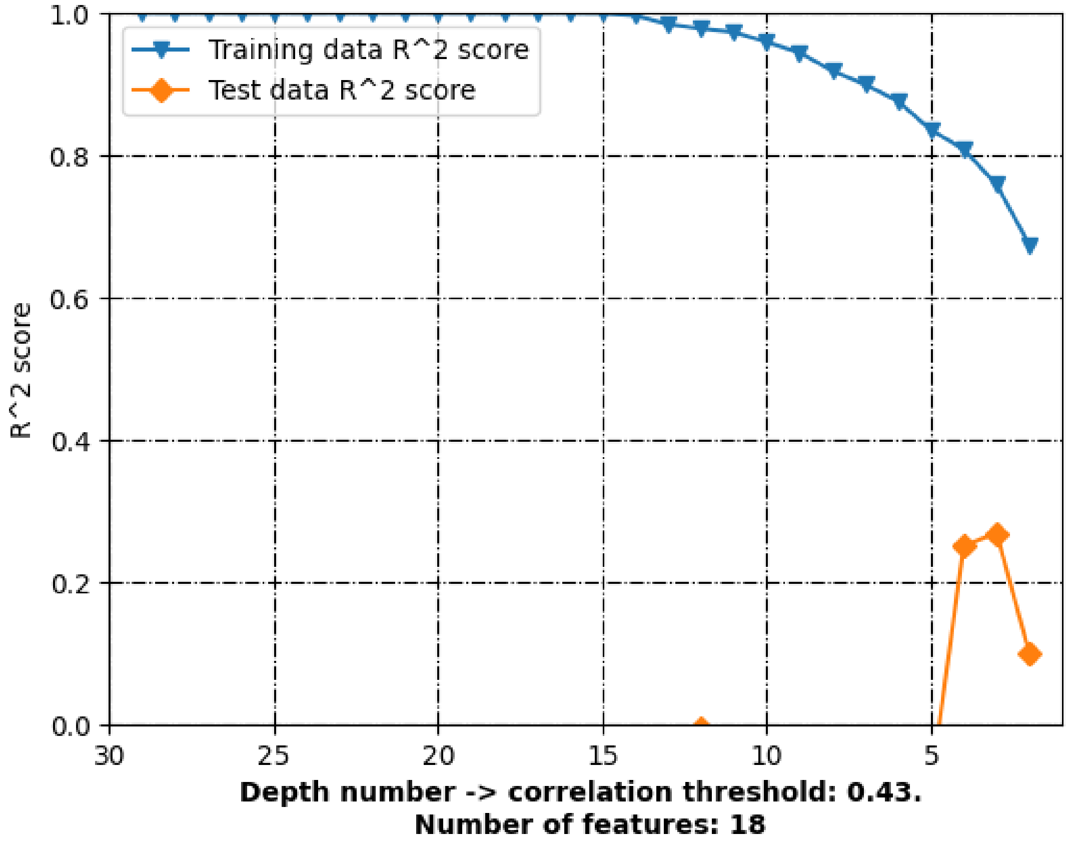

Training data R^2 score
Test data R^2 score
R^2 score
1.0
0.8
0.6
0.4
0.2
0.0
30
25
20
15
10
5
Depth number -> correlation threshold: 0.43.
Number of features: 18

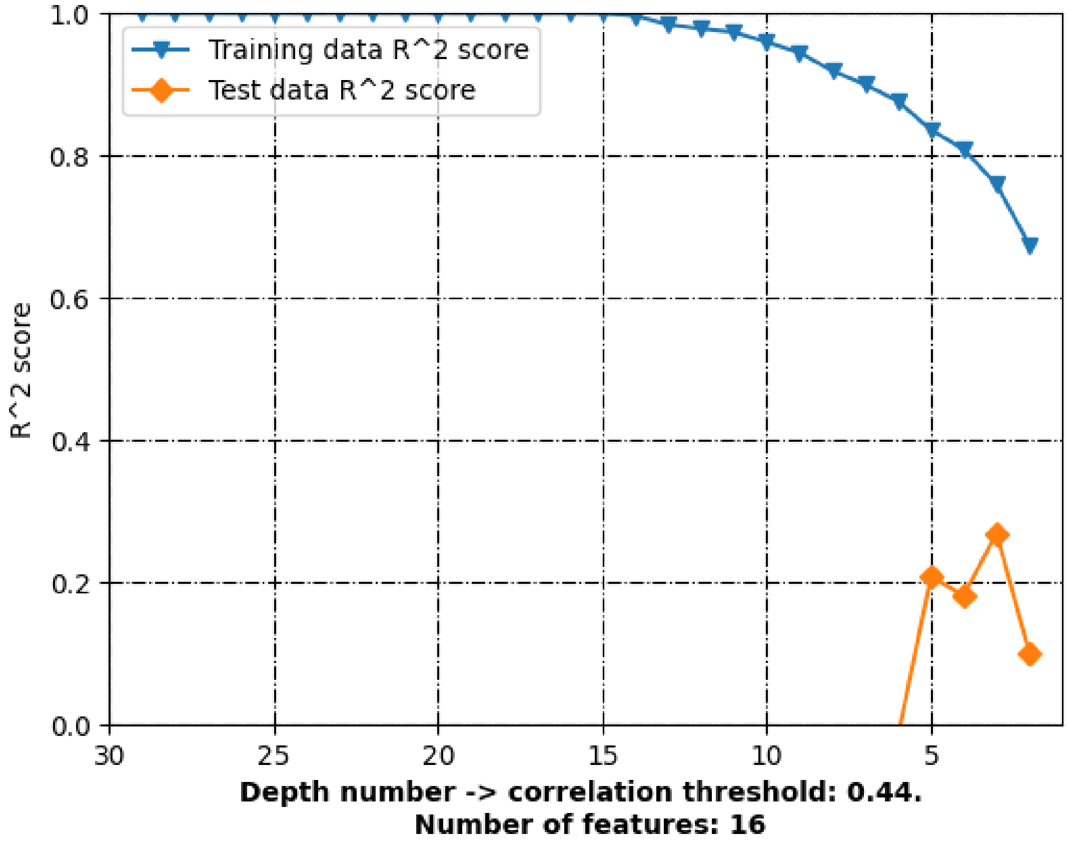

Training data R^2 score
Test data R^2 score
R^2 score
1.0
0.8
0.6
0.4
0.2
0.0
30
25
20
15
10
5
Depth number -> correlation threshold: 0.44.
Number of features: 16

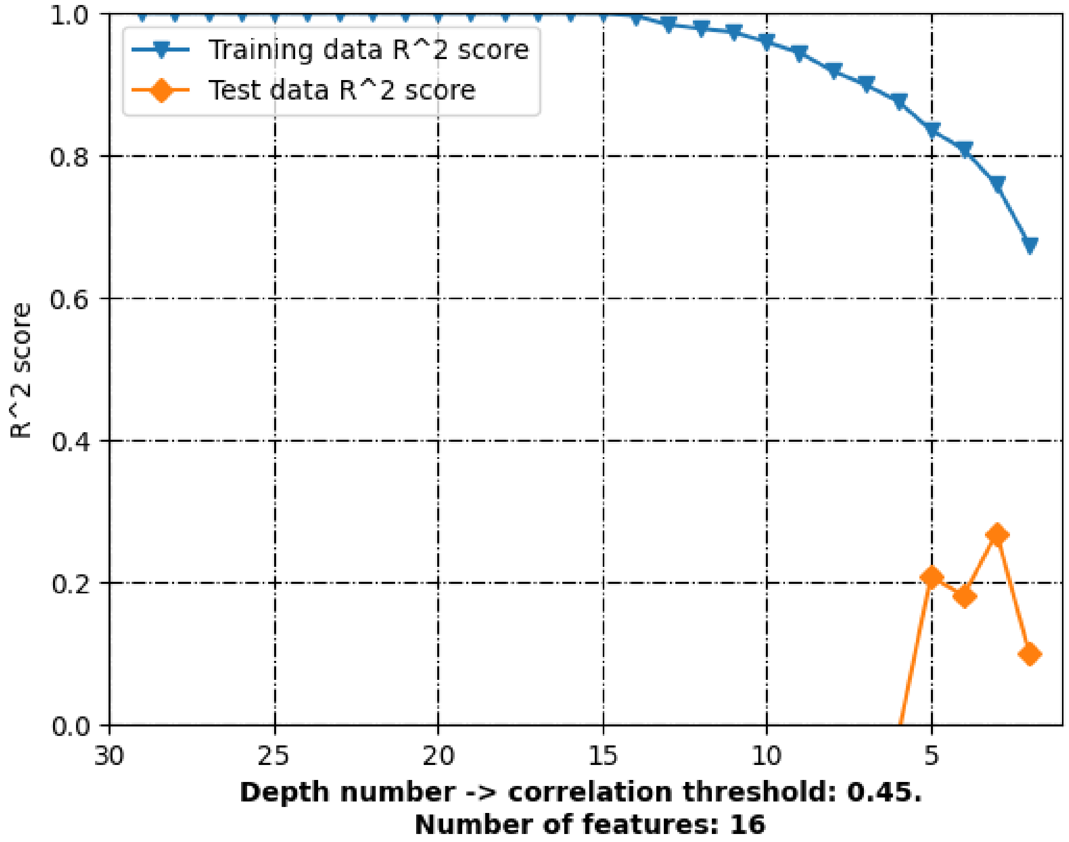
1.0
0.8
0.6
0.4
0.2
0.0
R^2 score
30
25
20
15
10
5
Training data R^2 score
Test data R^2 score
Depth number -> correlation threshold: 0.45.
Number of features: 16

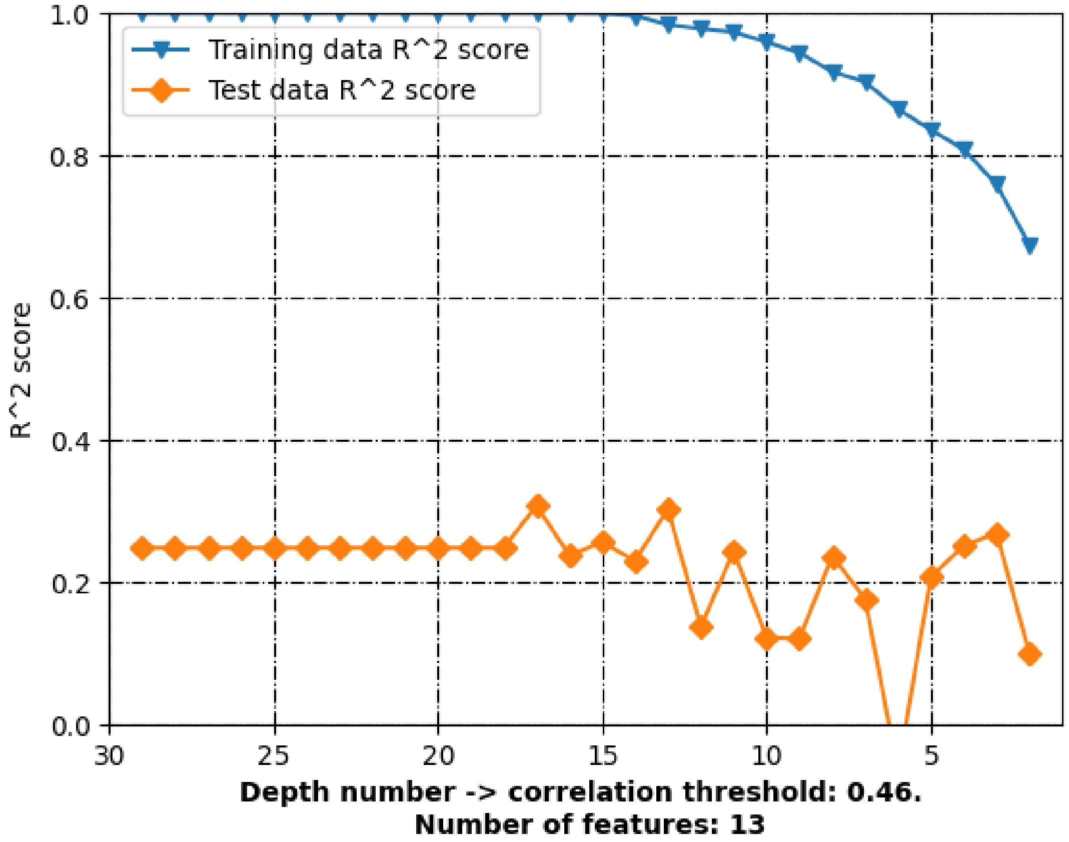

1.0
0.8
0.6
0.4
0.2
0.0
R^2 score
Training data R^2 score
Test data R^2 score
30
25
20
15
10
5
Depth number -> correlation threshold: 0.46.
Number of features: 13

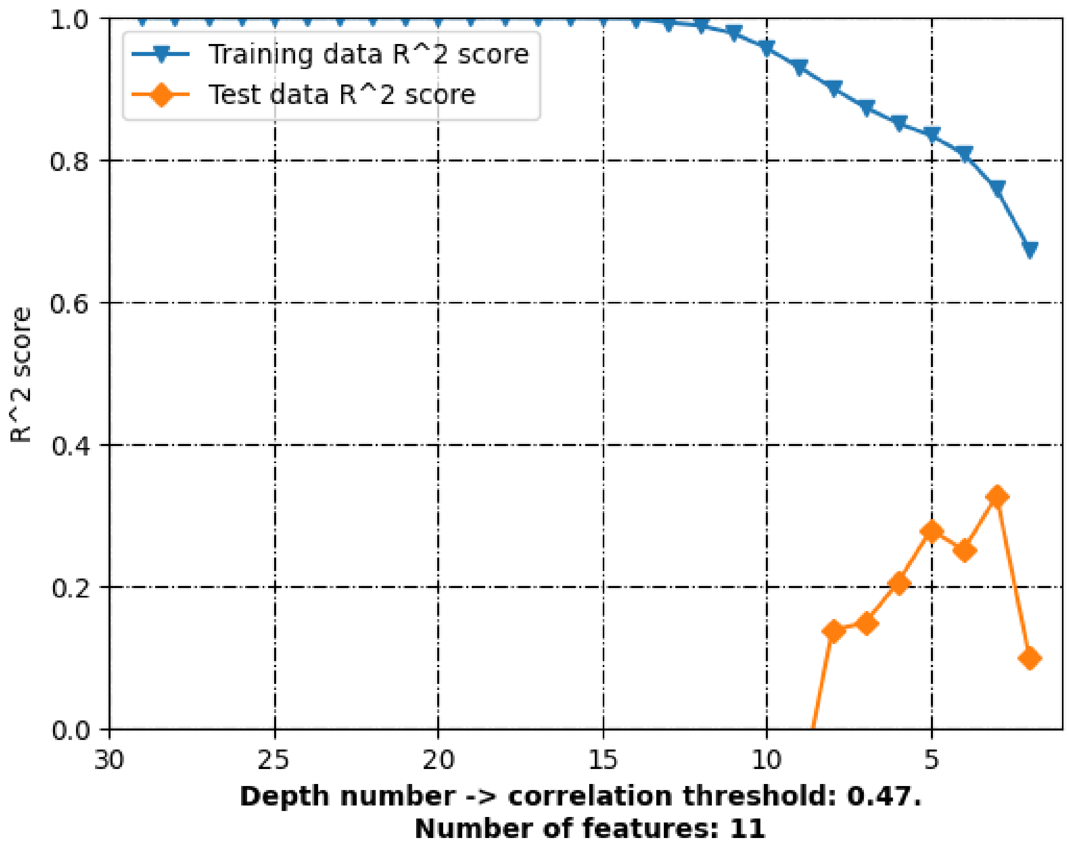
Training data R^2 score
Test data R^2 score
R^2 score
1.0
0.8
0.6
0.4
0.2
0.0
30
25
20
15
10
5
Depth number -> correlation threshold: 0.47.
Number of features: 11

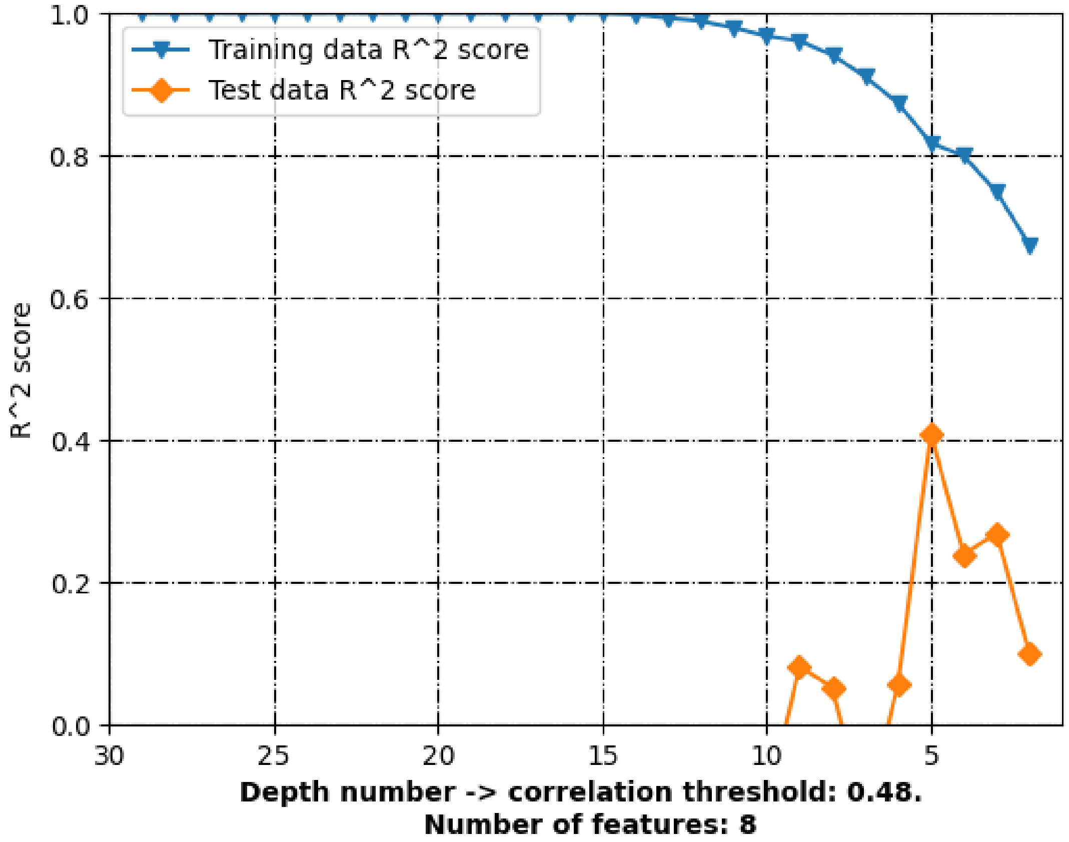

1.0
0.8
0.6
0.4
0.2
0.0
R^2 score
Training data R^2 score
Test data R^2 score
30
25
20
15
10
5
Depth number -> correlation threshold: 0.48.
Number of features: 8

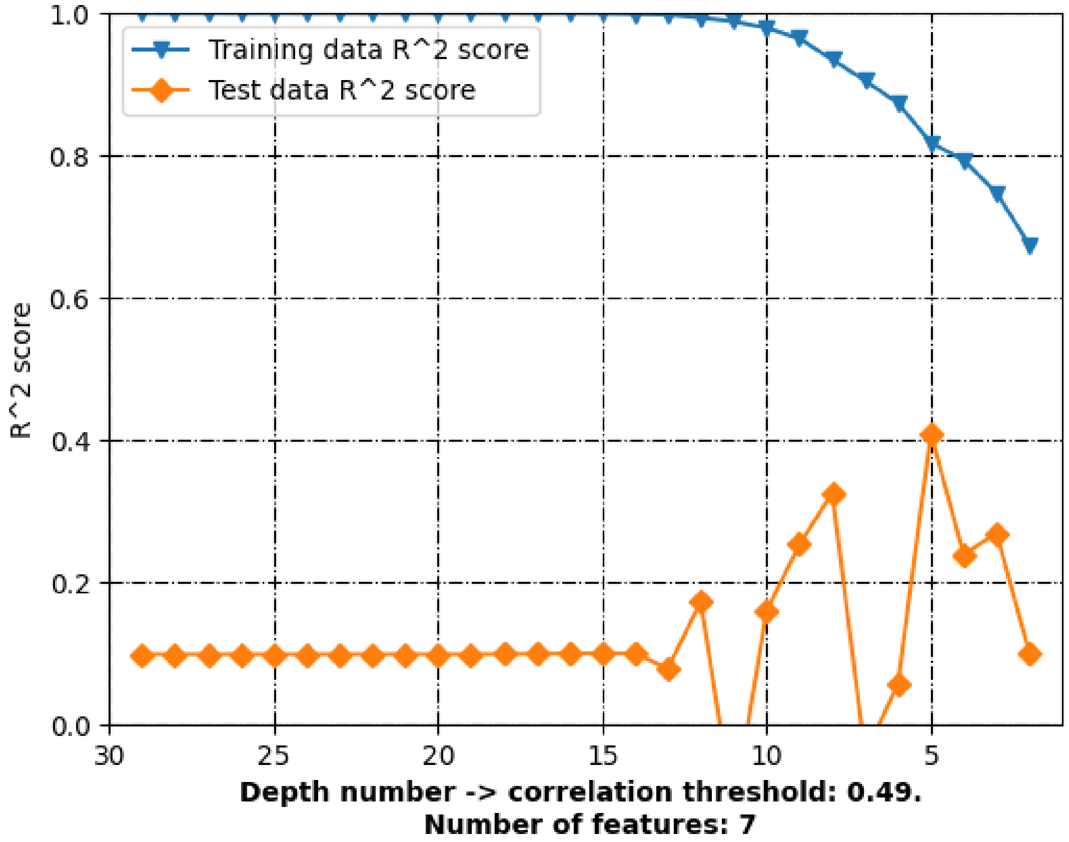

Training data R^2 score
Test data R^2 score
R^2 score
1.0
0.8
0.6
0.4
0.2
0.0
30
25
20
15
10
5
Depth number -> correlation threshold: 0.49.
Number of features: 7

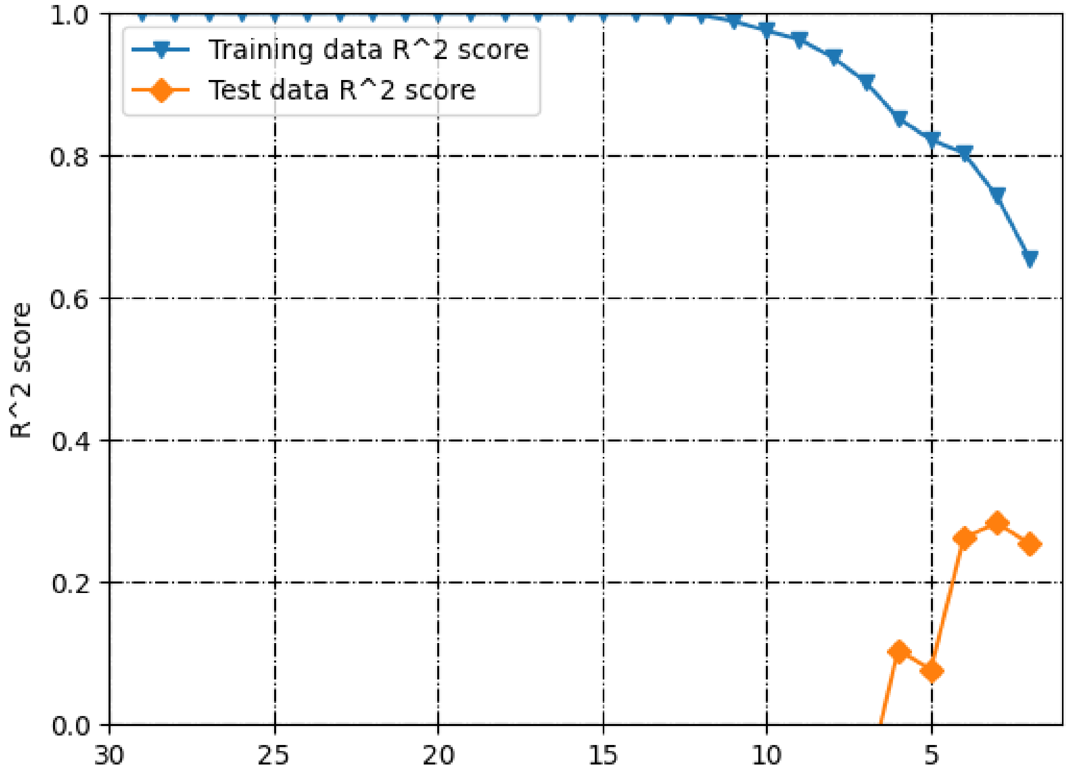

Depth number -> correlation threshold: 0.5.
Number of features: 5

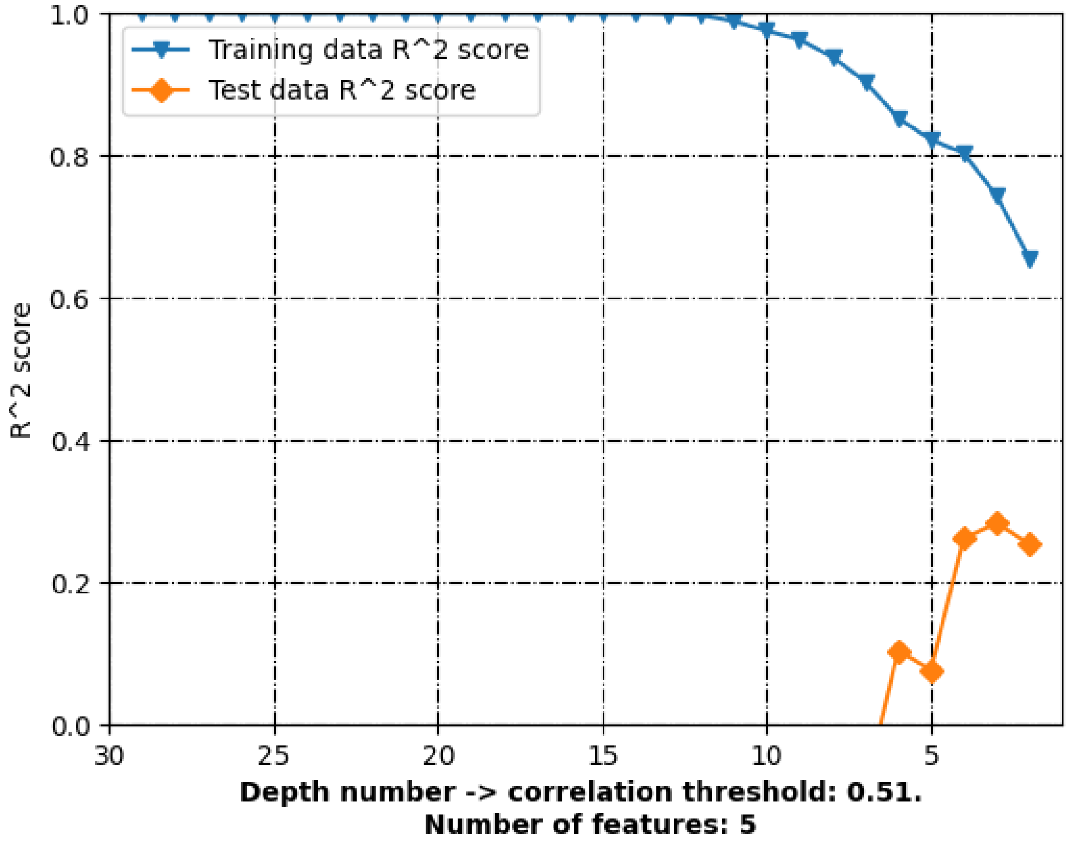
Training data R^2 score
Test data R^2 score
R^2 score
1.0
0.8
0.6
0.4
0.2
0.0
30
25
20
15
10
5
Depth number -> correlation threshold: 0.51.
Number of features: 5

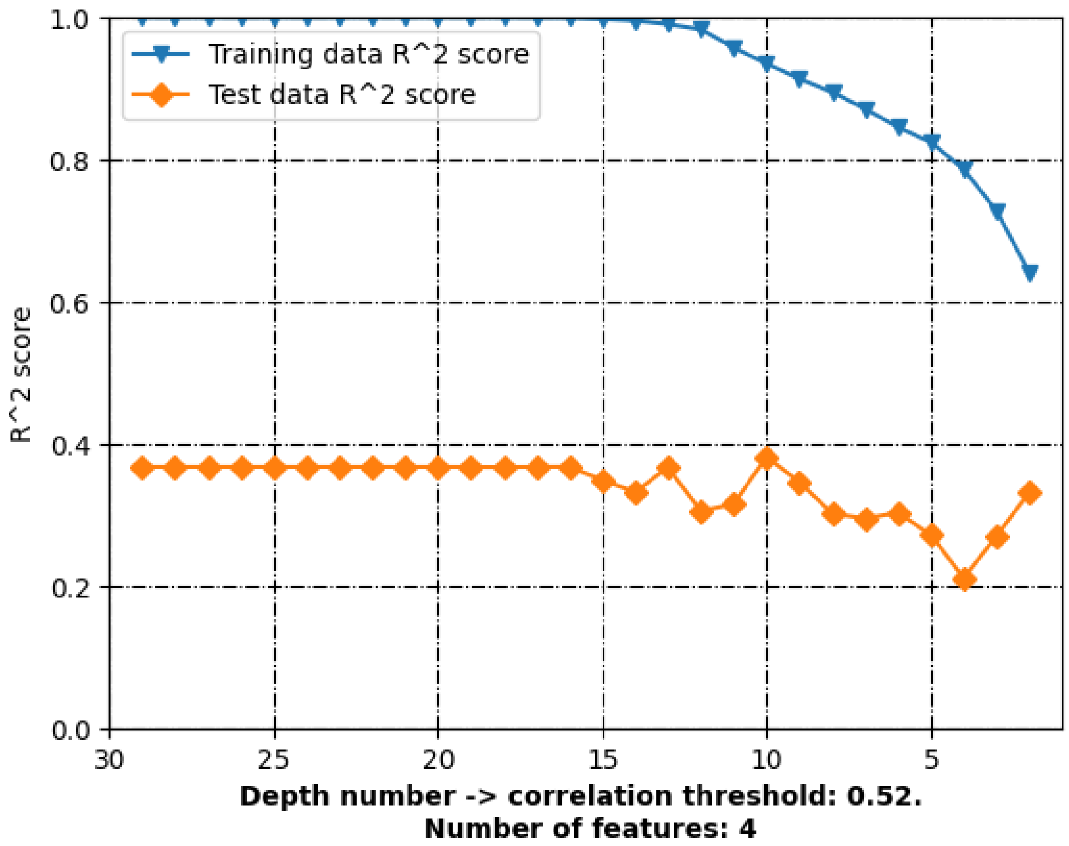

1.0
0.8
0.6
0.4
0.2
0.0
R^2 score
Training data R^2 score
Test data R^2 score
30
25
20
15
10
5
Depth number -> correlation threshold: 0.52.
Number of features: 4

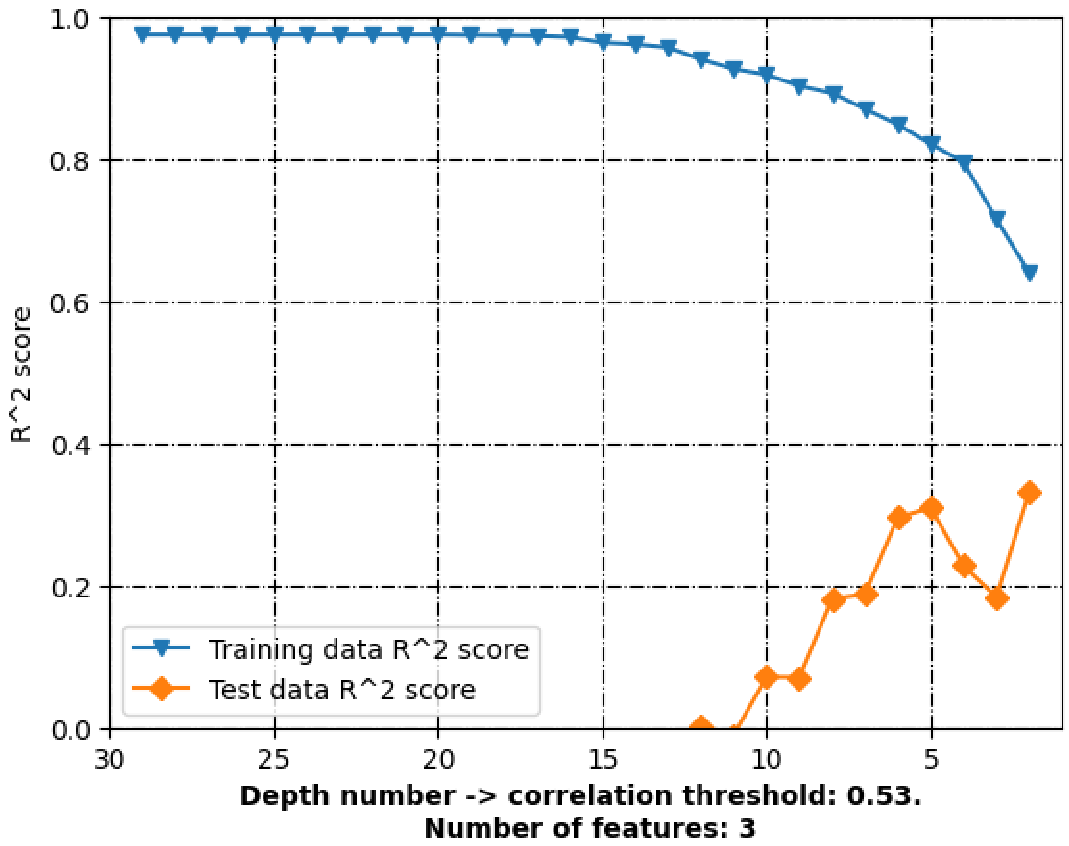

[20]:
```
plt.plot(df_without_standardization['Correlation threshold'],␣
  ↪df_without_standardization['Number of features'], label = "Number of␣
  ↪features", marker='.')
plt.legend()
plt.xlabel('Correlation threshold')
plt.ylabel('Number of features')
plt.show()
```

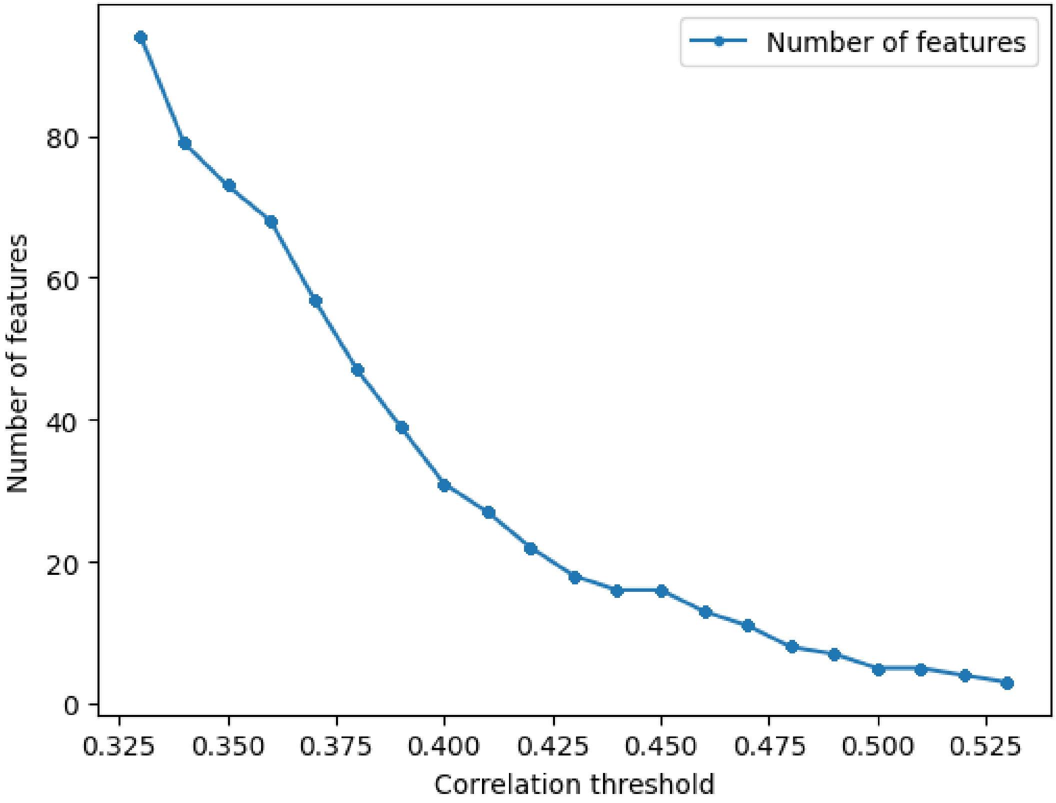


```
[ ]:
```

3 Random Forest

```
[21]: without_standardization, train_r2_, test_r2_, hist1, hist2, target_column_name,␣
      ↪training_data_RMSE, test_data_RMSE  = pred_model.
      ↪prepare_data_and_create_model(molecular_descriptors_df=data,
                                                                               ␣
      ↪                    correlation_threshold=0.50,
                                                                               ␣
      ↪                    standardization=False,
                                                                               ␣
      ↪                    model_type='RandomForestRegressor',
                                                                               ␣
      ↪                    n_estimators_=12,
                                                                               ␣
      ↪                    target_column_name = target,
                                                                               ␣
      ↪                    random_state=random_state,
```

```
↪                    train_test_split_=True,
↪                    verbose=True)
```

```
I am not doing standardization…
  molecular descriptor name
0                    AATS0Z
1                  AATS0are
2                    AATS0d
3                   AATS0dv
4                    AATS0i
  molecular descriptor name  corr_value
0                    AATS0Z   -0.022797
1                  AATS0are   -0.139064
2                    AATS0d    0.027592
3                   AATS0dv   -0.136049
4                    AATS0i    0.168958
  molecular descriptor name  corr_value  absolute correlation value
0                    AATS0Z   -0.022797                    0.022797
1                  AATS0are   -0.139064                    0.139064
2                    AATS0d    0.027592                    0.027592
3                   AATS0dv   -0.136049                    0.136049
4                    AATS0i    0.168958                    0.168958
     molecular descriptor name  corr_value  absolute correlation value
226                     AMID_O   -0.557717                    0.557717
505                EState_VSA5   -0.534748                    0.534748
791                   MDEO-12   -0.555724                    0.555724
921                      SaasC    0.513071                    0.513071
1091               VSA_EState5    0.526082                    0.526082
     molecular descriptor name  corr_value  absolute correlation value
226                     AMID_O   -0.557717                    0.557717
505                EState_VSA5   -0.534748                    0.534748
791                   MDEO-12   -0.555724                    0.555724
921                      SaasC    0.513071                    0.513071
1091               VSA_EState5    0.526082                    0.526082
The model used is: RandomForest…
Return the coefficient of determination of the prediction:
0.6518304025888859
R^2 score: 0.9434388899856951
Correlation coefficient: 0.9713078245261361
Test data - unseen during training:
R^2 score: 0.6518304025888859
Correlation coefficient: 0.8073601442905675
[7.9447878  5.83806583 7.48789076 6.61477061 7.42258424 8.23414219
 7.97322552 7.61315235 7.67534542 7.58053821 8.29502431 7.78406913
 7.83000603 7.24628922 8.23414219 6.60649711 7.22394916 6.8191525 ]
113    7.823909
```

```
35     6.053057
101    6.721246
36     6.221126
100    7.130768
13     7.966576
0      7.966576
114    7.920819
104    8.309804
96     7.102373
40     7.920819
103    8.397940
48     7.853872
39     8.356547
14     8.148742
117    6.031517
21     7.325139
9      6.818728
Name: A549, dtype: float64
Training Root Mean Square Error: 0.232296117071006l7
Testing Root Mean Square Error: 0.45852469814920027
```

[22]:
```
print(target_column_name+str('_transformed'))
print(hist1[target_column_name].hist())
```

```
A549_transformed
AxesSubplot(0.125,0.11;0.775x0.77)
```

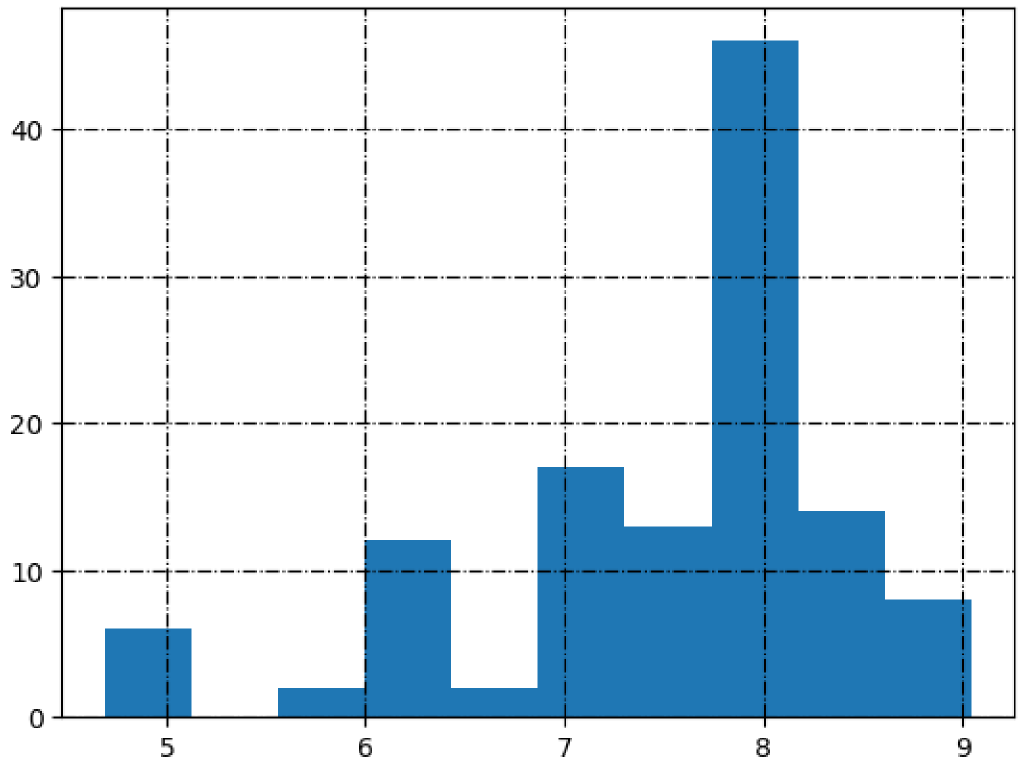

[23]:
```
with_standardization, train_r2_, test_r2_, hist1, hist2, target_column_name,␣
↪training_data_RMSE, test_data_RMSE  = pred_model.
↪prepare_data_and_create_model(molecular_descriptors_df=data,
                                                                              ␣
↪                          correlation_threshold=0.50,
                                                                              ␣
↪                          standardization=True,
                                                                              ␣
↪                          model_type='RandomForestRegressor',
                                                                              ␣
↪                          n_estimators_=12,
                                                                              ␣
↪                          target_column_name = target,
                                                                              ␣
↪                          random_state=random_state,
                                                                              ␣
↪                          train_test_split_=True,
                                                                              ␣
↪                          verbose=True)
```

```
I am doing standardization…
  molecular descriptor name
```

```
0                  AATS0Z
1                AATS0are
2                  AATS0d
3                 AATS0dv
4                  AATS0i
  molecular descriptor name  corr_value
0                  AATS0Z   -0.022797
1                AATS0are   -0.139064
2                  AATS0d    0.027592
3                 AATS0dv   -0.136049
4                  AATS0i    0.168958
  molecular descriptor name  corr_value  absolute correlation value
0                  AATS0Z   -0.022797                    0.022797
1                AATS0are   -0.139064                    0.139064
2                  AATS0d    0.027592                    0.027592
3                 AATS0dv   -0.136049                    0.136049
4                  AATS0i    0.168958                    0.168958
     molecular descriptor name  corr_value  absolute correlation value
226                     AMID_O   -0.557717                    0.557717
505                EState_VSA5   -0.534748                    0.534748
791                    MDEO-12   -0.555724                    0.555724
921                      SaasC    0.513071                    0.513071
1091               VSA_EState5    0.526082                    0.526082
```


```
     molecular descriptor name  corr_value  absolute correlation value
226                     AMID_O   -0.557717                    0.557717
505                EState_VSA5   -0.534748                    0.534748
791                    MDEO-12   -0.555724                    0.555724
921                      SaasC    0.513071                    0.513071
1091               VSA_EState5    0.526082                    0.526082
```


```
The model used is: RandomForest…
Return the coefficient of determination of the prediction:
0.6518304025888859
R^2 score: 0.9434388899856951
Correlation coefficient: 0.9713078245261361
Test data - unseen during training:
R^2 score: 0.6518304025888859
Correlation coefficient: 0.8073601442905675
[7.9447878  5.83806583 7.48789076 6.61477061 7.42258424 8.23414219
 7.97322552 7.61315235 7.67534542 7.58053821 8.29502431 7.78406913
 7.83000603 7.24628922 8.23414219 6.60649711 7.22394916 6.8191525 ]
113    7.823909
35     6.053057
101    6.721246
36     6.221126
100    7.130768
13     7.966576
0      7.966576
114    7.920819
```

```
104    8.309804
96     7.102373
40     7.920819
103    8.397940
48     7.853872
39     8.356547
14     8.148742
117    6.031517
21     7.325139
9      6.818728
Name: A549, dtype: float64
Training Root Mean Square Error: 0.23229611707100617
Testing Root Mean Square Error: 0.45852469814920027
```

3.1 Search inside correlation space

[24]:
```
step = 0.01
initial_step = corr_low
last_step = corr_high
first_list = [x / 100.0 for x in range(int(initial_step*100),␣
↪int(last_step*100), int(step*100))]
n_estimators = [range(2,21,1)]
corr_th = []
second_list = []
third_list = []
fourth_l = []
fifth_l = []
f_list = []
fif_list = []
for i in first_list:

    for estimator in n_estimators[0]:

        without_standardization, train_r2, test_r2, _, h_, target_column_name,␣
↪training_data_RMSE, test_data_RMSE = pred_model.
↪prepare_data_and_create_model(molecular_descriptors_df=data,
                                                                            ␣
↪                   correlation_threshold=i,
                                                                            ␣
↪                   standardization=False,
                                                                            ␣
↪                   model_type='RandomForestRegressor',
                                                                            ␣
↪                   n_estimators_=estimator,
                                                                            ␣
↪                   target_column_name = target,
```

```
                                                                          ␣
↪                    random_state=random_state,
                                                                          ␣
↪                    train_test_split_=True,
                                                                          ␣
↪                    verbose=False)
        corr_th.append(i)
        second_list.append(train_r2)
        third_list.append(test_r2)
        fourth_l.append(training_data_RMSE)
        fifth_l.append(test_data_RMSE)
        f_list.append(len(h_))
        fif_list.append(estimator)
```

[25]:
```
df_without_standardization = pd.DataFrame(data=corr_th, columns=["Correlation␣
↪threshold"])
df_without_standardization['Training data R^2 score'] = second_list
df_without_standardization['Test data R^2 score'] = third_list
df_without_standardization['Training RMSE'] = fourth_l
df_without_standardization['Test RMSE'] = fifth_l
df_without_standardization['Number of features'] = f_list
df_without_standardization['Number of estimators'] = fif_list
```

[26]:
```
df_random_forest = df_without_standardization.copy()
#df_without_standardization.to_excel('../Data/
↪A549_Random_forest_rs_'+str(random_state)+'.xlsx')
```

[27]:
```
corre_list = [x / 100.0 for x in range(int(initial_step*100),␣
↪int(last_step*100), int(0.01*100))]
for element in corre_list:
    element_ =␣
↪df_without_standardization[df_without_standardization['Correlation␣
↪threshold'] == float(element)]
    plt.plot(element_['Number of estimators'], element_['Training data R^2␣
↪score'], label = "Training data R^2 score", marker='v')
    plt.plot(element_['Number of estimators'], element_['Test data R^2 score'],␣
↪label = "Test data R^2 score", marker='D')
    plt.legend()
    plt.xlabel('Number of estimators -> correlation threshold: '+str(element)+'.
↪ \n Number of features: '+str(element_['Number of features'].iloc[0]))
    plt.xlim(max(element_['Number of estimators'])+1, min(element_['Number of␣
↪estimators'])-1)
    plt.ylabel('R^2 score')
    plt.ylim([0, 1])
    plt.rc('grid', linestyle="-.", color='black')
    plt.grid(True)
```

```
plt.show()
```

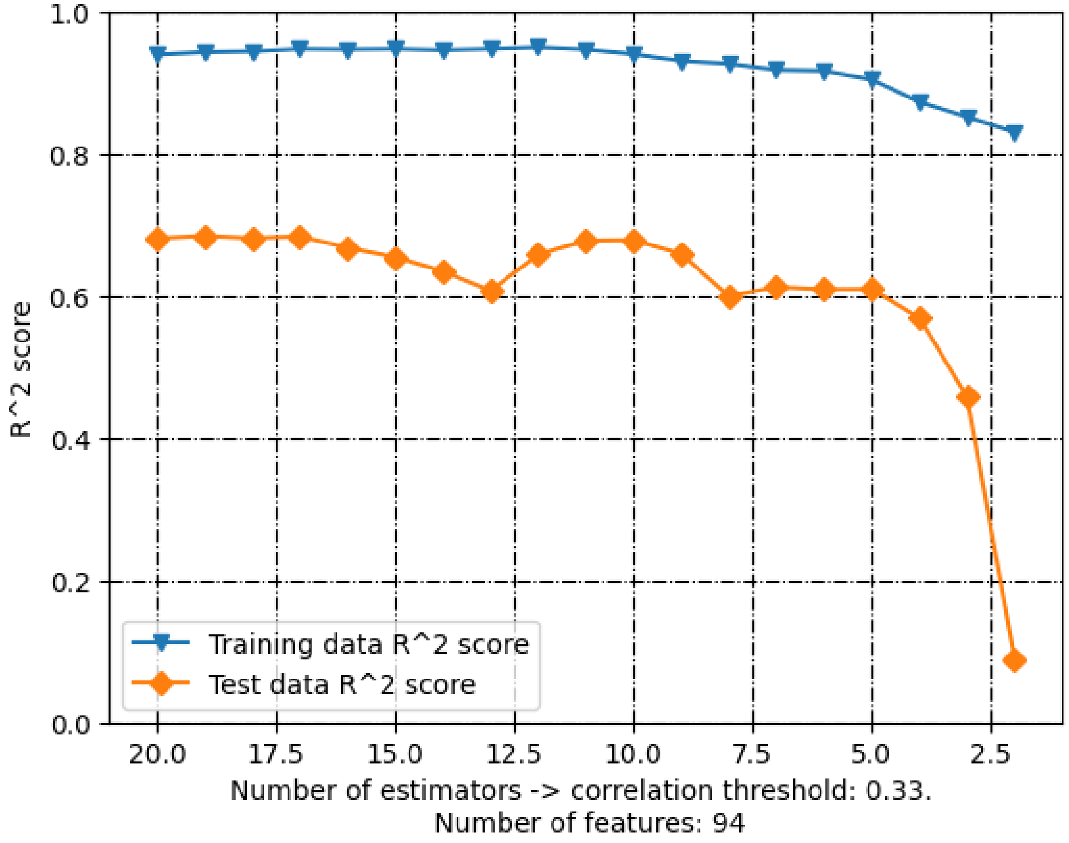

1.0
0.8
0.6
0.4
0.2
0.0
R^2 score
Training data R^2 score
Test data R^2 score
20.0
17.5
15.0
12.5
10.0
7.5
5.0
2.5
Number of estimators -> correlation threshold: 0.33.
Number of features: 94

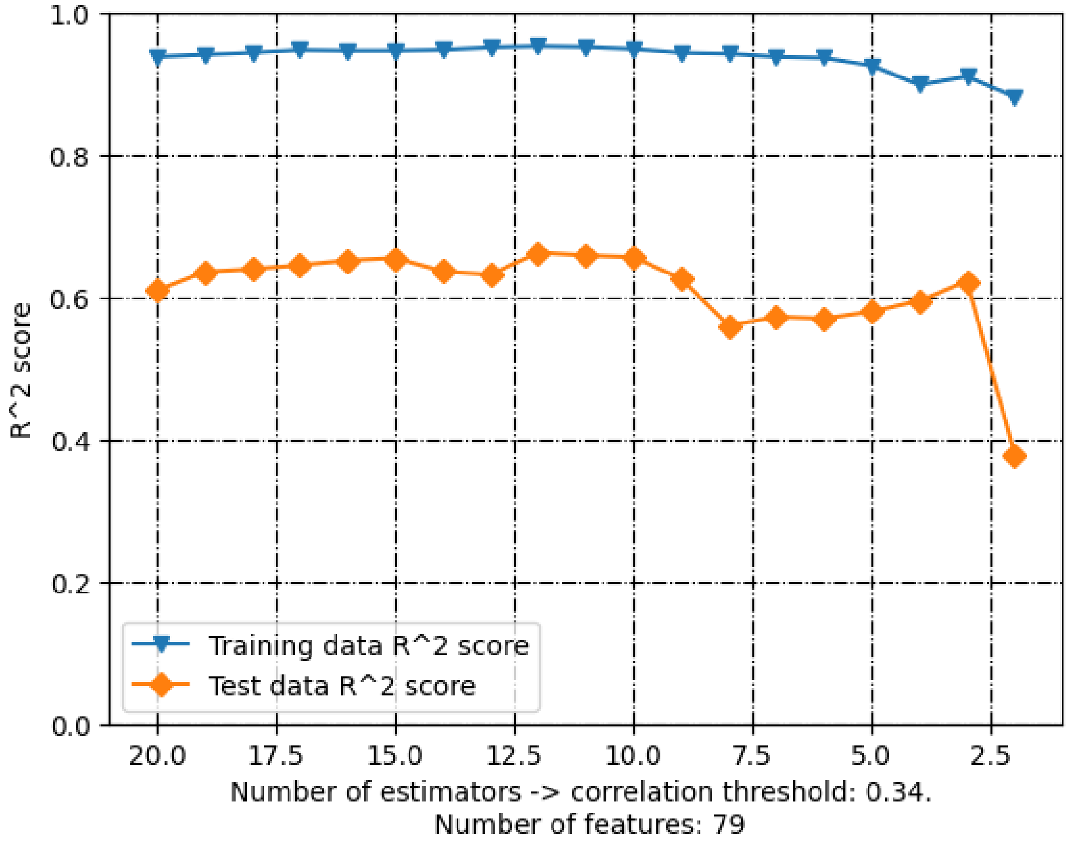
1.0
0.8
0.6
0.4
0.2
0.0
R^2 score
Training data R^2 score
Test data R^2 score
20.0
17.5
15.0
12.5
10.0
7.5
5.0
2.5
Number of estimators -> correlation threshold: 0.34.
Number of features: 79

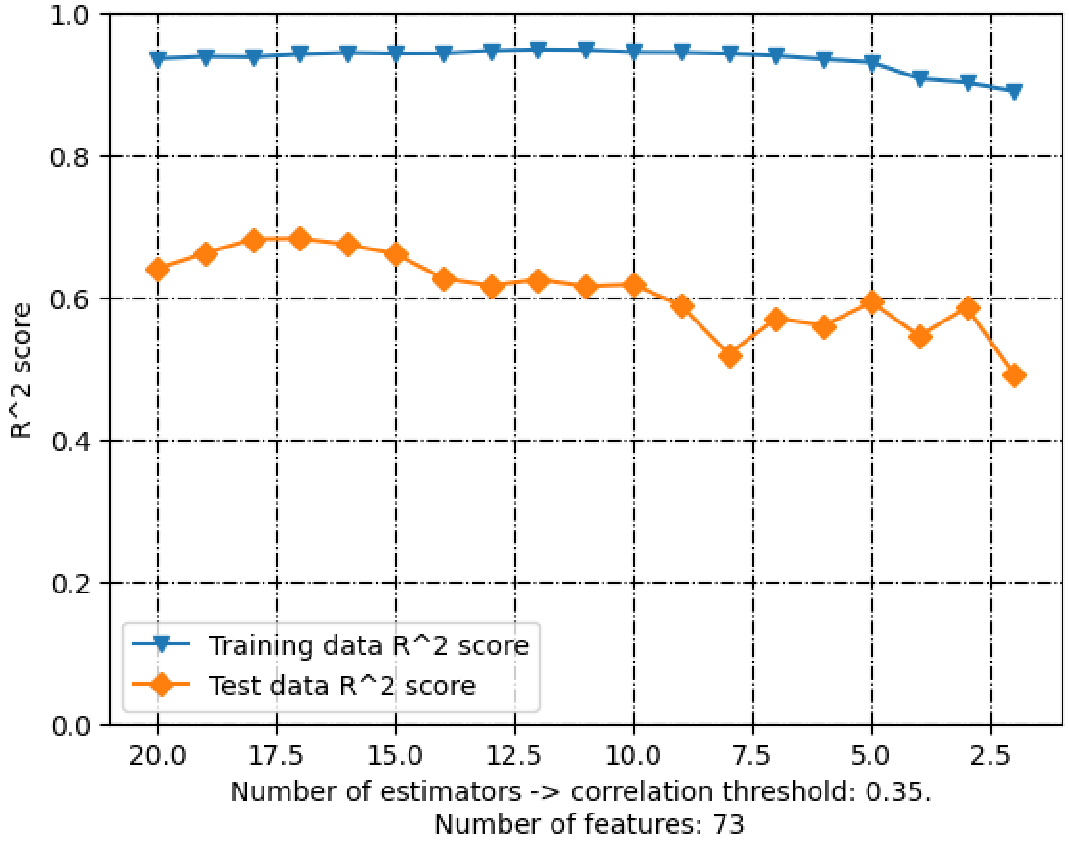

1.0
0.8
0.6
0.4
0.2
0.0
R^2 score
20.0
17.5
15.0
12.5
10.0
7.5
5.0
2.5
Number of estimators -> correlation threshold: 0.35.
Number of features: 73
Training data R^2 score
Test data R^2 score

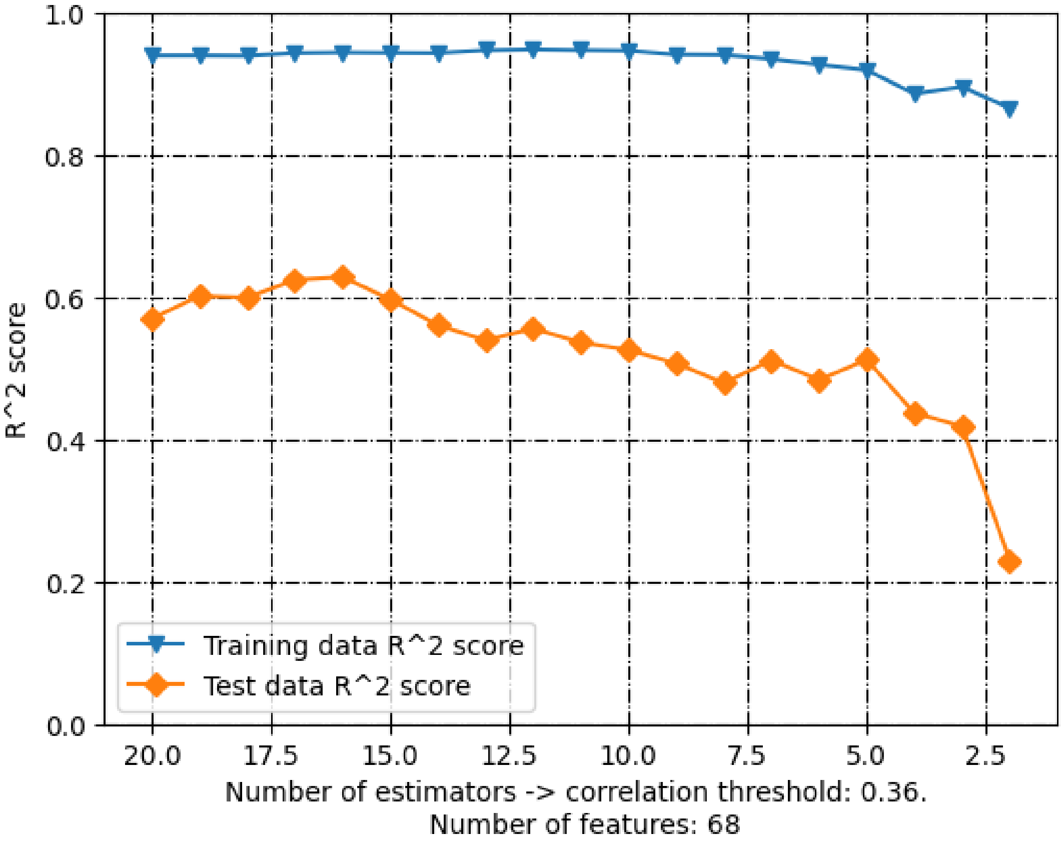

Training data R^2 score
Test data R^2 score
R^2 score
1.0
0.8
0.6
0.4
0.2
0.0
20.0
17.5
15.0
12.5
10.0
7.5
5.0
2.5
Number of estimators -> correlation threshold: 0.36.
Number of features: 68

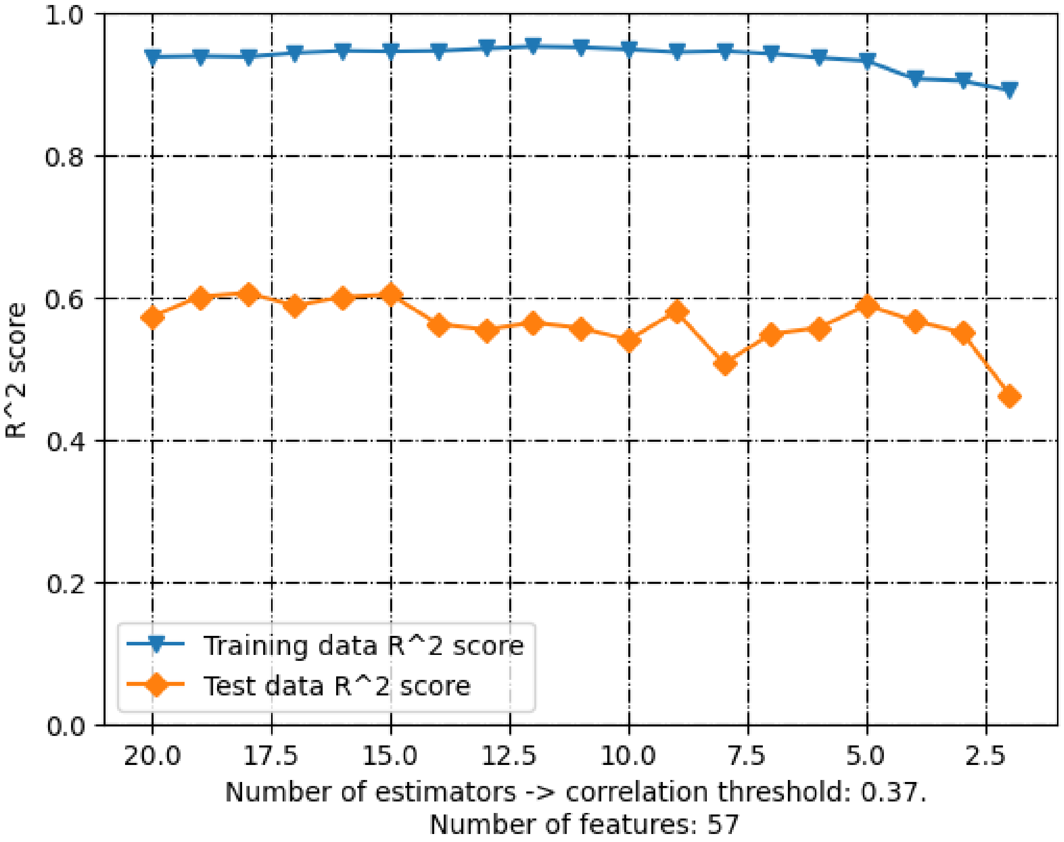
1.0
0.8
0.6
0.4
0.2
0.0
R^2 score
Training data R^2 score
Test data R^2 score
20.0
17.5
15.0
12.5
10.0
7.5
5.0
2.5
Number of estimators -> correlation threshold: 0.37.
Number of features: 57

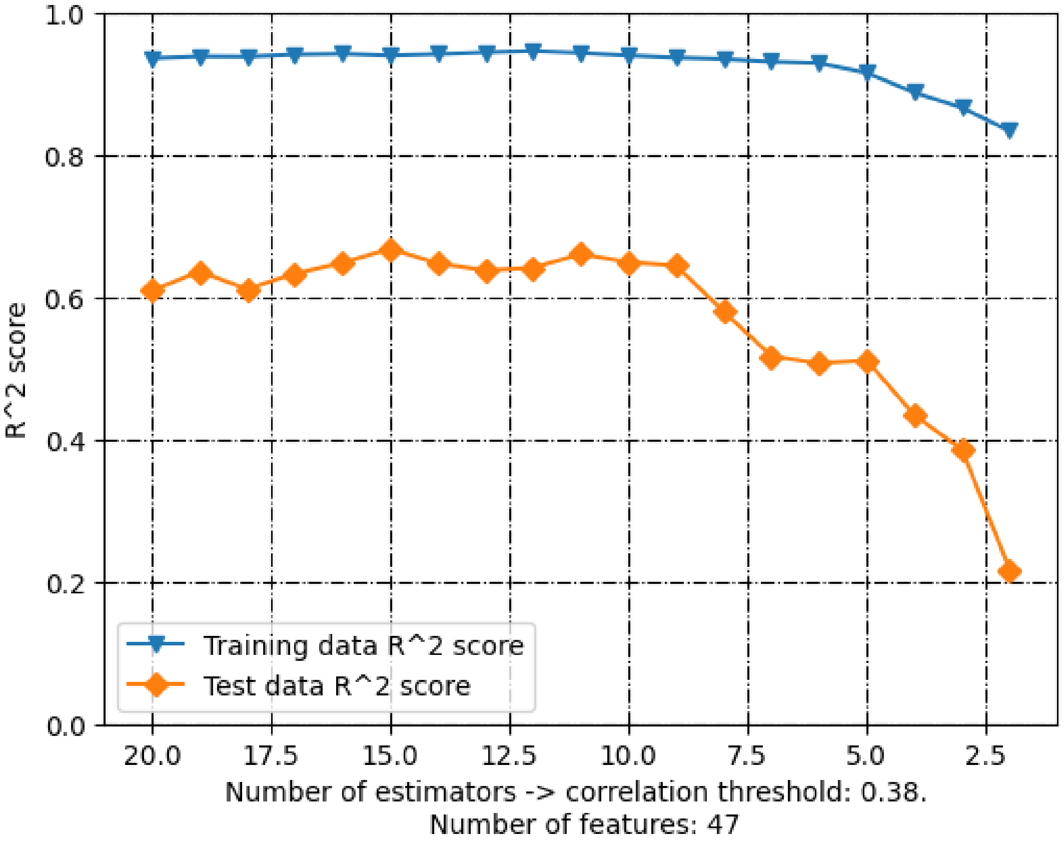
1.0
0.8
0.6
0.4
0.2
0.0
R^2 score
Training data R^2 score
Test data R^2 score
20.0
17.5
15.0
12.5
10.0
7.5
5.0
2.5
Number of estimators -> correlation threshold: 0.38.
Number of features: 47

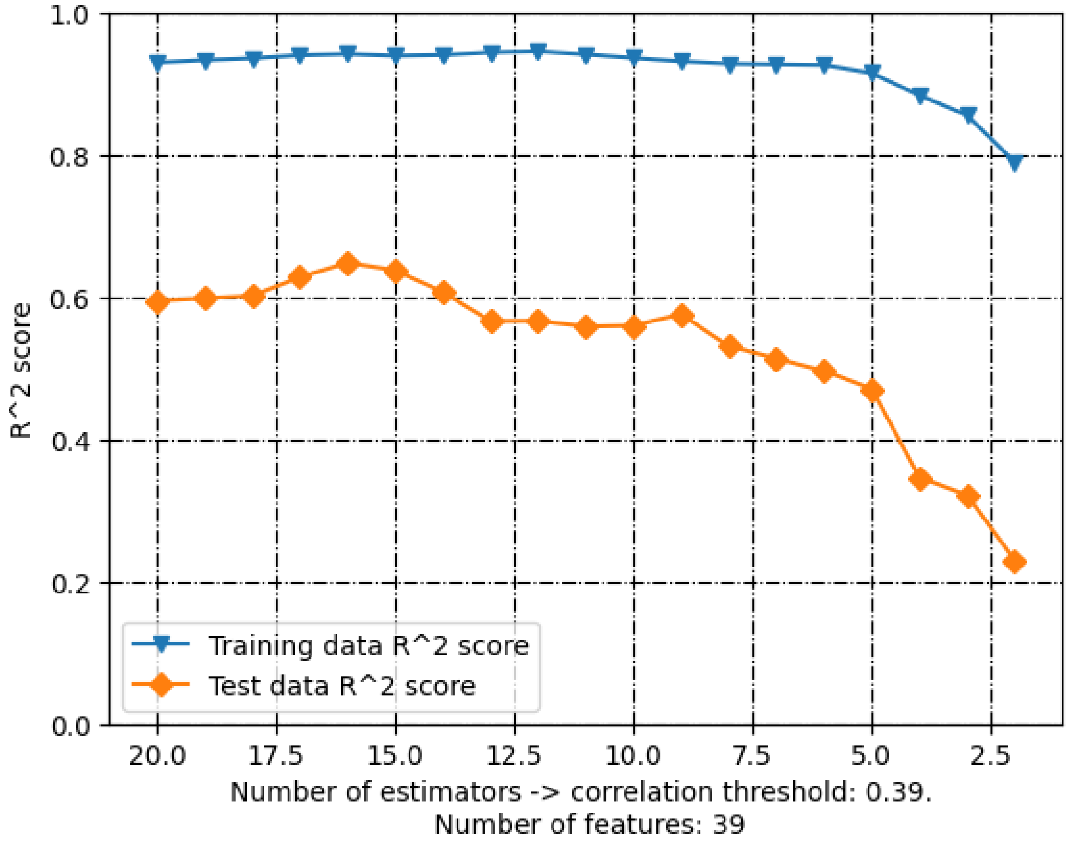

1.0
0.8
0.6
0.4
0.2
0.0
R^2 score
20.0
17.5
15.0
12.5
10.0
7.5
5.0
2.5
Training data R^2 score
Test data R^2 score
Number of estimators -> correlation threshold: 0.39.
Number of features: 39

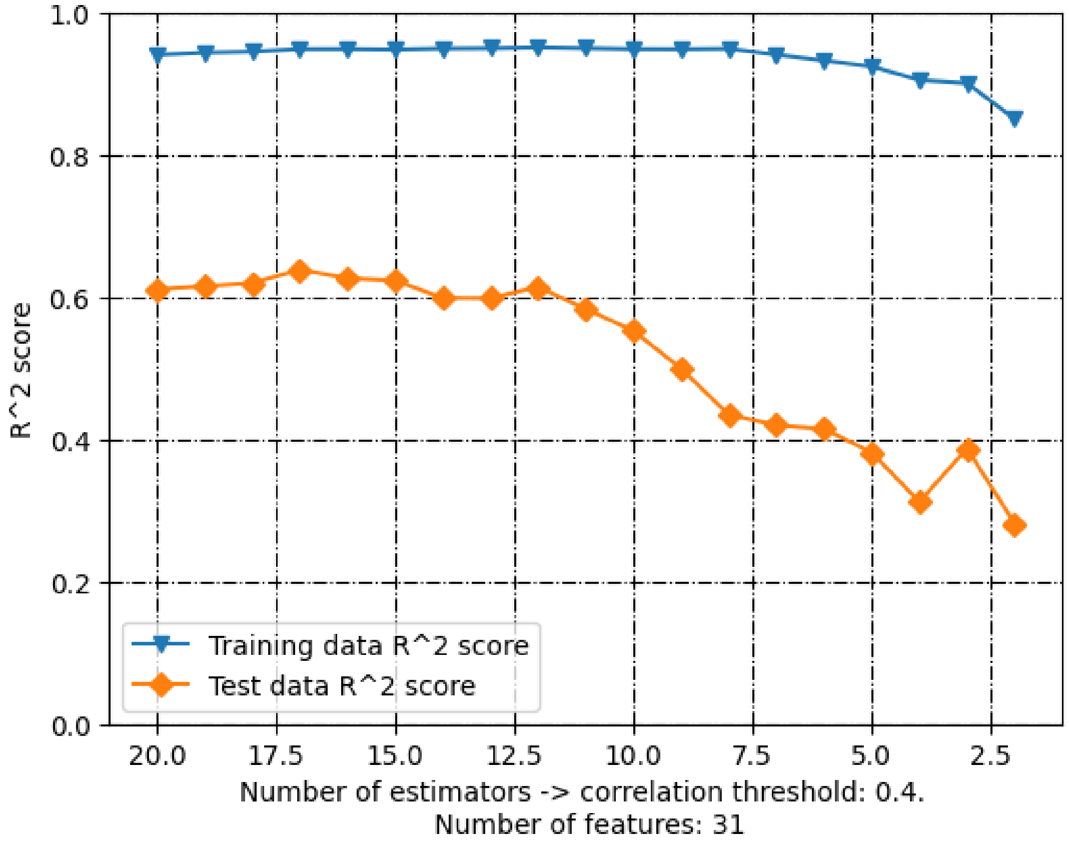
1.0
0.8
0.6
0.4
0.2
0.0
R^2 score
Training data R^2 score
Test data R^2 score
20.0
17.5
15.0
12.5
10.0
7.5
5.0
2.5
Number of estimators -> correlation threshold: 0.4.
Number of features: 31

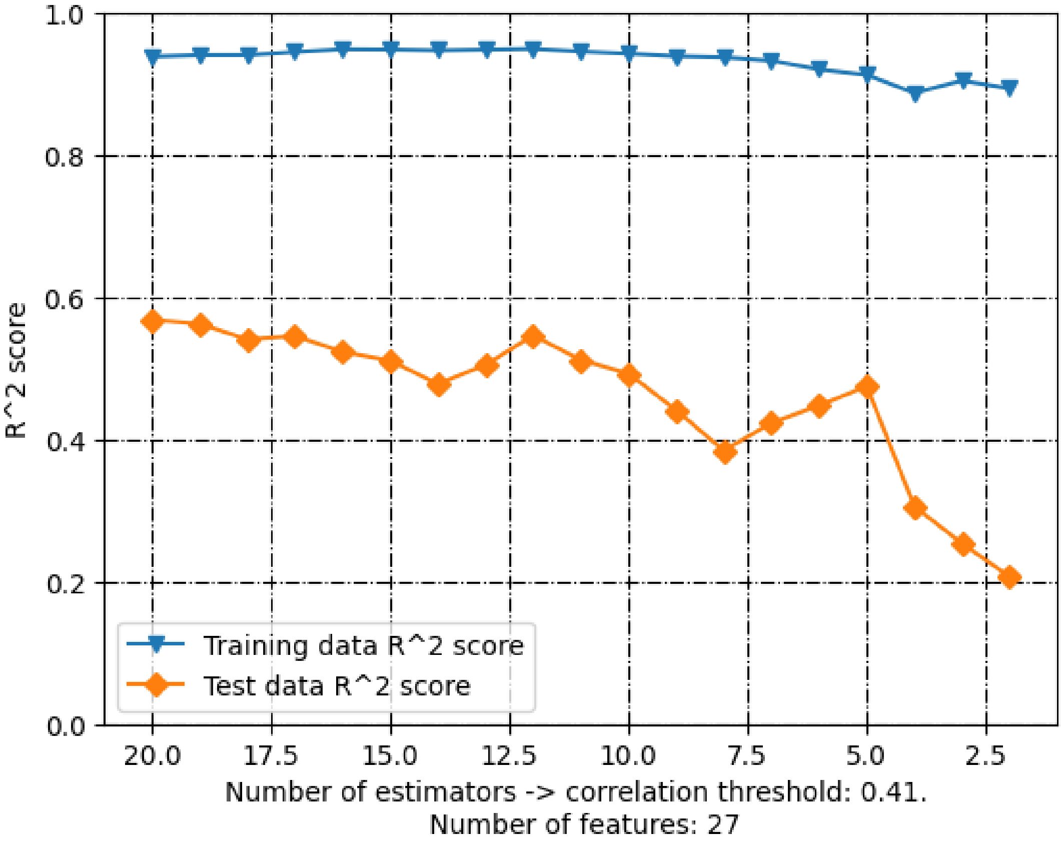

1.0
0.8
0.6
0.4
0.2
0.0
R^2 score
20.0
17.5
15.0
12.5
10.0
7.5
5.0
2.5
Number of estimators -> correlation threshold: 0.41.
Number of features: 27
Training data R^2 score
Test data R^2 score

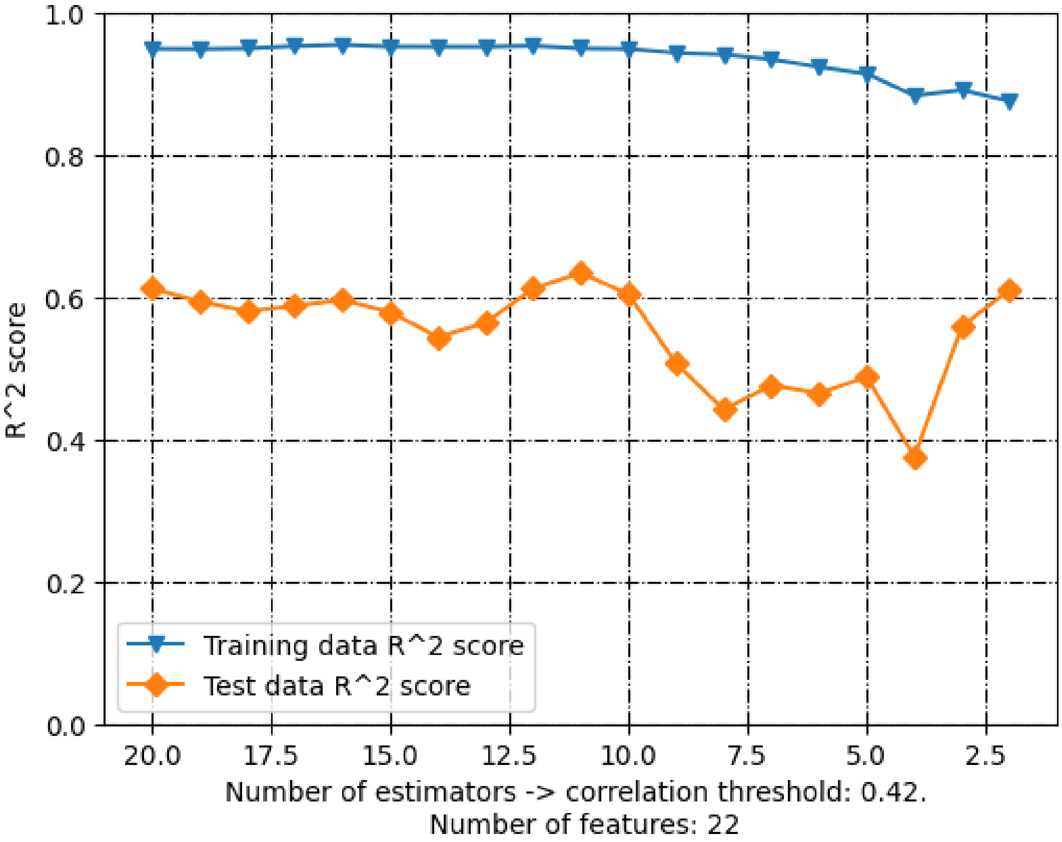

1.0
0.8
0.6
0.4
0.2
0.0
R^2 score
Training data R^2 score
Test data R^2 score
20.0
17.5
15.0
12.5
10.0
7.5
5.0
2.5
Number of estimators -> correlation threshold: 0.42.
Number of features: 22

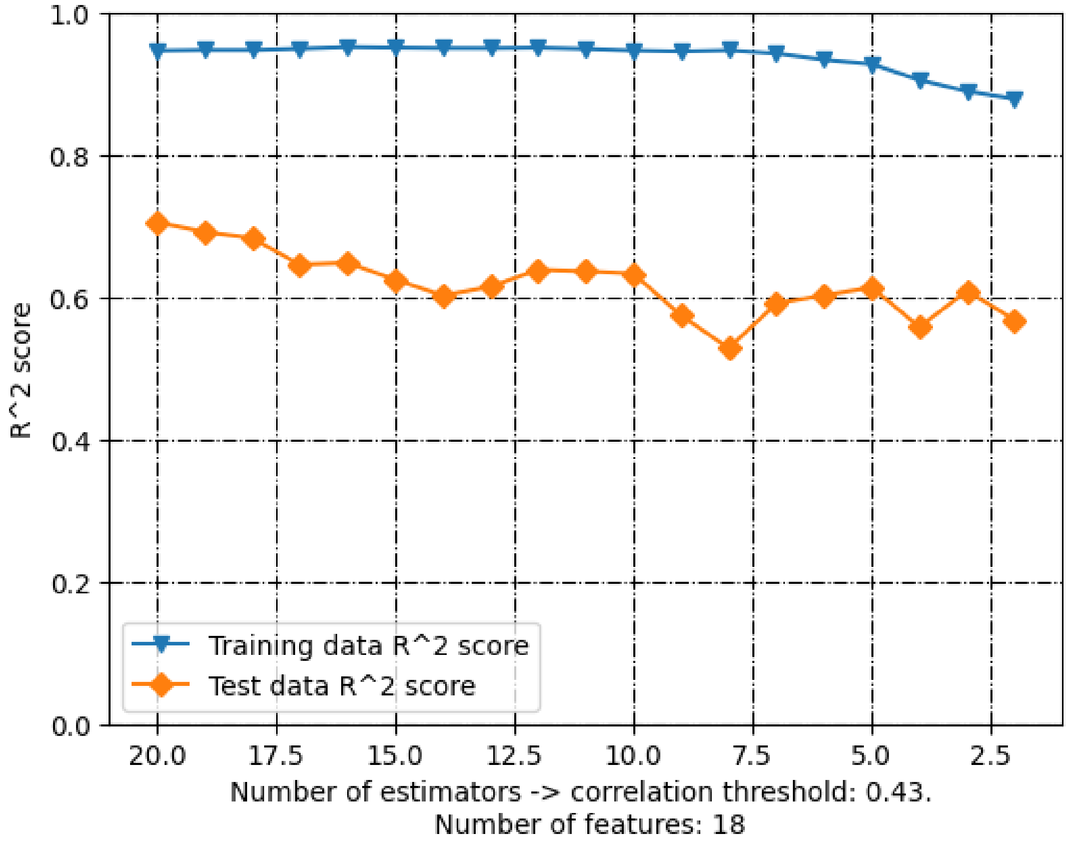
1.0
0.8
0.6
0.4
0.2
0.0
R^2 score
Training data R^2 score
Test data R^2 score
20.0
17.5
15.0
12.5
10.0
7.5
5.0
2.5
Number of estimators -> correlation threshold: 0.43.
Number of features: 18

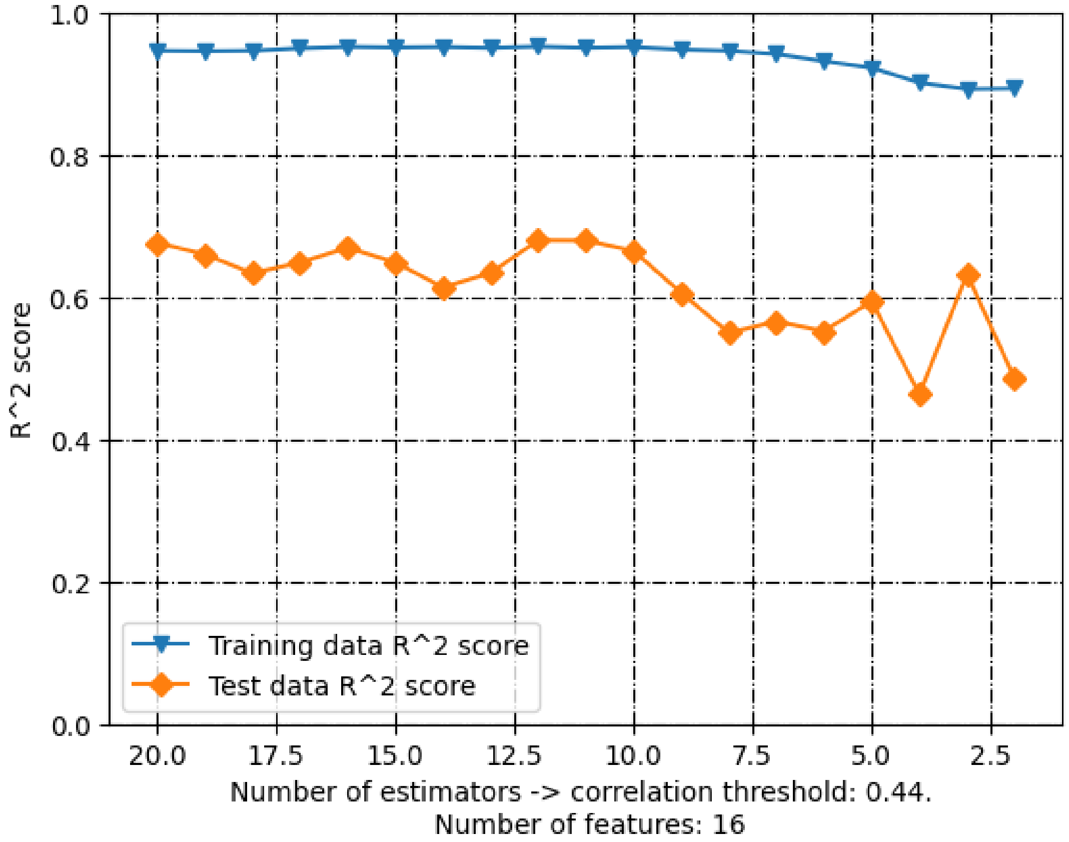

1.0
0.8
0.6
0.4
0.2
0.0
R^2 score
20.0
17.5
15.0
12.5
10.0
7.5
5.0
2.5
Training data R^2 score
Test data R^2 score
Number of estimators -> correlation threshold: 0.44.
Number of features: 16

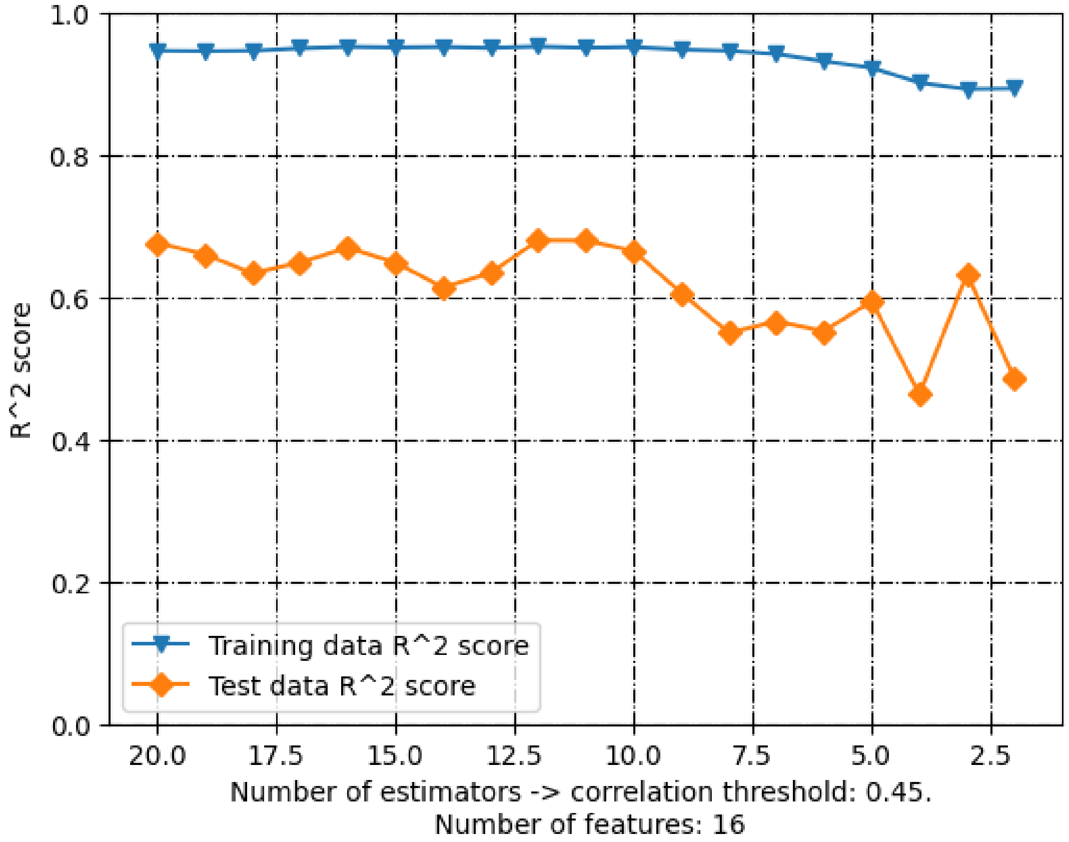
1.0
0.8
0.6
0.4
0.2
0.0
R^2 score
Training data R^2 score
Test data R^2 score
20.0
17.5
15.0
12.5
10.0
7.5
5.0
2.5
Number of estimators -> correlation threshold: 0.45.
Number of features: 16

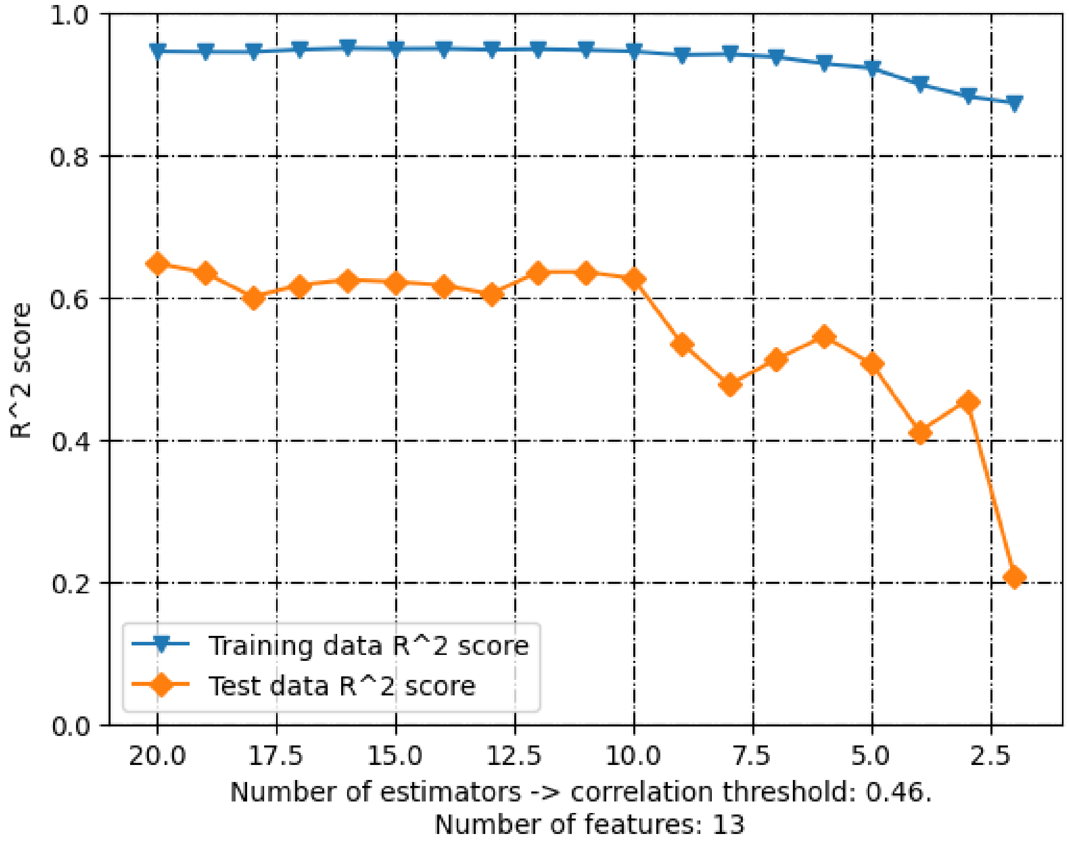
1.0
0.8
0.6
0.4
0.2
0.0
R^2 score
Training data R^2 score
Test data R^2 score
20.0
17.5
15.0
12.5
10.0
7.5
5.0
2.5
Number of estimators -> correlation threshold: 0.46.
Number of features: 13

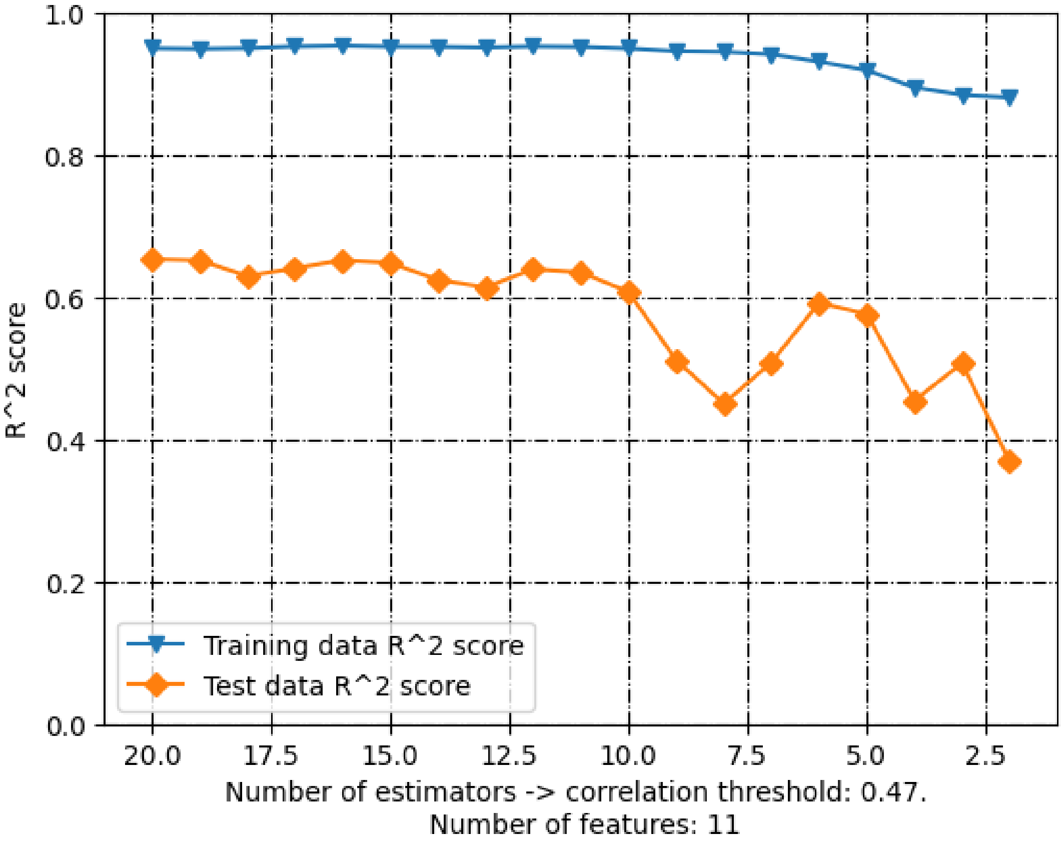

1.0
0.8
0.6
0.4
0.2
0.0
R^2 score
Training data R^2 score
Test data R^2 score
20.0
17.5
15.0
12.5
10.0
7.5
5.0
2.5
Number of estimators -> correlation threshold: 0.47.
Number of features: 11

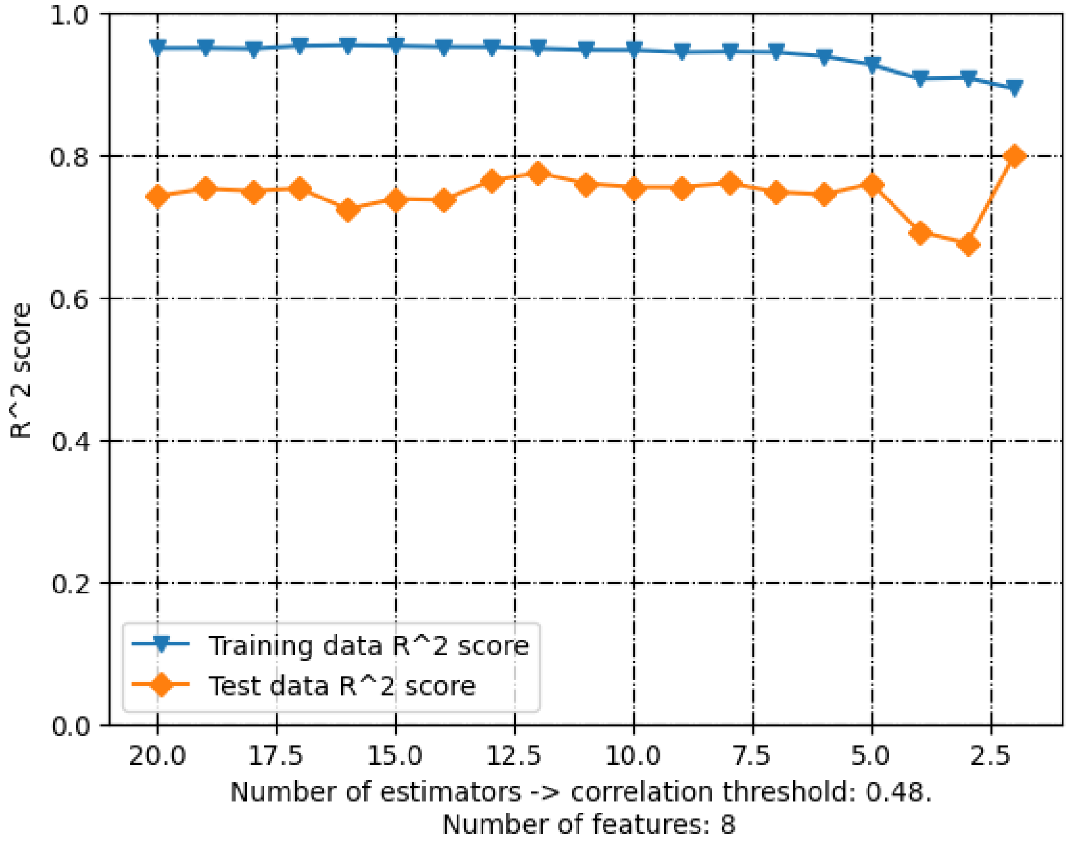

1.0
0.8
0.6
0.4
0.2
0.0
R^2 score
20.0
17.5
15.0
12.5
10.0
7.5
5.0
2.5
Training data R^2 score
Test data R^2 score
Number of estimators -> correlation threshold: 0.48.
Number of features: 8

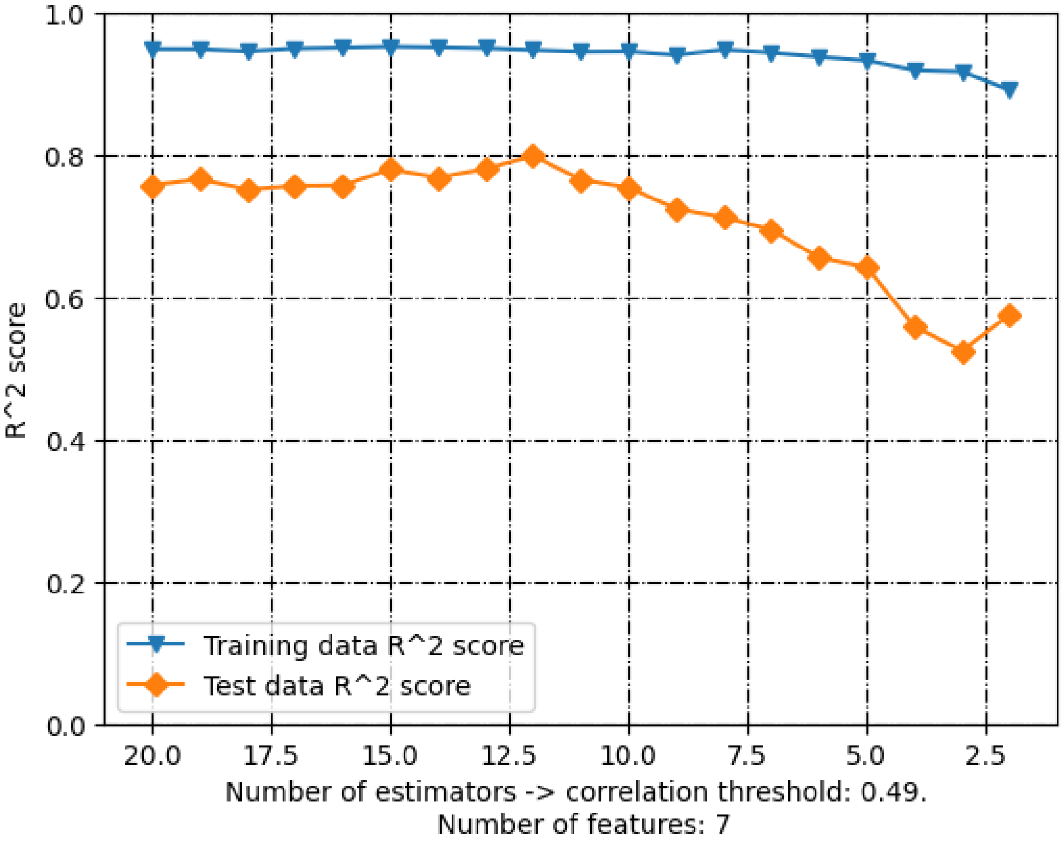
1.0
0.8
0.6
0.4
0.2
0.0
R^2 score
Training data R^2 score
Test data R^2 score
20.0
17.5
15.0
12.5
10.0
7.5
5.0
2.5
Number of estimators -> correlation threshold: 0.49.
Number of features: 7

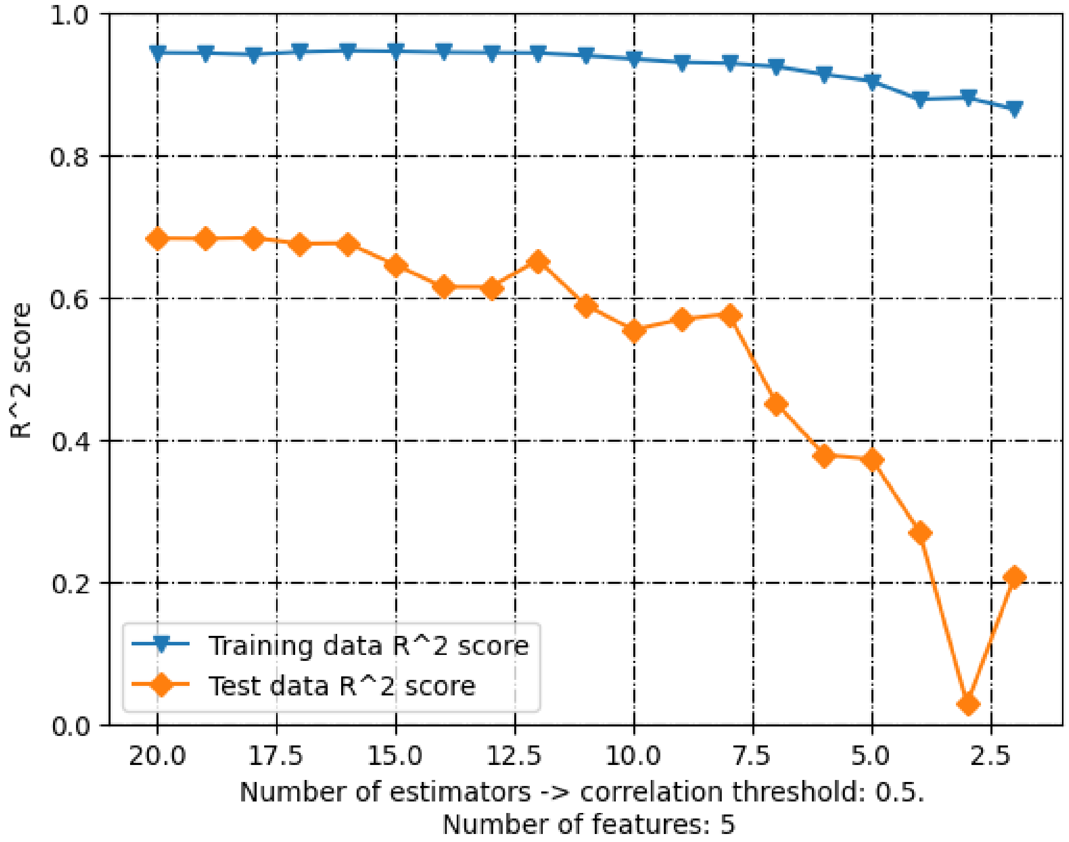
1.0
0.8
0.6
0.4
0.2
0.0
R^2 score
20.0
17.5
15.0
12.5
10.0
7.5
5.0
2.5
Training data R^2 score
Test data R^2 score
Number of estimators -> correlation threshold: 0.5.
Number of features: 5

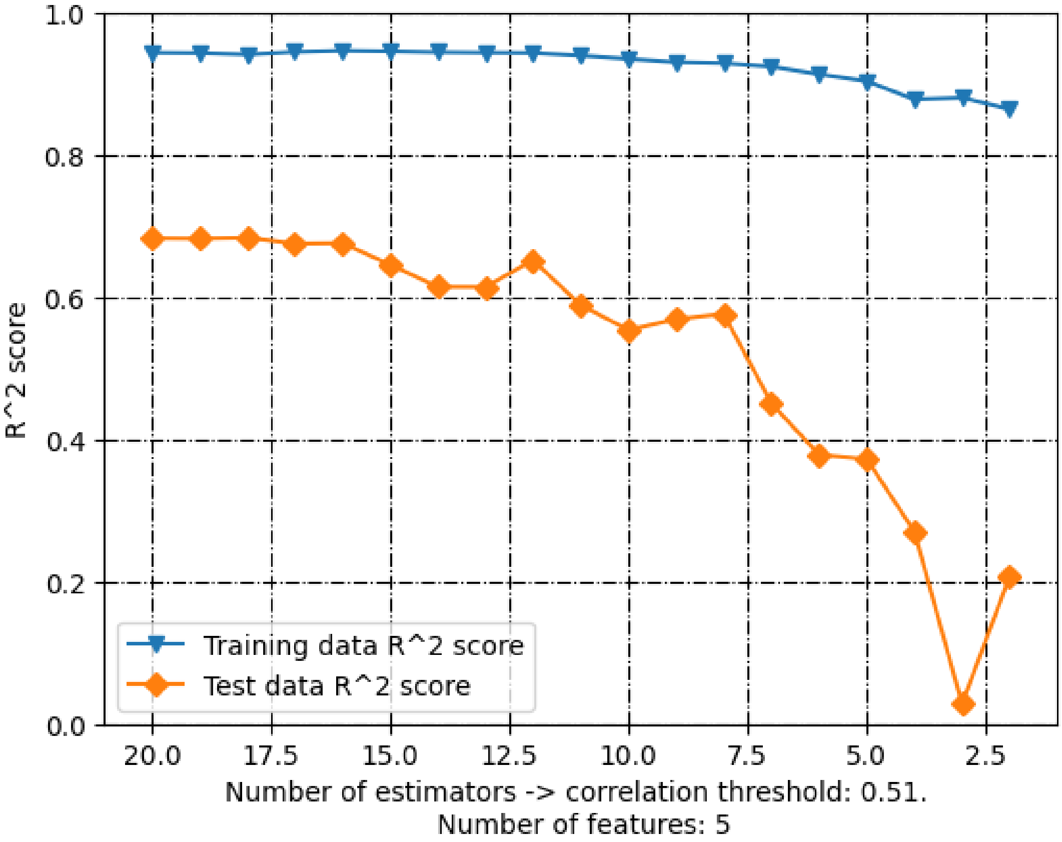

1.0
0.8
0.6
0.4
0.2
0.0
R^2 score
20.0
17.5
15.0
12.5
10.0
7.5
5.0
2.5
Number of estimators -> correlation threshold: 0.51.
Number of features: 5
Training data R^2 score
Test data R^2 score

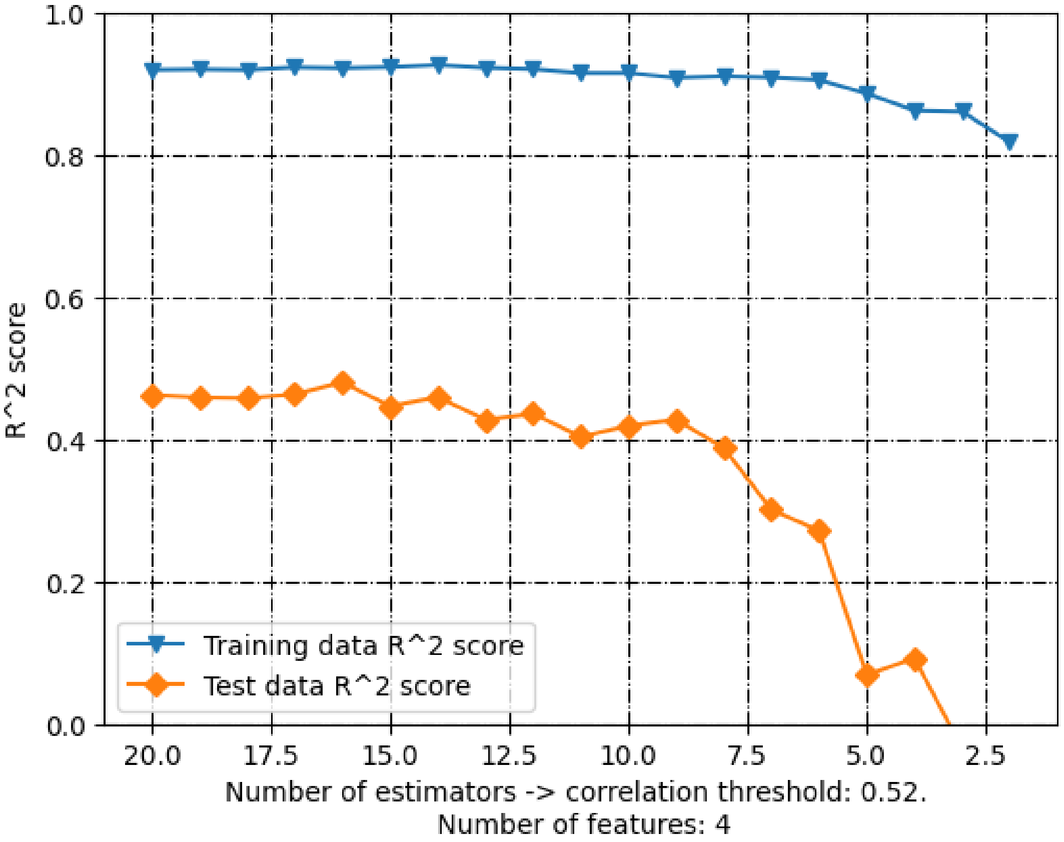

1.0
0.8
0.6
0.4
0.2
0.0
R^2 score
Training data R^2 score
Test data R^2 score
20.0
17.5
15.0
12.5
10.0
7.5
5.0
2.5
Number of estimators -> correlation threshold: 0.52.
Number of features: 4

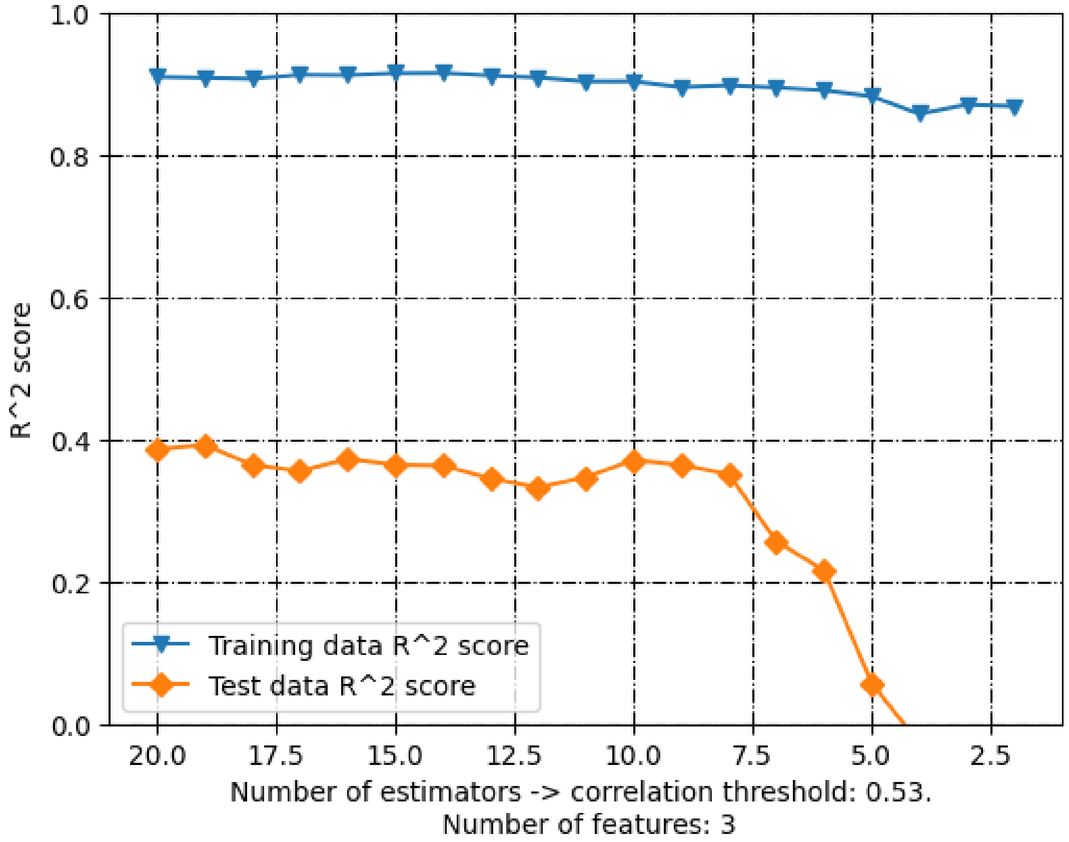

[28]:
```
plt.plot(df_without_standardization['Correlation threshold'],␣
  ↪df_without_standardization['Number of features'], label = "Number of␣
  ↪features", marker='.')
plt.legend()
plt.xlabel('Correlation threshold')
plt.ylabel('Number of features')
plt.show()
```

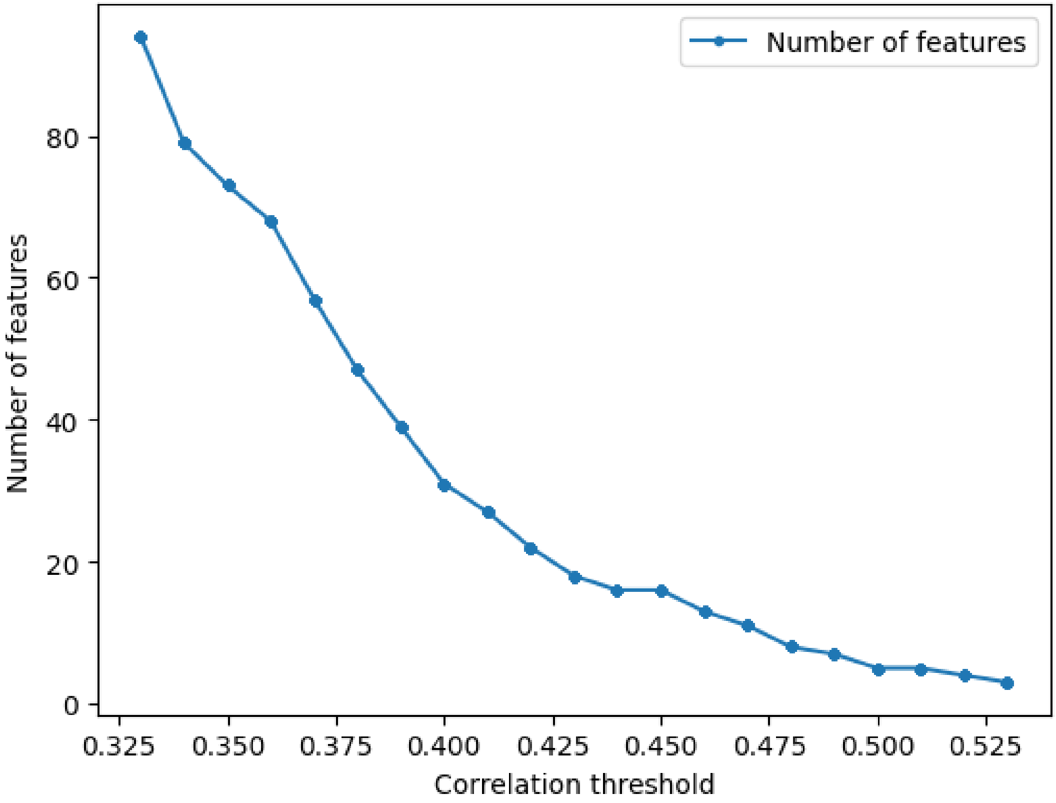

[ ]:

4 KNeighborsRegressor

[29]:
```
without_standardization, train_r2_, test_r2_, hist1, hist2, target_column_name,␣
↪training_data_RMSE, test_data_RMSE  = pred_model.
↪prepare_data_and_create_model(molecular_descriptors_df=data,
                                                                          ␣
↪                    correlation_threshold=0.50,
                                                                          ␣
↪                    standardization=False,
                                                                          ␣
↪                    model_type='KNeighborsRegressor',
                                                                          ␣
↪                    target_column_name = target,
                                                                          ␣
↪                    random_state=random_state,
                                                                          ␣
↪                    train_test_split_=True,
```

```
↪                     verbose=True)
```

```
I am not doing standardization…
  molecular descriptor name
0                    AATS0Z
1                  AATS0are
2                    AATS0d
3                   AATS0dv
4                    AATS0i
  molecular descriptor name  corr_value
0                    AATS0Z   -0.022797
1                  AATS0are   -0.139064
2                    AATS0d    0.027592
3                   AATS0dv   -0.136049
4                    AATS0i    0.168958
  molecular descriptor name  corr_value  absolute correlation value
0                    AATS0Z   -0.022797                    0.022797
1                  AATS0are   -0.139064                    0.139064
2                    AATS0d    0.027592                    0.027592
3                   AATS0dv   -0.136049                    0.136049
4                    AATS0i    0.168958                    0.168958
     molecular descriptor name  corr_value  absolute correlation value
226                     AMID_O   -0.557717                    0.557717
505                 EState_VSA5   -0.534748                    0.534748
791                    MDEO-12   -0.555724                    0.555724
921                      SaasC    0.513071                    0.513071
1091               VSA_EState5    0.526082                    0.526082
     molecular descriptor name  corr_value  absolute correlation value
226                     AMID_O   -0.557717                    0.557717
505                 EState_VSA5   -0.534748                    0.534748
791                    MDEO-12   -0.555724                    0.555724
921                      SaasC    0.513071                    0.513071
1091               VSA_EState5    0.526082                    0.526082
The model used is: KNeighbors…
Return the coefficient of determination of the prediction:
0.04184254994027059
R^2 score: 0.6331583890645831
Correlation coefficient: 0.7957125040267893
Test data - unseen during training:
R^2 score: 0.04184254994027059
Correlation coefficient: 0.2045545158149059
[7.82808927 5.36946488 7.66040754 5.36946488 7.36955586 7.94867293
 7.97341012 7.91497614 5.86629945 7.36955586 8.14630219 7.59157705
 7.62943875 8.0759456  7.94867293 7.15646789 7.62615027 6.93758129]
113    7.823909
35     6.053057
101    6.721246
```

```
36     6.221126
100    7.130768
13     7.966576
0      7.966576
114    7.920819
104    8.309804
96     7.102373
40     7.920819
103    8.397940
48     7.853872
39     8.356547
14     8.148742
117    6.031517
21     7.325139
9      6.818728
Name: A549, dtype: float64
Training Root Mean Square Error: 0.5915919890634596
Testing Root Mean Square Error: 0.7606515443663695
```

[30]:
```
print(target_column_name+str('_transformed'))
print(hist1[target_column_name].hist())
```

```
A549_transformed
AxesSubplot(0.125,0.11;0.775x0.77)
```

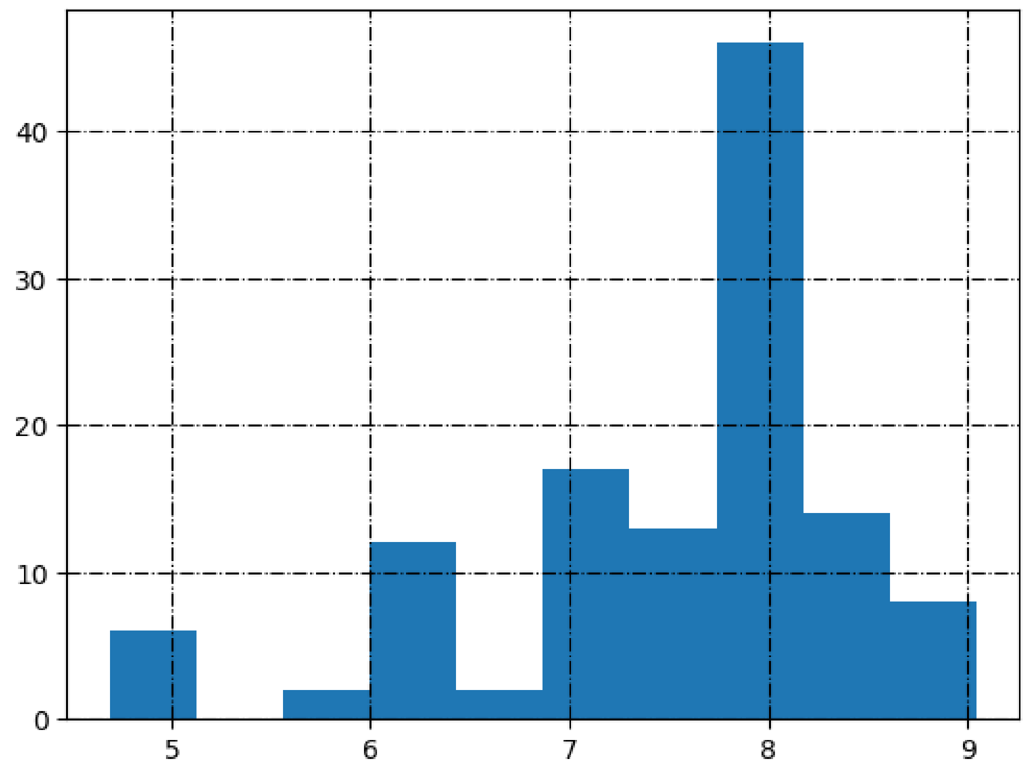

[31]:
```
with_standardization, train_r2_, test_r2_, hist1, hist2, target_column_name,␣
↪training_data_RMSE, test_data_RMSE  = pred_model.
↪prepare_data_and_create_model(molecular_descriptors_df=data,
                                                                          ␣
↪                    correlation_threshold=0.50,
                                                                          ␣
↪                    standardization=True,
                                                                          ␣
↪                    model_type='KNeighborsRegressor',
                                                                          ␣
↪                    target_column_name = target,
                                                                          ␣
↪                    random_state=random_state,
                                                                          ␣
↪                    train_test_split_=True,
                                                                          ␣
↪                    verbose=True)
```

```
I am doing standardization…
  molecular descriptor name
0                    AATS0Z
1                  AATS0are
2                    AATS0d
3                   AATS0dv
4                    AATS0i
  molecular descriptor name  corr_value
0                    AATS0Z   -0.022797
1                  AATS0are   -0.139064
2                    AATS0d    0.027592
3                   AATS0dv   -0.136049
4                    AATS0i    0.168958
  molecular descriptor name  corr_value  absolute correlation value
0                    AATS0Z   -0.022797                    0.022797
1                  AATS0are   -0.139064                    0.139064
2                    AATS0d    0.027592                    0.027592
3                   AATS0dv   -0.136049                    0.136049
4                    AATS0i    0.168958                    0.168958
     molecular descriptor name  corr_value  absolute correlation value
226                     AMID_O   -0.557717                    0.557717
505                 EState_VSA5   -0.534748                    0.534748
791                    MDEO-12   -0.555724                    0.555724
921                      SaasC    0.513071                    0.513071
1091                VSA_EState5    0.526082                    0.526082
     molecular descriptor name  corr_value  absolute correlation value
226                     AMID_O   -0.557717                    0.557717
505                 EState_VSA5   -0.534748                    0.534748
```

```
791                   MDEO-12   -0.555724                    0.555724
921                     SaasC    0.513071                    0.513071
1091                VSA_EState5    0.526082                    0.526082
The model used is: KNeighbors…
Return the coefficient of determination of the prediction:
0.04184254994027059
R^2 score: 0.6331583890645831
Correlation coefficient: 0.7957125040267893
Test data - unseen during training:
R^2 score: 0.04184254994027059
Correlation coefficient: 0.2045545158149059
[7.82808927 5.36946488 7.66040754 5.36946488 7.36955586 7.94867293
 7.97341012 7.91497614 5.86629945 7.36955586 8.14630219 7.59157705
 7.62943875 8.0759456  7.94867293 7.15646789 7.62615027 6.93758129]
113    7.823909
35     6.053057
101    6.721246
36     6.221126
100    7.130768
13     7.966576
0      7.966576
114    7.920819
104    8.309804
96     7.102373
40     7.920819
103    8.397940
48     7.853872
39     8.356547
14     8.148742
117    6.031517
21     7.325139
9      6.818728
Name: A549, dtype: float64
Training Root Mean Square Error: 0.5915919890634596
Testing Root Mean Square Error: 0.7606515443663695
```

4.1 Search inside correlation space

[32]:
```
step = 0.01
initial_step = corr_low
last_step = corr_high
first_list = [x / 100.0 for x in range(int(initial_step*100),␣
 ↪int(last_step*100), int(step*100))]
second_list = []
third_list = []
fourth_l = []
fifth_l = []
```

```
f_list = []
for i in first_list:
    without_standardization, train_r2, test_r2, _, h_, target_column_name,␣
 ↪training_data_RMSE, test_data_RMSE = pred_model.
 ↪prepare_data_and_create_model(molecular_descriptors_df = data,
                                                                             ␣
 ↪                    correlation_threshold = i,
                                                                             ␣
 ↪                    standardization = False,
                                                                             ␣
 ↪                    model_type = 'KNeighborsRegressor',
                                                                             ␣
 ↪                    target_column_name = target,
                                                                             ␣
 ↪                    random_state=random_state,
                                                                             ␣
 ↪                    train_test_split_ = True,
                                                                             ␣
 ↪                    verbose = False)
    second_list.append(train_r2)
    third_list.append(test_r2)
    fourth_l.append(training_data_RMSE)
    fifth_l.append(test_data_RMSE)
    f_list.append(len(h_))
```

```
  File "C:\Users\aleks\anaconda3\envs\cheminf_gpu\lib\site-
packages\joblib\externals\loky\backend\context.py", line 217, in
_count_physical_cores
    raise ValueError(
```

[33]:
```
df_without_standardization = pd.DataFrame(data=first_list,␣
 ↪columns=["Correlation threshold"])
df_without_standardization['Training data R^2 score'] = second_list
df_without_standardization['Test data R^2 score'] = third_list
df_without_standardization['Training RMSE'] = fourth_l
df_without_standardization['Test RMSE'] = fifth_l
df_without_standardization['Number of features'] = f_list
```

[34]:
```
df_k_nearest = df_without_standardization.copy()
df_without_standardization
```

[34]:

	Correlation threshold	Training data R^2 score	Test data R^2 score	\
0	0.33	0.420017	0.343014	
1	0.34	0.619363	-0.102421	
2	0.35	0.611502	-0.115525	
3	0.36	0.602405	-0.103076	
4	0.37	0.616706	-0.110209	

5	0.38	0.691690	0.281519
6	0.39	0.668597	0.292029
7	0.40	0.634598	0.193658
8	0.41	0.634598	0.193658
9	0.42	0.630124	0.184532
10	0.43	0.642788	0.224969
11	0.44	0.641698	0.224344
12	0.45	0.641698	0.224344
13	0.46	0.569757	-0.097736
14	0.47	0.555440	-0.040812
15	0.48	0.679077	0.100854
16	0.49	0.644649	0.064377
17	0.50	0.633158	0.041843
18	0.51	0.633158	0.041843
19	0.52	0.645061	-0.235118
20	0.53	0.624328	-0.270410

	Training RMSE	Test RMSE	Number of features
0	0.743859	0.629862	94
1	0.602613	0.815908	79
2	0.608804	0.820743	73
3	0.615890	0.816150	68
4	0.604712	0.818785	57
5	0.542346	0.658681	47
6	0.562291	0.653845	39
7	0.590430	0.697794	31
8	0.590430	0.697794	27
9	0.594033	0.701731	22
10	0.583776	0.684112	18
11	0.584666	0.684387	16
12	0.584666	0.684387	16
13	0.640678	0.814172	13
14	0.651251	0.792781	11
15	0.553329	0.736856	8
16	0.582253	0.751654	7
17	0.591592	0.760652	5
18	0.591592	0.760652	5
19	0.581916	0.863618	4
20	0.598670	0.875869	3

4.2 Plots

[35]:
```
plt.plot(df_without_standardization['Correlation threshold'],
    df_without_standardization['Training data R^2 score'], label = "Training
    data R^2 score", marker='v')
```

```
plt.plot(df_without_standardization['Correlation threshold'],␣
  ↪df_without_standardization['Test data R^2 score'], label = "Test data R^2␣
  ↪score", marker='D')
plt.legend()
plt.xlabel('Correlation threshold')
plt.ylabel('R^2 score')
plt.ylim([0, 1])
plt.show()
```

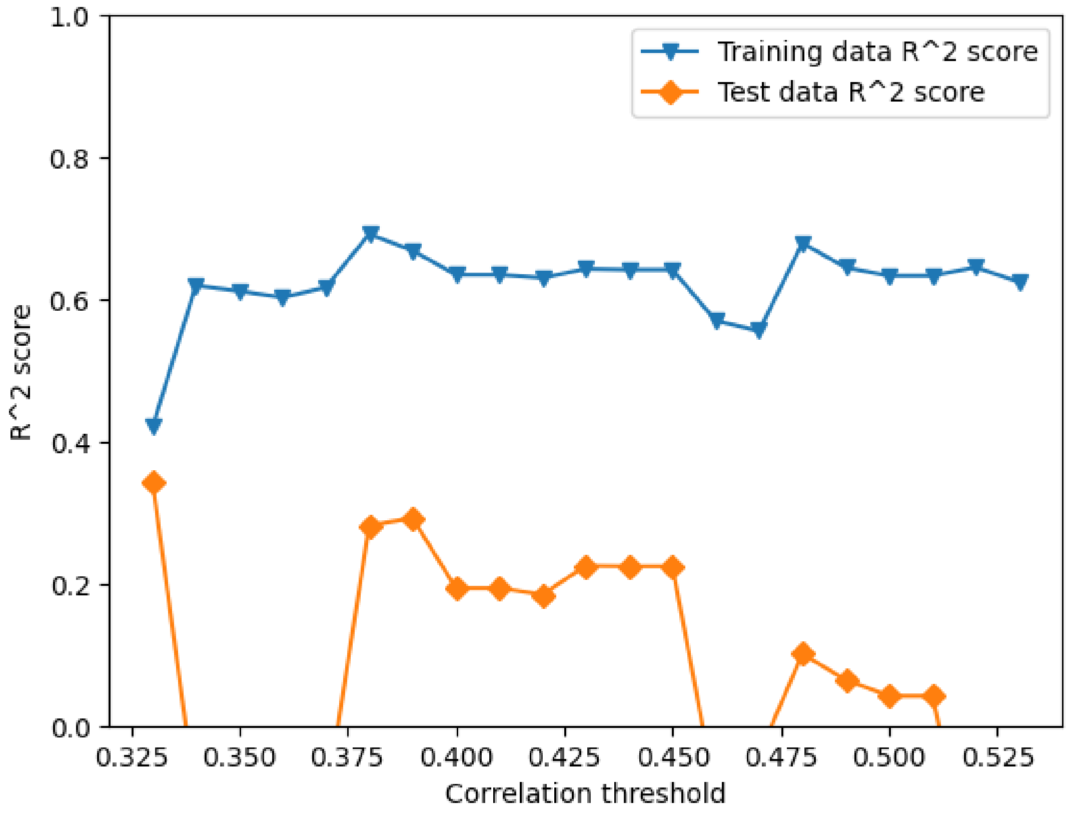

[36]:
```
plt.plot(df_without_standardization['Correlation threshold'],␣
  ↪df_without_standardization['Number of features'], label = "Number of␣
  ↪features", marker='.')
plt.legend()
plt.xlabel('Correlation threshold')
plt.ylabel('Number of features')
plt.show()
```

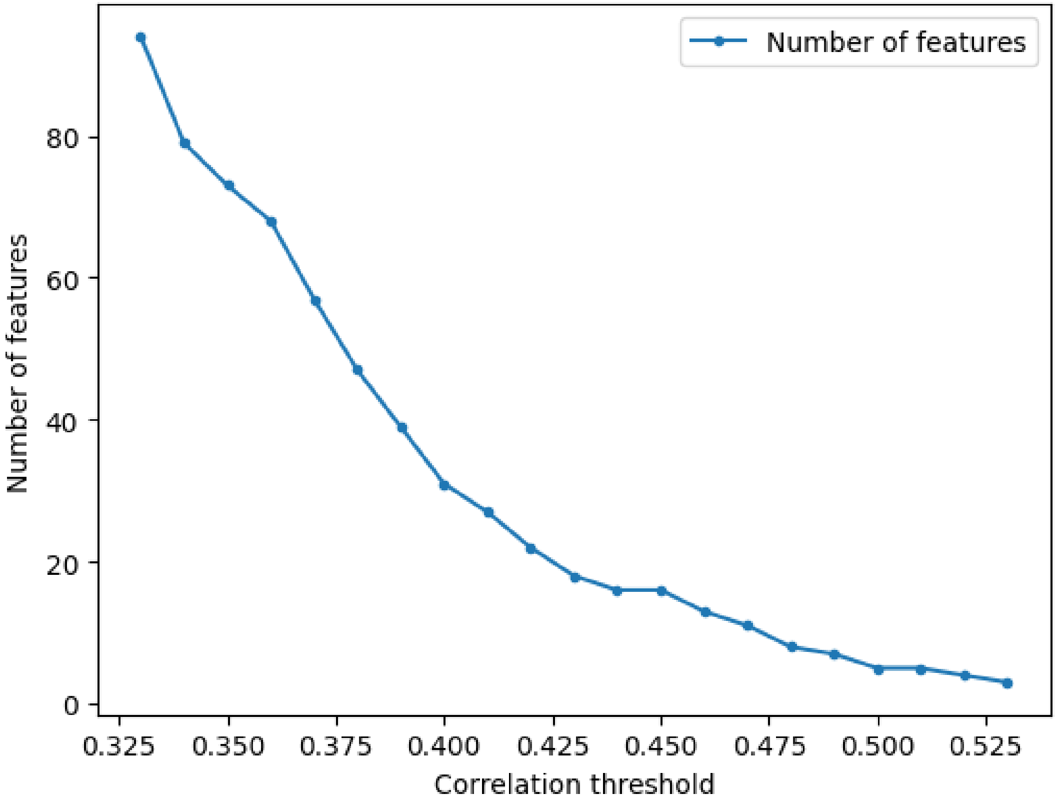

[ ]:

5 Support Vector Machines (SVM)

[37]:
```
without_standardization, train_r2_, test_r2_, hist1, hist2, target_column_name,␣
↪training_data_RMSE, test_data_RMSE  = pred_model.
↪prepare_data_and_create_model(molecular_descriptors_df=data,
                                                                              ␣
↪                          correlation_threshold=0.50,
                                                                              ␣
↪                          standardization=False,
                                                                              ␣
↪                          model_type='SVR',
                                                                              ␣
↪                          kernel_ = 'linear',
                                                                              ␣
↪                          gamma_ = 'auto',
                                                                              ␣
↪                          target_column_name = target,
```

```
                      random_state=random_state,
                      train_test_split_=True,
                      verbose=True)
```

```
I am not doing standardization…
  molecular descriptor name
0                    AATS0Z
1                  AATS0are
2                    AATS0d
3                   AATS0dv
4                    AATS0i
  molecular descriptor name  corr_value
0                    AATS0Z   -0.022797
1                  AATS0are   -0.139064
2                    AATS0d    0.027592
3                   AATS0dv   -0.136049
4                    AATS0i    0.168958
  molecular descriptor name  corr_value  absolute correlation value
0                    AATS0Z   -0.022797                    0.022797
1                  AATS0are   -0.139064                    0.139064
2                    AATS0d    0.027592                    0.027592
3                   AATS0dv   -0.136049                    0.136049
4                    AATS0i    0.168958                    0.168958
     molecular descriptor name  corr_value  absolute correlation value
226                     AMID_O   -0.557717                    0.557717
505                EState_VSA5   -0.534748                    0.534748
791                   MDEO-12   -0.555724                    0.555724
921                      SaasC    0.513071                    0.513071
1091               VSA_EState5    0.526082                    0.526082
     molecular descriptor name  corr_value  absolute correlation value
226                     AMID_O   -0.557717                    0.557717
505                EState_VSA5   -0.534748                    0.534748
791                   MDEO-12   -0.555724                    0.555724
921                      SaasC    0.513071                    0.513071
1091               VSA_EState5    0.526082                    0.526082
The model used is: SVR…
Return the coefficient of determination of the prediction:
0.32851122549444245
R^2 score: 0.45734753152418484
Correlation coefficient: 0.6762747455910096
Test data - unseen during training:
R^2 score: 0.32851122549444245
Correlation coefficient: 0.5731589879731822
[8.01131583 6.40143028 7.71342937 6.4509997  7.79496933 7.71369879
```

```
 7.8148241  8.15682258 6.70338719 7.77214303 7.96205722 7.91996377
 7.9676105  7.59625243 7.69566933 6.9732833  7.9711214  6.41709421]
113    7.823909
35     6.053057
101    6.721246
36     6.221126
100    7.130768
13     7.966576
0      7.966576
114    7.920819
104    8.309804
96     7.102373
40     7.920819
103    8.397940
48     7.853872
39     8.356547
14     8.148742
117    6.031517
21     7.325139
9      6.818728
Name: A549, dtype: float64
Training Root Mean Square Error: 0.7195216728556674
Testing Root Mean Square Error: 0.6367760041839747
```

[38]:

```python
print(target_column_name+str('_transformed'))
print(hist1[target_column_name].hist())
```

```
A549_transformed
AxesSubplot(0.125,0.11;0.775x0.77)
```

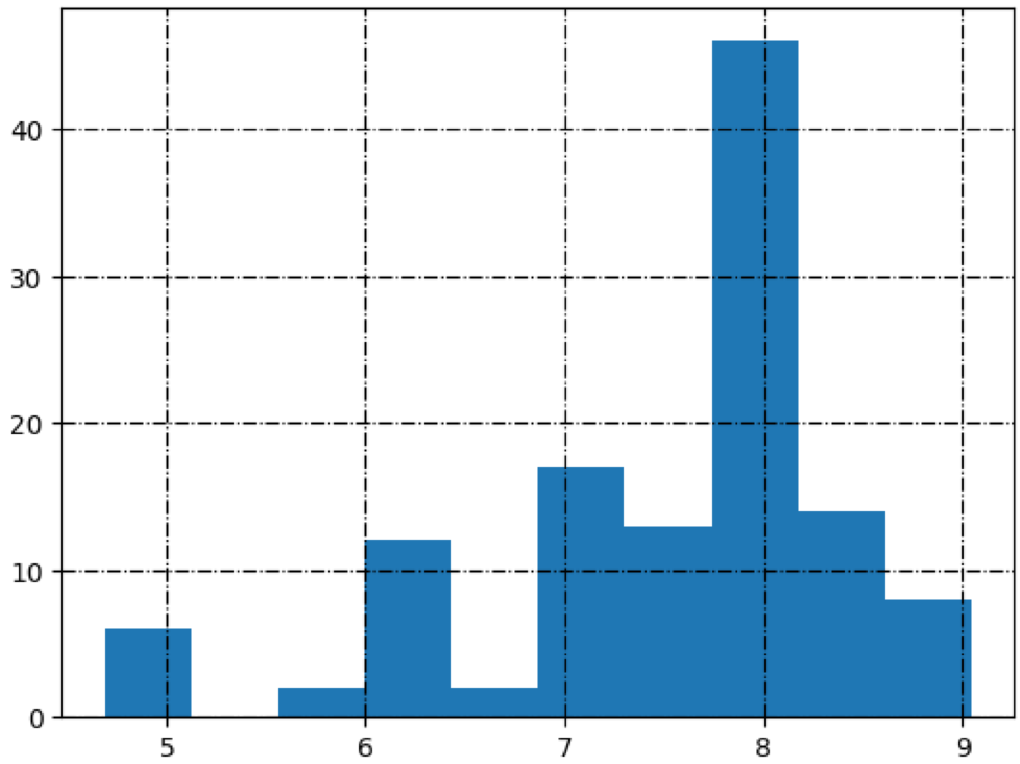

[39]:
```
with_standardization, train_r2_, test_r2_, hist1, hist2, target_column_name,␣
↪training_data_RMSE, test_data_RMSE  = pred_model.
↪prepare_data_and_create_model(molecular_descriptors_df=data,
                                                                              ␣
↪                         correlation_threshold=0.50,
                                                                              ␣
↪                         standardization=True,
                                                                              ␣
↪                         model_type='SVR',
                                                                              ␣
↪                         kernel_ = 'linear',
                                                                              ␣
↪                         gamma_ = 'auto',
                                                                              ␣
↪                         target_column_name = target,
                                                                              ␣
↪                         random_state=random_state,
                                                                              ␣
↪                         train_test_split_=True,
                                                                              ␣
↪                         verbose=True)
```

```
I am doing standardization…
  molecular descriptor name
0                    AATS0Z
1                  AATS0are
2                    AATS0d
3                   AATS0dv
4                    AATS0i
  molecular descriptor name  corr_value
0                    AATS0Z   -0.022797
1                  AATS0are   -0.139064
2                    AATS0d    0.027592
3                   AATS0dv   -0.136049
4                    AATS0i    0.168958
  molecular descriptor name  corr_value  absolute correlation value
0                    AATS0Z   -0.022797                    0.022797
1                  AATS0are   -0.139064                    0.139064
2                    AATS0d    0.027592                    0.027592
3                   AATS0dv   -0.136049                    0.136049
4                    AATS0i    0.168958                    0.168958
     molecular descriptor name  corr_value  absolute correlation value
226                     AMID_O   -0.557717                    0.557717
505                EState_VSA5   -0.534748                    0.534748
791                    MDEO-12   -0.555724                    0.555724
921                      SaasC    0.513071                    0.513071
1091               VSA_EState5    0.526082                    0.526082
     molecular descriptor name  corr_value  absolute correlation value
226                     AMID_O   -0.557717                    0.557717
505                EState_VSA5   -0.534748                    0.534748
791                    MDEO-12   -0.555724                    0.555724
921                      SaasC    0.513071                    0.513071
1091               VSA_EState5    0.526082                    0.526082
The model used is: SVR…
Return the coefficient of determination of the prediction:
0.32851122549444245
R^2 score: 0.45734753152418484
Correlation coefficient: 0.6762747455910096
Test data - unseen during training:
R^2 score: 0.32851122549444245
Correlation coefficient: 0.5731589879731822
[8.01131583 6.40143028 7.71342937 6.4509997  7.79496933 7.71369879
 7.8148241  8.15682258 6.70338719 7.77214303 7.96205722 7.91996377
 7.9676105  7.59625243 7.69566933 6.9732833  7.9711214  6.41709421]
113    7.823909
35     6.053057
101    6.721246
36     6.221126
100    7.130768
13     7.966576
```

```
0      7.966576
114    7.920819
104    8.309804
96     7.102373
40     7.920819
103    8.397940
48     7.853872
39     8.356547
14     8.148742
117    6.031517
21     7.325139
9      6.818728
Name: A549, dtype: float64
Training Root Mean Square Error: 0.7195216728556674
Testing Root Mean Square Error: 0.6367760041839747
```

5.1 Search inside correlation space

[40]:
```
step = 0.01
initial_step = corr_low
last_step = corr_high
first_list = [x / 100.0 for x in range(int(initial_step*100),␣
 ↪int(last_step*100), int(step*100))]
second_list = []
third_list = []
fourth_l = []
fifth_l = []
f_list = []
for i in first_list:
    without_standardization, train_r2, test_r2, _, h_, target_column_name,␣
 ↪training_data_RMSE, test_data_RMSE = pred_model.
 ↪prepare_data_and_create_model(molecular_descriptors_df = data,
                                                                           ␣
 ↪                      correlation_threshold = i,
                                                                           ␣
 ↪                      standardization = False,
                                                                           ␣
 ↪                      model_type = 'SVR',
                                                                           ␣
 ↪                      kernel_ = 'linear',
                                                                           ␣
 ↪                      gamma_ = 'auto',
                                                                           ␣
 ↪                      target_column_name = target,
                                                                           ␣
 ↪                      random_state=random_state,
```

```
                      train_test_split_ = True,
                      verbose = False)
    second_list.append(train_r2)
    third_list.append(test_r2)
    fourth_l.append(training_data_RMSE)
    fifth_l.append(test_data_RMSE)
    f_list.append(len(h_))
```

[41]:
```
df_without_standardization = pd.DataFrame(data=first_list, columns=["Correlation threshold"])
df_without_standardization['Training data R^2 score'] = second_list
df_without_standardization['Test data R^2 score'] = third_list
df_without_standardization['Training RMSE'] = fourth_l
df_without_standardization['Test RMSE'] = fifth_l
df_without_standardization['Number of features'] = f_list
```

[42]:
```
df_svm = df_without_standardization.copy()
df_without_standardization
```

[42]:
```
    Correlation threshold  Training data R^2 score  Test data R^2 score  \
0                    0.33                 0.815717            -0.376776
1                    0.34                 0.777201            -0.358594
2                    0.35                 0.777664            -0.306293
3                    0.36                 0.755687            -0.480017
4                    0.37                 0.744621            -0.513089
5                    0.38                 0.688560             0.099718
6                    0.39                 0.629787             0.282437
7                    0.40                 0.617305             0.228248
8                    0.41                 0.619835             0.206096
9                    0.42                 0.603365             0.214260
10                   0.43                 0.589163             0.291468
11                   0.44                 0.587385             0.361184
12                   0.45                 0.587385             0.361184
13                   0.46                 0.562318             0.382766
14                   0.47                 0.531856             0.481550
15                   0.48                 0.498493             0.408974
16                   0.49                 0.458901             0.351259
17                   0.50                 0.457348             0.328511
18                   0.51                 0.457348             0.328511
19                   0.52                 0.452975             0.285737
20                   0.53                 0.436320             0.284990

    Training RMSE  Test RMSE  Number of features
0        0.419301   0.911799                  94
1        0.461041   0.905758                  79
```

2	0.460561	0.888153	73
3	0.482788	0.945368	68
4	0.493600	0.955872	57
5	0.545092	0.737321	47
6	0.594304	0.658260	39
7	0.604240	0.682663	31
8	0.602239	0.692391	27
9	0.615147	0.688821	22
10	0.626062	0.654104	18
11	0.627416	0.621091	16
12	0.627416	0.621091	16
13	0.646193	0.610509	13
14	0.668302	0.559527	11
15	0.691706	0.597407	8
16	0.718491	0.625897	7
17	0.719522	0.636776	5
18	0.719522	0.636776	5
19	0.722415	0.656744	4
20	0.733330	0.657088	3

5.2 Plots

[43]:
```
plt.plot(df_without_standardization['Correlation threshold'],␣
 ↪df_without_standardization['Training data R^2 score'], label = "Training␣
 ↪data R^2 score", marker='v')
plt.plot(df_without_standardization['Correlation threshold'],␣
 ↪df_without_standardization['Test data R^2 score'], label = "Test data R^2␣
 ↪score", marker='D')
plt.legend()
plt.xlabel('Correlation threshold')
plt.ylabel('R^2 score')
plt.ylim([0, 1])
plt.show()
```

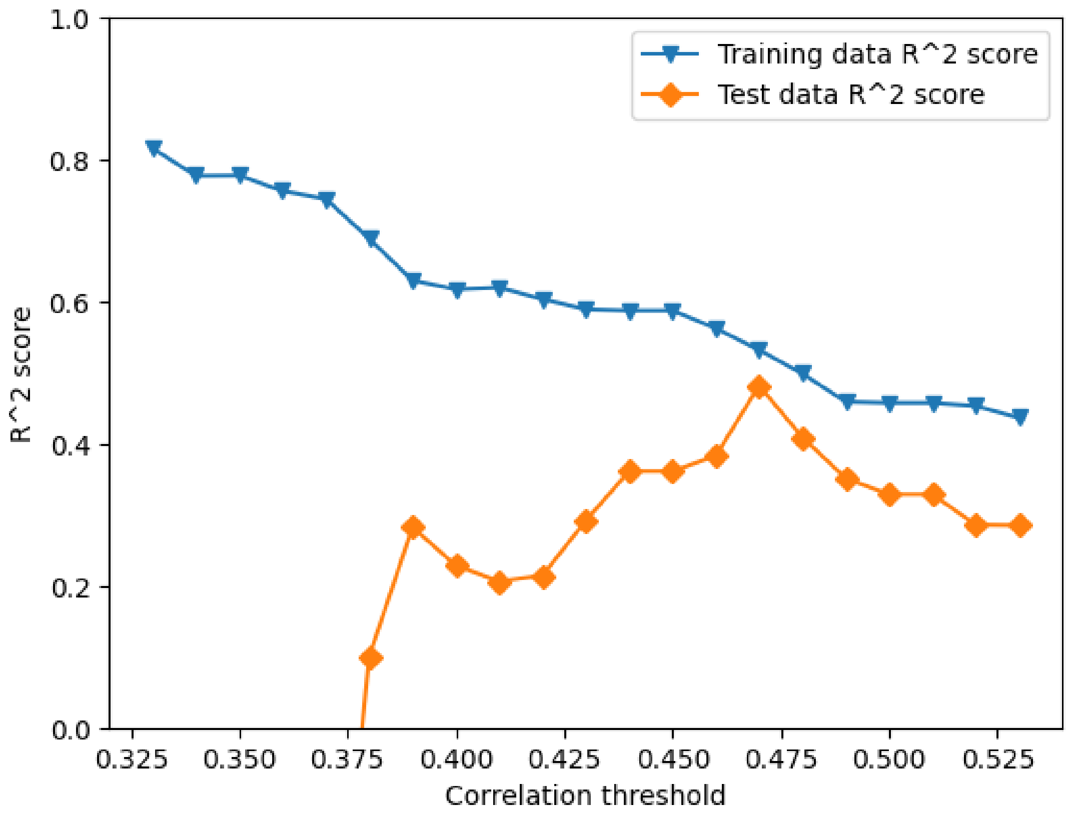

[44]:
```
plt.plot(df_without_standardization['Correlation threshold'],␣
↪df_without_standardization['Number of features'], label = "Number of␣
↪features", marker='.')
plt.legend()
plt.xlabel('Correlation threshold')
plt.ylabel('Number of features')
plt.show()
```

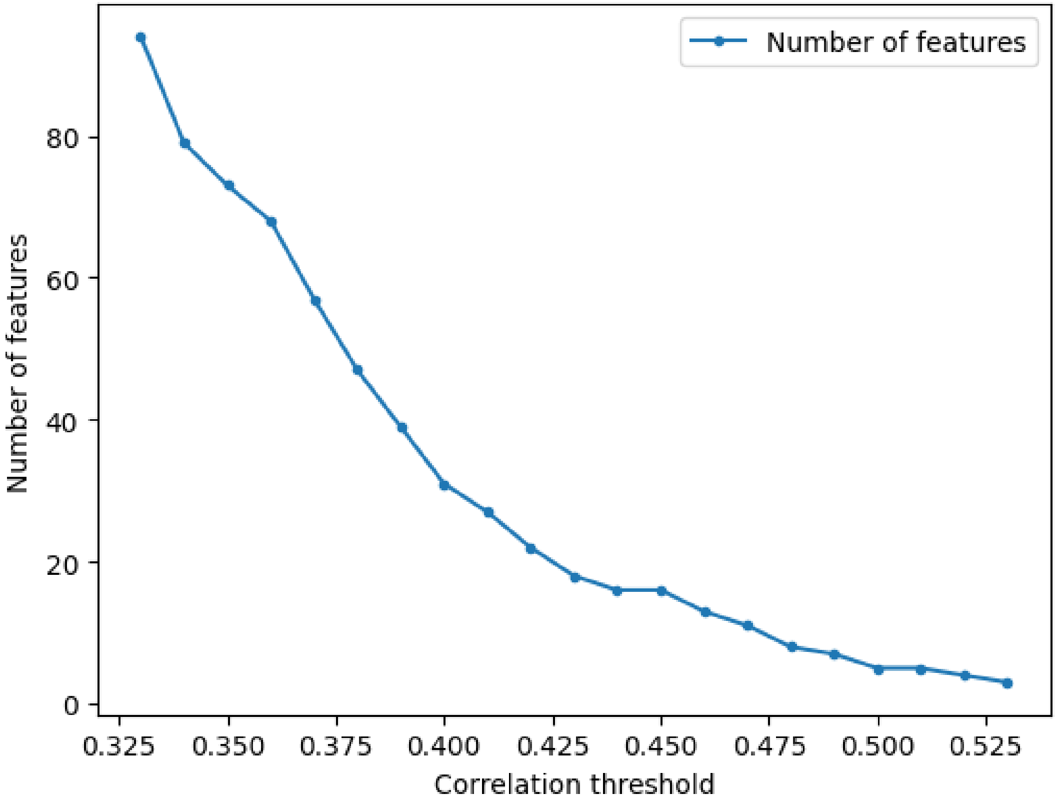

5.3 Store quality measurements

[45]:
```
with pd.ExcelWriter('../Data/Quality_'+str(target)+'_'+str(random_state)+'_.
 ↪xlsx') as writer:
    df_linear.to_excel(writer, sheet_name='MLR')
    df_decision_tree.to_excel(writer, sheet_name='DT')
    df_random_forest.to_excel(writer, sheet_name='RF')
    df_k_nearest.to_excel(writer, sheet_name='KNN')
    df_svm.to_excel(writer, sheet_name='SVM')
```

Notebook

January 18, 2024

1 File 17

[1]:
```
import sys
from pathlib import Path
prediction_mode_path = Path("../module")
sys.path.append(prediction_mode_path.as_posix())
import models_creation as pred_model

import pandas as pd
import numpy as np

from sklearn.metrics import r2_score

import matplotlib.pyplot as plt
from sklearn.tree import plot_tree

from matplotlib import pyplot as plt

import joblib

import warnings
warnings.filterwarnings('ignore')
```

[2]:
```
load_prepared_data = pd.read_excel('../Data/Kolchicyna_machine_learning.xlsx')
load_prepared_data = load_prepared_data.drop(columns=['Unnamed: 0'])

target = 'A549'

corr_low = 0.33
corr_high = 0.54

random_state = 42
```

[3]:
```
load_prepared_data.head()
```

[3]:
```
      AATS0Z  AATS0are    AATS0d   AATS0dv      AATS0i     AATS0m    AATS0p  \
0  25.090909  6.046518  3.109091  6.662626  160.775045  99.075564  1.555512
1  24.448276  6.008422  3.051724  6.386973  161.021674  96.473315  1.538471
```

```
2  23.868852  5.974074  3.000000  6.138434  161.244045  94.127025  1.523105
3  23.868852  5.974074  3.032787  6.171220  161.244045  94.127025  1.523105
4  23.343750  5.942945  2.953125  5.913194  161.445569  92.000700  1.509180

    AATS0pe    AATS0s   AATS0se  …  A549_float  MCF-7_float  LoVo_float  \
0  6.126680  3.435416  7.629859  …        10.8         10.3         6.5
1  6.088791  3.330998  7.596891  …        11.6         12.0         8.5
2  6.054630  3.236850  7.567166  …        10.9         12.2         8.8
3  6.054630  3.257798  7.567166  …        10.5         11.3         8.5
4  6.023670  3.151529  7.540228  …        89.5         92.7        52.8

   LoVo/DX_float  BALB/3T3_float  A549_float_transformed  \
0           54.9            10.2                7.966576
1           31.1            14.3                7.935542
2           17.9            11.7                7.962574
3           10.2            11.0                7.978811
4           77.8            99.4                7.048177

   MCF-7_float_transformed  LoVo_float_transformed  LoVo/DX_float_transformed  \
0                 7.987163                8.187087                   7.260428
1                 7.920819                8.070581                   7.507240
2                 7.913640                8.055517                   7.747147
3                 7.946922                8.070581                   7.991400
4                 7.032920                7.277366                   7.109020

   BALB/3T3_float_transformed
0                    7.991400
1                    7.844664
2                    7.931814
3                    7.958607
4                    7.002614

[5 rows x 1221 columns]
```

[4]:
```
data = load_prepared_data[list(load_prepared_data.columns)[0:-10]]
data[target] = load_prepared_data[load_prepared_data.columns[-5]]
data.head()
```

[4]:
```
      AATS0Z  AATS0are    AATS0d   AATS0dv      AATS0i     AATS0m    AATS0p  \
0  25.090909  6.046518  3.109091  6.662626  160.775045  99.075564  1.555512
1  24.448276  6.008422  3.051724  6.386973  161.021674  96.473315  1.538471
2  23.868852  5.974074  3.000000  6.138434  161.244045  94.127025  1.523105
3  23.868852  5.974074  3.032787  6.171220  161.244045  94.127025  1.523105
4  23.343750  5.942945  2.953125  5.913194  161.445569  92.000700  1.509180

    AATS0pe    AATS0s   AATS0se  …    piPC10     piPC2     piPC3     piPC4  \
0  6.126680  3.435416  7.629859  …  7.999655  4.266195  4.915408  5.516700
```

```
1  6.088791  3.330998  7.596891  …  8.018726  4.280132  4.922714  5.520711
2  6.054630  3.236850  7.567166  …  8.028799  4.293878  4.929967  5.524706
3  6.054630  3.257798  7.567166  …  8.037440  4.307438  4.929967  5.524706
4  6.023670  3.151529  7.540228  …  8.034892  4.307438  4.937168  5.528685

     piPC5     piPC6     piPC7     piPC8     piPC9      A549
0  6.098566  6.595759  6.979116  7.337313  7.681445  7.966576
1  6.103048  6.601209  6.985613  7.349442  7.695531  7.935542
2  6.105281  6.603923  6.989306  7.353932  7.704023  7.962574
3  6.107510  6.606629  6.992068  7.361425  7.709420  7.978811
4  6.107510  6.605277  6.991148  7.356489  7.707175  7.048177

[5 rows x 1212 columns]
```

2 Multiple Linear regression (MLR)

[5]:
```
without_standardization, train_r2_, test_r2_, hist1, hist2, target_column_name,␣
↪training_data_RMSE, test_data_RMSE  = pred_model.
↪prepare_data_and_create_model(molecular_descriptors_df=data,                                                                            ␣
↪                    correlation_threshold=0.5,                                                                            ␣
↪                    standardization=False,                                                                            ␣
↪                    model_type='linear_model',                                                                            ␣
↪                    target_column_name = str(target),                                                                            ␣
↪                    random_state=random_state,                                                                            ␣
↪                    train_test_split_=True,                                                                            ␣
↪                    verbose=True)
```

```
I am not doing standardization…
  molecular descriptor name
0                   AATS0Z
1                 AATS0are
2                   AATS0d
3                  AATS0dv
4                   AATS0i
  molecular descriptor name  corr_value
0                   AATS0Z   -0.022797
1                 AATS0are   -0.139064
2                   AATS0d    0.027592
3                  AATS0dv   -0.136049
```

```
4                   AATS0i    0.168958
  molecular descriptor name  corr_value  absolute correlation value
0                   AATS0Z   -0.022797                    0.022797
1                 AATS0are   -0.139064                    0.139064
2                   AATS0d    0.027592                    0.027592
3                  AATS0dv   -0.136049                    0.136049
4                   AATS0i    0.168958                    0.168958
     molecular descriptor name  corr_value  absolute correlation value
226                     AMID_O   -0.557717                    0.557717
505                EState_VSA5   -0.534748                    0.534748
791                    MDEO-12   -0.555724                    0.555724
921                      SaasC    0.513071                    0.513071
1091               VSA_EState5    0.526082                    0.526082
     molecular descriptor name  corr_value  absolute correlation value
226                     AMID_O   -0.557717                    0.557717
505                EState_VSA5   -0.534748                    0.534748
791                    MDEO-12   -0.555724                    0.555724
921                      SaasC    0.513071                    0.513071
1091               VSA_EState5    0.526082                    0.526082
The model used is: LinearReg…
Return the coefficient of determination of the prediction:
0.36959560986386164
R^2 score: 0.49290957430678395
Correlation coefficient: 0.7020751913483227
Test data - unseen during training:
R^2 score: 0.36959560986386164
Correlation coefficient: 0.607943755510213
[8.12901206 8.07870923 7.51604097 7.04078357 5.89815201 7.68595635
 7.96816567 7.80460535 7.64435354 7.87125241 7.84770827 6.77762909
 7.90563321 6.51297302 7.18652453 8.1847596  7.92634326 7.61620022]
44     8.193820
47     7.886057
4      7.048177
55     7.886057
26     4.904168
64     8.537602
73     7.657577
10     8.017729
40     7.920819
107    8.698970
18     6.994819
62     8.886057
11     7.962574
36     6.221126
89     8.022276
91     8.958607
109    7.886057
0      7.966576
```

```
Name: A549, dtype: float64
Training Root Mean Square Error: 0.6708383867358984
Testing Root Mean Square Error: 0.7555148852130892
```

[6]:
```
print(target_column_name+str('_transformed'))
print(hist1[target_column_name].hist())
```

```
A549_transformed
AxesSubplot(0.125,0.11;0.775x0.77)
```

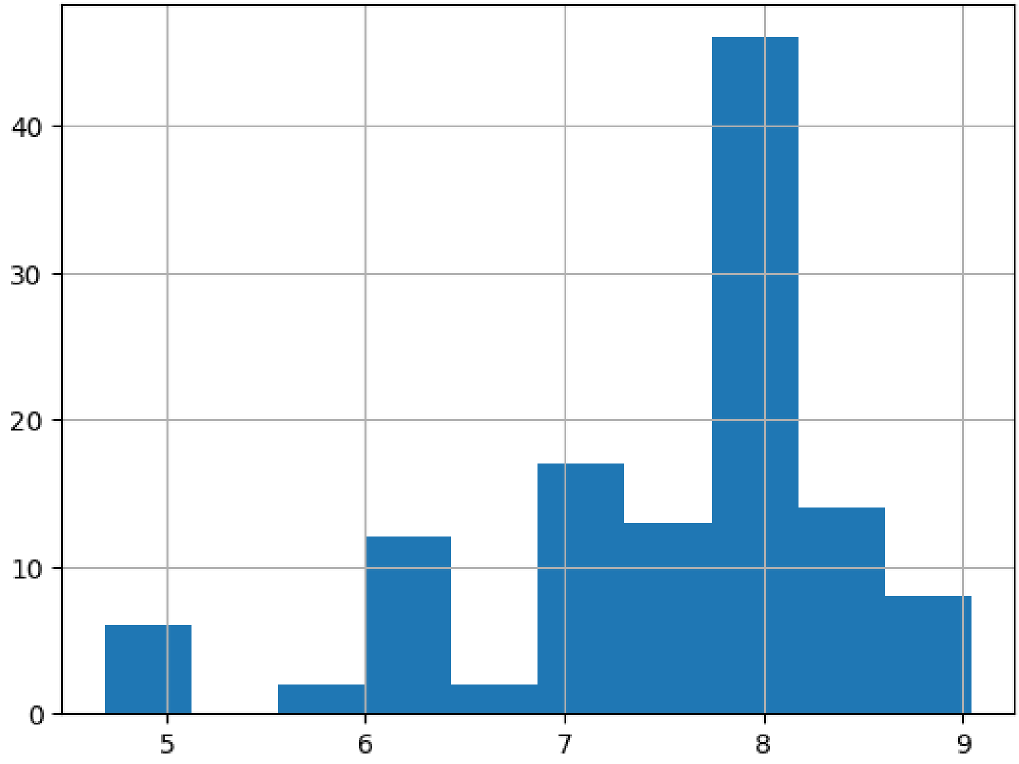

[7]:
```
with_standardization, train_r2_, test_r2_, hist1, hist2, target_column_name,␣
↪training_data_RMSE, test_data_RMSE  = pred_model.
↪prepare_data_and_create_model(molecular_descriptors_df=data,
                                                                              ␣
↪                       correlation_threshold=0.50,
                                                                              ␣
↪                       standardization=True,
                                                                              ␣
↪                       model_type='linear_model',
                                                                              ␣
↪                       target_column_name = target,
```

```
                        random_state=random_state,
                        train_test_split_=True,
                        verbose=True)
```

```
I am doing standardization…
  molecular descriptor name
0                    AATS0Z
1                  AATS0are
2                    AATS0d
3                   AATS0dv
4                    AATS0i
  molecular descriptor name  corr_value
0                    AATS0Z   -0.022797
1                  AATS0are   -0.139064
2                    AATS0d    0.027592
3                   AATS0dv   -0.136049
4                    AATS0i    0.168958
  molecular descriptor name  corr_value  absolute correlation value
0                    AATS0Z   -0.022797                    0.022797
1                  AATS0are   -0.139064                    0.139064
2                    AATS0d    0.027592                    0.027592
3                   AATS0dv   -0.136049                    0.136049
4                    AATS0i    0.168958                    0.168958
     molecular descriptor name  corr_value  absolute correlation value
226                     AMID_O   -0.557717                    0.557717
505                EState_VSA5   -0.534748                    0.534748
791                    MDEO-12   -0.555724                    0.555724
921                      SaasC    0.513071                    0.513071
1091               VSA_EState5    0.526082                    0.526082
     molecular descriptor name  corr_value  absolute correlation value
226                     AMID_O   -0.557717                    0.557717
505                EState_VSA5   -0.534748                    0.534748
791                    MDEO-12   -0.555724                    0.555724
921                      SaasC    0.513071                    0.513071
1091               VSA_EState5    0.526082                    0.526082
The model used is: LinearReg…
Return the coefficient of determination of the prediction:
0.36959560986386164
R^2 score: 0.49290957430678395
Correlation coefficient: 0.7020751913483227
Test data - unseen during training:
R^2 score: 0.36959560986386164
Correlation coefficient: 0.607943755510213
[8.12901206 8.07870923 7.51604097 7.04078357 5.89815201 7.68595635
```

```
 7.96816567 7.80460535 7.64435354 7.87125241 7.84770827 6.77762909
 7.90563321 6.51297302 7.18652453 8.1847596  7.92634326 7.61620022]
44     8.193820
47     7.886057
4      7.048177
55     7.886057
26     4.904168
64     8.537602
73     7.657577
10     8.017729
40     7.920819
107    8.698970
18     6.994819
62     8.886057
11     7.962574
36     6.221126
89     8.022276
91     8.958607
109    7.886057
0      7.966576
Name: A549, dtype: float64
Training Root Mean Square Error: 0.6708383867358984
Testing Root Mean Square Error: 0.7555148852130892
```

2.1 Search inside correlation space

[8]:
```
step = 0.01
initial_step = corr_low
last_step = corr_high
first_list = [x / 100.0 for x in range(int(initial_step*100),␣
 ↪int(last_step*100), int(step*100))]
second_list = []
third_list = []
fourth_l = []
fifth_l = []
f_list = []
for i in first_list:
    without_standardization, train_r2, test_r2, _, h_, target_column_name,␣
 ↪training_data_RMSE, test_data_RMSE = pred_model.
 ↪prepare_data_and_create_model(molecular_descriptors_df = data,
                                                                          ␣
 ↪                    correlation_threshold = i,
                                                                          ␣
 ↪                    standardization = False,
                                                                          ␣
 ↪                    model_type = 'linear_model',
```

```
                          target_column_name = target,
                          random_state=random_state,
                          train_test_split_ = True,
                          verbose = False)
    second_list.append(train_r2)
    third_list.append(test_r2)
    fourth_l.append(training_data_RMSE)
    fifth_l.append(test_data_RMSE)
    f_list.append(len(h_))
```

[9]:
```
df_without_standardization = pd.DataFrame(data=first_list,
 columns=["Correlation threshold"])
df_without_standardization['Training data R^2 score'] = second_list
df_without_standardization['Test data R^2 score'] = third_list
df_without_standardization['Training RMSE'] = fourth_l
df_without_standardization['Test RMSE'] = fifth_l
df_without_standardization['Number of features'] = f_list
```

[10]:
```
df_linear = df_without_standardization.copy()
df_without_standardization
```

[10]:

	Correlation threshold	Training data R^2 score	Test data R^2 score	\
0	0.33	0.993044	-4.544410	
1	0.34	0.964137	-6.199282	
2	0.35	0.945689	-3.017881	
3	0.36	0.934600	-3.136296	
4	0.37	0.908123	-0.841790	
5	0.38	0.865002	0.032270	
6	0.39	0.778480	0.486171	
7	0.40	0.676043	0.491690	
8	0.41	0.673892	0.474498	
9	0.42	0.649674	0.078386	
10	0.43	0.637643	0.204582	
11	0.44	0.613365	0.346221	
12	0.45	0.613365	0.346221	
13	0.46	0.590077	0.369409	
14	0.47	0.574384	0.351006	
15	0.48	0.524351	0.477630	
16	0.49	0.497891	0.417097	
17	0.50	0.492910	0.369596	
18	0.51	0.492910	0.369596	
19	0.52	0.490837	0.385044	
20	0.53	0.454814	0.492363	

```
    Training RMSE  Test RMSE  Number of features
0        0.078570   2.240583                  94
1        0.178402   2.553160                  79
2        0.219543   1.907357                  73
3        0.240914   1.935259                  68
4        0.285548   1.291378                  57
5        0.346130   0.936075                  47
6        0.443385   0.682092                  39
7        0.536190   0.678419                  31
8        0.537968   0.689796                  27
9        0.557585   0.913499                  22
10       0.567079   0.848655                  18
11       0.585769   0.769394                  16
12       0.585769   0.769394                  16
13       0.603151   0.755627                  13
14       0.614588   0.766573                  11
15       0.649709   0.687737                   8
16       0.667535   0.726494                   7
17       0.670838   0.755515                   5
18       0.670838   0.755515                   5
19       0.672208   0.746200                   4
20       0.695581   0.677969                   3
```

[ ]:

2.2 Plots

[11]:

```
plt.plot(df_without_standardization['Correlation threshold'],␣
  ↪df_without_standardization['Training data R^2 score'], label = "Training␣
  ↪data R^2 score", marker='v')
plt.plot(df_without_standardization['Correlation threshold'],␣
  ↪df_without_standardization['Test data R^2 score'], label = "Test data R^2␣
  ↪score", marker='D')
plt.legend()
plt.xlabel('Correlation threshold')
plt.ylabel('R^2 score')
plt.ylim([0, 1])
plt.show()
```

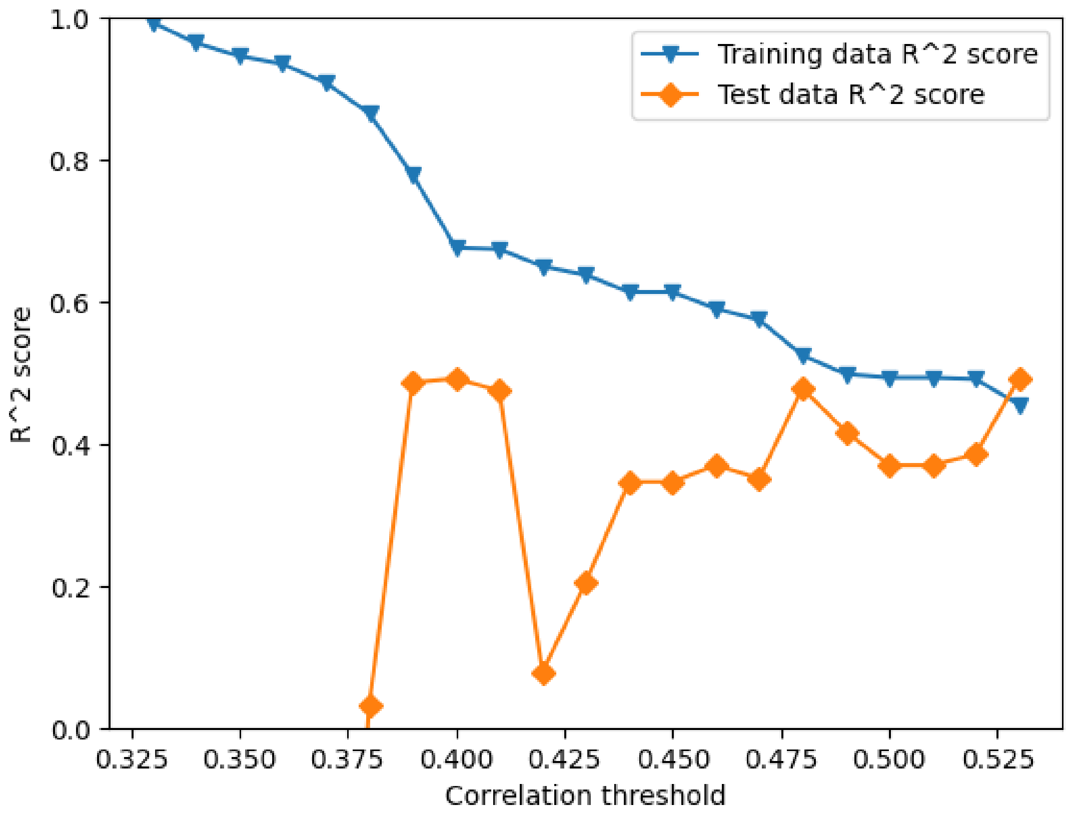

[12]:
```
plt.plot(df_without_standardization['Correlation threshold'],␣
  ↪df_without_standardization['Number of features'], label = "Number of␣
  ↪features", marker='.')
plt.legend()
plt.xlabel('Correlation threshold')
plt.ylabel('Number of features')
plt.show()
```

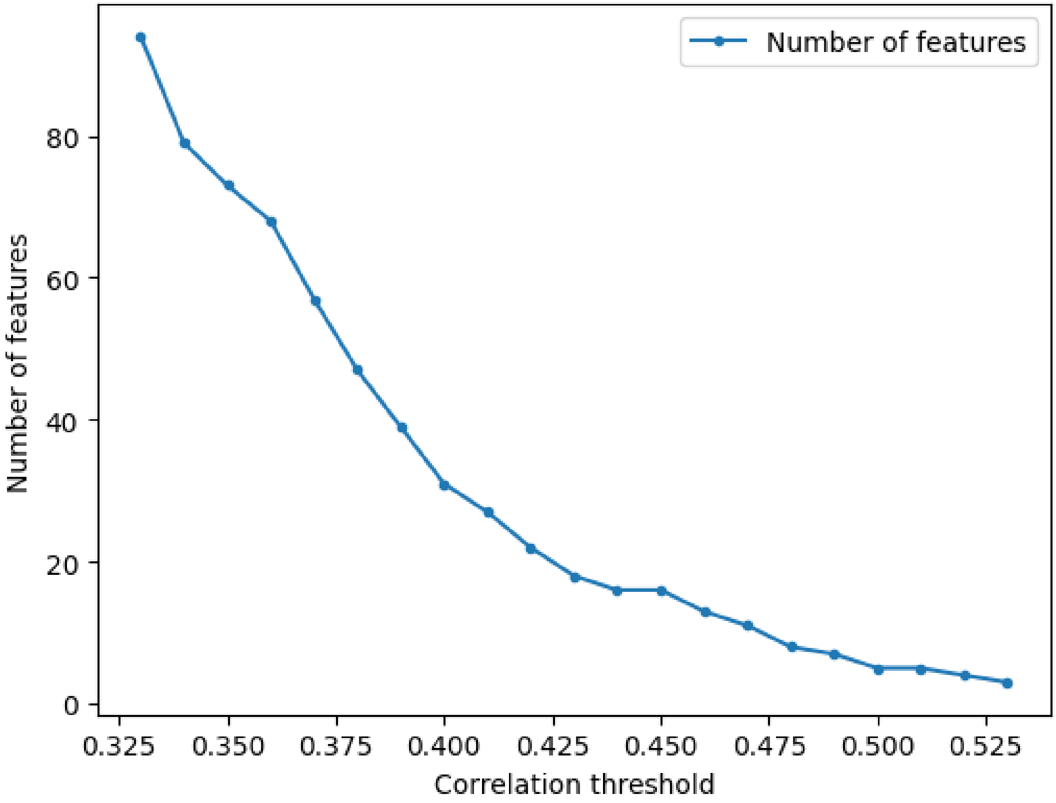

[ ]:

```

```

2.3 Decision Tree

[13]:

```
without_standardization, train_r2_, test_r2_, hist1, hist2, target_column_name,␣
↪training_data_RMSE, test_data_RMSE  = pred_model.
↪prepare_data_and_create_model(molecular_descriptors_df=data,
                                                                              ␣
↪                         correlation_threshold=0.50,
                                                                              ␣
↪                         standardization=False,
                                                                              ␣
↪                         model_type='DecisionTreeRegressor',
                                                                              ␣
↪                         max_depth=5,
                                                                              ␣
↪                         target_column_name = target,
                                                                              ␣
↪                         random_state=random_state,
```

```
↪                    train_test_split_=True,
↪                    verbose=True)
```

```
I am not doing standardization…
  molecular descriptor name
0                   AATS0Z
1                 AATS0are
2                   AATS0d
3                  AATS0dv
4                   AATS0i
  molecular descriptor name  corr_value
0                   AATS0Z   -0.022797
1                 AATS0are   -0.139064
2                   AATS0d    0.027592
3                  AATS0dv   -0.136049
4                   AATS0i    0.168958
  molecular descriptor name  corr_value  absolute correlation value
0                   AATS0Z   -0.022797                    0.022797
1                 AATS0are   -0.139064                    0.139064
2                   AATS0d    0.027592                    0.027592
3                  AATS0dv   -0.136049                    0.136049
4                   AATS0i    0.168958                    0.168958
     molecular descriptor name  corr_value  absolute correlation value
226                     AMID_O   -0.557717                    0.557717
505                EState_VSA5   -0.534748                    0.534748
791                    MDEO-12   -0.555724                    0.555724
921                      SaasC    0.513071                    0.513071
1091               VSA_EState5    0.526082                    0.526082
     molecular descriptor name  corr_value  absolute correlation value
226                     AMID_O   -0.557717                    0.557717
505                EState_VSA5   -0.534748                    0.534748
791                    MDEO-12   -0.555724                    0.555724
921                      SaasC    0.513071                    0.513071
1091               VSA_EState5    0.526082                    0.526082
The model used is: DecisionTree…
Return the coefficient of determination of the prediction:
0.45678797553100636
R^2 score: 0.9212015338337038
Correlation coefficient: 0.959792443100957
Test data - unseen during training:
R^2 score: 0.45678797553100636
Correlation coefficient: 0.6758609143388944
[7.63701524 7.93527443 7.93527443 7.79224678 6.12412387 8.1812164
 7.93527443 7.93527443 7.93527443 7.93527443 7.14451502 7.79224678
 7.93527443 6.0389332  7.06550155 7.14451502 7.93527443 7.93527443]
44     8.193820
```

```
47     7.886057
4      7.048177
55     7.886057
26     4.904168
64     8.537602
73     7.657577
10     8.017729
40     7.920819
107    8.698970
18     6.994819
62     8.886057
11     7.962574
36     6.221126
89     8.022276
91     8.958607
109    7.886057
0      7.966576
Name: A549, dtype: float64
Training Root Mean Square Error: 0.2644442220792924
Testing Root Mean Square Error: 0.7013230263965958
```

[14]:
```
print(target_column_name+str('_transformed'))
print(hist1[target_column_name].hist())
```

```
A549_transformed
AxesSubplot(0.125,0.11;0.775x0.77)
```

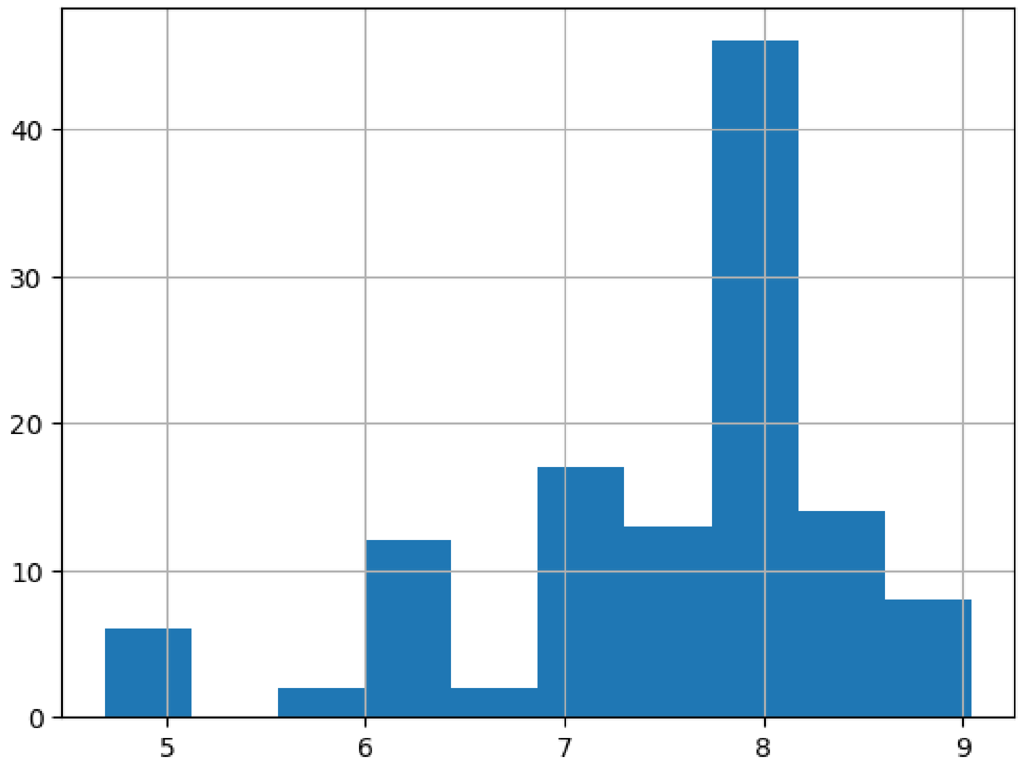

[15]:
```
with_standardization, train_r2_, test_r2_, hist1, hist2, target_column_name,␣
↪training_data_RMSE, test_data_RMSE  = pred_model.
↪prepare_data_and_create_model(molecular_descriptors_df=data,
                                                                              ␣
↪                          correlation_threshold=0.50,
                                                                              ␣
↪                          standardization=True,
                                                                              ␣
↪                          model_type='DecisionTreeRegressor',
                                                                              ␣
↪                          max_depth=5,
                                                                              ␣
↪                          target_column_name = target,
                                                                              ␣
↪                          random_state=random_state,
                                                                              ␣
↪                          train_test_split_=True,
                                                                              ␣
↪                          verbose=True)
```

```
I am doing standardization…
  molecular descriptor name
```

```
0                  AATS0Z
1                AATS0are
2                  AATS0d
3                 AATS0dv
4                  AATS0i
  molecular descriptor name  corr_value
0                  AATS0Z   -0.022797
1                AATS0are   -0.139064
2                  AATS0d    0.027592
3                 AATS0dv   -0.136049
4                  AATS0i    0.168958
  molecular descriptor name  corr_value  absolute correlation value
0                  AATS0Z   -0.022797                    0.022797
1                AATS0are   -0.139064                    0.139064
2                  AATS0d    0.027592                    0.027592
3                 AATS0dv   -0.136049                    0.136049
4                  AATS0i    0.168958                    0.168958
     molecular descriptor name  corr_value  absolute correlation value
226                     AMID_O   -0.557717                    0.557717
505                 EState_VSA5   -0.534748                    0.534748
791                    MDEO-12   -0.555724                    0.555724
921                      SaasC    0.513071                    0.513071
1091                VSA_EState5    0.526082                    0.526082
```


```
     molecular descriptor name  corr_value  absolute correlation value
226                     AMID_O   -0.557717                    0.557717
505                 EState_VSA5   -0.534748                    0.534748
791                    MDEO-12   -0.555724                    0.555724
921                      SaasC    0.513071                    0.513071
1091                VSA_EState5    0.526082                    0.526082
```


```
The model used is: DecisionTree…
Return the coefficient of determination of the prediction:
0.45678797553100636
R^2 score: 0.9212015338337038
Correlation coefficient: 0.959792443100957
Test data - unseen during training:
R^2 score: 0.45678797553100636
Correlation coefficient: 0.6758609143388944
[7.63701524 7.93527443 7.93527443 7.79224678 6.12412387 8.1812164
 7.93527443 7.93527443 7.93527443 7.93527443 7.14451502 7.79224678
 7.93527443 6.0389332  7.06550155 7.14451502 7.93527443 7.93527443]
44     8.193820
47     7.886057
4      7.048177
55     7.886057
26     4.904168
64     8.537602
73     7.657577
10     8.017729
```

```
40      7.920819
107     8.698970
18      6.994819
62      8.886057
11      7.962574
36      6.221126
89      8.022276
91      8.958607
109     7.886057
0       7.966576
Name: A549, dtype: float64
Training Root Mean Square Error: 0.2644442220792924
Testing Root Mean Square Error: 0.7013230263965958
```

2.4 Search inside correlation space

[16]:
```
step = 0.01
initial_step = corr_low
last_step = corr_high
first_list = [x / 100.0 for x in range(int(initial_step*100),␣
↪int(last_step*100), int(step*100))]
max_depth = [range(2, 30, 1)]
corr_th = []
second_list = []
third_list = []
fourth_l = []
fifth_l = []
f_list = []
fif_list = []
for i in first_list:
    for depth in max_depth[0]:

        without_standardization, train_r2, test_r2, _, h_, target_column_name,␣
↪training_data_RMSE, test_data_RMSE = pred_model.
↪prepare_data_and_create_model(molecular_descriptors_df=data,
                                                                              ␣
↪                    correlation_threshold=i,
                                                                              ␣
↪                    standardization=False,
                                                                              ␣
↪                    model_type='DecisionTreeRegressor',
                                                                              ␣
↪                    max_depth=depth,
                                                                              ␣
↪                    target_column_name = target,
                                                                              ␣
↪                    random_state=random_state,
```

```
↪                     train_test_split_=True,
↪                     verbose=False)
        corr_th.append(i)
        second_list.append(train_r2)
        third_list.append(test_r2)
        fourth_l.append(training_data_RMSE)
        fifth_l.append(test_data_RMSE)
        f_list.append(len(h_))
        fif_list.append(depth)
```

[17]:
```
df_without_standardization = pd.DataFrame(data=corr_th, columns=["Correlation␣
↪threshold"])
df_without_standardization['Training data R^2 score'] = second_list
df_without_standardization['Test data R^2 score'] = third_list
df_without_standardization['Training RMSE'] = fourth_l
df_without_standardization['Test RMSE'] = fifth_l
df_without_standardization['Number of features'] = f_list
df_without_standardization['Depth number'] = fif_list
```

[18]:
```
df_decision_tree = df_without_standardization.copy()
#df_without_standardization.to_excel('../Data/
↪A549_Decision_tree_rs_'+str(random_state)+'.xlsx')
```

2.5 Plots

[19]:
```
corre_list = [x / 100.0 for x in range(int(initial_step*100),␣
↪int(last_step*100), int(0.01*100))]
for element in corre_list:
    element_ =␣
↪df_without_standardization[df_without_standardization['Correlation␣
↪threshold'] == float(element)]
    plt.plot(element_['Depth number'], element_['Training data R^2 score'],␣
↪label = "Training data R^2 score", marker='v')
    plt.plot(element_['Depth number'], element_['Test data R^2 score'], label =␣
↪"Test data R^2 score", marker='D')
    plt.legend()
    plt.xlabel('Depth number -> correlation threshold: '+str(element)+'. \n␣
↪Number of features: '+str(element_['Number of features'].iloc[0]),␣
↪fontweight='bold')
    plt.xlim(max(element_['Depth number'])+1, min(element_['Depth number'])-1)
    plt.ylabel('R^2 score')
    plt.ylim([0, 1])
    plt.rc('grid', linestyle="-.", color='black')
    plt.grid(True)
    plt.show()
```

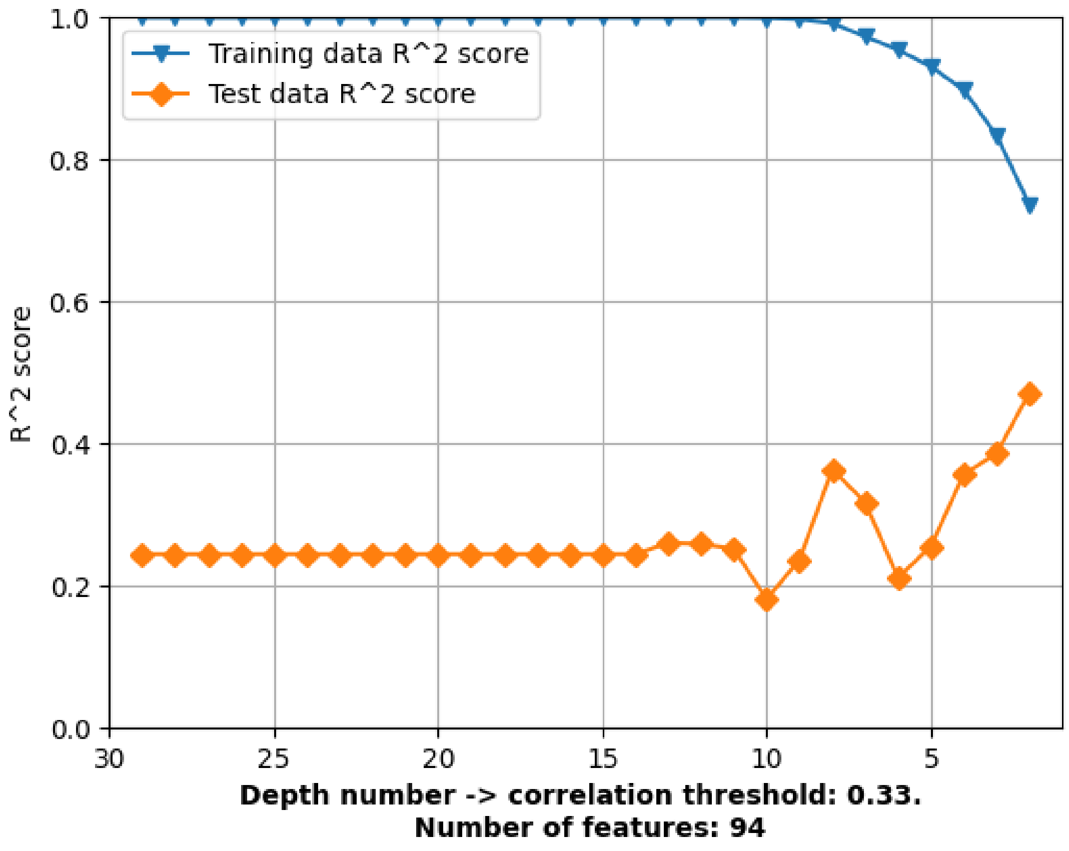

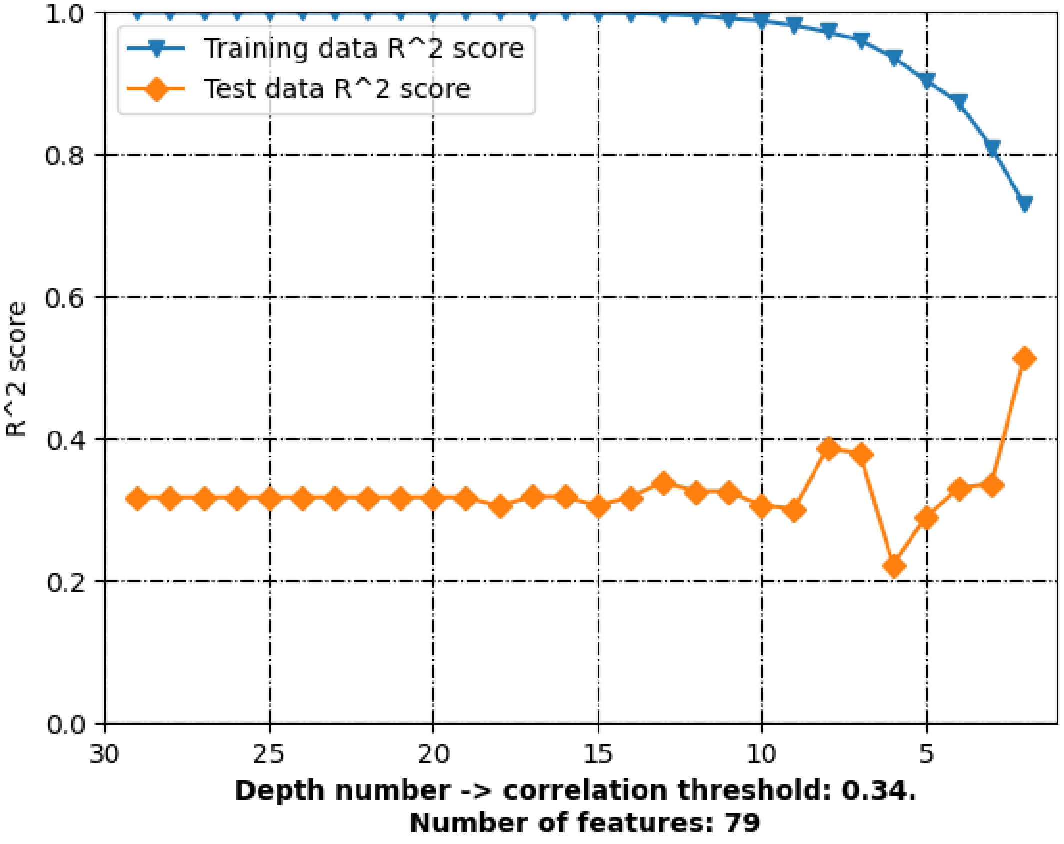
Training data R^2 score
Test data R^2 score
R^2 score
1.0
0.8
0.6
0.4
0.2
0.0
30
25
20
15
10
5
Depth number -> correlation threshold: 0.34.
Number of features: 79

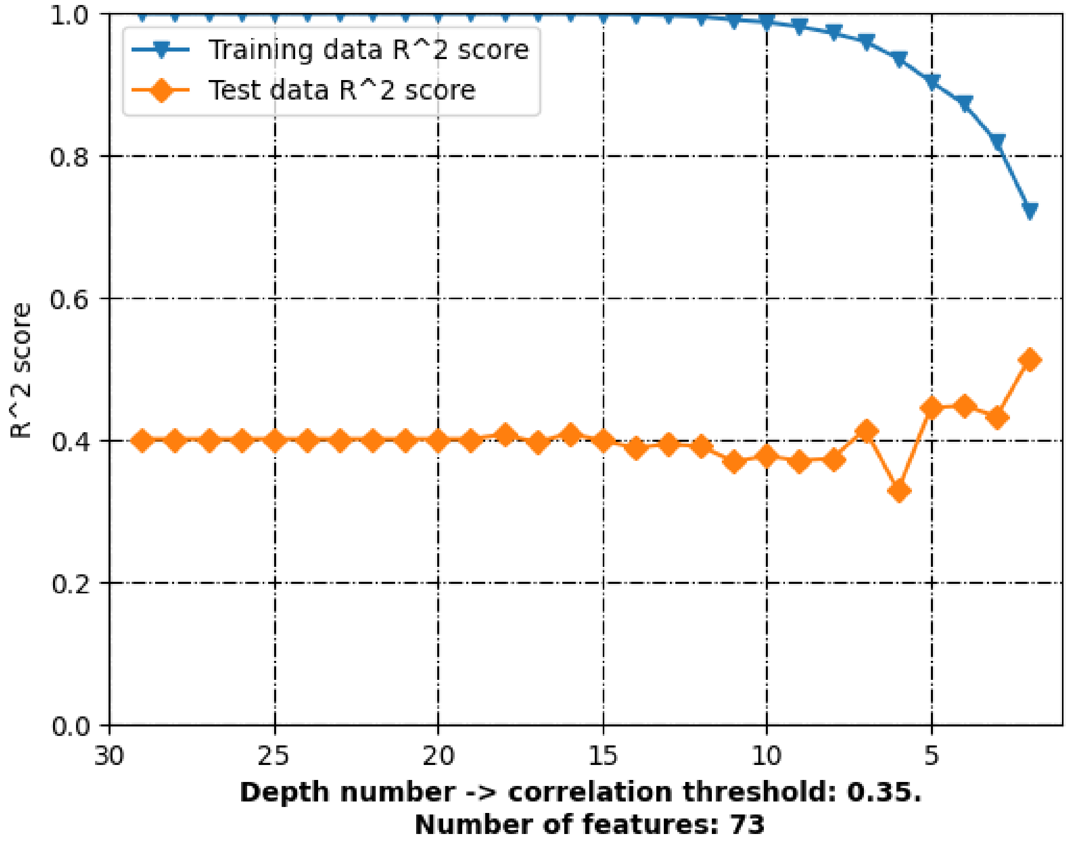
Training data R^2 score
Test data R^2 score
R^2 score
1.0
0.8
0.6
0.4
0.2
0.0
30
25
20
15
10
5
Depth number -> correlation threshold: 0.35.
Number of features: 73

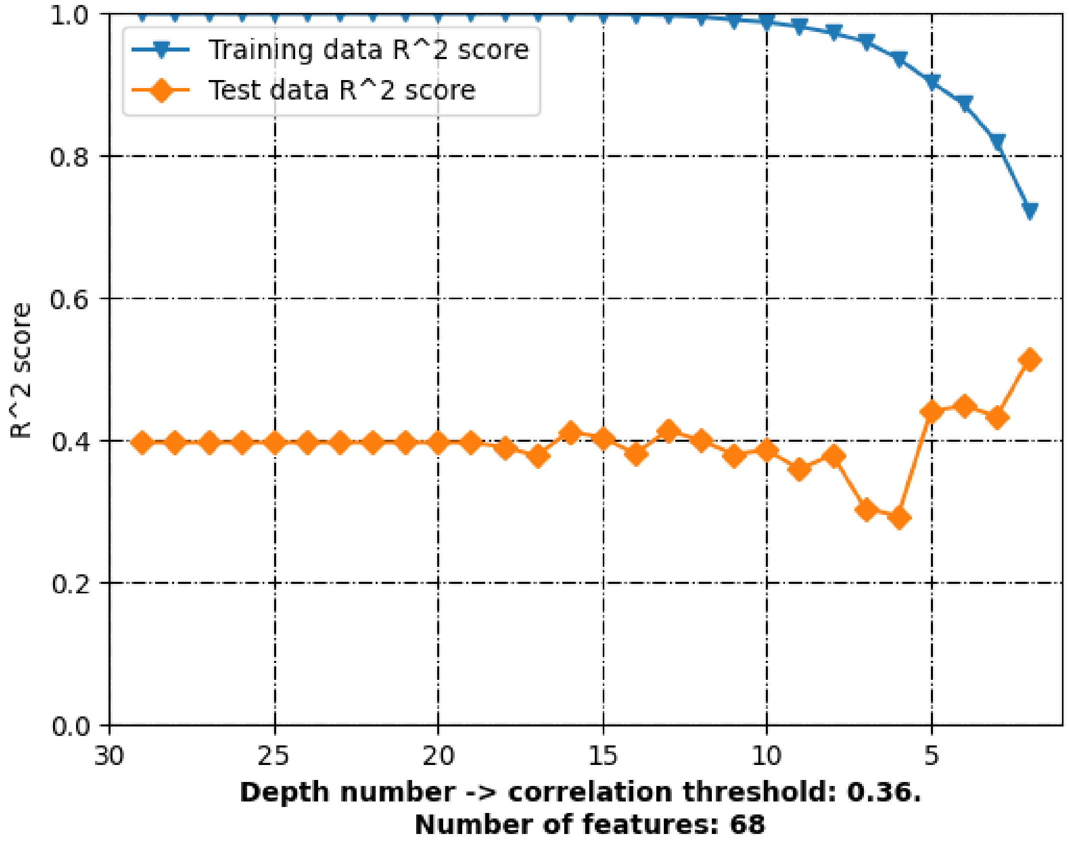
Training data R^2 score
Test data R^2 score
R^2 score
1.0
0.8
0.6
0.4
0.2
0.0
30
25
20
15
10
5
Depth number -> correlation threshold: 0.36.
Number of features: 68

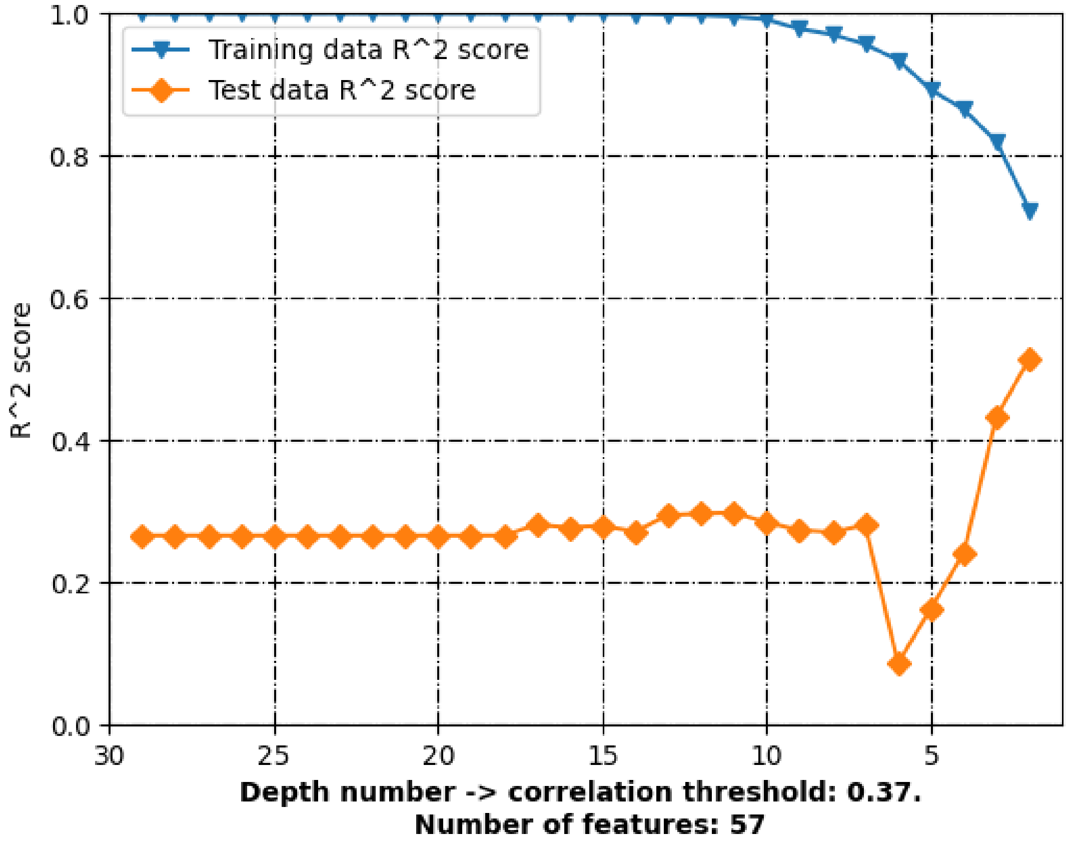

Training data R^2 score
Test data R^2 score
R^2 score
1.0
0.8
0.6
0.4
0.2
0.0
30
25
20
15
10
5
Depth number -> correlation threshold: 0.37.
Number of features: 57

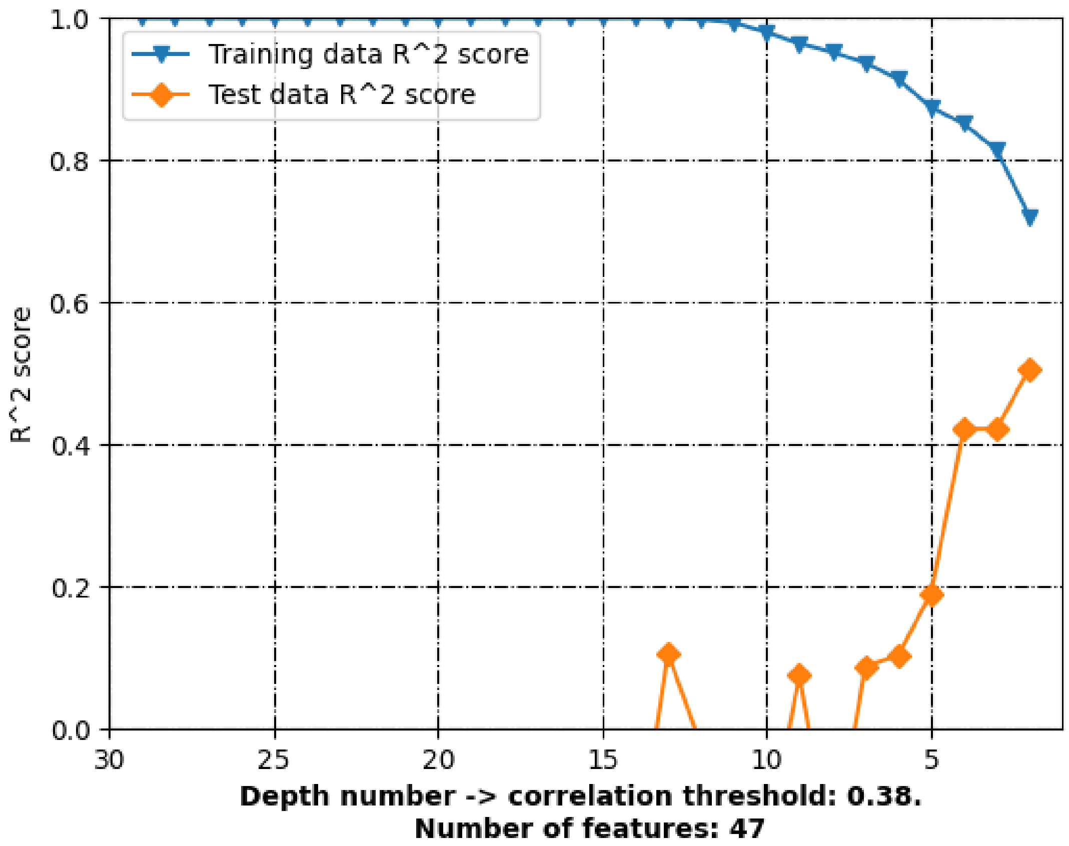

Training data R^2 score
Test data R^2 score
R^2 score
1.0
0.8
0.6
0.4
0.2
0.0
30
25
20
15
10
5
Depth number -> correlation threshold: 0.38.
Number of features: 47

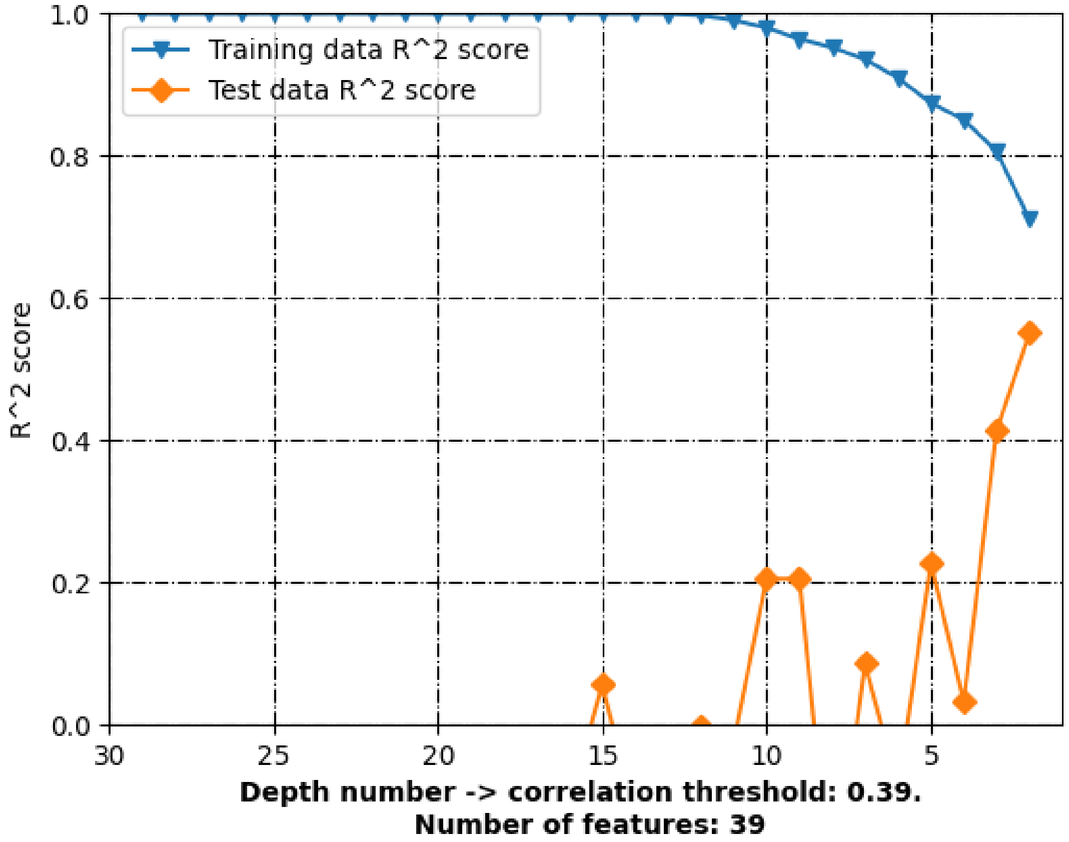
Training data R^2 score
Test data R^2 score
R^2 score
0.0
0.2
0.4
0.6
0.8
1.0
30
25
20
15
10
5
Depth number -> correlation threshold: 0.39.
Number of features: 39

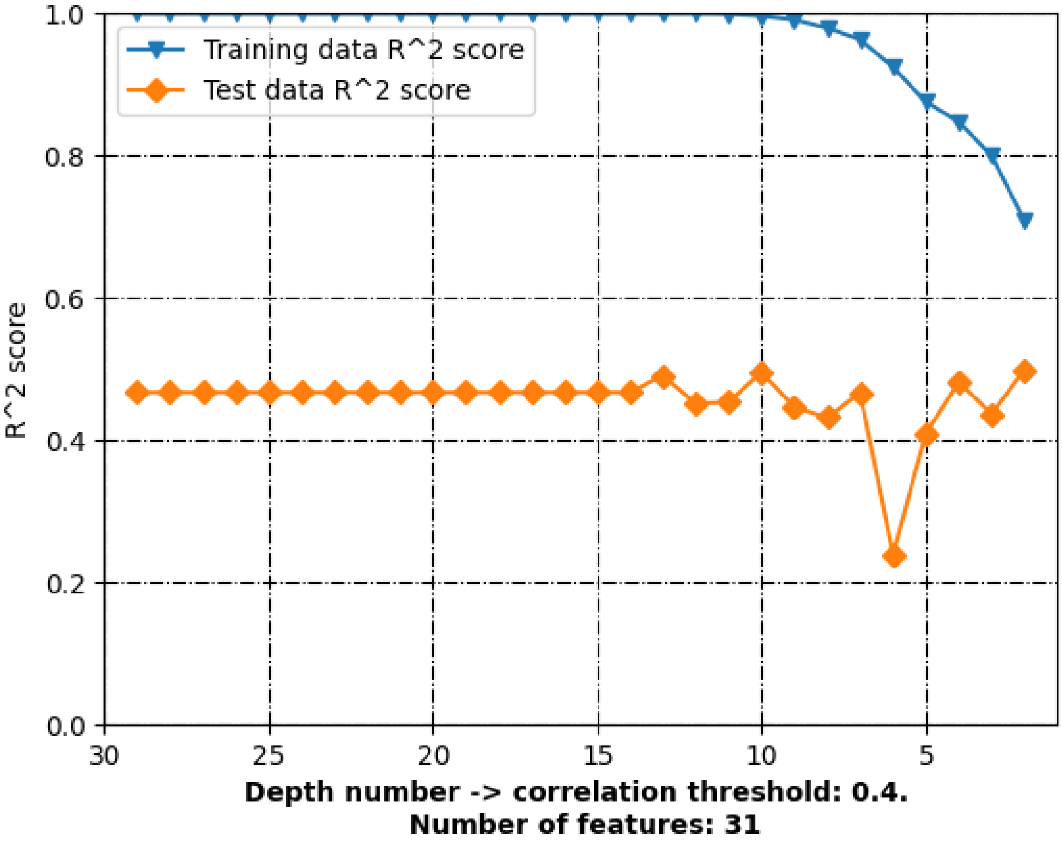
Training data R^2 score
Test data R^2 score
R^2 score
1.0
0.8
0.6
0.4
0.2
0.0
30
25
20
15
10
5
Depth number -> correlation threshold: 0.4.
Number of features: 31

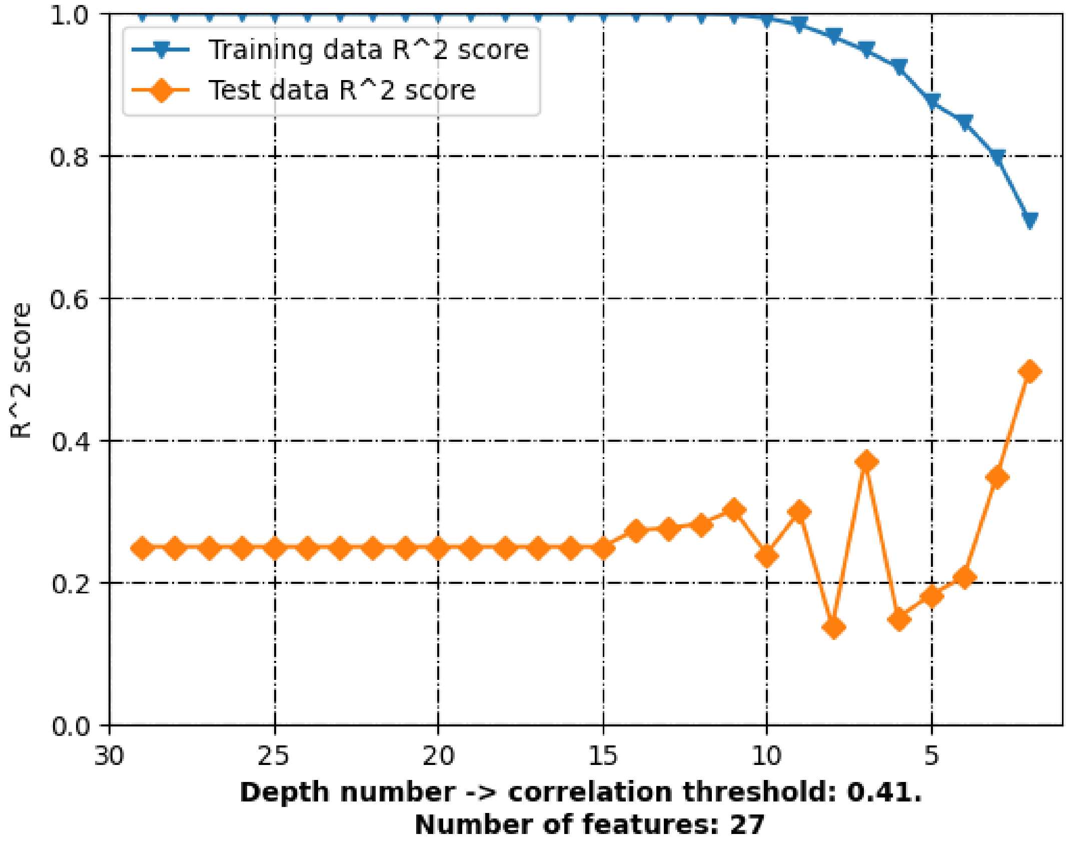

1.0
0.8
0.6
0.4
0.2
0.0
R^2 score
Training data R^2 score
Test data R^2 score
30
25
20
15
10
5
Depth number -> correlation threshold: 0.41.
Number of features: 27

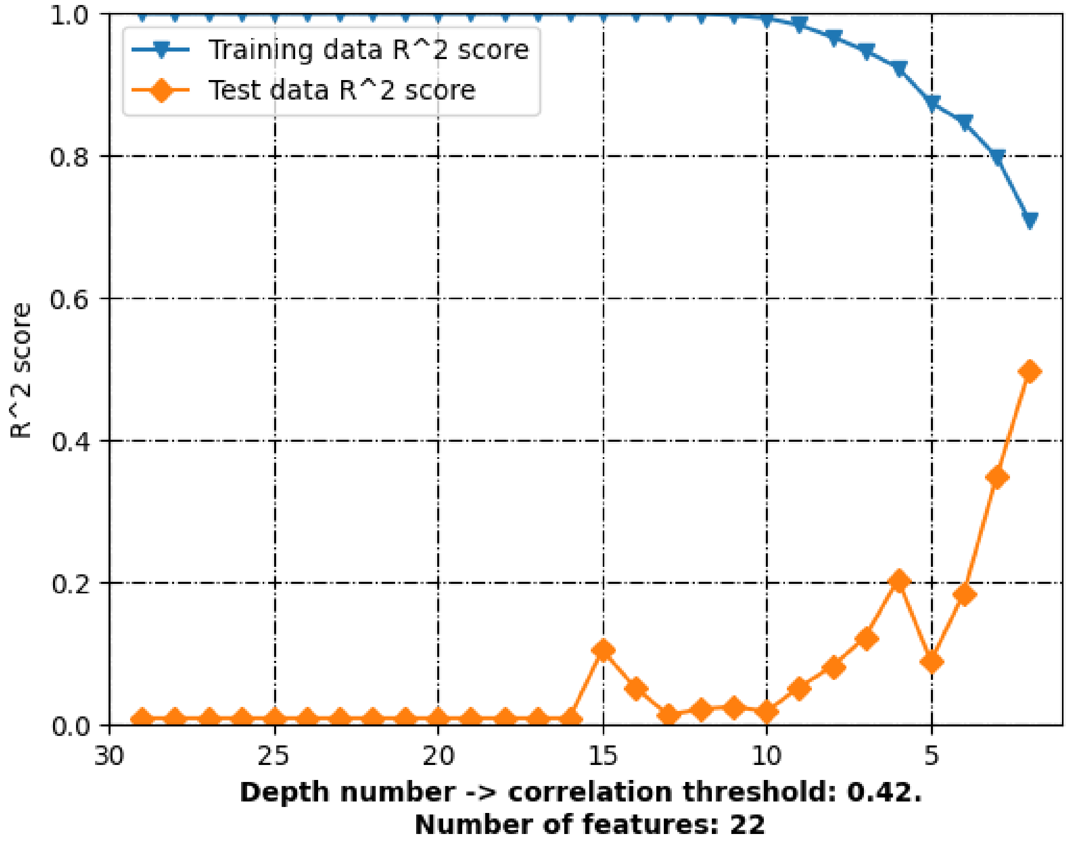

Training data R^2 score
Test data R^2 score
R^2 score
1.0
0.8
0.6
0.4
0.2
0.0
30
25
20
15
10
5
Depth number -> correlation threshold: 0.42.
Number of features: 22

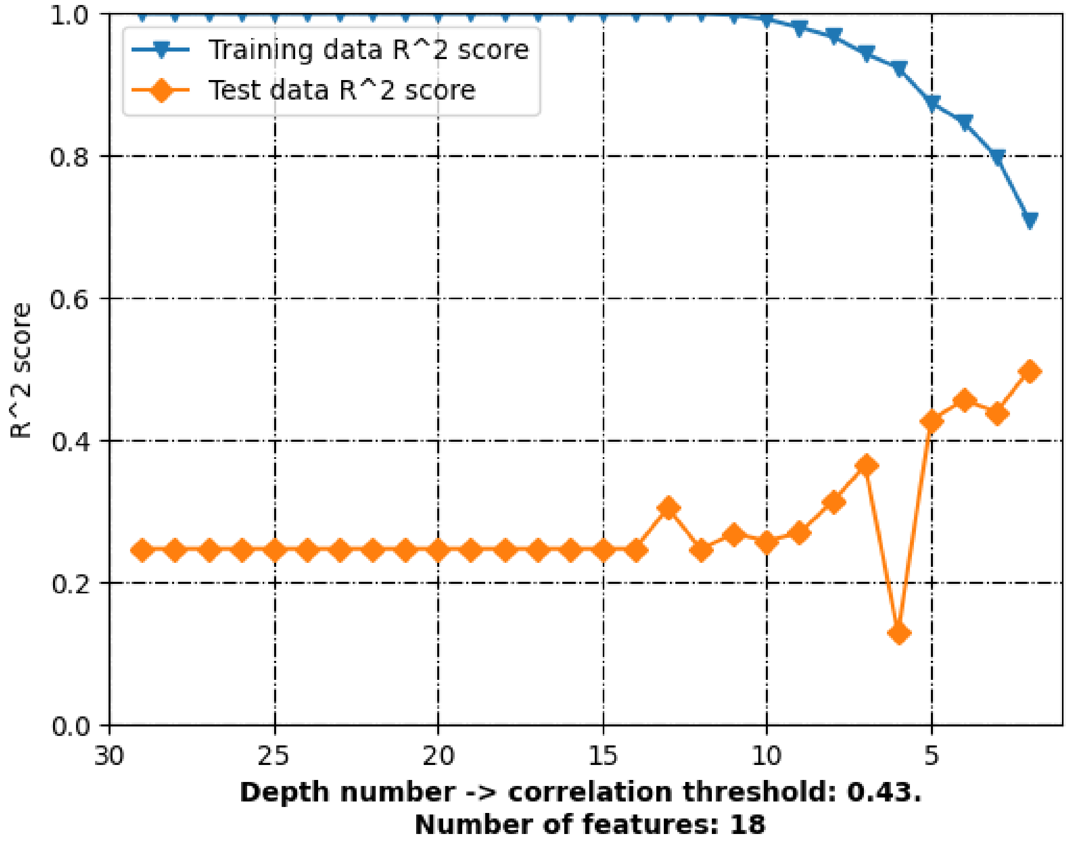

Training data R^2 score
Test data R^2 score
R^2 score
1.0
0.8
0.6
0.4
0.2
0.0
30
25
20
15
10
5
Depth number -> correlation threshold: 0.43.
Number of features: 18

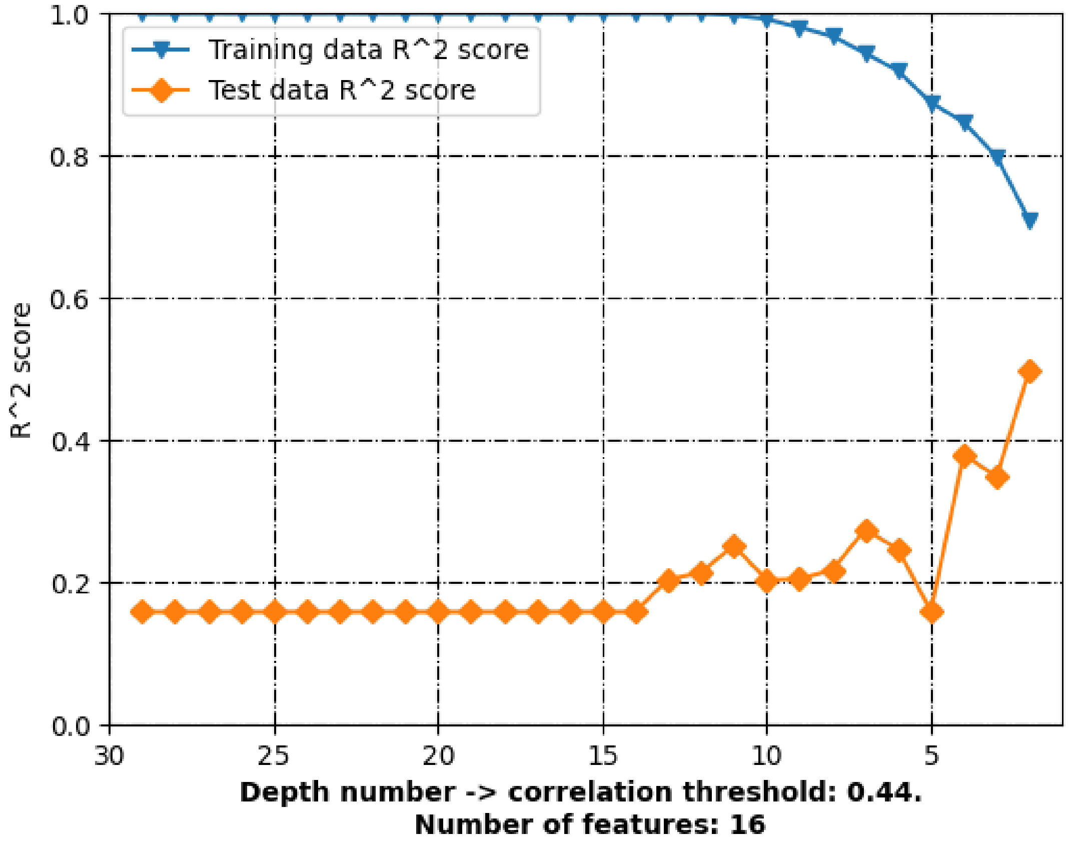

Training data R^2 score
Test data R^2 score
R^2 score
1.0
0.8
0.6
0.4
0.2
0.0
30
25
20
15
10
5
Depth number -> correlation threshold: 0.44.
Number of features: 16

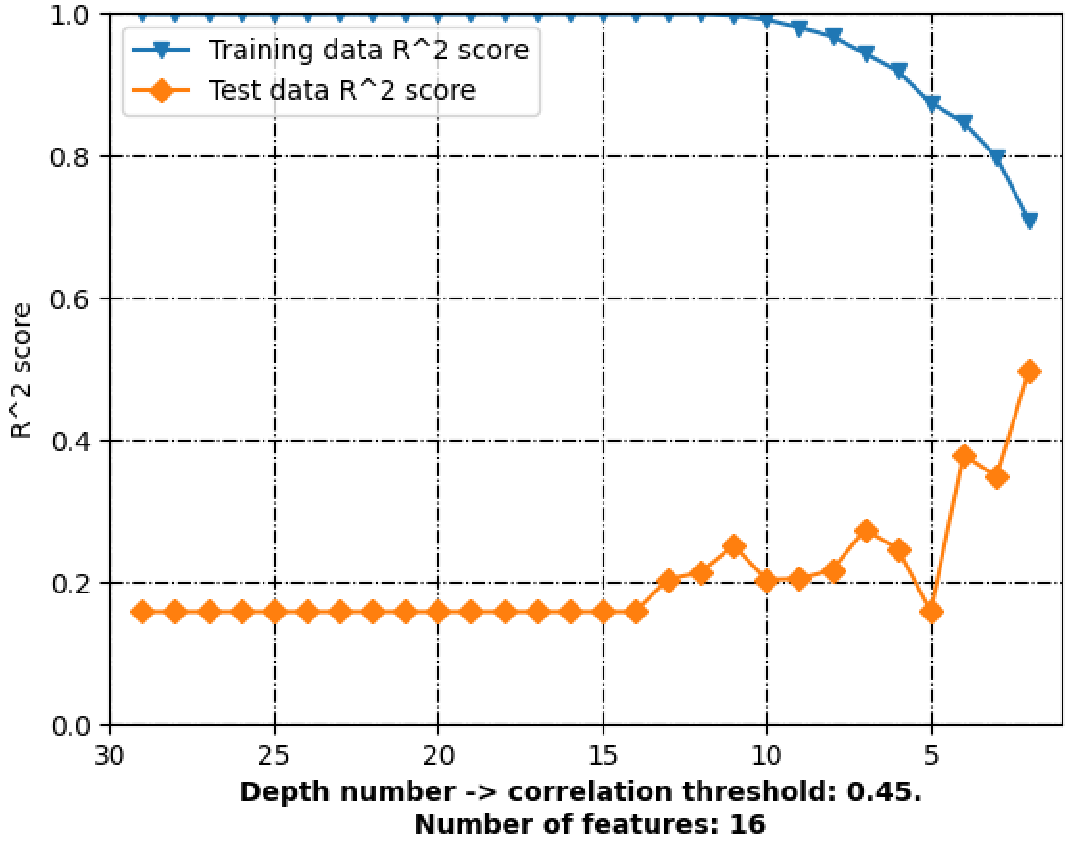
Training data R^2 score
Test data R^2 score
R^2 score
1.0
0.8
0.6
0.4
0.2
0.0
30
25
20
15
10
5
Depth number -> correlation threshold: 0.45.
Number of features: 16

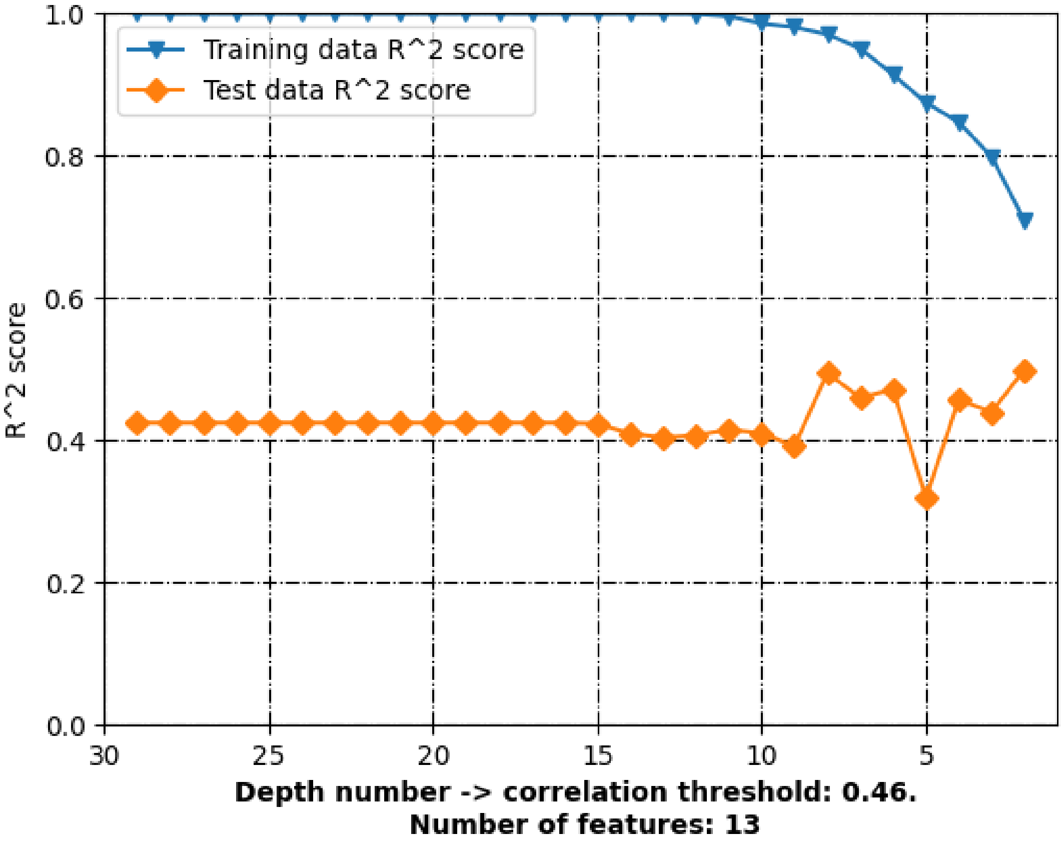
Training data R^2 score
Test data R^2 score
R^2 score
1.0
0.8
0.6
0.4
0.2
0.0
30
25
20
15
10
5
Depth number -> correlation threshold: 0.46.
Number of features: 13

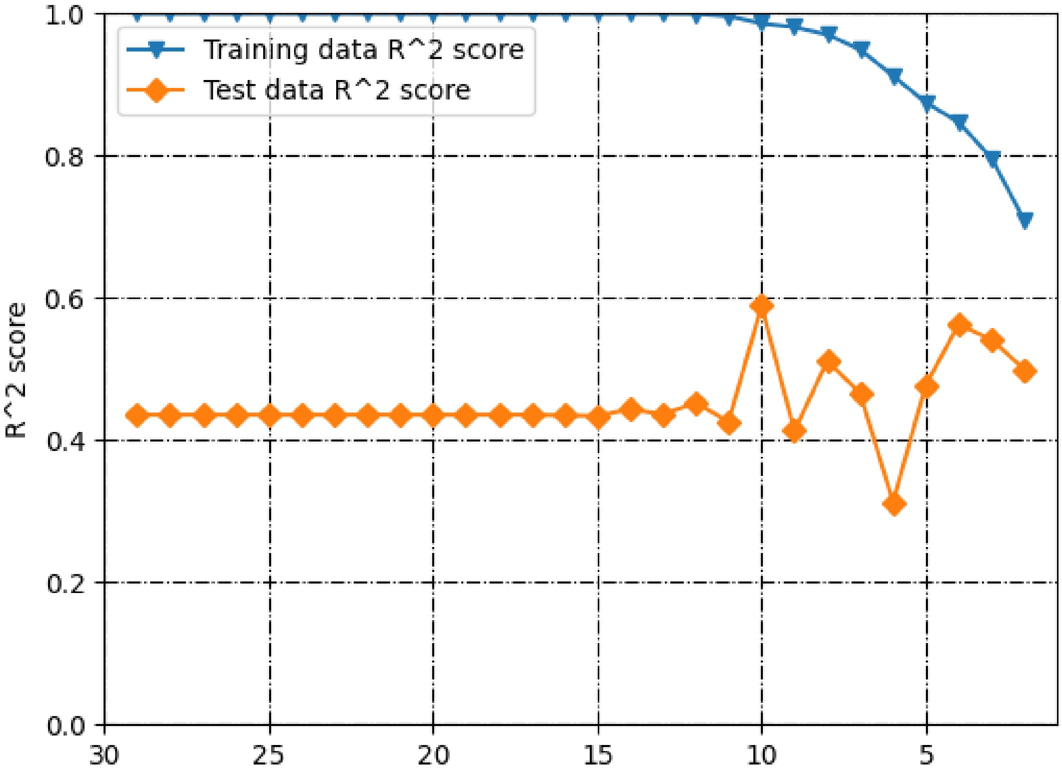

Depth number -> correlation threshold: 0.47.
Number of features: 11

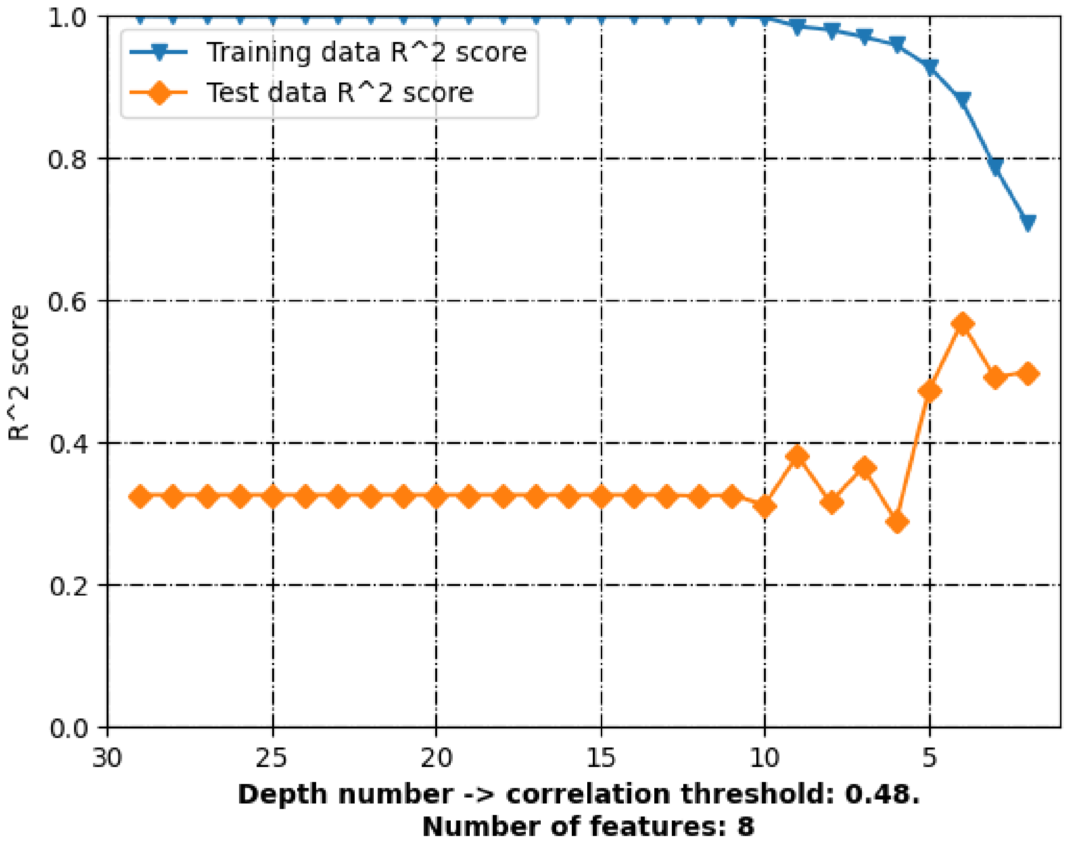
1.0
0.8
0.6
0.4
0.2
0.0
R^2 score
Training data R^2 score
Test data R^2 score
30
25
20
15
10
5
Depth number -> correlation threshold: 0.48.
Number of features: 8

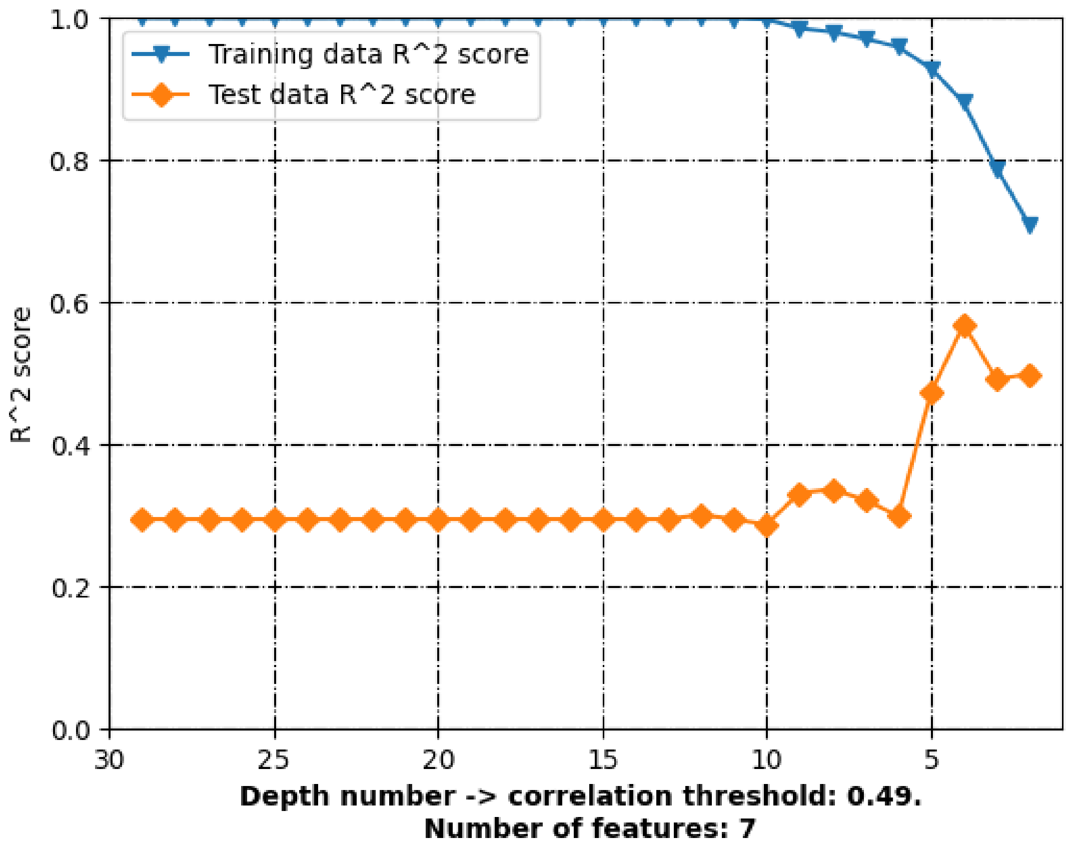

Training data R^2 score
Test data R^2 score
R^2 score
1.0
0.8
0.6
0.4
0.2
0.0
30
25
20
15
10
5
Depth number -> correlation threshold: 0.49.
Number of features: 7

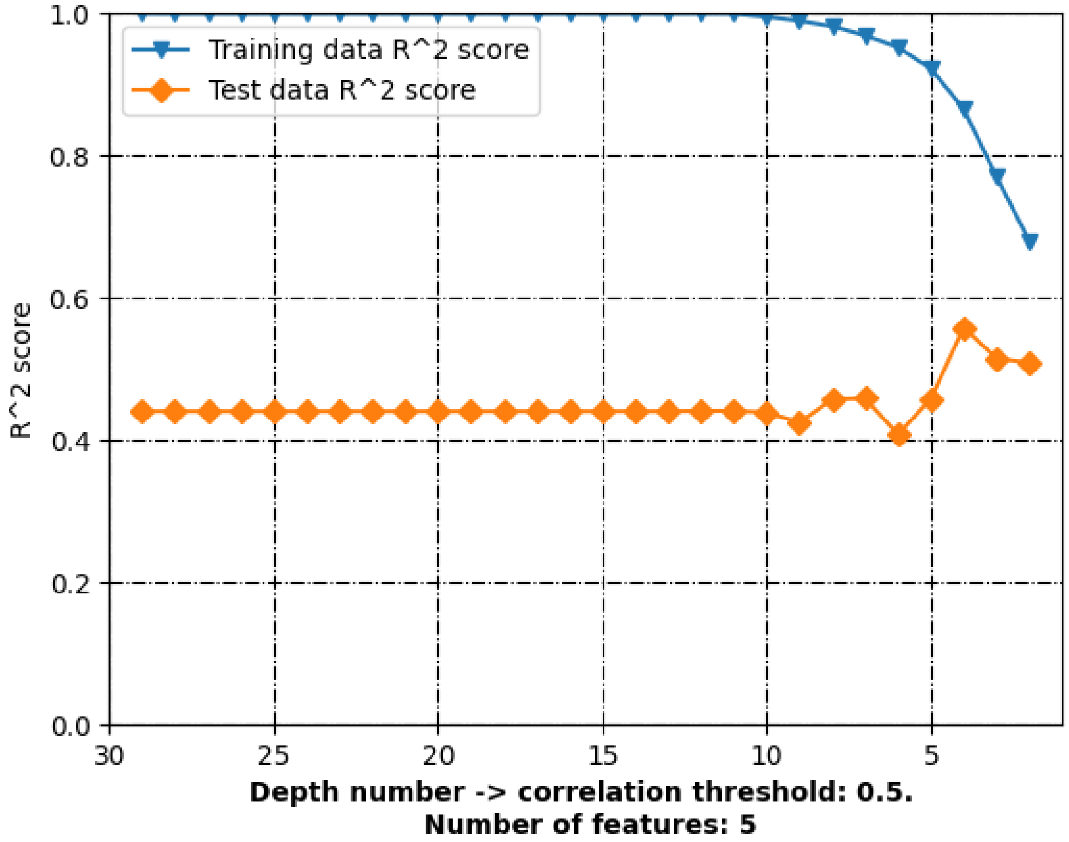

Training data R^2 score
Test data R^2 score
R^2 score
1.0
0.8
0.6
0.4
0.2
0.0
30
25
20
15
10
5
Depth number -> correlation threshold: 0.5.
Number of features: 5

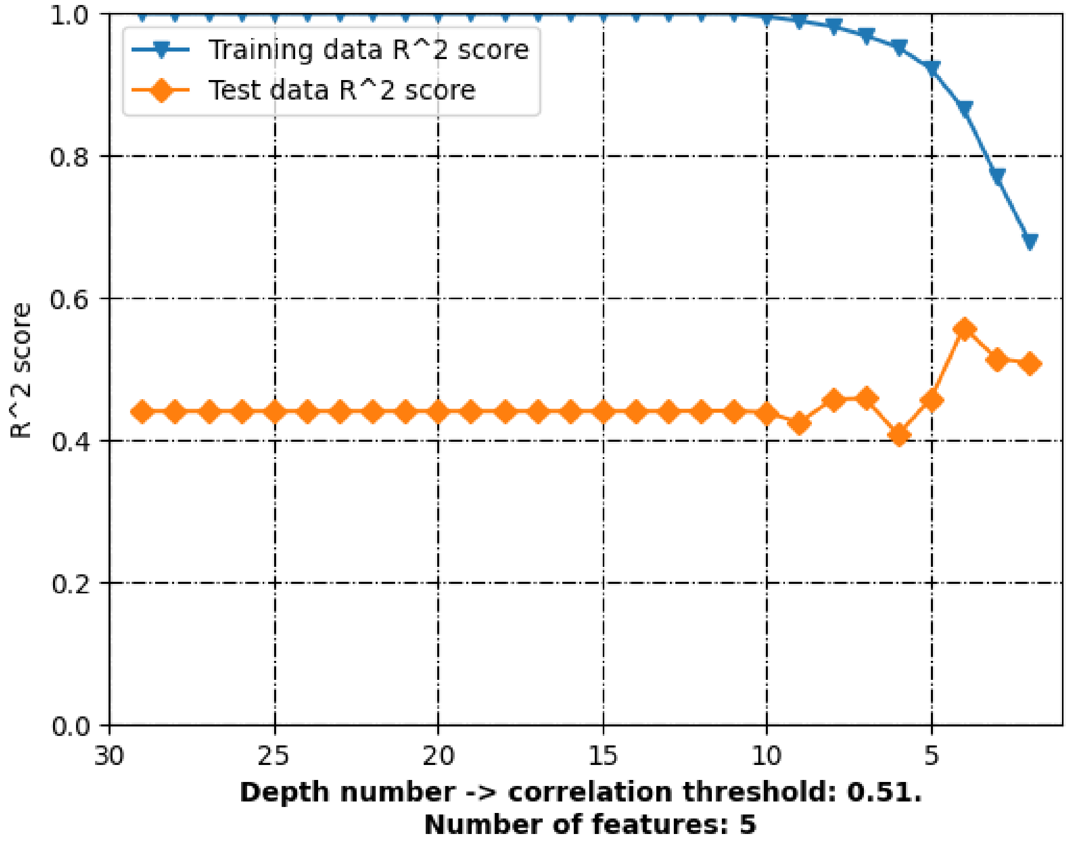

Training data R^2 score
Test data R^2 score
R^2 score
1.0
0.8
0.6
0.4
0.2
0.0
30
25
20
15
10
5
Depth number -> correlation threshold: 0.51.
Number of features: 5

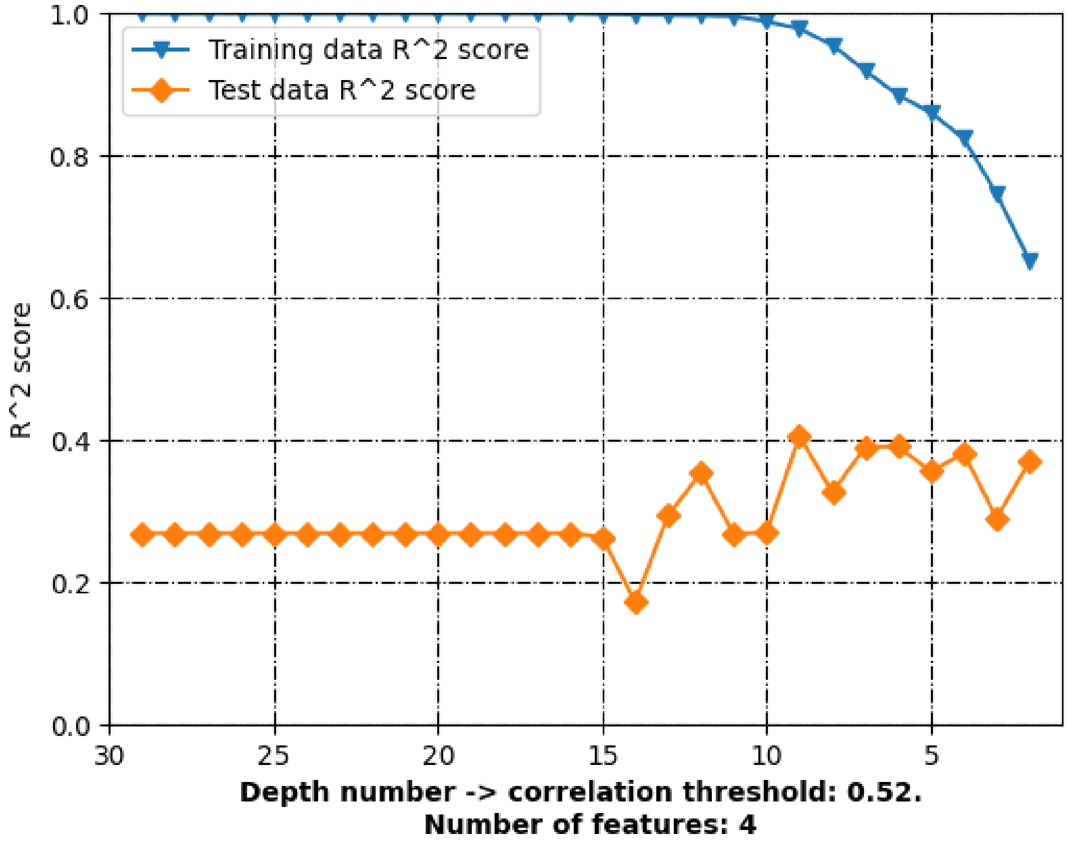

Training data R^2 score
Test data R^2 score
R^2 score
1.0
0.8
0.6
0.4
0.2
0.0
30
25
20
15
10
5
Depth number -> correlation threshold: 0.52.
Number of features: 4

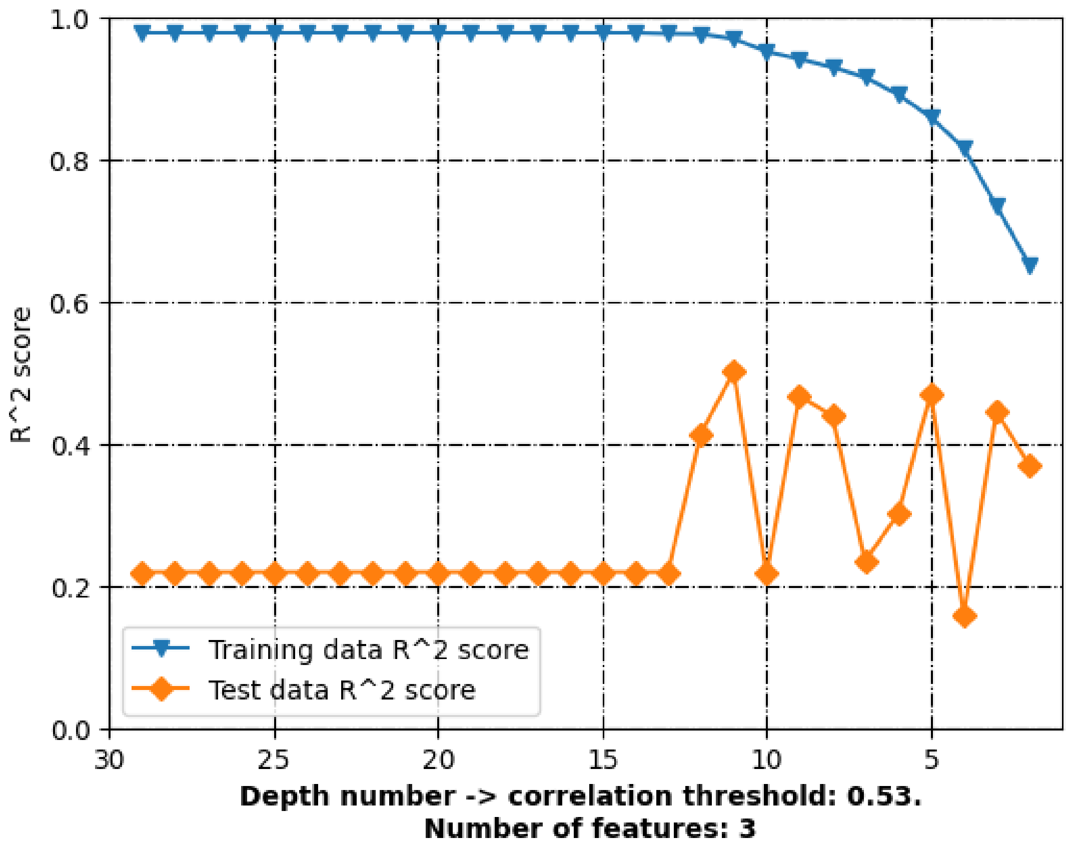

[20]:
```
plt.plot(df_without_standardization['Correlation threshold'],␣
  ↪df_without_standardization['Number of features'], label = "Number of␣
  ↪features", marker='.')
plt.legend()
plt.xlabel('Correlation threshold')
plt.ylabel('Number of features')
plt.show()
```

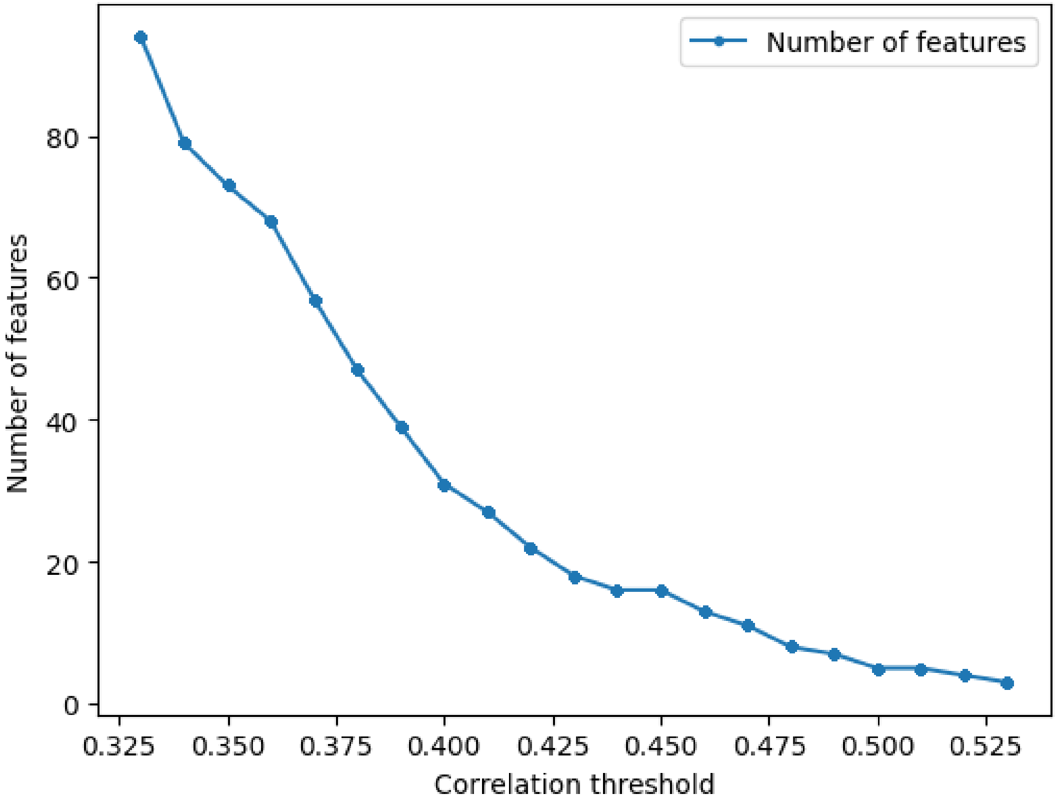

[ ]:

3 Random Forest

[21]:
```
without_standardization, train_r2_, test_r2_, hist1, hist2, target_column_name,␣
↪training_data_RMSE, test_data_RMSE  = pred_model.
↪prepare_data_and_create_model(molecular_descriptors_df=data,
                                                                              ␣
↪                          correlation_threshold=0.50,
                                                                              ␣
↪                          standardization=False,
                                                                              ␣
↪                          model_type='RandomForestRegressor',
                                                                              ␣
↪                          n_estimators_=12,
                                                                              ␣
↪                          target_column_name = target,
                                                                              ␣
↪                          random_state=random_state,
```

```
↪                     train_test_split_=True,
↪                     verbose=True)
```

```
I am not doing standardization…
  molecular descriptor name
0                    AATS0Z
1                  AATS0are
2                    AATS0d
3                   AATS0dv
4                    AATS0i
  molecular descriptor name  corr_value
0                    AATS0Z   -0.022797
1                  AATS0are   -0.139064
2                    AATS0d    0.027592
3                   AATS0dv   -0.136049
4                    AATS0i    0.168958
  molecular descriptor name  corr_value  absolute correlation value
0                    AATS0Z   -0.022797                    0.022797
1                  AATS0are   -0.139064                    0.139064
2                    AATS0d    0.027592                    0.027592
3                   AATS0dv   -0.136049                    0.136049
4                    AATS0i    0.168958                    0.168958
     molecular descriptor name  corr_value  absolute correlation value
226                     AMID_O   -0.557717                    0.557717
505                EState_VSA5   -0.534748                    0.534748
791                   MDEO-12   -0.555724                    0.555724
921                      SaasC    0.513071                    0.513071
1091               VSA_EState5    0.526082                    0.526082
     molecular descriptor name  corr_value  absolute correlation value
226                     AMID_O   -0.557717                    0.557717
505                EState_VSA5   -0.534748                    0.534748
791                   MDEO-12   -0.555724                    0.555724
921                      SaasC    0.513071                    0.513071
1091               VSA_EState5    0.526082                    0.526082
The model used is: RandomForest…
Return the coefficient of determination of the prediction:
0.5069352440802066
R^2 score: 0.9240354875228499
Correlation coefficient: 0.9612676461438042
Test data - unseen during training:
R^2 score: 0.5069352440802066
Correlation coefficient: 0.7119938511533696
[7.65137153 7.77327392 7.88934768 7.59369964 5.14608638 8.06568435
 7.93091305 7.87863225 8.06905679 8.04785191 7.2680587  7.62927442
 8.07574813 6.15268454 6.68421748 7.33463177 7.98931088 7.89716909]
44     8.193820
```

```
47     7.886057
4      7.048177
55     7.886057
26     4.904168
64     8.537602
73     7.657577
10     8.017729
40     7.920819
107    8.698970
18     6.994819
62     8.886057
11     7.962574
36     6.221126
89     8.022276
91     8.958607
109    7.886057
0      7.966576
Name: A549, dtype: float64
Training Root Mean Square Error: 0.2596453671959053
Testing Root Mean Square Error: 0.6681675669349053
```

[22]:
```
print(target_column_name+str('_transformed'))
print(hist1[target_column_name].hist())
```

```
A549_transformed
AxesSubplot(0.125,0.11;0.775x0.77)
```

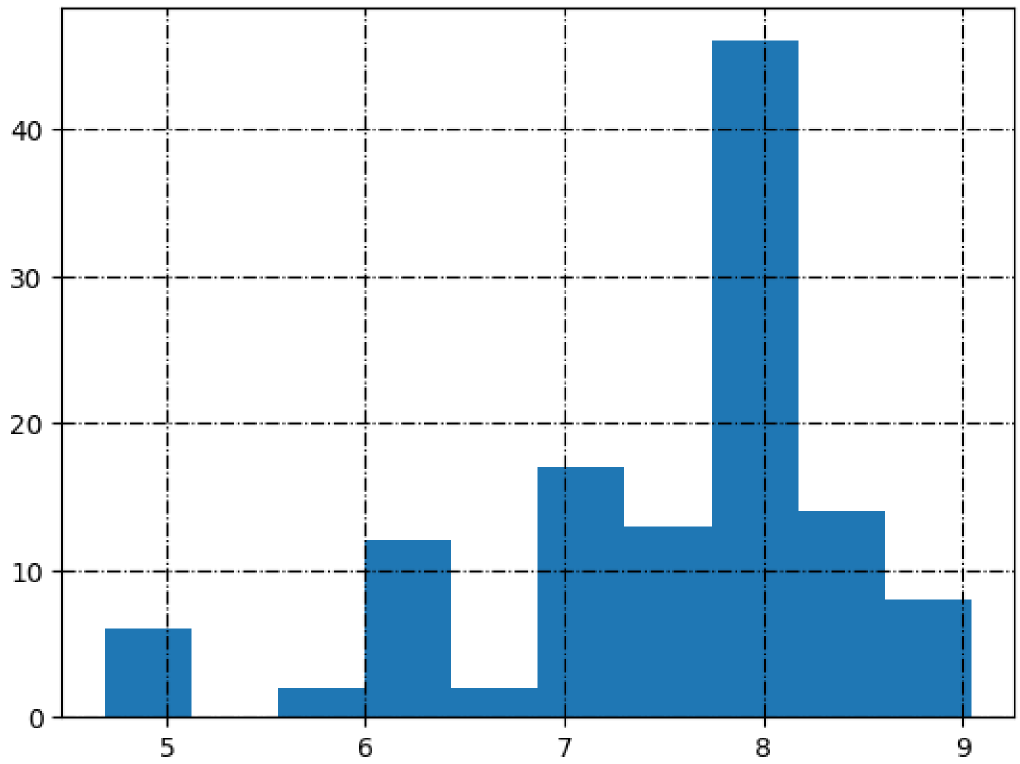

[23]:
```
with_standardization, train_r2_, test_r2_, hist1, hist2, target_column_name,␣
↪training_data_RMSE, test_data_RMSE  = pred_model.
↪prepare_data_and_create_model(molecular_descriptors_df=data,
                                                                             ␣
↪                         correlation_threshold=0.50,
                                                                             ␣
↪                         standardization=True,
                                                                             ␣
↪                         model_type='RandomForestRegressor',
                                                                             ␣
↪                         n_estimators_=12,
                                                                             ␣
↪                         target_column_name = target,
                                                                             ␣
↪                         random_state=random_state,
                                                                             ␣
↪                         train_test_split_=True,
                                                                             ␣
↪                         verbose=True)
```

```
I am doing standardization…
  molecular descriptor name
```

```
0                    AATS0Z
1                  AATS0are
2                    AATS0d
3                   AATS0dv
4                    AATS0i
  molecular descriptor name  corr_value
0                    AATS0Z   -0.022797
1                  AATS0are   -0.139064
2                    AATS0d    0.027592
3                   AATS0dv   -0.136049
4                    AATS0i    0.168958
  molecular descriptor name  corr_value  absolute correlation value
0                    AATS0Z   -0.022797                    0.022797
1                  AATS0are   -0.139064                    0.139064
2                    AATS0d    0.027592                    0.027592
3                   AATS0dv   -0.136049                    0.136049
4                    AATS0i    0.168958                    0.168958
     molecular descriptor name  corr_value  absolute correlation value
226                     AMID_O   -0.557717                    0.557717
505                 EState_VSA5  -0.534748                    0.534748
791                    MDEO-12   -0.555724                    0.555724
921                      SaasC    0.513071                    0.513071
1091               VSA_EState5    0.526082                    0.526082
     molecular descriptor name  corr_value  absolute correlation value
226                     AMID_O   -0.557717                    0.557717
505                 EState_VSA5  -0.534748                    0.534748
791                    MDEO-12   -0.555724                    0.555724
921                      SaasC    0.513071                    0.513071
1091               VSA_EState5    0.526082                    0.526082
The model used is: RandomForest…
Return the coefficient of determination of the prediction:
0.5069352440802066
R^2 score: 0.9240354875228499
Correlation coefficient: 0.9612676461438042
Test data - unseen during training:
R^2 score: 0.5069352440802066
Correlation coefficient: 0.7119938511533696
[7.65137153 7.77327392 7.88934768 7.59369964 5.14608638 8.06568435
 7.93091305 7.87863225 8.06905679 8.04785191 7.2680587  7.62927442
 8.07574813 6.15268454 6.68421748 7.33463177 7.98931088 7.89716909]
44     8.193820
47     7.886057
4      7.048177
55     7.886057
26     4.904168
64     8.537602
73     7.657577
10     8.017729
```

```
40      7.920819
107     8.698970
18      6.994819
62      8.886057
11      7.962574
36      6.221126
89      8.022276
91      8.958607
109     7.886057
0       7.966576
Name: A549, dtype: float64
Training Root Mean Square Error: 0.2596453671959053
Testing Root Mean Square Error: 0.6681675669349053
```

3.1 Search inside correlation space

[24]:
```
step = 0.01
initial_step = corr_low
last_step = corr_high
first_list = [x / 100.0 for x in range(int(initial_step*100),␣
↪int(last_step*100), int(step*100))]
n_estimators = [range(2,21,1)]
corr_th = []
second_list = []
third_list = []
fourth_l = []
fifth_l = []
f_list = []
fif_list = []
for i in first_list:

    for estimator in n_estimators[0]:

        without_standardization, train_r2, test_r2, _, h_, target_column_name,␣
↪training_data_RMSE, test_data_RMSE = pred_model.
↪prepare_data_and_create_model(molecular_descriptors_df=data,
                                                                                ␣
↪                       correlation_threshold=i,
                                                                                ␣
↪                       standardization=False,
                                                                                ␣
↪                       model_type='RandomForestRegressor',
                                                                                ␣
↪                       n_estimators_=estimator,
                                                                                ␣
↪                       target_column_name = target,
```

```
                                                                            ␣
↪                     random_state=random_state,
                                                                            ␣
↪                     train_test_split_=True,
                                                                            ␣
↪                     verbose=False)
        corr_th.append(i)
        second_list.append(train_r2)
        third_list.append(test_r2)
        fourth_l.append(training_data_RMSE)
        fifth_l.append(test_data_RMSE)
        f_list.append(len(h_))
        fif_list.append(estimator)
```

[25]:
```
df_without_standardization = pd.DataFrame(data=corr_th, columns=["Correlation␣
↪threshold"])
df_without_standardization['Training data R^2 score'] = second_list
df_without_standardization['Test data R^2 score'] = third_list
df_without_standardization['Training RMSE'] = fourth_l
df_without_standardization['Test RMSE'] = fifth_l
df_without_standardization['Number of features'] = f_list
df_without_standardization['Number of estimators'] = fif_list
```

[26]:
```
df_random_forest = df_without_standardization.copy()
#df_without_standardization.to_excel('../Data/
↪A549_Random_forest_rs_'+str(random_state)+'.xlsx')
```

[27]:
```
corre_list = [x / 100.0 for x in range(int(initial_step*100),␣
↪int(last_step*100), int(0.01*100))]
for element in corre_list:
    element_ =␣
↪df_without_standardization[df_without_standardization['Correlation␣
↪threshold'] == float(element)]
    plt.plot(element_['Number of estimators'], element_['Training data R^2␣
↪score'], label = "Training data R^2 score", marker='v')
    plt.plot(element_['Number of estimators'], element_['Test data R^2 score'],␣
↪label = "Test data R^2 score", marker='D')
    plt.legend()
    plt.xlabel('Number of estimators -> correlation threshold: '+str(element)+'.
↪ \n Number of features: '+str(element_['Number of features'].iloc[0]))
    plt.xlim(max(element_['Number of estimators'])+1, min(element_['Number of␣
↪estimators'])-1)
    plt.ylabel('R^2 score')
    plt.ylim([0, 1])
    plt.rc('grid', linestyle="-.", color='black')
    plt.grid(True)
```

```
plt.show()
```

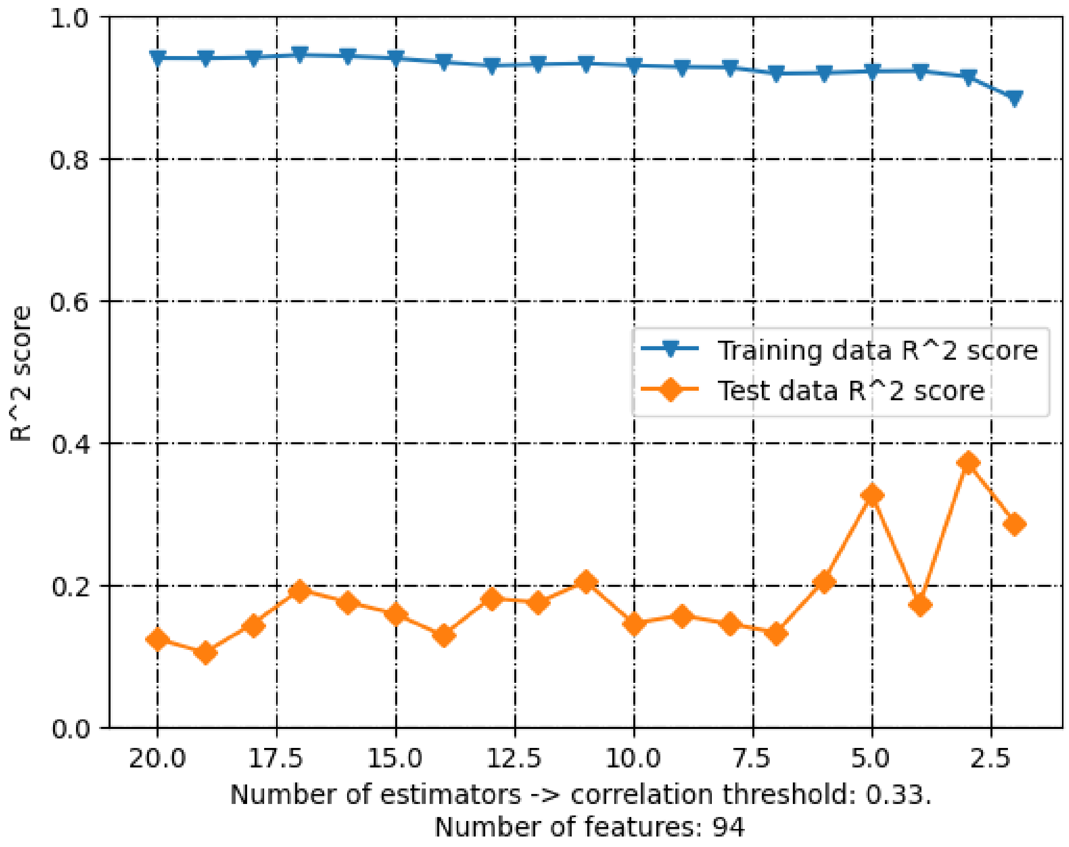

1.0
0.8
0.6
0.4
0.2
0.0
R^2 score
Training data R^2 score
Test data R^2 score
20.0
17.5
15.0
12.5
10.0
7.5
5.0
2.5
Number of estimators -> correlation threshold: 0.33.
Number of features: 94

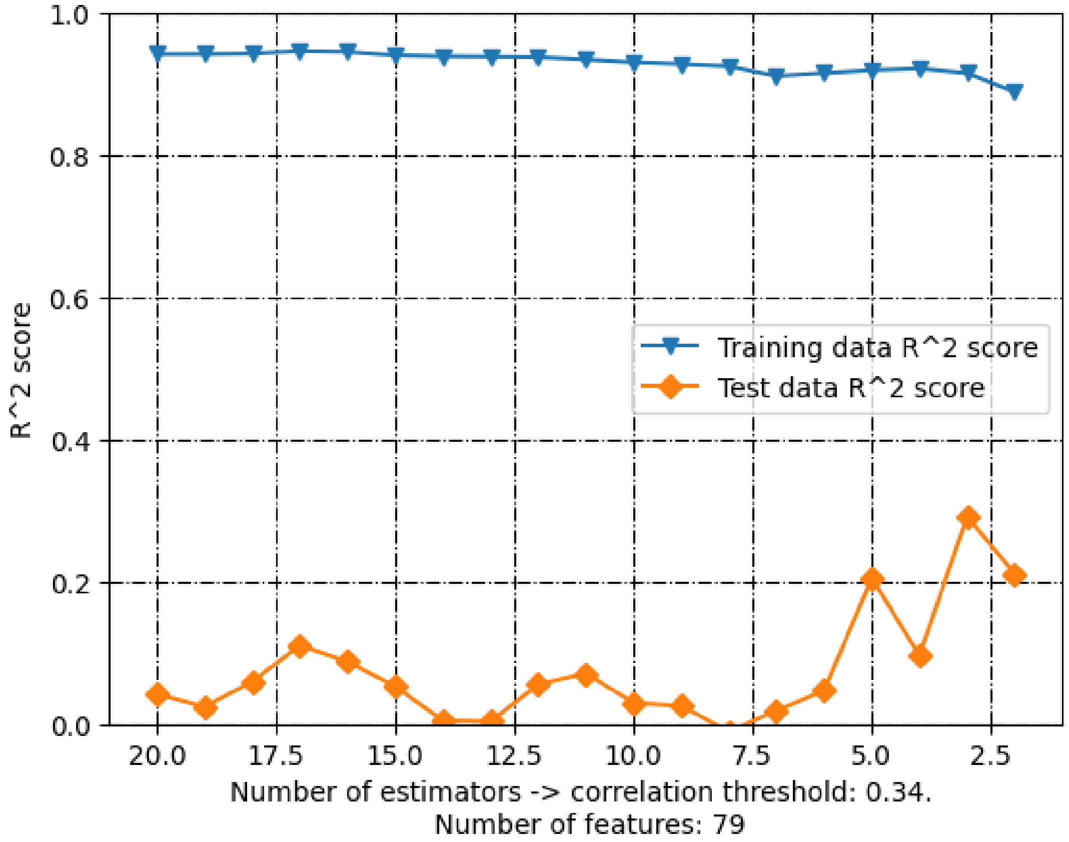
1.0
0.8
0.6
0.4
0.2
0.0
R^2 score
Training data R^2 score
Test data R^2 score
20.0
17.5
15.0
12.5
10.0
7.5
5.0
2.5
Number of estimators -> correlation threshold: 0.34.
Number of features: 79

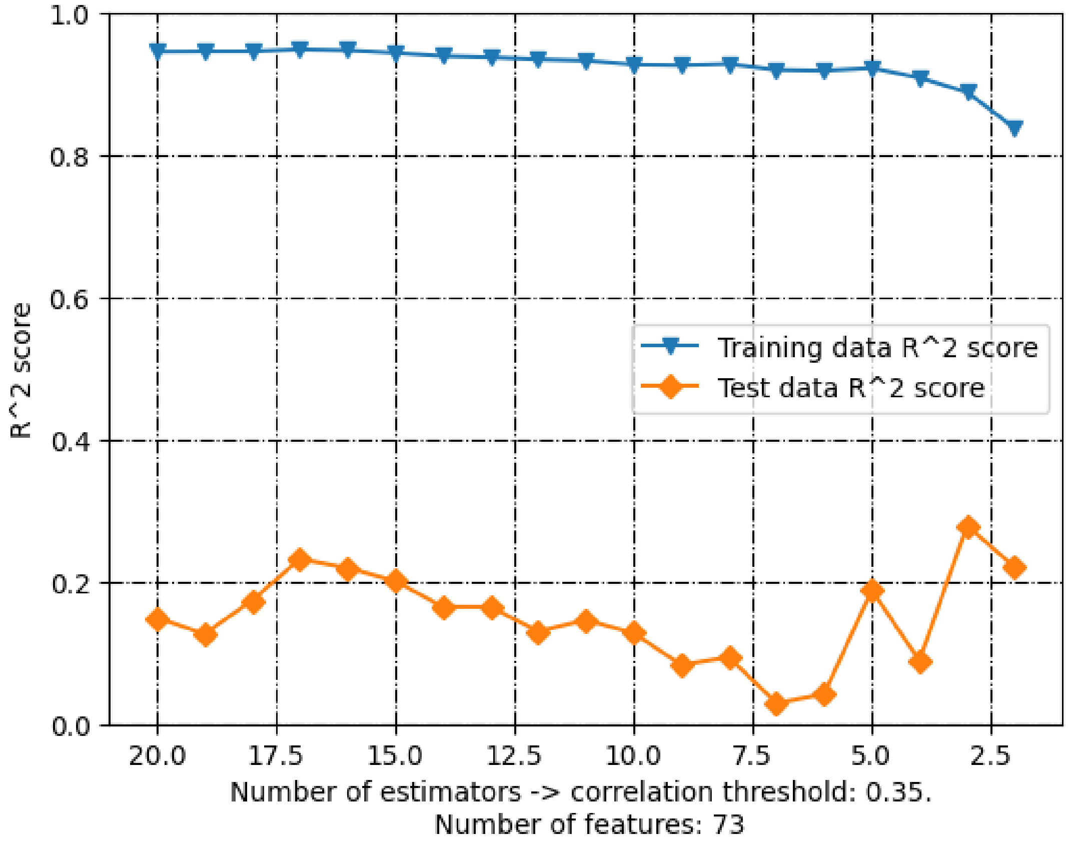

R^2 score
1.0
0.8
0.6
0.4
0.2
0.0
20.0
17.5
15.0
12.5
10.0
7.5
5.0
2.5
Training data R^2 score
Test data R^2 score
Number of estimators -> correlation threshold: 0.35.
Number of features: 73

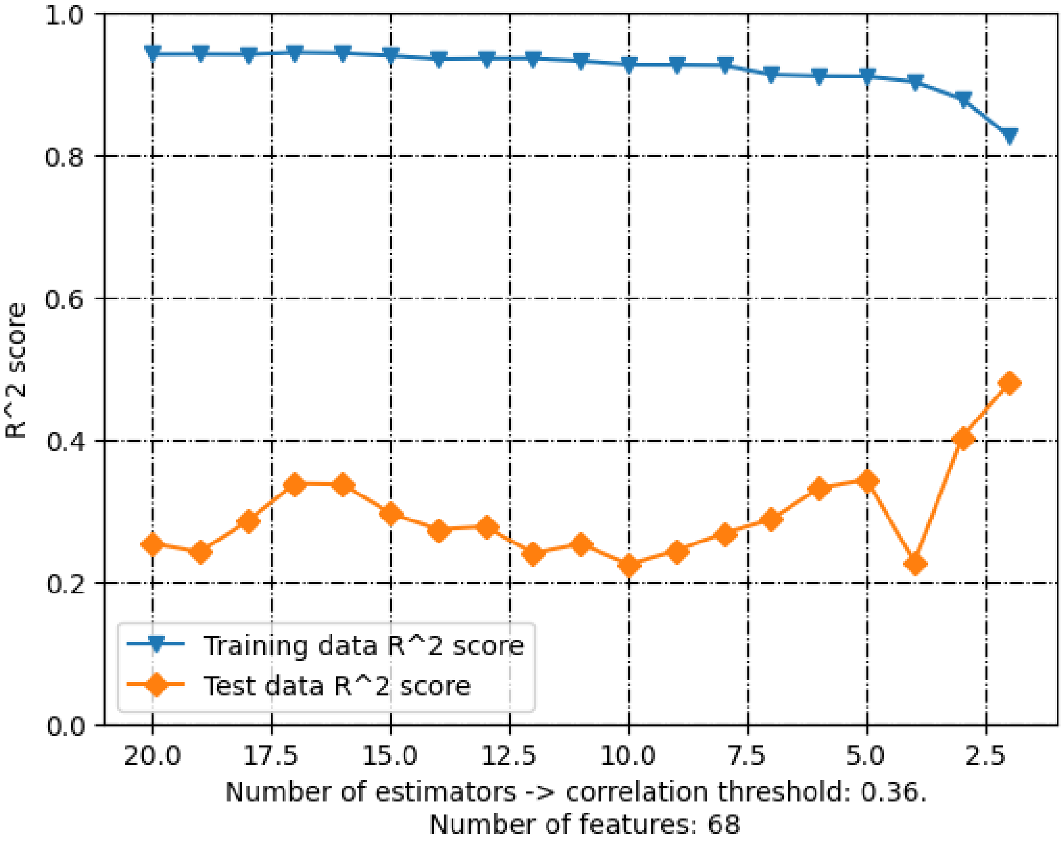

1.0
0.8
0.6
0.4
0.2
0.0
R^2 score
20.0
17.5
15.0
12.5
10.0
7.5
5.0
2.5
Number of estimators -> correlation threshold: 0.36.
Number of features: 68
Training data R^2 score
Test data R^2 score

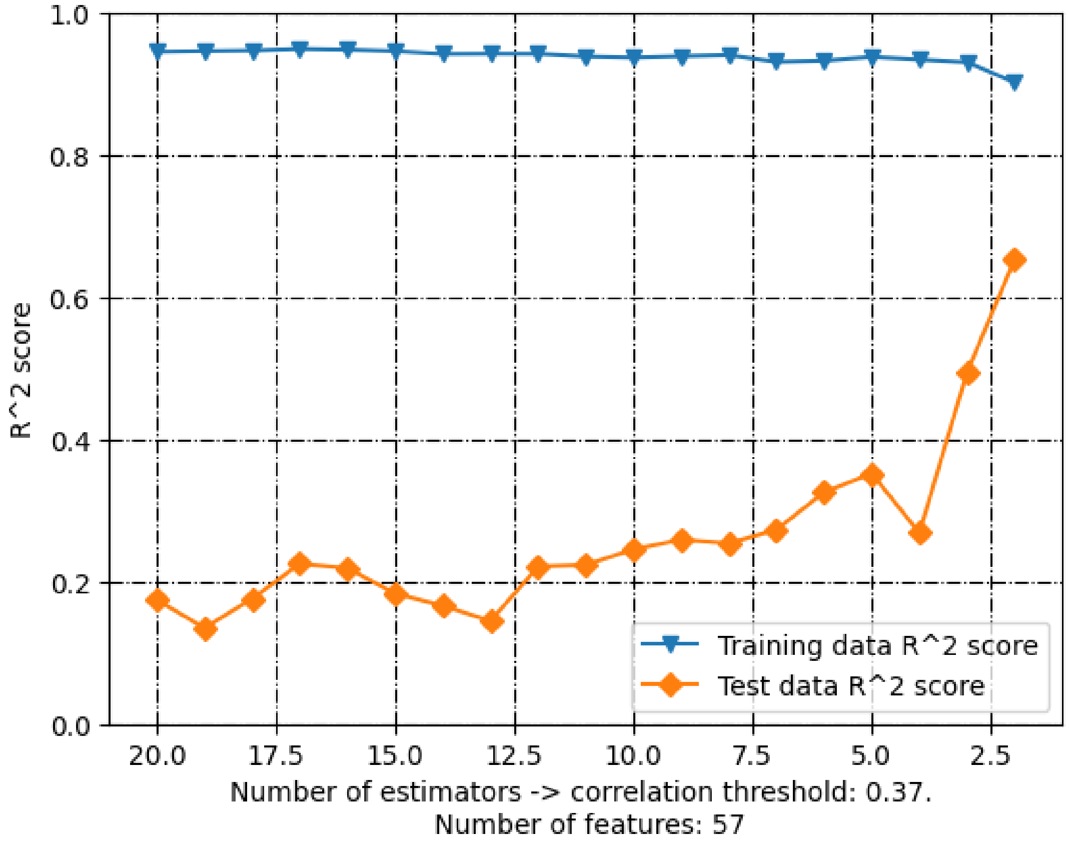

1.0
0.8
0.6
0.4
0.2
0.0
R^2 score
20.0
17.5
15.0
12.5
10.0
7.5
5.0
2.5
Number of estimators -> correlation threshold: 0.37.
Number of features: 57
Training data R^2 score
Test data R^2 score

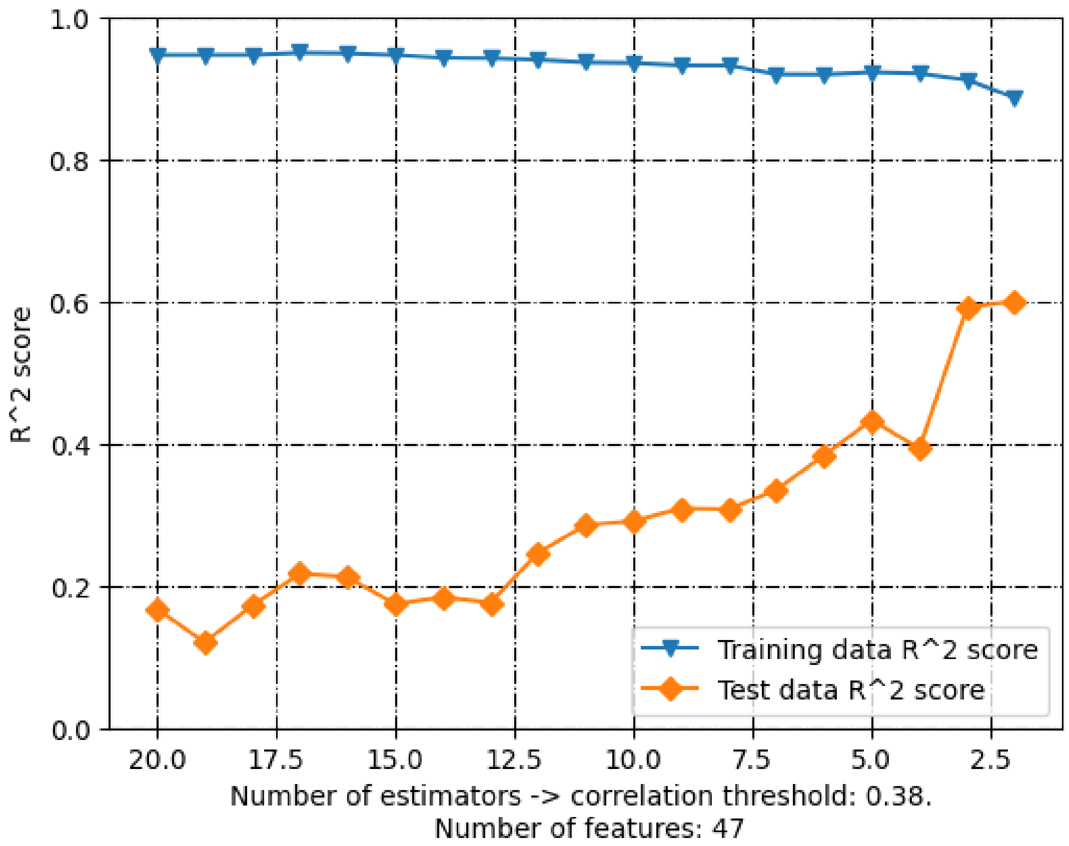

1.0
0.8
0.6
0.4
0.2
0.0
R^2 score
20.0
17.5
15.0
12.5
10.0
7.5
5.0
2.5
Training data R^2 score
Test data R^2 score
Number of estimators -> correlation threshold: 0.38.
Number of features: 47

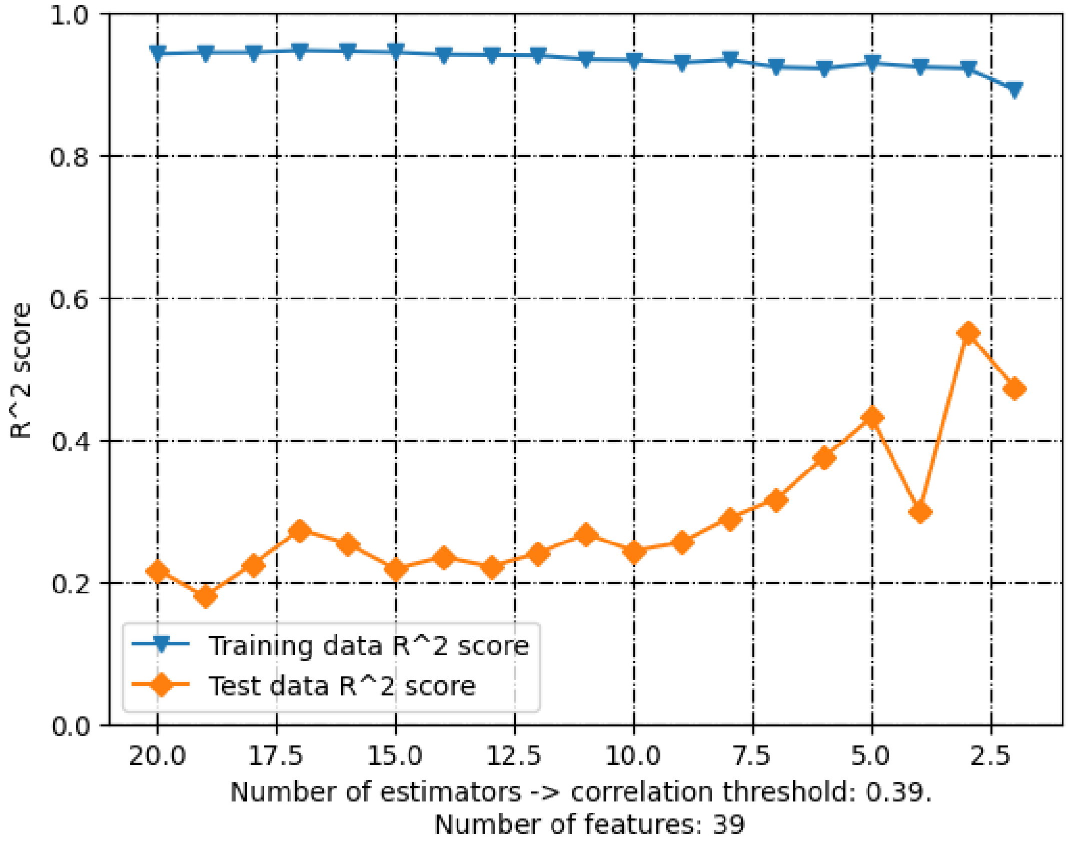

1.0
0.8
0.6
0.4
0.2
0.0
R^2 score
20.0
17.5
15.0
12.5
10.0
7.5
5.0
2.5
Number of estimators -> correlation threshold: 0.39.
Number of features: 39
Training data R^2 score
Test data R^2 score

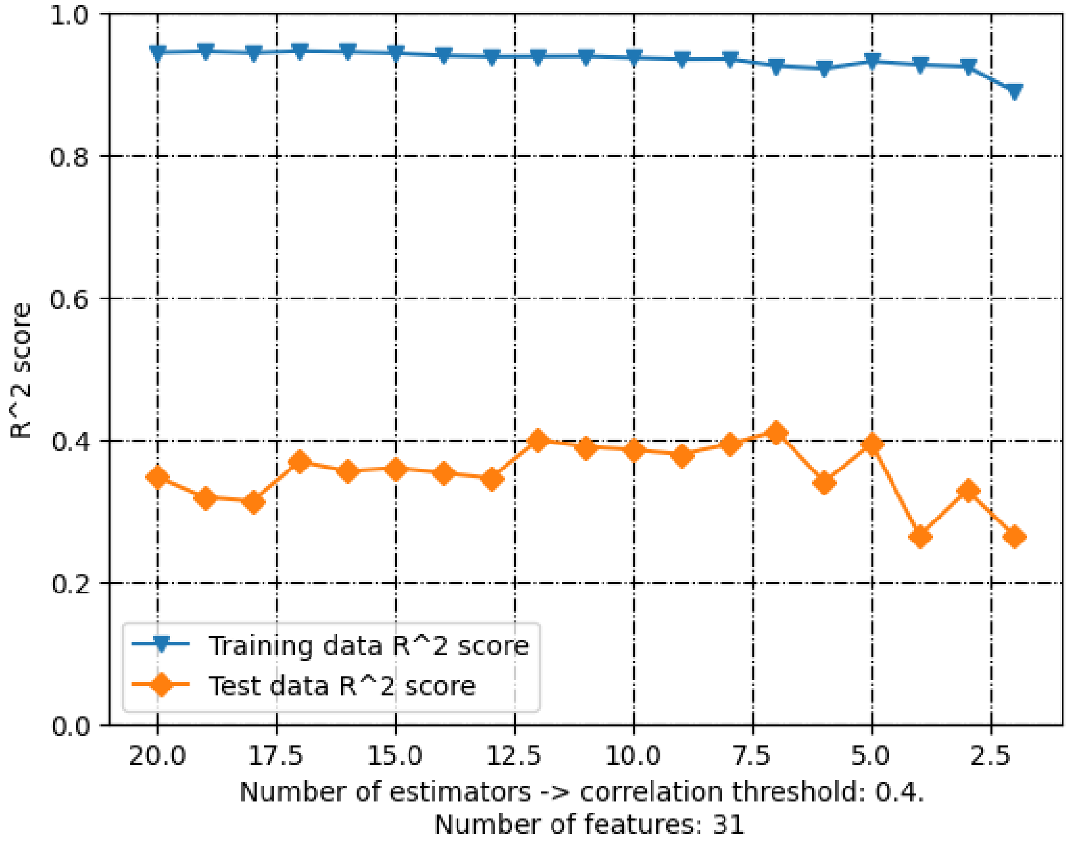
1.0
0.8
0.6
0.4
0.2
0.0
R^2 score
Training data R^2 score
Test data R^2 score
20.0
17.5
15.0
12.5
10.0
7.5
5.0
2.5
Number of estimators -> correlation threshold: 0.4.
Number of features: 31

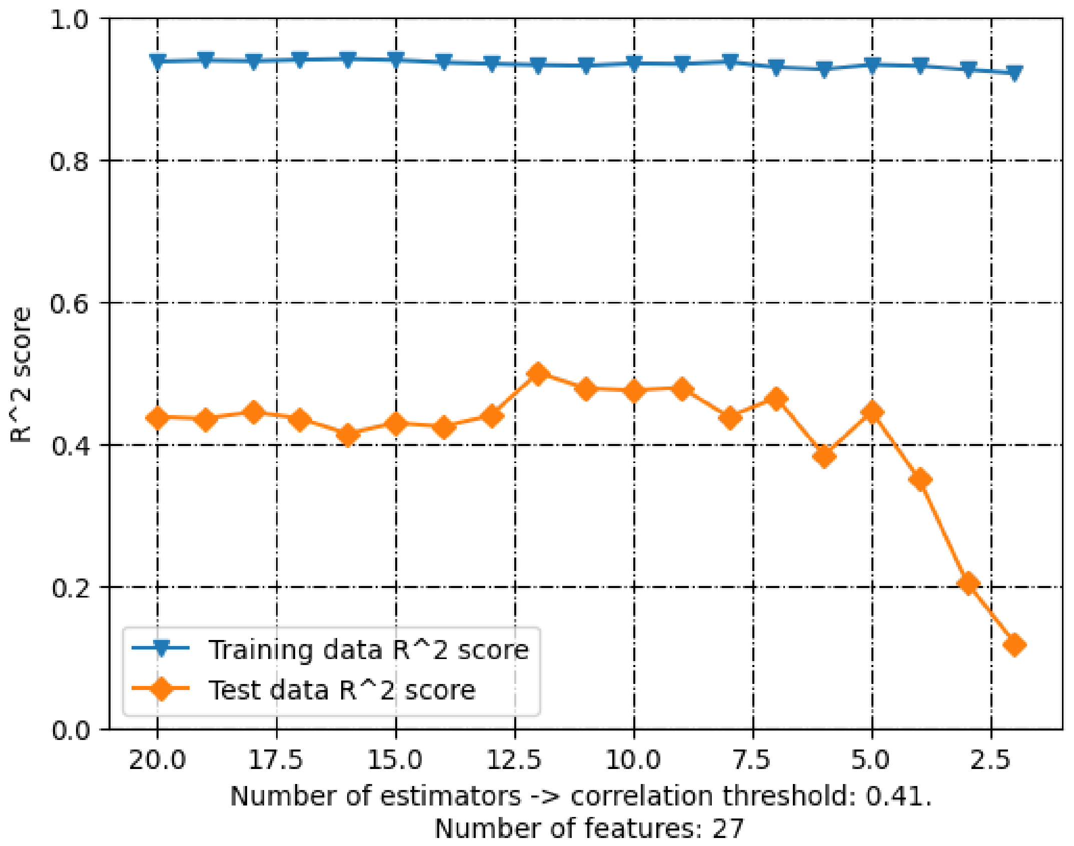
1.0
0.8
0.6
0.4
0.2
0.0
R^2 score
20.0
17.5
15.0
12.5
10.0
7.5
5.0
2.5
Training data R^2 score
Test data R^2 score
Number of estimators -> correlation threshold: 0.41.
Number of features: 27

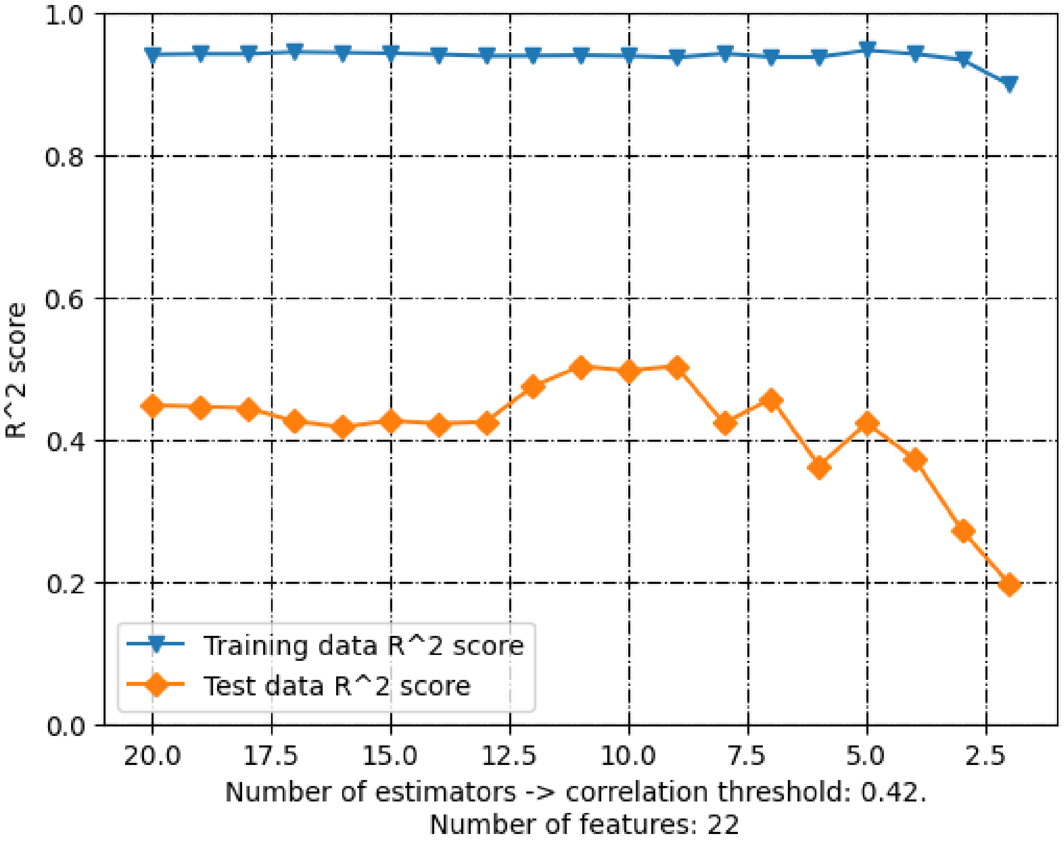
1.0
0.8
0.6
0.4
0.2
0.0
R^2 score
Training data R^2 score
Test data R^2 score
20.0
17.5
15.0
12.5
10.0
7.5
5.0
2.5
Number of estimators -> correlation threshold: 0.42.
Number of features: 22

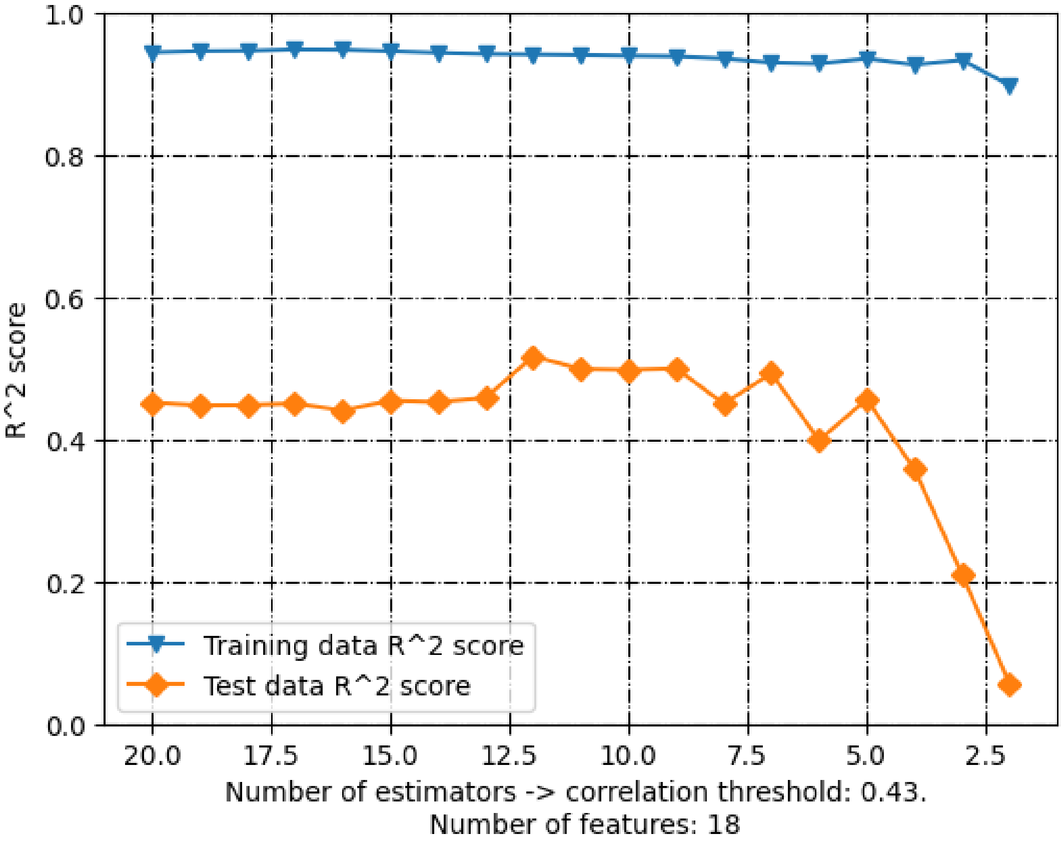

1.0
0.8
0.6
0.4
0.2
0.0
R^2 score
20.0
17.5
15.0
12.5
10.0
7.5
5.0
2.5
Training data R^2 score
Test data R^2 score
Number of estimators -> correlation threshold: 0.43.
Number of features: 18

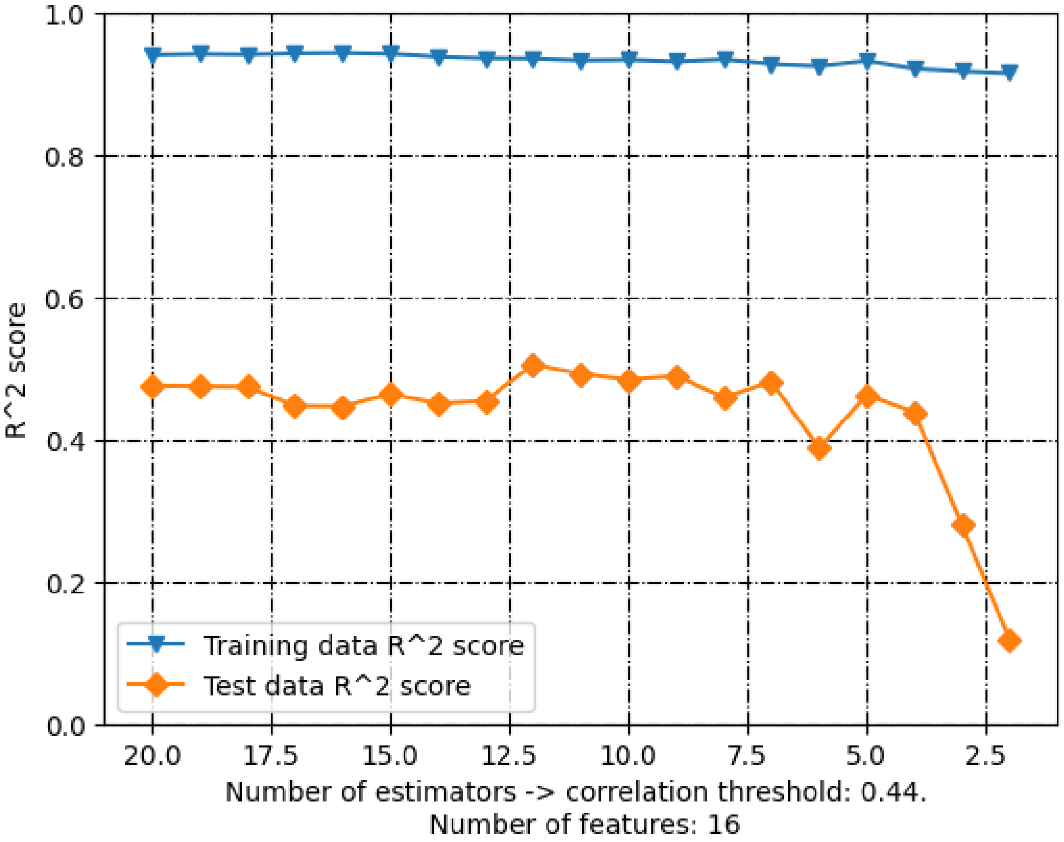
R^2 score
Training data R^2 score
Test data R^2 score
Number of estimators -> correlation threshold: 0.44.
Number of features: 16

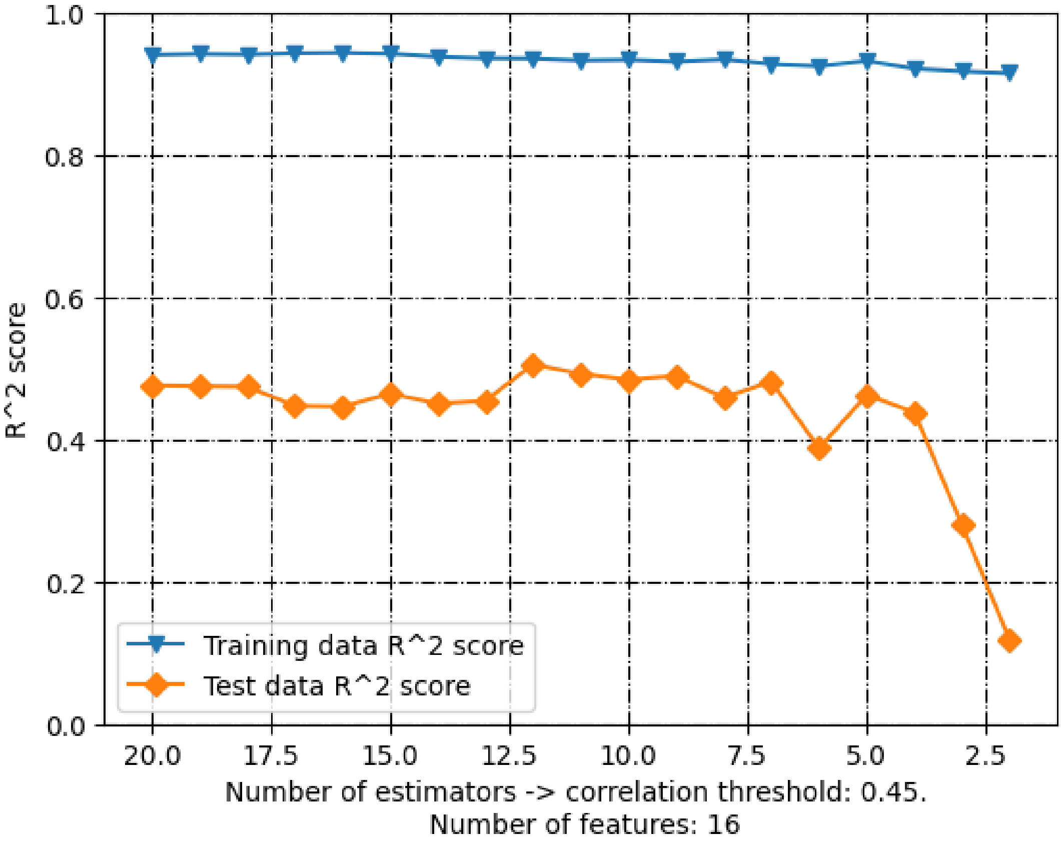

1.0
0.8
0.6
0.4
0.2
0.0
R^2 score
20.0
17.5
15.0
12.5
10.0
7.5
5.0
2.5
Training data R^2 score
Test data R^2 score
Number of estimators -> correlation threshold: 0.45.
Number of features: 16

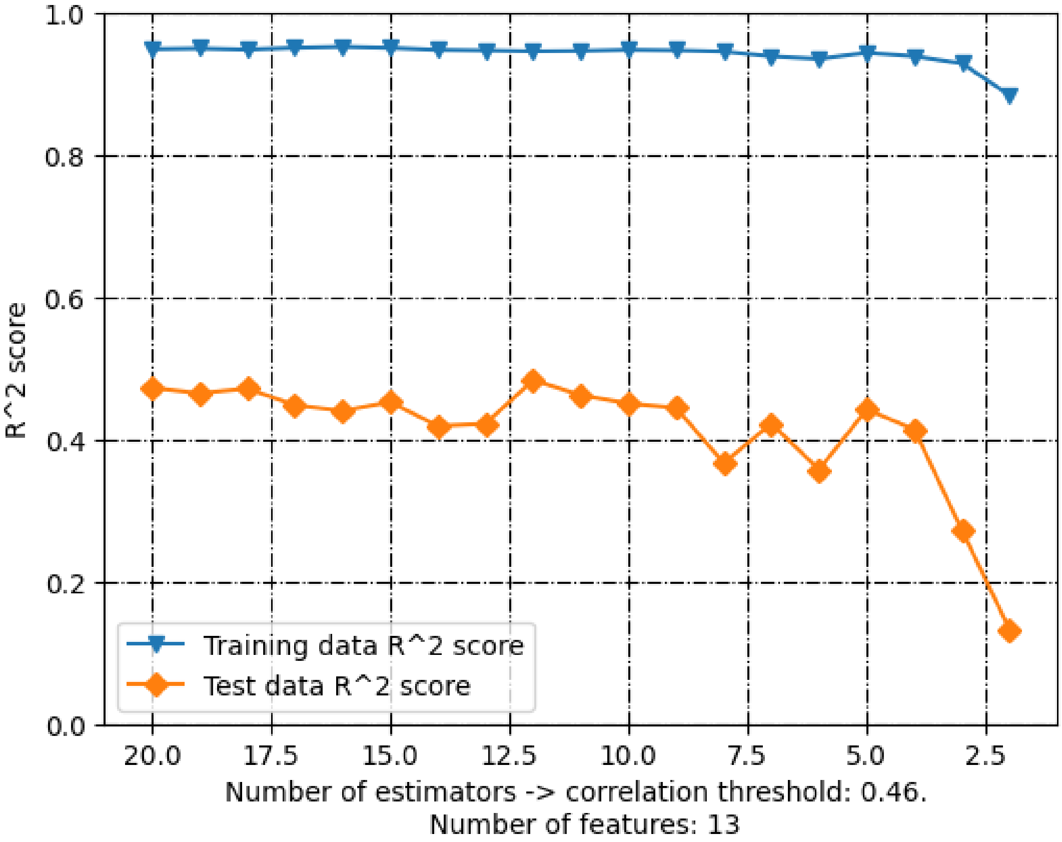

1.0
0.8
0.6
0.4
0.2
0.0
R^2 score
20.0
17.5
15.0
12.5
10.0
7.5
5.0
2.5
Training data R^2 score
Test data R^2 score
Number of estimators -> correlation threshold: 0.46.
Number of features: 13

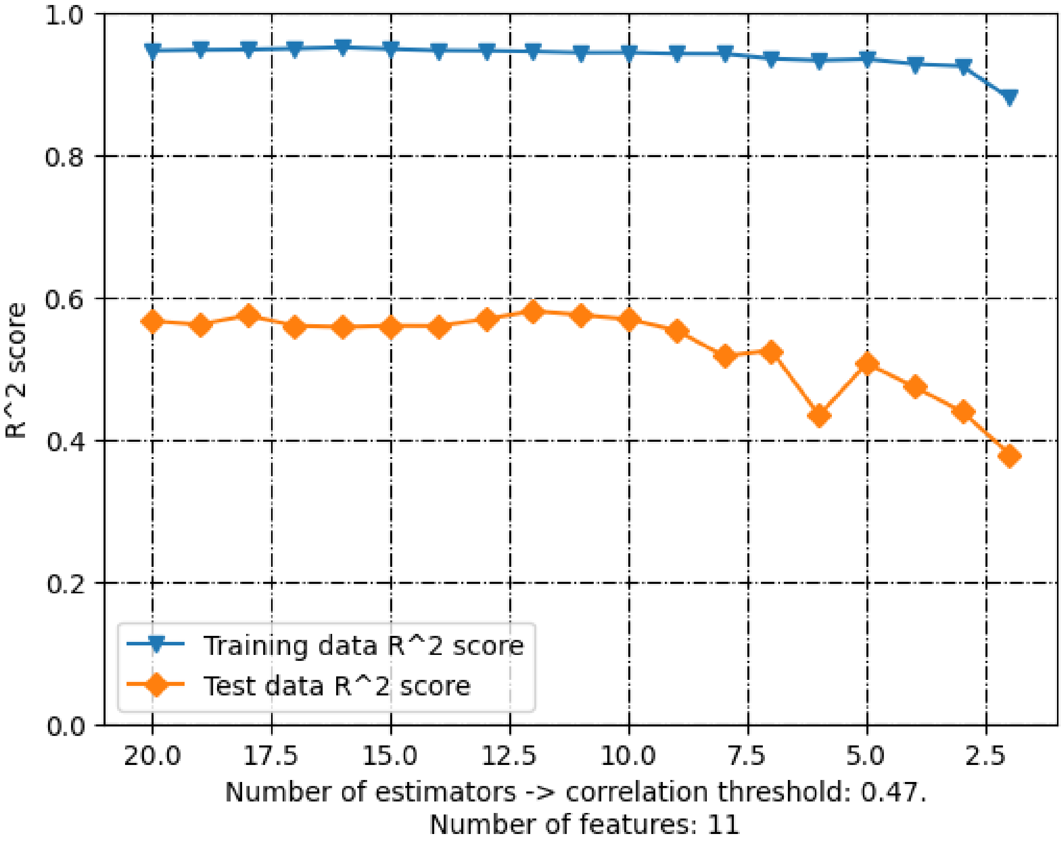

Training data R^2 score
Test data R^2 score
R^2 score
1.0
0.8
0.6
0.4
0.2
0.0
20.0
17.5
15.0
12.5
10.0
7.5
5.0
2.5
Number of estimators -> correlation threshold: 0.47.
Number of features: 11

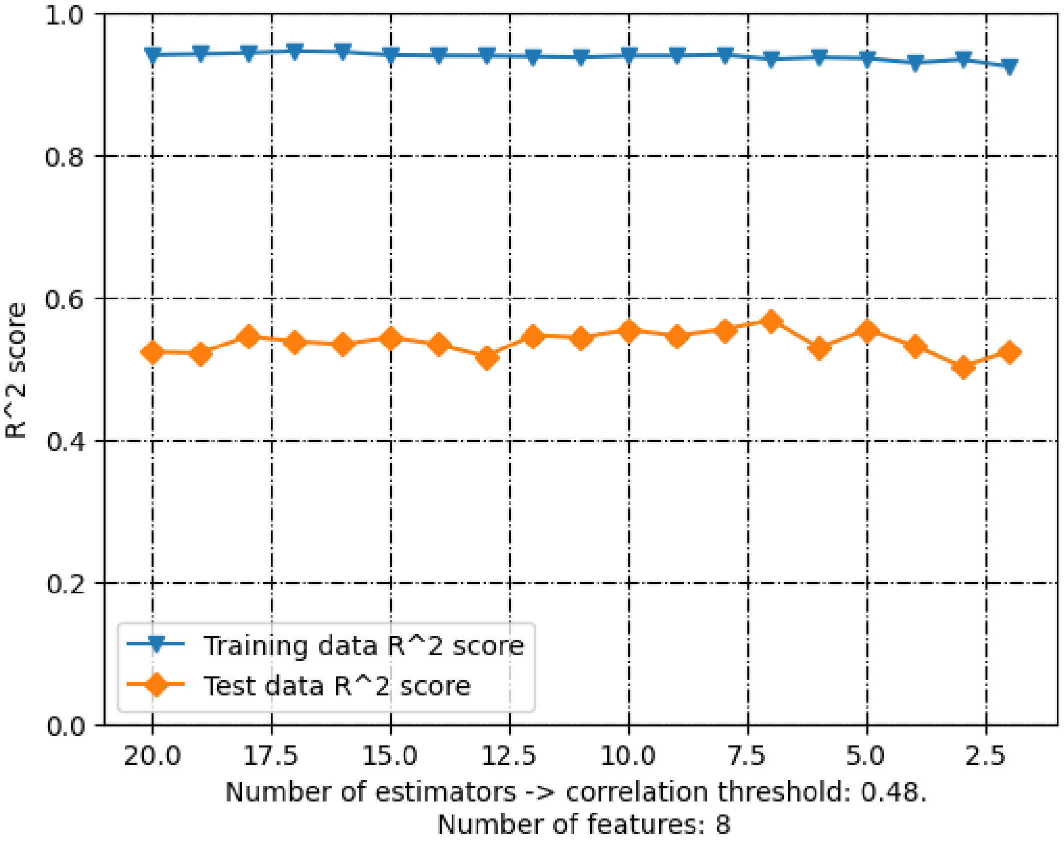
1.0
0.8
0.6
0.4
0.2
0.0
R^2 score
Training data R^2 score
Test data R^2 score
20.0
17.5
15.0
12.5
10.0
7.5
5.0
2.5
Number of estimators -> correlation threshold: 0.48.
Number of features: 8

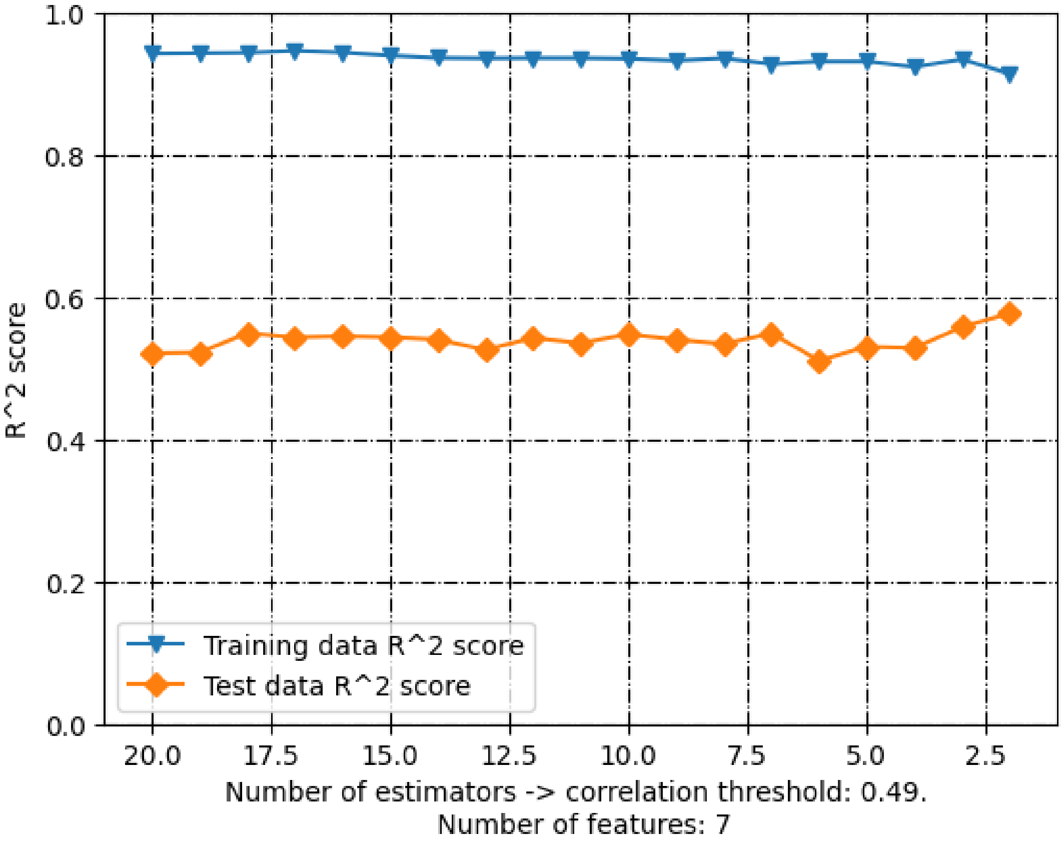
1.0
0.8
0.6
0.4
0.2
0.0
R^2 score
Training data R^2 score
Test data R^2 score
20.0
17.5
15.0
12.5
10.0
7.5
5.0
2.5
Number of estimators -> correlation threshold: 0.49.
Number of features: 7

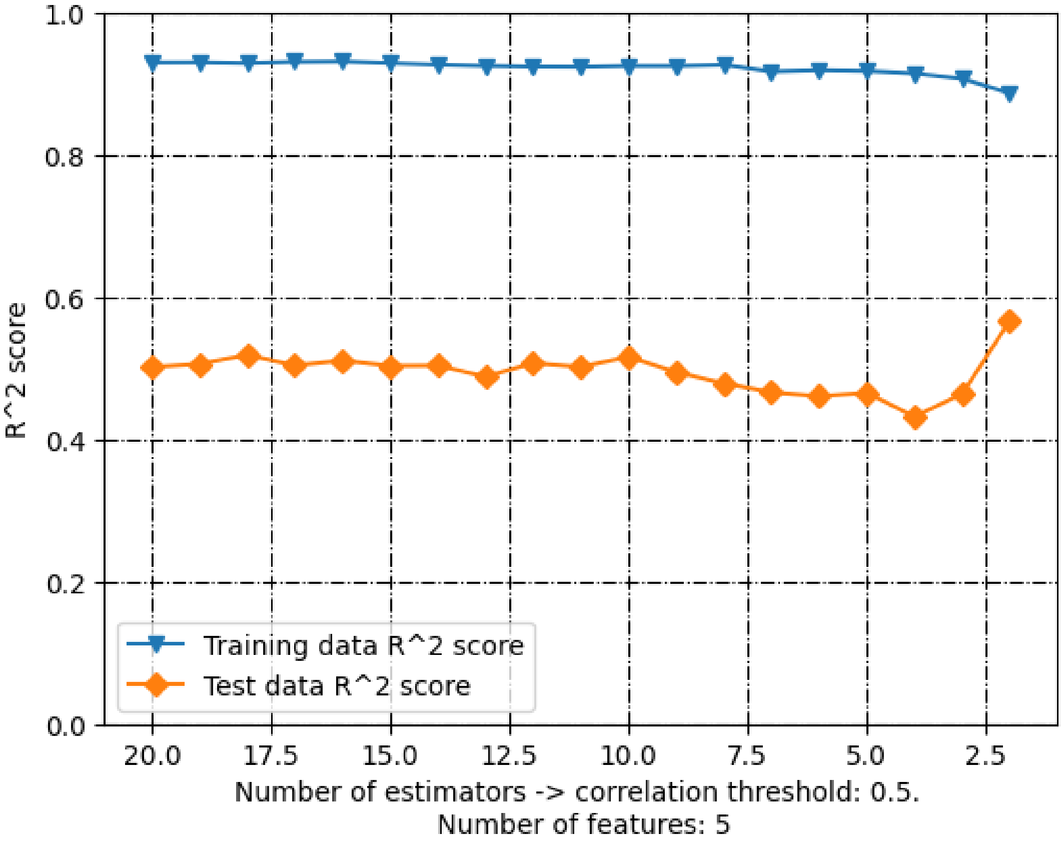

1.0
0.8
0.6
0.4
0.2
0.0
R^2 score
Training data R^2 score
Test data R^2 score
20.0
17.5
15.0
12.5
10.0
7.5
5.0
2.5
Number of estimators -> correlation threshold: 0.5.
Number of features: 5

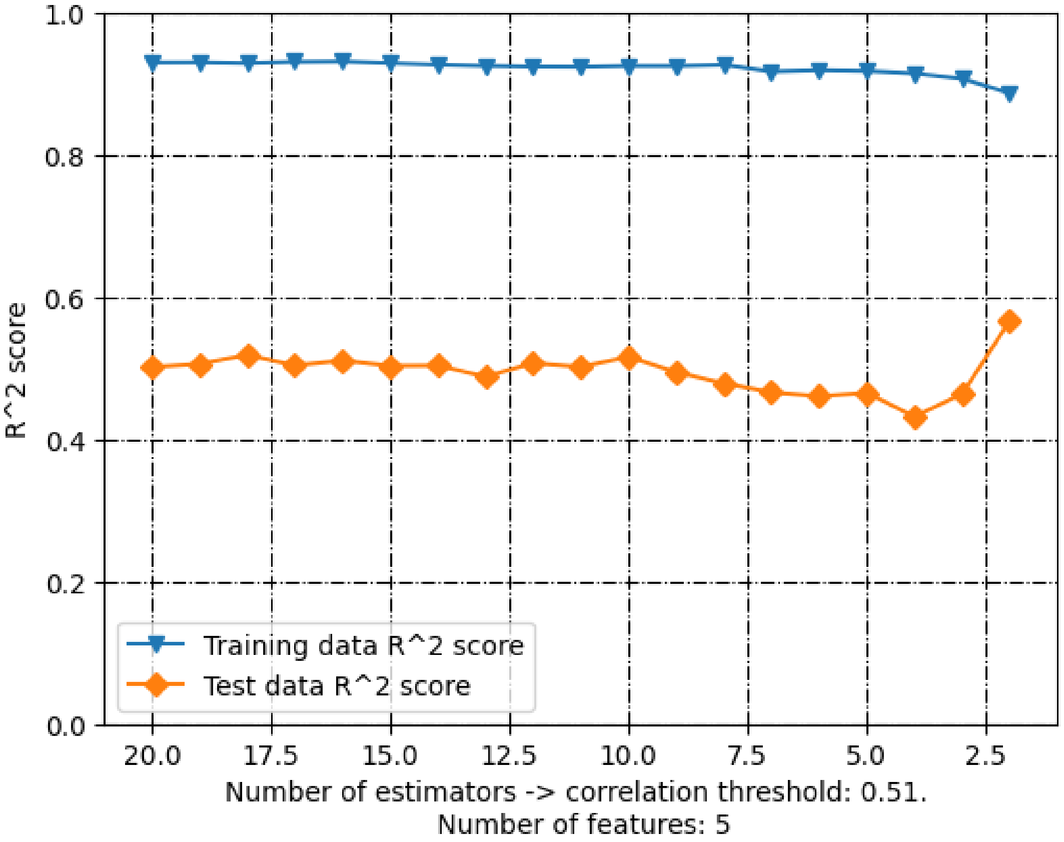

1.0
0.8
0.6
0.4
0.2
0.0
R^2 score
Training data R^2 score
Test data R^2 score
20.0
17.5
15.0
12.5
10.0
7.5
5.0
2.5
Number of estimators -> correlation threshold: 0.51.
Number of features: 5

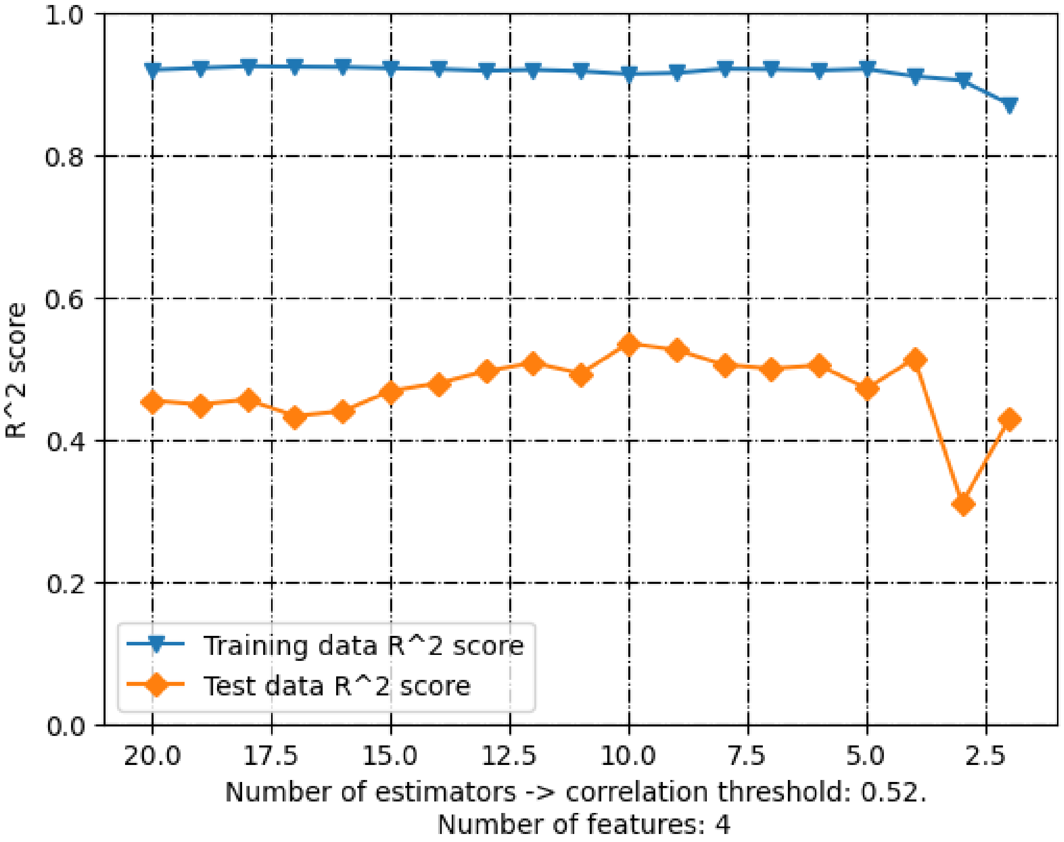

1.0
0.8
0.6
0.4
0.2
0.0
R^2 score
20.0
17.5
15.0
12.5
10.0
7.5
5.0
2.5
Number of estimators -> correlation threshold: 0.52.
Number of features: 4
Training data R^2 score
Test data R^2 score

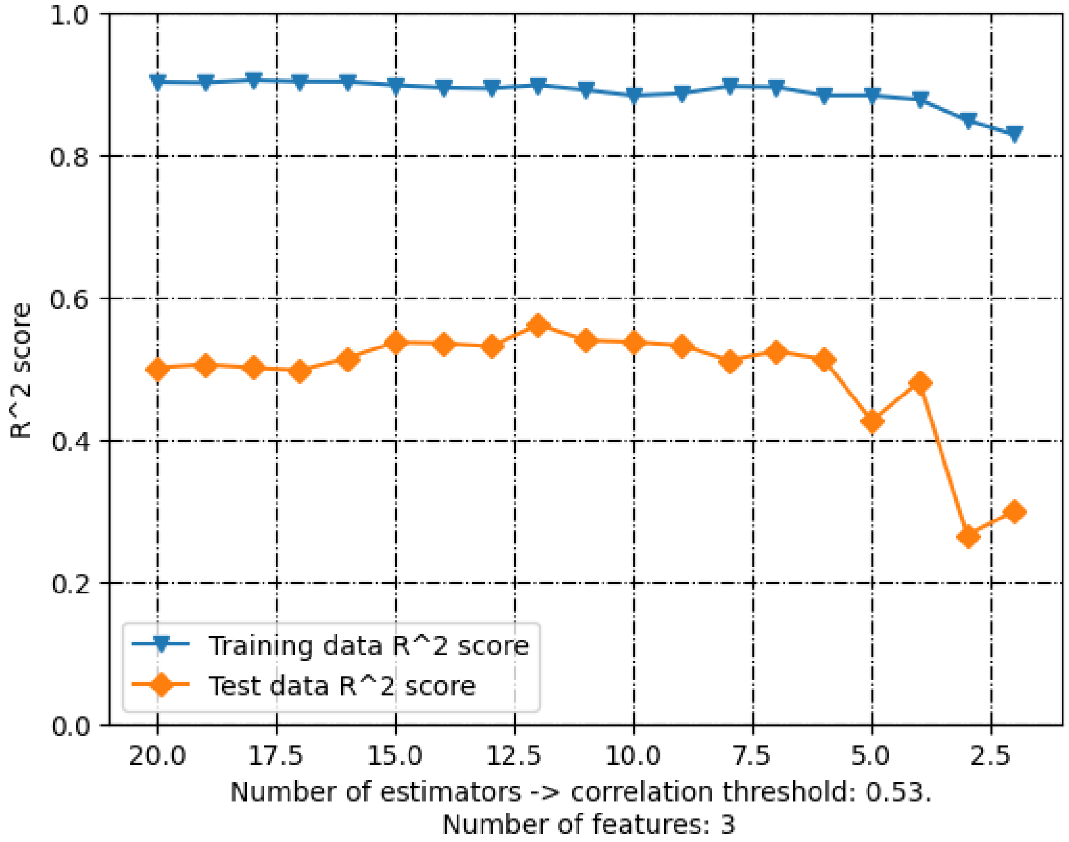

[28]:
```
plt.plot(df_without_standardization['Correlation threshold'],␣
  ↪df_without_standardization['Number of features'], label = "Number of␣
  ↪features", marker='.')
plt.legend()
plt.xlabel('Correlation threshold')
plt.ylabel('Number of features')
plt.show()
```

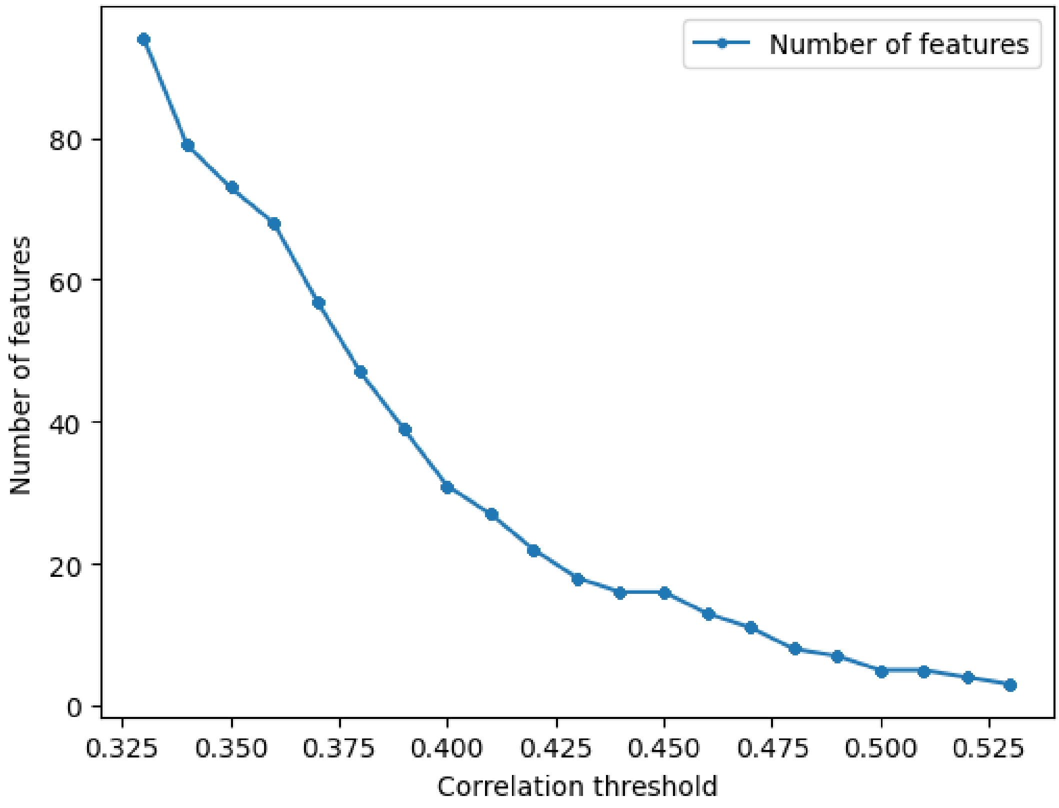

[ ]:

```

```

4 KNeighborsRegressor

[29]:

```
without_standardization, train_r2_, test_r2_, hist1, hist2, target_column_name,␣
↪training_data_RMSE, test_data_RMSE  = pred_model.
↪prepare_data_and_create_model(molecular_descriptors_df=data,
                                                                              ␣
↪                    correlation_threshold=0.50,
                                                                              ␣
↪                    standardization=False,
                                                                              ␣
↪                    model_type='KNeighborsRegressor',
                                                                              ␣
↪                    target_column_name = target,
                                                                              ␣
↪                    random_state=random_state,
                                                                              ␣
↪                    train_test_split_=True,
```

```
↪                    verbose=True)
```

```
I am not doing standardization…
  molecular descriptor name
0                   AATS0Z
1                 AATS0are
2                   AATS0d
3                  AATS0dv
4                   AATS0i
  molecular descriptor name  corr_value
0                   AATS0Z   -0.022797
1                 AATS0are   -0.139064
2                   AATS0d    0.027592
3                  AATS0dv   -0.136049
4                   AATS0i    0.168958
  molecular descriptor name  corr_value  absolute correlation value
0                   AATS0Z   -0.022797                    0.022797
1                 AATS0are   -0.139064                    0.139064
2                   AATS0d    0.027592                    0.027592
3                  AATS0dv   -0.136049                    0.136049
4                   AATS0i    0.168958                    0.168958
     molecular descriptor name  corr_value  absolute correlation value
226                     AMID_O   -0.557717                    0.557717
505                 EState_VSA5   -0.534748                    0.534748
791                    MDEO-12   -0.555724                    0.555724
921                      SaasC    0.513071                    0.513071
1091                VSA_EState5    0.526082                    0.526082
     molecular descriptor name  corr_value  absolute correlation value
226                     AMID_O   -0.557717                    0.557717
505                 EState_VSA5   -0.534748                    0.534748
791                    MDEO-12   -0.555724                    0.555724
921                      SaasC    0.513071                    0.513071
1091                VSA_EState5    0.526082                    0.526082
The model used is: KNeighbors…
Return the coefficient of determination of the prediction:
0.40201961974017886
R^2 score: 0.6398367295985287
Correlation coefficient: 0.7998979494901388
Test data - unseen during training:
R^2 score: 0.40201961974017886
Correlation coefficient: 0.6340501713115286
[7.86037589 7.85396571 7.36022595 7.97156198 6.54742656 8.12587932
 7.91433157 7.80354965 7.77853237 7.77853237 7.83649937 7.32741669
 7.49866334 5.36315619 7.32741669 7.91147259 7.77853237 7.97341012]
44     8.193820
47     7.886057
4      7.048177
```

```
55     7.886057
26     4.904168
64     8.537602
73     7.657577
10     8.017729
40     7.920819
107    8.698970
18     6.994819
62     8.886057
11     7.962574
36     6.221126
89     8.022276
91     8.958607
109    7.886057
0      7.966576
Name: A549, dtype: float64
Training Root Mean Square Error: 0.5653597823052801
Testing Root Mean Square Error: 0.7358289633292524
```

[30]:
```
print(target_column_name+str('_transformed'))
print(hist1[target_column_name].hist())
```

```
A549_transformed
AxesSubplot(0.125,0.11;0.775x0.77)
```

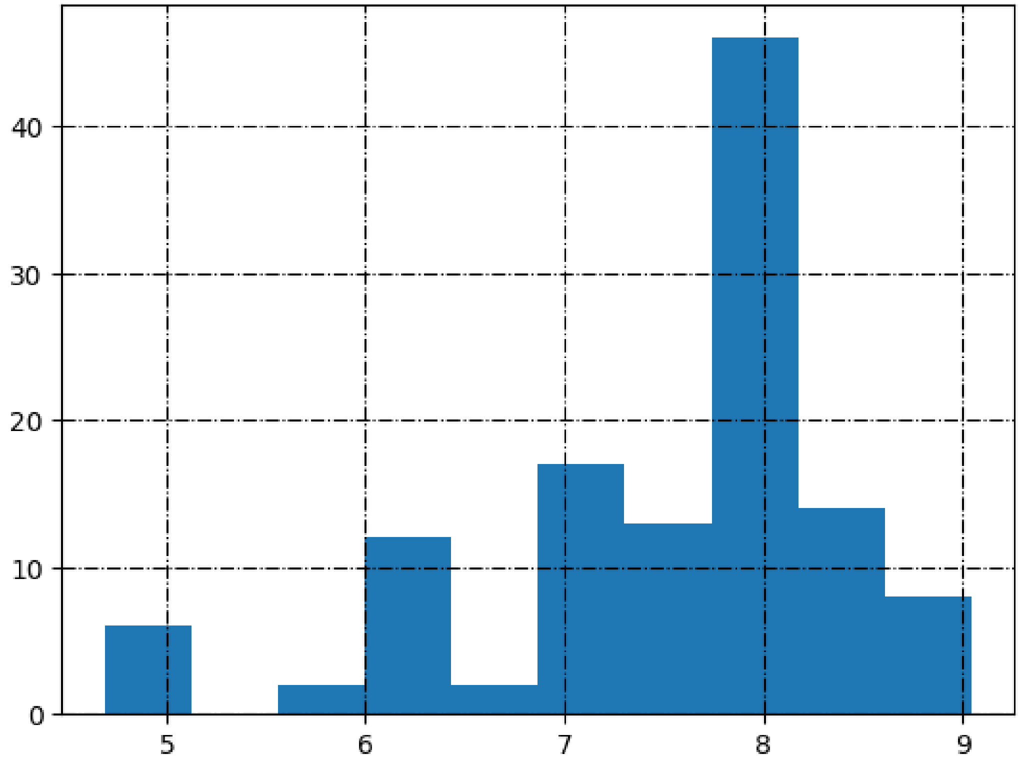

[31]:
```
with_standardization, train_r2_, test_r2_, hist1, hist2, target_column_name,␣
↪training_data_RMSE, test_data_RMSE  = pred_model.
↪prepare_data_and_create_model(molecular_descriptors_df=data,
                                                                              ␣
↪                        correlation_threshold=0.50,
                                                                              ␣
↪                        standardization=True,
                                                                              ␣
↪                        model_type='KNeighborsRegressor',
                                                                              ␣
↪                        target_column_name = target,
                                                                              ␣
↪                        random_state=random_state,
                                                                              ␣
↪                        train_test_split_=True,
                                                                              ␣
↪                        verbose=True)
```

```
I am doing standardization…
  molecular descriptor name
0                    AATS0Z
1                  AATS0are
2                    AATS0d
3                   AATS0dv
4                    AATS0i
  molecular descriptor name  corr_value
0                    AATS0Z   -0.022797
1                  AATS0are   -0.139064
2                    AATS0d    0.027592
3                   AATS0dv   -0.136049
4                    AATS0i    0.168958
  molecular descriptor name  corr_value  absolute correlation value
0                    AATS0Z   -0.022797                    0.022797
1                  AATS0are   -0.139064                    0.139064
2                    AATS0d    0.027592                    0.027592
3                   AATS0dv   -0.136049                    0.136049
4                    AATS0i    0.168958                    0.168958
     molecular descriptor name  corr_value  absolute correlation value
226                     AMID_O   -0.557717                    0.557717
505                 EState_VSA5   -0.534748                    0.534748
791                    MDEO-12   -0.555724                    0.555724
921                      SaasC    0.513071                    0.513071
1091               VSA_EState5    0.526082                    0.526082
     molecular descriptor name  corr_value  absolute correlation value
226                     AMID_O   -0.557717                    0.557717
505                 EState_VSA5   -0.534748                    0.534748
```

```
791                     MDEO-12   -0.555724                    0.555724
921                       SaasC    0.513071                    0.513071
1091                 VSA_EState5    0.526082                    0.526082
The model used is: KNeighbors…
Return the coefficient of determination of the prediction:
0.40201961974017886
R^2 score: 0.6398367295985287
Correlation coefficient: 0.7998979494901388
Test data - unseen during training:
R^2 score: 0.40201961974017886
Correlation coefficient: 0.6340501713115286
[7.86037589 7.85396571 7.36022595 7.97156198 6.54742656 8.12587932
 7.91433157 7.80354965 7.77853237 7.77853237 7.83649937 7.32741669
 7.49866334 5.36315619 7.32741669 7.91147259 7.77853237 7.97341012]
44     8.193820
47     7.886057
4      7.048177
55     7.886057
26     4.904168
64     8.537602
73     7.657577
10     8.017729
40     7.920819
107    8.698970
18     6.994819
62     8.886057
11     7.962574
36     6.221126
89     8.022276
91     8.958607
109    7.886057
0      7.966576
Name: A549, dtype: float64
Training Root Mean Square Error: 0.5653597823052801
Testing Root Mean Square Error: 0.7358289633292524
```

4.1 Search inside correlation space

[32]:
```
step = 0.01
initial_step = corr_low
last_step = corr_high
first_list = [x / 100.0 for x in range(int(initial_step*100),␣
  ↪int(last_step*100), int(step*100))]
second_list = []
third_list = []
fourth_l = []
fifth_l = []
```

```
f_list = []
for i in first_list:
    without_standardization, train_r2, test_r2, _, h_, target_column_name,␣
 ↪training_data_RMSE, test_data_RMSE = pred_model.
 ↪prepare_data_and_create_model(molecular_descriptors_df = data,
                                                                              ␣
 ↪                    correlation_threshold = i,
                                                                              ␣
 ↪                    standardization = False,
                                                                              ␣
 ↪                    model_type = 'KNeighborsRegressor',
                                                                              ␣
 ↪                    target_column_name = target,
                                                                              ␣
 ↪                    random_state=random_state,
                                                                              ␣
 ↪                    train_test_split_ = True,
                                                                              ␣
 ↪                    verbose = False)
    second_list.append(train_r2)
    third_list.append(test_r2)
    fourth_l.append(training_data_RMSE)
    fifth_l.append(test_data_RMSE)
    f_list.append(len(h_))
```

```
  File "C:\Users\aleks\anaconda3\envs\cheminf_gpu\lib\site-
packages\joblib\externals\loky\backend\context.py", line 217, in
_count_physical_cores
    raise ValueError(
```

[33]:

```
df_without_standardization = pd.DataFrame(data=first_list,␣
 ↪columns=["Correlation threshold"])
df_without_standardization['Training data R^2 score'] = second_list
df_without_standardization['Test data R^2 score'] = third_list
df_without_standardization['Training RMSE'] = fourth_l
df_without_standardization['Test RMSE'] = fifth_l
df_without_standardization['Number of features'] = f_list
```

[34]:

```
df_k_nearest = df_without_standardization.copy()
df_without_standardization
```

[34]:

```
   Correlation threshold  Training data R^2 score  Test data R^2 score  \
0                   0.33                 0.419236             0.110036
1                   0.34                 0.620736             0.281529
2                   0.35                 0.623656             0.281970
3                   0.36                 0.613087             0.259050
4                   0.37                 0.619949             0.270481
```

5	0.38	0.709012	0.388740
6	0.39	0.695405	0.345556
7	0.40	0.644268	0.429335
8	0.41	0.644268	0.429335
9	0.42	0.637618	0.407672
10	0.43	0.628703	0.490193
11	0.44	0.626614	0.473322
12	0.45	0.626614	0.473322
13	0.46	0.540127	0.140240
14	0.47	0.549909	0.126217
15	0.48	0.655201	0.389796
16	0.49	0.623380	0.381359
17	0.50	0.639837	0.402020
18	0.51	0.639837	0.402020
19	0.52	0.595532	0.426520
20	0.53	0.575237	0.454865

	Training RMSE	Test RMSE	Number of features
0	0.717918	0.897676	94
1	0.580158	0.806563	79
2	0.577920	0.806315	73
3	0.585979	0.819083	68
4	0.580759	0.812740	57
5	0.508174	0.743955	47
6	0.519920	0.769785	39
7	0.561871	0.718826	31
8	0.561871	0.718826	27
9	0.567098	0.732343	22
10	0.574032	0.679417	18
11	0.575644	0.690567	16
12	0.575644	0.690567	16
13	0.638843	0.882312	13
14	0.632012	0.889478	11
15	0.553169	0.743312	8
16	0.578132	0.748433	7
17	0.565360	0.735829	5
18	0.565360	0.735829	5
19	0.599125	0.720597	4
20	0.613972	0.702563	3

4.2 Plots

[35]:
```
plt.plot(df_without_standardization['Correlation threshold'],␣
↪df_without_standardization['Training data R^2 score'], label = "Training␣
↪data R^2 score", marker='v')
```

```
plt.plot(df_without_standardization['Correlation threshold'],␣
  ↪df_without_standardization['Test data R^2 score'], label = "Test data R^2␣
  ↪score", marker='D')
plt.legend()
plt.xlabel('Correlation threshold')
plt.ylabel('R^2 score')
plt.ylim([0, 1])
plt.show()
```

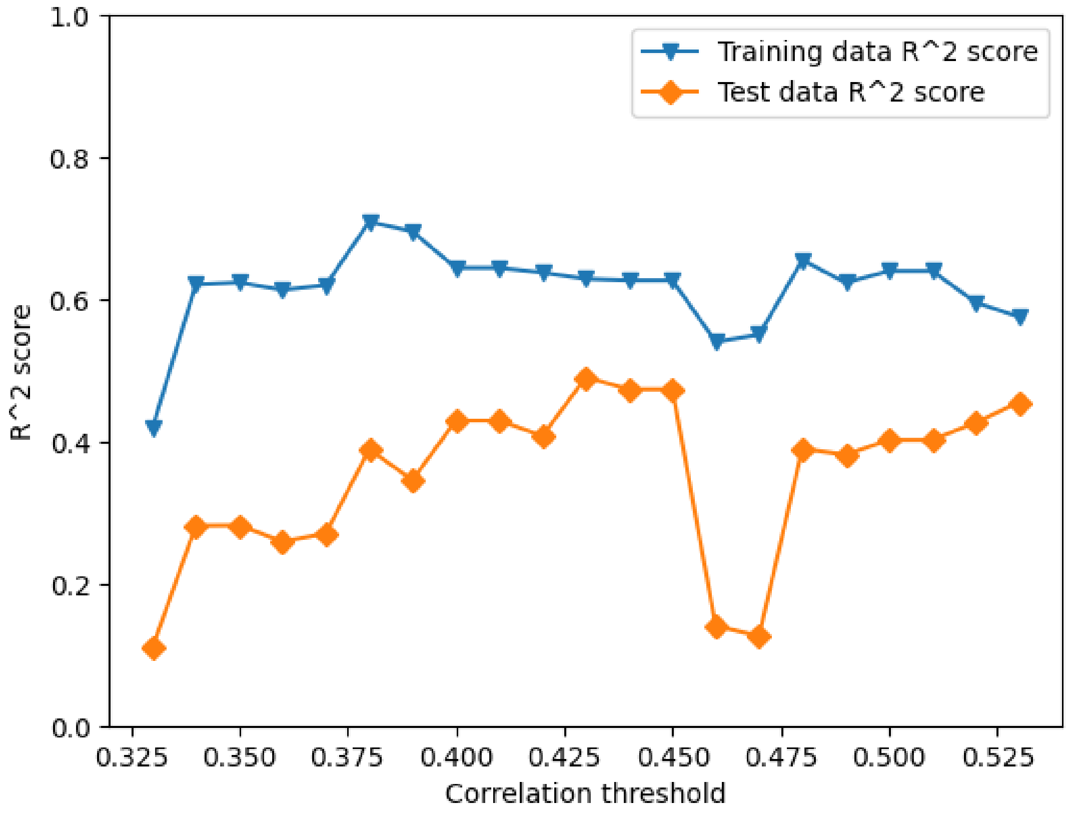

[36]:
```
plt.plot(df_without_standardization['Correlation threshold'],␣
  ↪df_without_standardization['Number of features'], label = "Number of␣
  ↪features", marker='.')
plt.legend()
plt.xlabel('Correlation threshold')
plt.ylabel('Number of features')
plt.show()
```

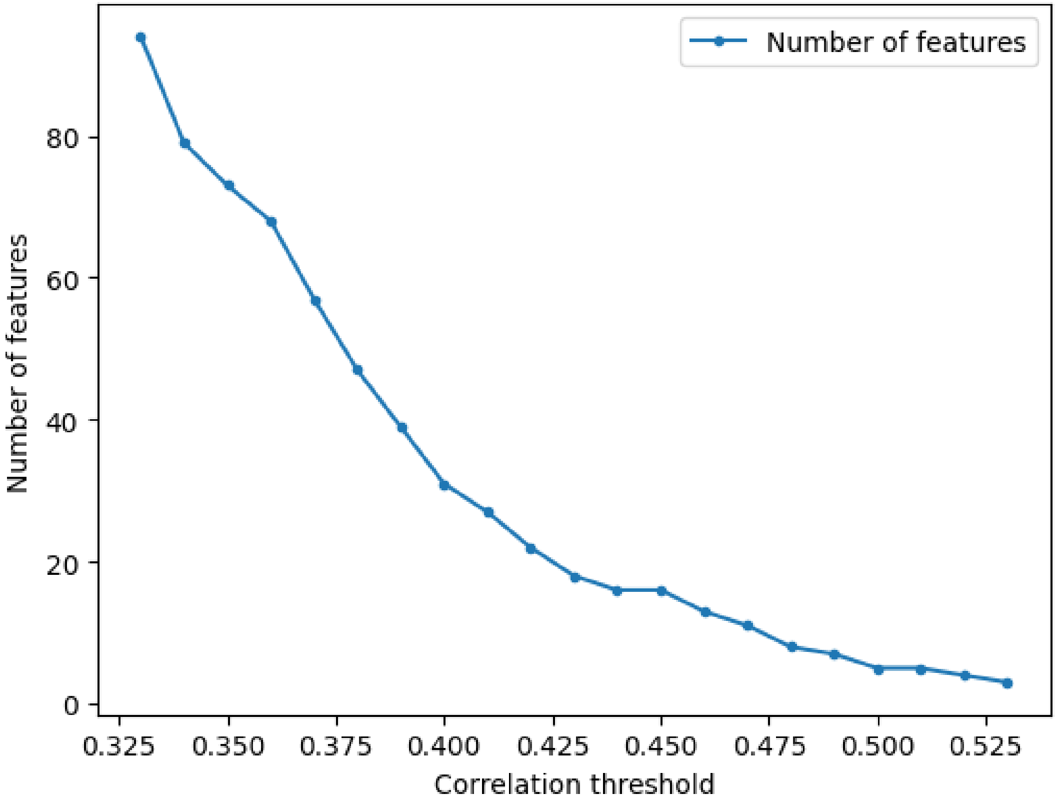

[ ]:

```

```

5 Support Vector Machines (SVM)

[37]:

```
without_standardization, train_r2_, test_r2_, hist1, hist2, target_column_name,␣
↪training_data_RMSE, test_data_RMSE  = pred_model.
↪prepare_data_and_create_model(molecular_descriptors_df=data,
                                                                              ␣
↪                       correlation_threshold=0.50,
                                                                              ␣
↪                       standardization=False,
                                                                              ␣
↪                       model_type='SVR',
                                                                              ␣
↪                       kernel_ = 'linear',
                                                                              ␣
↪                       gamma_ = 'auto',
                                                                              ␣
↪                       target_column_name = target,
```

```
↪                    random_state=random_state,
↪                    train_test_split_=True,
↪                    verbose=True)
```

```
I am not doing standardization…
  molecular descriptor name
0                    AATS0Z
1                  AATS0are
2                    AATS0d
3                   AATS0dv
4                    AATS0i
  molecular descriptor name  corr_value
0                    AATS0Z   -0.022797
1                  AATS0are   -0.139064
2                    AATS0d    0.027592
3                   AATS0dv   -0.136049
4                    AATS0i    0.168958
  molecular descriptor name  corr_value  absolute correlation value
0                    AATS0Z   -0.022797                    0.022797
1                  AATS0are   -0.139064                    0.139064
2                    AATS0d    0.027592                    0.027592
3                   AATS0dv   -0.136049                    0.136049
4                    AATS0i    0.168958                    0.168958
     molecular descriptor name  corr_value  absolute correlation value
226                     AMID_O   -0.557717                    0.557717
505                 EState_VSA5   -0.534748                    0.534748
791                     MDEO-12   -0.555724                    0.555724
921                       SaasC    0.513071                    0.513071
1091                VSA_EState5    0.526082                    0.526082
     molecular descriptor name  corr_value  absolute correlation value
226                     AMID_O   -0.557717                    0.557717
505                 EState_VSA5   -0.534748                    0.534748
791                     MDEO-12   -0.555724                    0.555724
921                       SaasC    0.513071                    0.513071
1091                VSA_EState5    0.526082                    0.526082
The model used is: SVR…
Return the coefficient of determination of the prediction:
0.2903936494024443
R^2 score: 0.46535014249070705
Correlation coefficient: 0.6821657734676426
Test data - unseen during training:
R^2 score: 0.2903936494024443
Correlation coefficient: 0.5388818510605495
[8.08842982 8.03970835 7.59466752 7.16843679 6.16607786 7.64730244
```

```
 7.86237616 7.91496845 7.87298659 7.8748025  8.07266322 6.68543785
 7.99377392 6.36729262 7.00073775 8.24618922 7.88604911 7.82525623]
44     8.193820
47     7.886057
4      7.048177
55     7.886057
26     4.904168
64     8.537602
73     7.657577
10     8.017729
40     7.920819
107    8.698970
18     6.994819
62     8.886057
11     7.962574
36     6.221126
89     8.022276
91     8.958607
109    7.886057
0      7.966576
Name: A549, dtype: float64
Training Root Mean Square Error: 0.6888266293809975
Testing Root Mean Square Error: 0.8015712973186128
```

[38]:

```python
print(target_column_name+str('_transformed'))
print(hist1[target_column_name].hist())
```

```
A549_transformed
AxesSubplot(0.125,0.11;0.775x0.77)
```

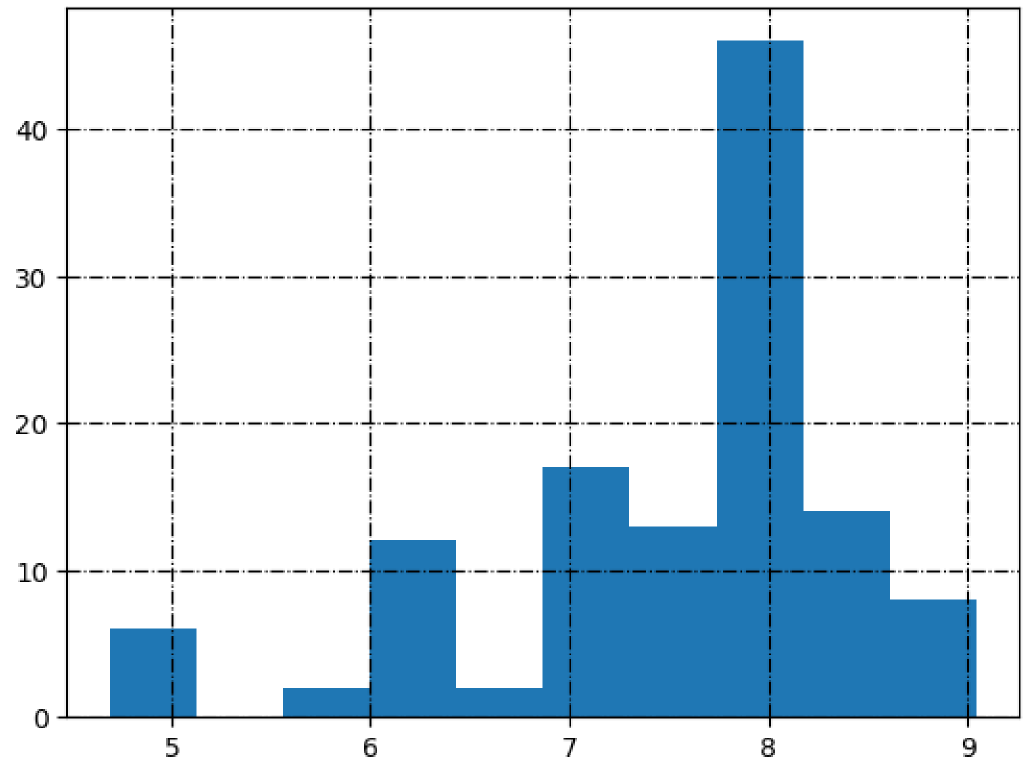

[39]:
```
with_standardization, train_r2_, test_r2_, hist1, hist2, target_column_name,␣
↪training_data_RMSE, test_data_RMSE  = pred_model.
↪prepare_data_and_create_model(molecular_descriptors_df=data,
                                                                                        ␣
↪                          correlation_threshold=0.50,
                                                                                        ␣
↪                          standardization=True,
                                                                                        ␣
↪                          model_type='SVR',
                                                                                        ␣
↪                          kernel_ = 'linear',
                                                                                        ␣
↪                          gamma_ = 'auto',
                                                                                        ␣
↪                          target_column_name = target,
                                                                                        ␣
↪                          random_state=random_state,
                                                                                        ␣
↪                          train_test_split_=True,
                                                                                        ␣
↪                          verbose=True)
```

```
I am doing standardization…
  molecular descriptor name
0                    AATS0Z
1                  AATS0are
2                    AATS0d
3                   AATS0dv
4                    AATS0i
  molecular descriptor name  corr_value
0                    AATS0Z   -0.022797
1                  AATS0are   -0.139064
2                    AATS0d    0.027592
3                   AATS0dv   -0.136049
4                    AATS0i    0.168958
  molecular descriptor name  corr_value  absolute correlation value
0                    AATS0Z   -0.022797                    0.022797
1                  AATS0are   -0.139064                    0.139064
2                    AATS0d    0.027592                    0.027592
3                   AATS0dv   -0.136049                    0.136049
4                    AATS0i    0.168958                    0.168958
     molecular descriptor name  corr_value  absolute correlation value
226                     AMID_O   -0.557717                    0.557717
505                EState_VSA5   -0.534748                    0.534748
791                    MDEO-12   -0.555724                    0.555724
921                      SaasC    0.513071                    0.513071
1091               VSA_EState5    0.526082                    0.526082
     molecular descriptor name  corr_value  absolute correlation value
226                     AMID_O   -0.557717                    0.557717
505                EState_VSA5   -0.534748                    0.534748
791                    MDEO-12   -0.555724                    0.555724
921                      SaasC    0.513071                    0.513071
1091               VSA_EState5    0.526082                    0.526082
The model used is: SVR…
Return the coefficient of determination of the prediction:
0.2903936494024443
R^2 score: 0.46535014249070705
Correlation coefficient: 0.6821657734676426
Test data - unseen during training:
R^2 score: 0.2903936494024443
Correlation coefficient: 0.5388818510605495
[8.08842982 8.03970835 7.59466752 7.16843679 6.16607786 7.64730244
 7.86237616 7.91496845 7.87298659 7.8748025  8.07266322 6.68543785
 7.99377392 6.36729262 7.00073775 8.24618922 7.88604911 7.82525623]
44    8.193820
47    7.886057
4     7.048177
55    7.886057
26    4.904168
64    8.537602
```

```
73     7.657577
10     8.017729
40     7.920819
107    8.698970
18     6.994819
62     8.886057
11     7.962574
36     6.221126
89     8.022276
91     8.958607
109    7.886057
0      7.966576
Name: A549, dtype: float64
Training Root Mean Square Error: 0.6888266293809975
Testing Root Mean Square Error: 0.8015712973186128
```

5.1 Search inside correlation space

[40]:

```
step = 0.01
initial_step = corr_low
last_step = corr_high
first_list = [x / 100.0 for x in range(int(initial_step*100),␣
 ↪int(last_step*100), int(step*100))]
second_list = []
third_list = []
fourth_l = []
fifth_l = []
f_list = []
for i in first_list:
    without_standardization, train_r2, test_r2, _, h_, target_column_name,␣
 ↪training_data_RMSE, test_data_RMSE = pred_model.
 ↪prepare_data_and_create_model(molecular_descriptors_df = data,
                                                                          ␣
 ↪                      correlation_threshold = i,
                                                                          ␣
 ↪                      standardization = False,
                                                                          ␣
 ↪                      model_type = 'SVR',
                                                                          ␣
 ↪                      kernel_ = 'linear',
                                                                          ␣
 ↪                      gamma_ = 'auto',
                                                                          ␣
 ↪                      target_column_name = target,
                                                                          ␣
 ↪                      random_state=random_state,
```

```
                        train_test_split_ = True,
                        verbose = False)
    second_list.append(train_r2)
    third_list.append(test_r2)
    fourth_l.append(training_data_RMSE)
    fifth_l.append(test_data_RMSE)
    f_list.append(len(h_))
```

[41]:
```
df_without_standardization = pd.DataFrame(data=first_list,
 columns=["Correlation threshold"])
df_without_standardization['Training data R^2 score'] = second_list
df_without_standardization['Test data R^2 score'] = third_list
df_without_standardization['Training RMSE'] = fourth_l
df_without_standardization['Test RMSE'] = fifth_l
df_without_standardization['Number of features'] = f_list
```

[42]:
```
df_svm = df_without_standardization.copy()
df_without_standardization
```

[42]:

	Correlation threshold	Training data R^2 score	Test data R^2 score
0	0.33	0.743433	0.679759
1	0.34	0.707639	0.683938
2	0.35	0.707945	0.687329
3	0.36	0.668367	0.546185
4	0.37	0.662693	0.493890
5	0.38	0.613721	0.336667
6	0.39	0.590237	0.042945
7	0.40	0.570338	0.251611
8	0.41	0.570390	0.270412
9	0.42	0.572385	0.242621
10	0.43	0.579096	0.345766
11	0.44	0.565823	0.394060
12	0.45	0.565823	0.394060
13	0.46	0.562645	0.380883
14	0.47	0.545067	0.409121
15	0.48	0.496560	0.415594
16	0.49	0.462930	0.313821
17	0.50	0.465350	0.290394
18	0.51	0.465350	0.290394
19	0.52	0.455708	0.318651
20	0.53	0.414234	0.445803

	Training RMSE	Test RMSE	Number of features
0	0.477173	0.538483	94
1	0.509372	0.534958	79

2	0.509105	0.532080	73
3	0.542506	0.641022	68
4	0.547126	0.676949	57
5	0.585499	0.774995	47
6	0.603034	0.930897	39
7	0.617502	0.823185	31
8	0.617465	0.812778	27
9	0.616030	0.828114	22
10	0.611177	0.769662	18
11	0.620738	0.740710	16
12	0.620738	0.740710	16
13	0.623006	0.748721	13
14	0.635403	0.731446	11
15	0.668419	0.727429	8
16	0.690384	0.788229	7
17	0.688827	0.801571	5
18	0.688827	0.801571	5
19	0.695010	0.785450	4
20	0.721003	0.708379	3

5.2 Plots

[43]:
```
plt.plot(df_without_standardization['Correlation threshold'],␣
  ↪df_without_standardization['Training data R^2 score'], label = "Training␣
  ↪data R^2 score", marker='v')
plt.plot(df_without_standardization['Correlation threshold'],␣
  ↪df_without_standardization['Test data R^2 score'], label = "Test data R^2␣
  ↪score", marker='D')
plt.legend()
plt.xlabel('Correlation threshold')
plt.ylabel('R^2 score')
plt.ylim([0, 1])
plt.show()
```

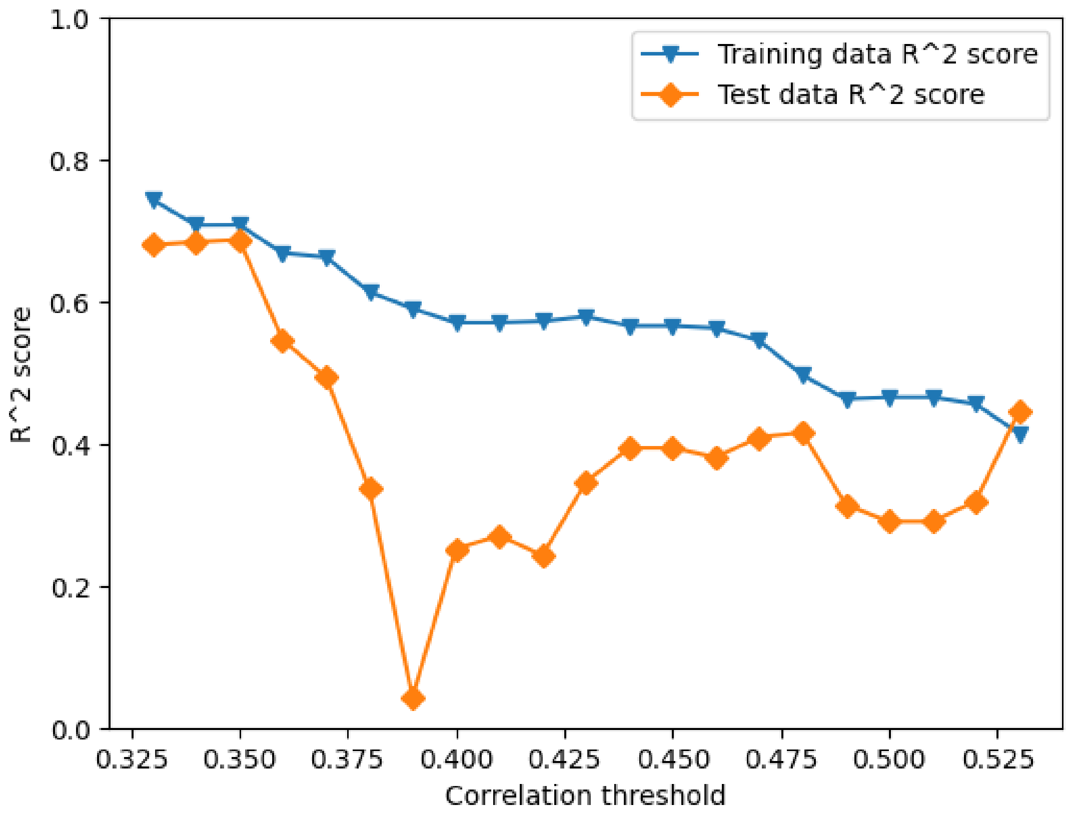

[44]:
```
plt.plot(df_without_standardization['Correlation threshold'],␣
    ↪df_without_standardization['Number of features'], label = "Number of␣
    ↪features", marker='.')
plt.legend()
plt.xlabel('Correlation threshold')
plt.ylabel('Number of features')
plt.show()
```

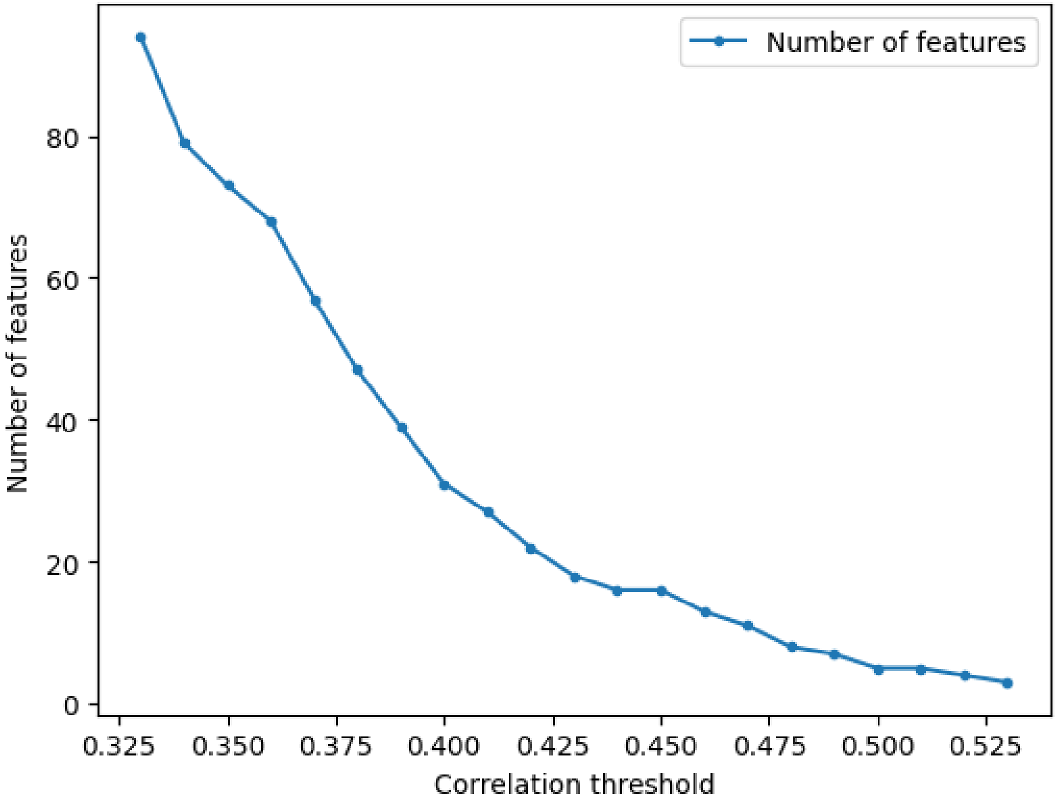

5.3 Store quality measurements

[45]:
```
with pd.ExcelWriter('../Data/Quality_'+str(target)+'_'+str(random_state)+'_.
↪xlsx') as writer:
    df_linear.to_excel(writer, sheet_name='MLR')
    df_decision_tree.to_excel(writer, sheet_name='DT')
    df_random_forest.to_excel(writer, sheet_name='RF')
    df_k_nearest.to_excel(writer, sheet_name='KNN')
    df_svm.to_excel(writer, sheet_name='SVM')
```

Notebook

January 18, 2024

1 File 18

[1]:
```
import sys
from pathlib import Path
prediction_mode_path = Path("../module")
sys.path.append(prediction_mode_path.as_posix())
import models_creation as pred_model

import pandas as pd
import numpy as np

from sklearn.metrics import r2_score

import matplotlib.pyplot as plt
from sklearn.tree import plot_tree

from matplotlib import pyplot as plt

import joblib

import warnings
warnings.filterwarnings('ignore')
```

[2]:
```
load_prepared_data = pd.read_excel('../Data/Kolchicyna_machine_learning.xlsx')
load_prepared_data = load_prepared_data.drop(columns=['Unnamed: 0'])

target = 'BALB/3T3'

corr_low = 0.33
corr_high = 0.58

random_state = 15
```

[3]:
```
load_prepared_data.head()
```

[3]:
```
      AATS0Z  AATS0are    AATS0d   AATS0dv      AATS0i     AATS0m    AATS0p  \
0  25.090909  6.046518  3.109091  6.662626  160.775045  99.075564  1.555512
1  24.448276  6.008422  3.051724  6.386973  161.021674  96.473315  1.538471
```

```
2  23.868852  5.974074  3.000000  6.138434  161.244045  94.127025  1.523105
3  23.868852  5.974074  3.032787  6.171220  161.244045  94.127025  1.523105
4  23.343750  5.942945  2.953125  5.913194  161.445569  92.000700  1.509180

    AATS0pe    AATS0s   AATS0se  …  A549_float  MCF-7_float  LoVo_float  \
0  6.126680  3.435416  7.629859  …        10.8         10.3         6.5
1  6.088791  3.330998  7.596891  …        11.6         12.0         8.5
2  6.054630  3.236850  7.567166  …        10.9         12.2         8.8
3  6.054630  3.257798  7.567166  …        10.5         11.3         8.5
4  6.023670  3.151529  7.540228  …        89.5         92.7        52.8

   LoVo/DX_float  BALB/3T3_float  A549_float_transformed  \
0           54.9            10.2                7.966576
1           31.1            14.3                7.935542
2           17.9            11.7                7.962574
3           10.2            11.0                7.978811
4           77.8            99.4                7.048177

   MCF-7_float_transformed  LoVo_float_transformed  LoVo/DX_float_transformed  \
0                 7.987163                8.187087                   7.260428
1                 7.920819                8.070581                   7.507240
2                 7.913640                8.055517                   7.747147
3                 7.946922                8.070581                   7.991400
4                 7.032920                7.277366                   7.109020

   BALB/3T3_float_transformed
0                    7.991400
1                    7.844664
2                    7.931814
3                    7.958607
4                    7.002614

[5 rows x 1221 columns]
```

[4]:
```python
data = load_prepared_data[list(load_prepared_data.columns)[0:-10]]
data[target] = load_prepared_data[load_prepared_data.columns[-1]]
data.head()
```

[4]:
```
      AATS0Z  AATS0are    AATS0d   AATS0dv      AATS0i     AATS0m    AATS0p  \
0  25.090909  6.046518  3.109091  6.662626  160.775045  99.075564  1.555512
1  24.448276  6.008422  3.051724  6.386973  161.021674  96.473315  1.538471
2  23.868852  5.974074  3.000000  6.138434  161.244045  94.127025  1.523105
3  23.868852  5.974074  3.032787  6.171220  161.244045  94.127025  1.523105
4  23.343750  5.942945  2.953125  5.913194  161.445569  92.000700  1.509180

    AATS0pe    AATS0s   AATS0se  …    piPC10     piPC2     piPC3     piPC4  \
0  6.126680  3.435416  7.629859  …  7.999655  4.266195  4.915408  5.516700
```

```
1  6.088791  3.330998  7.596891  …  8.018726  4.280132  4.922714  5.520711
2  6.054630  3.236850  7.567166  …  8.028799  4.293878  4.929967  5.524706
3  6.054630  3.257798  7.567166  …  8.037440  4.307438  4.929967  5.524706
4  6.023670  3.151529  7.540228  …  8.034892  4.307438  4.937168  5.528685

      piPC5     piPC6     piPC7     piPC8     piPC9  BALB/3T3
0  6.098566  6.595759  6.979116  7.337313  7.681445  7.991400
1  6.103048  6.601209  6.985613  7.349442  7.695531  7.844664
2  6.105281  6.603923  6.989306  7.353932  7.704023  7.931814
3  6.107510  6.606629  6.992068  7.361425  7.709420  7.958607
4  6.107510  6.605277  6.991148  7.356489  7.707175  7.002614

[5 rows x 1212 columns]
```

2 Multiple Linear regression (MLR)

[5]:
```python
without_standardization, train_r2_, test_r2_, hist1, hist2, target_column_name,␣
↪training_data_RMSE, test_data_RMSE  = pred_model.
↪prepare_data_and_create_model(molecular_descriptors_df=data,
                                                                                      ␣
↪                    correlation_threshold=0.5,
                                                                                      ␣
↪                    standardization=False,
                                                                                      ␣
↪                    model_type='linear_model',
                                                                                      ␣
↪                    target_column_name = str(target),
                                                                                      ␣
↪                    random_state=random_state,
                                                                                      ␣
↪                    train_test_split_=True,
                                                                                      ␣
↪                    verbose=True)
```

```
I am not doing standardization…
  molecular descriptor name
0                   AATS0Z
1                 AATS0are
2                   AATS0d
3                  AATS0dv
4                   AATS0i
  molecular descriptor name  corr_value
0                   AATS0Z   -0.020817
1                 AATS0are   -0.148257
2                   AATS0d    0.022999
3                  AATS0dv   -0.137980
```

```
4                  AATS0i    0.133257
  molecular descriptor name  corr_value  absolute correlation value
0                  AATS0Z   -0.020817                    0.020817
1                AATS0are   -0.148257                    0.148257
2                  AATS0d    0.022999                    0.022999
3                 AATS0dv   -0.137980                    0.137980
4                  AATS0i    0.133257                    0.133257
    molecular descriptor name  corr_value  absolute correlation value
226                    AMID_O   -0.546843                    0.546843
505                EState_VSA5   -0.588780                    0.588780
556                    GATS2c    0.511671                    0.511671
791                   MDEO-12   -0.582747                    0.582747
847                       NdO   -0.500462                    0.500462
    molecular descriptor name  corr_value  absolute correlation value
226                    AMID_O   -0.546843                    0.546843
505                EState_VSA5   -0.588780                    0.588780
556                    GATS2c    0.511671                    0.511671
791                   MDEO-12   -0.582747                    0.582747
847                       NdO   -0.500462                    0.500462
The model used is: LinearReg…
Return the coefficient of determination of the prediction:
0.6569499487893375
R^2 score: 0.5256266081134505
Correlation coefficient: 0.7250011090429107
Test data - unseen during training:
R^2 score: 0.6569499487893375
Correlation coefficient: 0.8105244899380509
[7.68442489 6.0649563  7.07776757 7.44460786 7.76758116 8.09501827
 8.03694771 7.82654221 7.91029739 7.46021396 6.76908355 7.56147833
 7.34978659 7.70817837 8.0526055  8.02027016 6.2746389  5.67578807]
102    7.698970
38     5.986741
8      5.984515
109    7.886057
11     7.962574
51     7.698970
88     8.229148
21     7.298432
82     8.065502
98     7.376751
58     7.602060
64     8.060481
74     7.853872
103    7.853872
91     8.346787
43     7.619789
25     4.989276
30     6.085657
```

```
Name: BALB/3T3, dtype: float64
Training Root Mean Square Error: 0.5976818108840668
Testing Root Mean Square Error: 0.5386939232139484
```

[6]:
```python
print(target_column_name+str('_transformed'))
print(hist1[target_column_name].hist())
```

```
BALB/3T3_transformed
AxesSubplot(0.125,0.11;0.775x0.77)
```

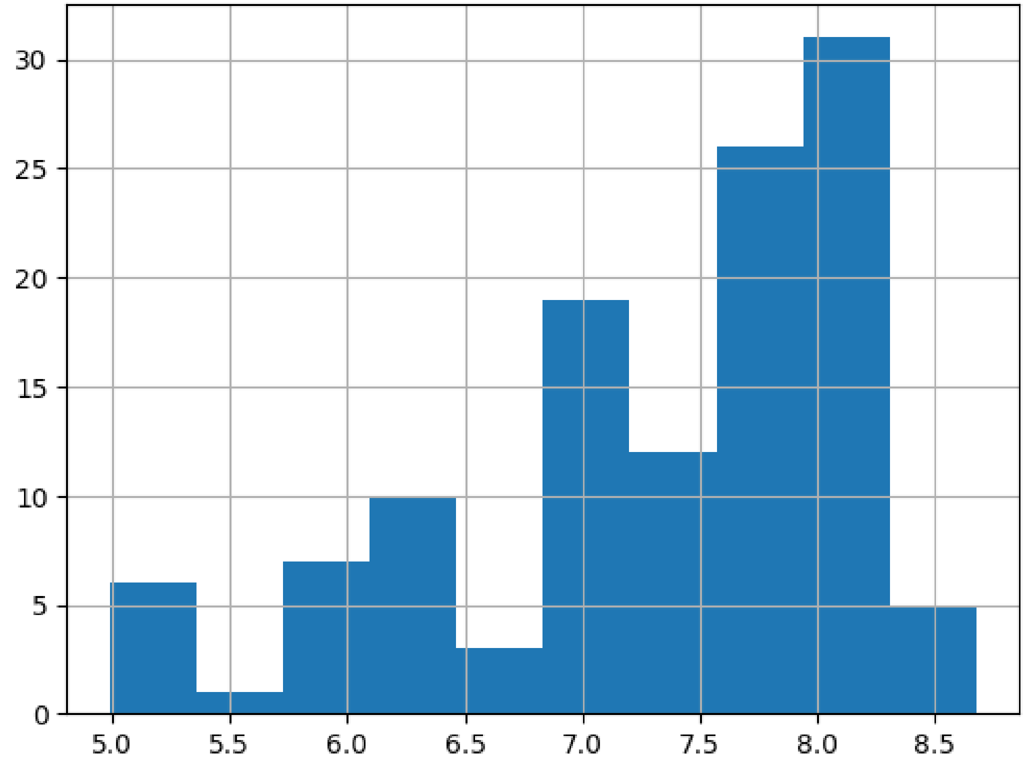

[7]:
```python
with_standardization, train_r2_, test_r2_, hist1, hist2, target_column_name,␣
↪training_data_RMSE, test_data_RMSE  = pred_model.
↪prepare_data_and_create_model(molecular_descriptors_df=data,
                                                                              ␣
↪                         correlation_threshold=0.50,
                                                                              ␣
↪                         standardization=True,
                                                                              ␣
↪                         model_type='linear_model',
                                                                              ␣
↪                         target_column_name = target,
```

```
↪                    random_state=random_state,
↪                    train_test_split_=True,
↪                    verbose=True)
```

```
I am doing standardization…
  molecular descriptor name
0                    AATS0Z
1                  AATS0are
2                    AATS0d
3                   AATS0dv
4                    AATS0i
  molecular descriptor name  corr_value
0                    AATS0Z   -0.020817
1                  AATS0are   -0.148257
2                    AATS0d    0.022999
3                   AATS0dv   -0.137980
4                    AATS0i    0.133257
  molecular descriptor name  corr_value  absolute correlation value
0                    AATS0Z   -0.020817                    0.020817
1                  AATS0are   -0.148257                    0.148257
2                    AATS0d    0.022999                    0.022999
3                   AATS0dv   -0.137980                    0.137980
4                    AATS0i    0.133257                    0.133257
    molecular descriptor name  corr_value  absolute correlation value
226                    AMID_O   -0.546843                    0.546843
505                EState_VSA5   -0.588780                    0.588780
556                    GATS2c    0.511671                    0.511671
791                  MDEO-12   -0.582747                    0.582747
847                       NdO   -0.500462                    0.500462
    molecular descriptor name  corr_value  absolute correlation value
226                    AMID_O   -0.546843                    0.546843
505                EState_VSA5   -0.588780                    0.588780
556                    GATS2c    0.511671                    0.511671
791                  MDEO-12   -0.582747                    0.582747
847                       NdO   -0.500462                    0.500462
The model used is: LinearReg…
Return the coefficient of determination of the prediction:
0.6569499487893375
R^2 score: 0.5256266081134505
Correlation coefficient: 0.7250011090429107
Test data - unseen during training:
R^2 score: 0.6569499487893375
Correlation coefficient: 0.8105244899380509
[7.68442489 6.0649563  7.07776757 7.44460786 7.76758116 8.09501827
```

```
 8.03694771 7.82654221 7.91029739 7.46021396 6.76908355 7.56147833
 7.34978659 7.70817837 8.0526055  8.02027016 6.2746389  5.67578807]
102    7.698970
38     5.986741
8      5.984515
109    7.886057
11     7.962574
51     7.698970
88     8.229148
21     7.298432
82     8.065502
98     7.376751
58     7.602060
64     8.060481
74     7.853872
103    7.853872
91     8.346787
43     7.619789
25     4.989276
30     6.085657
Name: BALB/3T3, dtype: float64
Training Root Mean Square Error: 0.5976818108840668
Testing Root Mean Square Error: 0.5386939232139484
```

2.1 Search inside correlation space

[8]:
```
step = 0.01
initial_step = corr_low
last_step = corr_high
first_list = [x / 100.0 for x in range(int(initial_step*100),␣
 ↪int(last_step*100), int(step*100))]
second_list = []
third_list = []
fourth_l = []
fifth_l = []
f_list = []
for i in first_list:
    without_standardization, train_r2, test_r2, _, h_, target_column_name,␣
 ↪training_data_RMSE, test_data_RMSE = pred_model.
 ↪prepare_data_and_create_model(molecular_descriptors_df = data,
                                                                           ␣
 ↪                     correlation_threshold = i,
                                                                           ␣
 ↪                     standardization = False,
                                                                           ␣
 ↪                     model_type = 'linear_model',
```

```
                        target_column_name = target,
                        random_state=random_state,
                        train_test_split_ = True,
                        verbose = False)
    second_list.append(train_r2)
    third_list.append(test_r2)
    fourth_l.append(training_data_RMSE)
    fifth_l.append(test_data_RMSE)
    f_list.append(len(h_))
```

[9]:
```
df_without_standardization = pd.DataFrame(data=first_list, columns=["Correlation threshold"])
df_without_standardization['Training data R^2 score'] = second_list
df_without_standardization['Test data R^2 score'] = third_list
df_without_standardization['Training RMSE'] = fourth_l
df_without_standardization['Test RMSE'] = fifth_l
df_without_standardization['Number of features'] = f_list
```

[10]:
```
df_linear = df_without_standardization.copy()
df_without_standardization
```

[10]:

	Correlation threshold	Training data R^2 score	Test data R^2 score	\
0	0.33	0.975137	0.067877	
1	0.34	0.927395	0.385673	
2	0.35	0.911816	0.807698	
3	0.36	0.902814	0.791372	
4	0.37	0.883339	0.764207	
5	0.38	0.837840	0.662093	
6	0.39	0.782597	0.642720	
7	0.40	0.765315	0.640995	
8	0.41	0.693176	0.790639	
9	0.42	0.646013	0.760448	
10	0.43	0.629671	0.739706	
11	0.44	0.629043	0.727664	
12	0.45	0.598451	0.683043	
13	0.46	0.598331	0.684987	
14	0.47	0.596049	0.683608	
15	0.48	0.590219	0.703128	
16	0.49	0.558784	0.684205	
17	0.50	0.525627	0.656950	
18	0.51	0.506715	0.653417	
19	0.52	0.506121	0.648511	
20	0.53	0.478976	0.639600	

```
21                    0.54              0.478976            0.639600
22                    0.55              0.468132            0.576754
23                    0.56              0.468132            0.576754

    Training RMSE  Test RMSE  Number of features
0        0.136831   0.887974                  83
1        0.233826   0.720880                  70
2        0.257694   0.403325                  64
3        0.270528   0.420097                  59
4        0.296396   0.446610                  55
5        0.349447   0.534640                  51
6        0.404615   0.549753                  46
7        0.420391   0.551078                  41
8        0.480678   0.420835                  32
9        0.516302   0.450156                  28
10       0.528085   0.469240                  23
11       0.528532   0.479972                  21
12       0.549895   0.517802                  17
13       0.549977   0.516212                  16
14       0.551536   0.517340                  15
15       0.555502   0.501127                  14
16       0.576415   0.516852                  10
17       0.597682   0.538694                   7
18       0.609479   0.541461                   6
19       0.609846   0.545280                   5
20       0.626381   0.552148                   3
21       0.626381   0.552148                   3
22       0.632866   0.598356                   2
23       0.632866   0.598356                   2
```

[ ]:

2.2 Plots

[11]:
```
plt.plot(df_without_standardization['Correlation threshold'],
 ↪df_without_standardization['Training data R^2 score'], label = "Training
 ↪data R^2 score", marker='v')
plt.plot(df_without_standardization['Correlation threshold'],
 ↪df_without_standardization['Test data R^2 score'], label = "Test data R^2
 ↪score", marker='D')
plt.legend()
plt.xlabel('Correlation threshold')
plt.ylabel('R^2 score')
plt.ylim([0, 1])
plt.show()
```

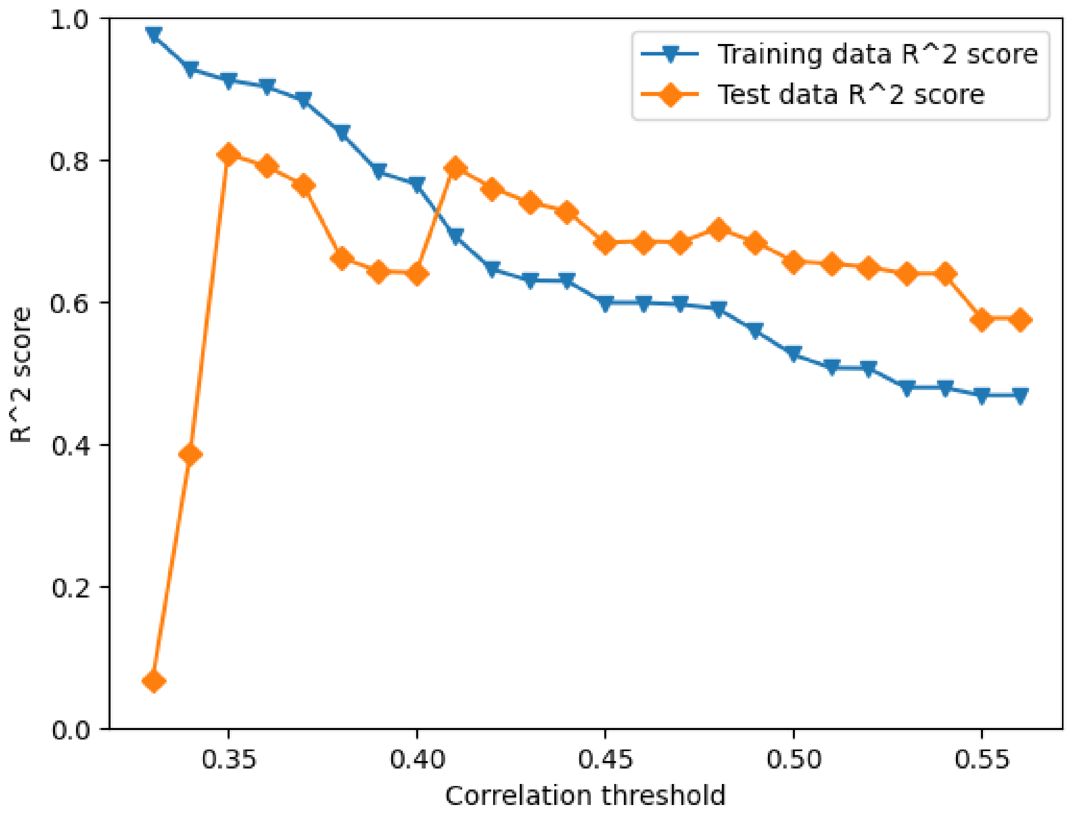

[12]:
```
plt.plot(df_without_standardization['Correlation threshold'],␣
 ↪df_without_standardization['Number of features'], label = "Number of␣
 ↪features", marker='.')
plt.legend()
plt.xlabel('Correlation threshold')
plt.ylabel('Number of features')
plt.show()
```

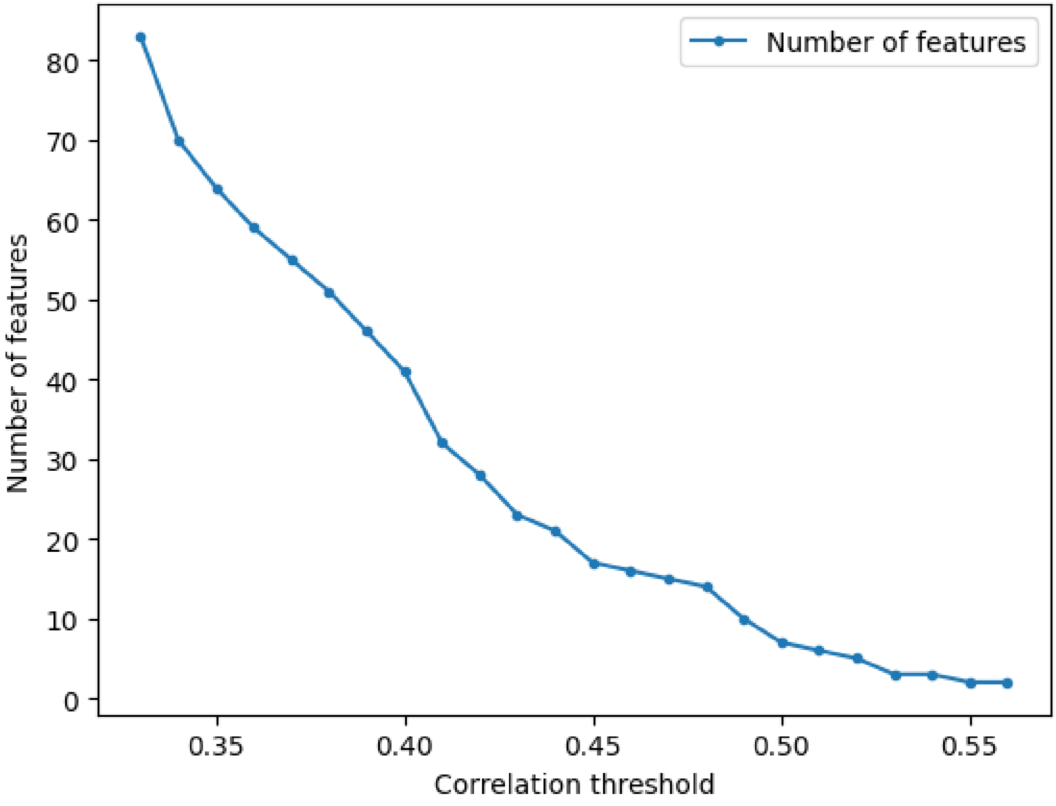

[ ]:

2.3 Decision Tree

[13]:
```
without_standardization, train_r2_, test_r2_, hist1, hist2, target_column_name,␣
↪training_data_RMSE, test_data_RMSE  = pred_model.
↪prepare_data_and_create_model(molecular_descriptors_df=data,
                                                                                   ␣
↪                        correlation_threshold=0.50,
                                                                                   ␣
↪                        standardization=False,
                                                                                   ␣
↪                        model_type='DecisionTreeRegressor',
                                                                                   ␣
↪                        max_depth=5,
                                                                                   ␣
↪                        target_column_name = target,
                                                                                   ␣
↪                        random_state=random_state,
```

```
↪                         train_test_split_=True,
↪                         verbose=True)
```

```
I am not doing standardization…
  molecular descriptor name
0                    AATS0Z
1                  AATS0are
2                    AATS0d
3                   AATS0dv
4                    AATS0i
  molecular descriptor name  corr_value
0                    AATS0Z   -0.020817
1                  AATS0are   -0.148257
2                    AATS0d    0.022999
3                   AATS0dv   -0.137980
4                    AATS0i    0.133257
  molecular descriptor name  corr_value  absolute correlation value
0                    AATS0Z   -0.020817                    0.020817
1                  AATS0are   -0.148257                    0.148257
2                    AATS0d    0.022999                    0.022999
3                   AATS0dv   -0.137980                    0.137980
4                    AATS0i    0.133257                    0.133257
    molecular descriptor name  corr_value  absolute correlation value
226                    AMID_O   -0.546843                    0.546843
505                EState_VSA5   -0.588780                    0.588780
556                    GATS2c    0.511671                    0.511671
791                   MDEO-12   -0.582747                    0.582747
847                       NdO   -0.500462                    0.500462
    molecular descriptor name  corr_value  absolute correlation value
226                    AMID_O   -0.546843                    0.546843
505                EState_VSA5   -0.588780                    0.588780
556                    GATS2c    0.511671                    0.511671
791                   MDEO-12   -0.582747                    0.582747
847                       NdO   -0.500462                    0.500462
The model used is: DecisionTree…
Return the coefficient of determination of the prediction:
0.44183643371267434
R^2 score: 0.8634311625290639
Correlation coefficient: 0.9292099668692022
Test data - unseen during training:
R^2 score: 0.44183643371267434
Correlation coefficient: 0.6647077806921432
[7.69361679 5.9034377  6.79428322 8.07200556 8.07200556 7.13946628
 8.07200556 7.13946628 8.07200556 7.69361679 7.87061249 7.69361679
 7.69361679 7.69361679 7.13946628 8.07200556 7.08460016 5.07789763]
102    7.698970
```

```
38     5.986741
8      5.984515
109    7.886057
11     7.962574
51     7.698970
88     8.229148
21     7.298432
82     8.065502
98     7.376751
58     7.602060
64     8.060481
74     7.853872
103    7.853872
91     8.346787
43     7.619789
25     4.989276
30     6.085657
Name: BALB/3T3, dtype: float64
Training Root Mean Square Error: 0.3206901974380038
Testing Root Mean Square Error: 0.6871381711144097
```

[14]:
```
print(target_column_name+str('_transformed'))
print(hist1[target_column_name].hist())
```

```
BALB/3T3_transformed
AxesSubplot(0.125,0.11;0.775x0.77)
```

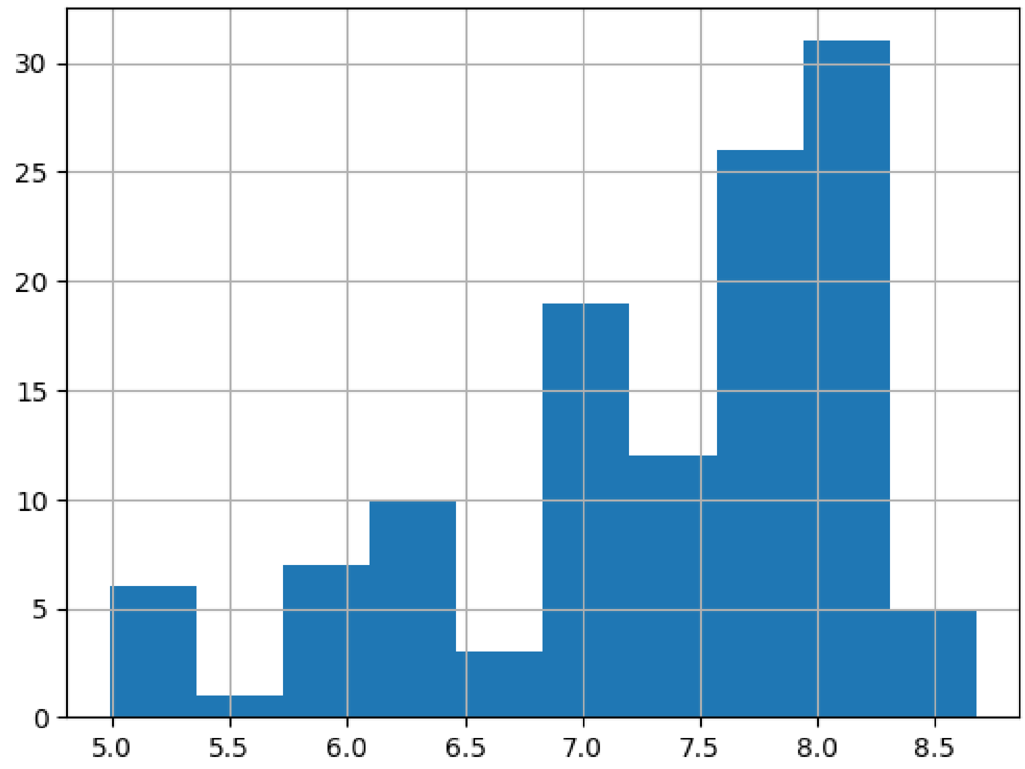

[15]:
```
with_standardization, train_r2_, test_r2_, hist1, hist2, target_column_name,␣
↪training_data_RMSE, test_data_RMSE  = pred_model.
↪prepare_data_and_create_model(molecular_descriptors_df=data,
                                                                              ␣
↪                          correlation_threshold=0.50,
                                                                              ␣
↪                          standardization=True,
                                                                              ␣
↪                          model_type='DecisionTreeRegressor',
                                                                              ␣
↪                          max_depth=5,
                                                                              ␣
↪                          target_column_name = target,
                                                                              ␣
↪                          random_state=random_state,
                                                                              ␣
↪                          train_test_split_=True,
                                                                              ␣
↪                          verbose=True)
```

```
I am doing standardization…
  molecular descriptor name
```

```
0                  AATS0Z
1                AATS0are
2                  AATS0d
3                 AATS0dv
4                  AATS0i
  molecular descriptor name  corr_value
0                  AATS0Z   -0.020817
1                AATS0are   -0.148257
2                  AATS0d    0.022999
3                 AATS0dv   -0.137980
4                  AATS0i    0.133257
  molecular descriptor name  corr_value  absolute correlation value
0                  AATS0Z   -0.020817                    0.020817
1                AATS0are   -0.148257                    0.148257
2                  AATS0d    0.022999                    0.022999
3                 AATS0dv   -0.137980                    0.137980
4                  AATS0i    0.133257                    0.133257
    molecular descriptor name  corr_value  absolute correlation value
226                    AMID_O   -0.546843                    0.546843
505               EState_VSA5   -0.588780                    0.588780
556                    GATS2c    0.511671                    0.511671
791                   MDEO-12   -0.582747                    0.582747
847                       NdO   -0.500462                    0.500462
    molecular descriptor name  corr_value  absolute correlation value
226                    AMID_O   -0.546843                    0.546843
505               EState_VSA5   -0.588780                    0.588780
556                    GATS2c    0.511671                    0.511671
791                   MDEO-12   -0.582747                    0.582747
847                       NdO   -0.500462                    0.500462
The model used is: DecisionTree…
Return the coefficient of determination of the prediction:
0.44183643371267434
R^2 score: 0.8634311625290639
Correlation coefficient: 0.9292099668692022
Test data - unseen during training:
R^2 score: 0.44183643371267434
Correlation coefficient: 0.6647077806921432
[7.69361679 5.9034377  6.79428322 8.07200556 8.07200556 7.13946628
 8.07200556 7.13946628 8.07200556 7.69361679 7.87061249 7.69361679
 7.69361679 7.69361679 7.13946628 8.07200556 7.08460016 5.07789763]
102    7.698970
38     5.986741
8      5.984515
109    7.886057
11     7.962574
51     7.698970
88     8.229148
21     7.298432
```

```
82      8.065502
98      7.376751
58      7.602060
64      8.060481
74      7.853872
103     7.853872
91      8.346787
43      7.619789
25      4.989276
30      6.085657
Name: BALB/3T3, dtype: float64
Training Root Mean Square Error: 0.3206901974380038
Testing Root Mean Square Error: 0.6871381711144097
```

2.4 Search inside correlation space

[16]:
```
step = 0.01
initial_step = corr_low
last_step = corr_high
first_list = [x / 100.0 for x in range(int(initial_step*100),␣
↪int(last_step*100), int(step*100))]
max_depth = [range(2, 30, 1)]
corr_th = []
second_list = []
third_list = []
fourth_l = []
fifth_l = []
f_list = []
fif_list = []
for i in first_list:
    for depth in max_depth[0]:

        without_standardization, train_r2, test_r2, _, h_, target_column_name,␣
↪training_data_RMSE, test_data_RMSE = pred_model.
↪prepare_data_and_create_model(molecular_descriptors_df=data,
                                                                                    ␣
↪                      correlation_threshold=i,
                                                                                    ␣
↪                      standardization=False,
                                                                                    ␣
↪                      model_type='DecisionTreeRegressor',
                                                                                    ␣
↪                      max_depth=depth,
                                                                                    ␣
↪                      target_column_name = target,
                                                                                    ␣
↪                      random_state=random_state,
```

```
↪                     train_test_split_=True,
↪                     verbose=False)
        corr_th.append(i)
        second_list.append(train_r2)
        third_list.append(test_r2)
        fourth_l.append(training_data_RMSE)
        fifth_l.append(test_data_RMSE)
        f_list.append(len(h_))
        fif_list.append(depth)
```

[17]:
```
df_without_standardization = pd.DataFrame(data=corr_th, columns=["Correlation␣
↪threshold"])
df_without_standardization['Training data R^2 score'] = second_list
df_without_standardization['Test data R^2 score'] = third_list
df_without_standardization['Training RMSE'] = fourth_l
df_without_standardization['Test RMSE'] = fifth_l
df_without_standardization['Number of features'] = f_list
df_without_standardization['Depth number'] = fif_list
```

[18]:
```
df_decision_tree = df_without_standardization.copy()
#df_without_standardization.to_excel('../Data/
↪A549_Decision_tree_rs_'+str(random_state)+'.xlsx')
```

2.5 Plots

[19]:

```
corre_list = [x / 100.0 for x in range(int(initial_step*100),␣
↪int(last_step*100), int(0.01*100))]
for element in corre_list:
    element_ =␣
↪df_without_standardization[df_without_standardization['Correlation␣
↪threshold'] == float(element)]
    plt.plot(element_['Depth number'], element_['Training data R^2 score'],␣
↪label = "Training data R^2 score", marker='v')
    plt.plot(element_['Depth number'], element_['Test data R^2 score'], label =␣
↪"Test data R^2 score", marker='D')
    plt.legend()
    plt.xlabel('Depth number -> correlation threshold: '+str(element)+'. \n␣
↪Number of features: '+str(element_['Number of features'].iloc[0]),␣
↪fontweight='bold')
    plt.xlim(max(element_['Depth number'])+1, min(element_['Depth number'])-1)
    plt.ylabel('R^2 score')
    plt.ylim([0, 1])
    plt.rc('grid', linestyle="-.", color='black')
    plt.grid(True)
    plt.show()
```

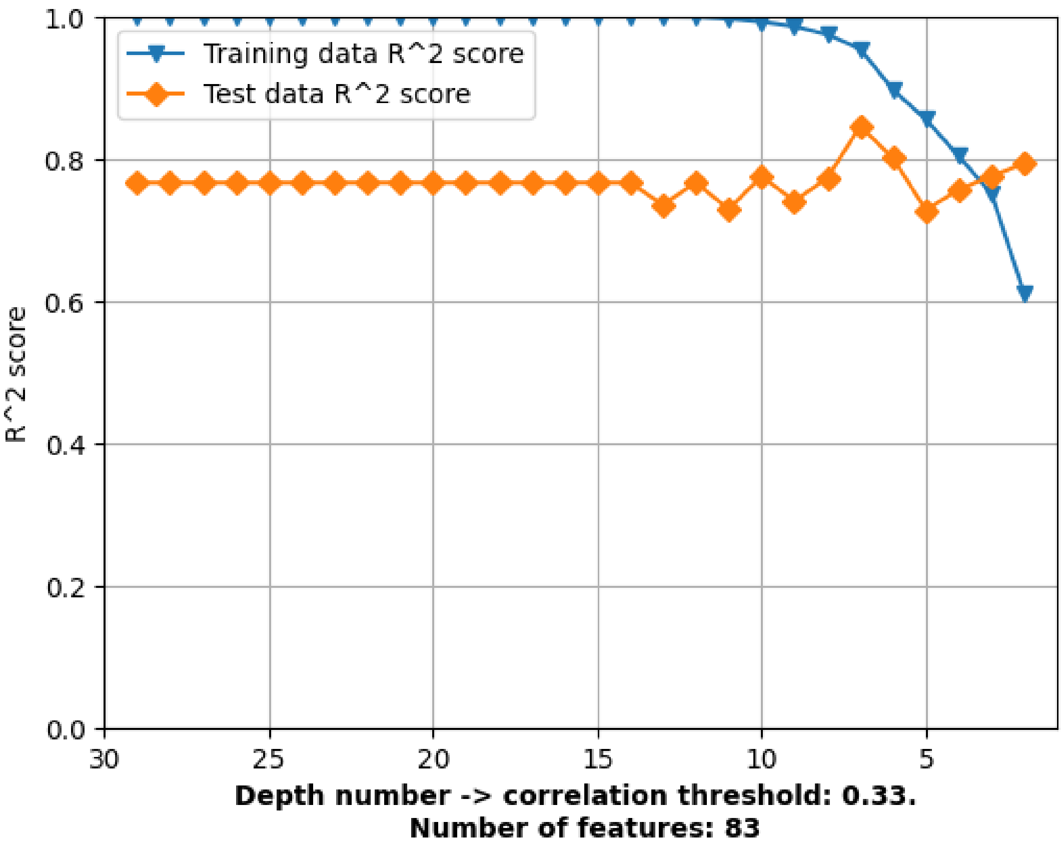

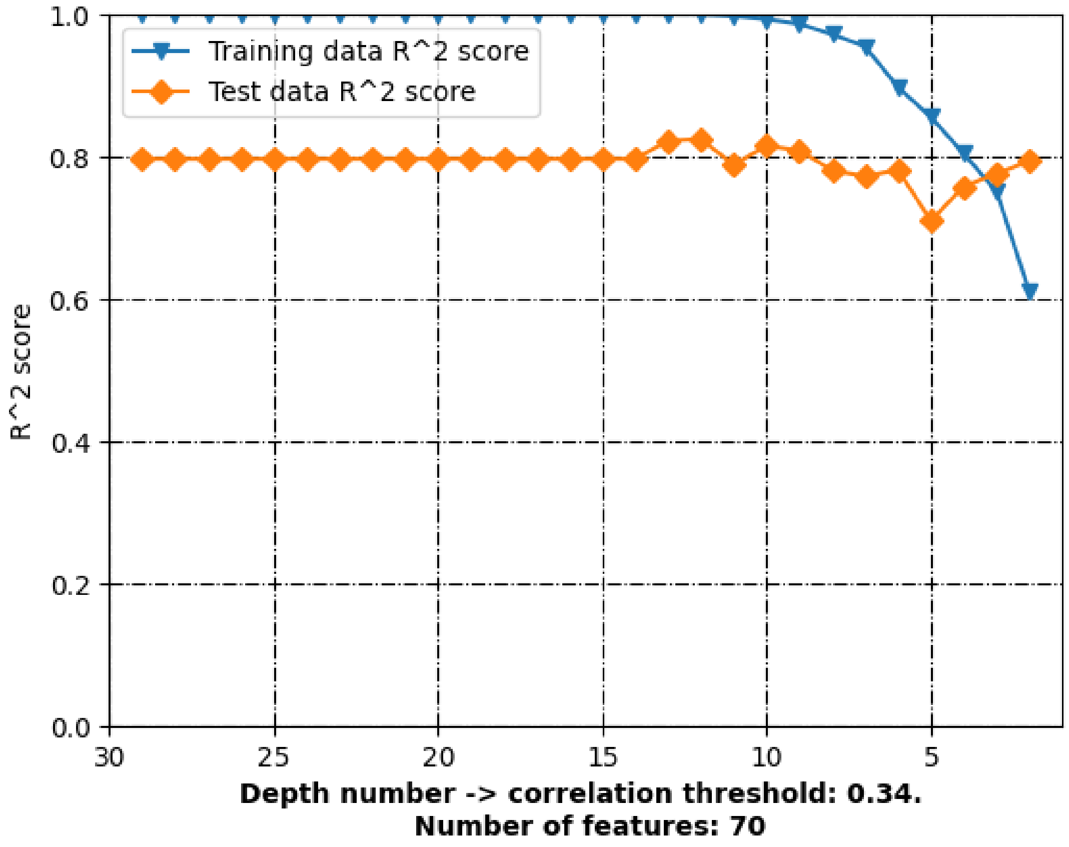

Training data R^2 score
Test data R^2 score
R^2 score
1.0
0.8
0.6
0.4
0.2
0.0
30
25
20
15
10
5
Depth number -> correlation threshold: 0.34.
Number of features: 70

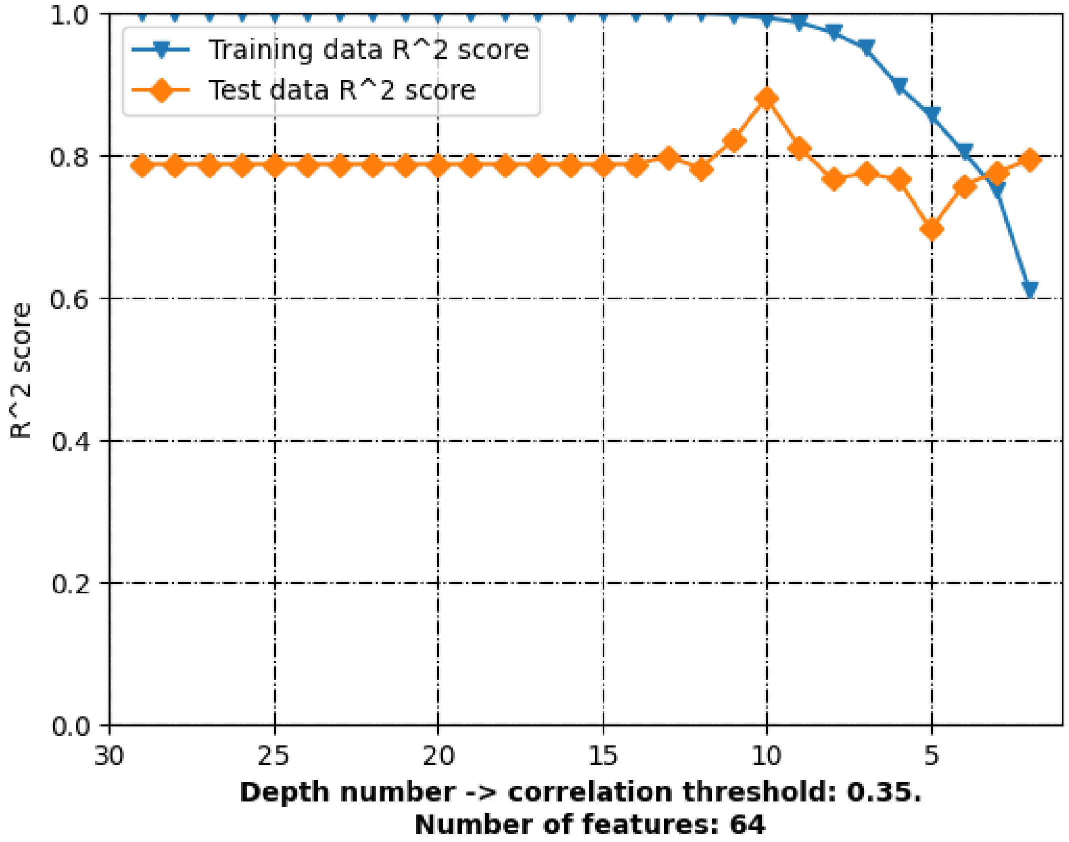

Training data R^2 score
Test data R^2 score
R^2 score
1.0
0.8
0.6
0.4
0.2
0.0
30
25
20
15
10
5
Depth number -> correlation threshold: 0.35.
Number of features: 64

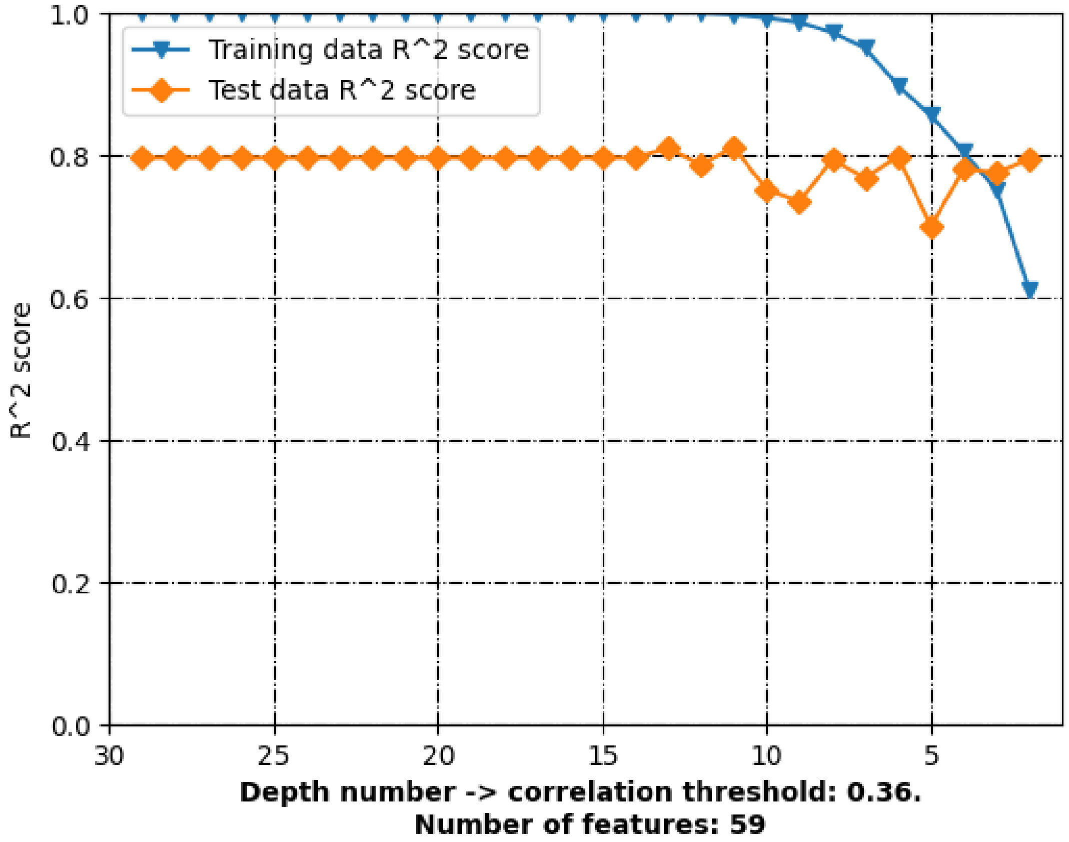
Training data R^2 score
Test data R^2 score
R^2 score
1.0
0.8
0.6
0.4
0.2
0.0
30
25
20
15
10
5
Depth number -> correlation threshold: 0.36.
Number of features: 59

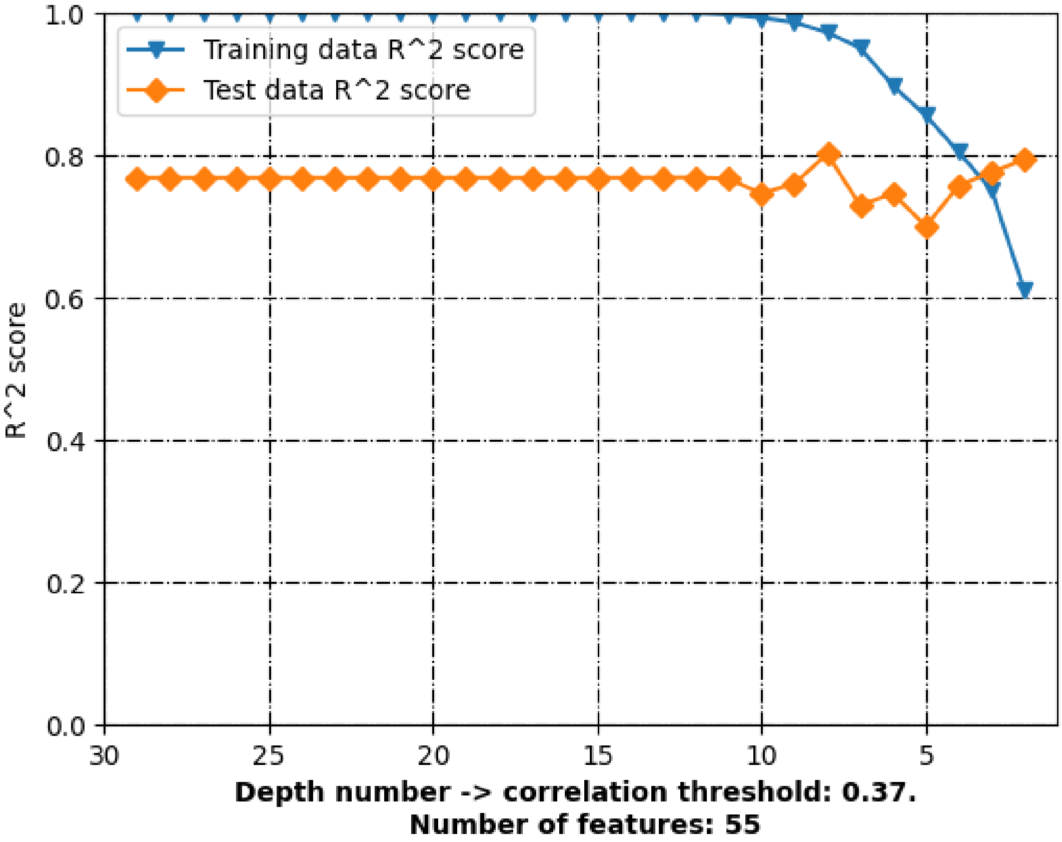
Training data R^2 score
Test data R^2 score
R^2 score
1.0
0.8
0.6
0.4
0.2
0.0
30
25
20
15
10
5
Depth number -> correlation threshold: 0.37.
Number of features: 55

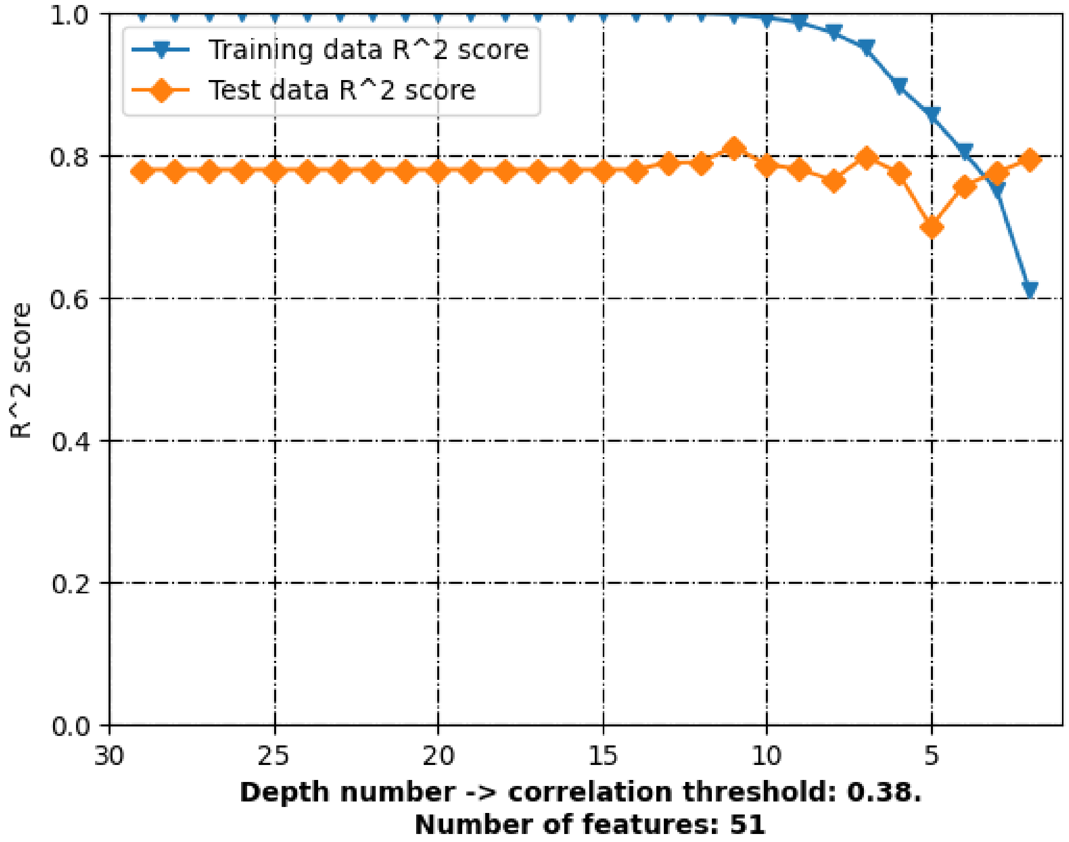
Training data R^2 score
Test data R^2 score
R^2 score
1.0
0.8
0.6
0.4
0.2
0.0
30
25
20
15
10
5
Depth number -> correlation threshold: 0.38.
Number of features: 51

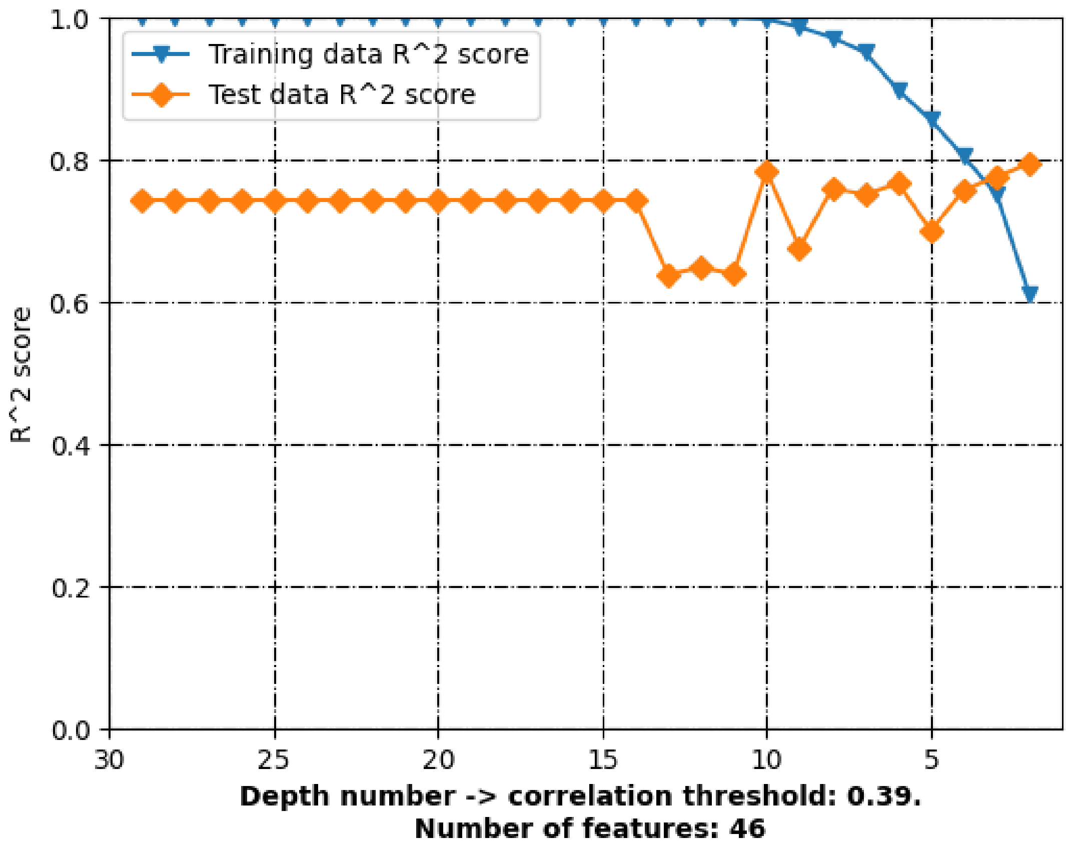

Training data R^2 score
Test data R^2 score
R^2 score
1.0
0.8
0.6
0.4
0.2
0.0
30
25
20
15
10
5
Depth number -> correlation threshold: 0.39.
Number of features: 46

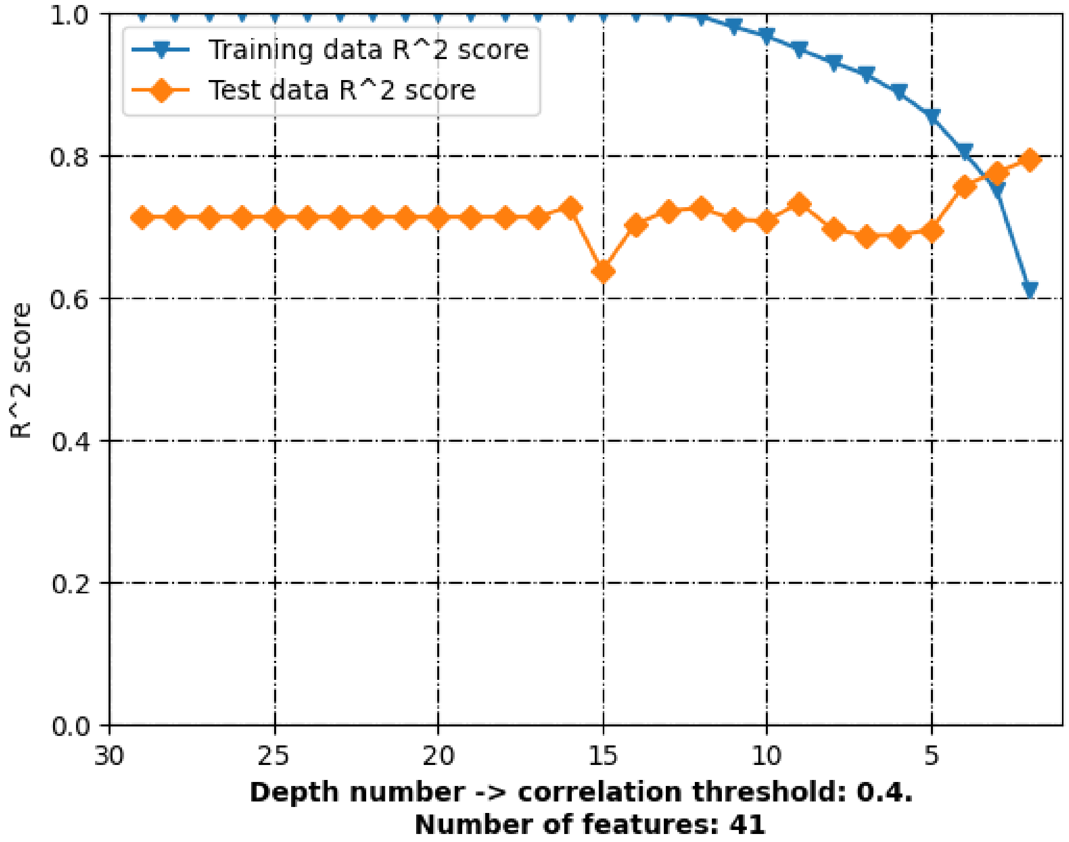

Training data R^2 score
Test data R^2 score
R^2 score
1.0
0.8
0.6
0.4
0.2
0.0
30
25
20
15
10
5
Depth number -> correlation threshold: 0.4.
Number of features: 41

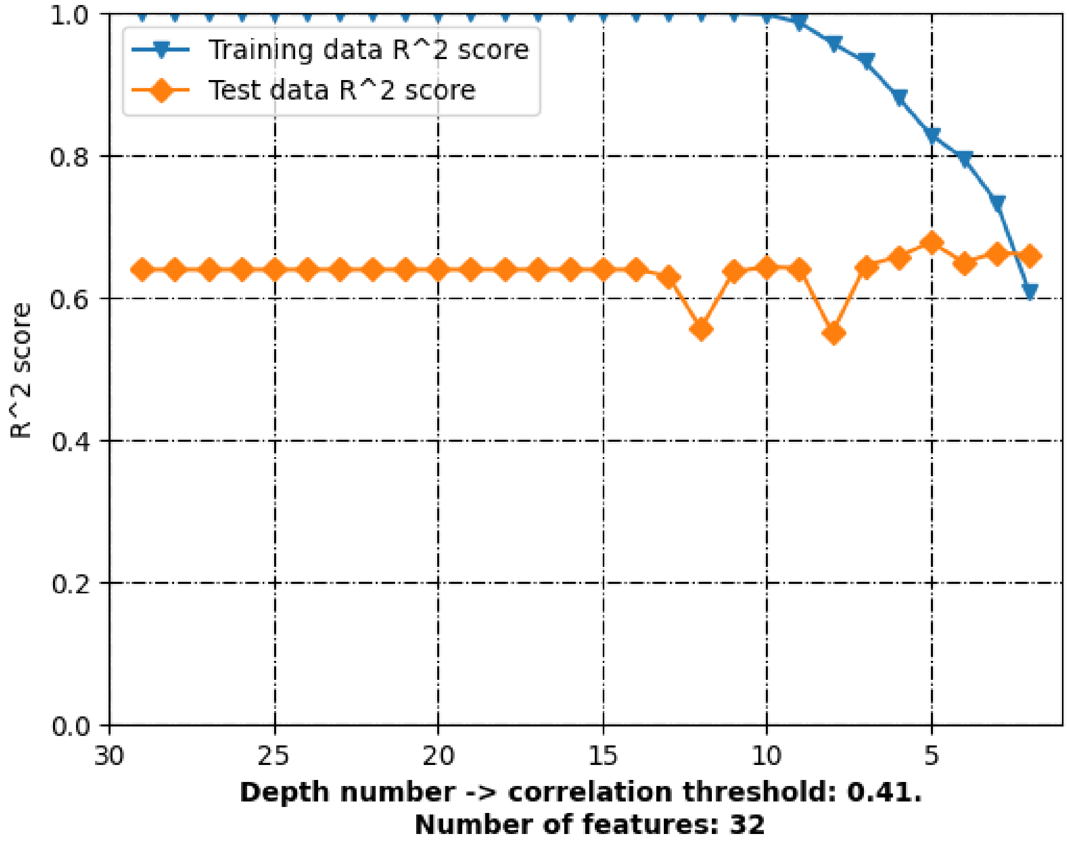

Training data R^2 score
Test data R^2 score
R^2 score
1.0
0.8
0.6
0.4
0.2
0.0
30
25
20
15
10
5
Depth number -> correlation threshold: 0.41.
Number of features: 32

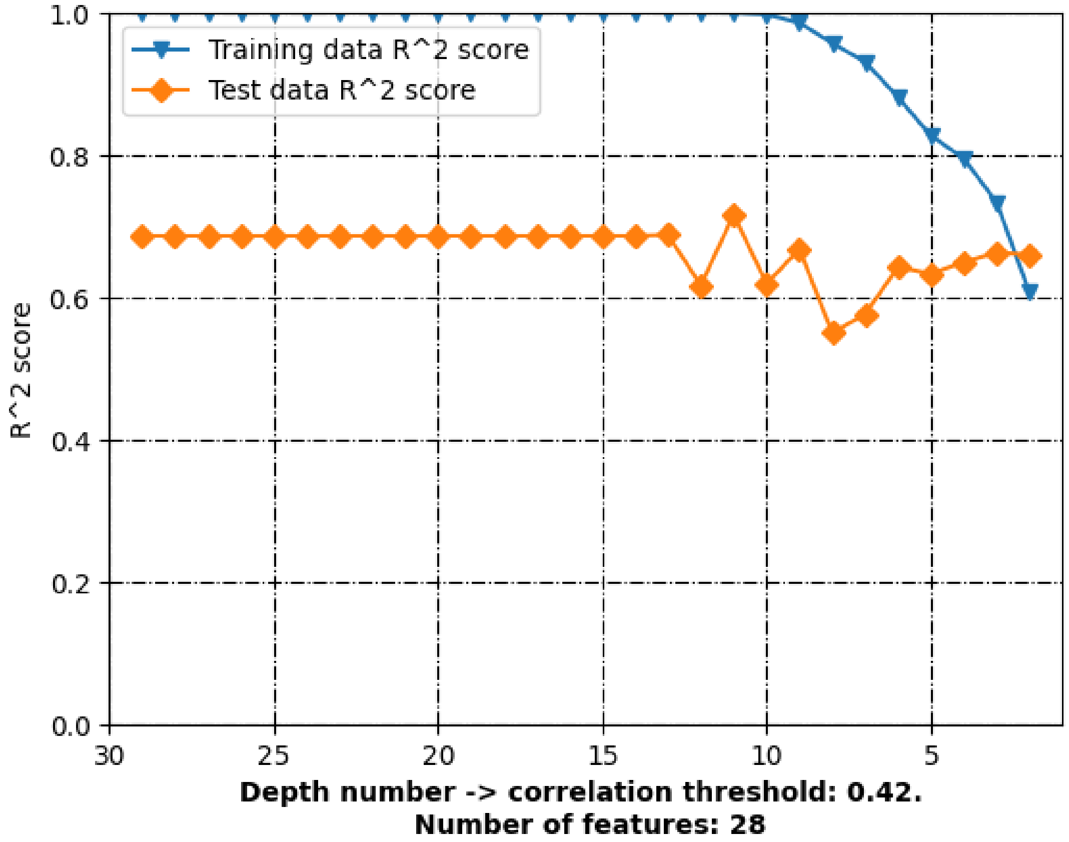

Training data R^2 score
Test data R^2 score
R^2 score
1.0
0.8
0.6
0.4
0.2
0.0
30
25
20
15
10
5
Depth number -> correlation threshold: 0.42.
Number of features: 28

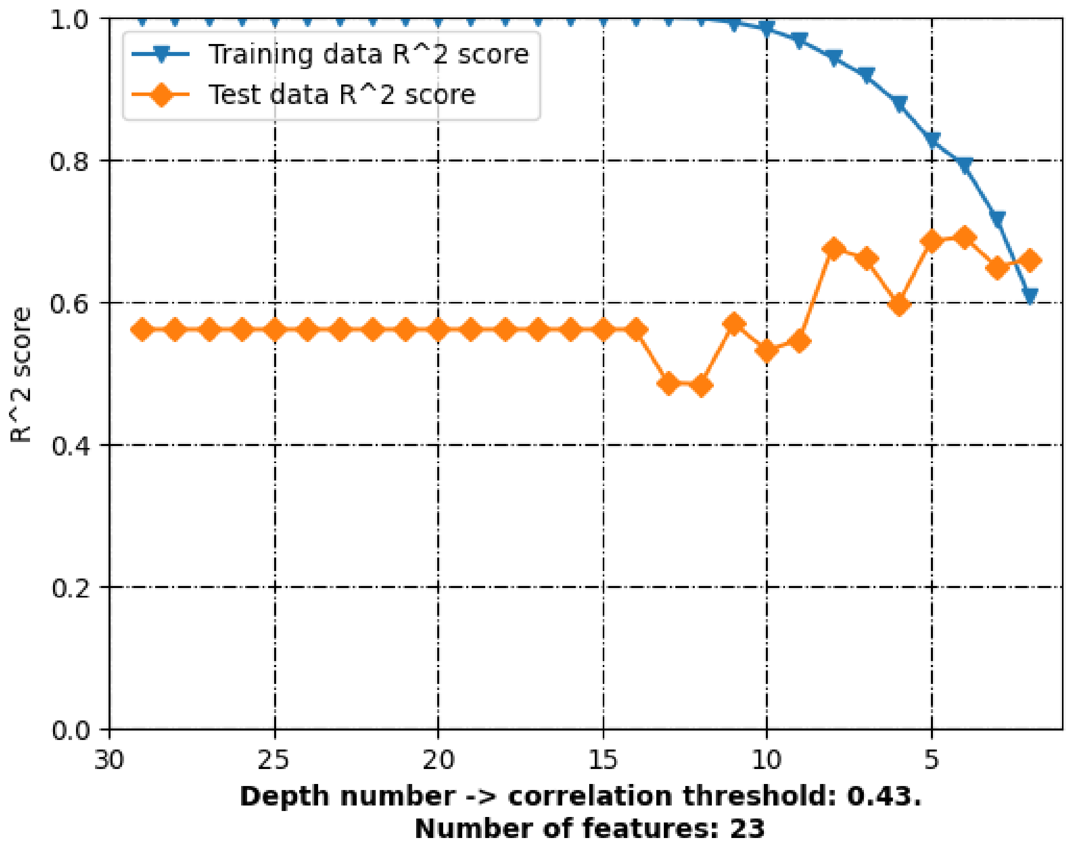

Training data R^2 score
Test data R^2 score
R^2 score
1.0
0.8
0.6
0.4
0.2
0.0
30
25
20
15
10
5
Depth number -> correlation threshold: 0.43.
Number of features: 23

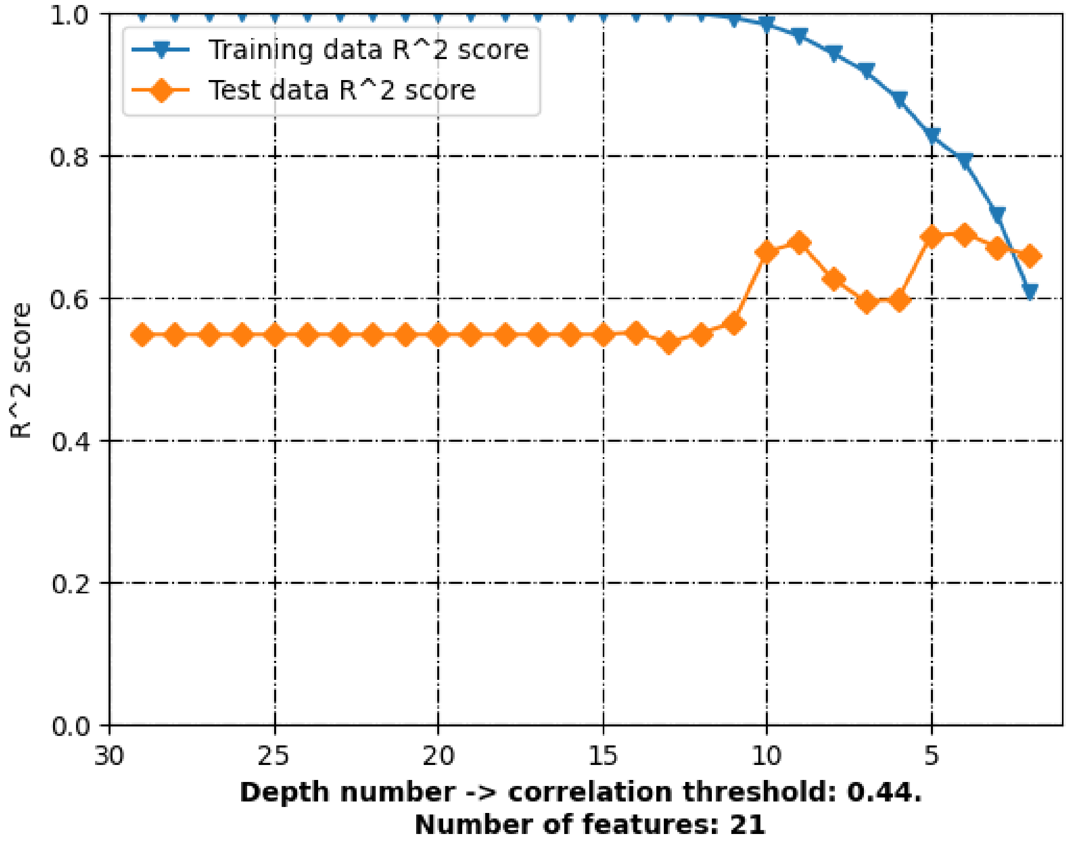

Training data R^2 score
Test data R^2 score
R^2 score
1.0
0.8
0.6
0.4
0.2
0.0
30
25
20
15
10
5
Depth number -> correlation threshold: 0.44.
Number of features: 21

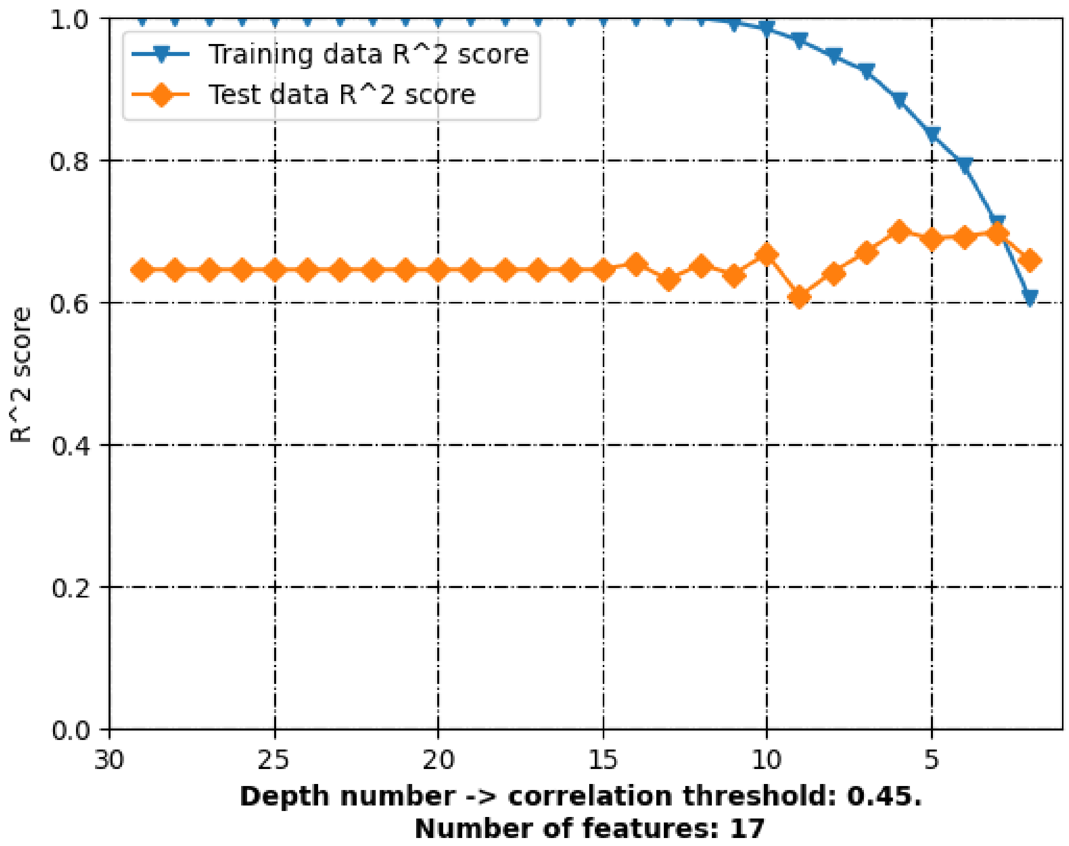

Training data R^2 score
Test data R^2 score
R^2 score
1.0
0.8
0.6
0.4
0.2
0.0
30
25
20
15
10
5
Depth number -> correlation threshold: 0.45.
Number of features: 17

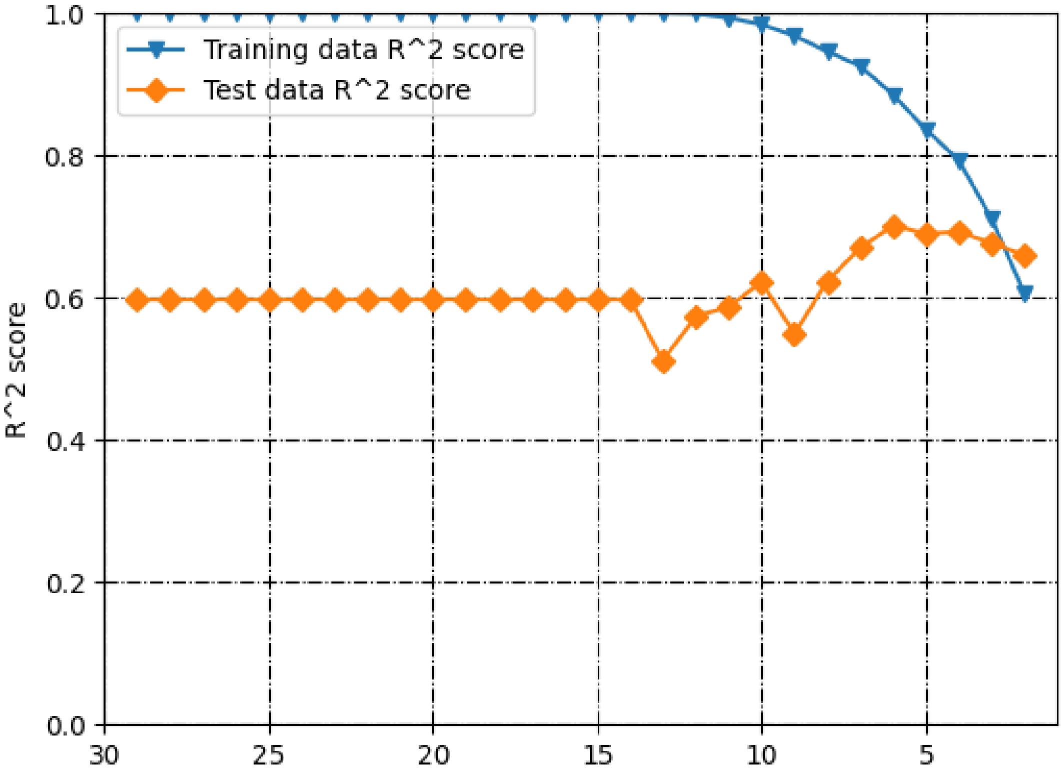

Depth number -> correlation threshold: 0.46.
Number of features: 16

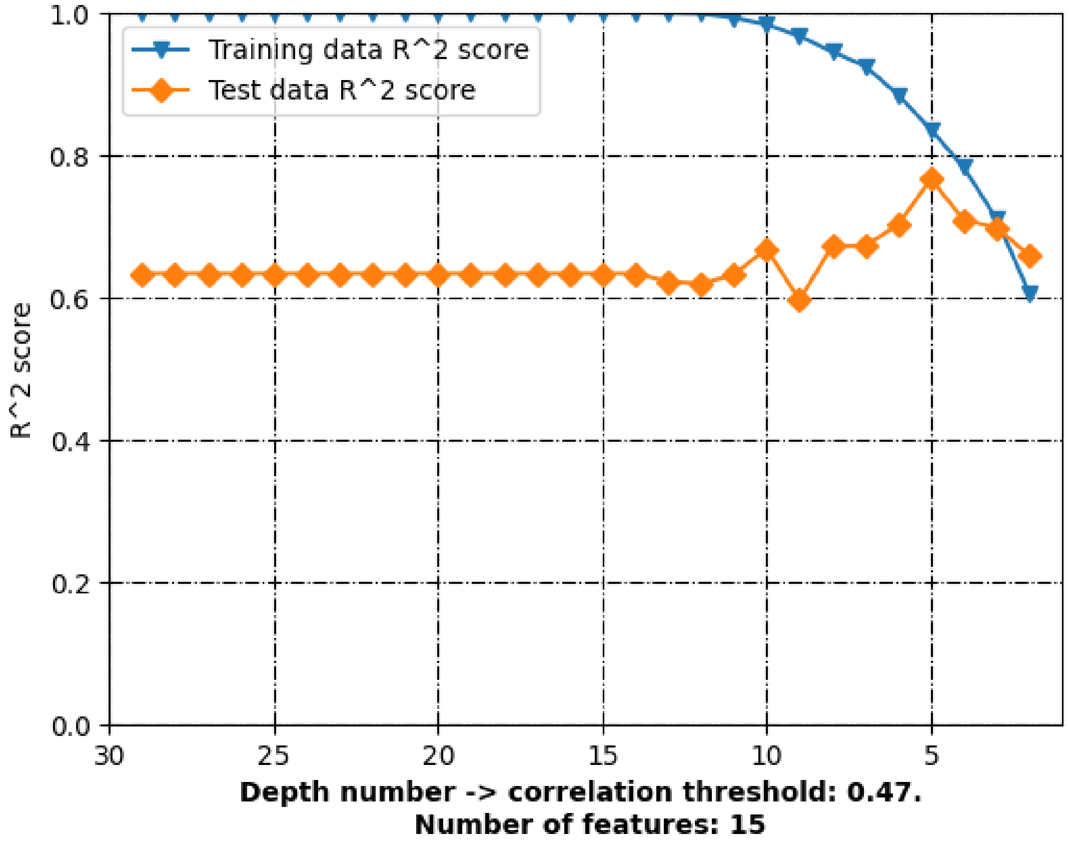
1.0
0.8
0.6
0.4
0.2
0.0
R^2 score
Training data R^2 score
Test data R^2 score
30
25
20
15
10
5
Depth number -> correlation threshold: 0.47.
Number of features: 15

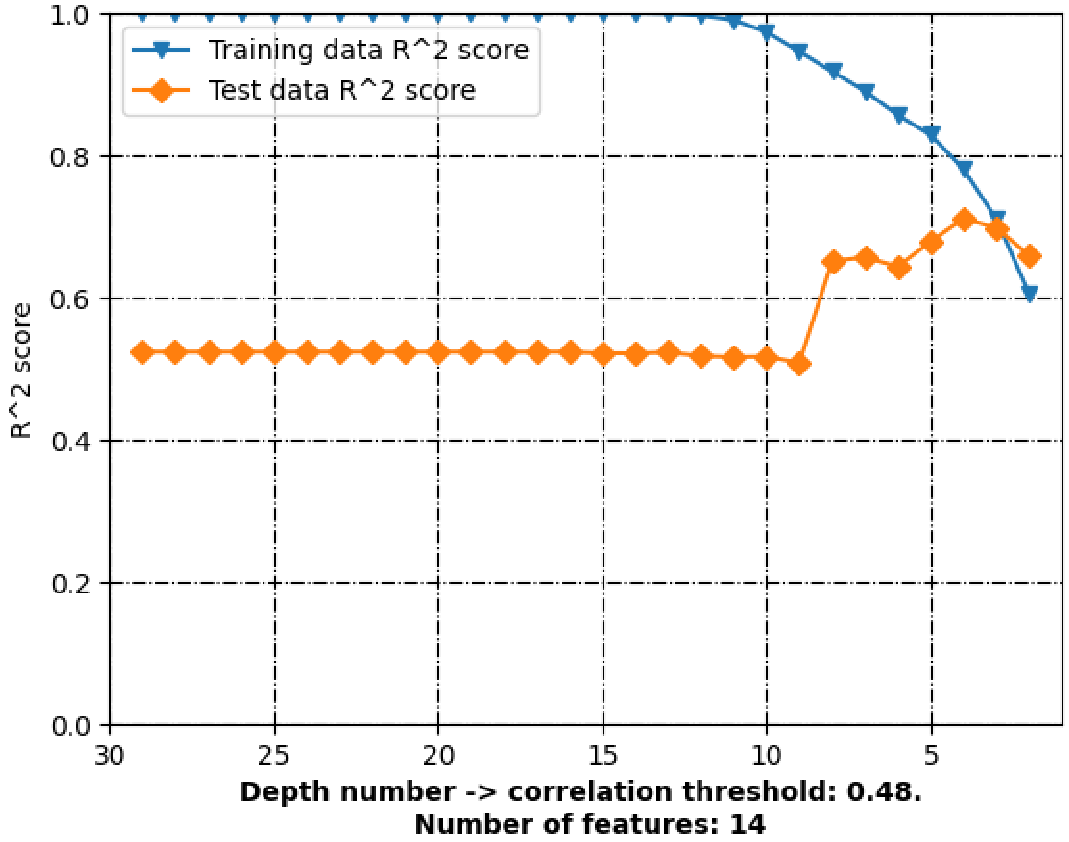

Training data R^2 score
Test data R^2 score
R^2 score
1.0
0.8
0.6
0.4
0.2
0.0
30
25
20
15
10
5
Depth number -> correlation threshold: 0.48.
Number of features: 14

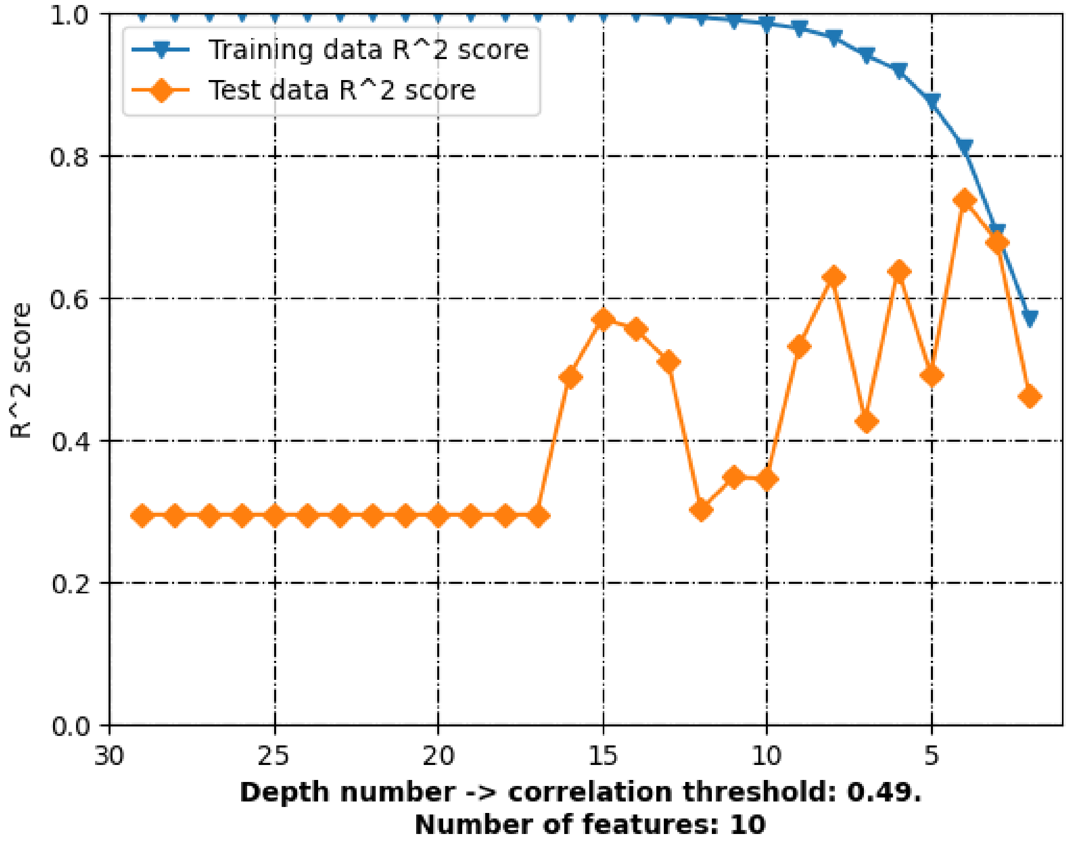

Training data R^2 score
Test data R^2 score
R^2 score
1.0
0.8
0.6
0.4
0.2
0.0
30
25
20
15
10
5
Depth number -> correlation threshold: 0.49.
Number of features: 10

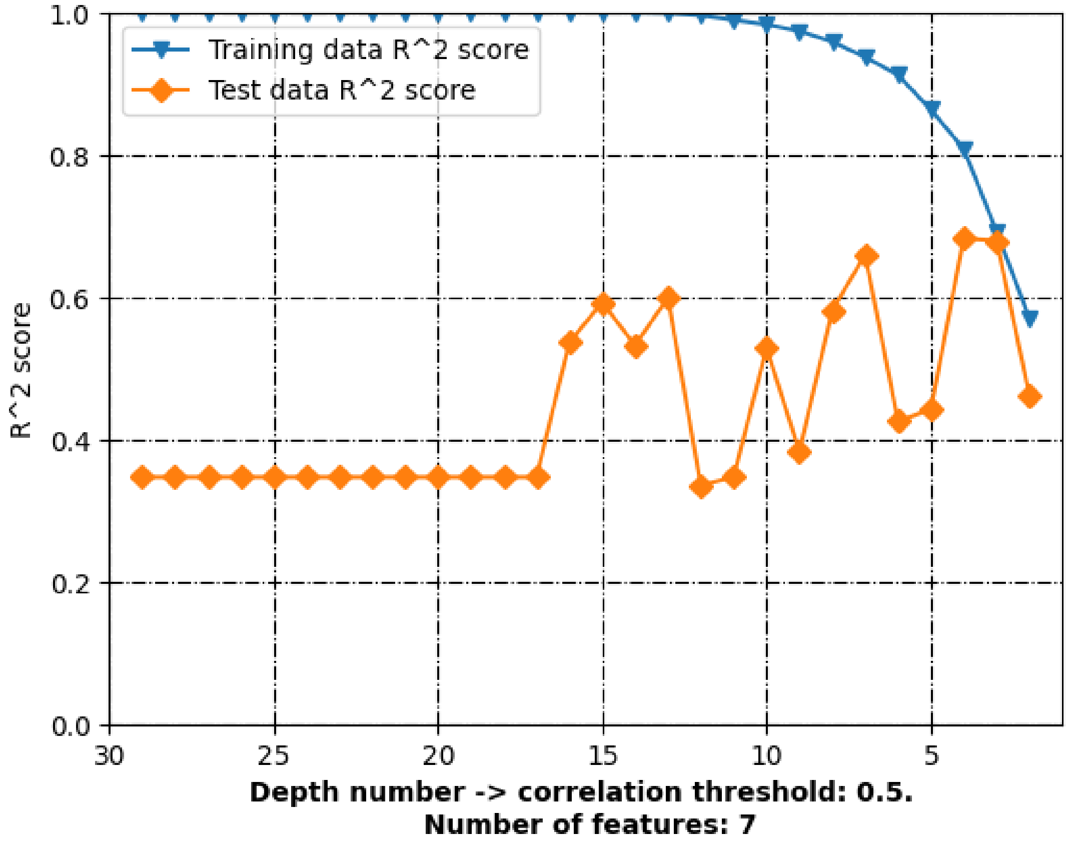

Training data R^2 score
Test data R^2 score
R^2 score
1.0
0.8
0.6
0.4
0.2
0.0
30
25
20
15
10
5
Depth number -> correlation threshold: 0.5.
Number of features: 7

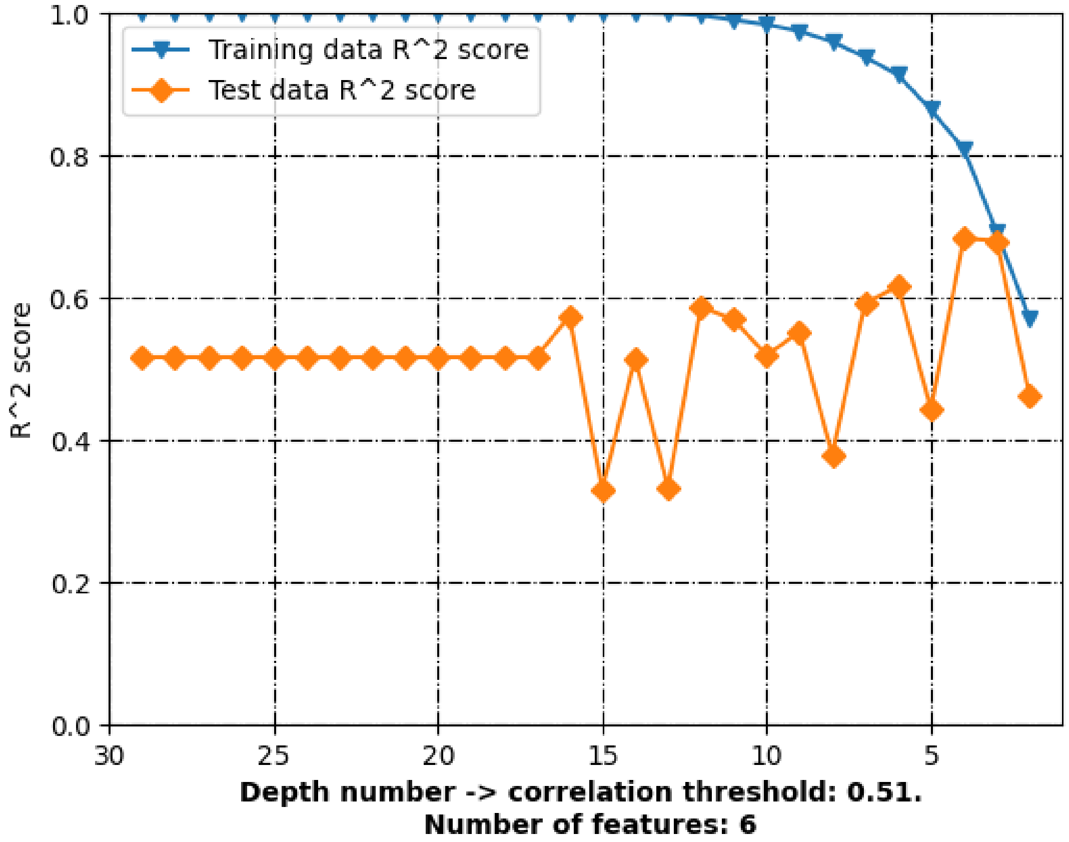

Training data R^2 score
Test data R^2 score
R^2 score
1.0
0.8
0.6
0.4
0.2
0.0
30
25
20
15
10
5
Depth number -> correlation threshold: 0.51.
Number of features: 6

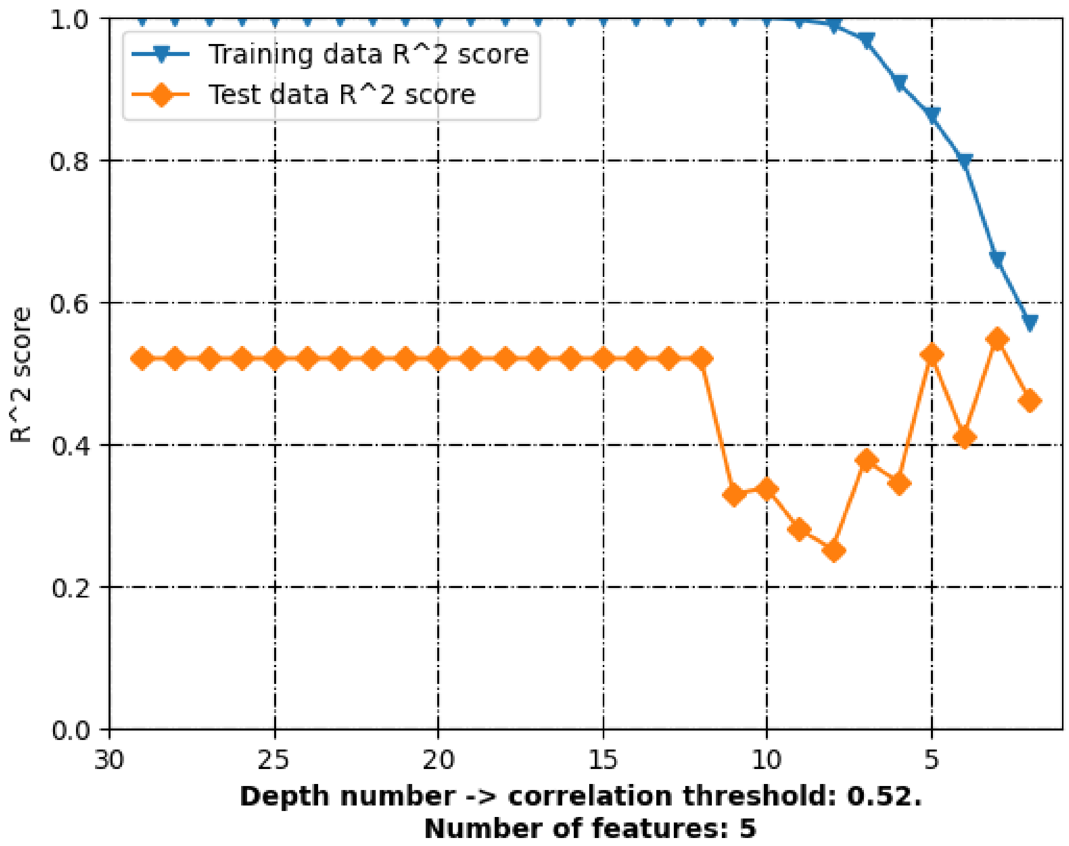

Training data R^2 score
Test data R^2 score
R^2 score
1.0
0.8
0.6
0.4
0.2
0.0
30
25
20
15
10
5
Depth number -> correlation threshold: 0.52.
Number of features: 5

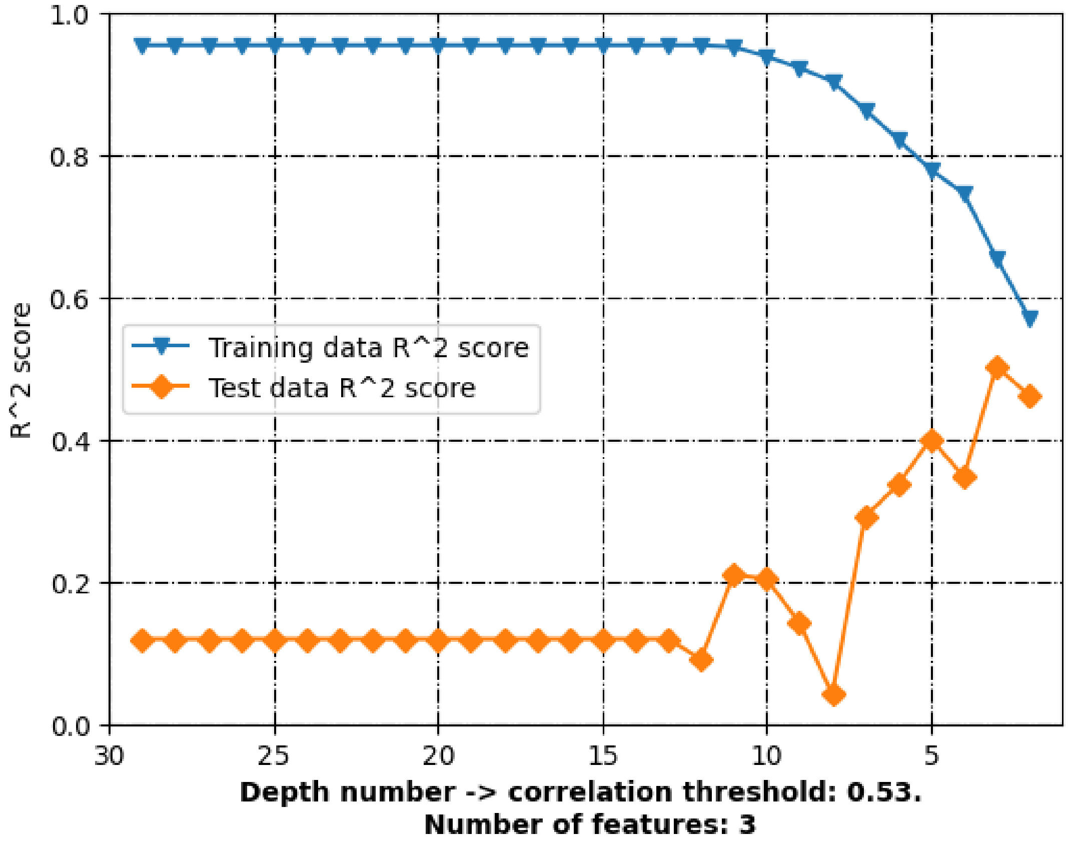
1.0
0.8
0.6
0.4
0.2
0.0
R^2 score
Training data R^2 score
Test data R^2 score
30
25
20
15
10
5
Depth number -> correlation threshold: 0.53.
Number of features: 3

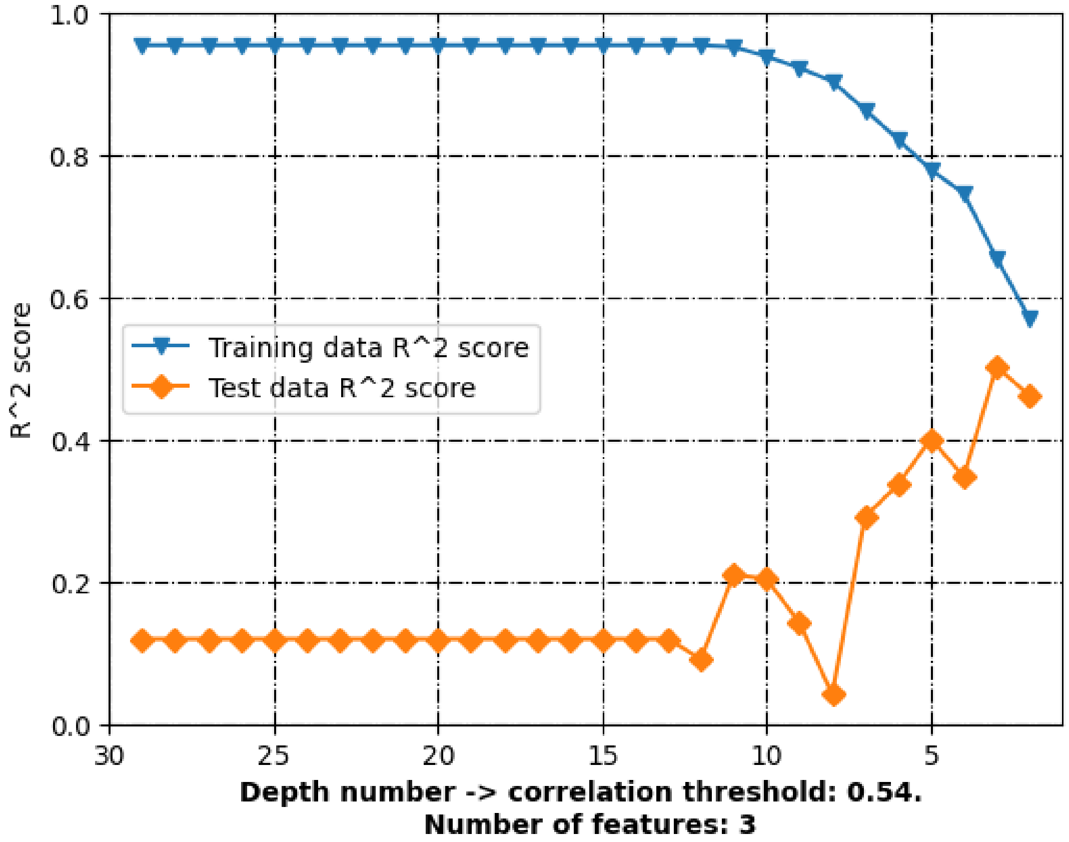

1.0
0.8
0.6
0.4
0.2
0.0
R^2 score
Training data R^2 score
Test data R^2 score
30
25
20
15
10
5
Depth number -> correlation threshold: 0.54.
Number of features: 3

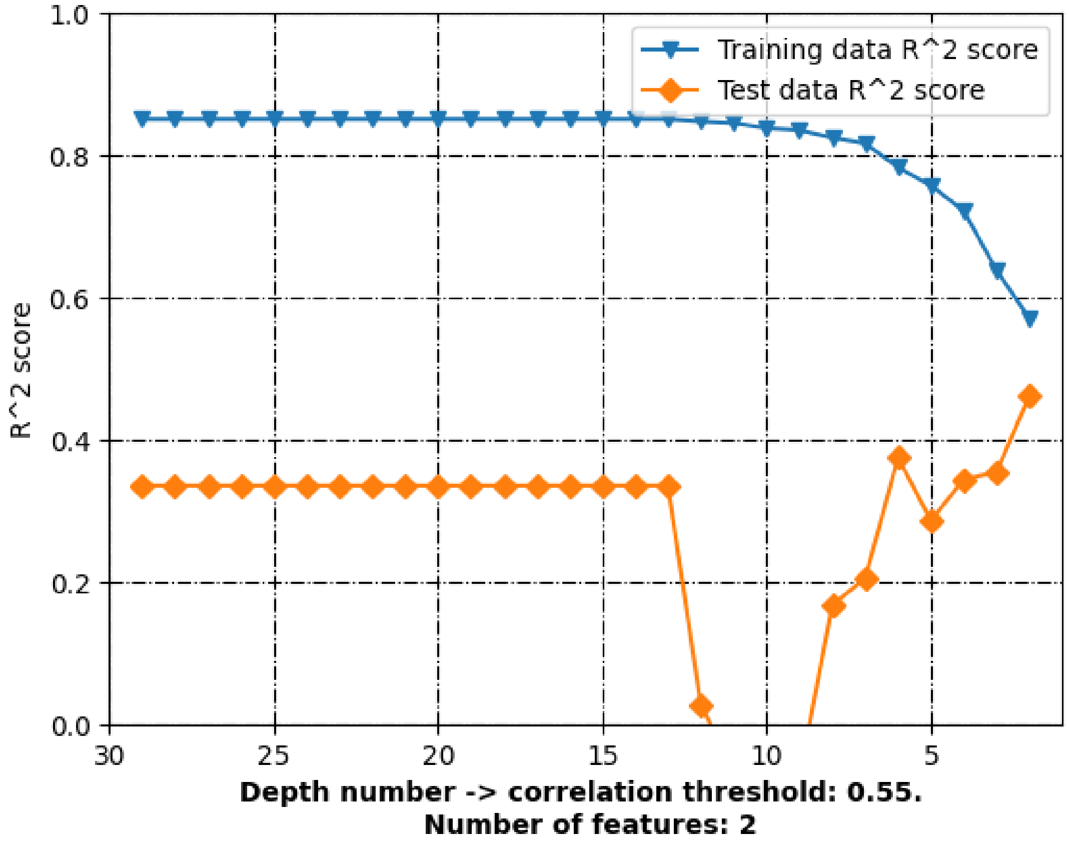

1.0
0.8
0.6
0.4
0.2
0.0
R^2 score
30
25
20
15
10
5
Training data R^2 score
Test data R^2 score
Depth number -> correlation threshold: 0.55.
Number of features: 2

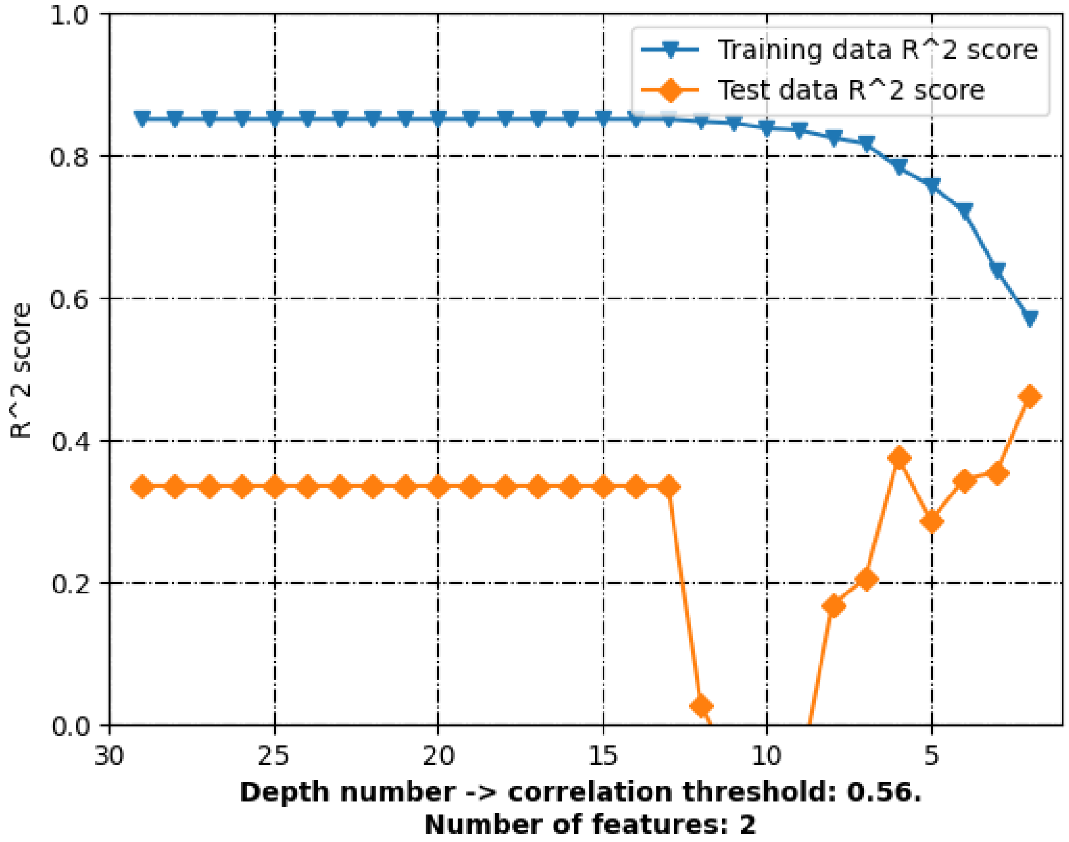

[20]:
```
plt.plot(df_without_standardization['Correlation threshold'],␣
  ↪df_without_standardization['Number of features'], label = "Number of␣
  ↪features", marker='.')
plt.legend()
plt.xlabel('Correlation threshold')
plt.ylabel('Number of features')
plt.show()
```

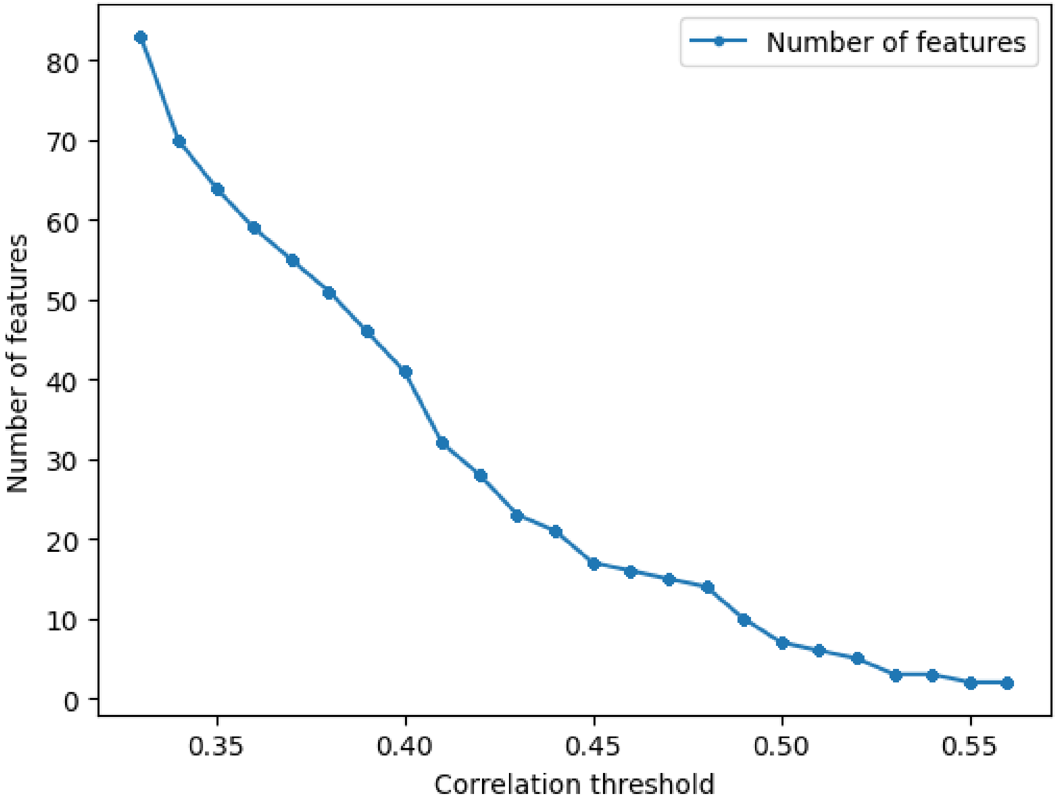

[ ]:

3 Random Forest

[21]:
```
without_standardization, train_r2_, test_r2_, hist1, hist2, target_column_name,␣
↪training_data_RMSE, test_data_RMSE  = pred_model.
↪prepare_data_and_create_model(molecular_descriptors_df=data,
                                                                              ␣
↪                         correlation_threshold=0.50,
                                                                              ␣
↪                         standardization=False,
                                                                              ␣
↪                         model_type='RandomForestRegressor',
                                                                              ␣
↪                         n_estimators_=12,
                                                                              ␣
↪                         target_column_name = target,
                                                                              ␣
↪                         random_state=random_state,
```

```
↪                       train_test_split_=True,
↪                       verbose=True)
```

```
I am not doing standardization…
  molecular descriptor name
0                    AATS0Z
1                  AATS0are
2                    AATS0d
3                   AATS0dv
4                    AATS0i
  molecular descriptor name  corr_value
0                    AATS0Z   -0.020817
1                  AATS0are   -0.148257
2                    AATS0d    0.022999
3                   AATS0dv   -0.137980
4                    AATS0i    0.133257
  molecular descriptor name  corr_value  absolute correlation value
0                    AATS0Z   -0.020817                    0.020817
1                  AATS0are   -0.148257                    0.148257
2                    AATS0d    0.022999                    0.022999
3                   AATS0dv   -0.137980                    0.137980
4                    AATS0i    0.133257                    0.133257
    molecular descriptor name  corr_value  absolute correlation value
226                    AMID_O   -0.546843                    0.546843
505               EState_VSA5   -0.588780                    0.588780
556                    GATS2c    0.511671                    0.511671
791                   MDEO-12   -0.582747                    0.582747
847                       NdO   -0.500462                    0.500462
    molecular descriptor name  corr_value  absolute correlation value
226                    AMID_O   -0.546843                    0.546843
505               EState_VSA5   -0.588780                    0.588780
556                    GATS2c    0.511671                    0.511671
791                   MDEO-12   -0.582747                    0.582747
847                       NdO   -0.500462                    0.500462
The model used is: RandomForest…
Return the coefficient of determination of the prediction:
0.6983472822308948
R^2 score: 0.9235215940349127
Correlation coefficient: 0.9610003090711848
Test data - unseen during training:
R^2 score: 0.6983472822308948
Correlation coefficient: 0.8356717550754571
[7.69834533 5.99736567 7.15163236 7.95490249 7.95796914 7.32280321
 7.97736763 7.1930782  7.99854953 7.86380064 7.25260105 7.86053838
 7.93833005 7.74918107 7.41523265 7.84509988 6.18122122 5.58676987]
102    7.698970
```

```
38     5.986741
8      5.984515
109    7.886057
11     7.962574
51     7.698970
88     8.229148
21     7.298432
82     8.065502
98     7.376751
58     7.602060
64     8.060481
74     7.853872
103    7.853872
91     8.346787
43     7.619789
25     4.989276
30     6.085657
Name: BALB/3T3, dtype: float64
Training Root Mean Square Error: 0.23998234483228884
Testing Root Mean Square Error: 0.5051460408205187
```

[22]:
```
print(target_column_name+str('_transformed'))
print(hist1[target_column_name].hist())
```

```
BALB/3T3_transformed
AxesSubplot(0.125,0.11;0.775x0.77)
```

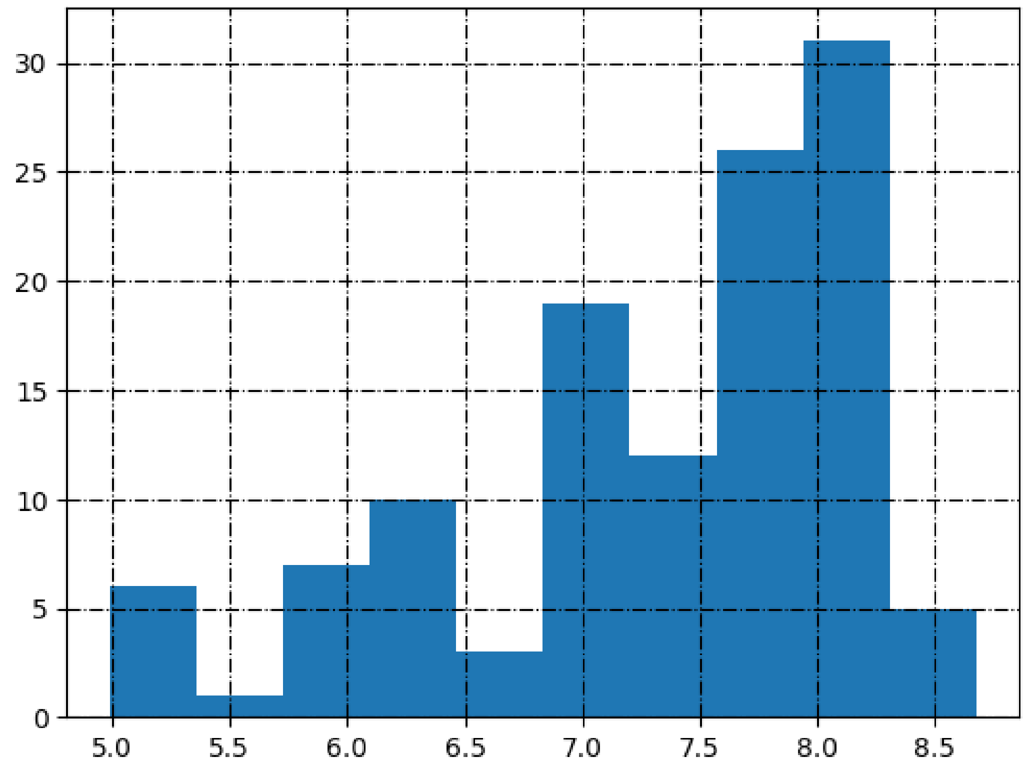

[23]:
```
with_standardization, train_r2_, test_r2_, hist1, hist2, target_column_name,␣
↪training_data_RMSE, test_data_RMSE  = pred_model.
↪prepare_data_and_create_model(molecular_descriptors_df=data,
                                                                              ␣
↪                         correlation_threshold=0.50,
                                                                              ␣
↪                         standardization=True,
                                                                              ␣
↪                         model_type='RandomForestRegressor',
                                                                              ␣
↪                         n_estimators_=12,
                                                                              ␣
↪                         target_column_name = target,
                                                                              ␣
↪                         random_state=random_state,
                                                                              ␣
↪                         train_test_split_=True,
                                                                              ␣
↪                         verbose=True)
```

```
I am doing standardization…
  molecular descriptor name
```

```
0                  AATS0Z
1                AATS0are
2                  AATS0d
3                 AATS0dv
4                  AATS0i
  molecular descriptor name  corr_value
0                  AATS0Z   -0.020817
1                AATS0are   -0.148257
2                  AATS0d    0.022999
3                 AATS0dv   -0.137980
4                  AATS0i    0.133257
  molecular descriptor name  corr_value  absolute correlation value
0                  AATS0Z   -0.020817                    0.020817
1                AATS0are   -0.148257                    0.148257
2                  AATS0d    0.022999                    0.022999
3                 AATS0dv   -0.137980                    0.137980
4                  AATS0i    0.133257                    0.133257
    molecular descriptor name  corr_value  absolute correlation value
226                    AMID_O   -0.546843                    0.546843
505               EState_VSA5   -0.588780                    0.588780
556                    GATS2c    0.511671                    0.511671
791                   MDEO-12   -0.582747                    0.582747
847                       NdO   -0.500462                    0.500462
```

```
    molecular descriptor name  corr_value  absolute correlation value
226                    AMID_O   -0.546843                    0.546843
505               EState_VSA5   -0.588780                    0.588780
556                    GATS2c    0.511671                    0.511671
791                   MDEO-12   -0.582747                    0.582747
847                       NdO   -0.500462                    0.500462
```

```
The model used is: RandomForest…
Return the coefficient of determination of the prediction:
0.6983472822308948
R^2 score: 0.9235215940349127
Correlation coefficient: 0.9610003090711848
Test data - unseen during training:
R^2 score: 0.6983472822308948
Correlation coefficient: 0.8356717550754571
[7.69834533 5.99736567 7.15163236 7.95490249 7.95796914 7.32280321
 7.97736763 7.1930782  7.99854953 7.86380064 7.25260105 7.86053838
 7.93833005 7.74918107 7.41523265 7.84509988 6.18122122 5.58676987]
102    7.698970
38     5.986741
8      5.984515
109    7.886057
11     7.962574
51     7.698970
88     8.229148
21     7.298432
```

```
82     8.065502
98     7.376751
58     7.602060
64     8.060481
74     7.853872
103    7.853872
91     8.346787
43     7.619789
25     4.989276
30     6.085657
Name: BALB/3T3, dtype: float64
Training Root Mean Square Error: 0.23998234483228884
Testing Root Mean Square Error: 0.5051460408205187
```

3.1 Search inside correlation space

[24]:
```
step = 0.01
initial_step = corr_low
last_step = corr_high
first_list = [x / 100.0 for x in range(int(initial_step*100),␣
 ↪int(last_step*100), int(step*100))]
n_estimators = [range(2,21,1)]
corr_th = []
second_list = []
third_list = []
fourth_l = []
fifth_l = []
f_list = []
fif_list = []
for i in first_list:

    for estimator in n_estimators[0]:

        without_standardization, train_r2, test_r2, _, h_, target_column_name,␣
 ↪training_data_RMSE, test_data_RMSE = pred_model.
 ↪prepare_data_and_create_model(molecular_descriptors_df=data,
                                                                                ␣
 ↪                   correlation_threshold=i,
                                                                                ␣
 ↪                   standardization=False,
                                                                                ␣
 ↪                   model_type='RandomForestRegressor',
                                                                                ␣
 ↪                   n_estimators_=estimator,
                                                                                ␣
 ↪                   target_column_name = target,
```

```
                                                                                  ␣
↪                   random_state=random_state,
                                                                                  ␣
↪                   train_test_split_=True,
                                                                                  ␣
↪                   verbose=False)
        corr_th.append(i)
        second_list.append(train_r2)
        third_list.append(test_r2)
        fourth_l.append(training_data_RMSE)
        fifth_l.append(test_data_RMSE)
        f_list.append(len(h_))
        fif_list.append(estimator)
```

[25]:

```
df_without_standardization = pd.DataFrame(data=corr_th, columns=["Correlation␣
↪threshold"])
df_without_standardization['Training data R^2 score'] = second_list
df_without_standardization['Test data R^2 score'] = third_list
df_without_standardization['Training RMSE'] = fourth_l
df_without_standardization['Test RMSE'] = fifth_l
df_without_standardization['Number of features'] = f_list
df_without_standardization['Number of estimators'] = fif_list
```

[26]:

```
df_random_forest = df_without_standardization.copy()
#df_without_standardization.to_excel('../Data/
↪A549_Random_forest_rs_'+str(random_state)+'.xlsx')
```

[27]:

```
corre_list = [x / 100.0 for x in range(int(initial_step*100),␣
↪int(last_step*100), int(0.01*100))]
for element in corre_list:
    element_ =␣
↪df_without_standardization[df_without_standardization['Correlation␣
↪threshold'] == float(element)]
    plt.plot(element_['Number of estimators'], element_['Training data R^2␣
↪score'], label = "Training data R^2 score", marker='v')
    plt.plot(element_['Number of estimators'], element_['Test data R^2 score'],␣
↪label = "Test data R^2 score", marker='D')
    plt.legend()
    plt.xlabel('Number of estimators -> correlation threshold: '+str(element)+'.
↪ \n Number of features: '+str(element_['Number of features'].iloc[0]))
    plt.xlim(max(element_['Number of estimators'])+1, min(element_['Number of␣
↪estimators'])-1)
    plt.ylabel('R^2 score')
    plt.ylim([0, 1])
    plt.rc('grid', linestyle="-.", color='black')
    plt.grid(True)
```

```
plt.show()
```

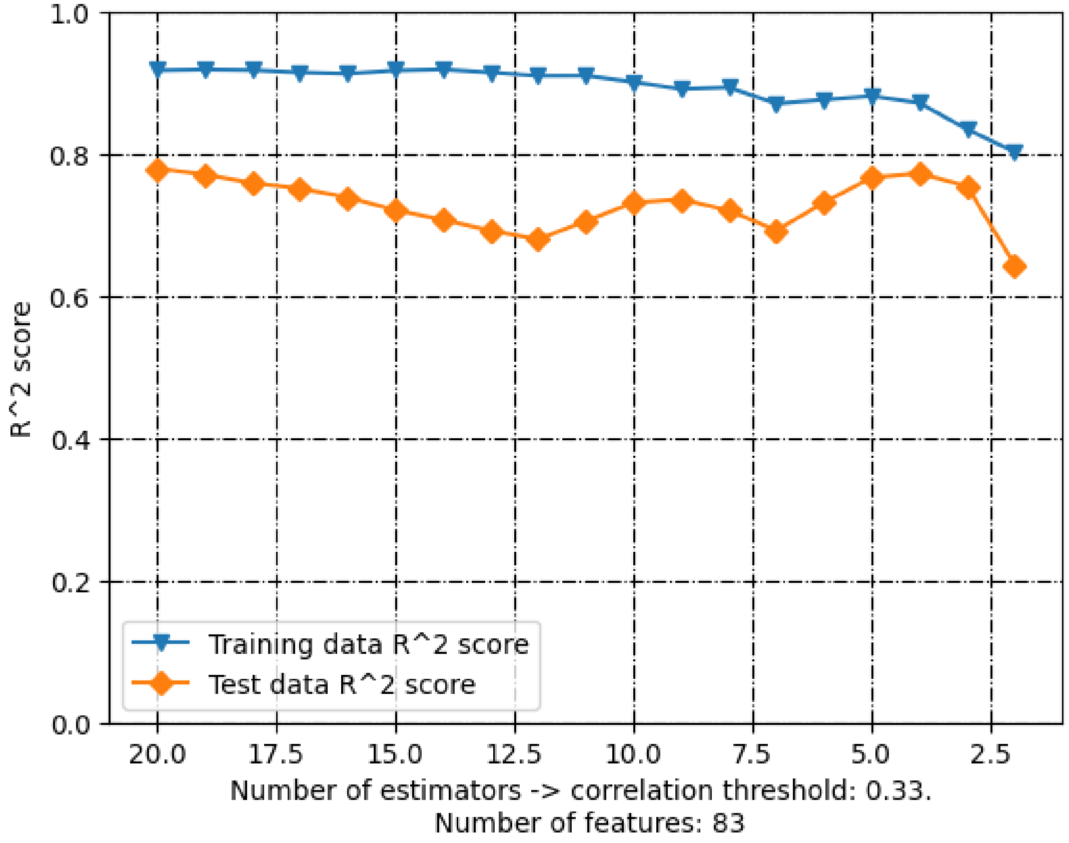

1.0
0.8
0.6
0.4
0.2
0.0
R^2 score
Training data R^2 score
Test data R^2 score
20.0
17.5
15.0
12.5
10.0
7.5
5.0
2.5
Number of estimators -> correlation threshold: 0.33.
Number of features: 83

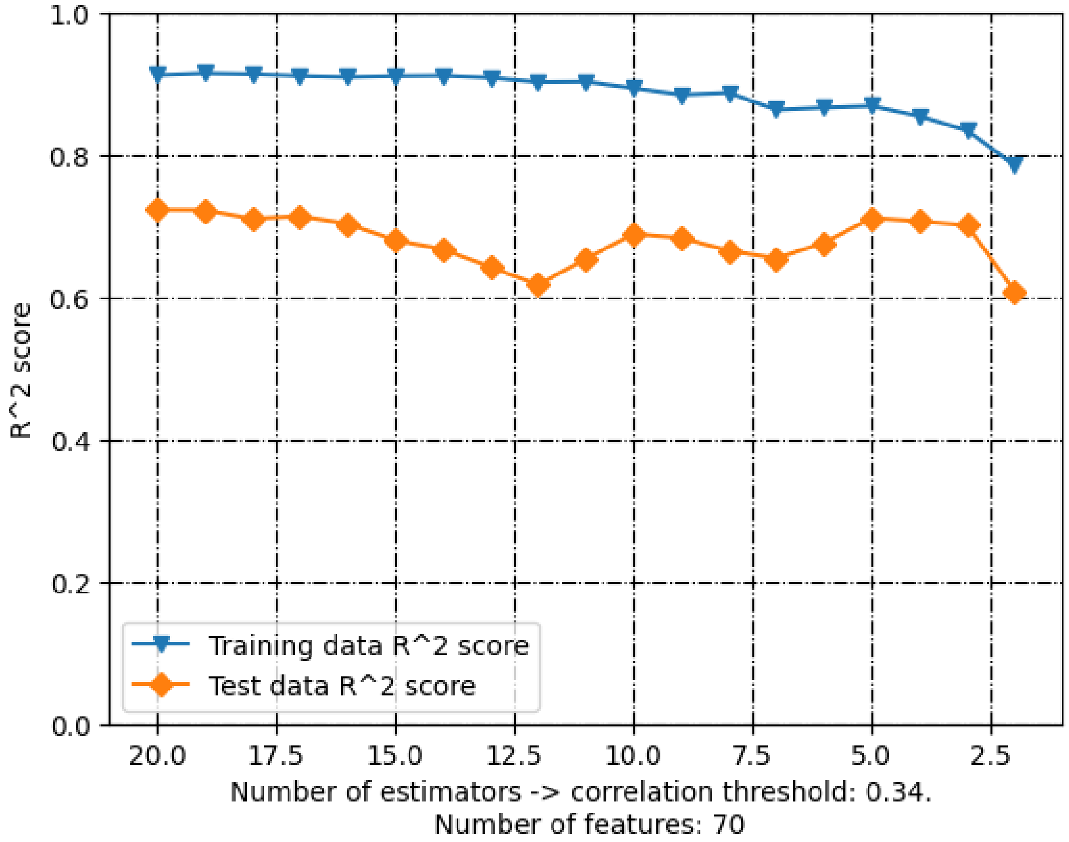

1.0
0.8
0.6
0.4
0.2
0.0
R^2 score
20.0
17.5
15.0
12.5
10.0
7.5
5.0
2.5
Training data R^2 score
Test data R^2 score
Number of estimators -> correlation threshold: 0.34.
Number of features: 70

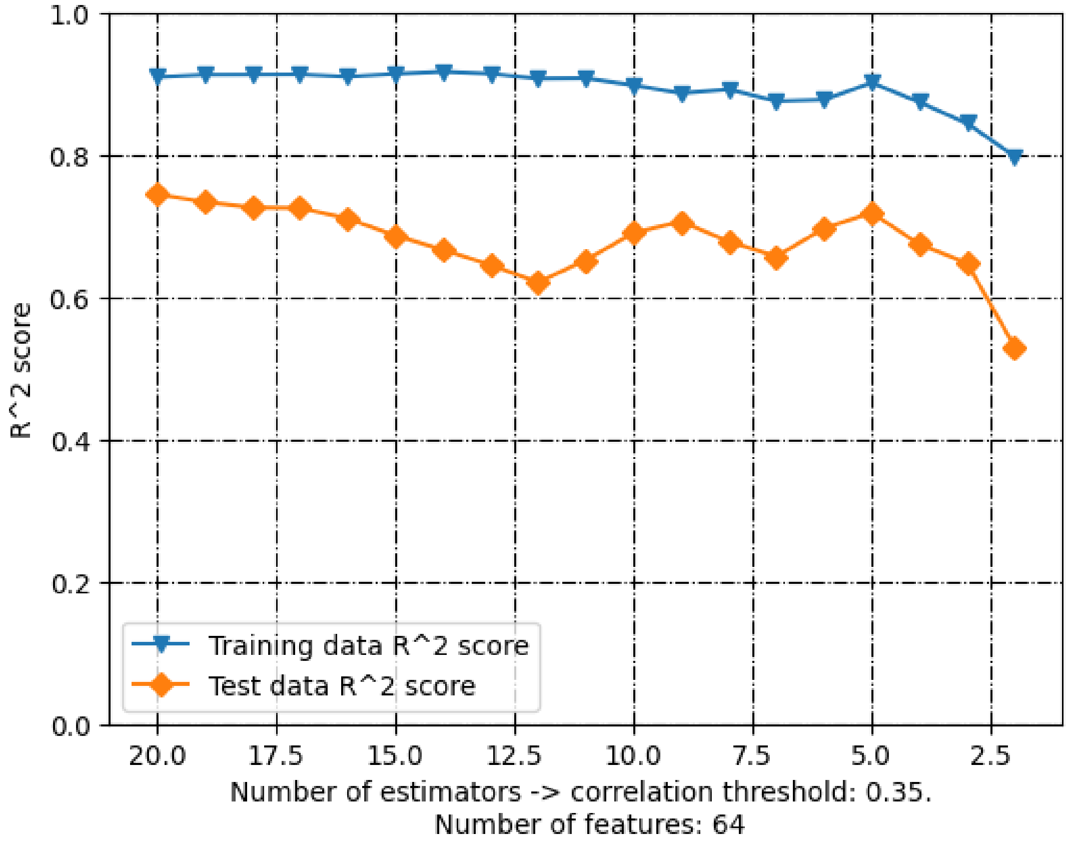

1.0
0.8
0.6
0.4
0.2
0.0
R^2 score
Training data R^2 score
Test data R^2 score
20.0
17.5
15.0
12.5
10.0
7.5
5.0
2.5
Number of estimators -> correlation threshold: 0.35.
Number of features: 64

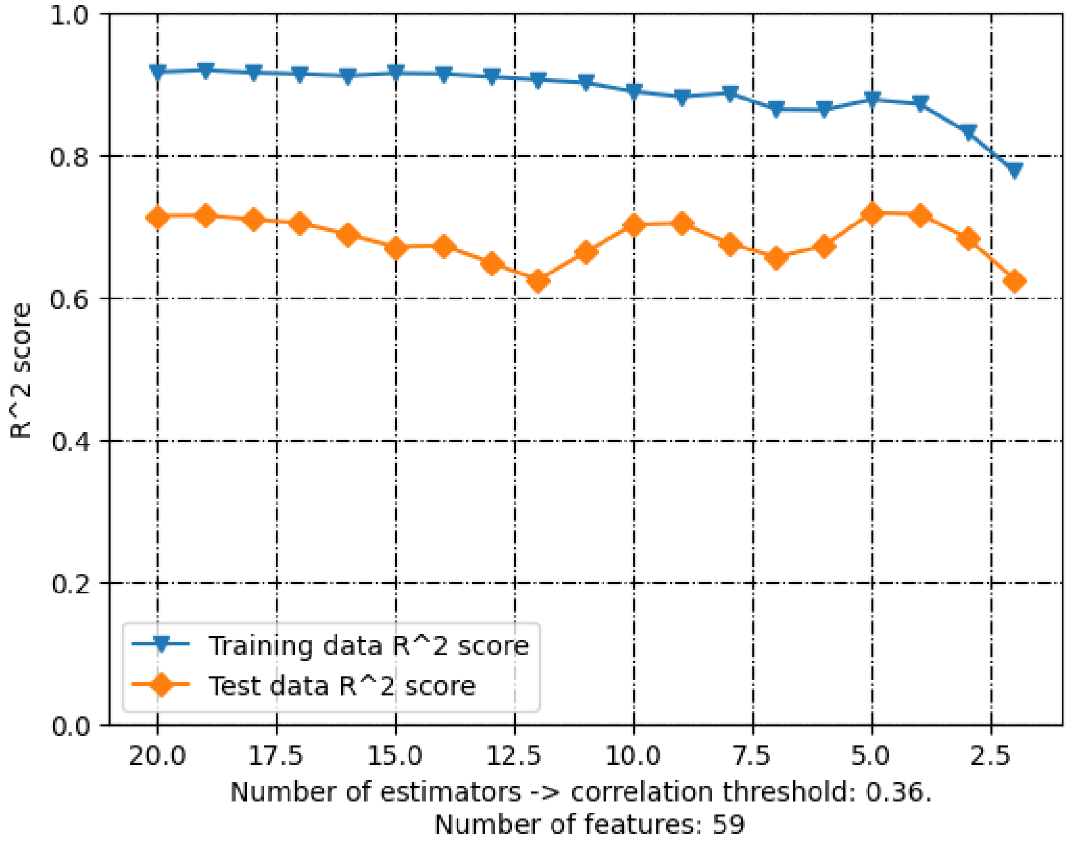
1.0
0.8
0.6
0.4
0.2
0.0
R^2 score
Training data R^2 score
Test data R^2 score
20.0
17.5
15.0
12.5
10.0
7.5
5.0
2.5
Number of estimators -> correlation threshold: 0.36.
Number of features: 59

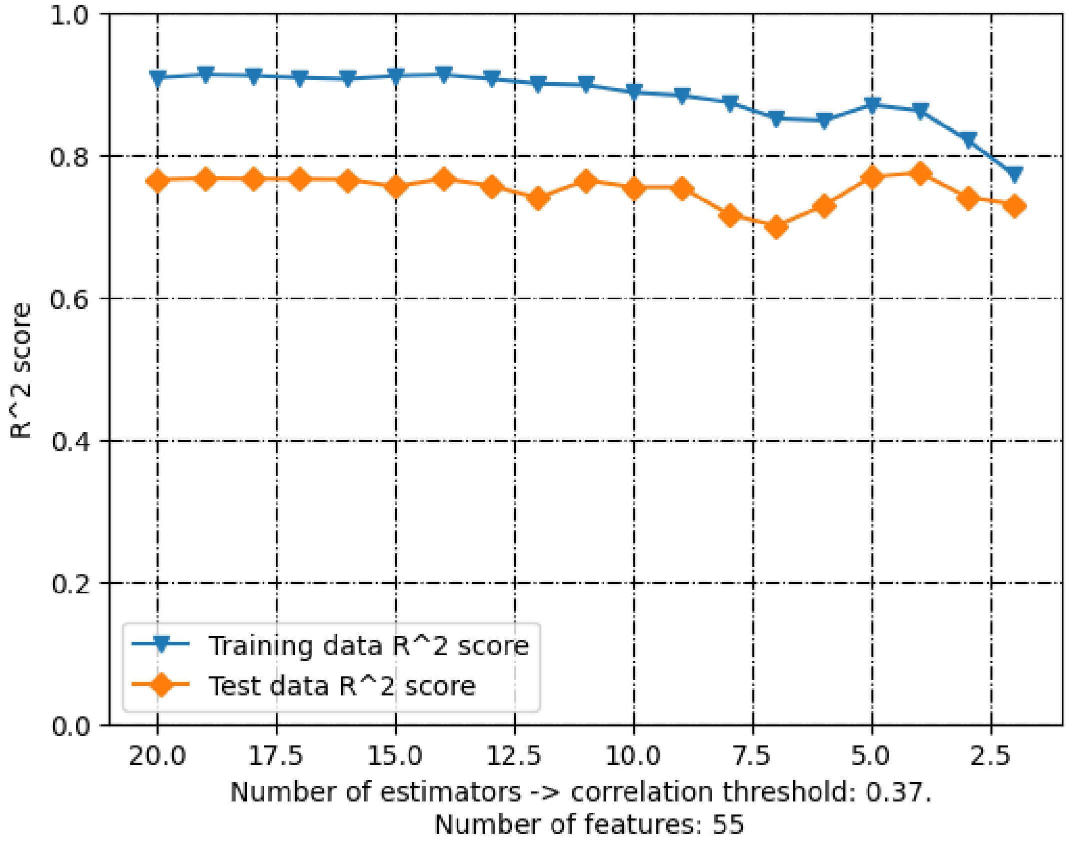
1.0
0.8
0.6
0.4
0.2
0.0
R^2 score
Training data R^2 score
Test data R^2 score
20.0
17.5
15.0
12.5
10.0
7.5
5.0
2.5
Number of estimators -> correlation threshold: 0.37.
Number of features: 55

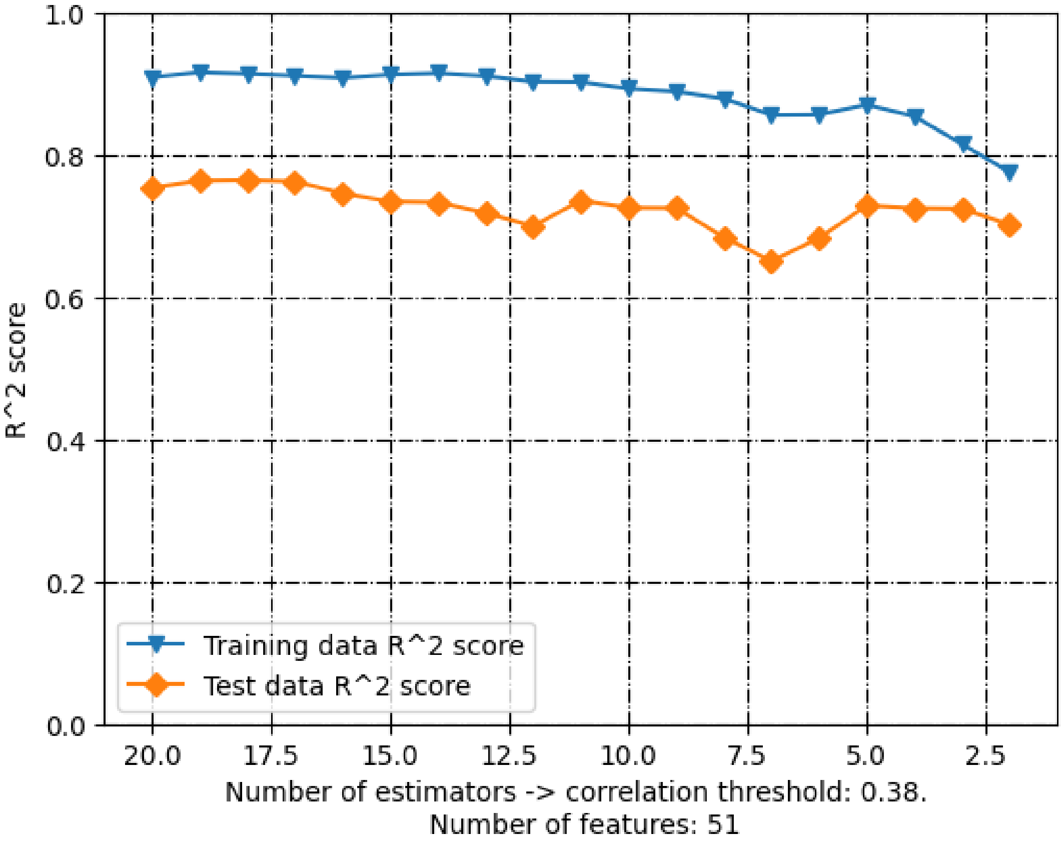
1.0
0.8
0.6
0.4
0.2
0.0
R^2 score
Training data R^2 score
Test data R^2 score
20.0
17.5
15.0
12.5
10.0
7.5
5.0
2.5
Number of estimators -> correlation threshold: 0.38.
Number of features: 51

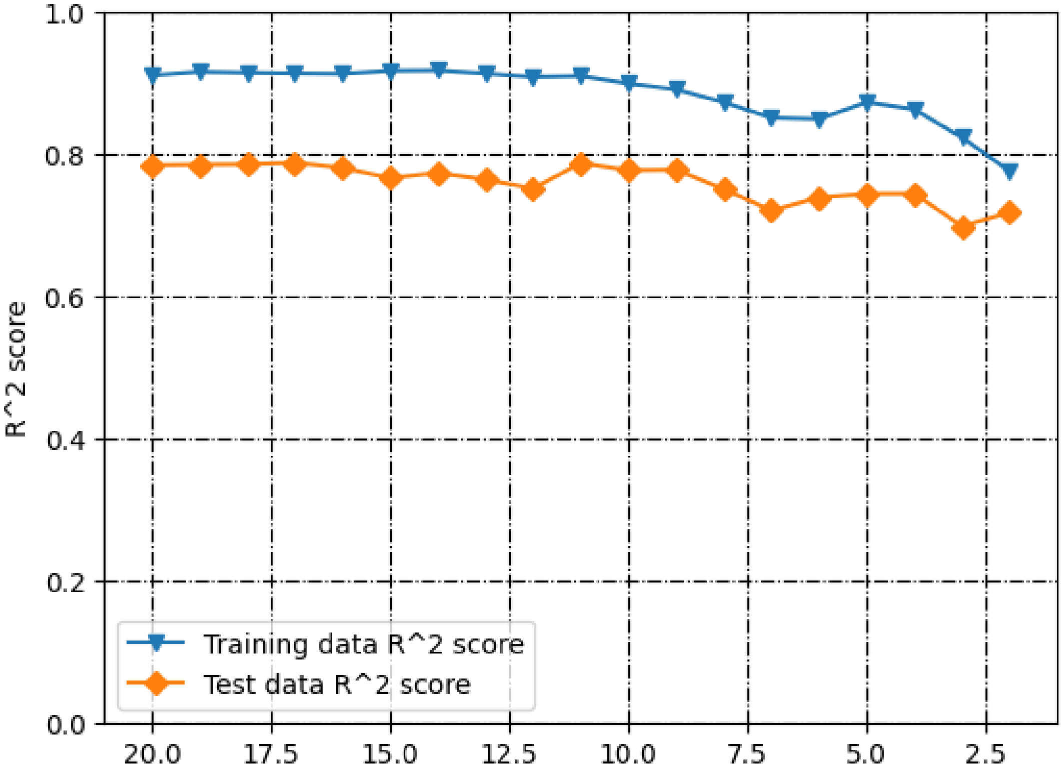

Number of estimators -> correlation threshold: 0.39.
Number of features: 46

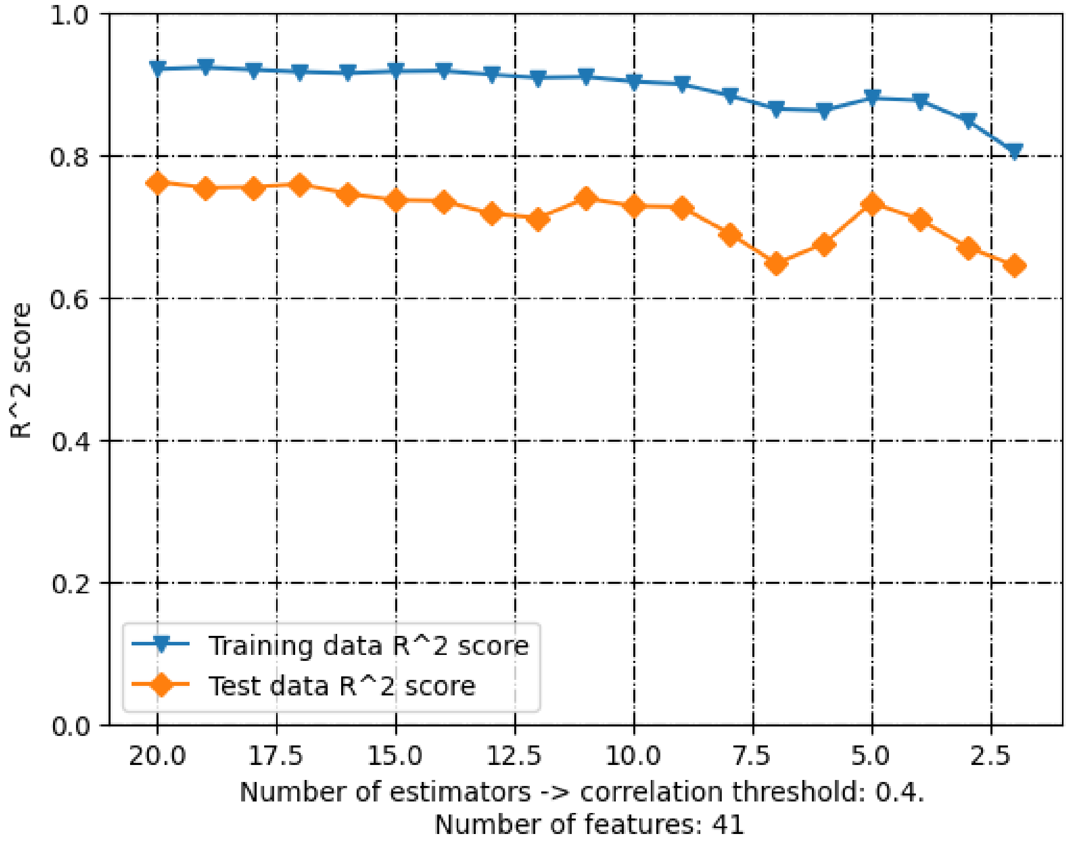

1.0
0.8
0.6
0.4
0.2
0.0
R^2 score
Training data R^2 score
Test data R^2 score
20.0
17.5
15.0
12.5
10.0
7.5
5.0
2.5
Number of estimators -> correlation threshold: 0.4.
Number of features: 41

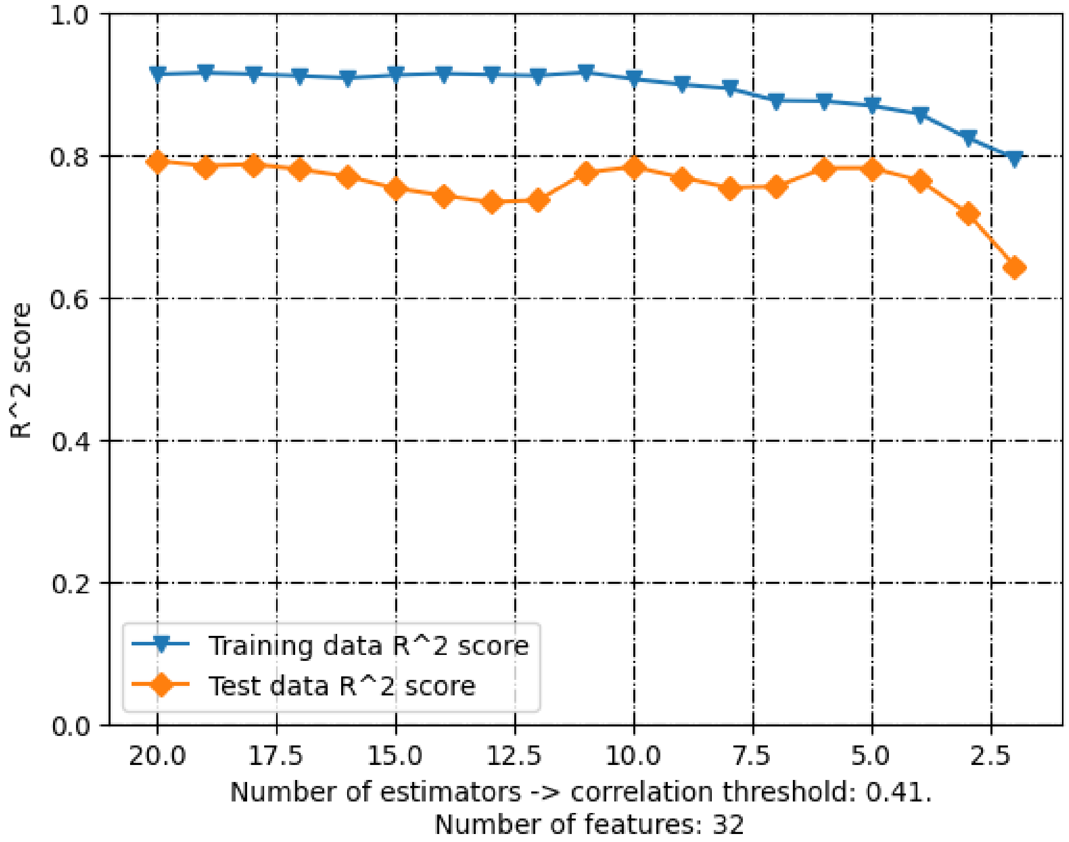
1.0
0.8
0.6
0.4
0.2
0.0
R^2 score
20.0
17.5
15.0
12.5
10.0
7.5
5.0
2.5
Number of estimators -> correlation threshold: 0.41.
Number of features: 32
Training data R^2 score
Test data R^2 score

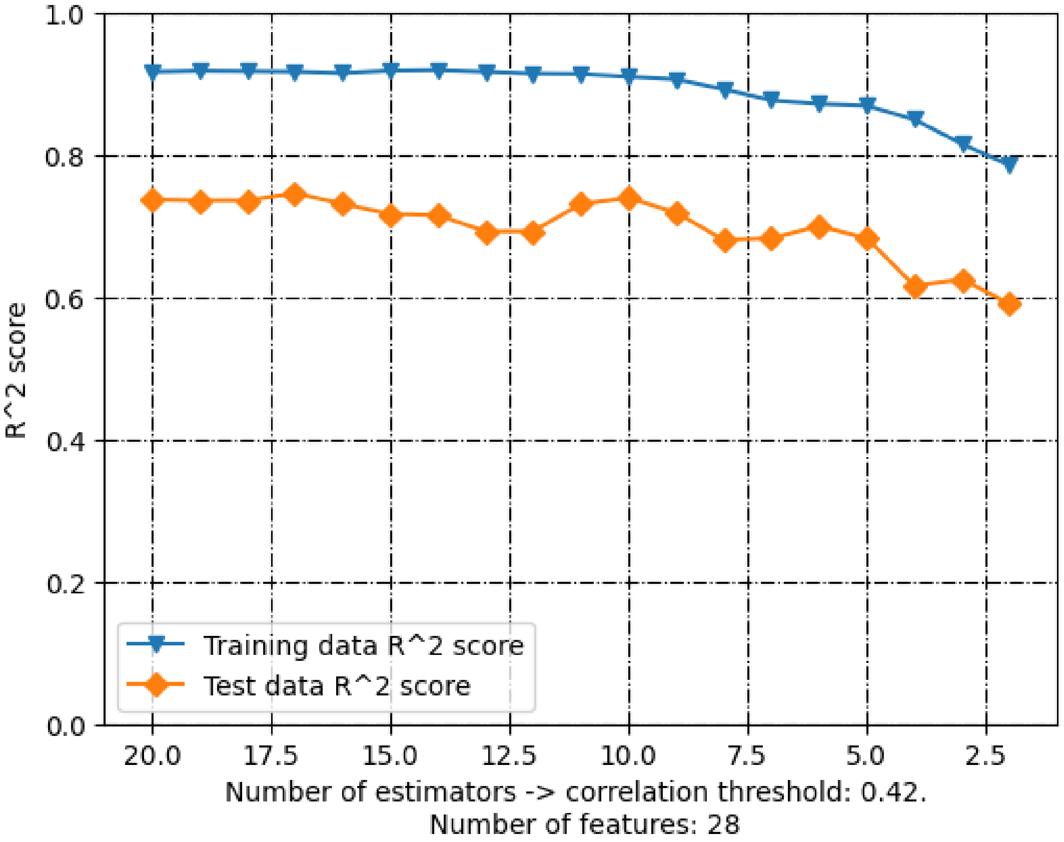

1.0
0.8
0.6
0.4
0.2
0.0
R^2 score
20.0
17.5
15.0
12.5
10.0
7.5
5.0
2.5
Number of estimators -> correlation threshold: 0.42.
Number of features: 28
Training data R^2 score
Test data R^2 score

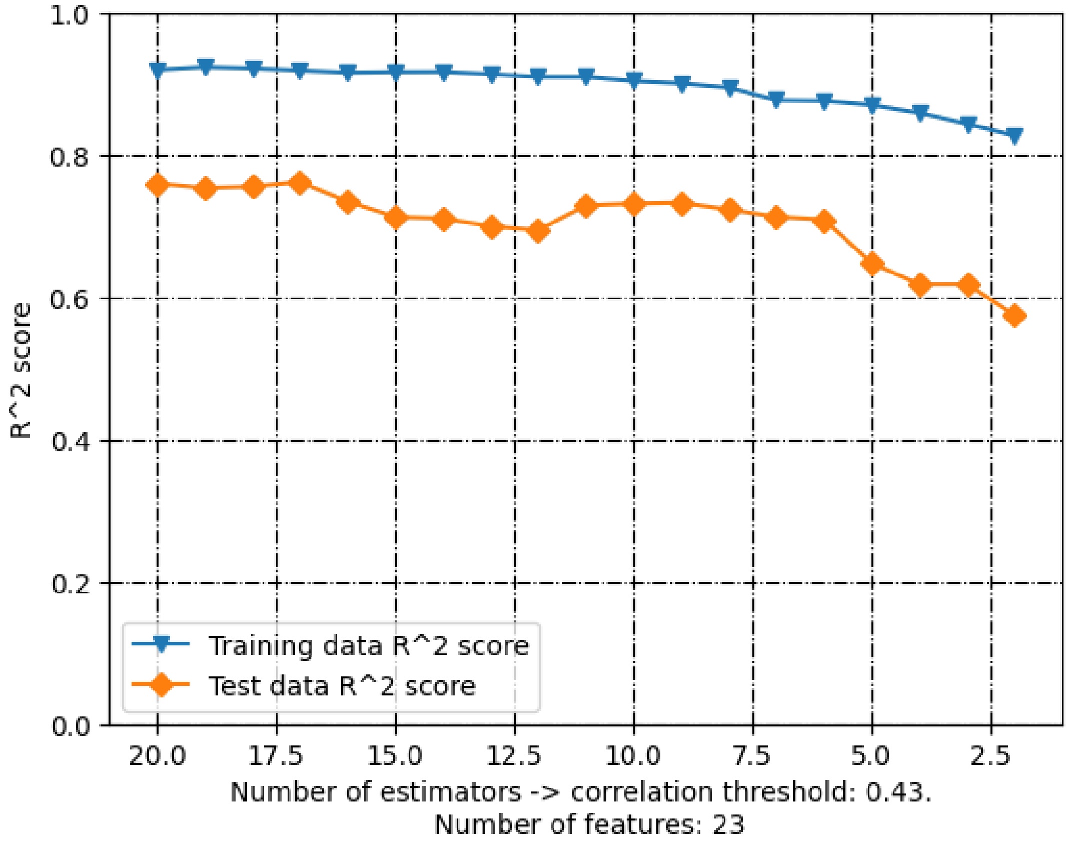

1.0
0.8
0.6
0.4
0.2
0.0
R^2 score
20.0
17.5
15.0
12.5
10.0
7.5
5.0
2.5
Number of estimators -> correlation threshold: 0.43.
Number of features: 23
Training data R^2 score
Test data R^2 score

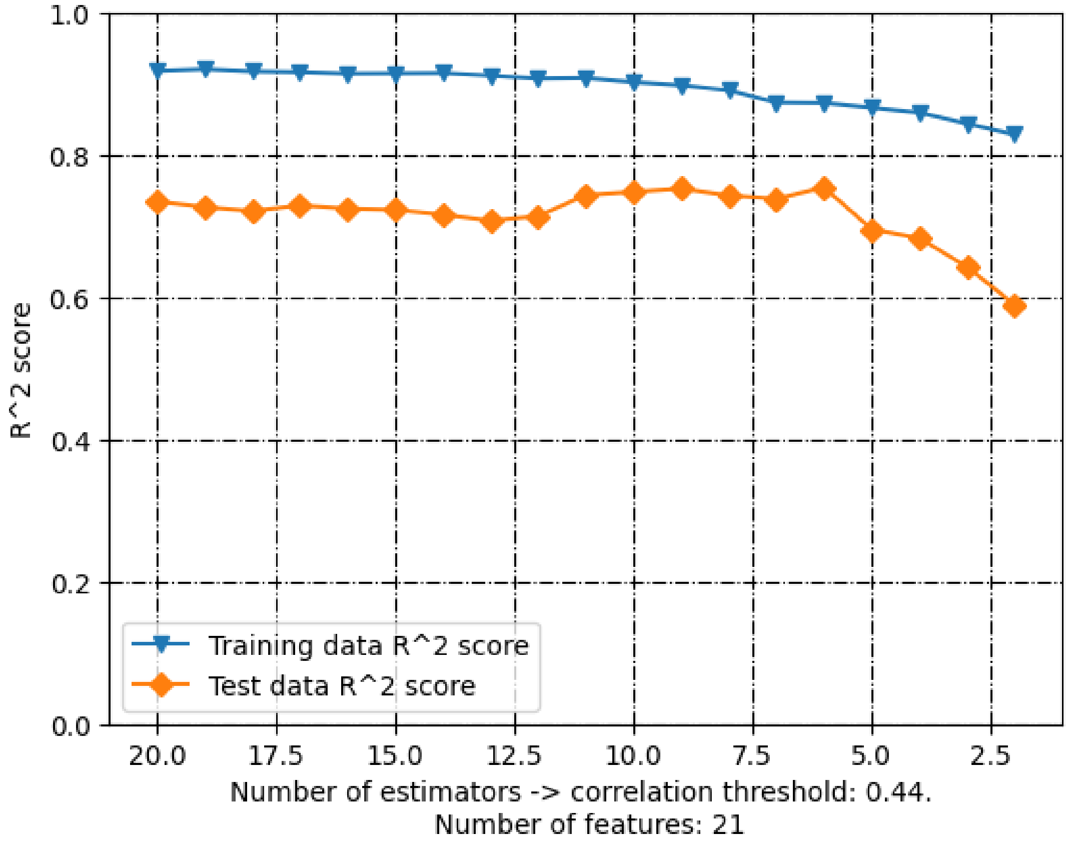
1.0
0.8
0.6
0.4
0.2
0.0
R^2 score
20.0
17.5
15.0
12.5
10.0
7.5
5.0
2.5
Number of estimators -> correlation threshold: 0.44.
Number of features: 21
Training data R^2 score
Test data R^2 score

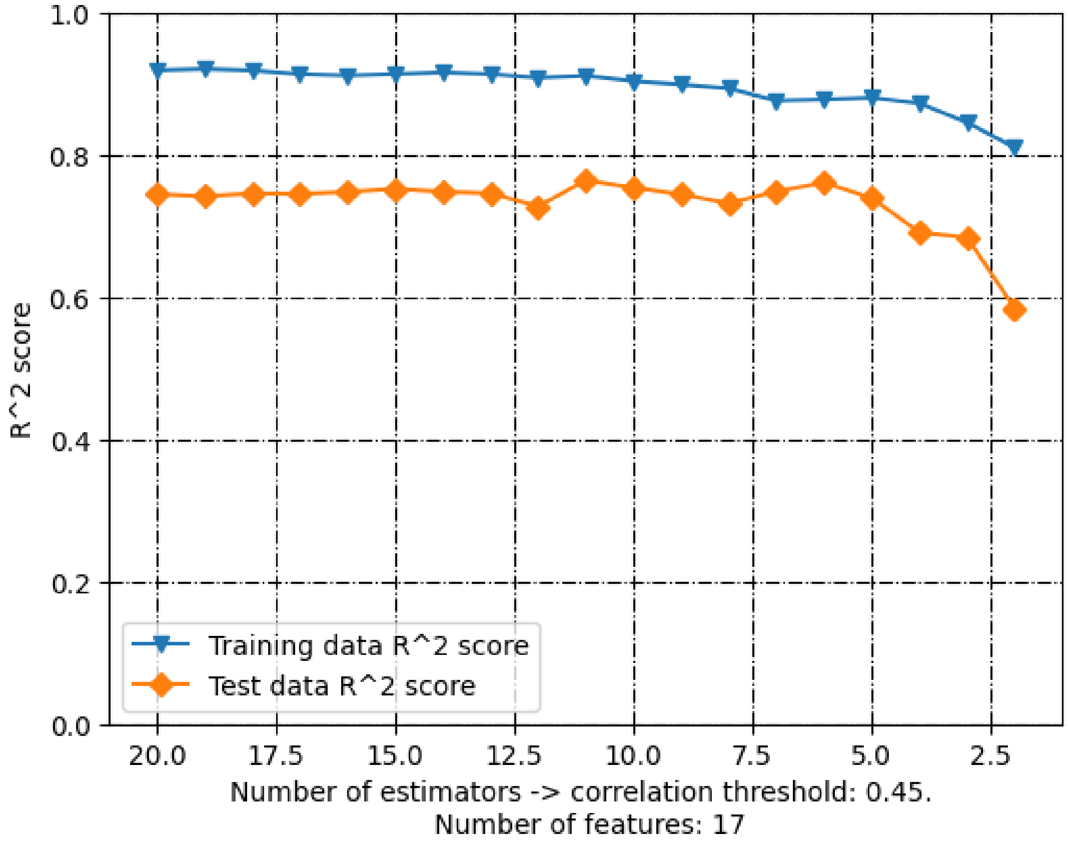
1.0
0.8
0.6
0.4
0.2
0.0
R^2 score
Training data R^2 score
Test data R^2 score
20.0
17.5
15.0
12.5
10.0
7.5
5.0
2.5
Number of estimators -> correlation threshold: 0.45.
Number of features: 17

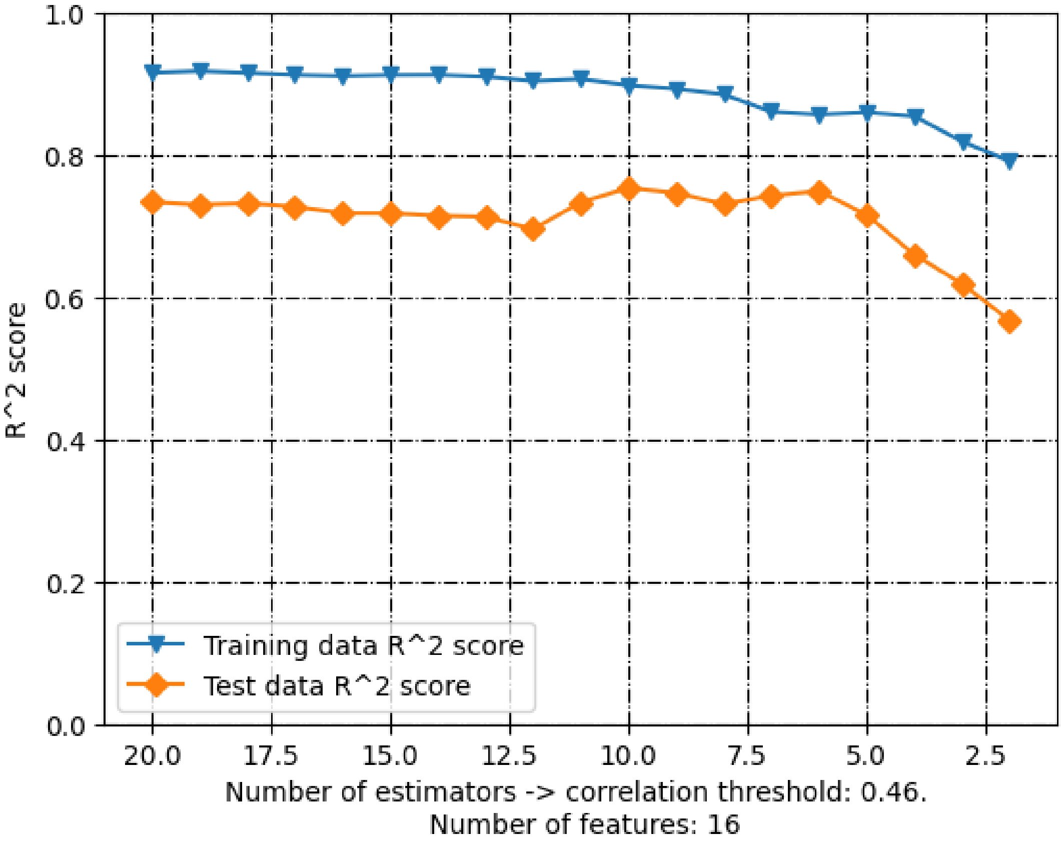
1.0
0.8
0.6
0.4
0.2
0.0
R^2 score
Training data R^2 score
Test data R^2 score
20.0
17.5
15.0
12.5
10.0
7.5
5.0
2.5
Number of estimators -> correlation threshold: 0.46.
Number of features: 16

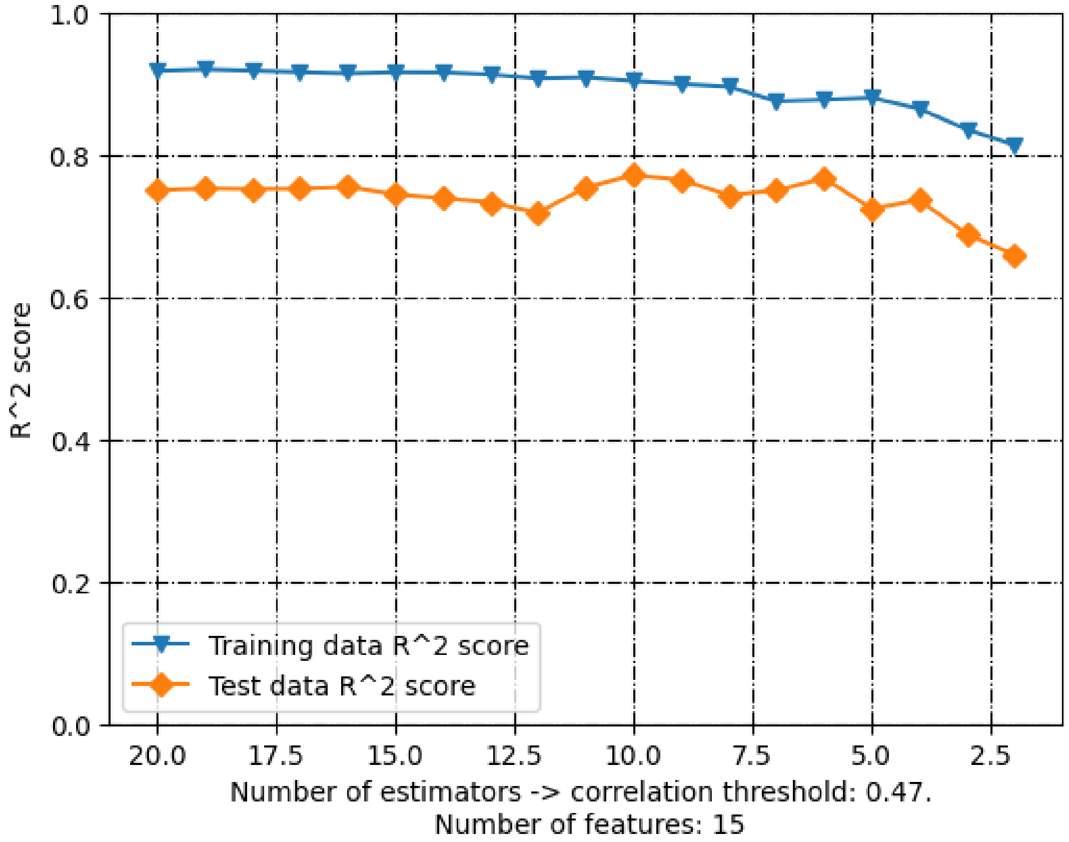

1.0
0.8
0.6
0.4
0.2
0.0
R^2 score
Training data R^2 score
Test data R^2 score
20.0
17.5
15.0
12.5
10.0
7.5
5.0
2.5
Number of estimators -> correlation threshold: 0.47.
Number of features: 15

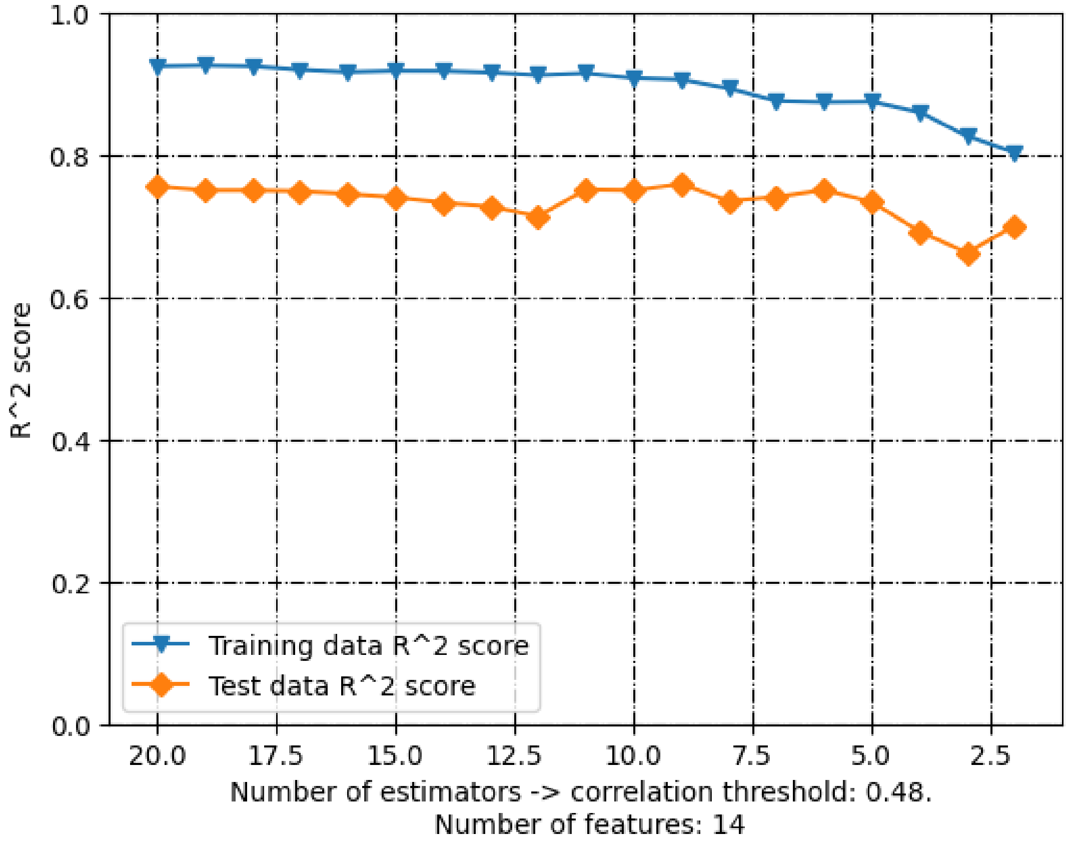

1.0
0.8
0.6
0.4
0.2
0.0
R^2 score
Training data R^2 score
Test data R^2 score
20.0
17.5
15.0
12.5
10.0
7.5
5.0
2.5
Number of estimators -> correlation threshold: 0.48.
Number of features: 14

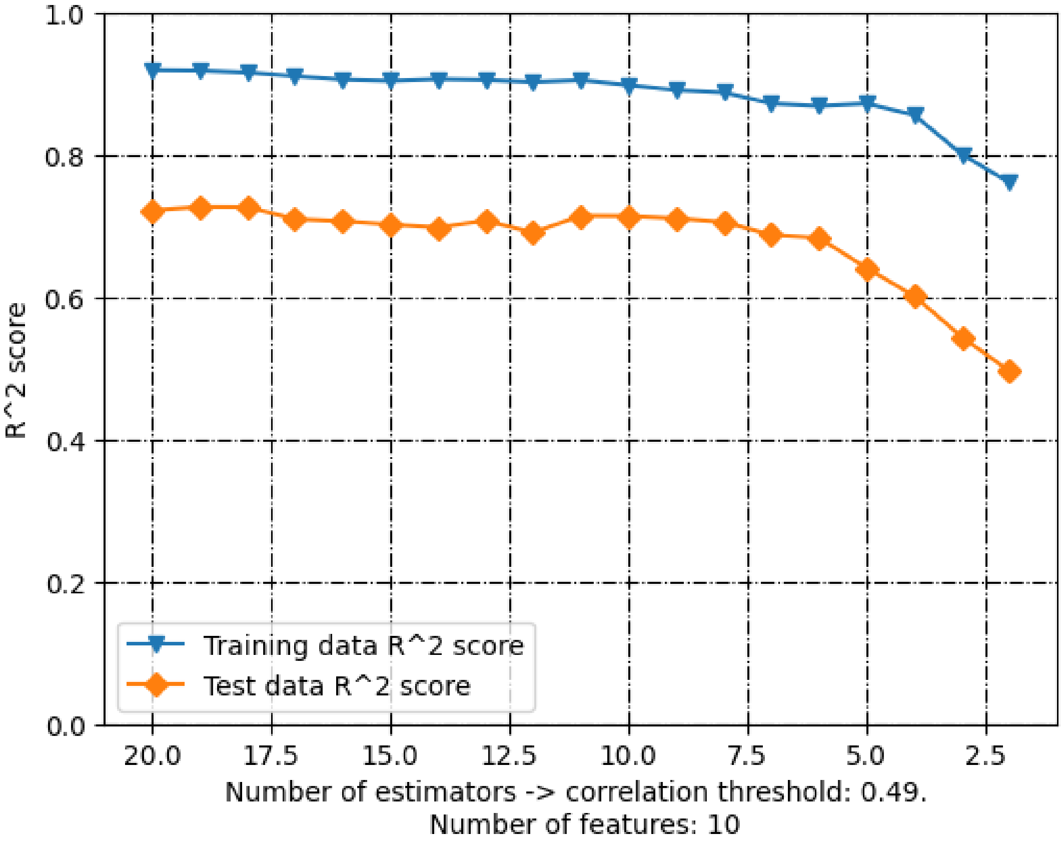
1.0
0.8
0.6
0.4
0.2
0.0
R^2 score
Training data R^2 score
Test data R^2 score
20.0
17.5
15.0
12.5
10.0
7.5
5.0
2.5
Number of estimators -> correlation threshold: 0.49.
Number of features: 10

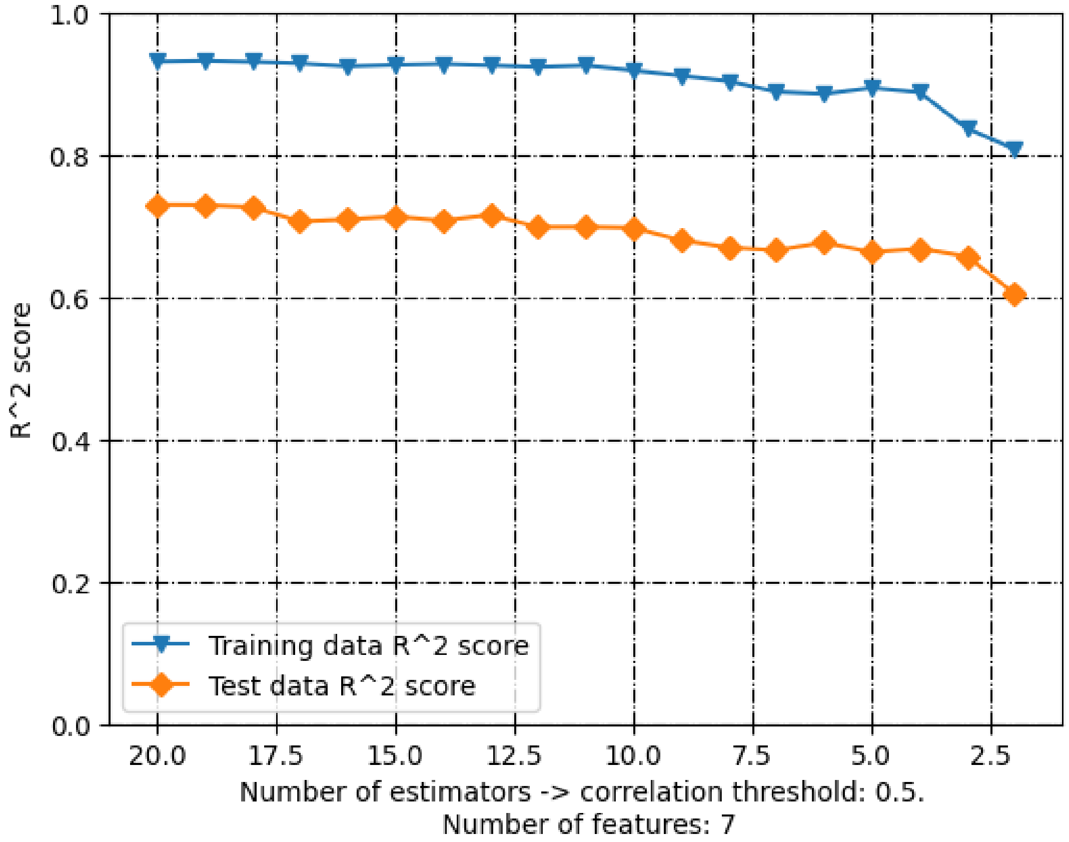

1.0
0.8
0.6
0.4
0.2
0.0
R^2 score
Training data R^2 score
Test data R^2 score
20.0
17.5
15.0
12.5
10.0
7.5
5.0
2.5
Number of estimators -> correlation threshold: 0.5.
Number of features: 7

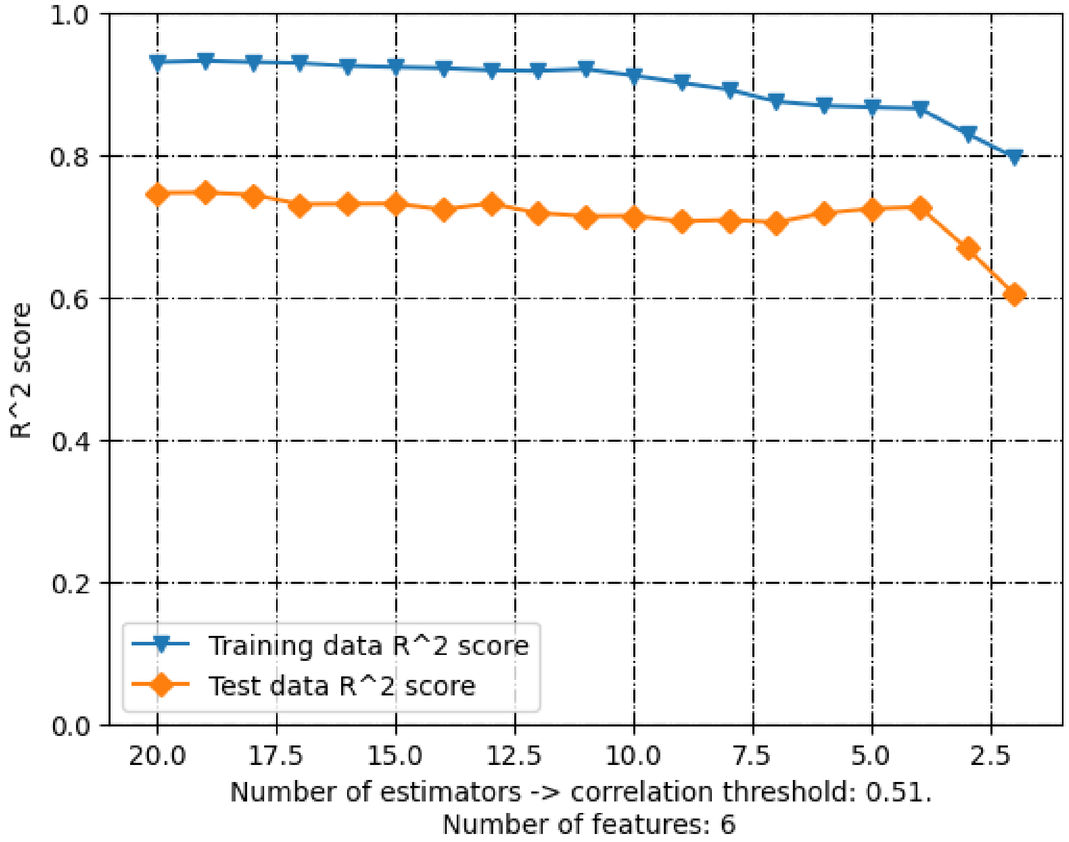

1.0
0.8
0.6
0.4
0.2
0.0
R^2 score
Training data R^2 score
Test data R^2 score
20.0
17.5
15.0
12.5
10.0
7.5
5.0
2.5
Number of estimators -> correlation threshold: 0.51.
Number of features: 6

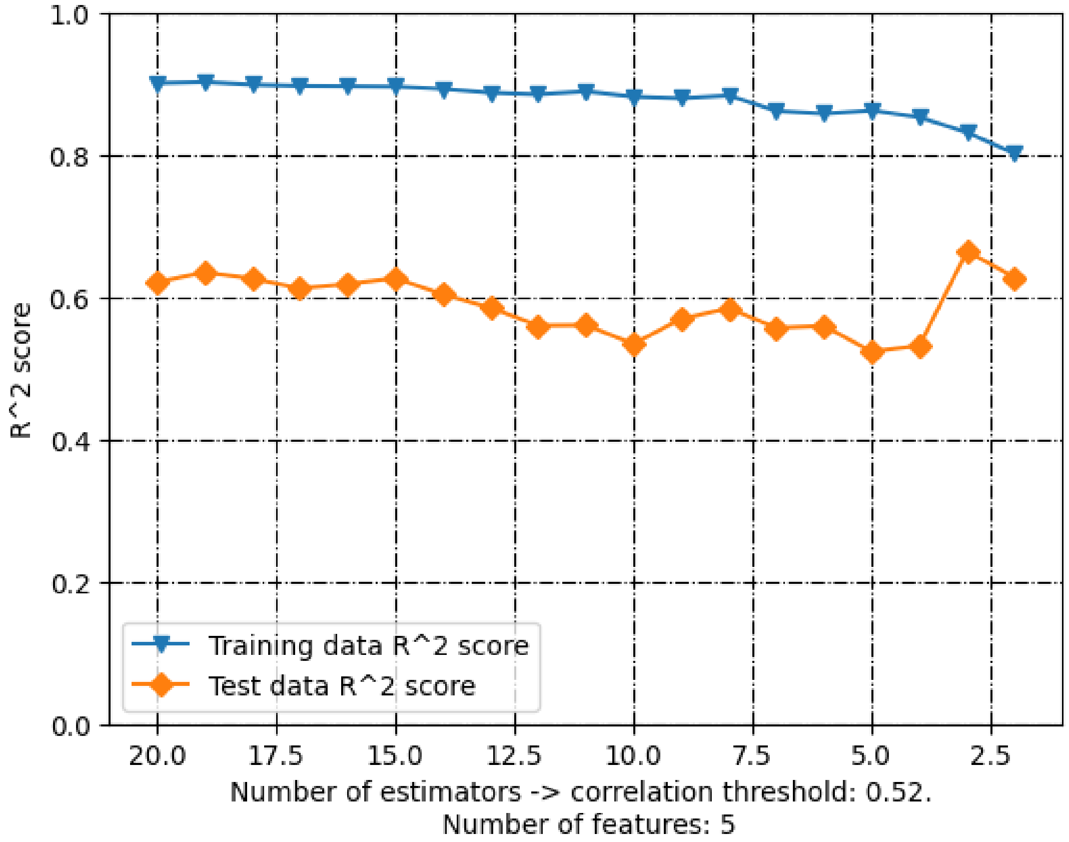

1.0
0.8
0.6
0.4
0.2
0.0
R^2 score
Training data R^2 score
Test data R^2 score
20.0
17.5
15.0
12.5
10.0
7.5
5.0
2.5
Number of estimators -> correlation threshold: 0.52.
Number of features: 5

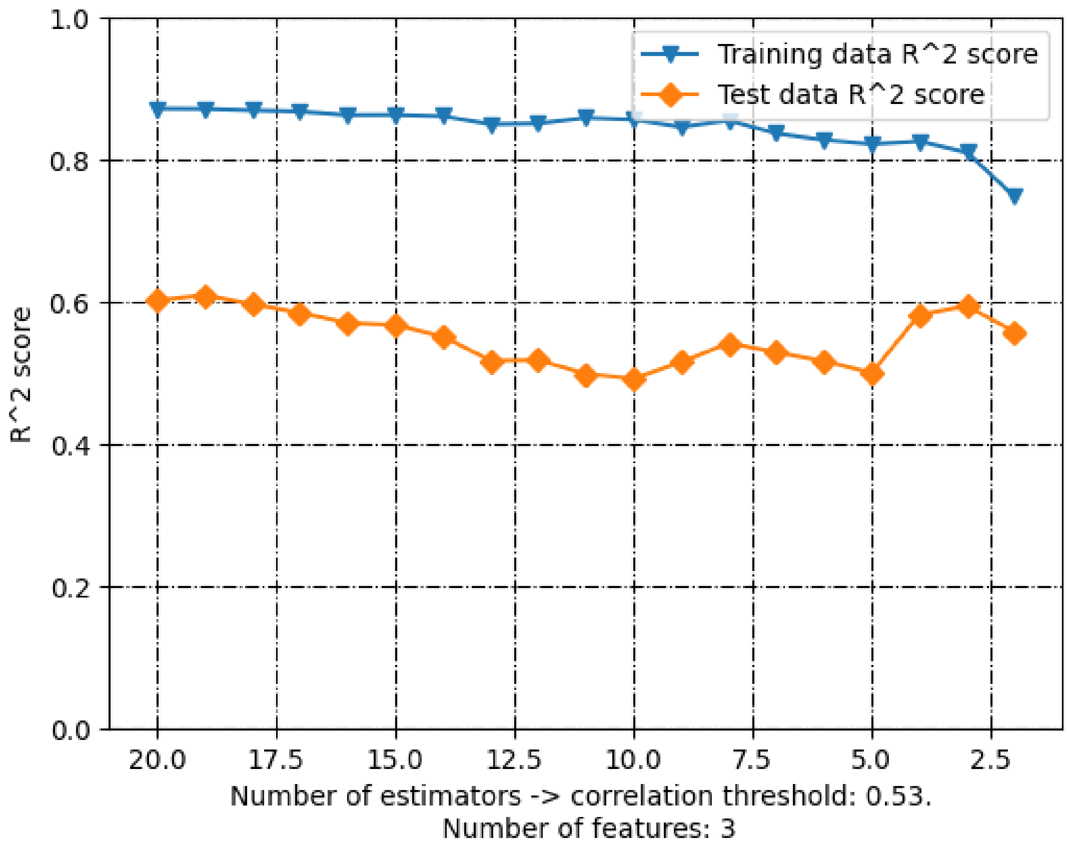
1.0
0.8
0.6
0.4
0.2
0.0
R^2 score
Training data R^2 score
Test data R^2 score
20.0
17.5
15.0
12.5
10.0
7.5
5.0
2.5
Number of estimators -> correlation threshold: 0.53.
Number of features: 3

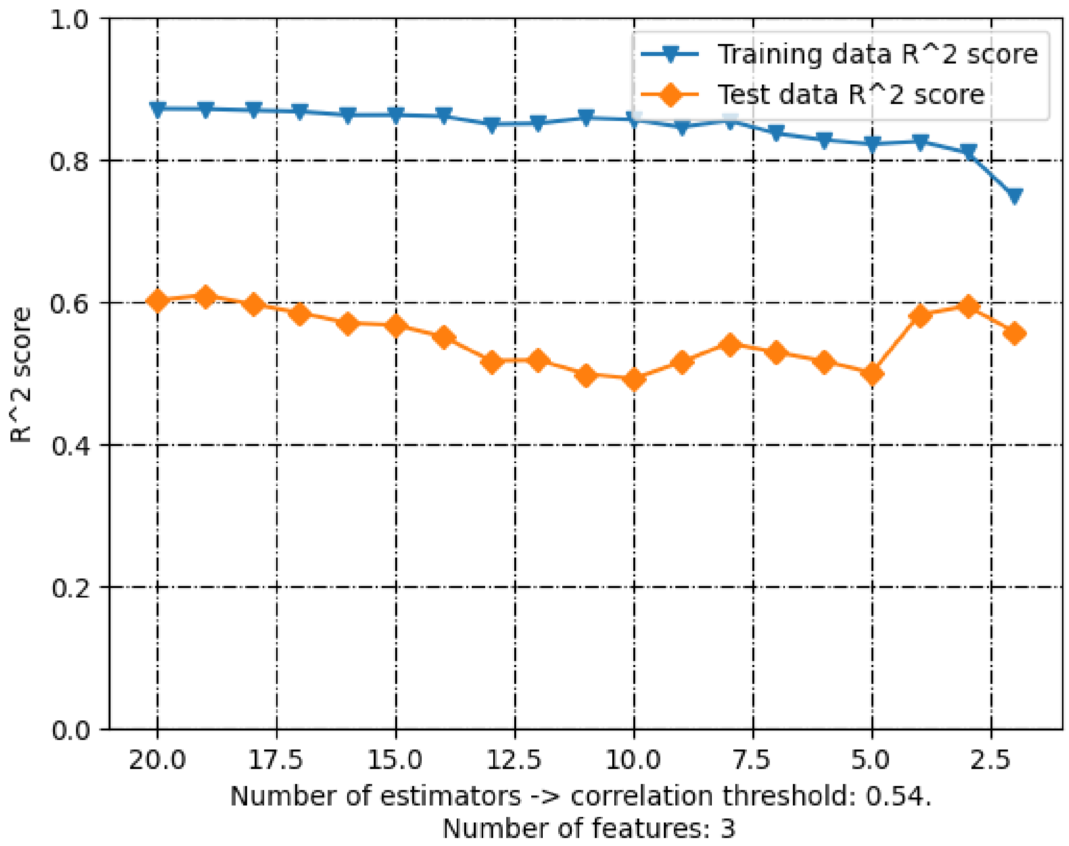
1.0
0.8
0.6
0.4
0.2
0.0
R^2 score
Training data R^2 score
Test data R^2 score
20.0
17.5
15.0
12.5
10.0
7.5
5.0
2.5
Number of estimators -> correlation threshold: 0.54.
Number of features: 3

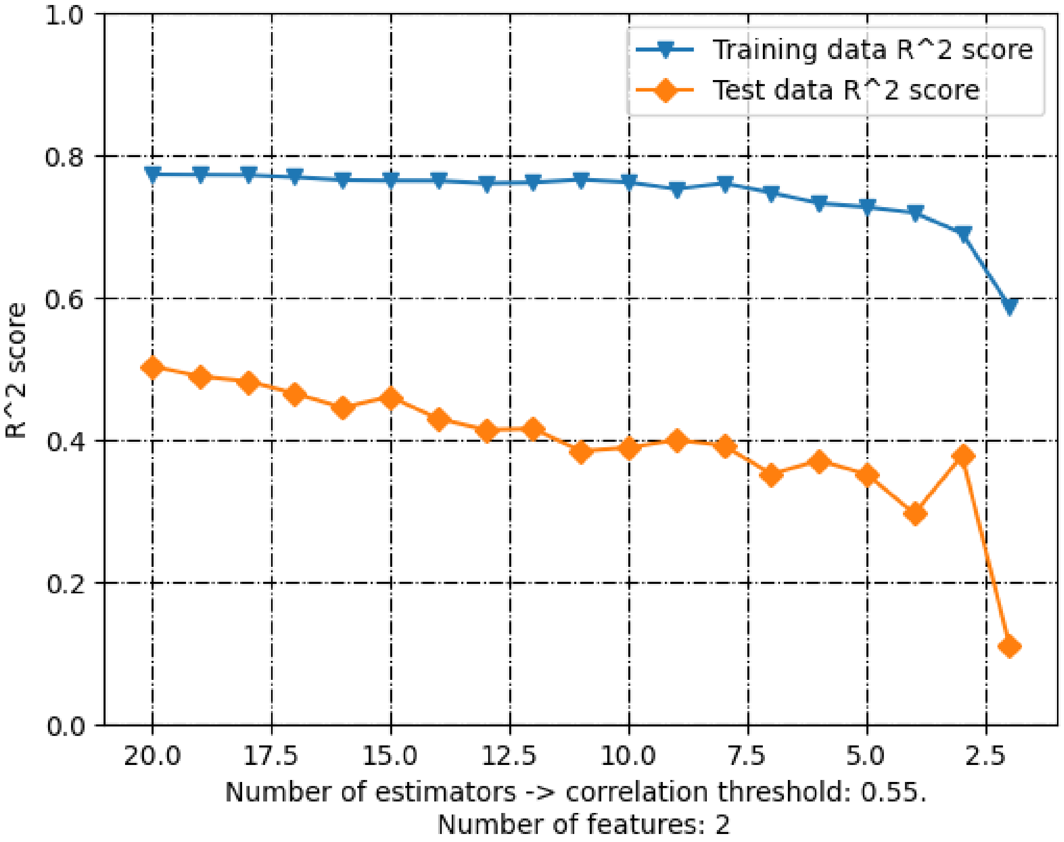

1.0
0.8
0.6
0.4
0.2
0.0
R^2 score
Training data R^2 score
Test data R^2 score
20.0
17.5
15.0
12.5
10.0
7.5
5.0
2.5
Number of estimators -> correlation threshold: 0.55.
Number of features: 2

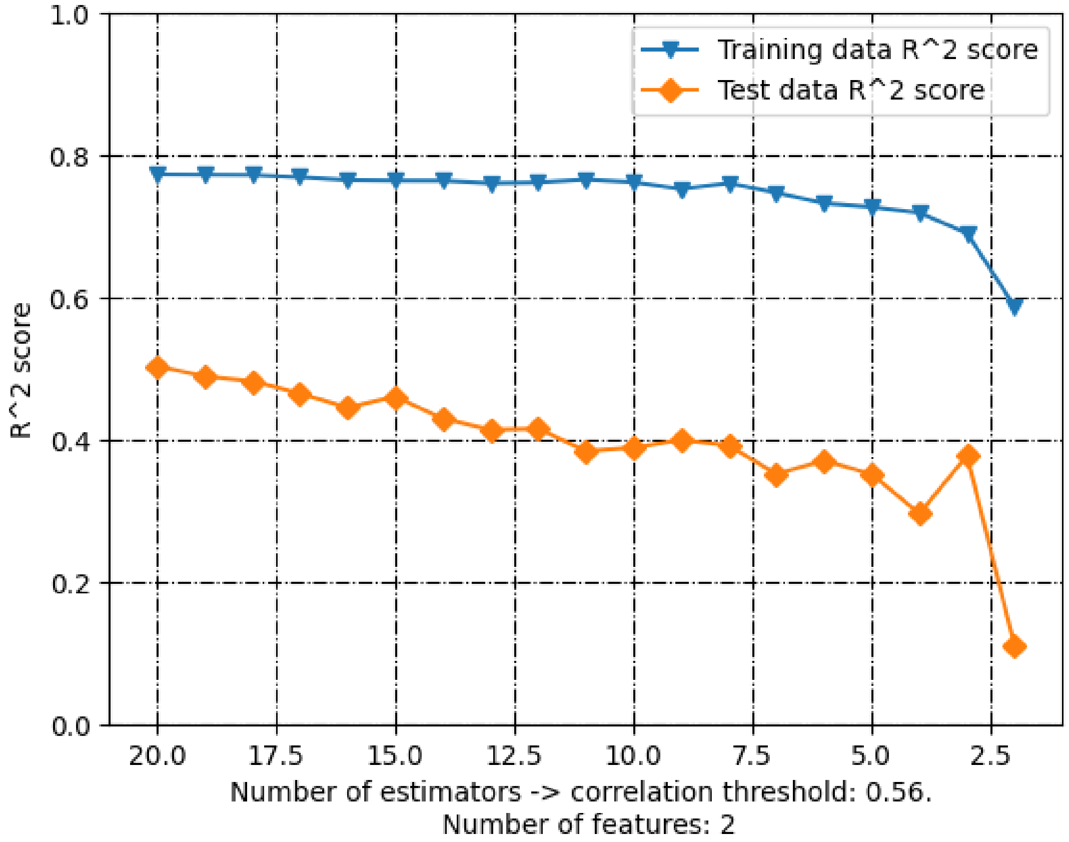

[28]:
```
plt.plot(df_without_standardization['Correlation threshold'],␣
  ↪df_without_standardization['Number of features'], label = "Number of␣
  ↪features", marker='.')
plt.legend()
plt.xlabel('Correlation threshold')
plt.ylabel('Number of features')
plt.show()
```

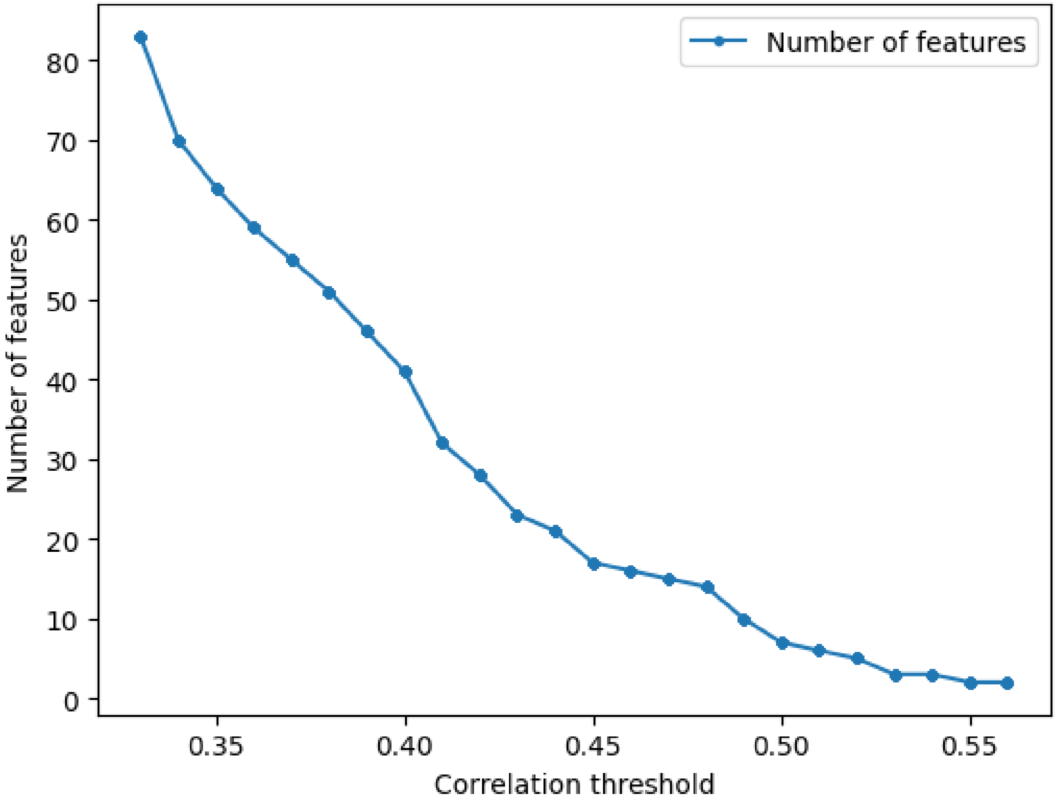


```
[ ]:
```

4 KNeighborsRegressor

```
[29]: without_standardization, train_r2_, test_r2_, hist1, hist2, target_column_name,␣
    ↪training_data_RMSE, test_data_RMSE  = pred_model.
    ↪prepare_data_and_create_model(molecular_descriptors_df=data,
                                                                                 ␣
    ↪                        correlation_threshold=0.50,
                                                                                 ␣
    ↪                        standardization=False,
                                                                                 ␣
    ↪                        model_type='KNeighborsRegressor',
                                                                                 ␣
    ↪                        target_column_name = target,
                                                                                 ␣
    ↪                        random_state=random_state,
                                                                                 ␣
    ↪                        train_test_split_=True,
```

```
↪                    verbose=True)
```

```
I am not doing standardization…
  molecular descriptor name
0                   AATS0Z
1                 AATS0are
2                   AATS0d
3                  AATS0dv
4                   AATS0i
  molecular descriptor name  corr_value
0                   AATS0Z   -0.020817
1                 AATS0are   -0.148257
2                   AATS0d    0.022999
3                  AATS0dv   -0.137980
4                   AATS0i    0.133257
  molecular descriptor name  corr_value  absolute correlation value
0                   AATS0Z   -0.020817                    0.020817
1                 AATS0are   -0.148257                    0.148257
2                   AATS0d    0.022999                    0.022999
3                  AATS0dv   -0.137980                    0.137980
4                   AATS0i    0.133257                    0.133257
    molecular descriptor name  corr_value  absolute correlation value
226                    AMID_O   -0.546843                    0.546843
505               EState_VSA5   -0.588780                    0.588780
556                    GATS2c    0.511671                    0.511671
791                  MDEO-12   -0.582747                    0.582747
847                       NdO   -0.500462                    0.500462
    molecular descriptor name  corr_value  absolute correlation value
226                    AMID_O   -0.546843                    0.546843
505               EState_VSA5   -0.588780                    0.588780
556                    GATS2c    0.511671                    0.511671
791                  MDEO-12   -0.582747                    0.582747
847                       NdO   -0.500462                    0.500462
The model used is: KNeighbors…
Return the coefficient of determination of the prediction:
0.7760027882370206
R^2 score: 0.5372579380668501
Correlation coefficient: 0.7329788114719621
Test data - unseen during training:
R^2 score: 0.7760027882370206
Correlation coefficient: 0.8809102044119029
[7.30313411 5.54474773 6.10879495 7.71347634 7.56570649 7.71206513
 8.07390425 7.56570649 8.07879901 8.01201206 6.52417123 7.84887537
 7.99442102 7.30313411 7.93705287 7.73919765 5.77329499 6.25872803]
102    7.698970
38     5.986741
8      5.984515
```

```
109    7.886057
11     7.962574
51     7.698970
88     8.229148
21     7.298432
82     8.065502
98     7.376751
58     7.602060
64     8.060481
74     7.853872
103    7.853872
91     8.346787
43     7.619789
25     4.989276
30     6.085657
Name: BALB/3T3, dtype: float64
Training Root Mean Square Error: 0.5903089494742803
Testing Root Mean Square Error: 0.435295961974470 46
```

[30]:

```python
print(target_column_name+str('_transformed'))
print(hist1[target_column_name].hist())
```

```
BALB/3T3_transformed
AxesSubplot(0.125,0.11;0.775x0.77)
```

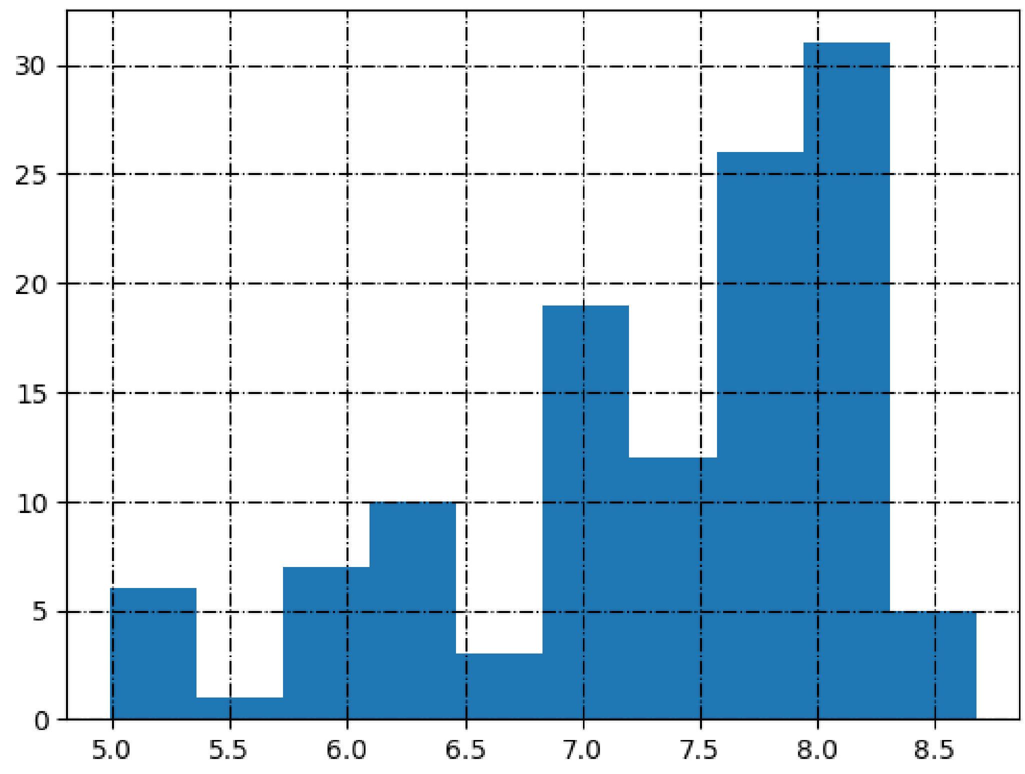

[31]:
```
with_standardization, train_r2_, test_r2_, hist1, hist2, target_column_name,␣
↪training_data_RMSE, test_data_RMSE  = pred_model.
↪prepare_data_and_create_model(molecular_descriptors_df=data,
                                                                              ␣
↪                    correlation_threshold=0.50,
                                                                              ␣
↪                    standardization=True,
                                                                              ␣
↪                    model_type='KNeighborsRegressor',
                                                                              ␣
↪                    target_column_name = target,
                                                                              ␣
↪                    random_state=random_state,
                                                                              ␣
↪                    train_test_split_=True,
                                                                              ␣
↪                    verbose=True)
```

```
I am doing standardization…
  molecular descriptor name
0                    AATS0Z
1                  AATS0are
2                    AATS0d
3                   AATS0dv
4                    AATS0i
  molecular descriptor name  corr_value
0                    AATS0Z   -0.020817
1                  AATS0are   -0.148257
2                    AATS0d    0.022999
3                   AATS0dv   -0.137980
4                    AATS0i    0.133257
  molecular descriptor name  corr_value  absolute correlation value
0                    AATS0Z   -0.020817                    0.020817
1                  AATS0are   -0.148257                    0.148257
2                    AATS0d    0.022999                    0.022999
3                   AATS0dv   -0.137980                    0.137980
4                    AATS0i    0.133257                    0.133257
    molecular descriptor name  corr_value  absolute correlation value
226                    AMID_O   -0.546843                    0.546843
505                EState_VSA5   -0.588780                    0.588780
556                    GATS2c    0.511671                    0.511671
791                   MDEO-12   -0.582747                    0.582747
847                       NdO   -0.500462                    0.500462
    molecular descriptor name  corr_value  absolute correlation value
226                    AMID_O   -0.546843                    0.546843
505                EState_VSA5   -0.588780                    0.588780
```

```
556                    GATS2c    0.511671                     0.511671
791                   MDEO-12   -0.582747                     0.582747
847                       NdO   -0.500462                     0.500462
The model used is: KNeighbors…
Return the coefficient of determination of the prediction:
0.7760027882370206
R^2 score: 0.5372579380668501
Correlation coefficient: 0.7329788114719621
Test data - unseen during training:
R^2 score: 0.7760027882370206
Correlation coefficient: 0.8809102044119029
[7.30313411 5.54474773 6.10879495 7.71347634 7.56570649 7.71206513
 8.07390425 7.56570649 8.07879901 8.01201206 6.52417123 7.84887537
 7.99442102 7.30313411 7.93705287 7.73919765 5.77329499 6.25872803]
102    7.698970
38     5.986741
8      5.984515
109    7.886057
11     7.962574
51     7.698970
88     8.229148
21     7.298432
82     8.065502
98     7.376751
58     7.602060
64     8.060481
74     7.853872
103    7.853872
91     8.346787
43     7.619789
25     4.989276
30     6.085657
Name: BALB/3T3, dtype: float64
Training Root Mean Square Error: 0.5903089494742803
Testing Root Mean Square Error: 0.43529596197447046
```

4.1 Search inside correlation space

[32]:
```python
step = 0.01
initial_step = corr_low
last_step = corr_high
first_list = [x / 100.0 for x in range(int(initial_step*100),␣
 ↪int(last_step*100), int(step*100))]
second_list = []
third_list = []
fourth_l = []
fifth_l = []
```

```
f_list = []
for i in first_list:
    without_standardization, train_r2, test_r2, _, h_, target_column_name,␣
 ↪training_data_RMSE, test_data_RMSE = pred_model.
 ↪prepare_data_and_create_model(molecular_descriptors_df = data,
                                                                             ␣
 ↪                    correlation_threshold = i,
                                                                             ␣
 ↪                    standardization = False,
                                                                             ␣
 ↪                    model_type = 'KNeighborsRegressor',
                                                                             ␣
 ↪                    target_column_name = target,
                                                                             ␣
 ↪                    random_state=random_state,
                                                                             ␣
 ↪                    train_test_split_ = True,
                                                                             ␣
 ↪                    verbose = False)
    second_list.append(train_r2)
    third_list.append(test_r2)
    fourth_l.append(training_data_RMSE)
    fifth_l.append(test_data_RMSE)
    f_list.append(len(h_))
```

```
  File "C:\Users\aleks\anaconda3\envs\cheminf_gpu\lib\site-
packages\joblib\externals\loky\backend\context.py", line 217, in
_count_physical_cores
    raise ValueError(
```

[33]:
```
df_without_standardization = pd.DataFrame(data=first_list,␣
 ↪columns=["Correlation threshold"])
df_without_standardization['Training data R^2 score'] = second_list
df_without_standardization['Test data R^2 score'] = third_list
df_without_standardization['Training RMSE'] = fourth_l
df_without_standardization['Test RMSE'] = fifth_l
df_without_standardization['Number of features'] = f_list
```

[34]:
```
df_k_nearest = df_without_standardization.copy()
df_without_standardization
```

[34]:
```
    Correlation threshold  Training data R^2 score  Test data R^2 score  \
0                    0.33                 0.551371             0.488642
1                    0.34                 0.510303             0.579362
2                    0.35                 0.510303             0.579362
3                    0.36                 0.510303             0.579362
4                    0.37                 0.510303             0.579362
```

5	0.38	0.510303	0.579362
6	0.39	0.638422	0.692804
7	0.40	0.638422	0.692804
8	0.41	0.591252	0.523025
9	0.42	0.594542	0.409736
10	0.43	0.590606	0.470853
11	0.44	0.595417	0.384114
12	0.45	0.595085	0.395033
13	0.46	0.597077	0.395033
14	0.47	0.596785	0.401560
15	0.48	0.602007	0.373963
16	0.49	0.600338	0.366093
17	0.50	0.537258	0.776003
18	0.51	0.542450	0.780535
19	0.52	0.537291	0.774699
20	0.53	0.534515	0.730942
21	0.54	0.534515	0.730942
22	0.55	0.478665	0.752177
23	0.56	0.478665	0.752177

	Training RMSE	Test RMSE	Number of features
0	0.581237	0.657697	83
1	0.607259	0.596510	70
2	0.607259	0.596510	64
3	0.607259	0.596510	59
4	0.607259	0.596510	55
5	0.607259	0.596510	51
6	0.521808	0.509766	46
7	0.521808	0.509766	41
8	0.554801	0.635201	32
9	0.552565	0.706621	28
10	0.555240	0.669039	23
11	0.551968	0.721794	21
12	0.552194	0.715367	17
13	0.550834	0.715367	16
14	0.551034	0.711498	15
15	0.547454	0.727718	14
16	0.548601	0.732278	10
17	0.590309	0.435296	7
18	0.586988	0.430870	6
19	0.590288	0.436561	5
20	0.592056	0.477075	3
21	0.592056	0.477075	3
22	0.626568	0.457861	2
23	0.626568	0.457861	2

4.2 Plots

[35]:
```
plt.plot(df_without_standardization['Correlation threshold'],␣
↪df_without_standardization['Training data R^2 score'], label = "Training␣
↪data R^2 score", marker='v')
plt.plot(df_without_standardization['Correlation threshold'],␣
↪df_without_standardization['Test data R^2 score'], label = "Test data R^2␣
↪score", marker='D')
plt.legend()
plt.xlabel('Correlation threshold')
plt.ylabel('R^2 score')
plt.ylim([0, 1])
plt.show()
```

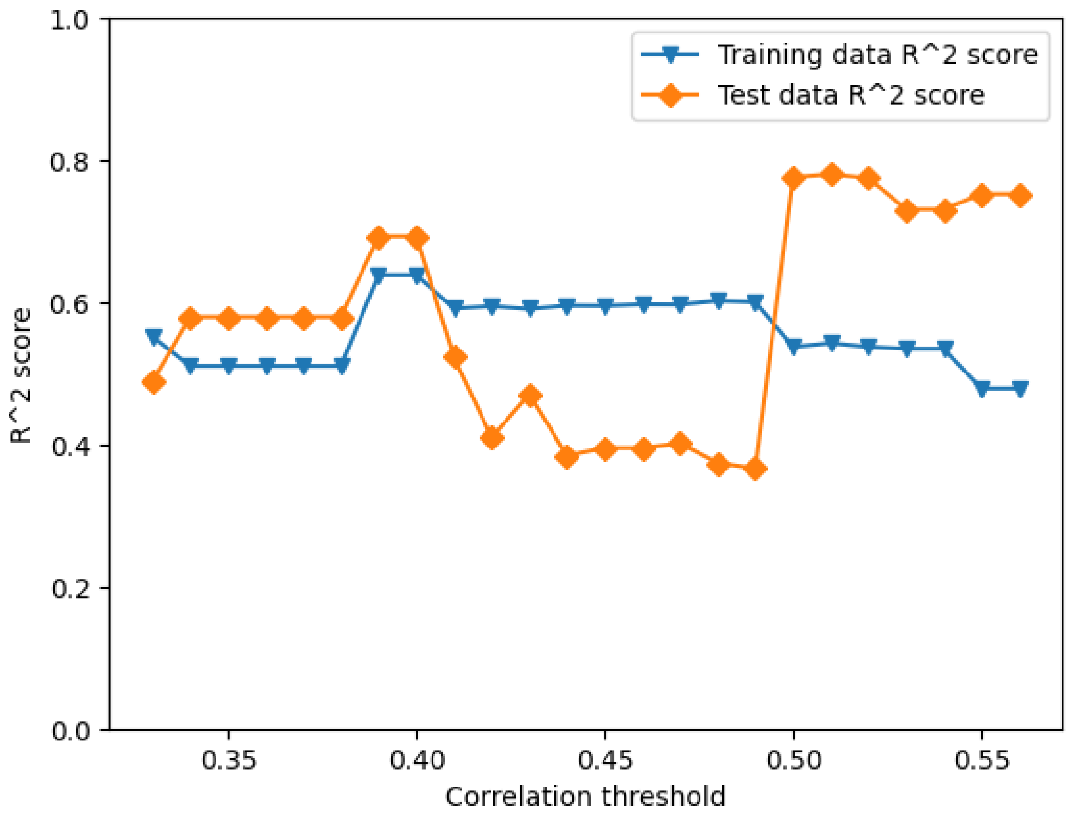

[36]:
```
plt.plot(df_without_standardization['Correlation threshold'],␣
↪df_without_standardization['Number of features'], label = "Number of␣
↪features", marker='.')
plt.legend()
plt.xlabel('Correlation threshold')
plt.ylabel('Number of features')
plt.show()
```

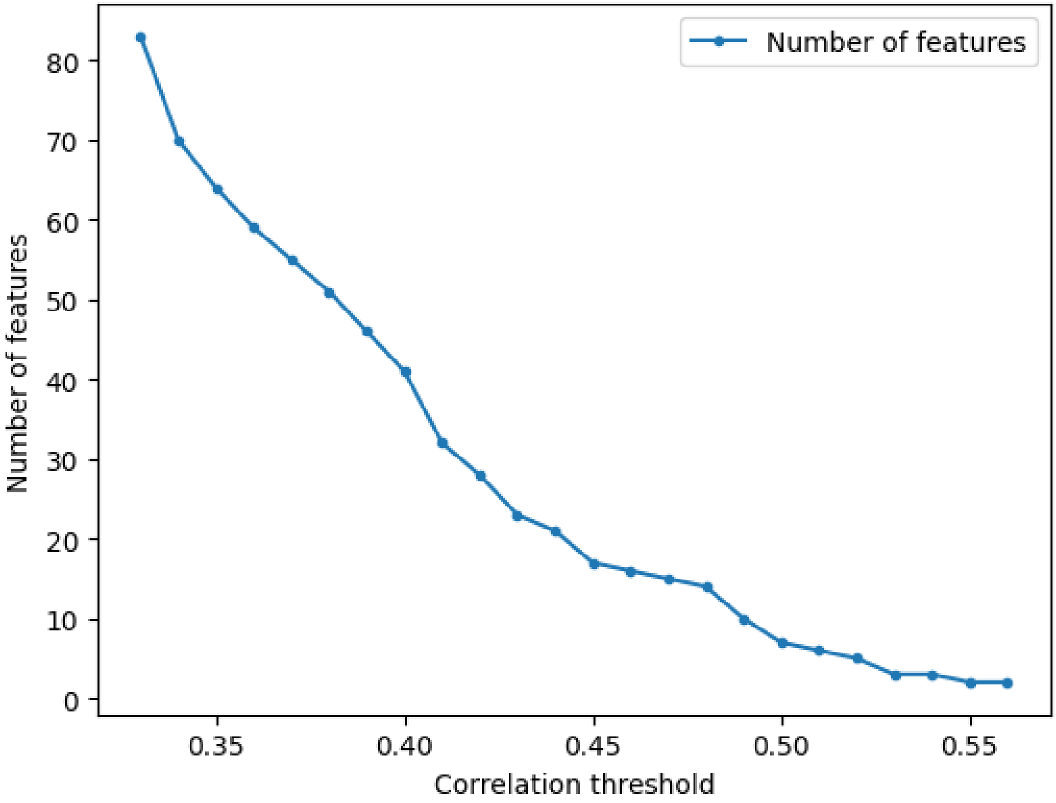


```
[ ]:
```

5 Support Vector Machines (SVM)

```
[37]: without_standardization, train_r2_, test_r2_, hist1, hist2, target_column_name,␣
      ↪training_data_RMSE, test_data_RMSE  = pred_model.
      ↪prepare_data_and_create_model(molecular_descriptors_df=data,
                                                                                ␣
      ↪                      correlation_threshold=0.50,
                                                                                ␣
      ↪                      standardization=False,
                                                                                ␣
      ↪                      model_type='SVR',
                                                                                ␣
      ↪                      kernel_ = 'linear',
                                                                                ␣
      ↪                      gamma_ = 'auto',
                                                                                ␣
      ↪                      target_column_name = target,
```

```
↪                    random_state=random_state,
↪                    train_test_split_=True,
↪                    verbose=True)
```

```
I am not doing standardization…
  molecular descriptor name
0                   AATS0Z
1                 AATS0are
2                   AATS0d
3                  AATS0dv
4                   AATS0i
  molecular descriptor name  corr_value
0                   AATS0Z   -0.020817
1                 AATS0are   -0.148257
2                   AATS0d    0.022999
3                  AATS0dv   -0.137980
4                   AATS0i    0.133257
  molecular descriptor name  corr_value  absolute correlation value
0                   AATS0Z   -0.020817                    0.020817
1                 AATS0are   -0.148257                    0.148257
2                   AATS0d    0.022999                    0.022999
3                  AATS0dv   -0.137980                    0.137980
4                   AATS0i    0.133257                    0.133257
    molecular descriptor name  corr_value  absolute correlation value
226                    AMID_O   -0.546843                    0.546843
505               EState_VSA5   -0.588780                    0.588780
556                    GATS2c    0.511671                    0.511671
791                   MDEO-12   -0.582747                    0.582747
847                       NdO   -0.500462                    0.500462
    molecular descriptor name  corr_value  absolute correlation value
226                    AMID_O   -0.546843                    0.546843
505               EState_VSA5   -0.588780                    0.588780
556                    GATS2c    0.511671                    0.511671
791                   MDEO-12   -0.582747                    0.582747
847                       NdO   -0.500462                    0.500462
The model used is: SVR…
Return the coefficient of determination of the prediction:
0.5844087903682672
R^2 score: 0.5038700283320365
Correlation coefficient: 0.7098380296462261
Test data - unseen during training:
R^2 score: 0.5844087903682672
Correlation coefficient: 0.764466343515702
[7.74409401 6.07894667 7.0773428  7.61926673 7.7602434  7.99556239
```

```
 8.15221908 7.76267828 8.0206463  7.61619051 6.59362476 7.43512479
 7.44585341 7.7392973  8.00563276 8.0043966  6.6259373  5.75141239]
102    7.698970
38     5.986741
8      5.984515
109    7.886057
11     7.962574
51     7.698970
88     8.229148
21     7.298432
82     8.065502
98     7.376751
58     7.602060
64     8.060481
74     7.853872
103    7.853872
91     8.346787
43     7.619789
25     4.989276
30     6.085657
Name: BALB/3T3, dtype: float64
Training Root Mean Square Error: 0.6112341502878809
Testing Root Mean Square Error: 0.5929205581098148
```

[38]:

```
print(target_column_name+str('_transformed'))
print(hist1[target_column_name].hist())
```

```
BALB/3T3_transformed
AxesSubplot(0.125,0.11;0.775x0.77)
```

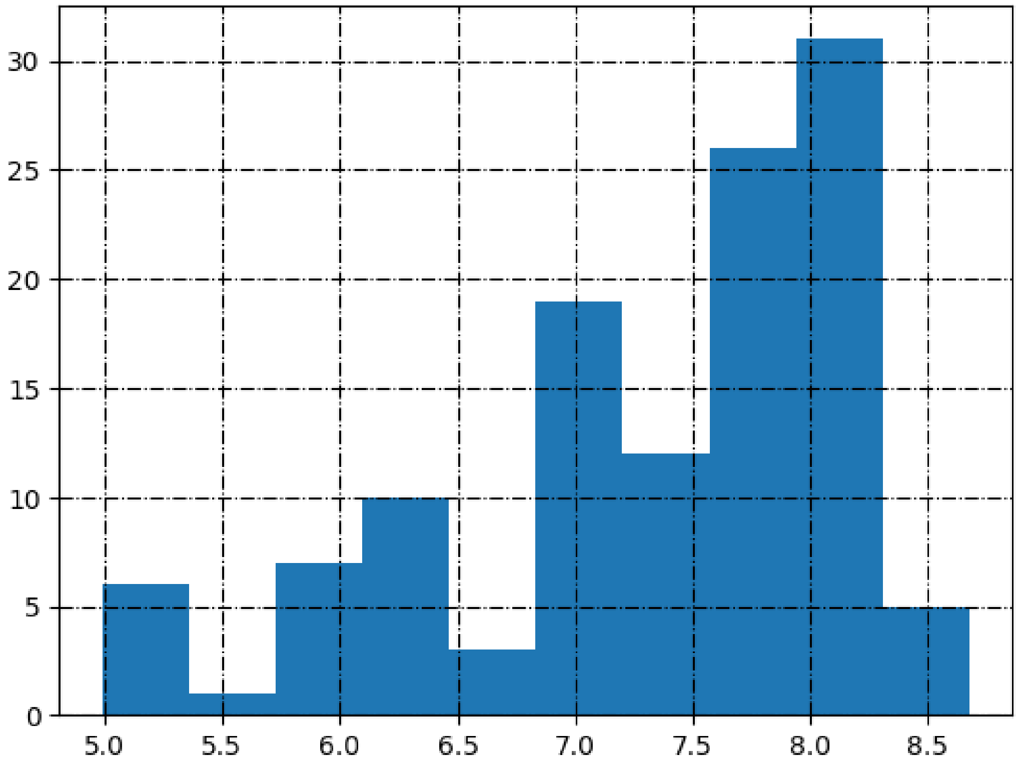

[39]:
```
with_standardization, train_r2_, test_r2_, hist1, hist2, target_column_name,␣
↪training_data_RMSE, test_data_RMSE  = pred_model.
↪prepare_data_and_create_model(molecular_descriptors_df=data,
                                                                              ␣
↪                     correlation_threshold=0.50,
                                                                              ␣
↪                     standardization=True,
                                                                              ␣
↪                     model_type='SVR',
                                                                              ␣
↪                     kernel_ = 'linear',
                                                                              ␣
↪                     gamma_ = 'auto',
                                                                              ␣
↪                     target_column_name = target,
                                                                              ␣
↪                     random_state=random_state,
                                                                              ␣
↪                     train_test_split_=True,
                                                                              ␣
↪                     verbose=True)
```

```
I am doing standardization…
  molecular descriptor name
0                    AATS0Z
1                  AATS0are
2                    AATS0d
3                   AATS0dv
4                    AATS0i
  molecular descriptor name  corr_value
0                    AATS0Z   -0.020817
1                  AATS0are   -0.148257
2                    AATS0d    0.022999
3                   AATS0dv   -0.137980
4                    AATS0i    0.133257
  molecular descriptor name  corr_value  absolute correlation value
0                    AATS0Z   -0.020817                    0.020817
1                  AATS0are   -0.148257                    0.148257
2                    AATS0d    0.022999                    0.022999
3                   AATS0dv   -0.137980                    0.137980
4                    AATS0i    0.133257                    0.133257
    molecular descriptor name  corr_value  absolute correlation value
226                    AMID_O   -0.546843                    0.546843
505                EState_VSA5   -0.588780                    0.588780
556                    GATS2c    0.511671                    0.511671
791                   MDEO-12   -0.582747                    0.582747
847                       NdO   -0.500462                    0.500462
    molecular descriptor name  corr_value  absolute correlation value
226                    AMID_O   -0.546843                    0.546843
505                EState_VSA5   -0.588780                    0.588780
556                    GATS2c    0.511671                    0.511671
791                   MDEO-12   -0.582747                    0.582747
847                       NdO   -0.500462                    0.500462
The model used is: SVR…
Return the coefficient of determination of the prediction:
0.5844087903682672
R^2 score: 0.5038700283320365
Correlation coefficient: 0.7098380296462261
Test data - unseen during training:
R^2 score: 0.5844087903682672
Correlation coefficient: 0.764466343515702
[7.74409401 6.07894667 7.0773428  7.61926673 7.7602434  7.99556239
 8.15221908 7.76267828 8.0206463  7.61619051 6.59362476 7.43512479
 7.44585341 7.7392973  8.00563276 8.0043966  6.6259373  5.75141239]
102    7.698970
38     5.986741
8      5.984515
109    7.886057
11     7.962574
51     7.698970
```

```
88     8.229148
21     7.298432
82     8.065502
98     7.376751
58     7.602060
64     8.060481
74     7.853872
103    7.853872
91     8.346787
43     7.619789
25     4.989276
30     6.085657
Name: BALB/3T3, dtype: float64
Training Root Mean Square Error: 0.6112341502878809
Testing Root Mean Square Error: 0.5929205581098148
```

5.1 Search inside correlation space

[40]:
```
step = 0.01
initial_step = corr_low
last_step = corr_high
first_list = [x / 100.0 for x in range(int(initial_step*100),␣
 ↪int(last_step*100), int(step*100))]
second_list = []
third_list = []
fourth_l = []
fifth_l = []
f_list = []
for i in first_list:
    without_standardization, train_r2, test_r2, _, h_, target_column_name,␣
 ↪training_data_RMSE, test_data_RMSE = pred_model.
 ↪prepare_data_and_create_model(molecular_descriptors_df = data,
                                                                                    ␣
 ↪                      correlation_threshold = i,
                                                                                    ␣
 ↪                      standardization = False,
                                                                                    ␣
 ↪                      model_type = 'SVR',
                                                                                    ␣
 ↪                      kernel_ = 'linear',
                                                                                    ␣
 ↪                      gamma_ = 'auto',
                                                                                    ␣
 ↪                      target_column_name = target,
                                                                                    ␣
 ↪                      random_state=random_state,
```

```
                        train_test_split_ = True,
                        verbose = False)
    second_list.append(train_r2)
    third_list.append(test_r2)
    fourth_l.append(training_data_RMSE)
    fifth_l.append(test_data_RMSE)
    f_list.append(len(h_))
```

[41]:
```
df_without_standardization = pd.DataFrame(data=first_list,
    columns=["Correlation threshold"])
df_without_standardization['Training data R^2 score'] = second_list
df_without_standardization['Test data R^2 score'] = third_list
df_without_standardization['Training RMSE'] = fourth_l
df_without_standardization['Test RMSE'] = fifth_l
df_without_standardization['Number of features'] = f_list
```

[42]:
```
df_svm = df_without_standardization.copy()
df_without_standardization
```

[42]:

	Correlation threshold	Training data R^2 score	Test data R^2 score	\
0	0.33	0.758706	0.762230	
1	0.34	0.673499	0.697317	
2	0.35	0.680598	0.680201	
3	0.36	0.685819	0.688987	
4	0.37	0.683062	0.702495	
5	0.38	0.673122	0.655446	
6	0.39	0.661432	0.665519	
7	0.40	0.654452	0.685718	
8	0.41	0.620802	0.726820	
9	0.42	0.597357	0.675935	
10	0.43	0.585465	0.676336	
11	0.44	0.578861	0.624502	
12	0.45	0.560153	0.583720	
13	0.46	0.560947	0.581063	
14	0.47	0.556380	0.559649	
15	0.48	0.539267	0.634048	
16	0.49	0.527182	0.626409	
17	0.50	0.503870	0.584409	
18	0.51	0.480646	0.561896	
19	0.52	0.483042	0.567314	
20	0.53	0.454180	0.543126	
21	0.54	0.454180	0.543126	
22	0.55	0.452892	0.538185	
23	0.56	0.452892	0.538185	

```
    Training RMSE  Test RMSE  Number of features
0        0.426269   0.448478                  83
1        0.495852   0.506008                  70
2        0.490432   0.520118                  64
3        0.486407   0.512923                  59
4        0.488536   0.501661                  55
5        0.496138   0.539873                  51
6        0.504932   0.531923                  46
7        0.510110   0.515612                  41
8        0.534371   0.480715                  32
9        0.550643   0.523576                  28
10       0.558715   0.523251                  23
11       0.563148   0.563595                  21
12       0.575520   0.593412                  17
13       0.575000   0.595302                  16
14       0.577983   0.610328                  15
15       0.589026   0.556385                  14
16       0.596701   0.562162                  10
17       0.611234   0.592921                   7
18       0.625377   0.608768                   6
19       0.623932   0.604992                   5
20       0.641113   0.621673                   3
21       0.641113   0.621673                   3
22       0.641869   0.625025                   2
23       0.641869   0.625025                   2
```

5.2 Plots

[43]:

```
plt.plot(df_without_standardization['Correlation threshold'],␣
  ↪df_without_standardization['Training data R^2 score'], label = "Training␣
  ↪data R^2 score", marker='v')
plt.plot(df_without_standardization['Correlation threshold'],␣
  ↪df_without_standardization['Test data R^2 score'], label = "Test data R^2␣
  ↪score", marker='D')
plt.legend()
plt.xlabel('Correlation threshold')
plt.ylabel('R^2 score')
plt.ylim([0, 1])
plt.show()
```

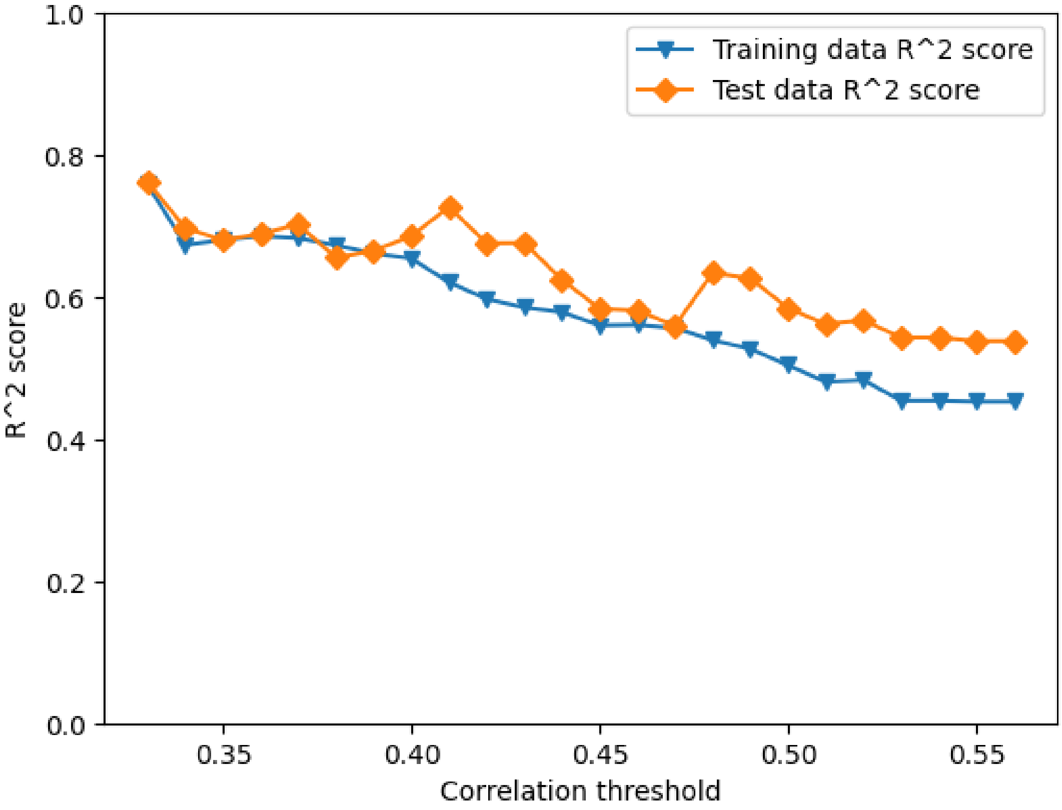

[44]:
```
plt.plot(df_without_standardization['Correlation threshold'],␣
 ↪df_without_standardization['Number of features'], label = "Number of␣
 ↪features", marker='.')
plt.legend()
plt.xlabel('Correlation threshold')
plt.ylabel('Number of features')
plt.show()
```

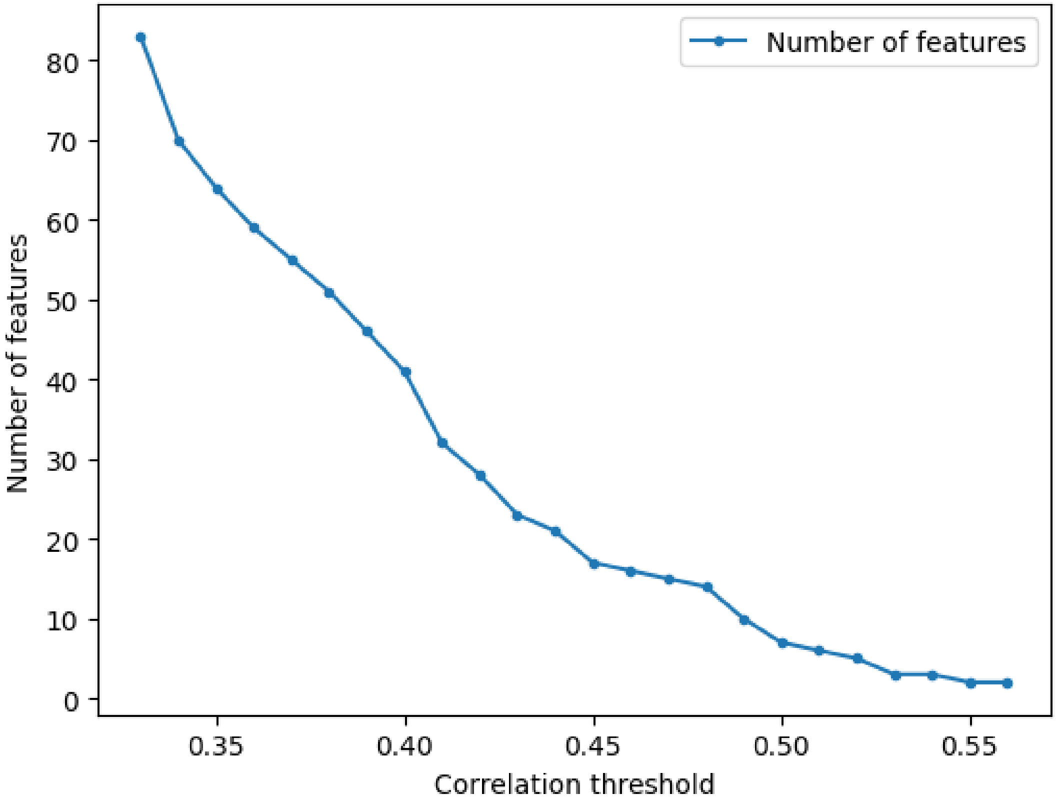

5.3 Store quality measurements

[45]:
```
with pd.ExcelWriter('../Data/Quality_'+'BALB_3T3'+'_'+str(random_state)+'_.
 ↪xlsx') as writer:
    df_linear.to_excel(writer, sheet_name='MLR')
    df_decision_tree.to_excel(writer, sheet_name='DT')
    df_random_forest.to_excel(writer, sheet_name='RF')
    df_k_nearest.to_excel(writer, sheet_name='KNN')
    df_svm.to_excel(writer, sheet_name='SVM')
```

Notebook

January 18, 2024

1 File 19

[1]:
```
import sys
from pathlib import Path
prediction_mode_path = Path("../module")
sys.path.append(prediction_mode_path.as_posix())
import models_creation as pred_model

import pandas as pd
import numpy as np

from sklearn.metrics import r2_score

import matplotlib.pyplot as plt
from sklearn.tree import plot_tree

from matplotlib import pyplot as plt

import joblib

import warnings
warnings.filterwarnings('ignore')
```

[2]:
```
load_prepared_data = pd.read_excel('../Data/Kolchicyna_machine_learning.xlsx')
load_prepared_data = load_prepared_data.drop(columns=['Unnamed: 0'])

target = 'BALB/3T3'

corr_low = 0.33
corr_high = 0.58

random_state = 28
```

[3]:
```
load_prepared_data.head()
```

[3]:
```
      AATS0Z  AATS0are    AATS0d   AATS0dv       AATS0i     AATS0m    AATS0p  \
0  25.090909  6.046518  3.109091  6.662626  160.775045  99.075564  1.555512
1  24.448276  6.008422  3.051724  6.386973  161.021674  96.473315  1.538471
```

```
2  23.868852  5.974074  3.000000  6.138434  161.244045  94.127025  1.523105
3  23.868852  5.974074  3.032787  6.171220  161.244045  94.127025  1.523105
4  23.343750  5.942945  2.953125  5.913194  161.445569  92.000700  1.509180

    AATS0pe    AATS0s   AATS0se  …  A549_float  MCF-7_float  LoVo_float  \
0  6.126680  3.435416  7.629859  …        10.8         10.3         6.5
1  6.088791  3.330998  7.596891  …        11.6         12.0         8.5
2  6.054630  3.236850  7.567166  …        10.9         12.2         8.8
3  6.054630  3.257798  7.567166  …        10.5         11.3         8.5
4  6.023670  3.151529  7.540228  …        89.5         92.7        52.8

   LoVo/DX_float  BALB/3T3_float  A549_float_transformed  \
0           54.9            10.2                7.966576
1           31.1            14.3                7.935542
2           17.9            11.7                7.962574
3           10.2            11.0                7.978811
4           77.8            99.4                7.048177

   MCF-7_float_transformed  LoVo_float_transformed  LoVo/DX_float_transformed  \
0                 7.987163                8.187087                   7.260428
1                 7.920819                8.070581                   7.507240
2                 7.913640                8.055517                   7.747147
3                 7.946922                8.070581                   7.991400
4                 7.032920                7.277366                   7.109020

   BALB/3T3_float_transformed
0                    7.991400
1                    7.844664
2                    7.931814
3                    7.958607
4                    7.002614

[5 rows x 1221 columns]
```

[4]:
```
data = load_prepared_data[list(load_prepared_data.columns)[0:-10]]
data[target] = load_prepared_data[load_prepared_data.columns[-1]]
data.head()
```

[4]:
```
      AATS0Z  AATS0are    AATS0d   AATS0dv      AATS0i     AATS0m    AATS0p  \
0  25.090909  6.046518  3.109091  6.662626  160.775045  99.075564  1.555512
1  24.448276  6.008422  3.051724  6.386973  161.021674  96.473315  1.538471
2  23.868852  5.974074  3.000000  6.138434  161.244045  94.127025  1.523105
3  23.868852  5.974074  3.032787  6.171220  161.244045  94.127025  1.523105
4  23.343750  5.942945  2.953125  5.913194  161.445569  92.000700  1.509180

    AATS0pe    AATS0s   AATS0se  …    piPC10     piPC2     piPC3     piPC4  \
0  6.126680  3.435416  7.629859  …  7.999655  4.266195  4.915408  5.516700
```

```
1  6.088791  3.330998  7.596891  …  8.018726  4.280132  4.922714  5.520711
2  6.054630  3.236850  7.567166  …  8.028799  4.293878  4.929967  5.524706
3  6.054630  3.257798  7.567166  …  8.037440  4.307438  4.929967  5.524706
4  6.023670  3.151529  7.540228  …  8.034892  4.307438  4.937168  5.528685

     piPC5     piPC6     piPC7     piPC8     piPC9  BALB/3T3
0  6.098566  6.595759  6.979116  7.337313  7.681445  7.991400
1  6.103048  6.601209  6.985613  7.349442  7.695531  7.844664
2  6.105281  6.603923  6.989306  7.353932  7.704023  7.931814
3  6.107510  6.606629  6.992068  7.361425  7.709420  7.958607
4  6.107510  6.605277  6.991148  7.356489  7.707175  7.002614

[5 rows x 1212 columns]
```

2 Multiple Linear regression (MLR)

[5]:
```
without_standardization, train_r2_, test_r2_, hist1, hist2, target_column_name,␣
↪training_data_RMSE, test_data_RMSE  = pred_model.
↪prepare_data_and_create_model(molecular_descriptors_df=data,
                                                                          ␣
↪                    correlation_threshold=0.5,
                                                                          ␣
↪                    standardization=False,
                                                                          ␣
↪                    model_type='linear_model',
                                                                          ␣
↪                    target_column_name = str(target),
                                                                          ␣
↪                    random_state=random_state,
                                                                          ␣
↪                    train_test_split_=True,
                                                                          ␣
↪                    verbose=True)
```

```
I am not doing standardization…
  molecular descriptor name
0                   AATS0Z
1                 AATS0are
2                   AATS0d
3                  AATS0dv
4                   AATS0i
  molecular descriptor name  corr_value
0                   AATS0Z   -0.020817
1                 AATS0are   -0.148257
2                   AATS0d    0.022999
3                  AATS0dv   -0.137980
```

```
4                   AATS0i    0.133257
  molecular descriptor name  corr_value  absolute correlation value
0                   AATS0Z   -0.020817                    0.020817
1                 AATS0are   -0.148257                    0.148257
2                   AATS0d    0.022999                    0.022999
3                  AATS0dv   -0.137980                    0.137980
4                   AATS0i    0.133257                    0.133257
    molecular descriptor name  corr_value  absolute correlation value
226                    AMID_O   -0.546843                    0.546843
505                EState_VSA5   -0.588780                    0.588780
556                    GATS2c    0.511671                    0.511671
791                   MDEO-12   -0.582747                    0.582747
847                       NdO   -0.500462                    0.500462
    molecular descriptor name  corr_value  absolute correlation value
226                    AMID_O   -0.546843                    0.546843
505                EState_VSA5   -0.588780                    0.588780
556                    GATS2c    0.511671                    0.511671
791                   MDEO-12   -0.582747                    0.582747
847                       NdO   -0.500462                    0.500462
The model used is: LinearReg…
Return the coefficient of determination of the prediction:
0.4531410949770688
R^2 score: 0.5600891898540288
Correlation coefficient: 0.7483910674600738
Test data - unseen during training:
R^2 score: 0.4531410949770688
Correlation coefficient: 0.6731575558344932
[7.54593608 6.20014013 7.43354052 6.38485025 7.69492011 7.32564568
 7.54054595 7.60386062 6.64019847 7.41310329 7.64890537 7.69020105
 7.7755592  7.20107755 7.33180425 6.22650972 7.86818438 6.4947382 ]
113    7.744727
35     6.149967
101    6.958607
36     6.346787
100    7.000000
13     7.966576
0      7.991400
114    7.443697
104    7.823909
96     7.677781
40     7.795880
103    7.853872
48     7.744727
39     8.221849
14     6.414314
117    5.886057
21     7.298432
9      5.971266
```

```
Name: BALB/3T3, dtype: float64
Training Root Mean Square Error: 0.5942890554179852
Testing Root Mean Square Error: 0.553300668506696
```

[6]:
```python
print(target_column_name+str('_transformed'))
print(hist1[target_column_name].hist())
```

```
BALB/3T3_transformed
AxesSubplot(0.125,0.11;0.775x0.77)
```

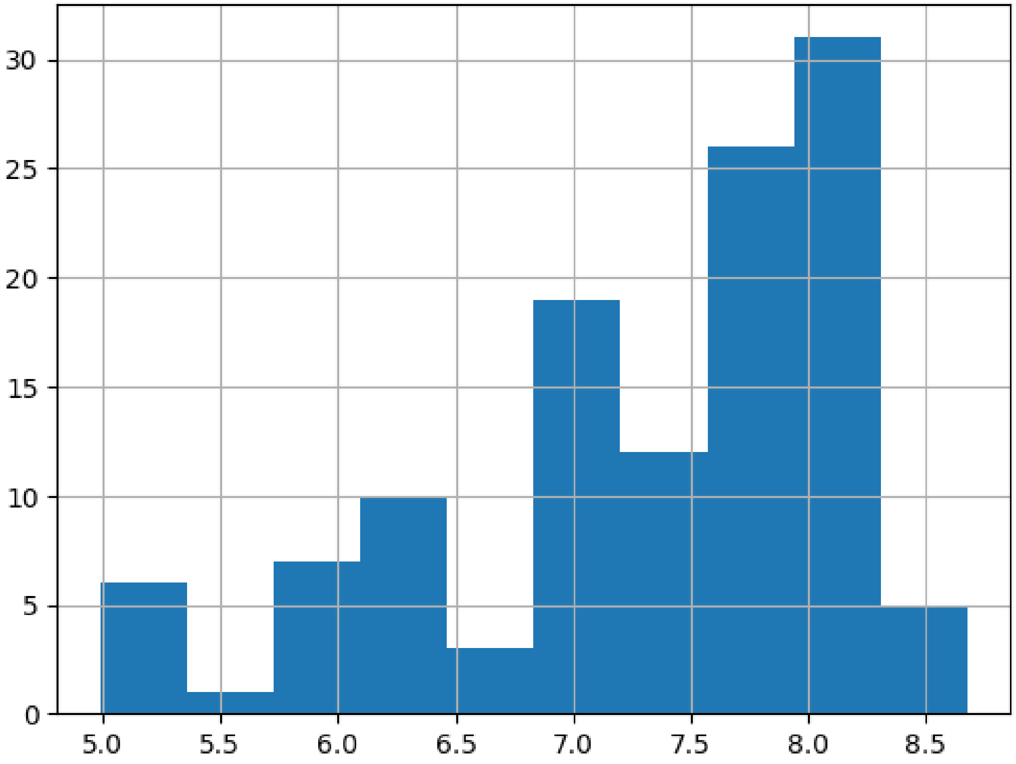

[7]:
```python
with_standardization, train_r2_, test_r2_, hist1, hist2, target_column_name,␣
↪training_data_RMSE, test_data_RMSE  = pred_model.
↪prepare_data_and_create_model(molecular_descriptors_df=data,
                                                                           ␣
↪                         correlation_threshold=0.50,
                                                                           ␣
↪                         standardization=True,
                                                                           ␣
↪                         model_type='linear_model',
                                                                           ␣
↪                         target_column_name = target,
```

```
↪                   random_state=random_state,
↪                   train_test_split_=True,
↪                   verbose=True)
```

```
I am doing standardization…
  molecular descriptor name
0                    AATS0Z
1                  AATS0are
2                    AATS0d
3                   AATS0dv
4                    AATS0i
  molecular descriptor name  corr_value
0                    AATS0Z   -0.020817
1                  AATS0are   -0.148257
2                    AATS0d    0.022999
3                   AATS0dv   -0.137980
4                    AATS0i    0.133257
  molecular descriptor name  corr_value  absolute correlation value
0                    AATS0Z   -0.020817                    0.020817
1                  AATS0are   -0.148257                    0.148257
2                    AATS0d    0.022999                    0.022999
3                   AATS0dv   -0.137980                    0.137980
4                    AATS0i    0.133257                    0.133257
    molecular descriptor name  corr_value  absolute correlation value
226                    AMID_O   -0.546843                    0.546843
505               EState_VSA5   -0.588780                    0.588780
556                    GATS2c    0.511671                    0.511671
791                   MDEO-12   -0.582747                    0.582747
847                       NdO   -0.500462                    0.500462
    molecular descriptor name  corr_value  absolute correlation value
226                    AMID_O   -0.546843                    0.546843
505               EState_VSA5   -0.588780                    0.588780
556                    GATS2c    0.511671                    0.511671
791                   MDEO-12   -0.582747                    0.582747
847                       NdO   -0.500462                    0.500462
The model used is: LinearReg…
Return the coefficient of determination of the prediction:
0.4531410949770688
R^2 score: 0.5600891898540288
Correlation coefficient: 0.7483910674600738
Test data - unseen during training:
R^2 score: 0.4531410949770688
Correlation coefficient: 0.6731575558344932
[7.54593608 6.20014013 7.43354052 6.38485025 7.69492011 7.32564568
```

```
 7.54054595 7.60386062 6.64019847 7.41310329 7.64890537 7.69020105
 7.7755592  7.20107755 7.33180425 6.22650972 7.86818438 6.4947382 ]
113    7.744727
35     6.149967
101    6.958607
36     6.346787
100    7.000000
13     7.966576
0      7.991400
114    7.443697
104    7.823909
96     7.677781
40     7.795880
103    7.853872
48     7.744727
39     8.221849
14     6.414314
117    5.886057
21     7.298432
9      5.971266
Name: BALB/3T3, dtype: float64
Training Root Mean Square Error: 0.5942890554179852
Testing Root Mean Square Error: 0.553300668506696
```

2.1 Search inside correlation space

[8]:
```
step = 0.01
initial_step = corr_low
last_step = corr_high
first_list = [x / 100.0 for x in range(int(initial_step*100),␣
 ↪int(last_step*100), int(step*100))]
second_list = []
third_list = []
fourth_l = []
fifth_l = []
f_list = []
for i in first_list:
    without_standardization, train_r2, test_r2, _, h_, target_column_name,␣
 ↪training_data_RMSE, test_data_RMSE = pred_model.
 ↪prepare_data_and_create_model(molecular_descriptors_df = data,
                                                                           ␣
 ↪                    correlation_threshold = i,
                                                                           ␣
 ↪                    standardization = False,
                                                                           ␣
 ↪                    model_type = 'linear_model',
```

```
                    target_column_name = target,
                    random_state=random_state,
                    train_test_split_ = True,
                    verbose = False)
    second_list.append(train_r2)
    third_list.append(test_r2)
    fourth_l.append(training_data_RMSE)
    fifth_l.append(test_data_RMSE)
    f_list.append(len(h_))
```

[9]:
```
df_without_standardization = pd.DataFrame(data=first_list, columns=["Correlation threshold"])
df_without_standardization['Training data R^2 score'] = second_list
df_without_standardization['Test data R^2 score'] = third_list
df_without_standardization['Training RMSE'] = fourth_l
df_without_standardization['Test RMSE'] = fifth_l
df_without_standardization['Number of features'] = f_list
```

[10]:
```
df_linear = df_without_standardization.copy()
df_without_standardization
```

[10]:

	Correlation threshold	Training data R^2 score	Test data R^2 score	\
0	0.33	0.986965	-5.823141	
1	0.34	0.947580	-3.372261	
2	0.35	0.937728	-0.239517	
3	0.36	0.931313	0.007546	
4	0.37	0.919148	-0.404108	
5	0.38	0.880120	-0.293754	
6	0.39	0.840043	-0.260312	
7	0.40	0.822495	-0.128123	
8	0.41	0.774976	-0.001020	
9	0.42	0.714224	0.121483	
10	0.43	0.688240	0.287600	
11	0.44	0.684284	0.294614	
12	0.45	0.644202	0.328652	
13	0.46	0.644094	0.330996	
14	0.47	0.643028	0.324173	
15	0.48	0.636882	0.374617	
16	0.49	0.601034	0.356643	
17	0.50	0.560089	0.453141	
18	0.51	0.551430	0.375287	
19	0.52	0.549509	0.374704	
20	0.53	0.514778	0.451642	

```
21                    0.54                0.514778            0.451642
22                    0.55                0.483583            0.522604
23                    0.56                0.483583            0.522604

    Training RMSE  Test RMSE  Number of features
0        0.102298   1.954410                  83
1        0.205146   1.564504                  70
2        0.223595   0.833009                  64
3        0.234829   0.745382                  59
4        0.254777   0.886592                  55
5        0.310234   0.851039                  51
6        0.358358   0.839968                  46
7        0.377504   0.794697                  41
8        0.425040   0.748592                  32
9        0.478992   0.701292                  28
10       0.500294   0.631518                  23
11       0.503459   0.628401                  21
12       0.534462   0.613052                  17
13       0.534543   0.611981                  16
14       0.535344   0.615094                  15
15       0.539932   0.591693                  14
16       0.565957   0.600136                  10
17       0.594289   0.553301                   7
18       0.600109   0.591376                   6
19       0.601393   0.591652                   5
20       0.624145   0.554058                   3
21       0.624145   0.554058                   3
22       0.643896   0.516967                   2
23       0.643896   0.516967                   2
```

[ ]:

2.2 Plots

[11]:
```
plt.plot(df_without_standardization['Correlation threshold'],
  df_without_standardization['Training data R^2 score'], label = "Training
  data R^2 score", marker='v')
plt.plot(df_without_standardization['Correlation threshold'],
  df_without_standardization['Test data R^2 score'], label = "Test data R^2
  score", marker='D')
plt.legend()
plt.xlabel('Correlation threshold')
plt.ylabel('R^2 score')
plt.ylim([0, 1])
plt.show()
```

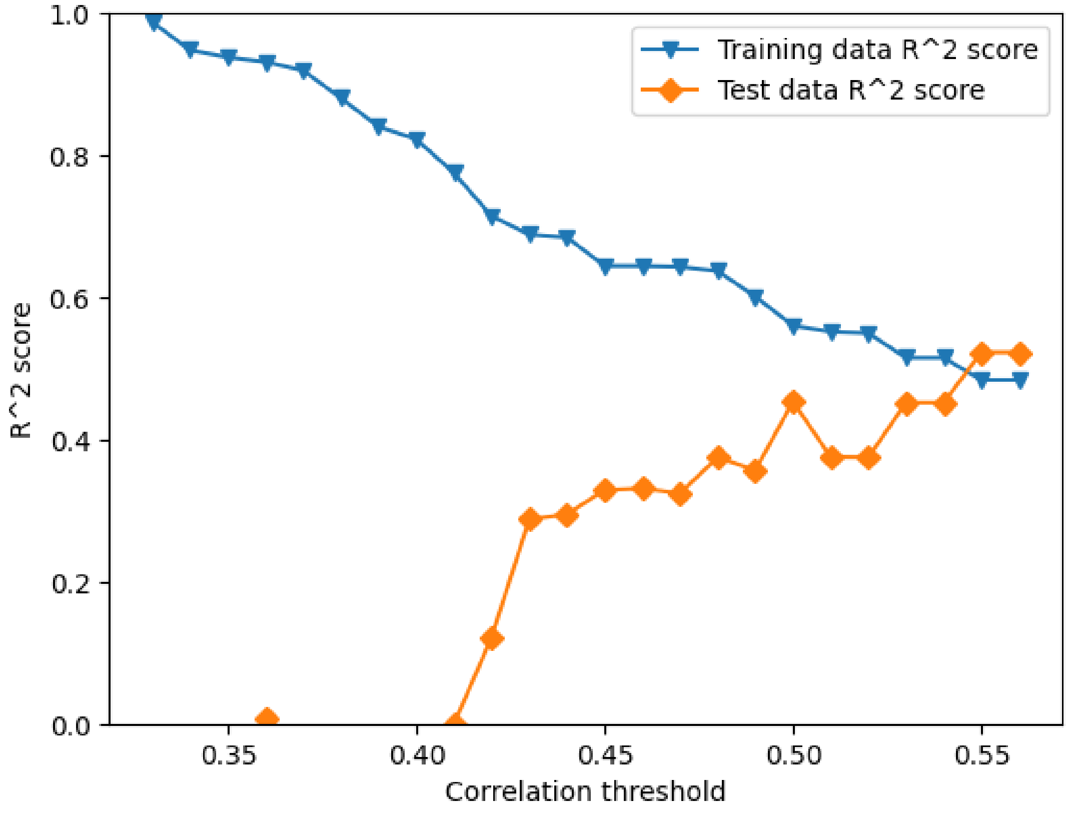

[12]:
```
plt.plot(df_without_standardization['Correlation threshold'],␣
 ↪df_without_standardization['Number of features'], label = "Number of␣
 ↪features", marker='.')
plt.legend()
plt.xlabel('Correlation threshold')
plt.ylabel('Number of features')
plt.show()
```

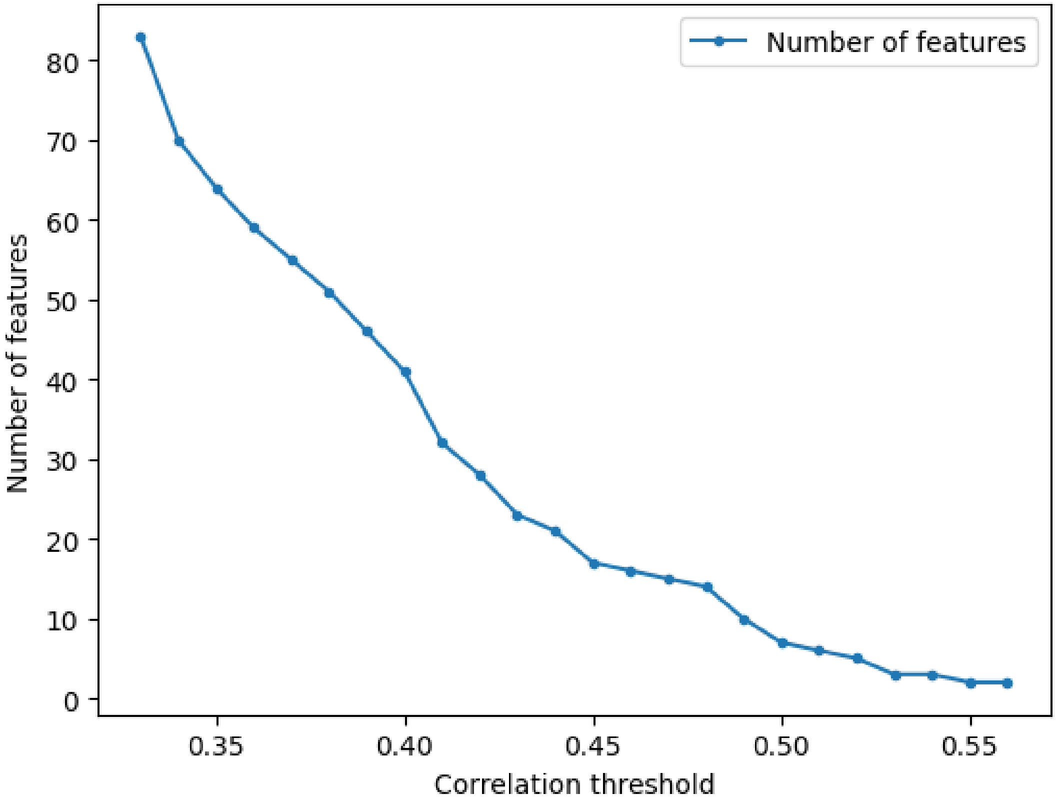

[ ]:

```

```

2.3 Decision Tree

[13]:

```
without_standardization, train_r2_, test_r2_, hist1, hist2, target_column_name,␣
↪training_data_RMSE, test_data_RMSE  = pred_model.
↪prepare_data_and_create_model(molecular_descriptors_df=data,
                                                                                                  ␣
↪                        correlation_threshold=0.50,
                                                                                                  ␣
↪                        standardization=False,
                                                                                                  ␣
↪                        model_type='DecisionTreeRegressor',
                                                                                                  ␣
↪                        max_depth=5,
                                                                                                  ␣
↪                        target_column_name = target,
                                                                                                  ␣
↪                        random_state=random_state,
```

```
↪                     train_test_split_=True,
↪                     verbose=True)
```

```
I am not doing standardization…
  molecular descriptor name
0                    AATS0Z
1                  AATS0are
2                    AATS0d
3                   AATS0dv
4                    AATS0i
  molecular descriptor name  corr_value
0                    AATS0Z   -0.020817
1                  AATS0are   -0.148257
2                    AATS0d    0.022999
3                   AATS0dv   -0.137980
4                    AATS0i    0.133257
  molecular descriptor name  corr_value  absolute correlation value
0                    AATS0Z   -0.020817                    0.020817
1                  AATS0are   -0.148257                    0.148257
2                    AATS0d    0.022999                    0.022999
3                   AATS0dv   -0.137980                    0.137980
4                    AATS0i    0.133257                    0.133257
    molecular descriptor name  corr_value  absolute correlation value
226                    AMID_O   -0.546843                    0.546843
505               EState_VSA5   -0.588780                    0.588780
556                    GATS2c    0.511671                    0.511671
791                  MDEO-12   -0.582747                    0.582747
847                       NdO   -0.500462                    0.500462
    molecular descriptor name  corr_value  absolute correlation value
226                    AMID_O   -0.546843                    0.546843
505               EState_VSA5   -0.588780                    0.588780
556                    GATS2c    0.511671                    0.511671
791                  MDEO-12   -0.582747                    0.582747
847                       NdO   -0.500462                    0.500462
The model used is: DecisionTree…
Return the coefficient of determination of the prediction:
0.6154217227335051
R^2 score: 0.8628508546216089
Correlation coefficient: 0.9288976556228403
Test data - unseen during training:
R^2 score: 0.6154217227335051
Correlation coefficient: 0.7844881915832164
[7.73440408 6.56863624 7.73440408 6.56863624 7.73440408 6.84343546
 8.01954532 7.40431209 7.75277606 7.73440408 7.73440408 7.73440408
 7.73440408 7.73440408 6.84343546 5.92081875 7.10968583 6.84343546]
113    7.744727
```

```
35     6.149967
101    6.958607
36     6.346787
100    7.000000
13     7.966576
0      7.991400
114    7.443697
104    7.823909
96     7.677781
40     7.795880
103    7.853872
48     7.744727
39     8.221849
14     6.414314
117    5.886057
21     7.298432
9      5.971266
Name: BALB/3T3, dtype: float64
Training Root Mean Square Error: 0.331827188967237
Testing Root Mean Square Error: 0.4639978043847089
```

[14]:

```
print(target_column_name+str('_transformed'))
print(hist1[target_column_name].hist())
```

```
BALB/3T3_transformed
AxesSubplot(0.125,0.11;0.775x0.77)
```

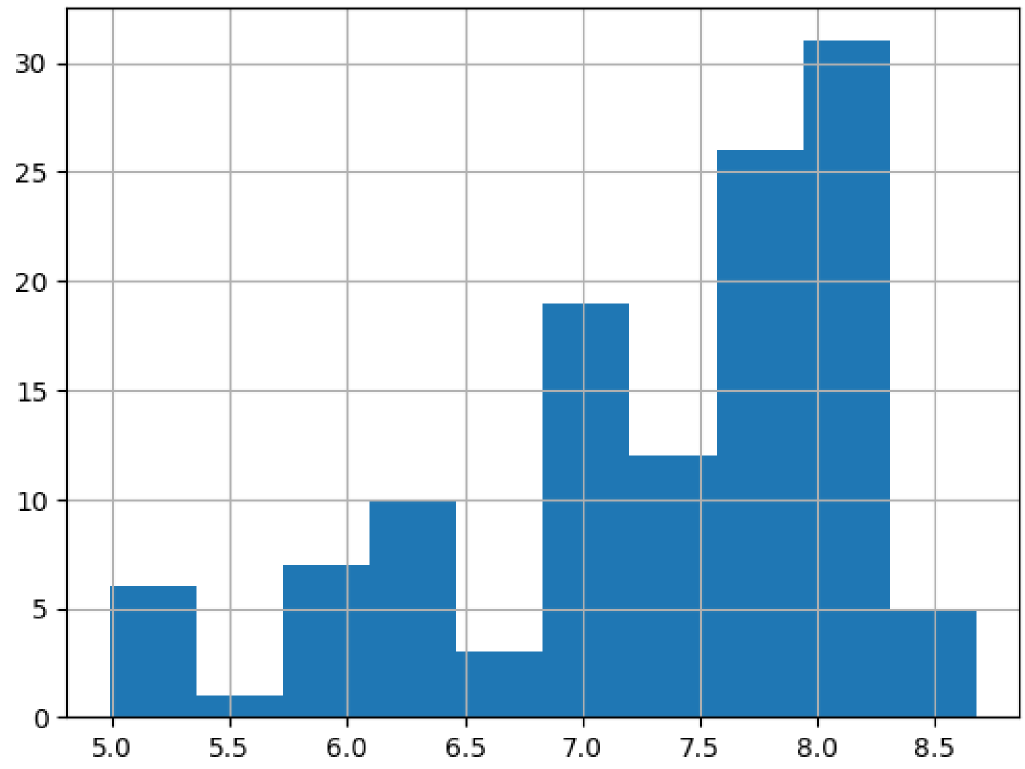

[15]:
```
with_standardization, train_r2_, test_r2_, hist1, hist2, target_column_name,␣
↪training_data_RMSE, test_data_RMSE  = pred_model.
↪prepare_data_and_create_model(molecular_descriptors_df=data,
                                                                              ␣
↪                          correlation_threshold=0.50,
                                                                              ␣
↪                          standardization=True,
                                                                              ␣
↪                          model_type='DecisionTreeRegressor',
                                                                              ␣
↪                          max_depth=5,
                                                                              ␣
↪                          target_column_name = target,
                                                                              ␣
↪                          random_state=random_state,
                                                                              ␣
↪                          train_test_split_=True,
                                                                              ␣
↪                          verbose=True)
```

```
I am doing standardization…
  molecular descriptor name
```

```
0                    AATS0Z
1                  AATS0are
2                    AATS0d
3                   AATS0dv
4                    AATS0i
  molecular descriptor name  corr_value
0                    AATS0Z   -0.020817
1                  AATS0are   -0.148257
2                    AATS0d    0.022999
3                   AATS0dv   -0.137980
4                    AATS0i    0.133257
  molecular descriptor name  corr_value  absolute correlation value
0                    AATS0Z   -0.020817                    0.020817
1                  AATS0are   -0.148257                    0.148257
2                    AATS0d    0.022999                    0.022999
3                   AATS0dv   -0.137980                    0.137980
4                    AATS0i    0.133257                    0.133257
    molecular descriptor name  corr_value  absolute correlation value
226                    AMID_O   -0.546843                    0.546843
505                EState_VSA5   -0.588780                    0.588780
556                    GATS2c    0.511671                    0.511671
791                   MDEO-12   -0.582747                    0.582747
847                       NdO   -0.500462                    0.500462
    molecular descriptor name  corr_value  absolute correlation value
226                    AMID_O   -0.546843                    0.546843
505                EState_VSA5   -0.588780                    0.588780
556                    GATS2c    0.511671                    0.511671
791                   MDEO-12   -0.582747                    0.582747
847                       NdO   -0.500462                    0.500462
The model used is: DecisionTree…
Return the coefficient of determination of the prediction:
0.6154217227335051
R^2 score: 0.8628508546216089
Correlation coefficient: 0.9288976556228403
Test data - unseen during training:
R^2 score: 0.6154217227335051
Correlation coefficient: 0.7844881915832164
[7.73440408 6.56863624 7.73440408 6.56863624 7.73440408 6.84343546
 8.01954532 7.40431209 7.75277606 7.73440408 7.73440408 7.73440408
 7.73440408 7.73440408 6.84343546 5.92081875 7.10968583 6.84343546]
113    7.744727
35     6.149967
101    6.958607
36     6.346787
100    7.000000
13     7.966576
0      7.991400
114    7.443697
```

```
104    7.823909
96     7.677781
40     7.795880
103    7.853872
48     7.744727
39     8.221849
14     6.414314
117    5.886057
21     7.298432
9      5.971266
Name: BALB/3T3, dtype: float64
Training Root Mean Square Error: 0.331827188967237
Testing Root Mean Square Error: 0.4639978043847089
```

2.4 Search inside correlation space

[16]:

```
step = 0.01
initial_step = corr_low
last_step = corr_high
first_list = [x / 100.0 for x in range(int(initial_step*100),␣
 ↪int(last_step*100), int(step*100))]
max_depth = [range(2, 30, 1)]
corr_th = []
second_list = []
third_list = []
fourth_l = []
fifth_l = []
f_list = []
fif_list = []
for i in first_list:
    for depth in max_depth[0]:

        without_standardization, train_r2, test_r2, _, h_, target_column_name,␣
 ↪training_data_RMSE, test_data_RMSE = pred_model.
 ↪prepare_data_and_create_model(molecular_descriptors_df=data,
                                                                              ␣
 ↪                        correlation_threshold=i,
                                                                              ␣
 ↪                        standardization=False,
                                                                              ␣
 ↪                        model_type='DecisionTreeRegressor',
                                                                              ␣
 ↪                        max_depth=depth,
                                                                              ␣
 ↪                        target_column_name = target,
                                                                              ␣
 ↪                        random_state=random_state,
```

```
                       train_test_split_=True,
                       verbose=False)
        corr_th.append(i)
        second_list.append(train_r2)
        third_list.append(test_r2)
        fourth_l.append(training_data_RMSE)
        fifth_l.append(test_data_RMSE)
        f_list.append(len(h_))
        fif_list.append(depth)
```

[17]:
```
df_without_standardization = pd.DataFrame(data=corr_th, columns=["Correlation threshold"])
df_without_standardization['Training data R^2 score'] = second_list
df_without_standardization['Test data R^2 score'] = third_list
df_without_standardization['Training RMSE'] = fourth_l
df_without_standardization['Test RMSE'] = fifth_l
df_without_standardization['Number of features'] = f_list
df_without_standardization['Depth number'] = fif_list
```

[18]:
```
df_decision_tree = df_without_standardization.copy()
#df_without_standardization.to_excel('../Data/A549_Decision_tree_rs_'+str(random_state)+'.xlsx')
```

2.5 Plots

[19]:

```
corre_list = [x / 100.0 for x in range(int(initial_step*100),␣
 ↪int(last_step*100), int(0.01*100))]
for element in corre_list:
    element_ =␣
 ↪df_without_standardization[df_without_standardization['Correlation␣
 ↪threshold'] == float(element)]
    plt.plot(element_['Depth number'], element_['Training data R^2 score'],␣
 ↪label = "Training data R^2 score", marker='v')
    plt.plot(element_['Depth number'], element_['Test data R^2 score'], label =␣
 ↪"Test data R^2 score", marker='D')
    plt.legend()
    plt.xlabel('Depth number -> correlation threshold: '+str(element)+'. \n␣
 ↪Number of features: '+str(element_['Number of features'].iloc[0]),␣
 ↪fontweight='bold')
    plt.xlim(max(element_['Depth number'])+1, min(element_['Depth number'])-1)
    plt.ylabel('R^2 score')
    plt.ylim([0, 1])
    plt.rc('grid', linestyle="-.", color='black')
    plt.grid(True)
    plt.show()
```

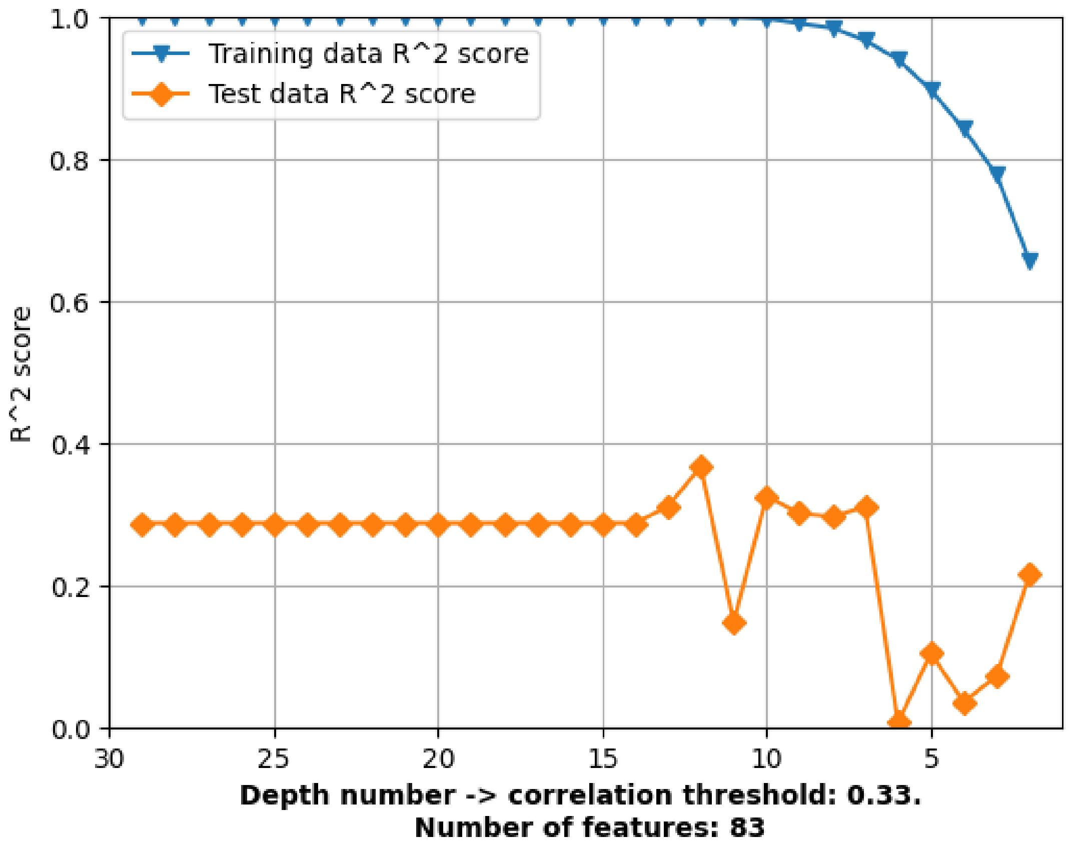

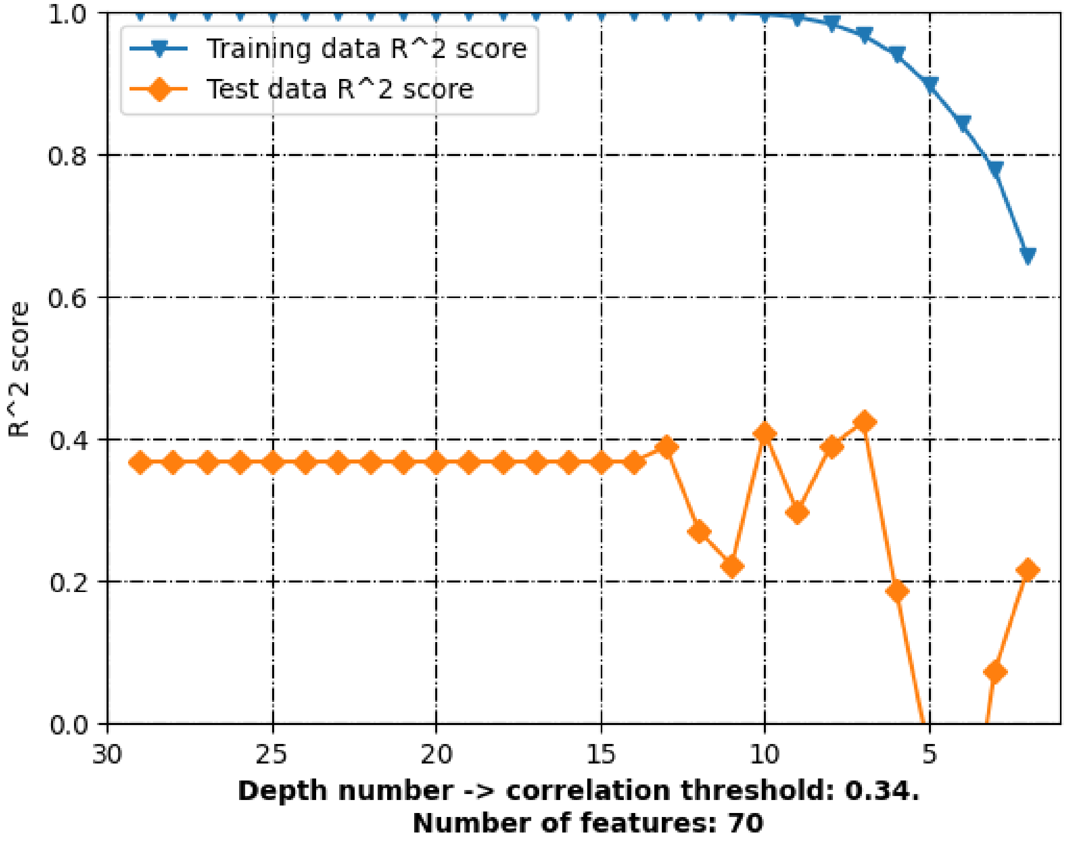

Training data R^2 score
Test data R^2 score
R^2 score
1.0
0.8
0.6
0.4
0.2
0.0
30
25
20
15
10
5
Depth number -> correlation threshold: 0.34.
Number of features: 70

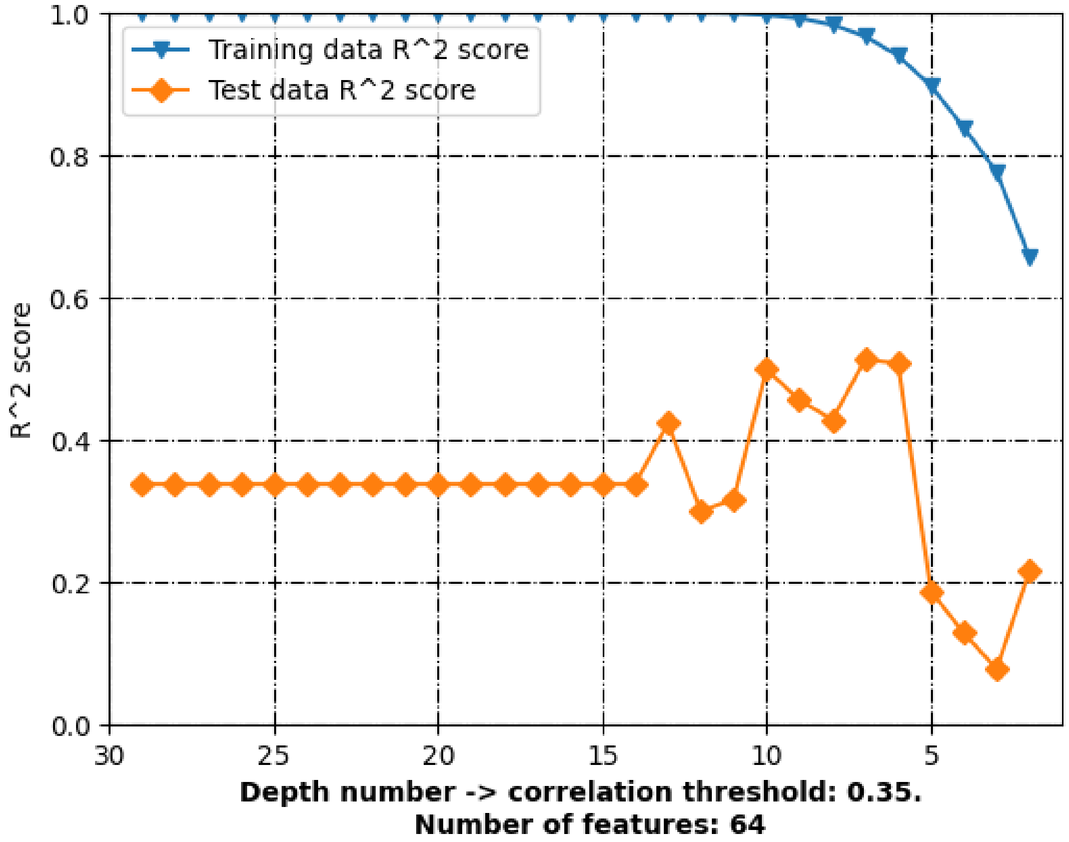

Training data R^2 score
Test data R^2 score
R^2 score
1.0
0.8
0.6
0.4
0.2
0.0
30
25
20
15
10
5
Depth number -> correlation threshold: 0.35.
Number of features: 64

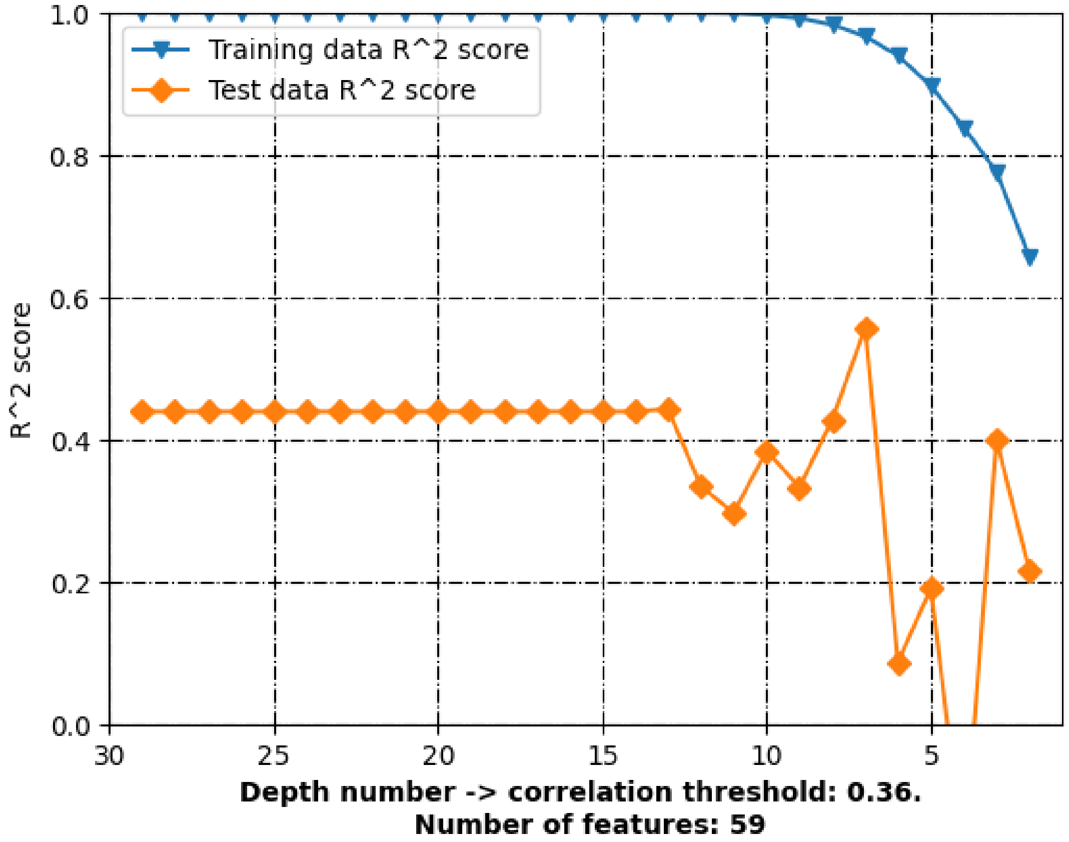

Training data R^2 score
Test data R^2 score
R^2 score
1.0
0.8
0.6
0.4
0.2
0.0
30
25
20
15
10
5
Depth number -> correlation threshold: 0.36.
Number of features: 59

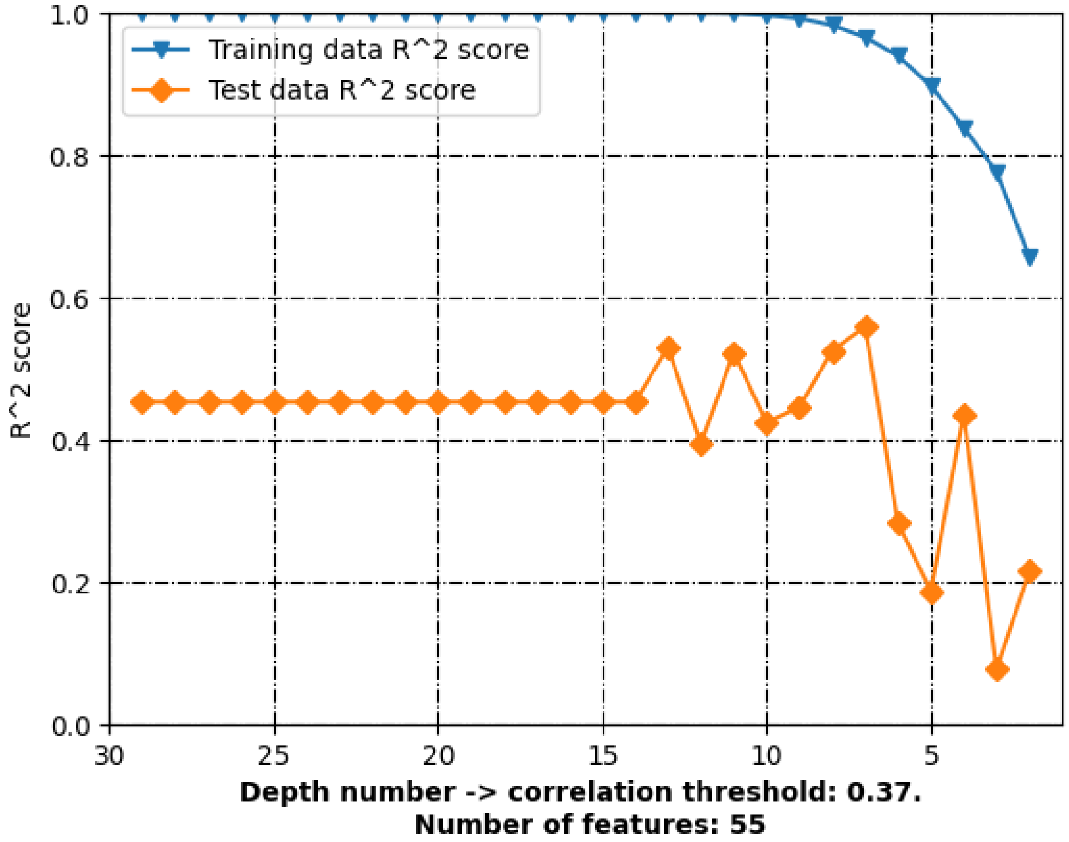
Training data R^2 score
Test data R^2 score
R^2 score
1.0
0.8
0.6
0.4
0.2
0.0
30
25
20
15
10
5
Depth number -> correlation threshold: 0.37.
Number of features: 55

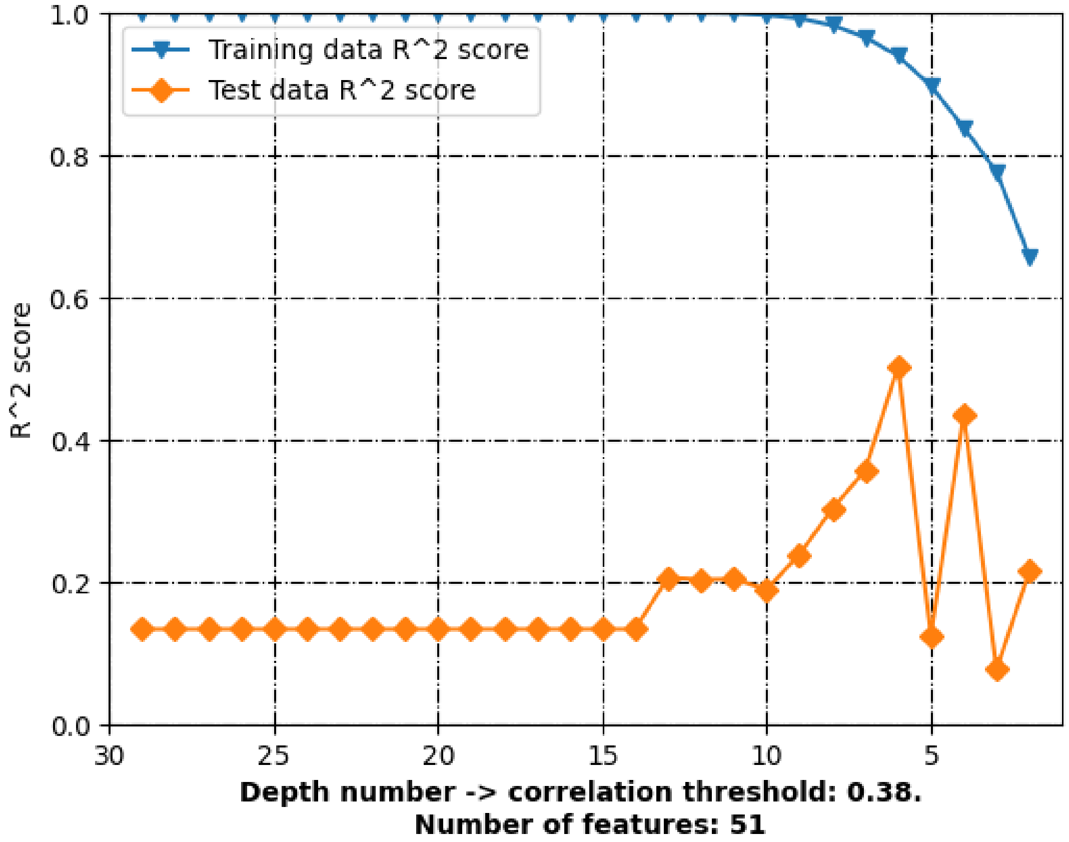
Training data R^2 score
Test data R^2 score
R^2 score
1.0
0.8
0.6
0.4
0.2
0.0
30
25
20
15
10
5
Depth number -> correlation threshold: 0.38.
Number of features: 51

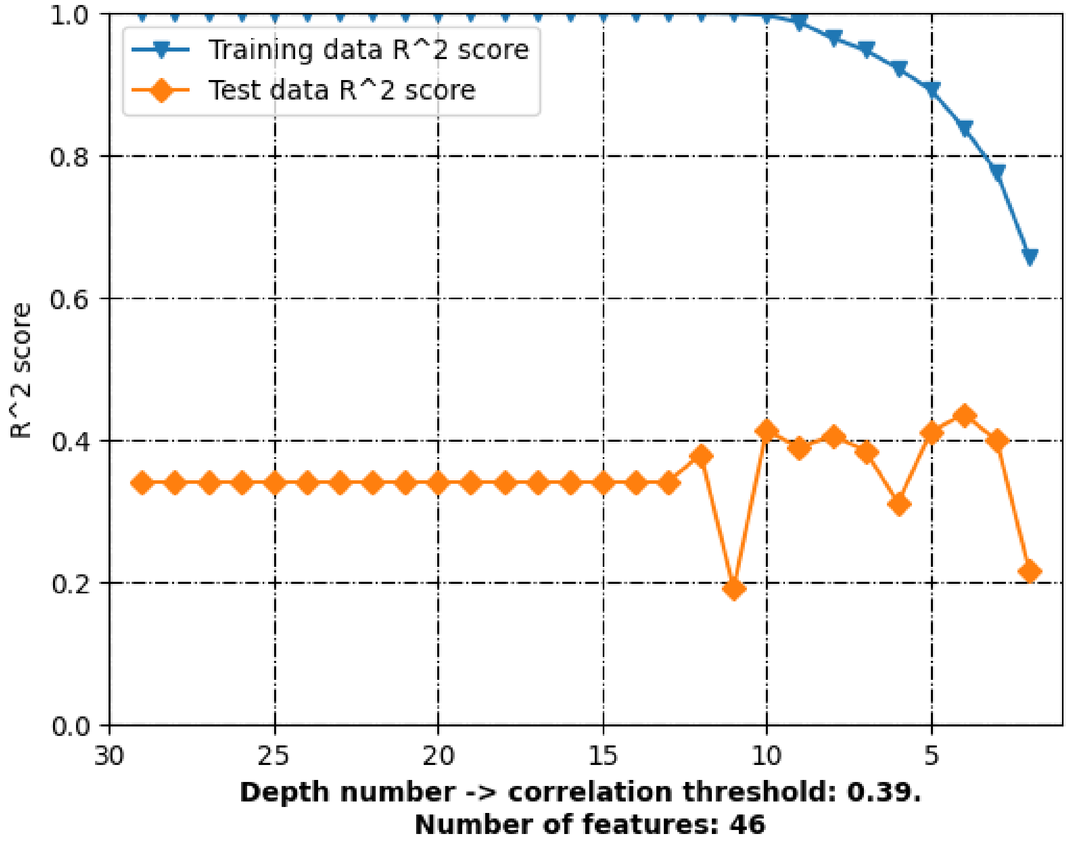

Training data R^2 score
Test data R^2 score
R^2 score
1.0
0.8
0.6
0.4
0.2
0.0
30
25
20
15
10
5
Depth number -> correlation threshold: 0.39.
Number of features: 46

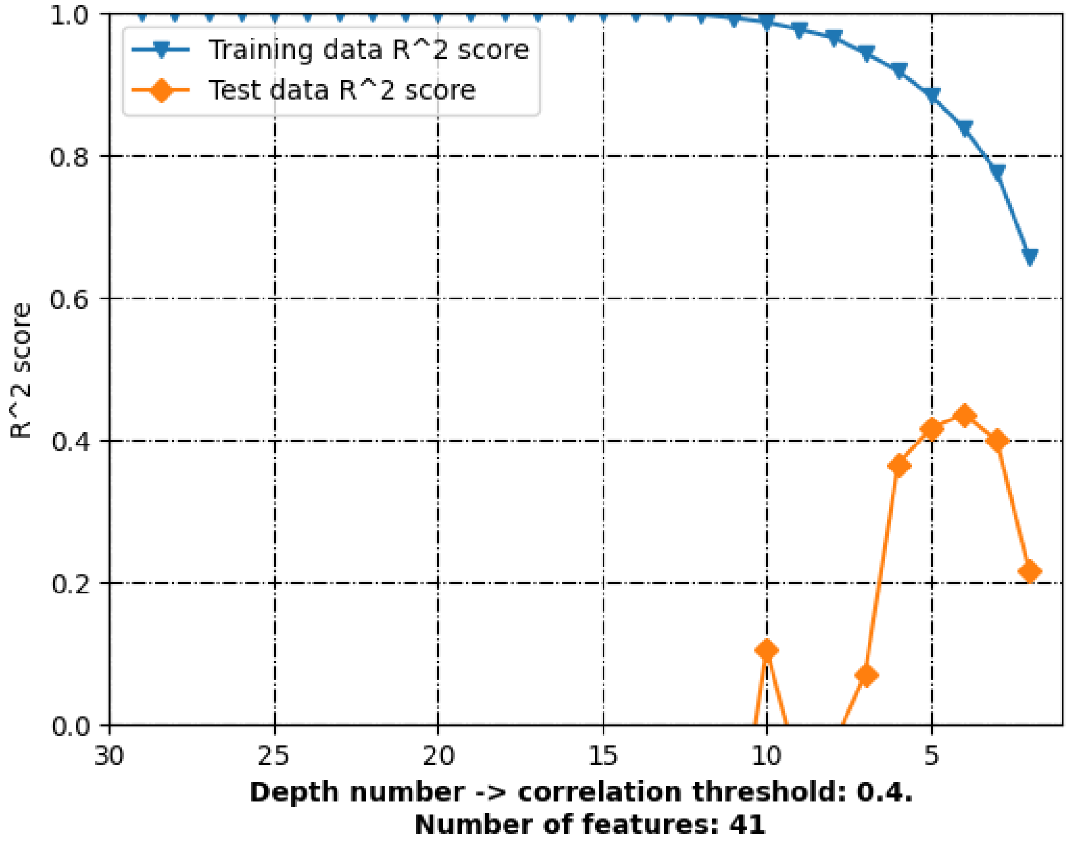
Training data R^2 score
Test data R^2 score
R^2 score
1.0
0.8
0.6
0.4
0.2
0.0
30
25
20
15
10
5
Depth number -> correlation threshold: 0.4.
Number of features: 41

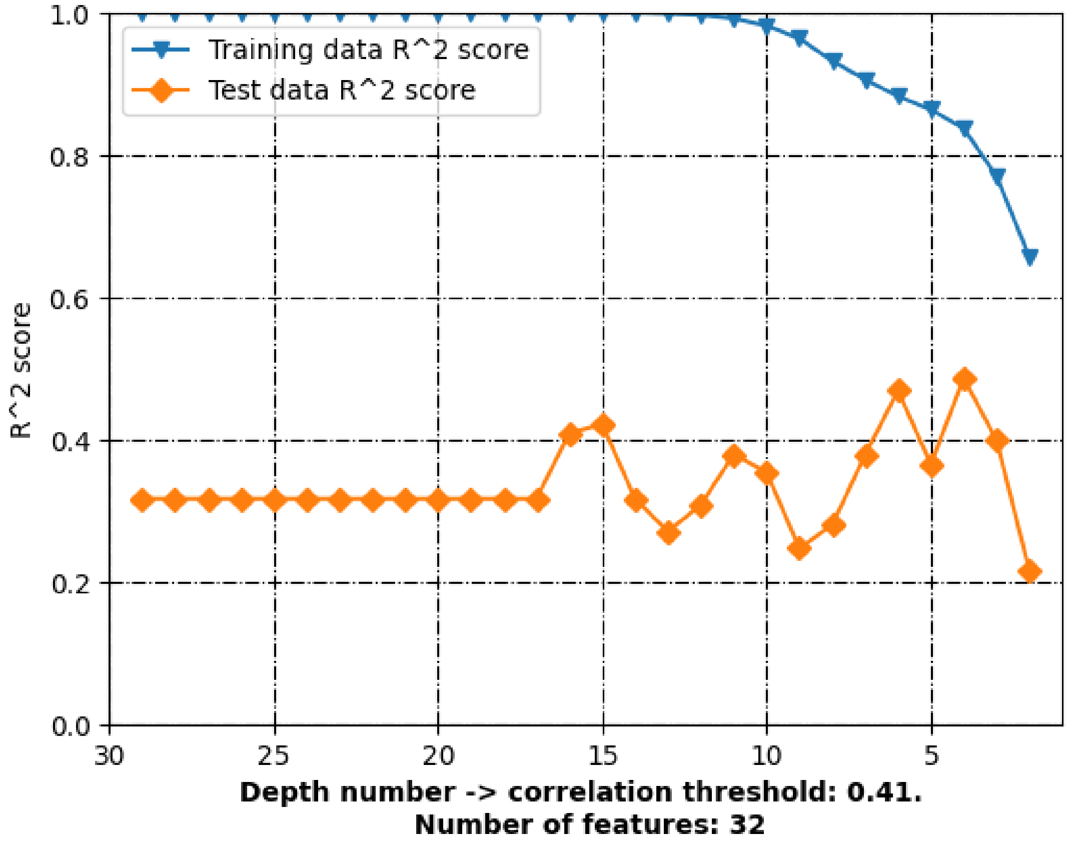

Training data R^2 score
Test data R^2 score
R^2 score
1.0
0.8
0.6
0.4
0.2
0.0
30
25
20
15
10
5
Depth number -> correlation threshold: 0.41.
Number of features: 32

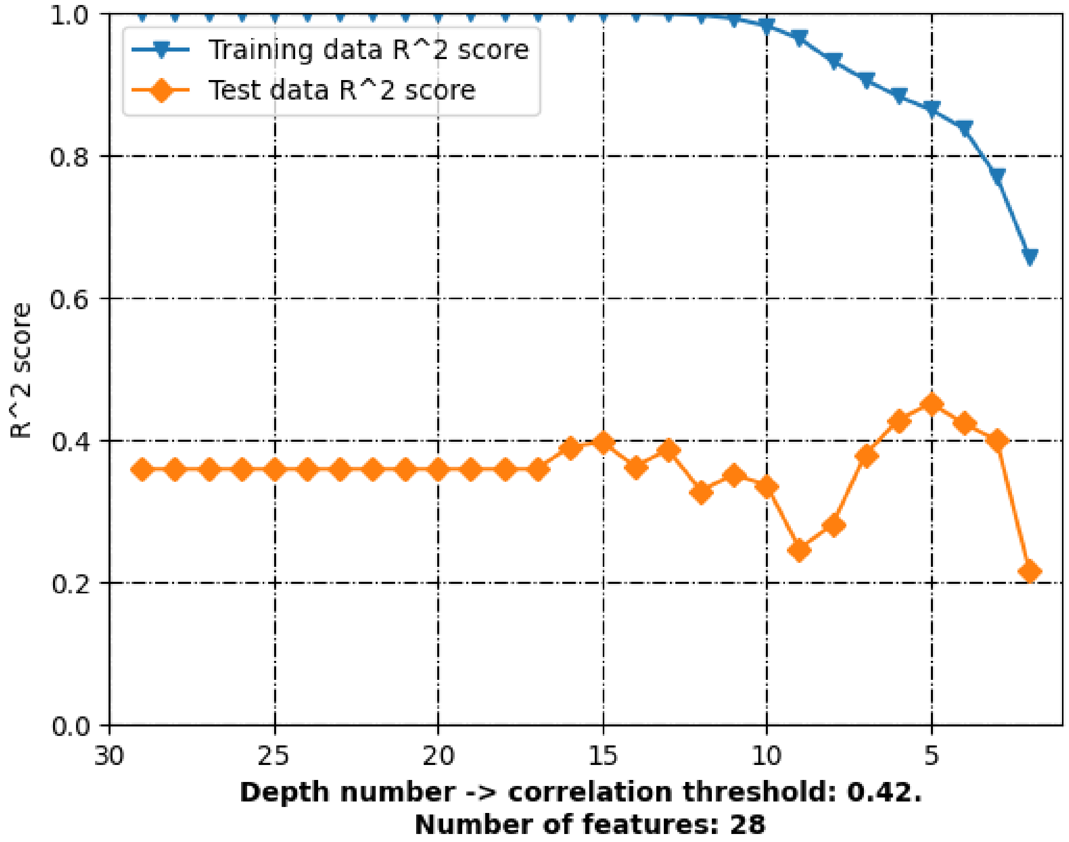
Training data R^2 score
Test data R^2 score
R^2 score
1.0
0.8
0.6
0.4
0.2
0.0
30
25
20
15
10
5
Depth number -> correlation threshold: 0.42.
Number of features: 28

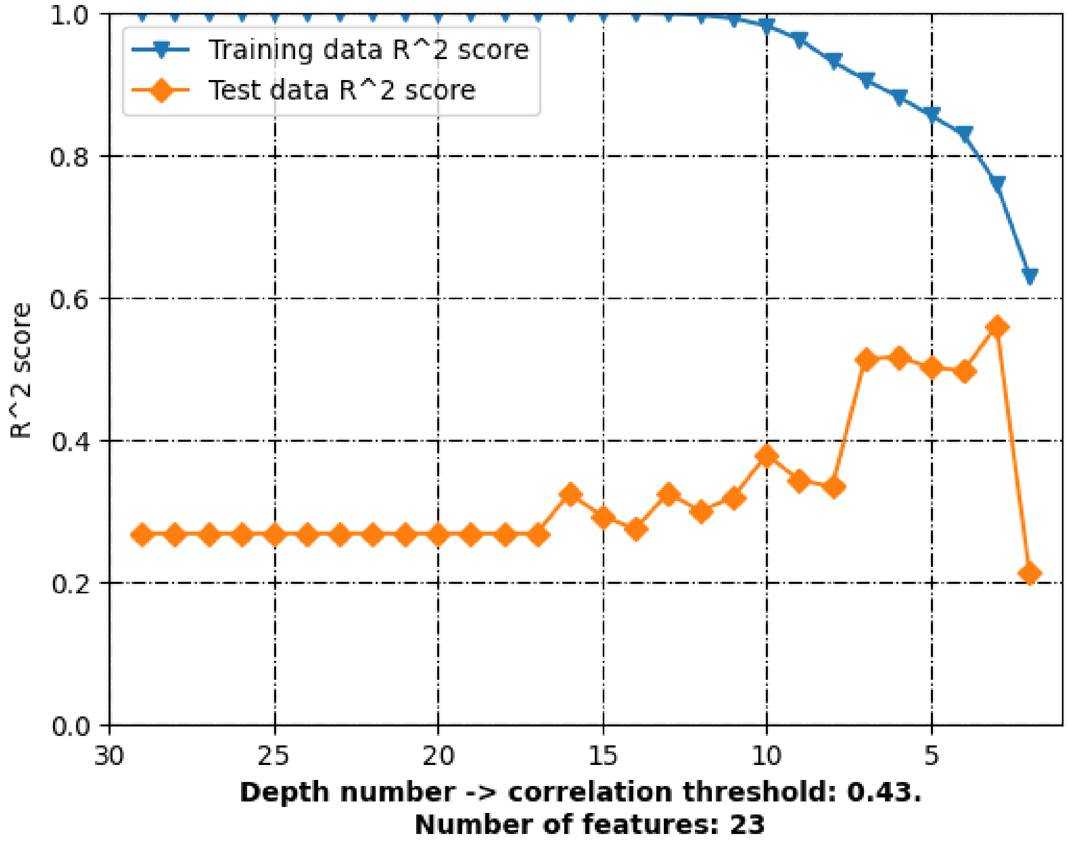

Training data R^2 score
Test data R^2 score
R^2 score
1.0
0.8
0.6
0.4
0.2
0.0
30
25
20
15
10
5
Depth number -> correlation threshold: 0.43.
Number of features: 23

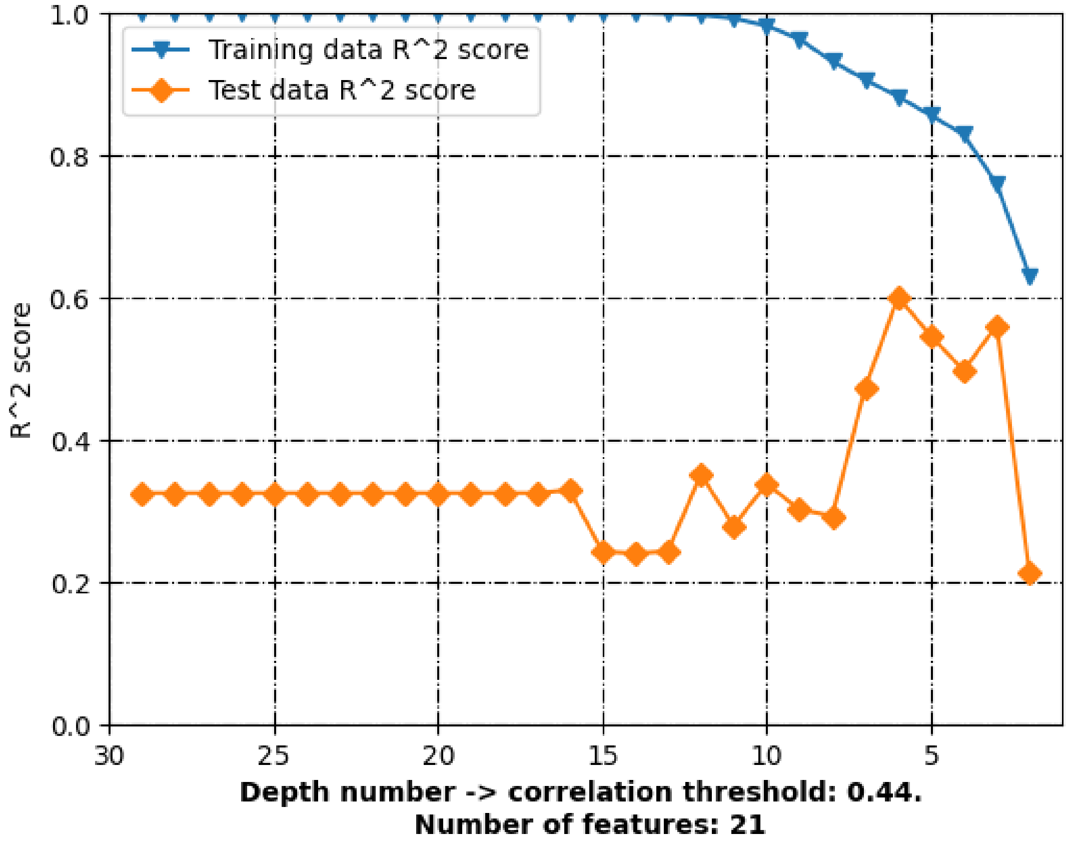
Training data R^2 score
Test data R^2 score
R^2 score
1.0
0.8
0.6
0.4
0.2
0.0
30
25
20
15
10
5
Depth number -> correlation threshold: 0.44.
Number of features: 21

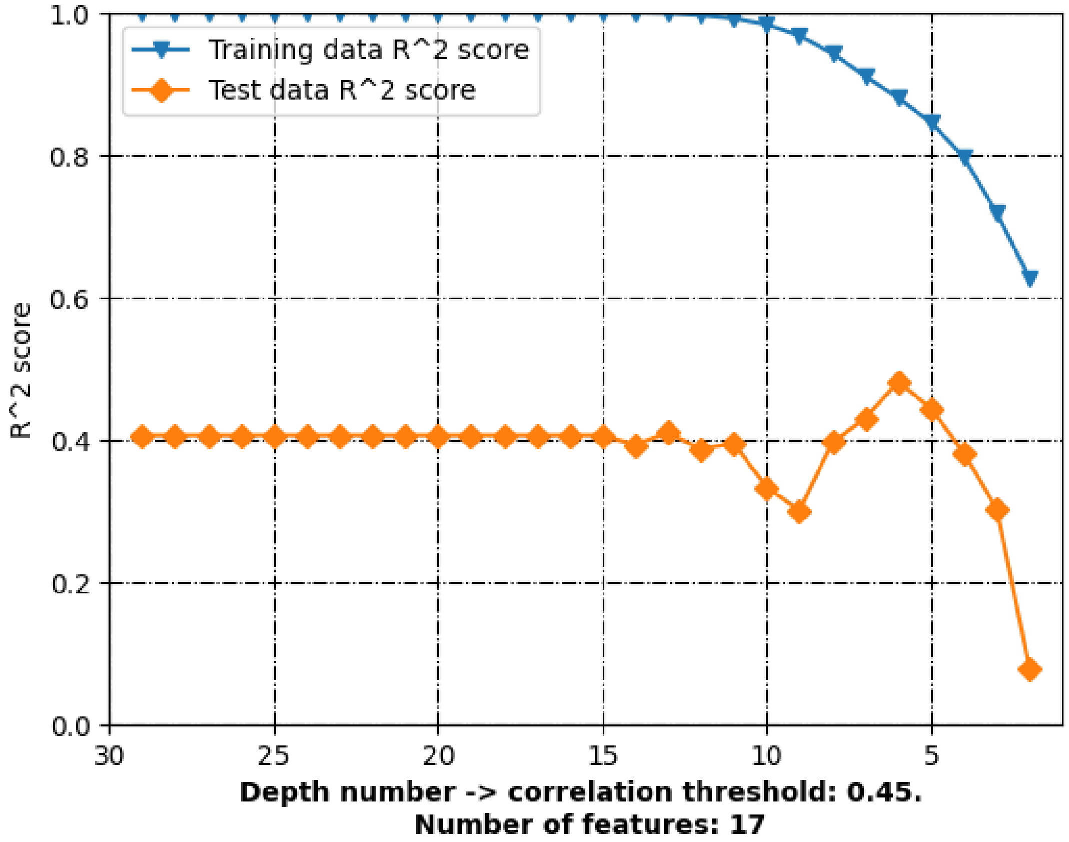

1.0
0.8
0.6
0.4
0.2
0.0
R^2 score
Training data R^2 score
Test data R^2 score
30
25
20
15
10
5
Depth number -> correlation threshold: 0.45.
Number of features: 17

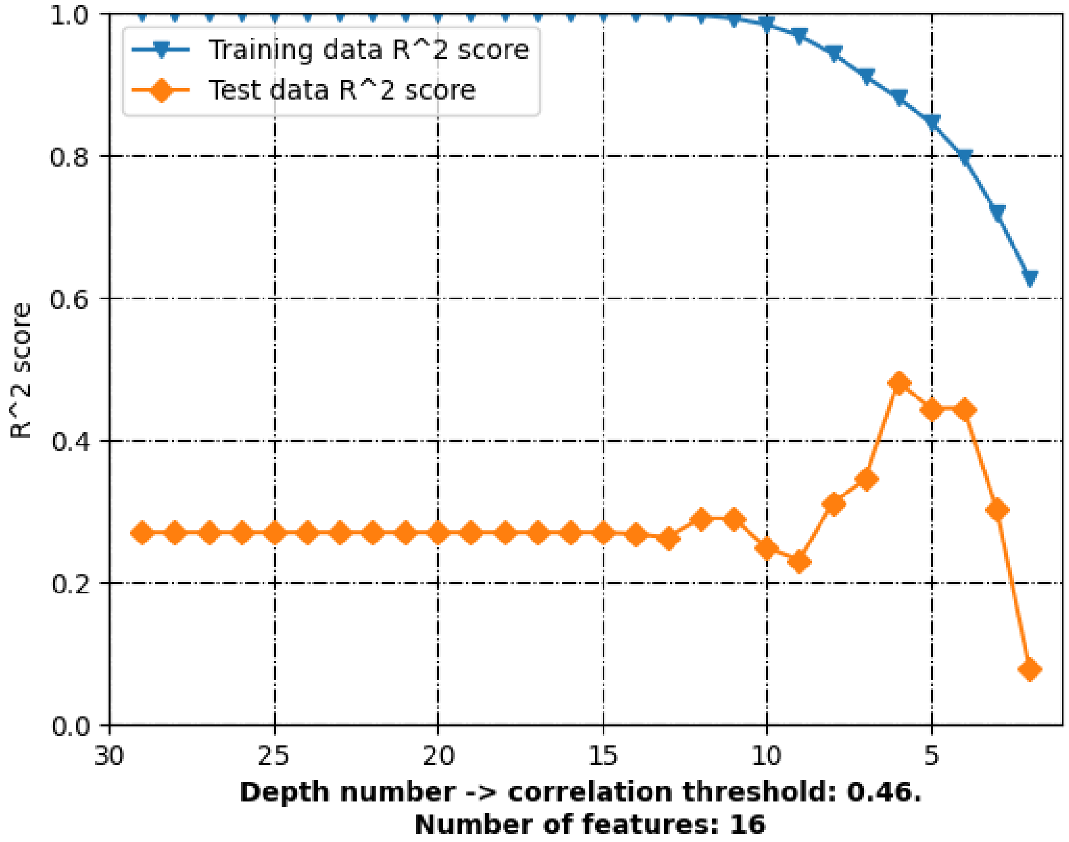
Training data R^2 score
Test data R^2 score
R^2 score
1.0
0.8
0.6
0.4
0.2
0.0
30
25
20
15
10
5
Depth number -> correlation threshold: 0.46.
Number of features: 16

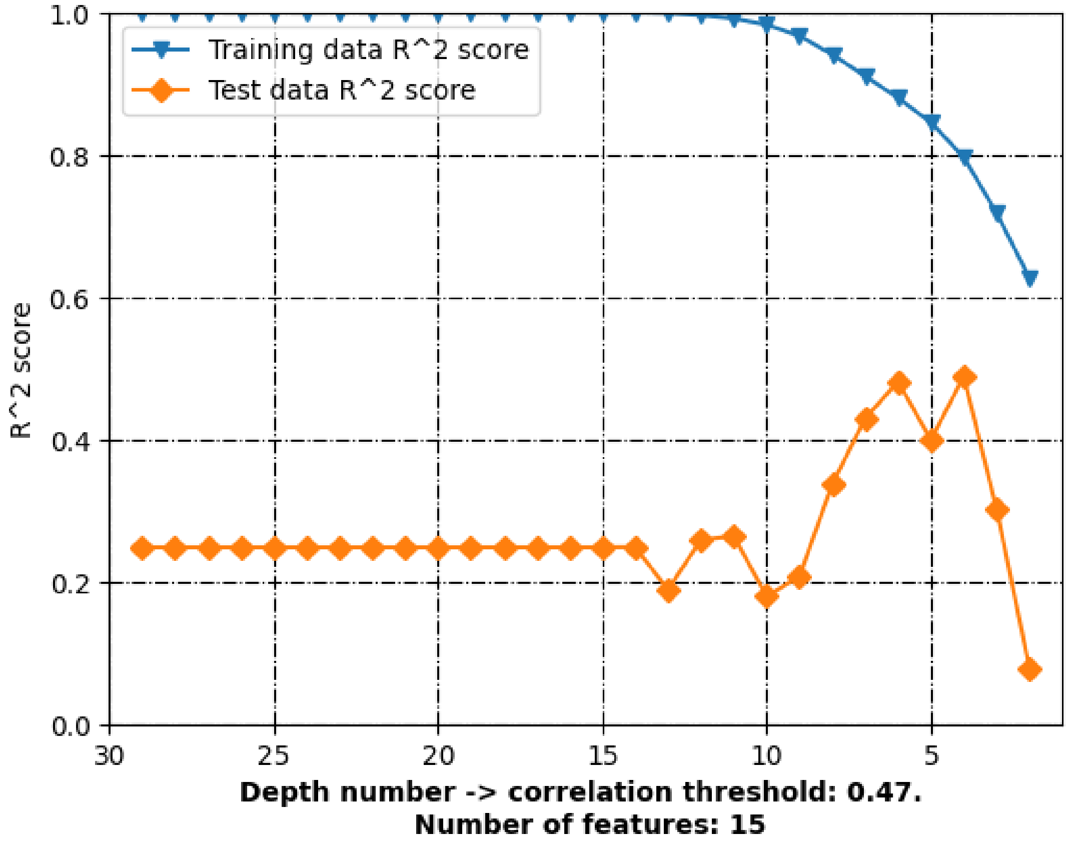
1.0
0.8
0.6
0.4
0.2
0.0
R^2 score
Training data R^2 score
Test data R^2 score
30
25
20
15
10
5
Depth number -> correlation threshold: 0.47.
Number of features: 15

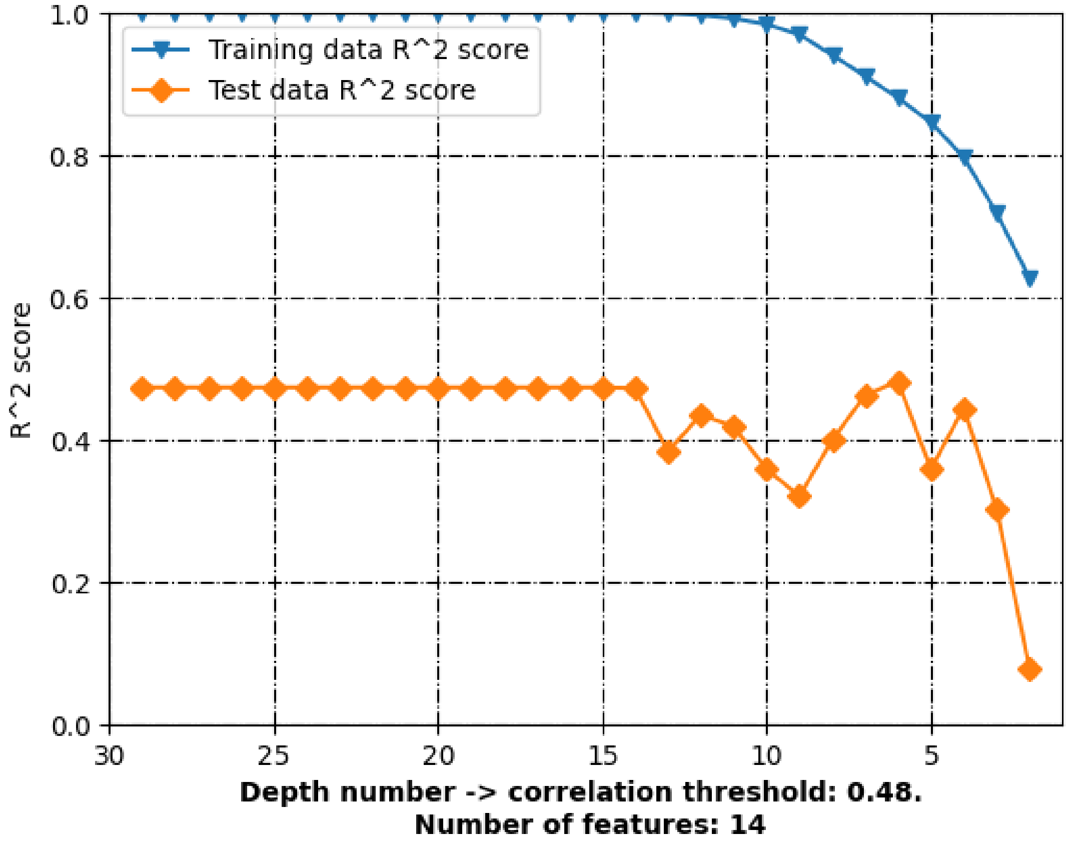

Training data R^2 score
Test data R^2 score
R^2 score
1.0
0.8
0.6
0.4
0.2
0.0
30
25
20
15
10
5
Depth number -> correlation threshold: 0.48.
Number of features: 14

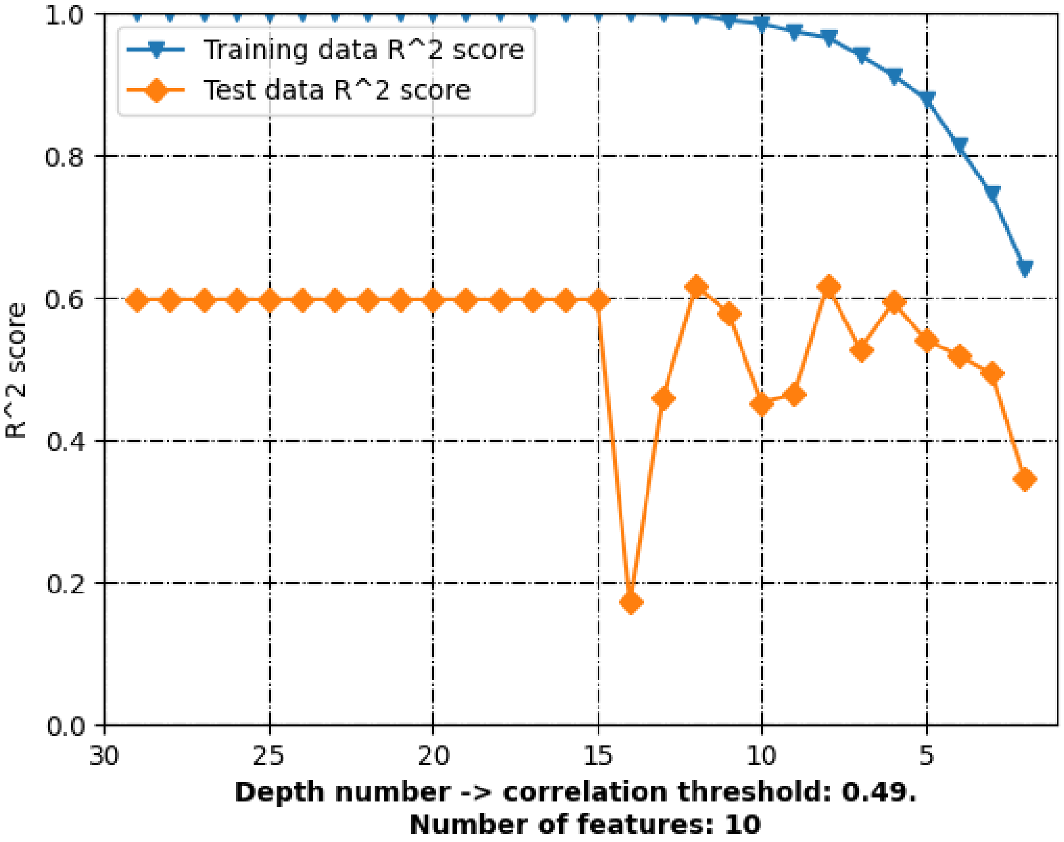
Training data R^2 score
Test data R^2 score
R^2 score
1.0
0.8
0.6
0.4
0.2
0.0
30
25
20
15
10
5
Depth number -> correlation threshold: 0.49.
Number of features: 10

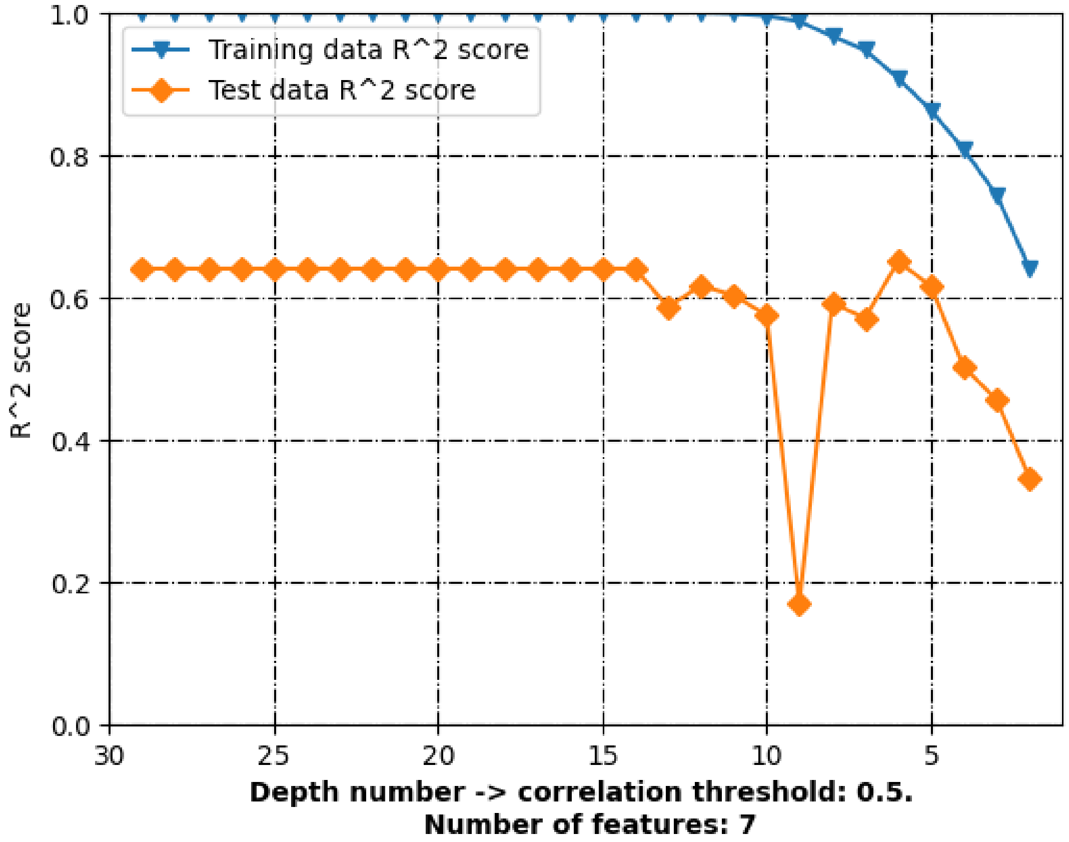
Training data R^2 score
Test data R^2 score
R^2 score
1.0
0.8
0.6
0.4
0.2
0.0
30
25
20
15
10
5
Depth number -> correlation threshold: 0.5.
Number of features: 7

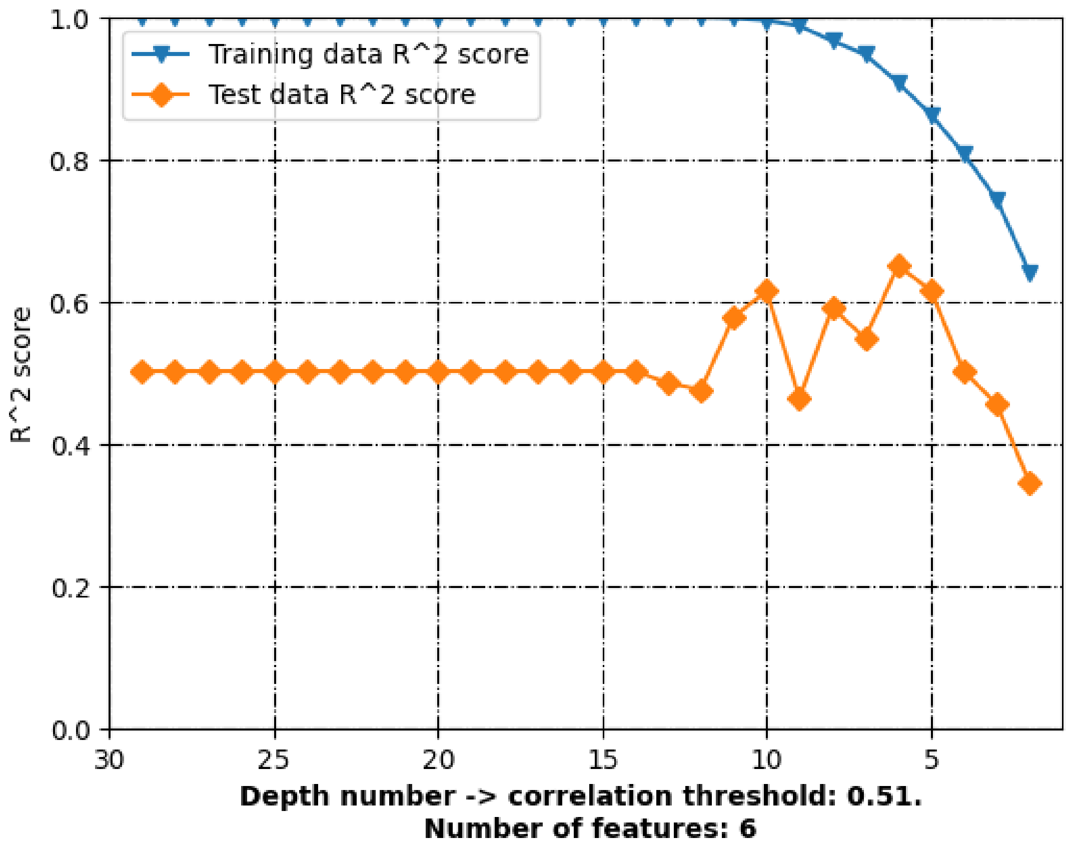
Training data R^2 score
Test data R^2 score
R^2 score
1.0
0.8
0.6
0.4
0.2
0.0
30
25
20
15
10
5
Depth number -> correlation threshold: 0.51.
Number of features: 6

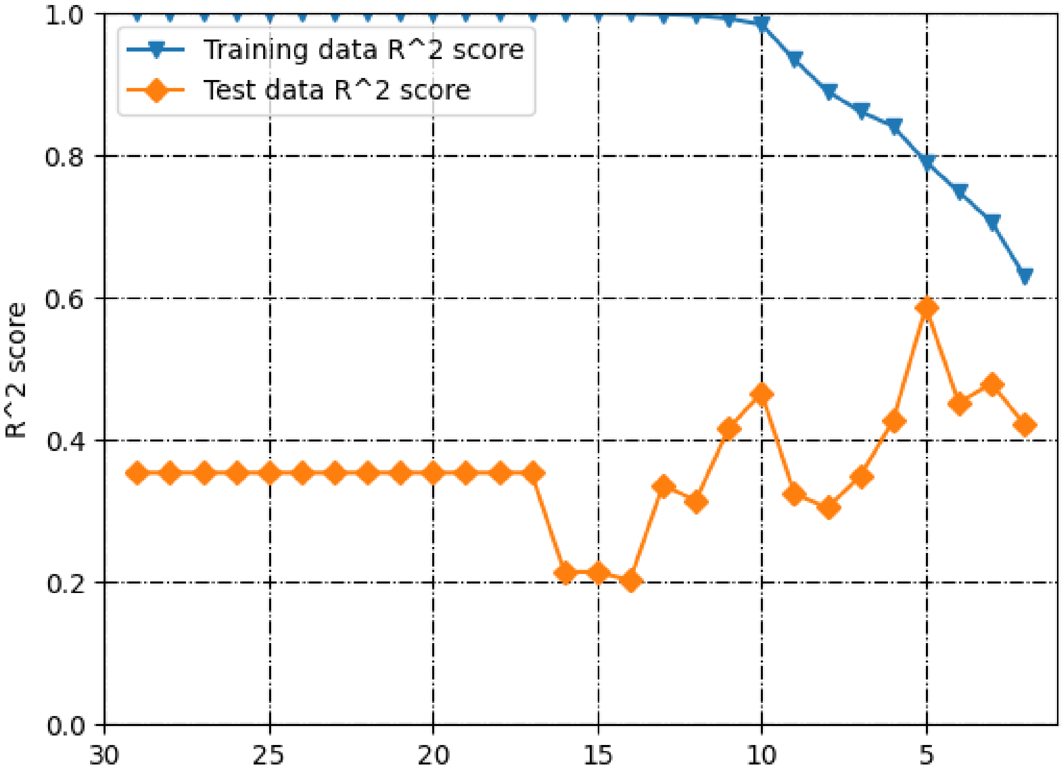

Depth number -> correlation threshold: 0.52.
Number of features: 5

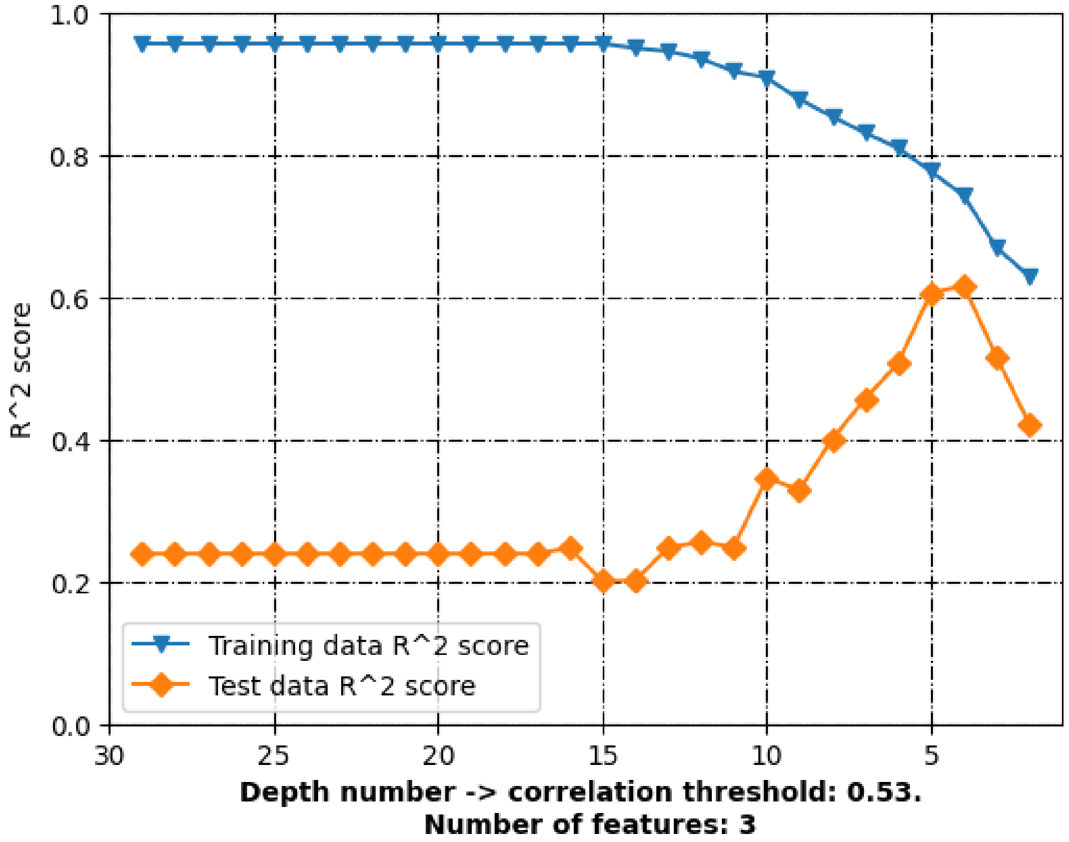

1.0
0.8
0.6
0.4
0.2
0.0
R^2 score
30
25
20
15
10
5
Training data R^2 score
Test data R^2 score
Depth number -> correlation threshold: 0.53.
Number of features: 3

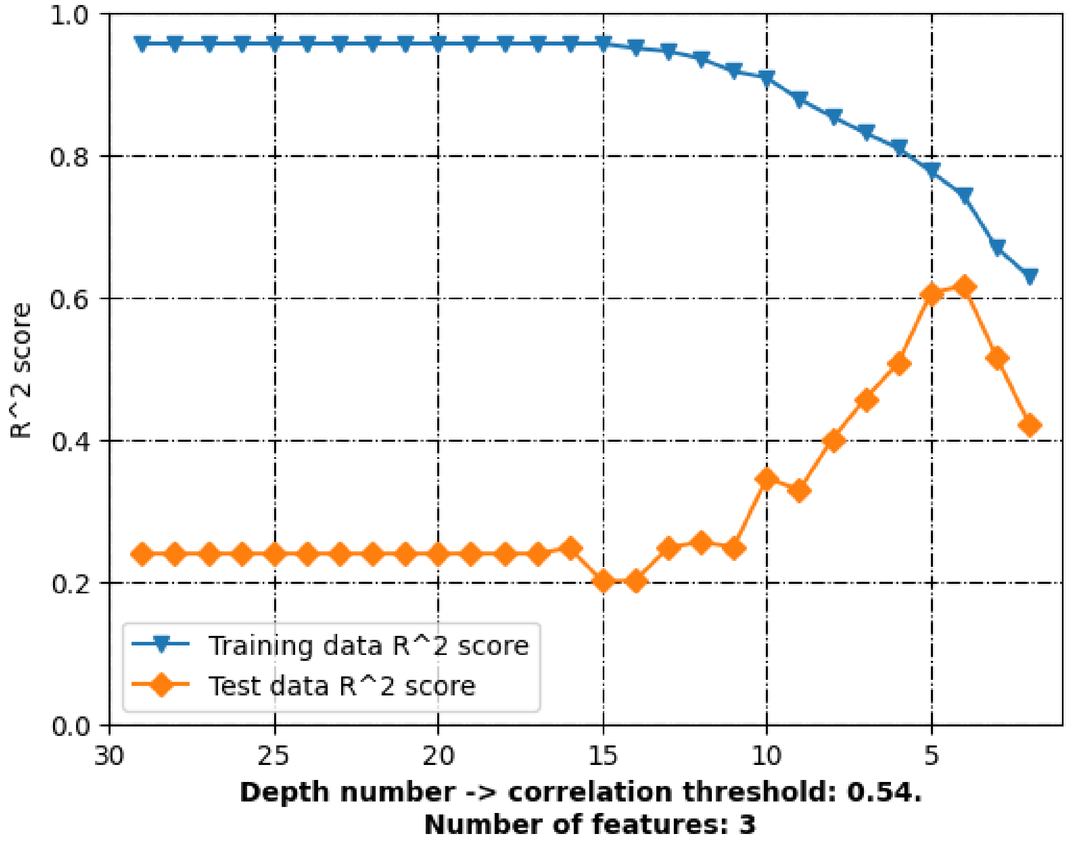
1.0
0.8
0.6
0.4
0.2
0.0
R^2 score
Training data R^2 score
Test data R^2 score
30
25
20
15
10
5
Depth number -> correlation threshold: 0.54.
Number of features: 3

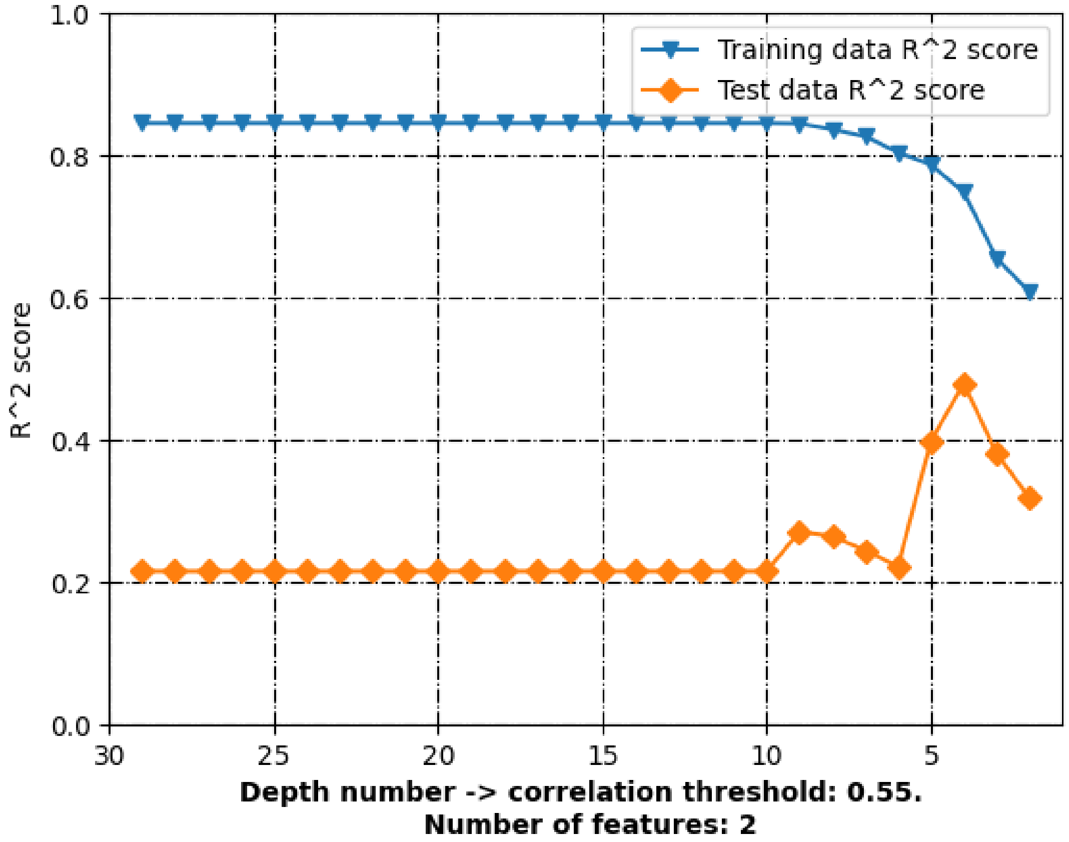

Training data R^2 score
Test data R^2 score
R^2 score
1.0
0.8
0.6
0.4
0.2
0.0
30
25
20
15
10
5
Depth number -> correlation threshold: 0.55.
Number of features: 2

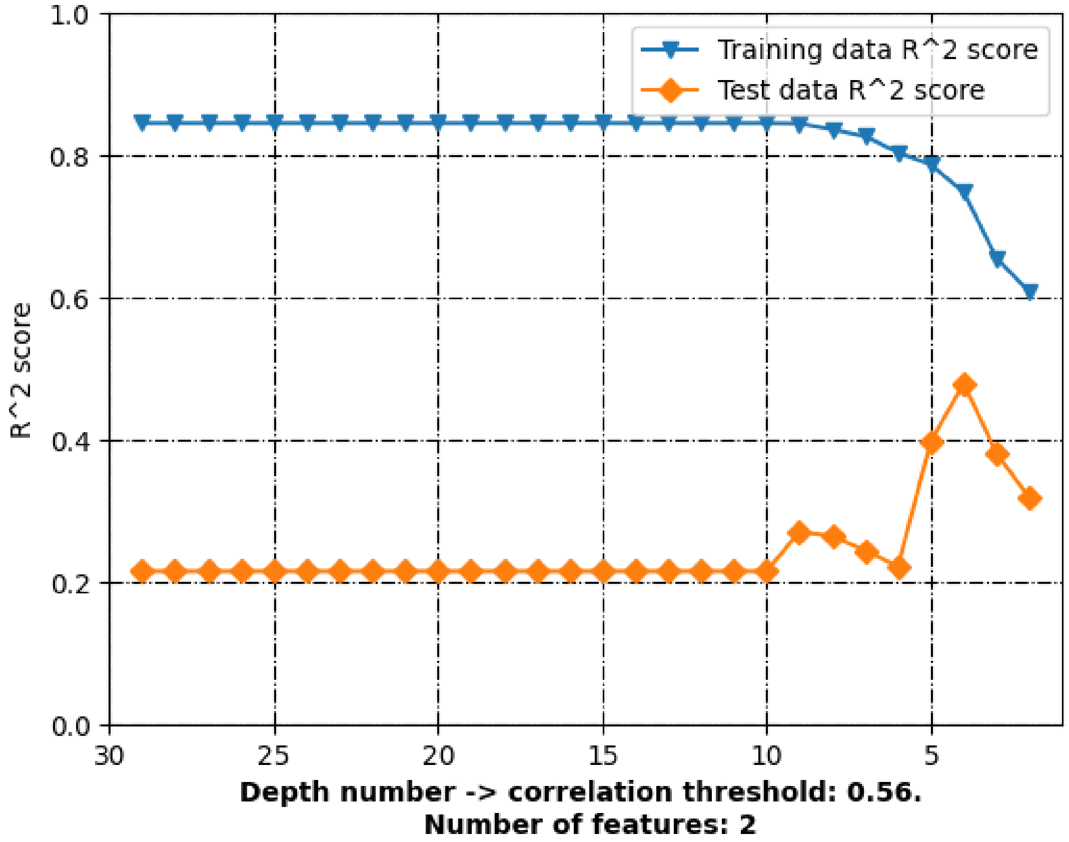

[20]:
```
plt.plot(df_without_standardization['Correlation threshold'],␣
 ↪df_without_standardization['Number of features'], label = "Number of␣
 ↪features", marker='.')
plt.legend()
plt.xlabel('Correlation threshold')
plt.ylabel('Number of features')
plt.show()
```

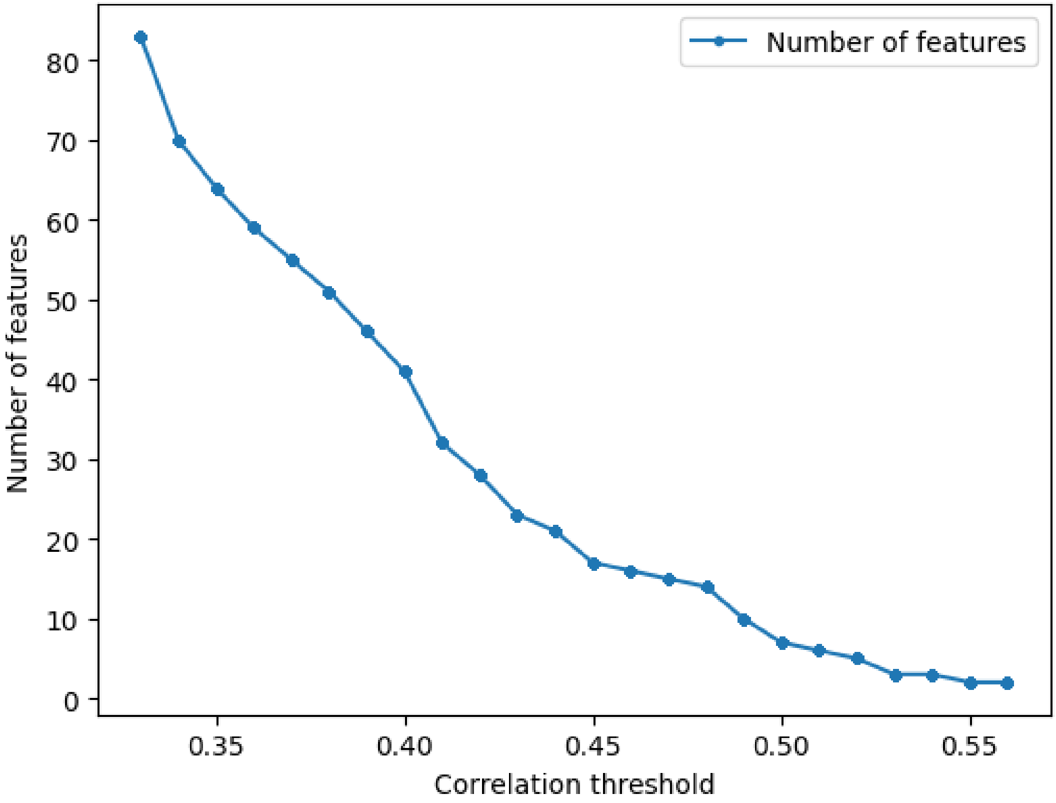

[ ]:

```

```

3 Random Forest

[21]:

```
without_standardization, train_r2_, test_r2_, hist1, hist2, target_column_name,␣
↪training_data_RMSE, test_data_RMSE  = pred_model.
↪prepare_data_and_create_model(molecular_descriptors_df=data,
                                                                                   ␣
↪                    correlation_threshold=0.50,
                                                                                   ␣
↪                    standardization=False,
                                                                                   ␣
↪                    model_type='RandomForestRegressor',
                                                                                   ␣
↪                    n_estimators_=12,
                                                                                   ␣
↪                    target_column_name = target,
                                                                                   ␣
↪                    random_state=random_state,
```

```
↪                    train_test_split_=True,
↪                    verbose=True)
```

```
I am not doing standardization…
  molecular descriptor name
0                    AATS0Z
1                  AATS0are
2                    AATS0d
3                   AATS0dv
4                    AATS0i
  molecular descriptor name  corr_value
0                    AATS0Z   -0.020817
1                  AATS0are   -0.148257
2                    AATS0d    0.022999
3                   AATS0dv   -0.137980
4                    AATS0i    0.133257
  molecular descriptor name  corr_value  absolute correlation value
0                    AATS0Z   -0.020817                    0.020817
1                  AATS0are   -0.148257                    0.148257
2                    AATS0d    0.022999                    0.022999
3                   AATS0dv   -0.137980                    0.137980
4                    AATS0i    0.133257                    0.133257
    molecular descriptor name  corr_value  absolute correlation value
226                    AMID_O   -0.546843                    0.546843
505               EState_VSA5   -0.588780                    0.588780
556                    GATS2c    0.511671                    0.511671
791                   MDEO-12   -0.582747                    0.582747
847                       NdO   -0.500462                    0.500462
    molecular descriptor name  corr_value  absolute correlation value
226                    AMID_O   -0.546843                    0.546843
505               EState_VSA5   -0.588780                    0.588780
556                    GATS2c    0.511671                    0.511671
791                   MDEO-12   -0.582747                    0.582747
847                       NdO   -0.500462                    0.500462
The model used is: RandomForest…
Return the coefficient of determination of the prediction:
0.6498034287135868
R^2 score: 0.9438562424636918
Correlation coefficient: 0.9715226412511917
Test data - unseen during training:
R^2 score: 0.6498034287135868
Correlation coefficient: 0.8061038572749709
[7.65944244 6.0454258  7.54703935 6.52903852 7.40701747 7.62433482
 7.89376655 7.2003814  7.00684143 7.77081841 8.07389022 7.8567583
 7.63415031 7.91175742 7.62433482 5.83522001 7.14831714 6.6258132 ]
113    7.744727
```

```
35     6.149967
101    6.958607
36     6.346787
100    7.000000
13     7.966576
0      7.991400
114    7.443697
104    7.823909
96     7.677781
40     7.795880
103    7.853872
48     7.744727
39     8.221849
14     6.414314
117    5.886057
21     7.298432
9      5.971266
Name: BALB/3T3, dtype: float64
Training Root Mean Square Error: 0.2123078991104117
Testing Root Mean Square Error: 0.4427713335651045
```

[22]:

```
print(target_column_name+str('_transformed'))
print(hist1[target_column_name].hist())
```

```
BALB/3T3_transformed
AxesSubplot(0.125,0.11;0.775x0.77)
```

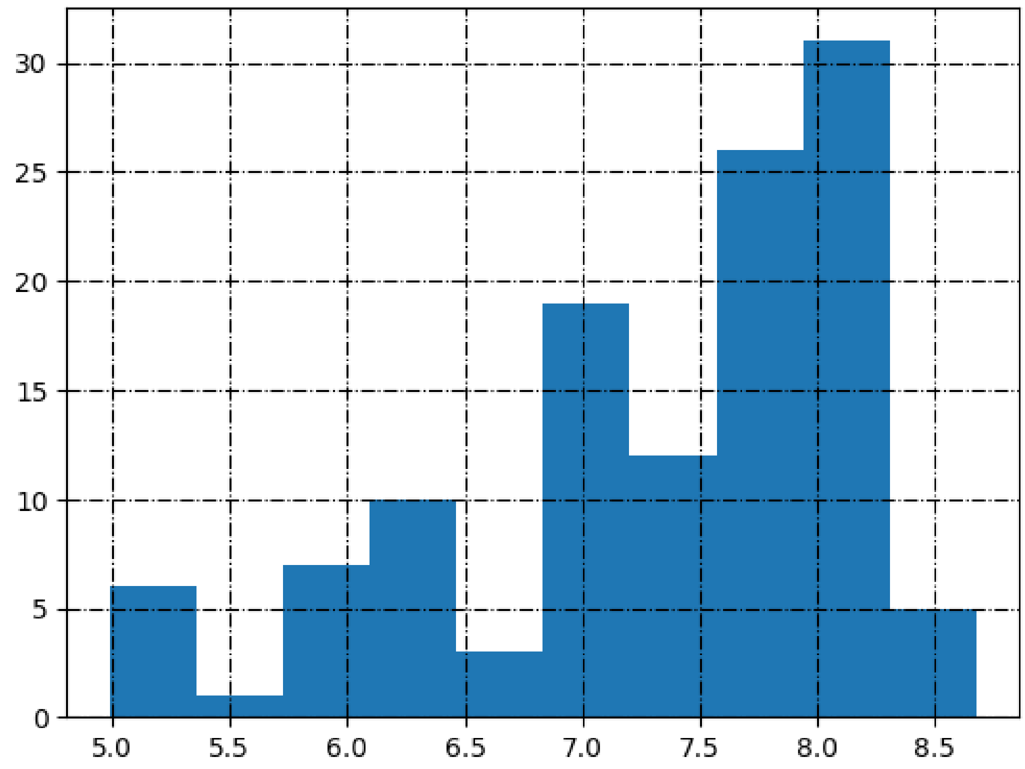

[23]:
```
with_standardization, train_r2_, test_r2_, hist1, hist2, target_column_name,␣
↪training_data_RMSE, test_data_RMSE  = pred_model.
↪prepare_data_and_create_model(molecular_descriptors_df=data,
                                                                        ␣
↪                        correlation_threshold=0.50,
                                                                        ␣
↪                        standardization=True,
                                                                        ␣
↪                        model_type='RandomForestRegressor',
                                                                        ␣
↪                        n_estimators_=12,
                                                                        ␣
↪                        target_column_name = target,
                                                                        ␣
↪                        random_state=random_state,
                                                                        ␣
↪                        train_test_split_=True,
                                                                        ␣
↪                        verbose=True)
```

```
I am doing standardization…
  molecular descriptor name
```

```
0                    AATS0Z
1                  AATS0are
2                    AATS0d
3                   AATS0dv
4                    AATS0i
  molecular descriptor name  corr_value
0                    AATS0Z   -0.020817
1                  AATS0are   -0.148257
2                    AATS0d    0.022999
3                   AATS0dv   -0.137980
4                    AATS0i    0.133257
  molecular descriptor name  corr_value  absolute correlation value
0                    AATS0Z   -0.020817                    0.020817
1                  AATS0are   -0.148257                    0.148257
2                    AATS0d    0.022999                    0.022999
3                   AATS0dv   -0.137980                    0.137980
4                    AATS0i    0.133257                    0.133257
    molecular descriptor name  corr_value  absolute correlation value
226                    AMID_O   -0.546843                    0.546843
505                EState_VSA5   -0.588780                    0.588780
556                    GATS2c    0.511671                    0.511671
791                   MDEO-12   -0.582747                    0.582747
847                       NdO   -0.500462                    0.500462
    molecular descriptor name  corr_value  absolute correlation value
226                    AMID_O   -0.546843                    0.546843
505                EState_VSA5   -0.588780                    0.588780
556                    GATS2c    0.511671                    0.511671
791                   MDEO-12   -0.582747                    0.582747
847                       NdO   -0.500462                    0.500462
The model used is: RandomForest…
Return the coefficient of determination of the prediction:
0.6498034287135868
R^2 score: 0.9438562424636918
Correlation coefficient: 0.9715226412511917
Test data - unseen during training:
R^2 score: 0.6498034287135868
Correlation coefficient: 0.8061038572749709
[7.65944244 6.0454258  7.54703935 6.52903852 7.40701747 7.62433482
 7.89376655 7.2003814  7.00684143 7.77081841 8.07389022 7.8567583
 7.63415031 7.91175742 7.62433482 5.83522001 7.14831714 6.6258132 ]
113    7.744727
35     6.149967
101    6.958607
36     6.346787
100    7.000000
13     7.966576
0      7.991400
114    7.443697
```

```
104    7.823909
96     7.677781
40     7.795880
103    7.853872
48     7.744727
39     8.221849
14     6.414314
117    5.886057
21     7.298432
9      5.971266
Name: BALB/3T3, dtype: float64
Training Root Mean Square Error: 0.2123078991104117
Testing Root Mean Square Error: 0.4427713335651045
```

3.1 Search inside correlation space

[24]:
```
step = 0.01
initial_step = corr_low
last_step = corr_high
first_list = [x / 100.0 for x in range(int(initial_step*100),␣
 ↪int(last_step*100), int(step*100))]
n_estimators = [range(2,21,1)]
corr_th = []
second_list = []
third_list = []
fourth_l = []
fifth_l = []
f_list = []
fif_list = []
for i in first_list:

    for estimator in n_estimators[0]:

        without_standardization, train_r2, test_r2, _, h_, target_column_name,␣
 ↪training_data_RMSE, test_data_RMSE = pred_model.
 ↪prepare_data_and_create_model(molecular_descriptors_df=data,
                                                                          ␣
 ↪                    correlation_threshold=i,
                                                                          ␣
 ↪                    standardization=False,
                                                                          ␣
 ↪                    model_type='RandomForestRegressor',
                                                                          ␣
 ↪                    n_estimators_=estimator,
                                                                          ␣
 ↪                    target_column_name = target,
```

```
                                                                                          ␣
↪                   random_state=random_state,
                                                                                          ␣
↪                   train_test_split_=True,
                                                                                          ␣
↪                   verbose=False)
        corr_th.append(i)
        second_list.append(train_r2)
        third_list.append(test_r2)
        fourth_l.append(training_data_RMSE)
        fifth_l.append(test_data_RMSE)
        f_list.append(len(h_))
        fif_list.append(estimator)
```

[25]:
```
df_without_standardization = pd.DataFrame(data=corr_th, columns=["Correlation␣
↪threshold"])
df_without_standardization['Training data R^2 score'] = second_list
df_without_standardization['Test data R^2 score'] = third_list
df_without_standardization['Training RMSE'] = fourth_l
df_without_standardization['Test RMSE'] = fifth_l
df_without_standardization['Number of features'] = f_list
df_without_standardization['Number of estimators'] = fif_list
```

[26]:
```
df_random_forest = df_without_standardization.copy()
#df_without_standardization.to_excel('../Data/
↪A549_Random_forest_rs_'+str(random_state)+'.xlsx')
```

[27]:
```
corre_list = [x / 100.0 for x in range(int(initial_step*100),␣
↪int(last_step*100), int(0.01*100))]
for element in corre_list:
    element_ =␣
↪df_without_standardization[df_without_standardization['Correlation␣
↪threshold'] == float(element)]
    plt.plot(element_['Number of estimators'], element_['Training data R^2␣
↪score'], label = "Training data R^2 score", marker='v')
    plt.plot(element_['Number of estimators'], element_['Test data R^2 score'],␣
↪label = "Test data R^2 score", marker='D')
    plt.legend()
    plt.xlabel('Number of estimators -> correlation threshold: '+str(element)+'.
↪ \n Number of features: '+str(element_['Number of features'].iloc[0]))
    plt.xlim(max(element_['Number of estimators'])+1, min(element_['Number of␣
↪estimators'])-1)
    plt.ylabel('R^2 score')
    plt.ylim([0, 1])
    plt.rc('grid', linestyle="-.", color='black')
    plt.grid(True)
```

```
plt.show()
```

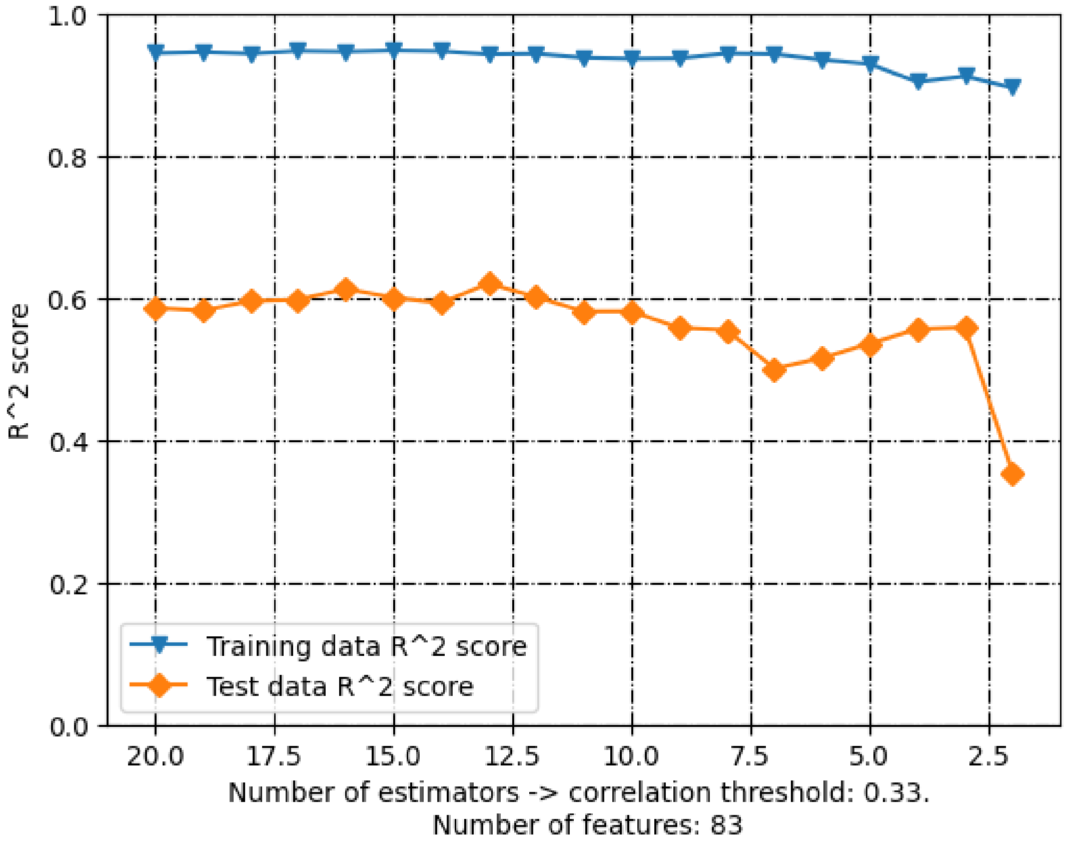

1.0
0.8
0.6
0.4
0.2
0.0
R^2 score
Training data R^2 score
Test data R^2 score
20.0
17.5
15.0
12.5
10.0
7.5
5.0
2.5
Number of estimators -> correlation threshold: 0.33.
Number of features: 83

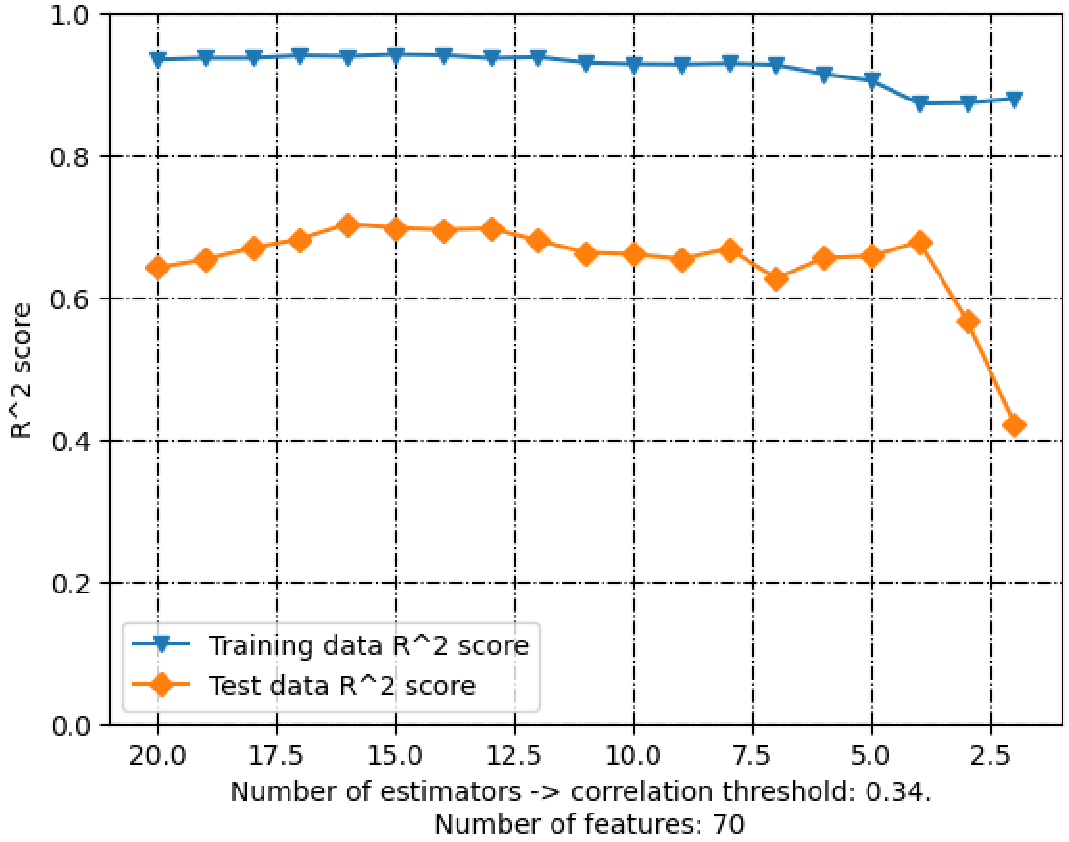
1.0
0.8
0.6
0.4
0.2
0.0
R^2 score
Training data R^2 score
Test data R^2 score
20.0
17.5
15.0
12.5
10.0
7.5
5.0
2.5
Number of estimators -> correlation threshold: 0.34.
Number of features: 70

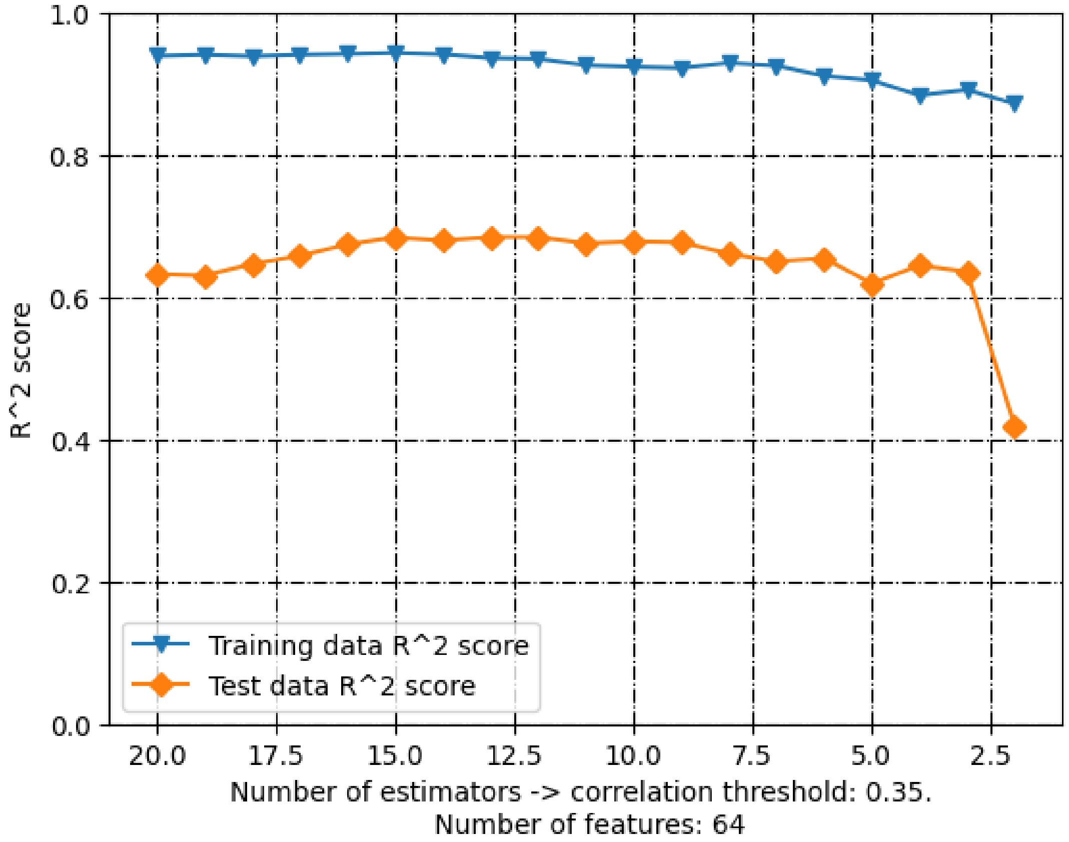
1.0
0.8
0.6
0.4
0.2
0.0
R^2 score
Training data R^2 score
Test data R^2 score
20.0
17.5
15.0
12.5
10.0
7.5
5.0
2.5
Number of estimators -> correlation threshold: 0.35.
Number of features: 64

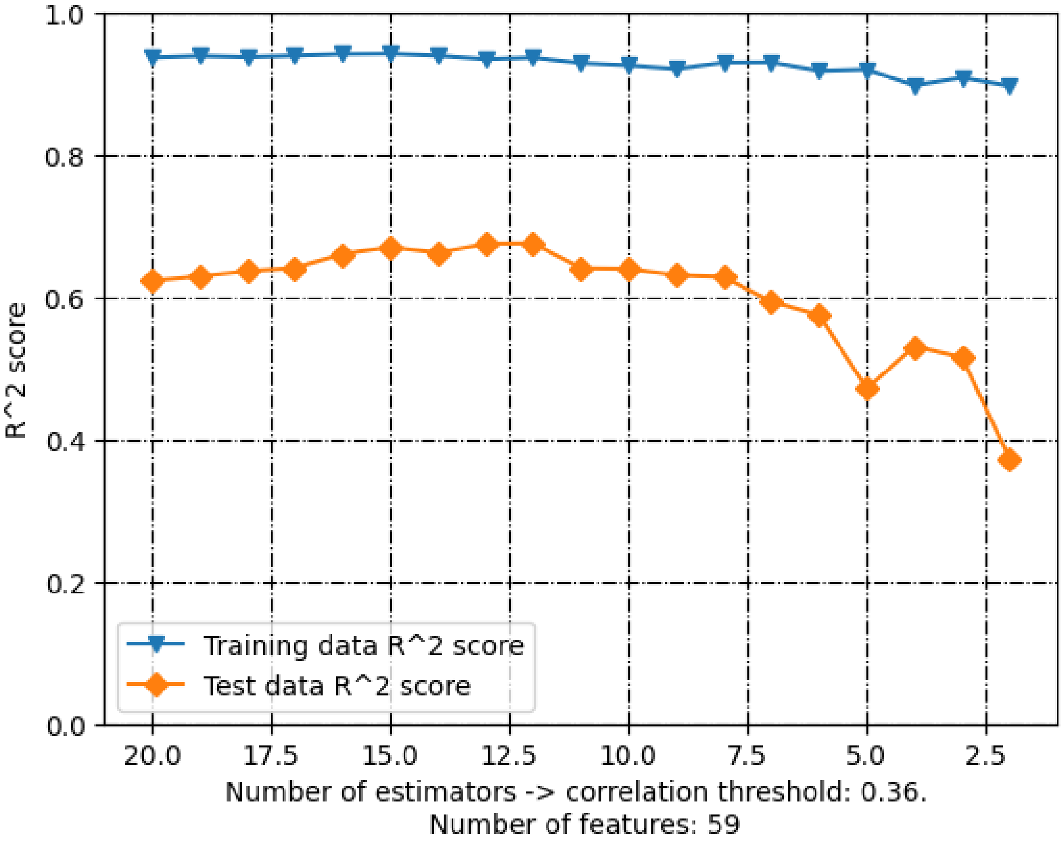
1.0
0.8
0.6
0.4
0.2
0.0
R^2 score
Training data R^2 score
Test data R^2 score
20.0
17.5
15.0
12.5
10.0
7.5
5.0
2.5
Number of estimators -> correlation threshold: 0.36.
Number of features: 59

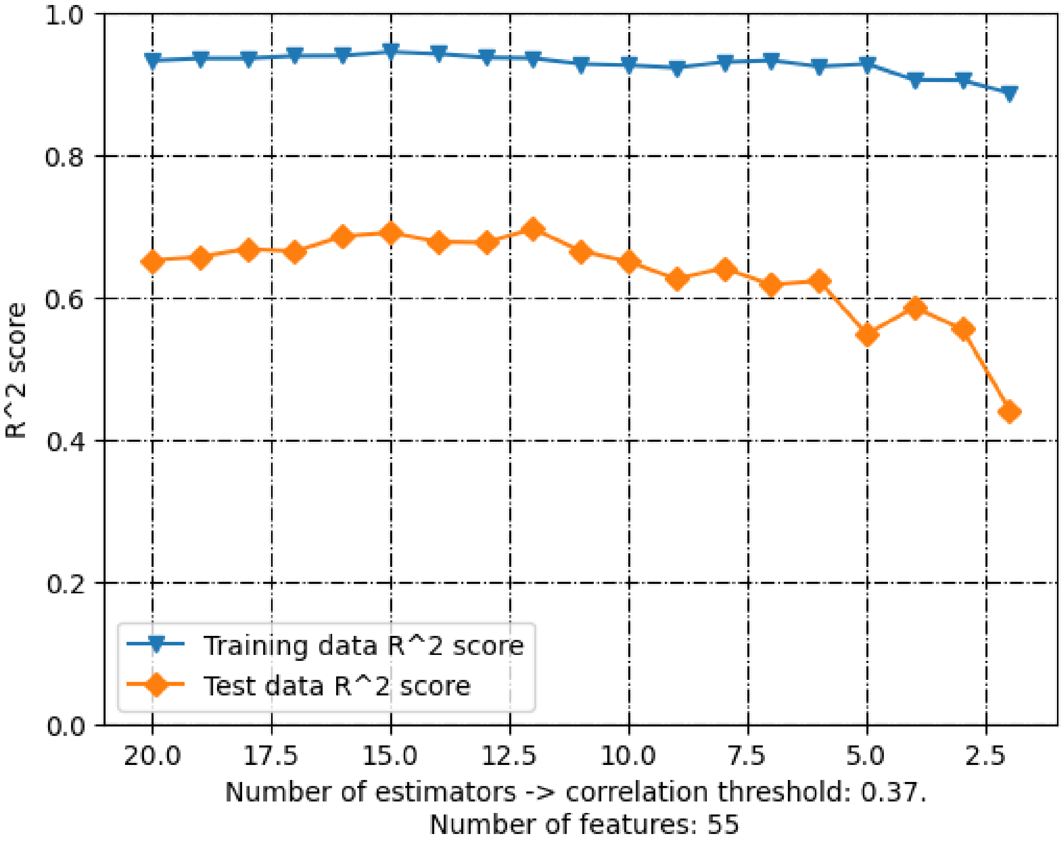

1.0
0.8
0.6
0.4
0.2
0.0
R^2 score
Training data R^2 score
Test data R^2 score
20.0
17.5
15.0
12.5
10.0
7.5
5.0
2.5
Number of estimators -> correlation threshold: 0.37.
Number of features: 55

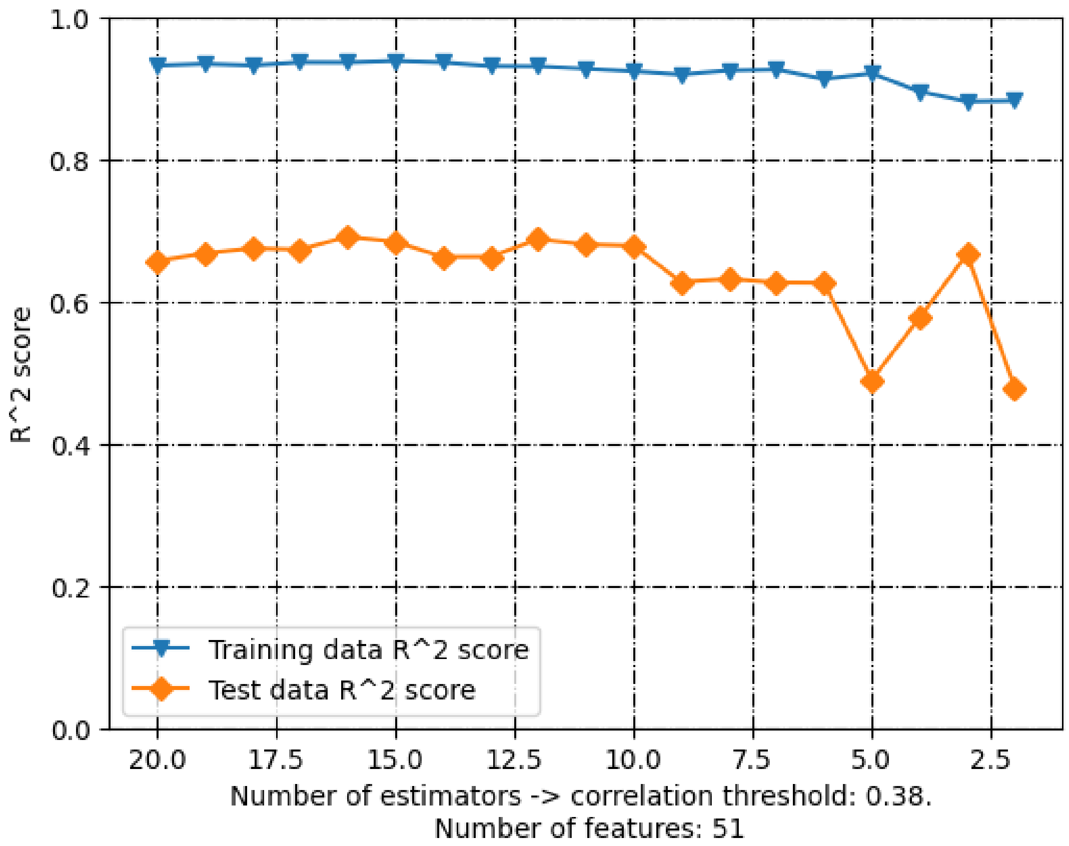
1.0
0.8
0.6
0.4
0.2
0.0
R^2 score
Training data R^2 score
Test data R^2 score
20.0
17.5
15.0
12.5
10.0
7.5
5.0
2.5
Number of estimators -> correlation threshold: 0.38.
Number of features: 51

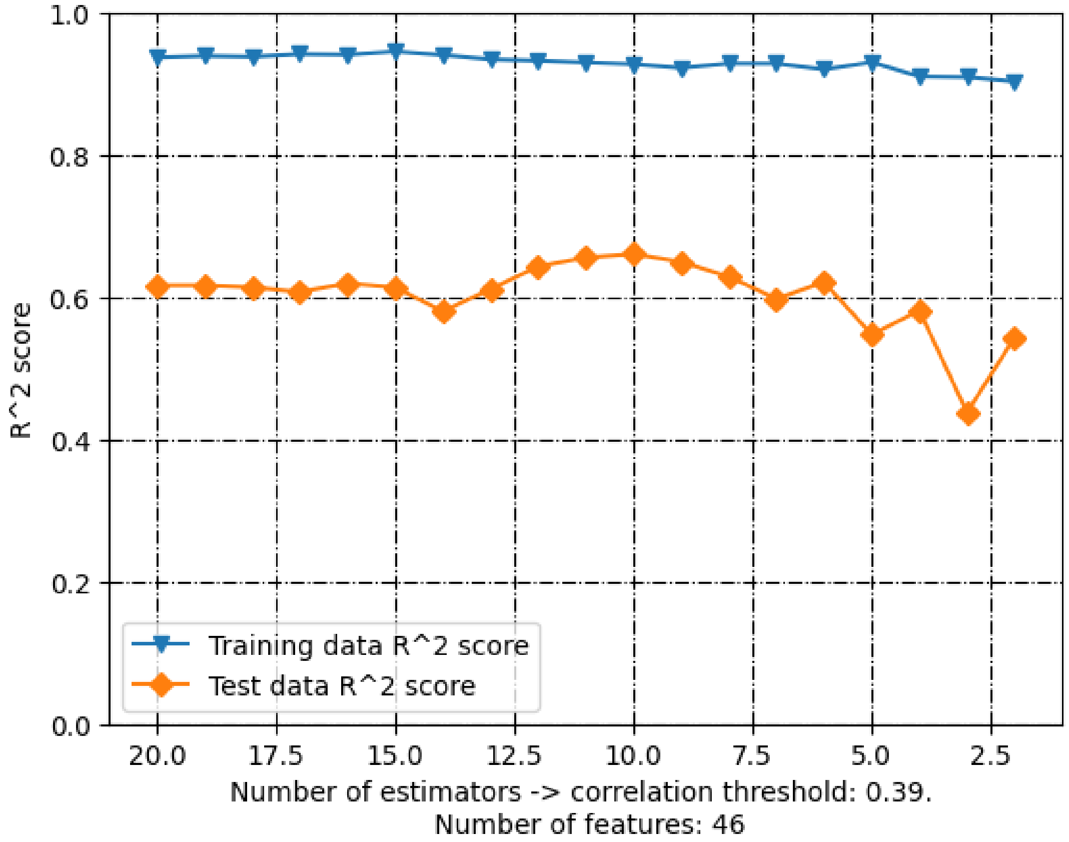

1.0
0.8
0.6
0.4
0.2
0.0
R^2 score
20.0
17.5
15.0
12.5
10.0
7.5
5.0
2.5
Training data R^2 score
Test data R^2 score
Number of estimators -> correlation threshold: 0.39.
Number of features: 46

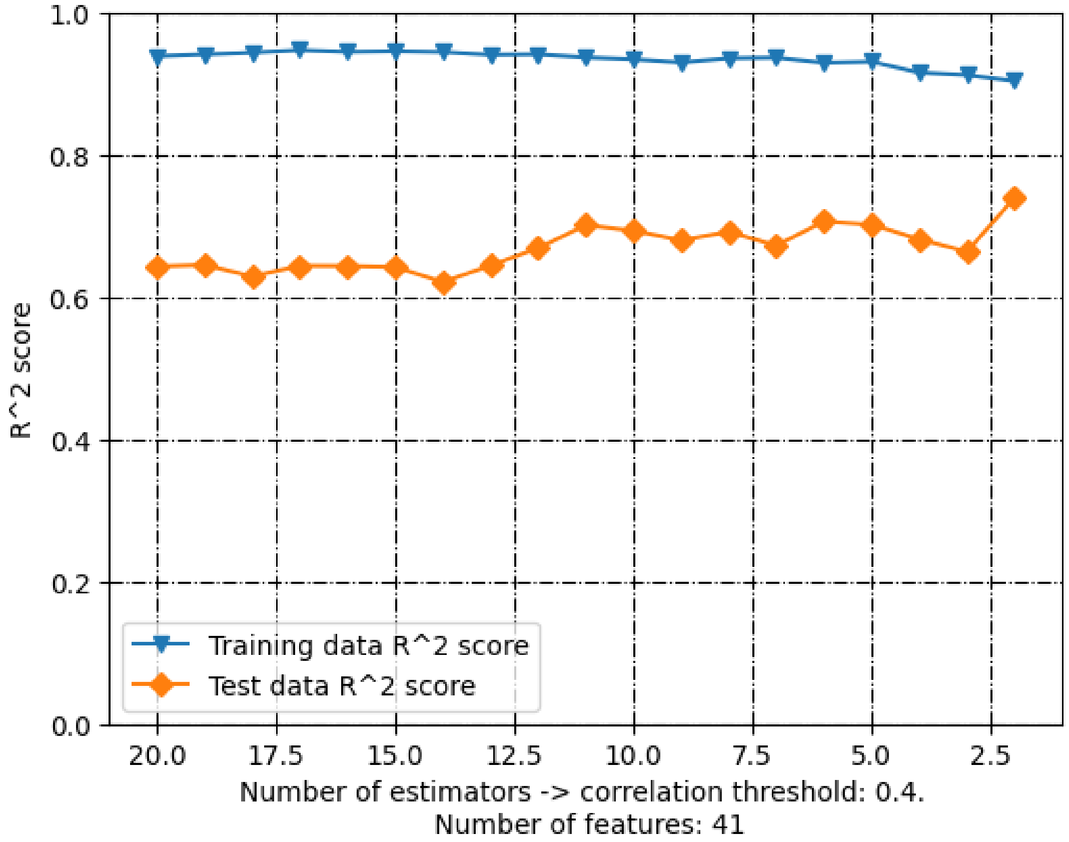
1.0
0.8
0.6
0.4
0.2
0.0
R^2 score
Training data R^2 score
Test data R^2 score
20.0
17.5
15.0
12.5
10.0
7.5
5.0
2.5
Number of estimators -> correlation threshold: 0.4.
Number of features: 41

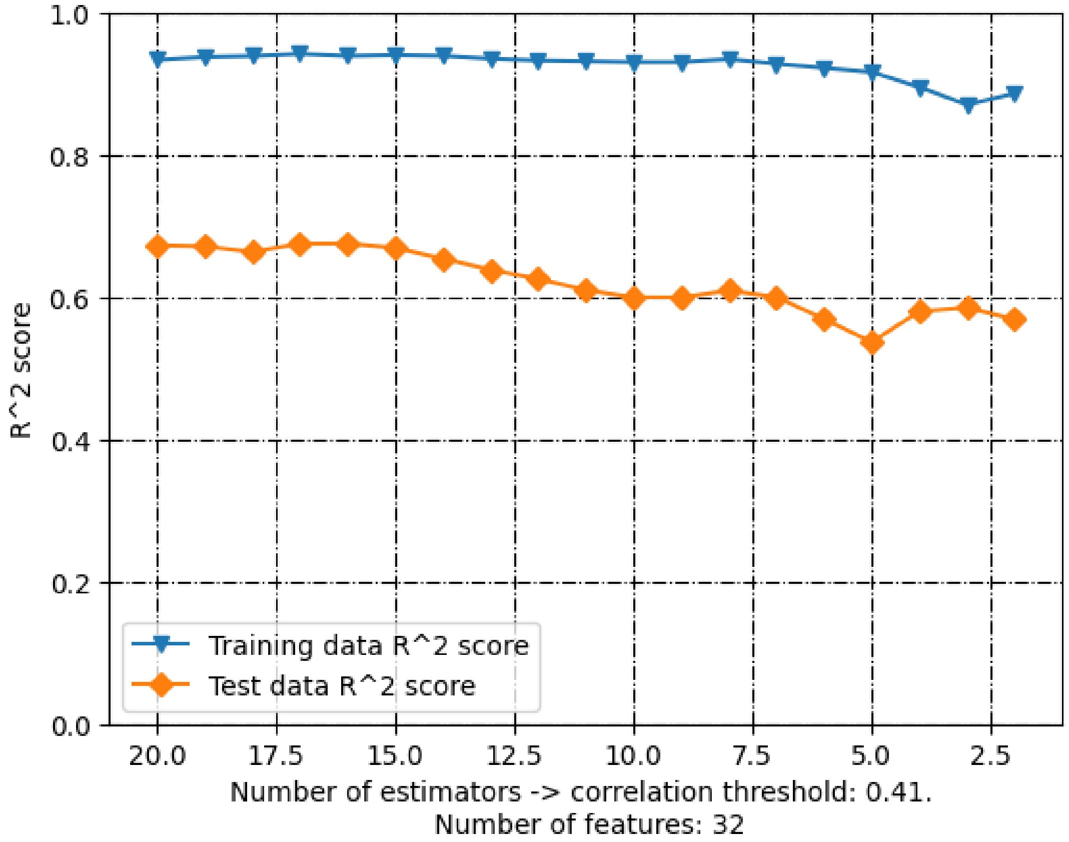

1.0
0.8
0.6
0.4
0.2
0.0
R^2 score
Training data R^2 score
Test data R^2 score
20.0
17.5
15.0
12.5
10.0
7.5
5.0
2.5
Number of estimators -> correlation threshold: 0.41.
Number of features: 32

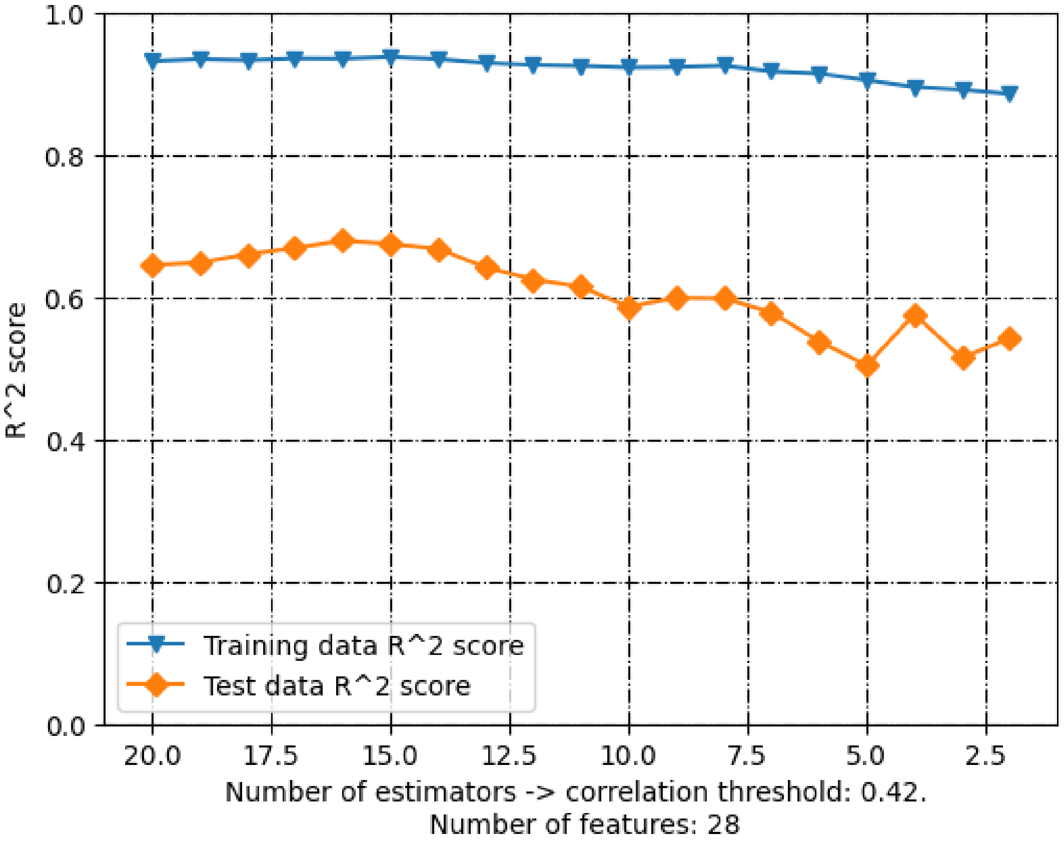

1.0
0.8
0.6
0.4
0.2
0.0
R^2 score
20.0
17.5
15.0
12.5
10.0
7.5
5.0
2.5
Number of estimators -> correlation threshold: 0.42.
Number of features: 28
Training data R^2 score
Test data R^2 score

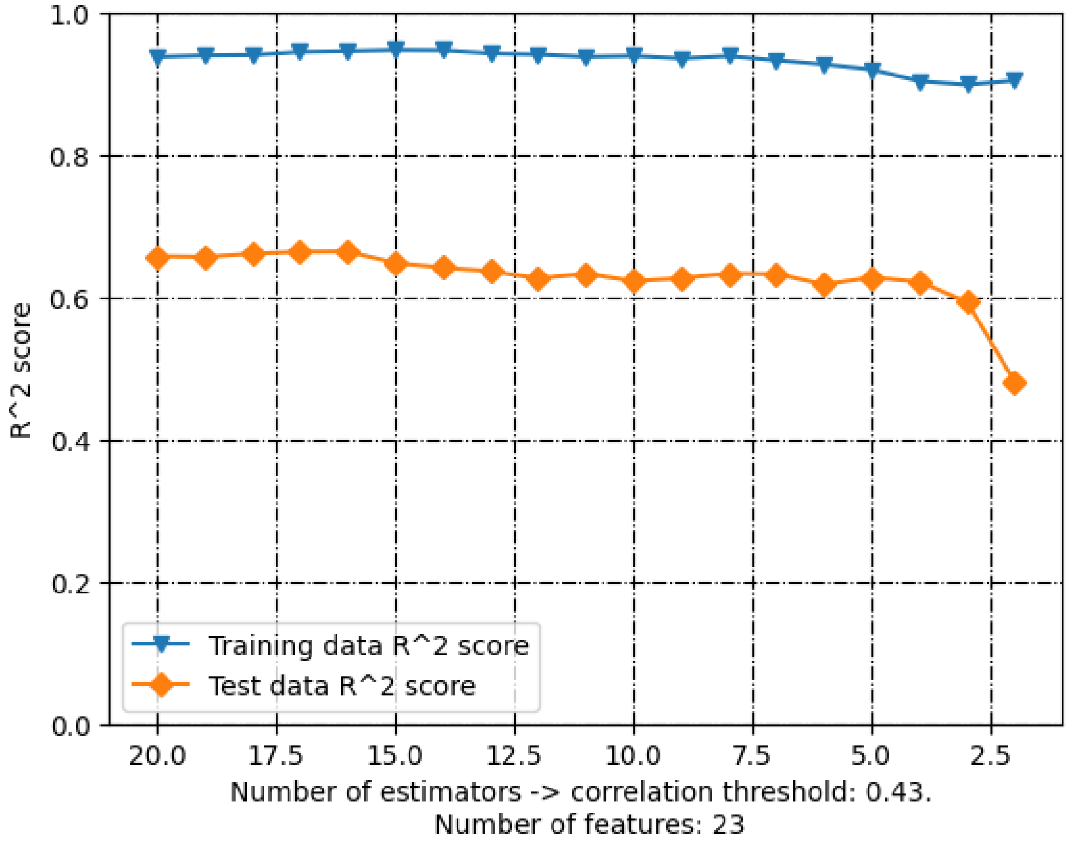
1.0
0.8
0.6
0.4
0.2
0.0
R^2 score
Training data R^2 score
Test data R^2 score
20.0
17.5
15.0
12.5
10.0
7.5
5.0
2.5
Number of estimators -> correlation threshold: 0.43.
Number of features: 23

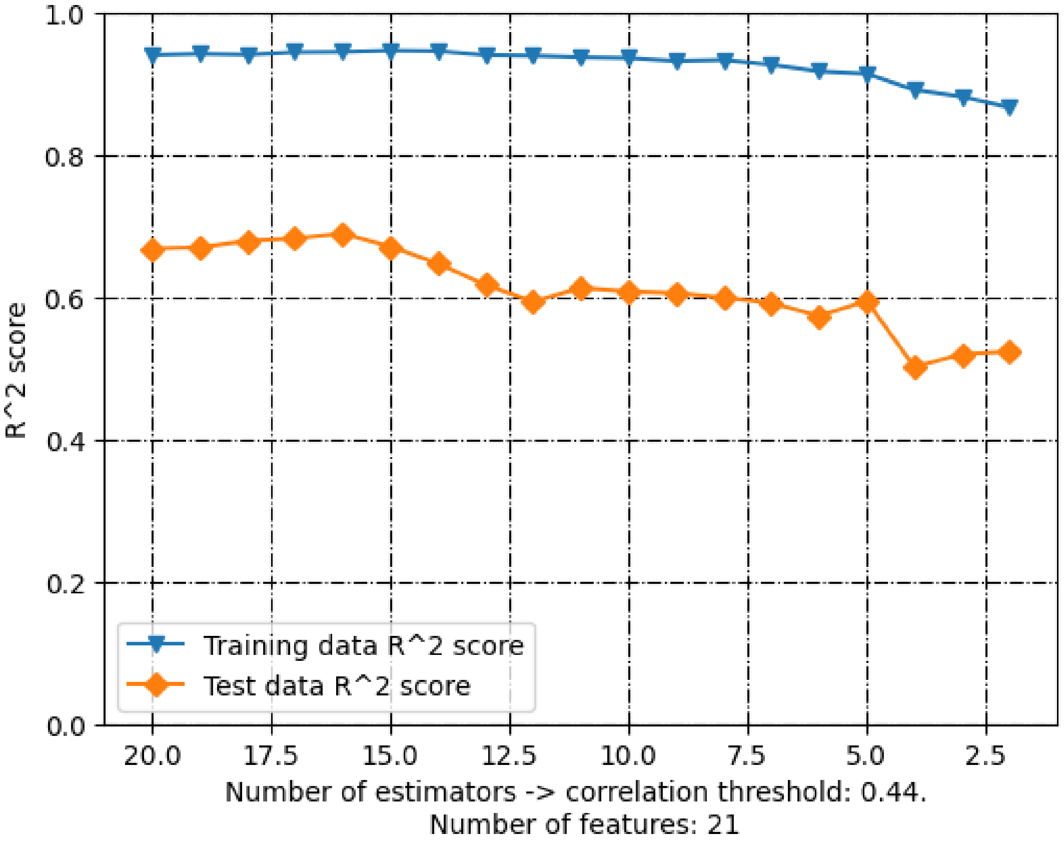
1.0
0.8
0.6
0.4
0.2
0.0
R^2 score
20.0
17.5
15.0
12.5
10.0
7.5
5.0
2.5
Number of estimators -> correlation threshold: 0.44.
Number of features: 21
Training data R^2 score
Test data R^2 score

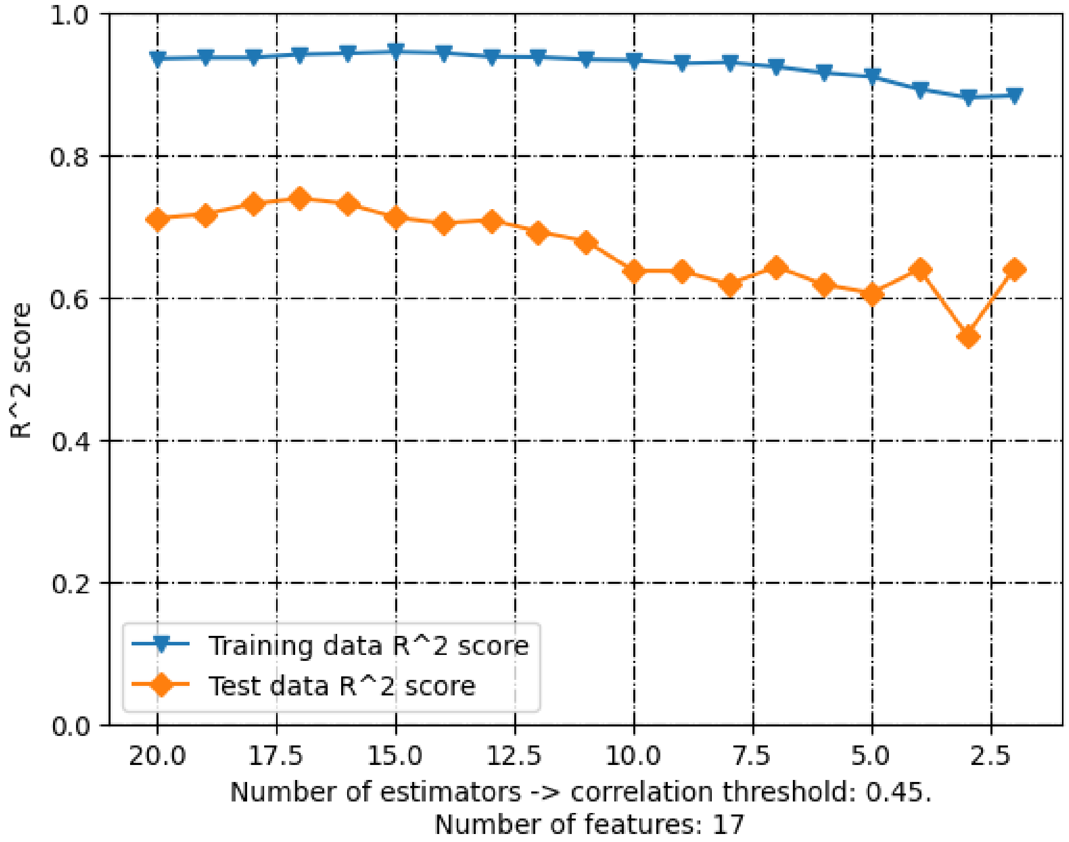
1.0
0.8
0.6
0.4
0.2
0.0
R^2 score
Training data R^2 score
Test data R^2 score
20.0
17.5
15.0
12.5
10.0
7.5
5.0
2.5
Number of estimators -> correlation threshold: 0.45.
Number of features: 17

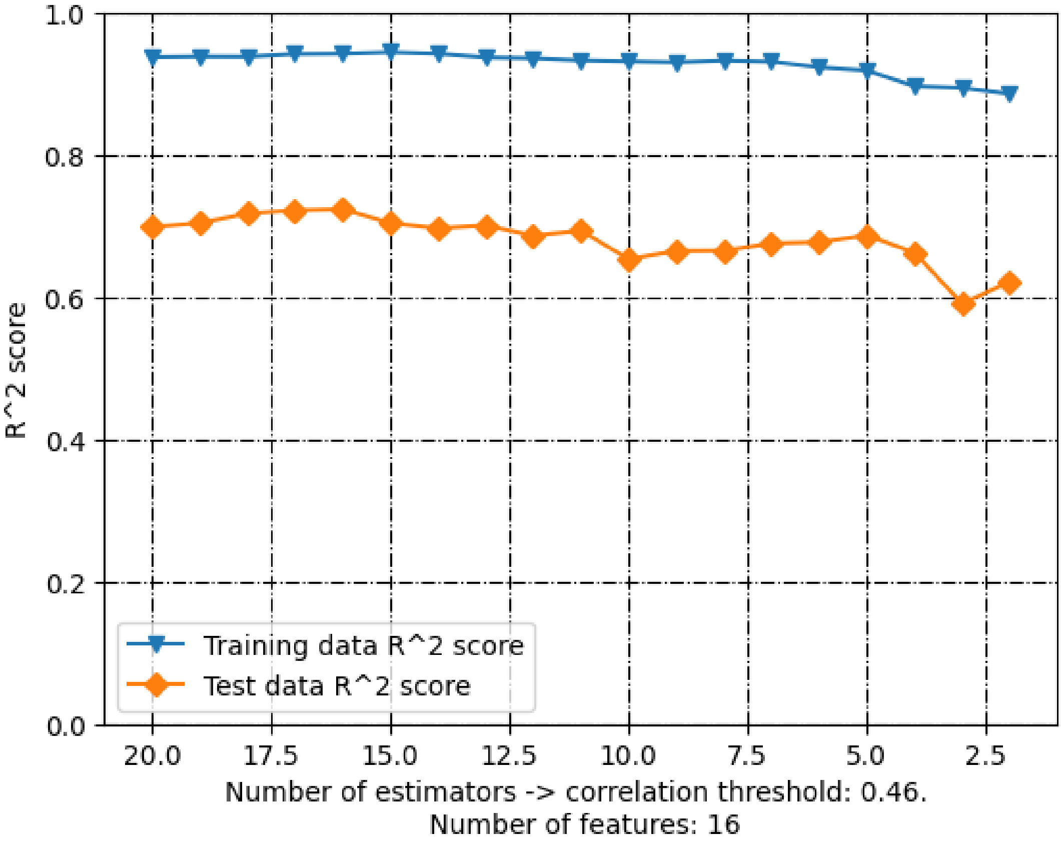
1.0
0.8
0.6
0.4
0.2
0.0
R^2 score
Training data R^2 score
Test data R^2 score
20.0
17.5
15.0
12.5
10.0
7.5
5.0
2.5
Number of estimators -> correlation threshold: 0.46.
Number of features: 16

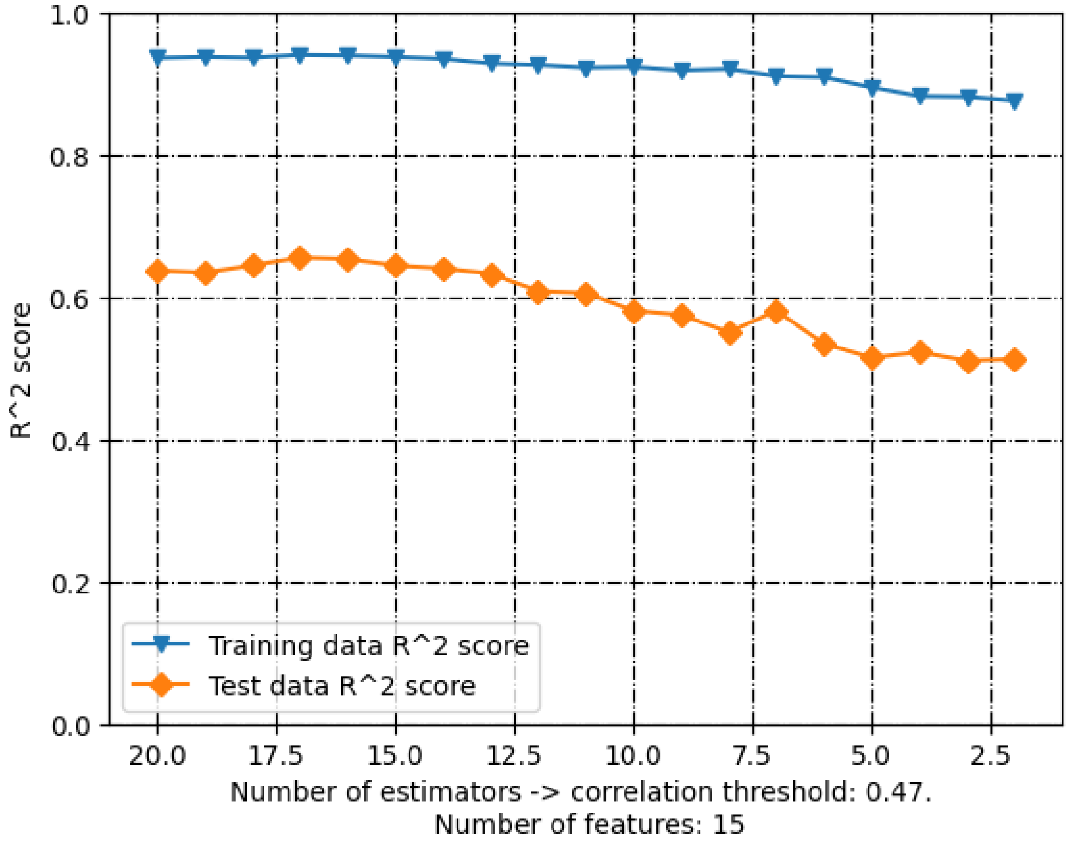

1.0
0.8
0.6
0.4
0.2
0.0
R^2 score
20.0
17.5
15.0
12.5
10.0
7.5
5.0
2.5
Training data R^2 score
Test data R^2 score
Number of estimators -> correlation threshold: 0.47.
Number of features: 15

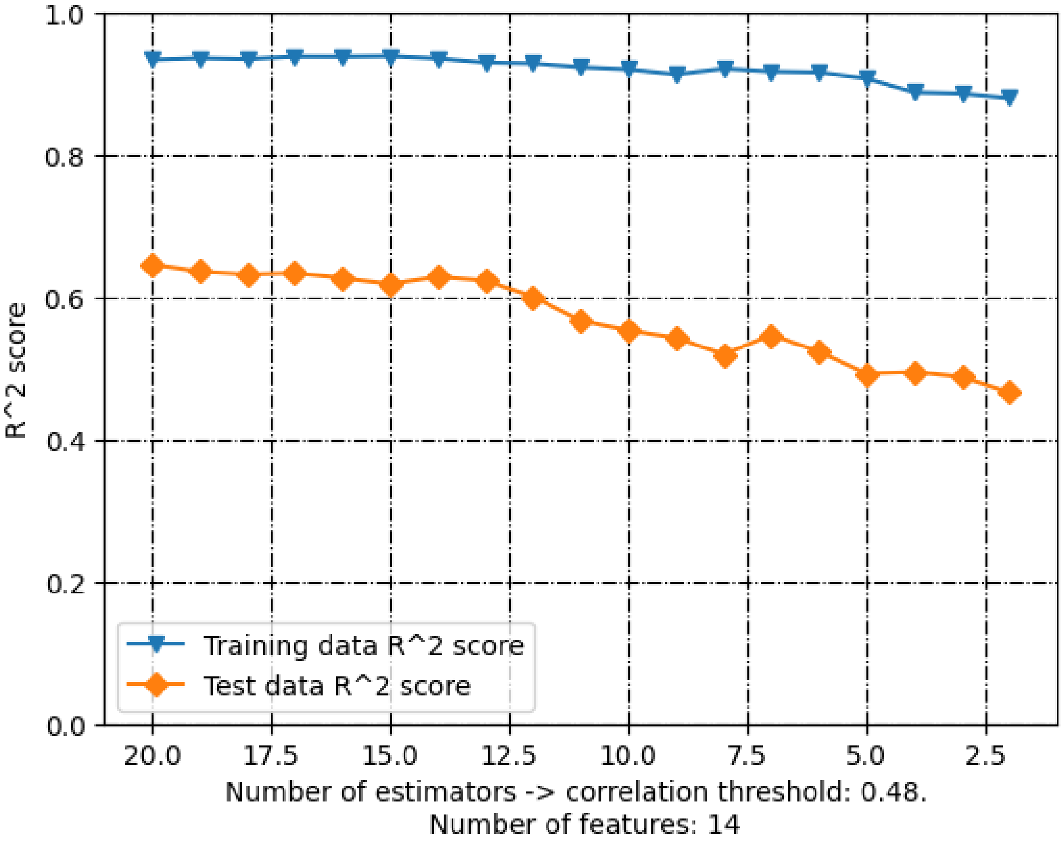
1.0
0.8
0.6
0.4
0.2
0.0
R^2 score
Training data R^2 score
Test data R^2 score
20.0
17.5
15.0
12.5
10.0
7.5
5.0
2.5
Number of estimators -> correlation threshold: 0.48.
Number of features: 14

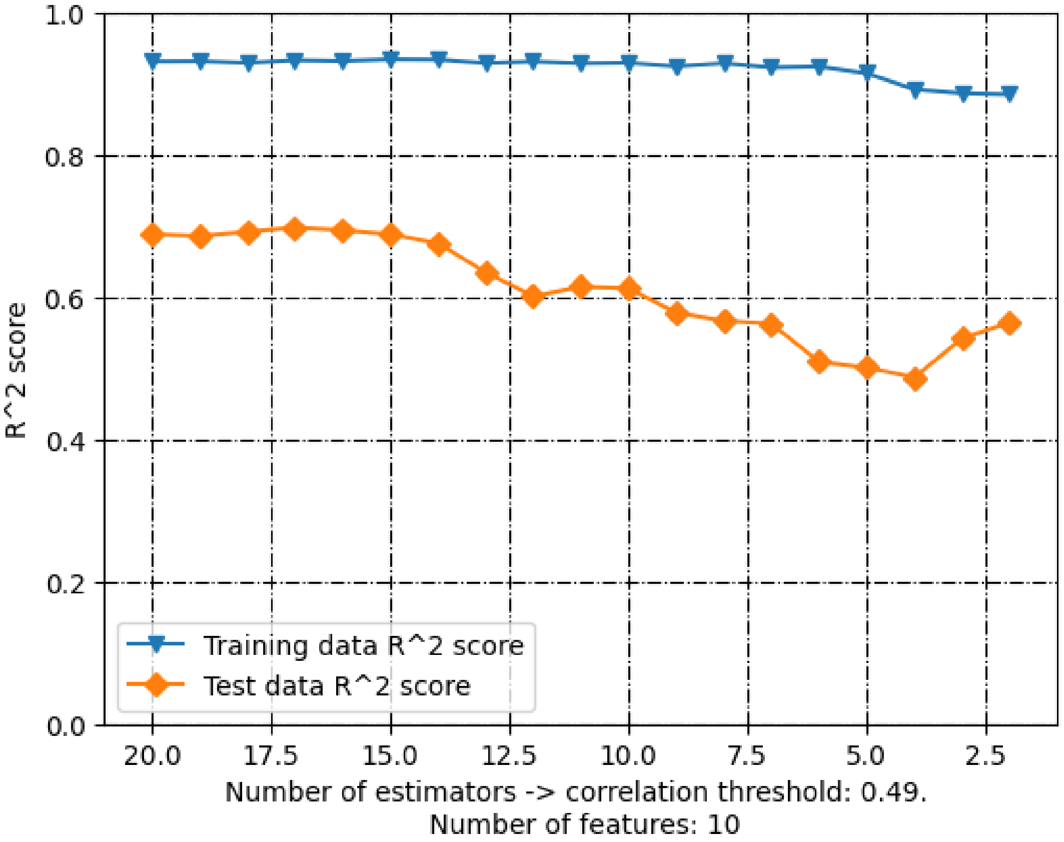
1.0
0.8
0.6
0.4
0.2
0.0
R^2 score
20.0
17.5
15.0
12.5
10.0
7.5
5.0
2.5
Number of estimators -> correlation threshold: 0.49.
Number of features: 10
Training data R^2 score
Test data R^2 score

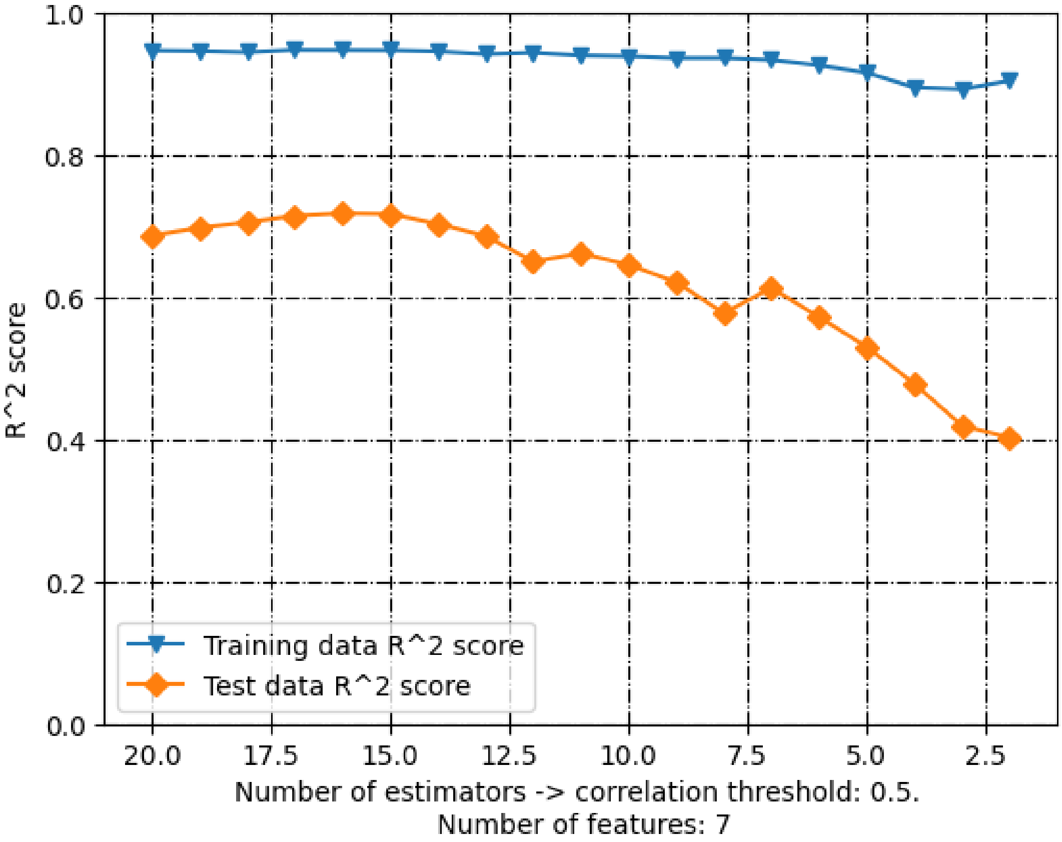

R^2 score
Number of estimators -> correlation threshold: 0.5.
Number of features: 7
Training data R^2 score
Test data R^2 score

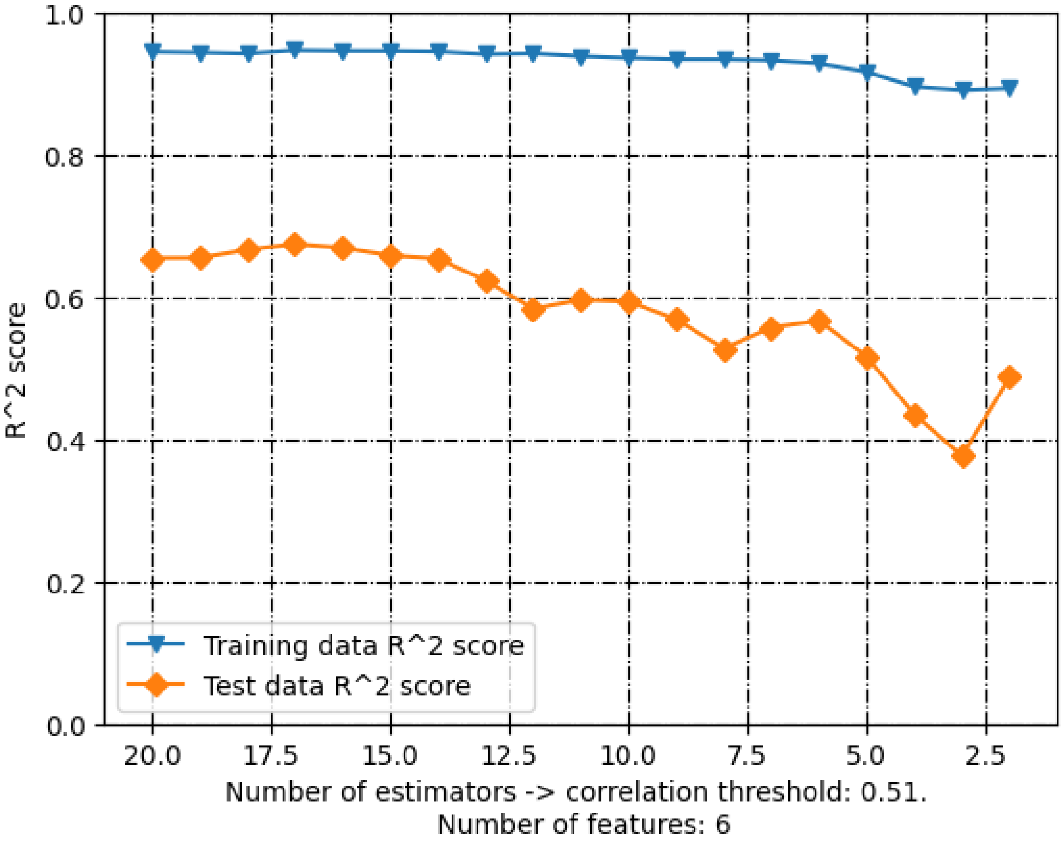

1.0
0.8
0.6
0.4
0.2
0.0
R^2 score
Training data R^2 score
Test data R^2 score
20.0
17.5
15.0
12.5
10.0
7.5
5.0
2.5
Number of estimators -> correlation threshold: 0.51.
Number of features: 6

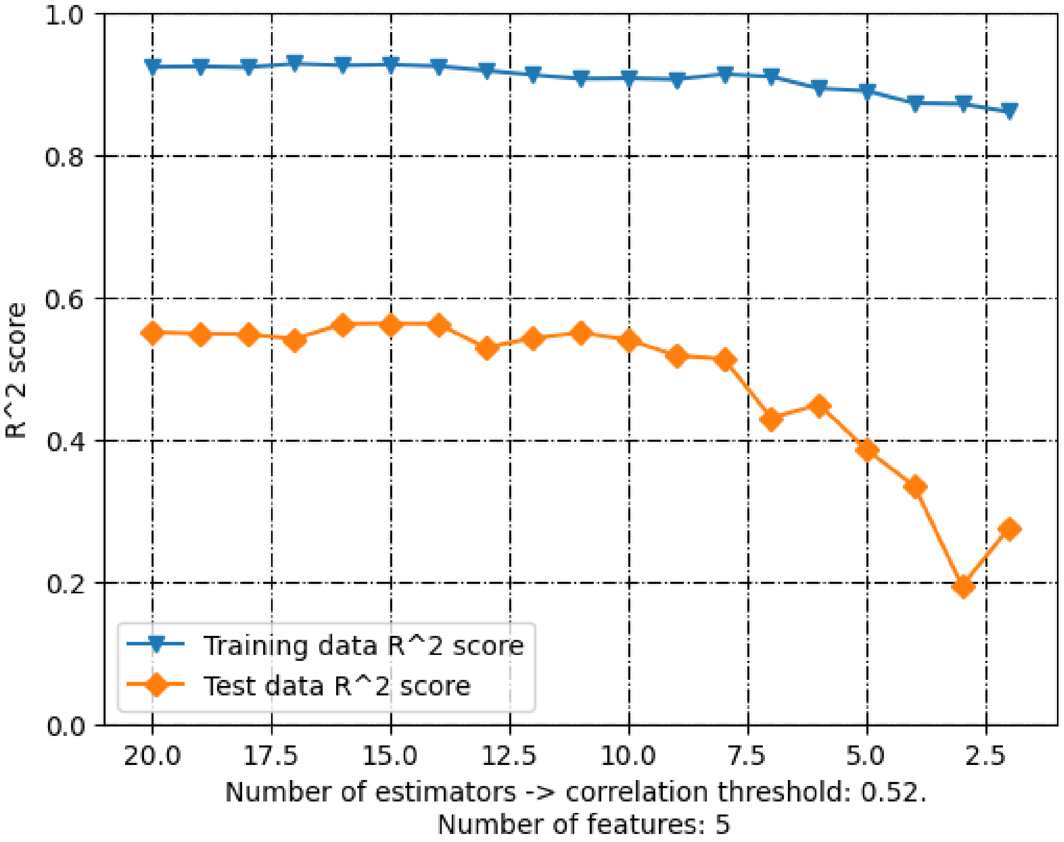
1.0
0.8
0.6
0.4
0.2
0.0
R^2 score
Training data R^2 score
Test data R^2 score
20.0
17.5
15.0
12.5
10.0
7.5
5.0
2.5
Number of estimators -> correlation threshold: 0.52.
Number of features: 5

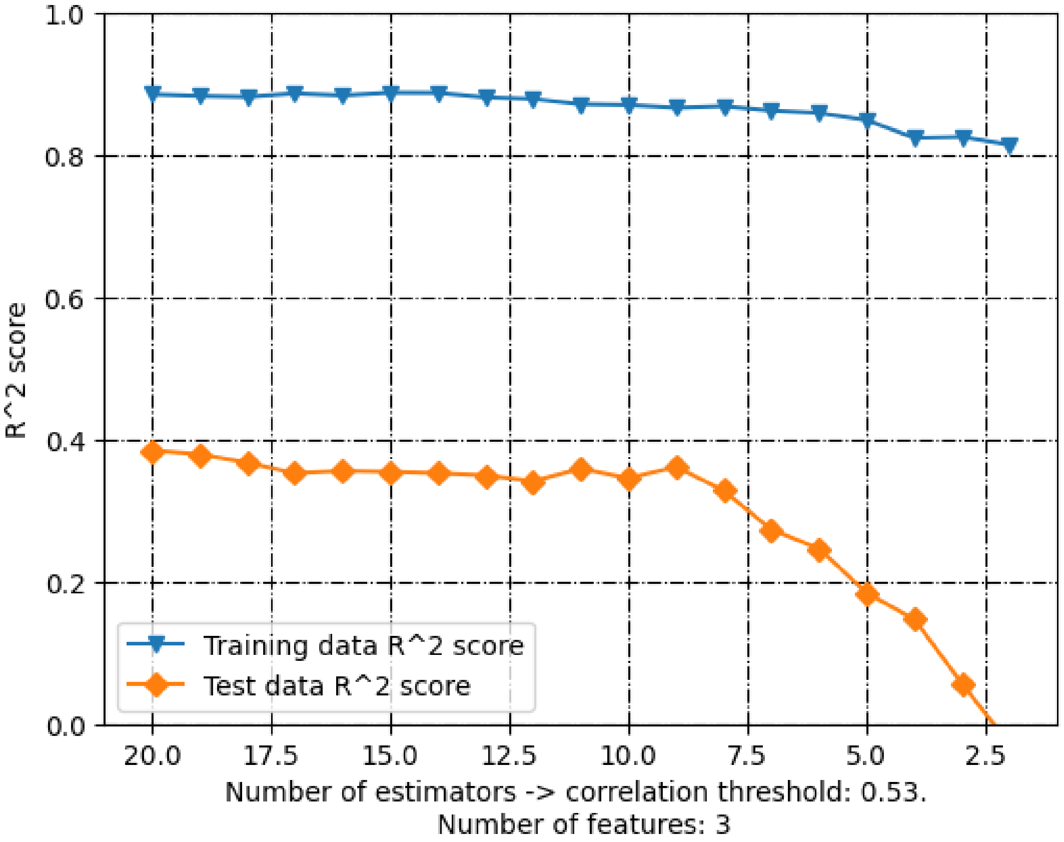
1.0
0.8
0.6
0.4
0.2
0.0
R^2 score
Training data R^2 score
Test data R^2 score
20.0
17.5
15.0
12.5
10.0
7.5
5.0
2.5
Number of estimators -> correlation threshold: 0.53.
Number of features: 3

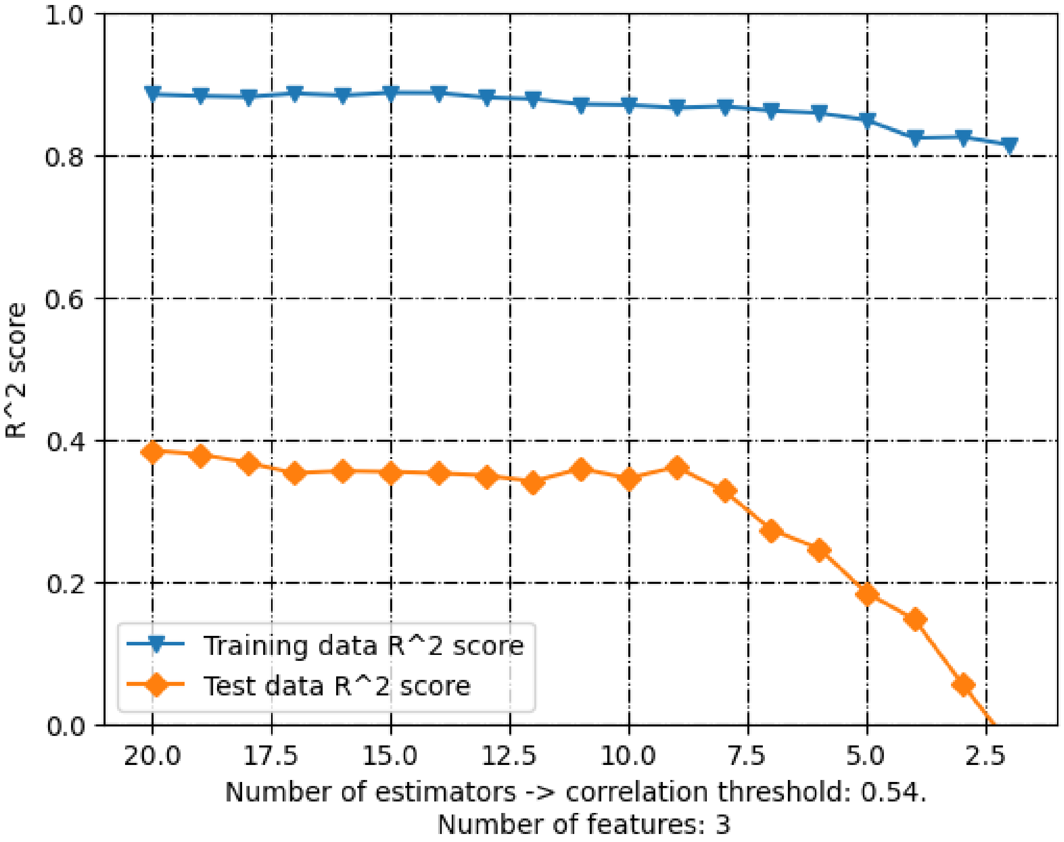

1.0
0.8
0.6
0.4
0.2
0.0
R^2 score
Training data R^2 score
Test data R^2 score
20.0
17.5
15.0
12.5
10.0
7.5
5.0
2.5
Number of estimators -> correlation threshold: 0.54.
Number of features: 3

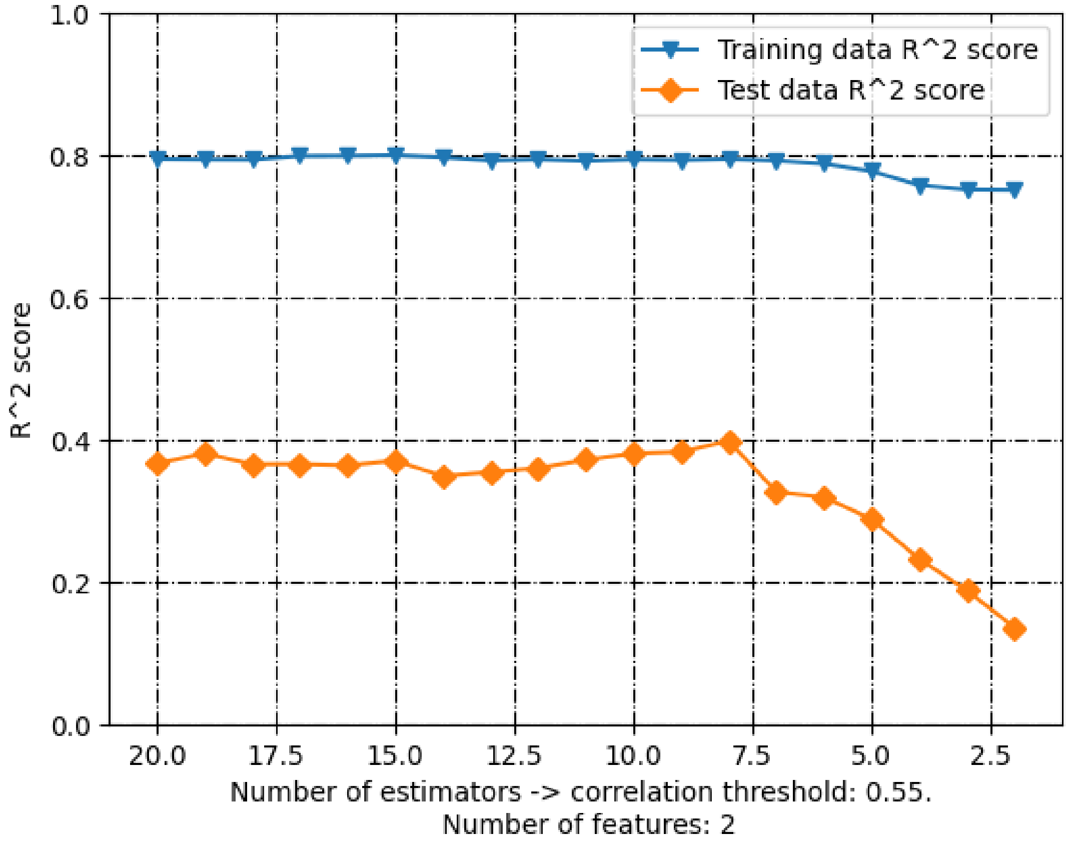
Training data R^2 score
Test data R^2 score
R^2 score
1.0
0.8
0.6
0.4
0.2
0.0
20.0
17.5
15.0
12.5
10.0
7.5
5.0
2.5
Number of estimators -> correlation threshold: 0.55.
Number of features: 2

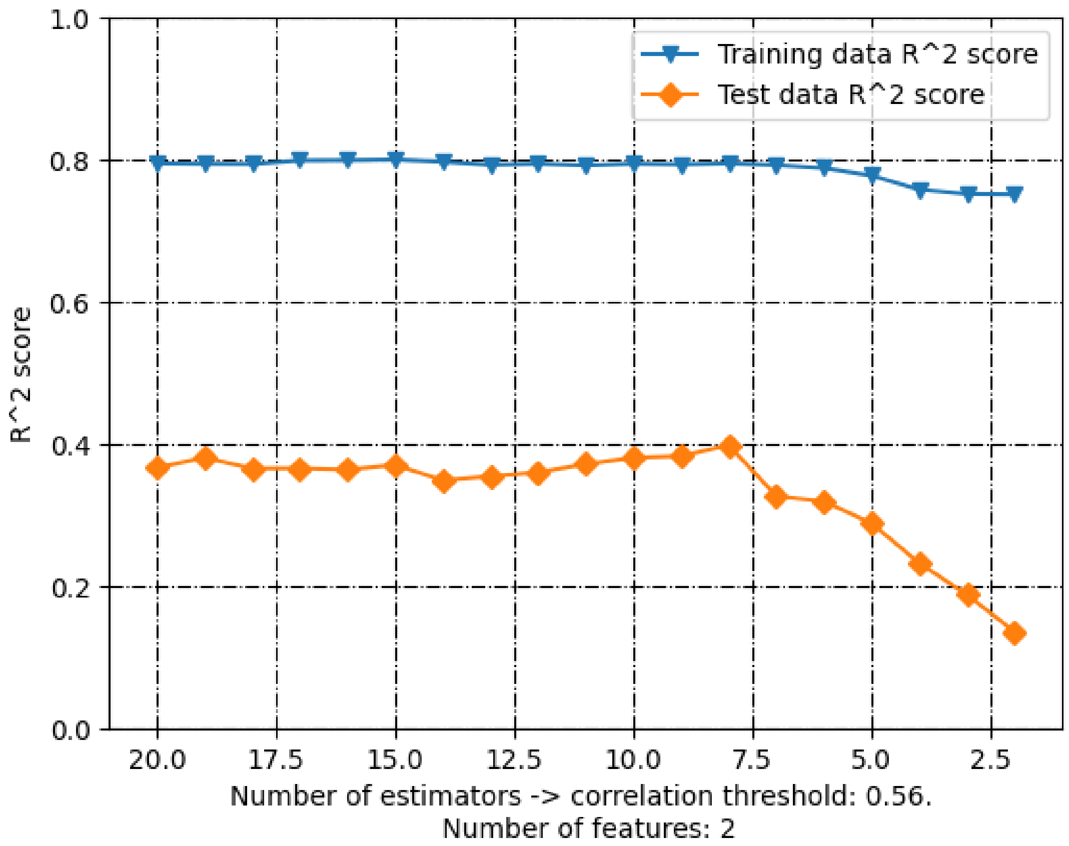

[28]:

```
plt.plot(df_without_standardization['Correlation threshold'],␣
  ↪df_without_standardization['Number of features'], label = "Number of␣
  ↪features", marker='.')
plt.legend()
plt.xlabel('Correlation threshold')
plt.ylabel('Number of features')
plt.show()
```

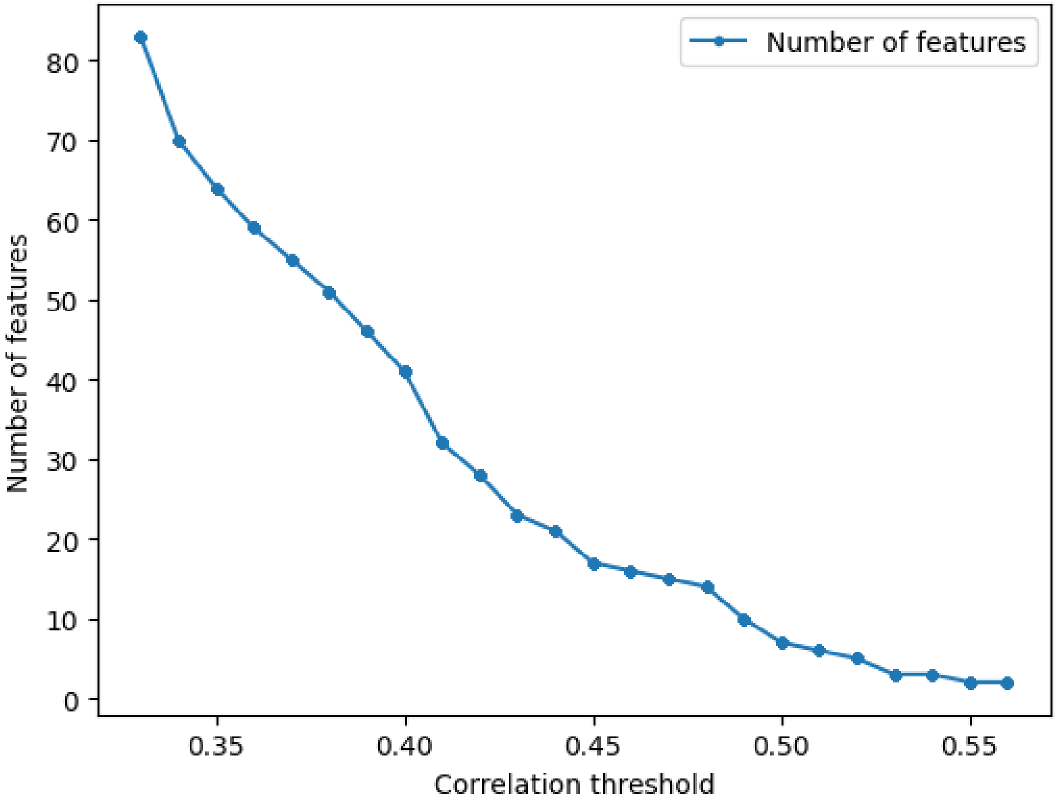

[ ]:

4 KNeighborsRegressor

[29]:
```
without_standardization, train_r2_, test_r2_, hist1, hist2, target_column_name,␣
↪training_data_RMSE, test_data_RMSE  = pred_model.
↪prepare_data_and_create_model(molecular_descriptors_df=data,
                                                                              ␣
↪                           correlation_threshold=0.50,
                                                                              ␣
↪                           standardization=False,
                                                                              ␣
↪                           model_type='KNeighborsRegressor',
                                                                              ␣
↪                           target_column_name = target,
                                                                              ␣
↪                           random_state=random_state,
                                                                              ␣
↪                           train_test_split_=True,
```

```
↪                    verbose=True)
```

```
I am not doing standardization…
  molecular descriptor name
0                    AATS0Z
1                  AATS0are
2                    AATS0d
3                   AATS0dv
4                    AATS0i
  molecular descriptor name  corr_value
0                    AATS0Z   -0.020817
1                  AATS0are   -0.148257
2                    AATS0d    0.022999
3                   AATS0dv   -0.137980
4                    AATS0i    0.133257
  molecular descriptor name  corr_value  absolute correlation value
0                    AATS0Z   -0.020817                    0.020817
1                  AATS0are   -0.148257                    0.148257
2                    AATS0d    0.022999                    0.022999
3                   AATS0dv   -0.137980                    0.137980
4                    AATS0i    0.133257                    0.133257
    molecular descriptor name  corr_value  absolute correlation value
226                    AMID_O   -0.546843                    0.546843
505                EState_VSA5   -0.588780                    0.588780
556                    GATS2c    0.511671                    0.511671
791                  MDEO-12   -0.582747                    0.582747
847                       NdO   -0.500462                    0.500462
    molecular descriptor name  corr_value  absolute correlation value
226                    AMID_O   -0.546843                    0.546843
505                EState_VSA5   -0.588780                    0.588780
556                    GATS2c    0.511671                    0.511671
791                  MDEO-12   -0.582747                    0.582747
847                       NdO   -0.500462                    0.500462
The model used is: KNeighbors…
Return the coefficient of determination of the prediction:
0.03596548905623609
R^2 score: 0.6394578454563543
Correlation coefficient: 0.7996610816191784
Test data - unseen during training:
R^2 score: 0.03596548905623609
Correlation coefficient: 0.18964569348191404
[7.74174218 5.4727385  7.98152154 5.4727385  7.49153572 7.49965533
 7.55994122 7.72931259 5.91107765 7.49153572 7.63092522 7.62109922
 7.63993908 7.92082567 7.49965533 6.9757706  7.55994122 6.60967766]
113    7.744727
35     6.149967
101    6.958607
```

```
36     6.346787
100    7.000000
13     7.966576
0      7.991400
114    7.443697
104    7.823909
96     7.677781
40     7.795880
103    7.853872
48     7.744727
39     8.221849
14     6.414314
117    5.886057
21     7.298432
9      5.971266
Name: BALB/3T3, dtype: float64
Training Root Mean Square Error: 0.5380138304158049
Testing Root Mean Square Error: 0.7346319988851434
```

[30]:
```
print(target_column_name+str('_transformed'))
print(hist1[target_column_name].hist())
```

```
BALB/3T3_transformed
AxesSubplot(0.125,0.11;0.775x0.77)
```

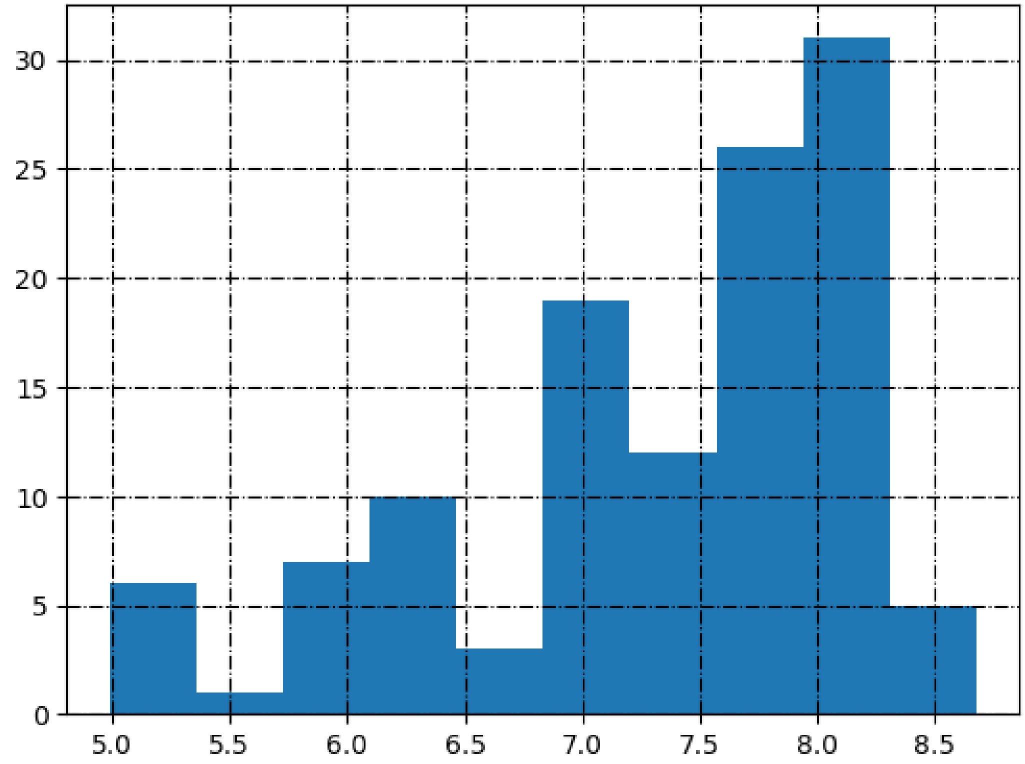

[31]:
```
with_standardization, train_r2_, test_r2_, hist1, hist2, target_column_name,␣
↪training_data_RMSE, test_data_RMSE  = pred_model.
↪prepare_data_and_create_model(molecular_descriptors_df=data,
                                                                            ␣
↪                    correlation_threshold=0.50,
                                                                            ␣
↪                    standardization=True,
                                                                            ␣
↪                    model_type='KNeighborsRegressor',
                                                                            ␣
↪                    target_column_name = target,
                                                                            ␣
↪                    random_state=random_state,
                                                                            ␣
↪                    train_test_split_=True,
                                                                            ␣
↪                    verbose=True)
```

```
I am doing standardization…
  molecular descriptor name
0                    AATS0Z
1                  AATS0are
2                    AATS0d
3                   AATS0dv
4                    AATS0i
  molecular descriptor name  corr_value
0                    AATS0Z   -0.020817
1                  AATS0are   -0.148257
2                    AATS0d    0.022999
3                   AATS0dv   -0.137980
4                    AATS0i    0.133257
  molecular descriptor name  corr_value  absolute correlation value
0                    AATS0Z   -0.020817                    0.020817
1                  AATS0are   -0.148257                    0.148257
2                    AATS0d    0.022999                    0.022999
3                   AATS0dv   -0.137980                    0.137980
4                    AATS0i    0.133257                    0.133257
    molecular descriptor name  corr_value  absolute correlation value
226                    AMID_O   -0.546843                    0.546843
505                EState_VSA5   -0.588780                    0.588780
556                    GATS2c    0.511671                    0.511671
791                  MDEO-12   -0.582747                    0.582747
847                       NdO   -0.500462                    0.500462
    molecular descriptor name  corr_value  absolute correlation value
226                    AMID_O   -0.546843                    0.546843
505                EState_VSA5   -0.588780                    0.588780
```

```
556                    GATS2c    0.511671                      0.511671
791                   MDEO-12   -0.582747                      0.582747
847                       NdO   -0.500462                      0.500462
The model used is: KNeighbors…
Return the coefficient of determination of the prediction:
0.03596548905623609
R^2 score: 0.6394578454563543
Correlation coefficient: 0.7996610816191784
Test data - unseen during training:
R^2 score: 0.03596548905623609
Correlation coefficient: 0.18964569348191404
[7.74174218 5.4727385  7.98152154 5.4727385  7.49153572 7.49965533
 7.55994122 7.72931259 5.91107765 7.49153572 7.63092522 7.62109922
 7.63993908 7.92082567 7.49965533 6.9757706  7.55994122 6.60967766]
113    7.744727
35     6.149967
101    6.958607
36     6.346787
100    7.000000
13     7.966576
0      7.991400
114    7.443697
104    7.823909
96     7.677781
40     7.795880
103    7.853872
48     7.744727
39     8.221849
14     6.414314
117    5.886057
21     7.298432
9      5.971266
Name: BALB/3T3, dtype: float64
Training Root Mean Square Error: 0.5380138304158049
Testing Root Mean Square Error: 0.7346319988851434
```

4.1 Search inside correlation space

[32]:
```python
step = 0.01
initial_step = corr_low
last_step = corr_high
first_list = [x / 100.0 for x in range(int(initial_step*100),␣
  ↪int(last_step*100), int(step*100))]
second_list = []
third_list = []
fourth_l = []
fifth_l = []
```

```
f_list = []
for i in first_list:
    without_standardization, train_r2, test_r2, _, h_, target_column_name,␣
 ↪training_data_RMSE, test_data_RMSE = pred_model.
 ↪prepare_data_and_create_model(molecular_descriptors_df = data,
                                                                            ␣
 ↪                    correlation_threshold = i,
                                                                            ␣
 ↪                    standardization = False,
                                                                            ␣
 ↪                    model_type = 'KNeighborsRegressor',
                                                                            ␣
 ↪                    target_column_name = target,
                                                                            ␣
 ↪                    random_state=random_state,
                                                                            ␣
 ↪                    train_test_split_ = True,
                                                                            ␣
 ↪                    verbose = False)
    second_list.append(train_r2)
    third_list.append(test_r2)
    fourth_l.append(training_data_RMSE)
    fifth_l.append(test_data_RMSE)
    f_list.append(len(h_))
```

```
  File "C:\Users\aleks\anaconda3\envs\cheminf_gpu\lib\site-
packages\joblib\externals\loky\backend\context.py", line 217, in
_count_physical_cores
    raise ValueError(
```

[33]:
```
df_without_standardization = pd.DataFrame(data=first_list,␣
 ↪columns=["Correlation threshold"])
df_without_standardization['Training data R^2 score'] = second_list
df_without_standardization['Test data R^2 score'] = third_list
df_without_standardization['Training RMSE'] = fourth_l
df_without_standardization['Test RMSE'] = fifth_l
df_without_standardization['Number of features'] = f_list
```

[34]:
```
df_k_nearest = df_without_standardization.copy()
df_without_standardization
```

[34]:
```
   Correlation threshold  Training data R^2 score  Test data R^2 score  \
0                   0.33                 0.629651            -0.036742
1                   0.34                 0.639095             0.139245
2                   0.35                 0.639095             0.139245
3                   0.36                 0.639095             0.139245
4                   0.37                 0.639095             0.139245
```

5	0.38	0.639095	0.139245
6	0.39	0.637122	0.238102
7	0.40	0.637122	0.238102
8	0.41	0.599270	0.114948
9	0.42	0.611799	0.176188
10	0.43	0.609663	0.076650
11	0.44	0.596976	-0.067617
12	0.45	0.603513	0.125988
13	0.46	0.601925	0.113482
14	0.47	0.605971	0.125852
15	0.48	0.610819	0.138037
16	0.49	0.614048	0.115808
17	0.50	0.639458	0.035965
18	0.51	0.633981	-0.015910
19	0.52	0.623860	-0.030642
20	0.53	0.589531	0.046000
21	0.54	0.589531	0.046000
22	0.55	0.557005	-0.019234
23	0.56	0.557005	-0.019234

	Training RMSE	Test RMSE	Number of features
0	0.545282	0.761831	83
1	0.538284	0.694166	70
2	0.538284	0.694166	64
3	0.538284	0.694166	59
4	0.538284	0.694166	55
5	0.538284	0.694166	51
6	0.539754	0.653088	46
7	0.539754	0.653088	41
8	0.567207	0.703895	32
9	0.558269	0.679106	28
10	0.559803	0.718963	23
11	0.568828	0.773092	21
12	0.564196	0.699491	17
13	0.565325	0.704478	16
14	0.562444	0.699546	15
15	0.558973	0.694653	14
16	0.556650	0.703553	10
17	0.538014	0.734632	7
18	0.542085	0.754139	6
19	0.549529	0.759587	5
20	0.574058	0.730799	3
21	0.574058	0.730799	3
22	0.596369	0.755371	2
23	0.596369	0.755371	2

4.2 Plots

[35]:
```
plt.plot(df_without_standardization['Correlation threshold'],␣
 ↪df_without_standardization['Training data R^2 score'], label = "Training␣
 ↪data R^2 score", marker='v')
plt.plot(df_without_standardization['Correlation threshold'],␣
 ↪df_without_standardization['Test data R^2 score'], label = "Test data R^2␣
 ↪score", marker='D')
plt.legend()
plt.xlabel('Correlation threshold')
plt.ylabel('R^2 score')
plt.ylim([0, 1])
plt.show()
```

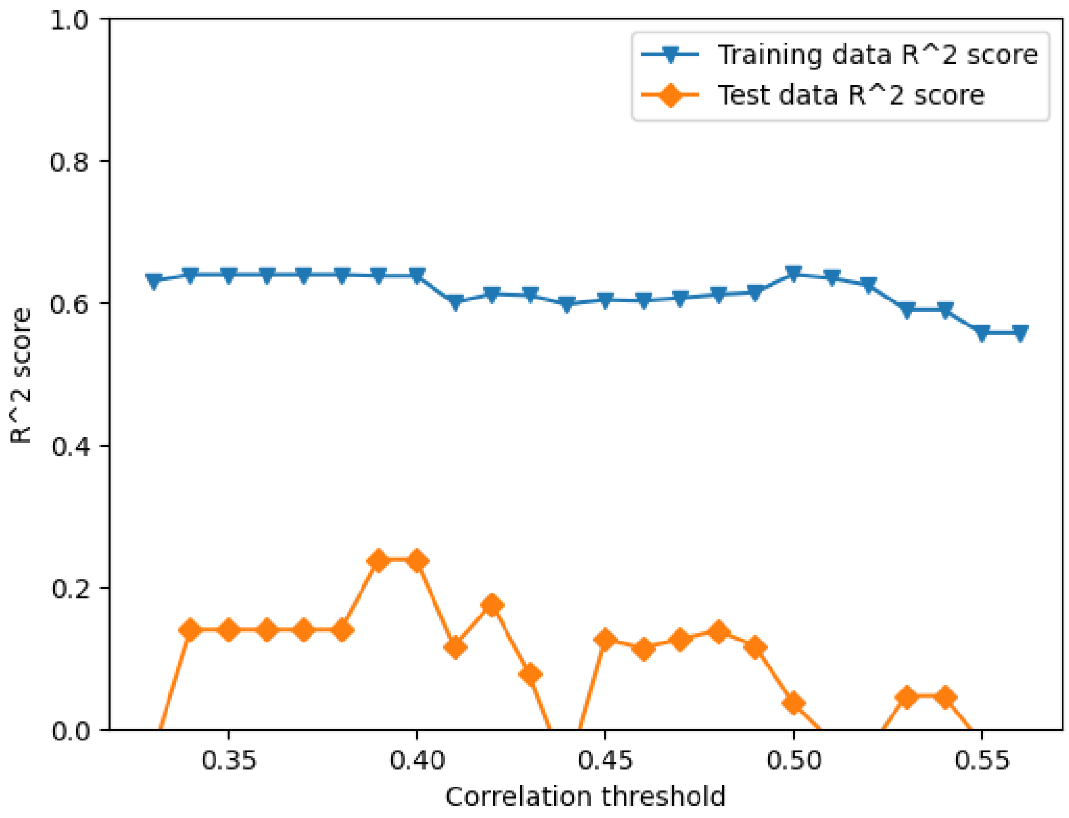

[36]:
```
plt.plot(df_without_standardization['Correlation threshold'],␣
 ↪df_without_standardization['Number of features'], label = "Number of␣
 ↪features", marker='.')
plt.legend()
plt.xlabel('Correlation threshold')
plt.ylabel('Number of features')
plt.show()
```

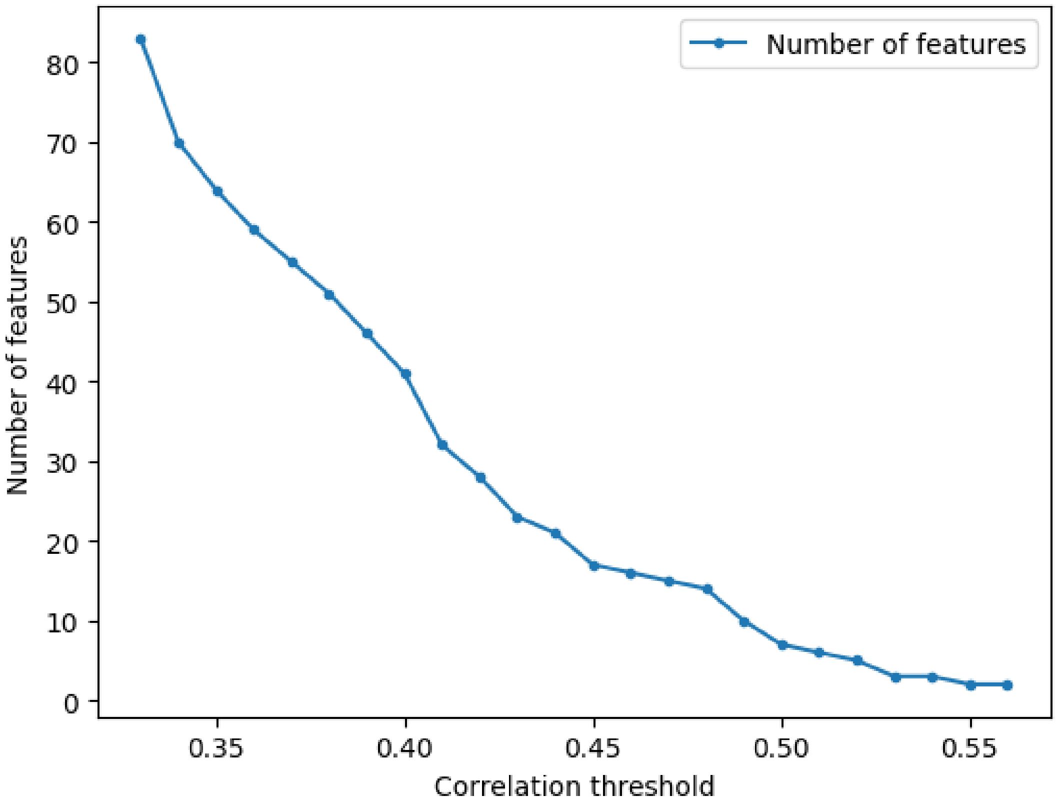


```
[ ]:
```

5 Support Vector Machines (SVM)

```
[37]: without_standardization, train_r2_, test_r2_, hist1, hist2, target_column_name,␣
       ↪training_data_RMSE, test_data_RMSE  = pred_model.
       ↪prepare_data_and_create_model(molecular_descriptors_df=data,
                                                                                      ␣
       ↪                         correlation_threshold=0.50,
                                                                                      ␣
       ↪                         standardization=False,
                                                                                      ␣
       ↪                         model_type='SVR',
                                                                                      ␣
       ↪                         kernel_ = 'linear',
                                                                                      ␣
       ↪                         gamma_ = 'auto',
                                                                                      ␣
       ↪                         target_column_name = target,
```

```
↪                    random_state=random_state,
↪                    train_test_split_=True,
↪                    verbose=True)
```

```
I am not doing standardization…
  molecular descriptor name
0                   AATS0Z
1                 AATS0are
2                   AATS0d
3                  AATS0dv
4                   AATS0i
  molecular descriptor name  corr_value
0                   AATS0Z   -0.020817
1                 AATS0are   -0.148257
2                   AATS0d    0.022999
3                  AATS0dv   -0.137980
4                   AATS0i    0.133257
  molecular descriptor name  corr_value  absolute correlation value
0                   AATS0Z   -0.020817                    0.020817
1                 AATS0are   -0.148257                    0.148257
2                   AATS0d    0.022999                    0.022999
3                  AATS0dv   -0.137980                    0.137980
4                   AATS0i    0.133257                    0.133257
    molecular descriptor name  corr_value  absolute correlation value
226                    AMID_O   -0.546843                    0.546843
505               EState_VSA5   -0.588780                    0.588780
556                    GATS2c    0.511671                    0.511671
791                   MDEO-12   -0.582747                    0.582747
847                       NdO   -0.500462                    0.500462
    molecular descriptor name  corr_value  absolute correlation value
226                    AMID_O   -0.546843                    0.546843
505               EState_VSA5   -0.588780                    0.588780
556                    GATS2c    0.511671                    0.511671
791                   MDEO-12   -0.582747                    0.582747
847                       NdO   -0.500462                    0.500462
The model used is: SVR…
Return the coefficient of determination of the prediction:
0.39713091168792347
R^2 score: 0.5120253005133564
Correlation coefficient: 0.7155594318526983
Test data - unseen during training:
R^2 score: 0.39713091168792347
Correlation coefficient: 0.630183236597042
[7.74774499 6.20132237 7.60965581 6.23184126 7.67800449 7.57637124
```

```
 7.79915899 7.72375861 6.70620896 7.65980561 8.02951732 7.65802784
 7.96384165 7.48307941 7.57147053 6.93264546 7.80689881 6.49916559]
113    7.744727
35     6.149967
101    6.958607
36     6.346787
100    7.000000
13     7.966576
0      7.991400
114    7.443697
104    7.823909
96     7.677781
40     7.795880
103    7.853872
48     7.744727
39     8.221849
14     6.414314
117    5.886057
21     7.298432
9      5.971266
Name: BALB/3T3, dtype: float64
Training Root Mean Square Error: 0.6259131383252285
Testing Root Mean Square Error: 0.5809450542874729
```

[38]:
```python
print(target_column_name+str('_transformed'))
print(hist1[target_column_name].hist())
```

```
BALB/3T3_transformed
AxesSubplot(0.125,0.11;0.775x0.77)
```

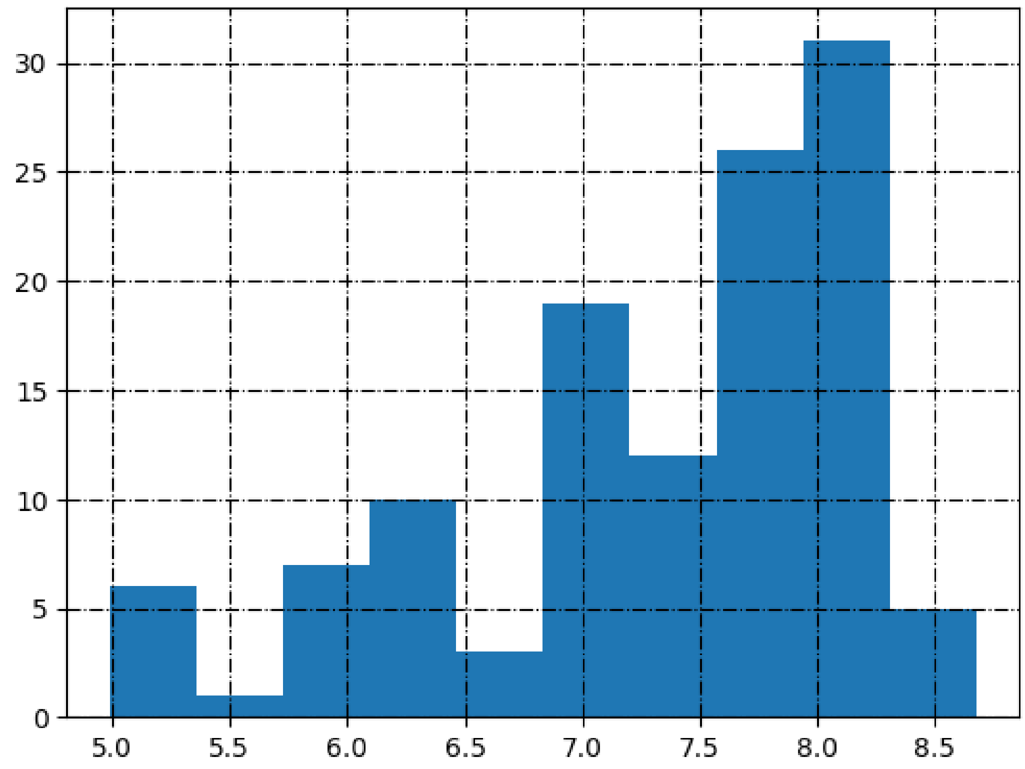

[39]:
```
with_standardization, train_r2_, test_r2_, hist1, hist2, target_column_name,␣
↪training_data_RMSE, test_data_RMSE  = pred_model.
↪prepare_data_and_create_model(molecular_descriptors_df=data,
                                                                             ␣
↪                        correlation_threshold=0.50,
                                                                             ␣
↪                        standardization=True,
                                                                             ␣
↪                        model_type='SVR',
                                                                             ␣
↪                        kernel_ = 'linear',
                                                                             ␣
↪                        gamma_ = 'auto',
                                                                             ␣
↪                        target_column_name = target,
                                                                             ␣
↪                        random_state=random_state,
                                                                             ␣
↪                        train_test_split_=True,
                                                                             ␣
↪                        verbose=True)
```

```
I am doing standardization…
  molecular descriptor name
0                   AATS0Z
1                 AATS0are
2                   AATS0d
3                  AATS0dv
4                   AATS0i
  molecular descriptor name  corr_value
0                   AATS0Z   -0.020817
1                 AATS0are   -0.148257
2                   AATS0d    0.022999
3                  AATS0dv   -0.137980
4                   AATS0i    0.133257
  molecular descriptor name  corr_value  absolute correlation value
0                   AATS0Z   -0.020817                    0.020817
1                 AATS0are   -0.148257                    0.148257
2                   AATS0d    0.022999                    0.022999
3                  AATS0dv   -0.137980                    0.137980
4                   AATS0i    0.133257                    0.133257
    molecular descriptor name  corr_value  absolute correlation value
226                    AMID_O   -0.546843                    0.546843
505                EState_VSA5   -0.588780                    0.588780
556                    GATS2c    0.511671                    0.511671
791                   MDEO-12   -0.582747                    0.582747
847                       NdO   -0.500462                    0.500462
    molecular descriptor name  corr_value  absolute correlation value
226                    AMID_O   -0.546843                    0.546843
505                EState_VSA5   -0.588780                    0.588780
556                    GATS2c    0.511671                    0.511671
791                   MDEO-12   -0.582747                    0.582747
847                       NdO   -0.500462                    0.500462
The model used is: SVR…
Return the coefficient of determination of the prediction:
0.39713091168792347
R^2 score: 0.5120253005133564
Correlation coefficient: 0.7155594318526983
Test data - unseen during training:
R^2 score: 0.39713091168792347
Correlation coefficient: 0.630183236597042
[7.74774499 6.20132237 7.60965581 6.23184126 7.67800449 7.57637124
 7.79915899 7.72375861 6.70620896 7.65980561 8.02951732 7.65802784
 7.96384165 7.48307941 7.57147053 6.93264546 7.80689881 6.49916559]
113    7.744727
35     6.149967
101    6.958607
36     6.346787
100    7.000000
13     7.966576
```

```
0      7.991400
114    7.443697
104    7.823909
96     7.677781
40     7.795880
103    7.853872
48     7.744727
39     8.221849
14     6.414314
117    5.886057
21     7.298432
9      5.971266
Name: BALB/3T3, dtype: float64
Training Root Mean Square Error: 0.6259131383252285
Testing Root Mean Square Error: 0.5809450542874729
```

5.1 Search inside correlation space

[40]:
```python
step = 0.01
initial_step = corr_low
last_step = corr_high
first_list = [x / 100.0 for x in range(int(initial_step*100),␣
 ↪int(last_step*100), int(step*100))]
second_list = []
third_list = []
fourth_l = []
fifth_l = []
f_list = []
for i in first_list:
    without_standardization, train_r2, test_r2, _, h_, target_column_name,␣
 ↪training_data_RMSE, test_data_RMSE = pred_model.
 ↪prepare_data_and_create_model(molecular_descriptors_df = data,
                                                                          ␣
 ↪                      correlation_threshold = i,
                                                                          ␣
 ↪                      standardization = False,
                                                                          ␣
 ↪                      model_type = 'SVR',
                                                                          ␣
 ↪                      kernel_ = 'linear',
                                                                          ␣
 ↪                      gamma_ = 'auto',
                                                                          ␣
 ↪                      target_column_name = target,
                                                                          ␣
 ↪                      random_state=random_state,
```

```
                             train_test_split_ = True,
                             verbose = False)
    second_list.append(train_r2)
    third_list.append(test_r2)
    fourth_l.append(training_data_RMSE)
    fifth_l.append(test_data_RMSE)
    f_list.append(len(h_))
```

[41]:
```
df_without_standardization = pd.DataFrame(data=first_list,
 ↪columns=["Correlation threshold"])
df_without_standardization['Training data R^2 score'] = second_list
df_without_standardization['Test data R^2 score'] = third_list
df_without_standardization['Training RMSE'] = fourth_l
df_without_standardization['Test RMSE'] = fifth_l
df_without_standardization['Number of features'] = f_list
```

[42]:
```
df_svm = df_without_standardization.copy()
df_without_standardization
```

[42]:

	Correlation threshold	Training data R^2 score	Test data R^2 score	\
0	0.33	0.833508	-0.317838	
1	0.34	0.767207	0.054930	
2	0.35	0.764112	-0.029497	
3	0.36	0.758894	0.017978	
4	0.37	0.752743	0.036937	
5	0.38	0.739794	-0.065670	
6	0.39	0.729804	-0.104285	
7	0.40	0.722749	-0.164598	
8	0.41	0.662256	0.407917	
9	0.42	0.641200	0.380035	
10	0.43	0.639518	0.383536	
11	0.44	0.617187	0.319788	
12	0.45	0.596438	0.355671	
13	0.46	0.596571	0.356252	
14	0.47	0.590345	0.396293	
15	0.48	0.587436	0.249061	
16	0.49	0.567418	0.301040	
17	0.50	0.512025	0.397131	
18	0.51	0.509541	0.387642	
19	0.52	0.508352	0.383807	
20	0.53	0.473627	0.495218	
21	0.54	0.473627	0.495218	
22	0.55	0.474450	0.499681	
23	0.56	0.474450	0.499681	

	Training RMSE	Test RMSE	Number of features
0	0.365605	0.858924	83
1	0.432315	0.727370	70
2	0.435180	0.759165	64
3	0.439966	0.741454	59
4	0.445543	0.734262	55
5	0.457061	0.772387	51
6	0.465752	0.786256	46
7	0.471794	0.807443	41
8	0.520726	0.575725	32
9	0.536712	0.589124	28
10	0.537969	0.587459	23
11	0.554381	0.617086	21
12	0.569208	0.600589	17
13	0.569114	0.600318	16
14	0.573488	0.581348	15
15	0.575521	0.648374	14
16	0.589318	0.625532	10
17	0.625913	0.580945	7
18	0.627505	0.585499	6
19	0.628265	0.587330	5
20	0.650073	0.531588	3
21	0.650073	0.531588	3
22	0.649565	0.529233	2
23	0.649565	0.529233	2

5.2 Plots

[43]:
```
plt.plot(df_without_standardization['Correlation threshold'],␣
  ↪df_without_standardization['Training data R^2 score'], label = "Training␣
  ↪data R^2 score", marker='v')
plt.plot(df_without_standardization['Correlation threshold'],␣
  ↪df_without_standardization['Test data R^2 score'], label = "Test data R^2␣
  ↪score", marker='D')
plt.legend()
plt.xlabel('Correlation threshold')
plt.ylabel('R^2 score')
plt.ylim([0, 1])
plt.show()
```

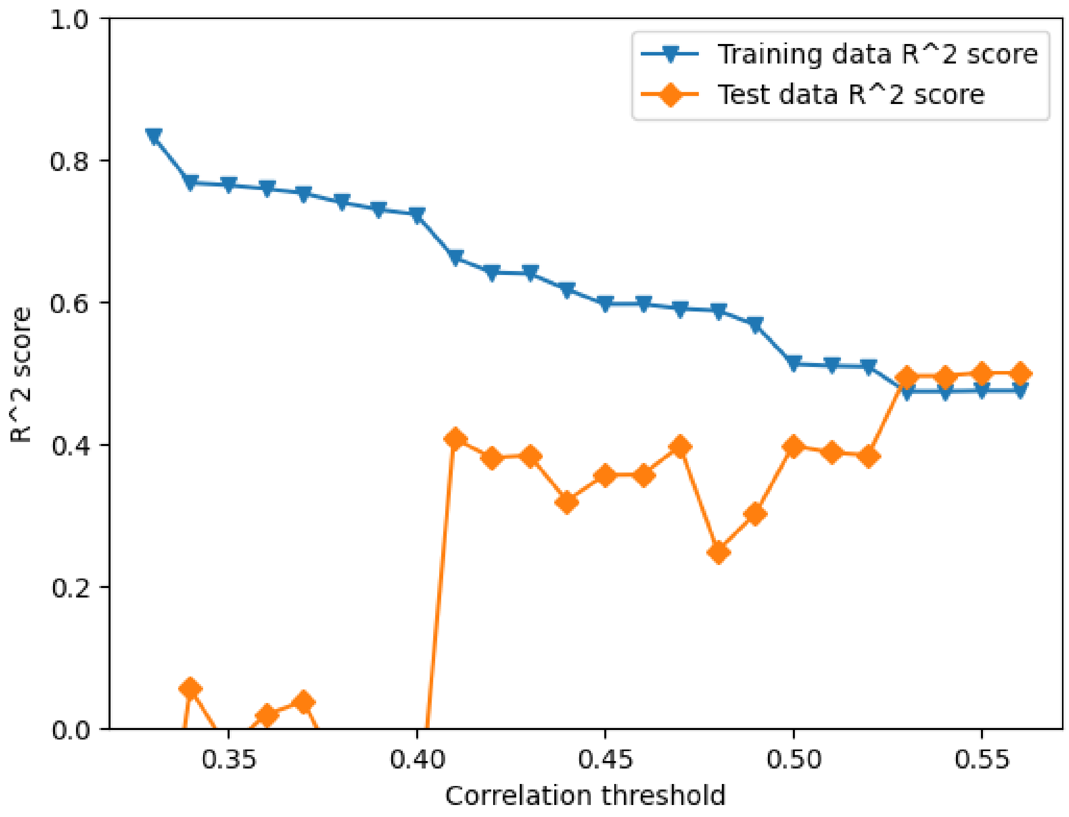

[44]:
```
plt.plot(df_without_standardization['Correlation threshold'],␣
 ↪df_without_standardization['Number of features'], label = "Number of␣
 ↪features", marker='.')
plt.legend()
plt.xlabel('Correlation threshold')
plt.ylabel('Number of features')
plt.show()
```

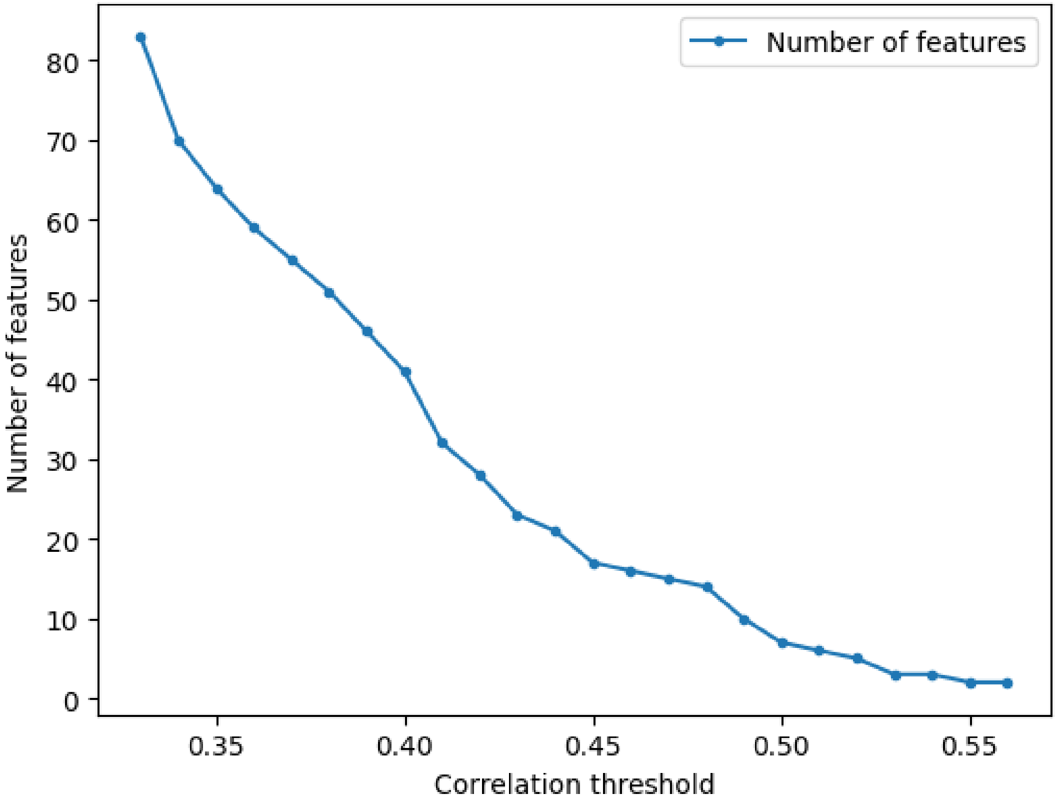

5.3 Store quality measurements

[45]:
```
with pd.ExcelWriter('../Data/Quality_'+'BALB_3T3'+'_'+str(random_state)+'_.
↪xlsx') as writer:
    df_linear.to_excel(writer, sheet_name='MLR')
    df_decision_tree.to_excel(writer, sheet_name='DT')
    df_random_forest.to_excel(writer, sheet_name='RF')
    df_k_nearest.to_excel(writer, sheet_name='KNN')
    df_svm.to_excel(writer, sheet_name='SVM')
```

Notebook

January 18, 2024

1 File 20

[1]:
```
import sys
from pathlib import Path
prediction_mode_path = Path("../module")
sys.path.append(prediction_mode_path.as_posix())
import models_creation as pred_model

import pandas as pd
import numpy as np

from sklearn.metrics import r2_score

import matplotlib.pyplot as plt
from sklearn.tree import plot_tree

from matplotlib import pyplot as plt

import joblib

import warnings
warnings.filterwarnings('ignore')
```

[2]:
```
load_prepared_data = pd.read_excel('../Data/Kolchicyna_machine_learning.xlsx')
load_prepared_data = load_prepared_data.drop(columns=['Unnamed: 0'])

target = 'BALB/3T3'

corr_low = 0.33
corr_high = 0.58

random_state = 42
```

[3]:
```
load_prepared_data.head()
```

[3]:
```
      AATS0Z  AATS0are    AATS0d   AATS0dv      AATS0i     AATS0m    AATS0p  \
0  25.090909  6.046518  3.109091  6.662626  160.775045  99.075564  1.555512
1  24.448276  6.008422  3.051724  6.386973  161.021674  96.473315  1.538471
```

```
2  23.868852  5.974074  3.000000  6.138434  161.244045  94.127025  1.523105
3  23.868852  5.974074  3.032787  6.171220  161.244045  94.127025  1.523105
4  23.343750  5.942945  2.953125  5.913194  161.445569  92.000700  1.509180

    AATS0pe    AATS0s   AATS0se  …  A549_float  MCF-7_float  LoVo_float  \
0  6.126680  3.435416  7.629859  …        10.8         10.3         6.5
1  6.088791  3.330998  7.596891  …        11.6         12.0         8.5
2  6.054630  3.236850  7.567166  …        10.9         12.2         8.8
3  6.054630  3.257798  7.567166  …        10.5         11.3         8.5
4  6.023670  3.151529  7.540228  …        89.5         92.7        52.8

   LoVo/DX_float  BALB/3T3_float  A549_float_transformed  \
0           54.9            10.2                7.966576
1           31.1            14.3                7.935542
2           17.9            11.7                7.962574
3           10.2            11.0                7.978811
4           77.8            99.4                7.048177

   MCF-7_float_transformed  LoVo_float_transformed  LoVo/DX_float_transformed  \
0                 7.987163                8.187087                   7.260428
1                 7.920819                8.070581                   7.507240
2                 7.913640                8.055517                   7.747147
3                 7.946922                8.070581                   7.991400
4                 7.032920                7.277366                   7.109020

   BALB/3T3_float_transformed
0                    7.991400
1                    7.844664
2                    7.931814
3                    7.958607
4                    7.002614

[5 rows x 1221 columns]
```

[4]:
```python
data = load_prepared_data[list(load_prepared_data.columns)[0:-10]]
data[target] = load_prepared_data[load_prepared_data.columns[-1]]
data.head()
```

[4]:
```
      AATS0Z  AATS0are    AATS0d   AATS0dv      AATS0i     AATS0m    AATS0p  \
0  25.090909  6.046518  3.109091  6.662626  160.775045  99.075564  1.555512
1  24.448276  6.008422  3.051724  6.386973  161.021674  96.473315  1.538471
2  23.868852  5.974074  3.000000  6.138434  161.244045  94.127025  1.523105
3  23.868852  5.974074  3.032787  6.171220  161.244045  94.127025  1.523105
4  23.343750  5.942945  2.953125  5.913194  161.445569  92.000700  1.509180

    AATS0pe    AATS0s   AATS0se  …    piPC10     piPC2     piPC3     piPC4  \
0  6.126680  3.435416  7.629859  …  7.999655  4.266195  4.915408  5.516700
```

```
1  6.088791  3.330998  7.596891  …  8.018726  4.280132  4.922714  5.520711
2  6.054630  3.236850  7.567166  …  8.028799  4.293878  4.929967  5.524706
3  6.054630  3.257798  7.567166  …  8.037440  4.307438  4.929967  5.524706
4  6.023670  3.151529  7.540228  …  8.034892  4.307438  4.937168  5.528685

      piPC5     piPC6     piPC7     piPC8     piPC9  BALB/3T3
0  6.098566  6.595759  6.979116  7.337313  7.681445  7.991400
1  6.103048  6.601209  6.985613  7.349442  7.695531  7.844664
2  6.105281  6.603923  6.989306  7.353932  7.704023  7.931814
3  6.107510  6.606629  6.992068  7.361425  7.709420  7.958607
4  6.107510  6.605277  6.991148  7.356489  7.707175  7.002614

[5 rows x 1212 columns]
```

2 Multiple Linear regression (MLR)

[5]:
```
without_standardization, train_r2_, test_r2_, hist1, hist2, target_column_name,␣
↪training_data_RMSE, test_data_RMSE  = pred_model.
↪prepare_data_and_create_model(molecular_descriptors_df=data,
                                                                                    ␣
↪                    correlation_threshold=0.5,
                                                                                    ␣
↪                    standardization=False,
                                                                                    ␣
↪                    model_type='linear_model',
                                                                                    ␣
↪                    target_column_name = str(target),
                                                                                    ␣
↪                    random_state=random_state,
                                                                                    ␣
↪                    train_test_split_=True,
                                                                                    ␣
↪                    verbose=True)
```

```
I am not doing standardization…
  molecular descriptor name
0                   AATS0Z
1                 AATS0are
2                   AATS0d
3                  AATS0dv
4                   AATS0i
  molecular descriptor name  corr_value
0                   AATS0Z   -0.020817
1                 AATS0are   -0.148257
2                   AATS0d    0.022999
3                  AATS0dv   -0.137980
```

```
4                   AATS0i    0.133257
  molecular descriptor name  corr_value  absolute correlation value
0                   AATS0Z   -0.020817                    0.020817
1                 AATS0are   -0.148257                    0.148257
2                   AATS0d    0.022999                    0.022999
3                  AATS0dv   -0.137980                    0.137980
4                   AATS0i    0.133257                    0.133257
    molecular descriptor name  corr_value  absolute correlation value
226                    AMID_O   -0.546843                    0.546843
505                EState_VSA5   -0.588780                    0.588780
556                    GATS2c    0.511671                    0.511671
791                   MDEO-12   -0.582747                    0.582747
847                       NdO   -0.500462                    0.500462
    molecular descriptor name  corr_value  absolute correlation value
226                    AMID_O   -0.546843                    0.546843
505                EState_VSA5   -0.588780                    0.588780
556                    GATS2c    0.511671                    0.511671
791                   MDEO-12   -0.582747                    0.582747
847                       NdO   -0.500462                    0.500462
The model used is: LinearReg…
Return the coefficient of determination of the prediction:
0.4009396151775283
R^2 score: 0.5596286877136032
Correlation coefficient: 0.7480833427590827
Test data - unseen during training:
R^2 score: 0.4009396151775283
Correlation coefficient: 0.6331979273319902
[8.01069718 8.00147704 7.32934201 7.05871281 5.8496097  7.6259535
 7.85353668 7.58094605 7.6505256  7.32688798 7.52250572 6.68303412
 7.70848619 6.37971395 7.08878708 8.02437081 7.42197046 7.52846533]
44     8.004365
47     7.886057
4      7.002614
55     7.698970
26     5.070734
64     8.060481
73     7.130768
10     8.008774
40     7.795880
107    8.086186
18     6.924818
62     8.173925
11     7.962574
36     6.346787
89     8.102373
91     8.346787
109    7.886057
0      7.991400
```

```
Name: BALB/3T3, dtype: float64
Training Root Mean Square Error: 0.5846600833873117
Testing Root Mean Square Error: 0.6162259719902169
```

[6]:
```
print(target_column_name+str('_transformed'))
print(hist1[target_column_name].hist())
```

```
BALB/3T3_transformed
AxesSubplot(0.125,0.11;0.775x0.77)
```

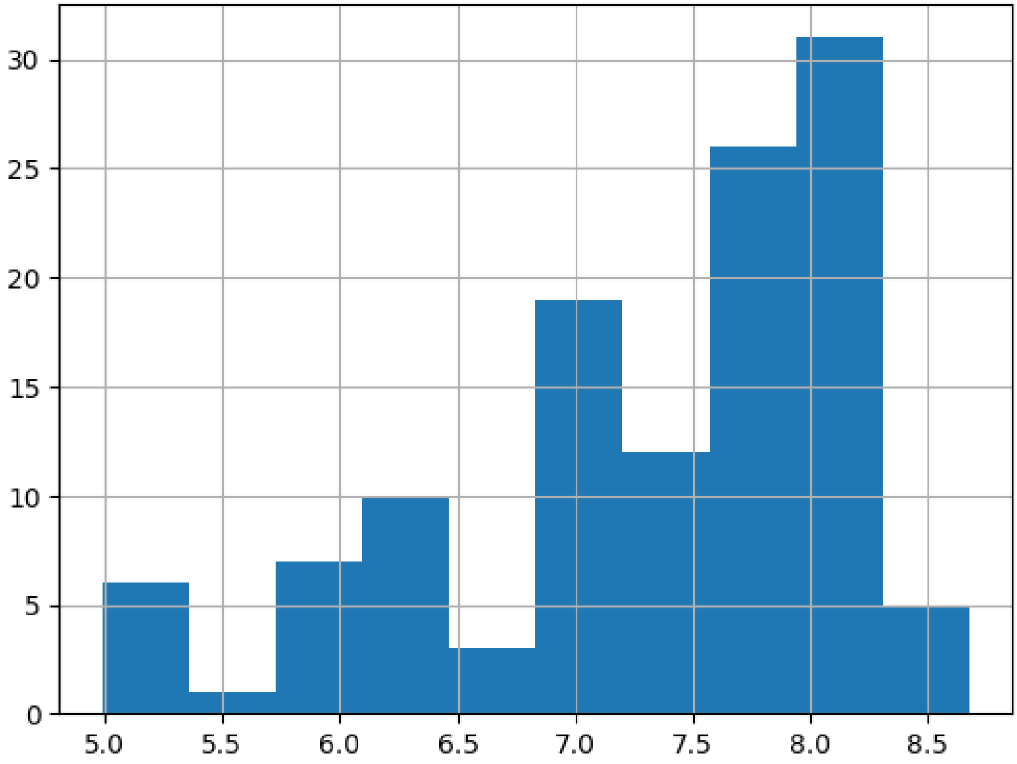

[7]:
```
with_standardization, train_r2_, test_r2_, hist1, hist2, target_column_name,␣
↪training_data_RMSE, test_data_RMSE  = pred_model.
↪prepare_data_and_create_model(molecular_descriptors_df=data,
                                                                              ␣
↪                       correlation_threshold=0.50,
                                                                              ␣
↪                       standardization=True,
                                                                              ␣
↪                       model_type='linear_model',
                                                                              ␣
↪                       target_column_name = target,
```

```
                        random_state=random_state,
                        train_test_split_=True,
                        verbose=True)
```

```
I am doing standardization…
  molecular descriptor name
0                    AATS0Z
1                  AATS0are
2                    AATS0d
3                   AATS0dv
4                    AATS0i
  molecular descriptor name  corr_value
0                    AATS0Z   -0.020817
1                  AATS0are   -0.148257
2                    AATS0d    0.022999
3                   AATS0dv   -0.137980
4                    AATS0i    0.133257
  molecular descriptor name  corr_value  absolute correlation value
0                    AATS0Z   -0.020817                    0.020817
1                  AATS0are   -0.148257                    0.148257
2                    AATS0d    0.022999                    0.022999
3                   AATS0dv   -0.137980                    0.137980
4                    AATS0i    0.133257                    0.133257
    molecular descriptor name  corr_value  absolute correlation value
226                    AMID_O   -0.546843                    0.546843
505               EState_VSA5   -0.588780                    0.588780
556                    GATS2c    0.511671                    0.511671
791                   MDEO-12   -0.582747                    0.582747
847                       NdO   -0.500462                    0.500462
    molecular descriptor name  corr_value  absolute correlation value
226                    AMID_O   -0.546843                    0.546843
505               EState_VSA5   -0.588780                    0.588780
556                    GATS2c    0.511671                    0.511671
791                   MDEO-12   -0.582747                    0.582747
847                       NdO   -0.500462                    0.500462
The model used is: LinearReg…
Return the coefficient of determination of the prediction:
0.4009396151775283
R^2 score: 0.5596286877136032
Correlation coefficient: 0.7480833427590827
Test data - unseen during training:
R^2 score: 0.4009396151775283
Correlation coefficient: 0.6331979273319902
[8.01069718 8.00147704 7.32934201 7.05871281 5.8496097  7.6259535
```

 7.85353668 7.58094605 7.6505256 7.32688798 7.52250572 6.68303412
 7.70848619 6.37971395 7.08878708 8.02437081 7.42197046 7.52846533]
44   8.004365
47   7.886057
4   7.002614
55   7.698970
26   5.070734
64   8.060481
73   7.130768
10   8.008774
40   7.795880
107   8.086186
18   6.924818
62   8.173925
11   7.962574
36   6.346787
89   8.102373
91   8.346787
109   7.886057
0   7.991400
Name: BALB/3T3, dtype: float64
Training Root Mean Square Error: 0.5846600833873117
Testing Root Mean Square Error: 0.6162259719902169

2.1 Search inside correlation space

[8]:
```
step = 0.01
initial_step = corr_low
last_step = corr_high
first_list = [x / 100.0 for x in range(int(initial_step*100),␣
 ↪int(last_step*100), int(step*100))]
second_list = []
third_list = []
fourth_l = []
fifth_l = []
f_list = []
for i in first_list:
    without_standardization, train_r2, test_r2, _, h_, target_column_name,␣
 ↪training_data_RMSE, test_data_RMSE = pred_model.
 ↪prepare_data_and_create_model(molecular_descriptors_df = data,
                                                                              ␣
 ↪                     correlation_threshold = i,
                                                                              ␣
 ↪                     standardization = False,
                                                                              ␣
 ↪                     model_type = 'linear_model',
```

```
                     target_column_name = target,
                     random_state=random_state,
                     train_test_split_ = True,
                     verbose = False)
    second_list.append(train_r2)
    third_list.append(test_r2)
    fourth_l.append(training_data_RMSE)
    fifth_l.append(test_data_RMSE)
    f_list.append(len(h_))
```

[9]:
```
df_without_standardization = pd.DataFrame(data=first_list,
 columns=["Correlation threshold"])
df_without_standardization['Training data R^2 score'] = second_list
df_without_standardization['Test data R^2 score'] = third_list
df_without_standardization['Training RMSE'] = fourth_l
df_without_standardization['Test RMSE'] = fifth_l
df_without_standardization['Number of features'] = f_list
```

[10]:
```
df_linear = df_without_standardization.copy()
df_without_standardization
```

[10]:

	Correlation threshold	Training data R^2 score	Test data R^2 score \
0	0.33	0.977568	-0.972190
1	0.34	0.920734	0.635875
2	0.35	0.915551	0.700364
3	0.36	0.908437	0.515587
4	0.37	0.895571	0.347253
5	0.38	0.858816	0.051724
6	0.39	0.809659	0.155354
7	0.40	0.794374	0.027209
8	0.41	0.725684	0.086462
9	0.42	0.656192	0.609335
10	0.43	0.633192	0.710255
11	0.44	0.630292	0.721657
12	0.45	0.611501	0.562029
13	0.46	0.611461	0.563456
14	0.47	0.611021	0.551893
15	0.48	0.609700	0.541209
16	0.49	0.588355	0.449447
17	0.50	0.559629	0.400940
18	0.51	0.533961	0.451107
19	0.52	0.531025	0.459570
20	0.53	0.499084	0.508269

```
21                     0.54                    0.499084                 0.508269
22                     0.55                    0.477930                 0.485309
23                     0.56                    0.477930                 0.485309

    Training RMSE  Test RMSE  Number of features
0        0.131955   1.118096                  83
1        0.248049   0.480430                  70
2        0.256030   0.435814                  64
3        0.266596   0.554131                  59
4        0.284712   0.643246                  55
5        0.331045   0.775304                  51
6        0.384380   0.731715                  46
7        0.399515   0.785262                  41
8        0.461444   0.760971                  32
9        0.516597   0.497631                  28
10       0.533597   0.428561                  23
11       0.535702   0.420044                  21
12       0.549147   0.526899                  17
13       0.549176   0.526040                  16
14       0.549486   0.532961                  15
15       0.550419   0.539277                  14
16       0.565269   0.590751                  10
17       0.584660   0.616226                   7
18       0.601457   0.589859                   6
19       0.603350   0.585295                   5
20       0.623557   0.558301                   3
21       0.623557   0.558301                   3
22       0.636588   0.571187                   2
23       0.636588   0.571187                   2
```

[ ]:

2.2 Plots

[11]:
```
plt.plot(df_without_standardization['Correlation threshold'],␣
  ↪df_without_standardization['Training data R^2 score'], label = "Training␣
  ↪data R^2 score", marker='v')
plt.plot(df_without_standardization['Correlation threshold'],␣
  ↪df_without_standardization['Test data R^2 score'], label = "Test data R^2␣
  ↪score", marker='D')
plt.legend()
plt.xlabel('Correlation threshold')
plt.ylabel('R^2 score')
plt.ylim([0, 1])
plt.show()
```

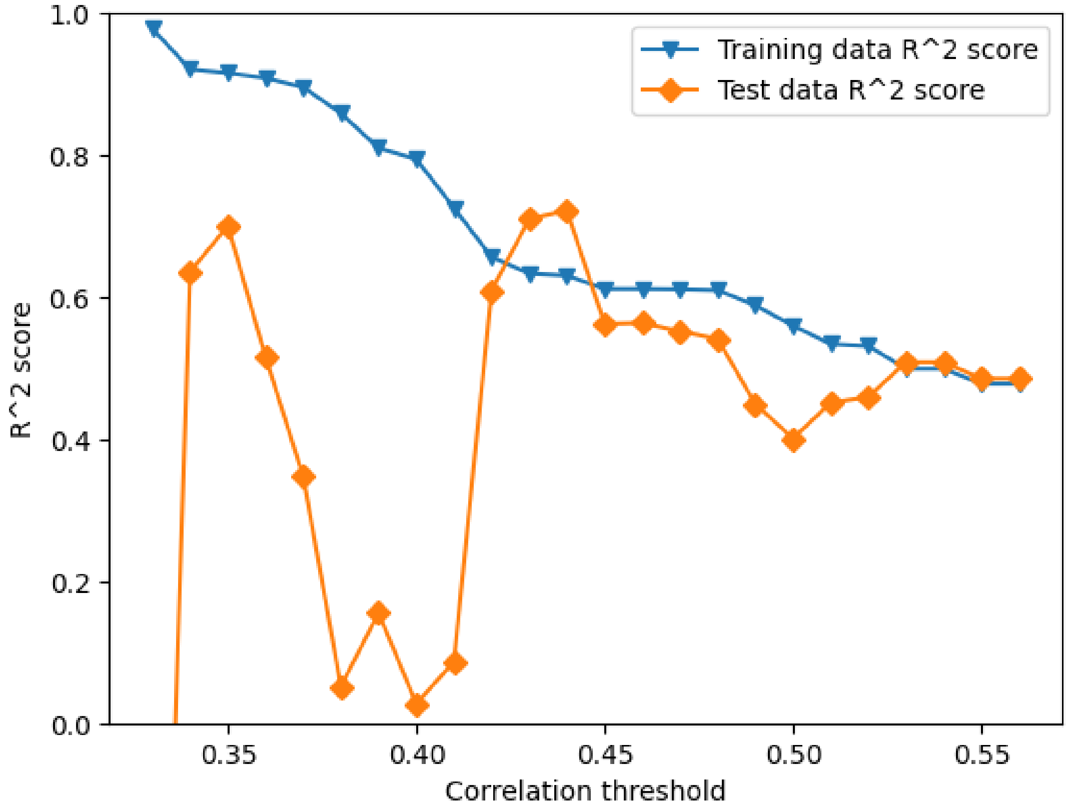

[12]:
```
plt.plot(df_without_standardization['Correlation threshold'],␣
 ↪df_without_standardization['Number of features'], label = "Number of␣
 ↪features", marker='.')
plt.legend()
plt.xlabel('Correlation threshold')
plt.ylabel('Number of features')
plt.show()
```

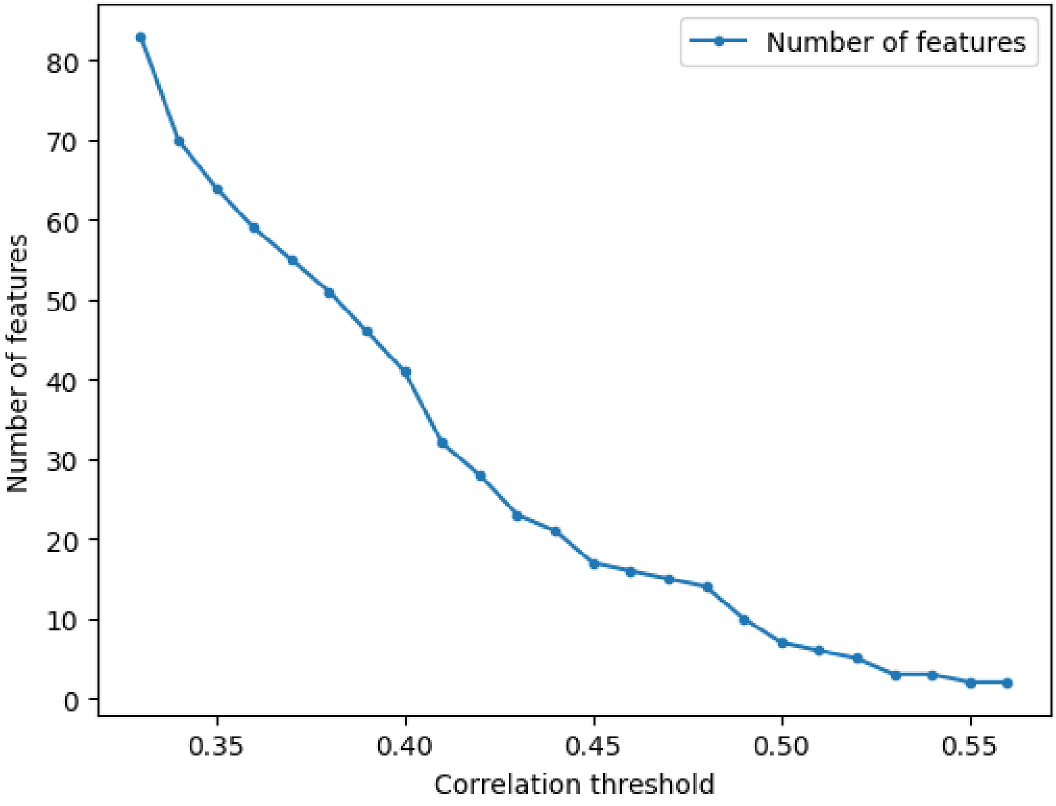


```
[ ]:
```

2.3 Decision Tree

```
[13]: without_standardization, train_r2_, test_r2_, hist1, hist2, target_column_name,␣
        ↪training_data_RMSE, test_data_RMSE  = pred_model.
        ↪prepare_data_and_create_model(molecular_descriptors_df=data,
                                                                                 ␣
        ↪                         correlation_threshold=0.50,
                                                                                 ␣
        ↪                         standardization=False,
                                                                                 ␣
        ↪                         model_type='DecisionTreeRegressor',
                                                                                 ␣
        ↪                         max_depth=5,
                                                                                 ␣
        ↪                         target_column_name = target,
                                                                                 ␣
        ↪                         random_state=random_state,
```

```
↪                     train_test_split_=True,
↪                     verbose=True)
```

```
I am not doing standardization…
  molecular descriptor name
0                    AATS0Z
1                  AATS0are
2                    AATS0d
3                   AATS0dv
4                    AATS0i
  molecular descriptor name  corr_value
0                    AATS0Z   -0.020817
1                  AATS0are   -0.148257
2                    AATS0d    0.022999
3                   AATS0dv   -0.137980
4                    AATS0i    0.133257
  molecular descriptor name  corr_value  absolute correlation value
0                    AATS0Z   -0.020817                    0.020817
1                  AATS0are   -0.148257                    0.148257
2                    AATS0d    0.022999                    0.022999
3                   AATS0dv   -0.137980                    0.137980
4                    AATS0i    0.133257                    0.133257
    molecular descriptor name  corr_value  absolute correlation value
226                    AMID_O   -0.546843                    0.546843
505               EState_VSA5   -0.588780                    0.588780
556                    GATS2c    0.511671                    0.511671
791                   MDEO-12   -0.582747                    0.582747
847                       NdO   -0.500462                    0.500462
    molecular descriptor name  corr_value  absolute correlation value
226                    AMID_O   -0.546843                    0.546843
505               EState_VSA5   -0.588780                    0.588780
556                    GATS2c    0.511671                    0.511671
791                   MDEO-12   -0.582747                    0.582747
847                       NdO   -0.500462                    0.500462
The model used is: DecisionTree…
Return the coefficient of determination of the prediction:
0.5592532851606207
R^2 score: 0.8558567162424137
Correlation coefficient: 0.9251252435440369
Test data - unseen during training:
R^2 score: 0.5592532851606207
Correlation coefficient: 0.7478323910881506
[7.1136393  7.84909171 7.31397077 7.00060493 5.12662126 7.84909171
 7.84909171 7.84909171 7.84909171 7.84909171 7.1136393  7.31397077
 7.84909171 6.56863624 7.31397077 7.1136393  7.84909171 7.84909171]
44     8.004365
```

```
47     7.886057
4      7.002614
55     7.698970
26     5.070734
64     8.060481
73     7.130768
10     8.008774
40     7.795880
107    8.086186
18     6.924818
62     8.173925
11     7.962574
36     6.346787
89     8.102373
91     8.346787
109    7.886057
0      7.991400
Name: BALB/3T3, dtype: float64
Training Root Mean Square Error: 0.33449616294891693
Testing Root Mean Square Error: 0.5285660197089223
```

[14]:
```python
print(target_column_name+str('_transformed'))
print(hist1[target_column_name].hist())
```

```
BALB/3T3_transformed
AxesSubplot(0.125,0.11;0.775x0.77)
```

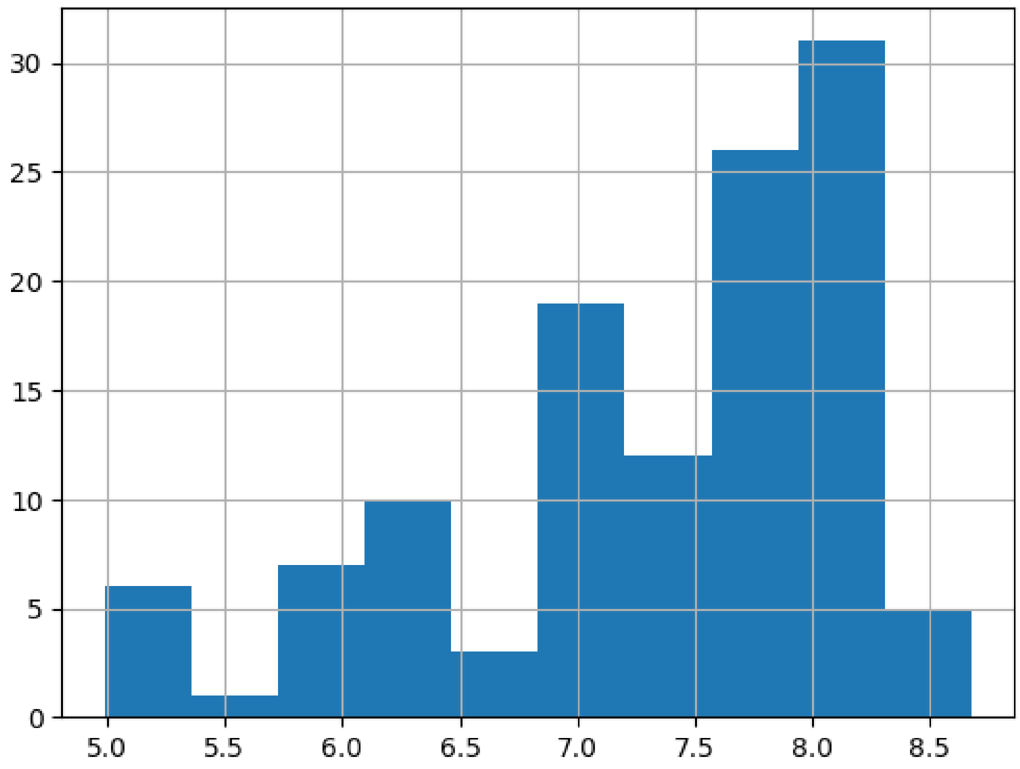

[15]:
```
with_standardization, train_r2_, test_r2_, hist1, hist2, target_column_name,␣
↪training_data_RMSE, test_data_RMSE  = pred_model.
↪prepare_data_and_create_model(molecular_descriptors_df=data,
                                                                                                    ␣
↪                        correlation_threshold=0.50,
                                                                                                    ␣
↪                        standardization=True,
                                                                                                    ␣
↪                        model_type='DecisionTreeRegressor',
                                                                                                    ␣
↪                        max_depth=5,
                                                                                                    ␣
↪                        target_column_name = target,
                                                                                                    ␣
↪                        random_state=random_state,
                                                                                                    ␣
↪                        train_test_split_=True,
                                                                                                    ␣
↪                        verbose=True)
```

```
I am doing standardization…
  molecular descriptor name
```

```
0                 AATS0Z
1               AATS0are
2                 AATS0d
3                AATS0dv
4                 AATS0i
  molecular descriptor name  corr_value
0                 AATS0Z   -0.020817
1               AATS0are   -0.148257
2                 AATS0d    0.022999
3                AATS0dv   -0.137980
4                 AATS0i    0.133257
  molecular descriptor name  corr_value  absolute correlation value
0                 AATS0Z   -0.020817                   0.020817
1               AATS0are   -0.148257                   0.148257
2                 AATS0d    0.022999                   0.022999
3                AATS0dv   -0.137980                   0.137980
4                 AATS0i    0.133257                   0.133257
    molecular descriptor name  corr_value  absolute correlation value
226                 AMID_O   -0.546843                   0.546843
505             EState_VSA5   -0.588780                   0.588780
556                 GATS2c    0.511671                   0.511671
791                MDEO-12   -0.582747                   0.582747
847                    NdO   -0.500462                   0.500462
    molecular descriptor name  corr_value  absolute correlation value
226                 AMID_O   -0.546843                   0.546843
505             EState_VSA5   -0.588780                   0.588780
556                 GATS2c    0.511671                   0.511671
791                MDEO-12   -0.582747                   0.582747
847                    NdO   -0.500462                   0.500462
The model used is: DecisionTree…
Return the coefficient of determination of the prediction:
0.5592532851606207
R^2 score: 0.8558567162424137
Correlation coefficient: 0.9251252435440369
Test data - unseen during training:
R^2 score: 0.5592532851606207
Correlation coefficient: 0.7478323910881506
[7.1136393  7.84909171 7.31397077 7.00060493 5.12662126 7.84909171
 7.84909171 7.84909171 7.84909171 7.84909171 7.1136393  7.31397077
 7.84909171 6.56863624 7.31397077 7.1136393  7.84909171 7.84909171]
44     8.004365
47     7.886057
4      7.002614
55     7.698970
26     5.070734
64     8.060481
73     7.130768
10     8.008774
```

```
40     7.795880
107    8.086186
18     6.924818
62     8.173925
11     7.962574
36     6.346787
89     8.102373
91     8.346787
109    7.886057
0      7.991400
Name: BALB/3T3, dtype: float64
Training Root Mean Square Error: 0.33449616294891693
Testing Root Mean Square Error: 0.5285660197089223
```

2.4 Search inside correlation space

[16]:
```
step = 0.01
initial_step = corr_low
last_step = corr_high
first_list = [x / 100.0 for x in range(int(initial_step*100),␣
 ↪int(last_step*100), int(step*100))]
max_depth = [range(2, 30, 1)]
corr_th = []
second_list = []
third_list = []
fourth_l = []
fifth_l = []
f_list = []
fif_list = []
for i in first_list:
    for depth in max_depth[0]:

        without_standardization, train_r2, test_r2, _, h_, target_column_name,␣
 ↪training_data_RMSE, test_data_RMSE = pred_model.
 ↪prepare_data_and_create_model(molecular_descriptors_df=data,
                                                                              ␣
 ↪                     correlation_threshold=i,
                                                                              ␣
 ↪                     standardization=False,
                                                                              ␣
 ↪                     model_type='DecisionTreeRegressor',
                                                                              ␣
 ↪                     max_depth=depth,
                                                                              ␣
 ↪                     target_column_name = target,
                                                                              ␣
 ↪                     random_state=random_state,
```

```
↪                       train_test_split_=True,
↪                       verbose=False)
        corr_th.append(i)
        second_list.append(train_r2)
        third_list.append(test_r2)
        fourth_l.append(training_data_RMSE)
        fifth_l.append(test_data_RMSE)
        f_list.append(len(h_))
        fif_list.append(depth)
```

[17]:
```
df_without_standardization = pd.DataFrame(data=corr_th, columns=["Correlation␣
↪threshold"])
df_without_standardization['Training data R^2 score'] = second_list
df_without_standardization['Test data R^2 score'] = third_list
df_without_standardization['Training RMSE'] = fourth_l
df_without_standardization['Test RMSE'] = fifth_l
df_without_standardization['Number of features'] = f_list
df_without_standardization['Depth number'] = fif_list
```

[18]:
```
df_decision_tree = df_without_standardization.copy()
#df_without_standardization.to_excel('../Data/
↪A549_Decision_tree_rs_'+str(random_state)+'.xlsx')
```

2.5 Plots

[19]:
```
corre_list = [x / 100.0 for x in range(int(initial_step*100),␣
↪int(last_step*100), int(0.01*100))]
for element in corre_list:
    element_ =␣
↪df_without_standardization[df_without_standardization['Correlation␣
↪threshold'] == float(element)]
    plt.plot(element_['Depth number'], element_['Training data R^2 score'],␣
↪label = "Training data R^2 score", marker='v')
    plt.plot(element_['Depth number'], element_['Test data R^2 score'], label =␣
↪"Test data R^2 score", marker='D')
    plt.legend()
    plt.xlabel('Depth number -> correlation threshold: '+str(element)+'. \n␣
↪Number of features: '+str(element_['Number of features'].iloc[0]),␣
↪fontweight='bold')
    plt.xlim(max(element_['Depth number'])+1, min(element_['Depth number'])-1)
    plt.ylabel('R^2 score')
    plt.ylim([0, 1])
    plt.rc('grid', linestyle="-.", color='black')
    plt.grid(True)
    plt.show()
```

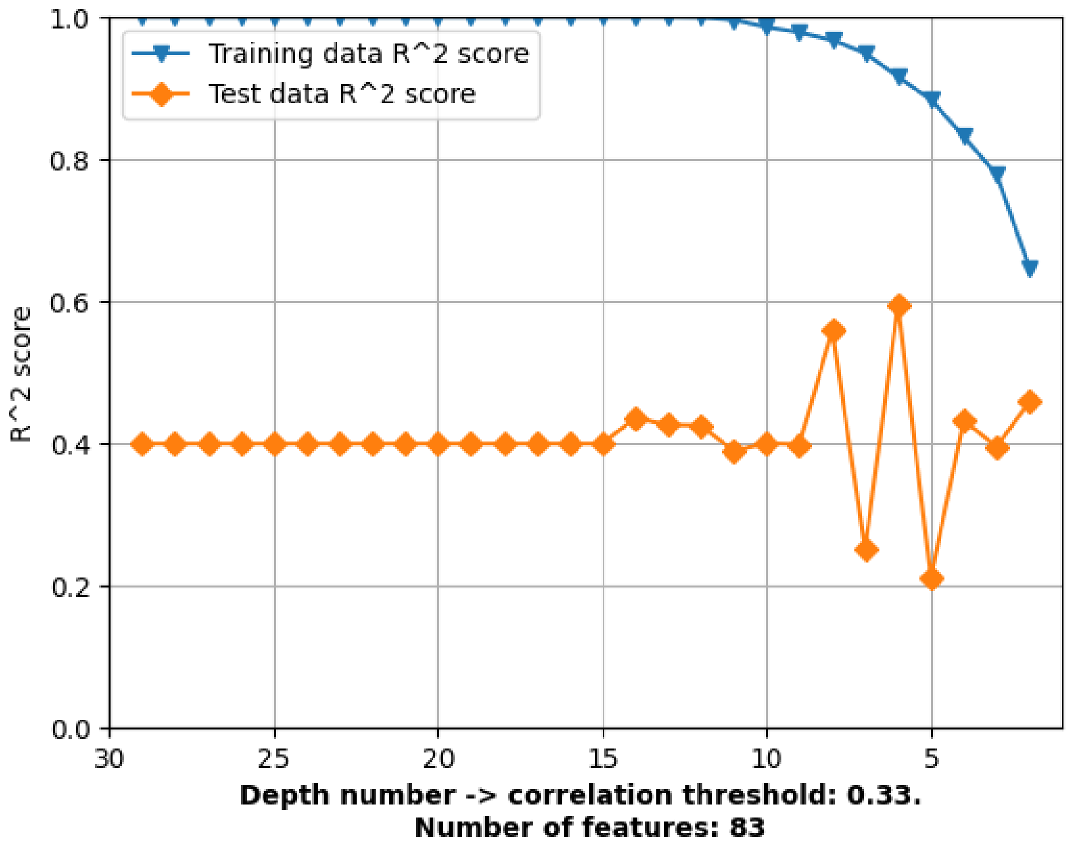

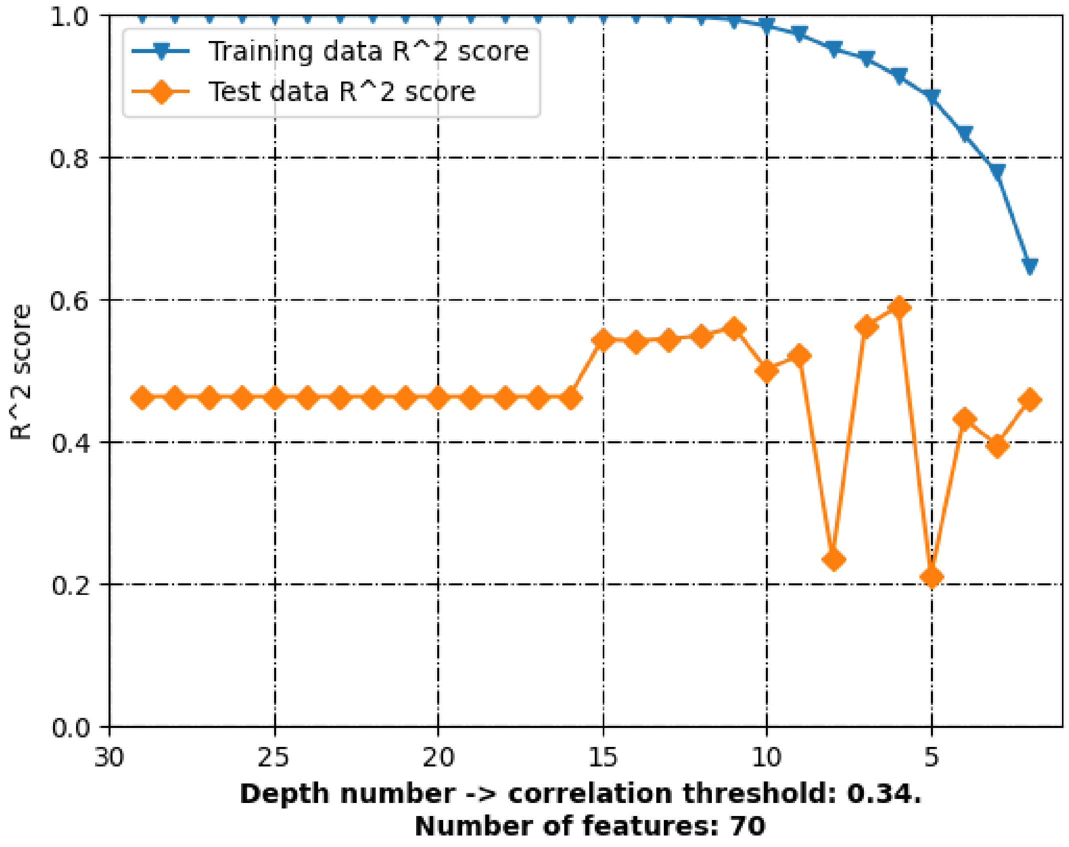

Training data R^2 score
Test data R^2 score
R^2 score
1.0
0.8
0.6
0.4
0.2
0.0
30
25
20
15
10
5
Depth number -> correlation threshold: 0.34.
Number of features: 70

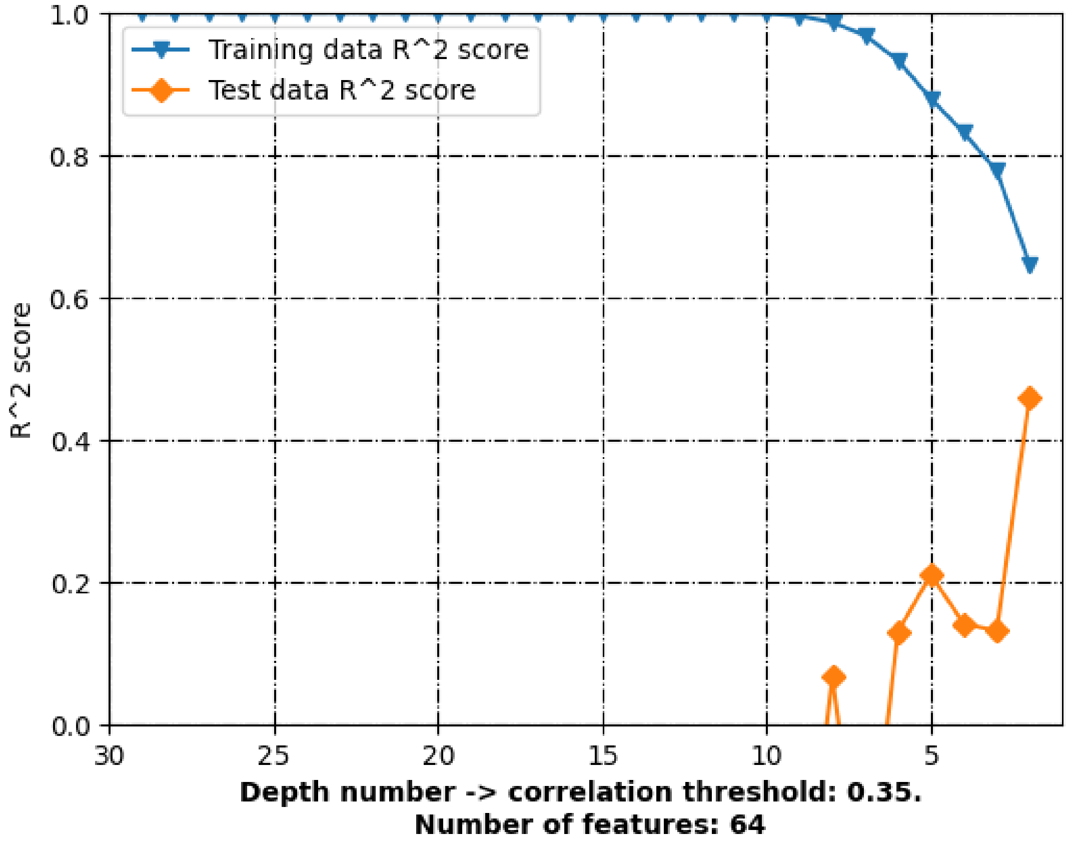

Training data R^2 score
Test data R^2 score
R^2 score
1.0
0.8
0.6
0.4
0.2
0.0
30
25
20
15
10
5
Depth number -> correlation threshold: 0.35.
Number of features: 64

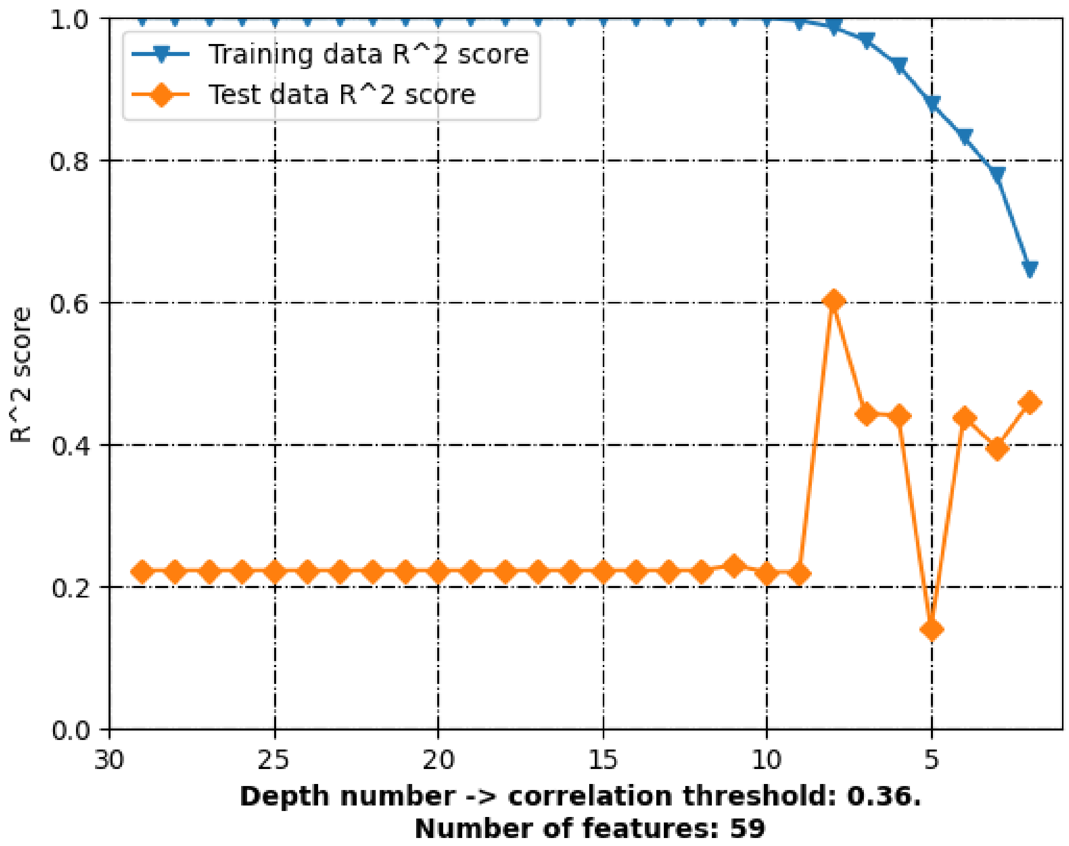

Training data R^2 score
Test data R^2 score
R^2 score
1.0
0.8
0.6
0.4
0.2
0.0
30
25
20
15
10
5
Depth number -> correlation threshold: 0.36.
Number of features: 59

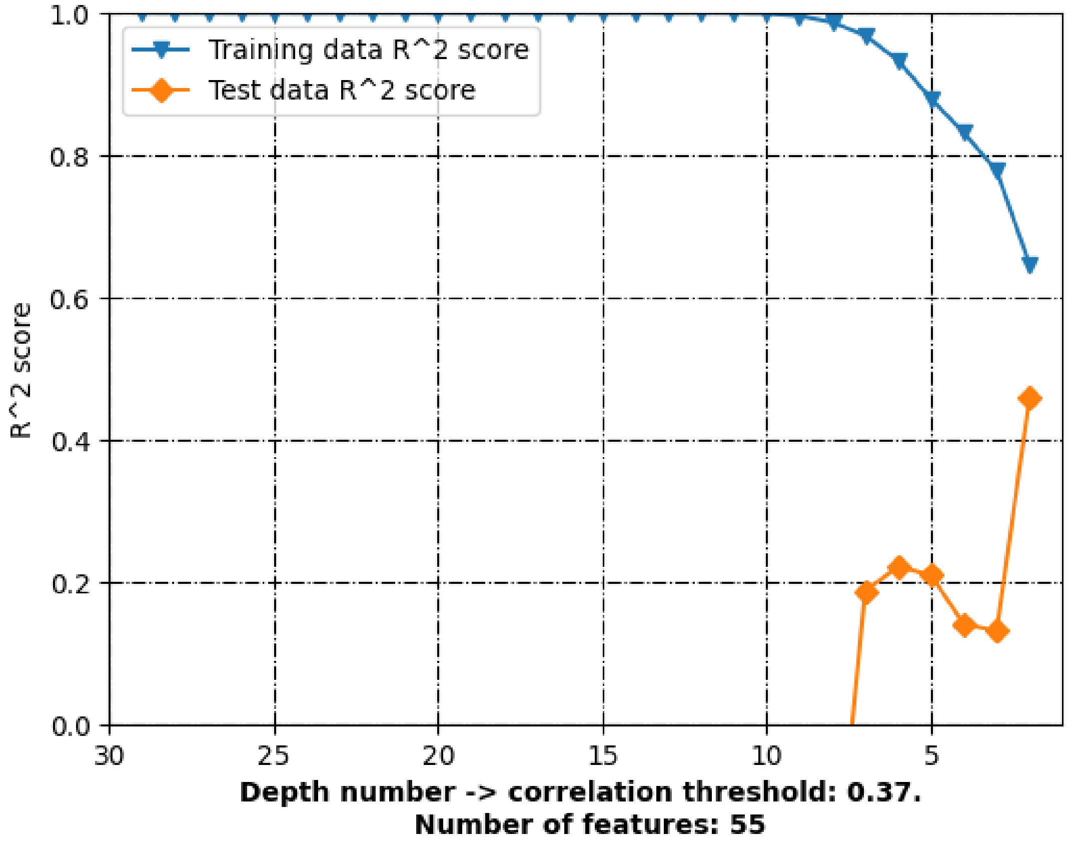
1.0
0.8
0.6
0.4
0.2
0.0
R^2 score
Training data R^2 score
Test data R^2 score
30
25
20
15
10
5
Depth number -> correlation threshold: 0.37.
Number of features: 55

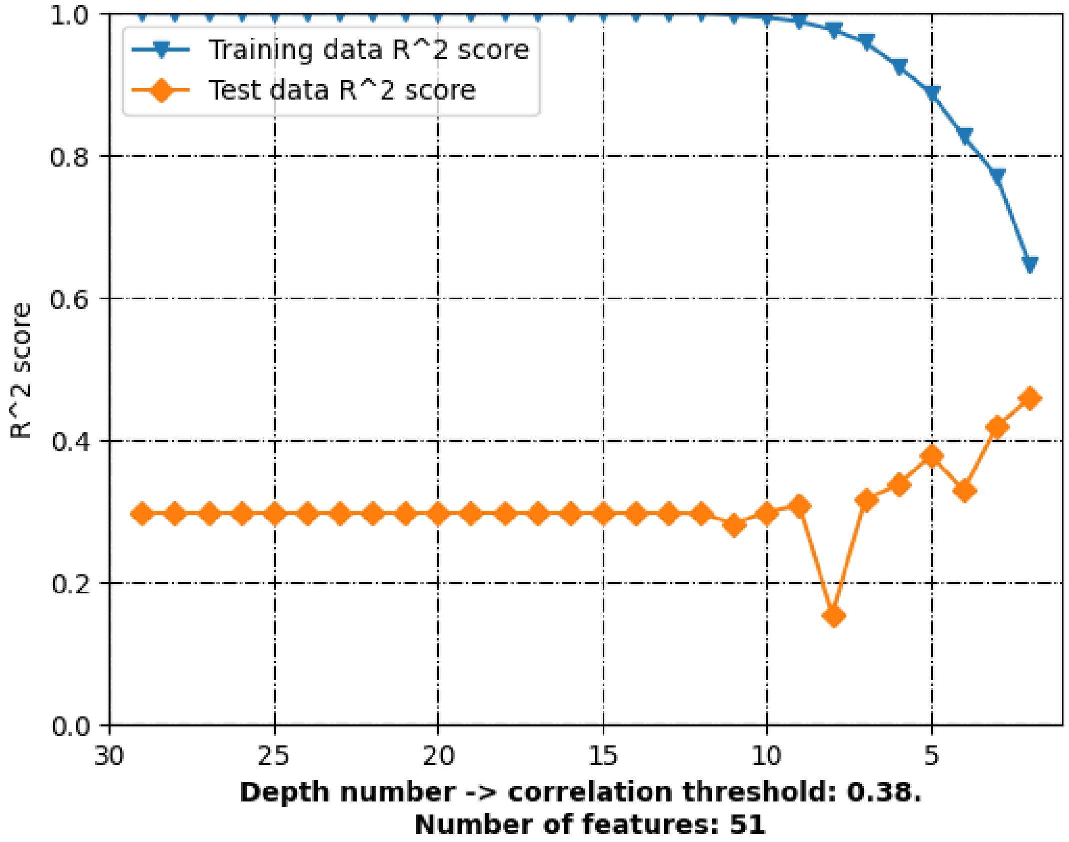

Training data R^2 score
Test data R^2 score
R^2 score
1.0
0.8
0.6
0.4
0.2
0.0
30
25
20
15
10
5
Depth number -> correlation threshold: 0.38.
Number of features: 51

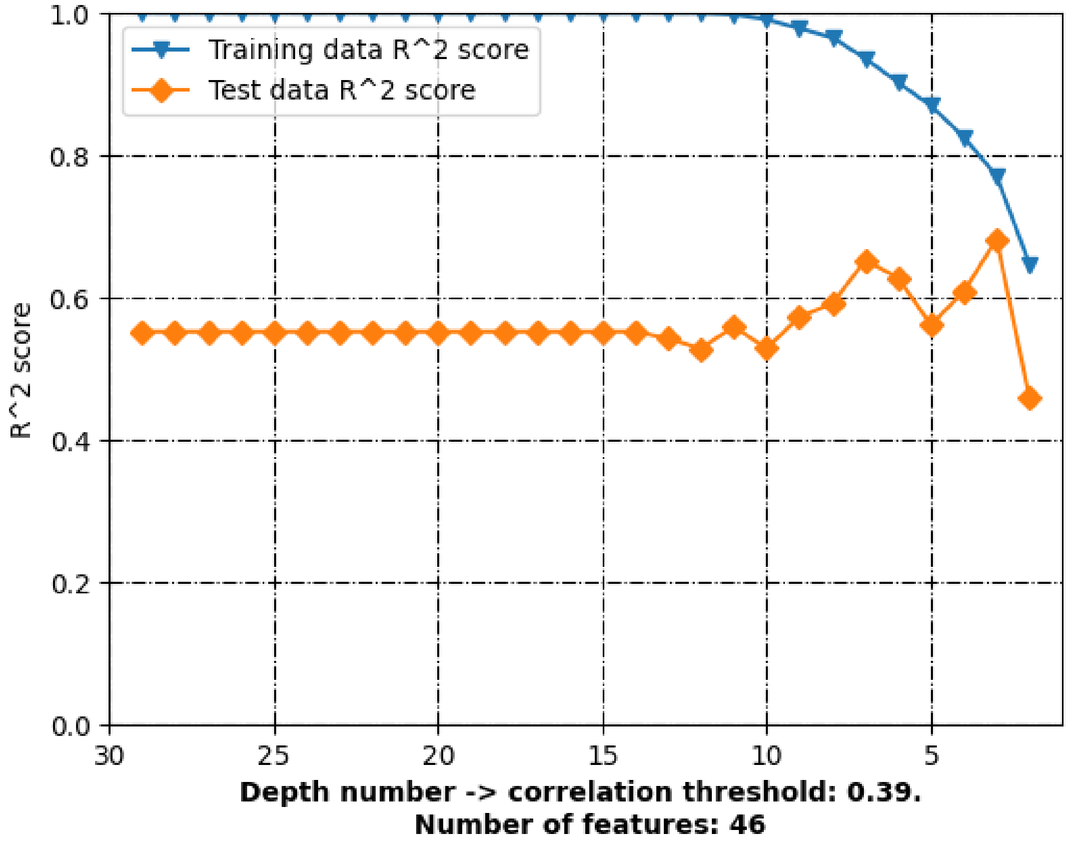
Training data R^2 score
Test data R^2 score
R^2 score
1.0
0.8
0.6
0.4
0.2
0.0
30
25
20
15
10
5
Depth number -> correlation threshold: 0.39.
Number of features: 46

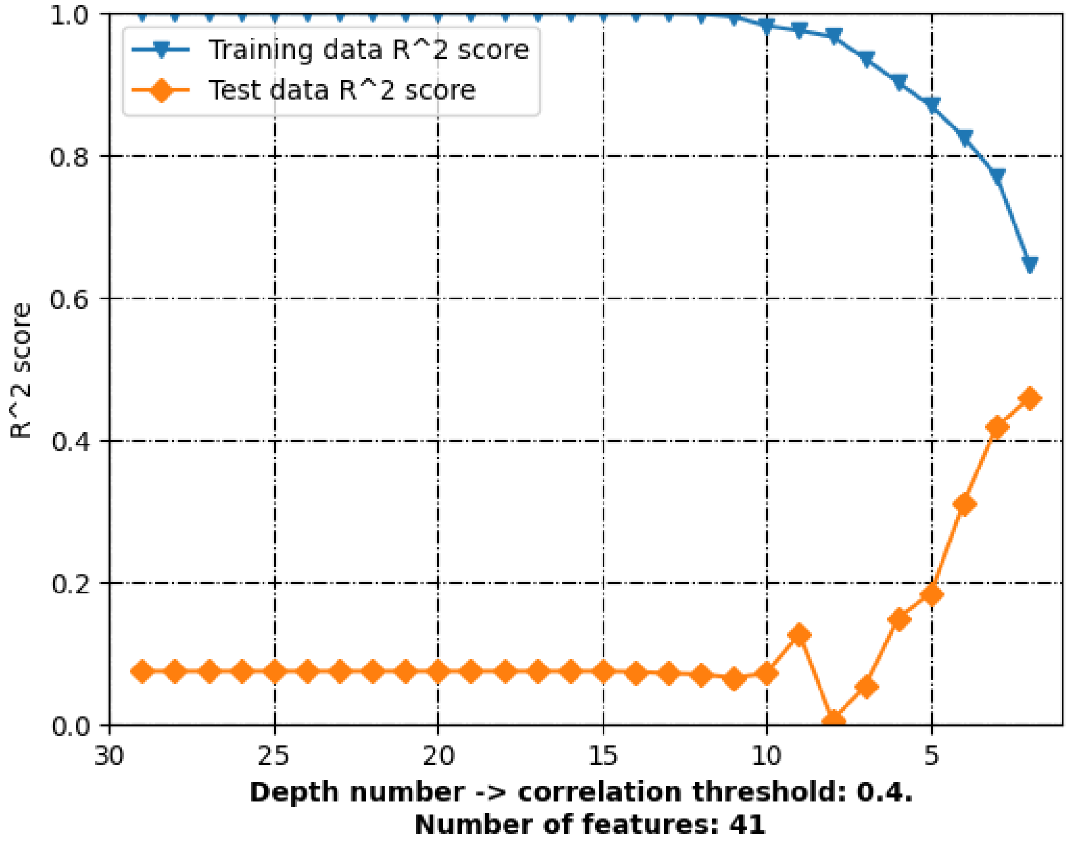

Training data R^2 score
Test data R^2 score
R^2 score
1.0
0.8
0.6
0.4
0.2
0.0
30
25
20
15
10
5
Depth number -> correlation threshold: 0.4.
Number of features: 41

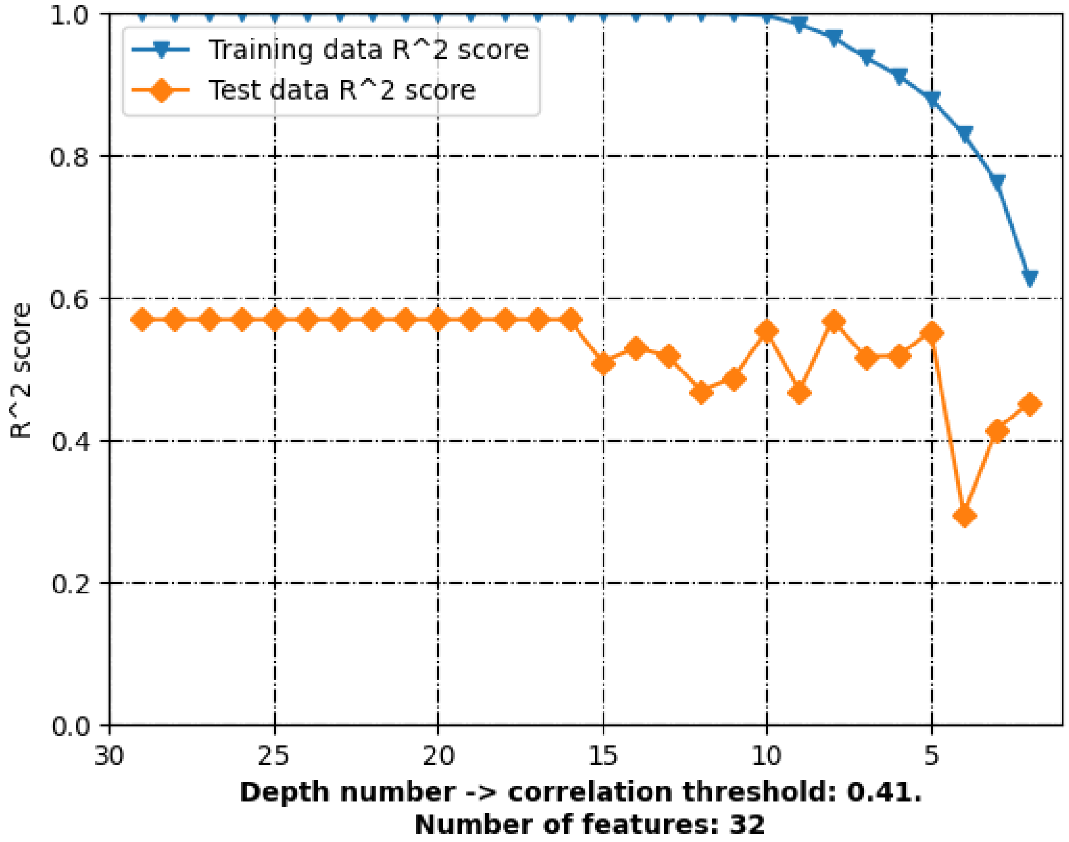
Training data R^2 score
Test data R^2 score
R^2 score
1.0
0.8
0.6
0.4
0.2
0.0
30
25
20
15
10
5
Depth number -> correlation threshold: 0.41.
Number of features: 32

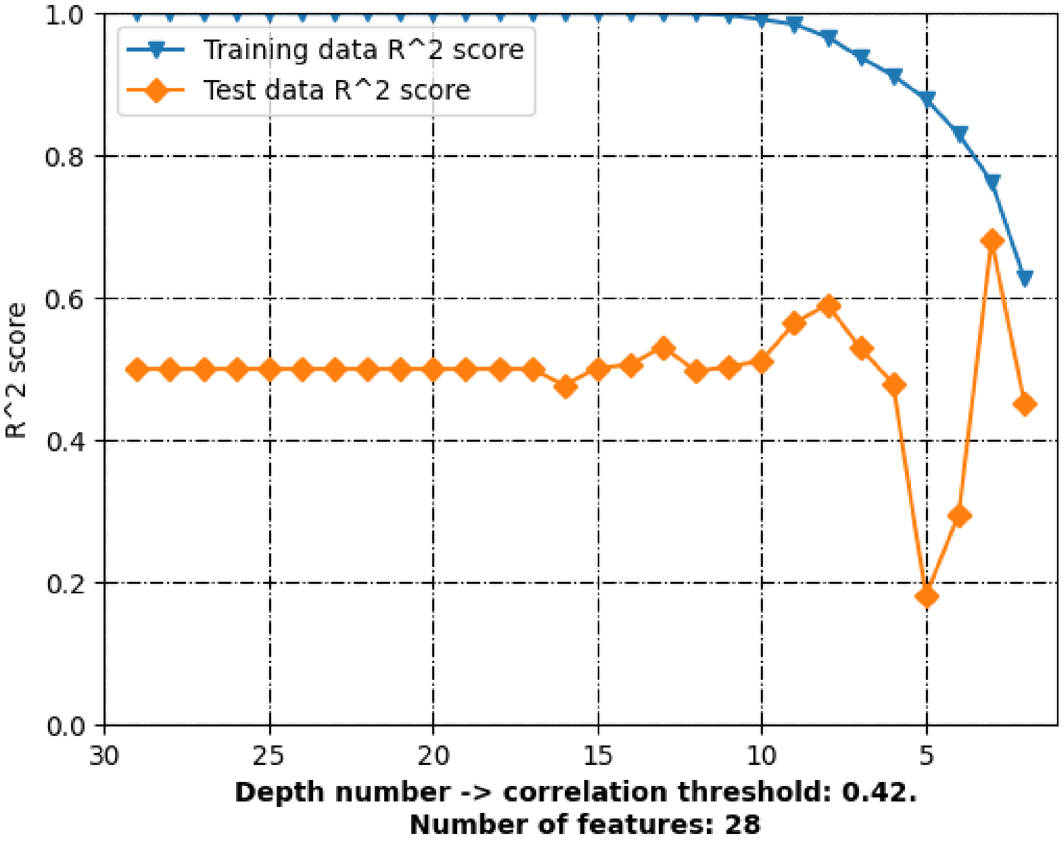
Training data R^2 score
Test data R^2 score
R^2 score
Depth number -> correlation threshold: 0.42.
Number of features: 28
1.0
0.8
0.6
0.4
0.2
0.0
30
25
20
15
10
5

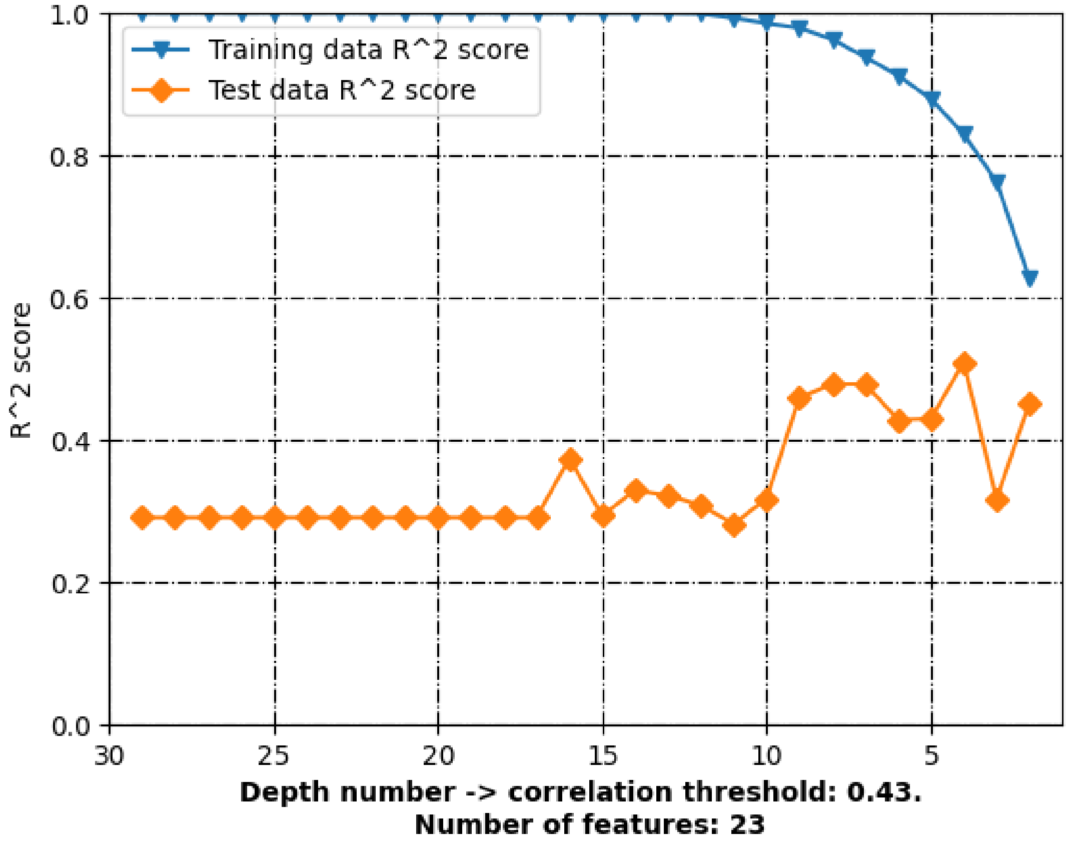

Training data R^2 score
Test data R^2 score
R^2 score
1.0
0.8
0.6
0.4
0.2
0.0
30
25
20
15
10
5
Depth number -> correlation threshold: 0.43.
Number of features: 23

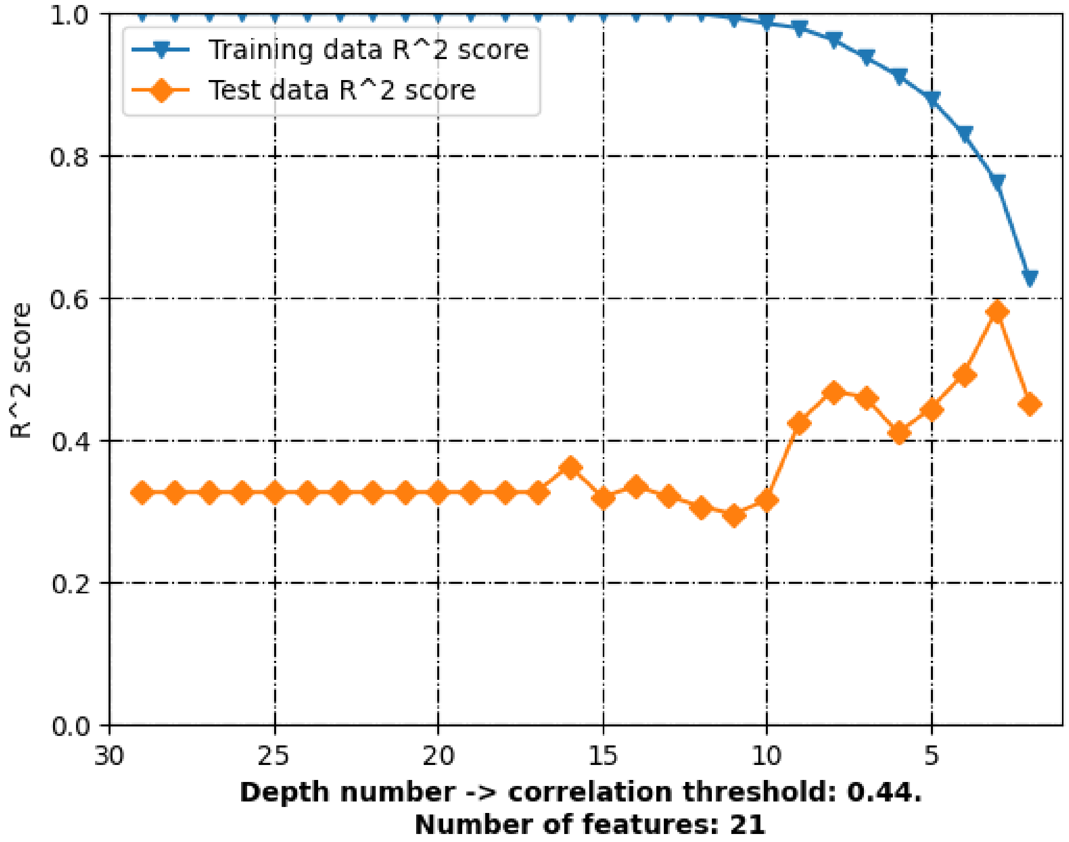

Training data R^2 score
Test data R^2 score
R^2 score
1.0
0.8
0.6
0.4
0.2
0.0
30
25
20
15
10
5
Depth number -> correlation threshold: 0.44.
Number of features: 21

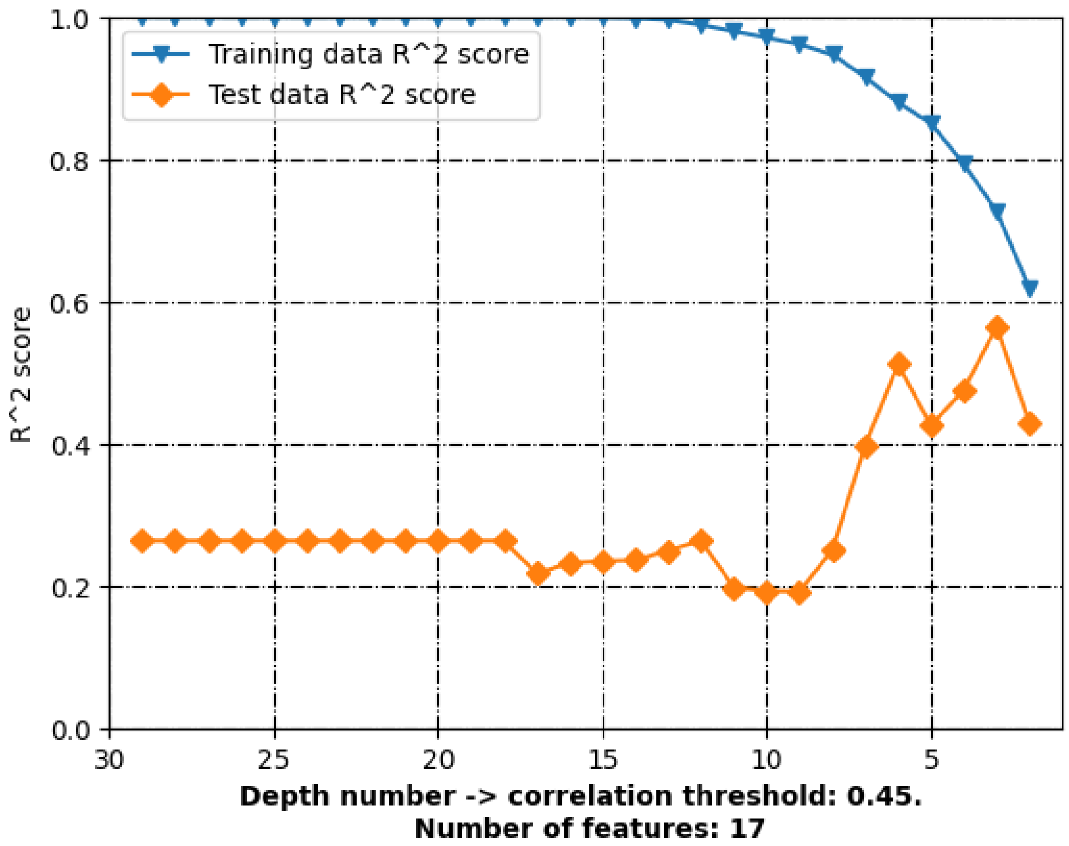

Training data R^2 score
Test data R^2 score
1.0
0.8
0.6
0.4
0.2
0.0
R^2 score
30
25
20
15
10
5
Depth number -> correlation threshold: 0.45.
Number of features: 17

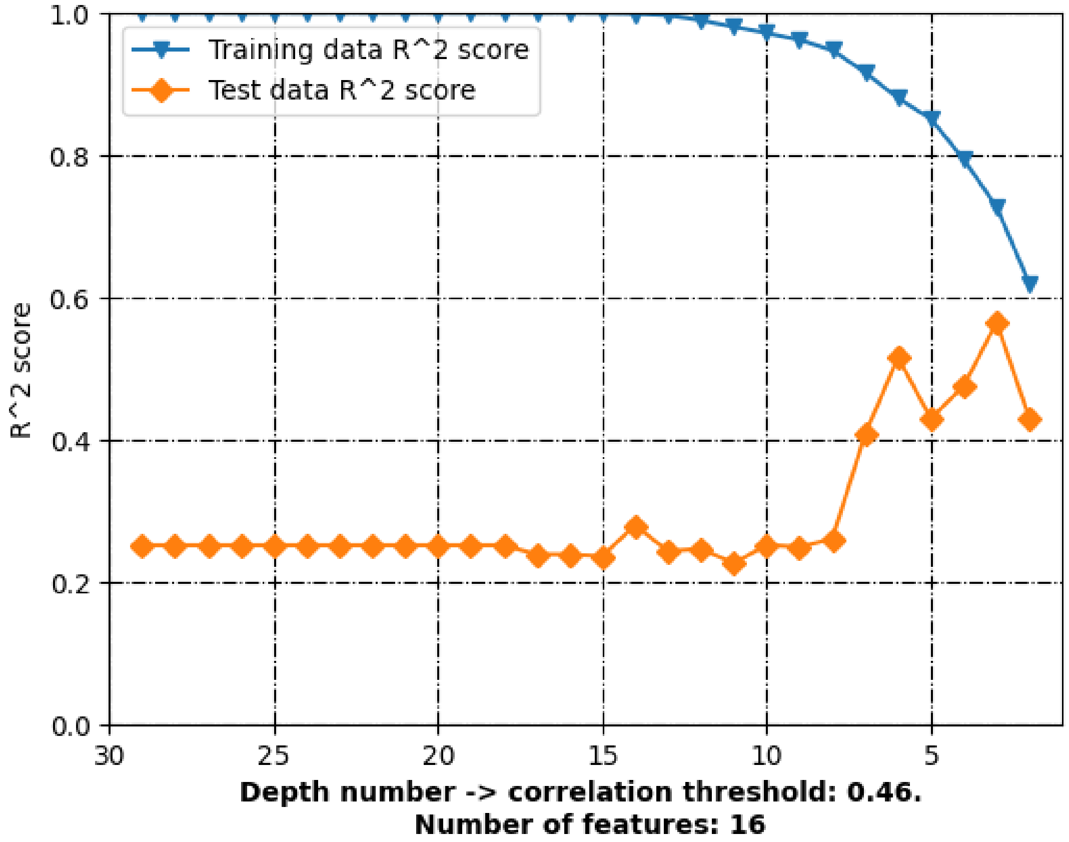

Training data R^2 score
Test data R^2 score
R^2 score
1.0
0.8
0.6
0.4
0.2
0.0
30
25
20
15
10
5
Depth number -> correlation threshold: 0.46.
Number of features: 16

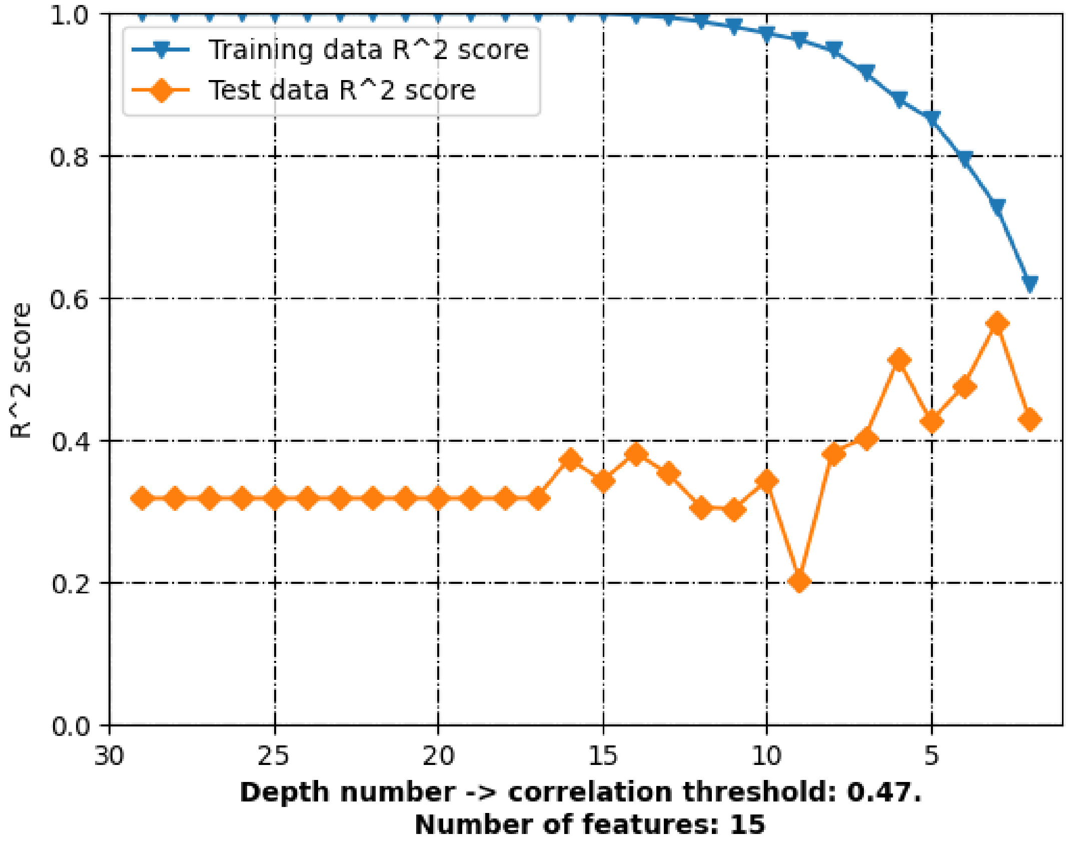

Training data R^2 score
Test data R^2 score
R^2 score
1.0
0.8
0.6
0.4
0.2
0.0
30
25
20
15
10
5
Depth number -> correlation threshold: 0.47.
Number of features: 15

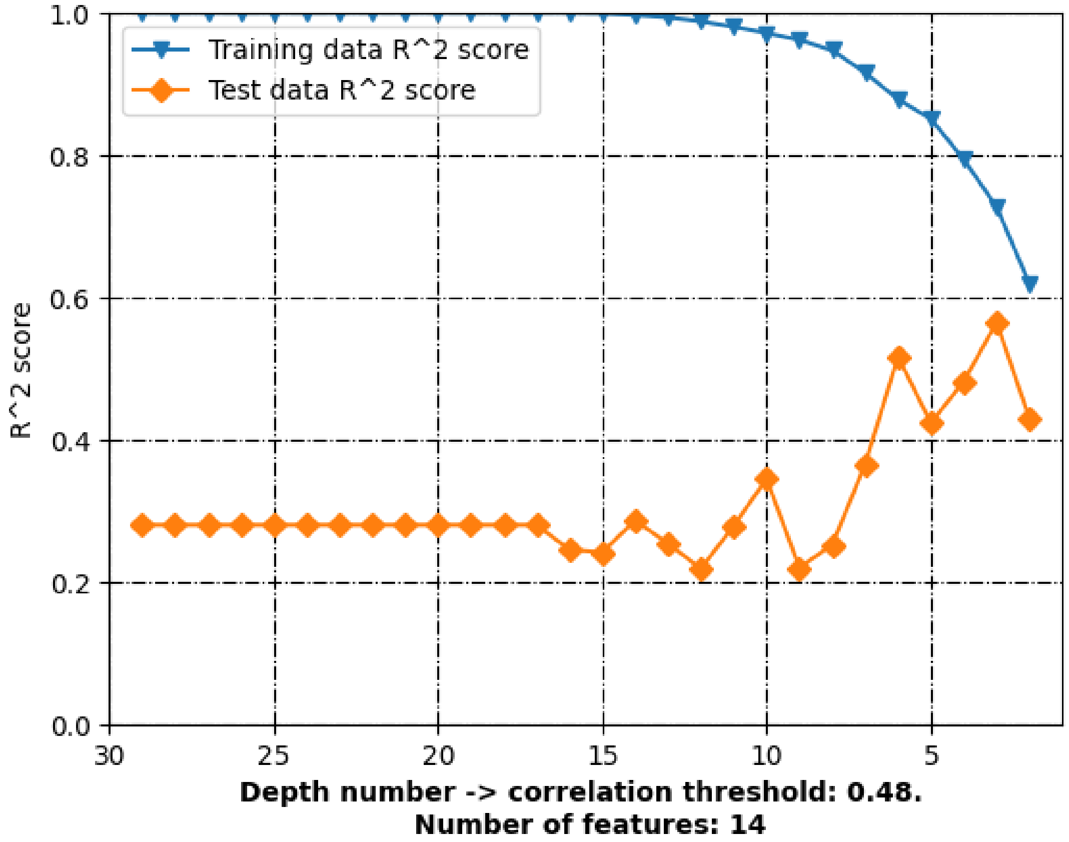
Training data R^2 score
Test data R^2 score
R^2 score
1.0
0.8
0.6
0.4
0.2
0.0
30
25
20
15
10
5
Depth number -> correlation threshold: 0.48.
Number of features: 14

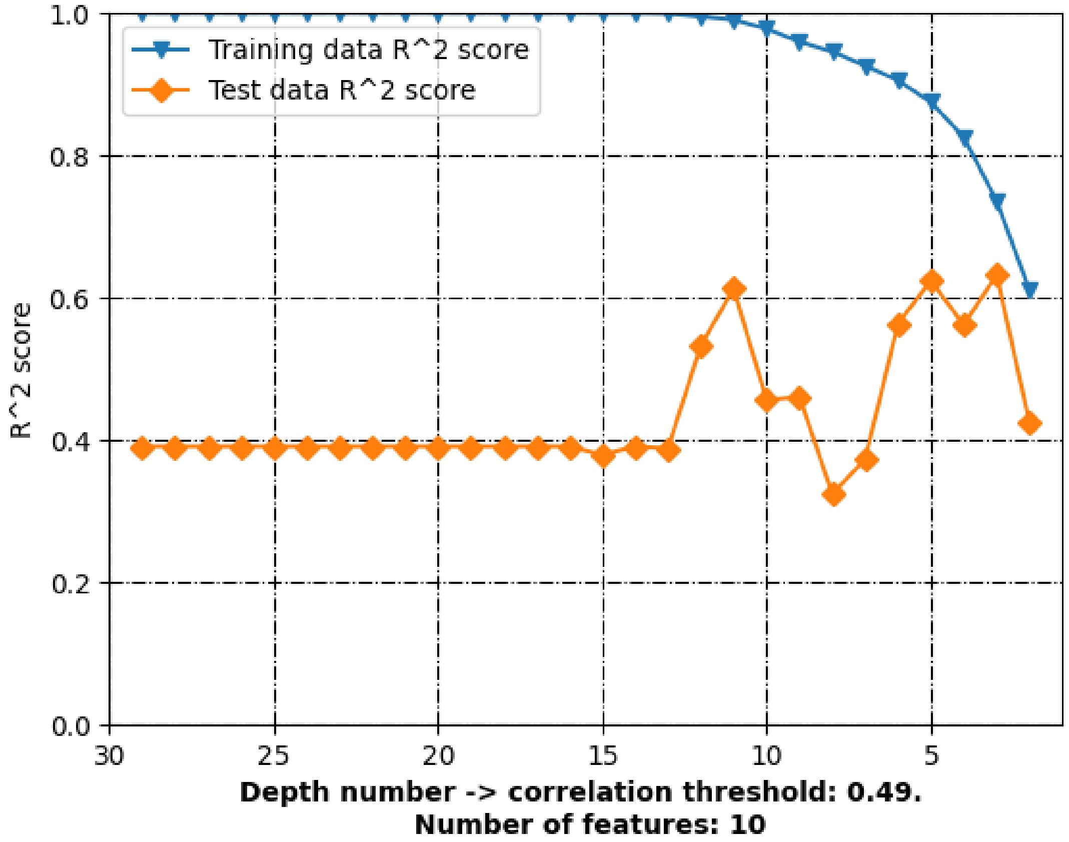
Training data R^2 score
Test data R^2 score
R^2 score
1.0
0.8
0.6
0.4
0.2
0.0
30
25
20
15
10
5
Depth number -> correlation threshold: 0.49.
Number of features: 10

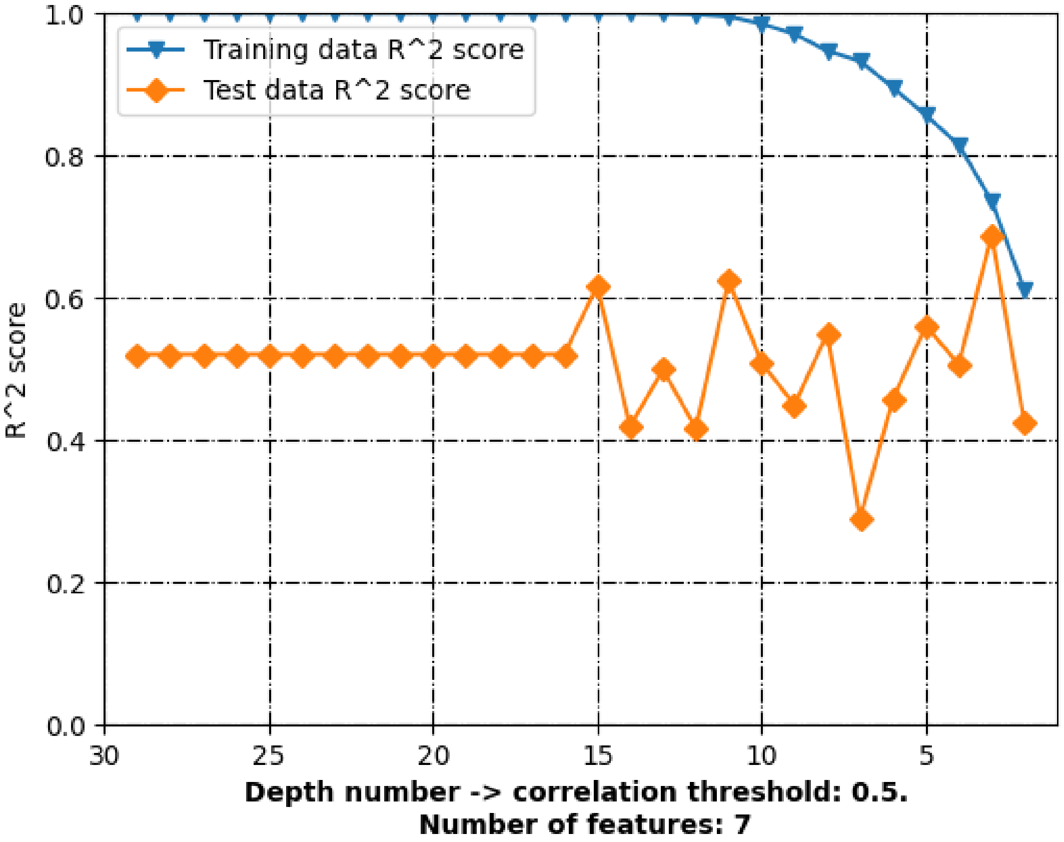
Training data R^2 score
Test data R^2 score
R^2 score
1.0
0.8
0.6
0.4
0.2
0.0
30
25
20
15
10
5
Depth number -> correlation threshold: 0.5.
Number of features: 7

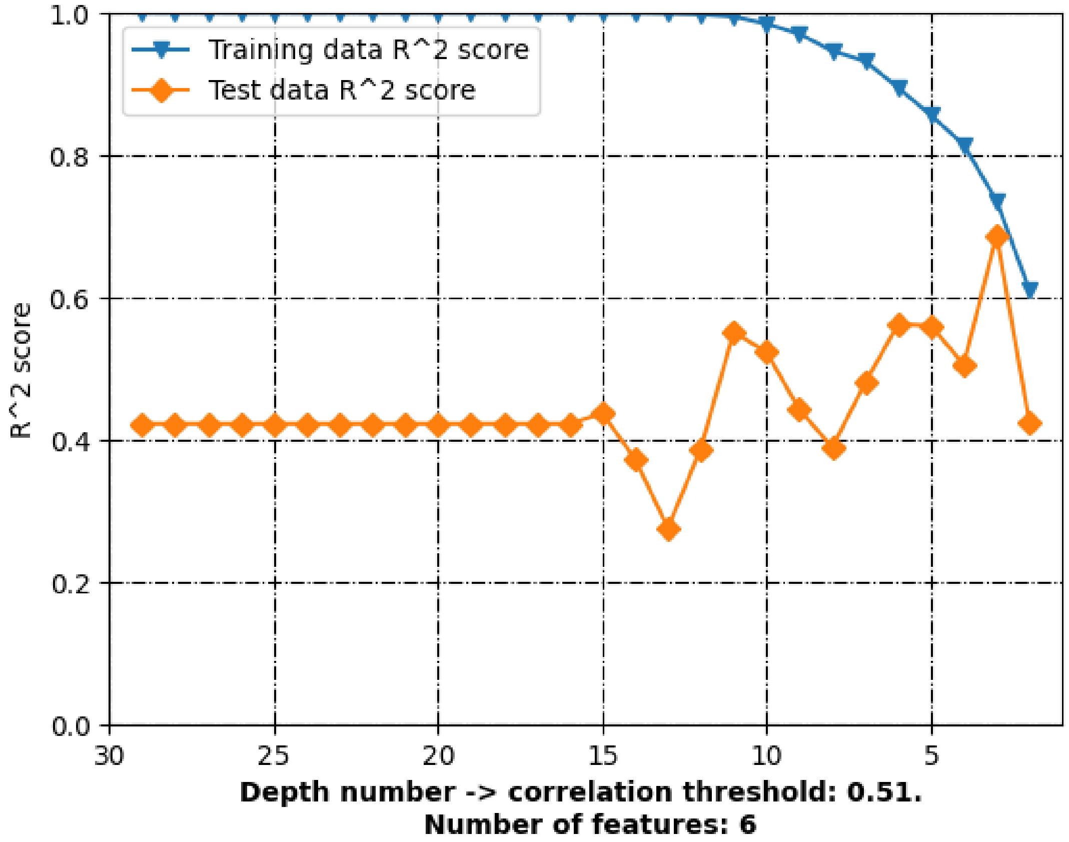

Training data R^2 score
Test data R^2 score
R^2 score
1.0
0.8
0.6
0.4
0.2
0.0
30
25
20
15
10
5
Depth number -> correlation threshold: 0.51.
Number of features: 6

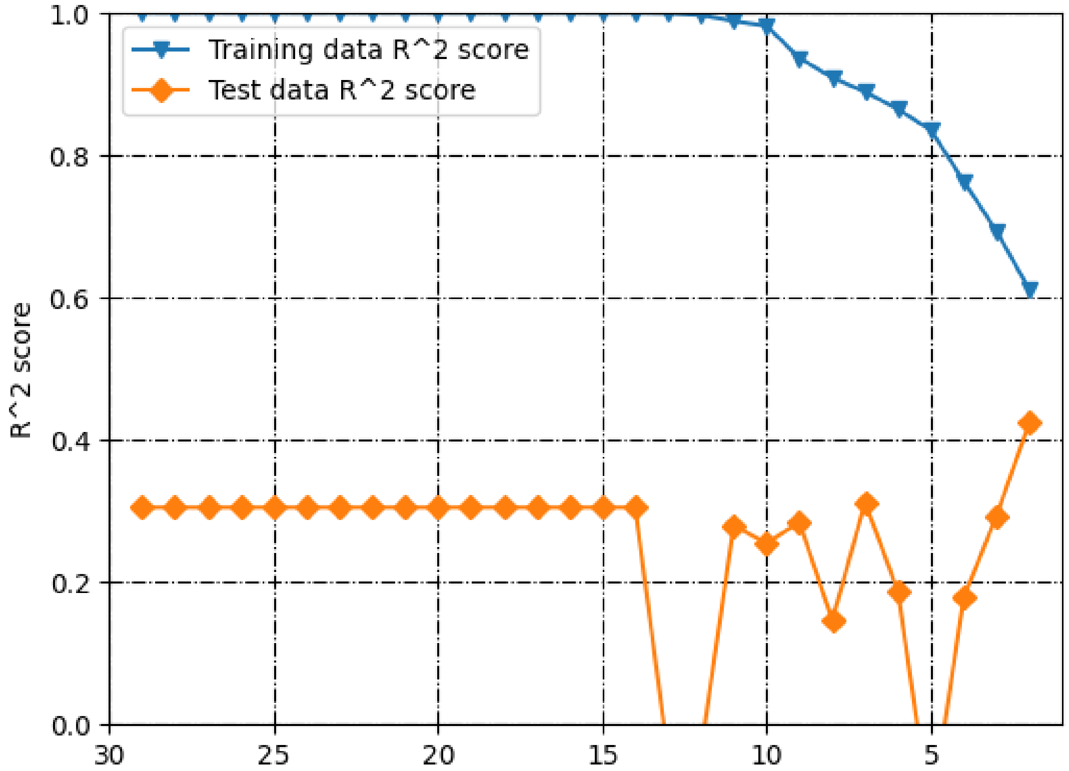

Depth number -> correlation threshold: 0.52.
Number of features: 5

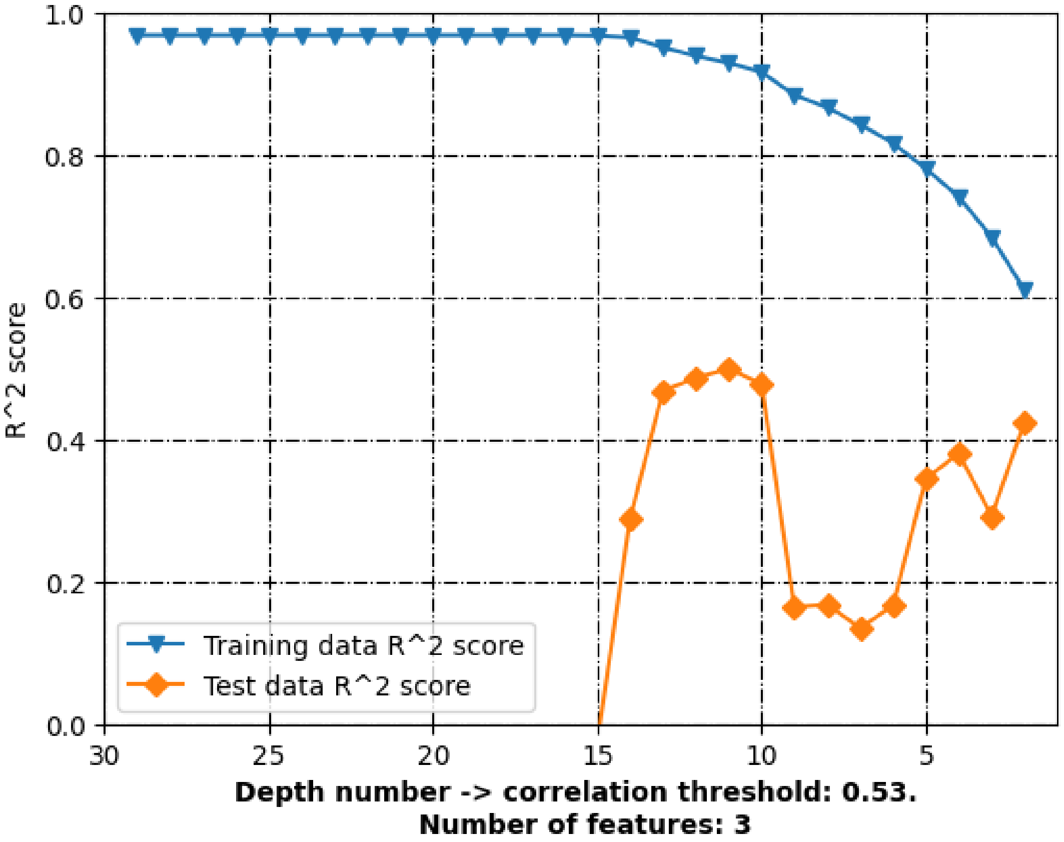
1.0
0.8
0.6
0.4
0.2
0.0
R^2 score
30
25
20
15
10
5
Training data R^2 score
Test data R^2 score
Depth number -> correlation threshold: 0.53.
Number of features: 3

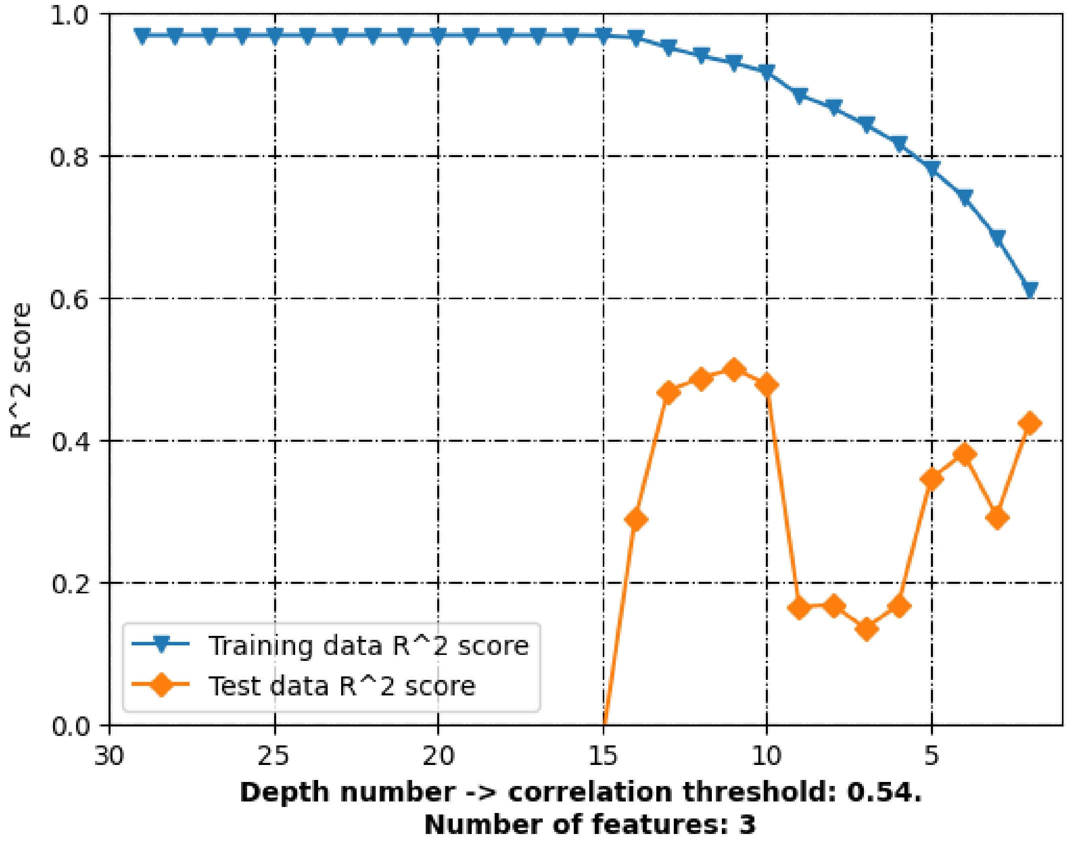

1.0
0.8
0.6
0.4
0.2
0.0
R^2 score
30
25
20
15
10
5
Depth number -> correlation threshold: 0.54.
Number of features: 3
Training data R^2 score
Test data R^2 score

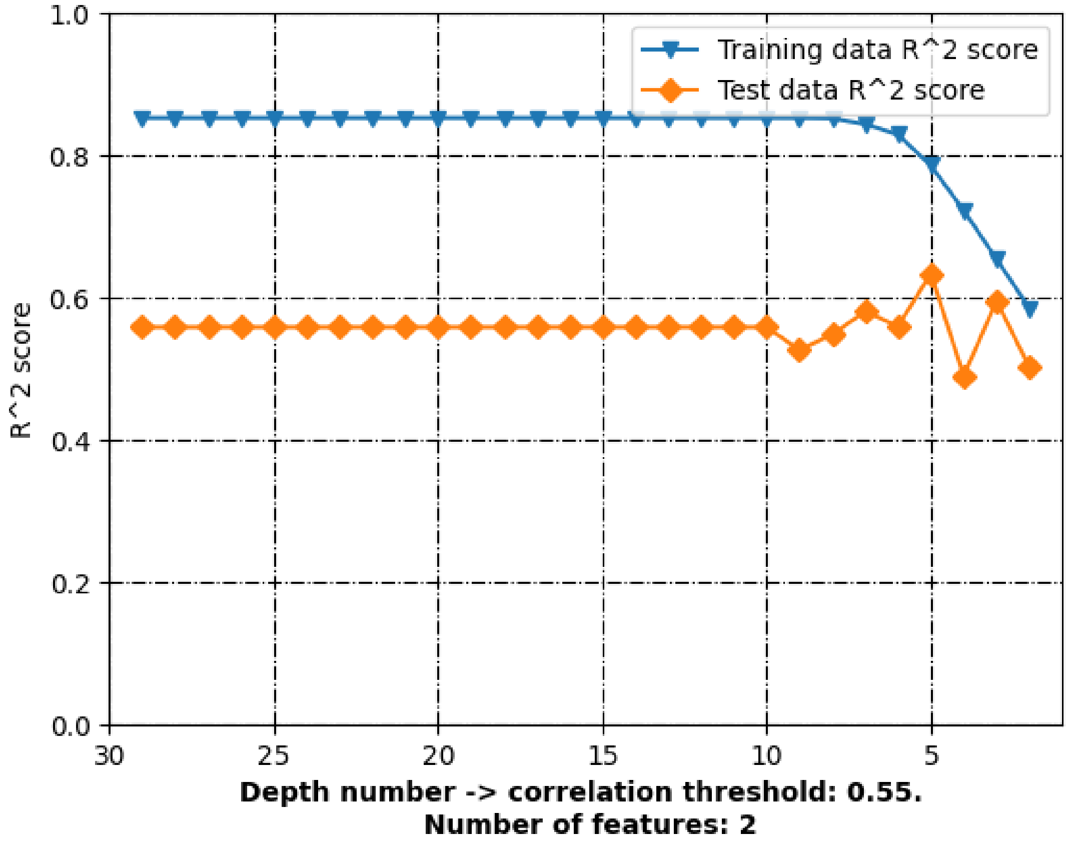

Training data R^2 score
Test data R^2 score
R^2 score
1.0
0.8
0.6
0.4
0.2
0.0
30
25
20
15
10
5
Depth number -> correlation threshold: 0.55.
Number of features: 2

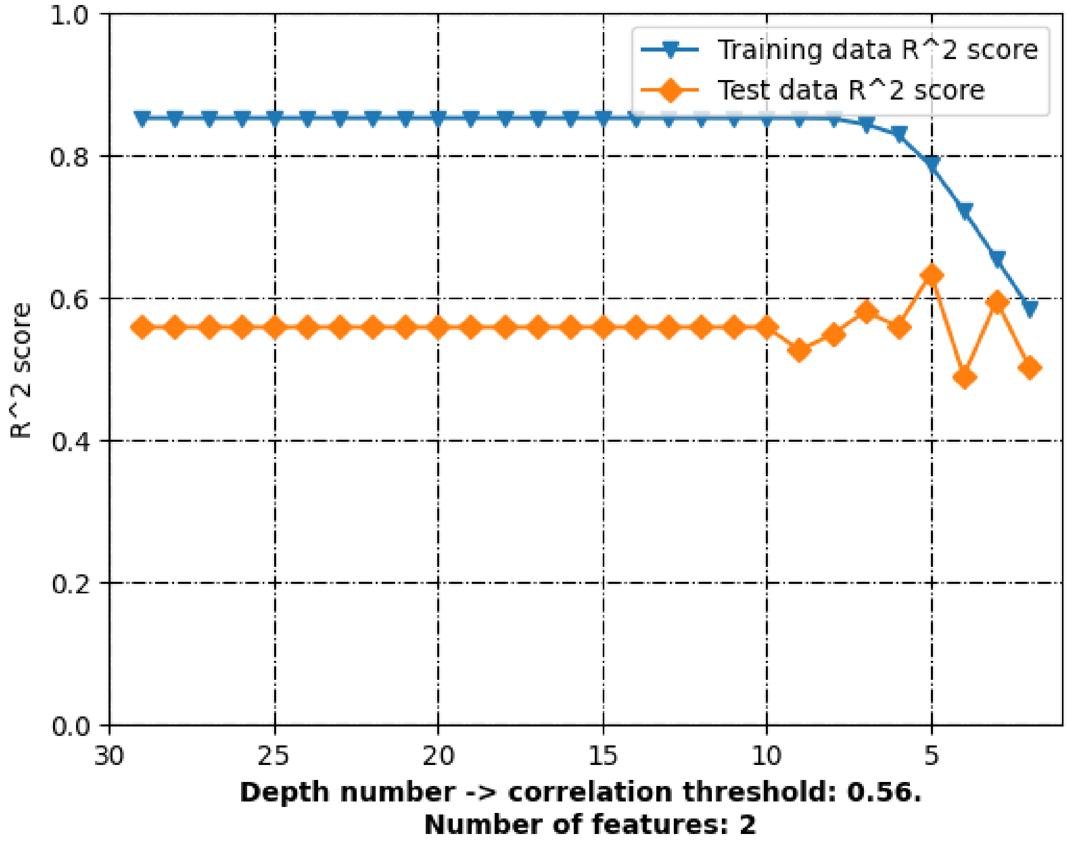

[20]:
```
plt.plot(df_without_standardization['Correlation threshold'],␣
  ↪df_without_standardization['Number of features'], label = "Number of␣
  ↪features", marker='.')
plt.legend()
plt.xlabel('Correlation threshold')
plt.ylabel('Number of features')
plt.show()
```

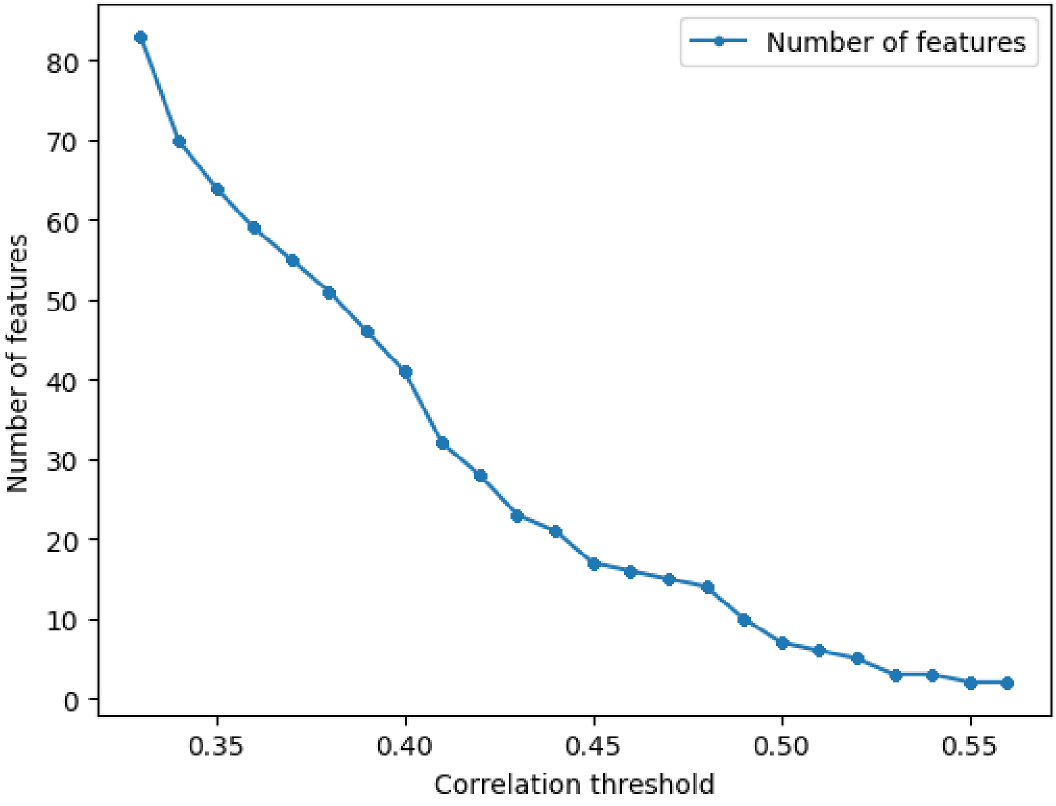

[ ]:

3 Random Forest

[21]:
```
without_standardization, train_r2_, test_r2_, hist1, hist2, target_column_name,␣
↪training_data_RMSE, test_data_RMSE  = pred_model.
↪prepare_data_and_create_model(molecular_descriptors_df=data,
                                                                              ␣
↪                    correlation_threshold=0.50,
                                                                              ␣
↪                    standardization=False,
                                                                              ␣
↪                    model_type='RandomForestRegressor',
                                                                              ␣
↪                    n_estimators_=12,
                                                                              ␣
↪                    target_column_name = target,
                                                                              ␣
↪                    random_state=random_state,
```

```
↪                    train_test_split_=True,
↪                    verbose=True)
```

```
I am not doing standardization…
  molecular descriptor name
0                    AATS0Z
1                  AATS0are
2                    AATS0d
3                   AATS0dv
4                    AATS0i
  molecular descriptor name  corr_value
0                    AATS0Z   -0.020817
1                  AATS0are   -0.148257
2                    AATS0d    0.022999
3                   AATS0dv   -0.137980
4                    AATS0i    0.133257
  molecular descriptor name  corr_value  absolute correlation value
0                    AATS0Z   -0.020817                    0.020817
1                  AATS0are   -0.148257                    0.148257
2                    AATS0d    0.022999                    0.022999
3                   AATS0dv   -0.137980                    0.137980
4                    AATS0i    0.133257                    0.133257
    molecular descriptor name  corr_value  absolute correlation value
226                    AMID_O   -0.546843                    0.546843
505               EState_VSA5   -0.588780                    0.588780
556                    GATS2c    0.511671                    0.511671
791                   MDEO-12   -0.582747                    0.582747
847                       NdO   -0.500462                    0.500462
    molecular descriptor name  corr_value  absolute correlation value
226                    AMID_O   -0.546843                    0.546843
505               EState_VSA5   -0.588780                    0.588780
556                    GATS2c    0.511671                    0.511671
791                   MDEO-12   -0.582747                    0.582747
847                       NdO   -0.500462                    0.500462
The model used is: RandomForest…
Return the coefficient of determination of the prediction:
0.7170418032396348
R^2 score: 0.9372587990141408
Correlation coefficient: 0.9681212728858616
Test data - unseen during training:
R^2 score: 0.7170418032396348
Correlation coefficient: 0.8467832091153171
[7.35211449 7.79122224 7.31824059 7.30021685 5.09868769 7.79530921
 7.45920973 7.63990371 7.92712578 7.62111312 7.12849658 7.43535266
 7.81811597 6.23860215 7.51009407 7.34459977 7.95622284 7.90608919]
44     8.004365
```

```
47     7.886057
4      7.002614
55     7.698970
26     5.070734
64     8.060481
73     7.130768
10     8.008774
40     7.795880
107    8.086186
18     6.924818
62     8.173925
11     7.962574
36     6.346787
89     8.102373
91     8.346787
109    7.886057
0      7.991400
Name: BALB/3T3, dtype: float64
Training Root Mean Square Error: 0.2206837465243046
Testing Root Mean Square Error: 0.4235121142472992
```

[22]:
```
print(target_column_name+str('_transformed'))
print(hist1[target_column_name].hist())
```

```
BALB/3T3_transformed
AxesSubplot(0.125,0.11;0.775x0.77)
```

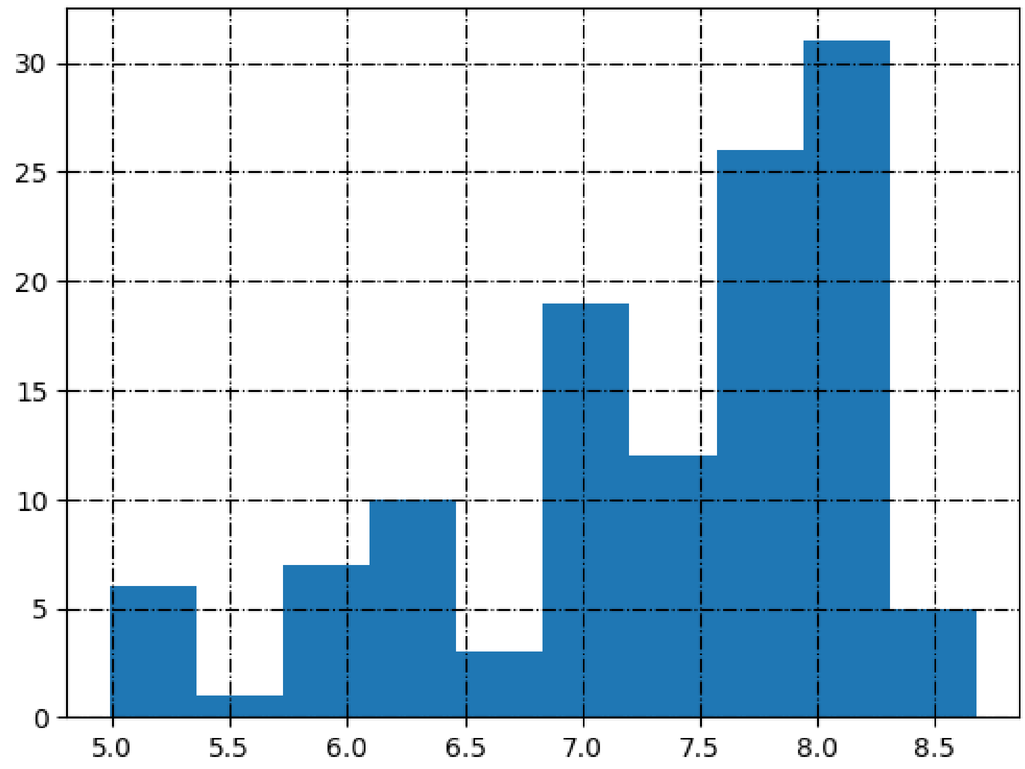

[23]:
```
with_standardization, train_r2_, test_r2_, hist1, hist2, target_column_name,␣
↪training_data_RMSE, test_data_RMSE  = pred_model.
↪prepare_data_and_create_model(molecular_descriptors_df=data,
                                                                            ␣
↪                       correlation_threshold=0.50,
                                                                            ␣
↪                       standardization=True,
                                                                            ␣
↪                       model_type='RandomForestRegressor',
                                                                            ␣
↪                       n_estimators_=12,
                                                                            ␣
↪                       target_column_name = target,
                                                                            ␣
↪                       random_state=random_state,
                                                                            ␣
↪                       train_test_split_=True,
                                                                            ␣
↪                       verbose=True)
```

```
I am doing standardization…
  molecular descriptor name
```

```
0                   AATS0Z
1                 AATS0are
2                   AATS0d
3                  AATS0dv
4                   AATS0i
  molecular descriptor name  corr_value
0                   AATS0Z   -0.020817
1                 AATS0are   -0.148257
2                   AATS0d    0.022999
3                  AATS0dv   -0.137980
4                   AATS0i    0.133257
  molecular descriptor name  corr_value  absolute correlation value
0                   AATS0Z   -0.020817                    0.020817
1                 AATS0are   -0.148257                    0.148257
2                   AATS0d    0.022999                    0.022999
3                  AATS0dv   -0.137980                    0.137980
4                   AATS0i    0.133257                    0.133257
    molecular descriptor name  corr_value  absolute correlation value
226                    AMID_O   -0.546843                    0.546843
505                EState_VSA5   -0.588780                    0.588780
556                    GATS2c    0.511671                    0.511671
791                   MDEO-12   -0.582747                    0.582747
847                       NdO   -0.500462                    0.500462
    molecular descriptor name  corr_value  absolute correlation value
226                    AMID_O   -0.546843                    0.546843
505                EState_VSA5   -0.588780                    0.588780
556                    GATS2c    0.511671                    0.511671
791                   MDEO-12   -0.582747                    0.582747
847                       NdO   -0.500462                    0.500462
The model used is: RandomForest…
Return the coefficient of determination of the prediction:
0.7170418032396348
R^2 score: 0.9372587990141408
Correlation coefficient: 0.9681212728858616
Test data - unseen during training:
R^2 score: 0.7170418032396348
Correlation coefficient: 0.8467832091153171
[7.35211449 7.79122224 7.31824059 7.30021685 5.09868769 7.79530921
 7.45920973 7.63990371 7.92712578 7.62111312 7.12849658 7.43535266
 7.81811597 6.23860215 7.51009407 7.34459977 7.95622284 7.90608919]
44     8.004365
47     7.886057
4      7.002614
55     7.698970
26     5.070734
64     8.060481
73     7.130768
10     8.008774
```

```
40     7.795880
107    8.086186
18     6.924818
62     8.173925
11     7.962574
36     6.346787
89     8.102373
91     8.346787
109    7.886057
0      7.991400
Name: BALB/3T3, dtype: float64
Training Root Mean Square Error: 0.2206837465243046
Testing Root Mean Square Error: 0.4235121142472992
```

3.1 Search inside correlation space

[24]:
```
step = 0.01
initial_step = corr_low
last_step = corr_high
first_list = [x / 100.0 for x in range(int(initial_step*100),␣
↪int(last_step*100), int(step*100))]
n_estimators = [range(2,21,1)]
corr_th = []
second_list = []
third_list = []
fourth_l = []
fifth_l = []
f_list = []
fif_list = []
for i in first_list:

    for estimator in n_estimators[0]:

        without_standardization, train_r2, test_r2, _, h_, target_column_name,␣
↪training_data_RMSE, test_data_RMSE = pred_model.
↪prepare_data_and_create_model(molecular_descriptors_df=data,
                                                                                ␣
↪                    correlation_threshold=i,
                                                                                ␣
↪                    standardization=False,
                                                                                ␣
↪                    model_type='RandomForestRegressor',
                                                                                ␣
↪                    n_estimators_=estimator,
                                                                                ␣
↪                    target_column_name = target,
```

```
                        random_state=random_state,
                        train_test_split_=True,
                        verbose=False)
        corr_th.append(i)
        second_list.append(train_r2)
        third_list.append(test_r2)
        fourth_l.append(training_data_RMSE)
        fifth_l.append(test_data_RMSE)
        f_list.append(len(h_))
        fif_list.append(estimator)
```

[25]:
```
df_without_standardization = pd.DataFrame(data=corr_th, columns=["Correlation threshold"])
df_without_standardization['Training data R^2 score'] = second_list
df_without_standardization['Test data R^2 score'] = third_list
df_without_standardization['Training RMSE'] = fourth_l
df_without_standardization['Test RMSE'] = fifth_l
df_without_standardization['Number of features'] = f_list
df_without_standardization['Number of estimators'] = fif_list
```

[26]:
```
df_random_forest = df_without_standardization.copy()
#df_without_standardization.to_excel('../Data/A549_Random_forest_rs_'+str(random_state)+'.xlsx')
```

[27]:
```
corre_list = [x / 100.0 for x in range(int(initial_step*100), int(last_step*100), int(0.01*100))]
for element in corre_list:
    element_ = df_without_standardization[df_without_standardization['Correlation threshold'] == float(element)]
    plt.plot(element_['Number of estimators'], element_['Training data R^2 score'], label = "Training data R^2 score", marker='v')
    plt.plot(element_['Number of estimators'], element_['Test data R^2 score'], label = "Test data R^2 score", marker='D')
    plt.legend()
    plt.xlabel('Number of estimators -> correlation threshold: '+str(element)+'. \n Number of features: '+str(element_['Number of features'].iloc[0]))
    plt.xlim(max(element_['Number of estimators'])+1, min(element_['Number of estimators'])-1)
    plt.ylabel('R^2 score')
    plt.ylim([0, 1])
    plt.rc('grid', linestyle="-.", color='black')
    plt.grid(True)
```

```
plt.show()
```

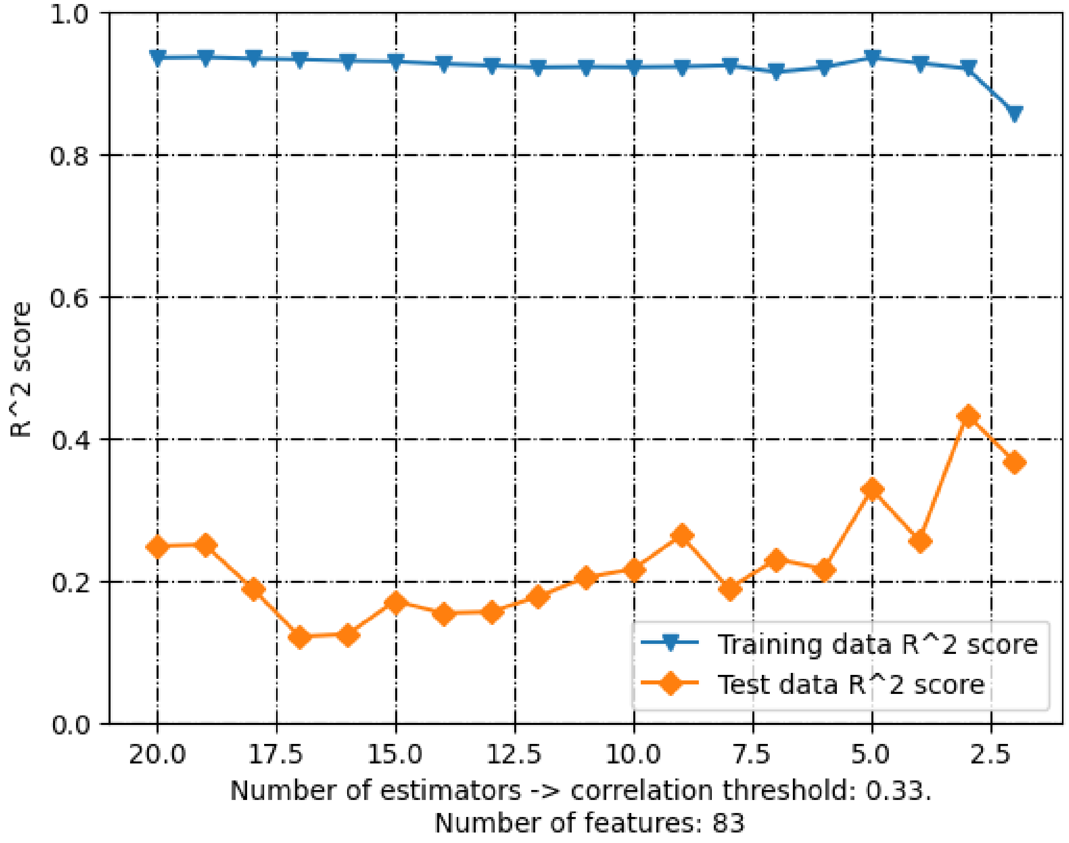
1.0
0.8
0.6
0.4
0.2
0.0
R^2 score
20.0
17.5
15.0
12.5
10.0
7.5
5.0
2.5
Number of estimators -> correlation threshold: 0.33.
Number of features: 83
Training data R^2 score
Test data R^2 score

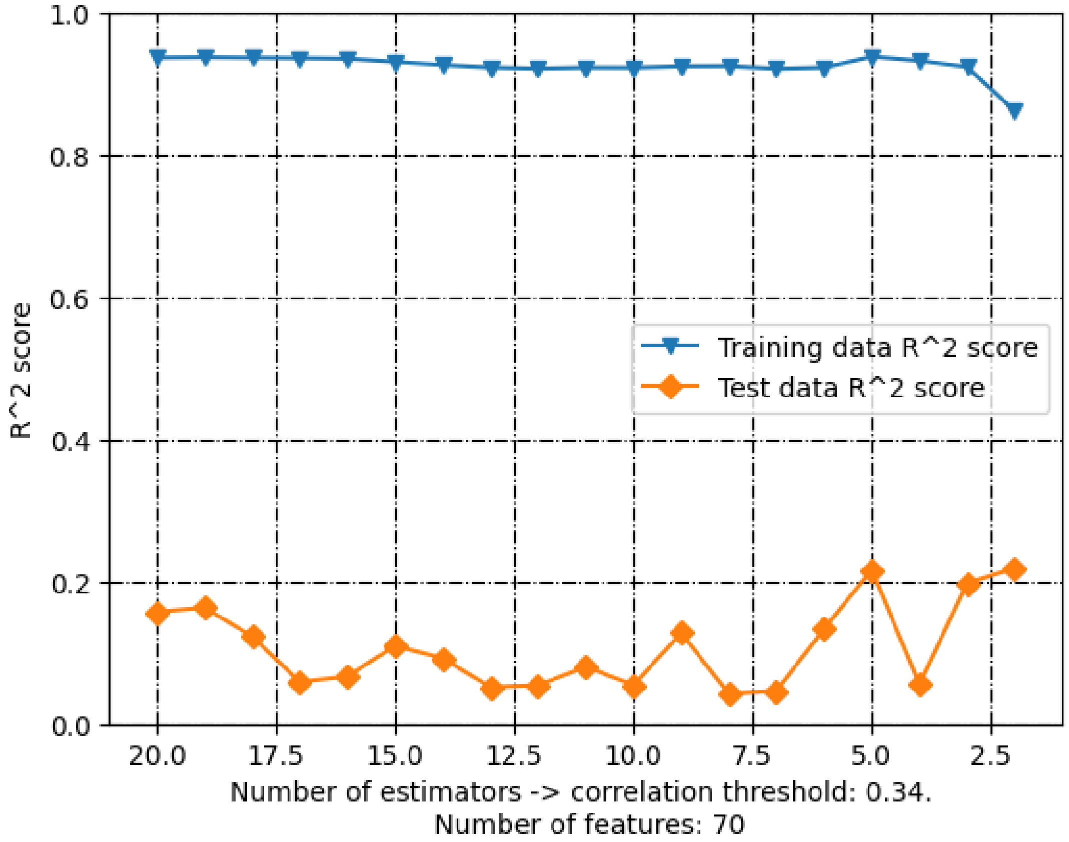

1.0
0.8
0.6
0.4
0.2
0.0
R^2 score
Training data R^2 score
Test data R^2 score
20.0
17.5
15.0
12.5
10.0
7.5
5.0
2.5
Number of estimators -> correlation threshold: 0.34.
Number of features: 70

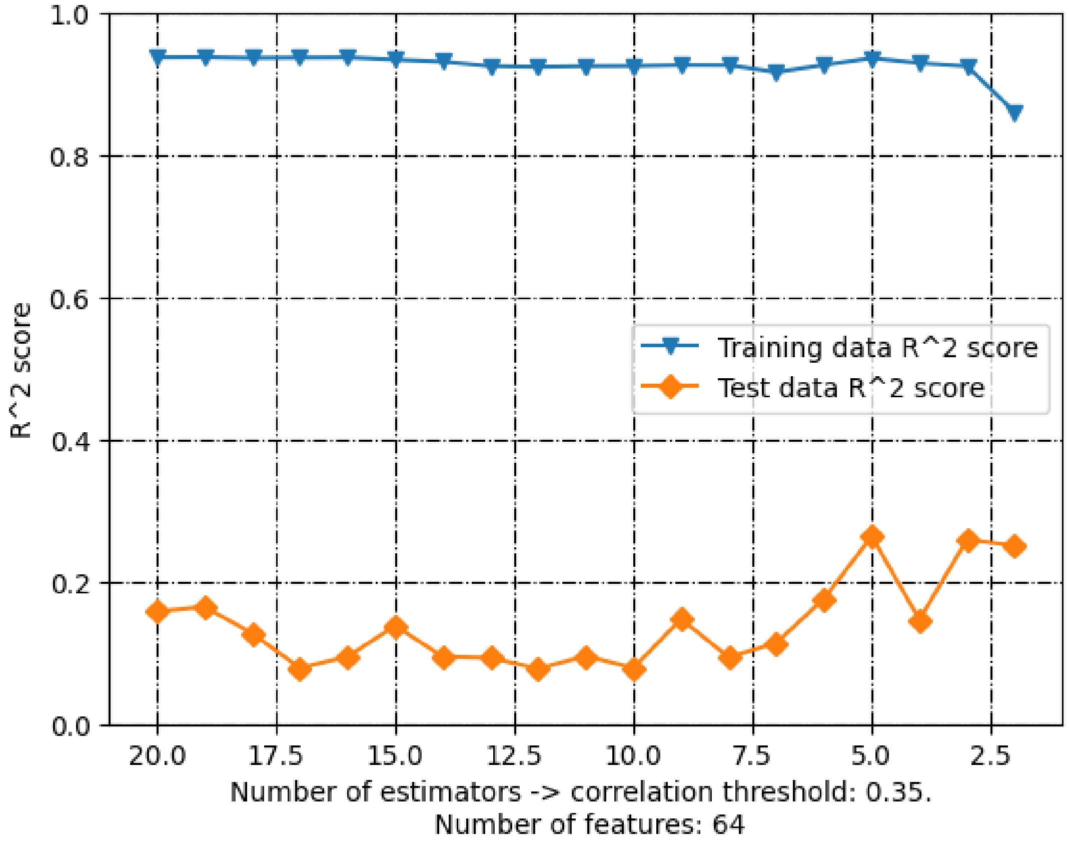

1.0
0.8
0.6
0.4
0.2
0.0
R^2 score
Training data R^2 score
Test data R^2 score
20.0
17.5
15.0
12.5
10.0
7.5
5.0
2.5
Number of estimators -> correlation threshold: 0.35.
Number of features: 64

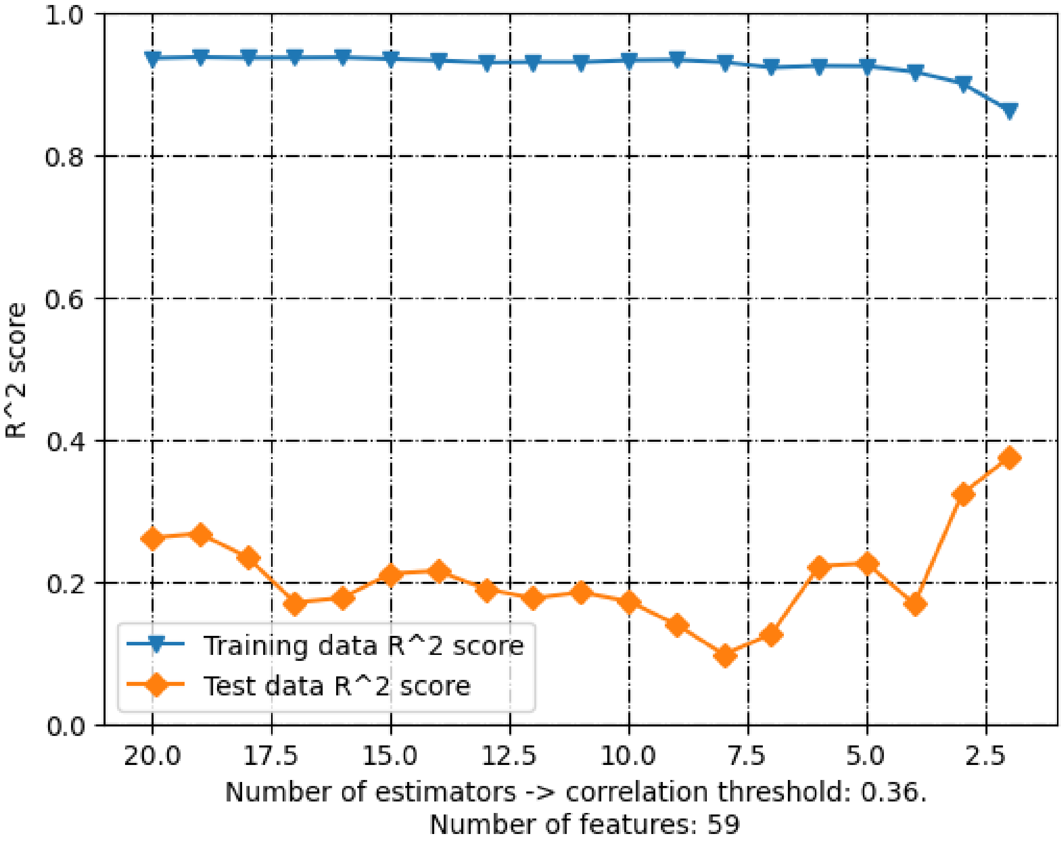
1.0
0.8
0.6
0.4
0.2
0.0
R^2 score
Training data R^2 score
Test data R^2 score
20.0
17.5
15.0
12.5
10.0
7.5
5.0
2.5
Number of estimators -> correlation threshold: 0.36.
Number of features: 59

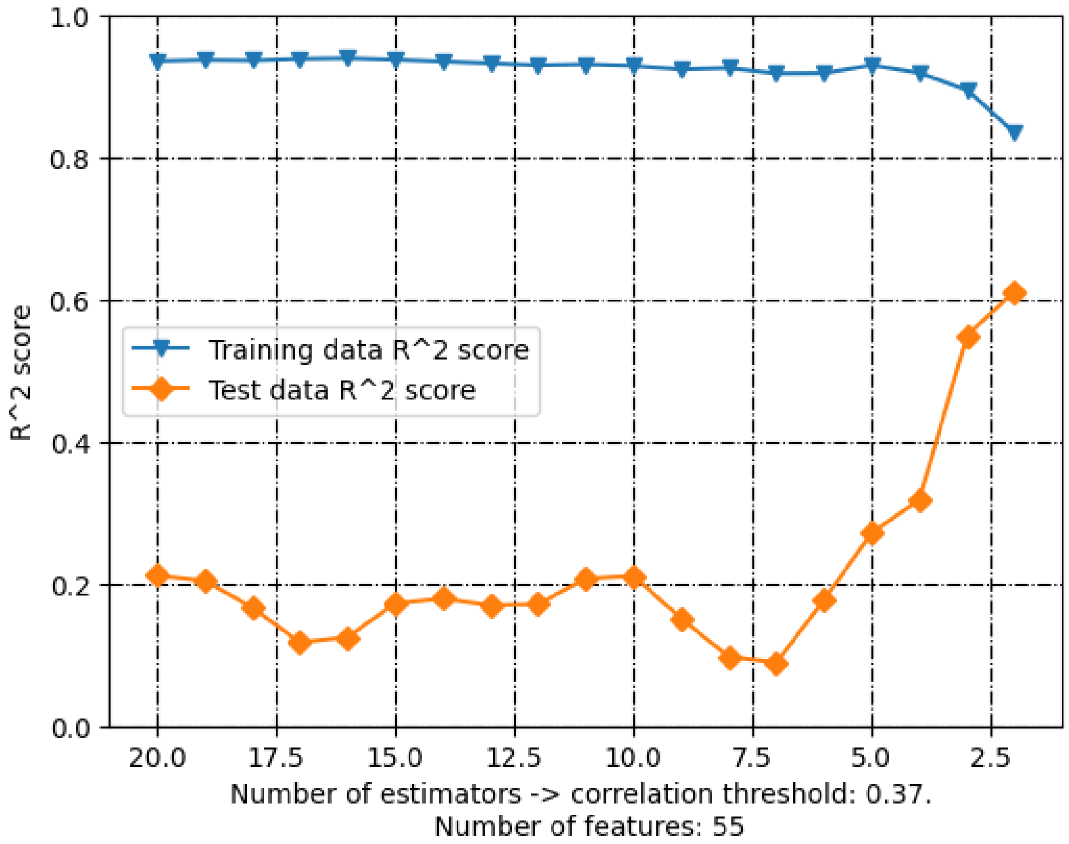
1.0
0.8
0.6
0.4
0.2
0.0
R^2 score
Training data R^2 score
Test data R^2 score
20.0
17.5
15.0
12.5
10.0
7.5
5.0
2.5
Number of estimators -> correlation threshold: 0.37.
Number of features: 55

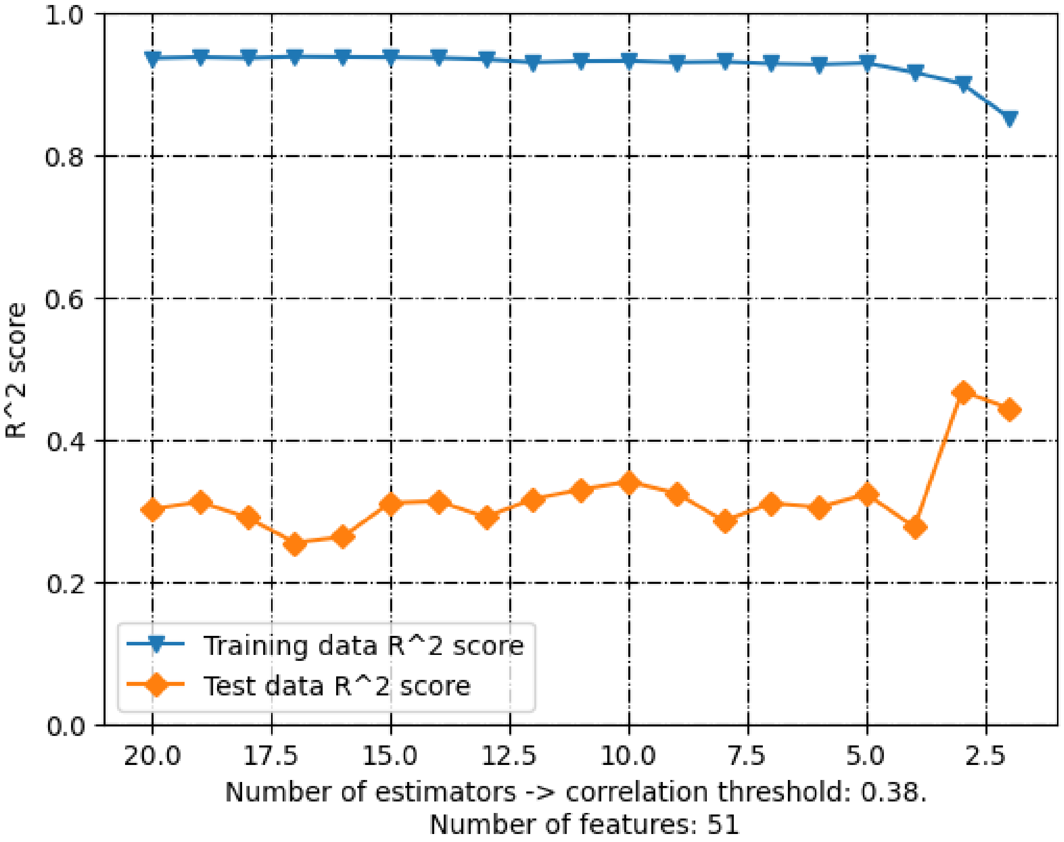
1.0
0.8
0.6
0.4
0.2
0.0
R^2 score
Training data R^2 score
Test data R^2 score
20.0
17.5
15.0
12.5
10.0
7.5
5.0
2.5
Number of estimators -> correlation threshold: 0.38.
Number of features: 51

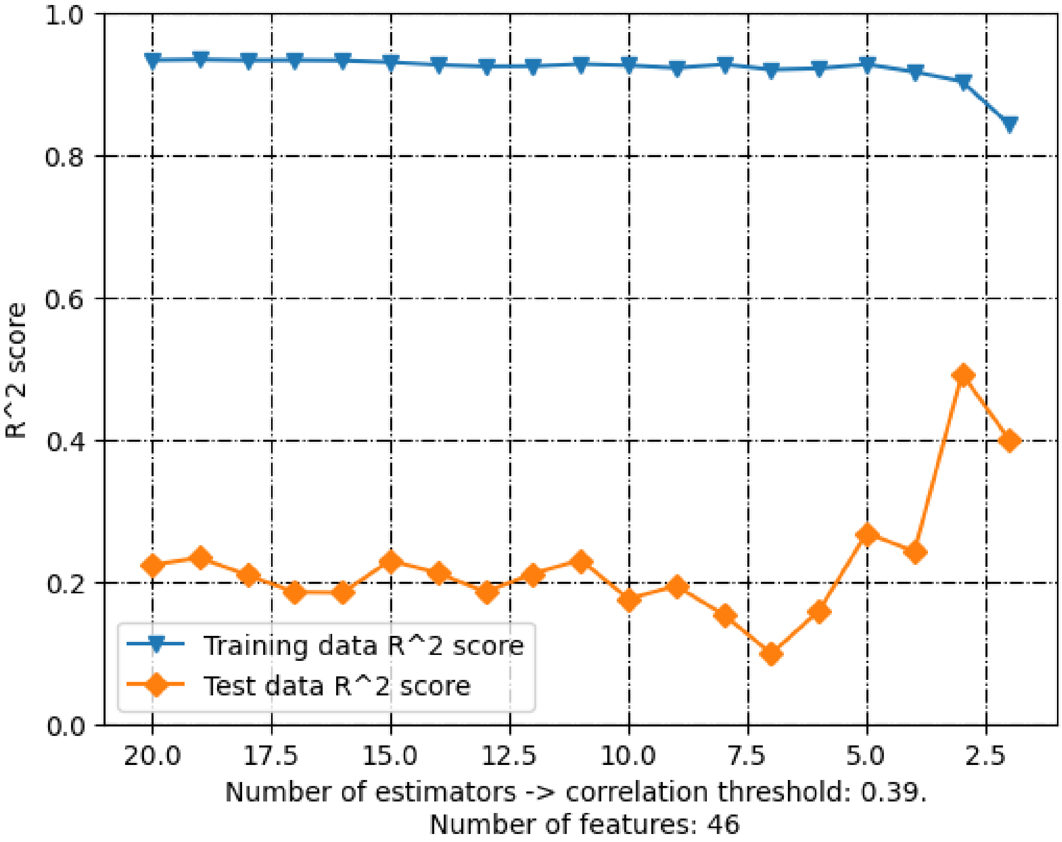

1.0
0.8
0.6
0.4
0.2
0.0
R^2 score
20.0
17.5
15.0
12.5
10.0
7.5
5.0
2.5
Number of estimators -> correlation threshold: 0.39.
Number of features: 46
Training data R^2 score
Test data R^2 score

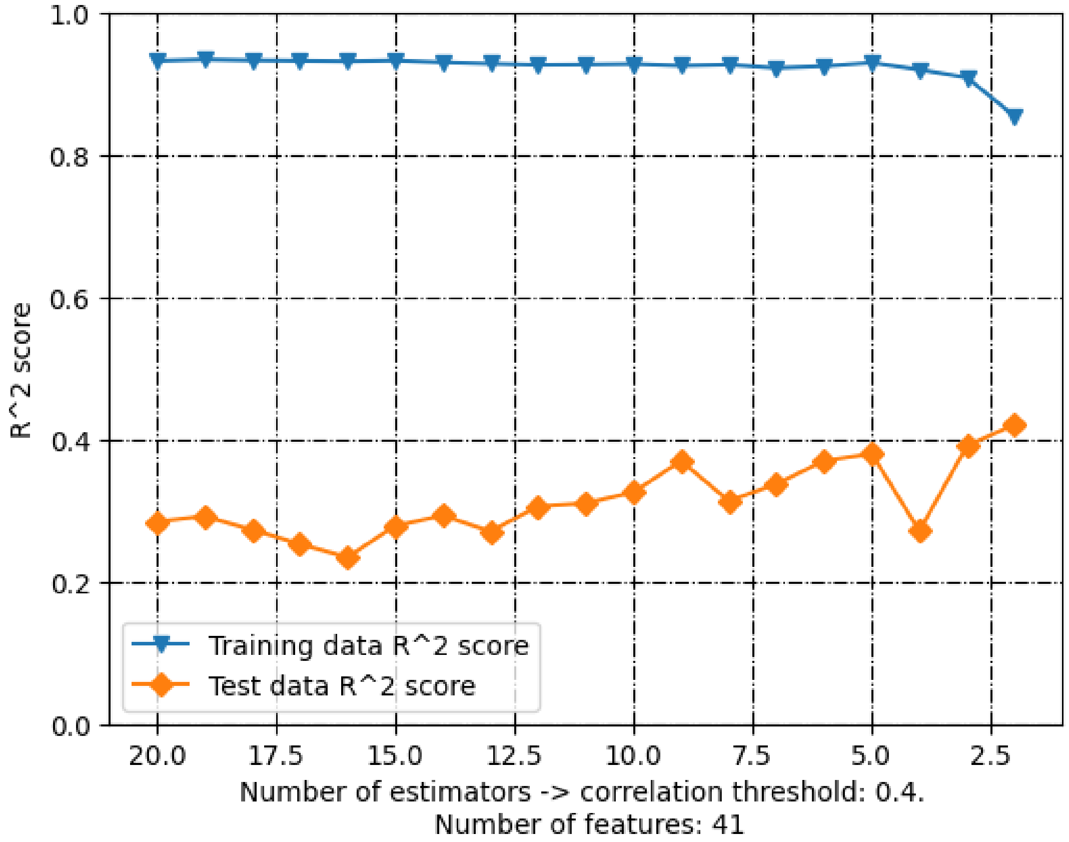
1.0
0.8
0.6
0.4
0.2
0.0
R^2 score
Training data R^2 score
Test data R^2 score
20.0
17.5
15.0
12.5
10.0
7.5
5.0
2.5
Number of estimators -> correlation threshold: 0.4.
Number of features: 41

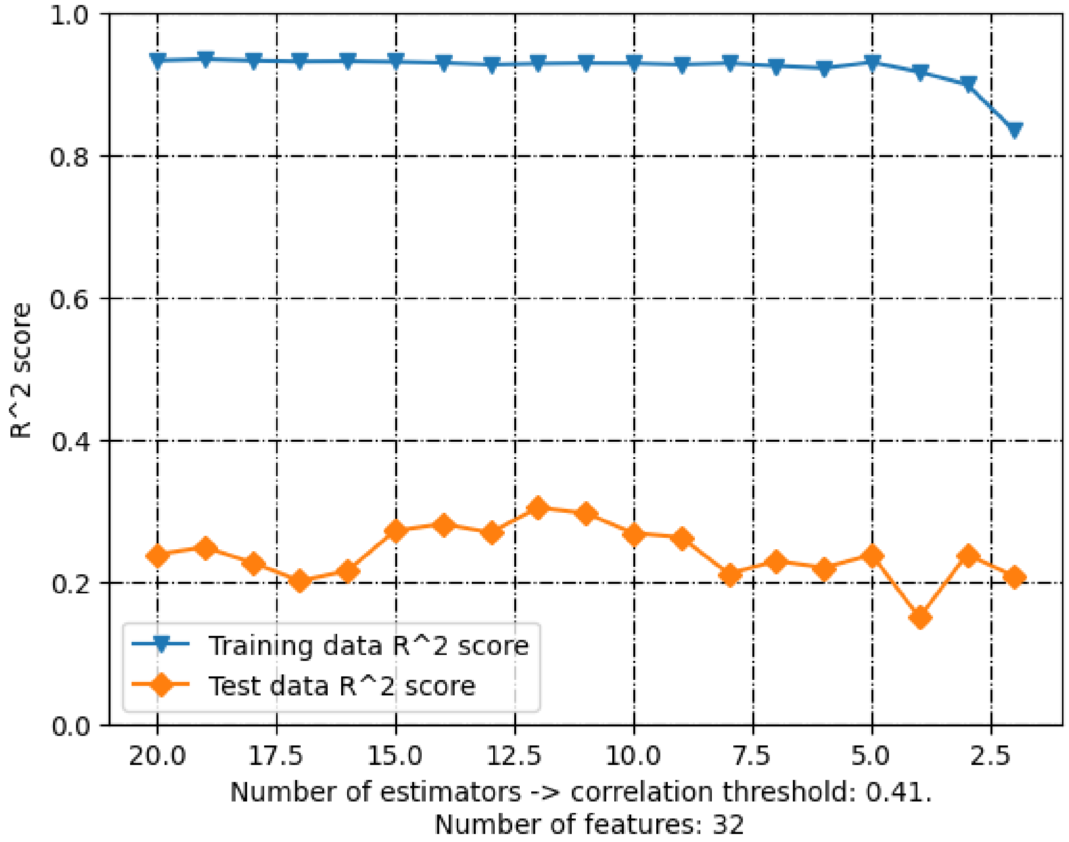

1.0
0.8
0.6
0.4
0.2
0.0
R^2 score
20.0
17.5
15.0
12.5
10.0
7.5
5.0
2.5
Number of estimators -> correlation threshold: 0.41.
Number of features: 32
Training data R^2 score
Test data R^2 score

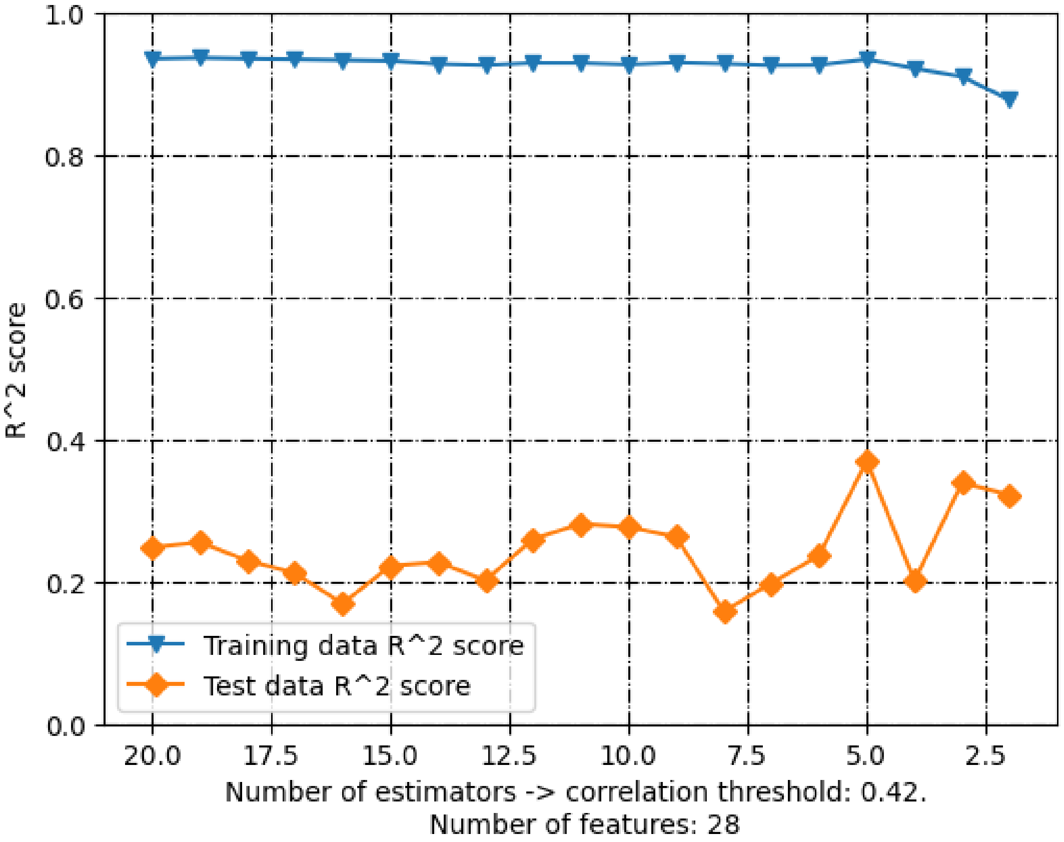

1.0
0.8
0.6
0.4
0.2
0.0
R^2 score
20.0
17.5
15.0
12.5
10.0
7.5
5.0
2.5
Number of estimators -> correlation threshold: 0.42.
Number of features: 28
Training data R^2 score
Test data R^2 score

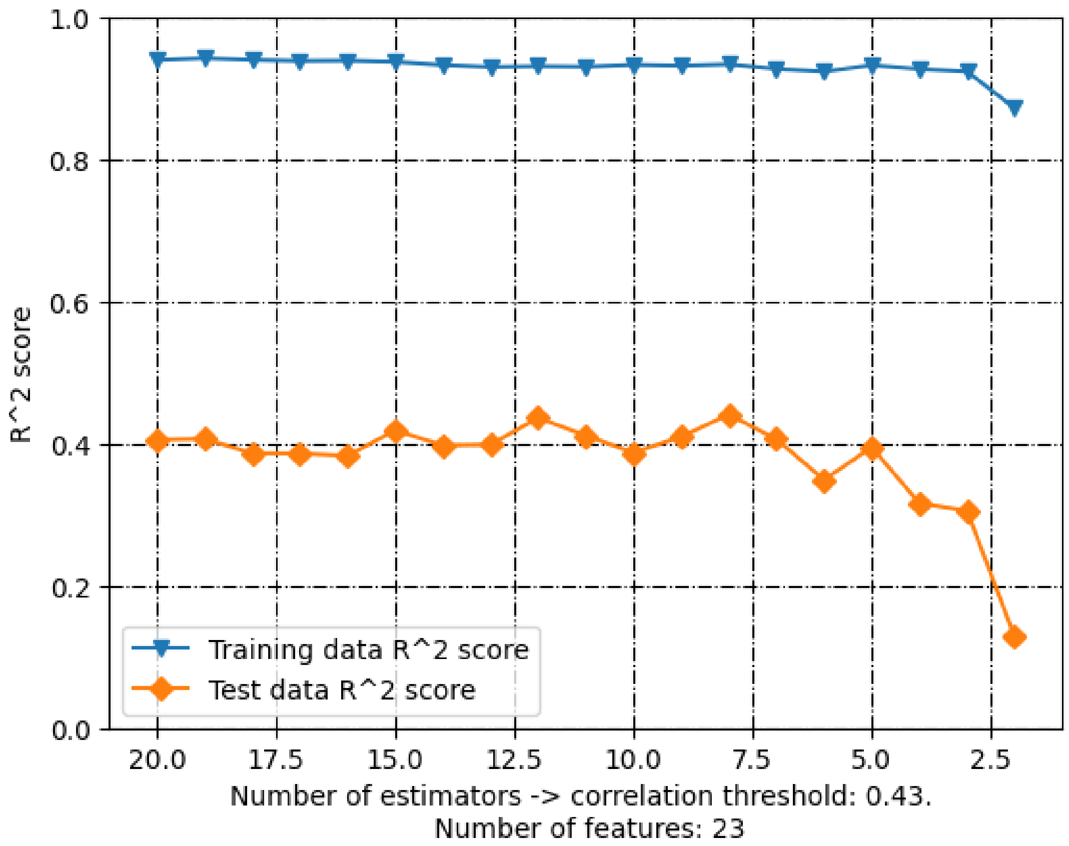

1.0
0.8
0.6
0.4
0.2
0.0
R^2 score
20.0
17.5
15.0
12.5
10.0
7.5
5.0
2.5
Training data R^2 score
Test data R^2 score
Number of estimators -> correlation threshold: 0.43.
Number of features: 23

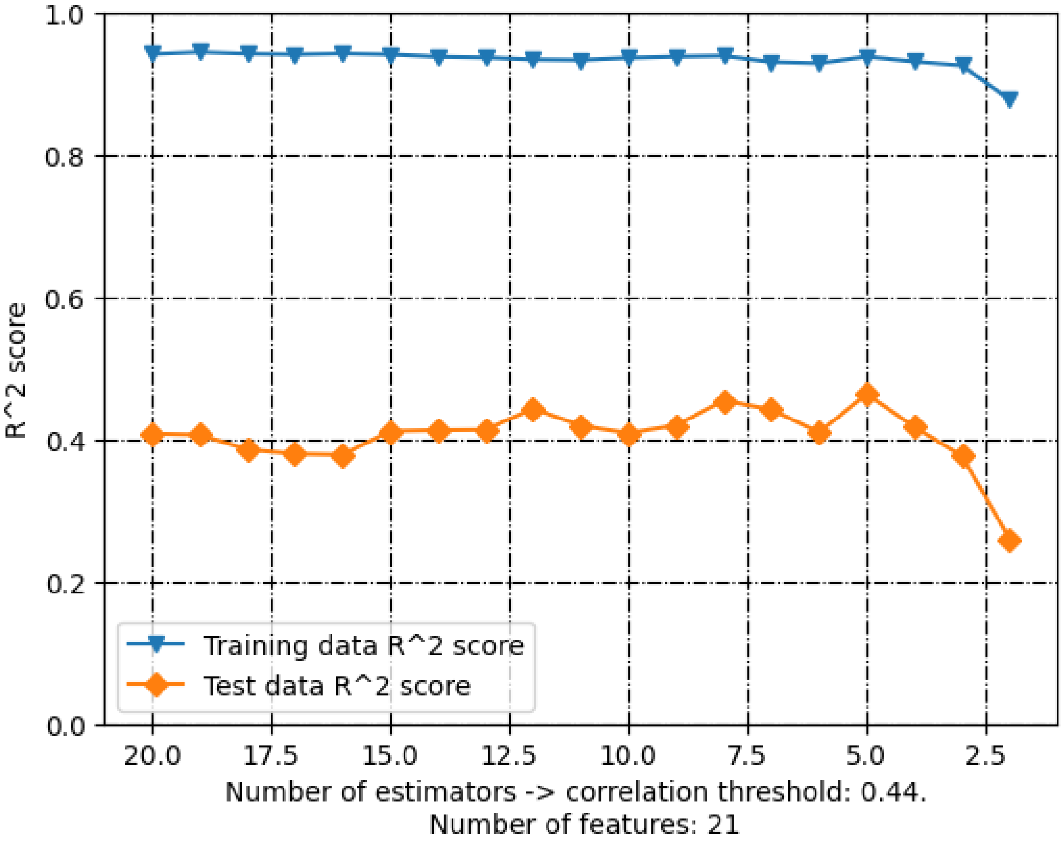

1.0
0.8
0.6
0.4
0.2
0.0
R^2 score
Training data R^2 score
Test data R^2 score
20.0
17.5
15.0
12.5
10.0
7.5
5.0
2.5
Number of estimators -> correlation threshold: 0.44.
Number of features: 21

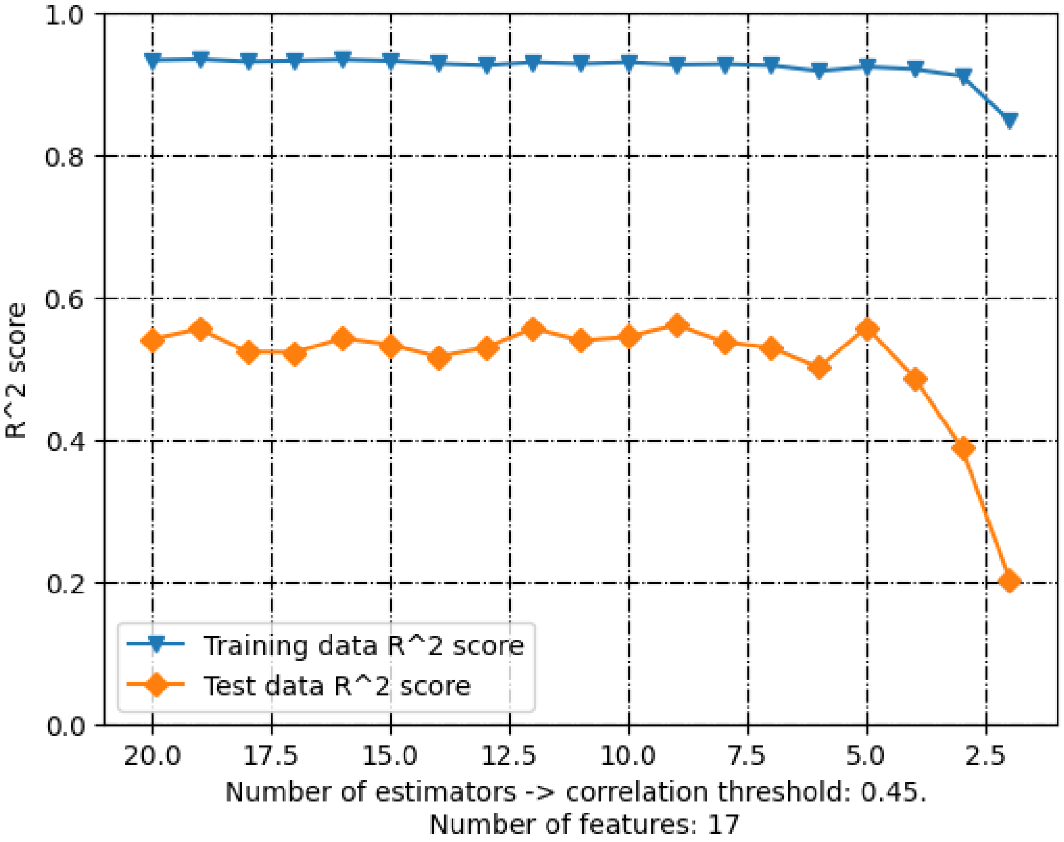
1.0
0.8
0.6
0.4
0.2
0.0
R^2 score
Training data R^2 score
Test data R^2 score
20.0
17.5
15.0
12.5
10.0
7.5
5.0
2.5
Number of estimators -> correlation threshold: 0.45.
Number of features: 17

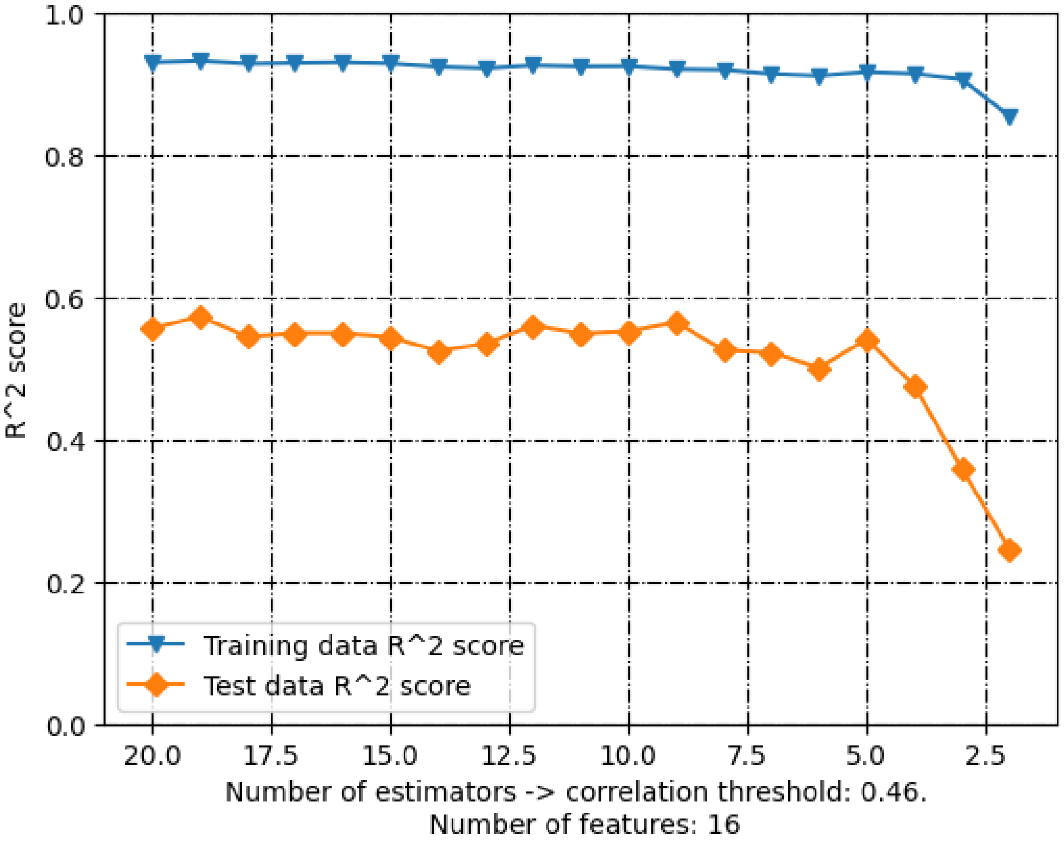
1.0
0.8
0.6
0.4
0.2
0.0
R^2 score
Training data R^2 score
Test data R^2 score
20.0
17.5
15.0
12.5
10.0
7.5
5.0
2.5
Number of estimators -> correlation threshold: 0.46.
Number of features: 16

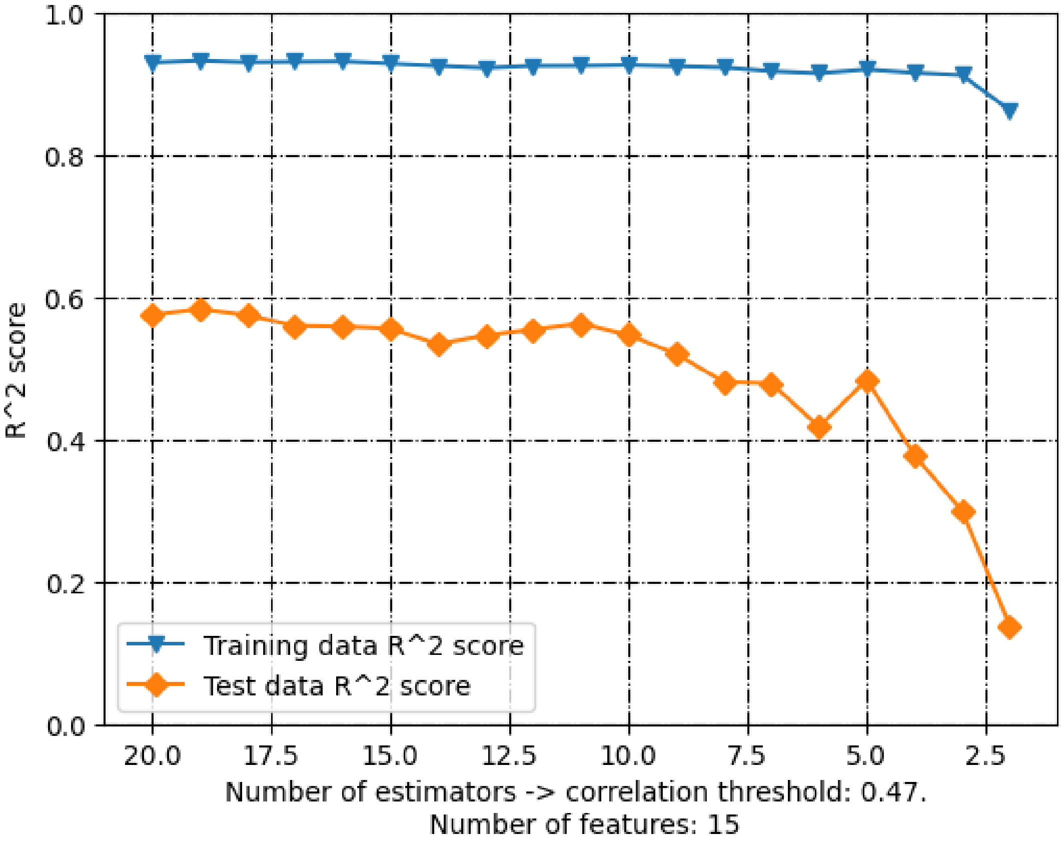

1.0
0.8
0.6
0.4
0.2
0.0
R^2 score
Training data R^2 score
Test data R^2 score
20.0
17.5
15.0
12.5
10.0
7.5
5.0
2.5
Number of estimators -> correlation threshold: 0.47.
Number of features: 15

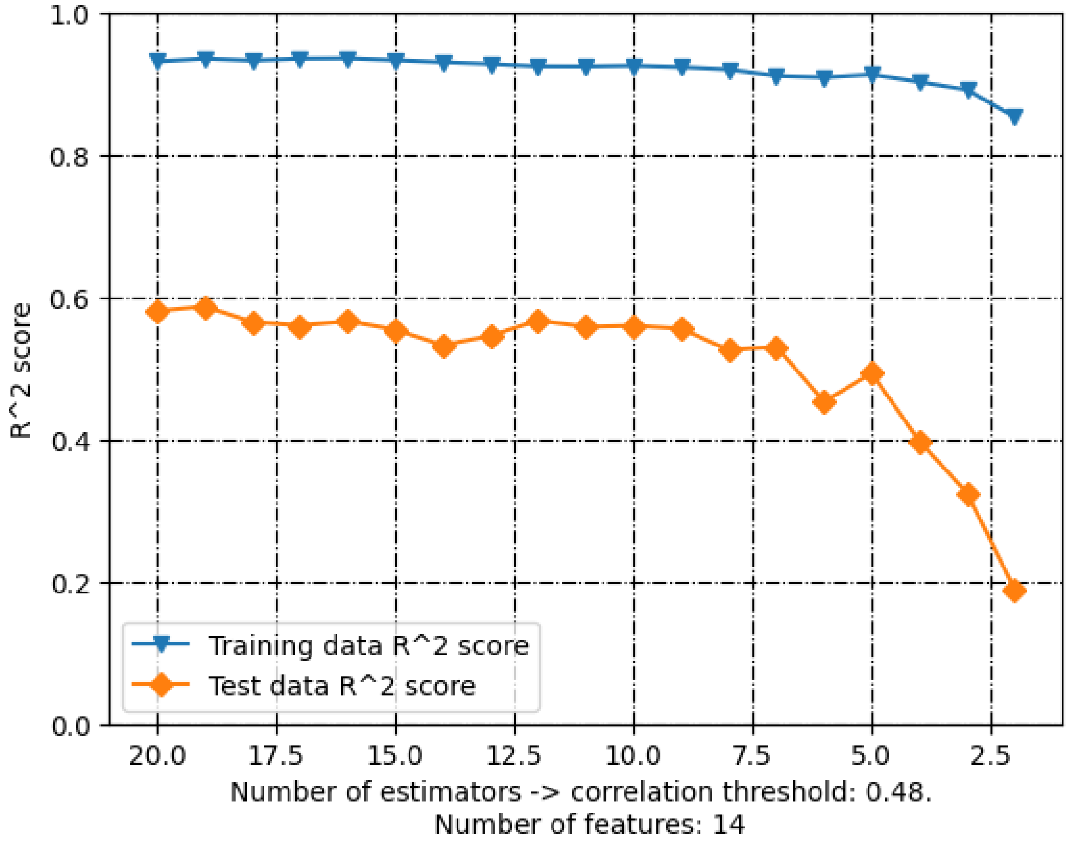

1.0
0.8
0.6
0.4
0.2
0.0
R^2 score
Training data R^2 score
Test data R^2 score
20.0
17.5
15.0
12.5
10.0
7.5
5.0
2.5
Number of estimators -> correlation threshold: 0.48.
Number of features: 14

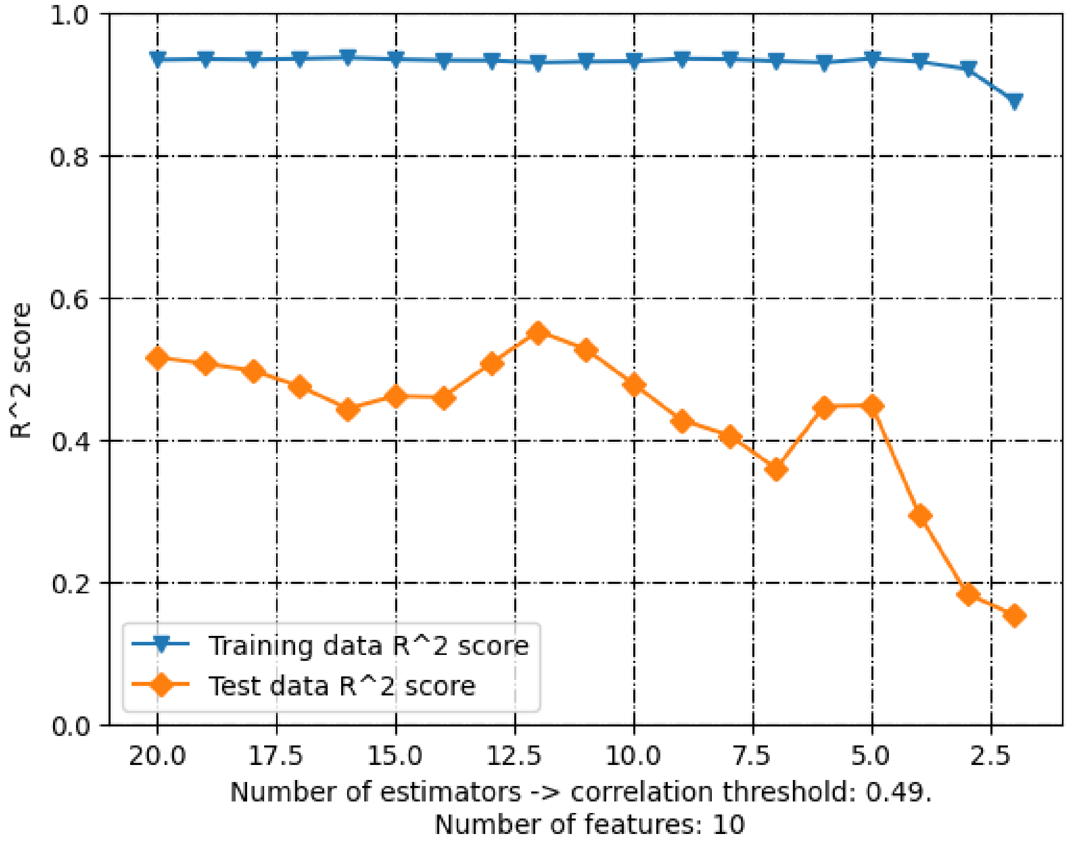

1.0
0.8
0.6
0.4
0.2
0.0
R^2 score
Training data R^2 score
Test data R^2 score
20.0
17.5
15.0
12.5
10.0
7.5
5.0
2.5
Number of estimators -> correlation threshold: 0.49.
Number of features: 10

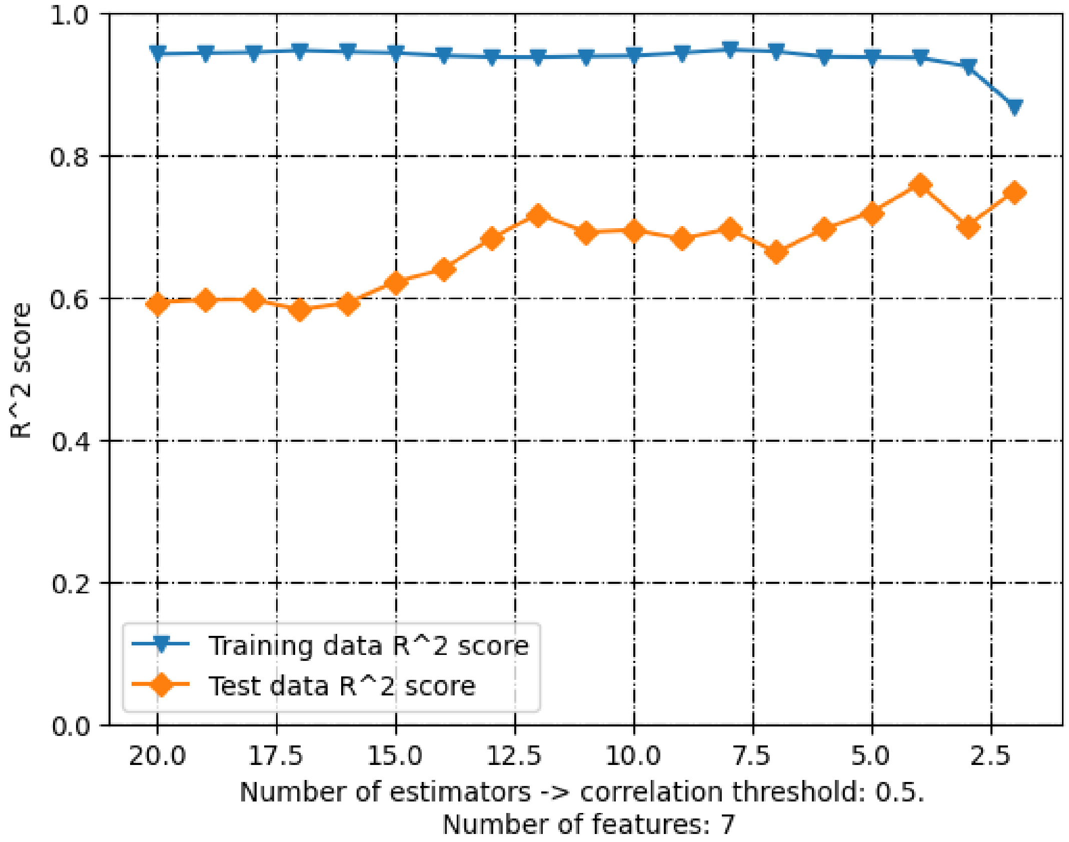

1.0
0.8
0.6
0.4
0.2
0.0
R^2 score
Training data R^2 score
Test data R^2 score
20.0
17.5
15.0
12.5
10.0
7.5
5.0
2.5
Number of estimators -> correlation threshold: 0.5.
Number of features: 7

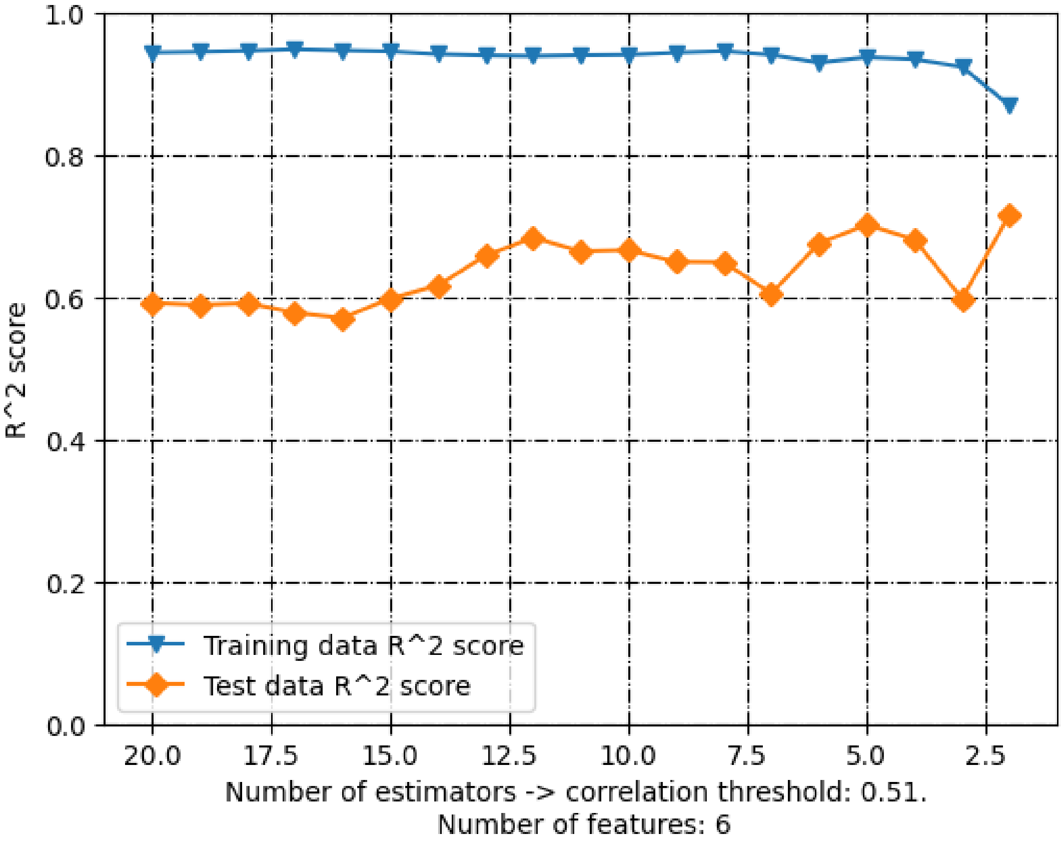
1.0
0.8
0.6
0.4
0.2
0.0
R^2 score
Training data R^2 score
Test data R^2 score
20.0
17.5
15.0
12.5
10.0
7.5
5.0
2.5
Number of estimators -> correlation threshold: 0.51.
Number of features: 6

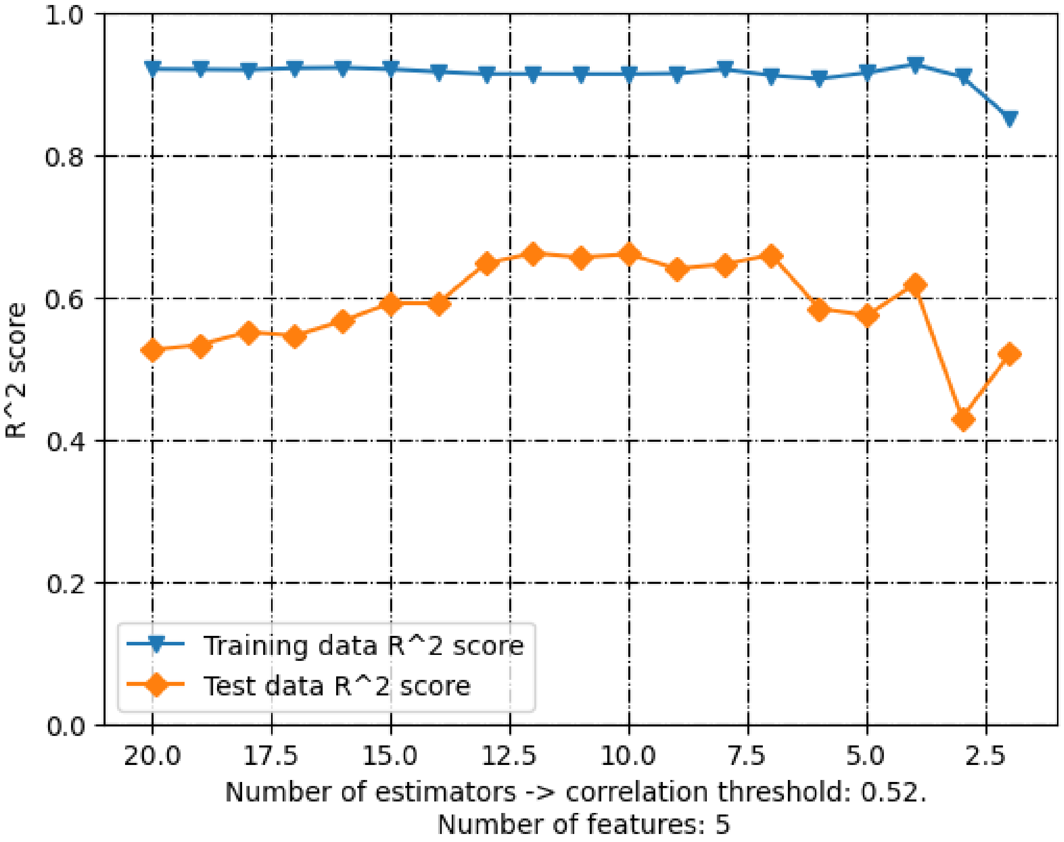
1.0
0.8
0.6
0.4
0.2
0.0
R^2 score
Training data R^2 score
Test data R^2 score
20.0
17.5
15.0
12.5
10.0
7.5
5.0
2.5
Number of estimators -> correlation threshold: 0.52.
Number of features: 5

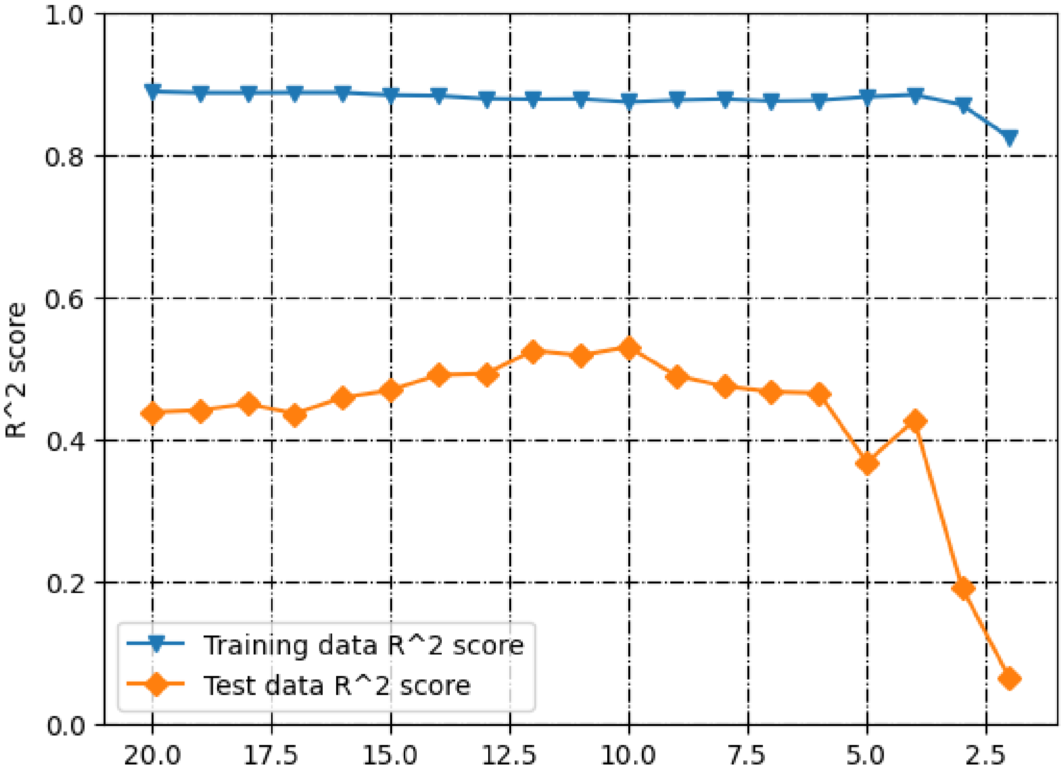

Number of estimators -> correlation threshold: 0.53.
Number of features: 3

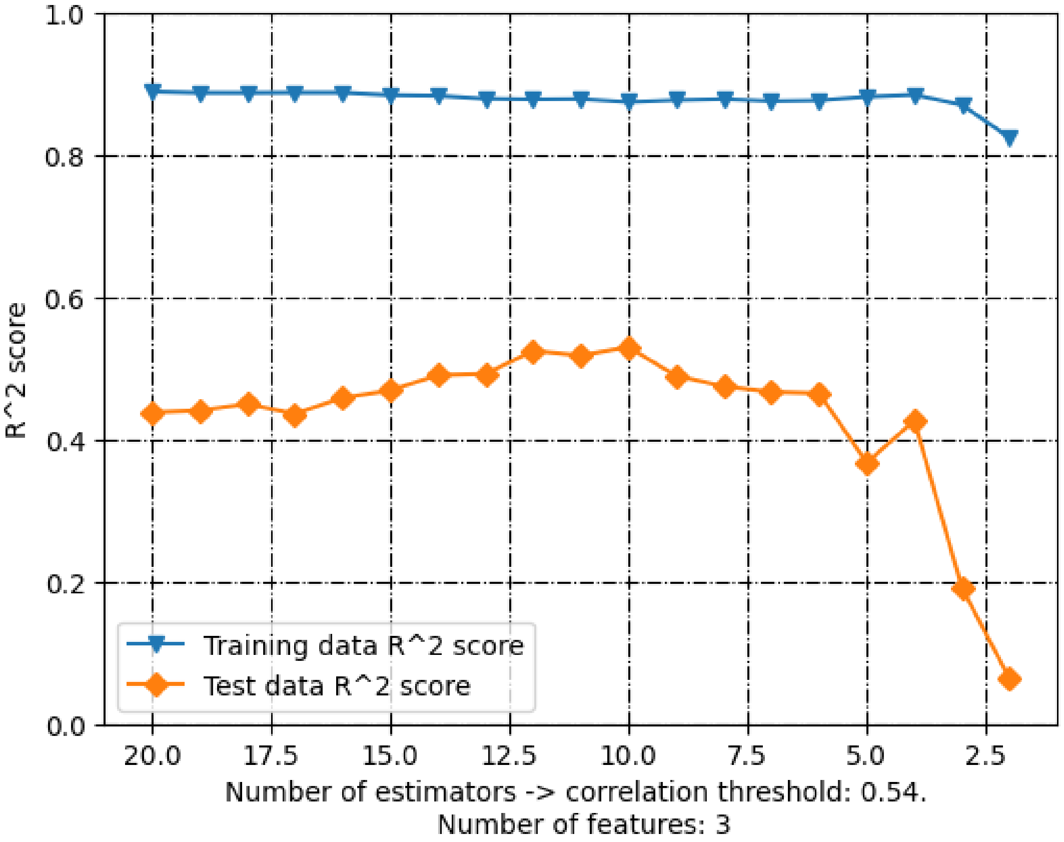
1.0
0.8
0.6
0.4
0.2
0.0
R^2 score
20.0
17.5
15.0
12.5
10.0
7.5
5.0
2.5
Number of estimators -> correlation threshold: 0.54.
Number of features: 3
Training data R^2 score
Test data R^2 score

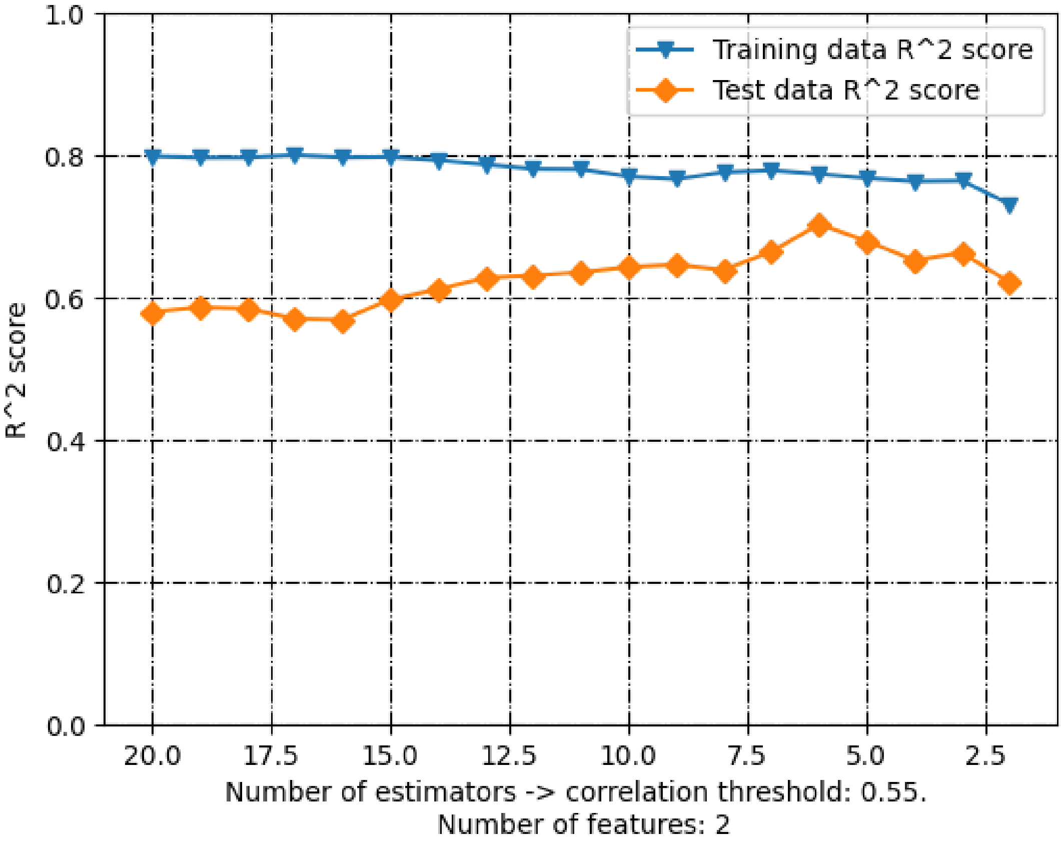

1.0
0.8
0.6
0.4
0.2
0.0
R^2 score
20.0
17.5
15.0
12.5
10.0
7.5
5.0
2.5
Training data R^2 score
Test data R^2 score
Number of estimators -> correlation threshold: 0.55.
Number of features: 2

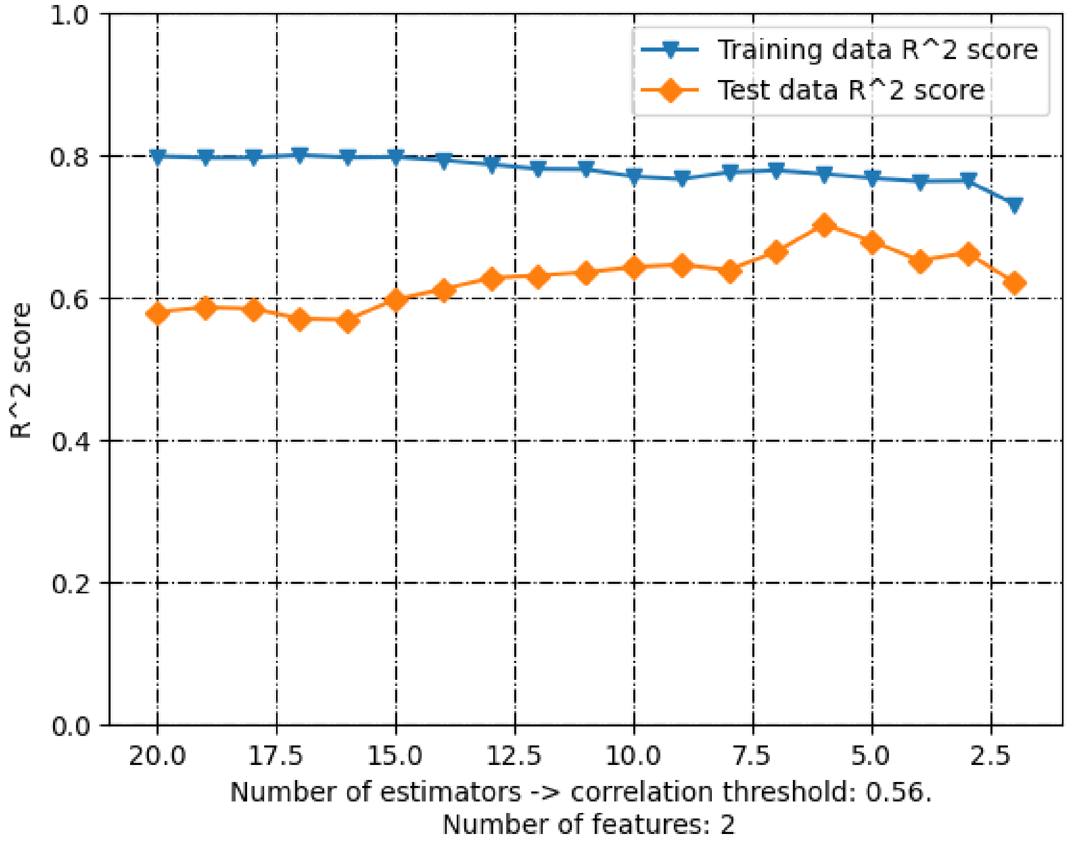

[28]:
```
plt.plot(df_without_standardization['Correlation threshold'],␣
  ↪df_without_standardization['Number of features'], label = "Number of␣
  ↪features", marker='.')
plt.legend()
plt.xlabel('Correlation threshold')
plt.ylabel('Number of features')
plt.show()
```

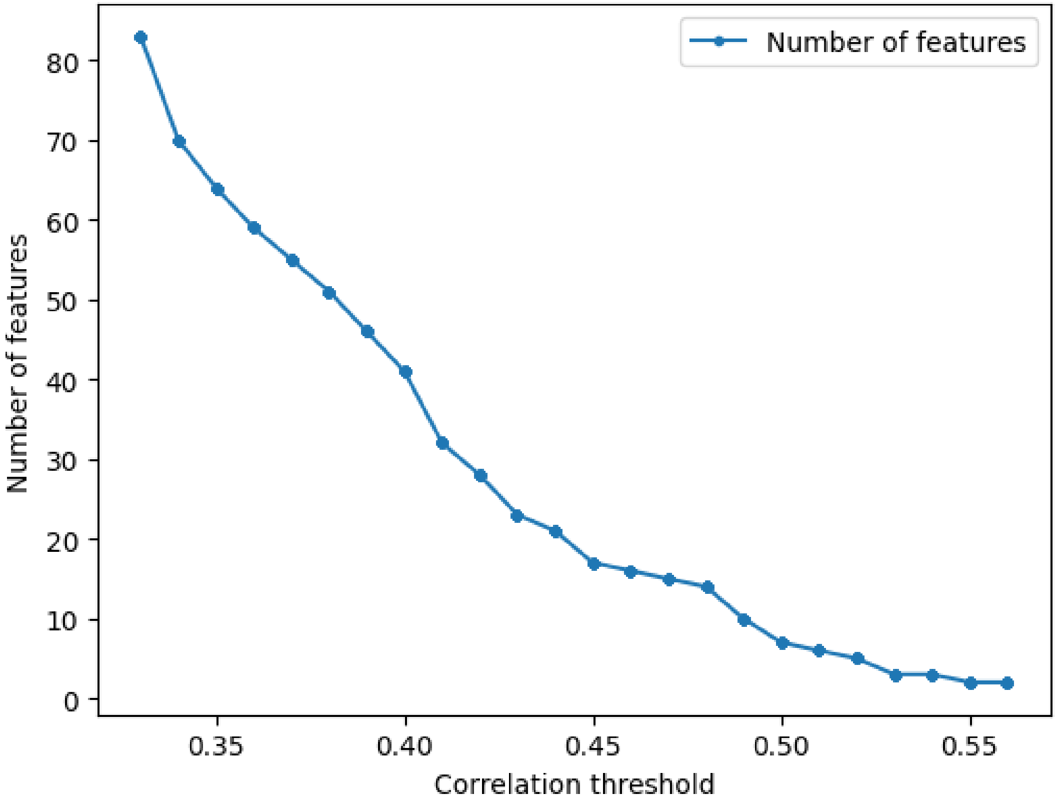

[ ]:

4 KNeighborsRegressor

[29]:
```
without_standardization, train_r2_, test_r2_, hist1, hist2, target_column_name,␣
↪training_data_RMSE, test_data_RMSE  = pred_model.
↪prepare_data_and_create_model(molecular_descriptors_df=data,
                                                                                 ␣
↪                          correlation_threshold=0.50,
                                                                                 ␣
↪                          standardization=False,
                                                                                 ␣
↪                          model_type='KNeighborsRegressor',
                                                                                 ␣
↪                          target_column_name = target,
                                                                                 ␣
↪                          random_state=random_state,
                                                                                 ␣
↪                          train_test_split_=True,
```

```
↪                    verbose=True)
```

```
I am not doing standardization…
  molecular descriptor name
0                    AATS0Z
1                  AATS0are
2                    AATS0d
3                   AATS0dv
4                    AATS0i
  molecular descriptor name  corr_value
0                    AATS0Z   -0.020817
1                  AATS0are   -0.148257
2                    AATS0d    0.022999
3                   AATS0dv   -0.137980
4                    AATS0i    0.133257
  molecular descriptor name  corr_value  absolute correlation value
0                    AATS0Z   -0.020817                    0.020817
1                  AATS0are   -0.148257                    0.148257
2                    AATS0d    0.022999                    0.022999
3                   AATS0dv   -0.137980                    0.137980
4                    AATS0i    0.133257                    0.133257
    molecular descriptor name  corr_value  absolute correlation value
226                    AMID_O   -0.546843                    0.546843
505                EState_VSA5   -0.588780                    0.588780
556                    GATS2c    0.511671                    0.511671
791                  MDEO-12   -0.582747                    0.582747
847                       NdO   -0.500462                    0.500462
    molecular descriptor name  corr_value  absolute correlation value
226                    AMID_O   -0.546843                    0.546843
505                EState_VSA5   -0.588780                    0.588780
556                    GATS2c    0.511671                    0.511671
791                  MDEO-12   -0.582747                    0.582747
847                       NdO   -0.500462                    0.500462
The model used is: KNeighbors…
Return the coefficient of determination of the prediction:
0.41213440641996724
R^2 score: 0.5930480323404299
Correlation coefficient: 0.7700961188971348
Test data - unseen during training:
R^2 score: 0.41213440641996724
Correlation coefficient: 0.6419769516267443
[7.92248844 8.11468799 7.01522868 7.41847691 6.46171252 7.88647346
 7.5584839  7.3571585  7.66228244 7.58497861 7.03594008 7.10633176
 7.42711292 5.50538359 7.10633176 7.92248844 7.58497861 7.42711292]
44     8.004365
47     7.886057
4      7.002614
```

```
55     7.698970
26     5.070734
64     8.060481
73     7.130768
10     8.008774
40     7.795880
107    8.086186
18     6.924818
62     8.173925
11     7.962574
36     6.346787
89     8.102373
91     8.346787
109    7.886057
0      7.991400
Name: BALB/3T3, dtype: float64
Training Root Mean Square Error: 0.5620377814804579
Testing Root Mean Square Error: 0.6104410339578933
```

[30]:
```python
print(target_column_name+str('_transformed'))
print(hist1[target_column_name].hist())
```

```
BALB/3T3_transformed
AxesSubplot(0.125,0.11;0.775x0.77)
```

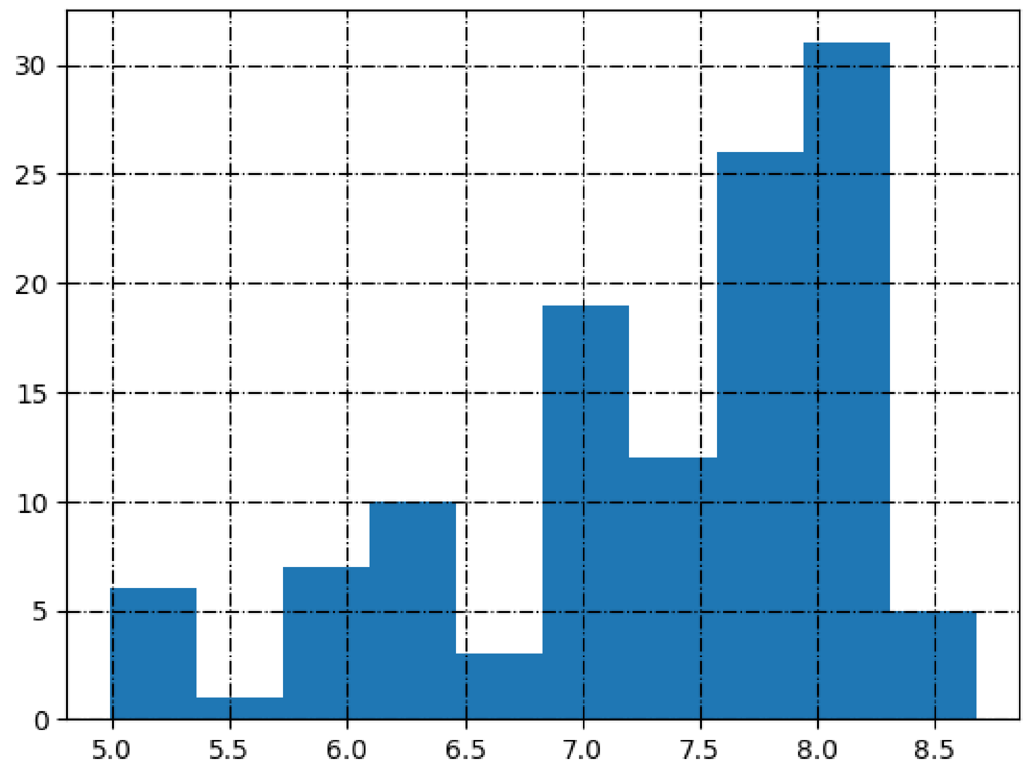

[31]:
```
with_standardization, train_r2_, test_r2_, hist1, hist2, target_column_name,␣
↪training_data_RMSE, test_data_RMSE  = pred_model.
↪prepare_data_and_create_model(molecular_descriptors_df=data,
                                                                              ␣
↪                    correlation_threshold=0.50,
                                                                              ␣
↪                    standardization=True,
                                                                              ␣
↪                    model_type='KNeighborsRegressor',
                                                                              ␣
↪                    target_column_name = target,
                                                                              ␣
↪                    random_state=random_state,
                                                                              ␣
↪                    train_test_split_=True,
                                                                              ␣
↪                    verbose=True)
```

```
I am doing standardization…
  molecular descriptor name
0                    AATS0Z
1                  AATS0are
2                    AATS0d
3                   AATS0dv
4                    AATS0i
  molecular descriptor name  corr_value
0                    AATS0Z   -0.020817
1                  AATS0are   -0.148257
2                    AATS0d    0.022999
3                   AATS0dv   -0.137980
4                    AATS0i    0.133257
  molecular descriptor name  corr_value  absolute correlation value
0                    AATS0Z   -0.020817                    0.020817
1                  AATS0are   -0.148257                    0.148257
2                    AATS0d    0.022999                    0.022999
3                   AATS0dv   -0.137980                    0.137980
4                    AATS0i    0.133257                    0.133257
    molecular descriptor name  corr_value  absolute correlation value
226                    AMID_O   -0.546843                    0.546843
505               EState_VSA5   -0.588780                    0.588780
556                   GATS2c    0.511671                    0.511671
791                  MDEO-12   -0.582747                    0.582747
847                      NdO   -0.500462                    0.500462
    molecular descriptor name  corr_value  absolute correlation value
226                    AMID_O   -0.546843                    0.546843
505               EState_VSA5   -0.588780                    0.588780
```

```
556                      GATS2c    0.511671                     0.511671
791                     MDEO-12   -0.582747                     0.582747
847                         NdO   -0.500462                     0.500462
The model used is: KNeighbors…
Return the coefficient of determination of the prediction:
0.41213440641996724
R^2 score: 0.5930480323404299
Correlation coefficient: 0.7700961188971348
Test data - unseen during training:
R^2 score: 0.41213440641996724
Correlation coefficient: 0.6419769516267443
[7.92248844 8.11468799 7.01522868 7.41847691 6.46171252 7.88647346
 7.5584839  7.3571585  7.66228244 7.58497861 7.03594008 7.10633176
 7.42711292 5.50538359 7.10633176 7.92248844 7.58497861 7.42711292]
44     8.004365
47     7.886057
4      7.002614
55     7.698970
26     5.070734
64     8.060481
73     7.130768
10     8.008774
40     7.795880
107    8.086186
18     6.924818
62     8.173925
11     7.962574
36     6.346787
89     8.102373
91     8.346787
109    7.886057
0      7.991400
Name: BALB/3T3, dtype: float64
Training Root Mean Square Error: 0.5620377814804579
Testing Root Mean Square Error: 0.6104410339578933
```

4.1 Search inside correlation space

[32]:
```
step = 0.01
initial_step = corr_low
last_step = corr_high
first_list = [x / 100.0 for x in range(int(initial_step*100),␣
  ↪int(last_step*100), int(step*100))]
second_list = []
third_list = []
fourth_l = []
fifth_l = []
```

```
f_list = []
for i in first_list:
    without_standardization, train_r2, test_r2, _, h_, target_column_name,␣
 ↪training_data_RMSE, test_data_RMSE = pred_model.
 ↪prepare_data_and_create_model(molecular_descriptors_df = data,
                                                                              ␣
 ↪                    correlation_threshold = i,
                                                                              ␣
 ↪                    standardization = False,
                                                                              ␣
 ↪                    model_type = 'KNeighborsRegressor',
                                                                              ␣
 ↪                    target_column_name = target,
                                                                              ␣
 ↪                    random_state=random_state,
                                                                              ␣
 ↪                    train_test_split_ = True,
                                                                              ␣
 ↪                    verbose = False)
    second_list.append(train_r2)
    third_list.append(test_r2)
    fourth_l.append(training_data_RMSE)
    fifth_l.append(test_data_RMSE)
    f_list.append(len(h_))
```

```
  File "C:\Users\aleks\anaconda3\envs\cheminf_gpu\lib\site-
packages\joblib\externals\loky\backend\context.py", line 217, in
_count_physical_cores
    raise ValueError(
```

[33]:
```
df_without_standardization = pd.DataFrame(data=first_list,␣
 ↪columns=["Correlation threshold"])
df_without_standardization['Training data R^2 score'] = second_list
df_without_standardization['Test data R^2 score'] = third_list
df_without_standardization['Training RMSE'] = fourth_l
df_without_standardization['Test RMSE'] = fifth_l
df_without_standardization['Number of features'] = f_list
```

[34]:
```
df_k_nearest = df_without_standardization.copy()
df_without_standardization
```

[34]:

	Correlation threshold	Training data R^2 score	Test data R^2 score	\
0	0.33	0.579637	0.195021	
1	0.34	0.612206	0.112471	
2	0.35	0.612206	0.112471	
3	0.36	0.612206	0.112471	
4	0.37	0.612206	0.112471	

5	0.38	0.612206	0.151377
6	0.39	0.654638	0.222194
7	0.40	0.654638	0.222194
8	0.41	0.607745	0.238325
9	0.42	0.591628	0.095145
10	0.43	0.595486	0.405419
11	0.44	0.578461	0.364451
12	0.45	0.589545	0.451468
13	0.46	0.592920	0.449529
14	0.47	0.597083	0.460702
15	0.48	0.591905	0.436552
16	0.49	0.581925	0.383116
17	0.50	0.593048	0.412134
18	0.51	0.585189	0.370109
19	0.52	0.578779	0.399724
20	0.53	0.579822	0.408287
21	0.54	0.579822	0.408287
22	0.55	0.542320	0.347880
23	0.56	0.542320	0.347880

	Training RMSE	Test RMSE	Number of features
0	0.571224	0.714327	83
1	0.548649	0.750060	70
2	0.548649	0.750060	64
3	0.548649	0.750060	59
4	0.548649	0.750060	55
5	0.548649	0.733436	51
6	0.517763	0.702167	46
7	0.517763	0.702167	41
8	0.551795	0.694848	32
9	0.563018	0.757346	28
10	0.560352	0.613918	23
11	0.572022	0.634716	21
12	0.564451	0.589665	17
13	0.562126	0.590707	16
14	0.559245	0.584681	15
15	0.562826	0.597629	14
16	0.569667	0.625326	10
17	0.562038	0.610441	7
18	0.567439	0.631884	6
19	0.571806	0.616851	5
20	0.571098	0.612435	3
21	0.571098	0.612435	3
22	0.596039	0.642937	2
23	0.596039	0.642937	2

4.2 Plots

[35]:
```
plt.plot(df_without_standardization['Correlation threshold'],␣
 ↪df_without_standardization['Training data R^2 score'], label = "Training␣
 ↪data R^2 score", marker='v')
plt.plot(df_without_standardization['Correlation threshold'],␣
 ↪df_without_standardization['Test data R^2 score'], label = "Test data R^2␣
 ↪score", marker='D')
plt.legend()
plt.xlabel('Correlation threshold')
plt.ylabel('R^2 score')
plt.ylim([0, 1])
plt.show()
```

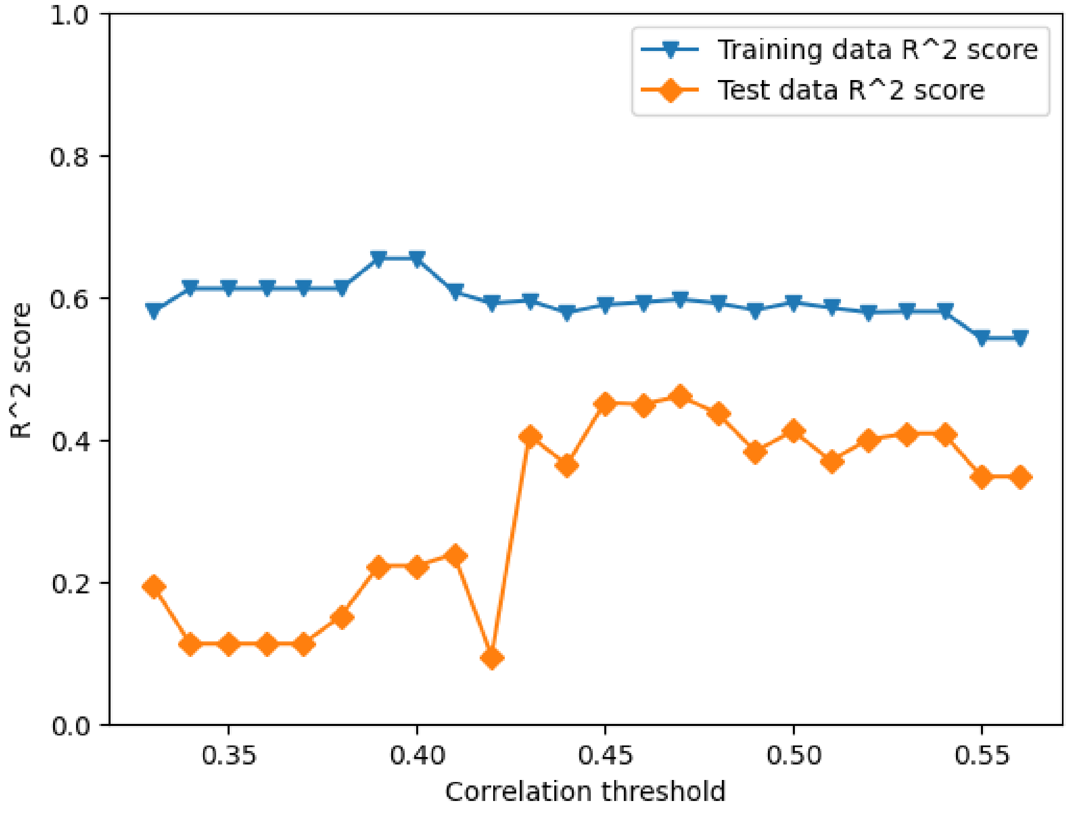

[36]:
```
plt.plot(df_without_standardization['Correlation threshold'],␣
 ↪df_without_standardization['Number of features'], label = "Number of␣
 ↪features", marker='.')
plt.legend()
plt.xlabel('Correlation threshold')
plt.ylabel('Number of features')
plt.show()
```

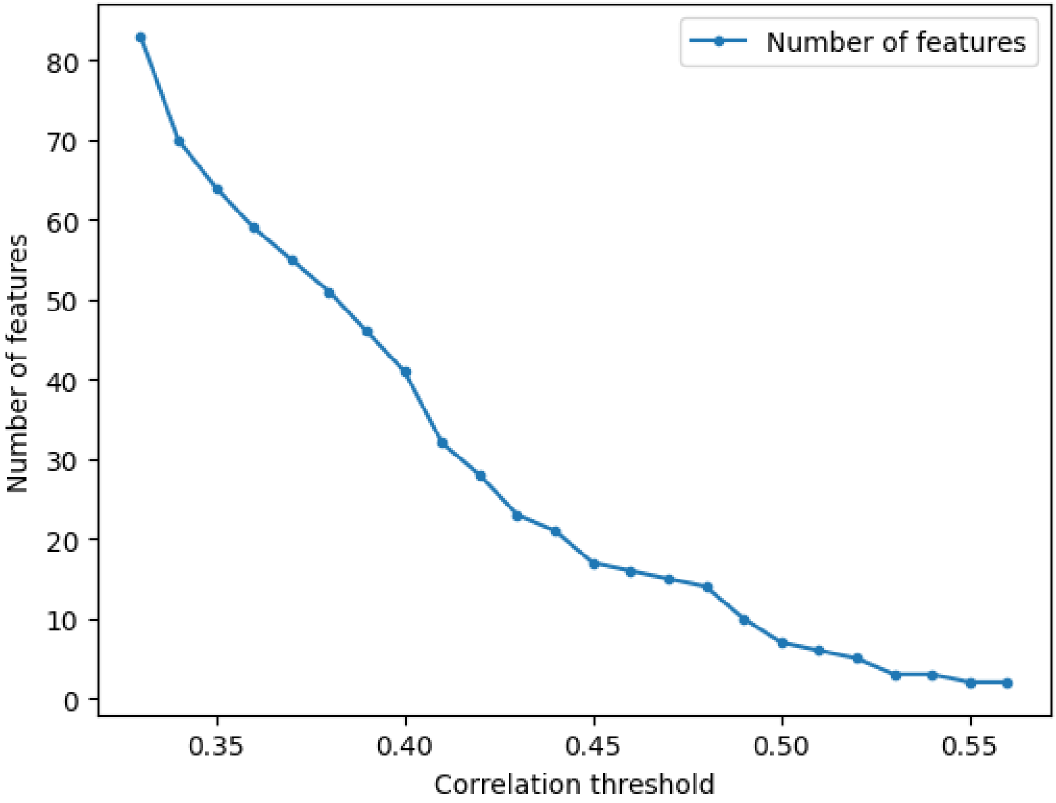


```
[ ]:
```

5 Support Vector Machines (SVM)

```
[37]: without_standardization, train_r2_, test_r2_, hist1, hist2, target_column_name,␣
      ↪training_data_RMSE, test_data_RMSE  = pred_model.
      ↪prepare_data_and_create_model(molecular_descriptors_df=data,
                                                                                     ␣
      ↪                     correlation_threshold=0.50,
                                                                                     ␣
      ↪                     standardization=False,
                                                                                     ␣
      ↪                     model_type='SVR',
                                                                                     ␣
      ↪                     kernel_ = 'linear',
                                                                                     ␣
      ↪                     gamma_ = 'auto',
                                                                                     ␣
      ↪                     target_column_name = target,
```

```
↪                   random_state=random_state,
↪                   train_test_split_=True,
↪                   verbose=True)
```

```
I am not doing standardization…
  molecular descriptor name
0                   AATS0Z
1                 AATS0are
2                   AATS0d
3                  AATS0dv
4                   AATS0i
  molecular descriptor name  corr_value
0                   AATS0Z   -0.020817
1                 AATS0are   -0.148257
2                   AATS0d    0.022999
3                  AATS0dv   -0.137980
4                   AATS0i    0.133257
  molecular descriptor name  corr_value  absolute correlation value
0                   AATS0Z   -0.020817                    0.020817
1                 AATS0are   -0.148257                    0.148257
2                   AATS0d    0.022999                    0.022999
3                  AATS0dv   -0.137980                    0.137980
4                   AATS0i    0.133257                    0.133257
    molecular descriptor name  corr_value  absolute correlation value
226                    AMID_O   -0.546843                    0.546843
505               EState_VSA5   -0.588780                    0.588780
556                   GATS2c    0.511671                    0.511671
791                  MDEO-12   -0.582747                    0.582747
847                      NdO   -0.500462                    0.500462
    molecular descriptor name  corr_value  absolute correlation value
226                    AMID_O   -0.546843                    0.546843
505               EState_VSA5   -0.588780                    0.588780
556                   GATS2c    0.511671                    0.511671
791                  MDEO-12   -0.582747                    0.582747
847                      NdO   -0.500462                    0.500462
The model used is: SVR…
Return the coefficient of determination of the prediction:
0.44709623399096254
R^2 score: 0.5159297330048184
Correlation coefficient: 0.7182824883044403
Test data - unseen during training:
R^2 score: 0.44709623399096254
Correlation coefficient: 0.6686525510240445
[8.00016581 8.05093548 7.42238496 7.14876453 6.07021375 7.5208028
```

```
 7.76394424 7.5899862  7.99109813 7.60464267 7.50180307 6.68220808
 7.72363523 6.28477308 7.21990624 8.00043516 7.60215619 7.73146765]
44     8.004365
47     7.886057
4      7.002614
55     7.698970
26     5.070734
64     8.060481
73     7.130768
10     8.008774
40     7.795880
107    8.086186
18     6.924818
62     8.173925
11     7.962574
36     6.346787
89     8.102373
91     8.346787
109    7.886057
0      7.991400
Name: BALB/3T3, dtype: float64
Training Root Mean Square Error: 0.6129825880081178
Testing Root Mean Square Error: 0.5920105851521211
```

[38]:
```
print(target_column_name+str('_transformed'))
print(hist1[target_column_name].hist())
```

```
BALB/3T3_transformed
AxesSubplot(0.125,0.11;0.775x0.77)
```

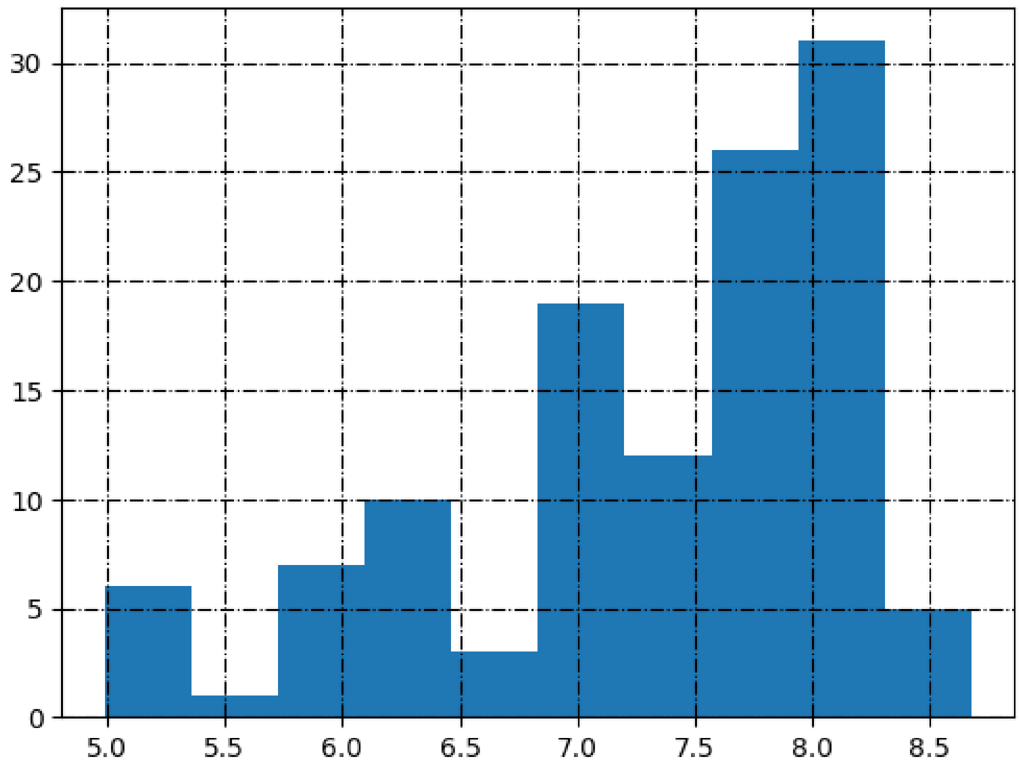

[39]:
```
with_standardization, train_r2_, test_r2_, hist1, hist2, target_column_name,␣
↪training_data_RMSE, test_data_RMSE  = pred_model.
↪prepare_data_and_create_model(molecular_descriptors_df=data,
                                                                                ␣
↪                          correlation_threshold=0.50,
                                                                                ␣
↪                          standardization=True,
                                                                                ␣
↪                          model_type='SVR',
                                                                                ␣
↪                          kernel_ = 'linear',
                                                                                ␣
↪                          gamma_ = 'auto',
                                                                                ␣
↪                          target_column_name = target,
                                                                                ␣
↪                          random_state=random_state,
                                                                                ␣
↪                          train_test_split_=True,
                                                                                ␣
↪                          verbose=True)
```

```
I am doing standardization…
  molecular descriptor name
0                    AATS0Z
1                  AATS0are
2                    AATS0d
3                   AATS0dv
4                    AATS0i
  molecular descriptor name  corr_value
0                    AATS0Z   -0.020817
1                  AATS0are   -0.148257
2                    AATS0d    0.022999
3                   AATS0dv   -0.137980
4                    AATS0i    0.133257
  molecular descriptor name  corr_value  absolute correlation value
0                    AATS0Z   -0.020817                    0.020817
1                  AATS0are   -0.148257                    0.148257
2                    AATS0d    0.022999                    0.022999
3                   AATS0dv   -0.137980                    0.137980
4                    AATS0i    0.133257                    0.133257
    molecular descriptor name  corr_value  absolute correlation value
226                    AMID_O   -0.546843                    0.546843
505                EState_VSA5   -0.588780                    0.588780
556                    GATS2c    0.511671                    0.511671
791                   MDEO-12   -0.582747                    0.582747
847                       NdO   -0.500462                    0.500462
    molecular descriptor name  corr_value  absolute correlation value
226                    AMID_O   -0.546843                    0.546843
505                EState_VSA5   -0.588780                    0.588780
556                    GATS2c    0.511671                    0.511671
791                   MDEO-12   -0.582747                    0.582747
847                       NdO   -0.500462                    0.500462
The model used is: SVR…
Return the coefficient of determination of the prediction:
0.44709623399096254
R^2 score: 0.5159297330048184
Correlation coefficient: 0.7182824883044403
Test data - unseen during training:
R^2 score: 0.44709623399096254
Correlation coefficient: 0.6686525510240445
[8.00016581 8.05093548 7.42238496 7.14876453 6.07021375 7.5208028
 7.76394424 7.5899862  7.99109813 7.60464267 7.50180307 6.68220808
 7.72363523 6.28477308 7.21990624 8.00043516 7.60215619 7.73146765]
44     8.004365
47     7.886057
4      7.002614
55     7.698970
26     5.070734
64     8.060481
```

```
73     7.130768
10     8.008774
40     7.795880
107    8.086186
18     6.924818
62     8.173925
11     7.962574
36     6.346787
89     8.102373
91     8.346787
109    7.886057
0      7.991400
Name: BALB/3T3, dtype: float64
Training Root Mean Square Error: 0.6129825880081178
Testing Root Mean Square Error: 0.5920105851521211
```

5.1 Search inside correlation space

[40]:
```
step = 0.01
initial_step = corr_low
last_step = corr_high
first_list = [x / 100.0 for x in range(int(initial_step*100),␣
 ↪int(last_step*100), int(step*100))]
second_list = []
third_list = []
fourth_l = []
fifth_l = []
f_list = []
for i in first_list:
    without_standardization, train_r2, test_r2, _, h_, target_column_name,␣
 ↪training_data_RMSE, test_data_RMSE = pred_model.
 ↪prepare_data_and_create_model(molecular_descriptors_df = data,
                                                                      ␣
 ↪                      correlation_threshold = i,
                                                                      ␣
 ↪                      standardization = False,
                                                                      ␣
 ↪                      model_type = 'SVR',
                                                                      ␣
 ↪                      kernel_ = 'linear',
                                                                      ␣
 ↪                      gamma_ = 'auto',
                                                                      ␣
 ↪                      target_column_name = target,
                                                                      ␣
 ↪                      random_state=random_state,
```

```
                    train_test_split_ = True,
                    verbose = False)
    second_list.append(train_r2)
    third_list.append(test_r2)
    fourth_l.append(training_data_RMSE)
    fifth_l.append(test_data_RMSE)
    f_list.append(len(h_))
```

[41]:
```
df_without_standardization = pd.DataFrame(data=first_list,
 columns=["Correlation threshold"])
df_without_standardization['Training data R^2 score'] = second_list
df_without_standardization['Test data R^2 score'] = third_list
df_without_standardization['Training RMSE'] = fourth_l
df_without_standardization['Test RMSE'] = fifth_l
df_without_standardization['Number of features'] = f_list
```

[42]:
```
df_svm = df_without_standardization.copy()
df_without_standardization
```

[42]:

	Correlation threshold	Training data R^2 score	Test data R^2 score	\
0	0.33	0.752351	0.638549	
1	0.34	0.679781	0.469111	
2	0.35	0.678435	0.413280	
3	0.36	0.675955	0.504552	
4	0.37	0.663070	0.324430	
5	0.38	0.660186	0.367016	
6	0.39	0.645235	0.453820	
7	0.40	0.644395	0.454880	
8	0.41	0.619180	0.617244	
9	0.42	0.582879	0.711228	
10	0.43	0.577743	0.709262	
11	0.44	0.556430	0.572454	
12	0.45	0.565542	0.588193	
13	0.46	0.565206	0.587915	
14	0.47	0.560389	0.550276	
15	0.48	0.563970	0.480963	
16	0.49	0.556276	0.462398	
17	0.50	0.515930	0.447096	
18	0.51	0.494480	0.480944	
19	0.52	0.495580	0.480057	
20	0.53	0.468545	0.512913	
21	0.54	0.468545	0.512913	
22	0.55	0.468300	0.511190	
23	0.56	0.468300	0.511190	

```
    Training RMSE  Test RMSE  Number of features
0        0.438442   0.478662                  83
1        0.498560   0.580105                  70
2        0.499607   0.609846                  64
3        0.501530   0.560407                  59
4        0.511404   0.654395                  55
5        0.513588   0.633433                  51
6        0.524764   0.588400                  46
7        0.525385   0.587829                  41
8        0.543693   0.492567                  32
9        0.569016   0.427841                  28
10       0.572509   0.429295                  23
11       0.586780   0.520590                  21
12       0.580722   0.510918                  17
13       0.580946   0.511091                  16
14       0.584155   0.533922                  15
15       0.581771   0.573593                  14
16       0.586882   0.583761                  10
17       0.612983   0.592011                   7
18       0.626417   0.573604                   6
19       0.625734   0.574093                   5
20       0.642284   0.555659                   3
21       0.642284   0.555659                   3
22       0.642432   0.556640                   2
23       0.642432   0.556640                   2
```

5.2 Plots

[43]:
```
plt.plot(df_without_standardization['Correlation threshold'],
 ↪df_without_standardization['Training data R^2 score'], label = "Training
 ↪data R^2 score", marker='v')
plt.plot(df_without_standardization['Correlation threshold'],
 ↪df_without_standardization['Test data R^2 score'], label = "Test data R^2
 ↪score", marker='D')
plt.legend()
plt.xlabel('Correlation threshold')
plt.ylabel('R^2 score')
plt.ylim([0, 1])
plt.show()
```

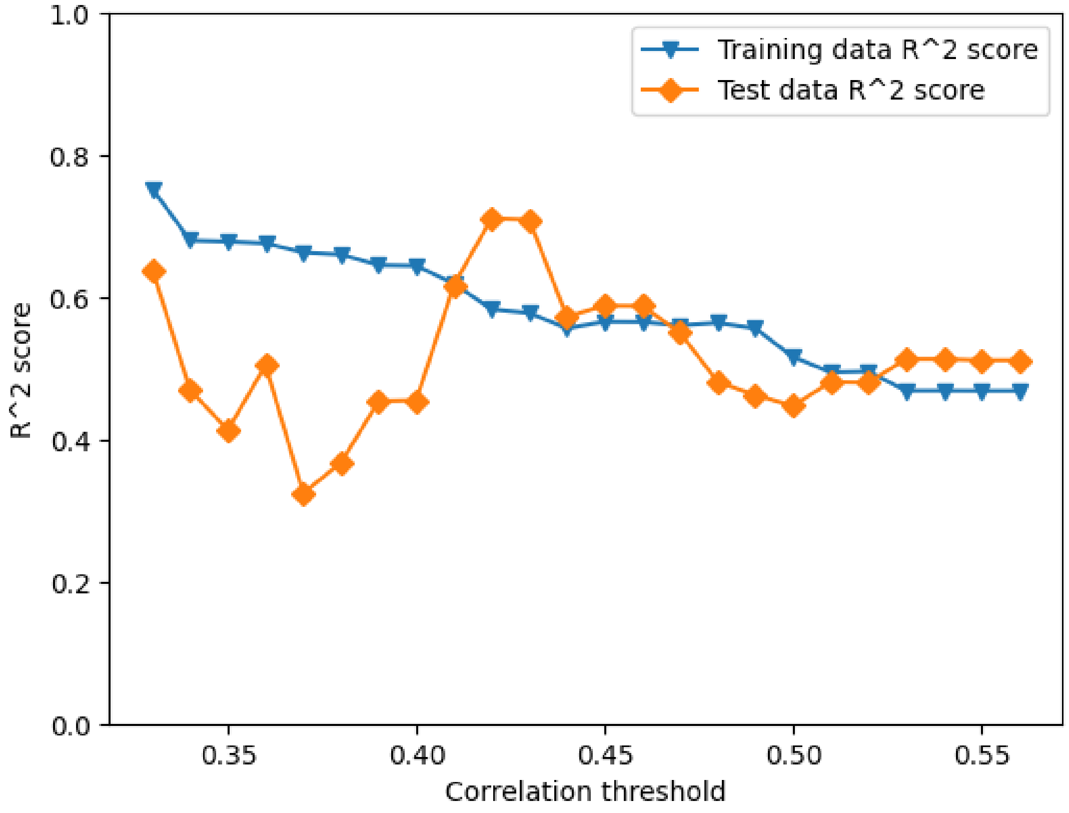

[44]:
```
plt.plot(df_without_standardization['Correlation threshold'],␣
↪df_without_standardization['Number of features'], label = "Number of␣
↪features", marker='.')
plt.legend()
plt.xlabel('Correlation threshold')
plt.ylabel('Number of features')
plt.show()
```

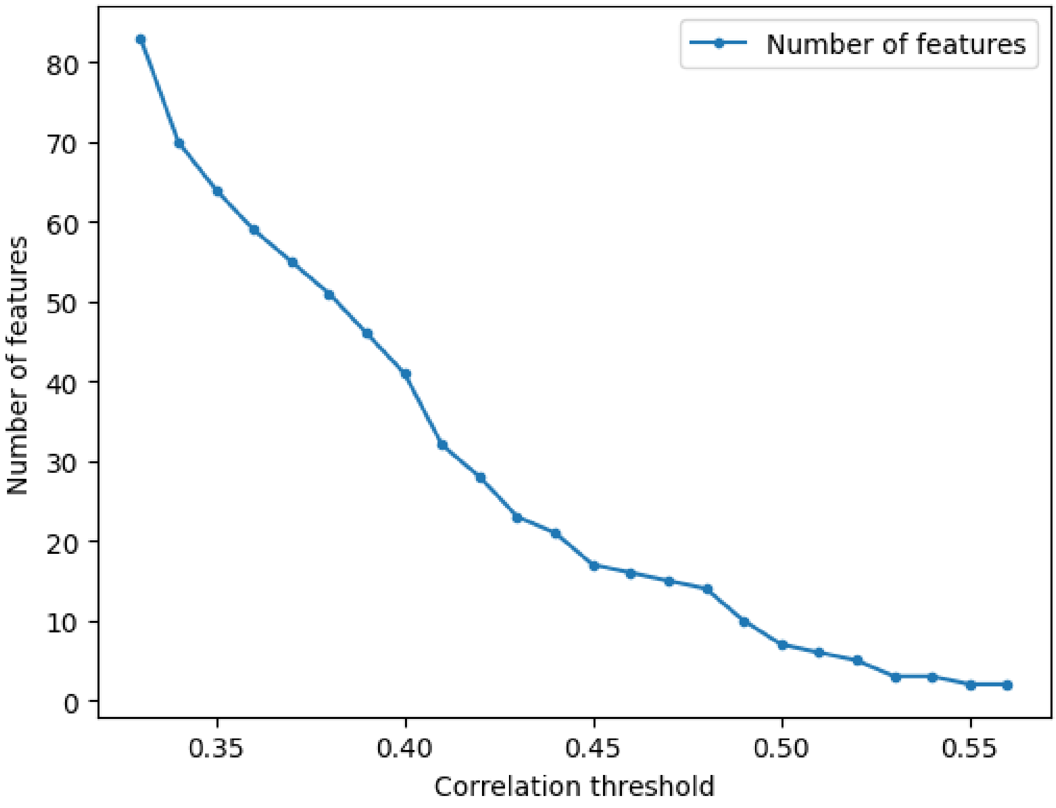

5.3 Store quality measurements

[45]:
```
with pd.ExcelWriter('../Data/Quality_'+'BALB_3T3'+'_'+str(random_state)+'_.
 ↪xlsx') as writer:
    df_linear.to_excel(writer, sheet_name='MLR')
    df_decision_tree.to_excel(writer, sheet_name='DT')
    df_random_forest.to_excel(writer, sheet_name='RF')
    df_k_nearest.to_excel(writer, sheet_name='KNN')
    df_svm.to_excel(writer, sheet_name='SVM')
```

Notebook

January 18, 2024

1 File 21

[1]:
```python
import sys
from pathlib import Path
prediction_mode_path = Path("../module")
sys.path.append(prediction_mode_path.as_posix())
import models_creation as pred_model

import pandas as pd
import numpy as np

from sklearn.metrics import r2_score

import matplotlib.pyplot as plt
from sklearn.tree import plot_tree

from matplotlib import pyplot as plt

import joblib

import warnings
warnings.filterwarnings('ignore')
```

[2]:
```python
load_prepared_data = pd.read_excel('../Data/Kolchicyna_machine_learning.xlsx')
load_prepared_data = load_prepared_data.drop(columns=['Unnamed: 0'])

target = 'LoVo/DX'

corr_low = 0.33
corr_high = 0.64

random_state = 15
```

[3]:
```python
load_prepared_data.head()
```

[3]:
```
      AATS0Z  AATS0are    AATS0d   AATS0dv      AATS0i     AATS0m    AATS0p  \
0  25.090909  6.046518  3.109091  6.662626  160.775045  99.075564  1.555512
1  24.448276  6.008422  3.051724  6.386973  161.021674  96.473315  1.538471
```

```
2  23.868852  5.974074  3.000000  6.138434  161.244045  94.127025  1.523105
3  23.868852  5.974074  3.032787  6.171220  161.244045  94.127025  1.523105
4  23.343750  5.942945  2.953125  5.913194  161.445569  92.000700  1.509180

    AATS0pe    AATS0s    AATS0se  …  A549_float  MCF-7_float  LoVo_float  \
0  6.126680  3.435416  7.629859  …        10.8         10.3         6.5
1  6.088791  3.330998  7.596891  …        11.6         12.0         8.5
2  6.054630  3.236850  7.567166  …        10.9         12.2         8.8
3  6.054630  3.257798  7.567166  …        10.5         11.3         8.5
4  6.023670  3.151529  7.540228  …        89.5         92.7        52.8

   LoVo/DX_float  BALB/3T3_float  A549_float_transformed  \
0           54.9            10.2                7.966576
1           31.1            14.3                7.935542
2           17.9            11.7                7.962574
3           10.2            11.0                7.978811
4           77.8            99.4                7.048177

   MCF-7_float_transformed  LoVo_float_transformed  LoVo/DX_float_transformed  \
0                 7.987163                8.187087                   7.260428
1                 7.920819                8.070581                   7.507240
2                 7.913640                8.055517                   7.747147
3                 7.946922                8.070581                   7.991400
4                 7.032920                7.277366                   7.109020

   BALB/3T3_float_transformed
0                    7.991400
1                    7.844664
2                    7.931814
3                    7.958607
4                    7.002614

[5 rows x 1221 columns]
```

[4]:
```
data = load_prepared_data[list(load_prepared_data.columns)[0:-10]]
data[target] = load_prepared_data[load_prepared_data.columns[-2]]
data.head()
```

[4]:
```
      AATS0Z  AATS0are    AATS0d   AATS0dv      AATS0i     AATS0m    AATS0p  \
0  25.090909  6.046518  3.109091  6.662626  160.775045  99.075564  1.555512
1  24.448276  6.008422  3.051724  6.386973  161.021674  96.473315  1.538471
2  23.868852  5.974074  3.000000  6.138434  161.244045  94.127025  1.523105
3  23.868852  5.974074  3.032787  6.171220  161.244045  94.127025  1.523105
4  23.343750  5.942945  2.953125  5.913194  161.445569  92.000700  1.509180

    AATS0pe    AATS0s    AATS0se  …    piPC10     piPC2     piPC3     piPC4  \
0  6.126680  3.435416  7.629859  …  7.999655  4.266195  4.915408  5.516700
```

```
1  6.088791  3.330998  7.596891  …  8.018726  4.280132  4.922714  5.520711
2  6.054630  3.236850  7.567166  …  8.028799  4.293878  4.929967  5.524706
3  6.054630  3.257798  7.567166  …  8.037440  4.307438  4.929967  5.524706
4  6.023670  3.151529  7.540228  …  8.034892  4.307438  4.937168  5.528685

      piPC5     piPC6     piPC7     piPC8     piPC9   LoVo/DX
0  6.098566  6.595759  6.979116  7.337313  7.681445  7.260428
1  6.103048  6.601209  6.985613  7.349442  7.695531  7.507240
2  6.105281  6.603923  6.989306  7.353932  7.704023  7.747147
3  6.107510  6.606629  6.992068  7.361425  7.709420  7.991400
4  6.107510  6.605277  6.991148  7.356489  7.707175  7.109020

[5 rows x 1212 columns]
```

[5]:
```
print(data.shape)
data = data.dropna()
data.shape
```

```
(120, 1212)
```

[5]:
```
(106, 1212)
```

2 Multiple Linear regression (MLR)

[6]:
```
without_standardization, train_r2_, test_r2_, hist1, hist2, target_column_name,␣
↪training_data_RMSE, test_data_RMSE  = pred_model.
↪prepare_data_and_create_model(molecular_descriptors_df=data,
                                                                                          ␣
↪                        correlation_threshold=0.5,
                                                                                          ␣
↪                        standardization=False,
                                                                                          ␣
↪                        model_type='linear_model',
                                                                                          ␣
↪                        target_column_name = str(target),
                                                                                          ␣
↪                        random_state=random_state,
                                                                                          ␣
↪                        train_test_split_=True,
                                                                                          ␣
↪                        verbose=True)
```

```
I am not doing standardization…
  molecular descriptor name
0                    AATS0Z
1                  AATS0are
2                    AATS0d
```

```
3                  AATS0dv
4                   AATS0i
  molecular descriptor name  corr_value
0                   AATS0Z    0.018677
1                 AATS0are   -0.341313
2                   AATS0d   -0.123443
3                  AATS0dv   -0.265670
4                   AATS0i   -0.428929
  molecular descriptor name  corr_value  absolute correlation value
0                   AATS0Z    0.018677                    0.018677
1                 AATS0are   -0.341313                    0.341313
2                   AATS0d   -0.123443                    0.123443
3                  AATS0dv   -0.265670                    0.265670
4                   AATS0i   -0.428929                    0.428929
    molecular descriptor name  corr_value  absolute correlation value
15                     AATS1i   -0.561620                    0.561620
113                   AATSC1c    0.565682                    0.565682
125                   AATSC2c   -0.543393                    0.543393
221                AETA_eta_R   -0.515778                    0.515778
224                    AMID_C    0.532458                    0.532458
    molecular descriptor name  corr_value  absolute correlation value
15                     AATS1i   -0.561620                    0.561620
113                   AATSC1c    0.565682                    0.565682
125                   AATSC2c   -0.543393                    0.543393
221                AETA_eta_R   -0.515778                    0.515778
224                    AMID_C    0.532458                    0.532458
The model used is: LinearReg…
Return the coefficient of determination of the prediction:
-1.0321044921399274
R^2 score: 0.923102060396964
Correlation coefficient: 0.960782004617574
Test data - unseen during training:
R^2 score: -1.0321044921399274
Correlation coefficient: nan
[6.46501046 7.15168096 5.6028327  5.08085315 6.81181054 4.25182797
 7.48467607 5.5582766  3.99921191 6.61153624 7.97920239 5.66537435
 7.06319213 4.96915563 7.01668286 6.14304559]
8      6.159455
110    6.065502
69     5.552842
95     5.200659
11     7.250264
104    6.000000
52     6.823909
112    6.853872
101    5.000000
50     5.769551
100    6.136677
```

```
103     6.000000
21      6.398375
60      5.537602
84      7.002177
39      6.638272
Name: LoVo/DX, dtype: float64
Training Root Mean Square Error: 0.2658491437480014
Testing Root Mean Square Error: 0.8972699205615916
```

[7]:
```
print(target_column_name+str('_transformed'))
print(hist1[target_column_name].hist())
```

```
LoVo/DX_transformed
AxesSubplot(0.125,0.11;0.775x0.77)
```

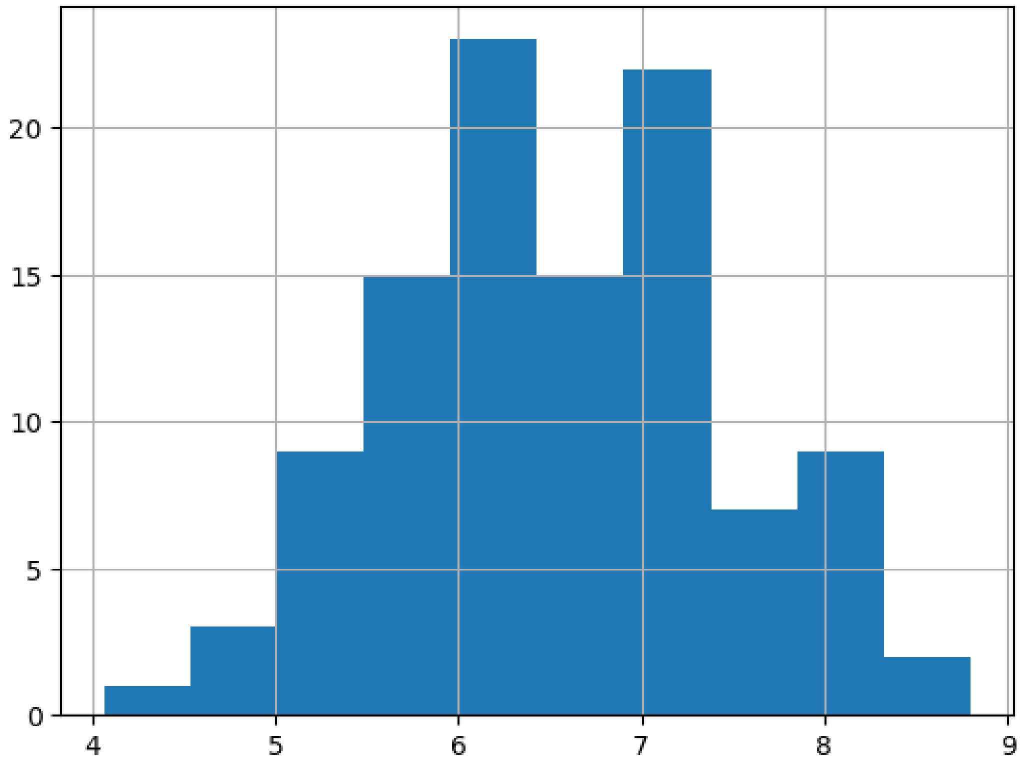

[8]:
```
with_standardization, train_r2_, test_r2_, hist1, hist2, target_column_name,␣
↪training_data_RMSE, test_data_RMSE  = pred_model.
↪prepare_data_and_create_model(molecular_descriptors_df=data,
                                                                              ␣
↪                       correlation_threshold=0.50,
                                                                              ␣
↪                       standardization=True,
```

```
↪                     model_type='linear_model',
↪                     target_column_name = target,
↪                     random_state=random_state,
↪                     train_test_split_=True,
↪                     verbose=True)
```

```
I am doing standardization…
  molecular descriptor name
0                    AATS0Z
1                  AATS0are
2                    AATS0d
3                   AATS0dv
4                    AATS0i
  molecular descriptor name  corr_value
0                    AATS0Z    0.018677
1                  AATS0are   -0.341313
2                    AATS0d   -0.123443
3                   AATS0dv   -0.265670
4                    AATS0i   -0.428929
  molecular descriptor name  corr_value  absolute correlation value
0                    AATS0Z    0.018677                    0.018677
1                  AATS0are   -0.341313                    0.341313
2                    AATS0d   -0.123443                    0.123443
3                   AATS0dv   -0.265670                    0.265670
4                    AATS0i   -0.428929                    0.428929
    molecular descriptor name  corr_value  absolute correlation value
15                     AATS1i   -0.561620                    0.561620
113                   AATSC1c    0.565682                    0.565682
125                   AATSC2c   -0.543393                    0.543393
221                AETA_eta_R   -0.515778                    0.515778
224                    AMID_C    0.532458                    0.532458
    molecular descriptor name  corr_value  absolute correlation value
15                     AATS1i   -0.561620                    0.561620
113                   AATSC1c    0.565682                    0.565682
125                   AATSC2c   -0.543393                    0.543393
221                AETA_eta_R   -0.515778                    0.515778
224                    AMID_C    0.532458                    0.532458
The model used is: LinearReg…
Return the coefficient of determination of the prediction:
-1.0321044921399274
R^2 score: 0.923102060396964
Correlation coefficient: 0.960782004617574
```

```
Test data - unseen during training:
R^2 score: -1.0321044921399274
Correlation coefficient: nan
[6.46501046 7.15168096 5.6028327  5.08085315 6.81181054 4.25182797
 7.48467607 5.5582766  3.99921191 6.61153624 7.97920239 5.66537435
 7.06319213 4.96915563 7.01668286 6.14304559]
8      6.159455
110    6.065502
69     5.552842
95     5.200659
11     7.250264
104    6.000000
52     6.823909
112    6.853872
101    5.000000
50     5.769551
100    6.136677
103    6.000000
21     6.398375
60     5.537602
84     7.002177
39     6.638272
Name: LoVo/DX, dtype: float64
Training Root Mean Square Error: 0.2658491437480014
Testing Root Mean Square Error: 0.8972699205615916
```

2.1 Search inside correlation space

[9]:
```
step = 0.01
initial_step = corr_low
last_step = corr_high
first_list = [x / 100.0 for x in range(int(initial_step*100),␣
 ↪int(last_step*100), int(step*100))]
second_list = []
third_list = []
fourth_l = []
fifth_l = []
f_list = []
for i in first_list:
    without_standardization, train_r2, test_r2, _, h_, target_column_name,␣
 ↪training_data_RMSE, test_data_RMSE = pred_model.
 ↪prepare_data_and_create_model(molecular_descriptors_df = data,
                                                                           ␣
 ↪                      correlation_threshold = i,
                                                                           ␣
 ↪                      standardization = False,
```

```
                    model_type = 'linear_model',
                    target_column_name = target,
                    random_state=random_state,
                    train_test_split_ = True,
                    verbose = False)
    second_list.append(train_r2)
    third_list.append(test_r2)
    fourth_l.append(training_data_RMSE)
    fifth_l.append(test_data_RMSE)
    f_list.append(len(h_))
```

[10]:
```
df_without_standardization = pd.DataFrame(data=first_list,
    columns=["Correlation threshold"])
df_without_standardization['Training data R^2 score'] = second_list
df_without_standardization['Test data R^2 score'] = third_list
df_without_standardization['Training RMSE'] = fourth_l
df_without_standardization['Test RMSE'] = fifth_l
df_without_standardization['Number of features'] = f_list
```

[11]:
```
df_linear = df_without_standardization.copy()
df_without_standardization
```

[11]:
```
    Correlation threshold  Training data R^2 score  Test data R^2 score  \
0                    0.33                 1.000000           -13.371015
1                    0.34                 1.000000           -13.804982
2                    0.35                 1.000000           -17.508581
3                    0.36                 1.000000           -18.173919
4                    0.37                 1.000000           -22.385675
5                    0.38                 1.000000           -19.327214
6                    0.39                 1.000000           -20.111861
7                    0.40                 1.000000           -12.284709
8                    0.41                 1.000000           -10.243588
9                    0.42                 1.000000            -9.687742
10                   0.43                 1.000000            -7.821218
11                   0.44                 1.000000           -15.222724
12                   0.45                 1.000000           -84.187281
13                   0.46                 1.000000         -4974.152161
14                   0.47                 0.994782           -14.155849
15                   0.48                 0.964264            -0.408703
16                   0.49                 0.954139            -0.588182
17                   0.50                 0.923102            -1.032104
18                   0.51                 0.917111            -0.675424
```

19	0.52	0.912945	-0.526957
20	0.53	0.880879	0.256343
21	0.54	0.803171	-0.304693
22	0.55	0.795778	-0.418417
23	0.56	0.792267	-0.277676
24	0.57	0.728309	0.001465
25	0.58	0.709122	0.031484
26	0.59	0.705202	0.048640
27	0.60	0.703033	0.036362
28	0.61	0.703033	0.036362
29	0.62	0.703033	0.036362
30	0.63	0.548666	-0.190446

	Training RMSE	Test RMSE	Number of features
0	8.219094e-13	2.386128	527
1	1.369763e-13	2.421888	512
2	3.272271e-13	2.707925	487
3	3.216461e-13	2.756167	455
4	5.639714e-13	3.043863	435
5	4.267848e-13	2.837847	399
6	8.725490e-13	2.892100	377
7	2.246411e-12	2.294173	343
8	6.586269e-12	2.110584	290
9	1.298848e-12	2.057752	245
10	2.231542e-12	1.869452	203
11	2.739645e-12	2.535199	143
12	1.981758e-11	5.809483	111
13	1.475365e-10	44.396964	94
14	6.924832e-02	2.450418	83
15	1.812313e-01	0.747067	69
16	2.053053e-01	0.793232	60
17	2.658491e-01	0.897270	50
18	2.760113e-01	0.814728	46
19	2.828624e-01	0.777792	41
20	3.308805e-01	0.542796	32
21	4.253269e-01	0.718959	25
22	4.332402e-01	0.749639	21
23	4.369484e-01	0.711476	16
24	4.997070e-01	0.628973	12
25	5.170507e-01	0.619446	9
26	5.205230e-01	0.613935	8
27	5.224345e-01	0.617884	7
28	5.224345e-01	0.617884	7
29	5.224345e-01	0.617884	7
30	6.440613e-01	0.686760	5

[ ]:

```

```

2.2 Plots

[12]:
```
plt.plot(df_without_standardization['Correlation threshold'],␣
 ↪df_without_standardization['Training data R^2 score'], label = "Training␣
 ↪data R^2 score", marker='v')
plt.plot(df_without_standardization['Correlation threshold'],␣
 ↪df_without_standardization['Test data R^2 score'], label = "Test data R^2␣
 ↪score", marker='D')
plt.legend()
plt.xlabel('Correlation threshold')
plt.ylabel('R^2 score')
plt.ylim([0, 1])
plt.show()
```

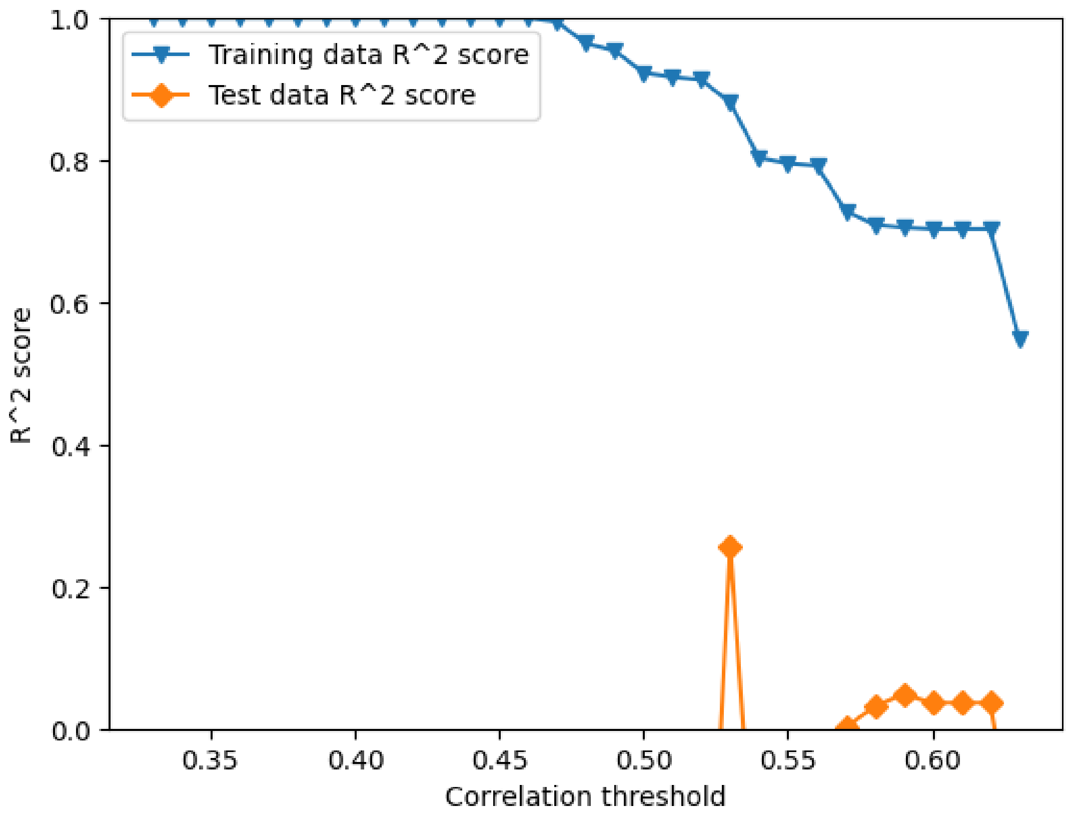

[13]:
```
plt.plot(df_without_standardization['Correlation threshold'],␣
 ↪df_without_standardization['Number of features'], label = "Number of␣
 ↪features", marker='.')
plt.legend()
plt.xlabel('Correlation threshold')
plt.ylabel('Number of features')
plt.show()
```

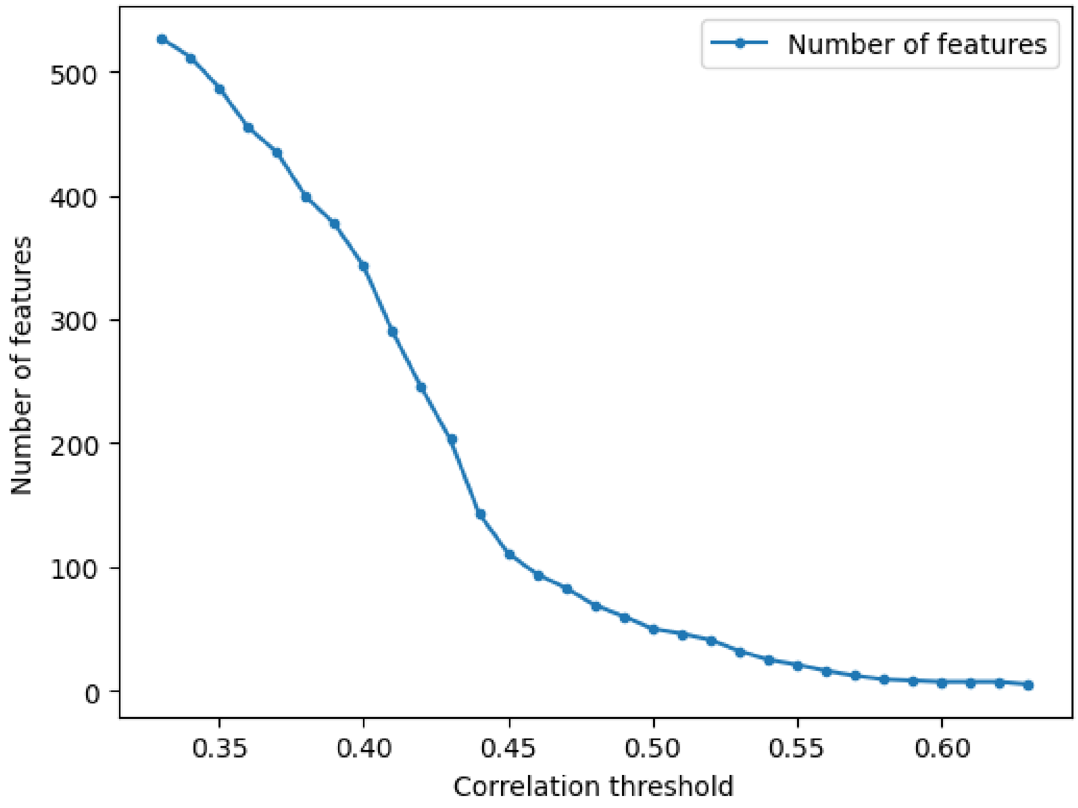

[ ]:

2.3 Decision Tree

[14]:
```
without_standardization, train_r2_, test_r2_, hist1, hist2, target_column_name,␣
↪training_data_RMSE, test_data_RMSE  = pred_model.
↪prepare_data_and_create_model(molecular_descriptors_df=data,
                                                                        ␣
↪                          correlation_threshold=0.50,
                                                                        ␣
↪                          standardization=False,
                                                                        ␣
↪                          model_type='DecisionTreeRegressor',
                                                                        ␣
↪                          max_depth=5,
                                                                        ␣
↪                          target_column_name = target,
                                                                        ␣
↪                          random_state=random_state,
```

```
↪                       train_test_split_=True,
↪                       verbose=True)
```

```
I am not doing standardization…
  molecular descriptor name
0                    AATS0Z
1                  AATS0are
2                    AATS0d
3                   AATS0dv
4                    AATS0i
  molecular descriptor name  corr_value
0                    AATS0Z    0.018677
1                  AATS0are   -0.341313
2                    AATS0d   -0.123443
3                   AATS0dv   -0.265670
4                    AATS0i   -0.428929
  molecular descriptor name  corr_value  absolute correlation value
0                    AATS0Z    0.018677                    0.018677
1                  AATS0are   -0.341313                    0.341313
2                    AATS0d   -0.123443                    0.123443
3                   AATS0dv   -0.265670                    0.265670
4                    AATS0i   -0.428929                    0.428929
    molecular descriptor name  corr_value  absolute correlation value
15                     AATS1i   -0.561620                    0.561620
113                   AATSC1c    0.565682                    0.565682
125                   AATSC2c   -0.543393                    0.543393
221                AETA_eta_R   -0.515778                    0.515778
224                    AMID_C    0.532458                    0.532458
    molecular descriptor name  corr_value  absolute correlation value
15                     AATS1i   -0.561620                    0.561620
113                   AATSC1c    0.565682                    0.565682
125                   AATSC2c   -0.543393                    0.543393
221                AETA_eta_R   -0.515778                    0.515778
224                    AMID_C    0.532458                    0.532458
The model used is: DecisionTree…
Return the coefficient of determination of the prediction:
0.06924294432206701
R^2 score: 0.942320762458848
Correlation coefficient: 0.9707320755279739
Test data - unseen during training:
R^2 score: 0.06924294432206701
Correlation coefficient: 0.26314054100815976
[7.1833148  6.69792506 5.69688833 6.24345954 7.8569852  5.69688833
 7.1833148  5.69688833 5.69688833 5.69688833 5.69688833 5.69688833
 6.6767479  6.24345954 7.15366289 6.84065953]
8      6.159455
```

```
110    6.065502
69     5.552842
95     5.200659
11     7.250264
104    6.000000
52     6.823909
112    6.853872
101    5.000000
50     5.769551
100    6.136677
103    6.000000
21     6.398375
60     5.537602
84     7.002177
39     6.638272
Name: LoVo/DX, dtype: float64
Training Root Mean Square Error: 0.2302436543011823
Testing Root Mean Square Error: 0.6072510293132874
```

[15]:
```
print(target_column_name+str('_transformed'))
print(hist1[target_column_name].hist())
```

```
LoVo/DX_transformed
AxesSubplot(0.125,0.11;0.775x0.77)
```

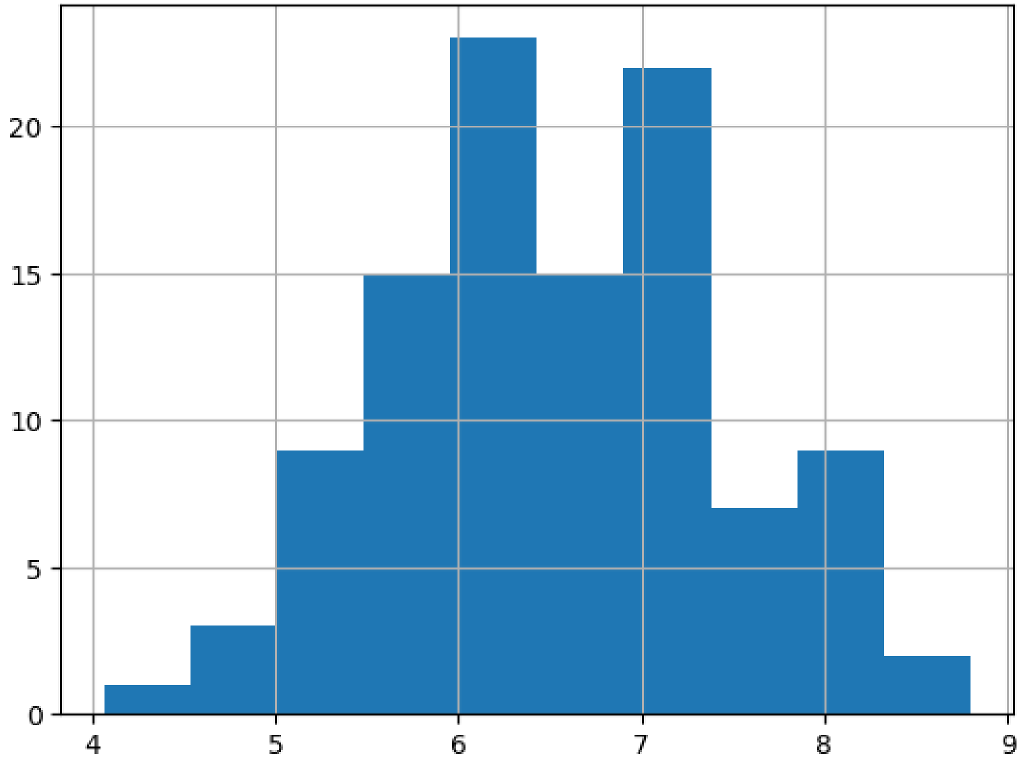

```
[16]: with_standardization, train_r2_, test_r2_, hist1, hist2, target_column_name,␣
      ↪training_data_RMSE, test_data_RMSE  = pred_model.
      ↪prepare_data_and_create_model(molecular_descriptors_df=data,
                                                                                    ␣
      ↪                    correlation_threshold=0.50,
                                                                                    ␣
      ↪                    standardization=True,
                                                                                    ␣
      ↪                    model_type='DecisionTreeRegressor',
                                                                                    ␣
      ↪                    max_depth=5,
                                                                                    ␣
      ↪                    target_column_name = target,
                                                                                    ␣
      ↪                    random_state=random_state,
                                                                                    ␣
      ↪                    train_test_split_=True,
                                                                                    ␣
      ↪                    verbose=True)
```

```
I am doing standardization…
  molecular descriptor name
0                    AATS0Z
1                  AATS0are
2                    AATS0d
3                   AATS0dv
4                    AATS0i
  molecular descriptor name  corr_value
0                    AATS0Z    0.018677
1                  AATS0are   -0.341313
2                    AATS0d   -0.123443
3                   AATS0dv   -0.265670
4                    AATS0i   -0.428929
  molecular descriptor name  corr_value  absolute correlation value
0                    AATS0Z    0.018677                    0.018677
1                  AATS0are   -0.341313                    0.341313
2                    AATS0d   -0.123443                    0.123443
3                   AATS0dv   -0.265670                    0.265670
4                    AATS0i   -0.428929                    0.428929
    molecular descriptor name  corr_value  absolute correlation value
15                     AATS1i   -0.561620                    0.561620
113                  AATSC1c    0.565682                    0.565682
125                  AATSC2c   -0.543393                    0.543393
221                AETA_eta_R   -0.515778                    0.515778
224                    AMID_C    0.532458                    0.532458
```

```
    molecular descriptor name  corr_value  absolute correlation value
15                     AATS1i   -0.561620                    0.561620
113                   AATSC1c    0.565682                    0.565682
125                   AATSC2c   -0.543393                    0.543393
221                AETA_eta_R   -0.515778                    0.515778
224                    AMID_C    0.532458                    0.532458
The model used is: DecisionTree…
Return the coefficient of determination of the prediction:
0.06924294432206701
R^2 score: 0.942320762458848
Correlation coefficient: 0.9707320755279739
Test data - unseen during training:
R^2 score: 0.06924294432206701
Correlation coefficient: 0.26314054100815976
[7.1833148  6.69792506 5.69688833 6.24345954 7.8569852  5.69688833
 7.1833148  5.69688833 5.69688833 5.69688833 5.69688833 5.69688833
 6.6767479  6.24345954 7.15366289 6.84065953]
8      6.159455
110    6.065502
69     5.552842
95     5.200659
11     7.250264
104    6.000000
52     6.823909
112    6.853872
101    5.000000
50     5.769551
100    6.136677
103    6.000000
21     6.398375
60     5.537602
84     7.002177
39     6.638272
Name: LoVo/DX, dtype: float64
Training Root Mean Square Error: 0.2302436543011823
Testing Root Mean Square Error: 0.6072510293132874
```

2.4 Search inside correlation space

[17]:
```
step = 0.01
initial_step = corr_low
last_step = corr_high
first_list = [x / 100.0 for x in range(int(initial_step*100),␣
 ↪int(last_step*100), int(step*100))]
max_depth = [range(2, 30, 1)]
corr_th = []
second_list = []
```

```
third_list = []
fourth_l = []
fifth_l = []
f_list = []
fif_list = []
for i in first_list:
    for depth in max_depth[0]:

        without_standardization, train_r2, test_r2, _, h_, target_column_name,␣
 ↪training_data_RMSE, test_data_RMSE = pred_model.
 ↪prepare_data_and_create_model(molecular_descriptors_df=data,
                                                                              ␣
 ↪                    correlation_threshold=i,
                                                                              ␣
 ↪                    standardization=False,
                                                                              ␣
 ↪                    model_type='DecisionTreeRegressor',
                                                                              ␣
 ↪                    max_depth=depth,
                                                                              ␣
 ↪                    target_column_name = target,
                                                                              ␣
 ↪                    random_state=random_state,
                                                                              ␣
 ↪                    train_test_split_=True,
                                                                              ␣
 ↪                    verbose=False)
        corr_th.append(i)
        second_list.append(train_r2)
        third_list.append(test_r2)
        fourth_l.append(training_data_RMSE)
        fifth_l.append(test_data_RMSE)
        f_list.append(len(h_))
        fif_list.append(depth)
```

[18]:
```
df_without_standardization = pd.DataFrame(data=corr_th, columns=["Correlation␣
 ↪threshold"])
df_without_standardization['Training data R^2 score'] = second_list
df_without_standardization['Test data R^2 score'] = third_list
df_without_standardization['Training RMSE'] = fourth_l
df_without_standardization['Test RMSE'] = fifth_l
df_without_standardization['Number of features'] = f_list
df_without_standardization['Depth number'] = fif_list
```

[19]:
```
df_decision_tree = df_without_standardization.copy()
```

```
#df_without_standardization.to_excel('../Data/
↪A549_Decision_tree_rs_'+str(random_state)+'.xlsx')
```

2.5 Plots

[ ]:
```
corre_list = [x / 100.0 for x in range(int(initial_step*100),␣
↪int(last_step*100), int(0.01*100))]
for element in corre_list:
    element_ =␣
↪df_without_standardization[df_without_standardization['Correlation␣
↪threshold'] == float(element)]
    plt.plot(element_['Depth number'], element_['Training data R^2 score'],␣
↪label = "Training data R^2 score", marker='v')
    plt.plot(element_['Depth number'], element_['Test data R^2 score'], label =␣
↪"Test data R^2 score", marker='D')
    plt.legend()
    plt.xlabel('Depth number -> correlation threshold: '+str(element)+'. \n␣
↪Number of features: '+str(element_['Number of features'].iloc[0]),␣
↪fontweight='bold')
    plt.xlim(max(element_['Depth number'])+1, min(element_['Depth number'])-1)
    plt.ylabel('R^2 score')
    plt.ylim([0, 1])
    plt.rc('grid', linestyle="-.", color='black')
    plt.grid(True)
    plt.show()
```

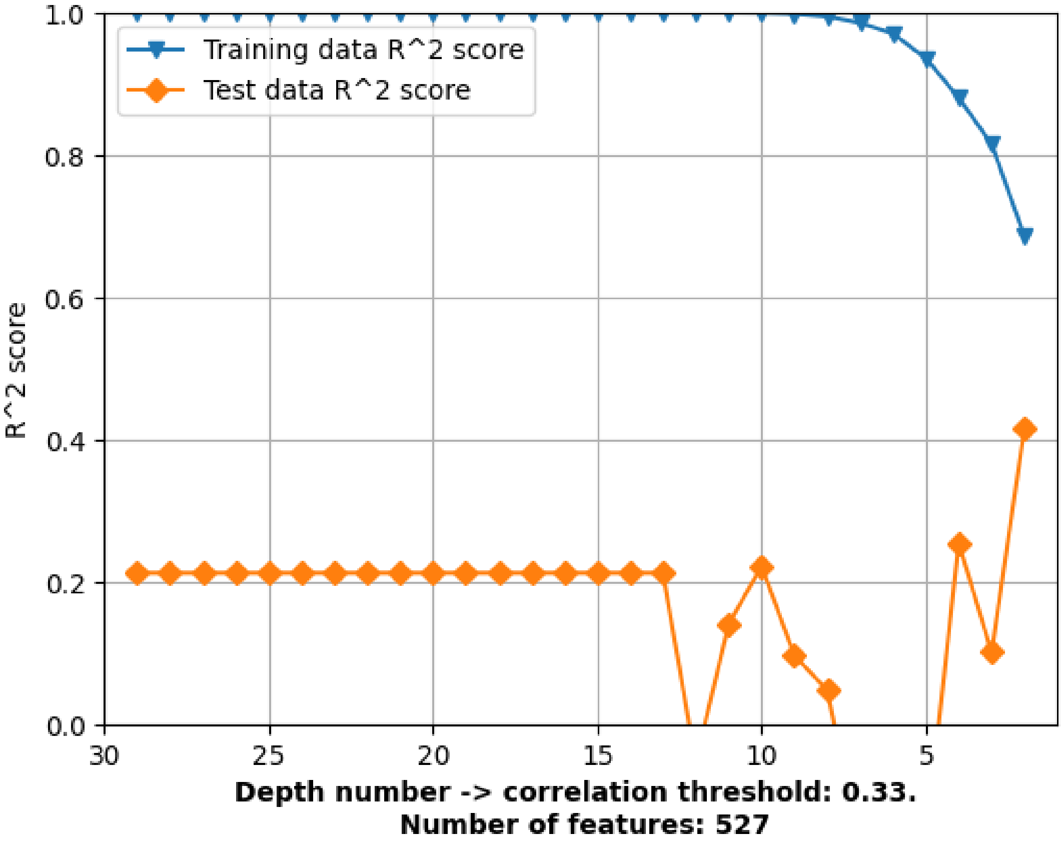
Training data R^2 score
Test data R^2 score
R^2 score
1.0
0.8
0.6
0.4
0.2
0.0
30
25
20
15
10
5
Depth number -> correlation threshold: 0.33.
Number of features: 527

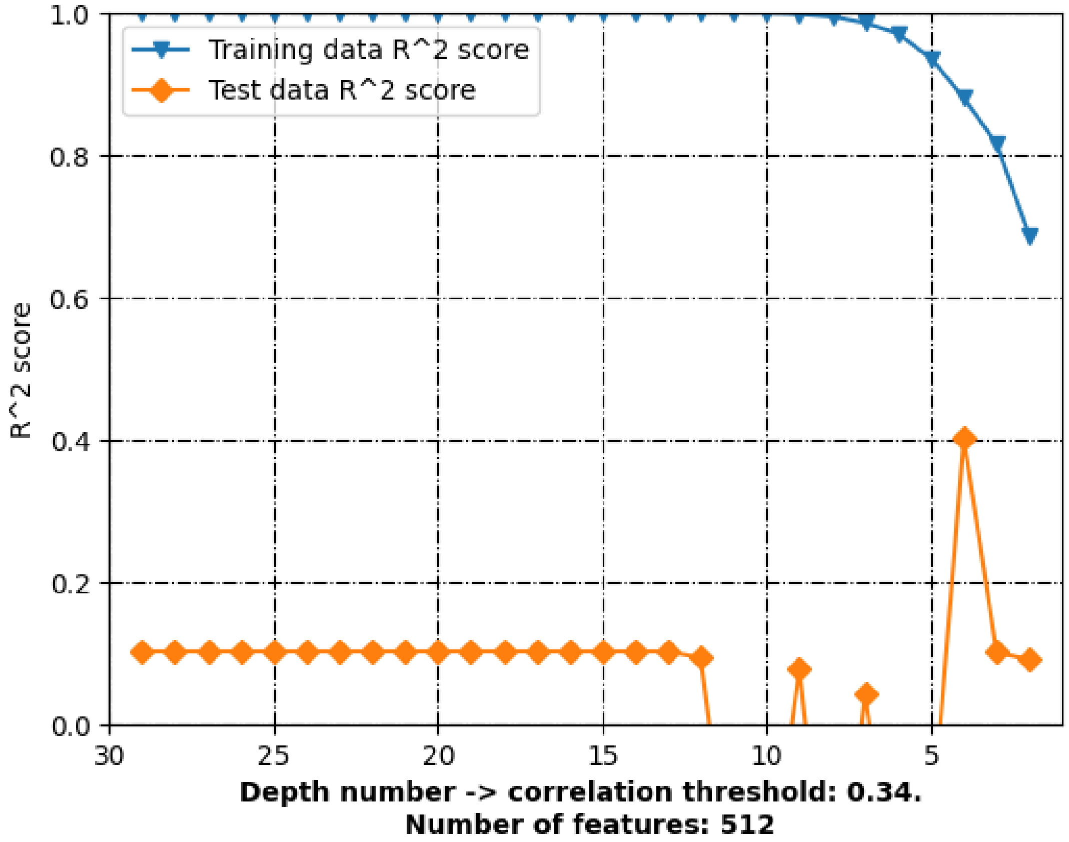

Training data R^2 score
Test data R^2 score
R^2 score
1.0
0.8
0.6
0.4
0.2
0.0
30
25
20
15
10
5
Depth number -> correlation threshold: 0.34.
Number of features: 512

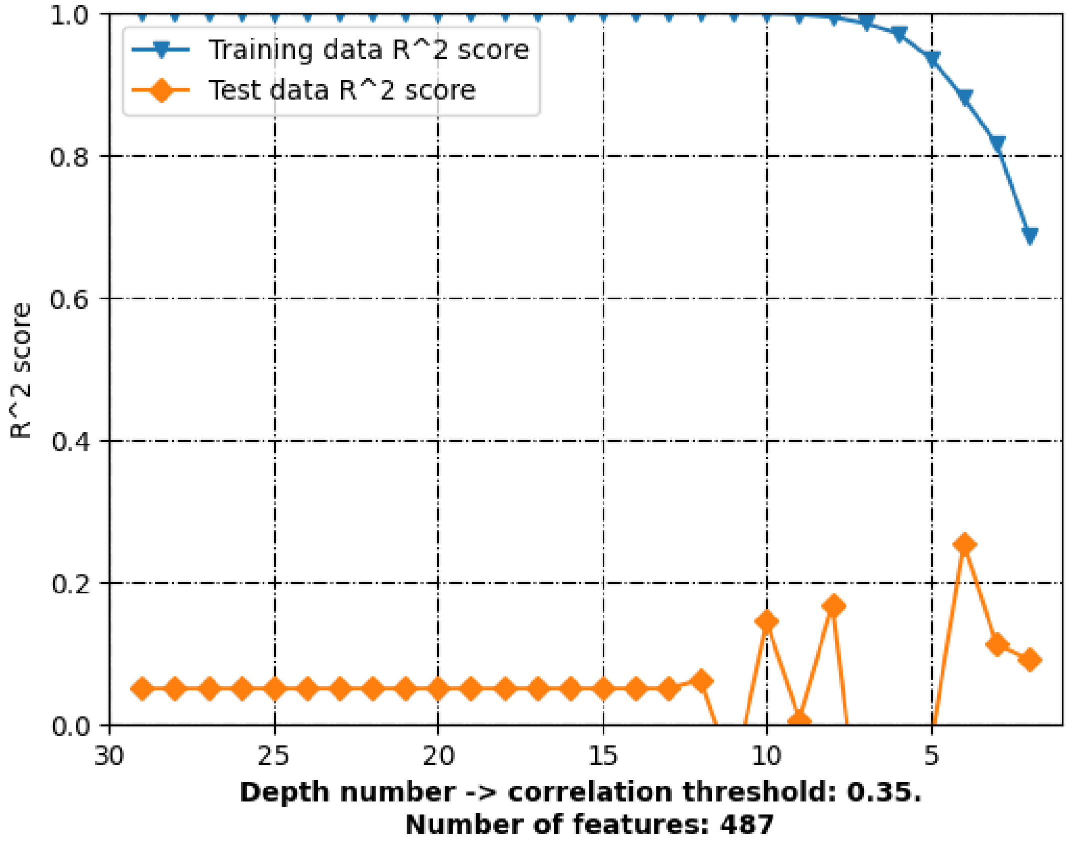
Training data R^2 score
Test data R^2 score
R^2 score
1.0
0.8
0.6
0.4
0.2
0.0
30
25
20
15
10
5
Depth number -> correlation threshold: 0.35.
Number of features: 487

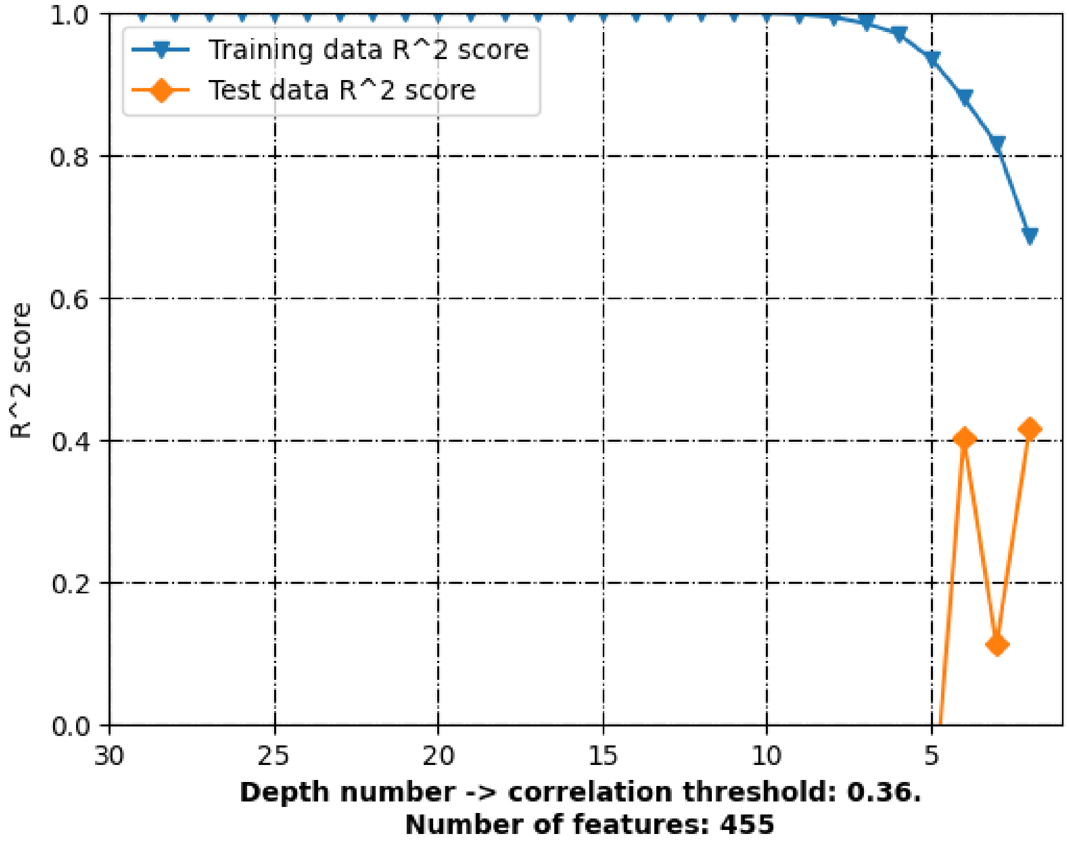
Training data R^2 score
Test data R^2 score
R^2 score
1.0
0.8
0.6
0.4
0.2
0.0
30
25
20
15
10
5
Depth number -> correlation threshold: 0.36.
Number of features: 455

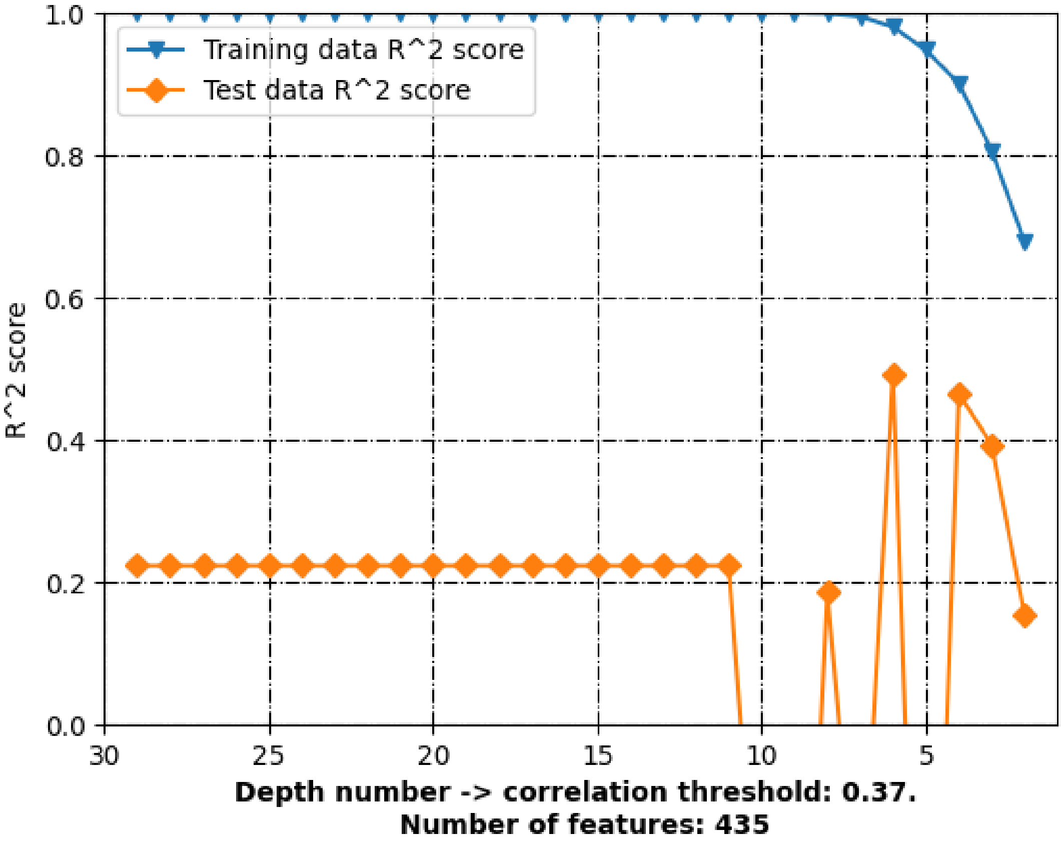
Training data R^2 score
Test data R^2 score
R^2 score
1.0
0.8
0.6
0.4
0.2
0.0
30
25
20
15
10
5
Depth number -> correlation threshold: 0.37.
Number of features: 435

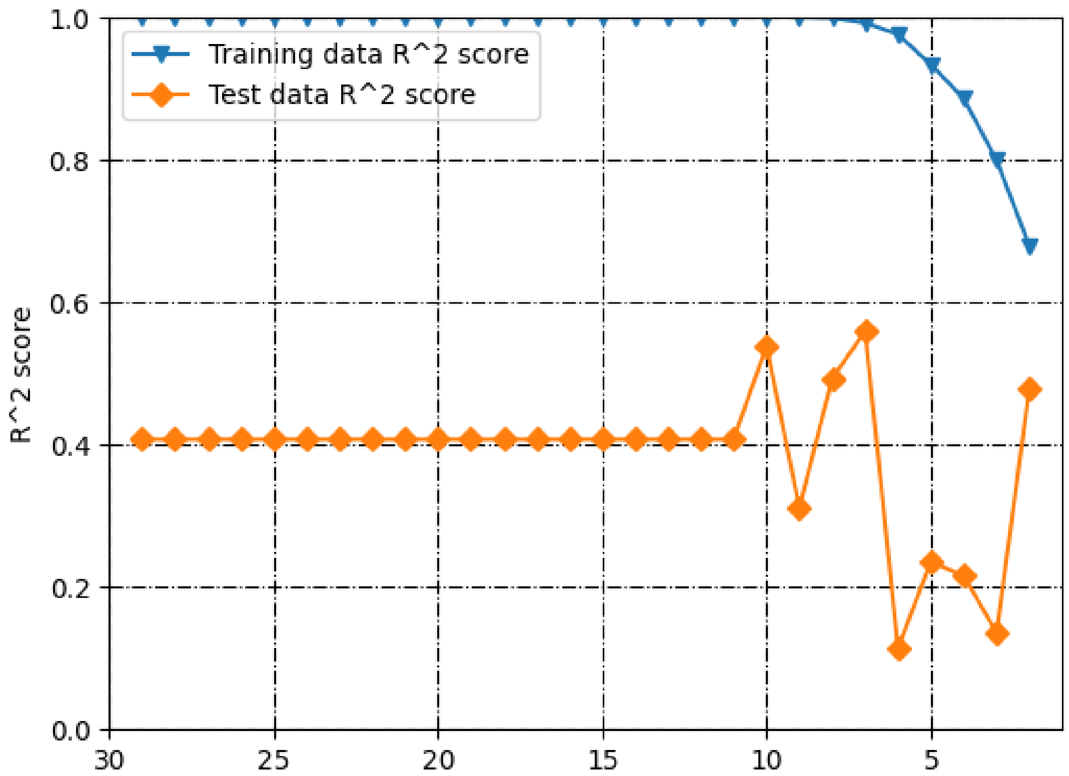

Depth number -> correlation threshold: 0.38.
Number of features: 399

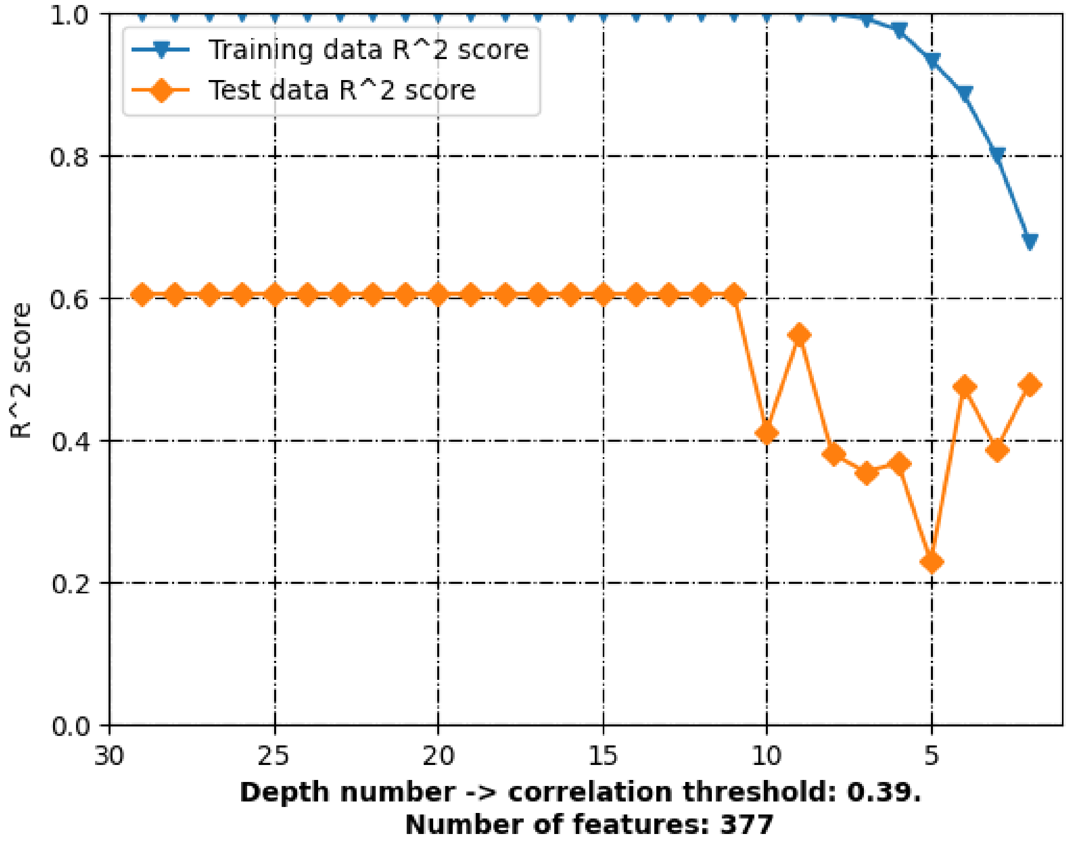
1.0
0.8
0.6
0.4
0.2
0.0
R^2 score
Training data R^2 score
Test data R^2 score
30
25
20
15
10
5
Depth number -> correlation threshold: 0.39.
Number of features: 377

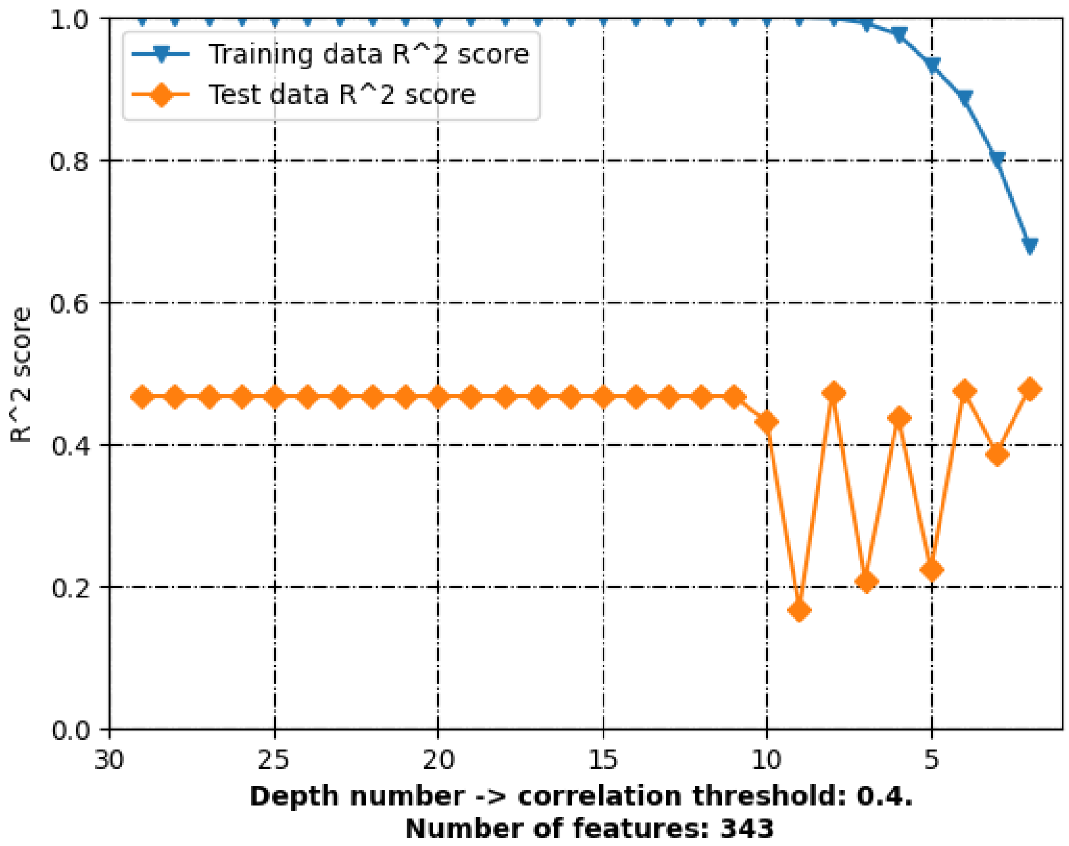

Training data R^2 score
Test data R^2 score
R^2 score
1.0
0.8
0.6
0.4
0.2
0.0
30
25
20
15
10
5
Depth number -> correlation threshold: 0.4.
Number of features: 343

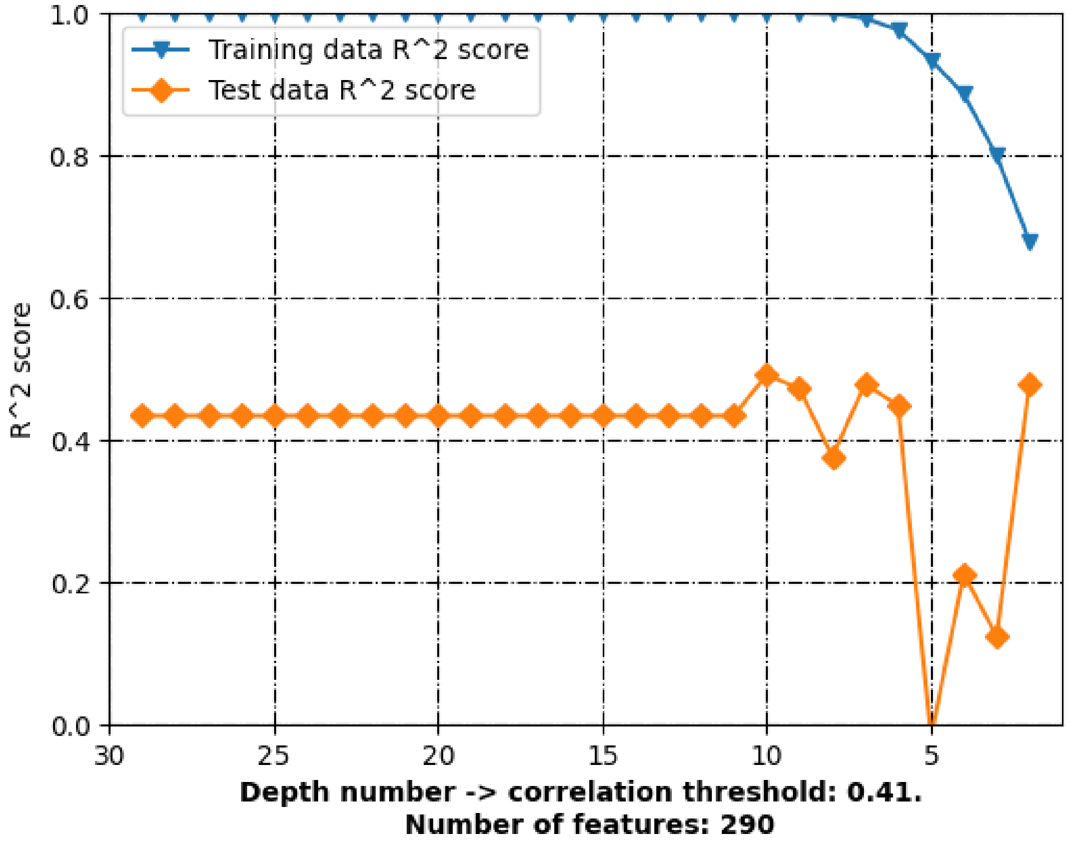

Training data R^2 score
Test data R^2 score
R^2 score
1.0
0.8
0.6
0.4
0.2
0.0
30
25
20
15
10
5
Depth number -> correlation threshold: 0.41.
Number of features: 290

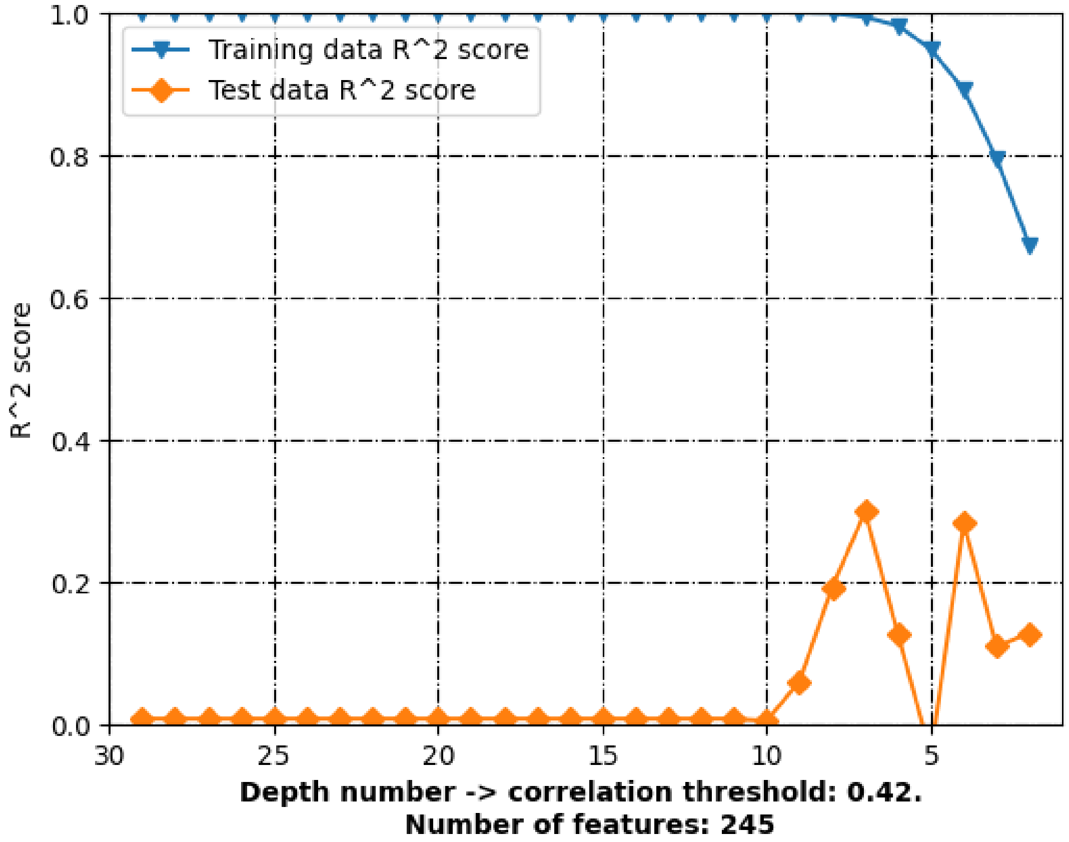

Training data R^2 score
Test data R^2 score
R^2 score
1.0
0.8
0.6
0.4
0.2
0.0
30
25
20
15
10
5
Depth number -> correlation threshold: 0.42.
Number of features: 245

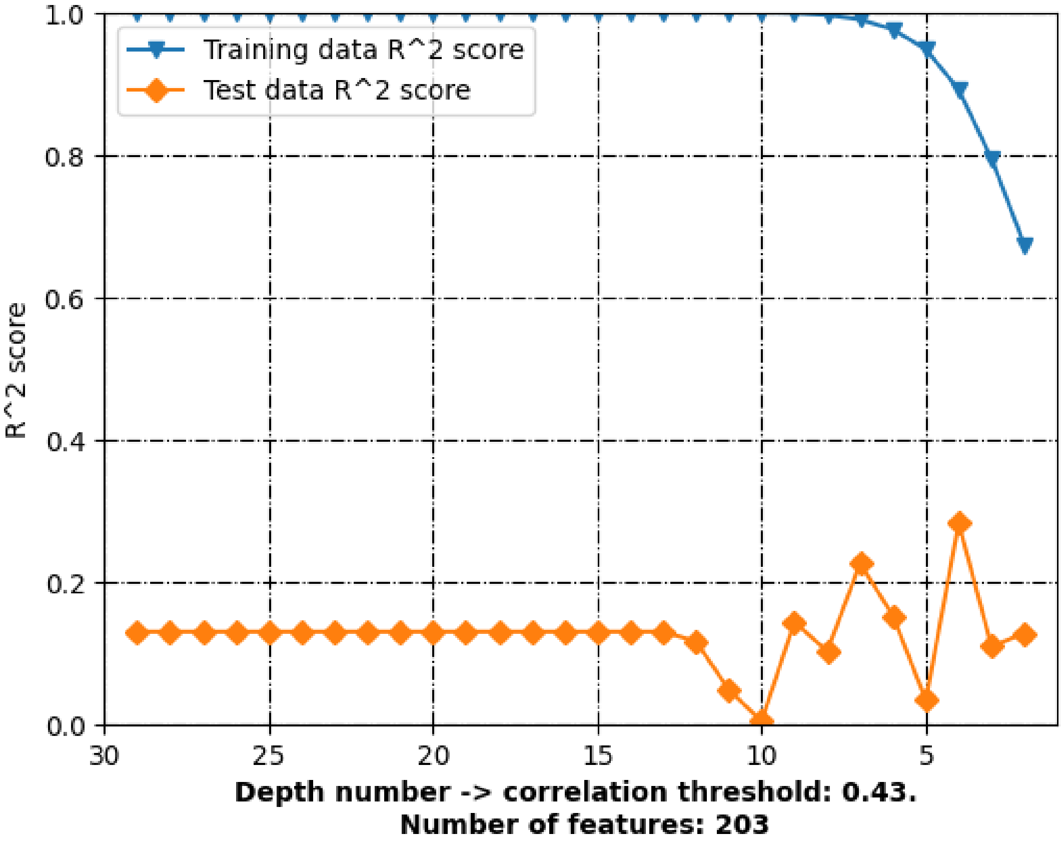
Training data R^2 score
Test data R^2 score
R^2 score
1.0
0.8
0.6
0.4
0.2
0.0
30
25
20
15
10
5
Depth number -> correlation threshold: 0.43.
Number of features: 203

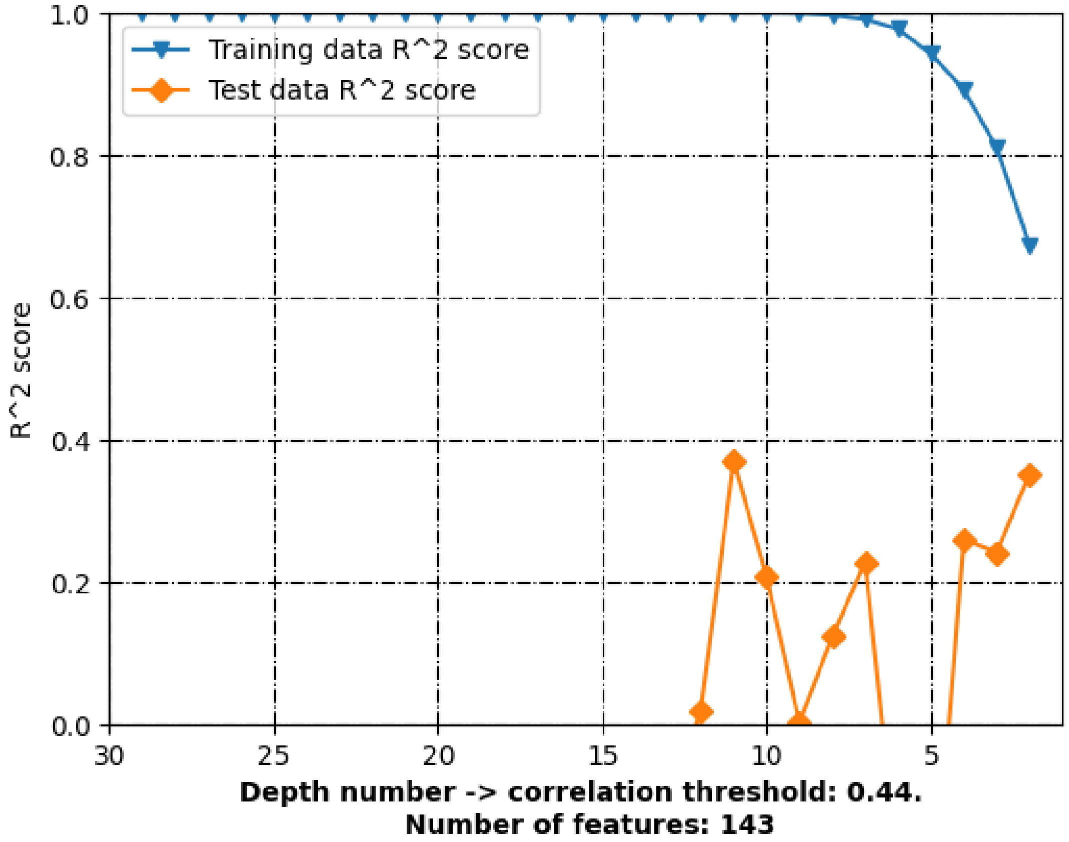
Training data R^2 score
Test data R^2 score
R^2 score
1.0
0.8
0.6
0.4
0.2
0.0
30
25
20
15
10
5
Depth number -> correlation threshold: 0.44.
Number of features: 143

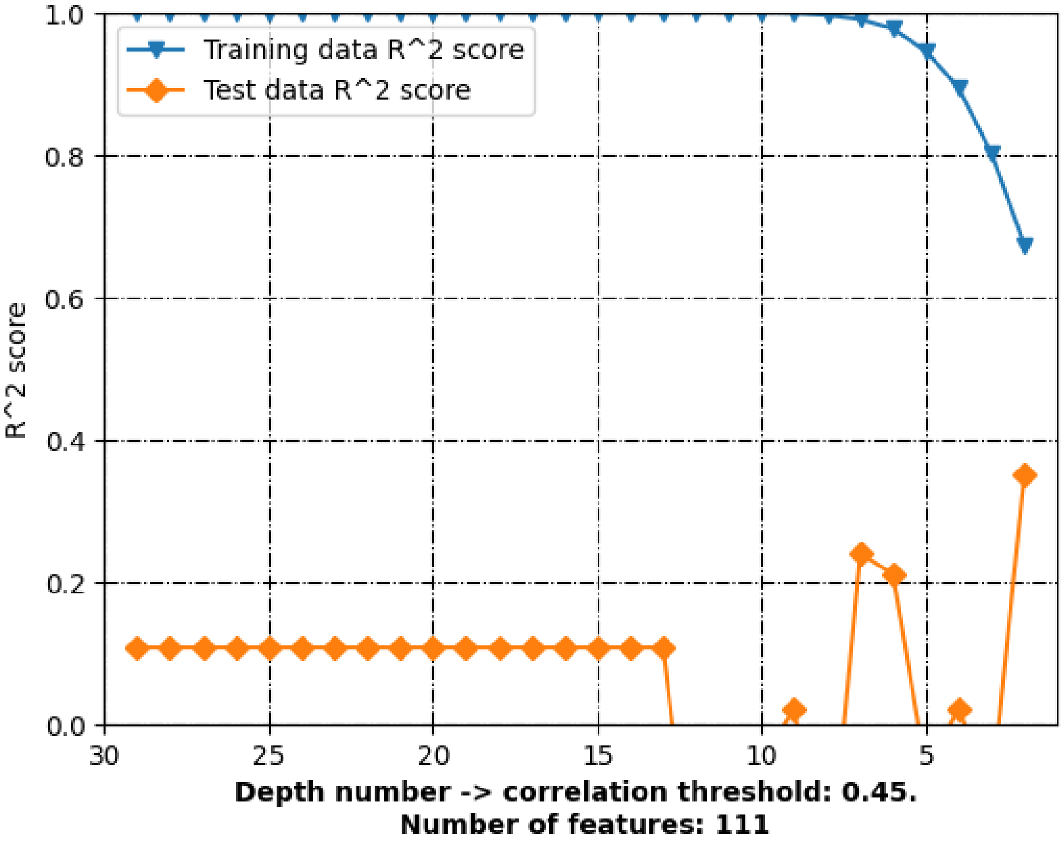

Training data R^2 score
Test data R^2 score
R^2 score
1.0
0.8
0.6
0.4
0.2
0.0
30
25
20
15
10
5
Depth number -> correlation threshold: 0.45.
Number of features: 111

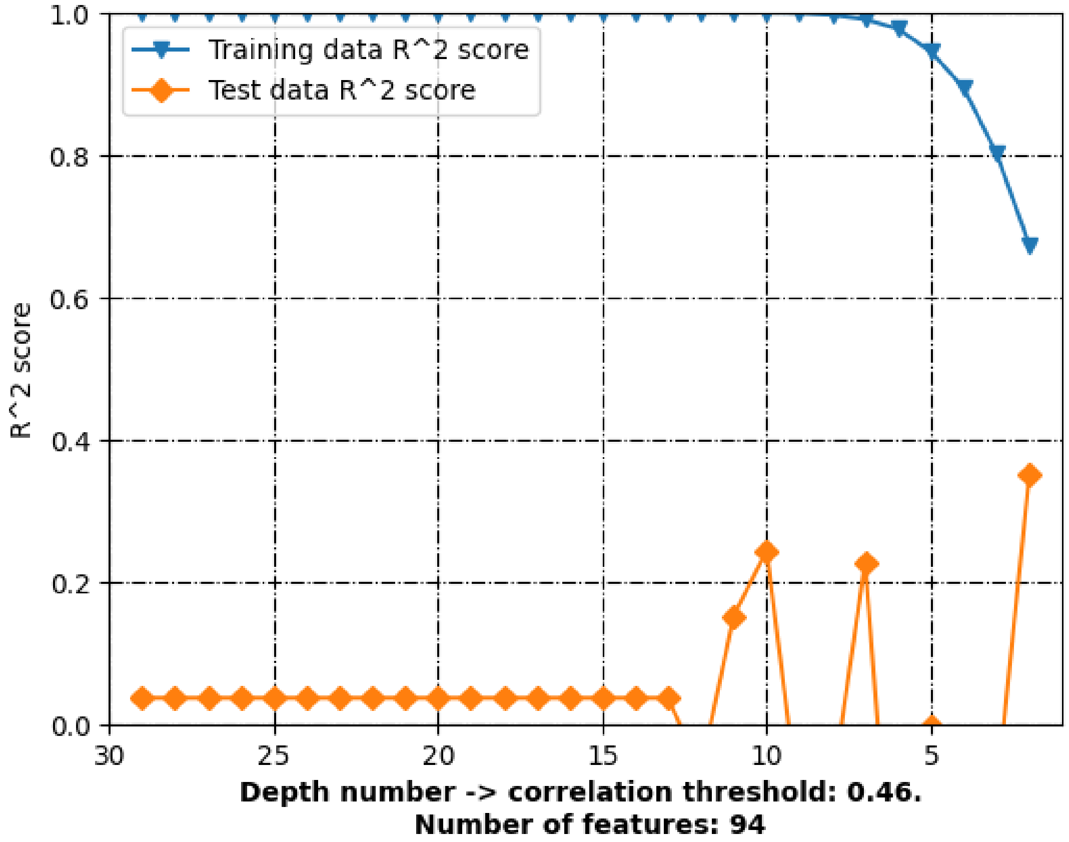
Training data R^2 score
Test data R^2 score
R^2 score
1.0
0.8
0.6
0.4
0.2
0.0
30
25
20
15
10
5
Depth number -> correlation threshold: 0.46.
Number of features: 94

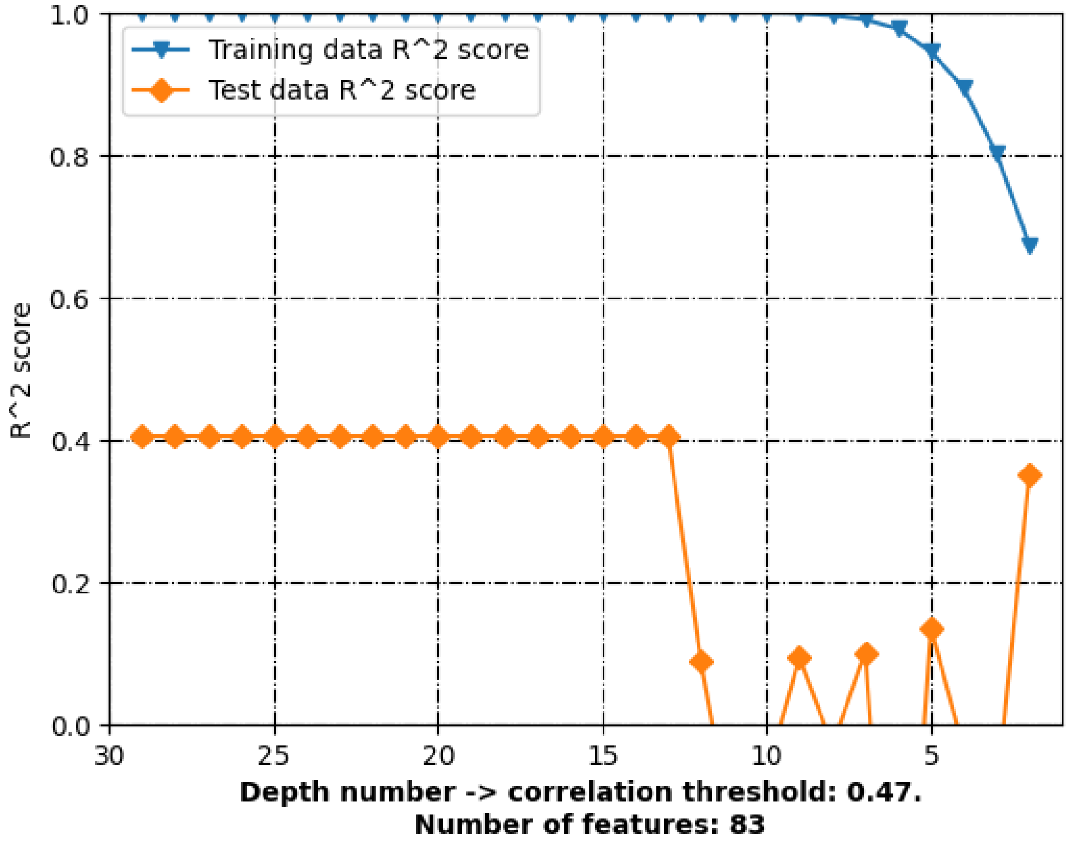
Training data R^2 score
Test data R^2 score
R^2 score
1.0
0.8
0.6
0.4
0.2
0.0
30
25
20
15
10
5
Depth number -> correlation threshold: 0.47.
Number of features: 83

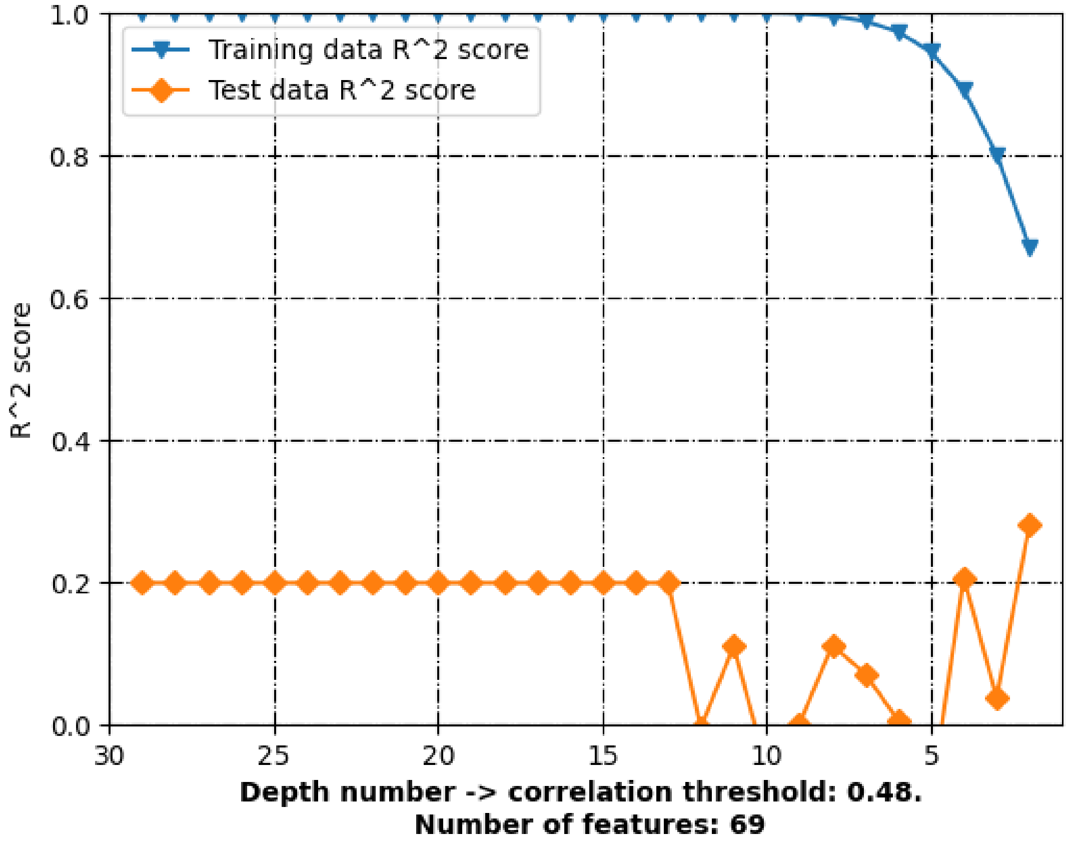

1.0
0.8
0.6
0.4
0.2
0.0
R^2 score
Training data R^2 score
Test data R^2 score
30
25
20
15
10
5
Depth number -> correlation threshold: 0.48.
Number of features: 69

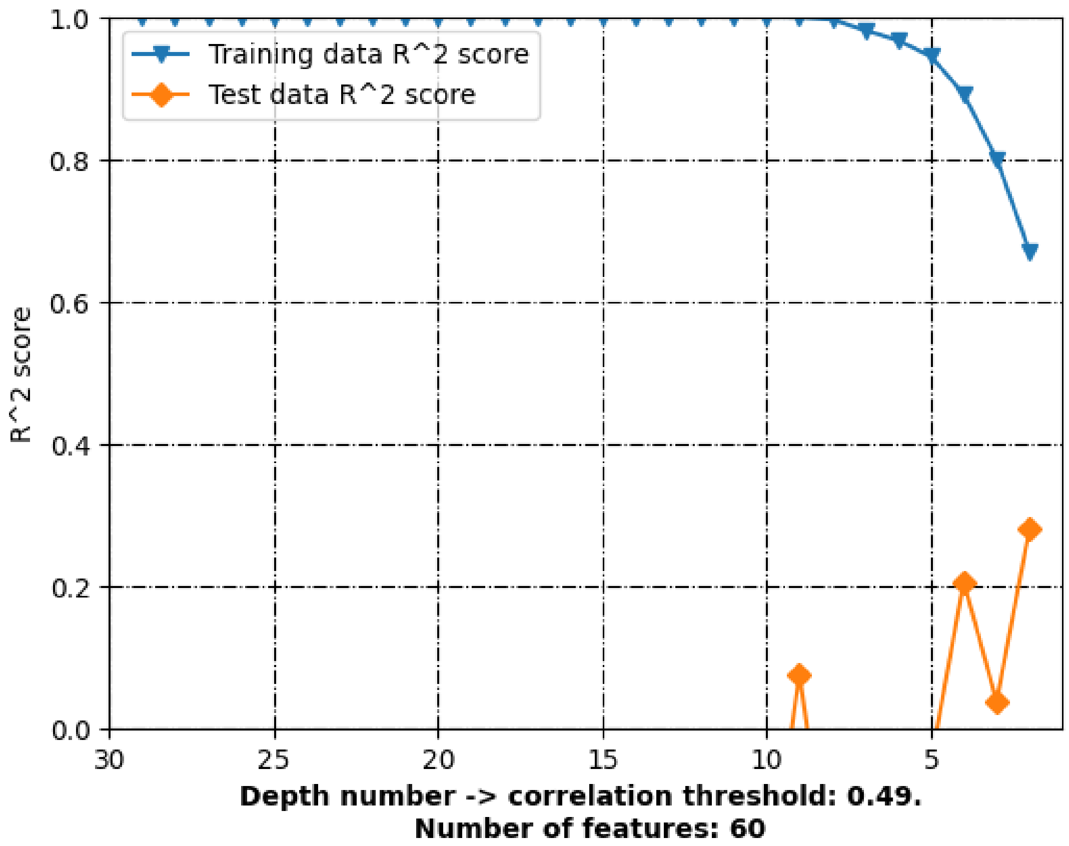
Training data R^2 score
Test data R^2 score
R^2 score
1.0
0.8
0.6
0.4
0.2
0.0
30
25
20
15
10
5
Depth number -> correlation threshold: 0.49.
Number of features: 60

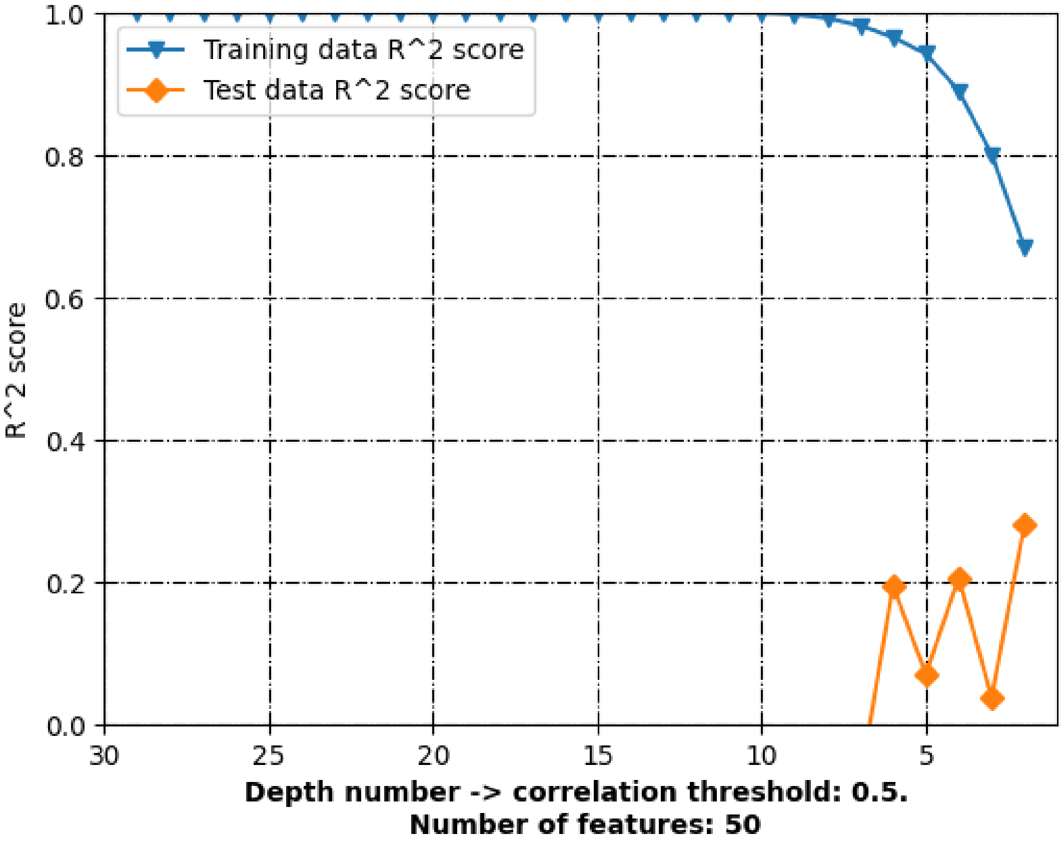

1.0
0.8
0.6
0.4
0.2
0.0
R^2 score
Training data R^2 score
Test data R^2 score
30
25
20
15
10
5
Depth number -> correlation threshold: 0.5.
Number of features: 50

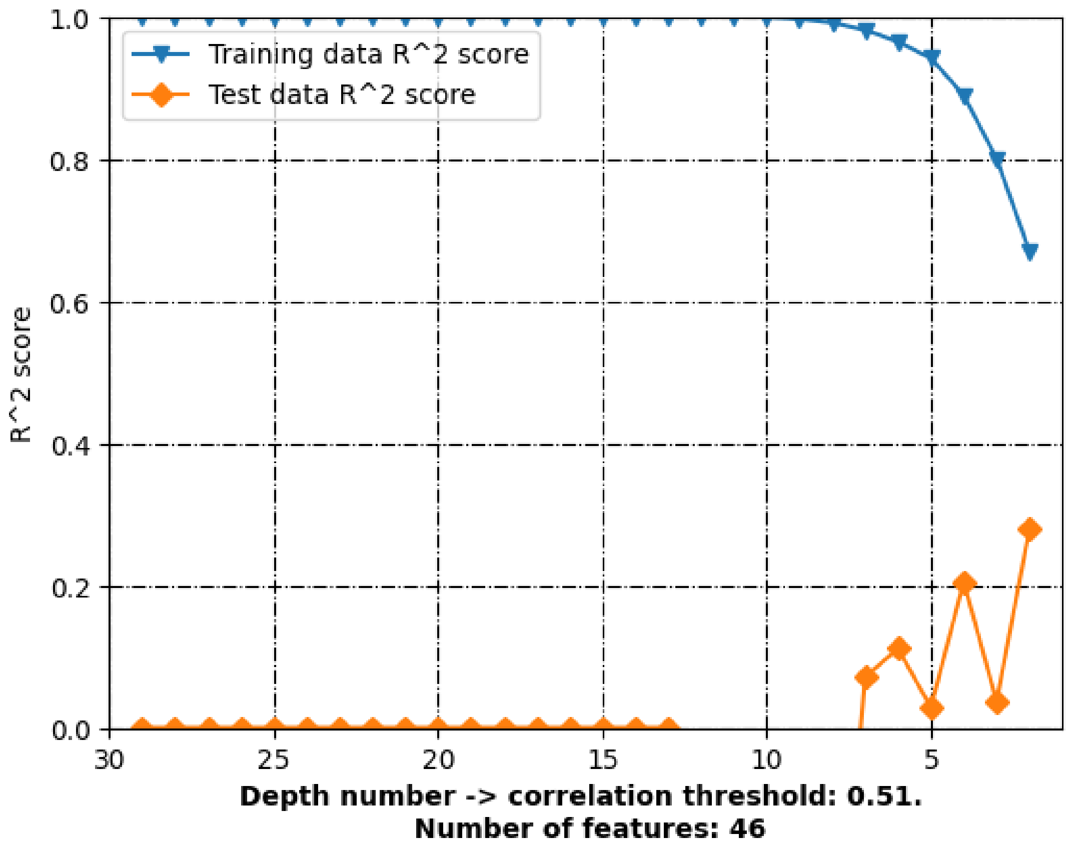

1.0
0.8
0.6
0.4
0.2
0.0
R^2 score
Training data R^2 score
Test data R^2 score
30
25
20
15
10
5
Depth number -> correlation threshold: 0.51.
Number of features: 46

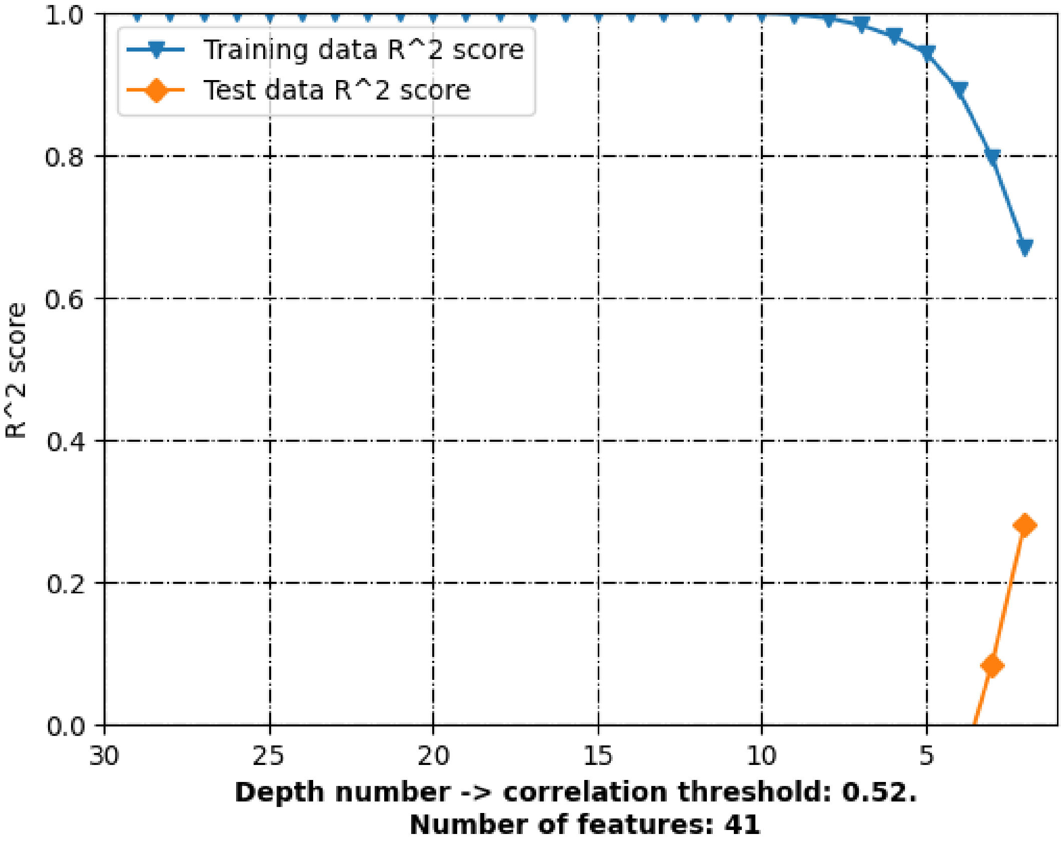
Training data R^2 score
Test data R^2 score
R^2 score
Depth number -> correlation threshold: 0.52.
Number of features: 41

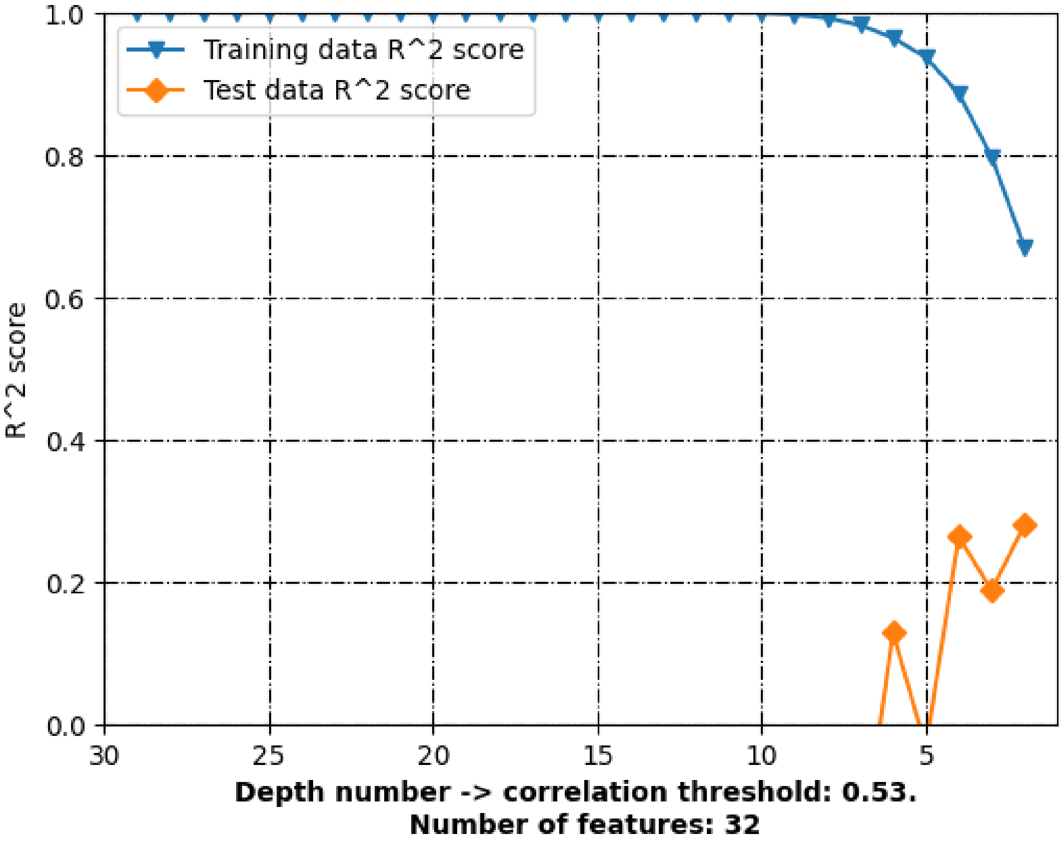
1.0
0.8
0.6
0.4
0.2
0.0
R^2 score
Training data R^2 score
Test data R^2 score
30
25
20
15
10
5
Depth number -> correlation threshold: 0.53.
Number of features: 32

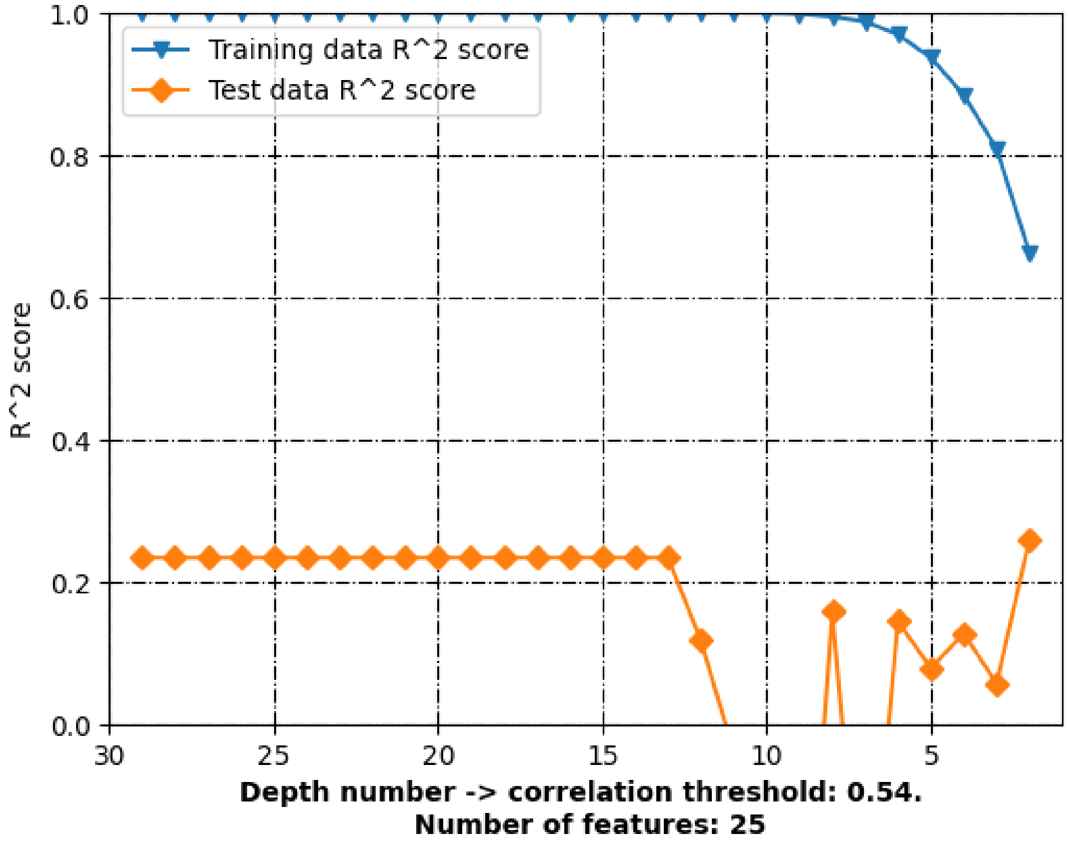
1.0
0.8
0.6
0.4
0.2
0.0
R^2 score
Training data R^2 score
Test data R^2 score
30
25
20
15
10
5
Depth number -> correlation threshold: 0.54.
Number of features: 25

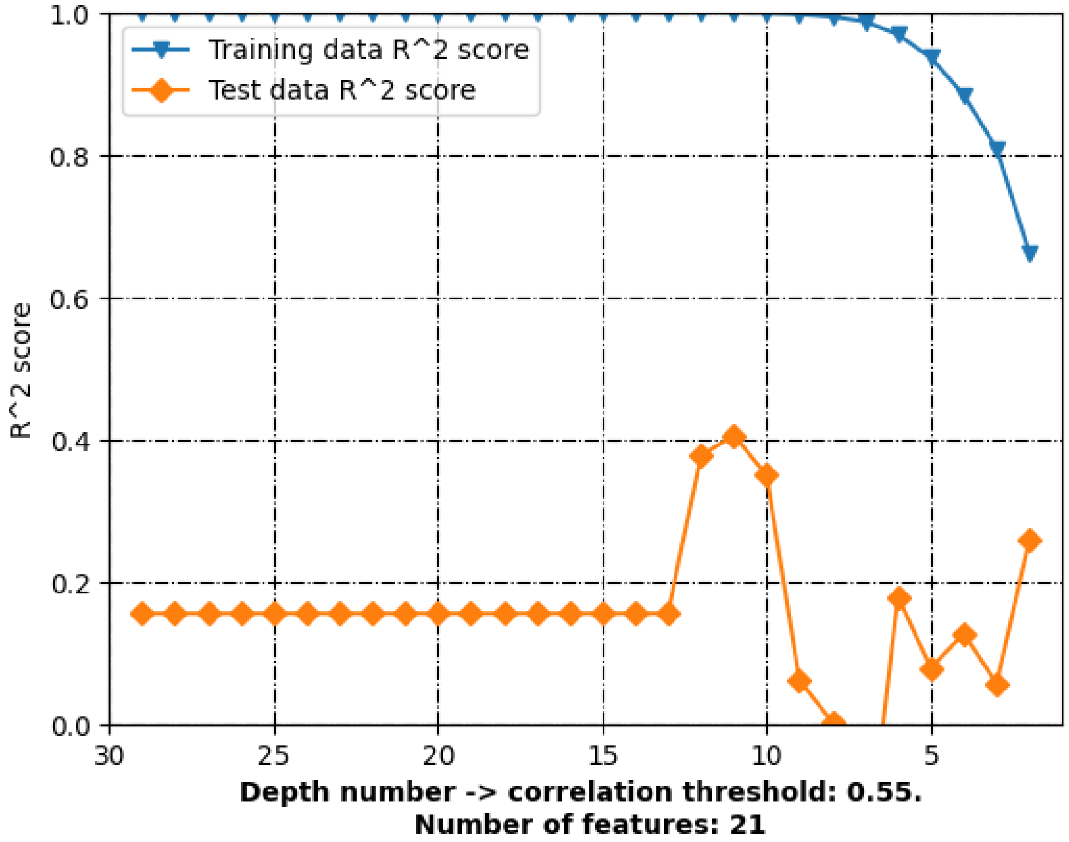

Training data R^2 score
Test data R^2 score
R^2 score
1.0
0.8
0.6
0.4
0.2
0.0
30
25
20
15
10
5
Depth number -> correlation threshold: 0.55.
Number of features: 21

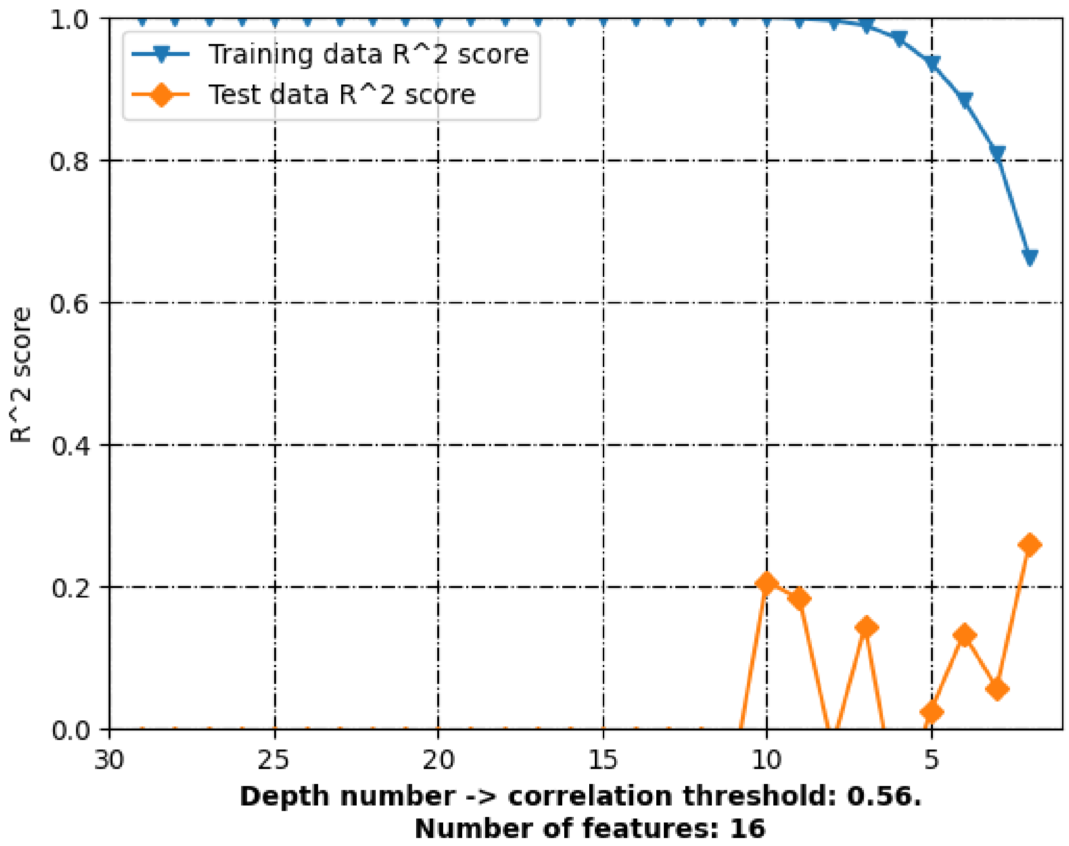

Training data R^2 score
Test data R^2 score
R^2 score
1.0
0.8
0.6
0.4
0.2
0.0
30
25
20
15
10
5
Depth number -> correlation threshold: 0.56.
Number of features: 16

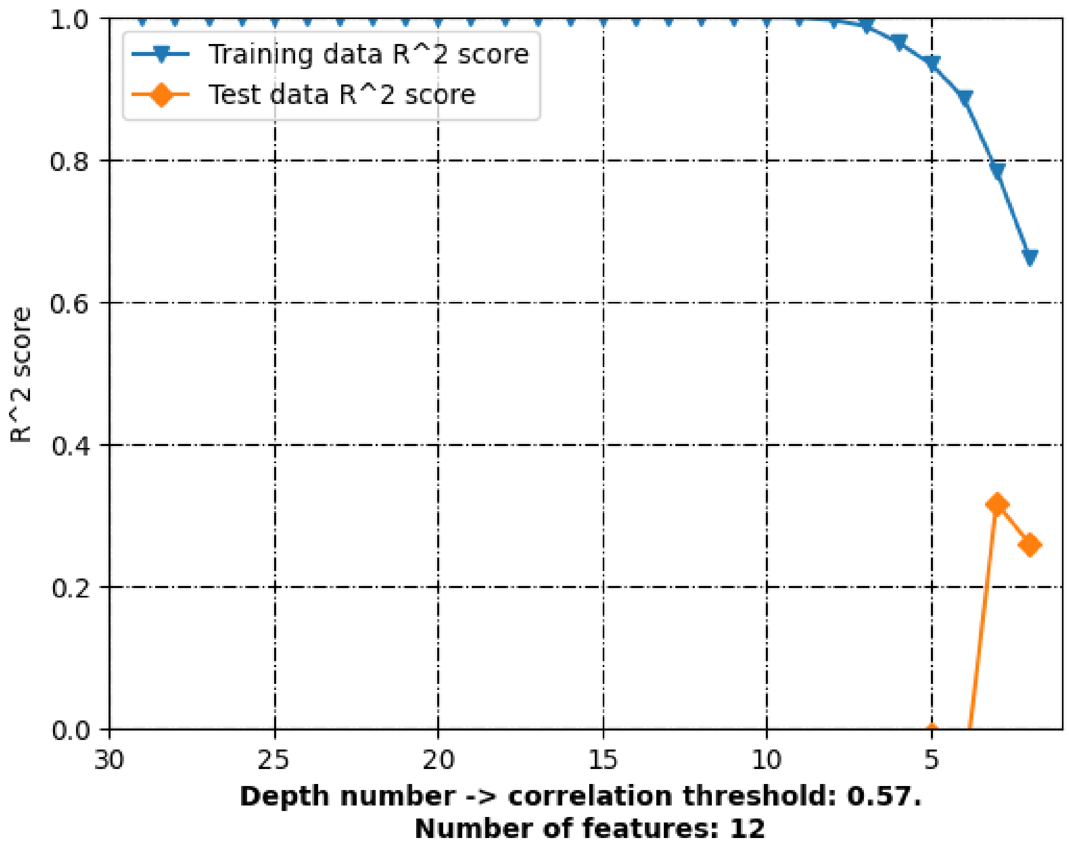


```
[ ]: plt.plot(df_without_standardization['Correlation threshold'],␣
     ↪df_without_standardization['Number of features'], label = "Number of␣
     ↪features", marker='.')
     plt.legend()
     plt.xlabel('Correlation threshold')
     plt.ylabel('Number of features')
     plt.show()
```

```
[ ]:
```

3 Random Forest

```
[ ]: without_standardization, train_r2_, test_r2_, hist1, hist2, target_column_name,␣
     ↪training_data_RMSE, test_data_RMSE  = pred_model.
     ↪prepare_data_and_create_model(molecular_descriptors_df=data,
                                                                                     ␣
     ↪                          correlation_threshold=0.50,
                                                                                     ␣
     ↪                          standardization=False,
```

```
                         model_type='RandomForestRegressor',
                         n_estimators_=12,
                         target_column_name = target,
                         random_state=random_state,
                         train_test_split_=True,
                         verbose=True)
```

[ ]:
```
print(target_column_name+str('_transformed'))
print(hist1[target_column_name].hist())
```

[ ]:
```
with_standardization, train_r2_, test_r2_, hist1, hist2, target_column_name, training_data_RMSE, test_data_RMSE  = pred_model.prepare_data_and_create_model(molecular_descriptors_df=data,
                         correlation_threshold=0.50,
                         standardization=True,
                         model_type='RandomForestRegressor',
                         n_estimators_=12,
                         target_column_name = target,
                         random_state=random_state,
                         train_test_split_=True,
                         verbose=True)
```

3.1 Search inside correlation space

[25]:
```
step = 0.01
initial_step = corr_low
last_step = corr_high
first_list = [x / 100.0 for x in range(int(initial_step*100), int(last_step*100), int(step*100))]
n_estimators = [range(2,21,1)]
corr_th = []
second_list = []
```

```
third_list = []
fourth_l = []
fifth_l = []
f_list = []
fif_list = []
for i in first_list:

    for estimator in n_estimators[0]:

        without_standardization, train_r2, test_r2, _, h_, target_column_name,␣
 ↪training_data_RMSE, test_data_RMSE = pred_model.
 ↪prepare_data_and_create_model(molecular_descriptors_df=data,
                                                                              ␣
 ↪                    correlation_threshold=i,
                                                                              ␣
 ↪                    standardization=False,
                                                                              ␣
 ↪                    model_type='RandomForestRegressor',
                                                                              ␣
 ↪                    n_estimators_=estimator,
                                                                              ␣
 ↪                    target_column_name = target,
                                                                              ␣
 ↪                    random_state=random_state,
                                                                              ␣
 ↪                    train_test_split_=True,
                                                                              ␣
 ↪                    verbose=False)
        corr_th.append(i)
        second_list.append(train_r2)
        third_list.append(test_r2)
        fourth_l.append(training_data_RMSE)
        fifth_l.append(test_data_RMSE)
        f_list.append(len(h_))
        fif_list.append(estimator)
```

[26]:

```
df_without_standardization = pd.DataFrame(data=corr_th, columns=["Correlation␣
 ↪threshold"])
df_without_standardization['Training data R^2 score'] = second_list
df_without_standardization['Test data R^2 score'] = third_list
df_without_standardization['Training RMSE'] = fourth_l
df_without_standardization['Test RMSE'] = fifth_l
df_without_standardization['Number of features'] = f_list
df_without_standardization['Number of estimators'] = fif_list
```

[27]:
```
df_random_forest = df_without_standardization.copy()
#df_without_standardization.to_excel('../Data/
↪A549_Random_forest_rs_'+str(random_state)+'.xlsx')
```

[28]:
```
corre_list = [x / 100.0 for x in range(int(initial_step*100),␣
↪int(last_step*100), int(0.01*100))]
for element in corre_list:
    element_ =␣
↪df_without_standardization[df_without_standardization['Correlation␣
↪threshold'] == float(element)]
    plt.plot(element_['Number of estimators'], element_['Training data R^2␣
↪score'], label = "Training data R^2 score", marker='v')
    plt.plot(element_['Number of estimators'], element_['Test data R^2 score'],␣
↪label = "Test data R^2 score", marker='D')
    plt.legend()
    plt.xlabel('Number of estimators -> correlation threshold: '+str(element)+'.
↪ \n Number of features: '+str(element_['Number of features'].iloc[0]))
    plt.xlim(max(element_['Number of estimators'])+1, min(element_['Number of␣
↪estimators'])-1)
    plt.ylabel('R^2 score')
    plt.ylim([0, 1])
    plt.rc('grid', linestyle="-.", color='black')
    plt.grid(True)
    plt.show()
```

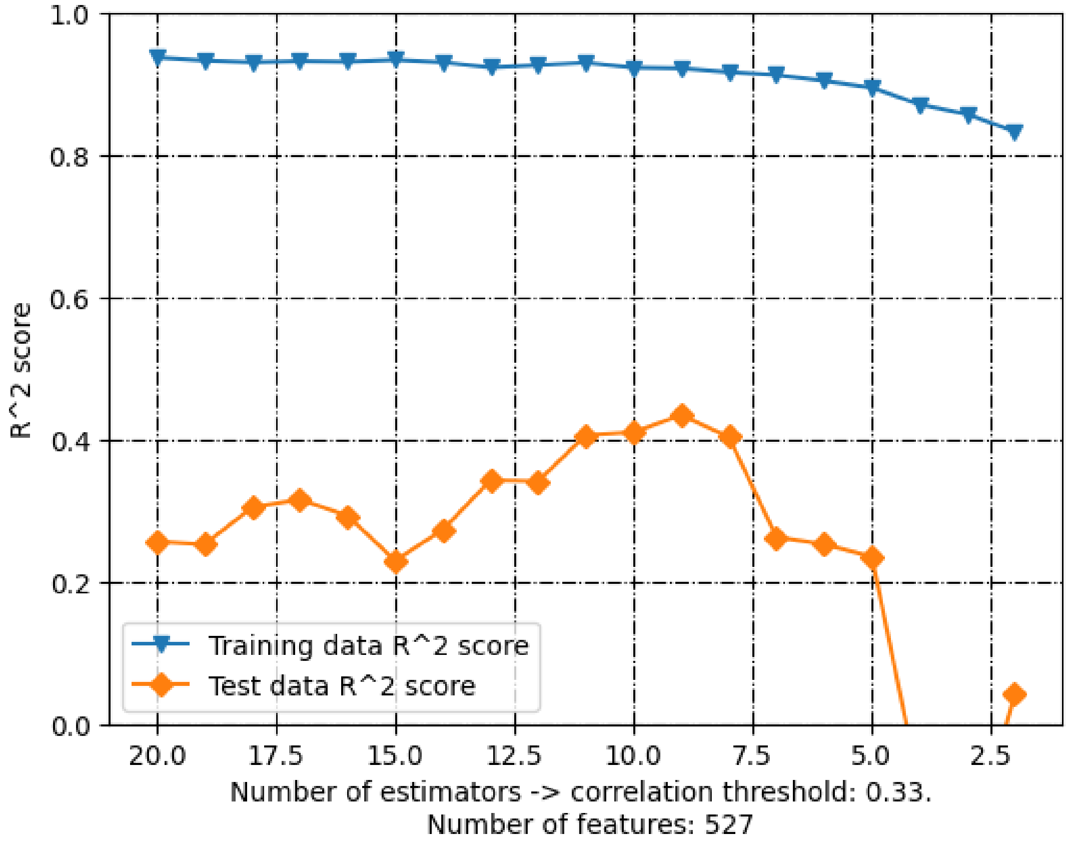
1.0
0.8
0.6
0.4
0.2
0.0
R^2 score
20.0
17.5
15.0
12.5
10.0
7.5
5.0
2.5
Training data R^2 score
Test data R^2 score
Number of estimators -> correlation threshold: 0.33.
Number of features: 527

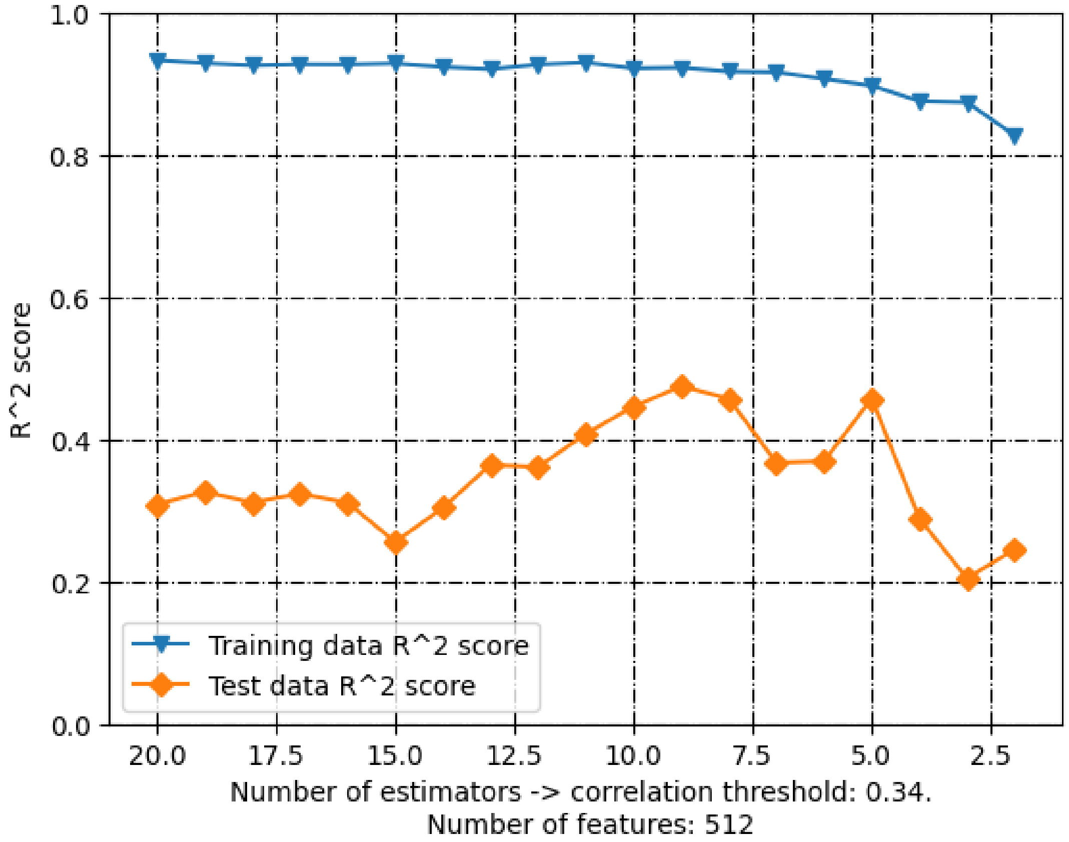

1.0
0.8
0.6
0.4
0.2
0.0
R^2 score
Training data R^2 score
Test data R^2 score
20.0
17.5
15.0
12.5
10.0
7.5
5.0
2.5
Number of estimators -> correlation threshold: 0.34.
Number of features: 512

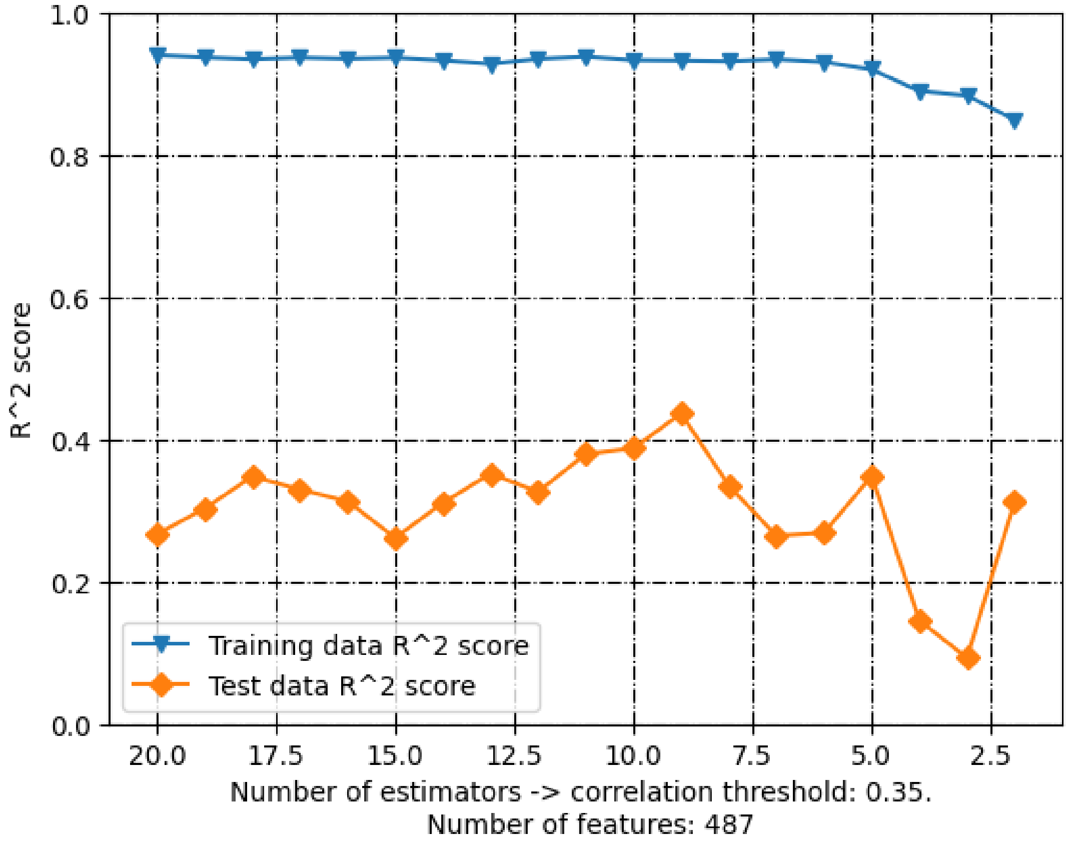
1.0
0.8
0.6
0.4
0.2
0.0
R^2 score
Training data R^2 score
Test data R^2 score
20.0
17.5
15.0
12.5
10.0
7.5
5.0
2.5
Number of estimators -> correlation threshold: 0.35.
Number of features: 487

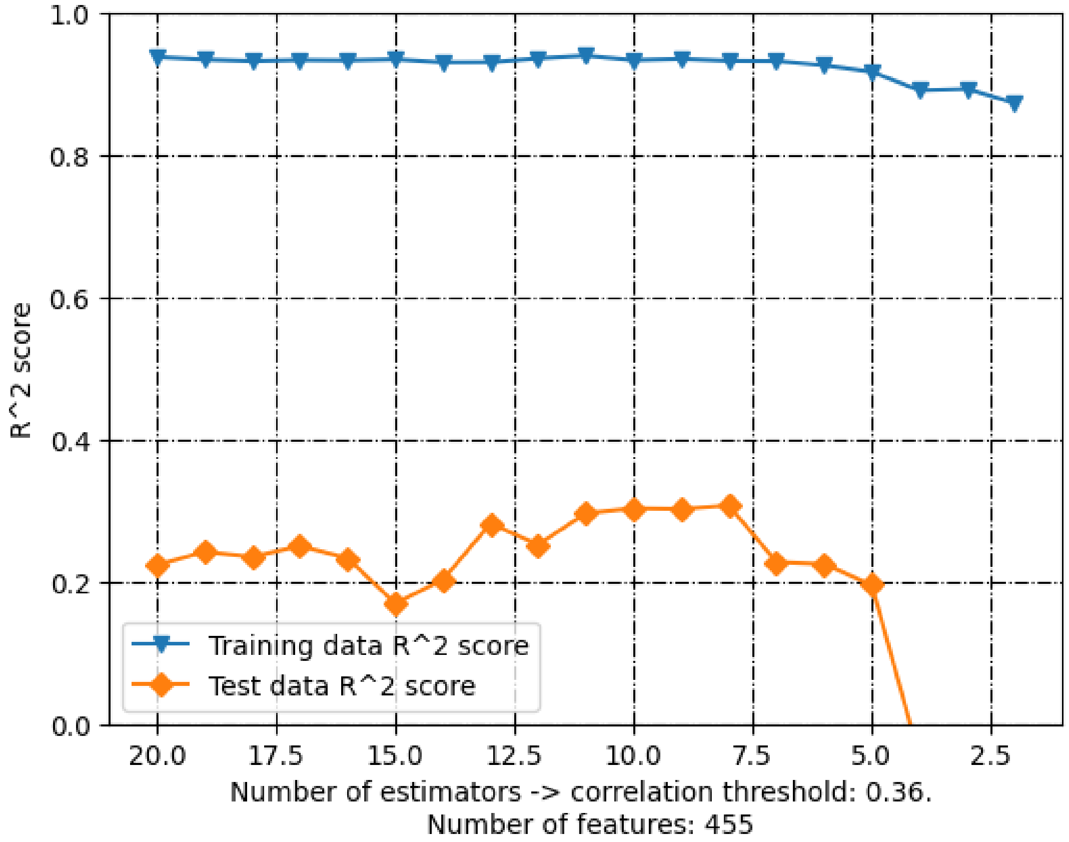

1.0
0.8
0.6
0.4
0.2
0.0
R^2 score
Training data R^2 score
Test data R^2 score
20.0
17.5
15.0
12.5
10.0
7.5
5.0
2.5
Number of estimators -> correlation threshold: 0.36.
Number of features: 455

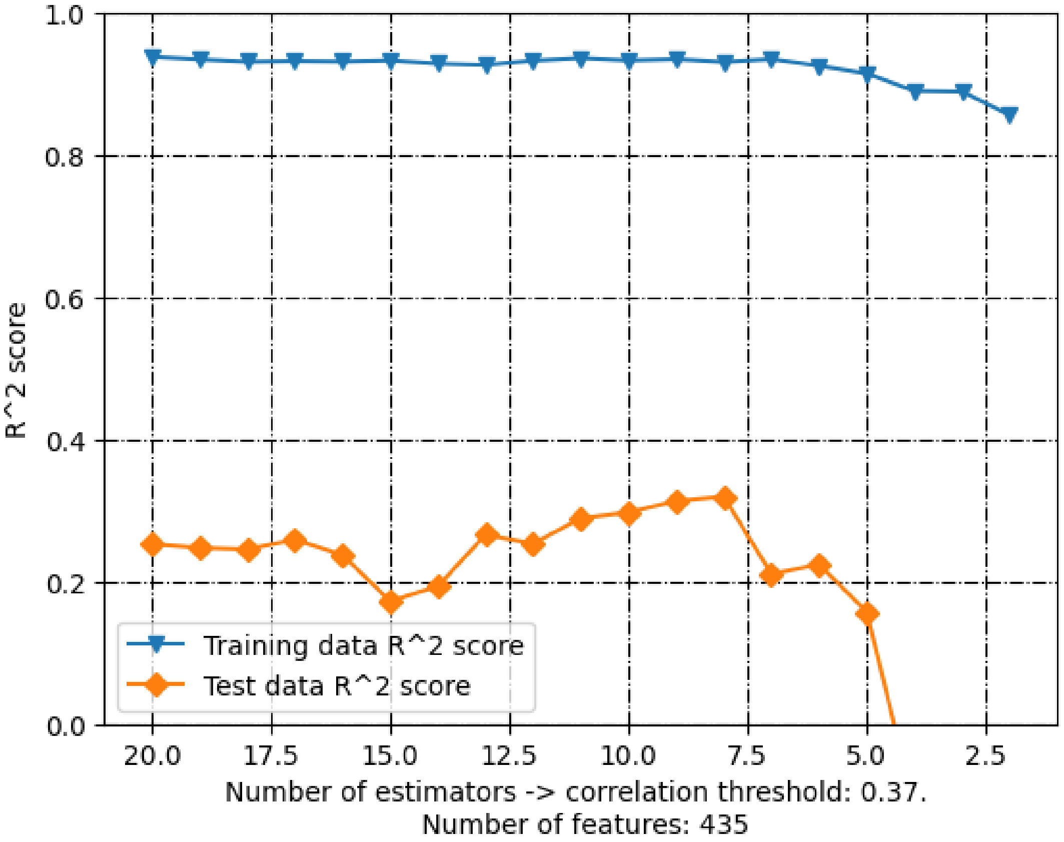
1.0
0.8
0.6
0.4
0.2
0.0
R^2 score
Training data R^2 score
Test data R^2 score
20.0
17.5
15.0
12.5
10.0
7.5
5.0
2.5
Number of estimators -> correlation threshold: 0.37.
Number of features: 435

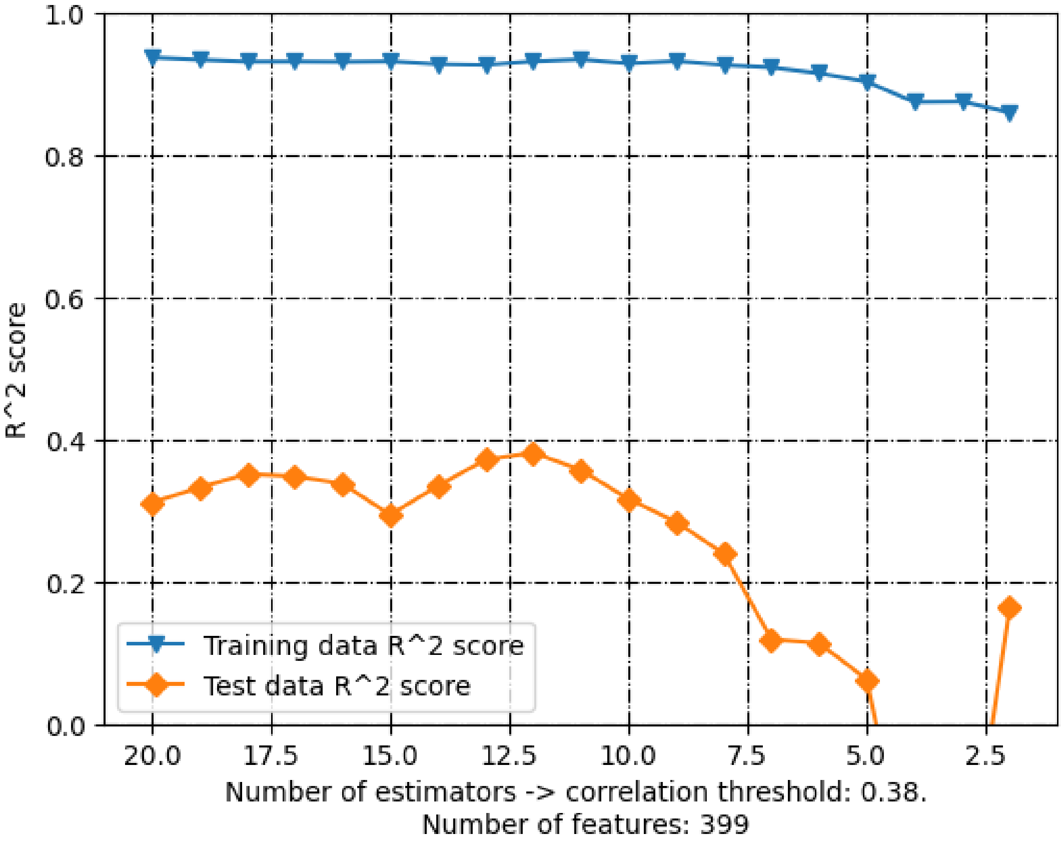
1.0
0.8
0.6
0.4
0.2
0.0
R^2 score
Training data R^2 score
Test data R^2 score
20.0
17.5
15.0
12.5
10.0
7.5
5.0
2.5
Number of estimators -> correlation threshold: 0.38.
Number of features: 399

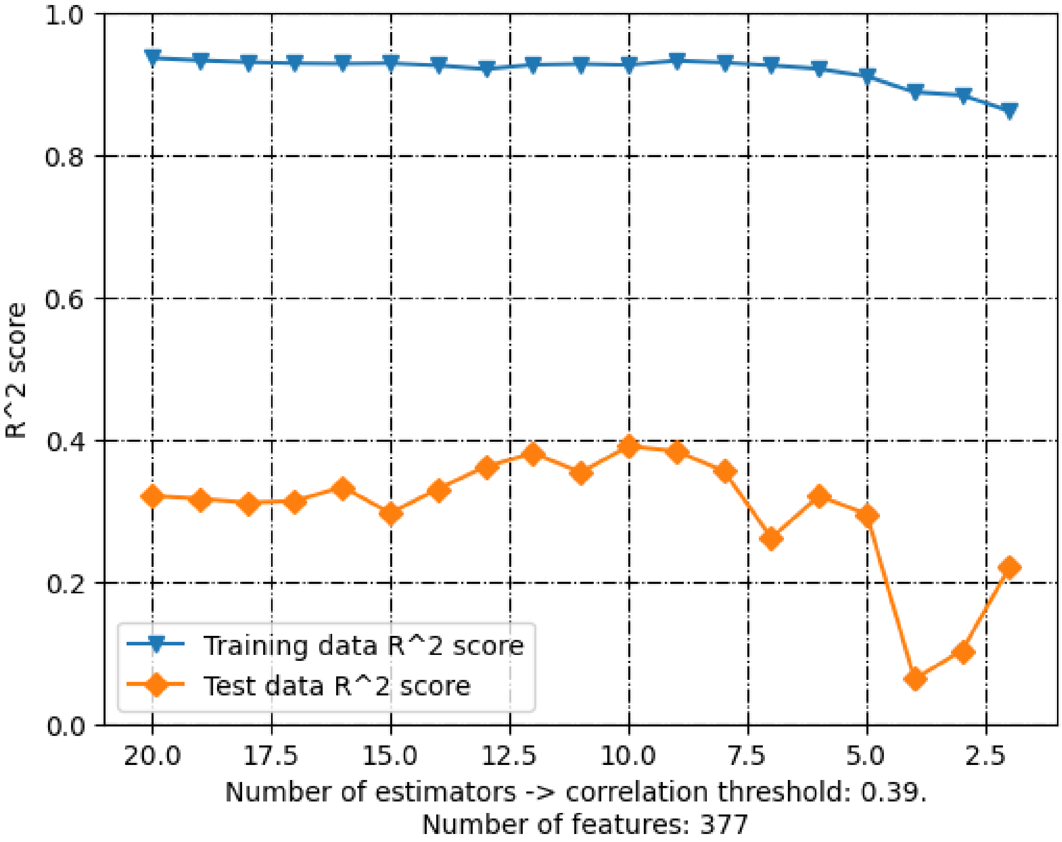
1.0
0.8
0.6
0.4
0.2
0.0
R^2 score
Training data R^2 score
Test data R^2 score
20.0
17.5
15.0
12.5
10.0
7.5
5.0
2.5
Number of estimators -> correlation threshold: 0.39.
Number of features: 377

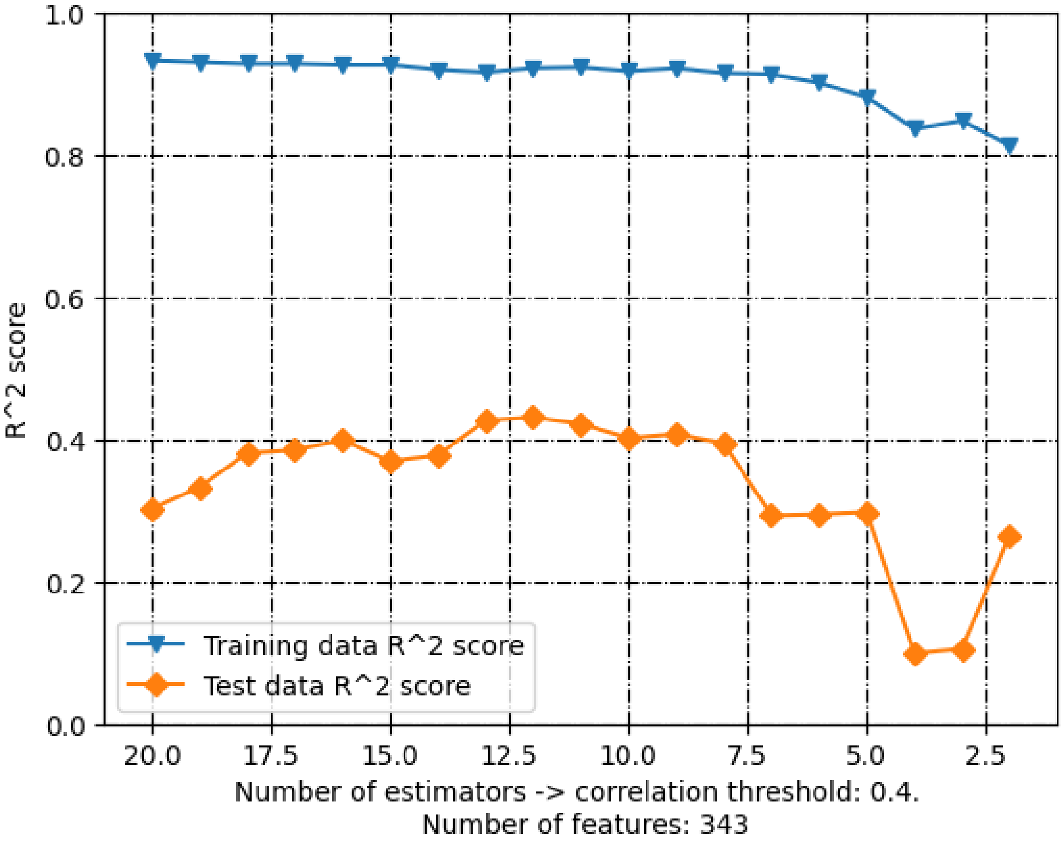
1.0
0.8
0.6
0.4
0.2
0.0
R^2 score
Training data R^2 score
Test data R^2 score
20.0
17.5
15.0
12.5
10.0
7.5
5.0
2.5
Number of estimators -> correlation threshold: 0.4.
Number of features: 343

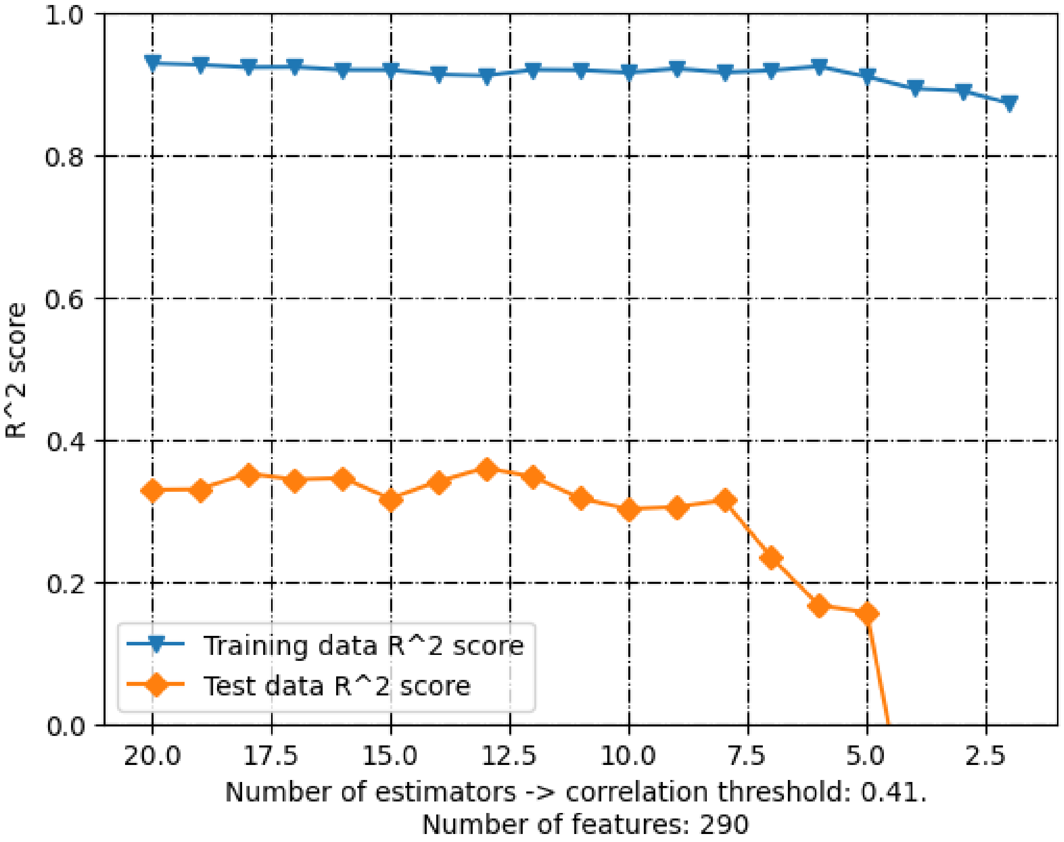

1.0
0.8
0.6
0.4
0.2
0.0
R^2 score
20.0
17.5
15.0
12.5
10.0
7.5
5.0
2.5
Number of estimators -> correlation threshold: 0.41.
Number of features: 290
Training data R^2 score
Test data R^2 score

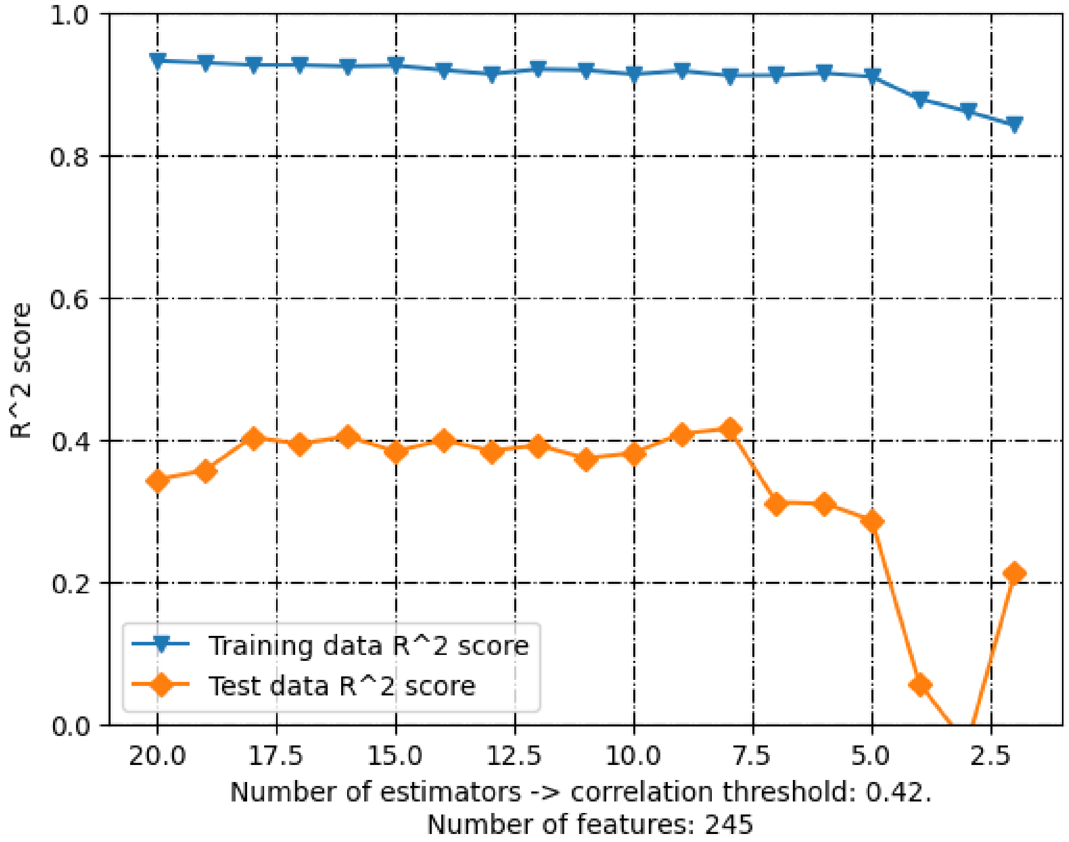
1.0
0.8
0.6
0.4
0.2
0.0
R^2 score
20.0
17.5
15.0
12.5
10.0
7.5
5.0
2.5
Training data R^2 score
Test data R^2 score
Number of estimators -> correlation threshold: 0.42.
Number of features: 245

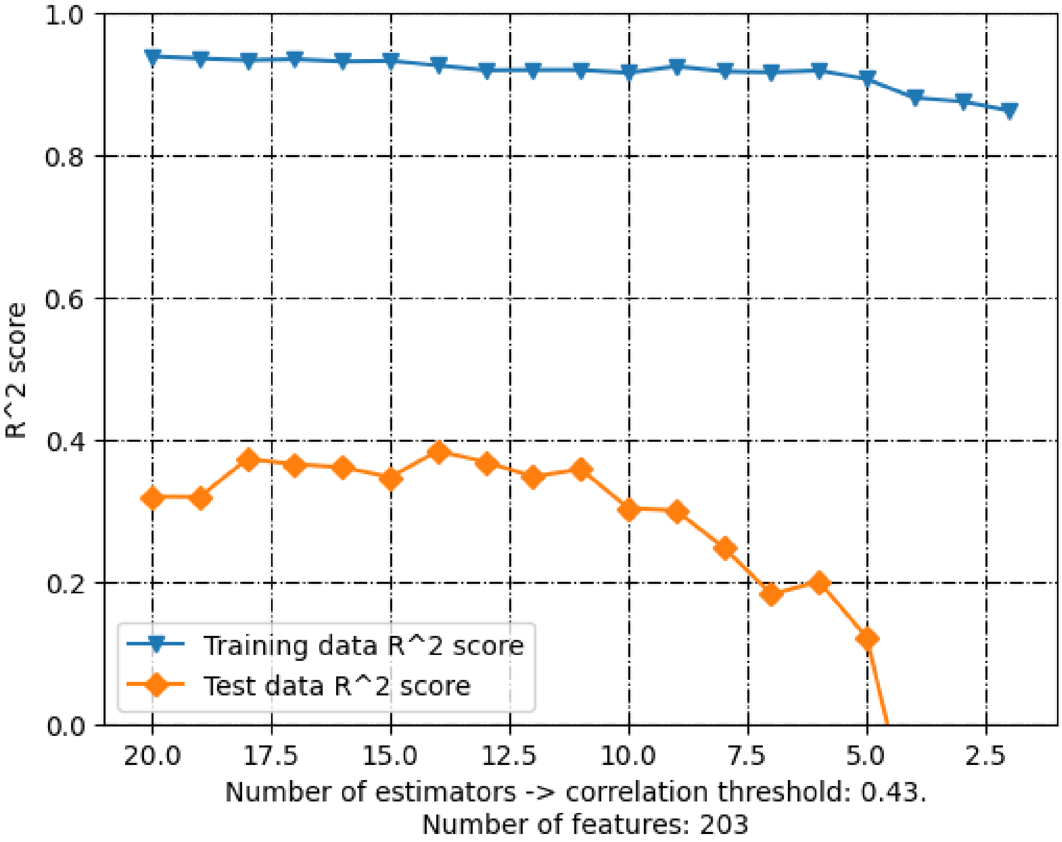
1.0
0.8
0.6
0.4
0.2
0.0
R^2 score
Training data R^2 score
Test data R^2 score
20.0
17.5
15.0
12.5
10.0
7.5
5.0
2.5
Number of estimators -> correlation threshold: 0.43.
Number of features: 203

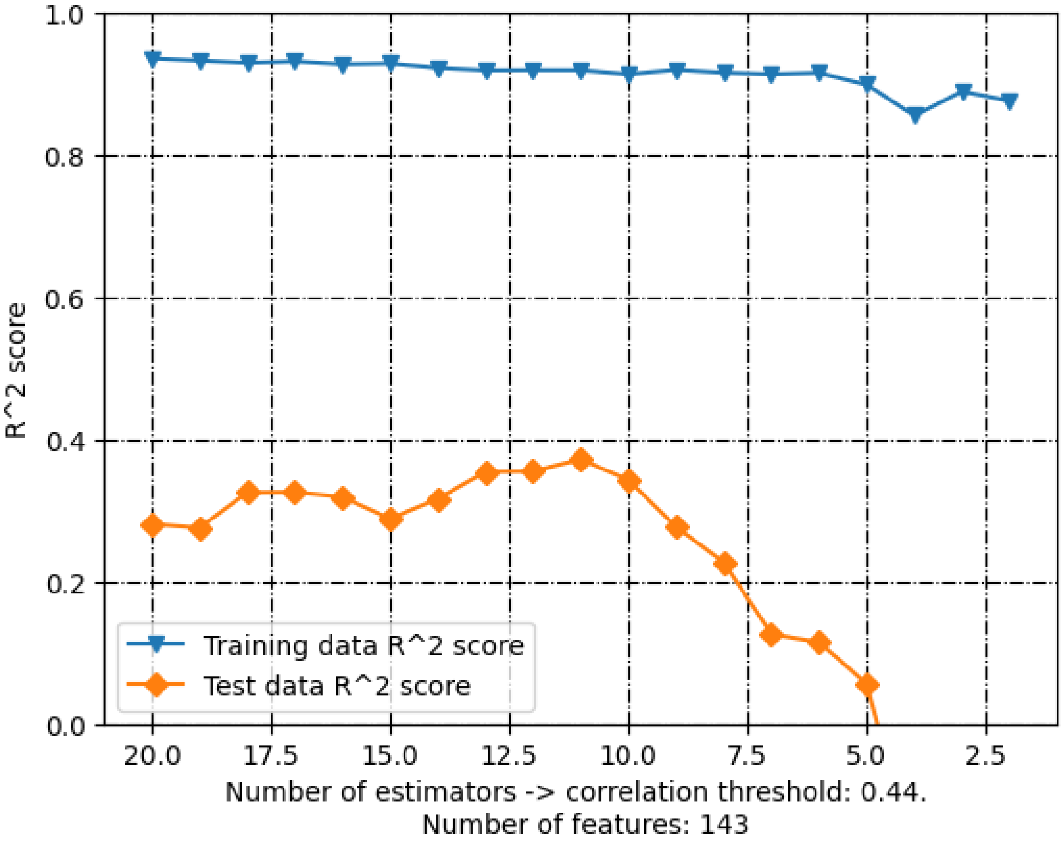
1.0
0.8
0.6
0.4
0.2
0.0
R^2 score
Training data R^2 score
Test data R^2 score
20.0
17.5
15.0
12.5
10.0
7.5
5.0
2.5
Number of estimators -> correlation threshold: 0.44.
Number of features: 143

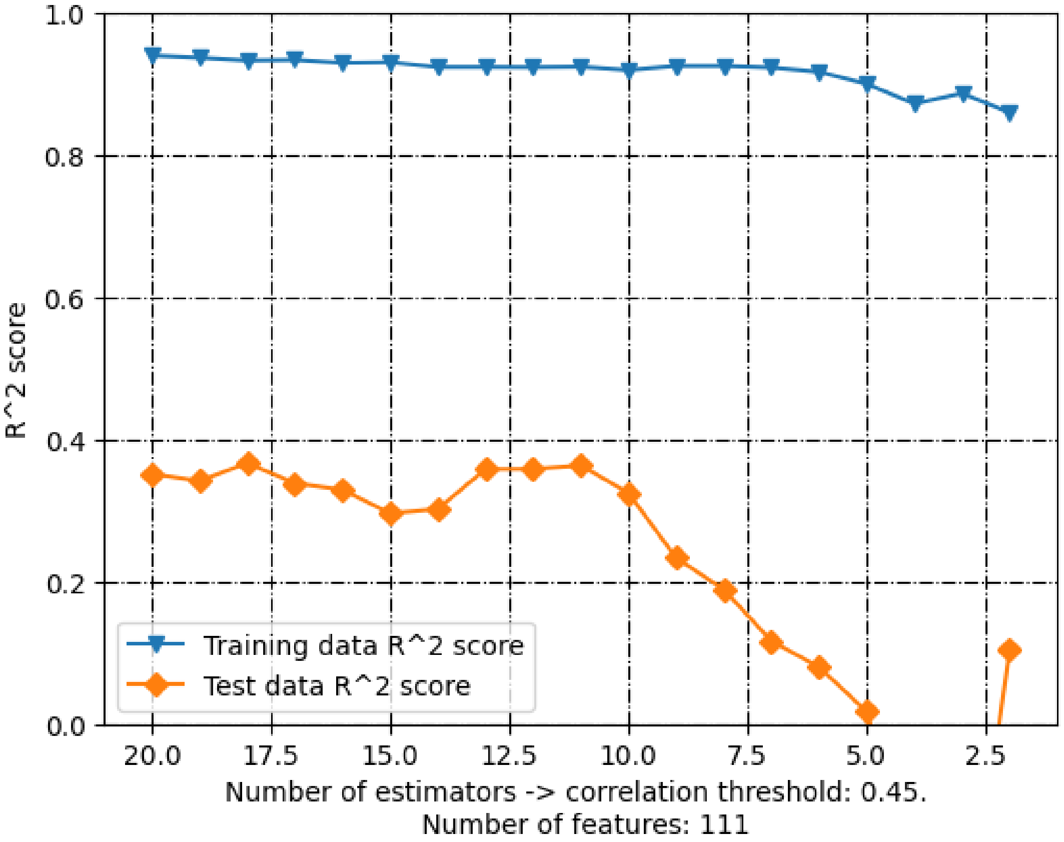
1.0
0.8
0.6
0.4
0.2
0.0
R^2 score
20.0
17.5
15.0
12.5
10.0
7.5
5.0
2.5
Training data R^2 score
Test data R^2 score
Number of estimators -> correlation threshold: 0.45.
Number of features: 111

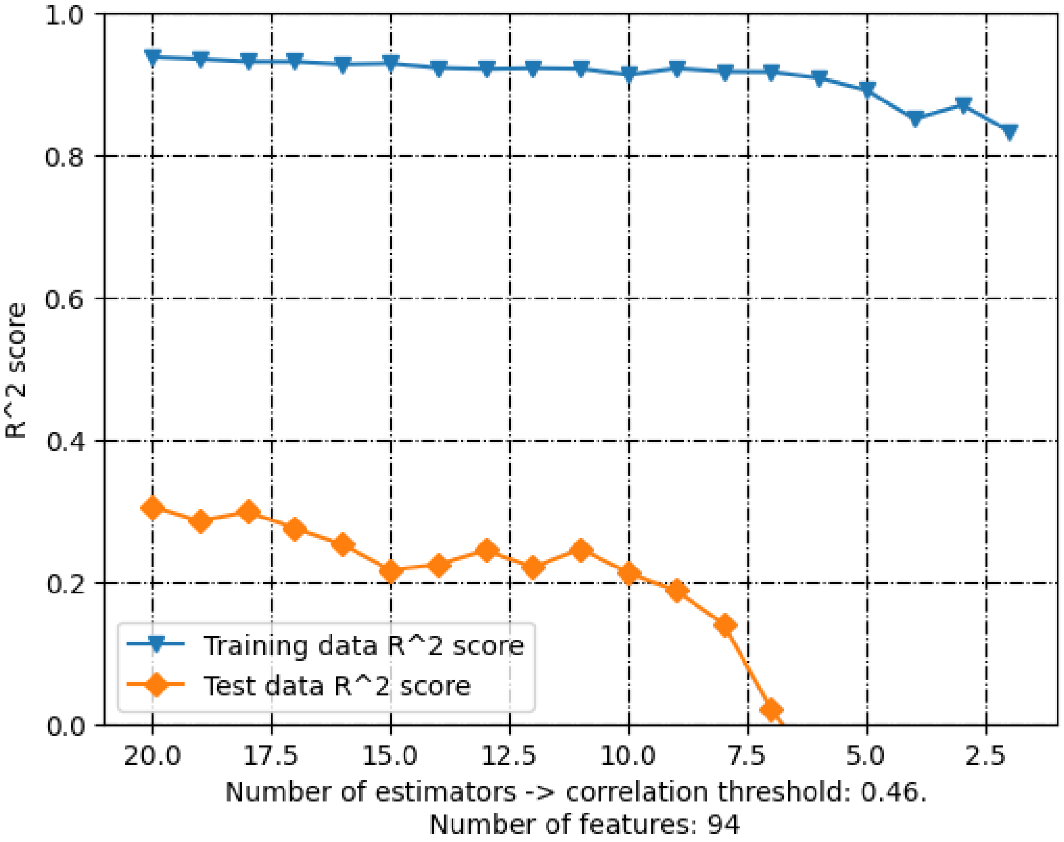

1.0
0.8
0.6
0.4
0.2
0.0
R^2 score
20.0
17.5
15.0
12.5
10.0
7.5
5.0
2.5
Training data R^2 score
Test data R^2 score
Number of estimators -> correlation threshold: 0.46.
Number of features: 94

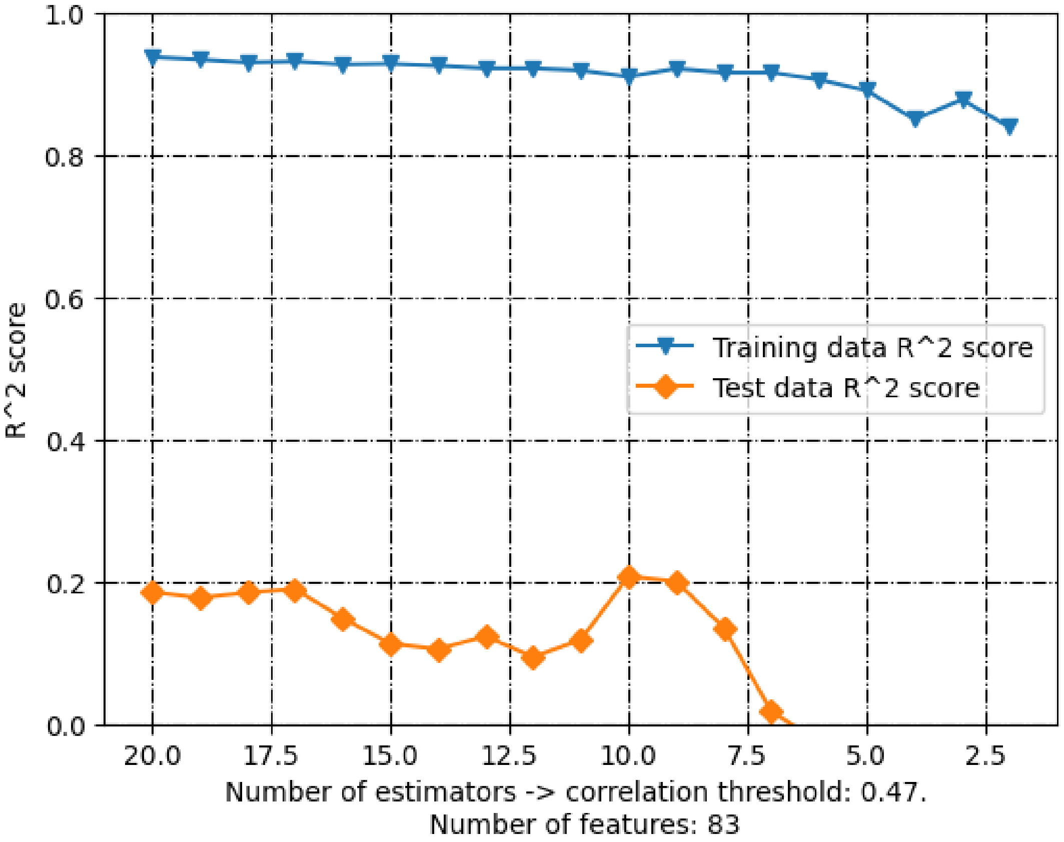
1.0
0.8
0.6
0.4
0.2
0.0
R^2 score
Training data R^2 score
Test data R^2 score
20.0
17.5
15.0
12.5
10.0
7.5
5.0
2.5
Number of estimators -> correlation threshold: 0.47.
Number of features: 83

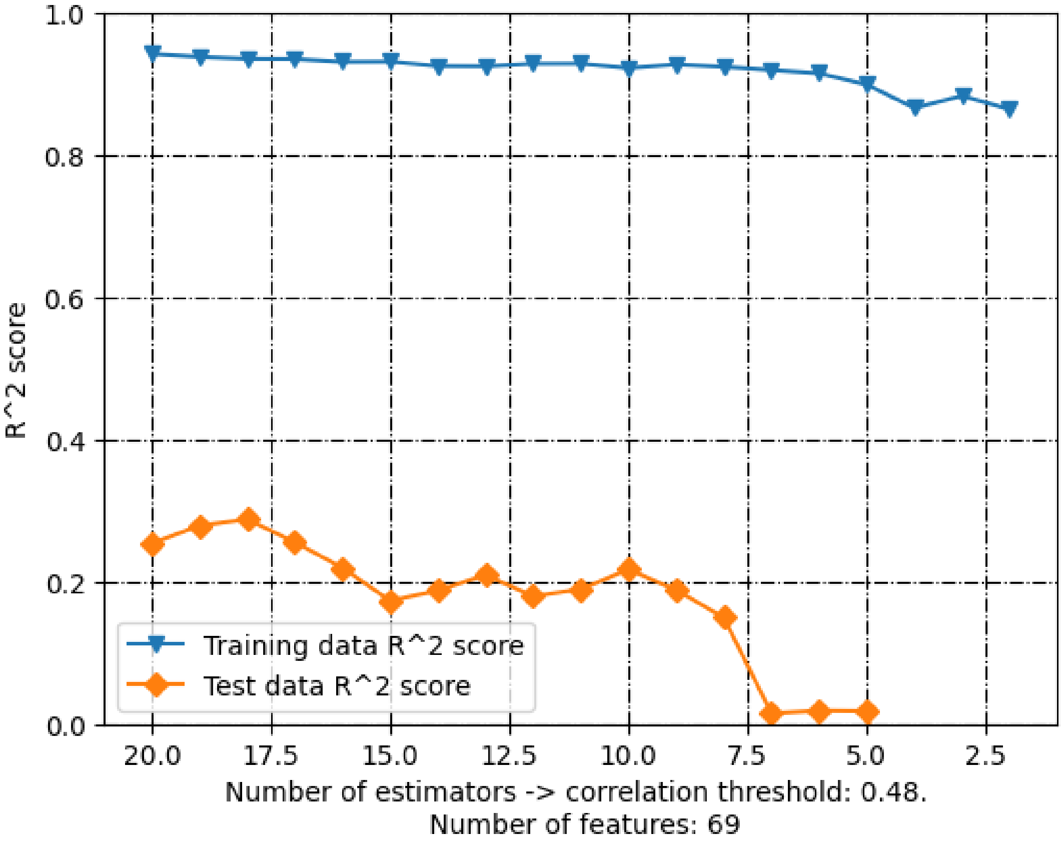

1.0
0.8
0.6
0.4
0.2
0.0
R^2 score
20.0
17.5
15.0
12.5
10.0
7.5
5.0
2.5
Number of estimators -> correlation threshold: 0.48.
Number of features: 69
Training data R^2 score
Test data R^2 score

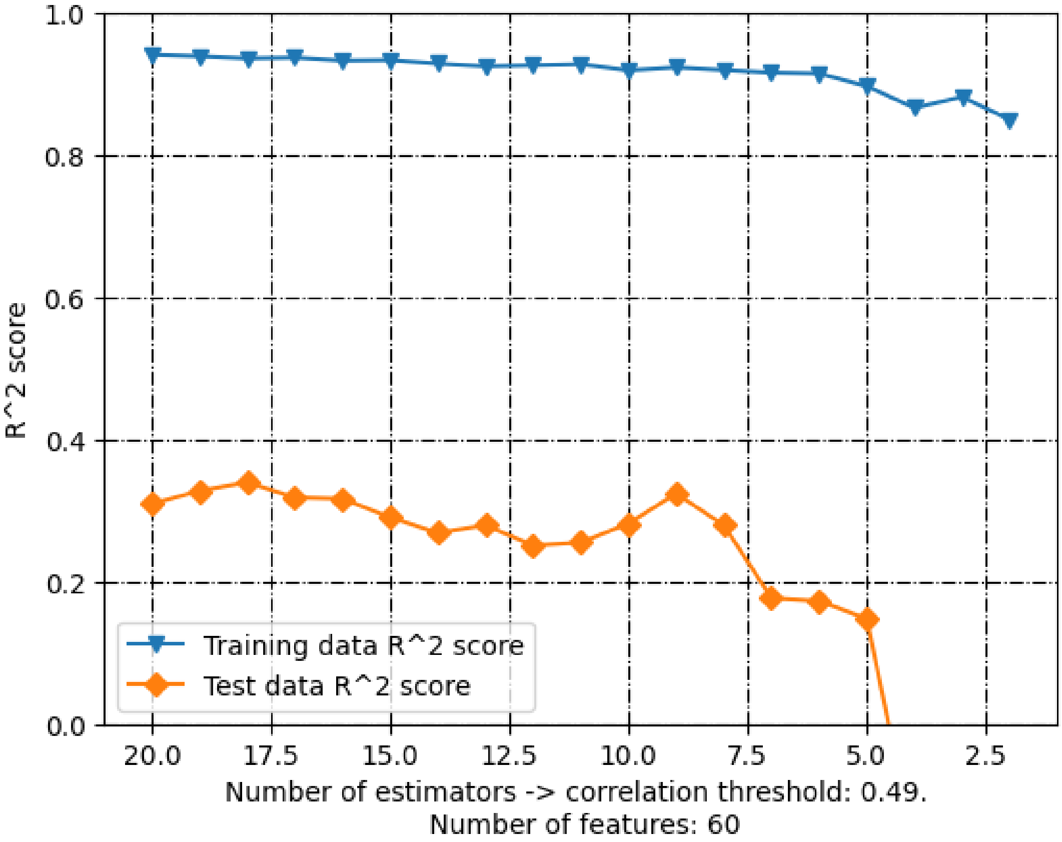

1.0
0.8
0.6
0.4
0.2
0.0
R^2 score
Training data R^2 score
Test data R^2 score
20.0
17.5
15.0
12.5
10.0
7.5
5.0
2.5
Number of estimators -> correlation threshold: 0.49.
Number of features: 60

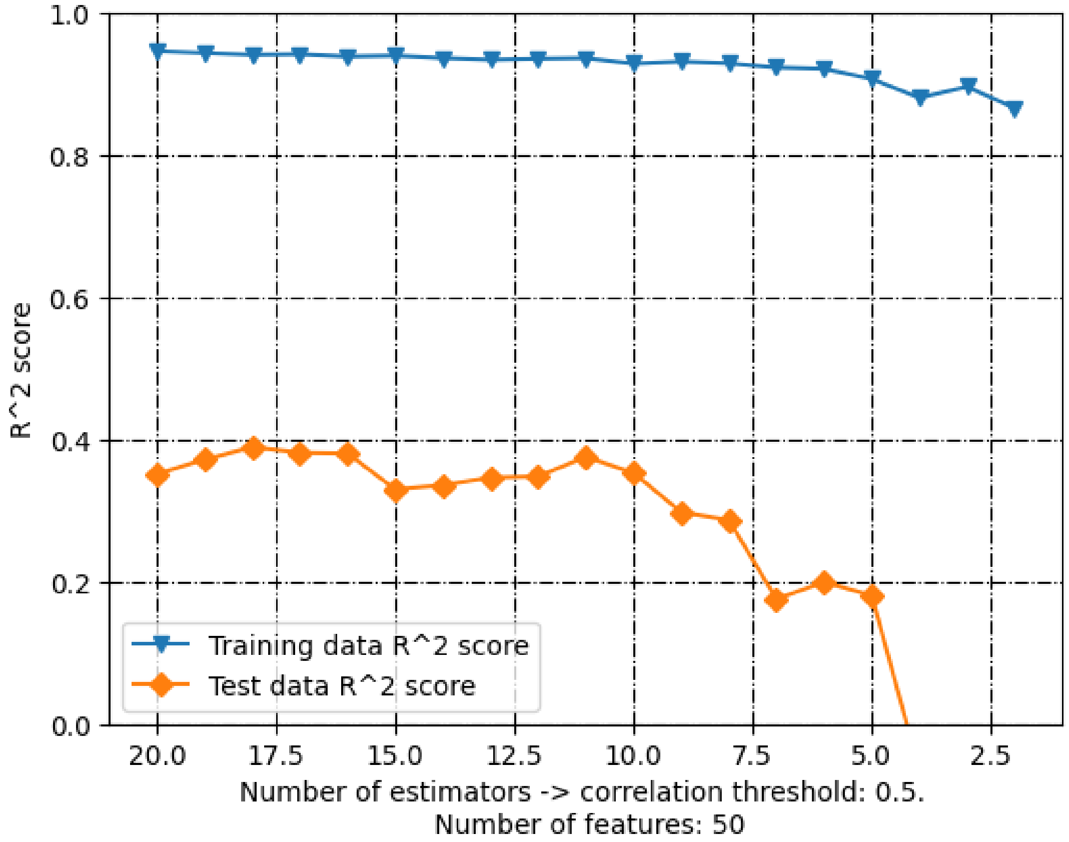
1.0
0.8
0.6
0.4
0.2
0.0
R^2 score
Training data R^2 score
Test data R^2 score
20.0
17.5
15.0
12.5
10.0
7.5
5.0
2.5
Number of estimators -> correlation threshold: 0.5.
Number of features: 50

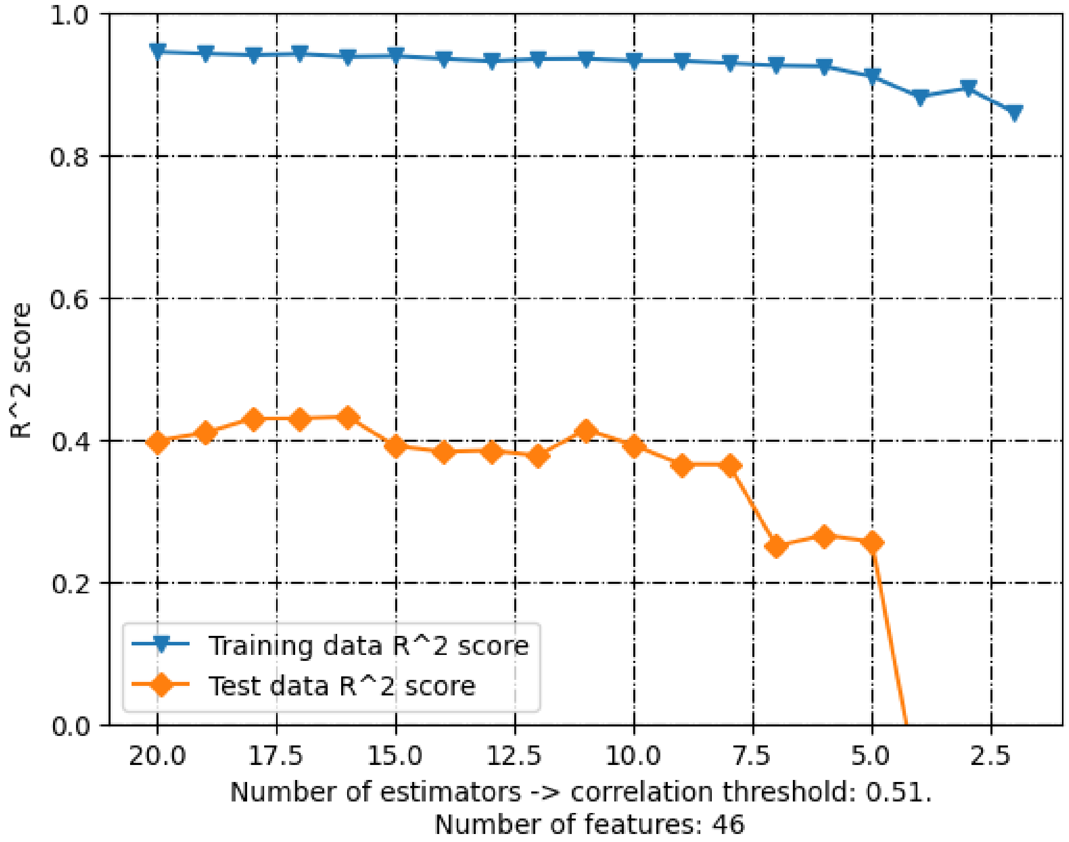

1.0
0.8
0.6
0.4
0.2
0.0
R^2 score
20.0
17.5
15.0
12.5
10.0
7.5
5.0
2.5
Number of estimators -> correlation threshold: 0.51.
Number of features: 46
Training data R^2 score
Test data R^2 score

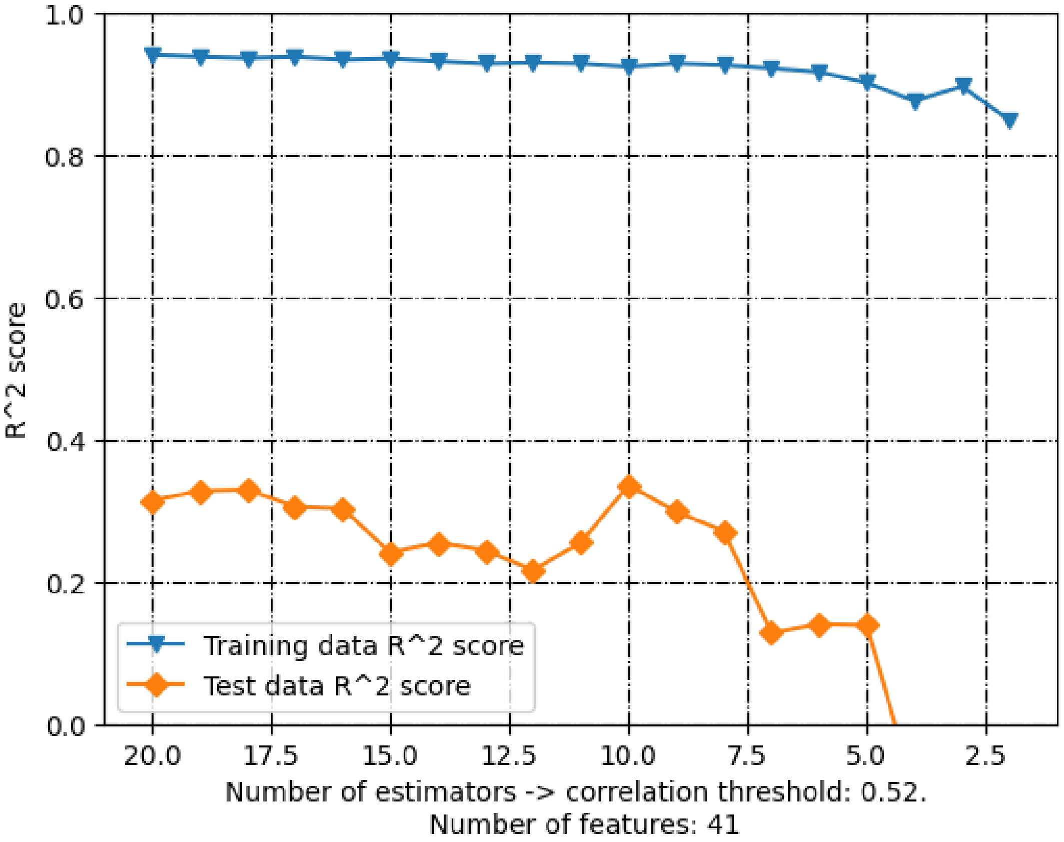
1.0
0.8
0.6
0.4
0.2
0.0
R^2 score
Training data R^2 score
Test data R^2 score
20.0
17.5
15.0
12.5
10.0
7.5
5.0
2.5
Number of estimators -> correlation threshold: 0.52.
Number of features: 41

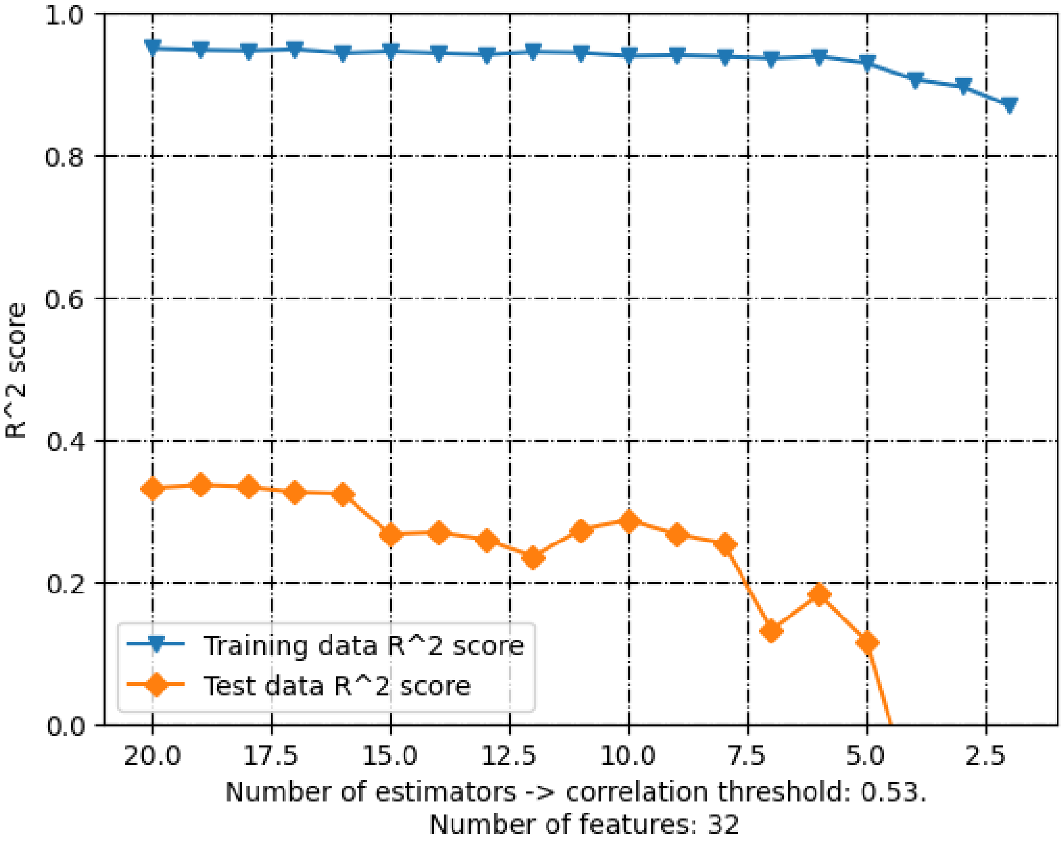
R^2 score
1.0
0.8
0.6
0.4
0.2
0.0
20.0
17.5
15.0
12.5
10.0
7.5
5.0
2.5
Training data R^2 score
Test data R^2 score
Number of estimators -> correlation threshold: 0.53.
Number of features: 32

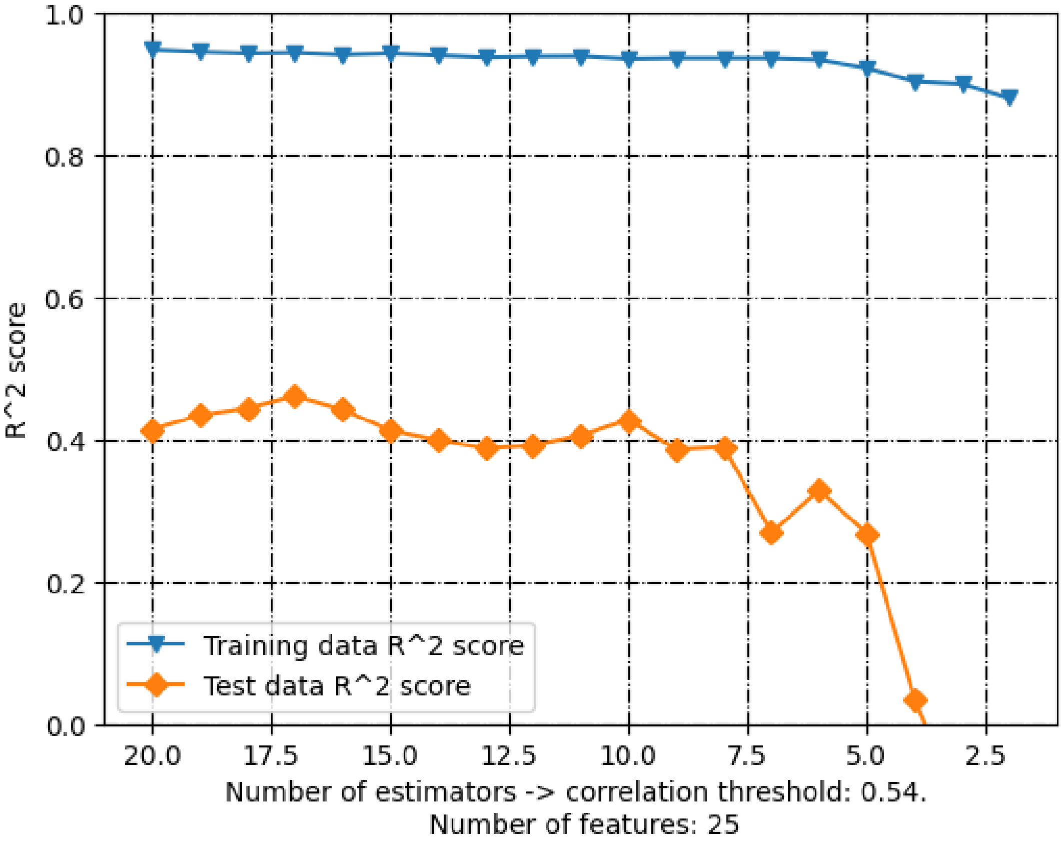
1.0
0.8
0.6
0.4
0.2
0.0
R^2 score
Training data R^2 score
Test data R^2 score
20.0
17.5
15.0
12.5
10.0
7.5
5.0
2.5
Number of estimators -> correlation threshold: 0.54.
Number of features: 25

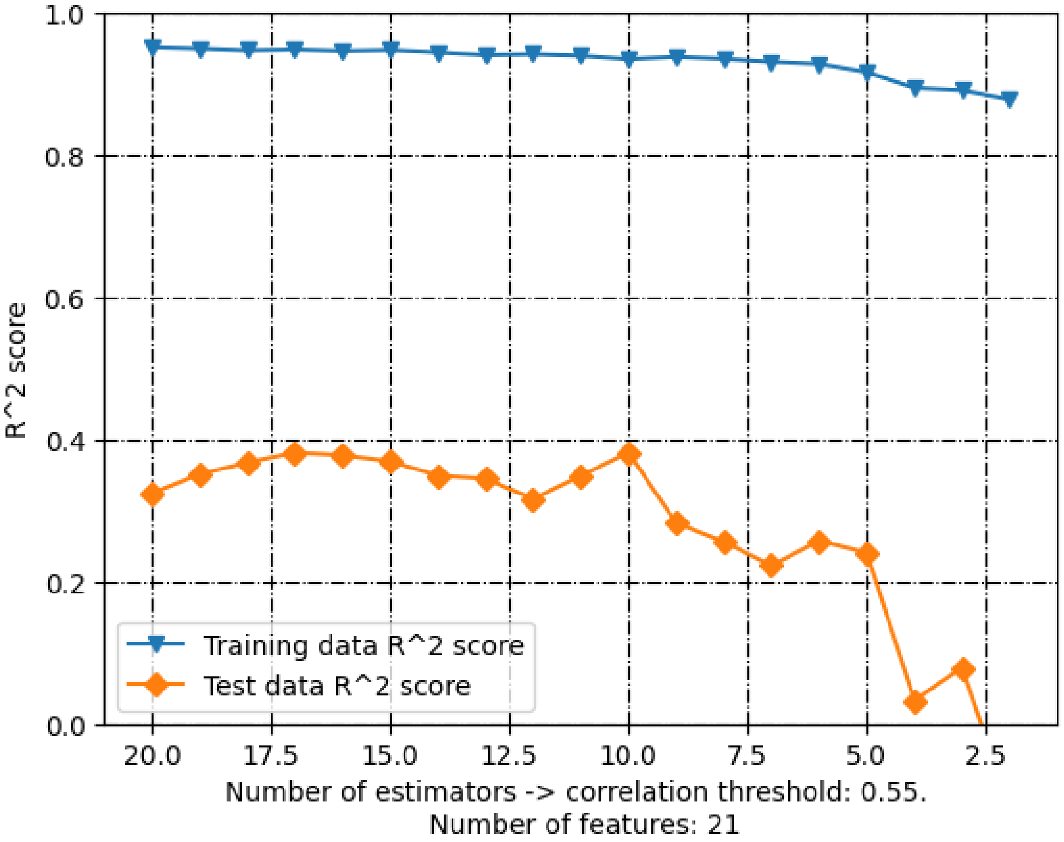

1.0
0.8
0.6
0.4
0.2
0.0
R^2 score
Training data R^2 score
Test data R^2 score
20.0
17.5
15.0
12.5
10.0
7.5
5.0
2.5
Number of estimators -> correlation threshold: 0.55.
Number of features: 21

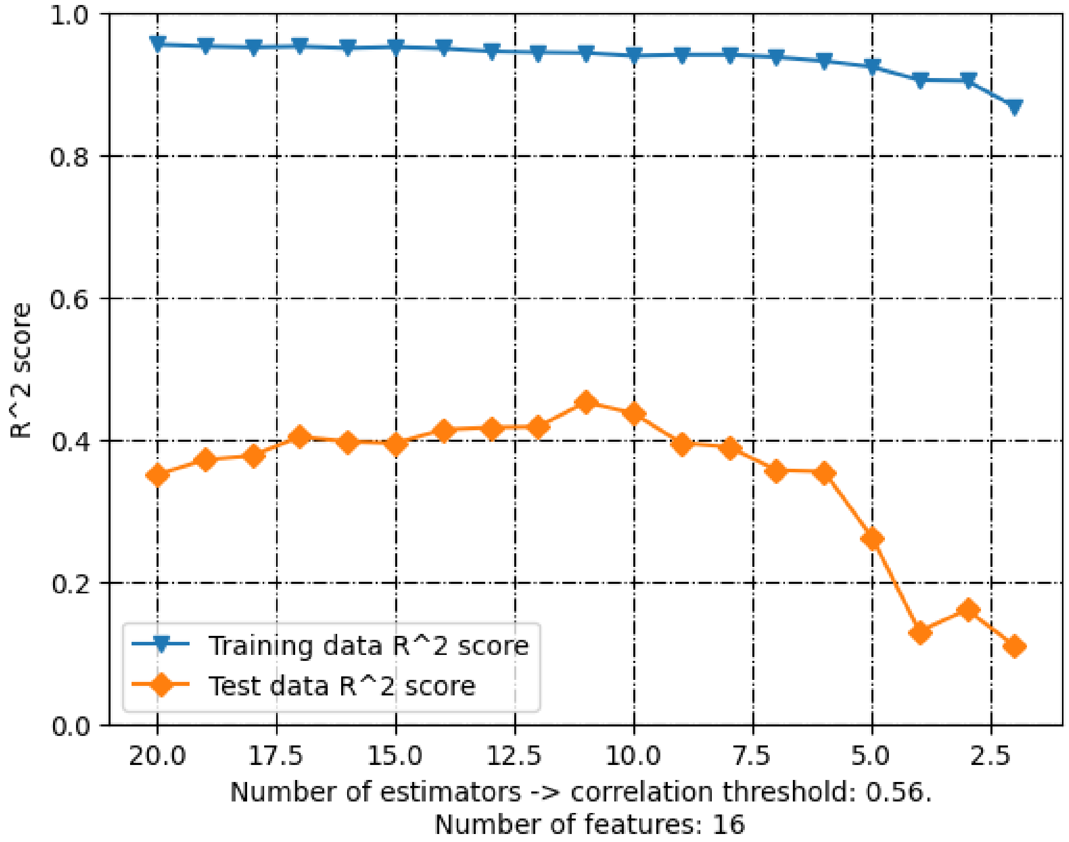

1.0
0.8
0.6
0.4
0.2
0.0
R^2 score
Training data R^2 score
Test data R^2 score
20.0
17.5
15.0
12.5
10.0
7.5
5.0
2.5
Number of estimators -> correlation threshold: 0.56.
Number of features: 16

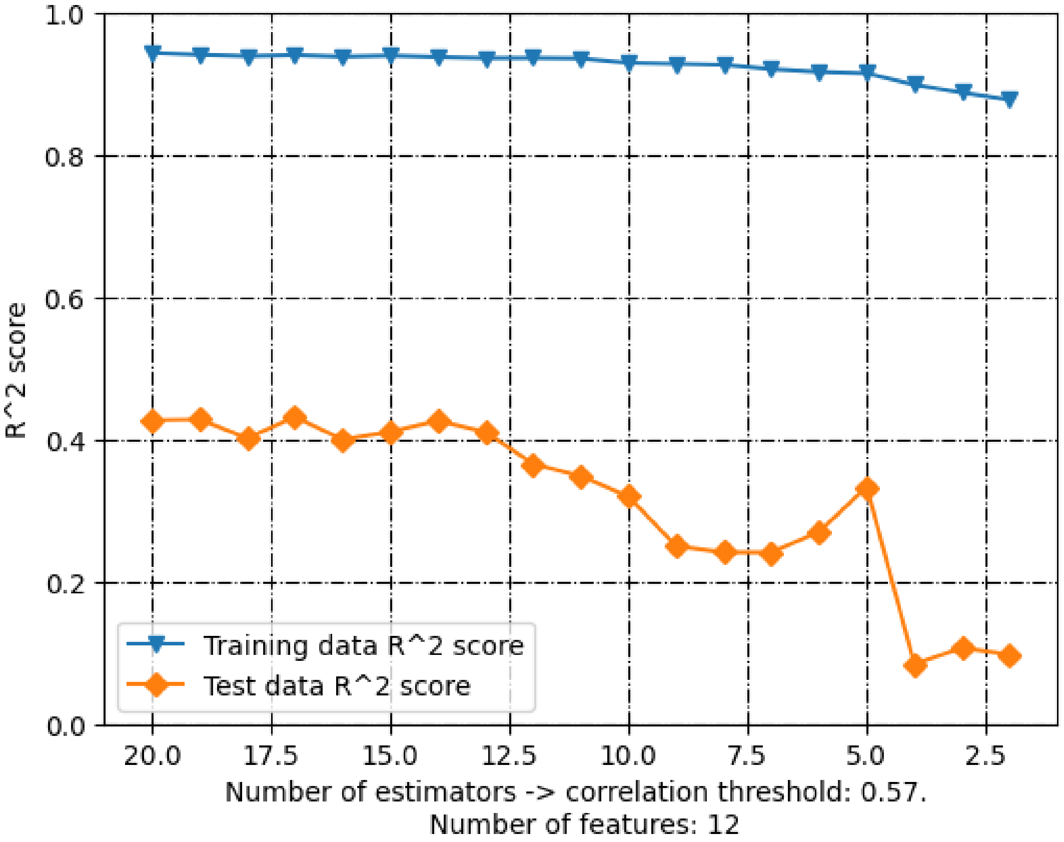
1.0
0.8
0.6
0.4
0.2
0.0
R^2 score
Training data R^2 score
Test data R^2 score
20.0
17.5
15.0
12.5
10.0
7.5
5.0
2.5
Number of estimators -> correlation threshold: 0.57.
Number of features: 12

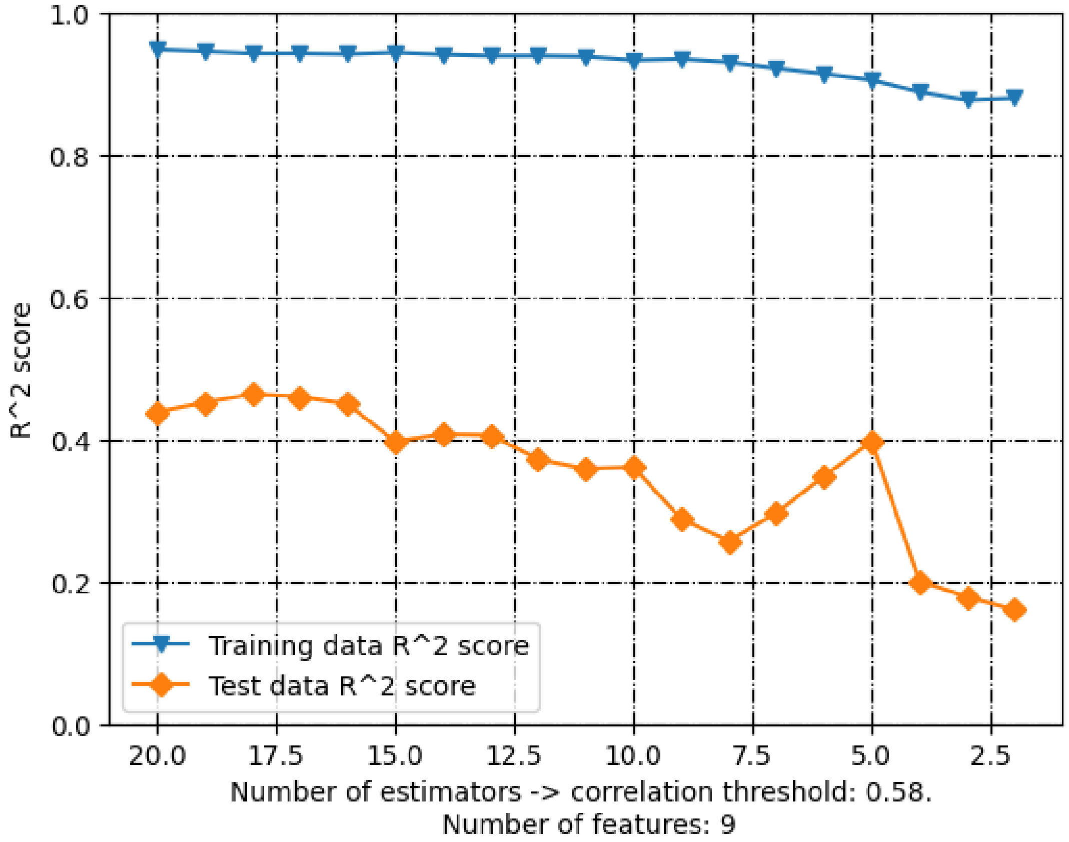
1.0
0.8
0.6
0.4
0.2
0.0
R^2 score
20.0
17.5
15.0
12.5
10.0
7.5
5.0
2.5
Number of estimators -> correlation threshold: 0.58.
Number of features: 9
Training data R^2 score
Test data R^2 score

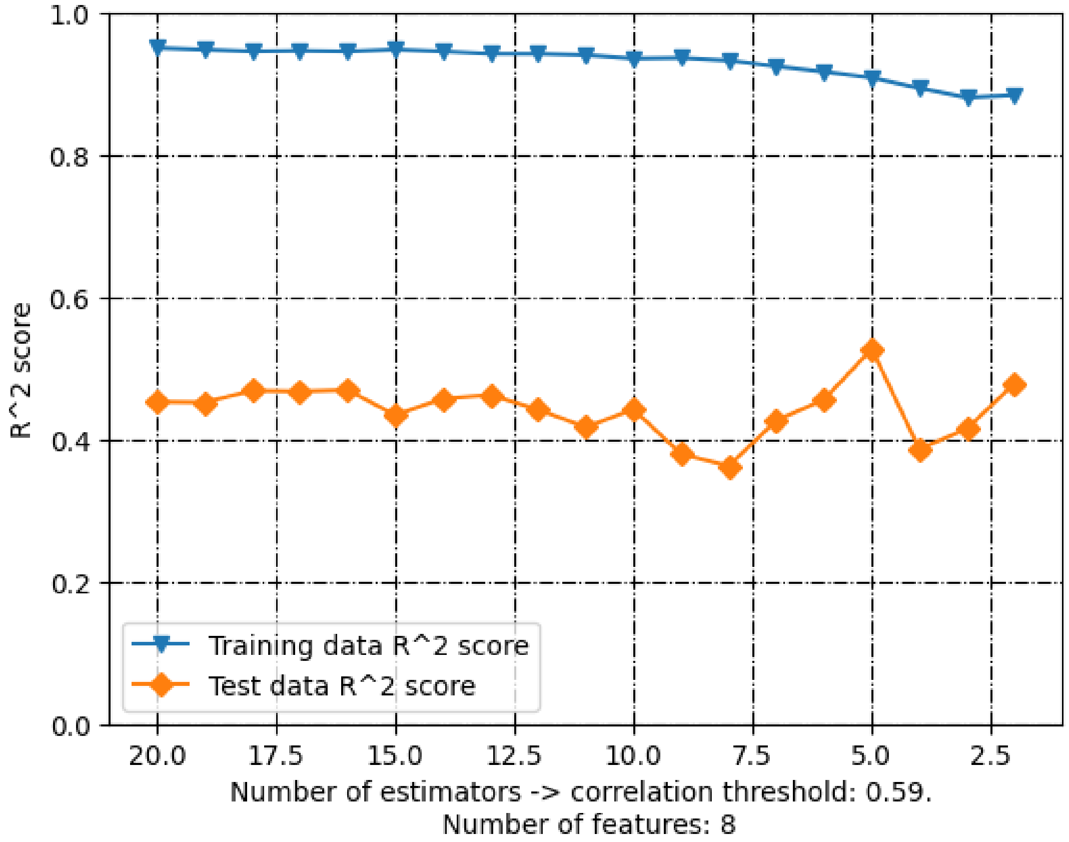
1.0
0.8
0.6
0.4
0.2
0.0
R^2 score
Training data R^2 score
Test data R^2 score
20.0
17.5
15.0
12.5
10.0
7.5
5.0
2.5
Number of estimators -> correlation threshold: 0.59.
Number of features: 8

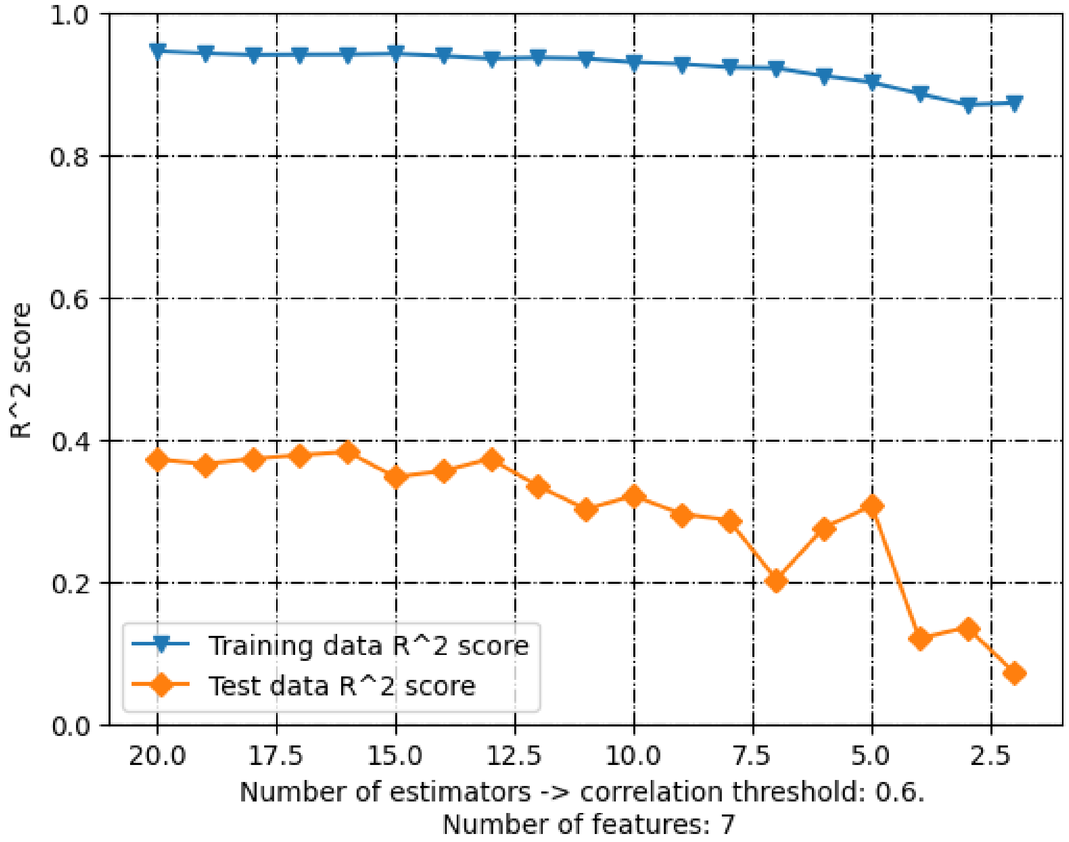

1.0
0.8
0.6
0.4
0.2
0.0
R^2 score
Training data R^2 score
Test data R^2 score
20.0
17.5
15.0
12.5
10.0
7.5
5.0
2.5
Number of estimators -> correlation threshold: 0.6.
Number of features: 7

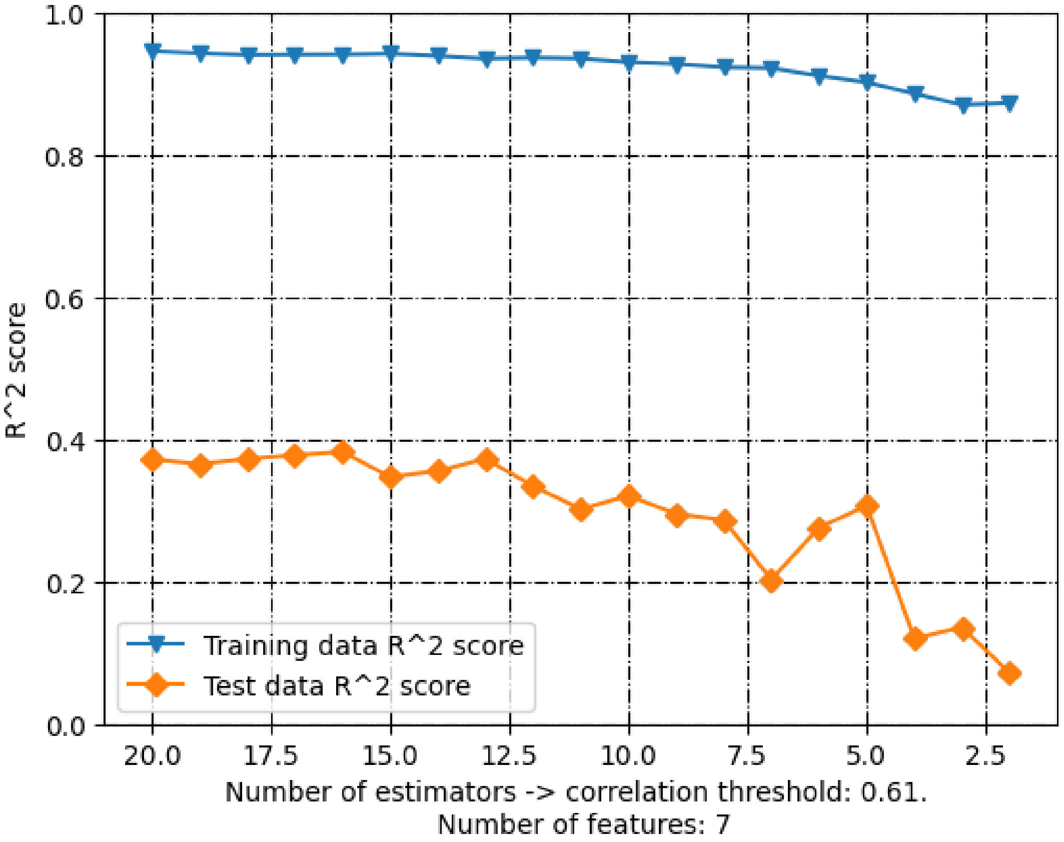
1.0
0.8
0.6
0.4
0.2
0.0
R^2 score
Training data R^2 score
Test data R^2 score
20.0
17.5
15.0
12.5
10.0
7.5
5.0
2.5
Number of estimators -> correlation threshold: 0.61.
Number of features: 7

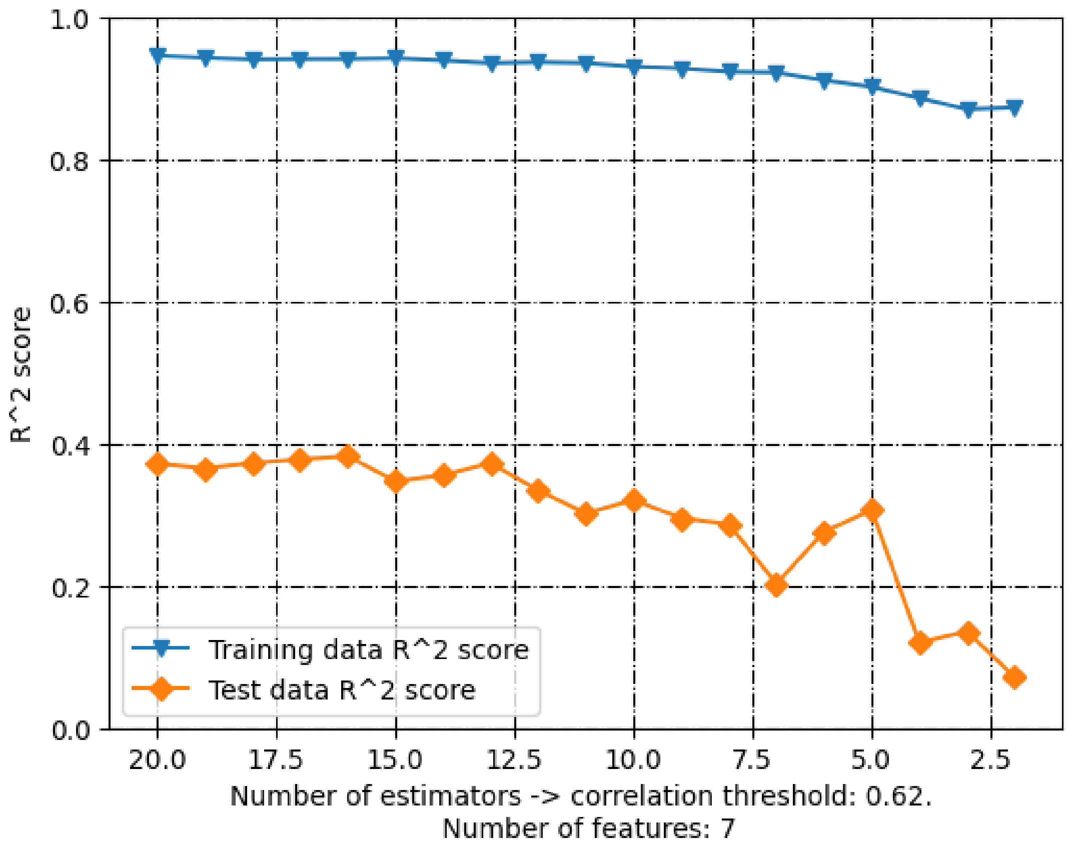
1.0
0.8
0.6
0.4
0.2
0.0
R^2 score
Training data R^2 score
Test data R^2 score
20.0
17.5
15.0
12.5
10.0
7.5
5.0
2.5
Number of estimators -> correlation threshold: 0.62.
Number of features: 7

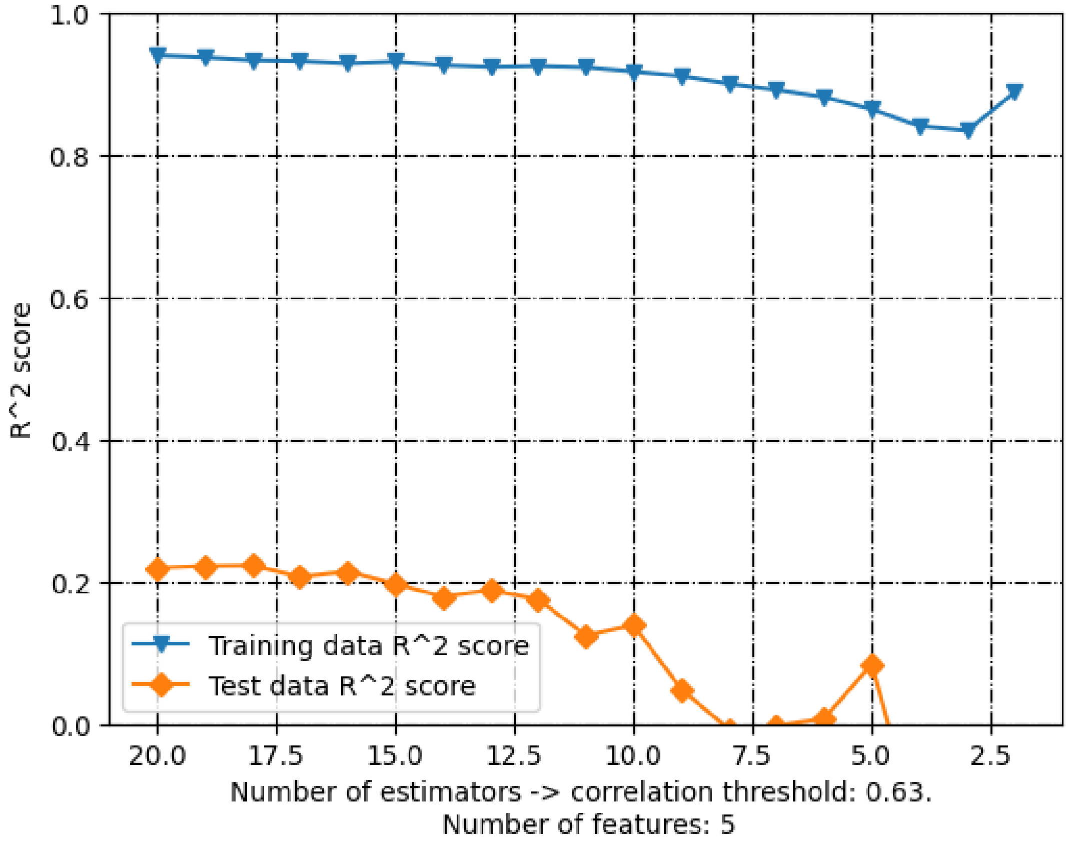

[29]:
```
plt.plot(df_without_standardization['Correlation threshold'],␣
 ↪df_without_standardization['Number of features'], label = "Number of␣
 ↪features", marker='.')
plt.legend()
plt.xlabel('Correlation threshold')
plt.ylabel('Number of features')
plt.show()
```

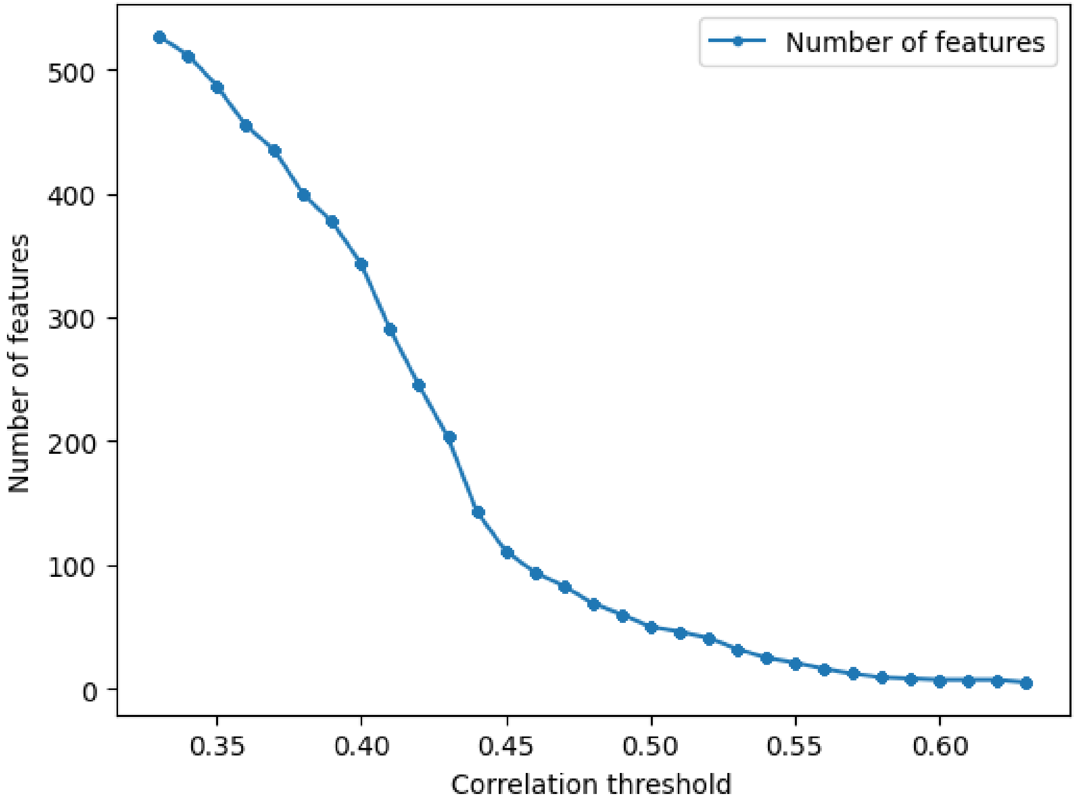


```
[ ]:
```

4 KNeighborsRegressor

```
[30]: without_standardization, train_r2_, test_r2_, hist1, hist2, target_column_name,␣
      ↪training_data_RMSE, test_data_RMSE  = pred_model.
      ↪prepare_data_and_create_model(molecular_descriptors_df=data,
                                                                               ␣
      ↪                    correlation_threshold=0.50,
                                                                               ␣
      ↪                    standardization=False,
                                                                               ␣
      ↪                    model_type='KNeighborsRegressor',
                                                                               ␣
      ↪                    target_column_name = target,
                                                                               ␣
      ↪                    random_state=random_state,
                                                                               ␣
      ↪                    train_test_split_=True,
```

```
↪                     verbose=True)
```

```
I am not doing standardization…
  molecular descriptor name
0                   AATS0Z
1                 AATS0are
2                   AATS0d
3                  AATS0dv
4                   AATS0i
  molecular descriptor name  corr_value
0                   AATS0Z    0.018677
1                 AATS0are   -0.341313
2                   AATS0d   -0.123443
3                  AATS0dv   -0.265670
4                   AATS0i   -0.428929
  molecular descriptor name  corr_value  absolute correlation value
0                   AATS0Z    0.018677                    0.018677
1                 AATS0are   -0.341313                    0.341313
2                   AATS0d   -0.123443                    0.123443
3                  AATS0dv   -0.265670                    0.265670
4                   AATS0i   -0.428929                    0.428929
    molecular descriptor name  corr_value  absolute correlation value
15                     AATS1i   -0.561620                    0.561620
113                   AATSC1c    0.565682                    0.565682
125                   AATSC2c   -0.543393                    0.543393
221                AETA_eta_R   -0.515778                    0.515778
224                    AMID_C    0.532458                    0.532458
    molecular descriptor name  corr_value  absolute correlation value
15                     AATS1i   -0.561620                    0.561620
113                   AATSC1c    0.565682                    0.565682
125                   AATSC2c   -0.543393                    0.543393
221                AETA_eta_R   -0.515778                    0.515778
224                    AMID_C    0.532458                    0.532458
The model used is: KNeighbors…
Return the coefficient of determination of the prediction:
0.005488987340492968
R^2 score: 0.5875467049770875
Correlation coefficient: 0.7665159522000097
Test data - unseen during training:
R^2 score: 0.005488987340492968
Correlation coefficient: 0.07408770033205896
[7.48268636 6.95799273 6.00648426 5.8661154  7.4119163  6.37290721
 6.70755163 6.02979336 5.77771093 6.07868985 5.73752796 6.4854175
 7.23551212 5.84727787 6.88998599 6.05037836]
8      6.159455
110    6.065502
69     5.552842
```

```
95     5.200659
11     7.250264
104    6.000000
52     6.823909
112    6.853872
101    5.000000
50     5.769551
100    6.136677
103    6.000000
21     6.398375
60     5.537602
84     7.002177
39     6.638272
Name: LoVo/DX, dtype: float64
Training Root Mean Square Error: 0.6156945408160687
Testing Root Mean Square Error: 0.6277039901858398
```

```
  File "C:\Users\aleks\anaconda3\envs\cheminf_gpu\lib\site-
packages\joblib\externals\loky\backend\context.py", line 217, in
_count_physical_cores
    raise ValueError(
```

[31]:
```
print(target_column_name+str('_transformed'))
print(hist1[target_column_name].hist())
```

```
LoVo/DX_transformed
AxesSubplot(0.125,0.11;0.775x0.77)
```

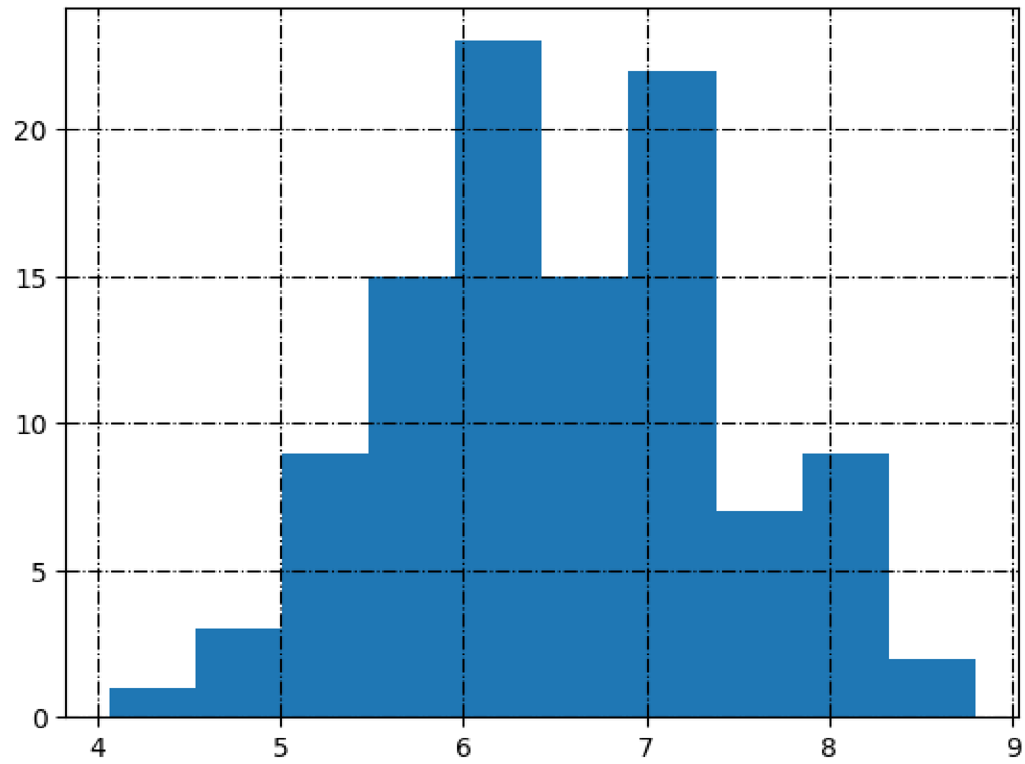

[32]:
```
with_standardization, train_r2_, test_r2_, hist1, hist2, target_column_name,␣
↪training_data_RMSE, test_data_RMSE  = pred_model.
↪prepare_data_and_create_model(molecular_descriptors_df=data,
                                                                            ␣
↪                        correlation_threshold=0.50,
                                                                            ␣
↪                        standardization=True,
                                                                            ␣
↪                        model_type='KNeighborsRegressor',
                                                                            ␣
↪                        target_column_name = target,
                                                                            ␣
↪                        random_state=random_state,
                                                                            ␣
↪                        train_test_split_=True,
                                                                            ␣
↪                        verbose=True)
```

```
I am doing standardization…
  molecular descriptor name
0                    AATS0Z
1                  AATS0are
```

```
2                   AATS0d
3                  AATS0dv
4                   AATS0i
  molecular descriptor name  corr_value
0                   AATS0Z    0.018677
1                 AATS0are   -0.341313
2                   AATS0d   -0.123443
3                  AATS0dv   -0.265670
4                   AATS0i   -0.428929
  molecular descriptor name  corr_value  absolute correlation value
0                   AATS0Z    0.018677                    0.018677
1                 AATS0are   -0.341313                    0.341313
2                   AATS0d   -0.123443                    0.123443
3                  AATS0dv   -0.265670                    0.265670
4                   AATS0i   -0.428929                    0.428929
    molecular descriptor name  corr_value  absolute correlation value
15                    AATS1i   -0.561620                    0.561620
113                  AATSC1c    0.565682                    0.565682
125                  AATSC2c   -0.543393                    0.543393
221               AETA_eta_R   -0.515778                    0.515778
224                   AMID_C    0.532458                    0.532458
    molecular descriptor name  corr_value  absolute correlation value
15                    AATS1i   -0.561620                    0.561620
113                  AATSC1c    0.565682                    0.565682
125                  AATSC2c   -0.543393                    0.543393
221               AETA_eta_R   -0.515778                    0.515778
224                   AMID_C    0.532458                    0.532458
The model used is: KNeighbors…
Return the coefficient of determination of the prediction:
0.005488987340492968
R^2 score: 0.5875467049770875
Correlation coefficient: 0.7665159522000097
Test data - unseen during training:
R^2 score: 0.005488987340492968
Correlation coefficient: 0.07408770033205896
[7.48268636 6.95799273 6.00648426 5.8661154  7.4119163  6.37290721
 6.70755163 6.02979336 5.77771093 6.07868985 5.73752796 6.4854175
 7.23551212 5.84727787 6.88998599 6.05037836]
8      6.159455
110    6.065502
69     5.552842
95     5.200659
11     7.250264
104    6.000000
52     6.823909
112    6.853872
101    5.000000
50     5.769551
```

```
100    6.136677
103    6.000000
21     6.398375
60     5.537602
84     7.002177
39     6.638272
Name: LoVo/DX, dtype: float64
Training Root Mean Square Error: 0.6156945408160687
Testing Root Mean Square Error: 0.6277039901858398
```

4.1 Search inside correlation space

[33]:
```python
step = 0.01
initial_step = corr_low
last_step = corr_high
first_list = [x / 100.0 for x in range(int(initial_step*100),␣
 ↪int(last_step*100), int(step*100))]
second_list = []
third_list = []
fourth_l = []
fifth_l = []
f_list = []
for i in first_list:
    without_standardization, train_r2, test_r2, _, h_, target_column_name,␣
 ↪training_data_RMSE, test_data_RMSE = pred_model.
 ↪prepare_data_and_create_model(molecular_descriptors_df = data,
                                                                            ␣
 ↪                      correlation_threshold = i,
                                                                            ␣
 ↪                      standardization = False,
                                                                            ␣
 ↪                      model_type = 'KNeighborsRegressor',
                                                                            ␣
 ↪                      target_column_name = target,
                                                                            ␣
 ↪                      random_state=random_state,
                                                                            ␣
 ↪                      train_test_split_ = True,
                                                                            ␣
 ↪                      verbose = False)
    second_list.append(train_r2)
    third_list.append(test_r2)
    fourth_l.append(training_data_RMSE)
    fifth_l.append(test_data_RMSE)
    f_list.append(len(h_))
```

[34]:
```
df_without_standardization = pd.DataFrame(data=first_list,␣
 ↪columns=["Correlation threshold"])
df_without_standardization['Training data R^2 score'] = second_list
df_without_standardization['Test data R^2 score'] = third_list
df_without_standardization['Training RMSE'] = fourth_l
df_without_standardization['Test RMSE'] = fifth_l
df_without_standardization['Number of features'] = f_list
```

[35]:
```
df_k_nearest = df_without_standardization.copy()
df_without_standardization
```

[35]:
```
    Correlation threshold  Training data R^2 score  Test data R^2 score  \
0                    0.33                 0.427920            -0.354151
1                    0.34                 0.393236            -0.610533
2                    0.35                 0.379229            -0.552617
3                    0.36                 0.374933            -0.728855
4                    0.37                 0.374933            -0.728855
5                    0.38                 0.454372            -0.467707
6                    0.39                 0.462046            -0.417494
7                    0.40                 0.480161            -0.515220
8                    0.41                 0.480910            -0.662761
9                    0.42                 0.466795            -0.542875
10                   0.43                 0.397237            -0.581741
11                   0.44                 0.400939            -0.200479
12                   0.45                 0.382070            -0.237902
13                   0.46                 0.414411            -0.345479
14                   0.47                 0.410059            -0.390075
15                   0.48                 0.593093            -0.182512
16                   0.49                 0.561020            -0.162826
17                   0.50                 0.587547             0.005489
18                   0.51                 0.534754             0.024275
19                   0.52                 0.542145             0.024275
20                   0.53                 0.603444             0.091865
21                   0.54                 0.623056             0.098882
22                   0.55                 0.631901             0.205681
23                   0.56                 0.674642             0.043061
24                   0.57                 0.666704             0.001119
25                   0.58                 0.666959            -0.241478
26                   0.59                 0.666648            -0.239143
27                   0.60                 0.670643            -0.094933
28                   0.61                 0.670643            -0.094933
29                   0.62                 0.670643            -0.094933
30                   0.63                 0.673705            -0.159111

    Training RMSE  Test RMSE  Number of features
0        0.725114   0.732460                 527
1        0.746771   0.798794                 512
```

2	0.755342	0.784300	487
3	0.757951	0.827617	455
4	0.757951	0.827617	435
5	0.708151	0.762553	399
6	0.703154	0.749395	377
7	0.691214	0.774797	343
8	0.690716	0.811643	290
9	0.700044	0.781836	245
10	0.744306	0.791622	203
11	0.742017	0.689648	143
12	0.753611	0.700315	111
13	0.733625	0.730110	94
14	0.736346	0.742112	83
15	0.611541	0.684468	69
16	0.635185	0.678746	60
17	0.615695	0.627704	50
18	0.653912	0.621747	46
19	0.648697	0.621747	41
20	0.603713	0.599826	32
21	0.588595	0.597504	25
22	0.581648	0.560980	21
23	0.546838	0.615733	16
24	0.553469	0.629081	12
25	0.553257	0.701326	9
26	0.553515	0.700666	8
27	0.550188	0.658633	7
28	0.550188	0.658633	7
29	0.550188	0.658633	7
30	0.547625	0.677661	5

4.2 Plots

[36]:
```
plt.plot(df_without_standardization['Correlation threshold'],␣
    ↪df_without_standardization['Training data R^2 score'], label = "Training␣
    ↪data R^2 score", marker='v')
plt.plot(df_without_standardization['Correlation threshold'],␣
    ↪df_without_standardization['Test data R^2 score'], label = "Test data R^2␣
    ↪score", marker='D')
plt.legend()
plt.xlabel('Correlation threshold')
plt.ylabel('R^2 score')
plt.ylim([0, 1])
plt.show()
```

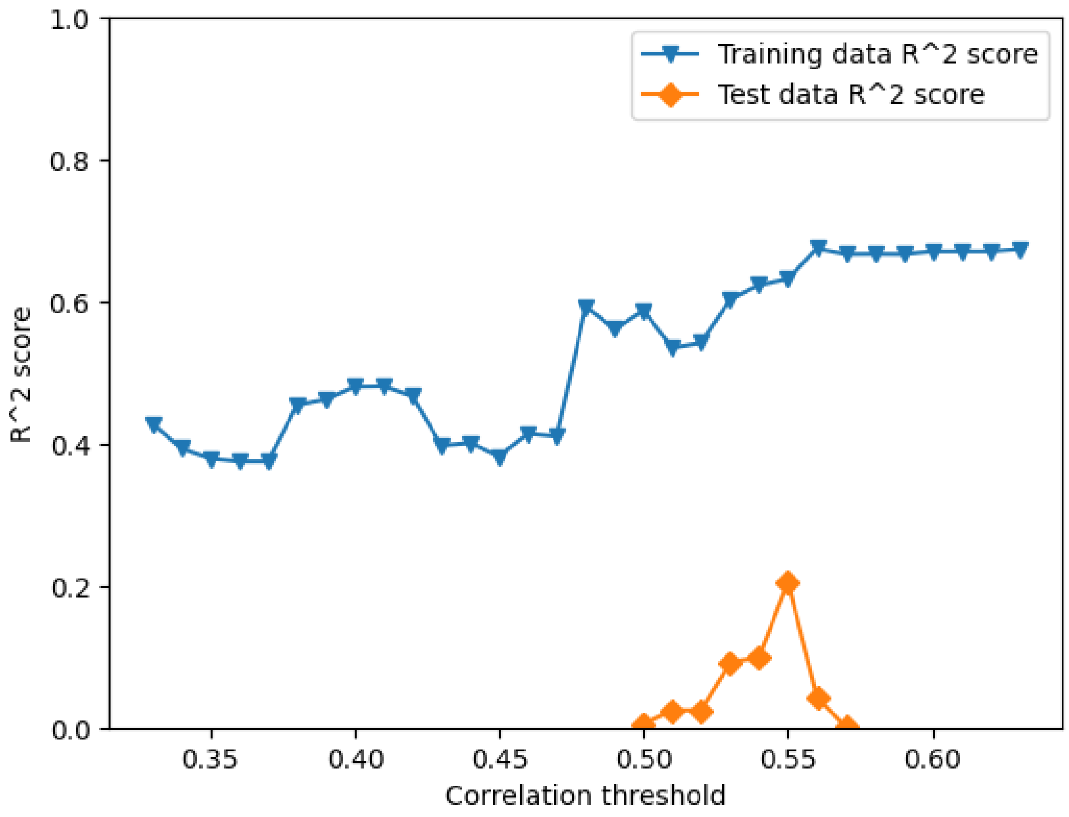

[37]:
```
plt.plot(df_without_standardization['Correlation threshold'],␣
↪df_without_standardization['Number of features'], label = "Number of␣
↪features", marker='.')
plt.legend()
plt.xlabel('Correlation threshold')
plt.ylabel('Number of features')
plt.show()
```

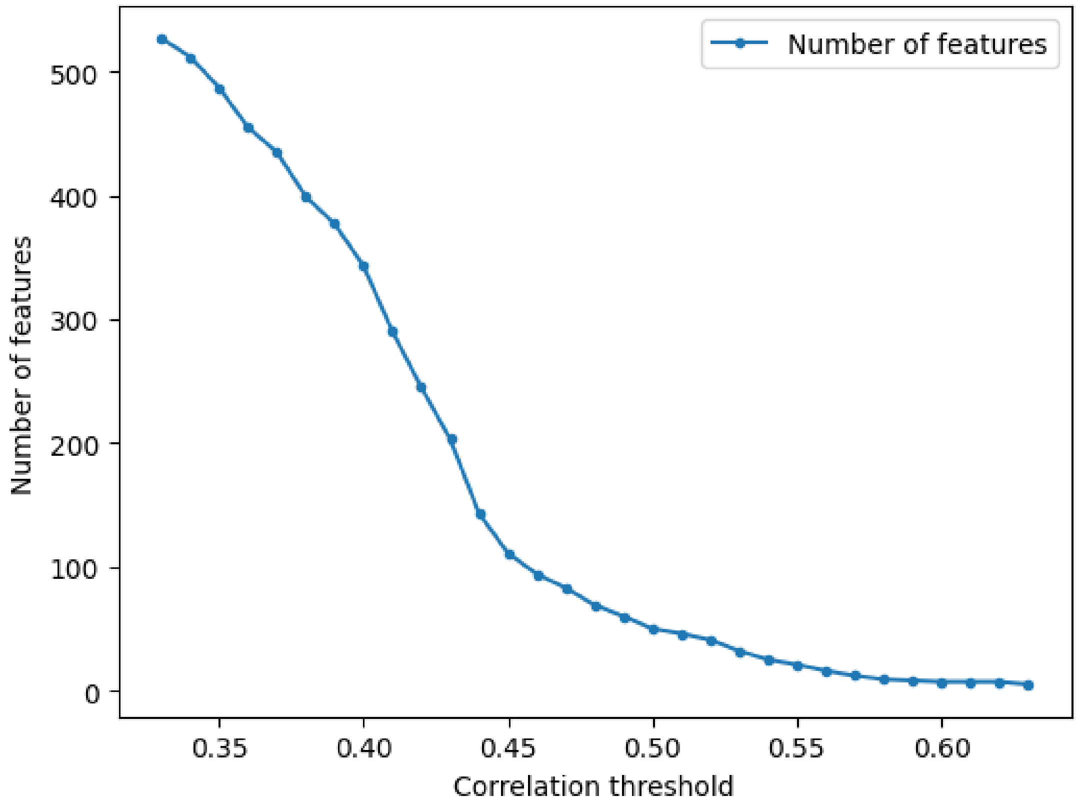

[ ]:

5 Support Vector Machines (SVM)

[38]:
```
without_standardization, train_r2_, test_r2_, hist1, hist2, target_column_name,␣
↪training_data_RMSE, test_data_RMSE  = pred_model.
↪prepare_data_and_create_model(molecular_descriptors_df=data,
                                                                              ␣
↪                        correlation_threshold=0.50,
                                                                              ␣
↪                        standardization=False,
                                                                              ␣
↪                        model_type='SVR',
                                                                              ␣
↪                        kernel_ = 'linear',
                                                                              ␣
↪                        gamma_ = 'auto',
                                                                              ␣
↪                        target_column_name = target,
```

```
↪                    random_state=random_state,
↪                    train_test_split_=True,
↪                    verbose=True)
```

```
I am not doing standardization…
  molecular descriptor name
0                    AATS0Z
1                  AATS0are
2                    AATS0d
3                   AATS0dv
4                    AATS0i
  molecular descriptor name  corr_value
0                    AATS0Z    0.018677
1                  AATS0are   -0.341313
2                    AATS0d   -0.123443
3                   AATS0dv   -0.265670
4                    AATS0i   -0.428929
  molecular descriptor name  corr_value  absolute correlation value
0                    AATS0Z    0.018677                    0.018677
1                  AATS0are   -0.341313                    0.341313
2                    AATS0d   -0.123443                    0.123443
3                   AATS0dv   -0.265670                    0.265670
4                    AATS0i   -0.428929                    0.428929
    molecular descriptor name  corr_value  absolute correlation value
15                     AATS1i   -0.561620                    0.561620
113                   AATSC1c    0.565682                    0.565682
125                   AATSC2c   -0.543393                    0.543393
221                AETA_eta_R   -0.515778                    0.515778
224                    AMID_C    0.532458                    0.532458
    molecular descriptor name  corr_value  absolute correlation value
15                     AATS1i   -0.561620                    0.561620
113                   AATSC1c    0.565682                    0.565682
125                   AATSC2c   -0.543393                    0.543393
221                AETA_eta_R   -0.515778                    0.515778
224                    AMID_C    0.532458                    0.532458
The model used is: SVR…
Return the coefficient of determination of the prediction:
-2.523256785292577
R^2 score: 0.4373249736417869
Correlation coefficient: 0.6613055070402687
Test data - unseen during training:
R^2 score: -2.523256785292577
Correlation coefficient: nan
[7.44854284 5.48027893 6.20963253 4.33885342 7.48930801 4.69193894
```

```
 6.77880036 3.46726043 4.49318457 6.99322789 5.63355736 5.47588549
 7.80390476 4.79277442 6.79565535 5.60811083]
8      6.159455
110    6.065502
69     5.552842
95     5.200659
11     7.250264
104    6.000000
52     6.823909
112    6.853872
101    5.000000
50     5.769551
100    6.136677
103    6.000000
21     6.398375
60     5.537602
84     7.002177
39     6.638272
Name: LoVo/DX, dtype: float64
Training Root Mean Square Error: 0.7191288863780737
Testing Root Mean Square Error: 1.1814687516044176
```

[39]:
```
print(target_column_name+str('_transformed'))
print(hist1[target_column_name].hist())
```

```
LoVo/DX_transformed
AxesSubplot(0.125,0.11;0.775x0.77)
```

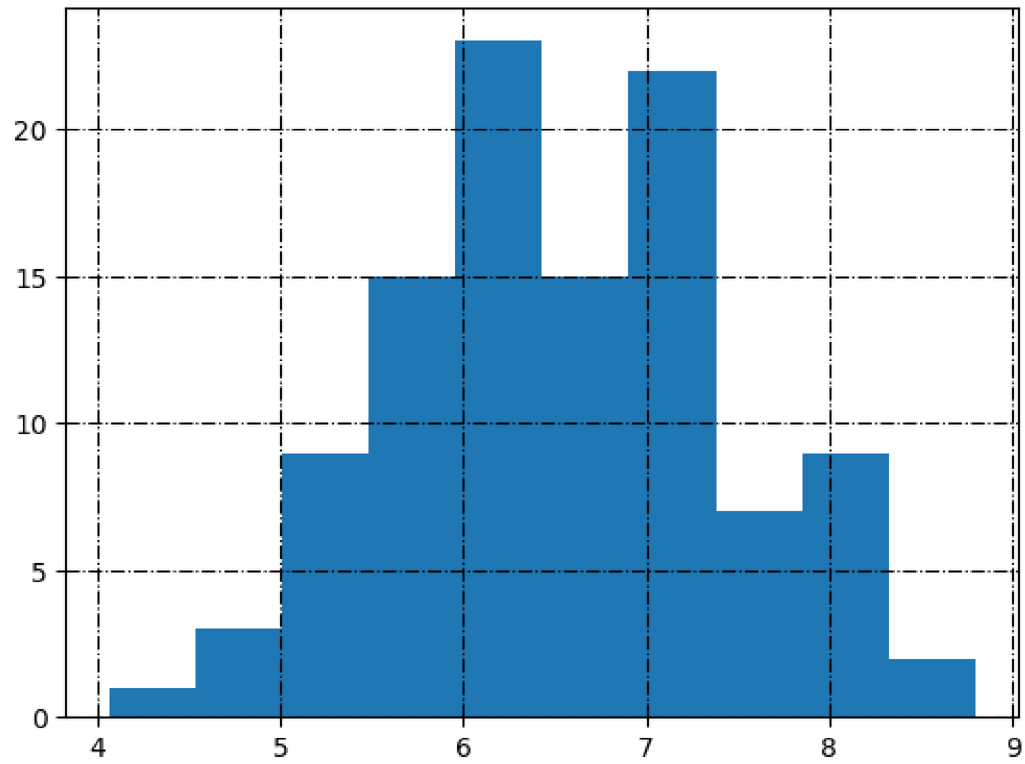

[40]:

```
with_standardization, train_r2_, test_r2_, hist1, hist2, target_column_name,␣
↪training_data_RMSE, test_data_RMSE  = pred_model.
↪prepare_data_and_create_model(molecular_descriptors_df=data,
                                                                                  ␣
↪                         correlation_threshold=0.50,
                                                                                  ␣
↪                         standardization=True,
                                                                                  ␣
↪                         model_type='SVR',
                                                                                  ␣
↪                         kernel_ = 'linear',
                                                                                  ␣
↪                         gamma_ = 'auto',
                                                                                  ␣
↪                         target_column_name = target,
                                                                                  ␣
↪                         random_state=random_state,
                                                                                  ␣
↪                         train_test_split_=True,
                                                                                  ␣
↪                         verbose=True)
```

```
I am doing standardization…
  molecular descriptor name
0                    AATS0Z
1                  AATS0are
2                    AATS0d
3                   AATS0dv
4                    AATS0i
  molecular descriptor name  corr_value
0                    AATS0Z    0.018677
1                  AATS0are   -0.341313
2                    AATS0d   -0.123443
3                   AATS0dv   -0.265670
4                    AATS0i   -0.428929
  molecular descriptor name  corr_value  absolute correlation value
0                    AATS0Z    0.018677                    0.018677
1                  AATS0are   -0.341313                    0.341313
2                    AATS0d   -0.123443                    0.123443
3                   AATS0dv   -0.265670                    0.265670
4                    AATS0i   -0.428929                    0.428929
    molecular descriptor name  corr_value  absolute correlation value
15                     AATS1i   -0.561620                    0.561620
113                   AATSC1c    0.565682                    0.565682
125                   AATSC2c   -0.543393                    0.543393
221                AETA_eta_R   -0.515778                    0.515778
224                    AMID_C    0.532458                    0.532458
    molecular descriptor name  corr_value  absolute correlation value
15                     AATS1i   -0.561620                    0.561620
113                   AATSC1c    0.565682                    0.565682
125                   AATSC2c   -0.543393                    0.543393
221                AETA_eta_R   -0.515778                    0.515778
224                    AMID_C    0.532458                    0.532458
The model used is: SVR…
Return the coefficient of determination of the prediction:
-2.523256785292577
R^2 score: 0.4373249736417869
Correlation coefficient: 0.6613055070402687
Test data - unseen during training:
R^2 score: -2.523256785292577
Correlation coefficient: nan
[7.44854284 5.48027893 6.20963253 4.33885342 7.48930801 4.69193894
 6.77880036 3.46726043 4.49318457 6.99322789 5.63355736 5.47588549
 7.80390476 4.79277442 6.79565535 5.60811083]
8      6.159455
110    6.065502
69     5.552842
95     5.200659
11     7.250264
104    6.000000
```

```
52     6.823909
112    6.853872
101    5.000000
50     5.769551
100    6.136677
103    6.000000
21     6.398375
60     5.537602
84     7.002177
39     6.638272
Name: LoVo/DX, dtype: float64
Training Root Mean Square Error: 0.7191288863780737
Testing Root Mean Square Error: 1.1814687516044176
```

5.1 Search inside correlation space

[41]:

```
step = 0.01
initial_step = corr_low
last_step = corr_high
first_list = [x / 100.0 for x in range(int(initial_step*100),␣
 ↪int(last_step*100), int(step*100))]
second_list = []
third_list = []
fourth_l = []
fifth_l = []
f_list = []
for i in first_list:
    without_standardization, train_r2, test_r2, _, h_, target_column_name,␣
 ↪training_data_RMSE, test_data_RMSE = pred_model.
 ↪prepare_data_and_create_model(molecular_descriptors_df = data,
                                                                            ␣
 ↪                    correlation_threshold = i,
                                                                            ␣
 ↪                    standardization = False,
                                                                            ␣
 ↪                    model_type = 'SVR',
                                                                            ␣
 ↪                    kernel_ = 'linear',
                                                                            ␣
 ↪                    gamma_ = 'auto',
                                                                            ␣
 ↪                    target_column_name = target,
                                                                            ␣
 ↪                    random_state=random_state,
                                                                            ␣
 ↪                    train_test_split_ = True,
```

```
                                verbose = False)
    second_list.append(train_r2)
    third_list.append(test_r2)
    fourth_l.append(training_data_RMSE)
    fifth_l.append(test_data_RMSE)
    f_list.append(len(h_))
```

[42]:
```
df_without_standardization = pd.DataFrame(data=first_list,
 columns=["Correlation threshold"])
df_without_standardization['Training data R^2 score'] = second_list
df_without_standardization['Test data R^2 score'] = third_list
df_without_standardization['Training RMSE'] = fourth_l
df_without_standardization['Test RMSE'] = fifth_l
df_without_standardization['Number of features'] = f_list
```

[43]:
```
df_svm = df_without_standardization.copy()
df_without_standardization
```

[43]:
```
    Correlation threshold  Training data R^2 score  Test data R^2 score  \
0                    0.33            -4.960511e+06        -1.795669e+07
1                    0.34            -4.592888e+06        -2.802811e+07
2                    0.35            -2.716580e+06        -1.402362e+07
3                    0.36            -1.101852e+06        -8.556822e+06
4                    0.37            -1.976878e+06        -7.256922e+06
5                    0.38            -7.107772e+05        -6.998006e+06
6                    0.39            -3.252029e+05        -1.124577e+06
7                    0.40            -7.844252e+05        -1.621615e+06
8                    0.41            -2.861919e+05        -1.117552e+06
9                    0.42            -1.170156e+04        -5.464849e+04
10                   0.43            -1.484455e+04        -3.463573e+04
11                   0.44            -7.682337e+03        -4.605611e+04
12                   0.45            -1.851702e+04        -3.703248e+04
13                   0.46            -2.978199e+03        -1.176859e+04
14                   0.47            -2.577781e+03        -6.833001e+03
15                   0.48            -2.392096e-01        -7.487001e+00
16                   0.49             2.651193e-01        -2.416547e+00
17                   0.50             4.373250e-01        -2.523257e+00
18                   0.51             6.085918e-01        -1.184967e+00
19                   0.52             4.782530e-01        -4.737919e-01
20                   0.53             6.339387e-01         2.596838e-01
21                   0.54             5.897526e-01        -1.577834e-01
22                   0.55             6.150073e-01         8.649615e-02
23                   0.56             5.926614e-01        -3.633828e-01
24                   0.57             5.822633e-01        -2.183953e-01
25                   0.58             5.622460e-01        -5.939987e-01
26                   0.59             5.591630e-01        -6.209359e-01
```

27	0.60	5.506998e-01	-4.375144e-01
28	0.61	5.506998e-01	-4.375144e-01
29	0.62	5.506998e-01	-4.375144e-01
30	0.63	5.261065e-01	-2.547599e-01

	Training RMSE	Test RMSE	Number of features
0	2135.212850	2667.247166	527
1	2054.569717	3332.322578	512
2	1580.117033	2357.111448	487
3	1006.328218	1841.222849	455
4	1347.932090	1695.611466	435
5	808.248633	1665.088300	399
6	546.708466	667.490194	377
7	849.090439	801.538002	343
8	512.870078	665.402160	290
9	103.709452	147.144200	245
10	116.808945	117.143590	203
11	84.033576	135.082296	143
12	130.459332	121.128789	111
13	52.327217	68.285852	94
14	48.683877	52.034048	83
15	1.067211	1.833695	69
16	0.821838	1.163439	60
17	0.719129	1.181469	50
18	0.599781	0.930406	46
19	0.692481	0.764132	41
20	0.580036	0.541575	32
21	0.614046	0.677273	25
22	0.594845	0.601596	21
23	0.611865	0.734952	16
24	0.619625	0.694775	12
25	0.634297	0.794683	9
26	0.636527	0.801370	8
27	0.642608	0.754668	7
28	0.642608	0.754668	7
29	0.642608	0.754668	7
30	0.659961	0.705067	5

5.2 Plots

[44]:

```
plt.plot(df_without_standardization['Correlation threshold'],␣
  ↪df_without_standardization['Training data R^2 score'], label = "Training␣
  ↪data R^2 score", marker='v')
plt.plot(df_without_standardization['Correlation threshold'],␣
  ↪df_without_standardization['Test data R^2 score'], label = "Test data R^2␣
  ↪score", marker='D')
plt.legend()
```

```
plt.xlabel('Correlation threshold')
plt.ylabel('R^2 score')
plt.ylim([0, 1])
plt.show()
```

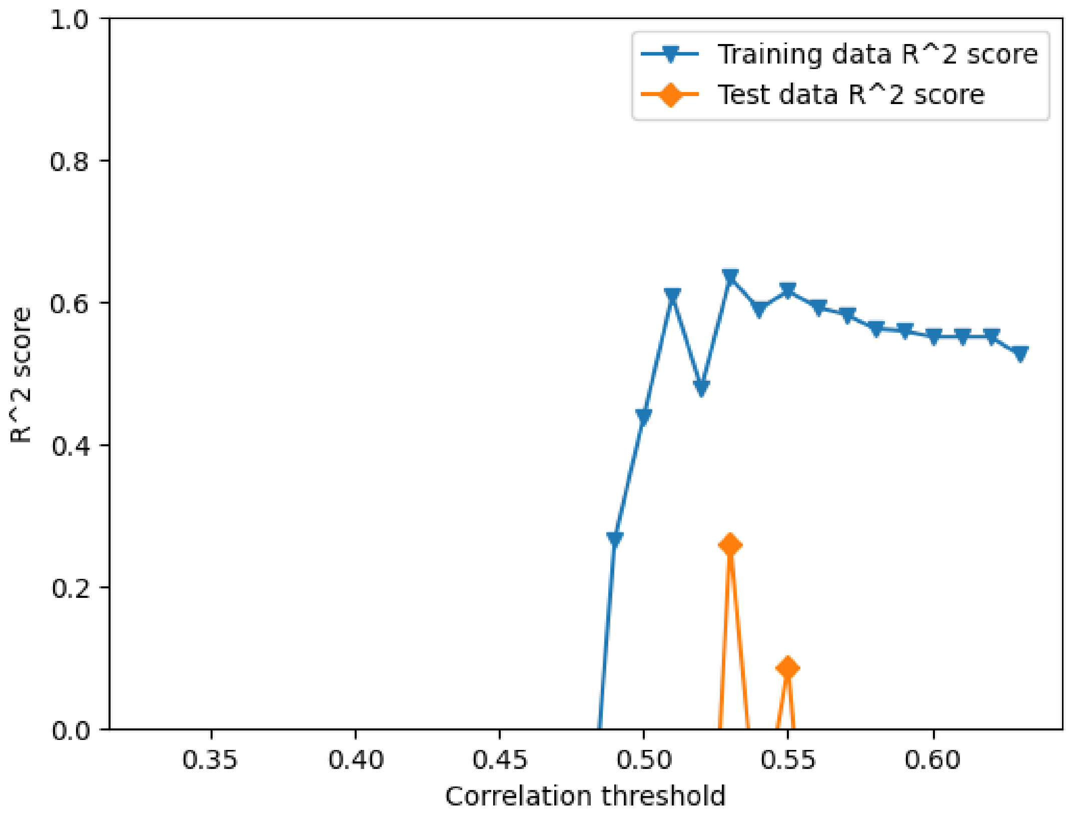

[45]:
```
plt.plot(df_without_standardization['Correlation threshold'],␣
↪df_without_standardization['Number of features'], label = "Number of␣
↪features", marker='.')
plt.legend()
plt.xlabel('Correlation threshold')
plt.ylabel('Number of features')
plt.show()
```

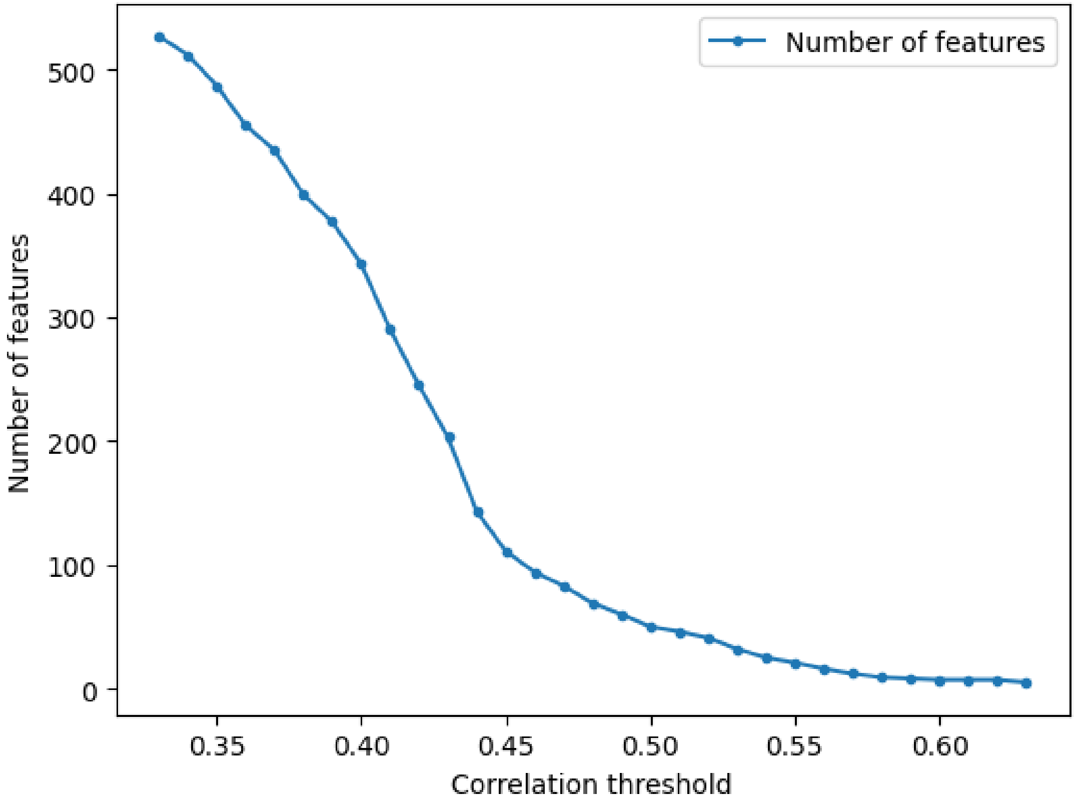

5.3 Store quality measurements

[46]:
```
with pd.ExcelWriter('../Data/Quality_'+'LoVo_DX'+'_'+str(random_state)+'_.
↪xlsx') as writer:
    df_linear.to_excel(writer, sheet_name='MLR')
    df_decision_tree.to_excel(writer, sheet_name='DT')
    df_random_forest.to_excel(writer, sheet_name='RF')
    df_k_nearest.to_excel(writer, sheet_name='KNN')
    df_svm.to_excel(writer, sheet_name='SVM')
```

Notebook

January 18, 2024

1 File 22

[1]:
```
import sys
from pathlib import Path
prediction_mode_path = Path("../module")
sys.path.append(prediction_mode_path.as_posix())
import models_creation as pred_model

import pandas as pd
import numpy as np

from sklearn.metrics import r2_score

import matplotlib.pyplot as plt
from sklearn.tree import plot_tree

from matplotlib import pyplot as plt

import joblib

import warnings
warnings.filterwarnings('ignore')
```

[2]:
```
load_prepared_data = pd.read_excel('../Data/Kolchicyna_machine_learning.xlsx')
load_prepared_data = load_prepared_data.drop(columns=['Unnamed: 0'])

target = 'LoVo/DX'

corr_low = 0.33
corr_high = 0.64

random_state = 28
```

[3]:
```
load_prepared_data.head()
```

[3]:
```
      AATS0Z  AATS0are    AATS0d   AATS0dv      AATS0i     AATS0m    AATS0p  \
0  25.090909  6.046518  3.109091  6.662626  160.775045  99.075564  1.555512
1  24.448276  6.008422  3.051724  6.386973  161.021674  96.473315  1.538471
```

```
2  23.868852  5.974074  3.000000  6.138434  161.244045  94.127025  1.523105
3  23.868852  5.974074  3.032787  6.171220  161.244045  94.127025  1.523105
4  23.343750  5.942945  2.953125  5.913194  161.445569  92.000700  1.509180

    AATS0pe    AATS0s   AATS0se  …  A549_float  MCF-7_float  LoVo_float  \
0  6.126680  3.435416  7.629859  …        10.8         10.3         6.5
1  6.088791  3.330998  7.596891  …        11.6         12.0         8.5
2  6.054630  3.236850  7.567166  …        10.9         12.2         8.8
3  6.054630  3.257798  7.567166  …        10.5         11.3         8.5
4  6.023670  3.151529  7.540228  …        89.5         92.7        52.8

   LoVo/DX_float  BALB/3T3_float  A549_float_transformed  \
0           54.9            10.2                7.966576
1           31.1            14.3                7.935542
2           17.9            11.7                7.962574
3           10.2            11.0                7.978811
4           77.8            99.4                7.048177

   MCF-7_float_transformed  LoVo_float_transformed  LoVo/DX_float_transformed  \
0                 7.987163                8.187087                   7.260428
1                 7.920819                8.070581                   7.507240
2                 7.913640                8.055517                   7.747147
3                 7.946922                8.070581                   7.991400
4                 7.032920                7.277366                   7.109020

   BALB/3T3_float_transformed
0                    7.991400
1                    7.844664
2                    7.931814
3                    7.958607
4                    7.002614

[5 rows x 1221 columns]
```

[4]:
```python
data = load_prepared_data[list(load_prepared_data.columns)[0:-10]]
data[target] = load_prepared_data[load_prepared_data.columns[-2]]
data.head()
```

[4]:
```
     AATS0Z  AATS0are   AATS0d   AATS0dv      AATS0i     AATS0m    AATS0p  \
0  25.090909  6.046518  3.109091  6.662626  160.775045  99.075564  1.555512
1  24.448276  6.008422  3.051724  6.386973  161.021674  96.473315  1.538471
2  23.868852  5.974074  3.000000  6.138434  161.244045  94.127025  1.523105
3  23.868852  5.974074  3.032787  6.171220  161.244045  94.127025  1.523105
4  23.343750  5.942945  2.953125  5.913194  161.445569  92.000700  1.509180

    AATS0pe    AATS0s   AATS0se  …    piPC10     piPC2     piPC3     piPC4  \
0  6.126680  3.435416  7.629859  …  7.999655  4.266195  4.915408  5.516700
```

```
1  6.088791  3.330998  7.596891  …  8.018726  4.280132  4.922714  5.520711
2  6.054630  3.236850  7.567166  …  8.028799  4.293878  4.929967  5.524706
3  6.054630  3.257798  7.567166  …  8.037440  4.307438  4.929967  5.524706
4  6.023670  3.151529  7.540228  …  8.034892  4.307438  4.937168  5.528685

      piPC5     piPC6     piPC7     piPC8     piPC9    LoVo/DX
0  6.098566  6.595759  6.979116  7.337313  7.681445  7.260428
1  6.103048  6.601209  6.985613  7.349442  7.695531  7.507240
2  6.105281  6.603923  6.989306  7.353932  7.704023  7.747147
3  6.107510  6.606629  6.992068  7.361425  7.709420  7.991400
4  6.107510  6.605277  6.991148  7.356489  7.707175  7.109020

[5 rows x 1212 columns]
```

[5]:
```python
print(data.shape)
data = data.dropna()
data.shape
```

```
(120, 1212)
```

[5]: (106, 1212)

2 Multiple Linear regression (MLR)

[6]:
```python
without_standardization, train_r2_, test_r2_, hist1, hist2, target_column_name,␣
↪training_data_RMSE, test_data_RMSE  = pred_model.
↪prepare_data_and_create_model(molecular_descriptors_df=data,
                                                                                    ␣
↪                          correlation_threshold=0.5,
                                                                                    ␣
↪                          standardization=False,
                                                                                    ␣
↪                          model_type='linear_model',
                                                                                    ␣
↪                          target_column_name = str(target),
                                                                                    ␣
↪                          random_state=random_state,
                                                                                    ␣
↪                          train_test_split_=True,
                                                                                    ␣
↪                          verbose=True)
```

```
I am not doing standardization…
  molecular descriptor name
0                    AATS0Z
1                  AATS0are
2                    AATS0d
```

```
3                    AATS0dv
4                     AATS0i
  molecular descriptor name  corr_value
0                     AATS0Z    0.018677
1                   AATS0are   -0.341313
2                     AATS0d   -0.123443
3                    AATS0dv   -0.265670
4                     AATS0i   -0.428929
  molecular descriptor name  corr_value  absolute correlation value
0                     AATS0Z    0.018677                    0.018677
1                   AATS0are   -0.341313                    0.341313
2                     AATS0d   -0.123443                    0.123443
3                    AATS0dv   -0.265670                    0.265670
4                     AATS0i   -0.428929                    0.428929
    molecular descriptor name  corr_value  absolute correlation value
15                     AATS1i   -0.561620                    0.561620
113                   AATSC1c    0.565682                    0.565682
125                   AATSC2c   -0.543393                    0.543393
221                AETA_eta_R   -0.515778                    0.515778
224                    AMID_C    0.532458                    0.532458
    molecular descriptor name  corr_value  absolute correlation value
15                     AATS1i   -0.561620                    0.561620
113                   AATSC1c    0.565682                    0.565682
125                   AATSC2c   -0.543393                    0.543393
221                AETA_eta_R   -0.515778                    0.515778
224                    AMID_C    0.532458                    0.532458
The model used is: LinearReg…
Return the coefficient of determination of the prediction:
0.38194140242409325
R^2 score: 0.913512128719289
Correlation coefficient: 0.9557782842894522
Test data - unseen during training:
R^2 score: 0.38194140242409325
Correlation coefficient: 0.6180140794707619
[7.97579884 7.0905308  5.36603678 5.89422187 7.28795085 7.20347708
 6.59870244 6.26151974 7.24574584 5.50629637 5.58010257 5.56370147
 6.57097459 6.86947166 7.10432563 7.53364675]
0      7.260428
21     6.398375
106    5.853872
68     6.080922
44     7.638272
14     7.067526
108    6.154902
62     6.455932
13     7.158641
9      5.702436
96     5.136677
```

```
49     5.619789
54     7.075721
114    6.026872
115    5.568636
87     7.288193
Name: LoVo/DX, dtype: float64
Training Root Mean Square Error: 0.282485586306591 93
Testing Root Mean Square Error: 0.5738467398009358
```

[7]:
```
print(target_column_name+str('_transformed'))
print(hist1[target_column_name].hist())
```

```
LoVo/DX_transformed
AxesSubplot(0.125,0.11;0.775x0.77)
```

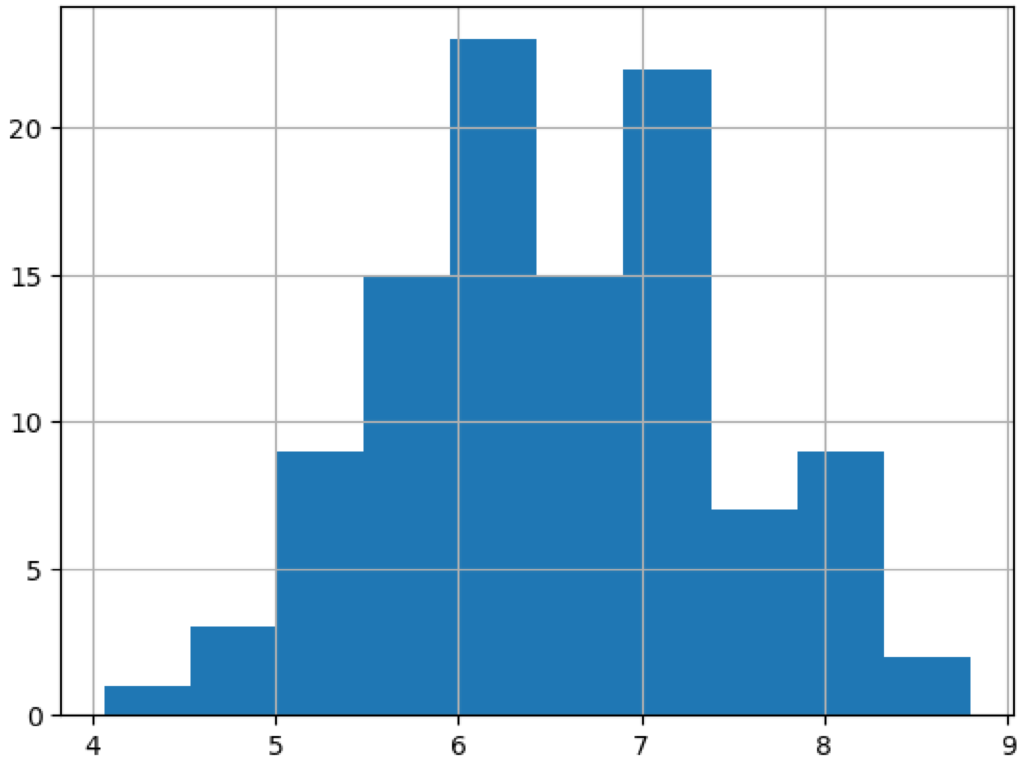

[8]:
```
with_standardization, train_r2_, test_r2_, hist1, hist2, target_column_name,␣
↪training_data_RMSE, test_data_RMSE  = pred_model.
↪prepare_data_and_create_model(molecular_descriptors_df=data,
                                                                                ␣
↪                      correlation_threshold=0.50,
                                                                                ␣
↪                      standardization=True,
```

```
↪                    model_type='linear_model',
↪                    target_column_name = target,
↪                    random_state=random_state,
↪                    train_test_split_=True,
↪                    verbose=True)
```

```
I am doing standardization…
  molecular descriptor name
0                    AATS0Z
1                  AATS0are
2                    AATS0d
3                   AATS0dv
4                    AATS0i
  molecular descriptor name  corr_value
0                    AATS0Z    0.018677
1                  AATS0are   -0.341313
2                    AATS0d   -0.123443
3                   AATS0dv   -0.265670
4                    AATS0i   -0.428929
  molecular descriptor name  corr_value  absolute correlation value
0                    AATS0Z    0.018677                    0.018677
1                  AATS0are   -0.341313                    0.341313
2                    AATS0d   -0.123443                    0.123443
3                   AATS0dv   -0.265670                    0.265670
4                    AATS0i   -0.428929                    0.428929
    molecular descriptor name  corr_value  absolute correlation value
15                     AATS1i   -0.561620                    0.561620
113                   AATSC1c    0.565682                    0.565682
125                   AATSC2c   -0.543393                    0.543393
221                AETA_eta_R   -0.515778                    0.515778
224                    AMID_C    0.532458                    0.532458
    molecular descriptor name  corr_value  absolute correlation value
15                     AATS1i   -0.561620                    0.561620
113                   AATSC1c    0.565682                    0.565682
125                   AATSC2c   -0.543393                    0.543393
221                AETA_eta_R   -0.515778                    0.515778
224                    AMID_C    0.532458                    0.532458
The model used is: LinearReg…
Return the coefficient of determination of the prediction:
0.38194140242409325
R^2 score: 0.913512128719289
Correlation coefficient: 0.9557782842894522
```

```
Test data - unseen during training:
R^2 score: 0.38194140242409325
Correlation coefficient: 0.6180140794707619
[7.97579884 7.0905308  5.36603678 5.89422187 7.28795085 7.20347708
 6.59870244 6.26151974 7.24574584 5.50629637 5.58010257 5.56370147
 6.57097459 6.86947166 7.10432563 7.53364675]
0      7.260428
21     6.398375
106    5.853872
68     6.080922
44     7.638272
14     7.067526
108    6.154902
62     6.455932
13     7.158641
9      5.702436
96     5.136677
49     5.619789
54     7.075721
114    6.026872
115    5.568636
87     7.288193
Name: LoVo/DX, dtype: float64
Training Root Mean Square Error: 0.28248558630659193
Testing Root Mean Square Error: 0.5738467398009358
```

2.1 Search inside correlation space

[9]:
```
step = 0.01
initial_step = corr_low
last_step = corr_high
first_list = [x / 100.0 for x in range(int(initial_step*100),
 ↪int(last_step*100), int(step*100))]
second_list = []
third_list = []
fourth_l = []
fifth_l = []
f_list = []
for i in first_list:
    without_standardization, train_r2, test_r2, _, h_, target_column_name,
 ↪training_data_RMSE, test_data_RMSE = pred_model.
 ↪prepare_data_and_create_model(molecular_descriptors_df = data,

 ↪                    correlation_threshold = i,

 ↪                    standardization = False,
```

```
↪                   model_type = 'linear_model',
↪                   target_column_name = target,
↪                   random_state=random_state,
↪                   train_test_split_ = True,
↪                   verbose = False)
    second_list.append(train_r2)
    third_list.append(test_r2)
    fourth_l.append(training_data_RMSE)
    fifth_l.append(test_data_RMSE)
    f_list.append(len(h_))
```

[10]:
```
df_without_standardization = pd.DataFrame(data=first_list,␣
↪columns=["Correlation threshold"])
df_without_standardization['Training data R^2 score'] = second_list
df_without_standardization['Test data R^2 score'] = third_list
df_without_standardization['Training RMSE'] = fourth_l
df_without_standardization['Test RMSE'] = fifth_l
df_without_standardization['Number of features'] = f_list
```

[11]:
```
df_linear = df_without_standardization.copy()
df_without_standardization
```

[11]:

	Correlation threshold	Training data R^2 score	Test data R^2 score \
0	0.33	0.999995	-7.717586
1	0.34	0.999995	-17.705554
2	0.35	0.999995	-12.720189
3	0.36	0.999995	-12.664929
4	0.37	0.999995	-20.965912
5	0.38	0.999995	-9.069472
6	0.39	0.999995	-21.678945
7	0.40	0.999995	-55.826234
8	0.41	0.999995	-32.687912
9	0.42	0.999995	-58.812134
10	0.43	0.999995	-52.472414
11	0.44	0.999995	-47.502059
12	0.45	0.999995	-166.634213
13	0.46	0.999995	-181.086352
14	0.47	0.993457	-18.140887
15	0.48	0.977801	-1.079957
16	0.49	0.970173	-1.786014
17	0.50	0.913512	0.381941
18	0.51	0.908500	0.456065

19	0.52	0.902100	0.436965
20	0.53	0.882822	0.413697
21	0.54	0.798473	0.274696
22	0.55	0.774569	0.328369
23	0.56	0.766363	0.375148
24	0.57	0.718837	0.351858
25	0.58	0.699977	0.364561
26	0.59	0.699240	0.346213
27	0.60	0.699234	0.349604
28	0.61	0.699234	0.349604
29	0.62	0.699234	0.349604
30	0.63	0.557435	0.094863

	Training RMSE	Test RMSE	Number of features
0	0.002233	2.155158	527
1	0.002233	3.156940	512
2	0.002233	2.703716	487
3	0.002233	2.698266	455
4	0.002233	3.421021	435
5	0.002233	2.316245	399
6	0.002233	3.476102	377
7	0.002233	5.502443	343
8	0.002233	4.236607	290
9	0.002233	5.645153	245
10	0.002233	5.337600	203
11	0.002233	5.083481	143
12	0.002233	9.450666	111
13	0.002233	9.849627	94
14	0.077700	3.193465	83
15	0.143115	1.052709	69
16	0.165892	1.218352	60
17	0.282486	0.573847	50
18	0.290556	0.538337	46
19	0.300545	0.547708	41
20	0.328807	0.558910	32
21	0.431206	0.621643	25
22	0.456063	0.598200	21
23	0.464290	0.576992	16
24	0.509328	0.587646	12
25	0.526133	0.581860	9
26	0.526779	0.590200	8
27	0.526785	0.588668	7
28	0.526785	0.588668	7
29	0.526785	0.588668	7
30	0.639009	0.694446	5

```
[ ]:
```

2.2 Plots

[12]:
```
plt.plot(df_without_standardization['Correlation threshold'],␣
 ↪df_without_standardization['Training data R^2 score'], label = "Training␣
 ↪data R^2 score", marker='v')
plt.plot(df_without_standardization['Correlation threshold'],␣
 ↪df_without_standardization['Test data R^2 score'], label = "Test data R^2␣
 ↪score", marker='D')
plt.legend()
plt.xlabel('Correlation threshold')
plt.ylabel('R^2 score')
plt.ylim([0, 1])
plt.show()
```

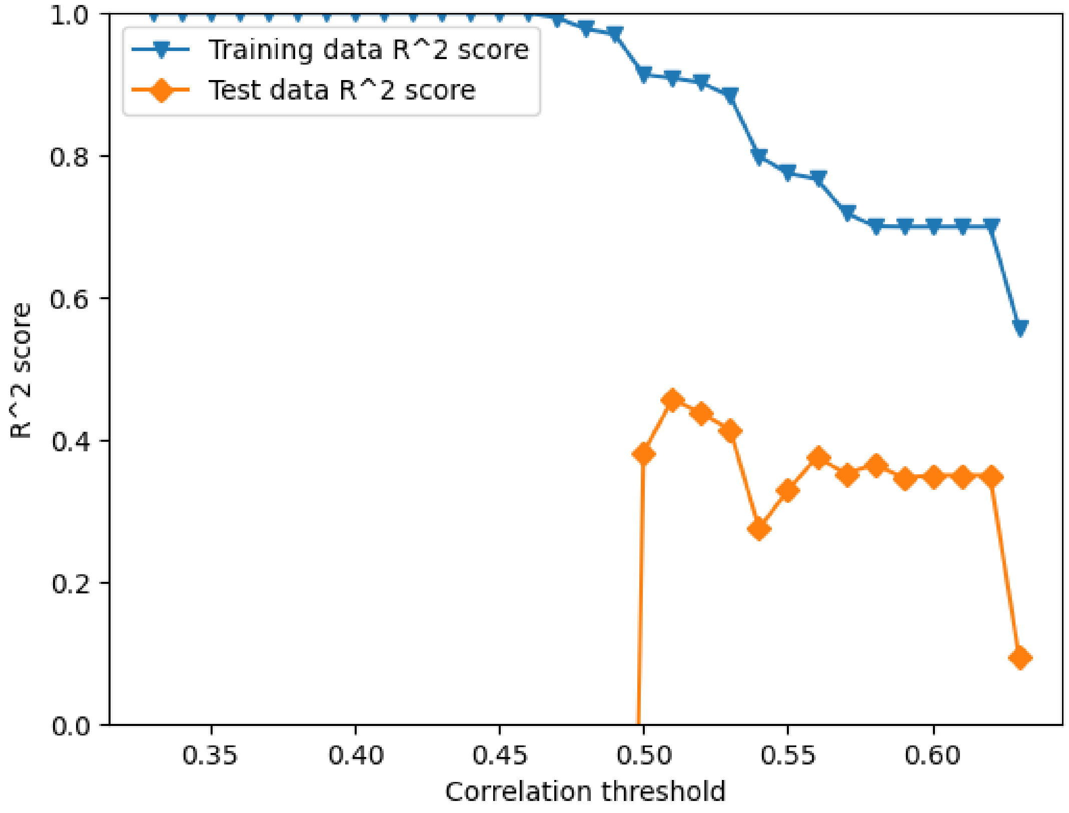

[13]:
```
plt.plot(df_without_standardization['Correlation threshold'],␣
 ↪df_without_standardization['Number of features'], label = "Number of␣
 ↪features", marker='.')
plt.legend()
plt.xlabel('Correlation threshold')
plt.ylabel('Number of features')
plt.show()
```

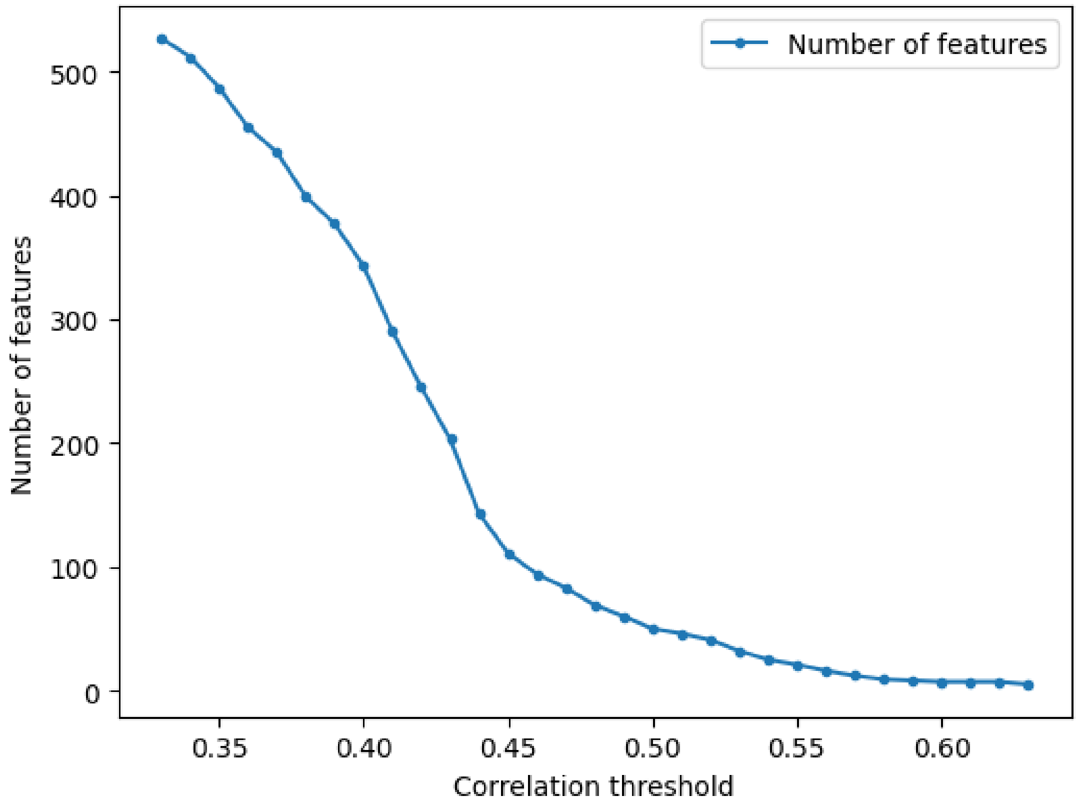


```
[ ]:
```

2.3 Decision Tree

```
[14]: without_standardization, train_r2_, test_r2_, hist1, hist2, target_column_name,␣
      ↪training_data_RMSE, test_data_RMSE  = pred_model.
      ↪prepare_data_and_create_model(molecular_descriptors_df=data,
                                                                                  ␣
      ↪                         correlation_threshold=0.50,
                                                                                  ␣
      ↪                         standardization=False,
                                                                                  ␣
      ↪                         model_type='DecisionTreeRegressor',
                                                                                  ␣
      ↪                         max_depth=5,
                                                                                  ␣
      ↪                         target_column_name = target,
                                                                                  ␣
      ↪                         random_state=random_state,
```

```
↪                       train_test_split_=True,
↪                       verbose=True)
```

```
I am not doing standardization…
  molecular descriptor name
0                    AATS0Z
1                  AATS0are
2                    AATS0d
3                   AATS0dv
4                    AATS0i
  molecular descriptor name  corr_value
0                    AATS0Z    0.018677
1                  AATS0are   -0.341313
2                    AATS0d   -0.123443
3                   AATS0dv   -0.265670
4                    AATS0i   -0.428929
  molecular descriptor name  corr_value  absolute correlation value
0                    AATS0Z    0.018677                    0.018677
1                  AATS0are   -0.341313                    0.341313
2                    AATS0d   -0.123443                    0.123443
3                   AATS0dv   -0.265670                    0.265670
4                    AATS0i   -0.428929                    0.428929
    molecular descriptor name  corr_value  absolute correlation value
15                     AATS1i   -0.561620                    0.561620
113                  AATSC1c    0.565682                    0.565682
125                  AATSC2c   -0.543393                    0.543393
221               AETA_eta_R   -0.515778                    0.515778
224                   AMID_C    0.532458                    0.532458
    molecular descriptor name  corr_value  absolute correlation value
15                     AATS1i   -0.561620                    0.561620
113                  AATSC1c    0.565682                    0.565682
125                  AATSC2c   -0.543393                    0.543393
221               AETA_eta_R   -0.515778                    0.515778
224                   AMID_C    0.532458                    0.532458
The model used is: DecisionTree…
Return the coefficient of determination of the prediction:
-0.23724984274096572
R^2 score: 0.9282999207105779
Correlation coefficient: 0.9634832228485236
Test data - unseen during training:
R^2 score: -0.23724984274096572
Correlation coefficient: nan
[7.68211241 6.87506153 5.64717667 6.64290682 6.87506153 7.37986395
 7.37986395 6.04720533 7.37986395 6.87506153 5.64717667 5.64717667
 6.64290682 7.37986395 7.37986395 7.96066227]
0      7.260428
```

```
21     6.398375
106    5.853872
68     6.080922
44     7.638272
14     7.067526
108    6.154902
62     6.455932
13     7.158641
9      5.702436
96     5.136677
49     5.619789
54     7.075721
114    6.026872
115    5.568636
87     7.288193
Name: LoVo/DX, dtype: float64
Training Root Mean Square Error: 0.2572044670564493
Testing Root Mean Square Error: 0.8119135627476753
```

[15]:
```
print(target_column_name+str('_transformed'))
print(hist1[target_column_name].hist())
```

```
LoVo/DX_transformed
AxesSubplot(0.125,0.11;0.775x0.77)
```

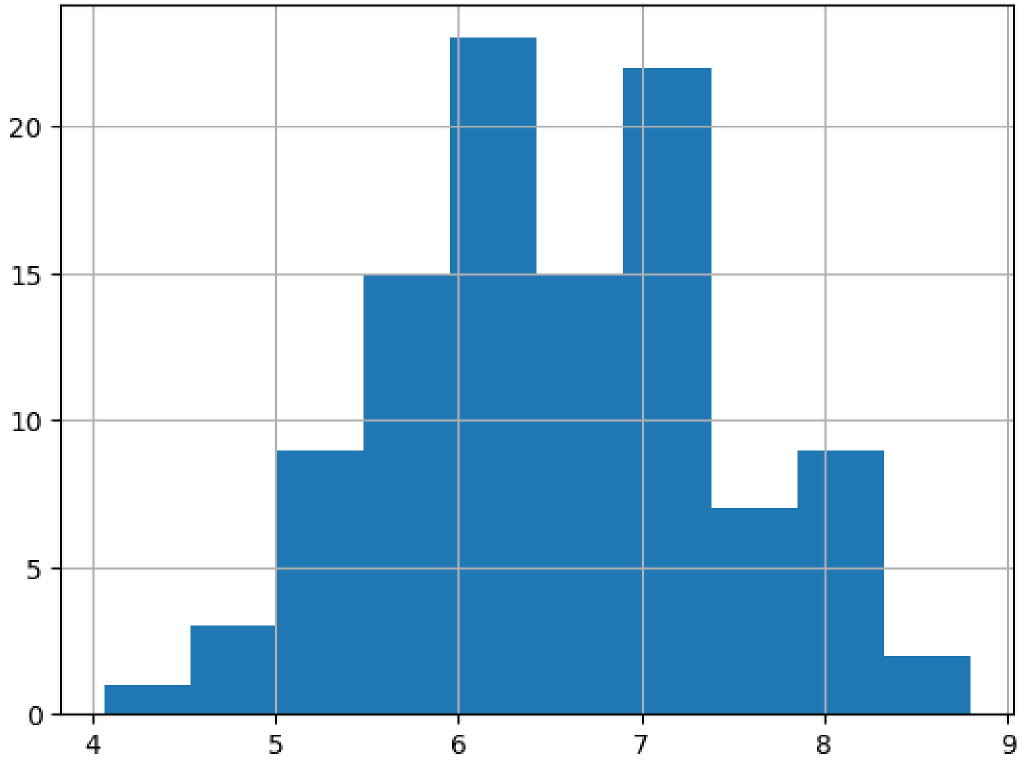

[16]:
```
with_standardization, train_r2_, test_r2_, hist1, hist2, target_column_name,␣
↪training_data_RMSE, test_data_RMSE  = pred_model.
↪prepare_data_and_create_model(molecular_descriptors_df=data,
                                                                              ␣
↪                    correlation_threshold=0.50,
                                                                              ␣
↪                    standardization=True,
                                                                              ␣
↪                    model_type='DecisionTreeRegressor',
                                                                              ␣
↪                    max_depth=5,
                                                                              ␣
↪                    target_column_name = target,
                                                                              ␣
↪                    random_state=random_state,
                                                                              ␣
↪                    train_test_split_=True,
                                                                              ␣
↪                    verbose=True)
```

```
I am doing standardization…
  molecular descriptor name
0                   AATS0Z
1                 AATS0are
2                   AATS0d
3                  AATS0dv
4                   AATS0i
  molecular descriptor name  corr_value
0                   AATS0Z    0.018677
1                 AATS0are   -0.341313
2                   AATS0d   -0.123443
3                  AATS0dv   -0.265670
4                   AATS0i   -0.428929
  molecular descriptor name  corr_value  absolute correlation value
0                   AATS0Z    0.018677                    0.018677
1                 AATS0are   -0.341313                    0.341313
2                   AATS0d   -0.123443                    0.123443
3                  AATS0dv   -0.265670                    0.265670
4                   AATS0i   -0.428929                    0.428929
    molecular descriptor name  corr_value  absolute correlation value
15                    AATS1i   -0.561620                    0.561620
113                  AATSC1c    0.565682                    0.565682
125                  AATSC2c   -0.543393                    0.543393
221                 AETA_eta_R   -0.515778                    0.515778
224                   AMID_C    0.532458                    0.532458
```

```
    molecular descriptor name  corr_value  absolute correlation value
15                     AATS1i   -0.561620                    0.561620
113                   AATSC1c    0.565682                    0.565682
125                   AATSC2c   -0.543393                    0.543393
221                AETA_eta_R   -0.515778                    0.515778
224                    AMID_C    0.532458                    0.532458
The model used is: DecisionTree…
Return the coefficient of determination of the prediction:
-0.23724984274096572
R^2 score: 0.9282999207105779
Correlation coefficient: 0.9634832228485236
Test data - unseen during training:
R^2 score: -0.23724984274096572
Correlation coefficient: nan
[7.68211241 6.87506153 5.64717667 6.64290682 6.87506153 7.37986395
 7.37986395 6.04720533 7.37986395 6.87506153 5.64717667 5.64717667
 6.64290682 7.37986395 7.37986395 7.96066227]
0      7.260428
21     6.398375
106    5.853872
68     6.080922
44     7.638272
14     7.067526
108    6.154902
62     6.455932
13     7.158641
9      5.702436
96     5.136677
49     5.619789
54     7.075721
114    6.026872
115    5.568636
87     7.288193
Name: LoVo/DX, dtype: float64
Training Root Mean Square Error: 0.2572044670564493
Testing Root Mean Square Error: 0.8119135627476753
```

2.4 Search inside correlation space

[17]:
```
step = 0.01
initial_step = corr_low
last_step = corr_high
first_list = [x / 100.0 for x in range(int(initial_step*100),␣
 ↪int(last_step*100), int(step*100))]
max_depth = [range(2, 30, 1)]
corr_th = []
second_list = []
```

```
third_list = []
fourth_l = []
fifth_l = []
f_list = []
fif_list = []
for i in first_list:
    for depth in max_depth[0]:

        without_standardization, train_r2, test_r2, _, h_, target_column_name,␣
 ↪training_data_RMSE, test_data_RMSE = pred_model.
 ↪prepare_data_and_create_model(molecular_descriptors_df=data,
                                                                              ␣
 ↪                      correlation_threshold=i,
                                                                              ␣
 ↪                      standardization=False,
                                                                              ␣
 ↪                      model_type='DecisionTreeRegressor',
                                                                              ␣
 ↪                      max_depth=depth,
                                                                              ␣
 ↪                      target_column_name = target,
                                                                              ␣
 ↪                      random_state=random_state,
                                                                              ␣
 ↪                      train_test_split_=True,
                                                                              ␣
 ↪                      verbose=False)
        corr_th.append(i)
        second_list.append(train_r2)
        third_list.append(test_r2)
        fourth_l.append(training_data_RMSE)
        fifth_l.append(test_data_RMSE)
        f_list.append(len(h_))
        fif_list.append(depth)
```

[18]:
```
df_without_standardization = pd.DataFrame(data=corr_th, columns=["Correlation␣
 ↪threshold"])
df_without_standardization['Training data R^2 score'] = second_list
df_without_standardization['Test data R^2 score'] = third_list
df_without_standardization['Training RMSE'] = fourth_l
df_without_standardization['Test RMSE'] = fifth_l
df_without_standardization['Number of features'] = f_list
df_without_standardization['Depth number'] = fif_list
```

[19]:
```
df_decision_tree = df_without_standardization.copy()
```

```
#df_without_standardization.to_excel('../Data/
↪A549_Decision_tree_rs_'+str(random_state)+'.xlsx')
```

2.5 Plots

[20]:
```
corre_list = [x / 100.0 for x in range(int(initial_step*100),␣
↪int(last_step*100), int(0.01*100))]
for element in corre_list:
    element_ =␣
↪df_without_standardization[df_without_standardization['Correlation␣
↪threshold'] == float(element)]
    plt.plot(element_['Depth number'], element_['Training data R^2 score'],␣
↪label = "Training data R^2 score", marker='v')
    plt.plot(element_['Depth number'], element_['Test data R^2 score'], label =␣
↪"Test data R^2 score", marker='D')
    plt.legend()
    plt.xlabel('Depth number -> correlation threshold: '+str(element)+'. \n␣
↪Number of features: '+str(element_['Number of features'].iloc[0]),␣
↪fontweight='bold')
    plt.xlim(max(element_['Depth number'])+1, min(element_['Depth number'])-1)
    plt.ylabel('R^2 score')
    plt.ylim([0, 1])
    plt.rc('grid', linestyle="-.", color='black')
    plt.grid(True)
    plt.show()
```

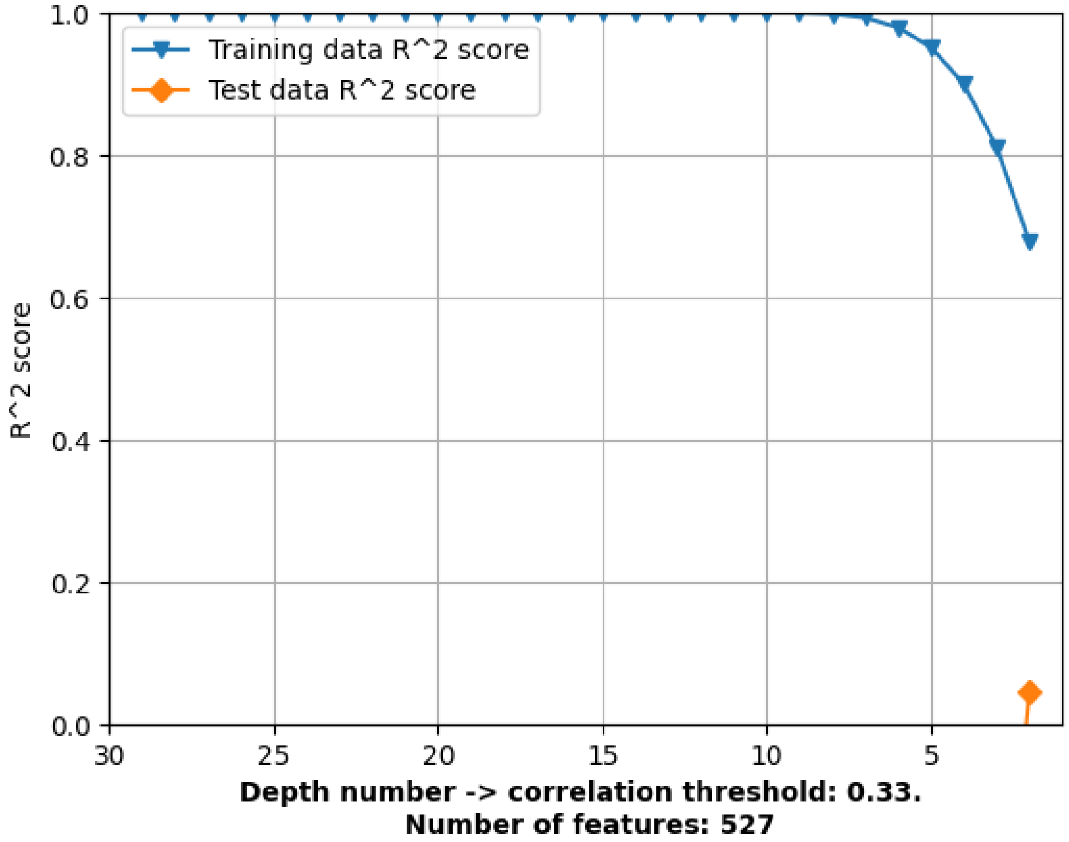
1.0
0.8
0.6
0.4
0.2
0.0
R^2 score
Training data R^2 score
Test data R^2 score
30
25
20
15
10
5
Depth number -> correlation threshold: 0.33.
Number of features: 527

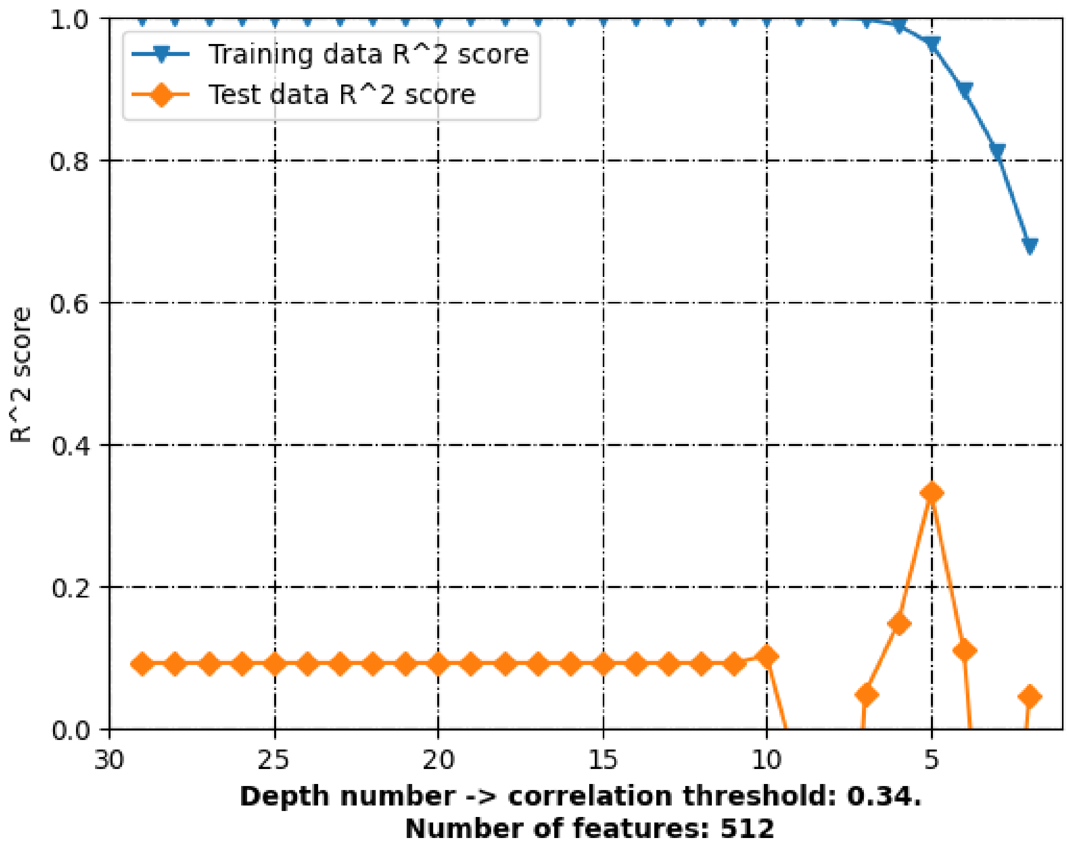

Training data R^2 score
Test data R^2 score
R^2 score
1.0
0.8
0.6
0.4
0.2
0.0
30
25
20
15
10
5
Depth number -> correlation threshold: 0.34.
Number of features: 512

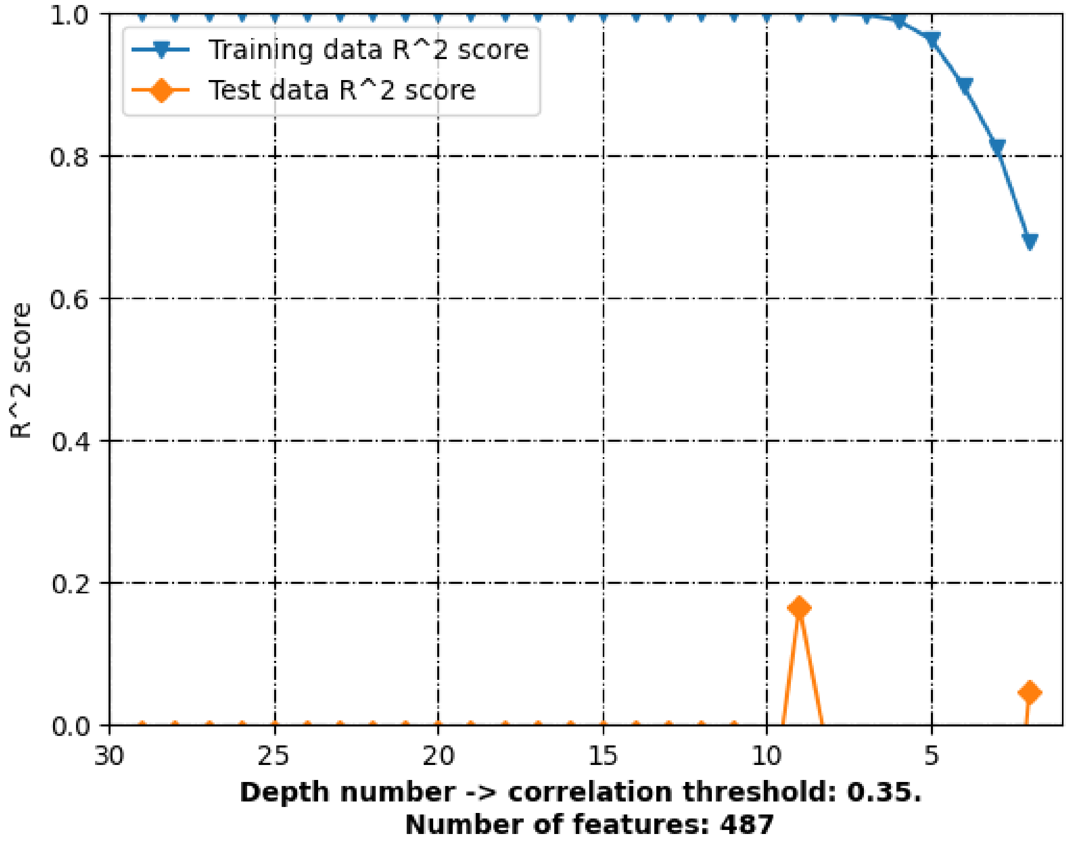

1.0
0.8
0.6
0.4
0.2
0.0
R^2 score
Training data R^2 score
Test data R^2 score
30
25
20
15
10
5
Depth number -> correlation threshold: 0.35.
Number of features: 487

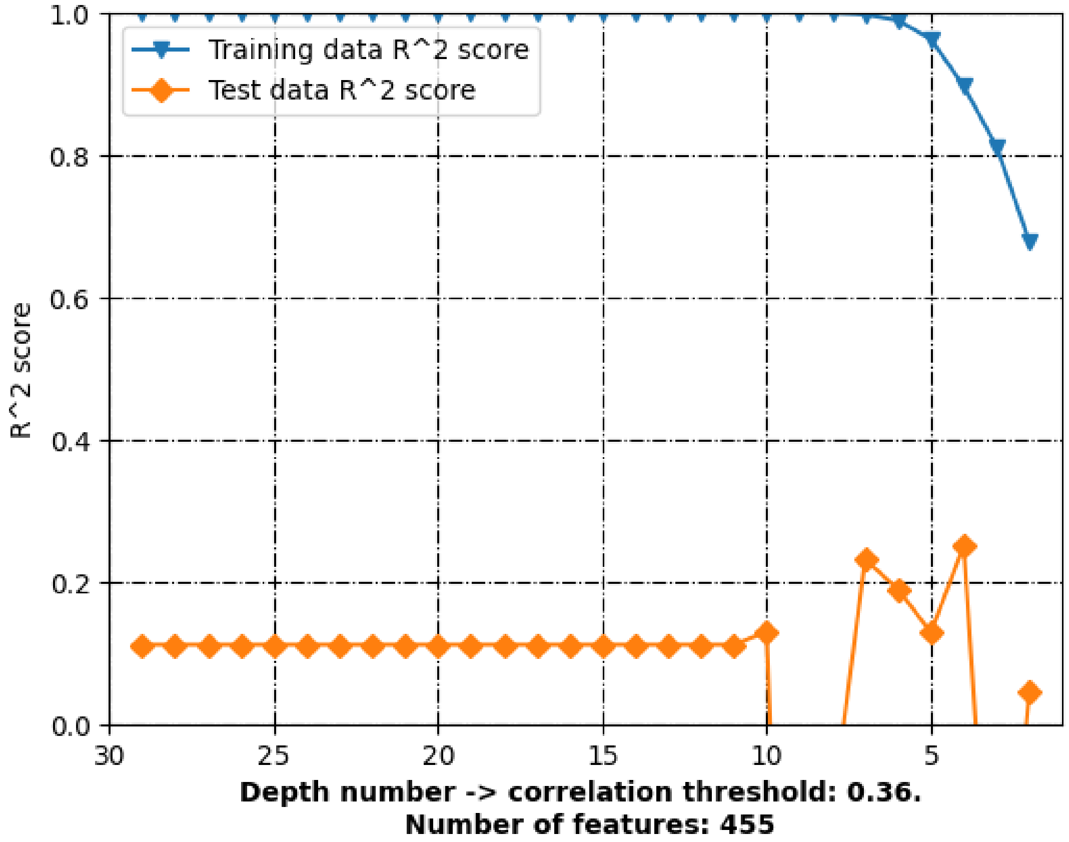
Training data R^2 score
Test data R^2 score
R^2 score
1.0
0.8
0.6
0.4
0.2
0.0
30
25
20
15
10
5
Depth number -> correlation threshold: 0.36.
Number of features: 455

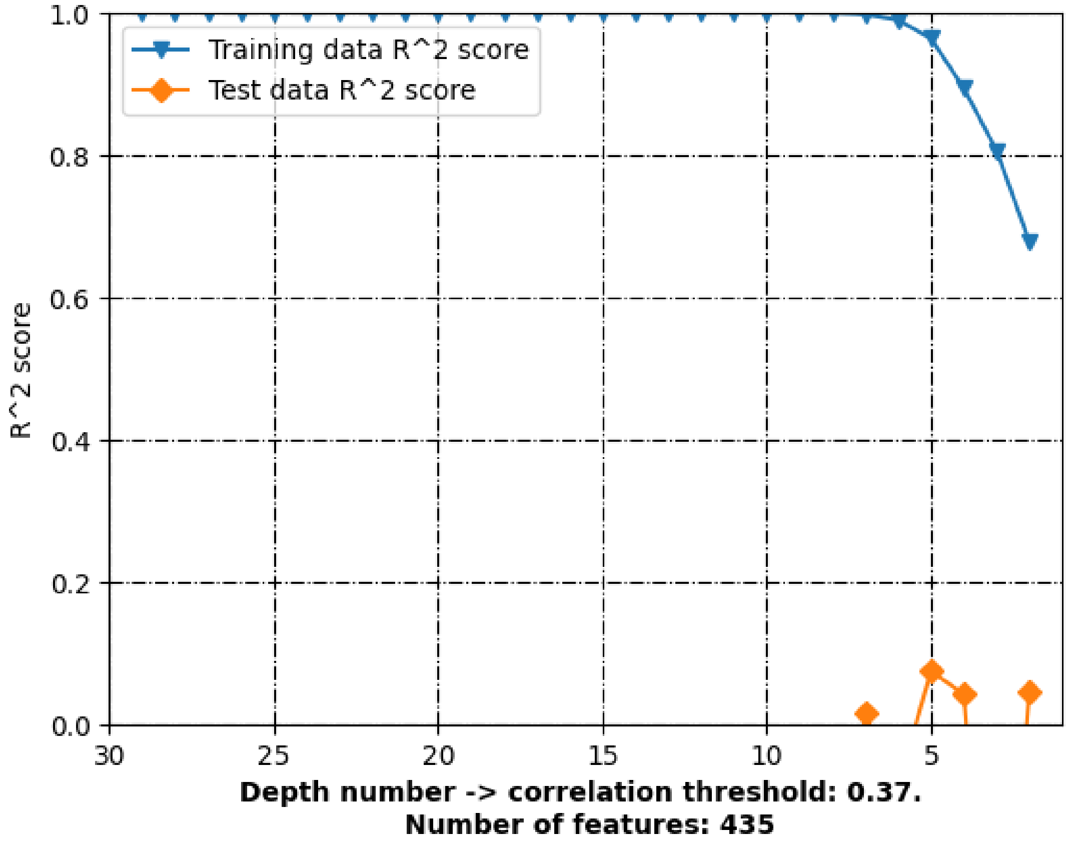
1.0
0.8
0.6
0.4
0.2
0.0
R^2 score
Training data R^2 score
Test data R^2 score
30
25
20
15
10
5
Depth number -> correlation threshold: 0.37.
Number of features: 435

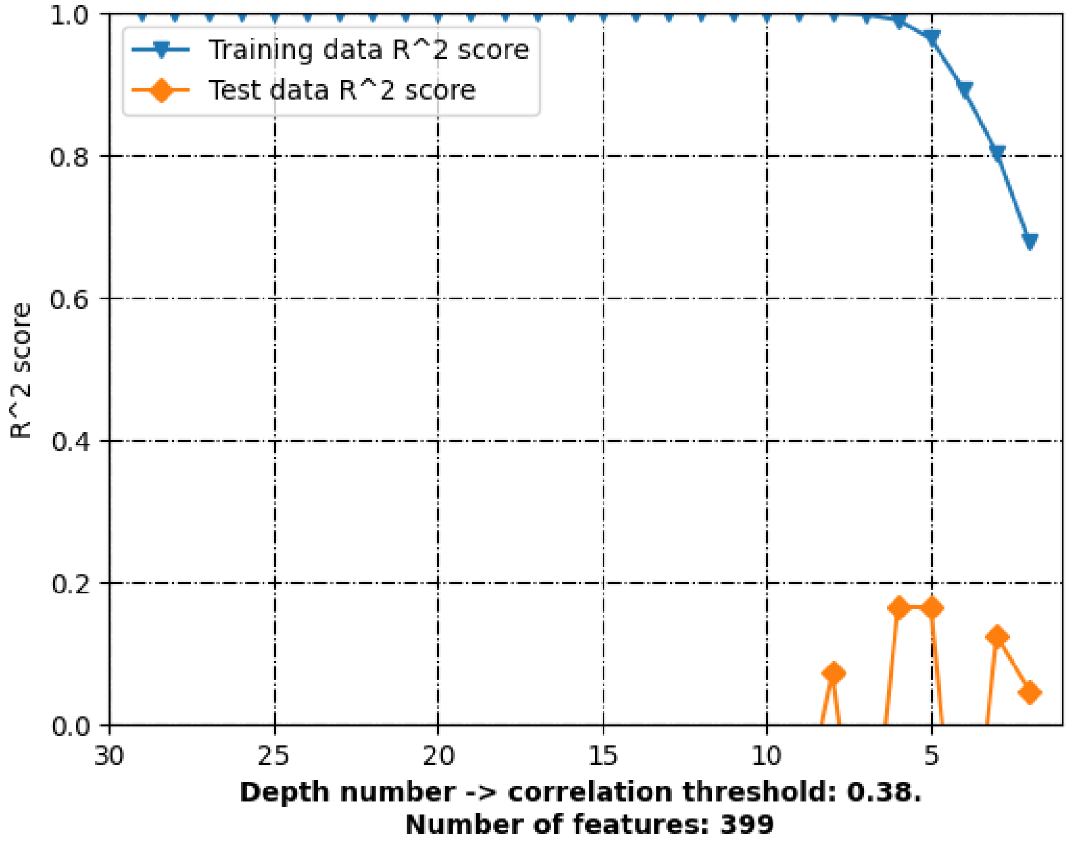
Training data R^2 score
Test data R^2 score
R^2 score
1.0
0.8
0.6
0.4
0.2
0.0
30
25
20
15
10
5
Depth number -> correlation threshold: 0.38.
Number of features: 399

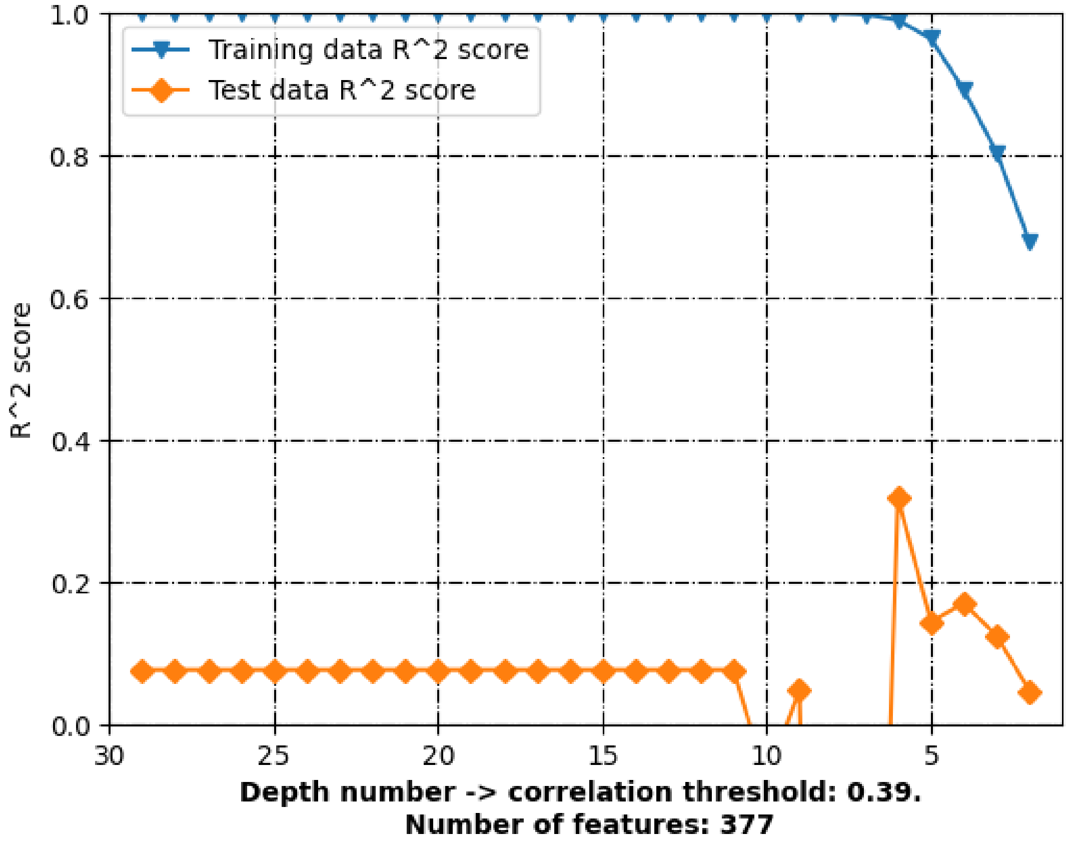
Training data R^2 score
Test data R^2 score
R^2 score
1.0
0.8
0.6
0.4
0.2
0.0
30
25
20
15
10
5
Depth number -> correlation threshold: 0.39.
Number of features: 377

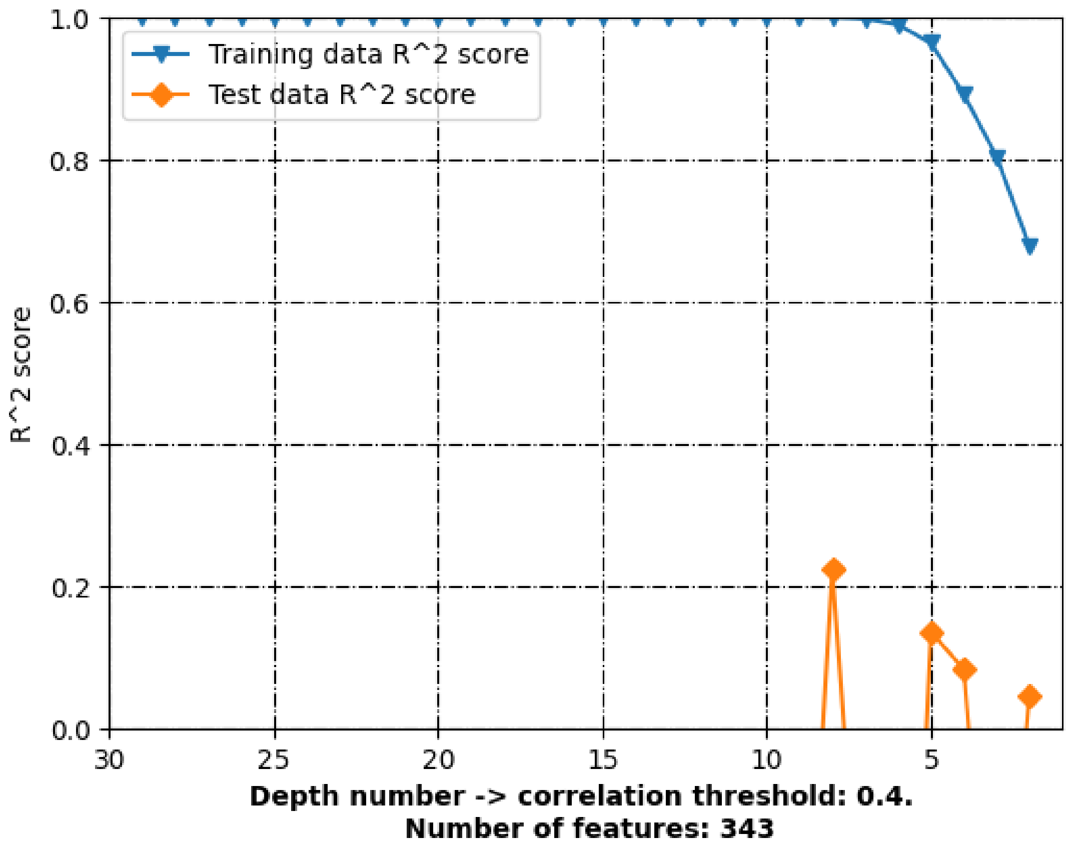
Training data R^2 score
Test data R^2 score
R^2 score
1.0
0.8
0.6
0.4
0.2
0.0
30
25
20
15
10
5
Depth number -> correlation threshold: 0.4.
Number of features: 343

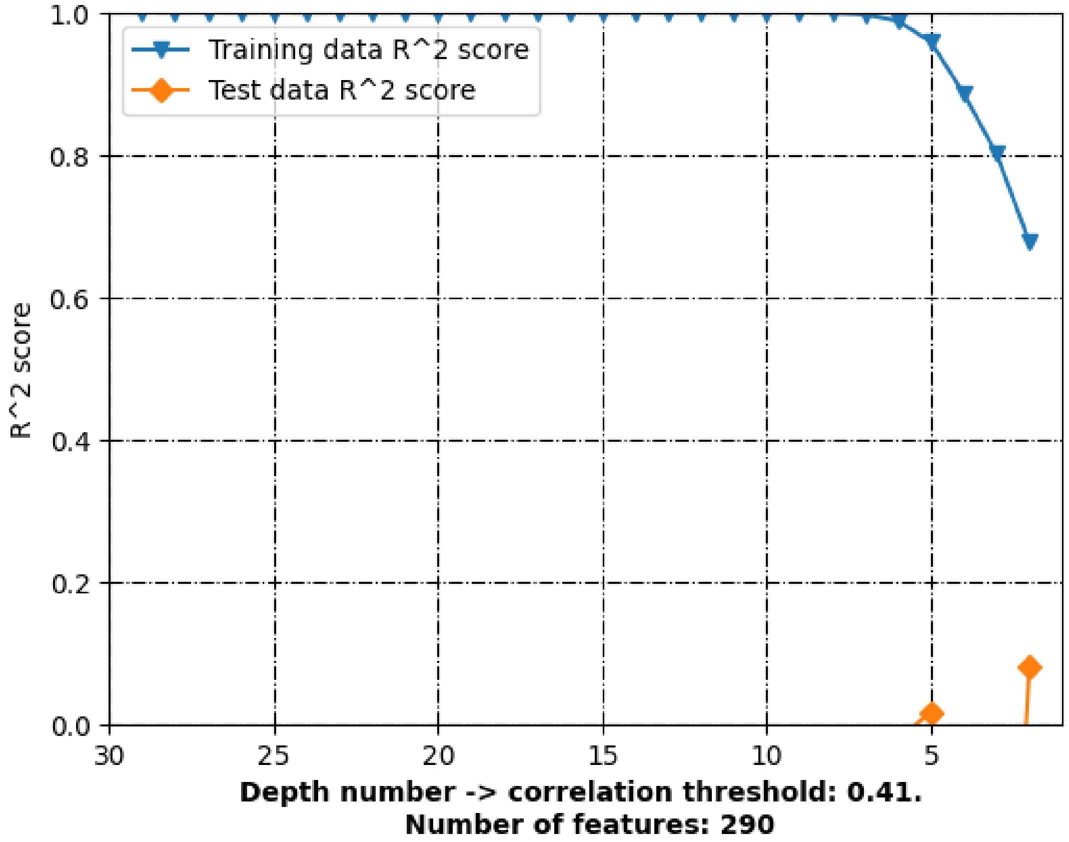
Training data R^2 score
Test data R^2 score
R^2 score
1.0
0.8
0.6
0.4
0.2
0.0
30
25
20
15
10
5
Depth number -> correlation threshold: 0.41.
Number of features: 290

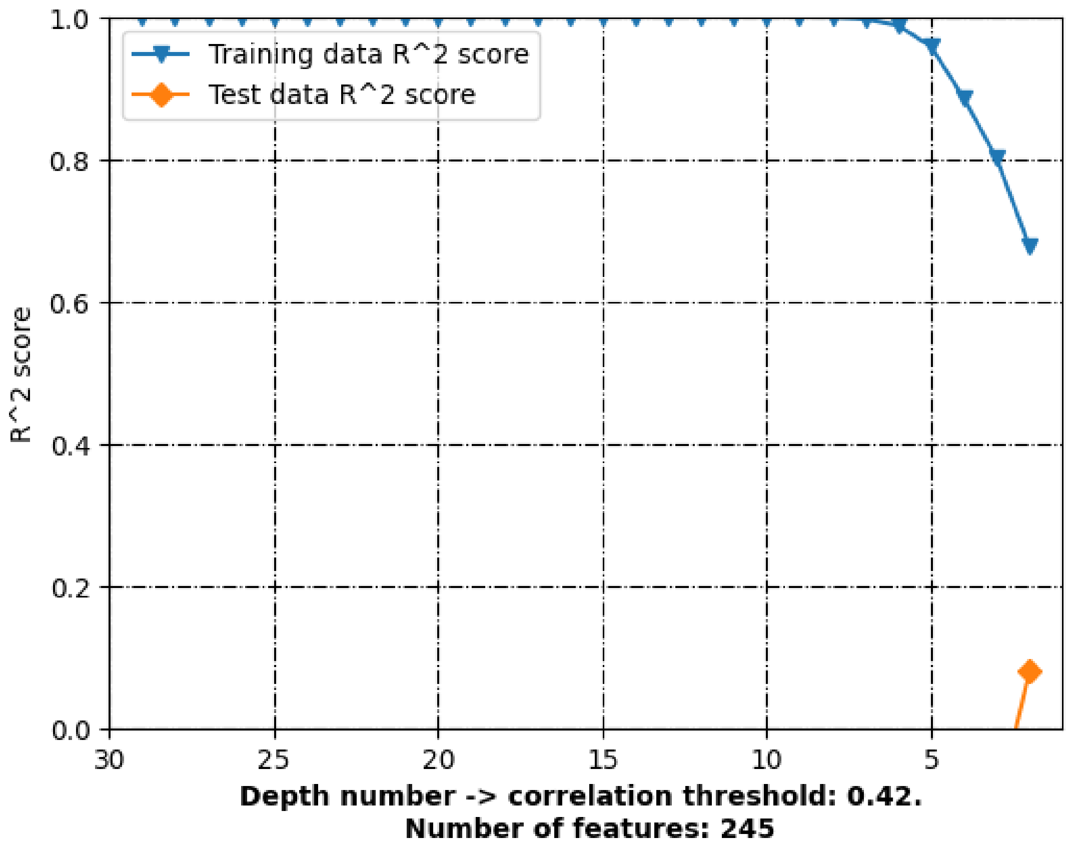

Training data R^2 score
Test data R^2 score
R^2 score
1.0
0.8
0.6
0.4
0.2
0.0
30
25
20
15
10
5
Depth number -> correlation threshold: 0.42.
Number of features: 245

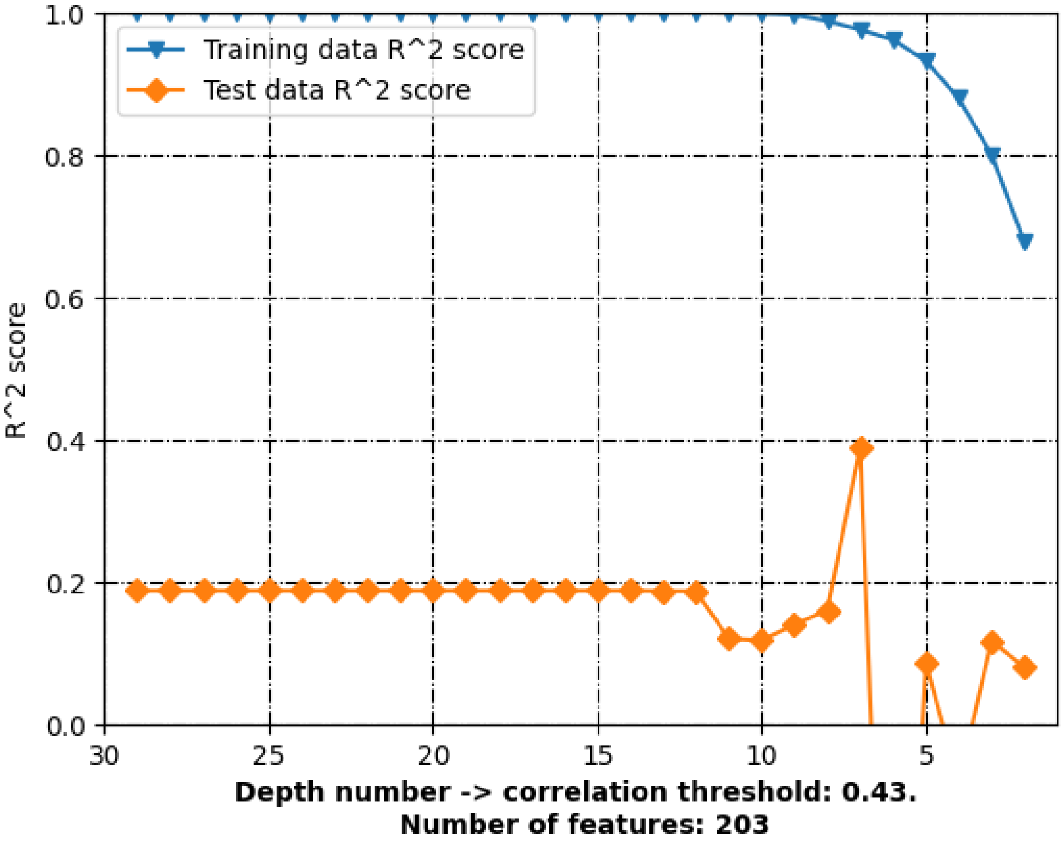
Training data R^2 score
Test data R^2 score
R^2 score
1.0
0.8
0.6
0.4
0.2
0.0
30
25
20
15
10
5
Depth number -> correlation threshold: 0.43.
Number of features: 203

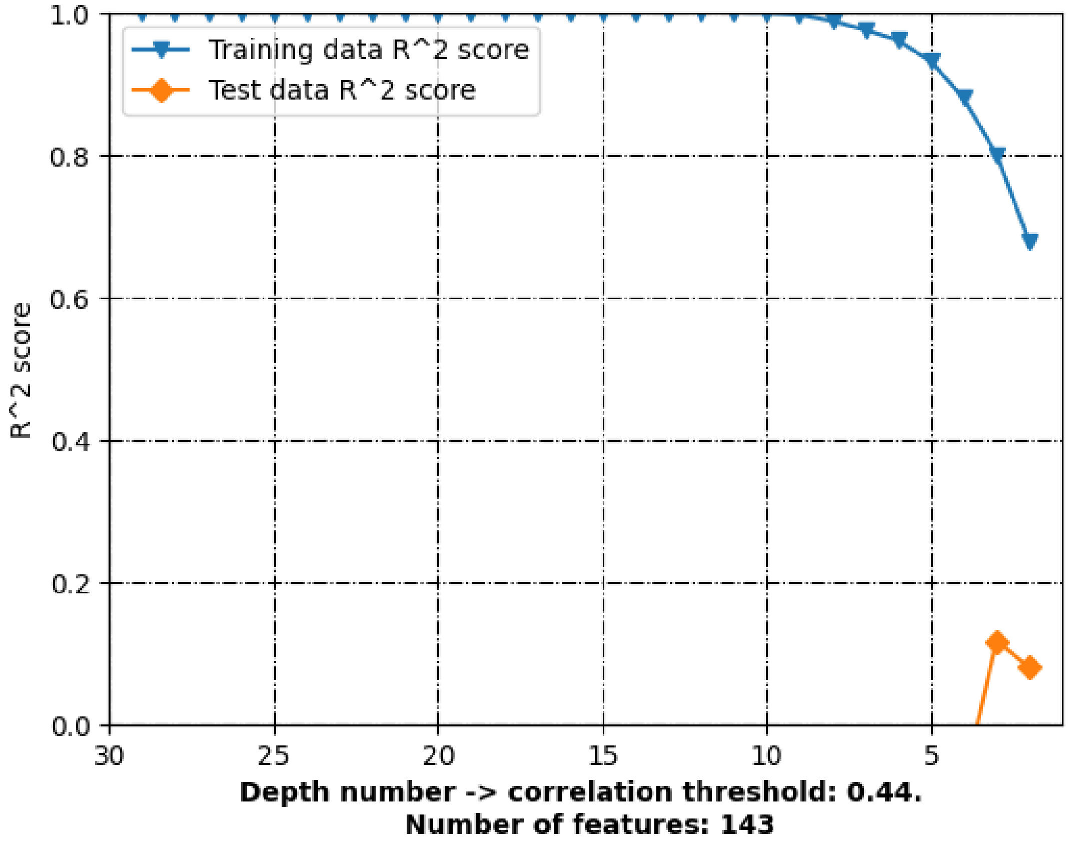

Training data R^2 score
Test data R^2 score
R^2 score
1.0
0.8
0.6
0.4
0.2
0.0
30
25
20
15
10
5
Depth number -> correlation threshold: 0.44.
Number of features: 143

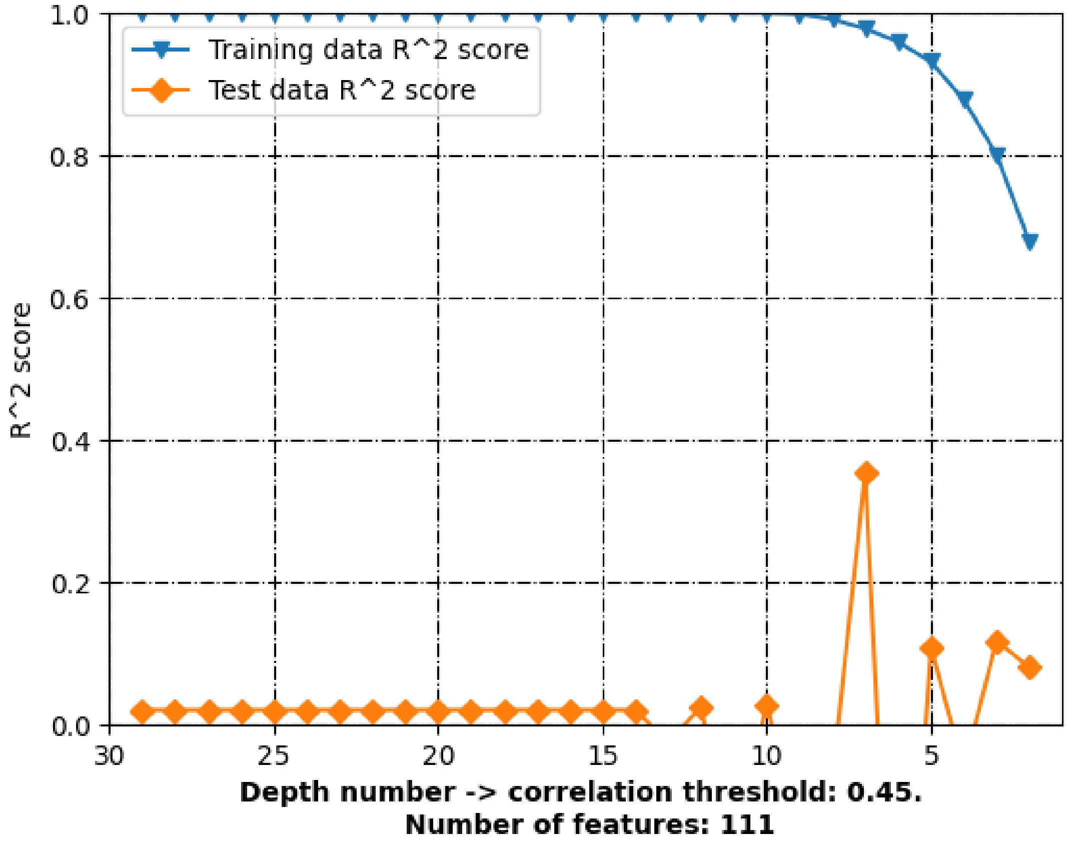
1.0
0.8
0.6
0.4
0.2
0.0
R^2 score
Training data R^2 score
Test data R^2 score
30
25
20
15
10
5
Depth number -> correlation threshold: 0.45.
Number of features: 111

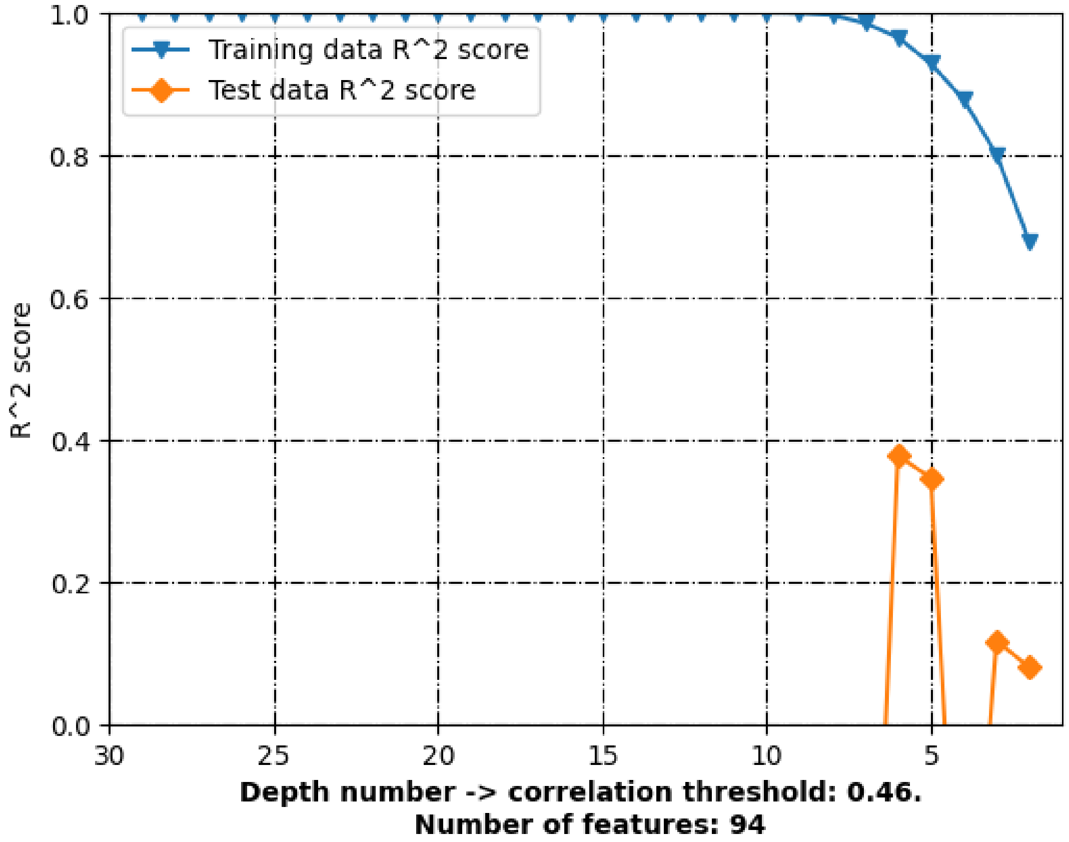

Training data R^2 score
Test data R^2 score
R^2 score
1.0
0.8
0.6
0.4
0.2
0.0
30
25
20
15
10
5
Depth number -> correlation threshold: 0.46.
Number of features: 94

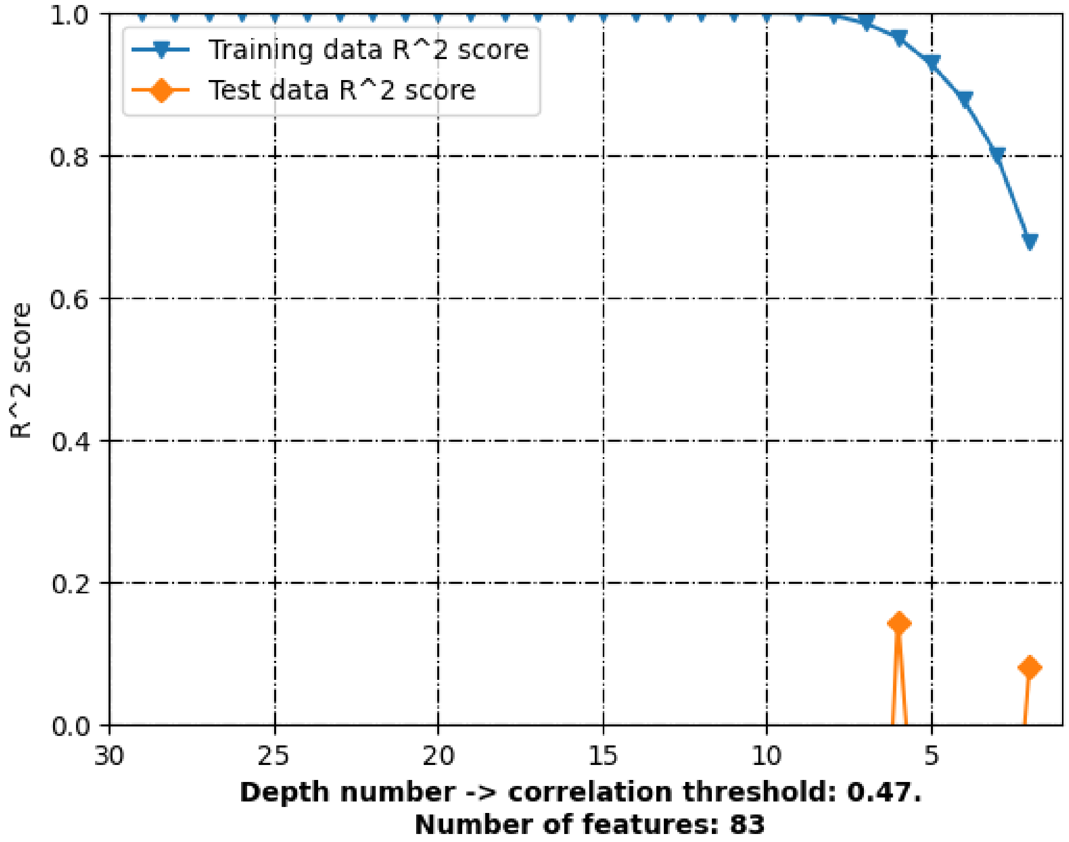
Training data R^2 score
Test data R^2 score
R^2 score
1.0
0.8
0.6
0.4
0.2
0.0
30
25
20
15
10
5
Depth number -> correlation threshold: 0.47.
Number of features: 83

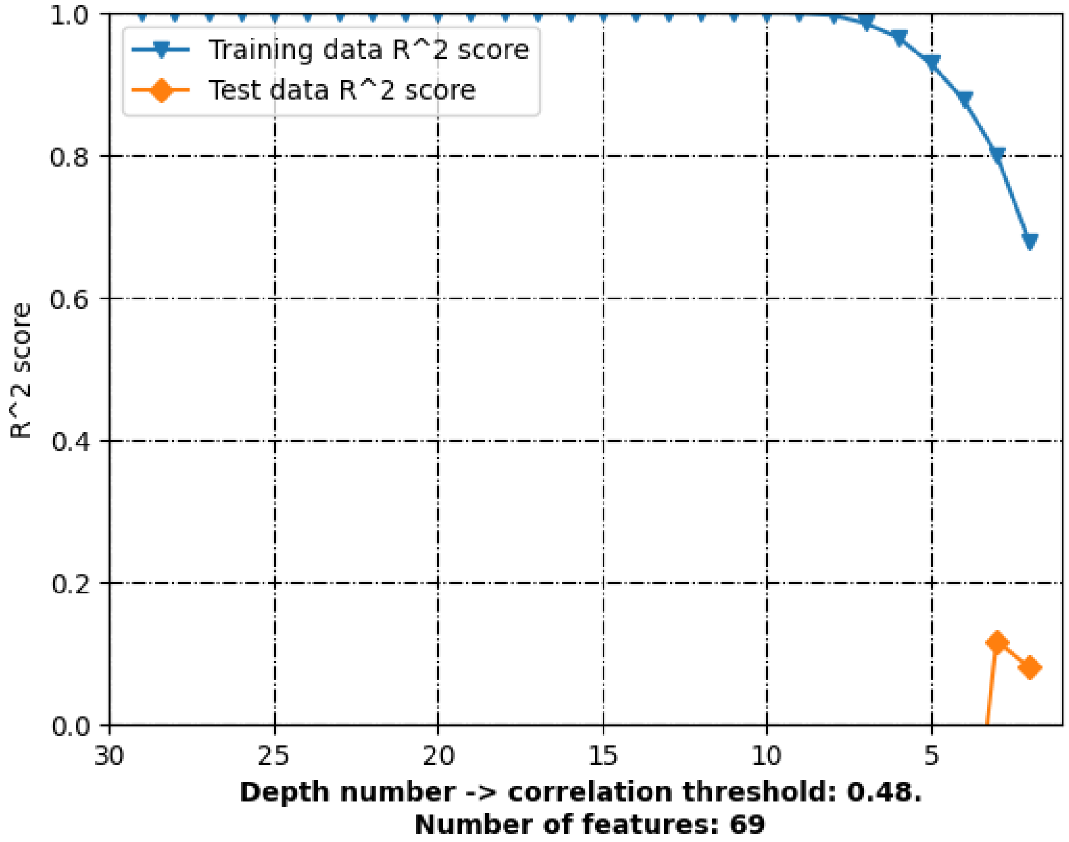

Training data R^2 score
Test data R^2 score
R^2 score
1.0
0.8
0.6
0.4
0.2
0.0
30
25
20
15
10
5
Depth number -> correlation threshold: 0.48.
Number of features: 69

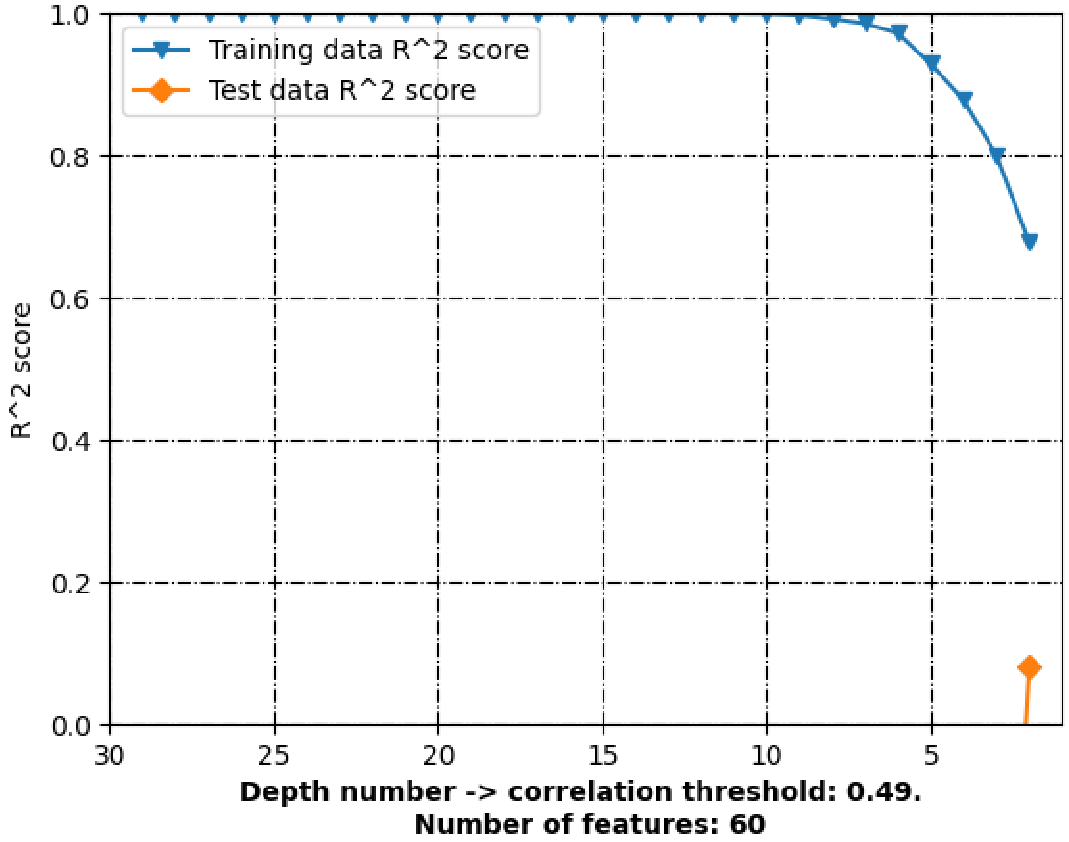
Training data R^2 score
Test data R^2 score
R^2 score
1.0
0.8
0.6
0.4
0.2
0.0
30
25
20
15
10
5
Depth number -> correlation threshold: 0.49.
Number of features: 60

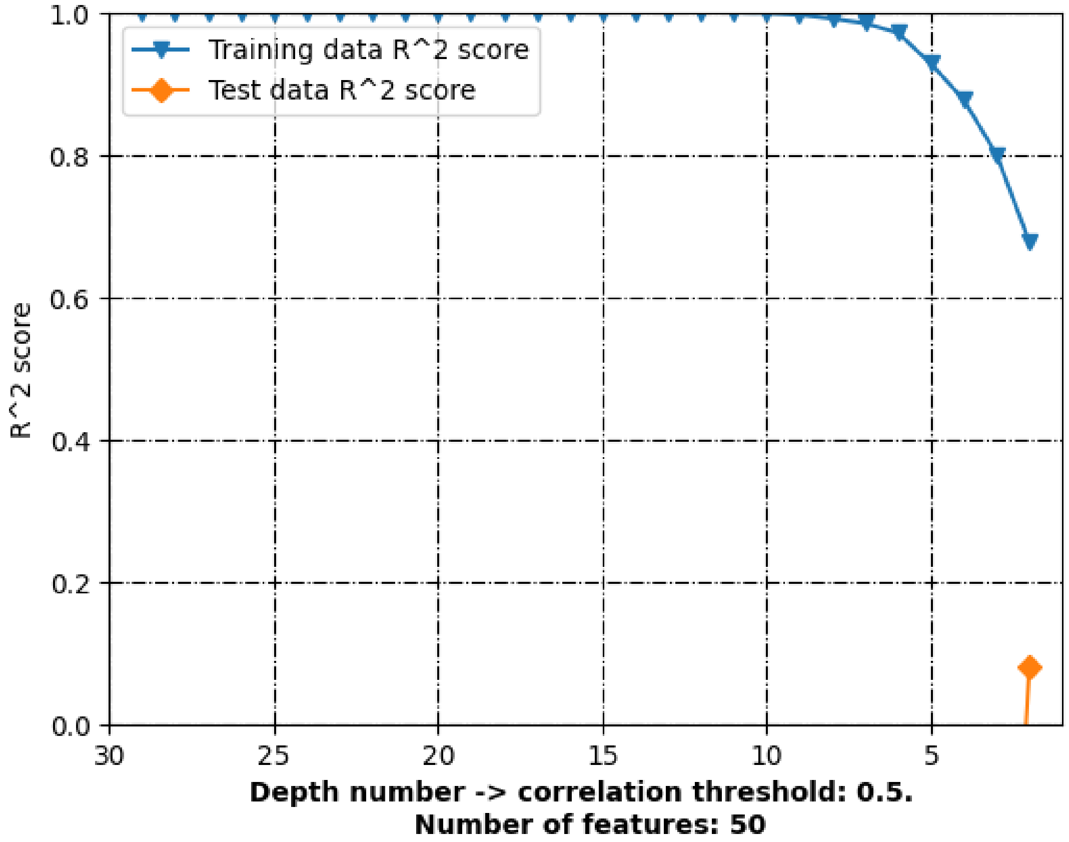

1.0
0.8
0.6
0.4
0.2
0.0
R^2 score
Training data R^2 score
Test data R^2 score
30
25
20
15
10
5
Depth number -> correlation threshold: 0.5.
Number of features: 50

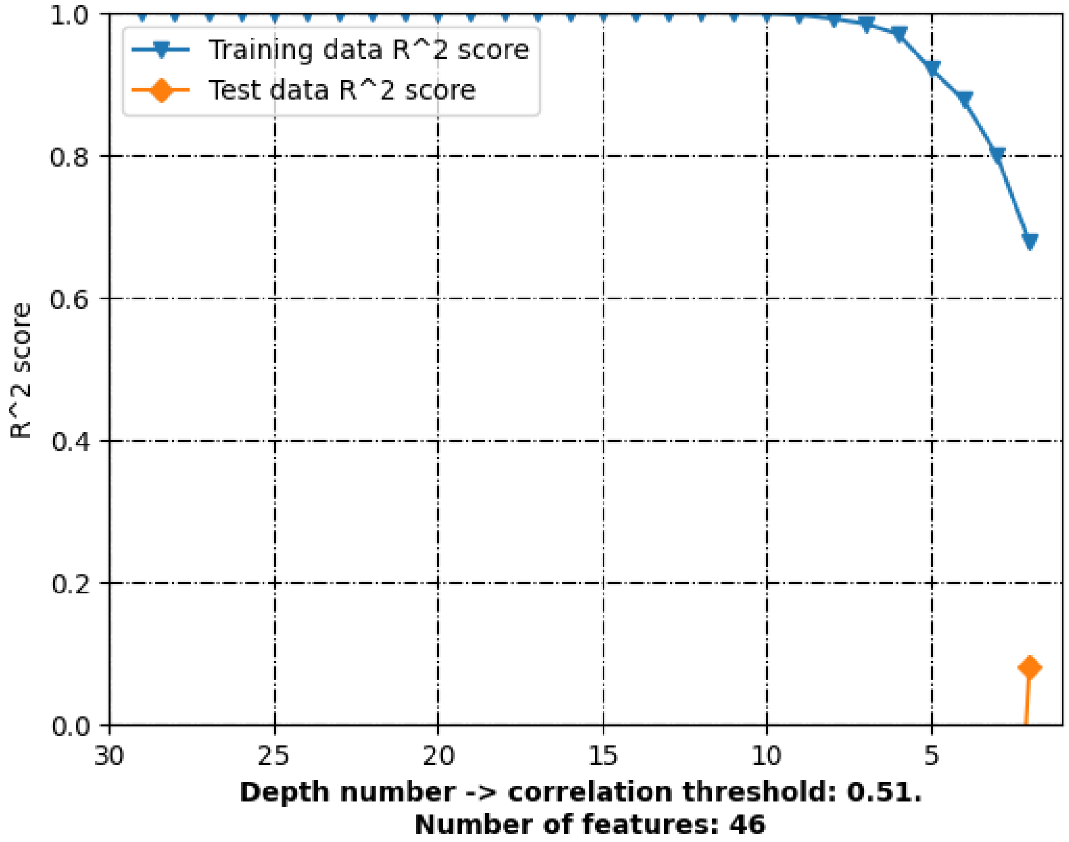
Training data R^2 score
Test data R^2 score
R^2 score
1.0
0.8
0.6
0.4
0.2
0.0
30
25
20
15
10
5
Depth number -> correlation threshold: 0.51.
Number of features: 46

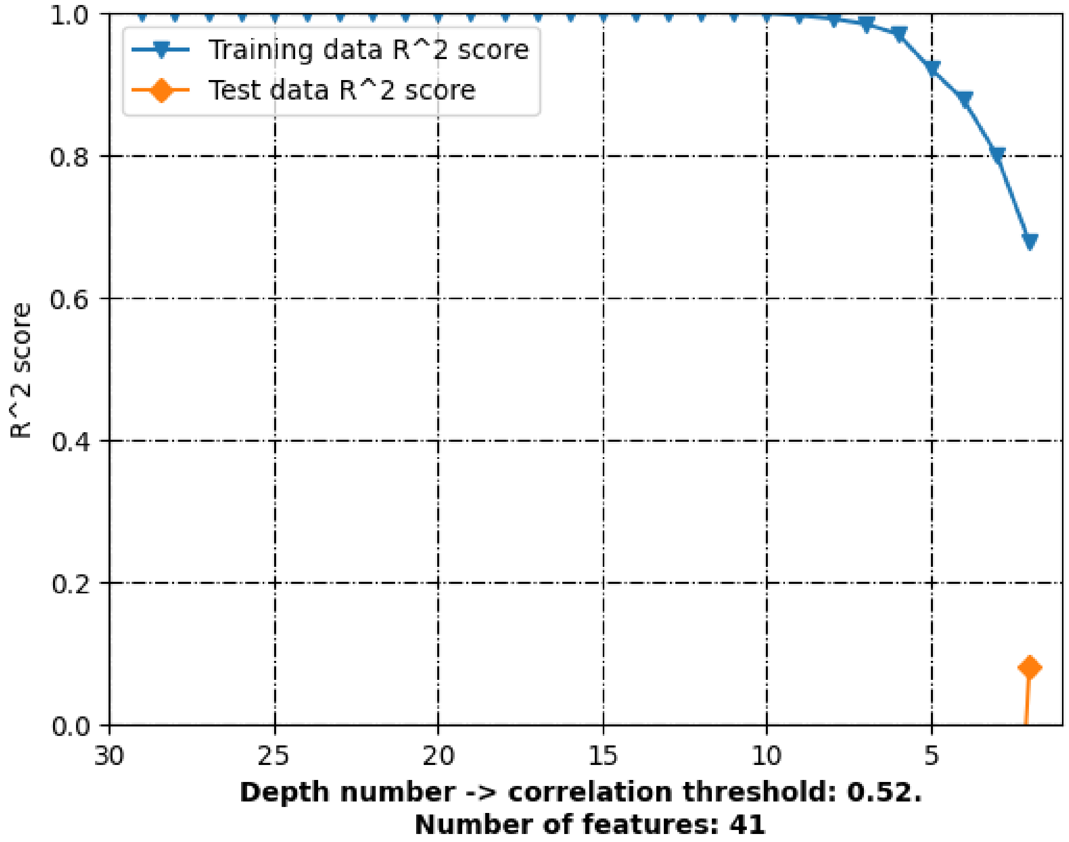

Training data R^2 score
Test data R^2 score
R^2 score
1.0
0.8
0.6
0.4
0.2
0.0
30
25
20
15
10
5
Depth number -> correlation threshold: 0.52.
Number of features: 41

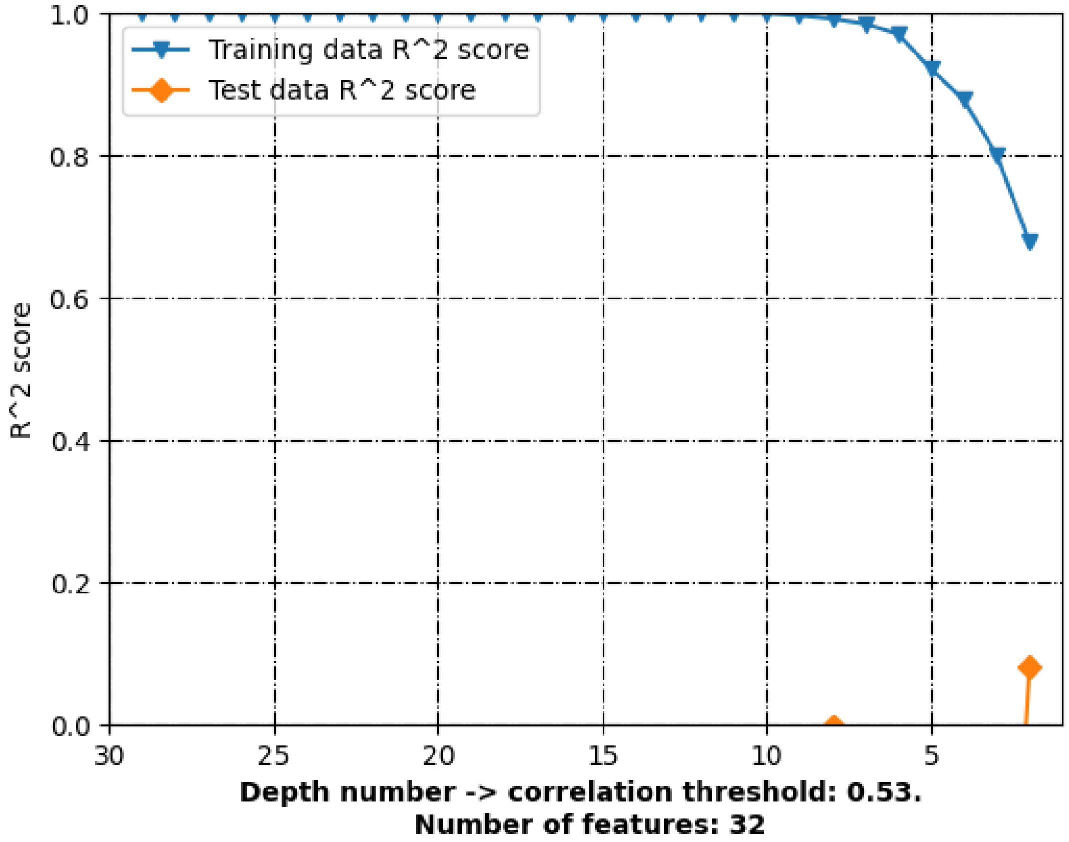
Training data R^2 score
Test data R^2 score
R^2 score
1.0
0.8
0.6
0.4
0.2
0.0
30
25
20
15
10
5
Depth number -> correlation threshold: 0.53.
Number of features: 32

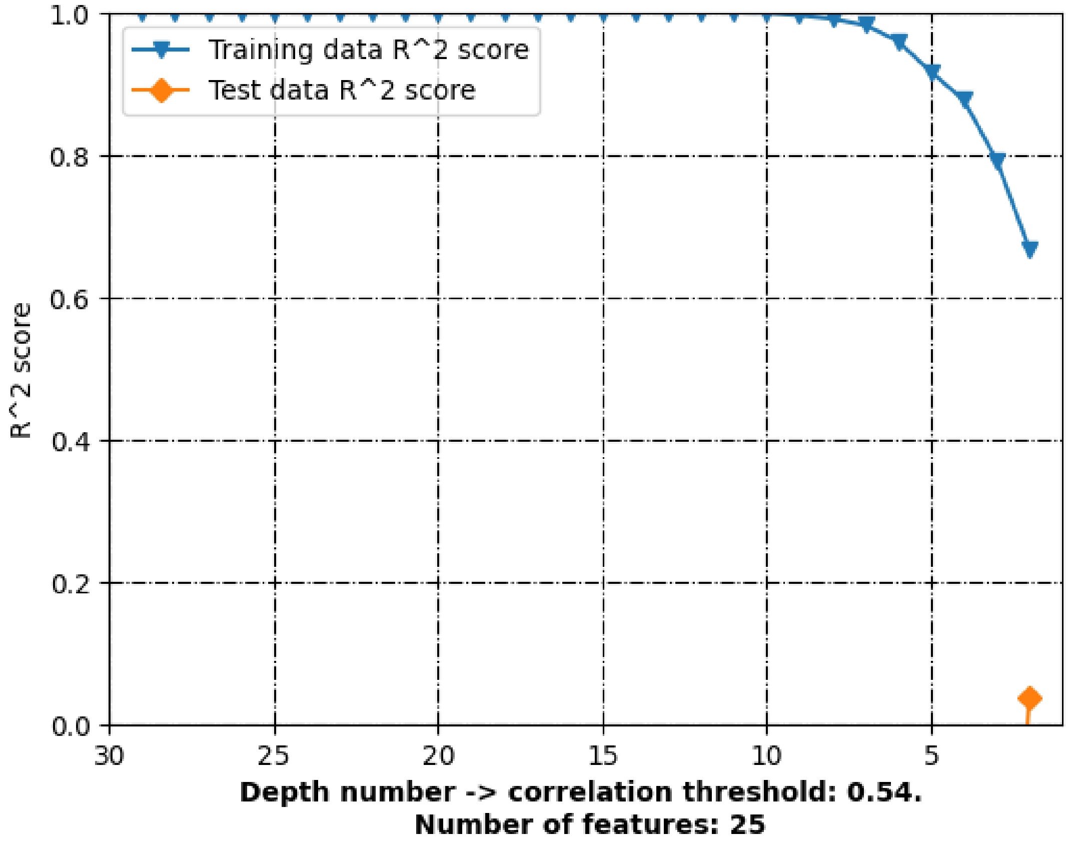

Training data R^2 score
Test data R^2 score
R^2 score
1.0
0.8
0.6
0.4
0.2
0.0
30
25
20
15
10
5
Depth number -> correlation threshold: 0.54.
Number of features: 25

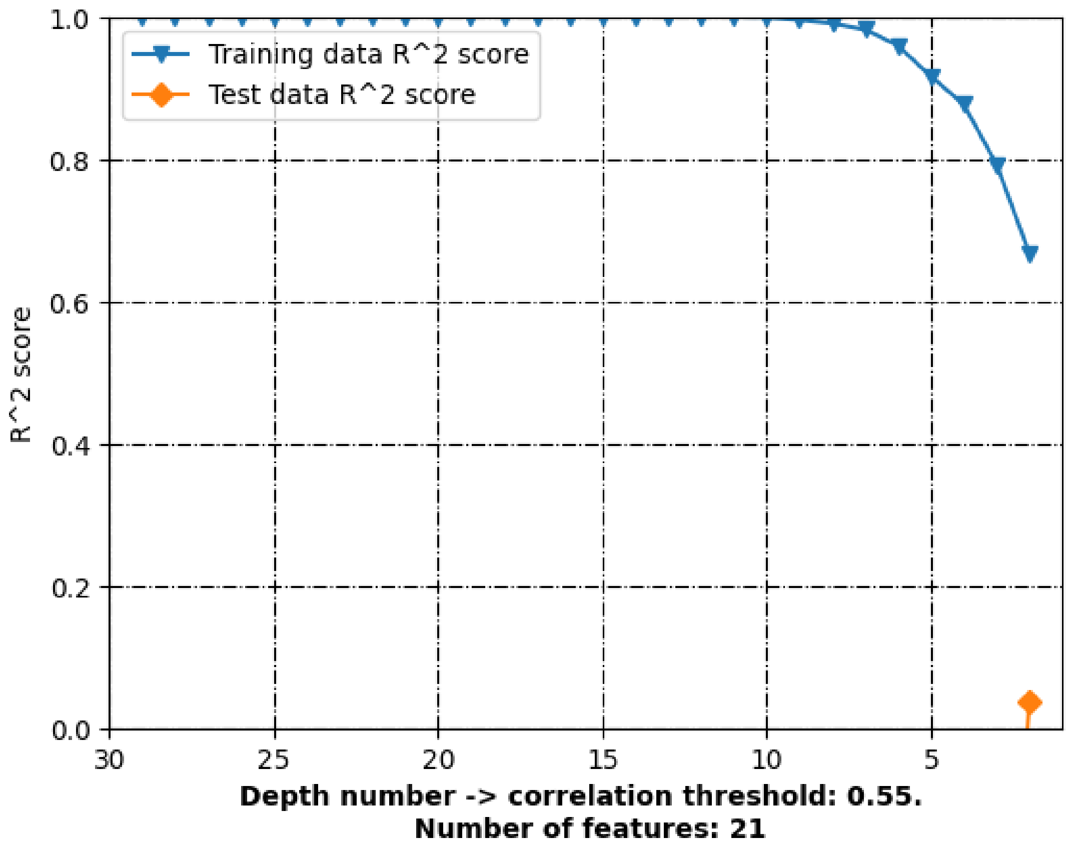
Training data R^2 score
Test data R^2 score
R^2 score
1.0
0.8
0.6
0.4
0.2
0.0
30
25
20
15
10
5
Depth number -> correlation threshold: 0.55.
Number of features: 21

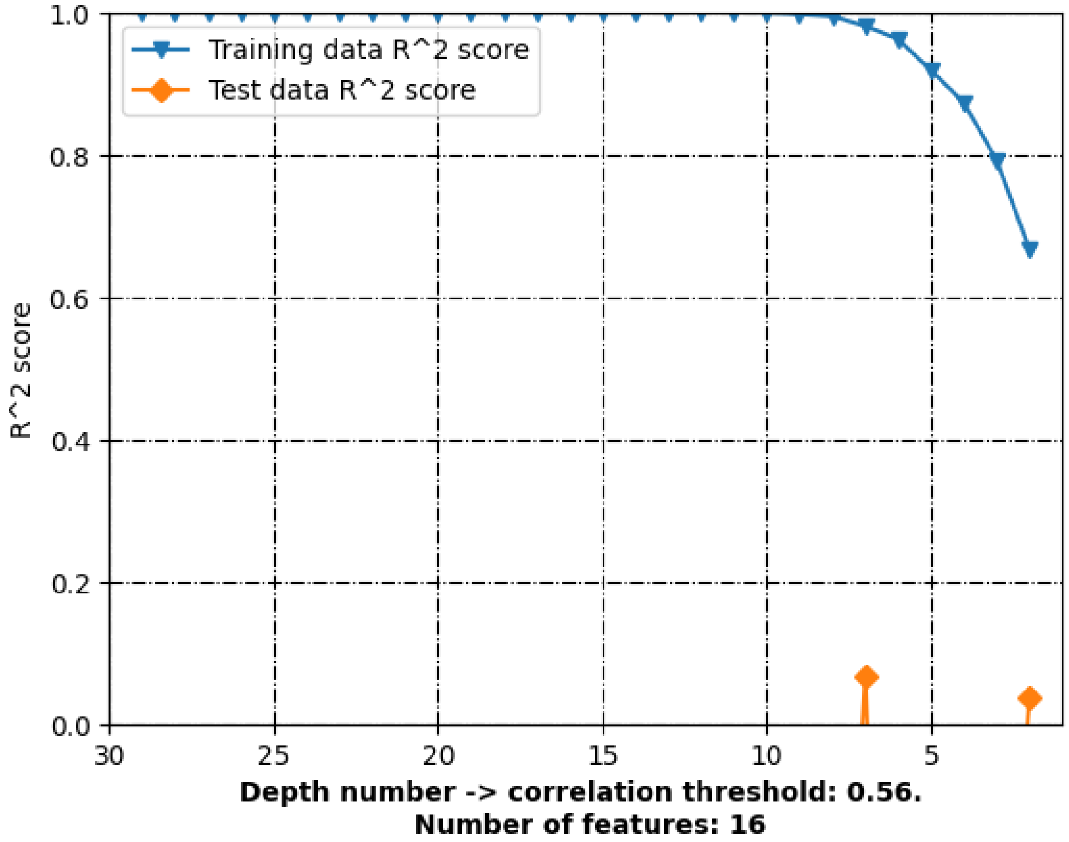

Training data R^2 score
Test data R^2 score
R^2 score
1.0
0.8
0.6
0.4
0.2
0.0
30
25
20
15
10
5
Depth number -> correlation threshold: 0.56.
Number of features: 16

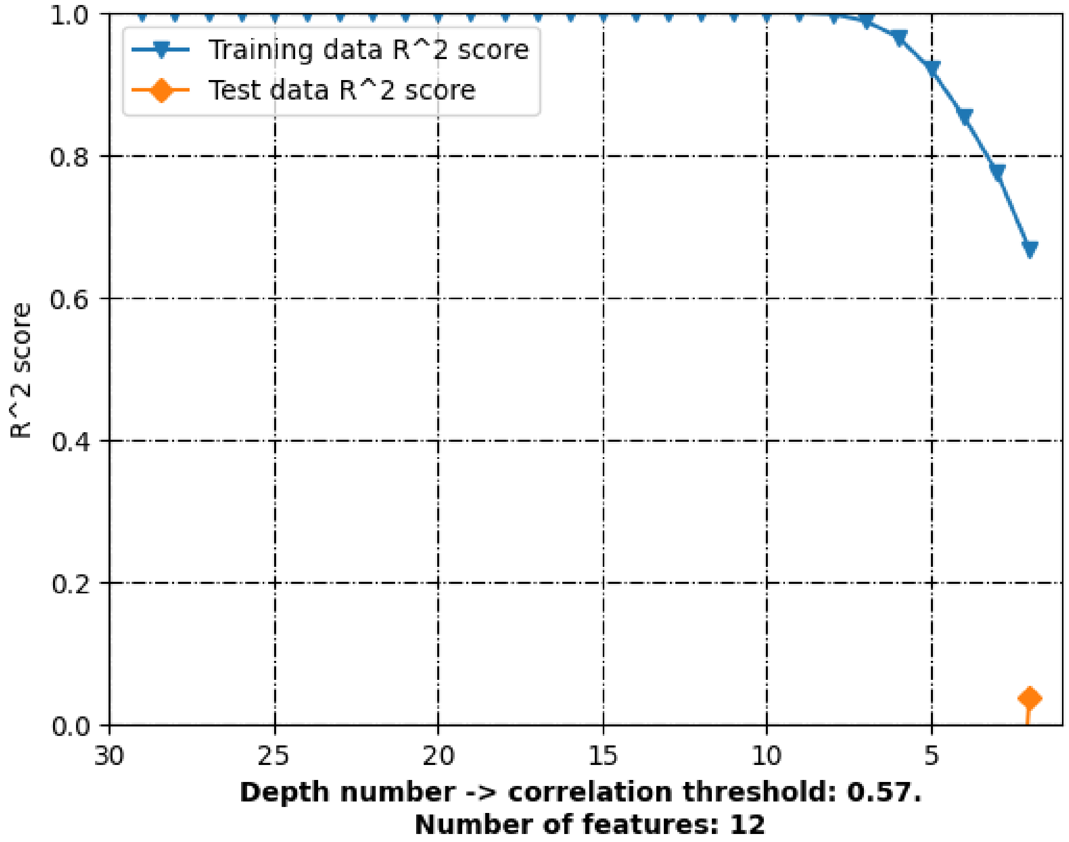
1.0
0.8
0.6
0.4
0.2
0.0
R^2 score
Training data R^2 score
Test data R^2 score
30
25
20
15
10
5
Depth number -> correlation threshold: 0.57.
Number of features: 12

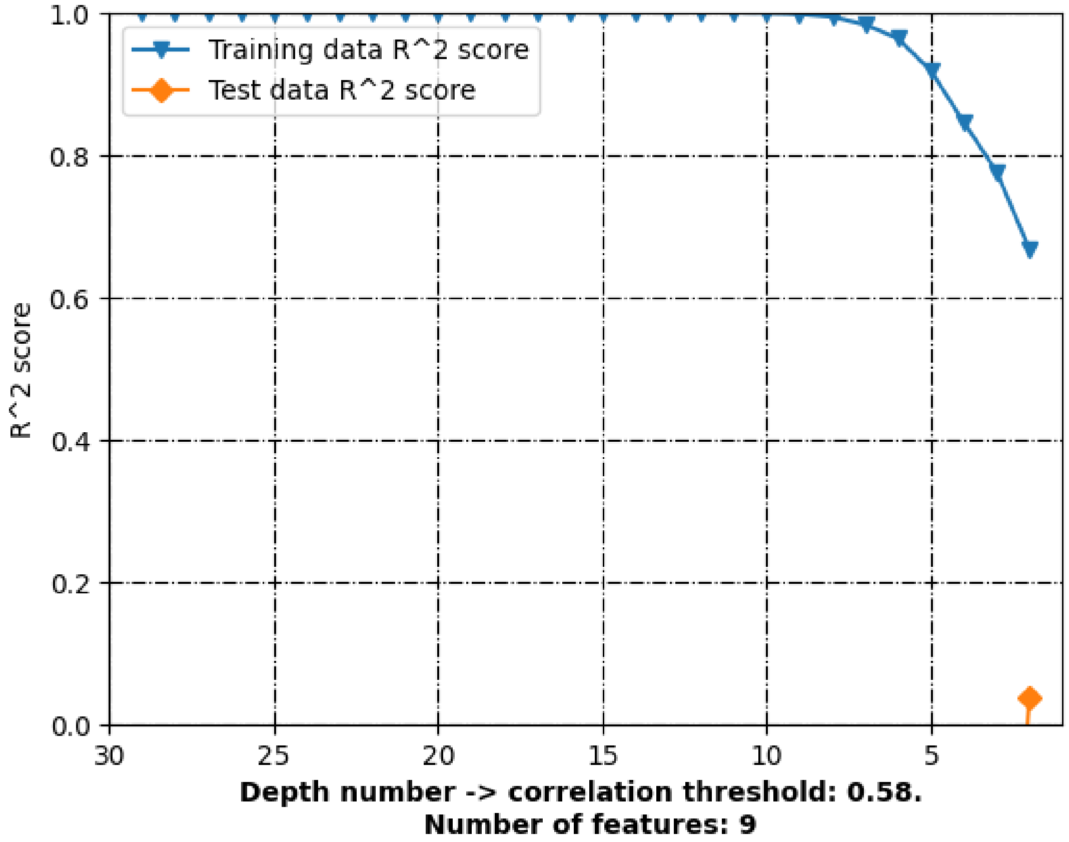
Training data R^2 score
Test data R^2 score
R^2 score
1.0
0.8
0.6
0.4
0.2
0.0
30
25
20
15
10
5
Depth number -> correlation threshold: 0.58.
Number of features: 9

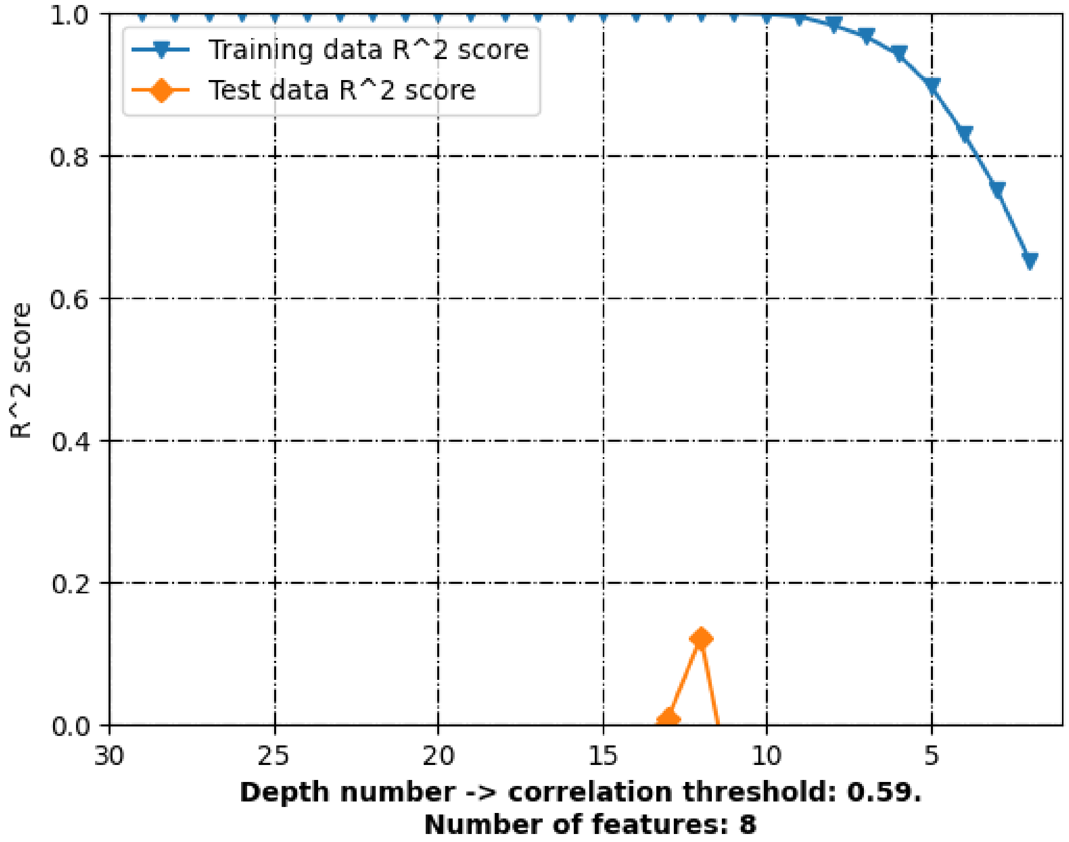

Training data R^2 score
Test data R^2 score
R^2 score
1.0
0.8
0.6
0.4
0.2
0.0
30
25
20
15
10
5
Depth number -> correlation threshold: 0.59.
Number of features: 8

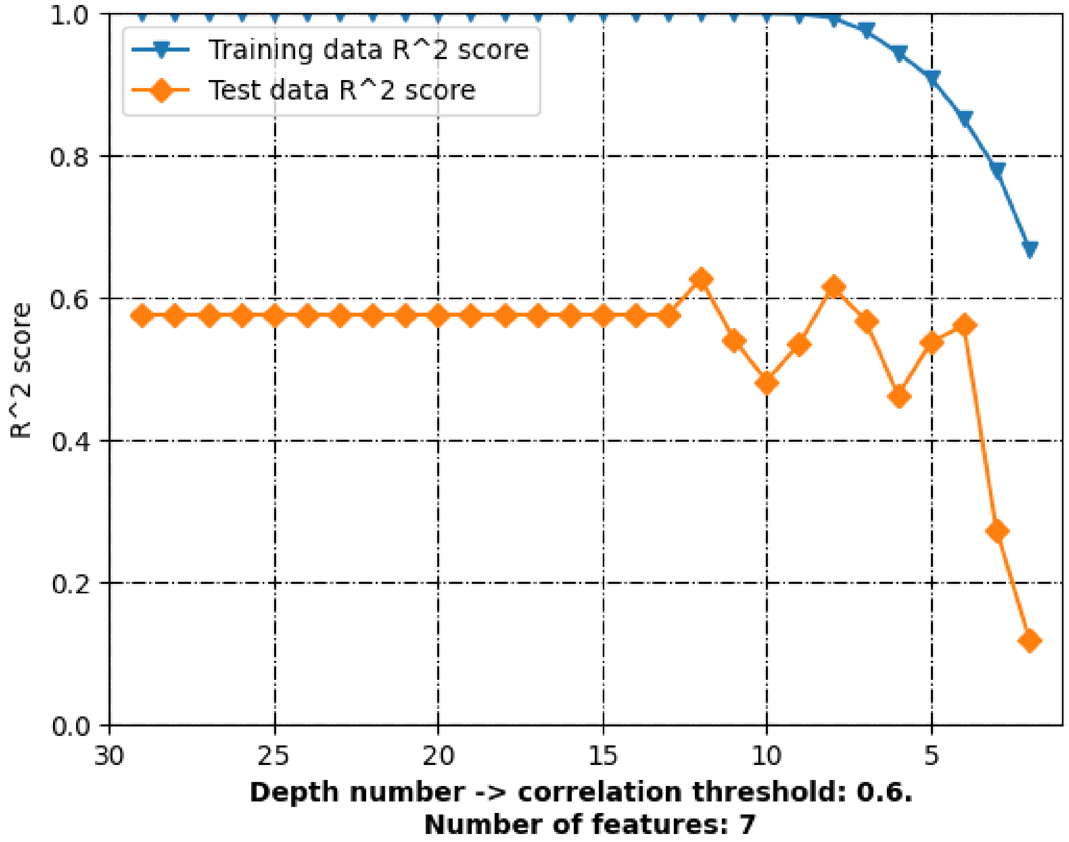

Training data R^2 score
Test data R^2 score
R^2 score
1.0
0.8
0.6
0.4
0.2
0.0
30
25
20
15
10
5
Depth number -> correlation threshold: 0.6.
Number of features: 7

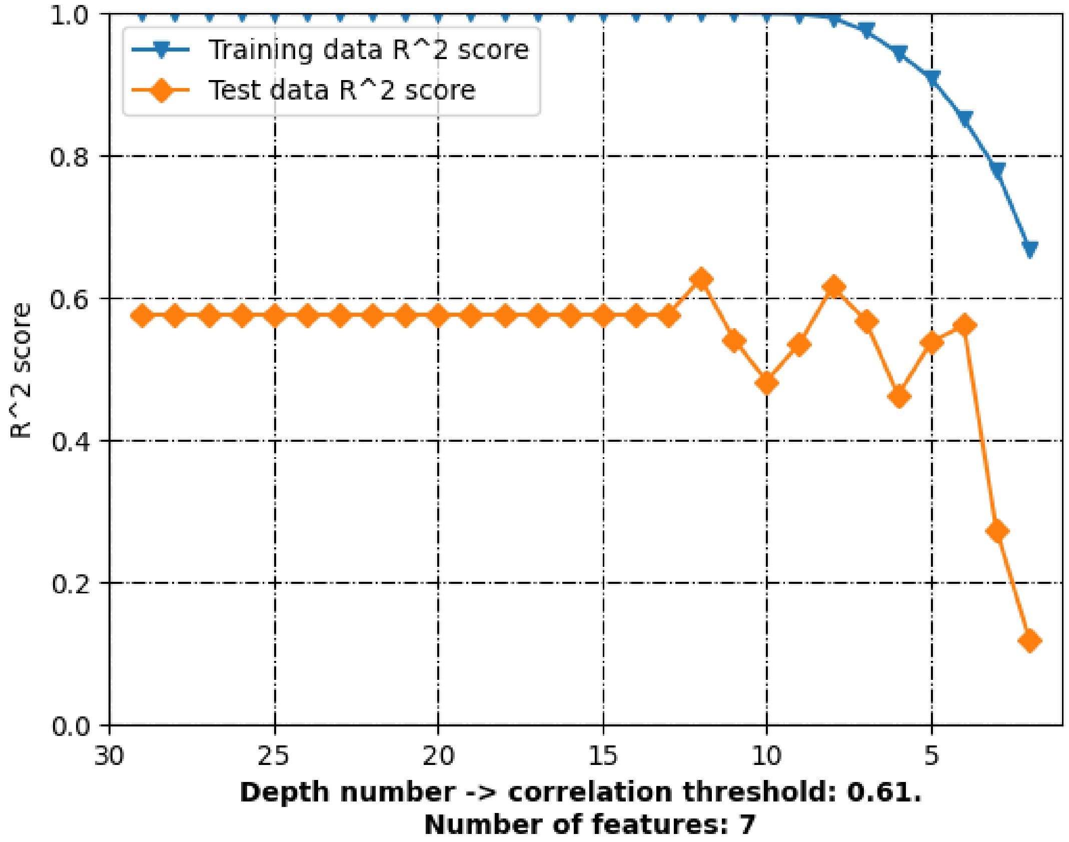

Training data R^2 score
Test data R^2 score
R^2 score
1.0
0.8
0.6
0.4
0.2
0.0
30
25
20
15
10
5
Depth number -> correlation threshold: 0.61.
Number of features: 7

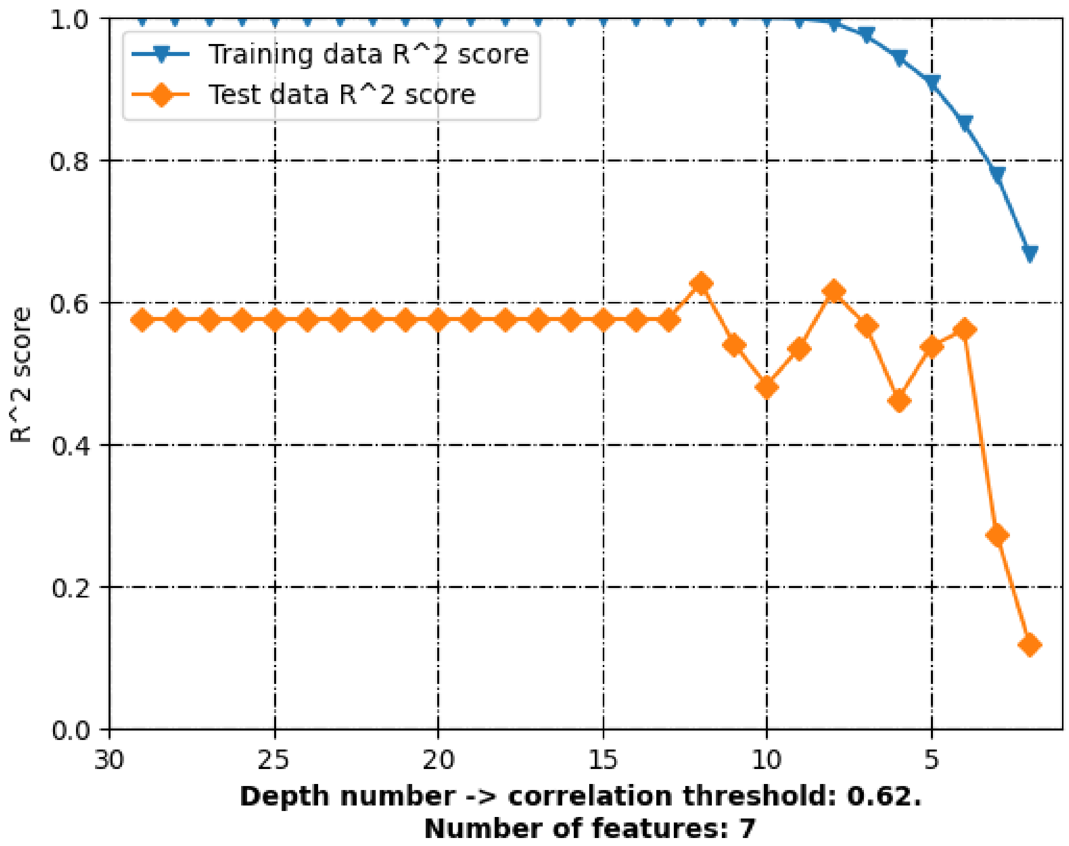
Training data R^2 score
Test data R^2 score
R^2 score
1.0
0.8
0.6
0.4
0.2
0.0
30
25
20
15
10
5
Depth number -> correlation threshold: 0.62.
Number of features: 7

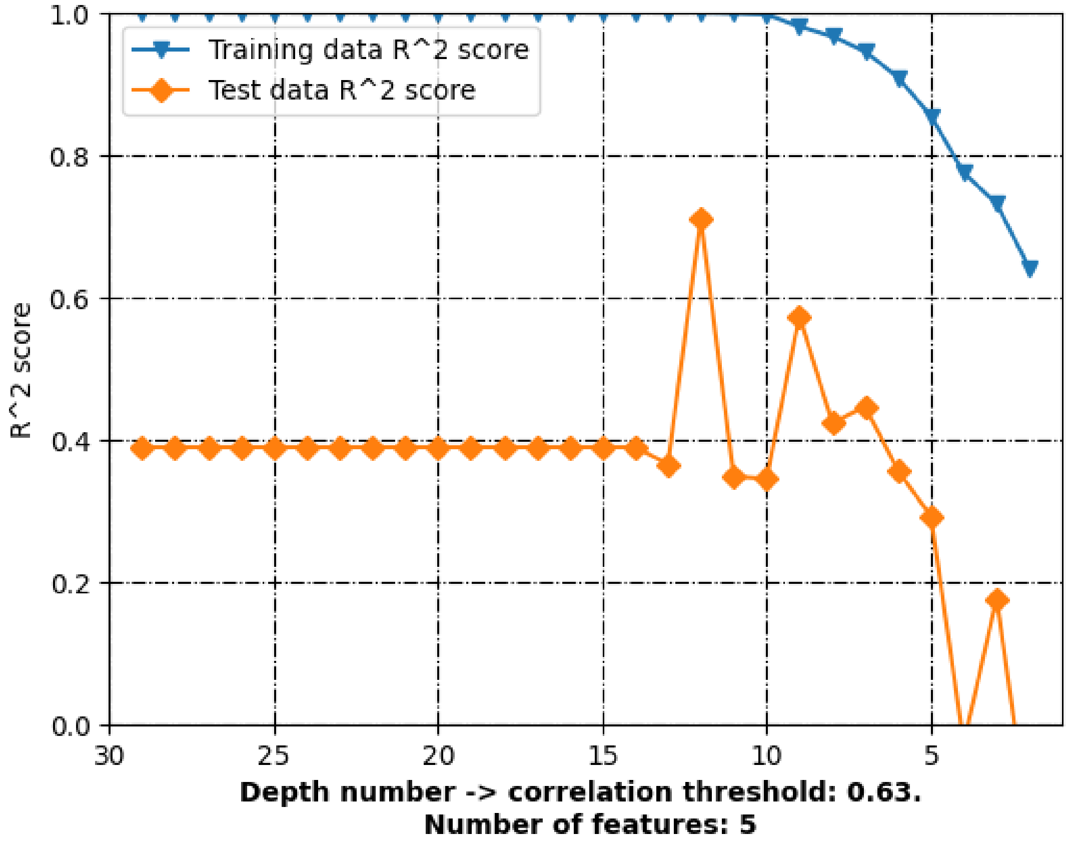

[21]:
```
plt.plot(df_without_standardization['Correlation threshold'],␣
  ↪df_without_standardization['Number of features'], label = "Number of␣
  ↪features", marker='.')
plt.legend()
plt.xlabel('Correlation threshold')
plt.ylabel('Number of features')
plt.show()
```

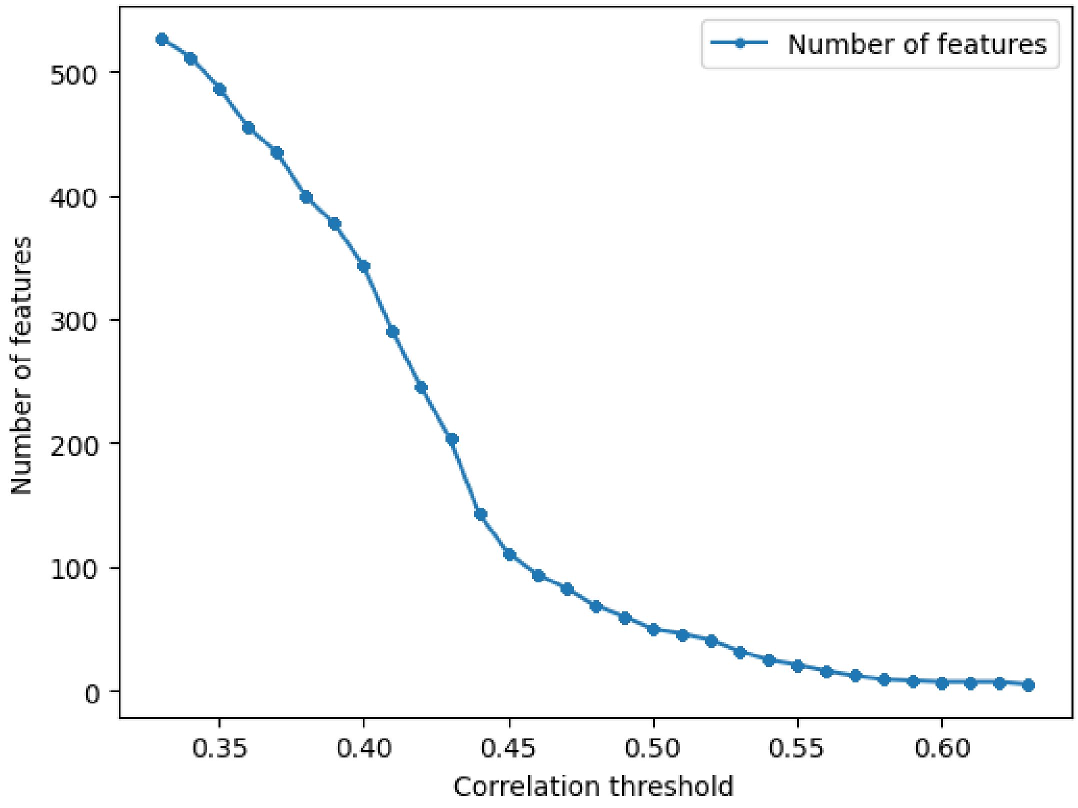

[ ]:

3 Random Forest

[22]:
```
without_standardization, train_r2_, test_r2_, hist1, hist2, target_column_name,␣
↪training_data_RMSE, test_data_RMSE  = pred_model.
↪prepare_data_and_create_model(molecular_descriptors_df=data,
                                                                               ␣
↪                        correlation_threshold=0.50,
                                                                               ␣
↪                        standardization=False,
                                                                               ␣
↪                        model_type='RandomForestRegressor',
                                                                               ␣
↪                        n_estimators_=12,
                                                                               ␣
↪                        target_column_name = target,
                                                                               ␣
↪                        random_state=random_state,
```

```
↪                      train_test_split_=True,
↪                      verbose=True)
```

```
I am not doing standardization…
  molecular descriptor name
0                    AATS0Z
1                  AATS0are
2                    AATS0d
3                   AATS0dv
4                    AATS0i
  molecular descriptor name  corr_value
0                    AATS0Z    0.018677
1                  AATS0are   -0.341313
2                    AATS0d   -0.123443
3                   AATS0dv   -0.265670
4                    AATS0i   -0.428929
  molecular descriptor name  corr_value  absolute correlation value
0                    AATS0Z    0.018677                    0.018677
1                  AATS0are   -0.341313                    0.341313
2                    AATS0d   -0.123443                    0.123443
3                   AATS0dv   -0.265670                    0.265670
4                    AATS0i   -0.428929                    0.428929
    molecular descriptor name  corr_value  absolute correlation value
15                     AATS1i   -0.561620                    0.561620
113                  AATSC1c    0.565682                    0.565682
125                  AATSC2c   -0.543393                    0.543393
221               AETA_eta_R   -0.515778                    0.515778
224                   AMID_C    0.532458                    0.532458
    molecular descriptor name  corr_value  absolute correlation value
15                     AATS1i   -0.561620                    0.561620
113                  AATSC1c    0.565682                    0.565682
125                  AATSC2c   -0.543393                    0.543393
221               AETA_eta_R   -0.515778                    0.515778
224                   AMID_C    0.532458                    0.532458
The model used is: RandomForest…
Return the coefficient of determination of the prediction:
0.4229517825255892
R^2 score: 0.9112439993057248
Correlation coefficient: 0.9545910115362101
Test data - unseen during training:
R^2 score: 0.4229517825255892
Correlation coefficient: 0.6503474321665222
[7.55581908 6.76701055 5.75119478 6.49932728 7.04485599 6.98625628
 6.20939289 5.47622337 7.15599556 6.92515496 5.79420297 5.94197432
 6.35947903 6.37983139 6.28160132 7.17954326]
0      7.260428
```

```
21      6.398375
106     5.853872
68      6.080922
44      7.638272
14      7.067526
108     6.154902
62      6.455932
13      7.158641
9       5.702436
96      5.136677
49      5.619789
54      7.075721
114     6.026872
115     5.568636
87      7.288193
Name: LoVo/DX, dtype: float64
Training Root Mean Square Error: 0.2861656827145399
Testing Root Mean Square Error: 0.554481606951121
```

[23]:

```
print(target_column_name+str('_transformed'))
print(hist1[target_column_name].hist())
```

```
LoVo/DX_transformed
AxesSubplot(0.125,0.11;0.775x0.77)
```

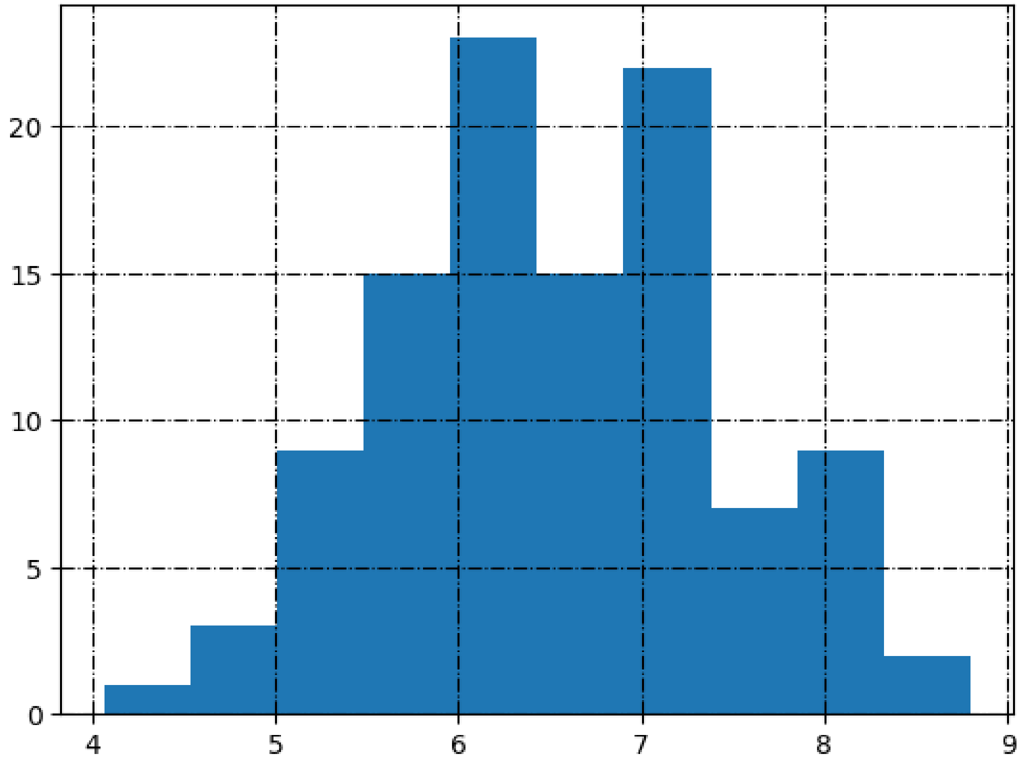

[24]:
```
with_standardization, train_r2_, test_r2_, hist1, hist2, target_column_name,␣
↪training_data_RMSE, test_data_RMSE  = pred_model.
↪prepare_data_and_create_model(molecular_descriptors_df=data,
                                                                        ␣
↪                    correlation_threshold=0.50,
                                                                        ␣
↪                    standardization=True,
                                                                        ␣
↪                    model_type='RandomForestRegressor',
                                                                        ␣
↪                    n_estimators_=12,
                                                                        ␣
↪                    target_column_name = target,
                                                                        ␣
↪                    random_state=random_state,
                                                                        ␣
↪                    train_test_split_=True,
                                                                        ␣
↪                    verbose=True)
```

```
I am doing standardization…
  molecular descriptor name
0                    AATS0Z
1                  AATS0are
2                    AATS0d
3                   AATS0dv
4                    AATS0i
  molecular descriptor name  corr_value
0                    AATS0Z    0.018677
1                  AATS0are   -0.341313
2                    AATS0d   -0.123443
3                   AATS0dv   -0.265670
4                    AATS0i   -0.428929
  molecular descriptor name  corr_value  absolute correlation value
0                    AATS0Z    0.018677                    0.018677
1                  AATS0are   -0.341313                    0.341313
2                    AATS0d   -0.123443                    0.123443
3                   AATS0dv   -0.265670                    0.265670
4                    AATS0i   -0.428929                    0.428929
    molecular descriptor name  corr_value  absolute correlation value
15                     AATS1i   -0.561620                    0.561620
113                   AATSC1c    0.565682                    0.565682
125                   AATSC2c   -0.543393                    0.543393
221                 AETA_eta_R   -0.515778                    0.515778
224                     AMID_C    0.532458                    0.532458
```

```
    molecular descriptor name  corr_value  absolute correlation value
15                     AATS1i   -0.561620                    0.561620
113                   AATSC1c    0.565682                    0.565682
125                   AATSC2c   -0.543393                    0.543393
221                AETA_eta_R   -0.515778                    0.515778
224                    AMID_C    0.532458                    0.532458
The model used is: RandomForest…
Return the coefficient of determination of the prediction:
0.4229517825255892
R^2 score: 0.9112439993057248
Correlation coefficient: 0.9545910115362101
Test data - unseen during training:
R^2 score: 0.4229517825255892
Correlation coefficient: 0.6503474321665222
[7.55581908 6.76701055 5.75119478 6.49932728 7.04485599 6.98625628
 6.20939289 5.47622337 7.15599556 6.92515496 5.79420297 5.94197432
 6.35947903 6.37983139 6.28160132 7.17954326]
0      7.260428
21     6.398375
106    5.853872
68     6.080922
44     7.638272
14     7.067526
108    6.154902
62     6.455932
13     7.158641
9      5.702436
96     5.136677
49     5.619789
54     7.075721
114    6.026872
115    5.568636
87     7.288193
Name: LoVo/DX, dtype: float64
Training Root Mean Square Error: 0.2861656827145399
Testing Root Mean Square Error: 0.554481606951121
```

3.1 Search inside correlation space

[25]:
```python
step = 0.01
initial_step = corr_low
last_step = corr_high
first_list = [x / 100.0 for x in range(int(initial_step*100),␣
 ↪int(last_step*100), int(step*100))]
n_estimators = [range(2,21,1)]
corr_th = []
second_list = []
```

```
third_list = []
fourth_l = []
fifth_l = []
f_list = []
fif_list = []
for i in first_list:

    for estimator in n_estimators[0]:

        without_standardization, train_r2, test_r2, _, h_, target_column_name,␣
↪training_data_RMSE, test_data_RMSE = pred_model.
↪prepare_data_and_create_model(molecular_descriptors_df=data,
                                                                                ␣
↪                    correlation_threshold=i,
                                                                                ␣
↪                    standardization=False,
                                                                                ␣
↪                    model_type='RandomForestRegressor',
                                                                                ␣
↪                    n_estimators_=estimator,
                                                                                ␣
↪                    target_column_name = target,
                                                                                ␣
↪                    random_state=random_state,
                                                                                ␣
↪                    train_test_split_=True,
                                                                                ␣
↪                    verbose=False)
        corr_th.append(i)
        second_list.append(train_r2)
        third_list.append(test_r2)
        fourth_l.append(training_data_RMSE)
        fifth_l.append(test_data_RMSE)
        f_list.append(len(h_))
        fif_list.append(estimator)
```

[26]:
```
df_without_standardization = pd.DataFrame(data=corr_th, columns=["Correlation␣
↪threshold"])
df_without_standardization['Training data R^2 score'] = second_list
df_without_standardization['Test data R^2 score'] = third_list
df_without_standardization['Training RMSE'] = fourth_l
df_without_standardization['Test RMSE'] = fifth_l
df_without_standardization['Number of features'] = f_list
df_without_standardization['Number of estimators'] = fif_list
```

[27]:
```
df_random_forest = df_without_standardization.copy()
#df_without_standardization.to_excel('../Data/
↪A549_Random_forest_rs_'+str(random_state)+'.xlsx')
```

[28]:
```
corre_list = [x / 100.0 for x in range(int(initial_step*100),␣
↪int(last_step*100), int(0.01*100))]
for element in corre_list:
    element_ =␣
↪df_without_standardization[df_without_standardization['Correlation␣
↪threshold'] == float(element)]
    plt.plot(element_['Number of estimators'], element_['Training data R^2␣
↪score'], label = "Training data R^2 score", marker='v')
    plt.plot(element_['Number of estimators'], element_['Test data R^2 score'],␣
↪label = "Test data R^2 score", marker='D')
    plt.legend()
    plt.xlabel('Number of estimators -> correlation threshold: '+str(element)+'.
↪ \n Number of features: '+str(element_['Number of features'].iloc[0]))
    plt.xlim(max(element_['Number of estimators'])+1, min(element_['Number of␣
↪estimators'])-1)
    plt.ylabel('R^2 score')
    plt.ylim([0, 1])
    plt.rc('grid', linestyle="-.", color='black')
    plt.grid(True)
    plt.show()
```

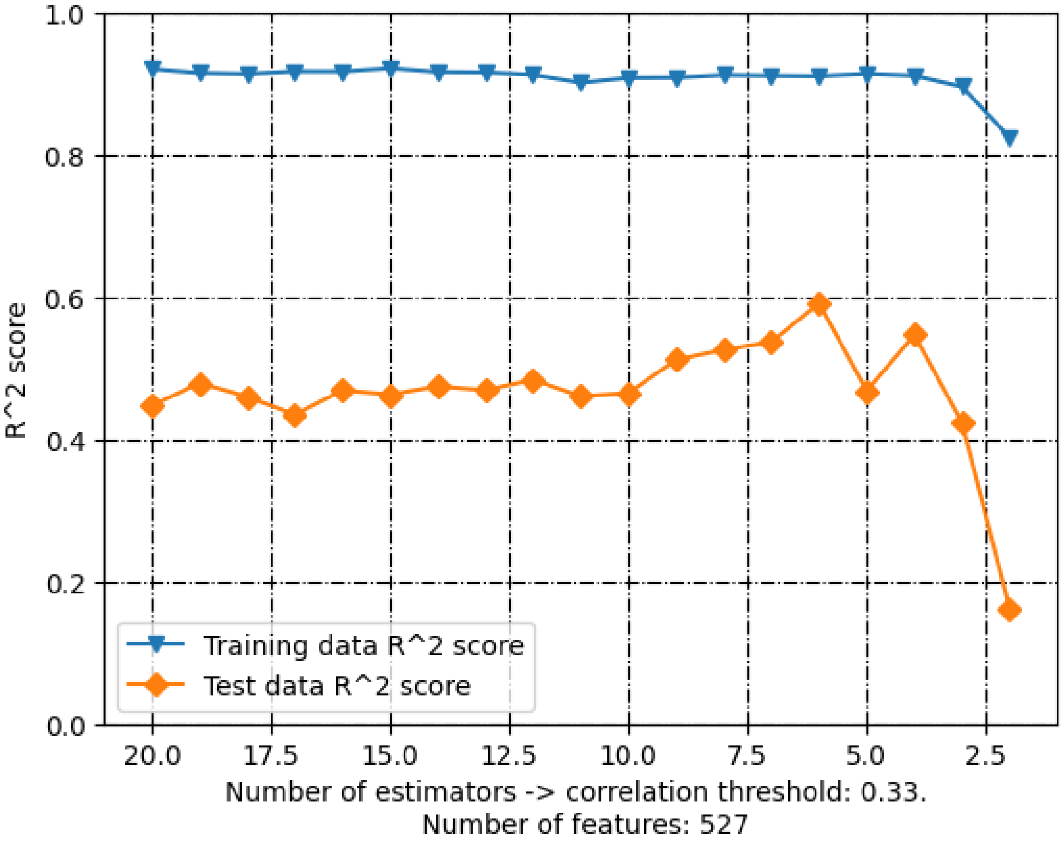

1.0
0.8
0.6
0.4
0.2
0.0
R^2 score
Training data R^2 score
Test data R^2 score
20.0
17.5
15.0
12.5
10.0
7.5
5.0
2.5
Number of estimators -> correlation threshold: 0.33.
Number of features: 527

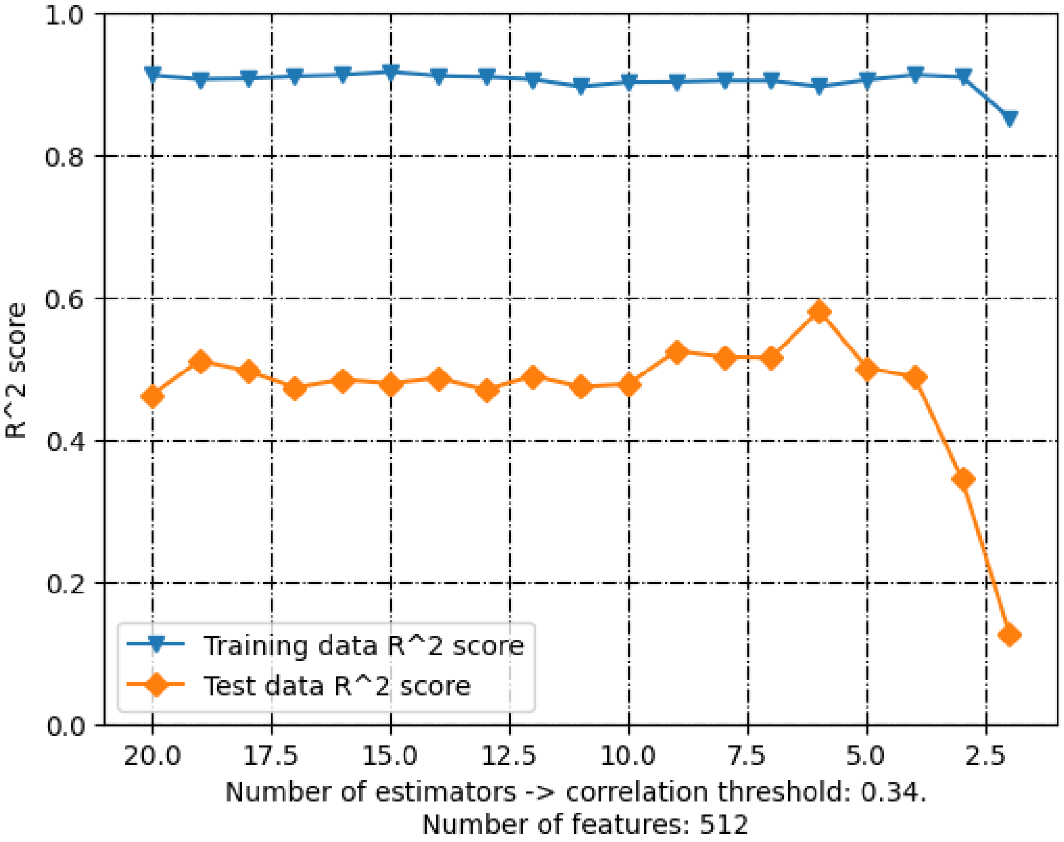

1.0
0.8
0.6
0.4
0.2
0.0
R^2 score
Training data R^2 score
Test data R^2 score
20.0
17.5
15.0
12.5
10.0
7.5
5.0
2.5
Number of estimators -> correlation threshold: 0.34.
Number of features: 512

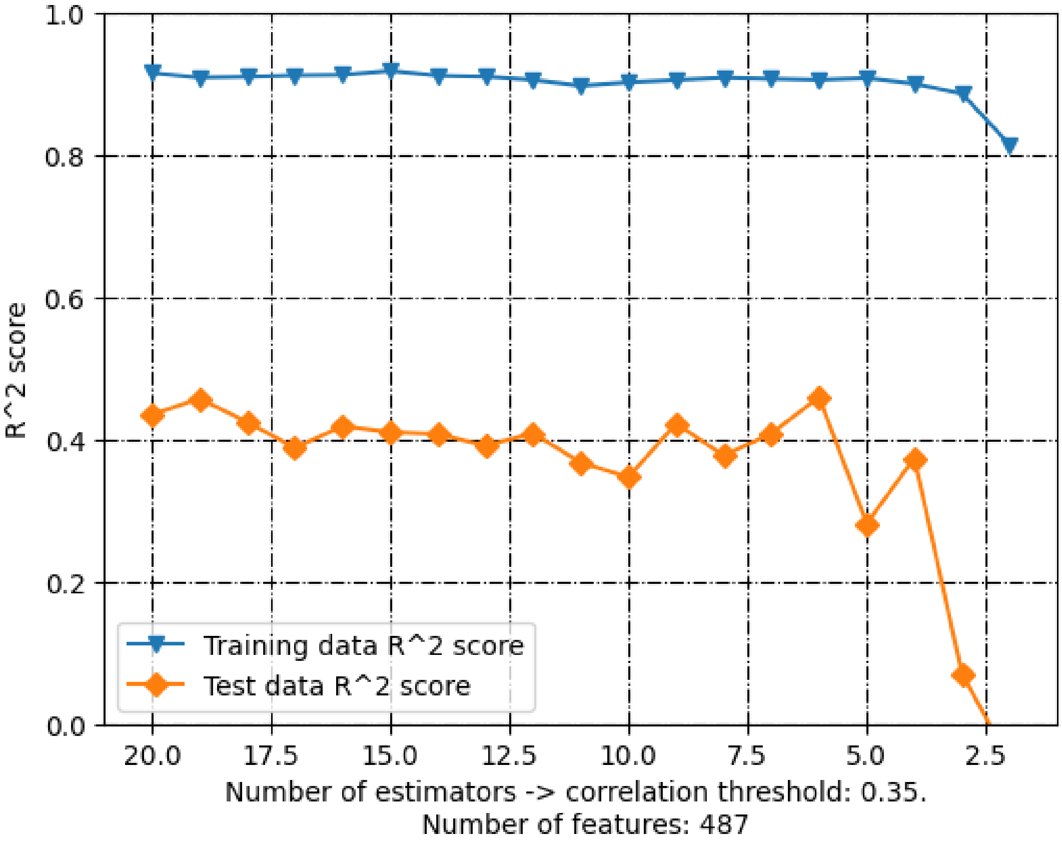

1.0
0.8
0.6
0.4
0.2
0.0
R^2 score
20.0
17.5
15.0
12.5
10.0
7.5
5.0
2.5
Training data R^2 score
Test data R^2 score
Number of estimators -> correlation threshold: 0.35.
Number of features: 487

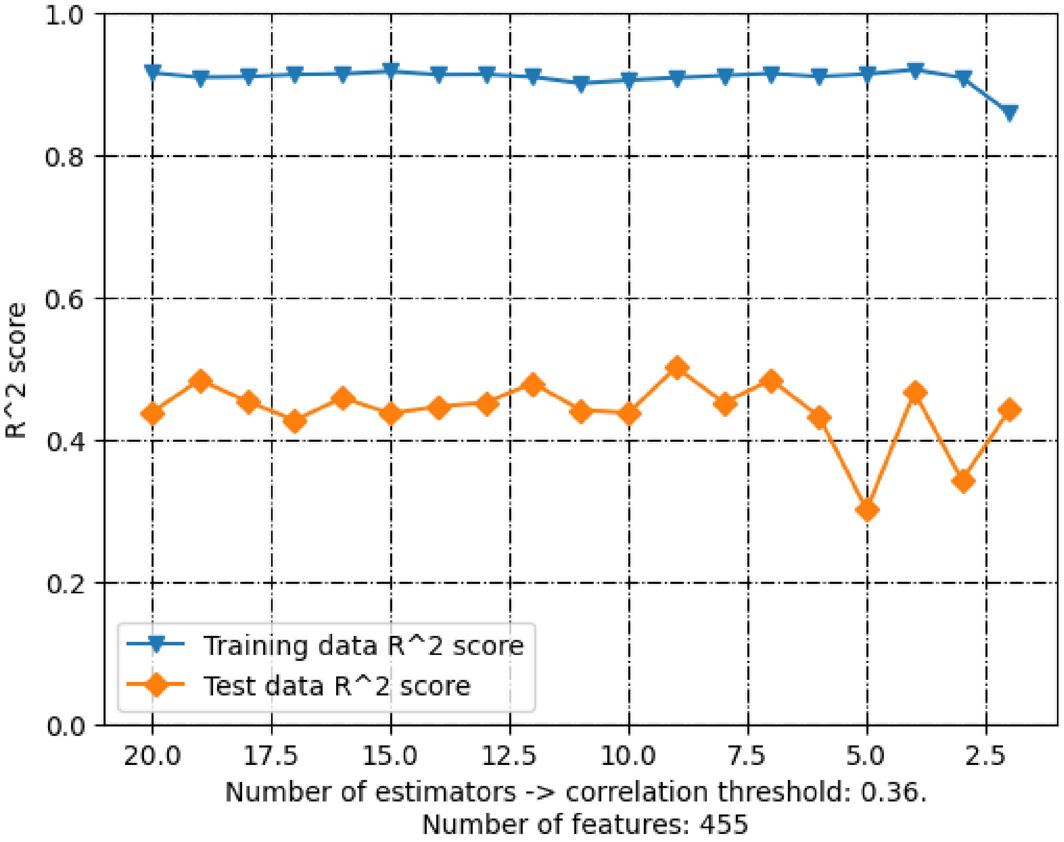
1.0
0.8
0.6
0.4
0.2
0.0
R^2 score
20.0
17.5
15.0
12.5
10.0
7.5
5.0
2.5
Training data R^2 score
Test data R^2 score
Number of estimators -> correlation threshold: 0.36.
Number of features: 455

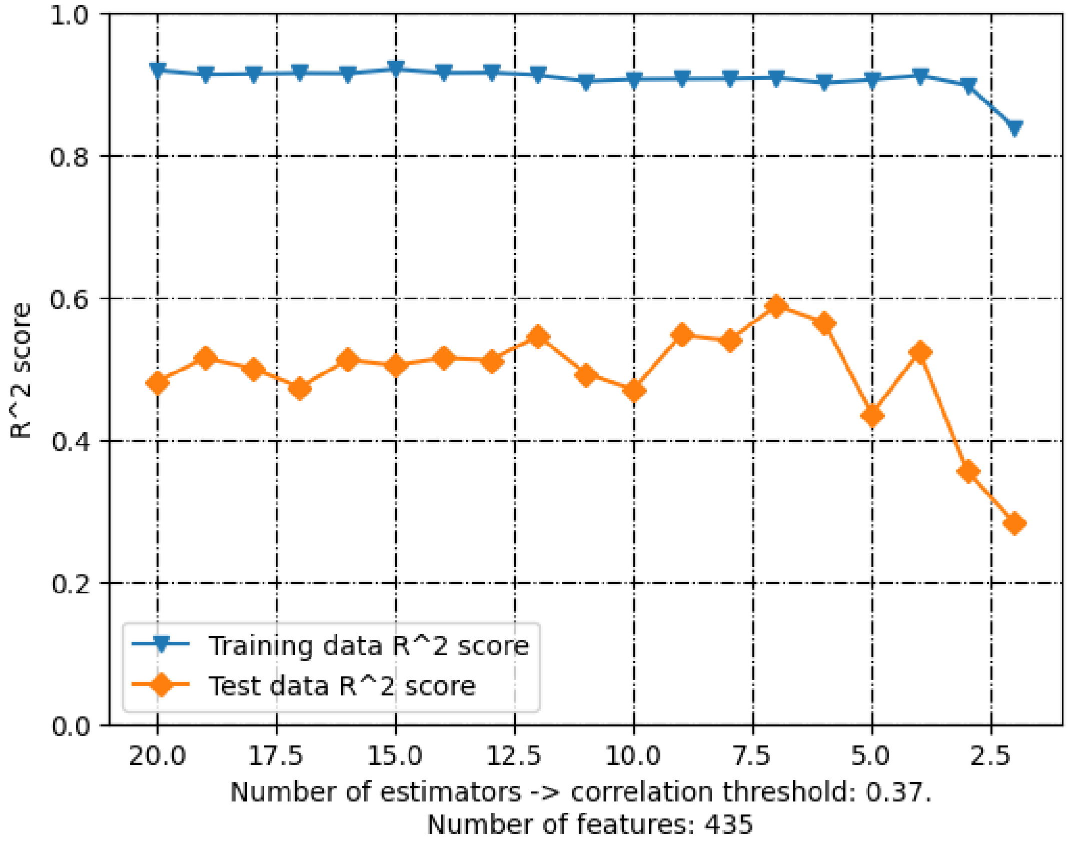
1.0
0.8
0.6
0.4
0.2
0.0
R^2 score
Training data R^2 score
Test data R^2 score
20.0
17.5
15.0
12.5
10.0
7.5
5.0
2.5
Number of estimators -> correlation threshold: 0.37.
Number of features: 435

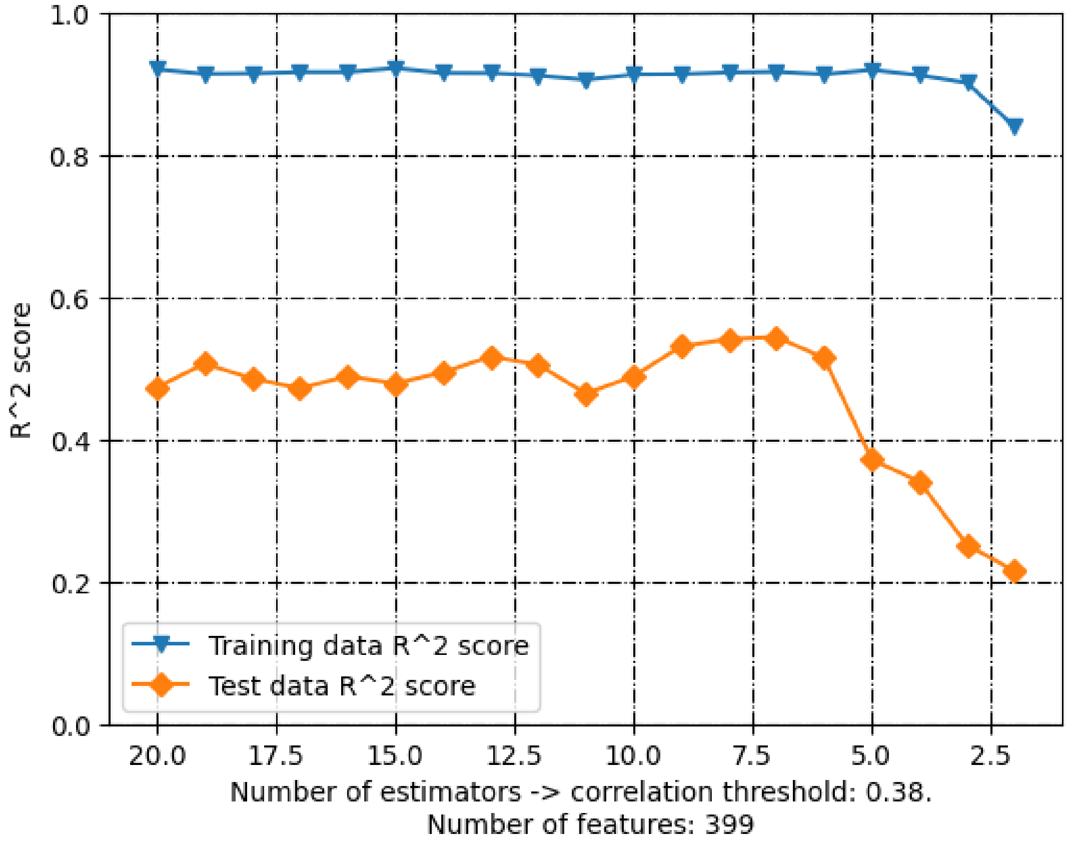
1.0
0.8
0.6
0.4
0.2
0.0
R^2 score
Training data R^2 score
Test data R^2 score
20.0
17.5
15.0
12.5
10.0
7.5
5.0
2.5
Number of estimators -> correlation threshold: 0.38.
Number of features: 399

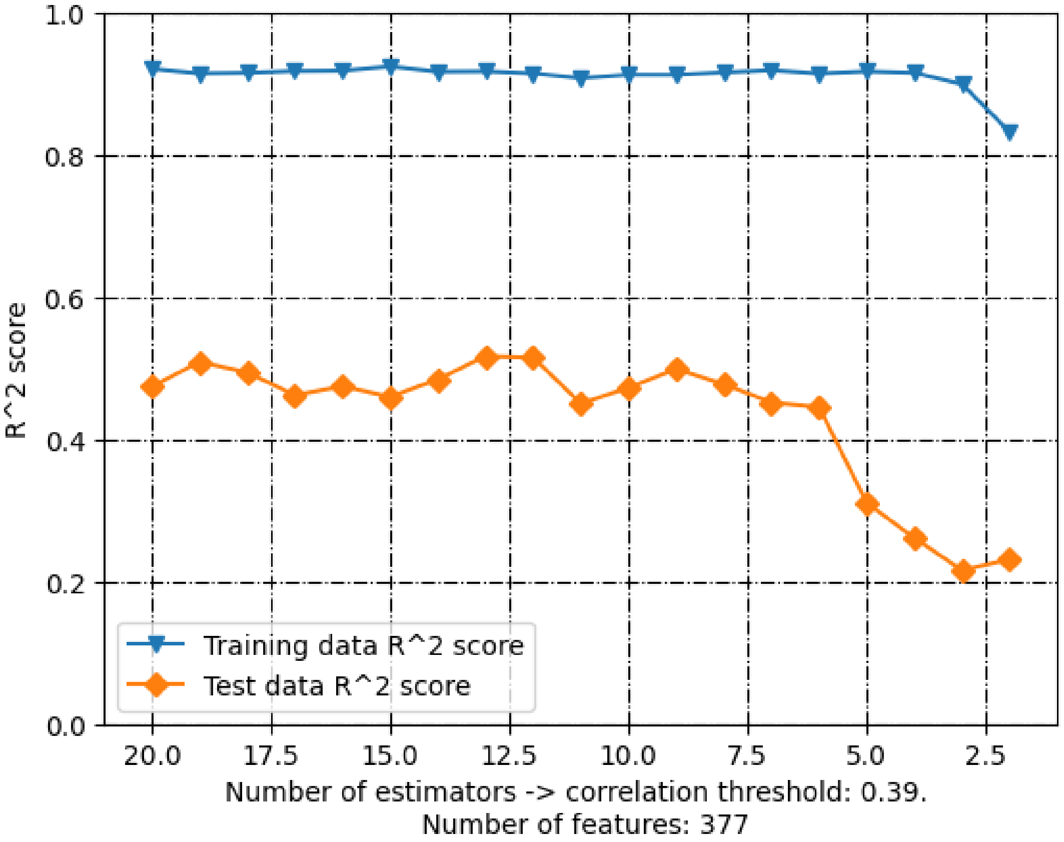
1.0
0.8
0.6
0.4
0.2
0.0
R^2 score
20.0
17.5
15.0
12.5
10.0
7.5
5.0
2.5
Number of estimators -> correlation threshold: 0.39.
Number of features: 377
Training data R^2 score
Test data R^2 score

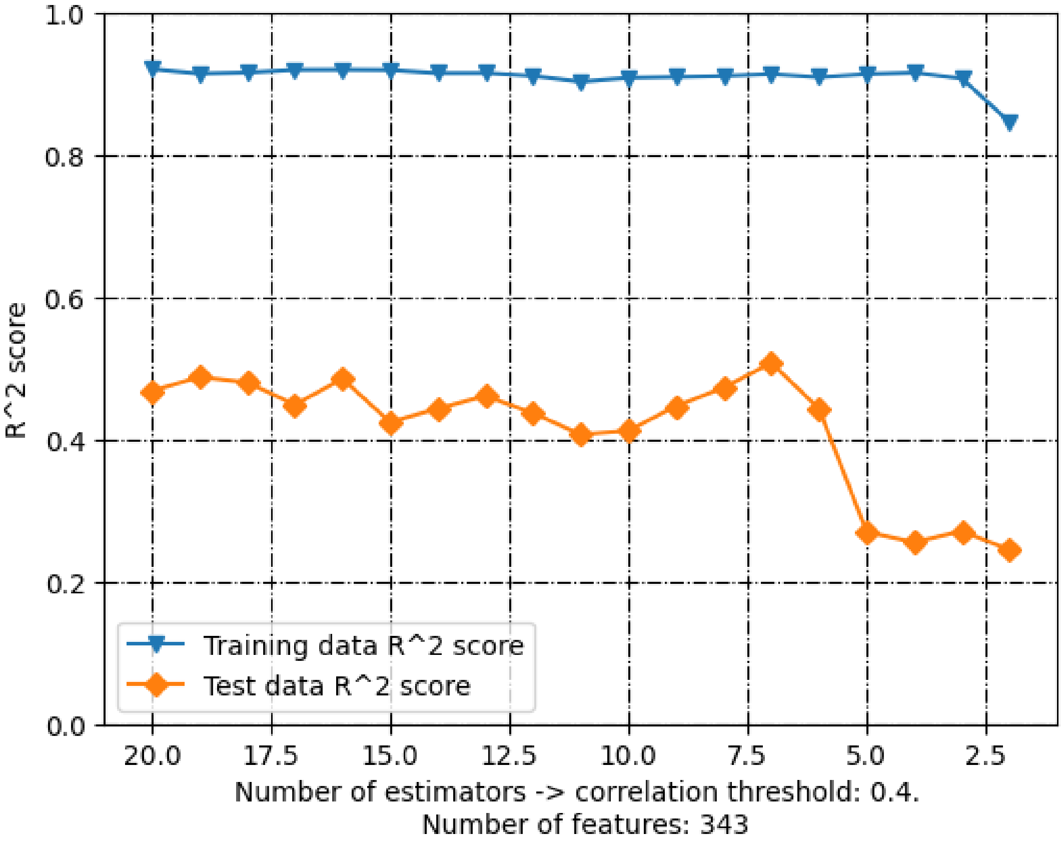
1.0
0.8
0.6
0.4
0.2
0.0
R^2 score
Training data R^2 score
Test data R^2 score
20.0
17.5
15.0
12.5
10.0
7.5
5.0
2.5
Number of estimators -> correlation threshold: 0.4.
Number of features: 343

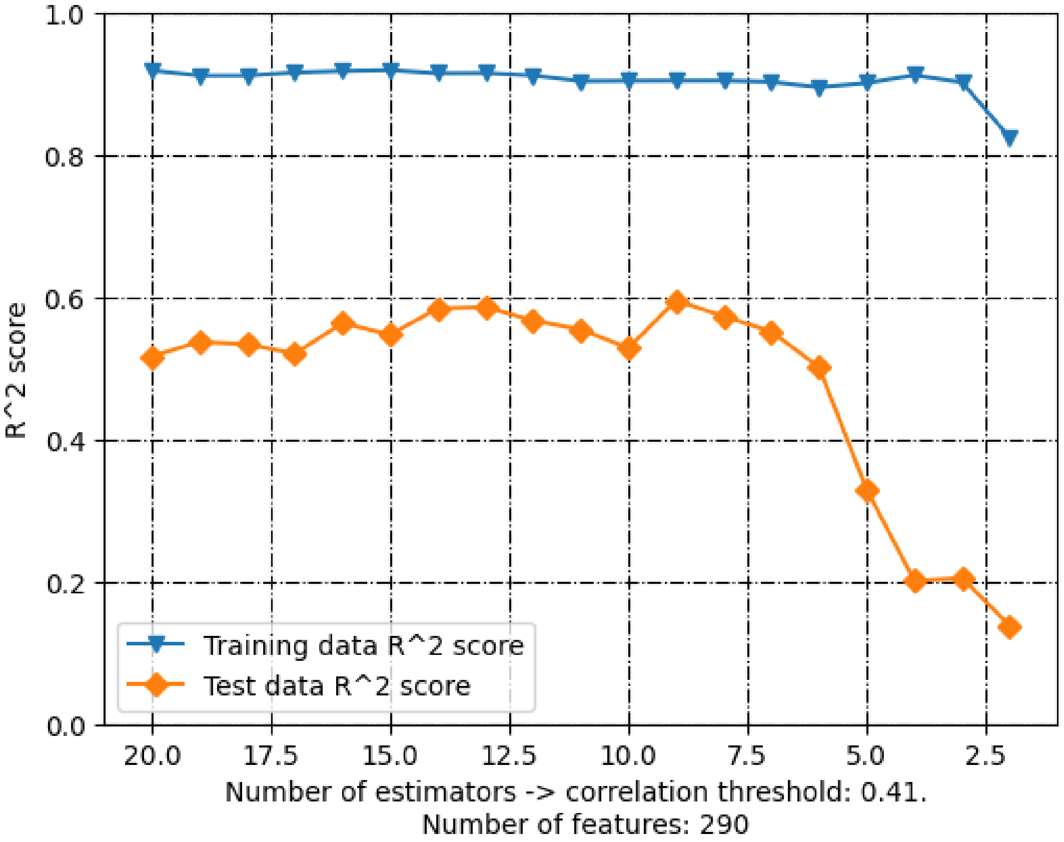
1.0
0.8
0.6
0.4
0.2
0.0
R^2 score
20.0
17.5
15.0
12.5
10.0
7.5
5.0
2.5
Number of estimators -> correlation threshold: 0.41.
Number of features: 290
Training data R^2 score
Test data R^2 score

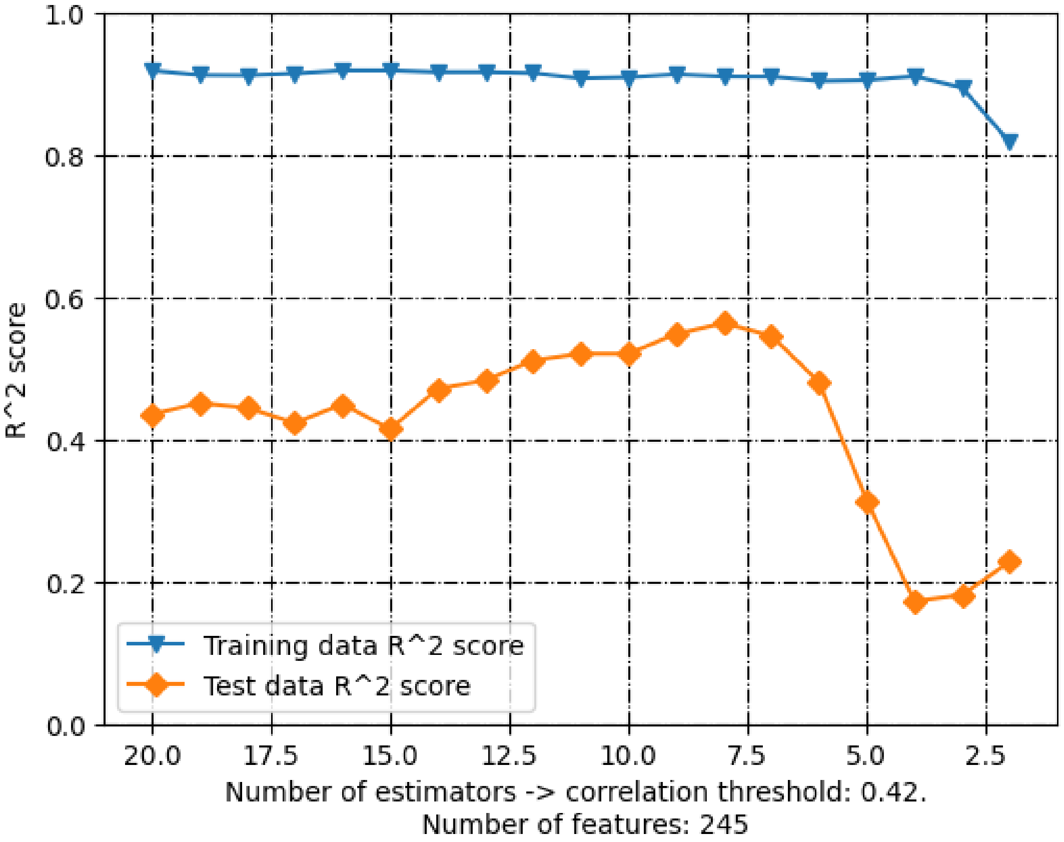
1.0
0.8
0.6
0.4
0.2
0.0
R^2 score
Training data R^2 score
Test data R^2 score
20.0
17.5
15.0
12.5
10.0
7.5
5.0
2.5
Number of estimators -> correlation threshold: 0.42.
Number of features: 245

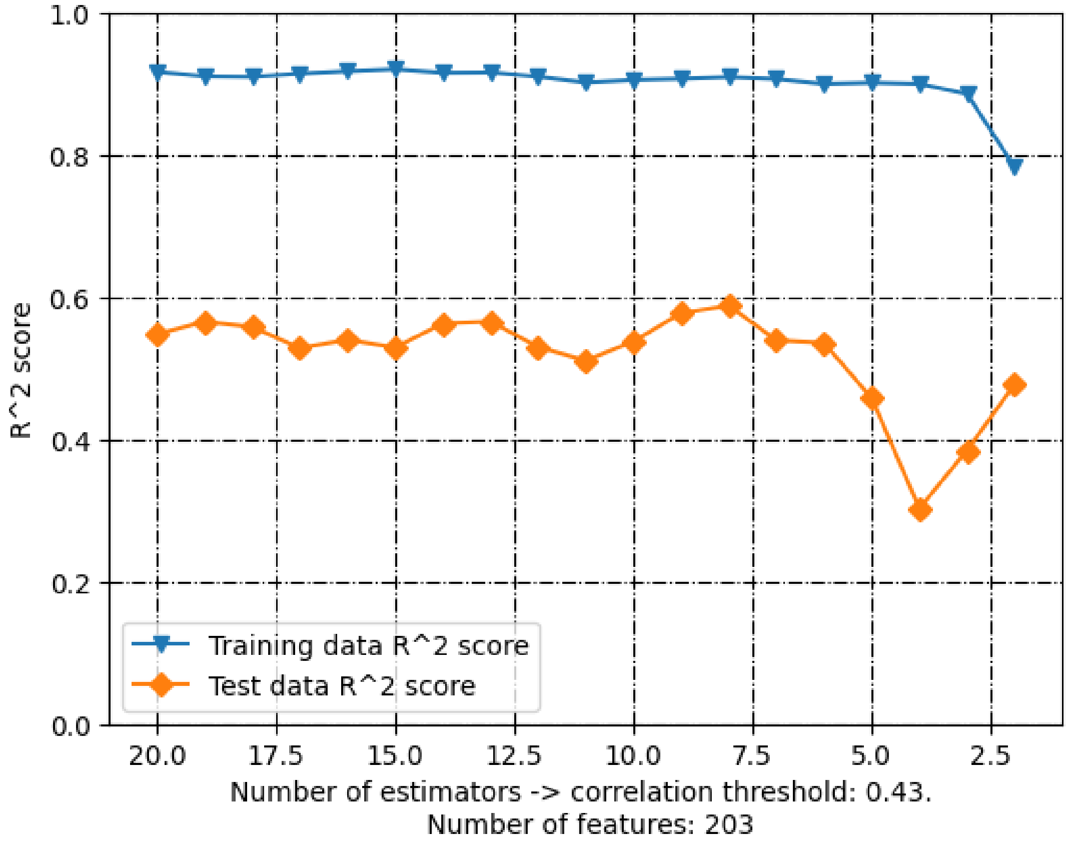

1.0
0.8
0.6
0.4
0.2
0.0
R^2 score
Training data R^2 score
Test data R^2 score
20.0
17.5
15.0
12.5
10.0
7.5
5.0
2.5
Number of estimators -> correlation threshold: 0.43.
Number of features: 203

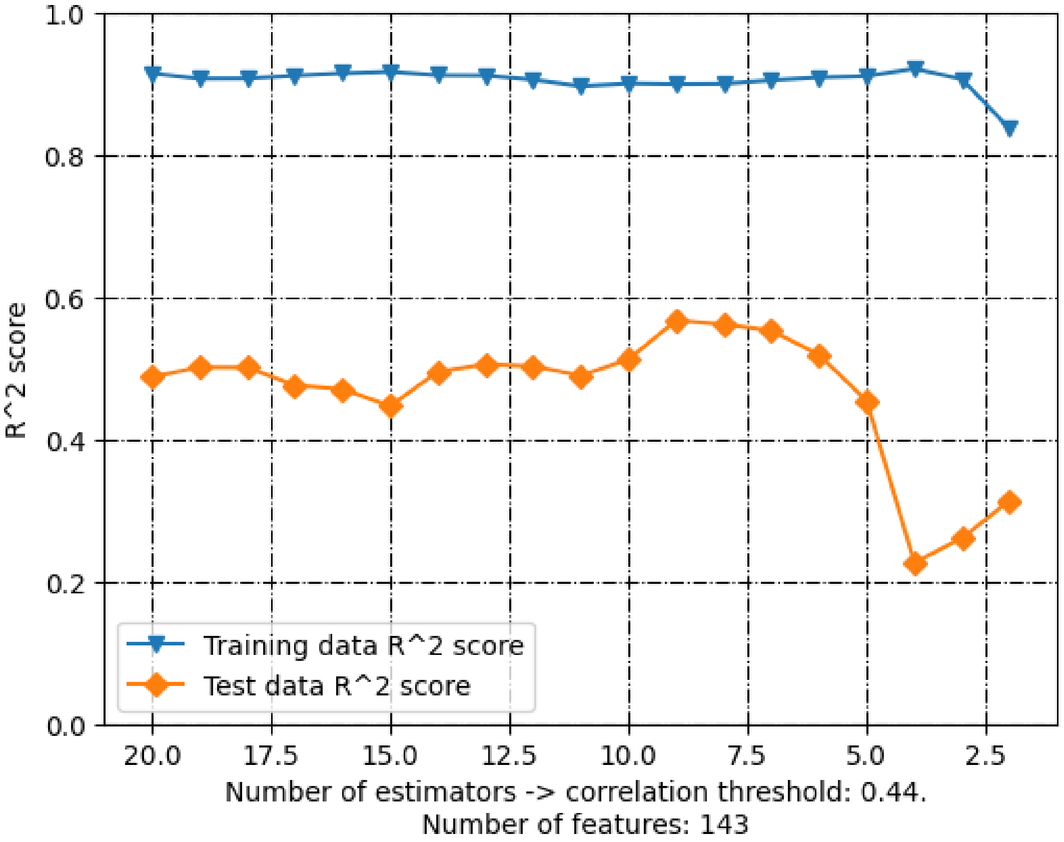
1.0
0.8
0.6
0.4
0.2
0.0
R^2 score
Training data R^2 score
Test data R^2 score
20.0
17.5
15.0
12.5
10.0
7.5
5.0
2.5
Number of estimators -> correlation threshold: 0.44.
Number of features: 143

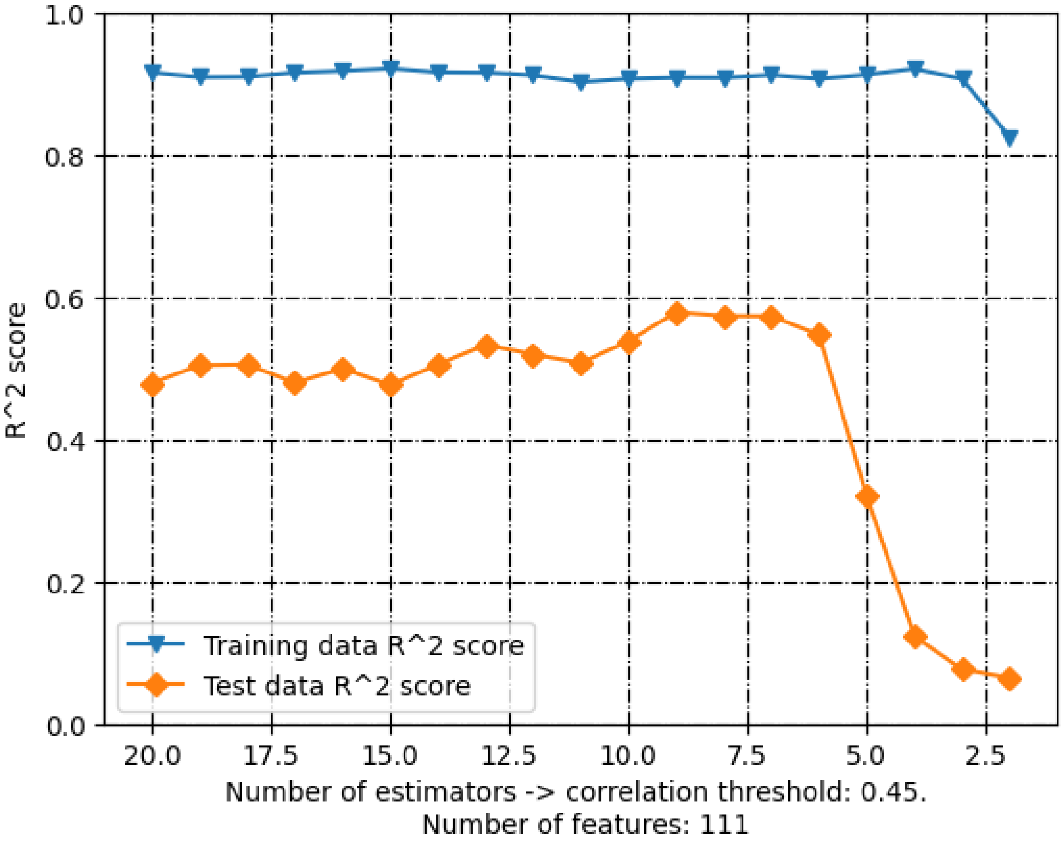
1.0
0.8
0.6
0.4
0.2
0.0
R^2 score
Training data R^2 score
Test data R^2 score
20.0
17.5
15.0
12.5
10.0
7.5
5.0
2.5
Number of estimators -> correlation threshold: 0.45.
Number of features: 111

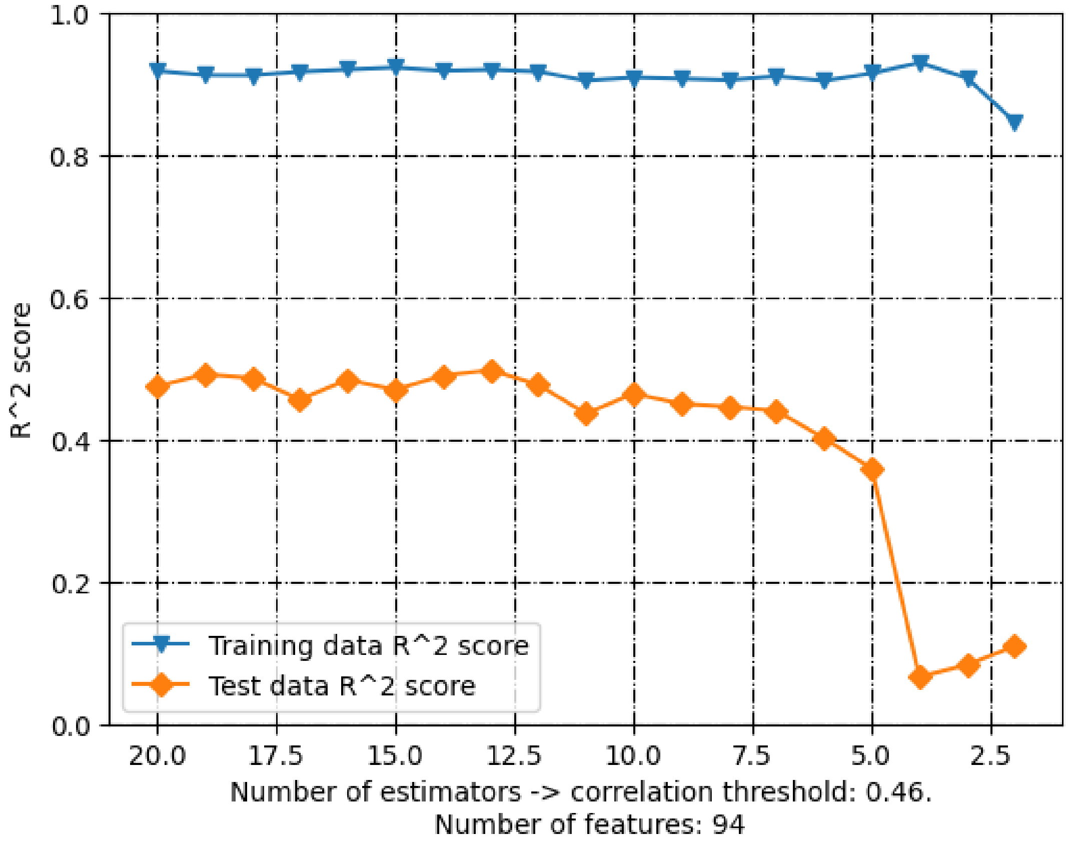

1.0
0.8
0.6
0.4
0.2
0.0
R^2 score
20.0
17.5
15.0
12.5
10.0
7.5
5.0
2.5
Training data R^2 score
Test data R^2 score
Number of estimators -> correlation threshold: 0.46.
Number of features: 94

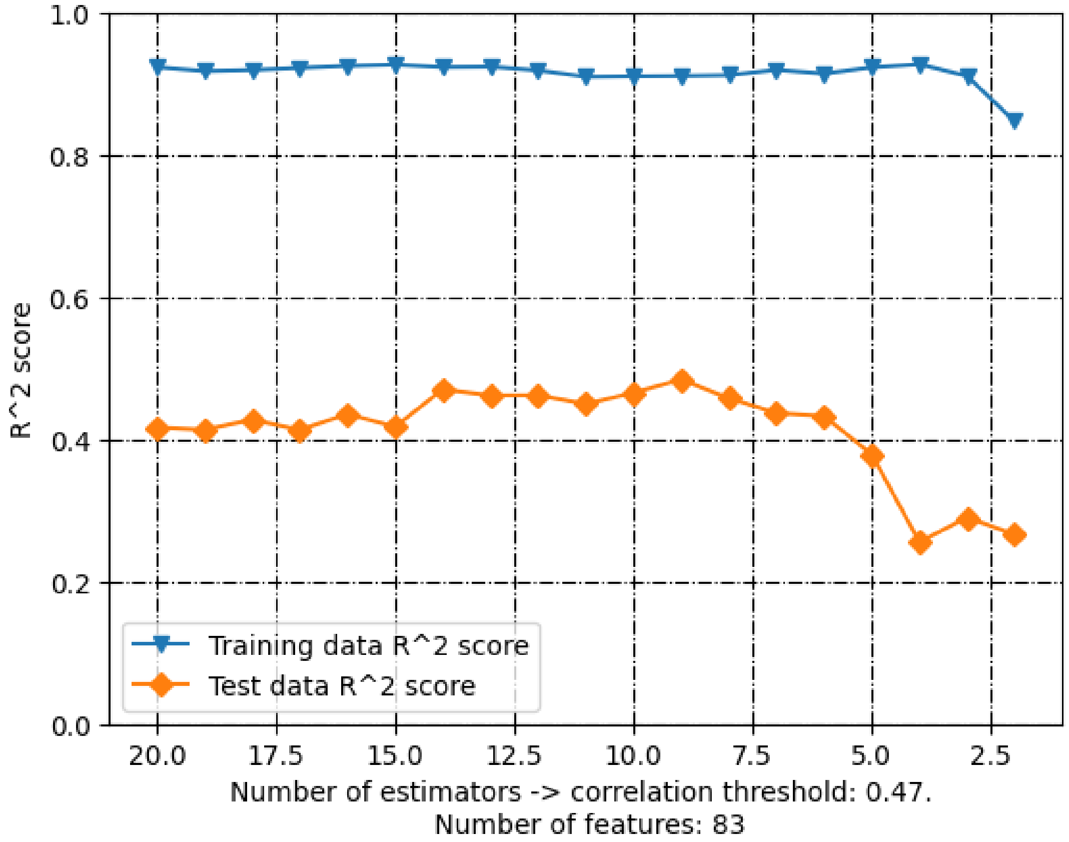
1.0
0.8
0.6
0.4
0.2
0.0
R^2 score
Training data R^2 score
Test data R^2 score
20.0
17.5
15.0
12.5
10.0
7.5
5.0
2.5
Number of estimators -> correlation threshold: 0.47.
Number of features: 83

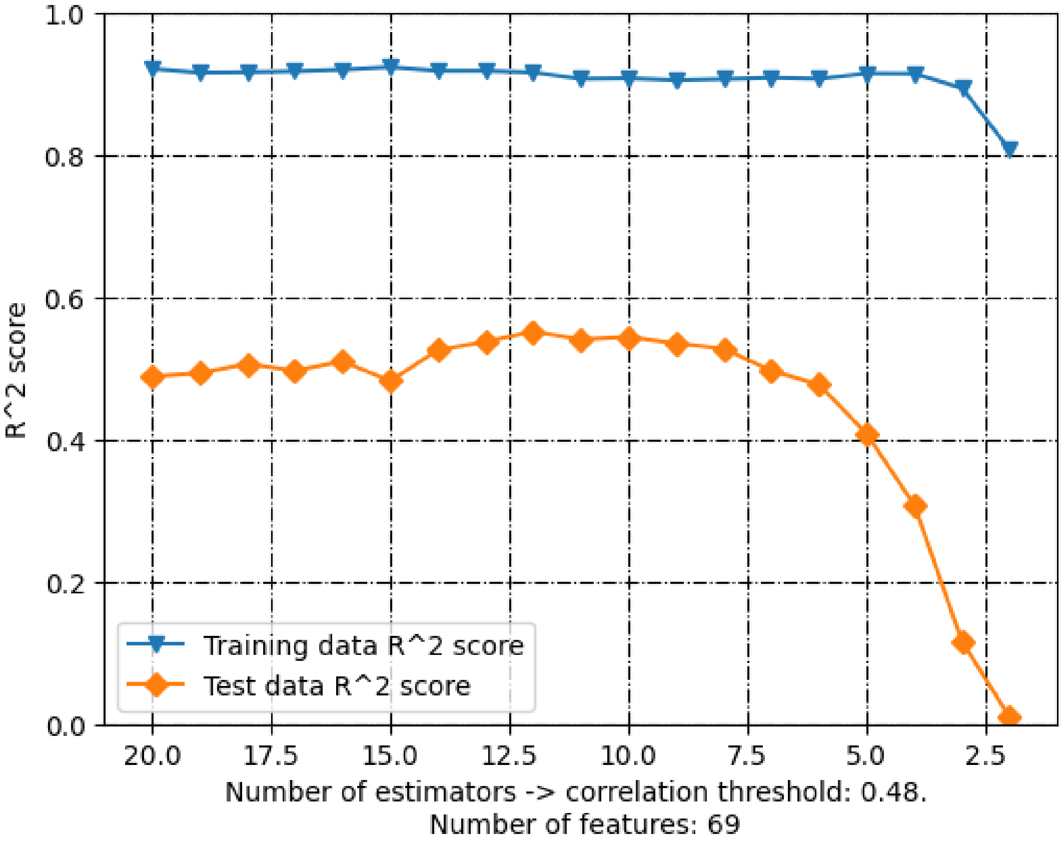

1.0
0.8
0.6
0.4
0.2
0.0
R^2 score
20.0
17.5
15.0
12.5
10.0
7.5
5.0
2.5
Training data R^2 score
Test data R^2 score
Number of estimators -> correlation threshold: 0.48.
Number of features: 69

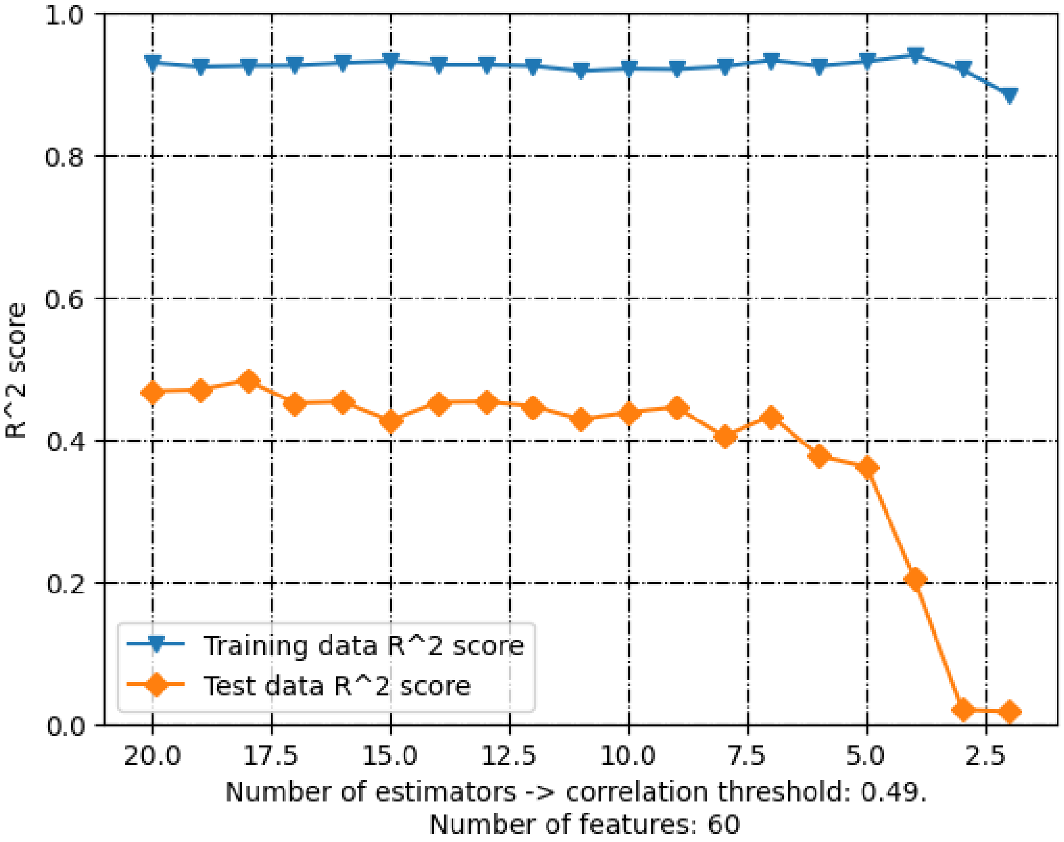
1.0
0.8
0.6
0.4
0.2
0.0
R^2 score
Training data R^2 score
Test data R^2 score
20.0
17.5
15.0
12.5
10.0
7.5
5.0
2.5
Number of estimators -> correlation threshold: 0.49.
Number of features: 60

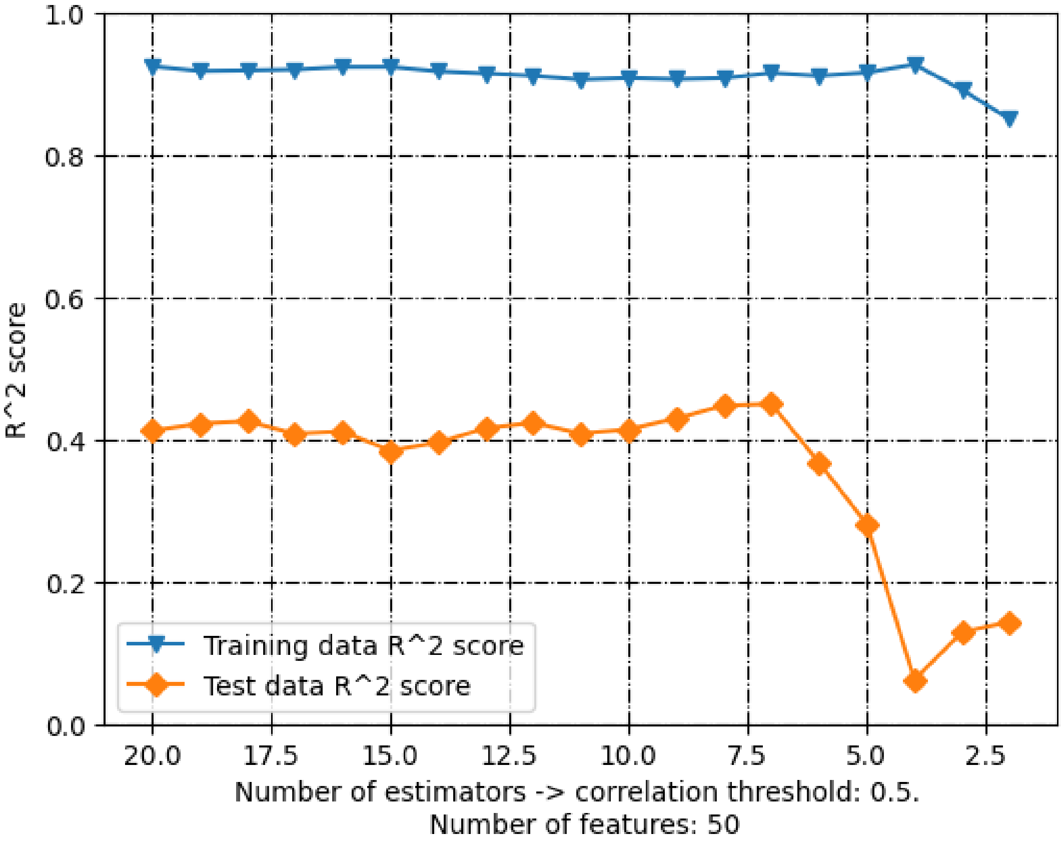
1.0
0.8
0.6
0.4
0.2
0.0
R^2 score
Training data R^2 score
Test data R^2 score
20.0
17.5
15.0
12.5
10.0
7.5
5.0
2.5
Number of estimators -> correlation threshold: 0.5.
Number of features: 50

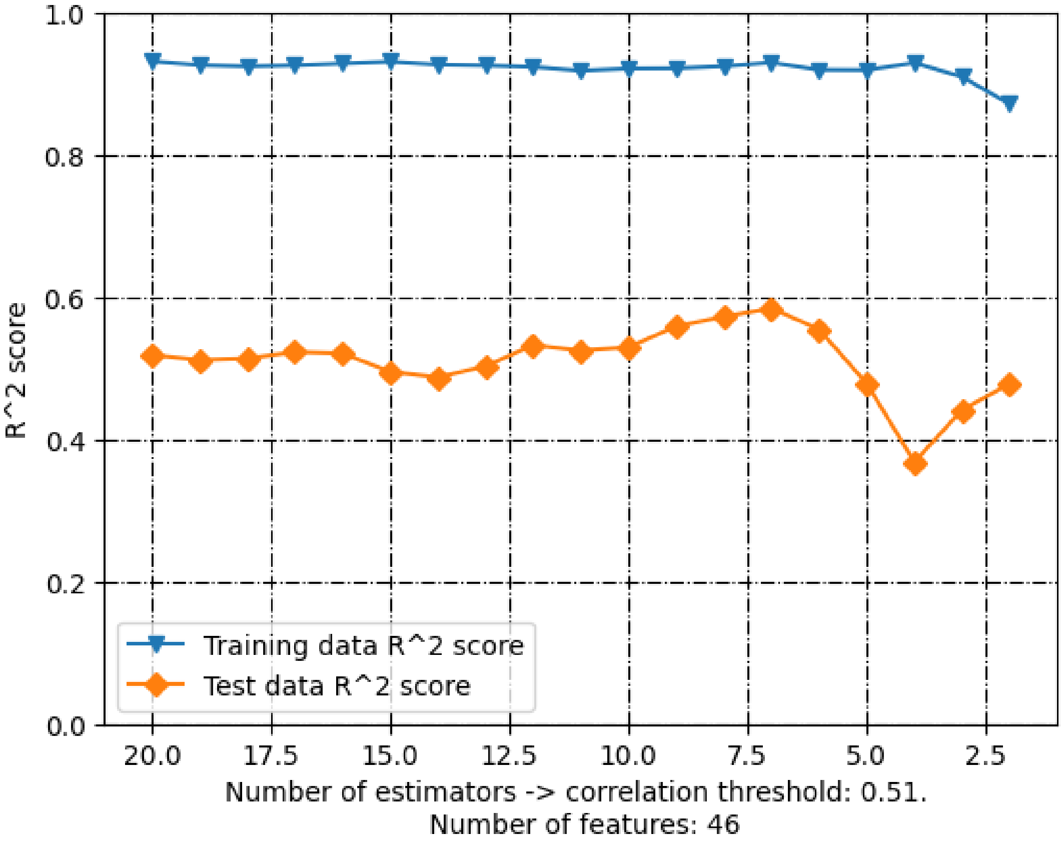

1.0
0.8
0.6
0.4
0.2
0.0
R^2 score
20.0
17.5
15.0
12.5
10.0
7.5
5.0
2.5
Number of estimators -> correlation threshold: 0.51.
Number of features: 46
Training data R^2 score
Test data R^2 score

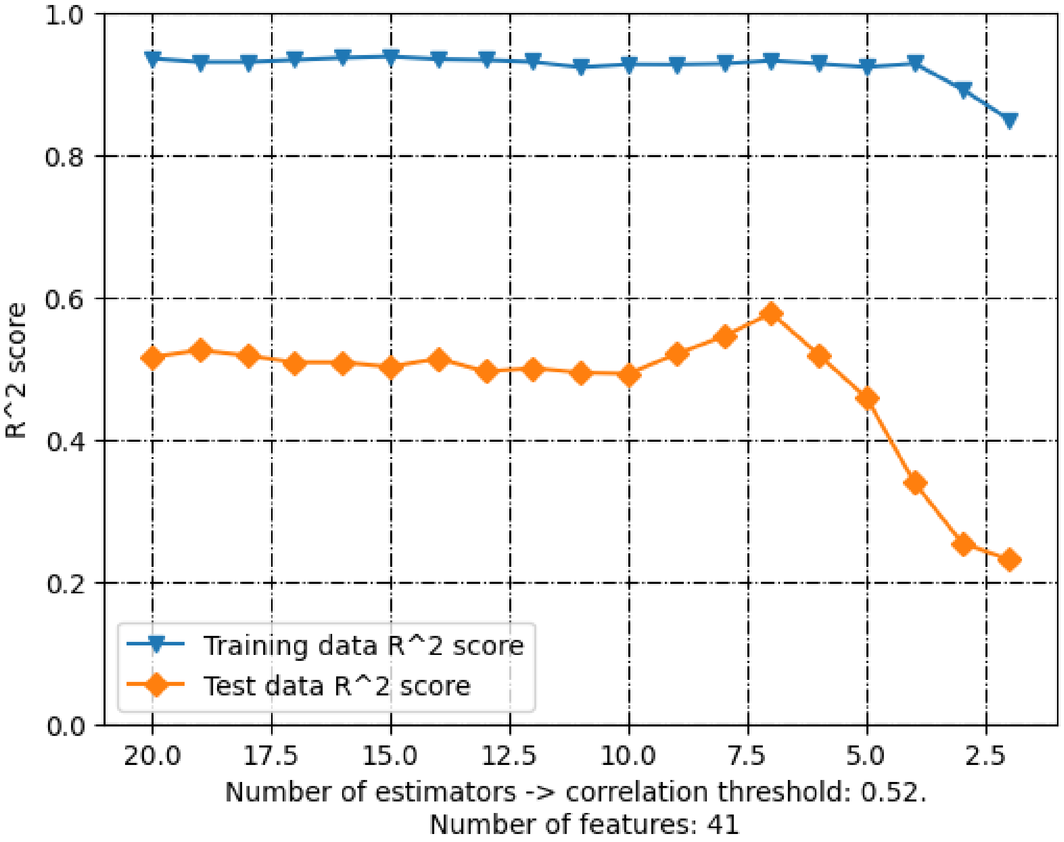
1.0
0.8
0.6
0.4
0.2
0.0
R^2 score
20.0
17.5
15.0
12.5
10.0
7.5
5.0
2.5
Number of estimators -> correlation threshold: 0.52.
Number of features: 41
Training data R^2 score
Test data R^2 score

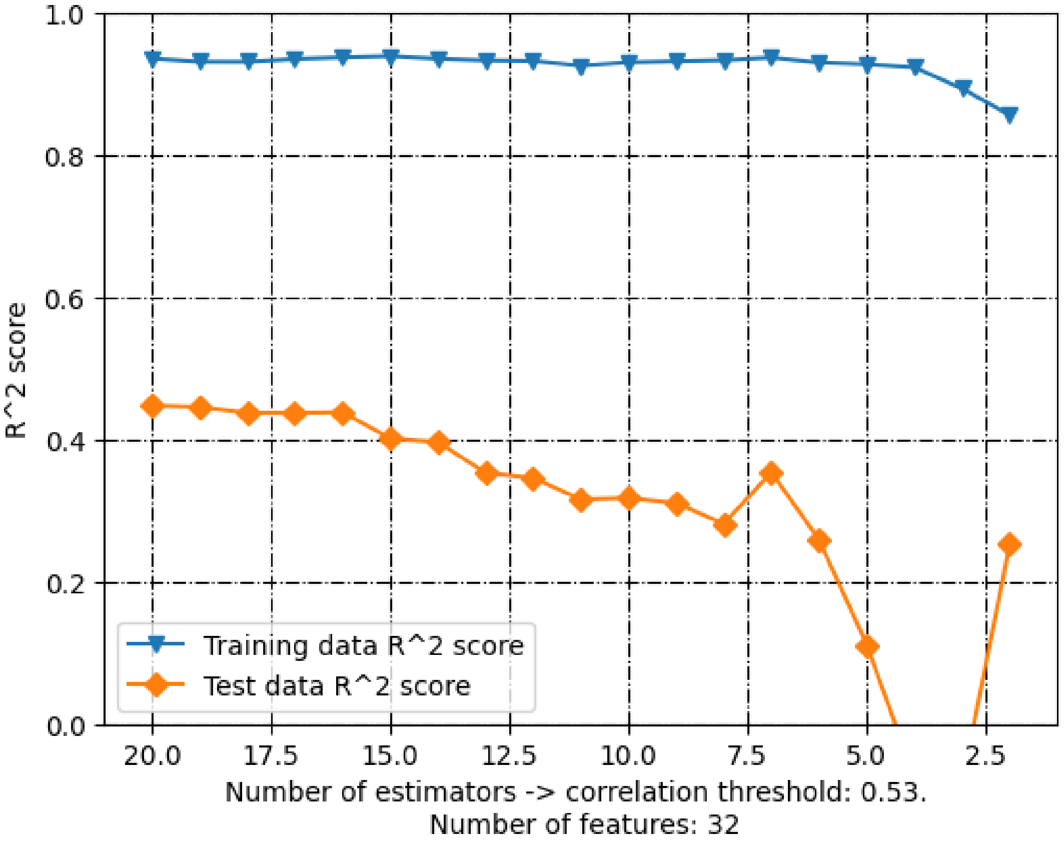
1.0
0.8
0.6
0.4
0.2
0.0
R^2 score
Training data R^2 score
Test data R^2 score
20.0
17.5
15.0
12.5
10.0
7.5
5.0
2.5
Number of estimators -> correlation threshold: 0.53.
Number of features: 32

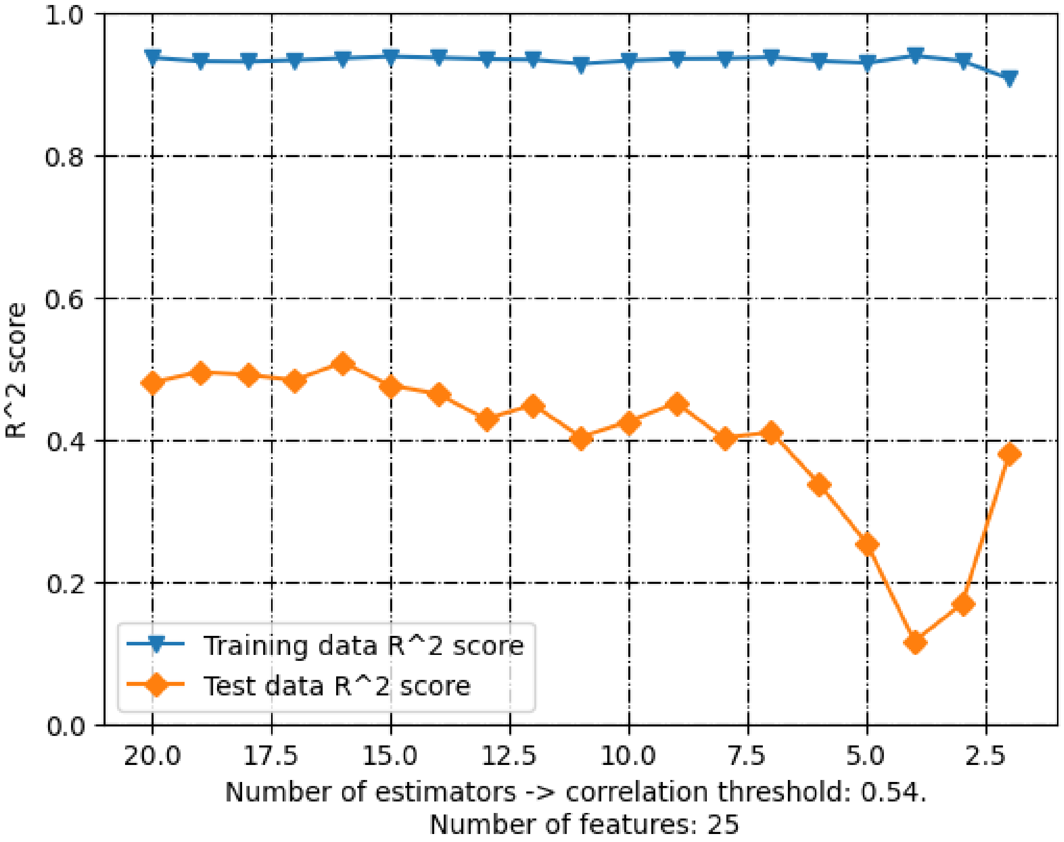

1.0
0.8
0.6
0.4
0.2
0.0
R^2 score
Training data R^2 score
Test data R^2 score
20.0
17.5
15.0
12.5
10.0
7.5
5.0
2.5
Number of estimators -> correlation threshold: 0.54.
Number of features: 25

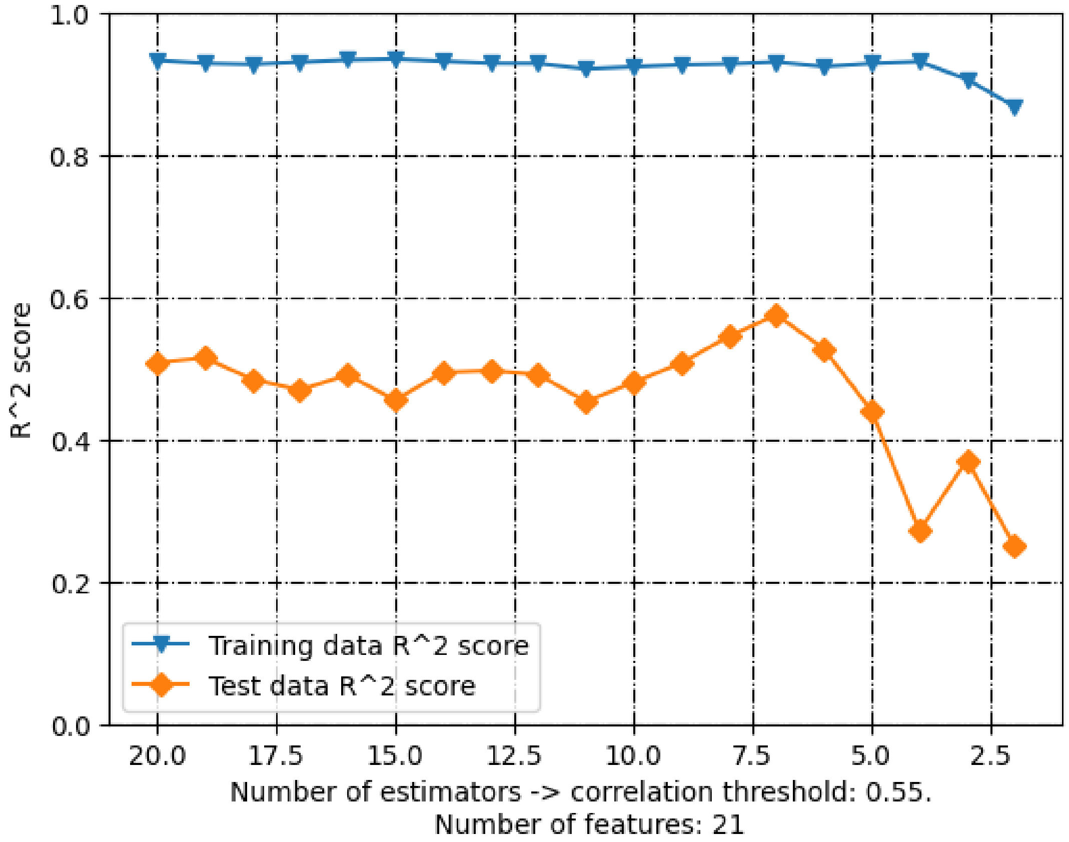
1.0
0.8
0.6
0.4
0.2
0.0
R^2 score
20.0
17.5
15.0
12.5
10.0
7.5
5.0
2.5
Number of estimators -> correlation threshold: 0.55.
Number of features: 21
Training data R^2 score
Test data R^2 score

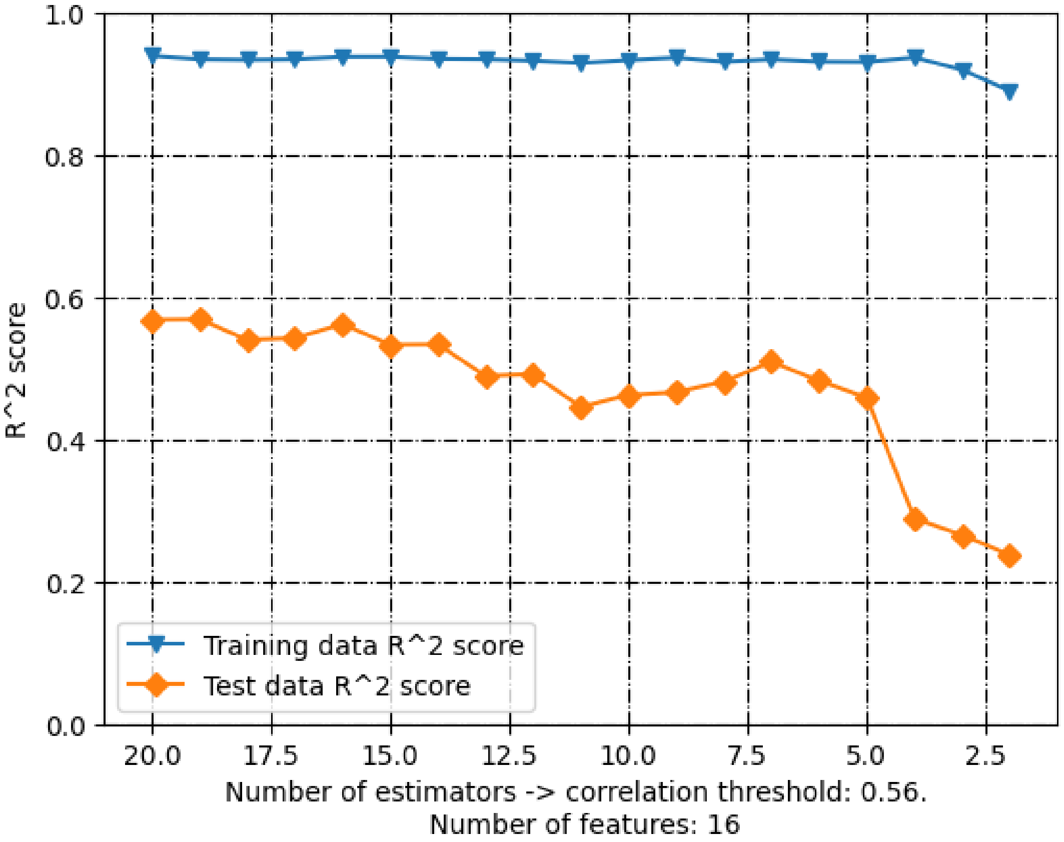

1.0
0.8
0.6
0.4
0.2
0.0
R^2 score
Training data R^2 score
Test data R^2 score
20.0
17.5
15.0
12.5
10.0
7.5
5.0
2.5
Number of estimators -> correlation threshold: 0.56.
Number of features: 16

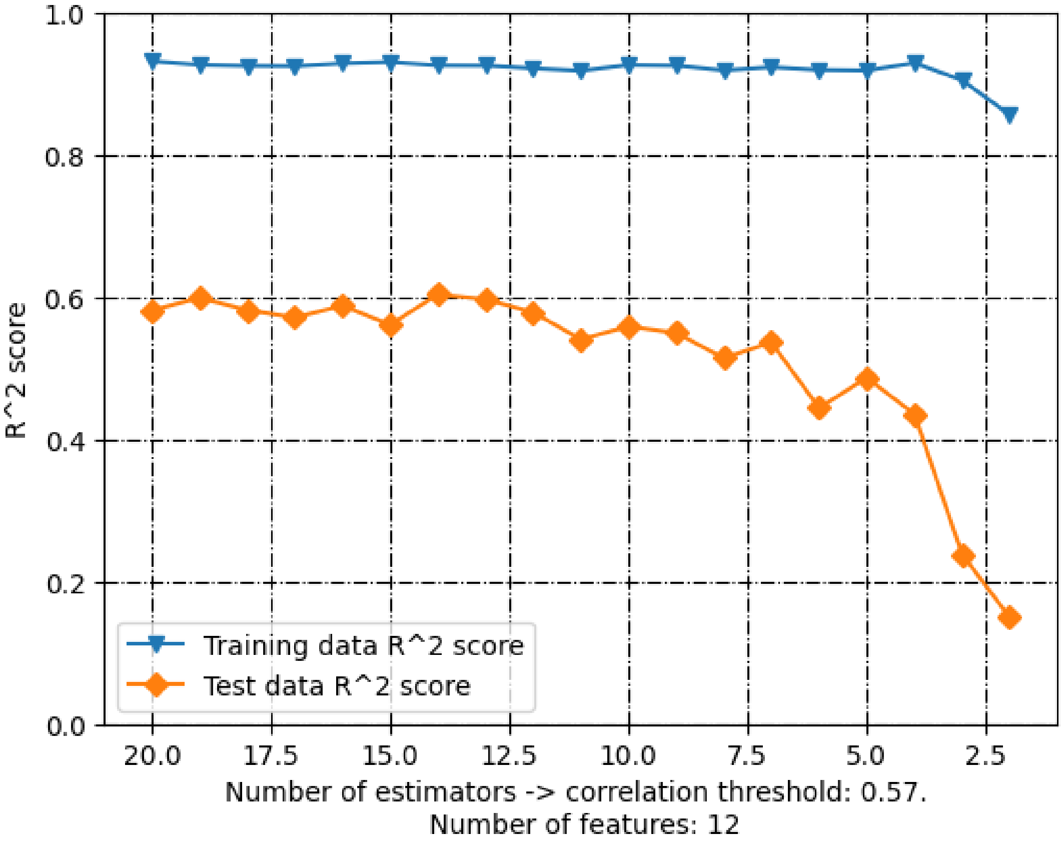

1.0
0.8
0.6
0.4
0.2
0.0
R^2 score
Training data R^2 score
Test data R^2 score
20.0
17.5
15.0
12.5
10.0
7.5
5.0
2.5
Number of estimators -> correlation threshold: 0.57.
Number of features: 12

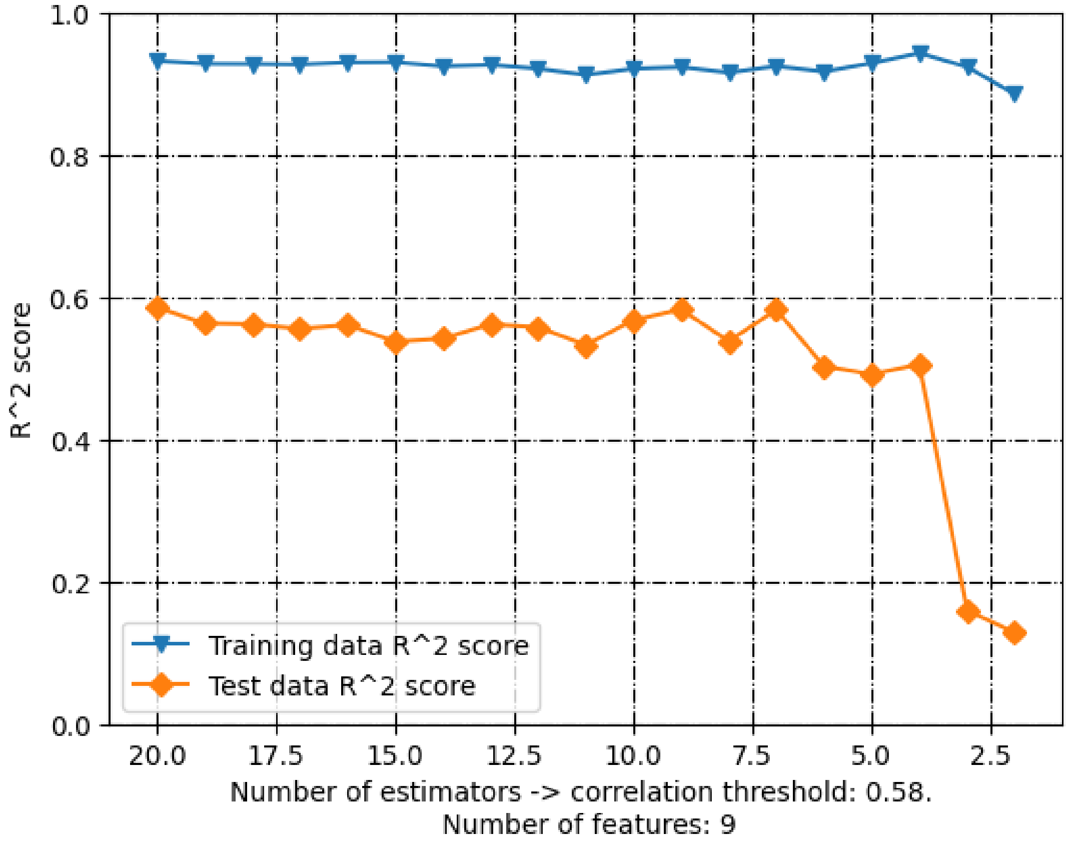
1.0
0.8
0.6
0.4
0.2
0.0
R^2 score
Training data R^2 score
Test data R^2 score
20.0
17.5
15.0
12.5
10.0
7.5
5.0
2.5
Number of estimators -> correlation threshold: 0.58.
Number of features: 9

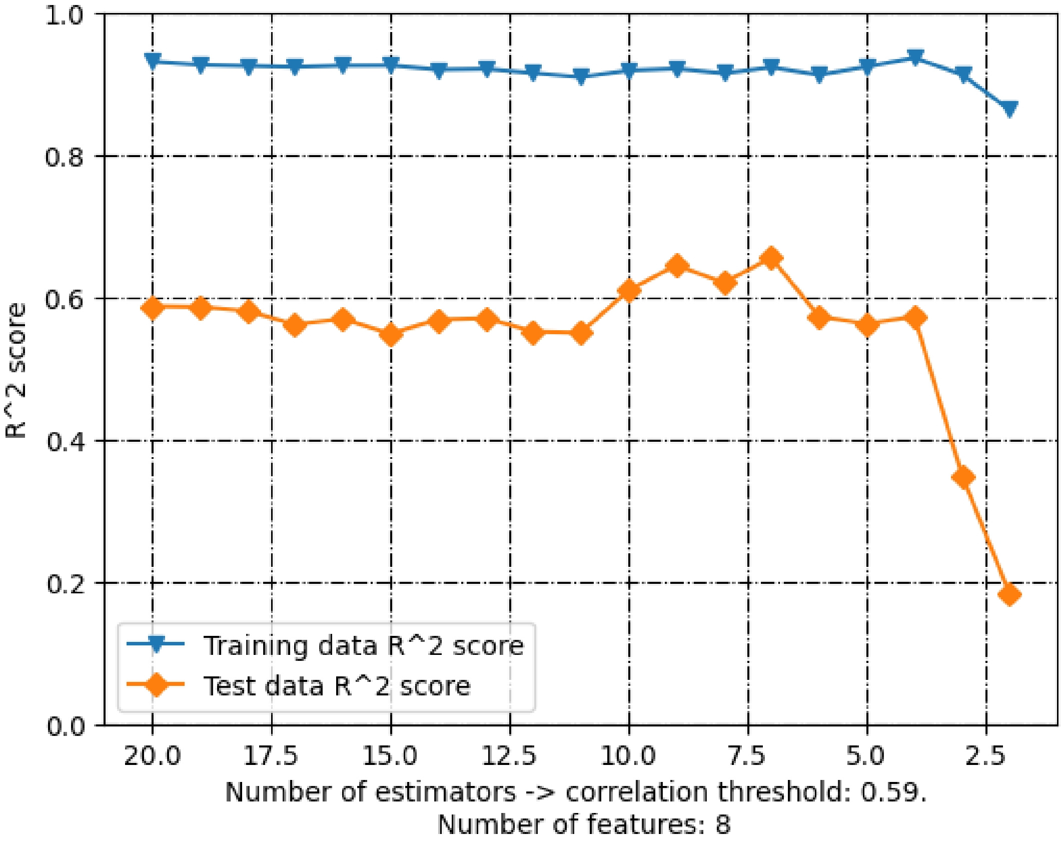

1.0
0.8
0.6
0.4
0.2
0.0
R^2 score
20.0
17.5
15.0
12.5
10.0
7.5
5.0
2.5
Training data R^2 score
Test data R^2 score
Number of estimators -> correlation threshold: 0.59.
Number of features: 8

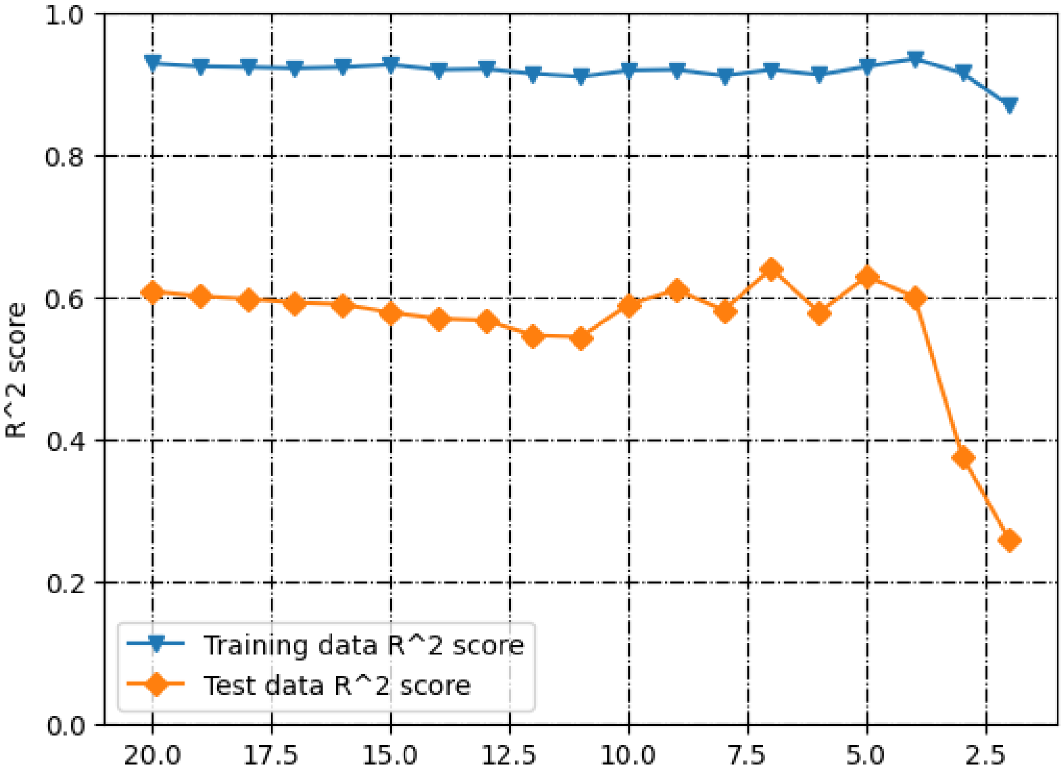

Number of estimators -> correlation threshold: 0.6.
Number of features: 7

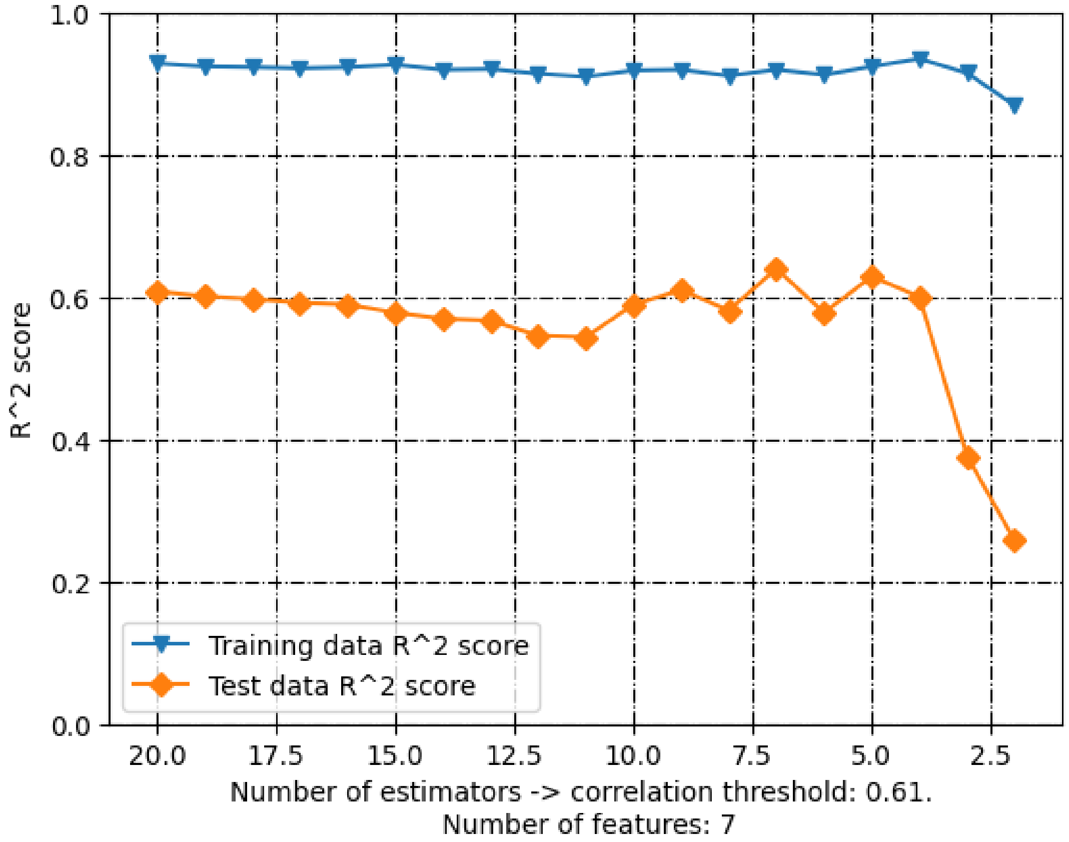
1.0
0.8
0.6
0.4
0.2
0.0
R^2 score
Training data R^2 score
Test data R^2 score
20.0
17.5
15.0
12.5
10.0
7.5
5.0
2.5
Number of estimators -> correlation threshold: 0.61.
Number of features: 7

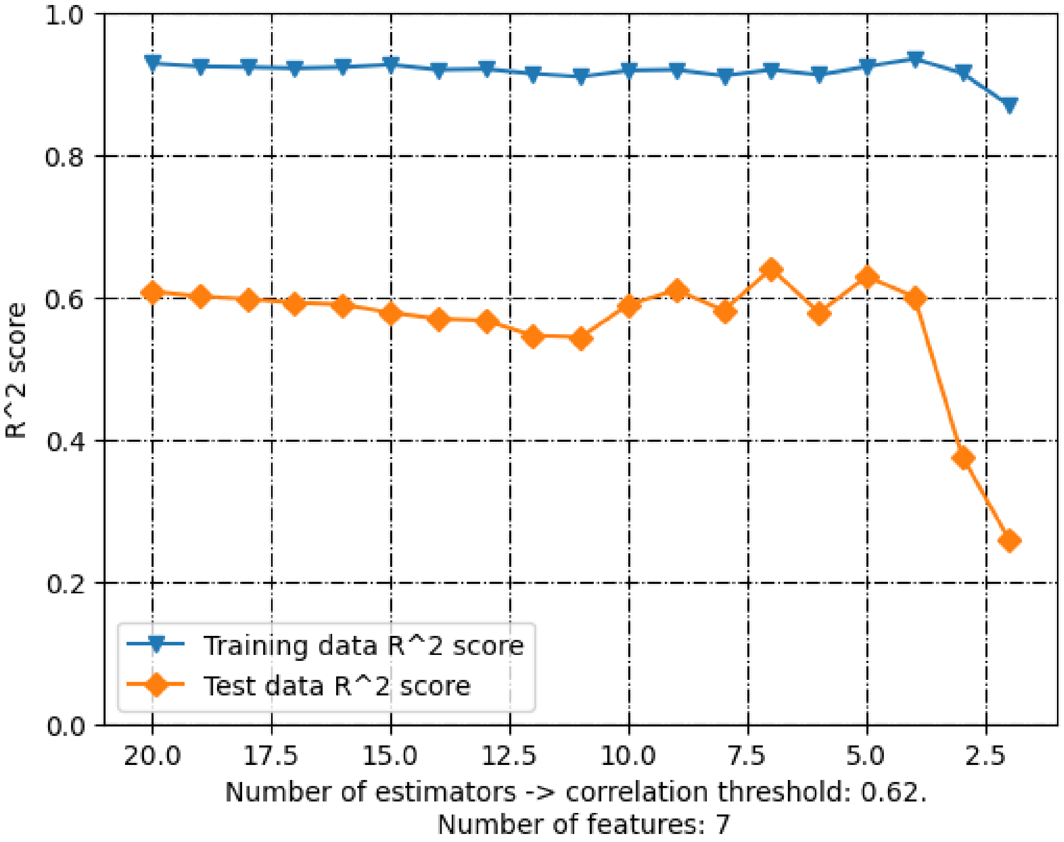

1.0
0.8
0.6
0.4
0.2
0.0
R^2 score
Training data R^2 score
Test data R^2 score
20.0
17.5
15.0
12.5
10.0
7.5
5.0
2.5
Number of estimators -> correlation threshold: 0.62.
Number of features: 7

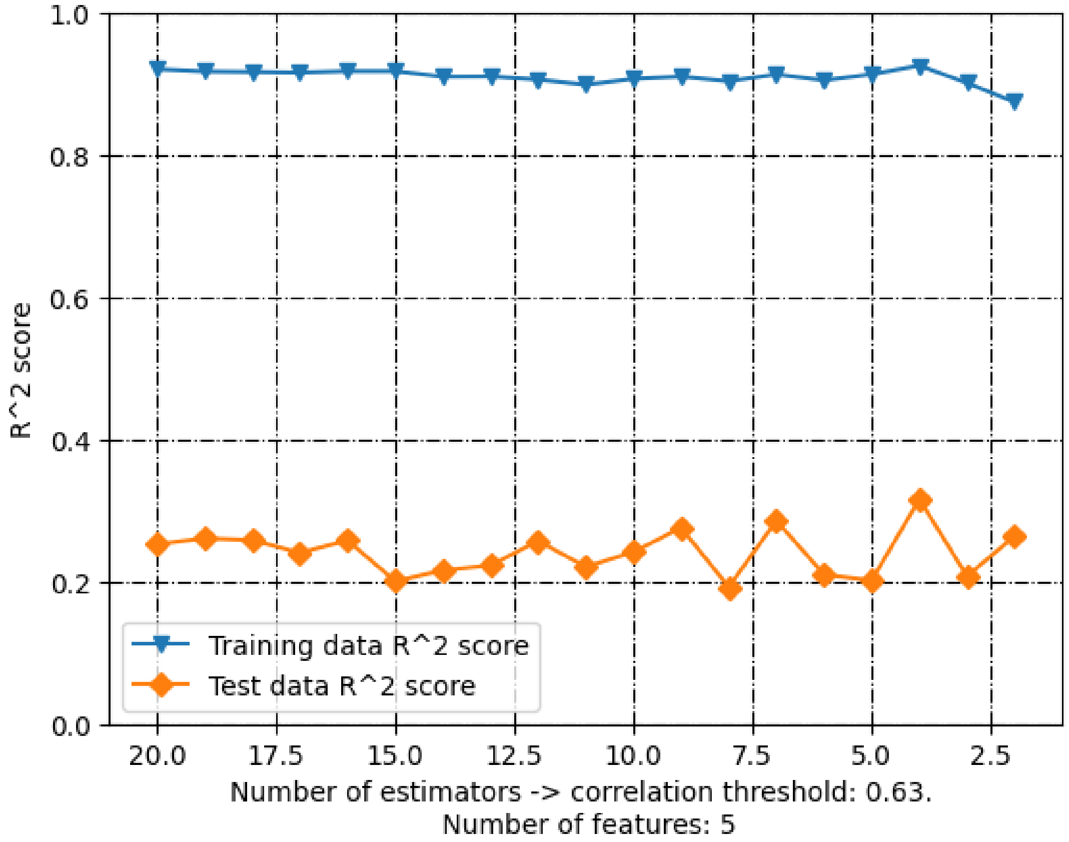

[29]:
```
plt.plot(df_without_standardization['Correlation threshold'],␣
  ↪df_without_standardization['Number of features'], label = "Number of␣
  ↪features", marker='.')
plt.legend()
plt.xlabel('Correlation threshold')
plt.ylabel('Number of features')
plt.show()
```

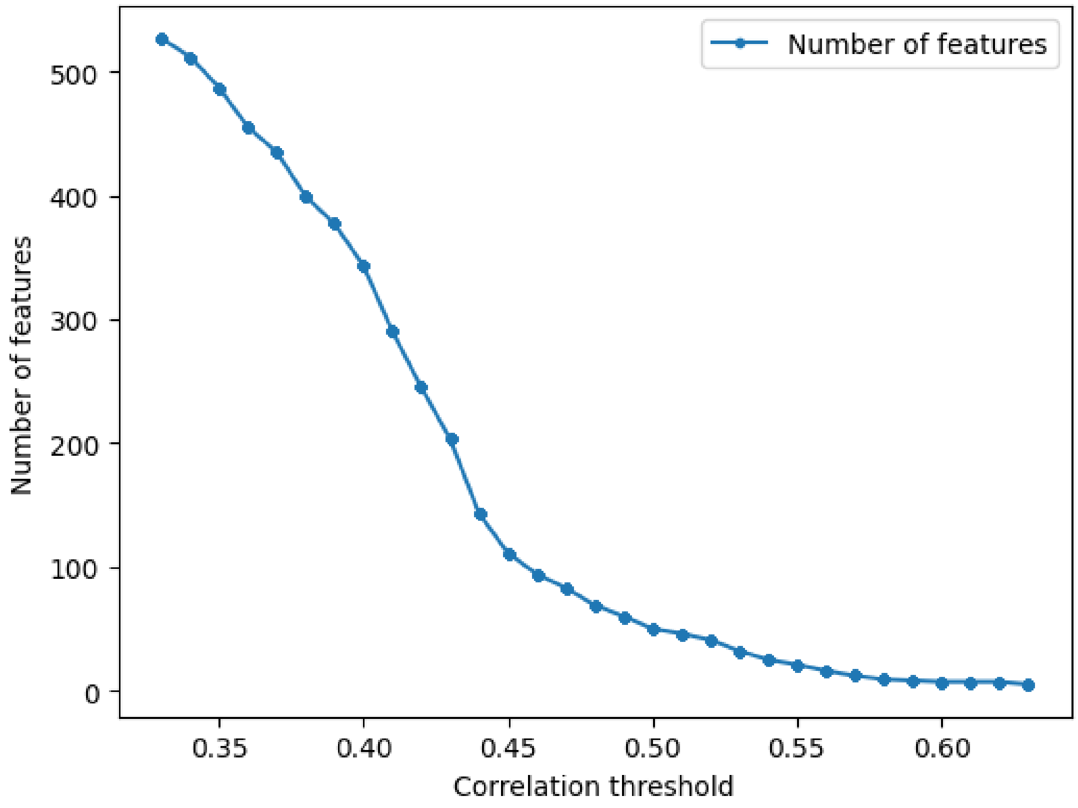

[ ]:

4 KNeighborsRegressor

[30]:
```
without_standardization, train_r2_, test_r2_, hist1, hist2, target_column_name,␣
↪training_data_RMSE, test_data_RMSE  = pred_model.
↪prepare_data_and_create_model(molecular_descriptors_df=data,
                                                                               ␣
↪                          correlation_threshold=0.50,
                                                                               ␣
↪                          standardization=False,
                                                                               ␣
↪                          model_type='KNeighborsRegressor',
                                                                               ␣
↪                          target_column_name = target,
                                                                               ␣
↪                          random_state=random_state,
                                                                               ␣
↪                          train_test_split_=True,
```

```
↪                        verbose=True)
```

```
I am not doing standardization…
  molecular descriptor name
0                    AATS0Z
1                  AATS0are
2                    AATS0d
3                   AATS0dv
4                    AATS0i
  molecular descriptor name  corr_value
0                    AATS0Z    0.018677
1                  AATS0are   -0.341313
2                    AATS0d   -0.123443
3                   AATS0dv   -0.265670
4                    AATS0i   -0.428929
  molecular descriptor name  corr_value  absolute correlation value
0                    AATS0Z    0.018677                    0.018677
1                  AATS0are   -0.341313                    0.341313
2                    AATS0d   -0.123443                    0.123443
3                   AATS0dv   -0.265670                    0.265670
4                    AATS0i   -0.428929                    0.428929
    molecular descriptor name  corr_value  absolute correlation value
15                     AATS1i   -0.561620                    0.561620
113                   AATSC1c    0.565682                    0.565682
125                   AATSC2c   -0.543393                    0.543393
221                AETA_eta_R   -0.515778                    0.515778
224                    AMID_C    0.532458                    0.532458
    molecular descriptor name  corr_value  absolute correlation value
15                     AATS1i   -0.561620                    0.561620
113                   AATSC1c    0.565682                    0.565682
125                   AATSC2c   -0.543393                    0.543393
221                AETA_eta_R   -0.515778                    0.515778
224                    AMID_C    0.532458                    0.532458
The model used is: KNeighbors…
Return the coefficient of determination of the prediction:
-0.5245164414023789
R^2 score: 0.641306136499944
Correlation coefficient: 0.8008159192348413
Test data - unseen during training:
R^2 score: -0.5245164414023789
Correlation coefficient: nan
[7.54260641 7.23551212 7.20230296 6.71036235 5.90869339 6.95171104
 7.00759247 5.47686477 6.95171104 7.01252472 5.94299531 6.12750954
 5.70879728 6.83491425 5.87714714 7.45272435]
0      7.260428
21     6.398375
106    5.853872
```

```
68     6.080922
44     7.638272
14     7.067526
108    6.154902
62     6.455932
13     7.158641
9      5.702436
96     5.136677
49     5.619789
54     7.075721
114    6.026872
115    5.568636
87     7.288193
Name: LoVo/DX, dtype: float64
Training Root Mean Square Error: 0.5752818369342935
Testing Root Mean Square Error: 0.9012538828554976
```

```
  File "C:\Users\aleks\anaconda3\envs\cheminf_gpu\lib\site-
packages\joblib\externals\loky\backend\context.py", line 217, in
_count_physical_cores
    raise ValueError(
```

[31]:
```
print(target_column_name+str('_transformed'))
print(hist1[target_column_name].hist())
```

```
LoVo/DX_transformed
AxesSubplot(0.125,0.11;0.775x0.77)
```

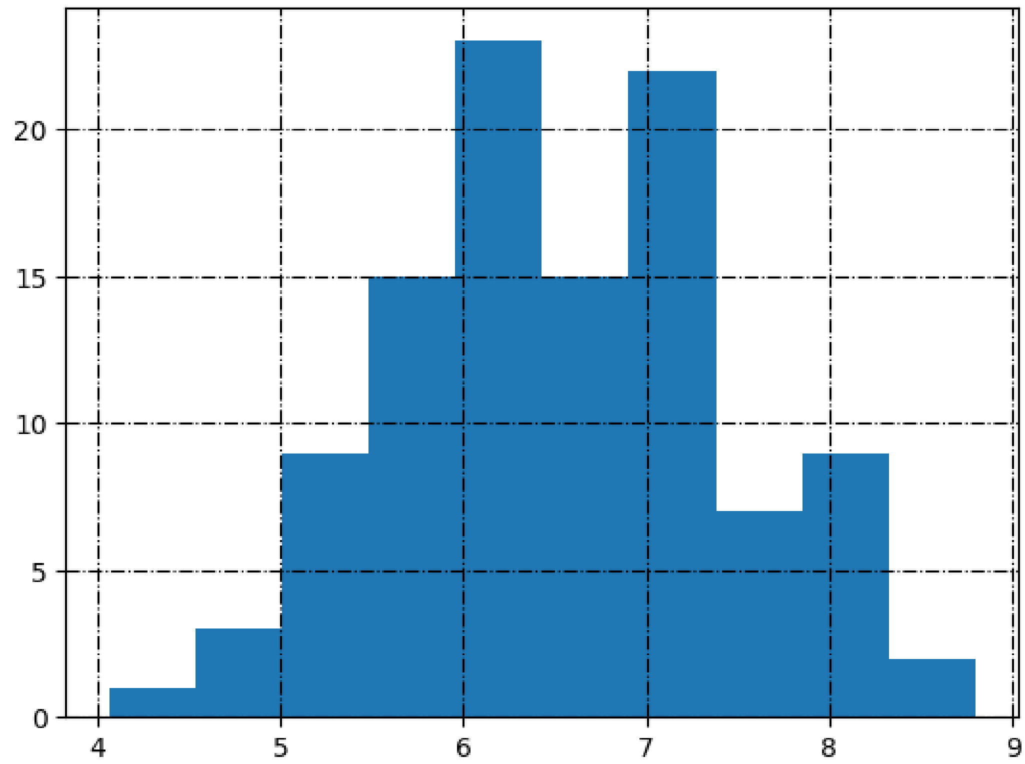


```
[32]: with_standardization, train_r2_, test_r2_, hist1, hist2, target_column_name,␣
      ↪training_data_RMSE, test_data_RMSE  = pred_model.
      ↪prepare_data_and_create_model(molecular_descriptors_df=data,
                                                                              ␣
      ↪                          correlation_threshold=0.50,
                                                                              ␣
      ↪                          standardization=True,
                                                                              ␣
      ↪                          model_type='KNeighborsRegressor',
                                                                              ␣
      ↪                          target_column_name = target,
                                                                              ␣
      ↪                          random_state=random_state,
                                                                              ␣
      ↪                          train_test_split_=True,
                                                                              ␣
      ↪                          verbose=True)
```

```
I am doing standardization…
  molecular descriptor name
0                    AATS0Z
1                  AATS0are
```

```
2                  AATS0d
3                 AATS0dv
4                  AATS0i
  molecular descriptor name  corr_value
0                  AATS0Z    0.018677
1                AATS0are   -0.341313
2                  AATS0d   -0.123443
3                 AATS0dv   -0.265670
4                  AATS0i   -0.428929
  molecular descriptor name  corr_value  absolute correlation value
0                  AATS0Z    0.018677                    0.018677
1                AATS0are   -0.341313                    0.341313
2                  AATS0d   -0.123443                    0.123443
3                 AATS0dv   -0.265670                    0.265670
4                  AATS0i   -0.428929                    0.428929
    molecular descriptor name  corr_value  absolute correlation value
15                     AATS1i   -0.561620                    0.561620
113                   AATSC1c    0.565682                    0.565682
125                   AATSC2c   -0.543393                    0.543393
221                AETA_eta_R   -0.515778                    0.515778
224                    AMID_C    0.532458                    0.532458
    molecular descriptor name  corr_value  absolute correlation value
15                     AATS1i   -0.561620                    0.561620
113                   AATSC1c    0.565682                    0.565682
125                   AATSC2c   -0.543393                    0.543393
221                AETA_eta_R   -0.515778                    0.515778
224                    AMID_C    0.532458                    0.532458
The model used is: KNeighbors…
Return the coefficient of determination of the prediction:
-0.5245164414023789
R^2 score: 0.641306136499944
Correlation coefficient: 0.8008159192348413
Test data - unseen during training:
R^2 score: -0.5245164414023789
Correlation coefficient: nan
[7.54260641 7.23551212 7.20230296 6.71036235 5.90869339 6.95171104
 7.00759247 5.47686477 6.95171104 7.01252472 5.94299531 6.12750954
 5.70879728 6.83491425 5.87714714 7.45272435]
0      7.260428
21     6.398375
106    5.853872
68     6.080922
44     7.638272
14     7.067526
108    6.154902
62     6.455932
13     7.158641
9      5.702436
```

```
96      5.136677
49      5.619789
54      7.075721
114     6.026872
115     5.568636
87      7.288193
Name: LoVo/DX, dtype: float64
Training Root Mean Square Error: 0.5752818369342935
Testing Root Mean Square Error: 0.9012538828554976
```

4.1 Search inside correlation space

[33]:
```
step = 0.01
initial_step = corr_low
last_step = corr_high
first_list = [x / 100.0 for x in range(int(initial_step*100),␣
 ↪int(last_step*100), int(step*100))]
second_list = []
third_list = []
fourth_l = []
fifth_l = []
f_list = []
for i in first_list:
    without_standardization, train_r2, test_r2, _, h_, target_column_name,␣
 ↪training_data_RMSE, test_data_RMSE = pred_model.
 ↪prepare_data_and_create_model(molecular_descriptors_df = data,
                                                                           ␣
 ↪                      correlation_threshold = i,
                                                                           ␣
 ↪                      standardization = False,
                                                                           ␣
 ↪                      model_type = 'KNeighborsRegressor',
                                                                           ␣
 ↪                      target_column_name = target,
                                                                           ␣
 ↪                      random_state=random_state,
                                                                           ␣
 ↪                      train_test_split_ = True,
                                                                           ␣
 ↪                      verbose = False)
    second_list.append(train_r2)
    third_list.append(test_r2)
    fourth_l.append(training_data_RMSE)
    fifth_l.append(test_data_RMSE)
    f_list.append(len(h_))
```

[34]:
```
df_without_standardization = pd.DataFrame(data=first_list,␣
↪columns=["Correlation threshold"])
df_without_standardization['Training data R^2 score'] = second_list
df_without_standardization['Test data R^2 score'] = third_list
df_without_standardization['Training RMSE'] = fourth_l
df_without_standardization['Test RMSE'] = fifth_l
df_without_standardization['Number of features'] = f_list
```

[35]:
```
df_k_nearest = df_without_standardization.copy()
df_without_standardization
```

[35]:
```
    Correlation threshold  Training data R^2 score  Test data R^2 score  \
0                    0.33                 0.479264            -0.091260
1                    0.34                 0.449586            -0.540179
2                    0.35                 0.474786            -0.434286
3                    0.36                 0.455877            -0.579715
4                    0.37                 0.455877            -0.579715
5                    0.38                 0.510462            -0.694617
6                    0.39                 0.515700            -0.472243
7                    0.40                 0.525052            -0.480476
8                    0.41                 0.510964            -0.428514
9                    0.42                 0.574199            -1.196495
10                   0.43                 0.543995            -1.125321
11                   0.44                 0.536471            -1.116713
12                   0.45                 0.502059            -1.147740
13                   0.46                 0.520034            -1.026238
14                   0.47                 0.510079            -1.026786
15                   0.48                 0.611073            -0.500654
16                   0.49                 0.625599            -0.495928
17                   0.50                 0.641306            -0.524516
18                   0.51                 0.604986            -0.279072
19                   0.52                 0.609748            -0.279072
20                   0.53                 0.676802            -0.104932
21                   0.54                 0.660569            -0.008270
22                   0.55                 0.694005            -0.119325
23                   0.56                 0.702567            -0.029016
24                   0.57                 0.697715             0.090609
25                   0.58                 0.696547             0.013541
26                   0.59                 0.696236             0.013541
27                   0.60                 0.704246             0.001790
28                   0.61                 0.704246             0.001790
29                   0.62                 0.704246             0.001790
30                   0.63                 0.696019            -0.060309

    Training RMSE  Test RMSE  Number of features
0        0.693150   0.762509                 527
1        0.712629   0.905872                 512
```

2	0.696124	0.874176	487
3	0.708545	0.917425	455
4	0.708545	0.917425	435
5	0.672066	0.950204	399
6	0.668461	0.885668	377
7	0.661975	0.888141	343
8	0.671721	0.872416	290
9	0.626790	1.081798	245
10	0.648640	1.064127	203
11	0.653969	1.061970	143
12	0.677809	1.069725	111
13	0.665463	1.039026	94
14	0.672329	1.039166	83
15	0.599036	0.894173	69
16	0.587743	0.892764	60
17	0.575282	0.901254	50
18	0.603705	0.825522	46
19	0.600055	0.825522	41
20	0.546076	0.767271	32
21	0.559621	0.732942	25
22	0.531344	0.772252	21
23	0.523857	0.740444	16
24	0.528113	0.696075	12
25	0.529133	0.724971	9
26	0.529403	0.724971	8
27	0.522377	0.729276	7
28	0.522377	0.729276	7
29	0.522377	0.729276	7
30	0.529592	0.751618	5

4.2 Plots

[36]:

```
plt.plot(df_without_standardization['Correlation threshold'],
 ↪df_without_standardization['Training data R^2 score'], label = "Training
 ↪data R^2 score", marker='v')
plt.plot(df_without_standardization['Correlation threshold'],
 ↪df_without_standardization['Test data R^2 score'], label = "Test data R^2
 ↪score", marker='D')
plt.legend()
plt.xlabel('Correlation threshold')
plt.ylabel('R^2 score')
plt.ylim([0, 1])
plt.show()
```

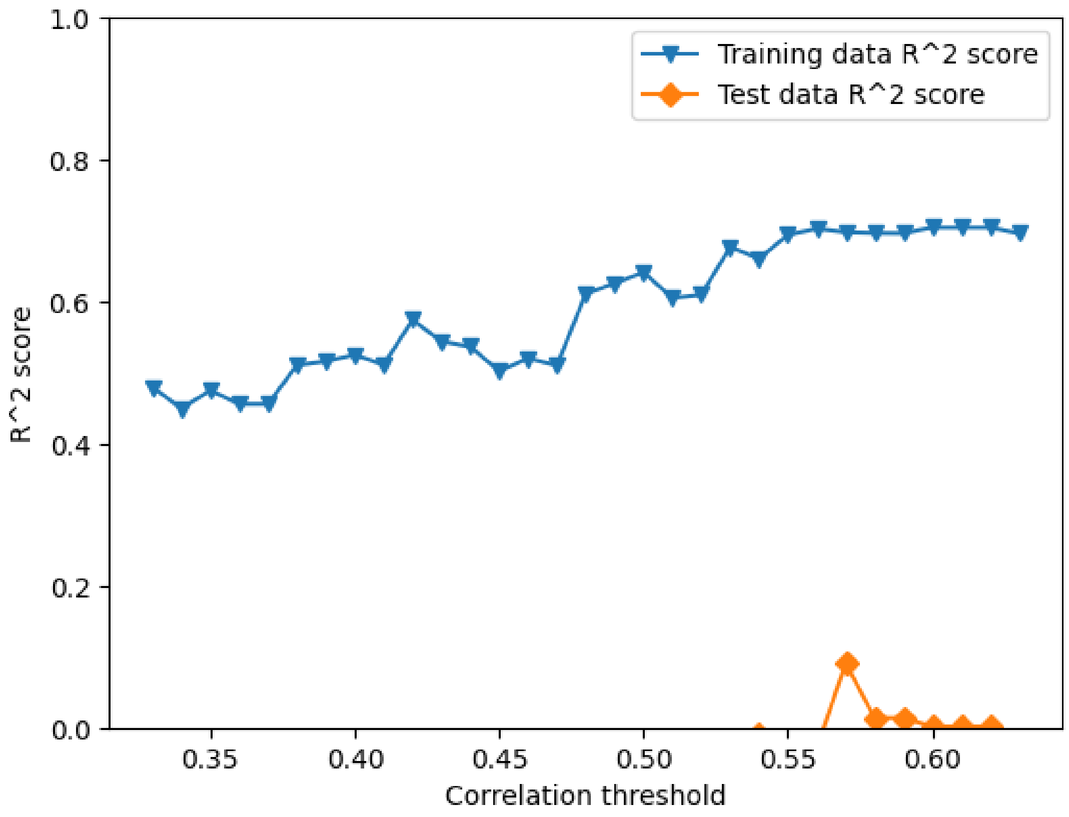

[37]:
```
plt.plot(df_without_standardization['Correlation threshold'],␣
  ↪df_without_standardization['Number of features'], label = "Number of␣
  ↪features", marker='.')
plt.legend()
plt.xlabel('Correlation threshold')
plt.ylabel('Number of features')
plt.show()
```

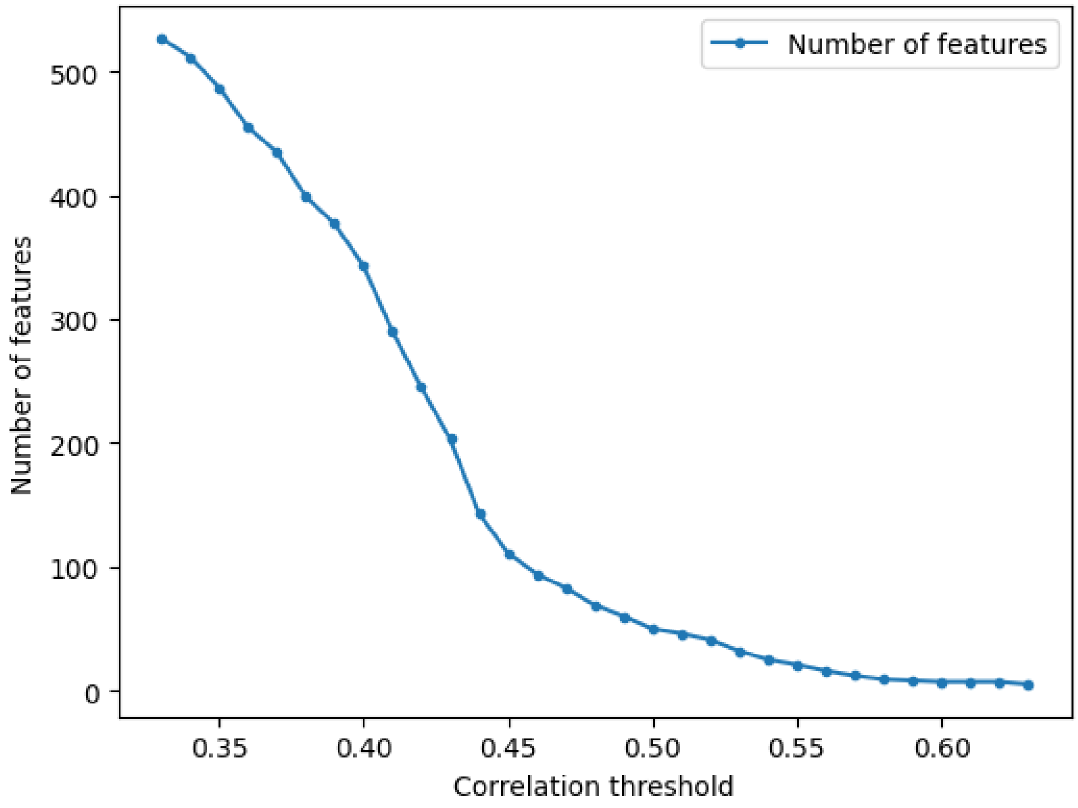

[ ]:

5 Support Vector Machines (SVM)

[38]:
```
without_standardization, train_r2_, test_r2_, hist1, hist2, target_column_name,␣
↪training_data_RMSE, test_data_RMSE  = pred_model.
↪prepare_data_and_create_model(molecular_descriptors_df=data,
                                                                               ␣
↪                        correlation_threshold=0.50,
                                                                               ␣
↪                        standardization=False,
                                                                               ␣
↪                        model_type='SVR',
                                                                               ␣
↪                        kernel_ = 'linear',
                                                                               ␣
↪                        gamma_ = 'auto',
                                                                               ␣
↪                        target_column_name = target,
```

```
↪                    random_state=random_state,
↪                    train_test_split_=True,
↪                    verbose=True)
```

```
I am not doing standardization…
  molecular descriptor name
0                    AATS0Z
1                  AATS0are
2                    AATS0d
3                   AATS0dv
4                    AATS0i
  molecular descriptor name  corr_value
0                    AATS0Z    0.018677
1                  AATS0are   -0.341313
2                    AATS0d   -0.123443
3                   AATS0dv   -0.265670
4                    AATS0i   -0.428929
  molecular descriptor name  corr_value  absolute correlation value
0                    AATS0Z    0.018677                    0.018677
1                  AATS0are   -0.341313                    0.341313
2                    AATS0d   -0.123443                    0.123443
3                   AATS0dv   -0.265670                    0.265670
4                    AATS0i   -0.428929                    0.428929
    molecular descriptor name  corr_value  absolute correlation value
15                     AATS1i   -0.561620                    0.561620
113                   AATSC1c    0.565682                    0.565682
125                   AATSC2c   -0.543393                    0.543393
221                AETA_eta_R   -0.515778                    0.515778
224                    AMID_C    0.532458                    0.532458
    molecular descriptor name  corr_value  absolute correlation value
15                     AATS1i   -0.561620                    0.561620
113                   AATSC1c    0.565682                    0.565682
125                   AATSC2c   -0.543393                    0.543393
221                AETA_eta_R   -0.515778                    0.515778
224                    AMID_C    0.532458                    0.532458
The model used is: SVR…
Return the coefficient of determination of the prediction:
-0.4496965939271831
R^2 score: 0.586153754648779
Correlation coefficient: 0.7656067885336303
Test data - unseen during training:
R^2 score: -0.4496965939271831
Correlation coefficient: nan
[7.64018302 7.85315872 5.78332399 5.95003031 6.88182489 7.33071639
```

```
 6.08875973 8.34772883 7.06686417 7.05142133 5.48633924 6.28322853
 6.90076177 6.1857832  7.30273529 7.91849526]
0      7.260428
21     6.398375
106    5.853872
68     6.080922
44     7.638272
14     7.067526
108    6.154902
62     6.455932
13     7.158641
9      5.702436
96     5.136677
49     5.619789
54     7.075721
114    6.026872
115    5.568636
87     7.288193
Name: LoVo/DX, dtype: float64
Training Root Mean Square Error: 0.6179284610346645
Testing Root Mean Square Error: 0.8788599052493705
```

[39]:

```
print(target_column_name+str('_transformed'))
print(hist1[target_column_name].hist())
```

```
LoVo/DX_transformed
AxesSubplot(0.125,0.11;0.775x0.77)
```

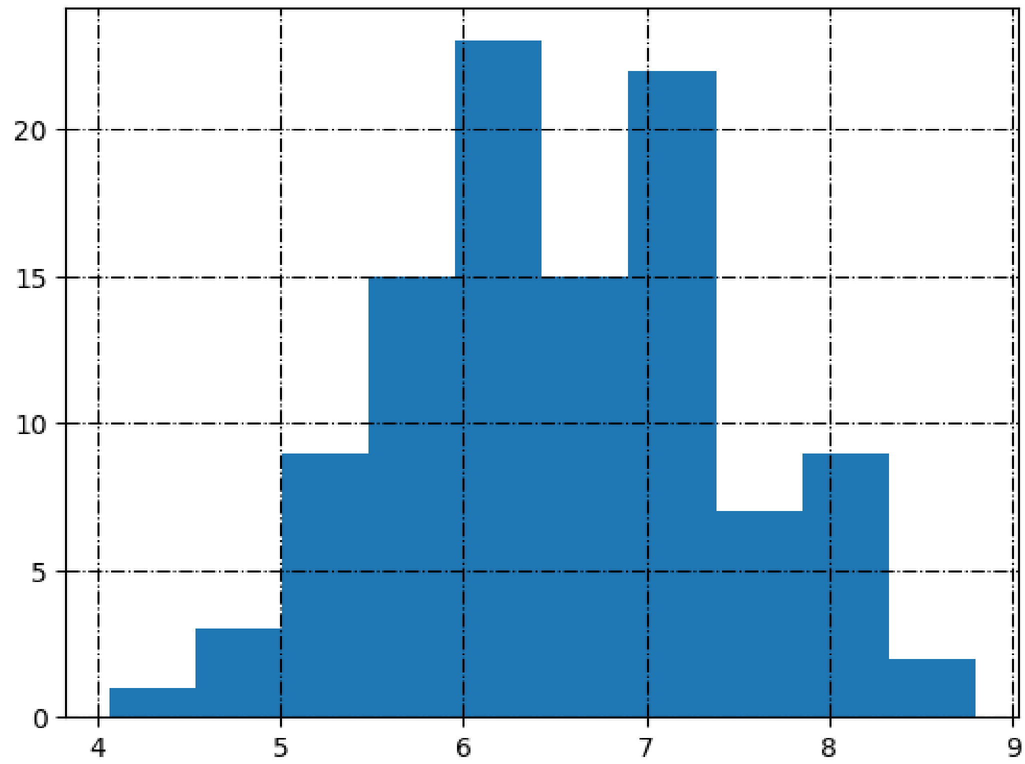

[40]:
```
with_standardization, train_r2_, test_r2_, hist1, hist2, target_column_name,␣
 ↪training_data_RMSE, test_data_RMSE  = pred_model.
 ↪prepare_data_and_create_model(molecular_descriptors_df=data,
                                                                                           ␣
 ↪                      correlation_threshold=0.50,
                                                                                           ␣
 ↪                      standardization=True,
                                                                                           ␣
 ↪                      model_type='SVR',
                                                                                           ␣
 ↪                      kernel_ = 'linear',
                                                                                           ␣
 ↪                      gamma_ = 'auto',
                                                                                           ␣
 ↪                      target_column_name = target,
                                                                                           ␣
 ↪                      random_state=random_state,
                                                                                           ␣
 ↪                      train_test_split_=True,
                                                                                           ␣
 ↪                      verbose=True)
```

```
I am doing standardization…
  molecular descriptor name
0                    AATS0Z
1                  AATS0are
2                    AATS0d
3                   AATS0dv
4                    AATS0i
  molecular descriptor name  corr_value
0                    AATS0Z    0.018677
1                  AATS0are   -0.341313
2                    AATS0d   -0.123443
3                   AATS0dv   -0.265670
4                    AATS0i   -0.428929
  molecular descriptor name  corr_value  absolute correlation value
0                    AATS0Z    0.018677                    0.018677
1                  AATS0are   -0.341313                    0.341313
2                    AATS0d   -0.123443                    0.123443
3                   AATS0dv   -0.265670                    0.265670
4                    AATS0i   -0.428929                    0.428929
    molecular descriptor name  corr_value  absolute correlation value
15                     AATS1i   -0.561620                    0.561620
113                   AATSC1c    0.565682                    0.565682
125                   AATSC2c   -0.543393                    0.543393
221                AETA_eta_R   -0.515778                    0.515778
224                    AMID_C    0.532458                    0.532458
    molecular descriptor name  corr_value  absolute correlation value
15                     AATS1i   -0.561620                    0.561620
113                   AATSC1c    0.565682                    0.565682
125                   AATSC2c   -0.543393                    0.543393
221                AETA_eta_R   -0.515778                    0.515778
224                    AMID_C    0.532458                    0.532458
The model used is: SVR…
Return the coefficient of determination of the prediction:
-0.4496965939271831
R^2 score: 0.586153754648779
Correlation coefficient: 0.7656067885336303
Test data - unseen during training:
R^2 score: -0.4496965939271831
Correlation coefficient: nan
[7.64018302 7.85315872 5.78332399 5.95003031 6.88182489 7.33071639
 6.08875973 8.34772883 7.06686417 7.05142133 5.48633924 6.28322853
 6.90076177 6.1857832  7.30273529 7.91849526]
0      7.260428
21     6.398375
106    5.853872
68     6.080922
44     7.638272
14     7.067526
```

```
108    6.154902
62     6.455932
13     7.158641
9      5.702436
96     5.136677
49     5.619789
54     7.075721
114    6.026872
115    5.568636
87     7.288193
Name: LoVo/DX, dtype: float64
Training Root Mean Square Error: 0.6179284610346645
Testing Root Mean Square Error: 0.8788599052493705
```

5.1 Search inside correlation space

[41]:
```
step = 0.01
initial_step = corr_low
last_step = corr_high
first_list = [x / 100.0 for x in range(int(initial_step*100),␣
↪int(last_step*100), int(step*100))]
second_list = []
third_list = []
fourth_l = []
fifth_l = []
f_list = []
for i in first_list:
    without_standardization, train_r2, test_r2, _, h_, target_column_name,␣
↪training_data_RMSE, test_data_RMSE = pred_model.
↪prepare_data_and_create_model(molecular_descriptors_df = data,
                                                                             ␣
↪                     correlation_threshold = i,
                                                                             ␣
↪                     standardization = False,
                                                                             ␣
↪                     model_type = 'SVR',
                                                                             ␣
↪                     kernel_ = 'linear',
                                                                             ␣
↪                     gamma_ = 'auto',
                                                                             ␣
↪                     target_column_name = target,
                                                                             ␣
↪                     random_state=random_state,
                                                                             ␣
↪                     train_test_split_ = True,
```

```
                              verbose = False)
    second_list.append(train_r2)
    third_list.append(test_r2)
    fourth_l.append(training_data_RMSE)
    fifth_l.append(test_data_RMSE)
    f_list.append(len(h_))
```

[42]:
```
df_without_standardization = pd.DataFrame(data=first_list,
 columns=["Correlation threshold"])
df_without_standardization['Training data R^2 score'] = second_list
df_without_standardization['Test data R^2 score'] = third_list
df_without_standardization['Training RMSE'] = fourth_l
df_without_standardization['Test RMSE'] = fifth_l
df_without_standardization['Number of features'] = f_list
```

[43]:
```
df_svm = df_without_standardization.copy()
df_without_standardization
```

[43]:
```
    Correlation threshold  Training data R^2 score  Test data R^2 score  \
0                    0.33            -4.576410e+06        -2.396690e+07
1                    0.34            -4.819789e+06        -1.459847e+07
2                    0.35            -5.239922e+06        -1.157864e+07
3                    0.36            -9.918947e+05        -8.869205e+06
4                    0.37            -1.825928e+06        -7.394667e+06
5                    0.38            -5.229611e+05        -1.336230e+06
6                    0.39            -6.852025e+05        -3.551945e+06
7                    0.40            -4.263337e+05        -1.897983e+06
8                    0.41            -3.609072e+05        -1.037173e+06
9                    0.42            -2.462539e+04        -1.582436e+05
10                   0.43            -1.053529e+04        -6.406834e+04
11                   0.44            -1.161727e+04        -1.516731e+05
12                   0.45            -1.151660e+04        -3.838399e+04
13                   0.46            -3.945495e+03        -1.845080e+04
14                   0.47            -5.015611e+03        -3.969418e+04
15                   0.48            -1.992983e-01        -1.352399e+01
16                   0.49            -1.050742e-01        -2.025419e+00
17                   0.50             5.861538e-01        -4.496966e-01
18                   0.51             5.035499e-01        -1.441652e-01
19                   0.52             4.283967e-01        -8.271995e-01
20                   0.53             6.363734e-01         1.174322e-01
21                   0.54             6.343838e-01         2.966910e-01
22                   0.55             6.132704e-01         2.045466e-01
23                   0.56             5.931617e-01         1.541282e-01
24                   0.57             5.813991e-01         1.130634e-01
25                   0.58             5.690961e-01         6.673427e-02
26                   0.59             5.666265e-01         5.261898e-02
```

27	0.60	5.497820e-01	1.336534e-02
28	0.61	5.497820e-01	1.336534e-02
29	0.62	5.497820e-01	1.336534e-02
30	0.63	5.427533e-01	2.935068e-02

	Training RMSE	Test RMSE	Number of features
0	2054.855077	3573.444324	527
1	2108.787075	2788.911760	512
2	2198.776688	2483.761668	487
3	956.646939	2173.819475	455
4	1297.957898	1984.908248	435
5	694.630410	843.765648	399
6	795.112328	1375.669975	377
7	627.182608	1005.604789	343
8	577.054823	743.373185	290
9	150.736735	290.365886	245
10	98.596749	184.759265	203
11	103.535533	284.273773	143
12	103.086013	143.008482	111
13	60.342664	99.151768	94
14	68.033674	145.428652	83
15	1.051919	2.781788	69
16	1.009751	1.269620	60
17	0.617928	0.878860	50
18	0.676794	0.780774	46
19	0.726216	0.986675	41
20	0.579224	0.685733	32
21	0.580806	0.612145	25
22	0.597341	0.651011	21
23	0.612674	0.671326	16
24	0.621468	0.687428	12
25	0.630535	0.705154	9
26	0.632339	0.710466	8
27	0.644511	0.725036	7
28	0.644511	0.725036	7
29	0.644511	0.725036	7
30	0.649522	0.719138	5

5.2 Plots

[44]:
```
plt.plot(df_without_standardization['Correlation threshold'],
    df_without_standardization['Training data R^2 score'], label = "Training
    data R^2 score", marker='v')
plt.plot(df_without_standardization['Correlation threshold'],
    df_without_standardization['Test data R^2 score'], label = "Test data R^2
    score", marker='D')
plt.legend()
```

```
plt.xlabel('Correlation threshold')
plt.ylabel('R^2 score')
plt.ylim([0, 1])
plt.show()
```

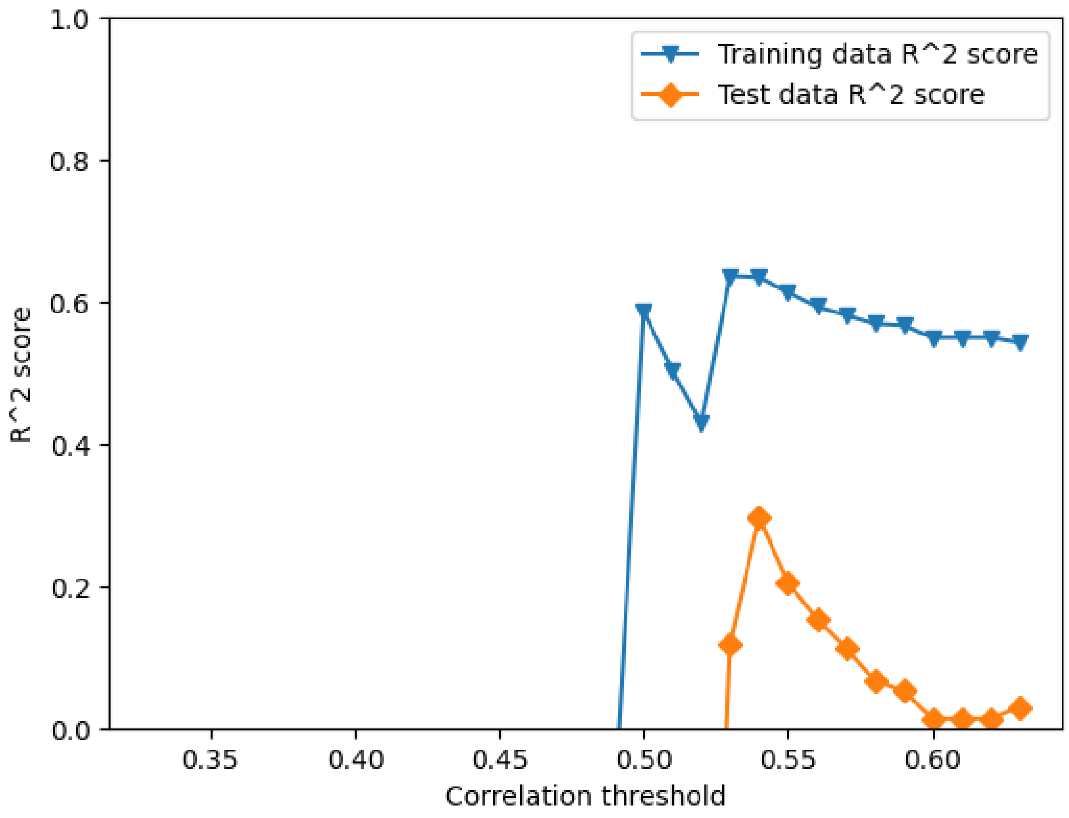

[45]:
```
plt.plot(df_without_standardization['Correlation threshold'],␣
 ↪df_without_standardization['Number of features'], label = "Number of␣
 ↪features", marker='.')
plt.legend()
plt.xlabel('Correlation threshold')
plt.ylabel('Number of features')
plt.show()
```

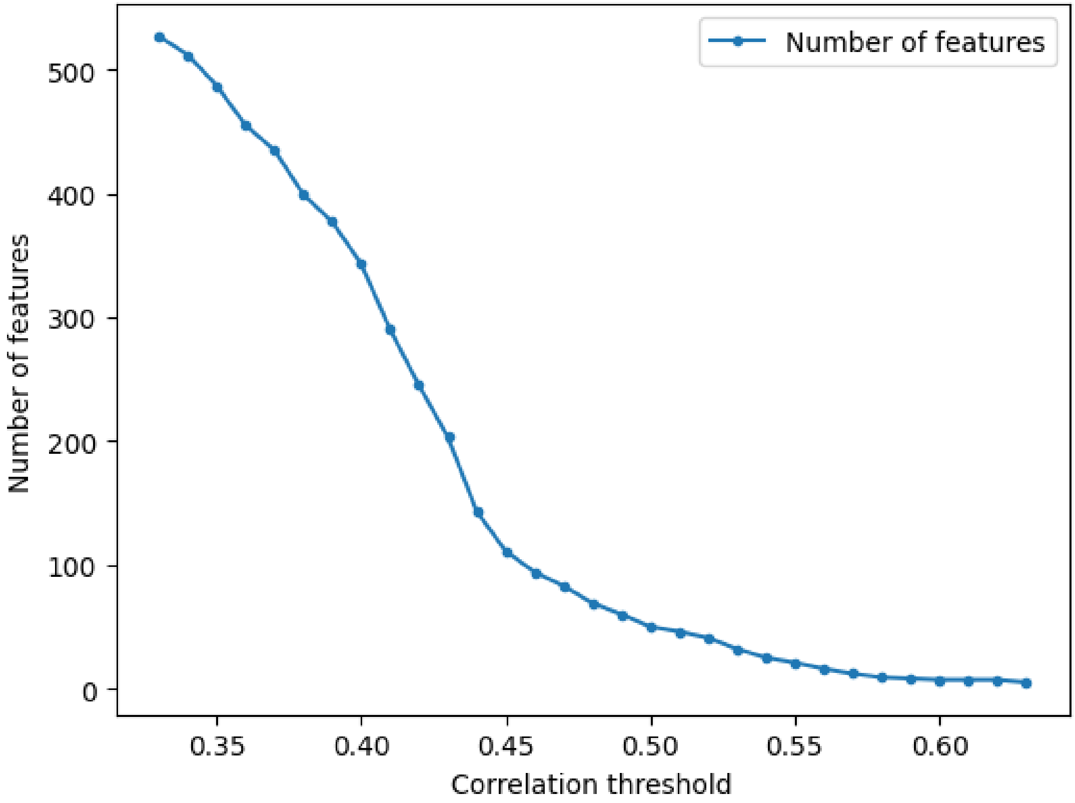

5.3 Store quality measurements

[46]:
```
with pd.ExcelWriter('../Data/Quality_'+'LoVo_DX'+'_'+str(random_state)+'_.
↪xlsx') as writer:
    df_linear.to_excel(writer, sheet_name='MLR')
    df_decision_tree.to_excel(writer, sheet_name='DT')
    df_random_forest.to_excel(writer, sheet_name='RF')
    df_k_nearest.to_excel(writer, sheet_name='KNN')
    df_svm.to_excel(writer, sheet_name='SVM')
```

Notebook

January 18, 2024

1 File 23

[1]:
```python
import sys
from pathlib import Path
prediction_mode_path = Path("../module")
sys.path.append(prediction_mode_path.as_posix())
import models_creation as pred_model

import pandas as pd
import numpy as np

from sklearn.metrics import r2_score

import matplotlib.pyplot as plt
from sklearn.tree import plot_tree

from matplotlib import pyplot as plt

import joblib

import warnings
warnings.filterwarnings('ignore')
```

[2]:
```python
load_prepared_data = pd.read_excel('../Data/Kolchicyna_machine_learning.xlsx')
load_prepared_data = load_prepared_data.drop(columns=['Unnamed: 0'])

target = 'LoVo/DX'

corr_low = 0.33
corr_high = 0.64

random_state = 42
```

[3]:
```python
load_prepared_data.head()
```

[3]:
```
      AATS0Z  AATS0are    AATS0d   AATS0dv       AATS0i     AATS0m    AATS0p  \
0  25.090909  6.046518  3.109091  6.662626  160.775045  99.075564  1.555512
1  24.448276  6.008422  3.051724  6.386973  161.021674  96.473315  1.538471
```

```
2  23.868852  5.974074  3.000000  6.138434  161.244045  94.127025  1.523105
3  23.868852  5.974074  3.032787  6.171220  161.244045  94.127025  1.523105
4  23.343750  5.942945  2.953125  5.913194  161.445569  92.000700  1.509180

    AATS0pe    AATS0s    AATS0se  …  A549_float  MCF-7_float  LoVo_float  \
0  6.126680  3.435416  7.629859  …        10.8         10.3         6.5
1  6.088791  3.330998  7.596891  …        11.6         12.0         8.5
2  6.054630  3.236850  7.567166  …        10.9         12.2         8.8
3  6.054630  3.257798  7.567166  …        10.5         11.3         8.5
4  6.023670  3.151529  7.540228  …        89.5         92.7        52.8

   LoVo/DX_float  BALB/3T3_float  A549_float_transformed  \
0           54.9            10.2                7.966576
1           31.1            14.3                7.935542
2           17.9            11.7                7.962574
3           10.2            11.0                7.978811
4           77.8            99.4                7.048177

   MCF-7_float_transformed  LoVo_float_transformed  LoVo/DX_float_transformed  \
0                 7.987163                8.187087                   7.260428
1                 7.920819                8.070581                   7.507240
2                 7.913640                8.055517                   7.747147
3                 7.946922                8.070581                   7.991400
4                 7.032920                7.277366                   7.109020

   BALB/3T3_float_transformed
0                    7.991400
1                    7.844664
2                    7.931814
3                    7.958607
4                    7.002614

[5 rows x 1221 columns]
```

[4]:
```
data = load_prepared_data[list(load_prepared_data.columns)[0:-10]]
data[target] = load_prepared_data[load_prepared_data.columns[-2]]
data.head()
```

[4]:
```
     AATS0Z  AATS0are   AATS0d   AATS0dv     AATS0i    AATS0m    AATS0p  \
0  25.090909  6.046518  3.109091  6.662626  160.775045  99.075564  1.555512
1  24.448276  6.008422  3.051724  6.386973  161.021674  96.473315  1.538471
2  23.868852  5.974074  3.000000  6.138434  161.244045  94.127025  1.523105
3  23.868852  5.974074  3.032787  6.171220  161.244045  94.127025  1.523105
4  23.343750  5.942945  2.953125  5.913194  161.445569  92.000700  1.509180

    AATS0pe    AATS0s    AATS0se  …    piPC10     piPC2     piPC3     piPC4  \
0  6.126680  3.435416  7.629859  …  7.999655  4.266195  4.915408  5.516700
```

```
1  6.088791  3.330998  7.596891  …  8.018726  4.280132  4.922714  5.520711
2  6.054630  3.236850  7.567166  …  8.028799  4.293878  4.929967  5.524706
3  6.054630  3.257798  7.567166  …  8.037440  4.307438  4.929967  5.524706
4  6.023670  3.151529  7.540228  …  8.034892  4.307438  4.937168  5.528685

      piPC5     piPC6     piPC7     piPC8     piPC9   LoVo/DX
0  6.098566  6.595759  6.979116  7.337313  7.681445  7.260428
1  6.103048  6.601209  6.985613  7.349442  7.695531  7.507240
2  6.105281  6.603923  6.989306  7.353932  7.704023  7.747147
3  6.107510  6.606629  6.992068  7.361425  7.709420  7.991400
4  6.107510  6.605277  6.991148  7.356489  7.707175  7.109020

[5 rows x 1212 columns]
```

[5]:
```
print(data.shape)
data = data.dropna()
data.shape
```

```
(120, 1212)
```

[5]: (106, 1212)

2 Multiple Linear regression (MLR)

[6]:
```
without_standardization, train_r2_, test_r2_, hist1, hist2, target_column_name,␣
↪training_data_RMSE, test_data_RMSE  = pred_model.
↪prepare_data_and_create_model(molecular_descriptors_df=data,
                                                                               ␣
↪                          correlation_threshold=0.5,
                                                                               ␣
↪                          standardization=False,
                                                                               ␣
↪                          model_type='linear_model',
                                                                               ␣
↪                          target_column_name = str(target),
                                                                               ␣
↪                          random_state=random_state,
                                                                               ␣
↪                          train_test_split_=True,
                                                                               ␣
↪                          verbose=True)
```

```
I am not doing standardization…
  molecular descriptor name
0                    AATS0Z
1                  AATS0are
2                    AATS0d
```

```
3                   AATS0dv
4                    AATS0i
  molecular descriptor name  corr_value
0                    AATS0Z    0.018677
1                  AATS0are   -0.341313
2                    AATS0d   -0.123443
3                   AATS0dv   -0.265670
4                    AATS0i   -0.428929
  molecular descriptor name  corr_value  absolute correlation value
0                    AATS0Z    0.018677                    0.018677
1                  AATS0are   -0.341313                    0.341313
2                    AATS0d   -0.123443                    0.123443
3                   AATS0dv   -0.265670                    0.265670
4                    AATS0i   -0.428929                    0.428929
    molecular descriptor name  corr_value  absolute correlation value
15                     AATS1i   -0.561620                    0.561620
113                   AATSC1c    0.565682                    0.565682
125                   AATSC2c   -0.543393                    0.543393
221                AETA_eta_R   -0.515778                    0.515778
224                    AMID_C    0.532458                    0.532458
```

```
    molecular descriptor name  corr_value  absolute correlation value
15                     AATS1i   -0.561620                    0.561620
113                   AATSC1c    0.565682                    0.565682
125                   AATSC2c   -0.543393                    0.543393
221                AETA_eta_R   -0.515778                    0.515778
224                    AMID_C    0.532458                    0.532458
```

```
The model used is: LinearReg…
Return the coefficient of determination of the prediction:
-5.938065076572442
R^2 score: 0.9071734240174797
Correlation coefficient: 0.9524565208015953
Test data - unseen during training:
R^2 score: -5.938065076572442
Correlation coefficient: nan
[ 6.43590962  7.477597    7.32156869  5.37406941  5.8892975   6.23573967
  7.33433266  6.57042651  6.12174257  6.86770113  6.24010271  6.53729444
  7.90575101  7.76599362 -0.71527592  5.40247701]
114    6.026872
10     7.856985
4      7.109020
95     5.200659
111    6.853872
79     6.327902
44     7.638272
47     7.022276
107    6.000000
11     7.250264
61     5.481486
```

```
56      6.657577
0       7.260428
92      7.141463
18      6.630970
78      6.244125
Name: LoVo/DX, dtype: float64
Training Root Mean Square Error: 0.292979841686242
Testing Root Mean Square Error: 1.9015661490887223
```

[7]:
```python
print(target_column_name+str('_transformed'))
print(hist1[target_column_name].hist())
```

```
LoVo/DX_transformed
AxesSubplot(0.125,0.11;0.775x0.77)
```

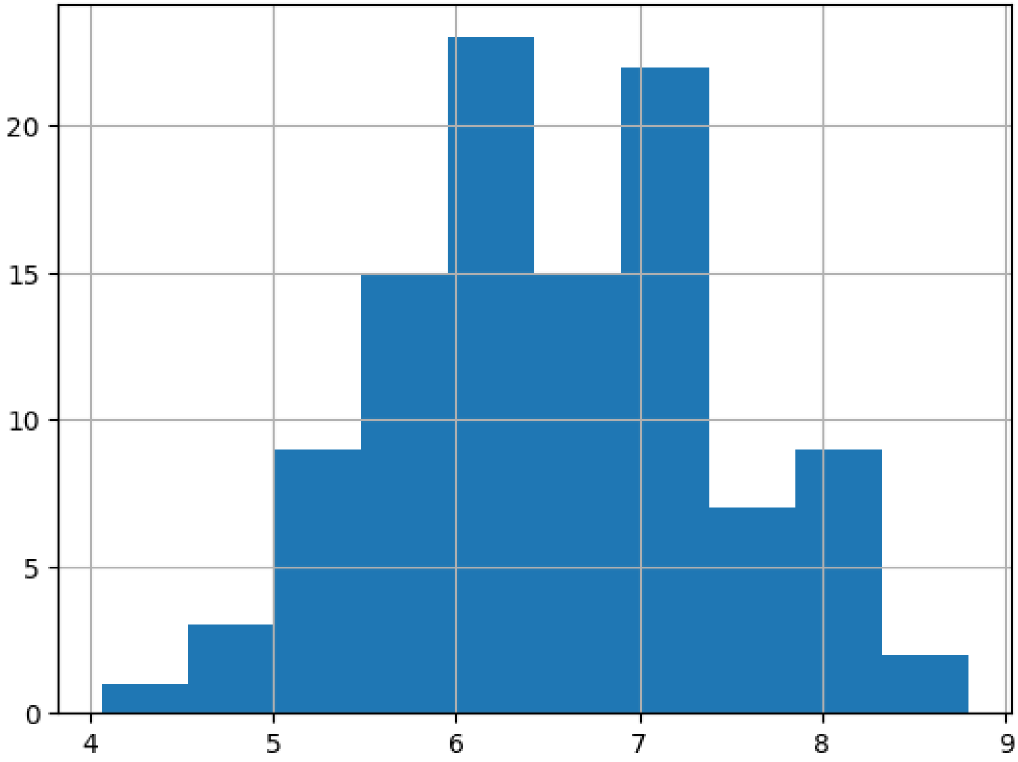

[8]:
```python
with_standardization, train_r2_, test_r2_, hist1, hist2, target_column_name,␣
↪training_data_RMSE, test_data_RMSE  = pred_model.
↪prepare_data_and_create_model(molecular_descriptors_df=data,
                                                                              ␣
↪                       correlation_threshold=0.50,
                                                                              ␣
↪                       standardization=True,
```

```
↪                    model_type='linear_model',
↪                    target_column_name = target,
↪                    random_state=random_state,
↪                    train_test_split_=True,
↪                    verbose=True)
```

```
I am doing standardization…
  molecular descriptor name
0                   AATS0Z
1                 AATS0are
2                   AATS0d
3                  AATS0dv
4                   AATS0i
  molecular descriptor name  corr_value
0                   AATS0Z    0.018677
1                 AATS0are   -0.341313
2                   AATS0d   -0.123443
3                  AATS0dv   -0.265670
4                   AATS0i   -0.428929
  molecular descriptor name  corr_value  absolute correlation value
0                   AATS0Z    0.018677                    0.018677
1                 AATS0are   -0.341313                    0.341313
2                   AATS0d   -0.123443                    0.123443
3                  AATS0dv   -0.265670                    0.265670
4                   AATS0i   -0.428929                    0.428929
    molecular descriptor name  corr_value  absolute correlation value
15                    AATS1i   -0.561620                    0.561620
113                  AATSC1c    0.565682                    0.565682
125                  AATSC2c   -0.543393                    0.543393
221               AETA_eta_R   -0.515778                    0.515778
224                   AMID_C    0.532458                    0.532458
    molecular descriptor name  corr_value  absolute correlation value
15                    AATS1i   -0.561620                    0.561620
113                  AATSC1c    0.565682                    0.565682
125                  AATSC2c   -0.543393                    0.543393
221               AETA_eta_R   -0.515778                    0.515778
224                   AMID_C    0.532458                    0.532458
The model used is: LinearReg…
Return the coefficient of determination of the prediction:
-5.938065076572442
R^2 score: 0.9071734240174797
Correlation coefficient: 0.9524565208015953
```

```
Test data - unseen during training:
R^2 score: -5.938065076572442
Correlation coefficient: nan
[ 6.43590962  7.477597    7.32156869  5.37406941  5.8892975   6.23573967
  7.33433266  6.57042651  6.12174257  6.86770113  6.24010271  6.53729444
  7.90575101  7.76599362 -0.71527592  5.40247701]
114    6.026872
10     7.856985
4      7.109020
95     5.200659
111    6.853872
79     6.327902
44     7.638272
47     7.022276
107    6.000000
11     7.250264
61     5.481486
56     6.657577
0      7.260428
92     7.141463
18     6.630970
78     6.244125
Name: LoVo/DX, dtype: float64
Training Root Mean Square Error: 0.292979841686242
Testing Root Mean Square Error: 1.9015661490887223
```

2.1 Search inside correlation space

[9]:
```
step = 0.01
initial_step = corr_low
last_step = corr_high
first_list = [x / 100.0 for x in range(int(initial_step*100),␣
 ↪int(last_step*100), int(step*100))]
second_list = []
third_list = []
fourth_l = []
fifth_l = []
f_list = []
for i in first_list:
    without_standardization, train_r2, test_r2, _, h_, target_column_name,␣
 ↪training_data_RMSE, test_data_RMSE = pred_model.
 ↪prepare_data_and_create_model(molecular_descriptors_df = data,
                                                                              ␣
 ↪                    correlation_threshold = i,
                                                                              ␣
 ↪                    standardization = False,
```

```
                        model_type = 'linear_model',
                        target_column_name = target,
                        random_state=random_state,
                        train_test_split_ = True,
                        verbose = False)
    second_list.append(train_r2)
    third_list.append(test_r2)
    fourth_l.append(training_data_RMSE)
    fifth_l.append(test_data_RMSE)
    f_list.append(len(h_))
```

[10]:
```
df_without_standardization = pd.DataFrame(data=first_list, columns=["Correlation threshold"])
df_without_standardization['Training data R^2 score'] = second_list
df_without_standardization['Test data R^2 score'] = third_list
df_without_standardization['Training RMSE'] = fourth_l
df_without_standardization['Test RMSE'] = fifth_l
df_without_standardization['Number of features'] = f_list
```

[11]:
```
df_linear = df_without_standardization.copy()
df_without_standardization
```

[11]:

	Correlation threshold	Training data R^2 score	Test data R^2 score	\
0	0.33	0.999529	-25.366214	
1	0.34	0.999529	-118.874703	
2	0.35	0.999529	-88.944337	
3	0.36	0.999529	-133.757490	
4	0.37	0.999529	-152.149817	
5	0.38	0.999529	-134.333640	
6	0.39	0.999529	-149.104334	
7	0.40	0.999529	-684.758130	
8	0.41	0.999529	-692.818706	
9	0.42	0.999529	-70.002815	
10	0.43	0.999529	-39.013138	
11	0.44	0.999529	-104.132516	
12	0.45	0.999529	-17.907666	
13	0.46	0.999529	-8126.913150	
14	0.47	0.990640	-5.805265	
15	0.48	0.973257	-3.025601	
16	0.49	0.956264	-7.382606	
17	0.50	0.907173	-5.938065	
18	0.51	0.902360	-4.308421	

19	0.52	0.897049	-2.564160
20	0.53	0.878136	-4.509620
21	0.54	0.806169	-0.191509
22	0.55	0.775066	0.161569
23	0.56	0.764378	0.393203
24	0.57	0.706754	0.503572
25	0.58	0.680843	0.585644
26	0.59	0.679485	0.577302
27	0.60	0.677151	0.568799
28	0.61	0.677151	0.568799
29	0.62	0.677151	0.568799
30	0.63	0.516627	0.527256

	Training RMSE	Test RMSE	Number of features
0	0.020869	3.706945	527
1	0.020869	7.904166	512
2	0.020869	6.846666	487
3	0.020869	8.380476	455
4	0.020869	8.934094	435
5	0.020869	8.398373	399
6	0.020869	8.844817	377
7	0.020869	18.905046	343
8	0.020869	19.015829	290
9	0.020869	6.083171	245
10	0.020869	4.566606	203
11	0.020869	7.402199	143
12	0.020869	3.139144	111
13	0.020869	65.085131	94
14	0.093034	1.883280	83
15	0.157256	1.448464	69
16	0.201104	2.090171	60
17	0.292980	1.901566	50
18	0.300480	1.663317	46
19	0.308544	1.362922	41
20	0.335691	1.694545	32
21	0.423363	0.788027	25
22	0.456067	0.661037	21
23	0.466777	0.562360	16
24	0.520736	0.508652	12
25	0.543255	0.464707	9
26	0.544409	0.469362	8
27	0.546388	0.474058	7
28	0.546388	0.474058	7
29	0.546388	0.474058	7
30	0.668564	0.496370	5

[ ]:

2.2 Plots

[12]:
```
plt.plot(df_without_standardization['Correlation threshold'],␣
  ↪df_without_standardization['Training data R^2 score'], label = "Training␣
  ↪data R^2 score", marker='v')
plt.plot(df_without_standardization['Correlation threshold'],␣
  ↪df_without_standardization['Test data R^2 score'], label = "Test data R^2␣
  ↪score", marker='D')
plt.legend()
plt.xlabel('Correlation threshold')
plt.ylabel('R^2 score')
plt.ylim([0, 1])
plt.show()
```

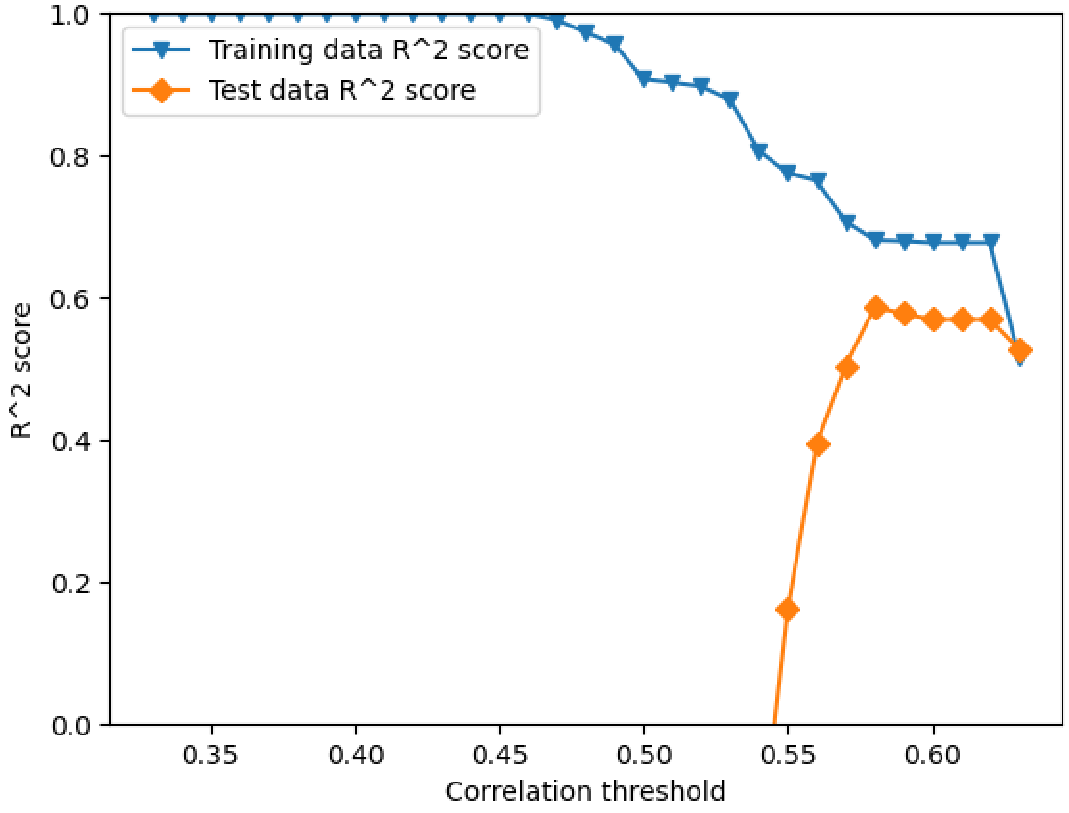

[13]:
```
plt.plot(df_without_standardization['Correlation threshold'],␣
  ↪df_without_standardization['Number of features'], label = "Number of␣
  ↪features", marker='.')
plt.legend()
plt.xlabel('Correlation threshold')
plt.ylabel('Number of features')
plt.show()
```

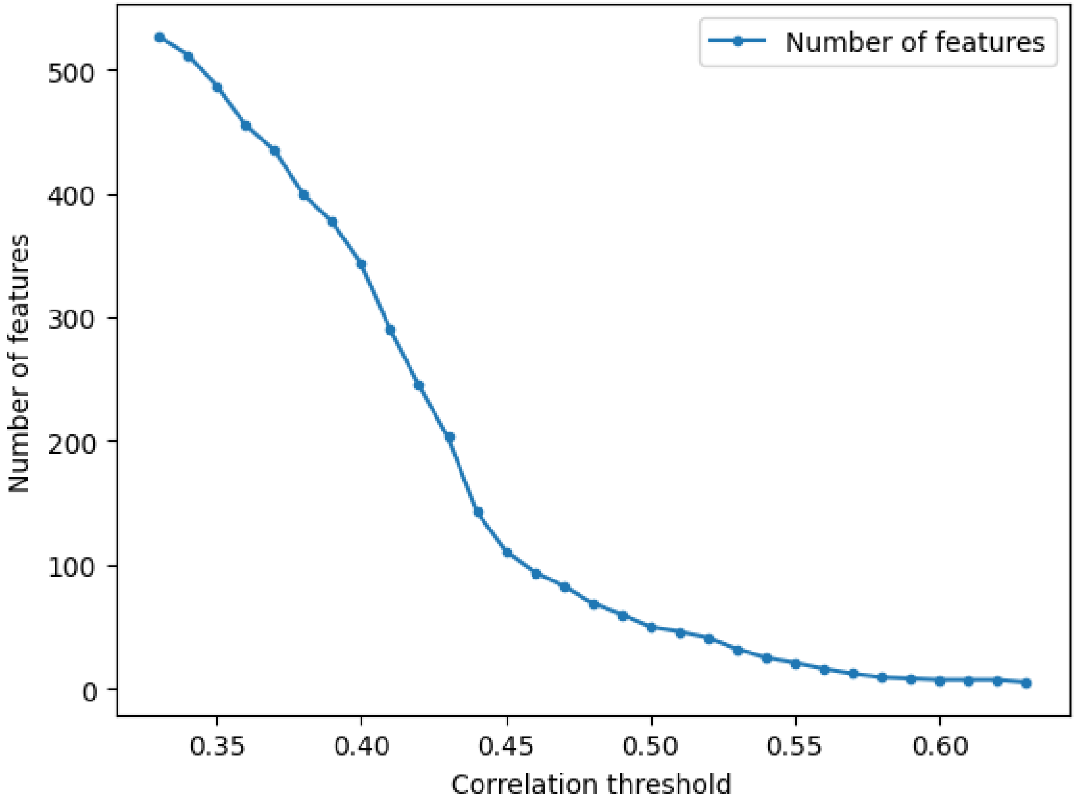

[ ]:

```

```

2.3 Decision Tree

[14]:

```
without_standardization, train_r2_, test_r2_, hist1, hist2, target_column_name,␣
↪training_data_RMSE, test_data_RMSE  = pred_model.
↪prepare_data_and_create_model(molecular_descriptors_df=data,
                                                                              ␣
↪                         correlation_threshold=0.50,
                                                                              ␣
↪                         standardization=False,
                                                                              ␣
↪                         model_type='DecisionTreeRegressor',
                                                                              ␣
↪                         max_depth=5,
                                                                              ␣
↪                         target_column_name = target,
                                                                              ␣
↪                         random_state=random_state,
```

```
↪                       train_test_split_=True,
↪                       verbose=True)
```

```
I am not doing standardization…
  molecular descriptor name
0                    AATS0Z
1                  AATS0are
2                    AATS0d
3                   AATS0dv
4                    AATS0i
  molecular descriptor name  corr_value
0                    AATS0Z    0.018677
1                  AATS0are   -0.341313
2                    AATS0d   -0.123443
3                   AATS0dv   -0.265670
4                    AATS0i   -0.428929
  molecular descriptor name  corr_value  absolute correlation value
0                    AATS0Z    0.018677                    0.018677
1                  AATS0are   -0.341313                    0.341313
2                    AATS0d   -0.123443                    0.123443
3                   AATS0dv   -0.265670                    0.265670
4                    AATS0i   -0.428929                    0.428929
    molecular descriptor name  corr_value  absolute correlation value
15                     AATS1i   -0.561620                    0.561620
113                   AATSC1c    0.565682                    0.565682
125                   AATSC2c   -0.543393                    0.543393
221                AETA_eta_R   -0.515778                    0.515778
224                    AMID_C    0.532458                    0.532458
    molecular descriptor name  corr_value  absolute correlation value
15                     AATS1i   -0.561620                    0.561620
113                   AATSC1c    0.565682                    0.565682
125                   AATSC2c   -0.543393                    0.543393
221                AETA_eta_R   -0.515778                    0.515778
224                    AMID_C    0.532458                    0.532458
The model used is: DecisionTree…
Return the coefficient of determination of the prediction:
0.33684316448779406
R^2 score: 0.9184499075482622
Correlation coefficient: 0.9583579224633467
Test data - unseen during training:
R^2 score: 0.33684316448779406
Correlation coefficient: 0.5803819126125435
[5.85304018 7.27468871 7.56156648 5.85304018 6.92324501 5.85304018
 7.17422529 8.78271555 5.85304018 7.27468871 5.85304018 6.24964472
 7.56156648 7.11308338 7.27468871 5.85304018]
114    6.026872
```

```
10     7.856985
4      7.109020
95     5.200659
111    6.853872
79     6.327902
44     7.638272
47     7.022276
107    6.000000
11     7.250264
61     5.481486
56     6.657577
0      7.260428
92     7.141463
18     6.630970
78     6.244125
Name: LoVo/DX, dtype: float64
Training Root Mean Square Error: 0.2746083908132104
Testing Root Mean Square Error: 0.5878958281921561
```

[15]:
```
print(target_column_name+str('_transformed'))
print(hist1[target_column_name].hist())
```

```
LoVo/DX_transformed
AxesSubplot(0.125,0.11;0.775x0.77)
```

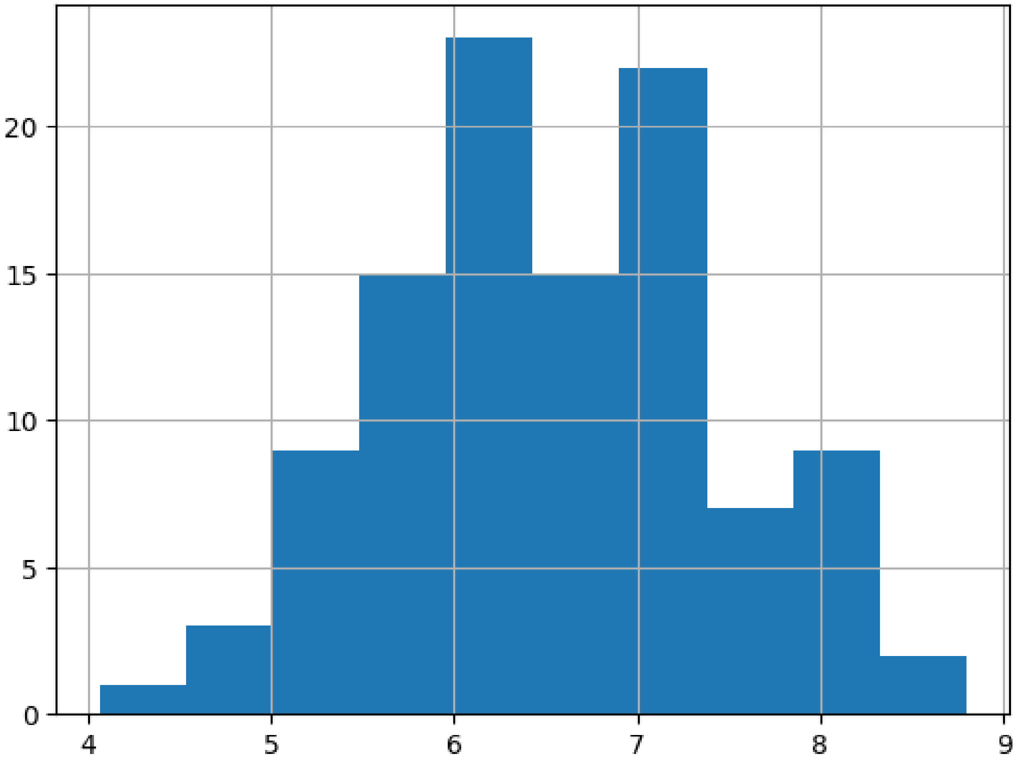

[16]:
```
with_standardization, train_r2_, test_r2_, hist1, hist2, target_column_name,␣
↪training_data_RMSE, test_data_RMSE  = pred_model.
↪prepare_data_and_create_model(molecular_descriptors_df=data,
                                                                              ␣
↪                    correlation_threshold=0.50,
                                                                              ␣
↪                    standardization=True,
                                                                              ␣
↪                    model_type='DecisionTreeRegressor',
                                                                              ␣
↪                    max_depth=5,
                                                                              ␣
↪                    target_column_name = target,
                                                                              ␣
↪                    random_state=random_state,
                                                                              ␣
↪                    train_test_split_=True,
                                                                              ␣
↪                    verbose=True)
```

```
I am doing standardization…
  molecular descriptor name
0                    AATS0Z
1                  AATS0are
2                    AATS0d
3                   AATS0dv
4                    AATS0i
  molecular descriptor name  corr_value
0                    AATS0Z    0.018677
1                  AATS0are   -0.341313
2                    AATS0d   -0.123443
3                   AATS0dv   -0.265670
4                    AATS0i   -0.428929
  molecular descriptor name  corr_value  absolute correlation value
0                    AATS0Z    0.018677                    0.018677
1                  AATS0are   -0.341313                    0.341313
2                    AATS0d   -0.123443                    0.123443
3                   AATS0dv   -0.265670                    0.265670
4                    AATS0i   -0.428929                    0.428929
    molecular descriptor name  corr_value  absolute correlation value
15                     AATS1i   -0.561620                    0.561620
113                  AATSC1c    0.565682                    0.565682
125                  AATSC2c   -0.543393                    0.543393
221                AETA_eta_R   -0.515778                    0.515778
224                    AMID_C    0.532458                    0.532458
```

```
    molecular descriptor name  corr_value  absolute correlation value
15                     AATS1i   -0.561620                    0.561620
113                   AATSC1c    0.565682                    0.565682
125                   AATSC2c   -0.543393                    0.543393
221                AETA_eta_R   -0.515778                    0.515778
224                    AMID_C    0.532458                    0.532458
The model used is: DecisionTree…
Return the coefficient of determination of the prediction:
0.33684316448779406
R^2 score: 0.9184499075482622
Correlation coefficient: 0.9583579224633467
Test data - unseen during training:
R^2 score: 0.33684316448779406
Correlation coefficient: 0.5803819126125435
[5.85304018 7.27468871 7.56156648 5.85304018 6.92324501 5.85304018
 7.17422529 8.78271555 5.85304018 7.27468871 5.85304018 6.24964472
 7.56156648 7.11308338 7.27468871 5.85304018]
114    6.026872
10     7.856985
4      7.109020
95     5.200659
111    6.853872
79     6.327902
44     7.638272
47     7.022276
107    6.000000
11     7.250264
61     5.481486
56     6.657577
0      7.260428
92     7.141463
18     6.630970
78     6.244125
Name: LoVo/DX, dtype: float64
Training Root Mean Square Error: 0.2746083908132104
Testing Root Mean Square Error: 0.5878958281921561
```

2.4 Search inside correlation space

[17]:
```
step = 0.01
initial_step = corr_low
last_step = corr_high
first_list = [x / 100.0 for x in range(int(initial_step*100),␣
 ↪int(last_step*100), int(step*100))]
max_depth = [range(2, 30, 1)]
corr_th = []
second_list = []
```

```
third_list = []
fourth_l = []
fifth_l = []
f_list = []
fif_list = []
for i in first_list:
    for depth in max_depth[0]:

        without_standardization, train_r2, test_r2, _, h_, target_column_name,␣
 ↪training_data_RMSE, test_data_RMSE = pred_model.
 ↪prepare_data_and_create_model(molecular_descriptors_df=data,
                                                                              ␣
 ↪                    correlation_threshold=i,
                                                                              ␣
 ↪                    standardization=False,
                                                                              ␣
 ↪                    model_type='DecisionTreeRegressor',
                                                                              ␣
 ↪                    max_depth=depth,
                                                                              ␣
 ↪                    target_column_name = target,
                                                                              ␣
 ↪                    random_state=random_state,
                                                                              ␣
 ↪                    train_test_split_=True,
                                                                              ␣
 ↪                    verbose=False)
        corr_th.append(i)
        second_list.append(train_r2)
        third_list.append(test_r2)
        fourth_l.append(training_data_RMSE)
        fifth_l.append(test_data_RMSE)
        f_list.append(len(h_))
        fif_list.append(depth)
```

[18]:
```
df_without_standardization = pd.DataFrame(data=corr_th, columns=["Correlation␣
 ↪threshold"])
df_without_standardization['Training data R^2 score'] = second_list
df_without_standardization['Test data R^2 score'] = third_list
df_without_standardization['Training RMSE'] = fourth_l
df_without_standardization['Test RMSE'] = fifth_l
df_without_standardization['Number of features'] = f_list
df_without_standardization['Depth number'] = fif_list
```

[19]:
```
df_decision_tree = df_without_standardization.copy()
```

```
#df_without_standardization.to_excel('../Data/
↪A549_Decision_tree_rs_'+str(random_state)+'.xlsx')
```

2.5 Plots

[20]:
```
corre_list = [x / 100.0 for x in range(int(initial_step*100),␣
↪int(last_step*100), int(0.01*100))]
for element in corre_list:
    element_ =␣
↪df_without_standardization[df_without_standardization['Correlation␣
↪threshold'] == float(element)]
    plt.plot(element_['Depth number'], element_['Training data R^2 score'],␣
↪label = "Training data R^2 score", marker='v')
    plt.plot(element_['Depth number'], element_['Test data R^2 score'], label =␣
↪"Test data R^2 score", marker='D')
    plt.legend()
    plt.xlabel('Depth number -> correlation threshold: '+str(element)+'. \n␣
↪Number of features: '+str(element_['Number of features'].iloc[0]),␣
↪fontweight='bold')
    plt.xlim(max(element_['Depth number'])+1, min(element_['Depth number'])-1)
    plt.ylabel('R^2 score')
    plt.ylim([0, 1])
    plt.rc('grid', linestyle="-.", color='black')
    plt.grid(True)
    plt.show()
```

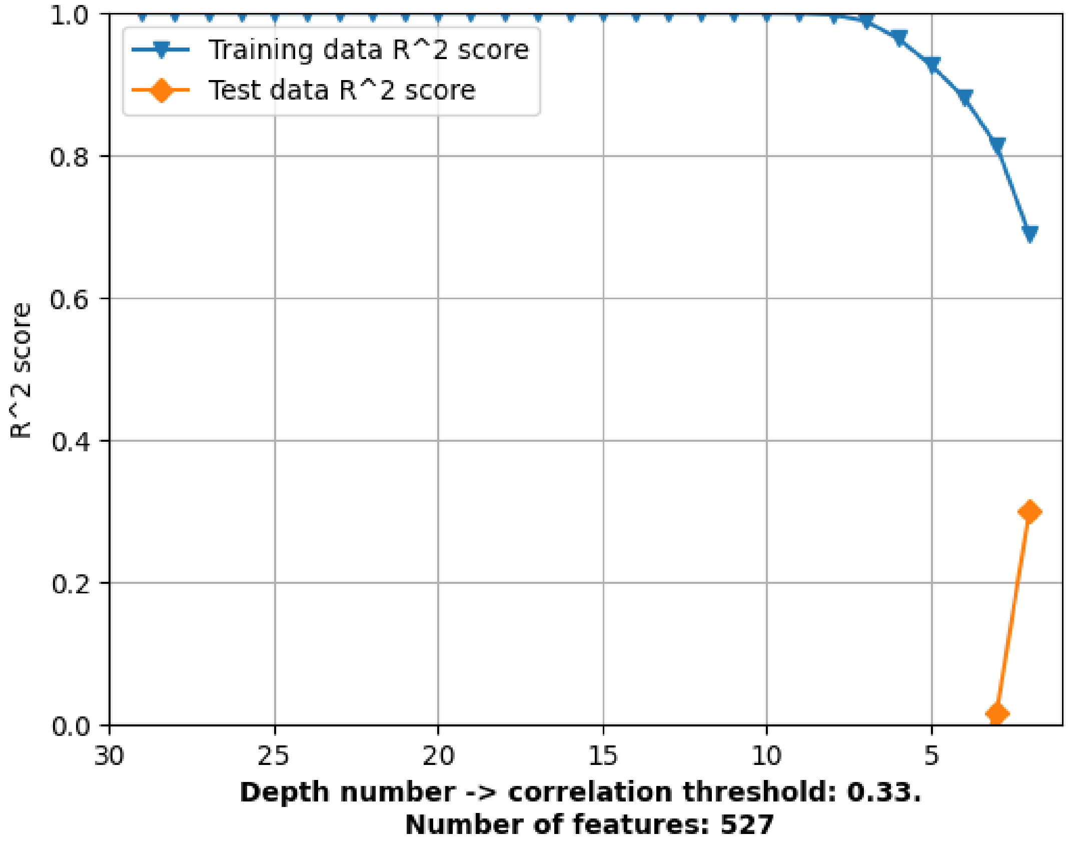
Training data R^2 score
Test data R^2 score
R^2 score
1.0
0.8
0.6
0.4
0.2
0.0
30
25
20
15
10
5
Depth number -> correlation threshold: 0.33.
Number of features: 527

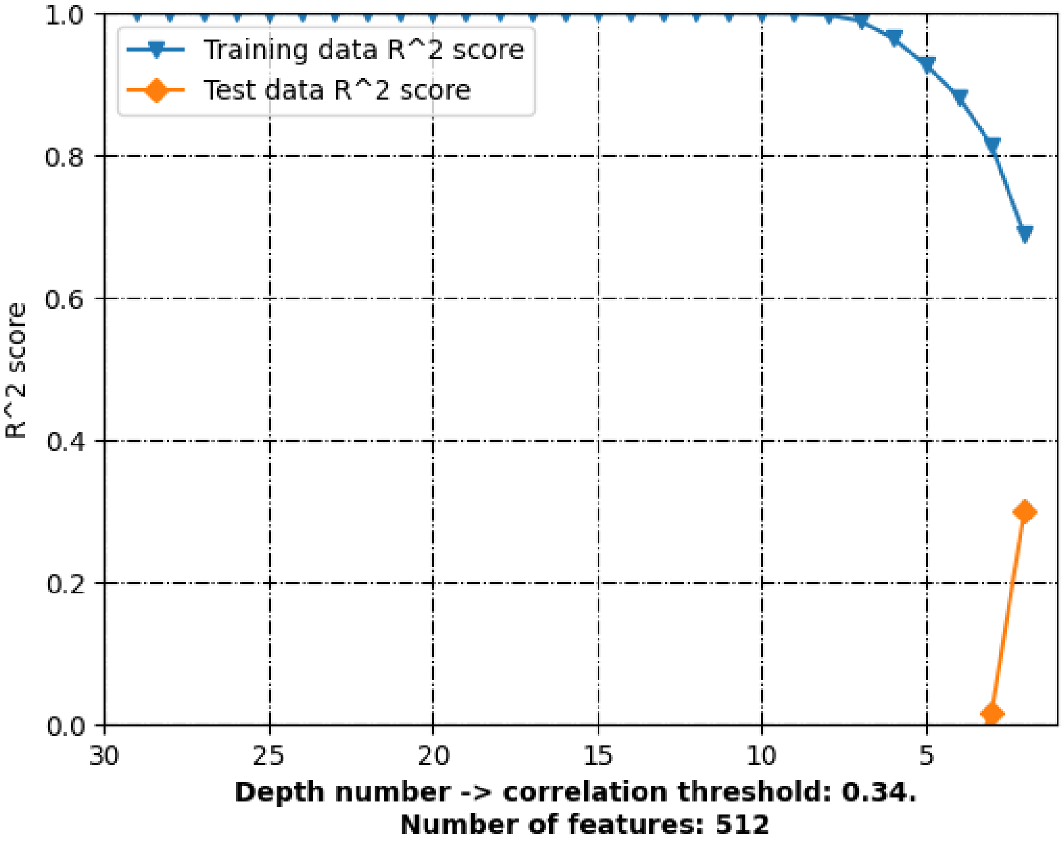

Training data R^2 score
Test data R^2 score
R^2 score
1.0
0.8
0.6
0.4
0.2
0.0
30
25
20
15
10
5
Depth number -> correlation threshold: 0.34.
Number of features: 512

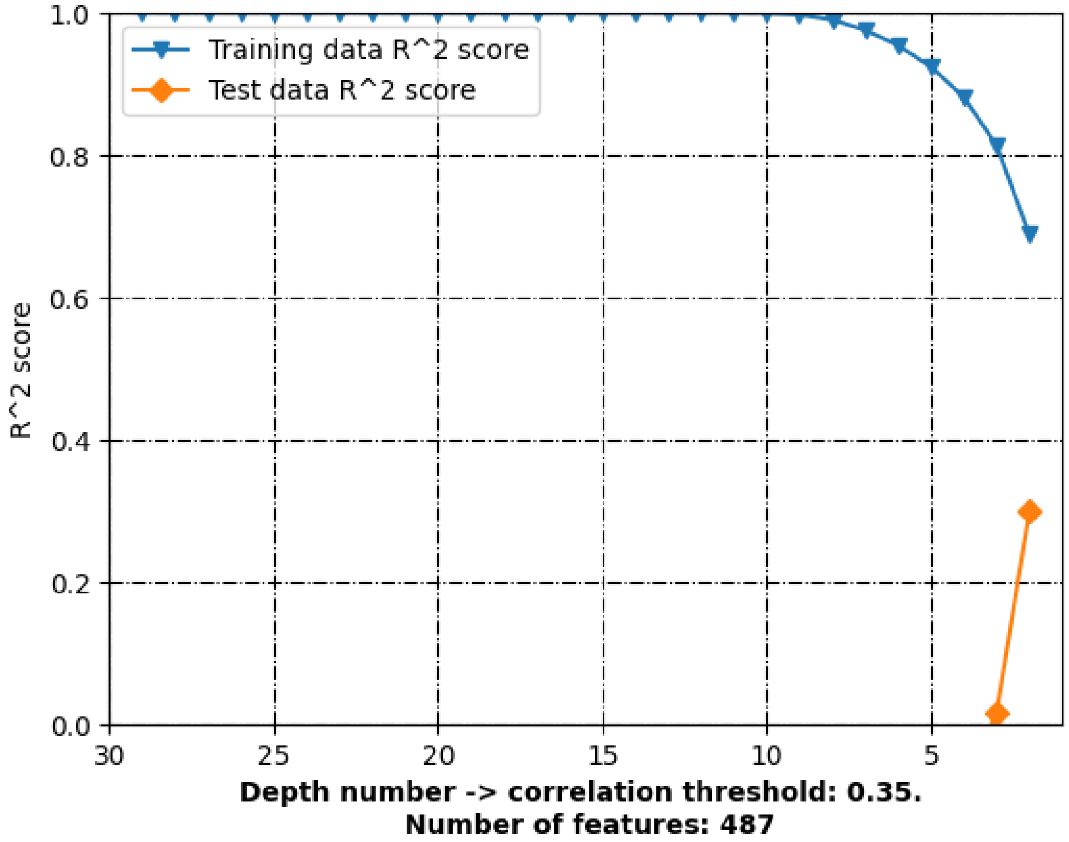

Training data R^2 score
Test data R^2 score
R^2 score
1.0
0.8
0.6
0.4
0.2
0.0
30
25
20
15
10
5
Depth number -> correlation threshold: 0.35.
Number of features: 487

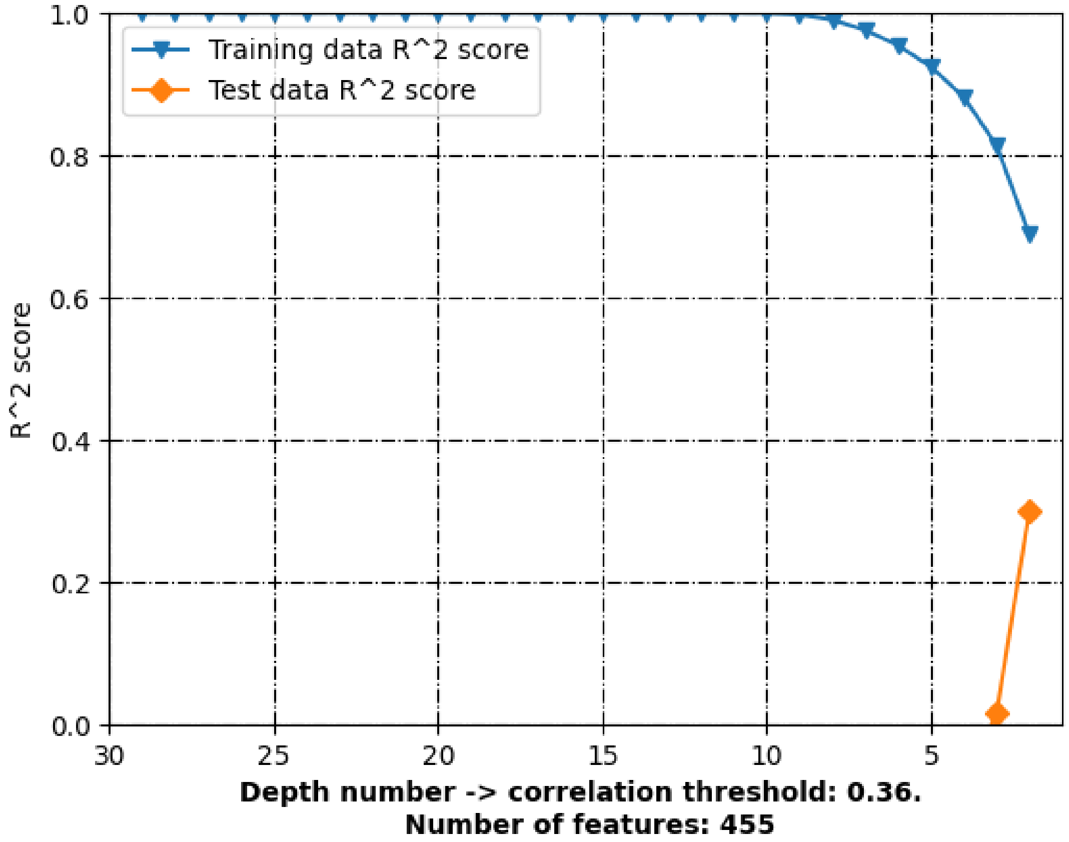

1.0
0.8
0.6
0.4
0.2
0.0
R^2 score
Training data R^2 score
Test data R^2 score
30
25
20
15
10
5
Depth number -> correlation threshold: 0.36.
Number of features: 455

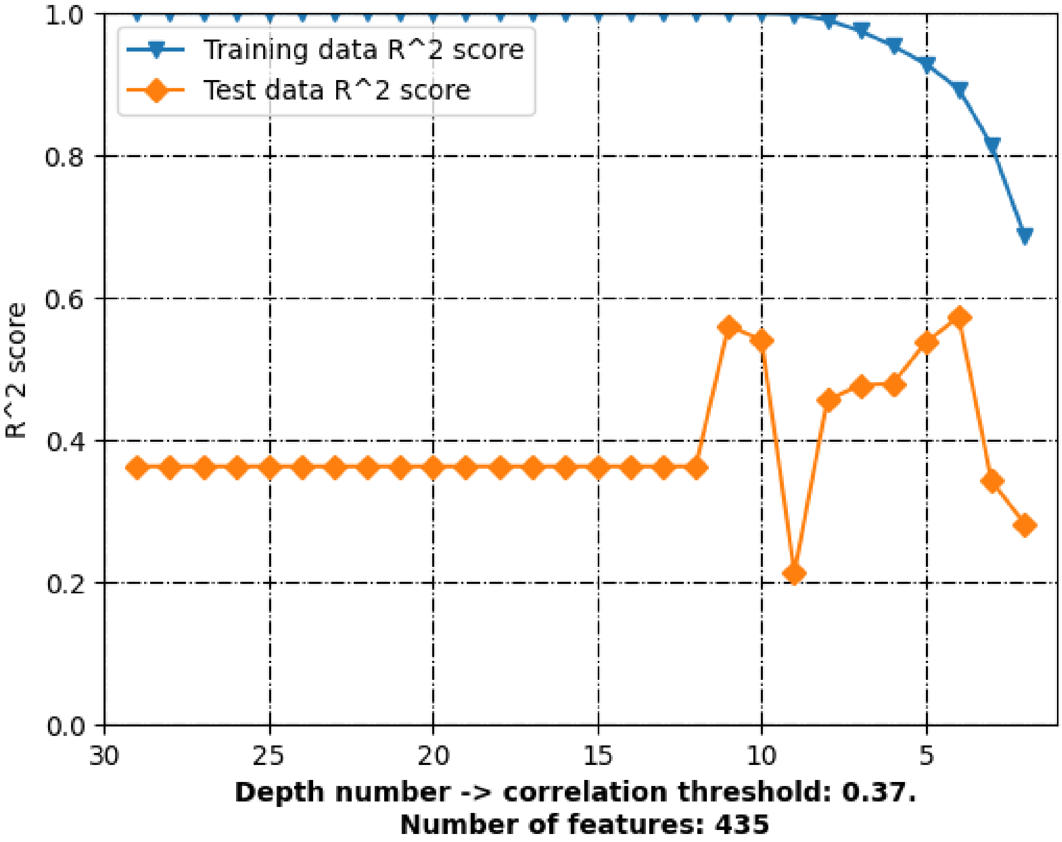
Training data R^2 score
Test data R^2 score
R^2 score
1.0
0.8
0.6
0.4
0.2
0.0
30
25
20
15
10
5
Depth number -> correlation threshold: 0.37.
Number of features: 435

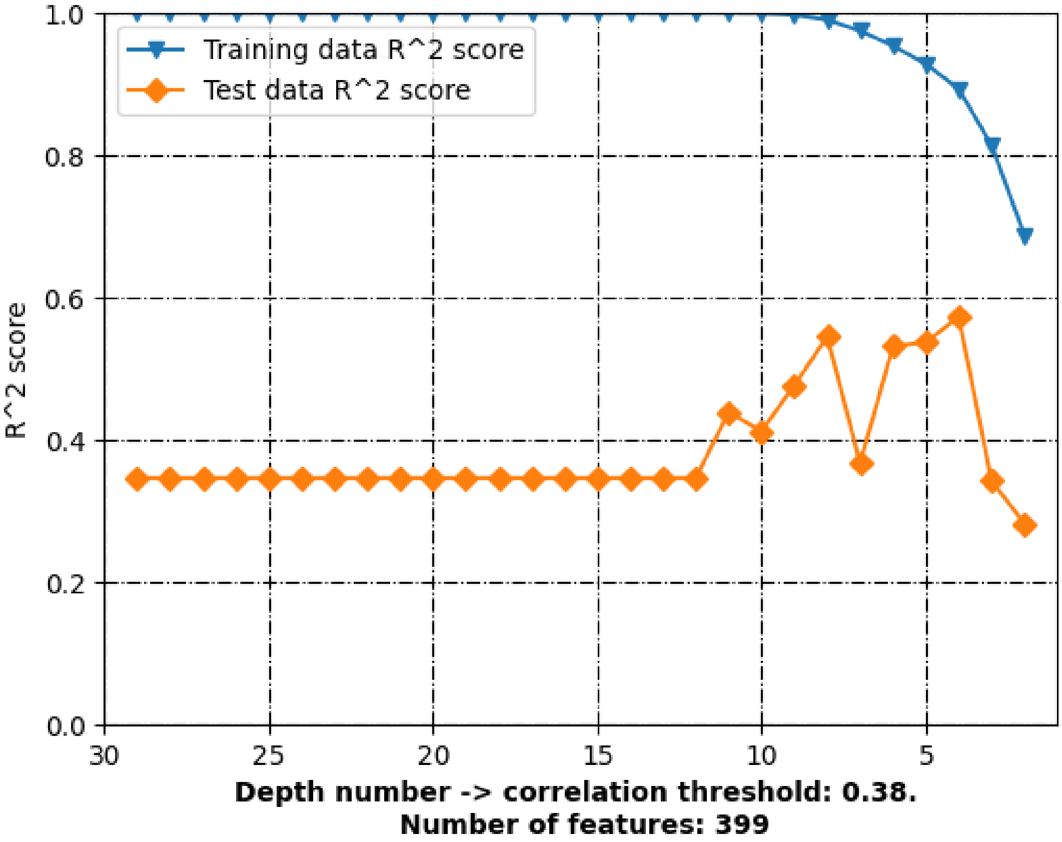

Training data R^2 score
Test data R^2 score
R^2 score
1.0
0.8
0.6
0.4
0.2
0.0
30
25
20
15
10
5
Depth number -> correlation threshold: 0.38.
Number of features: 399

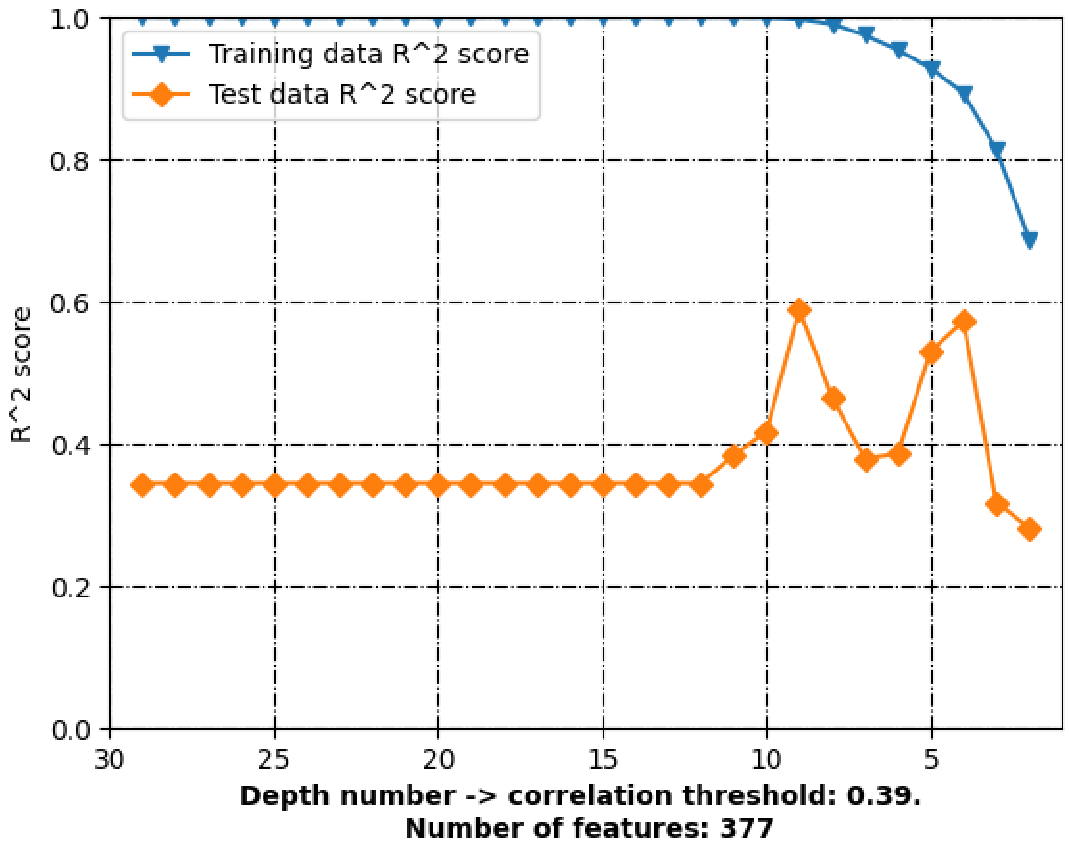
Training data R^2 score
Test data R^2 score
R^2 score
1.0
0.8
0.6
0.4
0.2
0.0
30
25
20
15
10
5
Depth number -> correlation threshold: 0.39.
Number of features: 377

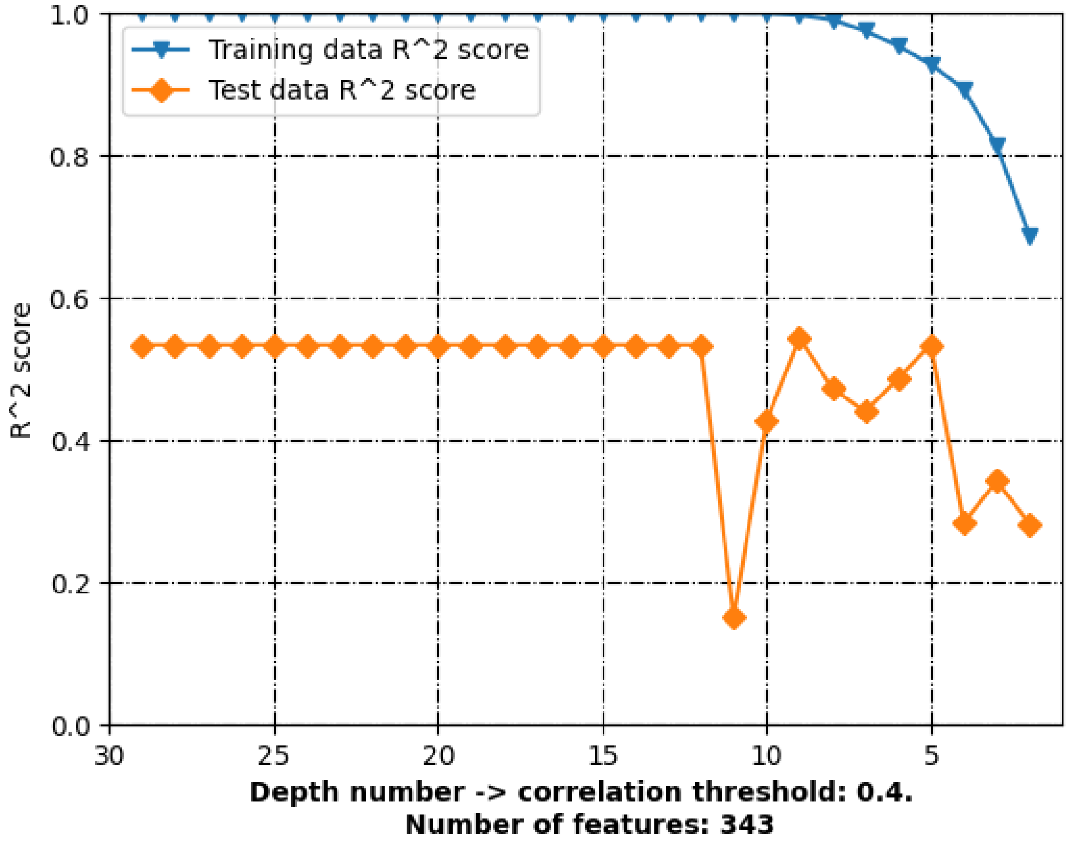
Training data R^2 score
Test data R^2 score
R^2 score
1.0
0.8
0.6
0.4
0.2
0.0
30
25
20
15
10
5
Depth number -> correlation threshold: 0.4.
Number of features: 343

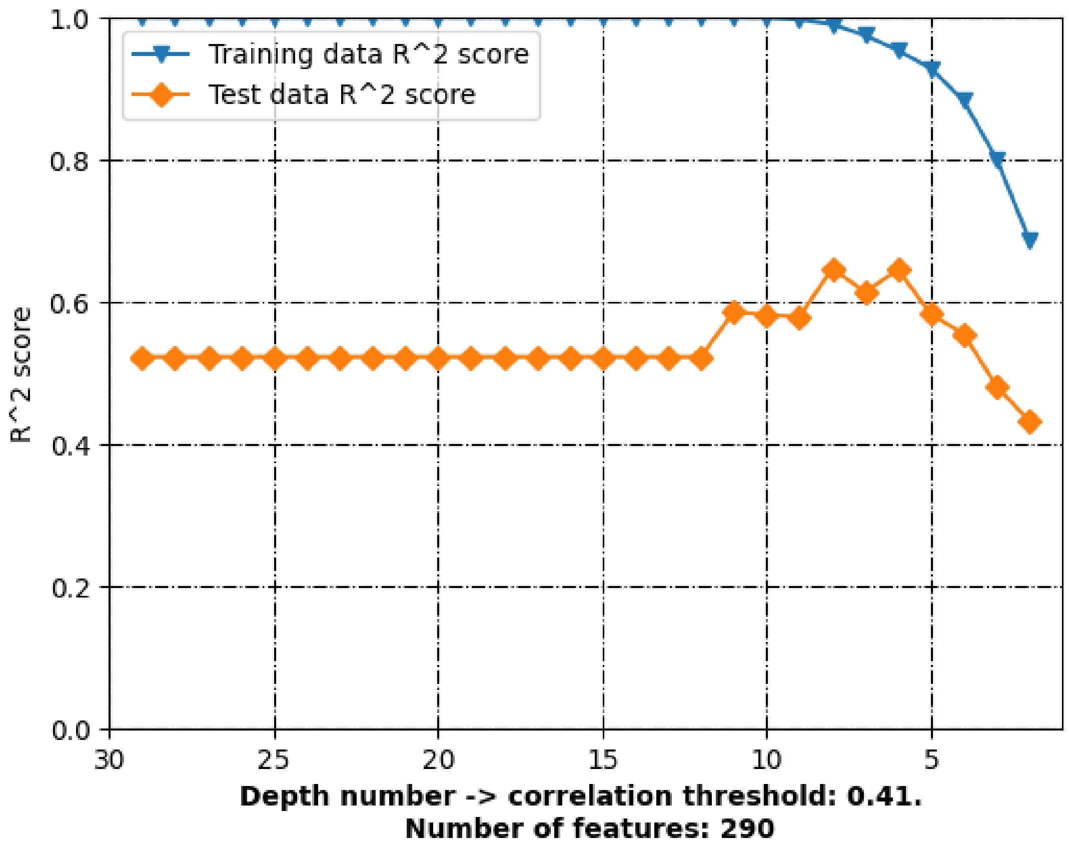
Training data R^2 score
Test data R^2 score
R^2 score
1.0
0.8
0.6
0.4
0.2
0.0
30
25
20
15
10
5
Depth number -> correlation threshold: 0.41.
Number of features: 290

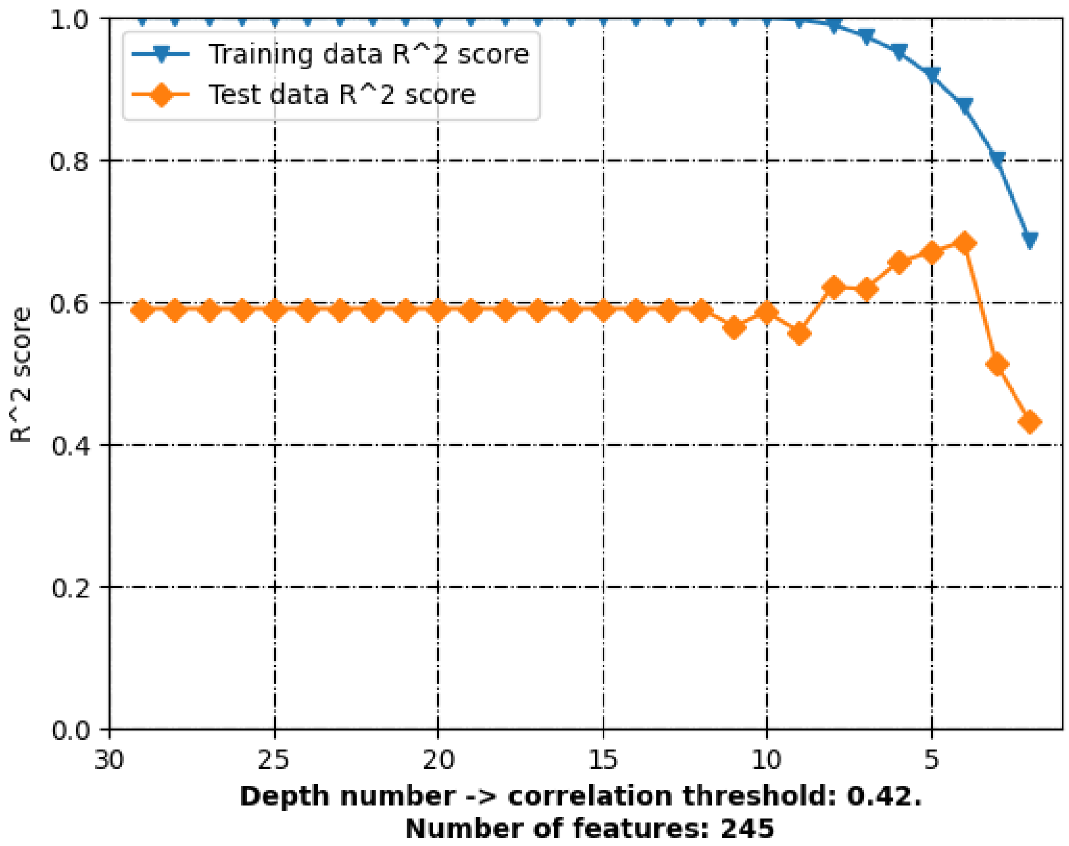

Training data R^2 score
Test data R^2 score
R^2 score
1.0
0.8
0.6
0.4
0.2
0.0
30
25
20
15
10
5
Depth number -> correlation threshold: 0.42.
Number of features: 245

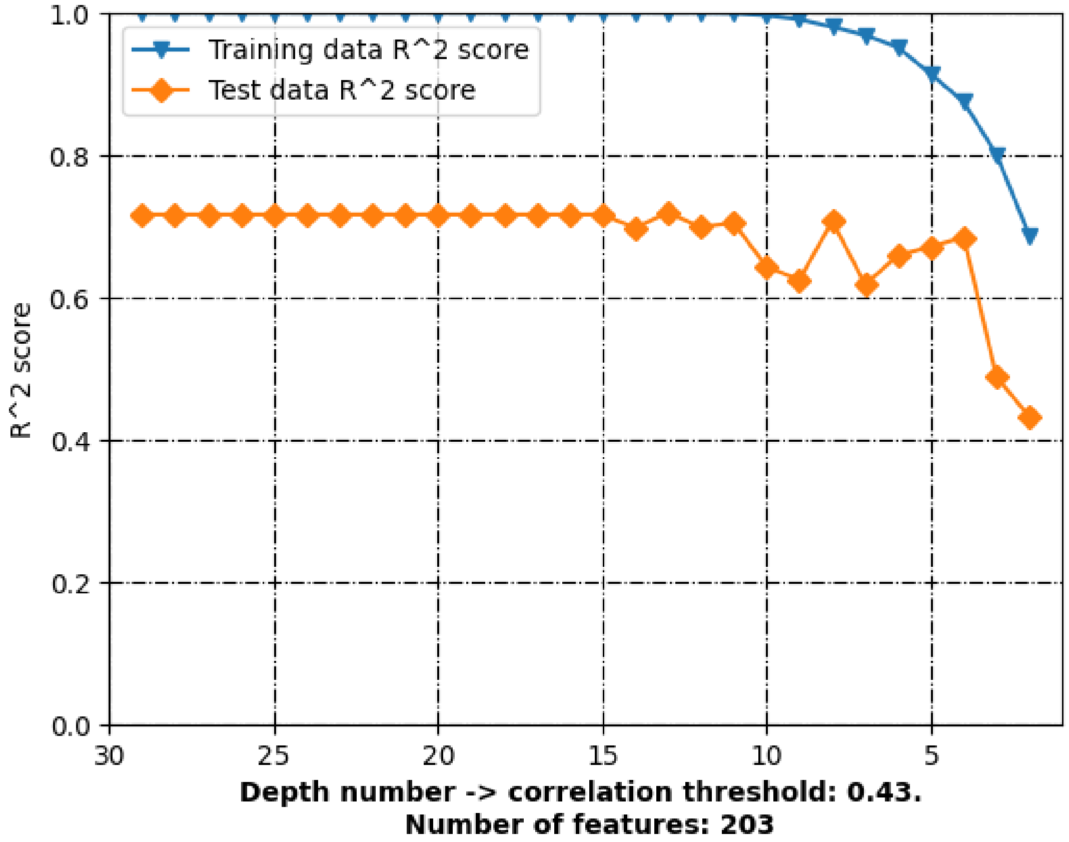

Training data R^2 score
Test data R^2 score
R^2 score
1.0
0.8
0.6
0.4
0.2
0.0
30
25
20
15
10
5
Depth number -> correlation threshold: 0.43.
Number of features: 203

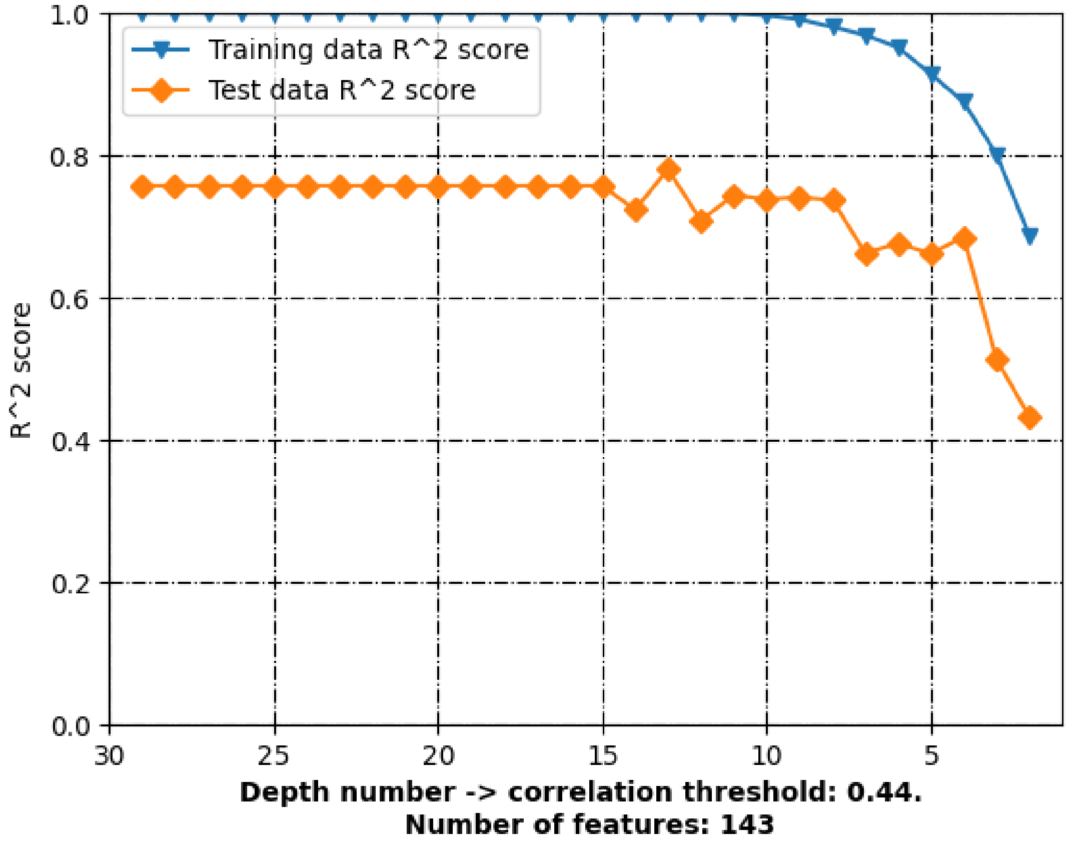

Training data R^2 score
Test data R^2 score
R^2 score
Depth number -> correlation threshold: 0.44.
Number of features: 143

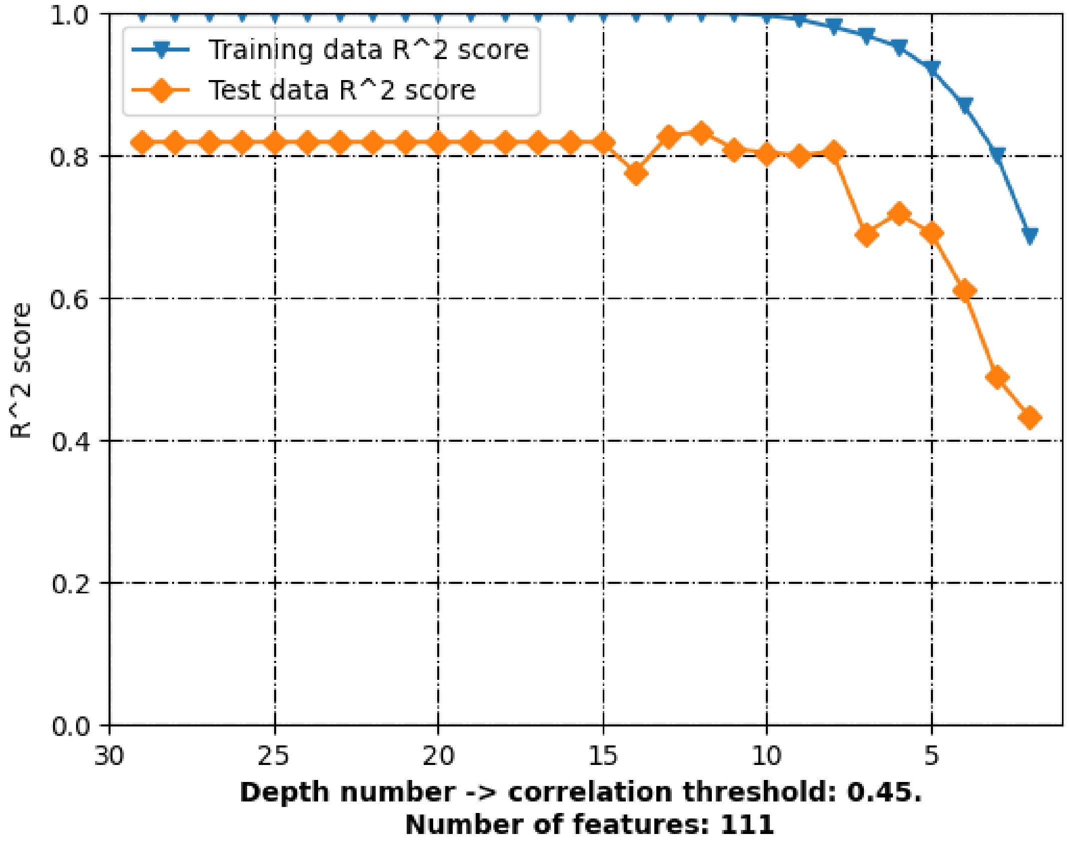
Training data R^2 score
Test data R^2 score
R^2 score
1.0
0.8
0.6
0.4
0.2
0.0
30
25
20
15
10
5
Depth number -> correlation threshold: 0.45.
Number of features: 111

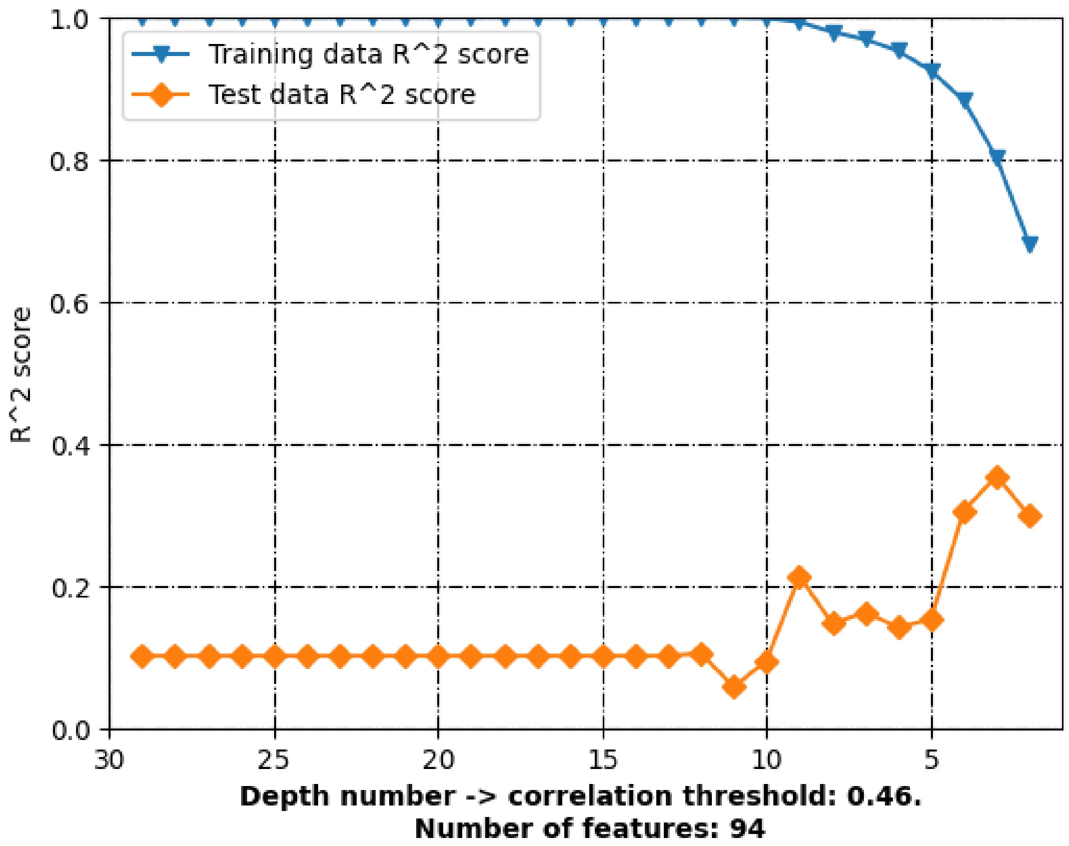

Training data R^2 score
Test data R^2 score
R^2 score
1.0
0.8
0.6
0.4
0.2
0.0
30
25
20
15
10
5
Depth number -> correlation threshold: 0.46.
Number of features: 94

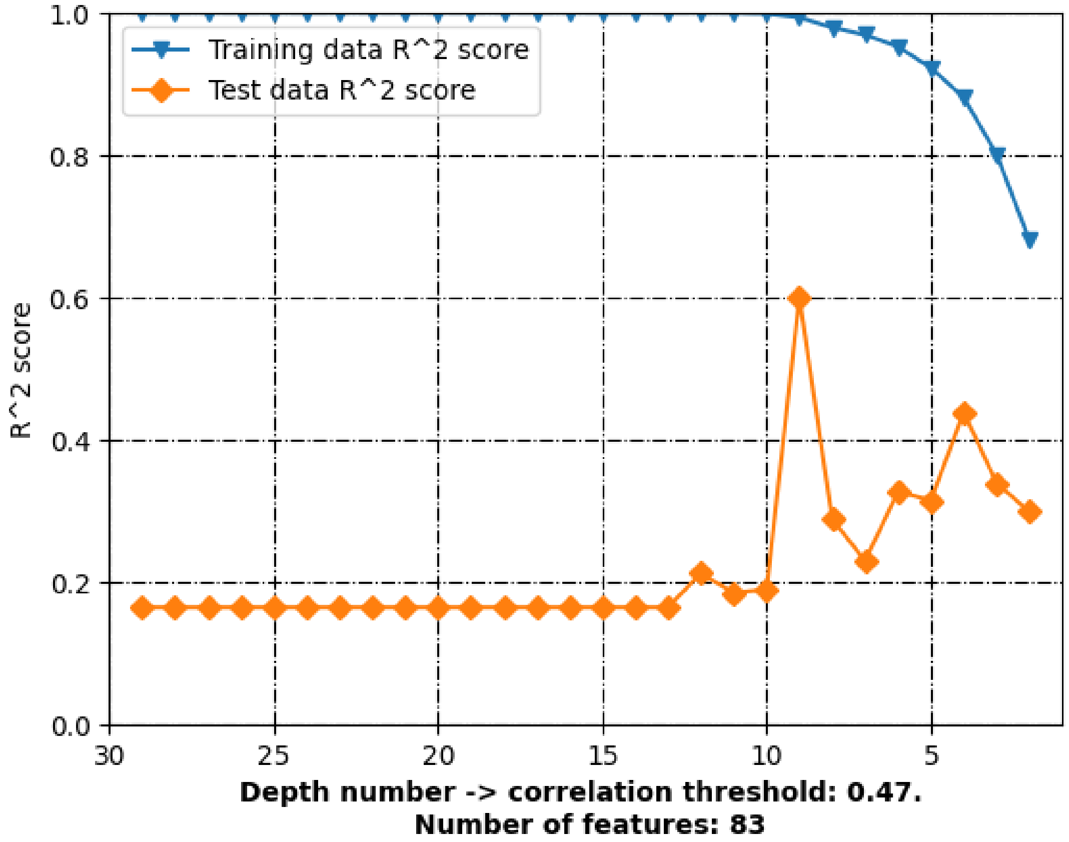
Training data R^2 score
Test data R^2 score
R^2 score
1.0
0.8
0.6
0.4
0.2
0.0
30
25
20
15
10
5
Depth number -> correlation threshold: 0.47.
Number of features: 83

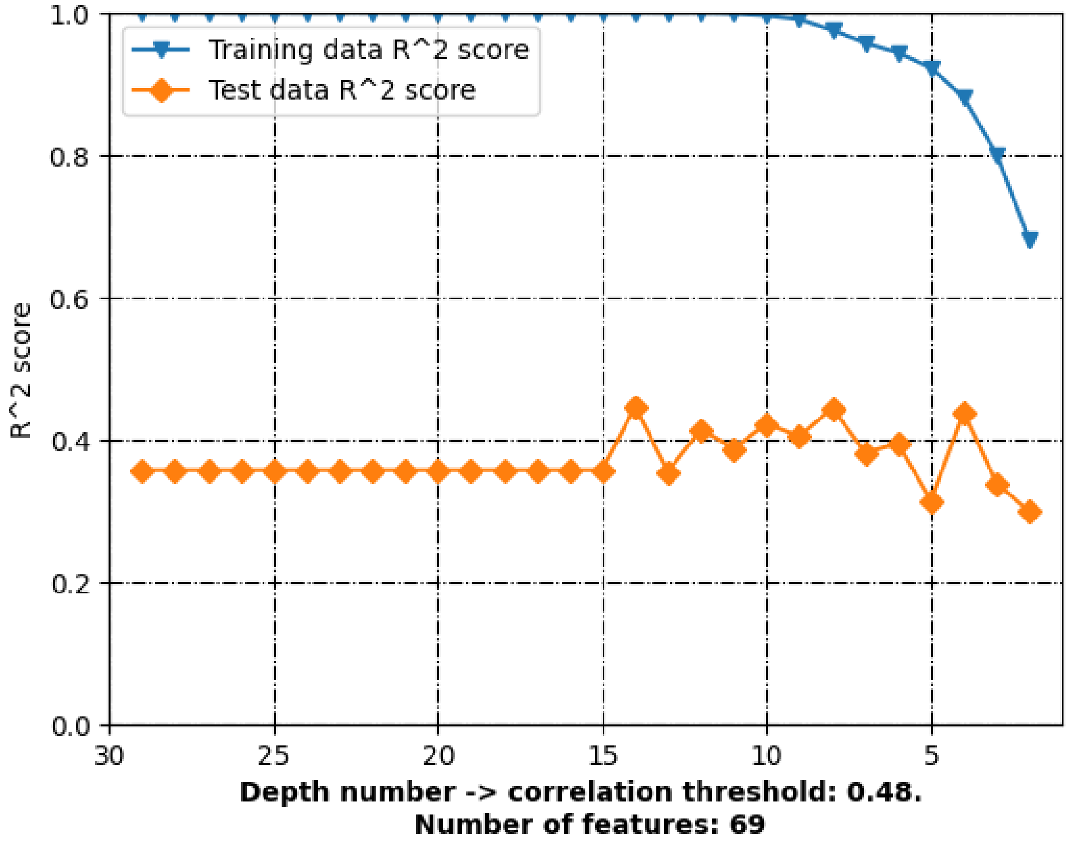

Training data R^2 score
Test data R^2 score
R^2 score
1.0
0.8
0.6
0.4
0.2
0.0
30
25
20
15
10
5
Depth number -> correlation threshold: 0.48.
Number of features: 69

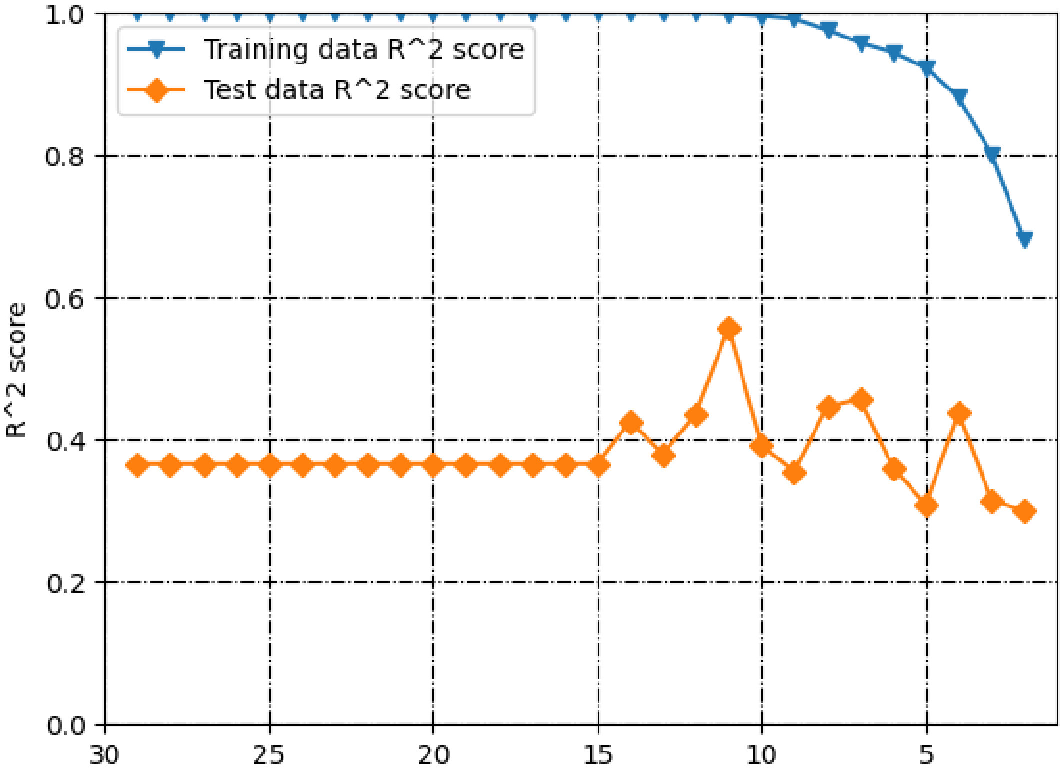

Depth number -> correlation threshold: 0.49.
Number of features: 60

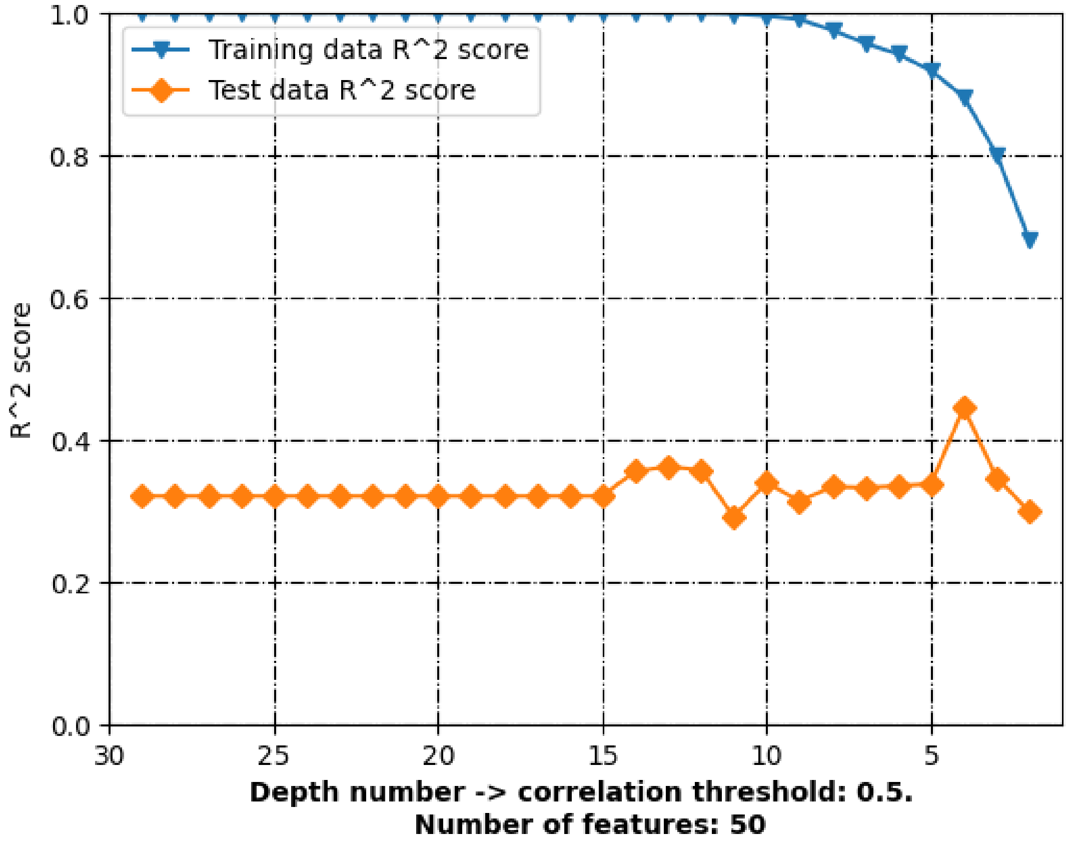
Training data R^2 score
Test data R^2 score
R^2 score
1.0
0.8
0.6
0.4
0.2
0.0
30
25
20
15
10
5
Depth number -> correlation threshold: 0.5.
Number of features: 50

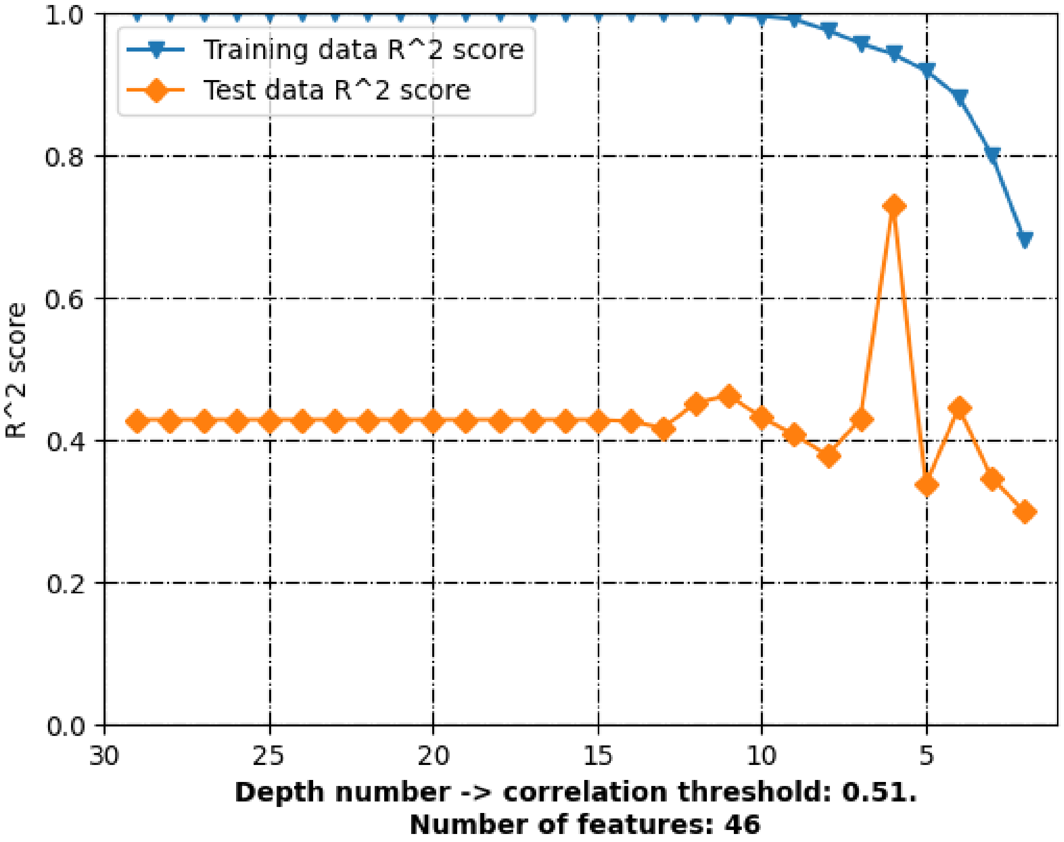

Training data R^2 score
Test data R^2 score
R^2 score
1.0
0.8
0.6
0.4
0.2
0.0
30
25
20
15
10
5
Depth number -> correlation threshold: 0.51.
Number of features: 46

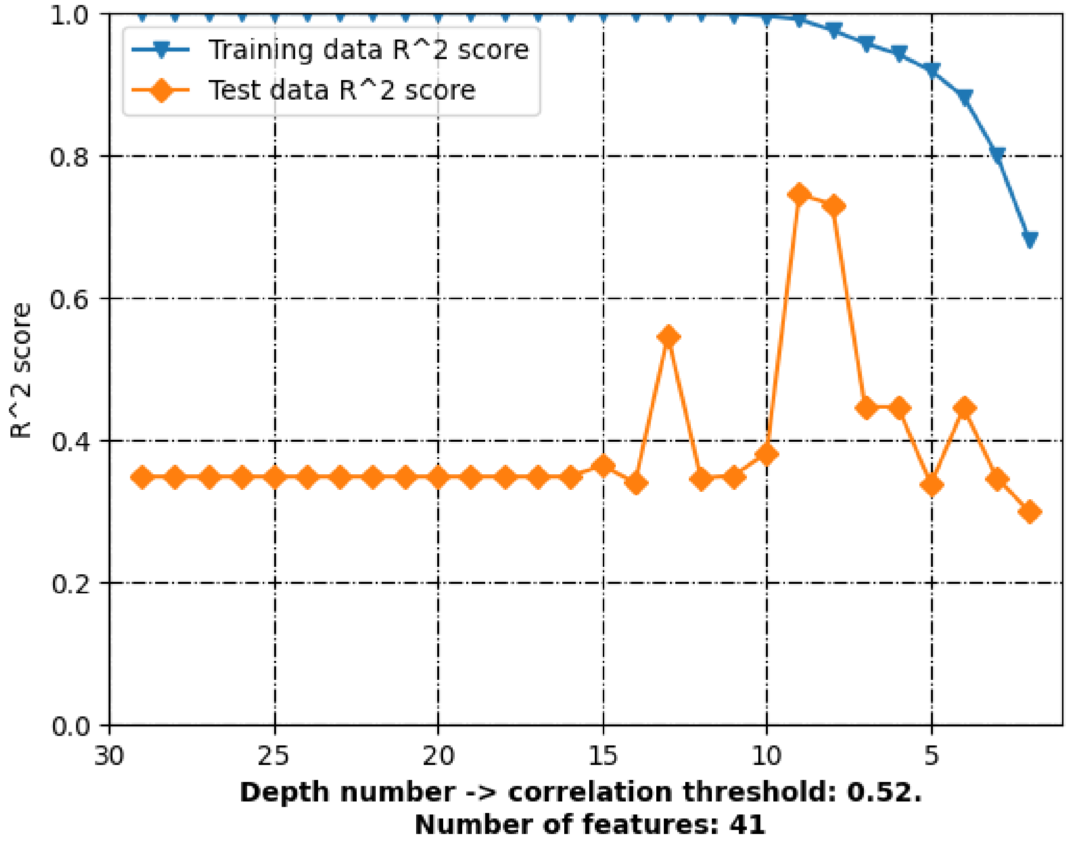

Training data R^2 score
Test data R^2 score
R^2 score
1.0
0.8
0.6
0.4
0.2
0.0
30
25
20
15
10
5
Depth number -> correlation threshold: 0.52.
Number of features: 41

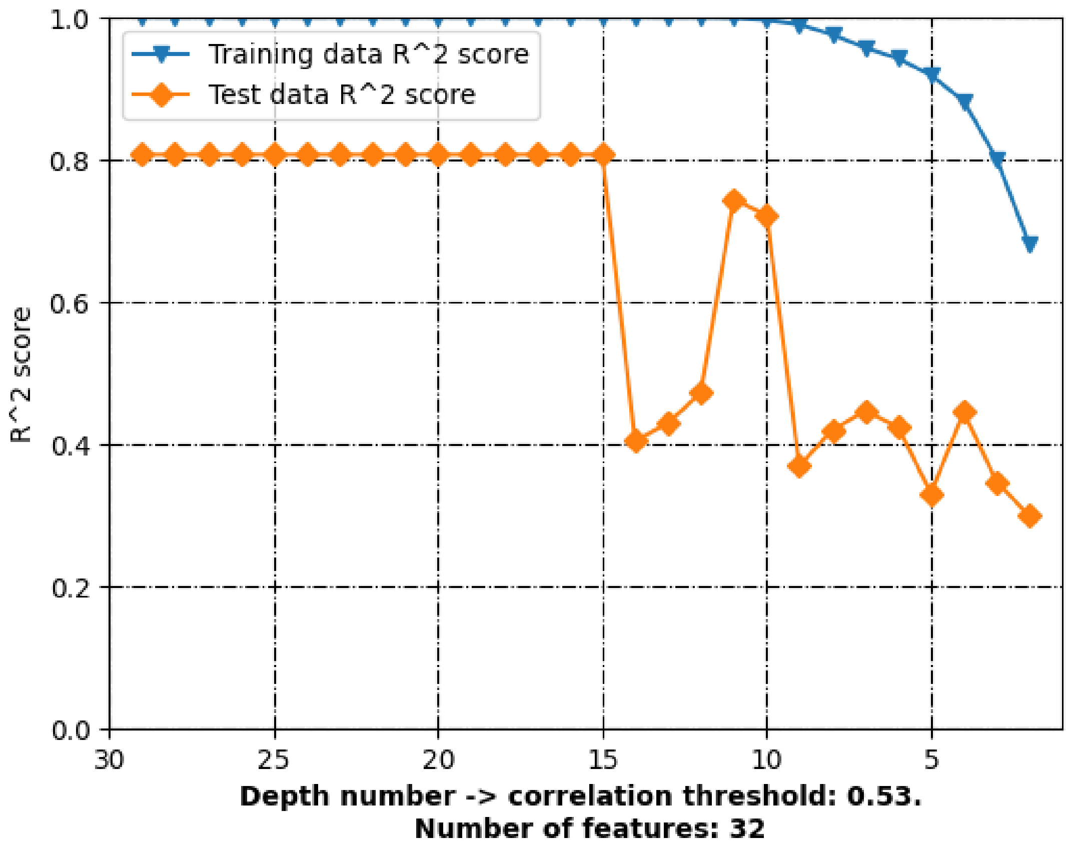

Training data R^2 score
Test data R^2 score
R^2 score
1.0
0.8
0.6
0.4
0.2
0.0
30
25
20
15
10
5
Depth number -> correlation threshold: 0.53.
Number of features: 32

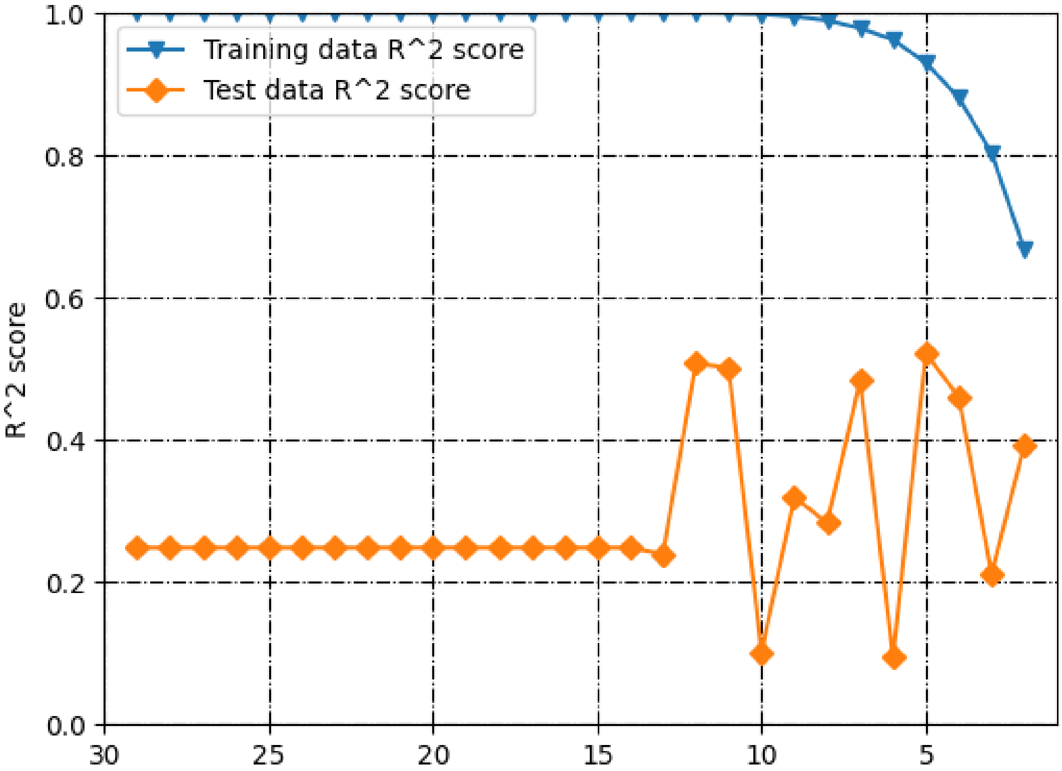

Depth number -> correlation threshold: 0.54.
Number of features: 25

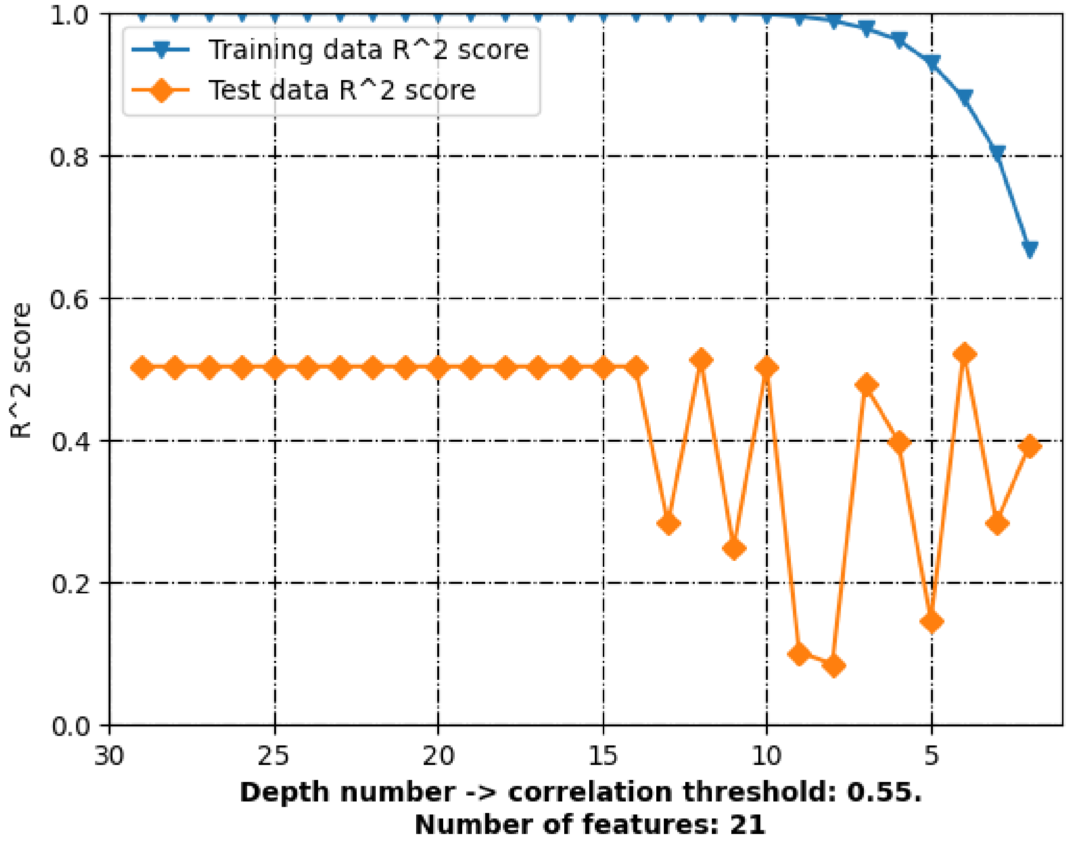

Training data R^2 score
Test data R^2 score
R^2 score
1.0
0.8
0.6
0.4
0.2
0.0
30
25
20
15
10
5
Depth number -> correlation threshold: 0.55.
Number of features: 21

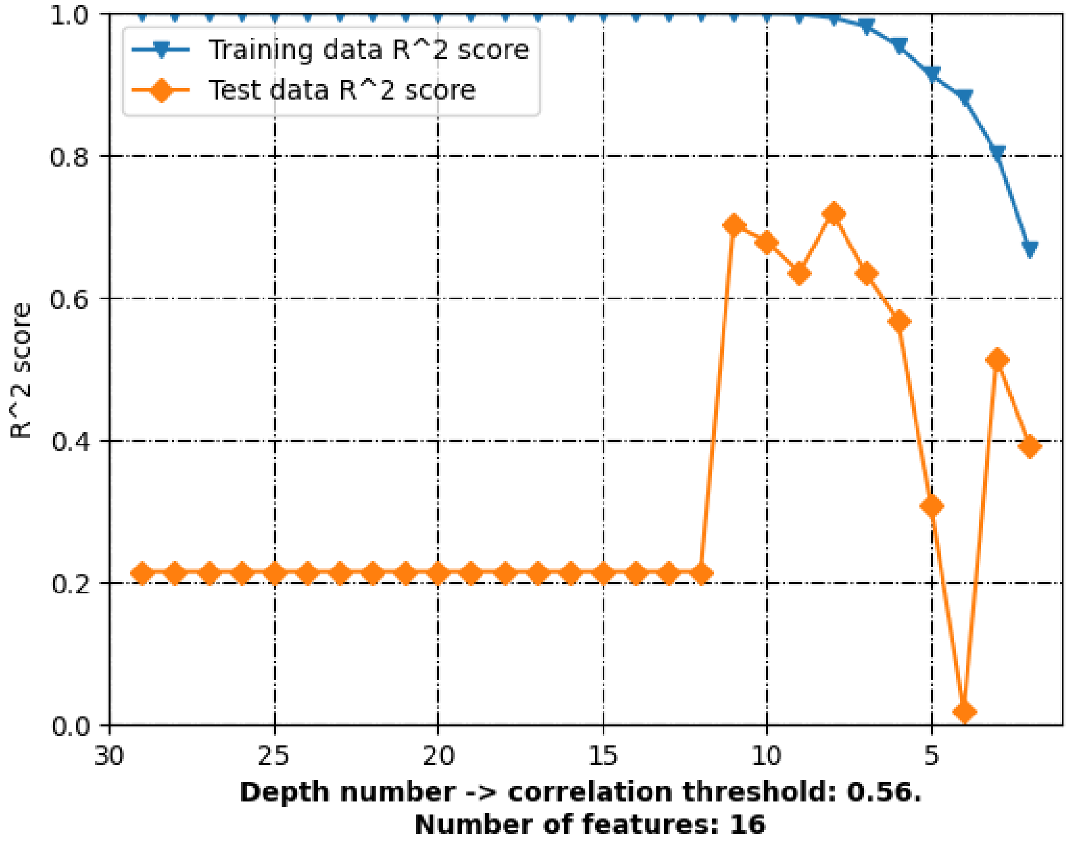
Training data R^2 score
Test data R^2 score
R^2 score
1.0
0.8
0.6
0.4
0.2
0.0
30
25
20
15
10
5
Depth number -> correlation threshold: 0.56.
Number of features: 16

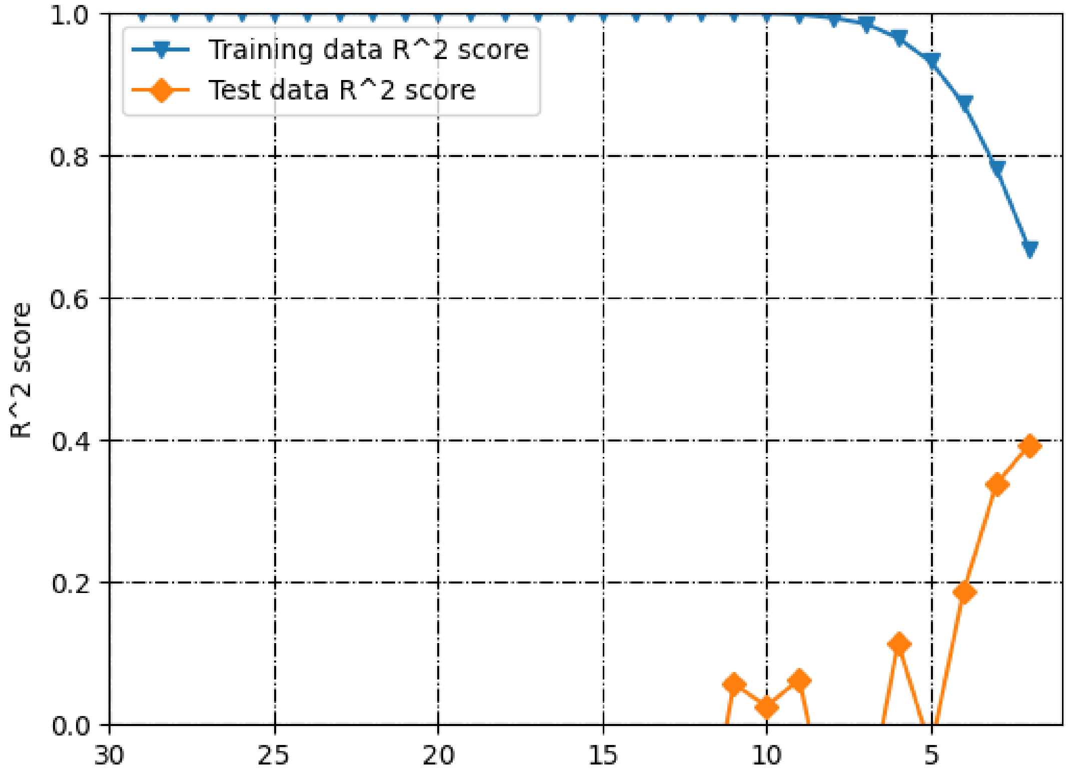

Depth number -> correlation threshold: 0.57.
Number of features: 12

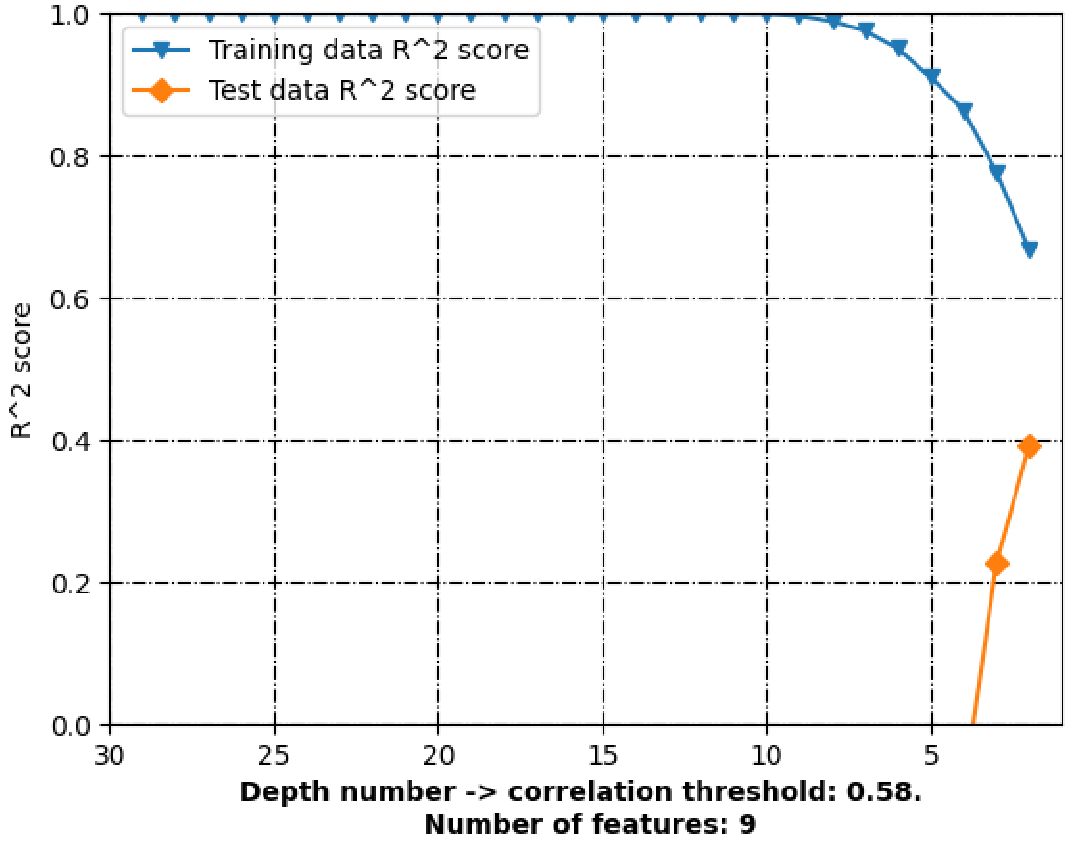

Training data R^2 score
Test data R^2 score
R^2 score
1.0
0.8
0.6
0.4
0.2
0.0
30
25
20
15
10
5
Depth number -> correlation threshold: 0.58.
Number of features: 9

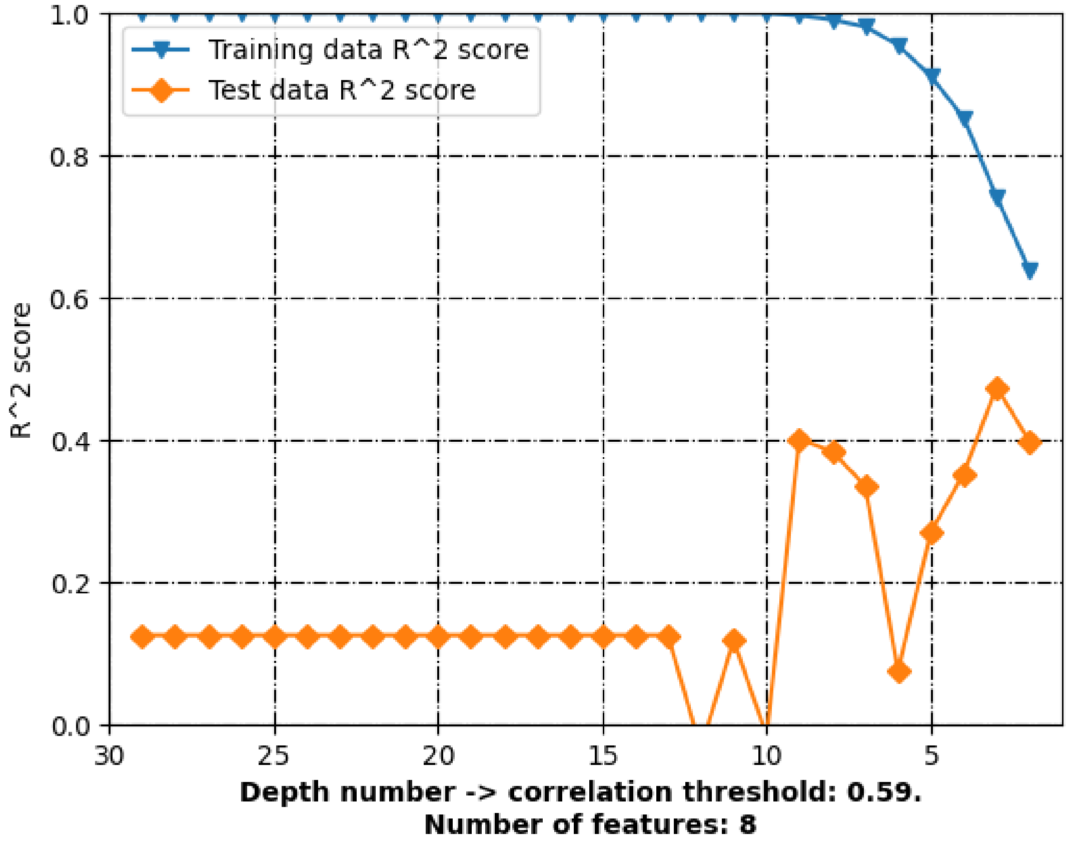
Training data R^2 score
Test data R^2 score
R^2 score
1.0
0.8
0.6
0.4
0.2
0.0
30
25
20
15
10
5
Depth number -> correlation threshold: 0.59.
Number of features: 8

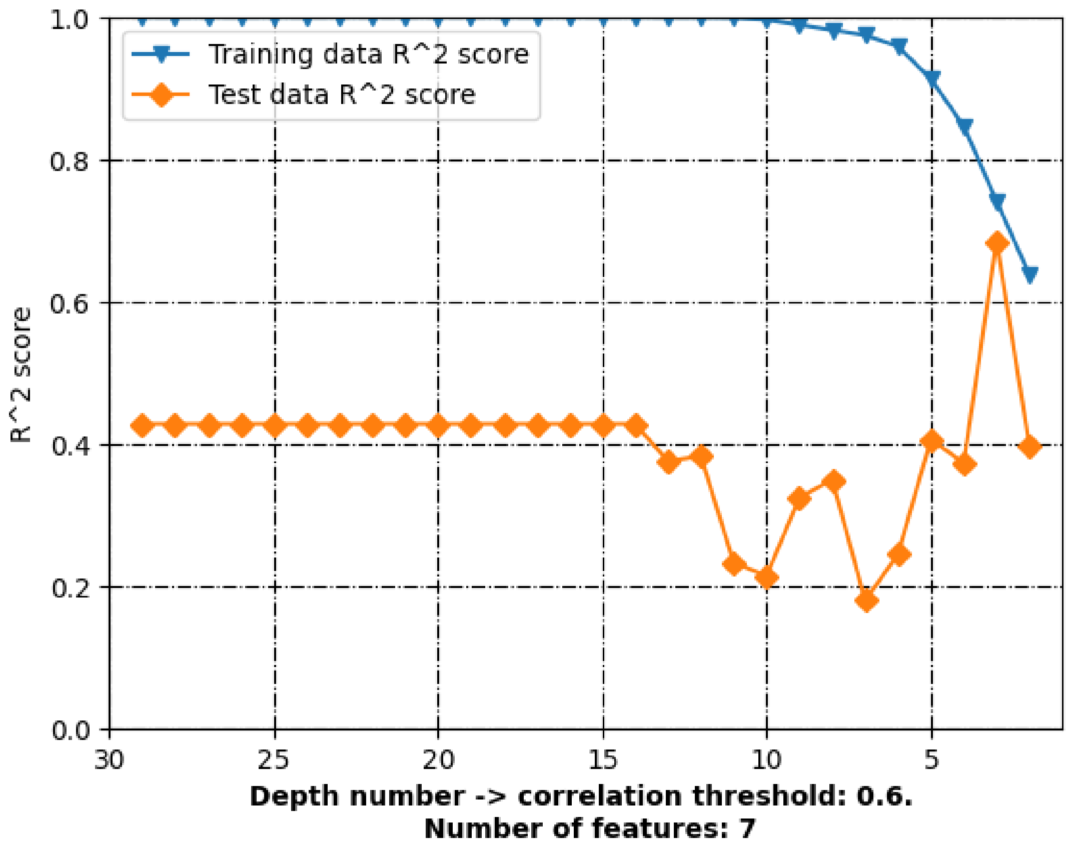

Training data R^2 score
Test data R^2 score
R^2 score
1.0
0.8
0.6
0.4
0.2
0.0
30
25
20
15
10
5
Depth number -> correlation threshold: 0.6.
Number of features: 7

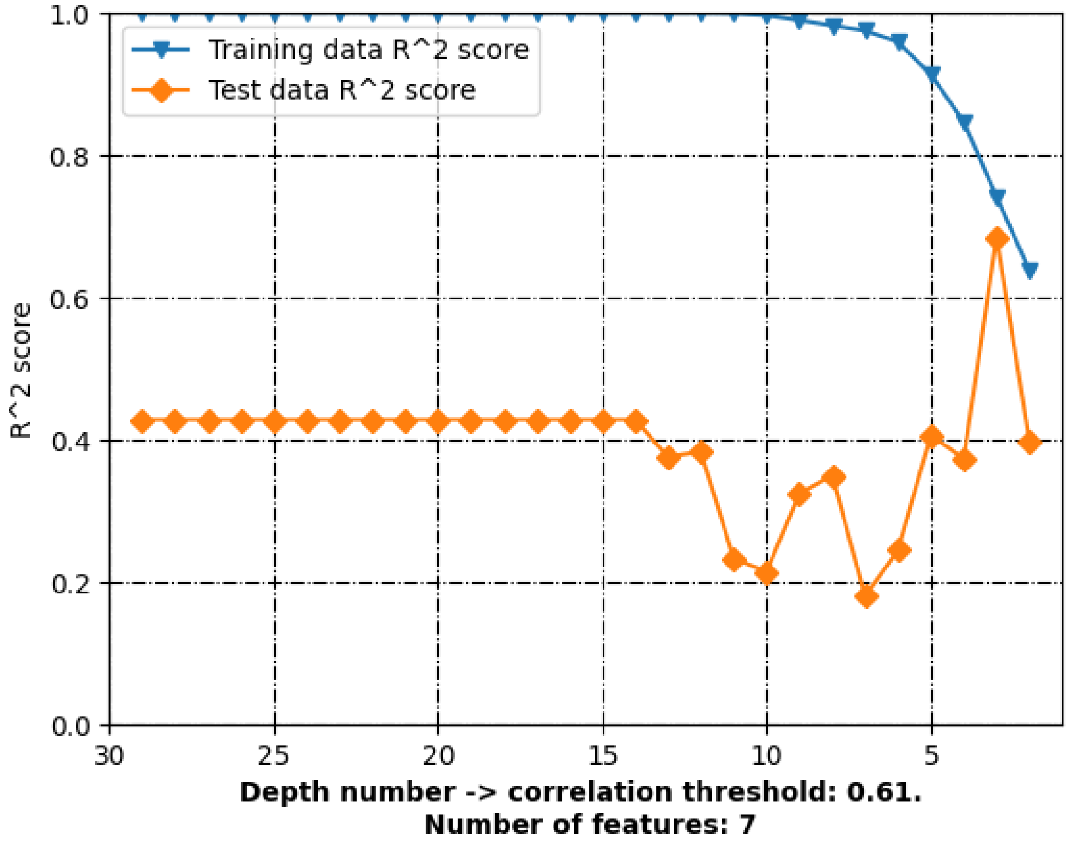
Training data R^2 score
Test data R^2 score
R^2 score
1.0
0.8
0.6
0.4
0.2
0.0
30
25
20
15
10
5
Depth number -> correlation threshold: 0.61.
Number of features: 7

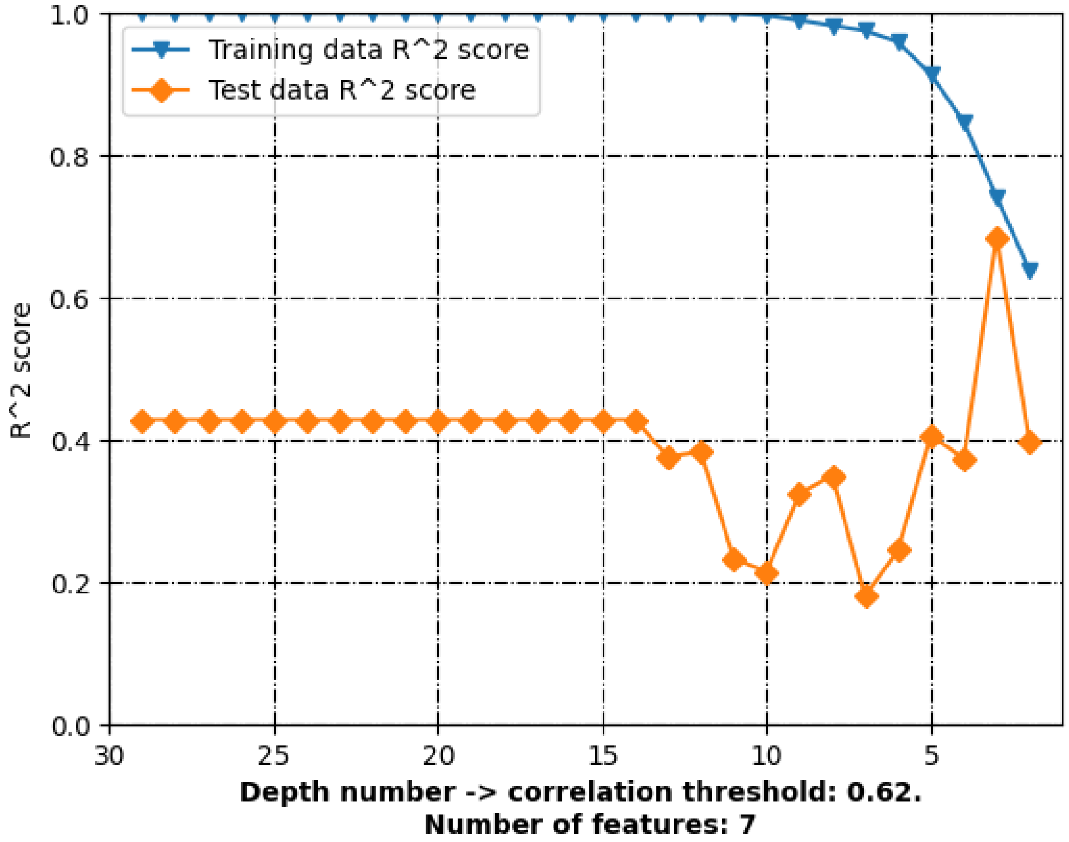
Training data R^2 score
Test data R^2 score
R^2 score
1.0
0.8
0.6
0.4
0.2
0.0
30
25
20
15
10
5
Depth number -> correlation threshold: 0.62.
Number of features: 7

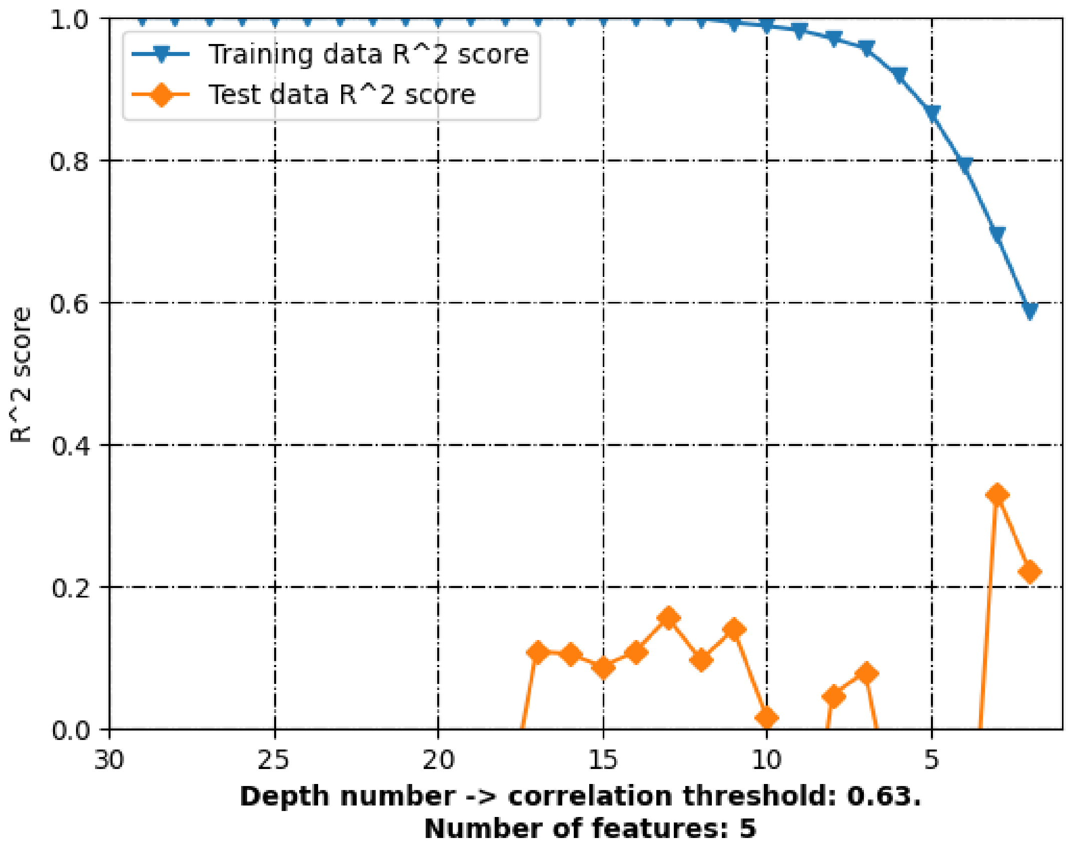

[21]:
```
plt.plot(df_without_standardization['Correlation threshold'],␣
  ↪df_without_standardization['Number of features'], label = "Number of␣
  ↪features", marker='.')
plt.legend()
plt.xlabel('Correlation threshold')
plt.ylabel('Number of features')
plt.show()
```

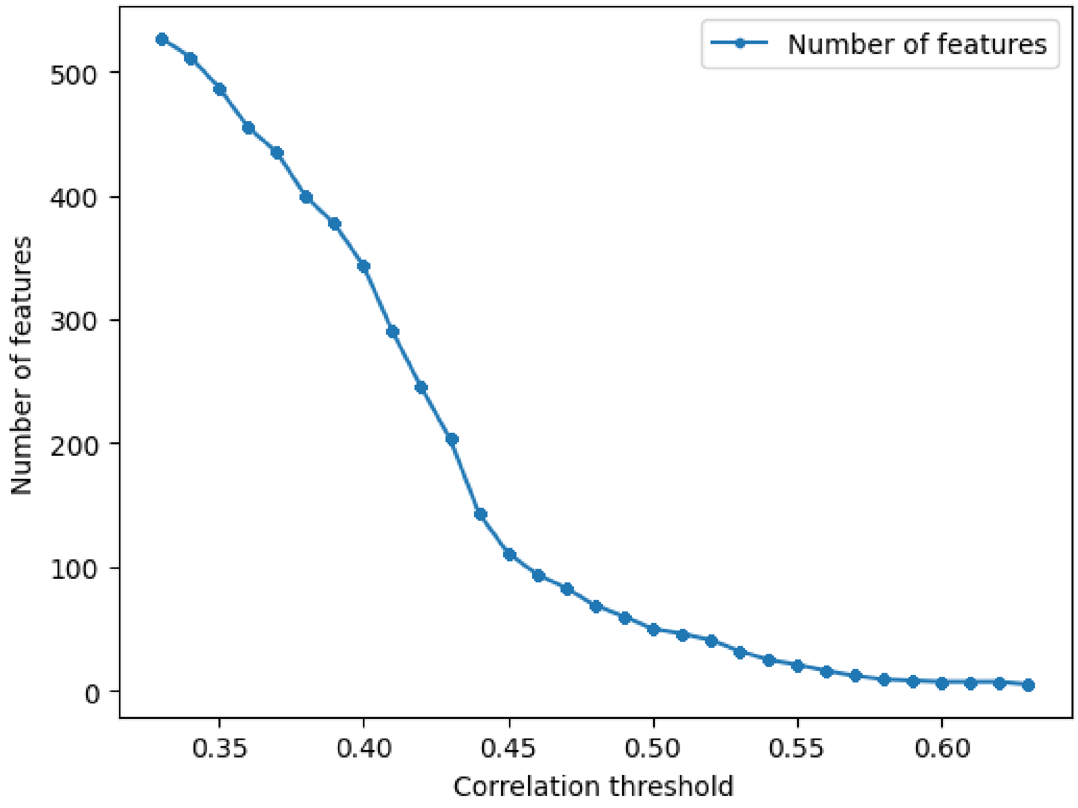


```
[ ]:
```

3 Random Forest

```
[22]: without_standardization, train_r2_, test_r2_, hist1, hist2, target_column_name,␣
↪training_data_RMSE, test_data_RMSE  = pred_model.
↪prepare_data_and_create_model(molecular_descriptors_df=data,
                                                                                ␣
↪                        correlation_threshold=0.50,
                                                                                ␣
↪                        standardization=False,
                                                                                ␣
↪                        model_type='RandomForestRegressor',
                                                                                ␣
↪                        n_estimators_=12,
                                                                                ␣
↪                        target_column_name = target,
                                                                                ␣
↪                        random_state=random_state,
```

```
↪                     train_test_split_=True,
↪                     verbose=True)
```

```
I am not doing standardization…
  molecular descriptor name
0                    AATS0Z
1                  AATS0are
2                    AATS0d
3                   AATS0dv
4                    AATS0i
  molecular descriptor name  corr_value
0                    AATS0Z    0.018677
1                  AATS0are   -0.341313
2                    AATS0d   -0.123443
3                   AATS0dv   -0.265670
4                    AATS0i   -0.428929
  molecular descriptor name  corr_value  absolute correlation value
0                    AATS0Z    0.018677                    0.018677
1                  AATS0are   -0.341313                    0.341313
2                    AATS0d   -0.123443                    0.123443
3                   AATS0dv   -0.265670                    0.265670
4                    AATS0i   -0.428929                    0.428929
    molecular descriptor name  corr_value  absolute correlation value
15                     AATS1i   -0.561620                    0.561620
113                  AATSC1c     0.565682                    0.565682
125                  AATSC2c    -0.543393                    0.543393
221               AETA_eta_R    -0.515778                    0.515778
224                   AMID_C     0.532458                    0.532458
    molecular descriptor name  corr_value  absolute correlation value
15                     AATS1i   -0.561620                    0.561620
113                  AATSC1c     0.565682                    0.565682
125                  AATSC2c    -0.543393                    0.543393
221               AETA_eta_R    -0.515778                    0.515778
224                   AMID_C     0.532458                    0.532458
The model used is: RandomForest…
Return the coefficient of determination of the prediction:
0.6510534913729729
R^2 score: 0.9182393349313648
Correlation coefficient: 0.9582480550104784
Test data - unseen during training:
R^2 score: 0.6510534913729729
Correlation coefficient: 0.8068788579291025
[6.49928877 7.342496   7.56932961 5.62596181 6.3811566  5.98596459
 6.95701087 6.88213329 6.53686289 7.40323582 5.71393145 6.06943759
 7.47467813 6.79355014 7.20231788 6.23555845]
114    6.026872
```

```
10     7.856985
4      7.109020
95     5.200659
111    6.853872
79     6.327902
44     7.638272
47     7.022276
107    6.000000
11     7.250264
61     5.481486
56     6.657577
0      7.260428
92     7.141463
18     6.630970
78     6.244125
Name: LoVo/DX, dtype: float64
Training Root Mean Square Error: 0.27496269898128745
Testing Root Mean Square Error: 0.42645349872036425
```

[23]:
```
print(target_column_name+str('_transformed'))
print(hist1[target_column_name].hist())
```

```
LoVo/DX_transformed
AxesSubplot(0.125,0.11;0.775x0.77)
```

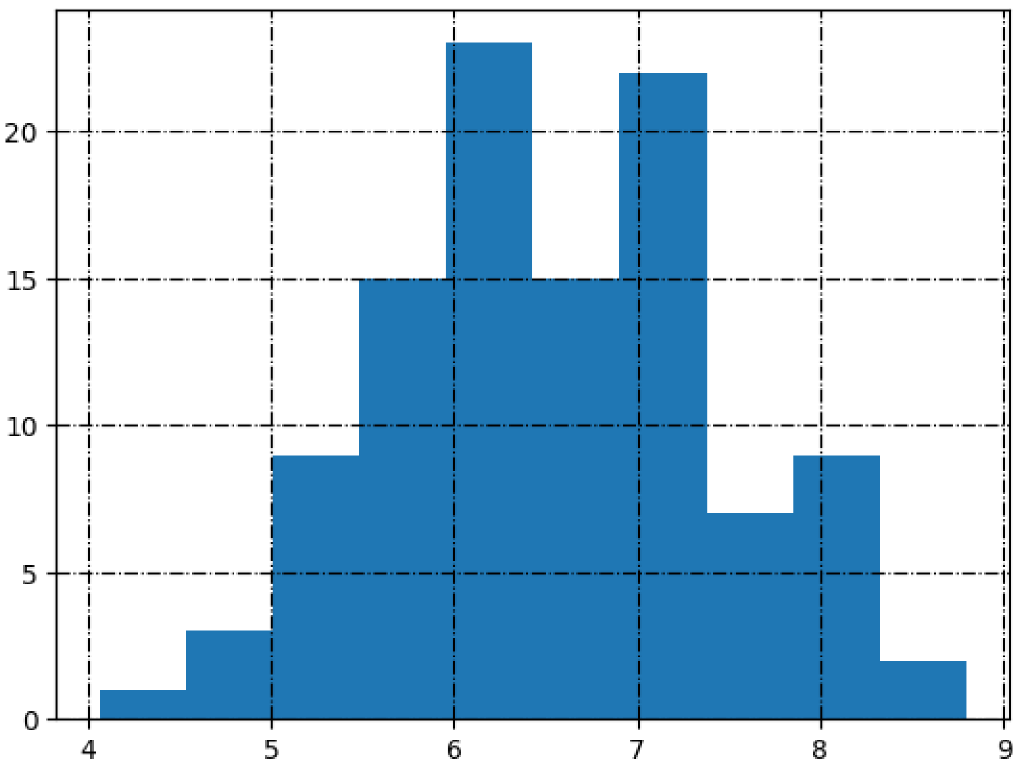

[24]:
```
with_standardization, train_r2_, test_r2_, hist1, hist2, target_column_name,␣
↪training_data_RMSE, test_data_RMSE  = pred_model.
↪prepare_data_and_create_model(molecular_descriptors_df=data,
                                                                              ␣
↪                    correlation_threshold=0.50,
                                                                              ␣
↪                    standardization=True,
                                                                              ␣
↪                    model_type='RandomForestRegressor',
                                                                              ␣
↪                    n_estimators_=12,
                                                                              ␣
↪                    target_column_name = target,
                                                                              ␣
↪                    random_state=random_state,
                                                                              ␣
↪                    train_test_split_=True,
                                                                              ␣
↪                    verbose=True)
```

```
I am doing standardization…
  molecular descriptor name
0                    AATS0Z
1                  AATS0are
2                    AATS0d
3                   AATS0dv
4                    AATS0i
  molecular descriptor name  corr_value
0                    AATS0Z    0.018677
1                  AATS0are   -0.341313
2                    AATS0d   -0.123443
3                   AATS0dv   -0.265670
4                    AATS0i   -0.428929
  molecular descriptor name  corr_value  absolute correlation value
0                    AATS0Z    0.018677                    0.018677
1                  AATS0are   -0.341313                    0.341313
2                    AATS0d   -0.123443                    0.123443
3                   AATS0dv   -0.265670                    0.265670
4                    AATS0i   -0.428929                    0.428929
    molecular descriptor name  corr_value  absolute correlation value
15                     AATS1i   -0.561620                    0.561620
113                   AATSC1c    0.565682                    0.565682
125                   AATSC2c   -0.543393                    0.543393
221                 AETA_eta_R   -0.515778                    0.515778
224                     AMID_C    0.532458                    0.532458
```

```
    molecular descriptor name  corr_value  absolute correlation value
15                     AATS1i   -0.561620                    0.561620
113                   AATSC1c    0.565682                    0.565682
125                   AATSC2c   -0.543393                    0.543393
221                AETA_eta_R   -0.515778                    0.515778
224                    AMID_C    0.532458                    0.532458
The model used is: RandomForest…
Return the coefficient of determination of the prediction:
0.6510534913729729
R^2 score: 0.9182393349313648
Correlation coefficient: 0.9582480550104784
Test data - unseen during training:
R^2 score: 0.6510534913729729
Correlation coefficient: 0.8068788579291025
[6.49928877 7.342496   7.56932961 5.62596181 6.3811566  5.98596459
 6.95701087 6.88213329 6.53686289 7.40323582 5.71393145 6.06943759
 7.47467813 6.79355014 7.20231788 6.23555845]
114    6.026872
10     7.856985
4      7.109020
95     5.200659
111    6.853872
79     6.327902
44     7.638272
47     7.022276
107    6.000000
11     7.250264
61     5.481486
56     6.657577
0      7.260428
92     7.141463
18     6.630970
78     6.244125
Name: LoVo/DX, dtype: float64
Training Root Mean Square Error: 0.27496269898128745
Testing Root Mean Square Error: 0.42645349872036425
```

3.1 Search inside correlation space

[25]:
```
step = 0.01
initial_step = corr_low
last_step = corr_high
first_list = [x / 100.0 for x in range(int(initial_step*100),
 ↪int(last_step*100), int(step*100))]
n_estimators = [range(2,21,1)]
corr_th = []
second_list = []
```

```
third_list = []
fourth_l = []
fifth_l = []
f_list = []
fif_list = []
for i in first_list:

    for estimator in n_estimators[0]:

        without_standardization, train_r2, test_r2, _, h_, target_column_name,␣
↪training_data_RMSE, test_data_RMSE = pred_model.
↪prepare_data_and_create_model(molecular_descriptors_df=data,
                                                                                 ␣
↪                    correlation_threshold=i,
                                                                                 ␣
↪                    standardization=False,
                                                                                 ␣
↪                    model_type='RandomForestRegressor',
                                                                                 ␣
↪                    n_estimators_=estimator,
                                                                                 ␣
↪                    target_column_name = target,
                                                                                 ␣
↪                    random_state=random_state,
                                                                                 ␣
↪                    train_test_split_=True,
                                                                                 ␣
↪                    verbose=False)
        corr_th.append(i)
        second_list.append(train_r2)
        third_list.append(test_r2)
        fourth_l.append(training_data_RMSE)
        fifth_l.append(test_data_RMSE)
        f_list.append(len(h_))
        fif_list.append(estimator)
```

[26]:

```
df_without_standardization = pd.DataFrame(data=corr_th, columns=["Correlation␣
↪threshold"])
df_without_standardization['Training data R^2 score'] = second_list
df_without_standardization['Test data R^2 score'] = third_list
df_without_standardization['Training RMSE'] = fourth_l
df_without_standardization['Test RMSE'] = fifth_l
df_without_standardization['Number of features'] = f_list
df_without_standardization['Number of estimators'] = fif_list
```

[27]:
```
df_random_forest = df_without_standardization.copy()
#df_without_standardization.to_excel('../Data/
↪A549_Random_forest_rs_'+str(random_state)+'.xlsx')
```

[28]:
```
corre_list = [x / 100.0 for x in range(int(initial_step*100),␣
↪int(last_step*100), int(0.01*100))]
for element in corre_list:
    element_ =␣
↪df_without_standardization[df_without_standardization['Correlation␣
↪threshold'] == float(element)]
    plt.plot(element_['Number of estimators'], element_['Training data R^2␣
↪score'], label = "Training data R^2 score", marker='v')
    plt.plot(element_['Number of estimators'], element_['Test data R^2 score'],␣
↪label = "Test data R^2 score", marker='D')
    plt.legend()
    plt.xlabel('Number of estimators -> correlation threshold: '+str(element)+'.
↪ \n Number of features: '+str(element_['Number of features'].iloc[0]))
    plt.xlim(max(element_['Number of estimators'])+1, min(element_['Number of␣
↪estimators'])-1)
    plt.ylabel('R^2 score')
    plt.ylim([0, 1])
    plt.rc('grid', linestyle="-.", color='black')
    plt.grid(True)
    plt.show()
```

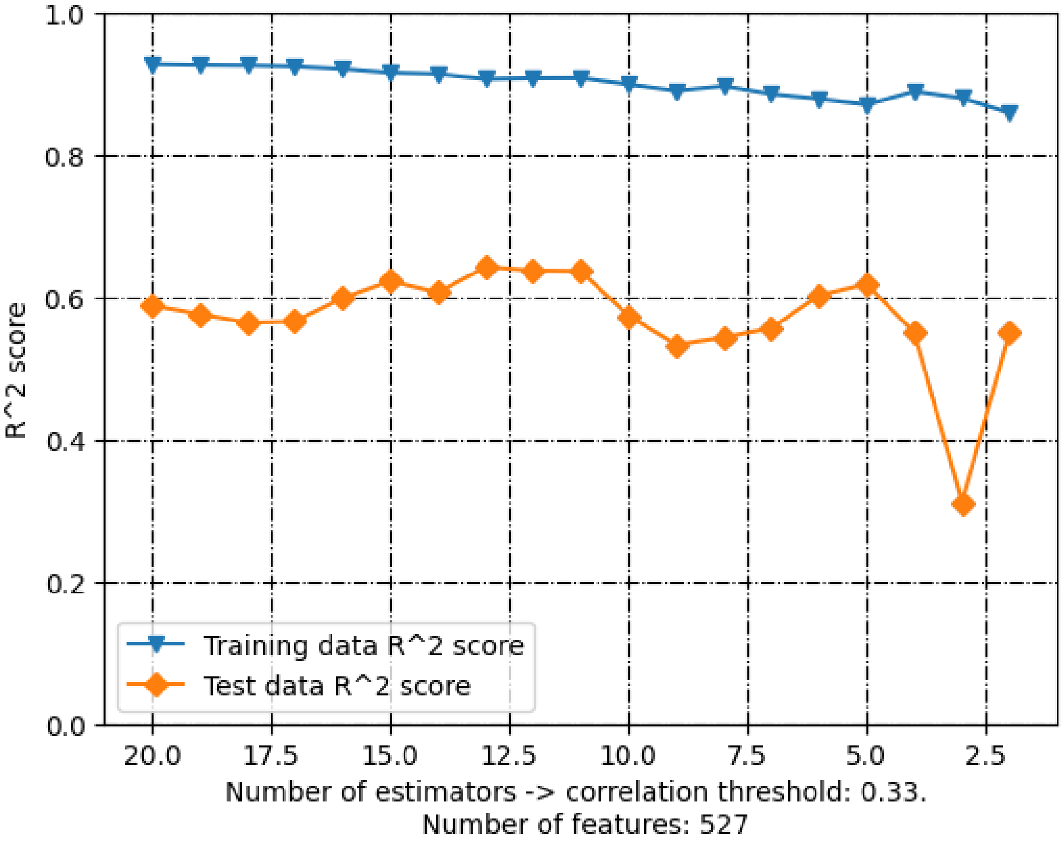

1.0
0.8
0.6
0.4
0.2
0.0
R^2 score
Training data R^2 score
Test data R^2 score
20.0
17.5
15.0
12.5
10.0
7.5
5.0
2.5
Number of estimators -> correlation threshold: 0.33.
Number of features: 527

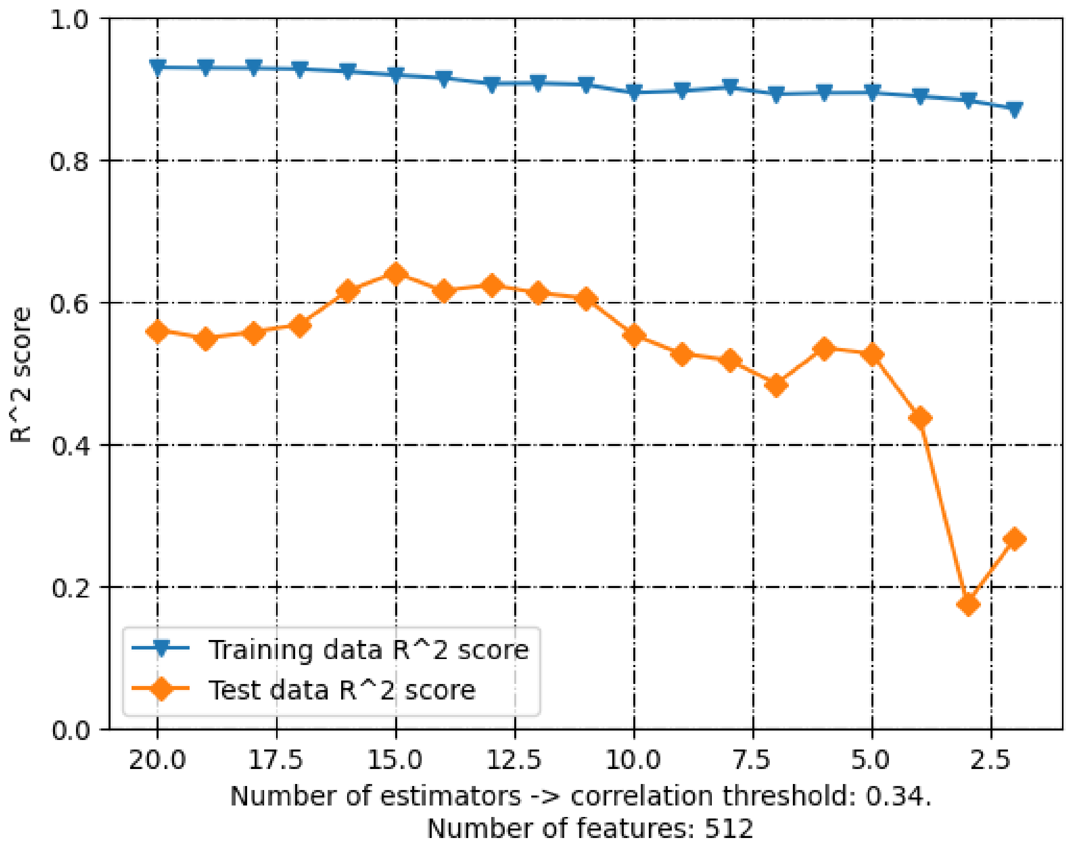

1.0
0.8
0.6
0.4
0.2
0.0
R^2 score
20.0
17.5
15.0
12.5
10.0
7.5
5.0
2.5
Training data R^2 score
Test data R^2 score
Number of estimators -> correlation threshold: 0.34.
Number of features: 512

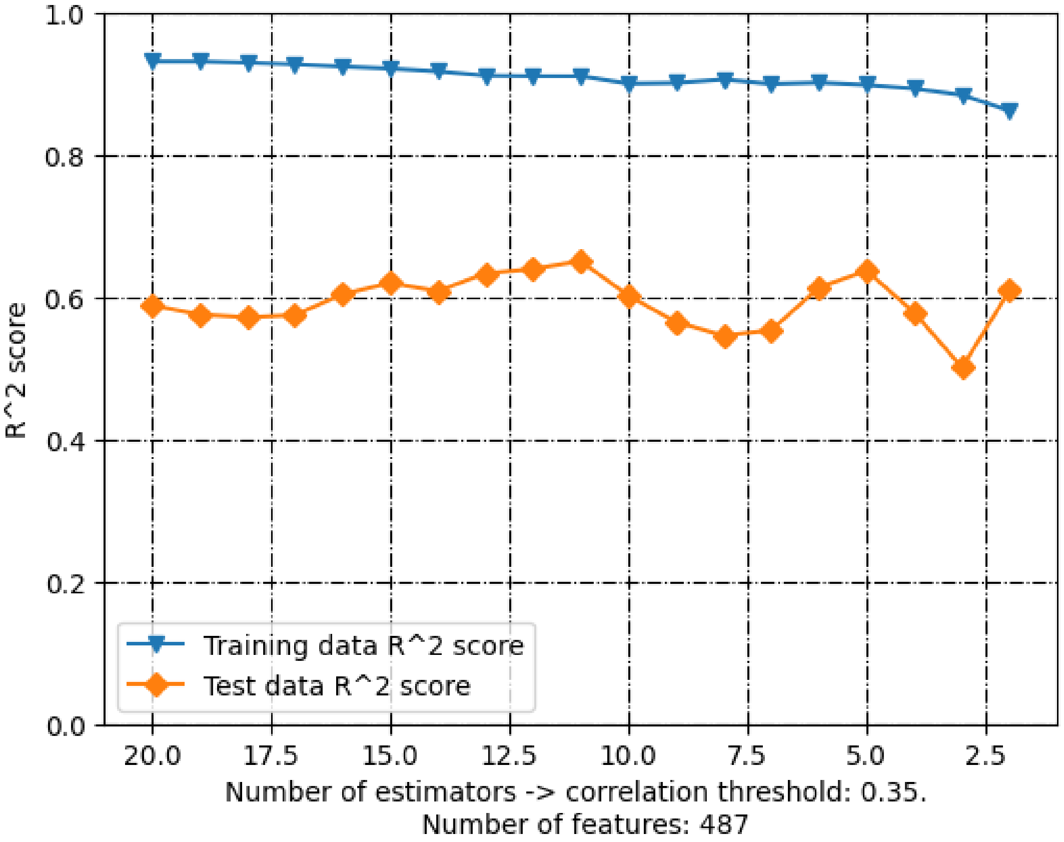
1.0
0.8
0.6
0.4
0.2
0.0
R^2 score
Training data R^2 score
Test data R^2 score
20.0
17.5
15.0
12.5
10.0
7.5
5.0
2.5
Number of estimators -> correlation threshold: 0.35.
Number of features: 487

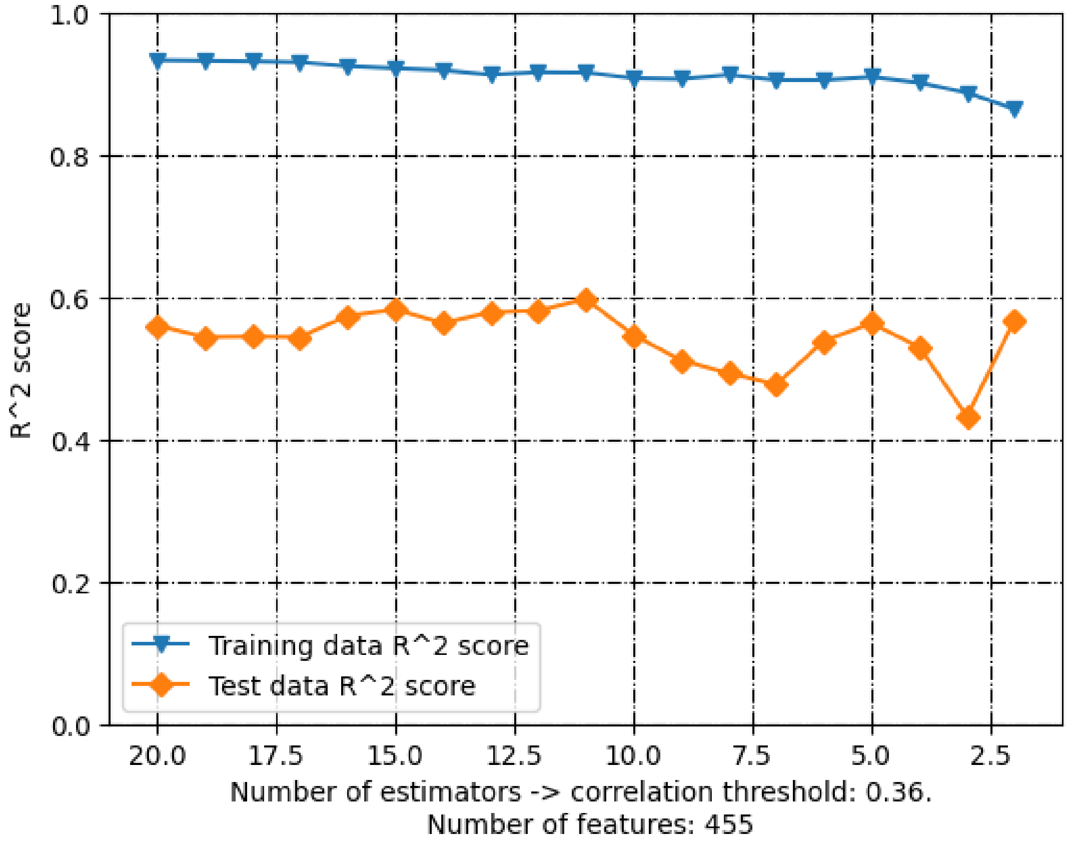

1.0
0.8
0.6
0.4
0.2
0.0
R^2 score
Training data R^2 score
Test data R^2 score
20.0
17.5
15.0
12.5
10.0
7.5
5.0
2.5
Number of estimators -> correlation threshold: 0.36.
Number of features: 455

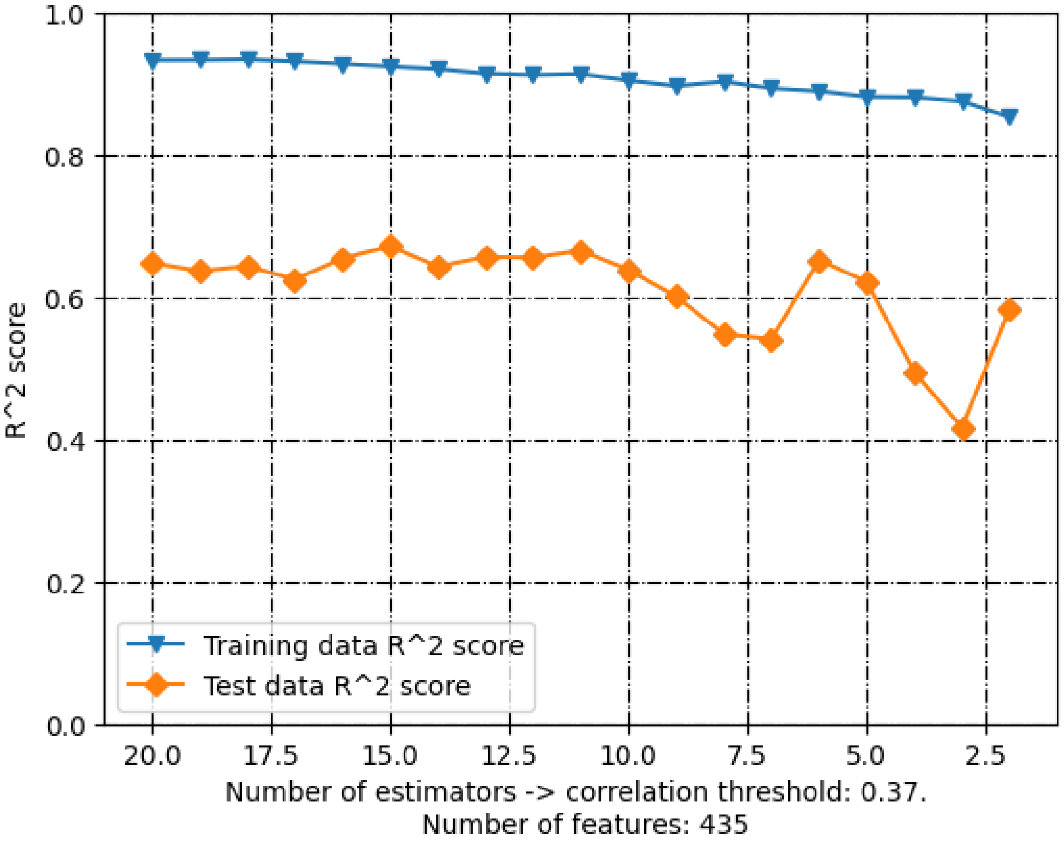

1.0
0.8
0.6
0.4
0.2
0.0
R^2 score
20.0
17.5
15.0
12.5
10.0
7.5
5.0
2.5
Number of estimators -> correlation threshold: 0.37.
Number of features: 435
Training data R^2 score
Test data R^2 score

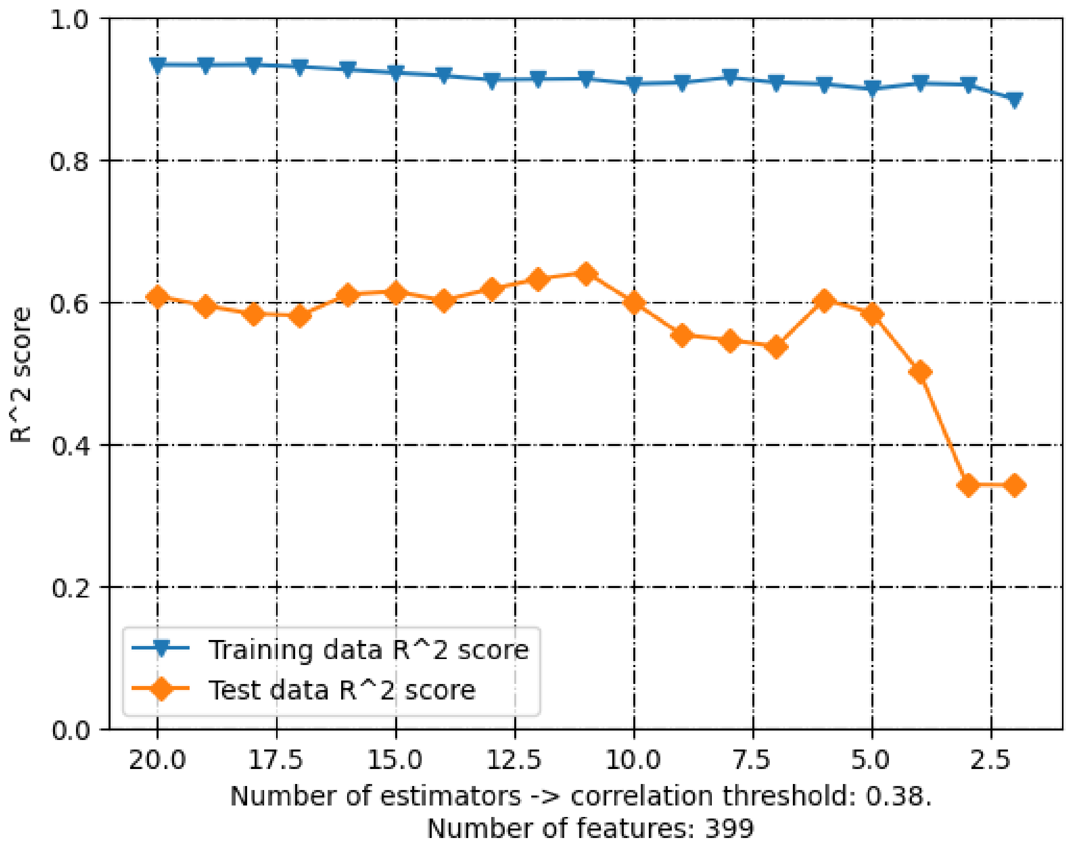
1.0
0.8
0.6
0.4
0.2
0.0
R^2 score
Training data R^2 score
Test data R^2 score
20.0
17.5
15.0
12.5
10.0
7.5
5.0
2.5
Number of estimators -> correlation threshold: 0.38.
Number of features: 399

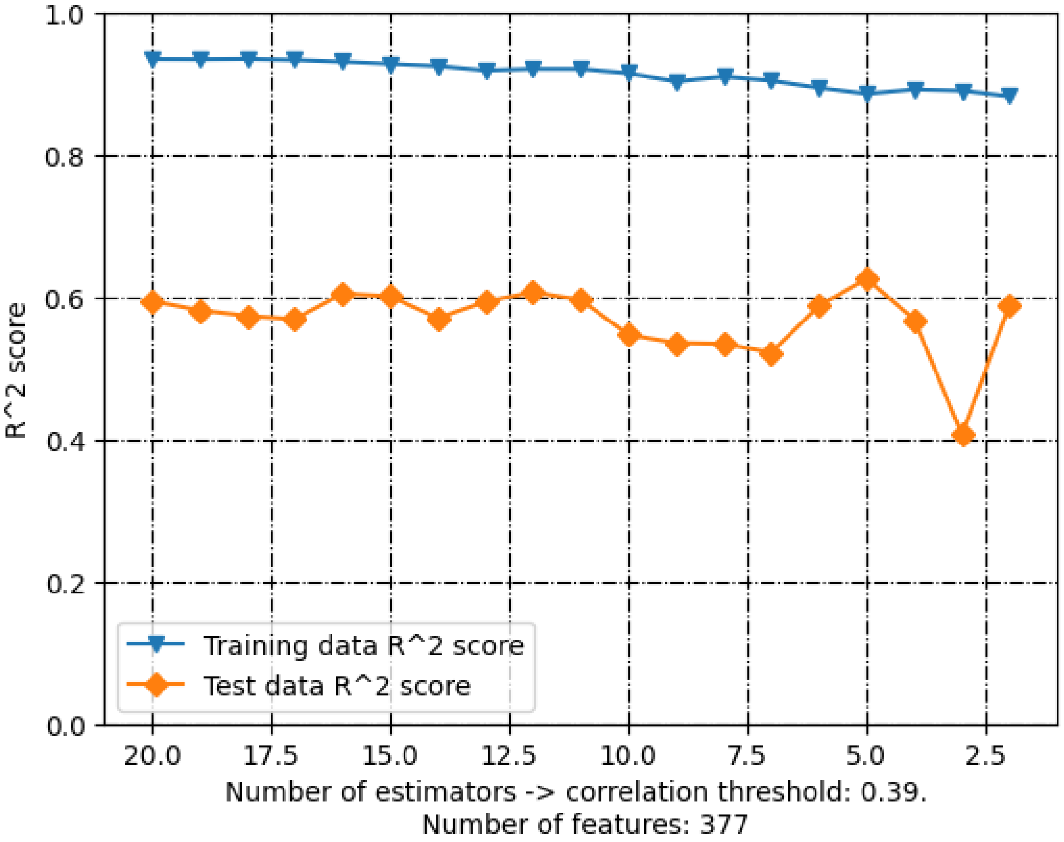
R^2 score
1.0
0.8
0.6
0.4
0.2
0.0
20.0
17.5
15.0
12.5
10.0
7.5
5.0
2.5
Training data R^2 score
Test data R^2 score
Number of estimators -> correlation threshold: 0.39.
Number of features: 377

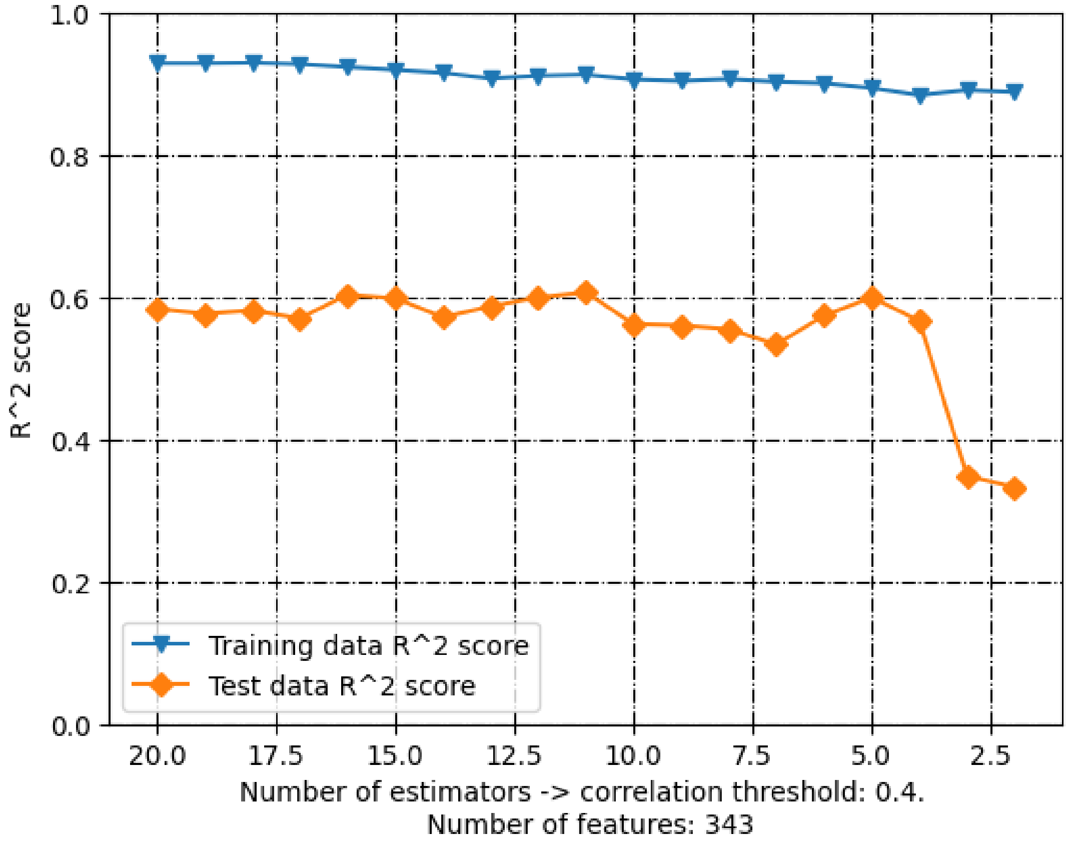
1.0
0.8
0.6
0.4
0.2
0.0
R^2 score
Training data R^2 score
Test data R^2 score
20.0
17.5
15.0
12.5
10.0
7.5
5.0
2.5
Number of estimators -> correlation threshold: 0.4.
Number of features: 343

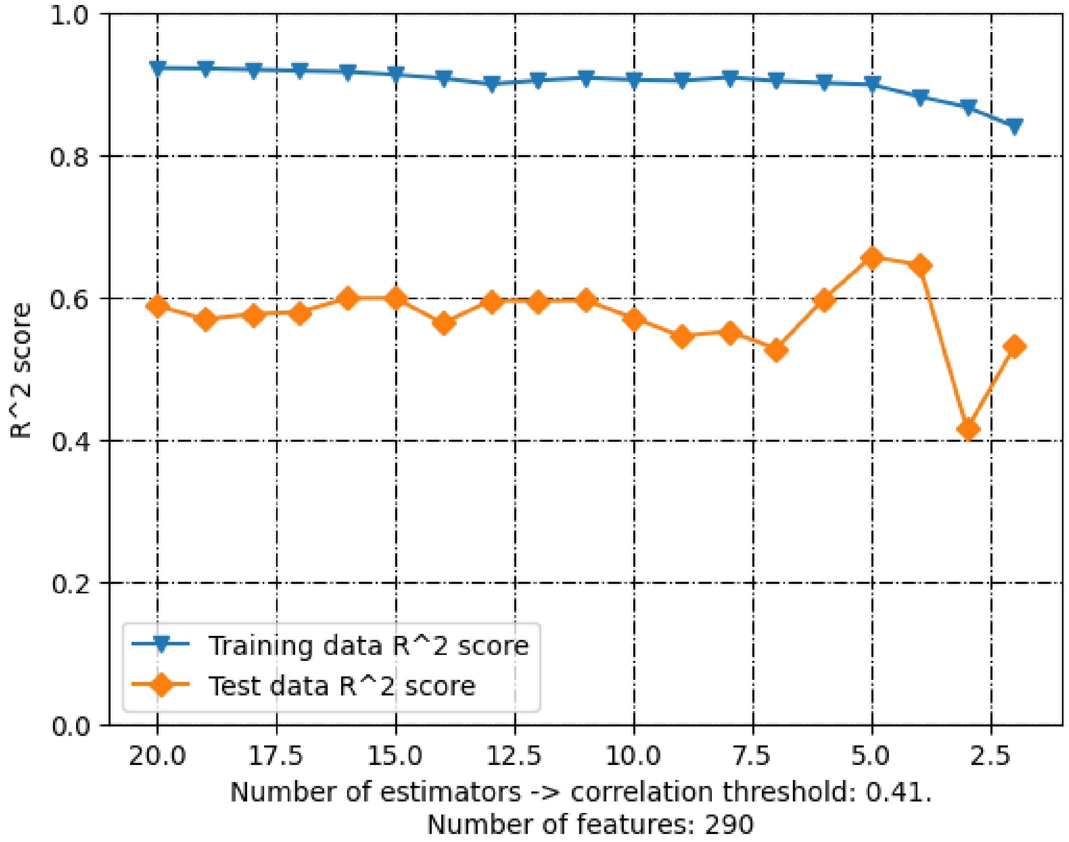

1.0
0.8
0.6
0.4
0.2
0.0
R^2 score
Training data R^2 score
Test data R^2 score
20.0
17.5
15.0
12.5
10.0
7.5
5.0
2.5
Number of estimators -> correlation threshold: 0.41.
Number of features: 290

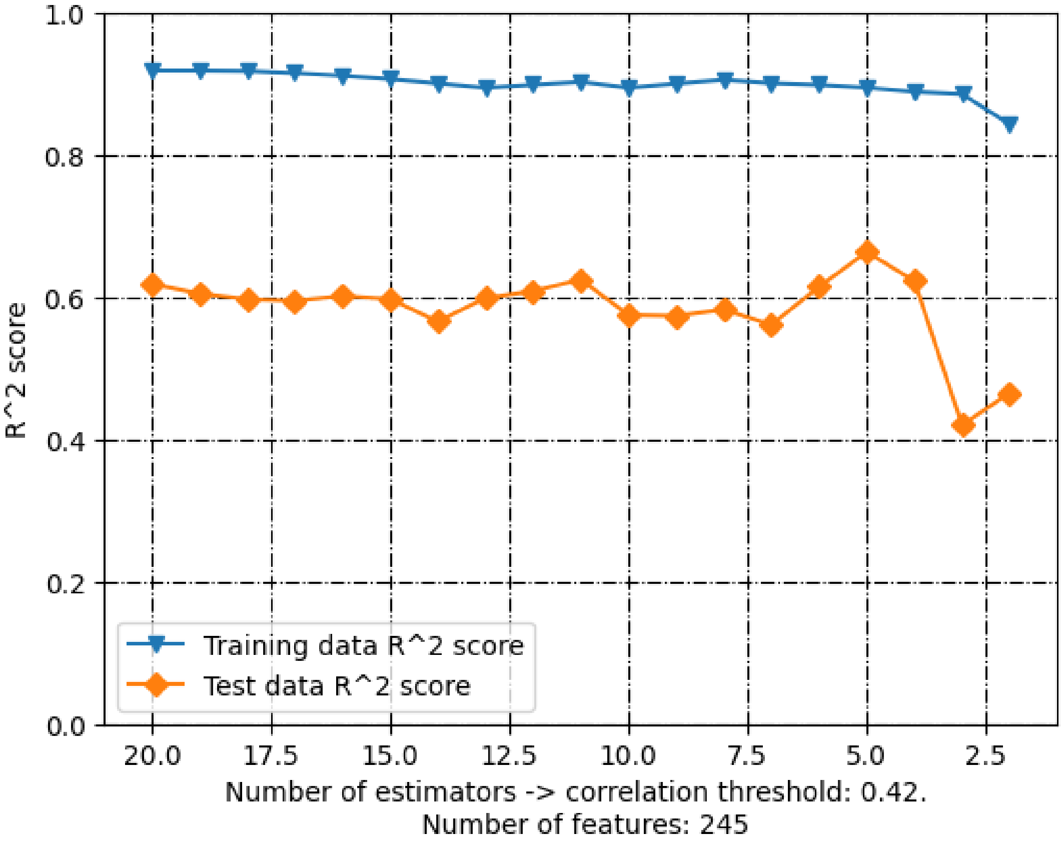
1.0
0.8
0.6
0.4
0.2
0.0
R^2 score
Training data R^2 score
Test data R^2 score
20.0
17.5
15.0
12.5
10.0
7.5
5.0
2.5
Number of estimators -> correlation threshold: 0.42.
Number of features: 245

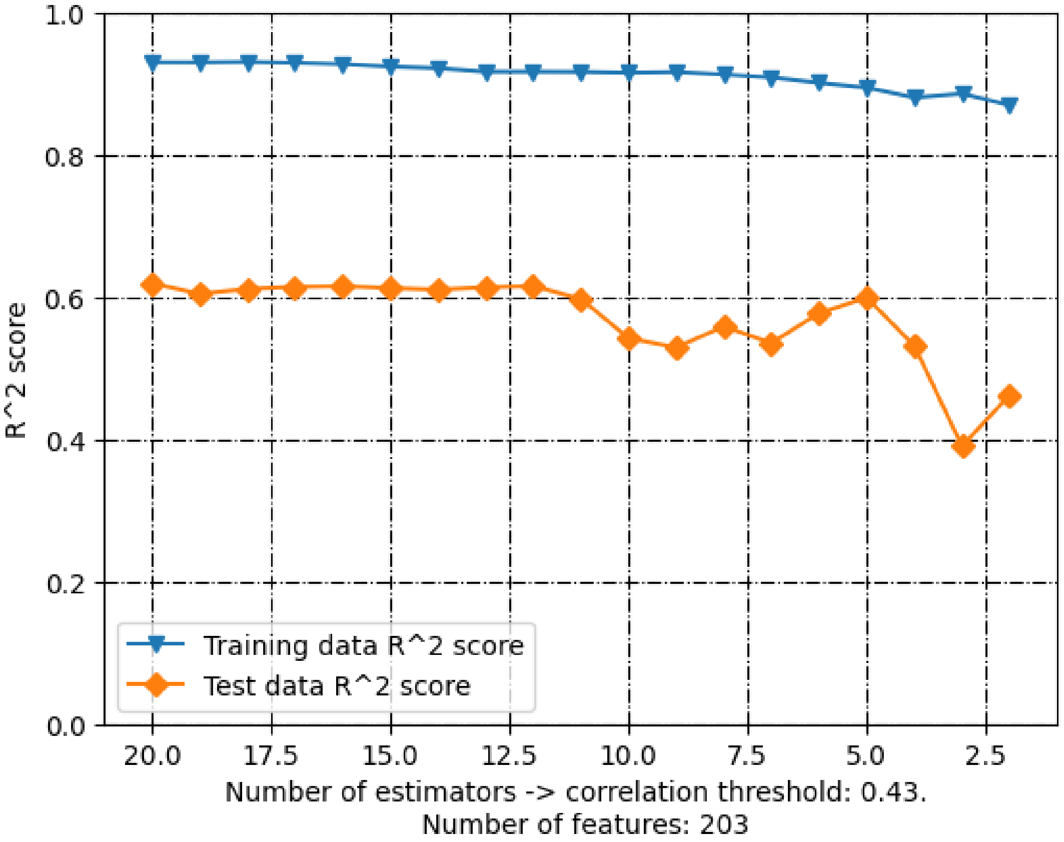
1.0
0.8
0.6
0.4
0.2
0.0
R^2 score
Training data R^2 score
Test data R^2 score
20.0
17.5
15.0
12.5
10.0
7.5
5.0
2.5
Number of estimators -> correlation threshold: 0.43.
Number of features: 203

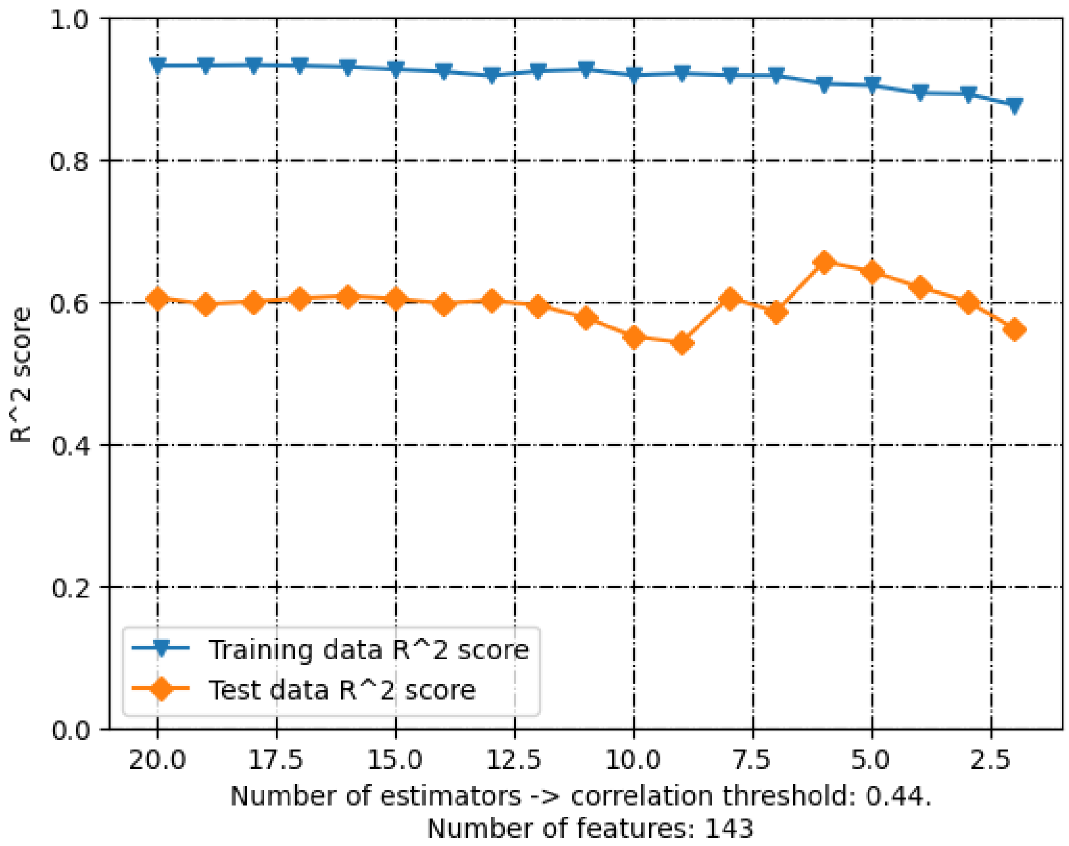

1.0
0.8
0.6
0.4
0.2
0.0
R^2 score
Training data R^2 score
Test data R^2 score
20.0
17.5
15.0
12.5
10.0
7.5
5.0
2.5
Number of estimators -> correlation threshold: 0.44.
Number of features: 143

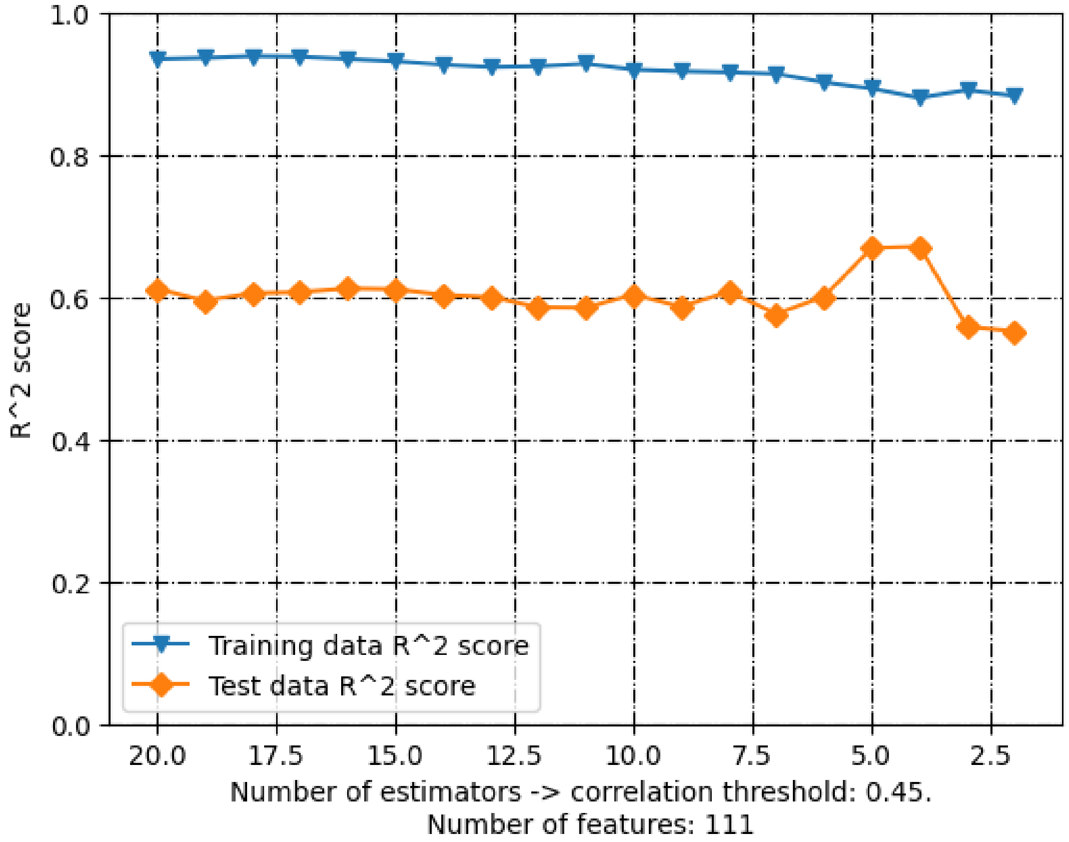
1.0
0.8
0.6
0.4
0.2
0.0
R^2 score
Training data R^2 score
Test data R^2 score
20.0
17.5
15.0
12.5
10.0
7.5
5.0
2.5
Number of estimators -> correlation threshold: 0.45.
Number of features: 111

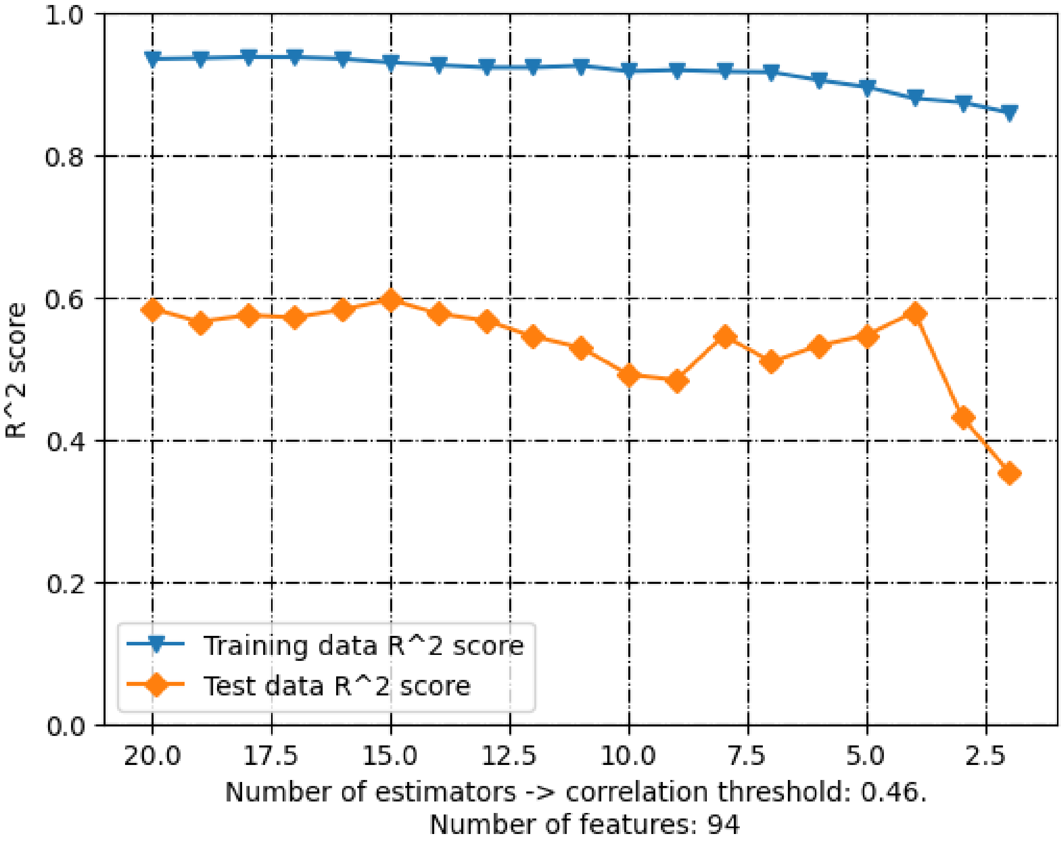
1.0
0.8
0.6
0.4
0.2
0.0
R^2 score
Training data R^2 score
Test data R^2 score
20.0
17.5
15.0
12.5
10.0
7.5
5.0
2.5
Number of estimators -> correlation threshold: 0.46.
Number of features: 94

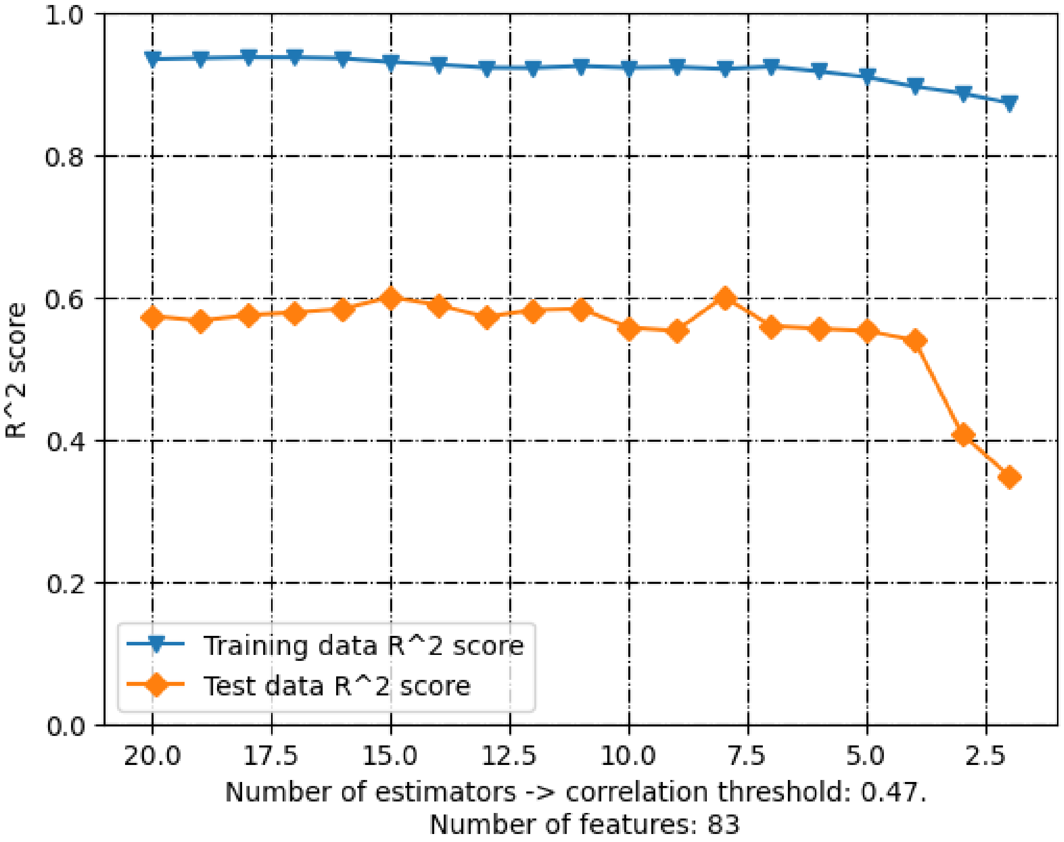
1.0
0.8
0.6
0.4
0.2
0.0
R^2 score
20.0
17.5
15.0
12.5
10.0
7.5
5.0
2.5
Number of estimators -> correlation threshold: 0.47.
Number of features: 83
Training data R^2 score
Test data R^2 score

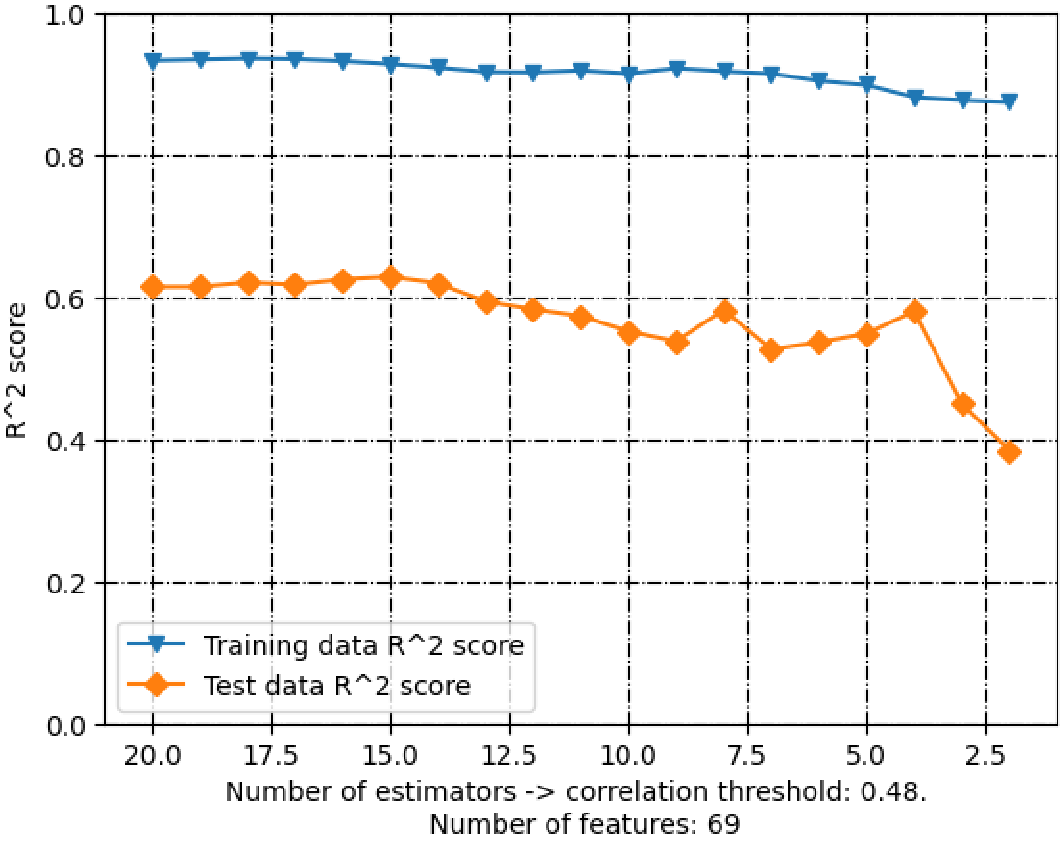

1.0
0.8
0.6
0.4
0.2
0.0
R^2 score
20.0
17.5
15.0
12.5
10.0
7.5
5.0
2.5
Number of estimators -> correlation threshold: 0.48.
Number of features: 69
Training data R^2 score
Test data R^2 score

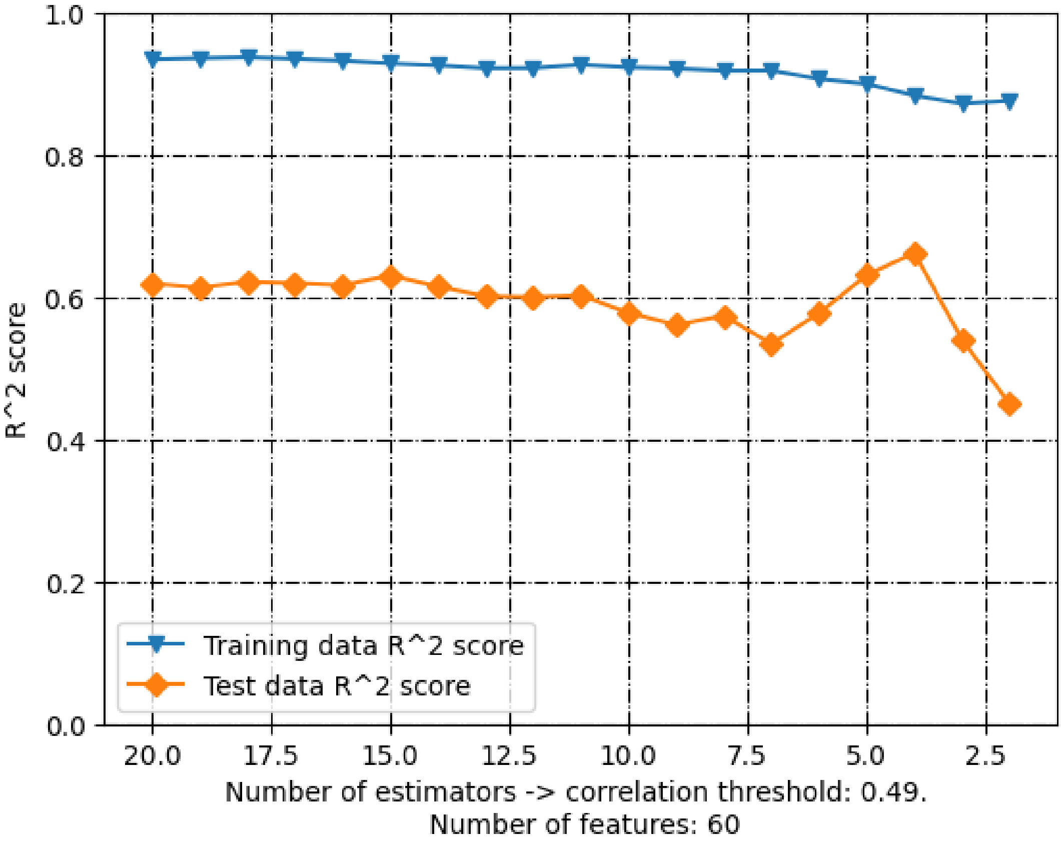
1.0
0.8
0.6
0.4
0.2
0.0
R^2 score
20.0
17.5
15.0
12.5
10.0
7.5
5.0
2.5
Number of estimators -> correlation threshold: 0.49.
Number of features: 60
Training data R^2 score
Test data R^2 score

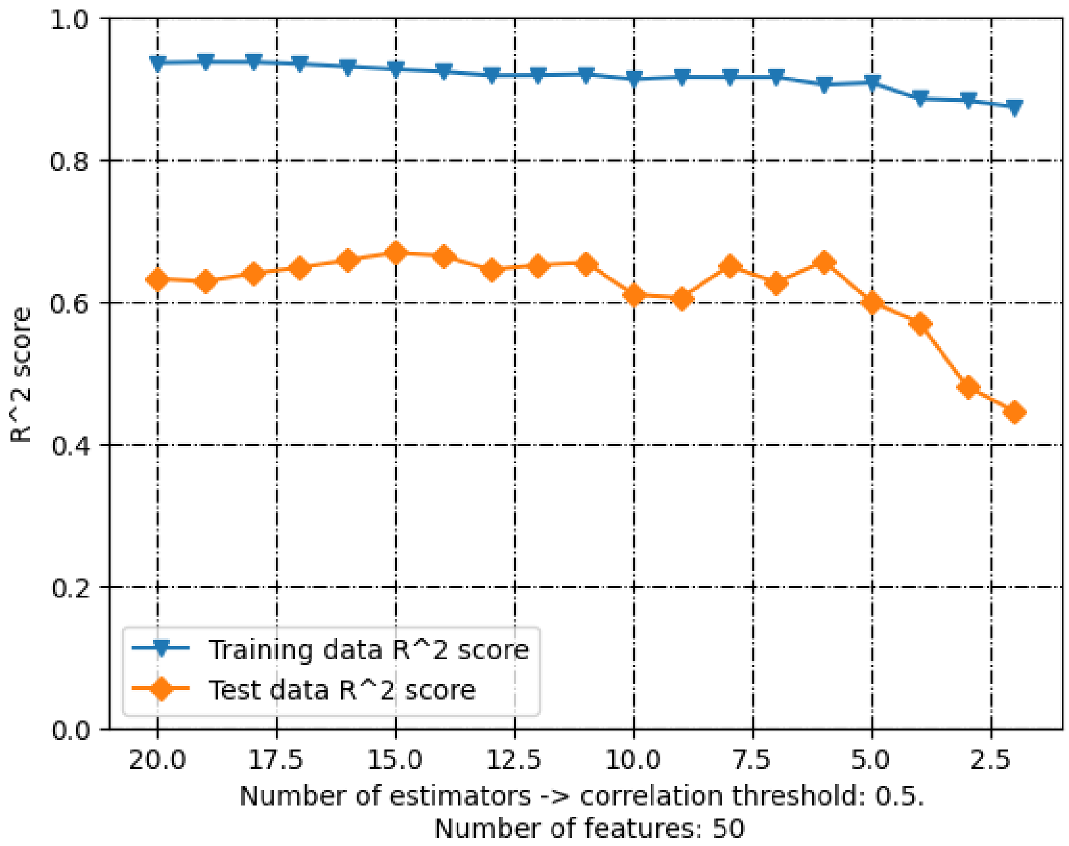
1.0
0.8
0.6
0.4
0.2
0.0
R^2 score
Training data R^2 score
Test data R^2 score
20.0
17.5
15.0
12.5
10.0
7.5
5.0
2.5
Number of estimators -> correlation threshold: 0.5.
Number of features: 50

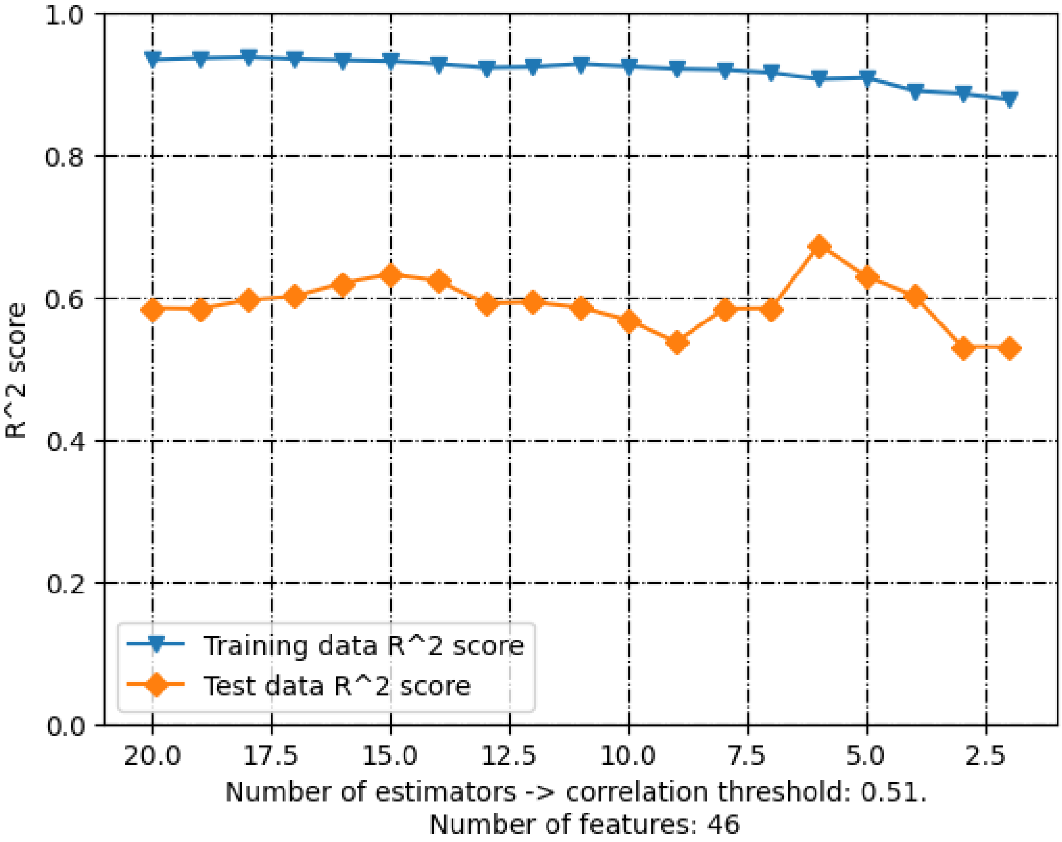
1.0
0.8
0.6
0.4
0.2
0.0
R^2 score
Training data R^2 score
Test data R^2 score
20.0
17.5
15.0
12.5
10.0
7.5
5.0
2.5
Number of estimators -> correlation threshold: 0.51.
Number of features: 46

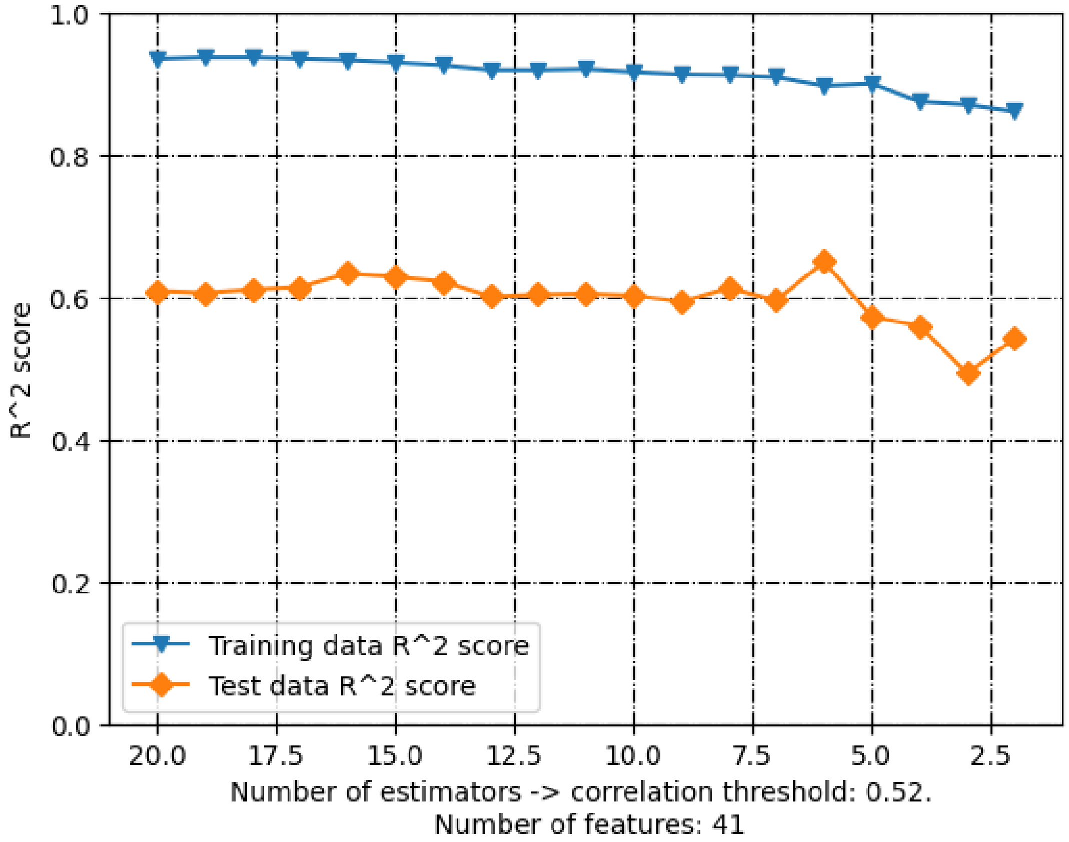

1.0
0.8
0.6
0.4
0.2
0.0
R^2 score
Training data R^2 score
Test data R^2 score
20.0
17.5
15.0
12.5
10.0
7.5
5.0
2.5
Number of estimators -> correlation threshold: 0.52.
Number of features: 41

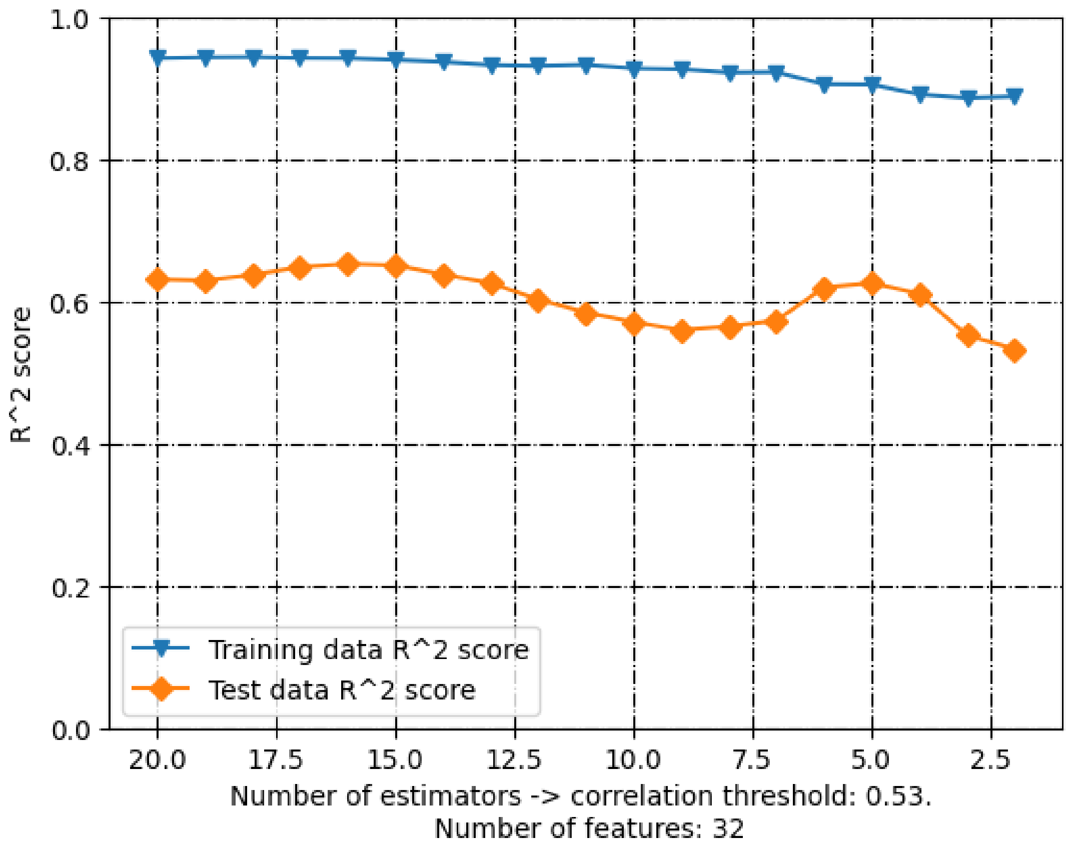
1.0
0.8
0.6
0.4
0.2
0.0
R^2 score
Training data R^2 score
Test data R^2 score
20.0
17.5
15.0
12.5
10.0
7.5
5.0
2.5
Number of estimators -> correlation threshold: 0.53.
Number of features: 32

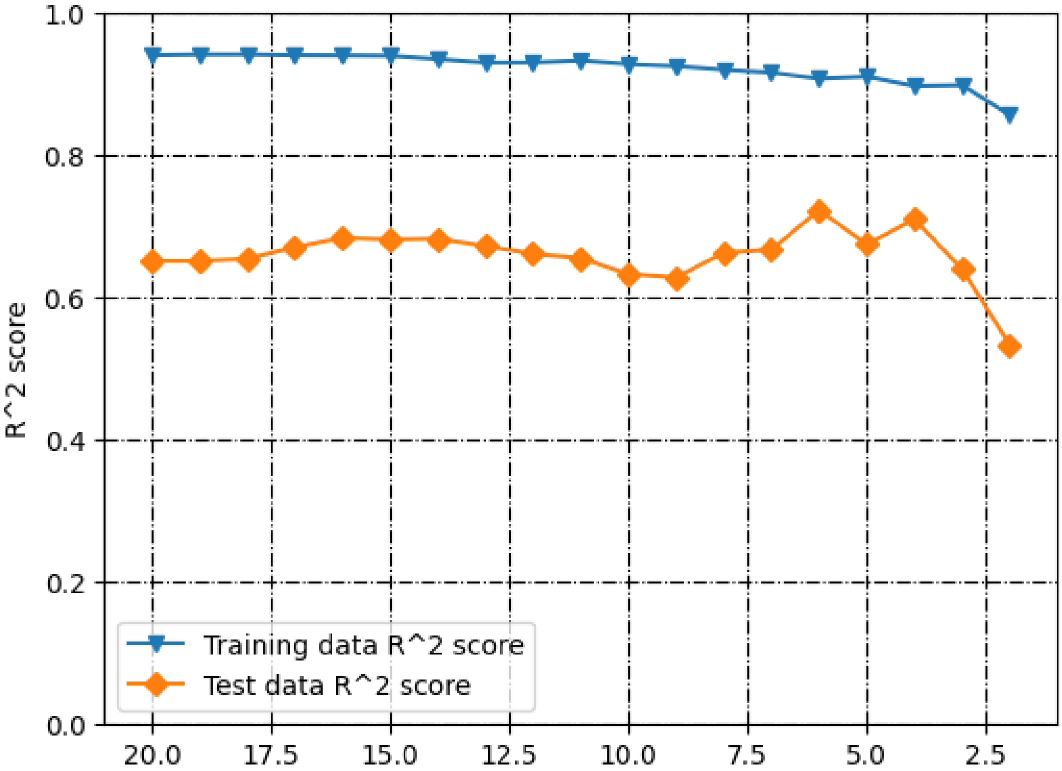

Number of estimators -> correlation threshold: 0.54.
Number of features: 25

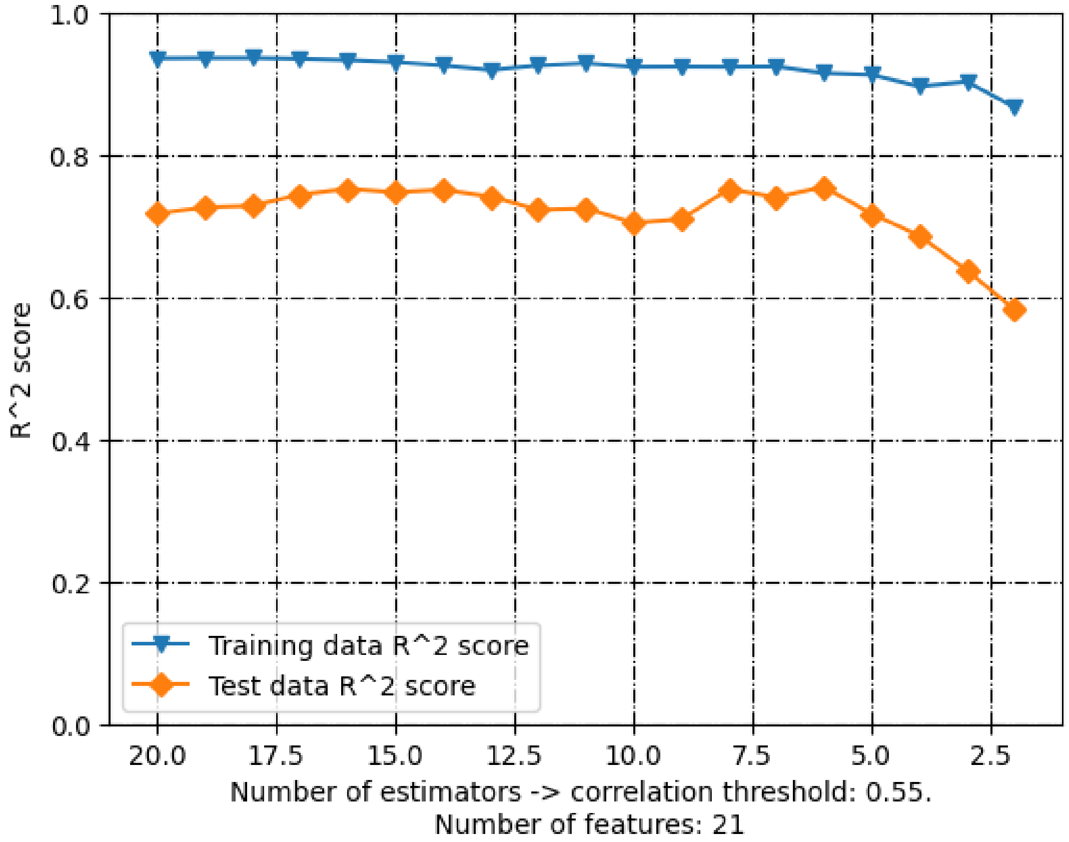

1.0
0.8
0.6
0.4
0.2
0.0
R^2 score
20.0
17.5
15.0
12.5
10.0
7.5
5.0
2.5
Training data R^2 score
Test data R^2 score
Number of estimators -> correlation threshold: 0.55.
Number of features: 21

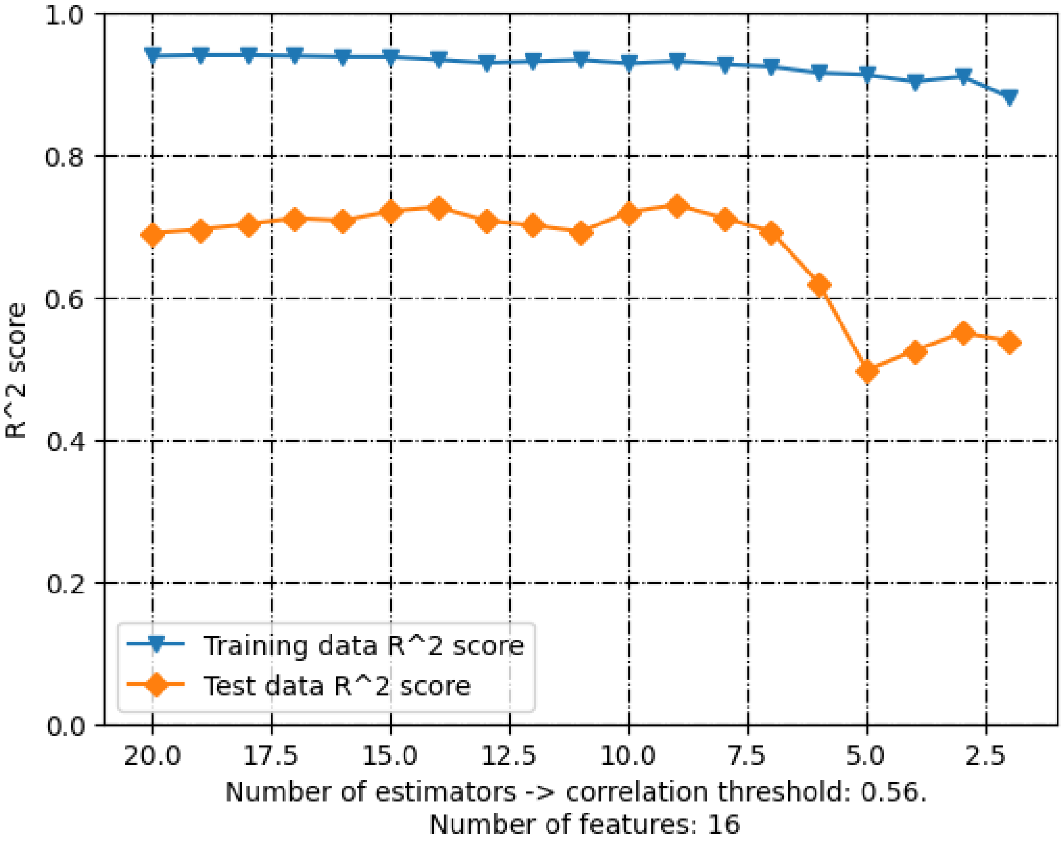

1.0
0.8
0.6
0.4
0.2
0.0
R^2 score
Training data R^2 score
Test data R^2 score
20.0
17.5
15.0
12.5
10.0
7.5
5.0
2.5
Number of estimators -> correlation threshold: 0.56.
Number of features: 16

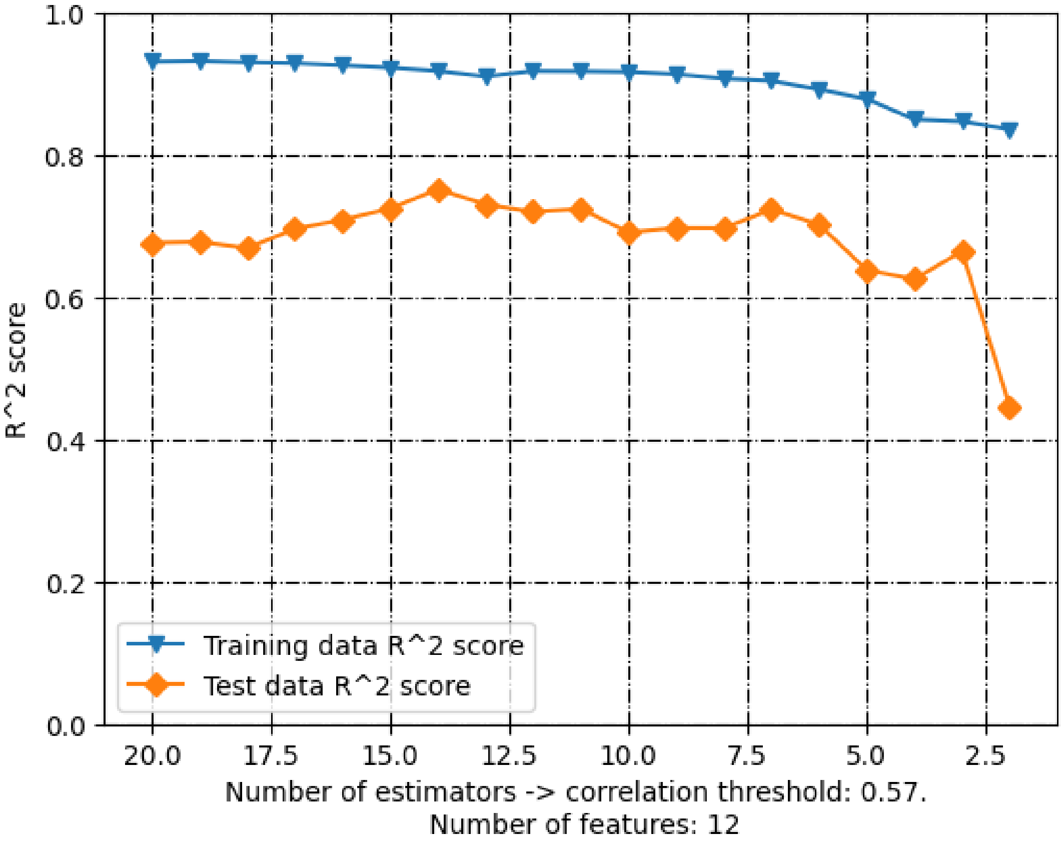
1.0
0.8
0.6
0.4
0.2
0.0
R^2 score
Training data R^2 score
Test data R^2 score
20.0
17.5
15.0
12.5
10.0
7.5
5.0
2.5
Number of estimators -> correlation threshold: 0.57.
Number of features: 12

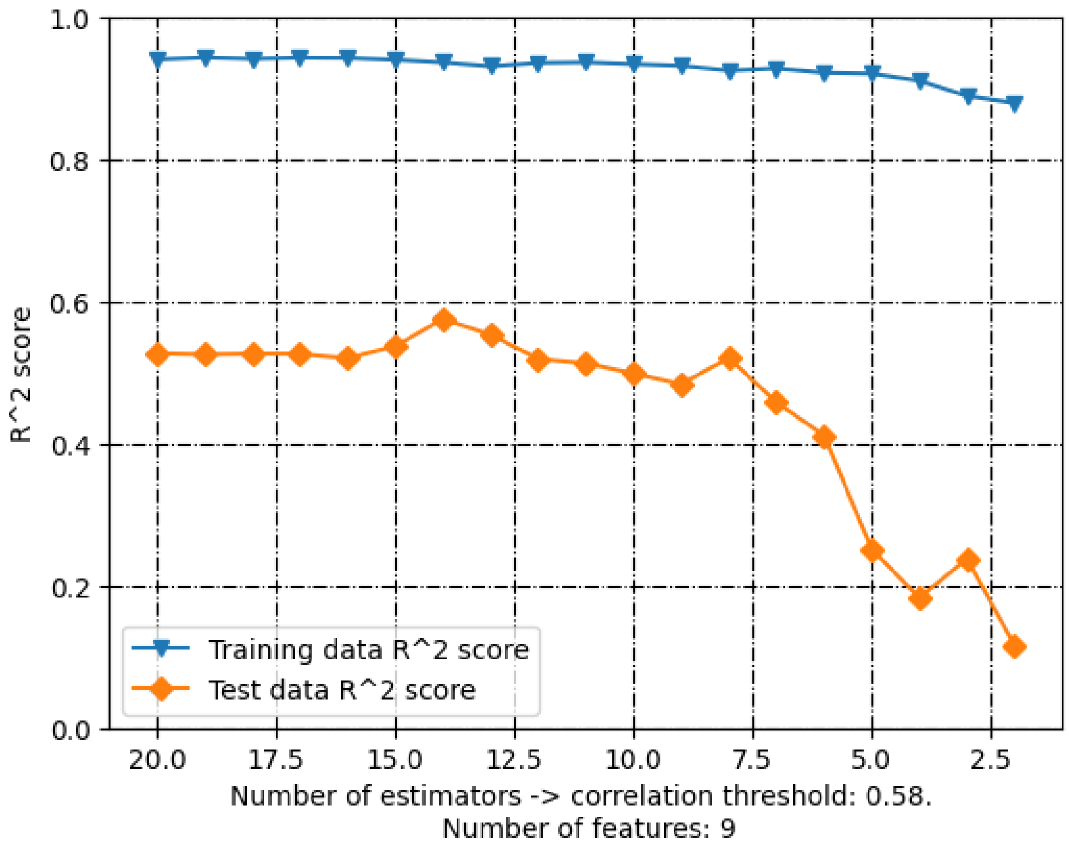
1.0
0.8
0.6
0.4
0.2
0.0
R^2 score
20.0
17.5
15.0
12.5
10.0
7.5
5.0
2.5
Number of estimators -> correlation threshold: 0.58.
Number of features: 9
Training data R^2 score
Test data R^2 score

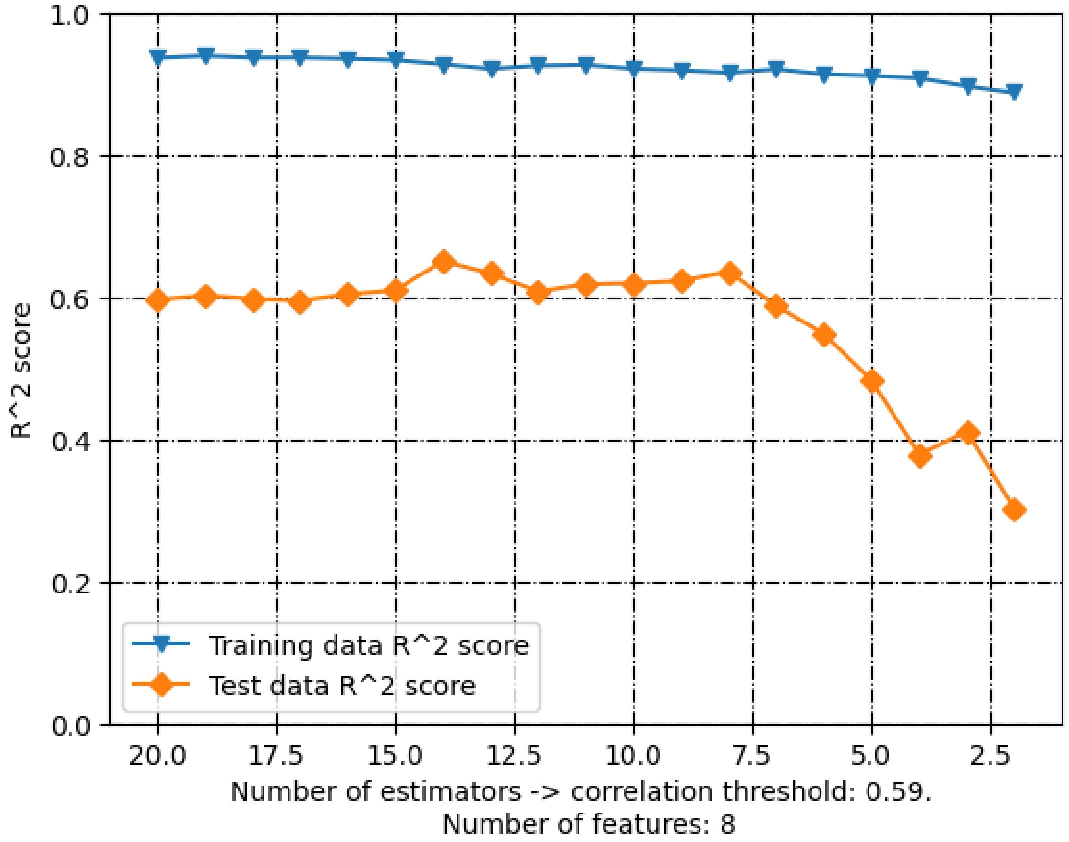
1.0
0.8
0.6
0.4
0.2
0.0
R^2 score
Training data R^2 score
Test data R^2 score
20.0
17.5
15.0
12.5
10.0
7.5
5.0
2.5
Number of estimators -> correlation threshold: 0.59.
Number of features: 8

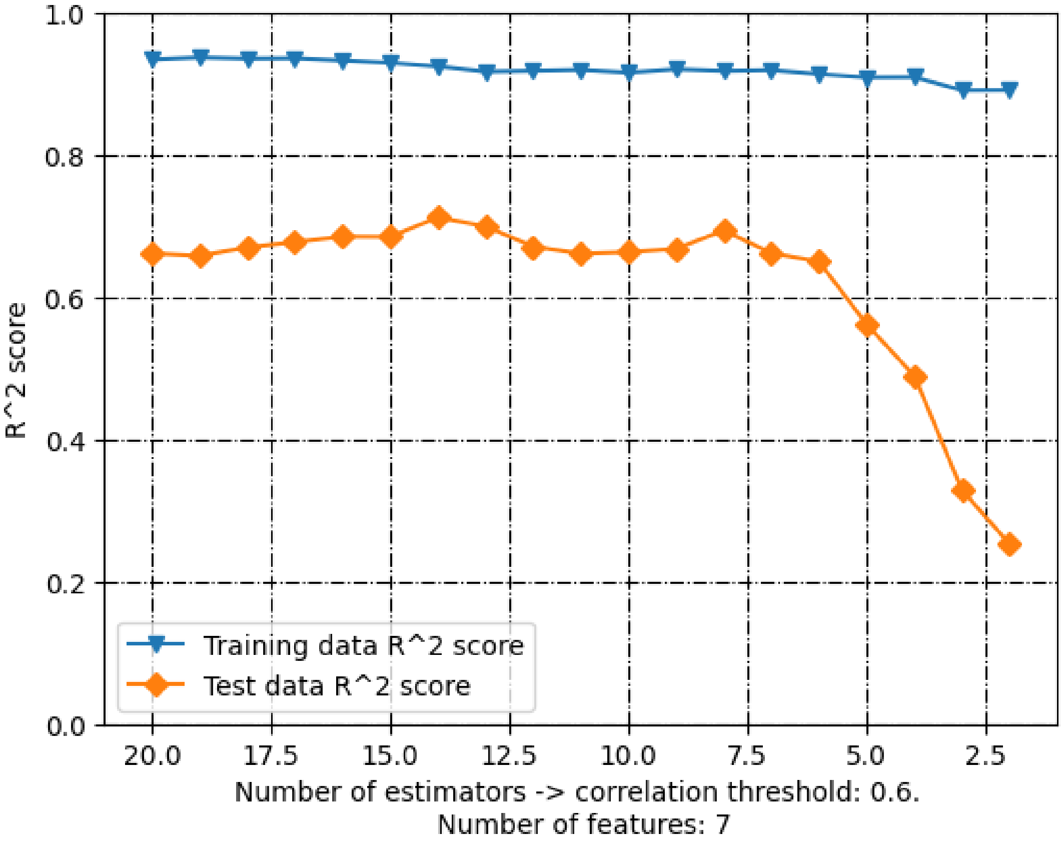

1.0
0.8
0.6
0.4
0.2
0.0
R^2 score
Training data R^2 score
Test data R^2 score
20.0
17.5
15.0
12.5
10.0
7.5
5.0
2.5
Number of estimators -> correlation threshold: 0.6.
Number of features: 7

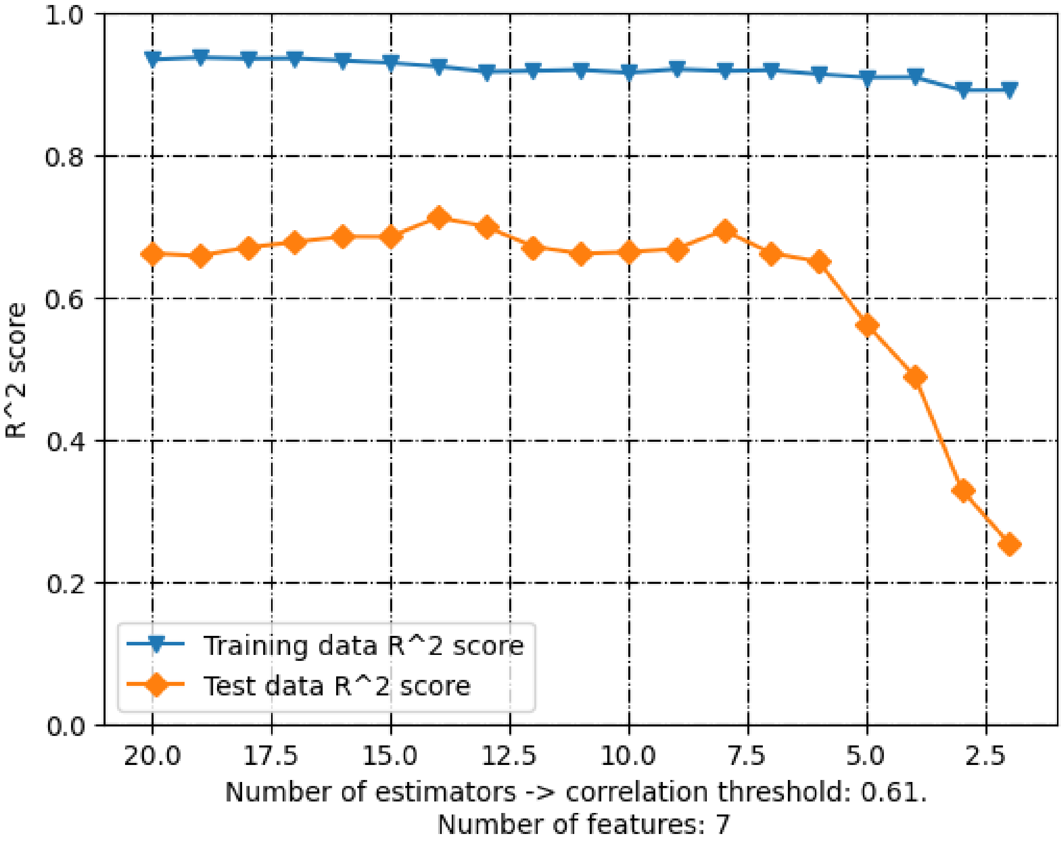
R^2 score
1.0
0.8
0.6
0.4
0.2
0.0
20.0
17.5
15.0
12.5
10.0
7.5
5.0
2.5
Training data R^2 score
Test data R^2 score
Number of estimators -> correlation threshold: 0.61.
Number of features: 7

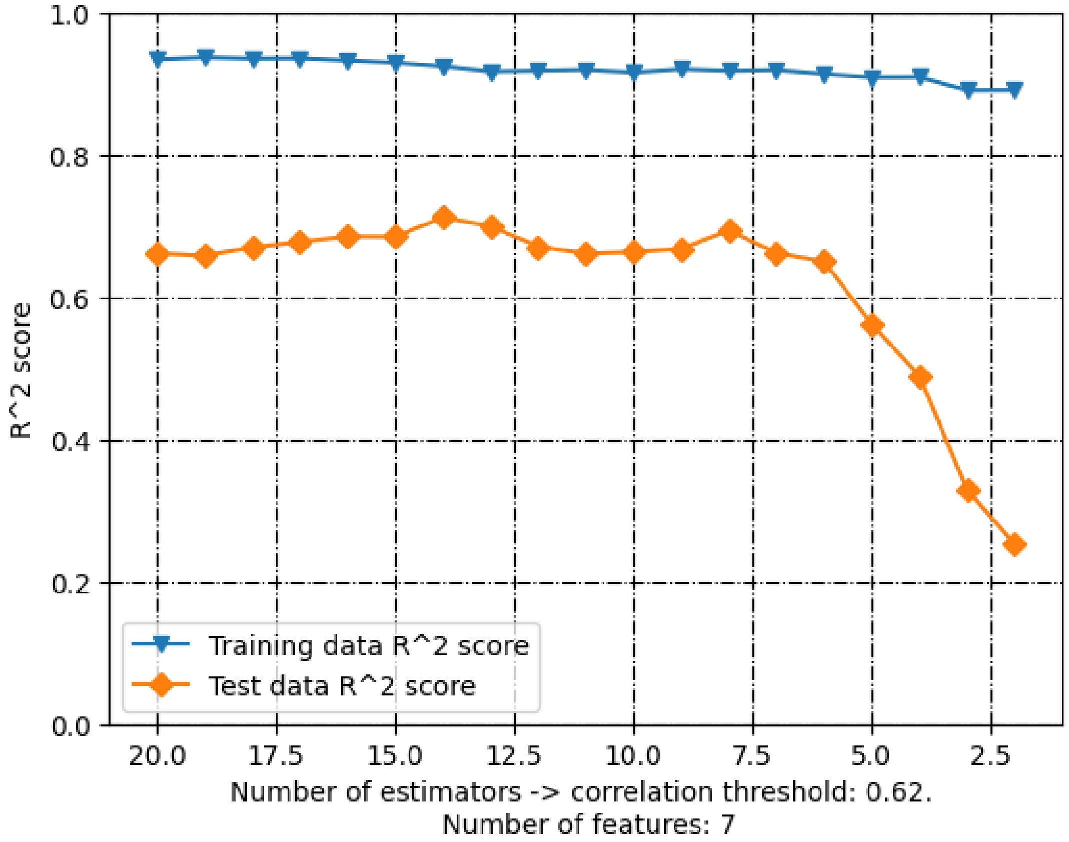

1.0
0.8
0.6
0.4
0.2
0.0
R^2 score
Training data R^2 score
Test data R^2 score
20.0
17.5
15.0
12.5
10.0
7.5
5.0
2.5
Number of estimators -> correlation threshold: 0.62.
Number of features: 7

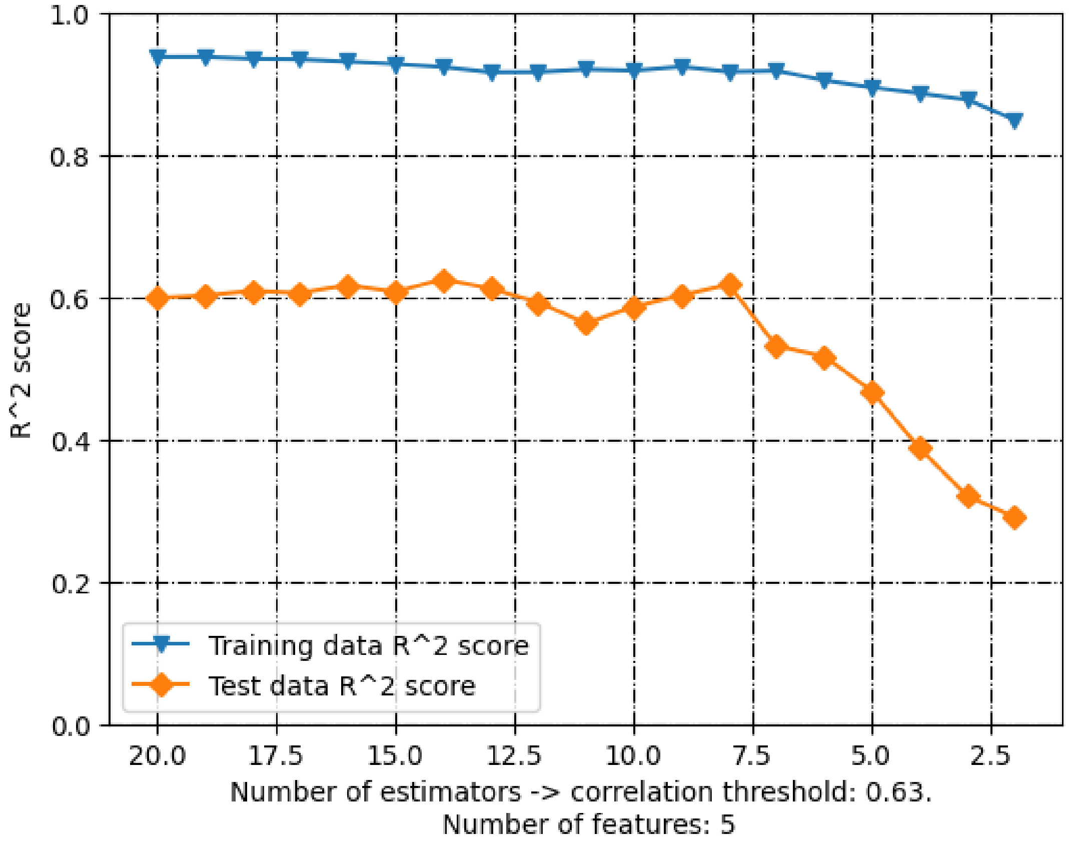

[29]:
```
plt.plot(df_without_standardization['Correlation threshold'],␣
  ↪df_without_standardization['Number of features'], label = "Number of␣
  ↪features", marker='.')
plt.legend()
plt.xlabel('Correlation threshold')
plt.ylabel('Number of features')
plt.show()
```

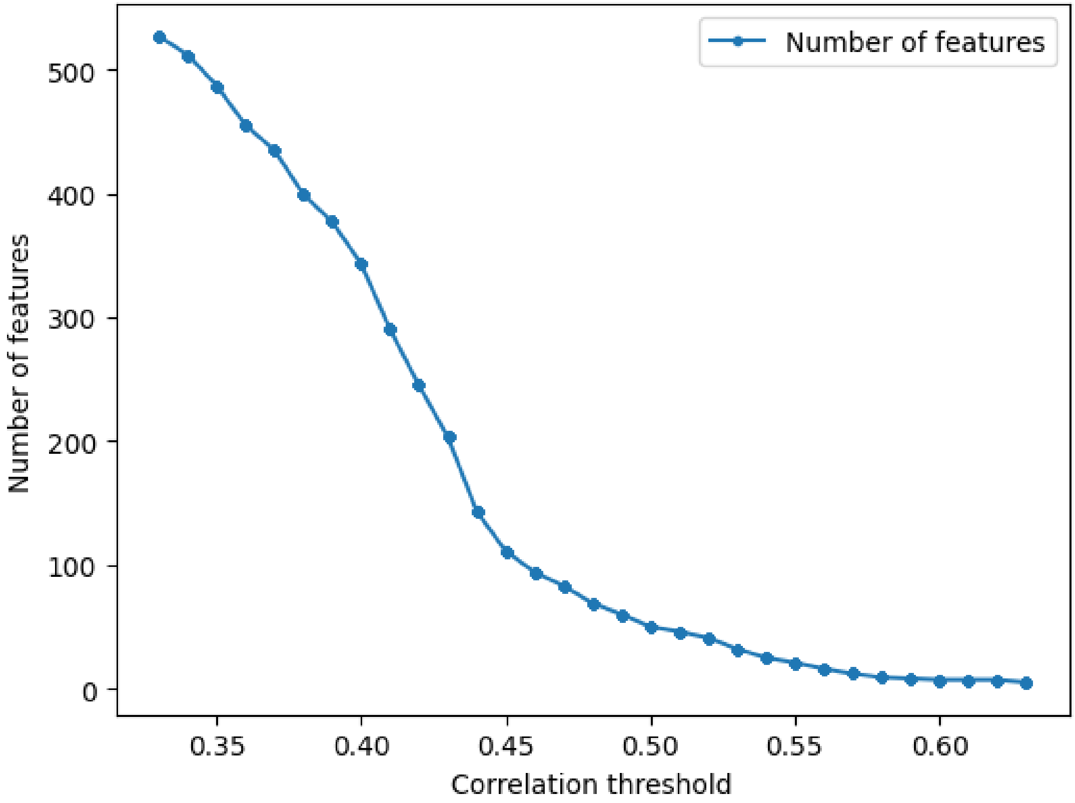


```
[ ]:
```

4 KNeighborsRegressor

```
[30]: without_standardization, train_r2_, test_r2_, hist1, hist2, target_column_name,␣
      ↪training_data_RMSE, test_data_RMSE  = pred_model.
      ↪prepare_data_and_create_model(molecular_descriptors_df=data,
                                                                                   ␣
      ↪                    correlation_threshold=0.50,
                                                                                   ␣
      ↪                    standardization=False,
                                                                                   ␣
      ↪                    model_type='KNeighborsRegressor',
                                                                                   ␣
      ↪                    target_column_name = target,
                                                                                   ␣
      ↪                    random_state=random_state,
                                                                                   ␣
      ↪                    train_test_split_=True,
```

```
↪                    verbose=True)
```

```
I am not doing standardization…
  molecular descriptor name
0                    AATS0Z
1                  AATS0are
2                    AATS0d
3                   AATS0dv
4                    AATS0i
  molecular descriptor name  corr_value
0                    AATS0Z    0.018677
1                  AATS0are   -0.341313
2                    AATS0d   -0.123443
3                   AATS0dv   -0.265670
4                    AATS0i   -0.428929
  molecular descriptor name  corr_value  absolute correlation value
0                    AATS0Z    0.018677                    0.018677
1                  AATS0are   -0.341313                    0.341313
2                    AATS0d   -0.123443                    0.123443
3                   AATS0dv   -0.265670                    0.265670
4                    AATS0i   -0.428929                    0.428929
    molecular descriptor name  corr_value  absolute correlation value
15                     AATS1i   -0.561620                    0.561620
113                   AATSC1c    0.565682                    0.565682
125                   AATSC2c   -0.543393                    0.543393
221                AETA_eta_R   -0.515778                    0.515778
224                    AMID_C    0.532458                    0.532458
    molecular descriptor name  corr_value  absolute correlation value
15                     AATS1i   -0.561620                    0.561620
113                   AATSC1c    0.565682                    0.565682
125                   AATSC2c   -0.543393                    0.543393
221                AETA_eta_R   -0.515778                    0.515778
224                    AMID_C    0.532458                    0.532458
The model used is: KNeighbors…
Return the coefficient of determination of the prediction:
0.05856826531689907
R^2 score: 0.6184964356115292
Correlation coefficient: 0.7864454435061146
Test data - unseen during training:
R^2 score: 0.05856826531689907
Correlation coefficient: 0.24200881247776715
[6.82235873 7.12934979 7.73367122 5.58412315 6.15570254 5.85942708
 5.90869339 6.16889939 6.92897264 7.13379421 5.37489126 6.07997088
 7.54260641 7.55618984 7.12934979 6.19135085]
114    6.026872
10     7.856985
4      7.109020
```

```
95     5.200659
111    6.853872
79     6.327902
44     7.638272
47     7.022276
107    6.000000
11     7.250264
61     5.481486
56     6.657577
0      7.260428
92     7.141463
18     6.630970
78     6.244125
Name: LoVo/DX, dtype: float64
Training Root Mean Square Error: 0.5939513507247756
Testing Root Mean Square Error: 0.7004653712674876
```

```
  File "C:\Users\aleks\anaconda3\envs\cheminf_gpu\lib\site-
packages\joblib\externals\loky\backend\context.py", line 217, in
_count_physical_cores
    raise ValueError(
```

[31]:

```
print(target_column_name+str('_transformed'))
print(hist1[target_column_name].hist())
```

```
LoVo/DX_transformed
AxesSubplot(0.125,0.11;0.775x0.77)
```

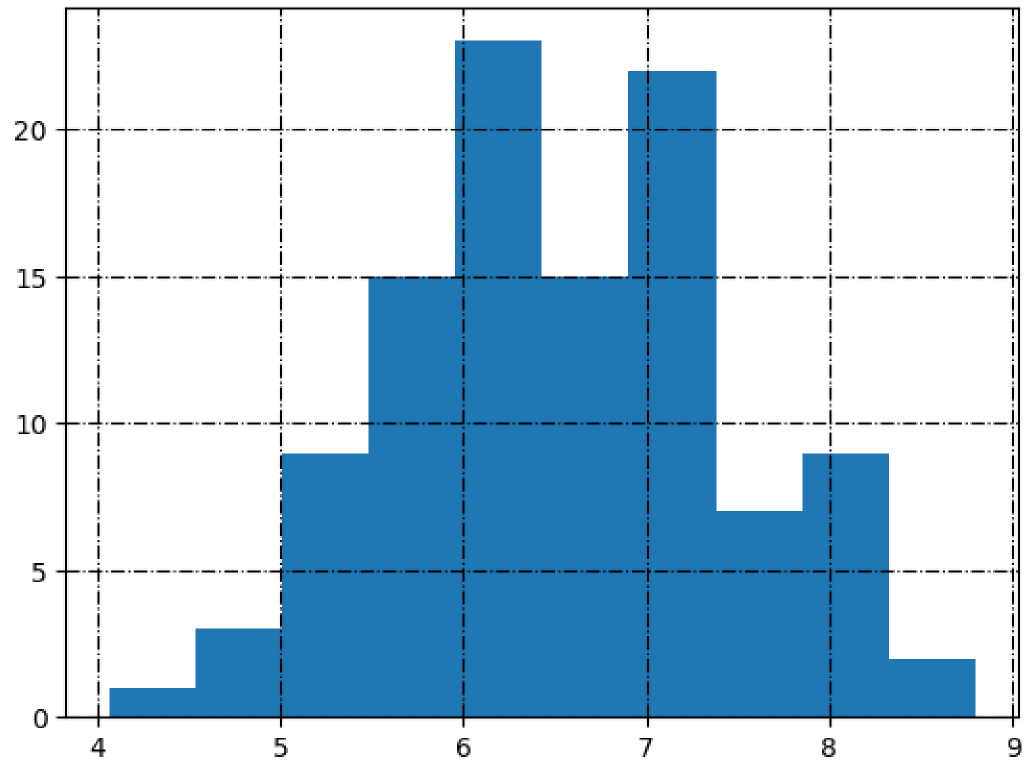

[32]:
```
with_standardization, train_r2_, test_r2_, hist1, hist2, target_column_name,␣
↪training_data_RMSE, test_data_RMSE  = pred_model.
↪prepare_data_and_create_model(molecular_descriptors_df=data,
                                                                              ␣
↪                         correlation_threshold=0.50,
                                                                              ␣
↪                         standardization=True,
                                                                              ␣
↪                         model_type='KNeighborsRegressor',
                                                                              ␣
↪                         target_column_name = target,
                                                                              ␣
↪                         random_state=random_state,
                                                                              ␣
↪                         train_test_split_=True,
                                                                              ␣
↪                         verbose=True)
```

```
I am doing standardization…
  molecular descriptor name
0                    AATS0Z
1                  AATS0are
```

```
2                  AATS0d
3                 AATS0dv
4                  AATS0i
  molecular descriptor name  corr_value
0                  AATS0Z    0.018677
1                AATS0are   -0.341313
2                  AATS0d   -0.123443
3                 AATS0dv   -0.265670
4                  AATS0i   -0.428929
  molecular descriptor name  corr_value  absolute correlation value
0                  AATS0Z    0.018677                    0.018677
1                AATS0are   -0.341313                    0.341313
2                  AATS0d   -0.123443                    0.123443
3                 AATS0dv   -0.265670                    0.265670
4                  AATS0i   -0.428929                    0.428929
    molecular descriptor name  corr_value  absolute correlation value
15                    AATS1i   -0.561620                    0.561620
113                  AATSC1c    0.565682                    0.565682
125                  AATSC2c   -0.543393                    0.543393
221               AETA_eta_R   -0.515778                    0.515778
224                   AMID_C    0.532458                    0.532458
    molecular descriptor name  corr_value  absolute correlation value
15                    AATS1i   -0.561620                    0.561620
113                  AATSC1c    0.565682                    0.565682
125                  AATSC2c   -0.543393                    0.543393
221               AETA_eta_R   -0.515778                    0.515778
224                   AMID_C    0.532458                    0.532458
The model used is: KNeighbors…
Return the coefficient of determination of the prediction:
0.05856826531689907
R^2 score: 0.6184964356115292
Correlation coefficient: 0.7864454435061146
Test data - unseen during training:
R^2 score: 0.05856826531689907
Correlation coefficient: 0.24200881247776715
[6.82235873 7.12934979 7.73367122 5.58412315 6.15570254 5.85942708
 5.90869339 6.16889939 6.92897264 7.13379421 5.37489126 6.07997088
 7.54260641 7.55618984 7.12934979 6.19135085]
114    6.026872
10     7.856985
4      7.109020
95     5.200659
111    6.853872
79     6.327902
44     7.638272
47     7.022276
107    6.000000
11     7.250264
```

```
61     5.481486
56     6.657577
0      7.260428
92     7.141463
18     6.630970
78     6.244125
Name: LoVo/DX, dtype: float64
Training Root Mean Square Error: 0.5939513507247756
Testing Root Mean Square Error: 0.7004653712674876
```

4.1 Search inside correlation space

[33]:
```
step = 0.01
initial_step = corr_low
last_step = corr_high
first_list = [x / 100.0 for x in range(int(initial_step*100),␣
 ↪int(last_step*100), int(step*100))]
second_list = []
third_list = []
fourth_l = []
fifth_l = []
f_list = []
for i in first_list:
    without_standardization, train_r2, test_r2, _, h_, target_column_name,␣
 ↪training_data_RMSE, test_data_RMSE = pred_model.
 ↪prepare_data_and_create_model(molecular_descriptors_df = data,
                                                                                ␣
 ↪                    correlation_threshold = i,
                                                                                ␣
 ↪                    standardization = False,
                                                                                ␣
 ↪                    model_type = 'KNeighborsRegressor',
                                                                                ␣
 ↪                    target_column_name = target,
                                                                                ␣
 ↪                    random_state=random_state,
                                                                                ␣
 ↪                    train_test_split_ = True,
                                                                                ␣
 ↪                    verbose = False)
    second_list.append(train_r2)
    third_list.append(test_r2)
    fourth_l.append(training_data_RMSE)
    fifth_l.append(test_data_RMSE)
    f_list.append(len(h_))
```

[34]:

```
df_without_standardization = pd.DataFrame(data=first_list,␣
 ↪columns=["Correlation threshold"])
df_without_standardization['Training data R^2 score'] = second_list
df_without_standardization['Test data R^2 score'] = third_list
df_without_standardization['Training RMSE'] = fourth_l
df_without_standardization['Test RMSE'] = fifth_l
df_without_standardization['Number of features'] = f_list
```

[35]:

```
df_k_nearest = df_without_standardization.copy()
df_without_standardization
```

[35]:

```
    Correlation threshold  Training data R^2 score  Test data R^2 score  \
0                    0.33                 0.480062             0.145765
1                    0.34                 0.412171            -0.066844
2                    0.35                 0.401596             0.070421
3                    0.36                 0.422658            -0.068587
4                    0.37                 0.422658            -0.068587
5                    0.38                 0.462916             0.053687
6                    0.39                 0.458547             0.034140
7                    0.40                 0.517999            -0.034205
8                    0.41                 0.502621            -0.073159
9                    0.42                 0.480991            -0.167617
10                   0.43                 0.450518            -0.056833
11                   0.44                 0.443118            -0.077436
12                   0.45                 0.428702            -0.133871
13                   0.46                 0.443945            -0.146117
14                   0.47                 0.431520            -0.074632
15                   0.48                 0.585454             0.058735
16                   0.49                 0.584182             0.057129
17                   0.50                 0.618496             0.058568
18                   0.51                 0.600802             0.094756
19                   0.52                 0.600802             0.138583
20                   0.53                 0.630536             0.301029
21                   0.54                 0.627198             0.340326
22                   0.55                 0.646752             0.453592
23                   0.56                 0.649685             0.556457
24                   0.57                 0.633248             0.414463
25                   0.58                 0.625208             0.336490
26                   0.59                 0.625089             0.336490
27                   0.60                 0.637140             0.391318
28                   0.61                 0.637140             0.391318
29                   0.62                 0.637140             0.391318
30                   0.63                 0.657269             0.318851

    Training RMSE  Test RMSE  Number of features
0        0.693390   0.667238                 527
1        0.737271   0.745663                 512
```

2	0.743873	0.696042	487
3	0.730665	0.746272	455
4	0.730665	0.746272	435
5	0.704730	0.702279	399
6	0.707591	0.709495	377
7	0.667614	0.734168	343
8	0.678180	0.747867	290
9	0.692770	0.780086	245
10	0.712817	0.742157	203
11	0.717601	0.749356	143
12	0.726830	0.768730	111
13	0.717068	0.772870	94
14	0.725035	0.748380	83
15	0.619139	0.700403	69
16	0.620088	0.701000	60
17	0.593951	0.700465	50
18	0.607569	0.686871	46
19	0.607569	0.670037	41
20	0.584504	0.603562	32
21	0.587139	0.586350	25
22	0.571533	0.533643	21
23	0.569155	0.480795	16
24	0.582355	0.552420	12
25	0.588704	0.588052	9
26	0.588797	0.588052	8
27	0.579257	0.563232	7
28	0.579257	0.563232	7
29	0.579257	0.563232	7
30	0.562961	0.595817	5

4.2 Plots

[36]:
```
plt.plot(df_without_standardization['Correlation threshold'],␣
↪df_without_standardization['Training data R^2 score'], label = "Training␣
↪data R^2 score", marker='v')
plt.plot(df_without_standardization['Correlation threshold'],␣
↪df_without_standardization['Test data R^2 score'], label = "Test data R^2␣
↪score", marker='D')
plt.legend()
plt.xlabel('Correlation threshold')
plt.ylabel('R^2 score')
plt.ylim([0, 1])
plt.show()
```

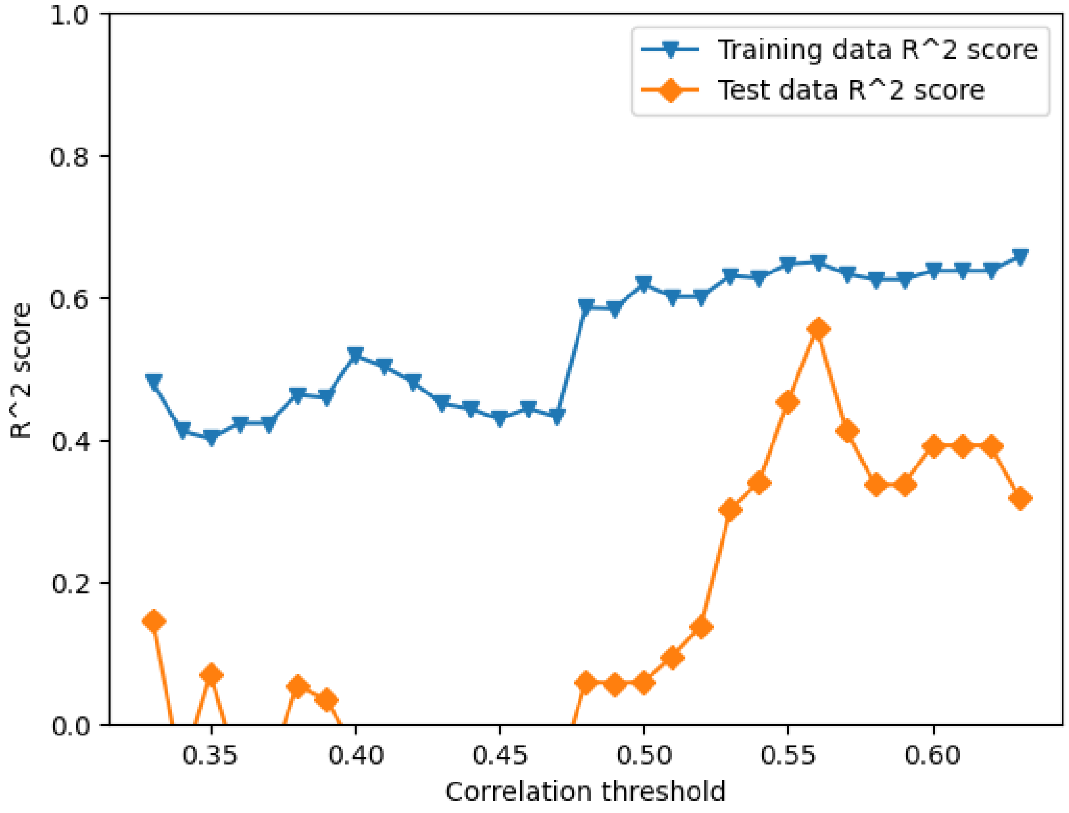

[37]:

```
plt.plot(df_without_standardization['Correlation threshold'],␣
    ↪df_without_standardization['Number of features'], label = "Number of␣
    ↪features", marker='.')
plt.legend()
plt.xlabel('Correlation threshold')
plt.ylabel('Number of features')
plt.show()
```

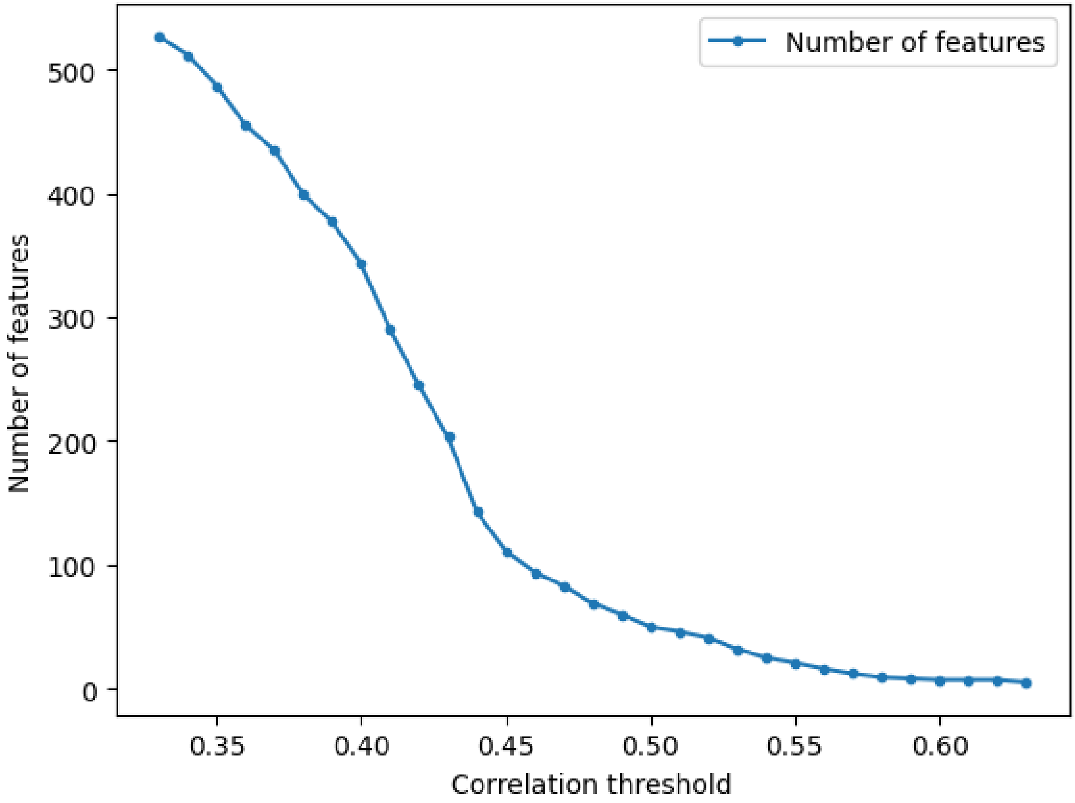

[ ]:

```

```

5 Support Vector Machines (SVM)

[38]:
```
without_standardization, train_r2_, test_r2_, hist1, hist2, target_column_name,␣
↪training_data_RMSE, test_data_RMSE  = pred_model.
↪prepare_data_and_create_model(molecular_descriptors_df=data,
                                                                                    ␣
↪                          correlation_threshold=0.50,
                                                                                    ␣
↪                          standardization=False,
                                                                                    ␣
↪                          model_type='SVR',
                                                                                    ␣
↪                          kernel_ = 'linear',
                                                                                    ␣
↪                          gamma_ = 'auto',
                                                                                    ␣
↪                          target_column_name = target,
```

```
↪                    random_state=random_state,
↪                    train_test_split_=True,
↪                    verbose=True)
```

```
I am not doing standardization…
  molecular descriptor name
0                    AATS0Z
1                  AATS0are
2                    AATS0d
3                   AATS0dv
4                    AATS0i
  molecular descriptor name  corr_value
0                    AATS0Z    0.018677
1                  AATS0are   -0.341313
2                    AATS0d   -0.123443
3                   AATS0dv   -0.265670
4                    AATS0i   -0.428929
  molecular descriptor name  corr_value  absolute correlation value
0                    AATS0Z    0.018677                    0.018677
1                  AATS0are   -0.341313                    0.341313
2                    AATS0d   -0.123443                    0.123443
3                   AATS0dv   -0.265670                    0.265670
4                    AATS0i   -0.428929                    0.428929
    molecular descriptor name  corr_value  absolute correlation value
15                     AATS1i   -0.561620                    0.561620
113                   AATSC1c    0.565682                    0.565682
125                   AATSC2c   -0.543393                    0.543393
221                AETA_eta_R   -0.515778                    0.515778
224                    AMID_C    0.532458                    0.532458
    molecular descriptor name  corr_value  absolute correlation value
15                     AATS1i   -0.561620                    0.561620
113                   AATSC1c    0.565682                    0.565682
125                   AATSC2c   -0.543393                    0.543393
221                AETA_eta_R   -0.515778                    0.515778
224                    AMID_C    0.532458                    0.532458
The model used is: SVR…
Return the coefficient of determination of the prediction:
-0.7067065714648701
R^2 score: 0.22680272300596427
Correlation coefficient: 0.47623809487058494
Test data - unseen during training:
R^2 score: -0.7067065714648701
Correlation coefficient: nan
[5.3191818  7.54606786 7.10433139 6.35575272 4.201683   5.89553052
```

```
 7.33731032 7.41729643 6.12151933 7.25899662 3.93371874 6.45051427
 7.60104524 7.71508202 7.42855583 5.10483484]
114    6.026872
10     7.856985
4      7.109020
95     5.200659
111    6.853872
79     6.327902
44     7.638272
47     7.022276
107    6.000000
11     7.250264
61     5.481486
56     6.657577
0      7.260428
92     7.141463
18     6.630970
78     6.244125
Name: LoVo/DX, dtype: float64
Training Root Mean Square Error: 0.8455644823555171
Testing Root Mean Square Error: 0.9431302691442349
```

[39]:
```
print(target_column_name+str('_transformed'))
print(hist1[target_column_name].hist())
```

```
LoVo/DX_transformed
AxesSubplot(0.125,0.11;0.775x0.77)
```

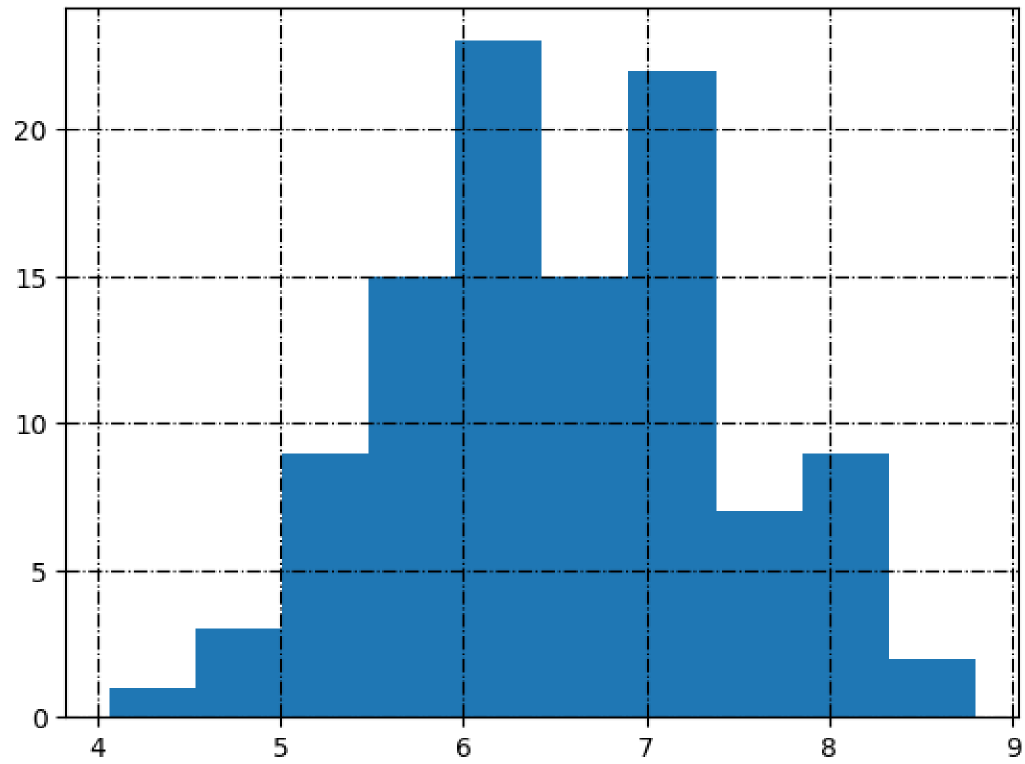

[40]:
```
with_standardization, train_r2_, test_r2_, hist1, hist2, target_column_name,␣
↪training_data_RMSE, test_data_RMSE  = pred_model.
↪prepare_data_and_create_model(molecular_descriptors_df=data,
                                                                              ␣
↪                      correlation_threshold=0.50,
                                                                              ␣
↪                      standardization=True,
                                                                              ␣
↪                      model_type='SVR',
                                                                              ␣
↪                      kernel_ = 'linear',
                                                                              ␣
↪                      gamma_ = 'auto',
                                                                              ␣
↪                      target_column_name = target,
                                                                              ␣
↪                      random_state=random_state,
                                                                              ␣
↪                      train_test_split_=True,
                                                                              ␣
↪                      verbose=True)
```

```
I am doing standardization…
  molecular descriptor name
0                    AATS0Z
1                  AATS0are
2                    AATS0d
3                   AATS0dv
4                    AATS0i
  molecular descriptor name  corr_value
0                    AATS0Z    0.018677
1                  AATS0are   -0.341313
2                    AATS0d   -0.123443
3                   AATS0dv   -0.265670
4                    AATS0i   -0.428929
  molecular descriptor name  corr_value  absolute correlation value
0                    AATS0Z    0.018677                    0.018677
1                  AATS0are   -0.341313                    0.341313
2                    AATS0d   -0.123443                    0.123443
3                   AATS0dv   -0.265670                    0.265670
4                    AATS0i   -0.428929                    0.428929
    molecular descriptor name  corr_value  absolute correlation value
15                     AATS1i   -0.561620                    0.561620
113                   AATSC1c    0.565682                    0.565682
125                   AATSC2c   -0.543393                    0.543393
221                AETA_eta_R   -0.515778                    0.515778
224                    AMID_C    0.532458                    0.532458
```

```
    molecular descriptor name  corr_value  absolute correlation value
15                     AATS1i   -0.561620                    0.561620
113                   AATSC1c    0.565682                    0.565682
125                   AATSC2c   -0.543393                    0.543393
221                AETA_eta_R   -0.515778                    0.515778
224                    AMID_C    0.532458                    0.532458
```

```
The model used is: SVR…
Return the coefficient of determination of the prediction:
-0.7067065714648701
R^2 score: 0.22680272300596427
Correlation coefficient: 0.47623809487058494
Test data - unseen during training:
R^2 score: -0.7067065714648701
Correlation coefficient: nan
[5.3191818  7.54606786 7.10433139 6.35575272 4.201683   5.89553052
 7.33731032 7.41729643 6.12151933 7.25899662 3.93371874 6.45051427
 7.60104524 7.71508202 7.42855583 5.10483484]
114    6.026872
10     7.856985
4      7.109020
95     5.200659
111    6.853872
79     6.327902
```

```
44     7.638272
47     7.022276
107    6.000000
11     7.250264
61     5.481486
56     6.657577
0      7.260428
92     7.141463
18     6.630970
78     6.244125
Name: LoVo/DX, dtype: float64
Training Root Mean Square Error: 0.8455644823555171
Testing Root Mean Square Error: 0.9431302691442349
```

5.1 Search inside correlation space

[41]:
```
step = 0.01
initial_step = corr_low
last_step = corr_high
first_list = [x / 100.0 for x in range(int(initial_step*100),␣
 ↪int(last_step*100), int(step*100))]
second_list = []
third_list = []
fourth_l = []
fifth_l = []
f_list = []
for i in first_list:
    without_standardization, train_r2, test_r2, _, h_, target_column_name,␣
 ↪training_data_RMSE, test_data_RMSE = pred_model.
 ↪prepare_data_and_create_model(molecular_descriptors_df = data,
                                                                                ␣
 ↪                   correlation_threshold = i,
                                                                                ␣
 ↪                   standardization = False,
                                                                                ␣
 ↪                   model_type = 'SVR',
                                                                                ␣
 ↪                   kernel_ = 'linear',
                                                                                ␣
 ↪                   gamma_ = 'auto',
                                                                                ␣
 ↪                   target_column_name = target,
                                                                                ␣
 ↪                   random_state=random_state,
                                                                                ␣
 ↪                   train_test_split_ = True,
```

```
                      verbose = False)
    second_list.append(train_r2)
    third_list.append(test_r2)
    fourth_l.append(training_data_RMSE)
    fifth_l.append(test_data_RMSE)
    f_list.append(len(h_))
```

[42]:
```
df_without_standardization = pd.DataFrame(data=first_list,
 ↪columns=["Correlation threshold"])
df_without_standardization['Training data R^2 score'] = second_list
df_without_standardization['Test data R^2 score'] = third_list
df_without_standardization['Training RMSE'] = fourth_l
df_without_standardization['Test RMSE'] = fifth_l
df_without_standardization['Number of features'] = f_list
```

[43]:
```
df_svm = df_without_standardization.copy()
df_without_standardization
```

[43]:
```
    Correlation threshold  Training data R^2 score  Test data R^2 score  \
0                    0.33            -3.967545e+06        -6.177944e+07
1                    0.34            -4.359720e+06        -1.763906e+07
2                    0.35            -4.169933e+06        -3.135337e+07
3                    0.36            -1.210090e+06        -1.721176e+07
4                    0.37            -1.031800e+06        -8.514192e+06
5                    0.38            -5.106398e+05        -1.623426e+06
6                    0.39            -4.992480e+05        -3.513337e+06
7                    0.40            -5.440960e+05        -1.187364e+07
8                    0.41            -2.560265e+05        -2.512385e+06
9                    0.42            -2.066783e+04        -1.002979e+05
10                   0.43            -1.392983e+04        -9.535859e+04
11                   0.44            -7.364753e+03        -2.221438e+04
12                   0.45            -1.139863e+04        -1.808719e+04
13                   0.46            -3.150243e+03        -2.527648e+04
14                   0.47            -3.908323e+03        -8.513583e+03
15                   0.48             1.550025e-01        -1.923098e+00
16                   0.49             1.518758e-01         1.193246e-01
17                   0.50             2.268027e-01        -7.067066e-01
18                   0.51             4.583327e-01        -6.995039e-01
19                   0.52             3.393138e-01         4.226412e-01
20                   0.53             6.062191e-01         5.354107e-01
21                   0.54             5.985621e-01         4.243816e-01
22                   0.55             5.913989e-01         4.135849e-01
23                   0.56             5.488128e-01         5.954298e-01
24                   0.57             5.307889e-01         6.487816e-01
25                   0.58             5.214640e-01         6.413845e-01
26                   0.59             5.185136e-01         6.367159e-01
```

27	0.60	4.984544e-01	6.327069e-01
28	0.61	4.984544e-01	6.327069e-01
29	0.62	4.984544e-01	6.327069e-01
30	0.63	4.852199e-01	6.049489e-01

	Training RMSE	Test RMSE	Number of features
0	1915.413525	5674.325327	527
1	2007.848476	3032.005082	512
2	1963.659483	4042.354371	487
3	1057.817080	2995.055521	455
4	976.786094	2106.512288	435
5	687.162275	919.832376	399
6	679.454180	1353.169588	377
7	709.315950	2487.620905	343
8	486.569522	1144.288300	290
9	138.248198	228.633766	245
10	113.498503	222.933045	203
11	82.529679	107.601689	143
12	102.670798	97.093419	111
13	53.981198	114.778130	94
14	60.124619	66.615293	83
15	0.883953	1.234281	69
16	0.885587	0.677486	60
17	0.845564	0.943130	50
18	0.707730	0.941138	46
19	0.781626	0.548549	41
20	0.603433	0.492070	32
21	0.609271	0.547721	25
22	0.614683	0.552834	21
23	0.645922	0.459187	16
24	0.658697	0.427840	12
25	0.665210	0.432321	9
26	0.667258	0.435126	8
27	0.681015	0.437521	7
28	0.681015	0.437521	7
29	0.681015	0.437521	7
30	0.689942	0.453752	5

5.2 Plots

[44]:
```
plt.plot(df_without_standardization['Correlation threshold'],␣
    ↪df_without_standardization['Training data R^2 score'], label = "Training␣
    ↪data R^2 score", marker='v')
plt.plot(df_without_standardization['Correlation threshold'],␣
    ↪df_without_standardization['Test data R^2 score'], label = "Test data R^2␣
    ↪score", marker='D')
plt.legend()
```

```
plt.xlabel('Correlation threshold')
plt.ylabel('R^2 score')
plt.ylim([0, 1])
plt.show()
```

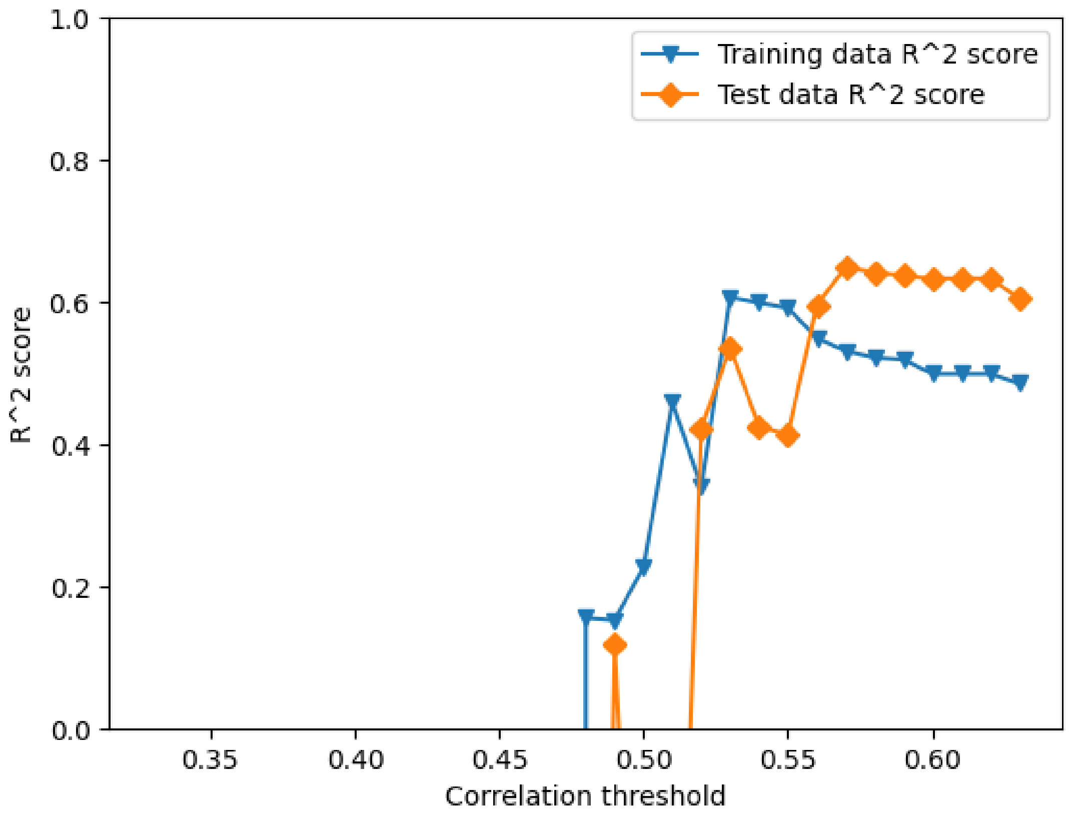

[45]:
```
plt.plot(df_without_standardization['Correlation threshold'],␣
  ↪df_without_standardization['Number of features'], label = "Number of␣
  ↪features", marker='.')
plt.legend()
plt.xlabel('Correlation threshold')
plt.ylabel('Number of features')
plt.show()
```

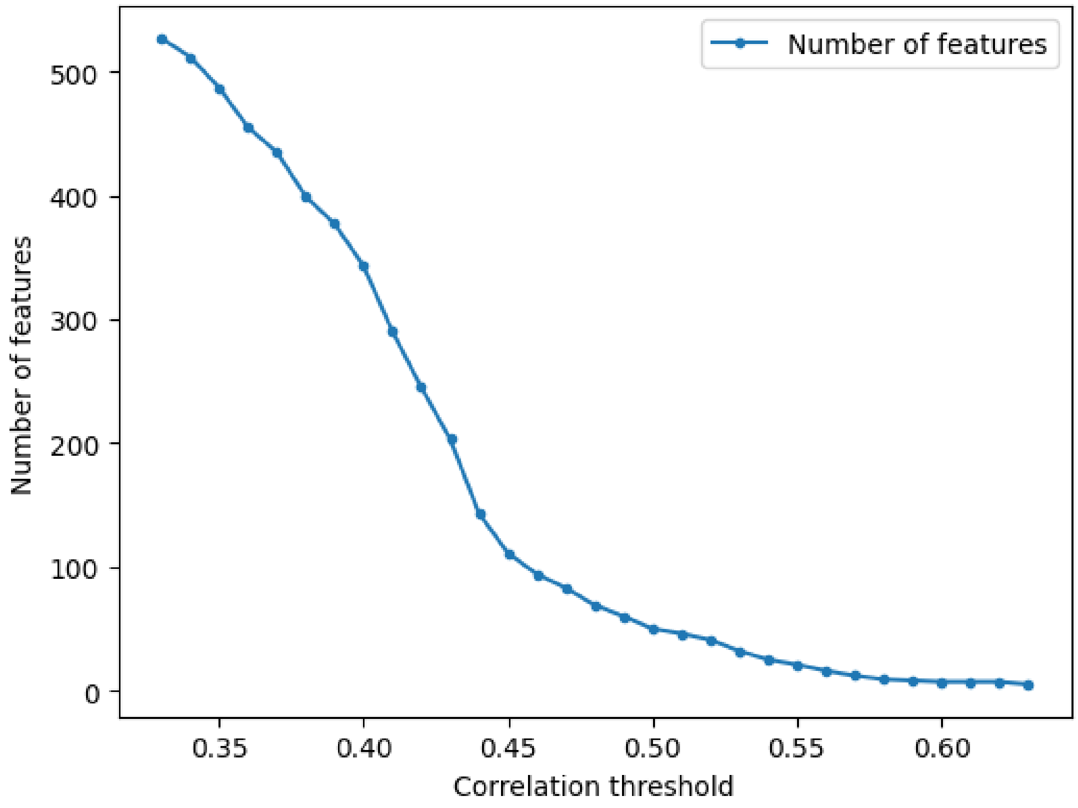

5.3 Store quality measurements

[46]:
```
with pd.ExcelWriter('../Data/Quality_'+'LoVo_DX'+'_'+str(random_state)+'_.
 ↪xlsx') as writer:
    df_linear.to_excel(writer, sheet_name='MLR')
    df_decision_tree.to_excel(writer, sheet_name='DT')
    df_random_forest.to_excel(writer, sheet_name='RF')
    df_k_nearest.to_excel(writer, sheet_name='KNN')
    df_svm.to_excel(writer, sheet_name='SVM')
```

Notebook

January 18, 2024

1 File 24

[1]:
```
import sys
from pathlib import Path
prediction_mode_path = Path("../module")
sys.path.append(prediction_mode_path.as_posix())
import models_creation as pred_model

import pandas as pd
import numpy as np

from sklearn.metrics import r2_score

import matplotlib.pyplot as plt
from sklearn.tree import plot_tree

from matplotlib import pyplot as plt

import joblib

import warnings
warnings.filterwarnings('ignore')
```

[2]:
```
load_prepared_data = pd.read_excel('../Data/Kolchicyna_machine_learning.xlsx')
load_prepared_data = load_prepared_data.drop(columns=['Unnamed: 0'])

target = 'LoVo'

corr_low = 0.33
corr_high = 0.55

random_state = 15
```

[3]:
```
load_prepared_data.head()
```

[3]:
```
      AATS0Z  AATS0are    AATS0d   AATS0dv      AATS0i     AATS0m    AATS0p  \
0  25.090909  6.046518  3.109091  6.662626  160.775045  99.075564  1.555512
1  24.448276  6.008422  3.051724  6.386973  161.021674  96.473315  1.538471
```

```
2  23.868852  5.974074  3.000000  6.138434  161.244045  94.127025  1.523105
3  23.868852  5.974074  3.032787  6.171220  161.244045  94.127025  1.523105
4  23.343750  5.942945  2.953125  5.913194  161.445569  92.000700  1.509180

    AATS0pe    AATS0s   AATS0se  …  A549_float  MCF-7_float  LoVo_float  \
0  6.126680  3.435416  7.629859  …        10.8         10.3         6.5
1  6.088791  3.330998  7.596891  …        11.6         12.0         8.5
2  6.054630  3.236850  7.567166  …        10.9         12.2         8.8
3  6.054630  3.257798  7.567166  …        10.5         11.3         8.5
4  6.023670  3.151529  7.540228  …        89.5         92.7        52.8

   LoVo/DX_float  BALB/3T3_float  A549_float_transformed  \
0           54.9            10.2                7.966576
1           31.1            14.3                7.935542
2           17.9            11.7                7.962574
3           10.2            11.0                7.978811
4           77.8            99.4                7.048177

   MCF-7_float_transformed  LoVo_float_transformed  LoVo/DX_float_transformed  \
0                 7.987163                8.187087                   7.260428
1                 7.920819                8.070581                   7.507240
2                 7.913640                8.055517                   7.747147
3                 7.946922                8.070581                   7.991400
4                 7.032920                7.277366                   7.109020

   BALB/3T3_float_transformed
0                    7.991400
1                    7.844664
2                    7.931814
3                    7.958607
4                    7.002614

[5 rows x 1221 columns]
```

[4]:
```python
data = load_prepared_data[list(load_prepared_data.columns)[0:-10]]
data[target] = load_prepared_data[load_prepared_data.columns[-3]]
data.head()
```

[4]:
```
      AATS0Z  AATS0are    AATS0d   AATS0dv      AATS0i     AATS0m    AATS0p  \
0  25.090909  6.046518  3.109091  6.662626  160.775045  99.075564  1.555512
1  24.448276  6.008422  3.051724  6.386973  161.021674  96.473315  1.538471
2  23.868852  5.974074  3.000000  6.138434  161.244045  94.127025  1.523105
3  23.868852  5.974074  3.032787  6.171220  161.244045  94.127025  1.523105
4  23.343750  5.942945  2.953125  5.913194  161.445569  92.000700  1.509180

    AATS0pe    AATS0s   AATS0se  …    piPC10     piPC2     piPC3     piPC4  \
0  6.126680  3.435416  7.629859  …  7.999655  4.266195  4.915408  5.516700
```

```
1  6.088791  3.330998  7.596891  …  8.018726  4.280132  4.922714  5.520711
2  6.054630  3.236850  7.567166  …  8.028799  4.293878  4.929967  5.524706
3  6.054630  3.257798  7.567166  …  8.037440  4.307438  4.929967  5.524706
4  6.023670  3.151529  7.540228  …  8.034892  4.307438  4.937168  5.528685

      piPC5     piPC6     piPC7     piPC8     piPC9      LoVo
0  6.098566  6.595759  6.979116  7.337313  7.681445  8.187087
1  6.103048  6.601209  6.985613  7.349442  7.695531  8.070581
2  6.105281  6.603923  6.989306  7.353932  7.704023  8.055517
3  6.107510  6.606629  6.992068  7.361425  7.709420  8.070581
4  6.107510  6.605277  6.991148  7.356489  7.707175  7.277366

[5 rows x 1212 columns]
```

2 Multiple Linear regression (MLR)

[5]:
```
without_standardization, train_r2_, test_r2_, hist1, hist2, target_column_name,␣
↪training_data_RMSE, test_data_RMSE  = pred_model.
↪prepare_data_and_create_model(molecular_descriptors_df=data,
                                                                                 ␣
↪                    correlation_threshold=0.5,
                                                                                 ␣
↪                    standardization=False,
                                                                                 ␣
↪                    model_type='linear_model',
                                                                                 ␣
↪                    target_column_name = str(target),
                                                                                 ␣
↪                    random_state=random_state,
                                                                                 ␣
↪                    train_test_split_=True,
                                                                                 ␣
↪                    verbose=True)
```

```
I am not doing standardization…
  molecular descriptor name
0                   AATS0Z
1                 AATS0are
2                   AATS0d
3                  AATS0dv
4                   AATS0i
  molecular descriptor name  corr_value
0                   AATS0Z   -0.027037
1                 AATS0are   -0.129823
2                   AATS0d    0.042740
3                  AATS0dv   -0.120173
```

```
4                   AATS0i    0.132395
  molecular descriptor name  corr_value  absolute correlation value
0                   AATS0Z   -0.027037                    0.027037
1                 AATS0are   -0.129823                    0.129823
2                   AATS0d    0.042740                    0.042740
3                  AATS0dv   -0.120173                    0.120173
4                   AATS0i    0.132395                    0.132395
     molecular descriptor name  corr_value  absolute correlation value
226                     AMID_O   -0.524157                    0.524157
505                EState_VSA5   -0.579664                    0.579664
791                    MDEO-12   -0.558727                    0.558727
851                      NdssC   -0.506855                    0.506855
1091               VSA_EState5    0.503578                    0.503578
     molecular descriptor name  corr_value  absolute correlation value
226                     AMID_O   -0.524157                    0.524157
505                EState_VSA5   -0.579664                    0.579664
791                    MDEO-12   -0.558727                    0.558727
851                      NdssC   -0.506855                    0.506855
1091               VSA_EState5    0.503578                    0.503578
The model used is: LinearReg…
Return the coefficient of determination of the prediction:
0.5938815939305324
R^2 score: 0.4715701900042242
Correlation coefficient: 0.6867096839307162
Test data - unseen during training:
R^2 score: 0.5938815939305324
Correlation coefficient: 0.7706371350580845
[7.89387822 6.432389   7.40284432 7.9722247  8.04392842 8.352564
 8.2751695  8.09665023 8.17323658 7.66460935 7.09914951 7.80818739
 7.60333465 7.91870918 8.31092654 8.28306099 6.64762499 6.08809477]
102     8.000000
38      6.104025
8       6.044360
109     7.886057
11      8.107905
51      8.000000
88      8.886057
21      7.554396
82      8.301030
98      7.568636
58      7.677781
64      8.327902
74      8.070581
103     8.136677
91     10.000000
43      7.886057
25      5.221704
30      6.315155
```

```
Name: LoVo, dtype: float64
Training Root Mean Square Error: 0.6300116411653003
Testing Root Mean Square Error: 0.6975864260393481
```

[6]:
```python
print(target_column_name+str('_transformed'))
print(hist1[target_column_name].hist())
```

```
LoVo_transformed
AxesSubplot(0.125,0.11;0.775x0.77)
```

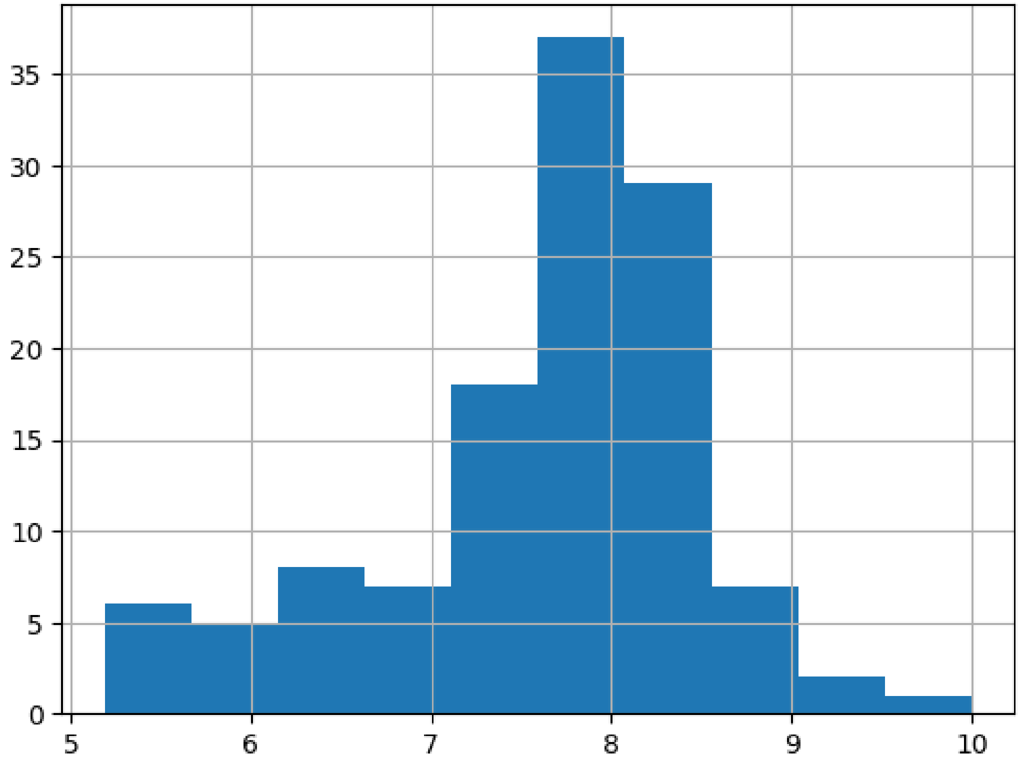

[7]:
```python
with_standardization, train_r2_, test_r2_, hist1, hist2, target_column_name,␣
 ↪training_data_RMSE, test_data_RMSE  = pred_model.
 ↪prepare_data_and_create_model(molecular_descriptors_df=data,
                                                                              ␣
 ↪                       correlation_threshold=0.50,
                                                                              ␣
 ↪                       standardization=True,
                                                                              ␣
 ↪                       model_type='linear_model',
                                                                              ␣
 ↪                       target_column_name = target,
```

```
↪                    random_state=random_state,
↪                    train_test_split_=True,
↪                    verbose=True)
```

```
I am doing standardization…
  molecular descriptor name
0                    AATS0Z
1                  AATS0are
2                    AATS0d
3                   AATS0dv
4                    AATS0i
  molecular descriptor name  corr_value
0                    AATS0Z   -0.027037
1                  AATS0are   -0.129823
2                    AATS0d    0.042740
3                   AATS0dv   -0.120173
4                    AATS0i    0.132395
  molecular descriptor name  corr_value  absolute correlation value
0                    AATS0Z   -0.027037                    0.027037
1                  AATS0are   -0.129823                    0.129823
2                    AATS0d    0.042740                    0.042740
3                   AATS0dv   -0.120173                    0.120173
4                    AATS0i    0.132395                    0.132395
     molecular descriptor name  corr_value  absolute correlation value
226                     AMID_O   -0.524157                    0.524157
505                 EState_VSA5   -0.579664                    0.579664
791                    MDEO-12   -0.558727                    0.558727
851                     NdssC   -0.506855                    0.506855
1091               VSA_EState5    0.503578                    0.503578
     molecular descriptor name  corr_value  absolute correlation value
226                     AMID_O   -0.524157                    0.524157
505                 EState_VSA5   -0.579664                    0.579664
791                    MDEO-12   -0.558727                    0.558727
851                     NdssC   -0.506855                    0.506855
1091               VSA_EState5    0.503578                    0.503578
The model used is: LinearReg…
Return the coefficient of determination of the prediction:
0.5938815939305324
R^2 score: 0.4715701900042242
Correlation coefficient: 0.6867096839307162
Test data - unseen during training:
R^2 score: 0.5938815939305324
Correlation coefficient: 0.7706371350580845
[7.89387822 6.432389   7.40284432 7.9722247  8.04392842 8.352564
```

```
 8.2751695  8.09665023 8.17323658 7.66460935 7.09914951 7.80818739
 7.60333465 7.91870918 8.31092654 8.28306099 6.64762499 6.08809477]
102     8.000000
38      6.104025
8       6.044360
109     7.886057
11      8.107905
51      8.000000
88      8.886057
21      7.554396
82      8.301030
98      7.568636
58      7.677781
64      8.327902
74      8.070581
103     8.136677
91     10.000000
43      7.886057
25      5.221704
30      6.315155
Name: LoVo, dtype: float64
Training Root Mean Square Error: 0.6300116411653003
Testing Root Mean Square Error: 0.6975864260393481
```

2.1 Search inside correlation space

[8]:
```
step = 0.01
initial_step = corr_low
last_step = corr_high
first_list = [x / 100.0 for x in range(int(initial_step*100),␣
 ↪int(last_step*100), int(step*100))]
second_list = []
third_list = []
fourth_l = []
fifth_l = []
f_list = []
for i in first_list:
    without_standardization, train_r2, test_r2, _, h_, target_column_name,␣
 ↪training_data_RMSE, test_data_RMSE = pred_model.
 ↪prepare_data_and_create_model(molecular_descriptors_df = data,
                                                                        ␣
 ↪                     correlation_threshold = i,
                                                                        ␣
 ↪                     standardization = False,
                                                                        ␣
 ↪                     model_type = 'linear_model',
```

```
                              target_column_name = target,
                              random_state=random_state,
                              train_test_split_ = True,
                              verbose = False)
    second_list.append(train_r2)
    third_list.append(test_r2)
    fourth_l.append(training_data_RMSE)
    fifth_l.append(test_data_RMSE)
    f_list.append(len(h_))
```

[9]:
```
df_without_standardization = pd.DataFrame(data=first_list, columns=["Correlation threshold"])
df_without_standardization['Training data R^2 score'] = second_list
df_without_standardization['Test data R^2 score'] = third_list
df_without_standardization['Training RMSE'] = fourth_l
df_without_standardization['Test RMSE'] = fifth_l
df_without_standardization['Number of features'] = f_list
```

[10]:
```
df_linear = df_without_standardization.copy()
df_without_standardization
```

[10]:

	Correlation threshold	Training data R^2 score	Test data R^2 score	\
0	0.33	0.901684	0.604189	
1	0.34	0.858631	0.550573	
2	0.35	0.850762	0.541115	
3	0.36	0.802103	0.482693	
4	0.37	0.727069	0.551007	
5	0.38	0.688721	0.662709	
6	0.39	0.659170	0.659592	
7	0.40	0.647360	0.630994	
8	0.41	0.647315	0.629807	
9	0.42	0.605225	0.567152	
10	0.43	0.605202	0.566682	
11	0.44	0.605202	0.566682	
12	0.45	0.583293	0.576643	
13	0.46	0.549192	0.597857	
14	0.47	0.484158	0.582995	
15	0.48	0.471582	0.594438	
16	0.49	0.471570	0.593882	
17	0.50	0.471570	0.593882	
18	0.51	0.448566	0.575939	
19	0.52	0.448566	0.575939	
20	0.53	0.439344	0.526231	

```
21                     0.54                  0.439344              0.526231

    Training RMSE  Test RMSE  Number of features
0        0.271749   0.688677                  63
1        0.325861   0.733840                  57
2        0.334807   0.741521                  52
3        0.385544   0.787310                  47
4        0.452774   0.733486                  39
5        0.483537   0.635732                  35
6        0.505969   0.638662                  27
7        0.514660   0.664949                  22
8        0.514693   0.666018                  21
9        0.544540   0.720177                  18
10       0.544556   0.720568                  17
11       0.544556   0.720568                  17
12       0.559462   0.712238                  14
13       0.581903   0.694164                  10
14       0.622463   0.706874                   7
15       0.630005   0.697108                   6
16       0.630012   0.697586                   5
17       0.630012   0.697586                   5
18       0.643579   0.712829                   3
19       0.643579   0.712829                   3
20       0.648938   0.753451                   2
21       0.648938   0.753451                   2
```

[ ]:

2.2 Plots

[11]:
```
plt.plot(df_without_standardization['Correlation threshold'],␣
 ↪df_without_standardization['Training data R^2 score'], label = "Training␣
 ↪data R^2 score", marker='v')
plt.plot(df_without_standardization['Correlation threshold'],␣
 ↪df_without_standardization['Test data R^2 score'], label = "Test data R^2␣
 ↪score", marker='D')
plt.legend()
plt.xlabel('Correlation threshold')
plt.ylabel('R^2 score')
plt.ylim([0, 1])
plt.show()
```

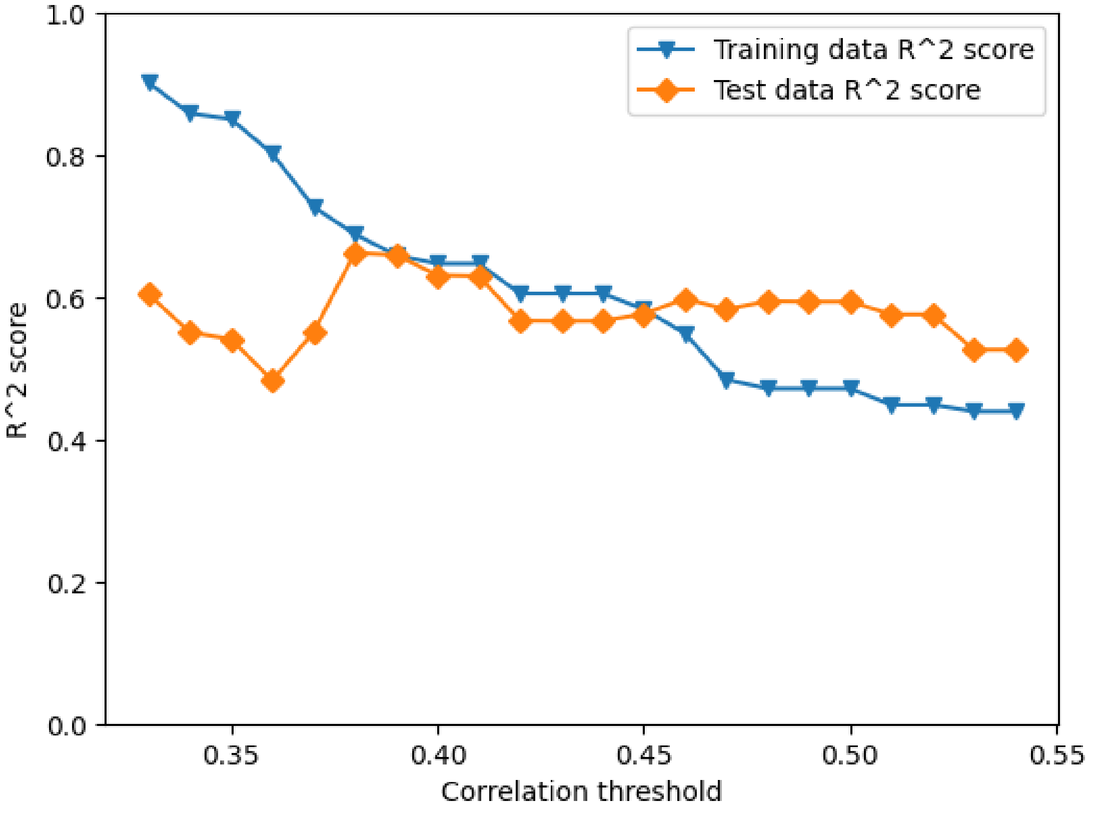

[12]:
```
plt.plot(df_without_standardization['Correlation threshold'],␣
↪df_without_standardization['Number of features'], label = "Number of␣
↪features", marker='.')
plt.legend()
plt.xlabel('Correlation threshold')
plt.ylabel('Number of features')
plt.show()
```

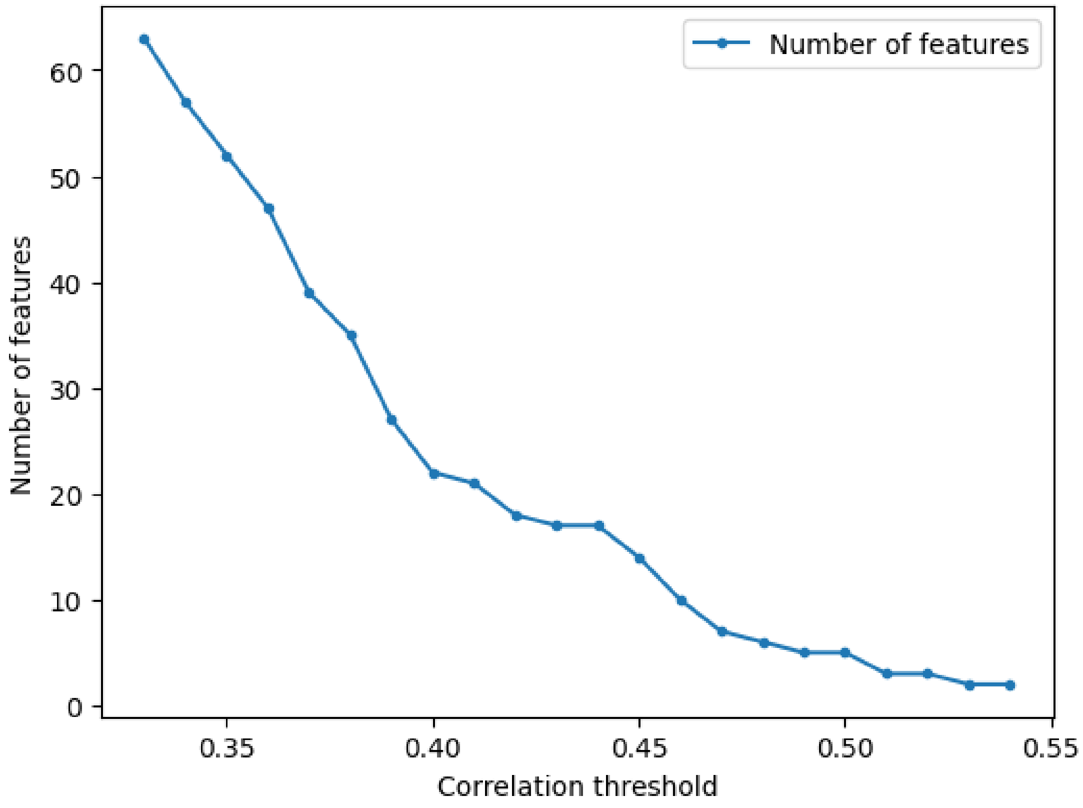

[ ]:

2.3 Decision Tree

[13]:
```
without_standardization, train_r2_, test_r2_, hist1, hist2, target_column_name,␣
↪training_data_RMSE, test_data_RMSE  = pred_model.
↪prepare_data_and_create_model(molecular_descriptors_df=data,
                                                                              ␣
↪                          correlation_threshold=0.50,
                                                                              ␣
↪                          standardization=False,
                                                                              ␣
↪                          model_type='DecisionTreeRegressor',
                                                                              ␣
↪                          max_depth=5,
                                                                              ␣
↪                          target_column_name = target,
                                                                              ␣
↪                          random_state=random_state,
```

```
                        train_test_split_=True,
                        verbose=True)
```

```
I am not doing standardization…
  molecular descriptor name
0                    AATS0Z
1                  AATS0are
2                    AATS0d
3                   AATS0dv
4                    AATS0i
  molecular descriptor name  corr_value
0                    AATS0Z   -0.027037
1                  AATS0are   -0.129823
2                    AATS0d    0.042740
3                   AATS0dv   -0.120173
4                    AATS0i    0.132395
  molecular descriptor name  corr_value  absolute correlation value
0                    AATS0Z   -0.027037                    0.027037
1                  AATS0are   -0.129823                    0.129823
2                    AATS0d    0.042740                    0.042740
3                   AATS0dv   -0.120173                    0.120173
4                    AATS0i    0.132395                    0.132395
     molecular descriptor name  corr_value  absolute correlation value
226                     AMID_O   -0.524157                    0.524157
505                EState_VSA5   -0.579664                    0.579664
791                    MDEO-12   -0.558727                    0.558727
851                      NdssC   -0.506855                    0.506855
1091               VSA_EState5    0.503578                    0.503578
     molecular descriptor name  corr_value  absolute correlation value
226                     AMID_O   -0.524157                    0.524157
505                EState_VSA5   -0.579664                    0.579664
791                    MDEO-12   -0.558727                    0.558727
851                      NdssC   -0.506855                    0.506855
1091               VSA_EState5    0.503578                    0.503578
The model used is: DecisionTree…
Return the coefficient of determination of the prediction:
0.31900598680762904
R^2 score: 0.8318545988389252
Correlation coefficient: 0.9120606333127887
Test data - unseen during training:
R^2 score: 0.31900598680762904
Correlation coefficient: 0.5648061497608087
[7.75784502 5.82126536 7.65111515 8.18929441 8.49920533 7.33370906
 8.18929441 7.33370906 8.18929441 8.49920533 7.04932534 7.75784502
 8.49920533 7.75784502 7.33370906 8.18929441 5.82126536 5.19341307]
102     8.000000
```

```
38      6.104025
8       6.044360
109     7.886057
11      8.107905
51      8.000000
88      8.886057
21      7.554396
82      8.301030
98      7.568636
58      7.677781
64      8.327902
74      8.070581
103     8.136677
91     10.000000
43      7.886057
25      5.221704
30      6.315155
Name: LoVo, dtype: float64
Training Root Mean Square Error: 0.3553836279194085
Testing Root Mean Square Error: 0.9033235482494083
```

[14]:
```
print(target_column_name+str('_transformed'))
print(hist1[target_column_name].hist())
```

```
LoVo_transformed
AxesSubplot(0.125,0.11;0.775x0.77)
```

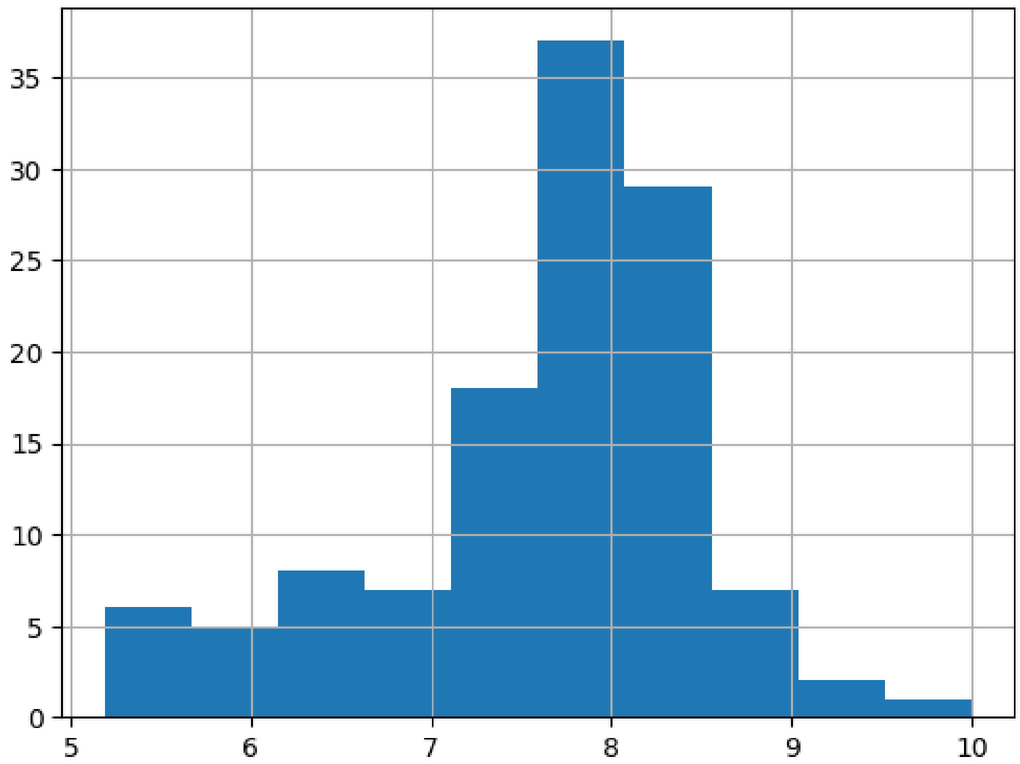


```
[15]: with_standardization, train_r2_, test_r2_, hist1, hist2, target_column_name,␣
  ↪training_data_RMSE, test_data_RMSE  = pred_model.
  ↪prepare_data_and_create_model(molecular_descriptors_df=data,                                                                                               ␣
  ↪                        correlation_threshold=0.50,                                                                                               ␣
  ↪                        standardization=True,                                                                                               ␣
  ↪                        model_type='DecisionTreeRegressor',                                                                                               ␣
  ↪                        max_depth=5,                                                                                               ␣
  ↪                        target_column_name = target,                                                                                               ␣
  ↪                        random_state=random_state,                                                                                               ␣
  ↪                        train_test_split_=True,                                                                                               ␣
  ↪                        verbose=True)
```

```
I am doing standardization…
  molecular descriptor name
```

```
0                   AATS0Z
1                 AATS0are
2                   AATS0d
3                  AATS0dv
4                   AATS0i
  molecular descriptor name  corr_value
0                   AATS0Z   -0.027037
1                 AATS0are   -0.129823
2                   AATS0d    0.042740
3                  AATS0dv   -0.120173
4                   AATS0i    0.132395
  molecular descriptor name  corr_value  absolute correlation value
0                   AATS0Z   -0.027037                    0.027037
1                 AATS0are   -0.129823                    0.129823
2                   AATS0d    0.042740                    0.042740
3                  AATS0dv   -0.120173                    0.120173
4                   AATS0i    0.132395                    0.132395
     molecular descriptor name  corr_value  absolute correlation value
226                     AMID_O   -0.524157                    0.524157
505                 EState_VSA5   -0.579664                    0.579664
791                    MDEO-12   -0.558727                    0.558727
851                      NdssC   -0.506855                    0.506855
1091               VSA_EState5    0.503578                    0.503578
     molecular descriptor name  corr_value  absolute correlation value
226                     AMID_O   -0.524157                    0.524157
505                 EState_VSA5   -0.579664                    0.579664
791                    MDEO-12   -0.558727                    0.558727
851                      NdssC   -0.506855                    0.506855
1091               VSA_EState5    0.503578                    0.503578
The model used is: DecisionTree…
Return the coefficient of determination of the prediction:
0.31900598680762904
R^2 score: 0.8318545988389252
Correlation coefficient: 0.9120606333127887
Test data - unseen during training:
R^2 score: 0.31900598680762904
Correlation coefficient: 0.5648061497608087
[7.75784502 5.82126536 7.65111515 8.18929441 8.49920533 7.33370906
 8.18929441 7.33370906 8.18929441 8.49920533 7.04932534 7.75784502
 8.49920533 7.75784502 7.33370906 8.18929441 5.82126536 5.19341307]
102     8.000000
38      6.104025
8       6.044360
109     7.886057
11      8.107905
51      8.000000
88      8.886057
21      7.554396
```

```
82      8.301030
98      7.568636
58      7.677781
64      8.327902
74      8.070581
103     8.136677
91     10.000000
43      7.886057
25      5.221704
30      6.315155
Name: LoVo, dtype: float64
Training Root Mean Square Error: 0.3553836279194085
Testing Root Mean Square Error: 0.9033235482494083
```

2.4 Search inside correlation space

[16]:
```
step = 0.01
initial_step = corr_low
last_step = corr_high
first_list = [x / 100.0 for x in range(int(initial_step*100),␣
 ↪int(last_step*100), int(step*100))]
max_depth = [range(2, 30, 1)]
corr_th = []
second_list = []
third_list = []
fourth_l = []
fifth_l = []
f_list = []
fif_list = []
for i in first_list:
    for depth in max_depth[0]:

        without_standardization, train_r2, test_r2, _, h_, target_column_name,␣
 ↪training_data_RMSE, test_data_RMSE = pred_model.
 ↪prepare_data_and_create_model(molecular_descriptors_df=data,
                                                                              ␣
 ↪                    correlation_threshold=i,
                                                                              ␣
 ↪                    standardization=False,
                                                                              ␣
 ↪                    model_type='DecisionTreeRegressor',
                                                                              ␣
 ↪                    max_depth=depth,
                                                                              ␣
 ↪                    target_column_name = target,
                                                                              ␣
 ↪                    random_state=random_state,
```

```
                      train_test_split_=True,
                      verbose=False)
        corr_th.append(i)
        second_list.append(train_r2)
        third_list.append(test_r2)
        fourth_l.append(training_data_RMSE)
        fifth_l.append(test_data_RMSE)
        f_list.append(len(h_))
        fif_list.append(depth)
```

[17]:
```
df_without_standardization = pd.DataFrame(data=corr_th, columns=["Correlation threshold"])
df_without_standardization['Training data R^2 score'] = second_list
df_without_standardization['Test data R^2 score'] = third_list
df_without_standardization['Training RMSE'] = fourth_l
df_without_standardization['Test RMSE'] = fifth_l
df_without_standardization['Number of features'] = f_list
df_without_standardization['Depth number'] = fif_list
```

[18]:
```
df_decision_tree = df_without_standardization.copy()
#df_without_standardization.to_excel('../Data/A549_Decision_tree_rs_'+str(random_state)+'.xlsx')
```

2.5 Plots

```
[19]: corre_list = [x / 100.0 for x in range(int(initial_step*100),␣
      ↪int(last_step*100), int(0.01*100))]
      for element in corre_list:
          element_ =␣
       ↪df_without_standardization[df_without_standardization['Correlation␣
       ↪threshold'] == float(element)]
          plt.plot(element_['Depth number'], element_['Training data R^2 score'],␣
       ↪label = "Training data R^2 score", marker='v')
          plt.plot(element_['Depth number'], element_['Test data R^2 score'], label =␣
       ↪"Test data R^2 score", marker='D')
          plt.legend()
          plt.xlabel('Depth number -> correlation threshold: '+str(element)+'. \n␣
       ↪Number of features: '+str(element_['Number of features'].iloc[0]),␣
       ↪fontweight='bold')
          plt.xlim(max(element_['Depth number'])+1, min(element_['Depth number'])-1)
          plt.ylabel('R^2 score')
          plt.ylim([0, 1])
          plt.rc('grid', linestyle="-.", color='black')
          plt.grid(True)
          plt.show()
```

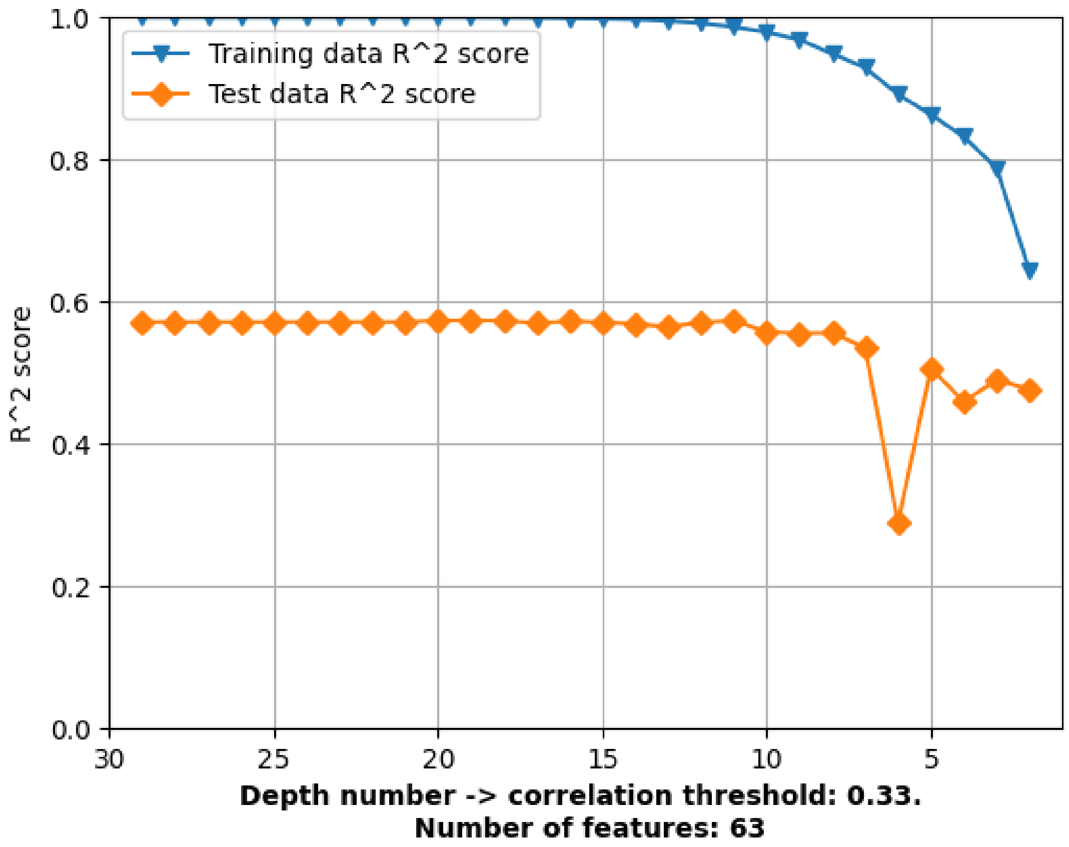

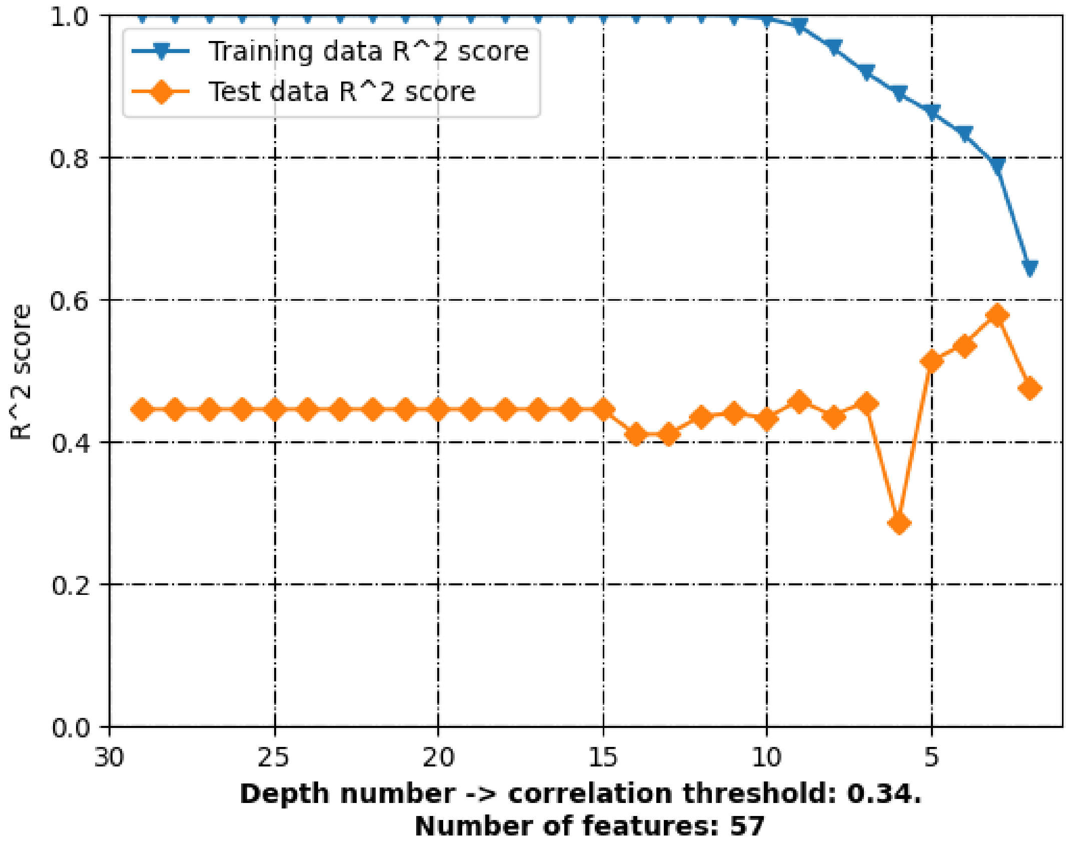

Training data R^2 score
Test data R^2 score
R^2 score
Depth number -> correlation threshold: 0.34.
Number of features: 57

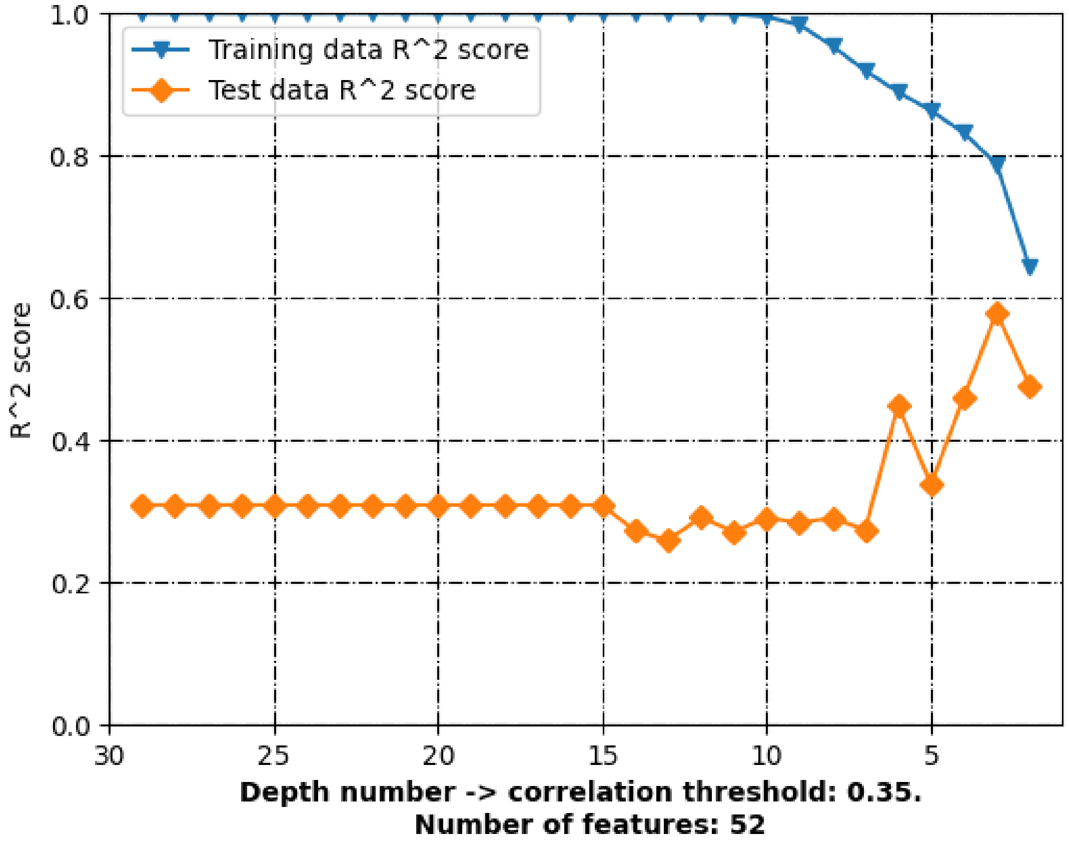

Training data R^2 score
Test data R^2 score
R^2 score
1.0
0.8
0.6
0.4
0.2
0.0
30
25
20
15
10
5
Depth number -> correlation threshold: 0.35.
Number of features: 52

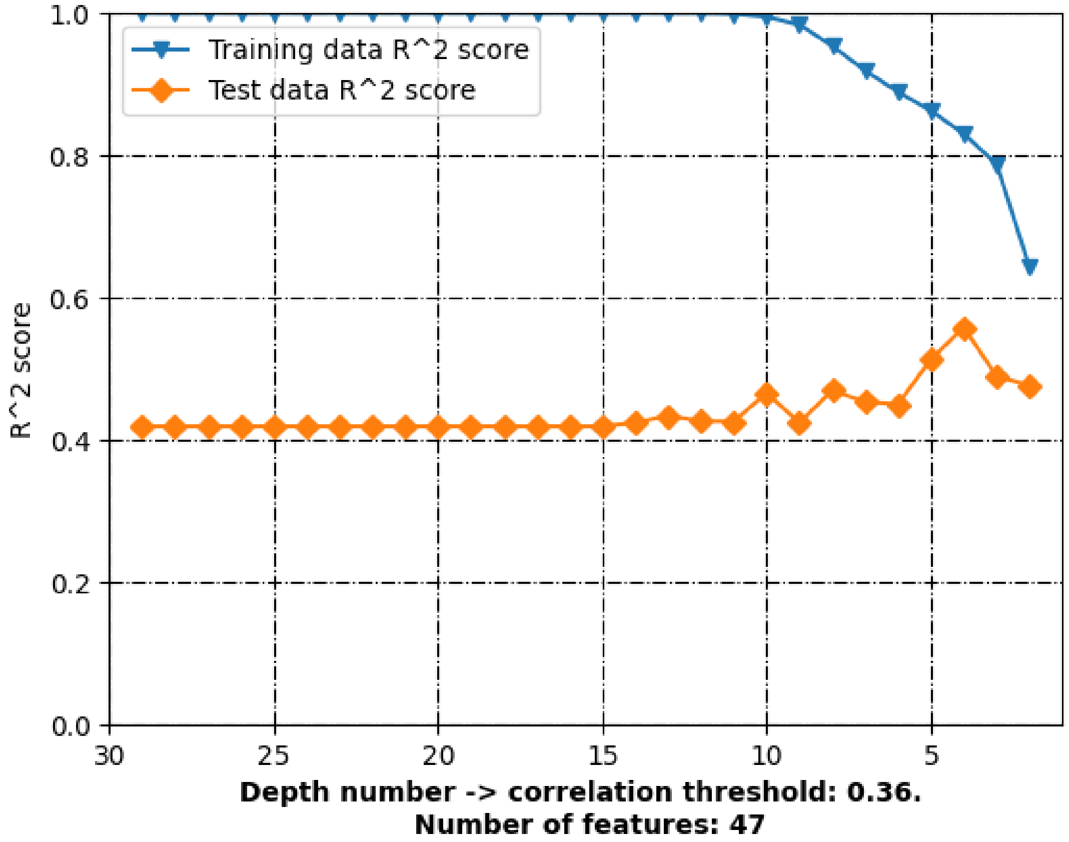

1.0
0.8
0.6
0.4
0.2
0.0
R^2 score
30
25
20
15
10
5
Training data R^2 score
Test data R^2 score
Depth number -> correlation threshold: 0.36.
Number of features: 47

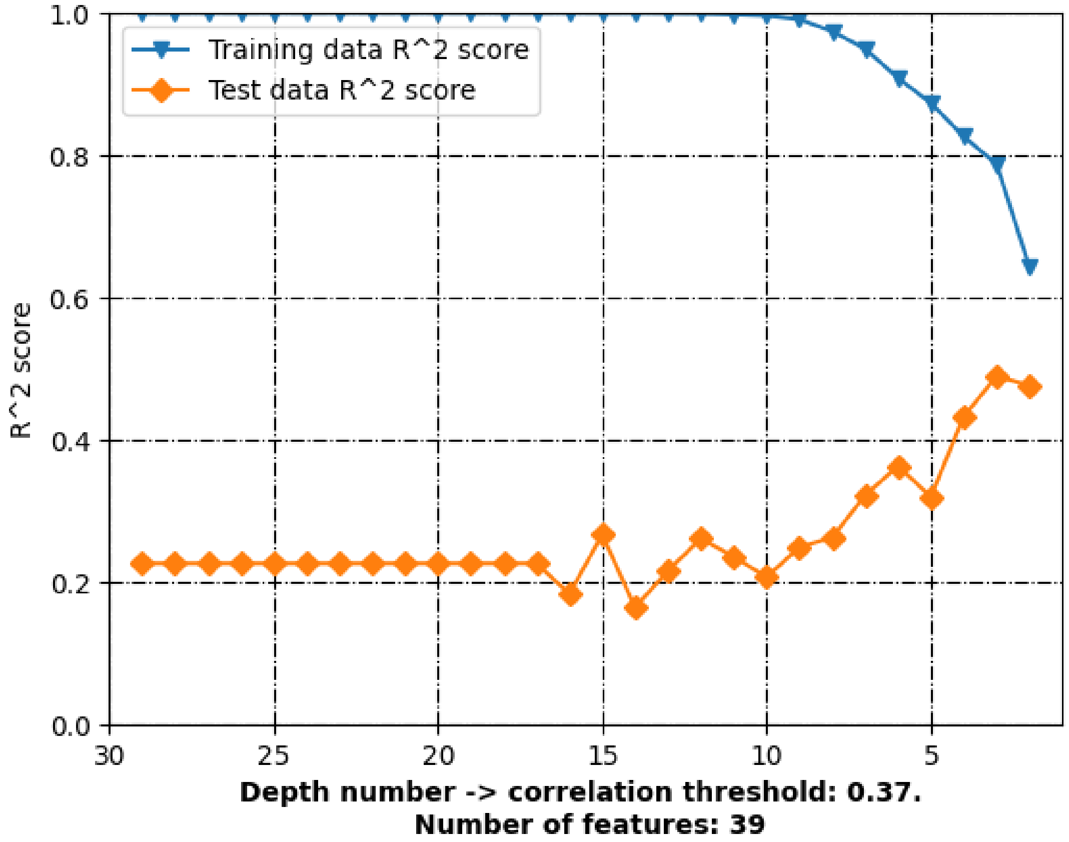

Training data R^2 score
Test data R^2 score
R^2 score
1.0
0.8
0.6
0.4
0.2
0.0
30
25
20
15
10
5
Depth number -> correlation threshold: 0.37.
Number of features: 39

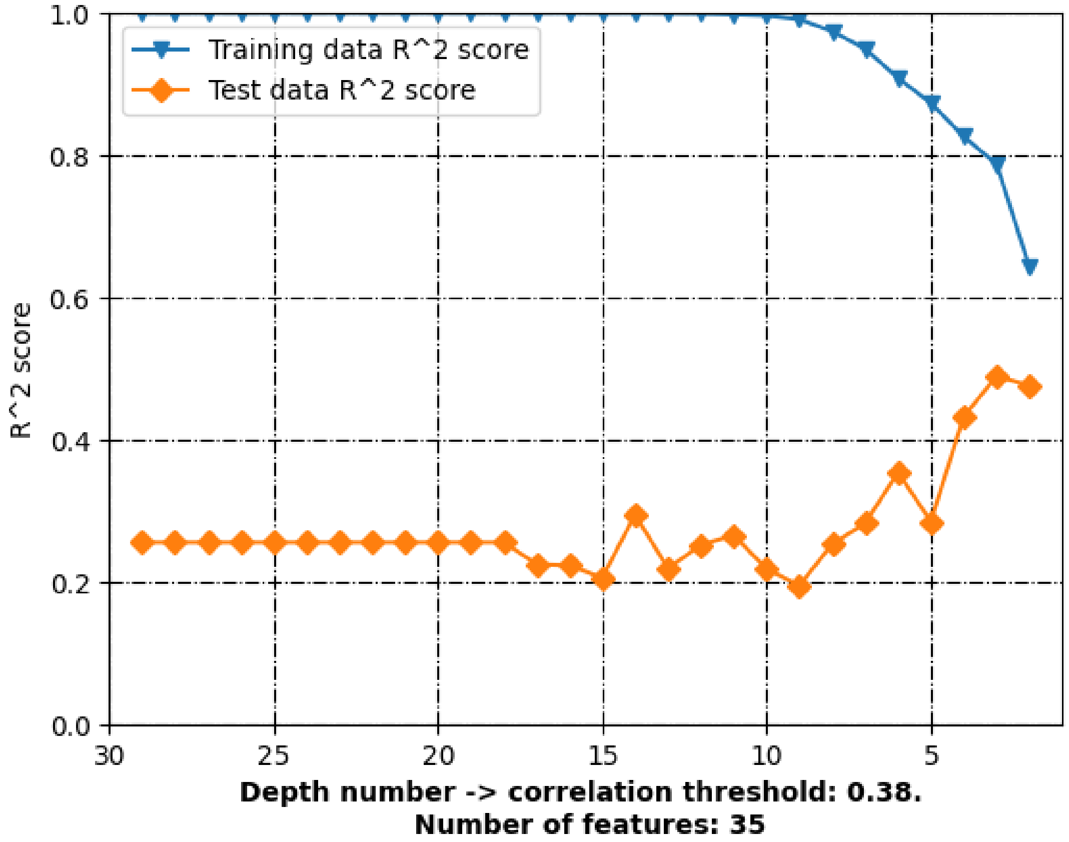
Training data R^2 score
Test data R^2 score
R^2 score
1.0
0.8
0.6
0.4
0.2
0.0
30
25
20
15
10
5
Depth number -> correlation threshold: 0.38.
Number of features: 35

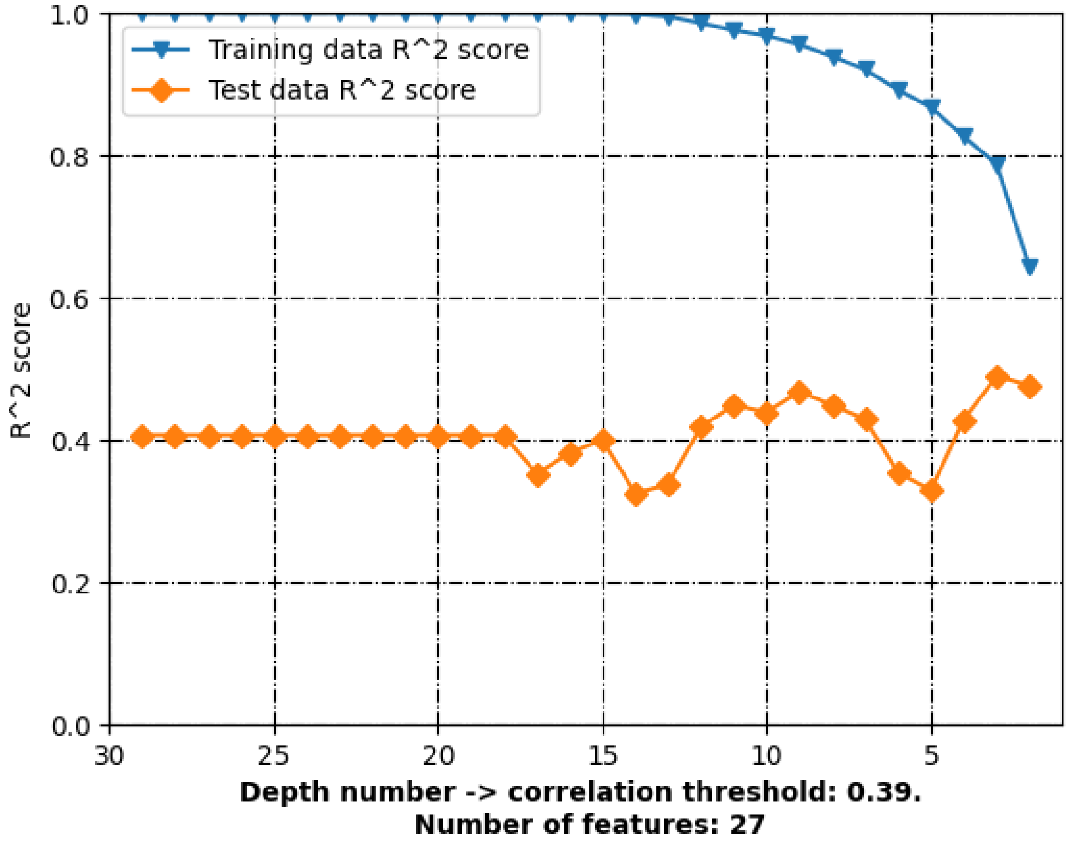
1.0
0.8
0.6
0.4
0.2
0.0
R^2 score
Training data R^2 score
Test data R^2 score
30
25
20
15
10
5
Depth number -> correlation threshold: 0.39.
Number of features: 27

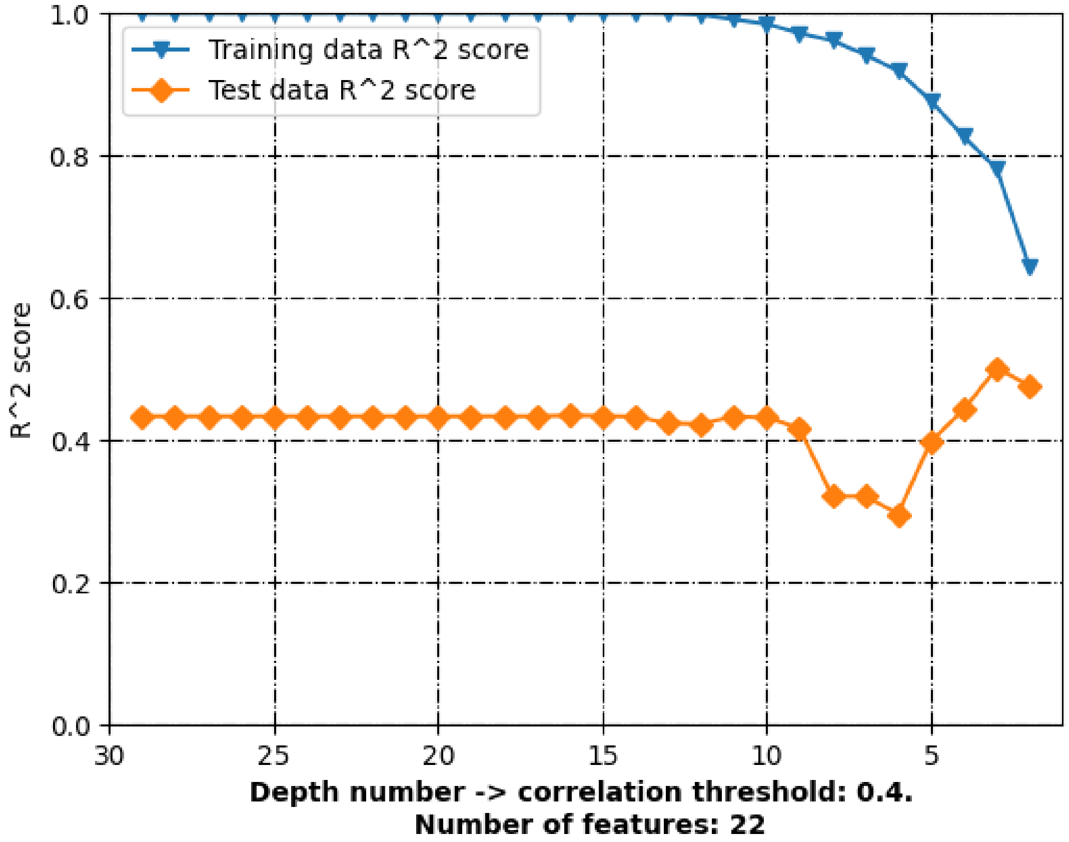
1.0
0.8
0.6
0.4
0.2
0.0
R^2 score
Training data R^2 score
Test data R^2 score
30
25
20
15
10
5
Depth number -> correlation threshold: 0.4.
Number of features: 22

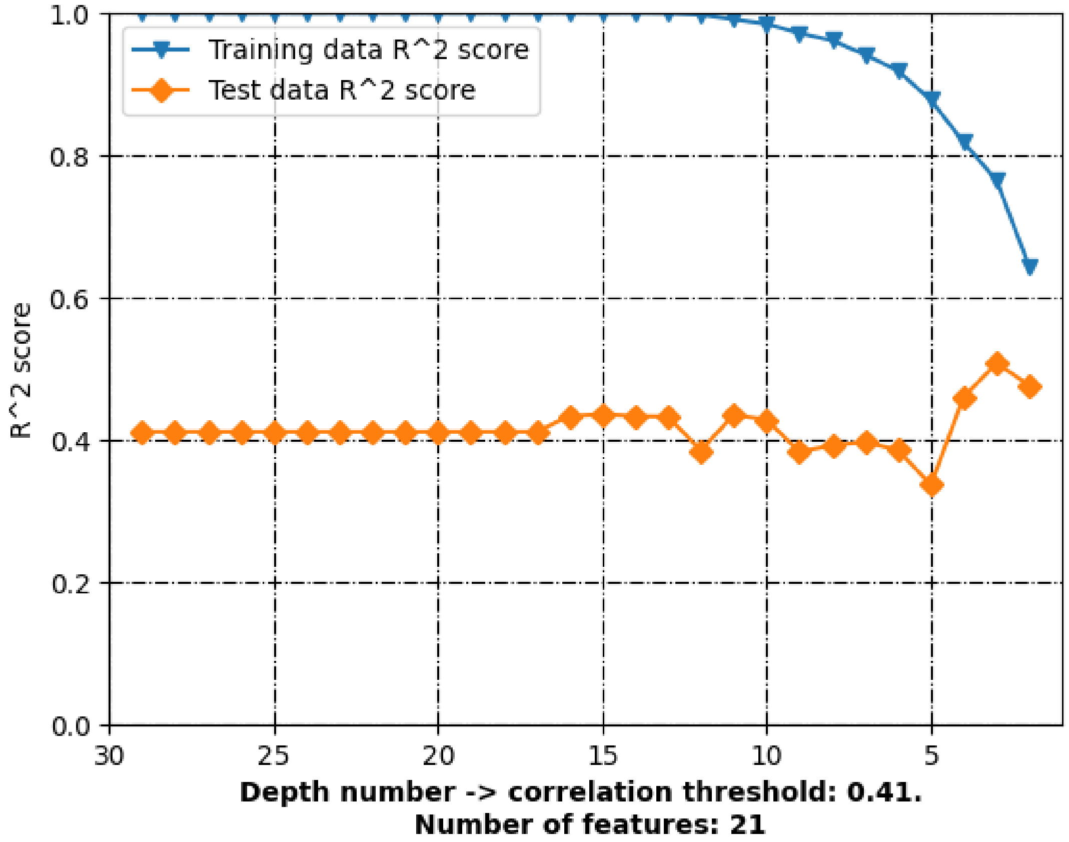

Training data R^2 score
Test data R^2 score
R^2 score
1.0
0.8
0.6
0.4
0.2
0.0
30
25
20
15
10
5
Depth number -> correlation threshold: 0.41.
Number of features: 21

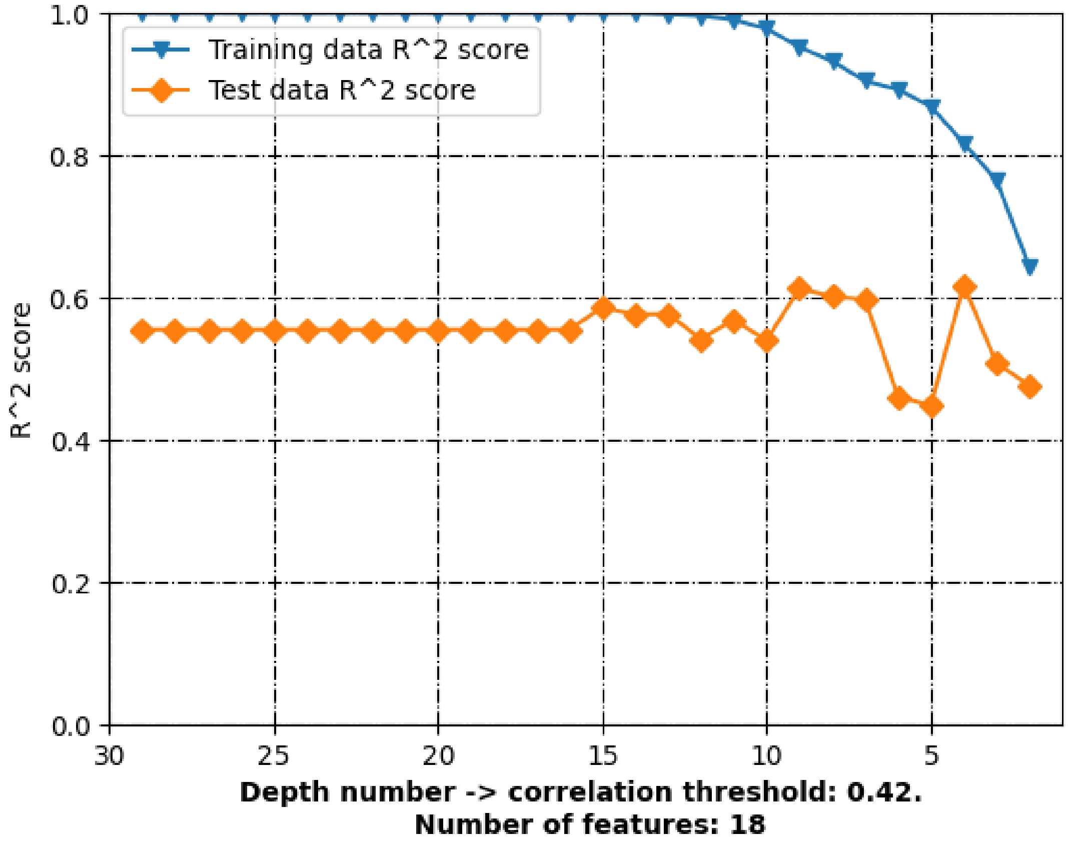
Training data R^2 score
Test data R^2 score
R^2 score
1.0
0.8
0.6
0.4
0.2
0.0
30
25
20
15
10
5
Depth number -> correlation threshold: 0.42.
Number of features: 18

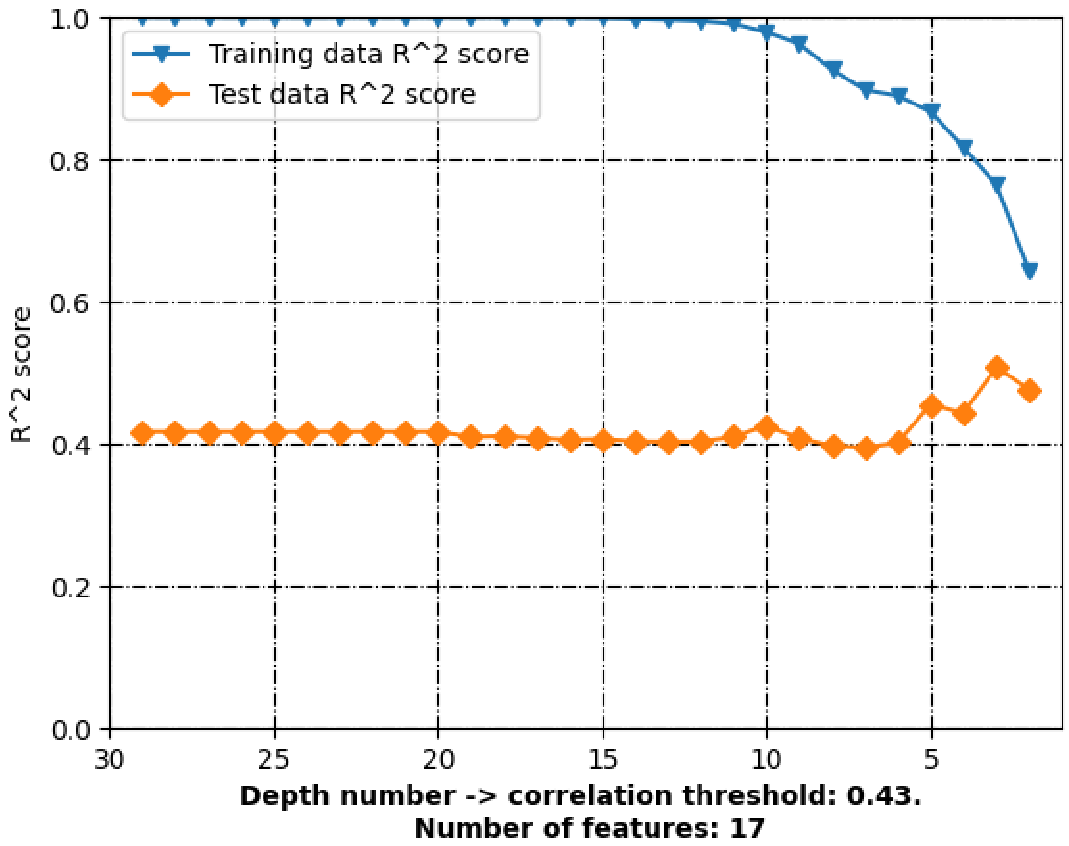
Training data R^2 score
Test data R^2 score
R^2 score
1.0
0.8
0.6
0.4
0.2
0.0
30
25
20
15
10
5
Depth number -> correlation threshold: 0.43.
Number of features: 17

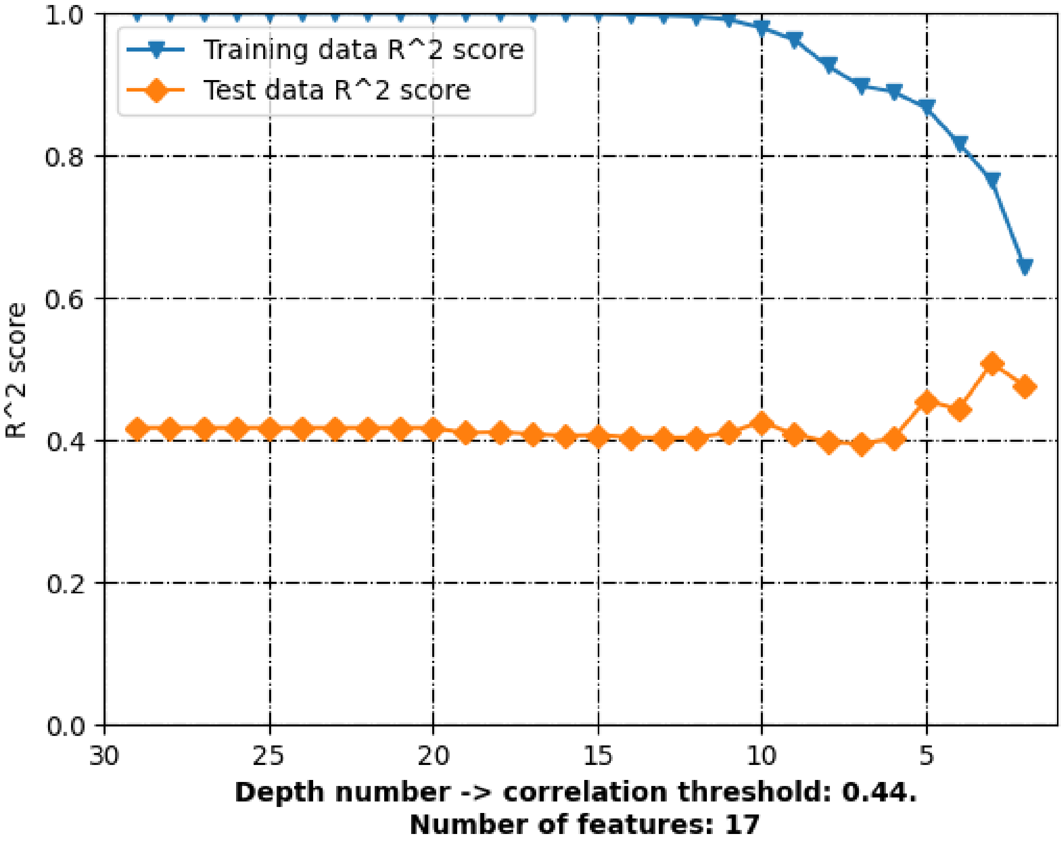

Training data R^2 score
Test data R^2 score
R^2 score
1.0
0.8
0.6
0.4
0.2
0.0
30
25
20
15
10
5
Depth number -> correlation threshold: 0.44.
Number of features: 17

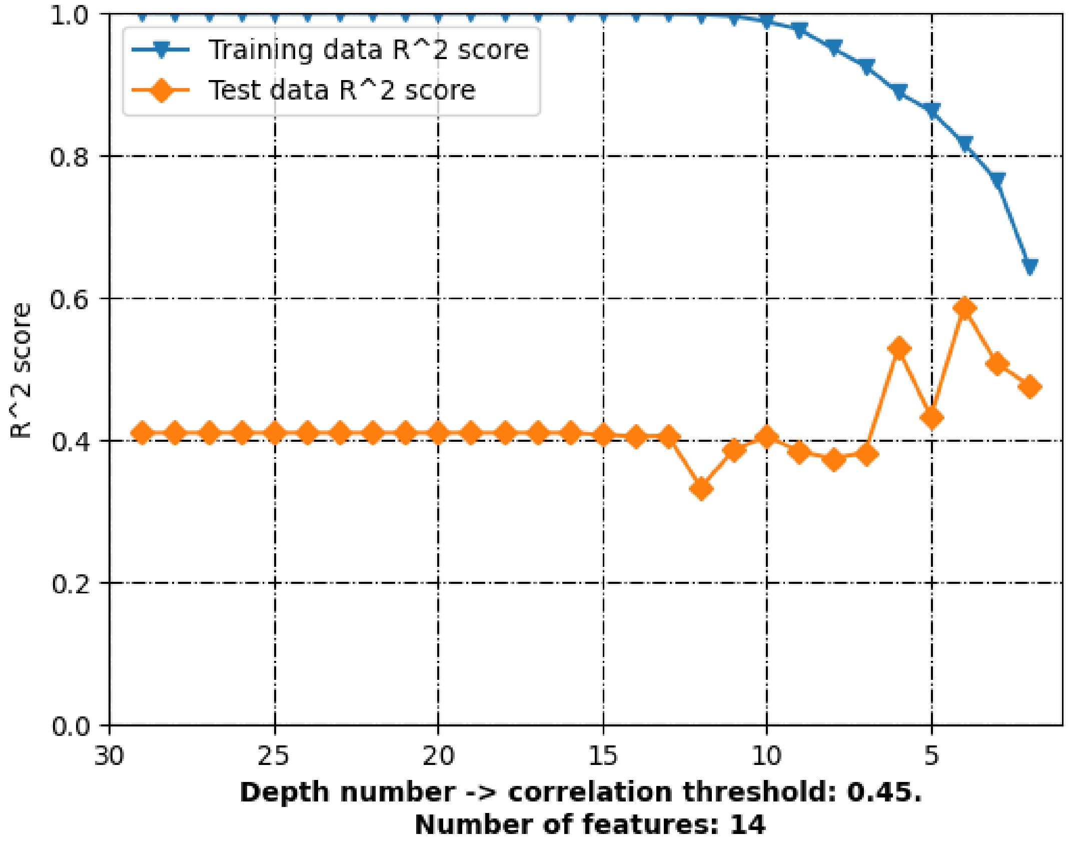
Training data R^2 score
Test data R^2 score
R^2 score
1.0
0.8
0.6
0.4
0.2
0.0
30
25
20
15
10
5
Depth number -> correlation threshold: 0.45.
Number of features: 14

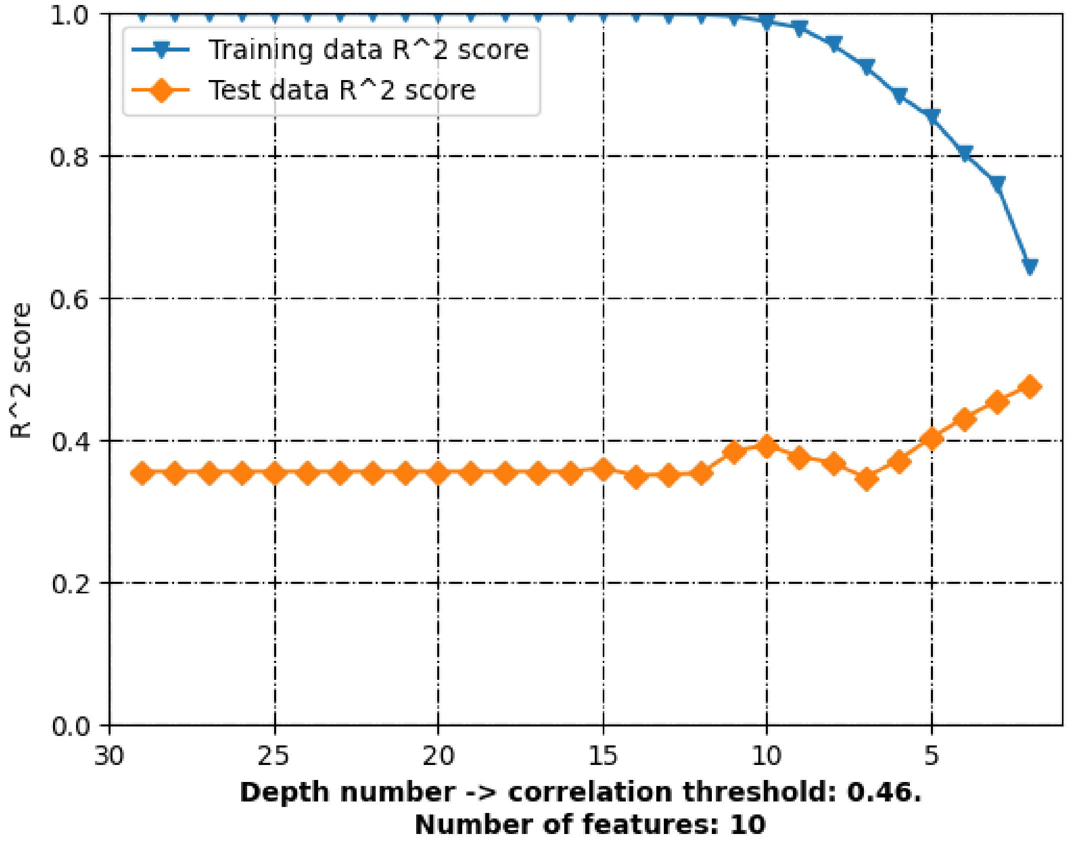

Training data R^2 score
Test data R^2 score
R^2 score
1.0
0.8
0.6
0.4
0.2
0.0
30
25
20
15
10
5
Depth number -> correlation threshold: 0.46.
Number of features: 10

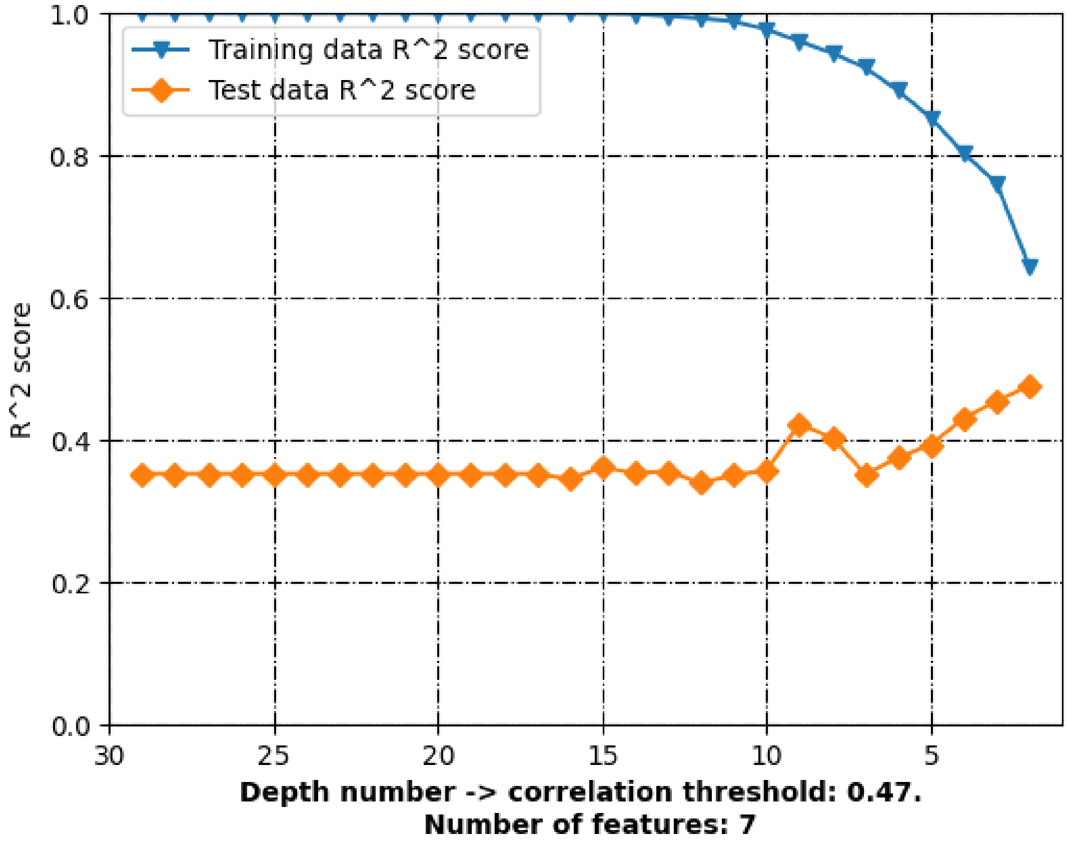
1.0
0.8
0.6
0.4
0.2
0.0
R^2 score
Training data R^2 score
Test data R^2 score
30
25
20
15
10
5
Depth number -> correlation threshold: 0.47.
Number of features: 7

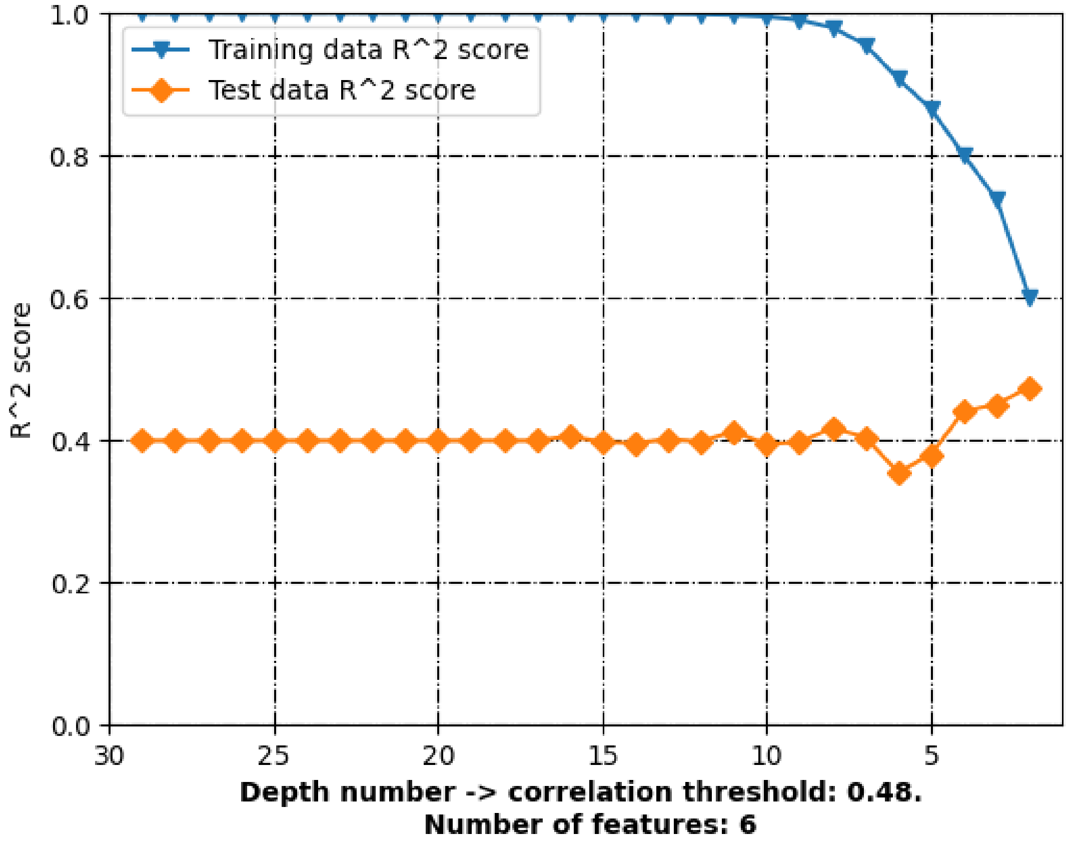

Training data R^2 score
Test data R^2 score
R^2 score
1.0
0.8
0.6
0.4
0.2
0.0
30
25
20
15
10
5
Depth number -> correlation threshold: 0.48.
Number of features: 6

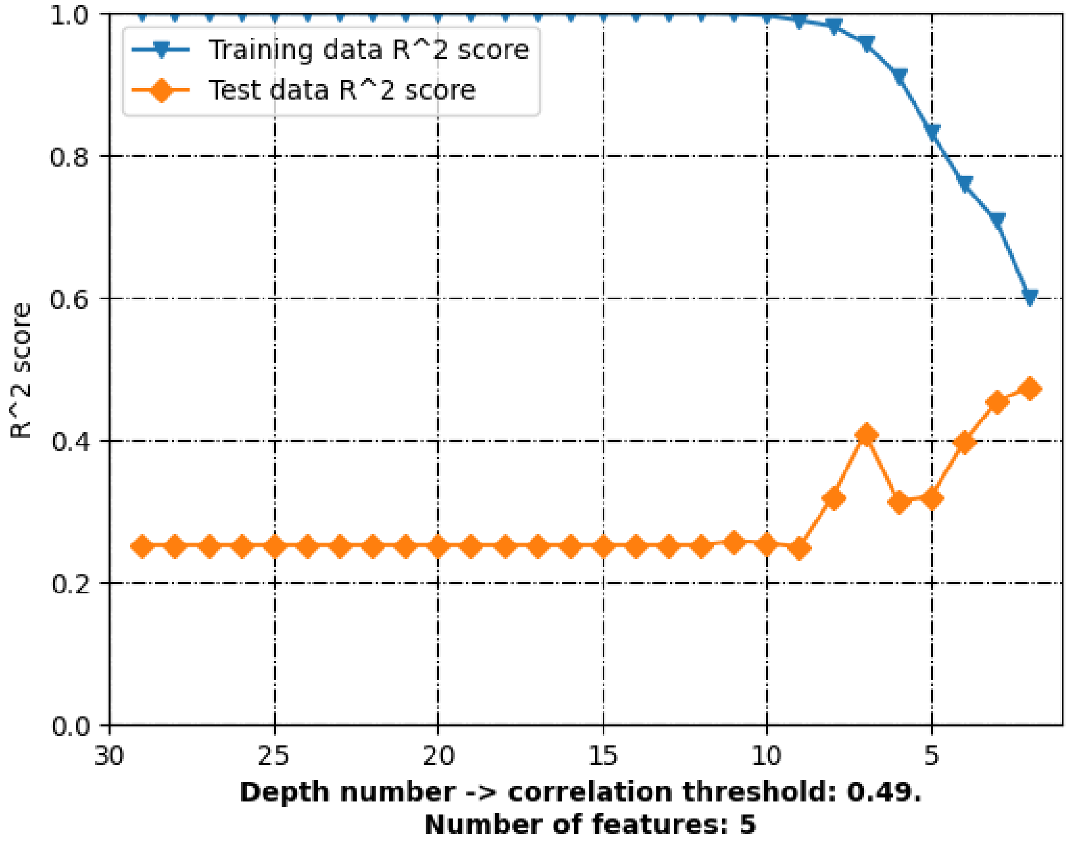
Training data R^2 score
Test data R^2 score
R^2 score
1.0
0.8
0.6
0.4
0.2
0.0
30
25
20
15
10
5
Depth number -> correlation threshold: 0.49.
Number of features: 5

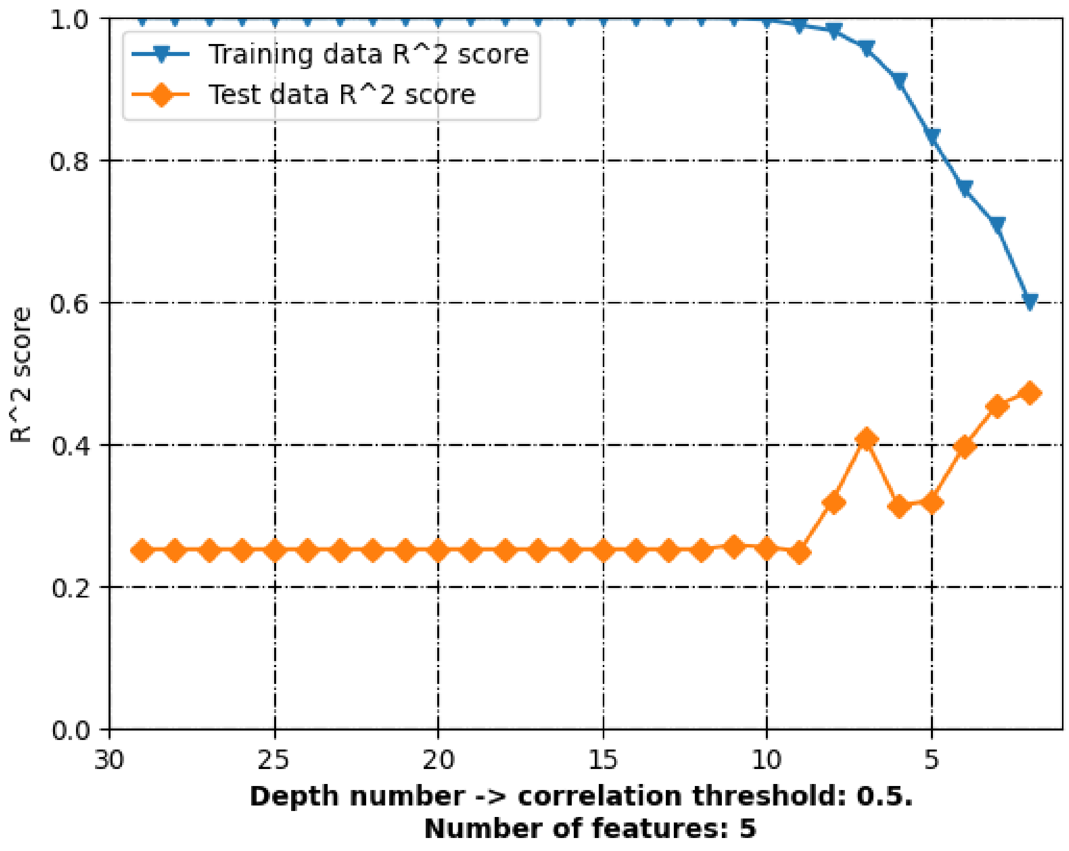

Training data R^2 score
Test data R^2 score
R^2 score
1.0
0.8
0.6
0.4
0.2
0.0
30
25
20
15
10
5
Depth number -> correlation threshold: 0.5.
Number of features: 5

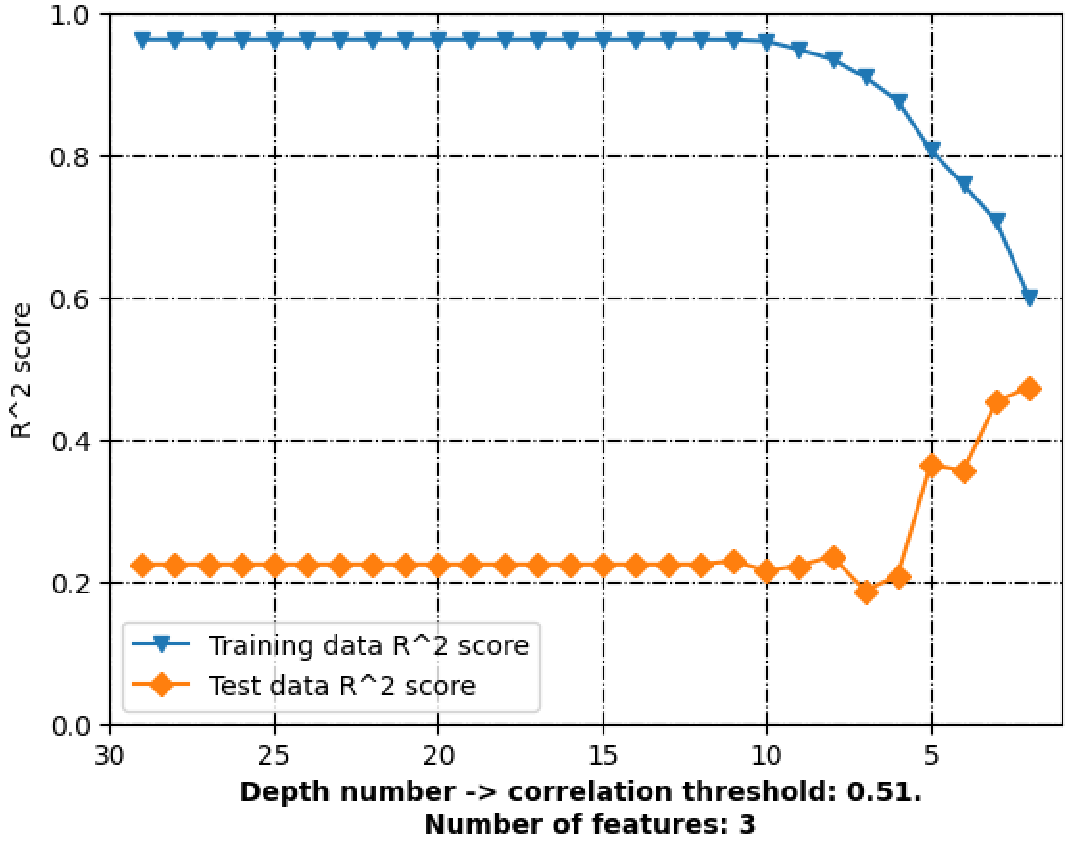
1.0
0.8
0.6
0.4
0.2
0.0
R^2 score
Training data R^2 score
Test data R^2 score
30
25
20
15
10
5
Depth number -> correlation threshold: 0.51.
Number of features: 3

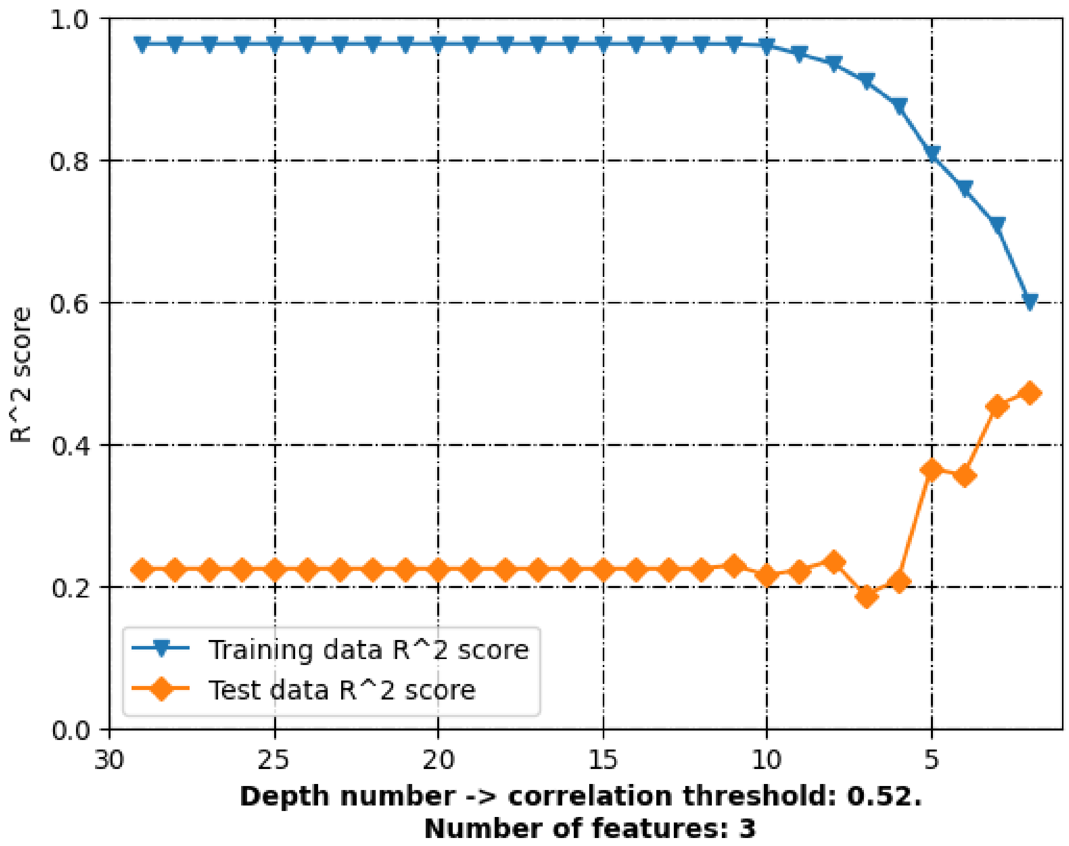
1.0
0.8
0.6
0.4
0.2
0.0
R^2 score
30
25
20
15
10
5
Training data R^2 score
Test data R^2 score
Depth number -> correlation threshold: 0.52.
Number of features: 3

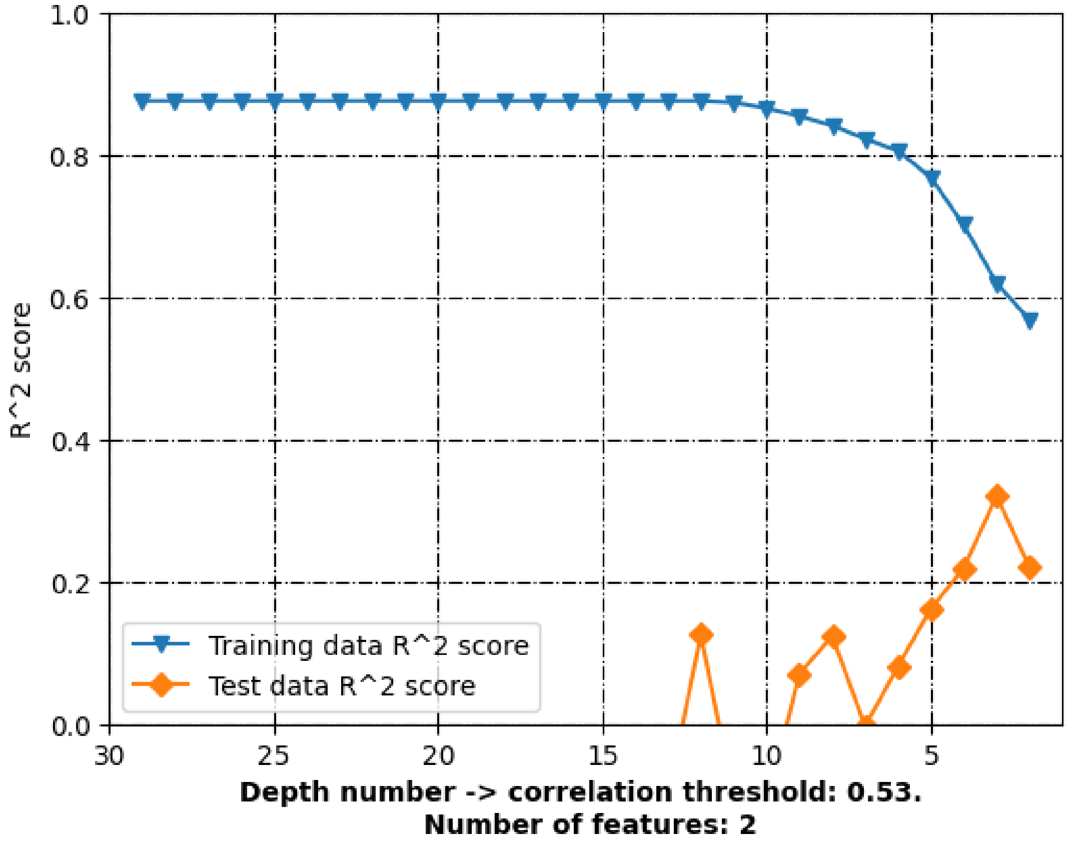
1.0
0.8
0.6
0.4
0.2
0.0
R^2 score
30
25
20
15
10
5
Training data R^2 score
Test data R^2 score
Depth number -> correlation threshold: 0.53.
Number of features: 2

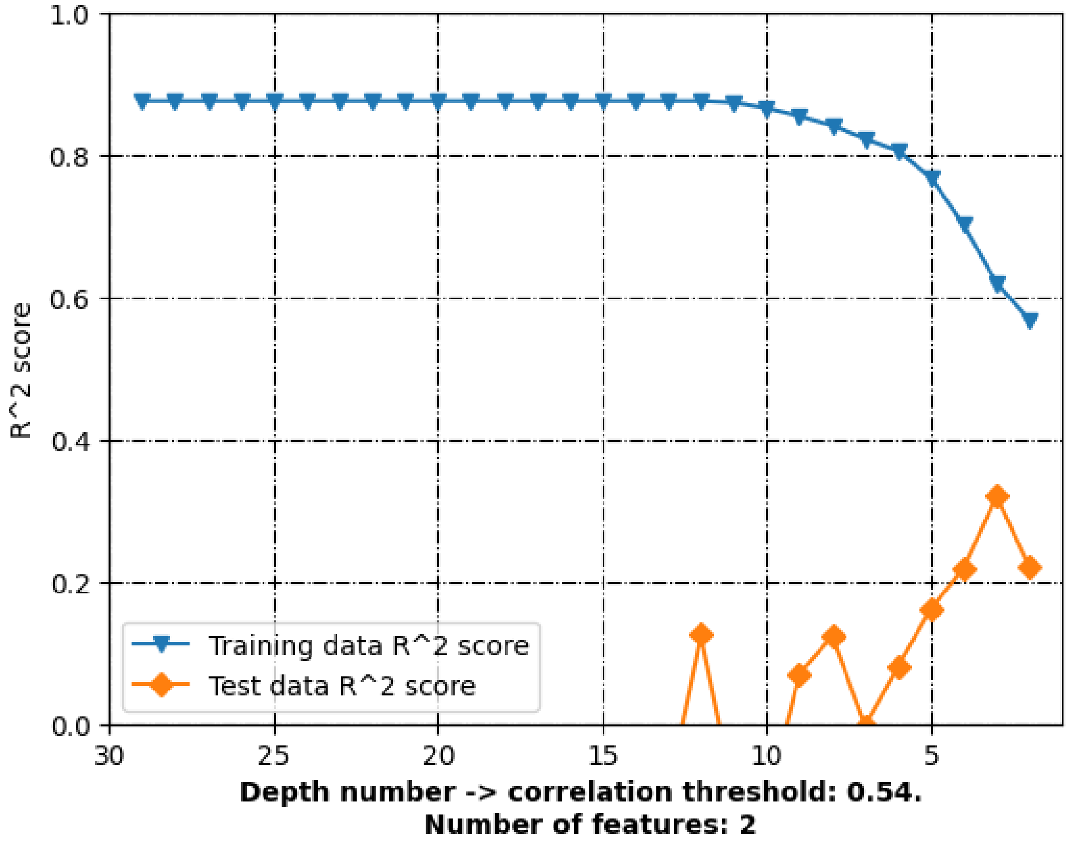

[20]:

```
plt.plot(df_without_standardization['Correlation threshold'],␣
  ↪df_without_standardization['Number of features'], label = "Number of␣
  ↪features", marker='.')
plt.legend()
plt.xlabel('Correlation threshold')
plt.ylabel('Number of features')
plt.show()
```

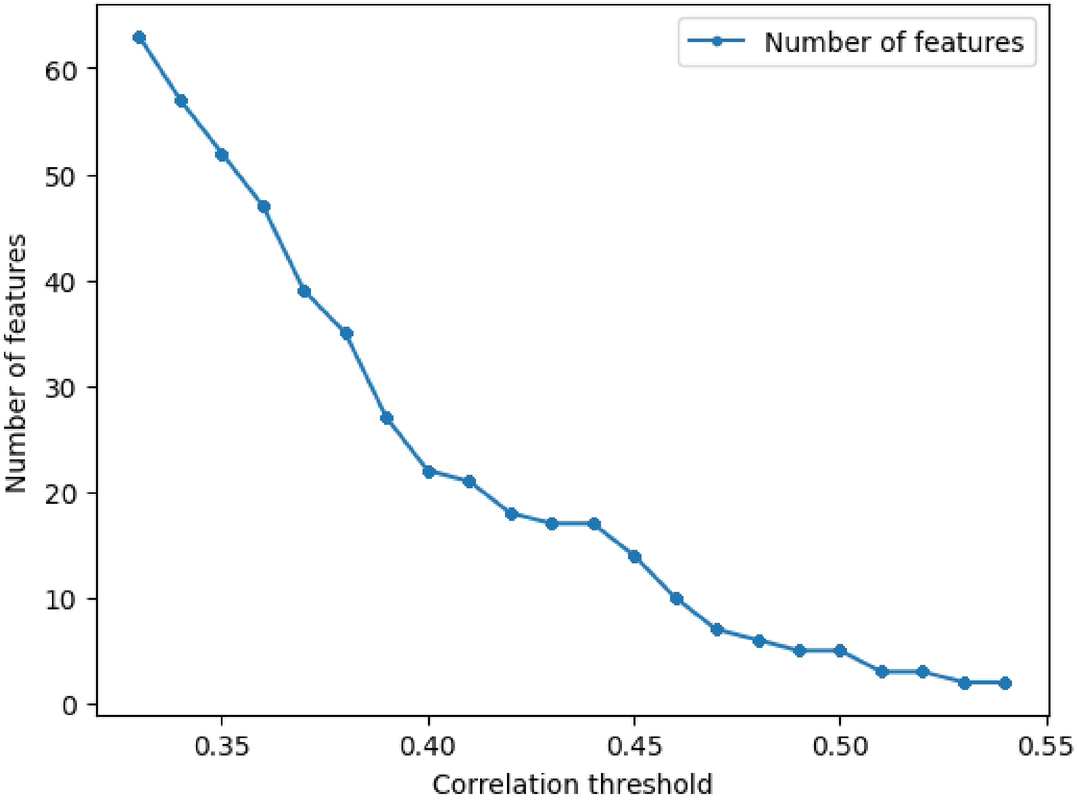

[ ]:

3 Random Forest

[21]:
```
without_standardization, train_r2_, test_r2_, hist1, hist2, target_column_name,␣
↪training_data_RMSE, test_data_RMSE  = pred_model.
↪prepare_data_and_create_model(molecular_descriptors_df=data,
                                                                              ␣
↪                      correlation_threshold=0.50,
                                                                              ␣
↪                      standardization=False,
                                                                              ␣
↪                      model_type='RandomForestRegressor',
                                                                              ␣
↪                      n_estimators_=12,
                                                                              ␣
↪                      target_column_name = target,
                                                                              ␣
↪                      random_state=random_state,
```

```
↪                    train_test_split_=True,
↪                    verbose=True)
```

```
I am not doing standardization…
  molecular descriptor name
0                    AATS0Z
1                  AATS0are
2                    AATS0d
3                   AATS0dv
4                    AATS0i
  molecular descriptor name  corr_value
0                    AATS0Z   -0.027037
1                  AATS0are   -0.129823
2                    AATS0d    0.042740
3                   AATS0dv   -0.120173
4                    AATS0i    0.132395
  molecular descriptor name  corr_value  absolute correlation value
0                    AATS0Z   -0.027037                    0.027037
1                  AATS0are   -0.129823                    0.129823
2                    AATS0d    0.042740                    0.042740
3                   AATS0dv   -0.120173                    0.120173
4                    AATS0i    0.132395                    0.132395
     molecular descriptor name  corr_value  absolute correlation value
226                     AMID_O   -0.524157                    0.524157
505                 EState_VSA5   -0.579664                    0.579664
791                    MDEO-12   -0.558727                    0.558727
851                      NdssC   -0.506855                    0.506855
1091               VSA_EState5    0.503578                    0.503578
     molecular descriptor name  corr_value  absolute correlation value
226                     AMID_O   -0.524157                    0.524157
505                 EState_VSA5   -0.579664                    0.579664
791                    MDEO-12   -0.558727                    0.558727
851                      NdssC   -0.506855                    0.506855
1091               VSA_EState5    0.503578                    0.503578
The model used is: RandomForest…
Return the coefficient of determination of the prediction:
0.4902931070613592
R^2 score: 0.9026786871770149
Correlation coefficient: 0.9500940412280329
Test data - unseen during training:
R^2 score: 0.4902931070613592
Correlation coefficient: 0.7002093308870992
[7.79645131 5.79278282 7.45510343 8.06465388 8.34997349 7.48588561
 8.07372575 7.34615379 7.92162209 8.08270908 7.89558771 8.2638769
 8.11810739 7.50814976 7.9828299  8.30277519 6.69346735 5.57950951]
102     8.000000
```

```
38       6.104025
8        6.044360
109      7.886057
11       8.107905
51       8.000000
88       8.886057
21       7.554396
82       8.301030
98       7.568636
58       7.677781
64       8.327902
74       8.070581
103      8.136677
91      10.000000
43       7.886057
25       5.221704
30       6.315155
Name: LoVo, dtype: float64
Training Root Mean Square Error: 0.2703702477820712
Testing Root Mean Square Error: 0.7815053189241797
```

[22]:

```
print(target_column_name+str('_transformed'))
print(hist1[target_column_name].hist())
```

```
LoVo_transformed
AxesSubplot(0.125,0.11;0.775x0.77)
```

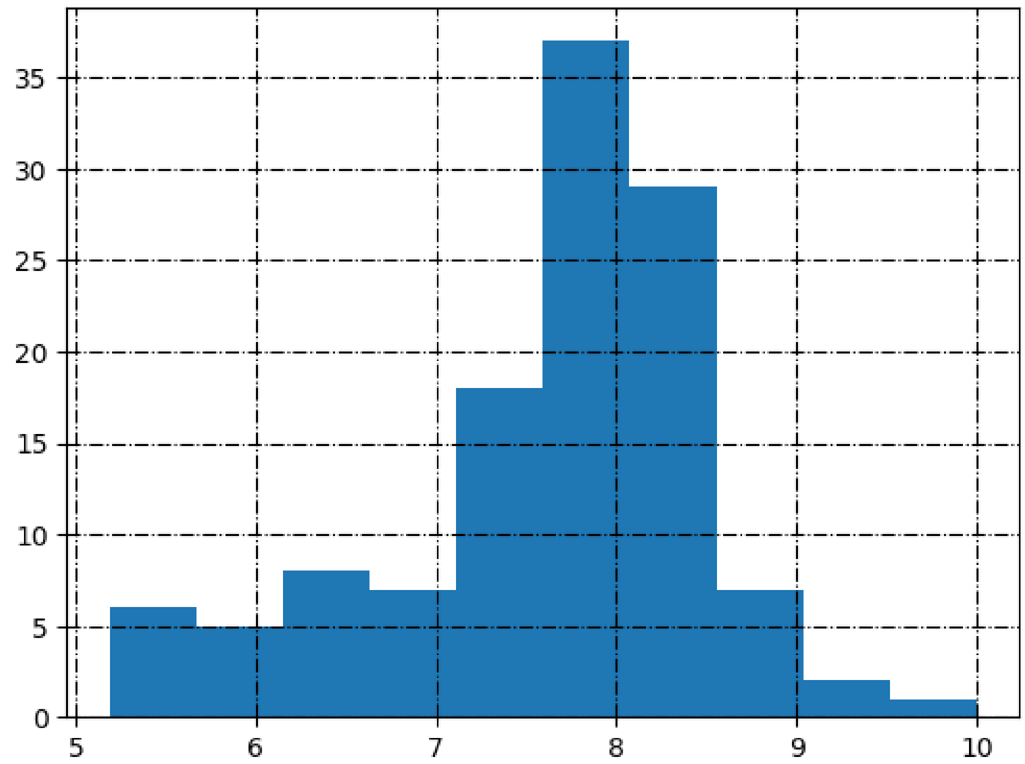

[23]:
```
with_standardization, train_r2_, test_r2_, hist1, hist2, target_column_name,␣
↪training_data_RMSE, test_data_RMSE  = pred_model.
↪prepare_data_and_create_model(molecular_descriptors_df=data,
                                                                              ␣
↪                        correlation_threshold=0.50,
                                                                              ␣
↪                        standardization=True,
                                                                              ␣
↪                        model_type='RandomForestRegressor',
                                                                              ␣
↪                        n_estimators_=12,
                                                                              ␣
↪                        target_column_name = target,
                                                                              ␣
↪                        random_state=random_state,
                                                                              ␣
↪                        train_test_split_=True,
                                                                              ␣
↪                        verbose=True)
```

```
I am doing standardization…
  molecular descriptor name
```

```
0                   AATS0Z
1                 AATS0are
2                   AATS0d
3                  AATS0dv
4                   AATS0i
  molecular descriptor name  corr_value
0                   AATS0Z   -0.027037
1                 AATS0are   -0.129823
2                   AATS0d    0.042740
3                  AATS0dv   -0.120173
4                   AATS0i    0.132395
  molecular descriptor name  corr_value  absolute correlation value
0                   AATS0Z   -0.027037                     0.027037
1                 AATS0are   -0.129823                     0.129823
2                   AATS0d    0.042740                     0.042740
3                  AATS0dv   -0.120173                     0.120173
4                   AATS0i    0.132395                     0.132395
     molecular descriptor name  corr_value  absolute correlation value
226                     AMID_O   -0.524157                     0.524157
505                 EState_VSA5   -0.579664                     0.579664
791                     MDEO-12   -0.558727                     0.558727
851                       NdssC   -0.506855                     0.506855
1091                VSA_EState5    0.503578                     0.503578
     molecular descriptor name  corr_value  absolute correlation value
226                     AMID_O   -0.524157                     0.524157
505                 EState_VSA5   -0.579664                     0.579664
791                     MDEO-12   -0.558727                     0.558727
851                       NdssC   -0.506855                     0.506855
1091                VSA_EState5    0.503578                     0.503578
The model used is: RandomForest…
Return the coefficient of determination of the prediction:
0.4902931070613592
R^2 score: 0.9026786871770149
Correlation coefficient: 0.9500940412280329
Test data - unseen during training:
R^2 score: 0.4902931070613592
Correlation coefficient: 0.7002093308870992
[7.79645131 5.79278282 7.45510343 8.06465388 8.34997349 7.48588561
 8.07372575 7.34615379 7.92162209 8.08270908 7.89558771 8.2638769
 8.11810739 7.50814976 7.9828299  8.30277519 6.69346735 5.57950951]
102     8.000000
38      6.104025
8       6.044360
109     7.886057
11      8.107905
51      8.000000
88      8.886057
21      7.554396
```

```
82      8.301030
98      7.568636
58      7.677781
64      8.327902
74      8.070581
103     8.136677
91     10.000000
43      7.886057
25      5.221704
30      6.315155
Name: LoVo, dtype: float64
Training Root Mean Square Error: 0.2703702477820712
Testing Root Mean Square Error: 0.7815053189241797
```

3.1 Search inside correlation space

[24]:

```
step = 0.01
initial_step = corr_low
last_step = corr_high
first_list = [x / 100.0 for x in range(int(initial_step*100),␣
↪int(last_step*100), int(step*100))]
n_estimators = [range(2,21,1)]
corr_th = []
second_list = []
third_list = []
fourth_l = []
fifth_l = []
f_list = []
fif_list = []
for i in first_list:

    for estimator in n_estimators[0]:

        without_standardization, train_r2, test_r2, _, h_, target_column_name,␣
↪training_data_RMSE, test_data_RMSE = pred_model.
↪prepare_data_and_create_model(molecular_descriptors_df=data,
                                                                                     ␣
↪                      correlation_threshold=i,
                                                                                     ␣
↪                      standardization=False,
                                                                                     ␣
↪                      model_type='RandomForestRegressor',
                                                                                     ␣
↪                      n_estimators_=estimator,
                                                                                     ␣
↪                      target_column_name = target,
```

```
                        random_state=random_state,
                        train_test_split_=True,
                        verbose=False)
        corr_th.append(i)
        second_list.append(train_r2)
        third_list.append(test_r2)
        fourth_l.append(training_data_RMSE)
        fifth_l.append(test_data_RMSE)
        f_list.append(len(h_))
        fif_list.append(estimator)
```

[25]:
```
df_without_standardization = pd.DataFrame(data=corr_th, columns=["Correlation threshold"])
df_without_standardization['Training data R^2 score'] = second_list
df_without_standardization['Test data R^2 score'] = third_list
df_without_standardization['Training RMSE'] = fourth_l
df_without_standardization['Test RMSE'] = fifth_l
df_without_standardization['Number of features'] = f_list
df_without_standardization['Number of estimators'] = fif_list
```

[26]:
```
df_random_forest = df_without_standardization.copy()
#df_without_standardization.to_excel('../Data/A549_Random_forest_rs_'+str(random_state)+'.xlsx')
```

[27]:
```
corre_list = [x / 100.0 for x in range(int(initial_step*100), int(last_step*100), int(0.01*100))]
for element in corre_list:
    element_ = df_without_standardization[df_without_standardization['Correlation threshold'] == float(element)]
    plt.plot(element_['Number of estimators'], element_['Training data R^2 score'], label = "Training data R^2 score", marker='v')
    plt.plot(element_['Number of estimators'], element_['Test data R^2 score'], label = "Test data R^2 score", marker='D')
    plt.legend()
    plt.xlabel('Number of estimators -> correlation threshold: '+str(element)+'. \n Number of features: '+str(element_['Number of features'].iloc[0]))
    plt.xlim(max(element_['Number of estimators'])+1, min(element_['Number of estimators'])-1)
    plt.ylabel('R^2 score')
    plt.ylim([0, 1])
    plt.rc('grid', linestyle="-.", color='black')
    plt.grid(True)
```

```
plt.show()
```

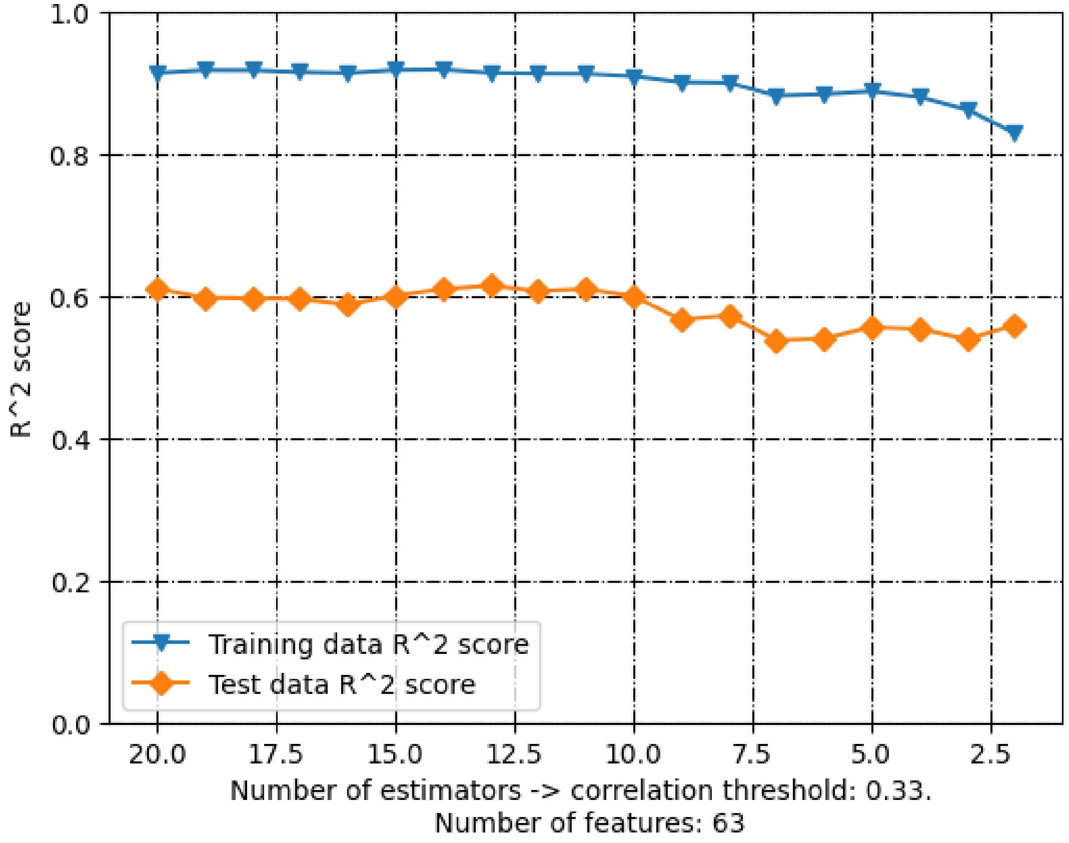

R^2 score
1.0
0.8
0.6
0.4
0.2
0.0
20.0
17.5
15.0
12.5
10.0
7.5
5.0
2.5
Number of estimators -> correlation threshold: 0.33.
Number of features: 63
Training data R^2 score
Test data R^2 score

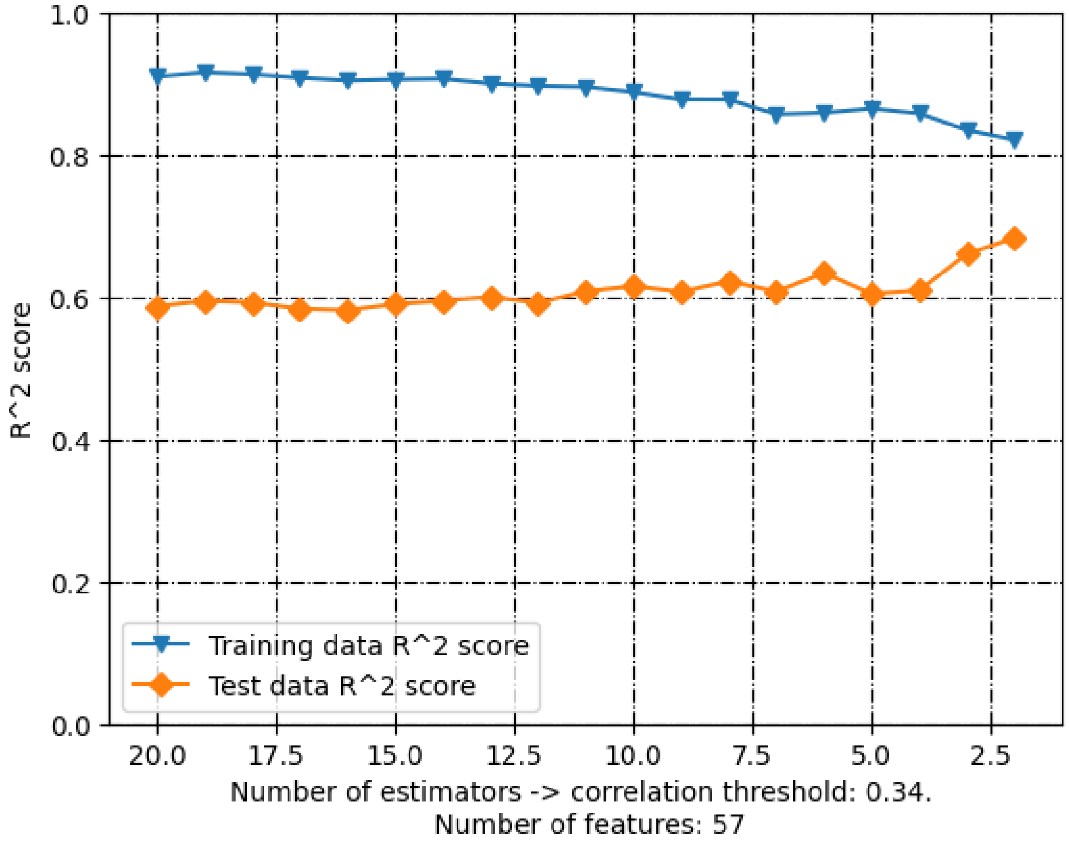

1.0
0.8
0.6
0.4
0.2
0.0
R^2 score
Training data R^2 score
Test data R^2 score
20.0
17.5
15.0
12.5
10.0
7.5
5.0
2.5
Number of estimators -> correlation threshold: 0.34.
Number of features: 57

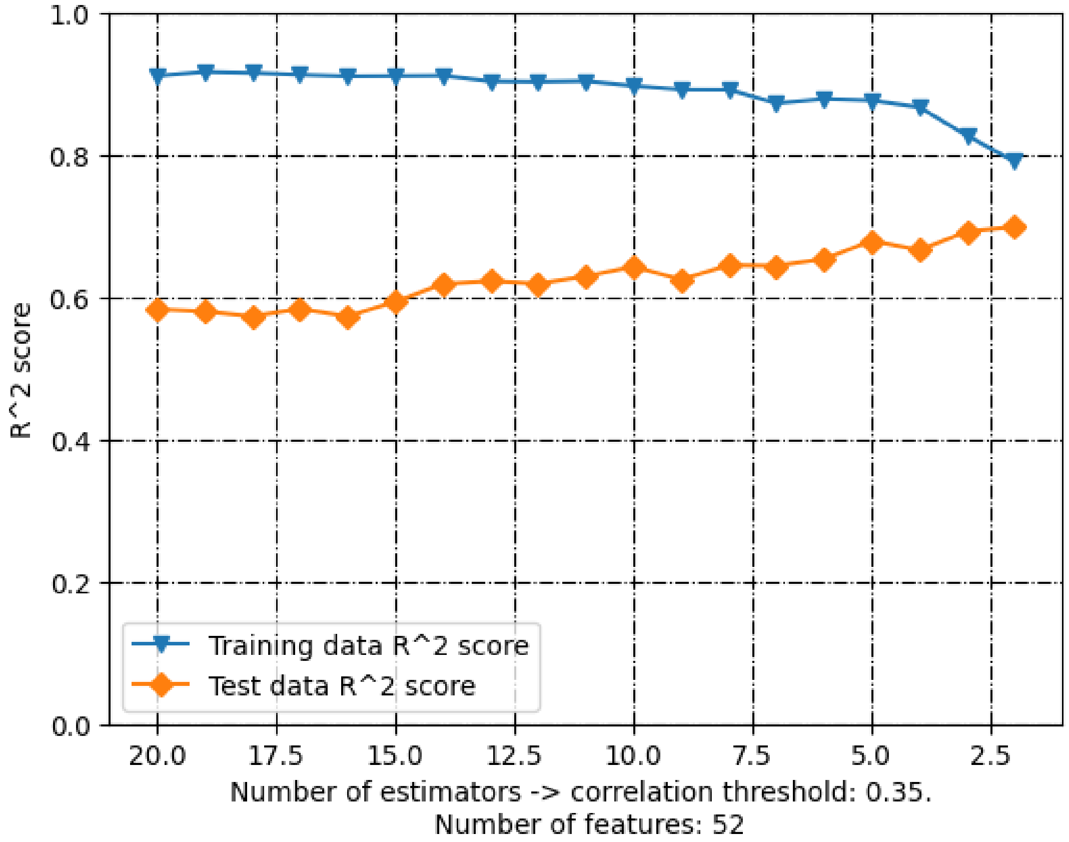
1.0
0.8
0.6
0.4
0.2
0.0
R^2 score
Training data R^2 score
Test data R^2 score
20.0
17.5
15.0
12.5
10.0
7.5
5.0
2.5
Number of estimators -> correlation threshold: 0.35.
Number of features: 52

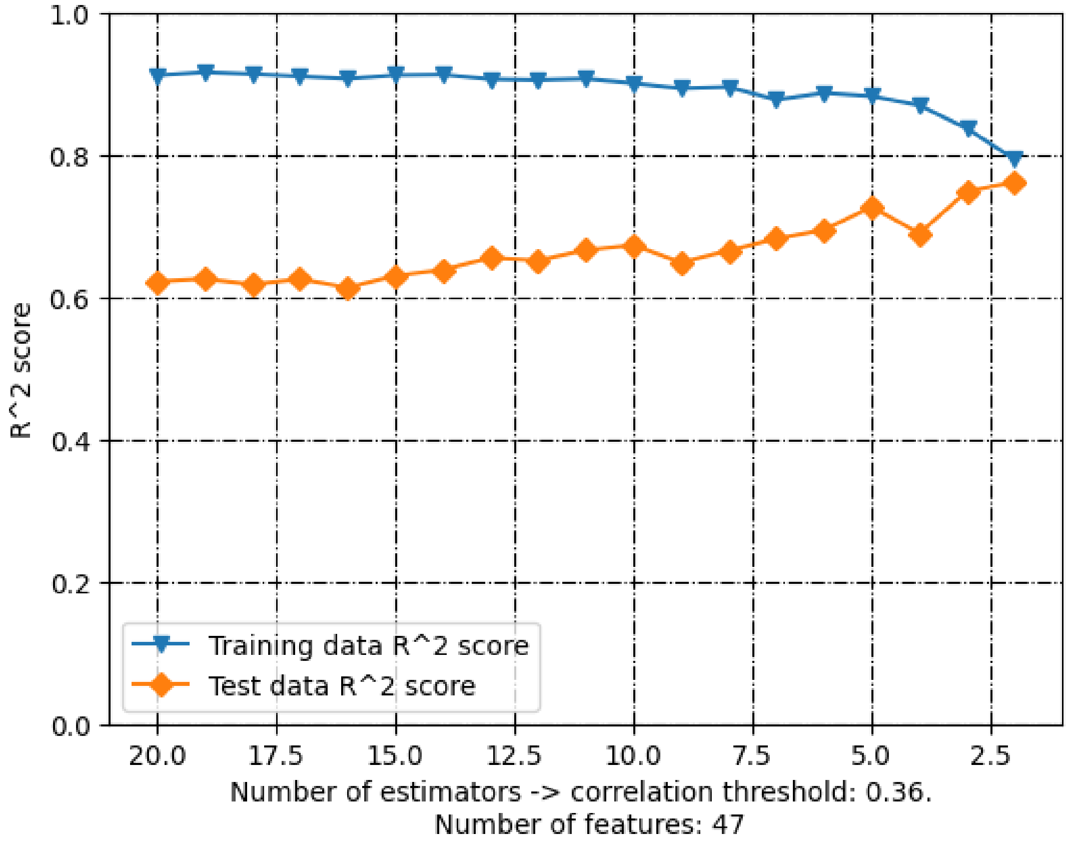

1.0
0.8
0.6
0.4
0.2
0.0
R^2 score
20.0
17.5
15.0
12.5
10.0
7.5
5.0
2.5
Training data R^2 score
Test data R^2 score
Number of estimators -> correlation threshold: 0.36.
Number of features: 47

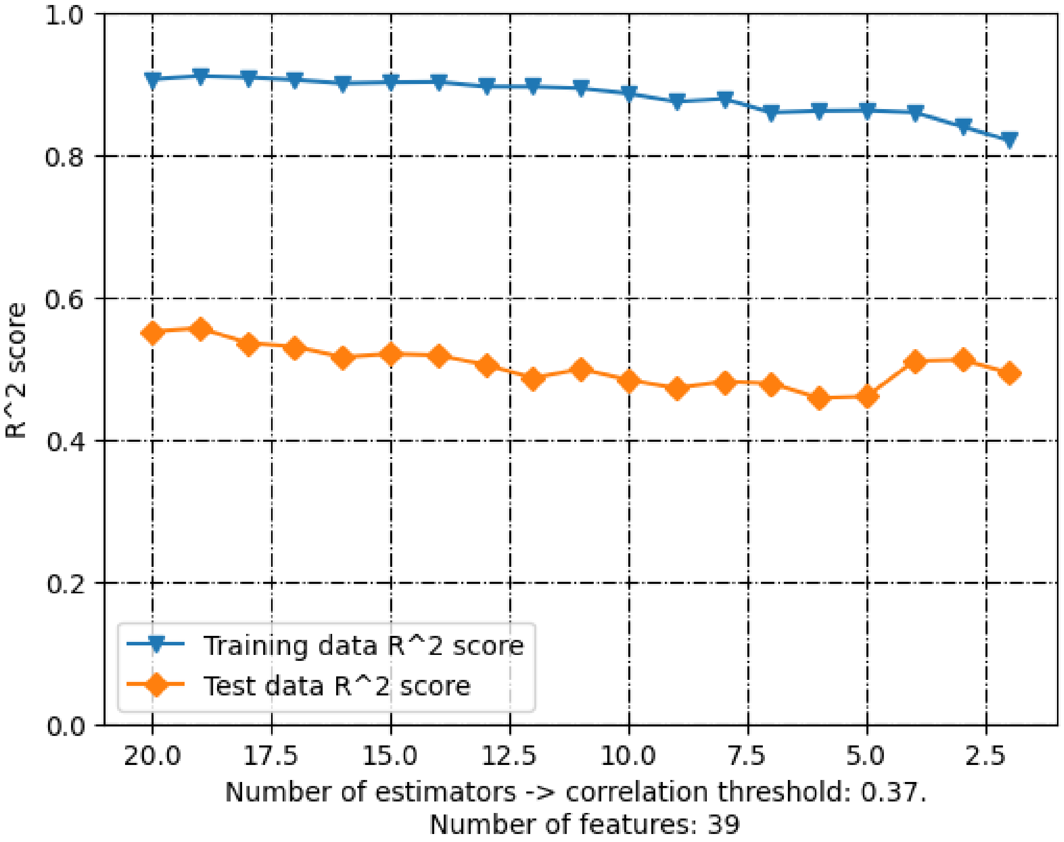

1.0
0.8
0.6
0.4
0.2
0.0
R^2 score
Training data R^2 score
Test data R^2 score
20.0
17.5
15.0
12.5
10.0
7.5
5.0
2.5
Number of estimators -> correlation threshold: 0.37.
Number of features: 39

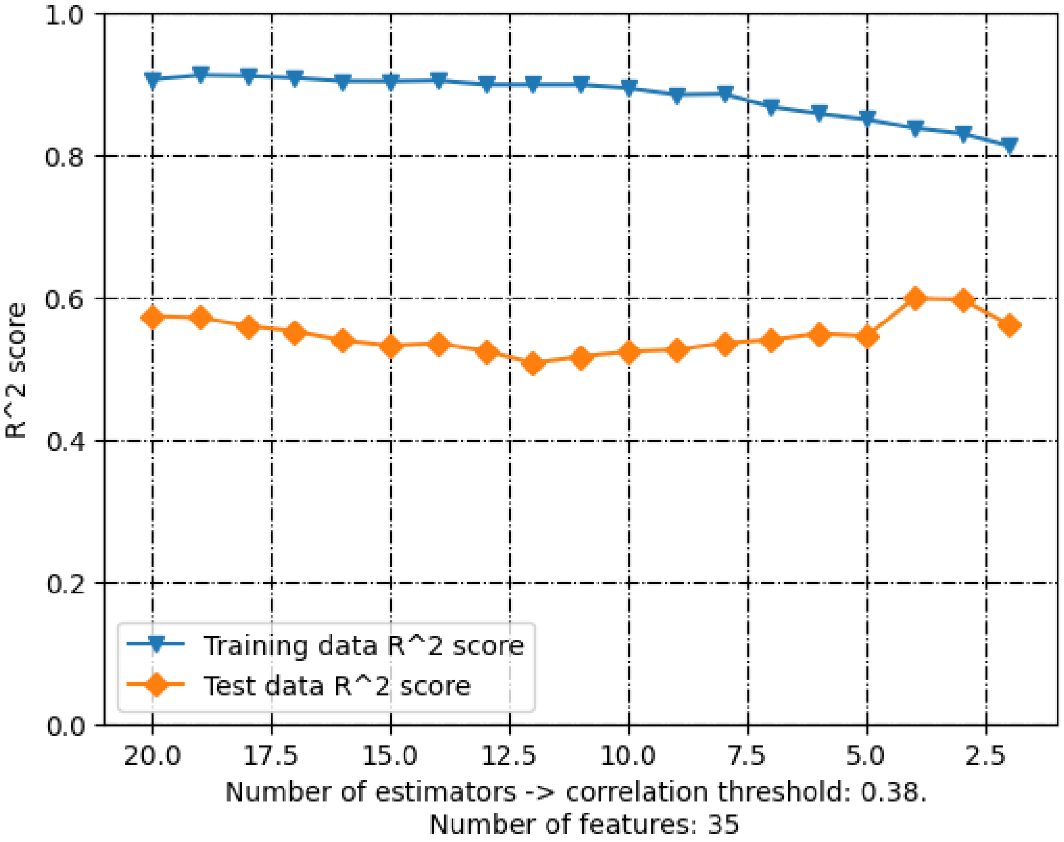
1.0
0.8
0.6
0.4
0.2
0.0
R^2 score
20.0
17.5
15.0
12.5
10.0
7.5
5.0
2.5
Number of estimators -> correlation threshold: 0.38.
Number of features: 35
Training data R^2 score
Test data R^2 score

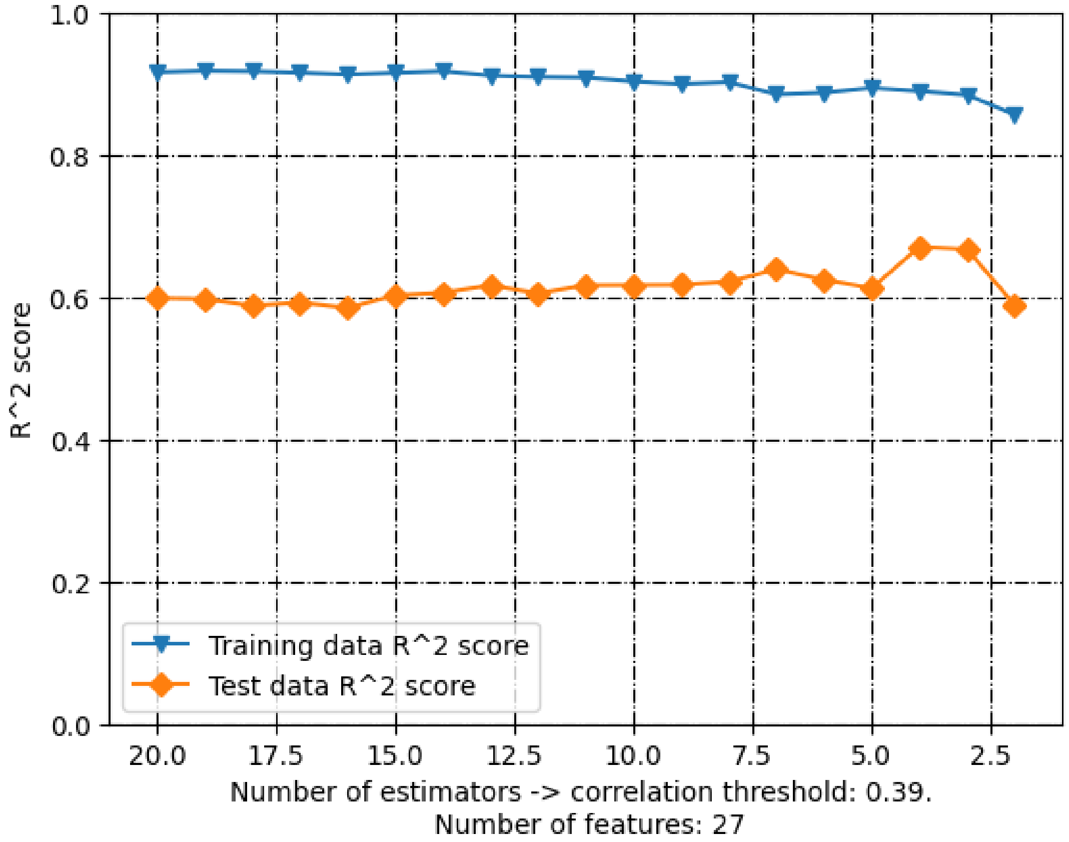
1.0
0.8
0.6
0.4
0.2
0.0
R^2 score
Training data R^2 score
Test data R^2 score
20.0
17.5
15.0
12.5
10.0
7.5
5.0
2.5
Number of estimators -> correlation threshold: 0.39.
Number of features: 27

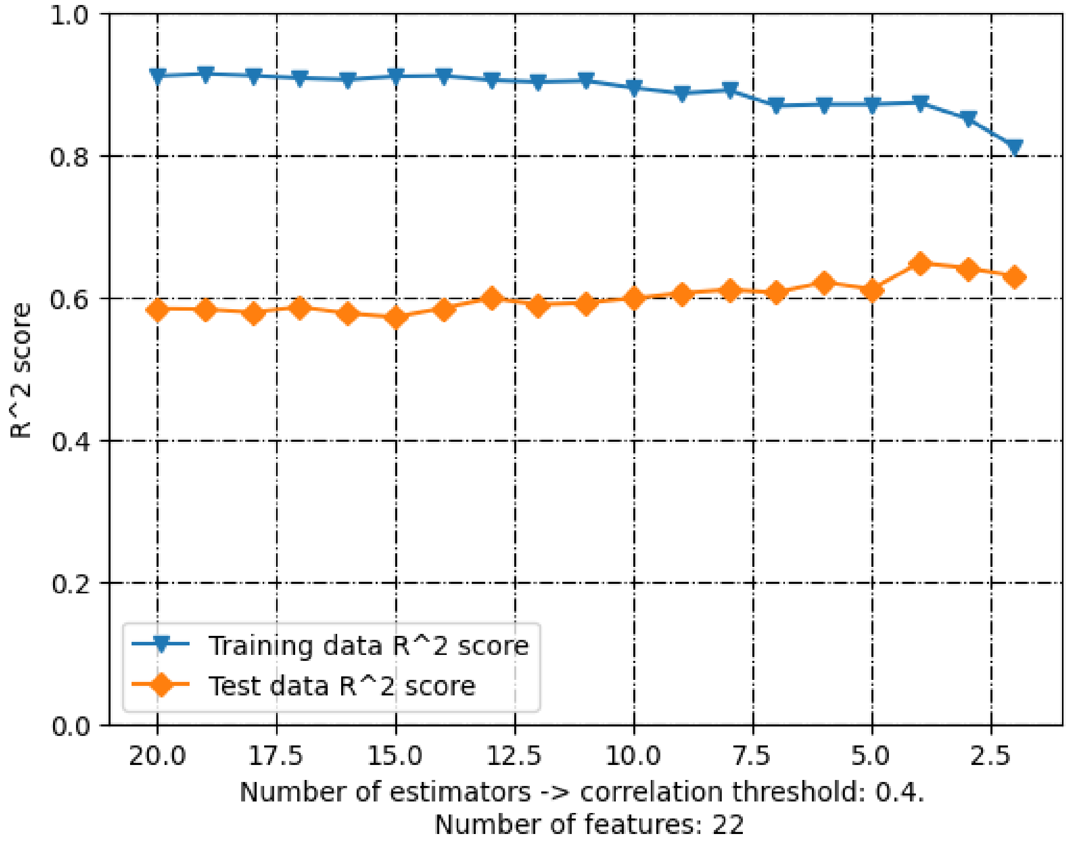

1.0
0.8
0.6
0.4
0.2
0.0
R^2 score
Training data R^2 score
Test data R^2 score
20.0
17.5
15.0
12.5
10.0
7.5
5.0
2.5
Number of estimators -> correlation threshold: 0.4.
Number of features: 22

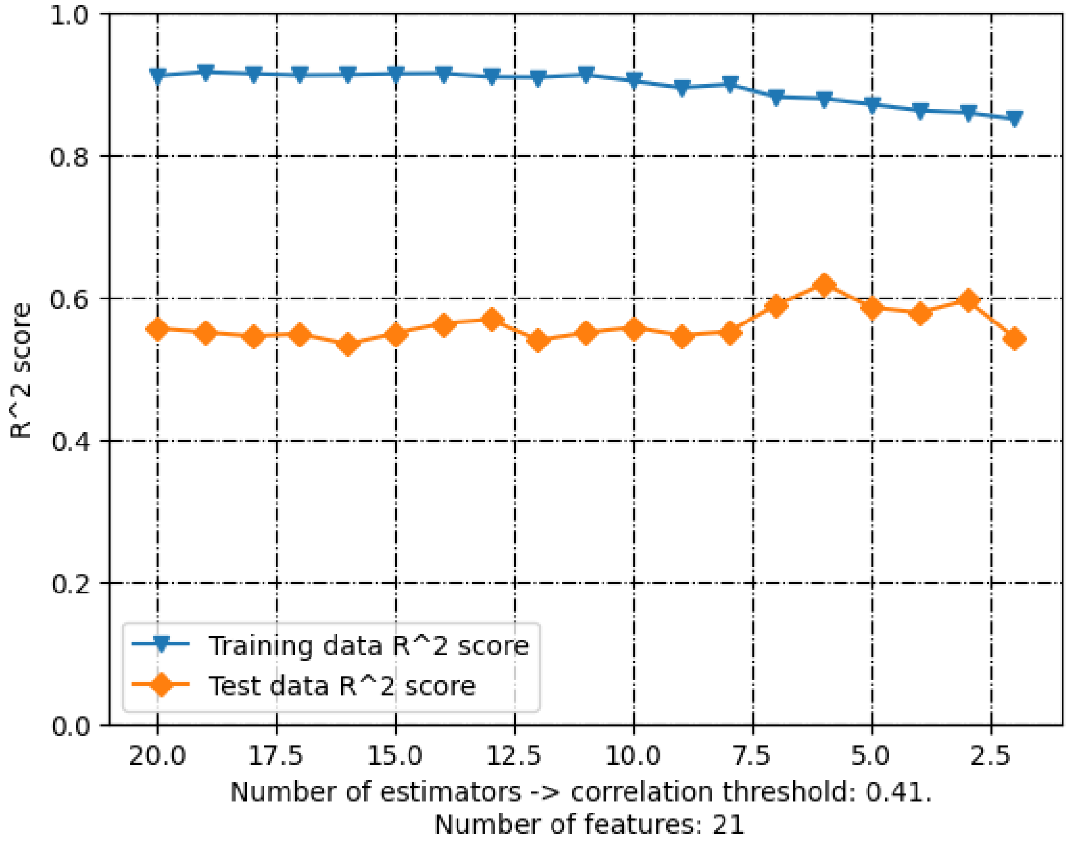
1.0
0.8
0.6
0.4
0.2
0.0
R^2 score
Training data R^2 score
Test data R^2 score
20.0
17.5
15.0
12.5
10.0
7.5
5.0
2.5
Number of estimators -> correlation threshold: 0.41.
Number of features: 21

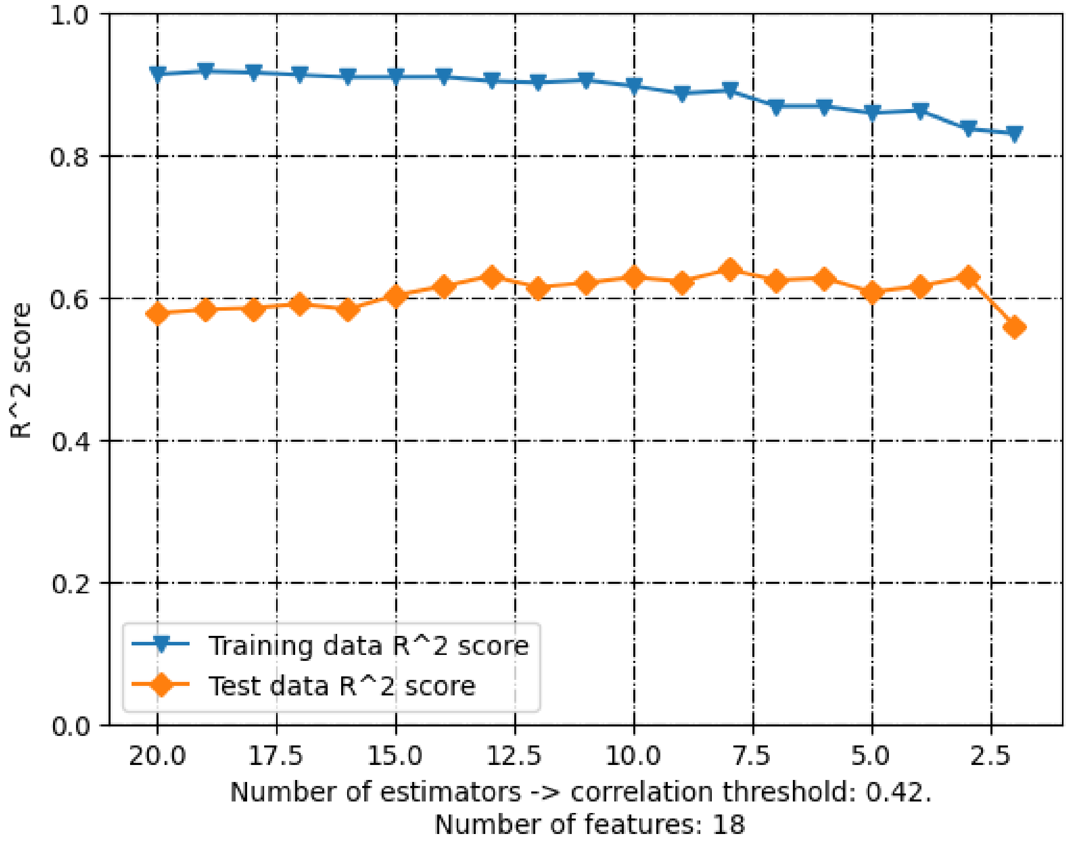

1.0
0.8
0.6
0.4
0.2
0.0
R^2 score
20.0
17.5
15.0
12.5
10.0
7.5
5.0
2.5
Training data R^2 score
Test data R^2 score
Number of estimators -> correlation threshold: 0.42.
Number of features: 18

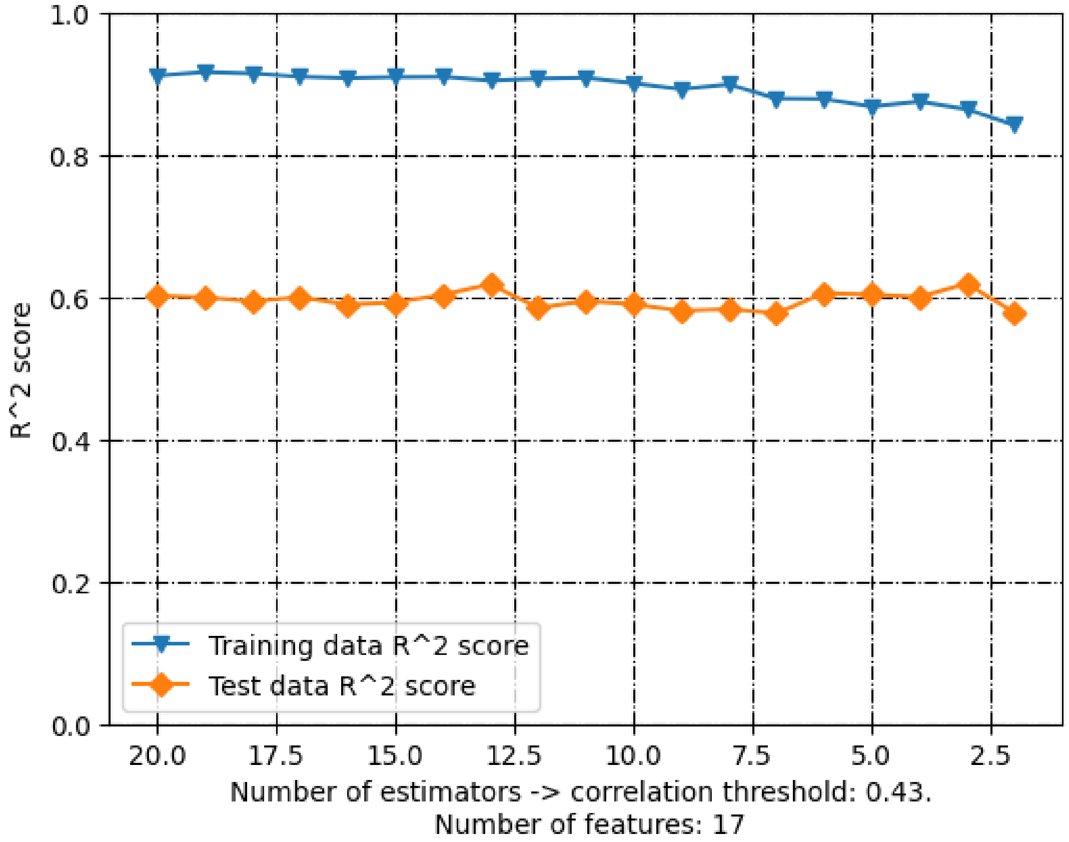

1.0
0.8
0.6
0.4
0.2
0.0
R^2 score
20.0
17.5
15.0
12.5
10.0
7.5
5.0
2.5
Training data R^2 score
Test data R^2 score
Number of estimators -> correlation threshold: 0.43.
Number of features: 17

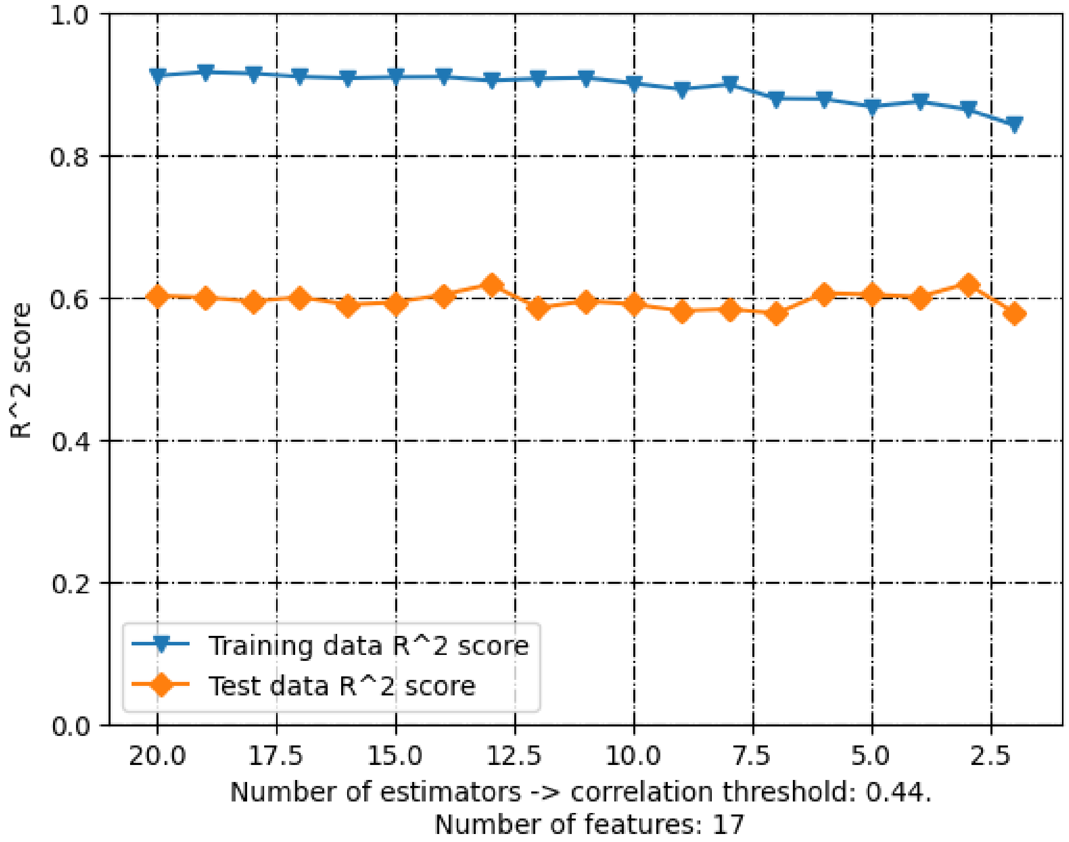
1.0
0.8
0.6
0.4
0.2
0.0
R^2 score
Training data R^2 score
Test data R^2 score
20.0
17.5
15.0
12.5
10.0
7.5
5.0
2.5
Number of estimators -> correlation threshold: 0.44.
Number of features: 17

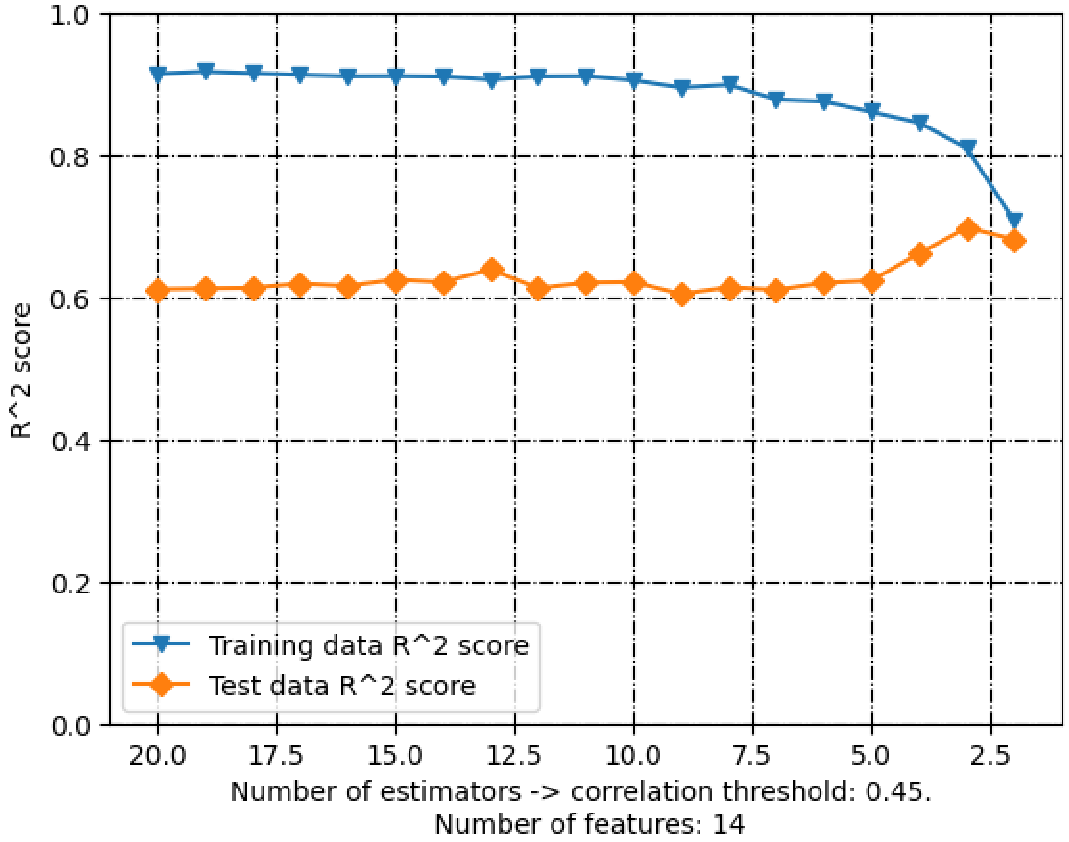
1.0
0.8
0.6
0.4
0.2
0.0
R^2 score
Training data R^2 score
Test data R^2 score
20.0
17.5
15.0
12.5
10.0
7.5
5.0
2.5
Number of estimators -> correlation threshold: 0.45.
Number of features: 14

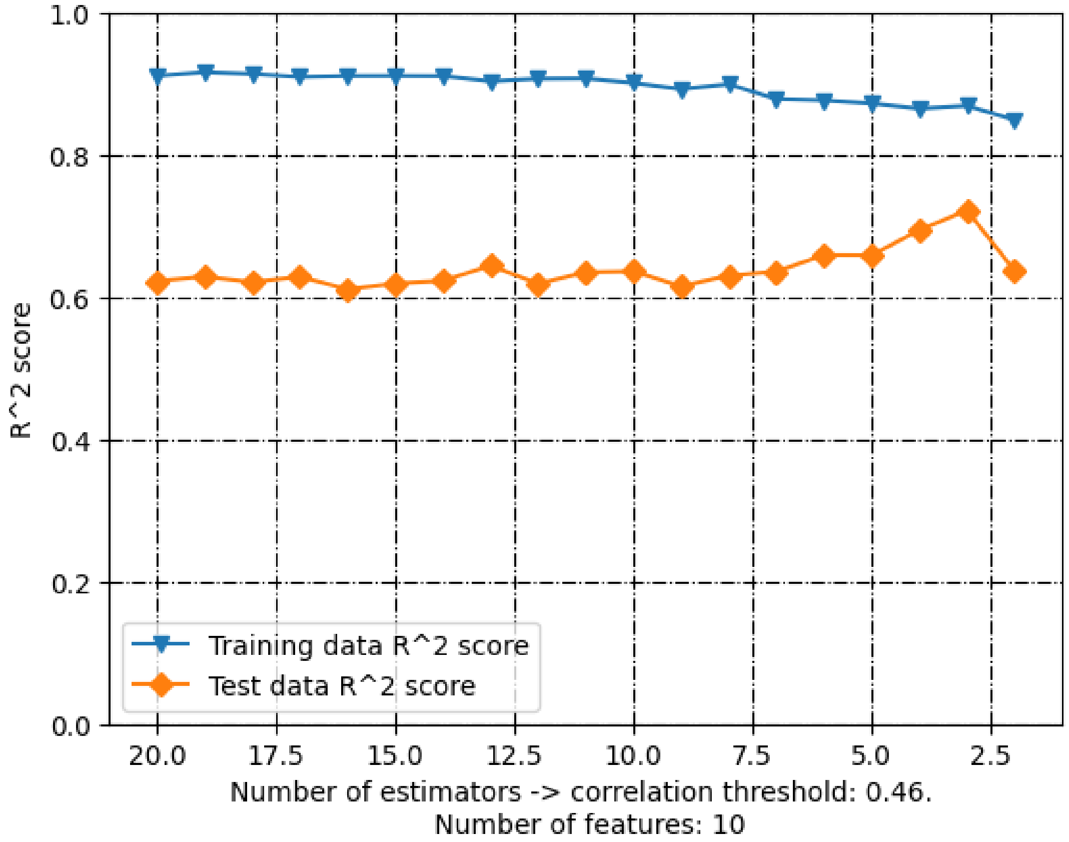
1.0
0.8
0.6
0.4
0.2
0.0
R^2 score
20.0
17.5
15.0
12.5
10.0
7.5
5.0
2.5
Number of estimators -> correlation threshold: 0.46.
Number of features: 10
Training data R^2 score
Test data R^2 score

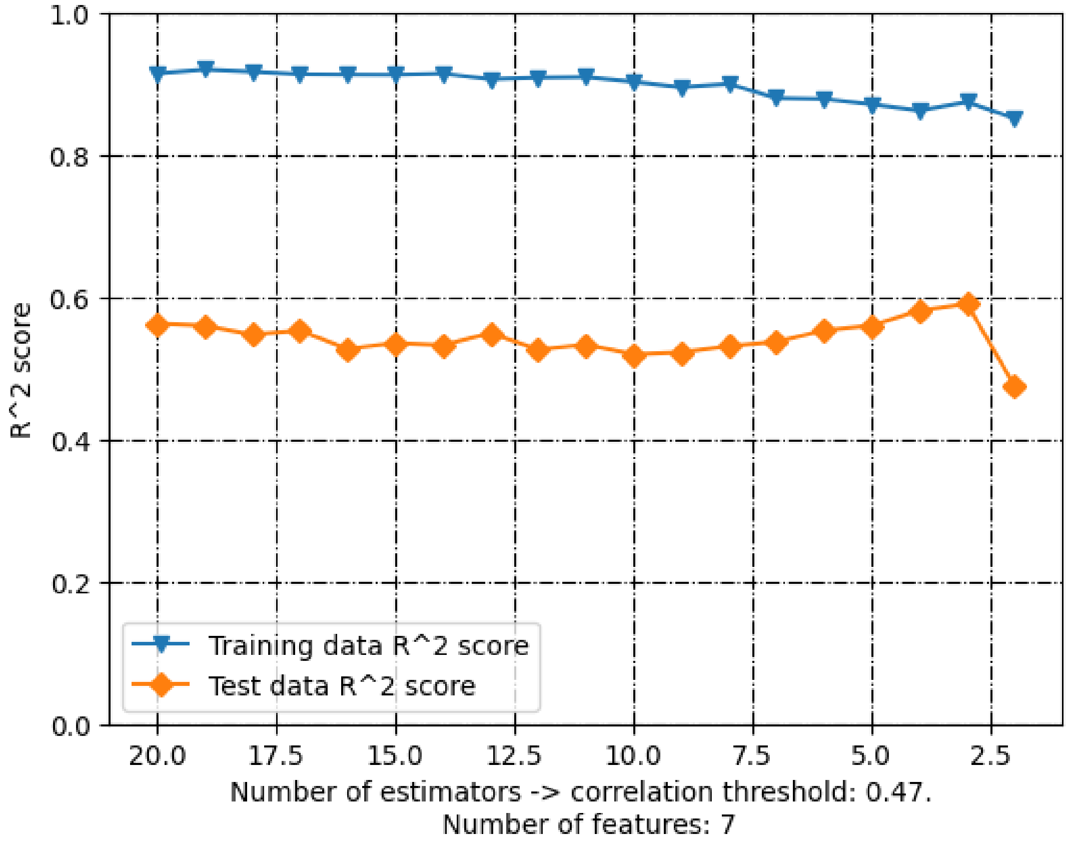
1.0
0.8
0.6
0.4
0.2
0.0
R^2 score
20.0
17.5
15.0
12.5
10.0
7.5
5.0
2.5
Number of estimators -> correlation threshold: 0.47.
Number of features: 7
Training data R^2 score
Test data R^2 score

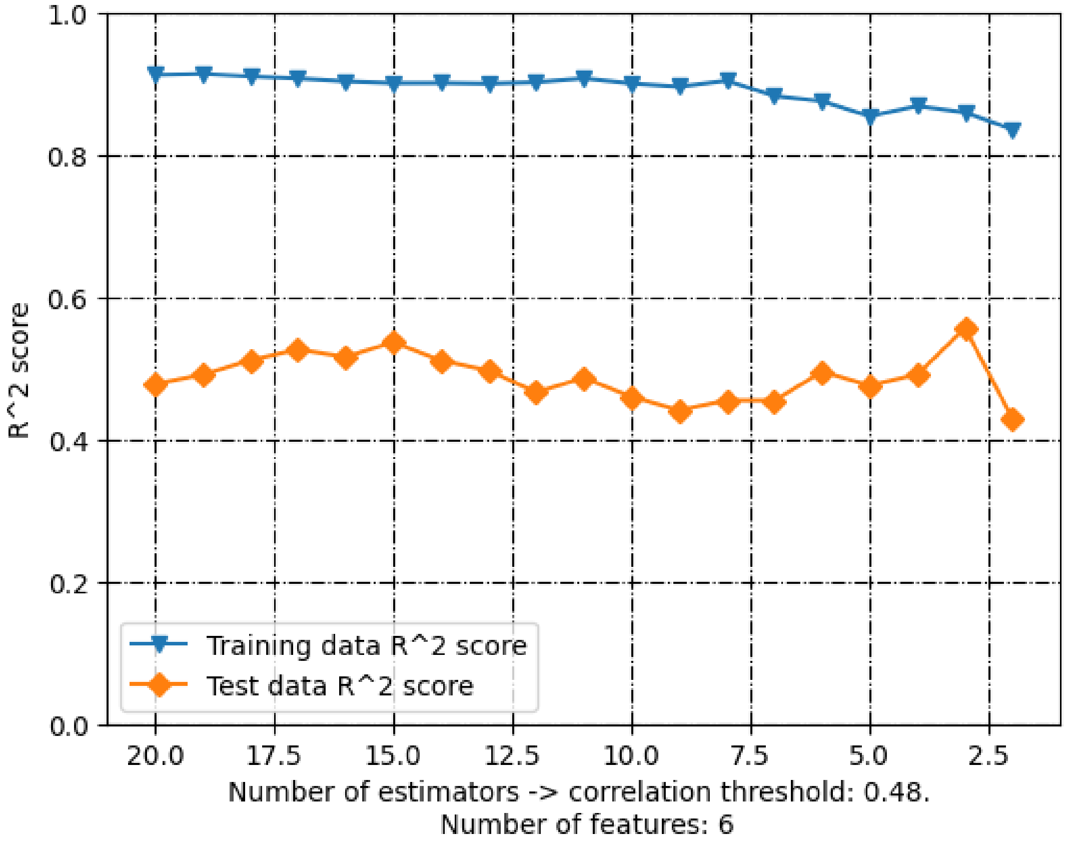
1.0
0.8
0.6
0.4
0.2
0.0
R^2 score
20.0
17.5
15.0
12.5
10.0
7.5
5.0
2.5
Training data R^2 score
Test data R^2 score
Number of estimators -> correlation threshold: 0.48.
Number of features: 6

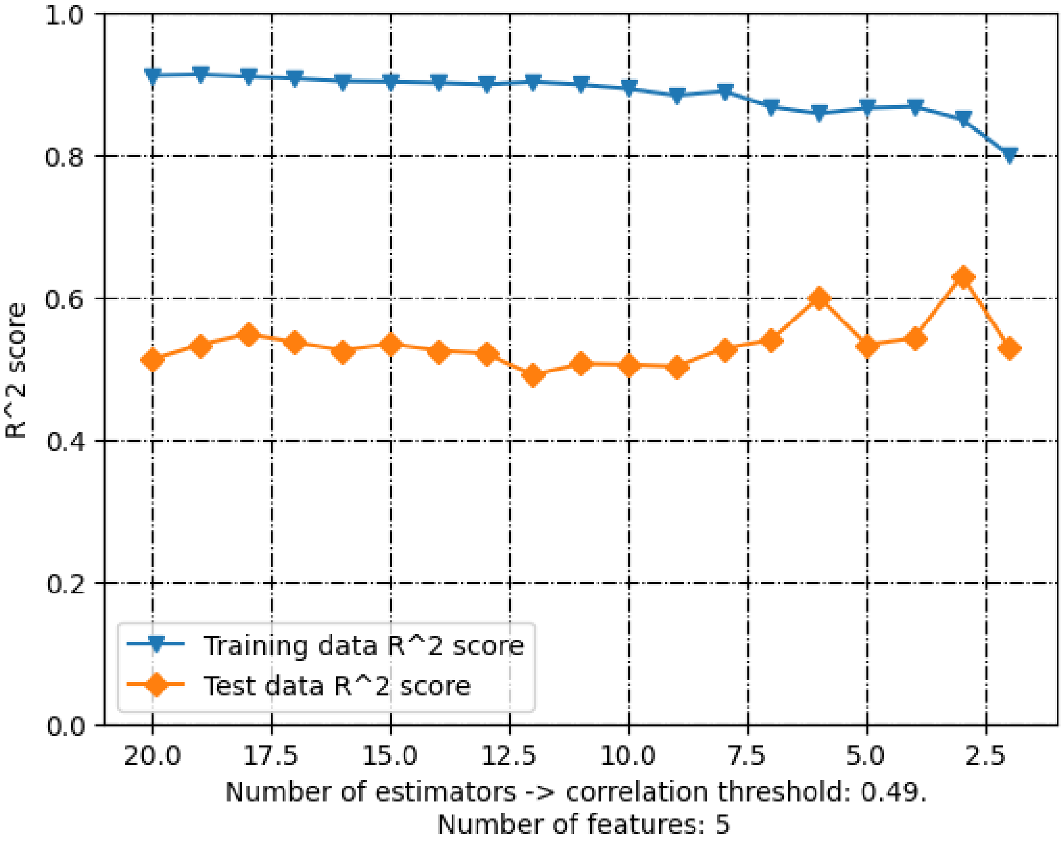
1.0
0.8
0.6
0.4
0.2
0.0
R^2 score
Training data R^2 score
Test data R^2 score
20.0
17.5
15.0
12.5
10.0
7.5
5.0
2.5
Number of estimators -> correlation threshold: 0.49.
Number of features: 5

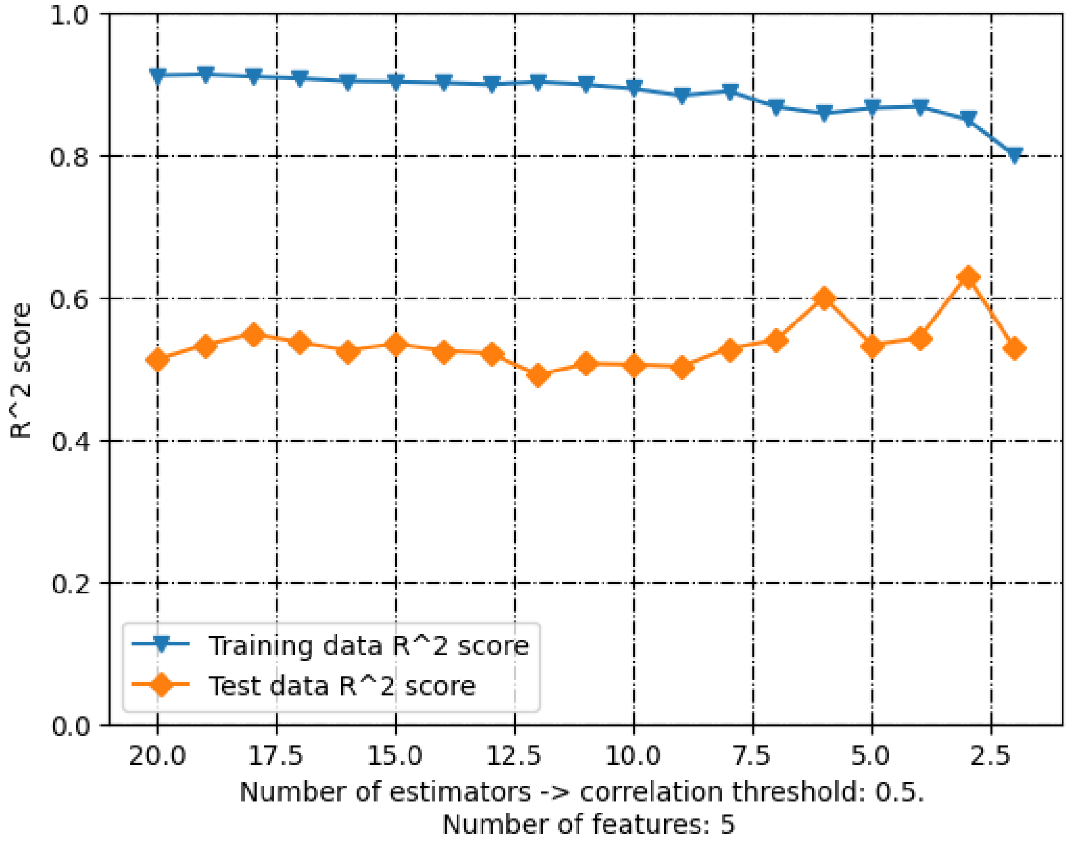
1.0
0.8
0.6
0.4
0.2
0.0
R^2 score
20.0
17.5
15.0
12.5
10.0
7.5
5.0
2.5
Training data R^2 score
Test data R^2 score
Number of estimators -> correlation threshold: 0.5.
Number of features: 5

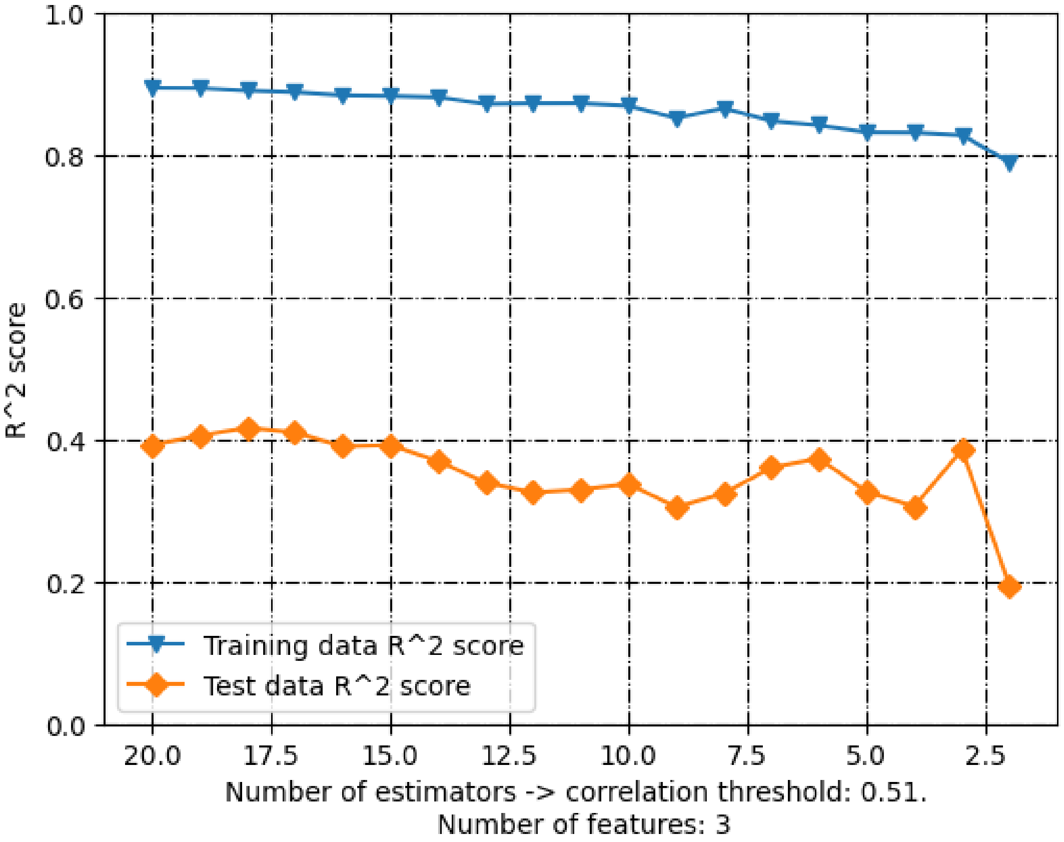
1.0
0.8
0.6
0.4
0.2
0.0
R^2 score
Training data R^2 score
Test data R^2 score
20.0
17.5
15.0
12.5
10.0
7.5
5.0
2.5
Number of estimators -> correlation threshold: 0.51.
Number of features: 3

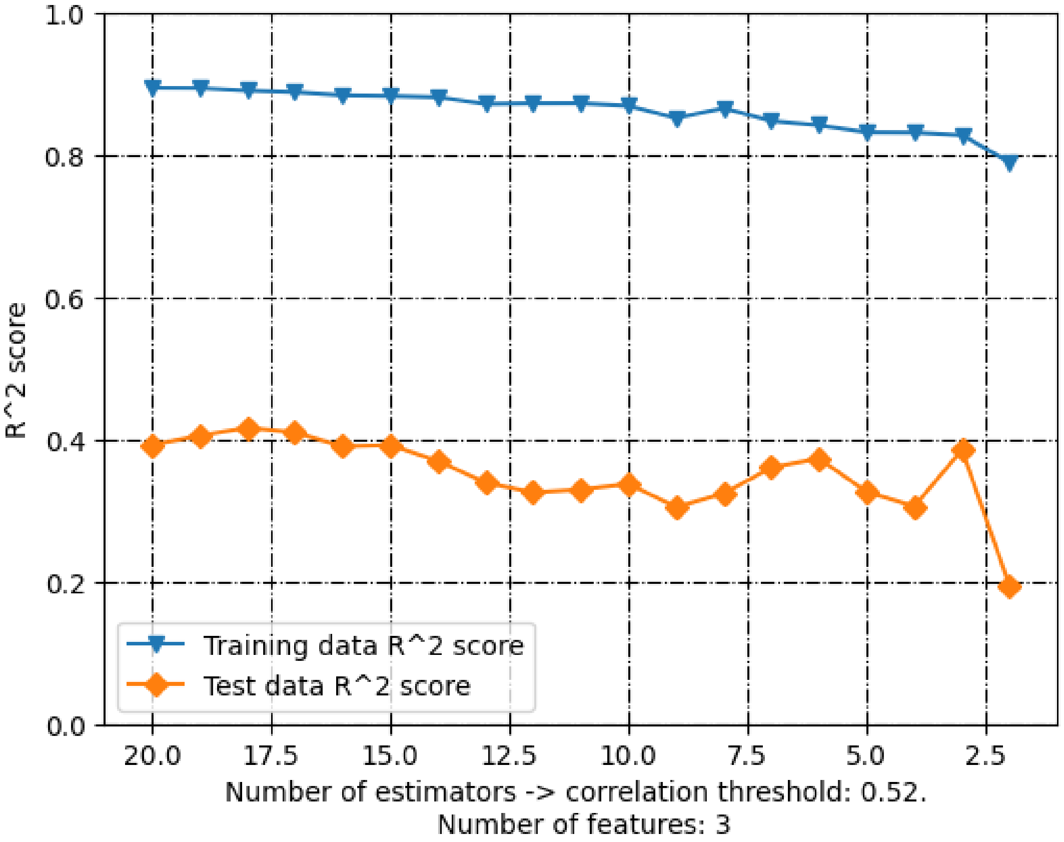

1.0
0.8
0.6
0.4
0.2
0.0
R^2 score
20.0
17.5
15.0
12.5
10.0
7.5
5.0
2.5
Number of estimators -> correlation threshold: 0.52.
Number of features: 3
Training data R^2 score
Test data R^2 score

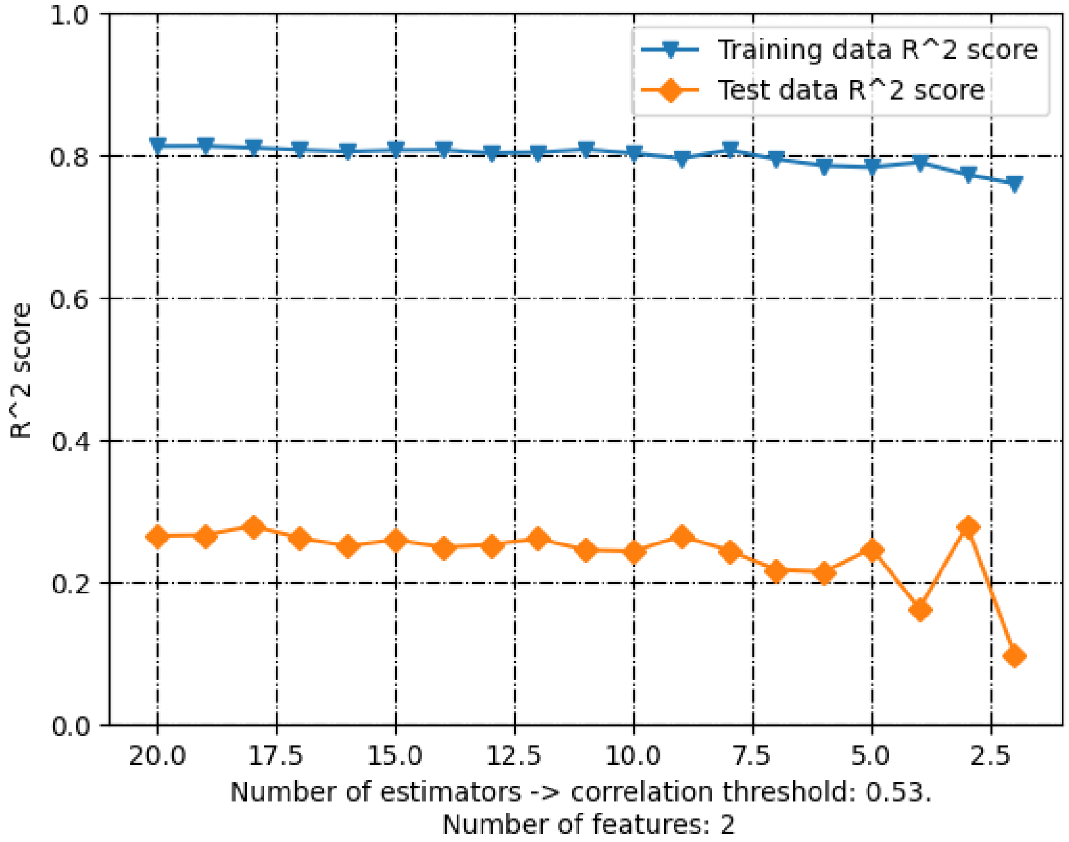
1.0
0.8
0.6
0.4
0.2
0.0
R^2 score
Training data R^2 score
Test data R^2 score
20.0
17.5
15.0
12.5
10.0
7.5
5.0
2.5
Number of estimators -> correlation threshold: 0.53.
Number of features: 2

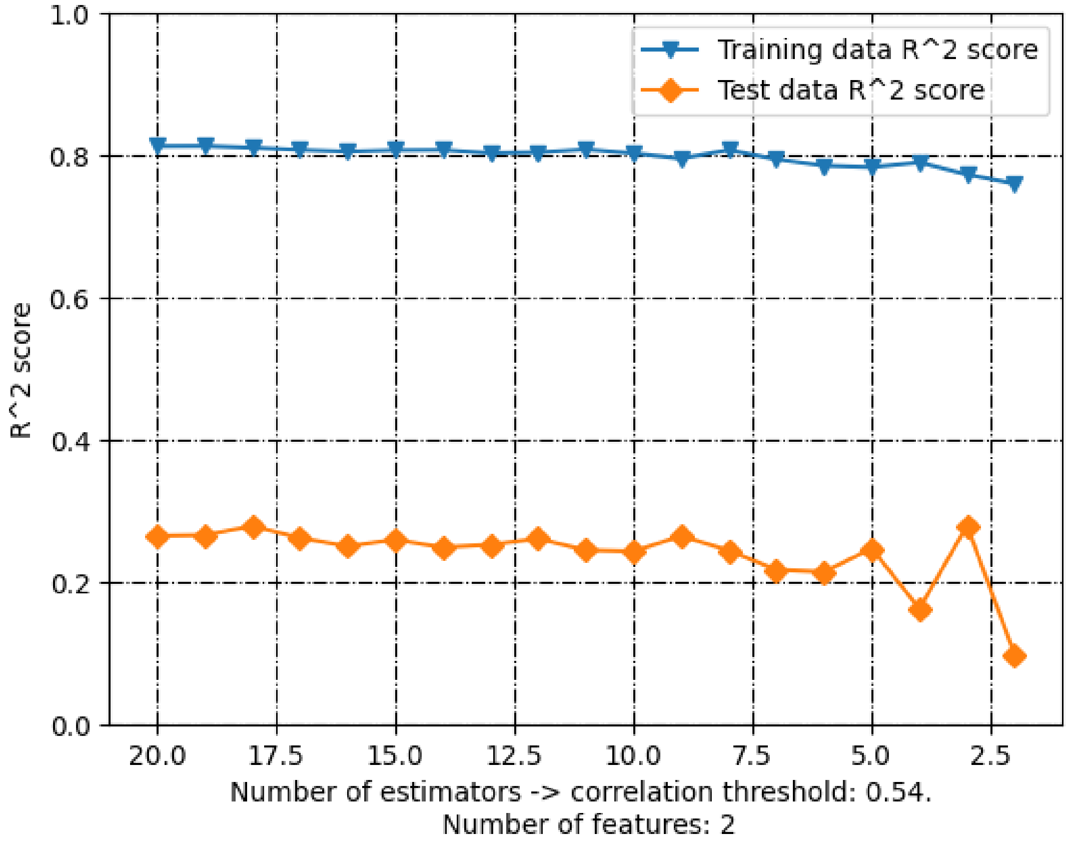

[28]:
```
plt.plot(df_without_standardization['Correlation threshold'],␣
    ↪df_without_standardization['Number of features'], label = "Number of␣
    ↪features", marker='.')
plt.legend()
plt.xlabel('Correlation threshold')
plt.ylabel('Number of features')
plt.show()
```

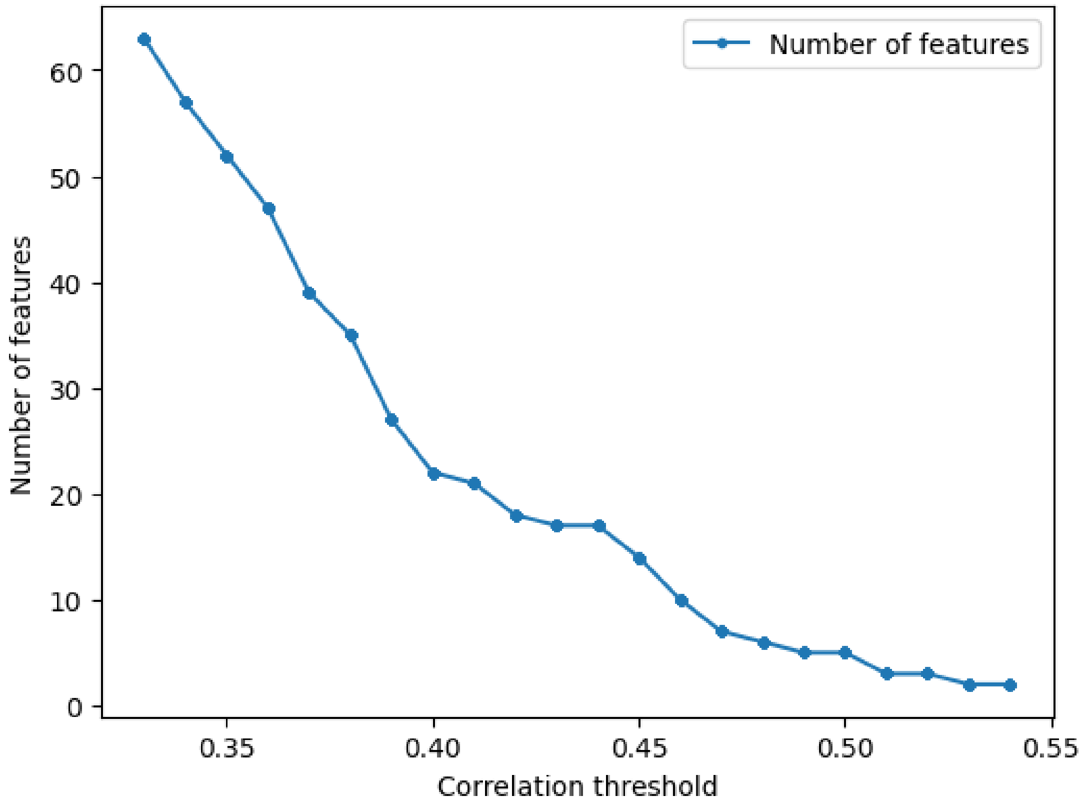

[ ]:

4 KNeighborsRegressor

[29]:
```
without_standardization, train_r2_, test_r2_, hist1, hist2, target_column_name,␣
↪training_data_RMSE, test_data_RMSE  = pred_model.
↪prepare_data_and_create_model(molecular_descriptors_df=data,
                                                                              ␣
↪                        correlation_threshold=0.50,
                                                                              ␣
↪                        standardization=False,
                                                                              ␣
↪                        model_type='KNeighborsRegressor',
                                                                              ␣
↪                        target_column_name = target,
                                                                              ␣
↪                        random_state=random_state,
                                                                              ␣
↪                        train_test_split_=True,
```

```
↪                    verbose=True)
```

```
I am not doing standardization…
  molecular descriptor name
0                    AATS0Z
1                  AATS0are
2                    AATS0d
3                   AATS0dv
4                    AATS0i
  molecular descriptor name  corr_value
0                    AATS0Z   -0.027037
1                  AATS0are   -0.129823
2                    AATS0d    0.042740
3                   AATS0dv   -0.120173
4                    AATS0i    0.132395
  molecular descriptor name  corr_value  absolute correlation value
0                    AATS0Z   -0.027037                    0.027037
1                  AATS0are   -0.129823                    0.129823
2                    AATS0d    0.042740                    0.042740
3                   AATS0dv   -0.120173                    0.120173
4                    AATS0i    0.132395                    0.132395
     molecular descriptor name  corr_value  absolute correlation value
226                     AMID_O   -0.524157                    0.524157
505                EState_VSA5   -0.579664                    0.579664
791                    MDEO-12   -0.558727                    0.558727
851                     NdssC   -0.506855                    0.506855
1091               VSA_EState5    0.503578                    0.503578
     molecular descriptor name  corr_value  absolute correlation value
226                     AMID_O   -0.524157                    0.524157
505                EState_VSA5   -0.579664                    0.579664
791                    MDEO-12   -0.558727                    0.558727
851                     NdssC   -0.506855                    0.506855
1091               VSA_EState5    0.503578                    0.503578
The model used is: KNeighbors…
Return the coefficient of determination of the prediction:
0.6586264226909913
R^2 score: 0.5303397396133361
Correlation coefficient: 0.728244285671598
Test data - unseen during training:
R^2 score: 0.6586264226909913
Correlation coefficient: 0.8115580217649205
[7.6432074  5.89053092 6.36533707 7.81601169 7.75558072 7.79160373
 8.11146067 7.76925163 8.11146067 8.68333077 6.87389833 8.18032549
 8.52770052 7.70025454 8.35880539 8.11945778 6.24208266 6.68016526]
102    8.000000
38     6.104025
8      6.044360
```

```
109     7.886057
11      8.107905
51      8.000000
88      8.886057
21      7.554396
82      8.301030
98      7.568636
58      7.677781
64      8.327902
74      8.070581
103     8.136677
91     10.000000
43      7.886057
25      5.221704
30      6.315155
Name: LoVo, dtype: float64
Training Root Mean Square Error: 0.5939458154815481
Testing Root Mean Square Error: 0.639567867727069
```

[30]:

```
print(target_column_name+str('_transformed'))
print(hist1[target_column_name].hist())
```

```
LoVo_transformed
AxesSubplot(0.125,0.11;0.775x0.77)
```

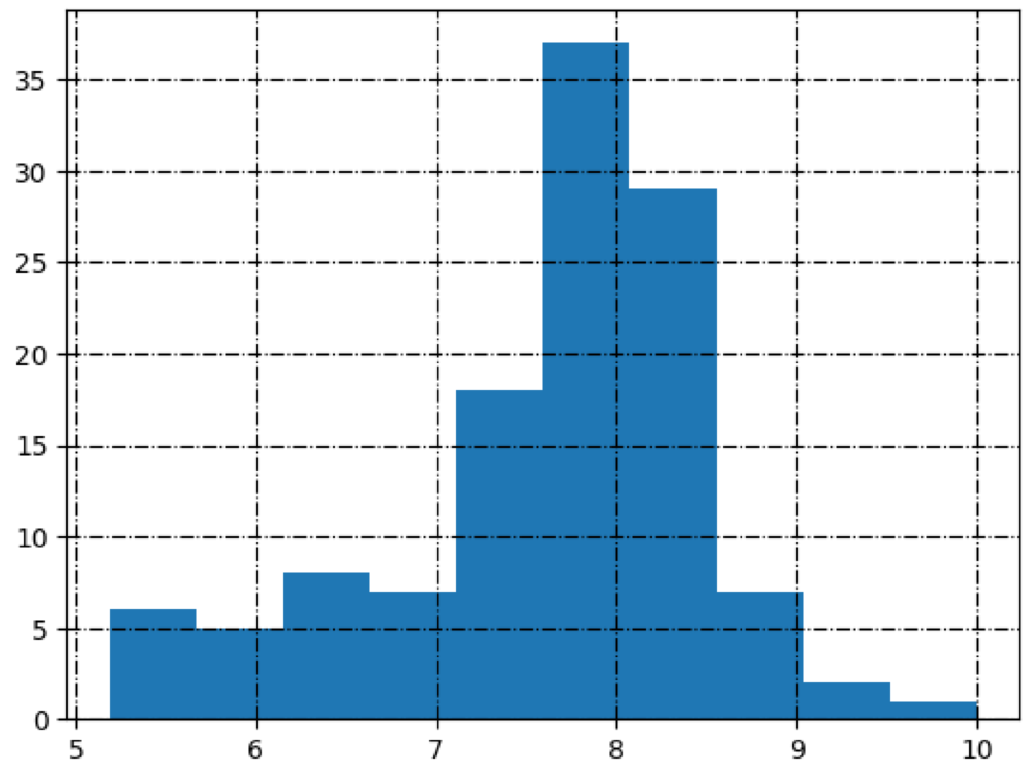

[31]:
```
with_standardization, train_r2_, test_r2_, hist1, hist2, target_column_name,␣
↪training_data_RMSE, test_data_RMSE  = pred_model.
↪prepare_data_and_create_model(molecular_descriptors_df=data,
                                                                              ␣
↪                    correlation_threshold=0.50,
                                                                              ␣
↪                    standardization=True,
                                                                              ␣
↪                    model_type='KNeighborsRegressor',
                                                                              ␣
↪                    target_column_name = target,
                                                                              ␣
↪                    random_state=random_state,
                                                                              ␣
↪                    train_test_split_=True,
                                                                              ␣
↪                    verbose=True)
```

```
I am doing standardization…
  molecular descriptor name
0                    AATS0Z
1                  AATS0are
2                    AATS0d
3                   AATS0dv
4                    AATS0i
  molecular descriptor name  corr_value
0                    AATS0Z   -0.027037
1                  AATS0are   -0.129823
2                    AATS0d    0.042740
3                   AATS0dv   -0.120173
4                    AATS0i    0.132395
  molecular descriptor name  corr_value  absolute correlation value
0                    AATS0Z   -0.027037                    0.027037
1                  AATS0are   -0.129823                    0.129823
2                    AATS0d    0.042740                    0.042740
3                   AATS0dv   -0.120173                    0.120173
4                    AATS0i    0.132395                    0.132395
     molecular descriptor name  corr_value  absolute correlation value
226                     AMID_O   -0.524157                    0.524157
505                EState_VSA5   -0.579664                    0.579664
791                    MDEO-12   -0.558727                    0.558727
851                      NdssC   -0.506855                    0.506855
1091               VSA_EState5    0.503578                    0.503578
     molecular descriptor name  corr_value  absolute correlation value
226                     AMID_O   -0.524157                    0.524157
505                EState_VSA5   -0.579664                    0.579664
```

```
791                    MDEO-12   -0.558727                    0.558727
851                      NdssC   -0.506855                    0.506855
1091               VSA_EState5    0.503578                    0.503578
The model used is: KNeighbors…
Return the coefficient of determination of the prediction:
0.6586264226909913
R^2 score: 0.5303397396133361
Correlation coefficient: 0.728244285671598
Test data - unseen during training:
R^2 score: 0.6586264226909913
Correlation coefficient: 0.8115580217649205
[7.6432074  5.89053092 6.36533707 7.81601169 7.75558072 7.79160373
 8.11146067 7.76925163 8.11146067 8.68333077 6.87389833 8.18032549
 8.52770052 7.70025454 8.35880539 8.11945778 6.24208266 6.68016526]
102     8.000000
38      6.104025
8       6.044360
109     7.886057
11      8.107905
51      8.000000
88      8.886057
21      7.554396
82      8.301030
98      7.568636
58      7.677781
64      8.327902
74      8.070581
103     8.136677
91     10.000000
43      7.886057
25      5.221704
30      6.315155
Name: LoVo, dtype: float64
Training Root Mean Square Error: 0.5939458154815481
Testing Root Mean Square Error: 0.639567867727069
```

4.1 Search inside correlation space

[32]:
```
step = 0.01
initial_step = corr_low
last_step = corr_high
first_list = [x / 100.0 for x in range(int(initial_step*100),␣
 ↪int(last_step*100), int(step*100))]
second_list = []
third_list = []
fourth_l = []
fifth_l = []
```

```
f_list = []
for i in first_list:
    without_standardization, train_r2, test_r2, _, h_, target_column_name,␣
 ↪training_data_RMSE, test_data_RMSE = pred_model.
 ↪prepare_data_and_create_model(molecular_descriptors_df = data,
                                                                              ␣
 ↪                    correlation_threshold = i,
                                                                              ␣
 ↪                    standardization = False,
                                                                              ␣
 ↪                    model_type = 'KNeighborsRegressor',
                                                                              ␣
 ↪                    target_column_name = target,
                                                                              ␣
 ↪                    random_state=random_state,
                                                                              ␣
 ↪                    train_test_split_ = True,
                                                                              ␣
 ↪                    verbose = False)
    second_list.append(train_r2)
    third_list.append(test_r2)
    fourth_l.append(training_data_RMSE)
    fifth_l.append(test_data_RMSE)
    f_list.append(len(h_))
```

```
  File "C:\Users\aleks\anaconda3\envs\cheminf_gpu\lib\site-
packages\joblib\externals\loky\backend\context.py", line 217, in
_count_physical_cores
    raise ValueError(
```

[33]:
```
df_without_standardization = pd.DataFrame(data=first_list,␣
 ↪columns=["Correlation threshold"])
df_without_standardization['Training data R^2 score'] = second_list
df_without_standardization['Test data R^2 score'] = third_list
df_without_standardization['Training RMSE'] = fourth_l
df_without_standardization['Test RMSE'] = fifth_l
df_without_standardization['Number of features'] = f_list
```

[34]:
```
df_k_nearest = df_without_standardization.copy()
df_without_standardization
```

[34]:
```
   Correlation threshold  Training data R^2 score  Test data R^2 score  \
0                   0.33                 0.471797             0.467040
1                   0.34                 0.471797             0.467040
2                   0.35                 0.621866             0.571927
3                   0.36                 0.621866             0.571927
4                   0.37                 0.591939             0.617980
```

5	0.38	0.602228	0.604400
6	0.39	0.595156	0.503138
7	0.40	0.616176	0.251338
8	0.41	0.616176	0.251338
9	0.42	0.626014	0.240570
10	0.43	0.621218	0.240034
11	0.44	0.621218	0.240034
12	0.45	0.606762	0.210415
13	0.46	0.573191	0.567609
14	0.47	0.547279	0.607128
15	0.48	0.531633	0.643283
16	0.49	0.530340	0.658626
17	0.50	0.530340	0.658626
18	0.51	0.523685	0.574992
19	0.52	0.523685	0.574992
20	0.53	0.474835	0.601116
21	0.54	0.474835	0.601116

	Training RMSE	Test RMSE	Number of features
0	0.629876	0.799133	63
1	0.629876	0.799133	57
2	0.532940	0.716194	52
3	0.532940	0.716194	47
4	0.553627	0.676573	39
5	0.546603	0.688493	35
6	0.551441	0.771595	27
7	0.536934	0.947141	22
8	0.536934	0.947141	21
9	0.530008	0.953928	18
10	0.533396	0.954264	17
11	0.533396	0.954264	17
12	0.543479	0.972683	14
13	0.566203	0.719797	10
14	0.583136	0.686116	7
15	0.593127	0.653783	6
16	0.593946	0.639568	5
17	0.593946	0.639568	5
18	0.598139	0.713625	3
19	0.598139	0.713625	3
20	0.628063	0.691345	2
21	0.628063	0.691345	2

4.2 Plots

[35]:
```
plt.plot(df_without_standardization['Correlation threshold'],␣
 ↪df_without_standardization['Training data R^2 score'], label = "Training␣
 ↪data R^2 score", marker='v')
plt.plot(df_without_standardization['Correlation threshold'],␣
 ↪df_without_standardization['Test data R^2 score'], label = "Test data R^2␣
 ↪score", marker='D')
plt.legend()
plt.xlabel('Correlation threshold')
plt.ylabel('R^2 score')
plt.ylim([0, 1])
plt.show()
```

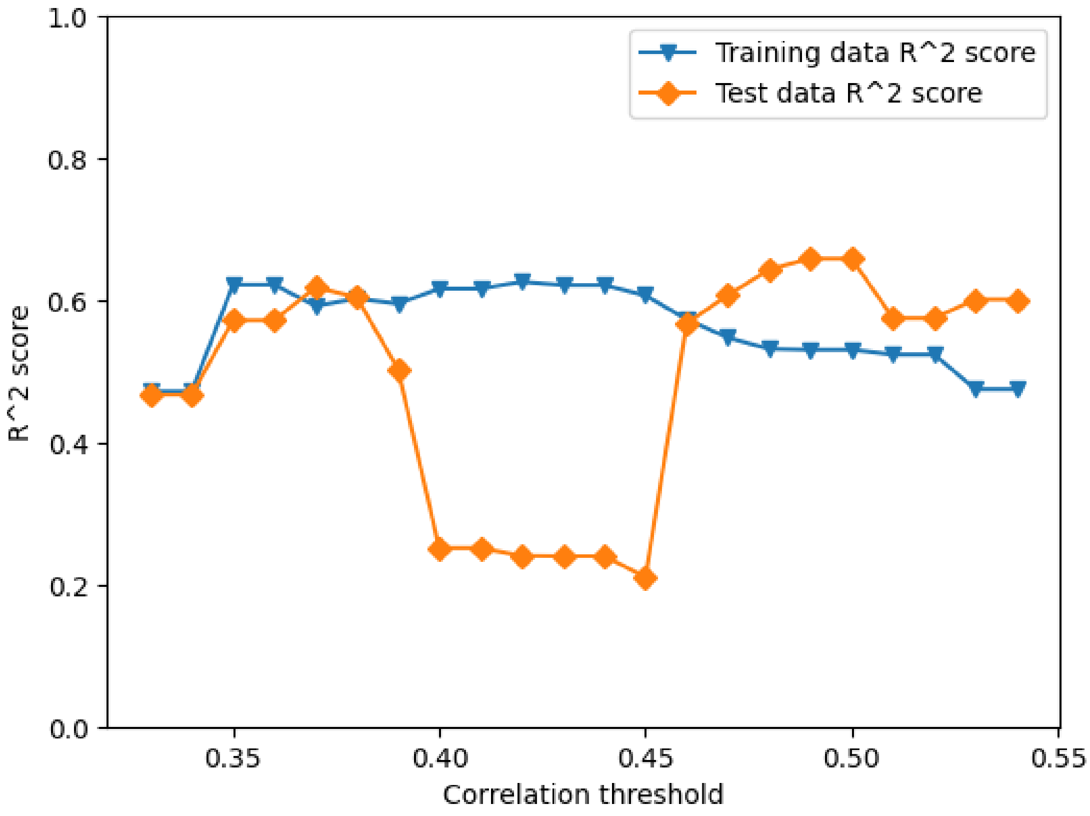

[36]:
```
plt.plot(df_without_standardization['Correlation threshold'],␣
 ↪df_without_standardization['Number of features'], label = "Number of␣
 ↪features", marker='.')
plt.legend()
plt.xlabel('Correlation threshold')
plt.ylabel('Number of features')
plt.show()
```

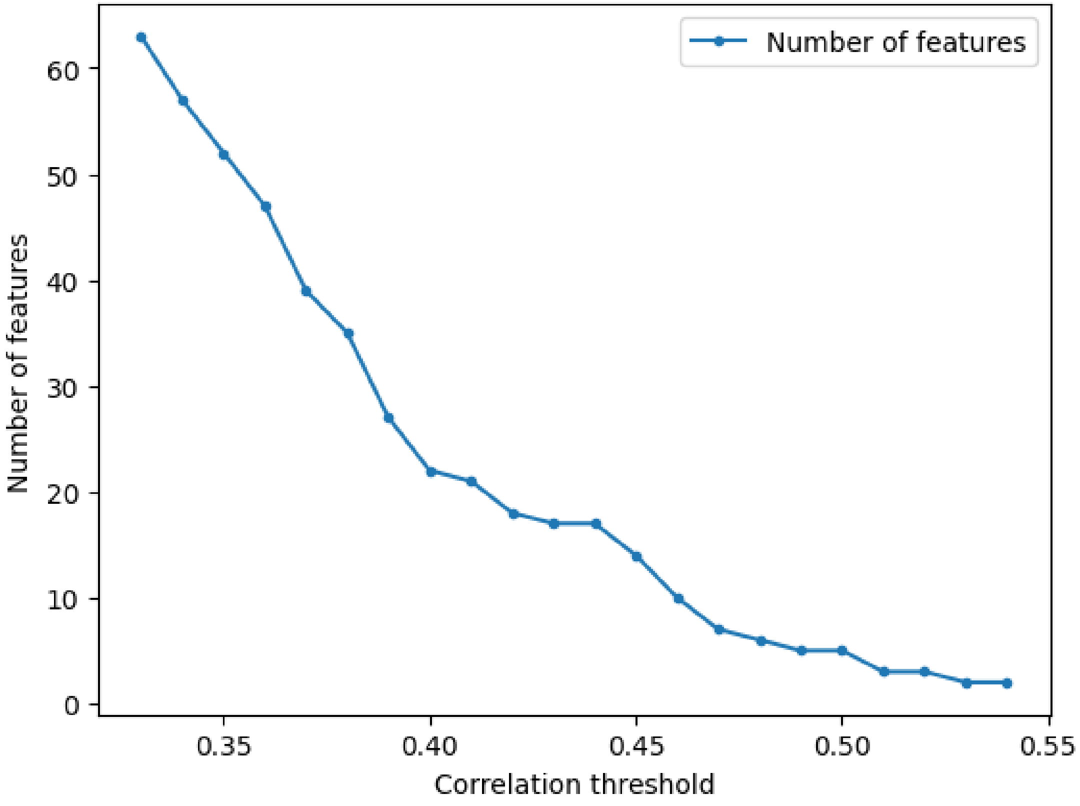

[ ]:

5 Support Vector Machines (SVM)

[37]:
```
without_standardization, train_r2_, test_r2_, hist1, hist2, target_column_name,␣
↪training_data_RMSE, test_data_RMSE  = pred_model.
↪prepare_data_and_create_model(molecular_descriptors_df=data,
                                                                                    ␣
↪                      correlation_threshold=0.50,
                                                                                    ␣
↪                      standardization=False,
                                                                                    ␣
↪                      model_type='SVR',
                                                                                    ␣
↪                      kernel_ = 'linear',
                                                                                    ␣
↪                      gamma_ = 'auto',
                                                                                    ␣
↪                      target_column_name = target,
```

```
                                                                    ␣
↪                   random_state=random_state,
                                                                    ␣
↪                   train_test_split_=True,
                                                                    ␣
↪                   verbose=True)
```

```
I am not doing standardization…
  molecular descriptor name
0                    AATS0Z
1                  AATS0are
2                    AATS0d
3                   AATS0dv
4                    AATS0i
  molecular descriptor name  corr_value
0                    AATS0Z   -0.027037
1                  AATS0are   -0.129823
2                    AATS0d    0.042740
3                   AATS0dv   -0.120173
4                    AATS0i    0.132395
  molecular descriptor name  corr_value  absolute correlation value
0                    AATS0Z   -0.027037                    0.027037
1                  AATS0are   -0.129823                    0.129823
2                    AATS0d    0.042740                    0.042740
3                   AATS0dv   -0.120173                    0.120173
4                    AATS0i    0.132395                    0.132395
     molecular descriptor name  corr_value  absolute correlation value
226                     AMID_O   -0.524157                    0.524157
505                 EState_VSA5   -0.579664                    0.579664
791                    MDEO-12   -0.558727                    0.558727
851                      NdssC   -0.506855                    0.506855
1091               VSA_EState5    0.503578                    0.503578
     molecular descriptor name  corr_value  absolute correlation value
226                     AMID_O   -0.524157                    0.524157
505                 EState_VSA5   -0.579664                    0.579664
791                    MDEO-12   -0.558727                    0.558727
851                      NdssC   -0.506855                    0.506855
1091               VSA_EState5    0.503578                    0.503578
The model used is: SVR…
Return the coefficient of determination of the prediction:
0.537072682454653
R^2 score: 0.45036618843522247
Correlation coefficient: 0.6710932784905705
Test data - unseen during training:
R^2 score: 0.537072682454653
Correlation coefficient: 0.7328524288386121
[7.85699147 6.58053324 7.30668225 7.92226268 7.95876083 8.18224156
```

```
 8.33410513 7.96378315 8.2076654  7.75689871 6.93246836 7.70691664
 7.69093605 7.85065507 8.19210208 8.18838247 6.95968616 6.46641962]
102     8.000000
38      6.104025
8       6.044360
109     7.886057
11      8.107905
51      8.000000
88      8.886057
21      7.554396
82      8.301030
98      7.568636
58      7.677781
64      8.327902
74      8.070581
103     8.136677
91     10.000000
43      7.886057
25      5.221704
30      6.315155
Name: LoVo, dtype: float64
Training Root Mean Square Error: 0.6425273818962154
Testing Root Mean Square Error: 0.744780143630552
```

[38]:

```
print(target_column_name+str('_transformed'))
print(hist1[target_column_name].hist())
```

```
LoVo_transformed
AxesSubplot(0.125,0.11;0.775x0.77)
```

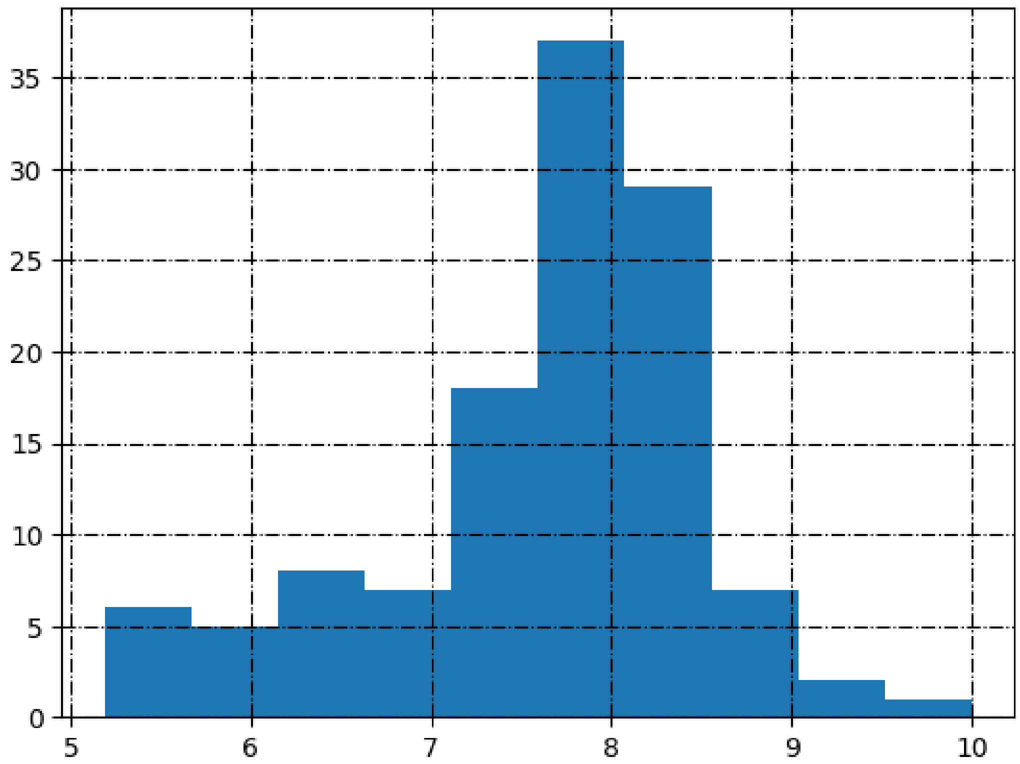

[39]:
```
with_standardization, train_r2_, test_r2_, hist1, hist2, target_column_name,␣
↪training_data_RMSE, test_data_RMSE  = pred_model.
↪prepare_data_and_create_model(molecular_descriptors_df=data,
                                                                              ␣
↪                          correlation_threshold=0.50,
                                                                              ␣
↪                          standardization=True,
                                                                              ␣
↪                          model_type='SVR',
                                                                              ␣
↪                          kernel_ = 'linear',
                                                                              ␣
↪                          gamma_ = 'auto',
                                                                              ␣
↪                          target_column_name = target,
                                                                              ␣
↪                          random_state=random_state,
                                                                              ␣
↪                          train_test_split_=True,
                                                                              ␣
↪                          verbose=True)
```

```
I am doing standardization…
  molecular descriptor name
0                    AATS0Z
1                  AATS0are
2                    AATS0d
3                   AATS0dv
4                    AATS0i
  molecular descriptor name  corr_value
0                    AATS0Z   -0.027037
1                  AATS0are   -0.129823
2                    AATS0d    0.042740
3                   AATS0dv   -0.120173
4                    AATS0i    0.132395
  molecular descriptor name  corr_value  absolute correlation value
0                    AATS0Z   -0.027037                    0.027037
1                  AATS0are   -0.129823                    0.129823
2                    AATS0d    0.042740                    0.042740
3                   AATS0dv   -0.120173                    0.120173
4                    AATS0i    0.132395                    0.132395
     molecular descriptor name  corr_value  absolute correlation value
226                     AMID_O   -0.524157                    0.524157
505                EState_VSA5   -0.579664                    0.579664
791                    MDEO-12   -0.558727                    0.558727
851                      NdssC   -0.506855                    0.506855
1091               VSA_EState5    0.503578                    0.503578
     molecular descriptor name  corr_value  absolute correlation value
226                     AMID_O   -0.524157                    0.524157
505                EState_VSA5   -0.579664                    0.579664
791                    MDEO-12   -0.558727                    0.558727
851                      NdssC   -0.506855                    0.506855
1091               VSA_EState5    0.503578                    0.503578
The model used is: SVR…
Return the coefficient of determination of the prediction:
0.537072682454653
R^2 score: 0.45036618843522247
Correlation coefficient: 0.6710932784905705
Test data - unseen during training:
R^2 score: 0.537072682454653
Correlation coefficient: 0.7328524288386121
[7.85699147 6.58053324 7.30668225 7.92226268 7.95876083 8.18224156
 8.33410513 7.96378315 8.2076654  7.75689871 6.93246836 7.70691664
 7.69093605 7.85065507 8.19210208 8.18838247 6.95968616 6.46641962]
102     8.000000
38      6.104025
8       6.044360
109     7.886057
11      8.107905
51      8.000000
```

```
88      8.886057
21      7.554396
82      8.301030
98      7.568636
58      7.677781
64      8.327902
74      8.070581
103     8.136677
91     10.000000
43      7.886057
25      5.221704
30      6.315155
Name: LoVo, dtype: float64
Training Root Mean Square Error: 0.6425273818962154
Testing Root Mean Square Error: 0.744780143630552
```

5.1 Search inside correlation space

[40]:
```python
step = 0.01
initial_step = corr_low
last_step = corr_high
first_list = [x / 100.0 for x in range(int(initial_step*100),␣
↪int(last_step*100), int(step*100))]
second_list = []
third_list = []
fourth_l = []
fifth_l = []
f_list = []
for i in first_list:
    without_standardization, train_r2, test_r2, _, h_, target_column_name,␣
↪training_data_RMSE, test_data_RMSE = pred_model.
↪prepare_data_and_create_model(molecular_descriptors_df = data,␣
↪                      correlation_threshold = i,␣
↪                      standardization = False,␣
↪                      model_type = 'SVR',␣
↪                      kernel_ = 'linear',␣
↪                      gamma_ = 'auto',␣
↪                      target_column_name = target,␣
↪                      random_state=random_state,
```

```
↪                       train_test_split_ = True,
↪                       verbose = False)
    second_list.append(train_r2)
    third_list.append(test_r2)
    fourth_l.append(training_data_RMSE)
    fifth_l.append(test_data_RMSE)
    f_list.append(len(h_))
```

[41]:
```
df_without_standardization = pd.DataFrame(data=first_list,␣
↪columns=["Correlation threshold"])
df_without_standardization['Training data R^2 score'] = second_list
df_without_standardization['Test data R^2 score'] = third_list
df_without_standardization['Training RMSE'] = fourth_l
df_without_standardization['Test RMSE'] = fifth_l
df_without_standardization['Number of features'] = f_list
```

[42]:
```
df_svm = df_without_standardization.copy()
df_without_standardization
```

[42]:
```
    Correlation threshold  Training data R^2 score  Test data R^2 score  \
0                    0.33                 0.656023             0.572259
1                    0.34                 0.661062             0.601702
2                    0.35                 0.649279             0.585295
3                    0.36                 0.654003             0.598627
4                    0.37                 0.608604             0.569823
5                    0.38                 0.613239             0.587956
6                    0.39                 0.585160             0.569465
7                    0.40                 0.570670             0.545900
8                    0.41                 0.568912             0.545411
9                    0.42                 0.542509             0.514666
10                   0.43                 0.542186             0.496898
11                   0.44                 0.542186             0.496898
12                   0.45                 0.515360             0.546672
13                   0.46                 0.499300             0.529713
14                   0.47                 0.450797             0.532164
15                   0.48                 0.451336             0.542205
16                   0.49                 0.450366             0.537073
17                   0.50                 0.450366             0.537073
18                   0.51                 0.431293             0.531133
19                   0.52                 0.431293             0.531133
20                   0.53                 0.430157             0.531358
21                   0.54                 0.430157             0.531358

    Training RMSE  Test RMSE  Number of features
0        0.508299   0.715916                  63
```

1	0.504562	0.690837	57
2	0.513258	0.704922	52
3	0.509790	0.693499	47
4	0.542204	0.717952	39
5	0.538984	0.702657	35
6	0.558207	0.718251	27
7	0.567872	0.737645	22
8	0.569033	0.738042	21
9	0.586201	0.762592	18
10	0.586407	0.776426	17
11	0.586407	0.776426	17
12	0.603343	0.737018	14
13	0.613259	0.750677	10
14	0.642276	0.748719	7
15	0.641960	0.740640	6
16	0.642527	0.744780	5
17	0.642527	0.744780	5
18	0.653581	0.749543	3
19	0.653581	0.749543	3
20	0.654233	0.749363	2
21	0.654233	0.749363	2

5.2 Plots

[43]:
```
plt.plot(df_without_standardization['Correlation threshold'], 
 ↪df_without_standardization['Training data R^2 score'], label = "Training 
 ↪data R^2 score", marker='v')
plt.plot(df_without_standardization['Correlation threshold'], 
 ↪df_without_standardization['Test data R^2 score'], label = "Test data R^2 
 ↪score", marker='D')
plt.legend()
plt.xlabel('Correlation threshold')
plt.ylabel('R^2 score')
plt.ylim([0, 1])
plt.show()
```

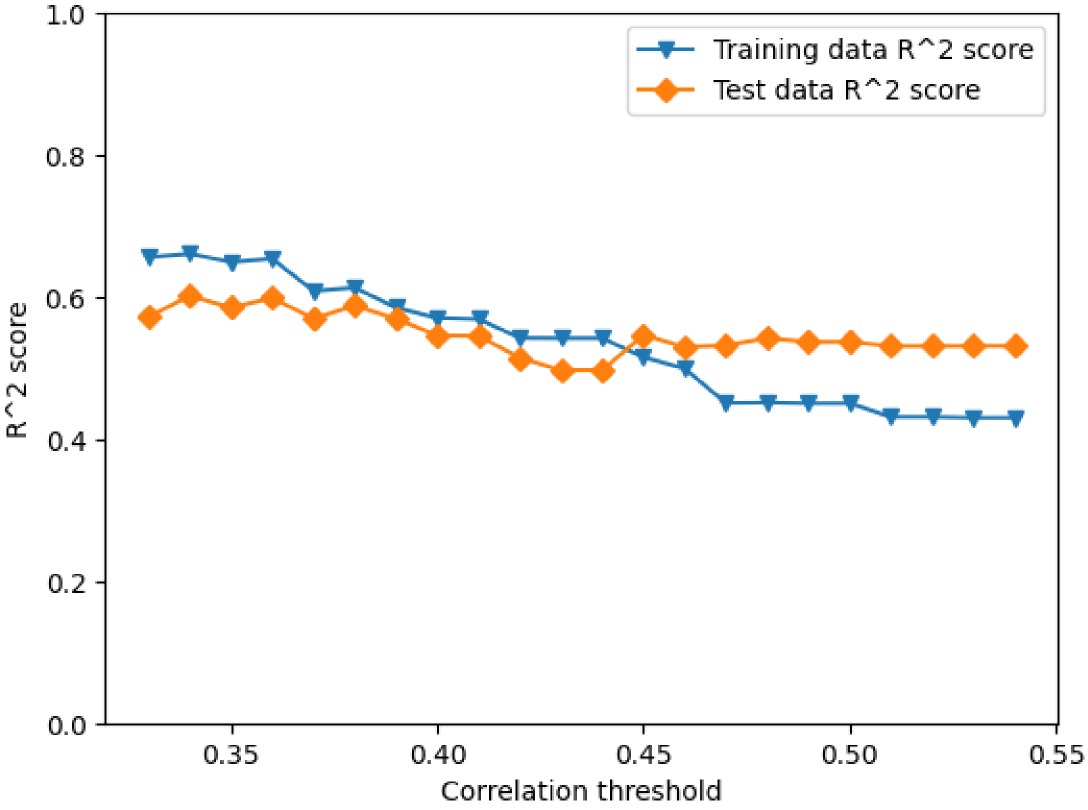

[44]:
```
plt.plot(df_without_standardization['Correlation threshold'],␣
↪df_without_standardization['Number of features'], label = "Number of␣
↪features", marker='.')
plt.legend()
plt.xlabel('Correlation threshold')
plt.ylabel('Number of features')
plt.show()
```

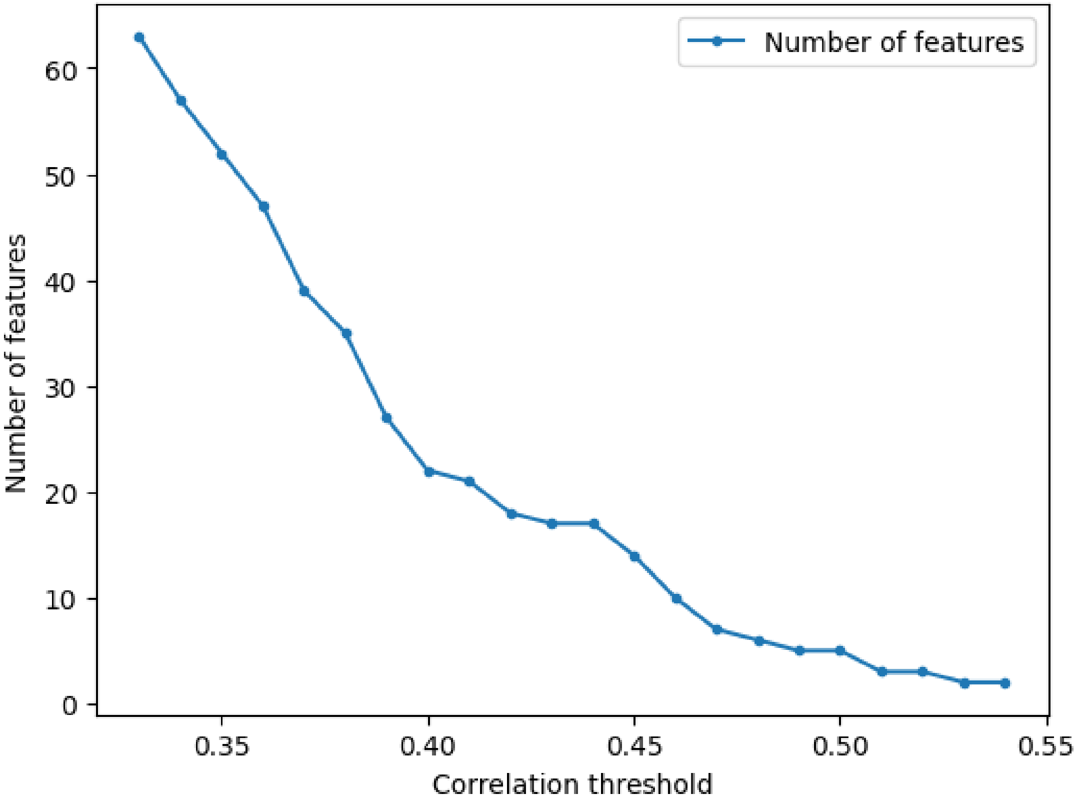

5.3 Store quality measurements

[45]:
```
with pd.ExcelWriter('../Data/Quality_'+str(target)+'_'+str(random_state)+'_.
 ↪xlsx') as writer:
    df_linear.to_excel(writer, sheet_name='MLR')
    df_decision_tree.to_excel(writer, sheet_name='DT')
    df_random_forest.to_excel(writer, sheet_name='RF')
    df_k_nearest.to_excel(writer, sheet_name='KNN')
    df_svm.to_excel(writer, sheet_name='SVM')
```

Notebook

January 18, 2024

1 File 25

[1]:
```
import sys
from pathlib import Path
prediction_mode_path = Path("../module")
sys.path.append(prediction_mode_path.as_posix())
import models_creation as pred_model

import pandas as pd
import numpy as np

from sklearn.metrics import r2_score

import matplotlib.pyplot as plt
from sklearn.tree import plot_tree

from matplotlib import pyplot as plt

import joblib

import warnings
warnings.filterwarnings('ignore')
```

[2]:
```
load_prepared_data = pd.read_excel('../Data/Kolchicyna_machine_learning.xlsx')
load_prepared_data = load_prepared_data.drop(columns=['Unnamed: 0'])

target = 'LoVo'

corr_low = 0.33
corr_high = 0.55

random_state = 28
```

[3]:
```
load_prepared_data.head()
```

[3]:
```
      AATS0Z  AATS0are    AATS0d   AATS0dv      AATS0i     AATS0m    AATS0p  \
0  25.090909  6.046518  3.109091  6.662626  160.775045  99.075564  1.555512
1  24.448276  6.008422  3.051724  6.386973  161.021674  96.473315  1.538471
```

```
2  23.868852  5.974074  3.000000  6.138434  161.244045  94.127025  1.523105
3  23.868852  5.974074  3.032787  6.171220  161.244045  94.127025  1.523105
4  23.343750  5.942945  2.953125  5.913194  161.445569  92.000700  1.509180

    AATS0pe    AATS0s    AATS0se  …  A549_float  MCF-7_float  LoVo_float  \
0  6.126680  3.435416  7.629859  …        10.8         10.3         6.5
1  6.088791  3.330998  7.596891  …        11.6         12.0         8.5
2  6.054630  3.236850  7.567166  …        10.9         12.2         8.8
3  6.054630  3.257798  7.567166  …        10.5         11.3         8.5
4  6.023670  3.151529  7.540228  …        89.5         92.7        52.8

   LoVo/DX_float  BALB/3T3_float  A549_float_transformed  \
0           54.9            10.2                7.966576
1           31.1            14.3                7.935542
2           17.9            11.7                7.962574
3           10.2            11.0                7.978811
4           77.8            99.4                7.048177

   MCF-7_float_transformed  LoVo_float_transformed  LoVo/DX_float_transformed  \
0                 7.987163                8.187087                   7.260428
1                 7.920819                8.070581                   7.507240
2                 7.913640                8.055517                   7.747147
3                 7.946922                8.070581                   7.991400
4                 7.032920                7.277366                   7.109020

   BALB/3T3_float_transformed
0                    7.991400
1                    7.844664
2                    7.931814
3                    7.958607
4                    7.002614

[5 rows x 1221 columns]
```

[4]:
```python
data = load_prepared_data[list(load_prepared_data.columns)[0:-10]]
data[target] = load_prepared_data[load_prepared_data.columns[-3]]
data.head()
```

[4]:
```
      AATS0Z  AATS0are    AATS0d   AATS0dv      AATS0i     AATS0m    AATS0p  \
0  25.090909  6.046518  3.109091  6.662626  160.775045  99.075564  1.555512
1  24.448276  6.008422  3.051724  6.386973  161.021674  96.473315  1.538471
2  23.868852  5.974074  3.000000  6.138434  161.244045  94.127025  1.523105
3  23.868852  5.974074  3.032787  6.171220  161.244045  94.127025  1.523105
4  23.343750  5.942945  2.953125  5.913194  161.445569  92.000700  1.509180

    AATS0pe    AATS0s    AATS0se  …    piPC10     piPC2     piPC3     piPC4  \
0  6.126680  3.435416  7.629859  …  7.999655  4.266195  4.915408  5.516700
```

```
1  6.088791  3.330998  7.596891  …  8.018726  4.280132  4.922714  5.520711
2  6.054630  3.236850  7.567166  …  8.028799  4.293878  4.929967  5.524706
3  6.054630  3.257798  7.567166  …  8.037440  4.307438  4.929967  5.524706
4  6.023670  3.151529  7.540228  …  8.034892  4.307438  4.937168  5.528685

     piPC5     piPC6     piPC7     piPC8     piPC9      LoVo
0  6.098566  6.595759  6.979116  7.337313  7.681445  8.187087
1  6.103048  6.601209  6.985613  7.349442  7.695531  8.070581
2  6.105281  6.603923  6.989306  7.353932  7.704023  8.055517
3  6.107510  6.606629  6.992068  7.361425  7.709420  8.070581
4  6.107510  6.605277  6.991148  7.356489  7.707175  7.277366

[5 rows x 1212 columns]
```

2 Multiple Linear regression (MLR)

[5]:
```
without_standardization, train_r2_, test_r2_, hist1, hist2, target_column_name,␣
↪training_data_RMSE, test_data_RMSE  = pred_model.
↪prepare_data_and_create_model(molecular_descriptors_df=data,
                                                                              ␣
↪                    correlation_threshold=0.5,
                                                                              ␣
↪                    standardization=False,
                                                                              ␣
↪                    model_type='linear_model',
                                                                              ␣
↪                    target_column_name = str(target),
                                                                              ␣
↪                    random_state=random_state,
                                                                              ␣
↪                    train_test_split_=True,
                                                                              ␣
↪                    verbose=True)
```

```
I am not doing standardization…
  molecular descriptor name
0                   AATS0Z
1                 AATS0are
2                   AATS0d
3                  AATS0dv
4                   AATS0i
  molecular descriptor name  corr_value
0                   AATS0Z   -0.027037
1                 AATS0are   -0.129823
2                   AATS0d    0.042740
3                  AATS0dv   -0.120173
```

```
4                  AATS0i    0.132395
  molecular descriptor name  corr_value  absolute correlation value
0                  AATS0Z   -0.027037                    0.027037
1                AATS0are   -0.129823                    0.129823
2                  AATS0d    0.042740                    0.042740
3                 AATS0dv   -0.120173                    0.120173
4                  AATS0i    0.132395                    0.132395
     molecular descriptor name  corr_value  absolute correlation value
226                     AMID_O   -0.524157                    0.524157
505                EState_VSA5   -0.579664                    0.579664
791                    MDEO-12   -0.558727                    0.558727
851                      NdssC   -0.506855                    0.506855
1091               VSA_EState5    0.503578                    0.503578
     molecular descriptor name  corr_value  absolute correlation value
226                     AMID_O   -0.524157                    0.524157
505                EState_VSA5   -0.579664                    0.579664
791                    MDEO-12   -0.558727                    0.558727
851                      NdssC   -0.506855                    0.506855
1091               VSA_EState5    0.503578                    0.503578
The model used is: LinearReg…
Return the coefficient of determination of the prediction:
0.337000351477726
R^2 score: 0.5162015514838987
Correlation coefficient: 0.7184716775794984
Test data - unseen during training:
R^2 score: 0.337000351477726
Correlation coefficient: 0.580517313676109
[8.04195487 6.44740649 7.70878358 6.62243668 7.96878809 7.73545677
 7.83197255 8.13941052 6.93948294 7.70520781 7.9984246  7.99982883
 8.16688408 7.4465517  7.74339443 6.94259984 8.14978125 6.74021135]
113    7.795880
35     6.254925
101    7.275724
36     7.017729
100    7.337242
13     8.055517
0      8.187087
114    7.638272
104    8.318759
96     7.769551
40     8.050610
103    8.136677
48     7.853872
39     8.267606
14     7.273273
117    6.167491
21     7.554396
9      6.343902
```

```
Name: LoVo, dtype: float64
Training Root Mean Square Error: 0.6528390819257617
Testing Root Mean Square Error: 0.5453533851862918
```

[6]:
```python
print(target_column_name+str('_transformed'))
print(hist1[target_column_name].hist())
```

```
LoVo_transformed
AxesSubplot(0.125,0.11;0.775x0.77)
```

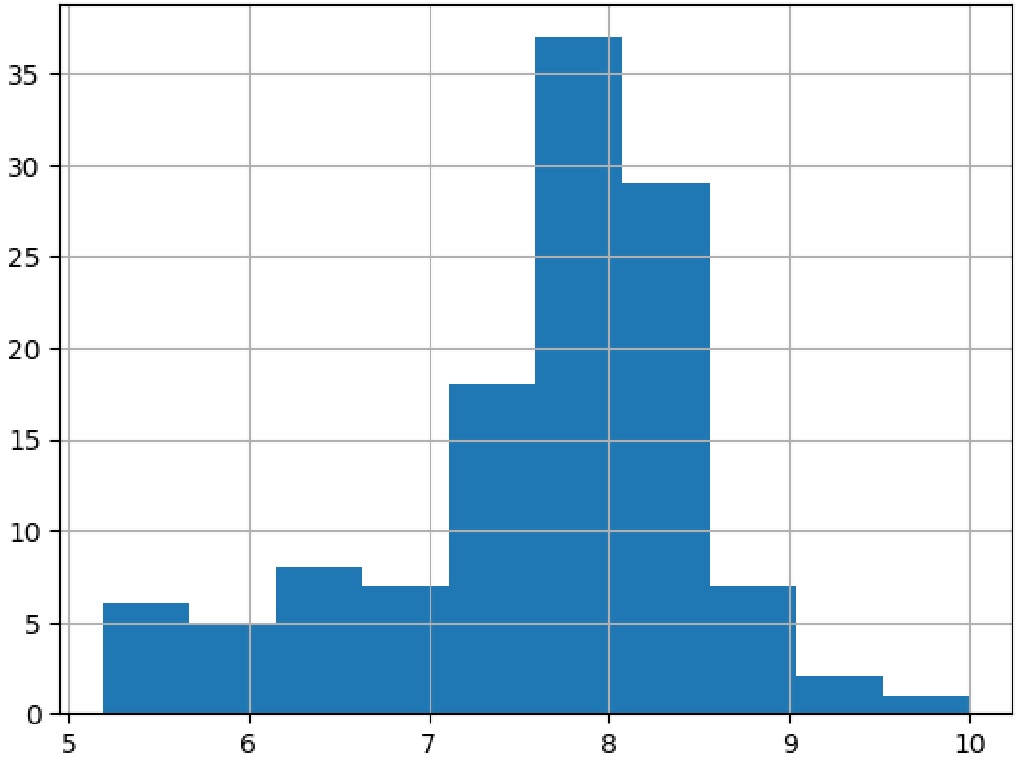

[7]:
```python
with_standardization, train_r2_, test_r2_, hist1, hist2, target_column_name,␣
↪training_data_RMSE, test_data_RMSE  = pred_model.
↪prepare_data_and_create_model(molecular_descriptors_df=data,
                                                                            ␣
↪                         correlation_threshold=0.50,
                                                                            ␣
↪                         standardization=True,
                                                                            ␣
↪                         model_type='linear_model',
                                                                            ␣
↪                         target_column_name = target,
```

```
↪                     random_state=random_state,
↪                     train_test_split_=True,
↪                     verbose=True)
```

```
I am doing standardization…
  molecular descriptor name
0                    AATS0Z
1                  AATS0are
2                    AATS0d
3                   AATS0dv
4                    AATS0i
  molecular descriptor name  corr_value
0                    AATS0Z   -0.027037
1                  AATS0are   -0.129823
2                    AATS0d    0.042740
3                   AATS0dv   -0.120173
4                    AATS0i    0.132395
  molecular descriptor name  corr_value  absolute correlation value
0                    AATS0Z   -0.027037                    0.027037
1                  AATS0are   -0.129823                    0.129823
2                    AATS0d    0.042740                    0.042740
3                   AATS0dv   -0.120173                    0.120173
4                    AATS0i    0.132395                    0.132395
     molecular descriptor name  corr_value  absolute correlation value
226                     AMID_O   -0.524157                    0.524157
505                EState_VSA5   -0.579664                    0.579664
791                    MDEO-12   -0.558727                    0.558727
851                     NdssC   -0.506855                    0.506855
1091               VSA_EState5    0.503578                    0.503578
     molecular descriptor name  corr_value  absolute correlation value
226                     AMID_O   -0.524157                    0.524157
505                EState_VSA5   -0.579664                    0.579664
791                    MDEO-12   -0.558727                    0.558727
851                     NdssC   -0.506855                    0.506855
1091               VSA_EState5    0.503578                    0.503578
The model used is: LinearReg…
Return the coefficient of determination of the prediction:
0.337000351477726
R^2 score: 0.5162015514838987
Correlation coefficient: 0.7184716775794984
Test data - unseen during training:
R^2 score: 0.337000351477726
Correlation coefficient: 0.580517313676109
[8.04195487 6.44740649 7.70878358 6.62243668 7.96878809 7.73545677
```

```
 7.83197255 8.13941052 6.93948294 7.70520781 7.9984246  7.99982883
 8.16688408 7.4465517  7.74339443 6.94259984 8.14978125 6.74021135]
113    7.795880
35     6.254925
101    7.275724
36     7.017729
100    7.337242
13     8.055517
0      8.187087
114    7.638272
104    8.318759
96     7.769551
40     8.050610
103    8.136677
48     7.853872
39     8.267606
14     7.273273
117    6.167491
21     7.554396
9      6.343902
Name: LoVo, dtype: float64
Training Root Mean Square Error: 0.6528390819257617
Testing Root Mean Square Error: 0.5453533851862918
```

2.1 Search inside correlation space

[8]:
```
step = 0.01
initial_step = corr_low
last_step = corr_high
first_list = [x / 100.0 for x in range(int(initial_step*100),␣
 ↪int(last_step*100), int(step*100))]
second_list = []
third_list = []
fourth_l = []
fifth_l = []
f_list = []
for i in first_list:
    without_standardization, train_r2, test_r2, _, h_, target_column_name,␣
 ↪training_data_RMSE, test_data_RMSE = pred_model.
 ↪prepare_data_and_create_model(molecular_descriptors_df = data,
                                                                          ␣
 ↪                    correlation_threshold = i,
                                                                          ␣
 ↪                    standardization = False,
                                                                          ␣
 ↪                    model_type = 'linear_model',
```

```
                        target_column_name = target,
                        random_state=random_state,
                        train_test_split_ = True,
                        verbose = False)
    second_list.append(train_r2)
    third_list.append(test_r2)
    fourth_l.append(training_data_RMSE)
    fifth_l.append(test_data_RMSE)
    f_list.append(len(h_))
```

[9]:
```
df_without_standardization = pd.DataFrame(data=first_list,
 columns=["Correlation threshold"])
df_without_standardization['Training data R^2 score'] = second_list
df_without_standardization['Test data R^2 score'] = third_list
df_without_standardization['Training RMSE'] = fourth_l
df_without_standardization['Test RMSE'] = fifth_l
df_without_standardization['Number of features'] = f_list
```

[10]:
```
df_linear = df_without_standardization.copy()
df_without_standardization
```

[10]:

	Correlation threshold	Training data R^2 score	Test data R^2 score	\
0	0.33	0.926564	-2.796318	
1	0.34	0.891545	-1.867318	
2	0.35	0.869952	-0.626826	
3	0.36	0.844732	-1.084081	
4	0.37	0.760465	-0.085131	
5	0.38	0.741120	-0.360039	
6	0.39	0.704287	-0.065653	
7	0.40	0.677245	0.250783	
8	0.41	0.677188	0.258607	
9	0.42	0.626303	0.289063	
10	0.43	0.626292	0.290803	
11	0.44	0.626292	0.290803	
12	0.45	0.598673	0.465834	
13	0.46	0.583054	0.247032	
14	0.47	0.525993	0.334133	
15	0.48	0.517159	0.331566	
16	0.49	0.516202	0.337000	
17	0.50	0.516202	0.337000	
18	0.51	0.489571	0.369378	
19	0.52	0.489571	0.369378	
20	0.53	0.464962	0.394784	

```
21                    0.54                0.464962            0.394784

    Training RMSE  Test RMSE  Number of features
0        0.254349   1.304976                  63
1        0.309100   1.134121                  57
2        0.338474   0.854264                  52
3        0.369840   0.966893                  47
4        0.459365   0.697690                  39
5        0.477554   0.781083                  35
6        0.510398   0.691400                  27
7        0.533225   0.579729                  22
8        0.533272   0.576694                  21
9        0.573765   0.564725                  18
10       0.573773   0.564033                  17
11       0.573773   0.564033                  17
12       0.594598   0.489507                  14
13       0.606058   0.581178                  10
14       0.646199   0.546531                   7
15       0.652192   0.547584                   6
16       0.652839   0.545353                   5
17       0.652839   0.545353                   5
18       0.670566   0.531871                   3
19       0.670566   0.531871                   3
20       0.686541   0.521047                   2
21       0.686541   0.521047                   2
```

[ ]:

```

```

2.2 Plots

[11]:

```
plt.plot(df_without_standardization['Correlation threshold'],␣
 ↪df_without_standardization['Training data R^2 score'], label = "Training␣
 ↪data R^2 score", marker='v')
plt.plot(df_without_standardization['Correlation threshold'],␣
 ↪df_without_standardization['Test data R^2 score'], label = "Test data R^2␣
 ↪score", marker='D')
plt.legend()
plt.xlabel('Correlation threshold')
plt.ylabel('R^2 score')
plt.ylim([0, 1])
plt.show()
```

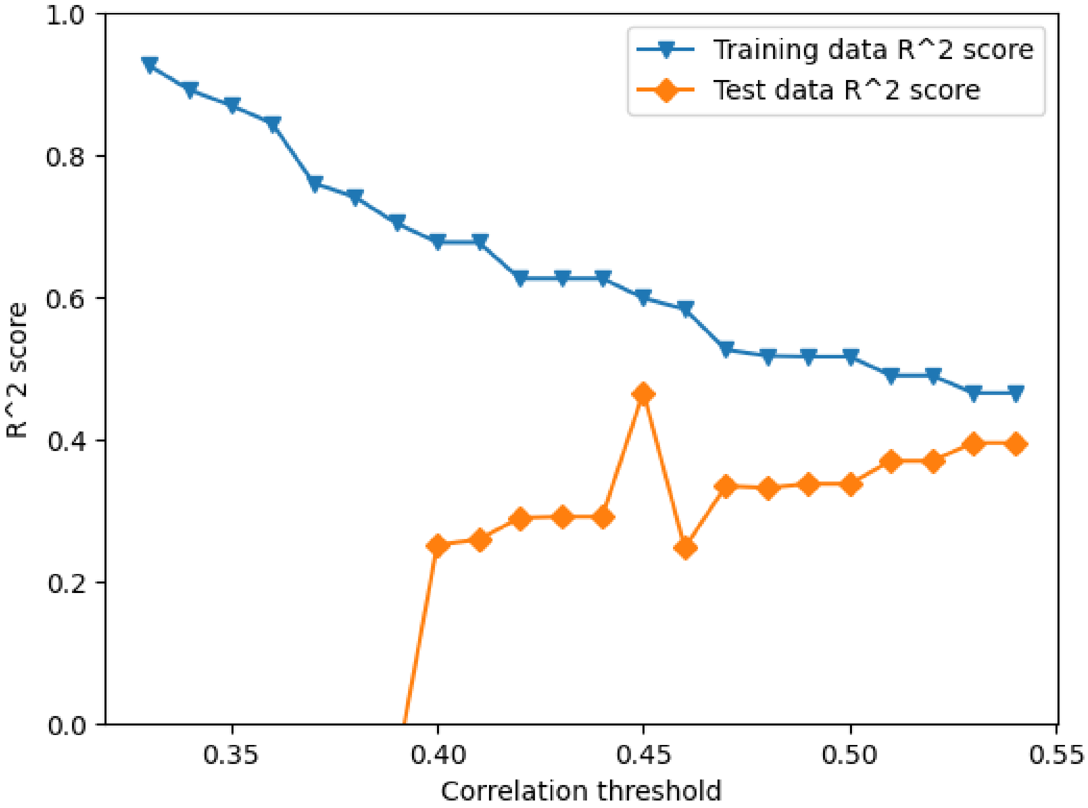

[12]:
```
plt.plot(df_without_standardization['Correlation threshold'],␣
 ↪df_without_standardization['Number of features'], label = "Number of␣
 ↪features", marker='.')
plt.legend()
plt.xlabel('Correlation threshold')
plt.ylabel('Number of features')
plt.show()
```

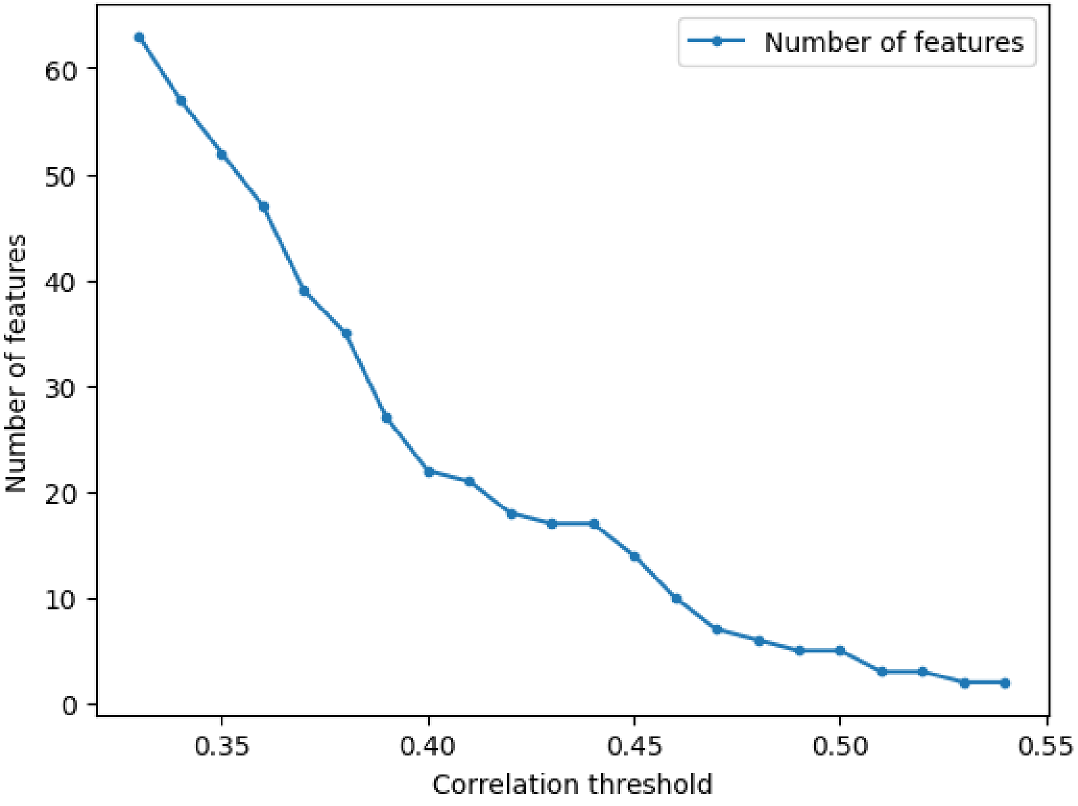

[ ]:

```
 
```

2.3 Decision Tree

[13]:

```
without_standardization, train_r2_, test_r2_, hist1, hist2, target_column_name,␣
↪training_data_RMSE, test_data_RMSE  = pred_model.
↪prepare_data_and_create_model(molecular_descriptors_df=data,
                                                                                 ␣
↪                          correlation_threshold=0.50,
                                                                                 ␣
↪                          standardization=False,
                                                                                 ␣
↪                          model_type='DecisionTreeRegressor',
                                                                                 ␣
↪                          max_depth=5,
                                                                                 ␣
↪                          target_column_name = target,
                                                                                 ␣
↪                          random_state=random_state,
```

```
↪                    train_test_split_=True,
↪                    verbose=True)
```

```
I am not doing standardization…
  molecular descriptor name
0                    AATS0Z
1                  AATS0are
2                    AATS0d
3                   AATS0dv
4                    AATS0i
  molecular descriptor name  corr_value
0                    AATS0Z   -0.027037
1                  AATS0are   -0.129823
2                    AATS0d    0.042740
3                   AATS0dv   -0.120173
4                    AATS0i    0.132395
  molecular descriptor name  corr_value  absolute correlation value
0                    AATS0Z   -0.027037                    0.027037
1                  AATS0are   -0.129823                    0.129823
2                    AATS0d    0.042740                    0.042740
3                   AATS0dv   -0.120173                    0.120173
4                    AATS0i    0.132395                    0.132395
     molecular descriptor name  corr_value  absolute correlation value
226                     AMID_O   -0.524157                    0.524157
505                 EState_VSA5   -0.579664                    0.579664
791                    MDEO-12   -0.558727                    0.558727
851                      NdssC   -0.506855                    0.506855
1091               VSA_EState5    0.503578                    0.503578
     molecular descriptor name  corr_value  absolute correlation value
226                     AMID_O   -0.524157                    0.524157
505                 EState_VSA5   -0.579664                    0.579664
791                    MDEO-12   -0.558727                    0.558727
851                      NdssC   -0.506855                    0.506855
1091               VSA_EState5    0.503578                    0.503578
The model used is: DecisionTree…
Return the coefficient of determination of the prediction:
0.49523366571868277
R^2 score: 0.7741874734750118
Correlation coefficient: 0.8798792380065641
Test data - unseen during training:
R^2 score: 0.49523366571868277
Correlation coefficient: 0.7037284033763898
[8.05782805 7.01392173 8.05782805 7.01392173 8.05782805 8.05782805
 8.05782805 7.58505897 7.59136896 8.05782805 8.05782805 8.05782805
 8.05782805 8.05782805 8.05782805 5.31054481 7.18444053 6.04436035]
113    7.795880
```

```
35     6.254925
101    7.275724
36     7.017729
100    7.337242
13     8.055517
0      8.187087
114    7.638272
104    8.318759
96     7.769551
40     8.050610
103    8.136677
48     7.853872
39     8.267606
14     7.273273
117    6.167491
21     7.554396
9      6.343902
Name: LoVo, dtype: float64
Training Root Mean Square Error: 0.4460134183437457
Testing Root Mean Square Error: 0.47584614471491754
```

[14]:
```
print(target_column_name+str('_transformed'))
print(hist1[target_column_name].hist())
```

```
LoVo_transformed
AxesSubplot(0.125,0.11;0.775x0.77)
```

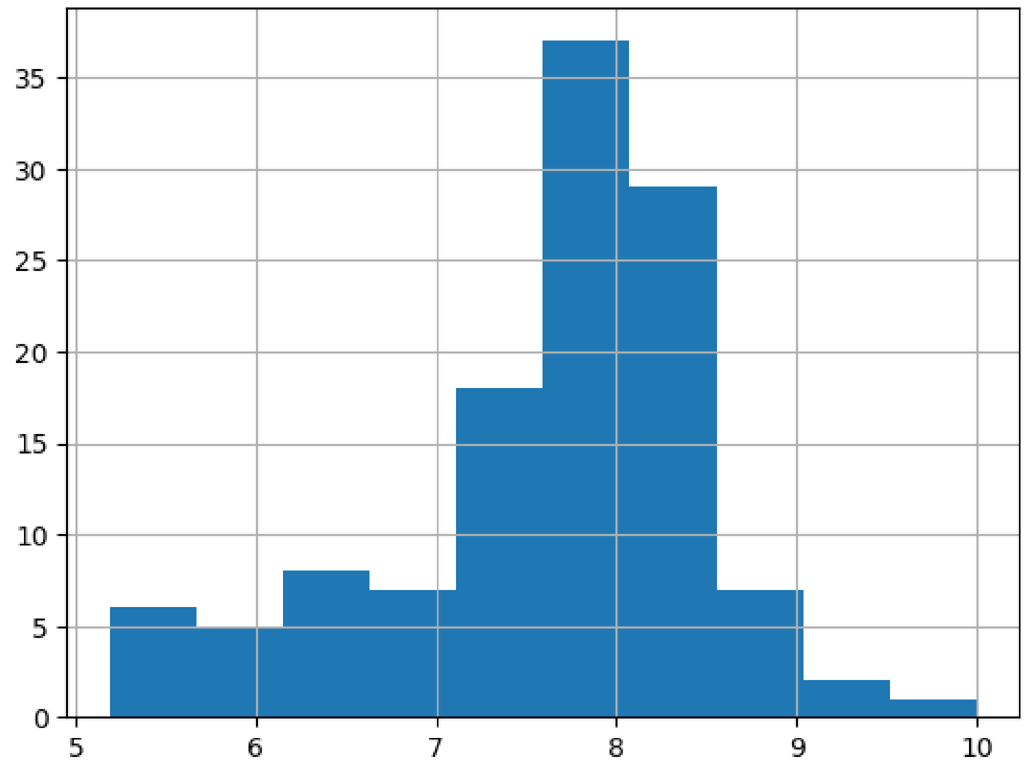

[15]:
```
with_standardization, train_r2_, test_r2_, hist1, hist2, target_column_name,␣
↪training_data_RMSE, test_data_RMSE  = pred_model.
↪prepare_data_and_create_model(molecular_descriptors_df=data,
                                                                                              ␣
↪                        correlation_threshold=0.50,
                                                                                              ␣
↪                        standardization=True,
                                                                                              ␣
↪                        model_type='DecisionTreeRegressor',
                                                                                              ␣
↪                        max_depth=5,
                                                                                              ␣
↪                        target_column_name = target,
                                                                                              ␣
↪                        random_state=random_state,
                                                                                              ␣
↪                        train_test_split_=True,
                                                                                              ␣
↪                        verbose=True)
```

```
I am doing standardization…
  molecular descriptor name
```

```
0                   AATS0Z
1                 AATS0are
2                   AATS0d
3                  AATS0dv
4                   AATS0i
  molecular descriptor name  corr_value
0                   AATS0Z   -0.027037
1                 AATS0are   -0.129823
2                   AATS0d    0.042740
3                  AATS0dv   -0.120173
4                   AATS0i    0.132395
  molecular descriptor name  corr_value  absolute correlation value
0                   AATS0Z   -0.027037                    0.027037
1                 AATS0are   -0.129823                    0.129823
2                   AATS0d    0.042740                    0.042740
3                  AATS0dv   -0.120173                    0.120173
4                   AATS0i    0.132395                    0.132395
     molecular descriptor name  corr_value  absolute correlation value
226                     AMID_O   -0.524157                    0.524157
505                 EState_VSA5   -0.579664                    0.579664
791                    MDEO-12   -0.558727                    0.558727
851                      NdssC   -0.506855                    0.506855
1091                VSA_EState5    0.503578                    0.503578
     molecular descriptor name  corr_value  absolute correlation value
226                     AMID_O   -0.524157                    0.524157
505                 EState_VSA5   -0.579664                    0.579664
791                    MDEO-12   -0.558727                    0.558727
851                      NdssC   -0.506855                    0.506855
1091                VSA_EState5    0.503578                    0.503578
The model used is: DecisionTree…
Return the coefficient of determination of the prediction:
0.49523366571868277
R^2 score: 0.7741874734750118
Correlation coefficient: 0.8798792380065641
Test data - unseen during training:
R^2 score: 0.49523366571868277
Correlation coefficient: 0.7037284033763898
[8.05782805 7.01392173 8.05782805 7.01392173 8.05782805 8.05782805
 8.05782805 7.58505897 7.59136896 8.05782805 8.05782805 8.05782805
 8.05782805 8.05782805 8.05782805 5.31054481 7.18444053 6.04436035]
113    7.795880
35     6.254925
101    7.275724
36     7.017729
100    7.337242
13     8.055517
0      8.187087
114    7.638272
```

```
104    8.318759
96     7.769551
40     8.050610
103    8.136677
48     7.853872
39     8.267606
14     7.273273
117    6.167491
21     7.554396
9      6.343902
Name: LoVo, dtype: float64
Training Root Mean Square Error: 0.4460134183437457
Testing Root Mean Square Error: 0.475846144714917 54
```

2.4 Search inside correlation space

[16]:
```
step = 0.01
initial_step = corr_low
last_step = corr_high
first_list = [x / 100.0 for x in range(int(initial_step*100),␣
 ↪int(last_step*100), int(step*100))]
max_depth = [range(2, 30, 1)]
corr_th = []
second_list = []
third_list = []
fourth_l = []
fifth_l = []
f_list = []
fif_list = []
for i in first_list:
    for depth in max_depth[0]:

        without_standardization, train_r2, test_r2, _, h_, target_column_name,␣
 ↪training_data_RMSE, test_data_RMSE = pred_model.
 ↪prepare_data_and_create_model(molecular_descriptors_df=data,
                                                                             ␣
 ↪                    correlation_threshold=i,
                                                                             ␣
 ↪                    standardization=False,
                                                                             ␣
 ↪                    model_type='DecisionTreeRegressor',
                                                                             ␣
 ↪                    max_depth=depth,
                                                                             ␣
 ↪                    target_column_name = target,
                                                                             ␣
 ↪                    random_state=random_state,
```

```
                                                                                ␣
↪                       train_test_split_=True,
                                                                                ␣
↪                       verbose=False)
        corr_th.append(i)
        second_list.append(train_r2)
        third_list.append(test_r2)
        fourth_l.append(training_data_RMSE)
        fifth_l.append(test_data_RMSE)
        f_list.append(len(h_))
        fif_list.append(depth)
```

[17]:
```
df_without_standardization = pd.DataFrame(data=corr_th, columns=["Correlation␣
 ↪threshold"])
df_without_standardization['Training data R^2 score'] = second_list
df_without_standardization['Test data R^2 score'] = third_list
df_without_standardization['Training RMSE'] = fourth_l
df_without_standardization['Test RMSE'] = fifth_l
df_without_standardization['Number of features'] = f_list
df_without_standardization['Depth number'] = fif_list
```

[18]:
```
df_decision_tree = df_without_standardization.copy()
#df_without_standardization.to_excel('../Data/
 ↪A549_Decision_tree_rs_'+str(random_state)+'.xlsx')
```

2.5 Plots

[19]:

```
corre_list = [x / 100.0 for x in range(int(initial_step*100),␣
↪int(last_step*100), int(0.01*100))]
for element in corre_list:
    element_ =␣
↪df_without_standardization[df_without_standardization['Correlation␣
↪threshold'] == float(element)]
    plt.plot(element_['Depth number'], element_['Training data R^2 score'],␣
↪label = "Training data R^2 score", marker='v')
    plt.plot(element_['Depth number'], element_['Test data R^2 score'], label =␣
↪"Test data R^2 score", marker='D')
    plt.legend()
    plt.xlabel('Depth number -> correlation threshold: '+str(element)+'. \n␣
↪Number of features: '+str(element_['Number of features'].iloc[0]),␣
↪fontweight='bold')
    plt.xlim(max(element_['Depth number'])+1, min(element_['Depth number'])-1)
    plt.ylabel('R^2 score')
    plt.ylim([0, 1])
    plt.rc('grid', linestyle="-.", color='black')
    plt.grid(True)
    plt.show()
```

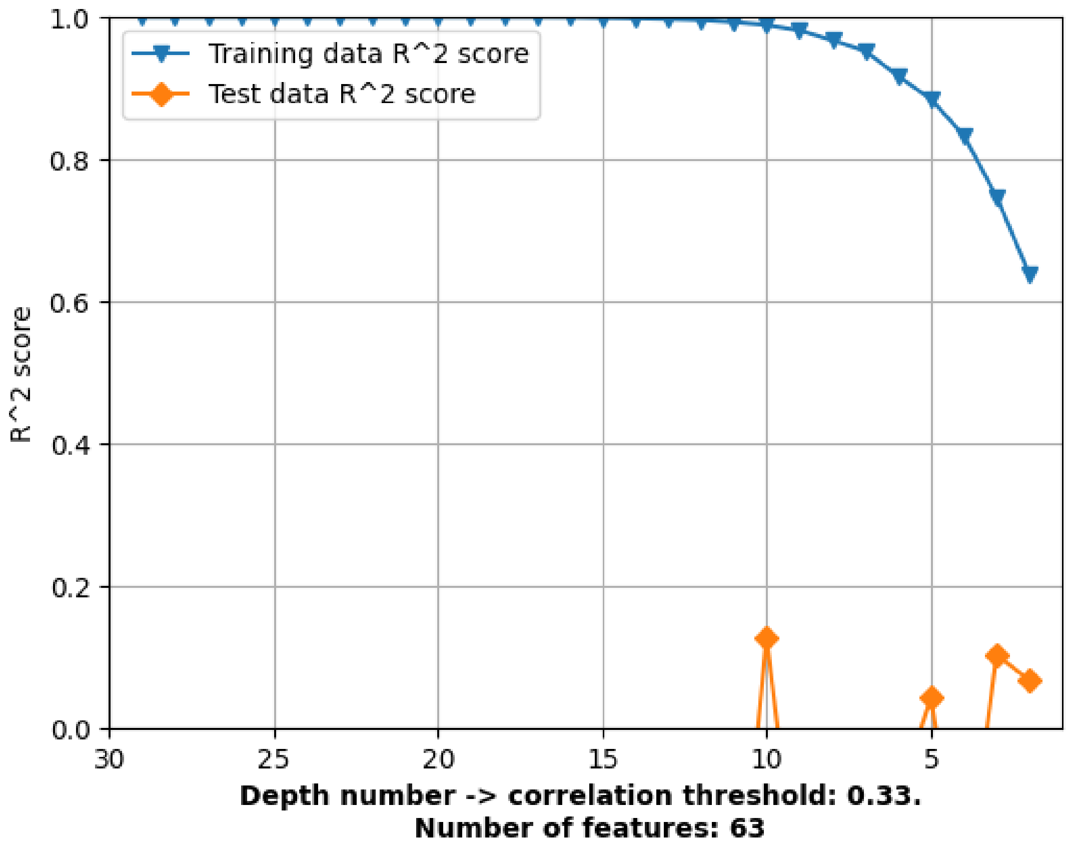

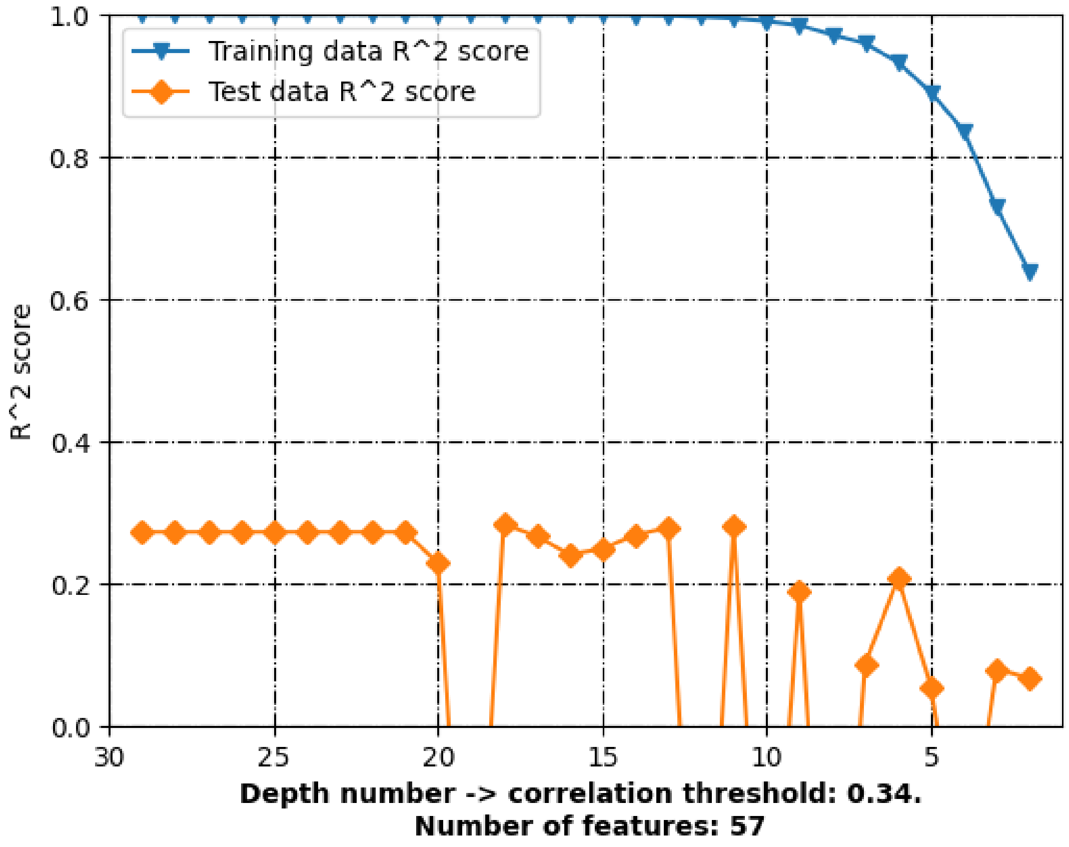

Training data R^2 score
Test data R^2 score
R^2 score
1.0
0.8
0.6
0.4
0.2
0.0
30
25
20
15
10
5
Depth number -> correlation threshold: 0.34.
Number of features: 57

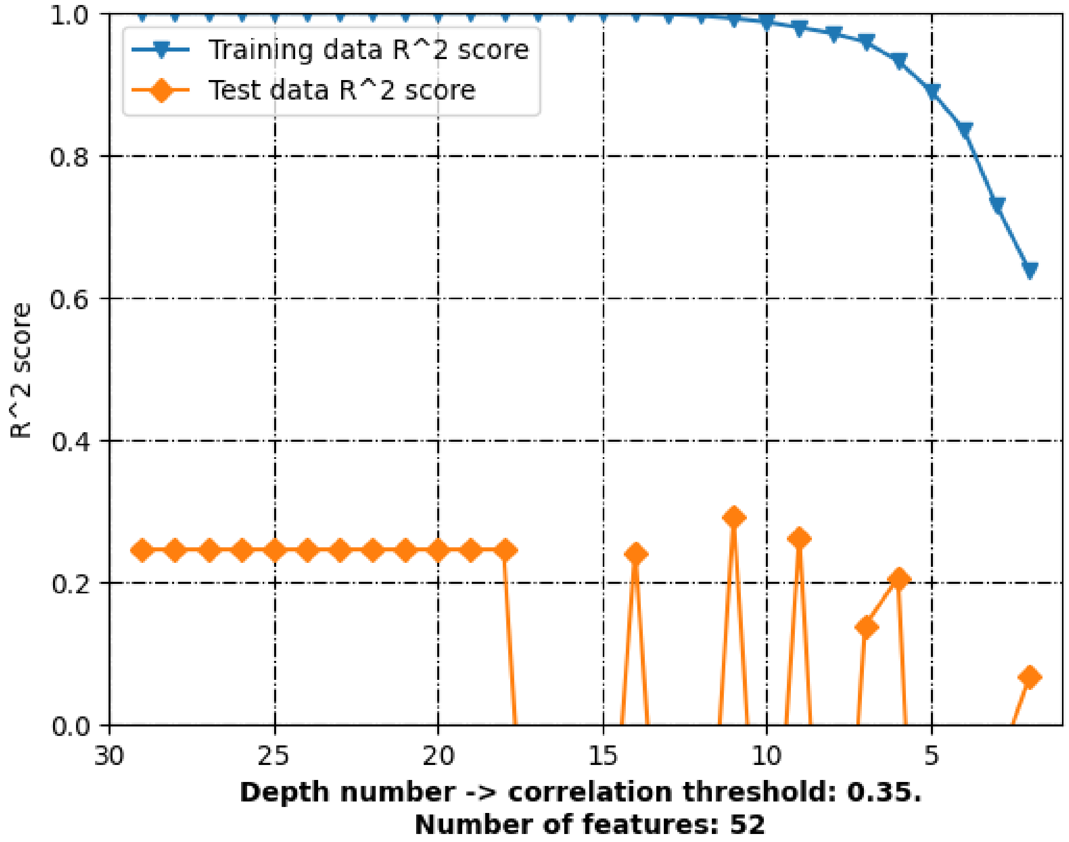

Training data R^2 score
Test data R^2 score
R^2 score
1.0
0.8
0.6
0.4
0.2
0.0
30
25
20
15
10
5
Depth number -> correlation threshold: 0.35.
Number of features: 52

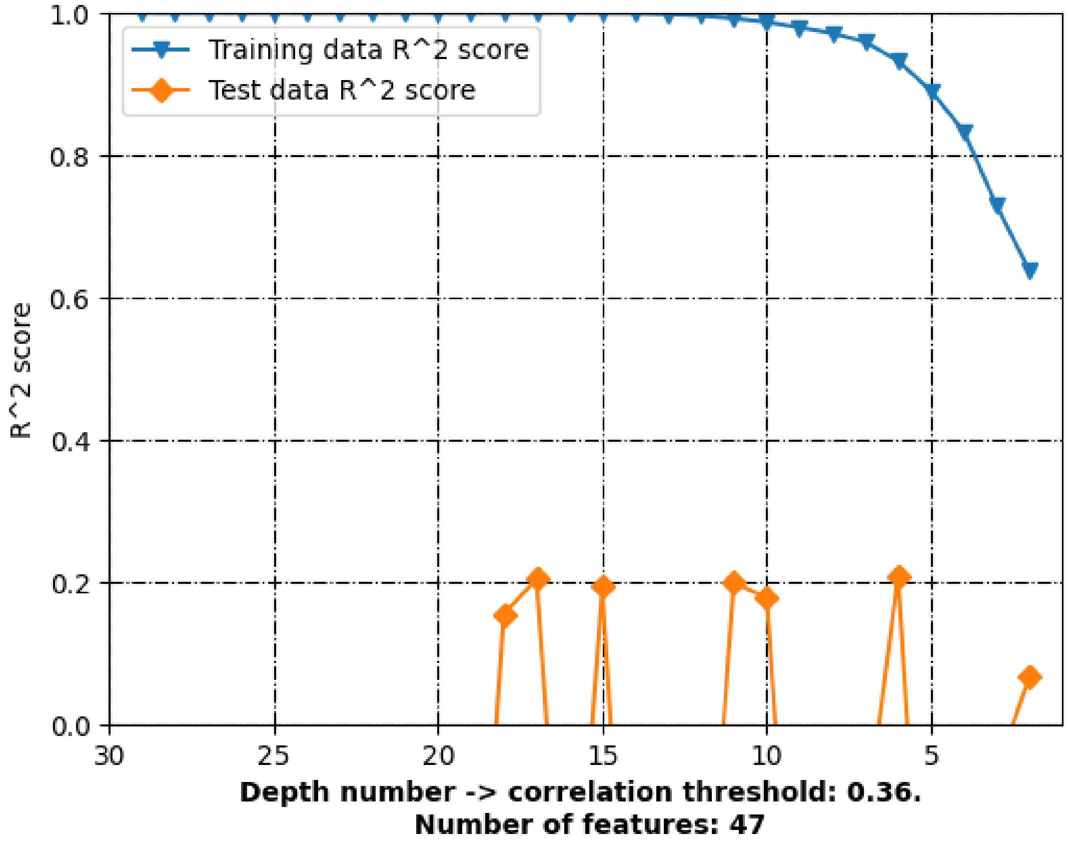

Training data R^2 score
Test data R^2 score
R^2 score
1.0
0.8
0.6
0.4
0.2
0.0
30
25
20
15
10
5
Depth number -> correlation threshold: 0.36.
Number of features: 47

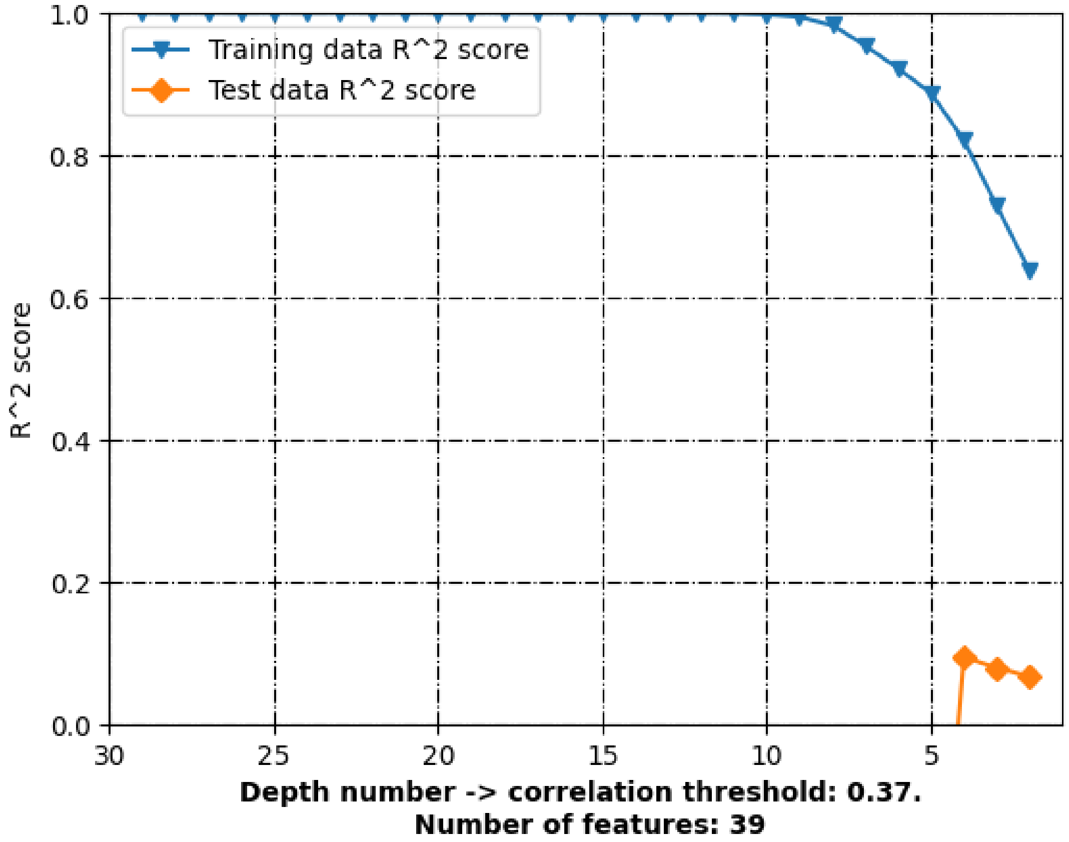
Training data R^2 score
Test data R^2 score
R^2 score
1.0
0.8
0.6
0.4
0.2
0.0
30
25
20
15
10
5
Depth number -> correlation threshold: 0.37.
Number of features: 39

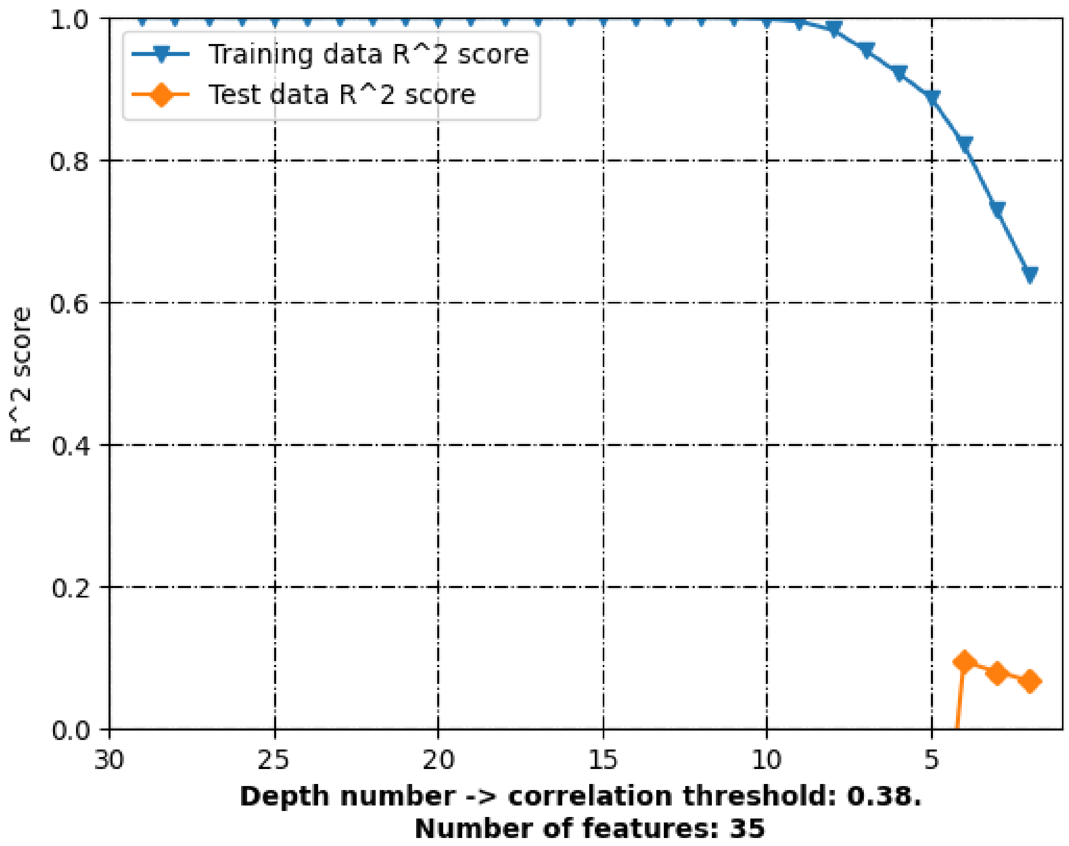
1.0
0.8
0.6
0.4
0.2
0.0
R^2 score
Training data R^2 score
Test data R^2 score
30
25
20
15
10
5
Depth number -> correlation threshold: 0.38.
Number of features: 35

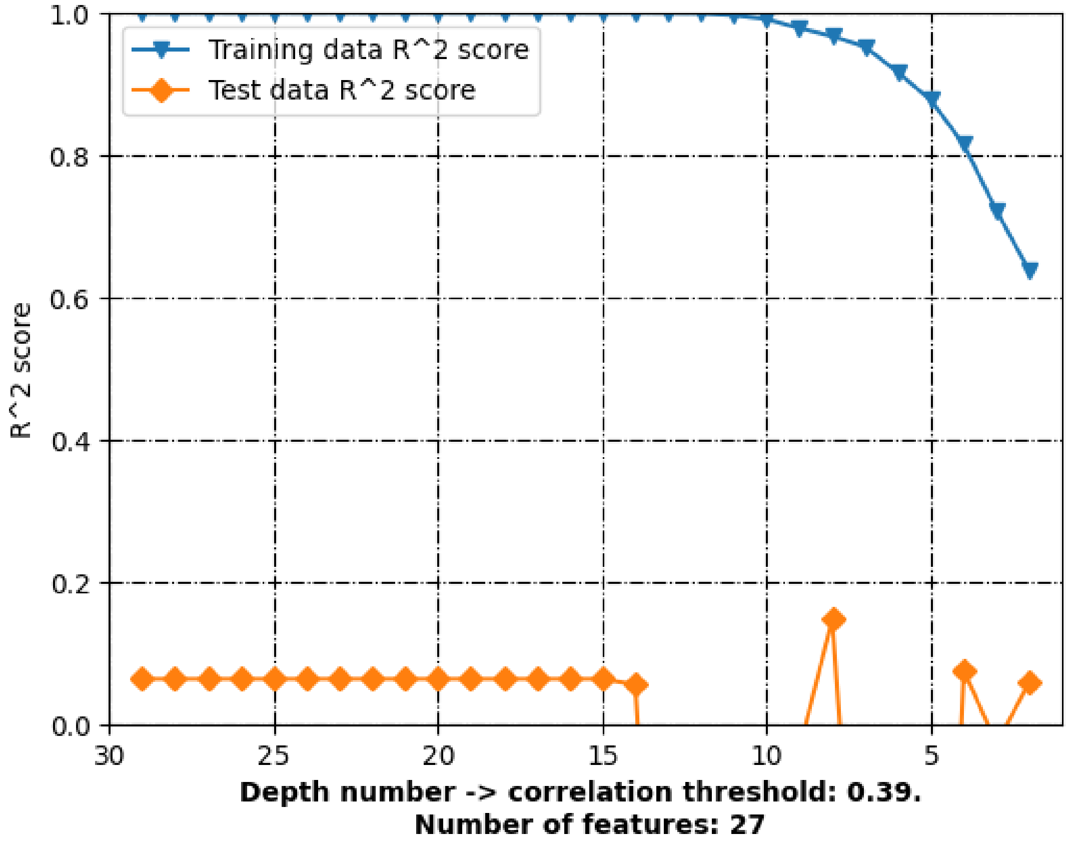
Training data R^2 score
Test data R^2 score
R^2 score
1.0
0.8
0.6
0.4
0.2
0.0
30
25
20
15
10
5
Depth number -> correlation threshold: 0.39.
Number of features: 27

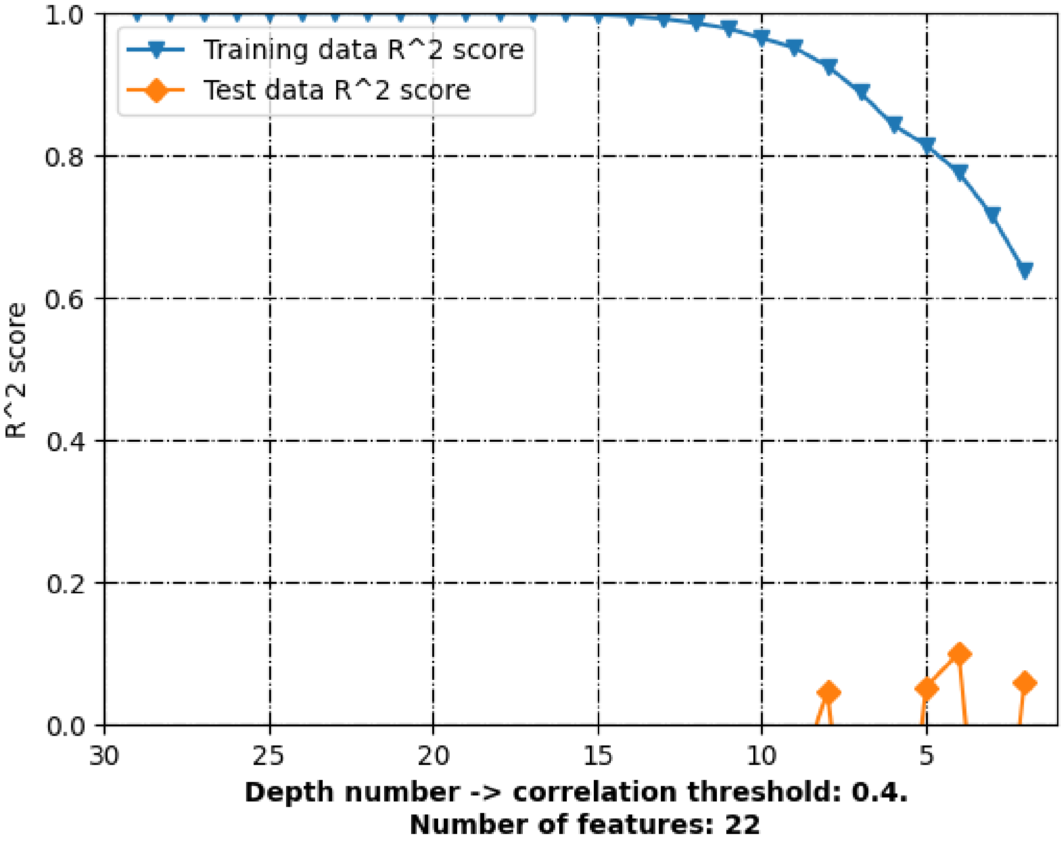

Training data R^2 score
Test data R^2 score
R^2 score
1.0
0.8
0.6
0.4
0.2
0.0
30
25
20
15
10
5
Depth number -> correlation threshold: 0.4.
Number of features: 22

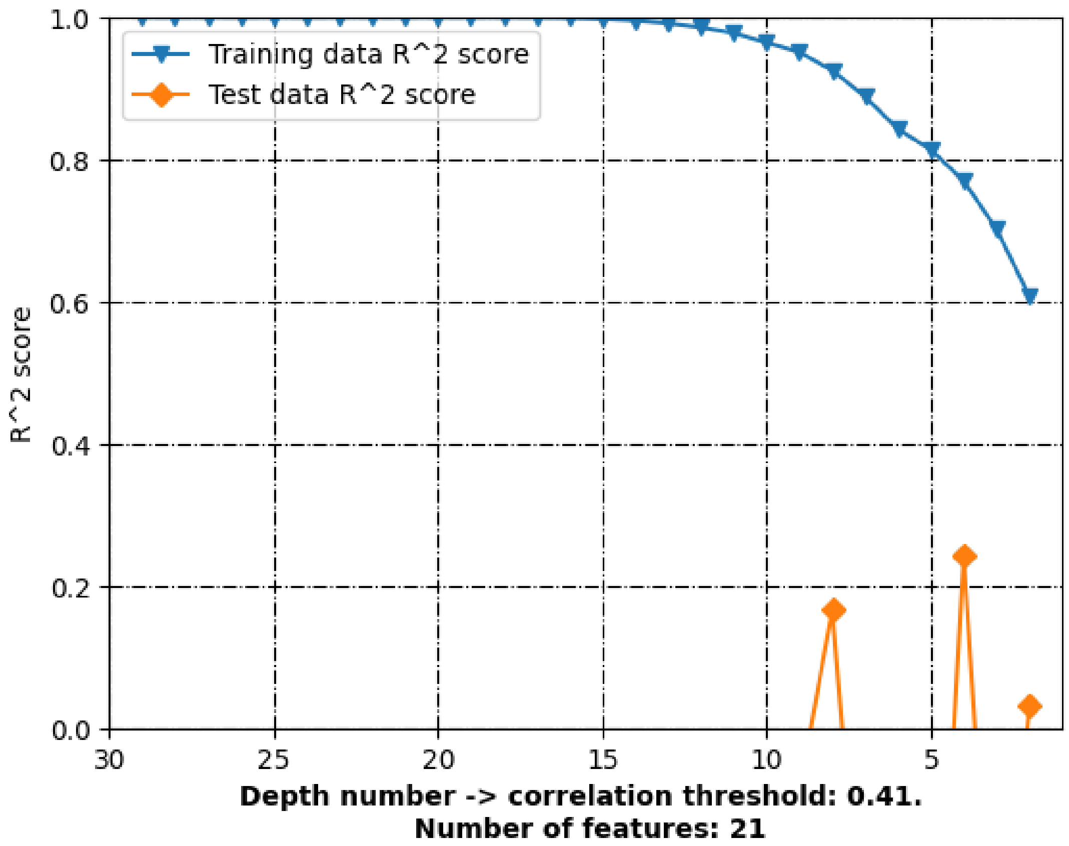

Training data R^2 score
Test data R^2 score
R^2 score
1.0
0.8
0.6
0.4
0.2
0.0
30
25
20
15
10
5
Depth number -> correlation threshold: 0.41.
Number of features: 21

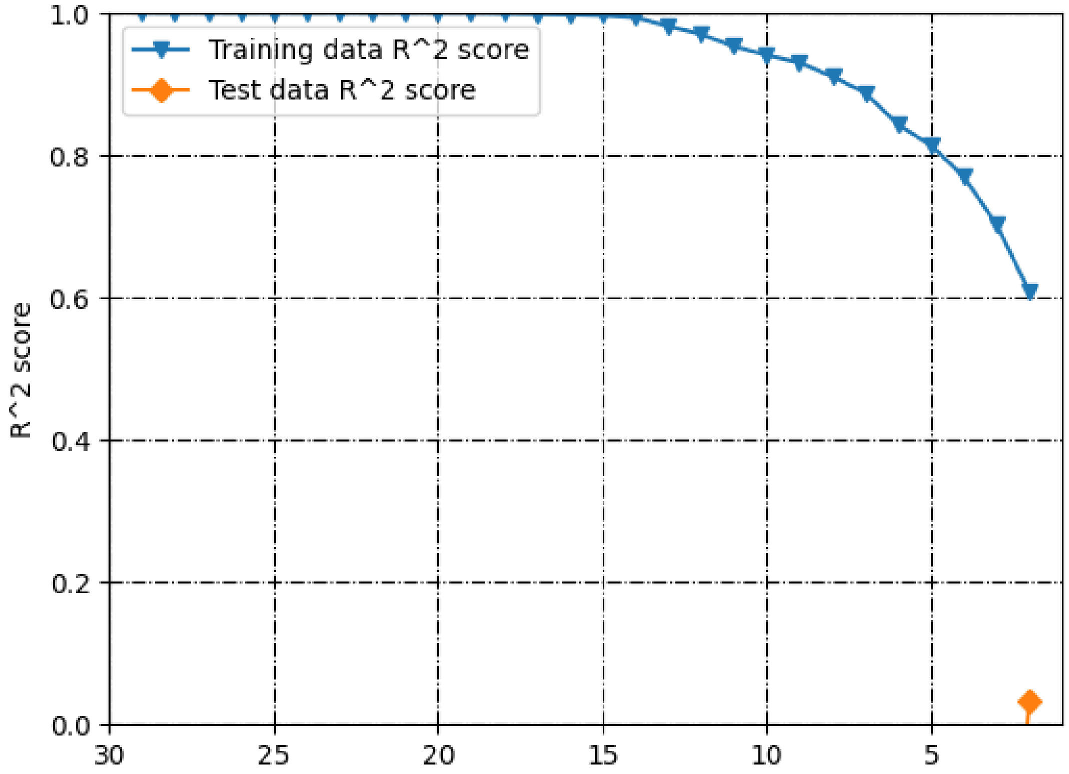

Depth number -> correlation threshold: 0.42.
Number of features: 18

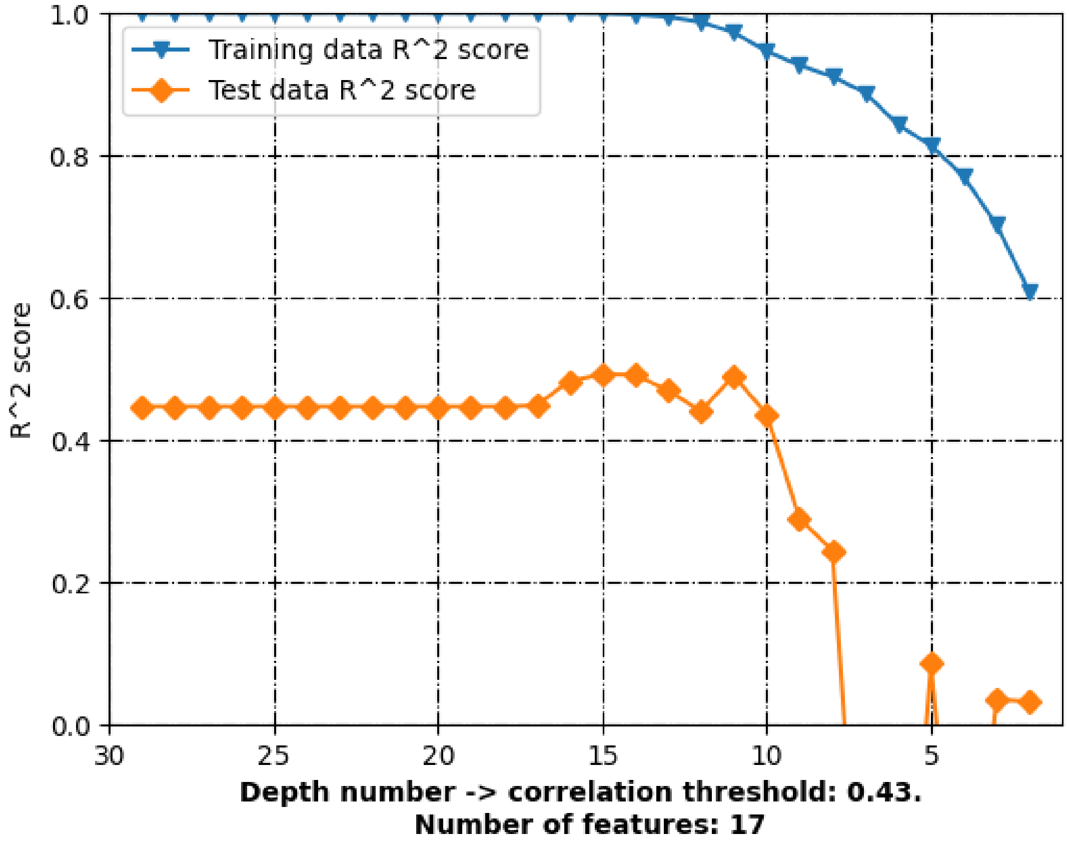
Training data R^2 score
Test data R^2 score
R^2 score
1.0
0.8
0.6
0.4
0.2
0.0
30
25
20
15
10
5
Depth number -> correlation threshold: 0.43.
Number of features: 17

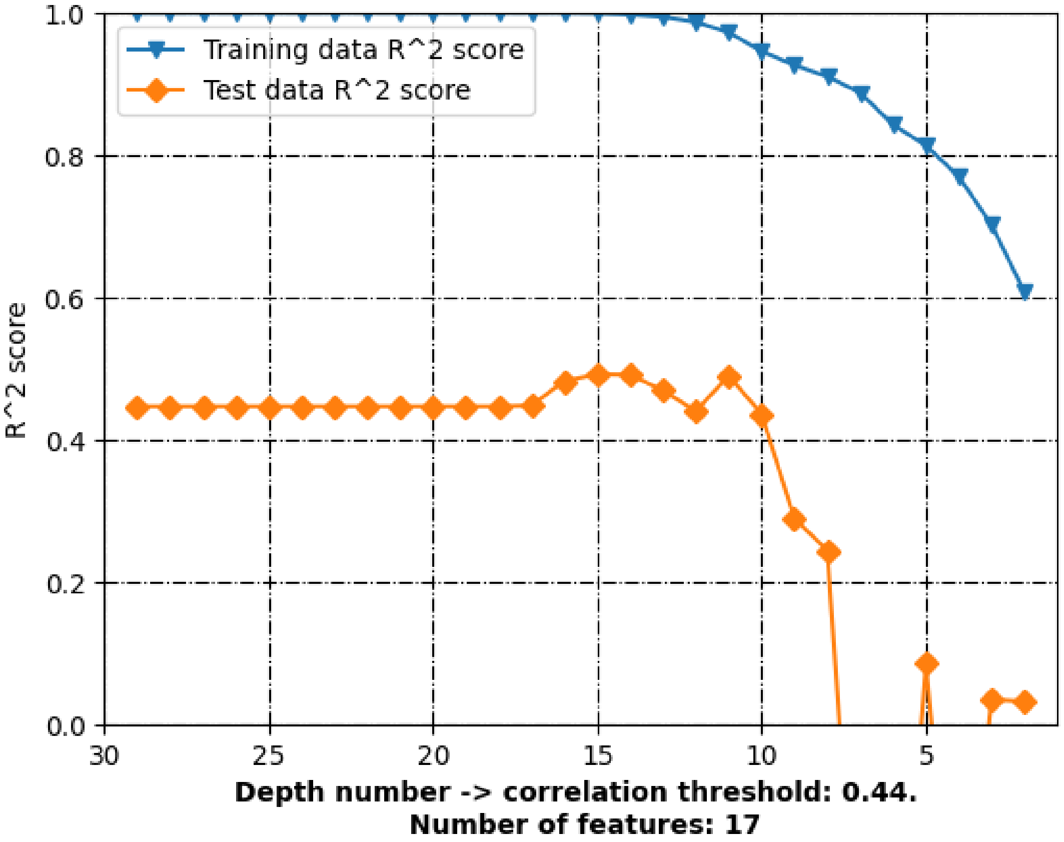

Training data R^2 score
Test data R^2 score
R^2 score
1.0
0.8
0.6
0.4
0.2
0.0
30
25
20
15
10
5
Depth number -> correlation threshold: 0.44.
Number of features: 17

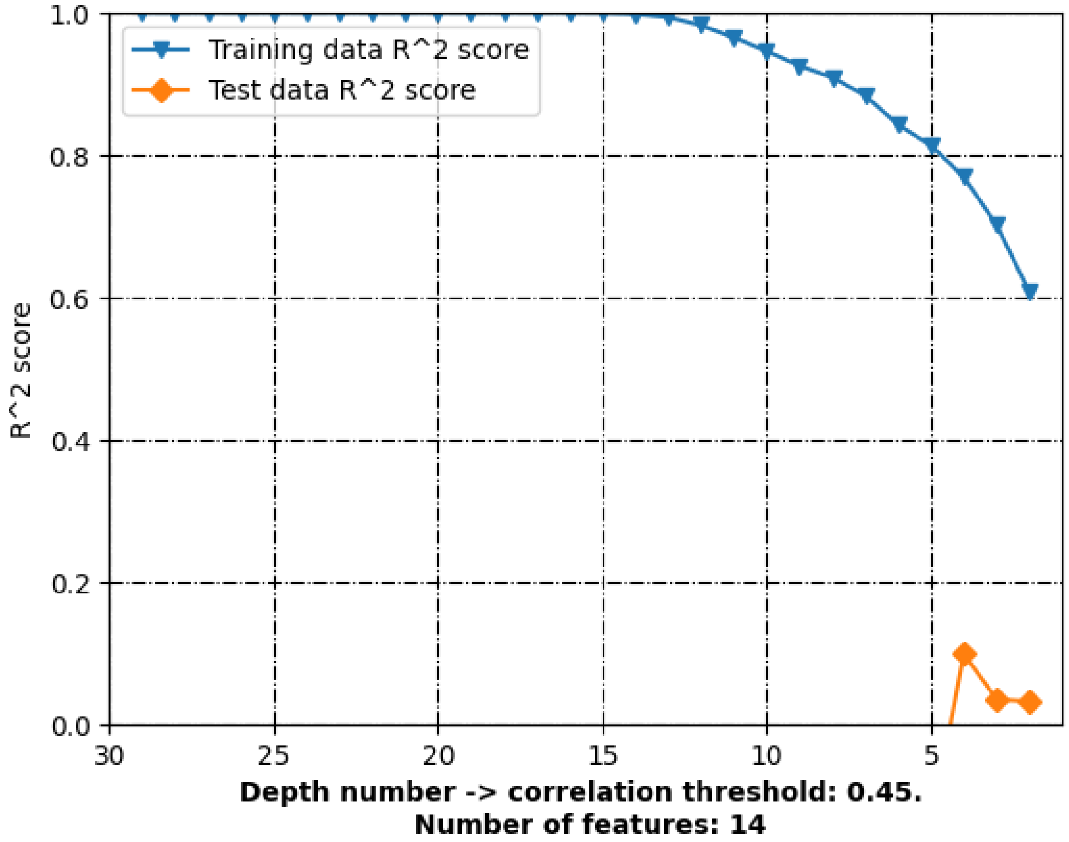

Training data R^2 score
Test data R^2 score
R^2 score
1.0
0.8
0.6
0.4
0.2
0.0
30
25
20
15
10
5
Depth number -> correlation threshold: 0.45.
Number of features: 14

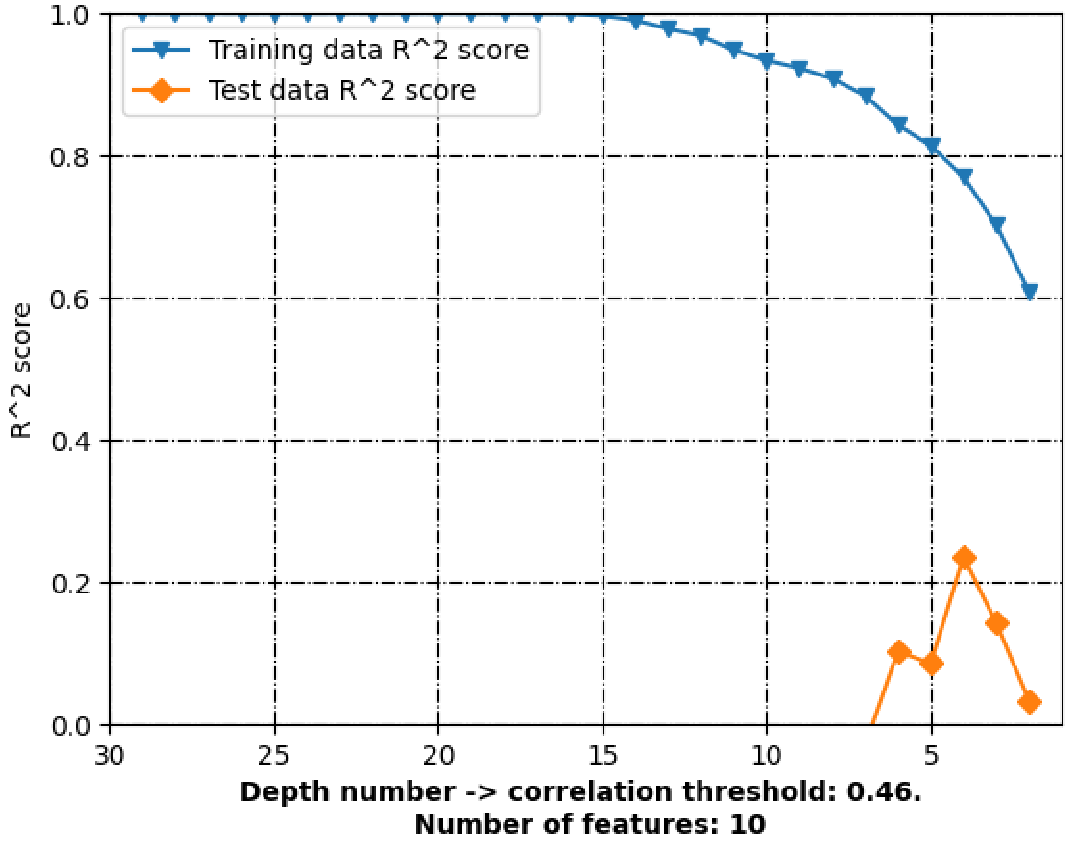
Training data R^2 score
Test data R^2 score
R^2 score
1.0
0.8
0.6
0.4
0.2
0.0
30
25
20
15
10
5
Depth number -> correlation threshold: 0.46.
Number of features: 10

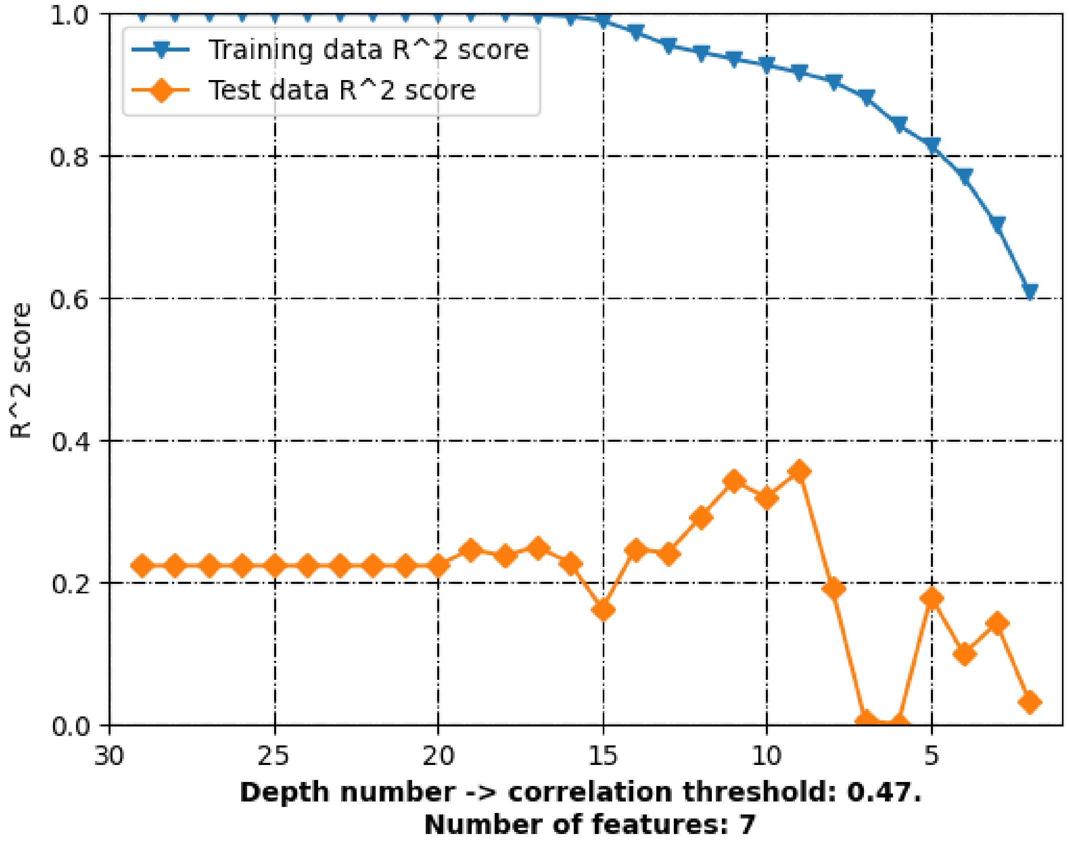

Training data R^2 score
Test data R^2 score
R^2 score
1.0
0.8
0.6
0.4
0.2
0.0
30
25
20
15
10
5
Depth number -> correlation threshold: 0.47.
Number of features: 7

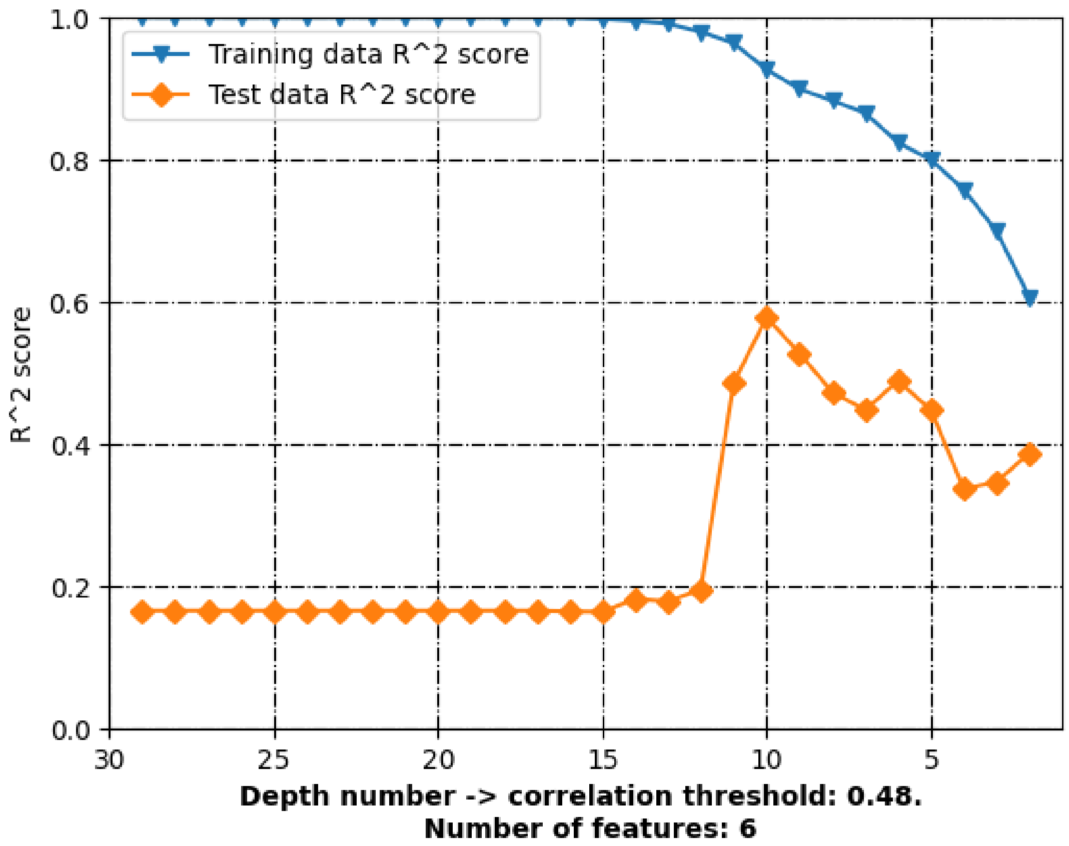
Training data R^2 score
Test data R^2 score
R^2 score
1.0
0.8
0.6
0.4
0.2
0.0
30
25
20
15
10
5
Depth number -> correlation threshold: 0.48.
Number of features: 6

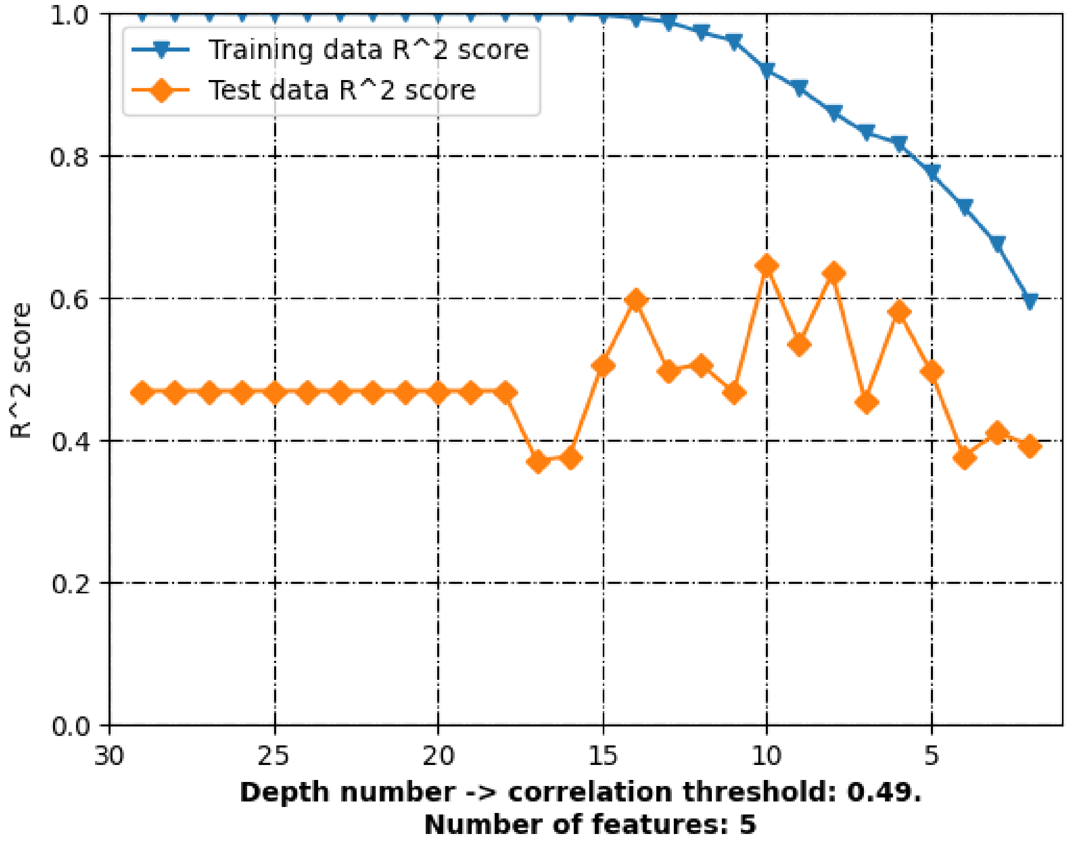
Training data R^2 score
Test data R^2 score
R^2 score
1.0
0.8
0.6
0.4
0.2
0.0
30
25
20
15
10
5
Depth number -> correlation threshold: 0.49.
Number of features: 5

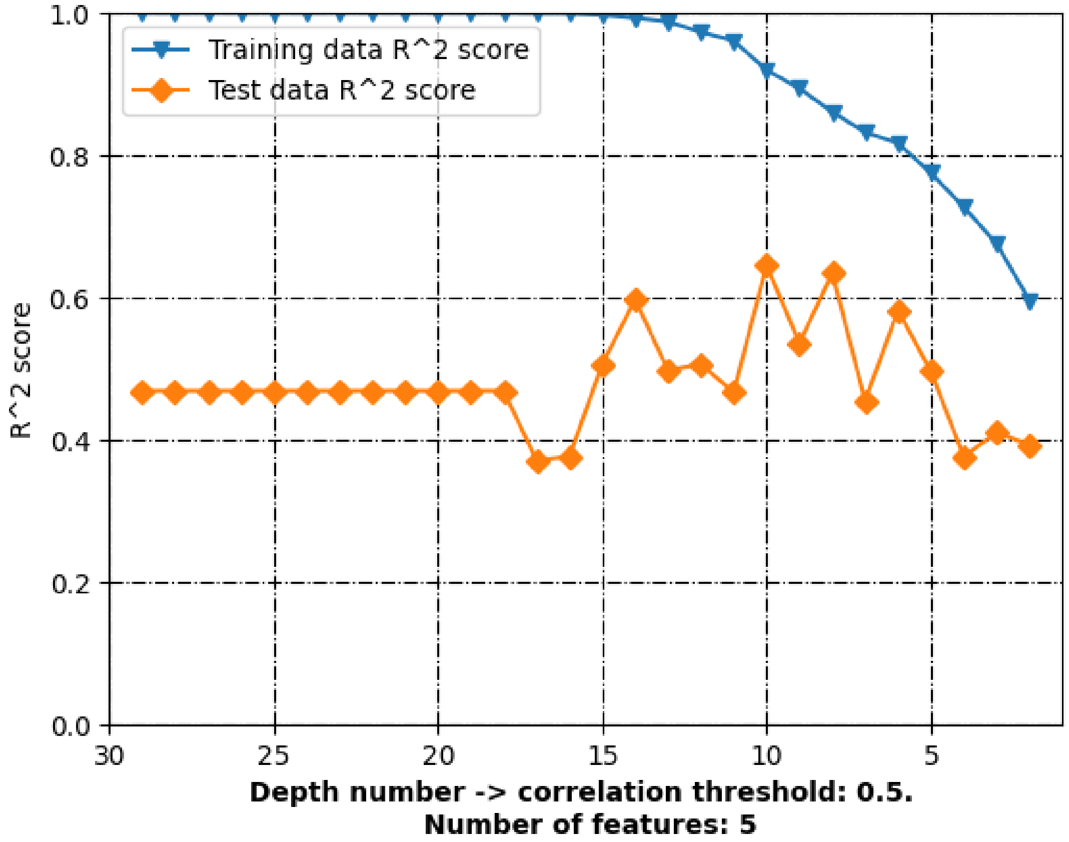
Training data R^2 score
Test data R^2 score
R^2 score
1.0
0.8
0.6
0.4
0.2
0.0
30
25
20
15
10
5
Depth number -> correlation threshold: 0.5.
Number of features: 5

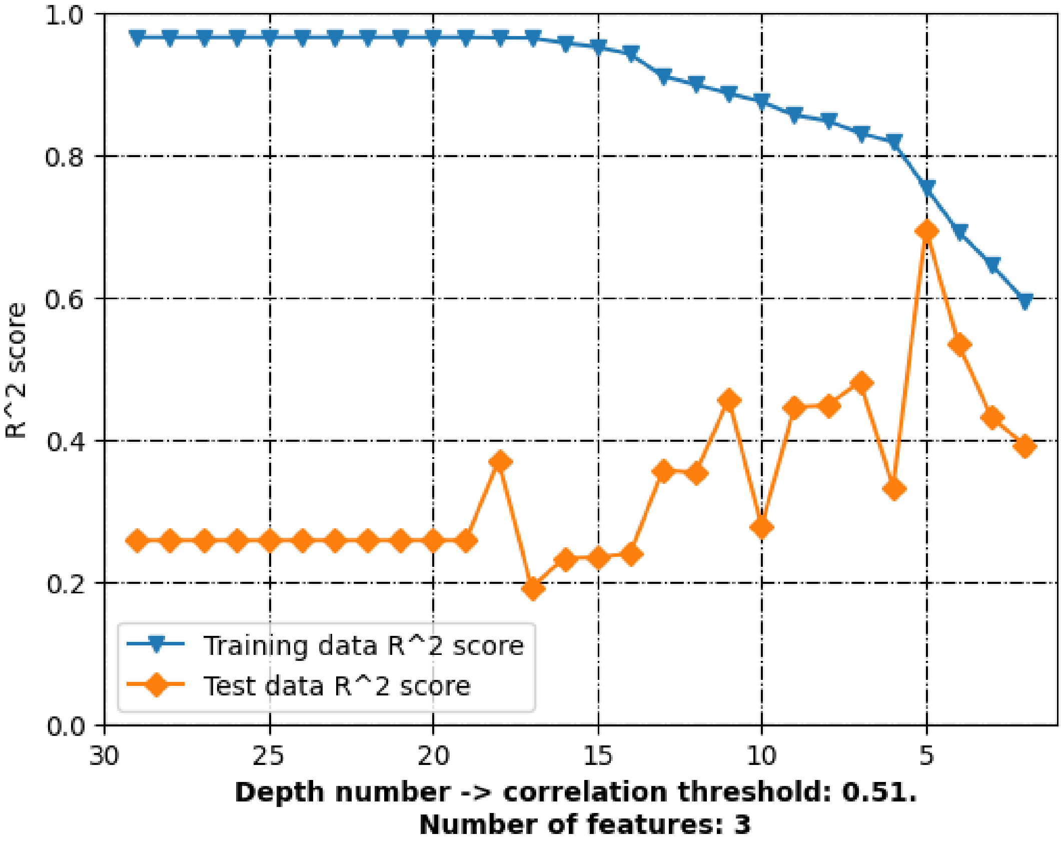
1.0
0.8
0.6
0.4
0.2
0.0
R^2 score
30
25
20
15
10
5
Training data R^2 score
Test data R^2 score
Depth number -> correlation threshold: 0.51.
Number of features: 3

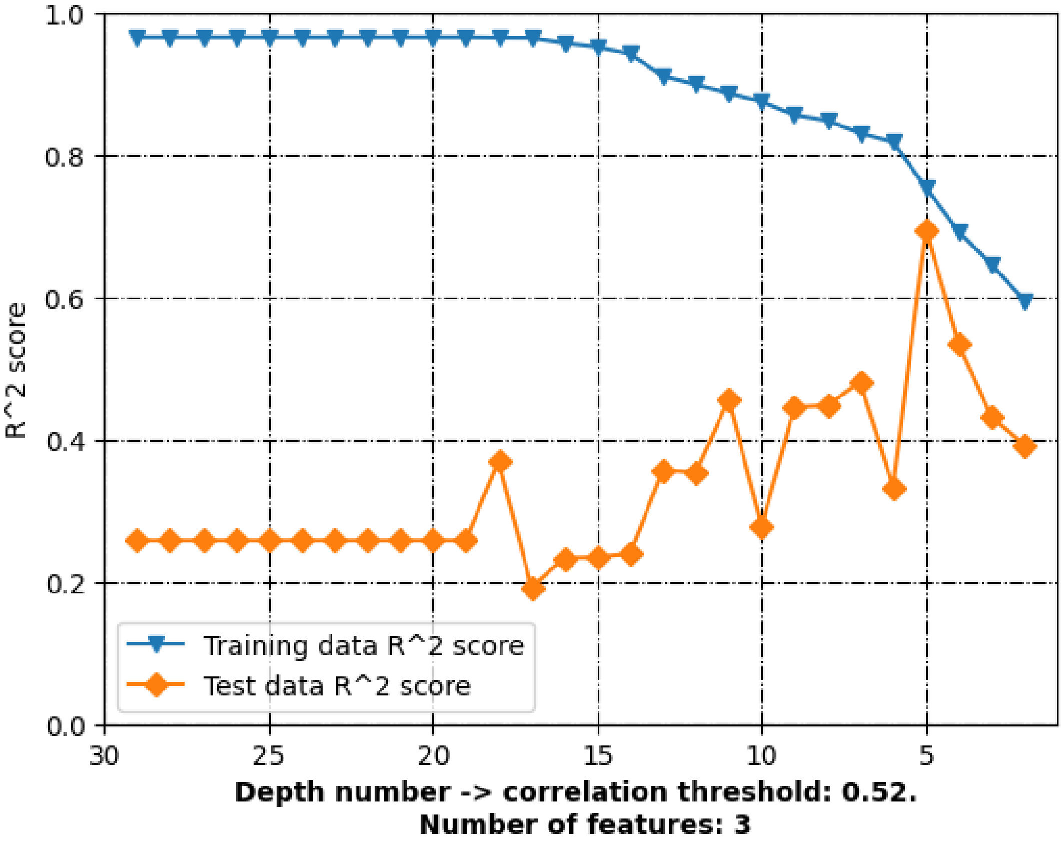
1.0
0.8
0.6
0.4
0.2
0.0
R^2 score
30
25
20
15
10
5
Training data R^2 score
Test data R^2 score
Depth number -> correlation threshold: 0.52.
Number of features: 3

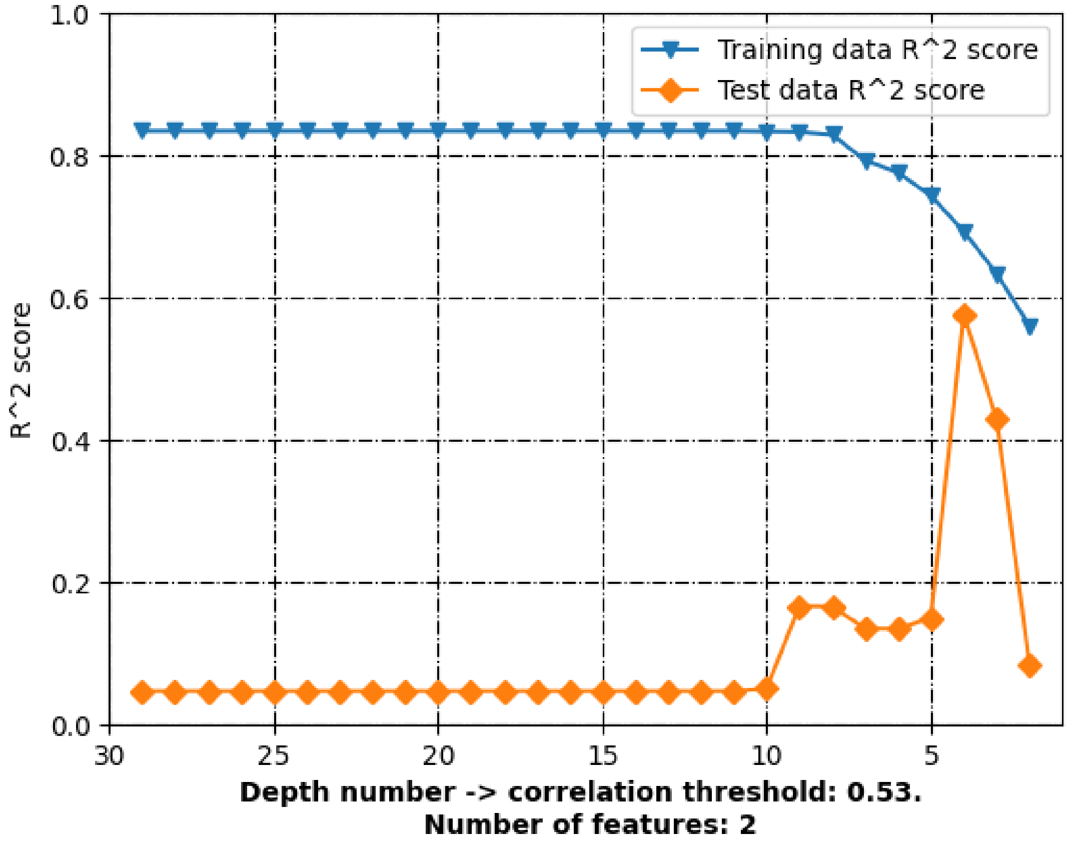

1.0
0.8
0.6
0.4
0.2
0.0
R^2 score
30
25
20
15
10
5
Training data R^2 score
Test data R^2 score
Depth number -> correlation threshold: 0.53.
Number of features: 2

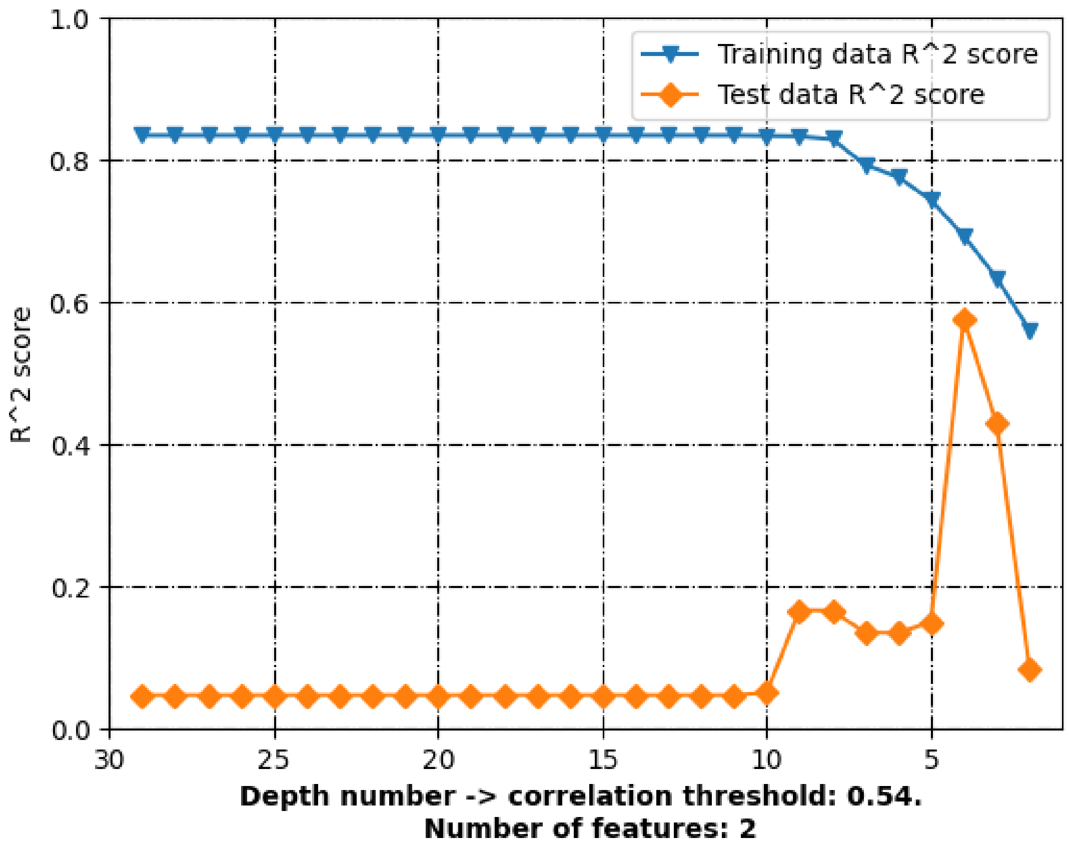

[20]:
```
plt.plot(df_without_standardization['Correlation threshold'],␣
  ↪df_without_standardization['Number of features'], label = "Number of␣
  ↪features", marker='.')
plt.legend()
plt.xlabel('Correlation threshold')
plt.ylabel('Number of features')
plt.show()
```

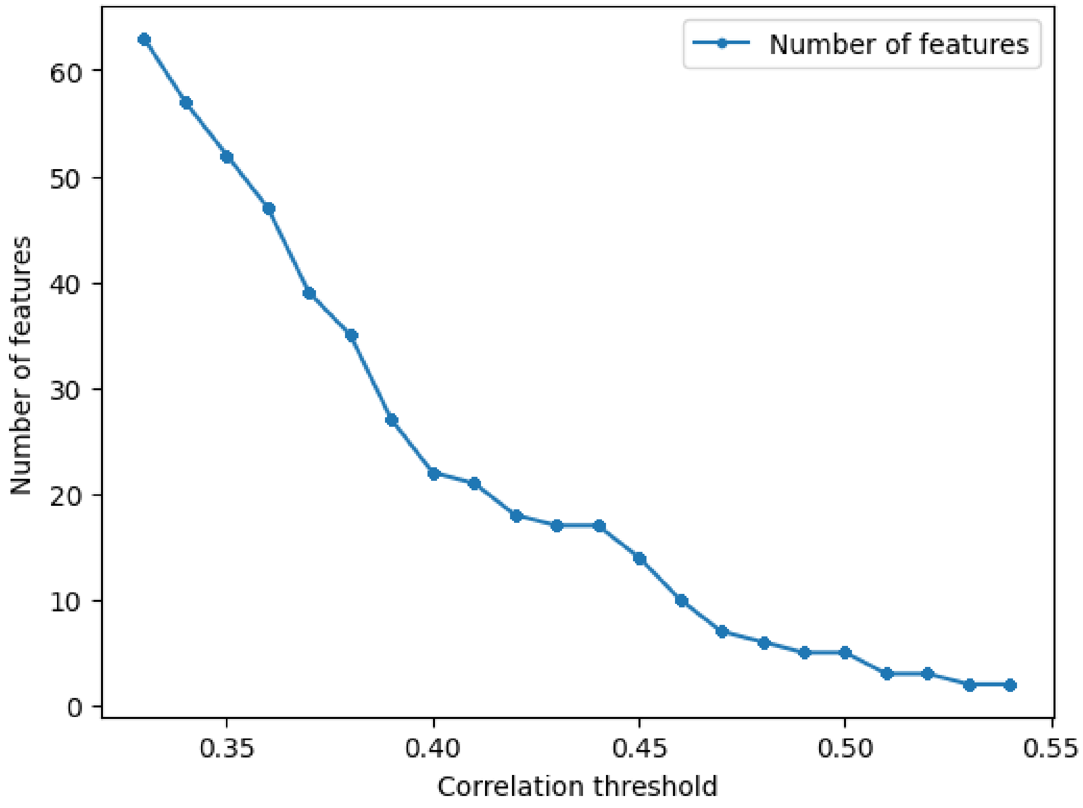

[ ]:

3 Random Forest

[21]:
```
without_standardization, train_r2_, test_r2_, hist1, hist2, target_column_name,␣
↪training_data_RMSE, test_data_RMSE  = pred_model.
↪prepare_data_and_create_model(molecular_descriptors_df=data,
                                                                                    ␣
↪                       correlation_threshold=0.50,
                                                                                    ␣
↪                       standardization=False,
                                                                                    ␣
↪                       model_type='RandomForestRegressor',
                                                                                    ␣
↪                       n_estimators_=12,
                                                                                    ␣
↪                       target_column_name = target,
                                                                                    ␣
↪                       random_state=random_state,
```

```
                        train_test_split_=True,
                        verbose=True)
```

```
I am not doing standardization…
  molecular descriptor name
0                    AATS0Z
1                  AATS0are
2                    AATS0d
3                   AATS0dv
4                    AATS0i
  molecular descriptor name  corr_value
0                    AATS0Z   -0.027037
1                  AATS0are   -0.129823
2                    AATS0d    0.042740
3                   AATS0dv   -0.120173
4                    AATS0i    0.132395
  molecular descriptor name  corr_value  absolute correlation value
0                    AATS0Z   -0.027037                    0.027037
1                  AATS0are   -0.129823                    0.129823
2                    AATS0d    0.042740                    0.042740
3                   AATS0dv   -0.120173                    0.120173
4                    AATS0i    0.132395                    0.132395
     molecular descriptor name  corr_value  absolute correlation value
226                     AMID_O   -0.524157                    0.524157
505                EState_VSA5   -0.579664                    0.579664
791                    MDEO-12   -0.558727                    0.558727
851                      NdssC   -0.506855                    0.506855
1091               VSA_EState5    0.503578                    0.503578
     molecular descriptor name  corr_value  absolute correlation value
226                     AMID_O   -0.524157                    0.524157
505                EState_VSA5   -0.579664                    0.579664
791                    MDEO-12   -0.558727                    0.558727
851                      NdssC   -0.506855                    0.506855
1091               VSA_EState5    0.503578                    0.503578
The model used is: RandomForest…
Return the coefficient of determination of the prediction:
0.5325679351654988
R^2 score: 0.8956653627358289
Correlation coefficient: 0.946395986221322
Test data - unseen during training:
R^2 score: 0.5325679351654988
Correlation coefficient: 0.7297725228901804
[8.18549545 6.43267746 7.90373055 7.07002075 7.91925871 8.28997064
 8.072342   7.49377386 7.18387349 7.91093882 7.97294975 8.01110989
 7.84943728 8.13009111 8.28997064 6.50109233 7.28812865 6.79273421]
113    7.795880
```

```
35     6.254925
101    7.275724
36     7.017729
100    7.337242
13     8.055517
0      8.187087
114    7.638272
104    8.318759
96     7.769551
40     8.050610
103    8.136677
48     7.853872
39     8.267606
14     7.273273
117    6.167491
21     7.554396
9      6.343902
Name: LoVo, dtype: float64
Training Root Mean Square Error: 0.30317135512403l5
Testing Root Mean Square Error: 0.4579105131310473
```

[22]:
```
print(target_column_name+str('_transformed'))
print(hist1[target_column_name].hist())
```

```
LoVo_transformed
AxesSubplot(0.125,0.11;0.775x0.77)
```

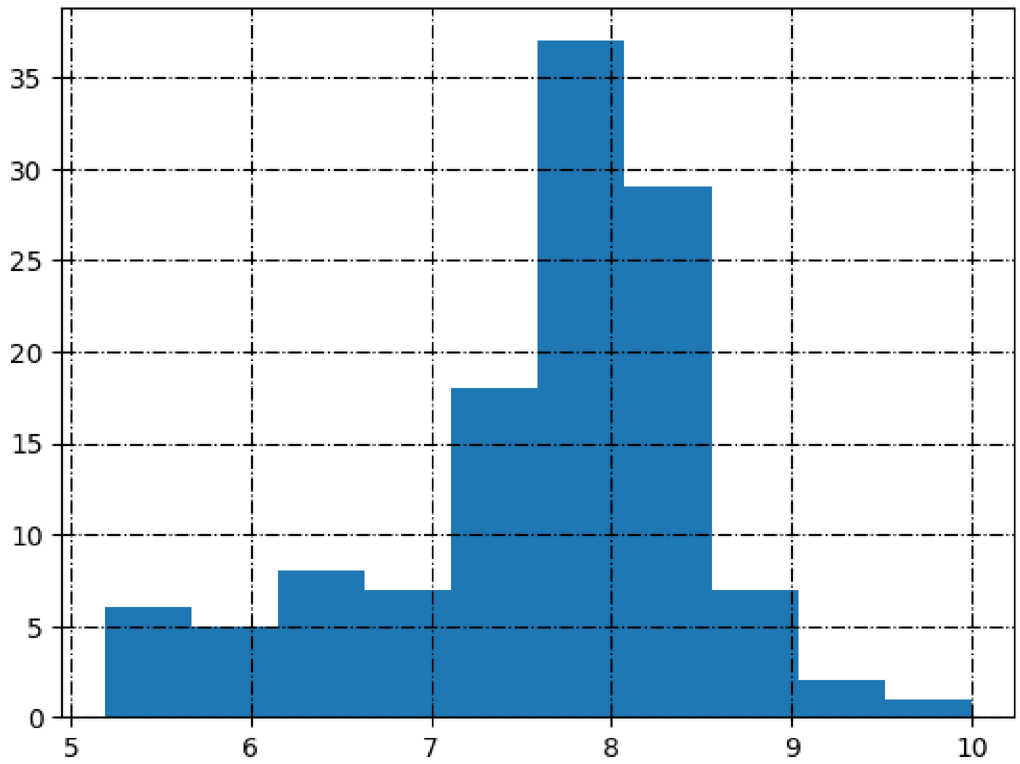


```
[23]: with_standardization, train_r2_, test_r2_, hist1, hist2, target_column_name,␣
    ↪training_data_RMSE, test_data_RMSE  = pred_model.
    ↪prepare_data_and_create_model(molecular_descriptors_df=data,
                                                                              ␣
    ↪                         correlation_threshold=0.50,
                                                                              ␣
    ↪                         standardization=True,
                                                                              ␣
    ↪                         model_type='RandomForestRegressor',
                                                                              ␣
    ↪                         n_estimators_=12,
                                                                              ␣
    ↪                         target_column_name = target,
                                                                              ␣
    ↪                         random_state=random_state,
                                                                              ␣
    ↪                         train_test_split_=True,
                                                                              ␣
    ↪                         verbose=True)
```

```
I am doing standardization…
  molecular descriptor name
```

```
0                  AATS0Z
1                AATS0are
2                  AATS0d
3                 AATS0dv
4                  AATS0i
  molecular descriptor name  corr_value
0                  AATS0Z   -0.027037
1                AATS0are   -0.129823
2                  AATS0d    0.042740
3                 AATS0dv   -0.120173
4                  AATS0i    0.132395
  molecular descriptor name  corr_value  absolute correlation value
0                  AATS0Z   -0.027037                    0.027037
1                AATS0are   -0.129823                    0.129823
2                  AATS0d    0.042740                    0.042740
3                 AATS0dv   -0.120173                    0.120173
4                  AATS0i    0.132395                    0.132395
     molecular descriptor name  corr_value  absolute correlation value
226                     AMID_O   -0.524157                    0.524157
505                EState_VSA5   -0.579664                    0.579664
791                    MDEO-12   -0.558727                    0.558727
851                      NdssC   -0.506855                    0.506855
1091               VSA_EState5    0.503578                    0.503578
     molecular descriptor name  corr_value  absolute correlation value
226                     AMID_O   -0.524157                    0.524157
505                EState_VSA5   -0.579664                    0.579664
791                    MDEO-12   -0.558727                    0.558727
851                      NdssC   -0.506855                    0.506855
1091               VSA_EState5    0.503578                    0.503578
The model used is: RandomForest…
Return the coefficient of determination of the prediction:
0.5325679351654988
R^2 score: 0.8956653627358289
Correlation coefficient: 0.946395986221322
Test data - unseen during training:
R^2 score: 0.5325679351654988
Correlation coefficient: 0.7297725228901804
[8.18549545 6.43267746 7.90373055 7.07002075 7.91925871 8.28997064
 8.072342   7.49377386 7.18387349 7.91093882 7.97294975 8.01110989
 7.84943728 8.13009111 8.28997064 6.50109233 7.28812865 6.79273421]
113    7.795880
35     6.254925
101    7.275724
36     7.017729
100    7.337242
13     8.055517
0      8.187087
114    7.638272
```

```
104    8.318759
96     7.769551
40     8.050610
103    8.136677
48     7.853872
39     8.267606
14     7.273273
117    6.167491
21     7.554396
9      6.343902
Name: LoVo, dtype: float64
Training Root Mean Square Error: 0.3031713551240315
Testing Root Mean Square Error: 0.4579105131310473
```

3.1 Search inside correlation space

[24]:
```
step = 0.01
initial_step = corr_low
last_step = corr_high
first_list = [x / 100.0 for x in range(int(initial_step*100),␣
 ↪int(last_step*100), int(step*100))]
n_estimators = [range(2,21,1)]
corr_th = []
second_list = []
third_list = []
fourth_l = []
fifth_l = []
f_list = []
fif_list = []
for i in first_list:

    for estimator in n_estimators[0]:

        without_standardization, train_r2, test_r2, _, h_, target_column_name,␣
 ↪training_data_RMSE, test_data_RMSE = pred_model.
 ↪prepare_data_and_create_model(molecular_descriptors_df=data,
                                                                                ␣
 ↪                     correlation_threshold=i,
                                                                                ␣
 ↪                     standardization=False,
                                                                                ␣
 ↪                     model_type='RandomForestRegressor',
                                                                                ␣
 ↪                     n_estimators_=estimator,
                                                                                ␣
 ↪                     target_column_name = target,
```

```
↪                    random_state=random_state,
↪                    train_test_split_=True,
↪                    verbose=False)
        corr_th.append(i)
        second_list.append(train_r2)
        third_list.append(test_r2)
        fourth_l.append(training_data_RMSE)
        fifth_l.append(test_data_RMSE)
        f_list.append(len(h_))
        fif_list.append(estimator)
```

[25]:
```
df_without_standardization = pd.DataFrame(data=corr_th, columns=["Correlation␣
↪threshold"])
df_without_standardization['Training data R^2 score'] = second_list
df_without_standardization['Test data R^2 score'] = third_list
df_without_standardization['Training RMSE'] = fourth_l
df_without_standardization['Test RMSE'] = fifth_l
df_without_standardization['Number of features'] = f_list
df_without_standardization['Number of estimators'] = fif_list
```

[26]:
```
df_random_forest = df_without_standardization.copy()
#df_without_standardization.to_excel('../Data/
↪A549_Random_forest_rs_'+str(random_state)+'.xlsx')
```

[27]:
```
corre_list = [x / 100.0 for x in range(int(initial_step*100),␣
↪int(last_step*100), int(0.01*100))]
for element in corre_list:
    element_ =␣
↪df_without_standardization[df_without_standardization['Correlation␣
↪threshold'] == float(element)]
    plt.plot(element_['Number of estimators'], element_['Training data R^2␣
↪score'], label = "Training data R^2 score", marker='v')
    plt.plot(element_['Number of estimators'], element_['Test data R^2 score'],␣
↪label = "Test data R^2 score", marker='D')
    plt.legend()
    plt.xlabel('Number of estimators -> correlation threshold: '+str(element)+'.
↪ \n Number of features: '+str(element_['Number of features'].iloc[0]))
    plt.xlim(max(element_['Number of estimators'])+1, min(element_['Number of␣
↪estimators'])-1)
    plt.ylabel('R^2 score')
    plt.ylim([0, 1])
    plt.rc('grid', linestyle="-.", color='black')
    plt.grid(True)
```

```
plt.show()
```

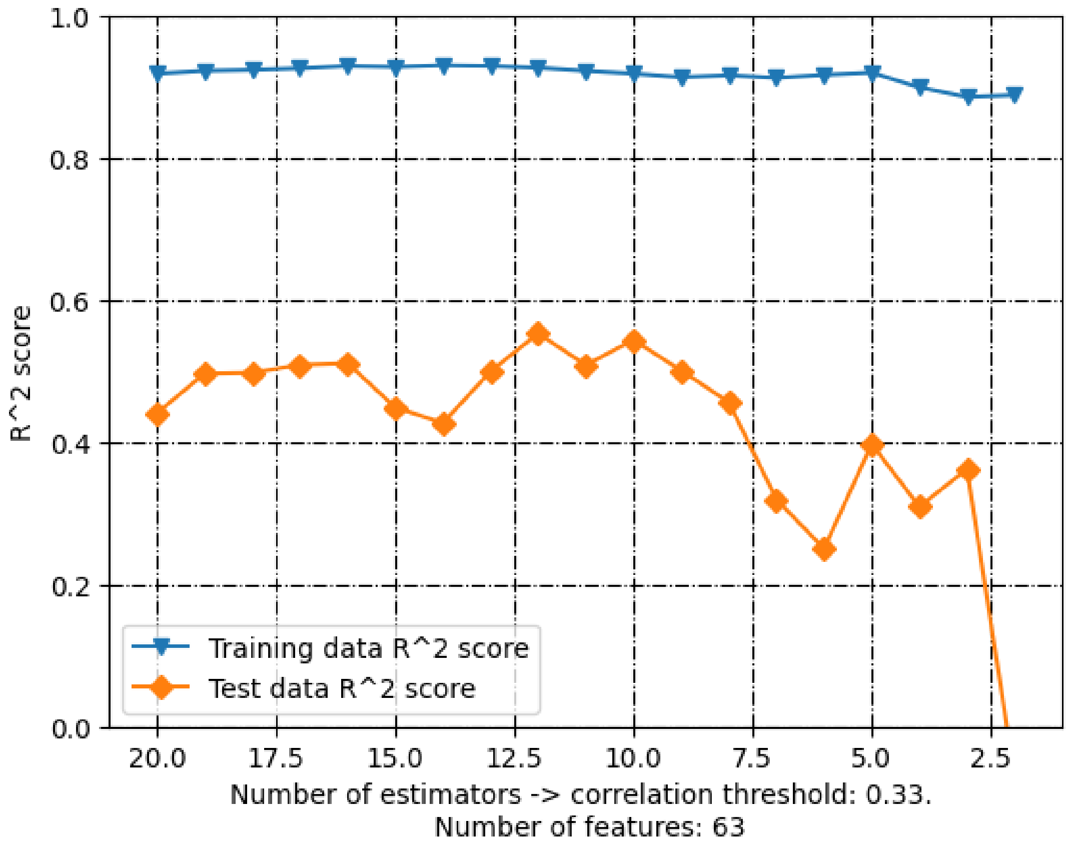

1.0
0.8
0.6
0.4
0.2
0.0
R^2 score
Training data R^2 score
Test data R^2 score
20.0
17.5
15.0
12.5
10.0
7.5
5.0
2.5
Number of estimators -> correlation threshold: 0.33.
Number of features: 63

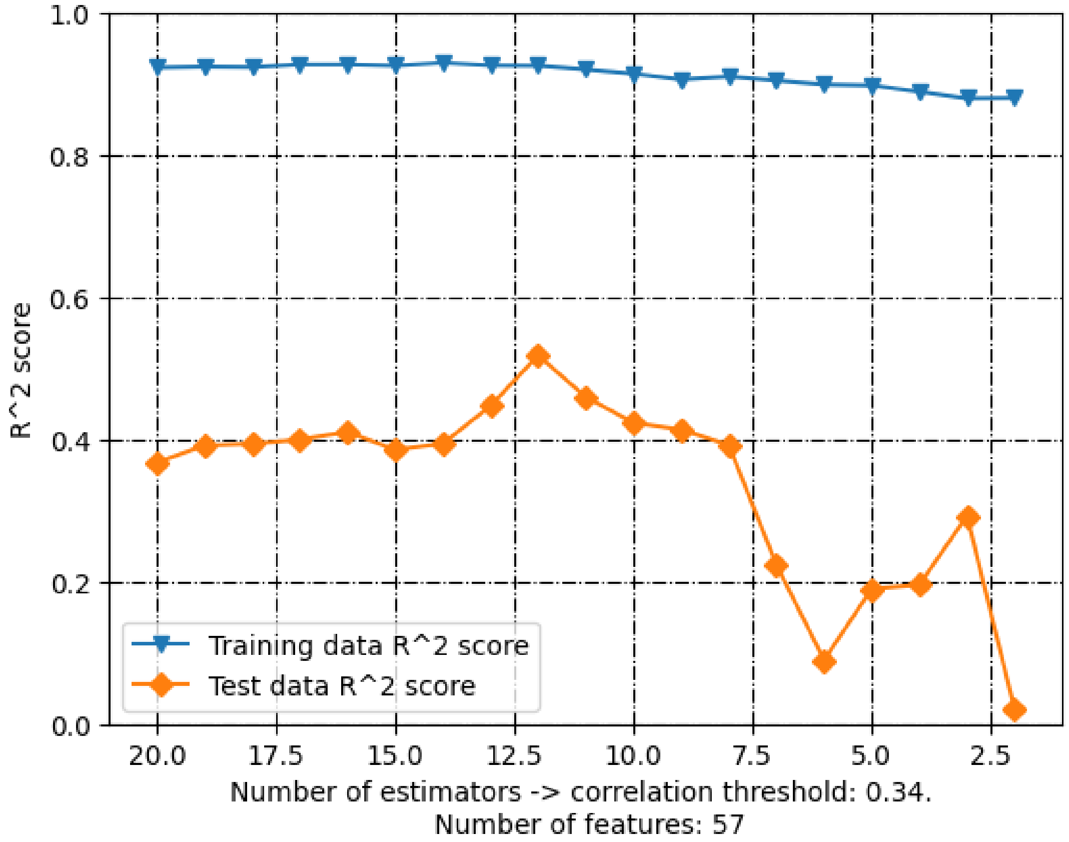

1.0
0.8
0.6
0.4
0.2
0.0
R^2 score
20.0
17.5
15.0
12.5
10.0
7.5
5.0
2.5
Training data R^2 score
Test data R^2 score
Number of estimators -> correlation threshold: 0.34.
Number of features: 57

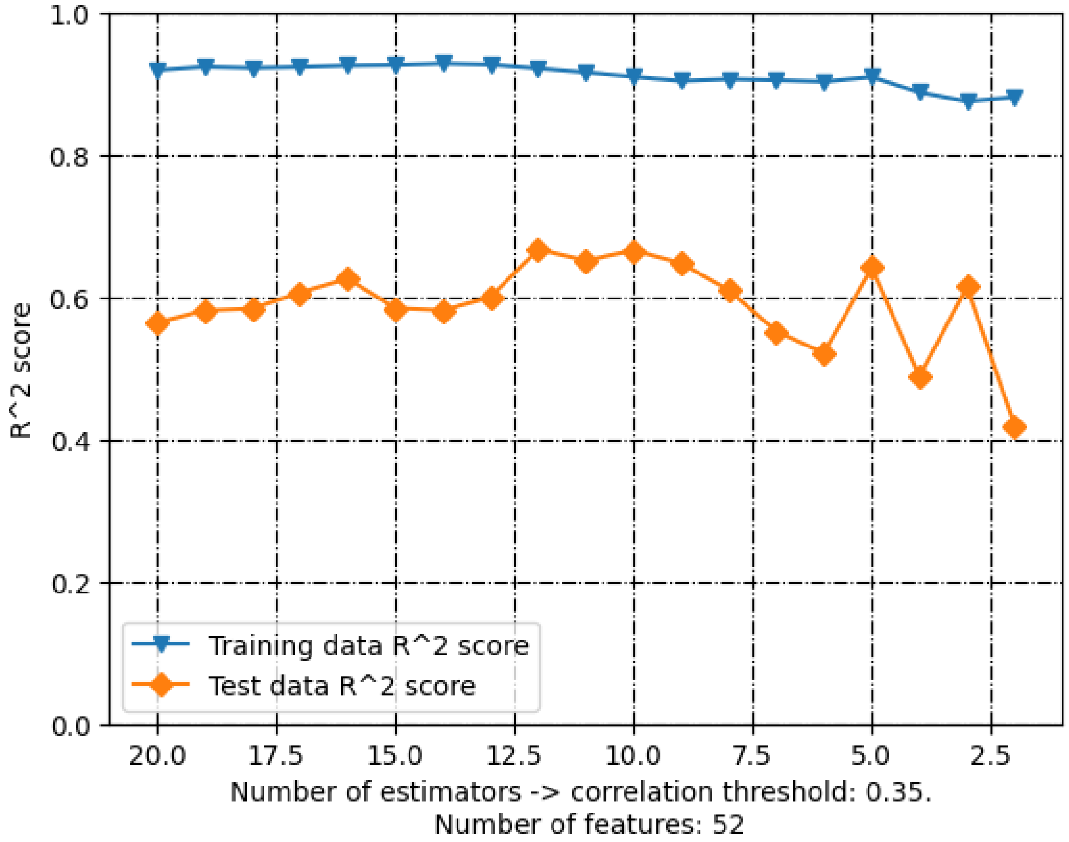
1.0
0.8
0.6
0.4
0.2
0.0
R^2 score
Training data R^2 score
Test data R^2 score
20.0
17.5
15.0
12.5
10.0
7.5
5.0
2.5
Number of estimators -> correlation threshold: 0.35.
Number of features: 52

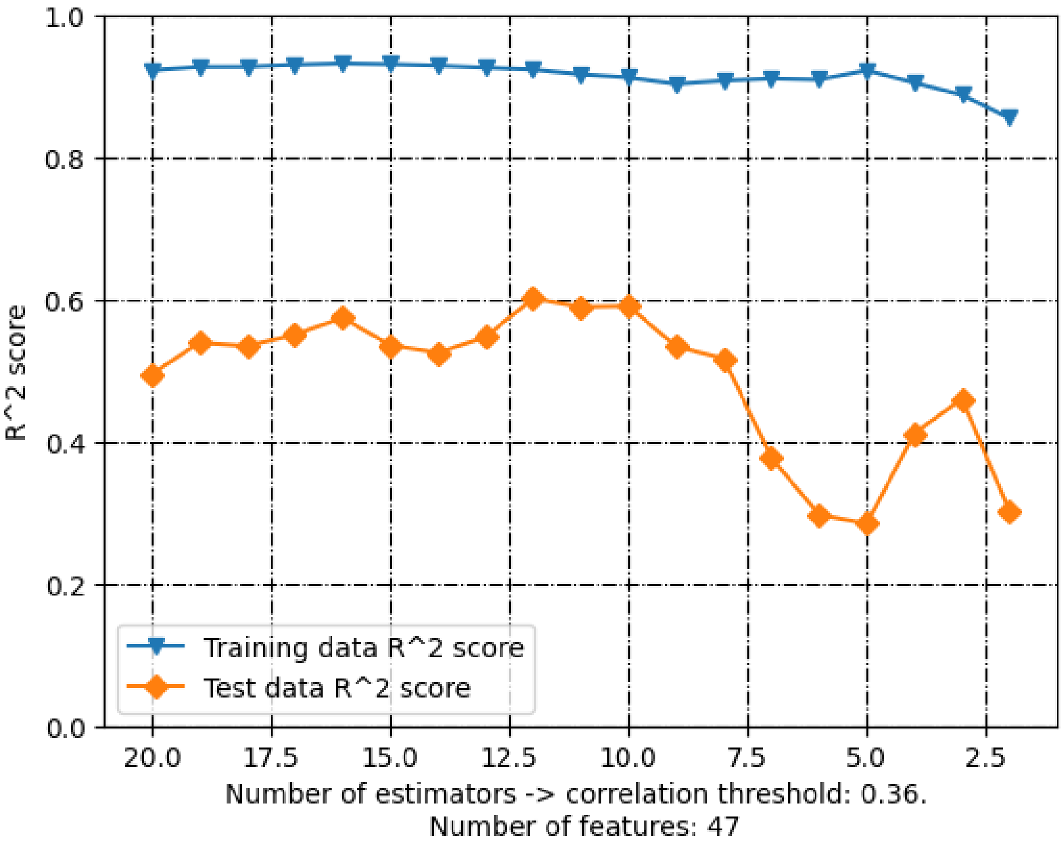
1.0
0.8
0.6
0.4
0.2
0.0
R^2 score
Training data R^2 score
Test data R^2 score
20.0
17.5
15.0
12.5
10.0
7.5
5.0
2.5
Number of estimators -> correlation threshold: 0.36.
Number of features: 47

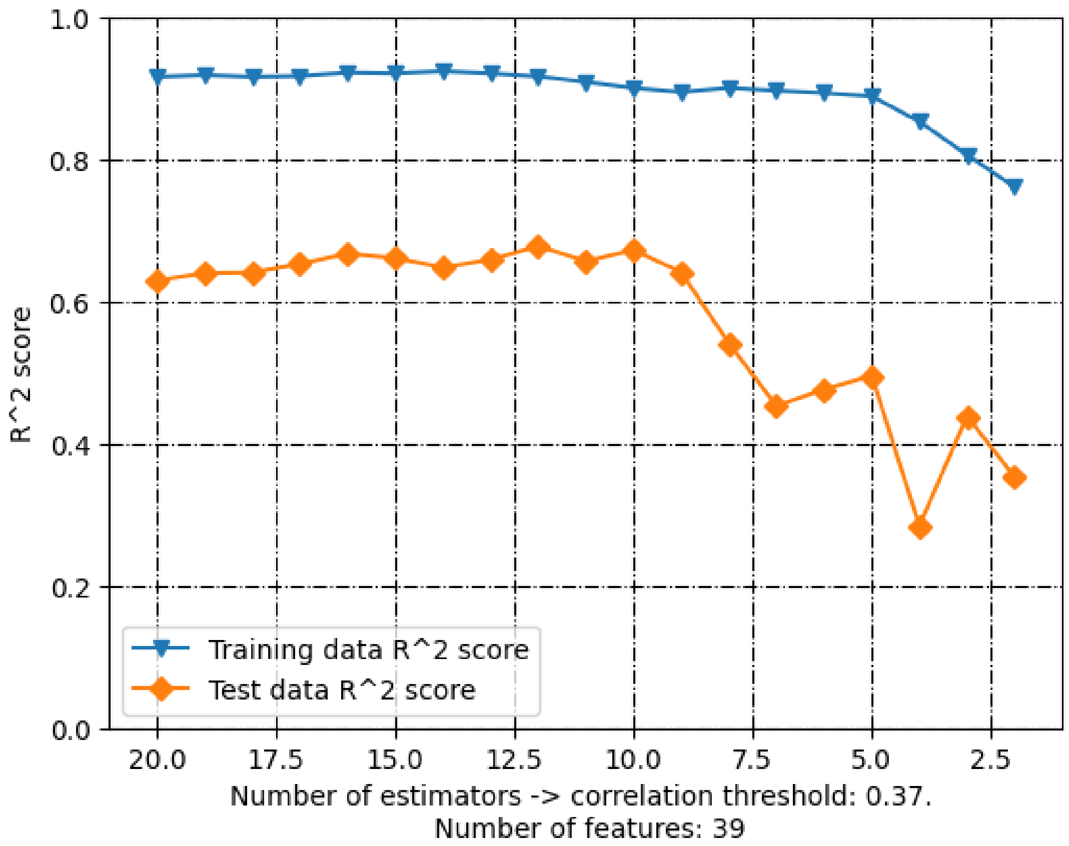

1.0
0.8
0.6
0.4
0.2
0.0
R^2 score
20.0
17.5
15.0
12.5
10.0
7.5
5.0
2.5
Training data R^2 score
Test data R^2 score
Number of estimators -> correlation threshold: 0.37.
Number of features: 39

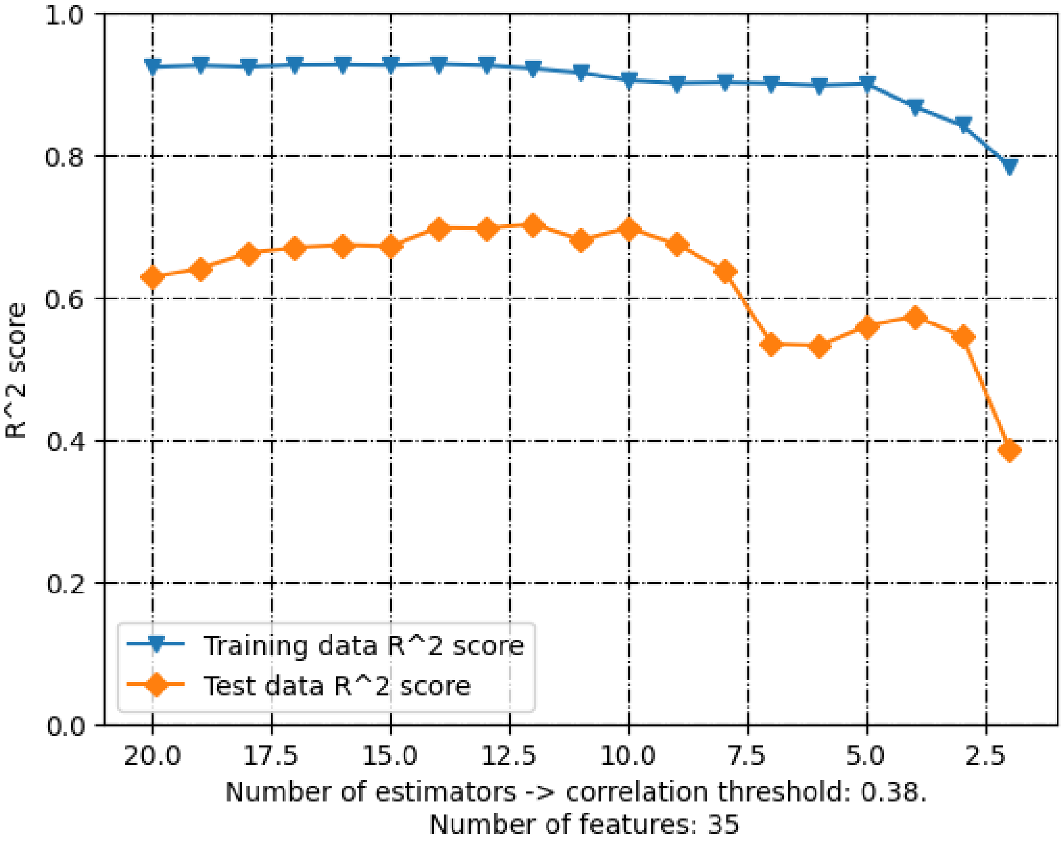
1.0
0.8
0.6
0.4
0.2
0.0
R^2 score
Training data R^2 score
Test data R^2 score
20.0
17.5
15.0
12.5
10.0
7.5
5.0
2.5
Number of estimators -> correlation threshold: 0.38.
Number of features: 35

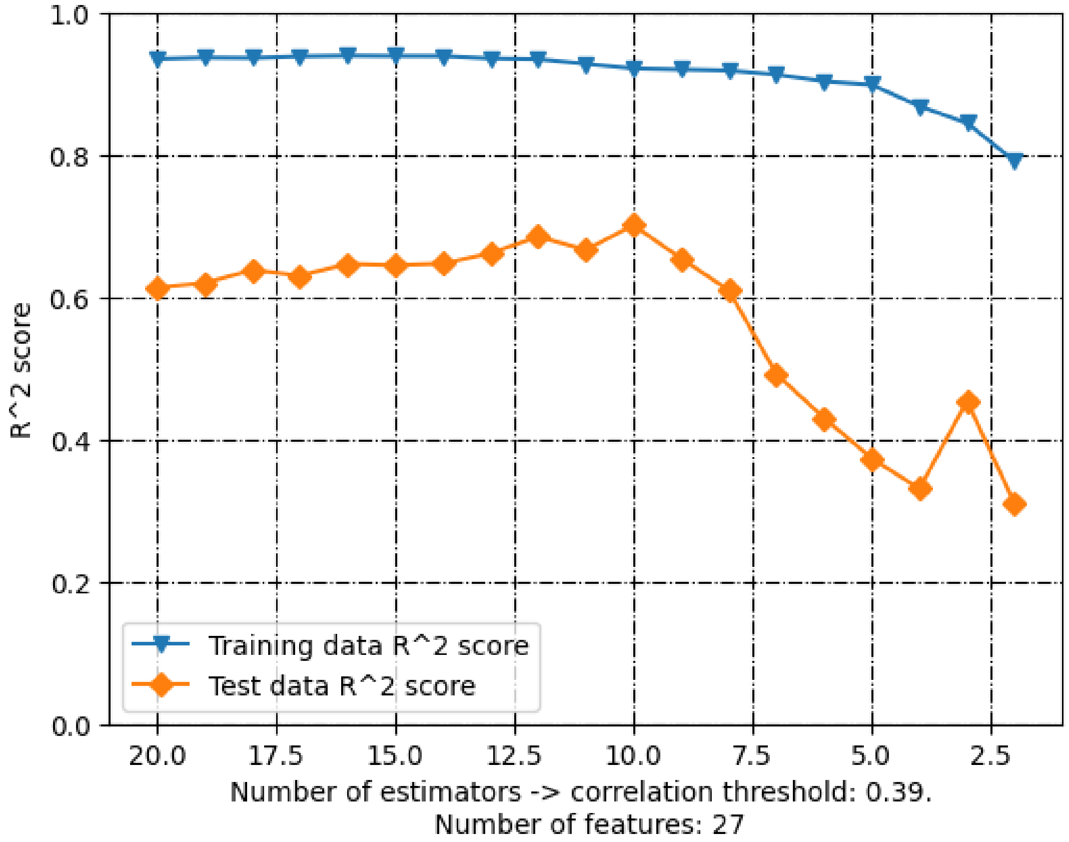

1.0
0.8
0.6
0.4
0.2
0.0
R^2 score
Training data R^2 score
Test data R^2 score
20.0
17.5
15.0
12.5
10.0
7.5
5.0
2.5
Number of estimators -> correlation threshold: 0.39.
Number of features: 27

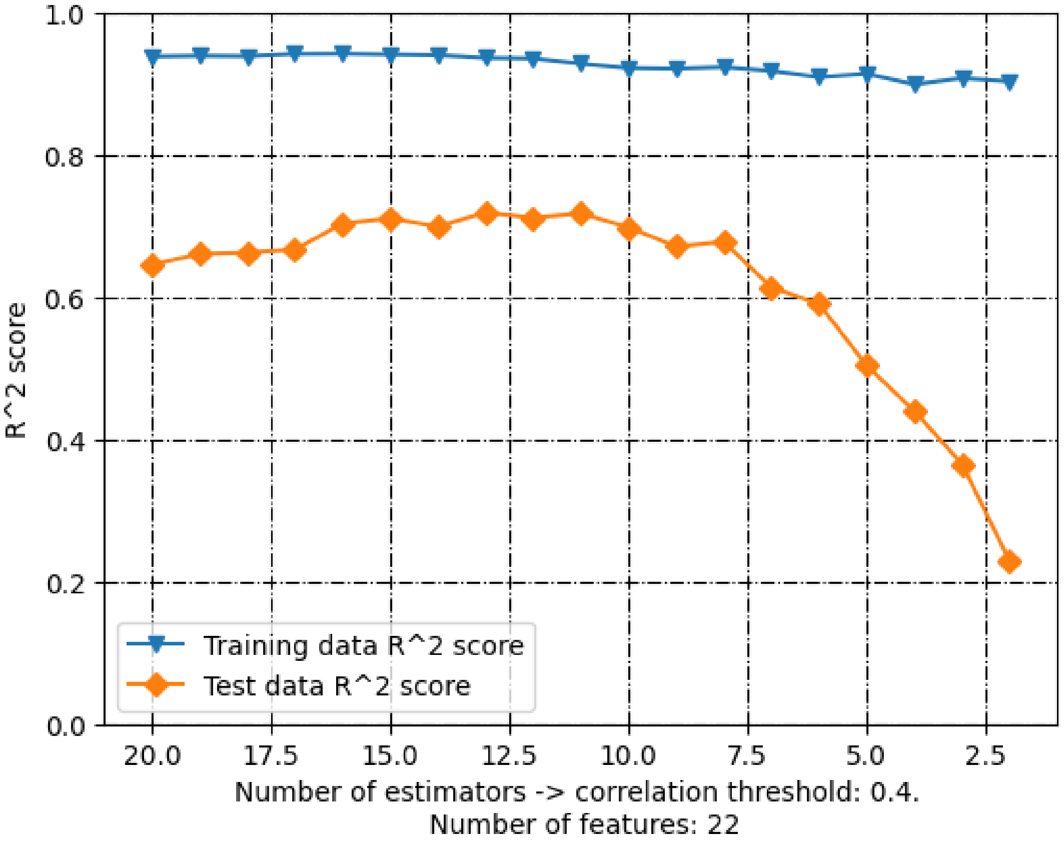
1.0
0.8
0.6
0.4
0.2
0.0
R^2 score
Training data R^2 score
Test data R^2 score
20.0
17.5
15.0
12.5
10.0
7.5
5.0
2.5
Number of estimators -> correlation threshold: 0.4.
Number of features: 22

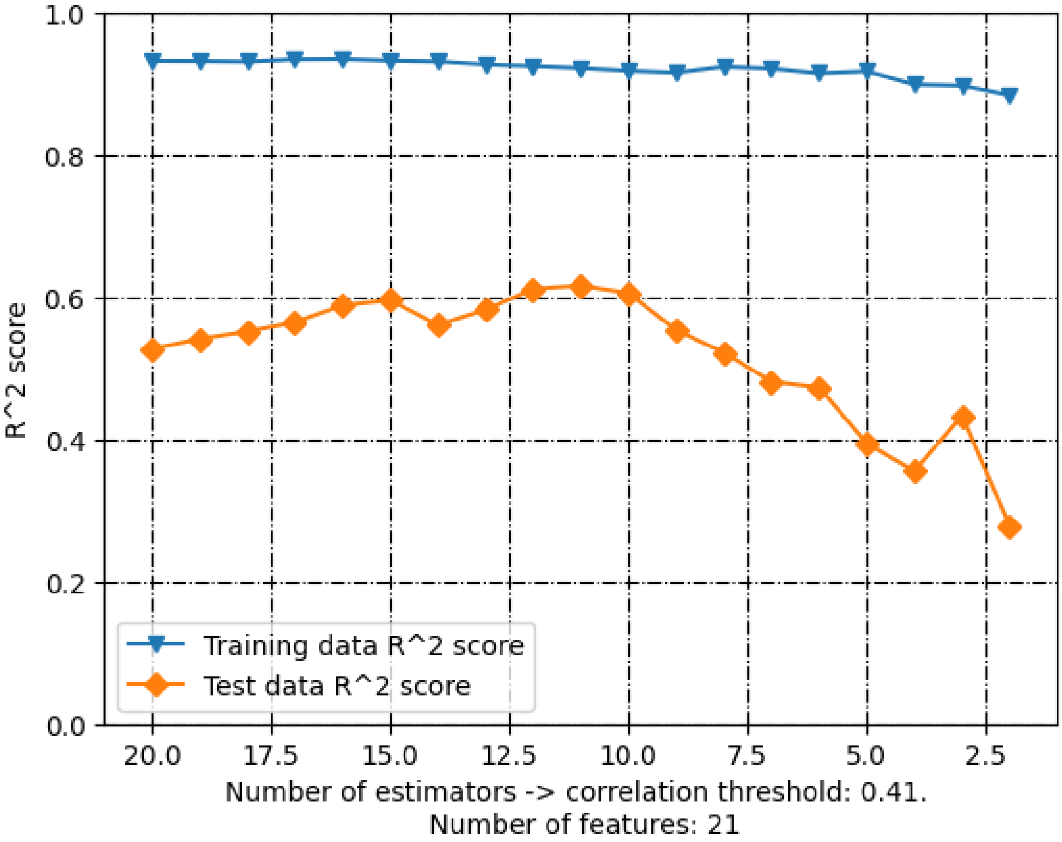

1.0
0.8
0.6
0.4
0.2
0.0
R^2 score
Training data R^2 score
Test data R^2 score
20.0
17.5
15.0
12.5
10.0
7.5
5.0
2.5
Number of estimators -> correlation threshold: 0.41.
Number of features: 21

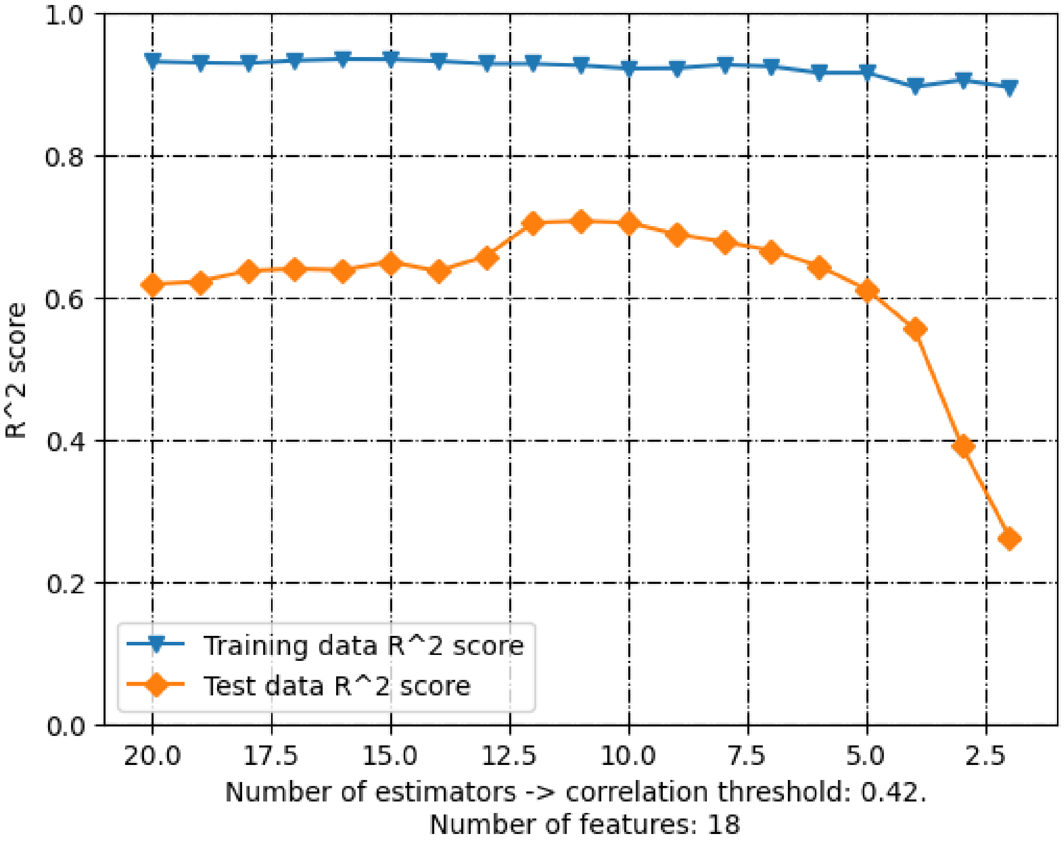

R^2 score
1.0
0.8
0.6
0.4
0.2
0.0
20.0
17.5
15.0
12.5
10.0
7.5
5.0
2.5
Training data R^2 score
Test data R^2 score
Number of estimators -> correlation threshold: 0.42.
Number of features: 18

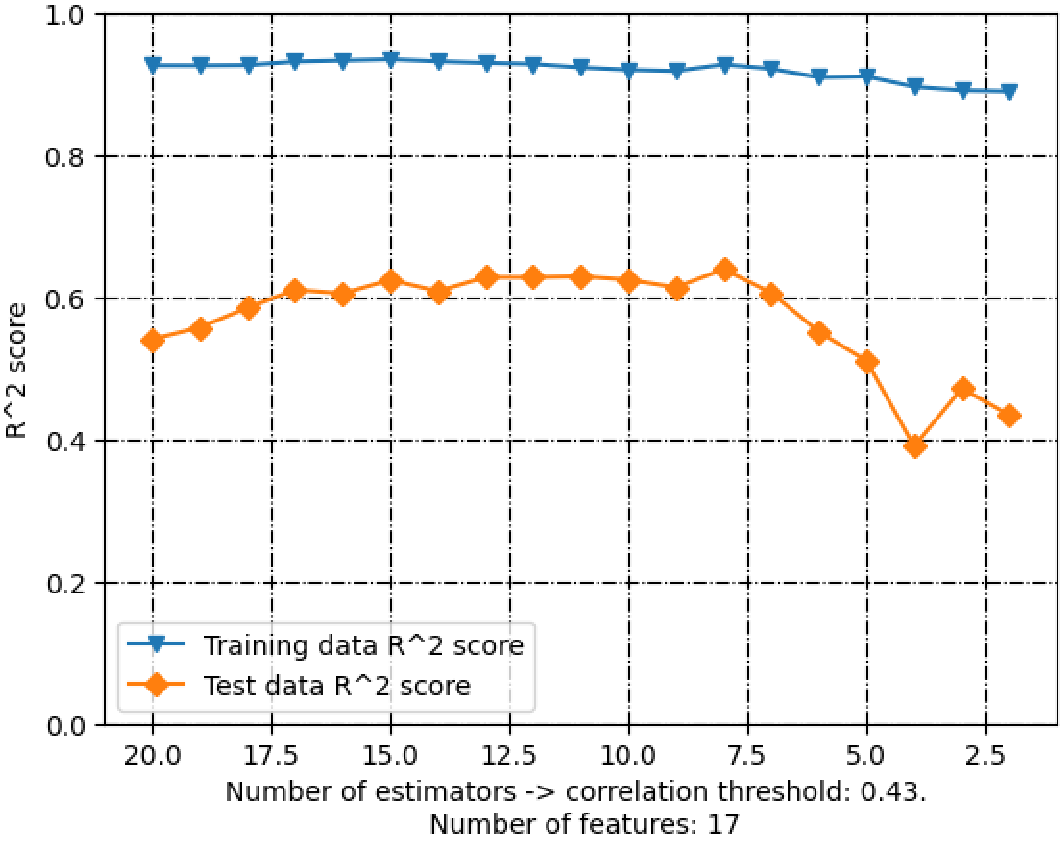
1.0
0.8
0.6
0.4
0.2
0.0
R^2 score
Training data R^2 score
Test data R^2 score
20.0
17.5
15.0
12.5
10.0
7.5
5.0
2.5
Number of estimators -> correlation threshold: 0.43.
Number of features: 17

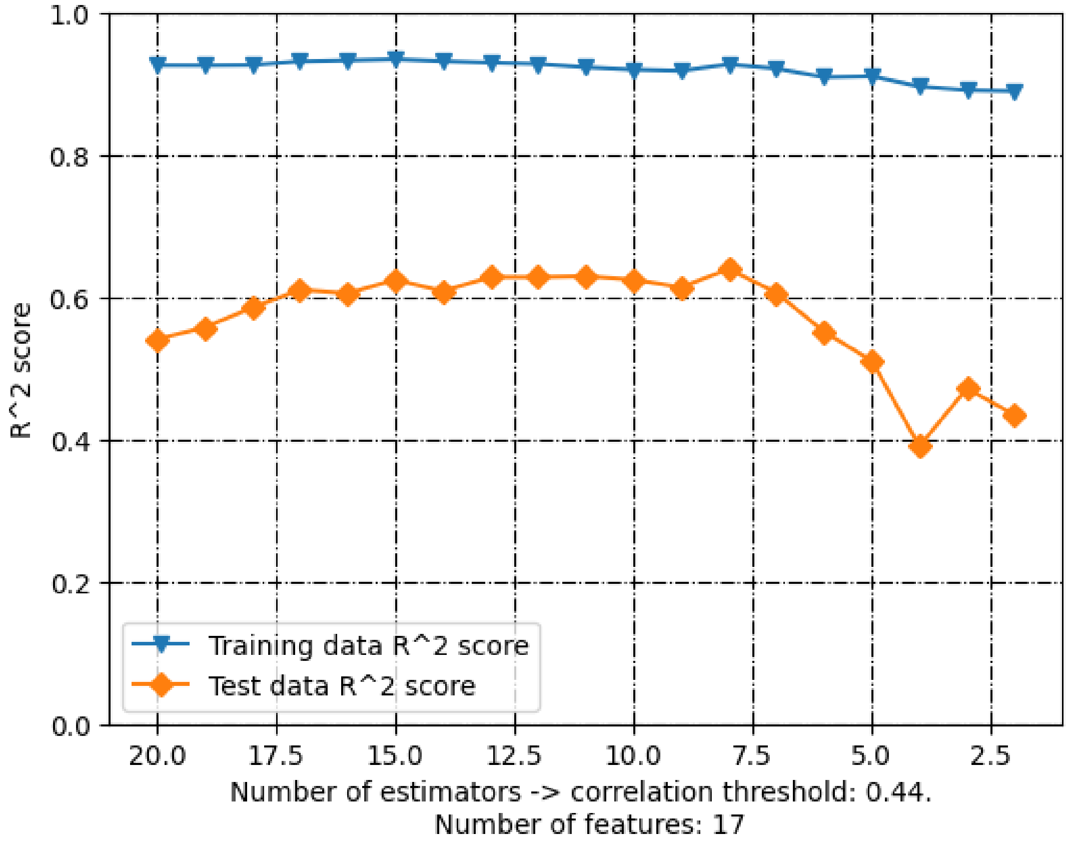
1.0
0.8
0.6
0.4
0.2
0.0
R^2 score
Training data R^2 score
Test data R^2 score
20.0
17.5
15.0
12.5
10.0
7.5
5.0
2.5
Number of estimators -> correlation threshold: 0.44.
Number of features: 17

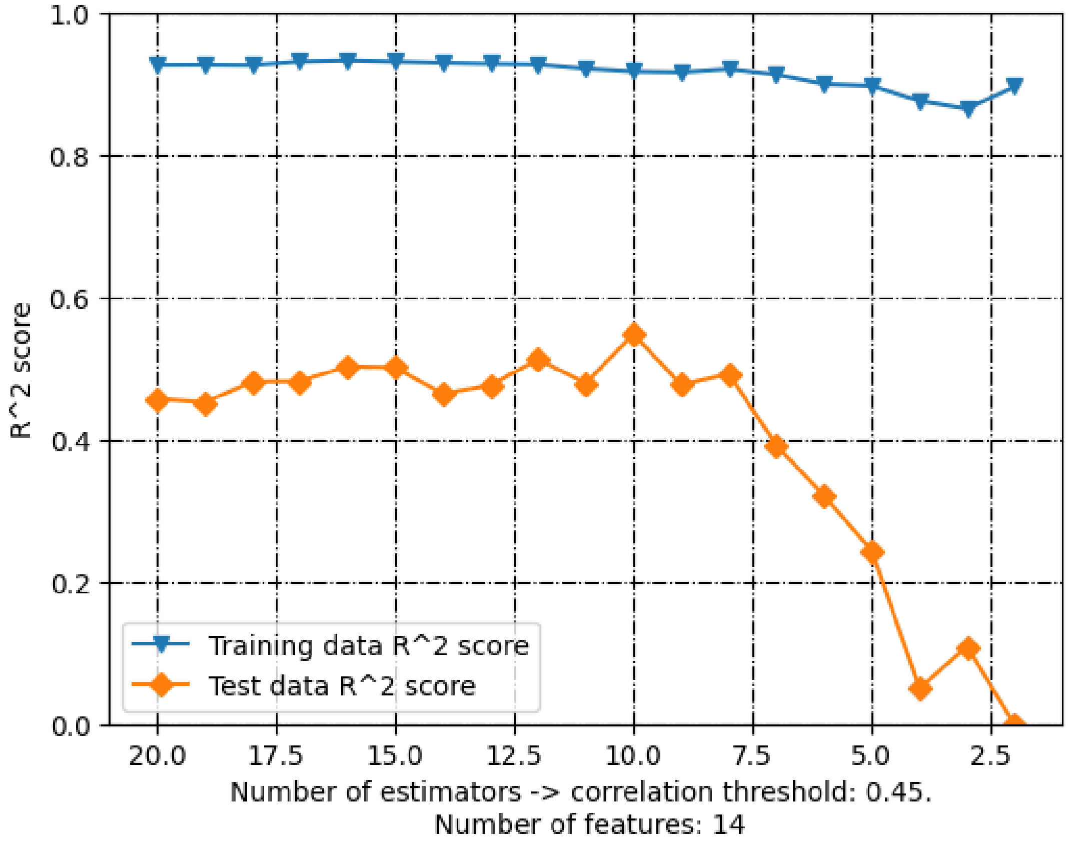
1.0
0.8
0.6
0.4
0.2
0.0
R^2 score
Training data R^2 score
Test data R^2 score
20.0
17.5
15.0
12.5
10.0
7.5
5.0
2.5
Number of estimators -> correlation threshold: 0.45.
Number of features: 14

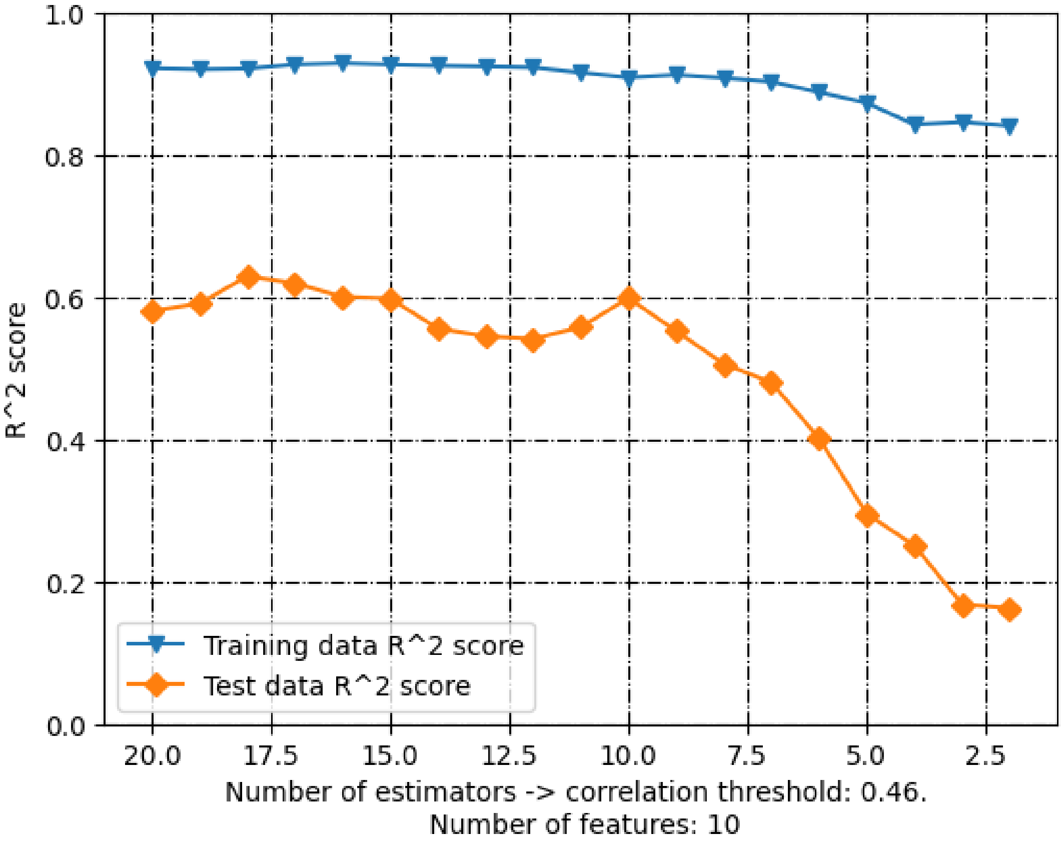
1.0
0.8
0.6
0.4
0.2
0.0
R^2 score
20.0
17.5
15.0
12.5
10.0
7.5
5.0
2.5
Number of estimators -> correlation threshold: 0.46.
Number of features: 10
Training data R^2 score
Test data R^2 score

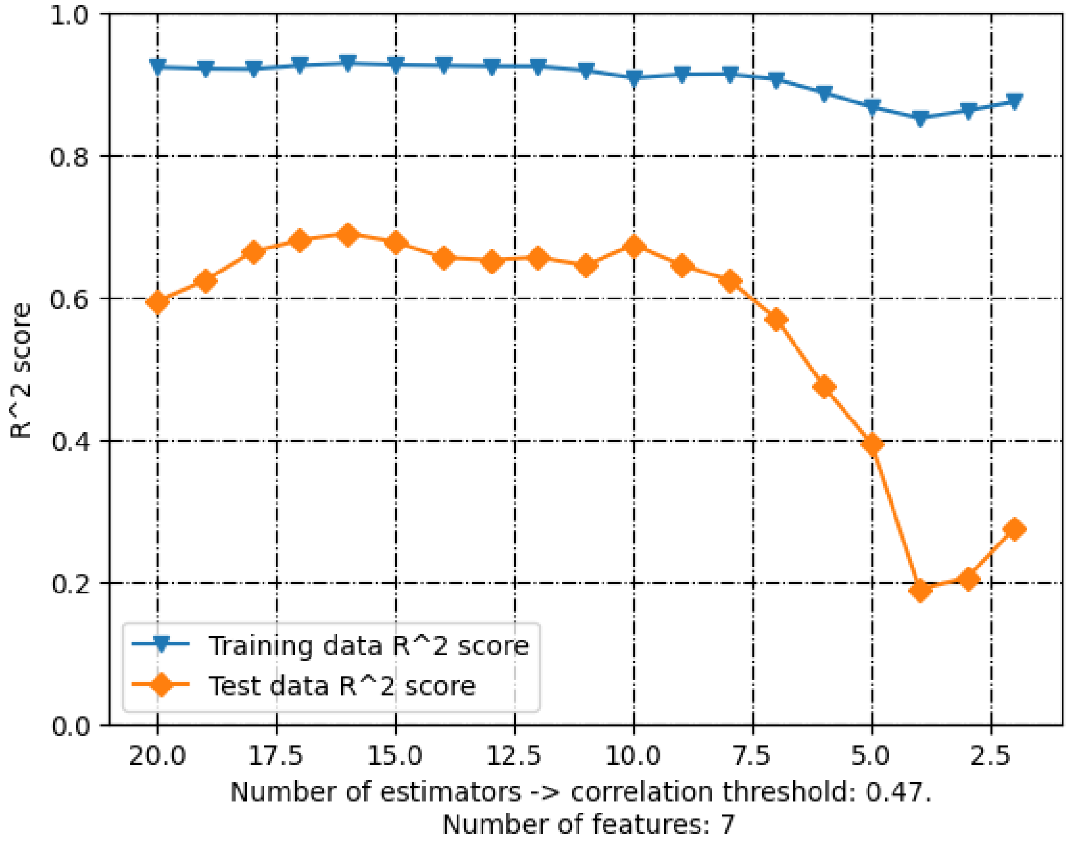

1.0
0.8
0.6
0.4
0.2
0.0
R^2 score
Training data R^2 score
Test data R^2 score
20.0
17.5
15.0
12.5
10.0
7.5
5.0
2.5
Number of estimators -> correlation threshold: 0.47.
Number of features: 7

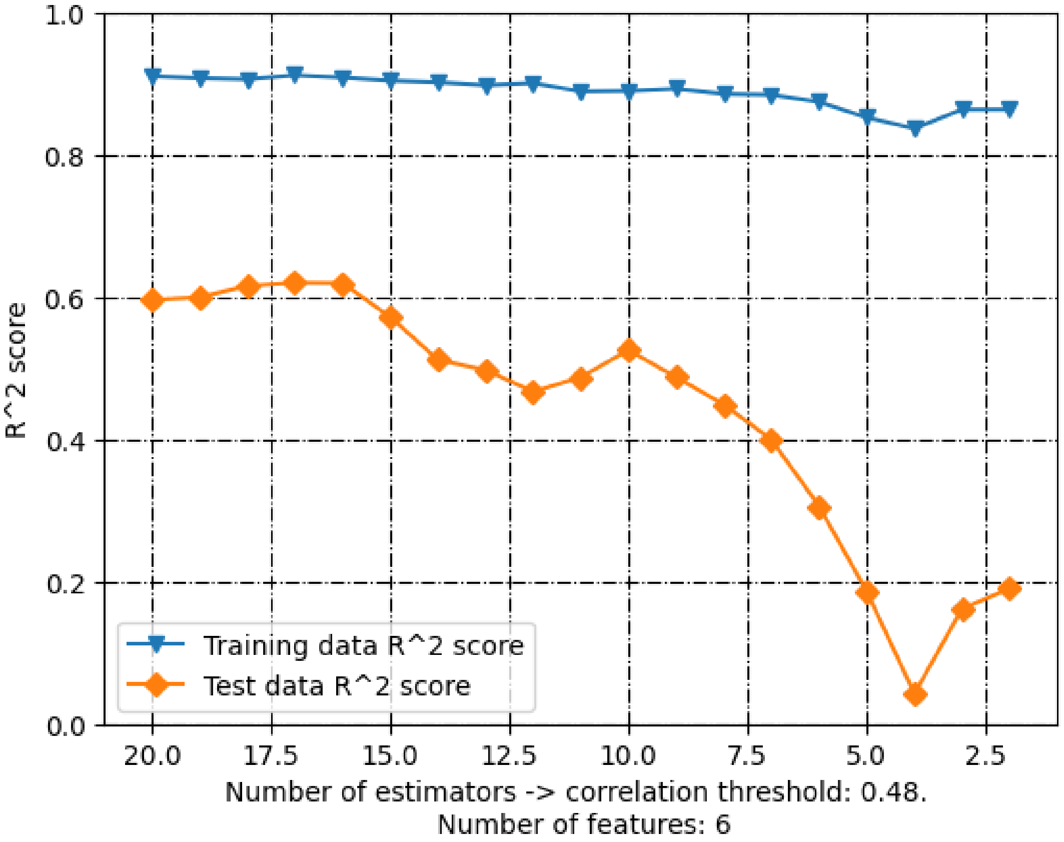

1.0
0.8
0.6
0.4
0.2
0.0
R^2 score
Training data R^2 score
Test data R^2 score
20.0
17.5
15.0
12.5
10.0
7.5
5.0
2.5
Number of estimators -> correlation threshold: 0.48.
Number of features: 6

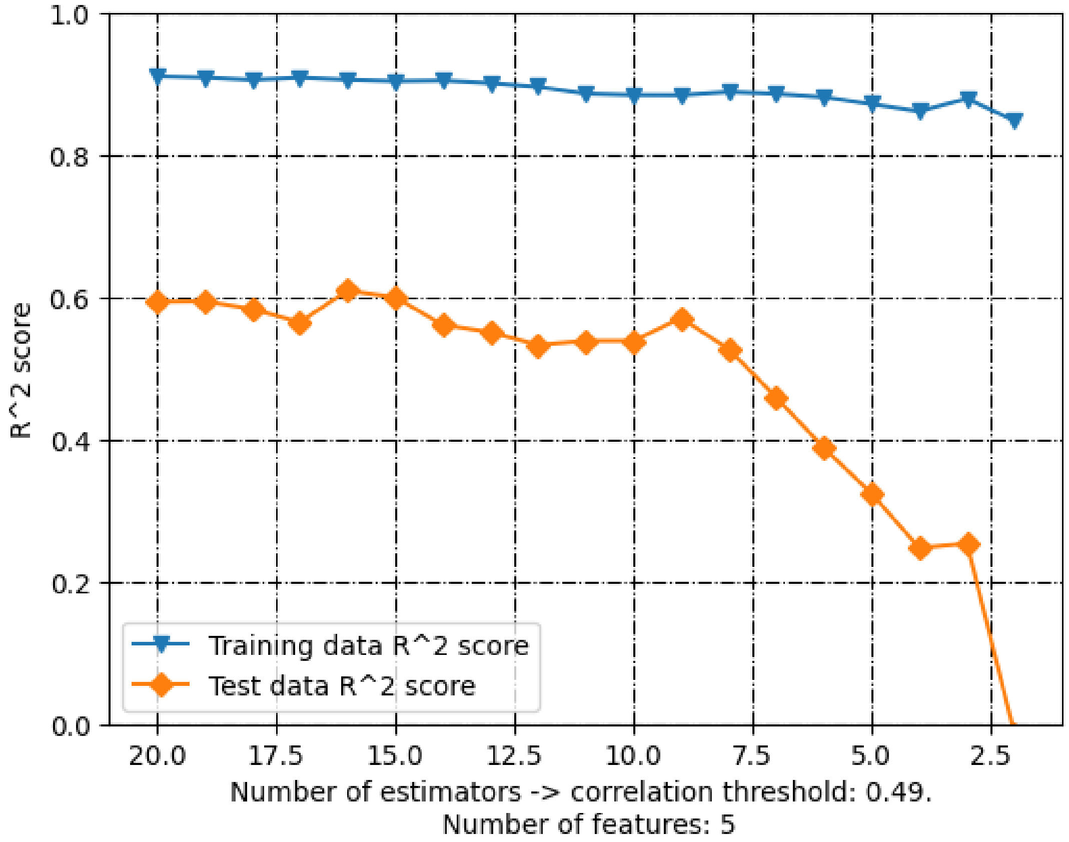
1.0
0.8
0.6
0.4
0.2
0.0
R^2 score
20.0
17.5
15.0
12.5
10.0
7.5
5.0
2.5
Number of estimators -> correlation threshold: 0.49.
Number of features: 5
Training data R^2 score
Test data R^2 score

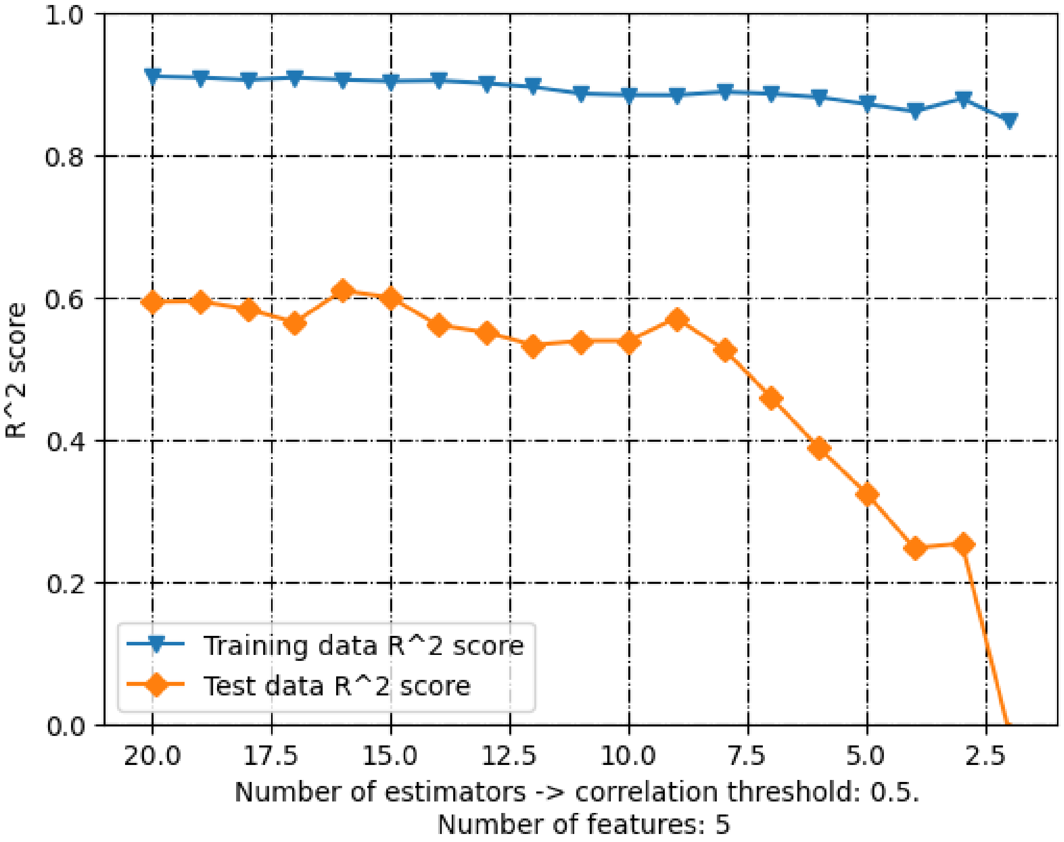

1.0
0.8
0.6
0.4
0.2
0.0
R^2 score
20.0
17.5
15.0
12.5
10.0
7.5
5.0
2.5
Training data R^2 score
Test data R^2 score
Number of estimators -> correlation threshold: 0.5.
Number of features: 5

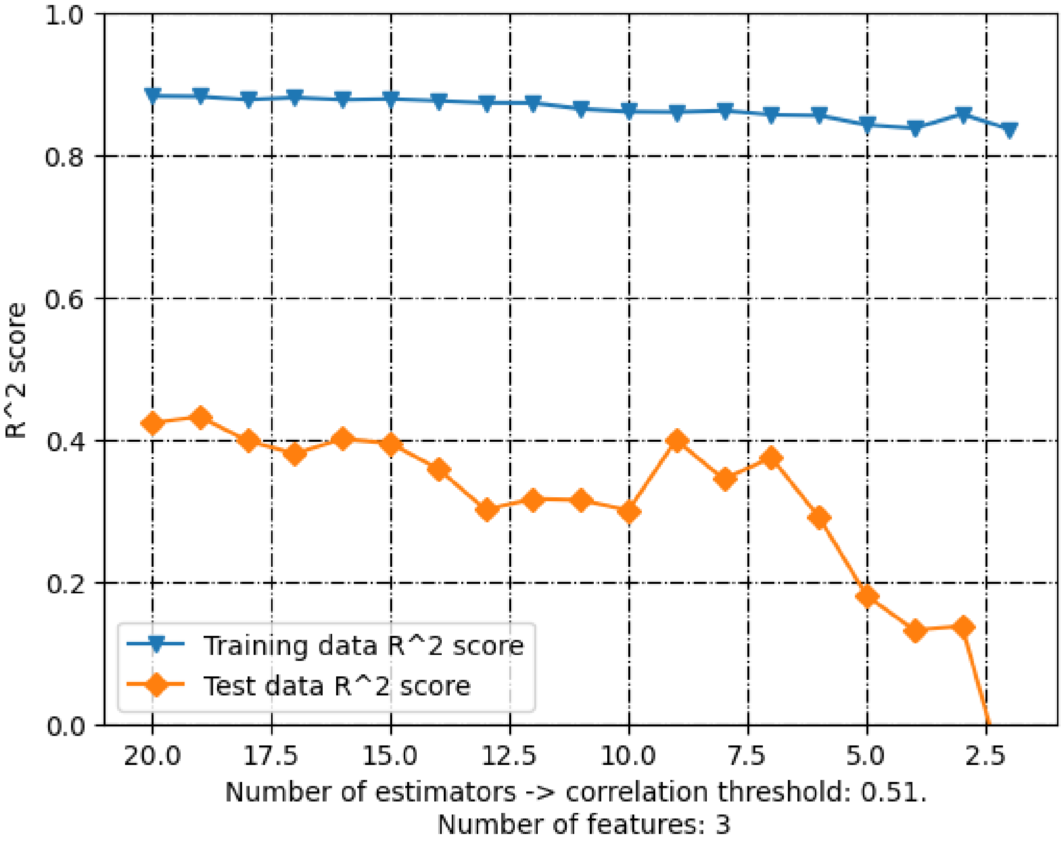
1.0
0.8
0.6
0.4
0.2
0.0
R^2 score
20.0
17.5
15.0
12.5
10.0
7.5
5.0
2.5
Training data R^2 score
Test data R^2 score
Number of estimators -> correlation threshold: 0.51.
Number of features: 3

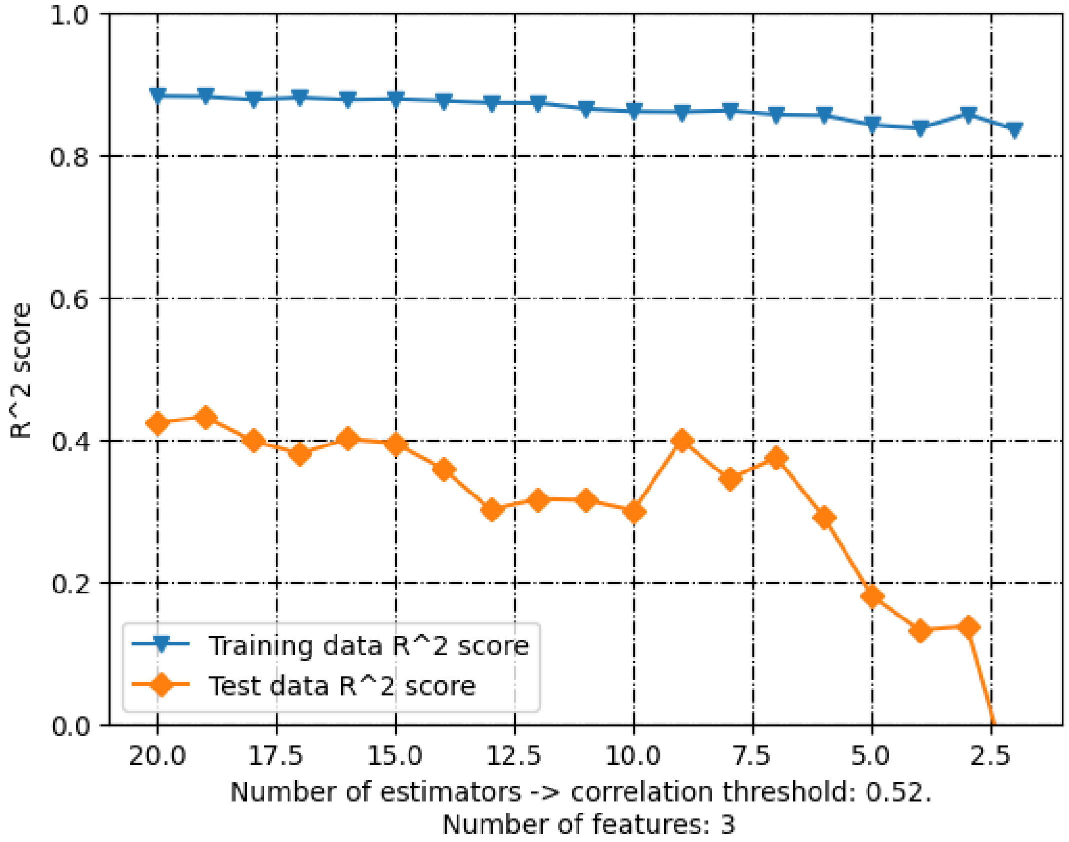

1.0
0.8
0.6
0.4
0.2
0.0
R^2 score
20.0
17.5
15.0
12.5
10.0
7.5
5.0
2.5
Training data R^2 score
Test data R^2 score
Number of estimators -> correlation threshold: 0.52.
Number of features: 3

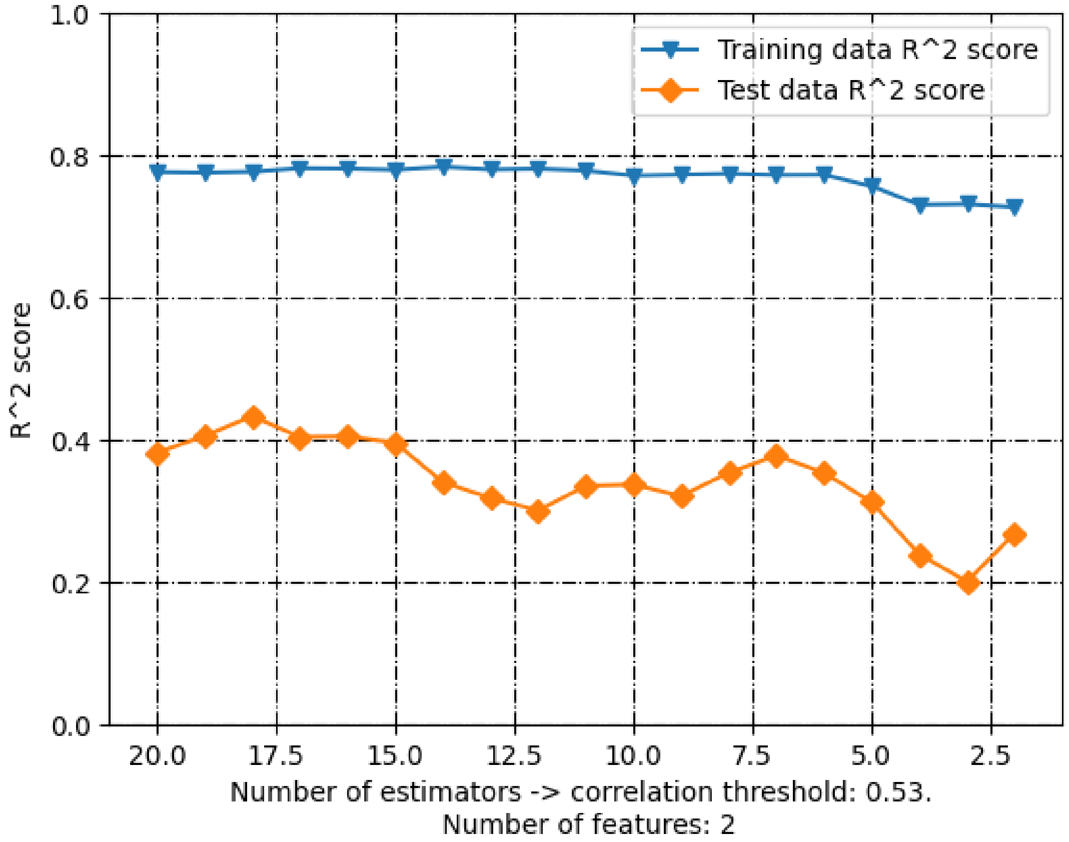

1.0
0.8
0.6
0.4
0.2
0.0
R^2 score
Training data R^2 score
Test data R^2 score
20.0
17.5
15.0
12.5
10.0
7.5
5.0
2.5
Number of estimators -> correlation threshold: 0.53.
Number of features: 2

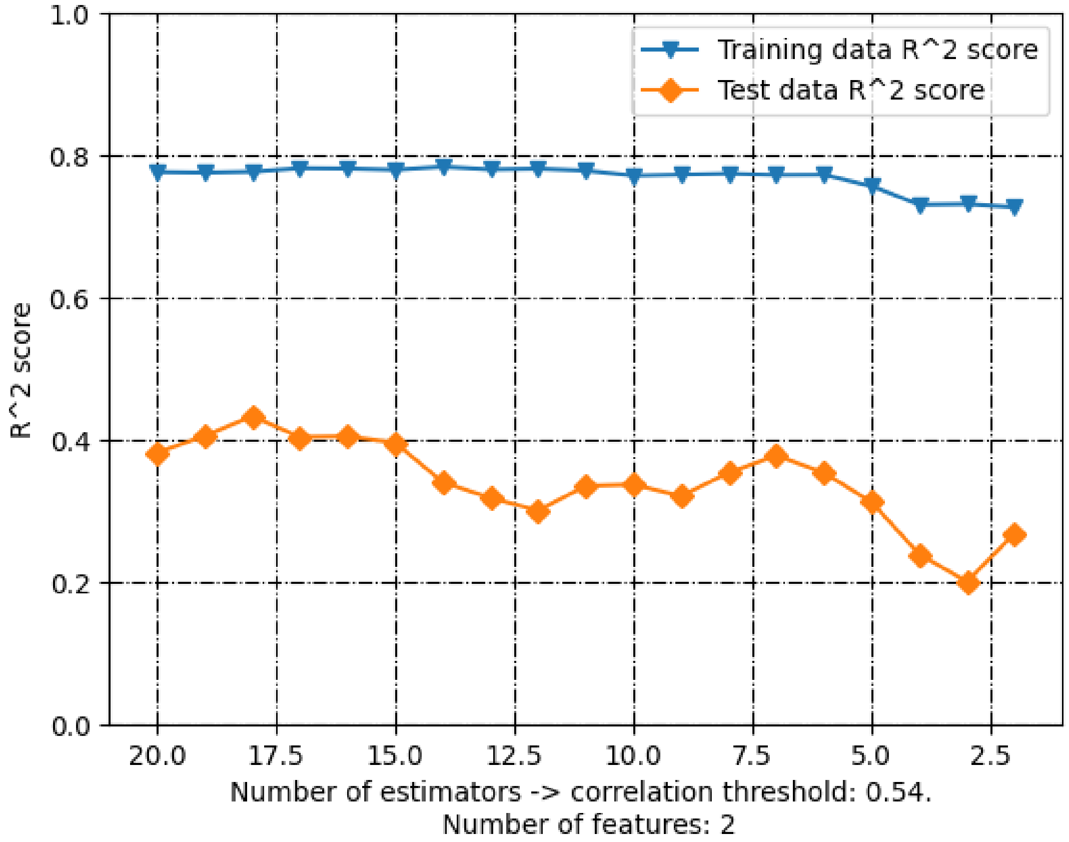

[28]:
```
plt.plot(df_without_standardization['Correlation threshold'],
    df_without_standardization['Number of features'], label = "Number of
    features", marker='.')
plt.legend()
plt.xlabel('Correlation threshold')
plt.ylabel('Number of features')
plt.show()
```

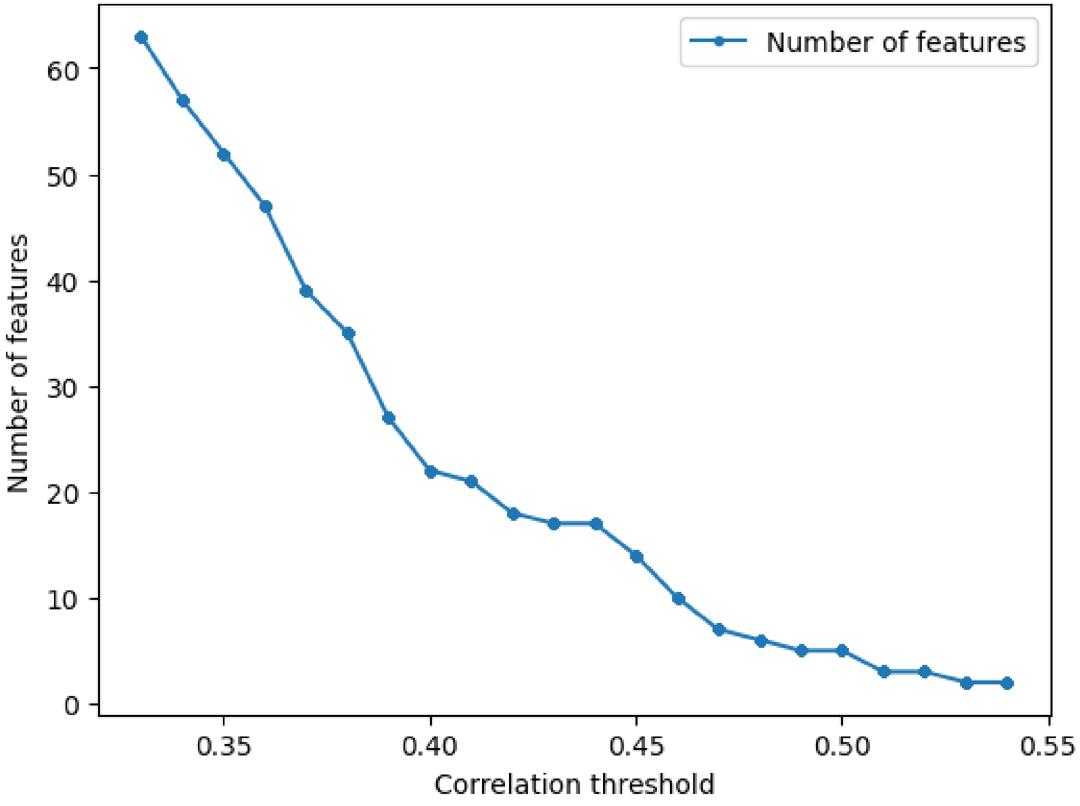

[ ]:

```

```

4 KNeighborsRegressor

[29]:

```
without_standardization, train_r2_, test_r2_, hist1, hist2, target_column_name,␣
↪training_data_RMSE, test_data_RMSE  = pred_model.
↪prepare_data_and_create_model(molecular_descriptors_df=data,
                                                                                ␣
↪                        correlation_threshold=0.50,
                                                                                ␣
↪                        standardization=False,
                                                                                ␣
↪                        model_type='KNeighborsRegressor',
                                                                                ␣
↪                        target_column_name = target,
                                                                                ␣
↪                        random_state=random_state,
                                                                                ␣
↪                        train_test_split_=True,
```

```
↪                   verbose=True)
```

```
I am not doing standardization…
  molecular descriptor name
0                   AATS0Z
1                 AATS0are
2                   AATS0d
3                  AATS0dv
4                   AATS0i
  molecular descriptor name  corr_value
0                   AATS0Z   -0.027037
1                 AATS0are   -0.129823
2                   AATS0d    0.042740
3                  AATS0dv   -0.120173
4                   AATS0i    0.132395
  molecular descriptor name  corr_value  absolute correlation value
0                   AATS0Z   -0.027037                    0.027037
1                 AATS0are   -0.129823                    0.129823
2                   AATS0d    0.042740                    0.042740
3                  AATS0dv   -0.120173                    0.120173
4                   AATS0i    0.132395                    0.132395
     molecular descriptor name  corr_value  absolute correlation value
226                     AMID_O   -0.524157                    0.524157
505                 EState_VSA5   -0.579664                    0.579664
791                    MDEO-12   -0.558727                    0.558727
851                      NdssC   -0.506855                    0.506855
1091               VSA_EState5    0.503578                    0.503578
     molecular descriptor name  corr_value  absolute correlation value
226                     AMID_O   -0.524157                    0.524157
505                 EState_VSA5   -0.579664                    0.579664
791                    MDEO-12   -0.558727                    0.558727
851                      NdssC   -0.506855                    0.506855
1091               VSA_EState5    0.503578                    0.503578
The model used is: KNeighbors…
Return the coefficient of determination of the prediction:
-0.3334799528711829
R^2 score: 0.6066454945879838
Correlation coefficient: 0.7788745050314484
Test data - unseen during training:
R^2 score: -0.3334799528711829
Correlation coefficient: nan
[7.83404702 5.70779022 8.23286856 5.70779022 7.79262314 8.04735833
 7.75341538 7.86659248 6.27944443 7.79262314 8.66909539 7.79262314
 7.87257549 8.48829548 8.04735833 7.52101879 7.75341538 6.79834213]
113    7.795880
35     6.254925
101    7.275724
```

```
36     7.017729
100    7.337242
13     8.055517
0      8.187087
114    7.638272
104    8.318759
96     7.769551
40     8.050610
103    8.136677
48     7.853872
39     8.267606
14     7.273273
117    6.167491
21     7.554396
9      6.343902
Name: LoVo, dtype: float64
Training Root Mean Square Error: 0.5886619627612601
Testing Root Mean Square Error: 0.7734185954981734
```

[30]:
```
print(target_column_name+str('_transformed'))
print(hist1[target_column_name].hist())
```

```
LoVo_transformed
AxesSubplot(0.125,0.11;0.775x0.77)
```

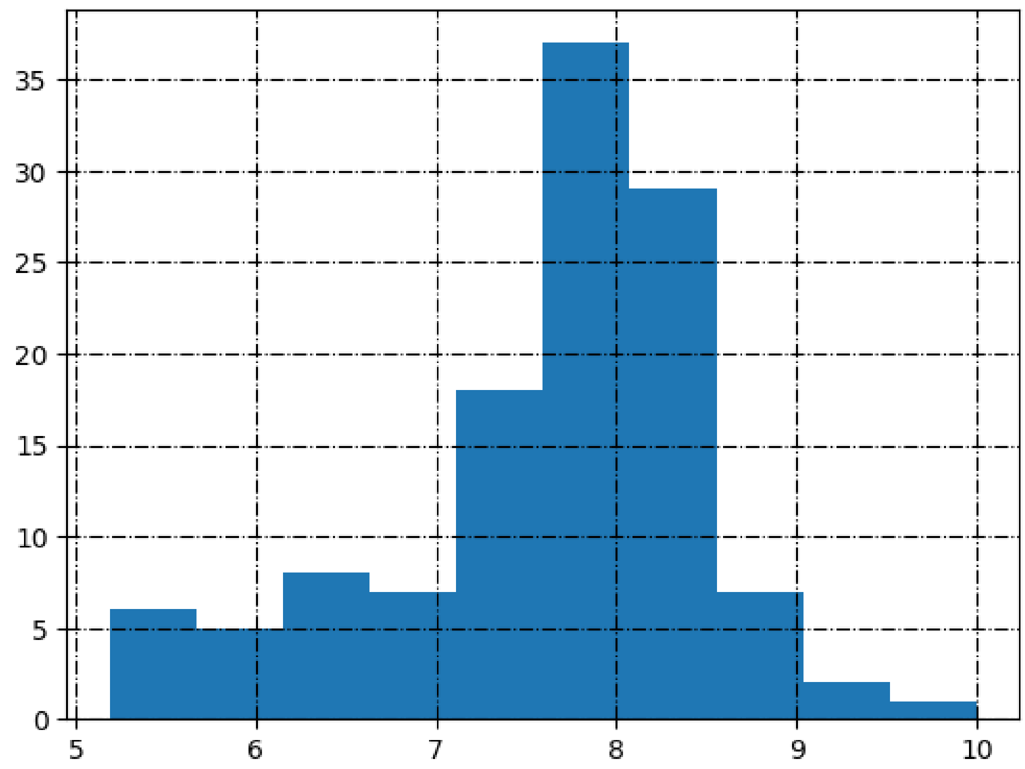

[31]:
```
with_standardization, train_r2_, test_r2_, hist1, hist2, target_column_name,␣
↪training_data_RMSE, test_data_RMSE  = pred_model.
↪prepare_data_and_create_model(molecular_descriptors_df=data,
                                                                               ␣
↪                    correlation_threshold=0.50,
                                                                               ␣
↪                    standardization=True,
                                                                               ␣
↪                    model_type='KNeighborsRegressor',
                                                                               ␣
↪                    target_column_name = target,
                                                                               ␣
↪                    random_state=random_state,
                                                                               ␣
↪                    train_test_split_=True,
                                                                               ␣
↪                    verbose=True)
```

```
I am doing standardization…
  molecular descriptor name
0                   AATS0Z
1                 AATS0are
2                   AATS0d
3                  AATS0dv
4                   AATS0i
  molecular descriptor name  corr_value
0                   AATS0Z   -0.027037
1                 AATS0are   -0.129823
2                   AATS0d    0.042740
3                  AATS0dv   -0.120173
4                   AATS0i    0.132395
  molecular descriptor name  corr_value  absolute correlation value
0                   AATS0Z   -0.027037                    0.027037
1                 AATS0are   -0.129823                    0.129823
2                   AATS0d    0.042740                    0.042740
3                  AATS0dv   -0.120173                    0.120173
4                   AATS0i    0.132395                    0.132395
     molecular descriptor name  corr_value  absolute correlation value
226                     AMID_O   -0.524157                    0.524157
505                 EState_VSA5   -0.579664                    0.579664
791                   MDEO-12   -0.558727                    0.558727
851                     NdssC   -0.506855                    0.506855
1091               VSA_EState5    0.503578                    0.503578
     molecular descriptor name  corr_value  absolute correlation value
226                     AMID_O   -0.524157                    0.524157
505                 EState_VSA5   -0.579664                    0.579664
```

```
791                    MDEO-12   -0.558727                    0.558727
851                      NdssC   -0.506855                    0.506855
1091               VSA_EState5    0.503578                    0.503578
The model used is: KNeighbors…
Return the coefficient of determination of the prediction:
-0.3334799528711829
R^2 score: 0.6066454945879838
Correlation coefficient: 0.7788745050314484
Test data - unseen during training:
R^2 score: -0.3334799528711829
Correlation coefficient: nan
[7.83404702 5.70779022 8.23286856 5.70779022 7.79262314 8.04735833
 7.75341538 7.86659248 6.27944443 7.79262314 8.66909539 7.79262314
 7.87257549 8.48829548 8.04735833 7.52101879 7.75341538 6.79834213]
113    7.795880
35     6.254925
101    7.275724
36     7.017729
100    7.337242
13     8.055517
0      8.187087
114    7.638272
104    8.318759
96     7.769551
40     8.050610
103    8.136677
48     7.853872
39     8.267606
14     7.273273
117    6.167491
21     7.554396
9      6.343902
Name: LoVo, dtype: float64
Training Root Mean Square Error: 0.5886619627612601
Testing Root Mean Square Error: 0.7734185954981734
```

4.1 Search inside correlation space

[32]:
```python
step = 0.01
initial_step = corr_low
last_step = corr_high
first_list = [x / 100.0 for x in range(int(initial_step*100),␣
 ↪int(last_step*100), int(step*100))]
second_list = []
third_list = []
fourth_l = []
fifth_l = []
```

```
f_list = []
for i in first_list:
    without_standardization, train_r2, test_r2, _, h_, target_column_name,␣
 ↪training_data_RMSE, test_data_RMSE = pred_model.
 ↪prepare_data_and_create_model(molecular_descriptors_df = data,
                                                                                        ␣
 ↪                    correlation_threshold = i,
                                                                                        ␣
 ↪                    standardization = False,
                                                                                        ␣
 ↪                    model_type = 'KNeighborsRegressor',
                                                                                        ␣
 ↪                    target_column_name = target,
                                                                                        ␣
 ↪                    random_state=random_state,
                                                                                        ␣
 ↪                    train_test_split_ = True,
                                                                                        ␣
 ↪                    verbose = False)
    second_list.append(train_r2)
    third_list.append(test_r2)
    fourth_l.append(training_data_RMSE)
    fifth_l.append(test_data_RMSE)
    f_list.append(len(h_))
```

```
  File "C:\Users\aleks\anaconda3\envs\cheminf_gpu\lib\site-
packages\joblib\externals\loky\backend\context.py", line 217, in
_count_physical_cores
    raise ValueError(
```

[33]:
```
df_without_standardization = pd.DataFrame(data=first_list,␣
 ↪columns=["Correlation threshold"])
df_without_standardization['Training data R^2 score'] = second_list
df_without_standardization['Test data R^2 score'] = third_list
df_without_standardization['Training RMSE'] = fourth_l
df_without_standardization['Test RMSE'] = fifth_l
df_without_standardization['Number of features'] = f_list
```

[34]:
```
df_k_nearest = df_without_standardization.copy()
df_without_standardization
```

[34]:

	Correlation threshold	Training data R^2 score	Test data R^2 score	\
0	0.33	0.576390	0.179466	
1	0.34	0.576390	0.179466	
2	0.35	0.629945	0.129927	
3	0.36	0.629945	0.129927	
4	0.37	0.623904	0.124725	

5	0.38	0.614863	0.185015
6	0.39	0.594616	0.072307
7	0.40	0.600934	-0.255977
8	0.41	0.600934	-0.255977
9	0.42	0.614689	-0.036872
10	0.43	0.616975	-0.034882
11	0.44	0.616975	-0.034882
12	0.45	0.608333	0.005907
13	0.46	0.628704	-0.173299
14	0.47	0.613090	-0.102667
15	0.48	0.612998	-0.285140
16	0.49	0.606645	-0.333480
17	0.50	0.606645	-0.333480
18	0.51	0.572722	-0.264338
19	0.52	0.572722	-0.264338
20	0.53	0.544344	-0.247045
21	0.54	0.544344	-0.247045

	Training RMSE	Test RMSE	Number of features
0	0.610882	0.606694	63
1	0.610882	0.606694	57
2	0.570962	0.624740	52
3	0.570962	0.624740	47
4	0.575603	0.626605	39
5	0.582481	0.604639	35
6	0.597595	0.645095	27
7	0.592920	0.750606	22
8	0.592920	0.750606	21
9	0.582612	0.681999	18
10	0.580881	0.681345	17
11	0.580881	0.681345	17
12	0.587398	0.667782	14
13	0.571919	0.725480	10
14	0.583820	0.703305	7
15	0.583890	0.759271	6
16	0.588662	0.773419	5
17	0.588662	0.773419	5
18	0.613521	0.753101	3
19	0.613521	0.753101	3
20	0.633567	0.747933	2
21	0.633567	0.747933	2

4.2 Plots

[35]:
```
plt.plot(df_without_standardization['Correlation threshold'],␣
 ↪df_without_standardization['Training data R^2 score'], label = "Training␣
 ↪data R^2 score", marker='v')
plt.plot(df_without_standardization['Correlation threshold'],␣
 ↪df_without_standardization['Test data R^2 score'], label = "Test data R^2␣
 ↪score", marker='D')
plt.legend()
plt.xlabel('Correlation threshold')
plt.ylabel('R^2 score')
plt.ylim([0, 1])
plt.show()
```

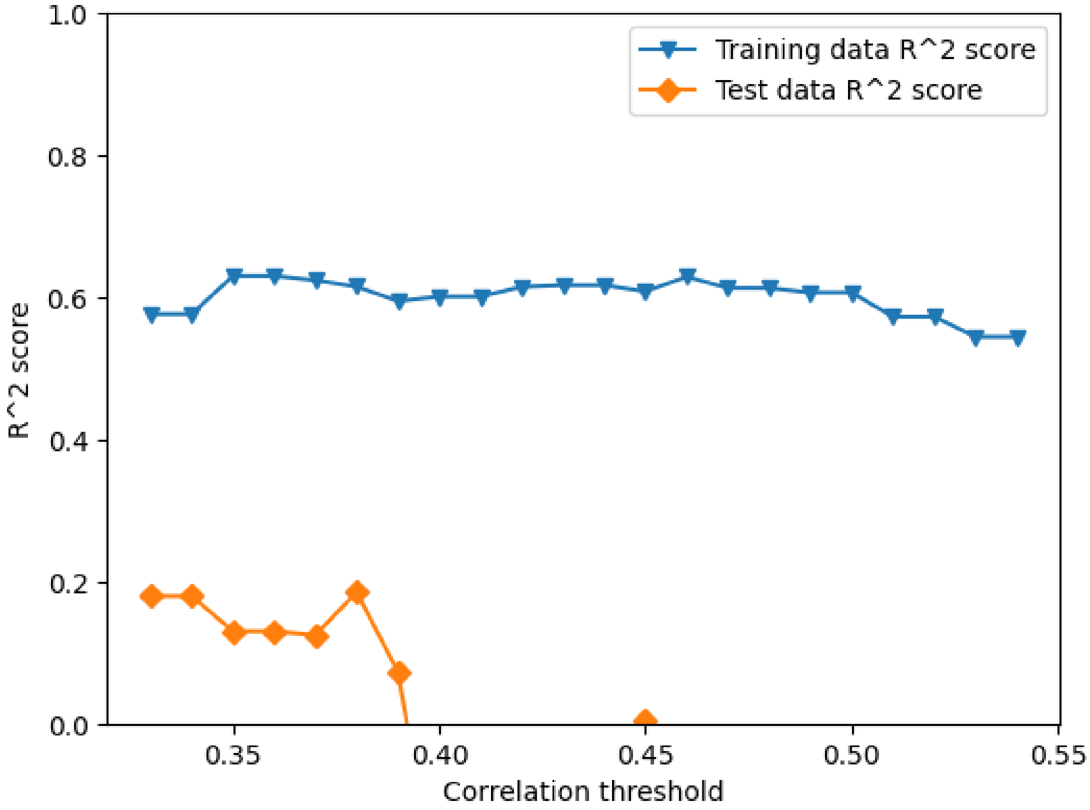

[36]:
```
plt.plot(df_without_standardization['Correlation threshold'],␣
 ↪df_without_standardization['Number of features'], label = "Number of␣
 ↪features", marker='.')
plt.legend()
plt.xlabel('Correlation threshold')
plt.ylabel('Number of features')
plt.show()
```

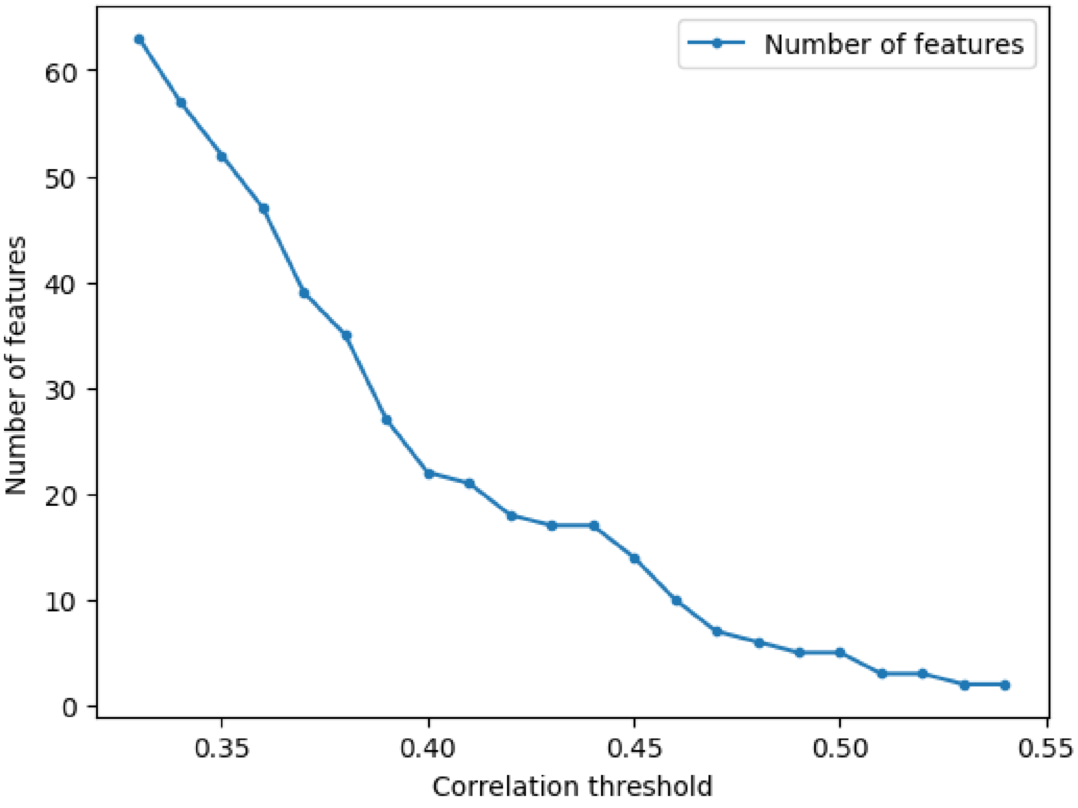


```
[ ]:
```

5 Support Vector Machines (SVM)

```
[37]: without_standardization, train_r2_, test_r2_, hist1, hist2, target_column_name,␣
      ↪training_data_RMSE, test_data_RMSE  = pred_model.
      ↪prepare_data_and_create_model(molecular_descriptors_df=data,
                                                                                 ␣
      ↪                     correlation_threshold=0.50,
                                                                                 ␣
      ↪                     standardization=False,
                                                                                 ␣
      ↪                     model_type='SVR',
                                                                                 ␣
      ↪                     kernel_ = 'linear',
                                                                                 ␣
      ↪                     gamma_ = 'auto',
                                                                                 ␣
      ↪                     target_column_name = target,
```

```
                         random_state=random_state,
                         train_test_split_=True,
                         verbose=True)
```

```
I am not doing standardization…
  molecular descriptor name
0                    AATS0Z
1                  AATS0are
2                    AATS0d
3                   AATS0dv
4                    AATS0i
  molecular descriptor name  corr_value
0                    AATS0Z   -0.027037
1                  AATS0are   -0.129823
2                    AATS0d    0.042740
3                   AATS0dv   -0.120173
4                    AATS0i    0.132395
  molecular descriptor name  corr_value  absolute correlation value
0                    AATS0Z   -0.027037                    0.027037
1                  AATS0are   -0.129823                    0.129823
2                    AATS0d    0.042740                    0.042740
3                   AATS0dv   -0.120173                    0.120173
4                    AATS0i    0.132395                    0.132395
     molecular descriptor name  corr_value  absolute correlation value
226                     AMID_O   -0.524157                    0.524157
505                EState_VSA5   -0.579664                    0.579664
791                   MDEO-12   -0.558727                    0.558727
851                     NdssC   -0.506855                    0.506855
1091               VSA_EState5    0.503578                    0.503578
     molecular descriptor name  corr_value  absolute correlation value
226                     AMID_O   -0.524157                    0.524157
505                EState_VSA5   -0.579664                    0.579664
791                   MDEO-12   -0.558727                    0.558727
851                     NdssC   -0.506855                    0.506855
1091               VSA_EState5    0.503578                    0.503578
The model used is: SVR…
Return the coefficient of determination of the prediction:
0.3110687450006334
R^2 score: 0.48759250346101435
Correlation coefficient: 0.698278242150659
Test data - unseen during training:
R^2 score: 0.3110687450006334
Correlation coefficient: 0.5577353718392204
[8.00534204 6.50259618 7.87937287 6.53792643 7.92664356 7.83744649
```

```
 7.97335513 7.98651677 6.94328241 7.90727454 8.20711195 7.91125276
 8.17092115 7.71680439 7.83433292 7.30834886 7.98259006 6.67742613]
113    7.795880
35     6.254925
101    7.275724
36     7.017729
100    7.337242
13     8.055517
0      8.187087
114    7.638272
104    8.318759
96     7.769551
40     8.050610
103    8.136677
48     7.853872
39     8.267606
14     7.273273
117    6.167491
21     7.554396
9      6.343902
Name: LoVo, dtype: float64
Training Root Mean Square Error: 0.6718644268665568
Testing Root Mean Square Error: 0.5559161720518064
```

[38]:

```
print(target_column_name+str('_transformed'))
print(hist1[target_column_name].hist())
```

```
LoVo_transformed
AxesSubplot(0.125,0.11;0.775x0.77)
```

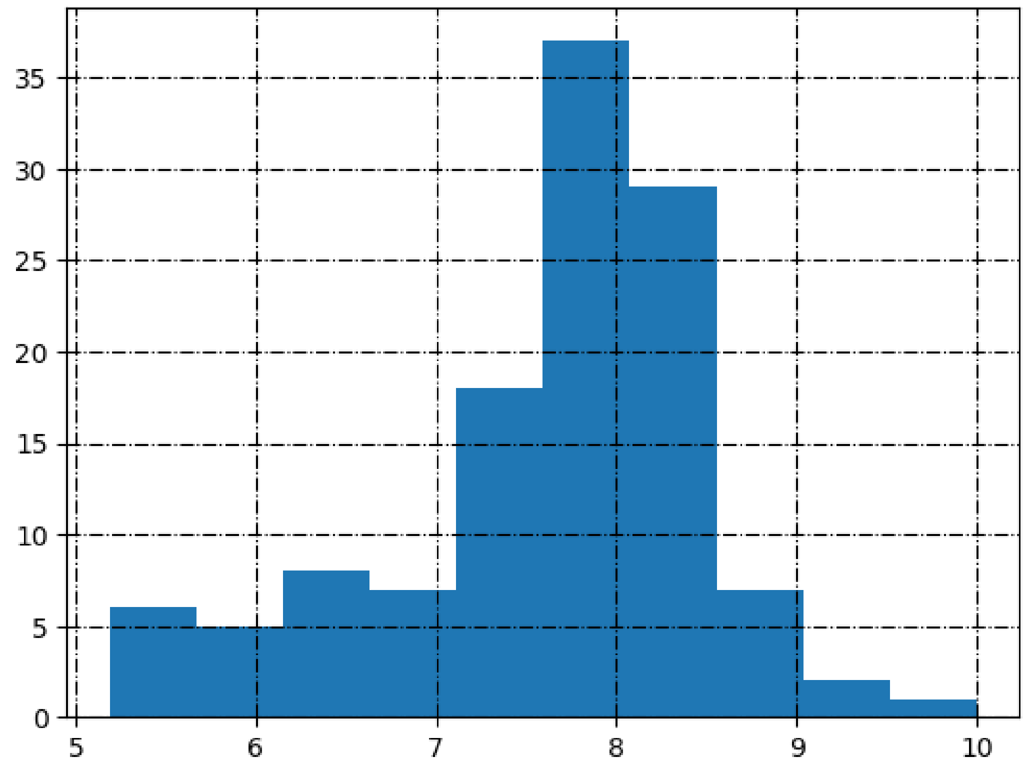

[39]:
```
with_standardization, train_r2_, test_r2_, hist1, hist2, target_column_name,␣
↪training_data_RMSE, test_data_RMSE  = pred_model.
↪prepare_data_and_create_model(molecular_descriptors_df=data,
                                                                                  ␣
↪                    correlation_threshold=0.50,
                                                                                  ␣
↪                    standardization=True,
                                                                                  ␣
↪                    model_type='SVR',
                                                                                  ␣
↪                    kernel_ = 'linear',
                                                                                  ␣
↪                    gamma_ = 'auto',
                                                                                  ␣
↪                    target_column_name = target,
                                                                                  ␣
↪                    random_state=random_state,
                                                                                  ␣
↪                    train_test_split_=True,
                                                                                  ␣
↪                    verbose=True)
```

```
I am doing standardization…
  molecular descriptor name
0                    AATS0Z
1                  AATS0are
2                    AATS0d
3                   AATS0dv
4                    AATS0i
  molecular descriptor name  corr_value
0                    AATS0Z   -0.027037
1                  AATS0are   -0.129823
2                    AATS0d    0.042740
3                   AATS0dv   -0.120173
4                    AATS0i    0.132395
  molecular descriptor name  corr_value  absolute correlation value
0                    AATS0Z   -0.027037                    0.027037
1                  AATS0are   -0.129823                    0.129823
2                    AATS0d    0.042740                    0.042740
3                   AATS0dv   -0.120173                    0.120173
4                    AATS0i    0.132395                    0.132395
     molecular descriptor name  corr_value  absolute correlation value
226                     AMID_O   -0.524157                    0.524157
505                 EState_VSA5   -0.579664                    0.579664
791                    MDEO-12   -0.558727                    0.558727
851                      NdssC   -0.506855                    0.506855
1091               VSA_EState5    0.503578                    0.503578
     molecular descriptor name  corr_value  absolute correlation value
226                     AMID_O   -0.524157                    0.524157
505                 EState_VSA5   -0.579664                    0.579664
791                    MDEO-12   -0.558727                    0.558727
851                      NdssC   -0.506855                    0.506855
1091               VSA_EState5    0.503578                    0.503578
The model used is: SVR…
Return the coefficient of determination of the prediction:
0.3110687450006334
R^2 score: 0.48759250346101435
Correlation coefficient: 0.698278242150659
Test data - unseen during training:
R^2 score: 0.3110687450006334
Correlation coefficient: 0.5577353718392204
[8.00534204 6.50259618 7.87937287 6.53792643 7.92664356 7.83744649
 7.97335513 7.98651677 6.94328241 7.90727454 8.20711195 7.91125276
 8.17092115 7.71680439 7.83433292 7.30834886 7.98259006 6.67742613]
113    7.795880
35     6.254925
101    7.275724
36     7.017729
100    7.337242
13     8.055517
```

```
0      8.187087
114    7.638272
104    8.318759
96     7.769551
40     8.050610
103    8.136677
48     7.853872
39     8.267606
14     7.273273
117    6.167491
21     7.554396
9      6.343902
Name: LoVo, dtype: float64
Training Root Mean Square Error: 0.6718644268665568
Testing Root Mean Square Error: 0.5559161720518064
```

5.1 Search inside correlation space

[40]:
```
step = 0.01
initial_step = corr_low
last_step = corr_high
first_list = [x / 100.0 for x in range(int(initial_step*100),␣
 ↪int(last_step*100), int(step*100))]
second_list = []
third_list = []
fourth_l = []
fifth_l = []
f_list = []
for i in first_list:
    without_standardization, train_r2, test_r2, _, h_, target_column_name,␣
 ↪training_data_RMSE, test_data_RMSE = pred_model.
 ↪prepare_data_and_create_model(molecular_descriptors_df = data,
                                                                              ␣
 ↪                      correlation_threshold = i,
                                                                              ␣
 ↪                      standardization = False,
                                                                              ␣
 ↪                      model_type = 'SVR',
                                                                              ␣
 ↪                      kernel_ = 'linear',
                                                                              ␣
 ↪                      gamma_ = 'auto',
                                                                              ␣
 ↪                      target_column_name = target,
                                                                              ␣
 ↪                      random_state=random_state,
```

```
                    train_test_split_ = True,
                    verbose = False)
    second_list.append(train_r2)
    third_list.append(test_r2)
    fourth_l.append(training_data_RMSE)
    fifth_l.append(test_data_RMSE)
    f_list.append(len(h_))
```

[41]:
```
df_without_standardization = pd.DataFrame(data=first_list,
 columns=["Correlation threshold"])
df_without_standardization['Training data R^2 score'] = second_list
df_without_standardization['Test data R^2 score'] = third_list
df_without_standardization['Training RMSE'] = fourth_l
df_without_standardization['Test RMSE'] = fifth_l
df_without_standardization['Number of features'] = f_list
```

[42]:
```
df_svm = df_without_standardization.copy()
df_without_standardization
```

[42]:
```
    Correlation threshold  Training data R^2 score  Test data R^2 score  \
0                    0.33                 0.732859            -0.295018
1                    0.34                 0.721125            -0.187894
2                    0.35                 0.696024            -0.190945
3                    0.36                 0.693197            -0.113206
4                    0.37                 0.660363             0.226054
5                    0.38                 0.659989             0.254549
6                    0.39                 0.646928             0.322221
7                    0.40                 0.595491             0.156139
8                    0.41                 0.595628             0.155251
9                    0.42                 0.569292             0.339228
10                   0.43                 0.563646             0.355258
11                   0.44                 0.563646             0.355258
12                   0.45                 0.548121             0.269939
13                   0.46                 0.531614             0.148373
14                   0.47                 0.487905             0.313219
15                   0.48                 0.488640             0.314526
16                   0.49                 0.487593             0.311069
17                   0.50                 0.487593             0.311069
18                   0.51                 0.452958             0.380862
19                   0.52                 0.452958             0.380862
20                   0.53                 0.451715             0.384150
21                   0.54                 0.451715             0.384150

    Training RMSE  Test RMSE  Number of features
0        0.485115   0.762183                  63
```

1	0.495654	0.729979	57
2	0.517480	0.730916	52
3	0.519881	0.706658	47
4	0.546992	0.589219	39
5	0.547294	0.578271	35
6	0.557706	0.551398	27
7	0.596950	0.615257	22
8	0.596849	0.615581	21
9	0.615978	0.544437	18
10	0.620003	0.537792	17
11	0.620003	0.537792	17
12	0.630936	0.572270	14
13	0.642356	0.618082	10
14	0.671660	0.555048	7
15	0.671177	0.554519	6
16	0.671864	0.555916	5
17	0.671864	0.555916	5
18	0.694199	0.527005	3
19	0.694199	0.527005	3
20	0.694988	0.525604	2
21	0.694988	0.525604	2

5.2 Plots

[43]:
```
plt.plot(df_without_standardization['Correlation threshold'], df_without_standardization['Training data R^2 score'], label = "Training data R^2 score", marker='v')
plt.plot(df_without_standardization['Correlation threshold'], df_without_standardization['Test data R^2 score'], label = "Test data R^2 score", marker='D')
plt.legend()
plt.xlabel('Correlation threshold')
plt.ylabel('R^2 score')
plt.ylim([0, 1])
plt.show()
```

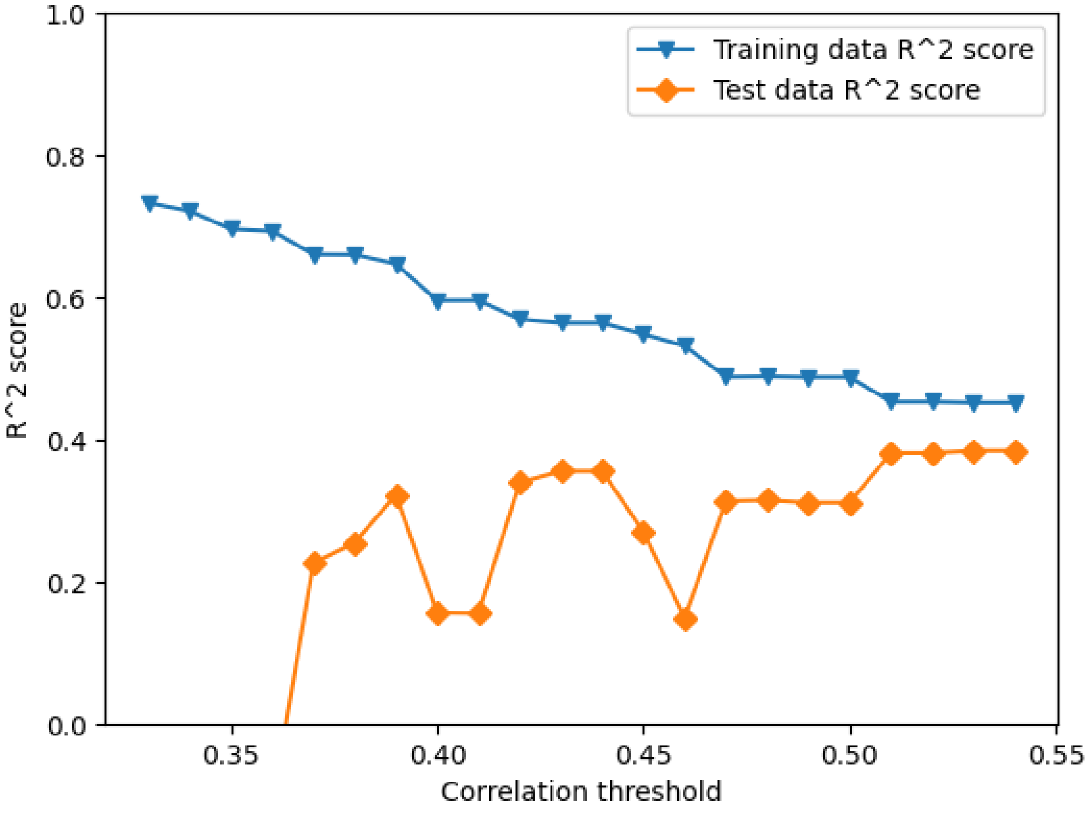

[44]:
```
plt.plot(df_without_standardization['Correlation threshold'],␣
 ↪df_without_standardization['Number of features'], label = "Number of␣
 ↪features", marker='.')
plt.legend()
plt.xlabel('Correlation threshold')
plt.ylabel('Number of features')
plt.show()
```

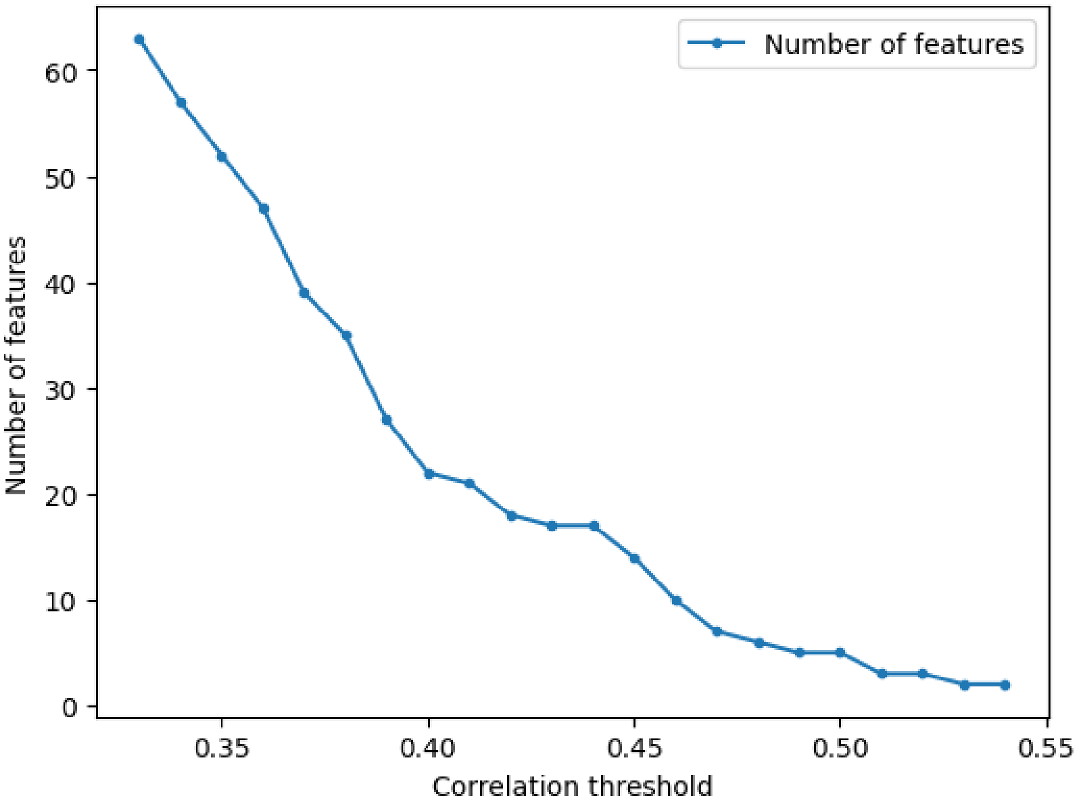

5.3 Store quality measurements

[45]:
```
with pd.ExcelWriter('../Data/Quality_'+str(target)+'_'+str(random_state)+'_.
 ↪xlsx') as writer:
    df_linear.to_excel(writer, sheet_name='MLR')
    df_decision_tree.to_excel(writer, sheet_name='DT')
    df_random_forest.to_excel(writer, sheet_name='RF')
    df_k_nearest.to_excel(writer, sheet_name='KNN')
    df_svm.to_excel(writer, sheet_name='SVM')
```

Notebook

January 18, 2024

1 File 26

[1]:
```
import sys
from pathlib import Path
prediction_mode_path = Path("../module")
sys.path.append(prediction_mode_path.as_posix())
import models_creation as pred_model

import pandas as pd
import numpy as np

from sklearn.metrics import r2_score

import matplotlib.pyplot as plt
from sklearn.tree import plot_tree

from matplotlib import pyplot as plt

import joblib

import warnings
warnings.filterwarnings('ignore')
```

[2]:
```
load_prepared_data = pd.read_excel('../Data/Kolchicyna_machine_learning.xlsx')
load_prepared_data = load_prepared_data.drop(columns=['Unnamed: 0'])

target = 'LoVo'

corr_low = 0.33
corr_high = 0.55

random_state = 42
```

[3]:
```
load_prepared_data.head()
```

[3]:
```
      AATS0Z   AATS0are    AATS0d   AATS0dv      AATS0i     AATS0m    AATS0p  \
0  25.090909  6.046518  3.109091  6.662626  160.775045  99.075564  1.555512
1  24.448276  6.008422  3.051724  6.386973  161.021674  96.473315  1.538471
```

```
2  23.868852  5.974074  3.000000  6.138434  161.244045  94.127025  1.523105
3  23.868852  5.974074  3.032787  6.171220  161.244045  94.127025  1.523105
4  23.343750  5.942945  2.953125  5.913194  161.445569  92.000700  1.509180

    AATS0pe    AATS0s    AATS0se  …  A549_float  MCF-7_float  LoVo_float  \
0  6.126680  3.435416  7.629859  …        10.8         10.3         6.5
1  6.088791  3.330998  7.596891  …        11.6         12.0         8.5
2  6.054630  3.236850  7.567166  …        10.9         12.2         8.8
3  6.054630  3.257798  7.567166  …        10.5         11.3         8.5
4  6.023670  3.151529  7.540228  …        89.5         92.7        52.8

   LoVo/DX_float  BALB/3T3_float  A549_float_transformed  \
0           54.9            10.2                7.966576
1           31.1            14.3                7.935542
2           17.9            11.7                7.962574
3           10.2            11.0                7.978811
4           77.8            99.4                7.048177

   MCF-7_float_transformed  LoVo_float_transformed  LoVo/DX_float_transformed  \
0                 7.987163                8.187087                   7.260428
1                 7.920819                8.070581                   7.507240
2                 7.913640                8.055517                   7.747147
3                 7.946922                8.070581                   7.991400
4                 7.032920                7.277366                   7.109020

   BALB/3T3_float_transformed
0                    7.991400
1                    7.844664
2                    7.931814
3                    7.958607
4                    7.002614

[5 rows x 1221 columns]
```

[4]:
```python
data = load_prepared_data[list(load_prepared_data.columns)[0:-10]]
data[target] = load_prepared_data[load_prepared_data.columns[-3]]
data.head()
```

[4]:
```
      AATS0Z  AATS0are    AATS0d   AATS0dv      AATS0i     AATS0m    AATS0p  \
0  25.090909  6.046518  3.109091  6.662626  160.775045  99.075564  1.555512
1  24.448276  6.008422  3.051724  6.386973  161.021674  96.473315  1.538471
2  23.868852  5.974074  3.000000  6.138434  161.244045  94.127025  1.523105
3  23.868852  5.974074  3.032787  6.171220  161.244045  94.127025  1.523105
4  23.343750  5.942945  2.953125  5.913194  161.445569  92.000700  1.509180

    AATS0pe    AATS0s    AATS0se  …    piPC10     piPC2     piPC3     piPC4  \
0  6.126680  3.435416  7.629859  …  7.999655  4.266195  4.915408  5.516700
```

```
1  6.088791  3.330998  7.596891  …  8.018726  4.280132  4.922714  5.520711
2  6.054630  3.236850  7.567166  …  8.028799  4.293878  4.929967  5.524706
3  6.054630  3.257798  7.567166  …  8.037440  4.307438  4.929967  5.524706
4  6.023670  3.151529  7.540228  …  8.034892  4.307438  4.937168  5.528685

      piPC5     piPC6     piPC7     piPC8     piPC9      LoVo
0  6.098566  6.595759  6.979116  7.337313  7.681445  8.187087
1  6.103048  6.601209  6.985613  7.349442  7.695531  8.070581
2  6.105281  6.603923  6.989306  7.353932  7.704023  8.055517
3  6.107510  6.606629  6.992068  7.361425  7.709420  8.070581
4  6.107510  6.605277  6.991148  7.356489  7.707175  7.277366

[5 rows x 1212 columns]
```

2 Multiple Linear regression (MLR)

[5]:
```
without_standardization, train_r2_, test_r2_, hist1, hist2, target_column_name,␣
↪training_data_RMSE, test_data_RMSE  = pred_model.
↪prepare_data_and_create_model(molecular_descriptors_df=data,
                                                                                ␣
↪                       correlation_threshold=0.5,
                                                                                ␣
↪                       standardization=False,
                                                                                ␣
↪                       model_type='linear_model',
                                                                                ␣
↪                       target_column_name = str(target),
                                                                                ␣
↪                       random_state=random_state,
                                                                                ␣
↪                       train_test_split_=True,
                                                                                ␣
↪                       verbose=True)
```

```
I am not doing standardization…
  molecular descriptor name
0                   AATS0Z
1                 AATS0are
2                   AATS0d
3                  AATS0dv
4                   AATS0i
  molecular descriptor name  corr_value
0                   AATS0Z   -0.027037
1                 AATS0are   -0.129823
2                   AATS0d    0.042740
3                  AATS0dv   -0.120173
```

```
4                   AATS0i    0.132395
  molecular descriptor name  corr_value  absolute correlation value
0                    AATS0Z   -0.027037                    0.027037
1                  AATS0are   -0.129823                    0.129823
2                    AATS0d    0.042740                    0.042740
3                   AATS0dv   -0.120173                    0.120173
4                    AATS0i    0.132395                    0.132395
     molecular descriptor name  corr_value  absolute correlation value
226                     AMID_O   -0.524157                    0.524157
505                EState_VSA5   -0.579664                    0.579664
791                    MDEO-12   -0.558727                    0.558727
851                     NdssC   -0.506855                    0.506855
1091               VSA_EState5    0.503578                    0.503578
     molecular descriptor name  corr_value  absolute correlation value
226                     AMID_O   -0.524157                    0.524157
505                EState_VSA5   -0.579664                    0.579664
791                    MDEO-12   -0.558727                    0.558727
851                     NdssC   -0.506855                    0.506855
1091               VSA_EState5    0.503578                    0.503578
The model used is: LinearReg…
Return the coefficient of determination of the prediction:
0.34262532352099506
R^2 score: 0.5124549074751447
Correlation coefficient: 0.7158595584855626
Test data - unseen during training:
R^2 score: 0.34262532352099506
Correlation coefficient: 0.5853420568530806
[8.25792779 8.2509851  7.61307432 7.26729303 6.09331676 7.79366094
 7.97875067 7.83583733 7.97290693 7.94455467 7.7775089  6.95407307
 7.9643469  6.55467624 7.44264866 8.25966546 7.98082096 7.80836735]
44      8.508638
47      7.920819
4       7.277366
55      7.920819
26      5.208801
64      8.327902
73      7.886057
10      8.102373
40      8.050610
107     8.017729
18      7.140261
62      8.657577
11      8.107905
36      7.017729
89      8.638272
91     10.000000
109     7.886057
0       8.187087
```

```
Name: LoVo, dtype: float64
Training Root Mean Square Error: 0.6222643149396536
Testing Root Mean Square Error: 0.7462571611515958
```

[6]:

```
print(target_column_name+str('_transformed'))
print(hist1[target_column_name].hist())
```

```
LoVo_transformed
AxesSubplot(0.125,0.11;0.775x0.77)
```

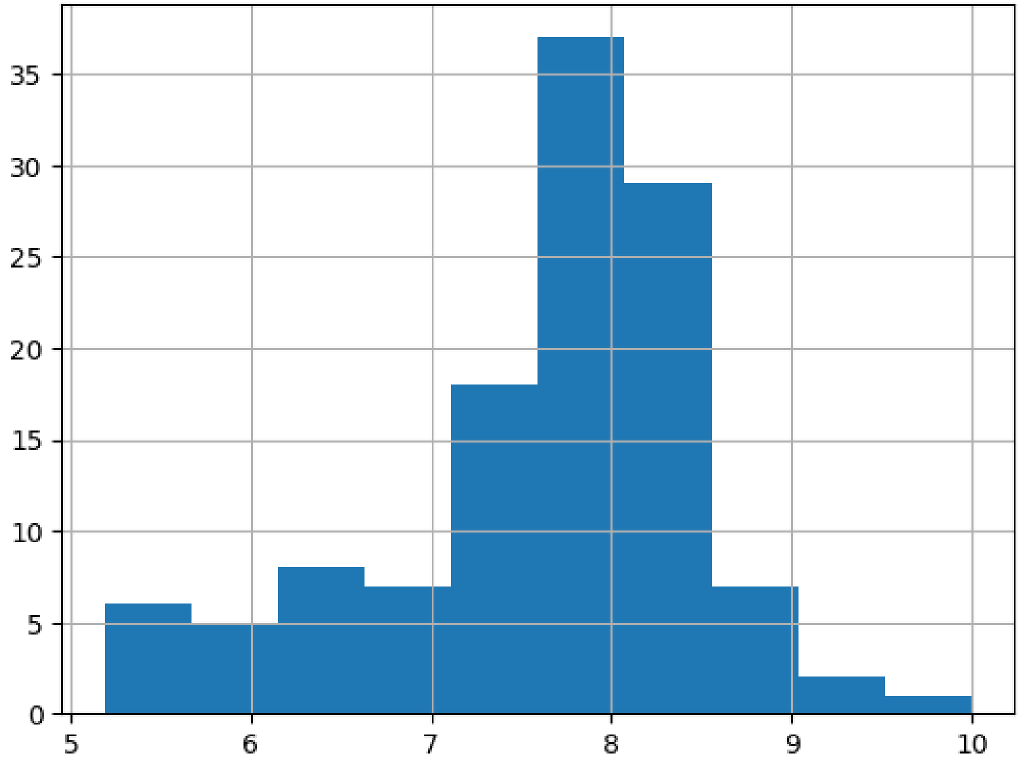

[7]:

```
with_standardization, train_r2_, test_r2_, hist1, hist2, target_column_name,␣
↪training_data_RMSE, test_data_RMSE  = pred_model.
↪prepare_data_and_create_model(molecular_descriptors_df=data,
                                                                              ␣
↪                         correlation_threshold=0.50,
                                                                              ␣
↪                         standardization=True,
                                                                              ␣
↪                         model_type='linear_model',
                                                                              ␣
↪                         target_column_name = target,
```

```
↪                       random_state=random_state,
↪                       train_test_split_=True,
↪                       verbose=True)
```

```
I am doing standardization…
  molecular descriptor name
0                    AATS0Z
1                  AATS0are
2                    AATS0d
3                   AATS0dv
4                    AATS0i
  molecular descriptor name  corr_value
0                    AATS0Z   -0.027037
1                  AATS0are   -0.129823
2                    AATS0d    0.042740
3                   AATS0dv   -0.120173
4                    AATS0i    0.132395
  molecular descriptor name  corr_value  absolute correlation value
0                    AATS0Z   -0.027037                    0.027037
1                  AATS0are   -0.129823                    0.129823
2                    AATS0d    0.042740                    0.042740
3                   AATS0dv   -0.120173                    0.120173
4                    AATS0i    0.132395                    0.132395
     molecular descriptor name  corr_value  absolute correlation value
226                     AMID_O   -0.524157                    0.524157
505                EState_VSA5   -0.579664                    0.579664
791                   MDEO-12   -0.558727                    0.558727
851                     NdssC   -0.506855                    0.506855
1091               VSA_EState5    0.503578                    0.503578
     molecular descriptor name  corr_value  absolute correlation value
226                     AMID_O   -0.524157                    0.524157
505                EState_VSA5   -0.579664                    0.579664
791                   MDEO-12   -0.558727                    0.558727
851                     NdssC   -0.506855                    0.506855
1091               VSA_EState5    0.503578                    0.503578
The model used is: LinearReg…
Return the coefficient of determination of the prediction:
0.34262532352099506
R^2 score: 0.5124549074751447
Correlation coefficient: 0.7158595584855626
Test data - unseen during training:
R^2 score: 0.34262532352099506
Correlation coefficient: 0.5853420568530806
[8.25792779 8.2509851  7.61307432 7.26729303 6.09331676 7.79366094
```

 7.97875067 7.83583733 7.97290693 7.94455467 7.7775089 6.95407307
 7.9643469 6.55467624 7.44264866 8.25966546 7.98082096 7.80836735]
44   8.508638
47   7.920819
4   7.277366
55   7.920819
26   5.208801
64   8.327902
73   7.886057
10   8.102373
40   8.050610
107   8.017729
18   7.140261
62   8.657577
11   8.107905
36   7.017729
89   8.638272
91   10.000000
109   7.886057
0   8.187087
Name: LoVo, dtype: float64
Training Root Mean Square Error: 0.6222643149396536
Testing Root Mean Square Error: 0.7462571611515958

2.1 Search inside correlation space

[8]:
```
step = 0.01
initial_step = corr_low
last_step = corr_high
first_list = [x / 100.0 for x in range(int(initial_step*100),␣
 ↪int(last_step*100), int(step*100))]
second_list = []
third_list = []
fourth_l = []
fifth_l = []
f_list = []
for i in first_list:
    without_standardization, train_r2, test_r2, _, h_, target_column_name,␣
 ↪training_data_RMSE, test_data_RMSE = pred_model.
 ↪prepare_data_and_create_model(molecular_descriptors_df = data,
                                                                            ␣
 ↪                    correlation_threshold = i,
                                                                            ␣
 ↪                    standardization = False,
                                                                            ␣
 ↪                    model_type = 'linear_model',
```

```
                         target_column_name = target,
                         random_state=random_state,
                         train_test_split_ = True,
                         verbose = False)
    second_list.append(train_r2)
    third_list.append(test_r2)
    fourth_l.append(training_data_RMSE)
    fifth_l.append(test_data_RMSE)
    f_list.append(len(h_))
```

[9]:
```
df_without_standardization = pd.DataFrame(data=first_list,
    columns=["Correlation threshold"])
df_without_standardization['Training data R^2 score'] = second_list
df_without_standardization['Test data R^2 score'] = third_list
df_without_standardization['Training RMSE'] = fourth_l
df_without_standardization['Test RMSE'] = fifth_l
df_without_standardization['Number of features'] = f_list
```

[10]:
```
df_linear = df_without_standardization.copy()
df_without_standardization
```

[10]:

	Correlation threshold	Training data R^2 score	Test data R^2 score	\
0	0.33	0.891403	0.349177	
1	0.34	0.854779	0.473705	
2	0.35	0.853467	0.435703	
3	0.36	0.814316	0.079623	
4	0.37	0.761190	0.250571	
5	0.38	0.703439	0.555031	
6	0.39	0.672921	0.553539	
7	0.40	0.653598	0.554680	
8	0.41	0.653597	0.554461	
9	0.42	0.623426	0.395490	
10	0.43	0.623143	0.401648	
11	0.44	0.623143	0.401648	
12	0.45	0.607865	0.394167	
13	0.46	0.586099	0.356643	
14	0.47	0.518802	0.353025	
15	0.48	0.515240	0.324260	
16	0.49	0.512455	0.342625	
17	0.50	0.512455	0.342625	
18	0.51	0.481620	0.400392	
19	0.52	0.481620	0.400392	
20	0.53	0.467784	0.348953	

```
21                    0.54                 0.467784             0.348953

    Training RMSE  Test RMSE  Number of features
0        0.293682   0.742529                  63
1        0.339612   0.667723                  57
2        0.341143   0.691410                  52
3        0.384020   0.883009                  47
4        0.435506   0.796796                  39
5        0.485316   0.613970                  35
6        0.509676   0.614998                  27
7        0.524515   0.614212                  22
8        0.524516   0.614363                  21
9        0.546881   0.715622                  18
10       0.547086   0.711968                  17
11       0.547086   0.711968                  17
12       0.558066   0.716405                  14
13       0.573344   0.738258                  10
14       0.618200   0.740331                   7
15       0.620485   0.756610                   6
16       0.622264   0.746257                   5
17       0.622264   0.746257                   5
18       0.641640   0.712715                   3
19       0.641640   0.712715                   3
20       0.650147   0.742657                   2
21       0.650147   0.742657                   2
```

[ ]:

2.2 Plots

[11]:
```
plt.plot(df_without_standardization['Correlation threshold'],␣
 ↪df_without_standardization['Training data R^2 score'], label = "Training␣
 ↪data R^2 score", marker='v')
plt.plot(df_without_standardization['Correlation threshold'],␣
 ↪df_without_standardization['Test data R^2 score'], label = "Test data R^2␣
 ↪score", marker='D')
plt.legend()
plt.xlabel('Correlation threshold')
plt.ylabel('R^2 score')
plt.ylim([0, 1])
plt.show()
```

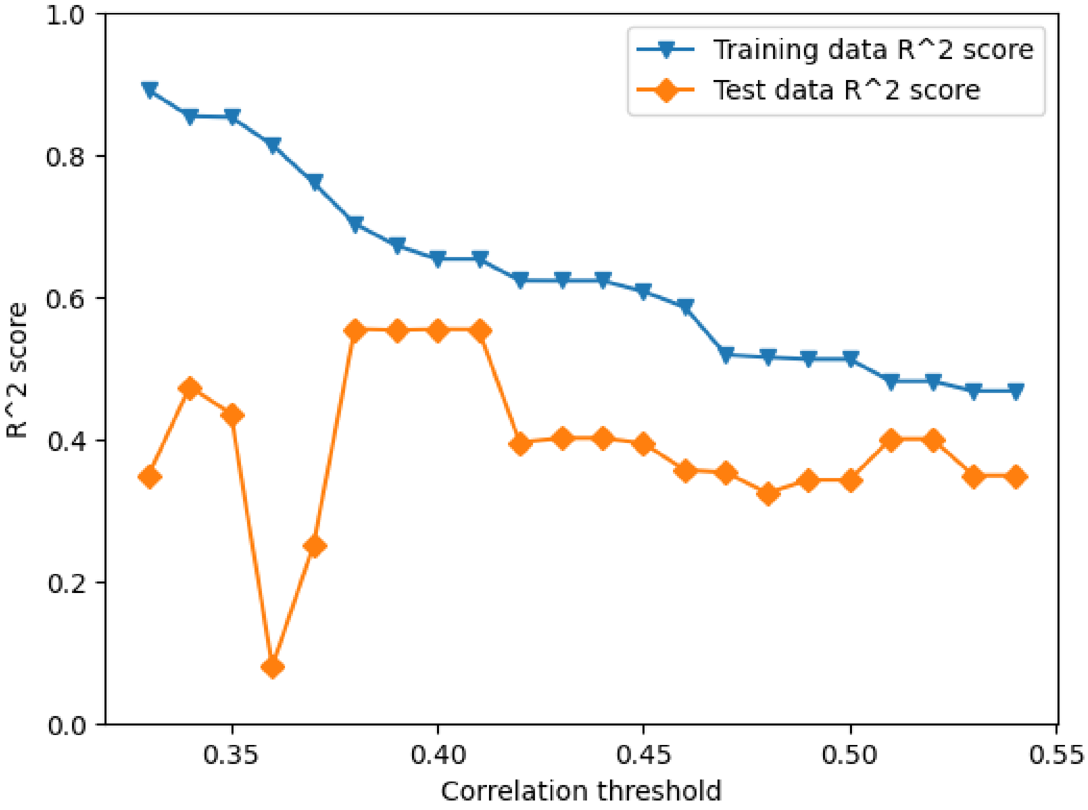

[12]:
```
plt.plot(df_without_standardization['Correlation threshold'],␣
 ↪df_without_standardization['Number of features'], label = "Number of␣
 ↪features", marker='.')
plt.legend()
plt.xlabel('Correlation threshold')
plt.ylabel('Number of features')
plt.show()
```

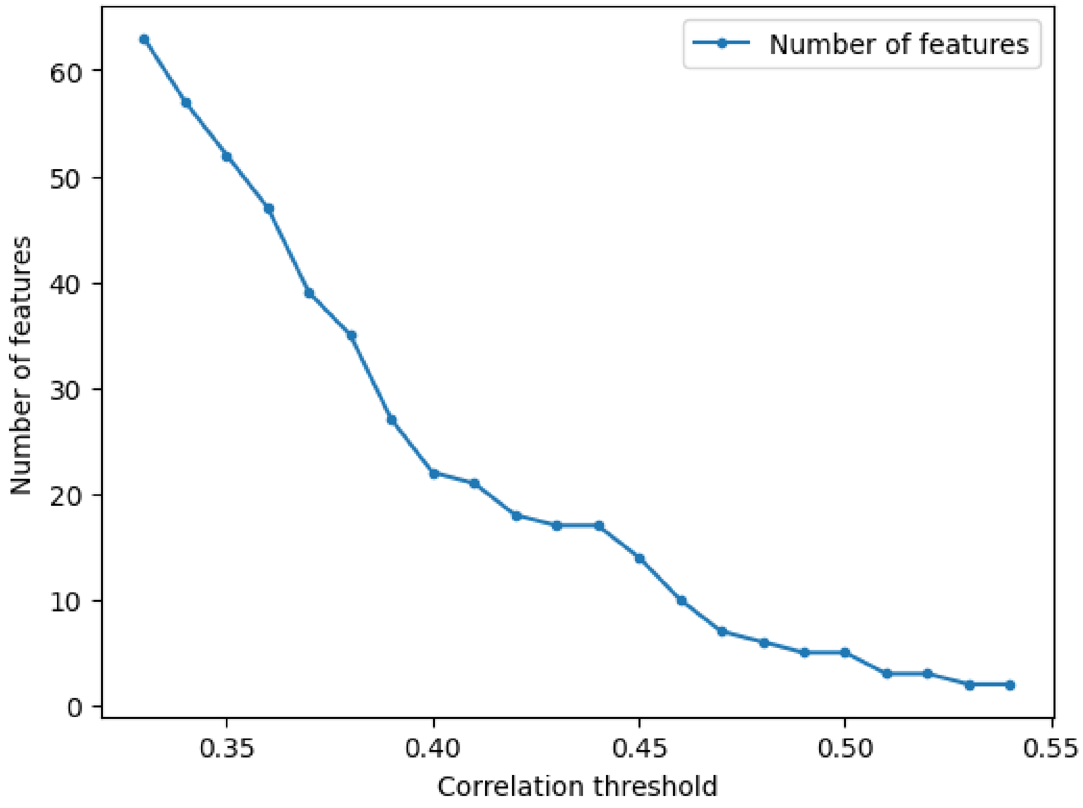

[ ]:

2.3 Decision Tree

[13]:
```
without_standardization, train_r2_, test_r2_, hist1, hist2, target_column_name,␣
↪training_data_RMSE, test_data_RMSE  = pred_model.
↪prepare_data_and_create_model(molecular_descriptors_df=data,
                                                                              ␣
↪                         correlation_threshold=0.50,
                                                                              ␣
↪                         standardization=False,
                                                                              ␣
↪                         model_type='DecisionTreeRegressor',
                                                                              ␣
↪                         max_depth=5,
                                                                              ␣
↪                         target_column_name = target,
                                                                              ␣
↪                         random_state=random_state,
```

```
↪                       train_test_split_=True,
↪                       verbose=True)
```

```
I am not doing standardization…
  molecular descriptor name
0                    AATS0Z
1                  AATS0are
2                    AATS0d
3                   AATS0dv
4                    AATS0i
  molecular descriptor name  corr_value
0                    AATS0Z   -0.027037
1                  AATS0are   -0.129823
2                    AATS0d    0.042740
3                   AATS0dv   -0.120173
4                    AATS0i    0.132395
  molecular descriptor name  corr_value  absolute correlation value
0                    AATS0Z   -0.027037                    0.027037
1                  AATS0are   -0.129823                    0.129823
2                    AATS0d    0.042740                    0.042740
3                   AATS0dv   -0.120173                    0.120173
4                    AATS0i    0.132395                    0.132395
     molecular descriptor name  corr_value  absolute correlation value
226                     AMID_O   -0.524157                    0.524157
505                EState_VSA5   -0.579664                    0.579664
791                    MDEO-12   -0.558727                    0.558727
851                      NdssC   -0.506855                    0.506855
1091               VSA_EState5    0.503578                    0.503578
     molecular descriptor name  corr_value  absolute correlation value
226                     AMID_O   -0.524157                    0.524157
505                EState_VSA5   -0.579664                    0.579664
791                    MDEO-12   -0.558727                    0.558727
851                      NdssC   -0.506855                    0.506855
1091               VSA_EState5    0.503578                    0.503578
The model used is: DecisionTree…
Return the coefficient of determination of the prediction:
0.16887026297895746
R^2 score: 0.8262751673389552
Correlation coefficient: 0.9089967917099352
Test data - unseen during training:
R^2 score: 0.16887026297895746
Correlation coefficient: 0.4109382714945853
[7.59570161 8.31317281 7.4413779  6.47839279 5.84400658 7.78096821
 7.78096821 7.95190315 8.31317281 8.31317281 6.16749109 7.67778071
 7.95190315 6.47839279 7.97010949 7.59570161 8.31317281 7.95190315]
44      8.508638
```

```
47      7.920819
4       7.277366
55      7.920819
26      5.208801
64      8.327902
73      7.886057
10      8.102373
40      8.050610
107     8.017729
18      7.140261
62      8.657577
11      8.107905
36      7.017729
89      8.638272
91     10.000000
109     7.886057
0       8.187087
Name: LoVo, dtype: float64
Training Root Mean Square Error: 0.3714483662429523
Testing Root Mean Square Error: 0.839105247916584
```

[14]:
```
print(target_column_name+str('_transformed'))
print(hist1[target_column_name].hist())
```

```
LoVo_transformed
AxesSubplot(0.125,0.11;0.775x0.77)
```

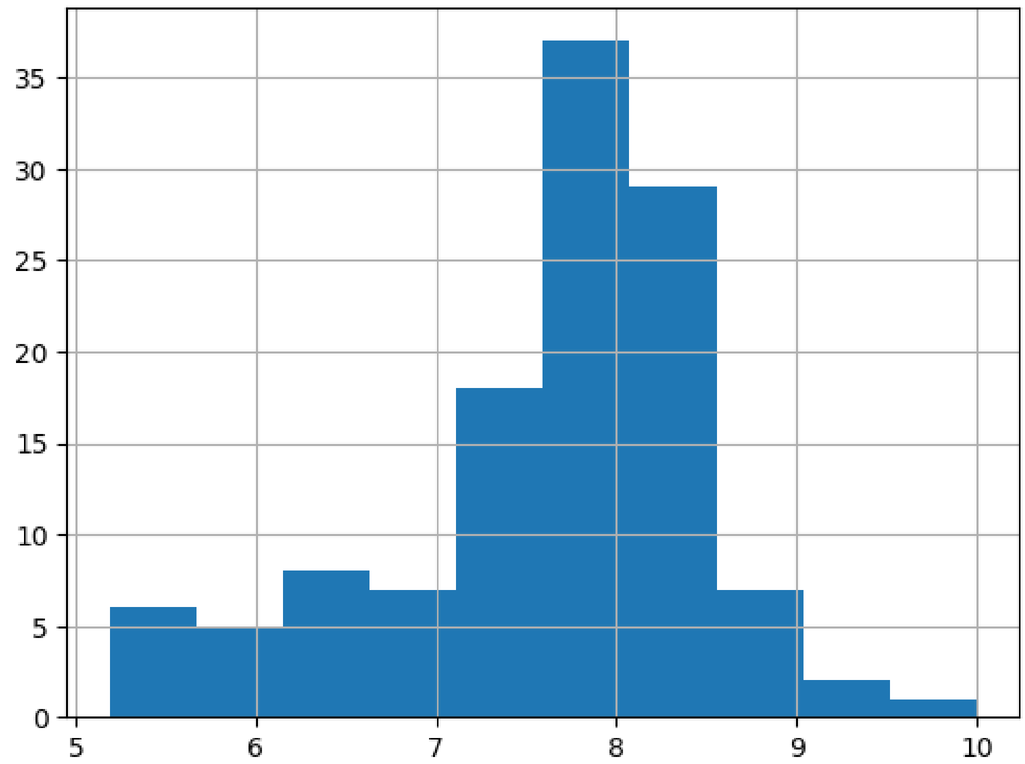

[15]:
```
with_standardization, train_r2_, test_r2_, hist1, hist2, target_column_name,␣
↪training_data_RMSE, test_data_RMSE  = pred_model.
↪prepare_data_and_create_model(molecular_descriptors_df=data,
                                                                           ␣
↪                         correlation_threshold=0.50,
                                                                           ␣
↪                         standardization=True,
                                                                           ␣
↪                         model_type='DecisionTreeRegressor',
                                                                           ␣
↪                         max_depth=5,
                                                                           ␣
↪                         target_column_name = target,
                                                                           ␣
↪                         random_state=random_state,
                                                                           ␣
↪                         train_test_split_=True,
                                                                           ␣
↪                         verbose=True)
```

```
I am doing standardization…
  molecular descriptor name
```

```
0                 AATS0Z
1               AATS0are
2                 AATS0d
3                AATS0dv
4                 AATS0i
  molecular descriptor name  corr_value
0                 AATS0Z   -0.027037
1               AATS0are   -0.129823
2                 AATS0d    0.042740
3                AATS0dv   -0.120173
4                 AATS0i    0.132395
  molecular descriptor name  corr_value  absolute correlation value
0                 AATS0Z   -0.027037                    0.027037
1               AATS0are   -0.129823                    0.129823
2                 AATS0d    0.042740                    0.042740
3                AATS0dv   -0.120173                    0.120173
4                 AATS0i    0.132395                    0.132395
     molecular descriptor name  corr_value  absolute correlation value
226                     AMID_O   -0.524157                    0.524157
505                 EState_VSA5   -0.579664                    0.579664
791                    MDEO-12   -0.558727                    0.558727
851                      NdssC   -0.506855                    0.506855
1091                VSA_EState5    0.503578                    0.503578
     molecular descriptor name  corr_value  absolute correlation value
226                     AMID_O   -0.524157                    0.524157
505                 EState_VSA5   -0.579664                    0.579664
791                    MDEO-12   -0.558727                    0.558727
851                      NdssC   -0.506855                    0.506855
1091                VSA_EState5    0.503578                    0.503578
The model used is: DecisionTree…
Return the coefficient of determination of the prediction:
0.16887026297895746
R^2 score: 0.8262751673389552
Correlation coefficient: 0.9089967917099352
Test data - unseen during training:
R^2 score: 0.16887026297895746
Correlation coefficient: 0.4109382714945853
[7.59570161 8.31317281 7.4413779  6.47839279 5.84400658 7.78096821
 7.78096821 7.95190315 8.31317281 8.31317281 6.16749109 7.67778071
 7.95190315 6.47839279 7.97010949 7.59570161 8.31317281 7.95190315]
44      8.508638
47      7.920819
4       7.277366
55      7.920819
26      5.208801
64      8.327902
73      7.886057
10      8.102373
```

```
40      8.050610
107     8.017729
18      7.140261
62      8.657577
11      8.107905
36      7.017729
89      8.638272
91     10.000000
109     7.886057
0       8.187087
Name: LoVo, dtype: float64
Training Root Mean Square Error: 0.3714483662429523
Testing Root Mean Square Error: 0.839105247916584
```

2.4 Search inside correlation space

[16]:
```
step = 0.01
initial_step = corr_low
last_step = corr_high
first_list = [x / 100.0 for x in range(int(initial_step*100),␣
 ↪int(last_step*100), int(step*100))]
max_depth = [range(2, 30, 1)]
corr_th = []
second_list = []
third_list = []
fourth_l = []
fifth_l = []
f_list = []
fif_list = []
for i in first_list:
    for depth in max_depth[0]:

        without_standardization, train_r2, test_r2, _, h_, target_column_name,␣
 ↪training_data_RMSE, test_data_RMSE = pred_model.
 ↪prepare_data_and_create_model(molecular_descriptors_df=data,
                                                                              ␣
 ↪                        correlation_threshold=i,
                                                                              ␣
 ↪                        standardization=False,
                                                                              ␣
 ↪                        model_type='DecisionTreeRegressor',
                                                                              ␣
 ↪                        max_depth=depth,
                                                                              ␣
 ↪                        target_column_name = target,
                                                                              ␣
 ↪                        random_state=random_state,
```

```
                          train_test_split_=True,
                          verbose=False)
        corr_th.append(i)
        second_list.append(train_r2)
        third_list.append(test_r2)
        fourth_l.append(training_data_RMSE)
        fifth_l.append(test_data_RMSE)
        f_list.append(len(h_))
        fif_list.append(depth)
```

[17]:
```
df_without_standardization = pd.DataFrame(data=corr_th, columns=["Correlation threshold"])
df_without_standardization['Training data R^2 score'] = second_list
df_without_standardization['Test data R^2 score'] = third_list
df_without_standardization['Training RMSE'] = fourth_l
df_without_standardization['Test RMSE'] = fifth_l
df_without_standardization['Number of features'] = f_list
df_without_standardization['Depth number'] = fif_list
```

[18]:
```
df_decision_tree = df_without_standardization.copy()
#df_without_standardization.to_excel('../Data/A549_Decision_tree_rs_'+str(random_state)+'.xlsx')
```

2.5 Plots

[19]:
```
corre_list = [x / 100.0 for x in range(int(initial_step*100),␣
↪int(last_step*100), int(0.01*100))]
for element in corre_list:
    element_ =␣
↪df_without_standardization[df_without_standardization['Correlation␣
↪threshold'] == float(element)]
    plt.plot(element_['Depth number'], element_['Training data R^2 score'],␣
↪label = "Training data R^2 score", marker='v')
    plt.plot(element_['Depth number'], element_['Test data R^2 score'], label =␣
↪"Test data R^2 score", marker='D')
    plt.legend()
    plt.xlabel('Depth number -> correlation threshold: '+str(element)+'. \n␣
↪Number of features: '+str(element_['Number of features'].iloc[0]),␣
↪fontweight='bold')
    plt.xlim(max(element_['Depth number'])+1, min(element_['Depth number'])-1)
    plt.ylabel('R^2 score')
    plt.ylim([0, 1])
    plt.rc('grid', linestyle="-.", color='black')
    plt.grid(True)
    plt.show()
```

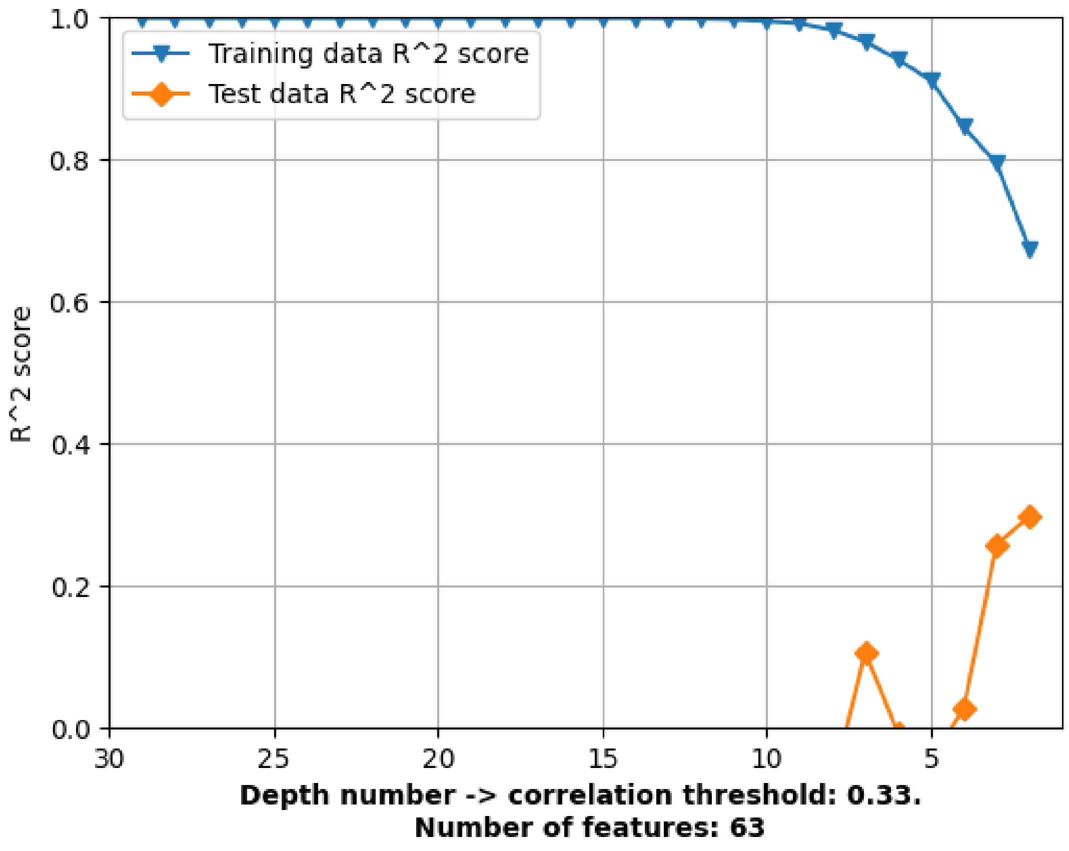

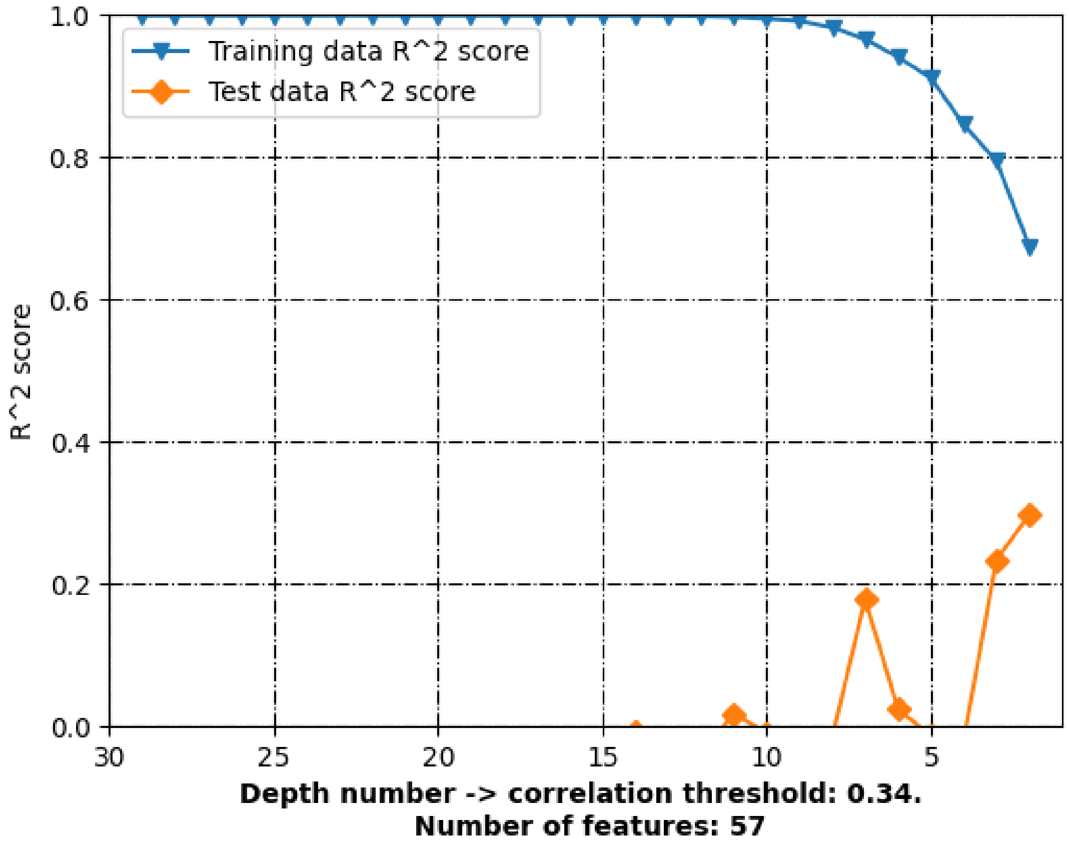
Training data R^2 score
Test data R^2 score
R^2 score
1.0
0.8
0.6
0.4
0.2
0.0
30
25
20
15
10
5
Depth number -> correlation threshold: 0.34.
Number of features: 57

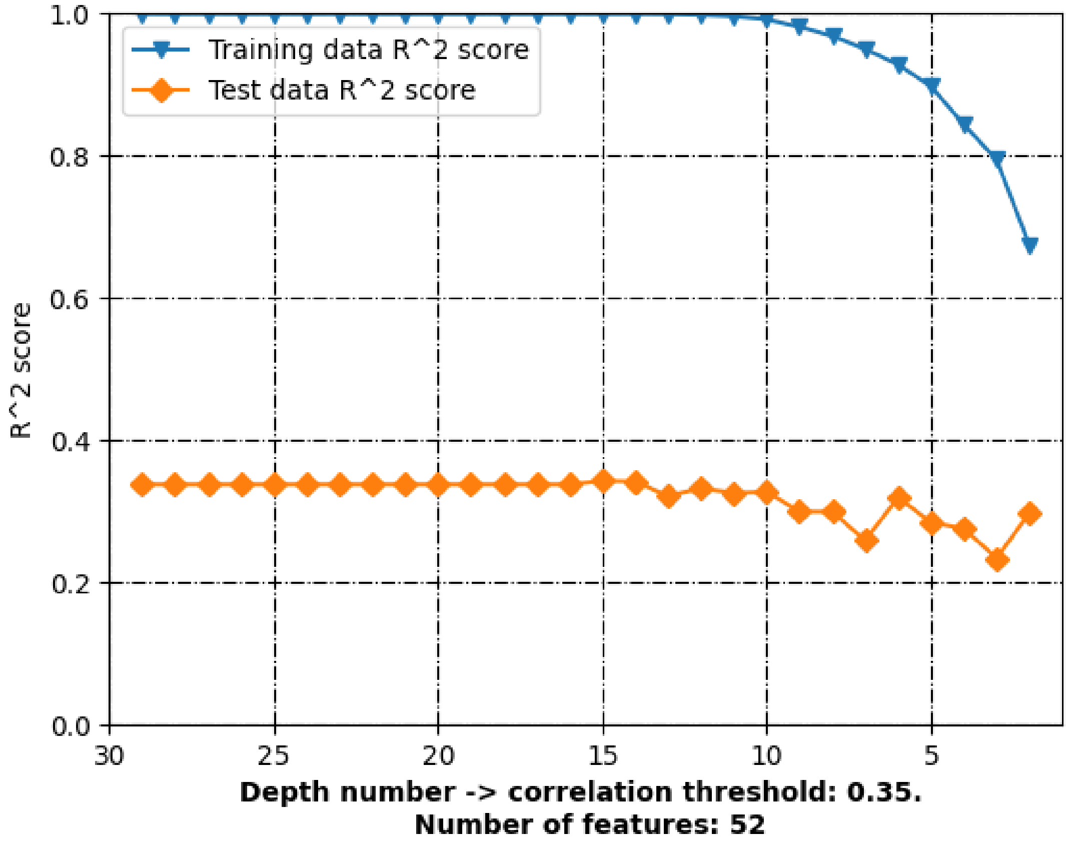

Training data R^2 score
Test data R^2 score
R^2 score
1.0
0.8
0.6
0.4
0.2
0.0
30
25
20
15
10
5
Depth number -> correlation threshold: 0.35.
Number of features: 52

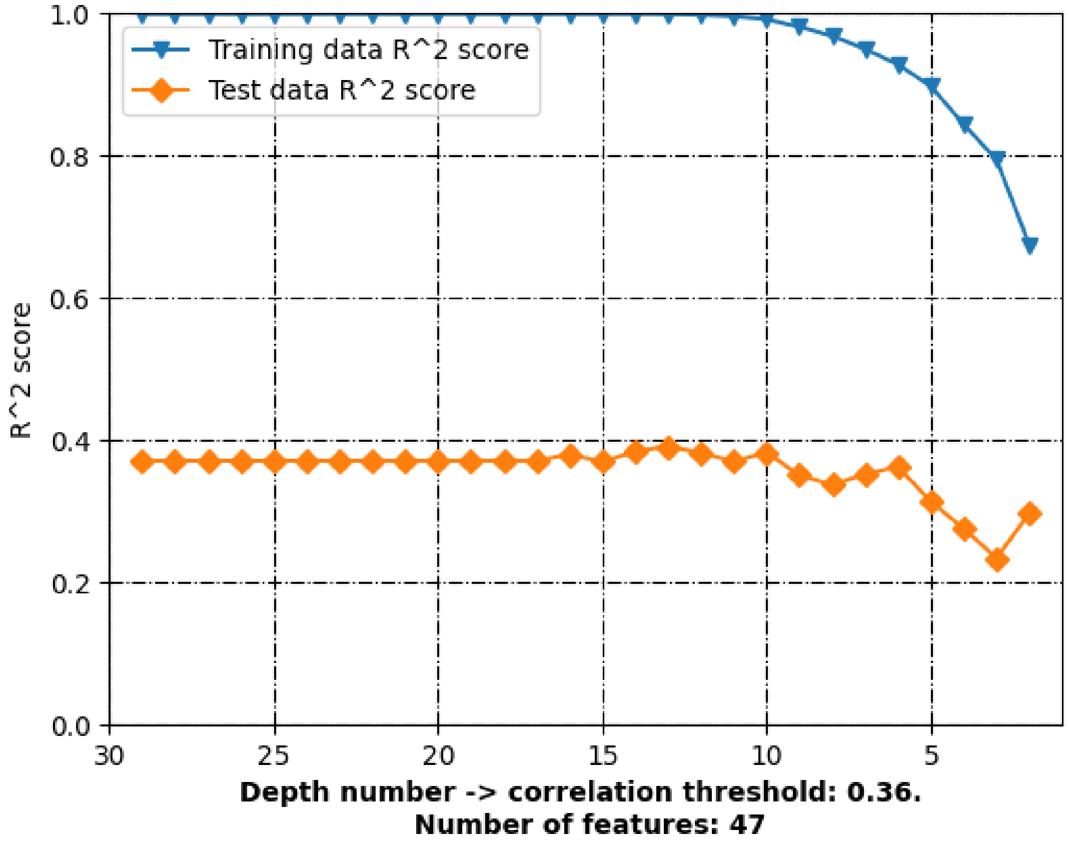
Training data R^2 score
Test data R^2 score
R^2 score
1.0
0.8
0.6
0.4
0.2
0.0
30
25
20
15
10
5
Depth number -> correlation threshold: 0.36.
Number of features: 47

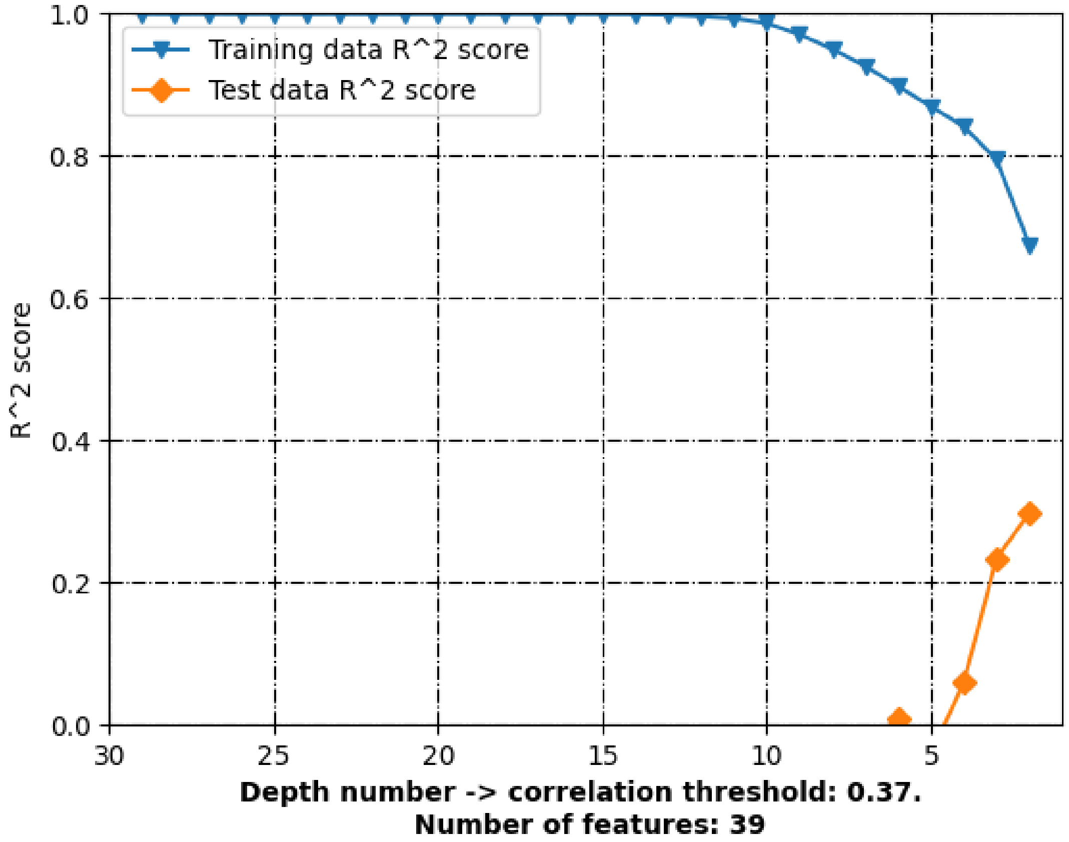

Training data R^2 score
Test data R^2 score
R^2 score
1.0
0.8
0.6
0.4
0.2
0.0
30
25
20
15
10
5
Depth number -> correlation threshold: 0.37.
Number of features: 39

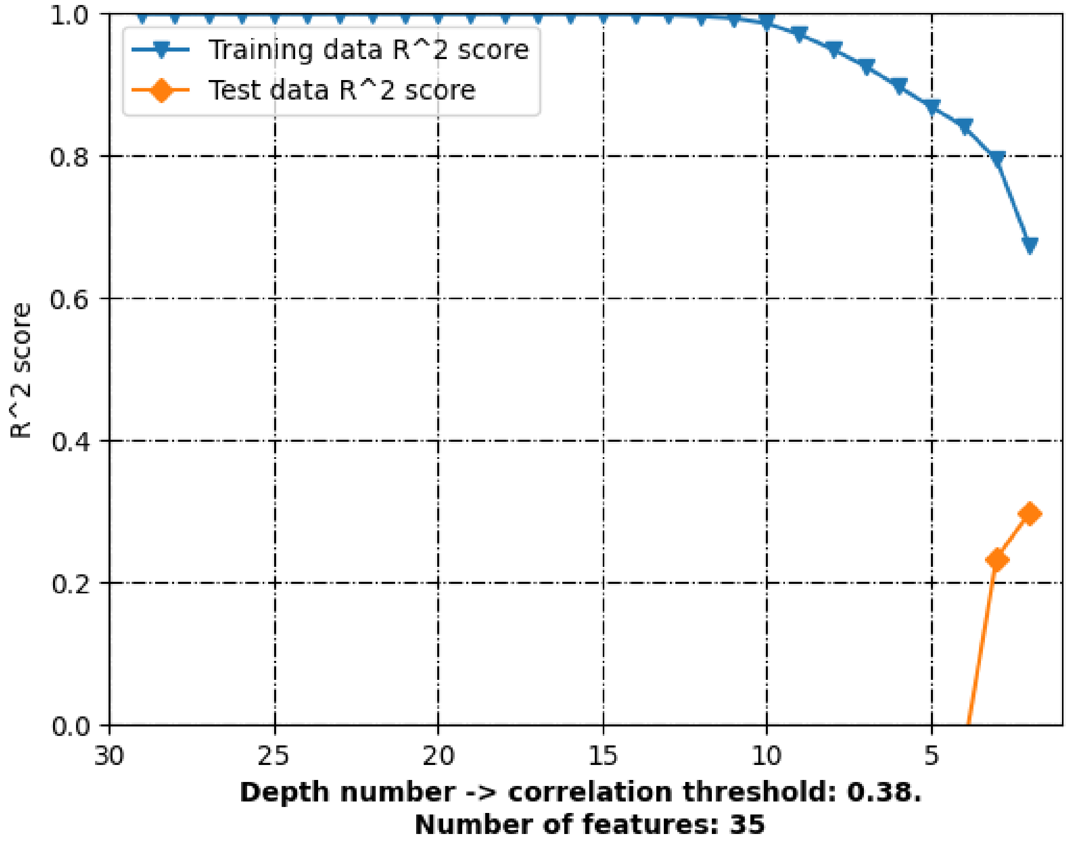

Training data R^2 score
Test data R^2 score
R^2 score
1.0
0.8
0.6
0.4
0.2
0.0
30
25
20
15
10
5
Depth number -> correlation threshold: 0.38.
Number of features: 35

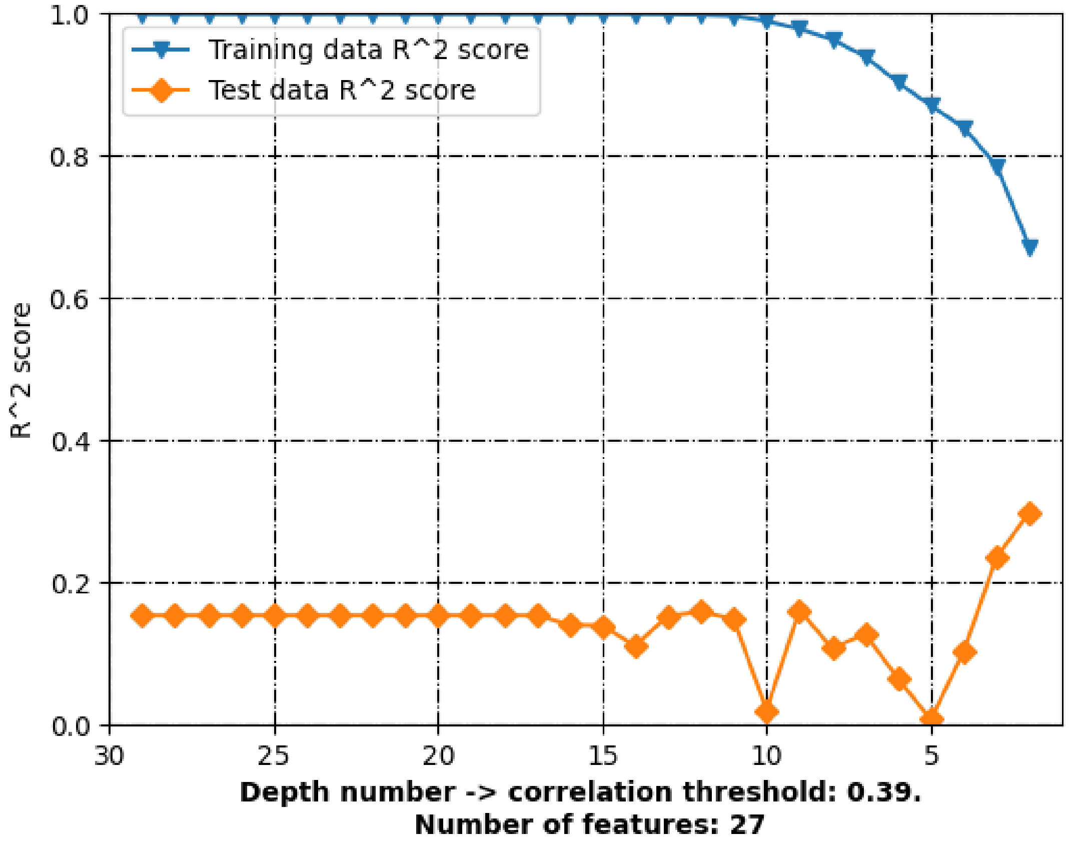
Training data R^2 score
Test data R^2 score
R^2 score
1.0
0.8
0.6
0.4
0.2
0.0
30
25
20
15
10
5
Depth number -> correlation threshold: 0.39.
Number of features: 27

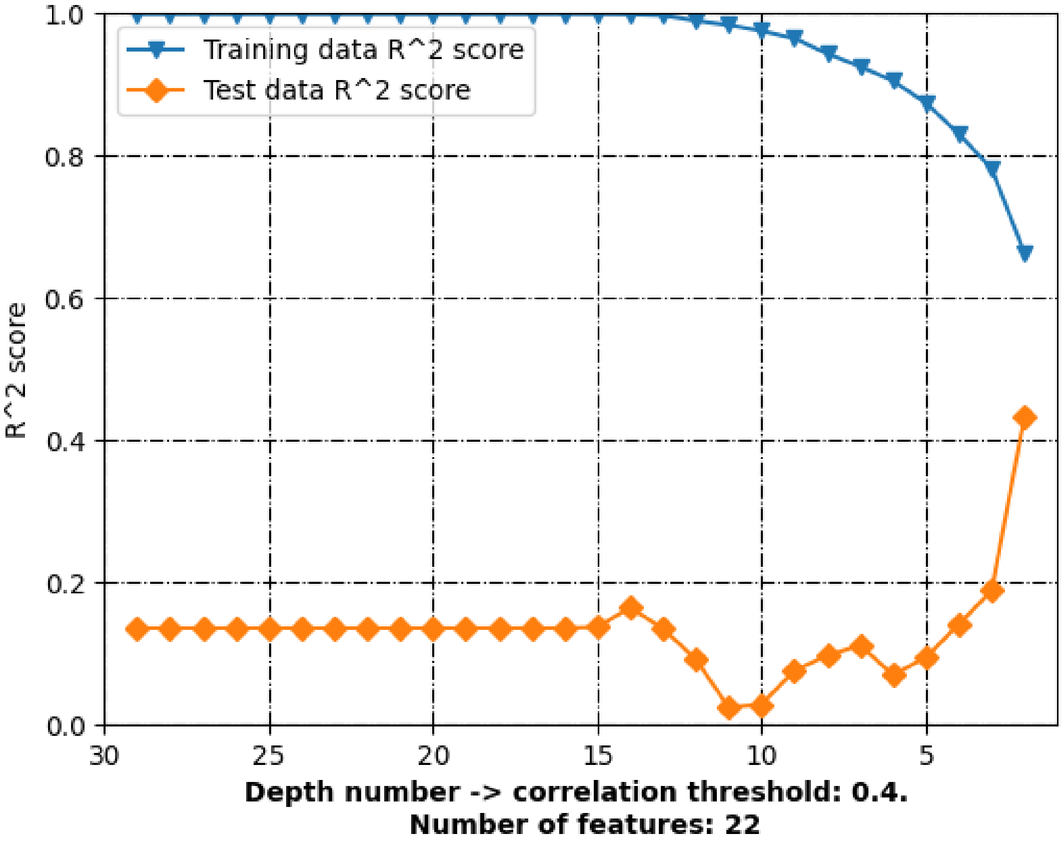
Training data R^2 score
Test data R^2 score
R^2 score
1.0
0.8
0.6
0.4
0.2
0.0
30
25
20
15
10
5
Depth number -> correlation threshold: 0.4.
Number of features: 22

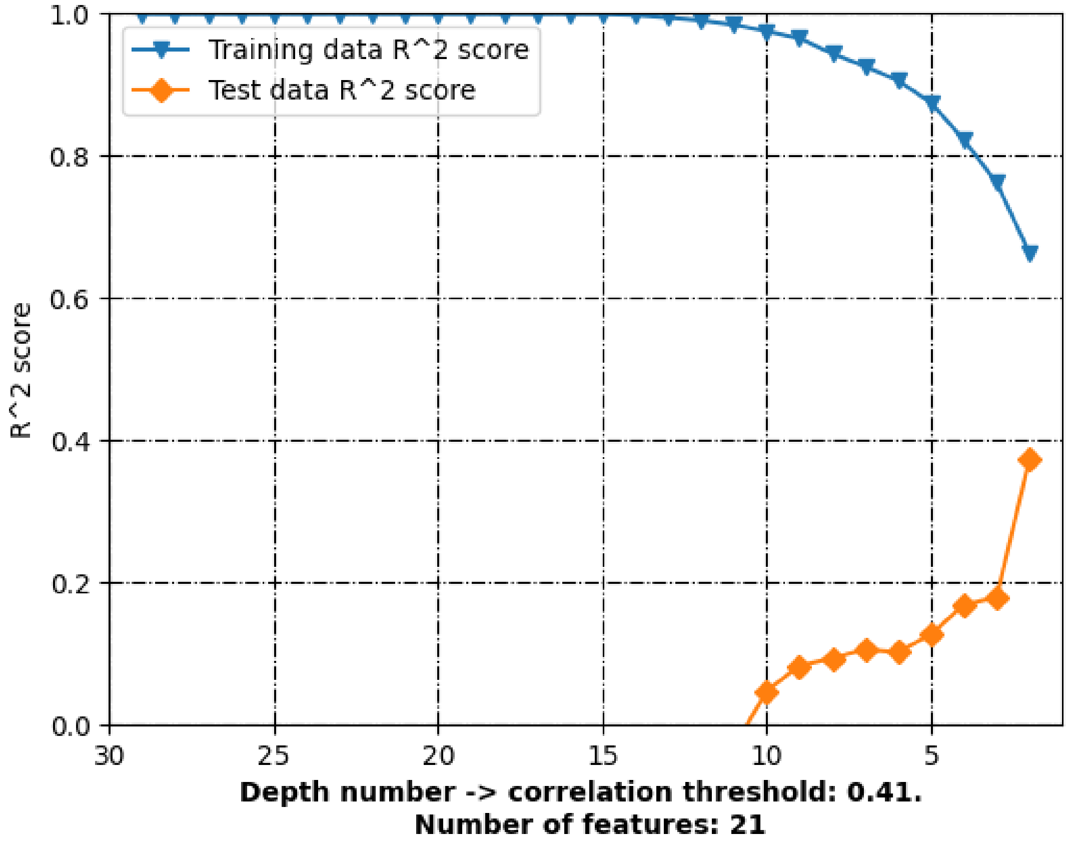

Training data R^2 score
Test data R^2 score
R^2 score
1.0
0.8
0.6
0.4
0.2
0.0
30
25
20
15
10
5
Depth number -> correlation threshold: 0.41.
Number of features: 21

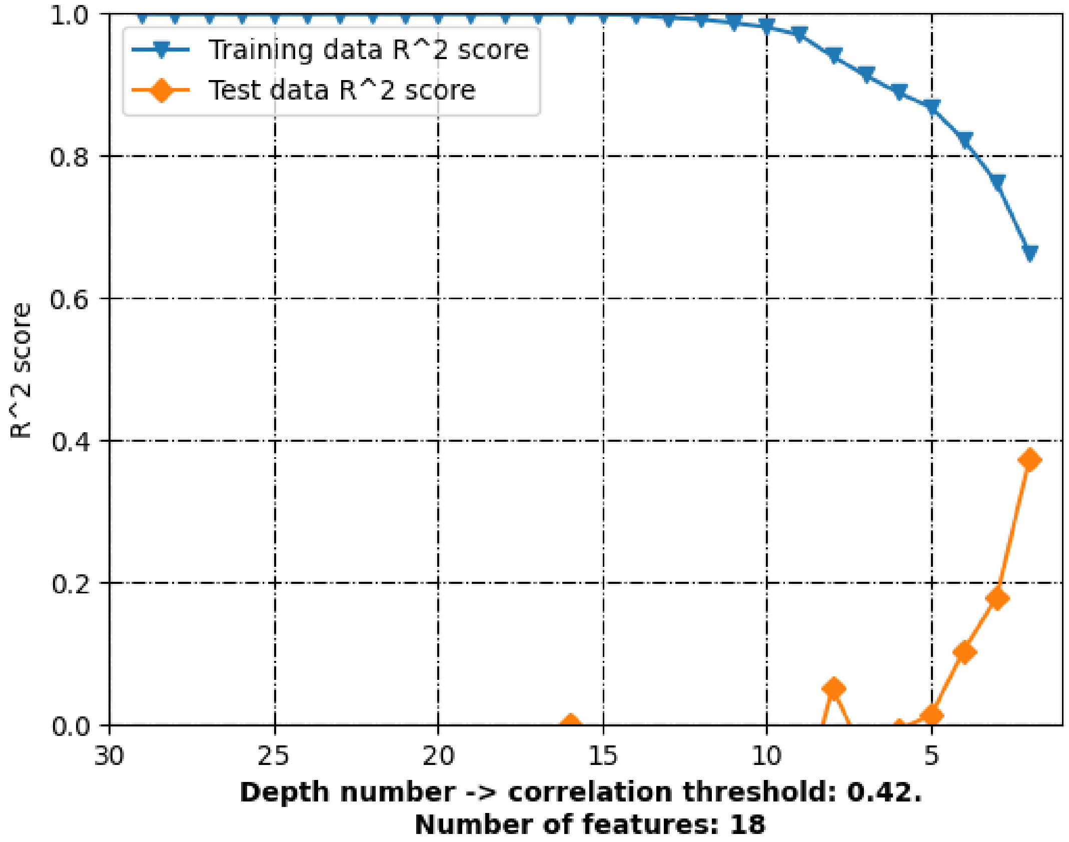
Training data R^2 score
Test data R^2 score
R^2 score
1.0
0.8
0.6
0.4
0.2
0.0
30
25
20
15
10
5
Depth number -> correlation threshold: 0.42.
Number of features: 18

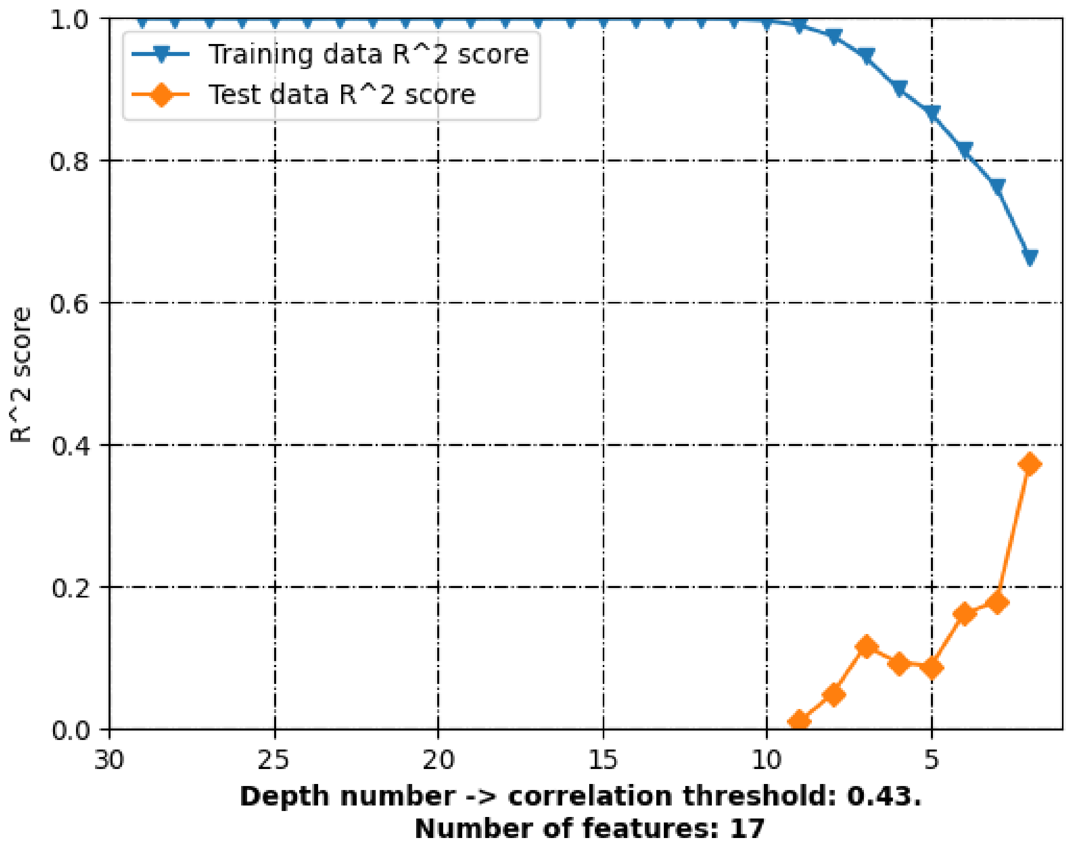

Training data R^2 score
Test data R^2 score
R^2 score
1.0
0.8
0.6
0.4
0.2
0.0
30
25
20
15
10
5
Depth number -> correlation threshold: 0.43.
Number of features: 17

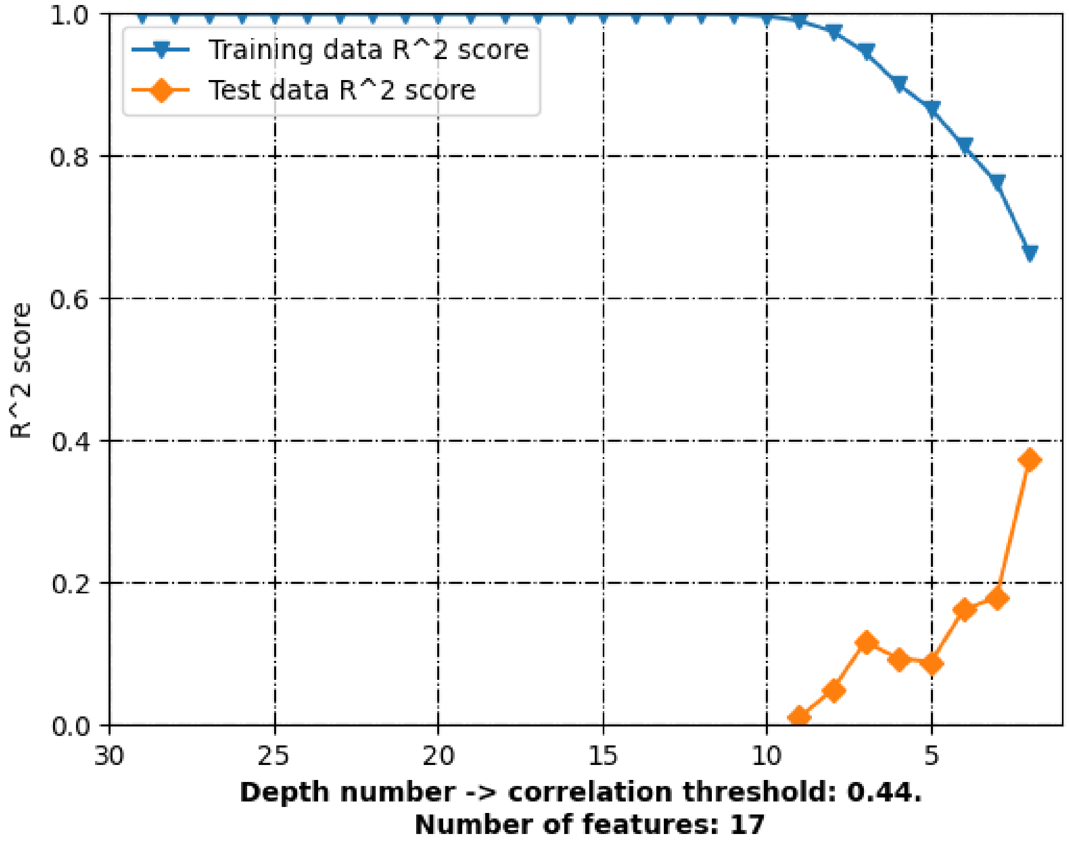

Training data R^2 score
Test data R^2 score
R^2 score
1.0
0.8
0.6
0.4
0.2
0.0
30
25
20
15
10
5
Depth number -> correlation threshold: 0.44.
Number of features: 17

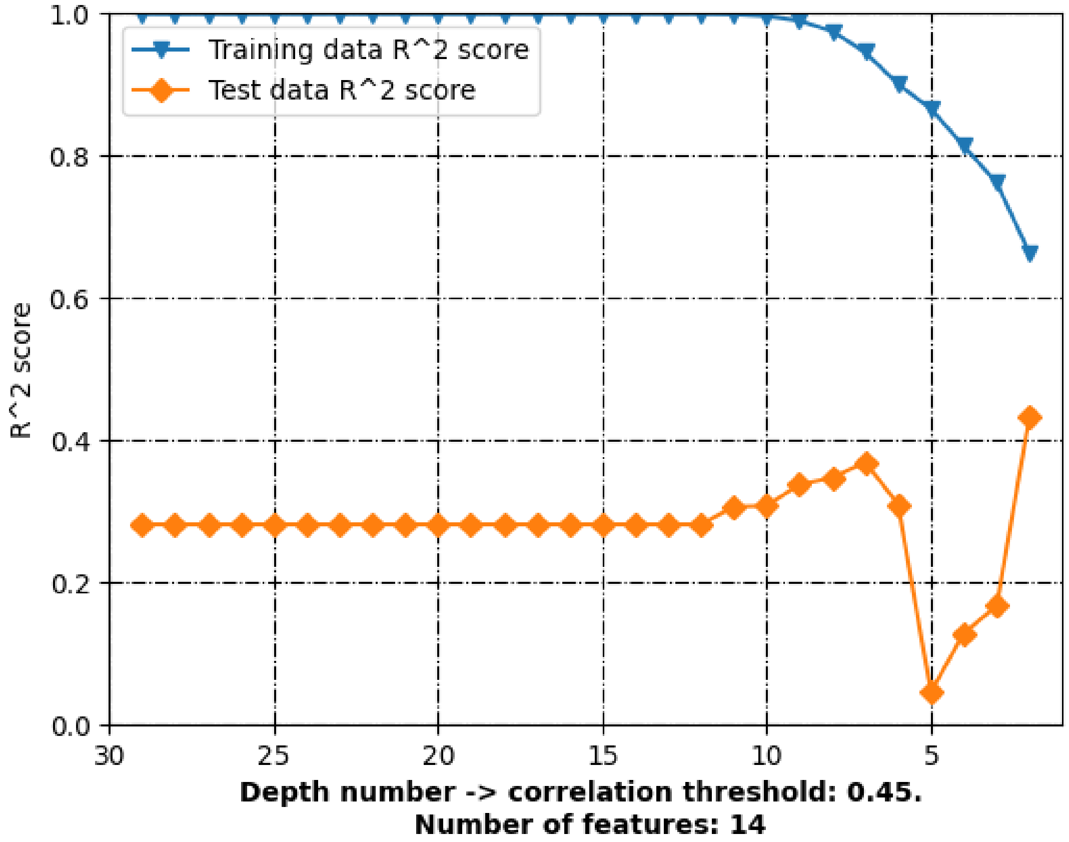

1.0
0.8
0.6
0.4
0.2
0.0
R^2 score
Training data R^2 score
Test data R^2 score
30
25
20
15
10
5
Depth number -> correlation threshold: 0.45.
Number of features: 14

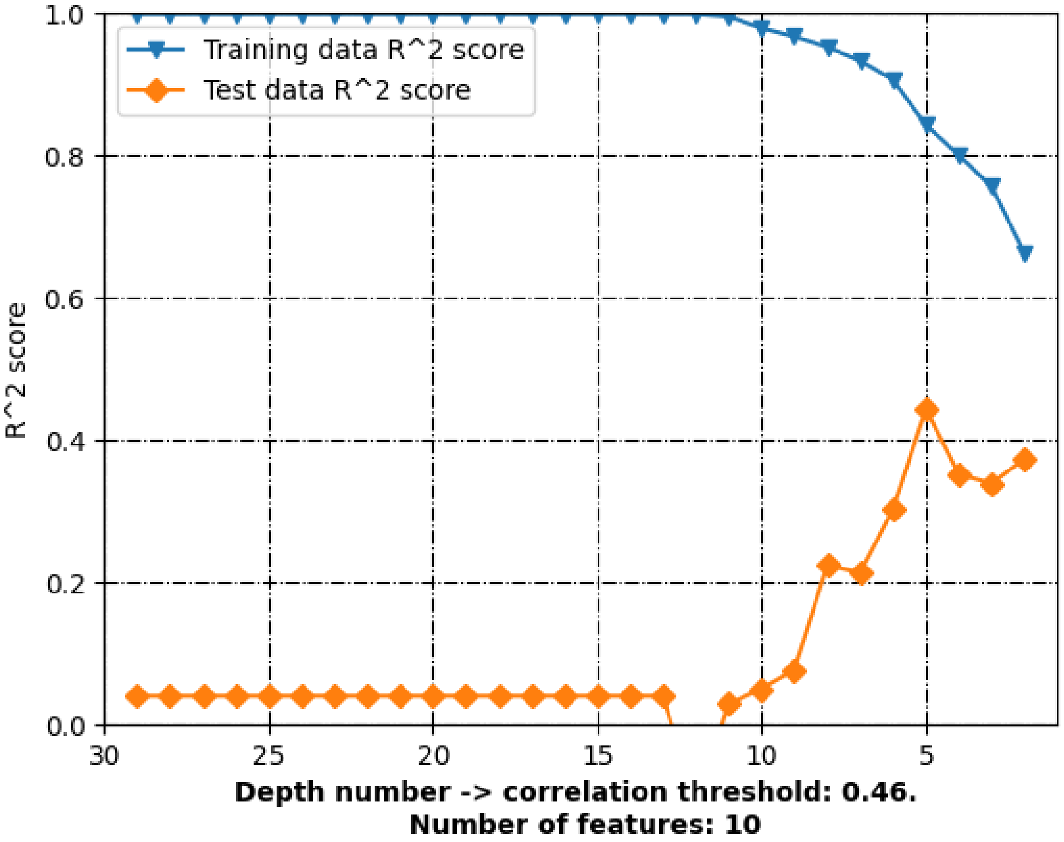
Training data R^2 score
Test data R^2 score
R^2 score
1.0
0.8
0.6
0.4
0.2
0.0
30
25
20
15
10
5
Depth number -> correlation threshold: 0.46.
Number of features: 10

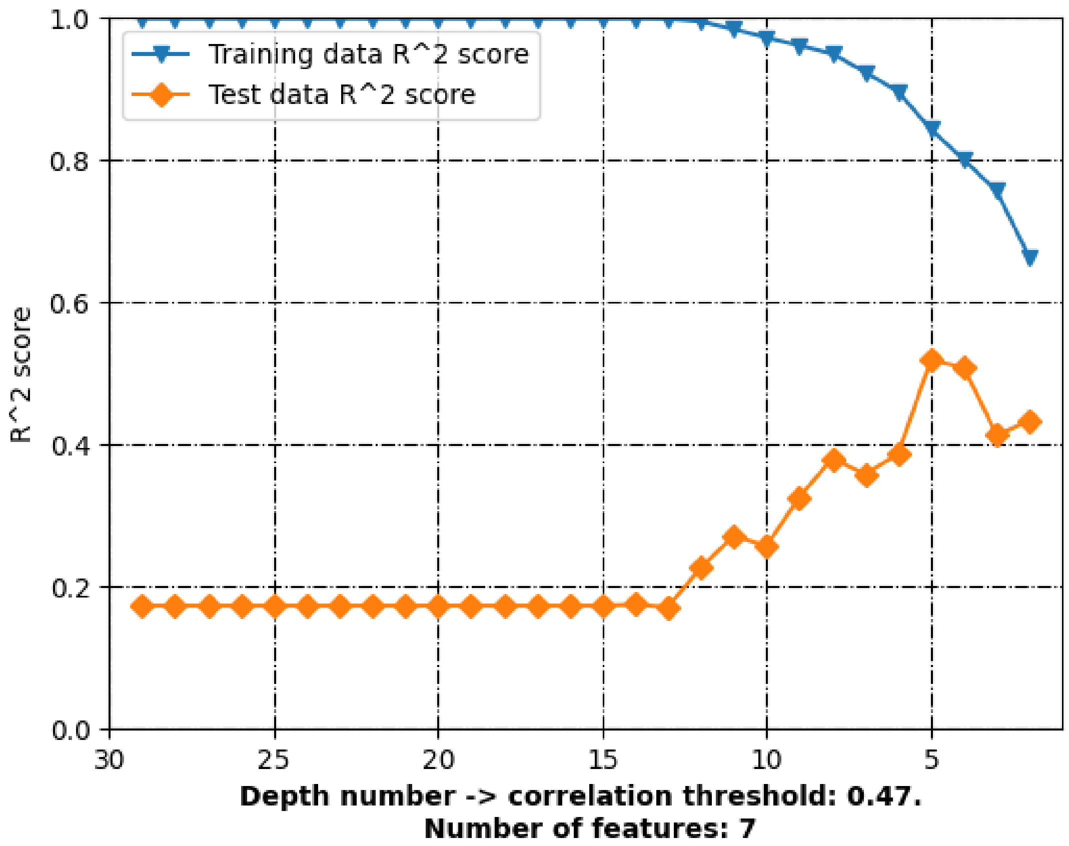

Training data R^2 score
Test data R^2 score
R^2 score
1.0
0.8
0.6
0.4
0.2
0.0
30
25
20
15
10
5
Depth number -> correlation threshold: 0.47.
Number of features: 7

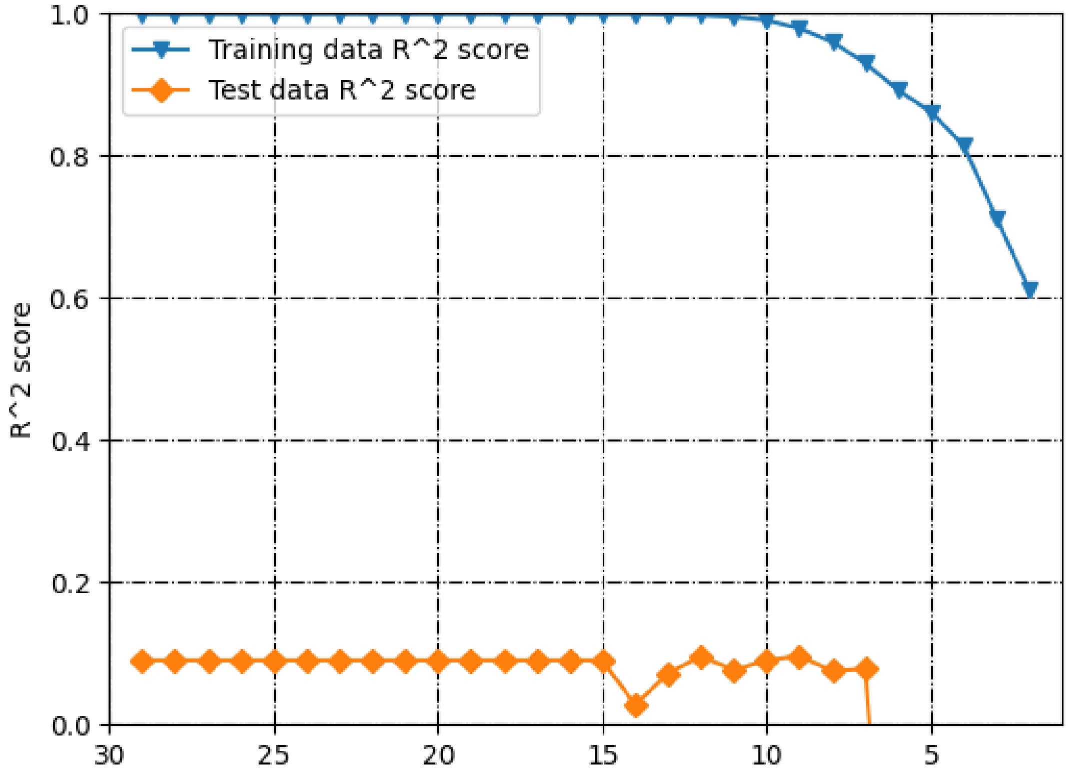

Depth number -> correlation threshold: 0.48.
Number of features: 6

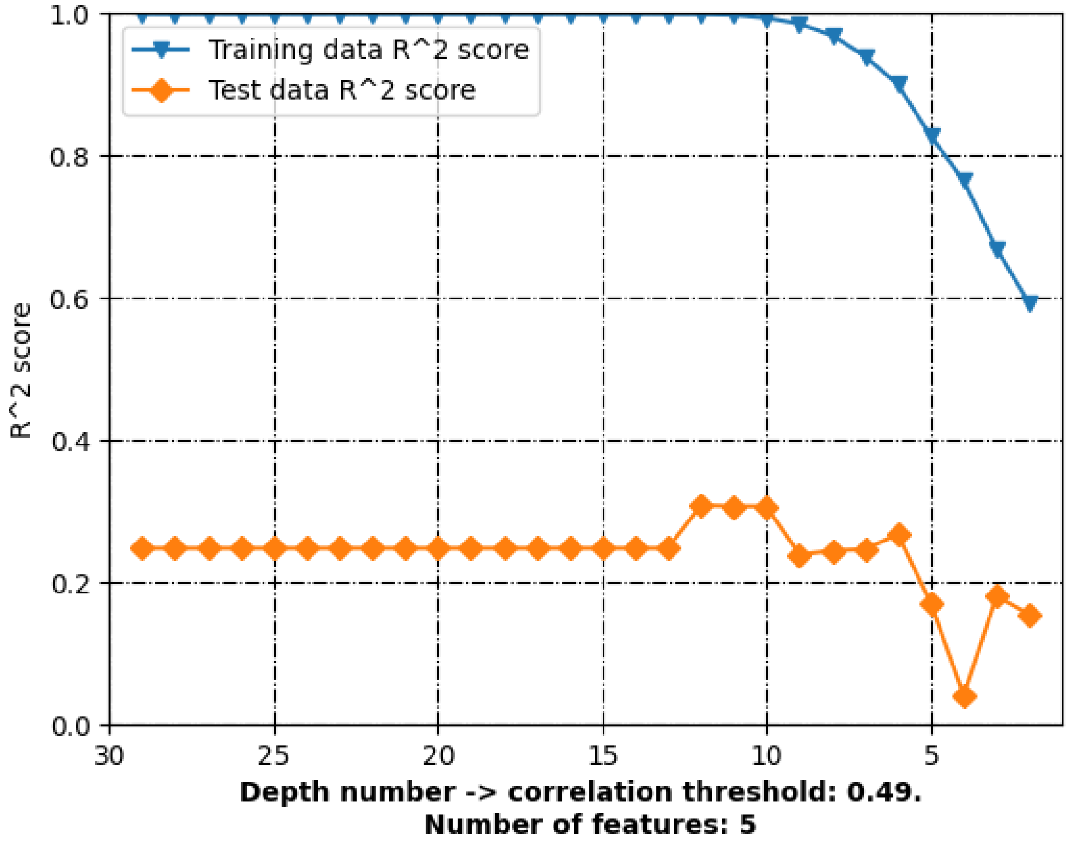
Training data R^2 score
Test data R^2 score
R^2 score
1.0
0.8
0.6
0.4
0.2
0.0
30
25
20
15
10
5
Depth number -> correlation threshold: 0.49.
Number of features: 5

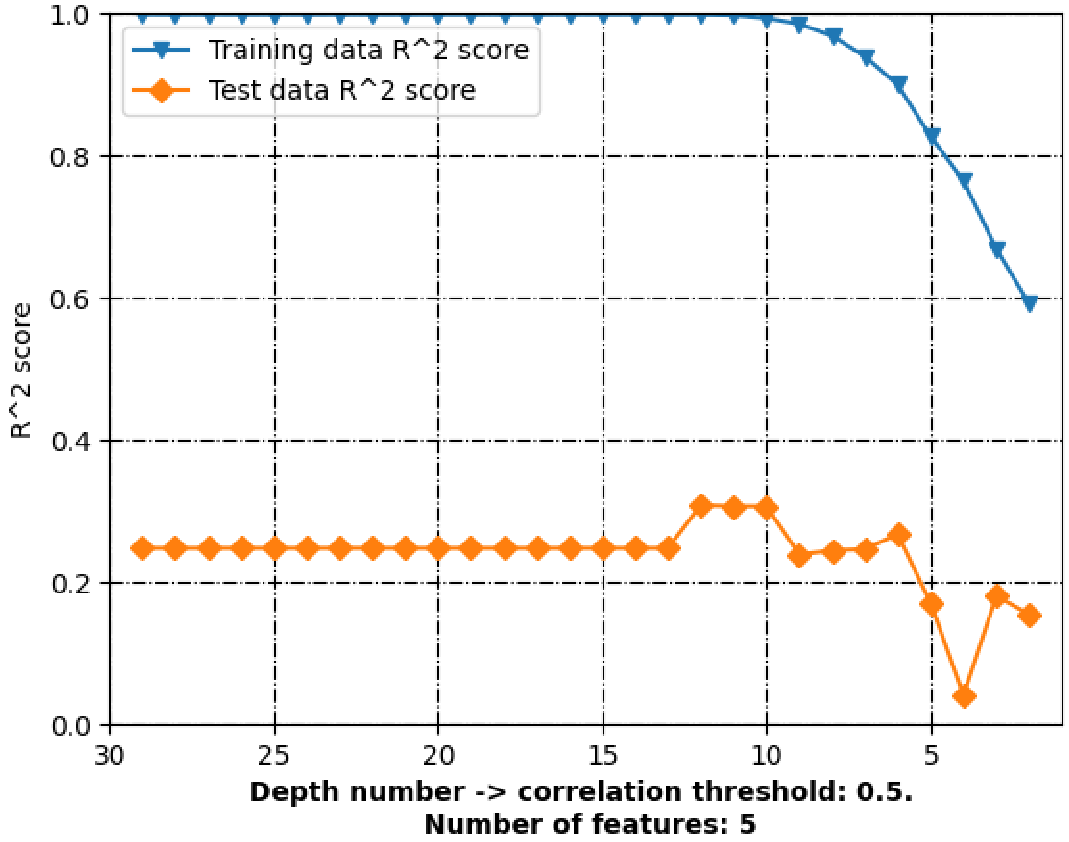

Training data R^2 score
Test data R^2 score
R^2 score
1.0
0.8
0.6
0.4
0.2
0.0
30
25
20
15
10
5
Depth number -> correlation threshold: 0.5.
Number of features: 5

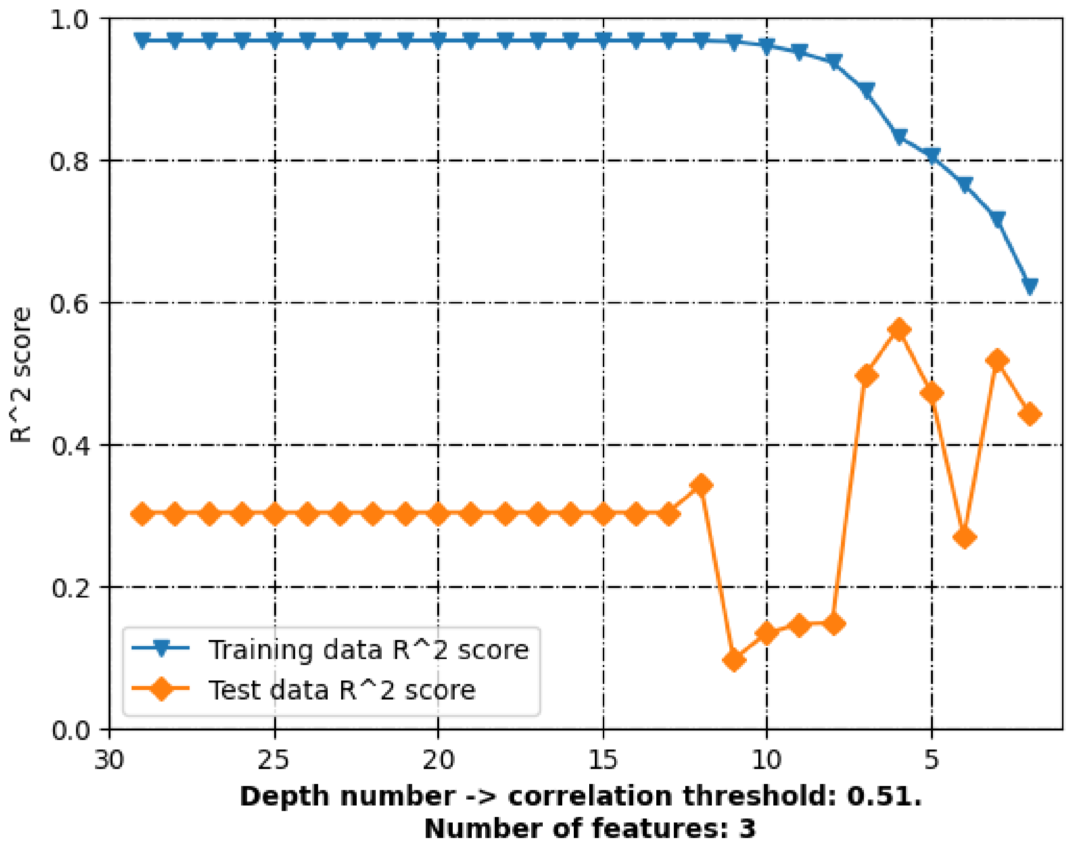

1.0
0.8
0.6
0.4
0.2
0.0
R^2 score
30
25
20
15
10
5
Training data R^2 score
Test data R^2 score
Depth number -> correlation threshold: 0.51.
Number of features: 3

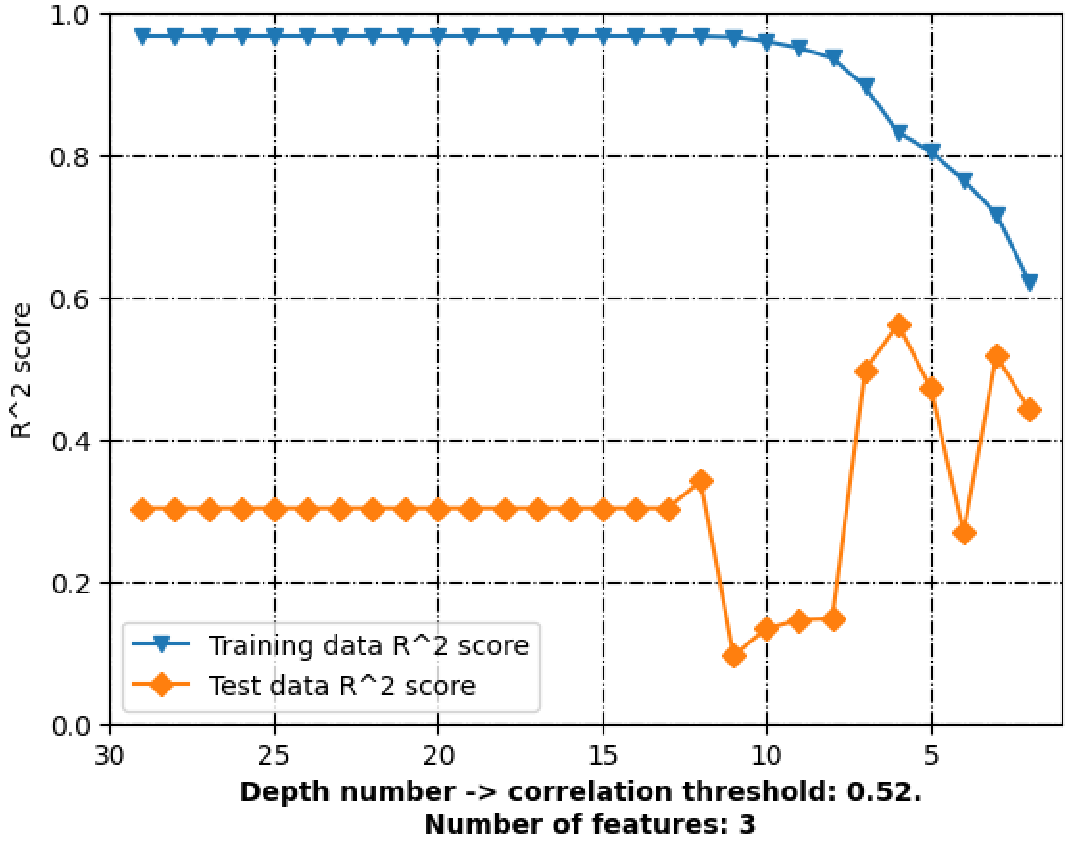
1.0
0.8
0.6
0.4
0.2
0.0
R^2 score
30
25
20
15
10
5
Depth number -> correlation threshold: 0.52.
Number of features: 3
Training data R^2 score
Test data R^2 score

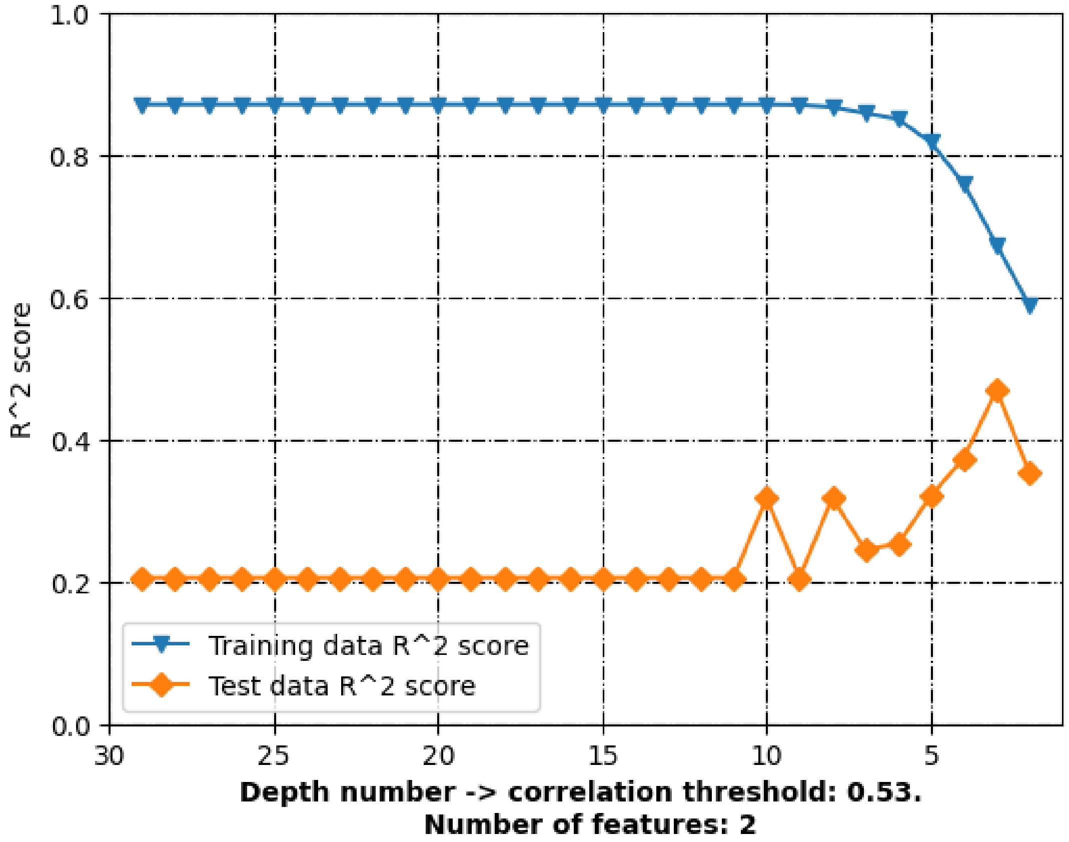
1.0
0.8
0.6
0.4
0.2
0.0
R^2 score
Training data R^2 score
Test data R^2 score
30
25
20
15
10
5
Depth number -> correlation threshold: 0.53.
Number of features: 2

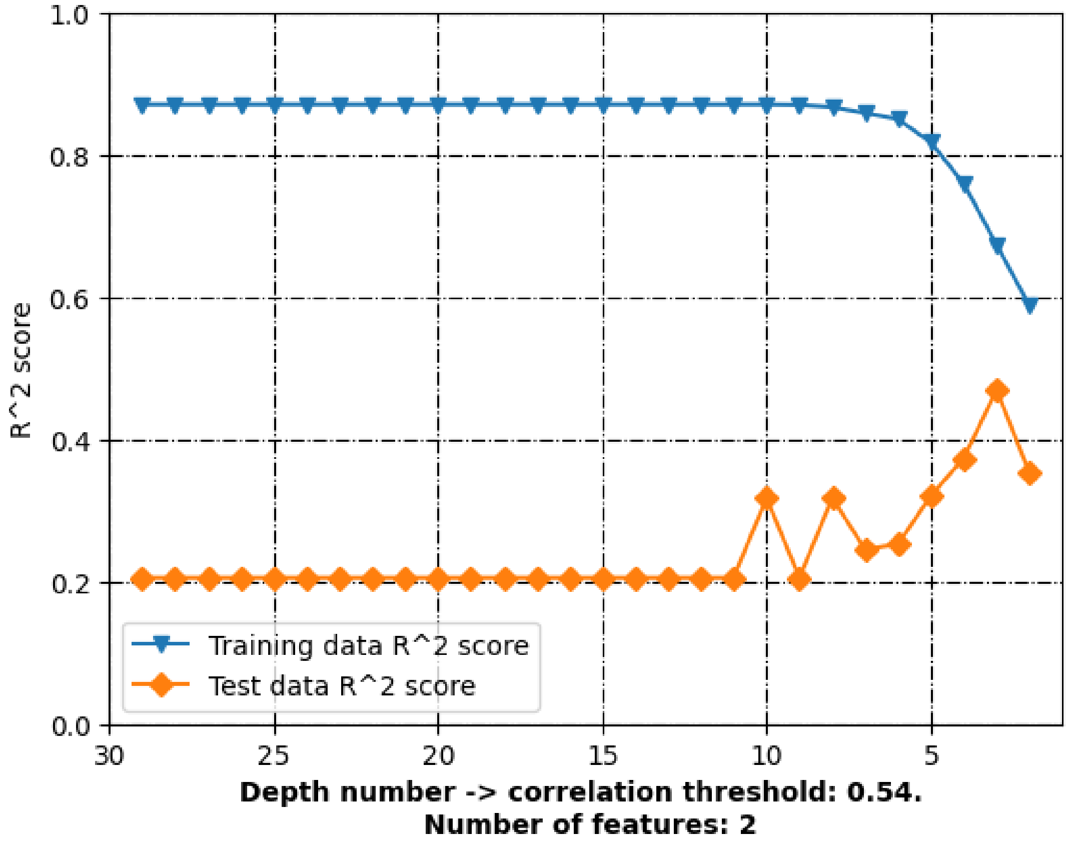

[20]:
```
plt.plot(df_without_standardization['Correlation threshold'],␣
  ↪df_without_standardization['Number of features'], label = "Number of␣
  ↪features", marker='.')
plt.legend()
plt.xlabel('Correlation threshold')
plt.ylabel('Number of features')
plt.show()
```

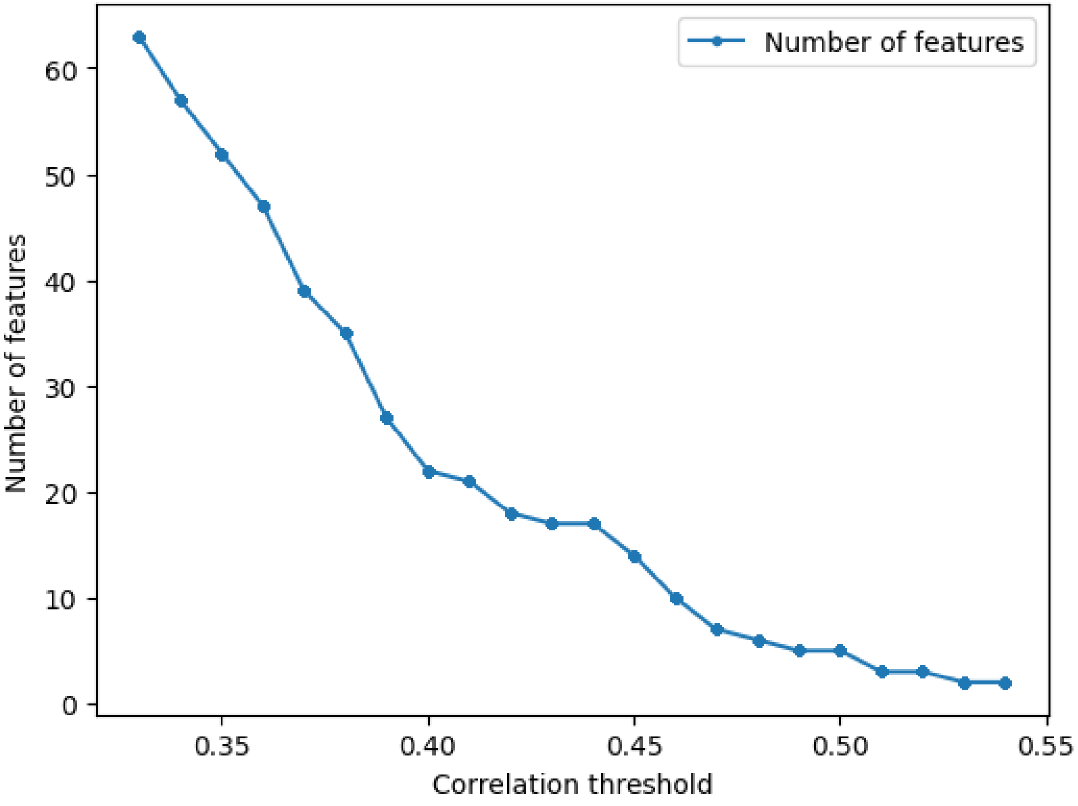


```
[ ]:
```

3 Random Forest

```
[21]: without_standardization, train_r2_, test_r2_, hist1, hist2, target_column_name, training_data_RMSE, test_data_RMSE  = pred_model.prepare_data_and_create_model(molecular_descriptors_df=data,
                              correlation_threshold=0.50,
                              standardization=False,
                              model_type='RandomForestRegressor',
                              n_estimators_=12,
                              target_column_name = target,
                              random_state=random_state,
```

```
                         train_test_split_=True,
                         verbose=True)
```

```
I am not doing standardization…
  molecular descriptor name
0                    AATS0Z
1                  AATS0are
2                    AATS0d
3                   AATS0dv
4                    AATS0i
  molecular descriptor name  corr_value
0                    AATS0Z   -0.027037
1                  AATS0are   -0.129823
2                    AATS0d    0.042740
3                   AATS0dv   -0.120173
4                    AATS0i    0.132395
  molecular descriptor name  corr_value  absolute correlation value
0                    AATS0Z   -0.027037                    0.027037
1                  AATS0are   -0.129823                    0.129823
2                    AATS0d    0.042740                    0.042740
3                   AATS0dv   -0.120173                    0.120173
4                    AATS0i    0.132395                    0.132395
     molecular descriptor name  corr_value  absolute correlation value
226                     AMID_O   -0.524157                    0.524157
505                 EState_VSA5   -0.579664                    0.579664
791                    MDEO-12   -0.558727                    0.558727
851                      NdssC   -0.506855                    0.506855
1091               VSA_EState5    0.503578                    0.503578
     molecular descriptor name  corr_value  absolute correlation value
226                     AMID_O   -0.524157                    0.524157
505                 EState_VSA5   -0.579664                    0.579664
791                    MDEO-12   -0.558727                    0.558727
851                      NdssC   -0.506855                    0.506855
1091               VSA_EState5    0.503578                    0.503578
The model used is: RandomForest…
Return the coefficient of determination of the prediction:
0.43879969172007904
R^2 score: 0.9116702784829944
Correlation coefficient: 0.9548142638665357
Test data - unseen during training:
R^2 score: 0.43879969172007904
Correlation coefficient: 0.6624195737748689
[7.78203756 8.2311727  7.86241599 7.58647733 5.55112428 8.22495905
 7.70238976 7.9122974  8.21435838 8.07512252 7.60532507 7.499756
 8.129522   6.87536406 7.8184975  7.78203756 8.14011191 8.05270232]
44      8.508638
```

```
47      7.920819
4       7.277366
55      7.920819
26      5.208801
64      8.327902
73      7.886057
10      8.102373
40      8.050610
107     8.017729
18      7.140261
62      8.657577
11      8.107905
36      7.017729
89      8.638272
91     10.000000
109     7.886057
0       8.187087
Name: LoVo, dtype: float64
Training Root Mean Square Error: 0.2648627793983205
Testing Root Mean Square Error: 0.6895106476870391
```

[22]:
```
print(target_column_name+str('_transformed'))
print(hist1[target_column_name].hist())
```

```
LoVo_transformed
AxesSubplot(0.125,0.11;0.775x0.77)
```

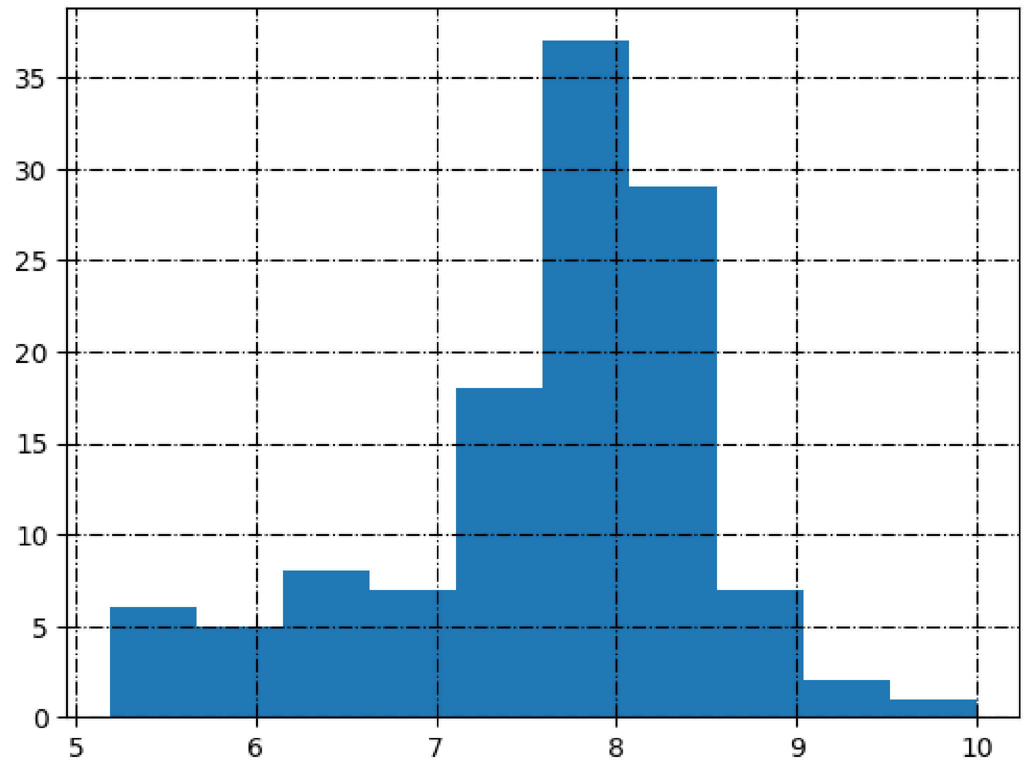

[23]:
```
with_standardization, train_r2_, test_r2_, hist1, hist2, target_column_name,␣
↪training_data_RMSE, test_data_RMSE  = pred_model.
↪prepare_data_and_create_model(molecular_descriptors_df=data,
                                                                              ␣
↪                         correlation_threshold=0.50,
                                                                              ␣
↪                         standardization=True,
                                                                              ␣
↪                         model_type='RandomForestRegressor',
                                                                              ␣
↪                         n_estimators_=12,
                                                                              ␣
↪                         target_column_name = target,
                                                                              ␣
↪                         random_state=random_state,
                                                                              ␣
↪                         train_test_split_=True,
                                                                              ␣
↪                         verbose=True)
```

```
I am doing standardization…
  molecular descriptor name
```

```
0                  AATS0Z
1                AATS0are
2                  AATS0d
3                 AATS0dv
4                  AATS0i
  molecular descriptor name  corr_value
0                  AATS0Z   -0.027037
1                AATS0are   -0.129823
2                  AATS0d    0.042740
3                 AATS0dv   -0.120173
4                  AATS0i    0.132395
  molecular descriptor name  corr_value  absolute correlation value
0                  AATS0Z   -0.027037                    0.027037
1                AATS0are   -0.129823                    0.129823
2                  AATS0d    0.042740                    0.042740
3                 AATS0dv   -0.120173                    0.120173
4                  AATS0i    0.132395                    0.132395
     molecular descriptor name  corr_value  absolute correlation value
226                     AMID_O   -0.524157                    0.524157
505                 EState_VSA5   -0.579664                    0.579664
791                    MDEO-12   -0.558727                    0.558727
851                      NdssC   -0.506855                    0.506855
1091                VSA_EState5    0.503578                    0.503578
     molecular descriptor name  corr_value  absolute correlation value
226                     AMID_O   -0.524157                    0.524157
505                 EState_VSA5   -0.579664                    0.579664
791                    MDEO-12   -0.558727                    0.558727
851                      NdssC   -0.506855                    0.506855
1091                VSA_EState5    0.503578                    0.503578
The model used is: RandomForest…
Return the coefficient of determination of the prediction:
0.43879969172007904
R^2 score: 0.9116702784829944
Correlation coefficient: 0.9548142638665357
Test data - unseen during training:
R^2 score: 0.43879969172007904
Correlation coefficient: 0.6624195737748689
[7.78203756 8.2311727  7.86241599 7.58647733 5.55112428 8.22495905
 7.70238976 7.9122974  8.21435838 8.07512252 7.60532507 7.499756
 8.129522   6.87536406 7.8184975  7.78203756 8.14011191 8.05270232]
44     8.508638
47     7.920819
4      7.277366
55     7.920819
26     5.208801
64     8.327902
73     7.886057
10     8.102373
```

```
40      8.050610
107     8.017729
18      7.140261
62      8.657577
11      8.107905
36      7.017729
89      8.638272
91     10.000000
109     7.886057
0       8.187087
Name: LoVo, dtype: float64
Training Root Mean Square Error: 0.2648627793983205
Testing Root Mean Square Error: 0.6895106476870391
```

3.1 Search inside correlation space

[24]:
```
step = 0.01
initial_step = corr_low
last_step = corr_high
first_list = [x / 100.0 for x in range(int(initial_step*100),␣
↪int(last_step*100), int(step*100))]
n_estimators = [range(2,21,1)]
corr_th = []
second_list = []
third_list = []
fourth_l = []
fifth_l = []
f_list = []
fif_list = []
for i in first_list:

    for estimator in n_estimators[0]:

        without_standardization, train_r2, test_r2, _, h_, target_column_name,␣
↪training_data_RMSE, test_data_RMSE = pred_model.
↪prepare_data_and_create_model(molecular_descriptors_df=data,
                                                                                  ␣
↪                   correlation_threshold=i,
                                                                                  ␣
↪                   standardization=False,
                                                                                  ␣
↪                   model_type='RandomForestRegressor',
                                                                                  ␣
↪                   n_estimators_=estimator,
                                                                                  ␣
↪                   target_column_name = target,
```

```
                                                                    ␣
↪                   random_state=random_state,
                                                                    ␣
↪                   train_test_split_=True,
                                                                    ␣
↪                   verbose=False)
        corr_th.append(i)
        second_list.append(train_r2)
        third_list.append(test_r2)
        fourth_l.append(training_data_RMSE)
        fifth_l.append(test_data_RMSE)
        f_list.append(len(h_))
        fif_list.append(estimator)
```

[25]:
```
df_without_standardization = pd.DataFrame(data=corr_th, columns=["Correlation␣
↪threshold"])
df_without_standardization['Training data R^2 score'] = second_list
df_without_standardization['Test data R^2 score'] = third_list
df_without_standardization['Training RMSE'] = fourth_l
df_without_standardization['Test RMSE'] = fifth_l
df_without_standardization['Number of features'] = f_list
df_without_standardization['Number of estimators'] = fif_list
```

[26]:
```
df_random_forest = df_without_standardization.copy()
#df_without_standardization.to_excel('../Data/
↪A549_Random_forest_rs_'+str(random_state)+'.xlsx')
```

[27]:
```
corre_list = [x / 100.0 for x in range(int(initial_step*100),␣
↪int(last_step*100), int(0.01*100))]
for element in corre_list:
    element_ =␣
↪df_without_standardization[df_without_standardization['Correlation␣
↪threshold'] == float(element)]
    plt.plot(element_['Number of estimators'], element_['Training data R^2␣
↪score'], label = "Training data R^2 score", marker='v')
    plt.plot(element_['Number of estimators'], element_['Test data R^2 score'],␣
↪label = "Test data R^2 score", marker='D')
    plt.legend()
    plt.xlabel('Number of estimators -> correlation threshold: '+str(element)+'.
↪ \n Number of features: '+str(element_['Number of features'].iloc[0]))
    plt.xlim(max(element_['Number of estimators'])+1, min(element_['Number of␣
↪estimators'])-1)
    plt.ylabel('R^2 score')
    plt.ylim([0, 1])
    plt.rc('grid', linestyle="-.", color='black')
    plt.grid(True)
```

```
plt.show()
```

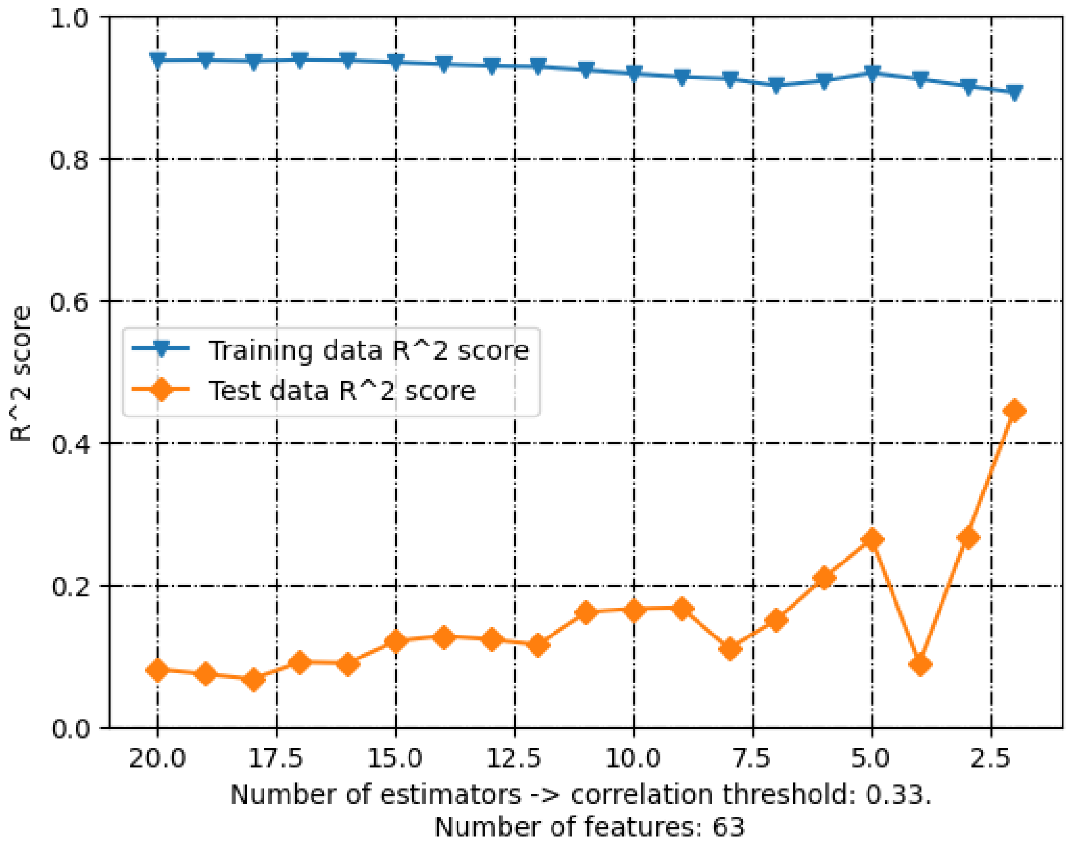

1.0
0.8
0.6
0.4
0.2
0.0
R^2 score
Training data R^2 score
Test data R^2 score
20.0
17.5
15.0
12.5
10.0
7.5
5.0
2.5
Number of estimators -> correlation threshold: 0.33.
Number of features: 63

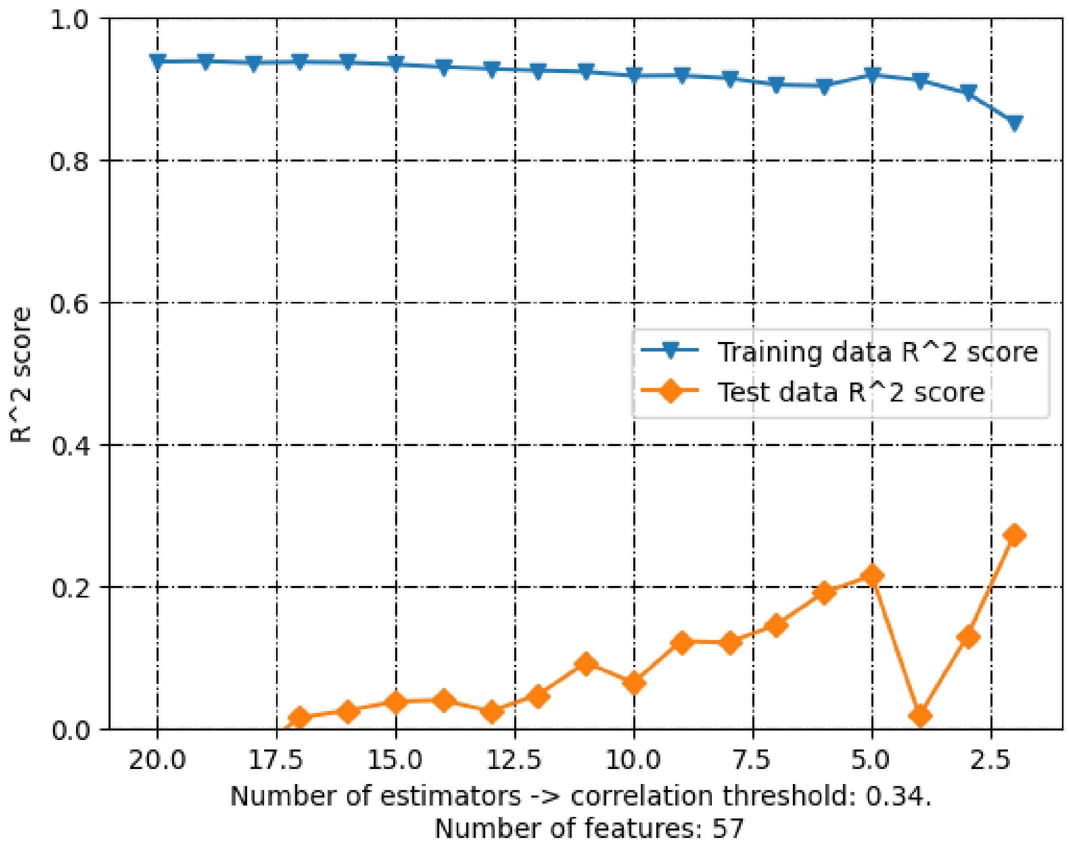
1.0
0.8
0.6
0.4
0.2
0.0
R^2 score
Training data R^2 score
Test data R^2 score
20.0
17.5
15.0
12.5
10.0
7.5
5.0
2.5
Number of estimators -> correlation threshold: 0.34.
Number of features: 57

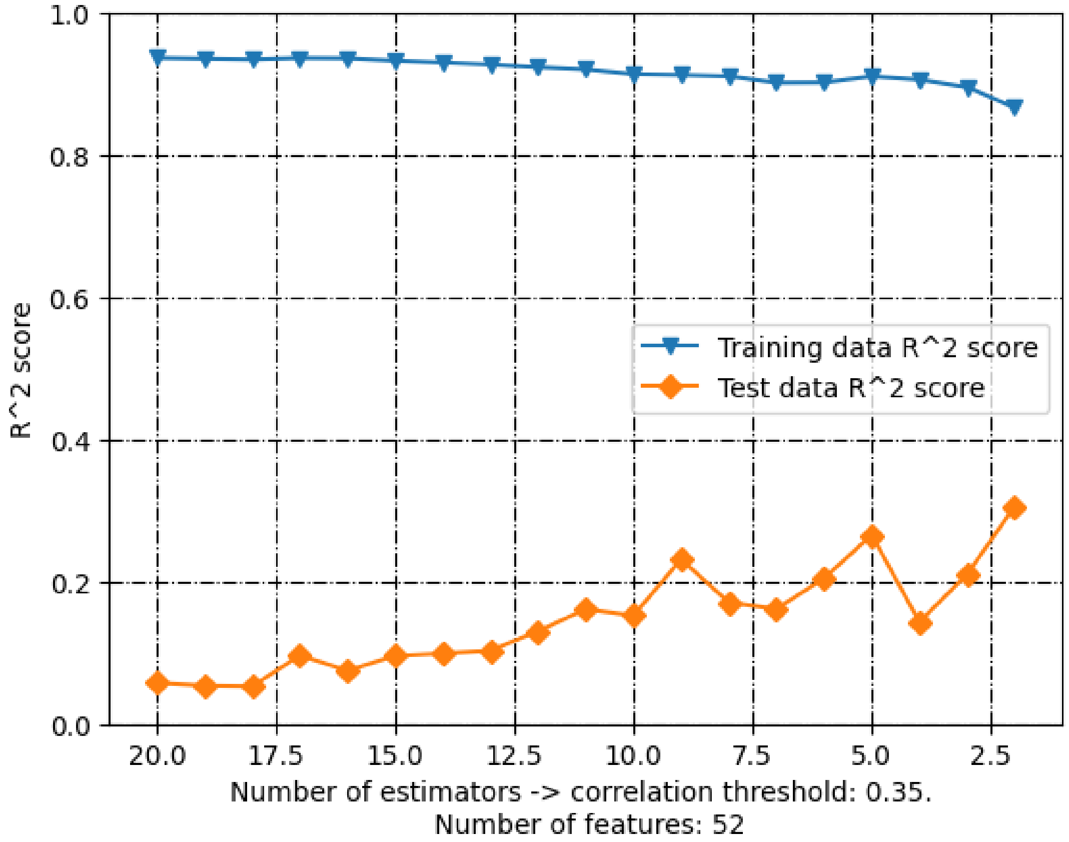
1.0
0.8
0.6
0.4
0.2
0.0
R^2 score
Training data R^2 score
Test data R^2 score
20.0
17.5
15.0
12.5
10.0
7.5
5.0
2.5
Number of estimators -> correlation threshold: 0.35.
Number of features: 52

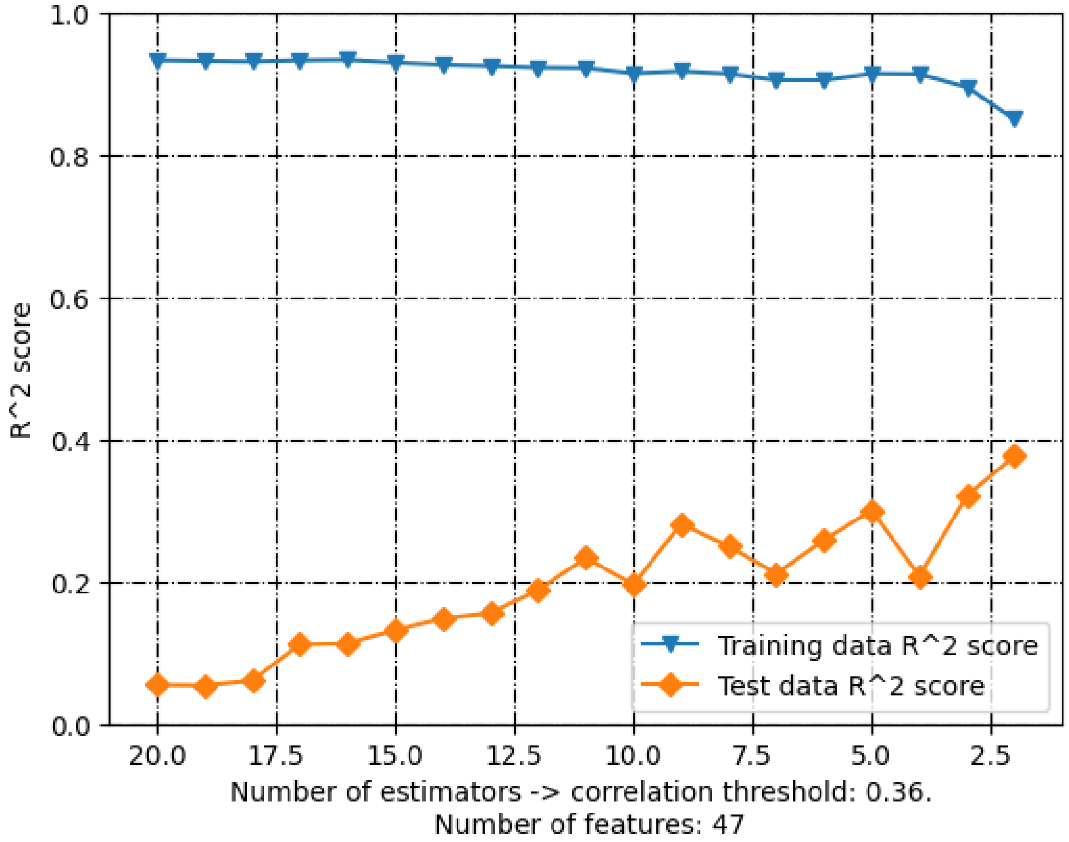
1.0
0.8
0.6
0.4
0.2
0.0
R^2 score
20.0
17.5
15.0
12.5
10.0
7.5
5.0
2.5
Number of estimators -> correlation threshold: 0.36.
Number of features: 47
Training data R^2 score
Test data R^2 score

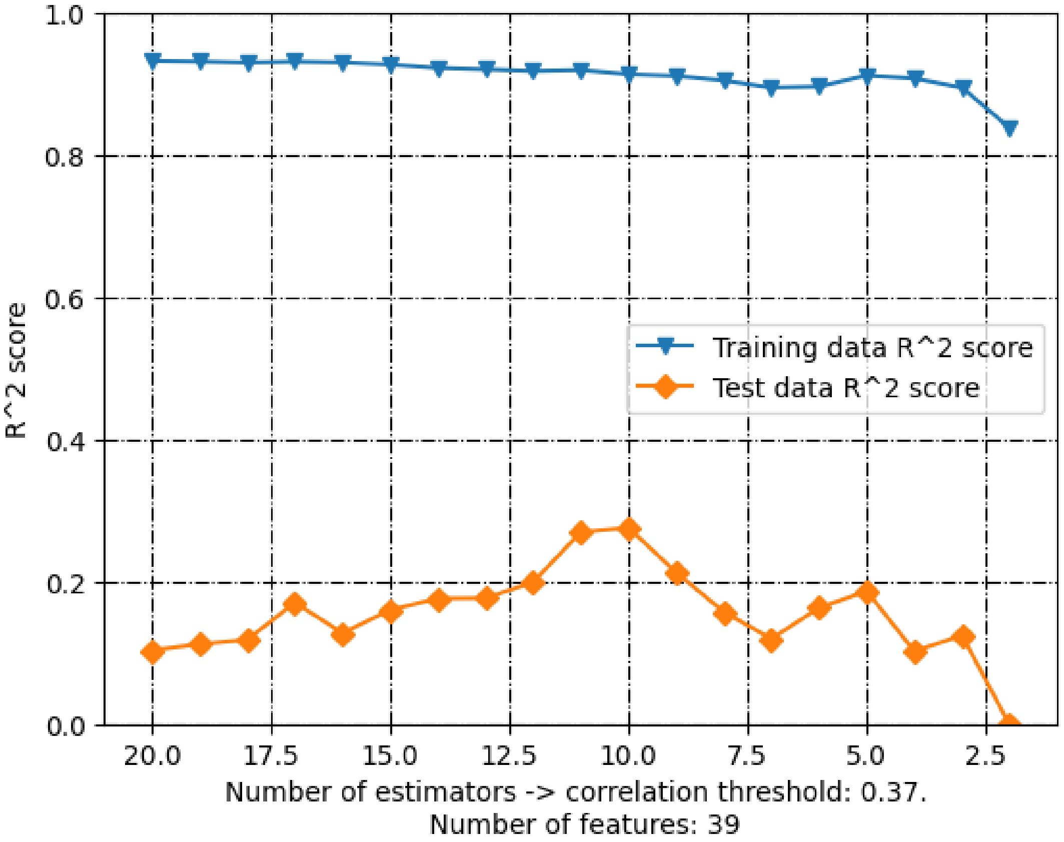

1.0
0.8
0.6
0.4
0.2
0.0
R^2 score
Training data R^2 score
Test data R^2 score
20.0
17.5
15.0
12.5
10.0
7.5
5.0
2.5
Number of estimators -> correlation threshold: 0.37.
Number of features: 39

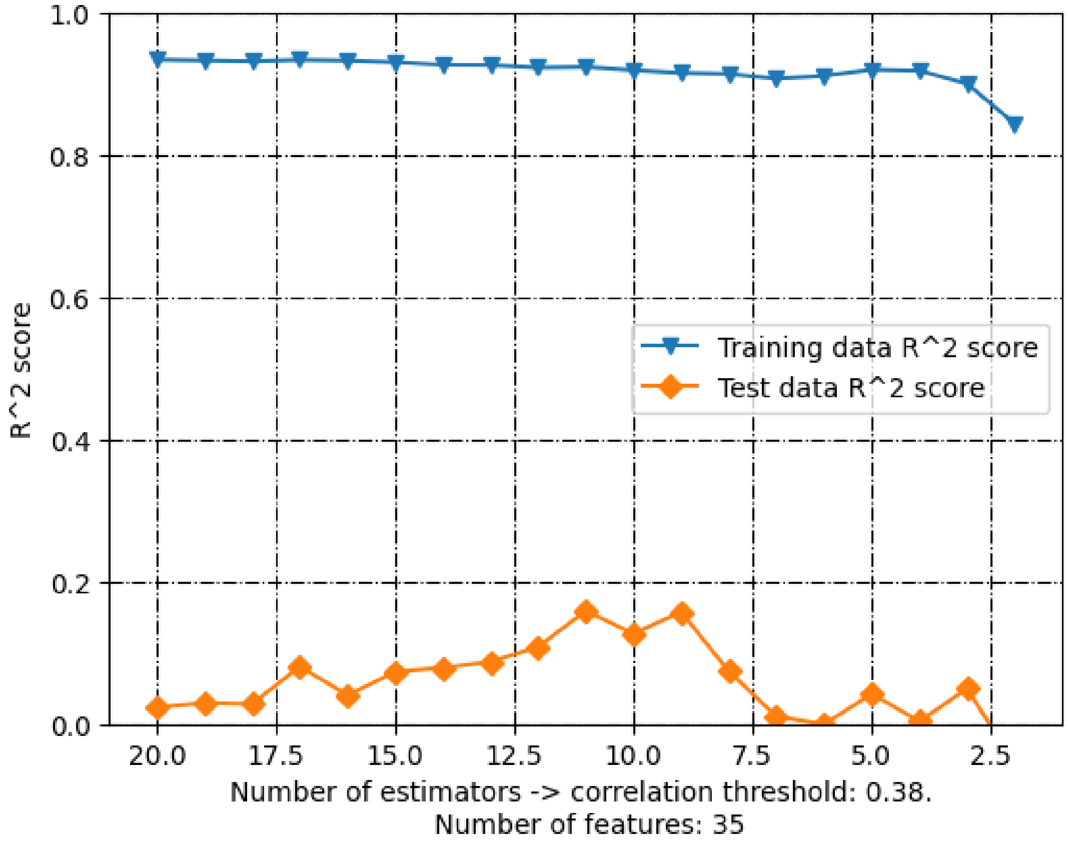
1.0
0.8
0.6
0.4
0.2
0.0
R^2 score
Training data R^2 score
Test data R^2 score
20.0
17.5
15.0
12.5
10.0
7.5
5.0
2.5
Number of estimators -> correlation threshold: 0.38.
Number of features: 35

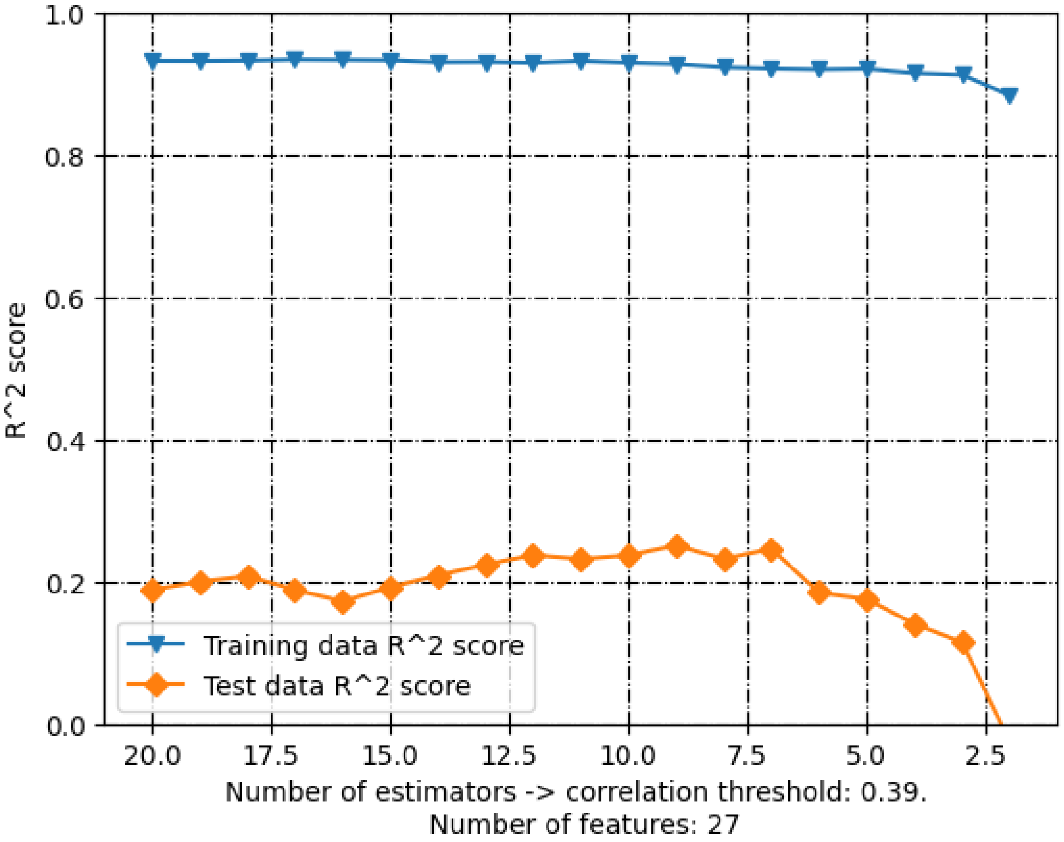
1.0
0.8
0.6
0.4
0.2
0.0
R^2 score
Training data R^2 score
Test data R^2 score
20.0
17.5
15.0
12.5
10.0
7.5
5.0
2.5
Number of estimators -> correlation threshold: 0.39.
Number of features: 27

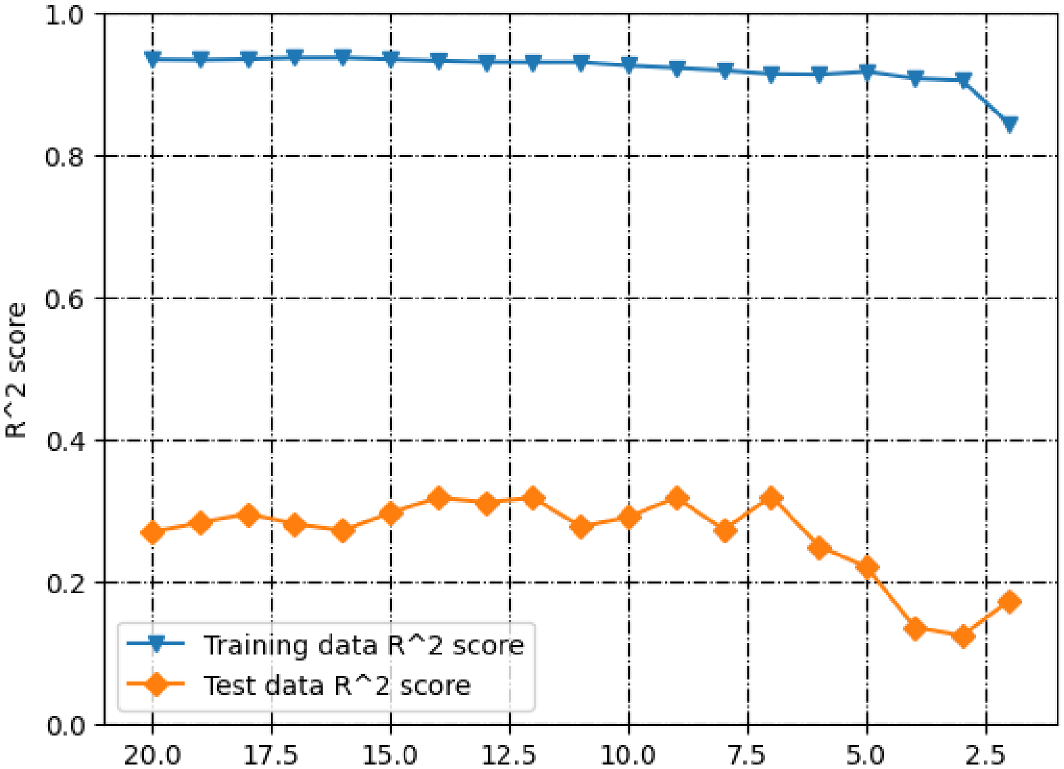

Number of estimators -> correlation threshold: 0.4.
Number of features: 22

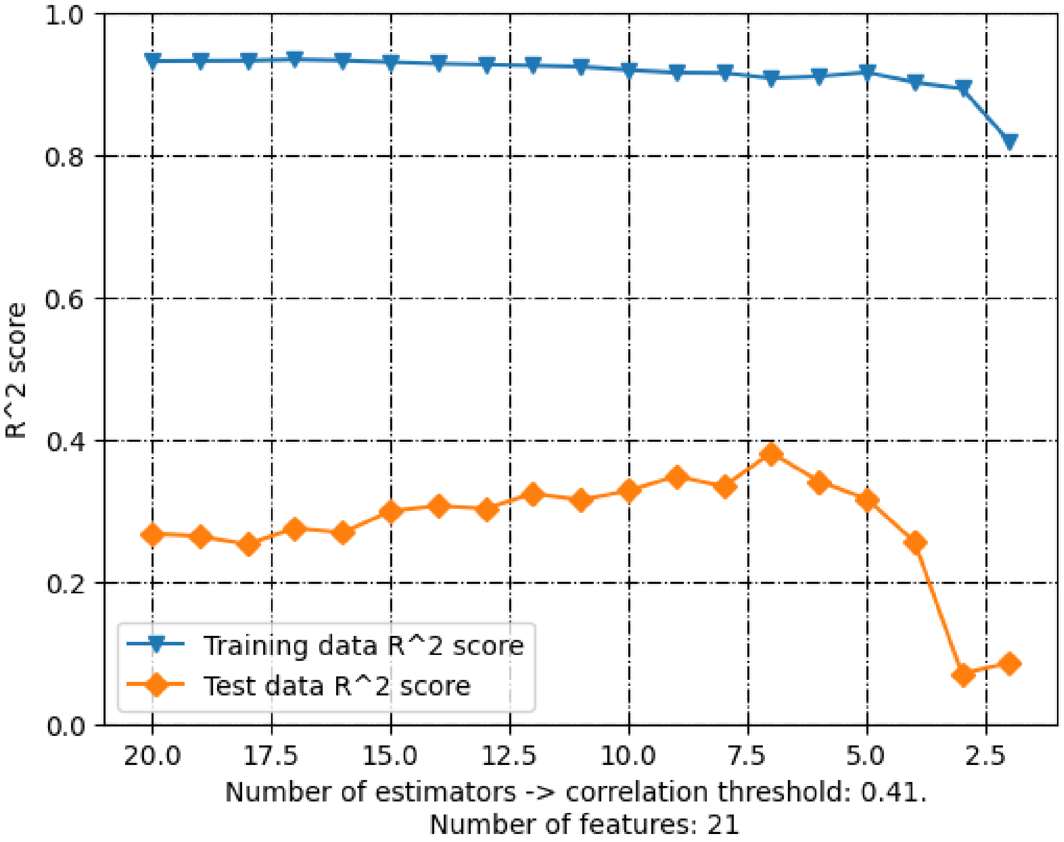
1.0
0.8
0.6
0.4
0.2
0.0
R^2 score
20.0
17.5
15.0
12.5
10.0
7.5
5.0
2.5
Training data R^2 score
Test data R^2 score
Number of estimators -> correlation threshold: 0.41.
Number of features: 21

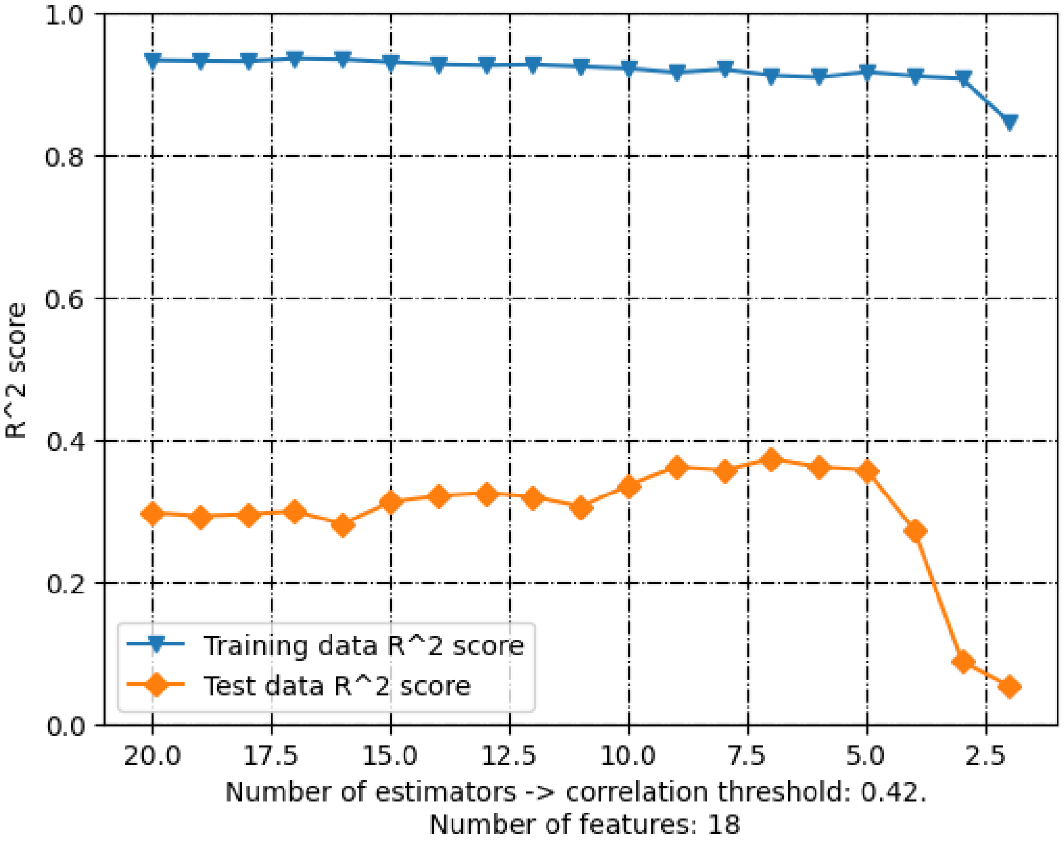
1.0
0.8
0.6
0.4
0.2
0.0
R^2 score
Training data R^2 score
Test data R^2 score
20.0
17.5
15.0
12.5
10.0
7.5
5.0
2.5
Number of estimators -> correlation threshold: 0.42.
Number of features: 18

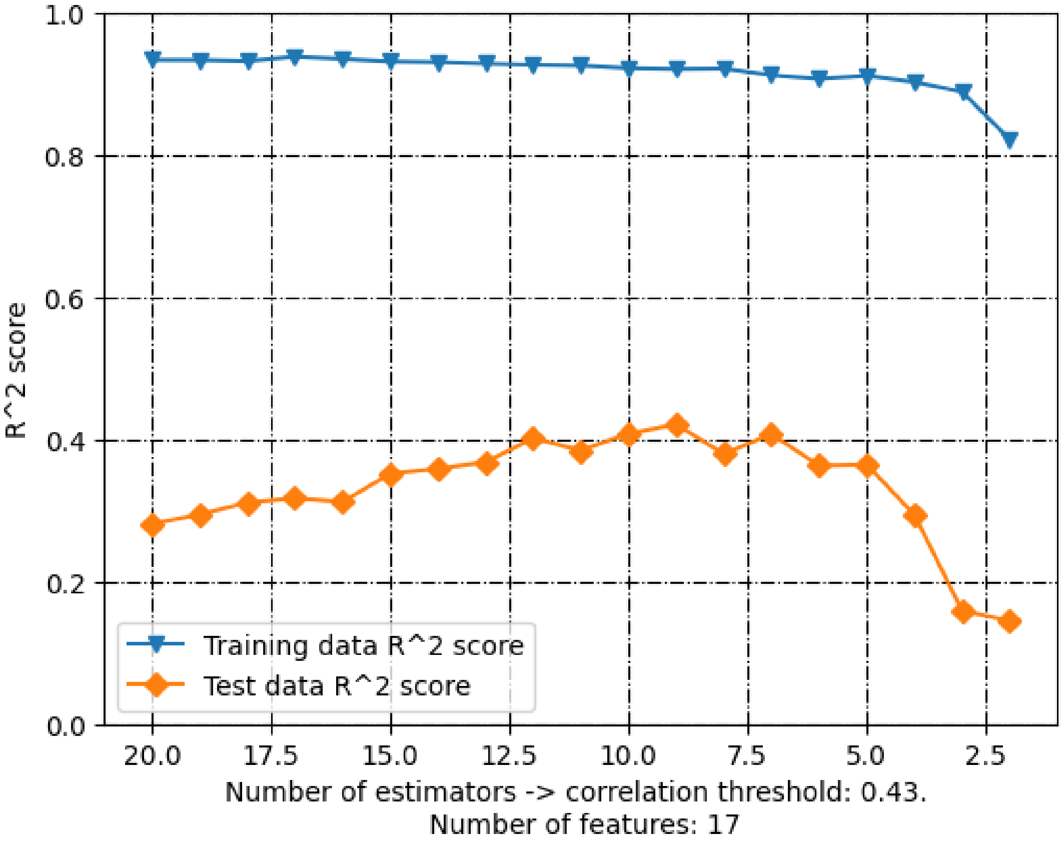

R^2 score
1.0
0.8
0.6
0.4
0.2
0.0
20.0
17.5
15.0
12.5
10.0
7.5
5.0
2.5
Training data R^2 score
Test data R^2 score
Number of estimators -> correlation threshold: 0.43.
Number of features: 17

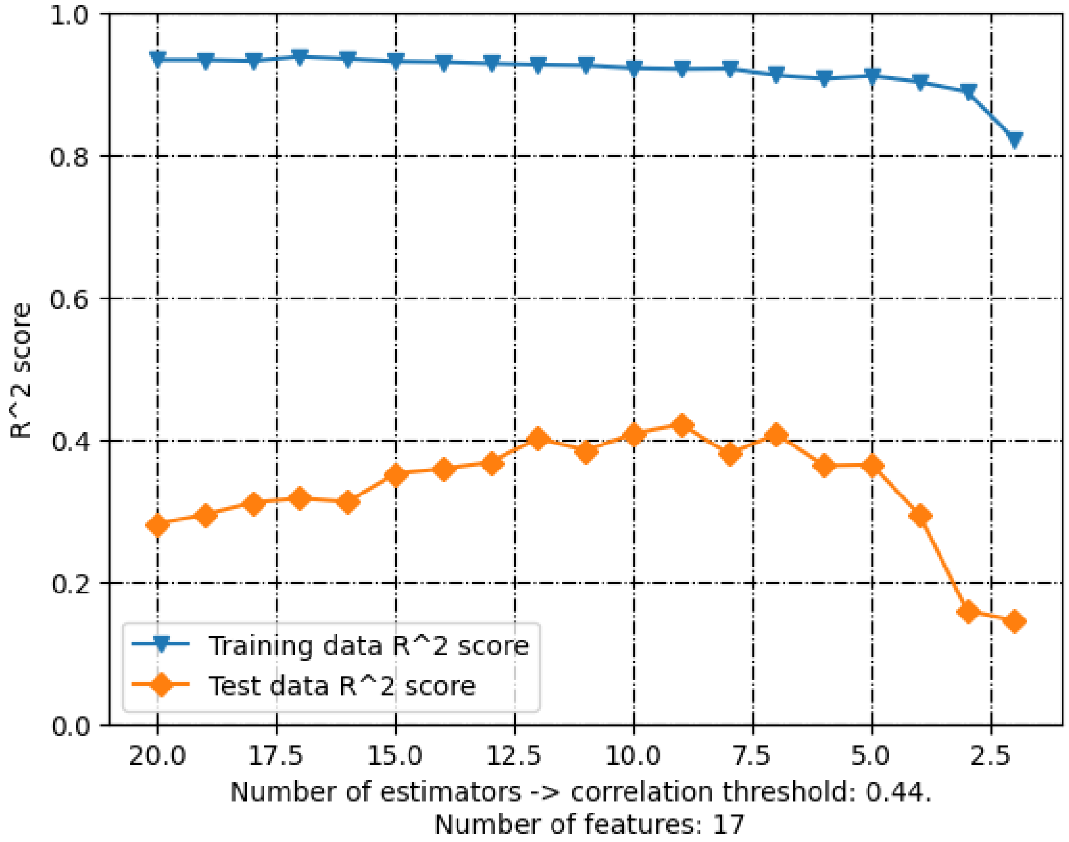
1.0
0.8
0.6
0.4
0.2
0.0
R^2 score
Training data R^2 score
Test data R^2 score
20.0
17.5
15.0
12.5
10.0
7.5
5.0
2.5
Number of estimators -> correlation threshold: 0.44.
Number of features: 17

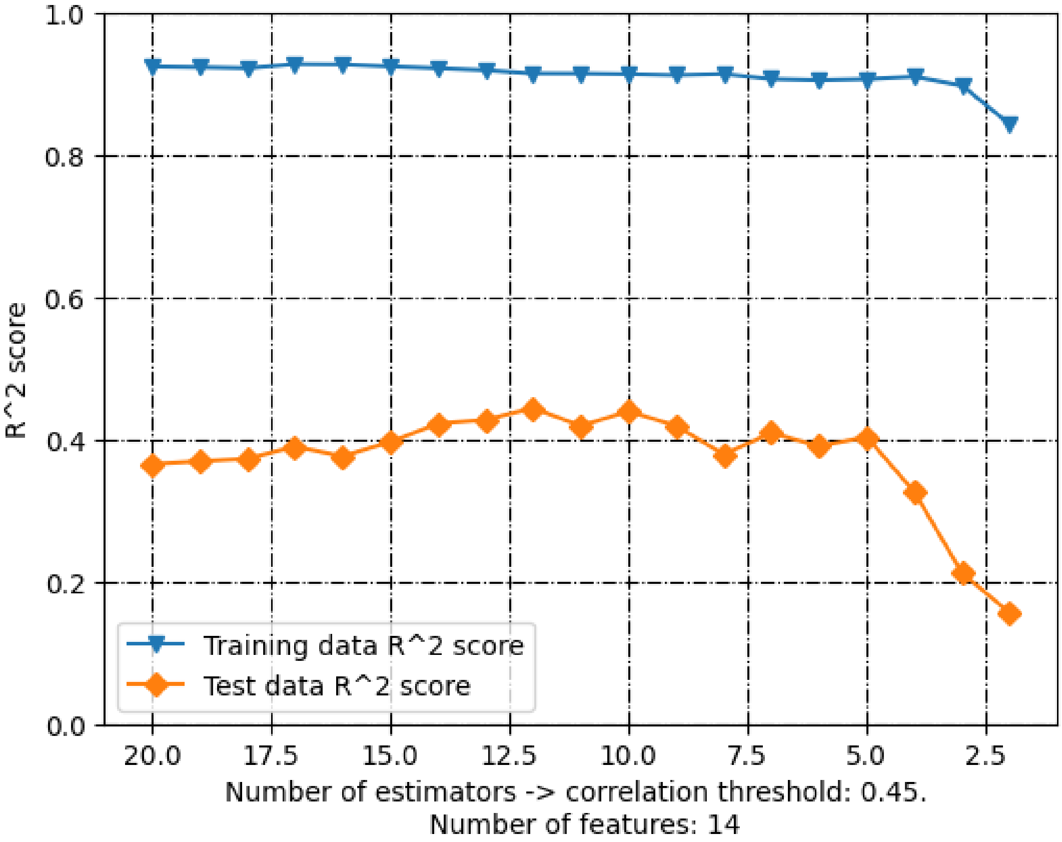

1.0
0.8
0.6
0.4
0.2
0.0
R^2 score
Training data R^2 score
Test data R^2 score
20.0
17.5
15.0
12.5
10.0
7.5
5.0
2.5
Number of estimators -> correlation threshold: 0.45.
Number of features: 14

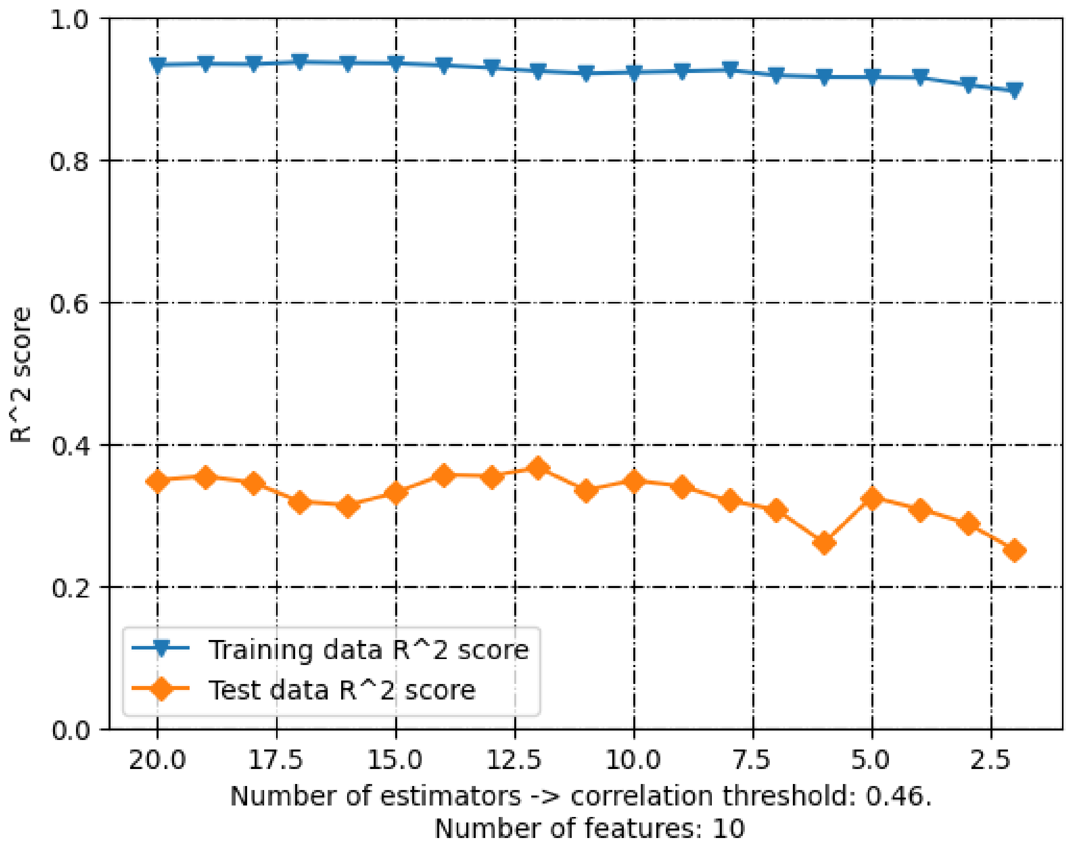

1.0
0.8
0.6
0.4
0.2
0.0
R^2 score
Training data R^2 score
Test data R^2 score
20.0
17.5
15.0
12.5
10.0
7.5
5.0
2.5
Number of estimators -> correlation threshold: 0.46.
Number of features: 10

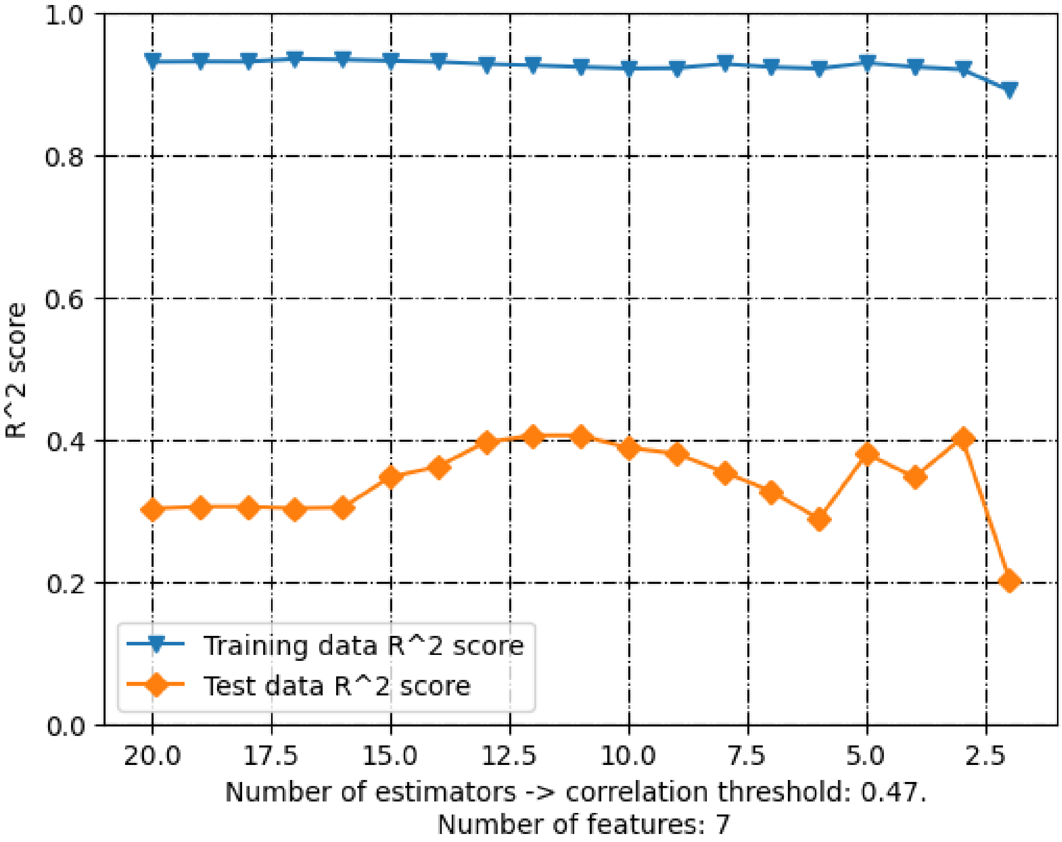
1.0
0.8
0.6
0.4
0.2
0.0
R^2 score
Training data R^2 score
Test data R^2 score
20.0
17.5
15.0
12.5
10.0
7.5
5.0
2.5
Number of estimators -> correlation threshold: 0.47.
Number of features: 7

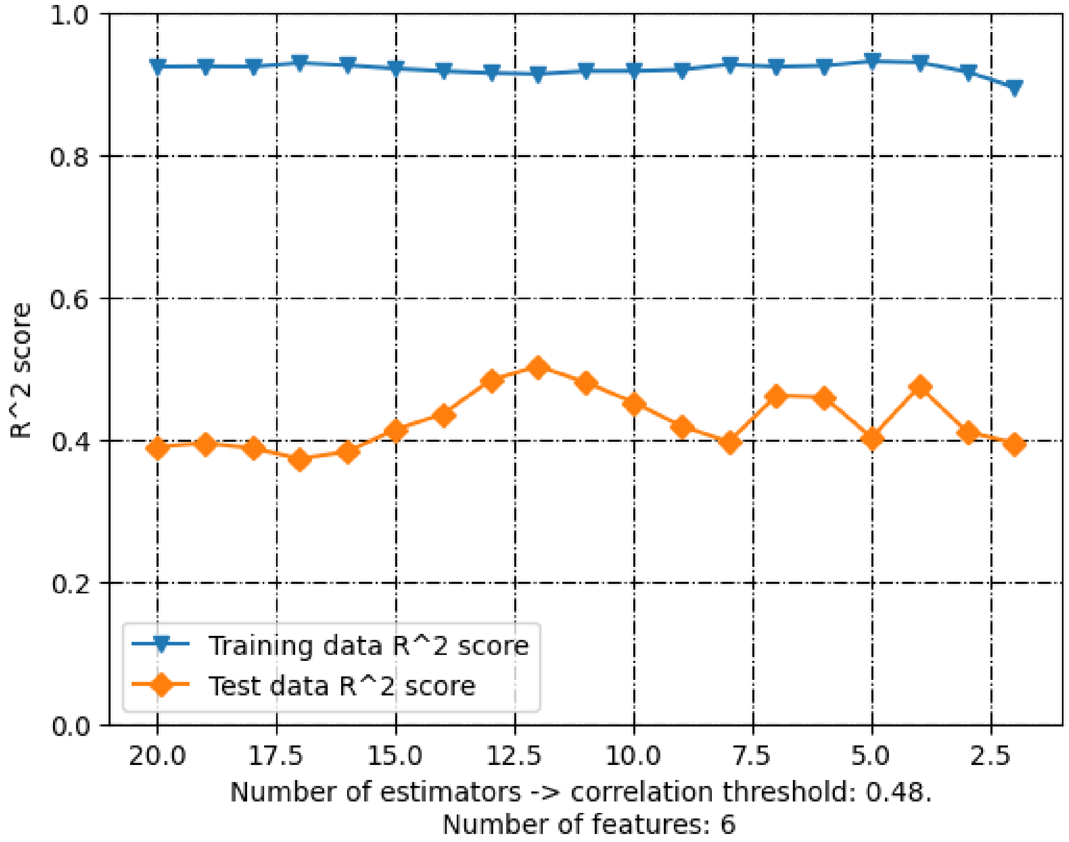
1.0
0.8
0.6
0.4
0.2
0.0
R^2 score
Training data R^2 score
Test data R^2 score
20.0
17.5
15.0
12.5
10.0
7.5
5.0
2.5
Number of estimators -> correlation threshold: 0.48.
Number of features: 6

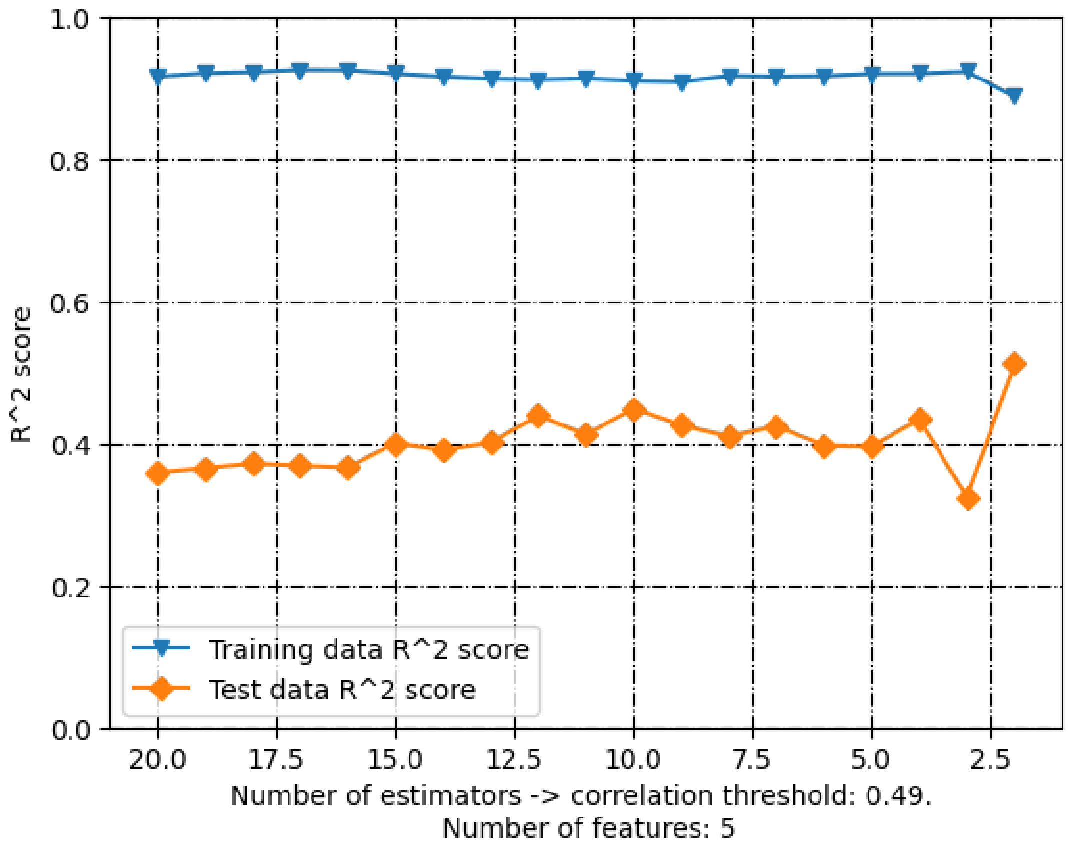
1.0
0.8
0.6
0.4
0.2
0.0
R^2 score
Training data R^2 score
Test data R^2 score
20.0
17.5
15.0
12.5
10.0
7.5
5.0
2.5
Number of estimators -> correlation threshold: 0.49.
Number of features: 5

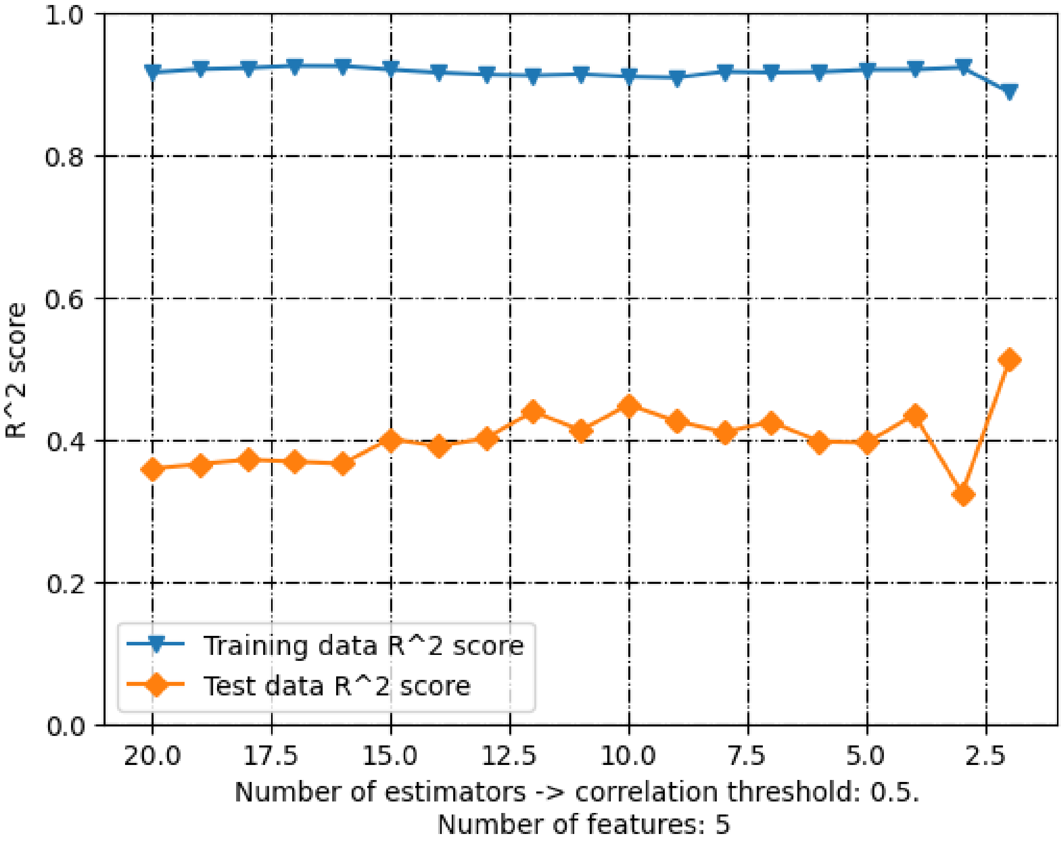

1.0
0.8
0.6
0.4
0.2
0.0
R^2 score
20.0
17.5
15.0
12.5
10.0
7.5
5.0
2.5
Training data R^2 score
Test data R^2 score
Number of estimators -> correlation threshold: 0.5.
Number of features: 5

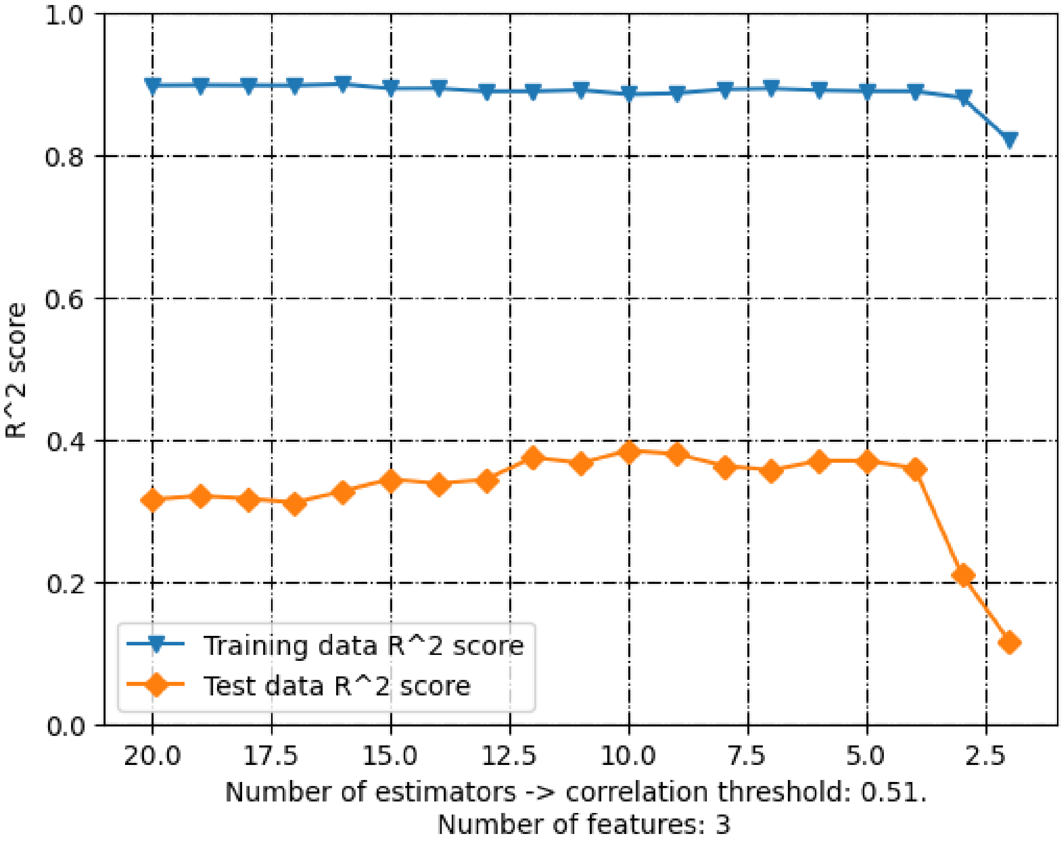

1.0
0.8
0.6
0.4
0.2
0.0
R^2 score
Training data R^2 score
Test data R^2 score
20.0
17.5
15.0
12.5
10.0
7.5
5.0
2.5
Number of estimators -> correlation threshold: 0.51.
Number of features: 3

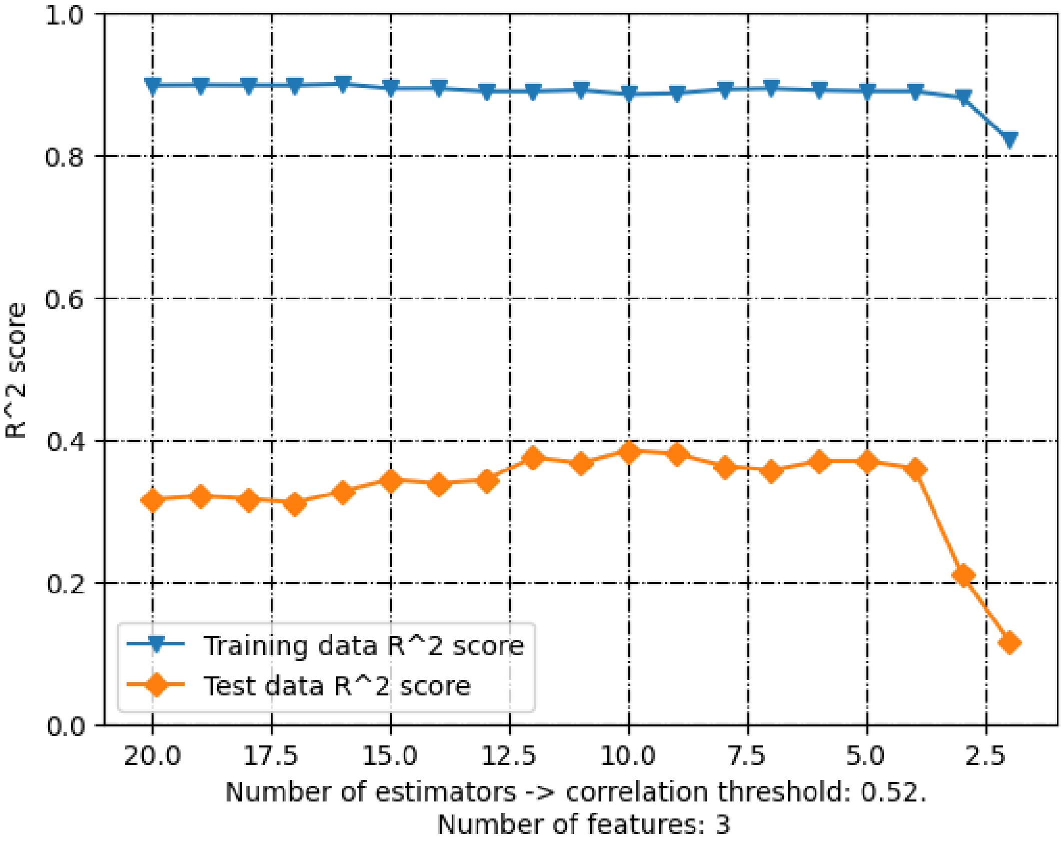
1.0
0.8
0.6
0.4
0.2
0.0
R^2 score
Training data R^2 score
Test data R^2 score
20.0
17.5
15.0
12.5
10.0
7.5
5.0
2.5
Number of estimators -> correlation threshold: 0.52.
Number of features: 3

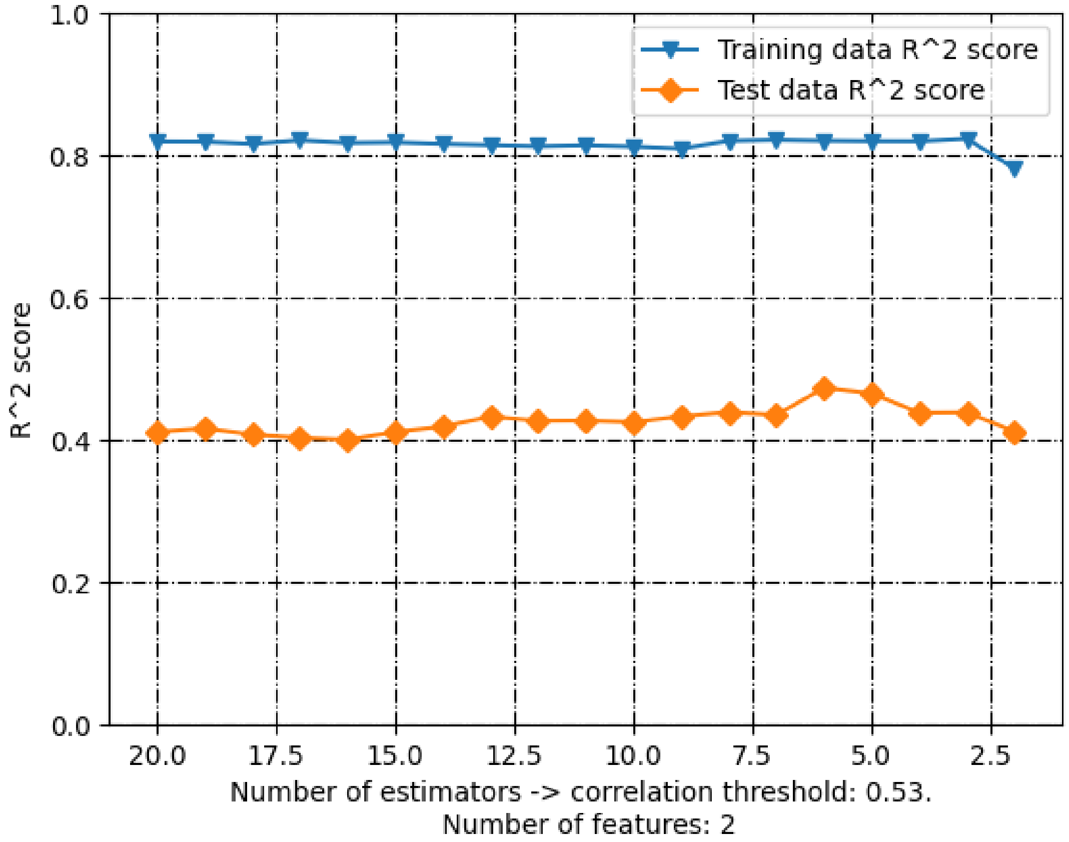

Training data R^2 score
Test data R^2 score
R^2 score
1.0
0.8
0.6
0.4
0.2
0.0
20.0
17.5
15.0
12.5
10.0
7.5
5.0
2.5
Number of estimators -> correlation threshold: 0.53.
Number of features: 2

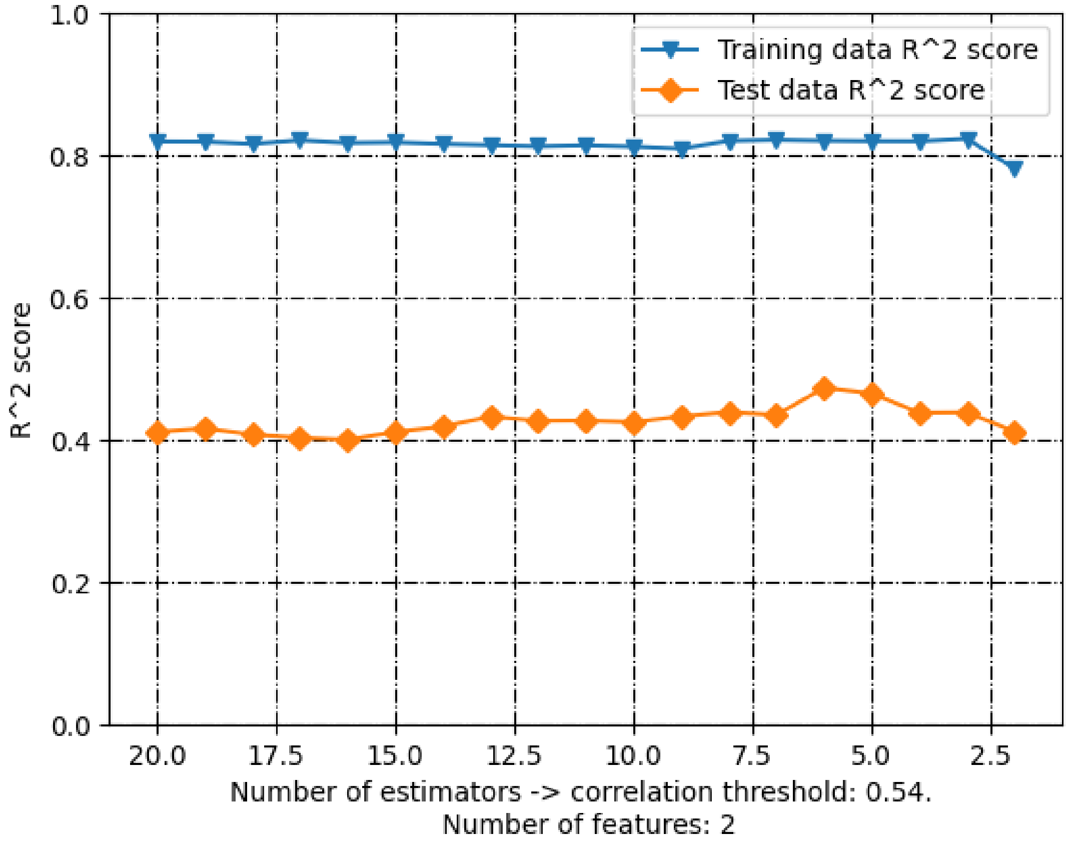

[28]:
```
plt.plot(df_without_standardization['Correlation threshold'],␣
  ↪df_without_standardization['Number of features'], label = "Number of␣
  ↪features", marker='.')
plt.legend()
plt.xlabel('Correlation threshold')
plt.ylabel('Number of features')
plt.show()
```

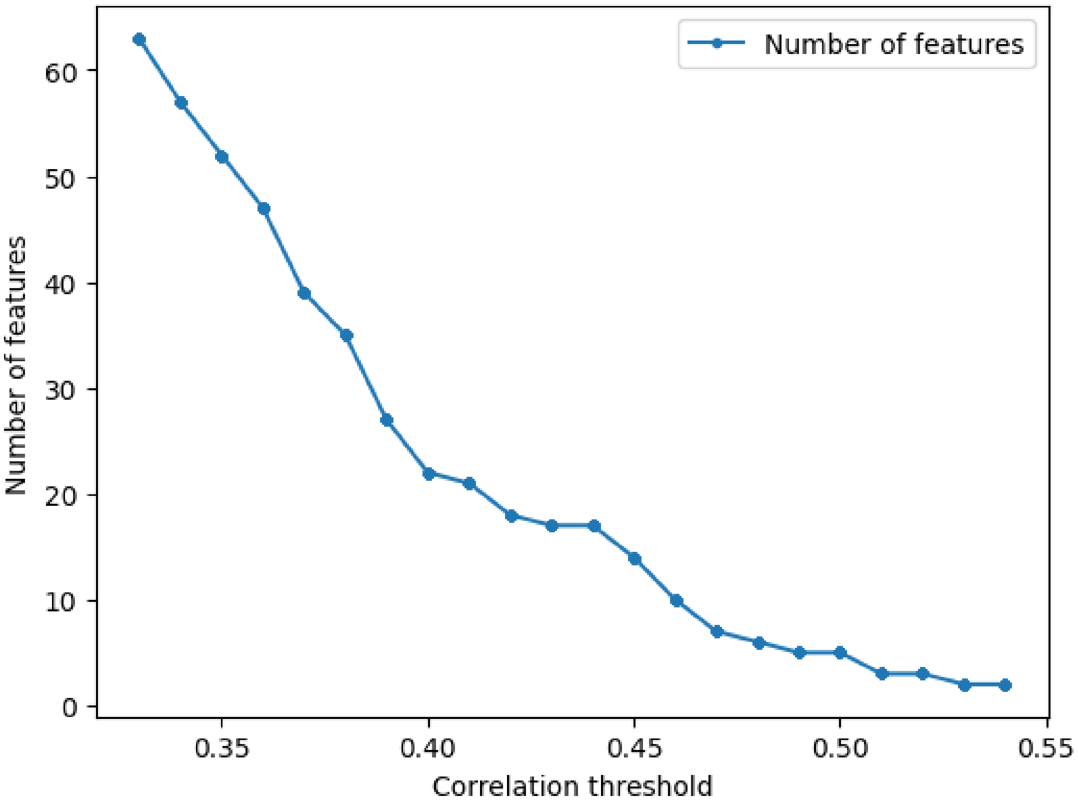


```
[ ]:
```

4 KNeighborsRegressor

```
[29]: without_standardization, train_r2_, test_r2_, hist1, hist2, target_column_name,␣
    ↪training_data_RMSE, test_data_RMSE  = pred_model.
    ↪prepare_data_and_create_model(molecular_descriptors_df=data,
                                                                              ␣
    ↪                       correlation_threshold=0.50,
                                                                              ␣
    ↪                       standardization=False,
                                                                              ␣
    ↪                       model_type='KNeighborsRegressor',
                                                                              ␣
    ↪                       target_column_name = target,
                                                                              ␣
    ↪                       random_state=random_state,
                                                                              ␣
    ↪                       train_test_split_=True,
```

```
↪                    verbose=True)
```

```
I am not doing standardization…
  molecular descriptor name
0                    AATS0Z
1                  AATS0are
2                    AATS0d
3                   AATS0dv
4                    AATS0i
  molecular descriptor name  corr_value
0                    AATS0Z   -0.027037
1                  AATS0are   -0.129823
2                    AATS0d    0.042740
3                   AATS0dv   -0.120173
4                    AATS0i    0.132395
  molecular descriptor name  corr_value  absolute correlation value
0                    AATS0Z   -0.027037                    0.027037
1                  AATS0are   -0.129823                    0.129823
2                    AATS0d    0.042740                    0.042740
3                   AATS0dv   -0.120173                    0.120173
4                    AATS0i    0.132395                    0.132395
     molecular descriptor name  corr_value  absolute correlation value
226                     AMID_O   -0.524157                    0.524157
505                 EState_VSA5  -0.579664                    0.579664
791                    MDEO-12   -0.558727                    0.558727
851                      NdssC   -0.506855                    0.506855
1091                VSA_EState5   0.503578                    0.503578
     molecular descriptor name  corr_value  absolute correlation value
226                     AMID_O   -0.524157                    0.524157
505                 EState_VSA5  -0.579664                    0.579664
791                    MDEO-12   -0.558727                    0.558727
851                      NdssC   -0.506855                    0.506855
1091                VSA_EState5   0.503578                    0.503578
The model used is: KNeighbors…
Return the coefficient of determination of the prediction:
0.21255820544251047
R^2 score: 0.5727423256167272
Correlation coefficient: 0.7567974138544127
Test data - unseen during training:
R^2 score: 0.21255820544251047
Correlation coefficient: 0.46104035120855796
[8.22416706 8.18750292 7.48521712 7.82052038 6.90143604 8.26160013
 7.64548415 7.64404502 7.99494145 7.8041874  7.3418408  7.37284186
 7.62904255 5.73797021 7.37284186 8.22416706 7.8041874  7.64271346]
44      8.508638
47      7.920819
4       7.277366
```

```
55      7.920819
26      5.208801
64      8.327902
73      7.886057
10      8.102373
40      8.050610
107     8.017729
18      7.140261
62      8.657577
11      8.107905
36      7.017729
89      8.638272
91     10.000000
109     7.886057
0       8.187087
Name: LoVo, dtype: float64
Training Root Mean Square Error: 0.5825221417711341
Testing Root Mean Square Error: 0.8167539764598583
```

[30]:

```
print(target_column_name+str('_transformed'))
print(hist1[target_column_name].hist())
```

```
LoVo_transformed
AxesSubplot(0.125,0.11;0.775x0.77)
```

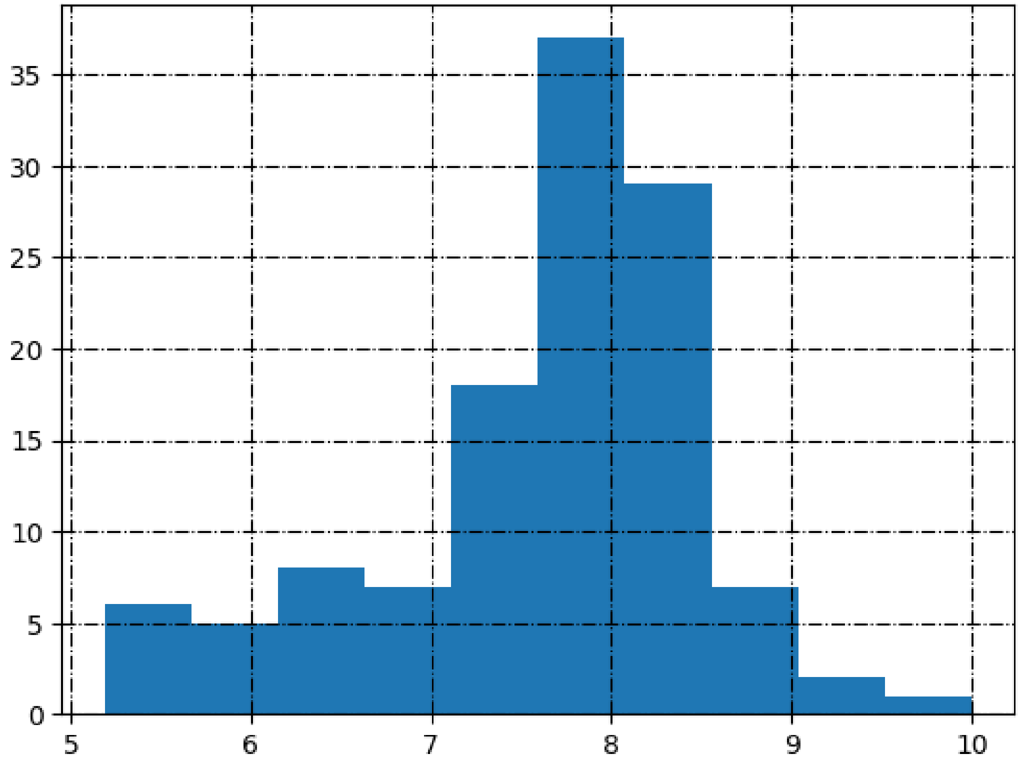

[31]:
```
with_standardization, train_r2_, test_r2_, hist1, hist2, target_column_name,␣
↪training_data_RMSE, test_data_RMSE  = pred_model.
↪prepare_data_and_create_model(molecular_descriptors_df=data,
                                                                              ␣
↪                     correlation_threshold=0.50,
                                                                              ␣
↪                     standardization=True,
                                                                              ␣
↪                     model_type='KNeighborsRegressor',
                                                                              ␣
↪                     target_column_name = target,
                                                                              ␣
↪                     random_state=random_state,
                                                                              ␣
↪                     train_test_split_=True,
                                                                              ␣
↪                     verbose=True)
```

```
I am doing standardization…
  molecular descriptor name
0                   AATS0Z
1                 AATS0are
2                   AATS0d
3                  AATS0dv
4                   AATS0i
  molecular descriptor name  corr_value
0                   AATS0Z   -0.027037
1                 AATS0are   -0.129823
2                   AATS0d    0.042740
3                  AATS0dv   -0.120173
4                   AATS0i    0.132395
  molecular descriptor name  corr_value  absolute correlation value
0                   AATS0Z   -0.027037                    0.027037
1                 AATS0are   -0.129823                    0.129823
2                   AATS0d    0.042740                    0.042740
3                  AATS0dv   -0.120173                    0.120173
4                   AATS0i    0.132395                    0.132395
     molecular descriptor name  corr_value  absolute correlation value
226                     AMID_O   -0.524157                    0.524157
505                 EState_VSA5   -0.579664                    0.579664
791                   MDEO-12   -0.558727                    0.558727
851                     NdssC   -0.506855                    0.506855
1091                VSA_EState5    0.503578                    0.503578
     molecular descriptor name  corr_value  absolute correlation value
226                     AMID_O   -0.524157                    0.524157
505                 EState_VSA5   -0.579664                    0.579664
```

```
791                    MDEO-12   -0.558727                    0.558727
851                      NdssC   -0.506855                    0.506855
1091                VSA_EState5    0.503578                    0.503578
The model used is: KNeighbors…
Return the coefficient of determination of the prediction:
0.21255820544251047
R^2 score: 0.5727423256167272
Correlation coefficient: 0.7567974138544127
Test data - unseen during training:
R^2 score: 0.21255820544251047
Correlation coefficient: 0.46104035120855796
[8.22416706 8.18750292 7.48521712 7.82052038 6.90143604 8.26160013
 7.64548415 7.64404502 7.99494145 7.8041874  7.3418408  7.37284186
 7.62904255 5.73797021 7.37284186 8.22416706 7.8041874  7.64271346]
44      8.508638
47      7.920819
4       7.277366
55      7.920819
26      5.208801
64      8.327902
73      7.886057
10      8.102373
40      8.050610
107     8.017729
18      7.140261
62      8.657577
11      8.107905
36      7.017729
89      8.638272
91     10.000000
109     7.886057
0       8.187087
Name: LoVo, dtype: float64
Training Root Mean Square Error: 0.5825221417711341
Testing Root Mean Square Error: 0.8167539764598583
```

4.1 Search inside correlation space

[32]:
```
step = 0.01
initial_step = corr_low
last_step = corr_high
first_list = [x / 100.0 for x in range(int(initial_step*100),␣
 ↪int(last_step*100), int(step*100))]
second_list = []
third_list = []
fourth_l = []
fifth_l = []
```

```
f_list = []
for i in first_list:
    without_standardization, train_r2, test_r2, _, h_, target_column_name,␣
 ↪training_data_RMSE, test_data_RMSE = pred_model.
 ↪prepare_data_and_create_model(molecular_descriptors_df = data,
                                                                            ␣
 ↪                    correlation_threshold = i,
                                                                            ␣
 ↪                    standardization = False,
                                                                            ␣
 ↪                    model_type = 'KNeighborsRegressor',
                                                                            ␣
 ↪                    target_column_name = target,
                                                                            ␣
 ↪                    random_state=random_state,
                                                                            ␣
 ↪                    train_test_split_ = True,
                                                                            ␣
 ↪                    verbose = False)
    second_list.append(train_r2)
    third_list.append(test_r2)
    fourth_l.append(training_data_RMSE)
    fifth_l.append(test_data_RMSE)
    f_list.append(len(h_))
```

```
  File "C:\Users\aleks\anaconda3\envs\cheminf_gpu\lib\site-
packages\joblib\externals\loky\backend\context.py", line 217, in
_count_physical_cores
    raise ValueError(
```

[33]:
```
df_without_standardization = pd.DataFrame(data=first_list,␣
 ↪columns=["Correlation threshold"])
df_without_standardization['Training data R^2 score'] = second_list
df_without_standardization['Test data R^2 score'] = third_list
df_without_standardization['Training RMSE'] = fourth_l
df_without_standardization['Test RMSE'] = fifth_l
df_without_standardization['Number of features'] = f_list
```

[34]:
```
df_k_nearest = df_without_standardization.copy()
df_without_standardization
```

[34]:
```
   Correlation threshold  Training data R^2 score  Test data R^2 score  \
0                   0.33                 0.593520             0.003552
1                   0.34                 0.593520             0.003552
2                   0.35                 0.648241             0.088554
3                   0.36                 0.648241             0.088554
4                   0.37                 0.662743             0.186736
```

5	0.38	0.617782	0.248856
6	0.39	0.578109	0.161662
7	0.40	0.615540	0.139525
8	0.41	0.615540	0.139525
9	0.42	0.618519	0.251357
10	0.43	0.623715	0.254735
11	0.44	0.623715	0.254735
12	0.45	0.617540	0.179787
13	0.46	0.626009	0.099528
14	0.47	0.612948	0.136080
15	0.48	0.580521	0.208204
16	0.49	0.572742	0.212558
17	0.50	0.572742	0.212558
18	0.51	0.568965	0.147240
19	0.52	0.568965	0.147240
20	0.53	0.535698	0.175973
21	0.54	0.535698	0.175973

	Training RMSE	Test RMSE	Number of features
0	0.568182	0.918775	63
1	0.568182	0.918775	57
2	0.528555	0.878714	52
3	0.528555	0.878714	47
4	0.517545	0.830038	39
5	0.550964	0.797708	35
6	0.578852	0.842736	27
7	0.552577	0.853790	22
8	0.552577	0.853790	21
9	0.550432	0.796378	18
10	0.546671	0.794580	17
11	0.546671	0.794580	17
12	0.551138	0.833577	14
13	0.545002	0.873408	10
14	0.554437	0.855497	7
15	0.577195	0.819009	6
16	0.582522	0.816754	5
17	0.582522	0.816754	5
18	0.585092	0.849954	3
19	0.585092	0.849954	3
20	0.607250	0.835512	2
21	0.607250	0.835512	2

4.2 Plots

[35]:
```
plt.plot(df_without_standardization['Correlation threshold'],␣
 ↪df_without_standardization['Training data R^2 score'], label = "Training␣
 ↪data R^2 score", marker='v')
plt.plot(df_without_standardization['Correlation threshold'],␣
 ↪df_without_standardization['Test data R^2 score'], label = "Test data R^2␣
 ↪score", marker='D')
plt.legend()
plt.xlabel('Correlation threshold')
plt.ylabel('R^2 score')
plt.ylim([0, 1])
plt.show()
```

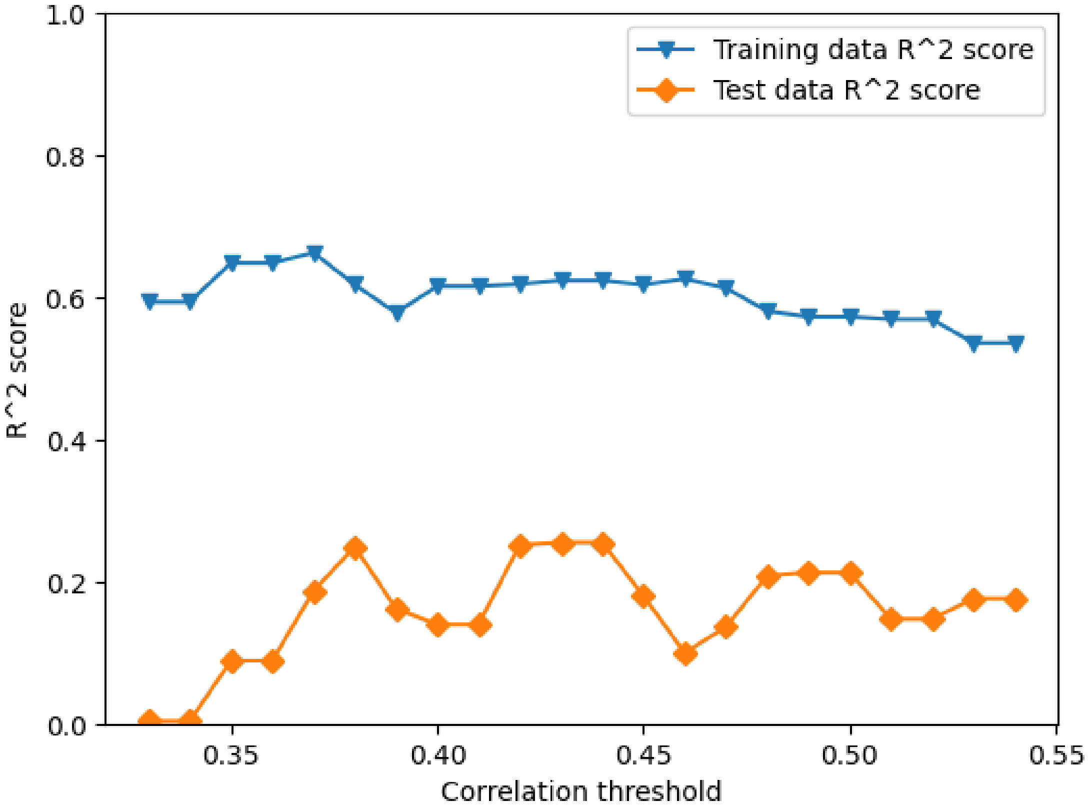

[36]:
```
plt.plot(df_without_standardization['Correlation threshold'],␣
 ↪df_without_standardization['Number of features'], label = "Number of␣
 ↪features", marker='.')
plt.legend()
plt.xlabel('Correlation threshold')
plt.ylabel('Number of features')
plt.show()
```

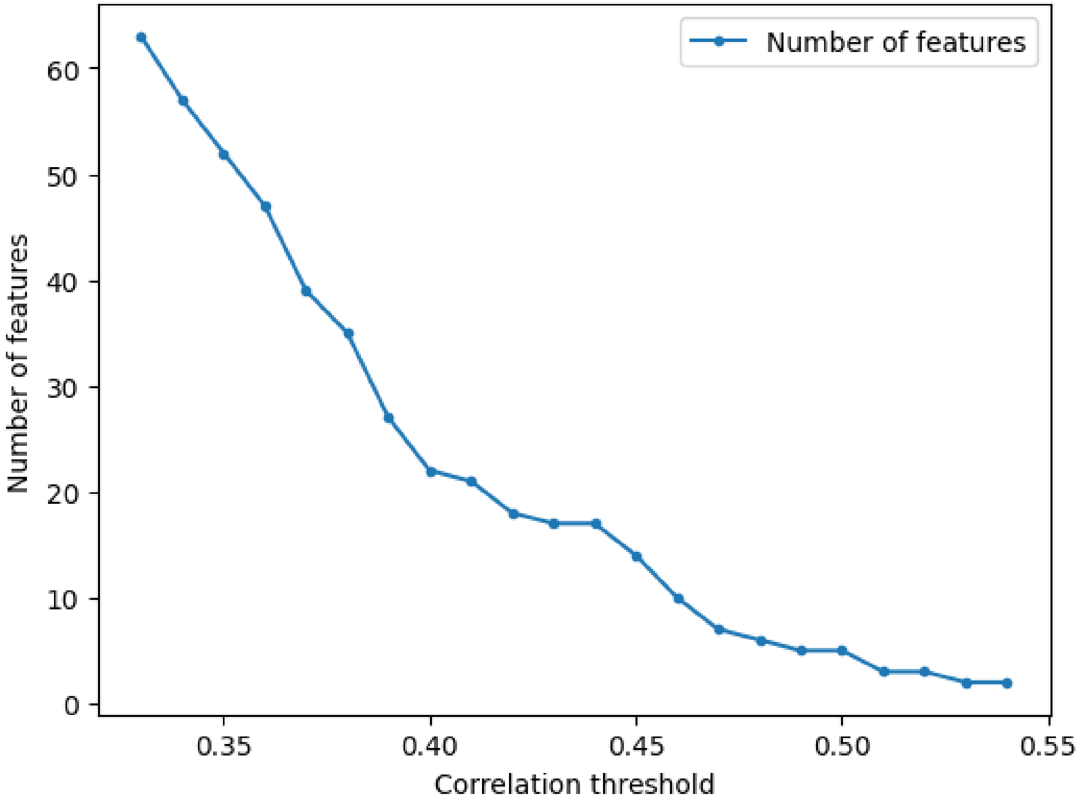

[ ]:

5 Support Vector Machines (SVM)

[37]:
```
without_standardization, train_r2_, test_r2_, hist1, hist2, target_column_name,␣
↪training_data_RMSE, test_data_RMSE  = pred_model.
↪prepare_data_and_create_model(molecular_descriptors_df=data,
                                                                                    ␣
↪                         correlation_threshold=0.50,
                                                                                    ␣
↪                         standardization=False,
                                                                                    ␣
↪                         model_type='SVR',
                                                                                    ␣
↪                         kernel_ = 'linear',
                                                                                    ␣
↪                         gamma_ = 'auto',
                                                                                    ␣
↪                         target_column_name = target,
```

```
↪                    random_state=random_state,
↪                    train_test_split_=True,
↪                    verbose=True)
```

```
I am not doing standardization…
  molecular descriptor name
0                    AATS0Z
1                  AATS0are
2                    AATS0d
3                   AATS0dv
4                    AATS0i
  molecular descriptor name  corr_value
0                    AATS0Z   -0.027037
1                  AATS0are   -0.129823
2                    AATS0d    0.042740
3                   AATS0dv   -0.120173
4                    AATS0i    0.132395
  molecular descriptor name  corr_value  absolute correlation value
0                    AATS0Z   -0.027037                    0.027037
1                  AATS0are   -0.129823                    0.129823
2                    AATS0d    0.042740                    0.042740
3                   AATS0dv   -0.120173                    0.120173
4                    AATS0i    0.132395                    0.132395
     molecular descriptor name  corr_value  absolute correlation value
226                     AMID_O   -0.524157                    0.524157
505                EState_VSA5   -0.579664                    0.579664
791                   MDEO-12   -0.558727                    0.558727
851                      NdssC   -0.506855                    0.506855
1091               VSA_EState5    0.503578                    0.503578
     molecular descriptor name  corr_value  absolute correlation value
226                     AMID_O   -0.524157                    0.524157
505                EState_VSA5   -0.579664                    0.579664
791                   MDEO-12   -0.558727                    0.558727
851                      NdssC   -0.506855                    0.506855
1091               VSA_EState5    0.503578                    0.503578
The model used is: SVR…
Return the coefficient of determination of the prediction:
0.29235327296971636
R^2 score: 0.4921032692822115
Correlation coefficient: 0.7015007265015565
Test data - unseen during training:
R^2 score: 0.29235327296971636
Correlation coefficient: 0.5406970251163921
[8.18453538 8.23239603 7.67600691 7.38657623 6.3760783  7.69978402
```

```
 7.83363999 7.82458324 8.17426745 7.94287493 7.74643631 6.94494391
 7.94177595 6.49276778 7.48384123 8.18609783 7.94631176 7.95017326]
44      8.508638
47      7.920819
4       7.277366
55      7.920819
26      5.208801
64      8.327902
73      7.886057
10      8.102373
40      8.050610
107     8.017729
18      7.140261
62      8.657577
11      8.107905
36      7.017729
89      8.638272
91     10.000000
109     7.886057
0       8.187087
Name: LoVo, dtype: float64
Training Root Mean Square Error: 0.63511915357598
Testing Root Mean Square Error: 0.7742661588653977
```

[38]:

```
print(target_column_name+str('_transformed'))
print(hist1[target_column_name].hist())
```

```
LoVo_transformed
AxesSubplot(0.125,0.11;0.775x0.77)
```

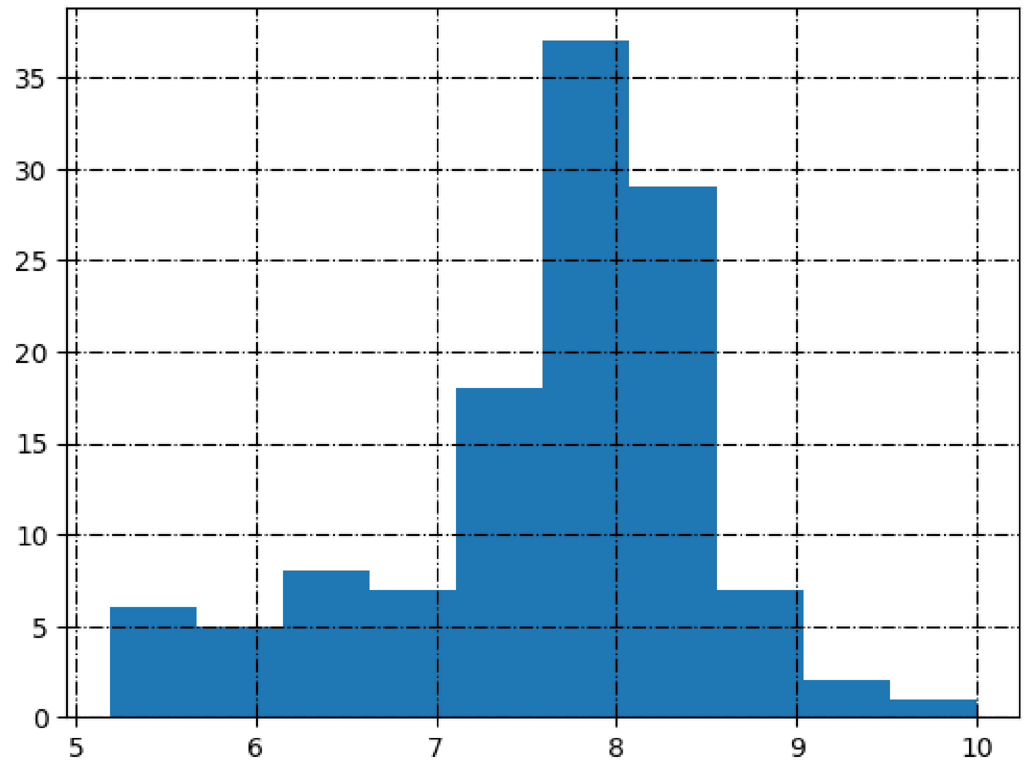


```
[39]: with_standardization, train_r2_, test_r2_, hist1, hist2, target_column_name,␣
      ↪training_data_RMSE, test_data_RMSE  = pred_model.
      ↪prepare_data_and_create_model(molecular_descriptors_df=data,
                                                                                 ␣
      ↪                         correlation_threshold=0.50,
                                                                                 ␣
      ↪                         standardization=True,
                                                                                 ␣
      ↪                         model_type='SVR',
                                                                                 ␣
      ↪                         kernel_ = 'linear',
                                                                                 ␣
      ↪                         gamma_ = 'auto',
                                                                                 ␣
      ↪                         target_column_name = target,
                                                                                 ␣
      ↪                         random_state=random_state,
                                                                                 ␣
      ↪                         train_test_split_=True,
                                                                                 ␣
      ↪                         verbose=True)
```

```
I am doing standardization…
  molecular descriptor name
0                    AATS0Z
1                  AATS0are
2                    AATS0d
3                   AATS0dv
4                    AATS0i
  molecular descriptor name  corr_value
0                    AATS0Z   -0.027037
1                  AATS0are   -0.129823
2                    AATS0d    0.042740
3                   AATS0dv   -0.120173
4                    AATS0i    0.132395
  molecular descriptor name  corr_value  absolute correlation value
0                    AATS0Z   -0.027037                    0.027037
1                  AATS0are   -0.129823                    0.129823
2                    AATS0d    0.042740                    0.042740
3                   AATS0dv   -0.120173                    0.120173
4                    AATS0i    0.132395                    0.132395
     molecular descriptor name  corr_value  absolute correlation value
226                     AMID_O   -0.524157                    0.524157
505                 EState_VSA5   -0.579664                    0.579664
791                    MDEO-12   -0.558727                    0.558727
851                      NdssC   -0.506855                    0.506855
1091                VSA_EState5    0.503578                    0.503578
     molecular descriptor name  corr_value  absolute correlation value
226                     AMID_O   -0.524157                    0.524157
505                 EState_VSA5   -0.579664                    0.579664
791                    MDEO-12   -0.558727                    0.558727
851                      NdssC   -0.506855                    0.506855
1091                VSA_EState5    0.503578                    0.503578
The model used is: SVR…
Return the coefficient of determination of the prediction:
0.29235327296971636
R^2 score: 0.4921032692822115
Correlation coefficient: 0.7015007265015565
Test data - unseen during training:
R^2 score: 0.29235327296971636
Correlation coefficient: 0.5406970251163921
[8.18453538 8.23239603 7.67600691 7.38657623 6.3760783  7.69978402
 7.83363999 7.82458324 8.17426745 7.94287493 7.74643631 6.94494391
 7.94177595 6.49276778 7.48384123 8.18609783 7.94631176 7.95017326]
44      8.508638
47      7.920819
4       7.277366
55      7.920819
26      5.208801
64      8.327902
```

```
73      7.886057
10      8.102373
40      8.050610
107     8.017729
18      7.140261
62      8.657577
11      8.107905
36      7.017729
89      8.638272
91     10.000000
109     7.886057
0       8.187087
Name: LoVo, dtype: float64
Training Root Mean Square Error: 0.6351191535957598
Testing Root Mean Square Error: 0.7742661588653977
```

5.1 Search inside correlation space

[40]:
```
step = 0.01
initial_step = corr_low
last_step = corr_high
first_list = [x / 100.0 for x in range(int(initial_step*100),␣
 ↪int(last_step*100), int(step*100))]
second_list = []
third_list = []
fourth_l = []
fifth_l = []
f_list = []
for i in first_list:
    without_standardization, train_r2, test_r2, _, h_, target_column_name,␣
 ↪training_data_RMSE, test_data_RMSE = pred_model.
 ↪prepare_data_and_create_model(molecular_descriptors_df = data,
                                                                                     ␣
 ↪                     correlation_threshold = i,
                                                                                     ␣
 ↪                     standardization = False,
                                                                                     ␣
 ↪                     model_type = 'SVR',
                                                                                     ␣
 ↪                     kernel_ = 'linear',
                                                                                     ␣
 ↪                     gamma_ = 'auto',
                                                                                     ␣
 ↪                     target_column_name = target,
                                                                                     ␣
 ↪                     random_state=random_state,
```

```
                          train_test_split_ = True,
                          verbose = False)
    second_list.append(train_r2)
    third_list.append(test_r2)
    fourth_l.append(training_data_RMSE)
    fifth_l.append(test_data_RMSE)
    f_list.append(len(h_))
```

[41]:
```
df_without_standardization = pd.DataFrame(data=first_list,
  columns=["Correlation threshold"])
df_without_standardization['Training data R^2 score'] = second_list
df_without_standardization['Test data R^2 score'] = third_list
df_without_standardization['Training RMSE'] = fourth_l
df_without_standardization['Test RMSE'] = fifth_l
df_without_standardization['Number of features'] = f_list
```

[42]:
```
df_svm = df_without_standardization.copy()
df_without_standardization
```

[42]:
```
    Correlation threshold  Training data R^2 score  Test data R^2 score  \
0                    0.33                 0.637913             0.404687
1                    0.34                 0.636394             0.344376
2                    0.35                 0.622530             0.504512
3                    0.36                 0.632914             0.500235
4                    0.37                 0.605979             0.403215
5                    0.38                 0.603589             0.404943
6                    0.39                 0.587512             0.429481
7                    0.40                 0.555427             0.215861
8                    0.41                 0.555160             0.214816
9                    0.42                 0.565297             0.318582
10                   0.43                 0.564830             0.317756
11                   0.44                 0.564830             0.317756
12                   0.45                 0.551738             0.321373
13                   0.46                 0.538088             0.283675
14                   0.47                 0.492210             0.290894
15                   0.48                 0.492340             0.293198
16                   0.49                 0.492103             0.292353
17                   0.50                 0.492103             0.292353
18                   0.51                 0.462275             0.366926
19                   0.52                 0.462275             0.366926
20                   0.53                 0.461516             0.367015
21                   0.54                 0.461516             0.367015

    Training RMSE  Test RMSE  Number of features
0        0.536258   0.710157                  63
```

1	0.537382	0.745263	57
2	0.547531	0.647886	52
3	0.539947	0.650676	47
4	0.559406	0.711035	39
5	0.561100	0.710005	35
6	0.572365	0.695212	27
7	0.594209	0.815039	22
8	0.594387	0.815582	21
9	0.587576	0.759782	18
10	0.587891	0.760242	17
11	0.587891	0.760242	17
12	0.596669	0.758224	14
13	0.605685	0.778999	10
14	0.635052	0.775064	7
15	0.634971	0.773804	6
16	0.635119	0.774266	5
17	0.635119	0.774266	5
18	0.653503	0.732334	3
19	0.653503	0.732334	3
20	0.653964	0.732283	2
21	0.653964	0.732283	2

5.2 Plots

[43]:
```
plt.plot(df_without_standardization['Correlation threshold'],␣
 ↪df_without_standardization['Training data R^2 score'], label = "Training␣
 ↪data R^2 score", marker='v')
plt.plot(df_without_standardization['Correlation threshold'],␣
 ↪df_without_standardization['Test data R^2 score'], label = "Test data R^2␣
 ↪score", marker='D')
plt.legend()
plt.xlabel('Correlation threshold')
plt.ylabel('R^2 score')
plt.ylim([0, 1])
plt.show()
```

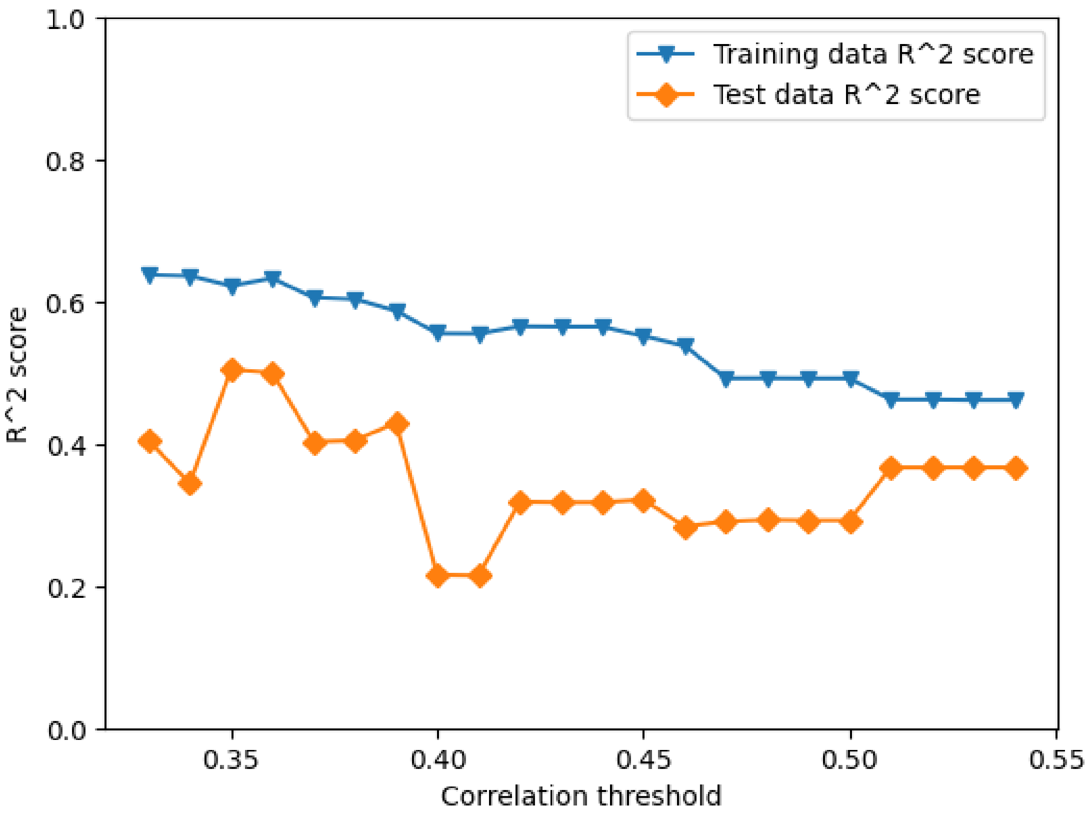

[44]:

```
plt.plot(df_without_standardization['Correlation threshold'],␣
↪df_without_standardization['Number of features'], label = "Number of␣
↪features", marker='.')
plt.legend()
plt.xlabel('Correlation threshold')
plt.ylabel('Number of features')
plt.show()
```

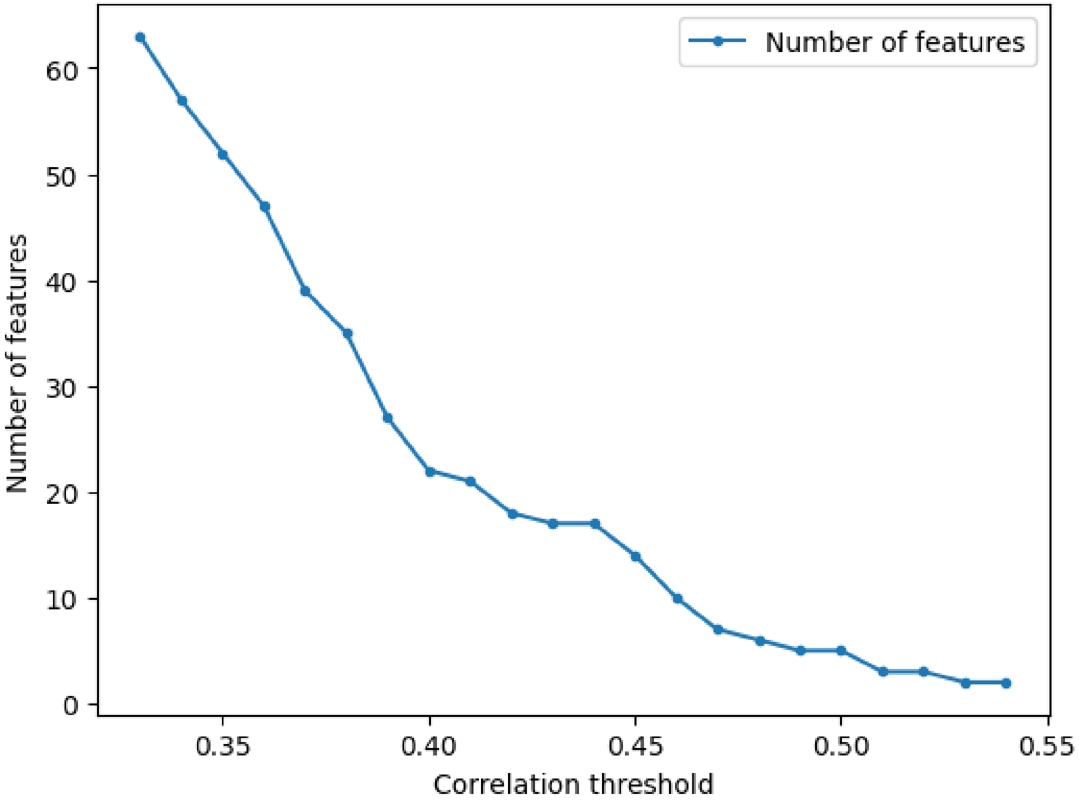

5.3 Store quality measurements

[45]:
```
with pd.ExcelWriter('../Data/Quality_'+str(target)+'_'+str(random_state)+'_.
 ↪xlsx') as writer:
    df_linear.to_excel(writer, sheet_name='MLR')
    df_decision_tree.to_excel(writer, sheet_name='DT')
    df_random_forest.to_excel(writer, sheet_name='RF')
    df_k_nearest.to_excel(writer, sheet_name='KNN')
    df_svm.to_excel(writer, sheet_name='SVM')
```

Notebook

January 18, 2024

1 File 27

[1]:
```
import sys
from pathlib import Path
prediction_mode_path = Path("../module")
sys.path.append(prediction_mode_path.as_posix())
import models_creation as pred_model

import pandas as pd
import numpy as np

from sklearn.metrics import r2_score

import matplotlib.pyplot as plt
from sklearn.tree import plot_tree

from matplotlib import pyplot as plt

import joblib

import warnings
warnings.filterwarnings('ignore')
```

[2]:
```
load_prepared_data = pd.read_excel('../Data/Kolchicyna_machine_learning.xlsx')
load_prepared_data = load_prepared_data.drop(columns=['Unnamed: 0'])

target = 'MCF-7'

corr_low = 0.33
corr_high = 0.525

random_state = 15
```

[3]:
```
load_prepared_data.head()
```

[3]:
```
     AATS0Z  AATS0are    AATS0d   AATS0dv      AATS0i     AATS0m    AATS0p  \
0  25.090909  6.046518  3.109091  6.662626  160.775045  99.075564  1.555512
1  24.448276  6.008422  3.051724  6.386973  161.021674  96.473315  1.538471
```

```
2  23.868852  5.974074  3.000000  6.138434  161.244045  94.127025  1.523105
3  23.868852  5.974074  3.032787  6.171220  161.244045  94.127025  1.523105
4  23.343750  5.942945  2.953125  5.913194  161.445569  92.000700  1.509180

    AATS0pe    AATS0s   AATS0se  …  A549_float  MCF-7_float  LoVo_float  \
0  6.126680  3.435416  7.629859  …        10.8         10.3         6.5
1  6.088791  3.330998  7.596891  …        11.6         12.0         8.5
2  6.054630  3.236850  7.567166  …        10.9         12.2         8.8
3  6.054630  3.257798  7.567166  …        10.5         11.3         8.5
4  6.023670  3.151529  7.540228  …        89.5         92.7        52.8

   LoVo/DX_float  BALB/3T3_float  A549_float_transformed  \
0           54.9            10.2                7.966576
1           31.1            14.3                7.935542
2           17.9            11.7                7.962574
3           10.2            11.0                7.978811
4           77.8            99.4                7.048177

   MCF-7_float_transformed  LoVo_float_transformed  LoVo/DX_float_transformed  \
0                 7.987163                8.187087                   7.260428
1                 7.920819                8.070581                   7.507240
2                 7.913640                8.055517                   7.747147
3                 7.946922                8.070581                   7.991400
4                 7.032920                7.277366                   7.109020

   BALB/3T3_float_transformed
0                    7.991400
1                    7.844664
2                    7.931814
3                    7.958607
4                    7.002614

[5 rows x 1221 columns]
```

[4]:

```
data = load_prepared_data[list(load_prepared_data.columns)[0:-10]]
data[target] = load_prepared_data[load_prepared_data.columns[-4]]
data.head()
```

[4]:

```
      AATS0Z  AATS0are    AATS0d   AATS0dv      AATS0i     AATS0m    AATS0p  \
0  25.090909  6.046518  3.109091  6.662626  160.775045  99.075564  1.555512
1  24.448276  6.008422  3.051724  6.386973  161.021674  96.473315  1.538471
2  23.868852  5.974074  3.000000  6.138434  161.244045  94.127025  1.523105
3  23.868852  5.974074  3.032787  6.171220  161.244045  94.127025  1.523105
4  23.343750  5.942945  2.953125  5.913194  161.445569  92.000700  1.509180

    AATS0pe    AATS0s   AATS0se  …    piPC10     piPC2     piPC3     piPC4  \
0  6.126680  3.435416  7.629859  …  7.999655  4.266195  4.915408  5.516700
```

```
1  6.088791  3.330998  7.596891  …  8.018726  4.280132  4.922714  5.520711
2  6.054630  3.236850  7.567166  …  8.028799  4.293878  4.929967  5.524706
3  6.054630  3.257798  7.567166  …  8.037440  4.307438  4.929967  5.524706
4  6.023670  3.151529  7.540228  …  8.034892  4.307438  4.937168  5.528685

      piPC5     piPC6     piPC7     piPC8     piPC9     MCF-7
0  6.098566  6.595759  6.979116  7.337313  7.681445  7.987163
1  6.103048  6.601209  6.985613  7.349442  7.695531  7.920819
2  6.105281  6.603923  6.989306  7.353932  7.704023  7.913640
3  6.107510  6.606629  6.992068  7.361425  7.709420  7.946922
4  6.107510  6.605277  6.991148  7.356489  7.707175  7.032920

[5 rows x 1212 columns]
```

2 Multiple Linear regression (MLR)

[5]:
```
without_standardization, train_r2_, test_r2_, hist1, hist2, target_column_name,␣
↪training_data_RMSE, test_data_RMSE  = pred_model.
↪prepare_data_and_create_model(molecular_descriptors_df=data,
                                                                                ␣
↪                    correlation_threshold=0.5,
                                                                                ␣
↪                    standardization=False,
                                                                                ␣
↪                    model_type='linear_model',
                                                                                ␣
↪                    target_column_name = str(target),
                                                                                ␣
↪                    random_state=random_state,
                                                                                ␣
↪                    train_test_split_=True,
                                                                                ␣
↪                    verbose=True)
```

```
I am not doing standardization…
  molecular descriptor name
0                   AATS0Z
1                 AATS0are
2                   AATS0d
3                  AATS0dv
4                   AATS0i
  molecular descriptor name  corr_value
0                   AATS0Z   -0.038502
1                 AATS0are   -0.120847
2                   AATS0d    0.041648
3                  AATS0dv   -0.115899
```

```
4                   AATS0i    0.191765
  molecular descriptor name  corr_value  absolute correlation value
0                   AATS0Z   -0.038502                    0.038502
1                 AATS0are   -0.120847                    0.120847
2                   AATS0d    0.041648                    0.041648
3                  AATS0dv   -0.115899                    0.115899
4                   AATS0i    0.191765                    0.191765
     molecular descriptor name  corr_value  absolute correlation value
226                     AMID_O   -0.520578                    0.520578
505                EState_VSA5   -0.578904                    0.578904
506                EState_VSA6    0.519794                    0.519794
791                    MDEO-12   -0.525441                    0.525441
1211                     MCF-7    1.000000                    1.000000
    molecular descriptor name  corr_value  absolute correlation value
226                    AMID_O   -0.520578                    0.520578
505               EState_VSA5   -0.578904                    0.578904
506               EState_VSA6    0.519794                    0.519794
791                   MDEO-12   -0.525441                    0.525441
The model used is: LinearReg…
Return the coefficient of determination of the prediction:
0.6355287275624781
R^2 score: 0.4907381530827223
Correlation coefficient: 0.7005270537835939
Test data - unseen during training:
R^2 score: 0.6355287275624781
Correlation coefficient: 0.7972005566747167
[7.74208566 5.90539287 7.24041213 8.06935086 7.94619102 8.0188816
 7.92716129 7.61131341 7.94011305 8.11595796 7.31547916 8.37382755
 7.78046818 8.34838516 7.88293042 7.89304006 6.60782536 5.21892825]
102    7.958607
38     6.022276
8      5.949079
109    8.086186
11     8.013228
51     8.000000
88     8.119186
21     8.113509
82     7.982967
98     7.795880
58     7.677781
64     8.619789
74     8.327902
103    8.376751
91     9.154902
43     7.958607
25     4.949659
30     6.010550
Name: MCF-7, dtype: float64
```

```
Training Root Mean Square Error: 0.6639506858888914
Testing Root Mean Square Error: 0.6505219513902984
```

[6]:
```
print(target_column_name+str('_transformed'))
print(hist1[target_column_name].hist())
```

```
MCF-7_transformed
AxesSubplot(0.125,0.11;0.775x0.77)
```

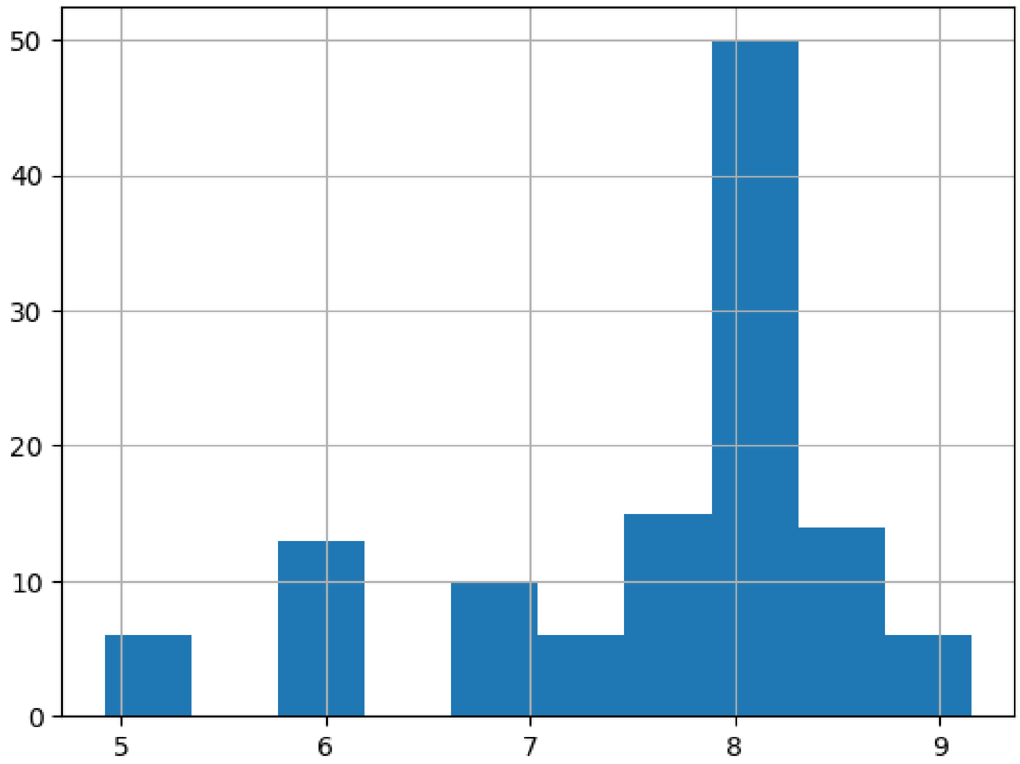

[7]:
```
with_standardization, train_r2_, test_r2_, hist1, hist2, target_column_name,␣
 ↪training_data_RMSE, test_data_RMSE  = pred_model.
 ↪prepare_data_and_create_model(molecular_descriptors_df=data,
                                                                            ␣
 ↪                       correlation_threshold=0.50,
                                                                            ␣
 ↪                       standardization=True,
                                                                            ␣
 ↪                       model_type='linear_model',
                                                                            ␣
 ↪                       target_column_name = target,
                                                                            ␣
 ↪                       random_state=random_state,
```

```
                    train_test_split_=True,
                    verbose=True)
```

```
I am doing standardization…
  molecular descriptor name
0                    AATS0Z
1                  AATS0are
2                    AATS0d
3                   AATS0dv
4                    AATS0i
  molecular descriptor name  corr_value
0                    AATS0Z   -0.038502
1                  AATS0are   -0.120847
2                    AATS0d    0.041648
3                   AATS0dv   -0.115899
4                    AATS0i    0.191765
  molecular descriptor name  corr_value  absolute correlation value
0                    AATS0Z   -0.038502                    0.038502
1                  AATS0are   -0.120847                    0.120847
2                    AATS0d    0.041648                    0.041648
3                   AATS0dv   -0.115899                    0.115899
4                    AATS0i    0.191765                    0.191765
     molecular descriptor name  corr_value  absolute correlation value
226                     AMID_O   -0.520578                    0.520578
505                EState_VSA5   -0.578904                    0.578904
506                EState_VSA6    0.519794                    0.519794
791                    MDEO-12   -0.525441                    0.525441
1211                     MCF-7    1.000000                    1.000000
    molecular descriptor name  corr_value  absolute correlation value
226                    AMID_O   -0.520578                    0.520578
505               EState_VSA5   -0.578904                    0.578904
506               EState_VSA6    0.519794                    0.519794
791                   MDEO-12   -0.525441                    0.525441
The model used is: LinearReg…
Return the coefficient of determination of the prediction:
0.6355287275624781
R^2 score: 0.4907381530827223
Correlation coefficient: 0.7005270537835939
Test data - unseen during training:
R^2 score: 0.6355287275624781
Correlation coefficient: 0.7972005566747167
[7.74208566 5.90539287 7.24041213 8.06935086 7.94619102 8.0188816
 7.92716129 7.61131341 7.94011305 8.11595796 7.31547916 8.37382755
 7.78046818 8.34838516 7.88293042 7.89304006 6.60782536 5.21892825]
102    7.958607
38     6.022276
```

```
8      5.949079
109    8.086186
11     8.013228
51     8.000000
88     8.119186
21     8.113509
82     7.982967
98     7.795880
58     7.677781
64     8.619789
74     8.327902
103    8.376751
91     9.154902
43     7.958607
25     4.949659
30     6.010550
Name: MCF-7, dtype: float64
Training Root Mean Square Error: 0.6639506858888914
Testing Root Mean Square Error: 0.6505219513902984
```

2.1 Search inside correlation space

[8]:
```
step = 0.01
initial_step = corr_low
last_step = corr_high
first_list = [x / 100.0 for x in range(int(initial_step*100),␣
↪int(last_step*100), int(step*100))]
second_list = []
third_list = []
fourth_l = []
fifth_l = []
f_list = []
for i in first_list:
    without_standardization, train_r2, test_r2, _, h_, target_column_name,␣
↪training_data_RMSE, test_data_RMSE = pred_model.
↪prepare_data_and_create_model(molecular_descriptors_df = data,
                                                                        ␣
↪                   correlation_threshold = i,
                                                                        ␣
↪                   standardization = False,
                                                                        ␣
↪                   model_type = 'linear_model',
                                                                        ␣
↪                   target_column_name = target,
                                                                        ␣
↪                   random_state=random_state,
```

```
                         train_test_split_ = True,
                         verbose = False)
    second_list.append(train_r2)
    third_list.append(test_r2)
    fourth_l.append(training_data_RMSE)
    fifth_l.append(test_data_RMSE)
    f_list.append(len(h_))
```

[9]:
```
df_without_standardization = pd.DataFrame(data=first_list,␣
 ↪columns=["Correlation threshold"])
df_without_standardization['Training data R^2 score'] = second_list
df_without_standardization['Test data R^2 score'] = third_list
df_without_standardization['Training RMSE'] = fourth_l
df_without_standardization['Test RMSE'] = fifth_l
df_without_standardization['Number of features'] = f_list
```

[10]:
```
df_linear = df_without_standardization.copy()
df_without_standardization
```

[10]:

	Correlation threshold	Training data R^2 score	Test data R^2 score	\
0	0.33	0.978516	-0.094412	
1	0.34	0.957841	0.477894	
2	0.35	0.929150	0.168513	
3	0.36	0.875844	0.523529	
4	0.37	0.807568	0.617056	
5	0.38	0.777231	0.661517	
6	0.39	0.685249	0.746559	
7	0.40	0.657338	0.712357	
8	0.41	0.657259	0.714074	
9	0.42	0.635363	0.677386	
10	0.43	0.550125	0.694509	
11	0.44	0.549982	0.690317	
12	0.45	0.546381	0.690199	
13	0.46	0.528483	0.659053	
14	0.47	0.527007	0.647292	
15	0.48	0.515703	0.646798	
16	0.49	0.510924	0.637683	
17	0.50	0.490738	0.635529	
18	0.51	0.490738	0.635529	

	Training RMSE	Test RMSE	Number of features
0	0.136373	1.127251	84
1	0.191034	0.778591	74
2	0.247648	0.982558	61
3	0.327831	0.743787	50

4	0.408135	0.666803	43
5	0.439130	0.626901	37
6	0.521974	0.542462	29
7	0.544625	0.577906	26
8	0.544689	0.576179	25
9	0.561818	0.612029	19
10	0.624038	0.595566	14
11	0.624138	0.599638	13
12	0.626630	0.599752	11
13	0.638872	0.629178	10
14	0.639871	0.639938	9
15	0.647472	0.640386	8
16	0.650659	0.648596	6
17	0.663951	0.650522	4
18	0.663951	0.650522	4

[ ]:

```

```

2.2 Plots

[11]:

```
plt.plot(df_without_standardization['Correlation threshold'],␣
 ↪df_without_standardization['Training data R^2 score'], label = "Training␣
 ↪data R^2 score", marker='v')
plt.plot(df_without_standardization['Correlation threshold'],␣
 ↪df_without_standardization['Test data R^2 score'], label = "Test data R^2␣
 ↪score", marker='D')
plt.legend()
plt.xlabel('Correlation threshold')
plt.ylabel('R^2 score')
plt.ylim([0, 1])
plt.show()
```

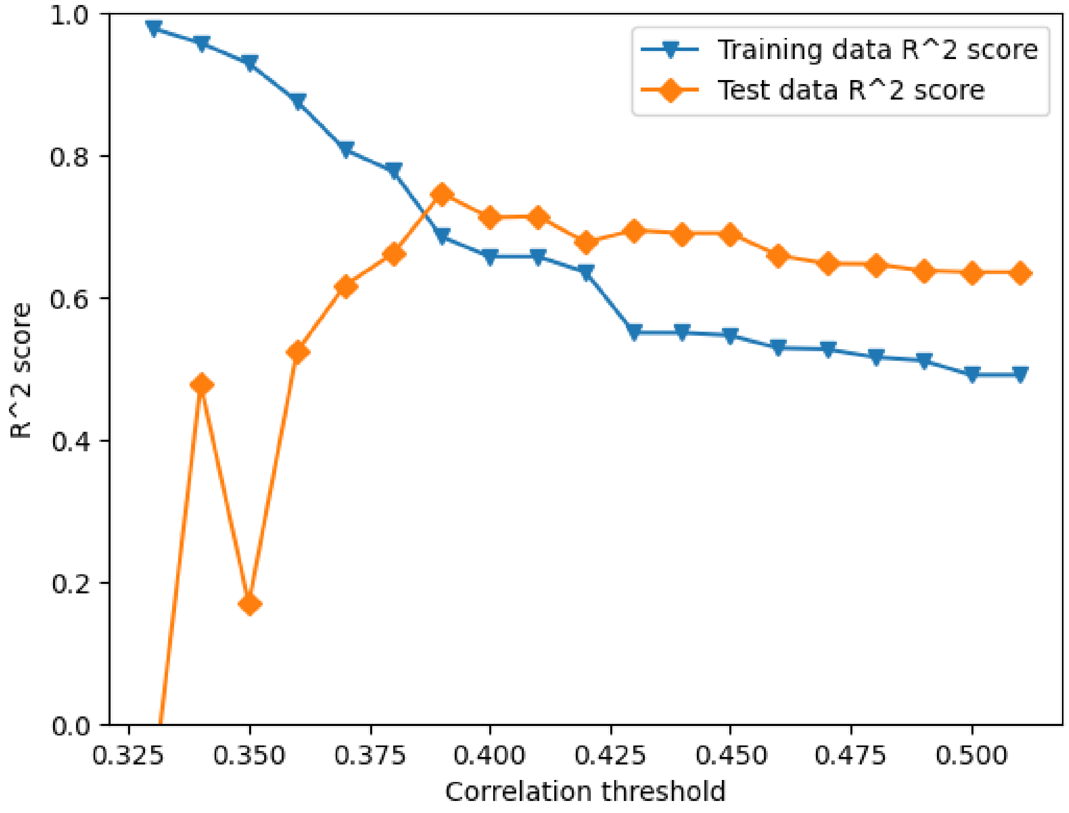

[12]:
```
plt.plot(df_without_standardization['Correlation threshold'],␣
↪df_without_standardization['Number of features'], label = "Number of␣
↪features", marker='.')
plt.legend()
plt.xlabel('Correlation threshold')
plt.ylabel('Number of features')
plt.show()
```

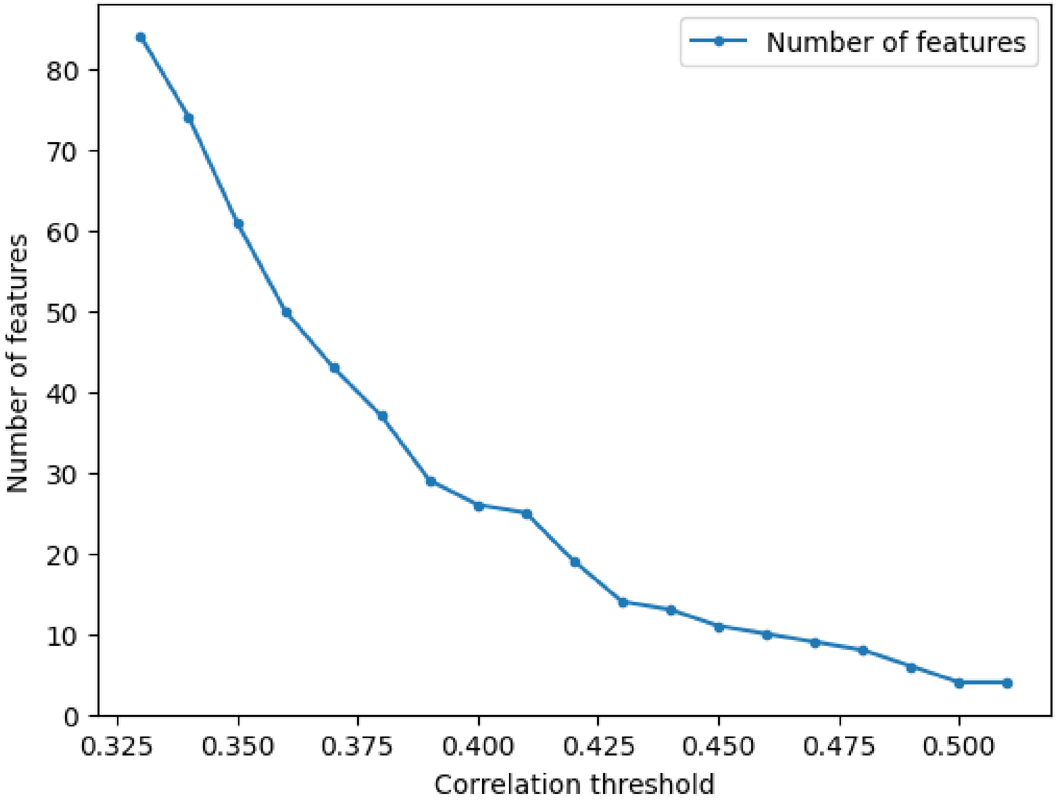


```
[ ]:
```

2.3 Decision Tree

```
[13]: without_standardization, train_r2_, test_r2_, hist1, hist2, target_column_name,␣
      ↪training_data_RMSE, test_data_RMSE  = pred_model.
      ↪prepare_data_and_create_model(molecular_descriptors_df=data,
                                                                          ␣
      ↪                        correlation_threshold=0.50,
                                                                          ␣
      ↪                        standardization=False,
                                                                          ␣
      ↪                        model_type='DecisionTreeRegressor',
                                                                          ␣
      ↪                        max_depth=5,
                                                                          ␣
      ↪                        target_column_name = target,
                                                                          ␣
      ↪                        random_state=random_state,
```

```
↪                        train_test_split_=True,
↪                        verbose=True)
```

```
I am not doing standardization…
  molecular descriptor name
0                    AATS0Z
1                  AATS0are
2                    AATS0d
3                   AATS0dv
4                    AATS0i
  molecular descriptor name  corr_value
0                    AATS0Z   -0.038502
1                  AATS0are   -0.120847
2                    AATS0d    0.041648
3                   AATS0dv   -0.115899
4                    AATS0i    0.191765
  molecular descriptor name  corr_value  absolute correlation value
0                    AATS0Z   -0.038502                    0.038502
1                  AATS0are   -0.120847                    0.120847
2                    AATS0d    0.041648                    0.041648
3                   AATS0dv   -0.115899                    0.115899
4                    AATS0i    0.191765                    0.191765
     molecular descriptor name  corr_value  absolute correlation value
226                     AMID_O   -0.520578                    0.520578
505                EState_VSA5   -0.578904                    0.578904
506                EState_VSA6    0.519794                    0.519794
791                    MDEO-12   -0.525441                    0.525441
1211                     MCF-7    1.000000                    1.000000
    molecular descriptor name  corr_value  absolute correlation value
226                    AMID_O   -0.520578                    0.520578
505               EState_VSA5   -0.578904                    0.578904
506               EState_VSA6    0.519794                    0.519794
791                   MDEO-12   -0.525441                    0.525441
The model used is: DecisionTree…
Return the coefficient of determination of the prediction:
0.48604135610978816
R^2 score: 0.8522633268113505
Correlation coefficient: 0.9231810910170065
Test data - unseen during training:
R^2 score: 0.48604135610978816
Correlation coefficient: 0.6971666630797747
[7.99044322 5.50688472 6.96137984 7.99044322 7.99044322 7.65055844
 7.99044322 7.35584115 7.99044322 7.99044322 8.39482702 8.39482702
 8.39482702 7.99044322 7.35584115 7.99044322 6.89807577 5.0424406 ]
102    7.958607
38     6.022276
```

```
8      5.949079
109    8.086186
11     8.013228
51     8.000000
88     8.119186
21     8.113509
82     7.982967
98     7.795880
58     7.677781
64     8.619789
74     8.327902
103    8.376751
91     9.154902
43     7.958607
25     4.949659
30     6.010550
Name: MCF-7, dtype: float64
Training Root Mean Square Error: 0.3576097994283484
Testing Root Mean Square Error: 0.7724927138832102
```

[14]:
```
print(target_column_name+str('_transformed'))
print(hist1[target_column_name].hist())
```

```
MCF-7_transformed
AxesSubplot(0.125,0.11;0.775x0.77)
```

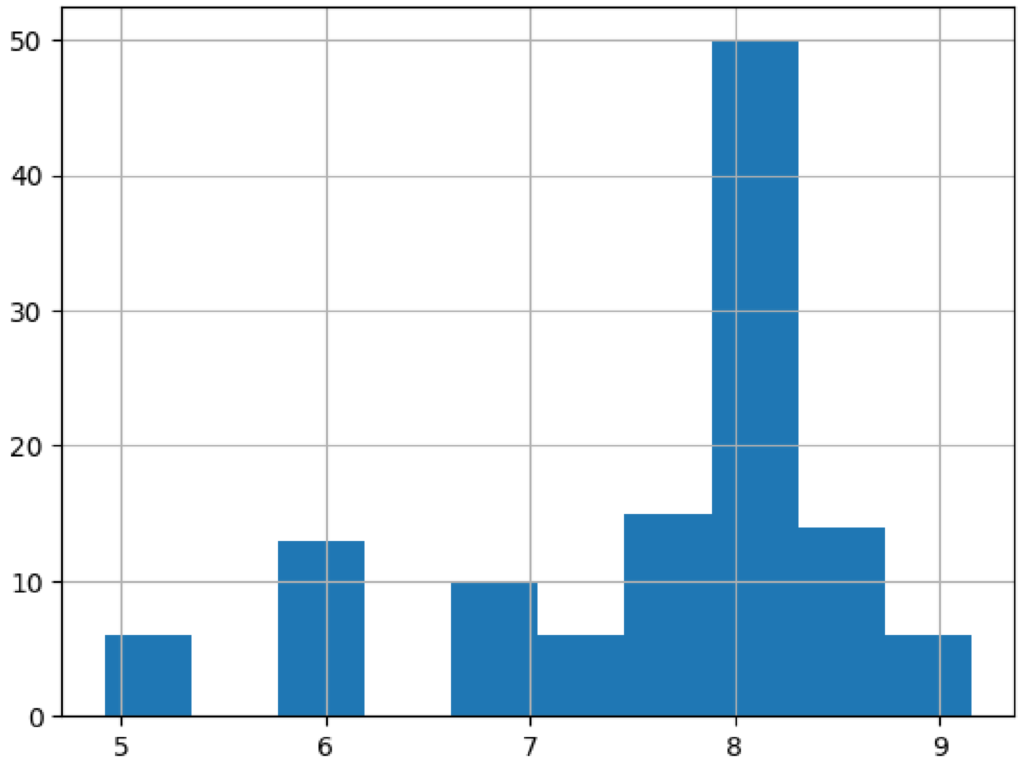

[15]:
```
with_standardization, train_r2_, test_r2_, hist1, hist2, target_column_name,␣
↪training_data_RMSE, test_data_RMSE  = pred_model.
↪prepare_data_and_create_model(molecular_descriptors_df=data,
                                                                              ␣
↪                    correlation_threshold=0.50,
                                                                              ␣
↪                    standardization=True,
                                                                              ␣
↪                    model_type='DecisionTreeRegressor',
                                                                              ␣
↪                    max_depth=5,
                                                                              ␣
↪                    target_column_name = target,
                                                                              ␣
↪                    random_state=random_state,
                                                                              ␣
↪                    train_test_split_=True,
                                                                              ␣
↪                    verbose=True)
```

```
I am doing standardization…
  molecular descriptor name
0                    AATS0Z
1                  AATS0are
2                    AATS0d
3                   AATS0dv
4                    AATS0i
  molecular descriptor name  corr_value
0                    AATS0Z   -0.038502
1                  AATS0are   -0.120847
2                    AATS0d    0.041648
3                   AATS0dv   -0.115899
4                    AATS0i    0.191765
  molecular descriptor name  corr_value  absolute correlation value
0                    AATS0Z   -0.038502                    0.038502
1                  AATS0are   -0.120847                    0.120847
2                    AATS0d    0.041648                    0.041648
3                   AATS0dv   -0.115899                    0.115899
4                    AATS0i    0.191765                    0.191765
     molecular descriptor name  corr_value  absolute correlation value
226                     AMID_O   -0.520578                    0.520578
505                 EState_VSA5   -0.578904                    0.578904
506                 EState_VSA6    0.519794                    0.519794
791                    MDEO-12   -0.525441                    0.525441
```

```
1211                     MCF-7    1.000000                    1.000000
    molecular descriptor name  corr_value  absolute correlation value
226                    AMID_O   -0.520578                    0.520578
505               EState_VSA5   -0.578904                    0.578904
506               EState_VSA6    0.519794                    0.519794
791                  MDEO-12   -0.525441                    0.525441
The model used is: DecisionTree…
Return the coefficient of determination of the prediction:
0.48604135610978816
R^2 score: 0.8522633268113505
Correlation coefficient: 0.9231810910170065
Test data - unseen during training:
R^2 score: 0.48604135610978816
Correlation coefficient: 0.6971666630797747
[7.99044322 5.50688472 6.96137984 7.99044322 7.99044322 7.65055844
 7.99044322 7.35584115 7.99044322 7.99044322 8.39482702 8.39482702
 8.39482702 7.99044322 7.35584115 7.99044322 6.89807577 5.0424406 ]
102    7.958607
38     6.022276
8      5.949079
109    8.086186
11     8.013228
51     8.000000
88     8.119186
21     8.113509
82     7.982967
98     7.795880
58     7.677781
64     8.619789
74     8.327902
103    8.376751
91     9.154902
43     7.958607
25     4.949659
30     6.010550
Name: MCF-7, dtype: float64
Training Root Mean Square Error: 0.3576097994283484
Testing Root Mean Square Error: 0.7724927138832102
```

2.4 Search inside correlation space

[16]:
```
step = 0.01
initial_step = corr_low
last_step = corr_high
first_list = [x / 100.0 for x in range(int(initial_step*100),␣
 ↪int(last_step*100), int(step*100))]
max_depth = [range(2, 30, 1)]
```

```
corr_th = []
second_list = []
third_list = []
fourth_l = []
fifth_l = []
f_list = []
fif_list = []
for i in first_list:
    for depth in max_depth[0]:

        without_standardization, train_r2, test_r2, _, h_, target_column_name, training_data_RMSE, test_data_RMSE = pred_model.prepare_data_and_create_model(molecular_descriptors_df=data,
                                    correlation_threshold=i,
                                    standardization=False,
                                    model_type='DecisionTreeRegressor',
                                    max_depth=depth,
                                    target_column_name = target,
                                    random_state=random_state,
                                    train_test_split_=True,
                                    verbose=False)
        corr_th.append(i)
        second_list.append(train_r2)
        third_list.append(test_r2)
        fourth_l.append(training_data_RMSE)
        fifth_l.append(test_data_RMSE)
        f_list.append(len(h_))
        fif_list.append(depth)
```

[17]:
```
df_without_standardization = pd.DataFrame(data=corr_th, columns=["Correlation threshold"])
df_without_standardization['Training data R^2 score'] = second_list
df_without_standardization['Test data R^2 score'] = third_list
df_without_standardization['Training RMSE'] = fourth_l
df_without_standardization['Test RMSE'] = fifth_l
df_without_standardization['Number of features'] = f_list
df_without_standardization['Depth number'] = fif_list
```

```
[18]: df_decision_tree = df_without_standardization.copy()
#df_without_standardization.to_excel('../Data/
↪A549_Decision_tree_rs_'+str(random_state)+'.xlsx')
```

2.5 Plots

```
[19]: corre_list = [x / 100.0 for x in range(int(initial_step*100),␣
↪int(last_step*100), int(0.01*100))]
for element in corre_list:
    element_ =␣
↪df_without_standardization[df_without_standardization['Correlation␣
↪threshold'] == float(element)]
    plt.plot(element_['Depth number'], element_['Training data R^2 score'],␣
↪label = "Training data R^2 score", marker='v')
    plt.plot(element_['Depth number'], element_['Test data R^2 score'], label =␣
↪"Test data R^2 score", marker='D')
    plt.legend()
    plt.xlabel('Depth number -> correlation threshold: '+str(element)+'. \n␣
↪Number of features: '+str(element_['Number of features'].iloc[0]),␣
↪fontweight='bold')
    plt.xlim(max(element_['Depth number'])+1, min(element_['Depth number'])-1)
    plt.ylabel('R^2 score')
    plt.ylim([0, 1])
    plt.rc('grid', linestyle="-.", color='black')
    plt.grid(True)
    plt.show()
```

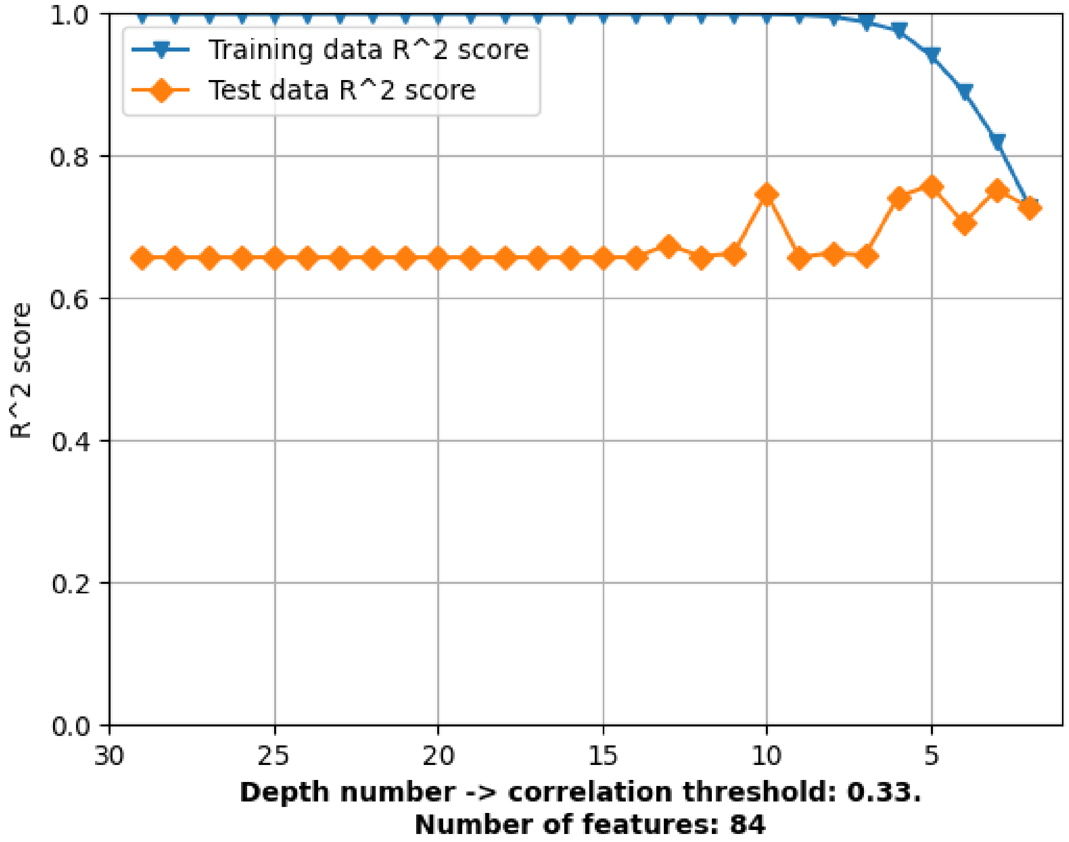

Training data R^2 score
Test data R^2 score
R^2 score
1.0
0.8
0.6
0.4
0.2
0.0
30
25
20
15
10
5
Depth number -> correlation threshold: 0.33.
Number of features: 84

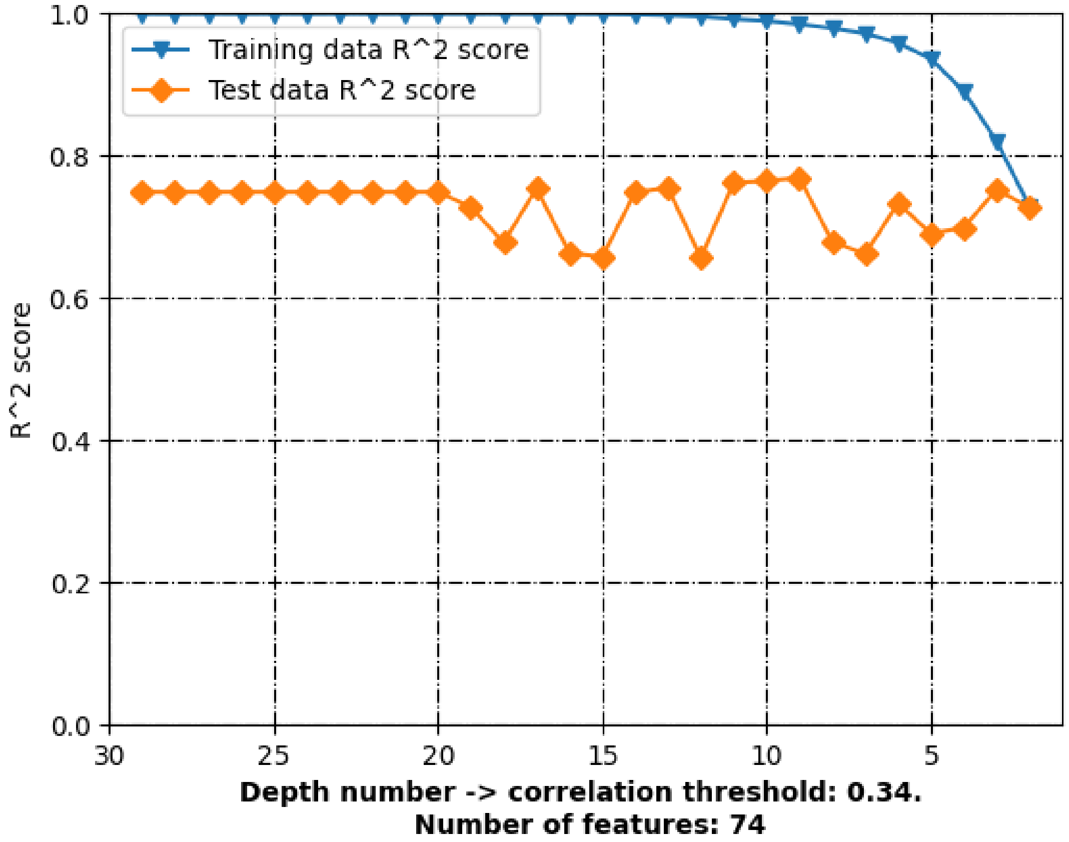

1.0
0.8
0.6
0.4
0.2
0.0
R^2 score
Training data R^2 score
Test data R^2 score
30
25
20
15
10
5
Depth number -> correlation threshold: 0.34.
Number of features: 74

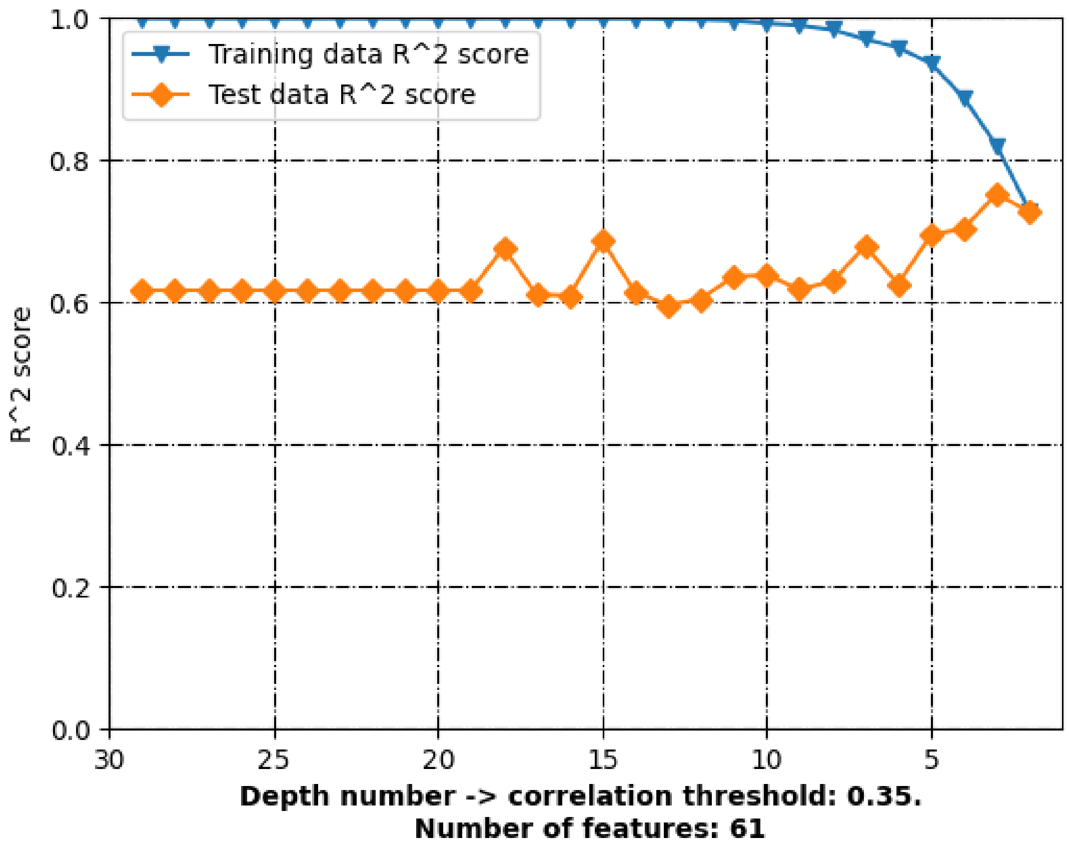
Training data R^2 score
Test data R^2 score
R^2 score
1.0
0.8
0.6
0.4
0.2
0.0
30
25
20
15
10
5
Depth number -> correlation threshold: 0.35.
Number of features: 61

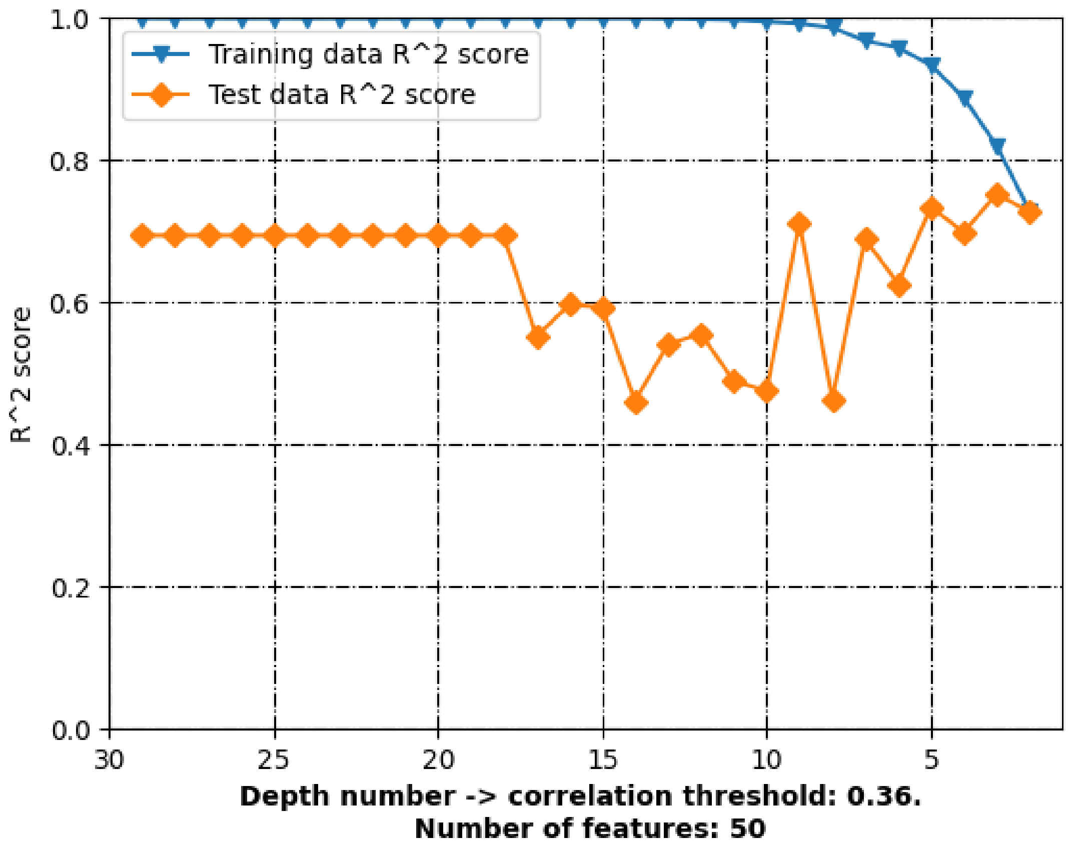

Training data R^2 score
Test data R^2 score
R^2 score
1.0
0.8
0.6
0.4
0.2
0.0
30
25
20
15
10
5
Depth number -> correlation threshold: 0.36.
Number of features: 50

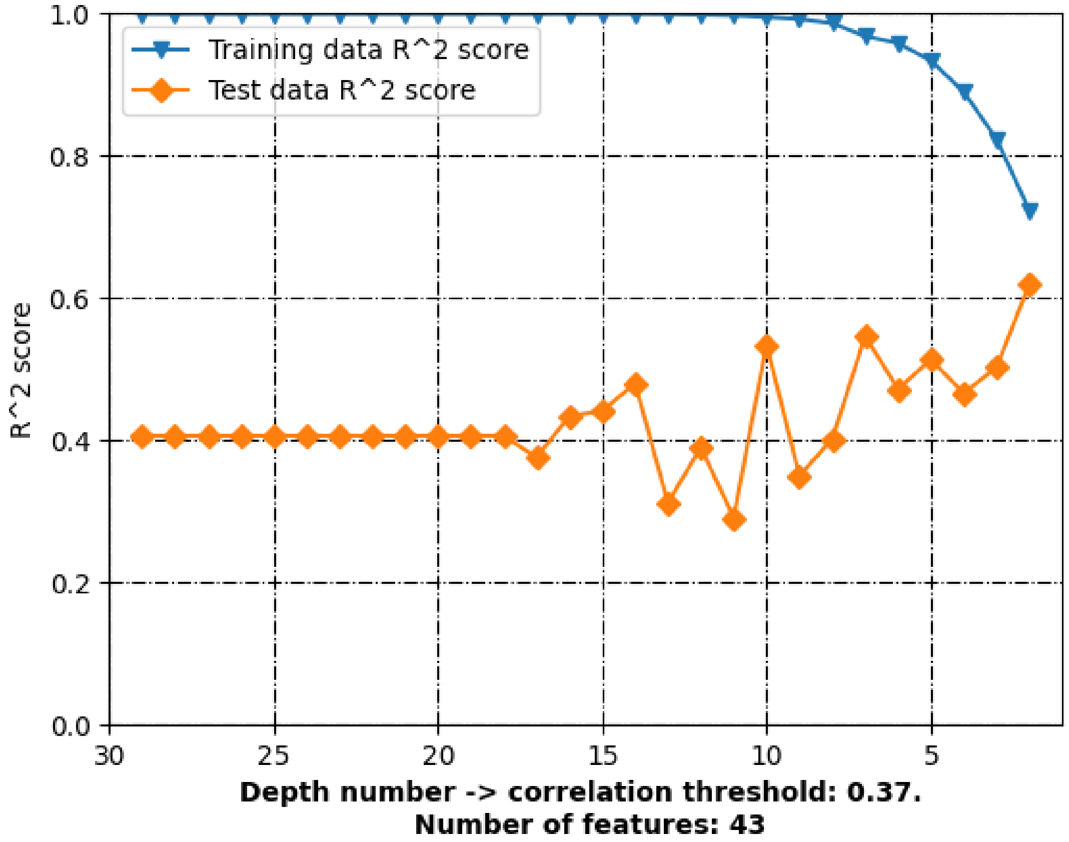
Training data R^2 score
Test data R^2 score
R^2 score
1.0
0.8
0.6
0.4
0.2
0.0
30
25
20
15
10
5
Depth number -> correlation threshold: 0.37.
Number of features: 43

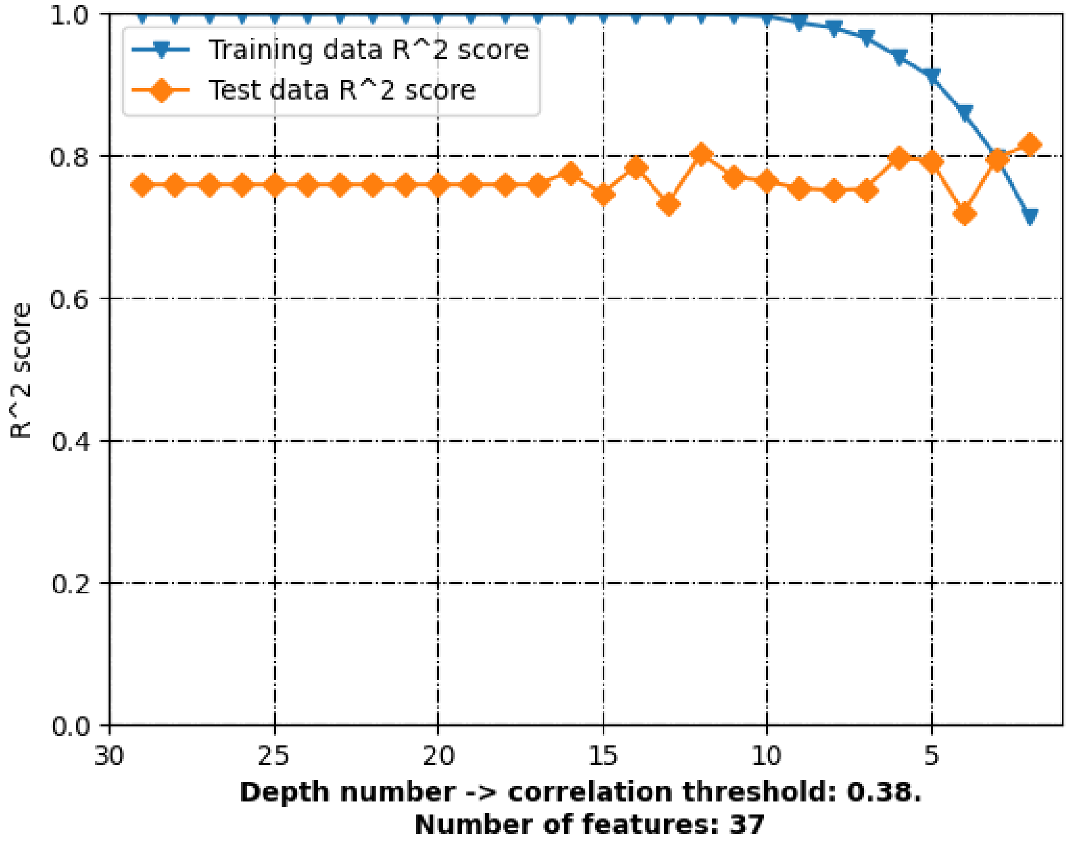

Training data R^2 score
Test data R^2 score
R^2 score
Depth number -> correlation threshold: 0.38.
Number of features: 37

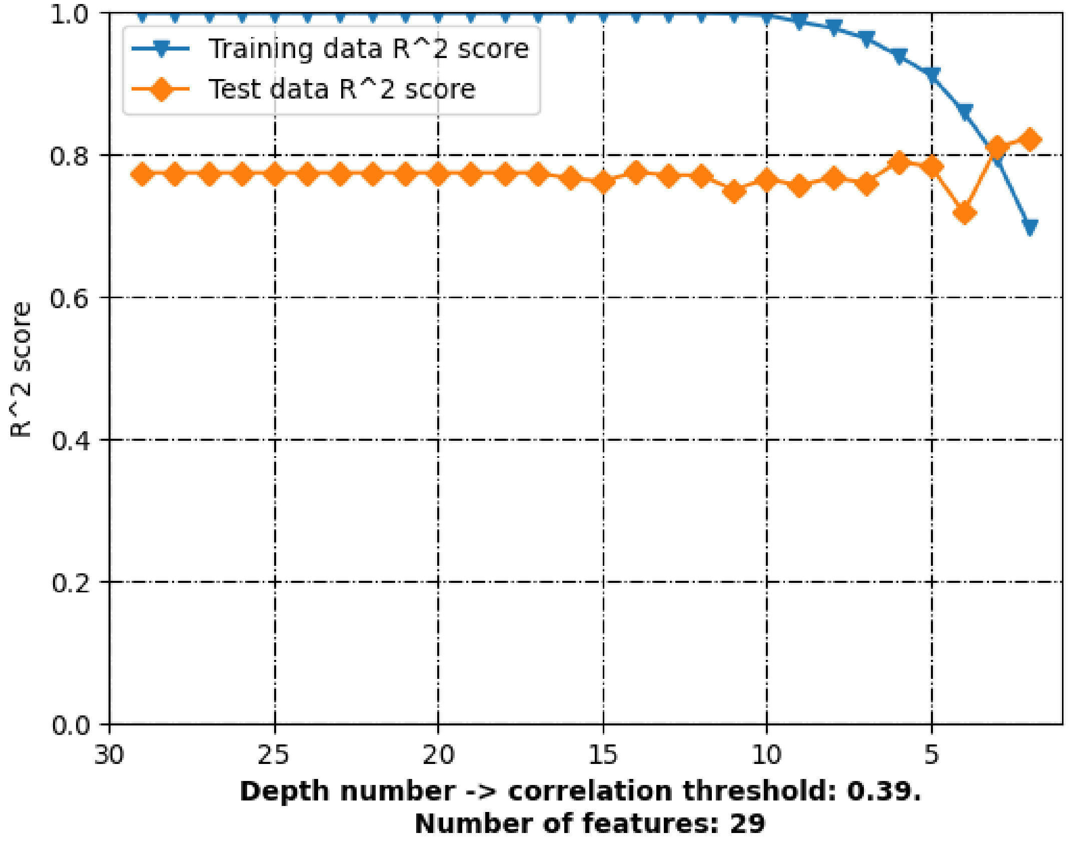

Training data R^2 score
Test data R^2 score
R^2 score
1.0
0.8
0.6
0.4
0.2
0.0
30
25
20
15
10
5
Depth number -> correlation threshold: 0.39.
Number of features: 29

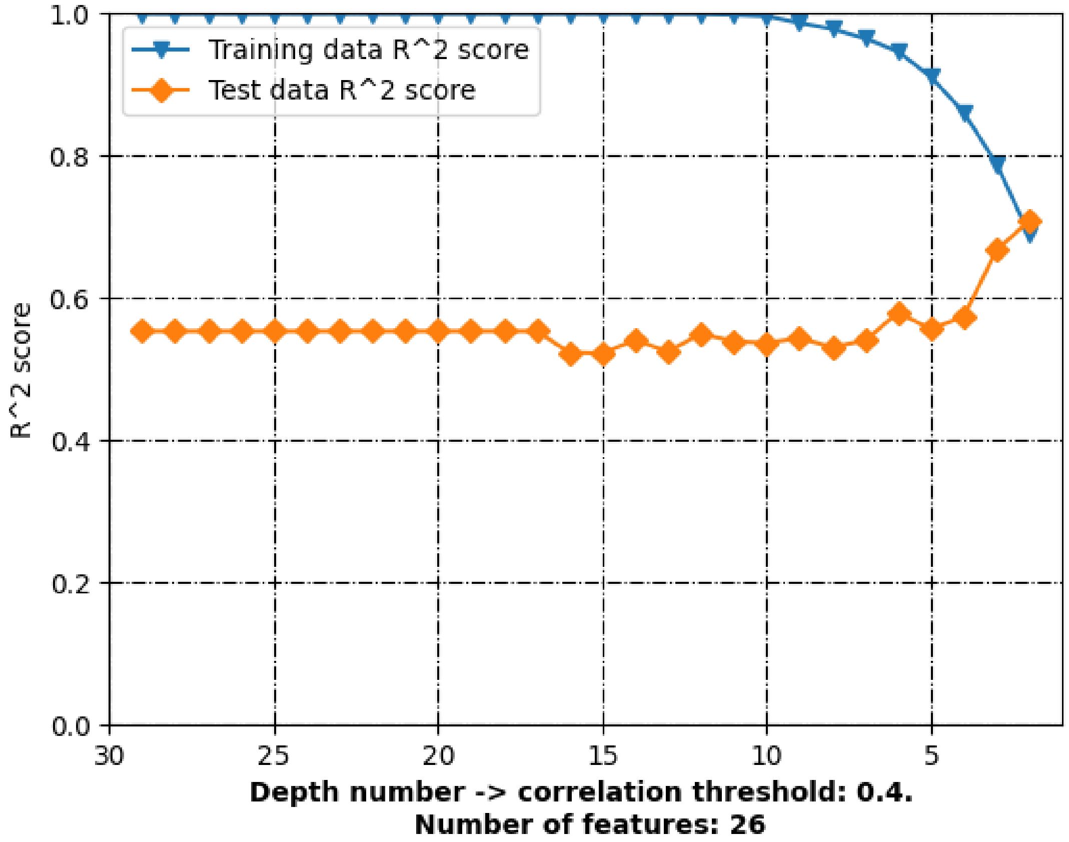
Training data R^2 score
Test data R^2 score
R^2 score
Depth number -> correlation threshold: 0.4.
Number of features: 26
1.0
0.8
0.6
0.4
0.2
0.0
30
25
20
15
10
5

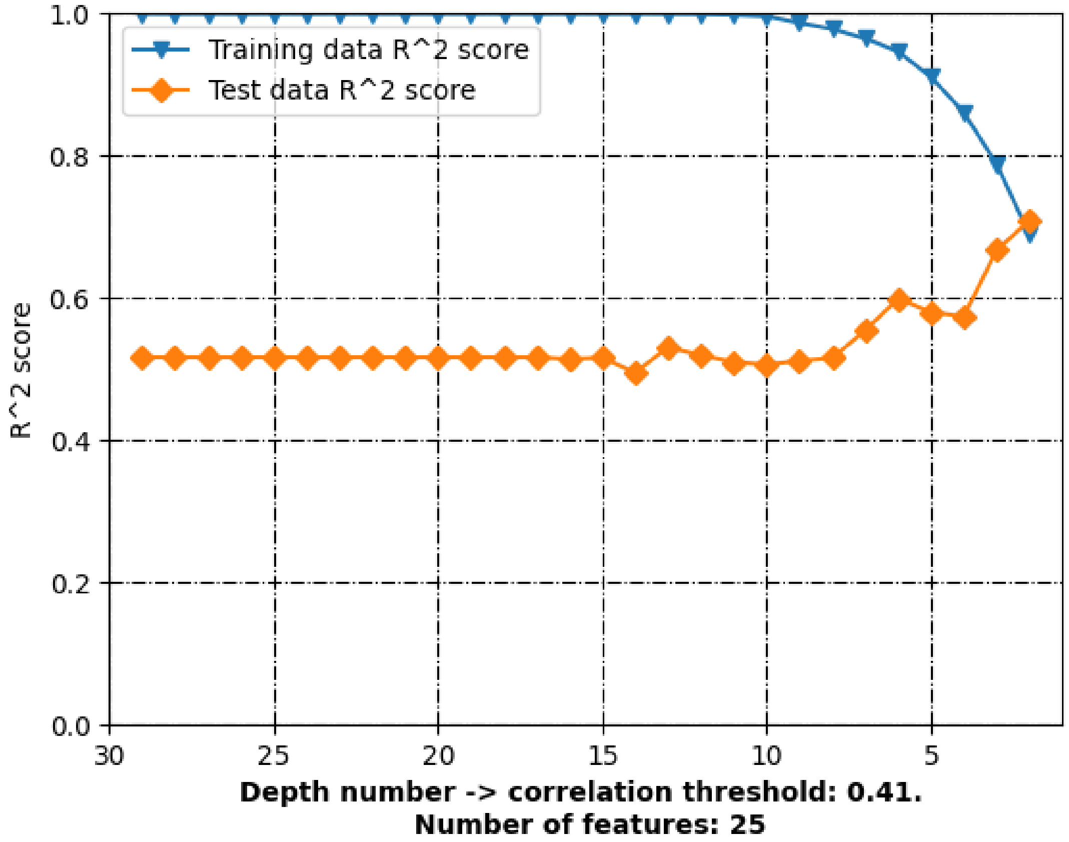
Training data R^2 score
Test data R^2 score
R^2 score
1.0
0.8
0.6
0.4
0.2
0.0
30
25
20
15
10
5
Depth number -> correlation threshold: 0.41.
Number of features: 25

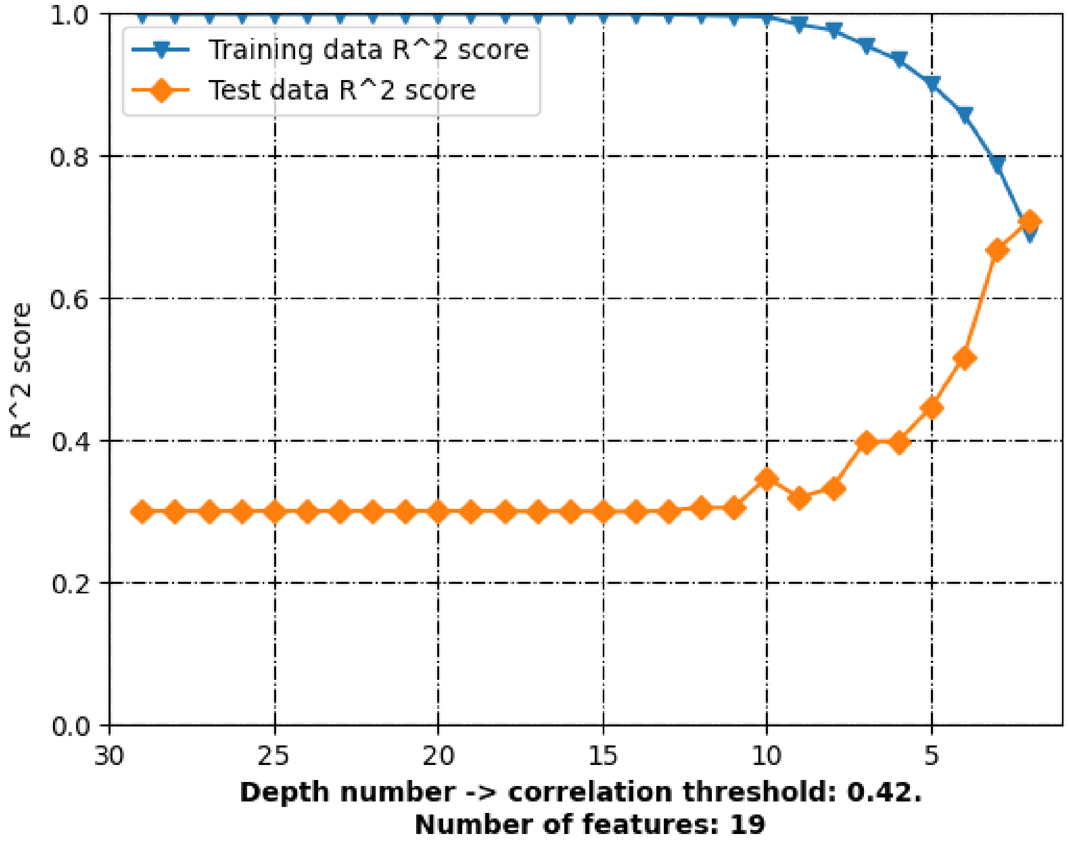
Training data R^2 score
Test data R^2 score
R^2 score
1.0
0.8
0.6
0.4
0.2
0.0
30
25
20
15
10
5
Depth number -> correlation threshold: 0.42.
Number of features: 19

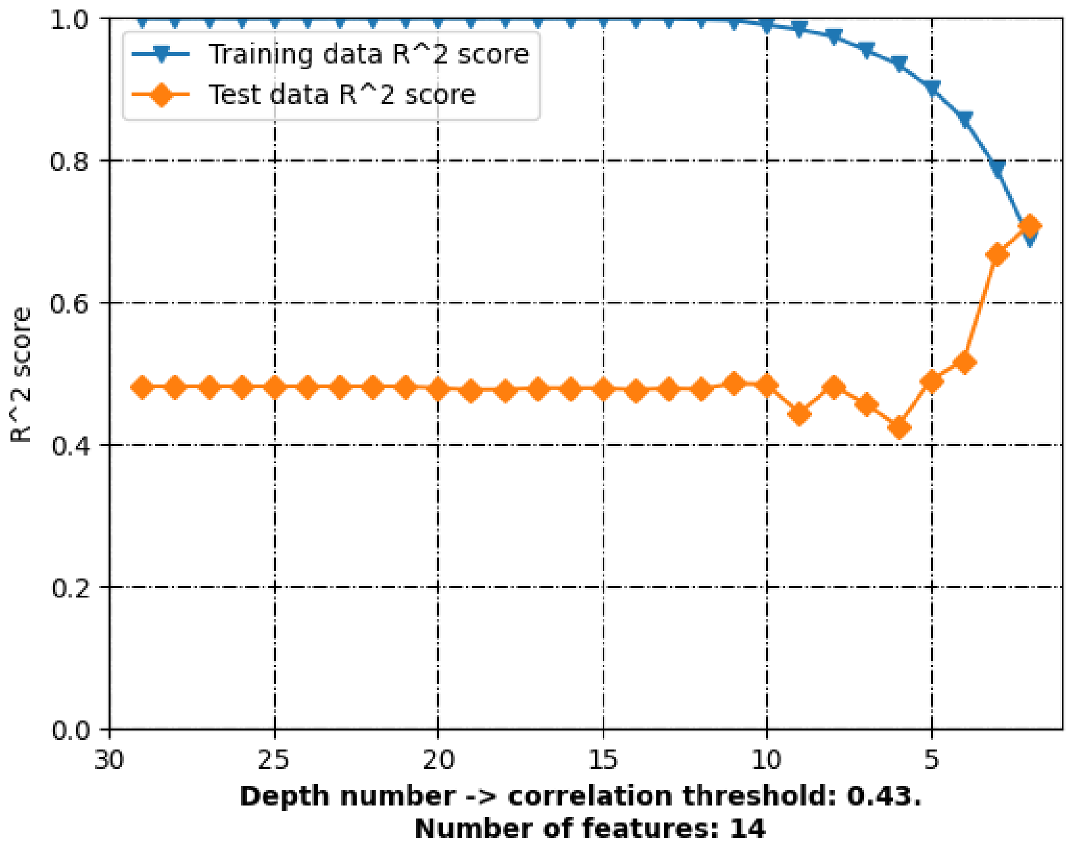

Training data R^2 score
Test data R^2 score
R^2 score
1.0
0.8
0.6
0.4
0.2
0.0
30
25
20
15
10
5
Depth number -> correlation threshold: 0.43.
Number of features: 14

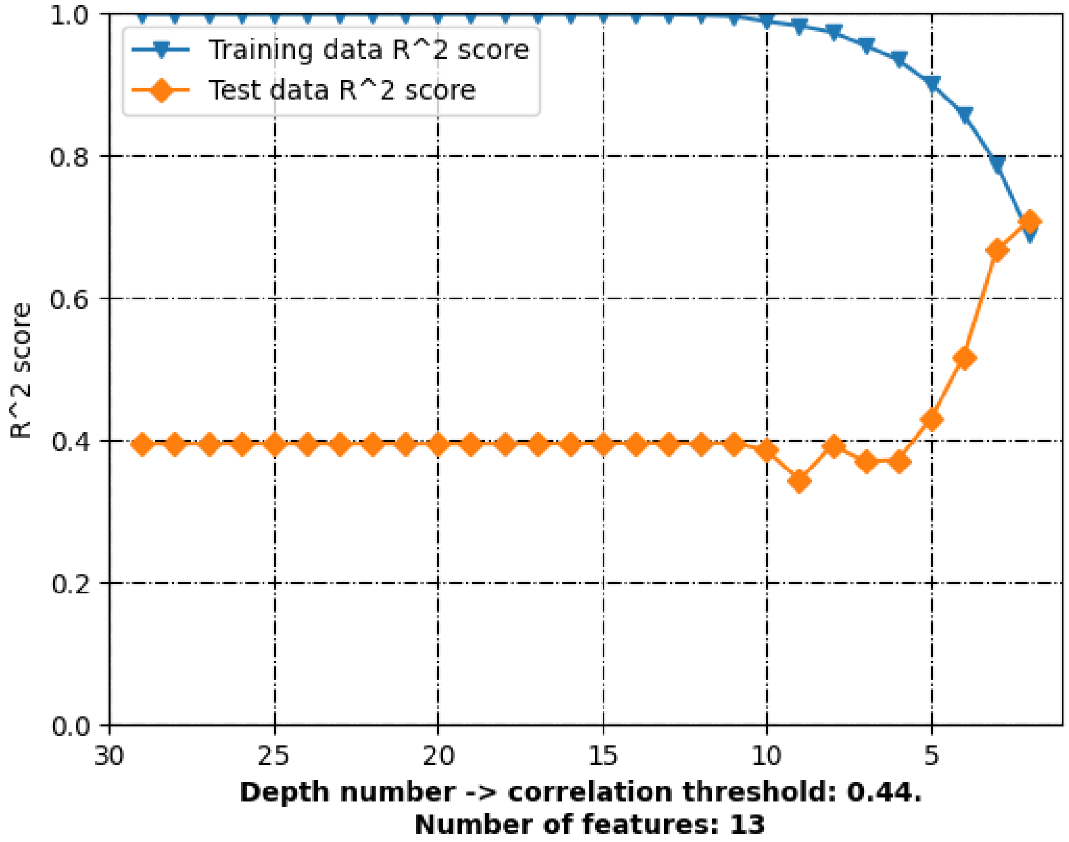

1.0
0.8
0.6
0.4
0.2
0.0
R^2 score
Training data R^2 score
Test data R^2 score
30
25
20
15
10
5
Depth number -> correlation threshold: 0.44.
Number of features: 13

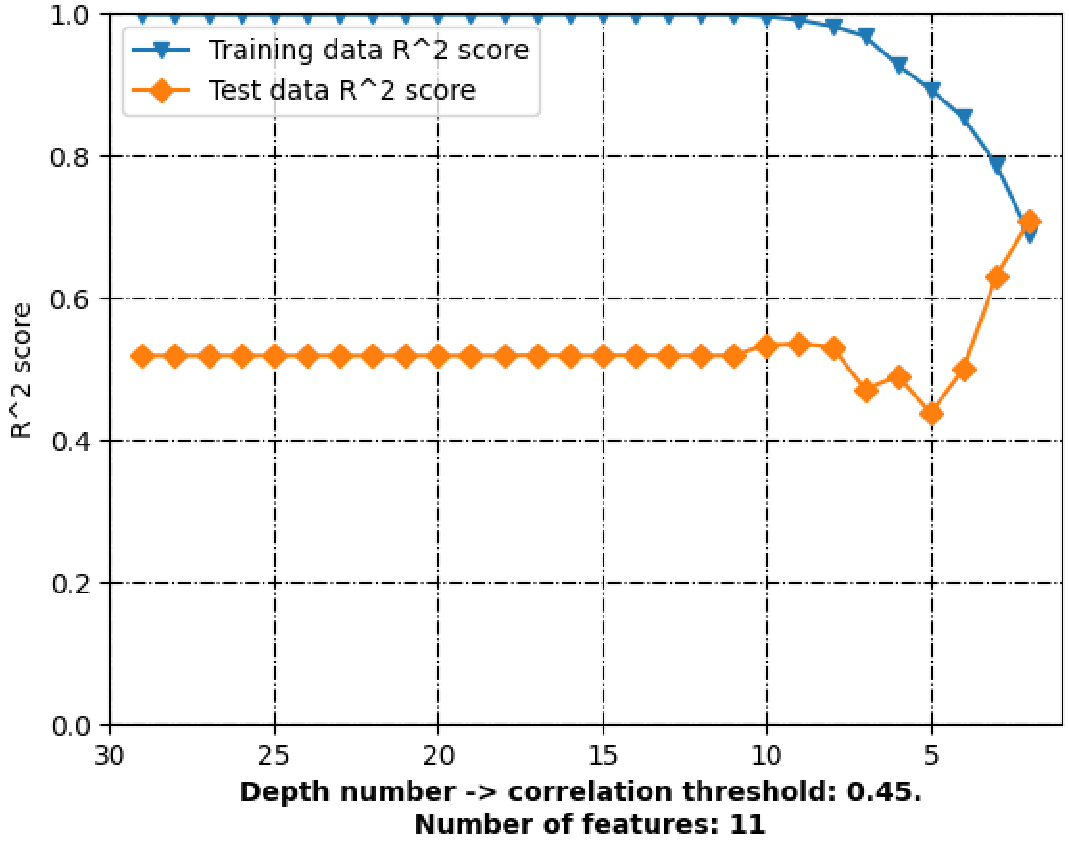
Training data R^2 score
Test data R^2 score
R^2 score
1.0
0.8
0.6
0.4
0.2
0.0
30
25
20
15
10
5
Depth number -> correlation threshold: 0.45.
Number of features: 11

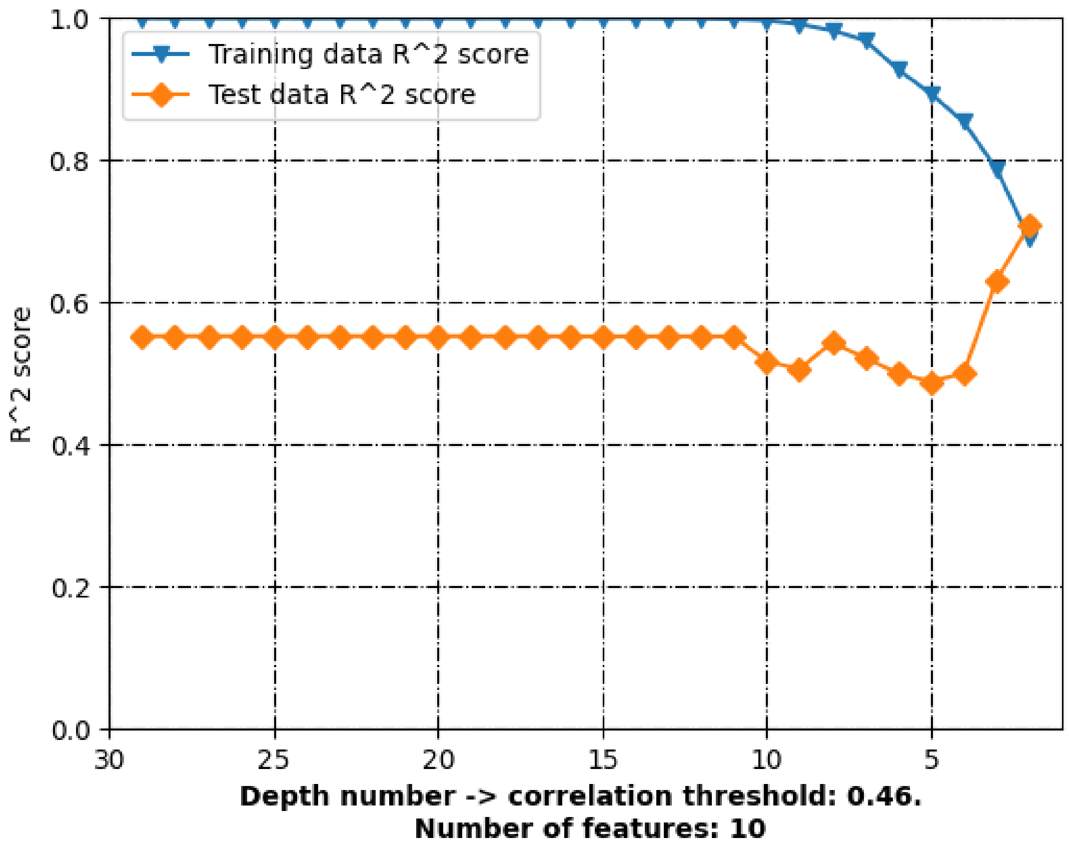

Training data R^2 score
Test data R^2 score
R^2 score
1.0
0.8
0.6
0.4
0.2
0.0
30
25
20
15
10
5
Depth number -> correlation threshold: 0.46.
Number of features: 10

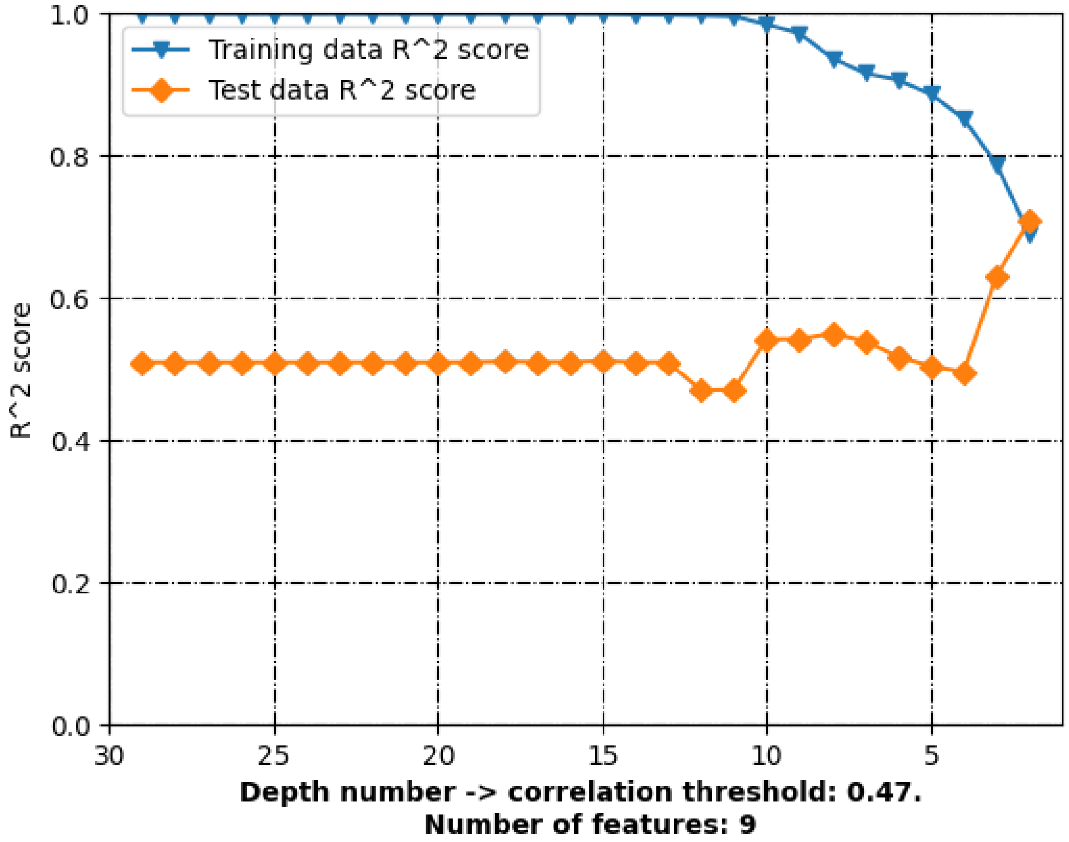
Training data R^2 score
Test data R^2 score
R^2 score
1.0
0.8
0.6
0.4
0.2
0.0
30
25
20
15
10
5
Depth number -> correlation threshold: 0.47.
Number of features: 9

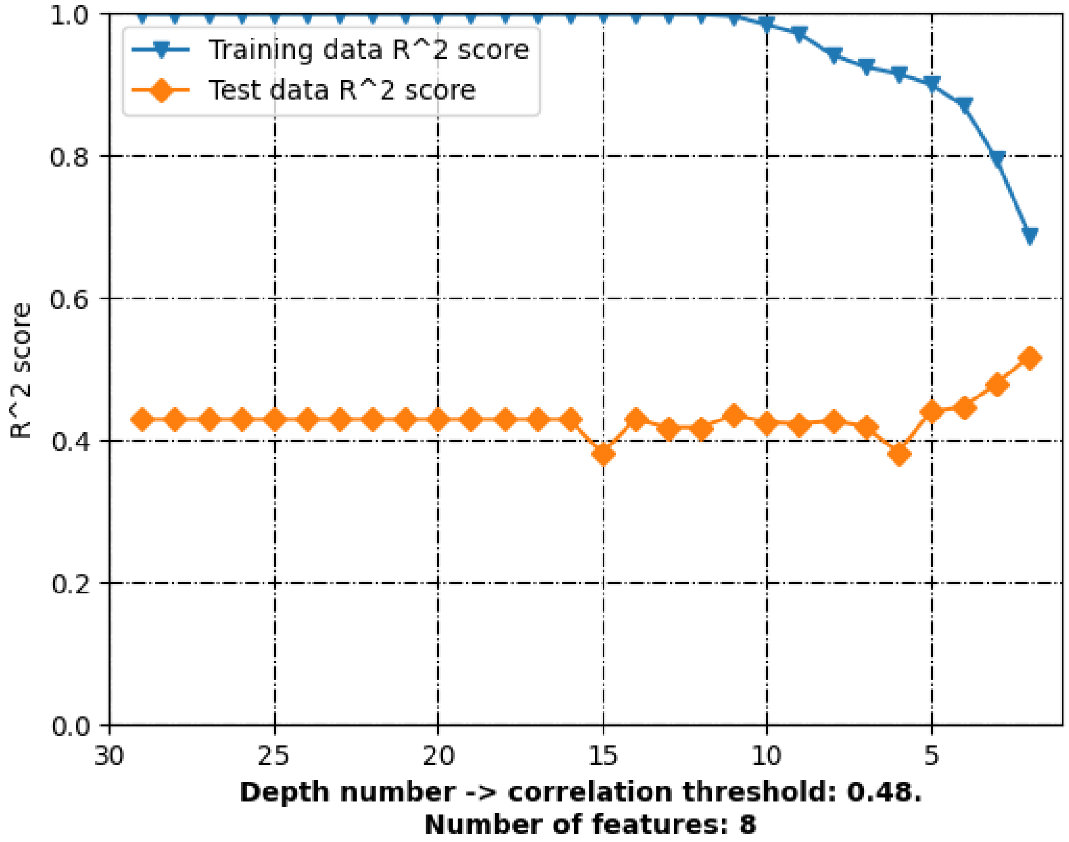
Training data R^2 score
Test data R^2 score
R^2 score
1.0
0.8
0.6
0.4
0.2
0.0
30
25
20
15
10
5
Depth number -> correlation threshold: 0.48.
Number of features: 8

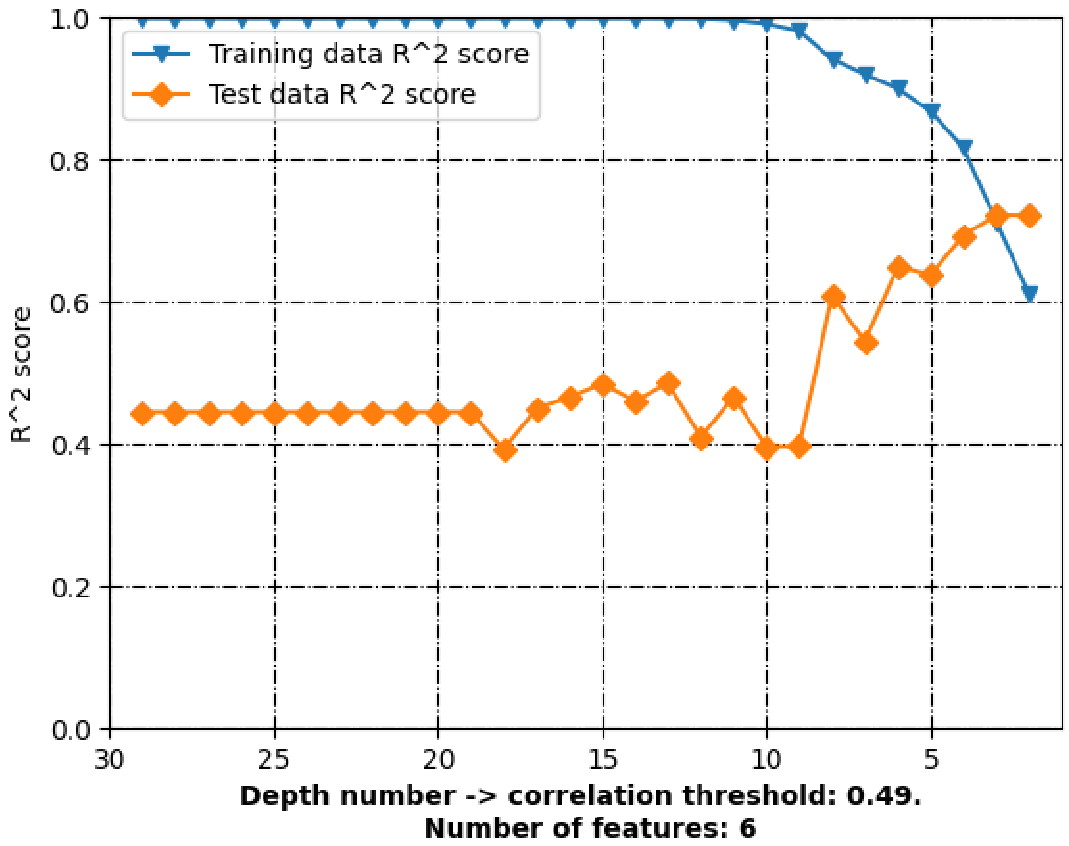

Training data R^2 score
Test data R^2 score
R^2 score
1.0
0.8
0.6
0.4
0.2
0.0
30
25
20
15
10
5
Depth number -> correlation threshold: 0.49.
Number of features: 6

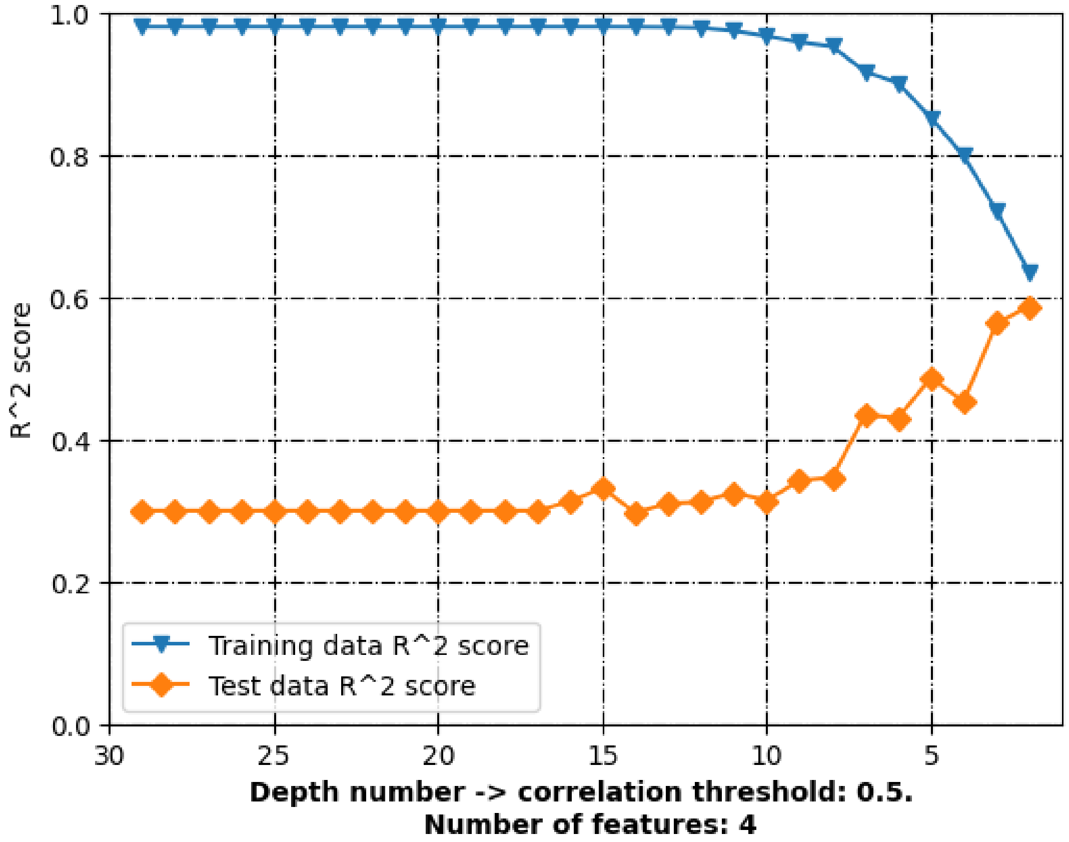

1.0
0.8
0.6
0.4
0.2
0.0
R^2 score
30
25
20
15
10
5
Training data R^2 score
Test data R^2 score
Depth number -> correlation threshold: 0.5.
Number of features: 4

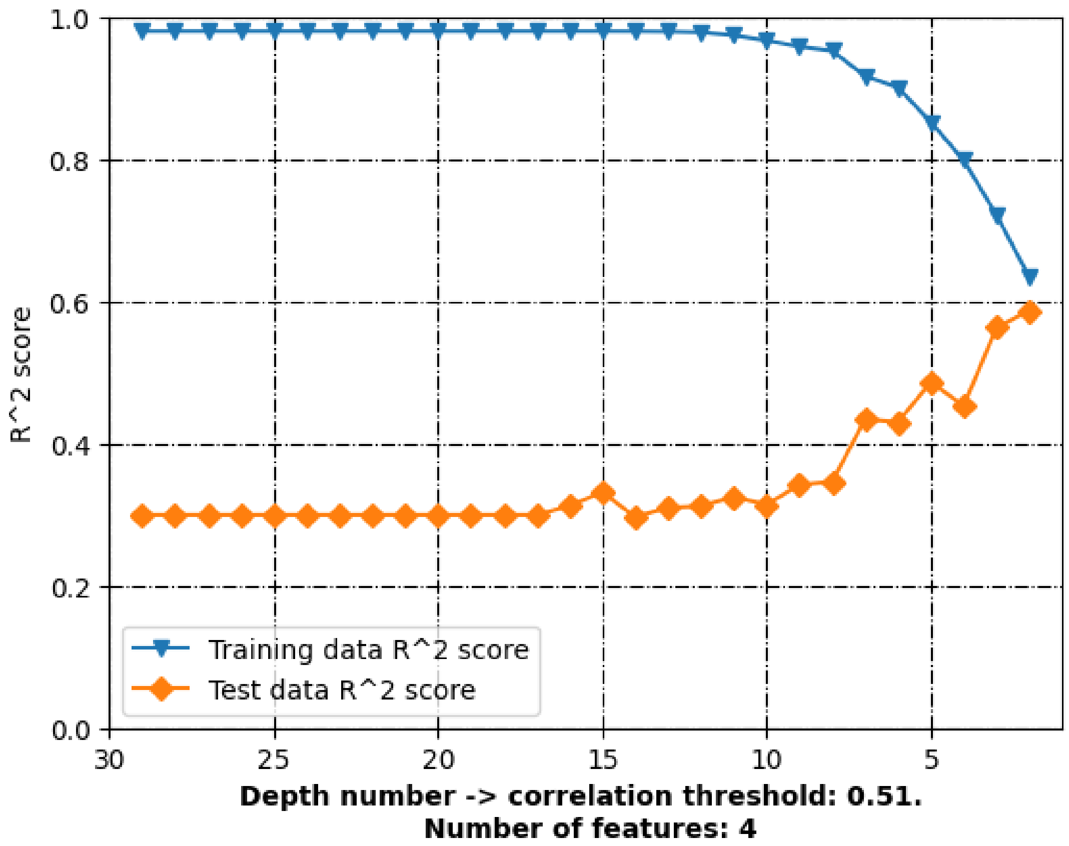

[20]:
```
plt.plot(df_without_standardization['Correlation threshold'],␣
  ↪df_without_standardization['Number of features'], label = "Number of␣
  ↪features", marker='.')
plt.legend()
plt.xlabel('Correlation threshold')
plt.ylabel('Number of features')
plt.show()
```

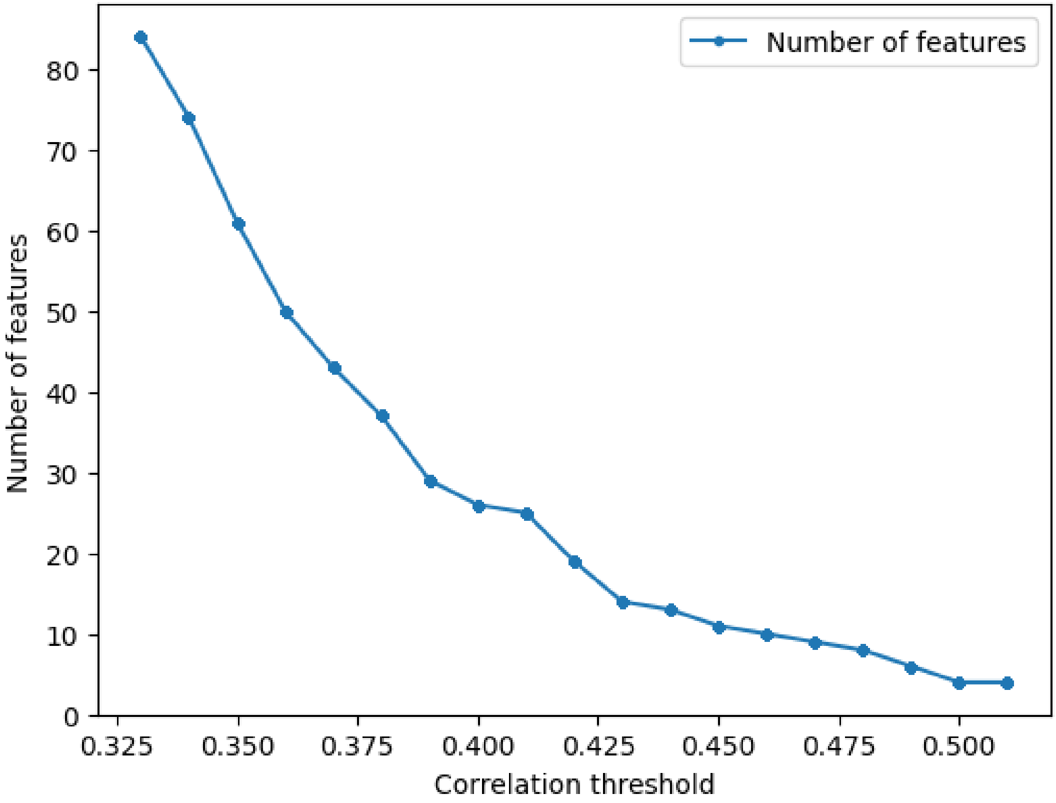


```
[ ]:
```

3 Random Forest

```
[21]: without_standardization, train_r2_, test_r2_, hist1, hist2, target_column_name,␣
    ↪training_data_RMSE, test_data_RMSE  = pred_model.
    ↪prepare_data_and_create_model(molecular_descriptors_df=data,
                                                                                    ␣
    ↪                         correlation_threshold=0.50,
                                                                                    ␣
    ↪                         standardization=False,
                                                                                    ␣
    ↪                         model_type='RandomForestRegressor',
                                                                                    ␣
    ↪                         n_estimators_=12,
                                                                                    ␣
    ↪                         target_column_name = target,
                                                                                    ␣
    ↪                         random_state=random_state,
```

```
↪                     train_test_split_=True,
↪                     verbose=True)
```

```
I am not doing standardization…
  molecular descriptor name
0                    AATS0Z
1                  AATS0are
2                    AATS0d
3                   AATS0dv
4                    AATS0i
  molecular descriptor name  corr_value
0                    AATS0Z   -0.038502
1                  AATS0are   -0.120847
2                    AATS0d    0.041648
3                   AATS0dv   -0.115899
4                    AATS0i    0.191765
  molecular descriptor name  corr_value  absolute correlation value
0                    AATS0Z   -0.038502                    0.038502
1                  AATS0are   -0.120847                    0.120847
2                    AATS0d    0.041648                    0.041648
3                   AATS0dv   -0.115899                    0.115899
4                    AATS0i    0.191765                    0.191765
     molecular descriptor name  corr_value  absolute correlation value
226                     AMID_O   -0.520578                    0.520578
505                EState_VSA5   -0.578904                    0.578904
506                EState_VSA6    0.519794                    0.519794
791                    MDEO-12   -0.525441                    0.525441
1211                     MCF-7    1.000000                    1.000000
    molecular descriptor name  corr_value  absolute correlation value
226                    AMID_O   -0.520578                    0.520578
505               EState_VSA5   -0.578904                    0.578904
506               EState_VSA6    0.519794                    0.519794
791                   MDEO-12   -0.525441                    0.525441
The model used is: RandomForest…
Return the coefficient of determination of the prediction:
0.45189820560775484
R^2 score: 0.8988911629700564
Correlation coefficient: 0.9480987095076422
Test data - unseen during training:
R^2 score: 0.45189820560775484
Correlation coefficient: 0.6722337432826136
[7.48904776 5.75768222 7.01387872 8.03604241 8.004483   7.59008558
 8.05858924 7.30810016 7.41942745 8.24788273 8.12758092 8.34192311
 8.44322142 7.6573918  7.53840752 8.03812066 7.16953157 5.43756702]
102    7.958607
38     6.022276
```

```
8      5.949079
109    8.086186
11     8.013228
51     8.000000
88     8.119186
21     8.113509
82     7.982967
98     7.795880
58     7.677781
64     8.619789
74     8.327902
103    8.376751
91     9.154902
43     7.958607
25     4.949659
30     6.010550
Name: MCF-7, dtype: float64
Training Root Mean Square Error: 0.295841987221968
Testing Root Mean Square Error: 0.7977391691942761
```

[22]:
```
print(target_column_name+str('_transformed'))
print(hist1[target_column_name].hist())
```

```
MCF-7_transformed
AxesSubplot(0.125,0.11;0.775x0.77)
```

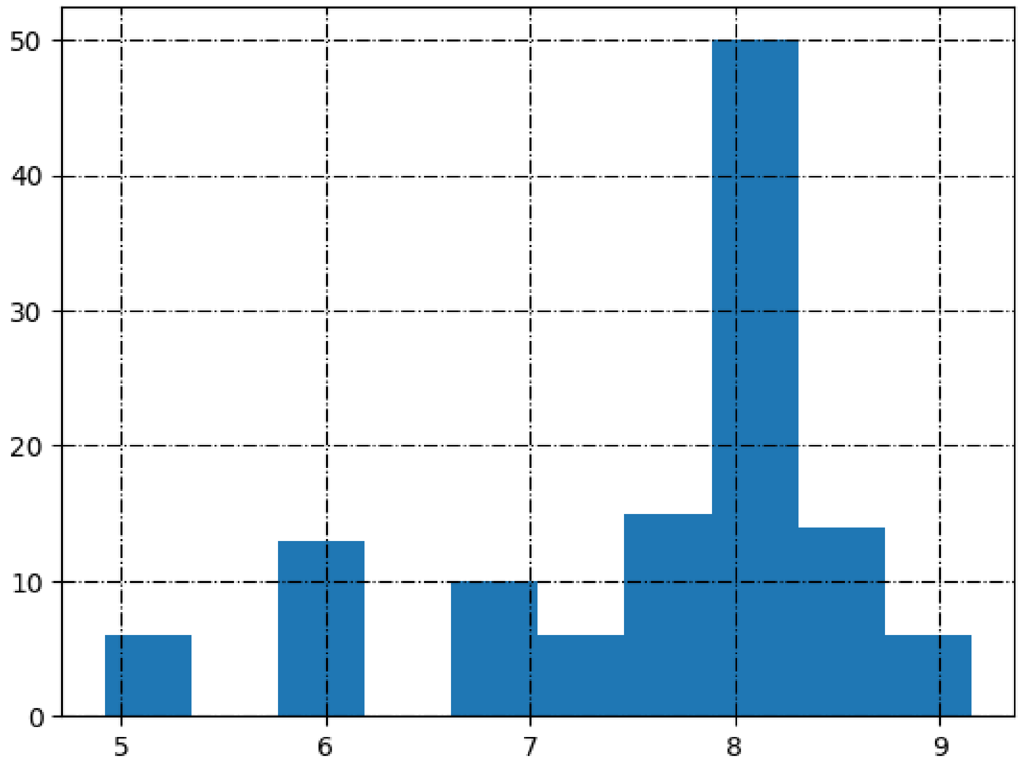

[23]:
```
with_standardization, train_r2_, test_r2_, hist1, hist2, target_column_name,␣
↪training_data_RMSE, test_data_RMSE  = pred_model.
↪prepare_data_and_create_model(molecular_descriptors_df=data,
                                                                               ␣
↪                    correlation_threshold=0.50,
                                                                               ␣
↪                    standardization=True,
                                                                               ␣
↪                    model_type='RandomForestRegressor',
                                                                               ␣
↪                    n_estimators_=12,
                                                                               ␣
↪                    target_column_name = target,
                                                                               ␣
↪                    random_state=random_state,
                                                                               ␣
↪                    train_test_split_=True,
                                                                               ␣
↪                    verbose=True)
```

```
I am doing standardization…
  molecular descriptor name
0                    AATS0Z
1                  AATS0are
2                    AATS0d
3                   AATS0dv
4                    AATS0i
  molecular descriptor name  corr_value
0                    AATS0Z   -0.038502
1                  AATS0are   -0.120847
2                    AATS0d    0.041648
3                   AATS0dv   -0.115899
4                    AATS0i    0.191765
  molecular descriptor name  corr_value  absolute correlation value
0                    AATS0Z   -0.038502                    0.038502
1                  AATS0are   -0.120847                    0.120847
2                    AATS0d    0.041648                    0.041648
3                   AATS0dv   -0.115899                    0.115899
4                    AATS0i    0.191765                    0.191765
     molecular descriptor name  corr_value  absolute correlation value
226                     AMID_O   -0.520578                    0.520578
505                EState_VSA5   -0.578904                    0.578904
506                EState_VSA6    0.519794                    0.519794
791                   MDEO-12   -0.525441                    0.525441
```

```
1211                     MCF-7    1.000000                     1.000000
    molecular descriptor name  corr_value  absolute correlation value
226                    AMID_O   -0.520578                    0.520578
505                EState_VSA5  -0.578904                    0.578904
506                EState_VSA6   0.519794                    0.519794
791                   MDEO-12   -0.525441                    0.525441
The model used is: RandomForest…
Return the coefficient of determination of the prediction:
0.45189820560775484
R^2 score: 0.8988911629700564
Correlation coefficient: 0.9480987095076422
Test data - unseen during training:
R^2 score: 0.45189820560775484
Correlation coefficient: 0.6722337432826136
[7.48904776 5.75768222 7.01387872 8.03604241 8.004483   7.59008558
 8.05858924 7.30810016 7.41942745 8.24788273 8.12758092 8.34192311
 8.44322142 7.6573918  7.53840752 8.03812066 7.16953157 5.43756702]
102    7.958607
38     6.022276
8      5.949079
109    8.086186
11     8.013228
51     8.000000
88     8.119186
21     8.113509
82     7.982967
98     7.795880
58     7.677781
64     8.619789
74     8.327902
103    8.376751
91     9.154902
43     7.958607
25     4.949659
30     6.010550
Name: MCF-7, dtype: float64
Training Root Mean Square Error: 0.295841987221968
Testing Root Mean Square Error: 0.7977391691942761
```

3.1 Search inside correlation space

[24]:
```
step = 0.01
initial_step = corr_low
last_step = corr_high
first_list = [x / 100.0 for x in range(int(initial_step*100),␣
 ↪int(last_step*100), int(step*100))]
n_estimators = [range(2,21,1)]
```

```
corr_th = []
second_list = []
third_list = []
fourth_l = []
fifth_l = []
f_list = []
fif_list = []
for i in first_list:

    for estimator in n_estimators[0]:

        without_standardization, train_r2, test_r2, _, h_, target_column_name, training_data_RMSE, test_data_RMSE = pred_model.prepare_data_and_create_model(molecular_descriptors_df=data,
                                correlation_threshold=i,
                                standardization=False,
                                model_type='RandomForestRegressor',
                                n_estimators_=estimator,
                                target_column_name = target,
                                random_state=random_state,
                                train_test_split_=True,
                                verbose=False)
        corr_th.append(i)
        second_list.append(train_r2)
        third_list.append(test_r2)
        fourth_l.append(training_data_RMSE)
        fifth_l.append(test_data_RMSE)
        f_list.append(len(h_))
        fif_list.append(estimator)
```

[25]:
```
df_without_standardization = pd.DataFrame(data=corr_th, columns=["Correlation threshold"])
df_without_standardization['Training data R^2 score'] = second_list
df_without_standardization['Test data R^2 score'] = third_list
df_without_standardization['Training RMSE'] = fourth_l
df_without_standardization['Test RMSE'] = fifth_l
df_without_standardization['Number of features'] = f_list
df_without_standardization['Number of estimators'] = fif_list
```

[26]:
```
df_random_forest = df_without_standardization.copy()
#df_without_standardization.to_excel('../Data/
↪A549_Random_forest_rs_'+str(random_state)+'.xlsx')
```

[27]:
```
corre_list = [x / 100.0 for x in range(int(initial_step*100),␣
↪int(last_step*100), int(0.01*100))]
for element in corre_list:
    element_ =␣
↪df_without_standardization[df_without_standardization['Correlation␣
↪threshold'] == float(element)]
    plt.plot(element_['Number of estimators'], element_['Training data R^2␣
↪score'], label = "Training data R^2 score", marker='v')
    plt.plot(element_['Number of estimators'], element_['Test data R^2 score'],␣
↪label = "Test data R^2 score", marker='D')
    plt.legend()
    plt.xlabel('Number of estimators -> correlation threshold: '+str(element)+'.
↪ \n Number of features: '+str(element_['Number of features'].iloc[0]))
    plt.xlim(max(element_['Number of estimators'])+1, min(element_['Number of␣
↪estimators'])-1)
    plt.ylabel('R^2 score')
    plt.ylim([0, 1])
    plt.rc('grid', linestyle="-.", color='black')
    plt.grid(True)
    plt.show()
```

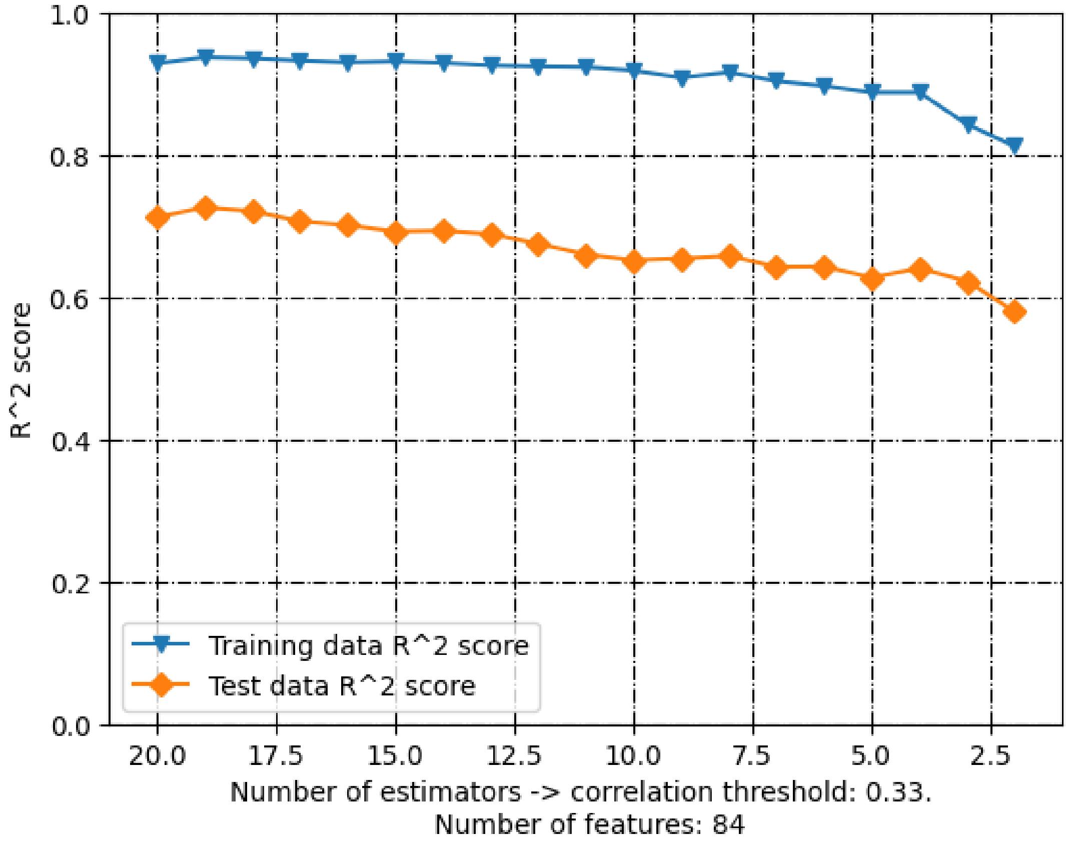

1.0
0.8
0.6
0.4
0.2
0.0
R^2 score
Training data R^2 score
Test data R^2 score
20.0
17.5
15.0
12.5
10.0
7.5
5.0
2.5
Number of estimators -> correlation threshold: 0.33.
Number of features: 84

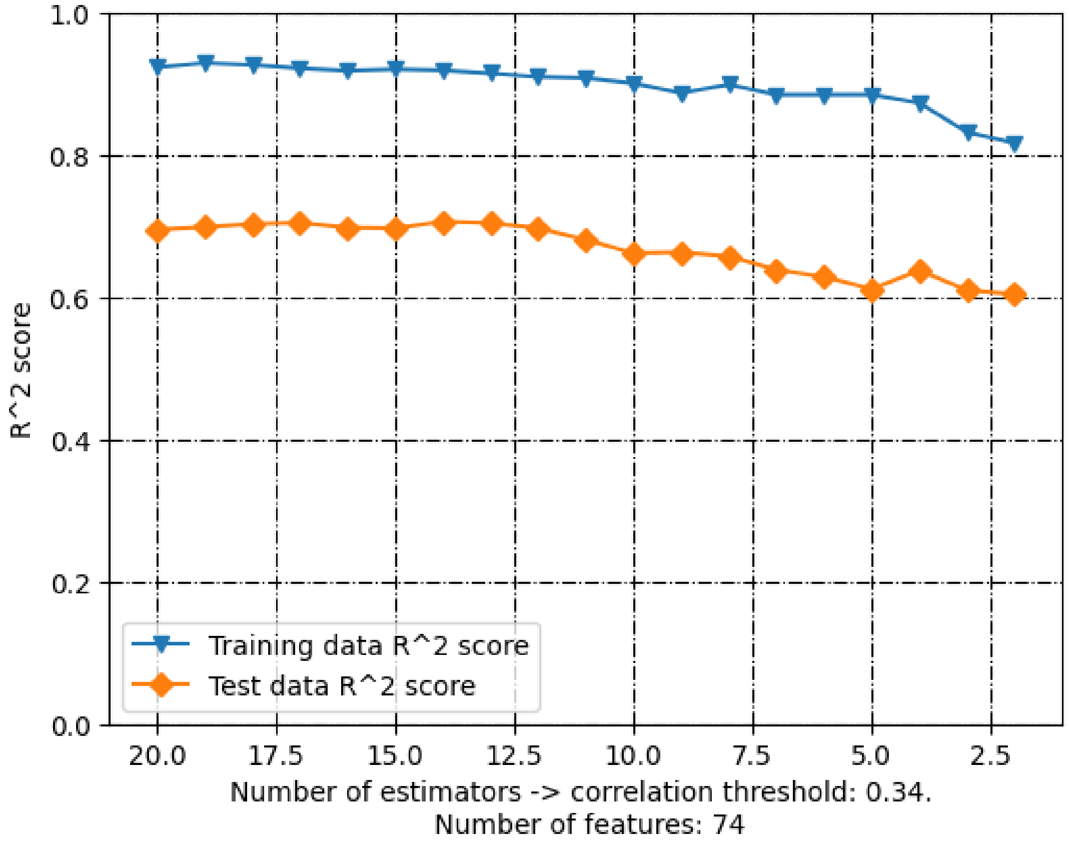
1.0
0.8
0.6
0.4
0.2
0.0
R^2 score
Training data R^2 score
Test data R^2 score
20.0
17.5
15.0
12.5
10.0
7.5
5.0
2.5
Number of estimators -> correlation threshold: 0.34.
Number of features: 74

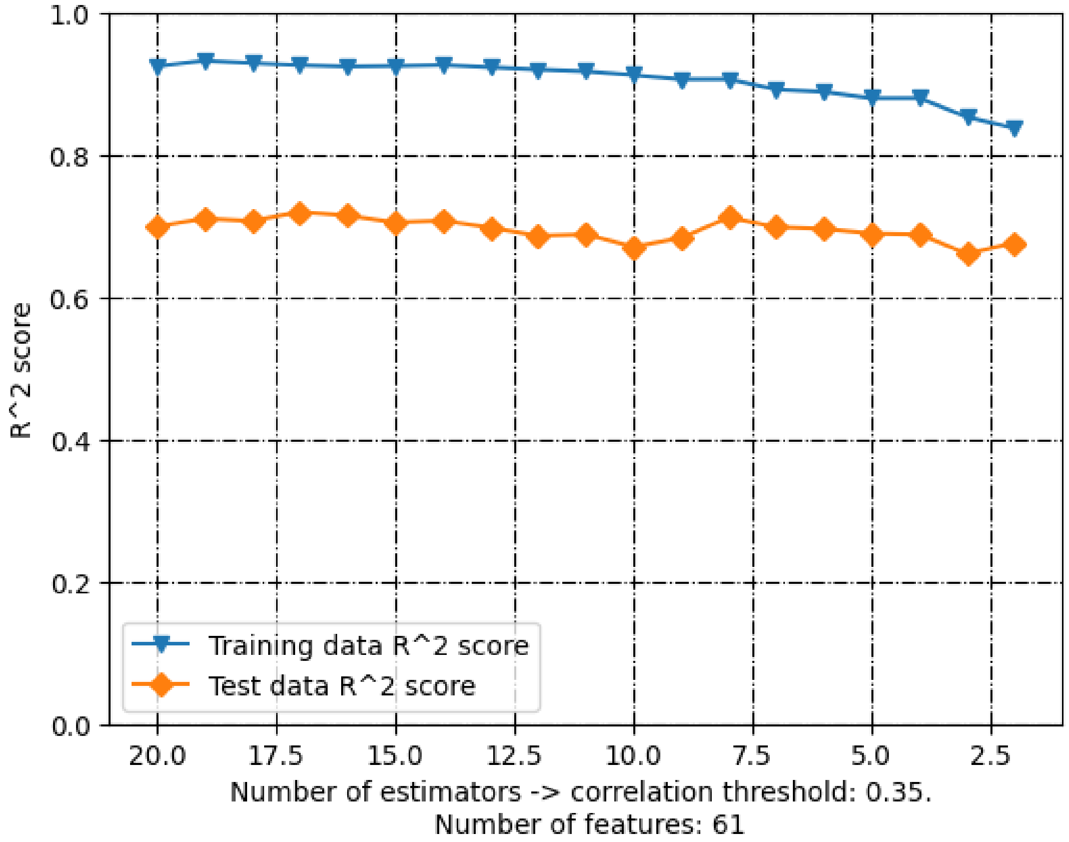
1.0
0.8
0.6
0.4
0.2
0.0
R^2 score
Training data R^2 score
Test data R^2 score
20.0
17.5
15.0
12.5
10.0
7.5
5.0
2.5
Number of estimators -> correlation threshold: 0.35.
Number of features: 61

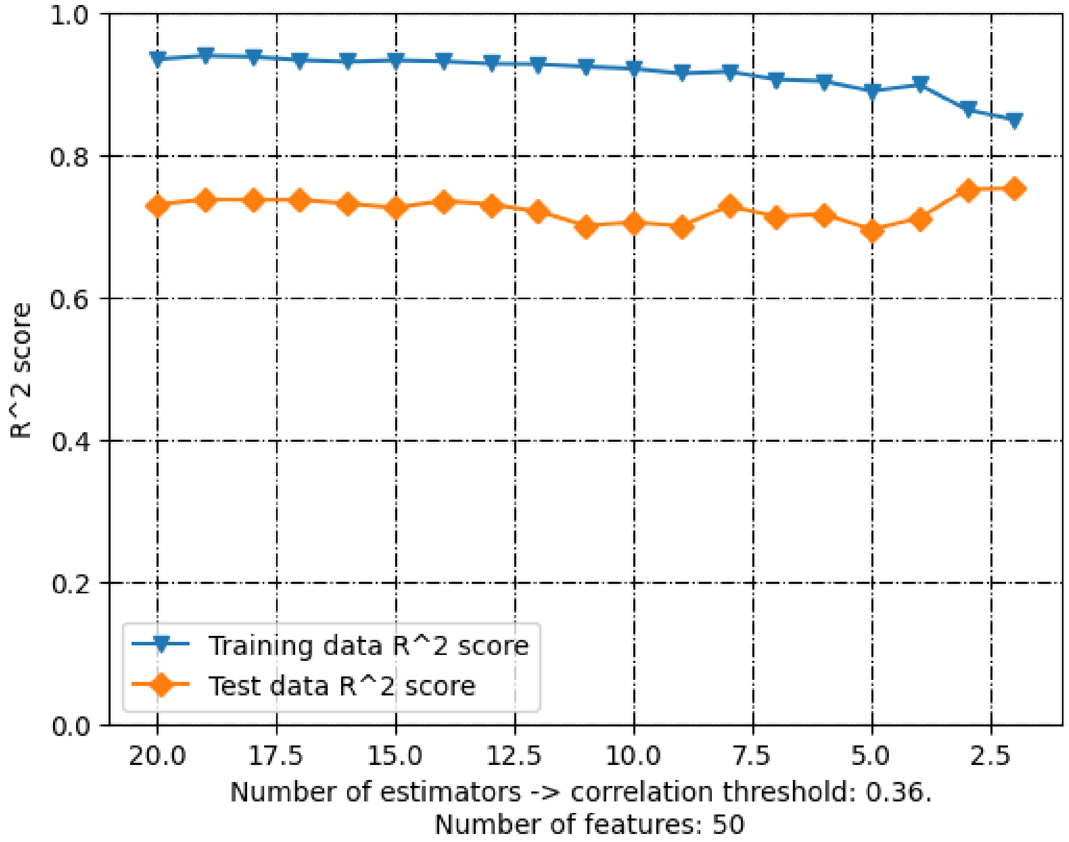

1.0
0.8
0.6
0.4
0.2
0.0
R^2 score
20.0
17.5
15.0
12.5
10.0
7.5
5.0
2.5
Number of estimators -> correlation threshold: 0.36.
Number of features: 50
Training data R^2 score
Test data R^2 score

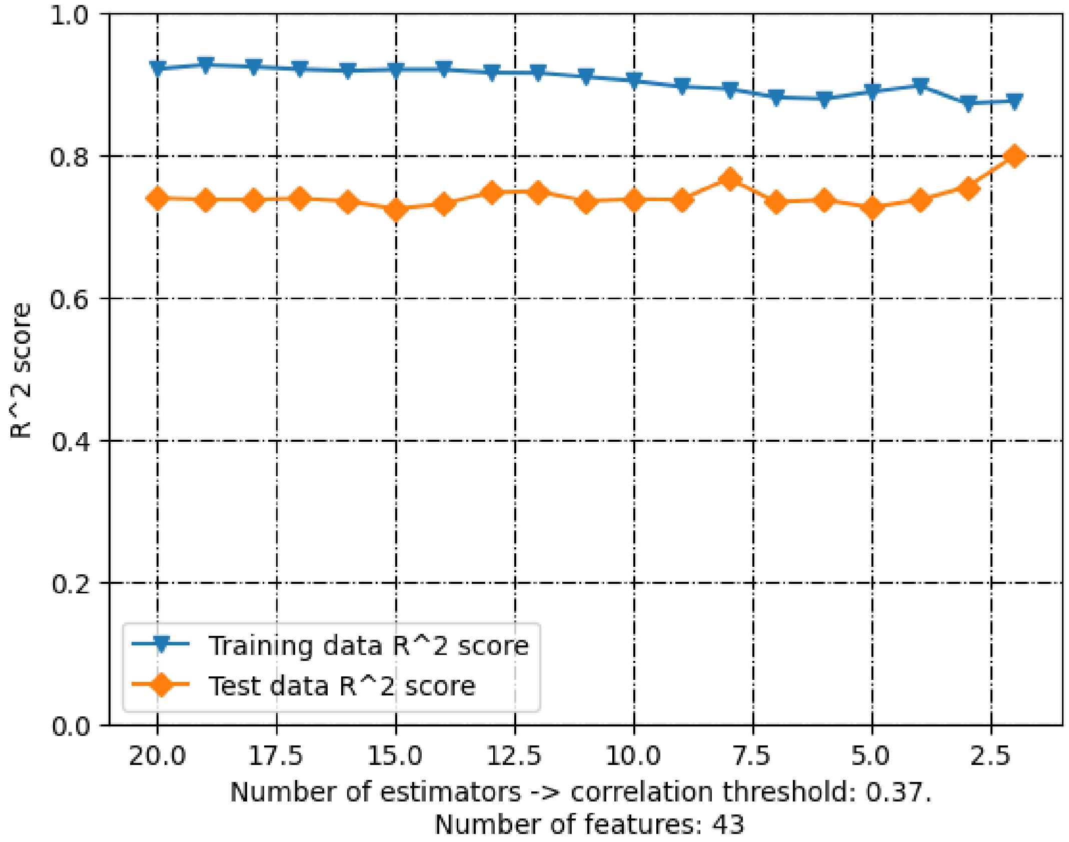
1.0
0.8
0.6
0.4
0.2
0.0
R^2 score
Training data R^2 score
Test data R^2 score
20.0
17.5
15.0
12.5
10.0
7.5
5.0
2.5
Number of estimators -> correlation threshold: 0.37.
Number of features: 43

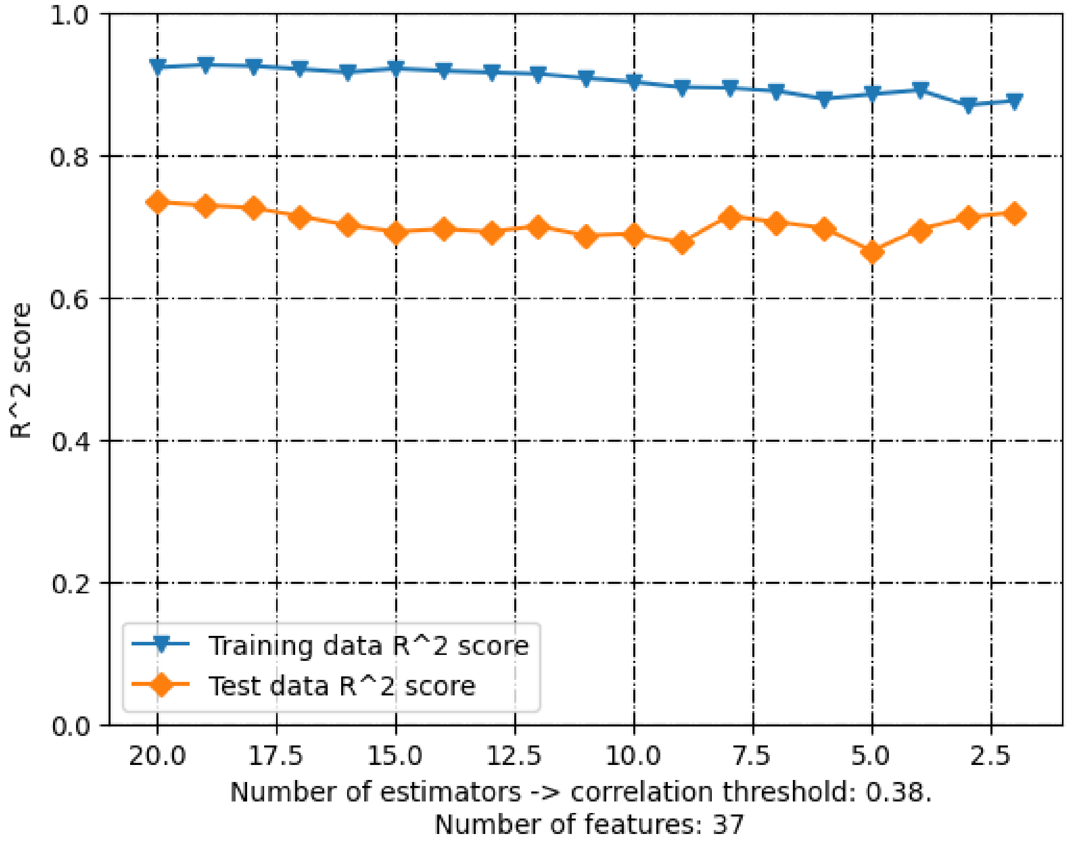

1.0
0.8
0.6
0.4
0.2
0.0
R^2 score
20.0
17.5
15.0
12.5
10.0
7.5
5.0
2.5
Number of estimators -> correlation threshold: 0.38.
Number of features: 37
Training data R^2 score
Test data R^2 score

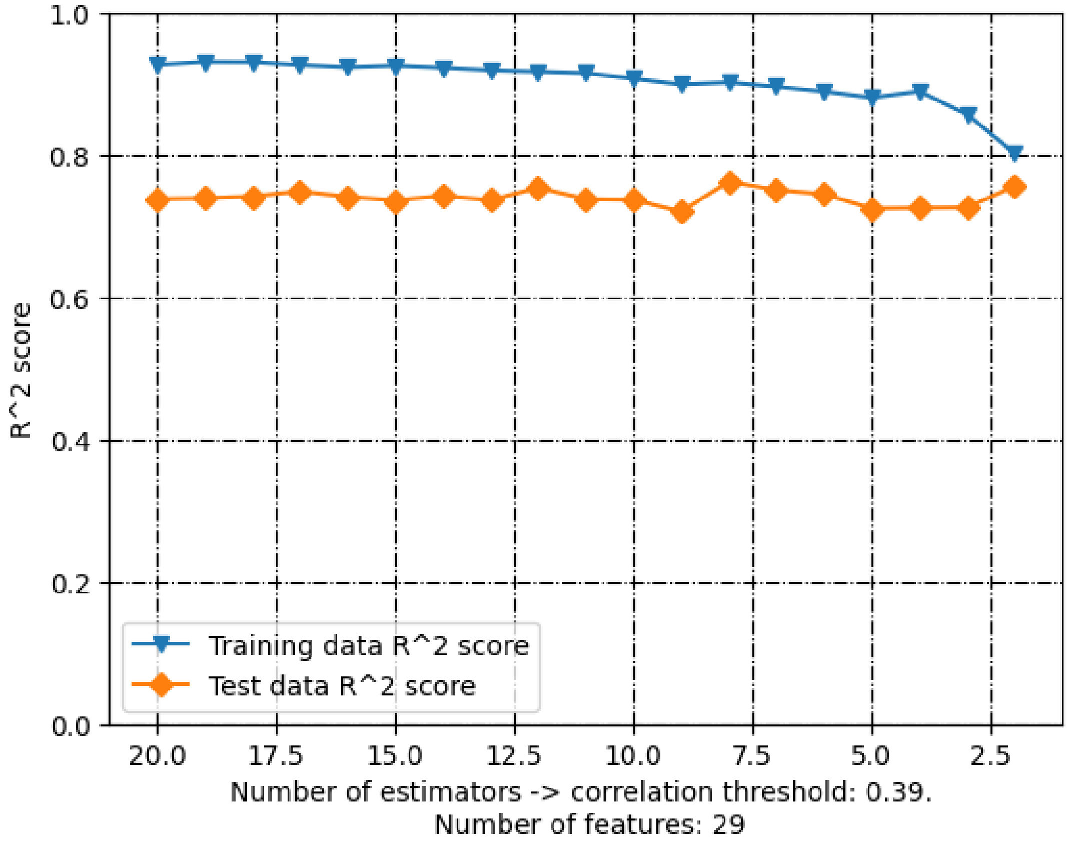
1.0
0.8
0.6
0.4
0.2
0.0
R^2 score
Training data R^2 score
Test data R^2 score
20.0
17.5
15.0
12.5
10.0
7.5
5.0
2.5
Number of estimators -> correlation threshold: 0.39.
Number of features: 29

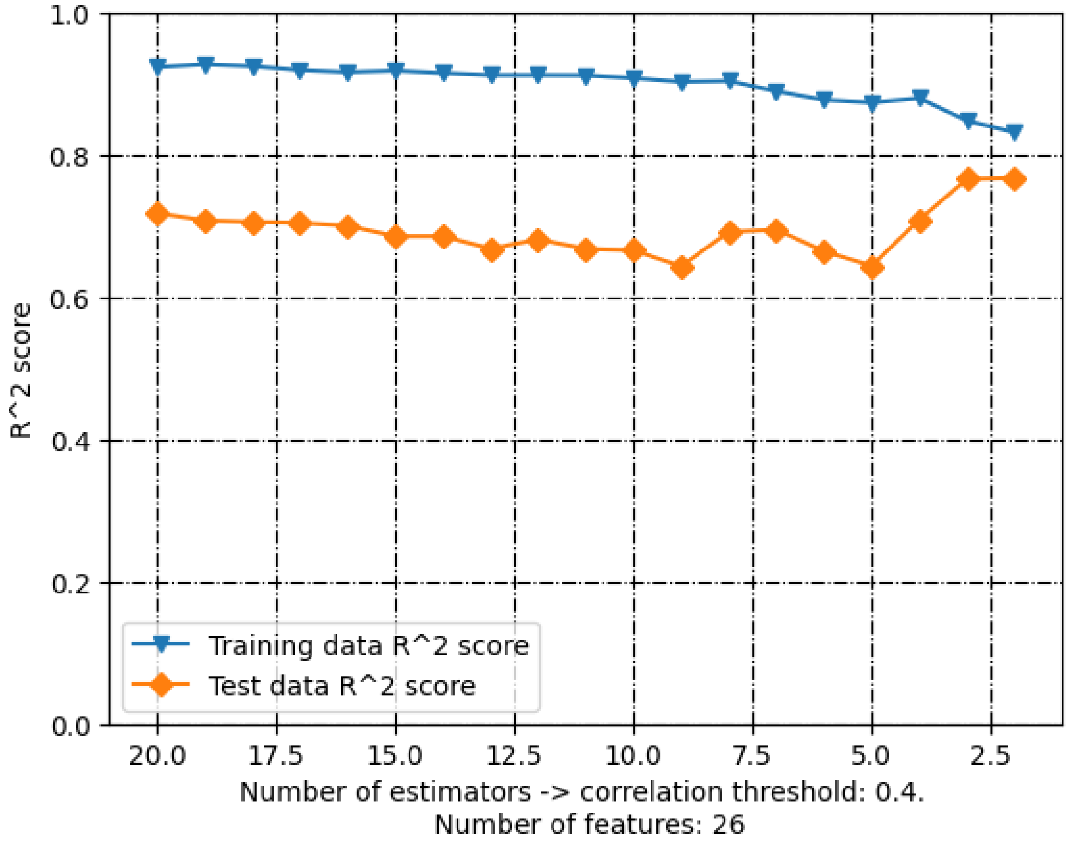
1.0
0.8
0.6
0.4
0.2
0.0
R^2 score
Training data R^2 score
Test data R^2 score
20.0
17.5
15.0
12.5
10.0
7.5
5.0
2.5
Number of estimators -> correlation threshold: 0.4.
Number of features: 26

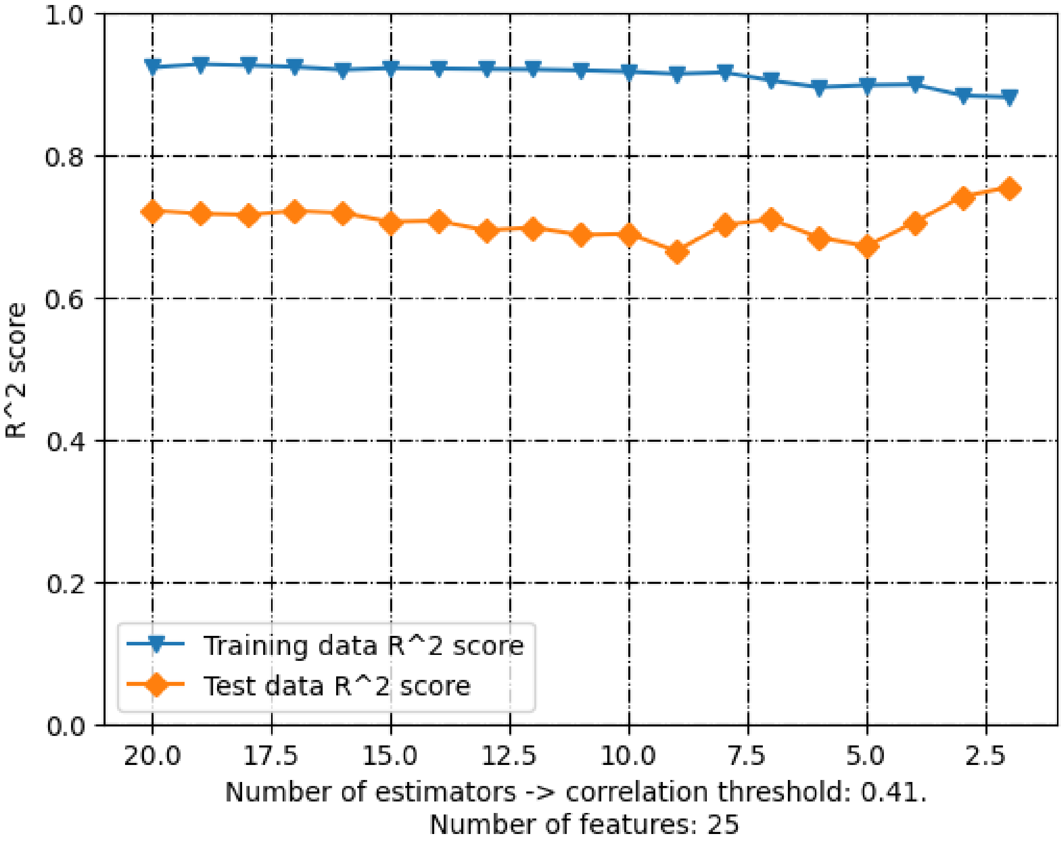

1.0
0.8
0.6
0.4
0.2
0.0
R^2 score
20.0
17.5
15.0
12.5
10.0
7.5
5.0
2.5
Number of estimators -> correlation threshold: 0.41.
Number of features: 25
Training data R^2 score
Test data R^2 score

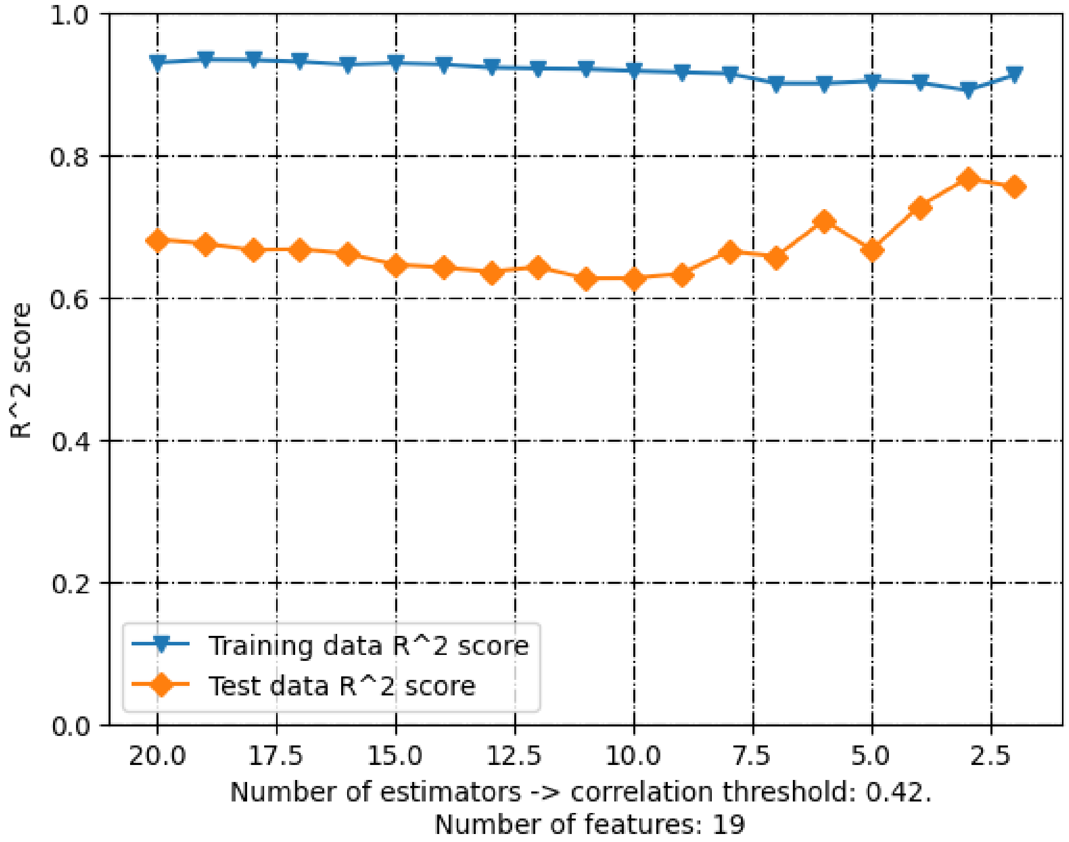
1.0
0.8
0.6
0.4
0.2
0.0
R^2 score
Training data R^2 score
Test data R^2 score
20.0
17.5
15.0
12.5
10.0
7.5
5.0
2.5
Number of estimators -> correlation threshold: 0.42.
Number of features: 19

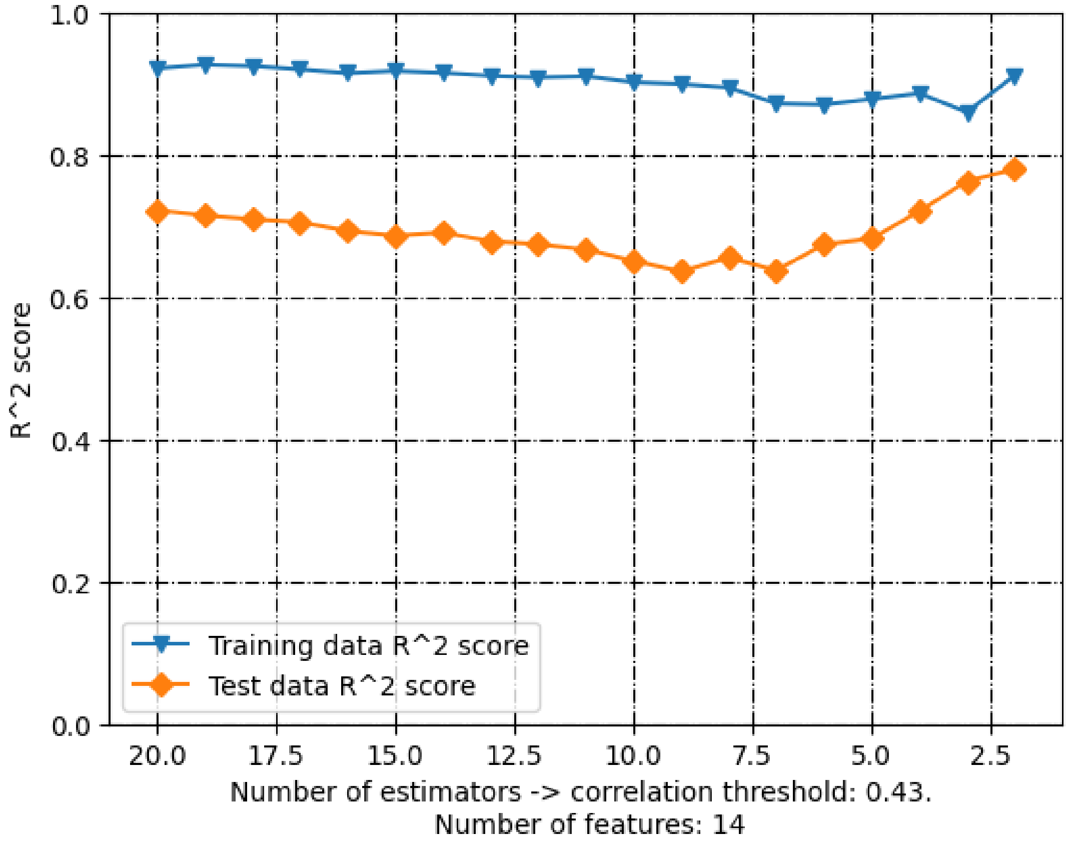

1.0
0.8
0.6
0.4
0.2
0.0
R^2 score
20.0
17.5
15.0
12.5
10.0
7.5
5.0
2.5
Number of estimators -> correlation threshold: 0.43.
Number of features: 14
Training data R^2 score
Test data R^2 score

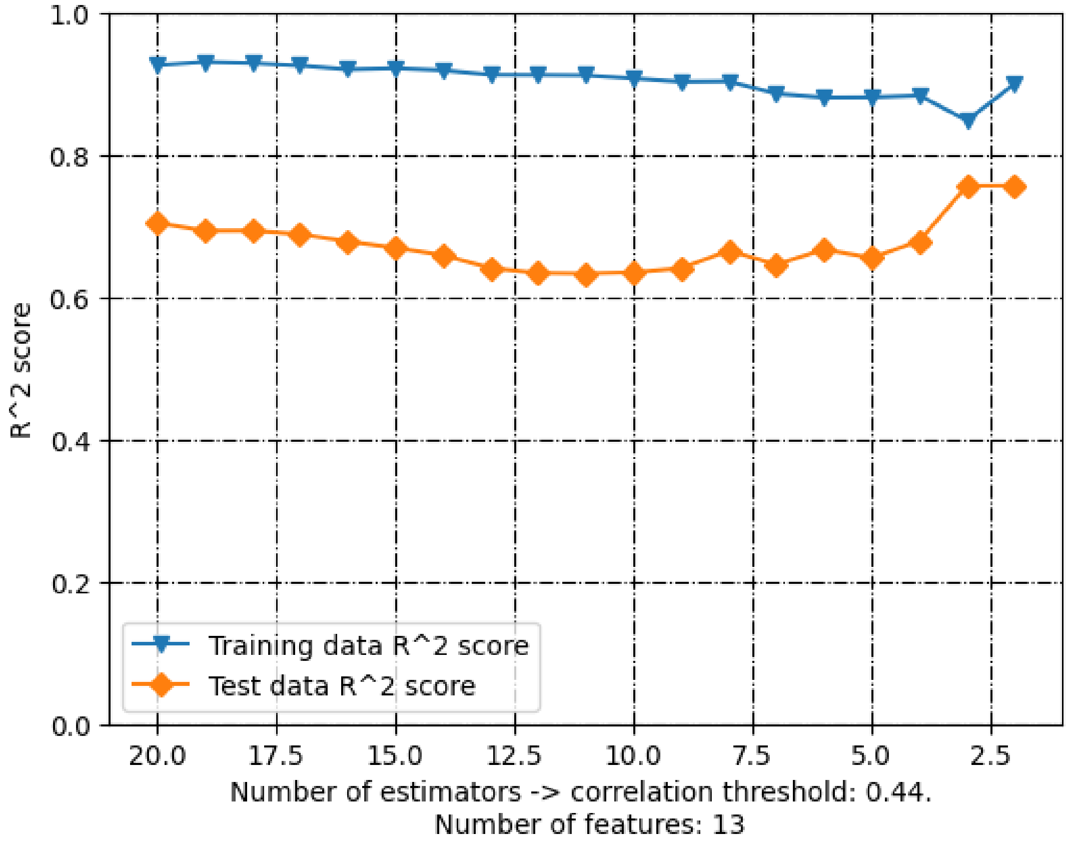
1.0
0.8
0.6
0.4
0.2
0.0
R^2 score
Training data R^2 score
Test data R^2 score
20.0
17.5
15.0
12.5
10.0
7.5
5.0
2.5
Number of estimators -> correlation threshold: 0.44.
Number of features: 13

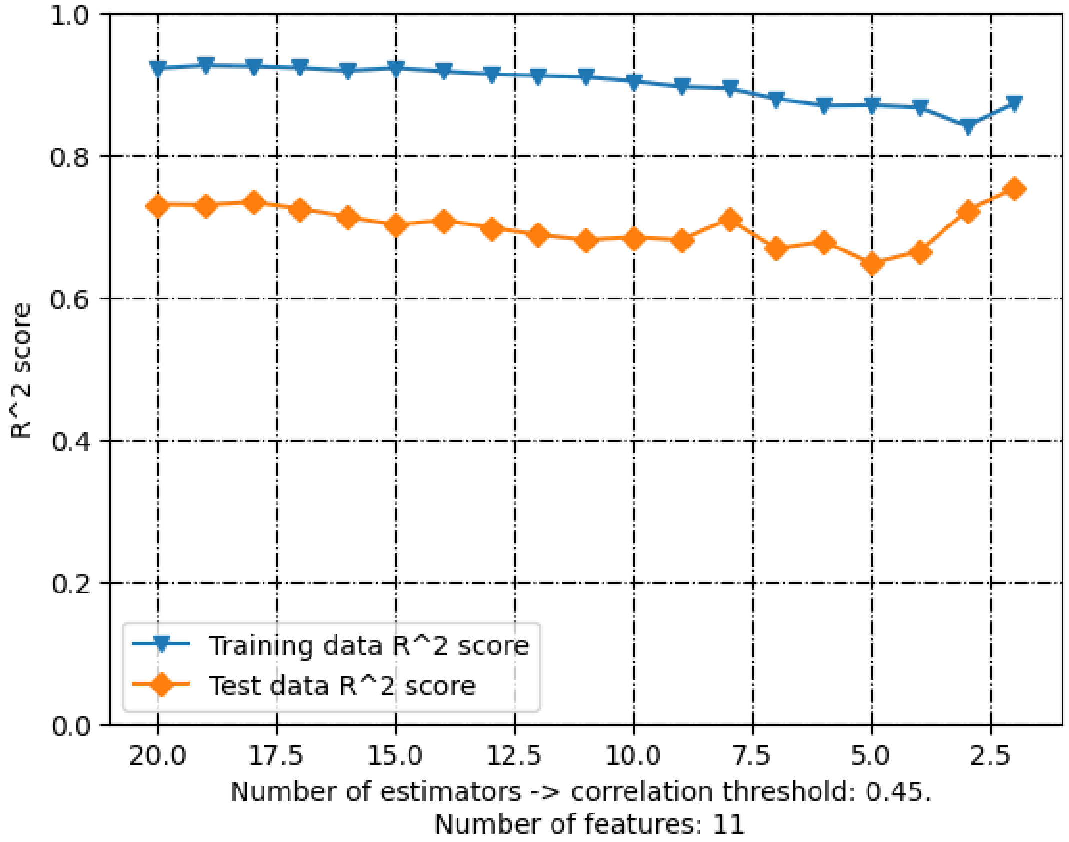

1.0
0.8
0.6
0.4
0.2
0.0
R^2 score
20.0
17.5
15.0
12.5
10.0
7.5
5.0
2.5
Number of estimators -> correlation threshold: 0.45.
Number of features: 11
Training data R^2 score
Test data R^2 score

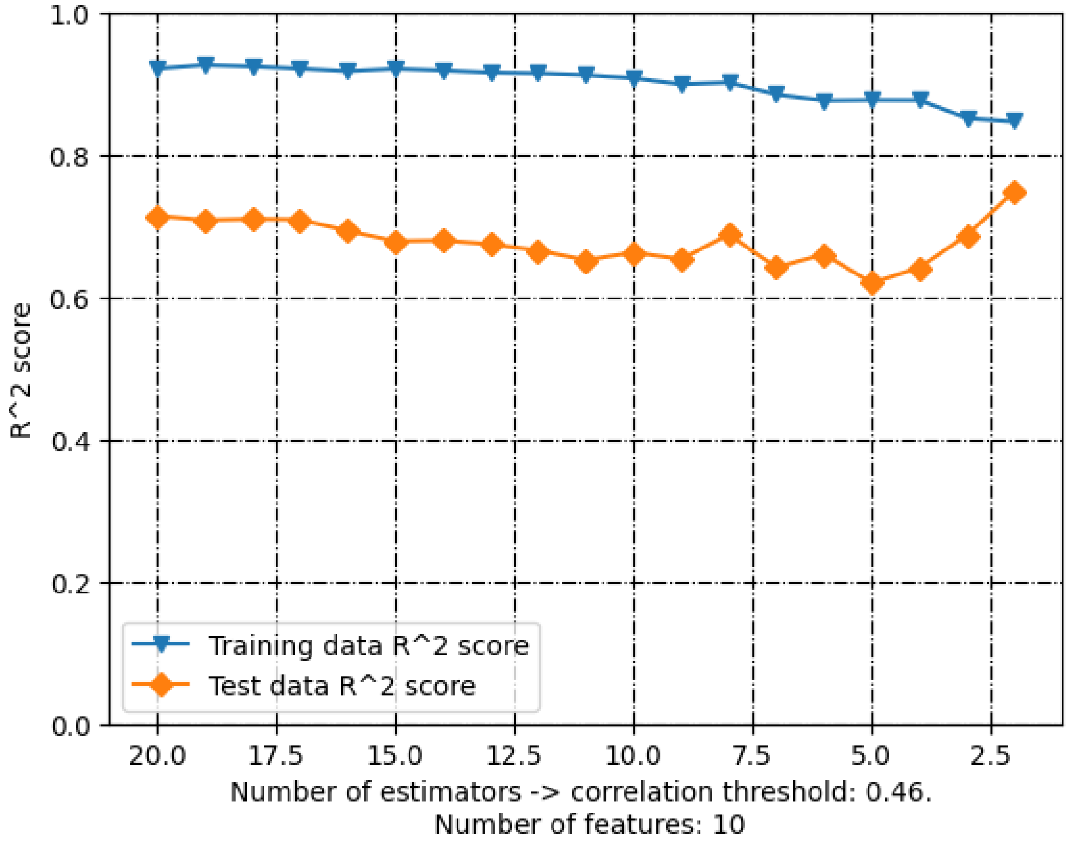
1.0
0.8
0.6
0.4
0.2
0.0
R^2 score
Training data R^2 score
Test data R^2 score
20.0
17.5
15.0
12.5
10.0
7.5
5.0
2.5
Number of estimators -> correlation threshold: 0.46.
Number of features: 10

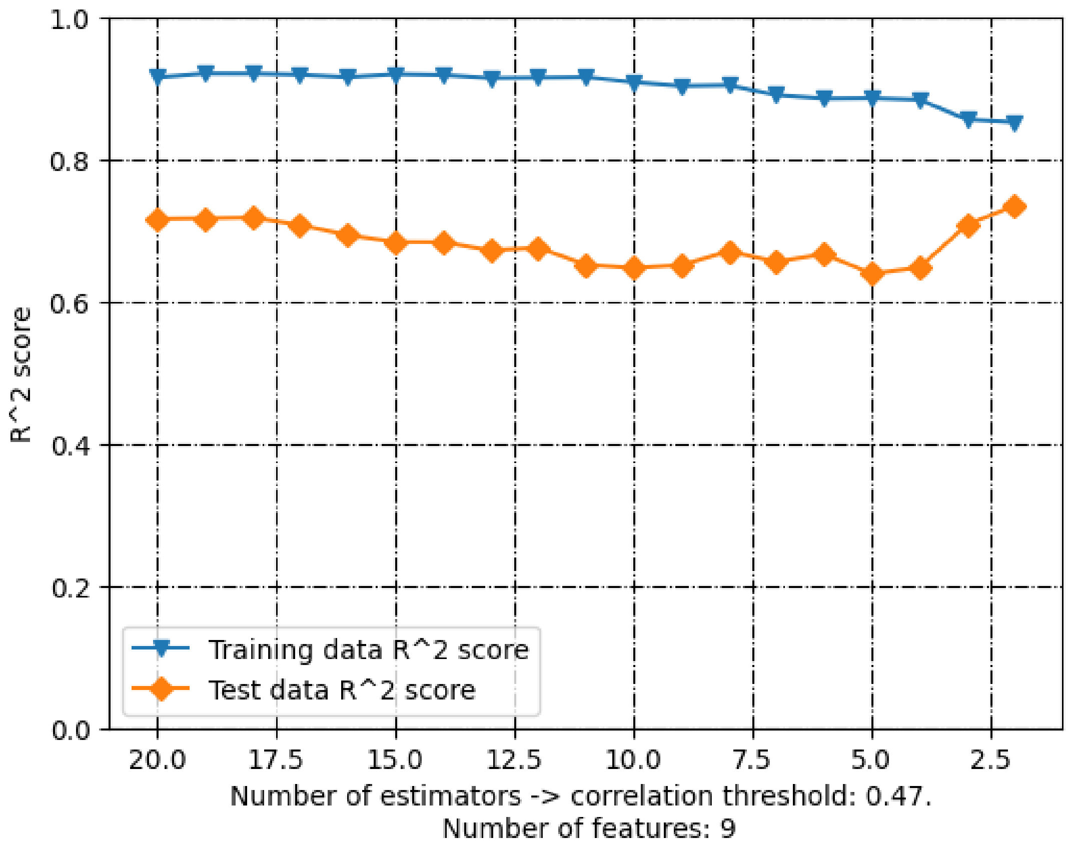

1.0
0.8
0.6
0.4
0.2
0.0
R^2 score
Training data R^2 score
Test data R^2 score
20.0
17.5
15.0
12.5
10.0
7.5
5.0
2.5
Number of estimators -> correlation threshold: 0.47.
Number of features: 9

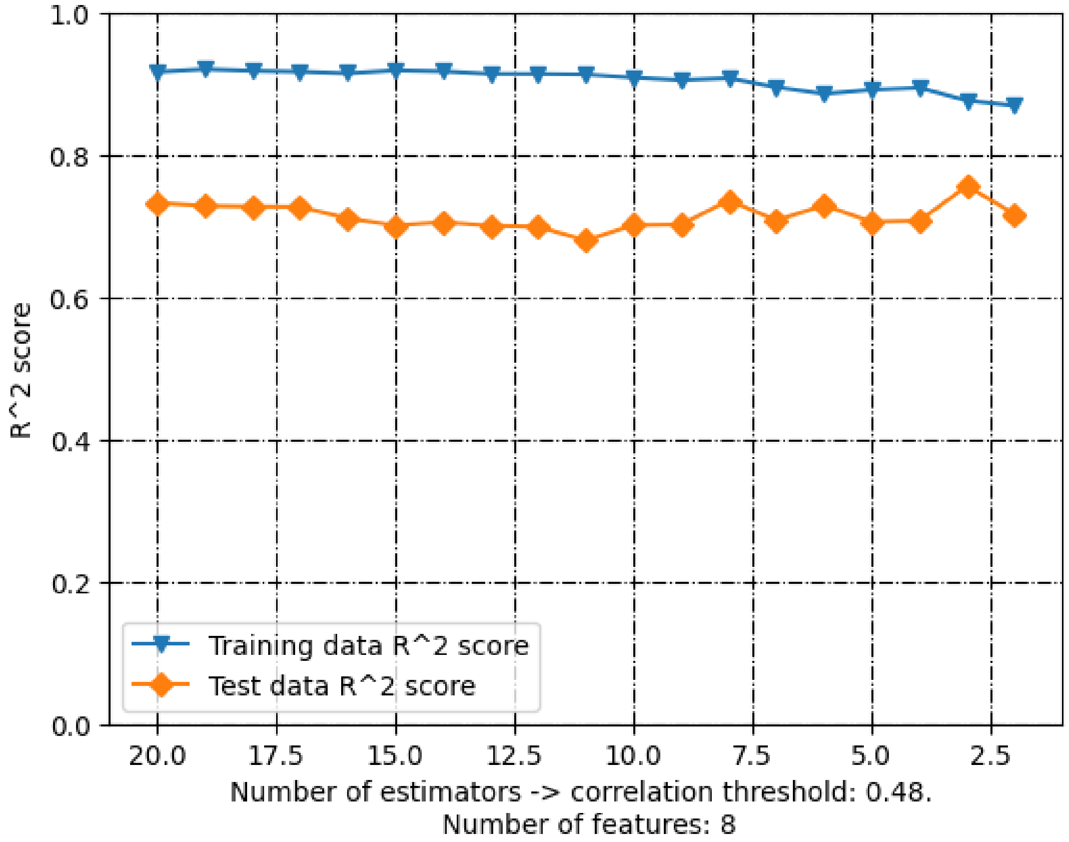
1.0
0.8
0.6
0.4
0.2
0.0
R^2 score
Training data R^2 score
Test data R^2 score
20.0
17.5
15.0
12.5
10.0
7.5
5.0
2.5
Number of estimators -> correlation threshold: 0.48.
Number of features: 8

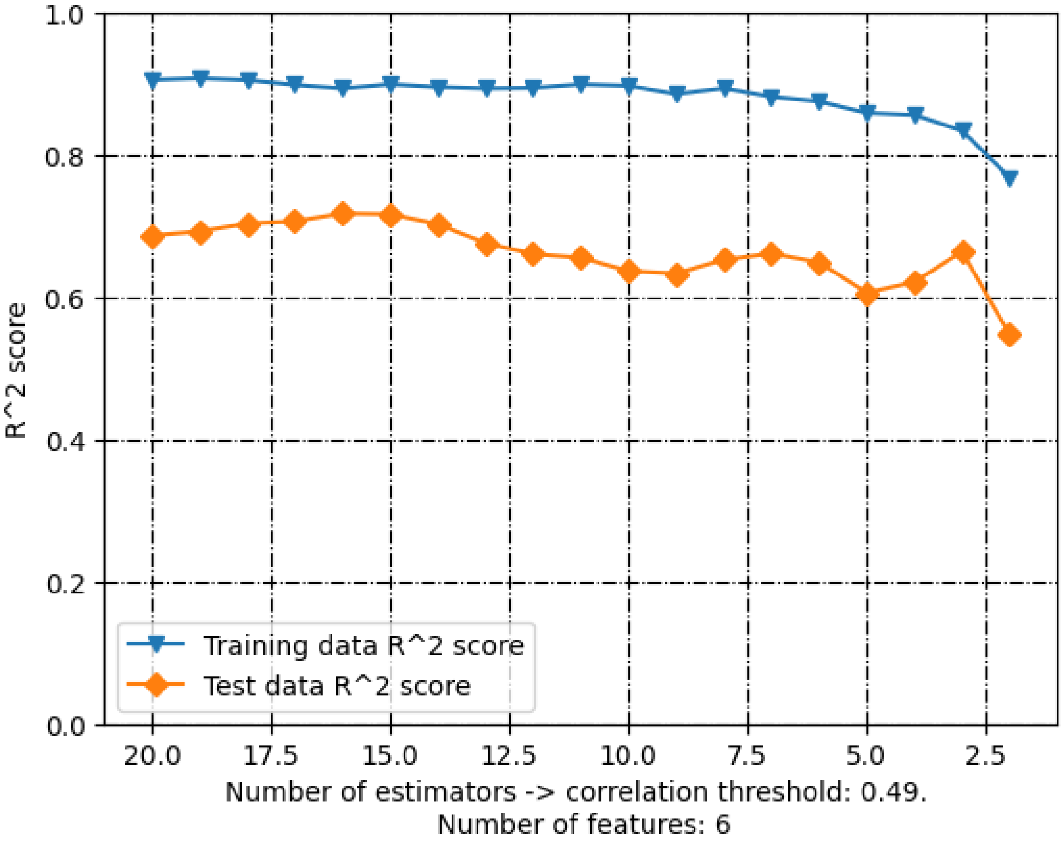

1.0
0.8
0.6
0.4
0.2
0.0
R^2 score
20.0
17.5
15.0
12.5
10.0
7.5
5.0
2.5
Training data R^2 score
Test data R^2 score
Number of estimators -> correlation threshold: 0.49.
Number of features: 6

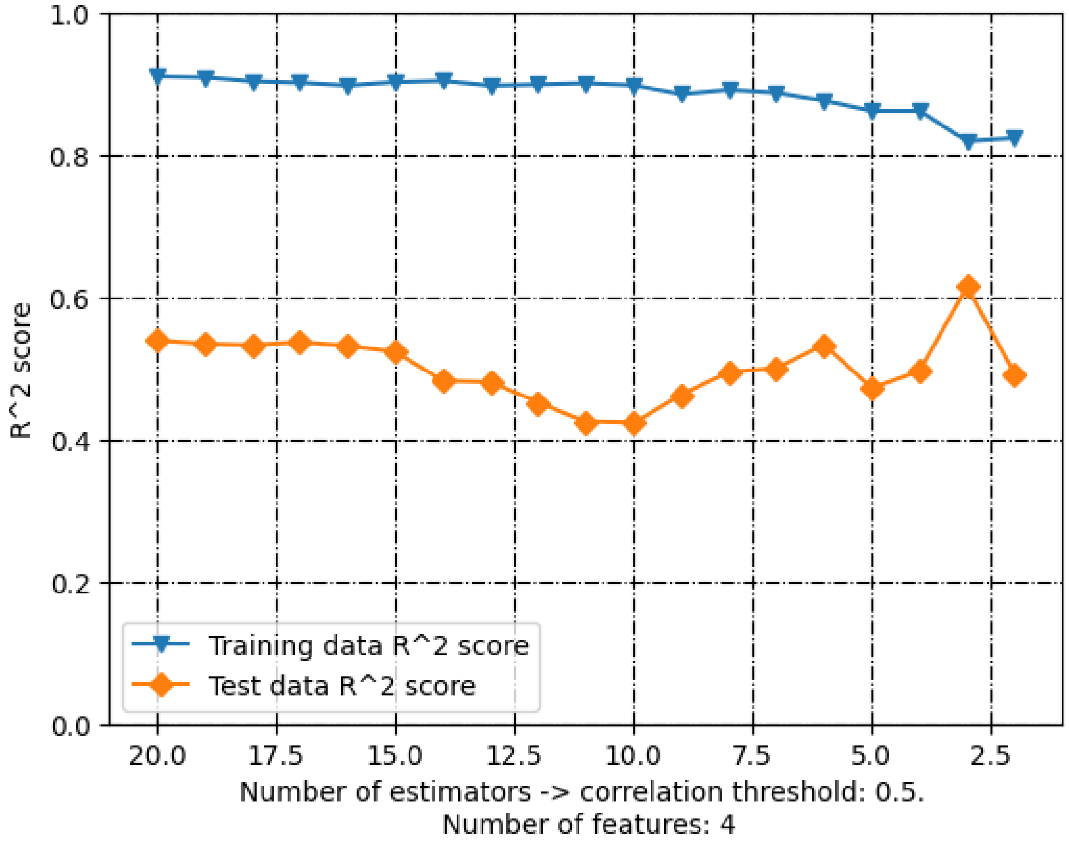

1.0
0.8
0.6
0.4
0.2
0.0
R^2 score
Training data R^2 score
Test data R^2 score
20.0
17.5
15.0
12.5
10.0
7.5
5.0
2.5
Number of estimators -> correlation threshold: 0.5.
Number of features: 4

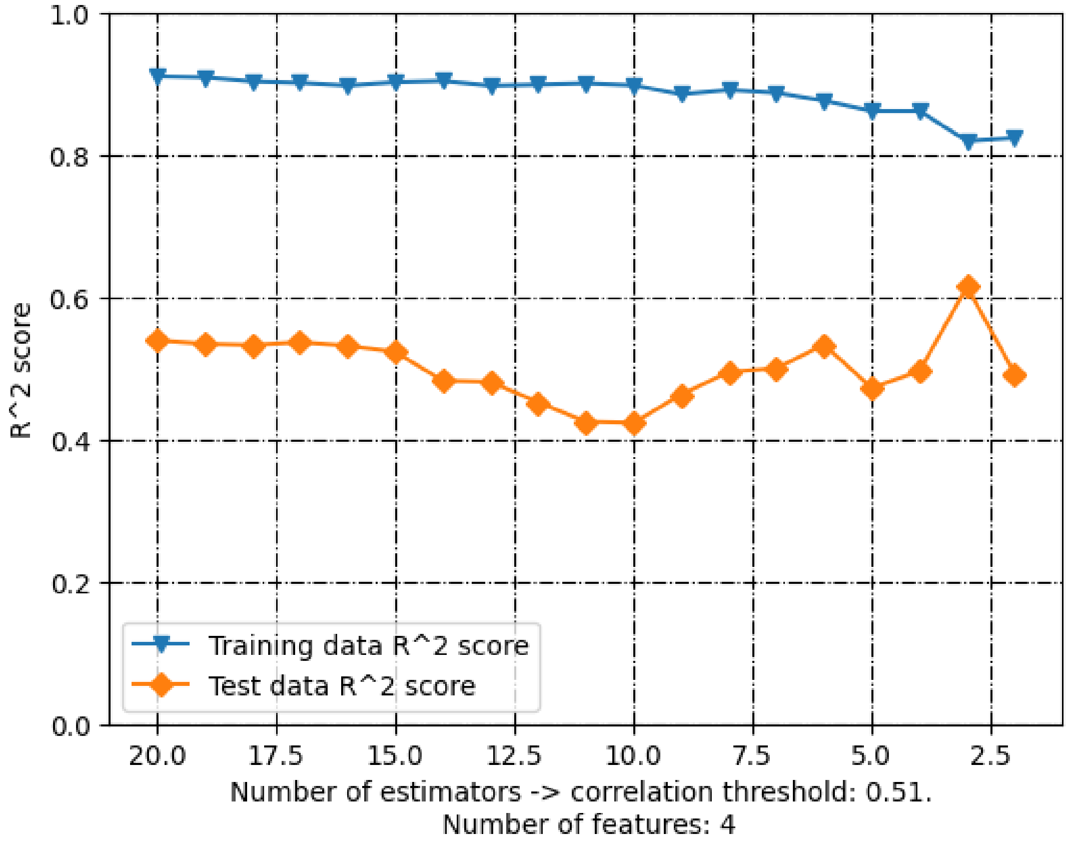

[28]:
```
plt.plot(df_without_standardization['Correlation threshold'],␣
 ↪df_without_standardization['Number of features'], label = "Number of␣
 ↪features", marker='.')
plt.legend()
plt.xlabel('Correlation threshold')
plt.ylabel('Number of features')
plt.show()
```

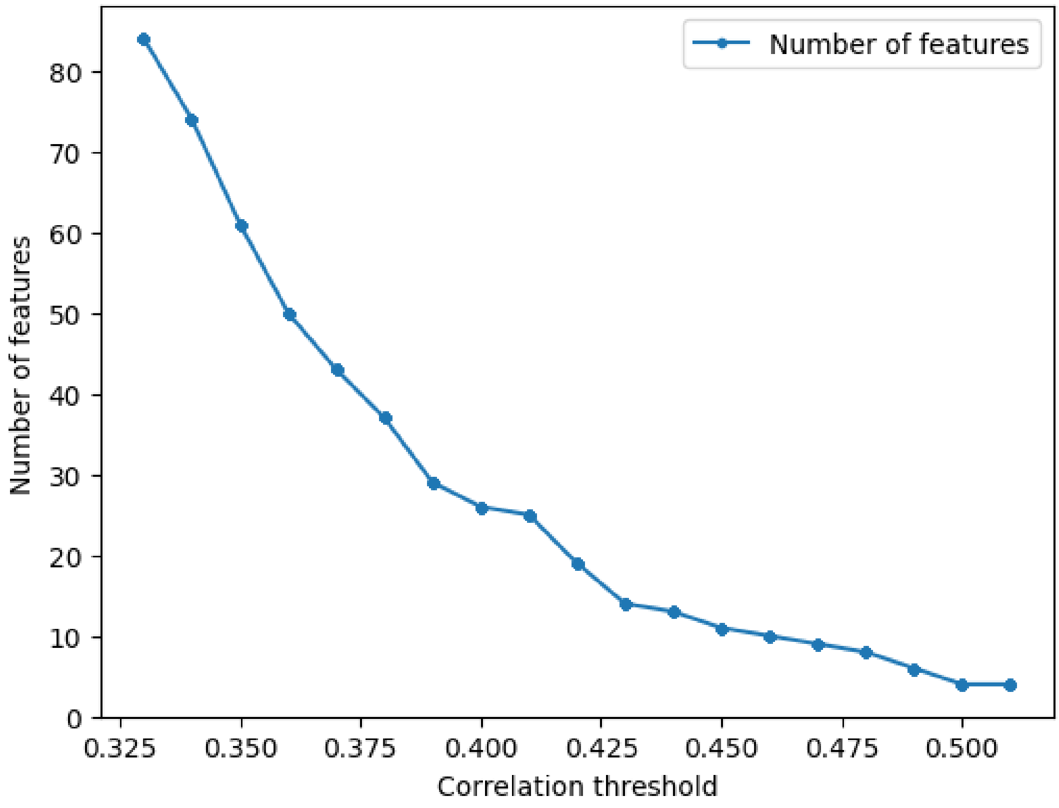


```
[ ]:
```

4 KNeighborsRegressor

```
[29]: without_standardization, train_r2_, test_r2_, hist1, hist2, target_column_name,␣
    ↪training_data_RMSE, test_data_RMSE  = pred_model.
    ↪prepare_data_and_create_model(molecular_descriptors_df=data,
                                                                                   ␣
    ↪                    correlation_threshold=0.50,
                                                                                   ␣
    ↪                    standardization=False,
                                                                                   ␣
    ↪                    model_type='KNeighborsRegressor',
                                                                                   ␣
    ↪                    target_column_name = target,
                                                                                   ␣
    ↪                    random_state=random_state,
                                                                                   ␣
    ↪                    train_test_split_=True,
```

```
↪                    verbose=True)
```

```
I am not doing standardization…
  molecular descriptor name
0                   AATS0Z
1                 AATS0are
2                   AATS0d
3                  AATS0dv
4                   AATS0i
  molecular descriptor name  corr_value
0                   AATS0Z   -0.038502
1                 AATS0are   -0.120847
2                   AATS0d    0.041648
3                  AATS0dv   -0.115899
4                   AATS0i    0.191765
  molecular descriptor name  corr_value  absolute correlation value
0                   AATS0Z   -0.038502                    0.038502
1                 AATS0are   -0.120847                    0.120847
2                   AATS0d    0.041648                    0.041648
3                  AATS0dv   -0.115899                    0.115899
4                   AATS0i    0.191765                    0.191765
     molecular descriptor name  corr_value  absolute correlation value
226                     AMID_O   -0.520578                    0.520578
505                EState_VSA5   -0.578904                    0.578904
506                EState_VSA6    0.519794                    0.519794
791                    MDEO-12   -0.525441                    0.525441
1211                     MCF-7    1.000000                    1.000000
    molecular descriptor name  corr_value  absolute correlation value
226                    AMID_O   -0.520578                    0.520578
505               EState_VSA5   -0.578904                    0.578904
506               EState_VSA6    0.519794                    0.519794
791                   MDEO-12   -0.525441                    0.525441
The model used is: KNeighbors…
Return the coefficient of determination of the prediction:
0.7633277638944249
R^2 score: 0.4926643581637682
Correlation coefficient: 0.701900532955894
Test data - unseen during training:
R^2 score: 0.7633277638944249
Correlation coefficient: 0.8736863074893786
[7.50043319 6.60652477 6.26345173 7.45309764 8.25480167 7.45309764
 8.03129323 7.58116176 8.03129323 8.03397726 6.85253149 8.27774543
 8.46138271 8.14325294 8.07271833 8.07271833 6.01407407 5.99312666]
102    7.958607
38     6.022276
8      5.949079
109    8.086186
```

```
11      8.013228
51      8.000000
88      8.119186
21      8.113509
82      7.982967
98      7.795880
58      7.677781
64      8.619789
74      8.327902
103     8.376751
91      9.154902
43      7.958607
25      4.949659
30      6.010550
Name: MCF-7, dtype: float64
Training Root Mean Square Error: 0.6626938503329243
Testing Root Mean Square Error: 0.5242083828149131
```

[30]:

```
print(target_column_name+str('_transformed'))
print(hist1[target_column_name].hist())
```

```
MCF-7_transformed
AxesSubplot(0.125,0.11;0.775x0.77)
```

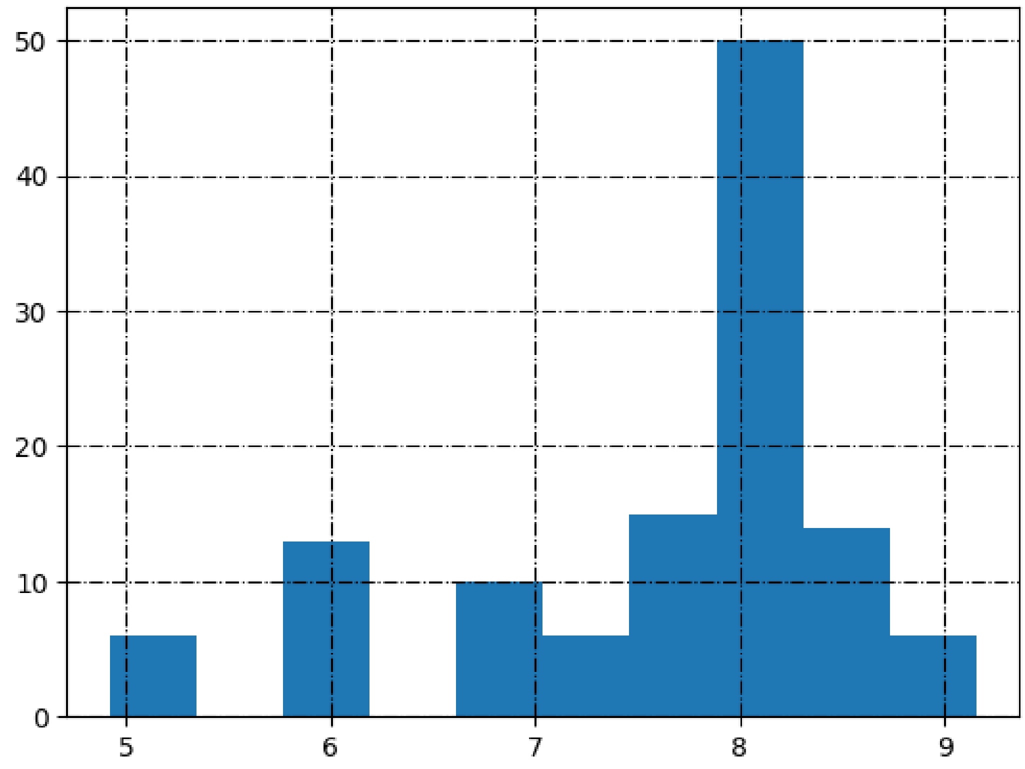

[31]:
```
with_standardization, train_r2_, test_r2_, hist1, hist2, target_column_name,␣
↪training_data_RMSE, test_data_RMSE  = pred_model.
↪prepare_data_and_create_model(molecular_descriptors_df=data,
                                                                              ␣
↪                    correlation_threshold=0.50,
                                                                              ␣
↪                    standardization=True,
                                                                              ␣
↪                    model_type='KNeighborsRegressor',
                                                                              ␣
↪                    target_column_name = target,
                                                                              ␣
↪                    random_state=random_state,
                                                                              ␣
↪                    train_test_split_=True,
                                                                              ␣
↪                    verbose=True)
```

```
I am doing standardization…
  molecular descriptor name
0                    AATS0Z
1                  AATS0are
2                    AATS0d
3                   AATS0dv
4                    AATS0i
  molecular descriptor name  corr_value
0                    AATS0Z   -0.038502
1                  AATS0are   -0.120847
2                    AATS0d    0.041648
3                   AATS0dv   -0.115899
4                    AATS0i    0.191765
  molecular descriptor name  corr_value  absolute correlation value
0                    AATS0Z   -0.038502                    0.038502
1                  AATS0are   -0.120847                    0.120847
2                    AATS0d    0.041648                    0.041648
3                   AATS0dv   -0.115899                    0.115899
4                    AATS0i    0.191765                    0.191765
     molecular descriptor name  corr_value  absolute correlation value
226                     AMID_O   -0.520578                    0.520578
505                EState_VSA5   -0.578904                    0.578904
506                EState_VSA6    0.519794                    0.519794
791                    MDEO-12   -0.525441                    0.525441
1211                     MCF-7    1.000000                    1.000000
    molecular descriptor name  corr_value  absolute correlation value
226                    AMID_O   -0.520578                    0.520578
505               EState_VSA5   -0.578904                    0.578904
506               EState_VSA6    0.519794                    0.519794
```

```
791                   MDEO-12   -0.525441                    0.525441
The model used is: KNeighbors…
Return the coefficient of determination of the prediction:
0.7633277638944249
R^2 score: 0.4926643581637682
Correlation coefficient: 0.701900532955894
Test data - unseen during training:
R^2 score: 0.7633277638944249
Correlation coefficient: 0.8736863074893786
[7.50043319 6.60652477 6.26345173 7.45309764 8.25480167 7.45309764
 8.03129323 7.58116176 8.03129323 8.03397726 6.85253149 8.27774543
 8.46138271 8.14325294 8.07271833 8.07271833 6.01407407 5.99312666]
102    7.958607
38     6.022276
8      5.949079
109    8.086186
11     8.013228
51     8.000000
88     8.119186
21     8.113509
82     7.982967
98     7.795880
58     7.677781
64     8.619789
74     8.327902
103    8.376751
91     9.154902
43     7.958607
25     4.949659
30     6.010550
Name: MCF-7, dtype: float64
Training Root Mean Square Error: 0.6626938503329243
Testing Root Mean Square Error: 0.5242083828149131
```

4.1 Search inside correlation space

[32]:
```python
step = 0.01
initial_step = corr_low
last_step = corr_high
first_list = [x / 100.0 for x in range(int(initial_step*100),␣
 ↪int(last_step*100), int(step*100))]
second_list = []
third_list = []
fourth_l = []
fifth_l = []
f_list = []
for i in first_list:
```

```
    without_standardization, train_r2, test_r2, _, h_, target_column_name,␣
↪training_data_RMSE, test_data_RMSE = pred_model.
↪prepare_data_and_create_model(molecular_descriptors_df = data,
                                                                          ␣
↪                    correlation_threshold = i,
                                                                          ␣
↪                    standardization = False,
                                                                          ␣
↪                    model_type = 'KNeighborsRegressor',
                                                                          ␣
↪                    target_column_name = target,
                                                                          ␣
↪                    random_state=random_state,
                                                                          ␣
↪                    train_test_split_ = True,
                                                                          ␣
↪                    verbose = False)
    second_list.append(train_r2)
    third_list.append(test_r2)
    fourth_l.append(training_data_RMSE)
    fifth_l.append(test_data_RMSE)
    f_list.append(len(h_))
```

```
  File "C:\Users\aleks\anaconda3\envs\cheminf_gpu\lib\site-
packages\joblib\externals\loky\backend\context.py", line 217, in
_count_physical_cores
    raise ValueError(
```

[33]:
```
df_without_standardization = pd.DataFrame(data=first_list,␣
↪columns=["Correlation threshold"])
df_without_standardization['Training data R^2 score'] = second_list
df_without_standardization['Test data R^2 score'] = third_list
df_without_standardization['Training RMSE'] = fourth_l
df_without_standardization['Test RMSE'] = fifth_l
df_without_standardization['Number of features'] = f_list
```

[34]:
```
df_k_nearest = df_without_standardization.copy()
df_without_standardization
```

[34]:
```
    Correlation threshold  Training data R^2 score  Test data R^2 score  \
0                    0.33                 0.570572             0.678929
1                    0.34                 0.572739             0.678929
2                    0.35                 0.572509             0.671096
3                    0.36                 0.574874             0.670597
4                    0.37                 0.561600             0.671880
5                    0.38                 0.659618             0.649241
6                    0.39                 0.663855             0.643022
```

7	0.40	0.658443	0.737529
8	0.41	0.665939	0.658555
9	0.42	0.559293	0.784286
10	0.43	0.531276	0.790893
11	0.44	0.531276	0.790893
12	0.45	0.531276	0.773425
13	0.46	0.538330	0.815329
14	0.47	0.538330	0.815329
15	0.48	0.538511	0.815329
16	0.49	0.519802	0.803034
17	0.50	0.492664	0.763328
18	0.51	0.492664	0.763328

	Training RMSE	Test RMSE	Number of features
0	0.609692	0.610564	84
1	0.608152	0.610564	74
2	0.608316	0.617966	61
3	0.606630	0.618435	50
4	0.616028	0.617230	43
5	0.542811	0.638168	37
6	0.539422	0.643800	29
7	0.543747	0.552041	26
8	0.537747	0.629637	25
9	0.617647	0.500460	19
10	0.636977	0.492736	14
11	0.636977	0.492736	13
12	0.636977	0.512904	11
13	0.632166	0.463052	10
14	0.632166	0.463052	9
15	0.632042	0.463052	8
16	0.644727	0.478218	6
17	0.662694	0.524208	4
18	0.662694	0.524208	4

4.2 Plots

[35]:
```
plt.plot(df_without_standardization['Correlation threshold'],␣
  ↪df_without_standardization['Training data R^2 score'], label = "Training␣
  ↪data R^2 score", marker='v')
plt.plot(df_without_standardization['Correlation threshold'],␣
  ↪df_without_standardization['Test data R^2 score'], label = "Test data R^2␣
  ↪score", marker='D')
plt.legend()
plt.xlabel('Correlation threshold')
plt.ylabel('R^2 score')
plt.ylim([0, 1])
plt.show()
```

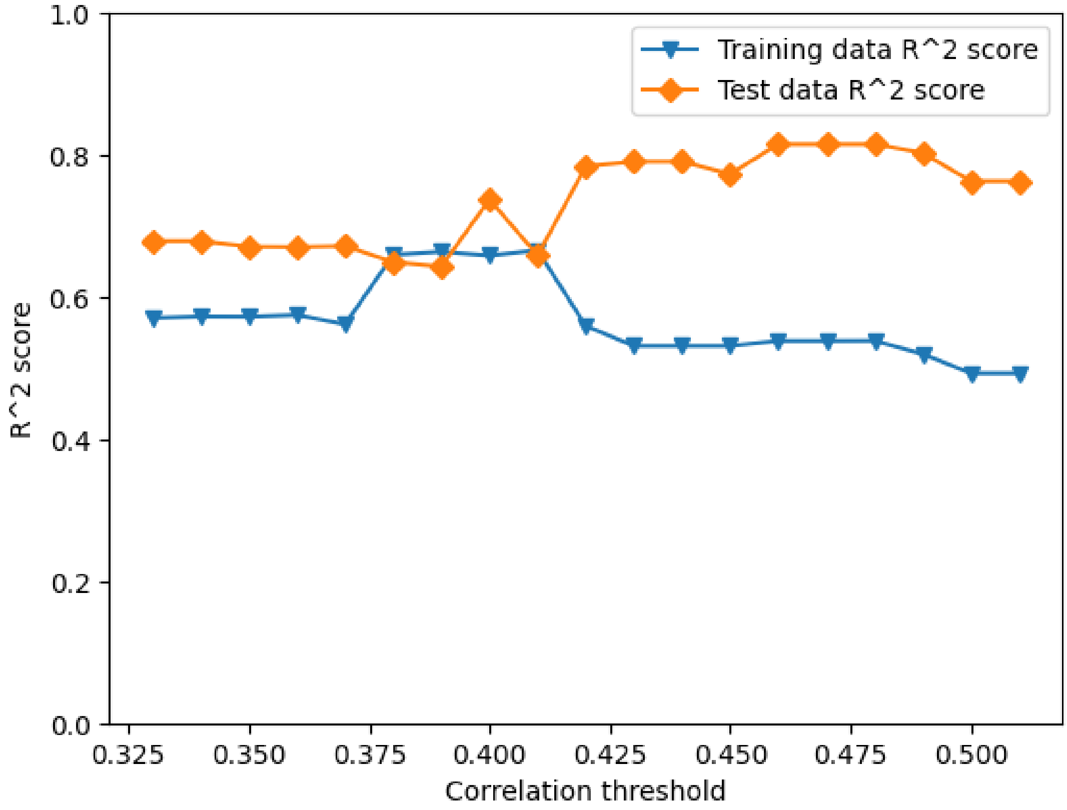

[36]:
```
plt.plot(df_without_standardization['Correlation threshold'],␣
  ↪df_without_standardization['Number of features'], label = "Number of␣
  ↪features", marker='.')
plt.legend()
plt.xlabel('Correlation threshold')
plt.ylabel('Number of features')
plt.show()
```

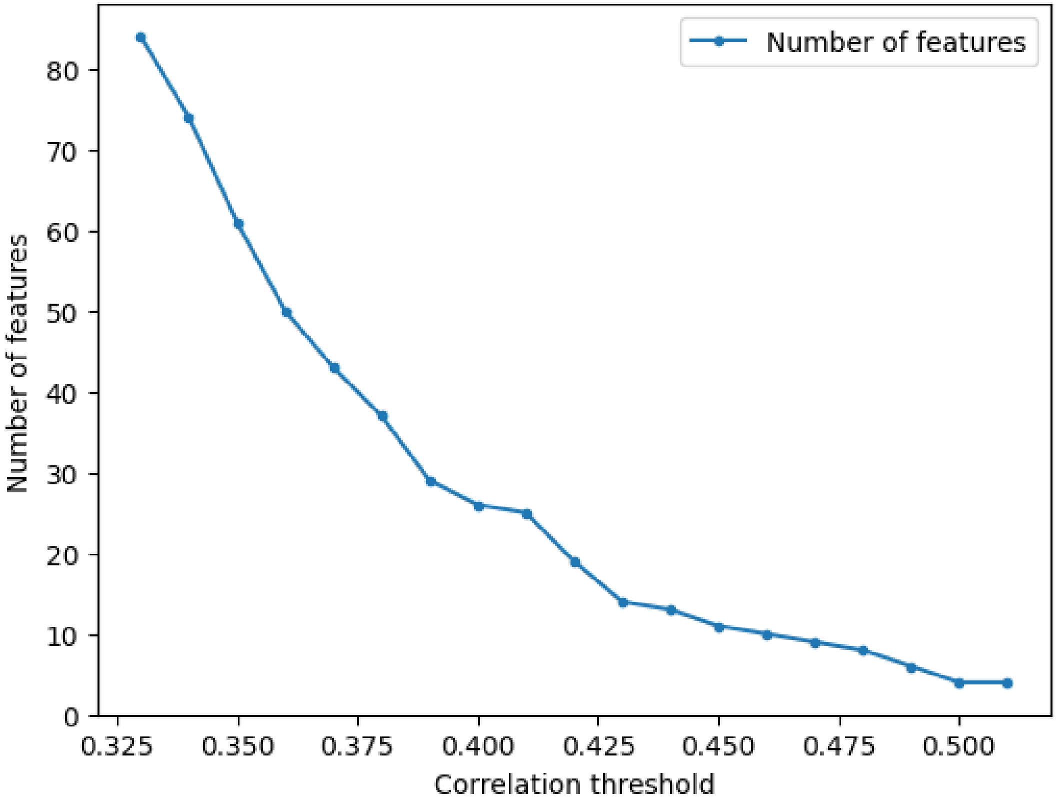


```
[ ]:
```

5 Support Vector Machines (SVM)

```
[37]: without_standardization, train_r2_, test_r2_, hist1, hist2, target_column_name,␣
      ↪training_data_RMSE, test_data_RMSE  = pred_model.
      ↪prepare_data_and_create_model(molecular_descriptors_df=data,
                                                                                    ␣
      ↪                    correlation_threshold=0.50,
                                                                                    ␣
      ↪                    standardization=False,
                                                                                    ␣
      ↪                    model_type='SVR',
                                                                                    ␣
      ↪                    kernel_ = 'linear',
                                                                                    ␣
      ↪                    gamma_ = 'auto',
                                                                                    ␣
      ↪                    target_column_name = target,
```

```
                   random_state=random_state,
                   train_test_split_=True,
                   verbose=True)
```

```
I am not doing standardization…
  molecular descriptor name
0                    AATS0Z
1                  AATS0are
2                    AATS0d
3                   AATS0dv
4                    AATS0i
  molecular descriptor name  corr_value
0                    AATS0Z   -0.038502
1                  AATS0are   -0.120847
2                    AATS0d    0.041648
3                   AATS0dv   -0.115899
4                    AATS0i    0.191765
  molecular descriptor name  corr_value  absolute correlation value
0                    AATS0Z   -0.038502                    0.038502
1                  AATS0are   -0.120847                    0.120847
2                    AATS0d    0.041648                    0.041648
3                   AATS0dv   -0.115899                    0.115899
4                    AATS0i    0.191765                    0.191765
     molecular descriptor name  corr_value  absolute correlation value
226                     AMID_O   -0.520578                    0.520578
505                EState_VSA5   -0.578904                    0.578904
506                EState_VSA6    0.519794                    0.519794
791                    MDEO-12   -0.525441                    0.525441
1211                     MCF-7    1.000000                    1.000000
    molecular descriptor name  corr_value  absolute correlation value
226                    AMID_O   -0.520578                    0.520578
505               EState_VSA5   -0.578904                    0.578904
506               EState_VSA6    0.519794                    0.519794
791                   MDEO-12   -0.525441                    0.525441
The model used is: SVR…
Return the coefficient of determination of the prediction:
0.6626155081380332
R^2 score: 0.4817882148371915
Correlation coefficient: 0.6941096562051211
Test data - unseen during training:
R^2 score: 0.6626155081380332
Correlation coefficient: 0.814011982797571
[7.83359725 6.16673987 7.24549289 8.09382641 7.95363952 8.08726971
 7.9732951  7.7016262  7.97497772 8.07261823 7.35817285 8.33987507
```

```
 7.8022178  8.32059965 7.96754886 7.96886225 6.67229675 5.5439242 ]
102    7.958607
38     6.022276
8      5.949079
109    8.086186
11     8.013228
51     8.000000
88     8.119186
21     8.113509
82     7.982967
98     7.795880
58     7.677781
64     8.619789
74     8.327902
103    8.376751
91     9.154902
43     7.958607
25     4.949659
30     6.010550
Name: MCF-7, dtype: float64
Training Root Mean Square Error: 0.6697595212937382
Testing Root Mean Square Error: 0.6258825778146967
```

[38]:

```
print(target_column_name+str('_transformed'))
print(hist1[target_column_name].hist())
```

```
MCF-7_transformed
AxesSubplot(0.125,0.11;0.775x0.77)
```

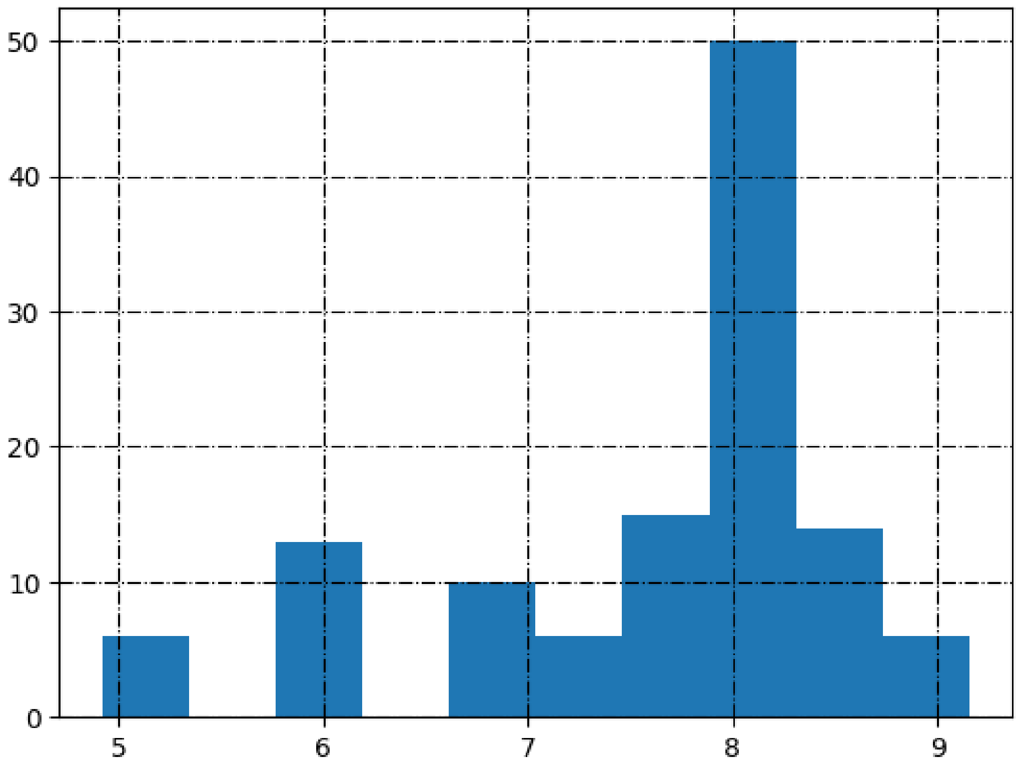

[39]:
```
with_standardization, train_r2_, test_r2_, hist1, hist2, target_column_name,␣
↪training_data_RMSE, test_data_RMSE  = pred_model.
↪prepare_data_and_create_model(molecular_descriptors_df=data,
                                                                                ␣
↪                         correlation_threshold=0.50,
                                                                                ␣
↪                         standardization=True,
                                                                                ␣
↪                         model_type='SVR',
                                                                                ␣
↪                         kernel_ = 'linear',
                                                                                ␣
↪                         gamma_ = 'auto',
                                                                                ␣
↪                         target_column_name = target,
                                                                                ␣
↪                         random_state=random_state,
                                                                                ␣
↪                         train_test_split_=True,
                                                                                ␣
↪                         verbose=True)
```

```
I am doing standardization…
  molecular descriptor name
0                    AATS0Z
1                  AATS0are
2                    AATS0d
3                   AATS0dv
4                    AATS0i
  molecular descriptor name  corr_value
0                    AATS0Z   -0.038502
1                  AATS0are   -0.120847
2                    AATS0d    0.041648
3                   AATS0dv   -0.115899
4                    AATS0i    0.191765
  molecular descriptor name  corr_value  absolute correlation value
0                    AATS0Z   -0.038502                    0.038502
1                  AATS0are   -0.120847                    0.120847
2                    AATS0d    0.041648                    0.041648
3                   AATS0dv   -0.115899                    0.115899
4                    AATS0i    0.191765                    0.191765
     molecular descriptor name  corr_value  absolute correlation value
226                     AMID_O   -0.520578                    0.520578
505                EState_VSA5   -0.578904                    0.578904
506                EState_VSA6    0.519794                    0.519794
791                    MDEO-12   -0.525441                    0.525441
1211                     MCF-7    1.000000                    1.000000
    molecular descriptor name  corr_value  absolute correlation value
226                    AMID_O   -0.520578                    0.520578
505               EState_VSA5   -0.578904                    0.578904
506               EState_VSA6    0.519794                    0.519794
791                   MDEO-12   -0.525441                    0.525441
The model used is: SVR…
Return the coefficient of determination of the prediction:
0.6626155081380332
R^2 score: 0.4817882148371915
Correlation coefficient: 0.6941096562051211
Test data - unseen during training:
R^2 score: 0.6626155081380332
Correlation coefficient: 0.814011982797571
[7.83359725 6.16673987 7.24549289 8.09382641 7.95363952 8.08726971
 7.9732951  7.7016262  7.97497772 8.07261823 7.35817285 8.33987507
 7.8022178  8.32059965 7.96754886 7.96886225 6.67229675 5.5439242 ]
102    7.958607
38     6.022276
8      5.949079
109    8.086186
11     8.013228
51     8.000000
88     8.119186
```

```
21     8.113509
82     7.982967
98     7.795880
58     7.677781
64     8.619789
74     8.327902
103    8.376751
91     9.154902
43     7.958607
25     4.949659
30     6.010550
Name: MCF-7, dtype: float64
Training Root Mean Square Error: 0.6697595212937382
Testing Root Mean Square Error: 0.6258825778146967
```

5.1 Search inside correlation space

[40]:
```
step = 0.01
initial_step = corr_low
last_step = corr_high
first_list = [x / 100.0 for x in range(int(initial_step*100),␣
 ↪int(last_step*100), int(step*100))]
second_list = []
third_list = []
fourth_l = []
fifth_l = []
f_list = []
for i in first_list:
    without_standardization, train_r2, test_r2, _, h_, target_column_name,␣
 ↪training_data_RMSE, test_data_RMSE = pred_model.
 ↪prepare_data_and_create_model(molecular_descriptors_df = data,
                                                                              ␣
 ↪                    correlation_threshold = i,
                                                                              ␣
 ↪                    standardization = False,
                                                                              ␣
 ↪                    model_type = 'SVR',
                                                                              ␣
 ↪                    kernel_ = 'linear',
                                                                              ␣
 ↪                    gamma_ = 'auto',
                                                                              ␣
 ↪                    target_column_name = target,
                                                                              ␣
 ↪                    random_state=random_state,
                                                                              ␣
 ↪                    train_test_split_ = True,
```

```
                                    verbose = False)
    second_list.append(train_r2)
    third_list.append(test_r2)
    fourth_l.append(training_data_RMSE)
    fifth_l.append(test_data_RMSE)
    f_list.append(len(h_))
```

[41]:
```
df_without_standardization = pd.DataFrame(data=first_list,
 columns=["Correlation threshold"])
df_without_standardization['Training data R^2 score'] = second_list
df_without_standardization['Test data R^2 score'] = third_list
df_without_standardization['Training RMSE'] = fourth_l
df_without_standardization['Test RMSE'] = fifth_l
df_without_standardization['Number of features'] = f_list
```

[42]:
```
df_svm = df_without_standardization.copy()
df_without_standardization
```

[42]:
```
    Correlation threshold  Training data R^2 score  Test data R^2 score  \
0                    0.33                 0.706721             0.711733
1                    0.34                 0.704253             0.718611
2                    0.35                 0.659778             0.599273
3                    0.36                 0.608444             0.529254
4                    0.37                 0.599551             0.541794
5                    0.38                 0.582890             0.595559
6                    0.39                 0.562359             0.600633
7                    0.40                 0.570156             0.616687
8                    0.41                 0.569599             0.616421
9                    0.42                 0.550841             0.558076
10                   0.43                 0.512317             0.624487
11                   0.44                 0.511461             0.622026
12                   0.45                 0.512574             0.622415
13                   0.46                 0.489160             0.620354
14                   0.47                 0.489987             0.624133
15                   0.48                 0.489131             0.626263
16                   0.49                 0.486902             0.597167
17                   0.50                 0.481788             0.662616
18                   0.51                 0.481788             0.662616

    Training RMSE  Test RMSE  Number of features
0        0.503855   0.578532                  84
1        0.505971   0.571589                  74
2        0.542684   0.682110                  61
3        0.582187   0.739305                  50
4        0.588761   0.729392                  43
5        0.600884   0.685264                  37
```

```
6         0.615495   0.680952                   29
7         0.609987   0.667125                   26
8         0.610383   0.667356                   25
9         0.623542   0.716315                   19
10        0.649732   0.660302                   14
11        0.650302   0.662463                   13
12        0.649560   0.662122                   11
13        0.664979   0.663926                   10
14        0.664440   0.660614                    9
15        0.664998   0.658739                    8
16        0.666447   0.683900                    6
17        0.669760   0.625883                    4
18        0.669760   0.625883                    4
```

5.2 Plots

[43]:
```
plt.plot(df_without_standardization['Correlation threshold'],␣
  ↪df_without_standardization['Training data R^2 score'], label = "Training␣
  ↪data R^2 score", marker='v')
plt.plot(df_without_standardization['Correlation threshold'],␣
  ↪df_without_standardization['Test data R^2 score'], label = "Test data R^2␣
  ↪score", marker='D')
plt.legend()
plt.xlabel('Correlation threshold')
plt.ylabel('R^2 score')
plt.ylim([0, 1])
plt.show()
```

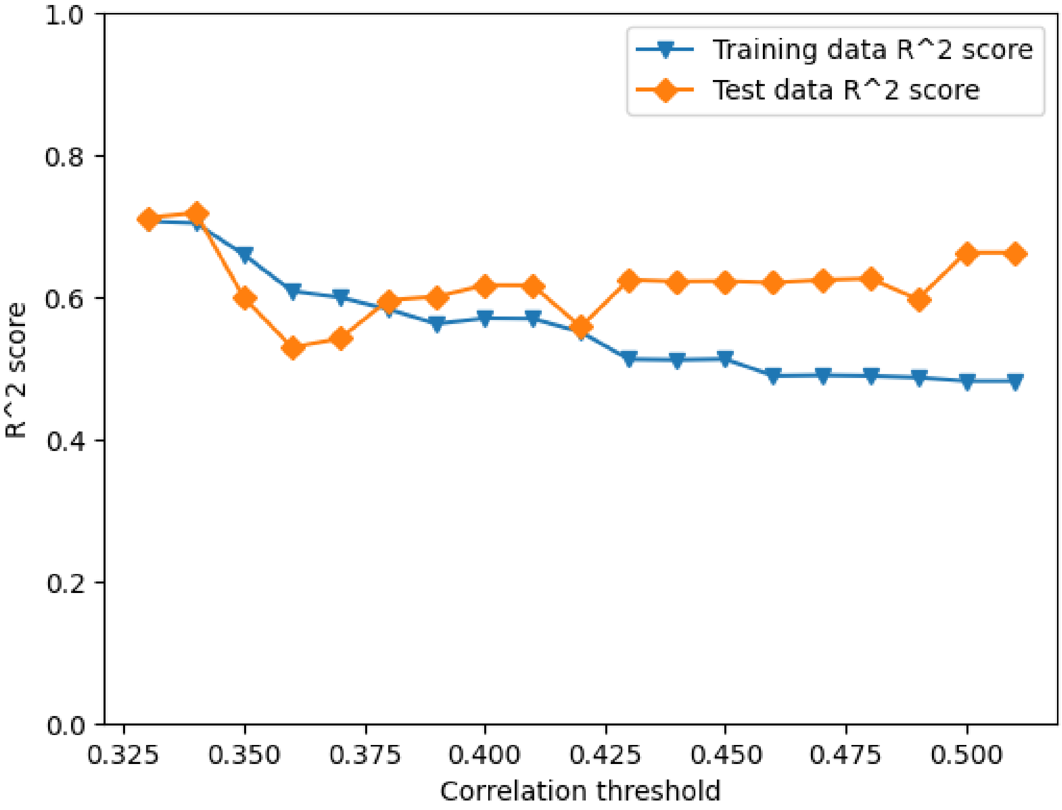

[44]:
```
plt.plot(df_without_standardization['Correlation threshold'],␣
  ↪df_without_standardization['Number of features'], label = "Number of␣
  ↪features", marker='.')
plt.legend()
plt.xlabel('Correlation threshold')
plt.ylabel('Number of features')
plt.show()
```

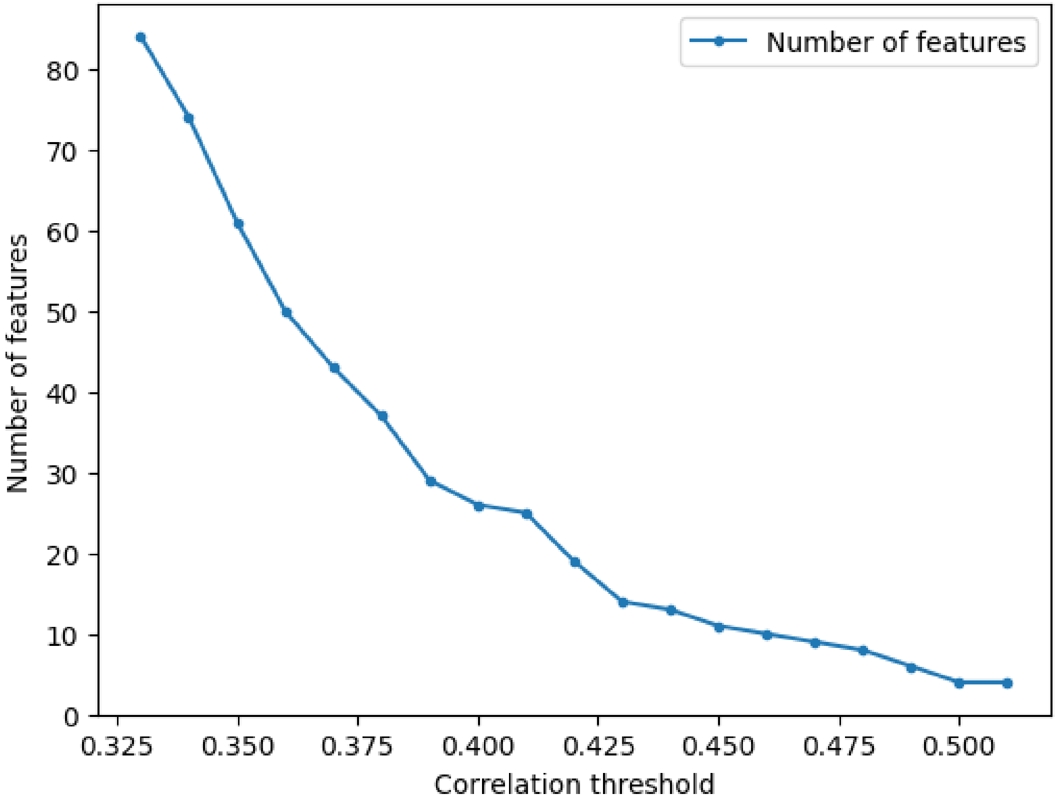

5.3 Store quality measurements

[45]:
```
with pd.ExcelWriter('../Data/Quality_'+str(target)+'_'+str(random_state)+'_.
↪xlsx') as writer:
    df_linear.to_excel(writer, sheet_name='MLR')
    df_decision_tree.to_excel(writer, sheet_name='DT')
    df_random_forest.to_excel(writer, sheet_name='RF')
    df_k_nearest.to_excel(writer, sheet_name='KNN')
    df_svm.to_excel(writer, sheet_name='SVM')
```

Notebook

January 18, 2024

1 File 28

[1]:
```
import sys
from pathlib import Path
prediction_mode_path = Path("../module")
sys.path.append(prediction_mode_path.as_posix())
import models_creation as pred_model

import pandas as pd
import numpy as np

from sklearn.metrics import r2_score

import matplotlib.pyplot as plt
from sklearn.tree import plot_tree

from matplotlib import pyplot as plt

import joblib

import warnings
warnings.filterwarnings('ignore')
```

[2]:
```
load_prepared_data = pd.read_excel('../Data/Kolchicyna_machine_learning.xlsx')
load_prepared_data = load_prepared_data.drop(columns=['Unnamed: 0'])

target = 'MCF-7'

corr_low = 0.33
corr_high = 0.525

random_state = 28
```

[3]:
```
load_prepared_data.head()
```

[3]:
```
      AATS0Z  AATS0are    AATS0d   AATS0dv      AATS0i     AATS0m    AATS0p  \
0  25.090909  6.046518  3.109091  6.662626  160.775045  99.075564  1.555512
1  24.448276  6.008422  3.051724  6.386973  161.021674  96.473315  1.538471
```

```
2  23.868852  5.974074  3.000000  6.138434  161.244045  94.127025  1.523105
3  23.868852  5.974074  3.032787  6.171220  161.244045  94.127025  1.523105
4  23.343750  5.942945  2.953125  5.913194  161.445569  92.000700  1.509180

   AATS0pe    AATS0s   AATS0se  …  A549_float  MCF-7_float  LoVo_float  \
0  6.126680  3.435416  7.629859  …        10.8         10.3         6.5
1  6.088791  3.330998  7.596891  …        11.6         12.0         8.5
2  6.054630  3.236850  7.567166  …        10.9         12.2         8.8
3  6.054630  3.257798  7.567166  …        10.5         11.3         8.5
4  6.023670  3.151529  7.540228  …        89.5         92.7        52.8

   LoVo/DX_float  BALB/3T3_float  A549_float_transformed  \
0           54.9            10.2                7.966576
1           31.1            14.3                7.935542
2           17.9            11.7                7.962574
3           10.2            11.0                7.978811
4           77.8            99.4                7.048177

   MCF-7_float_transformed  LoVo_float_transformed  LoVo/DX_float_transformed  \
0                 7.987163                8.187087                   7.260428
1                 7.920819                8.070581                   7.507240
2                 7.913640                8.055517                   7.747147
3                 7.946922                8.070581                   7.991400
4                 7.032920                7.277366                   7.109020

   BALB/3T3_float_transformed
0                    7.991400
1                    7.844664
2                    7.931814
3                    7.958607
4                    7.002614

[5 rows x 1221 columns]
```

[4]:
```
data = load_prepared_data[list(load_prepared_data.columns)[0:-10]]
data[target] = load_prepared_data[load_prepared_data.columns[-4]]
data.head()
```

[4]:
```
     AATS0Z  AATS0are    AATS0d   AATS0dv      AATS0i     AATS0m    AATS0p  \
0  25.090909  6.046518  3.109091  6.662626  160.775045  99.075564  1.555512
1  24.448276  6.008422  3.051724  6.386973  161.021674  96.473315  1.538471
2  23.868852  5.974074  3.000000  6.138434  161.244045  94.127025  1.523105
3  23.868852  5.974074  3.032787  6.171220  161.244045  94.127025  1.523105
4  23.343750  5.942945  2.953125  5.913194  161.445569  92.000700  1.509180

   AATS0pe    AATS0s   AATS0se  …    piPC10     piPC2     piPC3     piPC4  \
0  6.126680  3.435416  7.629859  …  7.999655  4.266195  4.915408  5.516700
```

```
1  6.088791  3.330998  7.596891  …  8.018726  4.280132  4.922714  5.520711
2  6.054630  3.236850  7.567166  …  8.028799  4.293878  4.929967  5.524706
3  6.054630  3.257798  7.567166  …  8.037440  4.307438  4.929967  5.524706
4  6.023670  3.151529  7.540228  …  8.034892  4.307438  4.937168  5.528685

     piPC5     piPC6     piPC7     piPC8     piPC9     MCF-7
0  6.098566  6.595759  6.979116  7.337313  7.681445  7.987163
1  6.103048  6.601209  6.985613  7.349442  7.695531  7.920819
2  6.105281  6.603923  6.989306  7.353932  7.704023  7.913640
3  6.107510  6.606629  6.992068  7.361425  7.709420  7.946922
4  6.107510  6.605277  6.991148  7.356489  7.707175  7.032920

[5 rows x 1212 columns]
```

2 Multiple Linear regression (MLR)

[5]:
```
without_standardization, train_r2_, test_r2_, hist1, hist2, target_column_name,␣
↪training_data_RMSE, test_data_RMSE  = pred_model.
↪prepare_data_and_create_model(molecular_descriptors_df=data,
                                                                                   ␣
↪                        correlation_threshold=0.5,
                                                                                   ␣
↪                        standardization=False,
                                                                                   ␣
↪                        model_type='linear_model',
                                                                                   ␣
↪                        target_column_name = str(target),
                                                                                   ␣
↪                        random_state=random_state,
                                                                                   ␣
↪                        train_test_split_=True,
                                                                                   ␣
↪                        verbose=True)
```

```
I am not doing standardization…
  molecular descriptor name
0                   AATS0Z
1                 AATS0are
2                   AATS0d
3                  AATS0dv
4                   AATS0i
  molecular descriptor name  corr_value
0                   AATS0Z   -0.038502
1                 AATS0are   -0.120847
2                   AATS0d    0.041648
3                  AATS0dv   -0.115899
```

```
4                   AATS0i    0.191765
  molecular descriptor name  corr_value  absolute correlation value
0                   AATS0Z   -0.038502                    0.038502
1                 AATS0are   -0.120847                    0.120847
2                   AATS0d    0.041648                    0.041648
3                  AATS0dv   -0.115899                    0.115899
4                   AATS0i    0.191765                    0.191765
     molecular descriptor name  corr_value  absolute correlation value
226                     AMID_O   -0.520578                    0.520578
505                EState_VSA5   -0.578904                    0.578904
506                EState_VSA6    0.519794                    0.519794
791                    MDEO-12   -0.525441                    0.525441
1211                     MCF-7    1.000000                    1.000000
    molecular descriptor name  corr_value  absolute correlation value
226                    AMID_O   -0.520578                    0.520578
505               EState_VSA5   -0.578904                    0.578904
506               EState_VSA6    0.519794                    0.519794
791                   MDEO-12   -0.525441                    0.525441
The model used is: LinearReg…
Return the coefficient of determination of the prediction:
0.5009760847434094
R^2 score: 0.5283510681142922
Correlation coefficient: 0.7268776156371114
Test data - unseen during training:
R^2 score: 0.5009760847434094
Correlation coefficient: 0.707796640811052
[8.00252575 6.60183757 7.65912481 6.88611169 7.8702033  7.23304372
 7.57064419 8.42411387 7.0697769  7.89024083 7.95174669 8.38076499
 7.96632403 7.61854434 7.3479345  6.45607766 7.72287088 6.79263447]
113    8.022276
35     6.066513
101    6.920819
36     6.108463
100    7.376751
13     7.987163
0      7.987163
114    7.823909
104    8.397940
96     7.920819
40     8.091515
103    8.376751
48     7.886057
39     8.455932
14     8.086186
117    5.920819
21     8.113509
9      6.143150
Name: MCF-7, dtype: float64
```

```
Training Root Mean Square Error: 0.66543954715775 22
Testing Root Mean Square Error: 0.6092268213393355
```

[6]:
```
print(target_column_name+str('_transformed'))
print(hist1[target_column_name].hist())
```

```
MCF-7_transformed
AxesSubplot(0.125,0.11;0.775x0.77)
```

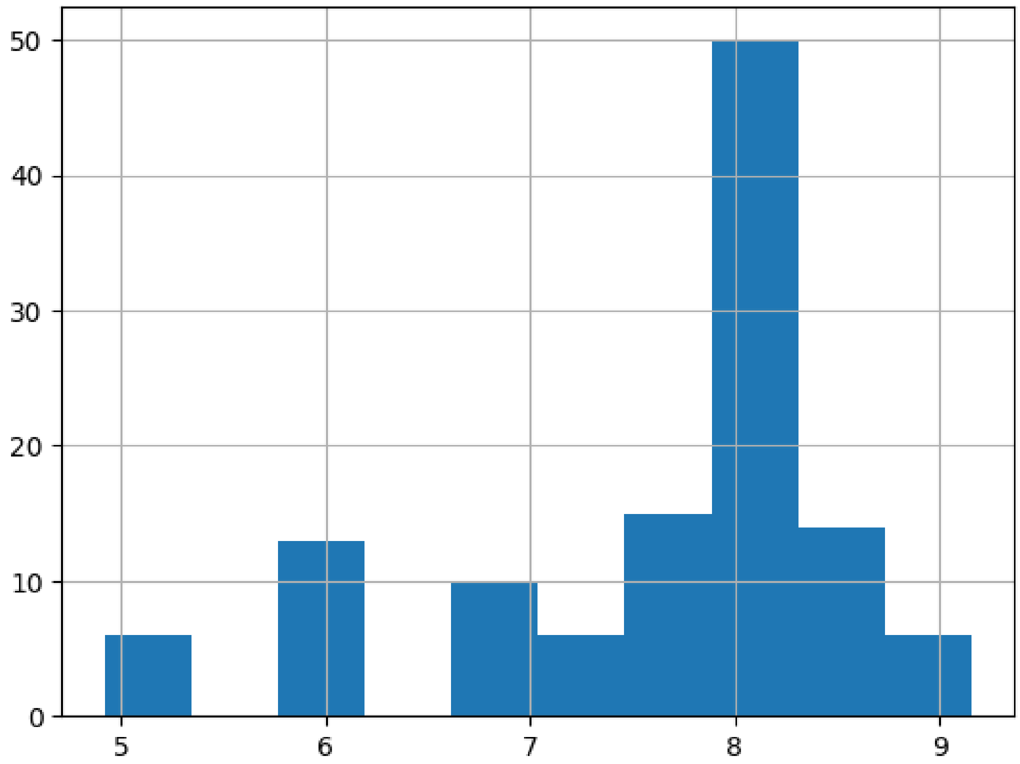

[7]:
```
with_standardization, train_r2_, test_r2_, hist1, hist2, target_column_name,␣
↪training_data_RMSE, test_data_RMSE  = pred_model.
↪prepare_data_and_create_model(molecular_descriptors_df=data,
                                                                           ␣
↪                    correlation_threshold=0.50,
                                                                           ␣
↪                    standardization=True,
                                                                           ␣
↪                    model_type='linear_model',
                                                                           ␣
↪                    target_column_name = target,
                                                                           ␣
↪                    random_state=random_state,
```

```
↪                       train_test_split_=True,
↪                       verbose=True)
```

```
I am doing standardization…
  molecular descriptor name
0                    AATS0Z
1                  AATS0are
2                    AATS0d
3                   AATS0dv
4                    AATS0i
  molecular descriptor name  corr_value
0                    AATS0Z   -0.038502
1                  AATS0are   -0.120847
2                    AATS0d    0.041648
3                   AATS0dv   -0.115899
4                    AATS0i    0.191765
  molecular descriptor name  corr_value  absolute correlation value
0                    AATS0Z   -0.038502                    0.038502
1                  AATS0are   -0.120847                    0.120847
2                    AATS0d    0.041648                    0.041648
3                   AATS0dv   -0.115899                    0.115899
4                    AATS0i    0.191765                    0.191765
     molecular descriptor name  corr_value  absolute correlation value
226                     AMID_O   -0.520578                    0.520578
505                EState_VSA5   -0.578904                    0.578904
506                EState_VSA6    0.519794                    0.519794
791                    MDEO-12   -0.525441                    0.525441
1211                     MCF-7    1.000000                    1.000000
    molecular descriptor name  corr_value  absolute correlation value
226                    AMID_O   -0.520578                    0.520578
505               EState_VSA5   -0.578904                    0.578904
506               EState_VSA6    0.519794                    0.519794
791                   MDEO-12   -0.525441                    0.525441
The model used is: LinearReg…
Return the coefficient of determination of the prediction:
0.5009760847434094
R^2 score: 0.5283510681142922
Correlation coefficient: 0.7268776156371114
Test data - unseen during training:
R^2 score: 0.5009760847434094
Correlation coefficient: 0.707796640811052
[8.00252575 6.60183757 7.65912481 6.88611169 7.8702033  7.23304372
 7.57064419 8.42411387 7.0697769  7.89024083 7.95174669 8.38076499
 7.96632403 7.61854434 7.3479345  6.45607766 7.72287088 6.79263447]
113    8.022276
35     6.066513
```

```
101    6.920819
36     6.108463
100    7.376751
13     7.987163
0      7.987163
114    7.823909
104    8.397940
96     7.920819
40     8.091515
103    8.376751
48     7.886057
39     8.455932
14     8.086186
117    5.920819
21     8.113509
9      6.143150
Name: MCF-7, dtype: float64
Training Root Mean Square Error: 0.6654395471577522
Testing Root Mean Square Error: 0.6092268213393355
```

2.1 Search inside correlation space

[8]:
```
step = 0.01
initial_step = corr_low
last_step = corr_high
first_list = [x / 100.0 for x in range(int(initial_step*100),␣
 ↪int(last_step*100), int(step*100))]
second_list = []
third_list = []
fourth_l = []
fifth_l = []
f_list = []
for i in first_list:
    without_standardization, train_r2, test_r2, _, h_, target_column_name,␣
 ↪training_data_RMSE, test_data_RMSE = pred_model.
 ↪prepare_data_and_create_model(molecular_descriptors_df = data,
                                                                                  ␣
 ↪                        correlation_threshold = i,
                                                                                  ␣
 ↪                        standardization = False,
                                                                                  ␣
 ↪                        model_type = 'linear_model',
                                                                                  ␣
 ↪                        target_column_name = target,
                                                                                  ␣
 ↪                        random_state=random_state,
```

```
                        train_test_split_ = True,
                        verbose = False)
    second_list.append(train_r2)
    third_list.append(test_r2)
    fourth_l.append(training_data_RMSE)
    fifth_l.append(test_data_RMSE)
    f_list.append(len(h_))
```

[9]:
```
df_without_standardization = pd.DataFrame(data=first_list,
 columns=["Correlation threshold"])
df_without_standardization['Training data R^2 score'] = second_list
df_without_standardization['Test data R^2 score'] = third_list
df_without_standardization['Training RMSE'] = fourth_l
df_without_standardization['Test RMSE'] = fifth_l
df_without_standardization['Number of features'] = f_list
```

[10]:
```
df_linear = df_without_standardization.copy()
df_without_standardization
```

[10]:

	Correlation threshold	Training data R^2 score	Test data R^2 score	\
0	0.33	0.983044	-9.346829	
1	0.34	0.963409	-2.228715	
2	0.35	0.919079	-0.202498	
3	0.36	0.893639	-0.095634	
4	0.37	0.843982	-0.466498	
5	0.38	0.817407	0.055234	
6	0.39	0.758880	-0.112670	
7	0.40	0.739958	-0.378030	
8	0.41	0.732273	-0.000031	
9	0.42	0.695477	0.127398	
10	0.43	0.629188	0.081366	
11	0.44	0.626877	0.080512	
12	0.45	0.621663	0.102429	
13	0.46	0.593073	0.174540	
14	0.47	0.592212	0.153668	
15	0.48	0.548178	0.553011	
16	0.49	0.540995	0.556265	
17	0.50	0.528351	0.500976	
18	0.51	0.528351	0.500976	

	Training RMSE	Test RMSE	Number of features
0	0.126172	2.774099	84
1	0.185349	1.549648	74
2	0.275632	0.945715	61
3	0.316002	0.902716	50

4	0.382724	1.044381	43
5	0.414038	0.838263	37
6	0.475791	0.909707	29
7	0.494107	1.012390	26
8	0.501355	0.862432	25
9	0.534698	0.805613	19
10	0.590032	0.826589	14
11	0.591868	0.826973	13
12	0.595990	0.817058	11
13	0.618098	0.783549	10
14	0.618752	0.793394	9
15	0.651302	0.576590	8
16	0.656459	0.574487	6
17	0.665440	0.609227	4
18	0.665440	0.609227	4

[ ]:

```
```

2.2 Plots

[11]:

```
plt.plot(df_without_standardization['Correlation threshold'],␣
↪df_without_standardization['Training data R^2 score'], label = "Training␣
↪data R^2 score", marker='v')
plt.plot(df_without_standardization['Correlation threshold'],␣
↪df_without_standardization['Test data R^2 score'], label = "Test data R^2␣
↪score", marker='D')
plt.legend()
plt.xlabel('Correlation threshold')
plt.ylabel('R^2 score')
plt.ylim([0, 1])
plt.show()
```

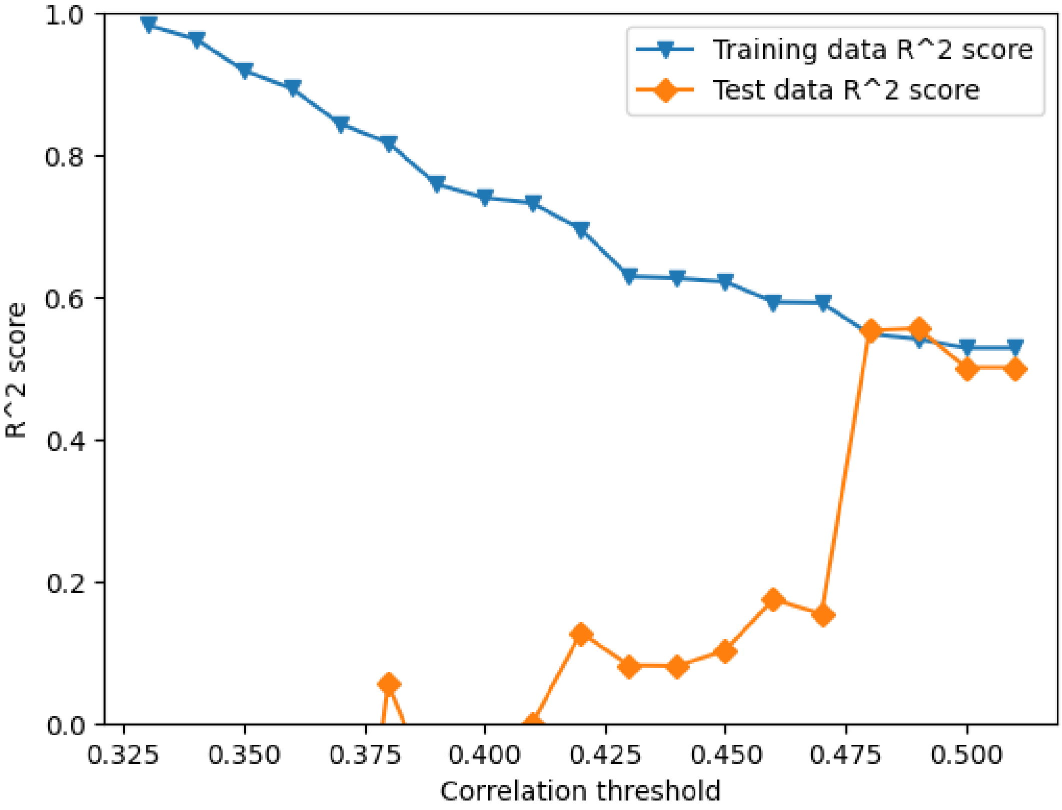

[12]:
```
plt.plot(df_without_standardization['Correlation threshold'],␣
 ↪df_without_standardization['Number of features'], label = "Number of␣
 ↪features", marker='.')
plt.legend()
plt.xlabel('Correlation threshold')
plt.ylabel('Number of features')
plt.show()
```

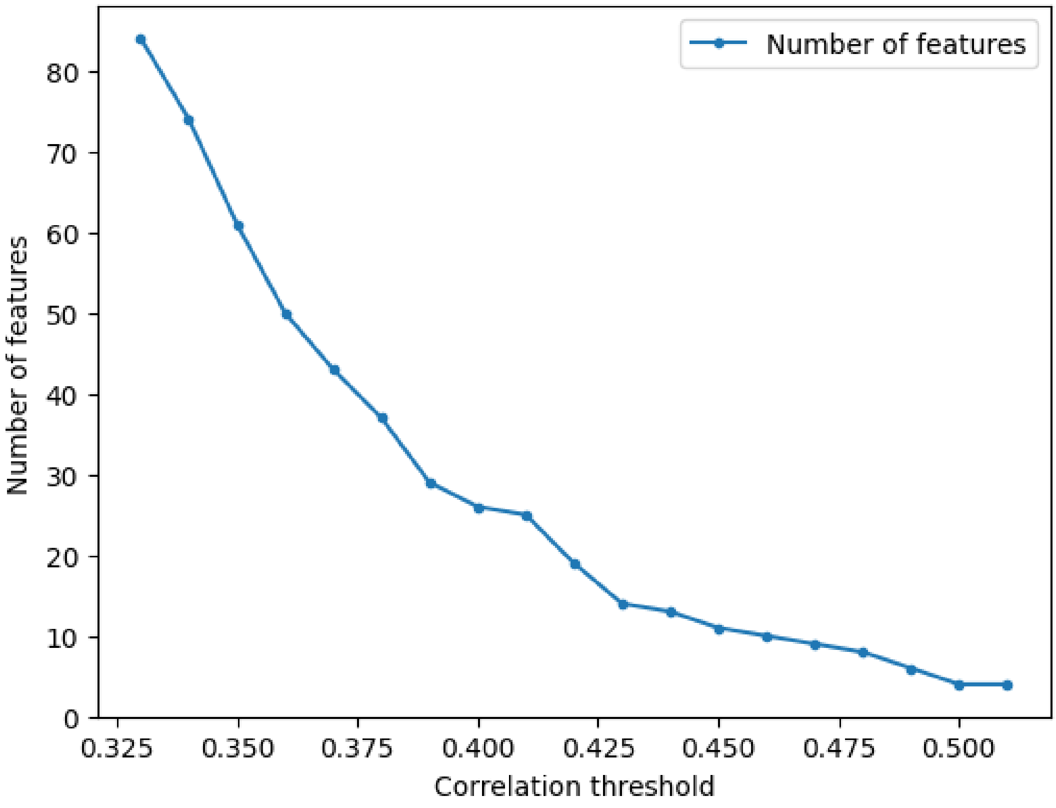


```
[ ]:
```

2.3 Decision Tree

```
[13]: without_standardization, train_r2_, test_r2_, hist1, hist2, target_column_name,␣
  ↪training_data_RMSE, test_data_RMSE  = pred_model.
  ↪prepare_data_and_create_model(molecular_descriptors_df=data,
                                                                                        ␣
  ↪                         correlation_threshold=0.50,
                                                                                        ␣
  ↪                         standardization=False,
                                                                                        ␣
  ↪                         model_type='DecisionTreeRegressor',
                                                                                        ␣
  ↪                         max_depth=5,
                                                                                        ␣
  ↪                         target_column_name = target,
                                                                                        ␣
  ↪                         random_state=random_state,
```

```
                    train_test_split_=True,
                    verbose=True)
```

```
I am not doing standardization…
  molecular descriptor name
0                    AATS0Z
1                  AATS0are
2                    AATS0d
3                   AATS0dv
4                    AATS0i
  molecular descriptor name  corr_value
0                    AATS0Z   -0.038502
1                  AATS0are   -0.120847
2                    AATS0d    0.041648
3                   AATS0dv   -0.115899
4                    AATS0i    0.191765
  molecular descriptor name  corr_value  absolute correlation value
0                    AATS0Z   -0.038502                    0.038502
1                  AATS0are   -0.120847                    0.120847
2                    AATS0d    0.041648                    0.041648
3                   AATS0dv   -0.115899                    0.115899
4                    AATS0i    0.191765                    0.191765
     molecular descriptor name  corr_value  absolute correlation value
226                     AMID_O   -0.520578                    0.520578
505                EState_VSA5   -0.578904                    0.578904
506                EState_VSA6    0.519794                    0.519794
791                    MDEO-12   -0.525441                    0.525441
1211                     MCF-7    1.000000                    1.000000
    molecular descriptor name  corr_value  absolute correlation value
226                    AMID_O   -0.520578                    0.520578
505               EState_VSA5   -0.578904                    0.578904
506               EState_VSA6    0.519794                    0.519794
791                   MDEO-12   -0.525441                    0.525441
The model used is: DecisionTree…
Return the coefficient of determination of the prediction:
-0.005104855722772195
R^2 score: 0.8657226333752897
Correlation coefficient: 0.9304421708925761
Test data - unseen during training:
R^2 score: -0.005104855722772195
Correlation coefficient: nan
[7.94564921 7.67778071 8.35232643 7.67778071 7.94564921 8.95860731
 7.94564921 7.69458125 7.20277093 7.94564921 7.94564921 7.94564921
 7.94564921 7.94564921 8.95860731 6.02227639 6.99012437 7.20277093]
113    8.022276
35     6.066513
```

```
101    6.920819
36     6.108463
100    7.376751
13     7.987163
0      7.987163
114    7.823909
104    8.397940
96     7.920819
40     8.091515
103    8.376751
48     7.886057
39     8.455932
14     8.086186
117    5.920819
21     8.113509
9      6.143150
Name: MCF-7, dtype: float64
Training Root Mean Square Error: 0.35505905070999344
Testing Root Mean Square Error: 0.8646174988464919
```

[14]:
```
print(target_column_name+str('_transformed'))
print(hist1[target_column_name].hist())
```

```
MCF-7_transformed
AxesSubplot(0.125,0.11;0.775x0.77)
```

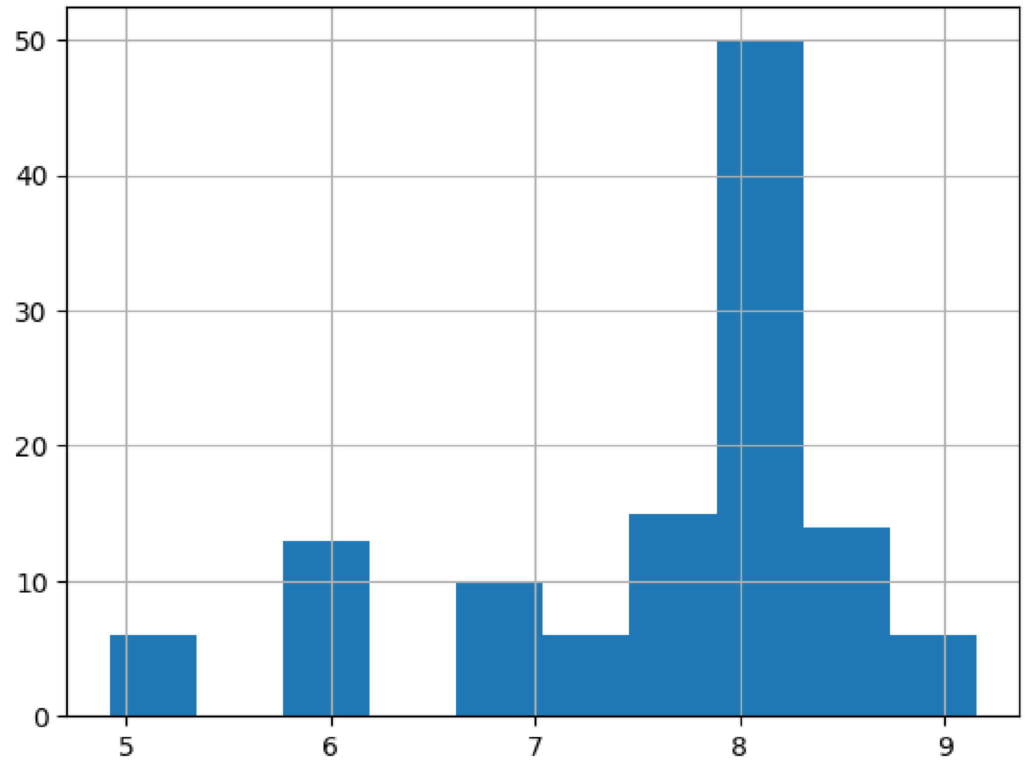

[15]:
```
with_standardization, train_r2_, test_r2_, hist1, hist2, target_column_name,␣
↪training_data_RMSE, test_data_RMSE  = pred_model.
↪prepare_data_and_create_model(molecular_descriptors_df=data,
                                                                              ␣
↪                     correlation_threshold=0.50,
                                                                              ␣
↪                     standardization=True,
                                                                              ␣
↪                     model_type='DecisionTreeRegressor',
                                                                              ␣
↪                     max_depth=5,
                                                                              ␣
↪                     target_column_name = target,
                                                                              ␣
↪                     random_state=random_state,
                                                                              ␣
↪                     train_test_split_=True,
                                                                              ␣
↪                     verbose=True)
```

```
I am doing standardization…
  molecular descriptor name
0                    AATS0Z
1                  AATS0are
2                    AATS0d
3                   AATS0dv
4                    AATS0i
  molecular descriptor name  corr_value
0                    AATS0Z   -0.038502
1                  AATS0are   -0.120847
2                    AATS0d    0.041648
3                   AATS0dv   -0.115899
4                    AATS0i    0.191765
  molecular descriptor name  corr_value  absolute correlation value
0                    AATS0Z   -0.038502                    0.038502
1                  AATS0are   -0.120847                    0.120847
2                    AATS0d    0.041648                    0.041648
3                   AATS0dv   -0.115899                    0.115899
4                    AATS0i    0.191765                    0.191765
    molecular descriptor name  corr_value  absolute correlation value
226                    AMID_O   -0.520578                    0.520578
505                EState_VSA5   -0.578904                    0.578904
506                EState_VSA6    0.519794                    0.519794
791                   MDEO-12   -0.525441                    0.525441
```

```
1211                      MCF-7    1.000000                    1.000000
    molecular descriptor name  corr_value  absolute correlation value
226                    AMID_O   -0.520578                    0.520578
505               EState_VSA5   -0.578904                    0.578904
506               EState_VSA6    0.519794                    0.519794
791                   MDEO-12   -0.525441                    0.525441
The model used is: DecisionTree…
Return the coefficient of determination of the prediction:
-0.005104855722772195
R^2 score: 0.8657226333752897
Correlation coefficient: 0.9304421708925761
Test data - unseen during training:
R^2 score: -0.005104855722772195
Correlation coefficient: nan
[7.94564921 7.67778071 8.35232643 7.67778071 7.94564921 8.95860731
 7.94564921 7.69458125 7.20277093 7.94564921 7.94564921 7.94564921
 7.94564921 7.94564921 8.95860731 6.02227639 6.99012437 7.20277093]
113    8.022276
35     6.066513
101    6.920819
36     6.108463
100    7.376751
13     7.987163
0      7.987163
114    7.823909
104    8.397940
96     7.920819
40     8.091515
103    8.376751
48     7.886057
39     8.455932
14     8.086186
117    5.920819
21     8.113509
9      6.143150
Name: MCF-7, dtype: float64
Training Root Mean Square Error: 0.35505905070999344
Testing Root Mean Square Error: 0.8646174988464919
```

2.4 Search inside correlation space

[16]:
```
step = 0.01
initial_step = corr_low
last_step = corr_high
first_list = [x / 100.0 for x in range(int(initial_step*100),␣
 ↪int(last_step*100), int(step*100))]
max_depth = [range(2, 30, 1)]
```

```
corr_th = []
second_list = []
third_list = []
fourth_l = []
fifth_l = []
f_list = []
fif_list = []
for i in first_list:
    for depth in max_depth[0]:

        without_standardization, train_r2, test_r2, _, h_, target_column_name,␣
 ↪training_data_RMSE, test_data_RMSE = pred_model.
 ↪prepare_data_and_create_model(molecular_descriptors_df=data,
                                                                                ␣
 ↪                    correlation_threshold=i,
                                                                                ␣
 ↪                    standardization=False,
                                                                                ␣
 ↪                    model_type='DecisionTreeRegressor',
                                                                                ␣
 ↪                    max_depth=depth,
                                                                                ␣
 ↪                    target_column_name = target,
                                                                                ␣
 ↪                    random_state=random_state,
                                                                                ␣
 ↪                    train_test_split_=True,
                                                                                ␣
 ↪                    verbose=False)
        corr_th.append(i)
        second_list.append(train_r2)
        third_list.append(test_r2)
        fourth_l.append(training_data_RMSE)
        fifth_l.append(test_data_RMSE)
        f_list.append(len(h_))
        fif_list.append(depth)
```

[17]:
```
df_without_standardization = pd.DataFrame(data=corr_th, columns=["Correlation␣
 ↪threshold"])
df_without_standardization['Training data R^2 score'] = second_list
df_without_standardization['Test data R^2 score'] = third_list
df_without_standardization['Training RMSE'] = fourth_l
df_without_standardization['Test RMSE'] = fifth_l
df_without_standardization['Number of features'] = f_list
df_without_standardization['Depth number'] = fif_list
```

[18]:
```
df_decision_tree = df_without_standardization.copy()
#df_without_standardization.to_excel('../Data/
↪A549_Decision_tree_rs_'+str(random_state)+'.xlsx')
```

2.5 Plots

[19]:
```
corre_list = [x / 100.0 for x in range(int(initial_step*100),␣
↪int(last_step*100), int(0.01*100))]
for element in corre_list:
    element_ =␣
↪df_without_standardization[df_without_standardization['Correlation␣
↪threshold'] == float(element)]
    plt.plot(element_['Depth number'], element_['Training data R^2 score'],␣
↪label = "Training data R^2 score", marker='v')
    plt.plot(element_['Depth number'], element_['Test data R^2 score'], label =␣
↪"Test data R^2 score", marker='D')
    plt.legend()
    plt.xlabel('Depth number -> correlation threshold: '+str(element)+'. \n␣
↪Number of features: '+str(element_['Number of features'].iloc[0]),␣
↪fontweight='bold')
    plt.xlim(max(element_['Depth number'])+1, min(element_['Depth number'])-1)
    plt.ylabel('R^2 score')
    plt.ylim([0, 1])
    plt.rc('grid', linestyle="-.", color='black')
    plt.grid(True)
    plt.show()
```

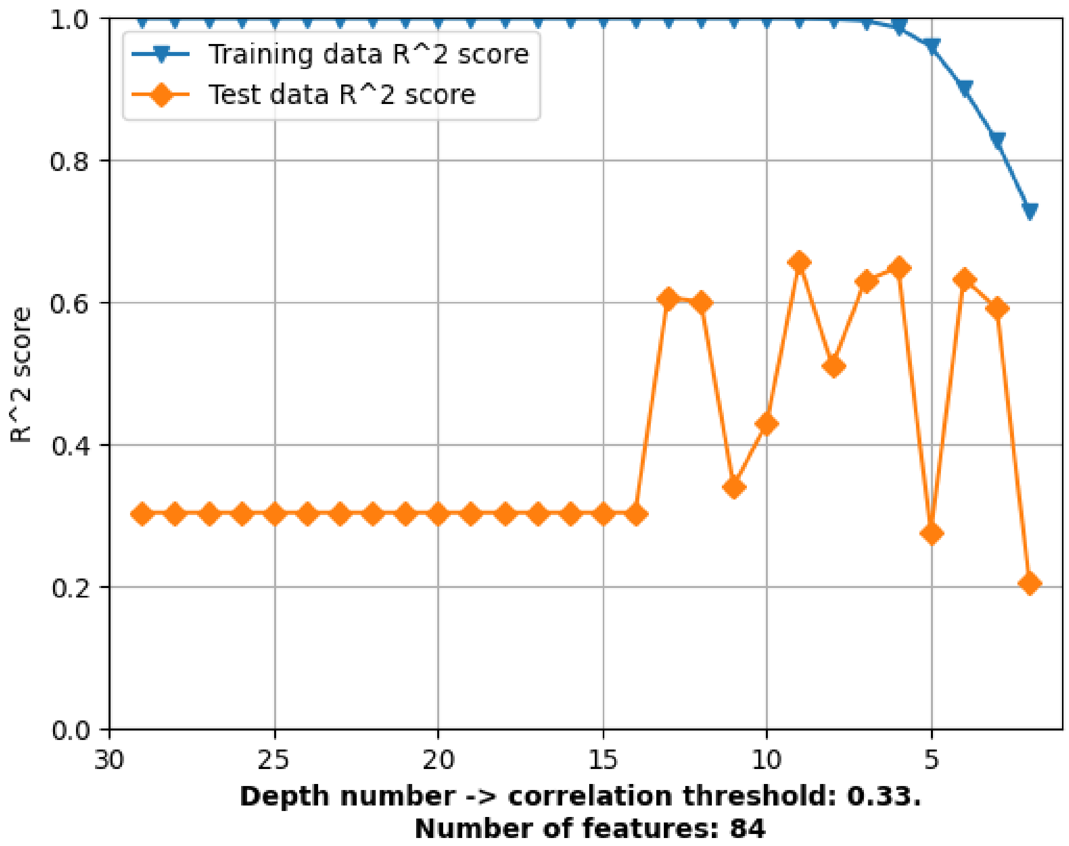

Training data R^2 score
Test data R^2 score
R^2 score
1.0
0.8
0.6
0.4
0.2
0.0
30
25
20
15
10
5
Depth number -> correlation threshold: 0.33.
Number of features: 84

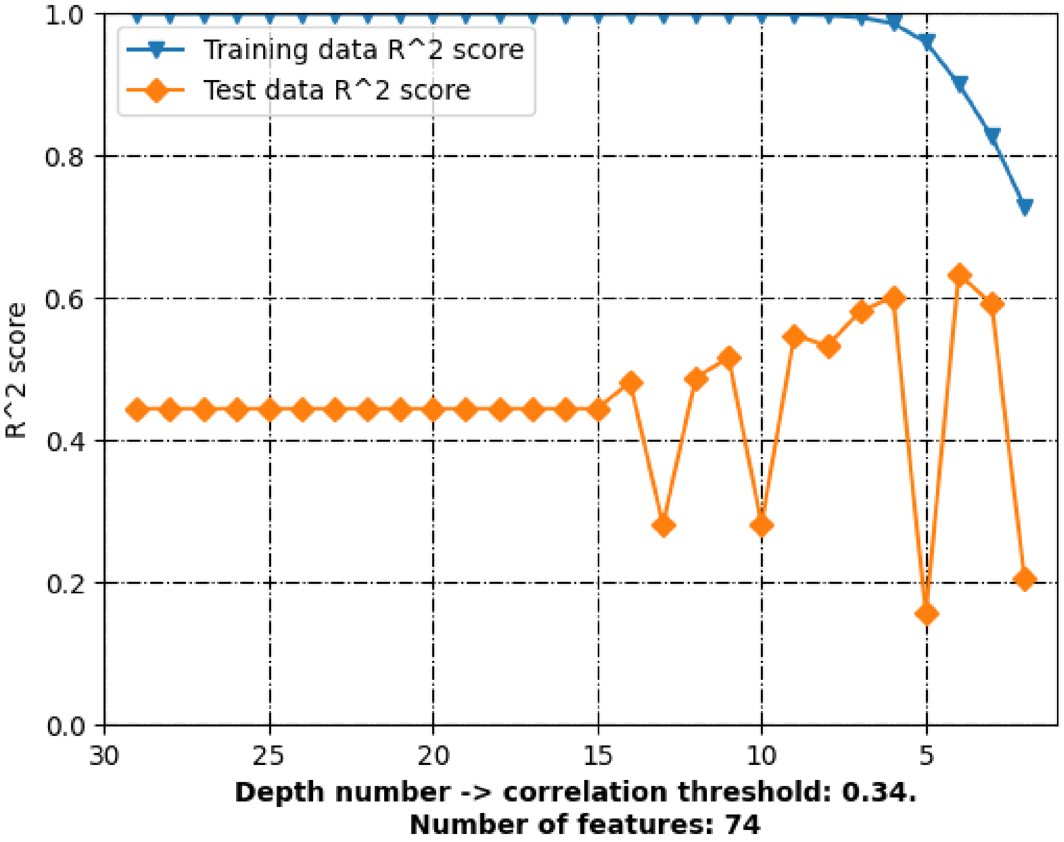

Training data R^2 score
Test data R^2 score
R^2 score
1.0
0.8
0.6
0.4
0.2
0.0
30
25
20
15
10
5
Depth number -> correlation threshold: 0.34.
Number of features: 74

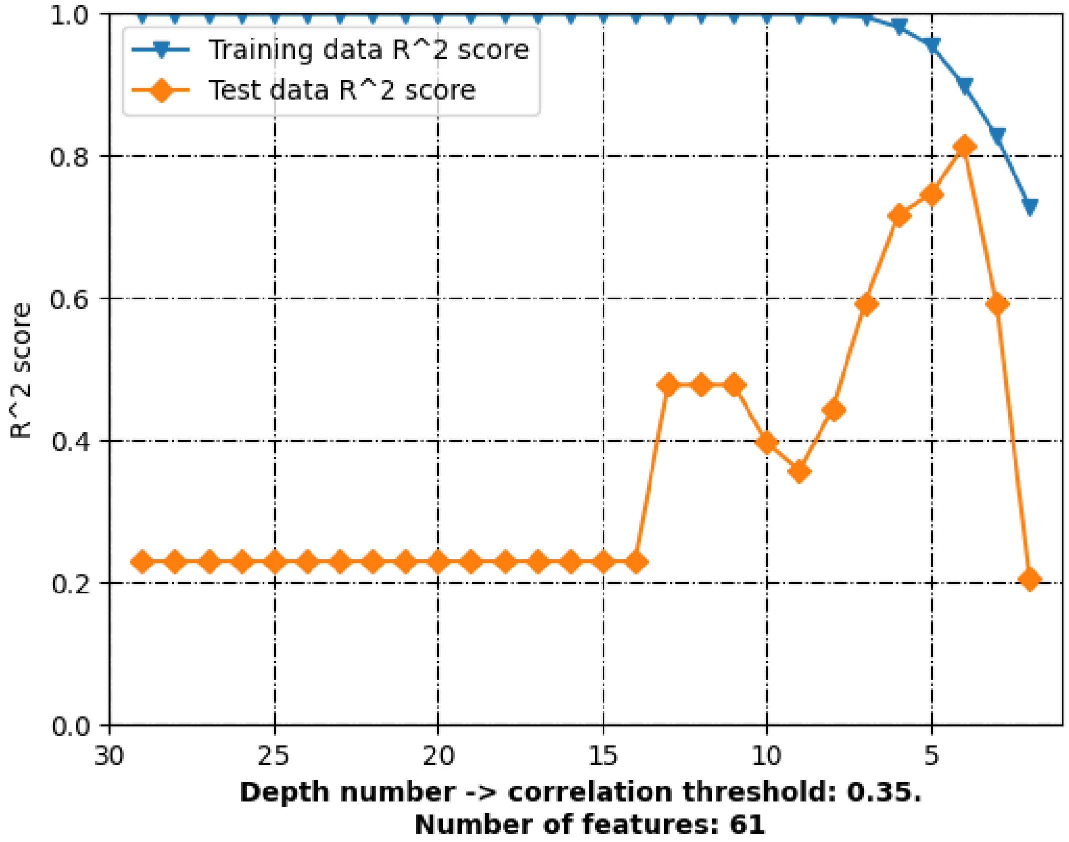

Training data R^2 score
Test data R^2 score
R^2 score
1.0
0.8
0.6
0.4
0.2
0.0
30
25
20
15
10
5
Depth number -> correlation threshold: 0.35.
Number of features: 61

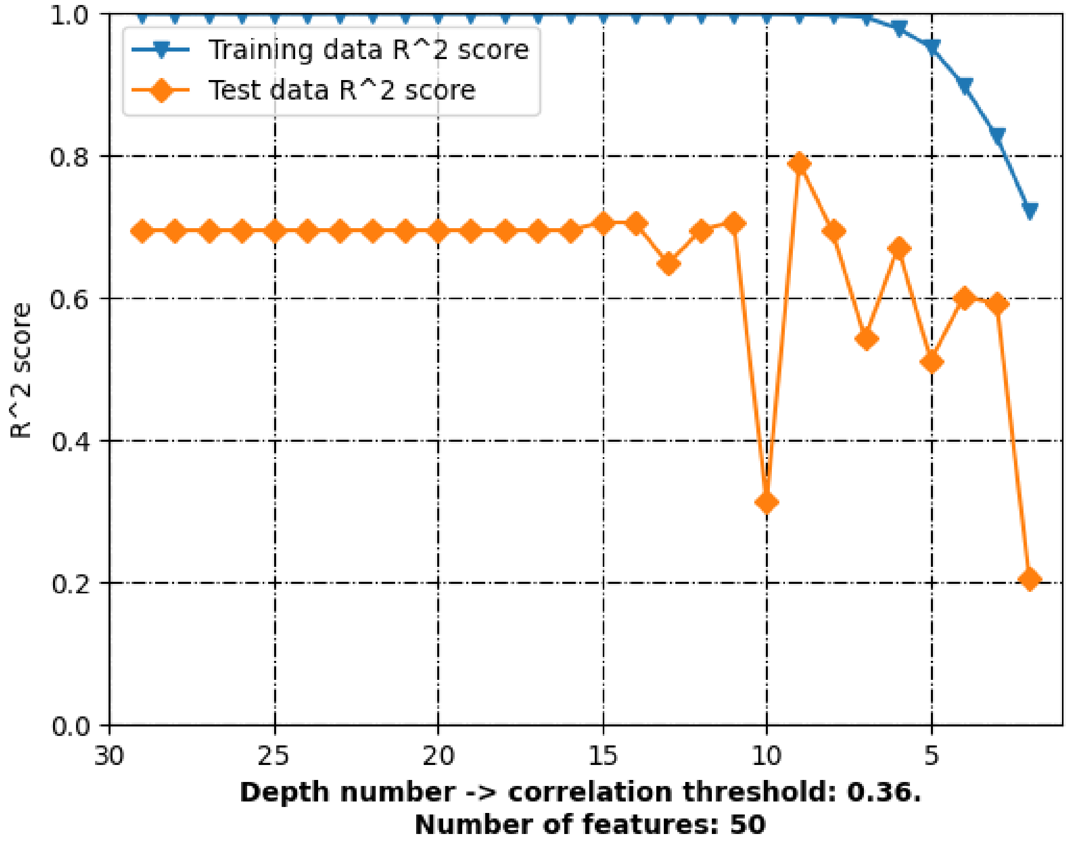
Training data R^2 score
Test data R^2 score
R^2 score
1.0
0.8
0.6
0.4
0.2
0.0
30
25
20
15
10
5
Depth number -> correlation threshold: 0.36.
Number of features: 50

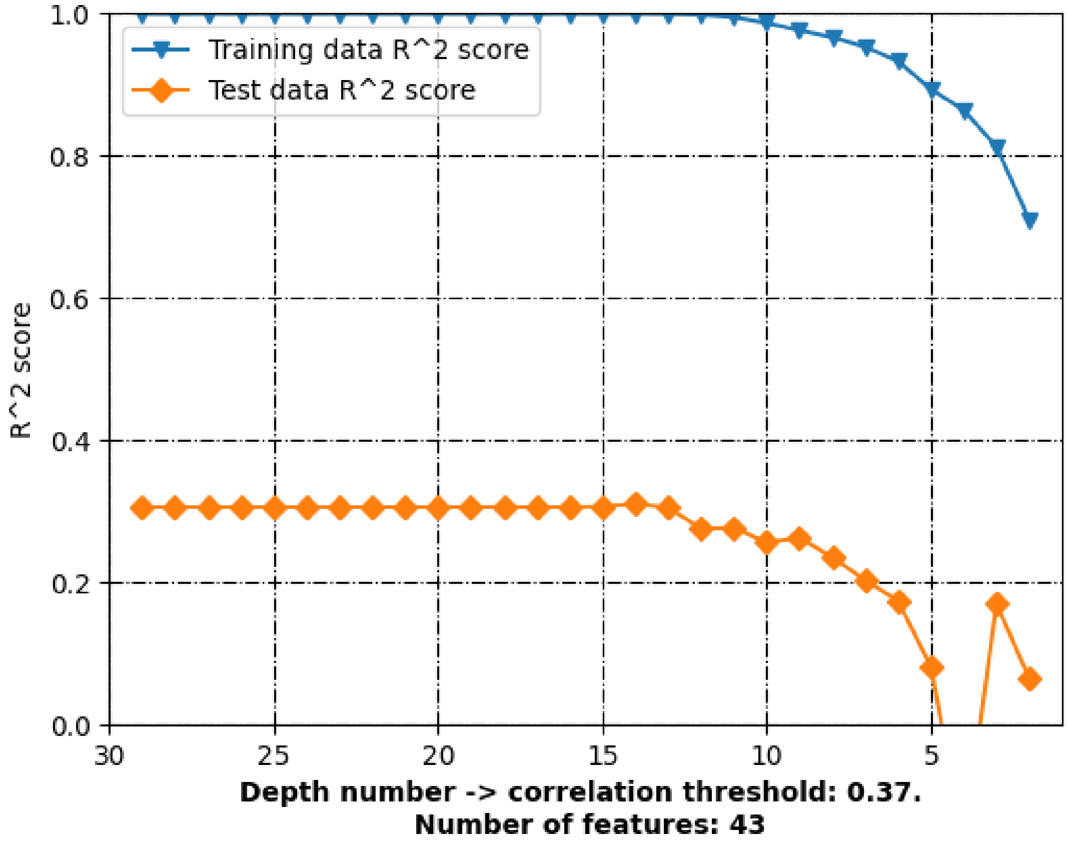
Training data R^2 score
Test data R^2 score
R^2 score
1.0
0.8
0.6
0.4
0.2
0.0
30
25
20
15
10
5
Depth number -> correlation threshold: 0.37.
Number of features: 43

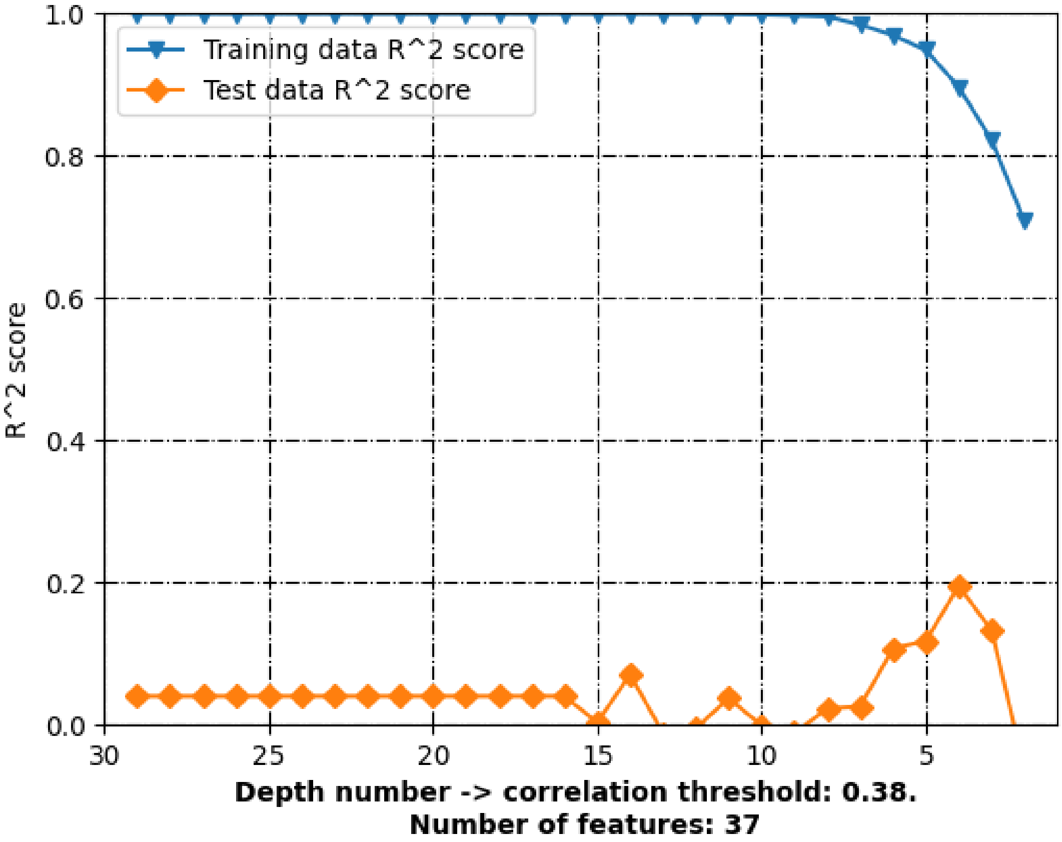

Training data R^2 score
Test data R^2 score
R^2 score
1.0
0.8
0.6
0.4
0.2
0.0
30
25
20
15
10
5
Depth number -> correlation threshold: 0.38.
Number of features: 37

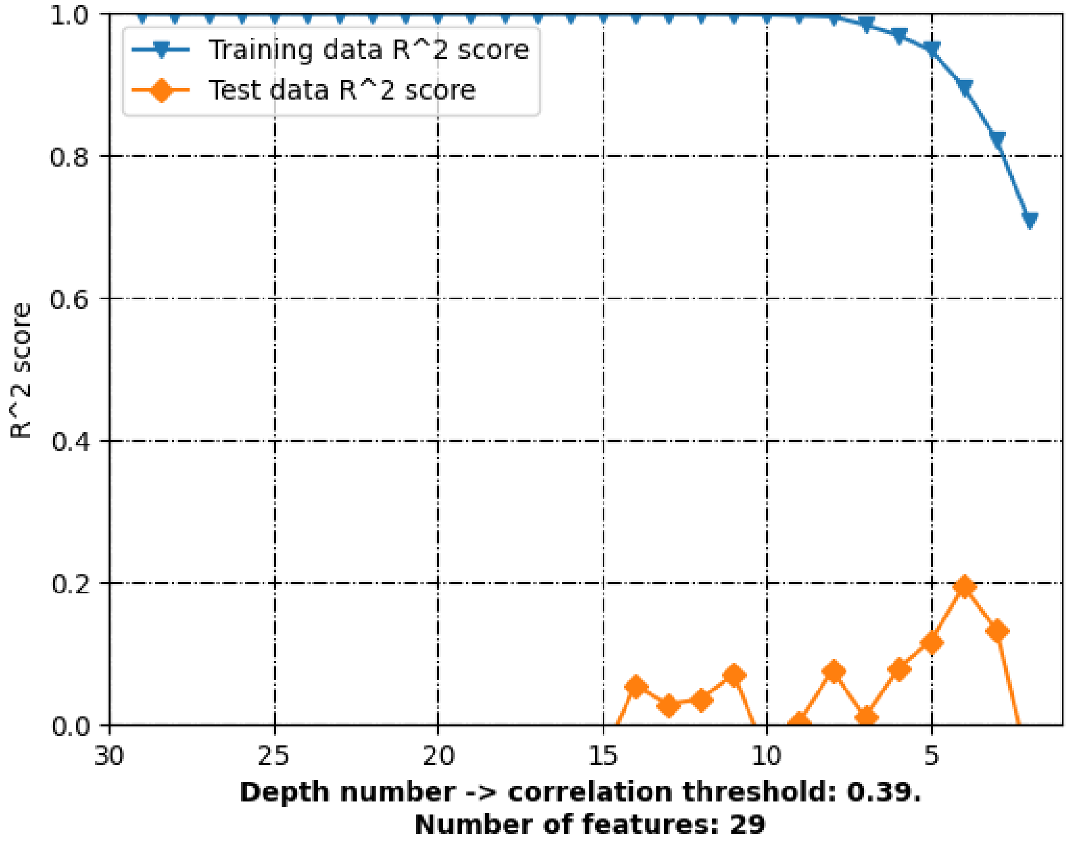
Training data R^2 score
Test data R^2 score
R^2 score
1.0
0.8
0.6
0.4
0.2
0.0
30
25
20
15
10
5
Depth number -> correlation threshold: 0.39.
Number of features: 29

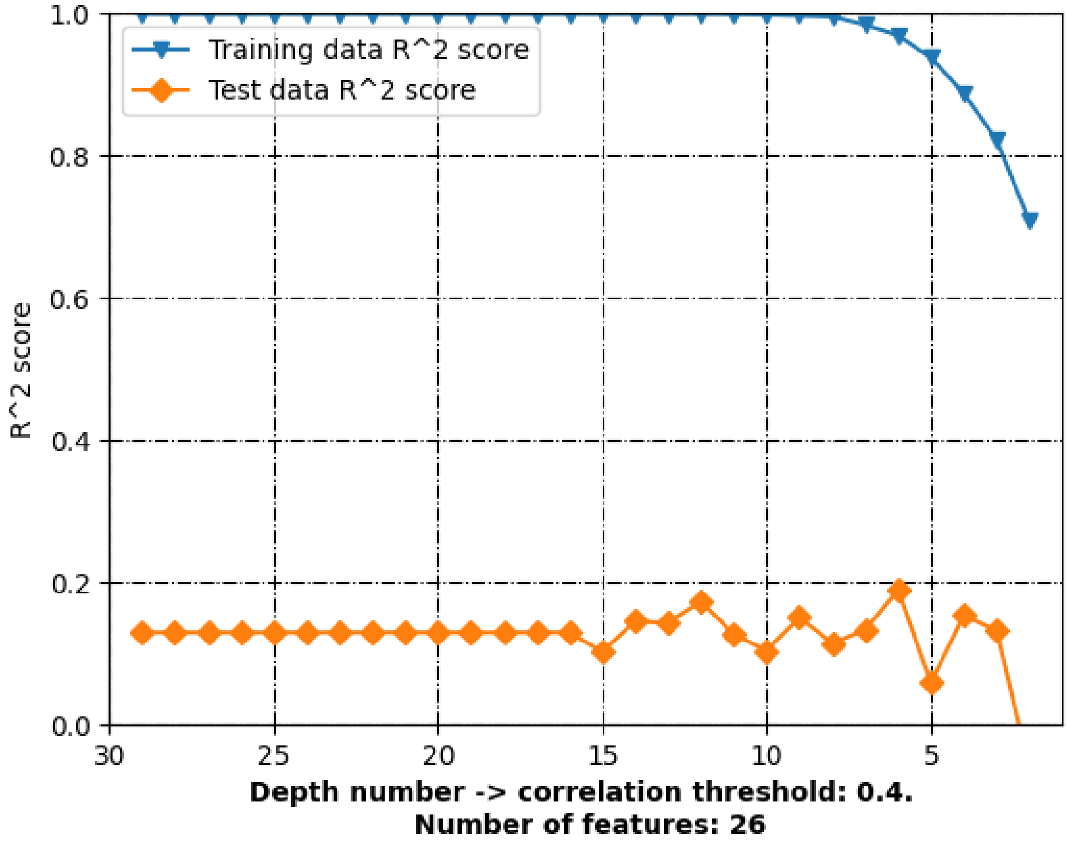

Training data R^2 score
Test data R^2 score
R^2 score
1.0
0.8
0.6
0.4
0.2
0.0
30
25
20
15
10
5
Depth number -> correlation threshold: 0.4.
Number of features: 26

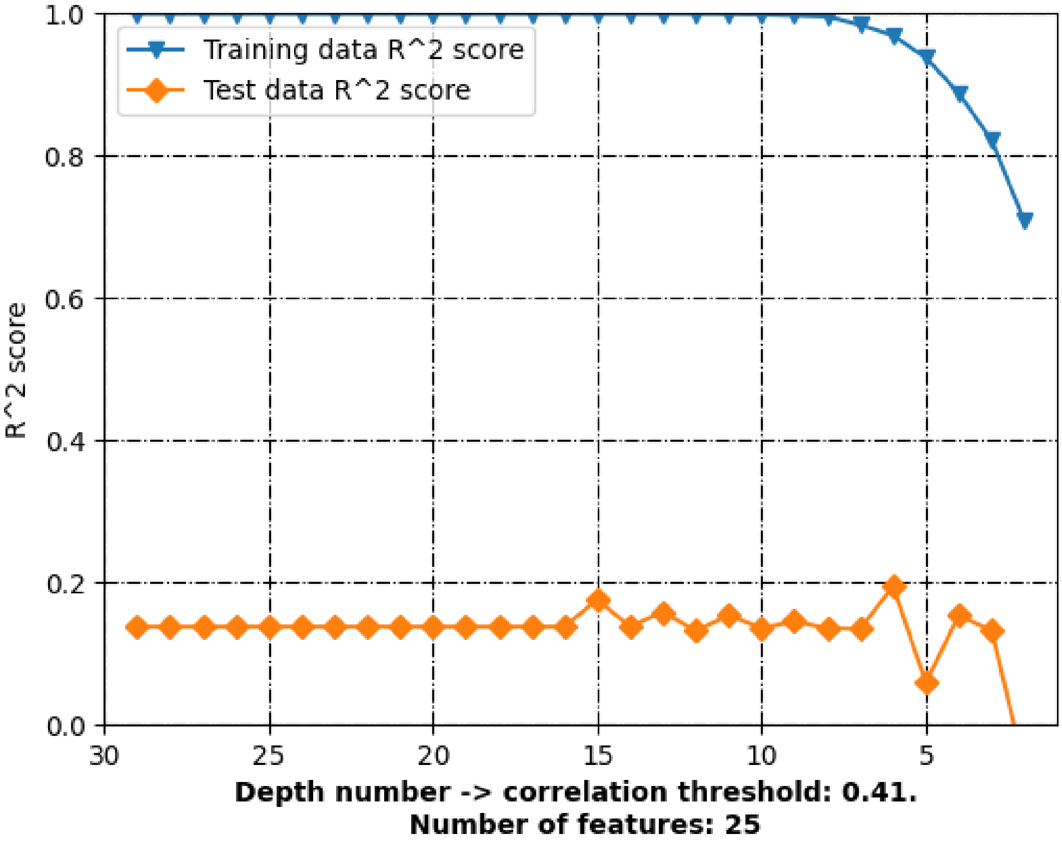
Training data R^2 score
Test data R^2 score
R^2 score
1.0
0.8
0.6
0.4
0.2
0.0
30
25
20
15
10
5
Depth number -> correlation threshold: 0.41.
Number of features: 25

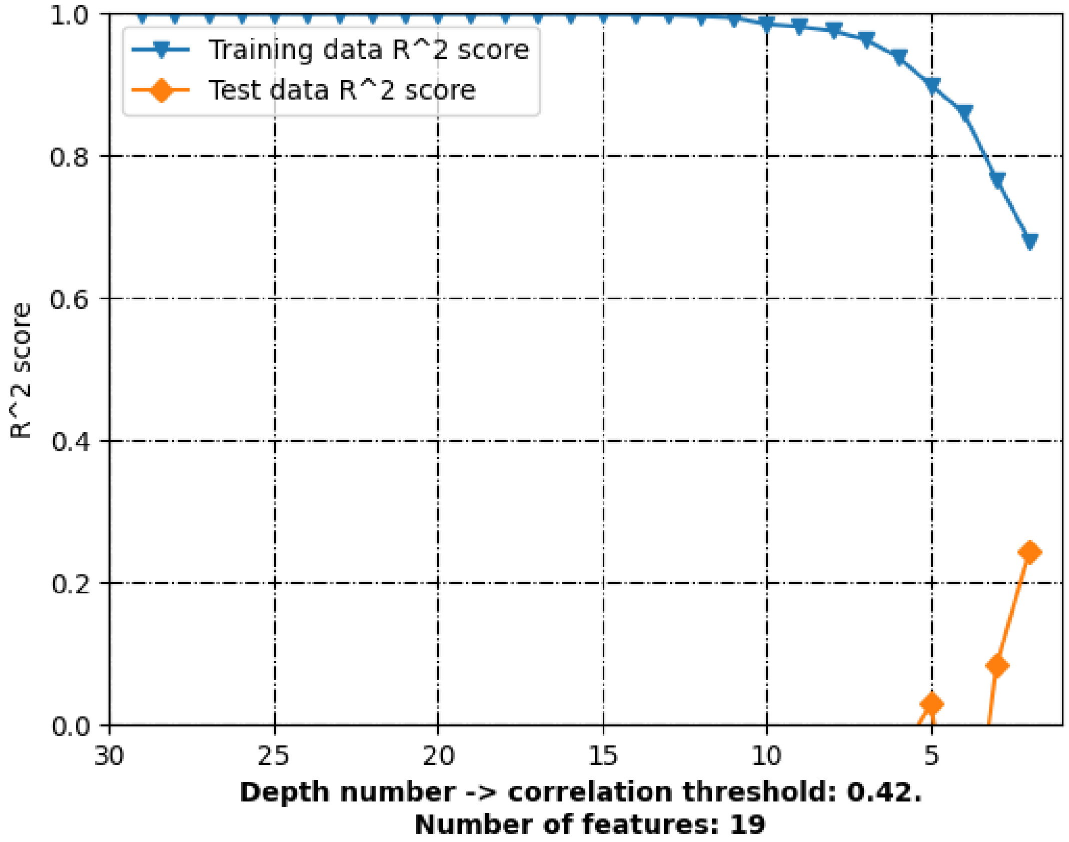
1.0
0.8
0.6
0.4
0.2
0.0
R^2 score
Training data R^2 score
Test data R^2 score
30
25
20
15
10
5
Depth number -> correlation threshold: 0.42.
Number of features: 19

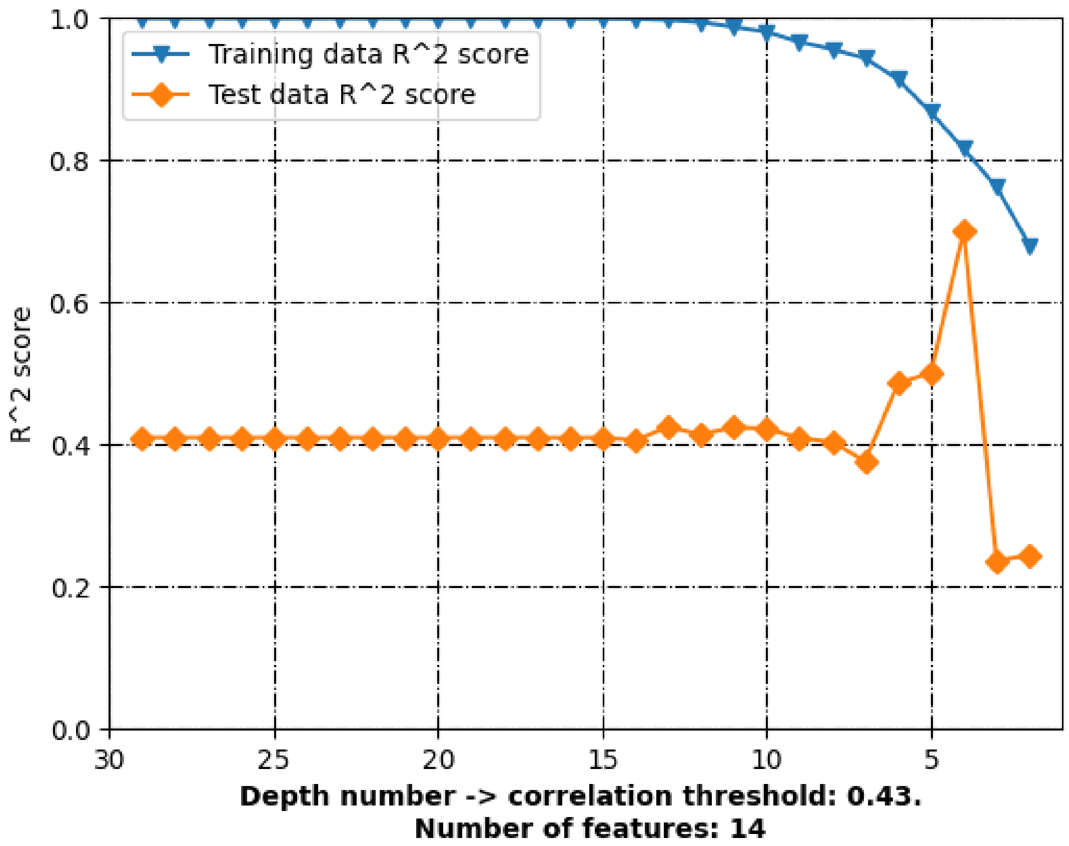

Training data R^2 score
Test data R^2 score
R^2 score
1.0
0.8
0.6
0.4
0.2
0.0
30
25
20
15
10
5
Depth number -> correlation threshold: 0.43.
Number of features: 14

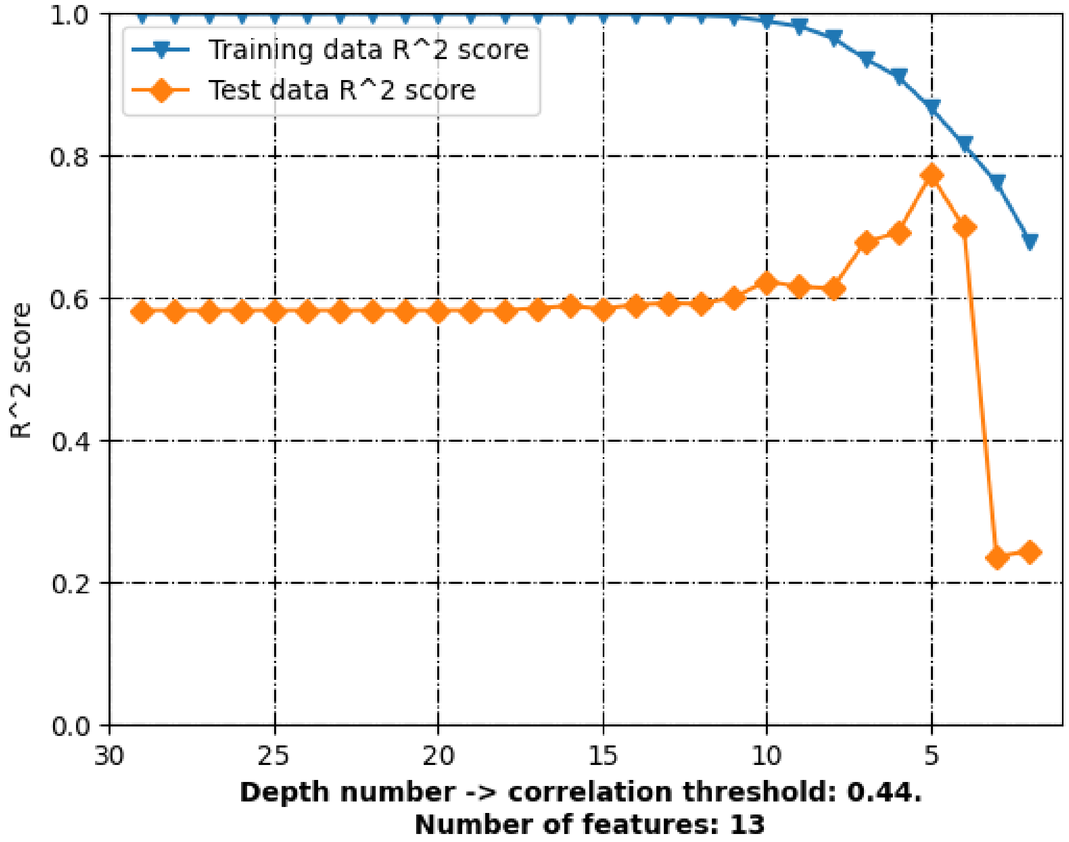
Training data R^2 score
Test data R^2 score
R^2 score
1.0
0.8
0.6
0.4
0.2
0.0
30
25
20
15
10
5
Depth number -> correlation threshold: 0.44.
Number of features: 13

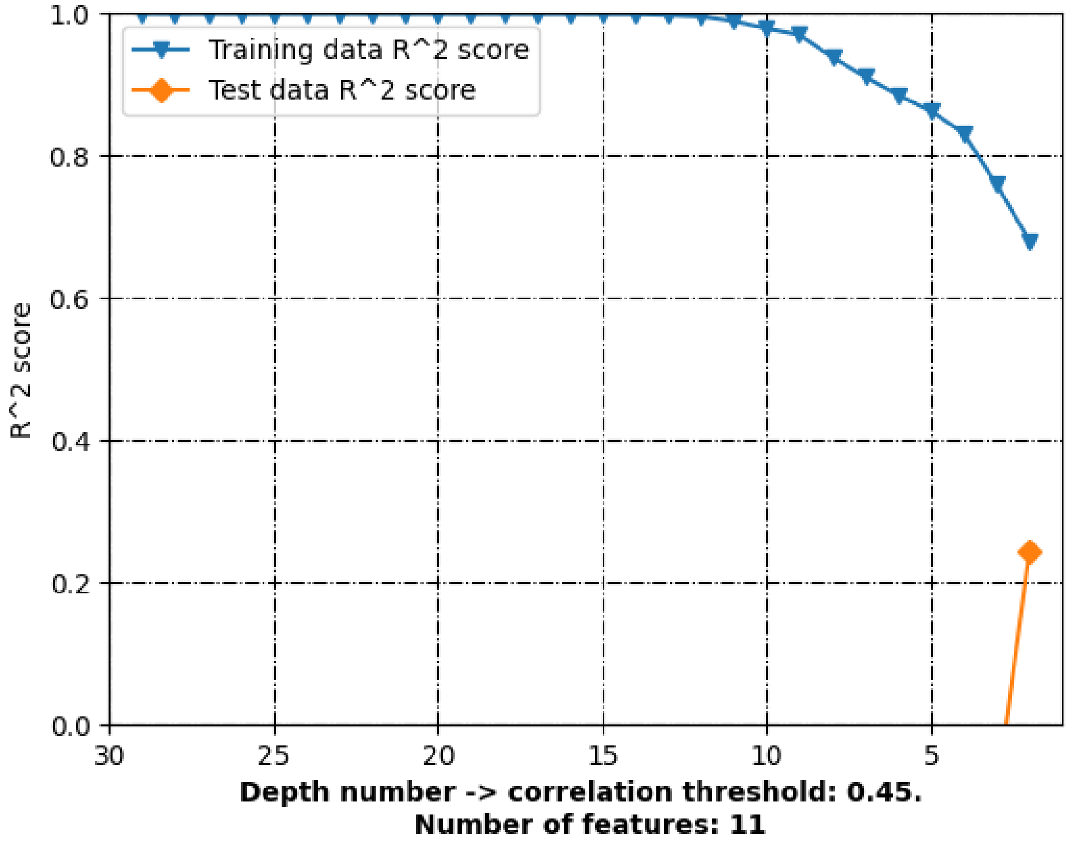
Training data R^2 score
Test data R^2 score
R^2 score
1.0
0.8
0.6
0.4
0.2
0.0
30
25
20
15
10
5
Depth number -> correlation threshold: 0.45.
Number of features: 11

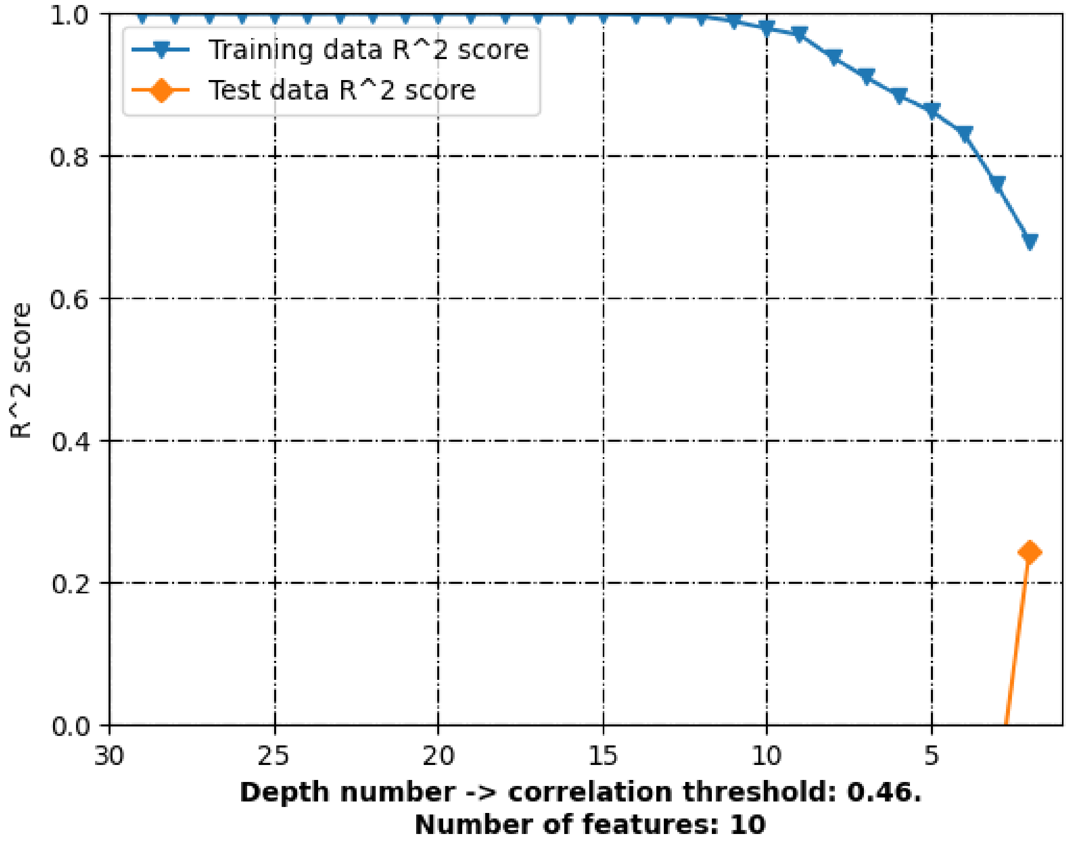
Training data R^2 score
Test data R^2 score
R^2 score
1.0
0.8
0.6
0.4
0.2
0.0
30
25
20
15
10
5
Depth number -> correlation threshold: 0.46.
Number of features: 10

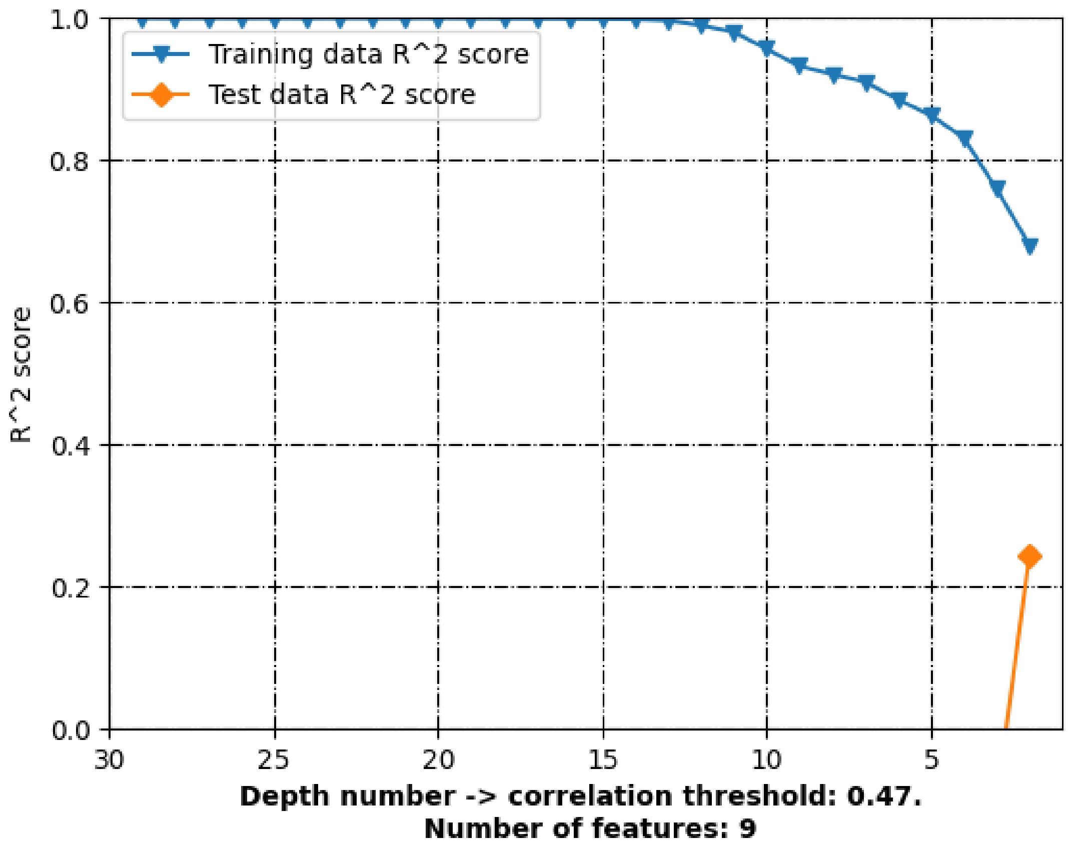

Training data R^2 score
Test data R^2 score
R^2 score
1.0
0.8
0.6
0.4
0.2
0.0
30
25
20
15
10
5
Depth number -> correlation threshold: 0.47.
Number of features: 9

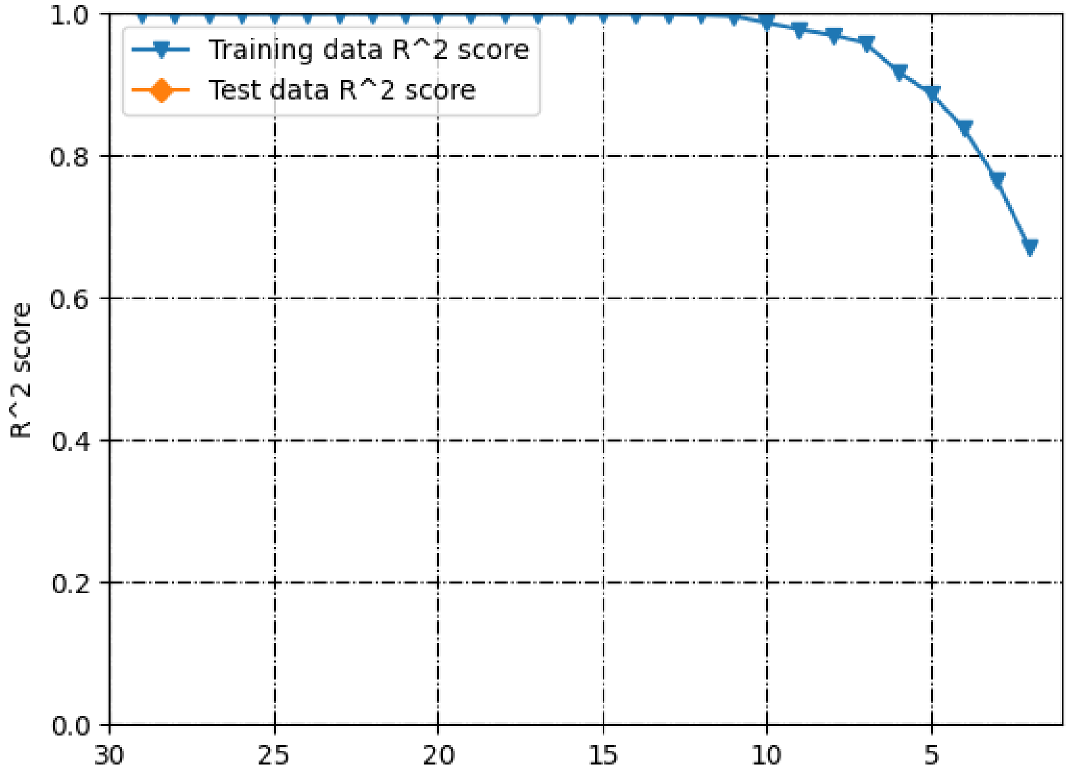

Depth number -> correlation threshold: 0.48.
Number of features: 8

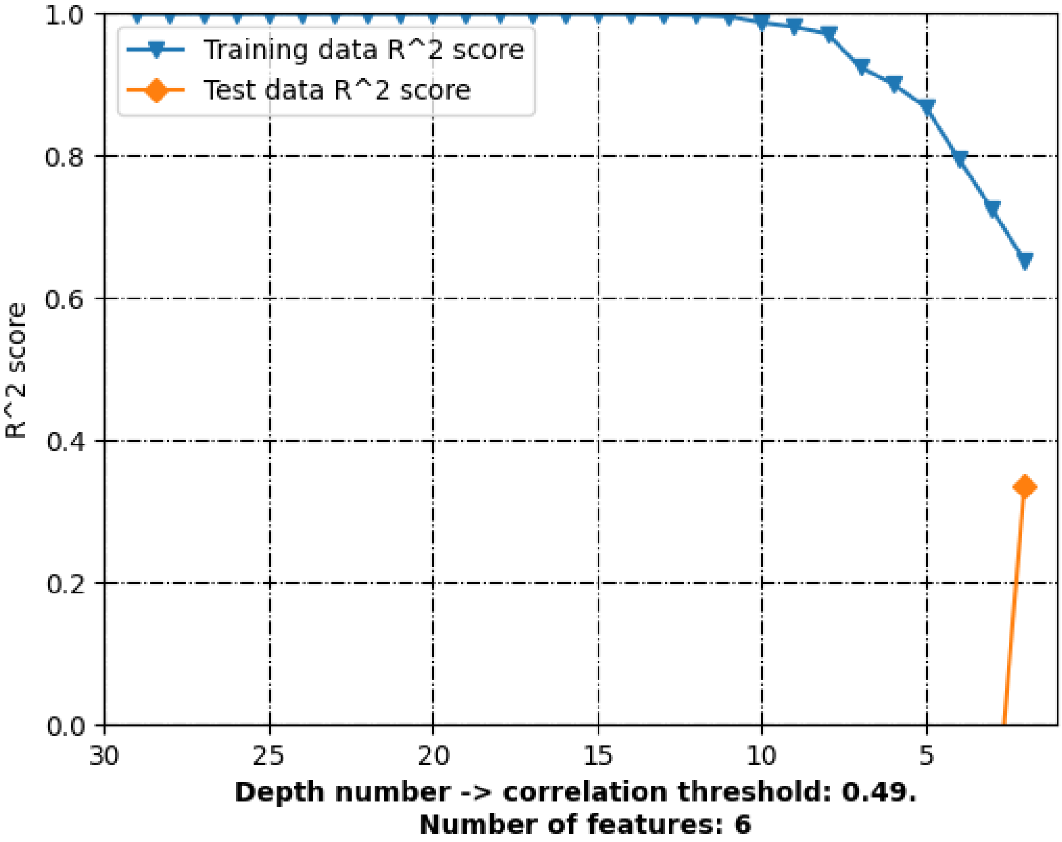
Training data R^2 score
Test data R^2 score
R^2 score
1.0
0.8
0.6
0.4
0.2
0.0
30
25
20
15
10
5
Depth number -> correlation threshold: 0.49.
Number of features: 6

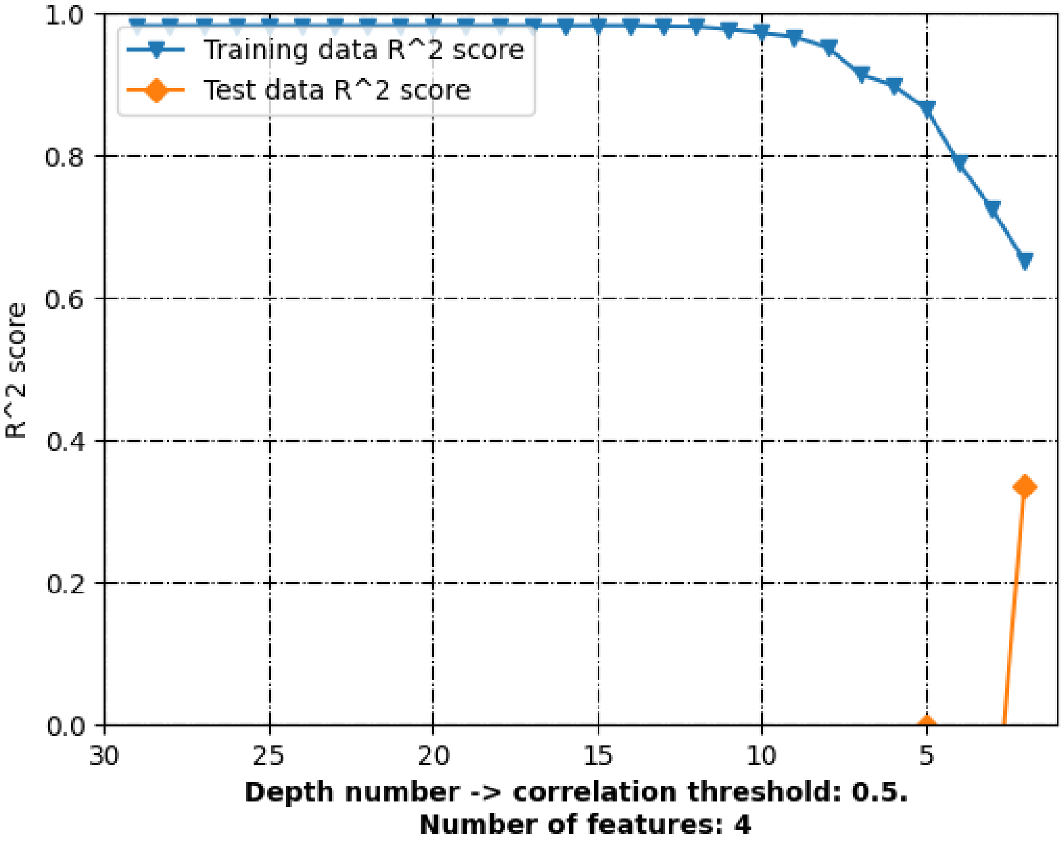

1.0
0.8
0.6
0.4
0.2
0.0
R^2 score
Training data R^2 score
Test data R^2 score
30
25
20
15
10
5
Depth number -> correlation threshold: 0.5.
Number of features: 4

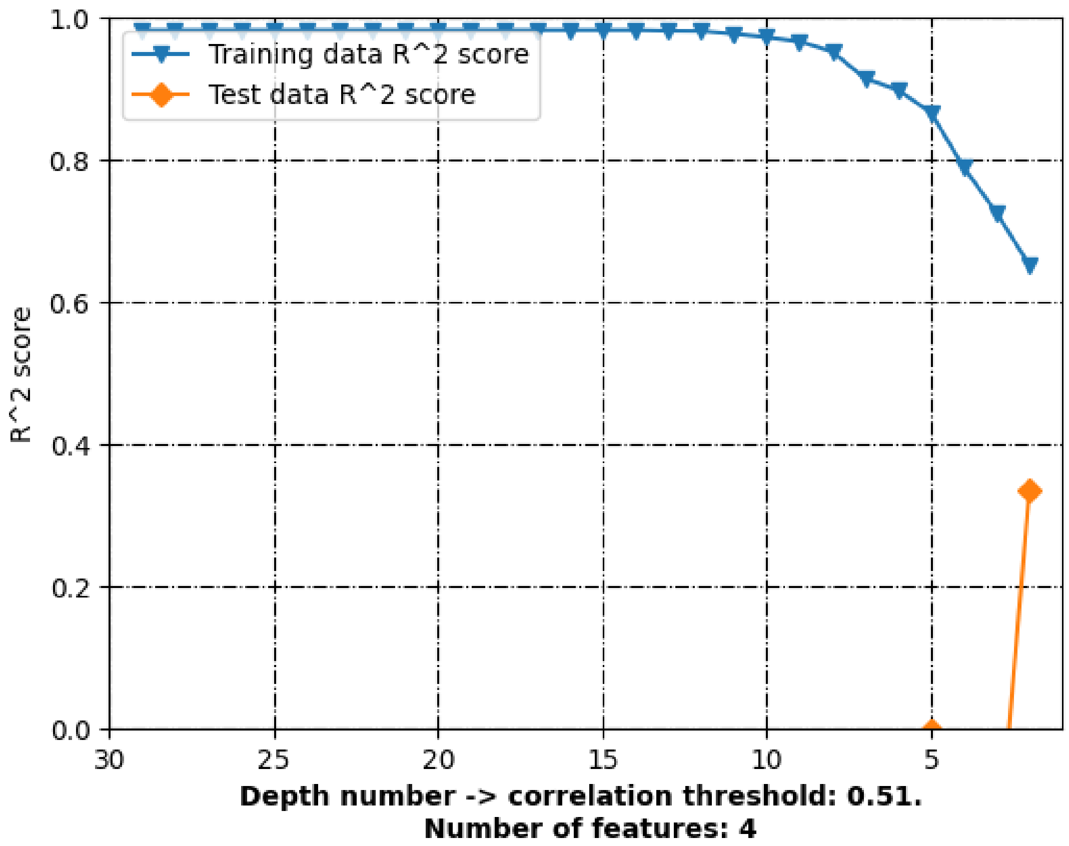

[20]:
```
plt.plot(df_without_standardization['Correlation threshold'],␣
  ↪df_without_standardization['Number of features'], label = "Number of␣
  ↪features", marker='.')
plt.legend()
plt.xlabel('Correlation threshold')
plt.ylabel('Number of features')
plt.show()
```

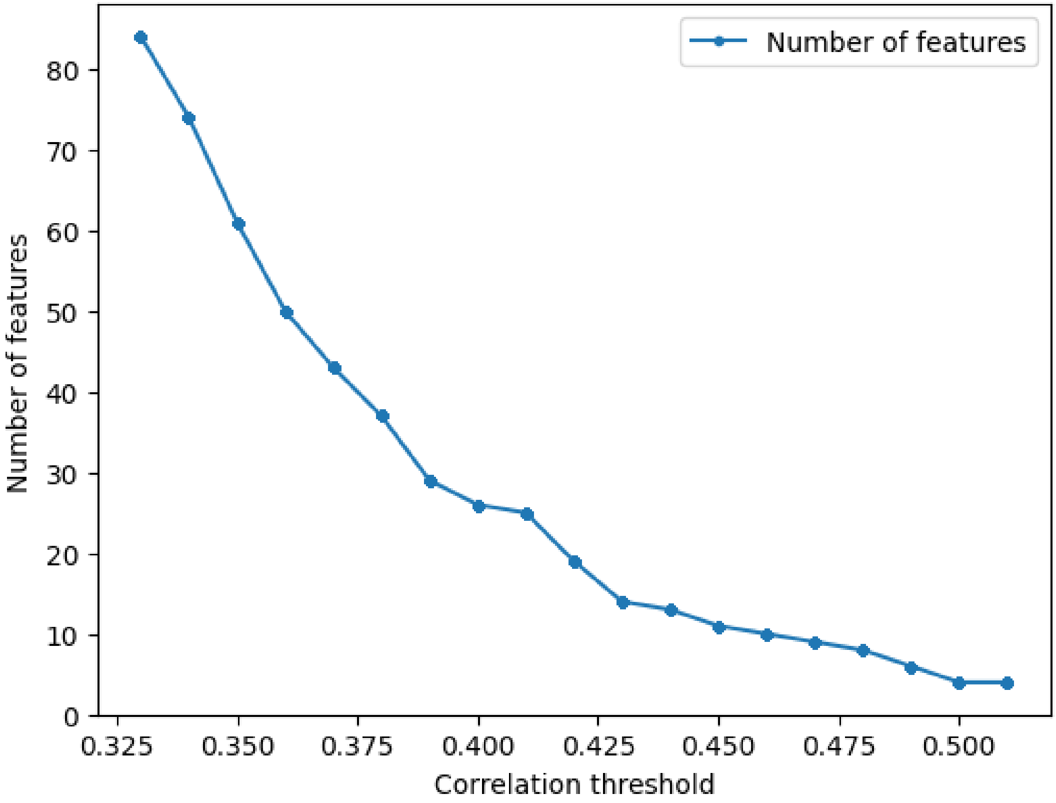


```
[ ]:
```

3 Random Forest

```
[21]: without_standardization, train_r2_, test_r2_, hist1, hist2, target_column_name,␣
      ↪training_data_RMSE, test_data_RMSE  = pred_model.
      ↪prepare_data_and_create_model(molecular_descriptors_df=data,
                                                                                   ␣
      ↪                          correlation_threshold=0.50,
                                                                                   ␣
      ↪                          standardization=False,
                                                                                   ␣
      ↪                          model_type='RandomForestRegressor',
                                                                                   ␣
      ↪                          n_estimators_=12,
                                                                                   ␣
      ↪                          target_column_name = target,
                                                                                   ␣
      ↪                          random_state=random_state,
```

```
                         train_test_split_=True,
                         verbose=True)
```

```
I am not doing standardization…
  molecular descriptor name
0                    AATS0Z
1                  AATS0are
2                    AATS0d
3                   AATS0dv
4                    AATS0i
  molecular descriptor name  corr_value
0                    AATS0Z   -0.038502
1                  AATS0are   -0.120847
2                    AATS0d    0.041648
3                   AATS0dv   -0.115899
4                    AATS0i    0.191765
  molecular descriptor name  corr_value  absolute correlation value
0                    AATS0Z   -0.038502                    0.038502
1                  AATS0are   -0.120847                    0.120847
2                    AATS0d    0.041648                    0.041648
3                   AATS0dv   -0.115899                    0.115899
4                    AATS0i    0.191765                    0.191765
     molecular descriptor name  corr_value  absolute correlation value
226                     AMID_O   -0.520578                    0.520578
505                EState_VSA5   -0.578904                    0.578904
506                EState_VSA6    0.519794                    0.519794
791                    MDEO-12   -0.525441                    0.525441
1211                     MCF-7    1.000000                    1.000000
    molecular descriptor name  corr_value  absolute correlation value
226                    AMID_O   -0.520578                    0.520578
505               EState_VSA5   -0.578904                    0.578904
506               EState_VSA6    0.519794                    0.519794
791                   MDEO-12   -0.525441                    0.525441
The model used is: RandomForest…
Return the coefficient of determination of the prediction:
0.4738764050596963
R^2 score: 0.9123281589088107
Correlation coefficient: 0.9551587087541058
Test data - unseen during training:
R^2 score: 0.4738764050596963
Correlation coefficient: 0.6883868135428629
[7.97408396 6.50095956 7.61650318 7.53828783 7.92073173 8.49185903
 8.18895309 7.68997471 7.25359702 7.85473599 8.04542871 7.37561052
 7.98444469 8.03751897 8.47521646 5.95134349 7.21864713 6.65187823]
113    8.022276
35     6.066513
```

```
101    6.920819
36     6.108463
100    7.376751
13     7.987163
0      7.987163
114    7.823909
104    8.397940
96     7.920819
40     8.091515
103    8.376751
48     7.886057
39     8.455932
14     8.086186
117    5.920819
21     8.113509
9      6.143150
Name: MCF-7, dtype: float64
Training Root Mean Square Error: 0.2868991378875584
Testing Root Mean Square Error: 0.625550282877547
```

[22]:

```
print(target_column_name+str('_transformed'))
print(hist1[target_column_name].hist())
```

```
MCF-7_transformed
AxesSubplot(0.125,0.11;0.775x0.77)
```

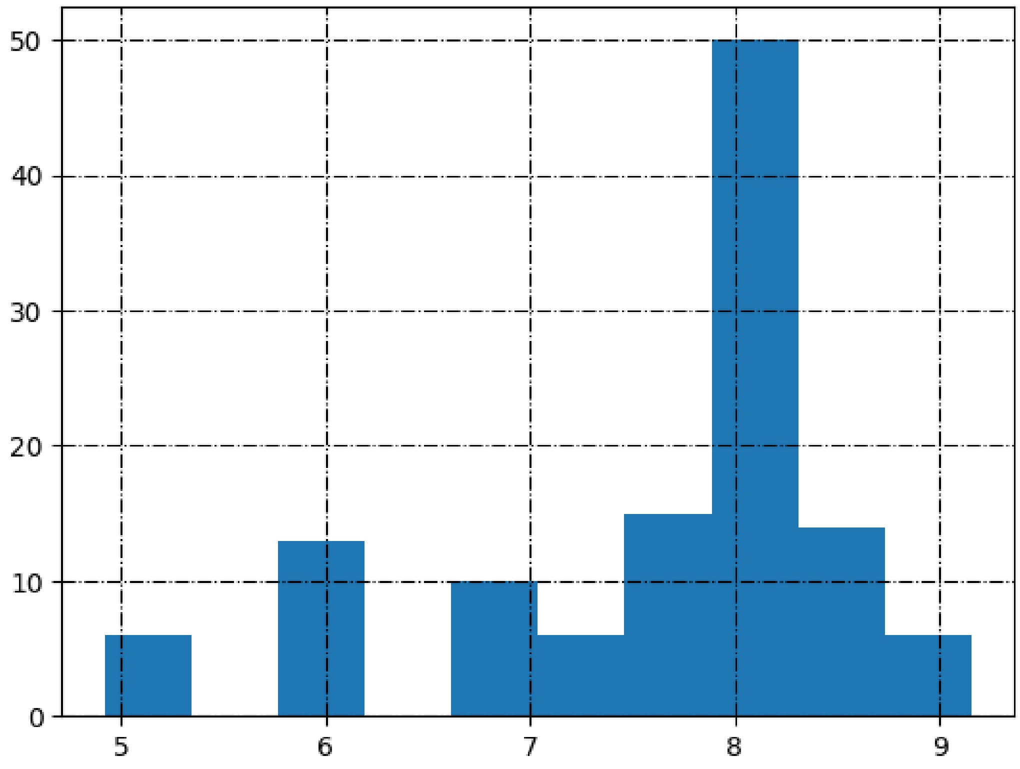

[23]:
```
with_standardization, train_r2_, test_r2_, hist1, hist2, target_column_name,␣
↪training_data_RMSE, test_data_RMSE  = pred_model.
↪prepare_data_and_create_model(molecular_descriptors_df=data,
                                                                                ␣
↪                    correlation_threshold=0.50,
                                                                                ␣
↪                    standardization=True,
                                                                                ␣
↪                    model_type='RandomForestRegressor',
                                                                                ␣
↪                    n_estimators_=12,
                                                                                ␣
↪                    target_column_name = target,
                                                                                ␣
↪                    random_state=random_state,
                                                                                ␣
↪                    train_test_split_=True,
                                                                                ␣
↪                    verbose=True)
```

```
I am doing standardization…
  molecular descriptor name
0                    AATS0Z
1                  AATS0are
2                    AATS0d
3                   AATS0dv
4                    AATS0i
  molecular descriptor name  corr_value
0                    AATS0Z   -0.038502
1                  AATS0are   -0.120847
2                    AATS0d    0.041648
3                   AATS0dv   -0.115899
4                    AATS0i    0.191765
  molecular descriptor name  corr_value  absolute correlation value
0                    AATS0Z   -0.038502                    0.038502
1                  AATS0are   -0.120847                    0.120847
2                    AATS0d    0.041648                    0.041648
3                   AATS0dv   -0.115899                    0.115899
4                    AATS0i    0.191765                    0.191765
     molecular descriptor name  corr_value  absolute correlation value
226                     AMID_O   -0.520578                    0.520578
505                 EState_VSA5   -0.578904                    0.578904
506                 EState_VSA6    0.519794                    0.519794
791                    MDEO-12   -0.525441                    0.525441
```

```
1211                     MCF-7    1.000000                    1.000000
    molecular descriptor name  corr_value  absolute correlation value
226                    AMID_O   -0.520578                    0.520578
505               EState_VSA5   -0.578904                    0.578904
506               EState_VSA6    0.519794                    0.519794
791                  MDEO-12   -0.525441                    0.525441
The model used is: RandomForest…
Return the coefficient of determination of the prediction:
0.4738764050596963
R^2 score: 0.9123281589088107
Correlation coefficient: 0.9551587087541058
Test data - unseen during training:
R^2 score: 0.4738764050596963
Correlation coefficient: 0.6883868135428629
[7.97408396 6.50095956 7.61650318 7.53828783 7.92073173 8.49185903
 8.18895309 7.68997471 7.25359702 7.85473599 8.04542871 7.37561052
 7.98444469 8.03751897 8.47521646 5.95134349 7.21864713 6.65187823]
113    8.022276
35     6.066513
101    6.920819
36     6.108463
100    7.376751
13     7.987163
0      7.987163
114    7.823909
104    8.397940
96     7.920819
40     8.091515
103    8.376751
48     7.886057
39     8.455932
14     8.086186
117    5.920819
21     8.113509
9      6.143150
Name: MCF-7, dtype: float64
Training Root Mean Square Error: 0.2868991378875584
Testing Root Mean Square Error: 0.625550282877547
```

3.1 Search inside correlation space

[24]:
```
step = 0.01
initial_step = corr_low
last_step = corr_high
first_list = [x / 100.0 for x in range(int(initial_step*100),␣
 ↪int(last_step*100), int(step*100))]
n_estimators = [range(2,21,1)]
```

```
corr_th = []
second_list = []
third_list = []
fourth_l = []
fifth_l = []
f_list = []
fif_list = []
for i in first_list:

    for estimator in n_estimators[0]:

        without_standardization, train_r2, test_r2, _, h_, target_column_name,␣
 ↪training_data_RMSE, test_data_RMSE = pred_model.
 ↪prepare_data_and_create_model(molecular_descriptors_df=data,
                                                                                ␣
 ↪                    correlation_threshold=i,
                                                                                ␣
 ↪                    standardization=False,
                                                                                ␣
 ↪                    model_type='RandomForestRegressor',
                                                                                ␣
 ↪                    n_estimators_=estimator,
                                                                                ␣
 ↪                    target_column_name = target,
                                                                                ␣
 ↪                    random_state=random_state,
                                                                                ␣
 ↪                    train_test_split_=True,
                                                                                ␣
 ↪                    verbose=False)
        corr_th.append(i)
        second_list.append(train_r2)
        third_list.append(test_r2)
        fourth_l.append(training_data_RMSE)
        fifth_l.append(test_data_RMSE)
        f_list.append(len(h_))
        fif_list.append(estimator)
```

[25]:
```
df_without_standardization = pd.DataFrame(data=corr_th, columns=["Correlation␣
 ↪threshold"])
df_without_standardization['Training data R^2 score'] = second_list
df_without_standardization['Test data R^2 score'] = third_list
df_without_standardization['Training RMSE'] = fourth_l
df_without_standardization['Test RMSE'] = fifth_l
df_without_standardization['Number of features'] = f_list
df_without_standardization['Number of estimators'] = fif_list
```

[26]:
```
df_random_forest = df_without_standardization.copy()
#df_without_standardization.to_excel('../Data/
↪A549_Random_forest_rs_'+str(random_state)+'.xlsx')
```

[27]:
```
corre_list = [x / 100.0 for x in range(int(initial_step*100),␣
↪int(last_step*100), int(0.01*100))]
for element in corre_list:
    element_ =␣
↪df_without_standardization[df_without_standardization['Correlation␣
↪threshold'] == float(element)]
    plt.plot(element_['Number of estimators'], element_['Training data R^2␣
↪score'], label = "Training data R^2 score", marker='v')
    plt.plot(element_['Number of estimators'], element_['Test data R^2 score'],␣
↪label = "Test data R^2 score", marker='D')
    plt.legend()
    plt.xlabel('Number of estimators -> correlation threshold: '+str(element)+'.
↪ \n Number of features: '+str(element_['Number of features'].iloc[0]))
    plt.xlim(max(element_['Number of estimators'])+1, min(element_['Number of␣
↪estimators'])-1)
    plt.ylabel('R^2 score')
    plt.ylim([0, 1])
    plt.rc('grid', linestyle="-.", color='black')
    plt.grid(True)
    plt.show()
```

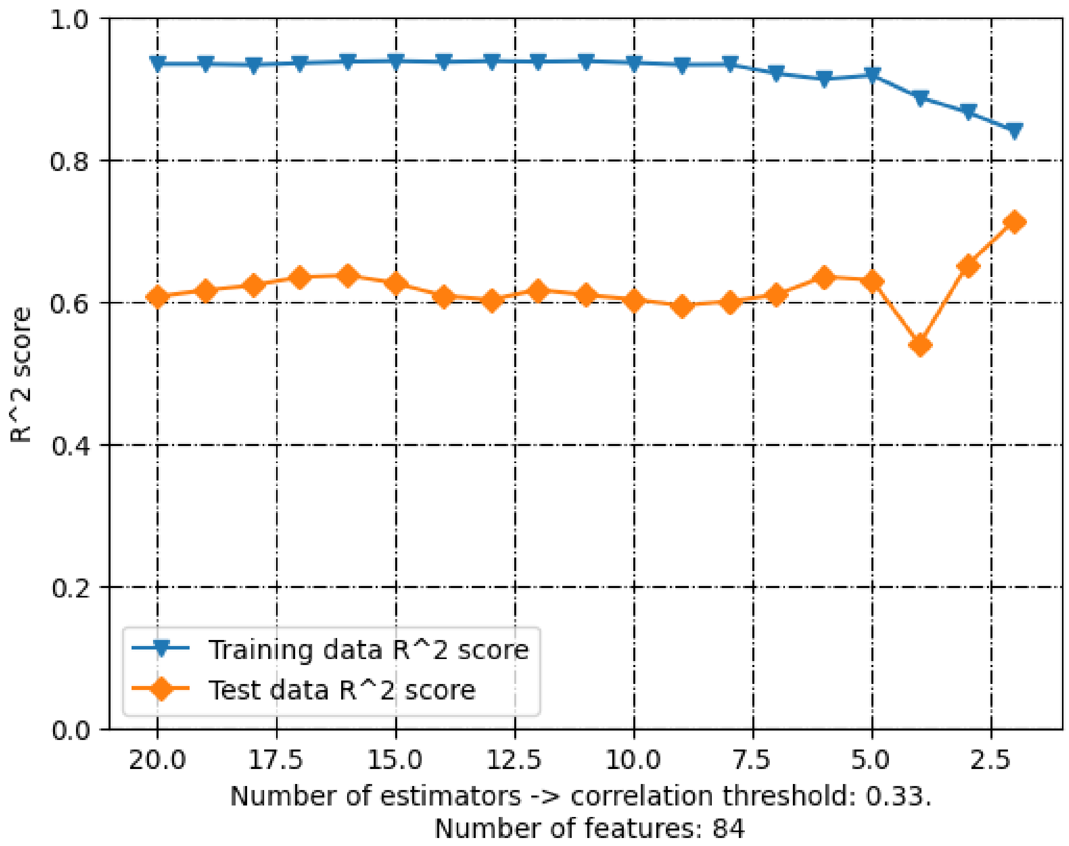
1.0
0.8
0.6
0.4
0.2
0.0
R^2 score
Training data R^2 score
Test data R^2 score
20.0
17.5
15.0
12.5
10.0
7.5
5.0
2.5
Number of estimators -> correlation threshold: 0.33.
Number of features: 84

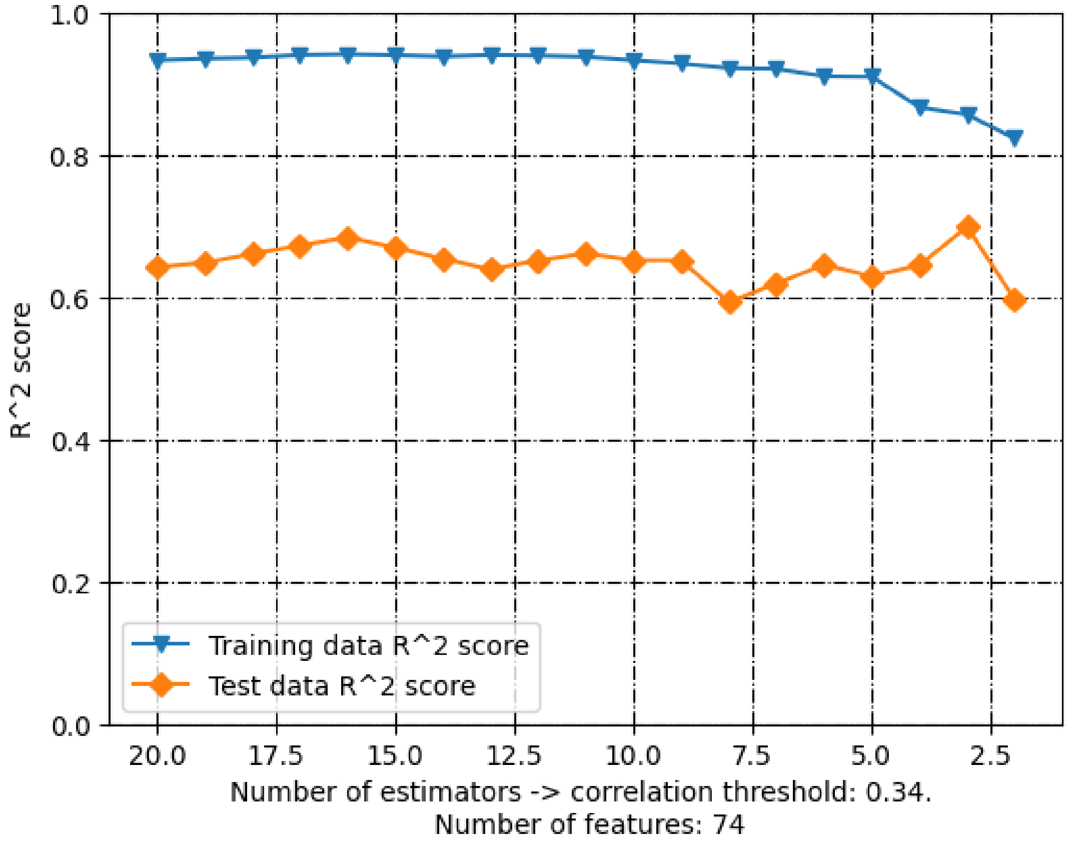
1.0
0.8
0.6
0.4
0.2
0.0
R^2 score
20.0
17.5
15.0
12.5
10.0
7.5
5.0
2.5
Number of estimators -> correlation threshold: 0.34.
Number of features: 74
Training data R^2 score
Test data R^2 score

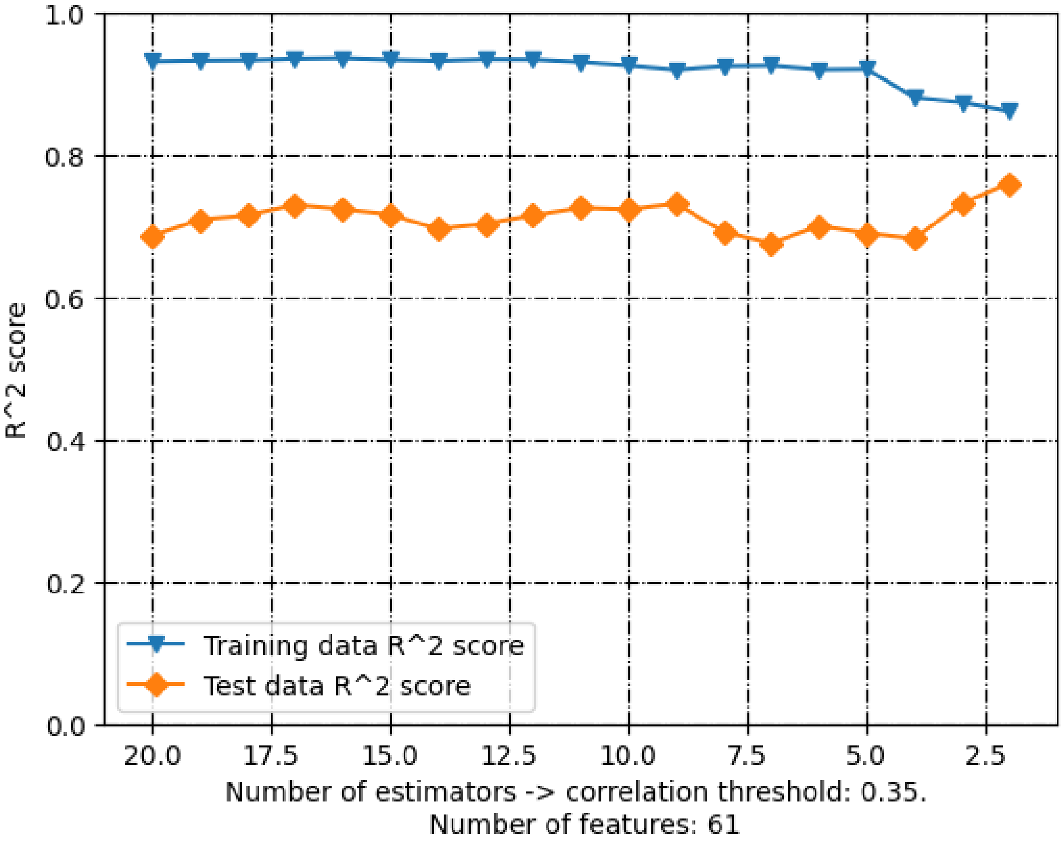

1.0
0.8
0.6
0.4
0.2
0.0
R^2 score
Training data R^2 score
Test data R^2 score
20.0
17.5
15.0
12.5
10.0
7.5
5.0
2.5
Number of estimators -> correlation threshold: 0.35.
Number of features: 61

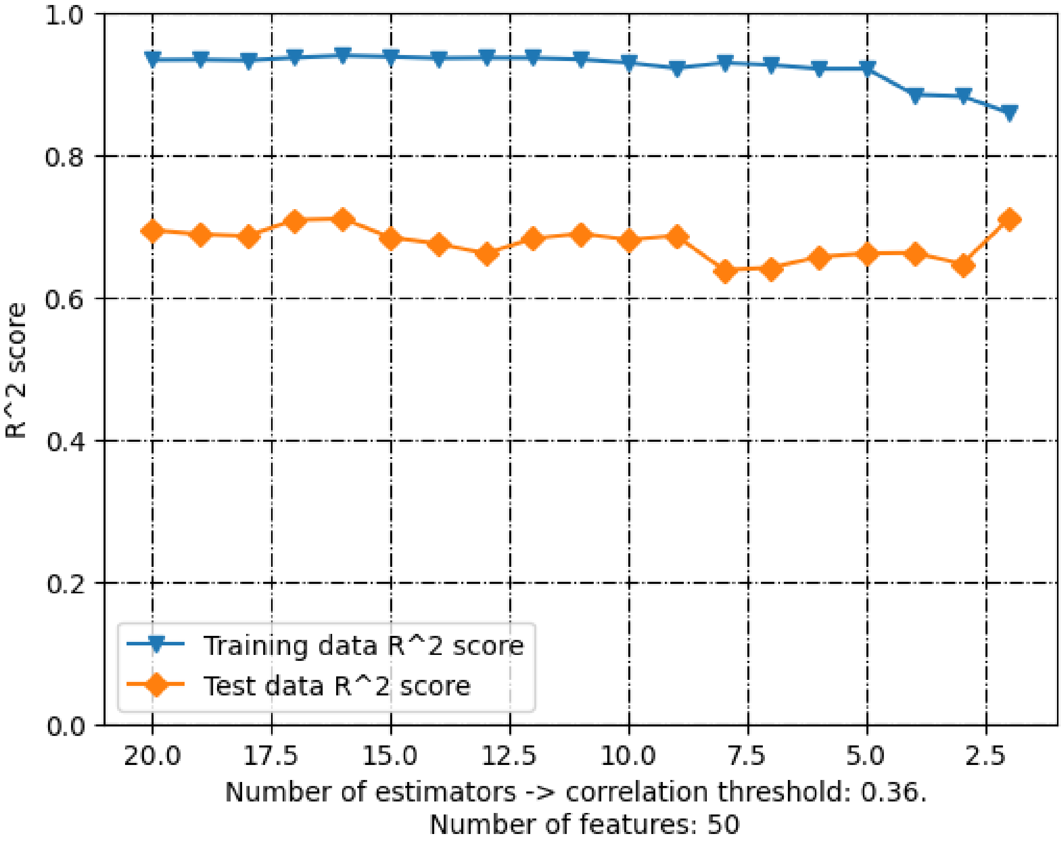
1.0
0.8
0.6
0.4
0.2
0.0
R^2 score
Training data R^2 score
Test data R^2 score
20.0
17.5
15.0
12.5
10.0
7.5
5.0
2.5
Number of estimators -> correlation threshold: 0.36.
Number of features: 50

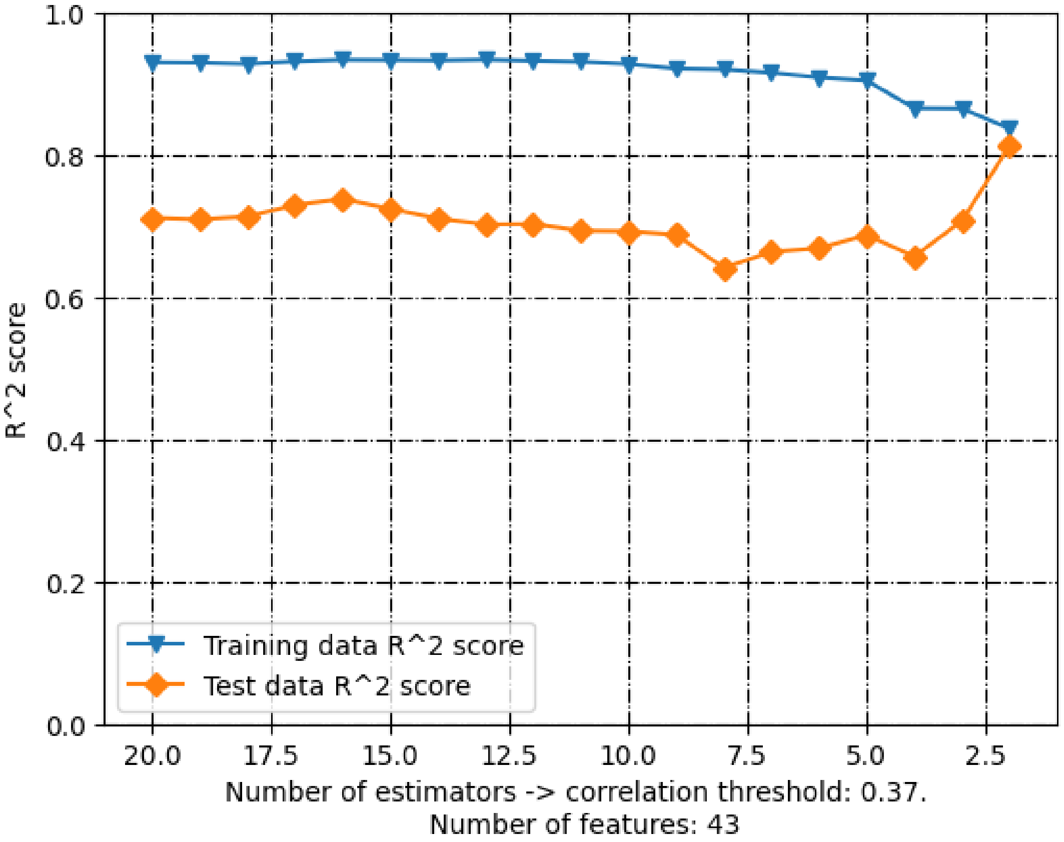
1.0
0.8
0.6
0.4
0.2
0.0
R^2 score
Training data R^2 score
Test data R^2 score
20.0
17.5
15.0
12.5
10.0
7.5
5.0
2.5
Number of estimators -> correlation threshold: 0.37.
Number of features: 43

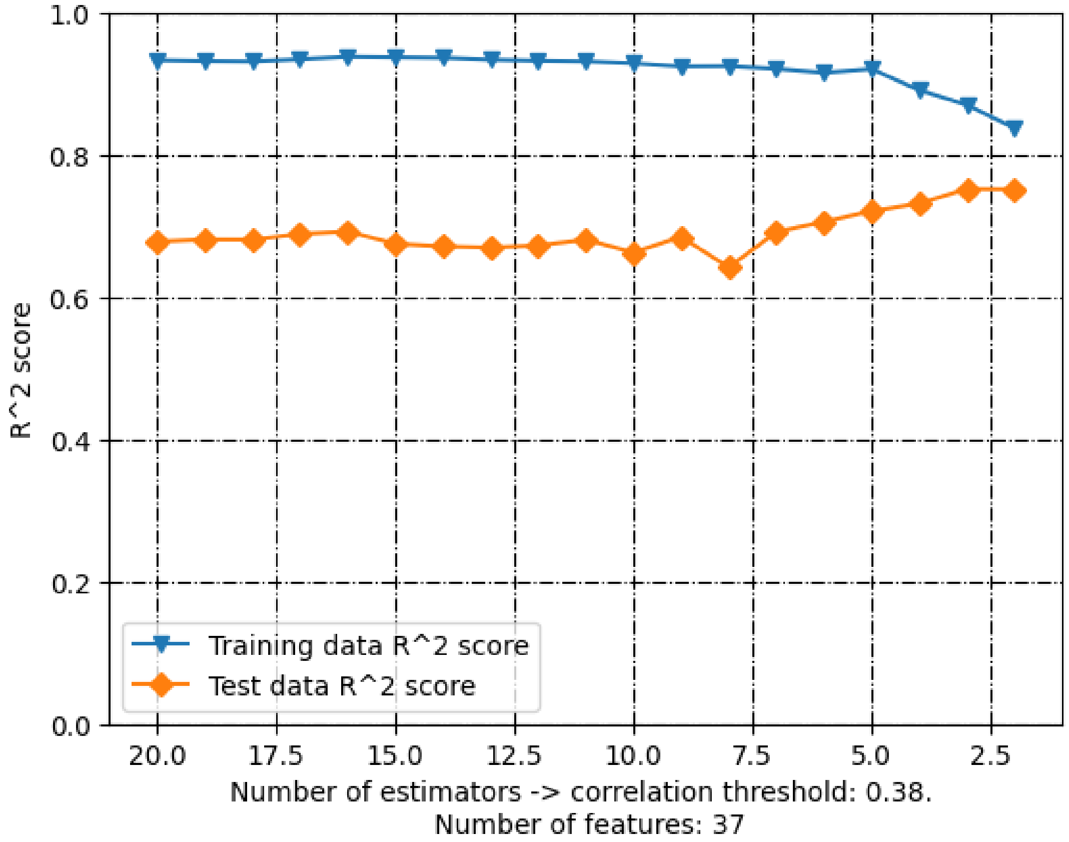

1.0
0.8
0.6
0.4
0.2
0.0
R^2 score
Training data R^2 score
Test data R^2 score
20.0
17.5
15.0
12.5
10.0
7.5
5.0
2.5
Number of estimators -> correlation threshold: 0.38.
Number of features: 37

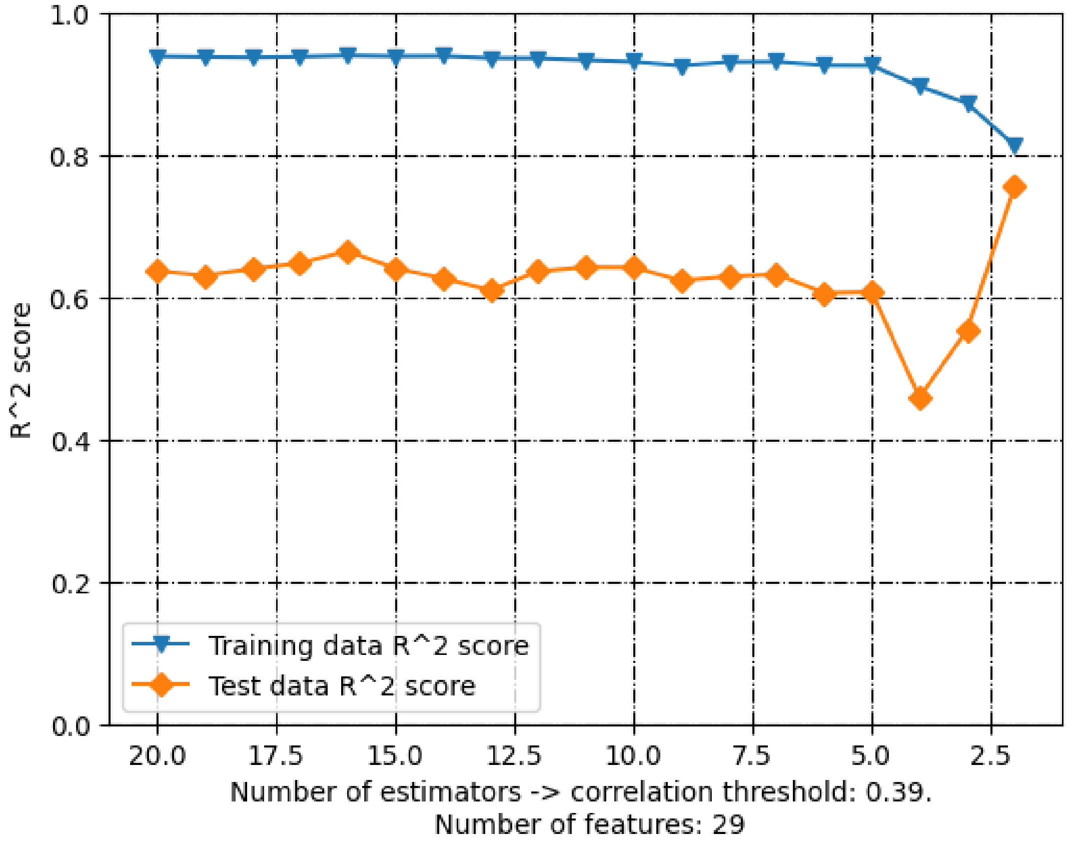
1.0
0.8
0.6
0.4
0.2
0.0
R^2 score
Training data R^2 score
Test data R^2 score
20.0
17.5
15.0
12.5
10.0
7.5
5.0
2.5
Number of estimators -> correlation threshold: 0.39.
Number of features: 29

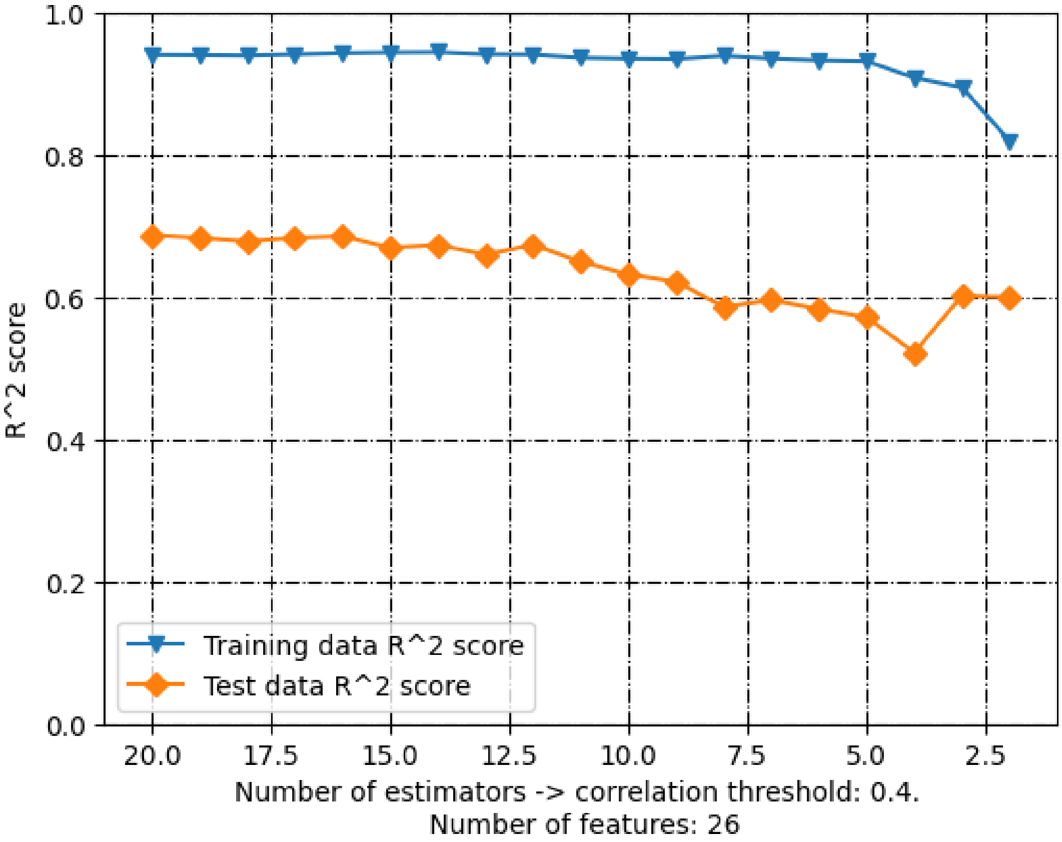
1.0
0.8
0.6
0.4
0.2
0.0
R^2 score
20.0
17.5
15.0
12.5
10.0
7.5
5.0
2.5
Number of estimators -> correlation threshold: 0.4.
Number of features: 26
Training data R^2 score
Test data R^2 score

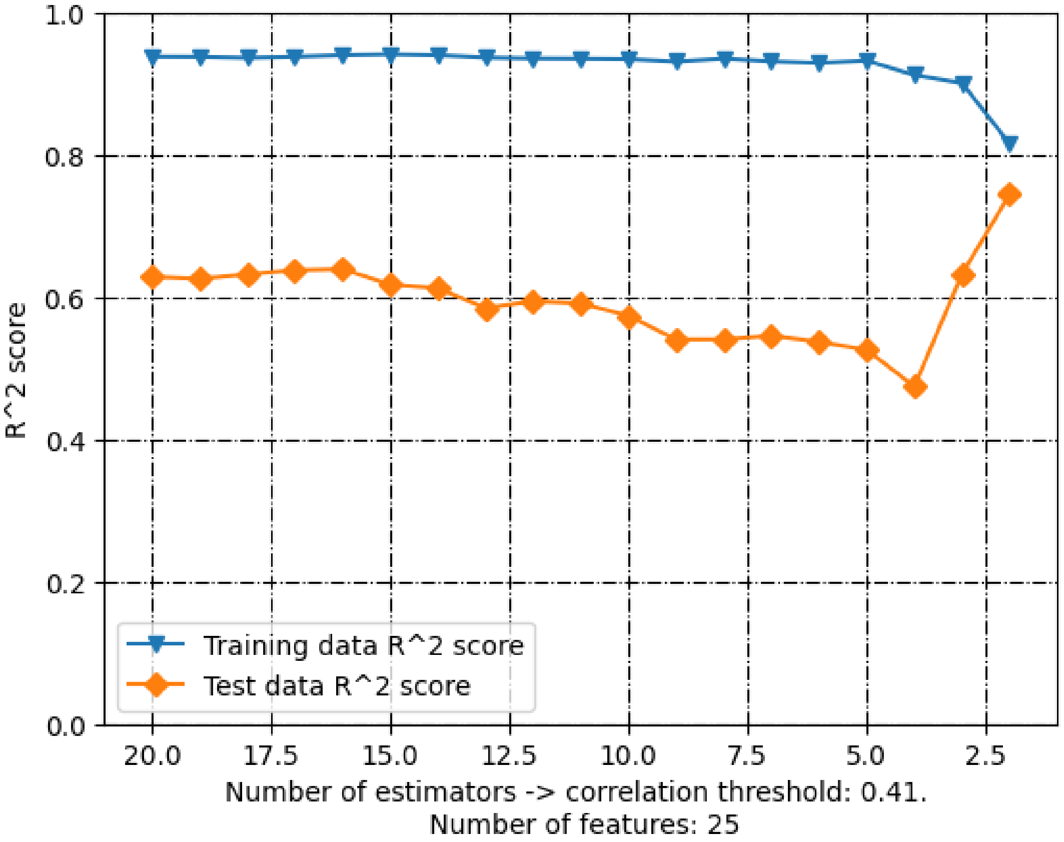
1.0
0.8
0.6
0.4
0.2
0.0
R^2 score
Training data R^2 score
Test data R^2 score
20.0
17.5
15.0
12.5
10.0
7.5
5.0
2.5
Number of estimators -> correlation threshold: 0.41.
Number of features: 25

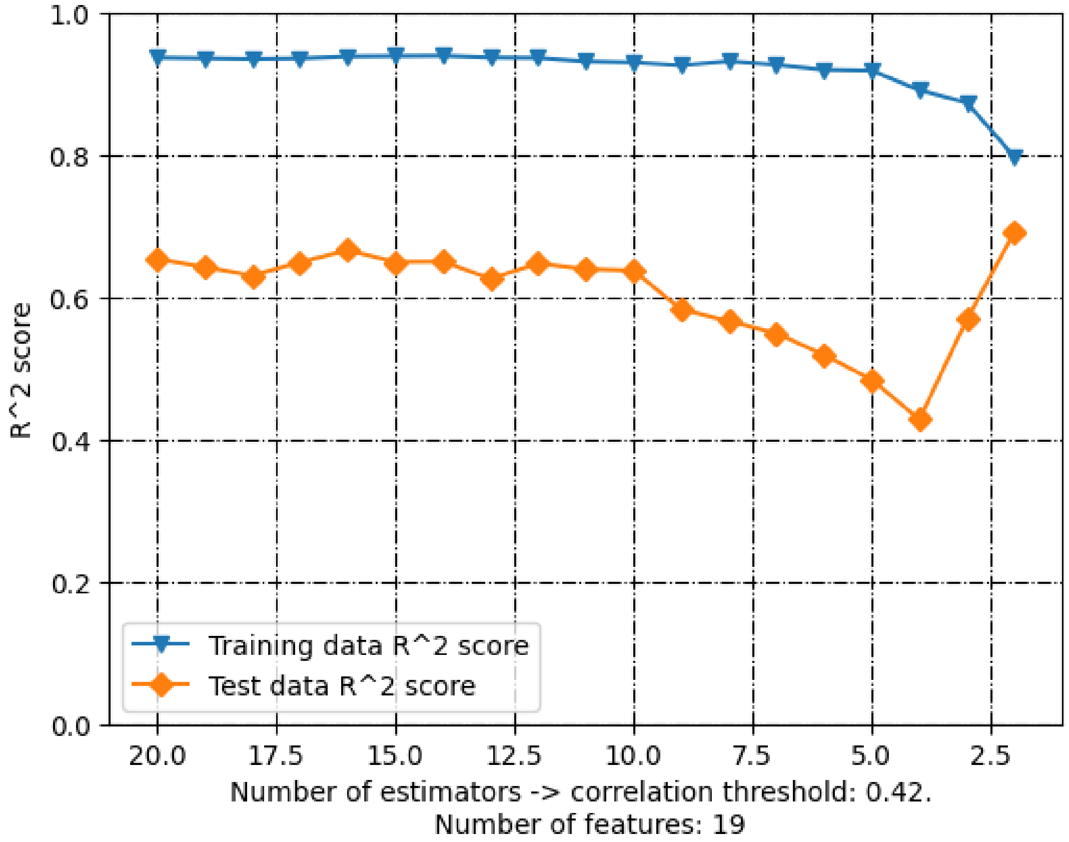

1.0
0.8
0.6
0.4
0.2
0.0
R^2 score
20.0
17.5
15.0
12.5
10.0
7.5
5.0
2.5
Number of estimators -> correlation threshold: 0.42.
Number of features: 19
Training data R^2 score
Test data R^2 score

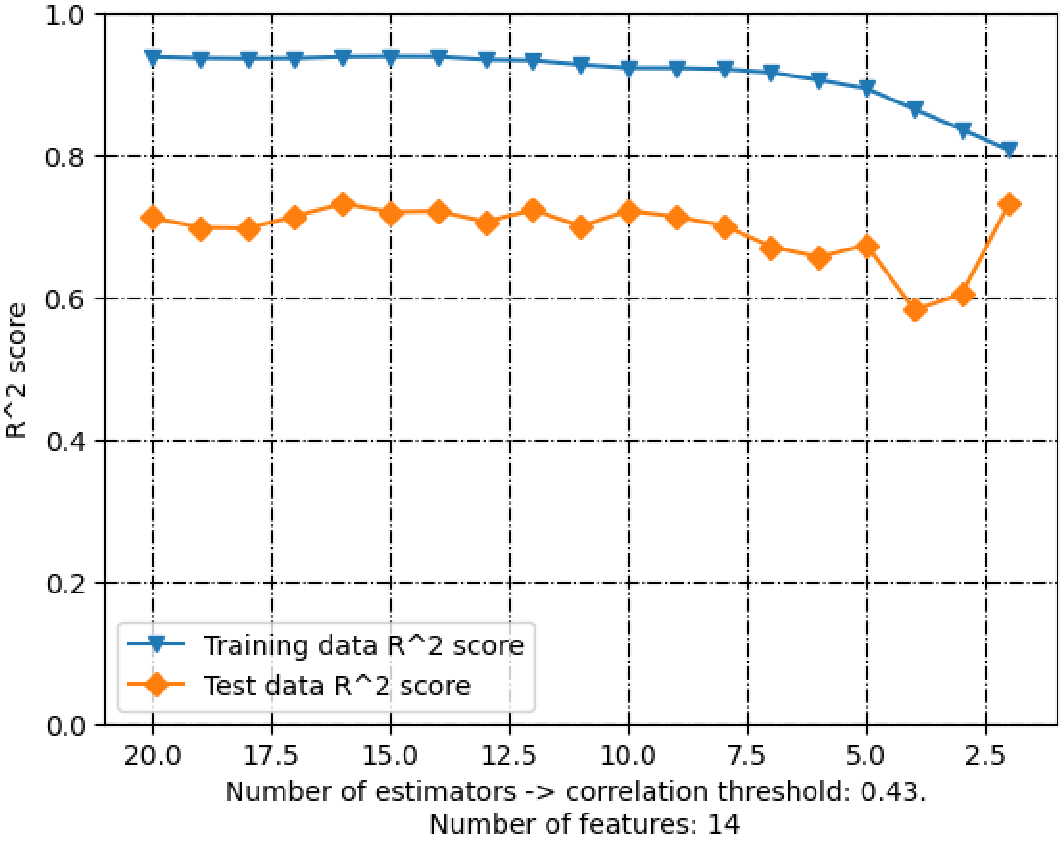

1.0
0.8
0.6
0.4
0.2
0.0
R^2 score
20.0
17.5
15.0
12.5
10.0
7.5
5.0
2.5
Number of estimators -> correlation threshold: 0.43.
Number of features: 14
Training data R^2 score
Test data R^2 score

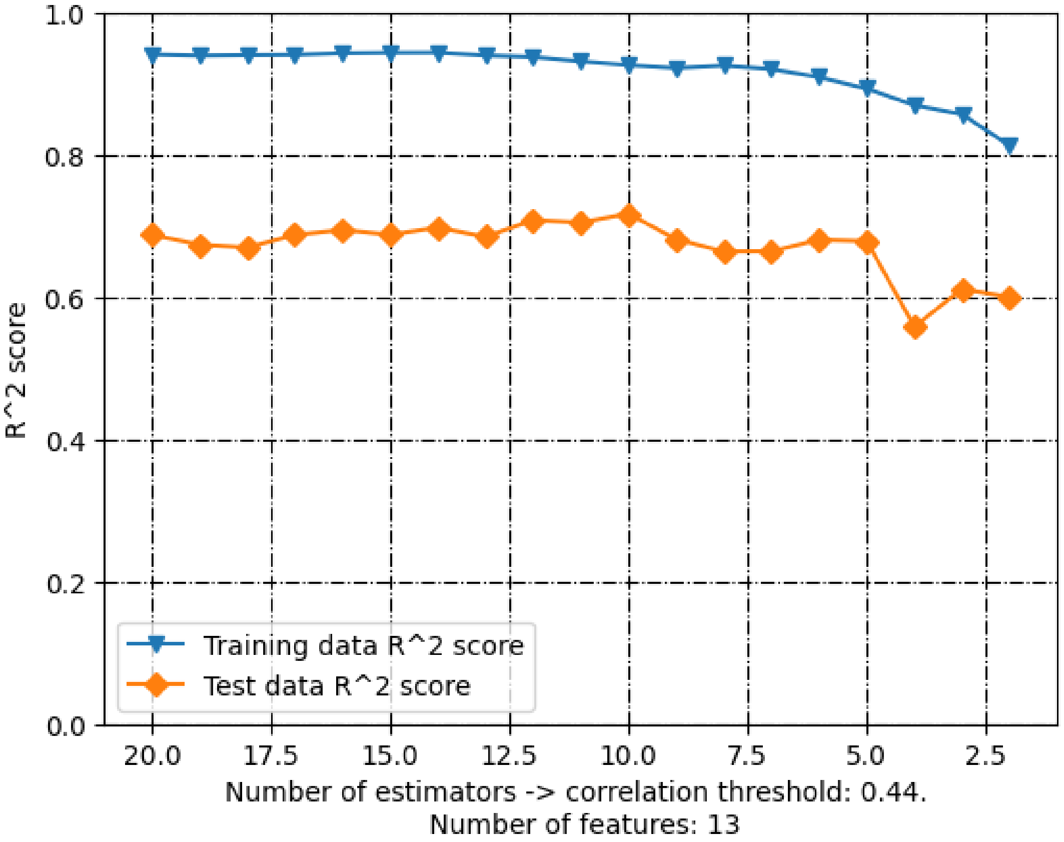
1.0
0.8
0.6
0.4
0.2
0.0
R^2 score
Training data R^2 score
Test data R^2 score
20.0
17.5
15.0
12.5
10.0
7.5
5.0
2.5
Number of estimators -> correlation threshold: 0.44.
Number of features: 13

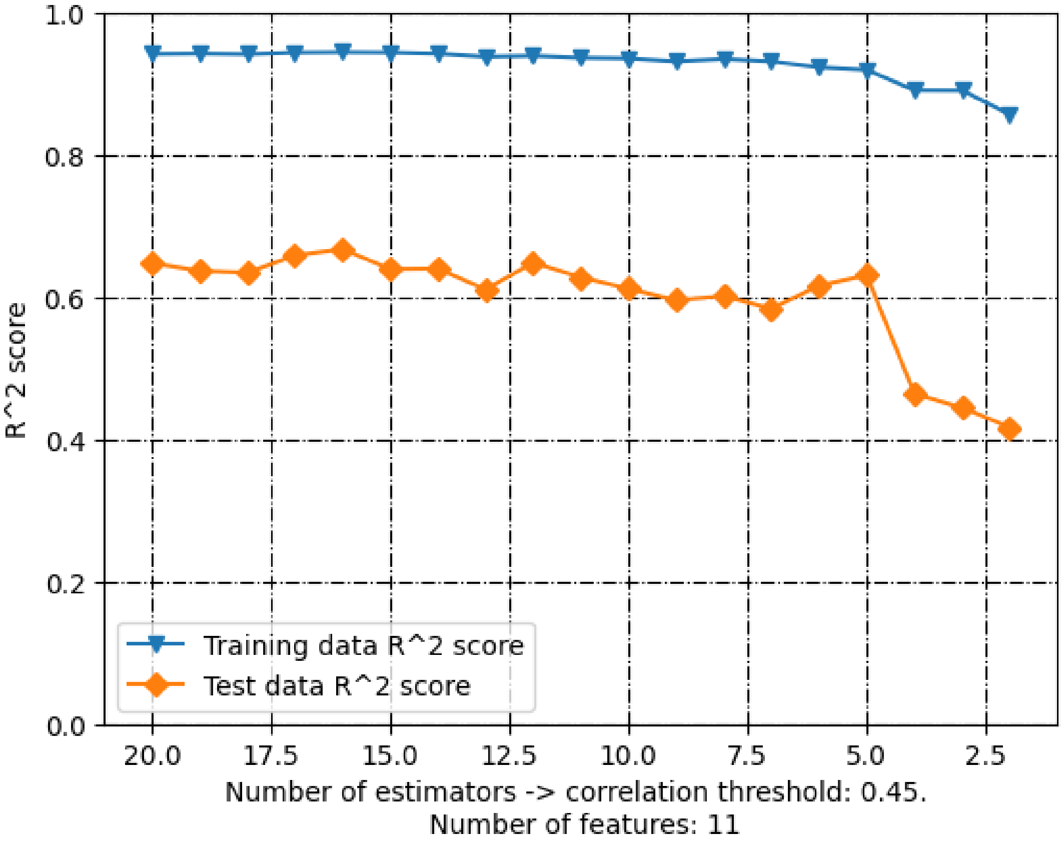
1.0
0.8
0.6
0.4
0.2
0.0
R^2 score
Training data R^2 score
Test data R^2 score
20.0
17.5
15.0
12.5
10.0
7.5
5.0
2.5
Number of estimators -> correlation threshold: 0.45.
Number of features: 11

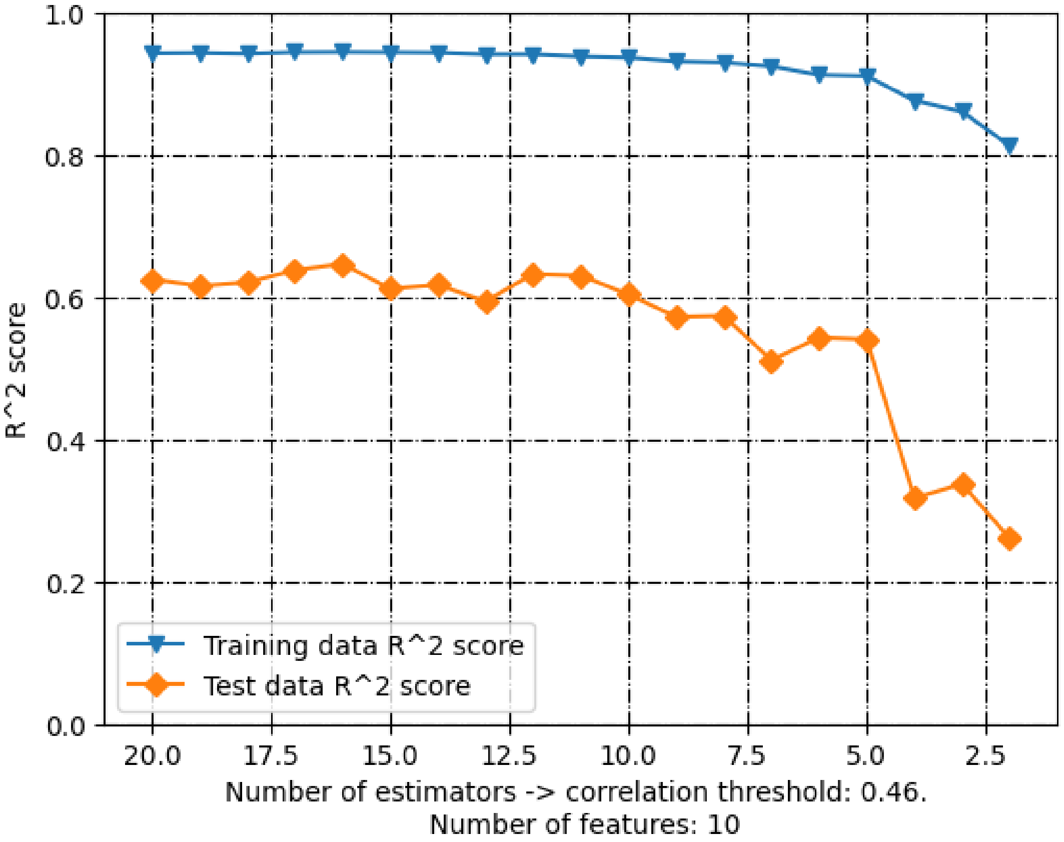

1.0
0.8
0.6
0.4
0.2
0.0
R^2 score
20.0
17.5
15.0
12.5
10.0
7.5
5.0
2.5
Training data R^2 score
Test data R^2 score
Number of estimators -> correlation threshold: 0.46.
Number of features: 10

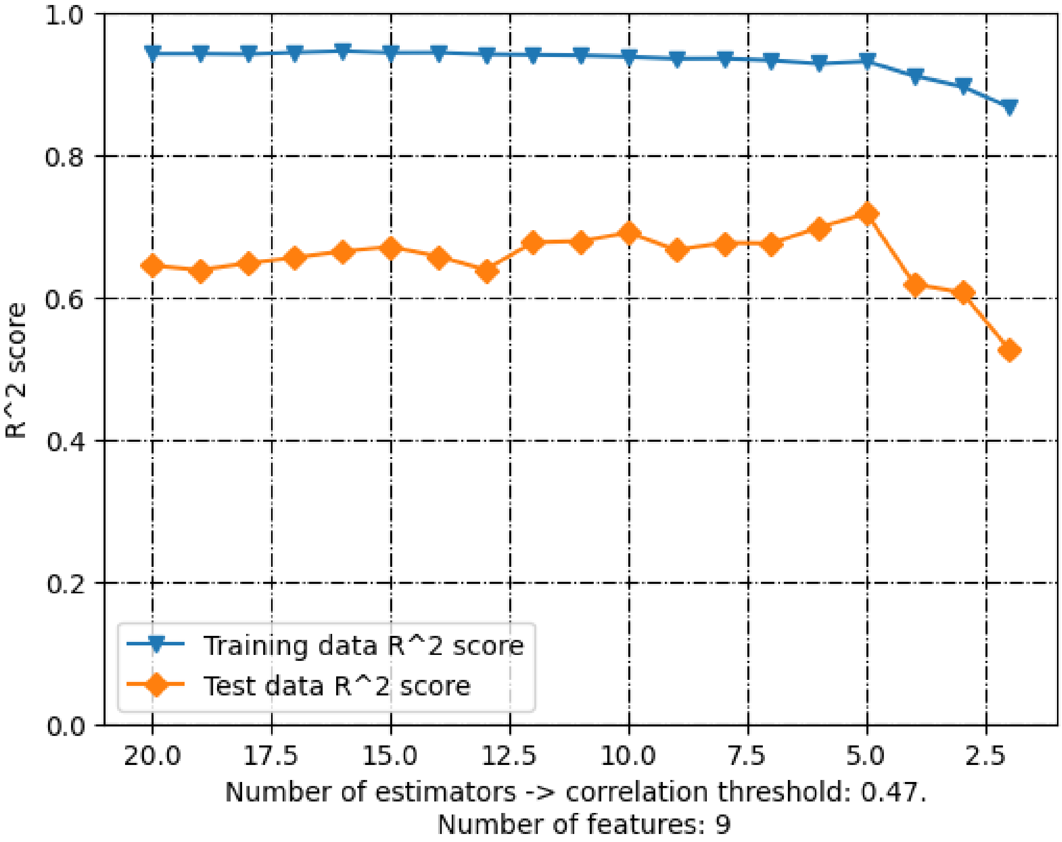
1.0
0.8
0.6
0.4
0.2
0.0
R^2 score
Training data R^2 score
Test data R^2 score
20.0
17.5
15.0
12.5
10.0
7.5
5.0
2.5
Number of estimators -> correlation threshold: 0.47.
Number of features: 9

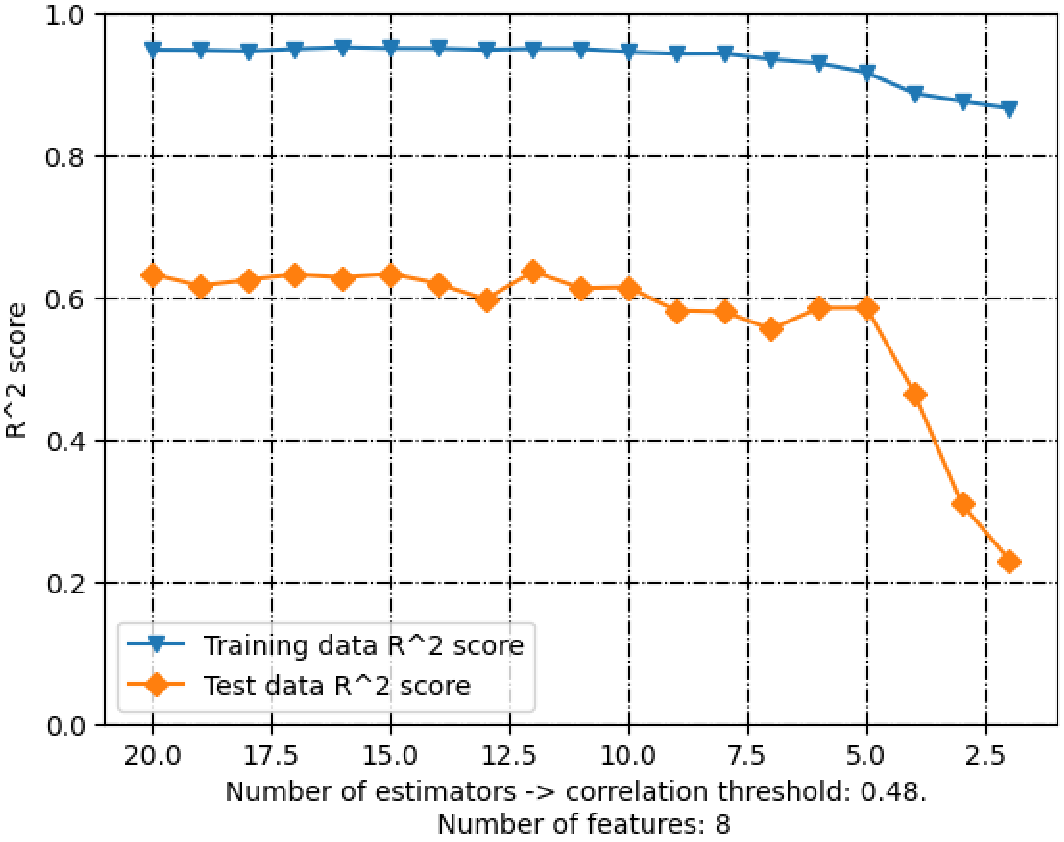

1.0
0.8
0.6
0.4
0.2
0.0
R^2 score
Training data R^2 score
Test data R^2 score
20.0
17.5
15.0
12.5
10.0
7.5
5.0
2.5
Number of estimators -> correlation threshold: 0.48.
Number of features: 8

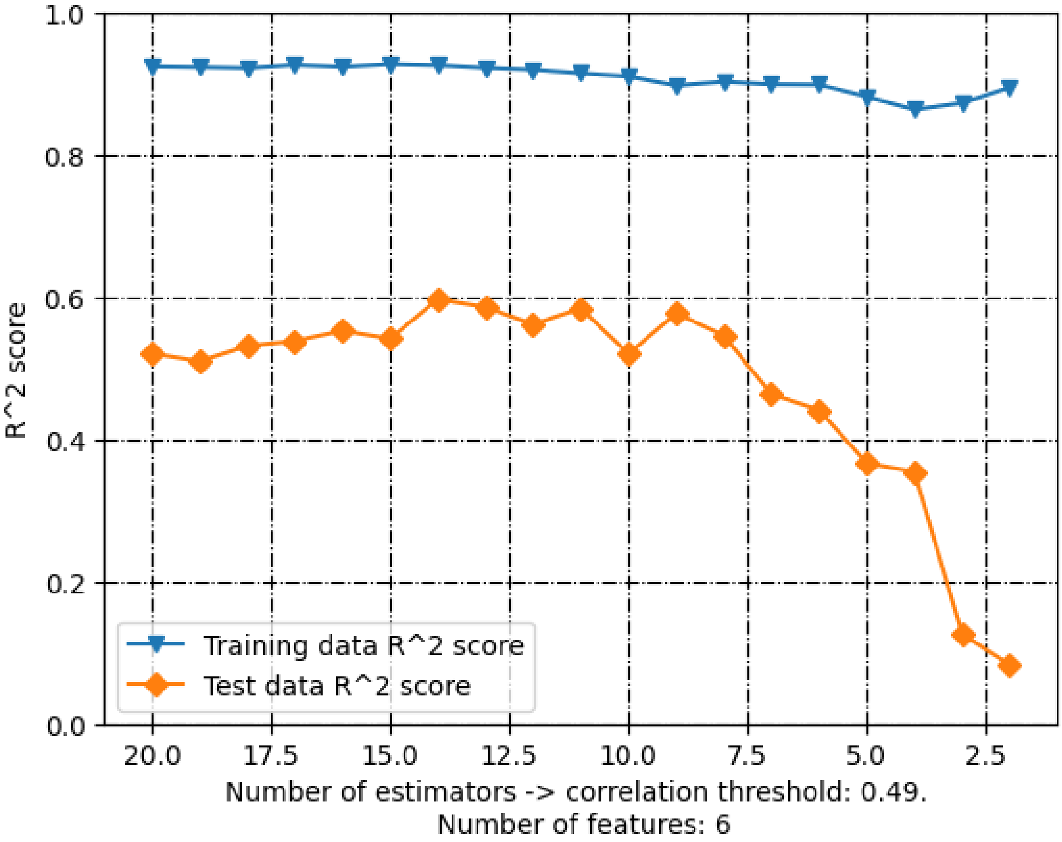
1.0
0.8
0.6
0.4
0.2
0.0
R^2 score
20.0
17.5
15.0
12.5
10.0
7.5
5.0
2.5
Training data R^2 score
Test data R^2 score
Number of estimators -> correlation threshold: 0.49.
Number of features: 6

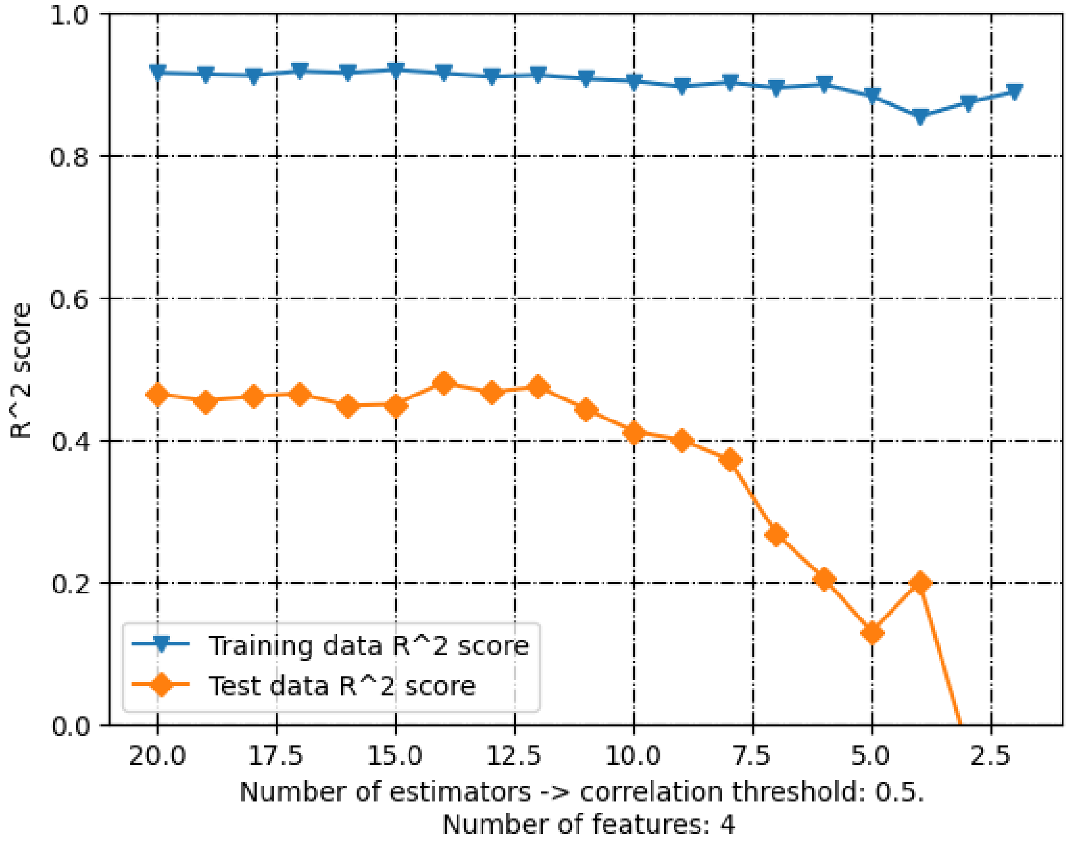

1.0
0.8
0.6
0.4
0.2
0.0
R^2 score
20.0
17.5
15.0
12.5
10.0
7.5
5.0
2.5
Training data R^2 score
Test data R^2 score
Number of estimators -> correlation threshold: 0.5.
Number of features: 4

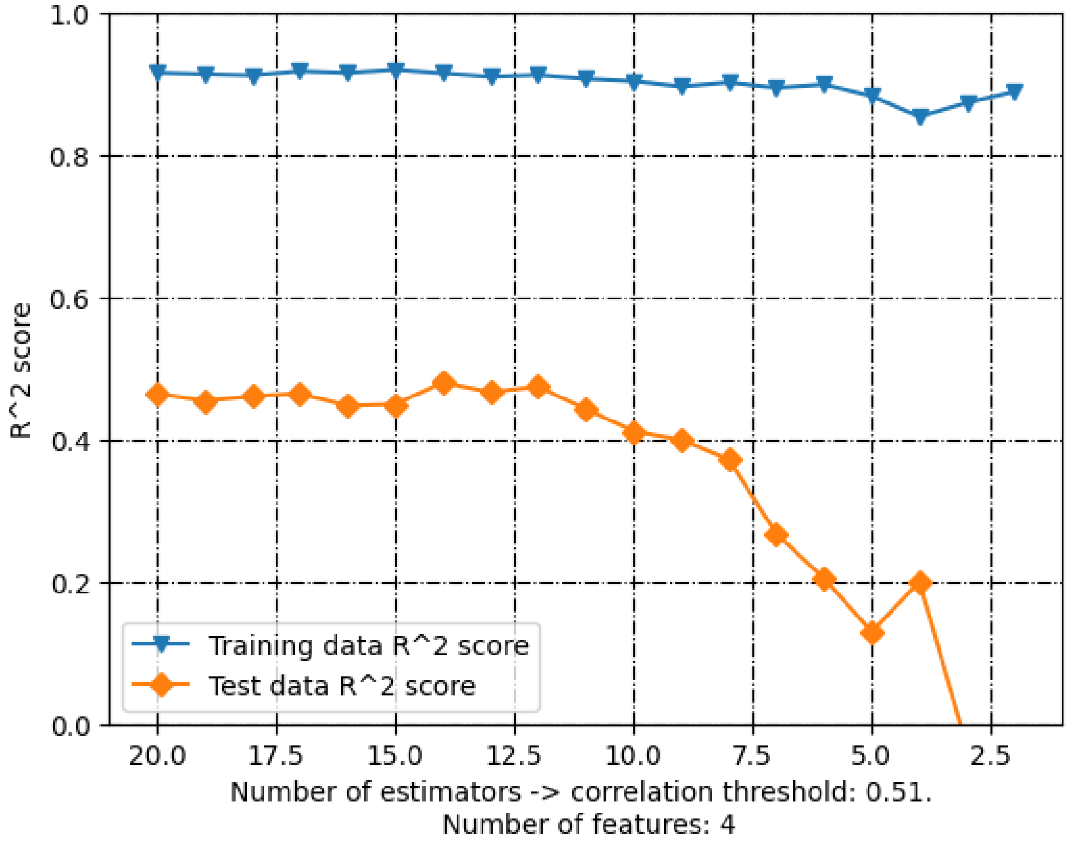

[28]:
```
plt.plot(df_without_standardization['Correlation threshold'],␣
 ↪df_without_standardization['Number of features'], label = "Number of␣
 ↪features", marker='.')
plt.legend()
plt.xlabel('Correlation threshold')
plt.ylabel('Number of features')
plt.show()
```

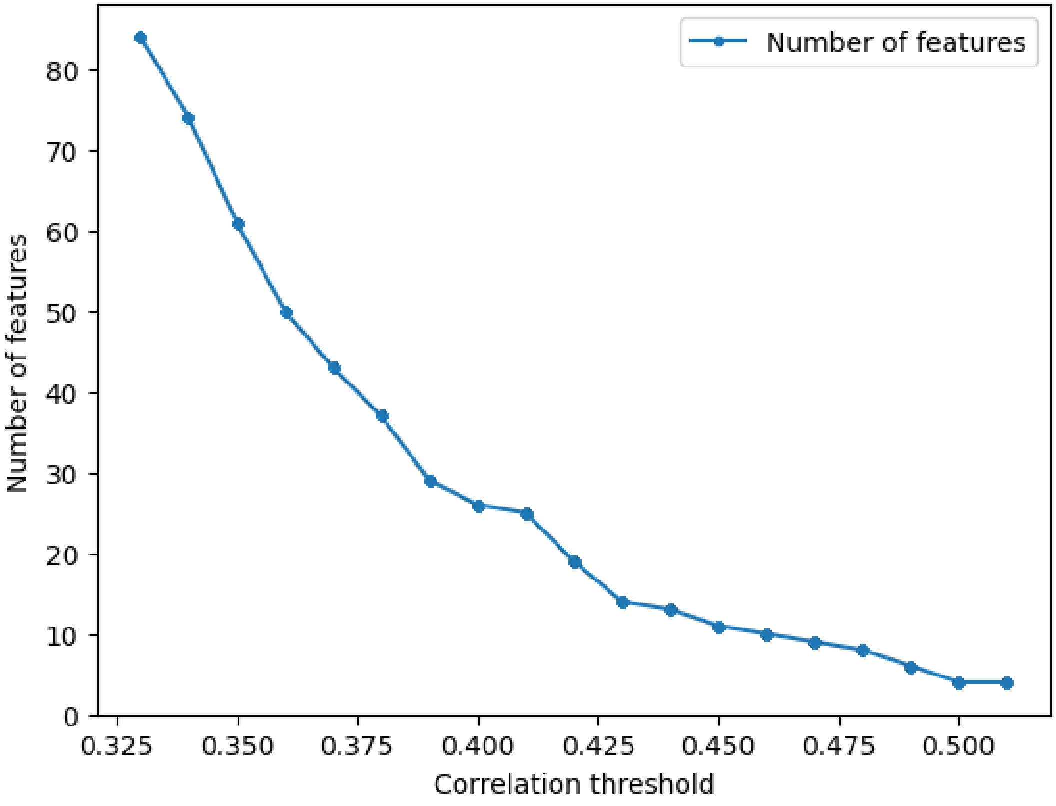

[ ]:

4 KNeighborsRegressor

[29]:
```
without_standardization, train_r2_, test_r2_, hist1, hist2, target_column_name,␣
↪training_data_RMSE, test_data_RMSE  = pred_model.
↪prepare_data_and_create_model(molecular_descriptors_df=data,
                                                                            ␣
↪                    correlation_threshold=0.50,
                                                                            ␣
↪                    standardization=False,
                                                                            ␣
↪                    model_type='KNeighborsRegressor',
                                                                            ␣
↪                    target_column_name = target,
                                                                            ␣
↪                    random_state=random_state,
                                                                            ␣
↪                    train_test_split_=True,
```

```
↪                    verbose=True)
```

```
I am not doing standardization…
  molecular descriptor name
0                   AATS0Z
1                 AATS0are
2                   AATS0d
3                  AATS0dv
4                   AATS0i
  molecular descriptor name  corr_value
0                   AATS0Z   -0.038502
1                 AATS0are   -0.120847
2                   AATS0d    0.041648
3                  AATS0dv   -0.115899
4                   AATS0i    0.191765
  molecular descriptor name  corr_value  absolute correlation value
0                   AATS0Z   -0.038502                    0.038502
1                 AATS0are   -0.120847                    0.120847
2                   AATS0d    0.041648                    0.041648
3                  AATS0dv   -0.115899                    0.115899
4                   AATS0i    0.191765                    0.191765
     molecular descriptor name  corr_value  absolute correlation value
226                     AMID_O   -0.520578                    0.520578
505                EState_VSA5   -0.578904                    0.578904
506                EState_VSA6    0.519794                    0.519794
791                   MDEO-12   -0.525441                    0.525441
1211                     MCF-7    1.000000                    1.000000
    molecular descriptor name  corr_value  absolute correlation value
226                    AMID_O   -0.520578                    0.520578
505               EState_VSA5   -0.578904                    0.578904
506               EState_VSA6    0.519794                    0.519794
791                  MDEO-12   -0.525441                    0.525441
The model used is: KNeighbors…
Return the coefficient of determination of the prediction:
0.17887598286330386
R^2 score: 0.5373267326789486
Correlation coefficient: 0.7330257380740111
Test data - unseen during training:
R^2 score: 0.17887598286330386
Correlation coefficient: 0.4229373273468586
[8.27886876 6.39366104 7.6963417  6.53369885 7.95214323 7.55661724
 7.94489513 8.26275858 6.18458136 7.8150608  7.57914848 8.14325294
 7.83929455 8.13033937 7.35856211 7.57972486 7.58116176 6.79653745]
113    8.022276
35     6.066513
101    6.920819
36     6.108463
```

```
100    7.376751
13     7.987163
0      7.987163
114    7.823909
104    8.397940
96     7.920819
40     8.091515
103    8.376751
48     7.886057
39     8.455932
14     8.086186
117    5.920819
21     8.113509
9      6.143150
Name: MCF-7, dtype: float64
Training Root Mean Square Error: 0.659077344888441
Testing Root Mean Square Error: 0.7814887183280818
```

[30]:

```
print(target_column_name+str('_transformed'))
print(hist1[target_column_name].hist())
```

```
MCF-7_transformed
AxesSubplot(0.125,0.11;0.775x0.77)
```

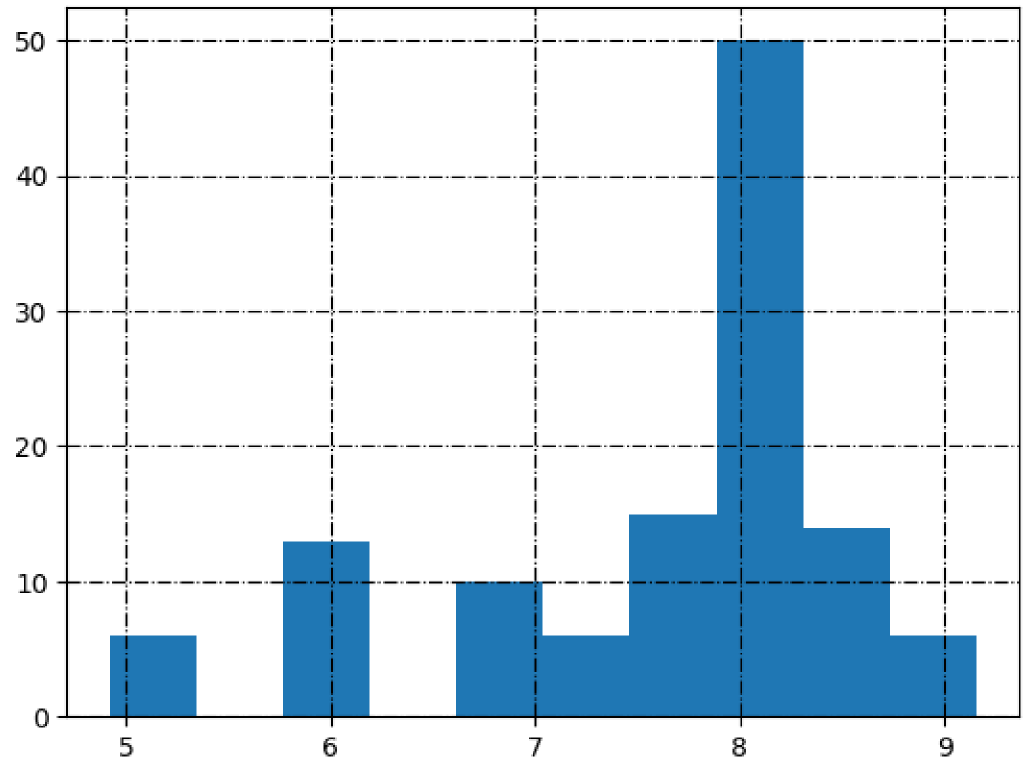

[31]:
```
with_standardization, train_r2_, test_r2_, hist1, hist2, target_column_name,␣
↪training_data_RMSE, test_data_RMSE  = pred_model.
↪prepare_data_and_create_model(molecular_descriptors_df=data,
                                                                                ␣
↪                    correlation_threshold=0.50,
                                                                                ␣
↪                    standardization=True,
                                                                                ␣
↪                    model_type='KNeighborsRegressor',
                                                                                ␣
↪                    target_column_name = target,
                                                                                ␣
↪                    random_state=random_state,
                                                                                ␣
↪                    train_test_split_=True,
                                                                                ␣
↪                    verbose=True)
```

```
I am doing standardization…
  molecular descriptor name
0                    AATS0Z
1                  AATS0are
2                    AATS0d
3                   AATS0dv
4                    AATS0i
  molecular descriptor name  corr_value
0                    AATS0Z   -0.038502
1                  AATS0are   -0.120847
2                    AATS0d    0.041648
3                   AATS0dv   -0.115899
4                    AATS0i    0.191765
  molecular descriptor name  corr_value  absolute correlation value
0                    AATS0Z   -0.038502                    0.038502
1                  AATS0are   -0.120847                    0.120847
2                    AATS0d    0.041648                    0.041648
3                   AATS0dv   -0.115899                    0.115899
4                    AATS0i    0.191765                    0.191765
     molecular descriptor name  corr_value  absolute correlation value
226                     AMID_O   -0.520578                    0.520578
505                EState_VSA5   -0.578904                    0.578904
506                EState_VSA6    0.519794                    0.519794
791                    MDEO-12   -0.525441                    0.525441
1211                     MCF-7    1.000000                    1.000000
    molecular descriptor name  corr_value  absolute correlation value
226                    AMID_O   -0.520578                    0.520578
505               EState_VSA5   -0.578904                    0.578904
506               EState_VSA6    0.519794                    0.519794
```

```
791                    MDEO-12   -0.525441                    0.525441
The model used is: KNeighbors…
Return the coefficient of determination of the prediction:
0.17887598286330386
R^2 score: 0.5373267326789486
Correlation coefficient: 0.7330257380740111
Test data - unseen during training:
R^2 score: 0.17887598286330386
Correlation coefficient: 0.4229373273468586
[8.27886876 6.39366104 7.6963417  6.53369885 7.95214323 7.55661724
 7.94489513 8.26275858 6.18458136 7.8150608  7.57914848 8.14325294
 7.83929455 8.13033937 7.35856211 7.57972486 7.58116176 6.79653745]
113    8.022276
35     6.066513
101    6.920819
36     6.108463
100    7.376751
13     7.987163
0      7.987163
114    7.823909
104    8.397940
96     7.920819
40     8.091515
103    8.376751
48     7.886057
39     8.455932
14     8.086186
117    5.920819
21     8.113509
9      6.143150
Name: MCF-7, dtype: float64
Training Root Mean Square Error: 0.659077344888441
Testing Root Mean Square Error: 0.7814887183280818
```

4.1 Search inside correlation space

[32]:
```
step = 0.01
initial_step = corr_low
last_step = corr_high
first_list = [x / 100.0 for x in range(int(initial_step*100),
 ↪int(last_step*100), int(step*100))]
second_list = []
third_list = []
fourth_l = []
fifth_l = []
f_list = []
for i in first_list:
```

```
    without_standardization, train_r2, test_r2, _, h_, target_column_name,␣
↪training_data_RMSE, test_data_RMSE = pred_model.
↪prepare_data_and_create_model(molecular_descriptors_df = data,
                                                                                ␣
↪                    correlation_threshold = i,
                                                                                ␣
↪                    standardization = False,
                                                                                ␣
↪                    model_type = 'KNeighborsRegressor',
                                                                                ␣
↪                    target_column_name = target,
                                                                                ␣
↪                    random_state=random_state,
                                                                                ␣
↪                    train_test_split_ = True,
                                                                                ␣
↪                    verbose = False)
    second_list.append(train_r2)
    third_list.append(test_r2)
    fourth_l.append(training_data_RMSE)
    fifth_l.append(test_data_RMSE)
    f_list.append(len(h_))
```

```
  File "C:\Users\aleks\anaconda3\envs\cheminf_gpu\lib\site-
packages\joblib\externals\loky\backend\context.py", line 217, in
_count_physical_cores
    raise ValueError(
```

[33]:
```
df_without_standardization = pd.DataFrame(data=first_list,␣
↪columns=["Correlation threshold"])
df_without_standardization['Training data R^2 score'] = second_list
df_without_standardization['Test data R^2 score'] = third_list
df_without_standardization['Training RMSE'] = fourth_l
df_without_standardization['Test RMSE'] = fifth_l
df_without_standardization['Number of features'] = f_list
```

[34]:
```
df_k_nearest = df_without_standardization.copy()
df_without_standardization
```

[34]:
```
    Correlation threshold  Training data R^2 score  Test data R^2 score  \
0                    0.33                 0.608638             0.016137
1                    0.34                 0.609763             0.016137
2                    0.35                 0.617607             0.005978
3                    0.36                 0.636317             0.004067
4                    0.37                 0.629161             0.003452
5                    0.38                 0.720982             0.334651
6                    0.39                 0.715501             0.334651
```

7	0.40	0.710436	0.334651
8	0.41	0.698984	0.324017
9	0.42	0.617632	0.109773
10	0.43	0.568566	0.070934
11	0.44	0.568547	0.070934
12	0.45	0.565701	0.070934
13	0.46	0.569946	0.112757
14	0.47	0.569946	0.112757
15	0.48	0.569946	0.112892
16	0.49	0.554510	0.181851
17	0.50	0.537327	0.178876
18	0.51	0.537327	0.178876

	Training RMSE	Test RMSE	Number of features
0	0.606162	0.855432	84
1	0.605289	0.855432	74
2	0.599175	0.859838	61
3	0.584333	0.860664	50
4	0.590055	0.860929	43
5	0.511818	0.703466	37
6	0.516821	0.703466	29
7	0.521400	0.703466	26
8	0.531611	0.709066	25
9	0.599156	0.813708	19
10	0.636438	0.831269	14
11	0.636453	0.831269	13
12	0.638548	0.831269	11
13	0.635420	0.812343	10
14	0.635420	0.812343	9
15	0.635420	0.812281	8
16	0.646723	0.780072	6
17	0.659077	0.781489	4
18	0.659077	0.781489	4

4.2 Plots

[35]:
```
plt.plot(df_without_standardization['Correlation threshold'], df_without_standardization['Training data R^2 score'], label = "Training data R^2 score", marker='v')
plt.plot(df_without_standardization['Correlation threshold'], df_without_standardization['Test data R^2 score'], label = "Test data R^2 score", marker='D')
plt.legend()
plt.xlabel('Correlation threshold')
plt.ylabel('R^2 score')
plt.ylim([0, 1])
plt.show()
```

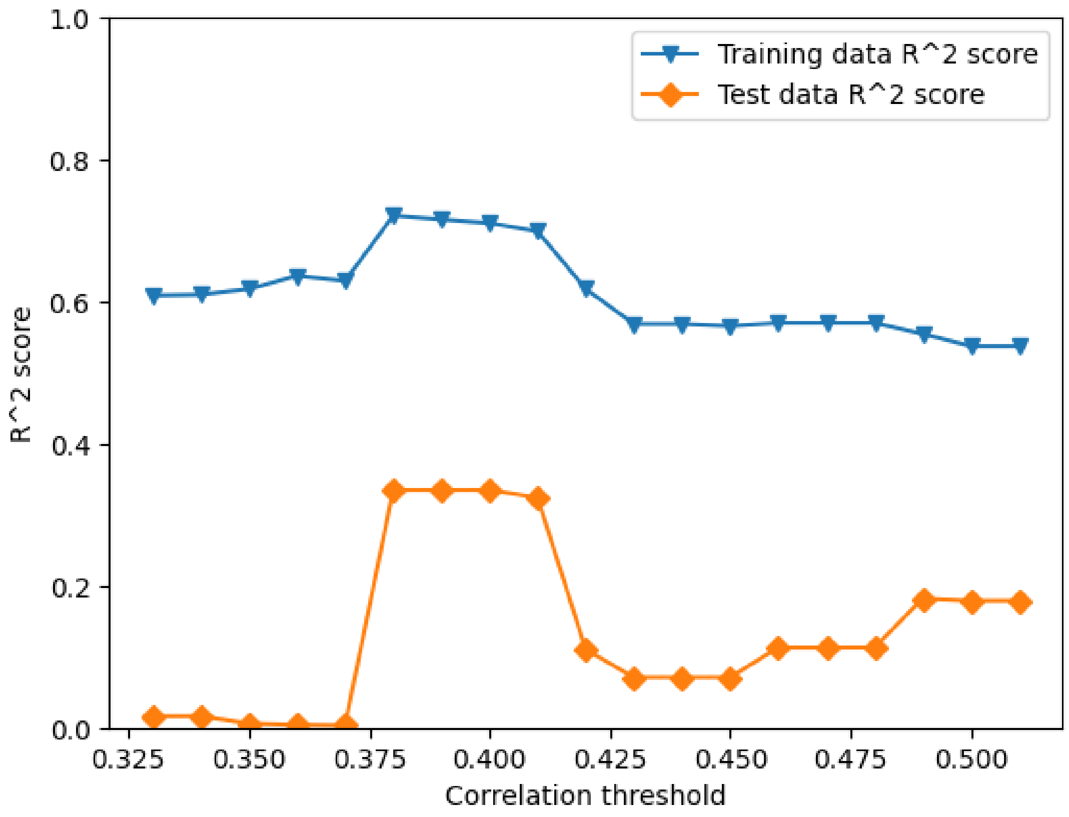

[36]:
```
plt.plot(df_without_standardization['Correlation threshold'],␣
 ↪df_without_standardization['Number of features'], label = "Number of␣
 ↪features", marker='.')
plt.legend()
plt.xlabel('Correlation threshold')
plt.ylabel('Number of features')
plt.show()
```

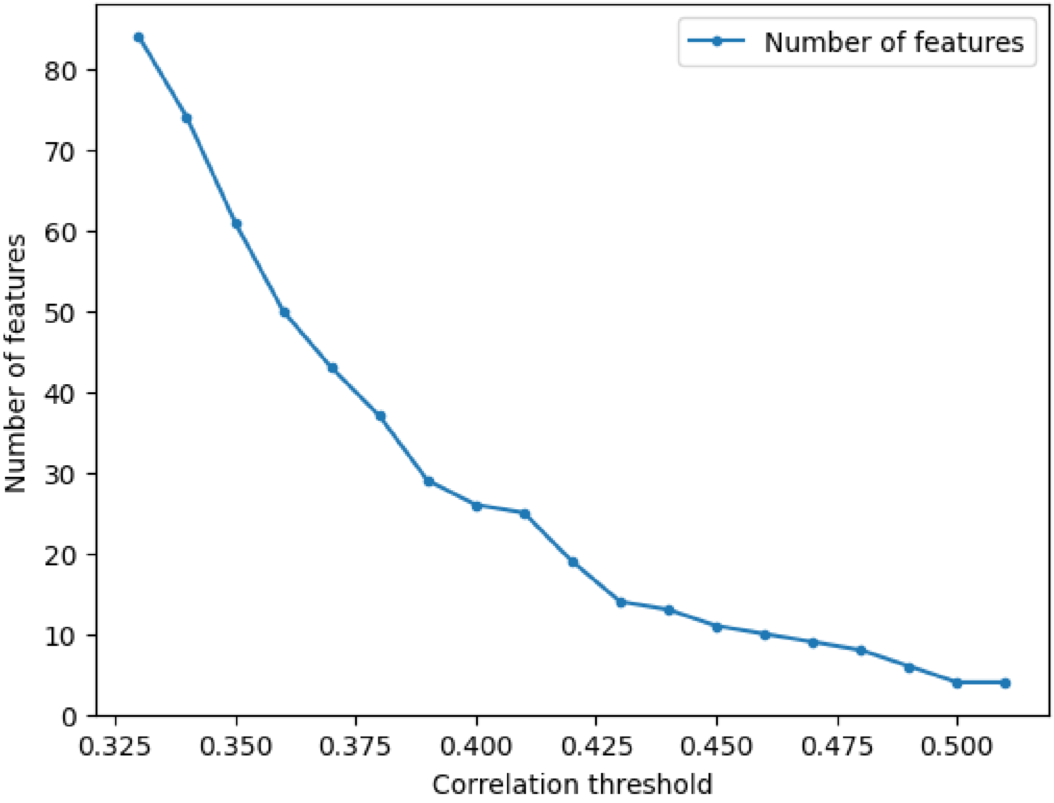

[ ]:

5 Support Vector Machines (SVM)

[37]:
```
without_standardization, train_r2_, test_r2_, hist1, hist2, target_column_name,␣
↪training_data_RMSE, test_data_RMSE  = pred_model.
↪prepare_data_and_create_model(molecular_descriptors_df=data,
                                                                              ␣
↪                    correlation_threshold=0.50,
                                                                              ␣
↪                    standardization=False,
                                                                              ␣
↪                    model_type='SVR',
                                                                              ␣
↪                    kernel_ = 'linear',
                                                                              ␣
↪                    gamma_ = 'auto',
                                                                              ␣
↪                    target_column_name = target,
```

```
                     random_state=random_state,
                     train_test_split_=True,
                     verbose=True)
```

```
I am not doing standardization…
  molecular descriptor name
0                    AATS0Z
1                  AATS0are
2                    AATS0d
3                   AATS0dv
4                    AATS0i
  molecular descriptor name  corr_value
0                    AATS0Z   -0.038502
1                  AATS0are   -0.120847
2                    AATS0d    0.041648
3                   AATS0dv   -0.115899
4                    AATS0i    0.191765
  molecular descriptor name  corr_value  absolute correlation value
0                    AATS0Z   -0.038502                    0.038502
1                  AATS0are   -0.120847                    0.120847
2                    AATS0d    0.041648                    0.041648
3                   AATS0dv   -0.115899                    0.115899
4                    AATS0i    0.191765                    0.191765
     molecular descriptor name  corr_value  absolute correlation value
226                     AMID_O   -0.520578                    0.520578
505                EState_VSA5   -0.578904                    0.578904
506                EState_VSA6    0.519794                    0.519794
791                    MDEO-12   -0.525441                    0.525441
1211                     MCF-7    1.000000                    1.000000
    molecular descriptor name  corr_value  absolute correlation value
226                    AMID_O   -0.520578                    0.520578
505               EState_VSA5   -0.578904                    0.578904
506               EState_VSA6    0.519794                    0.519794
791                   MDEO-12   -0.525441                    0.525441
The model used is: SVR…
Return the coefficient of determination of the prediction:
0.4743557839935354
R^2 score: 0.5020352037550295
Correlation coefficient: 0.7085444260983425
Test data - unseen during training:
R^2 score: 0.4743557839935354
Correlation coefficient: 0.6887349156195984
[7.96654481 6.82669237 7.83448445 7.03699901 7.89478809 7.40737585
 7.67935617 8.33257718 7.17327682 8.0339569  8.08511125 8.38190303
```

```
 8.04334248 7.80748886 7.5192305  6.8313205  7.68209688 6.85506111]
113    8.022276
35     6.066513
101    6.920819
36     6.108463
100    7.376751
13     7.987163
0      7.987163
114    7.823909
104    8.397940
96     7.920819
40     8.091515
103    8.376751
48     7.886057
39     8.455932
14     8.086186
117    5.920819
21     8.113509
9      6.143150
Name: MCF-7, dtype: float64
Training Root Mean Square Error: 0.6837518281038438
Testing Root Mean Square Error: 0.625265232017053
```

[38]:

```
print(target_column_name+str('_transformed'))
print(hist1[target_column_name].hist())
```

```
MCF-7_transformed
AxesSubplot(0.125,0.11;0.775x0.77)
```

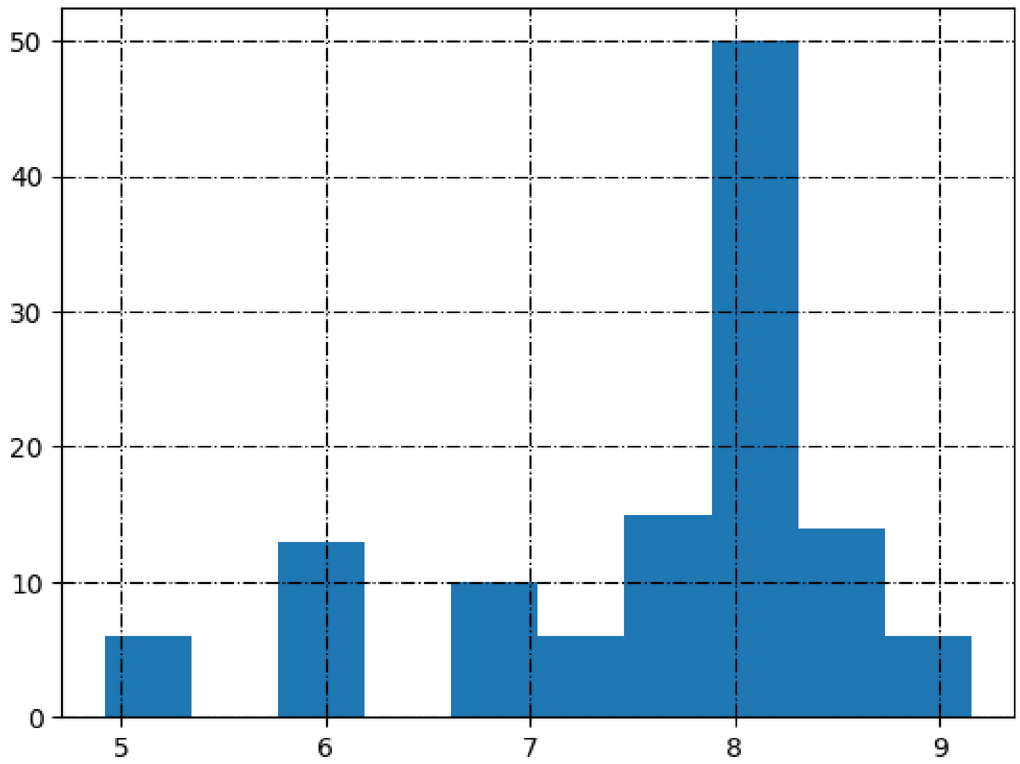

[39]:
```
with_standardization, train_r2_, test_r2_, hist1, hist2, target_column_name,␣
↪training_data_RMSE, test_data_RMSE  = pred_model.
↪prepare_data_and_create_model(molecular_descriptors_df=data,
                                                                            ␣
↪                          correlation_threshold=0.50,
                                                                            ␣
↪                          standardization=True,
                                                                            ␣
↪                          model_type='SVR',
                                                                            ␣
↪                          kernel_ = 'linear',
                                                                            ␣
↪                          gamma_ = 'auto',
                                                                            ␣
↪                          target_column_name = target,
                                                                            ␣
↪                          random_state=random_state,
                                                                            ␣
↪                          train_test_split_=True,
                                                                            ␣
↪                          verbose=True)
```

```
I am doing standardization…
  molecular descriptor name
0                    AATS0Z
1                  AATS0are
2                    AATS0d
3                   AATS0dv
4                    AATS0i
  molecular descriptor name  corr_value
0                    AATS0Z   -0.038502
1                  AATS0are   -0.120847
2                    AATS0d    0.041648
3                   AATS0dv   -0.115899
4                    AATS0i    0.191765
  molecular descriptor name  corr_value  absolute correlation value
0                    AATS0Z   -0.038502                    0.038502
1                  AATS0are   -0.120847                    0.120847
2                    AATS0d    0.041648                    0.041648
3                   AATS0dv   -0.115899                    0.115899
4                    AATS0i    0.191765                    0.191765
     molecular descriptor name  corr_value  absolute correlation value
226                     AMID_O   -0.520578                    0.520578
505                EState_VSA5   -0.578904                    0.578904
506                EState_VSA6    0.519794                    0.519794
791                    MDEO-12   -0.525441                    0.525441
1211                     MCF-7    1.000000                    1.000000
    molecular descriptor name  corr_value  absolute correlation value
226                    AMID_O   -0.520578                    0.520578
505               EState_VSA5   -0.578904                    0.578904
506               EState_VSA6    0.519794                    0.519794
791                   MDEO-12   -0.525441                    0.525441
The model used is: SVR…
Return the coefficient of determination of the prediction:
0.4743557839935354
R^2 score: 0.5020352037550295
Correlation coefficient: 0.7085444260983425
Test data - unseen during training:
R^2 score: 0.4743557839935354
Correlation coefficient: 0.6887349156195984
[7.96654481 6.82669237 7.83448445 7.03699901 7.89478809 7.40737585
 7.67935617 8.33257718 7.17327682 8.0339569  8.08511125 8.38190303
 8.04334248 7.80748886 7.5192305  6.8313205  7.68209688 6.85506111]
113    8.022276
35     6.066513
101    6.920819
36     6.108463
100    7.376751
13     7.987163
0      7.987163
```

```
114    7.823909
104    8.397940
96     7.920819
40     8.091515
103    8.376751
48     7.886057
39     8.455932
14     8.086186
117    5.920819
21     8.113509
9      6.143150
Name: MCF-7, dtype: float64
Training Root Mean Square Error: 0.6837518281038438
Testing Root Mean Square Error: 0.625265232017053
```

5.1 Search inside correlation space

[40]:
```python
step = 0.01
initial_step = corr_low
last_step = corr_high
first_list = [x / 100.0 for x in range(int(initial_step*100),␣
 ↪int(last_step*100), int(step*100))]
second_list = []
third_list = []
fourth_l = []
fifth_l = []
f_list = []
for i in first_list:
    without_standardization, train_r2, test_r2, _, h_, target_column_name,␣
 ↪training_data_RMSE, test_data_RMSE = pred_model.
 ↪prepare_data_and_create_model(molecular_descriptors_df = data,
                                                                              ␣
 ↪                    correlation_threshold = i,
                                                                              ␣
 ↪                    standardization = False,
                                                                              ␣
 ↪                    model_type = 'SVR',
                                                                              ␣
 ↪                    kernel_ = 'linear',
                                                                              ␣
 ↪                    gamma_ = 'auto',
                                                                              ␣
 ↪                    target_column_name = target,
                                                                              ␣
 ↪                    random_state=random_state,
                                                                              ␣
 ↪                    train_test_split_ = True,
```

```
                          verbose = False)
    second_list.append(train_r2)
    third_list.append(test_r2)
    fourth_l.append(training_data_RMSE)
    fifth_l.append(test_data_RMSE)
    f_list.append(len(h_))
```

[41]:
```
df_without_standardization = pd.DataFrame(data=first_list,
 ↪columns=["Correlation threshold"])
df_without_standardization['Training data R^2 score'] = second_list
df_without_standardization['Test data R^2 score'] = third_list
df_without_standardization['Training RMSE'] = fourth_l
df_without_standardization['Test RMSE'] = fifth_l
df_without_standardization['Number of features'] = f_list
```

[42]:
```
df_svm = df_without_standardization.copy()
df_without_standardization
```

[42]:
```
    Correlation threshold  Training data R^2 score  Test data R^2 score  \
0                    0.33                 0.784206             0.012930
1                    0.34                 0.784504             0.010467
2                    0.35                 0.761037            -0.176505
3                    0.36                 0.670546            -0.039968
4                    0.37                 0.668193             0.031631
5                    0.38                 0.647471             0.228751
6                    0.39                 0.629387             0.190286
7                    0.40                 0.632083             0.125215
8                    0.41                 0.614130             0.264660
9                    0.42                 0.594259             0.327494
10                   0.43                 0.580193             0.251954
11                   0.44                 0.579996             0.250150
12                   0.45                 0.580285             0.265700
13                   0.46                 0.540404             0.379934
14                   0.47                 0.540529             0.380586
15                   0.48                 0.528432             0.529576
16                   0.49                 0.525676             0.522684
17                   0.50                 0.502035             0.474356
18                   0.51                 0.502035             0.474356

    Training RMSE  Test RMSE  Number of features
0        0.450111   0.856825                  84
1        0.449799   0.857894                  74
2        0.473658   0.935439                  61
3        0.556156   0.879485                  50
4        0.558139   0.848670                  43
5        0.575303   0.757383                  37
```

6	0.589874	0.776040	29
7	0.587725	0.806620	26
8	0.601893	0.739541	25
9	0.617197	0.707240	19
10	0.627804	0.745903	14
11	0.627951	0.746802	13
12	0.627735	0.739018	11
13	0.656882	0.679106	10
14	0.656793	0.678748	9
15	0.665383	0.591511	8
16	0.667324	0.595829	6
17	0.683752	0.625265	4
18	0.683752	0.625265	4

5.2 Plots

[43]:
```
plt.plot(df_without_standardization['Correlation threshold'],␣
 ↪df_without_standardization['Training data R^2 score'], label = "Training␣
 ↪data R^2 score", marker='v')
plt.plot(df_without_standardization['Correlation threshold'],␣
 ↪df_without_standardization['Test data R^2 score'], label = "Test data R^2␣
 ↪score", marker='D')
plt.legend()
plt.xlabel('Correlation threshold')
plt.ylabel('R^2 score')
plt.ylim([0, 1])
plt.show()
```

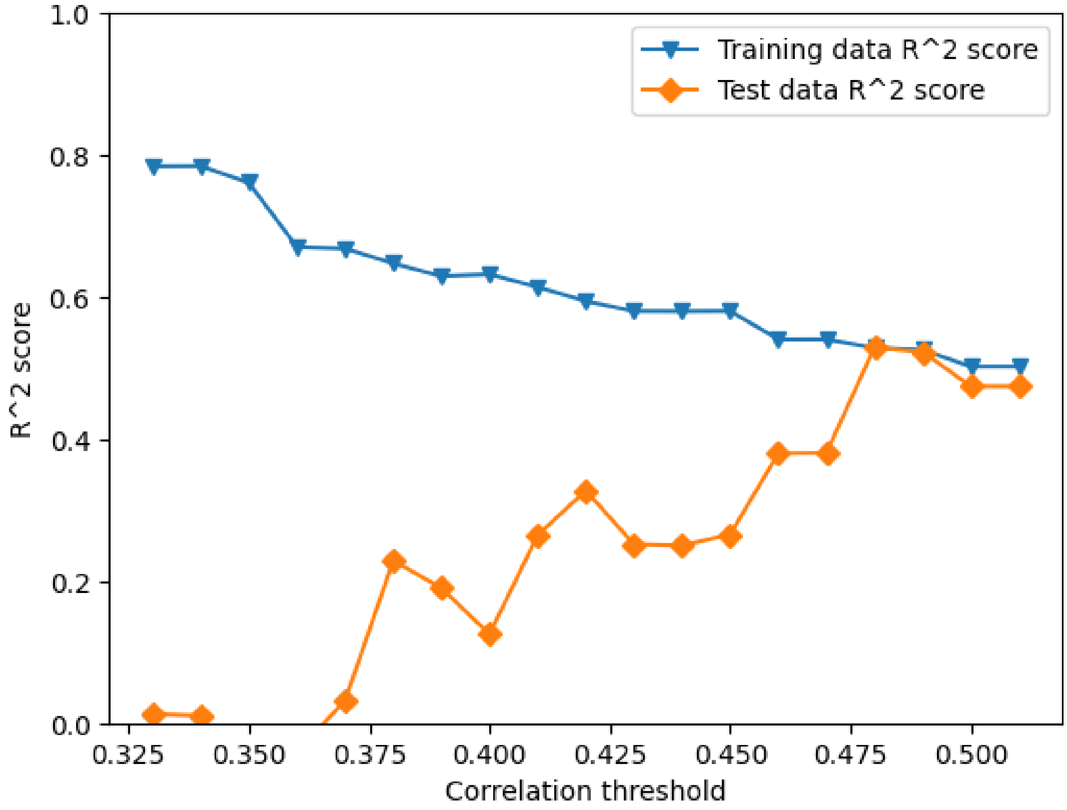

[44]:
```
plt.plot(df_without_standardization['Correlation threshold'],␣
↪df_without_standardization['Number of features'], label = "Number of␣
↪features", marker='.')
plt.legend()
plt.xlabel('Correlation threshold')
plt.ylabel('Number of features')
plt.show()
```

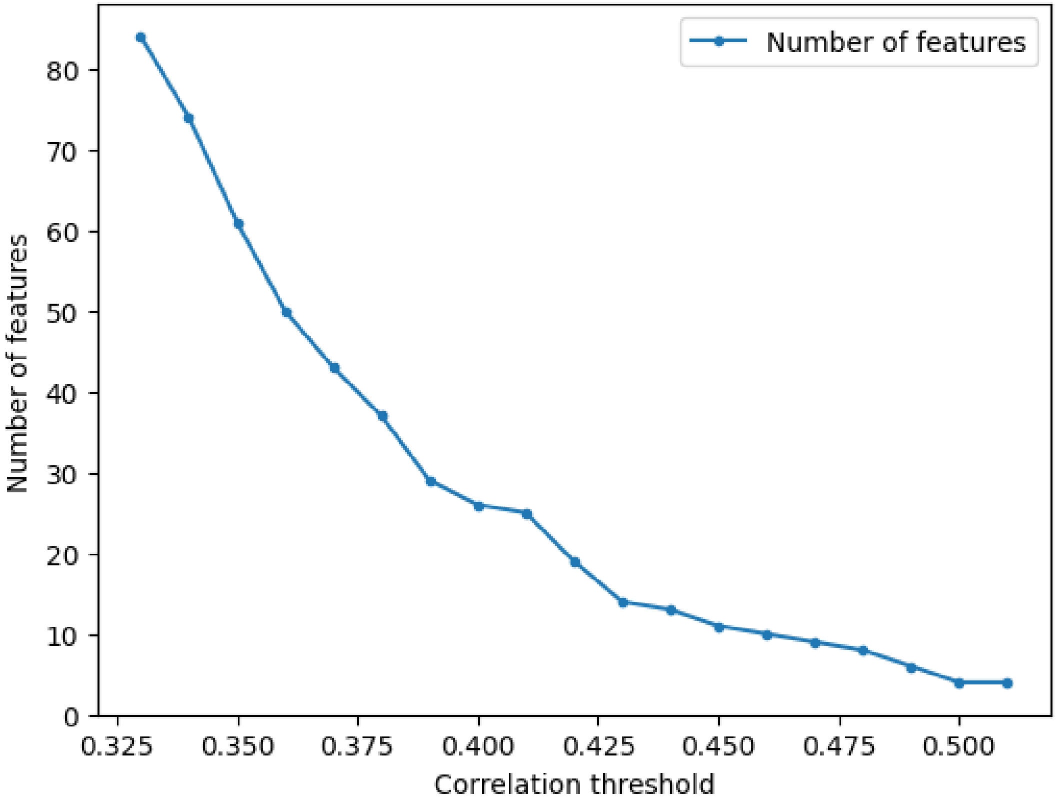

5.3 Store quality measurements

[45]:
```
with pd.ExcelWriter('../Data/Quality_'+str(target)+'_'+str(random_state)+'_.
↪xlsx') as writer:
    df_linear.to_excel(writer, sheet_name='MLR')
    df_decision_tree.to_excel(writer, sheet_name='DT')
    df_random_forest.to_excel(writer, sheet_name='RF')
    df_k_nearest.to_excel(writer, sheet_name='KNN')
    df_svm.to_excel(writer, sheet_name='SVM')
```

Notebook

December 19, 2023

1 File 28

[1]:
```
import sys
from pathlib import Path
prediction_mode_path = Path("../module")
sys.path.append(prediction_mode_path.as_posix())
import models_creation as pred_model

import pandas as pd
import numpy as np

from sklearn.metrics import r2_score

import matplotlib.pyplot as plt
from sklearn.tree import plot_tree

from matplotlib import pyplot as plt

import joblib

import warnings
warnings.filterwarnings('ignore')
```

[2]:
```
load_prepared_data = pd.read_excel('../Data/Kolchicyna_machine_learning.xlsx')
load_prepared_data = load_prepared_data.drop(columns=['Unnamed: 0'])

target = 'MCF-7'

corr_low = 0.33
corr_high = 0.525

random_state = 28
```

[3]:
```
load_prepared_data.head()
```

[3]:
```
     AATS0Z  AATS0are    AATS0d   AATS0dv      AATS0i     AATS0m    AATS0p  \
0  25.090909  6.046518  3.109091  6.662626  160.775045  99.075564  1.555512
1  24.448276  6.008422  3.051724  6.386973  161.021674  96.473315  1.538471
```

```
2  23.868852  5.974074  3.000000  6.138434  161.244045  94.127025  1.523105
3  23.868852  5.974074  3.032787  6.171220  161.244045  94.127025  1.523105
4  23.343750  5.942945  2.953125  5.913194  161.445569  92.000700  1.509180

    AATS0pe    AATS0s    AATS0se  …  A549_float  MCF-7_float  LoVo_float  \
0  6.126680  3.435416  7.629859  …        10.8         10.3         6.5
1  6.088791  3.330998  7.596891  …        11.6         12.0         8.5
2  6.054630  3.236850  7.567166  …        10.9         12.2         8.8
3  6.054630  3.257798  7.567166  …        10.5         11.3         8.5
4  6.023670  3.151529  7.540228  …        89.5         92.7        52.8

   LoVo/DX_float  BALB/3T3_float  A549_float_transformed  \
0           54.9            10.2                7.966576
1           31.1            14.3                7.935542
2           17.9            11.7                7.962574
3           10.2            11.0                7.978811
4           77.8            99.4                7.048177

   MCF-7_float_transformed  LoVo_float_transformed  LoVo/DX_float_transformed  \
0                 7.987163                8.187087                   7.260428
1                 7.920819                8.070581                   7.507240
2                 7.913640                8.055517                   7.747147
3                 7.946922                8.070581                   7.991400
4                 7.032920                7.277366                   7.109020

   BALB/3T3_float_transformed
0                    7.991400
1                    7.844664
2                    7.931814
3                    7.958607
4                    7.002614

[5 rows x 1221 columns]
```

[4]:
```
data = load_prepared_data[list(load_prepared_data.columns)[0:-10]]
data[target] = load_prepared_data[load_prepared_data.columns[-4]]
data.head()
```

[4]:
```
      AATS0Z  AATS0are    AATS0d   AATS0dv      AATS0i     AATS0m    AATS0p  \
0  25.090909  6.046518  3.109091  6.662626  160.775045  99.075564  1.555512
1  24.448276  6.008422  3.051724  6.386973  161.021674  96.473315  1.538471
2  23.868852  5.974074  3.000000  6.138434  161.244045  94.127025  1.523105
3  23.868852  5.974074  3.032787  6.171220  161.244045  94.127025  1.523105
4  23.343750  5.942945  2.953125  5.913194  161.445569  92.000700  1.509180

    AATS0pe    AATS0s    AATS0se  …    piPC10     piPC2     piPC3     piPC4  \
0  6.126680  3.435416  7.629859  …  7.999655  4.266195  4.915408  5.516700
```

```
1  6.088791  3.330998  7.596891  …  8.018726  4.280132  4.922714  5.520711
2  6.054630  3.236850  7.567166  …  8.028799  4.293878  4.929967  5.524706
3  6.054630  3.257798  7.567166  …  8.037440  4.307438  4.929967  5.524706
4  6.023670  3.151529  7.540228  …  8.034892  4.307438  4.937168  5.528685

      piPC5     piPC6     piPC7     piPC8     piPC9     MCF-7
0  6.098566  6.595759  6.979116  7.337313  7.681445  7.987163
1  6.103048  6.601209  6.985613  7.349442  7.695531  7.920819
2  6.105281  6.603923  6.989306  7.353932  7.704023  7.913640
3  6.107510  6.606629  6.992068  7.361425  7.709420  7.946922
4  6.107510  6.605277  6.991148  7.356489  7.707175  7.032920

[5 rows x 1212 columns]
```

2 Multiple Linear regression (MLR)

[5]:
```
without_standardization, train_r2_, test_r2_, hist1, hist2, target_column_name,␣
↪training_data_RMSE, test_data_RMSE  = pred_model.
↪prepare_data_and_create_model(molecular_descriptors_df=data,
                                                                                ␣
↪                    correlation_threshold=0.5,
                                                                                ␣
↪                    standardization=False,
                                                                                ␣
↪                    model_type='linear_model',
                                                                                ␣
↪                    target_column_name = str(target),
                                                                                ␣
↪                    random_state=random_state,
                                                                                ␣
↪                    train_test_split_=True,
                                                                                ␣
↪                    verbose=True)
```

```
I am not doing standardization…
  molecular descriptor name
0                   AATS0Z
1                 AATS0are
2                   AATS0d
3                  AATS0dv
4                   AATS0i
  molecular descriptor name  corr_value
0                   AATS0Z   -0.038502
1                 AATS0are   -0.120847
2                   AATS0d    0.041648
3                  AATS0dv   -0.115899
```

```
4                   AATS0i    0.191765
  molecular descriptor name  corr_value  absolute correlation value
0                   AATS0Z   -0.038502                    0.038502
1                 AATS0are   -0.120847                    0.120847
2                   AATS0d    0.041648                    0.041648
3                  AATS0dv   -0.115899                    0.115899
4                   AATS0i    0.191765                    0.191765
     molecular descriptor name  corr_value  absolute correlation value
226                     AMID_O   -0.520578                    0.520578
505                EState_VSA5   -0.578904                    0.578904
506                EState_VSA6    0.519794                    0.519794
791                    MDEO-12   -0.525441                    0.525441
1211                     MCF-7    1.000000                    1.000000
    molecular descriptor name  corr_value  absolute correlation value
226                    AMID_O   -0.520578                    0.520578
505               EState_VSA5   -0.578904                    0.578904
506               EState_VSA6    0.519794                    0.519794
791                   MDEO-12   -0.525441                    0.525441
The model used is: LinearReg…
Return the coefficient of determination of the prediction:
0.5009760847434094
R^2 score: 0.5283510681142922
Correlation coefficient: 0.7268776156371114
Test data - unseen during training:
R^2 score: 0.5009760847434094
Correlation coefficient: 0.707796640811052
[8.00252575 6.60183757 7.65912481 6.88611169 7.8702033  7.23304372
 7.57064419 8.42411387 7.0697769  7.89024083 7.95174669 8.38076499
 7.96632403 7.61854434 7.3479345  6.45607766 7.72287088 6.79263447]
113    8.022276
35     6.066513
101    6.920819
36     6.108463
100    7.376751
13     7.987163
0      7.987163
114    7.823909
104    8.397940
96     7.920819
40     8.091515
103    8.376751
48     7.886057
39     8.455932
14     8.086186
117    5.920819
21     8.113509
9      6.143150
Name: MCF-7, dtype: float64
```

```
Training Root Mean Square Error: 0.6654395471577522
Testing Root Mean Square Error: 0.6092268213393355
```

[6]:
```python
print(target_column_name+str('_transformed'))
print(hist1[target_column_name].hist())
```

```
MCF-7_transformed
AxesSubplot(0.125,0.11;0.775x0.77)
```

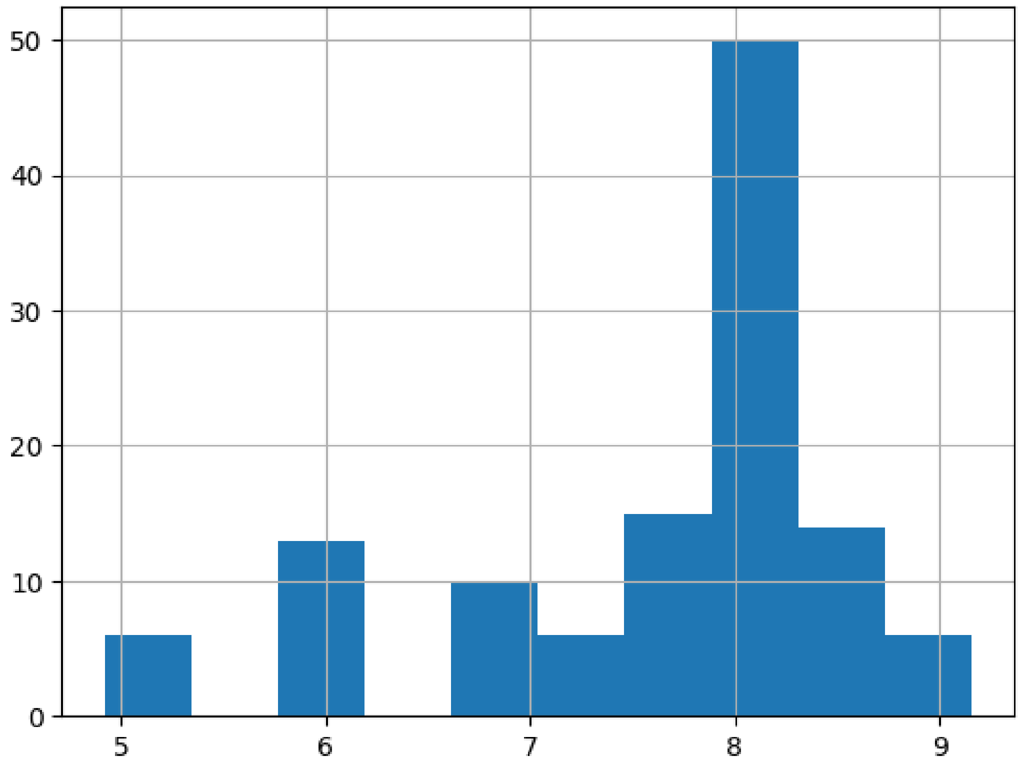

[7]:
```python
with_standardization, train_r2_, test_r2_, hist1, hist2, target_column_name,␣
↪training_data_RMSE, test_data_RMSE  = pred_model.
↪prepare_data_and_create_model(molecular_descriptors_df=data,
                                                                               ␣
↪                    correlation_threshold=0.50,
                                                                               ␣
↪                    standardization=True,
                                                                               ␣
↪                    model_type='linear_model',
                                                                               ␣
↪                    target_column_name = target,
                                                                               ␣
↪                    random_state=random_state,
```

```
↪                    train_test_split_=True,
↪                    verbose=True)
```

```
I am doing standardization…
  molecular descriptor name
0                    AATS0Z
1                  AATS0are
2                    AATS0d
3                   AATS0dv
4                    AATS0i
  molecular descriptor name  corr_value
0                    AATS0Z   -0.038502
1                  AATS0are   -0.120847
2                    AATS0d    0.041648
3                   AATS0dv   -0.115899
4                    AATS0i    0.191765
  molecular descriptor name  corr_value  absolute correlation value
0                    AATS0Z   -0.038502                    0.038502
1                  AATS0are   -0.120847                    0.120847
2                    AATS0d    0.041648                    0.041648
3                   AATS0dv   -0.115899                    0.115899
4                    AATS0i    0.191765                    0.191765
     molecular descriptor name  corr_value  absolute correlation value
226                     AMID_O   -0.520578                    0.520578
505                EState_VSA5   -0.578904                    0.578904
506                EState_VSA6    0.519794                    0.519794
791                    MDEO-12   -0.525441                    0.525441
1211                     MCF-7    1.000000                    1.000000
    molecular descriptor name  corr_value  absolute correlation value
226                    AMID_O   -0.520578                    0.520578
505               EState_VSA5   -0.578904                    0.578904
506               EState_VSA6    0.519794                    0.519794
791                   MDEO-12   -0.525441                    0.525441
The model used is: LinearReg…
Return the coefficient of determination of the prediction:
0.5009760847434094
R^2 score: 0.5283510681142922
Correlation coefficient: 0.7268776156371114
Test data - unseen during training:
R^2 score: 0.5009760847434094
Correlation coefficient: 0.707796640811052
[8.00252575 6.60183757 7.65912481 6.88611169 7.8702033  7.23304372
 7.57064419 8.42411387 7.0697769  7.89024083 7.95174669 8.38076499
 7.96632403 7.61854434 7.3479345  6.45607766 7.72287088 6.79263447]
113    8.022276
35     6.066513
```

```
101    6.920819
36     6.108463
100    7.376751
13     7.987163
0      7.987163
114    7.823909
104    8.397940
96     7.920819
40     8.091515
103    8.376751
48     7.886057
39     8.455932
14     8.086186
117    5.920819
21     8.113509
9      6.143150
Name: MCF-7, dtype: float64
Training Root Mean Square Error: 0.6654395471577522
Testing Root Mean Square Error: 0.6092268213393355
```

2.1 Search inside correlation space

[8]:
```
step = 0.01
initial_step = corr_low
last_step = corr_high
first_list = [x / 100.0 for x in range(int(initial_step*100),␣
 ↪int(last_step*100), int(step*100))]
second_list = []
third_list = []
fourth_l = []
fifth_l = []
f_list = []
for i in first_list:
    without_standardization, train_r2, test_r2, _, h_, target_column_name,␣
 ↪training_data_RMSE, test_data_RMSE = pred_model.
 ↪prepare_data_and_create_model(molecular_descriptors_df = data,
                                                                            ␣
 ↪                    correlation_threshold = i,
                                                                            ␣
 ↪                    standardization = False,
                                                                            ␣
 ↪                    model_type = 'linear_model',
                                                                            ␣
 ↪                    target_column_name = target,
                                                                            ␣
 ↪                    random_state=random_state,
```

```
                                                                          ␣
↪                   train_test_split_ = True,
                                                                          ␣
↪                   verbose = False)
    second_list.append(train_r2)
    third_list.append(test_r2)
    fourth_l.append(training_data_RMSE)
    fifth_l.append(test_data_RMSE)
    f_list.append(len(h_))
```

[9]:

```
df_without_standardization = pd.DataFrame(data=first_list,␣
 ↪columns=["Correlation threshold"])
df_without_standardization['Training data R^2 score'] = second_list
df_without_standardization['Test data R^2 score'] = third_list
df_without_standardization['Training RMSE'] = fourth_l
df_without_standardization['Test RMSE'] = fifth_l
df_without_standardization['Number of features'] = f_list
```

[10]:

```
df_linear = df_without_standardization.copy()
df_without_standardization
```

[10]:

```
    Correlation threshold  Training data R^2 score  Test data R^2 score  \
0                    0.33                 0.983044            -0.320708
1                    0.34                 0.963409            -0.189420
2                    0.35                 0.919079            -0.503844
3                    0.36                 0.893639            -0.336284
4                    0.37                 0.843982             0.194976
5                    0.38                 0.817407            -0.008212
6                    0.39                 0.758880            -0.872243
7                    0.40                 0.739958            -1.044879
8                    0.41                 0.732273            -0.597421
9                    0.42                 0.695477            -0.513998
10                   0.43                 0.629188            -0.828754
11                   0.44                 0.626877            -0.804570
12                   0.45                 0.621663            -0.940585
13                   0.46                 0.593073            -0.827574
14                   0.47                 0.592212            -0.859618
15                   0.48                 0.548178             0.082669
16                   0.49                 0.540995             0.051643
17                   0.50                 0.528351            -0.166067
18                   0.51                 0.528351            -0.166067

    Training RMSE  Test RMSE  Number of features
0        0.126172   2.774099                  84
1        0.185349   1.549648                  74
2        0.275632   0.945715                  61
3        0.316002   0.902716                  50
```

4	0.382724	1.044381	43
5	0.414038	0.838263	37
6	0.475791	0.909707	29
7	0.494107	1.012390	26
8	0.501355	0.862432	25
9	0.534698	0.805613	19
10	0.590032	0.826589	14
11	0.591868	0.826973	13
12	0.595990	0.817058	11
13	0.618098	0.783549	10
14	0.618752	0.793394	9
15	0.651302	0.576590	8
16	0.656459	0.574487	6
17	0.665440	0.609227	4
18	0.665440	0.609227	4

[ ]:

```

```

2.2 Plots

[11]:

```
plt.plot(df_without_standardization['Correlation threshold'],␣
↪df_without_standardization['Training data R^2 score'], label = "Training␣
↪data R^2 score", marker='v')
plt.plot(df_without_standardization['Correlation threshold'],␣
↪df_without_standardization['Test data R^2 score'], label = "Test data R^2␣
↪score", marker='D')
plt.legend()
plt.xlabel('Correlation threshold')
plt.ylabel('R^2 score')
plt.ylim([0, 1])
plt.show()
```

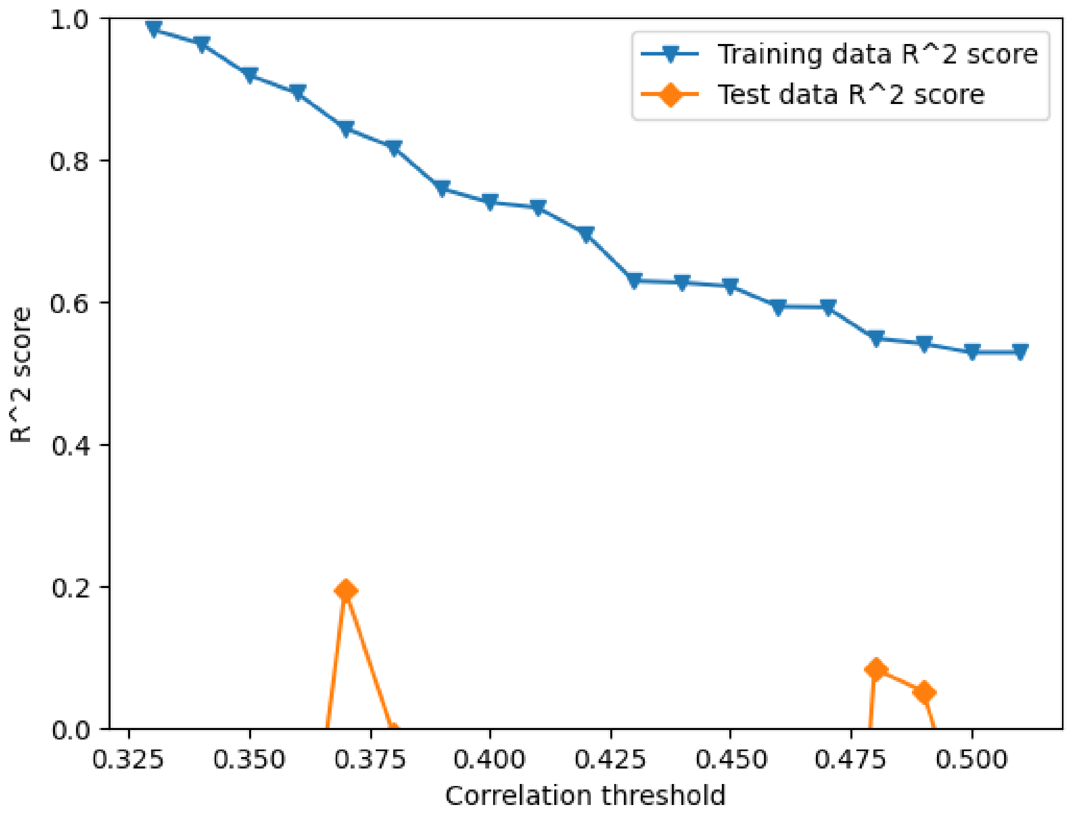

[12]:
```
plt.plot(df_without_standardization['Correlation threshold'],␣
  ↪df_without_standardization['Number of features'], label = "Number of␣
  ↪features", marker='.')
plt.legend()
plt.xlabel('Correlation threshold')
plt.ylabel('Number of features')
plt.show()
```

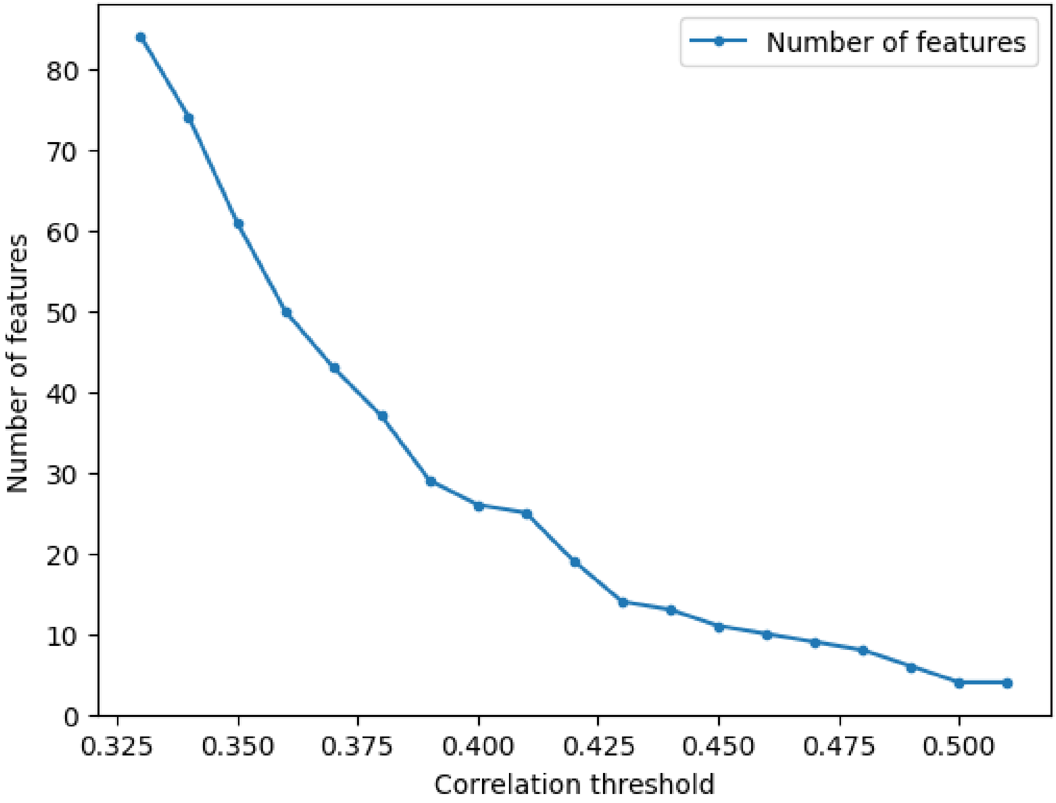


```
[ ]:
```

2.3 Decision Tree

```
[13]: without_standardization, train_r2_, test_r2_, hist1, hist2, target_column_name,␣
       ↪training_data_RMSE, test_data_RMSE  = pred_model.
       ↪prepare_data_and_create_model(molecular_descriptors_df=data,
                                                                                    ␣
       ↪                        correlation_threshold=0.50,
                                                                                    ␣
       ↪                        standardization=False,
                                                                                    ␣
       ↪                        model_type='DecisionTreeRegressor',
                                                                                    ␣
       ↪                        max_depth=5,
                                                                                    ␣
       ↪                        target_column_name = target,
                                                                                    ␣
       ↪                        random_state=random_state,
```

```
                                                                                        ␣
↪                    train_test_split_=True,
                                                                                        ␣
↪                    verbose=True)
```

```
I am not doing standardization…
  molecular descriptor name
0                    AATS0Z
1                  AATS0are
2                    AATS0d
3                   AATS0dv
4                    AATS0i
  molecular descriptor name  corr_value
0                    AATS0Z   -0.038502
1                  AATS0are   -0.120847
2                    AATS0d    0.041648
3                   AATS0dv   -0.115899
4                    AATS0i    0.191765
  molecular descriptor name  corr_value  absolute correlation value
0                    AATS0Z   -0.038502                    0.038502
1                  AATS0are   -0.120847                    0.120847
2                    AATS0d    0.041648                    0.041648
3                   AATS0dv   -0.115899                    0.115899
4                    AATS0i    0.191765                    0.191765
     molecular descriptor name  corr_value  absolute correlation value
226                     AMID_O   -0.520578                    0.520578
505                EState_VSA5   -0.578904                    0.578904
506                EState_VSA6    0.519794                    0.519794
791                    MDEO-12   -0.525441                    0.525441
1211                     MCF-7    1.000000                    1.000000
    molecular descriptor name  corr_value  absolute correlation value
226                    AMID_O   -0.520578                    0.520578
505               EState_VSA5   -0.578904                    0.578904
506               EState_VSA6    0.519794                    0.519794
791                   MDEO-12   -0.525441                    0.525441
The model used is: DecisionTree…
Return the coefficient of determination of the prediction:
-0.005104855722772195
R^2 score: 0.8657226333752897
Correlation coefficient: 0.9304421708925761
Test data - unseen during training:
R^2 score: -0.005104855722772195
Correlation coefficient: nan
[7.94564921 7.67778071 8.35232643 7.67778071 7.94564921 8.95860731
 7.94564921 7.69458125 7.20277093 7.94564921 7.94564921 7.94564921
 7.94564921 7.94564921 8.95860731 6.02227639 6.99012437 7.20277093]
113    8.022276
35     6.066513
```

```
101    6.920819
36     6.108463
100    7.376751
13     7.987163
0      7.987163
114    7.823909
104    8.397940
96     7.920819
40     8.091515
103    8.376751
48     7.886057
39     8.455932
14     8.086186
117    5.920819
21     8.113509
9      6.143150
Name: MCF-7, dtype: float64
Training Root Mean Square Error: 0.35505905070999344
Testing Root Mean Square Error: 0.8646174988464919
```

[14]:

```
print(target_column_name+str('_transformed'))
print(hist1[target_column_name].hist())
```

```
MCF-7_transformed
AxesSubplot(0.125,0.11;0.775x0.77)
```

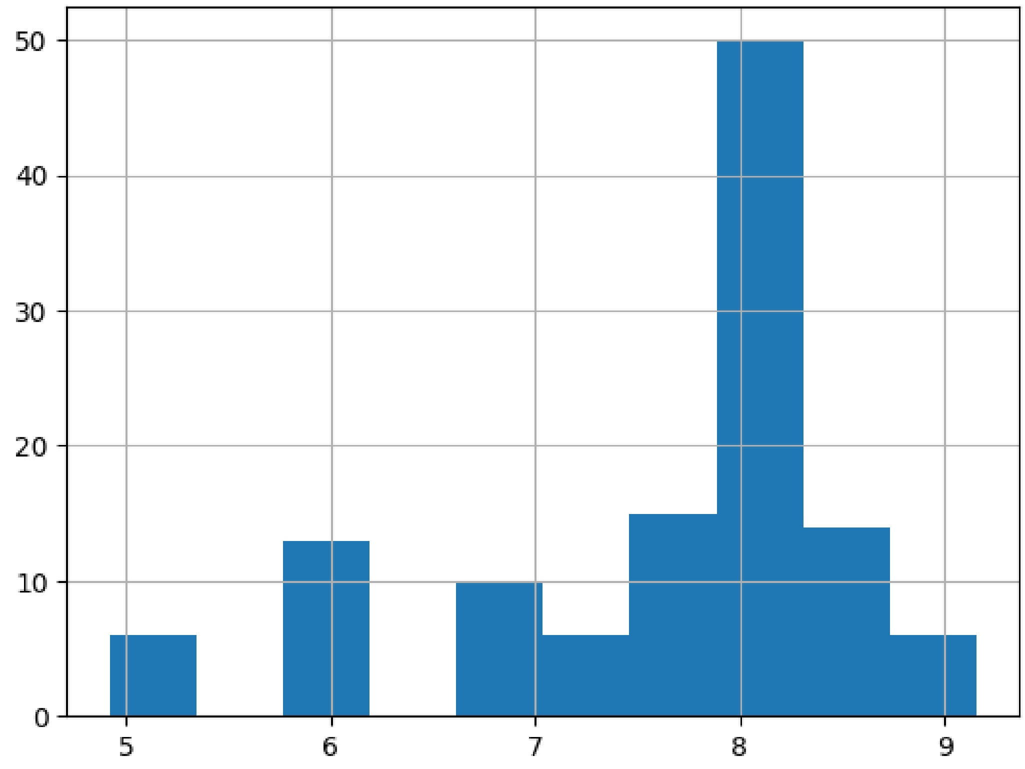

[15]:
```
with_standardization, train_r2_, test_r2_, hist1, hist2, target_column_name,
 ↪training_data_RMSE, test_data_RMSE  = pred_model.
 ↪prepare_data_and_create_model(molecular_descriptors_df=data,
 ↪                    correlation_threshold=0.50,
 ↪                    standardization=True,
 ↪                    model_type='DecisionTreeRegressor',
 ↪                    max_depth=5,
 ↪                    target_column_name = target,
 ↪                    random_state=random_state,
 ↪                    train_test_split_=True,
 ↪                    verbose=True)
```

```
I am doing standardization…
  molecular descriptor name
0                    AATS0Z
1                  AATS0are
2                    AATS0d
3                   AATS0dv
4                    AATS0i
  molecular descriptor name  corr_value
0                    AATS0Z   -0.038502
1                  AATS0are   -0.120847
2                    AATS0d    0.041648
3                   AATS0dv   -0.115899
4                    AATS0i    0.191765
  molecular descriptor name  corr_value  absolute correlation value
0                    AATS0Z   -0.038502                    0.038502
1                  AATS0are   -0.120847                    0.120847
2                    AATS0d    0.041648                    0.041648
3                   AATS0dv   -0.115899                    0.115899
4                    AATS0i    0.191765                    0.191765
     molecular descriptor name  corr_value  absolute correlation value
226                     AMID_O   -0.520578                    0.520578
505                 EState_VSA5   -0.578904                    0.578904
506                 EState_VSA6    0.519794                    0.519794
791                     MDEO-12   -0.525441                    0.525441
```

```
1211                     MCF-7    1.000000                    1.000000
    molecular descriptor name  corr_value  absolute correlation value
226                    AMID_O   -0.520578                    0.520578
505               EState_VSA5   -0.578904                    0.578904
506               EState_VSA6    0.519794                    0.519794
791                  MDEO-12   -0.525441                    0.525441
The model used is: DecisionTree…
Return the coefficient of determination of the prediction:
-0.005104855722772195
R^2 score: 0.8657226333752897
Correlation coefficient: 0.9304421708925761
Test data - unseen during training:
R^2 score: -0.005104855722772195
Correlation coefficient: nan
[7.94564921 7.67778071 8.35232643 7.67778071 7.94564921 8.95860731
 7.94564921 7.69458125 7.20277093 7.94564921 7.94564921 7.94564921
 7.94564921 7.94564921 8.95860731 6.02227639 6.99012437 7.20277093]
113    8.022276
35     6.066513
101    6.920819
36     6.108463
100    7.376751
13     7.987163
0      7.987163
114    7.823909
104    8.397940
96     7.920819
40     8.091515
103    8.376751
48     7.886057
39     8.455932
14     8.086186
117    5.920819
21     8.113509
9      6.143150
Name: MCF-7, dtype: float64
Training Root Mean Square Error: 0.35505905070999344
Testing Root Mean Square Error: 0.8646174988464919
```

2.4 Search inside correlation space

[16]:
```
step = 0.01
initial_step = corr_low
last_step = corr_high
first_list = [x / 100.0 for x in range(int(initial_step*100),␣
 ↪int(last_step*100), int(step*100))]
max_depth = [range(2, 30, 1)]
```

```
corr_th = []
second_list = []
third_list = []
fourth_l = []
fifth_l = []
f_list = []
fif_list = []
for i in first_list:
    for depth in max_depth[0]:

        without_standardization, train_r2, test_r2, _, h_, target_column_name,␣
 ↪training_data_RMSE, test_data_RMSE = pred_model.
 ↪prepare_data_and_create_model(molecular_descriptors_df=data,
                                                                                ␣
 ↪                    correlation_threshold=i,
                                                                                ␣
 ↪                    standardization=False,
                                                                                ␣
 ↪                    model_type='DecisionTreeRegressor',
                                                                                ␣
 ↪                    max_depth=depth,
                                                                                ␣
 ↪                    target_column_name = target,
                                                                                ␣
 ↪                    random_state=random_state,
                                                                                ␣
 ↪                    train_test_split_=True,
                                                                                ␣
 ↪                    verbose=False)
        corr_th.append(i)
        second_list.append(train_r2)
        third_list.append(test_r2)
        fourth_l.append(training_data_RMSE)
        fifth_l.append(test_data_RMSE)
        f_list.append(len(h_))
        fif_list.append(depth)
```

[17]:
```
df_without_standardization = pd.DataFrame(data=corr_th, columns=["Correlation␣
 ↪threshold"])
df_without_standardization['Training data R^2 score'] = second_list
df_without_standardization['Test data R^2 score'] = third_list
df_without_standardization['Training RMSE'] = fourth_l
df_without_standardization['Test RMSE'] = fifth_l
df_without_standardization['Number of features'] = f_list
df_without_standardization['Depth number'] = fif_list
```

[18]:
```
df_decision_tree = df_without_standardization.copy()
#df_without_standardization.to_excel('../Data/
↪A549_Decision_tree_rs_'+str(random_state)+'.xlsx')
```

2.5 Plots

[ ]:
```
corre_list = [x / 100.0 for x in range(int(initial_step*100),␣
↪int(last_step*100), int(0.01*100))]
for element in corre_list:
    element_ =␣
↪df_without_standardization[df_without_standardization['Correlation␣
↪threshold'] == float(element)]
    plt.plot(element_['Depth number'], element_['Training data R^2 score'],␣
↪label = "Training data R^2 score", marker='v')
    plt.plot(element_['Depth number'], element_['Test data R^2 score'], label =␣
↪"Test data R^2 score", marker='D')
    plt.legend()
    plt.xlabel('Depth number -> correlation threshold: '+str(element)+'. \n␣
↪Number of features: '+str(element_['Number of features'].iloc[0]),␣
↪fontweight='bold')
    plt.xlim(max(element_['Depth number'])+1, min(element_['Depth number'])-1)
    plt.ylabel('R^2 score')
    plt.ylim([0, 1])
    plt.rc('grid', linestyle="-.", color='black')
    plt.grid(True)
    plt.show()
```

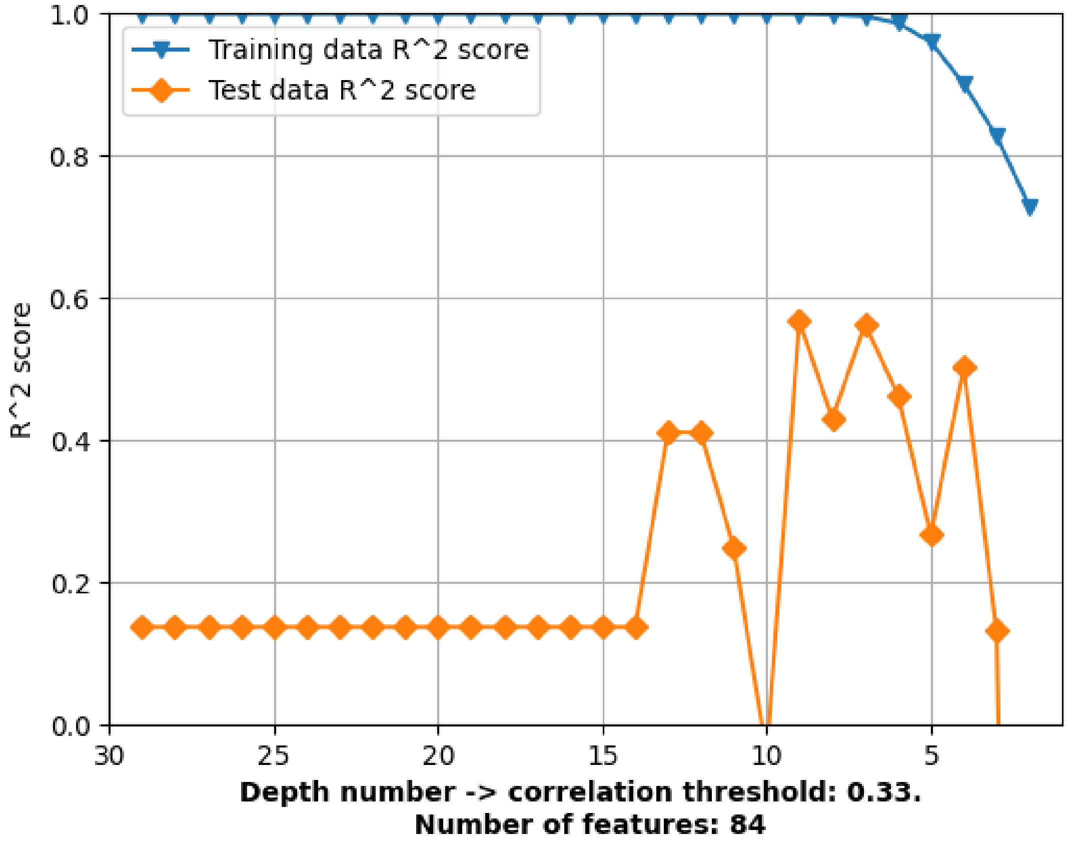

Training data R^2 score
Test data R^2 score
R^2 score
1.0
0.8
0.6
0.4
0.2
0.0
30
25
20
15
10
5
Depth number -> correlation threshold: 0.33.
Number of features: 84

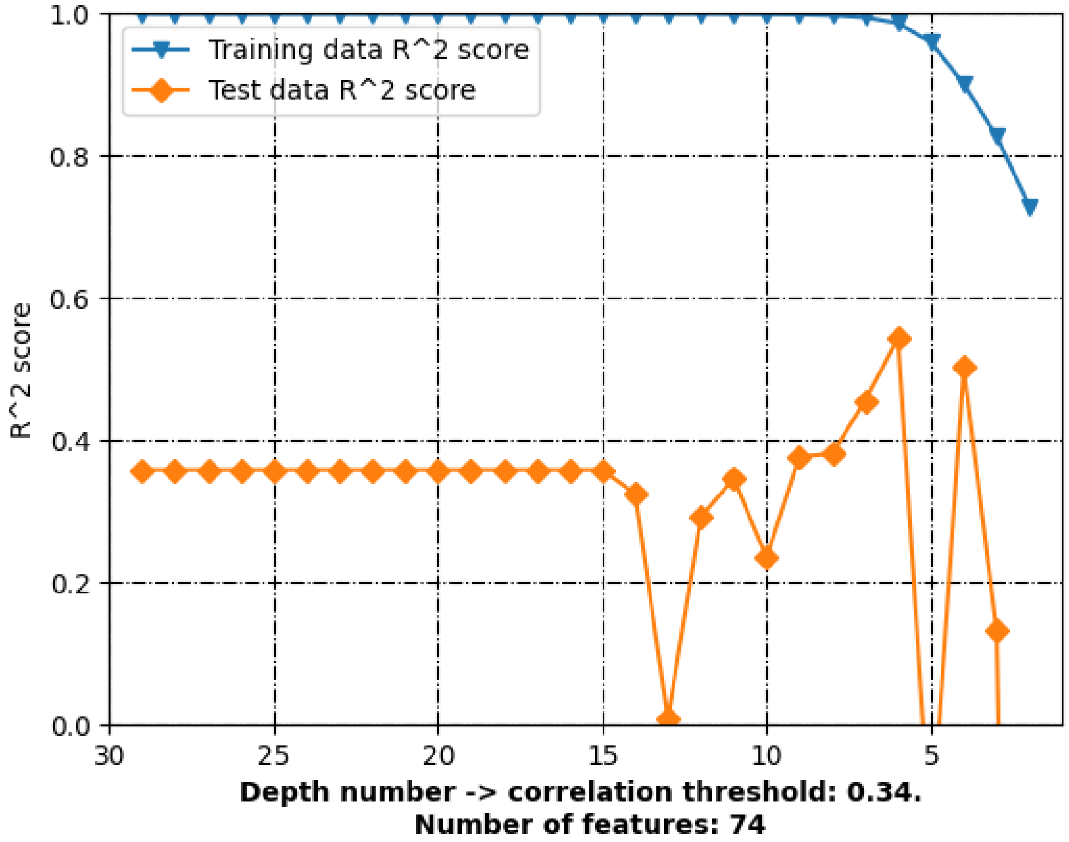

Training data R^2 score
Test data R^2 score
R^2 score
1.0
0.8
0.6
0.4
0.2
0.0
30
25
20
15
10
5
Depth number -> correlation threshold: 0.34.
Number of features: 74

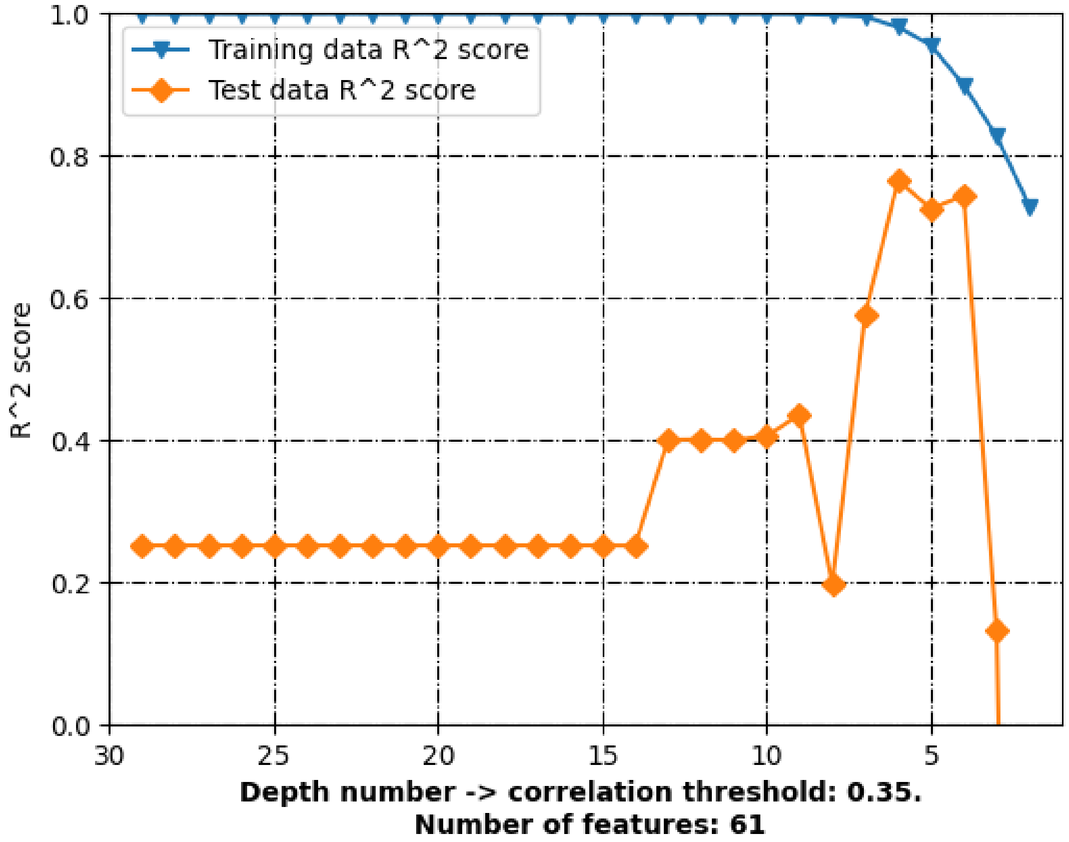

Training data R^2 score
Test data R^2 score
R^2 score
1.0
0.8
0.6
0.4
0.2
0.0
30
25
20
15
10
5
Depth number -> correlation threshold: 0.35.
Number of features: 61

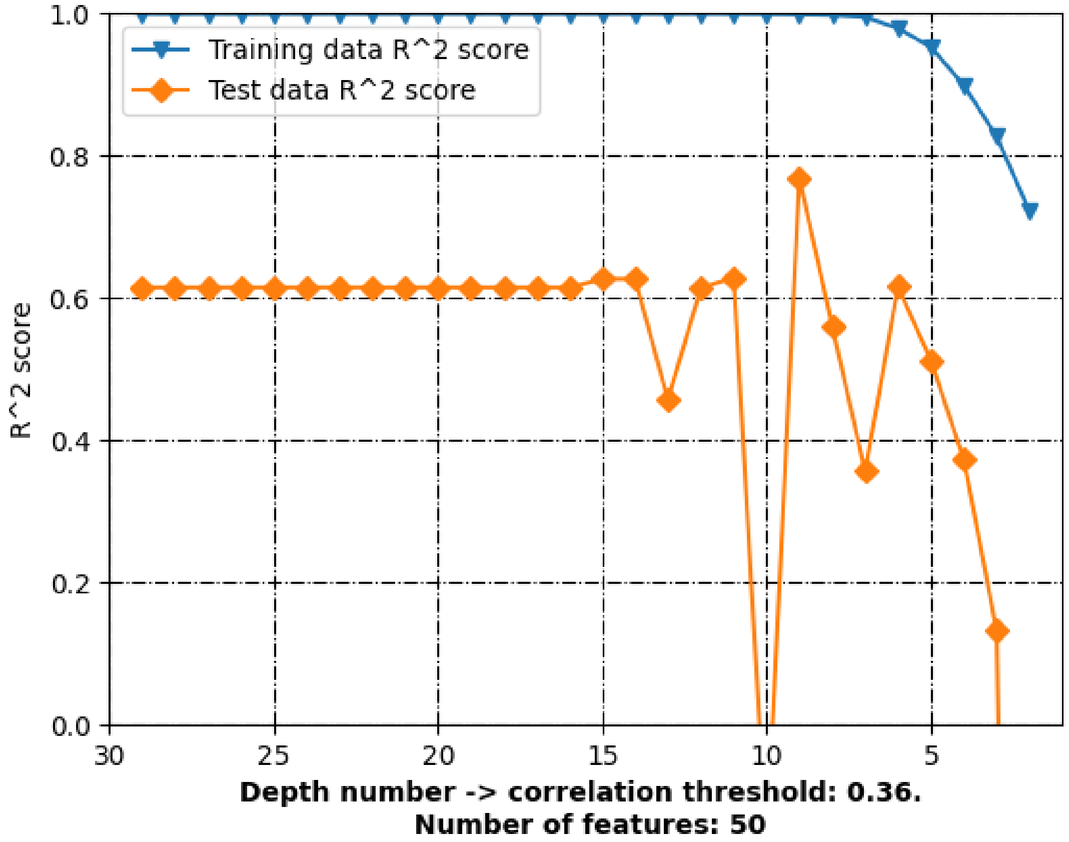
1.0
0.8
0.6
0.4
0.2
0.0
R^2 score
Training data R^2 score
Test data R^2 score
30
25
20
15
10
5
Depth number -> correlation threshold: 0.36.
Number of features: 50

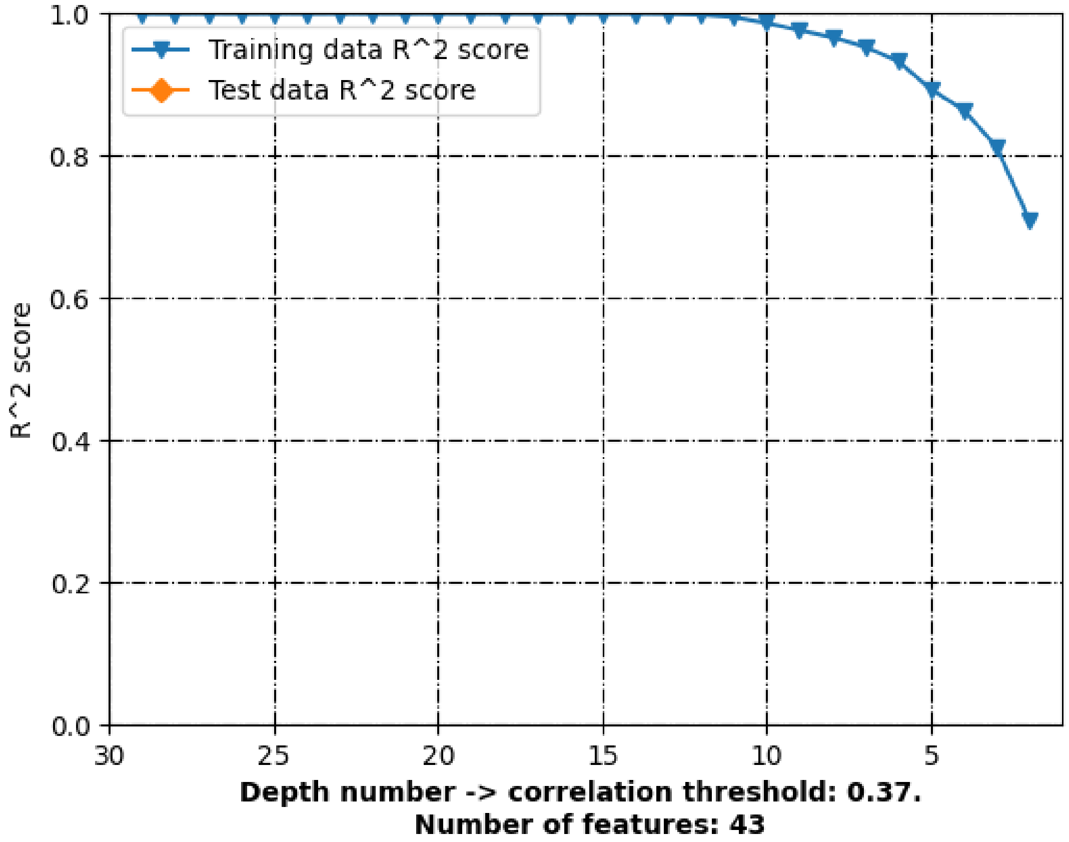
Training data R^2 score
Test data R^2 score
R^2 score
1.0
0.8
0.6
0.4
0.2
0.0
30
25
20
15
10
5
Depth number -> correlation threshold: 0.37.
Number of features: 43

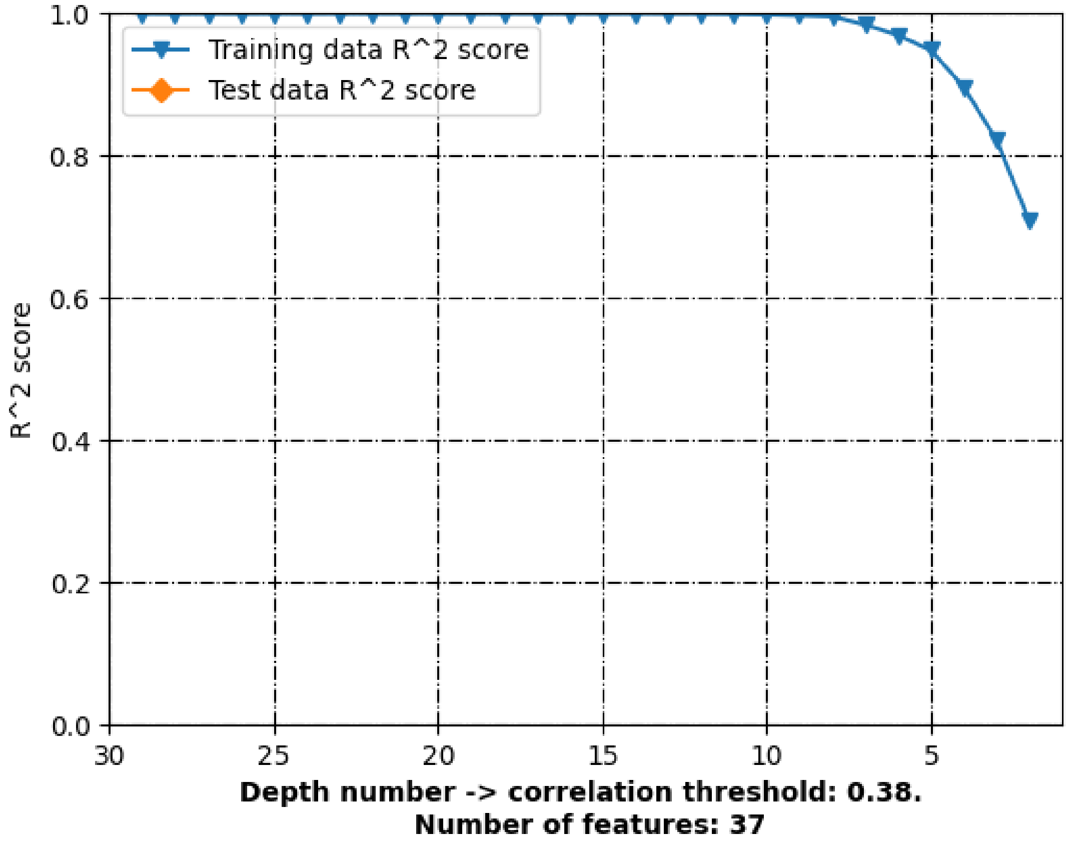

Training data R^2 score
Test data R^2 score
R^2 score
1.0
0.8
0.6
0.4
0.2
0.0
30
25
20
15
10
5
Depth number -> correlation threshold: 0.38.
Number of features: 37

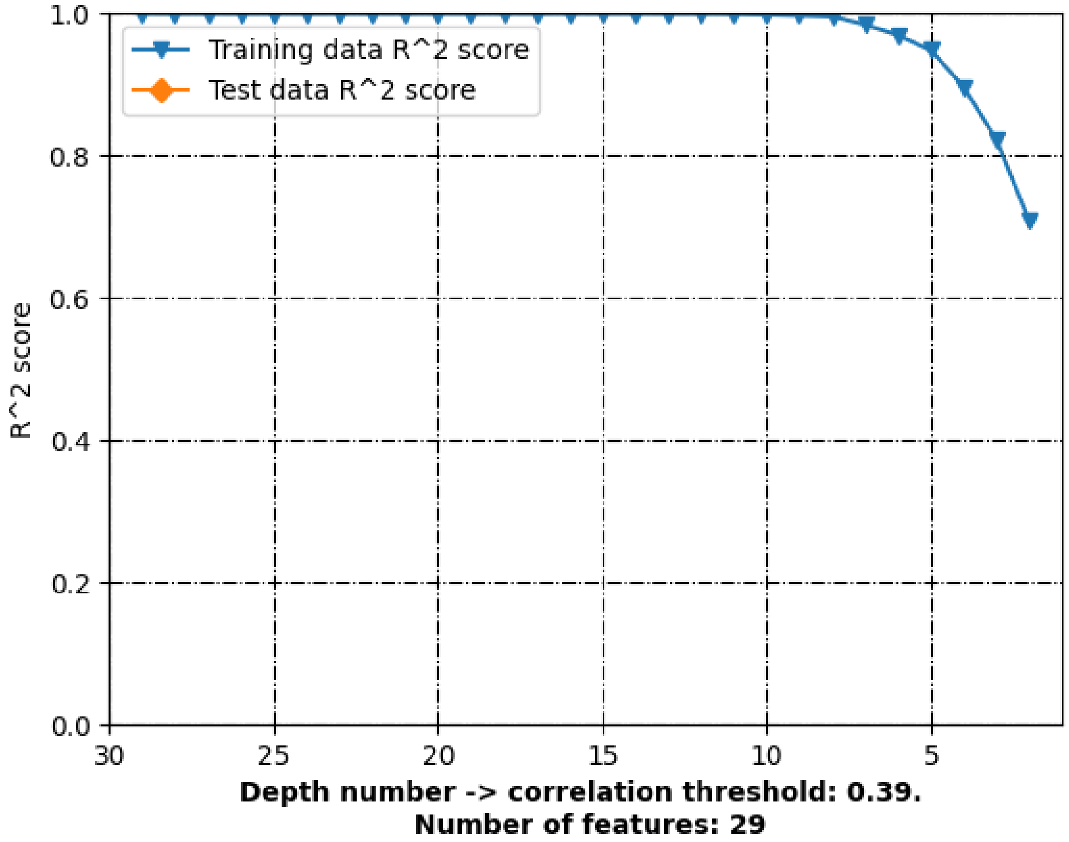
1.0
0.8
0.6
0.4
0.2
0.0
R^2 score
Training data R^2 score
Test data R^2 score
30
25
20
15
10
5
Depth number -> correlation threshold: 0.39.
Number of features: 29

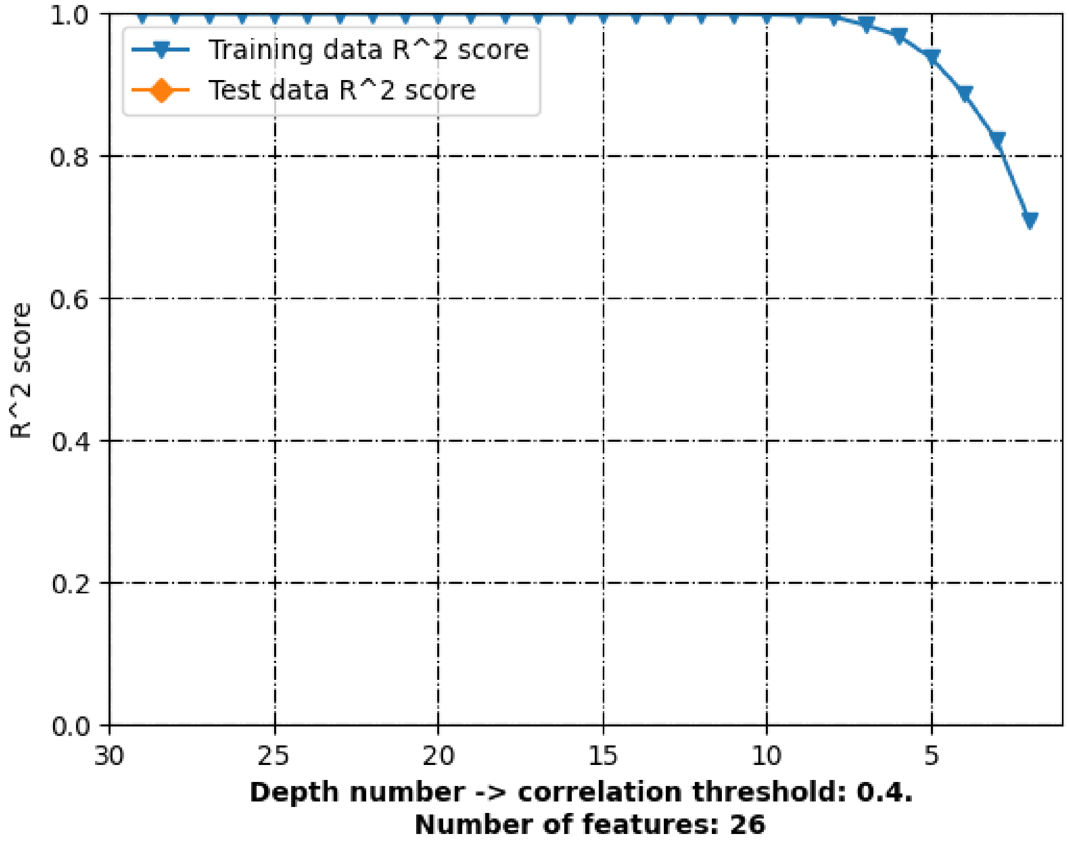
1.0
0.8
0.6
0.4
0.2
0.0
R^2 score
Training data R^2 score
Test data R^2 score
30
25
20
15
10
5
Depth number -> correlation threshold: 0.4.
Number of features: 26

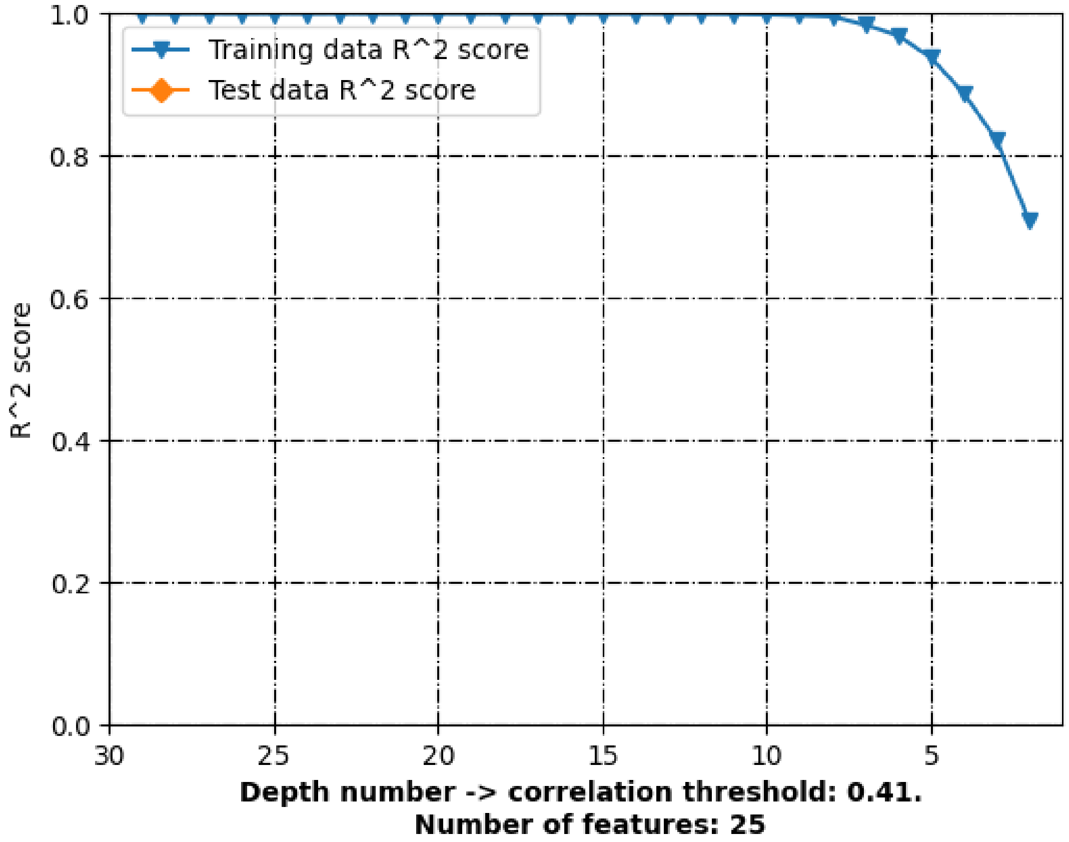

1.0
0.8
0.6
0.4
0.2
0.0
R^2 score
Training data R^2 score
Test data R^2 score
30
25
20
15
10
5
Depth number -> correlation threshold: 0.41.
Number of features: 25

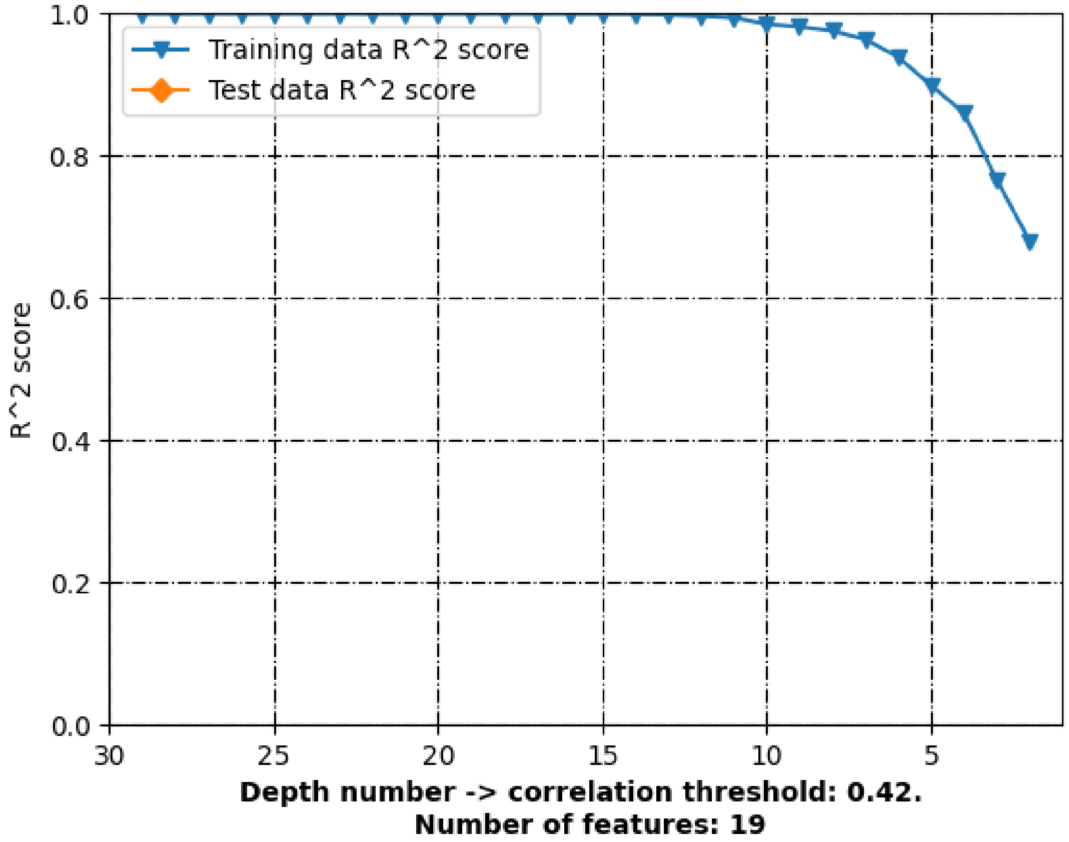
1.0
0.8
0.6
0.4
0.2
0.0
R^2 score
Training data R^2 score
Test data R^2 score
30
25
20
15
10
5
Depth number -> correlation threshold: 0.42.
Number of features: 19

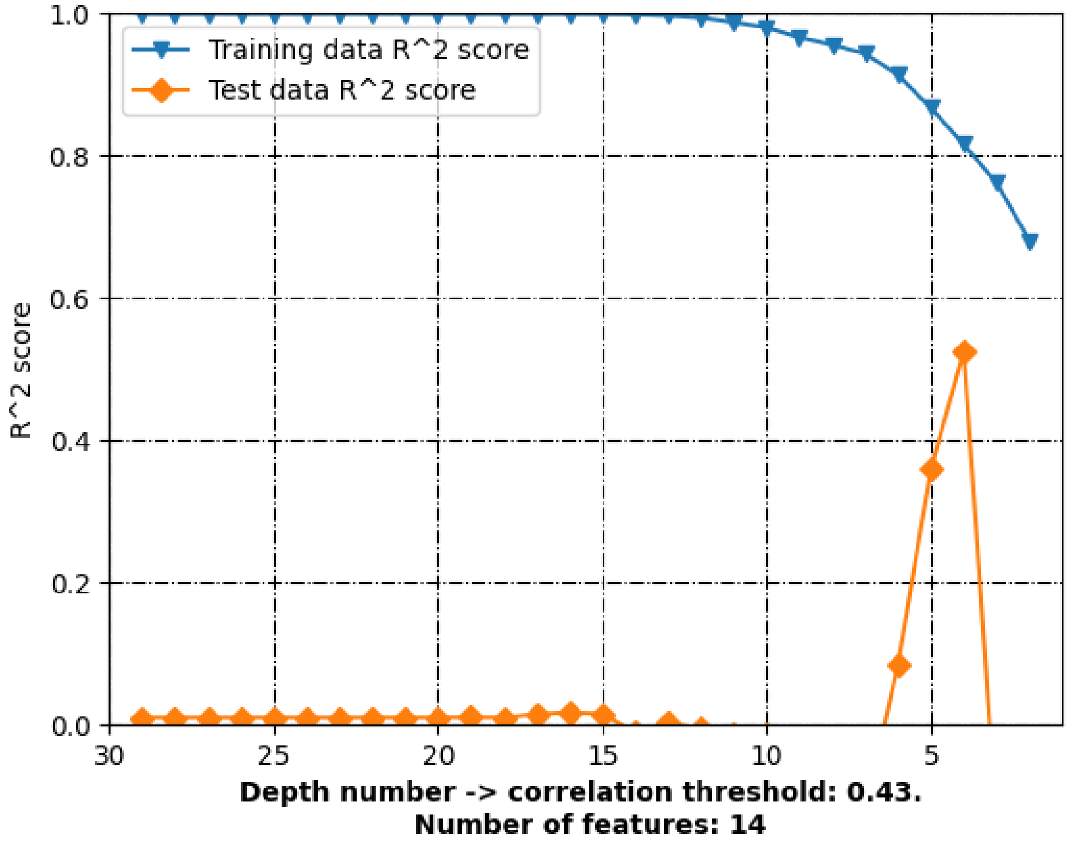

Training data R^2 score
Test data R^2 score
R^2 score
1.0
0.8
0.6
0.4
0.2
0.0
30
25
20
15
10
5
Depth number -> correlation threshold: 0.43.
Number of features: 14

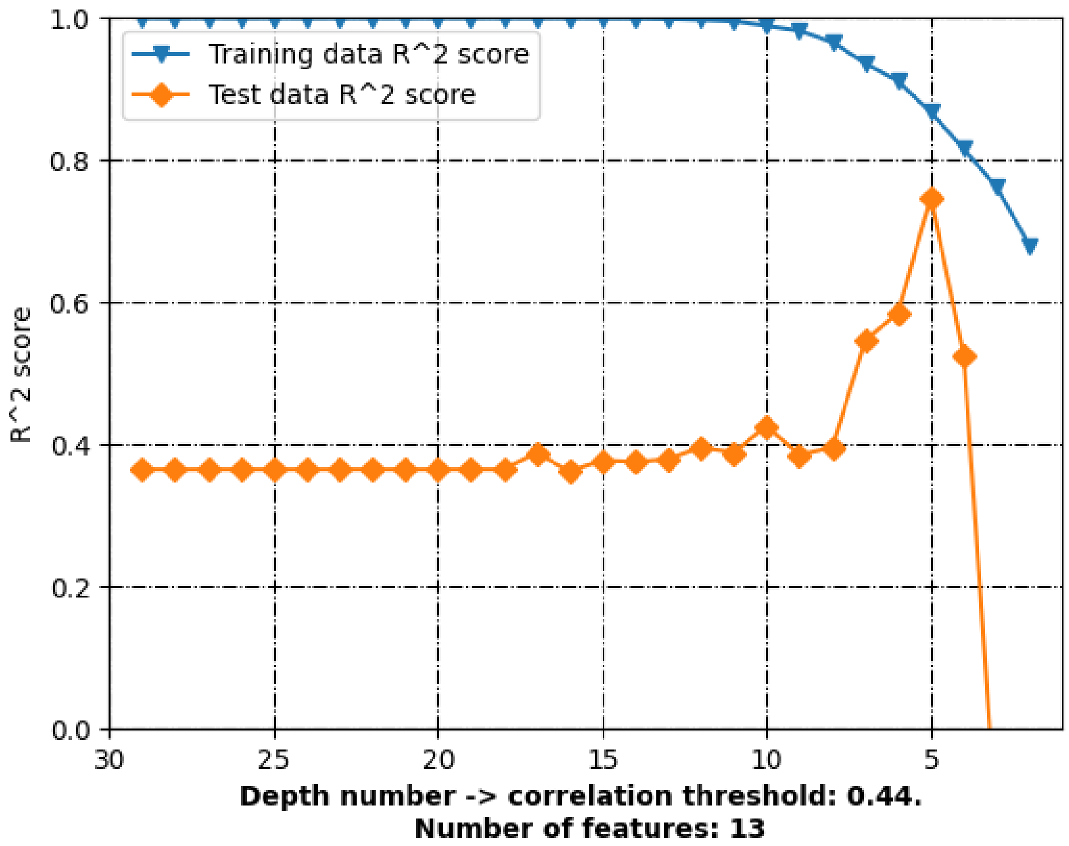

[ ]:
```
plt.plot(df_without_standardization['Correlation threshold'],␣
↪df_without_standardization['Number of features'], label = "Number of␣
↪features", marker='.')
plt.legend()
plt.xlabel('Correlation threshold')
plt.ylabel('Number of features')
plt.show()
```

[ ]:
```

```

3 Random Forest

[ ]:
```
without_standardization, train_r2_, test_r2_, hist1, hist2, target_column_name,␣
↪training_data_RMSE, test_data_RMSE  = pred_model.
↪prepare_data_and_create_model(molecular_descriptors_df=data,
                                                                               ␣
↪                        correlation_threshold=0.50,
                                                                               ␣
↪                        standardization=False,
```

```
                              model_type='RandomForestRegressor',
                              n_estimators_=12,
                              target_column_name = target,
                              random_state=random_state,
                              train_test_split_=True,
                              verbose=True)
```

[ ]:
```
print(target_column_name+str('_transformed'))
print(hist1[target_column_name].hist())
```

[ ]:
```
with_standardization, train_r2_, test_r2_, hist1, hist2, target_column_name, training_data_RMSE, test_data_RMSE  = pred_model.prepare_data_and_create_model(molecular_descriptors_df=data,
                              correlation_threshold=0.50,
                              standardization=True,
                              model_type='RandomForestRegressor',
                              n_estimators_=12,
                              target_column_name = target,
                              random_state=random_state,
                              train_test_split_=True,
                              verbose=True)
```

3.1 Search inside correlation space

[24]:
```
step = 0.01
initial_step = corr_low
last_step = corr_high
first_list = [x / 100.0 for x in range(int(initial_step*100), int(last_step*100), int(step*100))]
n_estimators = [range(2,21,1)]
corr_th = []
second_list = []
```

```
third_list = []
fourth_l = []
fifth_l = []
f_list = []
fif_list = []
for i in first_list:

    for estimator in n_estimators[0]:

        without_standardization, train_r2, test_r2, _, h_, target_column_name,␣
 ↪training_data_RMSE, test_data_RMSE = pred_model.
 ↪prepare_data_and_create_model(molecular_descriptors_df=data,
                                                                                 ␣
 ↪                      correlation_threshold=i,
                                                                                 ␣
 ↪                      standardization=False,
                                                                                 ␣
 ↪                      model_type='RandomForestRegressor',
                                                                                 ␣
 ↪                      n_estimators_=estimator,
                                                                                 ␣
 ↪                      target_column_name = target,
                                                                                 ␣
 ↪                      random_state=random_state,
                                                                                 ␣
 ↪                      train_test_split_=True,
                                                                                 ␣
 ↪                      verbose=False)
        corr_th.append(i)
        second_list.append(train_r2)
        third_list.append(test_r2)
        fourth_l.append(training_data_RMSE)
        fifth_l.append(test_data_RMSE)
        f_list.append(len(h_))
        fif_list.append(estimator)
```

[25]:
```
df_without_standardization = pd.DataFrame(data=corr_th, columns=["Correlation␣
 ↪threshold"])
df_without_standardization['Training data R^2 score'] = second_list
df_without_standardization['Test data R^2 score'] = third_list
df_without_standardization['Training RMSE'] = fourth_l
df_without_standardization['Test RMSE'] = fifth_l
df_without_standardization['Number of features'] = f_list
df_without_standardization['Number of estimators'] = fif_list
```

[26]:
```
df_random_forest = df_without_standardization.copy()
#df_without_standardization.to_excel('../Data/
↪A549_Random_forest_rs_'+str(random_state)+'.xlsx')
```

[27]:
```
corre_list = [x / 100.0 for x in range(int(initial_step*100),␣
↪int(last_step*100), int(0.01*100))]
for element in corre_list:
    element_ =␣
↪df_without_standardization[df_without_standardization['Correlation␣
↪threshold'] == float(element)]
    plt.plot(element_['Number of estimators'], element_['Training data R^2␣
↪score'], label = "Training data R^2 score", marker='v')
    plt.plot(element_['Number of estimators'], element_['Test data R^2 score'],␣
↪label = "Test data R^2 score", marker='D')
    plt.legend()
    plt.xlabel('Number of estimators -> correlation threshold: '+str(element)+'.
↪ \n Number of features: '+str(element_['Number of features'].iloc[0]))
    plt.xlim(max(element_['Number of estimators'])+1, min(element_['Number of␣
↪estimators'])-1)
    plt.ylabel('R^2 score')
    plt.ylim([0, 1])
    plt.rc('grid', linestyle="-.", color='black')
    plt.grid(True)
    plt.show()
```

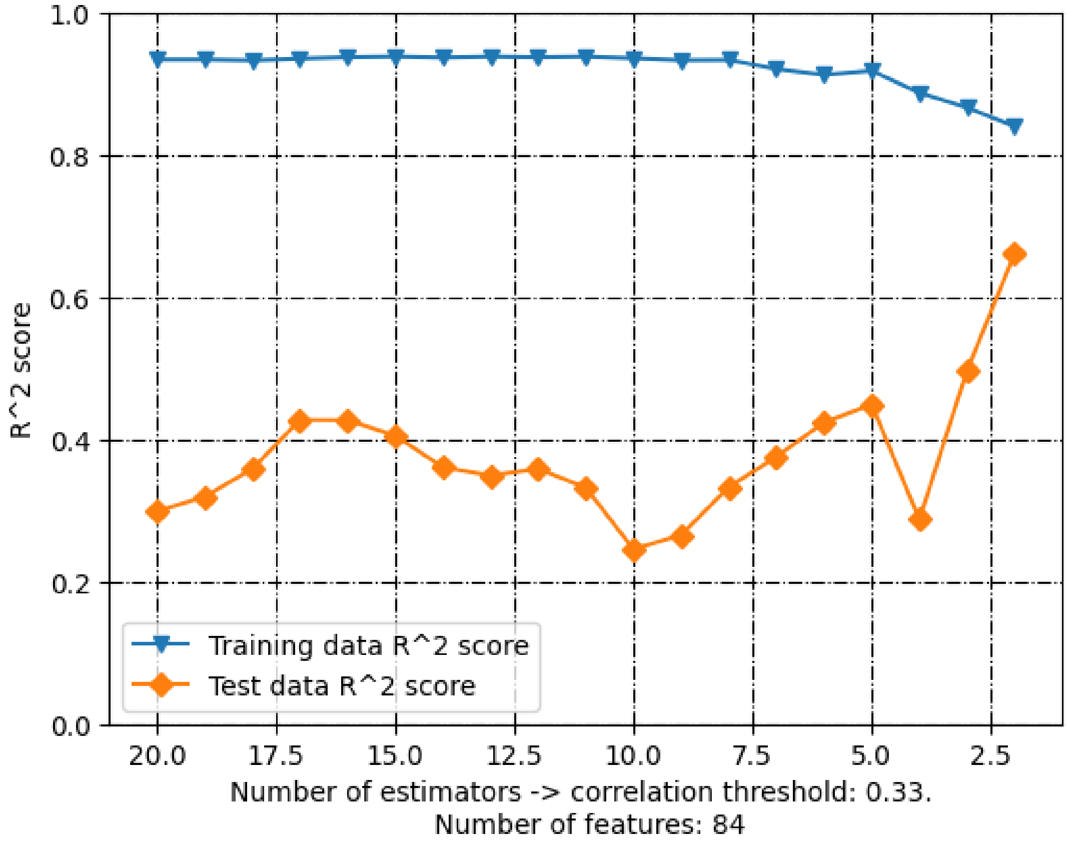
1.0
0.8
0.6
0.4
0.2
0.0
R^2 score
Training data R^2 score
Test data R^2 score
20.0
17.5
15.0
12.5
10.0
7.5
5.0
2.5
Number of estimators -> correlation threshold: 0.33.
Number of features: 84

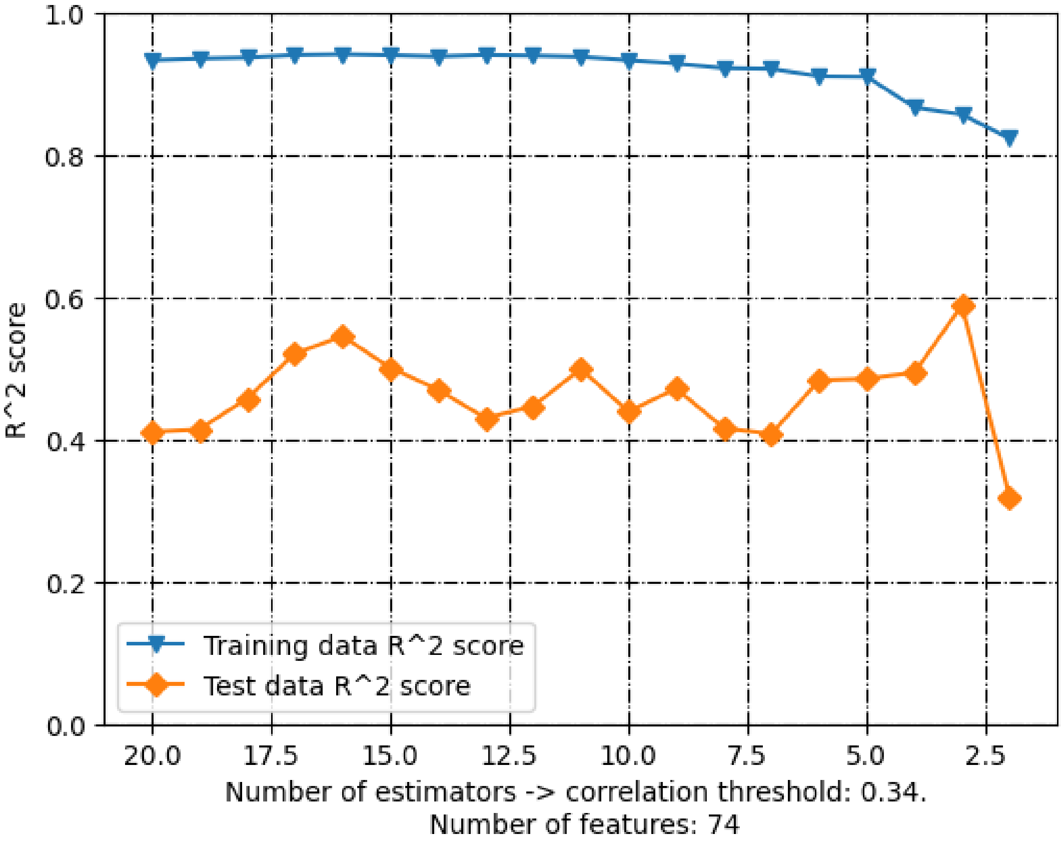

1.0
0.8
0.6
0.4
0.2
0.0
R^2 score
20.0
17.5
15.0
12.5
10.0
7.5
5.0
2.5
Training data R^2 score
Test data R^2 score
Number of estimators -> correlation threshold: 0.34.
Number of features: 74

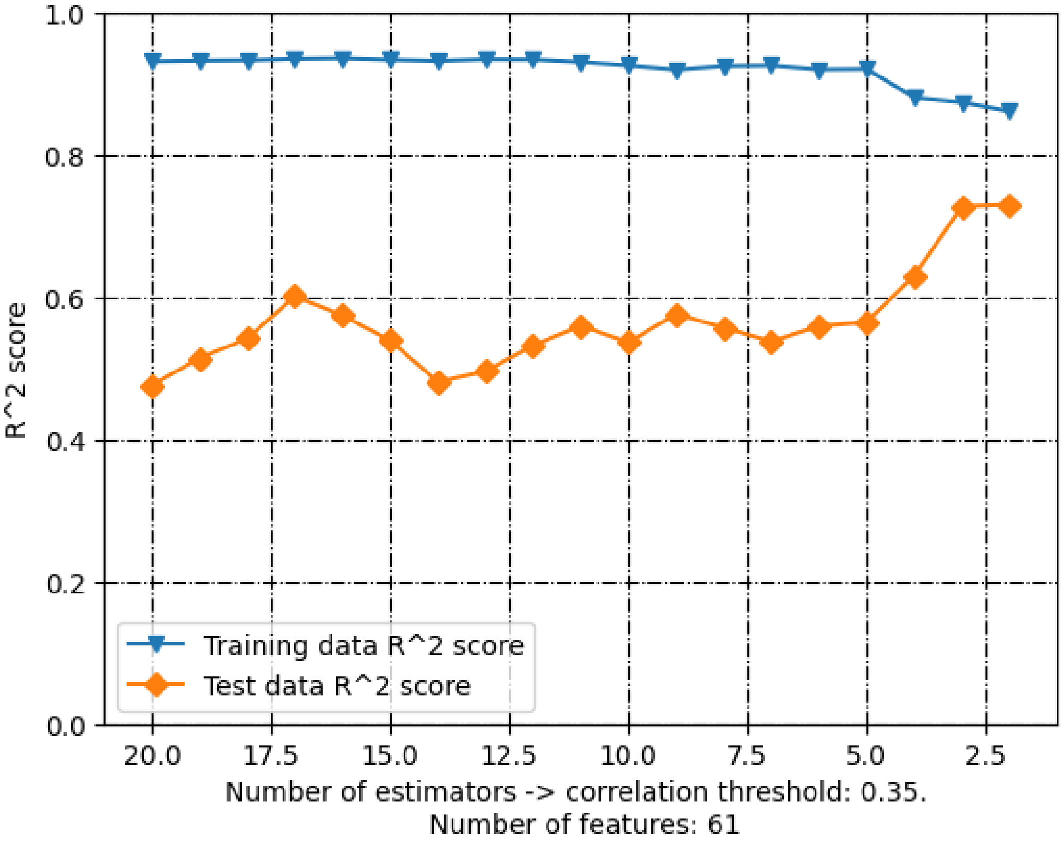
1.0
0.8
0.6
0.4
0.2
0.0
R^2 score
Training data R^2 score
Test data R^2 score
20.0
17.5
15.0
12.5
10.0
7.5
5.0
2.5
Number of estimators -> correlation threshold: 0.35.
Number of features: 61

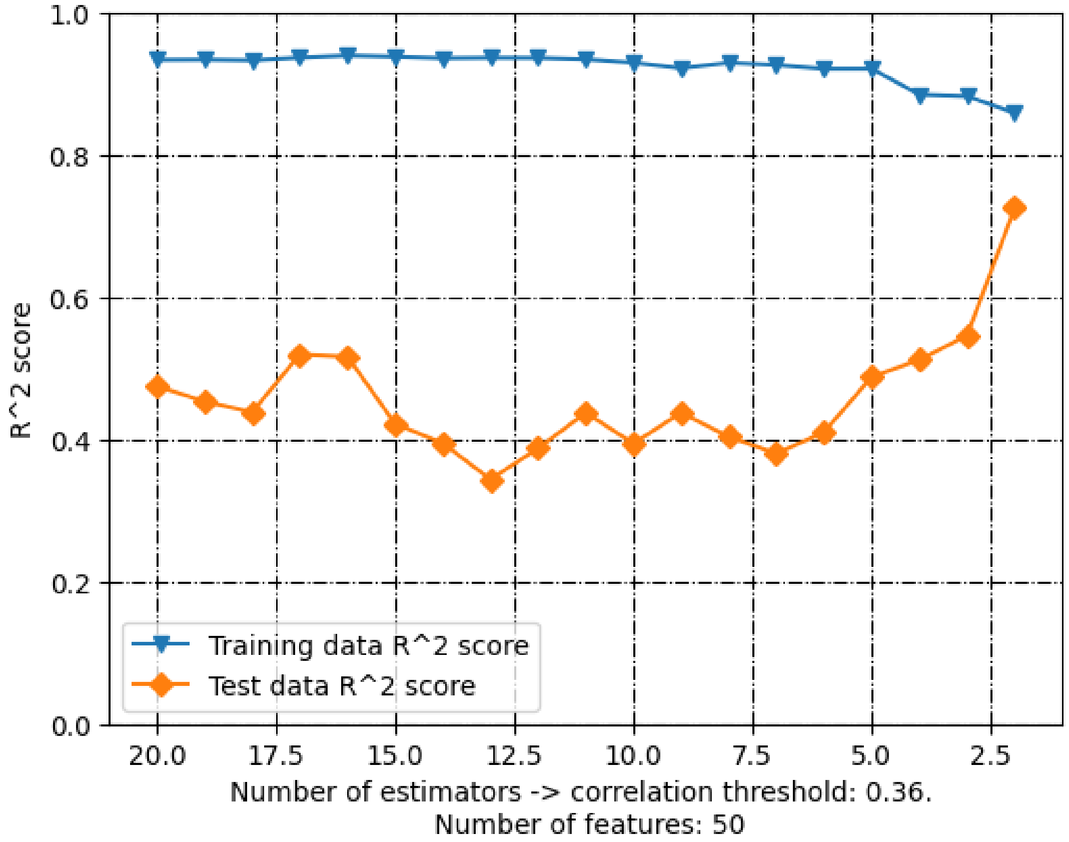

1.0
0.8
0.6
0.4
0.2
0.0
R^2 score
Training data R^2 score
Test data R^2 score
20.0
17.5
15.0
12.5
10.0
7.5
5.0
2.5
Number of estimators -> correlation threshold: 0.36.
Number of features: 50

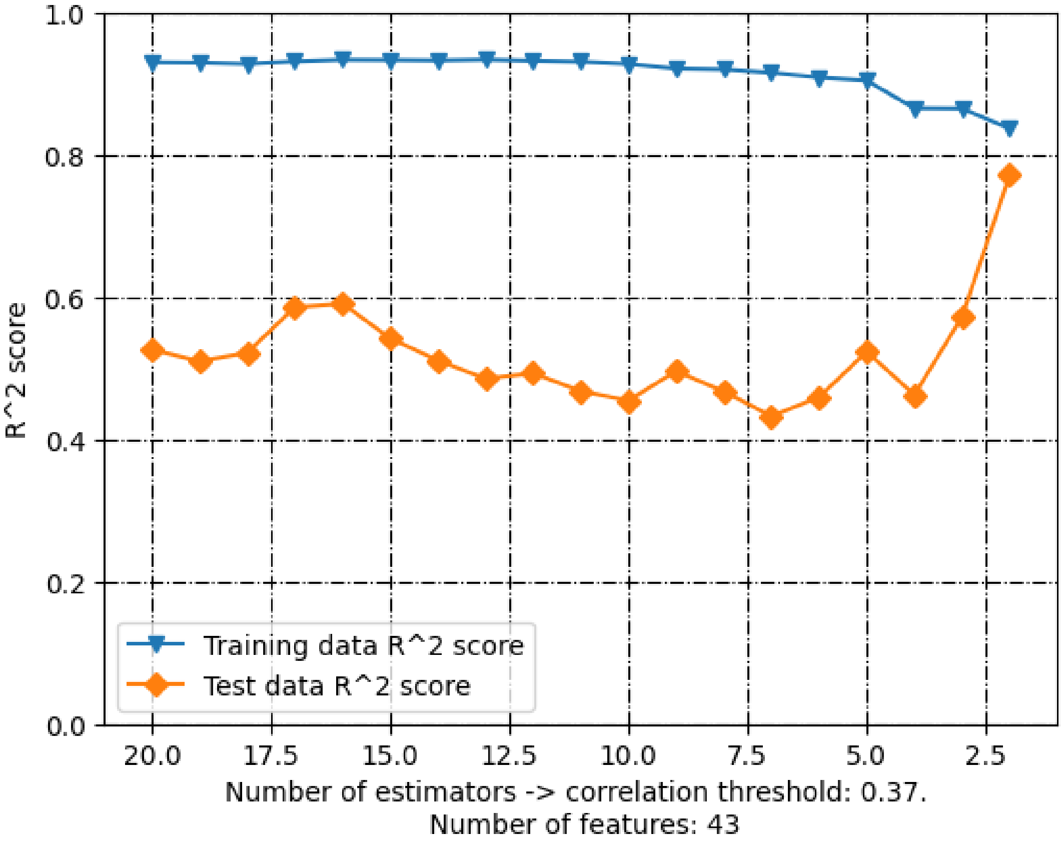
1.0
0.8
0.6
0.4
0.2
0.0
R^2 score
20.0
17.5
15.0
12.5
10.0
7.5
5.0
2.5
Training data R^2 score
Test data R^2 score
Number of estimators -> correlation threshold: 0.37.
Number of features: 43

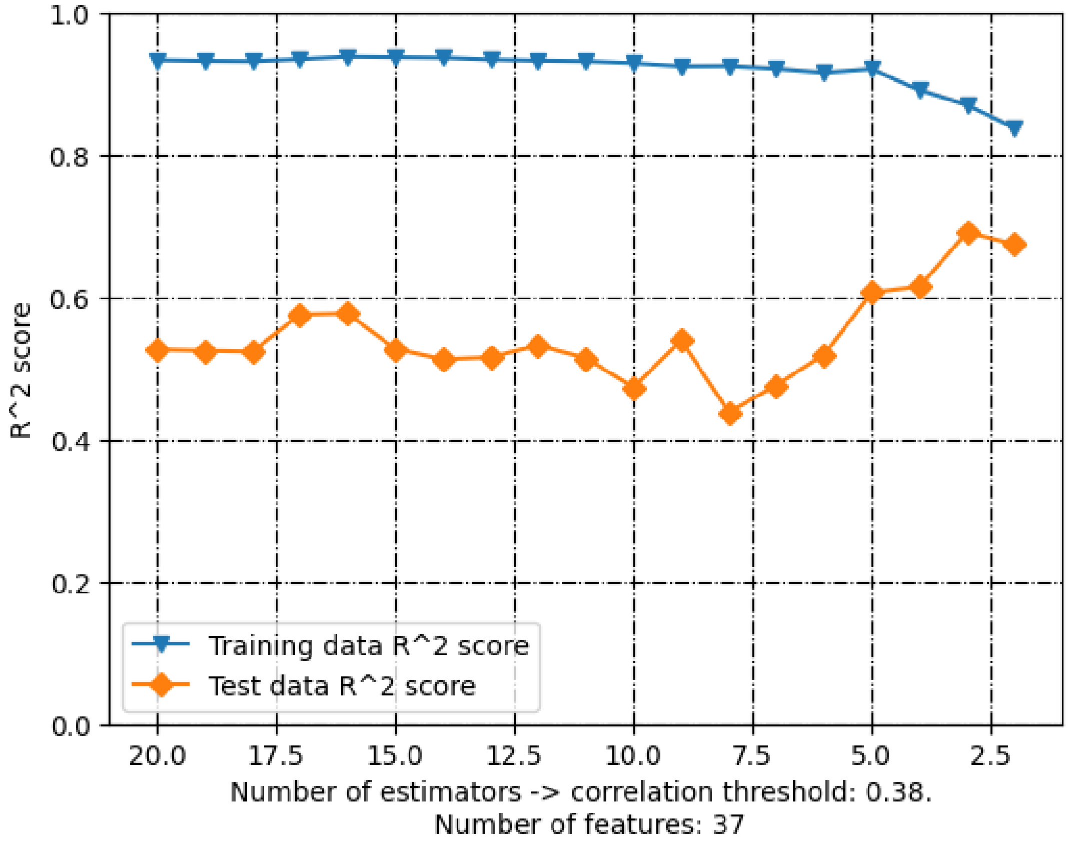
1.0
0.8
0.6
0.4
0.2
0.0
R^2 score
Training data R^2 score
Test data R^2 score
20.0
17.5
15.0
12.5
10.0
7.5
5.0
2.5
Number of estimators -> correlation threshold: 0.38.
Number of features: 37

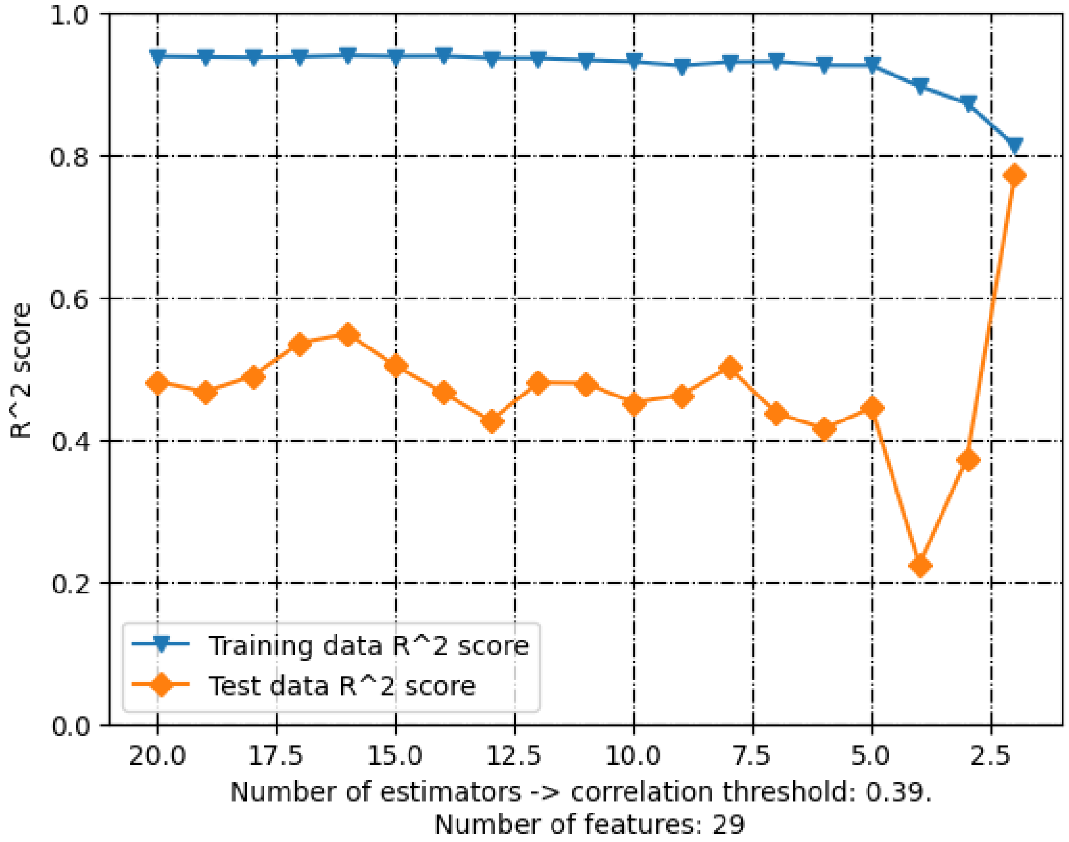
1.0
0.8
0.6
0.4
0.2
0.0
R^2 score
Training data R^2 score
Test data R^2 score
20.0
17.5
15.0
12.5
10.0
7.5
5.0
2.5
Number of estimators -> correlation threshold: 0.39.
Number of features: 29

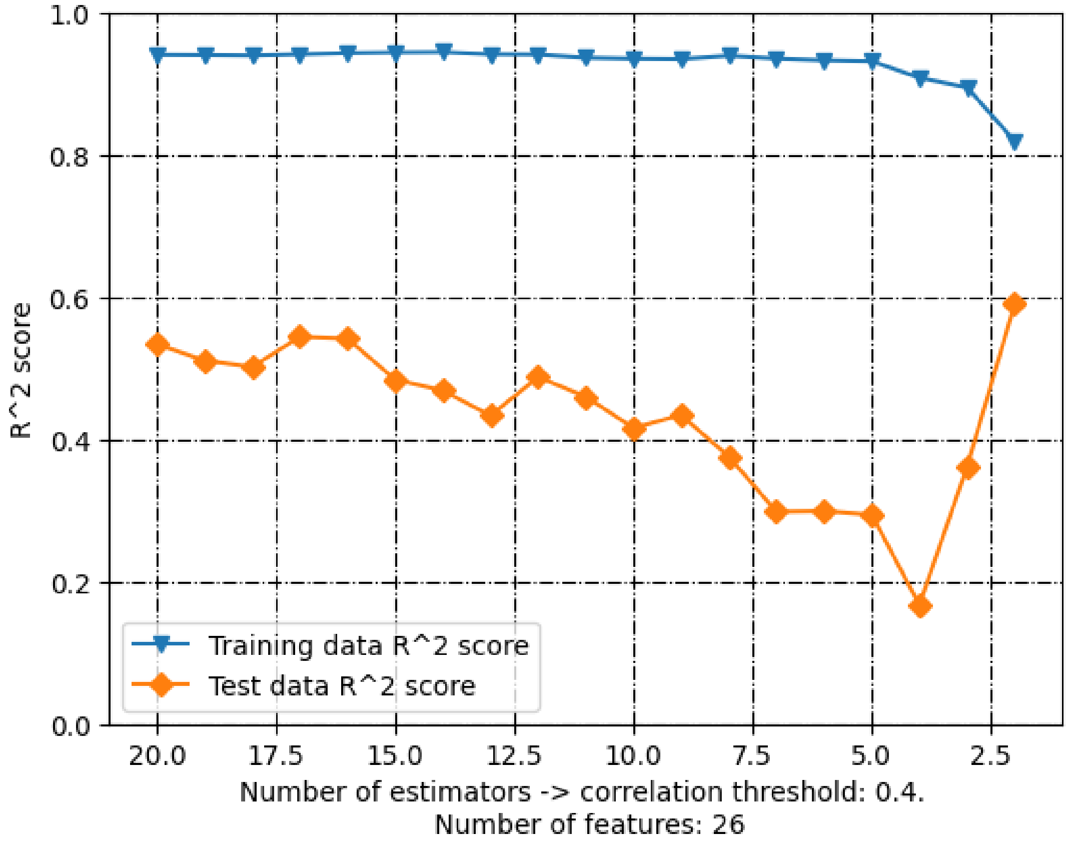

1.0
0.8
0.6
0.4
0.2
0.0
R^2 score
Training data R^2 score
Test data R^2 score
20.0
17.5
15.0
12.5
10.0
7.5
5.0
2.5
Number of estimators -> correlation threshold: 0.4.
Number of features: 26

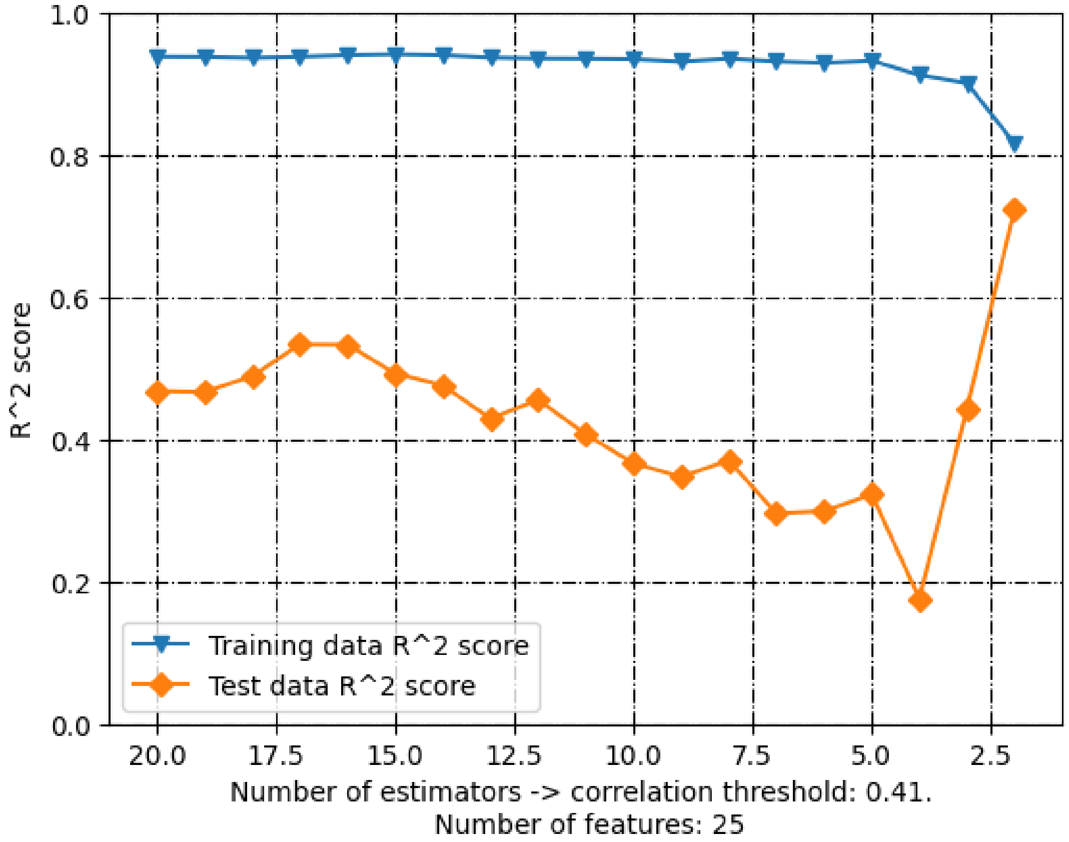

1.0
0.8
0.6
0.4
0.2
0.0
R^2 score
Training data R^2 score
Test data R^2 score
20.0
17.5
15.0
12.5
10.0
7.5
5.0
2.5
Number of estimators -> correlation threshold: 0.41.
Number of features: 25

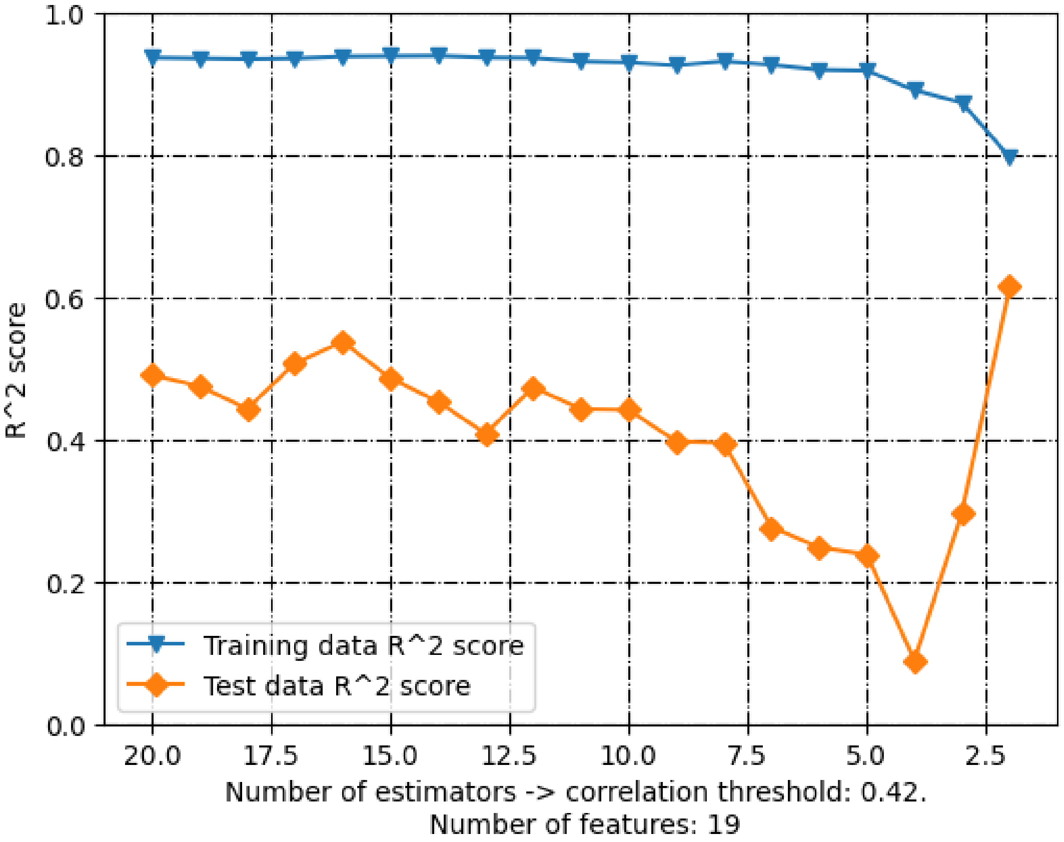
1.0
0.8
0.6
0.4
0.2
0.0
R^2 score
Training data R^2 score
Test data R^2 score
20.0
17.5
15.0
12.5
10.0
7.5
5.0
2.5
Number of estimators -> correlation threshold: 0.42.
Number of features: 19

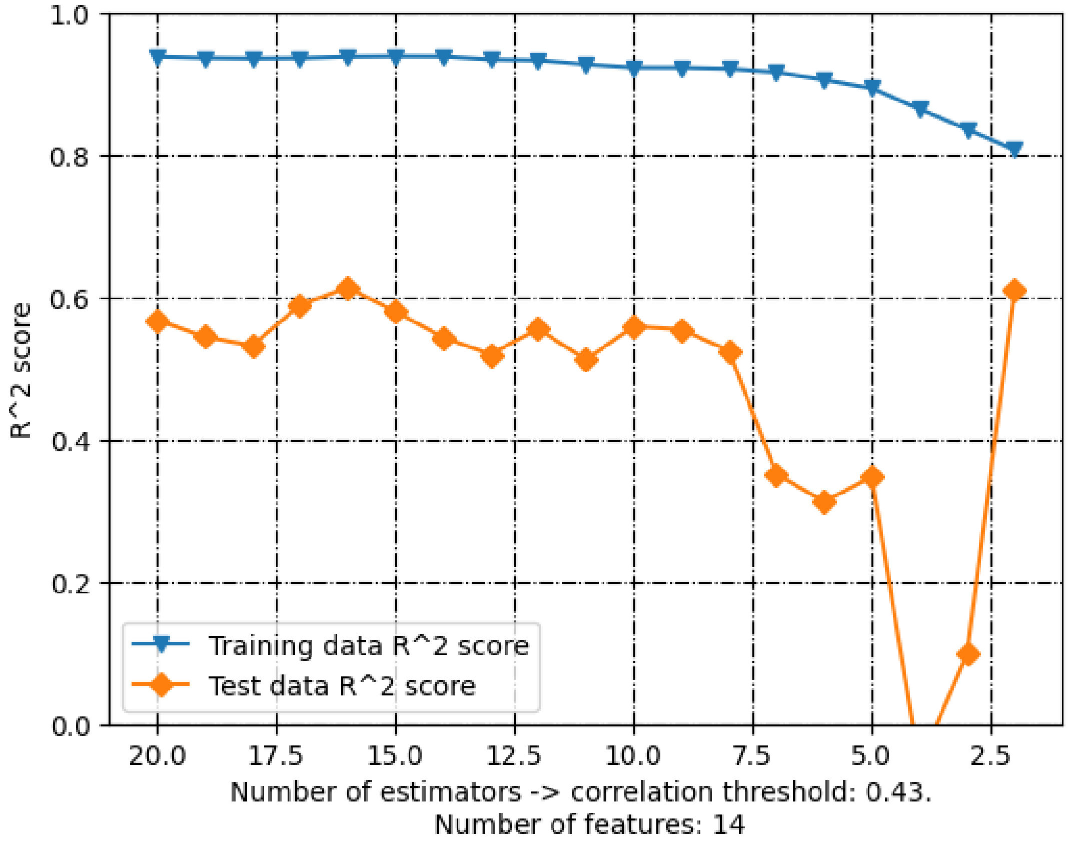

1.0
0.8
0.6
0.4
0.2
0.0
R^2 score
Training data R^2 score
Test data R^2 score
20.0
17.5
15.0
12.5
10.0
7.5
5.0
2.5
Number of estimators -> correlation threshold: 0.43.
Number of features: 14

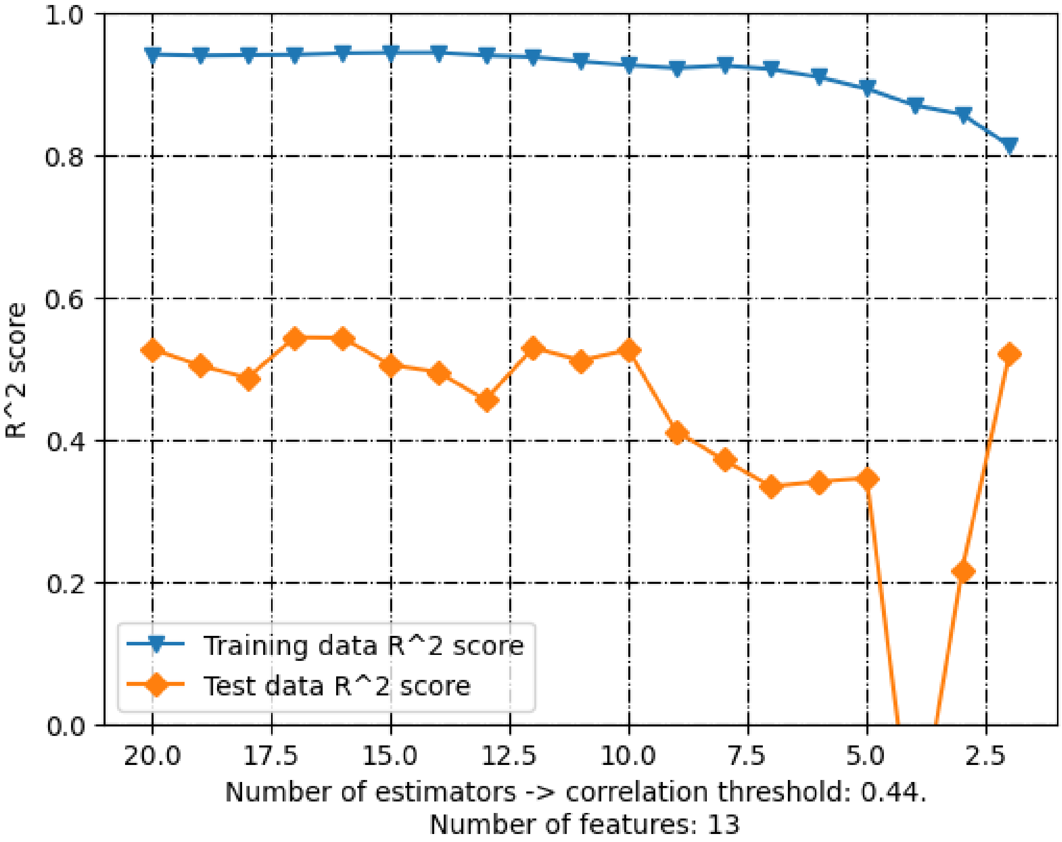

1.0
0.8
0.6
0.4
0.2
0.0
R^2 score
Training data R^2 score
Test data R^2 score
20.0
17.5
15.0
12.5
10.0
7.5
5.0
2.5
Number of estimators -> correlation threshold: 0.44.
Number of features: 13

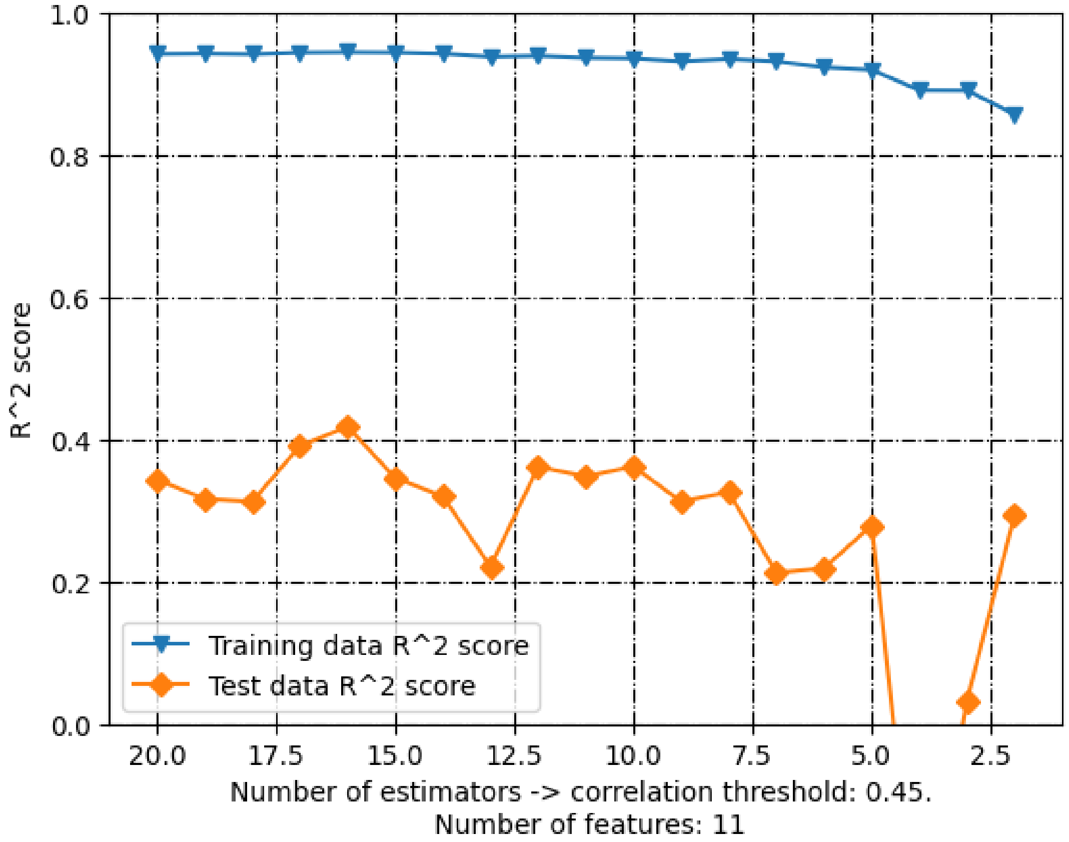

1.0
0.8
0.6
0.4
0.2
0.0
R^2 score
Training data R^2 score
Test data R^2 score
20.0
17.5
15.0
12.5
10.0
7.5
5.0
2.5
Number of estimators -> correlation threshold: 0.45.
Number of features: 11

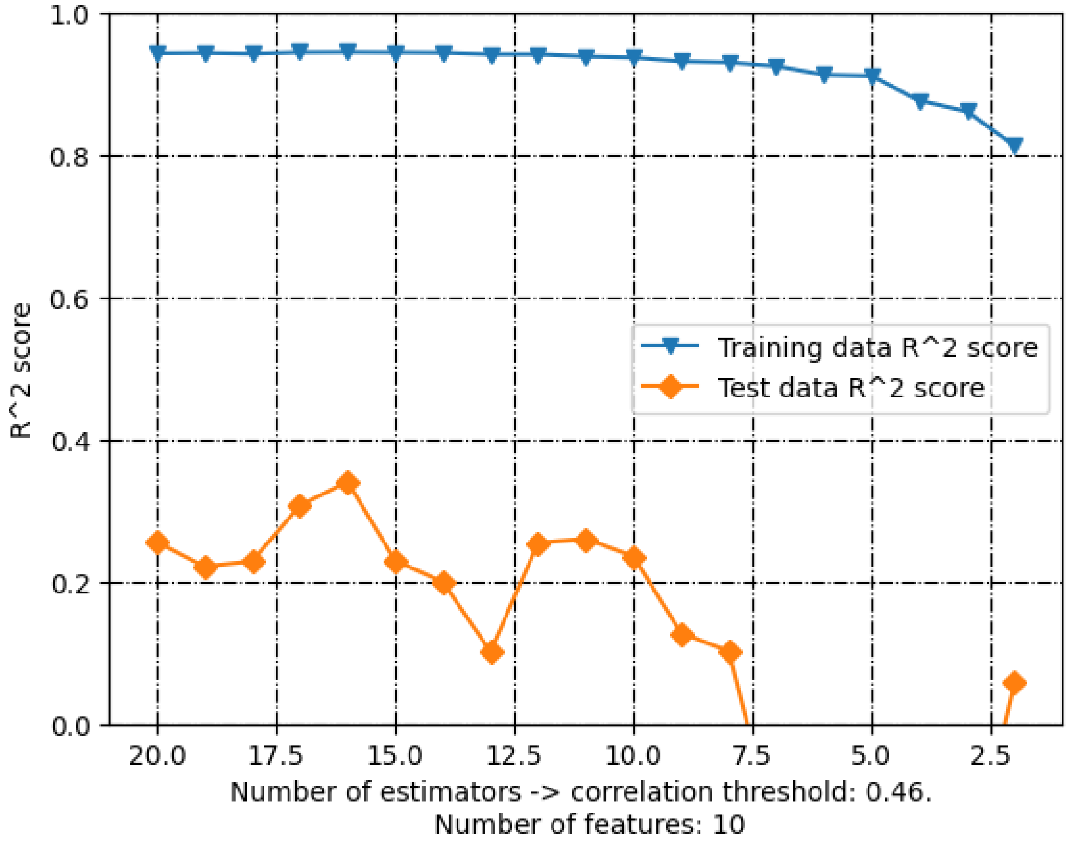

1.0
0.8
0.6
0.4
0.2
0.0
R^2 score
Training data R^2 score
Test data R^2 score
20.0
17.5
15.0
12.5
10.0
7.5
5.0
2.5
Number of estimators -> correlation threshold: 0.46.
Number of features: 10

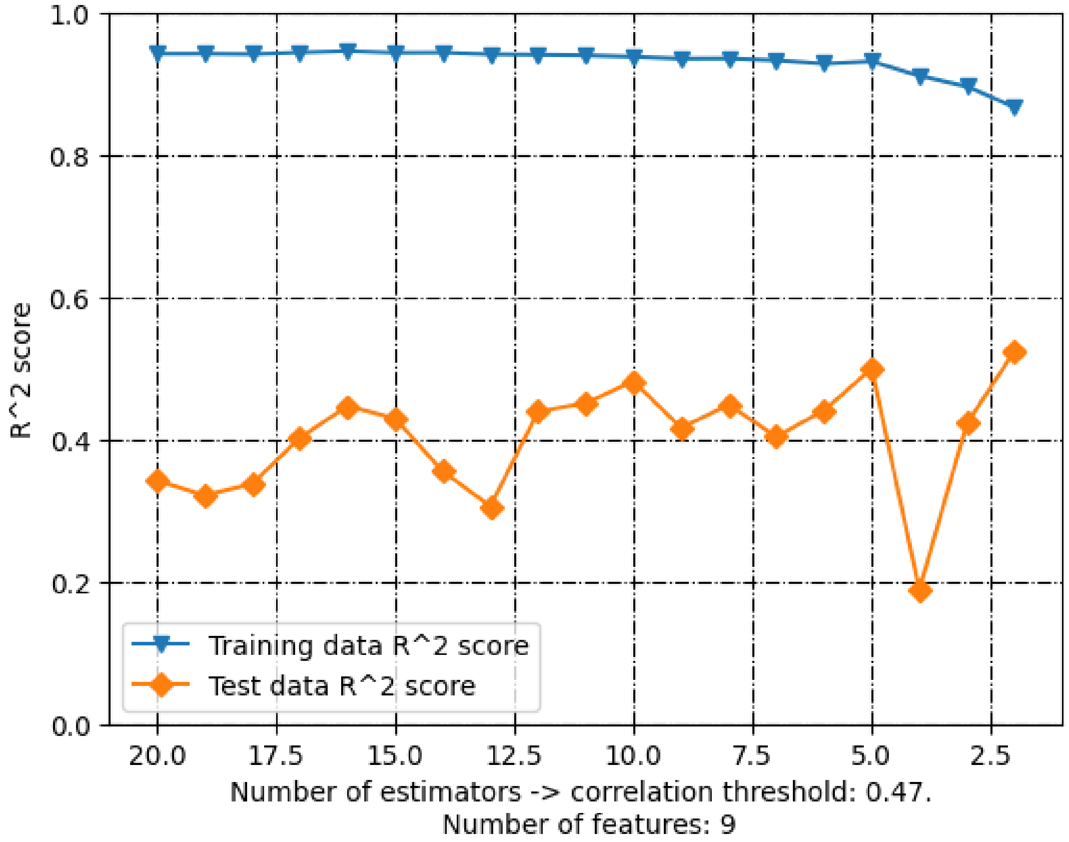
1.0
0.8
0.6
0.4
0.2
0.0
R^2 score
Training data R^2 score
Test data R^2 score
20.0
17.5
15.0
12.5
10.0
7.5
5.0
2.5
Number of estimators -> correlation threshold: 0.47.
Number of features: 9

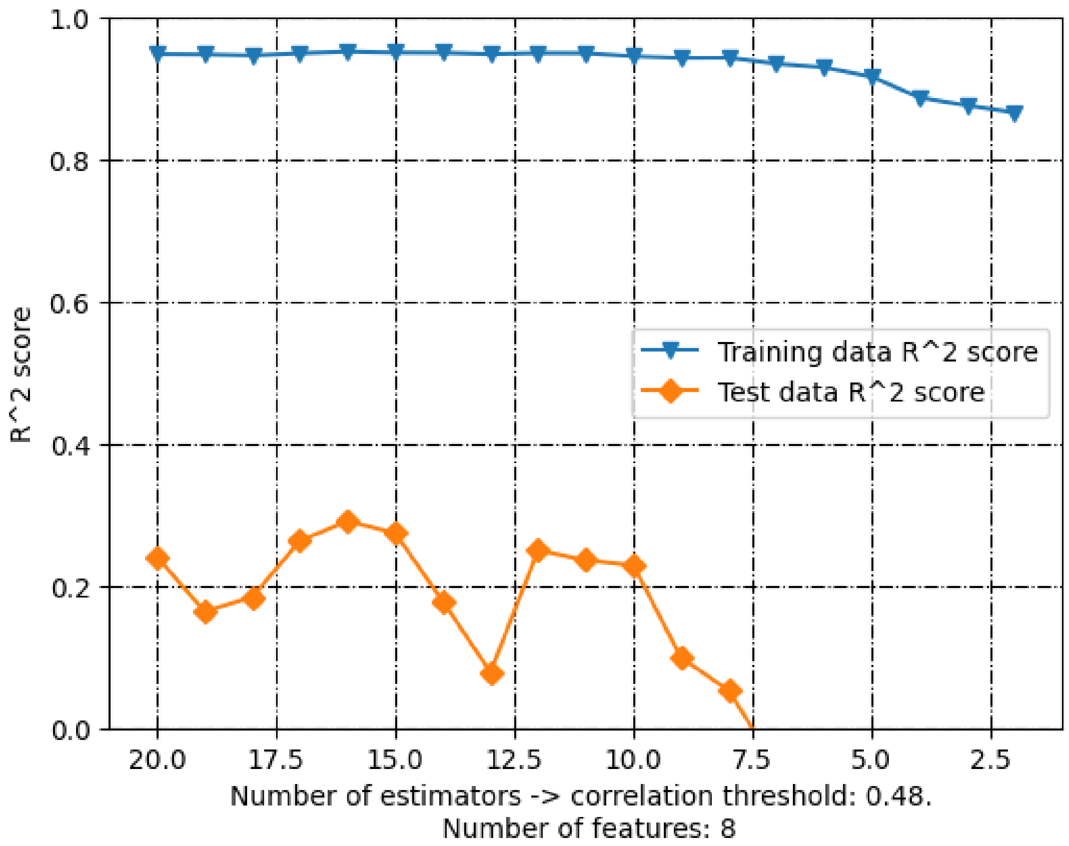
1.0
0.8
0.6
0.4
0.2
0.0
R^2 score
Training data R^2 score
Test data R^2 score
20.0
17.5
15.0
12.5
10.0
7.5
5.0
2.5
Number of estimators -> correlation threshold: 0.48.
Number of features: 8

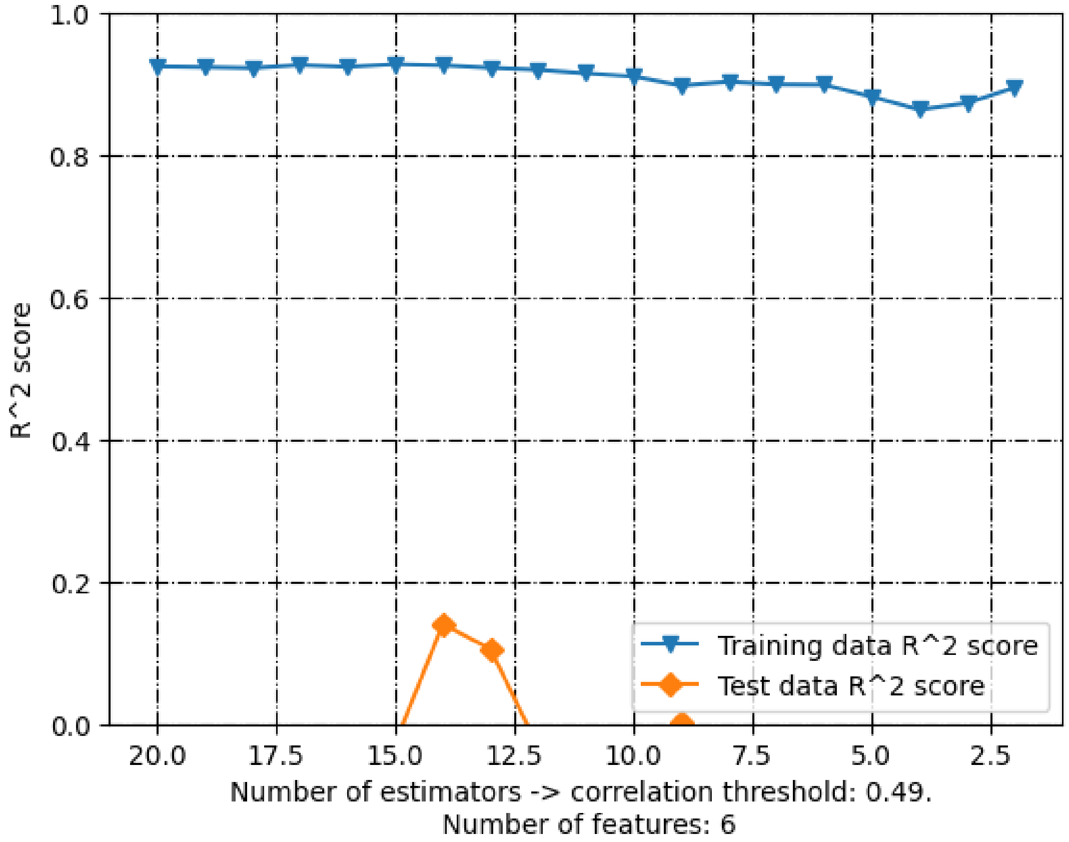
1.0
0.8
0.6
0.4
0.2
0.0
R^2 score
20.0
17.5
15.0
12.5
10.0
7.5
5.0
2.5
Number of estimators -> correlation threshold: 0.49.
Number of features: 6
Training data R^2 score
Test data R^2 score

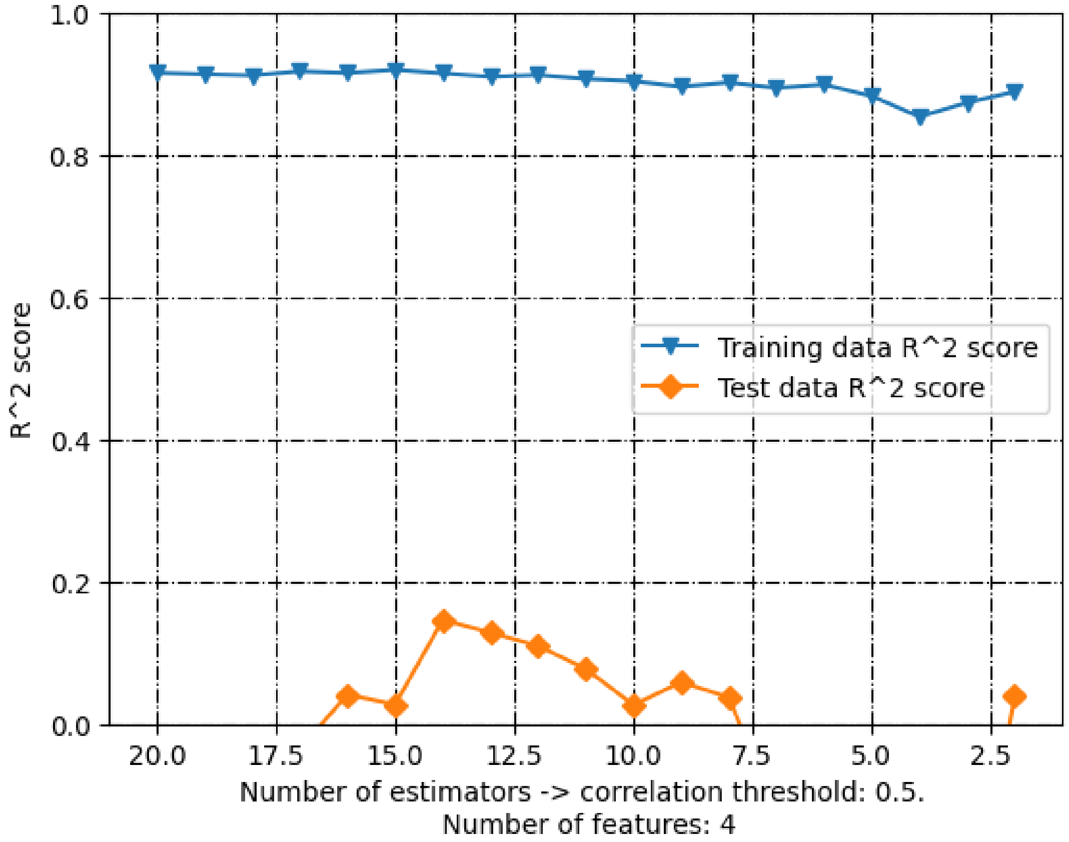

1.0
0.8
0.6
0.4
0.2
0.0
R^2 score
Training data R^2 score
Test data R^2 score
20.0
17.5
15.0
12.5
10.0
7.5
5.0
2.5
Number of estimators -> correlation threshold: 0.5.
Number of features: 4

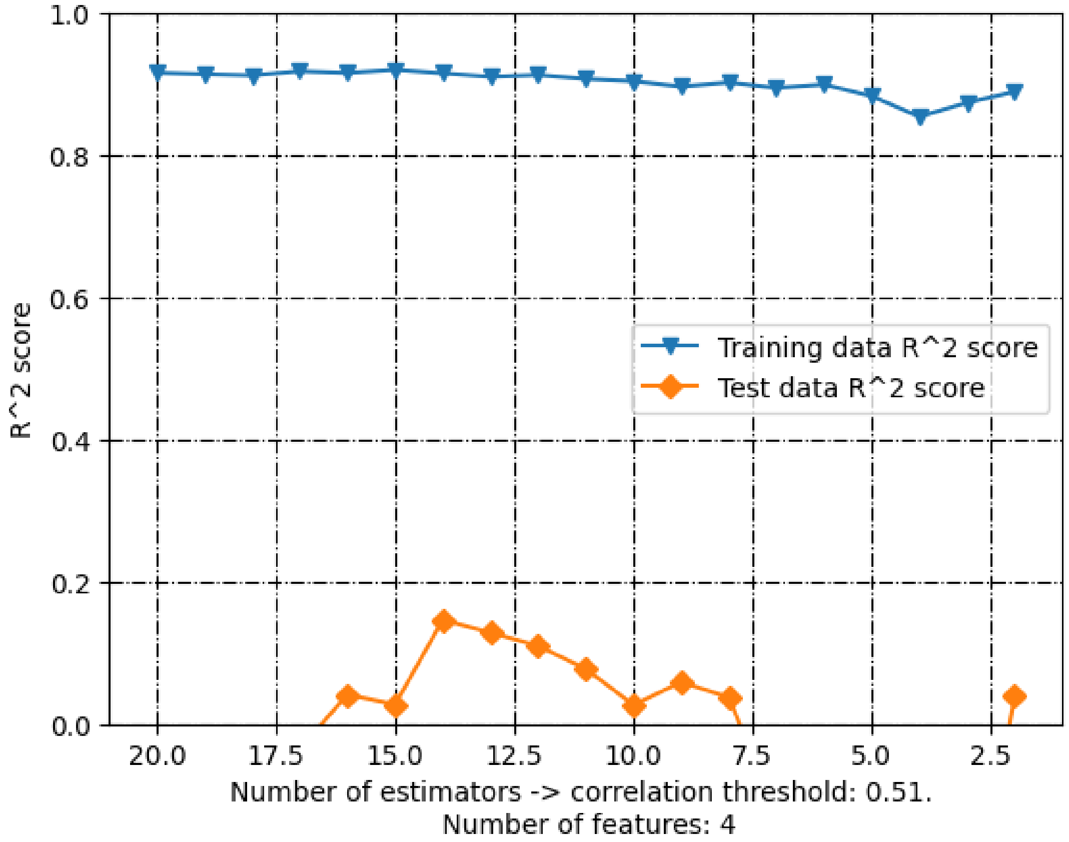

[28]:
```
plt.plot(df_without_standardization['Correlation threshold'],␣
  ↪df_without_standardization['Number of features'], label = "Number of␣
  ↪features", marker='.')
plt.legend()
plt.xlabel('Correlation threshold')
plt.ylabel('Number of features')
plt.show()
```

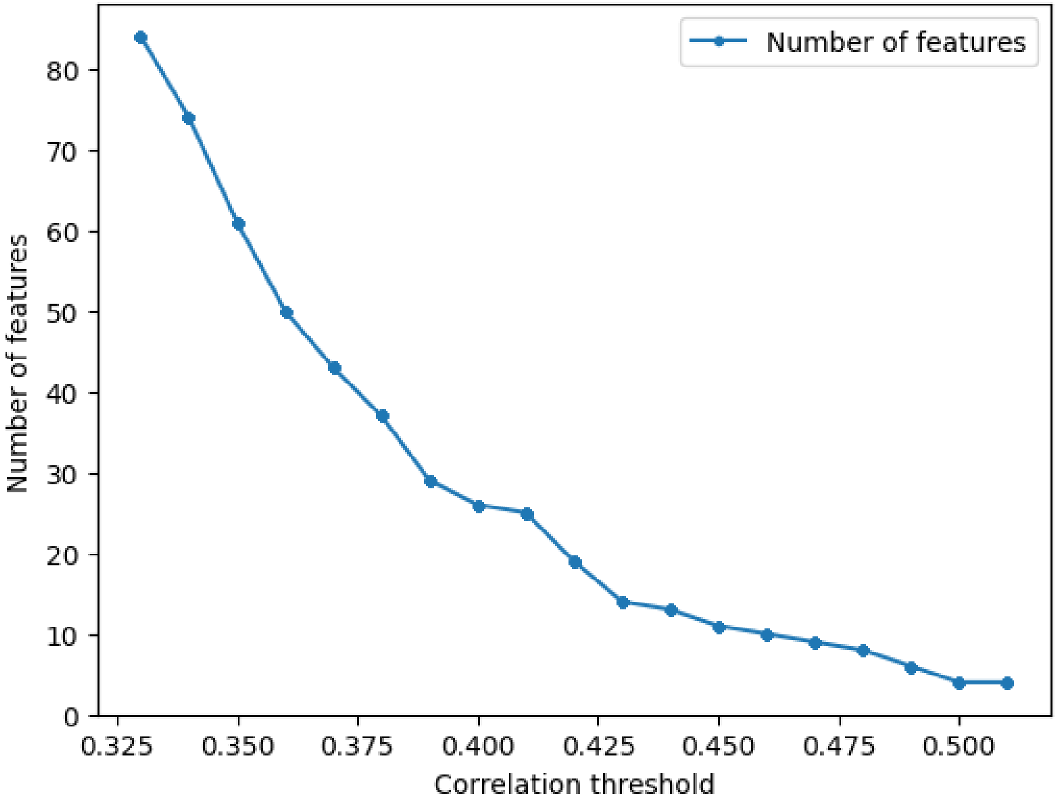

[ ]:

```

```

4 KNeighborsRegressor

[29]:

```
without_standardization, train_r2_, test_r2_, hist1, hist2, target_column_name,␣
↪training_data_RMSE, test_data_RMSE  = pred_model.
↪prepare_data_and_create_model(molecular_descriptors_df=data,
                                                                                         ␣
↪                     correlation_threshold=0.50,
                                                                                         ␣
↪                     standardization=False,
                                                                                         ␣
↪                     model_type='KNeighborsRegressor',
                                                                                         ␣
↪                     target_column_name = target,
                                                                                         ␣
↪                     random_state=random_state,
                                                                                         ␣
↪                     train_test_split_=True,
```

```
↪                    verbose=True)
```

```
I am not doing standardization…
  molecular descriptor name
0                    AATS0Z
1                  AATS0are
2                    AATS0d
3                   AATS0dv
4                    AATS0i
  molecular descriptor name  corr_value
0                    AATS0Z   -0.038502
1                  AATS0are   -0.120847
2                    AATS0d    0.041648
3                   AATS0dv   -0.115899
4                    AATS0i    0.191765
  molecular descriptor name  corr_value  absolute correlation value
0                    AATS0Z   -0.038502                    0.038502
1                  AATS0are   -0.120847                    0.120847
2                    AATS0d    0.041648                    0.041648
3                   AATS0dv   -0.115899                    0.115899
4                    AATS0i    0.191765                    0.191765
     molecular descriptor name  corr_value  absolute correlation value
226                     AMID_O   -0.520578                    0.520578
505                 EState_VSA5   -0.578904                    0.578904
506                 EState_VSA6    0.519794                    0.519794
791                    MDEO-12   -0.525441                    0.525441
1211                     MCF-7    1.000000                    1.000000
    molecular descriptor name  corr_value  absolute correlation value
226                    AMID_O   -0.520578                    0.520578
505                EState_VSA5   -0.578904                    0.578904
506                EState_VSA6    0.519794                    0.519794
791                   MDEO-12   -0.525441                    0.525441
The model used is: KNeighbors…
Return the coefficient of determination of the prediction:
0.17887598286330386
R^2 score: 0.5373267326789486
Correlation coefficient: 0.7330257380740111
Test data - unseen during training:
R^2 score: 0.17887598286330386
Correlation coefficient: 0.4229373273468586
[8.27886876 6.39366104 7.6963417  6.53369885 7.95214323 7.55661724
 7.94489513 8.26275858 6.18458136 7.8150608  7.57914848 8.14325294
 7.83929455 8.13033937 7.35856211 7.57972486 7.58116176 6.79653745]
113    8.022276
35     6.066513
101    6.920819
36     6.108463
```

```
100    7.376751
13     7.987163
0      7.987163
114    7.823909
104    8.397940
96     7.920819
40     8.091515
103    8.376751
48     7.886057
39     8.455932
14     8.086186
117    5.920819
21     8.113509
9      6.143150
Name: MCF-7, dtype: float64
Training Root Mean Square Error: 0.659077344888441
Testing Root Mean Square Error: 0.7814887183280818
```

[30]:
```
print(target_column_name+str('_transformed'))
print(hist1[target_column_name].hist())
```

```
MCF-7_transformed
AxesSubplot(0.125,0.11;0.775x0.77)
```

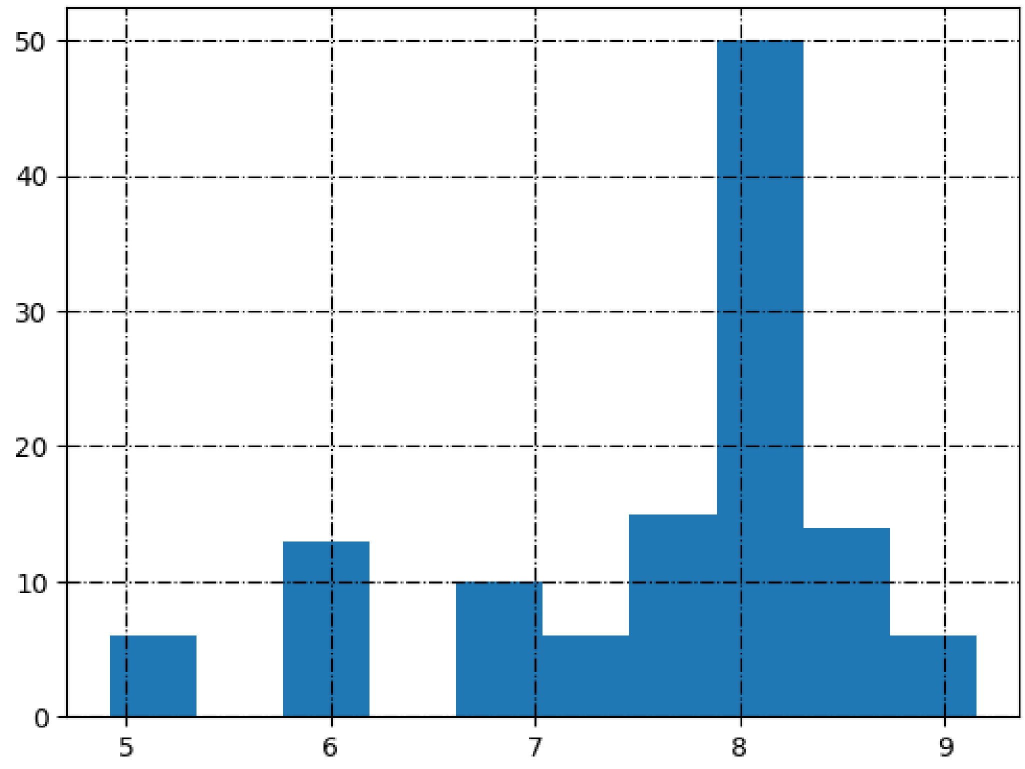

[31]:
```
with_standardization, train_r2_, test_r2_, hist1, hist2, target_column_name,␣
↪training_data_RMSE, test_data_RMSE  = pred_model.
↪prepare_data_and_create_model(molecular_descriptors_df=data,
                                                                              ␣
↪                    correlation_threshold=0.50,
                                                                              ␣
↪                    standardization=True,
                                                                              ␣
↪                    model_type='KNeighborsRegressor',
                                                                              ␣
↪                    target_column_name = target,
                                                                              ␣
↪                    random_state=random_state,
                                                                              ␣
↪                    train_test_split_=True,
                                                                              ␣
↪                    verbose=True)
```

```
I am doing standardization…
  molecular descriptor name
0                   AATS0Z
1                 AATS0are
2                   AATS0d
3                  AATS0dv
4                   AATS0i
  molecular descriptor name  corr_value
0                   AATS0Z   -0.038502
1                 AATS0are   -0.120847
2                   AATS0d    0.041648
3                  AATS0dv   -0.115899
4                   AATS0i    0.191765
  molecular descriptor name  corr_value  absolute correlation value
0                   AATS0Z   -0.038502                    0.038502
1                 AATS0are   -0.120847                    0.120847
2                   AATS0d    0.041648                    0.041648
3                  AATS0dv   -0.115899                    0.115899
4                   AATS0i    0.191765                    0.191765
     molecular descriptor name  corr_value  absolute correlation value
226                     AMID_O   -0.520578                    0.520578
505                EState_VSA5   -0.578904                    0.578904
506                EState_VSA6    0.519794                    0.519794
791                    MDEO-12   -0.525441                    0.525441
1211                     MCF-7    1.000000                    1.000000
    molecular descriptor name  corr_value  absolute correlation value
226                    AMID_O   -0.520578                    0.520578
505               EState_VSA5   -0.578904                    0.578904
506               EState_VSA6    0.519794                    0.519794
```

```
791                   MDEO-12   -0.525441                   0.525441
The model used is: KNeighbors…
Return the coefficient of determination of the prediction:
0.17887598286330386
R^2 score: 0.5373267326789486
Correlation coefficient: 0.7330257380740111
Test data - unseen during training:
R^2 score: 0.17887598286330386
Correlation coefficient: 0.4229373273468586
[8.27886876 6.39366104 7.6963417  6.53369885 7.95214323 7.55661724
 7.94489513 8.26275858 6.18458136 7.8150608  7.57914848 8.14325294
 7.83929455 8.13033937 7.35856211 7.57972486 7.58116176 6.79653745]
113    8.022276
35     6.066513
101    6.920819
36     6.108463
100    7.376751
13     7.987163
0      7.987163
114    7.823909
104    8.397940
96     7.920819
40     8.091515
103    8.376751
48     7.886057
39     8.455932
14     8.086186
117    5.920819
21     8.113509
9      6.143150
Name: MCF-7, dtype: float64
Training Root Mean Square Error: 0.659077344888441
Testing Root Mean Square Error: 0.7814887183280818
```

4.1 Search inside correlation space

[32]:
```
step = 0.01
initial_step = corr_low
last_step = corr_high
first_list = [x / 100.0 for x in range(int(initial_step*100),
 ↪int(last_step*100), int(step*100))]
second_list = []
third_list = []
fourth_l = []
fifth_l = []
f_list = []
for i in first_list:
```

```
    without_standardization, train_r2, test_r2, _, h_, target_column_name,␣
↪training_data_RMSE, test_data_RMSE = pred_model.
↪prepare_data_and_create_model(molecular_descriptors_df = data,
                                                                              ␣
↪                    correlation_threshold = i,
                                                                              ␣
↪                    standardization = False,
                                                                              ␣
↪                    model_type = 'KNeighborsRegressor',
                                                                              ␣
↪                    target_column_name = target,
                                                                              ␣
↪                    random_state=random_state,
                                                                              ␣
↪                    train_test_split_ = True,
                                                                              ␣
↪                    verbose = False)
    second_list.append(train_r2)
    third_list.append(test_r2)
    fourth_l.append(training_data_RMSE)
    fifth_l.append(test_data_RMSE)
    f_list.append(len(h_))
```

```
  File "C:\Users\aleks\anaconda3\envs\cheminf_gpu\lib\site-
packages\joblib\externals\loky\backend\context.py", line 217, in
_count_physical_cores
    raise ValueError(
```

[33]:
```
df_without_standardization = pd.DataFrame(data=first_list,␣
↪columns=["Correlation threshold"])
df_without_standardization['Training data R^2 score'] = second_list
df_without_standardization['Test data R^2 score'] = third_list
df_without_standardization['Training RMSE'] = fourth_l
df_without_standardization['Test RMSE'] = fifth_l
df_without_standardization['Number of features'] = f_list
```

[34]:
```
df_k_nearest = df_without_standardization.copy()
df_without_standardization
```

[34]:

	Correlation threshold	Training data R^2 score	Test data R^2 score	\
0	0.33	0.608638	-0.586847	
1	0.34	0.609763	-0.586847	
2	0.35	0.617607	-0.786457	
3	0.36	0.636317	-0.782164	
4	0.37	0.629161	-0.781532	
5	0.38	0.720982	0.252254	
6	0.39	0.715501	0.252254	

7	0.40	0.710436	0.252254
8	0.41	0.698984	0.253543
9	0.42	0.617632	-0.270316
10	0.43	0.568566	-0.753812
11	0.44	0.568547	-0.753812
12	0.45	0.565701	-0.753812
13	0.46	0.569946	-0.715307
14	0.47	0.569946	-0.715307
15	0.48	0.569946	-0.713488
16	0.49	0.554510	-0.565701
17	0.50	0.537327	-0.557452
18	0.51	0.537327	-0.557452

	Training RMSE	Test RMSE	Number of features
0	0.606162	0.855432	84
1	0.605289	0.855432	74
2	0.599175	0.859838	61
3	0.584333	0.860664	50
4	0.590055	0.860929	43
5	0.511818	0.703466	37
6	0.516821	0.703466	29
7	0.521400	0.703466	26
8	0.531611	0.709066	25
9	0.599156	0.813708	19
10	0.636438	0.831269	14
11	0.636453	0.831269	13
12	0.638548	0.831269	11
13	0.635420	0.812343	10
14	0.635420	0.812343	9
15	0.635420	0.812281	8
16	0.646723	0.780072	6
17	0.659077	0.781489	4
18	0.659077	0.781489	4

4.2 Plots

[35]:

```
plt.plot(df_without_standardization['Correlation threshold'],␣
 ↪df_without_standardization['Training data R^2 score'], label = "Training␣
 ↪data R^2 score", marker='v')
plt.plot(df_without_standardization['Correlation threshold'],␣
 ↪df_without_standardization['Test data R^2 score'], label = "Test data R^2␣
 ↪score", marker='D')
plt.legend()
plt.xlabel('Correlation threshold')
plt.ylabel('R^2 score')
plt.ylim([0, 1])
plt.show()
```

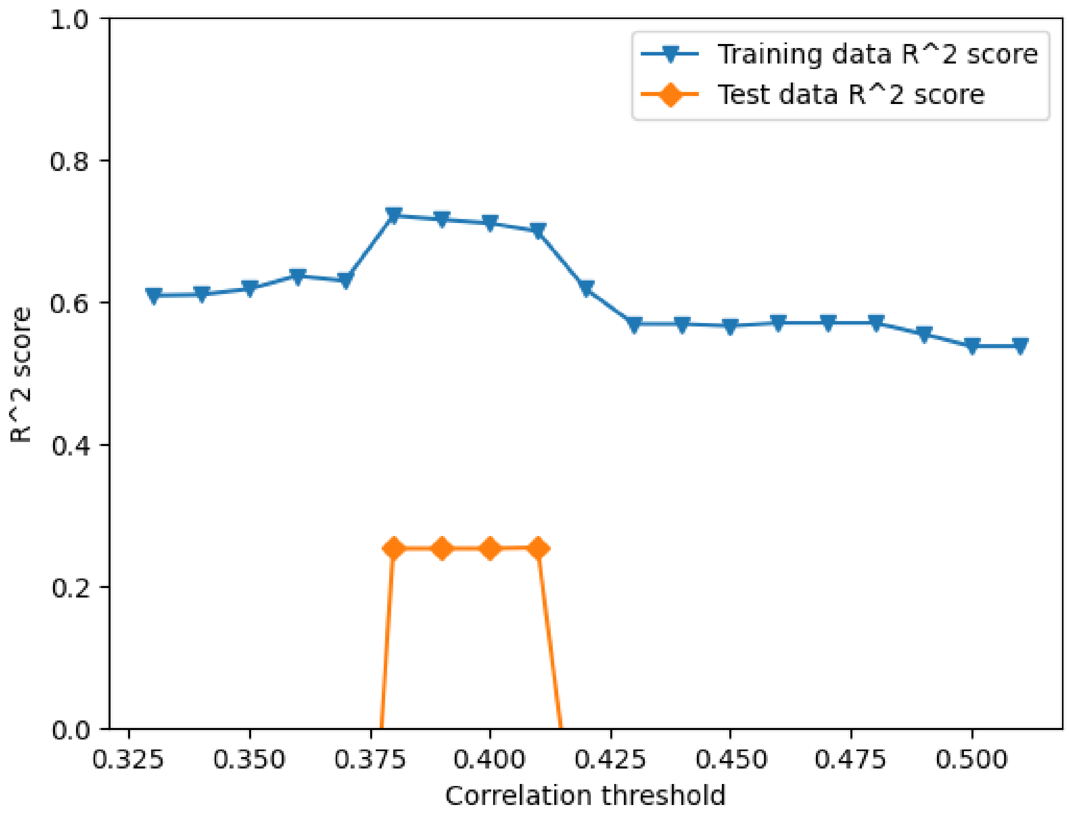

[36]:
```
plt.plot(df_without_standardization['Correlation threshold'],␣
  ↪df_without_standardization['Number of features'], label = "Number of␣
  ↪features", marker='.')
plt.legend()
plt.xlabel('Correlation threshold')
plt.ylabel('Number of features')
plt.show()
```

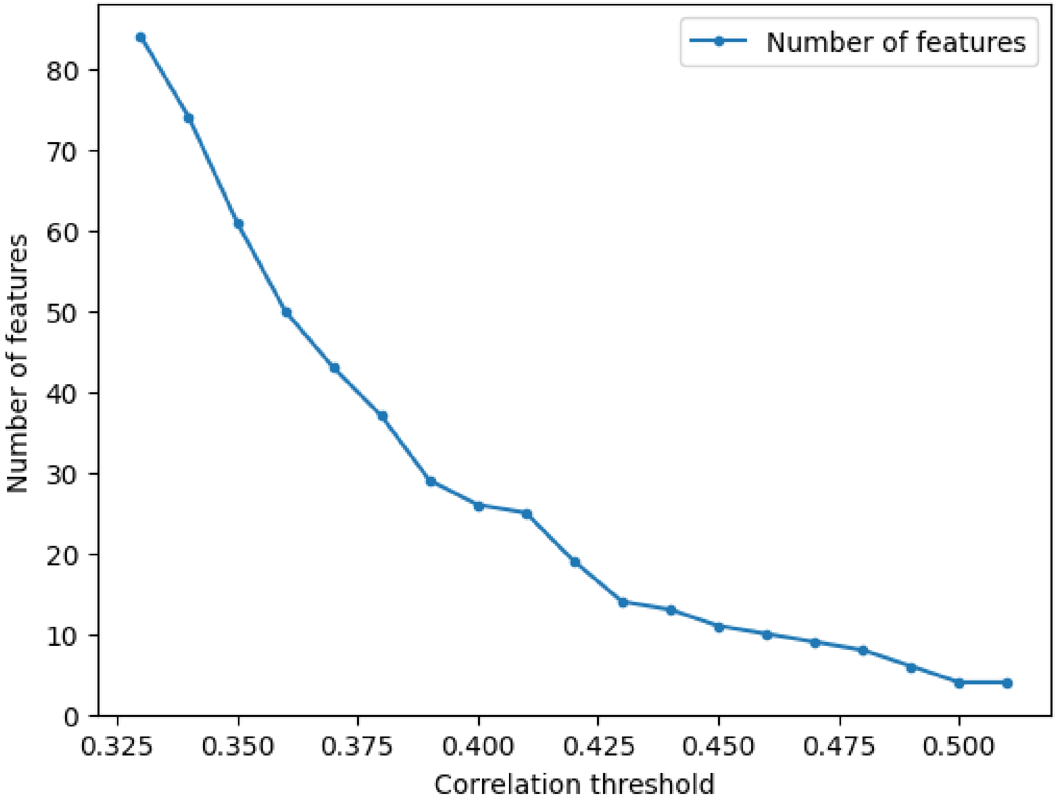


```
[ ]:
```

5 Support Vector Machines (SVM)

```
[37]: without_standardization, train_r2_, test_r2_, hist1, hist2, target_column_name,␣
      ↪training_data_RMSE, test_data_RMSE  = pred_model.
      ↪prepare_data_and_create_model(molecular_descriptors_df=data,
                                                                                 ␣
      ↪                        correlation_threshold=0.50,
                                                                                 ␣
      ↪                        standardization=False,
                                                                                 ␣
      ↪                        model_type='SVR',
                                                                                 ␣
      ↪                        kernel_ = 'linear',
                                                                                 ␣
      ↪                        gamma_ = 'auto',
                                                                                 ␣
      ↪                        target_column_name = target,
```

```
↪                    random_state=random_state,
↪                    train_test_split_=True,
↪                    verbose=True)
```

```
I am not doing standardization…
  molecular descriptor name
0                    AATS0Z
1                  AATS0are
2                    AATS0d
3                   AATS0dv
4                    AATS0i
  molecular descriptor name  corr_value
0                    AATS0Z   -0.038502
1                  AATS0are   -0.120847
2                    AATS0d    0.041648
3                   AATS0dv   -0.115899
4                    AATS0i    0.191765
  molecular descriptor name  corr_value  absolute correlation value
0                    AATS0Z   -0.038502                    0.038502
1                  AATS0are   -0.120847                    0.120847
2                    AATS0d    0.041648                    0.041648
3                   AATS0dv   -0.115899                    0.115899
4                    AATS0i    0.191765                    0.191765
     molecular descriptor name  corr_value  absolute correlation value
226                     AMID_O   -0.520578                    0.520578
505                EState_VSA5   -0.578904                    0.578904
506                EState_VSA6    0.519794                    0.519794
791                    MDEO-12   -0.525441                    0.525441
1211                     MCF-7    1.000000                    1.000000
    molecular descriptor name  corr_value  absolute correlation value
226                    AMID_O   -0.520578                    0.520578
505               EState_VSA5   -0.578904                    0.578904
506               EState_VSA6    0.519794                    0.519794
791                   MDEO-12   -0.525441                    0.525441
The model used is: SVR…
Return the coefficient of determination of the prediction:
0.4743557839935354
R^2 score: 0.5020352037550295
Correlation coefficient: 0.7085444260983425
Test data - unseen during training:
R^2 score: 0.4743557839935354
Correlation coefficient: 0.6887349156195984
[7.96654481 6.82669237 7.83448845 7.03699901 7.89478809 7.40737585
 7.67935617 8.33257718 7.17327682 8.0339569  8.08511125 8.38190303
```

```
 8.04334248 7.80748886 7.5192305  6.8313205  7.68209688 6.85506111]
113    8.022276
35     6.066513
101    6.920819
36     6.108463
100    7.376751
13     7.987163
0      7.987163
114    7.823909
104    8.397940
96     7.920819
40     8.091515
103    8.376751
48     7.886057
39     8.455932
14     8.086186
117    5.920819
21     8.113509
9      6.143150
Name: MCF-7, dtype: float64
Training Root Mean Square Error: 0.6837518281038438
Testing Root Mean Square Error: 0.625265232017053
```

[38]:

```
print(target_column_name+str('_transformed'))
print(hist1[target_column_name].hist())
```

```
MCF-7_transformed
AxesSubplot(0.125,0.11;0.775x0.77)
```

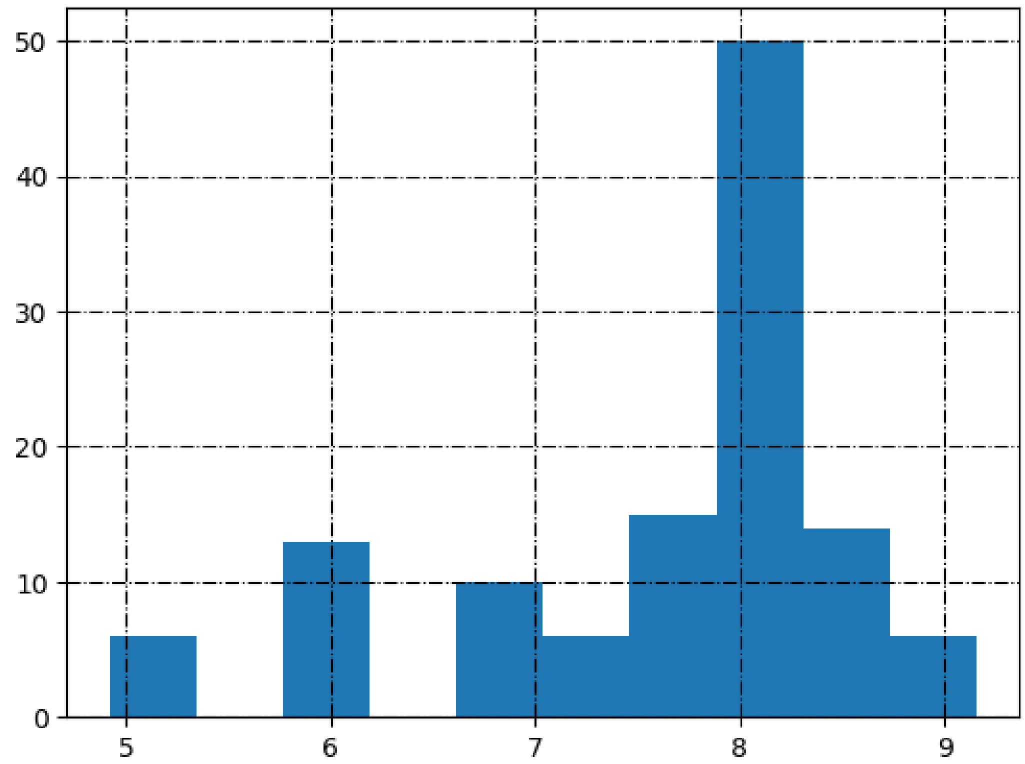

[39]:
```
with_standardization, train_r2_, test_r2_, hist1, hist2, target_column_name,␣
↪training_data_RMSE, test_data_RMSE  = pred_model.
↪prepare_data_and_create_model(molecular_descriptors_df=data,
                                                                              ␣
↪                         correlation_threshold=0.50,
                                                                              ␣
↪                         standardization=True,
                                                                              ␣
↪                         model_type='SVR',
                                                                              ␣
↪                         kernel_ = 'linear',
                                                                              ␣
↪                         gamma_ = 'auto',
                                                                              ␣
↪                         target_column_name = target,
                                                                              ␣
↪                         random_state=random_state,
                                                                              ␣
↪                         train_test_split_=True,
                                                                              ␣
↪                         verbose=True)
```

```
I am doing standardization…
  molecular descriptor name
0                    AATS0Z
1                  AATS0are
2                    AATS0d
3                   AATS0dv
4                    AATS0i
  molecular descriptor name  corr_value
0                    AATS0Z   -0.038502
1                  AATS0are   -0.120847
2                    AATS0d    0.041648
3                   AATS0dv   -0.115899
4                    AATS0i    0.191765
  molecular descriptor name  corr_value  absolute correlation value
0                    AATS0Z   -0.038502                    0.038502
1                  AATS0are   -0.120847                    0.120847
2                    AATS0d    0.041648                    0.041648
3                   AATS0dv   -0.115899                    0.115899
4                    AATS0i    0.191765                    0.191765
     molecular descriptor name  corr_value  absolute correlation value
226                     AMID_O   -0.520578                    0.520578
505                EState_VSA5   -0.578904                    0.578904
506                EState_VSA6    0.519794                    0.519794
791                    MDEO-12   -0.525441                    0.525441
1211                     MCF-7    1.000000                    1.000000
    molecular descriptor name  corr_value  absolute correlation value
226                    AMID_O   -0.520578                    0.520578
505               EState_VSA5   -0.578904                    0.578904
506               EState_VSA6    0.519794                    0.519794
791                   MDEO-12   -0.525441                    0.525441
The model used is: SVR…
Return the coefficient of determination of the prediction:
0.4743557839935354
R^2 score: 0.5020352037550295
Correlation coefficient: 0.7085444260983425
Test data - unseen during training:
R^2 score: 0.4743557839935354
Correlation coefficient: 0.6887349156195984
[7.96654481 6.82669237 7.83448445 7.03699901 7.89478809 7.40737585
 7.67935617 8.33257718 7.17327682 8.0339569  8.08511125 8.38190303
 8.04334248 7.80748886 7.5192305  6.8313205  7.68209688 6.85506111]
113    8.022276
35     6.066513
101    6.920819
36     6.108463
100    7.376751
13     7.987163
0      7.987163
```

```
114    7.823909
104    8.397940
96     7.920819
40     8.091515
103    8.376751
48     7.886057
39     8.455932
14     8.086186
117    5.920819
21     8.113509
9      6.143150
Name: MCF-7, dtype: float64
Training Root Mean Square Error: 0.6837518281038438
Testing Root Mean Square Error: 0.625265232017053
```

5.1 Search inside correlation space

[40]:
```
step = 0.01
initial_step = corr_low
last_step = corr_high
first_list = [x / 100.0 for x in range(int(initial_step*100),␣
↪int(last_step*100), int(step*100))]
second_list = []
third_list = []
fourth_l = []
fifth_l = []
f_list = []
for i in first_list:
    without_standardization, train_r2, test_r2, _, h_, target_column_name,␣
↪training_data_RMSE, test_data_RMSE = pred_model.
↪prepare_data_and_create_model(molecular_descriptors_df = data,
                                                                        ␣
↪                      correlation_threshold = i,
                                                                        ␣
↪                      standardization = False,
                                                                        ␣
↪                      model_type = 'SVR',
                                                                        ␣
↪                      kernel_ = 'linear',
                                                                        ␣
↪                      gamma_ = 'auto',
                                                                        ␣
↪                      target_column_name = target,
                                                                        ␣
↪                      random_state=random_state,
                                                                        ␣
↪                      train_test_split_ = True,
```

```
                                                                            ␣
↪                     verbose = False)
    second_list.append(train_r2)
    third_list.append(test_r2)
    fourth_l.append(training_data_RMSE)
    fifth_l.append(test_data_RMSE)
    f_list.append(len(h_))
```

[41]:
```
df_without_standardization = pd.DataFrame(data=first_list,␣
 ↪columns=["Correlation threshold"])
df_without_standardization['Training data R^2 score'] = second_list
df_without_standardization['Test data R^2 score'] = third_list
df_without_standardization['Training RMSE'] = fourth_l
df_without_standardization['Test RMSE'] = fifth_l
df_without_standardization['Number of features'] = f_list
```

[42]:
```
df_svm = df_without_standardization.copy()
df_without_standardization
```

[42]:
```
    Correlation threshold  Training data R^2 score  Test data R^2 score  \
0                    0.33                 0.784206            -0.273797
1                    0.34                 0.784504            -0.307622
2                    0.35                 0.761037            -0.433487
3                    0.36                 0.670546            -1.413869
4                    0.37                 0.668193            -1.200970
5                    0.38                 0.647471            -0.489896
6                    0.39                 0.629387            -0.710704
7                    0.40                 0.632083            -0.934216
8                    0.41                 0.614130            -0.474560
9                    0.42                 0.594259            -0.593504
10                   0.43                 0.580193            -1.003881
11                   0.44                 0.579996            -1.009983
12                   0.45                 0.580285            -0.976854
13                   0.46                 0.540404            -0.970051
14                   0.47                 0.540529            -0.979198
15                   0.48                 0.528432            -0.281578
16                   0.49                 0.525676            -0.251255
17                   0.50                 0.502035            -0.611223
18                   0.51                 0.502035            -0.611223

    Training RMSE  Test RMSE  Number of features
0        0.450111   0.856825                  84
1        0.449799   0.857894                  74
2        0.473658   0.935439                  61
3        0.556156   0.879485                  50
4        0.558139   0.848670                  43
5        0.575303   0.757383                  37
```

6	0.589874	0.776040	29
7	0.587725	0.806620	26
8	0.601893	0.739541	25
9	0.617197	0.707240	19
10	0.627804	0.745903	14
11	0.627951	0.746802	13
12	0.627735	0.739018	11
13	0.656882	0.679106	10
14	0.656793	0.678748	9
15	0.665383	0.591511	8
16	0.667324	0.595829	6
17	0.683752	0.625265	4
18	0.683752	0.625265	4

5.2 Plots

[43]:
```
plt.plot(df_without_standardization['Correlation threshold'],␣
  ↪df_without_standardization['Training data R^2 score'], label = "Training␣
  ↪data R^2 score", marker='v')
plt.plot(df_without_standardization['Correlation threshold'],␣
  ↪df_without_standardization['Test data R^2 score'], label = "Test data R^2␣
  ↪score", marker='D')
plt.legend()
plt.xlabel('Correlation threshold')
plt.ylabel('R^2 score')
plt.ylim([0, 1])
plt.show()
```

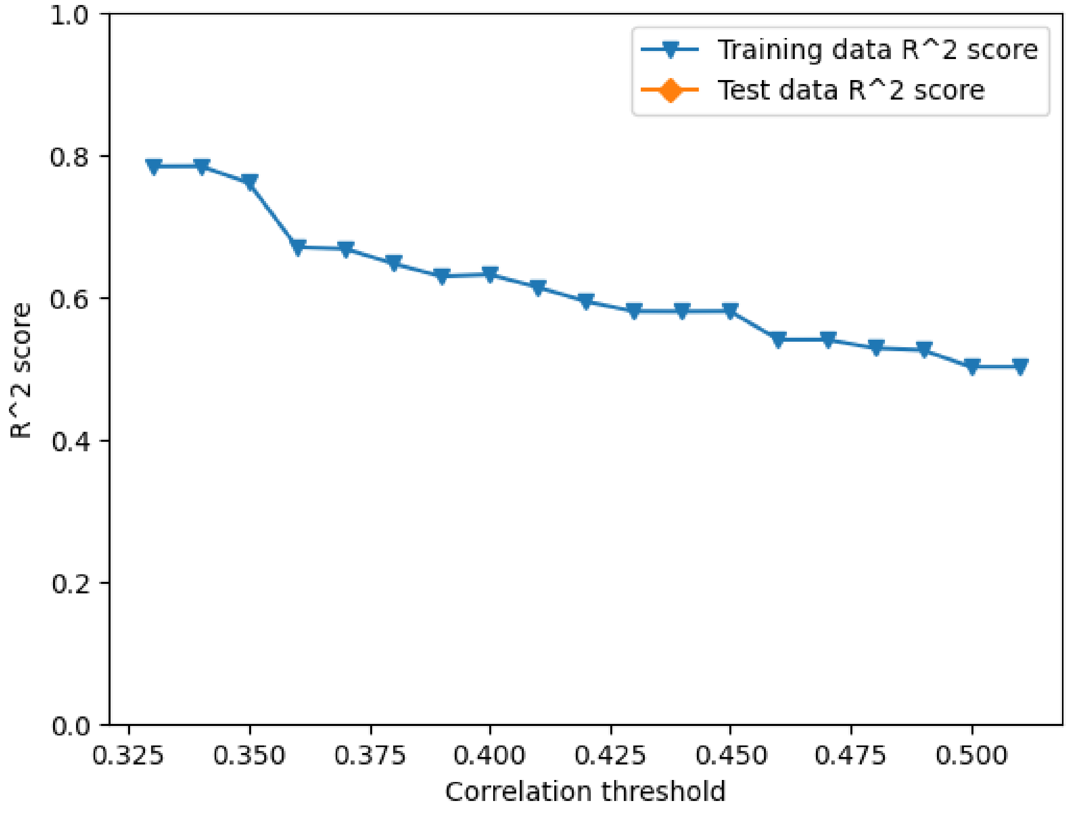

[44]:
```
plt.plot(df_without_standardization['Correlation threshold'],␣
  ↪df_without_standardization['Number of features'], label = "Number of␣
  ↪features", marker='.')
plt.legend()
plt.xlabel('Correlation threshold')
plt.ylabel('Number of features')
plt.show()
```

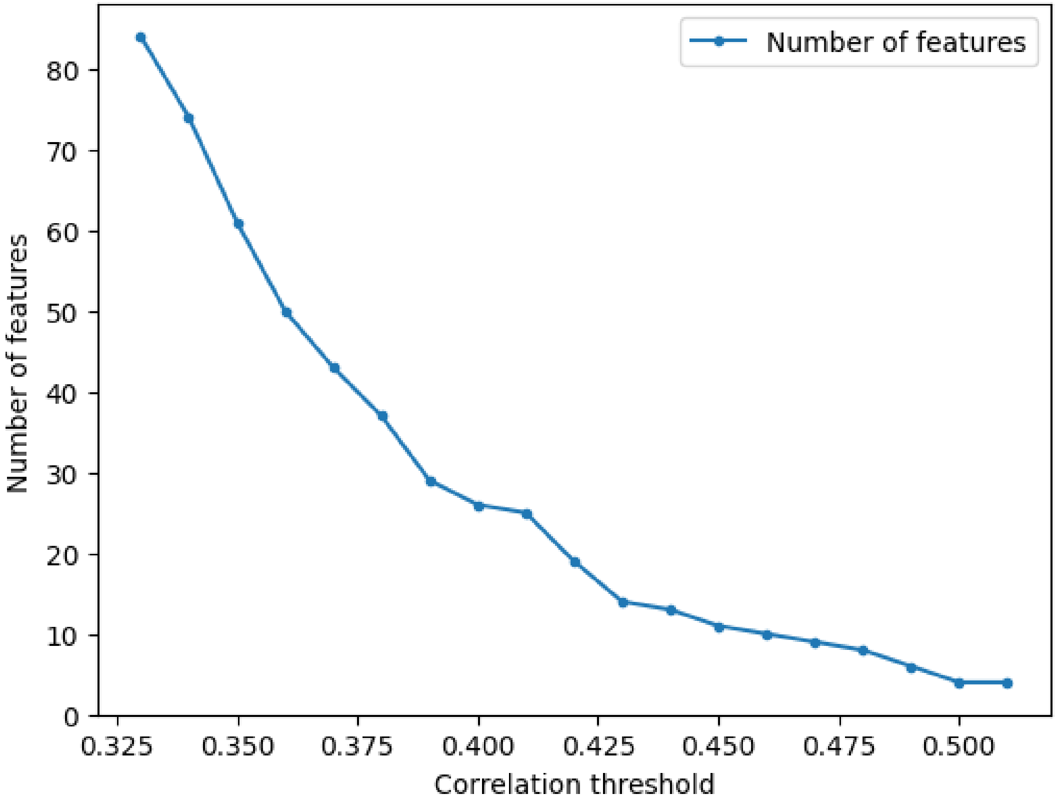

5.3 Store quality measurements

[45]:
```
with pd.ExcelWriter('../Data/Quality_'+str(target)+'_'+str(random_state)+'_.
 ↪xlsx') as writer:
    df_linear.to_excel(writer, sheet_name='MLR')
    df_decision_tree.to_excel(writer, sheet_name='DT')
    df_random_forest.to_excel(writer, sheet_name='RF')
    df_k_nearest.to_excel(writer, sheet_name='KNN')
    df_svm.to_excel(writer, sheet_name='SVM')
```

Notebook

January 18, 2024

1 File 29

[1]:
```
import sys
from pathlib import Path
prediction_mode_path = Path("../module")
sys.path.append(prediction_mode_path.as_posix())
import models_creation as pred_model

import pandas as pd
import numpy as np

from sklearn.metrics import r2_score

import matplotlib.pyplot as plt
from sklearn.tree import plot_tree

from matplotlib import pyplot as plt

import joblib

import warnings
warnings.filterwarnings('ignore')
```

[2]:
```
load_prepared_data = pd.read_excel('../Data/Kolchicyna_machine_learning.xlsx')
load_prepared_data = load_prepared_data.drop(columns=['Unnamed: 0'])

target = 'MCF-7'

corr_low = 0.33
corr_high = 0.525

random_state = 42
```

[3]:
```
load_prepared_data.head()
```

[3]:
```
     AATS0Z  AATS0are    AATS0d   AATS0dv      AATS0i     AATS0m    AATS0p  \
0  25.090909  6.046518  3.109091  6.662626  160.775045  99.075564  1.555512
1  24.448276  6.008422  3.051724  6.386973  161.021674  96.473315  1.538471
```

```
2  23.868852  5.974074  3.000000  6.138434  161.244045  94.127025  1.523105
3  23.868852  5.974074  3.032787  6.171220  161.244045  94.127025  1.523105
4  23.343750  5.942945  2.953125  5.913194  161.445569  92.000700  1.509180

    AATS0pe    AATS0s    AATS0se  …  A549_float  MCF-7_float  LoVo_float  \
0  6.126680  3.435416  7.629859  …        10.8         10.3         6.5
1  6.088791  3.330998  7.596891  …        11.6         12.0         8.5
2  6.054630  3.236850  7.567166  …        10.9         12.2         8.8
3  6.054630  3.257798  7.567166  …        10.5         11.3         8.5
4  6.023670  3.151529  7.540228  …        89.5         92.7        52.8

   LoVo/DX_float  BALB/3T3_float  A549_float_transformed  \
0           54.9            10.2                7.966576
1           31.1            14.3                7.935542
2           17.9            11.7                7.962574
3           10.2            11.0                7.978811
4           77.8            99.4                7.048177

   MCF-7_float_transformed  LoVo_float_transformed  LoVo/DX_float_transformed  \
0                 7.987163                8.187087                   7.260428
1                 7.920819                8.070581                   7.507240
2                 7.913640                8.055517                   7.747147
3                 7.946922                8.070581                   7.991400
4                 7.032920                7.277366                   7.109020

   BALB/3T3_float_transformed
0                    7.991400
1                    7.844664
2                    7.931814
3                    7.958607
4                    7.002614

[5 rows x 1221 columns]
```

[4]:
```python
data = load_prepared_data[list(load_prepared_data.columns)[0:-10]]
data[target] = load_prepared_data[load_prepared_data.columns[-4]]
data.head()
```

[4]:
```
      AATS0Z  AATS0are    AATS0d   AATS0dv      AATS0i     AATS0m    AATS0p  \
0  25.090909  6.046518  3.109091  6.662626  160.775045  99.075564  1.555512
1  24.448276  6.008422  3.051724  6.386973  161.021674  96.473315  1.538471
2  23.868852  5.974074  3.000000  6.138434  161.244045  94.127025  1.523105
3  23.868852  5.974074  3.032787  6.171220  161.244045  94.127025  1.523105
4  23.343750  5.942945  2.953125  5.913194  161.445569  92.000700  1.509180

    AATS0pe    AATS0s    AATS0se  …    piPC10     piPC2     piPC3     piPC4  \
0  6.126680  3.435416  7.629859  …  7.999655  4.266195  4.915408  5.516700
```

```
1  6.088791  3.330998  7.596891  …  8.018726  4.280132  4.922714  5.520711
2  6.054630  3.236850  7.567166  …  8.028799  4.293878  4.929967  5.524706
3  6.054630  3.257798  7.567166  …  8.037440  4.307438  4.929967  5.524706
4  6.023670  3.151529  7.540228  …  8.034892  4.307438  4.937168  5.528685

     piPC5     piPC6     piPC7     piPC8     piPC9     MCF-7
0  6.098566  6.595759  6.979116  7.337313  7.681445  7.987163
1  6.103048  6.601209  6.985613  7.349442  7.695531  7.920819
2  6.105281  6.603923  6.989306  7.353932  7.704023  7.913640
3  6.107510  6.606629  6.992068  7.361425  7.709420  7.946922
4  6.107510  6.605277  6.991148  7.356489  7.707175  7.032920

[5 rows x 1212 columns]
```

2 Multiple Linear regression (MLR)

[5]:
```
without_standardization, train_r2_, test_r2_, hist1, hist2, target_column_name,␣
↪training_data_RMSE, test_data_RMSE  = pred_model.
↪prepare_data_and_create_model(molecular_descriptors_df=data,
                                                                            ␣
↪                    correlation_threshold=0.5,
                                                                            ␣
↪                    standardization=False,
                                                                            ␣
↪                    model_type='linear_model',
                                                                            ␣
↪                    target_column_name = str(target),
                                                                            ␣
↪                    random_state=random_state,
                                                                            ␣
↪                    train_test_split_=True,
                                                                            ␣
↪                    verbose=True)
```

```
I am not doing standardization…
  molecular descriptor name
0                   AATS0Z
1                 AATS0are
2                   AATS0d
3                  AATS0dv
4                   AATS0i
  molecular descriptor name  corr_value
0                   AATS0Z   -0.038502
1                 AATS0are   -0.120847
2                   AATS0d    0.041648
3                  AATS0dv   -0.115899
```

```
4                   AATS0i    0.191765
  molecular descriptor name  corr_value  absolute correlation value
0                   AATS0Z   -0.038502                    0.038502
1                 AATS0are   -0.120847                    0.120847
2                   AATS0d    0.041648                    0.041648
3                  AATS0dv   -0.115899                    0.115899
4                   AATS0i    0.191765                    0.191765
     molecular descriptor name  corr_value  absolute correlation value
226                     AMID_O   -0.520578                    0.520578
505                EState_VSA5   -0.578904                    0.578904
506                EState_VSA6    0.519794                    0.519794
791                    MDEO-12   -0.525441                    0.525441
1211                     MCF-7    1.000000                    1.000000
    molecular descriptor name  corr_value  absolute correlation value
226                     AMID_O   -0.520578                    0.520578
505                EState_VSA5   -0.578904                    0.578904
506                EState_VSA6    0.519794                    0.519794
791                    MDEO-12   -0.525441                    0.525441
The model used is: LinearReg…
Return the coefficient of determination of the prediction:
0.383792023626658
R^2 score: 0.5417842357199687
Correlation coefficient: 0.7360599403037559
Test data - unseen during training:
R^2 score: 0.383792023626658
Correlation coefficient: 0.619509502450655
[7.87972672 8.17008106 7.28223576 7.52208649 5.90557033 8.51256076
 8.81684659 7.51552279 8.05723629 8.23684872 7.4774442  6.85294947
 7.90367018 6.81630238 7.19449679 7.87971906 8.04668352 7.58172269]
44     8.267606
47     8.065502
4      7.032920
55     7.920819
26     4.998569
64     8.619789
73     7.920819
10     7.987163
40     8.091515
107    7.795880
18     7.074688
62     8.958607
11     8.013228
36     6.108463
89     8.045757
91     9.154902
109    8.086186
0      7.987163
Name: MCF-7, dtype: float64
```

```
Training Root Mean Square Error: 0.6431444619819195
Testing Root Mean Square Error: 0.7477130559497913
```

[6]:
```
print(target_column_name+str('_transformed'))
print(hist1[target_column_name].hist())
```

```
MCF-7_transformed
AxesSubplot(0.125,0.11;0.775x0.77)
```

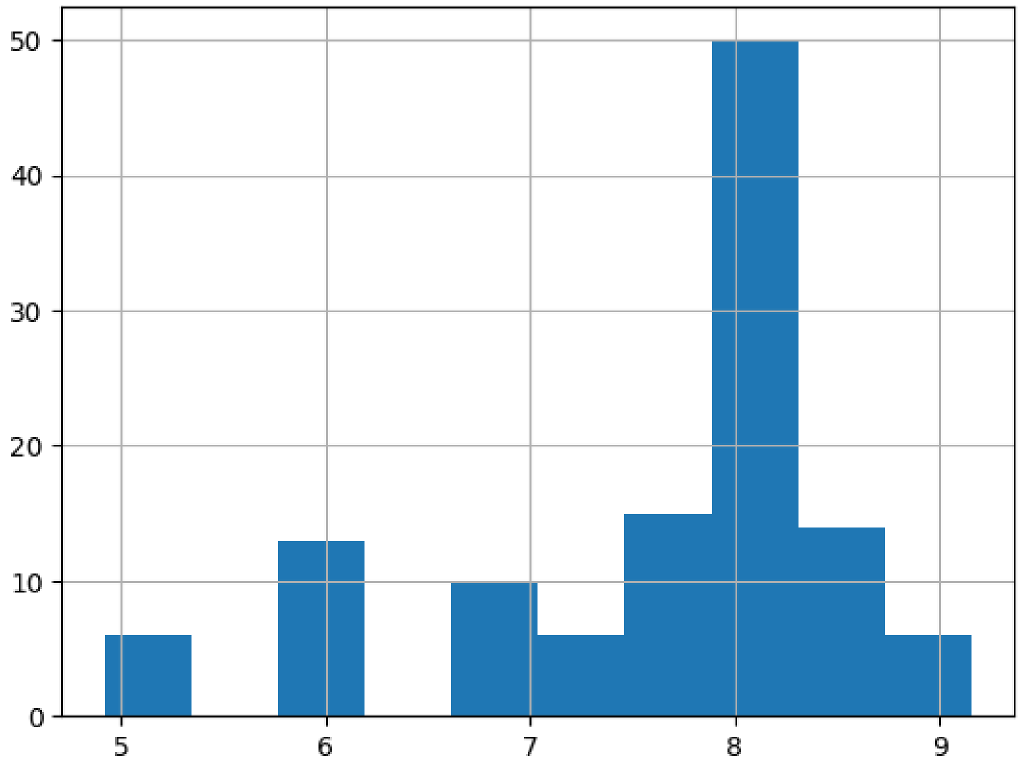

[7]:
```
with_standardization, train_r2_, test_r2_, hist1, hist2, target_column_name,␣
↪training_data_RMSE, test_data_RMSE  = pred_model.
↪prepare_data_and_create_model(molecular_descriptors_df=data,
                                                                          ␣
↪                       correlation_threshold=0.50,
                                                                          ␣
↪                       standardization=True,
                                                                          ␣
↪                       model_type='linear_model',
                                                                          ␣
↪                       target_column_name = target,
                                                                          ␣
↪                       random_state=random_state,
```

```
                     train_test_split_=True,
                     verbose=True)
```

```
I am doing standardization…
  molecular descriptor name
0                    AATS0Z
1                  AATS0are
2                    AATS0d
3                   AATS0dv
4                    AATS0i
  molecular descriptor name  corr_value
0                    AATS0Z   -0.038502
1                  AATS0are   -0.120847
2                    AATS0d    0.041648
3                   AATS0dv   -0.115899
4                    AATS0i    0.191765
  molecular descriptor name  corr_value  absolute correlation value
0                    AATS0Z   -0.038502                    0.038502
1                  AATS0are   -0.120847                    0.120847
2                    AATS0d    0.041648                    0.041648
3                   AATS0dv   -0.115899                    0.115899
4                    AATS0i    0.191765                    0.191765
     molecular descriptor name  corr_value  absolute correlation value
226                     AMID_O   -0.520578                    0.520578
505                EState_VSA5   -0.578904                    0.578904
506                EState_VSA6    0.519794                    0.519794
791                    MDEO-12   -0.525441                    0.525441
1211                     MCF-7    1.000000                    1.000000
    molecular descriptor name  corr_value  absolute correlation value
226                    AMID_O   -0.520578                    0.520578
505               EState_VSA5   -0.578904                    0.578904
506               EState_VSA6    0.519794                    0.519794
791                   MDEO-12   -0.525441                    0.525441
The model used is: LinearReg…
Return the coefficient of determination of the prediction:
0.383792023626658
R^2 score: 0.5417842357199687
Correlation coefficient: 0.7360599403037559
Test data - unseen during training:
R^2 score: 0.383792023626658
Correlation coefficient: 0.619509502450655
[7.87972672 8.17008106 7.28223576 7.52208649 5.90557033 8.51256076
 8.81684659 7.51552279 8.05723629 8.23684872 7.4774442  6.85294947
 7.90367018 6.81630238 7.19449679 7.87971906 8.04668352 7.58172269]
44     8.267606
47     8.065502
```

```
4      7.032920
55     7.920819
26     4.998569
64     8.619789
73     7.920819
10     7.987163
40     8.091515
107    7.795880
18     7.074688
62     8.958607
11     8.013228
36     6.108463
89     8.045757
91     9.154902
109    8.086186
0      7.987163
Name: MCF-7, dtype: float64
Training Root Mean Square Error: 0.6431444619819195
Testing Root Mean Square Error: 0.7477130559497913
```

2.1 Search inside correlation space

[8]:
```
step = 0.01
initial_step = corr_low
last_step = corr_high
first_list = [x / 100.0 for x in range(int(initial_step*100),␣
 ↪int(last_step*100), int(step*100))]
second_list = []
third_list = []
fourth_l = []
fifth_l = []
f_list = []
for i in first_list:
    without_standardization, train_r2, test_r2, _, h_, target_column_name,␣
 ↪training_data_RMSE, test_data_RMSE = pred_model.
 ↪prepare_data_and_create_model(molecular_descriptors_df = data,
                                                                          ␣
 ↪                    correlation_threshold = i,
                                                                          ␣
 ↪                    standardization = False,
                                                                          ␣
 ↪                    model_type = 'linear_model',
                                                                          ␣
 ↪                    target_column_name = target,
                                                                          ␣
 ↪                    random_state=random_state,
```

```
↪                    train_test_split_ = True,
                                                                          ␣
↪                    verbose = False)
    second_list.append(train_r2)
    third_list.append(test_r2)
    fourth_l.append(training_data_RMSE)
    fifth_l.append(test_data_RMSE)
    f_list.append(len(h_))
```

[9]:
```
df_without_standardization = pd.DataFrame(data=first_list,␣
 ↪columns=["Correlation threshold"])
df_without_standardization['Training data R^2 score'] = second_list
df_without_standardization['Test data R^2 score'] = third_list
df_without_standardization['Training RMSE'] = fourth_l
df_without_standardization['Test RMSE'] = fifth_l
df_without_standardization['Number of features'] = f_list
```

[10]:
```
df_linear = df_without_standardization.copy()
df_without_standardization
```

[10]:

	Correlation threshold	Training data R^2 score	Test data R^2 score	\
0	0.33	0.972334	-3.223339	
1	0.34	0.952769	-1.218524	
2	0.35	0.916550	-2.014334	
3	0.36	0.872997	0.302088	
4	0.37	0.798351	0.699391	
5	0.38	0.781694	0.659297	
6	0.39	0.720369	0.377002	
7	0.40	0.709253	0.036152	
8	0.41	0.707612	0.102063	
9	0.42	0.663994	0.440694	
10	0.43	0.593379	0.374782	
11	0.44	0.593227	0.385626	
12	0.45	0.583763	0.449877	
13	0.46	0.577016	0.379662	
14	0.47	0.569403	0.403736	
15	0.48	0.567419	0.364270	
16	0.49	0.564575	0.344717	
17	0.50	0.541784	0.383792	
18	0.51	0.541784	0.383792	

	Training RMSE	Test RMSE	Number of features
0	0.158033	1.957489	84
1	0.206484	1.418742	74
2	0.274465	1.653739	61
3	0.338595	0.795741	50

4	0.426649	0.522243	43
5	0.443922	0.555980	37
6	0.502419	0.751821	29
7	0.512308	0.935138	26
8	0.513752	0.902598	25
9	0.550741	0.712354	19
10	0.605855	0.753160	14
11	0.605968	0.746599	13
12	0.612977	0.706482	11
13	0.617925	0.750215	10
14	0.623460	0.735513	9
15	0.624895	0.759465	8
16	0.626946	0.771056	6
17	0.643144	0.747713	4
18	0.643144	0.747713	4

[ ]:

```
```

2.2 Plots

[11]:
```
plt.plot(df_without_standardization['Correlation threshold'],␣
 ↪df_without_standardization['Training data R^2 score'], label = "Training␣
 ↪data R^2 score", marker='v')
plt.plot(df_without_standardization['Correlation threshold'],␣
 ↪df_without_standardization['Test data R^2 score'], label = "Test data R^2␣
 ↪score", marker='D')
plt.legend()
plt.xlabel('Correlation threshold')
plt.ylabel('R^2 score')
plt.ylim([0, 1])
plt.show()
```

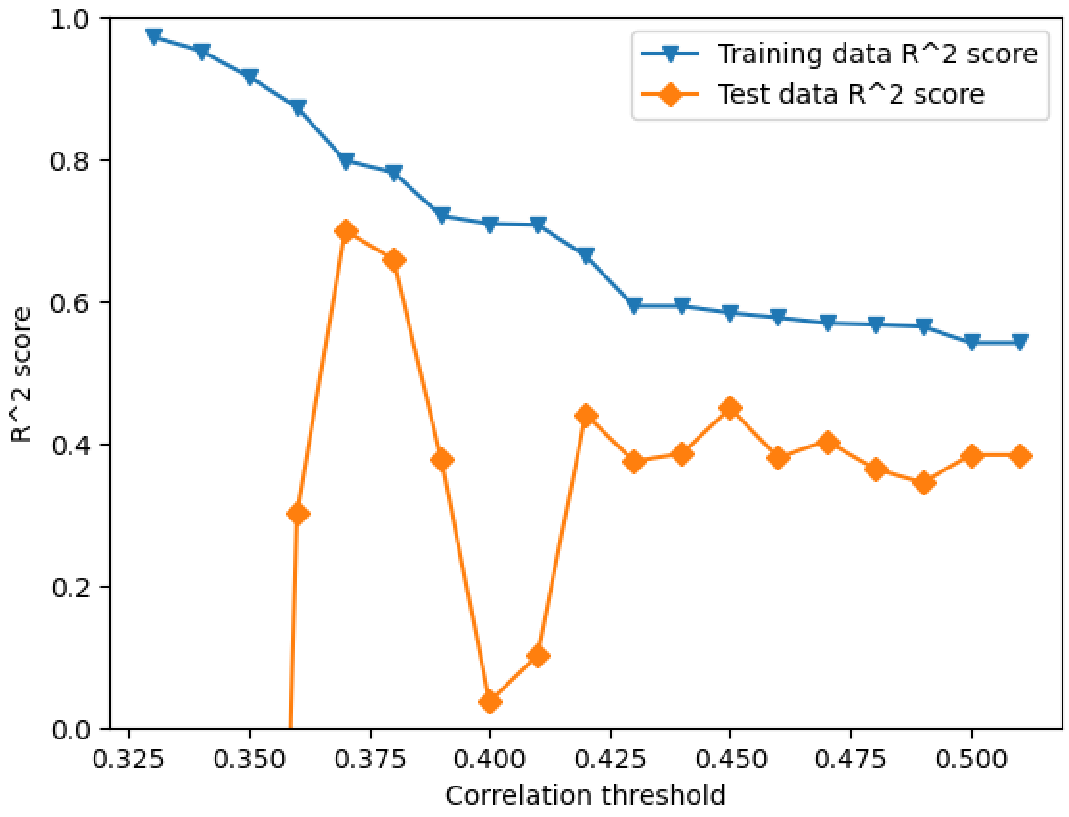

[12]:
```
plt.plot(df_without_standardization['Correlation threshold'],␣
 ↪df_without_standardization['Number of features'], label = "Number of␣
 ↪features", marker='.')
plt.legend()
plt.xlabel('Correlation threshold')
plt.ylabel('Number of features')
plt.show()
```

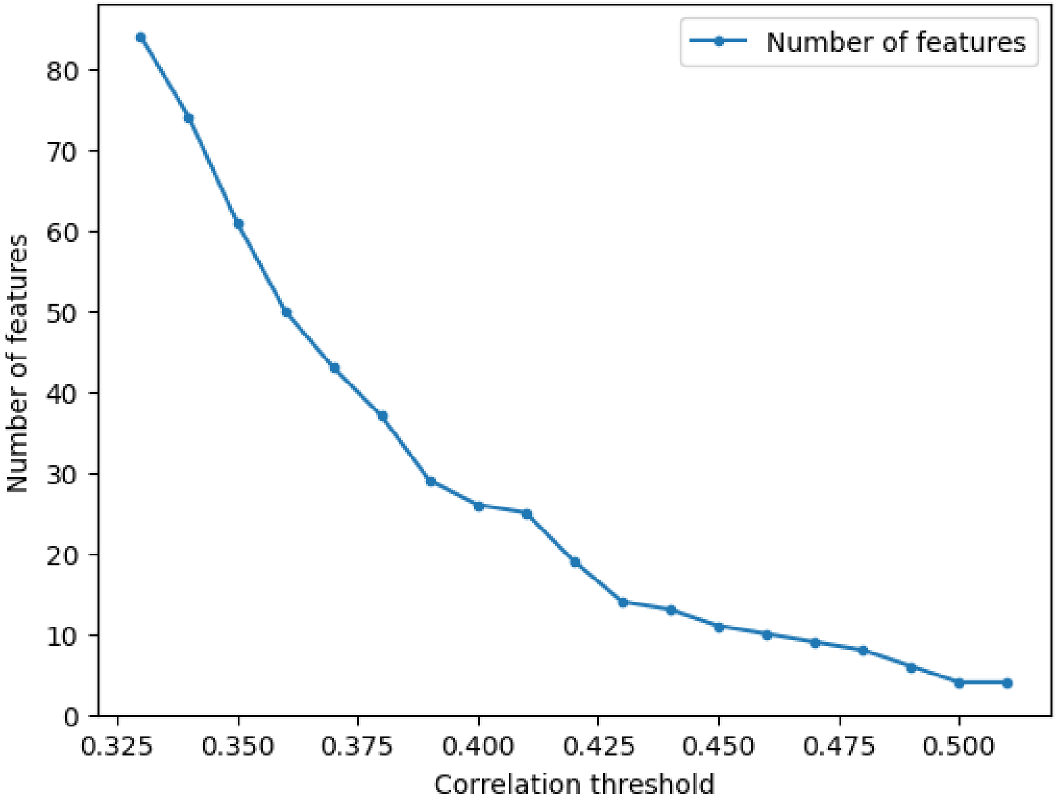


```
[ ]:
```

2.3 Decision Tree

```
[13]: without_standardization, train_r2_, test_r2_, hist1, hist2, target_column_name,␣
 ↪training_data_RMSE, test_data_RMSE  = pred_model.
 ↪prepare_data_and_create_model(molecular_descriptors_df=data,
                                                                    ␣
 ↪                        correlation_threshold=0.50,
                                                                    ␣
 ↪                        standardization=False,
                                                                    ␣
 ↪                        model_type='DecisionTreeRegressor',
                                                                    ␣
 ↪                        max_depth=5,
                                                                    ␣
 ↪                        target_column_name = target,
                                                                    ␣
 ↪                        random_state=random_state,
```

```
                    train_test_split_=True,
                    verbose=True)
```

```
I am not doing standardization…
  molecular descriptor name
0                    AATS0Z
1                  AATS0are
2                    AATS0d
3                   AATS0dv
4                    AATS0i
  molecular descriptor name  corr_value
0                    AATS0Z   -0.038502
1                  AATS0are   -0.120847
2                    AATS0d    0.041648
3                   AATS0dv   -0.115899
4                    AATS0i    0.191765
  molecular descriptor name  corr_value  absolute correlation value
0                    AATS0Z   -0.038502                    0.038502
1                  AATS0are   -0.120847                    0.120847
2                    AATS0d    0.041648                    0.041648
3                   AATS0dv   -0.115899                    0.115899
4                    AATS0i    0.191765                    0.191765
     molecular descriptor name  corr_value  absolute correlation value
226                     AMID_O   -0.520578                    0.520578
505                EState_VSA5   -0.578904                    0.578904
506                EState_VSA6    0.519794                    0.519794
791                    MDEO-12   -0.525441                    0.525441
1211                     MCF-7    1.000000                    1.000000
    molecular descriptor name  corr_value  absolute correlation value
226                    AMID_O   -0.520578                    0.520578
505               EState_VSA5   -0.578904                    0.578904
506               EState_VSA6    0.519794                    0.519794
791                   MDEO-12   -0.525441                    0.525441
The model used is: DecisionTree…
Return the coefficient of determination of the prediction:
0.08565969682925101
R^2 score: 0.885567607231033
Correlation coefficient: 0.9410460175948002
Test data - unseen during training:
R^2 score: 0.08565969682925101
Correlation coefficient: 0.29267677876669856
[7.54525818 7.81436625 7.74958    5.67780021 5.08184454 8.08153751
 8.37723827 8.1007251  7.81436625 7.81436625 7.01472228 7.74958
 8.1007251  8.1007251  7.74958    7.54525818 7.81436625 8.1007251 ]
44     8.267606
47     8.065502
```

```
4      7.032920
55     7.920819
26     4.998569
64     8.619789
73     7.920819
10     7.987163
40     8.091515
107    7.795880
18     7.074688
62     8.958607
11     8.013228
36     6.108463
89     8.045757
91     9.154902
109    8.086186
0      7.987163
Name: MCF-7, dtype: float64
Training Root Mean Square Error: 0.3214015824224863
Testing Root Mean Square Error: 0.9108046473050505
```

[14]:
```
print(target_column_name+str('_transformed'))
print(hist1[target_column_name].hist())
```

```
MCF-7_transformed
AxesSubplot(0.125,0.11;0.775x0.77)
```

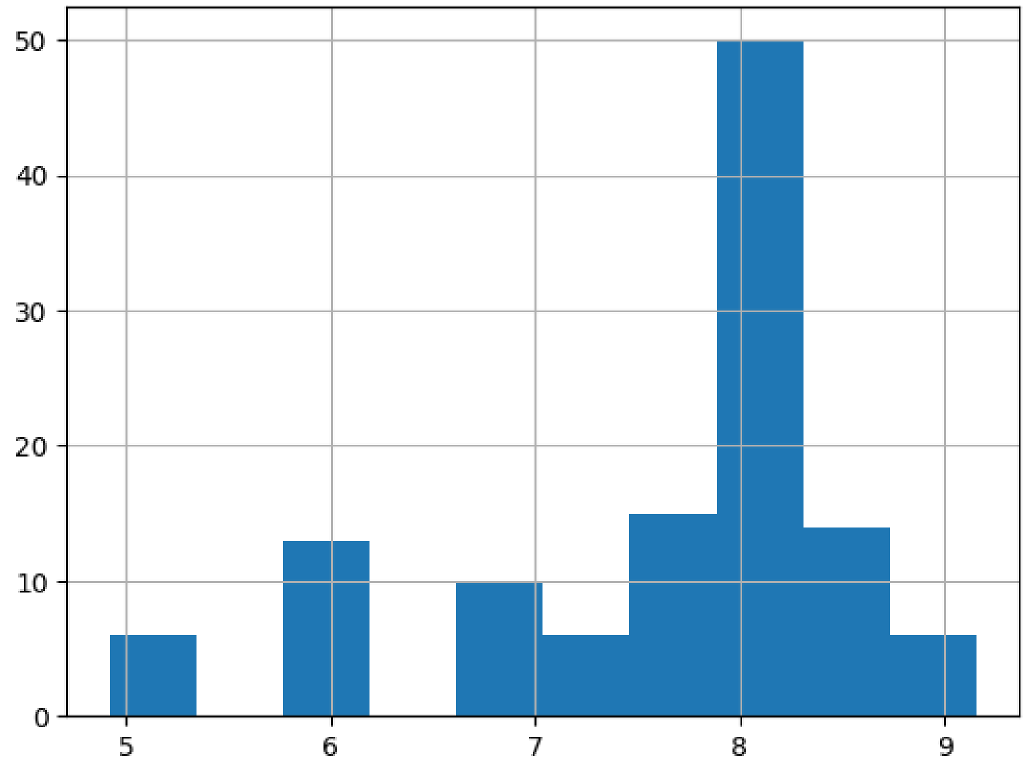

[15]:
```
with_standardization, train_r2_, test_r2_, hist1, hist2, target_column_name,␣
↪training_data_RMSE, test_data_RMSE  = pred_model.
↪prepare_data_and_create_model(molecular_descriptors_df=data,
                                                                              ␣
↪                      correlation_threshold=0.50,
                                                                              ␣
↪                      standardization=True,
                                                                              ␣
↪                      model_type='DecisionTreeRegressor',
                                                                              ␣
↪                      max_depth=5,
                                                                              ␣
↪                      target_column_name = target,
                                                                              ␣
↪                      random_state=random_state,
                                                                              ␣
↪                      train_test_split_=True,
                                                                              ␣
↪                      verbose=True)
```

```
I am doing standardization…
  molecular descriptor name
0                    AATS0Z
1                  AATS0are
2                    AATS0d
3                   AATS0dv
4                    AATS0i
  molecular descriptor name  corr_value
0                    AATS0Z   -0.038502
1                  AATS0are   -0.120847
2                    AATS0d    0.041648
3                   AATS0dv   -0.115899
4                    AATS0i    0.191765
  molecular descriptor name  corr_value  absolute correlation value
0                    AATS0Z   -0.038502                    0.038502
1                  AATS0are   -0.120847                    0.120847
2                    AATS0d    0.041648                    0.041648
3                   AATS0dv   -0.115899                    0.115899
4                    AATS0i    0.191765                    0.191765
     molecular descriptor name  corr_value  absolute correlation value
226                     AMID_O   -0.520578                    0.520578
505                 EState_VSA5   -0.578904                    0.578904
506                 EState_VSA6    0.519794                    0.519794
791                     MDEO-12   -0.525441                    0.525441
```

```
1211                     MCF-7    1.000000                    1.000000
    molecular descriptor name  corr_value  absolute correlation value
226                    AMID_O   -0.520578                    0.520578
505               EState_VSA5   -0.578904                    0.578904
506               EState_VSA6    0.519794                    0.519794
791                   MDEO-12   -0.525441                    0.525441
The model used is: DecisionTree…
Return the coefficient of determination of the prediction:
0.08565969682925101
R^2 score: 0.885567607231033
Correlation coefficient: 0.9410460175948002
Test data - unseen during training:
R^2 score: 0.08565969682925101
Correlation coefficient: 0.29267677876669856
[7.54525818 7.81436625 7.74958    5.67780021 5.08184454 8.08153751
 8.37723827 8.1007251  7.81436625 7.81436625 7.01472228 7.74958
 8.1007251  8.1007251  7.74958    7.54525818 7.81436625 8.1007251 ]
44     8.267606
47     8.065502
4      7.032920
55     7.920819
26     4.998569
64     8.619789
73     7.920819
10     7.987163
40     8.091515
107    7.795880
18     7.074688
62     8.958607
11     8.013228
36     6.108463
89     8.045757
91     9.154902
109    8.086186
0      7.987163
Name: MCF-7, dtype: float64
Training Root Mean Square Error: 0.3214015824224863
Testing Root Mean Square Error: 0.9108046473050505
```

2.4 Search inside correlation space

[16]:
```
step = 0.01
initial_step = corr_low
last_step = corr_high
first_list = [x / 100.0 for x in range(int(initial_step*100),␣
 ↪int(last_step*100), int(step*100))]
max_depth = [range(2, 30, 1)]
```

```
corr_th = []
second_list = []
third_list = []
fourth_l = []
fifth_l = []
f_list = []
fif_list = []
for i in first_list:
    for depth in max_depth[0]:

        without_standardization, train_r2, test_r2, _, h_, target_column_name,␣
↪training_data_RMSE, test_data_RMSE = pred_model.
↪prepare_data_and_create_model(molecular_descriptors_df=data,
                                                                              ␣
↪                    correlation_threshold=i,
                                                                              ␣
↪                    standardization=False,
                                                                              ␣
↪                    model_type='DecisionTreeRegressor',
                                                                              ␣
↪                    max_depth=depth,
                                                                              ␣
↪                    target_column_name = target,
                                                                              ␣
↪                    random_state=random_state,
                                                                              ␣
↪                    train_test_split_=True,
                                                                              ␣
↪                    verbose=False)
        corr_th.append(i)
        second_list.append(train_r2)
        third_list.append(test_r2)
        fourth_l.append(training_data_RMSE)
        fifth_l.append(test_data_RMSE)
        f_list.append(len(h_))
        fif_list.append(depth)
```

[17]:
```
df_without_standardization = pd.DataFrame(data=corr_th, columns=["Correlation␣
↪threshold"])
df_without_standardization['Training data R^2 score'] = second_list
df_without_standardization['Test data R^2 score'] = third_list
df_without_standardization['Training RMSE'] = fourth_l
df_without_standardization['Test RMSE'] = fifth_l
df_without_standardization['Number of features'] = f_list
df_without_standardization['Depth number'] = fif_list
```

[18]:
```
df_decision_tree = df_without_standardization.copy()
#df_without_standardization.to_excel('../Data/
↪A549_Decision_tree_rs_'+str(random_state)+'.xlsx')
```

2.5 Plots

[19]:
```
corre_list = [x / 100.0 for x in range(int(initial_step*100),␣
↪int(last_step*100), int(0.01*100))]
for element in corre_list:
    element_ =␣
↪df_without_standardization[df_without_standardization['Correlation␣
↪threshold'] == float(element)]
    plt.plot(element_['Depth number'], element_['Training data R^2 score'],␣
↪label = "Training data R^2 score", marker='v')
    plt.plot(element_['Depth number'], element_['Test data R^2 score'], label =␣
↪"Test data R^2 score", marker='D')
    plt.legend()
    plt.xlabel('Depth number -> correlation threshold: '+str(element)+'. \n␣
↪Number of features: '+str(element_['Number of features'].iloc[0]),␣
↪fontweight='bold')
    plt.xlim(max(element_['Depth number'])+1, min(element_['Depth number'])-1)
    plt.ylabel('R^2 score')
    plt.ylim([0, 1])
    plt.rc('grid', linestyle="-.", color='black')
    plt.grid(True)
    plt.show()
```

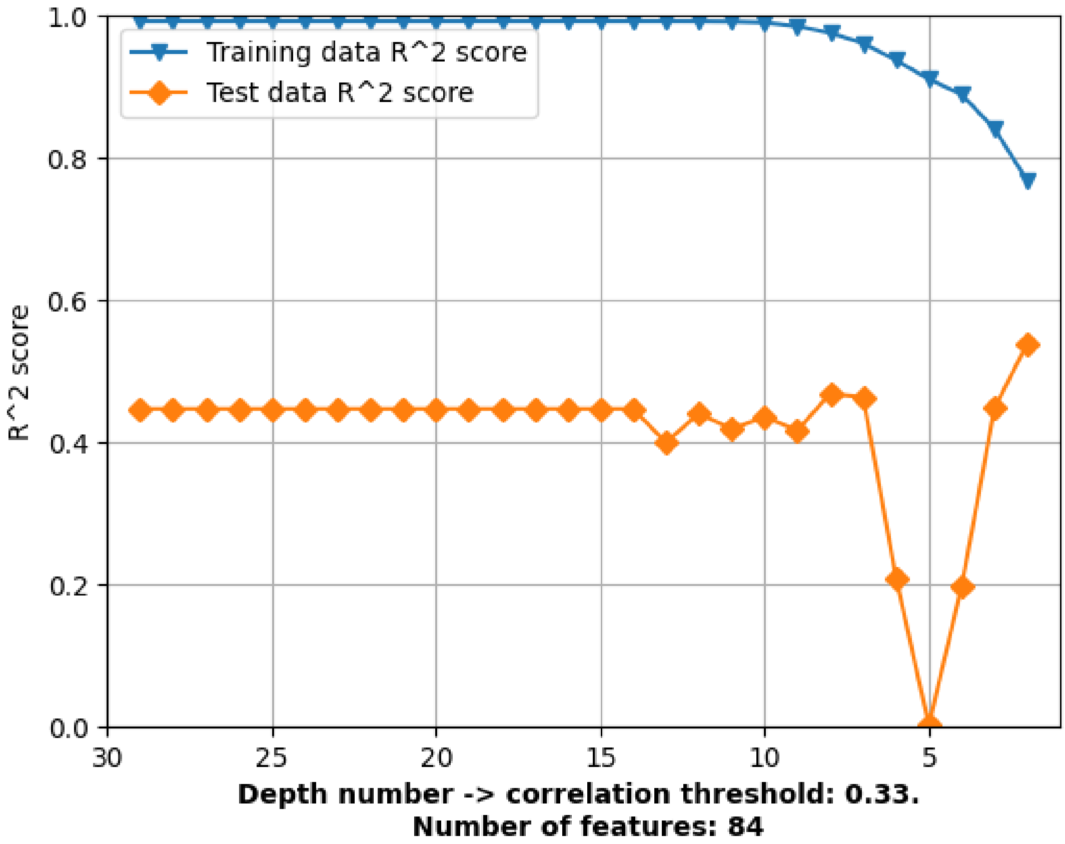

Training data R^2 score
Test data R^2 score
R^2 score
1.0
0.8
0.6
0.4
0.2
0.0
30
25
20
15
10
5
Depth number -> correlation threshold: 0.33.
Number of features: 84

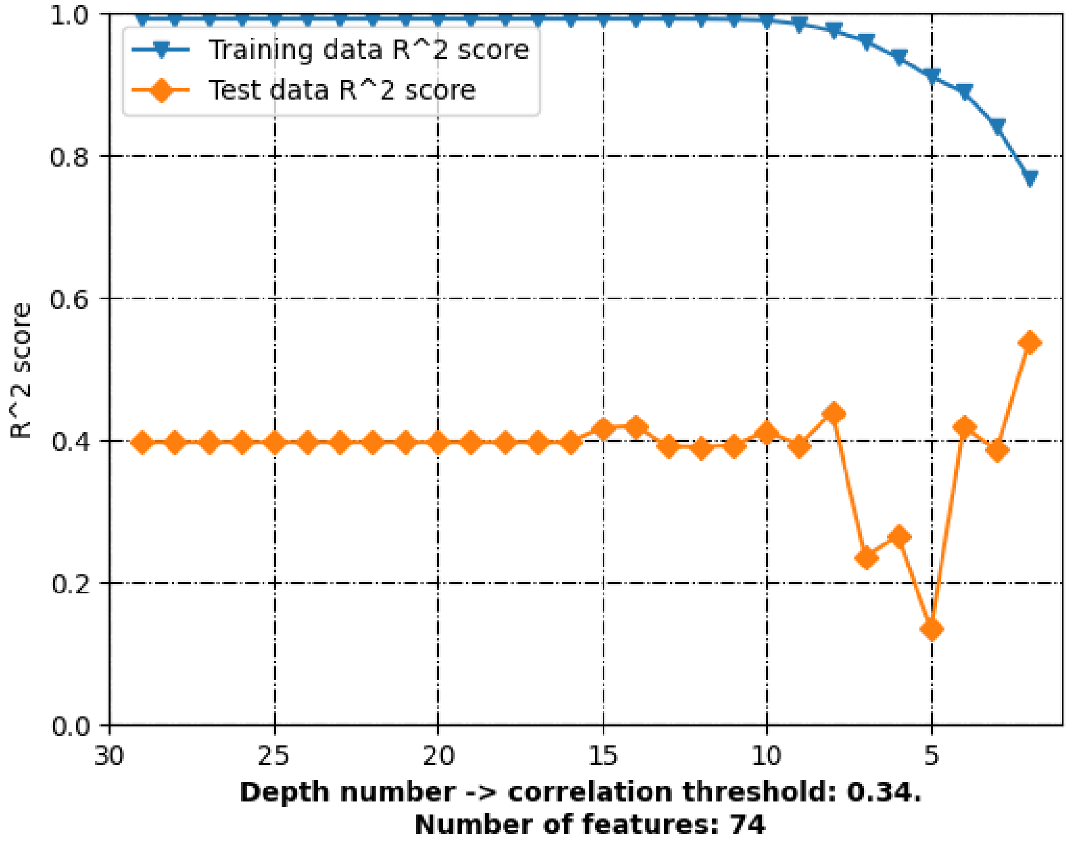

Training data R^2 score
Test data R^2 score
R^2 score
Depth number -> correlation threshold: 0.34.
Number of features: 74

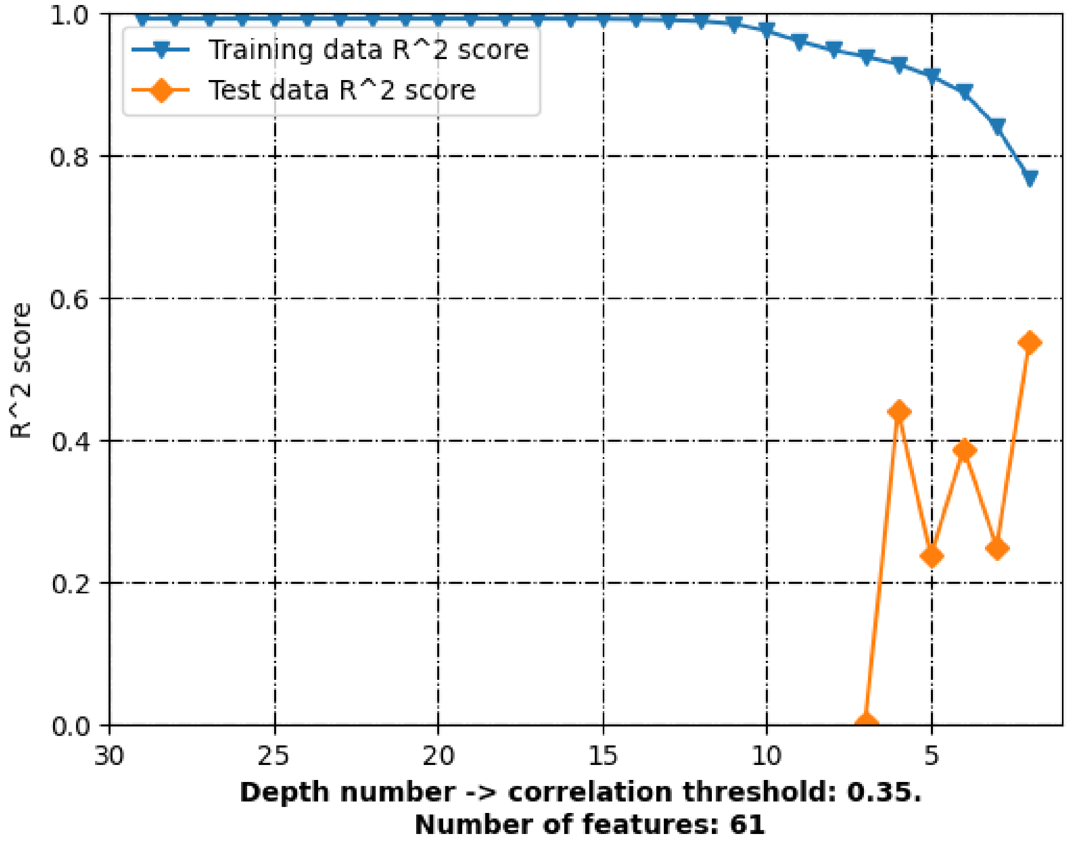
Training data R^2 score
Test data R^2 score
R^2 score
1.0
0.8
0.6
0.4
0.2
0.0
30
25
20
15
10
5
Depth number -> correlation threshold: 0.35.
Number of features: 61

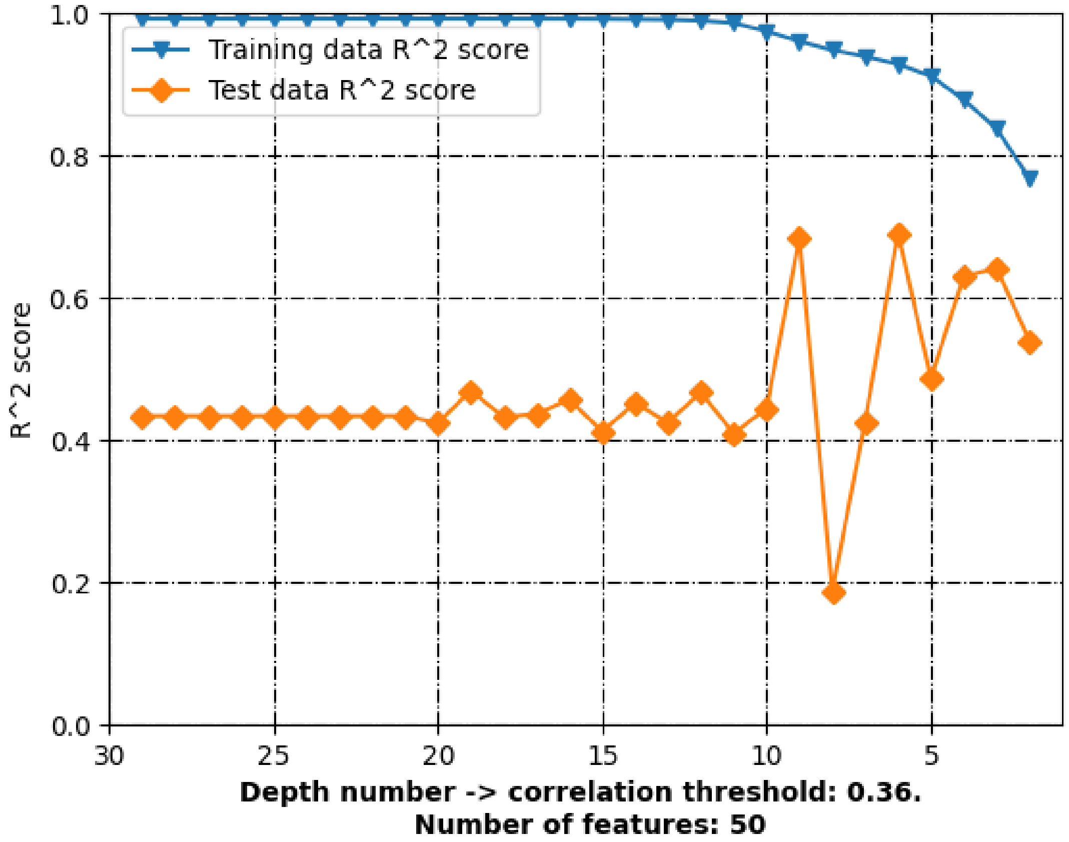

1.0
0.8
0.6
0.4
0.2
0.0
R^2 score
Training data R^2 score
Test data R^2 score
30
25
20
15
10
5
Depth number -> correlation threshold: 0.36.
Number of features: 50

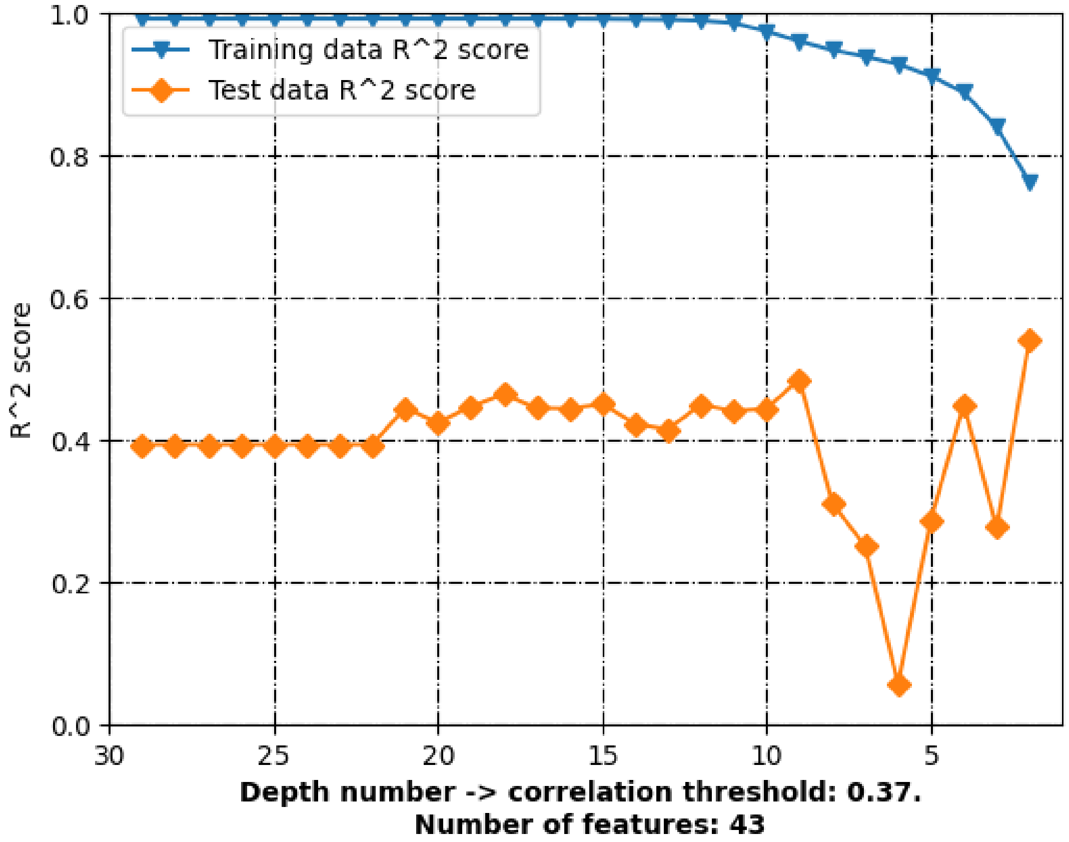

Training data R^2 score
Test data R^2 score
R^2 score
1.0
0.8
0.6
0.4
0.2
0.0
30
25
20
15
10
5
Depth number -> correlation threshold: 0.37.
Number of features: 43

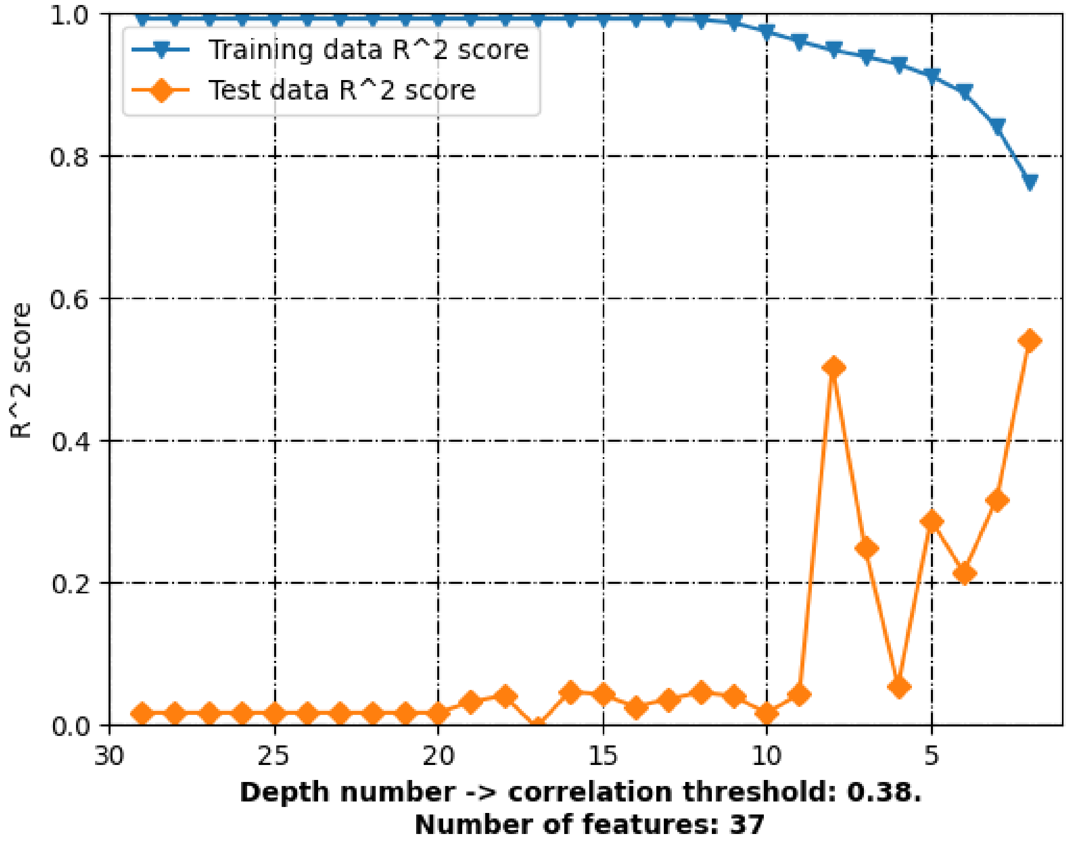

Training data R^2 score
Test data R^2 score
R^2 score
1.0
0.8
0.6
0.4
0.2
0.0
30
25
20
15
10
5
Depth number -> correlation threshold: 0.38.
Number of features: 37

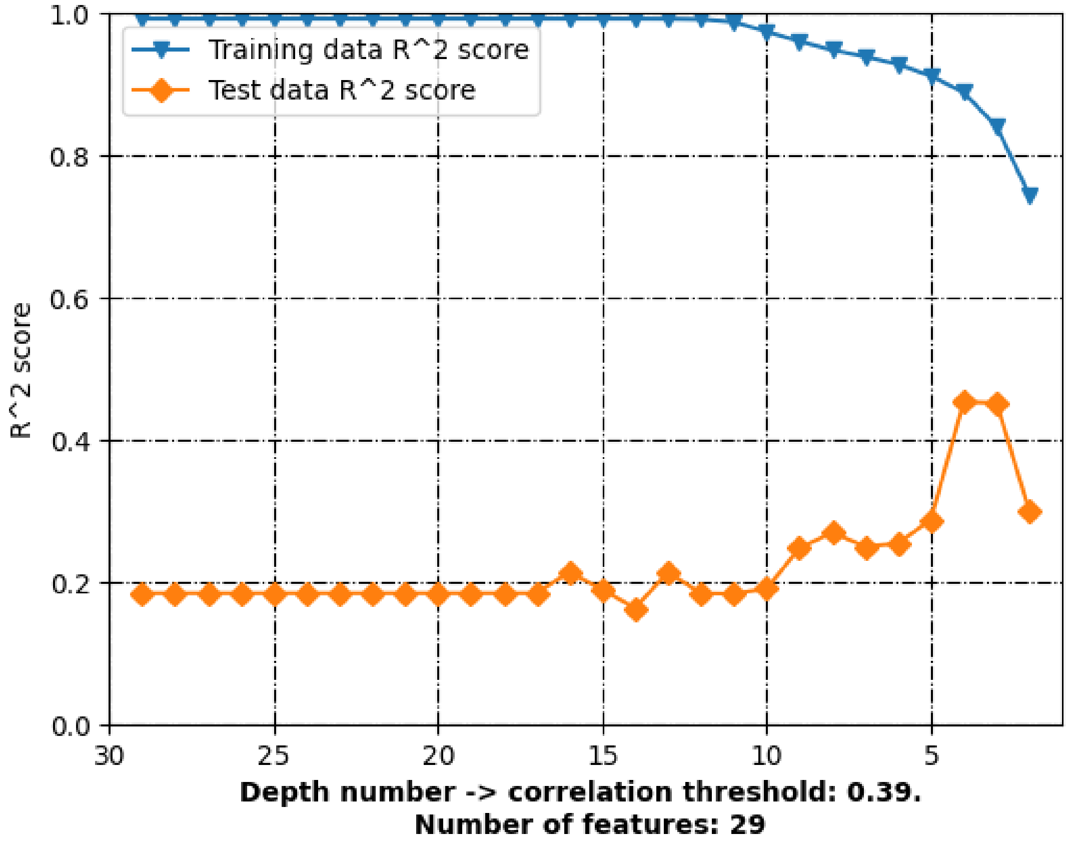

Training data R^2 score
Test data R^2 score
R^2 score
1.0
0.8
0.6
0.4
0.2
0.0
30
25
20
15
10
5
Depth number -> correlation threshold: 0.39.
Number of features: 29

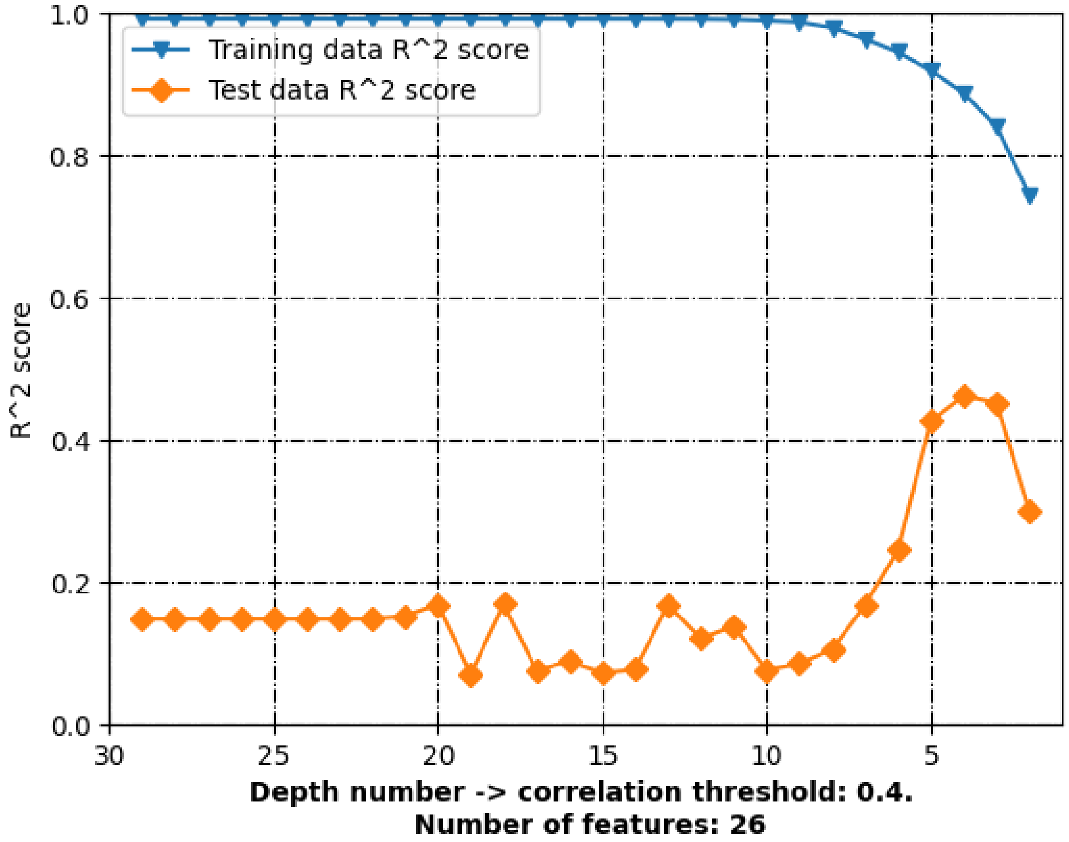
1.0
0.8
0.6
0.4
0.2
0.0
R^2 score
Training data R^2 score
Test data R^2 score
30
25
20
15
10
5
Depth number -> correlation threshold: 0.4.
Number of features: 26

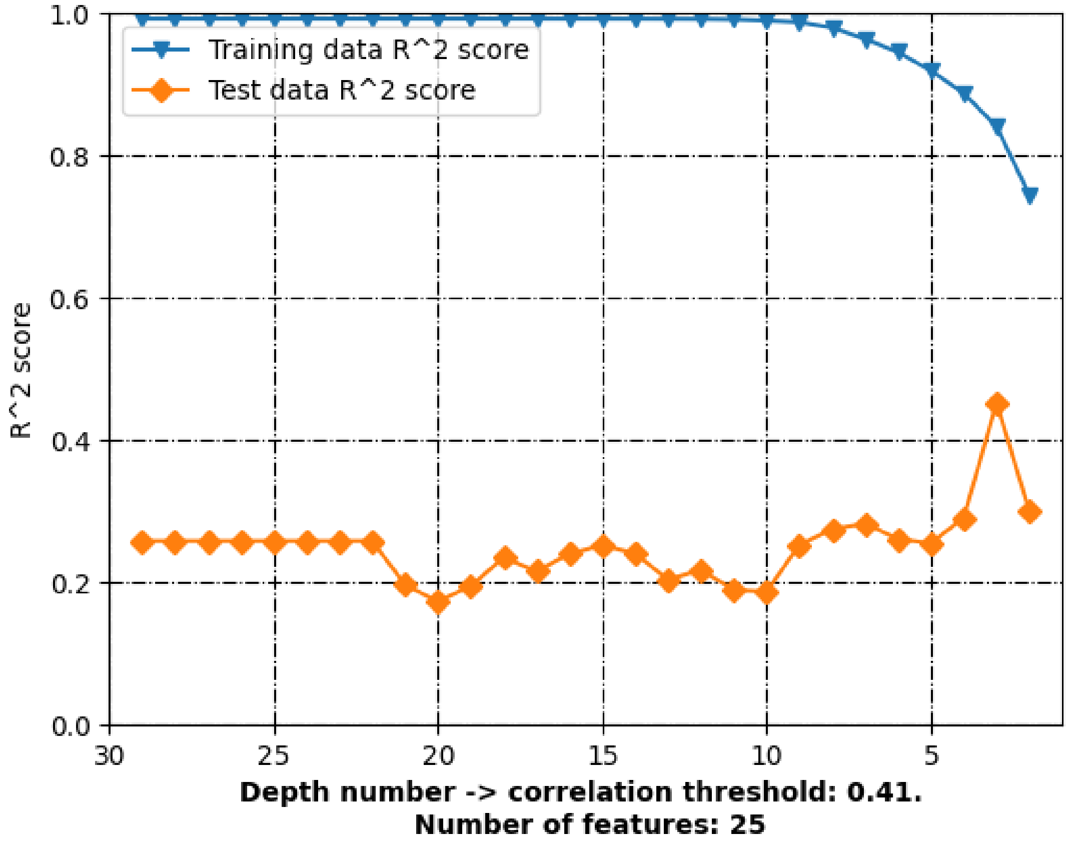
1.0
0.8
0.6
0.4
0.2
0.0
R^2 score
Training data R^2 score
Test data R^2 score
30
25
20
15
10
5
Depth number -> correlation threshold: 0.41.
Number of features: 25

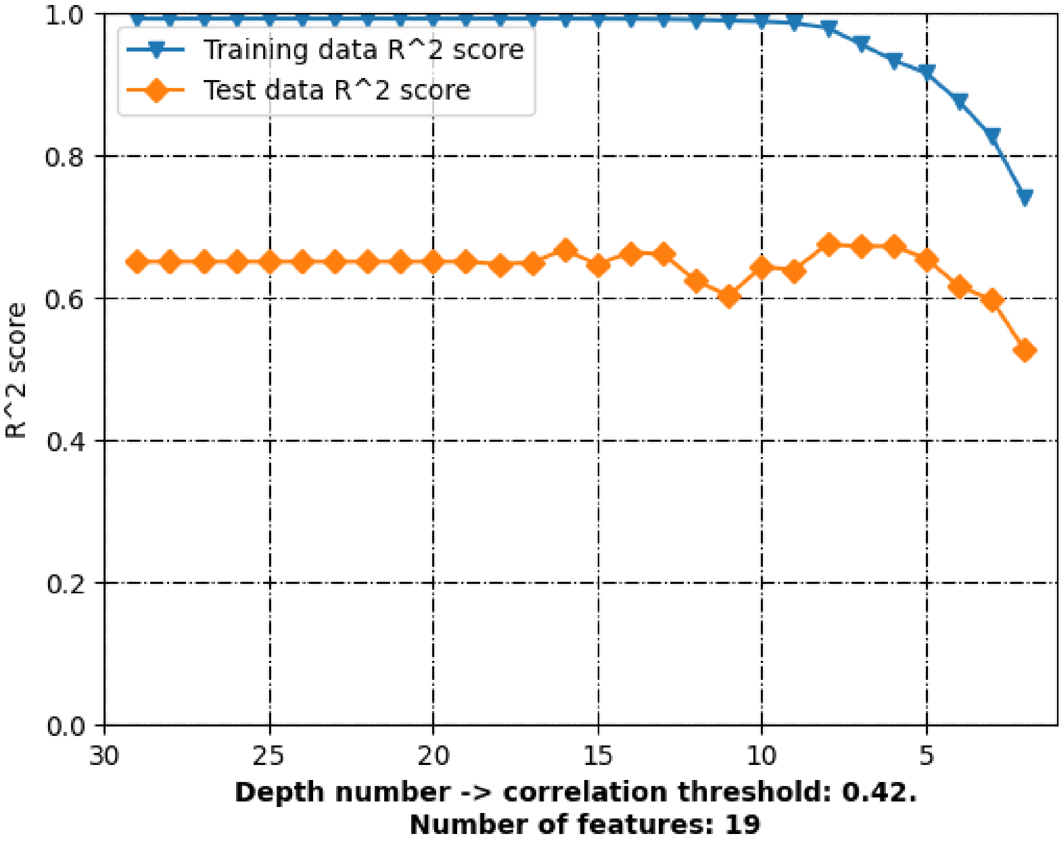
Training data R^2 score
Test data R^2 score
R^2 score
1.0
0.8
0.6
0.4
0.2
0.0
30
25
20
15
10
5
Depth number -> correlation threshold: 0.42.
Number of features: 19

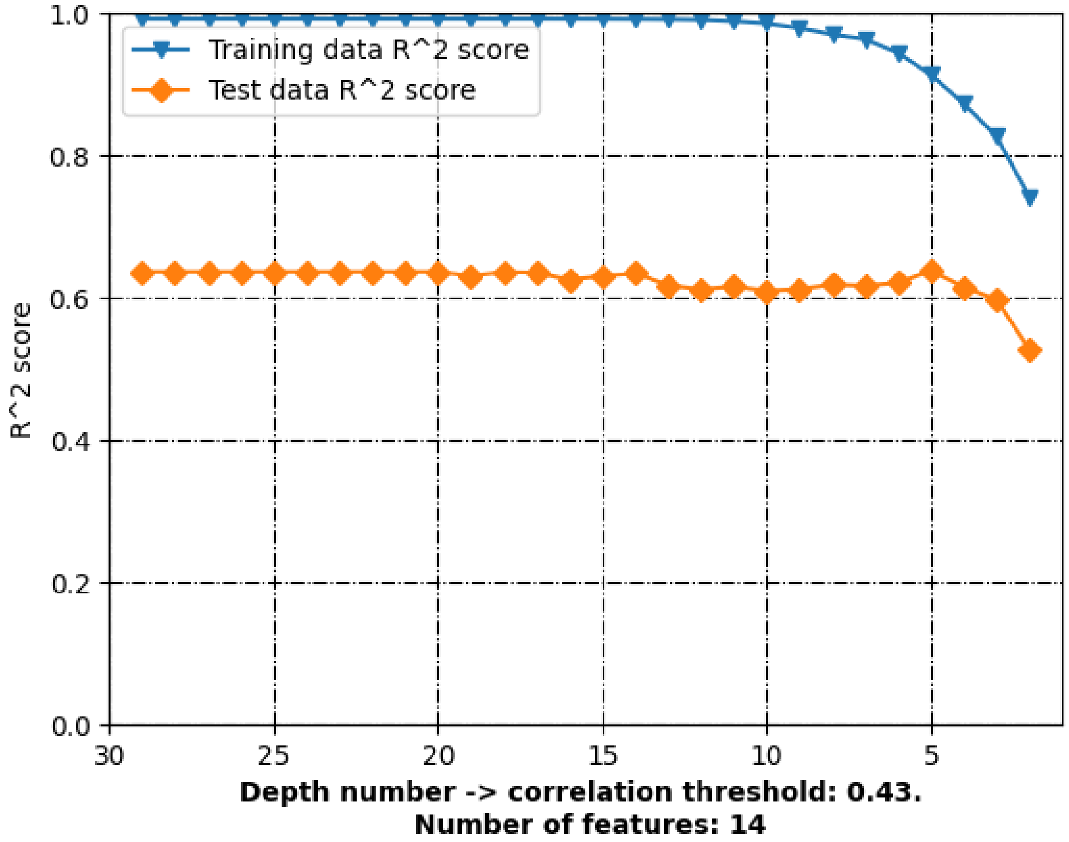
Training data R^2 score
Test data R^2 score
R^2 score
1.0
0.8
0.6
0.4
0.2
0.0
30
25
20
15
10
5
Depth number -> correlation threshold: 0.43.
Number of features: 14

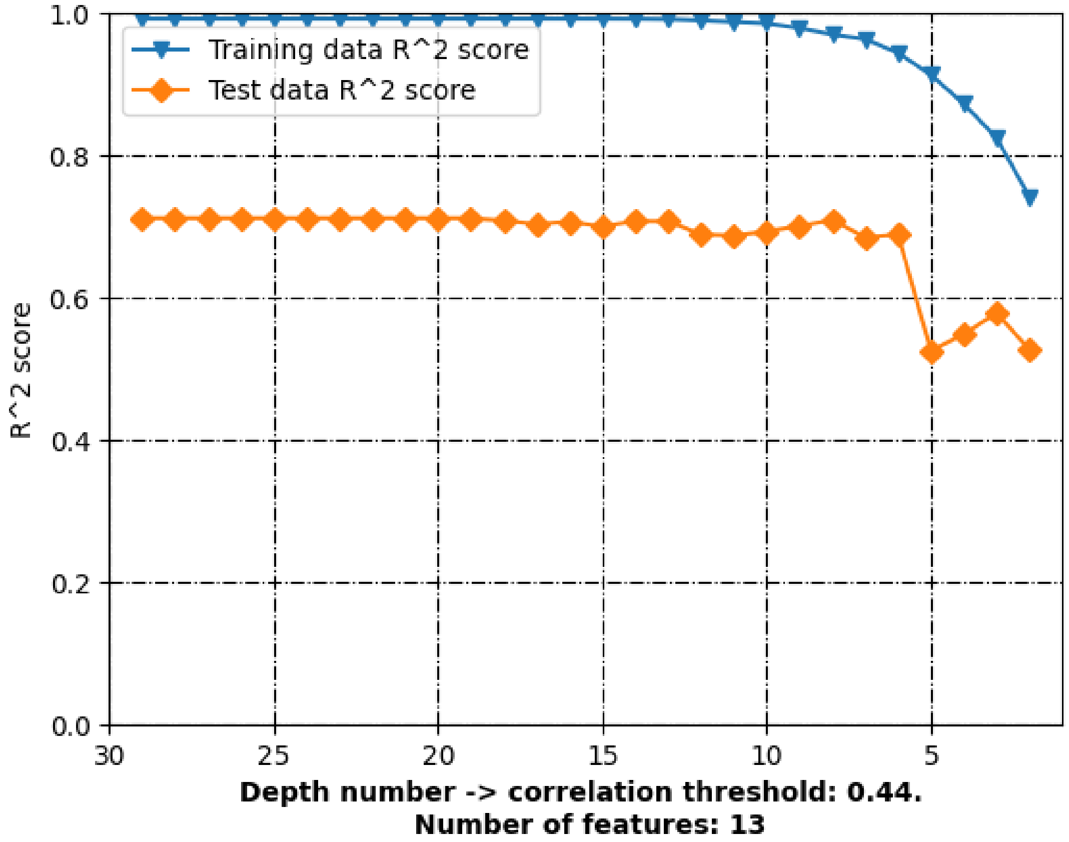

Training data R^2 score
Test data R^2 score
R^2 score
1.0
0.8
0.6
0.4
0.2
0.0
30
25
20
15
10
5
Depth number -> correlation threshold: 0.44.
Number of features: 13

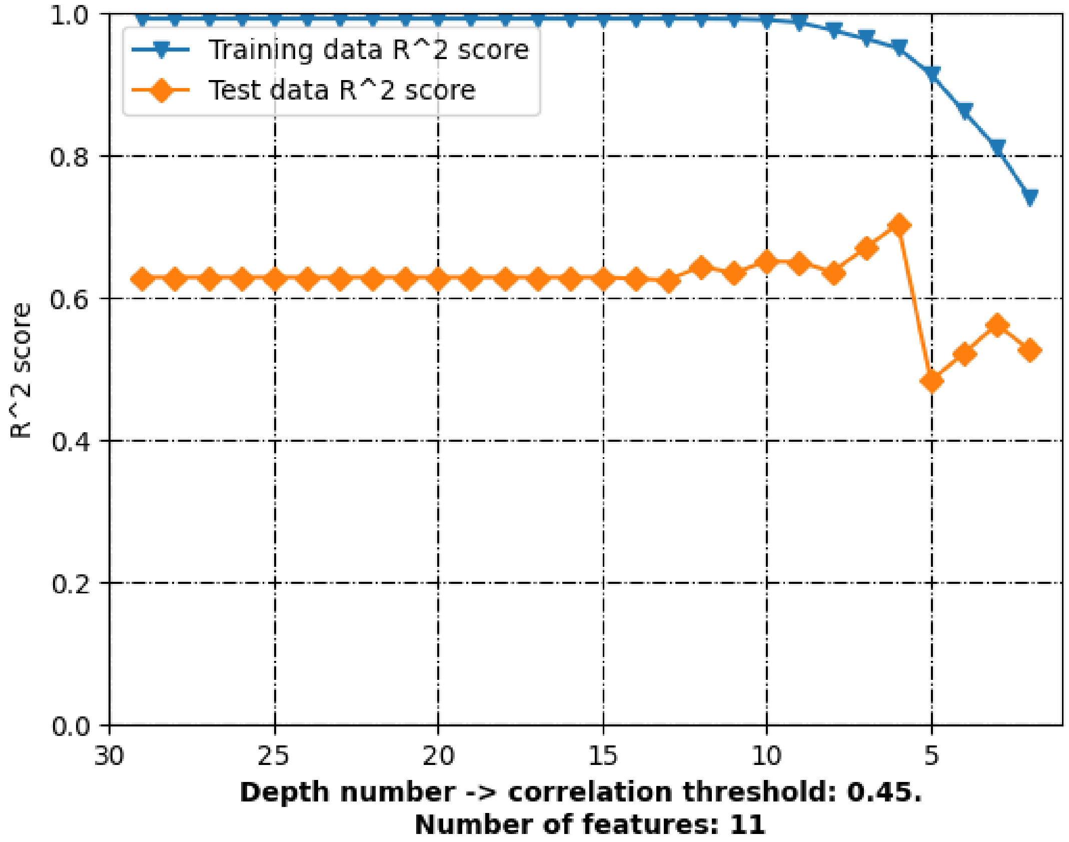
Training data R^2 score
Test data R^2 score
R^2 score
1.0
0.8
0.6
0.4
0.2
0.0
30
25
20
15
10
5
Depth number -> correlation threshold: 0.45.
Number of features: 11

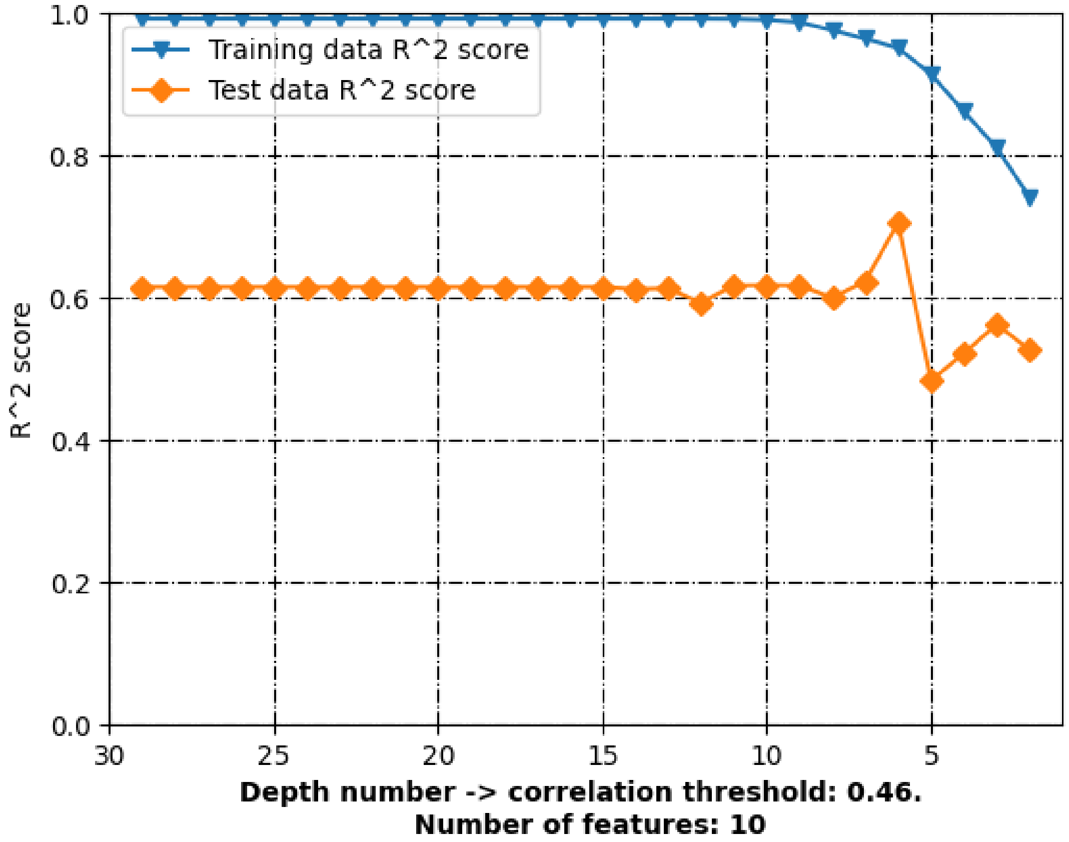

1.0
0.8
0.6
0.4
0.2
0.0
R^2 score
Training data R^2 score
Test data R^2 score
30
25
20
15
10
5
Depth number -> correlation threshold: 0.46.
Number of features: 10

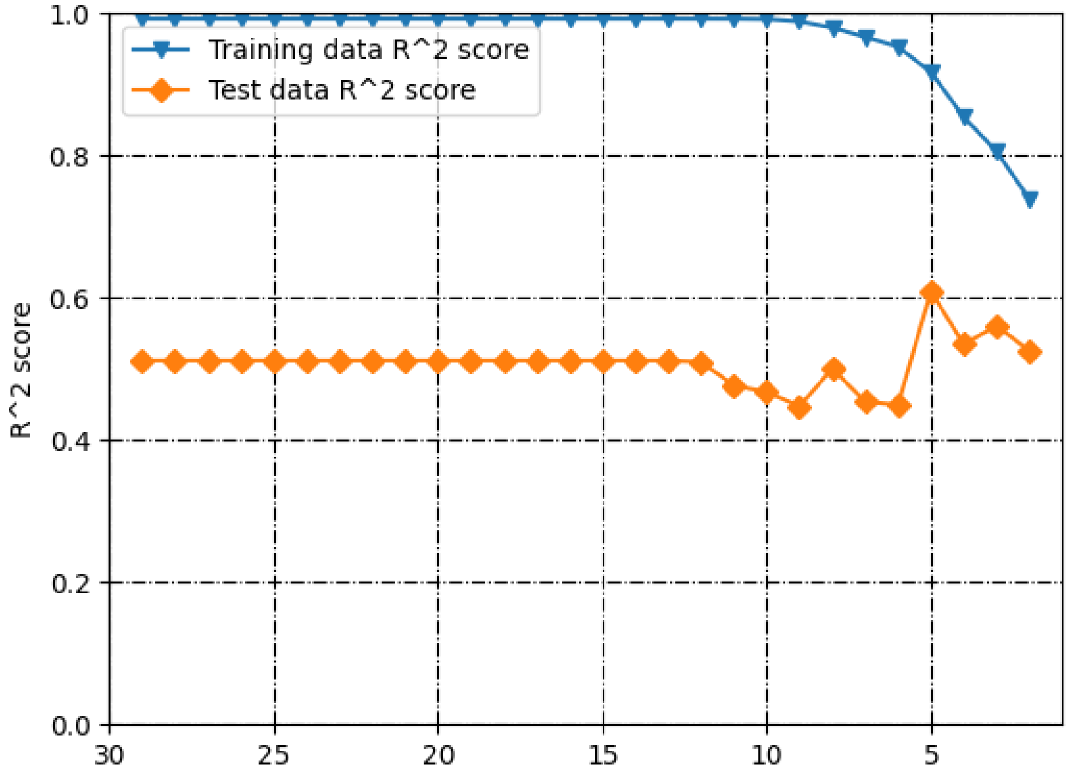

Depth number -> correlation threshold: 0.47.
Number of features: 9

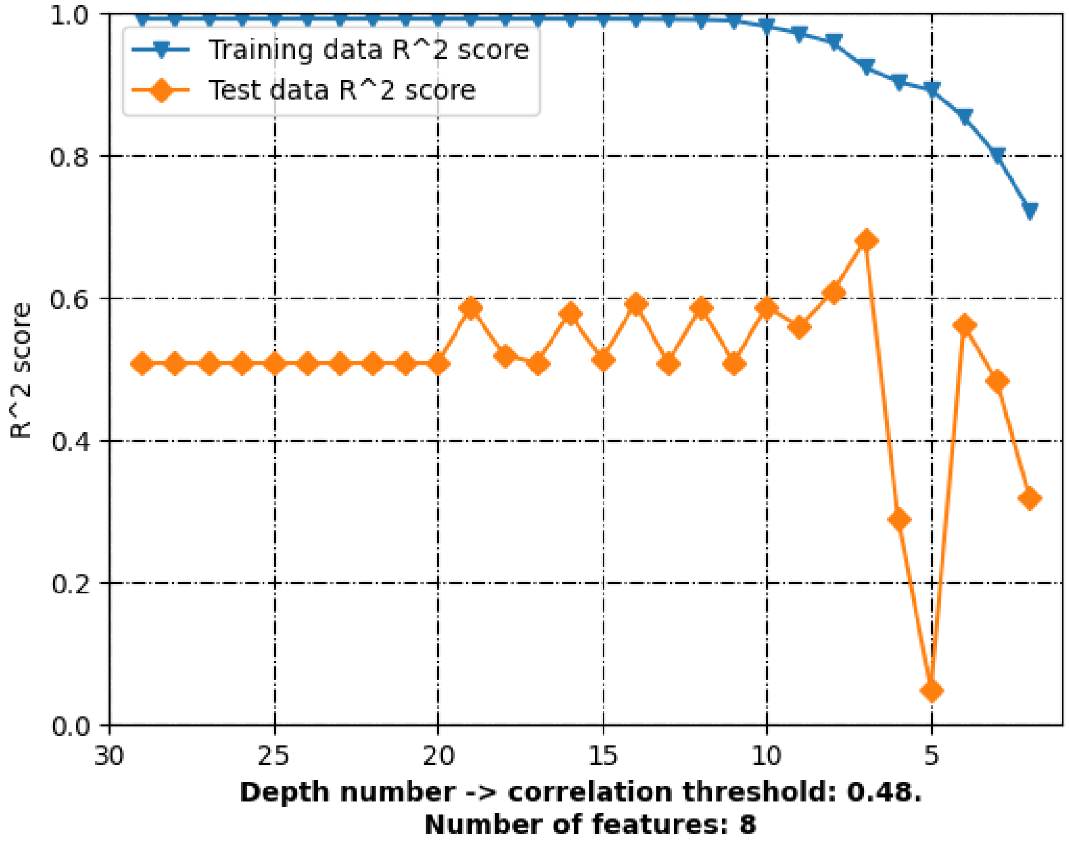
Training data R^2 score
Test data R^2 score
R^2 score
1.0
0.8
0.6
0.4
0.2
0.0
30
25
20
15
10
5
Depth number -> correlation threshold: 0.48.
Number of features: 8

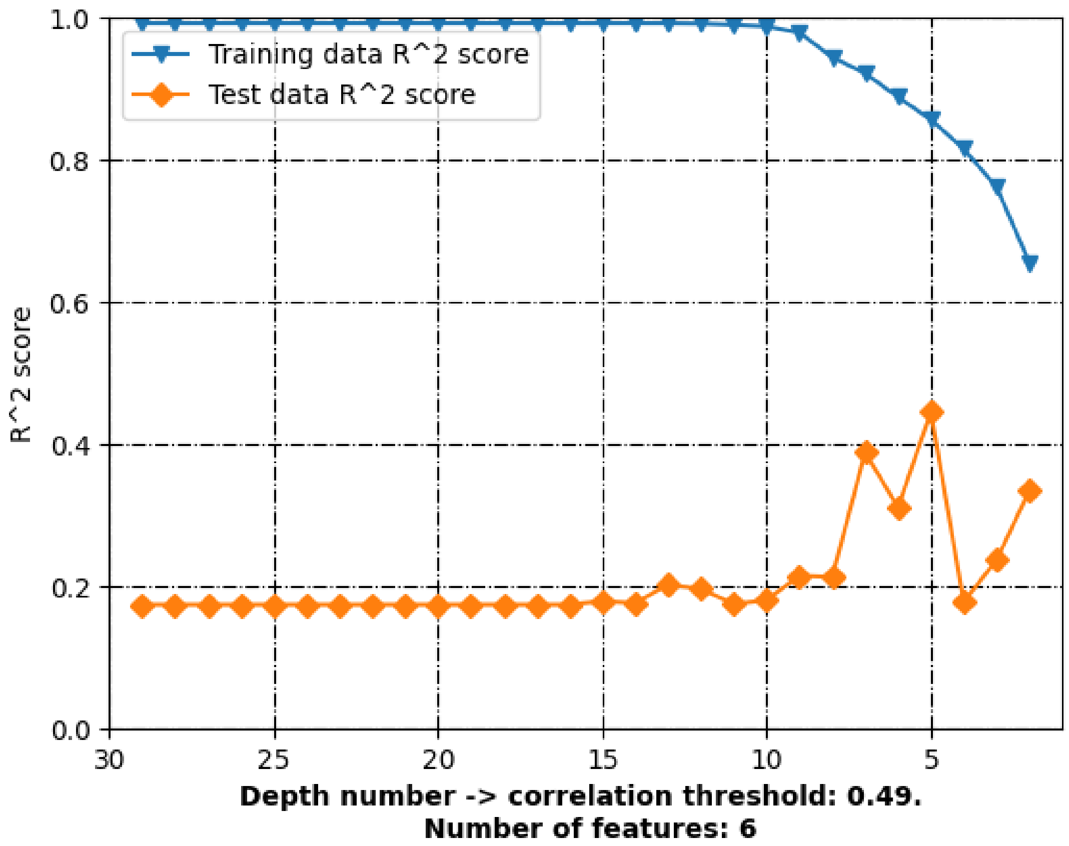

Training data R^2 score
Test data R^2 score
R^2 score
1.0
0.8
0.6
0.4
0.2
0.0
30
25
20
15
10
5
Depth number -> correlation threshold: 0.49.
Number of features: 6

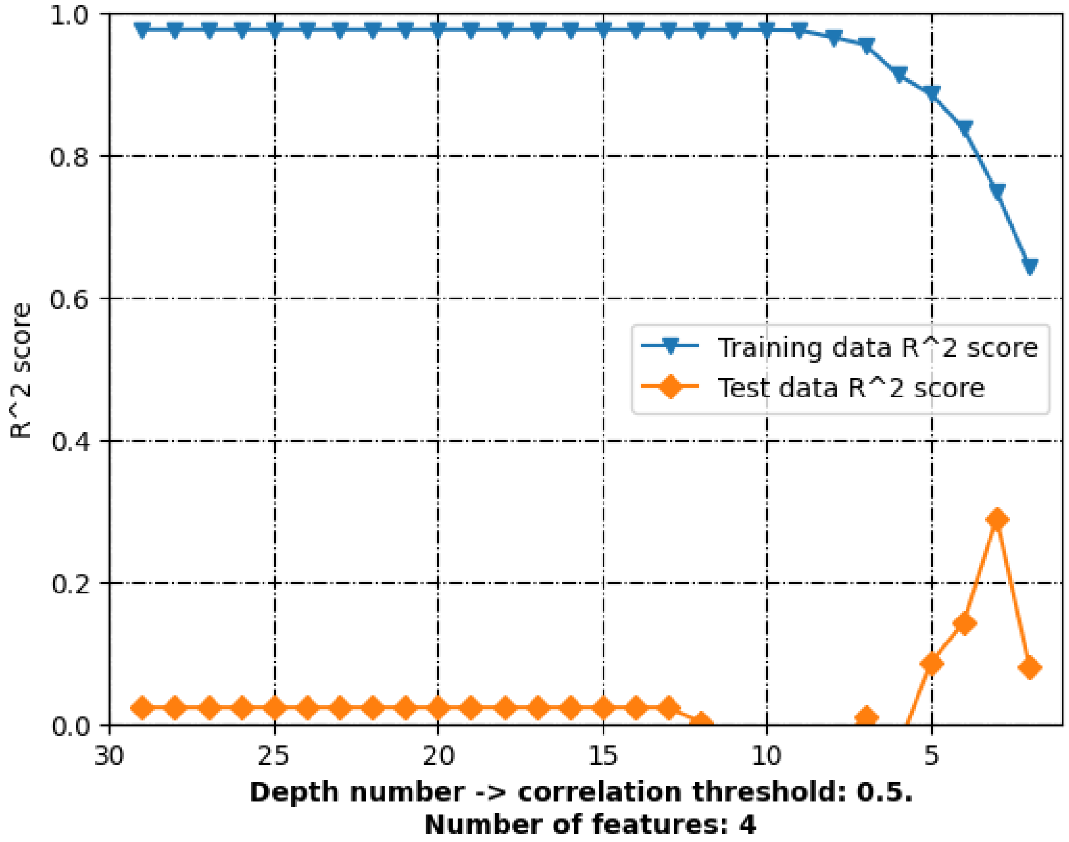

1.0
0.8
0.6
0.4
0.2
0.0
R^2 score
30
25
20
15
10
5
Training data R^2 score
Test data R^2 score
Depth number -> correlation threshold: 0.5.
Number of features: 4

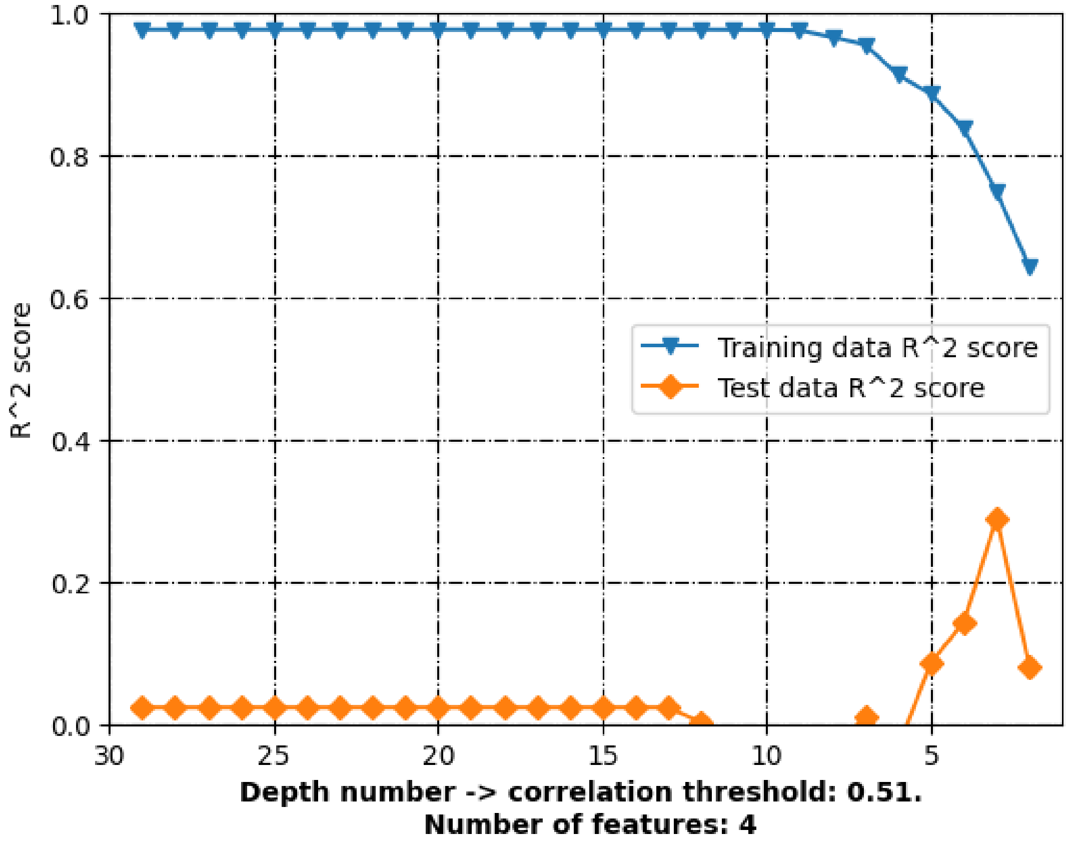

[20]:
```
plt.plot(df_without_standardization['Correlation threshold'],␣
  ↪df_without_standardization['Number of features'], label = "Number of␣
  ↪features", marker='.')
plt.legend()
plt.xlabel('Correlation threshold')
plt.ylabel('Number of features')
plt.show()
```

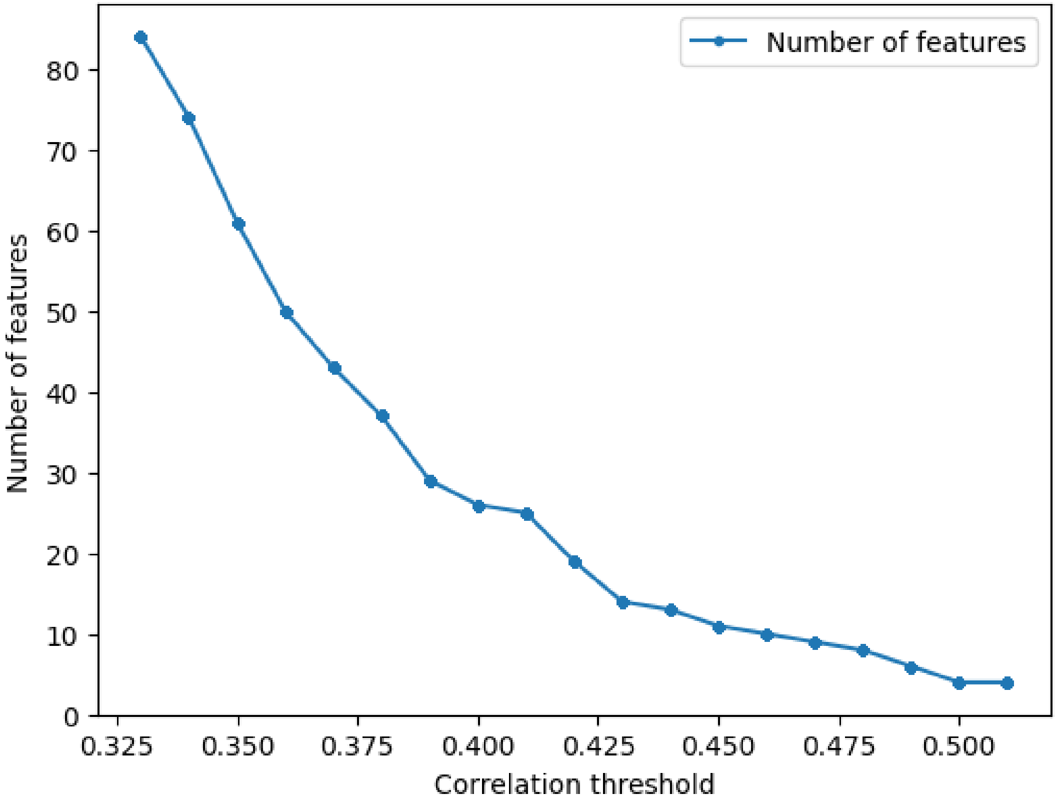

[ ]:

```

```

3 Random Forest

[21]:
```
without_standardization, train_r2_, test_r2_, hist1, hist2, target_column_name,␣
↪training_data_RMSE, test_data_RMSE  = pred_model.
↪prepare_data_and_create_model(molecular_descriptors_df=data,
                                                                               ␣
↪                       correlation_threshold=0.50,
                                                                               ␣
↪                       standardization=False,
                                                                               ␣
↪                       model_type='RandomForestRegressor',
                                                                               ␣
↪                       n_estimators_=12,
                                                                               ␣
↪                       target_column_name = target,
                                                                               ␣
↪                       random_state=random_state,
```

```
                        train_test_split_=True,
                        verbose=True)
```

```
I am not doing standardization…
  molecular descriptor name
0                    AATS0Z
1                  AATS0are
2                    AATS0d
3                   AATS0dv
4                    AATS0i
  molecular descriptor name  corr_value
0                    AATS0Z   -0.038502
1                  AATS0are   -0.120847
2                    AATS0d    0.041648
3                   AATS0dv   -0.115899
4                    AATS0i    0.191765
  molecular descriptor name  corr_value  absolute correlation value
0                    AATS0Z   -0.038502                    0.038502
1                  AATS0are   -0.120847                    0.120847
2                    AATS0d    0.041648                    0.041648
3                   AATS0dv   -0.115899                    0.115899
4                    AATS0i    0.191765                    0.191765
     molecular descriptor name  corr_value  absolute correlation value
226                     AMID_O   -0.520578                    0.520578
505                EState_VSA5   -0.578904                    0.578904
506                EState_VSA6    0.519794                    0.519794
791                    MDEO-12   -0.525441                    0.525441
1211                     MCF-7    1.000000                    1.000000
    molecular descriptor name  corr_value  absolute correlation value
226                    AMID_O   -0.520578                    0.520578
505               EState_VSA5   -0.578904                    0.578904
506               EState_VSA6    0.519794                    0.519794
791                   MDEO-12   -0.525441                    0.525441
The model used is: RandomForest…
Return the coefficient of determination of the prediction:
0.3896895830895062
R^2 score: 0.9161168599597713
Correlation coefficient: 0.9571399375011844
Test data - unseen during training:
R^2 score: 0.3896895830895062
Correlation coefficient: 0.6242512179319366
[7.7026269  7.66385111 7.28090766 7.13067235 5.29752234 8.36053117
 8.31454573 7.72841569 7.89518196 8.02037119 7.26759299 7.36357456
 8.0252565  7.89142517 7.4779577  7.7026269  7.81209996 8.24428702]
44     8.267606
47     8.065502
```

```
4       7.032920
55      7.920819
26      4.998569
64      8.619789
73      7.920819
10      7.987163
40      8.091515
107     7.795880
18      7.074688
62      8.958607
11      8.013228
36      6.108463
89      8.045757
91      9.154902
109     8.086186
0       7.987163
Name: MCF-7, dtype: float64
Training Root Mean Square Error: 0.2751761947609772
Testing Root Mean Square Error: 0.7441263740659375
```

[22]:

```
print(target_column_name+str('_transformed'))
print(hist1[target_column_name].hist())
```

```
MCF-7_transformed
AxesSubplot(0.125,0.11;0.775x0.77)
```

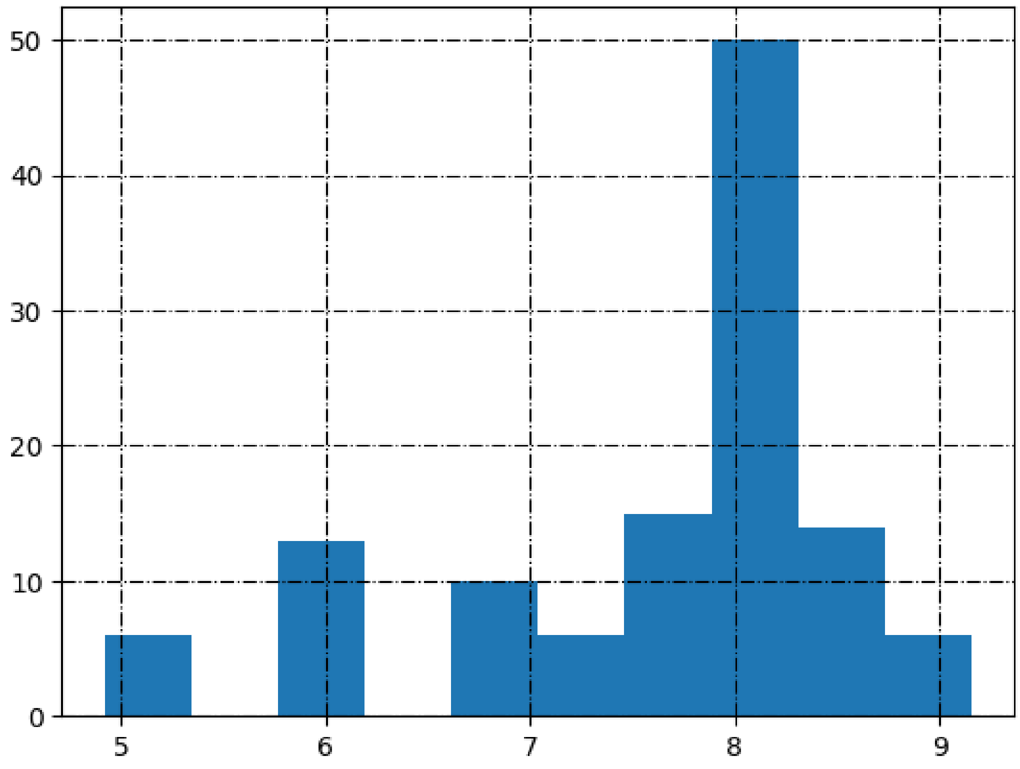

[23]:
```python
with_standardization, train_r2_, test_r2_, hist1, hist2, target_column_name,␣
↪training_data_RMSE, test_data_RMSE  = pred_model.
↪prepare_data_and_create_model(molecular_descriptors_df=data,
                                                                              ␣
↪                    correlation_threshold=0.50,
                                                                              ␣
↪                    standardization=True,
                                                                              ␣
↪                    model_type='RandomForestRegressor',
                                                                              ␣
↪                    n_estimators_=12,
                                                                              ␣
↪                    target_column_name = target,
                                                                              ␣
↪                    random_state=random_state,
                                                                              ␣
↪                    train_test_split_=True,
                                                                              ␣
↪                    verbose=True)
```

```
I am doing standardization…
  molecular descriptor name
0                    AATS0Z
1                  AATS0are
2                    AATS0d
3                   AATS0dv
4                    AATS0i
  molecular descriptor name  corr_value
0                    AATS0Z   -0.038502
1                  AATS0are   -0.120847
2                    AATS0d    0.041648
3                   AATS0dv   -0.115899
4                    AATS0i    0.191765
  molecular descriptor name  corr_value  absolute correlation value
0                    AATS0Z   -0.038502                    0.038502
1                  AATS0are   -0.120847                    0.120847
2                    AATS0d    0.041648                    0.041648
3                   AATS0dv   -0.115899                    0.115899
4                    AATS0i    0.191765                    0.191765
     molecular descriptor name  corr_value  absolute correlation value
226                     AMID_O   -0.520578                    0.520578
505                 EState_VSA5   -0.578904                    0.578904
506                 EState_VSA6    0.519794                    0.519794
791                   MDEO-12   -0.525441                    0.525441
```

```
1211                     MCF-7    1.000000                    1.000000
    molecular descriptor name  corr_value  absolute correlation value
226                    AMID_O   -0.520578                    0.520578
505               EState_VSA5   -0.578904                    0.578904
506               EState_VSA6    0.519794                    0.519794
791                  MDEO-12   -0.525441                    0.525441
The model used is: RandomForest…
Return the coefficient of determination of the prediction:
0.3896895830895062
R^2 score: 0.9161168599597713
Correlation coefficient: 0.9571399375011844
Test data - unseen during training:
R^2 score: 0.3896895830895062
Correlation coefficient: 0.6242512179319366
[7.7026269  7.66385111 7.28090766 7.13067235 5.29752234 8.36053117
 8.31454573 7.72841569 7.89518196 8.02037119 7.26759299 7.36357456
 8.0252565  7.89142517 7.4779577  7.7026269  7.81209996 8.24428702]
44     8.267606
47     8.065502
4      7.032920
55     7.920819
26     4.998569
64     8.619789
73     7.920819
10     7.987163
40     8.091515
107    7.795880
18     7.074688
62     8.958607
11     8.013228
36     6.108463
89     8.045757
91     9.154902
109    8.086186
0      7.987163
Name: MCF-7, dtype: float64
Training Root Mean Square Error: 0.2751761947609772
Testing Root Mean Square Error: 0.7441263740659375
```

3.1 Search inside correlation space

[24]:
```
step = 0.01
initial_step = corr_low
last_step = corr_high
first_list = [x / 100.0 for x in range(int(initial_step*100),
 ↪int(last_step*100), int(step*100))]
n_estimators = [range(2,21,1)]
```

```
corr_th = []
second_list = []
third_list = []
fourth_l = []
fifth_l = []
f_list = []
fif_list = []
for i in first_list:

    for estimator in n_estimators[0]:

        without_standardization, train_r2, test_r2, _, h_, target_column_name,␣
↪training_data_RMSE, test_data_RMSE = pred_model.
↪prepare_data_and_create_model(molecular_descriptors_df=data,
                                                                                ␣
↪                    correlation_threshold=i,
                                                                                ␣
↪                    standardization=False,
                                                                                ␣
↪                    model_type='RandomForestRegressor',
                                                                                ␣
↪                    n_estimators_=estimator,
                                                                                ␣
↪                    target_column_name = target,
                                                                                ␣
↪                    random_state=random_state,
                                                                                ␣
↪                    train_test_split_=True,
                                                                                ␣
↪                    verbose=False)
        corr_th.append(i)
        second_list.append(train_r2)
        third_list.append(test_r2)
        fourth_l.append(training_data_RMSE)
        fifth_l.append(test_data_RMSE)
        f_list.append(len(h_))
        fif_list.append(estimator)
```

[25]:
```
df_without_standardization = pd.DataFrame(data=corr_th, columns=["Correlation␣
↪threshold"])
df_without_standardization['Training data R^2 score'] = second_list
df_without_standardization['Test data R^2 score'] = third_list
df_without_standardization['Training RMSE'] = fourth_l
df_without_standardization['Test RMSE'] = fifth_l
df_without_standardization['Number of features'] = f_list
df_without_standardization['Number of estimators'] = fif_list
```

[26]:
```
df_random_forest = df_without_standardization.copy()
#df_without_standardization.to_excel('../Data/
↪A549_Random_forest_rs_'+str(random_state)+'.xlsx')
```

[27]:
```
corre_list = [x / 100.0 for x in range(int(initial_step*100),␣
↪int(last_step*100), int(0.01*100))]
for element in corre_list:
    element_ =␣
↪df_without_standardization[df_without_standardization['Correlation␣
↪threshold'] == float(element)]
    plt.plot(element_['Number of estimators'], element_['Training data R^2␣
↪score'], label = "Training data R^2 score", marker='v')
    plt.plot(element_['Number of estimators'], element_['Test data R^2 score'],␣
↪label = "Test data R^2 score", marker='D')
    plt.legend()
    plt.xlabel('Number of estimators -> correlation threshold: '+str(element)+'.
↪ \n Number of features: '+str(element_['Number of features'].iloc[0]))
    plt.xlim(max(element_['Number of estimators'])+1, min(element_['Number of␣
↪estimators'])-1)
    plt.ylabel('R^2 score')
    plt.ylim([0, 1])
    plt.rc('grid', linestyle="-.", color='black')
    plt.grid(True)
    plt.show()
```

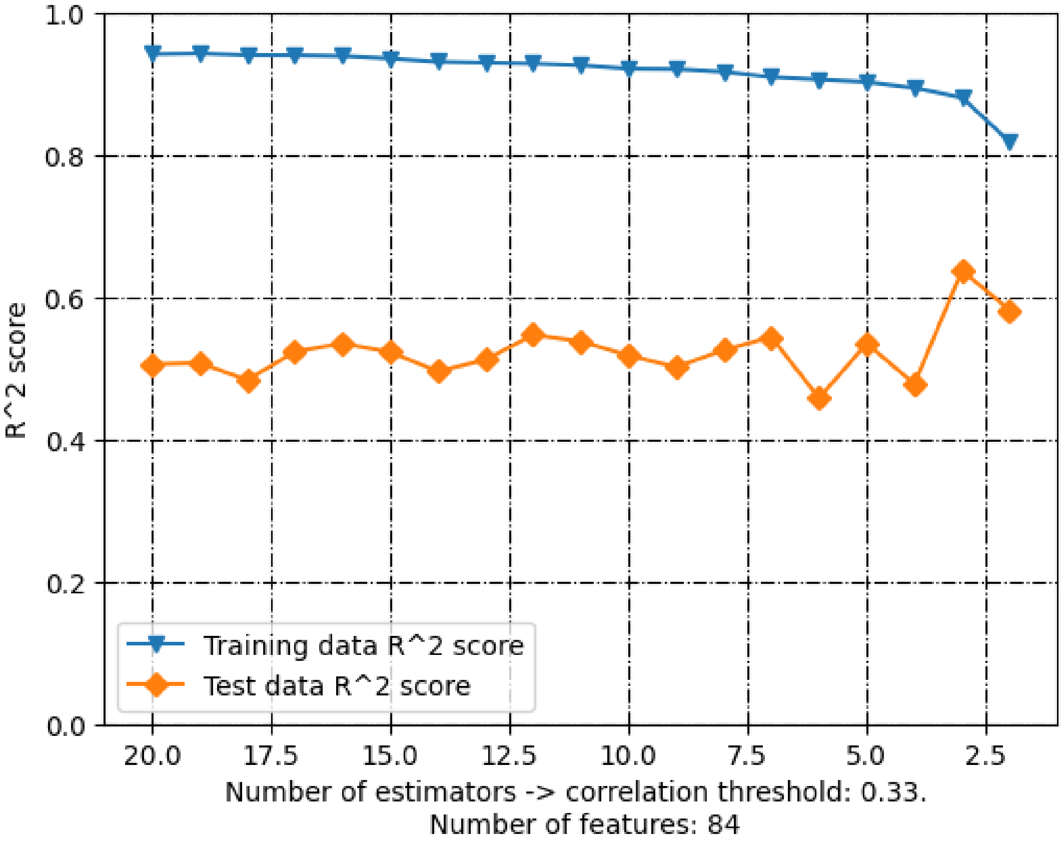
1.0
0.8
0.6
0.4
0.2
0.0
R^2 score
Training data R^2 score
Test data R^2 score
20.0
17.5
15.0
12.5
10.0
7.5
5.0
2.5
Number of estimators -> correlation threshold: 0.33.
Number of features: 84

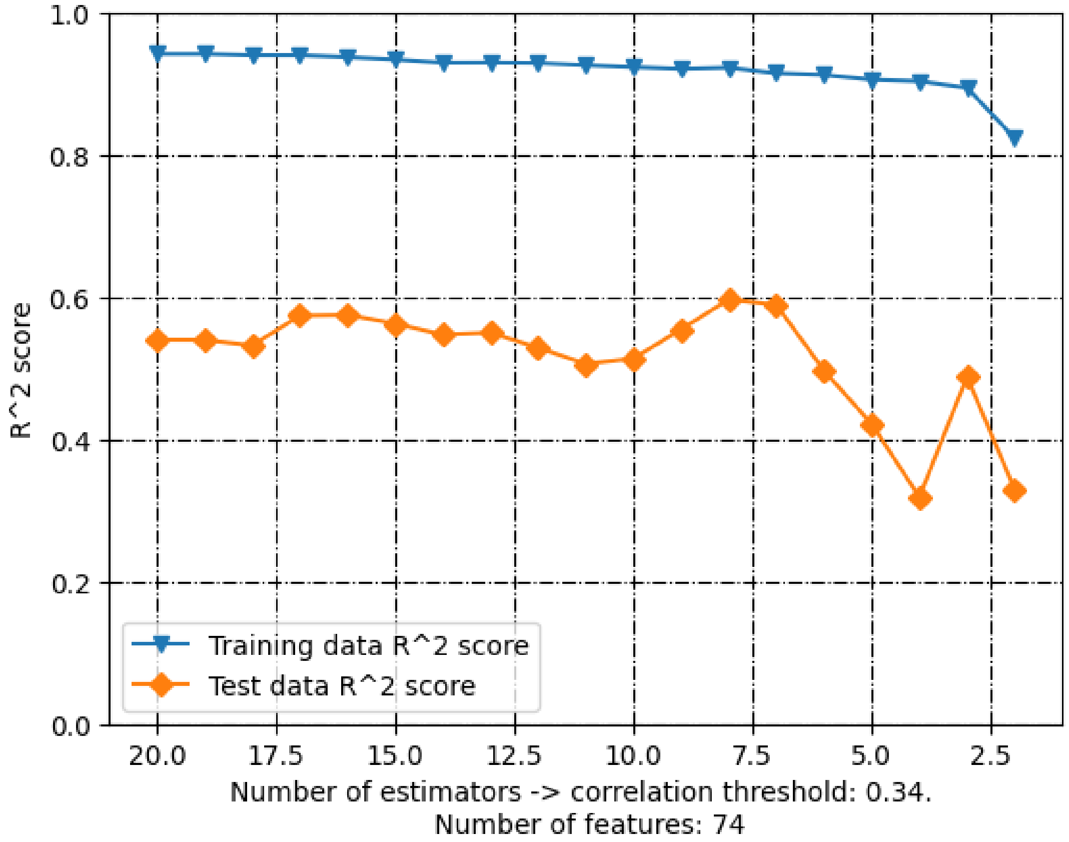

1.0
0.8
0.6
0.4
0.2
0.0
R^2 score
Training data R^2 score
Test data R^2 score
20.0
17.5
15.0
12.5
10.0
7.5
5.0
2.5
Number of estimators -> correlation threshold: 0.34.
Number of features: 74

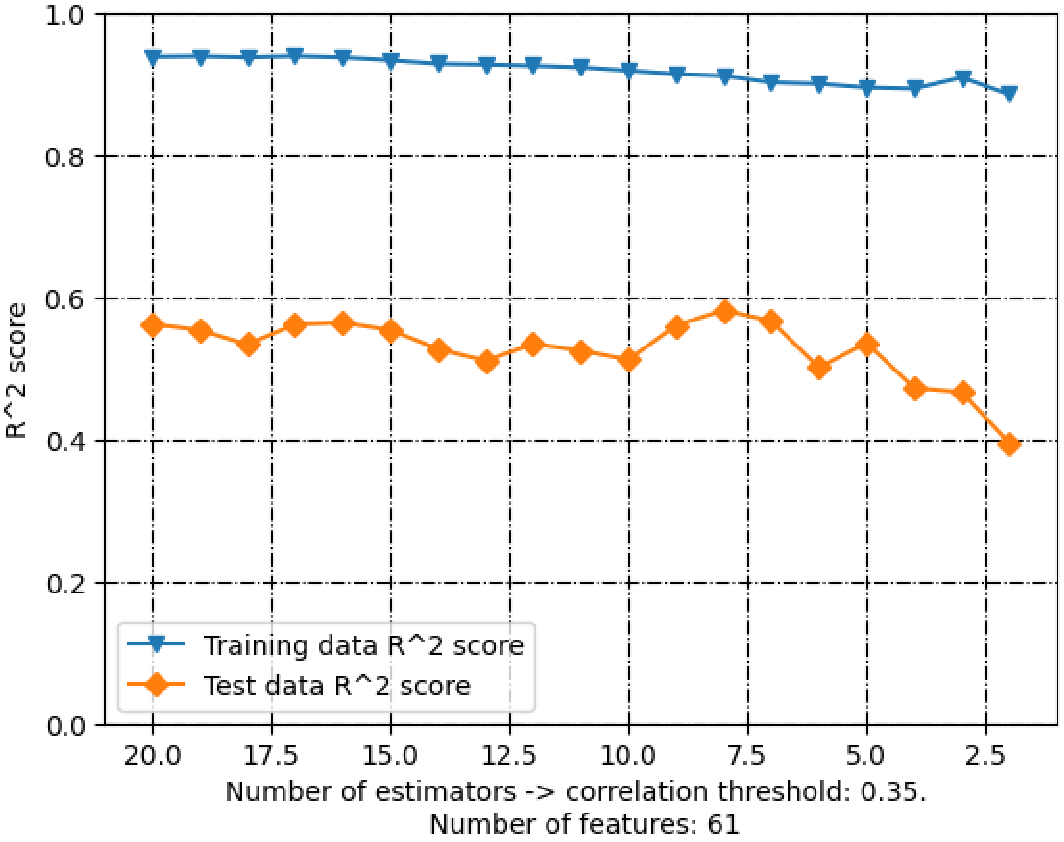
1.0
0.8
0.6
0.4
0.2
0.0
R^2 score
Training data R^2 score
Test data R^2 score
20.0
17.5
15.0
12.5
10.0
7.5
5.0
2.5
Number of estimators -> correlation threshold: 0.35.
Number of features: 61

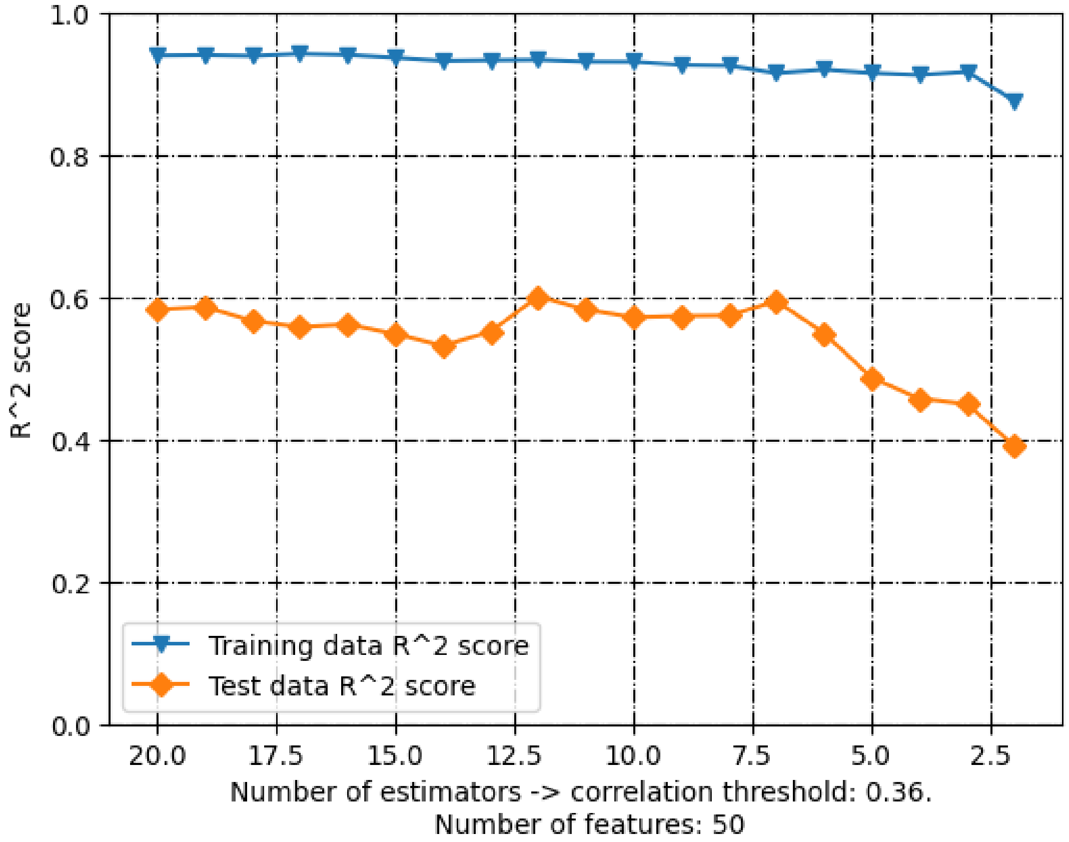
1.0
0.8
0.6
0.4
0.2
0.0
R^2 score
Training data R^2 score
Test data R^2 score
20.0
17.5
15.0
12.5
10.0
7.5
5.0
2.5
Number of estimators -> correlation threshold: 0.36.
Number of features: 50

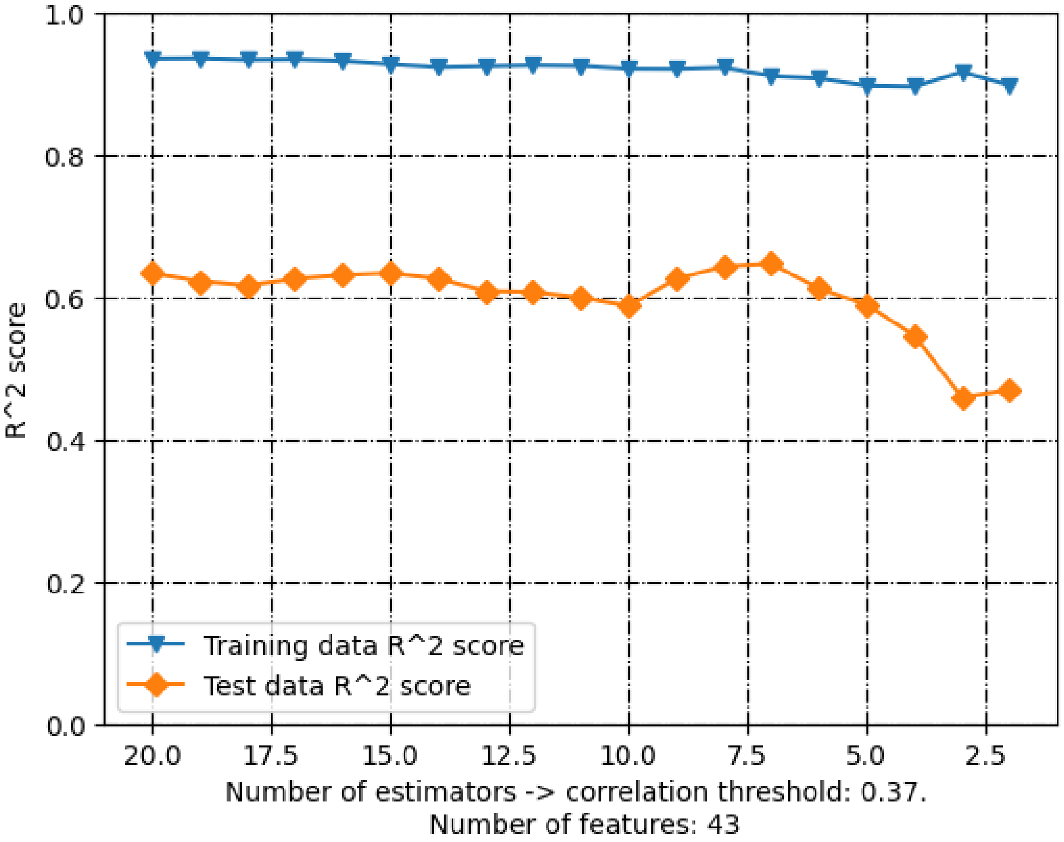

1.0
0.8
0.6
0.4
0.2
0.0
R^2 score
Training data R^2 score
Test data R^2 score
20.0
17.5
15.0
12.5
10.0
7.5
5.0
2.5
Number of estimators -> correlation threshold: 0.37.
Number of features: 43

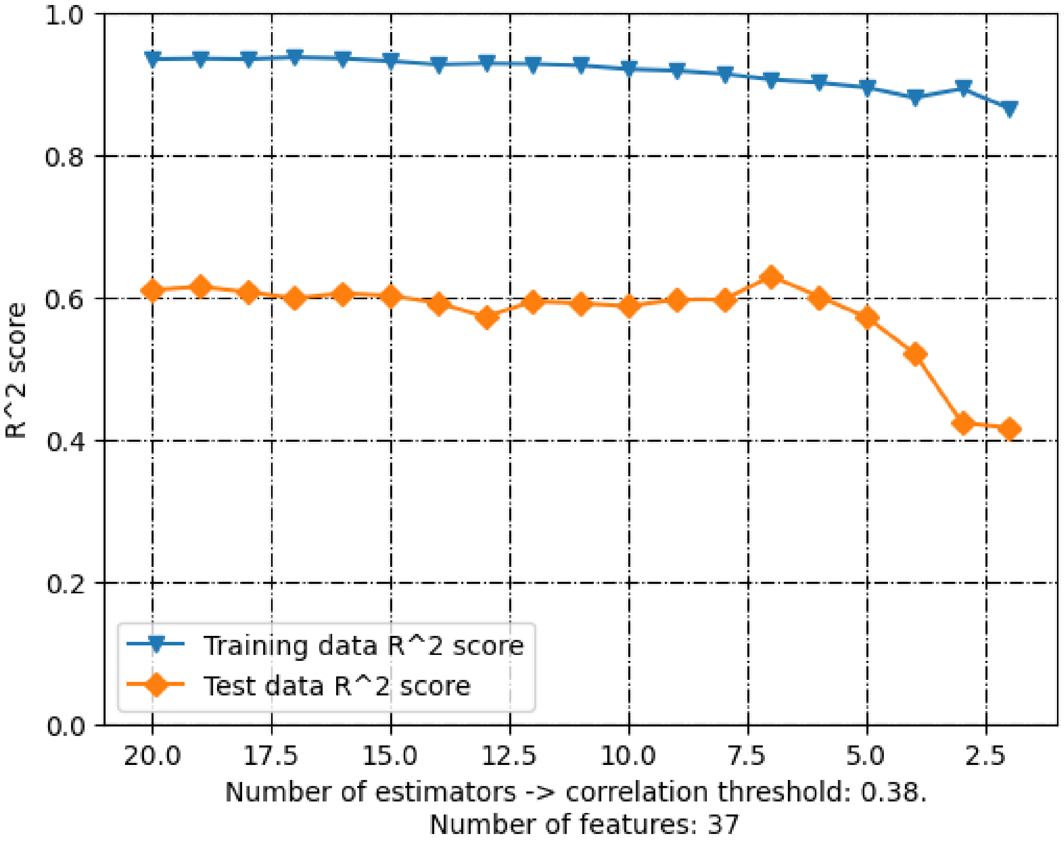

1.0
0.8
0.6
0.4
0.2
0.0
R^2 score
Training data R^2 score
Test data R^2 score
20.0
17.5
15.0
12.5
10.0
7.5
5.0
2.5
Number of estimators -> correlation threshold: 0.38.
Number of features: 37

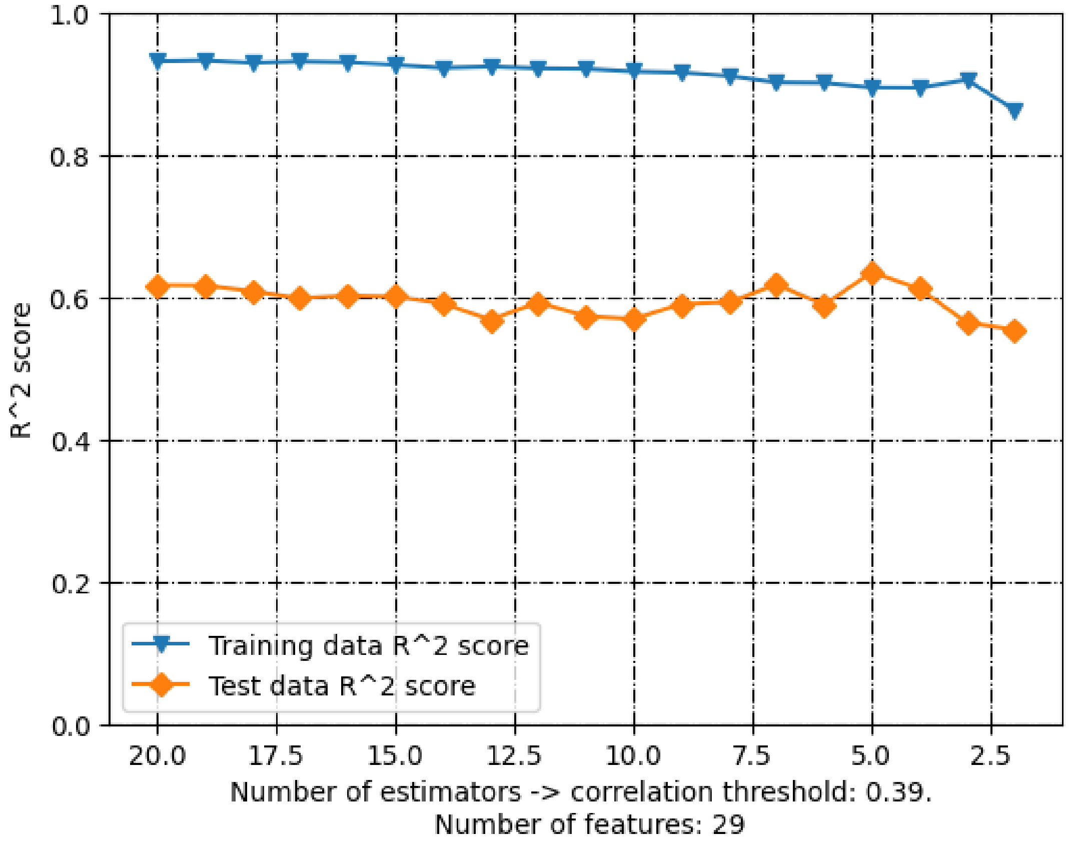

1.0
0.8
0.6
0.4
0.2
0.0
R^2 score
20.0
17.5
15.0
12.5
10.0
7.5
5.0
2.5
Training data R^2 score
Test data R^2 score
Number of estimators -> correlation threshold: 0.39.
Number of features: 29

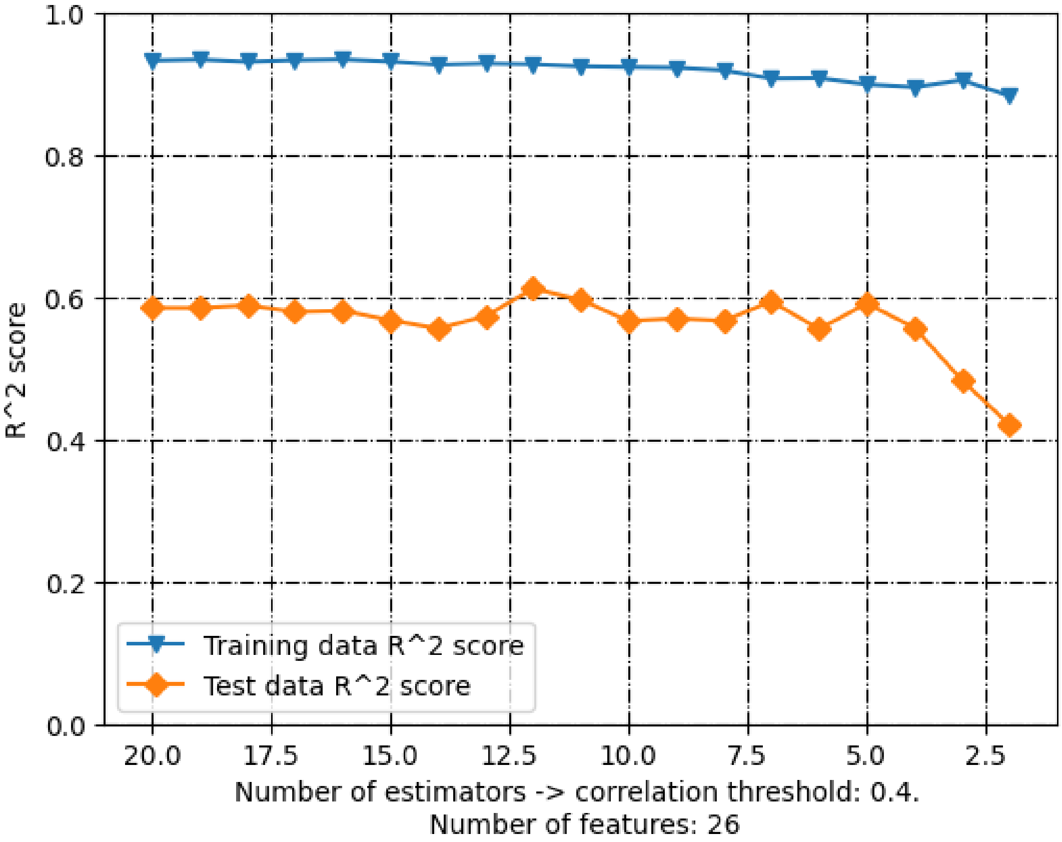
1.0
0.8
0.6
0.4
0.2
0.0
R^2 score
Training data R^2 score
Test data R^2 score
20.0
17.5
15.0
12.5
10.0
7.5
5.0
2.5
Number of estimators -> correlation threshold: 0.4.
Number of features: 26

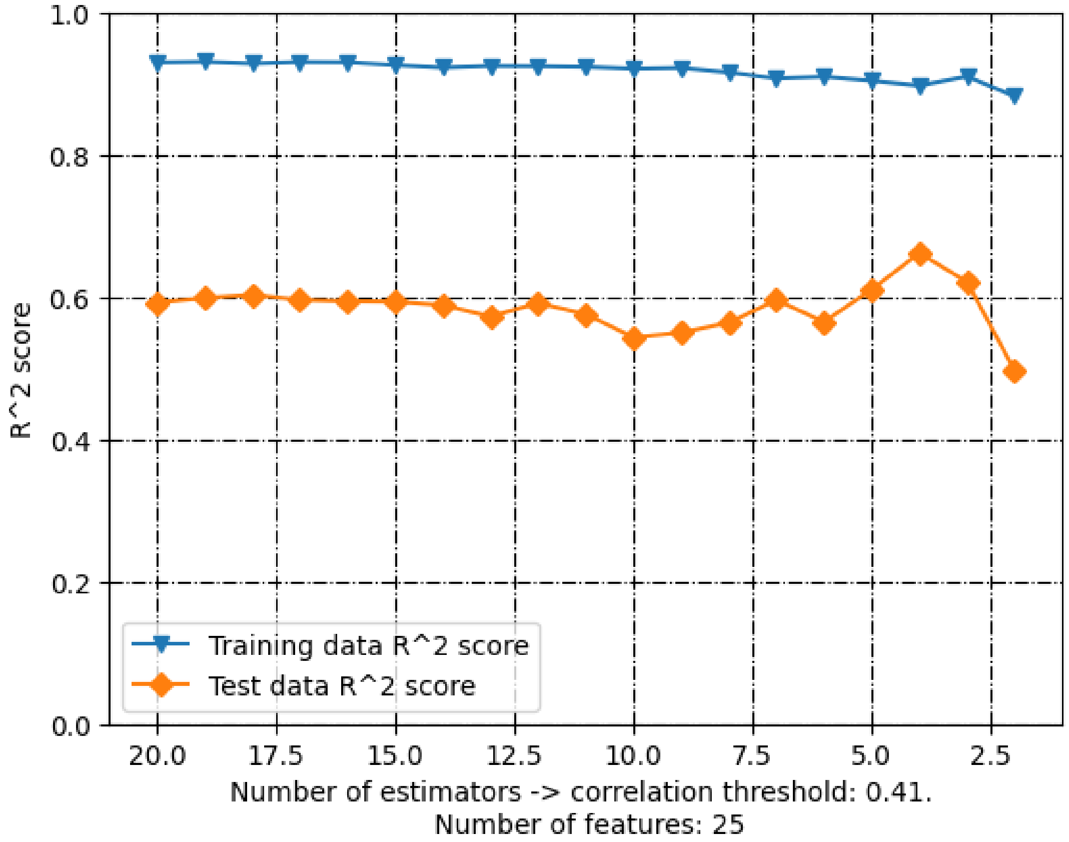

1.0
0.8
0.6
0.4
0.2
0.0
R^2 score
Training data R^2 score
Test data R^2 score
20.0
17.5
15.0
12.5
10.0
7.5
5.0
2.5
Number of estimators -> correlation threshold: 0.41.
Number of features: 25

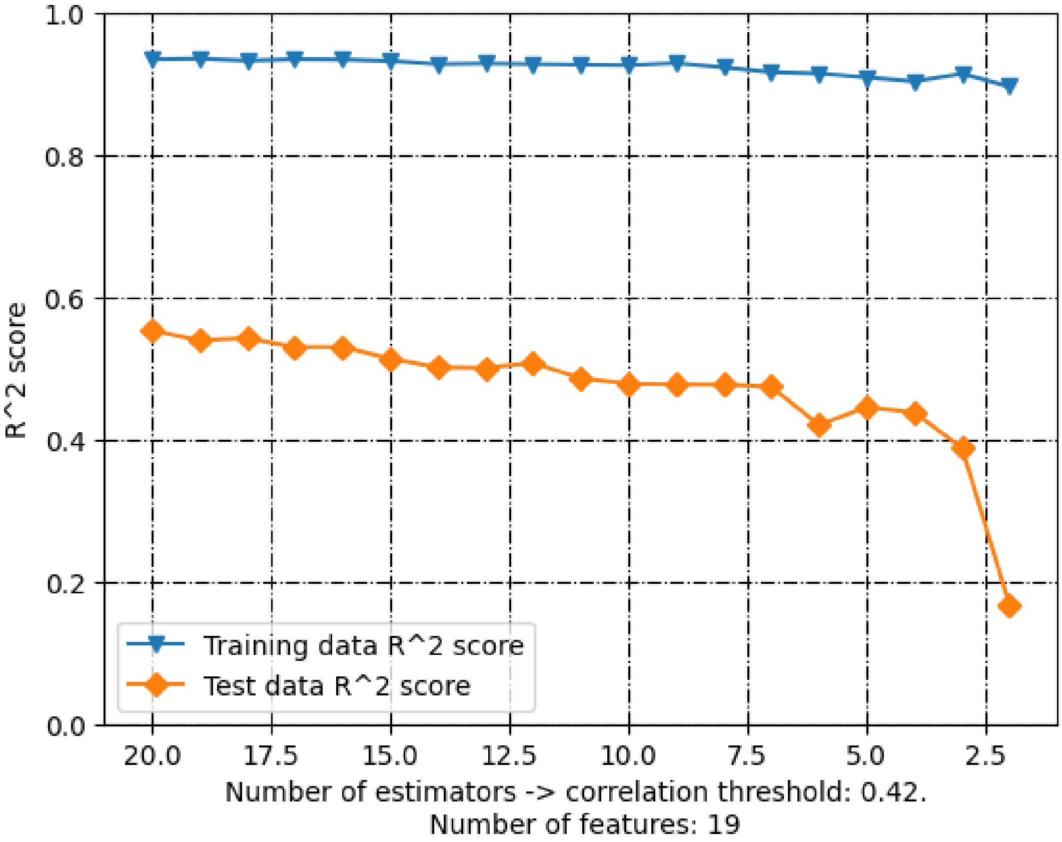

1.0
0.8
0.6
0.4
0.2
0.0
R^2 score
Training data R^2 score
Test data R^2 score
20.0
17.5
15.0
12.5
10.0
7.5
5.0
2.5
Number of estimators -> correlation threshold: 0.42.
Number of features: 19

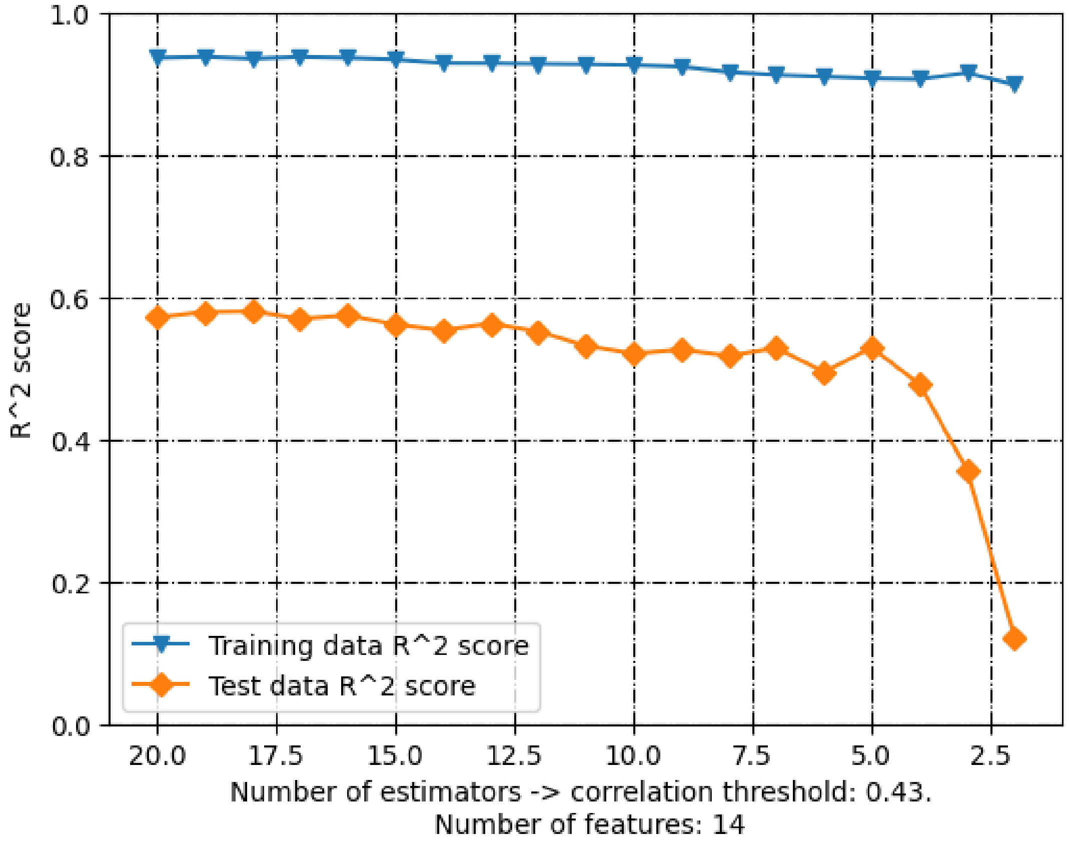
1.0
0.8
0.6
0.4
0.2
0.0
R^2 score
Training data R^2 score
Test data R^2 score
20.0
17.5
15.0
12.5
10.0
7.5
5.0
2.5
Number of estimators -> correlation threshold: 0.43.
Number of features: 14

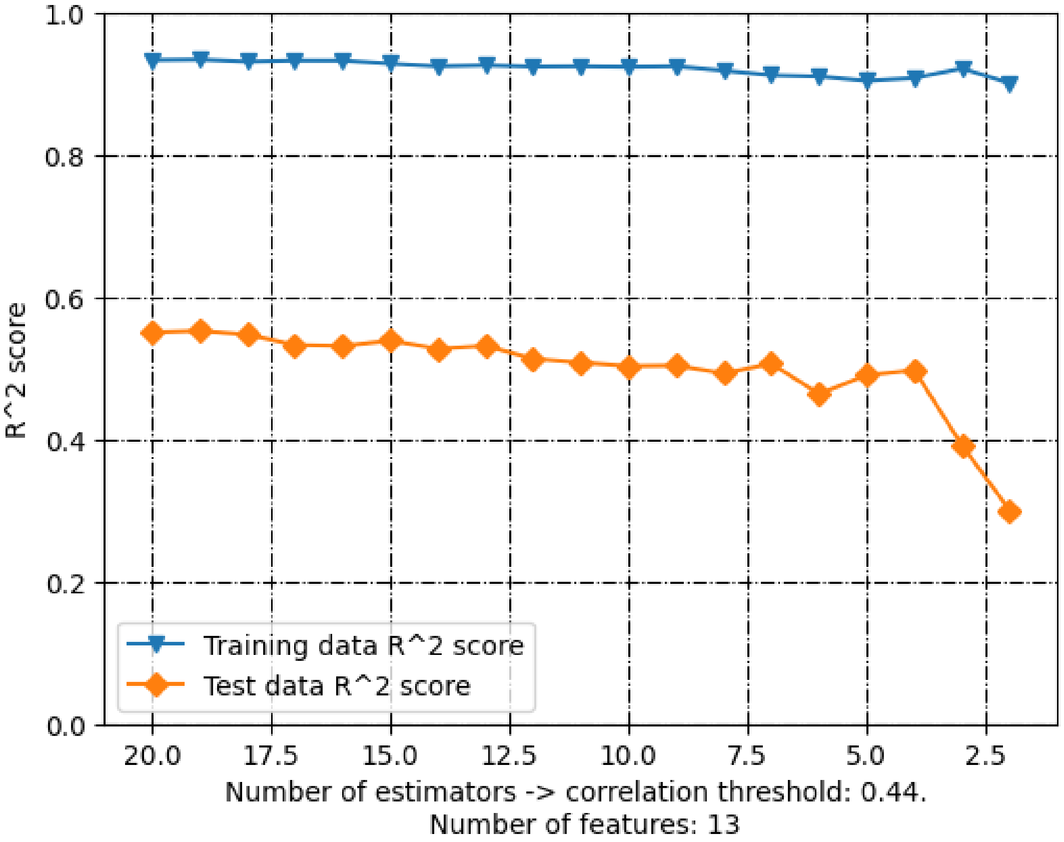

1.0
0.8
0.6
0.4
0.2
0.0
R^2 score
Training data R^2 score
Test data R^2 score
20.0
17.5
15.0
12.5
10.0
7.5
5.0
2.5
Number of estimators -> correlation threshold: 0.44.
Number of features: 13

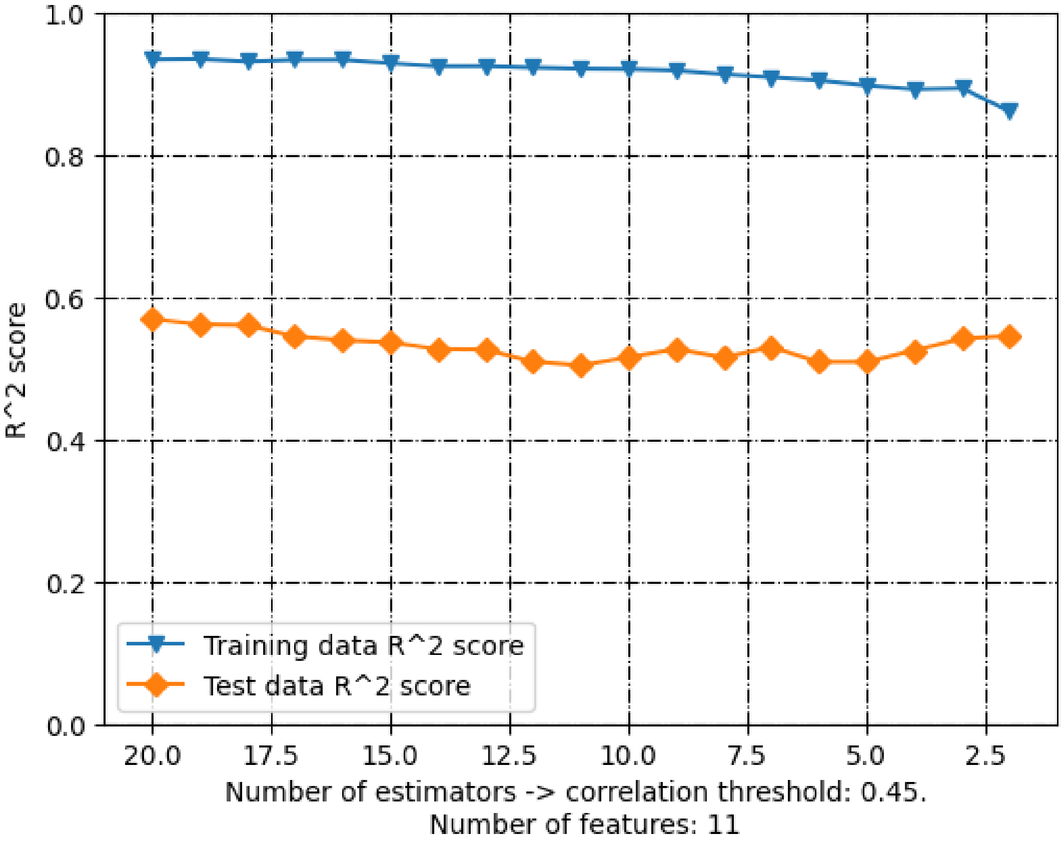

1.0
0.8
0.6
0.4
0.2
0.0
R^2 score
20.0
17.5
15.0
12.5
10.0
7.5
5.0
2.5
Training data R^2 score
Test data R^2 score
Number of estimators -> correlation threshold: 0.45.
Number of features: 11

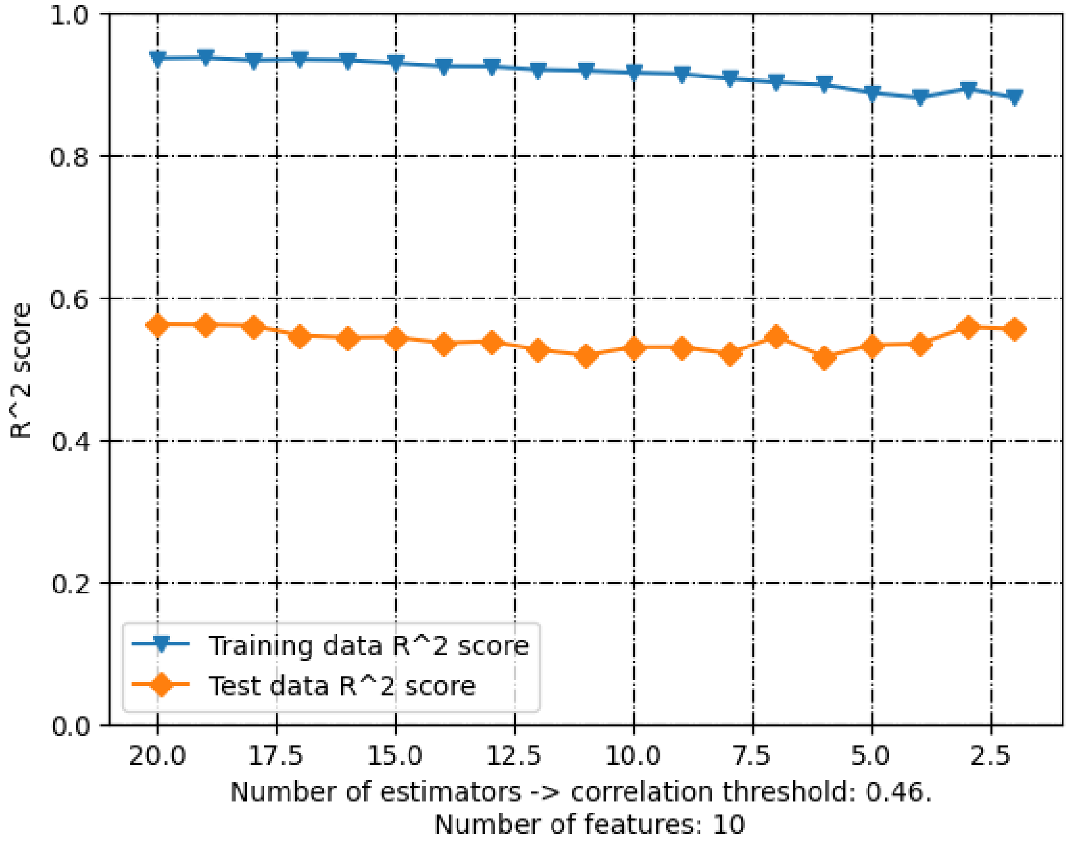
1.0
0.8
0.6
0.4
0.2
0.0
R^2 score
Training data R^2 score
Test data R^2 score
20.0
17.5
15.0
12.5
10.0
7.5
5.0
2.5
Number of estimators -> correlation threshold: 0.46.
Number of features: 10

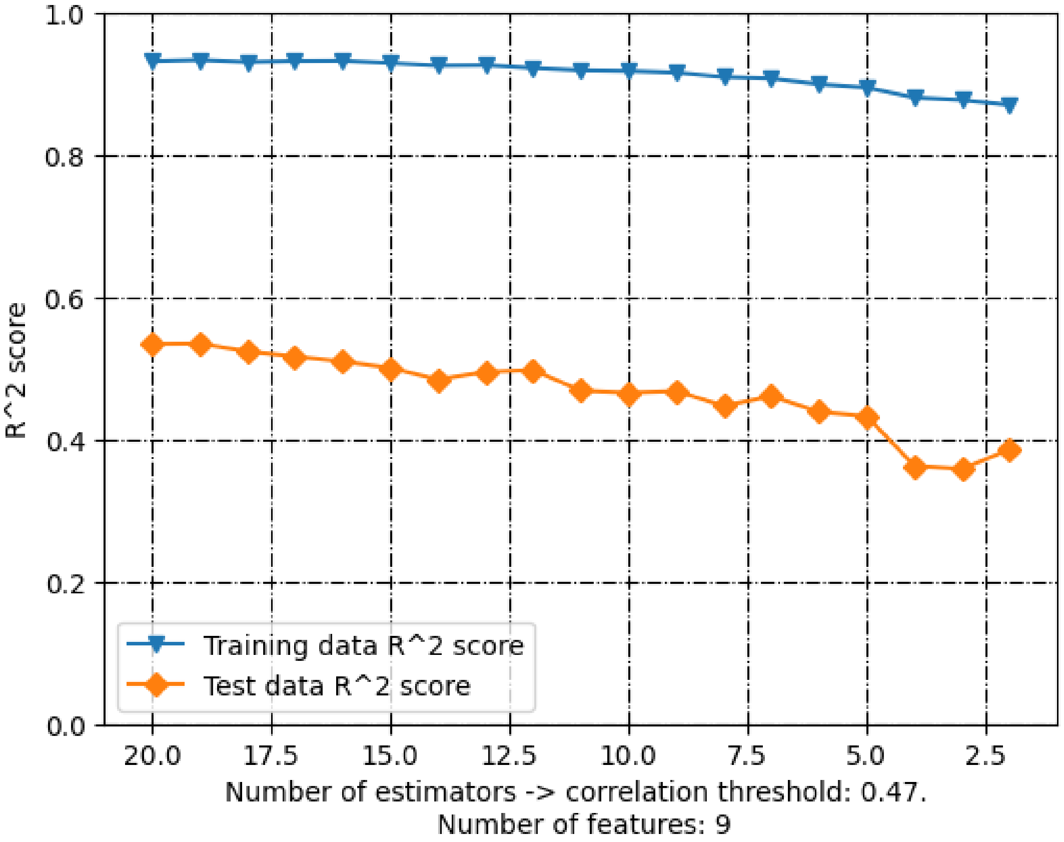

1.0
0.8
0.6
0.4
0.2
0.0
R^2 score
20.0
17.5
15.0
12.5
10.0
7.5
5.0
2.5
Training data R^2 score
Test data R^2 score
Number of estimators -> correlation threshold: 0.47.
Number of features: 9

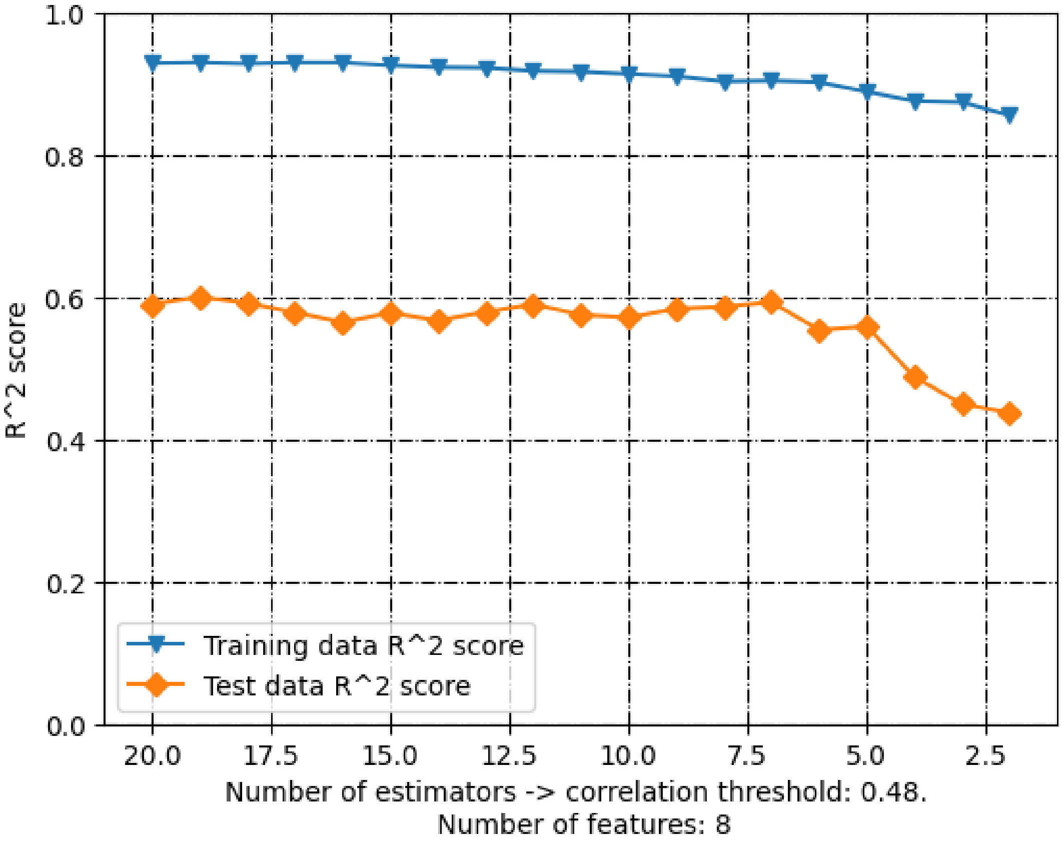
1.0
0.8
0.6
0.4
0.2
0.0
R^2 score
20.0
17.5
15.0
12.5
10.0
7.5
5.0
2.5
Training data R^2 score
Test data R^2 score
Number of estimators -> correlation threshold: 0.48.
Number of features: 8

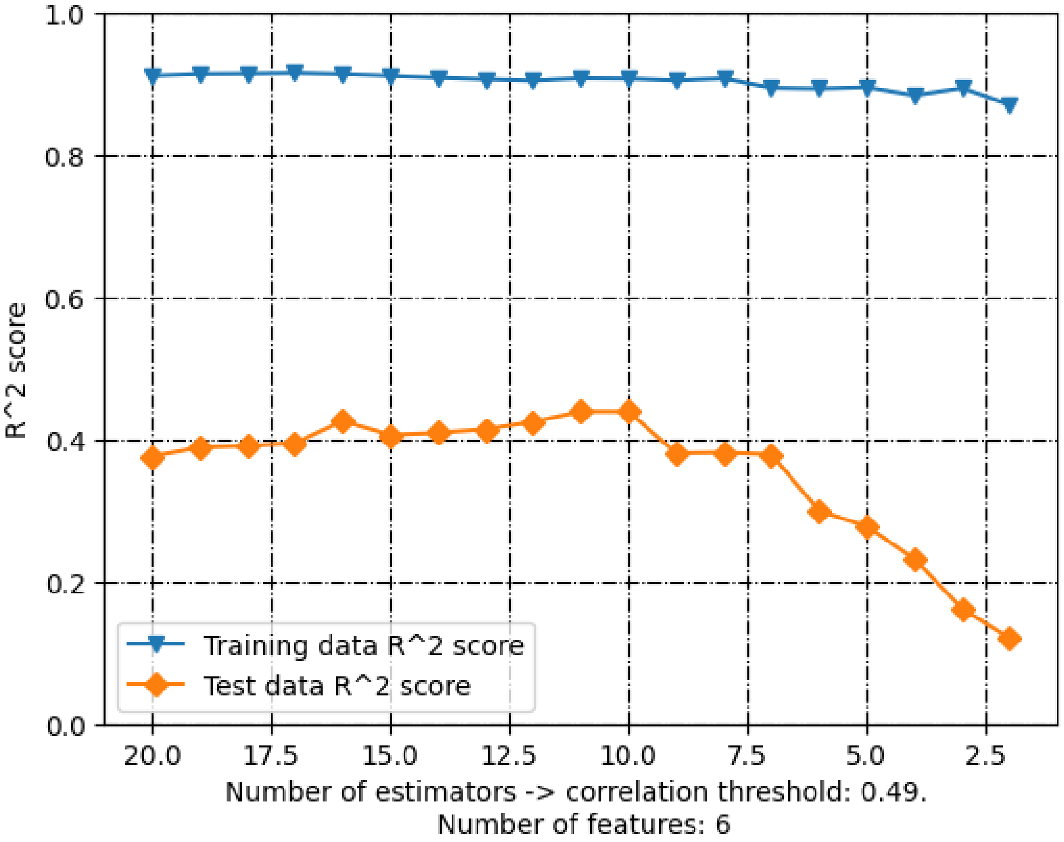

1.0
0.8
0.6
0.4
0.2
0.0
R^2 score
Training data R^2 score
Test data R^2 score
20.0
17.5
15.0
12.5
10.0
7.5
5.0
2.5
Number of estimators -> correlation threshold: 0.49.
Number of features: 6

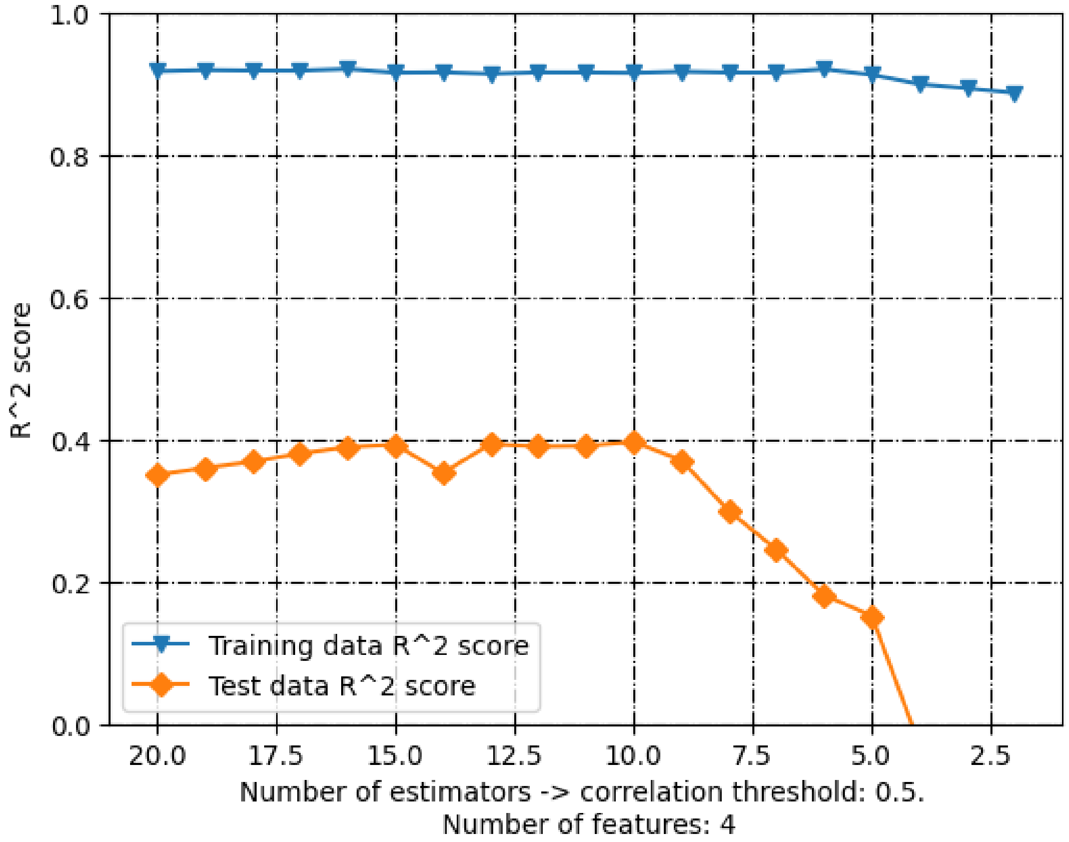
1.0
0.8
0.6
0.4
0.2
0.0
R^2 score
Training data R^2 score
Test data R^2 score
20.0
17.5
15.0
12.5
10.0
7.5
5.0
2.5
Number of estimators -> correlation threshold: 0.5.
Number of features: 4

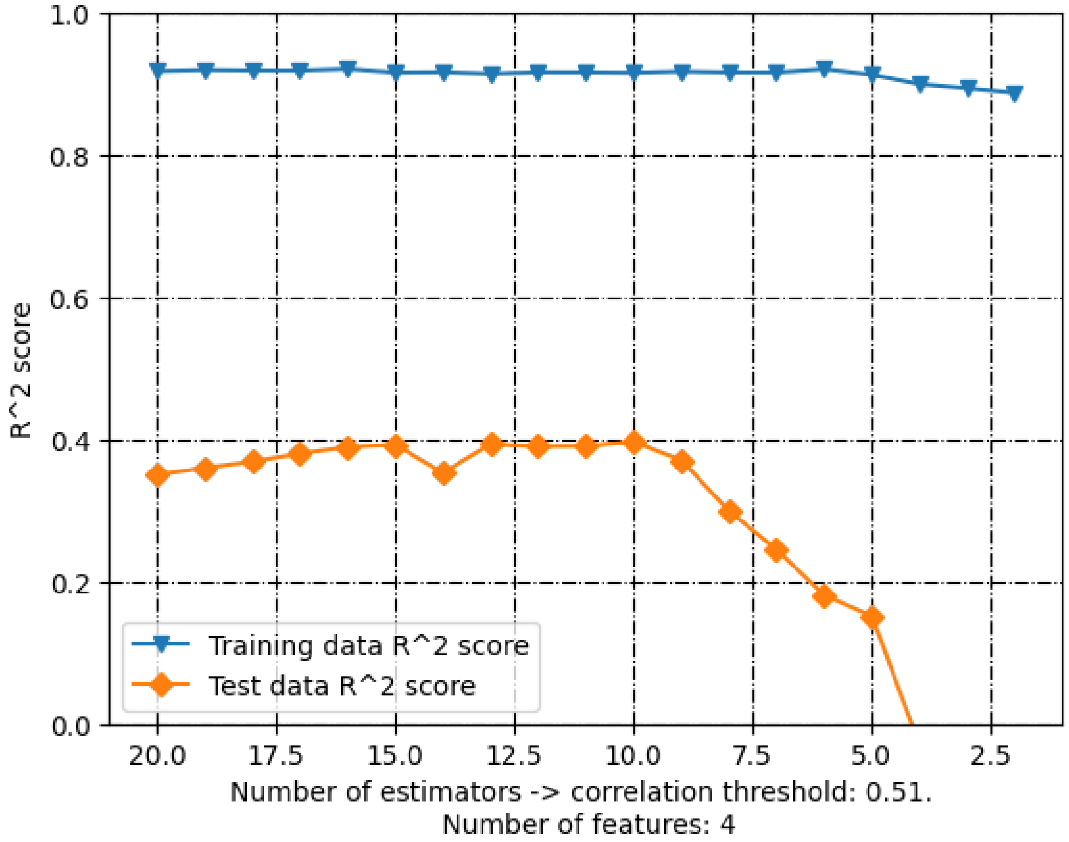

[28]:
```
plt.plot(df_without_standardization['Correlation threshold'],␣
 ↪df_without_standardization['Number of features'], label = "Number of␣
 ↪features", marker='.')
plt.legend()
plt.xlabel('Correlation threshold')
plt.ylabel('Number of features')
plt.show()
```

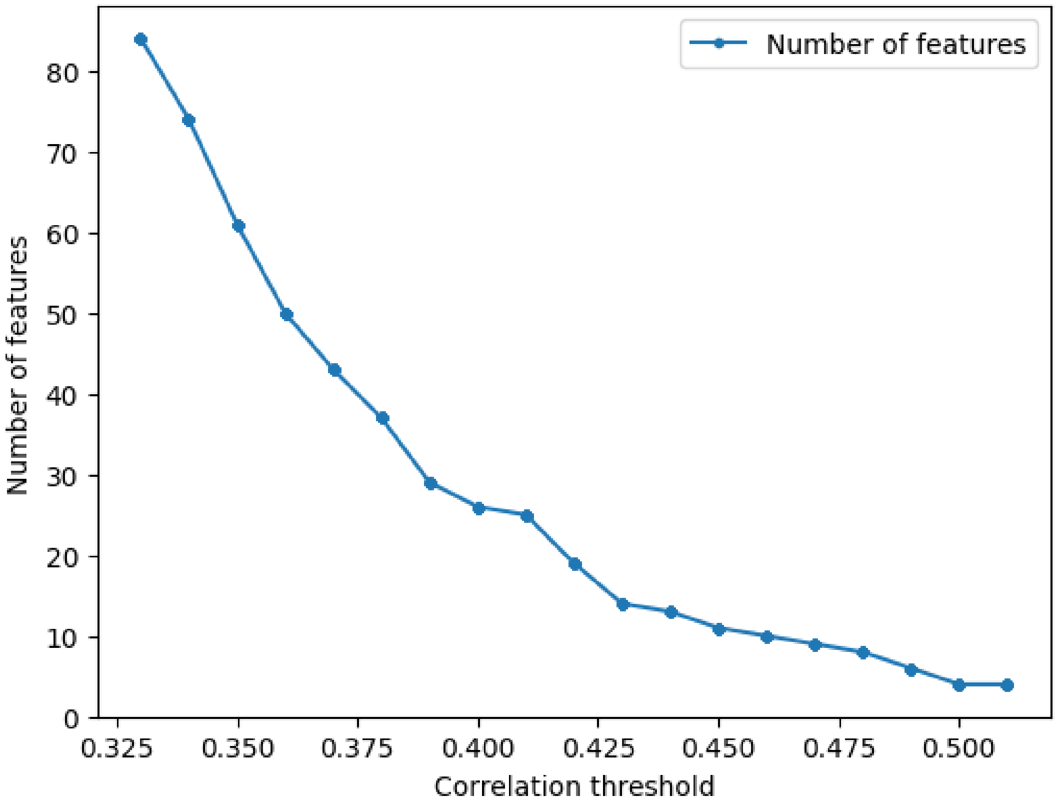

[ ]:

```

```

4 KNeighborsRegressor

[29]:

```
without_standardization, train_r2_, test_r2_, hist1, hist2, target_column_name,␣
↪training_data_RMSE, test_data_RMSE  = pred_model.
↪prepare_data_and_create_model(molecular_descriptors_df=data,
                                                                              ␣
↪                    correlation_threshold=0.50,
                                                                              ␣
↪                    standardization=False,
                                                                              ␣
↪                    model_type='KNeighborsRegressor',
                                                                              ␣
↪                    target_column_name = target,
                                                                              ␣
↪                    random_state=random_state,
                                                                              ␣
↪                    train_test_split_=True,
```

```
↪                     verbose=True)
```

```
I am not doing standardization…
  molecular descriptor name
0                    AATS0Z
1                  AATS0are
2                    AATS0d
3                   AATS0dv
4                    AATS0i
  molecular descriptor name  corr_value
0                    AATS0Z   -0.038502
1                  AATS0are   -0.120847
2                    AATS0d    0.041648
3                   AATS0dv   -0.115899
4                    AATS0i    0.191765
  molecular descriptor name  corr_value  absolute correlation value
0                    AATS0Z   -0.038502                    0.038502
1                  AATS0are   -0.120847                    0.120847
2                    AATS0d    0.041648                    0.041648
3                   AATS0dv   -0.115899                    0.115899
4                    AATS0i    0.191765                    0.191765
     molecular descriptor name  corr_value  absolute correlation value
226                     AMID_O   -0.520578                    0.520578
505                EState_VSA5   -0.578904                    0.578904
506                EState_VSA6    0.519794                    0.519794
791                    MDEO-12   -0.525441                    0.525441
1211                     MCF-7    1.000000                    1.000000
    molecular descriptor name  corr_value  absolute correlation value
226                    AMID_O   -0.520578                    0.520578
505               EState_VSA5   -0.578904                    0.578904
506               EState_VSA6    0.519794                    0.519794
791                   MDEO-12   -0.525441                    0.525441
The model used is: KNeighbors…
Return the coefficient of determination of the prediction:
0.2808374764517442
R^2 score: 0.5177433694804979
Correlation coefficient: 0.7195438620963269
Test data - unseen during training:
R^2 score: 0.2808374764517442
Correlation coefficient: 0.5299410122379133
[8.02265968 7.9714522  6.72714186 8.17774001 5.97853696 8.48530314
 8.48530314 7.80625019 7.56191125 7.84004855 7.76355315 6.75628066
 8.22981393 6.53369885 6.37453322 8.02265968 7.56191125 7.94489513]
44     8.267606
47     8.065502
4      7.032920
55     7.920819
```

```
26     4.998569
64     8.619789
73     7.920819
10     7.987163
40     8.091515
107    7.795880
18     7.074688
62     8.958607
11     8.013228
36     6.108463
89     8.045757
91     9.154902
109    8.086186
0      7.987163
Name: MCF-7, dtype: float64
Training Root Mean Square Error: 0.6598004765201537
Testing Root Mean Square Error: 0.8077646252541788
```

[30]:
```
print(target_column_name+str('_transformed'))
print(hist1[target_column_name].hist())
```

```
MCF-7_transformed
AxesSubplot(0.125,0.11;0.775x0.77)
```

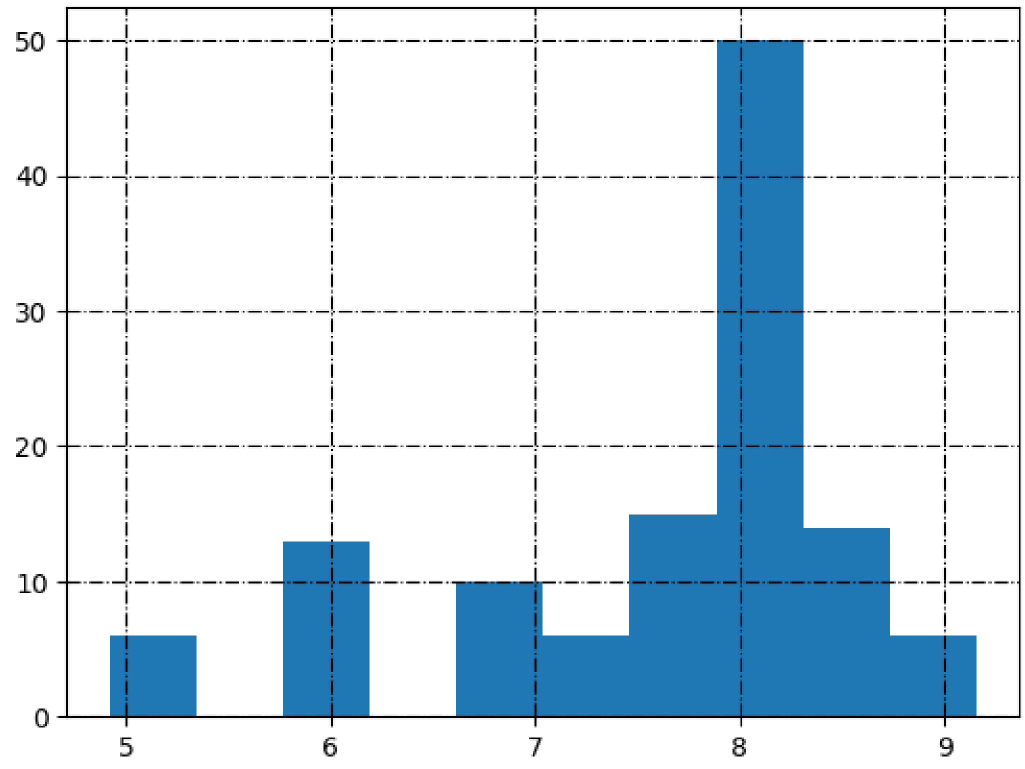

[31]:
```python
with_standardization, train_r2_, test_r2_, hist1, hist2, target_column_name,␣
↪training_data_RMSE, test_data_RMSE  = pred_model.
↪prepare_data_and_create_model(molecular_descriptors_df=data,
                                                                        ␣
↪                    correlation_threshold=0.50,
                                                                        ␣
↪                    standardization=True,
                                                                        ␣
↪                    model_type='KNeighborsRegressor',
                                                                        ␣
↪                    target_column_name = target,
                                                                        ␣
↪                    random_state=random_state,
                                                                        ␣
↪                    train_test_split_=True,
                                                                        ␣
↪                    verbose=True)
```

```
I am doing standardization…
  molecular descriptor name
0                    AATS0Z
1                  AATS0are
2                    AATS0d
3                   AATS0dv
4                    AATS0i
  molecular descriptor name  corr_value
0                    AATS0Z   -0.038502
1                  AATS0are   -0.120847
2                    AATS0d    0.041648
3                   AATS0dv   -0.115899
4                    AATS0i    0.191765
  molecular descriptor name  corr_value  absolute correlation value
0                    AATS0Z   -0.038502                    0.038502
1                  AATS0are   -0.120847                    0.120847
2                    AATS0d    0.041648                    0.041648
3                   AATS0dv   -0.115899                    0.115899
4                    AATS0i    0.191765                    0.191765
     molecular descriptor name  corr_value  absolute correlation value
226                     AMID_O   -0.520578                    0.520578
505                EState_VSA5   -0.578904                    0.578904
506                EState_VSA6    0.519794                    0.519794
791                    MDEO-12   -0.525441                    0.525441
1211                     MCF-7    1.000000                    1.000000
    molecular descriptor name  corr_value  absolute correlation value
226                    AMID_O   -0.520578                    0.520578
505               EState_VSA5   -0.578904                    0.578904
506               EState_VSA6    0.519794                    0.519794
```

```
791                    MDEO-12   -0.525441                    0.525441
The model used is: KNeighbors…
Return the coefficient of determination of the prediction:
0.2808374764517442
R^2 score: 0.5177433694804979
Correlation coefficient: 0.7195438620963269
Test data - unseen during training:
R^2 score: 0.2808374764517442
Correlation coefficient: 0.5299410122379133
[8.02265968 7.9714522  6.72714186 8.17774001 5.97853696 8.48530314
 8.48530314 7.80625019 7.56191125 7.84004855 7.76355315 6.75628066
 8.22981393 6.53369885 6.37453322 8.02265968 7.56191125 7.94489513]
44     8.267606
47     8.065502
4      7.032920
55     7.920819
26     4.998569
64     8.619789
73     7.920819
10     7.987163
40     8.091515
107    7.795880
18     7.074688
62     8.958607
11     8.013228
36     6.108463
89     8.045757
91     9.154902
109    8.086186
0      7.987163
Name: MCF-7, dtype: float64
Training Root Mean Square Error: 0.6598004765201537
Testing Root Mean Square Error: 0.8077646252541788
```

4.1 Search inside correlation space

[32]:
```
step = 0.01
initial_step = corr_low
last_step = corr_high
first_list = [x / 100.0 for x in range(int(initial_step*100),
 ↪int(last_step*100), int(step*100))]
second_list = []
third_list = []
fourth_l = []
fifth_l = []
f_list = []
for i in first_list:
```

```
    without_standardization, train_r2, test_r2, _, h_, target_column_name,␣
↪training_data_RMSE, test_data_RMSE = pred_model.
↪prepare_data_and_create_model(molecular_descriptors_df = data,
                                                                          ␣
↪                    correlation_threshold = i,
                                                                          ␣
↪                    standardization = False,
                                                                          ␣
↪                    model_type = 'KNeighborsRegressor',
                                                                          ␣
↪                    target_column_name = target,
                                                                          ␣
↪                    random_state=random_state,
                                                                          ␣
↪                    train_test_split_ = True,
                                                                          ␣
↪                    verbose = False)
    second_list.append(train_r2)
    third_list.append(test_r2)
    fourth_l.append(training_data_RMSE)
    fifth_l.append(test_data_RMSE)
    f_list.append(len(h_))
```

```
  File "C:\Users\aleks\anaconda3\envs\cheminf_gpu\lib\site-
packages\joblib\externals\loky\backend\context.py", line 217, in
_count_physical_cores
    raise ValueError(
```

[33]:
```
df_without_standardization = pd.DataFrame(data=first_list,␣
↪columns=["Correlation threshold"])
df_without_standardization['Training data R^2 score'] = second_list
df_without_standardization['Test data R^2 score'] = third_list
df_without_standardization['Training RMSE'] = fourth_l
df_without_standardization['Test RMSE'] = fifth_l
df_without_standardization['Number of features'] = f_list
```

[34]:
```
df_k_nearest = df_without_standardization.copy()
df_without_standardization
```

[34]:
```
    Correlation threshold  Training data R^2 score  Test data R^2 score  \
0                    0.33                 0.621822             0.246351
1                    0.34                 0.623312             0.246351
2                    0.35                 0.616957             0.243414
3                    0.36                 0.619734             0.310537
4                    0.37                 0.618726             0.338837
5                    0.38                 0.691970             0.601192
6                    0.39                 0.688301             0.599861
```

7	0.40	0.688301	0.592646
8	0.41	0.683917	0.592893
9	0.42	0.577840	0.266260
10	0.43	0.537940	0.159817
11	0.44	0.537920	0.159817
12	0.45	0.537920	0.136157
13	0.46	0.537023	0.162161
14	0.47	0.537023	0.162161
15	0.48	0.535527	0.162161
16	0.49	0.531239	0.296566
17	0.50	0.517743	0.280837
18	0.51	0.517743	0.280837

	Training RMSE	Test RMSE	Number of features
0	0.584281	0.826906	84
1	0.583129	0.826906	74
2	0.588027	0.828515	61
3	0.585892	0.790910	50
4	0.586668	0.774507	43
5	0.527315	0.601524	37
6	0.530446	0.602527	29
7	0.530446	0.607935	26
8	0.534163	0.607751	25
9	0.617323	0.815910	19
10	0.645837	0.873088	14
11	0.645851	0.873088	13
12	0.645851	0.885297	11
13	0.646477	0.871869	10
14	0.646477	0.871869	9
15	0.647521	0.871869	8
16	0.650503	0.798883	6
17	0.659800	0.807765	4
18	0.659800	0.807765	4

4.2 Plots

[35]:
```
plt.plot(df_without_standardization['Correlation threshold'],␣
 ↪df_without_standardization['Training data R^2 score'], label = "Training␣
 ↪data R^2 score", marker='v')
plt.plot(df_without_standardization['Correlation threshold'],␣
 ↪df_without_standardization['Test data R^2 score'], label = "Test data R^2␣
 ↪score", marker='D')
plt.legend()
plt.xlabel('Correlation threshold')
plt.ylabel('R^2 score')
plt.ylim([0, 1])
plt.show()
```

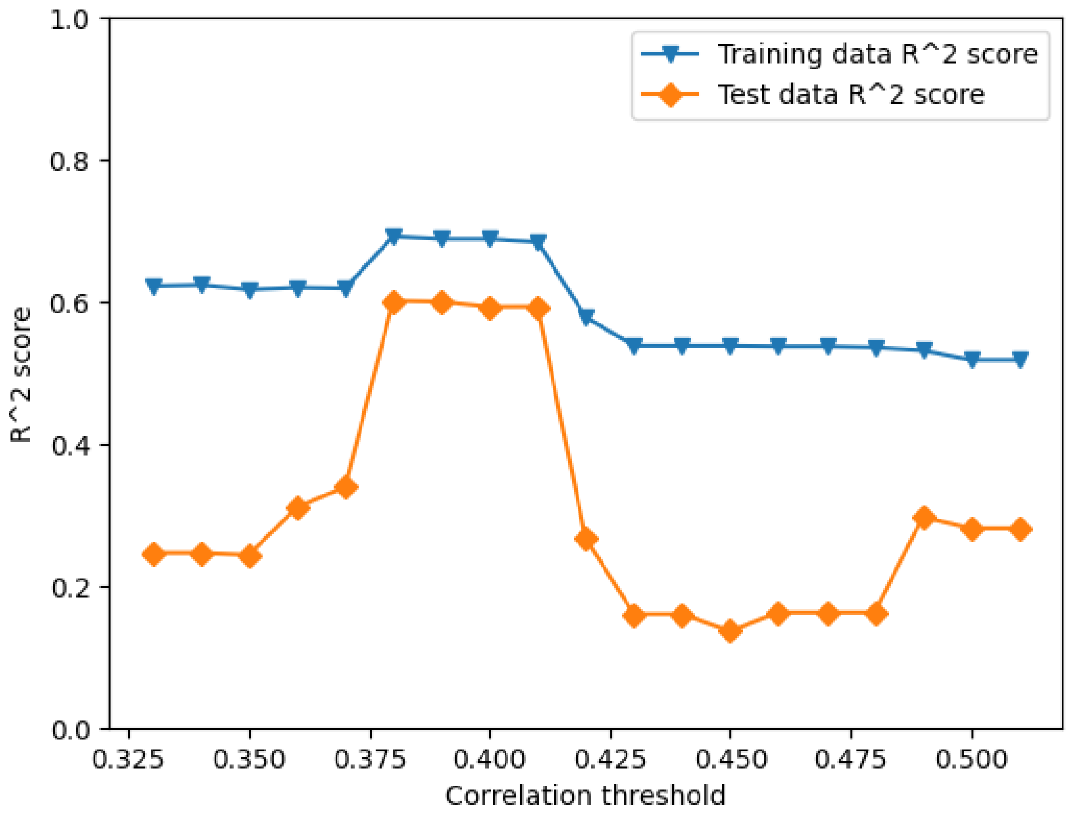

[36]:
```
plt.plot(df_without_standardization['Correlation threshold'],␣
  ↪df_without_standardization['Number of features'], label = "Number of␣
  ↪features", marker='.')
plt.legend()
plt.xlabel('Correlation threshold')
plt.ylabel('Number of features')
plt.show()
```

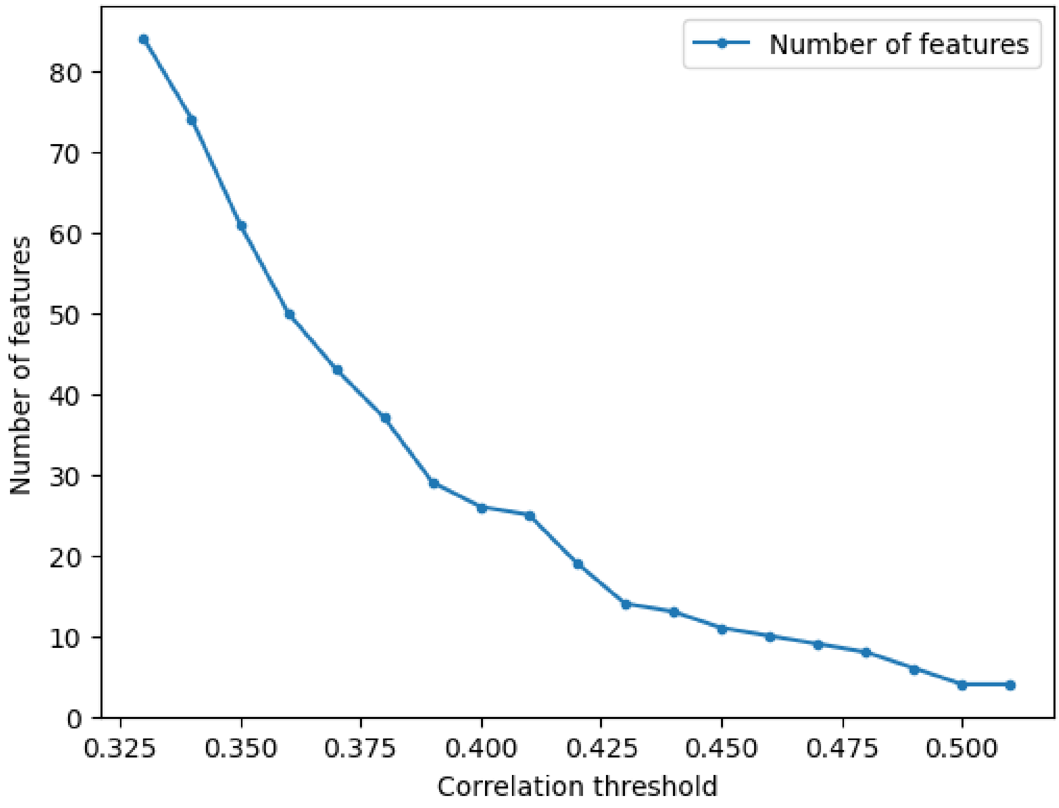

[ ]:

```

```

5 Support Vector Machines (SVM)

[37]:

```
without_standardization, train_r2_, test_r2_, hist1, hist2, target_column_name,␣
↪training_data_RMSE, test_data_RMSE  = pred_model.
↪prepare_data_and_create_model(molecular_descriptors_df=data,
                                                                                ␣
↪                         correlation_threshold=0.50,
                                                                                ␣
↪                         standardization=False,
                                                                                ␣
↪                         model_type='SVR',
                                                                                ␣
↪                         kernel_ = 'linear',
                                                                                ␣
↪                         gamma_ = 'auto',
                                                                                ␣
↪                         target_column_name = target,
```

```
↪                    random_state=random_state,
↪                    train_test_split_=True,
↪                    verbose=True)
```

```
I am not doing standardization…
  molecular descriptor name
0                    AATS0Z
1                  AATS0are
2                    AATS0d
3                   AATS0dv
4                    AATS0i
  molecular descriptor name  corr_value
0                    AATS0Z   -0.038502
1                  AATS0are   -0.120847
2                    AATS0d    0.041648
3                   AATS0dv   -0.115899
4                    AATS0i    0.191765
  molecular descriptor name  corr_value  absolute correlation value
0                    AATS0Z   -0.038502                    0.038502
1                  AATS0are   -0.120847                    0.120847
2                    AATS0d    0.041648                    0.041648
3                   AATS0dv   -0.115899                    0.115899
4                    AATS0i    0.191765                    0.191765
     molecular descriptor name  corr_value  absolute correlation value
226                     AMID_O   -0.520578                    0.520578
505                EState_VSA5   -0.578904                    0.578904
506                EState_VSA6    0.519794                    0.519794
791                    MDEO-12   -0.525441                    0.525441
1211                     MCF-7    1.000000                    1.000000
    molecular descriptor name  corr_value  absolute correlation value
226                    AMID_O   -0.520578                    0.520578
505               EState_VSA5   -0.578904                    0.578904
506               EState_VSA6    0.519794                    0.519794
791                   MDEO-12   -0.525441                    0.525441
The model used is: SVR…
Return the coefficient of determination of the prediction:
0.4161606958939972
R^2 score: 0.5301711041437702
Correlation coefficient: 0.7281284942534321
Test data - unseen during training:
R^2 score: 0.4161606958939972
Correlation coefficient: 0.6451051820393301
[7.95710679 8.19420823 7.37587573 7.58725734 6.13752633 8.45431951
 8.71877874 7.59489641 8.09716398 8.24770467 7.55125979 6.99273128
```

```
 7.93355344 6.92876717 7.29438534 7.95710329 8.09233701 7.6660344 ]
44     8.267606
47     8.065502
4      7.032920
55     7.920819
26     4.998569
64     8.619789
73     7.920819
10     7.987163
40     8.091515
107    7.795880
18     7.074688
62     8.958607
11     8.013228
36     6.108463
89     8.045757
91     9.154902
109    8.086186
0      7.987163
Name: MCF-7, dtype: float64
Training Root Mean Square Error: 0.6512434719413053
Testing Root Mean Square Error: 0.7278099194508179
```

[38]:

```
print(target_column_name+str('_transformed'))
print(hist1[target_column_name].hist())
```

```
MCF-7_transformed
AxesSubplot(0.125,0.11;0.775x0.77)
```

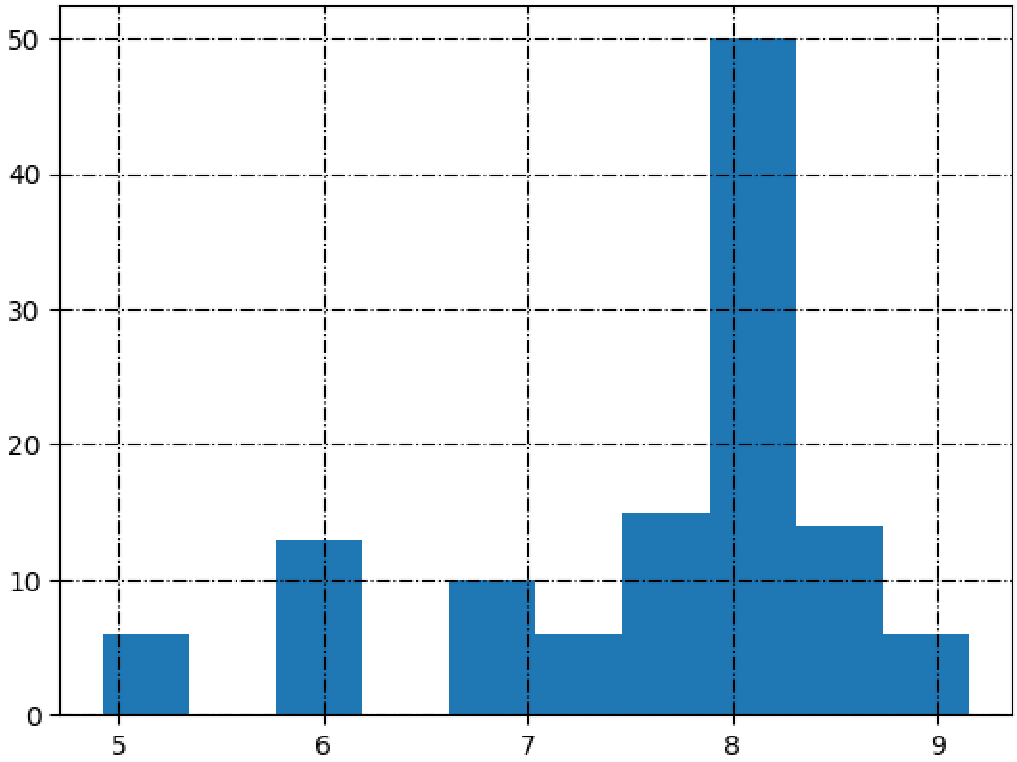

[39]:
```
with_standardization, train_r2_, test_r2_, hist1, hist2, target_column_name,␣
↪training_data_RMSE, test_data_RMSE  = pred_model.
↪prepare_data_and_create_model(molecular_descriptors_df=data,
                                                                                ␣
↪                    correlation_threshold=0.50,
                                                                                ␣
↪                    standardization=True,
                                                                                ␣
↪                    model_type='SVR',
                                                                                ␣
↪                    kernel_ = 'linear',
                                                                                ␣
↪                    gamma_ = 'auto',
                                                                                ␣
↪                    target_column_name = target,
                                                                                ␣
↪                    random_state=random_state,
                                                                                ␣
↪                    train_test_split_=True,
                                                                                ␣
↪                    verbose=True)
```

```
I am doing standardization…
  molecular descriptor name
0                    AATS0Z
1                  AATS0are
2                    AATS0d
3                   AATS0dv
4                    AATS0i
  molecular descriptor name  corr_value
0                    AATS0Z   -0.038502
1                  AATS0are   -0.120847
2                    AATS0d    0.041648
3                   AATS0dv   -0.115899
4                    AATS0i    0.191765
  molecular descriptor name  corr_value  absolute correlation value
0                    AATS0Z   -0.038502                    0.038502
1                  AATS0are   -0.120847                    0.120847
2                    AATS0d    0.041648                    0.041648
3                   AATS0dv   -0.115899                    0.115899
4                    AATS0i    0.191765                    0.191765
     molecular descriptor name  corr_value  absolute correlation value
226                     AMID_O   -0.520578                    0.520578
505                EState_VSA5   -0.578904                    0.578904
506                EState_VSA6    0.519794                    0.519794
791                    MDEO-12   -0.525441                    0.525441
1211                     MCF-7    1.000000                    1.000000
    molecular descriptor name  corr_value  absolute correlation value
226                     AMID_O   -0.520578                    0.520578
505                EState_VSA5   -0.578904                    0.578904
506                EState_VSA6    0.519794                    0.519794
791                    MDEO-12   -0.525441                    0.525441
The model used is: SVR…
Return the coefficient of determination of the prediction:
0.4161606958939972
R^2 score: 0.5301711041437702
Correlation coefficient: 0.7281284942534321
Test data - unseen during training:
R^2 score: 0.4161606958939972
Correlation coefficient: 0.6451051820393301
[7.95710679 8.19420823 7.37587573 7.58725734 6.13752633 8.45431951
 8.71877874 7.59489641 8.09716398 8.24770467 7.55125979 6.99273128
 7.93355344 6.92876717 7.29438534 7.95710329 8.09233701 7.6660344 ]
44     8.267606
47     8.065502
4      7.032920
55     7.920819
26     4.998569
64     8.619789
73     7.920819
```

```
10     7.987163
40     8.091515
107    7.795880
18     7.074688
62     8.958607
11     8.013228
36     6.108463
89     8.045757
91     9.154902
109    8.086186
0      7.987163
Name: MCF-7, dtype: float64
Training Root Mean Square Error: 0.6512434719413053
Testing Root Mean Square Error: 0.7278099194508179
```

5.1 Search inside correlation space

[40]:
```
step = 0.01
initial_step = corr_low
last_step = corr_high
first_list = [x / 100.0 for x in range(int(initial_step*100),␣
 ↪int(last_step*100), int(step*100))]
second_list = []
third_list = []
fourth_l = []
fifth_l = []
f_list = []
for i in first_list:
    without_standardization, train_r2, test_r2, _, h_, target_column_name,␣
 ↪training_data_RMSE, test_data_RMSE = pred_model.
 ↪prepare_data_and_create_model(molecular_descriptors_df = data,
                                                                         ␣
 ↪                    correlation_threshold = i,
                                                                         ␣
 ↪                    standardization = False,
                                                                         ␣
 ↪                    model_type = 'SVR',
                                                                         ␣
 ↪                    kernel_ = 'linear',
                                                                         ␣
 ↪                    gamma_ = 'auto',
                                                                         ␣
 ↪                    target_column_name = target,
                                                                         ␣
 ↪                    random_state=random_state,
                                                                         ␣
 ↪                    train_test_split_ = True,
```

```
                         verbose = False)
    second_list.append(train_r2)
    third_list.append(test_r2)
    fourth_l.append(training_data_RMSE)
    fifth_l.append(test_data_RMSE)
    f_list.append(len(h_))
```

[41]:
```
df_without_standardization = pd.DataFrame(data=first_list,
 columns=["Correlation threshold"])
df_without_standardization['Training data R^2 score'] = second_list
df_without_standardization['Test data R^2 score'] = third_list
df_without_standardization['Training RMSE'] = fourth_l
df_without_standardization['Test RMSE'] = fifth_l
df_without_standardization['Number of features'] = f_list
```

[42]:
```
df_svm = df_without_standardization.copy()
df_without_standardization
```

[42]:

	Correlation threshold	Training data R^2 score	Test data R^2 score
0	0.33	0.699678	0.703930
1	0.34	0.698681	0.714338
2	0.35	0.653574	0.598970
3	0.36	0.619117	0.232309
4	0.37	0.591768	0.076782
5	0.38	0.589794	0.234486
6	0.39	0.584302	0.231231
7	0.40	0.588370	0.317306
8	0.41	0.587545	0.434813
9	0.42	0.560240	0.453842
10	0.43	0.558524	0.438035
11	0.44	0.558364	0.437311
12	0.45	0.553869	0.472323
13	0.46	0.546483	0.405577
14	0.47	0.545600	0.408849
15	0.48	0.547666	0.407742
16	0.49	0.555221	0.381005
17	0.50	0.530171	0.416161
18	0.51	0.530171	0.416161

	Training RMSE	Test RMSE	Number of features
0	0.520676	0.518285	84
1	0.521539	0.509093	74
2	0.559215	0.603198	61
3	0.586367	0.834573	50
4	0.607053	0.915216	43
5	0.608520	0.833389	37

6	0.612579	0.835159	29
7	0.609575	0.787018	26
8	0.610185	0.716090	25
9	0.630059	0.703932	19
10	0.631287	0.714046	14
11	0.631402	0.714505	13
12	0.634607	0.691919	11
13	0.639839	0.734377	10
14	0.640461	0.732353	9
15	0.639003	0.733038	8
16	0.633644	0.749402	6
17	0.651243	0.727810	4
18	0.651243	0.727810	4

5.2 Plots

[43]:
```
plt.plot(df_without_standardization['Correlation threshold'],␣
  ↪df_without_standardization['Training data R^2 score'], label = "Training␣
  ↪data R^2 score", marker='v')
plt.plot(df_without_standardization['Correlation threshold'],␣
  ↪df_without_standardization['Test data R^2 score'], label = "Test data R^2␣
  ↪score", marker='D')
plt.legend()
plt.xlabel('Correlation threshold')
plt.ylabel('R^2 score')
plt.ylim([0, 1])
plt.show()
```

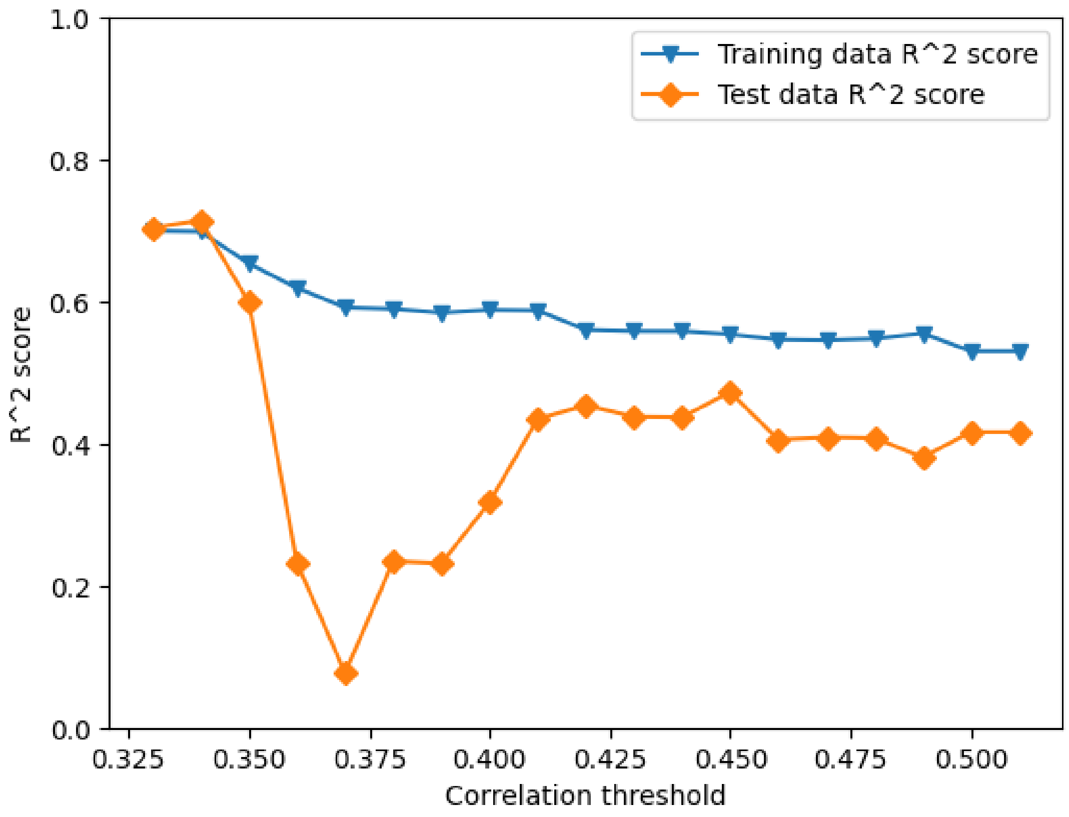

[44]:
```
plt.plot(df_without_standardization['Correlation threshold'],␣
↪df_without_standardization['Number of features'], label = "Number of␣
↪features", marker='.')
plt.legend()
plt.xlabel('Correlation threshold')
plt.ylabel('Number of features')
plt.show()
```

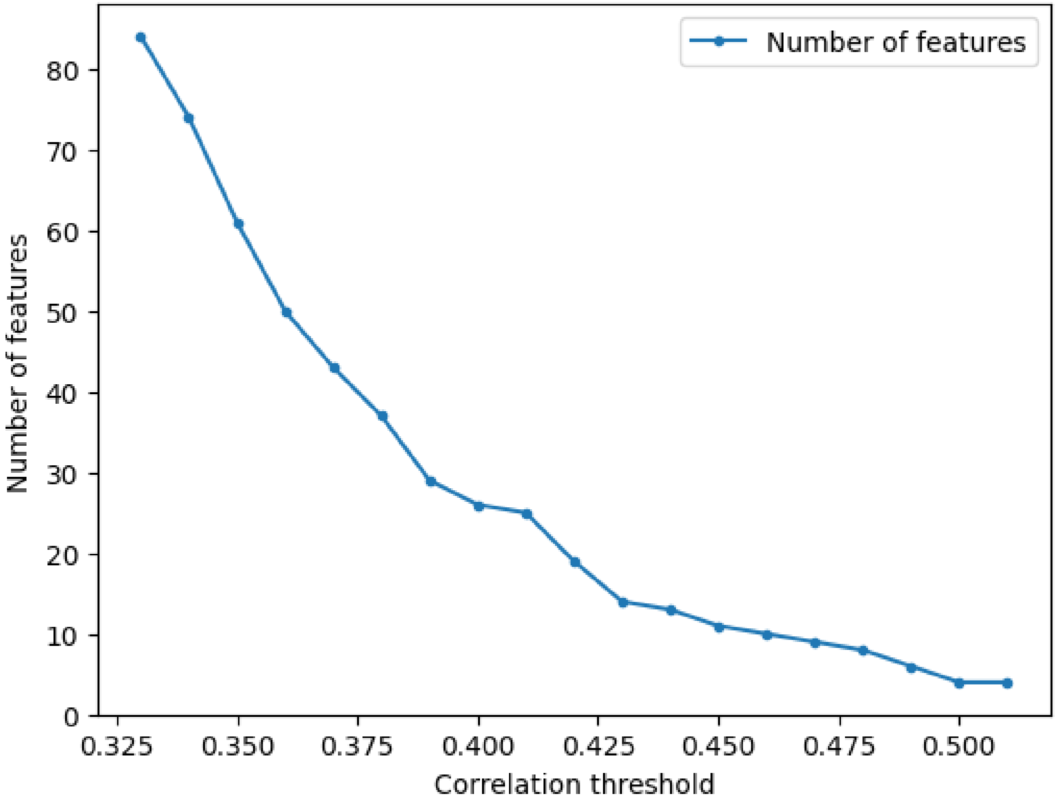

5.3 Store quality measurements

[45]:
```
with pd.ExcelWriter('../Data/Quality_'+str(target)+'_'+str(random_state)+'_.
 ↪xlsx') as writer:
    df_linear.to_excel(writer, sheet_name='MLR')
    df_decision_tree.to_excel(writer, sheet_name='DT')
    df_random_forest.to_excel(writer, sheet_name='RF')
    df_k_nearest.to_excel(writer, sheet_name='KNN')
    df_svm.to_excel(writer, sheet_name='SVM')
```

Notebook

January 18, 2024

1 File 30

[1]:
```
import glob
import pandas as pd
import matplotlib.pyplot as plt
import numpy as np
SMALL_SIZE = 16
MEDIUM_SIZE = 18
BIGGER_SIZE = 20

plt.rc('font', size=SMALL_SIZE)          # controls default text sizes
plt.rc('axes', titlesize=SMALL_SIZE)     # fontsize of the axes title
plt.rc('axes', labelsize=MEDIUM_SIZE)    # fontsize of the x and y labels
plt.rc('xtick', labelsize=SMALL_SIZE)    # fontsize of the tick labels
plt.rc('ytick', labelsize=SMALL_SIZE)    # fontsize of the tick labels
plt.rc('legend', fontsize=SMALL_SIZE)    # legend fontsize
plt.rc('figure', titlesize=BIGGER_SIZE)  # fontsize of the figure title
```

[2]:
```
sheet_names = ['MLR', 'DT', 'RF', 'KNN', 'SVM']
x_ticks = ['MLR train R', 'MLR test R', 'DT train R', 'DT test R', 'RF train␣
↪R', 'RF test R', 'KNN train R', 'KNN test R', 'SVM train R', 'SVM test R']
random_states = [15, 28, 42]
```

[3]:
```
def prepare_histogram_data(target_name, max_number_of_features):

    excel_files = []

    for name in glob.glob('../Data/Quality_'+str(target_name)+'*'):
        excel_files.append(name)

    random_state_15 = []
    random_state_28 = []
    random_state_42 = []
    features_15 = []
    features_28 = []
    features_42 = []
    random_states = [15, 28, 42]
```

```
    for random_state in range(len(random_states)):
        tmp = []
        to_plot = []
        values = []
        number_of_features = []
        for sheet in sheet_names:
            tmp.append(pd.read_excel(excel_files[random_state],␣
↪sheet_name=sheet))
        for data in tmp:
            temp = data[data['Number of features'] <= max_number_of_features]
            to_plot.append(temp[temp['Test data R^2 score'] == max(temp['Test␣
↪data R^2 score'])])
        for value in to_plot:
            values.append(value['Training data R^2 score'].tail(1))
            values.append(value['Test data R^2 score'].tail(1))
            number_of_features.append(value['Number of features'].tail(1))
        for element in values:
            if random_state == 0:
                random_state_15.append(float(element))
            elif random_state == 1:
                random_state_28.append(float(element))
            elif random_state == 2:
                random_state_42.append(float(element))
            else:
                print("Error with conditions...")

        for features in number_of_features:
            if random_state == 0:
                features_15.append(float(features))
            elif random_state == 1:
                features_28.append(float(features))
            elif random_state == 2:
                features_42.append(float(features))
            else:
                print("Error with conditions...")


    return  random_state_15, random_state_28, random_state_42, features_15,␣
↪features_28, features_42
```

[4]:
```
def handle_negative_corr(list_):
    res = []
    for element in list_:
        if element < 0:
            res.append(0.01)
        else:
```

```
            res.append(np.sqrt(element))
    return res
```

[5]:
```
def prepare_plot(data, name, max_number_of_features):

    fig = plt.figure(figsize=(20,10))
    X = x_ticks
    y_15 = handle_negative_corr(data[0])
    y_28 = handle_negative_corr(data[1])
    y_42 = handle_negative_corr(data[2])

    X_axis = np.arange(len(X))
    y_ax = [x/100 for x in range(0, 105, 5)]
    plt.yticks(y_ax)
    plt.bar(X_axis - 0.2, y_15, 0.2, label = 'Random state 15')
    plt.bar(X_axis + 0.0, y_28, 0.2, label = 'Random state 28')
    plt.bar(X_axis + 0.2, y_42, 0.2, label = 'Random state 42')

    plt.xticks(X_axis, X, weight='bold', fontsize=14)
    plt.xlabel("Methodology", fontsize=20, weight='bold')
    plt.ylabel("Correlation coefficient", fontsize=18, weight='bold')
    plt.ylim([0.0, 1.0])
    plt.title("Quality of each methodology - "+str(name)+' '+str('max number of␣
↪features: '+str(max_number_of_features)), fontsize=24, weight='bold')
    plt.grid(visible=True)
    plt.legend()
    plt.savefig('Figures/'+'_'+str(name)+'_'+str(max_number_of_features)+'_'+'.
↪pdf', bbox_inches='tight') #new line
    plt.show()
    for i, state in enumerate(random_states):
        print('Random state - '+str(state)+'␣
↪'+str(update_description(sheet_names, data[i+3])))
```

[6]:
```
def update_description(x_axis_labels, number_of_features):

    MLR = str(x_axis_labels[0])+' - number of features used:␣
↪'+str(number_of_features[0])
    DT = str(x_axis_labels[1])+' - number of features used:␣
↪'+str(number_of_features[1])
    RF = str(x_axis_labels[2])+' - number of features used:␣
↪'+str(number_of_features[2])
    KNN = str(x_axis_labels[3])+' - number of features used:␣
↪'+str(number_of_features[3])
    SVM = str(x_axis_labels[4])+' - number of features used:␣
↪'+str(number_of_features[4])
```

```
    return MLR, DT, RF, KNN, SVM
```

1.1 A549

[7]:
```
a549 = prepare_histogram_data('A549', 8)
```

[8]:
```
a549
```

[8]:
```
([0.4280888527210002,
  0.6266334128725354,
  0.8466334342142815,
  0.7153113378079368,
  0.9249742021818756,
  0.7525559593594799,
  0.5819621881074415,
  0.8307821665232054,
  0.4153462878154097,
  0.5885571370707737],
 [0.538457470613332,
  0.4261368142722509,
  0.8170806259589886,
  0.4076872049316587,
  0.8930162932196327,
  0.800771797336556,
  0.6790766967915924,
  0.1008539619720636,
  0.4984934257197747,
  0.4089742856265142],
 [0.4548140725424477,
  0.4923632130931052,
  0.8796761209043098,
  0.5671139561031637,
  0.9141127094423355,
  0.5770141099401139,
  0.575237291235299,
  0.4548654061537126,
  0.4142337951213418,
  0.4458030446072588],
 [3.0, 5.0, 8.0, 8.0, 5.0],
 [8.0, 7.0, 8.0, 8.0, 8.0],
 [3.0, 8.0, 7.0, 3.0, 3.0])
```

[9]:
```
for i in range(8, 3, -1):
    a549 = prepare_histogram_data('A549', i)
    prepare_plot(a549, 'A549', i)
```

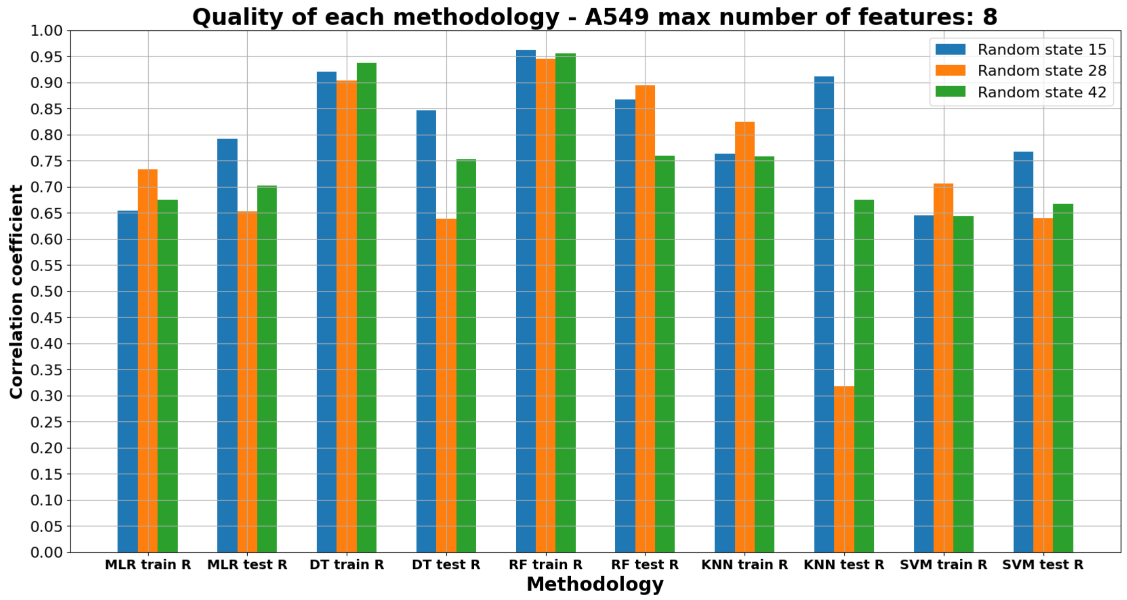


```
Random state - 15 ('MLR - number of features used: 3.0', 'DT - number of
features used: 5.0', 'RF - number of features used: 8.0', 'KNN - number of
features used: 8.0', 'SVM - number of features used: 5.0')
Random state - 28 ('MLR - number of features used: 8.0', 'DT - number of
features used: 7.0', 'RF - number of features used: 8.0', 'KNN - number of
features used: 8.0', 'SVM - number of features used: 8.0')
Random state - 42 ('MLR - number of features used: 3.0', 'DT - number of
features used: 8.0', 'RF - number of features used: 7.0', 'KNN - number of
features used: 3.0', 'SVM - number of features used: 3.0')
```

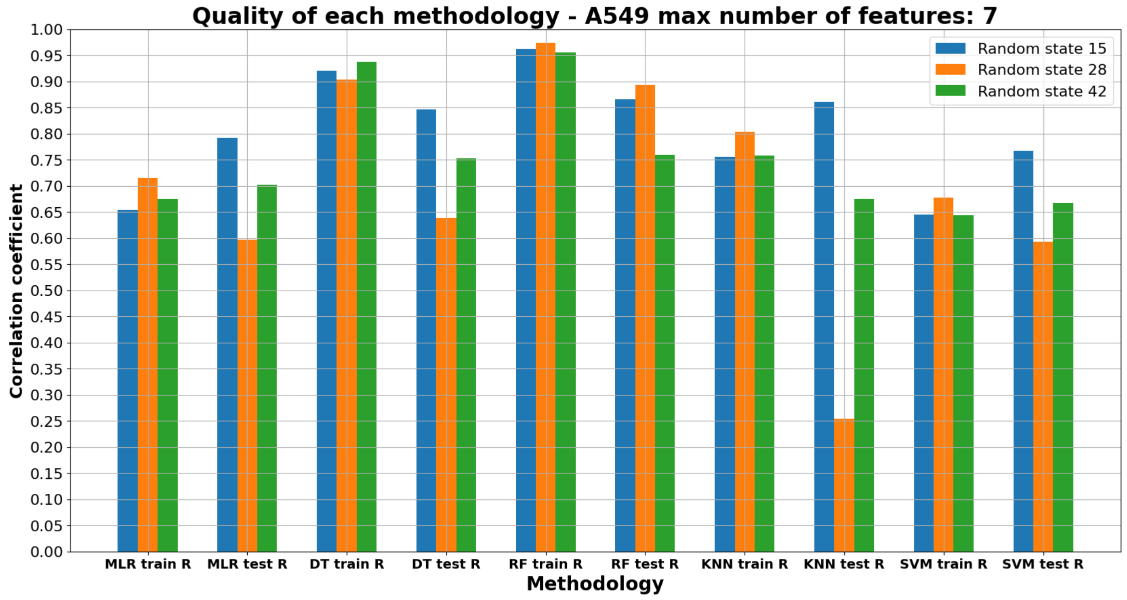

Random state - 15 ('MLR - number of features used: 3.0', 'DT - number of features used: 5.0', 'RF - number of features used: 7.0', 'KNN - number of features used: 7.0', 'SVM - number of features used: 5.0')
Random state - 28 ('MLR - number of features used: 7.0', 'DT - number of features used: 7.0', 'RF - number of features used: 7.0', 'KNN - number of features used: 7.0', 'SVM - number of features used: 7.0')
Random state - 42 ('MLR - number of features used: 3.0', 'DT - number of features used: 7.0', 'RF - number of features used: 7.0', 'KNN - number of features used: 3.0', 'SVM - number of features used: 3.0')

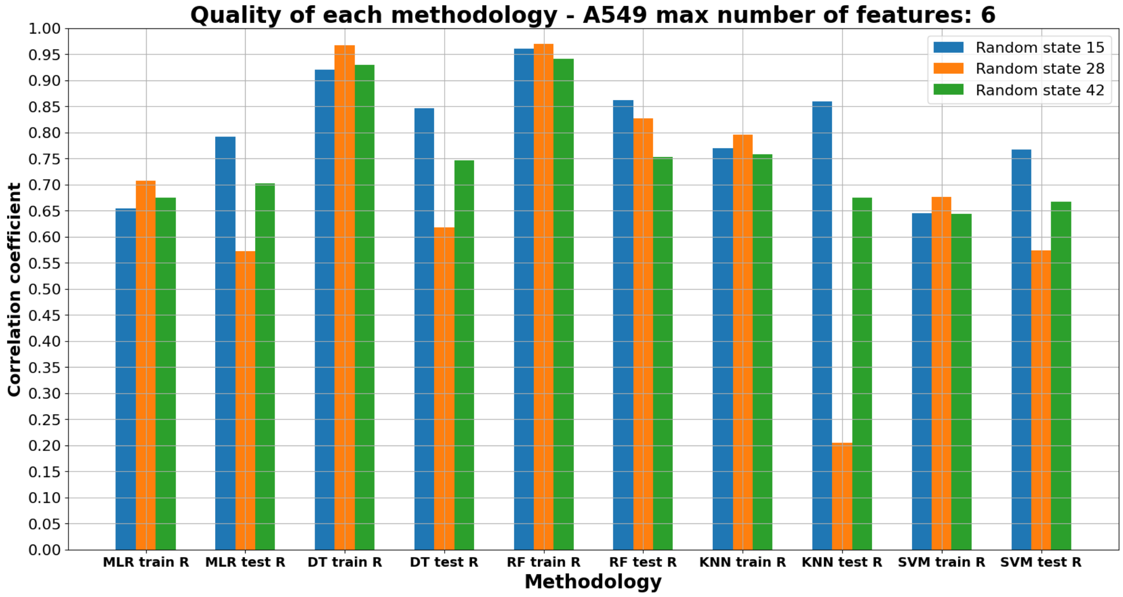

Random state - 15 ('MLR - number of features used: 3.0', 'DT - number of features used: 5.0', 'RF - number of features used: 5.0', 'KNN - number of features used: 5.0', 'SVM - number of features used: 5.0')
Random state - 28 ('MLR - number of features used: 4.0', 'DT - number of features used: 4.0', 'RF - number of features used: 5.0', 'KNN - number of features used: 5.0', 'SVM - number of features used: 5.0')
Random state - 42 ('MLR - number of features used: 3.0', 'DT - number of features used: 5.0', 'RF - number of features used: 5.0', 'KNN - number of features used: 3.0', 'SVM - number of features used: 3.0')

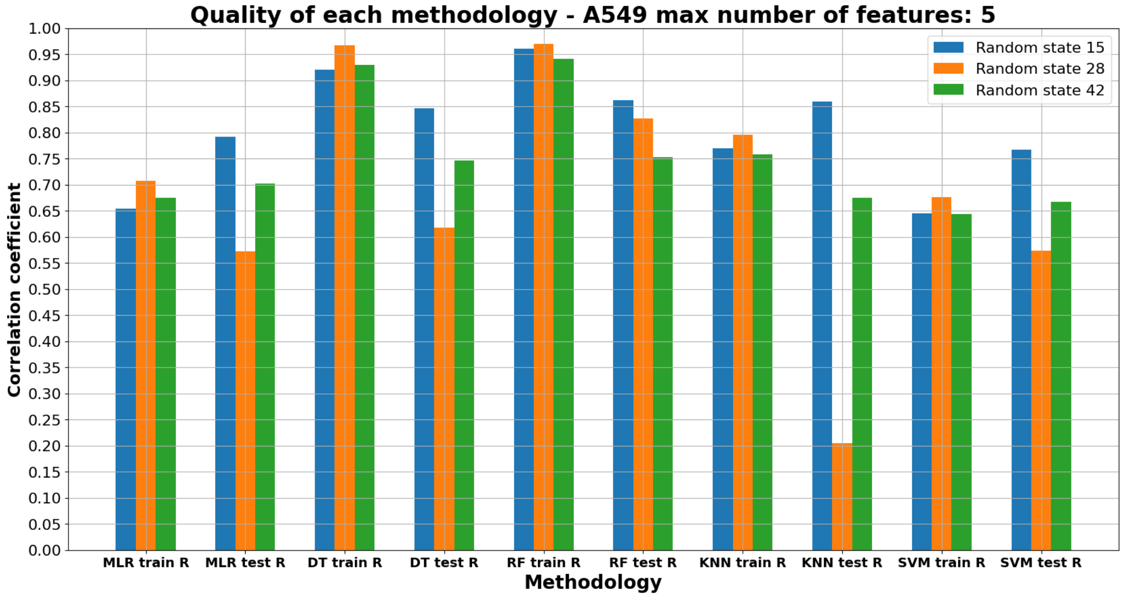

Random state - 15 ('MLR - number of features used: 3.0', 'DT - number of features used: 5.0', 'RF - number of features used: 5.0', 'KNN - number of features used: 5.0', 'SVM - number of features used: 5.0')
Random state - 28 ('MLR - number of features used: 4.0', 'DT - number of features used: 4.0', 'RF - number of features used: 5.0', 'KNN - number of features used: 5.0', 'SVM - number of features used: 5.0')
Random state - 42 ('MLR - number of features used: 3.0', 'DT - number of features used: 5.0', 'RF - number of features used: 5.0', 'KNN - number of features used: 3.0', 'SVM - number of features used: 3.0')

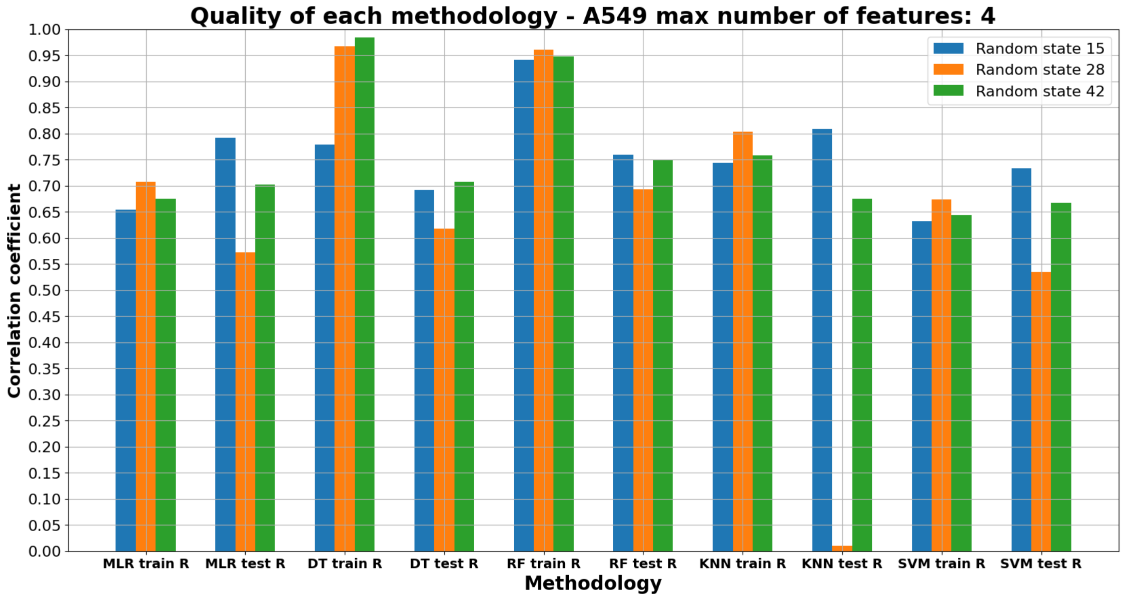

Random state - 15 ('MLR - number of features used: 3.0', 'DT - number of features used: 3.0', 'RF - number of features used: 4.0', 'KNN - number of features used: 3.0', 'SVM - number of features used: 3.0')
Random state - 28 ('MLR - number of features used: 4.0', 'DT - number of features used: 4.0', 'RF - number of features used: 4.0', 'KNN - number of features used: 4.0', 'SVM - number of features used: 4.0')
Random state - 42 ('MLR - number of features used: 3.0', 'DT - number of features used: 3.0', 'RF - number of features used: 3.0', 'KNN - number of features used: 3.0', 'SVM - number of features used: 3.0')

[ ]:

1.2 MCF-7

[10]:
```
for i in range(8, 3, -1):
    mcf7 = prepare_histogram_data('MCF-7', i)
    prepare_plot(mcf7, 'MCF-7', i)
```

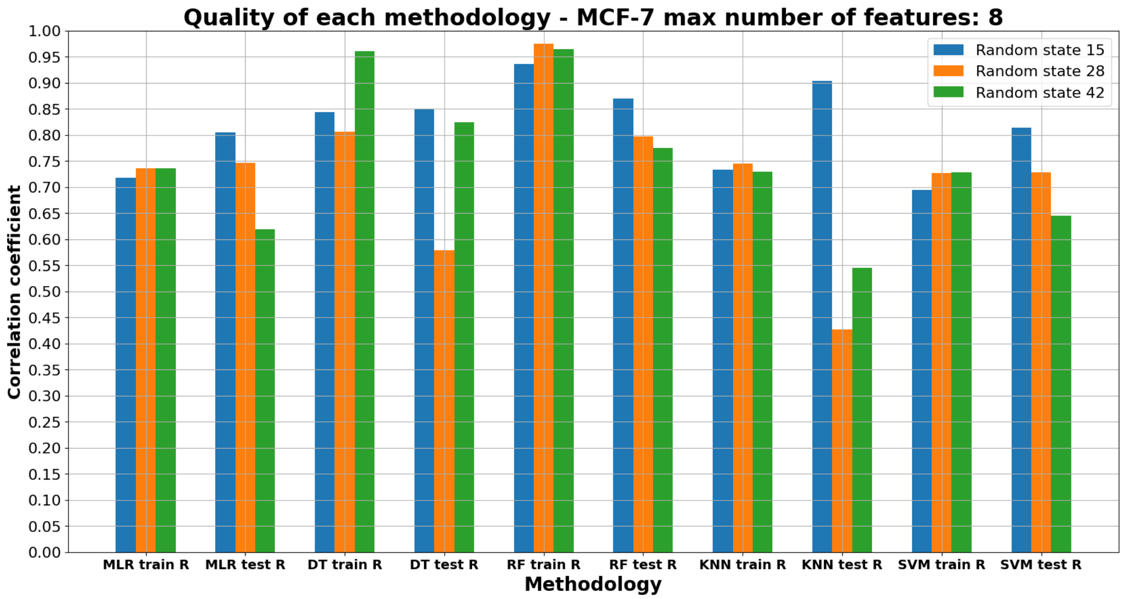

Random state - 15 ('MLR - number of features used: 8.0', 'DT - number of features used: 6.0', 'RF - number of features used: 8.0', 'KNN - number of features used: 8.0', 'SVM - number of features used: 4.0')
Random state - 28 ('MLR - number of features used: 6.0', 'DT - number of features used: 4.0', 'RF - number of features used: 8.0', 'KNN - number of features used: 6.0', 'SVM - number of features used: 8.0')
Random state - 42 ('MLR - number of features used: 4.0', 'DT - number of features used: 8.0', 'RF - number of features used: 8.0', 'KNN - number of features used: 6.0', 'SVM - number of features used: 4.0')

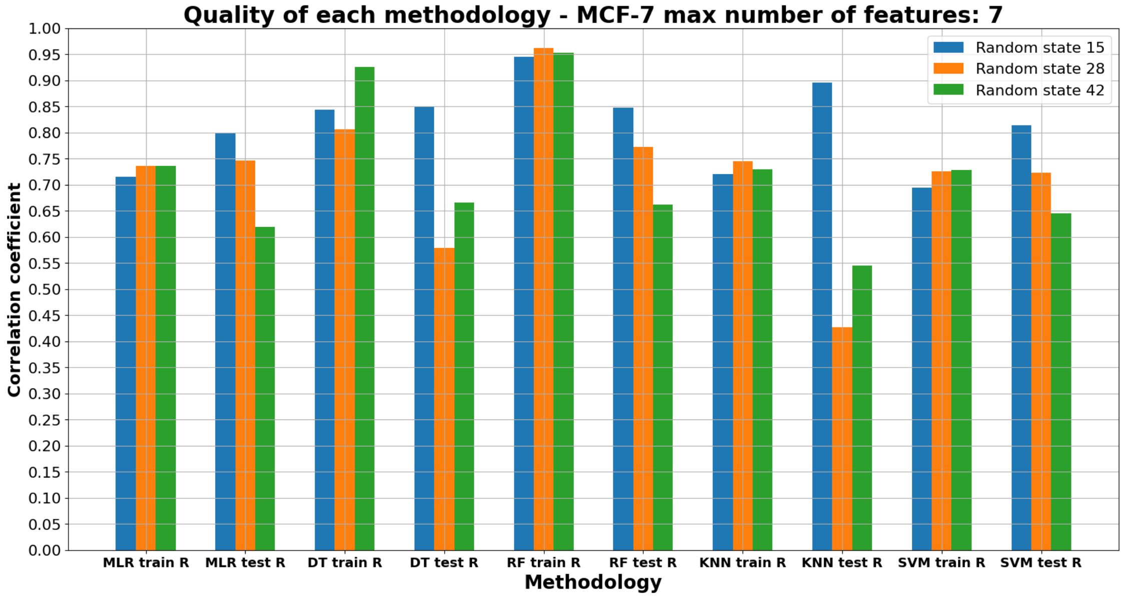


```
Random state - 15 ('MLR - number of features used: 6.0', 'DT - number of
features used: 6.0', 'RF - number of features used: 6.0', 'KNN - number of
features used: 6.0', 'SVM - number of features used: 4.0')
Random state - 28 ('MLR - number of features used: 6.0', 'DT - number of
features used: 4.0', 'RF - number of features used: 6.0', 'KNN - number of
features used: 6.0', 'SVM - number of features used: 6.0')
Random state - 42 ('MLR - number of features used: 4.0', 'DT - number of
features used: 6.0', 'RF - number of features used: 6.0', 'KNN - number of
features used: 6.0', 'SVM - number of features used: 4.0')
```

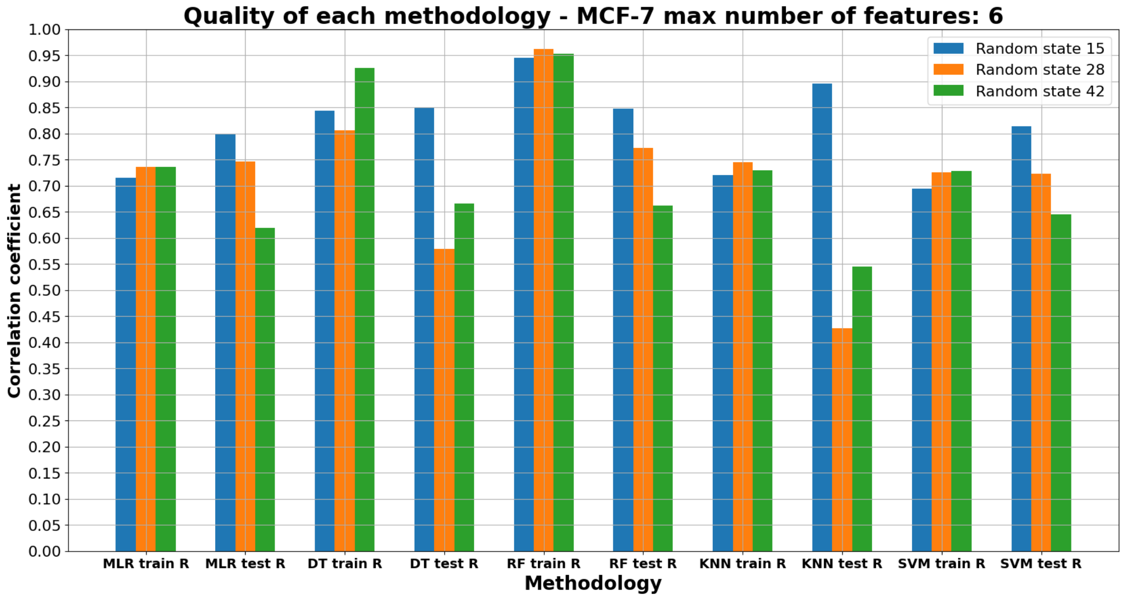

```
Random state - 15 ('MLR - number of features used: 6.0', 'DT - number of
features used: 6.0', 'RF - number of features used: 6.0', 'KNN - number of
features used: 6.0', 'SVM - number of features used: 4.0')
Random state - 28 ('MLR - number of features used: 6.0', 'DT - number of
features used: 4.0', 'RF - number of features used: 6.0', 'KNN - number of
features used: 6.0', 'SVM - number of features used: 6.0')
Random state - 42 ('MLR - number of features used: 4.0', 'DT - number of
features used: 6.0', 'RF - number of features used: 6.0', 'KNN - number of
features used: 6.0', 'SVM - number of features used: 4.0')
```

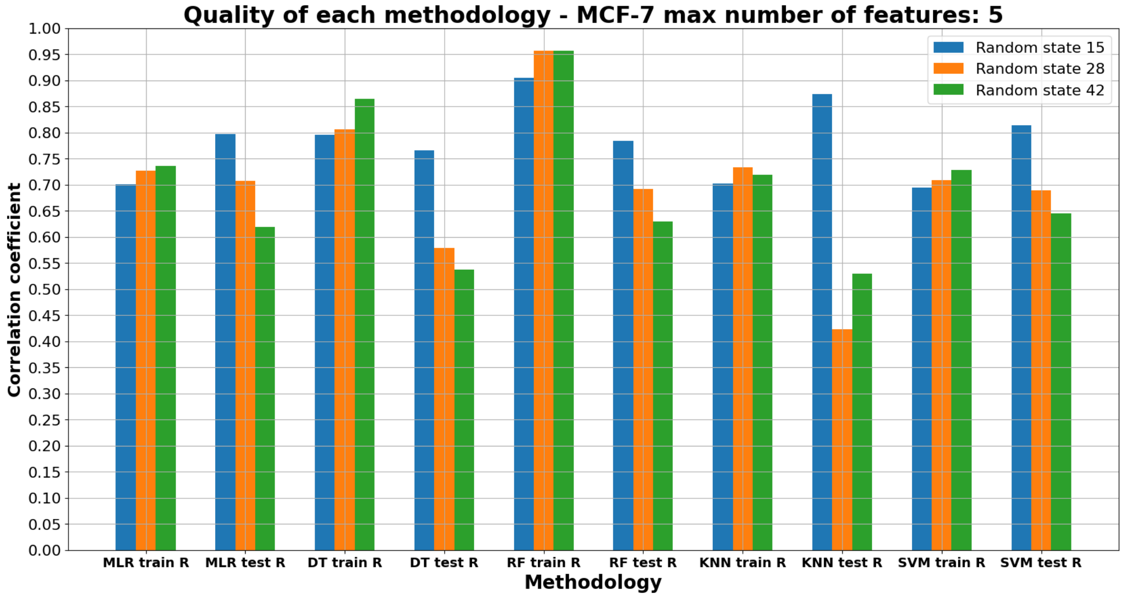


```
Random state - 15 ('MLR - number of features used: 4.0', 'DT - number of
features used: 4.0', 'RF - number of features used: 4.0', 'KNN - number of
features used: 4.0', 'SVM - number of features used: 4.0')
Random state - 28 ('MLR - number of features used: 4.0', 'DT - number of
features used: 4.0', 'RF - number of features used: 4.0', 'KNN - number of
features used: 4.0', 'SVM - number of features used: 4.0')
Random state - 42 ('MLR - number of features used: 4.0', 'DT - number of
features used: 4.0', 'RF - number of features used: 4.0', 'KNN - number of
features used: 4.0', 'SVM - number of features used: 4.0')
```

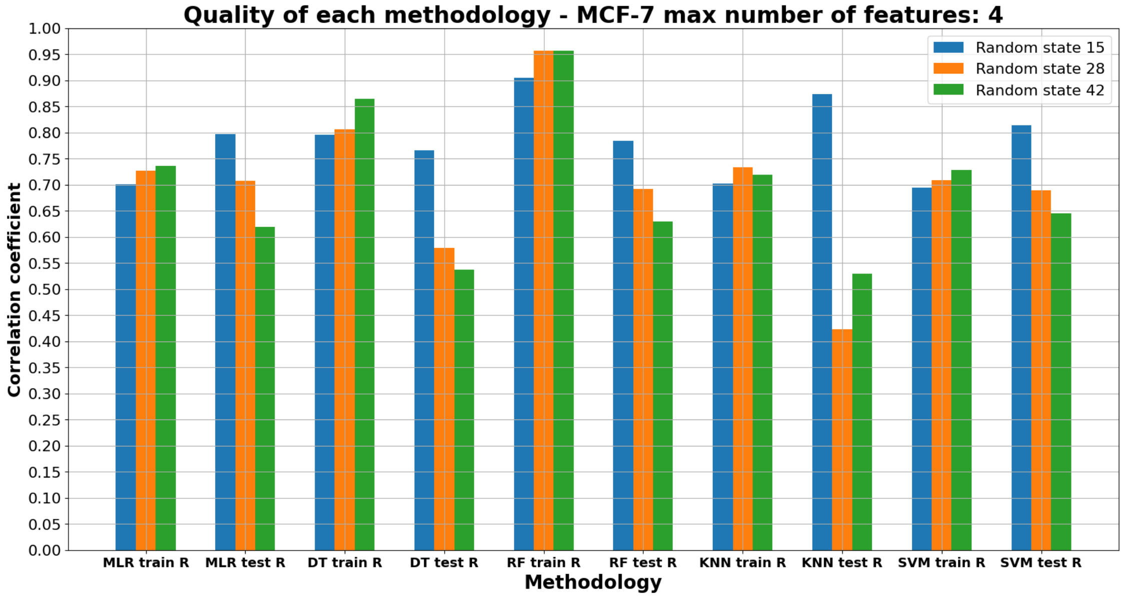


```
Random state - 15 ('MLR - number of features used: 4.0', 'DT - number of
features used: 4.0', 'RF - number of features used: 4.0', 'KNN - number of
features used: 4.0', 'SVM - number of features used: 4.0')
Random state - 28 ('MLR - number of features used: 4.0', 'DT - number of
features used: 4.0', 'RF - number of features used: 4.0', 'KNN - number of
features used: 4.0', 'SVM - number of features used: 4.0')
Random state - 42 ('MLR - number of features used: 4.0', 'DT - number of
features used: 4.0', 'RF - number of features used: 4.0', 'KNN - number of
features used: 4.0', 'SVM - number of features used: 4.0')
```

[ ]:

```
```

1.3 LoVo

[11]:

```
for i in range(8, 1, -1): # 2 -> 1
    lovo = prepare_histogram_data('LoVo', i)
    prepare_plot(lovo, 'LoVo', i)
```

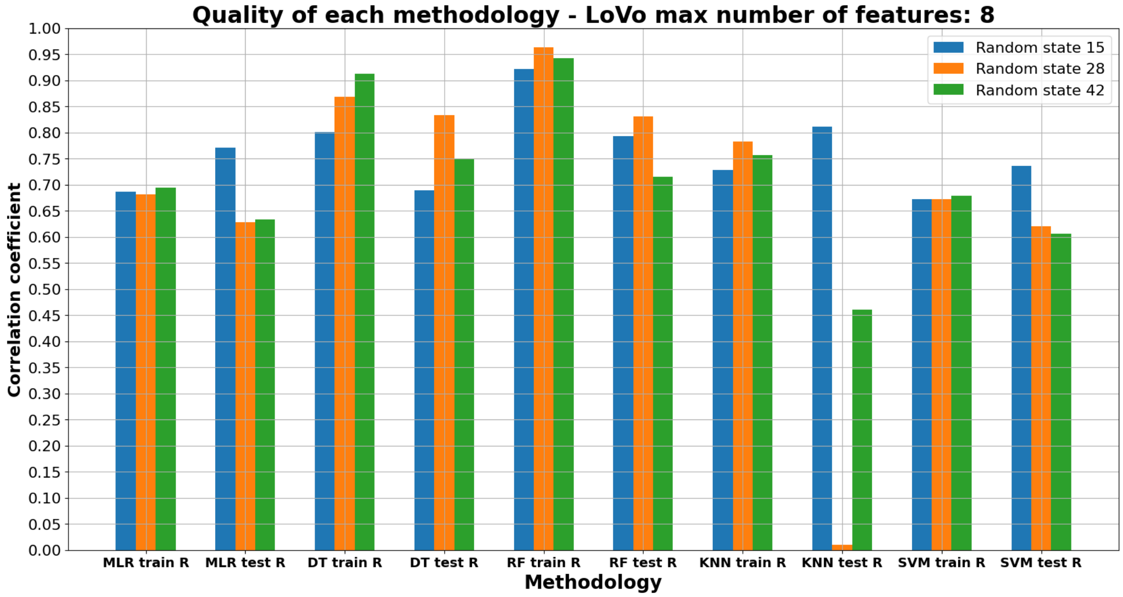

Random state - 15 ('MLR - number of features used: 6.0', 'DT - number of features used: 7.0', 'RF - number of features used: 5.0', 'KNN - number of features used: 5.0', 'SVM - number of features used: 6.0')
Random state - 28 ('MLR - number of features used: 2.0', 'DT - number of features used: 3.0', 'RF - number of features used: 7.0', 'KNN - number of features used: 7.0', 'SVM - number of features used: 2.0')
Random state - 42 ('MLR - number of features used: 3.0', 'DT - number of features used: 3.0', 'RF - number of features used: 5.0', 'KNN - number of features used: 5.0', 'SVM - number of features used: 2.0')

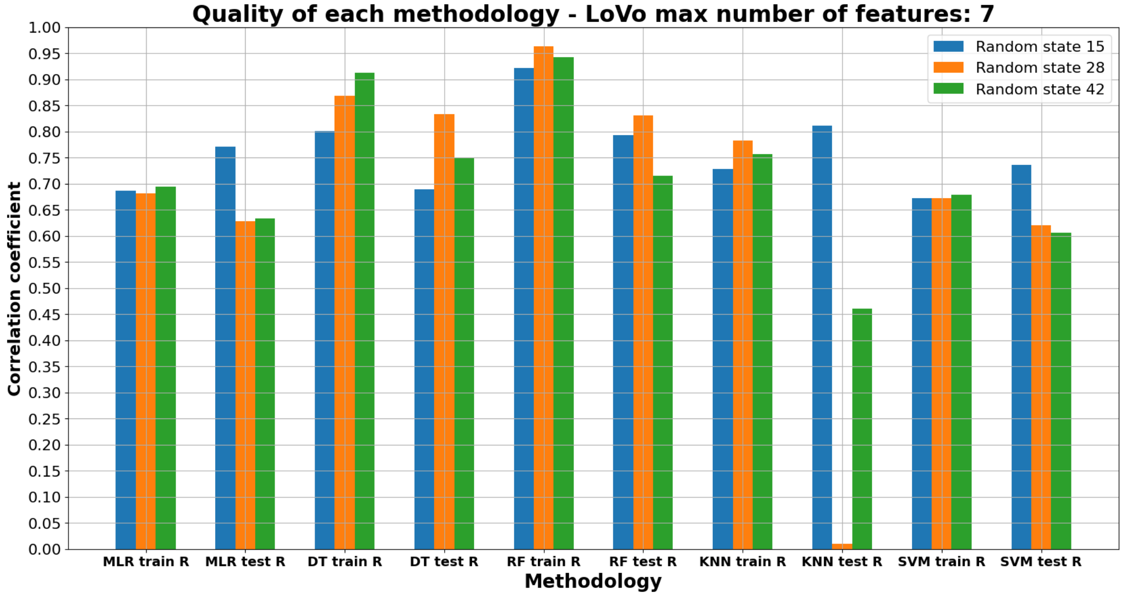

Random state - 15 ('MLR - number of features used: 6.0', 'DT - number of features used: 7.0', 'RF - number of features used: 5.0', 'KNN - number of features used: 5.0', 'SVM - number of features used: 6.0')
Random state - 28 ('MLR - number of features used: 2.0', 'DT - number of features used: 3.0', 'RF - number of features used: 7.0', 'KNN - number of features used: 7.0', 'SVM - number of features used: 2.0')
Random state - 42 ('MLR - number of features used: 3.0', 'DT - number of features used: 3.0', 'RF - number of features used: 5.0', 'KNN - number of features used: 5.0', 'SVM - number of features used: 2.0')

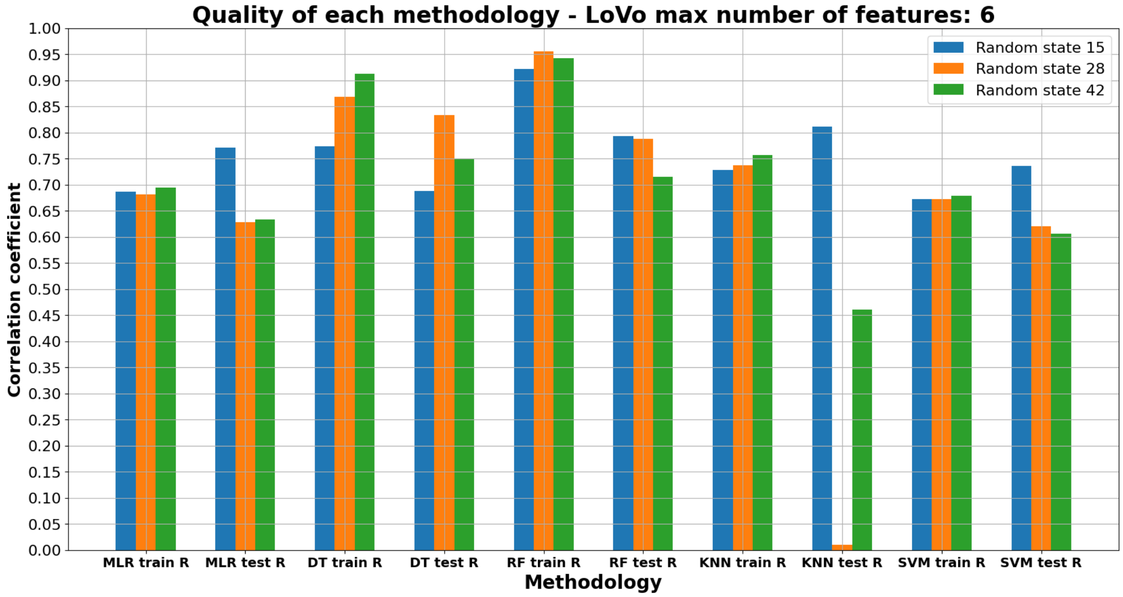

Random state - 15 ('MLR - number of features used: 6.0', 'DT - number of features used: 6.0', 'RF - number of features used: 5.0', 'KNN - number of features used: 5.0', 'SVM - number of features used: 6.0')
Random state - 28 ('MLR - number of features used: 2.0', 'DT - number of features used: 3.0', 'RF - number of features used: 6.0', 'KNN - number of features used: 2.0', 'SVM - number of features used: 2.0')
Random state - 42 ('MLR - number of features used: 3.0', 'DT - number of features used: 3.0', 'RF - number of features used: 5.0', 'KNN - number of features used: 5.0', 'SVM - number of features used: 2.0')

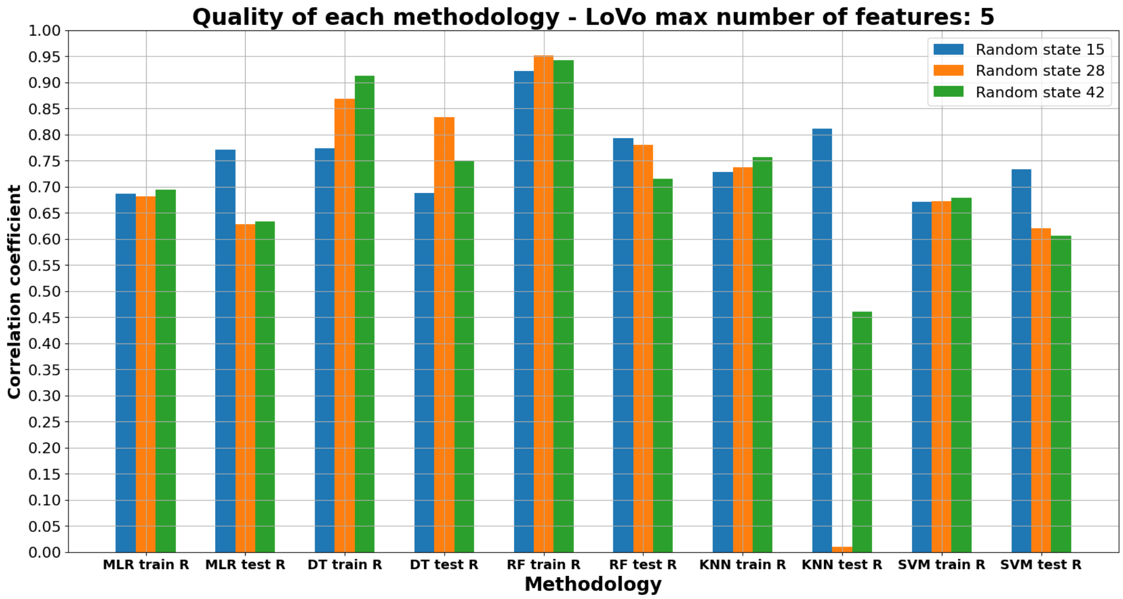

Random state - 15 ('MLR - number of features used: 5.0', 'DT - number of features used: 5.0', 'RF - number of features used: 5.0', 'KNN - number of features used: 5.0', 'SVM - number of features used: 5.0')
Random state - 28 ('MLR - number of features used: 2.0', 'DT - number of features used: 3.0', 'RF - number of features used: 5.0', 'KNN - number of features used: 2.0', 'SVM - number of features used: 2.0')
Random state - 42 ('MLR - number of features used: 3.0', 'DT - number of features used: 3.0', 'RF - number of features used: 5.0', 'KNN - number of features used: 5.0', 'SVM - number of features used: 2.0')

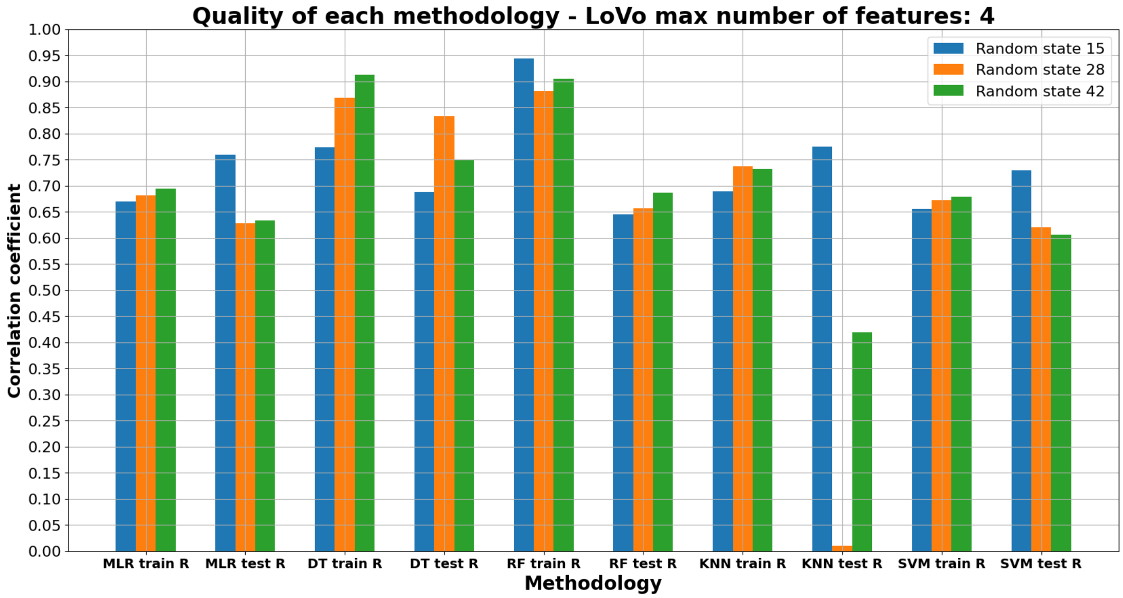

```
Random state - 15 ('MLR - number of features used: 3.0', 'DT - number of
features used: 3.0', 'RF - number of features used: 3.0', 'KNN - number of
features used: 2.0', 'SVM - number of features used: 2.0')
Random state - 28 ('MLR - number of features used: 2.0', 'DT - number of
features used: 3.0', 'RF - number of features used: 2.0', 'KNN - number of
features used: 2.0', 'SVM - number of features used: 2.0')
Random state - 42 ('MLR - number of features used: 3.0', 'DT - number of
features used: 3.0', 'RF - number of features used: 2.0', 'KNN - number of
features used: 2.0', 'SVM - number of features used: 2.0')
```

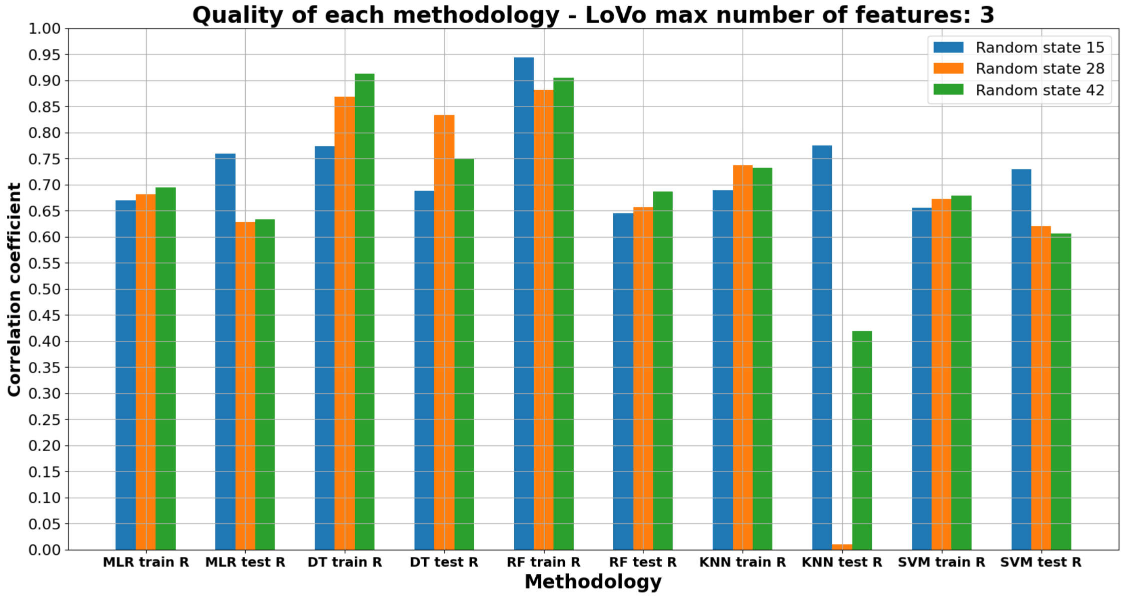


```
Random state - 15 ('MLR - number of features used: 3.0', 'DT - number of
features used: 3.0', 'RF - number of features used: 3.0', 'KNN - number of
features used: 2.0', 'SVM - number of features used: 2.0')
Random state - 28 ('MLR - number of features used: 2.0', 'DT - number of
features used: 3.0', 'RF - number of features used: 2.0', 'KNN - number of
features used: 2.0', 'SVM - number of features used: 2.0')
Random state - 42 ('MLR - number of features used: 3.0', 'DT - number of
features used: 3.0', 'RF - number of features used: 2.0', 'KNN - number of
features used: 2.0', 'SVM - number of features used: 2.0')
```

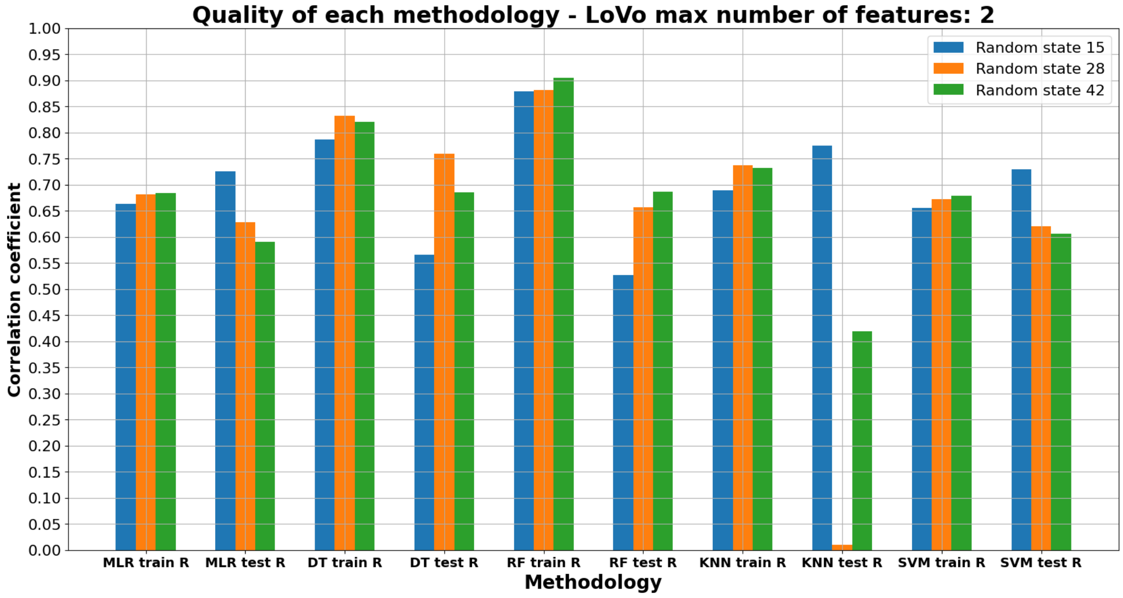


```
Random state - 15 ('MLR - number of features used: 2.0', 'DT - number of
features used: 2.0', 'RF - number of features used: 2.0', 'KNN - number of
features used: 2.0', 'SVM - number of features used: 2.0')
Random state - 28 ('MLR - number of features used: 2.0', 'DT - number of
features used: 2.0', 'RF - number of features used: 2.0', 'KNN - number of
features used: 2.0', 'SVM - number of features used: 2.0')
Random state - 42 ('MLR - number of features used: 2.0', 'DT - number of
features used: 2.0', 'RF - number of features used: 2.0', 'KNN - number of
features used: 2.0', 'SVM - number of features used: 2.0')
```

[ ]:

1.4 LoVo/DX

[12]:
```
for i in range(8, 4, -1): # 5 -> 4
    lovo_dx = prepare_histogram_data('LoVo_DX', i)
    prepare_plot(lovo_dx, 'LoVo_DX', i)
```

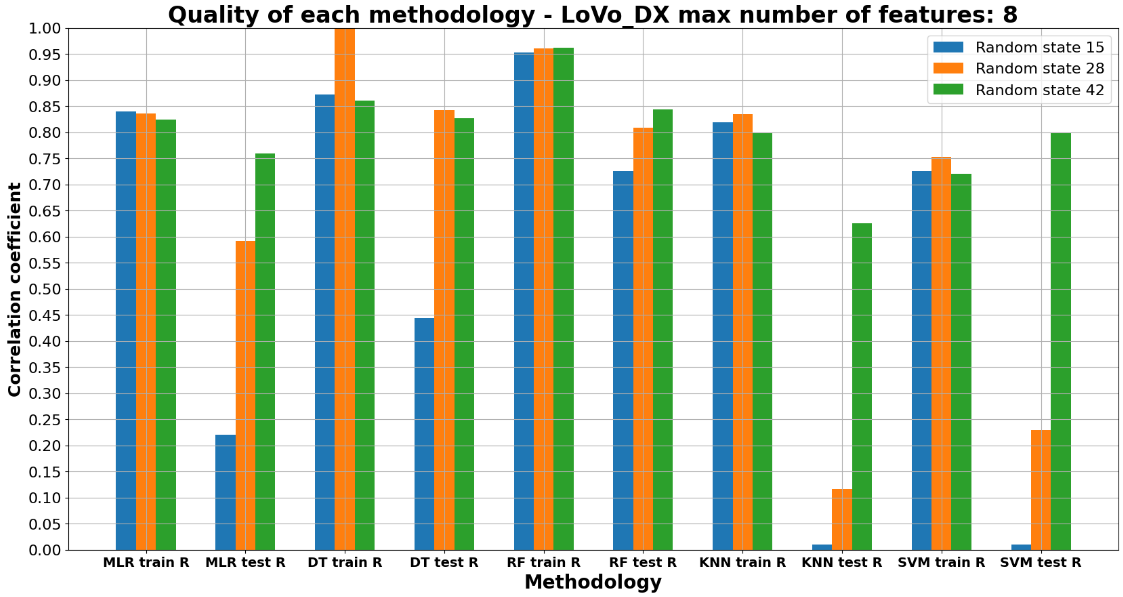

Random state - 15 ('MLR - number of features used: 8.0', 'DT - number of features used: 7.0', 'RF - number of features used: 8.0', 'KNN - number of features used: 7.0', 'SVM - number of features used: 5.0')
Random state - 28 ('MLR - number of features used: 7.0', 'DT - number of features used: 5.0', 'RF - number of features used: 8.0', 'KNN - number of features used: 8.0', 'SVM - number of features used: 8.0')
Random state - 42 ('MLR - number of features used: 8.0', 'DT - number of features used: 7.0', 'RF - number of features used: 7.0', 'KNN - number of features used: 7.0', 'SVM - number of features used: 8.0')

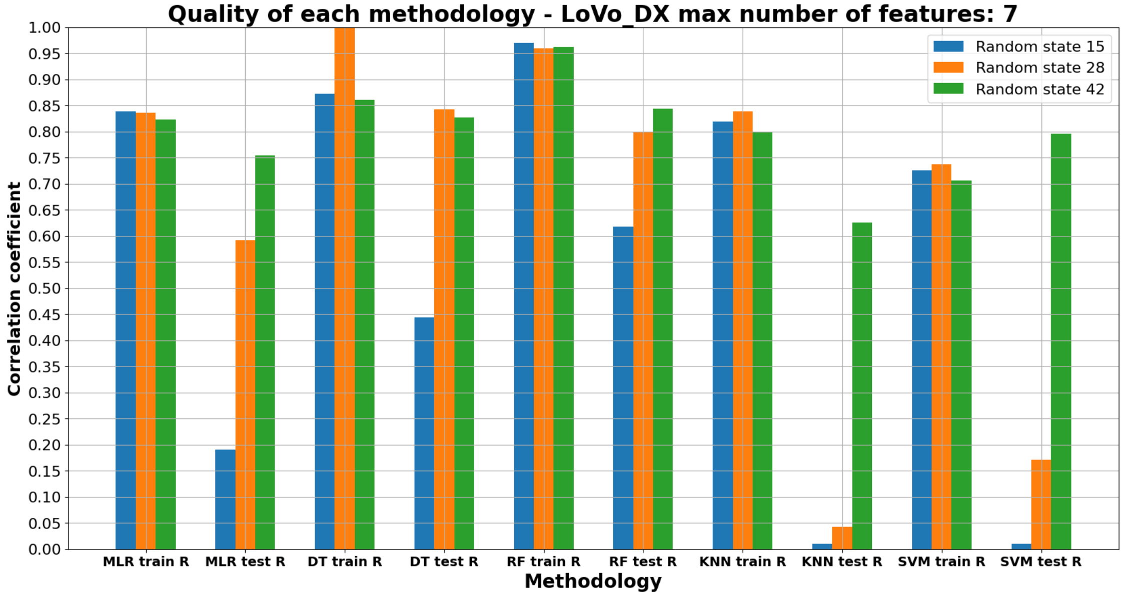

```
Random state - 15 ('MLR - number of features used: 7.0', 'DT - number of
features used: 7.0', 'RF - number of features used: 7.0', 'KNN - number of
features used: 7.0', 'SVM - number of features used: 5.0')
Random state - 28 ('MLR - number of features used: 7.0', 'DT - number of
features used: 5.0', 'RF - number of features used: 7.0', 'KNN - number of
features used: 7.0', 'SVM - number of features used: 5.0')
Random state - 42 ('MLR - number of features used: 7.0', 'DT - number of
features used: 7.0', 'RF - number of features used: 7.0', 'KNN - number of
features used: 7.0', 'SVM - number of features used: 7.0')
```

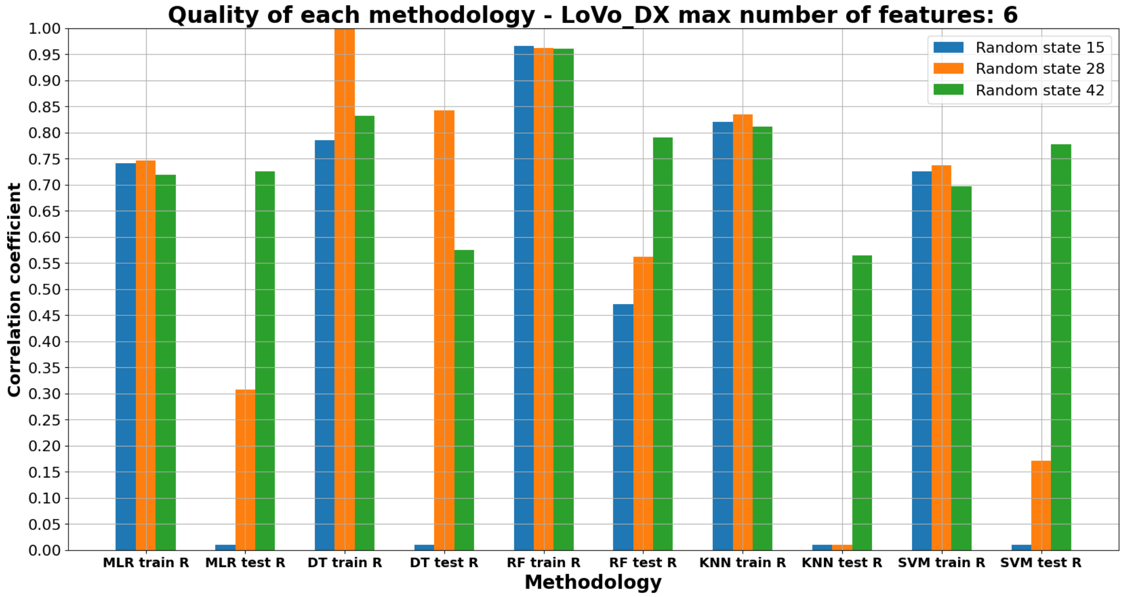


```
Random state - 15 ('MLR - number of features used: 5.0', 'DT - number of
features used: 5.0', 'RF - number of features used: 5.0', 'KNN - number of
features used: 5.0', 'SVM - number of features used: 5.0')
Random state - 28 ('MLR - number of features used: 5.0', 'DT - number of
features used: 5.0', 'RF - number of features used: 5.0', 'KNN - number of
features used: 5.0', 'SVM - number of features used: 5.0')
Random state - 42 ('MLR - number of features used: 5.0', 'DT - number of
features used: 5.0', 'RF - number of features used: 5.0', 'KNN - number of
features used: 5.0', 'SVM - number of features used: 5.0')
```

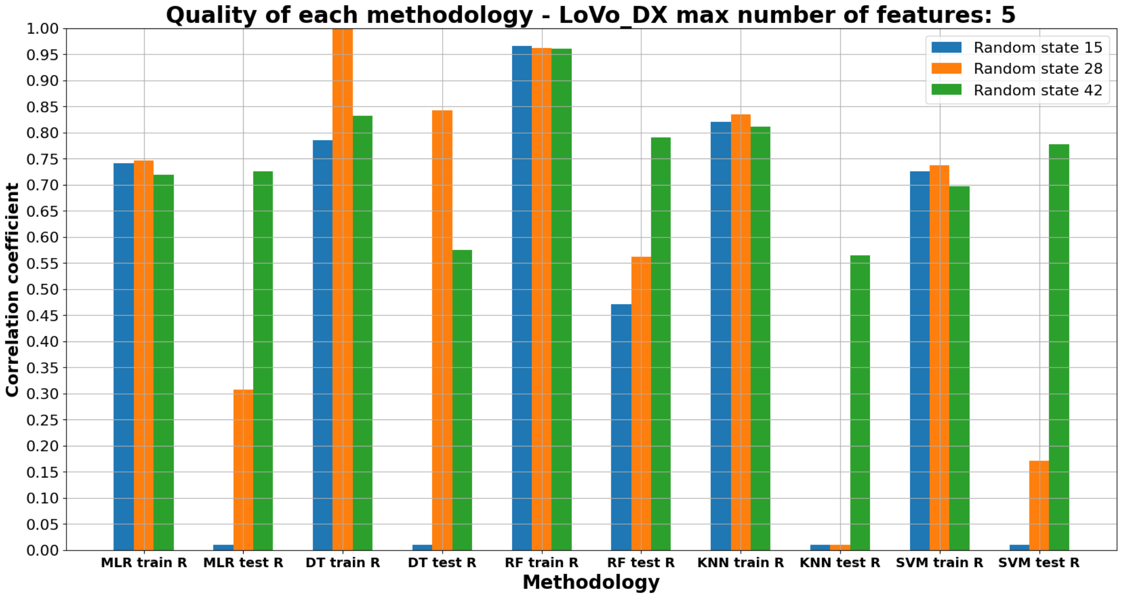


```
Random state - 15 ('MLR - number of features used: 5.0', 'DT - number of
features used: 5.0', 'RF - number of features used: 5.0', 'KNN - number of
features used: 5.0', 'SVM - number of features used: 5.0')
Random state - 28 ('MLR - number of features used: 5.0', 'DT - number of
features used: 5.0', 'RF - number of features used: 5.0', 'KNN - number of
features used: 5.0', 'SVM - number of features used: 5.0')
Random state - 42 ('MLR - number of features used: 5.0', 'DT - number of
features used: 5.0', 'RF - number of features used: 5.0', 'KNN - number of
features used: 5.0', 'SVM - number of features used: 5.0')
```

1.5 BALB/3T3

[13]:
```
for i in range(8, 2, -1):
    balb = prepare_histogram_data('BALB_3T3', i)
    prepare_plot(balb, 'BALB_3T3', i)
```

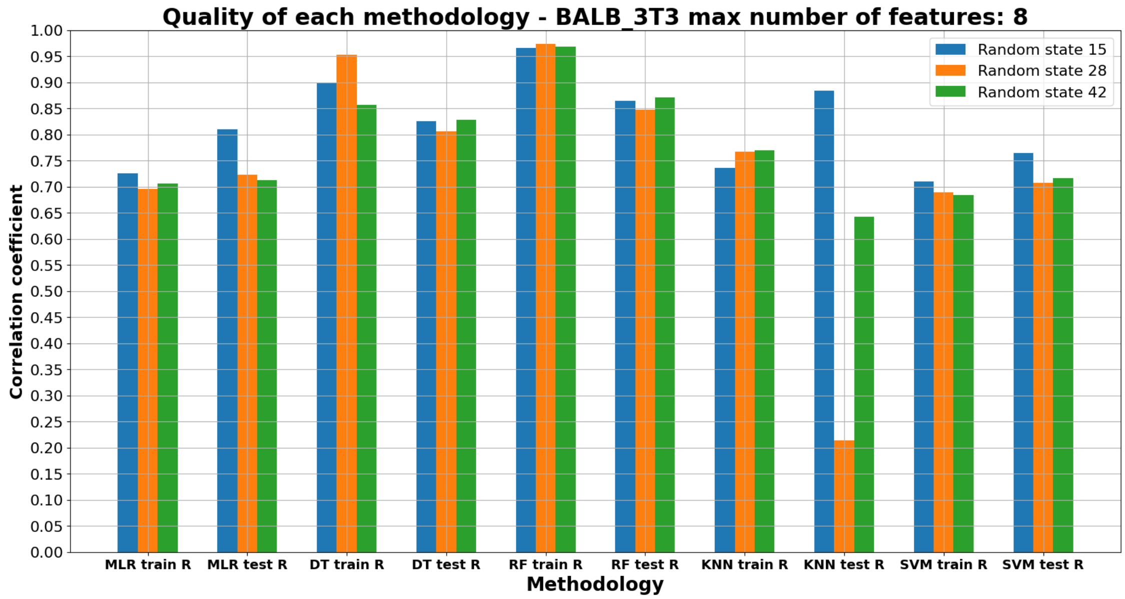

Random state - 15 ('MLR - number of features used: 7.0', 'DT - number of features used: 6.0', 'RF - number of features used: 6.0', 'KNN - number of features used: 6.0', 'SVM - number of features used: 7.0')
Random state - 28 ('MLR - number of features used: 2.0', 'DT - number of features used: 6.0', 'RF - number of features used: 7.0', 'KNN - number of features used: 3.0', 'SVM - number of features used: 2.0')
Random state - 42 ('MLR - number of features used: 3.0', 'DT - number of features used: 6.0', 'RF - number of features used: 7.0', 'KNN - number of features used: 7.0', 'SVM - number of features used: 3.0')

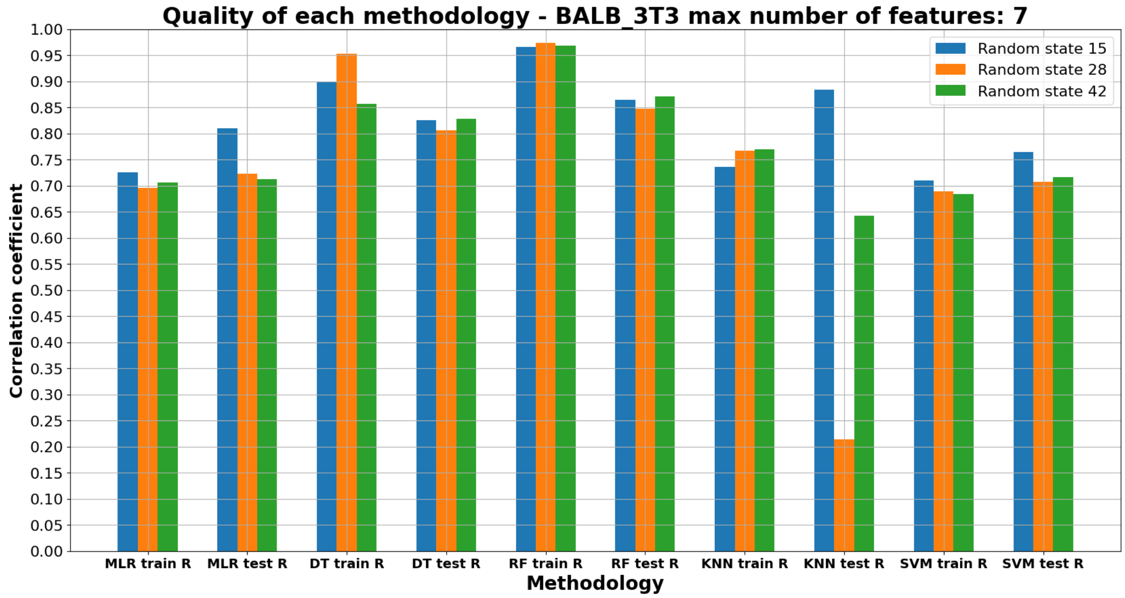

Random state - 15 ('MLR - number of features used: 7.0', 'DT - number of features used: 6.0', 'RF - number of features used: 6.0', 'KNN - number of features used: 6.0', 'SVM - number of features used: 7.0')
Random state - 28 ('MLR - number of features used: 2.0', 'DT - number of features used: 6.0', 'RF - number of features used: 7.0', 'KNN - number of features used: 3.0', 'SVM - number of features used: 2.0')
Random state - 42 ('MLR - number of features used: 3.0', 'DT - number of features used: 6.0', 'RF - number of features used: 7.0', 'KNN - number of features used: 7.0', 'SVM - number of features used: 3.0')

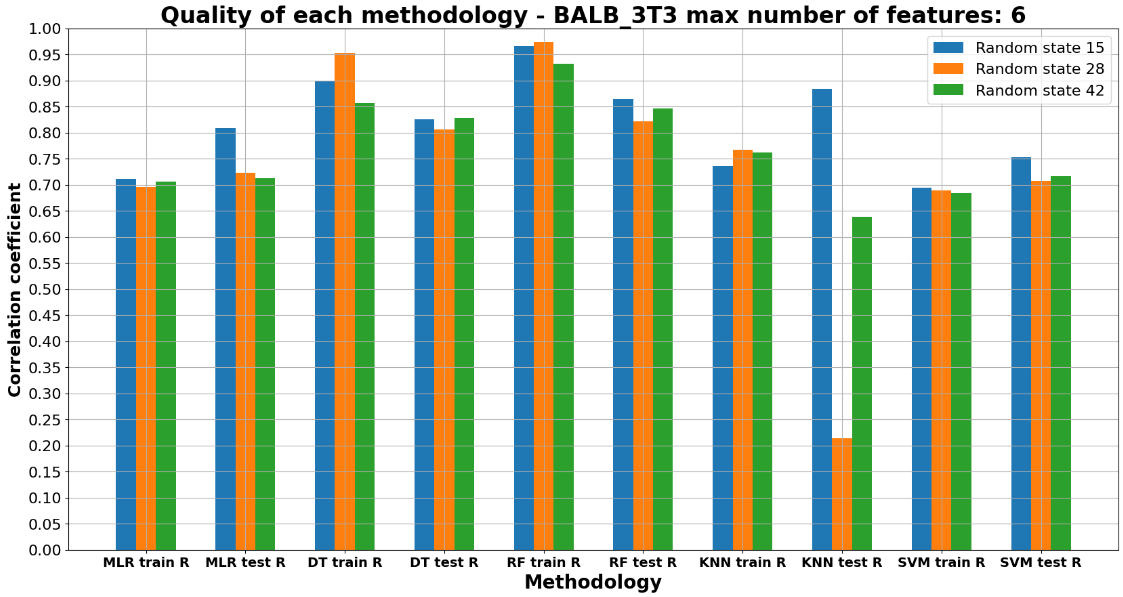

Random state - 15 ('MLR - number of features used: 6.0', 'DT - number of features used: 6.0', 'RF - number of features used: 6.0', 'KNN - number of features used: 6.0', 'SVM - number of features used: 5.0')
Random state - 28 ('MLR - number of features used: 2.0', 'DT - number of features used: 6.0', 'RF - number of features used: 6.0', 'KNN - number of features used: 3.0', 'SVM - number of features used: 2.0')
Random state - 42 ('MLR - number of features used: 3.0', 'DT - number of features used: 6.0', 'RF - number of features used: 6.0', 'KNN - number of features used: 3.0', 'SVM - number of features used: 3.0')

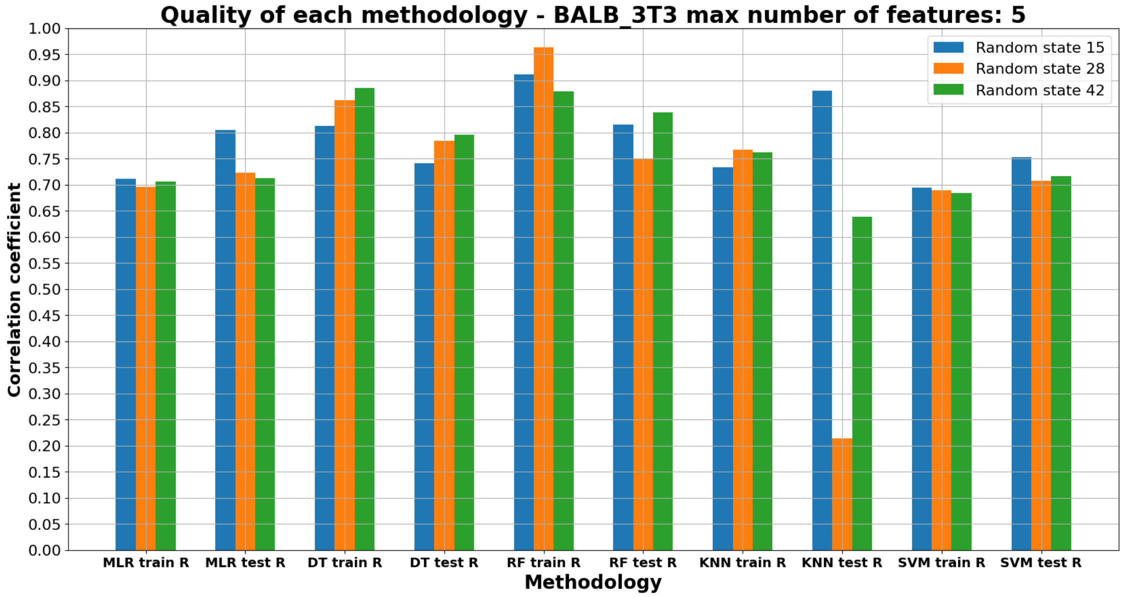

Random state - 15 ('MLR - number of features used: 5.0', 'DT - number of features used: 5.0', 'RF - number of features used: 5.0', 'KNN - number of features used: 5.0', 'SVM - number of features used: 5.0')
Random state - 28 ('MLR - number of features used: 2.0', 'DT - number of features used: 3.0', 'RF - number of features used: 5.0', 'KNN - number of features used: 3.0', 'SVM - number of features used: 2.0')
Random state - 42 ('MLR - number of features used: 3.0', 'DT - number of features used: 2.0', 'RF - number of features used: 2.0', 'KNN - number of features used: 3.0', 'SVM - number of features used: 3.0')

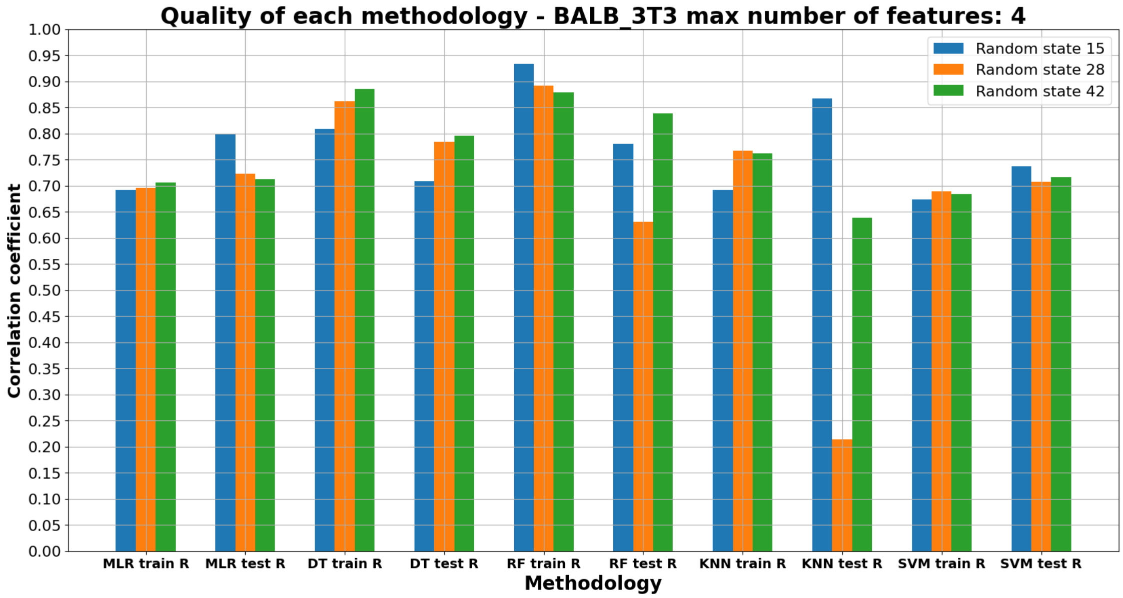

```
Random state - 15 ('MLR - number of features used: 3.0', 'DT - number of
features used: 3.0', 'RF - number of features used: 3.0', 'KNN - number of
features used: 2.0', 'SVM - number of features used: 3.0')
Random state - 28 ('MLR - number of features used: 2.0', 'DT - number of
features used: 3.0', 'RF - number of features used: 2.0', 'KNN - number of
features used: 3.0', 'SVM - number of features used: 2.0')
Random state - 42 ('MLR - number of features used: 3.0', 'DT - number of
features used: 2.0', 'RF - number of features used: 2.0', 'KNN - number of
features used: 3.0', 'SVM - number of features used: 3.0')
```

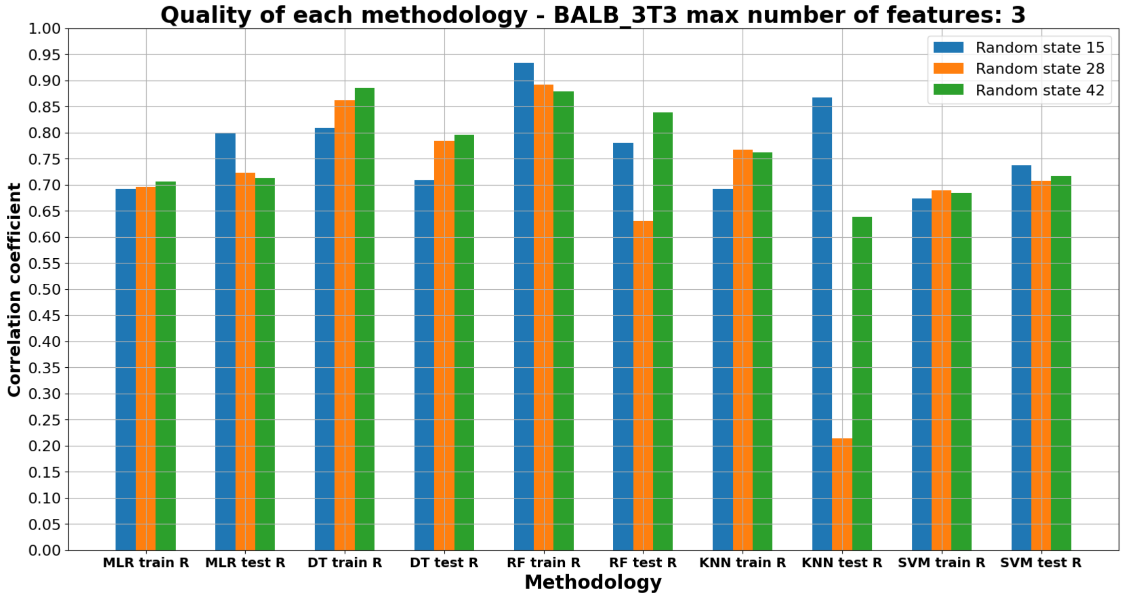


```
Random state - 15 ('MLR - number of features used: 3.0', 'DT - number of
features used: 3.0', 'RF - number of features used: 3.0', 'KNN - number of
features used: 2.0', 'SVM - number of features used: 3.0')
Random state - 28 ('MLR - number of features used: 2.0', 'DT - number of
features used: 3.0', 'RF - number of features used: 2.0', 'KNN - number of
features used: 3.0', 'SVM - number of features used: 2.0')
Random state - 42 ('MLR - number of features used: 3.0', 'DT - number of
features used: 2.0', 'RF - number of features used: 2.0', 'KNN - number of
features used: 3.0', 'SVM - number of features used: 3.0')
```

[ ]:

[ ]:

1.6 Best models selection

[14]:
```
def prepare_best_plot(target_name, max_number_of_features):

    excel_files = []

    for name in glob.glob('../Data/Quality_'+str(target_name)+'*'):
        excel_files.append(name)

    random_state_15 = []
    random_state_28 = []
    random_state_42 = []
    features_15 = []
    features_28 = []
    features_42 = []
    random_states = [15, 28, 42]

    for random_state in range(len(random_states)):
        tmp = []
        to_plot = []
        values = []
        number_of_features = []
        for sheet in sheet_names:
            tmp.append(pd.read_excel(excel_files[random_state],␣
↪sheet_name=sheet))
        for data in tmp:
            temp = data[data['Number of features'] <= max_number_of_features]
            to_plot.append(temp[temp['Test data R^2 score'] == max(temp['Test␣
↪data R^2 score'])])
        for value in to_plot:
            values.append(value['Training data R^2 score'].tail(1))
            values.append(value['Test data R^2 score'].tail(1))
            number_of_features.append(value['Number of features'].tail(1))
        for element in values:
            if random_state == 0:
                random_state_15.append(float(element))
            elif random_state == 1:
                random_state_28.append(float(element))
            elif random_state == 2:
                random_state_42.append(float(element))
            else:
                print("Error with conditions...")

        for features in number_of_features:
            if random_state == 0:
                features_15.append(float(features))
            elif random_state == 1:
```

```
                features_28.append(float(features))
            elif random_state == 2:
                features_42.append(float(features))
            else:
                print("Error with conditions...")


    return  random_state_15, random_state_28, random_state_42, features_15,␣
↪features_28, features_42
```

[15]:
```
def prepare_plot(data, name, max_number_of_features):
    fig = plt.figure(figsize=(20,10))
    X = x_ticks
    y_15 = handle_negative_corr(data[0])
    y_28 = handle_negative_corr(data[1])
    y_42 = handle_negative_corr(data[2])

    X_axis = np.arange(len(X))
    y_ax = [x/100 for x in range(0, 105, 5)]
    plt.yticks(y_ax)
    plt.bar(X_axis - 0.2, y_15, 0.2, label = 'Random state 15')
    plt.bar(X_axis + 0.0, y_28, 0.2, label = 'Random state 28')
    plt.bar(X_axis + 0.2, y_42, 0.2, label = 'Random state 42')
    #plt.plot(X_axis, y_15, color='r', label='Random state 15')
    #plt.plot(X_axis, y_28, color='g', label='Random state 28')
    #plt.plot(X_axis, y_42, color='b', label='Random state 42')

    plt.xticks(X_axis, X)
    plt.xlabel("Methodology", fontsize=20, weight='bold')
    plt.ylabel("Correlation coefficient", fontsize=18, weight='bold')
    plt.ylim([0.0, 1.0])
    plt.title("Quality of each methodology - "+str(name)+' '+str('max number of␣
↪features: '+str(max_number_of_features)), fontsize=24, weight='bold')
    plt.grid(visible=True)
    plt.legend()
    plt.show()
    for i, state in enumerate(random_states):
        print('Random state - '+str(state)+'␣
↪'+str(update_description(sheet_names, data[i+3])))
```

[16]:
```
def update_description(x_axis_labels, number_of_features):

    DT = str(x_axis_labels[0])+' - number of features used:␣
↪'+str(number_of_features[0])
    RF = str(x_axis_labels[1])+' - number of features used:␣
↪'+str(number_of_features[1])
```

```
    return DT, RF
```

[ ]:

```

```

1.6.1 A549

[17]:

```
sheet_names = ['DT', 'RF']
```

[18]:

```
x_ticks = ['DT train R', 'DT test R', 'RF train R', 'RF test R']
```

[19]:

```
for i in range(8, 3, -1):
    a549 = prepare_best_plot('A549', i)
    prepare_plot(a549, 'A549', i)
    print(handle_negative_corr(a549[0]))
    print(handle_negative_corr(a549[1]))
    print(handle_negative_corr(a549[2]))
```

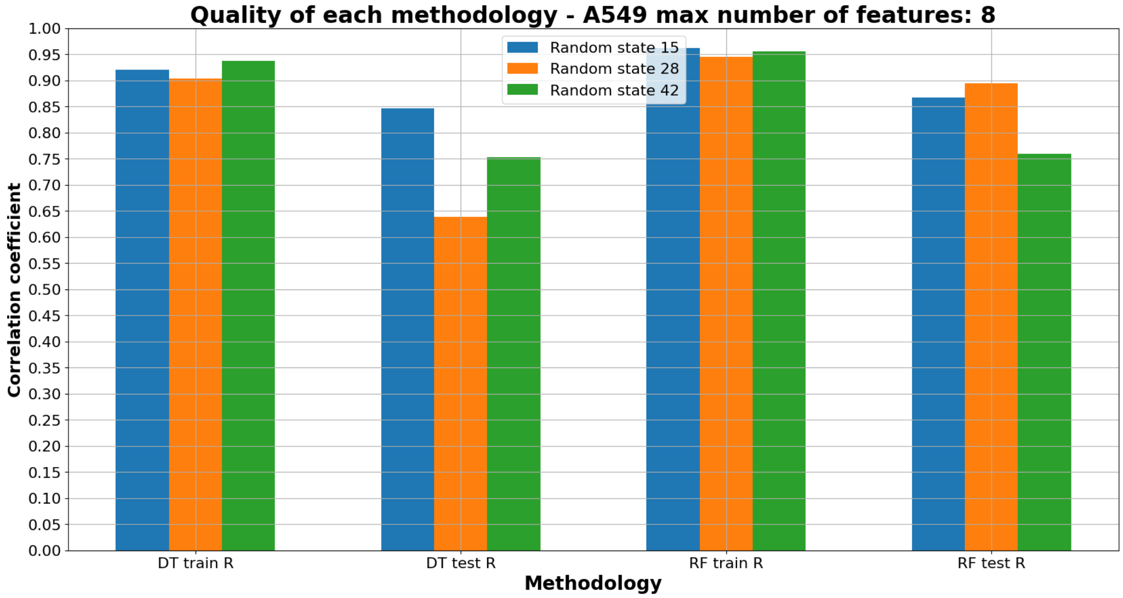


```
Random state - 15 ('DT - number of features used: 5.0', 'RF - number of features
used: 8.0')
Random state - 28 ('DT - number of features used: 7.0', 'RF - number of features
used: 8.0')
Random state - 42 ('DT - number of features used: 8.0', 'RF - number of features
used: 7.0')
[0.9201268576746804, 0.8457608041331407, 0.9617557913430392, 0.8674998324838339]
[0.9039251218762473, 0.6385038801226338, 0.9449953932266721, 0.8948585348179655]
[0.9379105079400218, 0.7530696887427908, 0.9560924167894731, 0.7596144482170636]
```

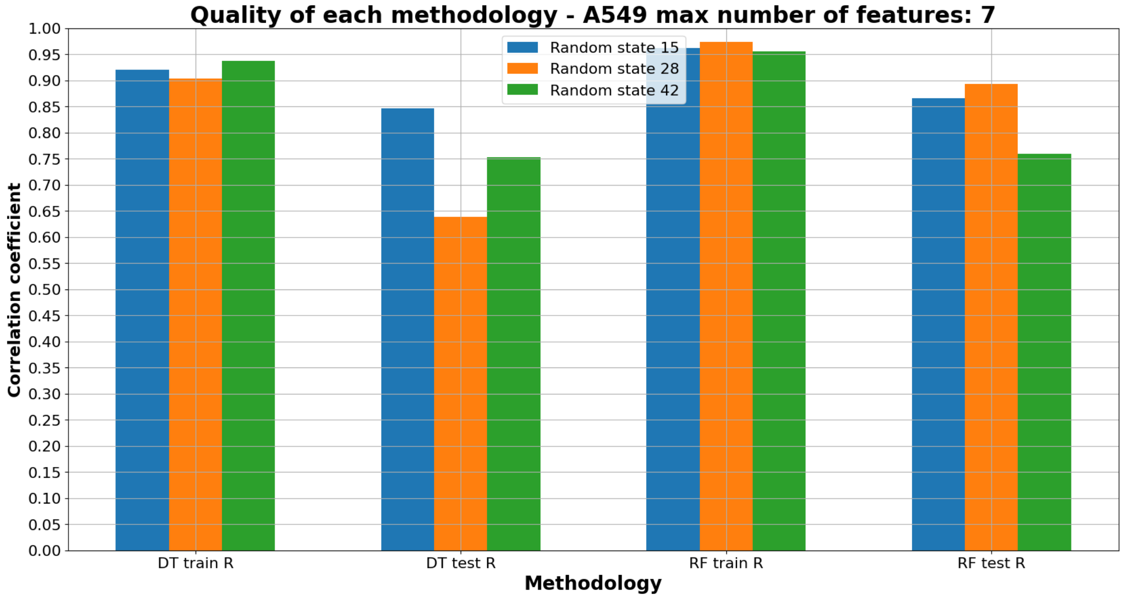


```
Random state - 15 ('DT - number of features used: 5.0', 'RF - number of features
used: 7.0')
Random state - 28 ('DT - number of features used: 7.0', 'RF - number of features
used: 7.0')
Random state - 42 ('DT - number of features used: 7.0', 'RF - number of features
used: 7.0')
[0.9201268576746804, 0.8457608041331407, 0.9617502720280803, 0.865633475359066]
[0.9039251218762473, 0.6385038801226338, 0.9733717517208741, 0.8936683566006186]
[0.9379105079400218, 0.7530696887427905, 0.9560924167894731, 0.7596144482170636]
```

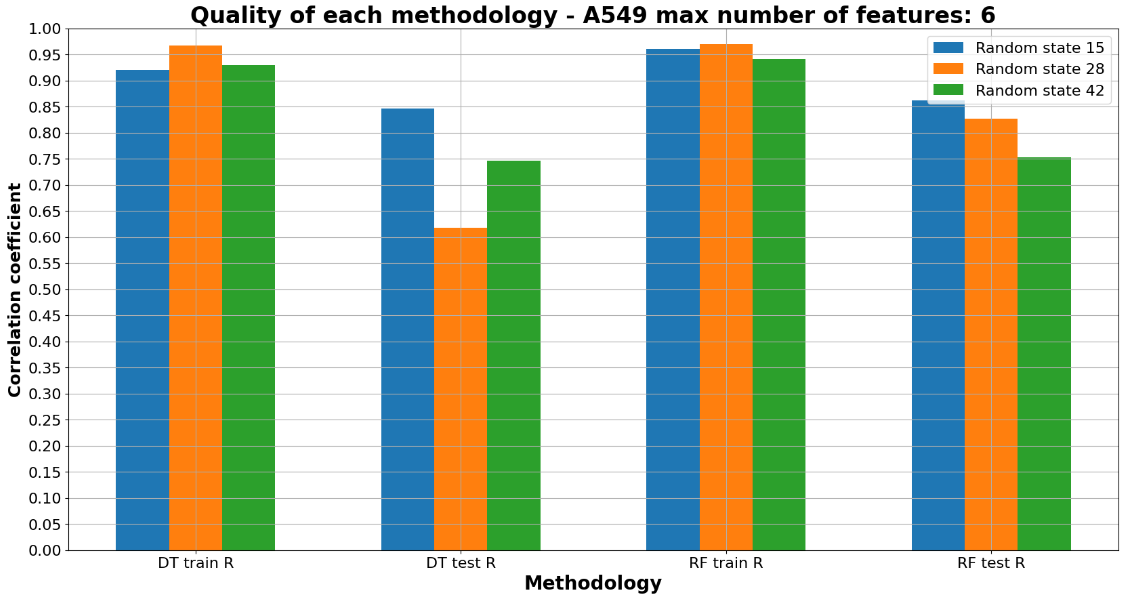

```
Random state - 15 ('DT - number of features used: 5.0', 'RF - number of features
used: 5.0')
Random state - 28 ('DT - number of features used: 4.0', 'RF - number of features
used: 5.0')
Random state - 42 ('DT - number of features used: 5.0', 'RF - number of features
used: 5.0')
[0.9201268576746804, 0.8457608041331407, 0.960591935079034, 0.8620618597285548]
[0.9669220010492233, 0.6172800607391895, 0.9701820261061275, 0.8267270635721486]
[0.9298029234842533, 0.746434872641246, 0.9417342321043257, 0.7526447589514621]
```

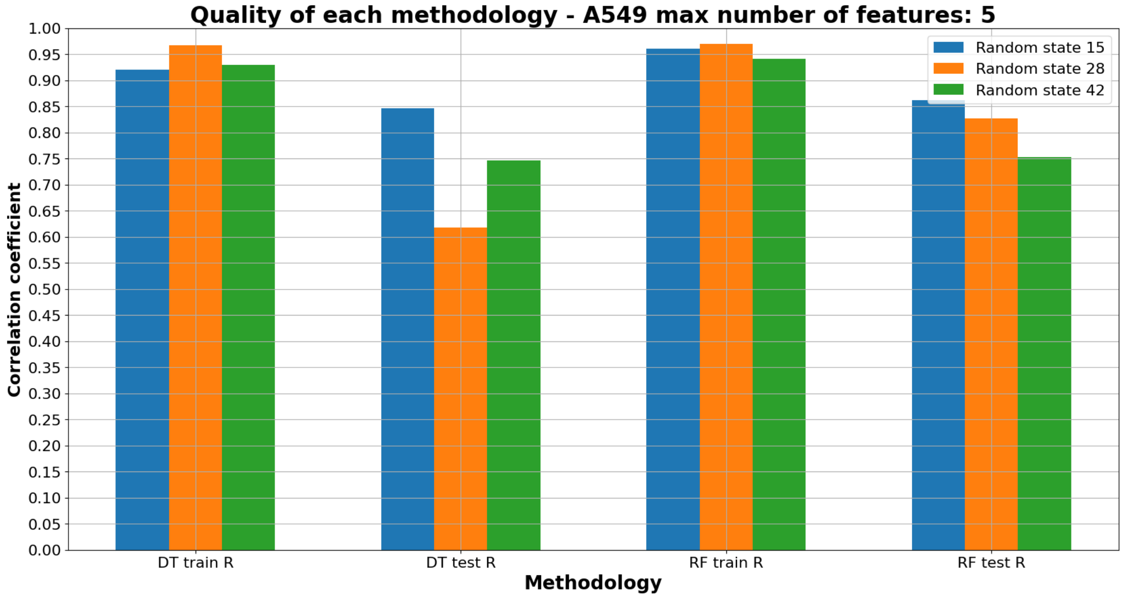


```
Random state - 15 ('DT - number of features used: 5.0', 'RF - number of features
used: 5.0')
Random state - 28 ('DT - number of features used: 4.0', 'RF - number of features
used: 5.0')
Random state - 42 ('DT - number of features used: 5.0', 'RF - number of features
used: 5.0')
[0.9201268576746804, 0.8457608041331407, 0.960591935079034, 0.8620618597285548]
[0.9669220010492233, 0.6172800607391895, 0.9701820261061275, 0.8267270635721486]
[0.9298029234842533, 0.746434872641246, 0.9417342321043257, 0.7526447589514621]
```

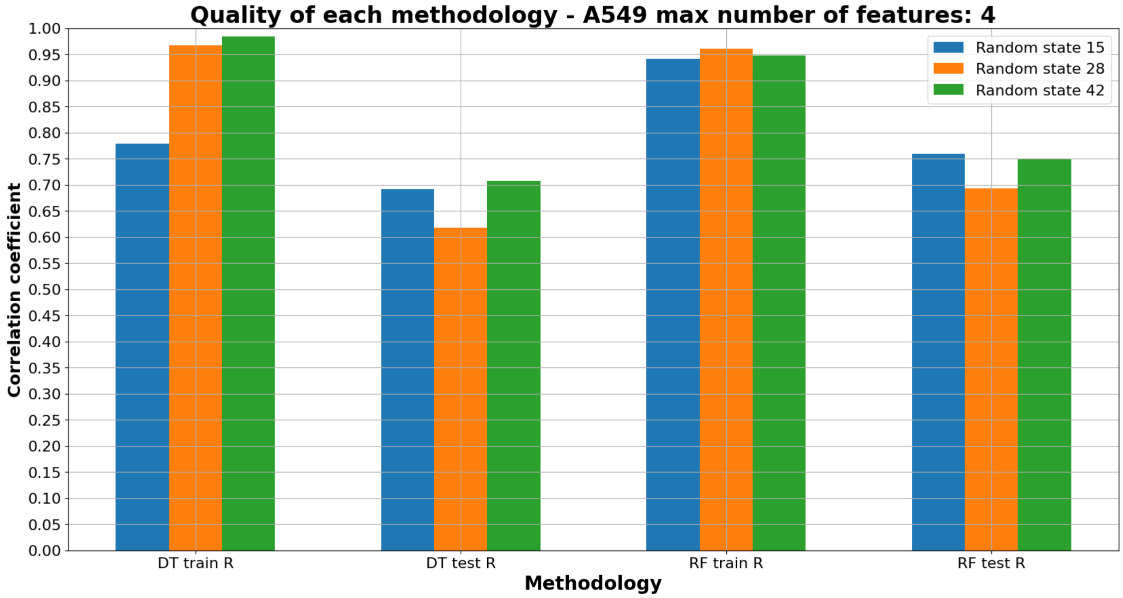


```
Random state - 15 ('DT - number of features used: 3.0', 'RF - number of features
used: 4.0')
Random state - 28 ('DT - number of features used: 4.0', 'RF - number of features
used: 4.0')
Random state - 42 ('DT - number of features used: 3.0', 'RF - number of features
used: 3.0')
[0.7787561234847591, 0.6915475851498438, 0.94133348297971, 0.7594733568417315]
[0.9669220010492233, 0.6172800607391895, 0.9601878273802564, 0.6926815941771752]
[0.9846195227501177, 0.70753906802074, 0.9477169502251152, 0.7487968593395388]
```

[ ]:

[20]:
```
for i in range(8, 2, -1):
    x = prepare_best_plot('BALB_3T3', i)
    prepare_plot(x, 'BALB_3T3', i)
    print(handle_negative_corr(x[0]))
    print(handle_negative_corr(x[1]))
    print(handle_negative_corr(x[2]))
```

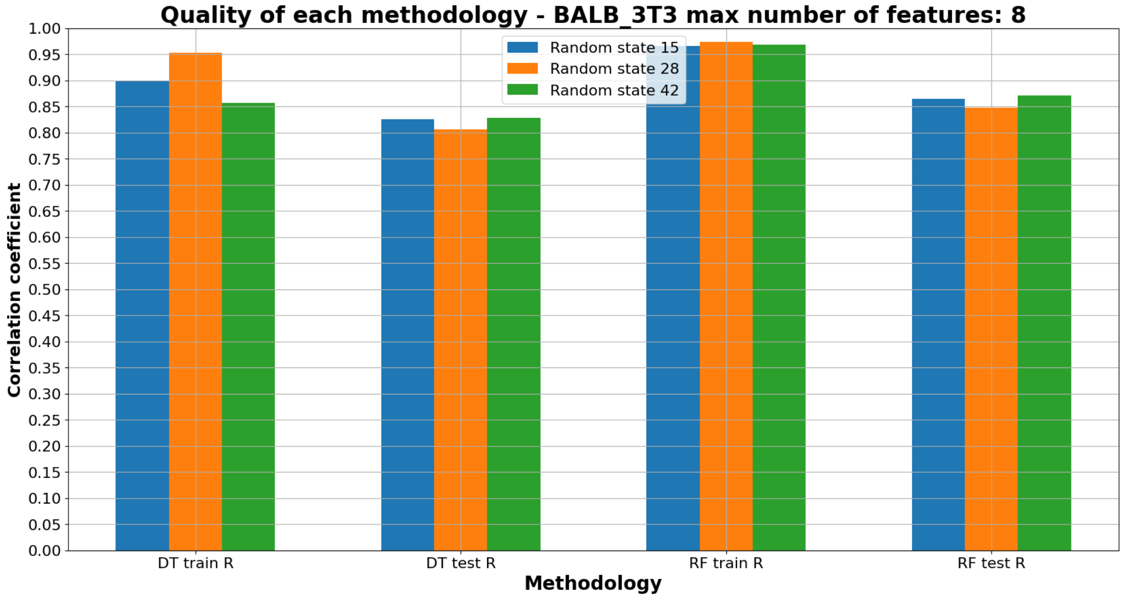

Random state - 15 ('DT - number of features used: 6.0', 'RF - number of features used: 6.0')
Random state - 28 ('DT - number of features used: 6.0', 'RF - number of features used: 7.0')
Random state - 42 ('DT - number of features used: 6.0', 'RF - number of features used: 7.0')
[0.8991491467263336, 0.8261911693918914, 0.9656538230216772, 0.8642992780332693]
[0.9528404289708712, 0.8063470063121128, 0.9733753371944485, 0.8472660678286129]
[0.8574233855704518, 0.8283658235151035, 0.9679324070733115, 0.8709265604132982]

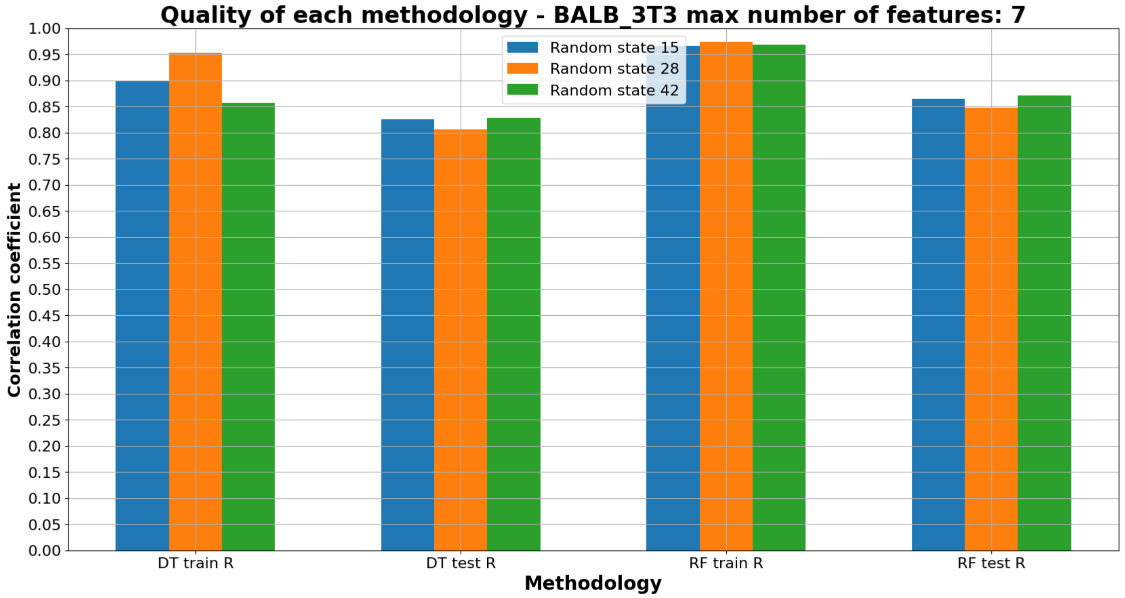

```
Random state - 15 ('DT - number of features used: 6.0', 'RF - number of features
used: 6.0')
Random state - 28 ('DT - number of features used: 6.0', 'RF - number of features
used: 7.0')
Random state - 42 ('DT - number of features used: 6.0', 'RF - number of features
used: 7.0')
[0.8991491467263336, 0.8261911693918914, 0.9656538230216772, 0.8642992780332693]
[0.9528404289708712, 0.8063470063121128, 0.9733753371944485, 0.8472660678286129]
[0.8574233855704518, 0.8283658235151035, 0.9679324070733115, 0.8709265604132982]
```

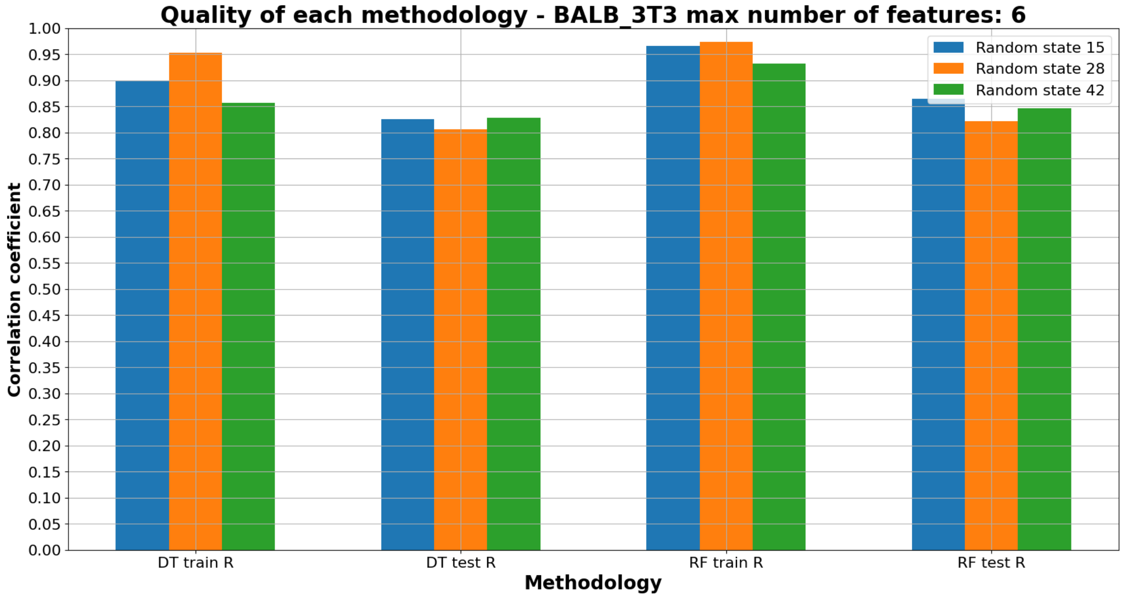


```
Random state - 15 ('DT - number of features used: 6.0', 'RF - number of features
used: 6.0')
Random state - 28 ('DT - number of features used: 6.0', 'RF - number of features
used: 6.0')
Random state - 42 ('DT - number of features used: 6.0', 'RF - number of features
used: 6.0')
[0.8991491467263336, 0.8261911693918914, 0.9656538230216772, 0.8642992780332693]
[0.9528404289708712, 0.8063470063121128, 0.9732431671159815, 0.8213095110427073]
[0.8574233855704518, 0.8283658235151035, 0.9323265067533627, 0.8462778707935902]
```

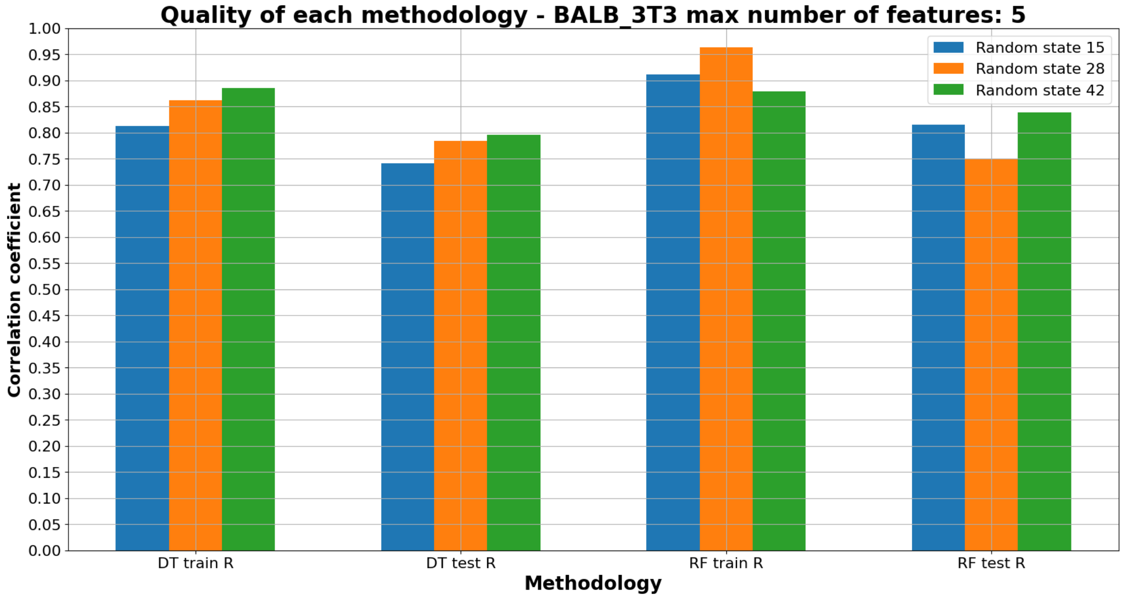


```
Random state - 15 ('DT - number of features used: 5.0', 'RF - number of features
used: 5.0')
Random state - 28 ('DT - number of features used: 3.0', 'RF - number of features
used: 5.0')
Random state - 42 ('DT - number of features used: 2.0', 'RF - number of features
used: 2.0')
[0.8124040437986895, 0.7410808070808154, 0.9118596336958118, 0.8149483739246309]
[0.8618249162158247, 0.7846752040880232, 0.9628242677281924, 0.7501347353760348]
[0.885912746009139, 0.7955365930788652, 0.8792917450164119, 0.8379661052032461]
```

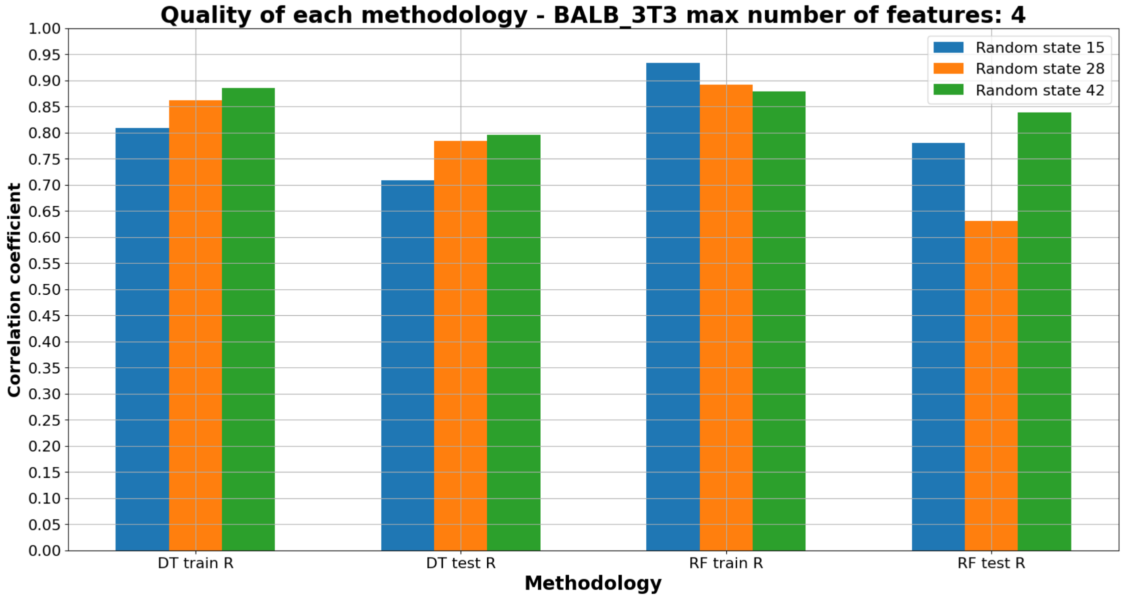

```
Random state - 15 ('DT - number of features used: 3.0', 'RF - number of features
used: 3.0')
Random state - 28 ('DT - number of features used: 3.0', 'RF - number of features
used: 2.0')
Random state - 42 ('DT - number of features used: 2.0', 'RF - number of features
used: 2.0')
[0.8089681087052601, 0.7083667815152337, 0.9331579969232232, 0.780272449227771]
[0.8618249162158247, 0.7846752040880232, 0.8911999290100625, 0.6303440412443166]
[0.885912746009139, 0.7955365930788652, 0.8792917450164119, 0.8379661052032461]
```

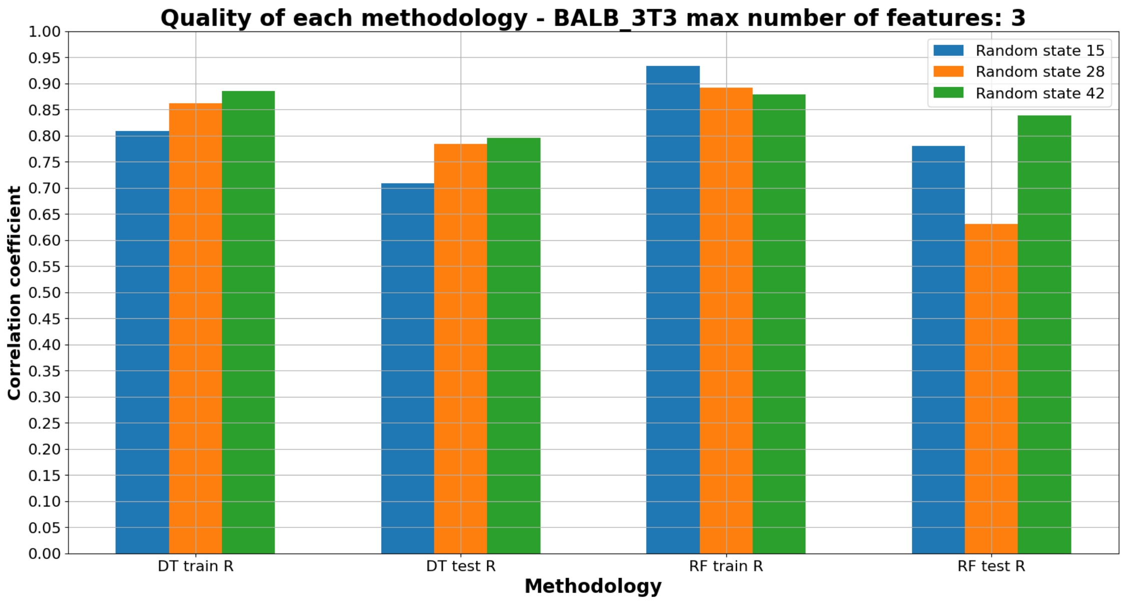


```
Random state - 15 ('DT - number of features used: 3.0', 'RF - number of features
used: 3.0')
Random state - 28 ('DT - number of features used: 3.0', 'RF - number of features
used: 2.0')
Random state - 42 ('DT - number of features used: 2.0', 'RF - number of features
used: 2.0')
[0.8089681087052601, 0.7083667815152337, 0.9331579969232232, 0.780272449227771]
[0.8618249162158247, 0.7846752040880232, 0.8911999290100625, 0.6303440412443166]
[0.885912746009139, 0.7955365930788652, 0.8792917450164119, 0.8379661052032461]
```


[ ]:

[21]:
```
for i in range(8, 2, -1):
    x = prepare_best_plot('LoVo', i)
    prepare_plot(x, 'LoVo', i)
    print(handle_negative_corr(x[0]))
```

```
print(handle_negative_corr(x[1]))
print(handle_negative_corr(x[2]))
```

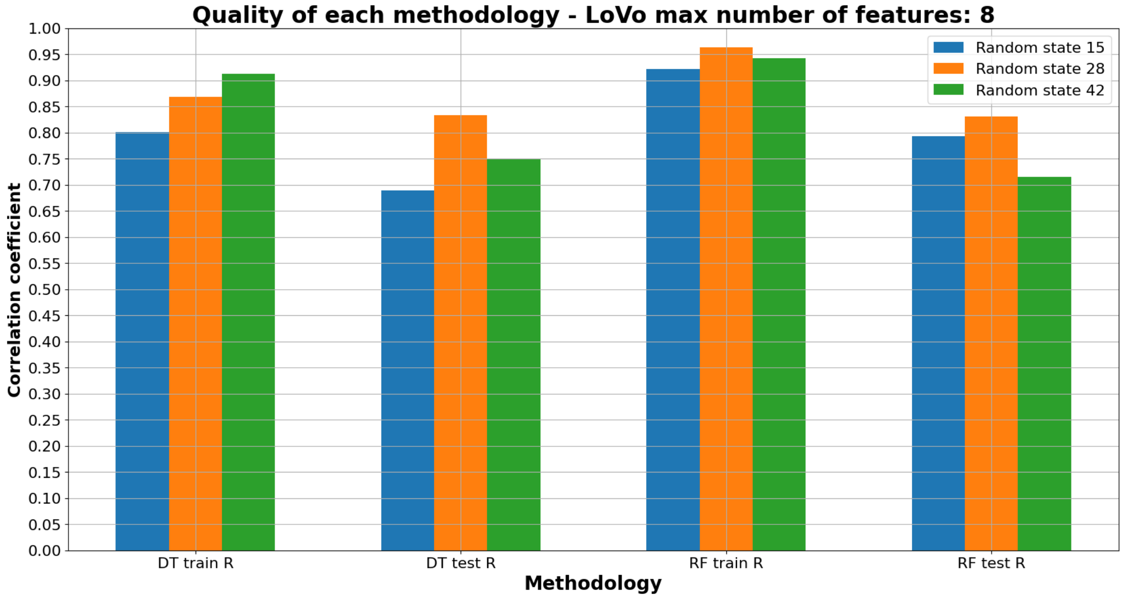


```
Random state - 15 ('DT - number of features used: 7.0', 'RF - number of features
used: 5.0')
Random state - 28 ('DT - number of features used: 3.0', 'RF - number of features
used: 7.0')
Random state - 42 ('DT - number of features used: 3.0', 'RF - number of features
used: 5.0')
[0.8012547599591731, 0.6895731340974959, 0.9218472144749, 0.7937730901899727]
[0.8682412855125715, 0.8337432078795763, 0.9636653219744876, 0.8305193093908672]
[0.9121710628859598, 0.7499171098832387, 0.9423441595215349, 0.7153725648027842]
```

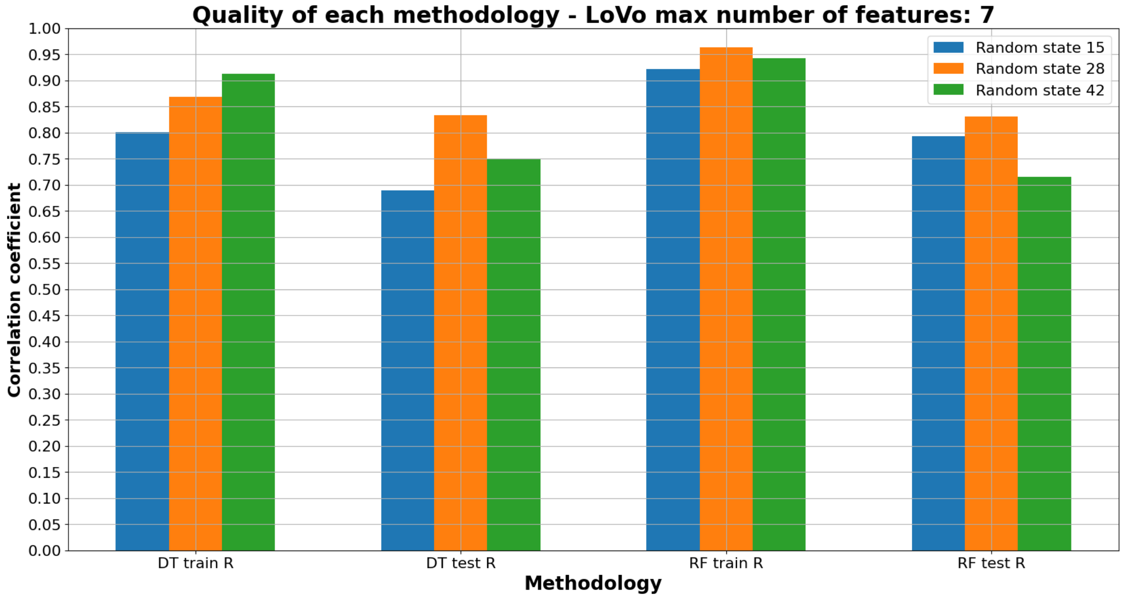


```
Random state - 15 ('DT - number of features used: 7.0', 'RF - number of features
used: 5.0')
Random state - 28 ('DT - number of features used: 3.0', 'RF - number of features
used: 7.0')
Random state - 42 ('DT - number of features used: 3.0', 'RF - number of features
used: 5.0')
[0.8012547599591731, 0.6895731340974959, 0.9218472144749, 0.7937730901899727]
[0.8682412855125715, 0.8337432078795763, 0.9636653219744876, 0.8305193093908672]
[0.9121710628859598, 0.7499171098832387, 0.9423441595215349, 0.7153725648027842]
```

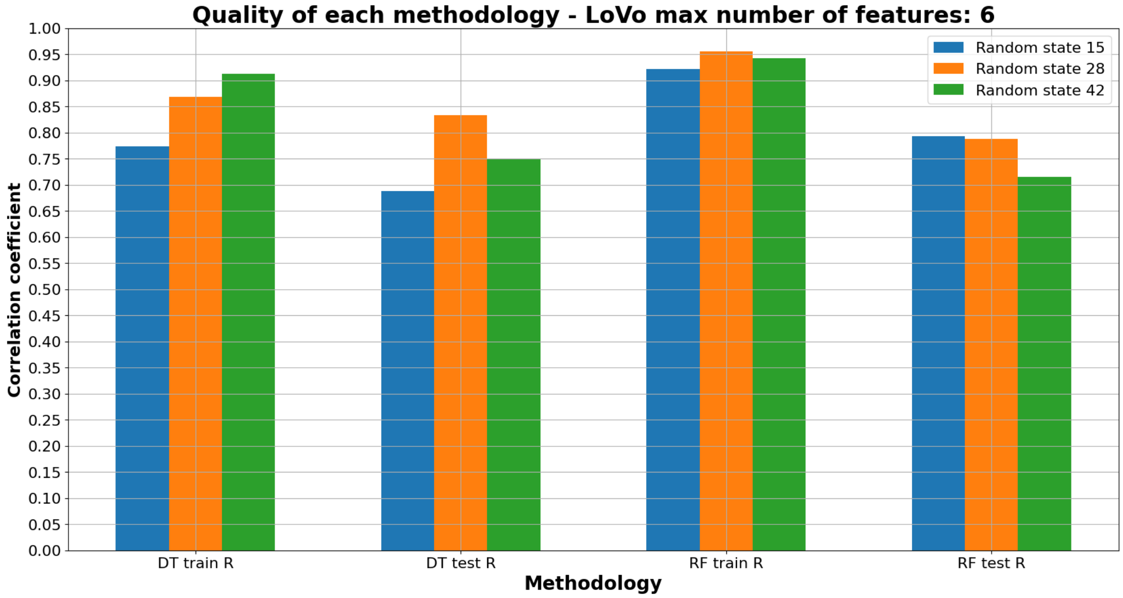

```
Random state - 15 ('DT - number of features used: 6.0', 'RF - number of features
used: 5.0')
Random state - 28 ('DT - number of features used: 3.0', 'RF - number of features
used: 6.0')
Random state - 42 ('DT - number of features used: 3.0', 'RF - number of features
used: 5.0')
[0.7738495519932647, 0.6881453471088331, 0.9218472144749, 0.7937730901899727]
[0.8682412855125715, 0.8337432078795763, 0.9549618967523424, 0.7874289926703334]
[0.9121710628859598, 0.7499171098832387, 0.9423441595215349, 0.7153725648027842]
```

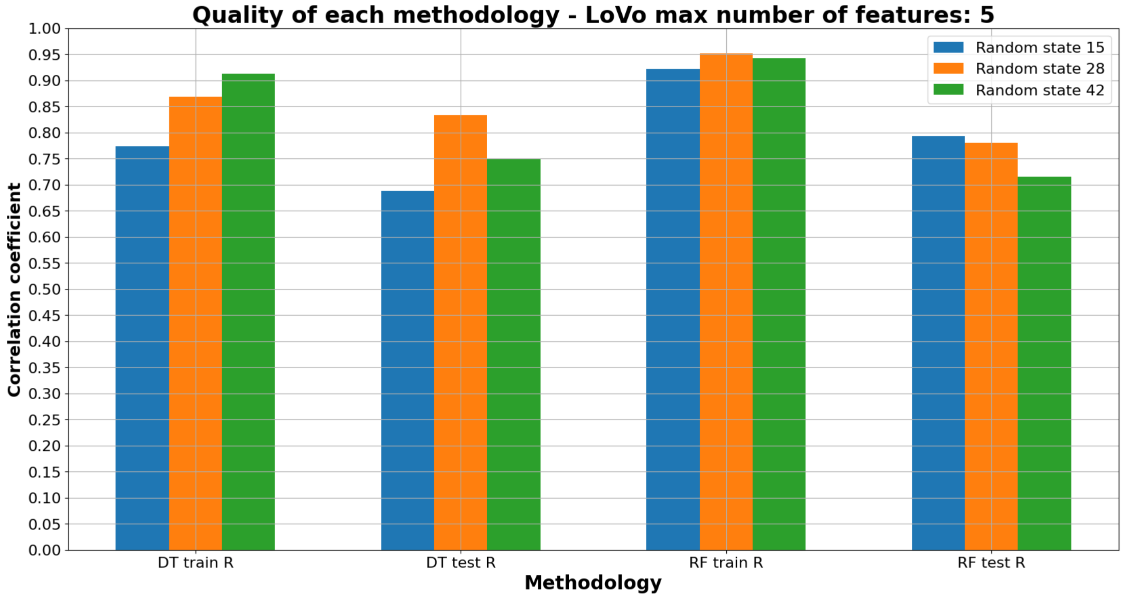


```
Random state - 15 ('DT - number of features used: 5.0', 'RF - number of features
used: 5.0')
Random state - 28 ('DT - number of features used: 3.0', 'RF - number of features
used: 5.0')
Random state - 42 ('DT - number of features used: 3.0', 'RF - number of features
used: 5.0')
[0.7738495519932644, 0.688145347108833, 0.9218472144749, 0.7937730901899727]
[0.8682412855125715, 0.8337432078795763, 0.95161256233916, 0.780370388796789]
[0.9121710628859598, 0.7499171098832387, 0.9423441595215349, 0.7153725648027842]
```

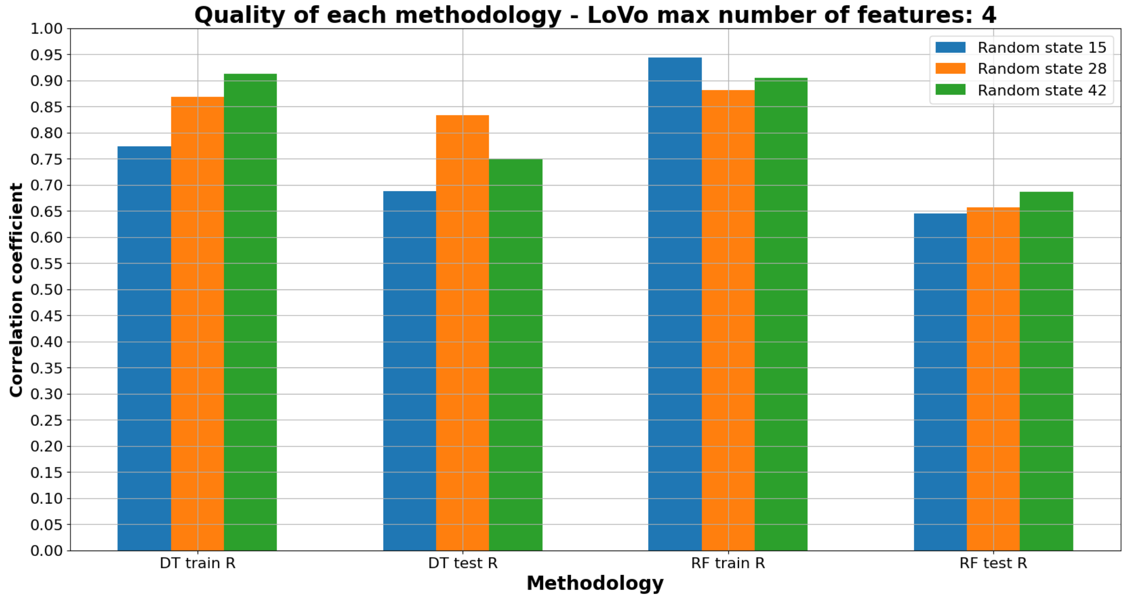


```
Random state - 15 ('DT - number of features used: 3.0', 'RF - number of features
used: 3.0')
Random state - 28 ('DT - number of features used: 3.0', 'RF - number of features
used: 2.0')
Random state - 42 ('DT - number of features used: 3.0', 'RF - number of features
used: 2.0')
[0.7738495519932647, 0.6881453471088325, 0.9435896757858103, 0.6450408235184336]
[0.8682412855125715, 0.8337432078795763, 0.8811267812945673, 0.6572630628008767]
[0.9121710628859598, 0.7499171098832387, 0.9053674539315217, 0.6872057325646179]
```

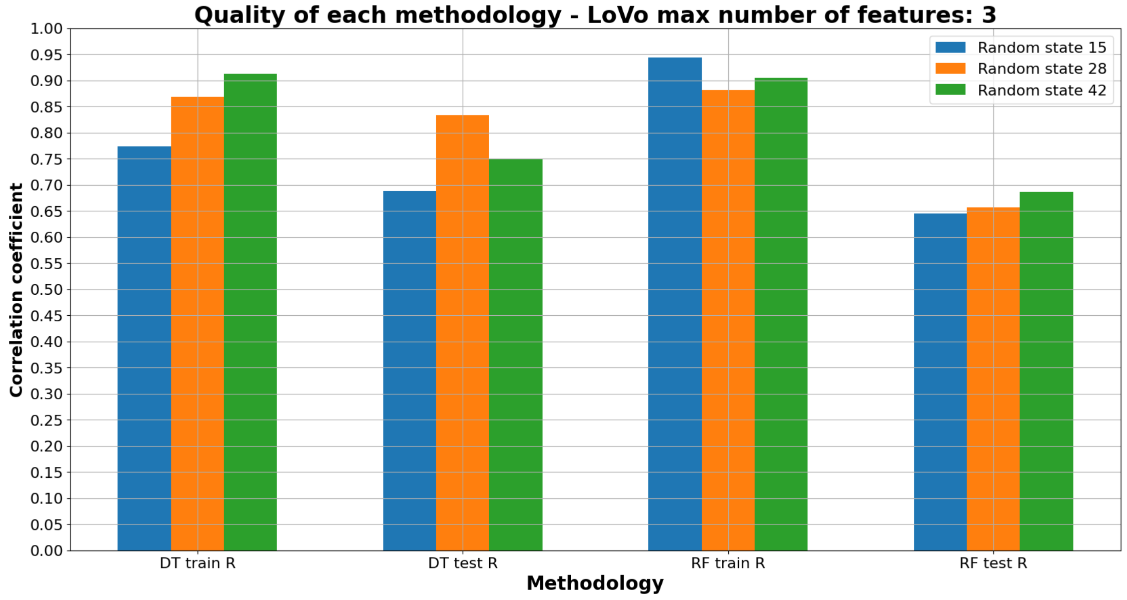

```
Random state - 15 ('DT - number of features used: 3.0', 'RF - number of features
used: 3.0')
Random state - 28 ('DT - number of features used: 3.0', 'RF - number of features
used: 2.0')
Random state - 42 ('DT - number of features used: 3.0', 'RF - number of features
used: 2.0')
[0.7738495519932647, 0.6881453471088325, 0.9435896757858103, 0.6450408235184336]
[0.8682412855125715, 0.8337432078795763, 0.8811267812945673, 0.6572630628008767]
[0.9121710628859598, 0.7499171098832387, 0.9053674539315217, 0.6872057325646179]
```

[22]:

```
for i in range(8, 5, -1):
    x = prepare_best_plot('LoVo_DX', i)
    prepare_plot(x, 'LoVo_DX', i)
    print(handle_negative_corr(x[0]))
    print(handle_negative_corr(x[1]))
    print(handle_negative_corr(x[2]))
```

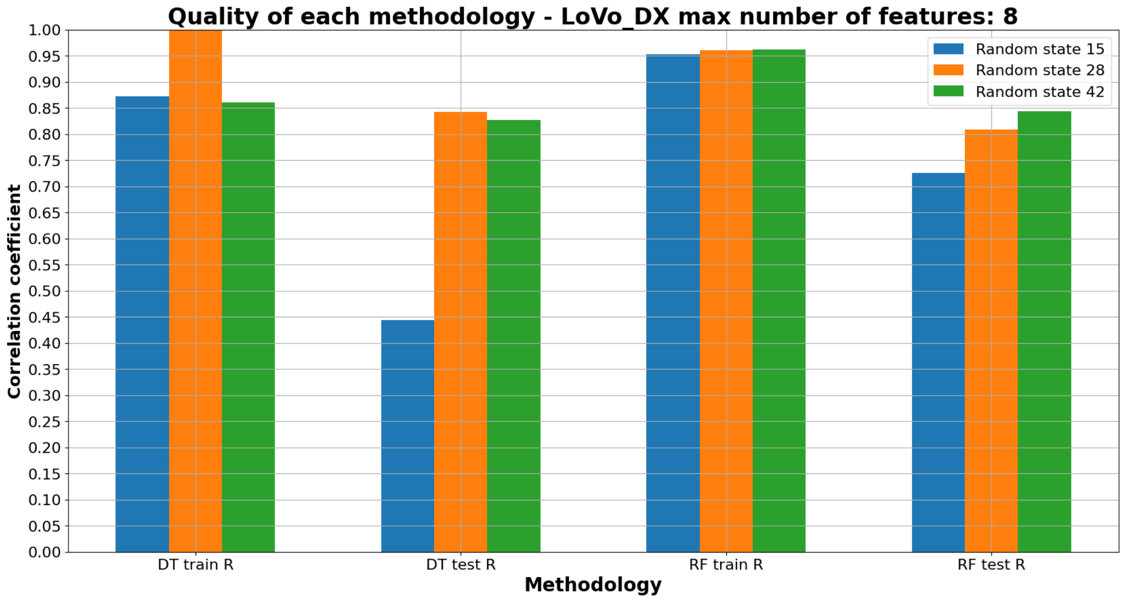


```
Random state - 15 ('DT - number of features used: 7.0', 'RF - number of features
used: 8.0')
Random state - 28 ('DT - number of features used: 5.0', 'RF - number of features
used: 8.0')
Random state - 42 ('DT - number of features used: 7.0', 'RF - number of features
used: 7.0')
[0.8724994277322107, 0.44416679666061354, 0.9531465584369622,
0.7258930436657147]
[0.9998881595139364, 0.8430877655864321, 0.9608248729709339, 0.8093430351511413]
[0.8607935732823858, 0.8271783219542648, 0.9614894965852848, 0.8437279214475684]
```

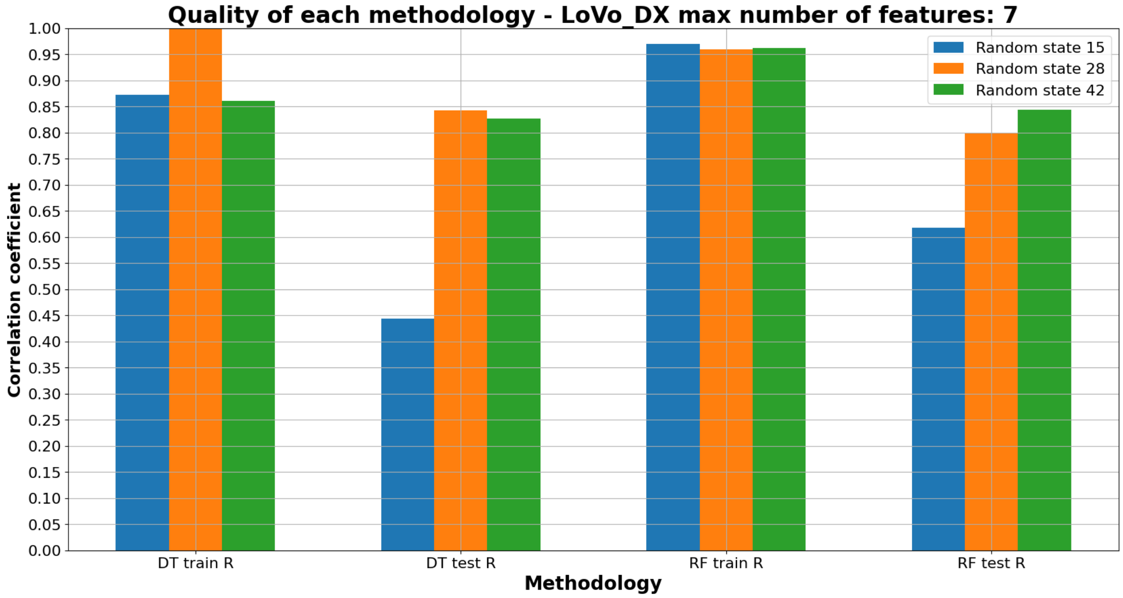


```
Random state - 15 ('DT - number of features used: 7.0', 'RF - number of features
used: 7.0')
Random state - 28 ('DT - number of features used: 5.0', 'RF - number of features
used: 7.0')
Random state - 42 ('DT - number of features used: 7.0', 'RF - number of features
used: 7.0')
[0.8724994277322107, 0.44416679666061354, 0.9701730613127154,
0.6181792070920507]
[0.9998881595139364, 0.8430877655864321, 0.95913927288201, 0.7997866699043976]
[0.8607935732823858, 0.8271783219542648, 0.9614894965852848, 0.8437279214475684]
```

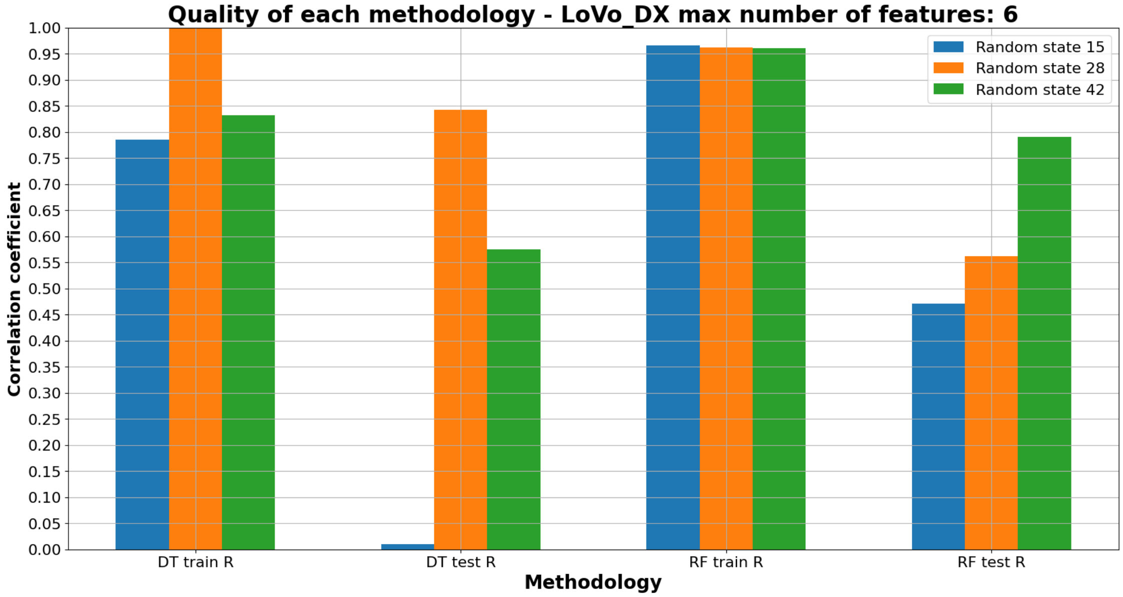

```
Random state - 15 ('DT - number of features used: 5.0', 'RF - number of features
used: 5.0')
Random state - 28 ('DT - number of features used: 5.0', 'RF - number of features
used: 5.0')
Random state - 42 ('DT - number of features used: 5.0', 'RF - number of features
used: 5.0')
[0.7851394944455857, 0.01, 0.965938844054454, 0.4715560592926372]
[0.9998881595139364, 0.8430877655864321, 0.9619512068981969, 0.5614693906239112]
[0.832498347961302, 0.5745589174162089, 0.9610074984048745, 0.790562123545645]
```

[ ]:

[23]:
```
for i in range(8, 4, -1):
    x = prepare_best_plot('MCF-7', i)
    prepare_plot(x, 'MCF-7', i)
    print(handle_negative_corr(x[0]))
    print(handle_negative_corr(x[1]))
    print(handle_negative_corr(x[2]))
```

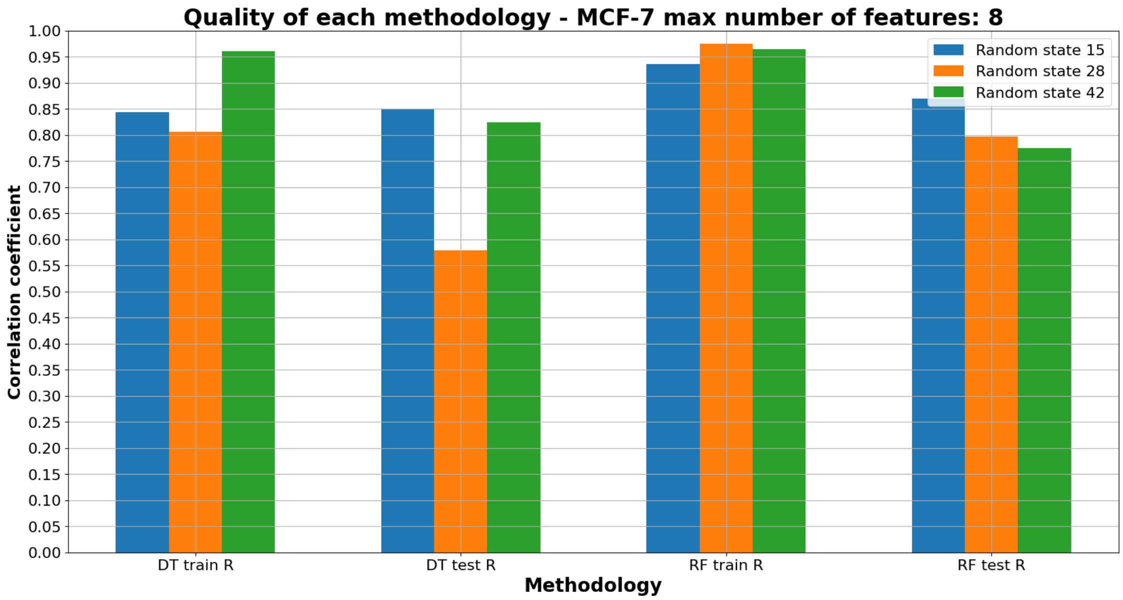


```
Random state - 15 ('DT - number of features used: 6.0', 'RF - number of features
used: 8.0')
Random state - 28 ('DT - number of features used: 4.0', 'RF - number of features
used: 8.0')
Random state - 42 ('DT - number of features used: 8.0', 'RF - number of features
used: 8.0')
[0.8435310621038742, 0.8490631015009857, 0.9361673240230982, 0.8695068587619961]
```

[0.8067266275260266, 0.5788863720704619, 0.9745022739926398, 0.7974479201634584]
[0.9607100088789956, 0.8246123913884399, 0.9643673177108916, 0.7746407763544272]

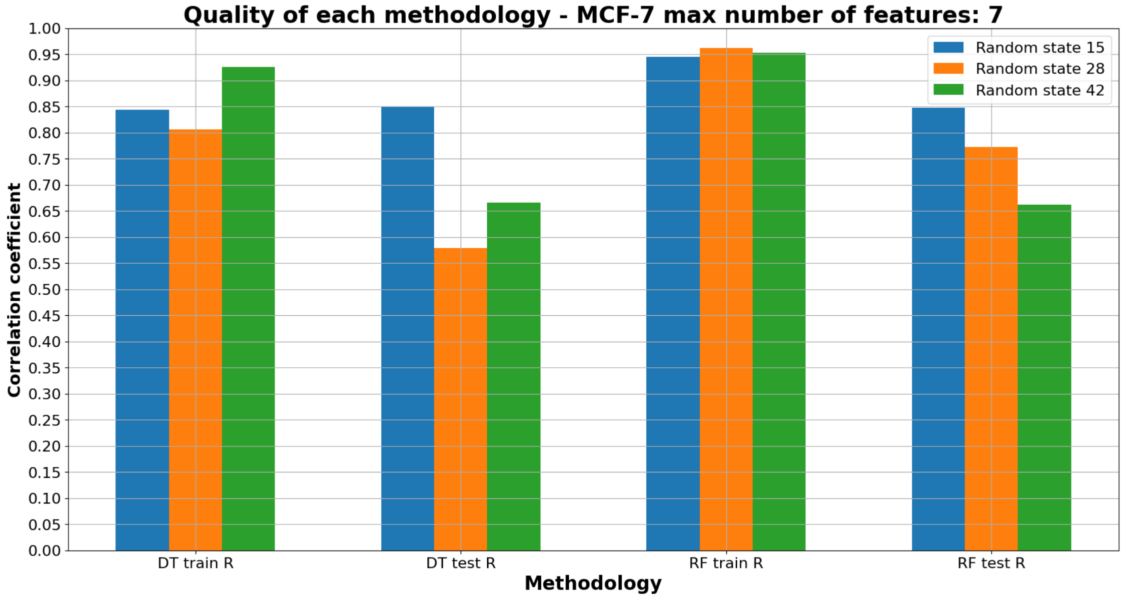

Random state - 15 ('DT - number of features used: 6.0', 'RF - number of features used: 6.0')
Random state - 28 ('DT - number of features used: 4.0', 'RF - number of features used: 6.0')
Random state - 42 ('DT - number of features used: 6.0', 'RF - number of features used: 6.0')
[0.8435310621038742, 0.8490631015009857, 0.945156244145722, 0.8470828152088354]
[0.8067266275260266, 0.5788863720704619, 0.9622709929006865, 0.7725823523082598]
[0.9250974432855168, 0.6663870801375169, 0.9527501485389981, 0.6624039809877339]

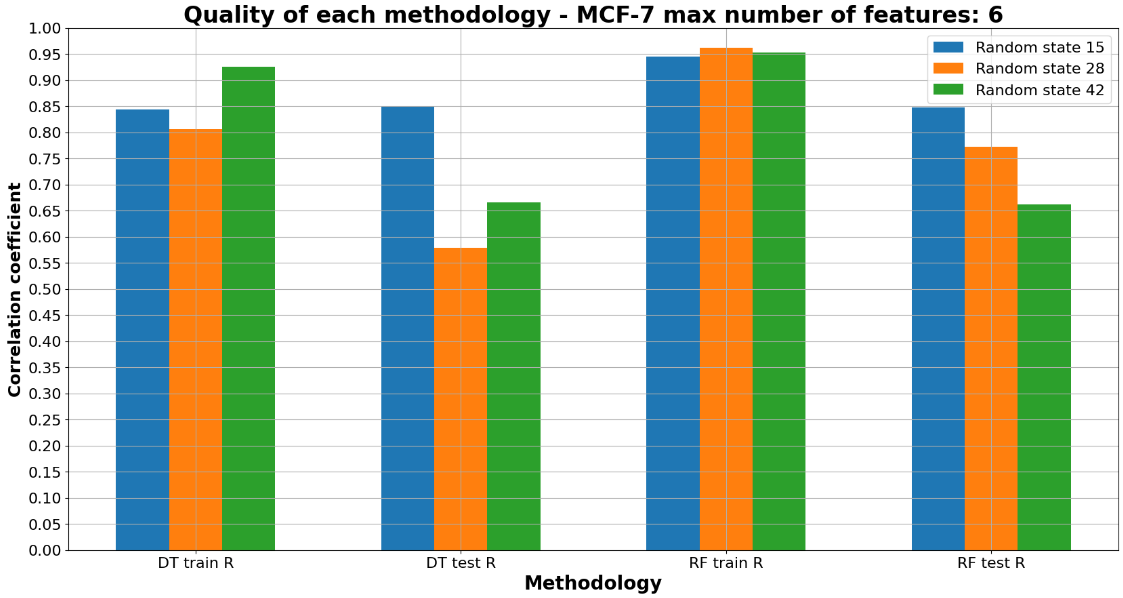


```
Random state - 15 ('DT - number of features used: 6.0', 'RF - number of features
used: 6.0')
Random state - 28 ('DT - number of features used: 4.0', 'RF - number of features
used: 6.0')
Random state - 42 ('DT - number of features used: 6.0', 'RF - number of features
used: 6.0')
[0.8435310621038742, 0.8490631015009857, 0.945156244145722, 0.8470828152088354]
[0.8067266275260266, 0.5788863720704619, 0.9622709929006865, 0.7725823523082598]
[0.9250974432855168, 0.6663870801375169, 0.9527501485389981, 0.6624039809877339]
```

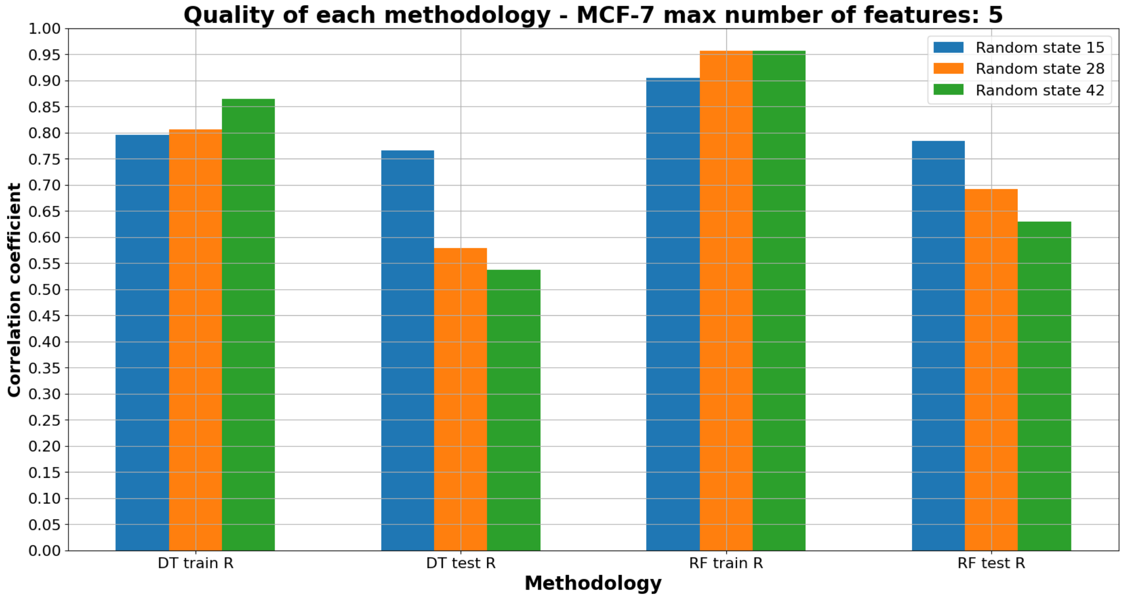

```
Random state - 15 ('DT - number of features used: 4.0', 'RF - number of features
used: 4.0')
Random state - 28 ('DT - number of features used: 4.0', 'RF - number of features
used: 4.0')
Random state - 42 ('DT - number of features used: 4.0', 'RF - number of features
used: 4.0')
[0.7959369681181857, 0.7660302989047363, 0.9051955244679118, 0.7837627386732194]
[0.8067266275260266, 0.5788863720704619, 0.9563828487646977, 0.6924899356899538]
[0.8646854500902919, 0.5371299141302905, 0.956767076965924, 0.6292292538599767]
```

[ ]:

[ ]:

1.7 Best models plot

Comments: (see different random states for each model)

- A549 -> random state 15, 5 features DT, 5 features RF

1. MLR (0.65, 0.55) -> 3 features
2. DT (0.92, 0.84) -> 5 features
3. RF (0.92, 0.82) -> 5 features
4. KNN (0.77, 0.83) -> 5 features
5. SVM (0.64, 0.18) -> 3 features

- BALB/3T3 -> random state 42, 6 features RF

1. MLR (0.73, 0.01) -> 3 features
2. DT (0.86, 0.75) -> 6 features
3. RF (0.93, 0.85) -> 6 features
4. KNN (0.77, 0.22) -> 3 features
5. SVM (0.68, 0.17) -> 3 features

- LoVo -> random state 28, 2 features RF

1. MLR (0.69, 0.40) -> 3 features
2. DT (0.94, 0.82) -> 5 features
3. RF (0.85, 0.70) -> 2 features
4. KNN (0.73, 0.38) -> 5 features
5. SVM (0.70, 0.01) -> 5 features

- LoVo/DX -> random state 42, 5 features RF

1. MLR (0.82, 0.78) -> 7 features
2. DT (0.86, 0.84) -> 7 features
3. RF (0.96, 0.83) -> 5 features
4. KNN (0.81, 0.01) -> 5 features
5. SVM (0.70, 0.63) -> 5 features

- MCF-7 -> random state 15, 4 features RF

1. MLR (0.70, 0.61) -> 4 features
2. DT (0.92, 0.53) -> 4 features
3. RF (0.91, 0.65) -> 4 features
4. KNN (0.70, 0.75) -> 4 features
5. SVM (0.69, 0.53) -> 4 features

[24]:
```
df_a549 = pd.read_excel('../Data/Quality_A549_15_.xlsx', sheet_name='RF')
df_a549 = df_a549[df_a549['Number of features'] == 5]
df_a549['Training data R score'] = handle_negative_corr(df_a549['Training data␣
↪R^2 score'])
df_a549['Test data R score'] = handle_negative_corr(df_a549['Test data R^2␣
↪score'])
df_a549
```

[24]:
```
     Unnamed: 0  Correlation threshold  Training data R^2 score  \
323         323                   0.50                 0.852952
324         324                   0.50                 0.860201
325         325                   0.50                 0.865749
326         326                   0.50                 0.887848
327         327                   0.50                 0.888773
328         328                   0.50                 0.892504
329         329                   0.50                 0.908859
330         330                   0.50                 0.909170
331         331                   0.50                 0.913283
332         332                   0.50                 0.918630
333         333                   0.50                 0.917600
334         334                   0.50                 0.915991
335         335                   0.50                 0.921486
336         336                   0.50                 0.924538
337         337                   0.50                 0.921465
338         338                   0.50                 0.922737
339         339                   0.50                 0.924854
340         340                   0.50                 0.927070
341         341                   0.50                 0.920711
342         342                   0.51                 0.852952
343         343                   0.51                 0.860201
344         344                   0.51                 0.865749
345         345                   0.51                 0.887848
346         346                   0.51                 0.888773
347         347                   0.51                 0.892504
348         348                   0.51                 0.908859
349         349                   0.51                 0.909170
350         350                   0.51                 0.913283
351         351                   0.51                 0.918630
352         352                   0.51                 0.917600
353         353                   0.51                 0.915991
354         354                   0.51                 0.921486
```

355	355	0.51	0.924538
356	356	0.51	0.921465
357	357	0.51	0.922737
358	358	0.51	0.924854
359	359	0.51	0.927070
360	360	0.51	0.920711

	Test data R^2 score	Training RMSE	Test RMSE	Number of features \
323	0.693940	0.357987	0.573479	5
324	0.699356	0.349052	0.568382	5
325	0.668966	0.342057	0.596418	5
326	0.623941	0.312638	0.635685	5
327	0.648644	0.311347	0.614452	5
328	0.672695	0.306080	0.593049	5
329	0.683983	0.281835	0.582732	5
330	0.668412	0.281354	0.596917	5
331	0.710288	0.274910	0.557952	5
332	0.703868	0.266300	0.564101	5
333	0.707403	0.267980	0.560723	5
334	0.732924	0.270583	0.535712	5
335	0.736283	0.261585	0.532333	5
336	0.734897	0.256449	0.533730	5
337	0.729516	0.261619	0.539119	5
338	0.743151	0.259492	0.525356	5
339	0.739241	0.255913	0.529339	5
340	0.734946	0.252110	0.533681	5
341	0.737105	0.262872	0.531502	5
342	0.693940	0.357987	0.573479	5
343	0.699356	0.349052	0.568382	5
344	0.668966	0.342057	0.596418	5
345	0.623941	0.312638	0.635685	5
346	0.648644	0.311347	0.614452	5
347	0.672695	0.306080	0.593049	5
348	0.683983	0.281835	0.582732	5
349	0.668412	0.281354	0.596917	5
350	0.710288	0.274910	0.557952	5
351	0.703868	0.266300	0.564101	5
352	0.707403	0.267980	0.560723	5
353	0.732924	0.270583	0.535712	5
354	0.736283	0.261585	0.532333	5
355	0.734897	0.256449	0.533730	5
356	0.729516	0.261619	0.539119	5
357	0.743151	0.259492	0.525356	5
358	0.739241	0.255913	0.529339	5
359	0.734946	0.252110	0.533681	5
360	0.737105	0.262872	0.531502	5

	Number of estimators	Training data R score	Test data R score
323	2	0.923554	0.833031
324	3	0.927470	0.836275
325	4	0.930456	0.817903
326	5	0.942257	0.789899
327	6	0.942747	0.805384
328	7	0.944724	0.820180
329	8	0.953341	0.827033
330	9	0.953504	0.817564
331	10	0.955658	0.842786
332	11	0.958452	0.838968
333	12	0.957914	0.841073
334	13	0.957074	0.856110
335	14	0.959941	0.858069
336	15	0.961529	0.857261
337	16	0.959930	0.854117
338	17	0.960592	0.862062
339	18	0.961693	0.859791
340	19	0.962845	0.857290
341	20	0.959537	0.858548
342	2	0.923554	0.833031
343	3	0.927470	0.836275
344	4	0.930456	0.817903
345	5	0.942257	0.789899
346	6	0.942747	0.805384
347	7	0.944724	0.820180
348	8	0.953341	0.827033
349	9	0.953504	0.817564
350	10	0.955658	0.842786
351	11	0.958452	0.838968
352	12	0.957914	0.841073
353	13	0.957074	0.856110
354	14	0.959941	0.858069
355	15	0.961529	0.857261
356	16	0.959930	0.854117
357	17	0.960592	0.862062
358	18	0.961693	0.859791
359	19	0.962845	0.857290
360	20	0.959537	0.858548

[25]:
```
df_ = pd.read_excel('../Data/Quality_BALB_3T3_42_.xlsx', sheet_name='RF')
df_ = df_[df_['Number of features'] == 6]
df_['Training data R score'] = handle_negative_corr(df_['Training data R^2␣
↪score'])
df_['Test data R score'] = handle_negative_corr(df_['Test data R^2 score'])
df_
```

[25]:

	Unnamed: 0	Correlation threshold	Training data R^2 score	\
342	342	0.51	0.869233	
343	343	0.51	0.923683	
344	344	0.51	0.934524	
345	345	0.51	0.937457	
346	346	0.51	0.929701	
347	347	0.51	0.940844	
348	348	0.51	0.945992	
349	349	0.51	0.943862	
350	350	0.51	0.940881	
351	351	0.51	0.940214	
352	352	0.51	0.939011	
353	353	0.51	0.939990	
354	354	0.51	0.941761	
355	355	0.51	0.945607	
356	356	0.51	0.946995	
357	357	0.51	0.948830	
358	358	0.51	0.946417	
359	359	0.51	0.944971	
360	360	0.51	0.943980	

	Test data R^2 score	Training RMSE	Test RMSE	Number of features	\
342	0.716186	0.318598	0.424152	6	
343	0.597886	0.243391	0.504870	6	
344	0.681066	0.225443	0.449630	6	
345	0.701507	0.220334	0.434983	6	
346	0.675674	0.233598	0.453414	6	
347	0.604822	0.214286	0.500497	6	
348	0.648896	0.204749	0.471762	6	
349	0.649432	0.208749	0.471401	6	
350	0.665910	0.214219	0.460189	6	
351	0.664213	0.215424	0.461356	6	
352	0.683600	0.217581	0.447840	6	
353	0.657975	0.215827	0.465622	6	
354	0.616112	0.212619	0.493295	6	
355	0.597845	0.205478	0.504895	6	
356	0.570470	0.202840	0.521797	6	
357	0.577984	0.199298	0.517213	6	
358	0.591313	0.203942	0.508979	6	
359	0.588724	0.206677	0.510589	6	
360	0.591825	0.208528	0.508661	6	

	Number of estimators	Training data R score	Test data R score
342	2	0.932327	0.846278
343	3	0.961084	0.773231
344	4	0.966708	0.825268
345	5	0.968224	0.837560

346	6	0.964210	0.821994
347	7	0.969971	0.777703
348	8	0.972621	0.805541
349	9	0.971525	0.805873
350	10	0.969990	0.816033
351	11	0.969646	0.814993
352	12	0.969026	0.826801
353	13	0.969531	0.811157
354	14	0.970444	0.784928
355	15	0.972423	0.773204
356	16	0.973136	0.755294
357	17	0.974079	0.760253
358	18	0.972840	0.768969
359	19	0.972096	0.767284
360	20	0.971586	0.769301

[ ]:

[26]:
```
df_ = pd.read_excel('../Data/Quality_LoVo_28_.xlsx', sheet_name='RF')
df_ = df_[df_['Number of features'] == 2]
df_['Training data R score'] = handle_negative_corr(df_['Training data R^2␣
↪score'])
df_['Test data R score'] = handle_negative_corr(df_['Test data R^2 score'])
df_
```

[26]:

	Unnamed: 0	Correlation threshold	Training data R^2 score \
380	380	0.53	0.726452
381	381	0.53	0.730699
382	382	0.53	0.729826
383	383	0.53	0.755753
384	384	0.53	0.772016
385	385	0.53	0.771714
386	386	0.53	0.773504
387	387	0.53	0.772194
388	388	0.53	0.770797
389	389	0.53	0.777861
390	390	0.53	0.780498
391	391	0.53	0.779444
392	392	0.53	0.783158
393	393	0.53	0.778982
394	394	0.53	0.780691
395	395	0.53	0.781139
396	396	0.53	0.776384
397	397	0.53	0.775154
398	398	0.53	0.775951
399	399	0.54	0.726452
400	400	0.54	0.730699

401	401	0.54	0.729826
402	402	0.54	0.755753
403	403	0.54	0.772016
404	404	0.54	0.771714
405	405	0.54	0.773504
406	406	0.54	0.772194
407	407	0.54	0.770797
408	408	0.54	0.777861
409	409	0.54	0.780498
410	410	0.54	0.779444
411	411	0.54	0.783158
412	412	0.54	0.778982
413	413	0.54	0.780691
414	414	0.54	0.781139
415	415	0.54	0.776384
416	416	0.54	0.775154
417	417	0.54	0.775951

	Test data R^2 score	Training RMSE	Test RMSE	Number of features \
380	0.267403	0.490898	0.573263	2
381	0.199666	0.487072	0.599180	2
382	0.237774	0.487861	0.584741	2
383	0.311228	0.463862	0.555852	2
384	0.352617	0.448153	0.538893	2
385	0.376864	0.448449	0.528704	2
386	0.352702	0.446688	0.538857	2
387	0.319981	0.447978	0.552309	2
388	0.336020	0.449349	0.545757	2
389	0.334133	0.442371	0.546531	2
390	0.299672	0.439737	0.560496	2
391	0.317294	0.440791	0.553399	2
392	0.339342	0.437064	0.544390	2
393	0.395078	0.441253	0.520920	2
394	0.403927	0.439544	0.517096	2
395	0.403071	0.439095	0.517467	2
396	0.431995	0.443838	0.504775	2
397	0.404526	0.445058	0.516836	2
398	0.380702	0.444269	0.527074	2
399	0.267403	0.490898	0.573263	2
400	0.199666	0.487072	0.599180	2
401	0.237774	0.487861	0.584741	2
402	0.311228	0.463862	0.555852	2
403	0.352617	0.448153	0.538893	2
404	0.376864	0.448449	0.528704	2
405	0.352702	0.446688	0.538857	2
406	0.319981	0.447978	0.552309	2
407	0.336020	0.449349	0.545757	2

408	0.334133	0.442371	0.546531	2
409	0.299672	0.439737	0.560496	2
410	0.317294	0.440791	0.553399	2
411	0.339342	0.437064	0.544390	2
412	0.395078	0.441253	0.520920	2
413	0.403927	0.439544	0.517096	2
414	0.403071	0.439095	0.517467	2
415	0.431995	0.443838	0.504775	2
416	0.404526	0.445058	0.516836	2
417	0.380702	0.444269	0.527074	2

	Number of estimators	Training data R score	Test data R score
380	2	0.852321	0.517110
381	3	0.854809	0.446839
382	4	0.854298	0.487620
383	5	0.869341	0.557879
384	6	0.878644	0.593815
385	7	0.878473	0.613892
386	8	0.879491	0.593887
387	9	0.878746	0.565669
388	10	0.877951	0.579672
389	11	0.881964	0.578042
390	12	0.883458	0.547423
391	13	0.882861	0.563289
392	14	0.884962	0.582531
393	15	0.882600	0.628552
394	16	0.883567	0.635553
395	17	0.883821	0.634879
396	18	0.881127	0.657263
397	19	0.880428	0.636024
398	20	0.880881	0.617010
399	2	0.852321	0.517110
400	3	0.854809	0.446839
401	4	0.854298	0.487620
402	5	0.869341	0.557879
403	6	0.878644	0.593815
404	7	0.878473	0.613892
405	8	0.879491	0.593887
406	9	0.878746	0.565669
407	10	0.877951	0.579672
408	11	0.881964	0.578042
409	12	0.883458	0.547423
410	13	0.882861	0.563289
411	14	0.884962	0.582531
412	15	0.882600	0.628552
413	16	0.883567	0.635553
414	17	0.883821	0.634879

```
415                  18            0.881127        0.657263
416                  19            0.880428        0.636024
417                  20            0.880881        0.617010
```

[ ]:

```

```

[27]:

```
df_ = pd.read_excel('../Data/Quality_LoVo_DX_42_.xlsx', sheet_name='RF')
df_ = df_[df_['Number of features'] == 5]
df_['Training data R score'] = handle_negative_corr(df_['Training data R^2␣
 ↪score'])
df_['Test data R score'] = handle_negative_corr(df_['Test data R^2 score'])
df_
```

[27]:

```
     Unnamed: 0  Correlation threshold  Training data R^2 score  \
570         570                   0.63                 0.849392
571         571                   0.63                 0.877695
572         572                   0.63                 0.886607
573         573                   0.63                 0.894969
574         574                   0.63                 0.904921
575         575                   0.63                 0.918333
576         576                   0.63                 0.916942
577         577                   0.63                 0.923954
578         578                   0.63                 0.918480
579         579                   0.63                 0.920255
580         580                   0.63                 0.916592
581         581                   0.63                 0.916344
582         582                   0.63                 0.923535
583         583                   0.63                 0.927928
584         584                   0.63                 0.931174
585         585                   0.63                 0.934467
586         586                   0.63                 0.935167
587         587                   0.63                 0.937938
588         588                   0.63                 0.937747

     Test data R^2 score  Training RMSE  Test RMSE  Number of features  \
570             0.290146       0.373186   0.608242                   5
571             0.320146       0.336297   0.595251                   5
572             0.387461       0.323813   0.565014                   5
573             0.467702       0.311645   0.526707                   5
574             0.516674       0.296513   0.501895                   5
575             0.530785       0.274806   0.494514                   5
576             0.617333       0.277136   0.446583                   5
577             0.602381       0.265179   0.455225                   5
578             0.586091       0.274558   0.464456                   5
579             0.563115       0.271552   0.477173                   5
580             0.592591       0.277719   0.460795                   5
581             0.612007       0.278132   0.449681                   5
```

```
582          0.624988     0.265908   0.442094                5
583          0.607313     0.258158   0.452393                5
584          0.616693     0.252278   0.446957                5
585          0.605643     0.246168   0.453354                5
586          0.608711     0.244850   0.451587                5
587          0.602155     0.239559   0.455354                5
588          0.598890     0.239929   0.457218                5

     Number of estimators  Training data R score  Test data R score
570                     2               0.921625           0.538652
571                     3               0.936854           0.565815
572                     4               0.941598           0.622463
573                     5               0.946028           0.683888
574                     6               0.951273           0.718800
575                     7               0.958297           0.728550
576                     8               0.957571           0.785706
577                     9               0.961225           0.776132
578                    10               0.958374           0.765566
579                    11               0.959299           0.750410
580                    12               0.957388           0.769800
581                    13               0.957258           0.782309
582                    14               0.961007           0.790562
583                    15               0.963290           0.779303
584                    16               0.964973           0.785298
585                    17               0.966678           0.778231
586                    18               0.967040           0.780199
587                    19               0.968472           0.775987
588                    20               0.968373           0.773880
```

[ ]:

[28]:
```
df_ = pd.read_excel('../Data/Quality_MCF-7_15_.xlsx', sheet_name='RF')
df_ = df_[df_['Number of features'] == 4]
df_['Training data R score'] = handle_negative_corr(df_['Training data R^2␣
 ↪score'])
df_['Test data R score'] = handle_negative_corr(df_['Test data R^2 score'])
df_
```

[28]:
```
     Unnamed: 0  Correlation threshold  Training data R^2 score  \
323         323                   0.50                 0.823725
324         324                   0.50                 0.819379
325         325                   0.50                 0.861725
326         326                   0.50                 0.861772
327         327                   0.50                 0.876076
328         328                   0.50                 0.887302
329         329                   0.50                 0.891312
330         330                   0.50                 0.885151
```

331	331	0.50	0.897883
332	332	0.50	0.900694
333	333	0.50	0.898891
334	334	0.50	0.897355
335	335	0.50	0.903932
336	336	0.50	0.902161
337	337	0.50	0.897602
338	338	0.50	0.901345
339	339	0.50	0.903325
340	340	0.50	0.909007
341	341	0.50	0.910494
342	342	0.51	0.823725
343	343	0.51	0.819379
344	344	0.51	0.861725
345	345	0.51	0.861772
346	346	0.51	0.876076
347	347	0.51	0.887302
348	348	0.51	0.891312
349	349	0.51	0.885151
350	350	0.51	0.897883
351	351	0.51	0.900694
352	352	0.51	0.898891
353	353	0.51	0.897355
354	354	0.51	0.903932
355	355	0.51	0.902161
356	356	0.51	0.897602
357	357	0.51	0.901345
358	358	0.51	0.903325
359	359	0.51	0.909007
360	360	0.51	0.910494

	Test data R^2 score	Training RMSE	Test RMSE	Number of features \
323	0.492083	0.390625	0.767939	4
324	0.614284	0.395412	0.669213	4
325	0.495651	0.345969	0.765237	4
326	0.472072	0.345911	0.782920	4
327	0.532895	0.327523	0.736440	4
328	0.499692	0.312337	0.762165	4
329	0.494367	0.306729	0.766211	4
330	0.462619	0.315304	0.789899	4
331	0.422784	0.297314	0.818652	4
332	0.424585	0.293192	0.817374	4
333	0.451898	0.295842	0.797739	4
334	0.480492	0.298081	0.776652	4
335	0.482197	0.288373	0.775377	4
336	0.522720	0.291019	0.744418	4
337	0.531393	0.297722	0.737623	4

338	0.536501	0.292231	0.733593	4
339	0.532348	0.289282	0.736871	4
340	0.534079	0.280653	0.735506	4
341	0.539028	0.278349	0.731589	4
342	0.492083	0.390625	0.767939	4
343	0.614284	0.395412	0.669213	4
344	0.495651	0.345969	0.765237	4
345	0.472072	0.345911	0.782920	4
346	0.532895	0.327523	0.736440	4
347	0.499692	0.312337	0.762165	4
348	0.494367	0.306729	0.766211	4
349	0.462619	0.315304	0.789899	4
350	0.422784	0.297314	0.818652	4
351	0.424585	0.293192	0.817374	4
352	0.451898	0.295842	0.797739	4
353	0.480492	0.298081	0.776652	4
354	0.482197	0.288373	0.775377	4
355	0.522720	0.291019	0.744418	4
356	0.531393	0.297722	0.737623	4
357	0.536501	0.292231	0.733593	4
358	0.532348	0.289282	0.736871	4
359	0.534079	0.280653	0.735506	4
360	0.539028	0.278349	0.731589	4

	Number of estimators	Training data R score	Test data R score
323	2	0.907593	0.701486
324	3	0.905196	0.783763
325	4	0.928291	0.704025
326	5	0.928317	0.687075
327	6	0.935989	0.729997
328	7	0.941967	0.706889
329	8	0.944093	0.703112
330	9	0.940824	0.680161
331	10	0.947567	0.650219
332	11	0.949049	0.651602
333	12	0.948099	0.672234
334	13	0.947288	0.693176
335	14	0.950753	0.694404
336	15	0.949821	0.722994
337	16	0.947419	0.728967
338	17	0.949392	0.732462
339	18	0.950434	0.729622
340	19	0.953419	0.730807
341	20	0.954198	0.734186
342	2	0.907593	0.701486
343	3	0.905196	0.783763
344	4	0.928291	0.704025

345	5	0.928317	0.687075
346	6	0.935989	0.729997
347	7	0.941967	0.706889
348	8	0.944093	0.703112
349	9	0.940824	0.680161
350	10	0.947567	0.650219
351	11	0.949049	0.651602
352	12	0.948099	0.672234
353	13	0.947288	0.693176
354	14	0.950753	0.694404
355	15	0.949821	0.722994
356	16	0.947419	0.728967
357	17	0.949392	0.732462
358	18	0.950434	0.729622
359	19	0.953419	0.730807
360	20	0.954198	0.734186

[ ]:

```

```

Notebook

January 18, 2024

1 File 30a

[1]:
```
import glob
import pandas as pd
import matplotlib.pyplot as plt
import numpy as np
SMALL_SIZE = 16
MEDIUM_SIZE = 18
BIGGER_SIZE = 20

plt.rc('font', size=SMALL_SIZE)          # controls default text sizes
plt.rc('axes', titlesize=SMALL_SIZE)     # fontsize of the axes title
plt.rc('axes', labelsize=MEDIUM_SIZE)    # fontsize of the x and y labels
plt.rc('xtick', labelsize=SMALL_SIZE)    # fontsize of the tick labels
plt.rc('ytick', labelsize=SMALL_SIZE)    # fontsize of the tick labels
plt.rc('legend', fontsize=SMALL_SIZE)    # legend fontsize
plt.rc('figure', titlesize=BIGGER_SIZE)  # fontsize of the figure title
```

[2]:
```
sheet_names = ['MLR', 'DT', 'RF', 'KNN', 'SVM']
x_ticks = ['MLR train', 'MLR test', 'DT train', 'DT test', 'RF train', 'RF␣
 ↪test', 'KNN train', 'KNN test', 'SVM train', 'SVM test']
random_states = [15, 28, 42]
```

[3]:
```
def prepare_histogram_data(target_name, max_number_of_features):

    excel_files = []

    for name in glob.glob('../Data/Quality_'+str(target_name)+'*'):
        excel_files.append(name)

    random_state_15 = []
    random_state_28 = []
    random_state_42 = []
    features_15 = []
    features_28 = []
    features_42 = []
    random_states = [15, 28, 42]
```

```
    for random_state in range(len(random_states)):
        tmp = []
        to_plot = []
        values = []
        number_of_features = []
        for sheet in sheet_names:
            tmp.append(pd.read_excel(excel_files[random_state],␣
↪sheet_name=sheet))
        for data in tmp:
            temp = data[data['Number of features'] <= max_number_of_features]
            to_plot.append(temp[temp['Test RMSE'] == min(temp['Test RMSE'])])
        for value in to_plot:
            values.append(value['Training RMSE'].tail(1))
            values.append(value['Test RMSE'].tail(1))
            number_of_features.append(value['Number of features'].tail(1))
            #values.append(value['Training RMSE'].head(1))
            #values.append(value['Test RMSE'].head(1))
            #number_of_features.append(value['Number of features'].head(1))
        for element in values:
            if random_state == 0:
                random_state_15.append(float(element))
            elif random_state == 1:
                random_state_28.append(float(element))
            elif random_state == 2:
                random_state_42.append(float(element))
            else:
                print("Error with conditions...")

        for features in number_of_features:
            if random_state == 0:
                features_15.append(float(features))
            elif random_state == 1:
                features_28.append(float(features))
            elif random_state == 2:
                features_42.append(float(features))
            else:
                print("Error with conditions...")


    return  random_state_15, random_state_28, random_state_42, features_15,␣
↪features_28, features_42
```

[4]:
```
def handle_negative_corr(list_):
    res = []
    for element in list_:
        if element < 0:
```

```
            res.append(0.01)
        else:
            res.append(np.sqrt(element))
    return res
```

[5]:
```
def prepare_plot(data, name, max_number_of_features):

    fig = plt.figure(figsize=(20,10))
    X = x_ticks
    y_15 = data[0] #handle_negative_corr(data[0])
    y_28 = data[1] #handle_negative_corr(data[1])
    y_42 = data[2] #handle_negative_corr(data[2])

    X_axis = np.arange(len(X))
    #y_ax = [x/100 for x in range(0, 105, 5)]
    #plt.yticks(y_ax)
    plt.bar(X_axis - 0.2, y_15, 0.2, label = 'Random state 15')
    plt.bar(X_axis + 0.0, y_28, 0.2, label = 'Random state 28')
    plt.bar(X_axis + 0.2, y_42, 0.2, label = 'Random state 42')

    plt.xticks(X_axis, X, weight='bold', fontsize=14)
    plt.xlabel("Methodology", fontsize=20, weight='bold')
    plt.ylabel("RMSE", fontsize=18, weight='bold')
    #plt.ylim([0.0, 1.0])
    plt.title("Quality of each methodology - "+str(name)+' '+str('max number of␣
↪features: '+str(max_number_of_features)), fontsize=24, weight='bold')
    plt.grid(visible=True)
    plt.legend()
    plt.savefig('Figures/'+'_'+str(name)+'_'+str(max_number_of_features)+'_'+'.
↪pdf', bbox_inches='tight') #new line
    plt.show()
    for i, state in enumerate(random_states):
        print('Random state - '+str(state)+'␣
↪'+str(update_description(sheet_names, data[i+3])))
```

[6]:
```
def update_description(x_axis_labels, number_of_features):

    MLR = str(x_axis_labels[0])+' - number of features used:␣
↪'+str(number_of_features[0])
    DT = str(x_axis_labels[1])+' - number of features used:␣
↪'+str(number_of_features[1])
    RF = str(x_axis_labels[2])+' - number of features used:␣
↪'+str(number_of_features[2])
    KNN = str(x_axis_labels[3])+' - number of features used:␣
↪'+str(number_of_features[3])
    SVM = str(x_axis_labels[4])+' - number of features used:␣
↪'+str(number_of_features[4])
```

```
    return MLR, DT, RF, KNN, SVM
```

1.1 A549

[7]:
```
a549 = prepare_histogram_data('A549', 8)
```

[8]:
```
a549
```

[8]:
```
([0.7059969481770844,
  0.6334055419392647,
  0.365598065751709,
  0.5530943469658123,
  0.255707728380049,
  0.5156471637607167,
  0.6035959622033769,
  0.4264196399135305,
  0.7138186632786595,
  0.6649192410798268],
 [0.6635732209289072,
  0.5886694438974678,
  0.4177465209814143,
  0.5980573995745231,
  0.3194786268521958,
  0.3468508151363199,
  0.5533292333013828,
  0.736855667889074,
  0.6917056192160755,
  0.5974072644132012],
 [0.6955806931760296,
  0.6779691838954662,
  0.3267767226460304,
  0.6260661211419981,
  0.2760829912510313,
  0.6188656116433753,
  0.6139717534296958,
  0.7025630129820909,
  0.7210034726109186,
  0.7083786747537733],
 [3.0, 5.0, 8.0, 8.0, 5.0],
 [8.0, 7.0, 8.0, 8.0, 8.0],
 [3.0, 8.0, 7.0, 3.0, 3.0])
```

[9]:
```
for i in range(8, 3, -1):
    a549 = prepare_histogram_data('A549', i)
    prepare_plot(a549, 'A549_RMSE', i)
```

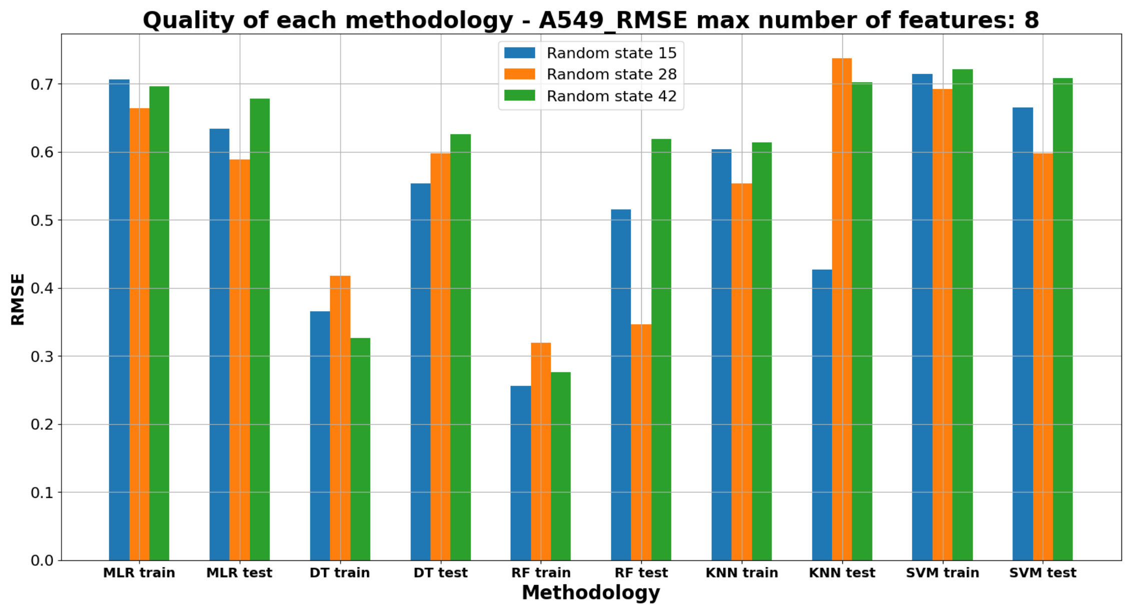


```
Random state - 15 ('MLR - number of features used: 3.0', 'DT - number of
features used: 5.0', 'RF - number of features used: 8.0', 'KNN - number of
features used: 8.0', 'SVM - number of features used: 5.0')
Random state - 28 ('MLR - number of features used: 8.0', 'DT - number of
features used: 7.0', 'RF - number of features used: 8.0', 'KNN - number of
features used: 8.0', 'SVM - number of features used: 8.0')
Random state - 42 ('MLR - number of features used: 3.0', 'DT - number of
features used: 8.0', 'RF - number of features used: 7.0', 'KNN - number of
features used: 3.0', 'SVM - number of features used: 3.0')
```

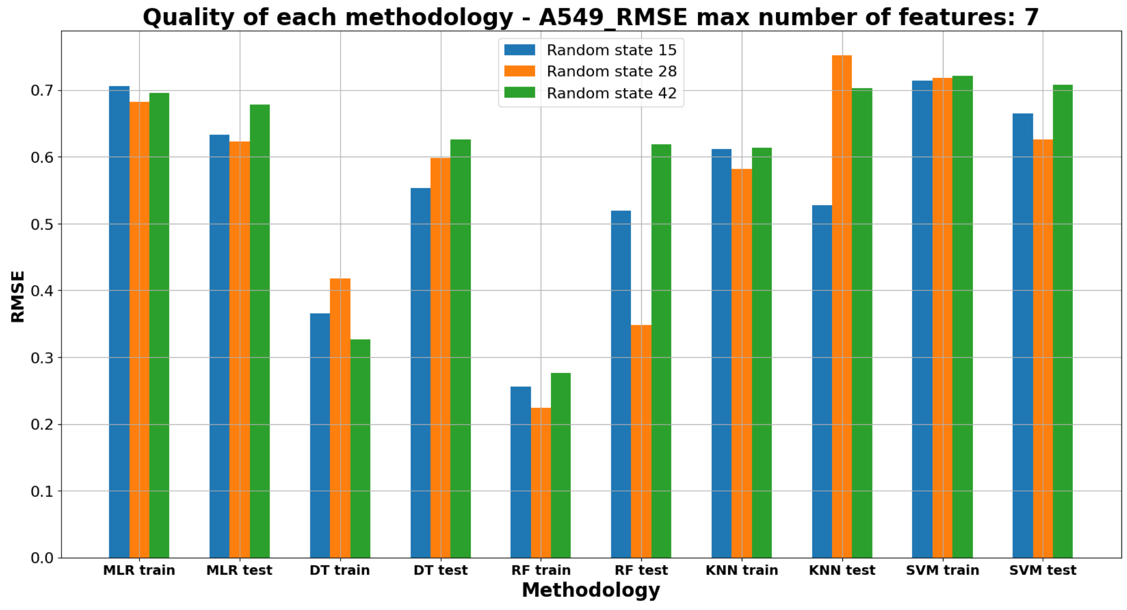

```
Random state - 15 ('MLR - number of features used: 3.0', 'DT - number of
features used: 5.0', 'RF - number of features used: 7.0', 'KNN - number of
features used: 7.0', 'SVM - number of features used: 5.0')
Random state - 28 ('MLR - number of features used: 7.0', 'DT - number of
features used: 7.0', 'RF - number of features used: 7.0', 'KNN - number of
features used: 7.0', 'SVM - number of features used: 7.0')
Random state - 42 ('MLR - number of features used: 3.0', 'DT - number of
features used: 7.0', 'RF - number of features used: 7.0', 'KNN - number of
features used: 3.0', 'SVM - number of features used: 3.0')
```

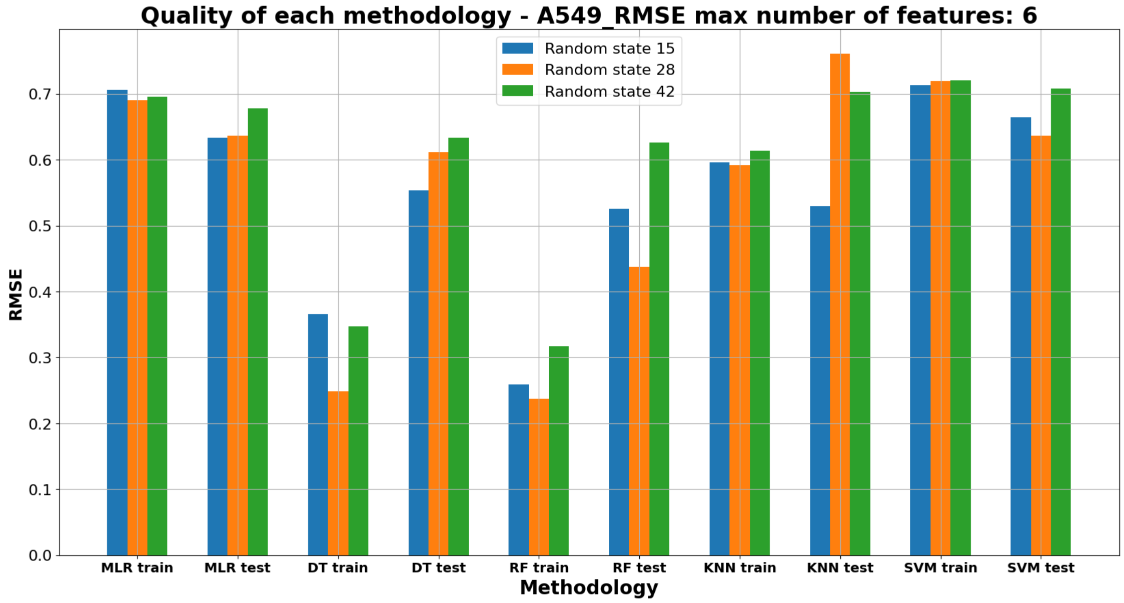


```
Random state - 15 ('MLR - number of features used: 3.0', 'DT - number of
features used: 5.0', 'RF - number of features used: 5.0', 'KNN - number of
features used: 5.0', 'SVM - number of features used: 5.0')
Random state - 28 ('MLR - number of features used: 4.0', 'DT - number of
features used: 4.0', 'RF - number of features used: 5.0', 'KNN - number of
features used: 5.0', 'SVM - number of features used: 5.0')
Random state - 42 ('MLR - number of features used: 3.0', 'DT - number of
features used: 5.0', 'RF - number of features used: 5.0', 'KNN - number of
features used: 3.0', 'SVM - number of features used: 3.0')
```

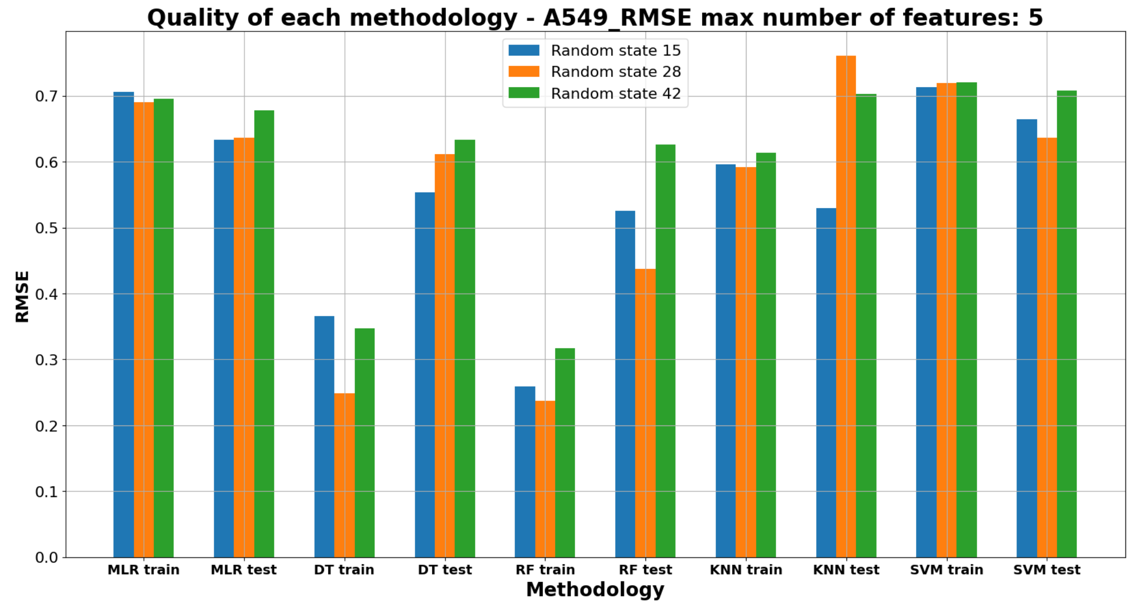


```
Random state - 15 ('MLR - number of features used: 3.0', 'DT - number of
features used: 5.0', 'RF - number of features used: 5.0', 'KNN - number of
features used: 5.0', 'SVM - number of features used: 5.0')
Random state - 28 ('MLR - number of features used: 4.0', 'DT - number of
features used: 4.0', 'RF - number of features used: 5.0', 'KNN - number of
features used: 5.0', 'SVM - number of features used: 5.0')
Random state - 42 ('MLR - number of features used: 3.0', 'DT - number of
features used: 5.0', 'RF - number of features used: 5.0', 'KNN - number of
features used: 3.0', 'SVM - number of features used: 3.0')
```

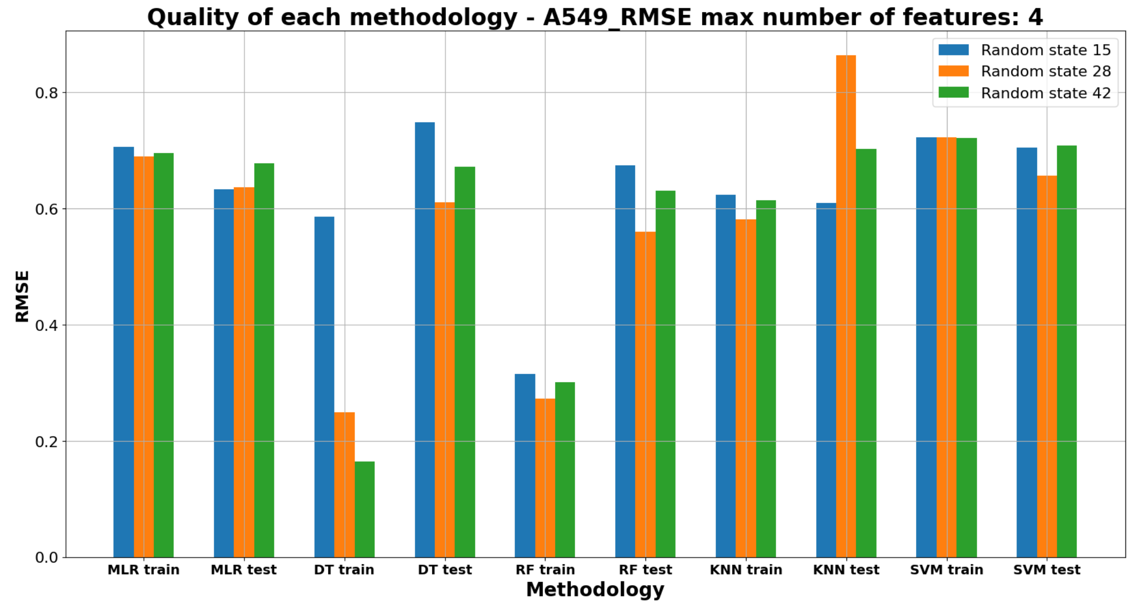

Random state - 15 ('MLR - number of features used: 3.0', 'DT - number of features used: 3.0', 'RF - number of features used: 4.0', 'KNN - number of features used: 3.0', 'SVM - number of features used: 3.0')
Random state - 28 ('MLR - number of features used: 4.0', 'DT - number of features used: 4.0', 'RF - number of features used: 4.0', 'KNN - number of features used: 4.0', 'SVM - number of features used: 4.0')
Random state - 42 ('MLR - number of features used: 3.0', 'DT - number of features used: 3.0', 'RF - number of features used: 3.0', 'KNN - number of features used: 3.0', 'SVM - number of features used: 3.0')

[ ]:

1.2 MCF-7

[10]:
```
for i in range(8, 3, -1):
    mcf7 = prepare_histogram_data('MCF-7', i)
    prepare_plot(mcf7, 'MCF-7_RMSE', i)
```

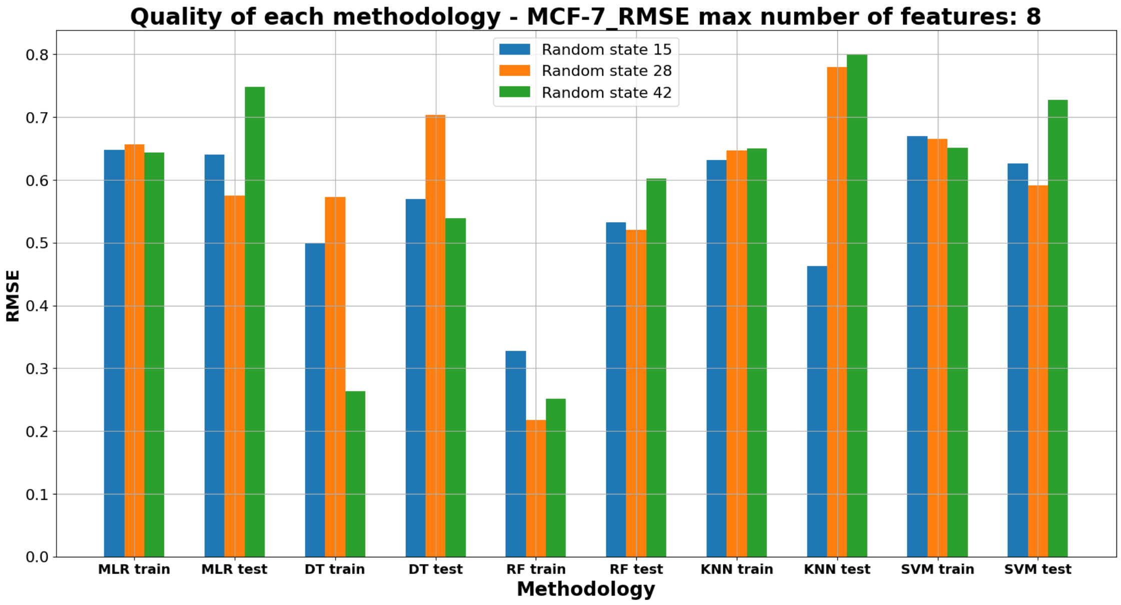

Random state - 15 ('MLR - number of features used: 8.0', 'DT - number of features used: 6.0', 'RF - number of features used: 8.0', 'KNN - number of features used: 8.0', 'SVM - number of features used: 4.0')
Random state - 28 ('MLR - number of features used: 6.0', 'DT - number of features used: 4.0', 'RF - number of features used: 8.0', 'KNN - number of features used: 6.0', 'SVM - number of features used: 8.0')
Random state - 42 ('MLR - number of features used: 4.0', 'DT - number of

features used: 8.0', 'RF - number of features used: 8.0', 'KNN - number of features used: 6.0', 'SVM - number of features used: 4.0')

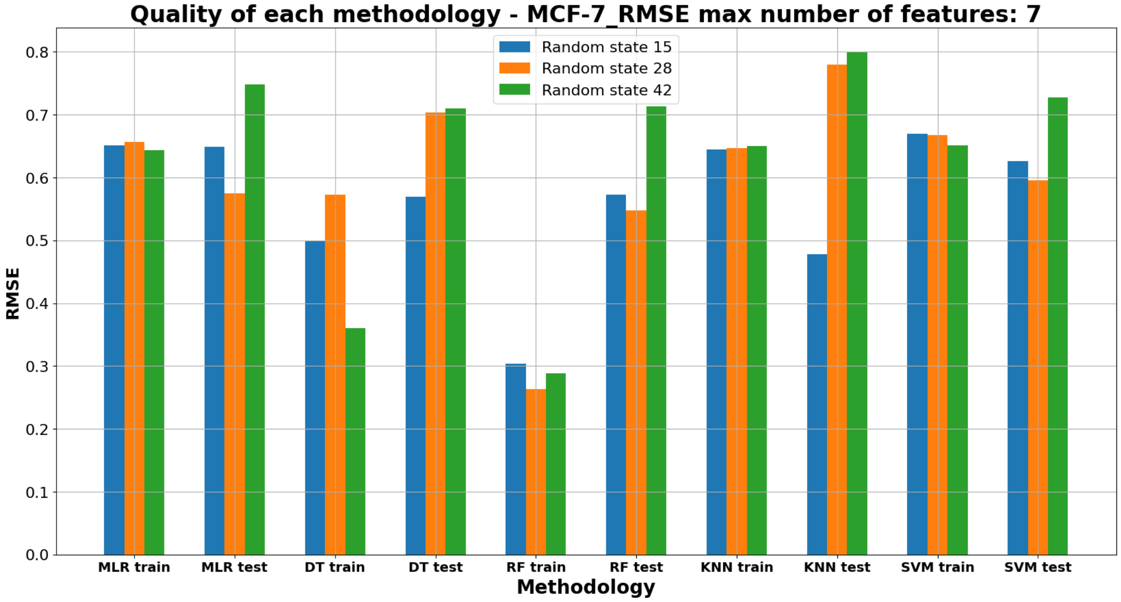

Random state - 15 ('MLR - number of features used: 6.0', 'DT - number of features used: 6.0', 'RF - number of features used: 6.0', 'KNN - number of features used: 6.0', 'SVM - number of features used: 4.0')
Random state - 28 ('MLR - number of features used: 6.0', 'DT - number of features used: 4.0', 'RF - number of features used: 6.0', 'KNN - number of features used: 6.0', 'SVM - number of features used: 6.0')
Random state - 42 ('MLR - number of features used: 4.0', 'DT - number of features used: 6.0', 'RF - number of features used: 6.0', 'KNN - number of features used: 6.0', 'SVM - number of features used: 4.0')

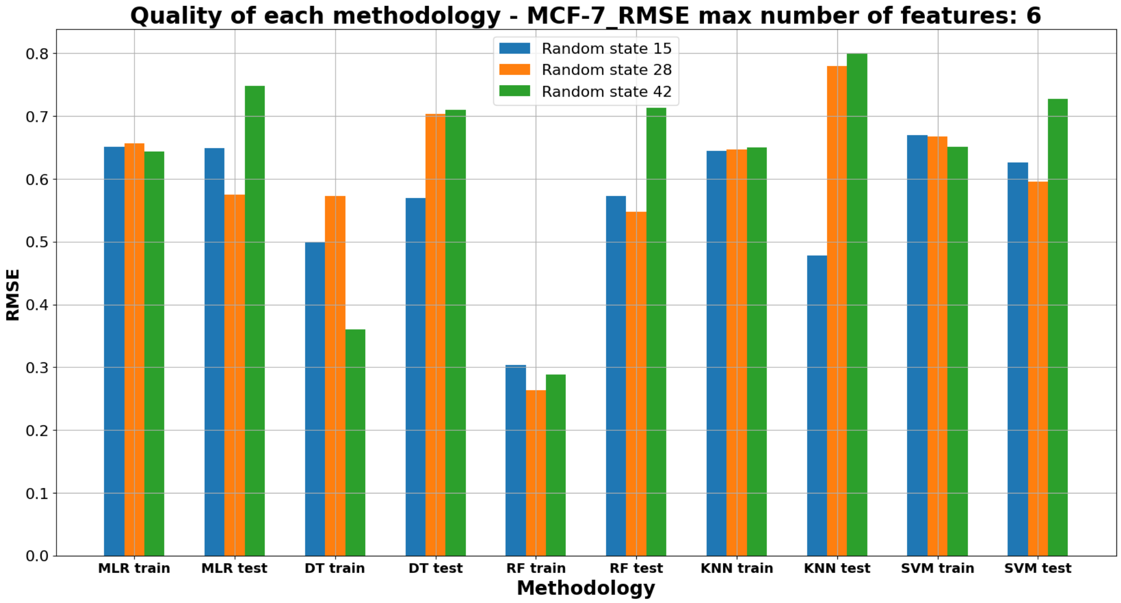


```
Random state - 15 ('MLR - number of features used: 6.0', 'DT - number of
features used: 6.0', 'RF - number of features used: 6.0', 'KNN - number of
features used: 6.0', 'SVM - number of features used: 4.0')
Random state - 28 ('MLR - number of features used: 6.0', 'DT - number of
features used: 4.0', 'RF - number of features used: 6.0', 'KNN - number of
features used: 6.0', 'SVM - number of features used: 6.0')
Random state - 42 ('MLR - number of features used: 4.0', 'DT - number of
features used: 6.0', 'RF - number of features used: 6.0', 'KNN - number of
features used: 6.0', 'SVM - number of features used: 4.0')
```

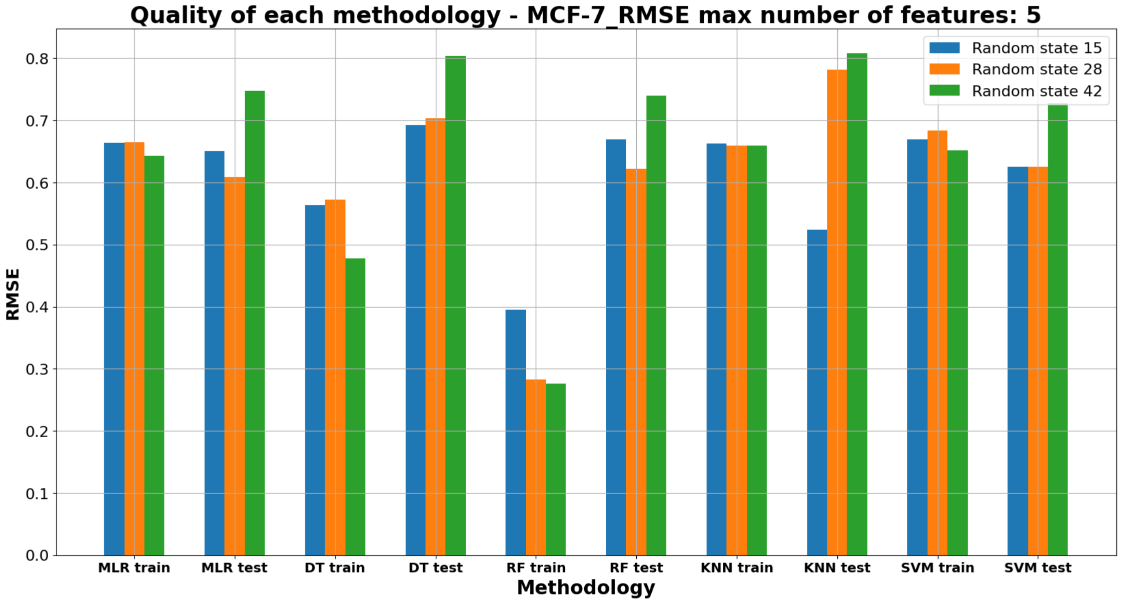

```
Random state - 15 ('MLR - number of features used: 4.0', 'DT - number of
features used: 4.0', 'RF - number of features used: 4.0', 'KNN - number of
features used: 4.0', 'SVM - number of features used: 4.0')
Random state - 28 ('MLR - number of features used: 4.0', 'DT - number of
features used: 4.0', 'RF - number of features used: 4.0', 'KNN - number of
features used: 4.0', 'SVM - number of features used: 4.0')
Random state - 42 ('MLR - number of features used: 4.0', 'DT - number of
features used: 4.0', 'RF - number of features used: 4.0', 'KNN - number of
features used: 4.0', 'SVM - number of features used: 4.0')
```

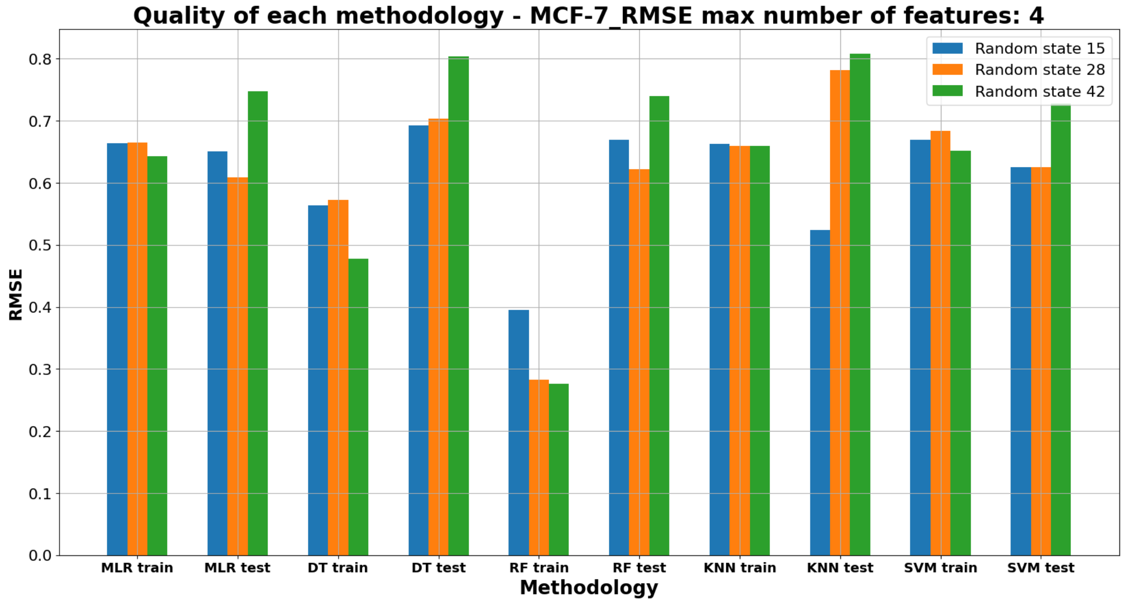


```
Random state - 15 ('MLR - number of features used: 4.0', 'DT - number of
features used: 4.0', 'RF - number of features used: 4.0', 'KNN - number of
features used: 4.0', 'SVM - number of features used: 4.0')
Random state - 28 ('MLR - number of features used: 4.0', 'DT - number of
features used: 4.0', 'RF - number of features used: 4.0', 'KNN - number of
features used: 4.0', 'SVM - number of features used: 4.0')
Random state - 42 ('MLR - number of features used: 4.0', 'DT - number of
features used: 4.0', 'RF - number of features used: 4.0', 'KNN - number of
features used: 4.0', 'SVM - number of features used: 4.0')
```

[ ]:

1.3 LoVo

[11]:
```
for i in range(8, 1, -1): # 2 -> 1
    lovo = prepare_histogram_data('LoVo', i)
    prepare_plot(lovo, 'LoVo_RMSE', i)
```

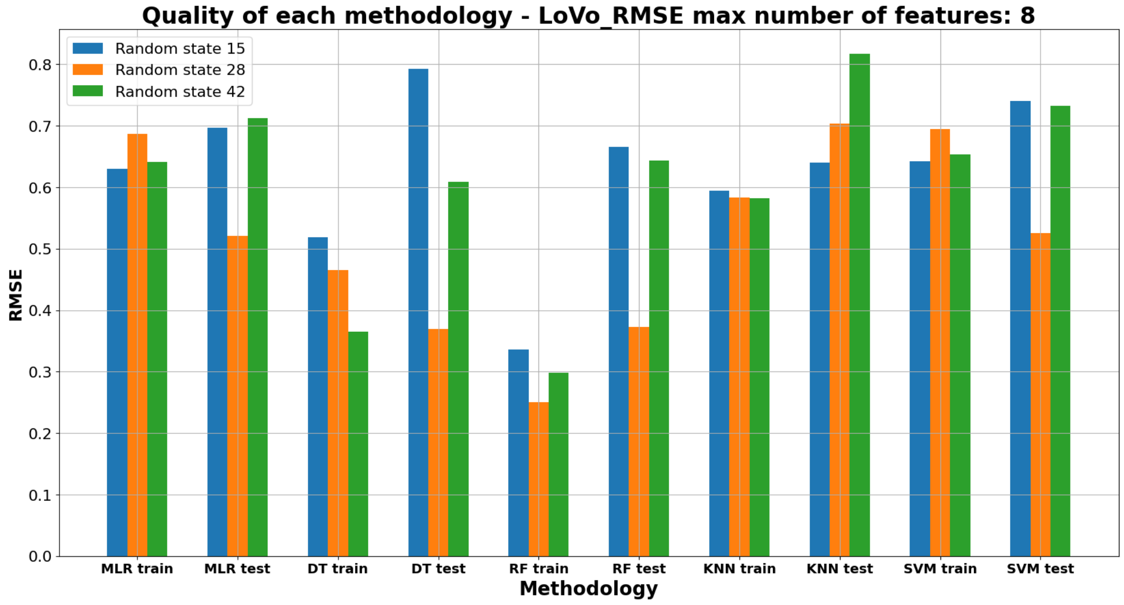


```
Random state - 15 ('MLR - number of features used: 6.0', 'DT - number of
features used: 7.0', 'RF - number of features used: 5.0', 'KNN - number of
features used: 5.0', 'SVM - number of features used: 6.0')
Random state - 28 ('MLR - number of features used: 2.0', 'DT - number of
features used: 3.0', 'RF - number of features used: 7.0', 'KNN - number of
features used: 7.0', 'SVM - number of features used: 2.0')
Random state - 42 ('MLR - number of features used: 3.0', 'DT - number of
features used: 3.0', 'RF - number of features used: 5.0', 'KNN - number of
features used: 5.0', 'SVM - number of features used: 2.0')
```

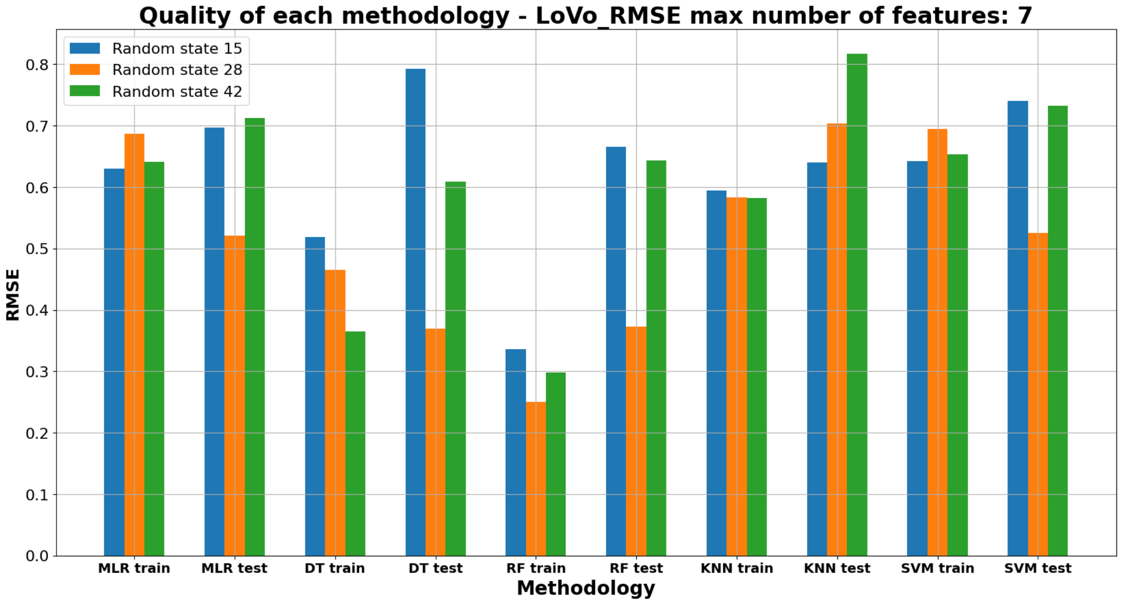

Random state - 15 ('MLR - number of features used: 6.0', 'DT - number of features used: 7.0', 'RF - number of features used: 5.0', 'KNN - number of features used: 5.0', 'SVM - number of features used: 6.0')
Random state - 28 ('MLR - number of features used: 2.0', 'DT - number of features used: 3.0', 'RF - number of features used: 7.0', 'KNN - number of features used: 7.0', 'SVM - number of features used: 2.0')
Random state - 42 ('MLR - number of features used: 3.0', 'DT - number of features used: 3.0', 'RF - number of features used: 5.0', 'KNN - number of features used: 5.0', 'SVM - number of features used: 2.0')

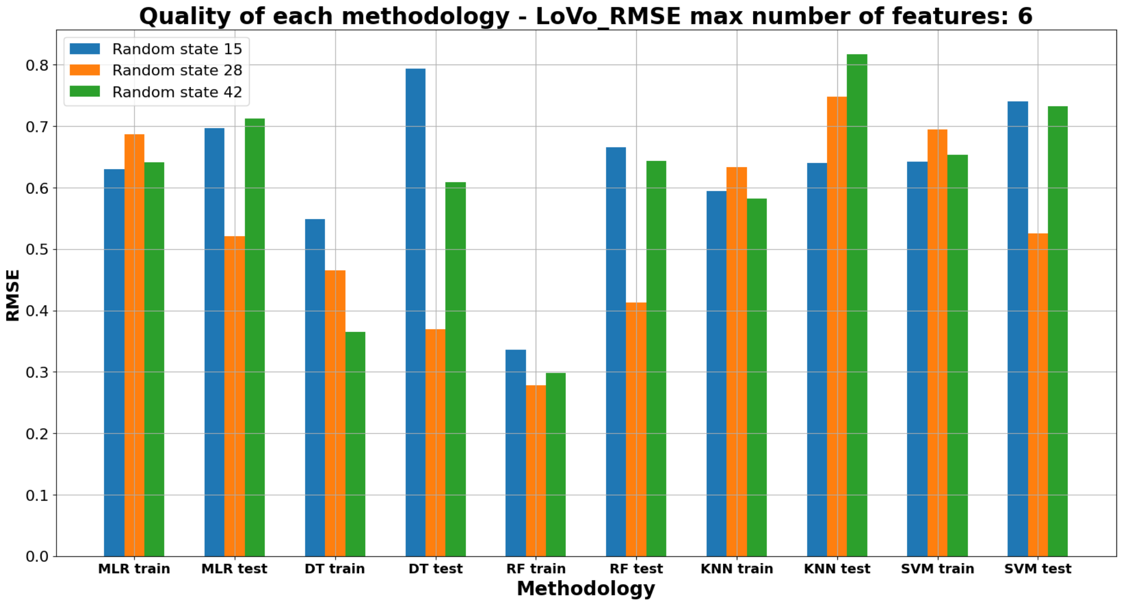

Random state - 15 ('MLR - number of features used: 6.0', 'DT - number of features used: 6.0', 'RF - number of features used: 5.0', 'KNN - number of features used: 5.0', 'SVM - number of features used: 6.0')
Random state - 28 ('MLR - number of features used: 2.0', 'DT - number of features used: 3.0', 'RF - number of features used: 6.0', 'KNN - number of features used: 2.0', 'SVM - number of features used: 2.0')
Random state - 42 ('MLR - number of features used: 3.0', 'DT - number of features used: 3.0', 'RF - number of features used: 5.0', 'KNN - number of features used: 5.0', 'SVM - number of features used: 2.0')

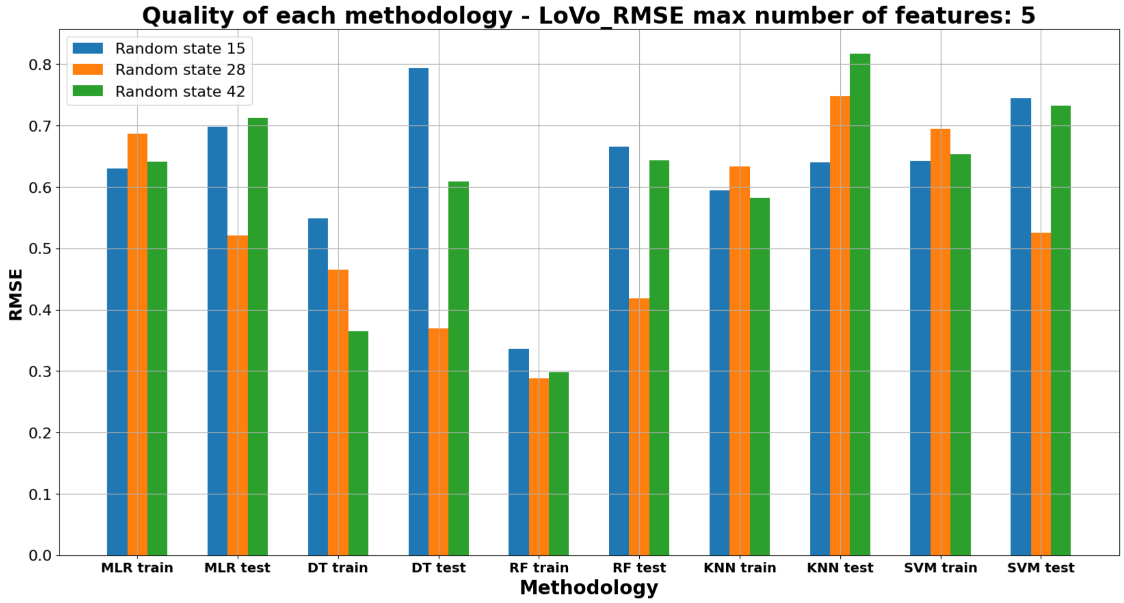

Random state - 15 ('MLR - number of features used: 5.0', 'DT - number of features used: 5.0', 'RF - number of features used: 5.0', 'KNN - number of features used: 5.0', 'SVM - number of features used: 5.0')
Random state - 28 ('MLR - number of features used: 2.0', 'DT - number of features used: 3.0', 'RF - number of features used: 5.0', 'KNN - number of features used: 2.0', 'SVM - number of features used: 2.0')
Random state - 42 ('MLR - number of features used: 3.0', 'DT - number of features used: 3.0', 'RF - number of features used: 5.0', 'KNN - number of features used: 5.0', 'SVM - number of features used: 2.0')

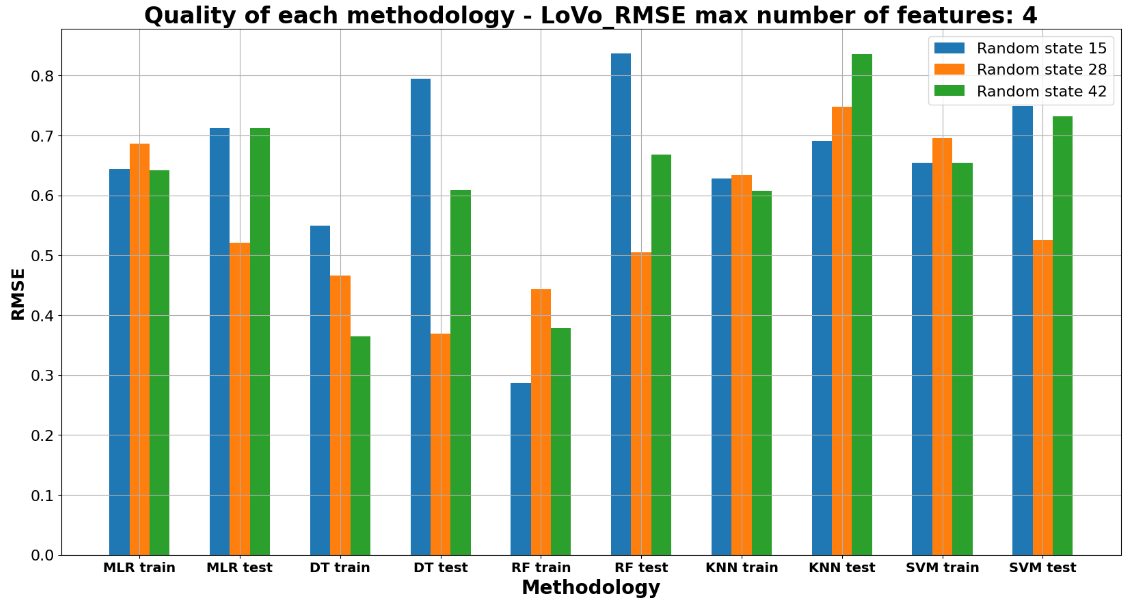


```
Random state - 15 ('MLR - number of features used: 3.0', 'DT - number of
features used: 3.0', 'RF - number of features used: 3.0', 'KNN - number of
features used: 2.0', 'SVM - number of features used: 2.0')
Random state - 28 ('MLR - number of features used: 2.0', 'DT - number of
features used: 3.0', 'RF - number of features used: 2.0', 'KNN - number of
features used: 2.0', 'SVM - number of features used: 2.0')
Random state - 42 ('MLR - number of features used: 3.0', 'DT - number of
features used: 3.0', 'RF - number of features used: 2.0', 'KNN - number of
features used: 2.0', 'SVM - number of features used: 2.0')
```

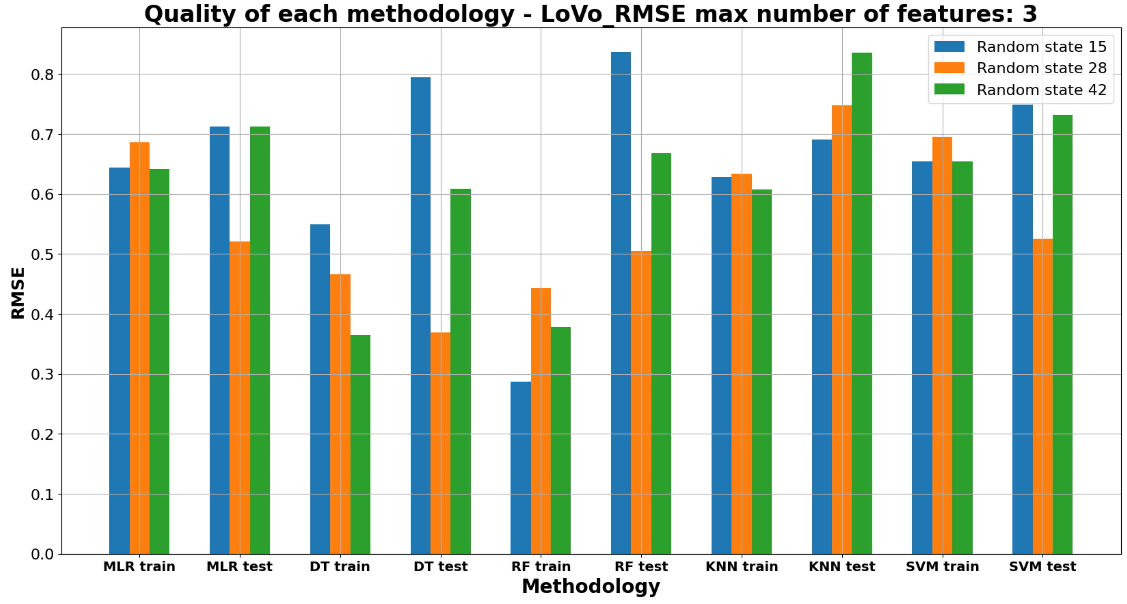

```
Random state - 15 ('MLR - number of features used: 3.0', 'DT - number of
features used: 3.0', 'RF - number of features used: 3.0', 'KNN - number of
features used: 2.0', 'SVM - number of features used: 2.0')
Random state - 28 ('MLR - number of features used: 2.0', 'DT - number of
features used: 3.0', 'RF - number of features used: 2.0', 'KNN - number of
features used: 2.0', 'SVM - number of features used: 2.0')
Random state - 42 ('MLR - number of features used: 3.0', 'DT - number of
features used: 3.0', 'RF - number of features used: 2.0', 'KNN - number of
features used: 2.0', 'SVM - number of features used: 2.0')
```

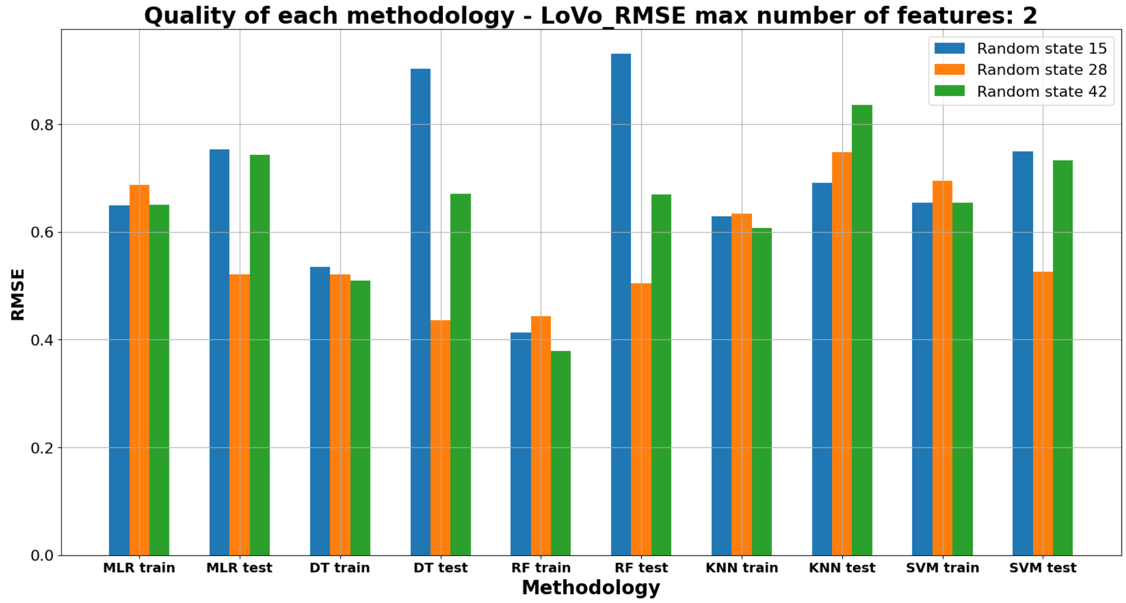


```
Random state - 15 ('MLR - number of features used: 2.0', 'DT - number of
features used: 2.0', 'RF - number of features used: 2.0', 'KNN - number of
features used: 2.0', 'SVM - number of features used: 2.0')
Random state - 28 ('MLR - number of features used: 2.0', 'DT - number of
features used: 2.0', 'RF - number of features used: 2.0', 'KNN - number of
features used: 2.0', 'SVM - number of features used: 2.0')
Random state - 42 ('MLR - number of features used: 2.0', 'DT - number of
features used: 2.0', 'RF - number of features used: 2.0', 'KNN - number of
features used: 2.0', 'SVM - number of features used: 2.0')
```

[ ]:

1.4 LoVo/DX

[12]:
```
for i in range(8, 4, -1): # 5 -> 4
    lovo_dx = prepare_histogram_data('LoVo_DX', i)
    prepare_plot(lovo_dx, 'LoVo_DX_RMSE', i)
```

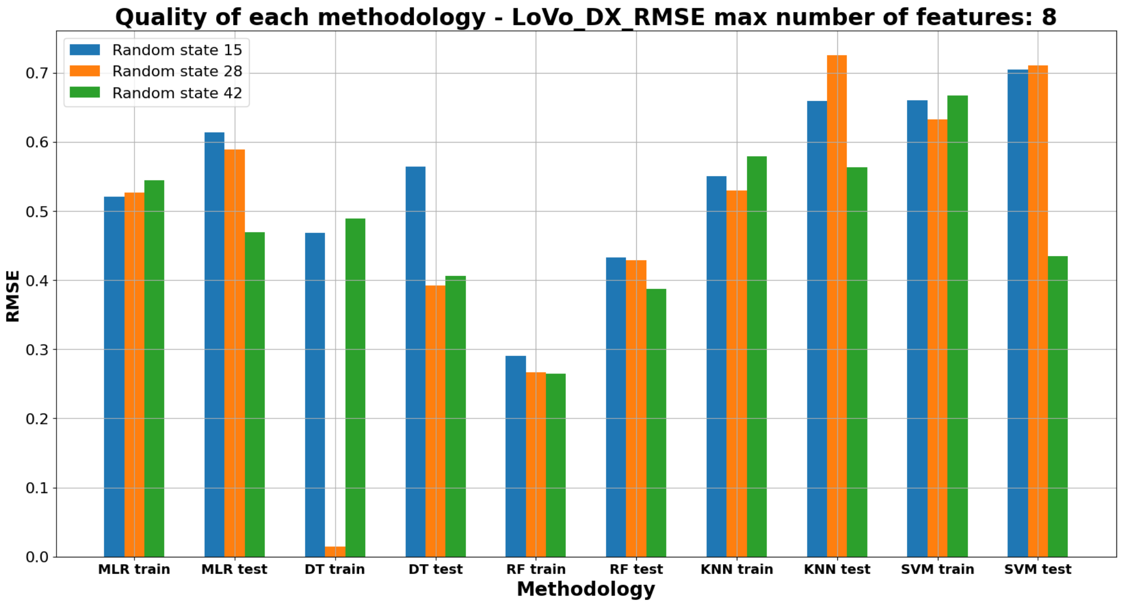


```
Random state - 15 ('MLR - number of features used: 8.0', 'DT - number of
features used: 7.0', 'RF - number of features used: 8.0', 'KNN - number of
features used: 7.0', 'SVM - number of features used: 5.0')
Random state - 28 ('MLR - number of features used: 7.0', 'DT - number of
features used: 5.0', 'RF - number of features used: 8.0', 'KNN - number of
features used: 8.0', 'SVM - number of features used: 8.0')
Random state - 42 ('MLR - number of features used: 8.0', 'DT - number of
features used: 7.0', 'RF - number of features used: 7.0', 'KNN - number of
features used: 7.0', 'SVM - number of features used: 8.0')
```

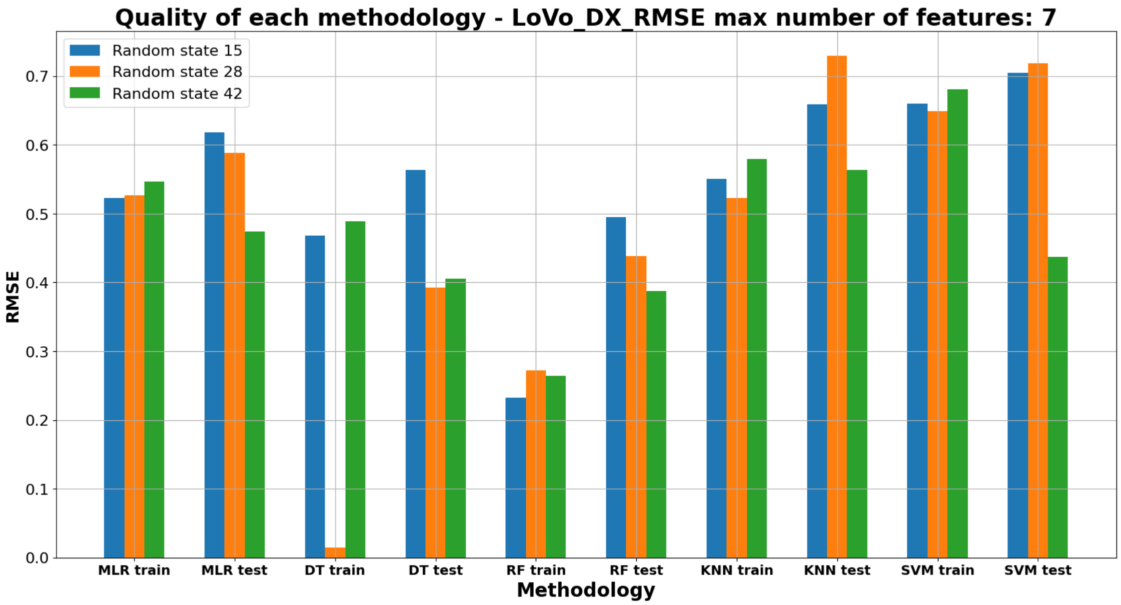

Random state - 15 ('MLR - number of features used: 7.0', 'DT - number of features used: 7.0', 'RF - number of features used: 7.0', 'KNN - number of features used: 7.0', 'SVM - number of features used: 5.0')
Random state - 28 ('MLR - number of features used: 7.0', 'DT - number of features used: 5.0', 'RF - number of features used: 7.0', 'KNN - number of features used: 7.0', 'SVM - number of features used: 5.0')
Random state - 42 ('MLR - number of features used: 7.0', 'DT - number of features used: 7.0', 'RF - number of features used: 7.0', 'KNN - number of features used: 7.0', 'SVM - number of features used: 7.0')

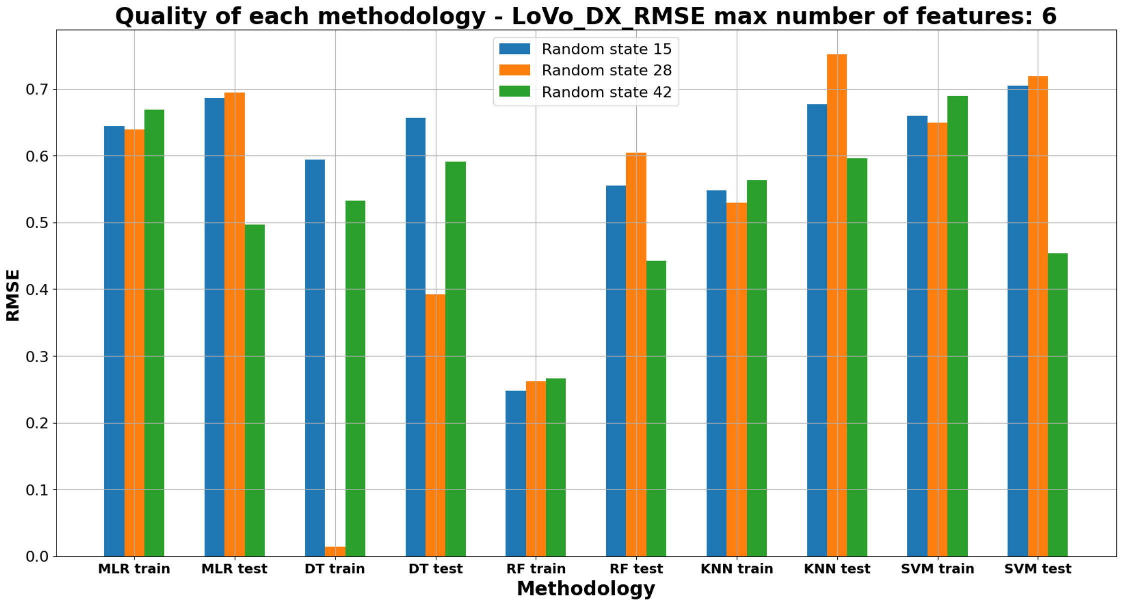

Random state - 15 ('MLR - number of features used: 5.0', 'DT - number of features used: 5.0', 'RF - number of features used: 5.0', 'KNN - number of features used: 5.0', 'SVM - number of features used: 5.0')
Random state - 28 ('MLR - number of features used: 5.0', 'DT - number of features used: 5.0', 'RF - number of features used: 5.0', 'KNN - number of features used: 5.0', 'SVM - number of features used: 5.0')
Random state - 42 ('MLR - number of features used: 5.0', 'DT - number of features used: 5.0', 'RF - number of features used: 5.0', 'KNN - number of features used: 5.0', 'SVM - number of features used: 5.0')

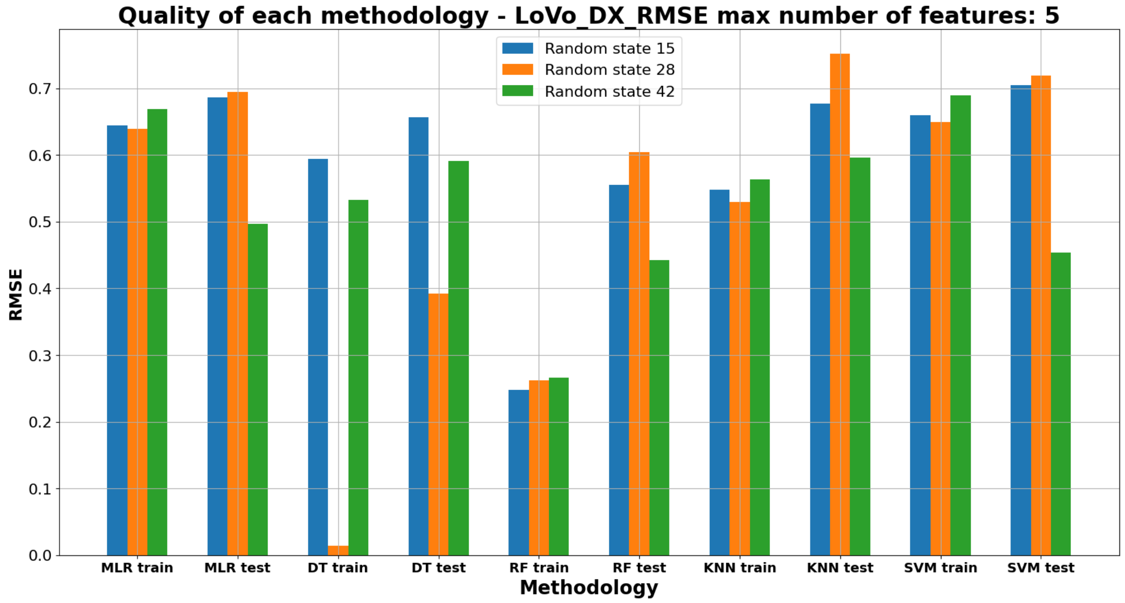

Random state - 15 ('MLR - number of features used: 5.0', 'DT - number of features used: 5.0', 'RF - number of features used: 5.0', 'KNN - number of features used: 5.0', 'SVM - number of features used: 5.0')
Random state - 28 ('MLR - number of features used: 5.0', 'DT - number of features used: 5.0', 'RF - number of features used: 5.0', 'KNN - number of features used: 5.0', 'SVM - number of features used: 5.0')
Random state - 42 ('MLR - number of features used: 5.0', 'DT - number of features used: 5.0', 'RF - number of features used: 5.0', 'KNN - number of features used: 5.0', 'SVM - number of features used: 5.0')

1.5 BALB/3T3

[13]:
```
for i in range(8, 2, -1):
    balb = prepare_histogram_data('BALB_3T3', i)
    prepare_plot(balb, 'BALB_3T3_RMSE', i)
```

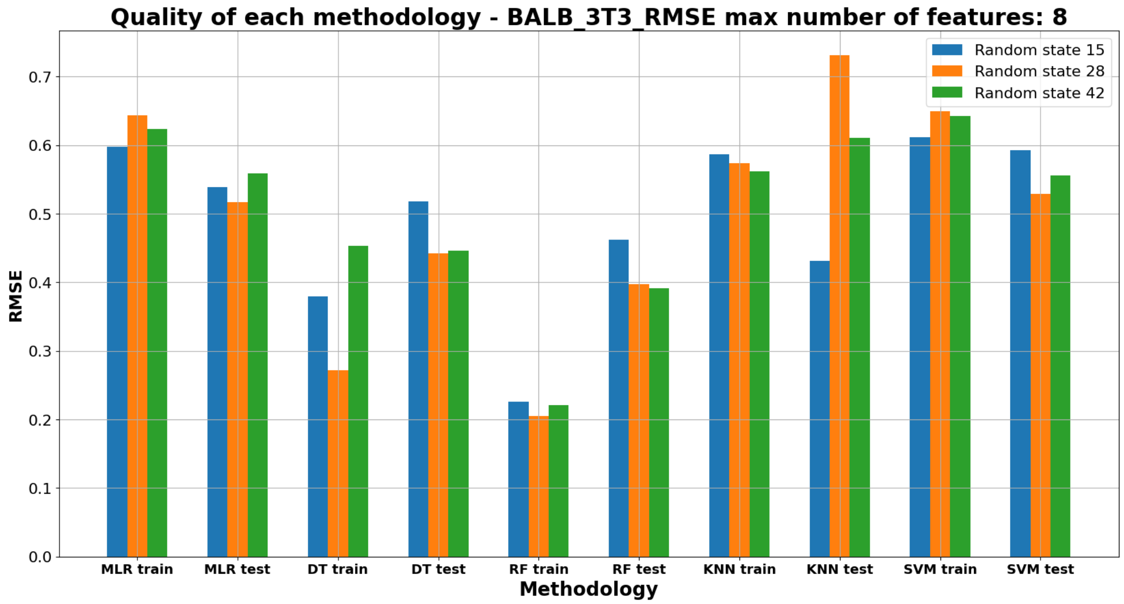


```
Random state - 15 ('MLR - number of features used: 7.0', 'DT - number of
features used: 6.0', 'RF - number of features used: 6.0', 'KNN - number of
features used: 6.0', 'SVM - number of features used: 7.0')
Random state - 28 ('MLR - number of features used: 2.0', 'DT - number of
features used: 6.0', 'RF - number of features used: 7.0', 'KNN - number of
features used: 3.0', 'SVM - number of features used: 2.0')
Random state - 42 ('MLR - number of features used: 3.0', 'DT - number of
features used: 6.0', 'RF - number of features used: 7.0', 'KNN - number of
features used: 7.0', 'SVM - number of features used: 3.0')
```

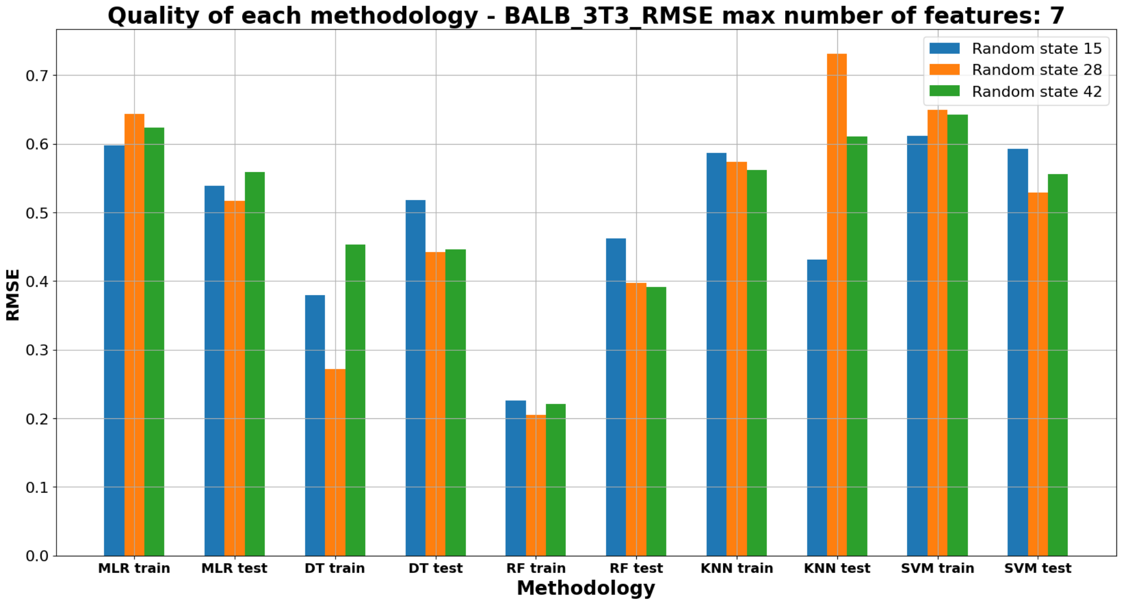

Random state - 15 ('MLR - number of features used: 7.0', 'DT - number of features used: 6.0', 'RF - number of features used: 6.0', 'KNN - number of features used: 6.0', 'SVM - number of features used: 7.0')
Random state - 28 ('MLR - number of features used: 2.0', 'DT - number of features used: 6.0', 'RF - number of features used: 7.0', 'KNN - number of features used: 3.0', 'SVM - number of features used: 2.0')
Random state - 42 ('MLR - number of features used: 3.0', 'DT - number of features used: 6.0', 'RF - number of features used: 7.0', 'KNN - number of features used: 7.0', 'SVM - number of features used: 3.0')

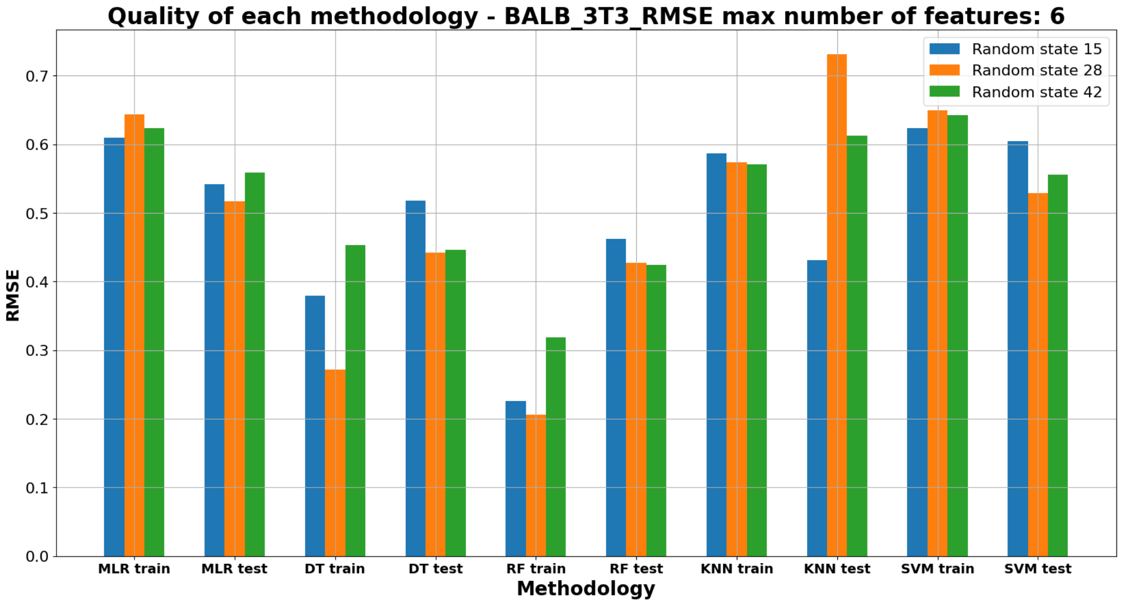

Random state - 15 ('MLR - number of features used: 6.0', 'DT - number of features used: 6.0', 'RF - number of features used: 6.0', 'KNN - number of features used: 6.0', 'SVM - number of features used: 5.0')
Random state - 28 ('MLR - number of features used: 2.0', 'DT - number of features used: 6.0', 'RF - number of features used: 6.0', 'KNN - number of features used: 3.0', 'SVM - number of features used: 2.0')
Random state - 42 ('MLR - number of features used: 3.0', 'DT - number of features used: 6.0', 'RF - number of features used: 6.0', 'KNN - number of features used: 3.0', 'SVM - number of features used: 3.0')

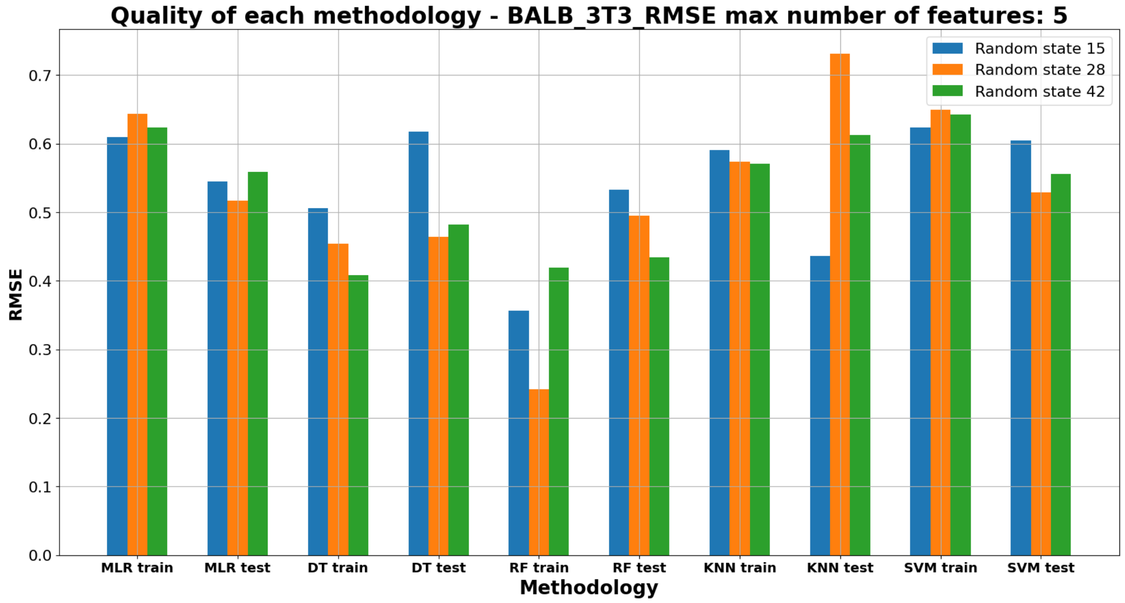

Random state - 15 ('MLR - number of features used: 5.0', 'DT - number of features used: 5.0', 'RF - number of features used: 5.0', 'KNN - number of features used: 5.0', 'SVM - number of features used: 5.0')
Random state - 28 ('MLR - number of features used: 2.0', 'DT - number of features used: 3.0', 'RF - number of features used: 5.0', 'KNN - number of features used: 3.0', 'SVM - number of features used: 2.0')
Random state - 42 ('MLR - number of features used: 3.0', 'DT - number of features used: 2.0', 'RF - number of features used: 2.0', 'KNN - number of features used: 3.0', 'SVM - number of features used: 3.0')

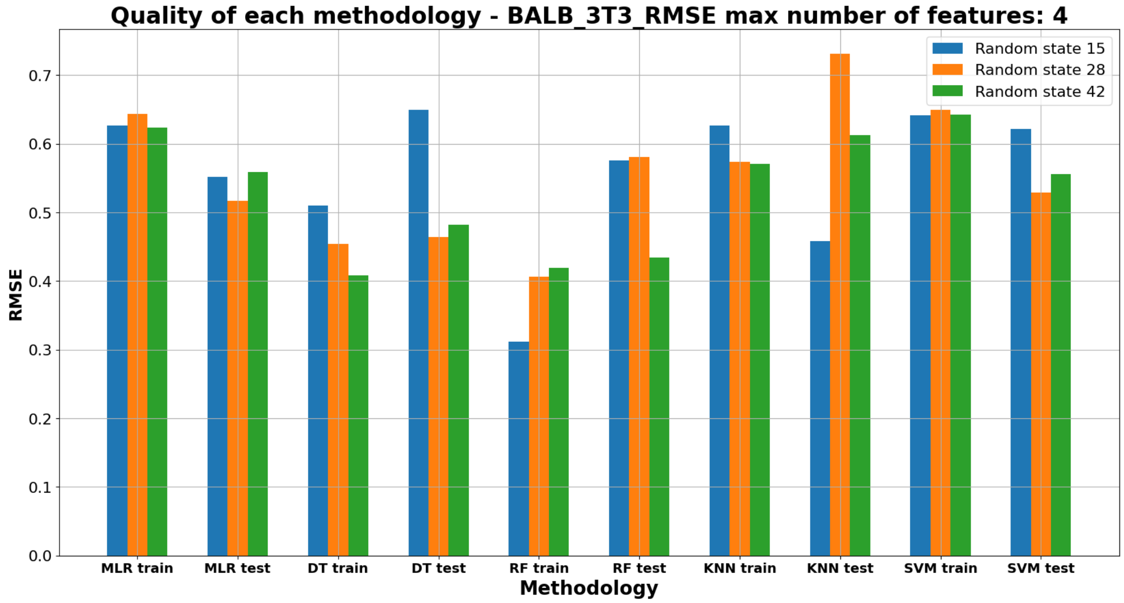

Random state - 15 ('MLR - number of features used: 3.0', 'DT - number of features used: 3.0', 'RF - number of features used: 3.0', 'KNN - number of features used: 2.0', 'SVM - number of features used: 3.0')
Random state - 28 ('MLR - number of features used: 2.0', 'DT - number of features used: 3.0', 'RF - number of features used: 2.0', 'KNN - number of features used: 3.0', 'SVM - number of features used: 2.0')
Random state - 42 ('MLR - number of features used: 3.0', 'DT - number of features used: 2.0', 'RF - number of features used: 2.0', 'KNN - number of features used: 3.0', 'SVM - number of features used: 3.0')

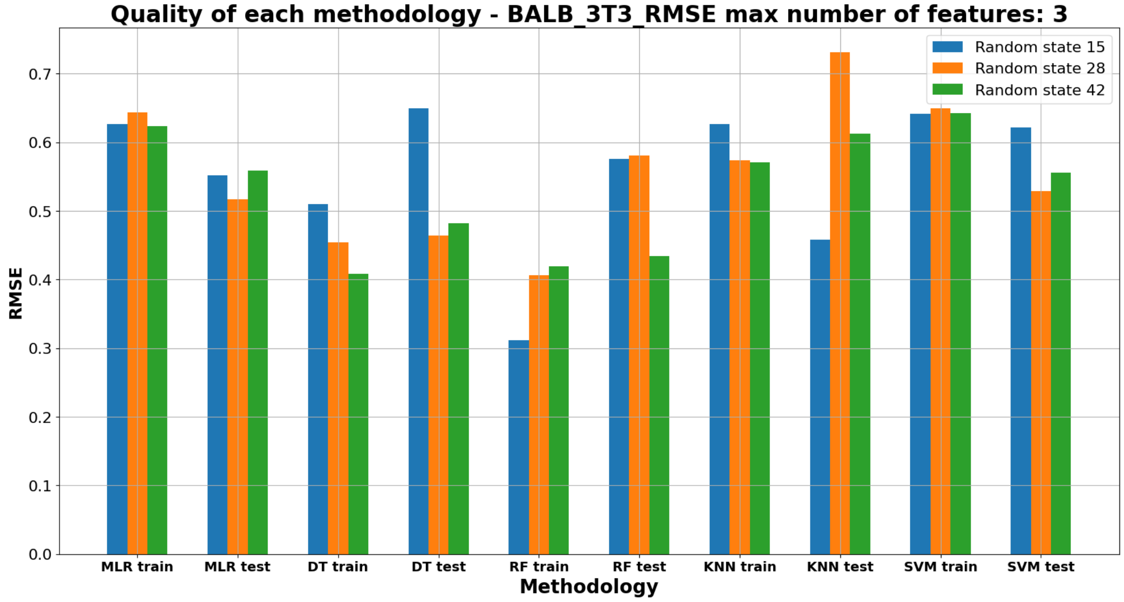

```
Random state - 15 ('MLR - number of features used: 3.0', 'DT - number of
features used: 3.0', 'RF - number of features used: 3.0', 'KNN - number of
features used: 2.0', 'SVM - number of features used: 3.0')
Random state - 28 ('MLR - number of features used: 2.0', 'DT - number of
features used: 3.0', 'RF - number of features used: 2.0', 'KNN - number of
features used: 3.0', 'SVM - number of features used: 2.0')
Random state - 42 ('MLR - number of features used: 3.0', 'DT - number of
features used: 2.0', 'RF - number of features used: 2.0', 'KNN - number of
features used: 3.0', 'SVM - number of features used: 3.0')
```

[ ]:

[ ]:

1.6 Best models selection

[14]:
```
def prepare_best_plot(target_name, max_number_of_features):

    excel_files = []

    for name in glob.glob('../Data/Quality_'+str(target_name)+'*'):
        excel_files.append(name)

    random_state_15 = []
    random_state_28 = []
    random_state_42 = []
    features_15 = []
    features_28 = []
    features_42 = []
    random_states = [15, 28, 42]

    for random_state in range(len(random_states)):
        tmp = []
        to_plot = []
        values = []
        number_of_features = []
        for sheet in sheet_names:
            tmp.append(pd.read_excel(excel_files[random_state],␣
 ↪sheet_name=sheet))
        for data in tmp:
            temp = data[data['Number of features'] <= max_number_of_features]
            to_plot.append(temp[temp['Test data R^2 score'] == max(temp['Test␣
 ↪data R^2 score'])])
        for value in to_plot:
            values.append(value['Training data R^2 score'].tail(1))
```

```
            values.append(value['Test data R^2 score'].tail(1))
            number_of_features.append(value['Number of features'].tail(1))
        for element in values:
            if random_state == 0:
                random_state_15.append(float(element))
            elif random_state == 1:
                random_state_28.append(float(element))
            elif random_state == 2:
                random_state_42.append(float(element))
            else:
                print("Error with conditions...")

        for features in number_of_features:
            if random_state == 0:
                features_15.append(float(features))
            elif random_state == 1:
                features_28.append(float(features))
            elif random_state == 2:
                features_42.append(float(features))
            else:
                print("Error with conditions...")


    return  random_state_15, random_state_28, random_state_42, features_15,␣
↪features_28, features_42
```

```
[15]: def prepare_plot(data, name, max_number_of_features):
    fig = plt.figure(figsize=(20,10))
    X = x_ticks
    y_15 = handle_negative_corr(data[0])
    y_28 = handle_negative_corr(data[1])
    y_42 = handle_negative_corr(data[2])

    X_axis = np.arange(len(X))
    y_ax = [x/100 for x in range(0, 105, 5)]
    plt.yticks(y_ax)
    plt.bar(X_axis - 0.2, y_15, 0.2, label = 'Random state 15')
    plt.bar(X_axis + 0.0, y_28, 0.2, label = 'Random state 28')
    plt.bar(X_axis + 0.2, y_42, 0.2, label = 'Random state 42')
    #plt.plot(X_axis, y_15, color='r', label='Random state 15')
    #plt.plot(X_axis, y_28, color='g', label='Random state 28')
    #plt.plot(X_axis, y_42, color='b', label='Random state 42')

    plt.xticks(X_axis, X)
    plt.xlabel("Methodology", fontsize=20, weight='bold')
    plt.ylabel("Correlation coefficient", fontsize=18, weight='bold')
    plt.ylim([0.0, 1.0])
```

```
    plt.title("Quality of each methodology - "+str(name)+' '+str('max number of␣
↪features: '+str(max_number_of_features)), fontsize=24, weight='bold')
    plt.grid(visible=True)
    plt.legend()
    plt.show()
    for i, state in enumerate(random_states):
        print('Random state - '+str(state)+'␣
↪'+str(update_description(sheet_names, data[i+3])))
```

[16]:
```
def update_description(x_axis_labels, number_of_features):

    DT = str(x_axis_labels[0])+' - number of features used:␣
↪'+str(number_of_features[0])
    RF = str(x_axis_labels[1])+' - number of features used:␣
↪'+str(number_of_features[1])

    return DT, RF
```

[ ]:
```

```

1.6.1 A549

[17]:
```
sheet_names = ['DT', 'RF']
```

[18]:
```
x_ticks = ['DT train R', 'DT test R', 'RF train R', 'RF test R']
```

[19]:
```
for i in range(8, 3, -1):
    a549 = prepare_best_plot('A549', i)
    prepare_plot(a549, 'A549', i)
    print(handle_negative_corr(a549[0]))
    print(handle_negative_corr(a549[1]))
    print(handle_negative_corr(a549[2]))
```

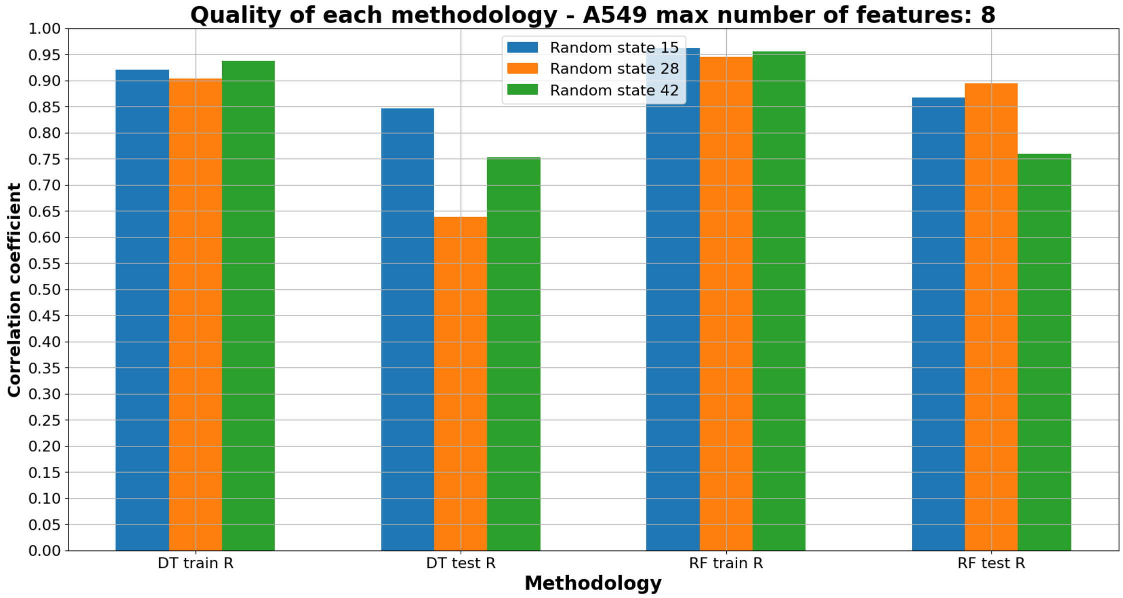

Random state - 15 ('DT - number of features used: 5.0', 'RF - number of features used: 8.0')
Random state - 28 ('DT - number of features used: 7.0', 'RF - number of features used: 8.0')
Random state - 42 ('DT - number of features used: 8.0', 'RF - number of features used: 7.0')
[0.9201268576746804, 0.8457608041331407, 0.9617557913430392, 0.8674998324838339]
[0.9039251218762473, 0.6385038801226338, 0.9449953932266721, 0.8948585348179655]
[0.9379105079400218, 0.7530696887427908, 0.9560924167894731, 0.7596144482170636]

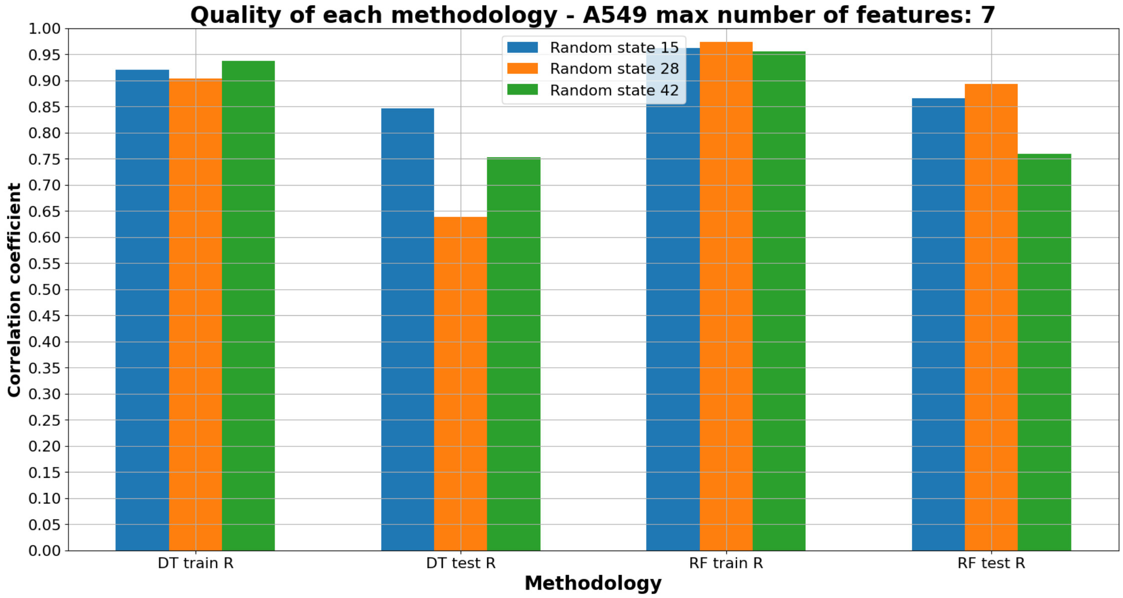

```
Random state - 15 ('DT - number of features used: 5.0', 'RF - number of features
used: 7.0')
Random state - 28 ('DT - number of features used: 7.0', 'RF - number of features
used: 7.0')
Random state - 42 ('DT - number of features used: 7.0', 'RF - number of features
used: 7.0')
[0.9201268576746804, 0.8457608041331407, 0.9617502720280803, 0.865633475359066]
[0.9039251218762473, 0.6385038801226338, 0.9733717517208741, 0.8936683566006186]
[0.9379105079400218, 0.7530696887427905, 0.9560924167894731, 0.7596144482170636]
```

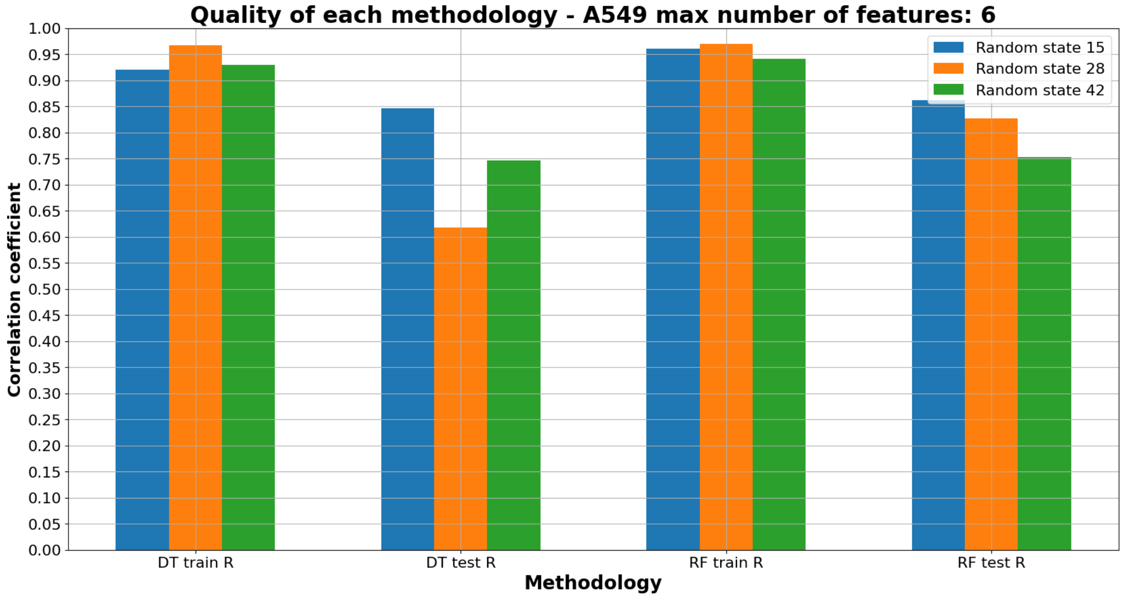


```
Random state - 15 ('DT - number of features used: 5.0', 'RF - number of features
used: 5.0')
Random state - 28 ('DT - number of features used: 4.0', 'RF - number of features
used: 5.0')
Random state - 42 ('DT - number of features used: 5.0', 'RF - number of features
used: 5.0')
[0.9201268576746804, 0.8457608041331407, 0.960591935079034, 0.8620618597285548]
[0.9669220010492233, 0.6172800607391895, 0.9701820261061275, 0.8267270635721486]
[0.9298029234842533, 0.746434872641246, 0.9417342321043257, 0.7526447589514621]
```

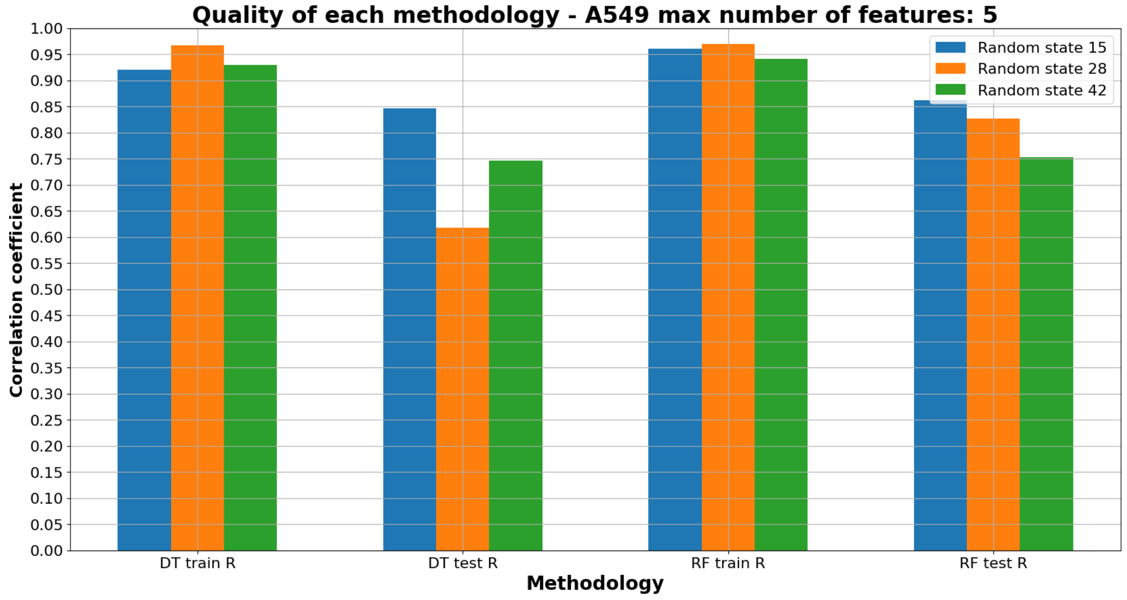


```
Random state - 15 ('DT - number of features used: 5.0', 'RF - number of features
used: 5.0')
Random state - 28 ('DT - number of features used: 4.0', 'RF - number of features
used: 5.0')
Random state - 42 ('DT - number of features used: 5.0', 'RF - number of features
used: 5.0')
[0.9201268576746804, 0.8457608041331407, 0.960591935079034, 0.8620618597285548]
[0.9669220010492233, 0.6172800607391895, 0.9701820261061275, 0.8267270635721486]
[0.9298029234842533, 0.746434872641246, 0.9417342321043257, 0.7526447589514621]
```

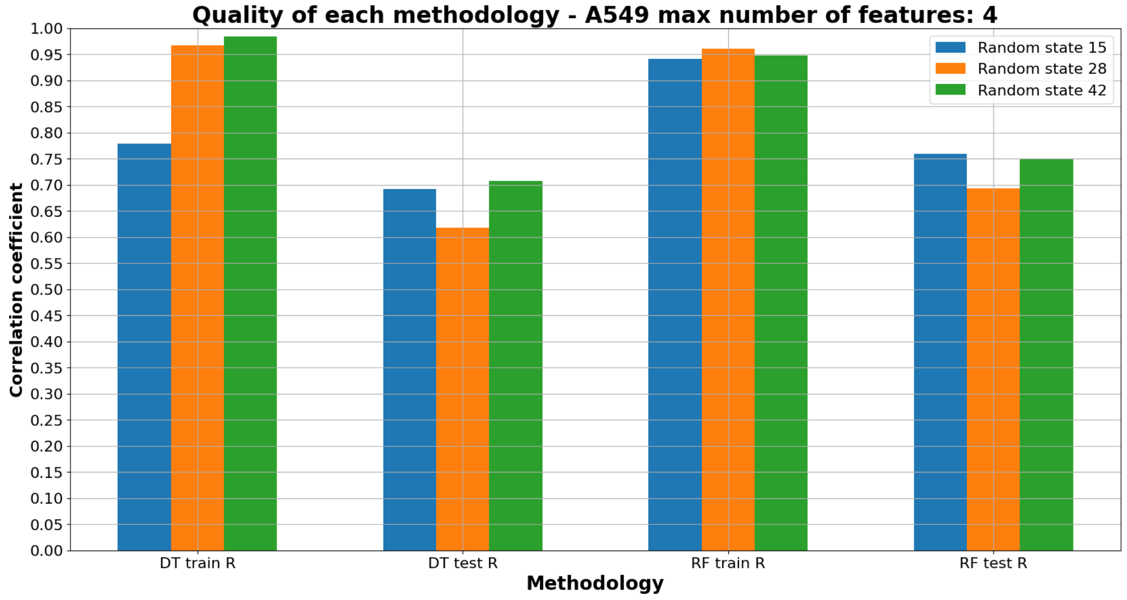

```
Random state - 15 ('DT - number of features used: 3.0', 'RF - number of features
used: 4.0')
Random state - 28 ('DT - number of features used: 4.0', 'RF - number of features
used: 4.0')
Random state - 42 ('DT - number of features used: 3.0', 'RF - number of features
used: 3.0')
[0.7787561234847591, 0.6915475851498438, 0.94133348297971, 0.7594733568417315]
[0.9669220010492233, 0.6172800607391895, 0.9601878273802564, 0.6926815941771752]
[0.9846195227501177, 0.70753906802074, 0.9477169502251152, 0.7487968593395388]
```

[ ]:

[20]:
```
for i in range(8, 2, -1):
    x = prepare_best_plot('BALB_3T3', i)
    prepare_plot(x, 'BALB_3T3', i)
    print(handle_negative_corr(x[0]))
    print(handle_negative_corr(x[1]))
    print(handle_negative_corr(x[2]))
```

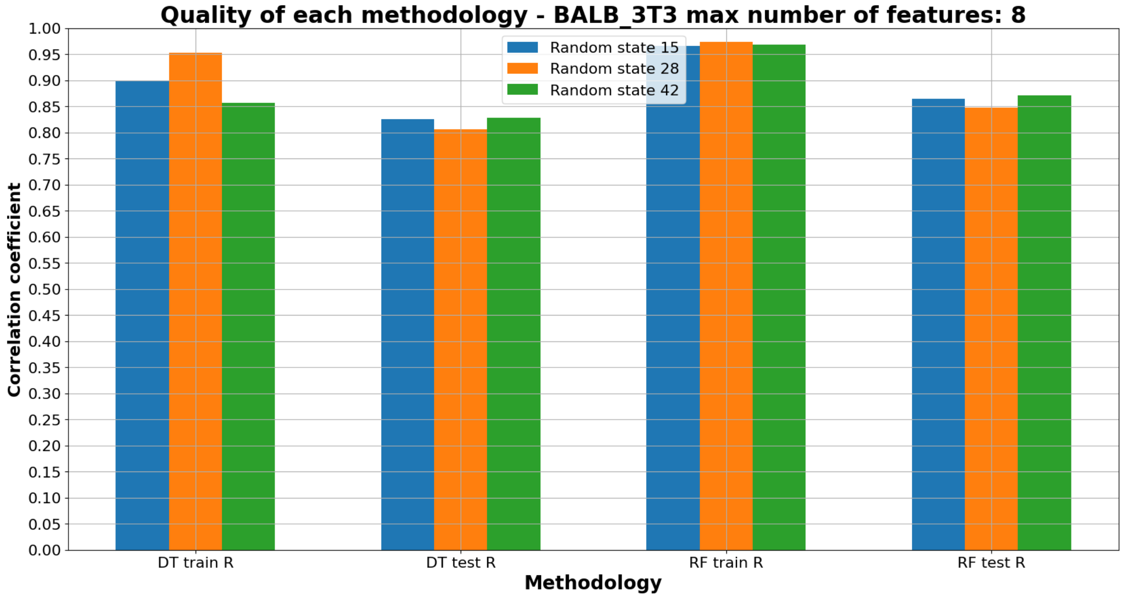


```
Random state - 15 ('DT - number of features used: 6.0', 'RF - number of features
used: 6.0')
Random state - 28 ('DT - number of features used: 6.0', 'RF - number of features
used: 7.0')
Random state - 42 ('DT - number of features used: 6.0', 'RF - number of features
used: 7.0')
[0.8991491467263336, 0.8261911693918914, 0.9656538230216772, 0.8642992780332693]
[0.9528404289708712, 0.8063470063121128, 0.9733753371944485, 0.8472660678286129]
```

[0.8574233855704518, 0.8283658235151035, 0.9679324070733115, 0.8709265604132982]

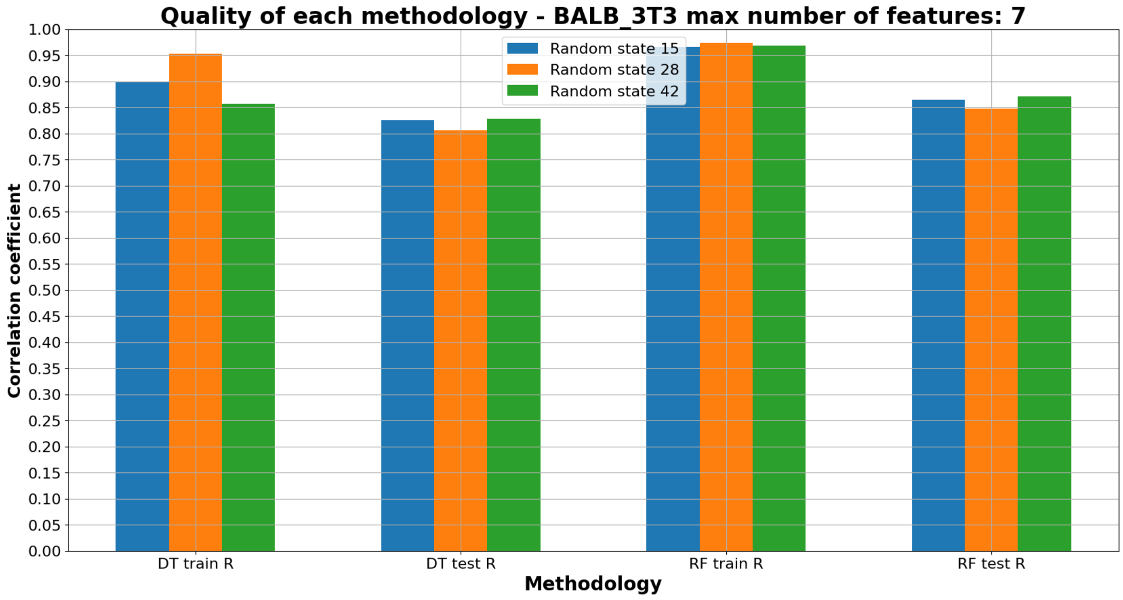

Random state - 15 ('DT - number of features used: 6.0', 'RF - number of features used: 6.0')
Random state - 28 ('DT - number of features used: 6.0', 'RF - number of features used: 7.0')
Random state - 42 ('DT - number of features used: 6.0', 'RF - number of features used: 7.0')
[0.8991491467263336, 0.8261911693918914, 0.9656538230216772, 0.8642992780332693]
[0.9528404289708712, 0.8063470063121128, 0.9733753371944485, 0.8472660678286129]
[0.8574233855704518, 0.8283658235151035, 0.9679324070733115, 0.8709265604132982]

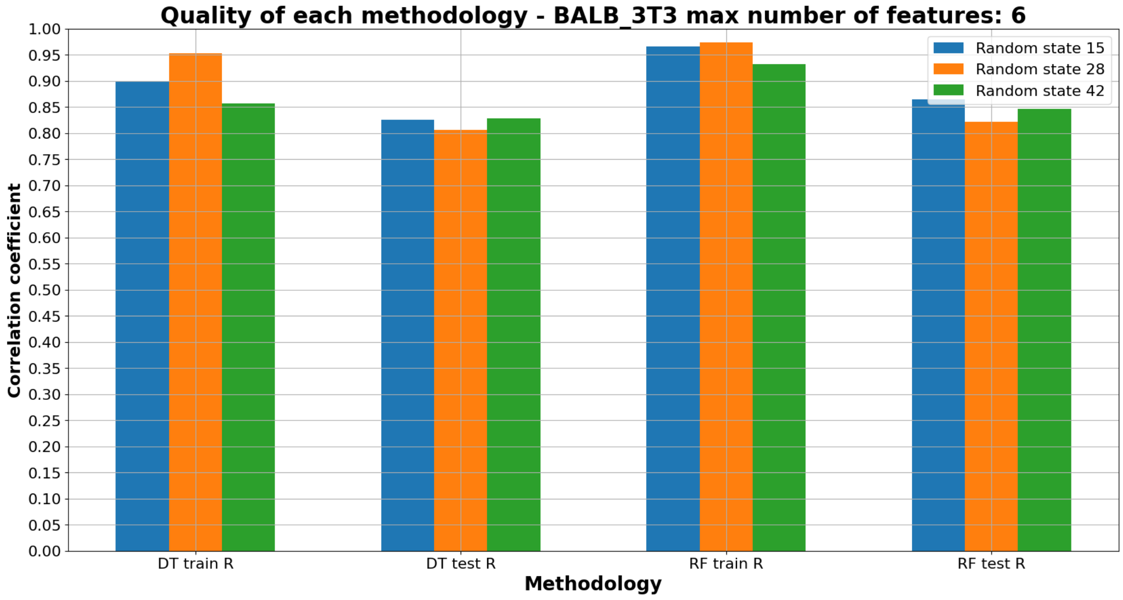

```
Random state - 15 ('DT - number of features used: 6.0', 'RF - number of features
used: 6.0')
Random state - 28 ('DT - number of features used: 6.0', 'RF - number of features
used: 6.0')
Random state - 42 ('DT - number of features used: 6.0', 'RF - number of features
used: 6.0')
[0.8991491467263336, 0.8261911693918914, 0.9656538230216772, 0.8642992780332693]
[0.9528404289708712, 0.8063470063121128, 0.9732431671159815, 0.8213095110427073]
[0.8574233855704518, 0.8283658235151035, 0.9323265067533627, 0.8462778707935902]
```

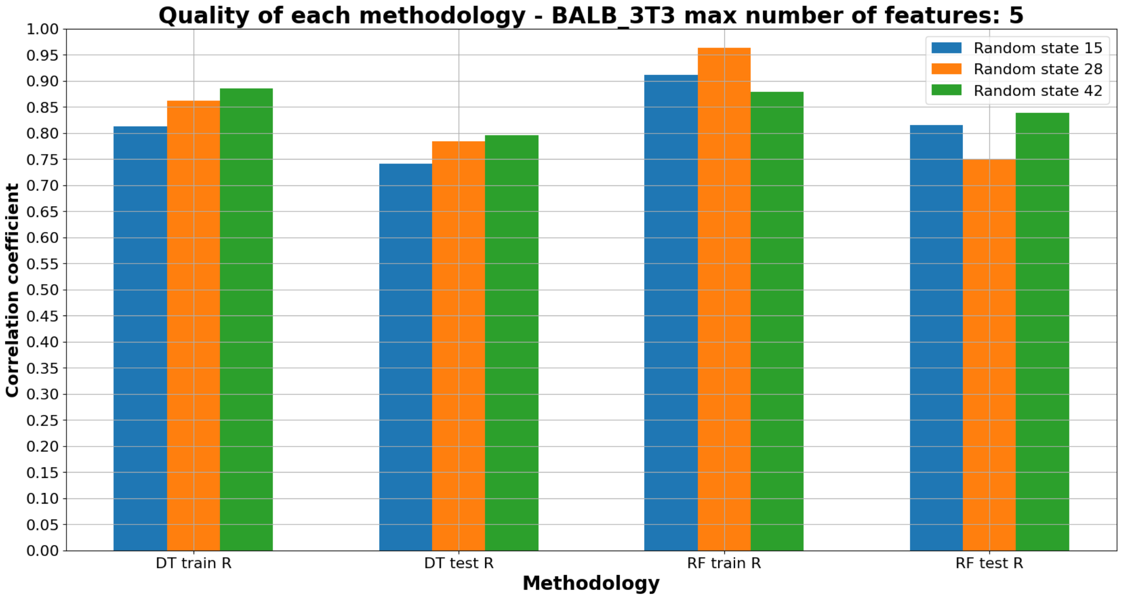


```
Random state - 15 ('DT - number of features used: 5.0', 'RF - number of features
used: 5.0')
Random state - 28 ('DT - number of features used: 3.0', 'RF - number of features
used: 5.0')
Random state - 42 ('DT - number of features used: 2.0', 'RF - number of features
used: 2.0')
[0.8124040437986895, 0.7410808070808154, 0.9118596336958118, 0.8149483739246309]
[0.8618249162158247, 0.7846752040880232, 0.9628242677281924, 0.7501347353760348]
[0.885912746009139, 0.7955365930788652, 0.8792917450164119, 0.837966105203246
1]
```

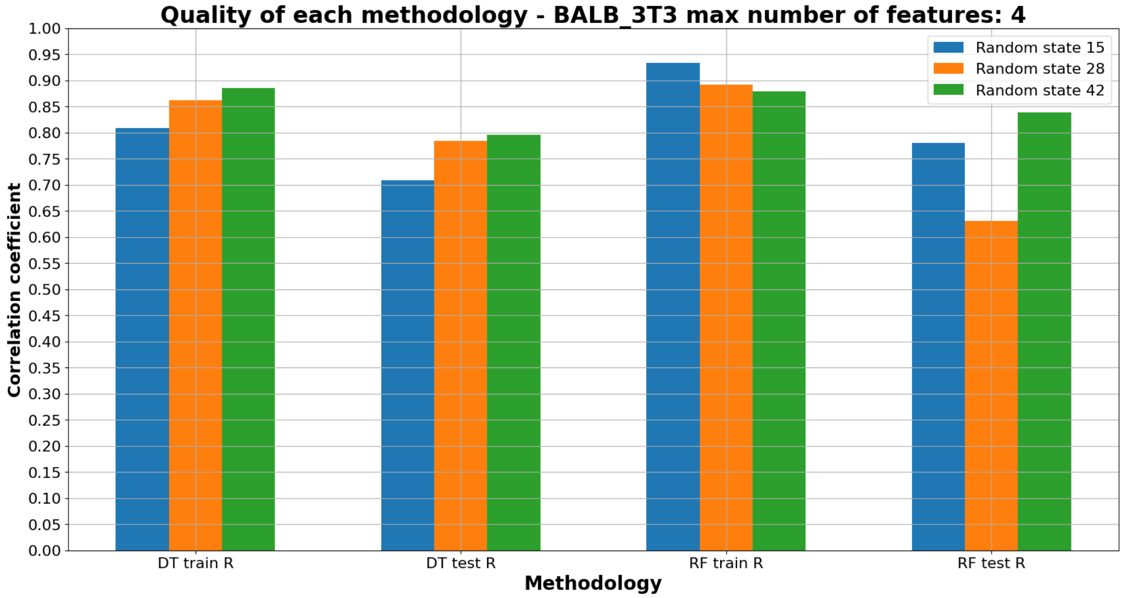


```
Random state - 15 ('DT - number of features used: 3.0', 'RF - number of features
used: 3.0')
Random state - 28 ('DT - number of features used: 3.0', 'RF - number of features
used: 2.0')
Random state - 42 ('DT - number of features used: 2.0', 'RF - number of features
used: 2.0')
[0.8089681087052601, 0.7083667815152337, 0.9331579969232232, 0.780272449227771]
[0.8618249162158247, 0.7846752040880232, 0.8911999290100625, 0.6303440412443166]
[0.885912746009139, 0.7955365930788652, 0.8792917450164119, 0.8379661052032461]
```

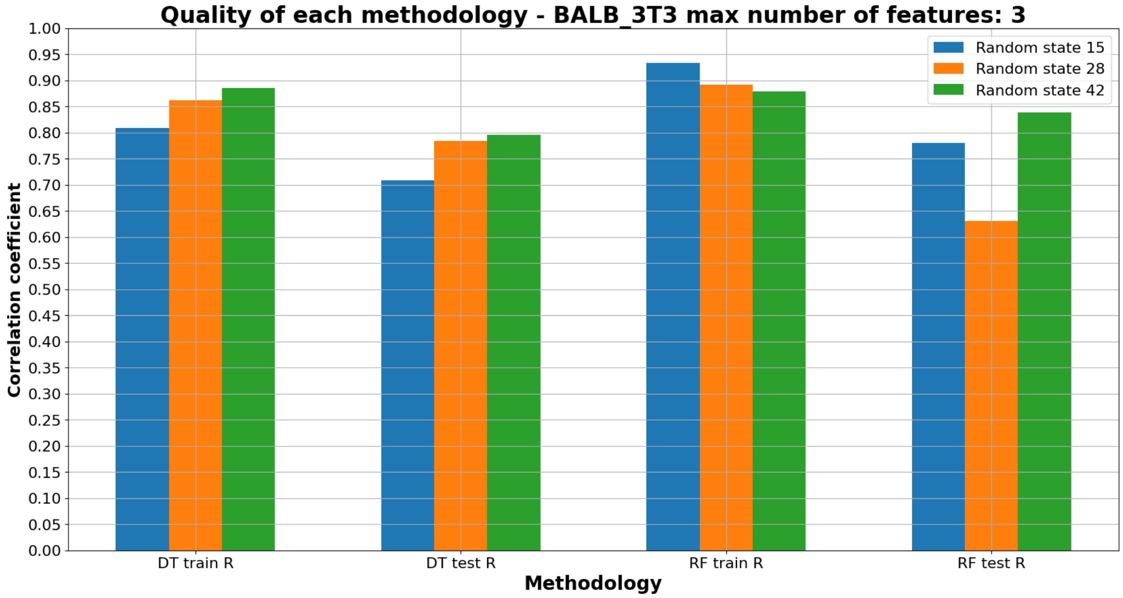

```
Random state - 15 ('DT - number of features used: 3.0', 'RF - number of features
used: 3.0')
Random state - 28 ('DT - number of features used: 3.0', 'RF - number of features
used: 2.0')
Random state - 42 ('DT - number of features used: 2.0', 'RF - number of features
used: 2.0')
[0.8089681087052601, 0.7083667815152337, 0.9331579969232232, 0.780272449227771]
[0.8618249162158247, 0.7846752040880232, 0.8911999290100625, 0.6303440412443166]
[0.885912746009139, 0.7955365930788652, 0.8792917450164119, 0.8379661052032461]
```

[ ]:

[21]:
```python
for i in range(8, 2, -1):
    x = prepare_best_plot('LoVo', i)
    prepare_plot(x, 'LoVo', i)
    print(handle_negative_corr(x[0]))
    print(handle_negative_corr(x[1]))
    print(handle_negative_corr(x[2]))
```

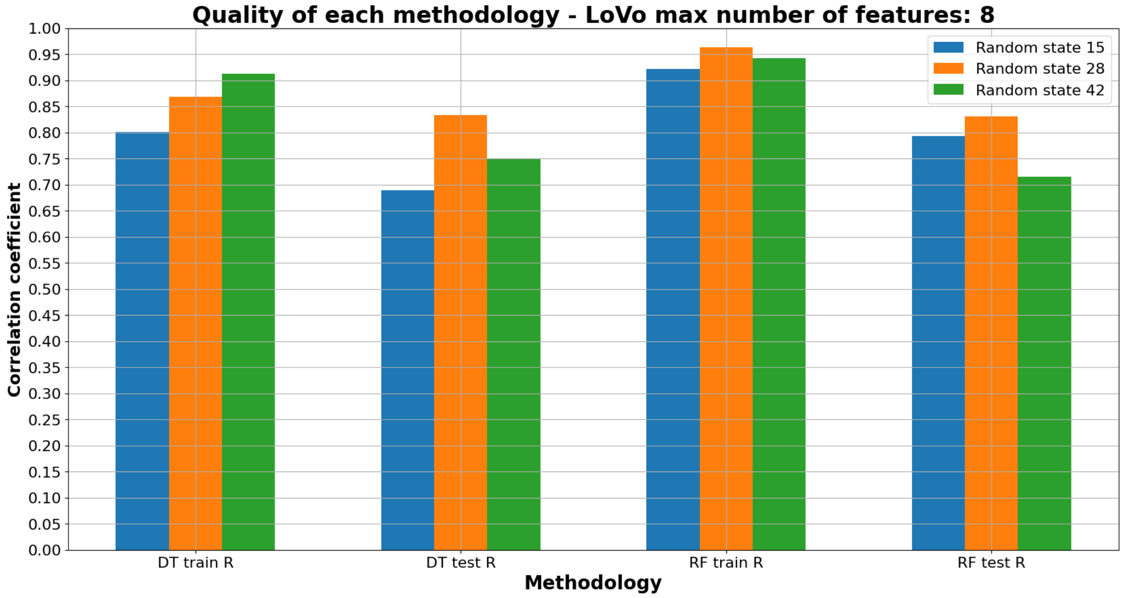


```
Random state - 15 ('DT - number of features used: 7.0', 'RF - number of features
used: 5.0')
Random state - 28 ('DT - number of features used: 3.0', 'RF - number of features
used: 7.0')
Random state - 42 ('DT - number of features used: 3.0', 'RF - number of features
used: 5.0')
[0.8012547599591731, 0.6895731340974959, 0.9218472144749, 0.7937730901899727]
[0.8682412855125715, 0.8337432078795763, 0.9636653219744876, 0.8305193093908672]
```

[0.9121710628859598, 0.7499171098832387, 0.9423441595215349, 0.7153725648027842]

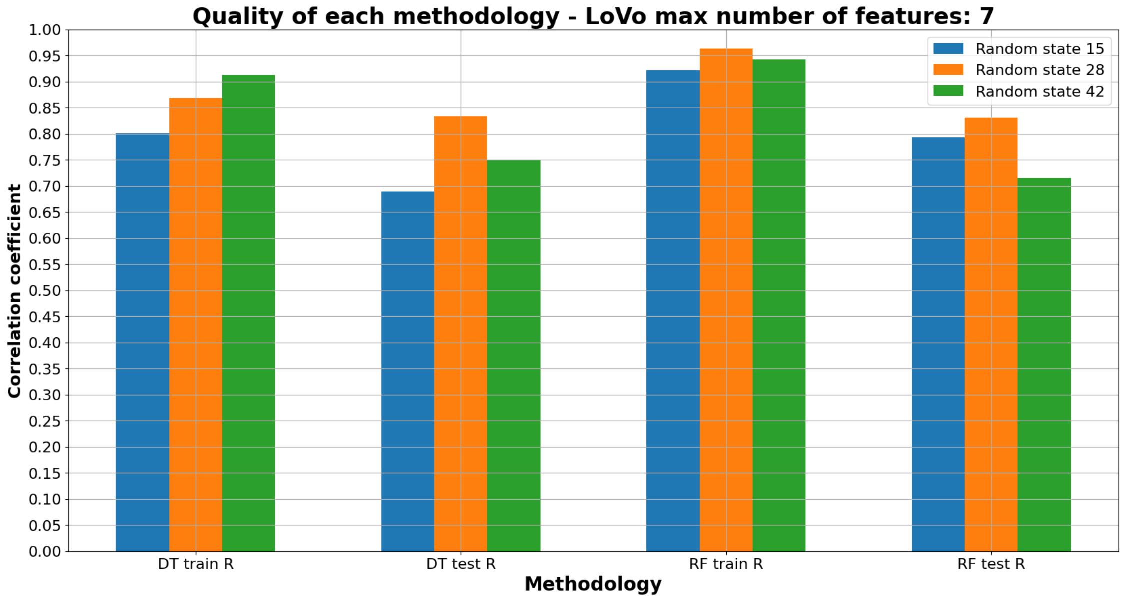

Random state - 15 ('DT - number of features used: 7.0', 'RF - number of features used: 5.0')
Random state - 28 ('DT - number of features used: 3.0', 'RF - number of features used: 7.0')
Random state - 42 ('DT - number of features used: 3.0', 'RF - number of features used: 5.0')
[0.8012547599591731, 0.6895731340974959, 0.9218472144749, 0.7937730901899727]
[0.8682412855125715, 0.8337432078795763, 0.9636653219744876, 0.8305193093908672]
[0.9121710628859598, 0.7499171098832387, 0.9423441595215349, 0.7153725648027842]

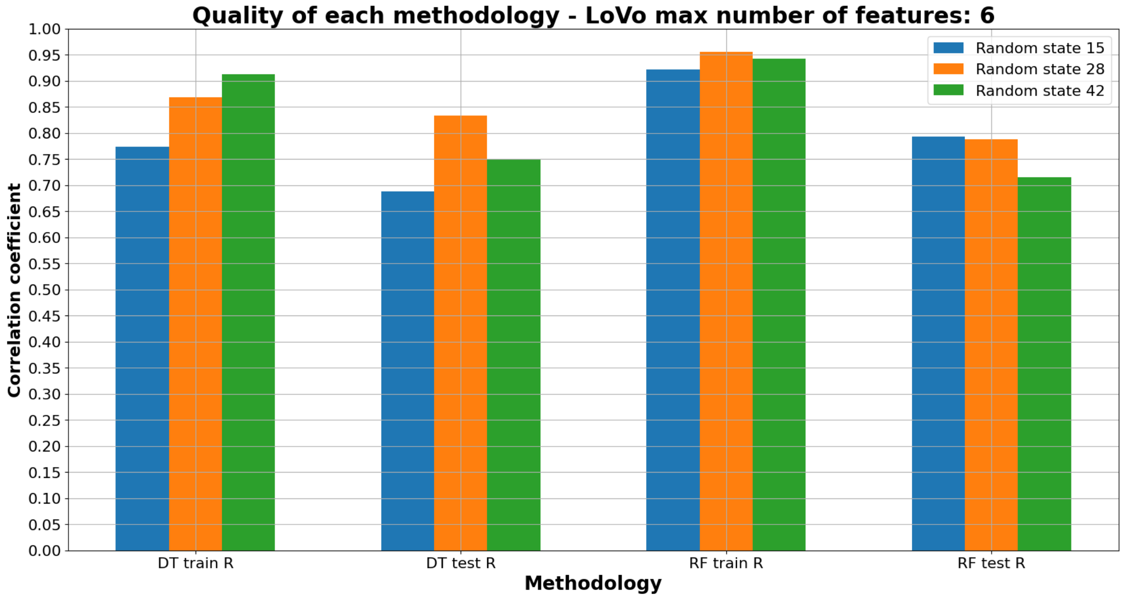

Random state - 15 ('DT - number of features used: 6.0', 'RF - number of features used: 5.0')
Random state - 28 ('DT - number of features used: 3.0', 'RF - number of features used: 6.0')
Random state - 42 ('DT - number of features used: 3.0', 'RF - number of features used: 5.0')
[0.7738495519932647, 0.6881453471088331, 0.9218472144749, 0.7937730901899727]
[0.8682412855125715, 0.8337432078795763, 0.9549618967523424, 0.7874289926703334]
[0.9121710628859598, 0.7499171098832387, 0.9423441595215349, 0.7153725648027842]

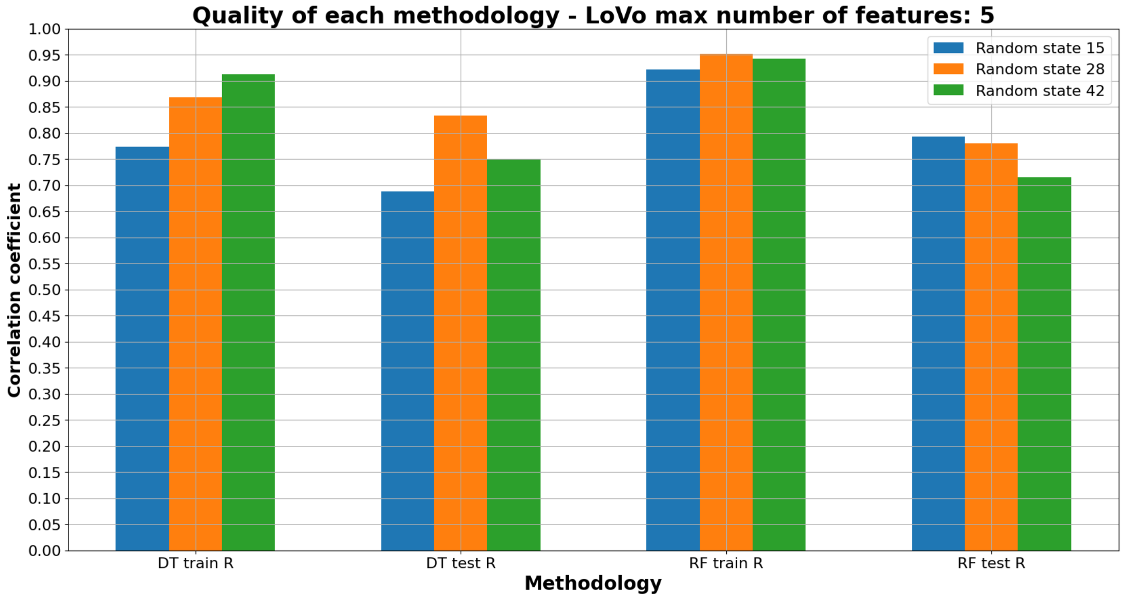

Random state - 15 ('DT - number of features used: 5.0', 'RF - number of features used: 5.0')
Random state - 28 ('DT - number of features used: 3.0', 'RF - number of features used: 5.0')
Random state - 42 ('DT - number of features used: 3.0', 'RF - number of features used: 5.0')
[0.7738495519932644, 0.688145347108833, 0.9218472144749, 0.7937730901899727]
[0.8682412855125715, 0.8337432078795763, 0.95161256233916, 0.780370388796789]
[0.9121710628859598, 0.7499171098832387, 0.9423441595215349, 0.7153725648027842]

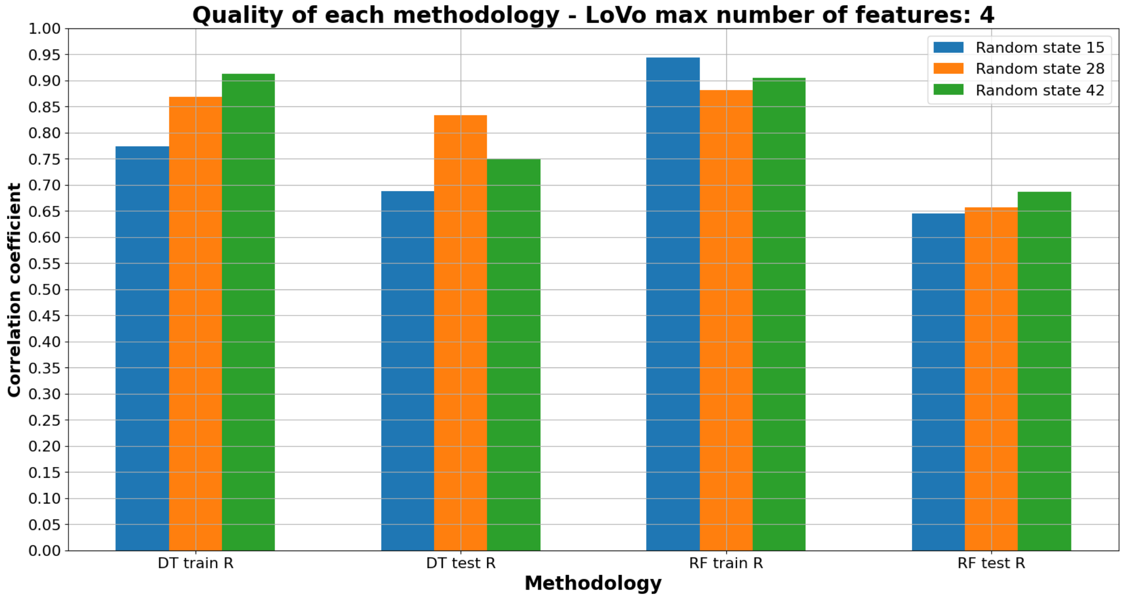

Random state - 15 ('DT - number of features used: 3.0', 'RF - number of features used: 3.0')
Random state - 28 ('DT - number of features used: 3.0', 'RF - number of features used: 2.0')
Random state - 42 ('DT - number of features used: 3.0', 'RF - number of features used: 2.0')
[0.7738495519932647, 0.6881453471088325, 0.9435896757858103, 0.6450408235184336]
[0.8682412855125715, 0.8337432078795763, 0.8811267812945673, 0.6572630628008767]
[0.9121710628859598, 0.7499171098832387, 0.9053674539315217, 0.6872057325646179]

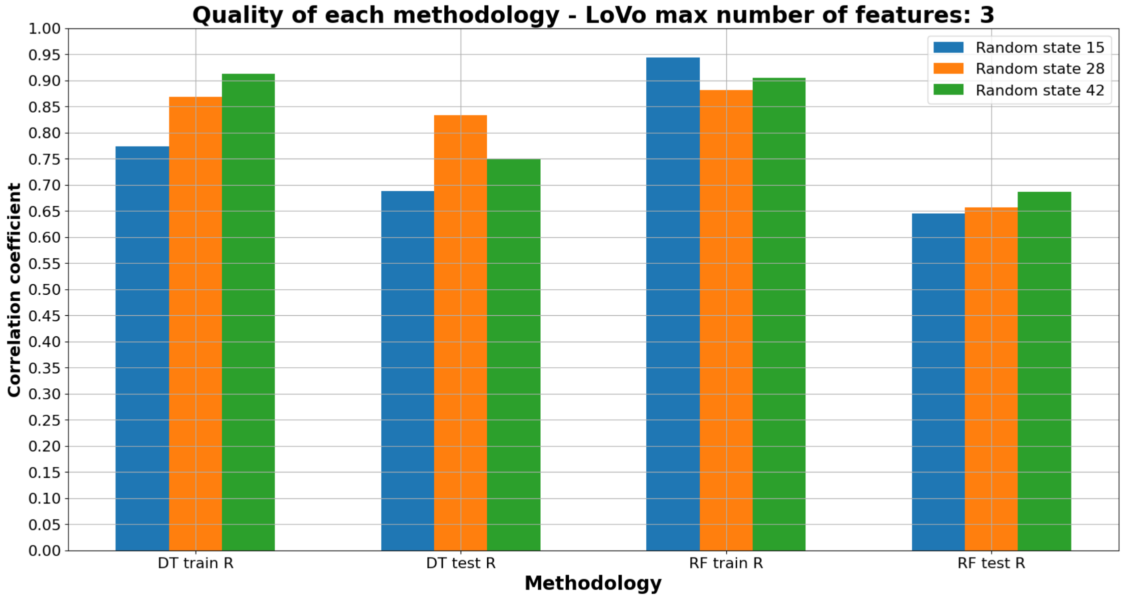

Random state - 15 ('DT - number of features used: 3.0', 'RF - number of features used: 3.0')
Random state - 28 ('DT - number of features used: 3.0', 'RF - number of features used: 2.0')
Random state - 42 ('DT - number of features used: 3.0', 'RF - number of features used: 2.0')
[0.7738495519932647, 0.6881453471088325, 0.9435896757858103, 0.6450408235184336]
[0.8682412855125715, 0.8337432078795763, 0.8811267812945673, 0.6572630628008767]
[0.9121710628859598, 0.7499171098832387, 0.9053674539315217, 0.6872057325646179]

[22]:
```
for i in range(8, 5, -1):
    x = prepare_best_plot('LoVo_DX', i)
    prepare_plot(x, 'LoVo_DX', i)
    print(handle_negative_corr(x[0]))
    print(handle_negative_corr(x[1]))
    print(handle_negative_corr(x[2]))
```

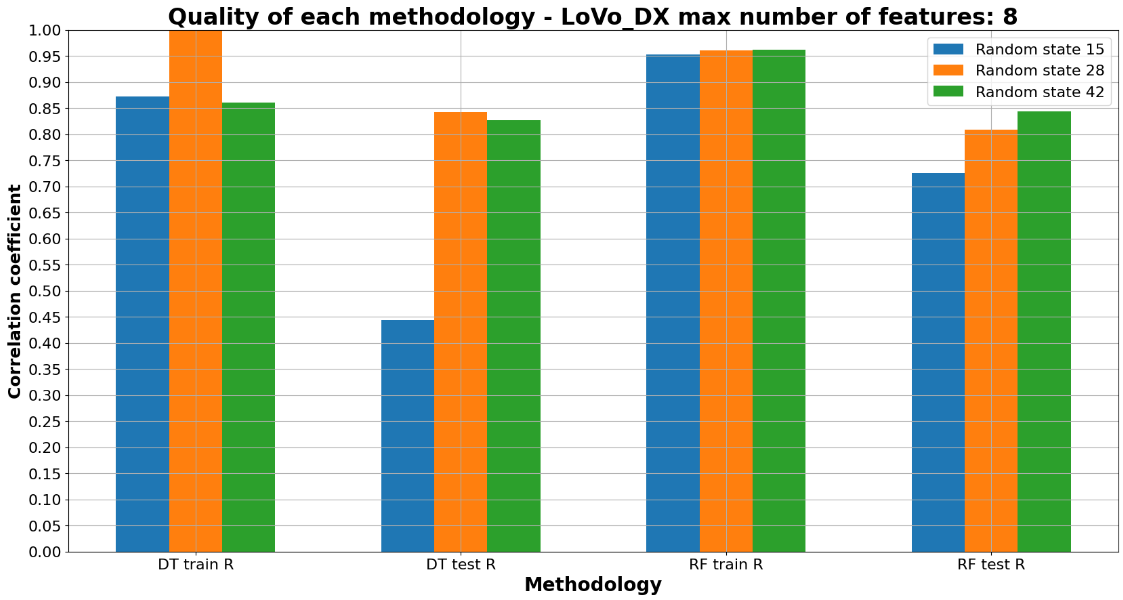

Random state - 15 ('DT - number of features used: 7.0', 'RF - number of features used: 8.0')
Random state - 28 ('DT - number of features used: 5.0', 'RF - number of features used: 8.0')
Random state - 42 ('DT - number of features used: 7.0', 'RF - number of features used: 7.0')
[0.8724994277322107, 0.44416679666061354, 0.9531465584369622, 0.7258930436657147]
[0.9998881595139364, 0.8430877655864321, 0.9608248729709339, 0.8093430351511413]
[0.8607935732823858, 0.8271783219542648, 0.9614894965852848, 0.8437279214475684]

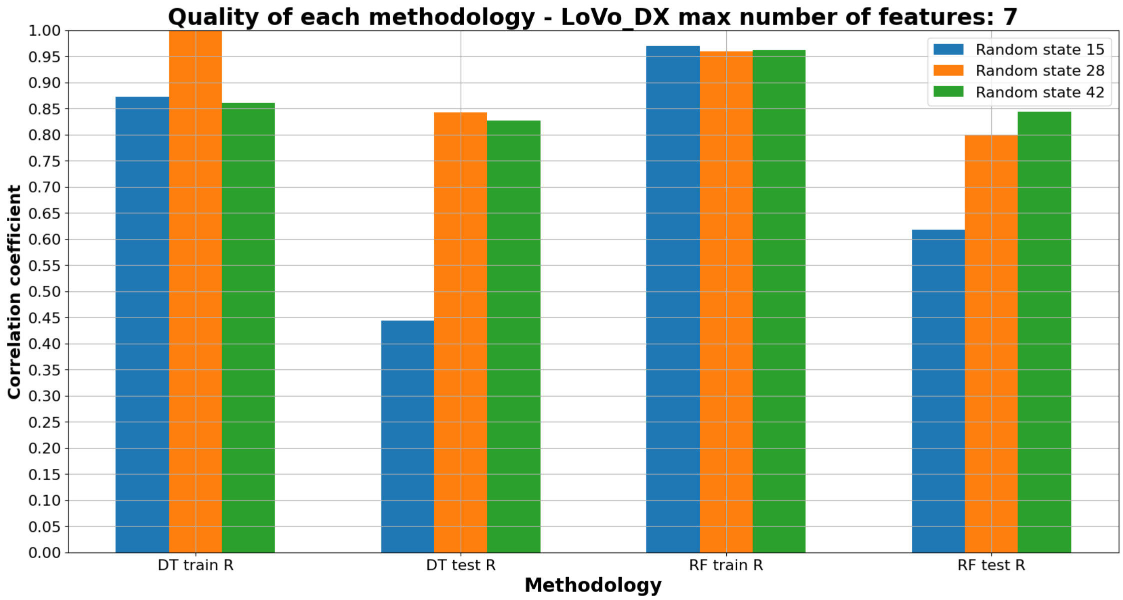


```
Random state - 15 ('DT - number of features used: 7.0', 'RF - number of features
used: 7.0')
Random state - 28 ('DT - number of features used: 5.0', 'RF - number of features
used: 7.0')
Random state - 42 ('DT - number of features used: 7.0', 'RF - number of features
used: 7.0')
[0.8724994277322107, 0.44416679666061354, 0.9701730613127154,
0.6181792070920507]
[0.9998881595139364, 0.8430877655864321, 0.95913927288201, 0.7997866699043976]
[0.8607935732823858, 0.8271783219542648, 0.9614894965852848, 0.8437279214475684]
```

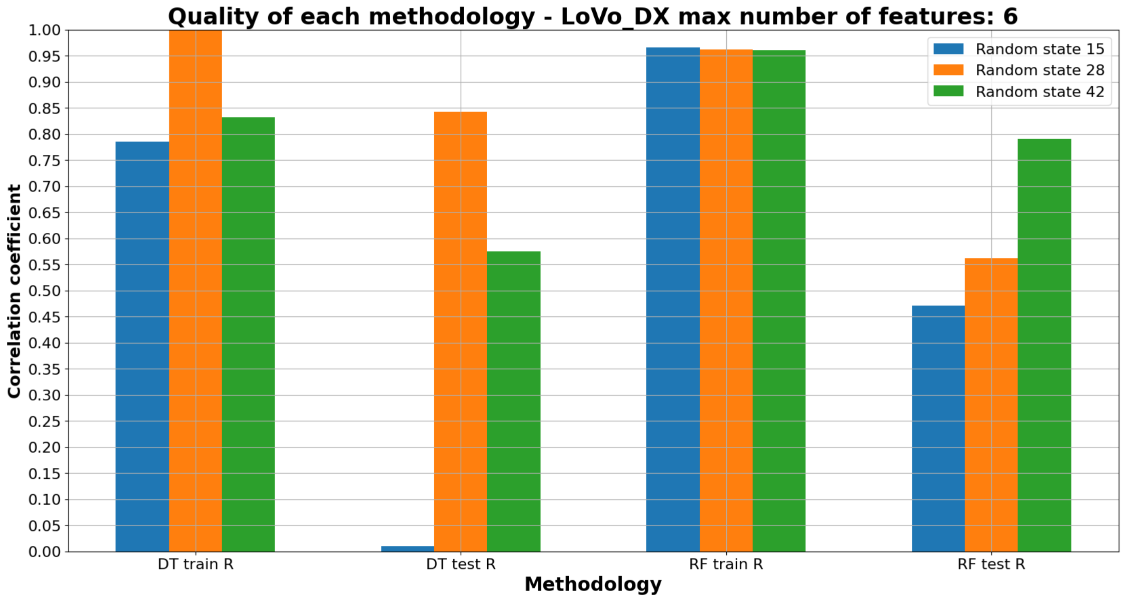

```
Random state - 15 ('DT - number of features used: 5.0', 'RF - number of features
used: 5.0')
Random state - 28 ('DT - number of features used: 5.0', 'RF - number of features
used: 5.0')
Random state - 42 ('DT - number of features used: 5.0', 'RF - number of features
used: 5.0')
[0.7851394944455857, 0.01, 0.965938844054454, 0.4715560592926372]
[0.9998881595139364, 0.8430877655864321, 0.9619512068981969, 0.5614693906239112]
[0.832498347961302, 0.5745589174162089, 0.9610074984048745, 0.790562123545645]
```

[ ]:

[23]:
```
for i in range(8, 4, -1):
    x = prepare_best_plot('MCF-7', i)
    prepare_plot(x, 'MCF-7', i)
    print(handle_negative_corr(x[0]))
    print(handle_negative_corr(x[1]))
    print(handle_negative_corr(x[2]))
```

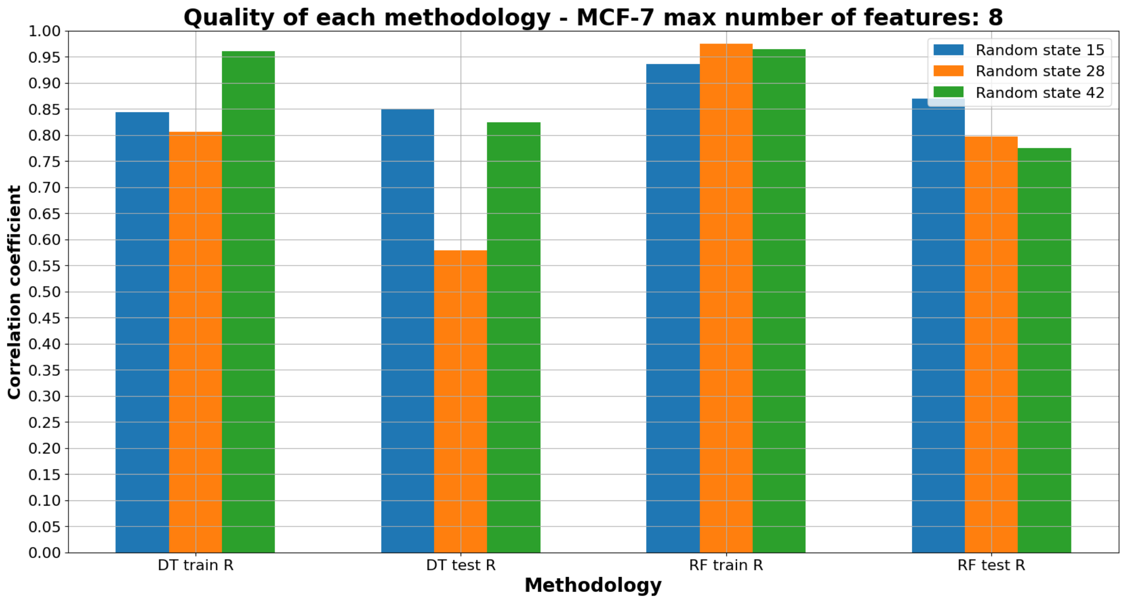


```
Random state - 15 ('DT - number of features used: 6.0', 'RF - number of features
used: 8.0')
Random state - 28 ('DT - number of features used: 4.0', 'RF - number of features
used: 8.0')
Random state - 42 ('DT - number of features used: 8.0', 'RF - number of features
used: 8.0')
[0.8435310621038742, 0.8490631015009857, 0.9361673240230982, 0.8695068587619961]
```

```
[0.8067266275260266, 0.5788863720704619, 0.9745022739926398, 0.7974479201634584]
[0.9607100088789956, 0.8246123913884399, 0.9643673177108916, 0.7746407763544272]
```

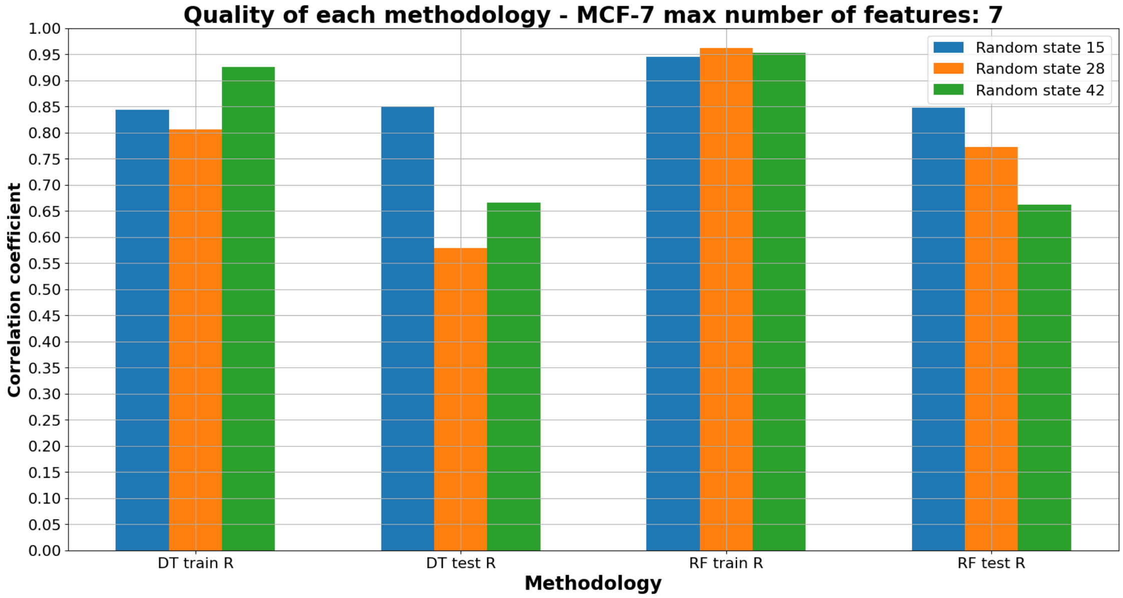


```
Random state - 15 ('DT - number of features used: 6.0', 'RF - number of features
used: 6.0')
Random state - 28 ('DT - number of features used: 4.0', 'RF - number of features
used: 6.0')
Random state - 42 ('DT - number of features used: 6.0', 'RF - number of features
used: 6.0')
[0.8435310621038742, 0.8490631015009857, 0.945156244145722, 0.8470828152088354]
[0.8067266275260266, 0.5788863720704619, 0.9622709929006865, 0.7725823523082598]
[0.9250974432855168, 0.6663870801375169, 0.9527501485389981, 0.6624039809877339]
```

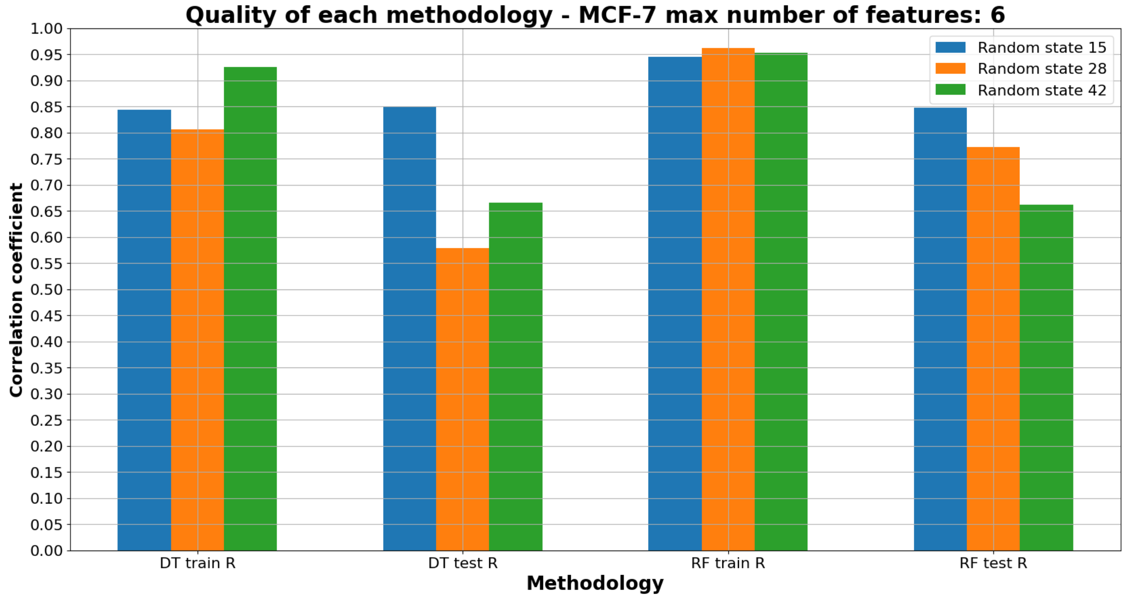

Random state - 15 ('DT - number of features used: 6.0', 'RF - number of features used: 6.0')
Random state - 28 ('DT - number of features used: 4.0', 'RF - number of features used: 6.0')
Random state - 42 ('DT - number of features used: 6.0', 'RF - number of features used: 6.0')
[0.8435310621038742, 0.8490631015009857, 0.945156244145722, 0.8470828152088354]
[0.8067266275260266, 0.5788863720704619, 0.9622709929006865, 0.7725823523082598]
[0.9250974432855168, 0.6663870801375169, 0.9527501485389981, 0.6624039809877339]

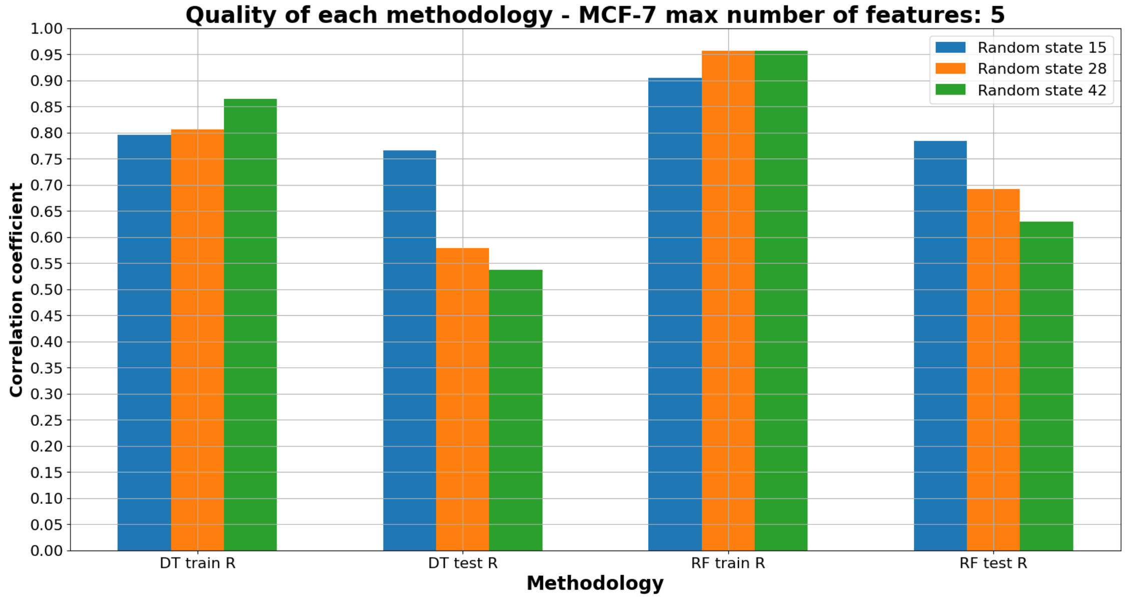

```
Random state - 15 ('DT - number of features used: 4.0', 'RF - number of features
used: 4.0')
Random state - 28 ('DT - number of features used: 4.0', 'RF - number of features
used: 4.0')
Random state - 42 ('DT - number of features used: 4.0', 'RF - number of features
used: 4.0')
[0.7959369681181857, 0.7660302989047363, 0.9051955244679118, 0.7837627386732194]
[0.8067266275260266, 0.5788863720704619, 0.9563828487646977, 0.6924899356899538]
[0.8646854500902919, 0.5371299141302905, 0.956767076965924, 0.6292292538599767]
```

[ ]:

[ ]:

1.7 Best models plot

Comments: (see different random states for each model)

- A549 -> random state 15, 5 features DT, 5 features RF

1. MLR (0.65, 0.55) -> 3 features
2. DT (0.92, 0.84) -> 5 features
3. RF (0.92, 0.82) -> 5 features
4. KNN (0.77, 0.83) -> 5 features
5. SVM (0.64, 0.18) -> 3 features

- BALB/3T3 -> random state 42, 6 features RF

1. MLR (0.73, 0.01) -> 3 features
2. DT (0.86, 0.75) -> 6 features
3. RF (0.93, 0.85) -> 6 features
4. KNN (0.77, 0.22) -> 3 features
5. SVM (0.68, 0.17) -> 3 features

- LoVo -> random state 28, 2 features RF

1. MLR (0.69, 0.40) -> 3 features
2. DT (0.94, 0.82) -> 5 features
3. RF (0.85, 0.70) -> 2 features
4. KNN (0.73, 0.38) -> 5 features
5. SVM (0.70, 0.01) -> 5 features

- LoVo/DX -> random state 42, 5 features RF

1. MLR (0.82, 0.78) -> 7 features
2. DT (0.86, 0.84) -> 7 features
3. RF (0.96, 0.83) -> 5 features
4. KNN (0.81, 0.01) -> 5 features
5. SVM (0.70, 0.63) -> 5 features

- MCF-7 -> random state 15, 4 features RF

1. MLR (0.70, 0.61) -> 4 features
2. DT (0.92, 0.53) -> 4 features
3. RF (0.91, 0.65) -> 4 features
4. KNN (0.70, 0.75) -> 4 features
5. SVM (0.69, 0.53) -> 4 features

[24]:
```
df_a549 = pd.read_excel('../Data/Quality_A549_15_.xlsx', sheet_name='RF')
df_a549 = df_a549[df_a549['Number of features'] == 5]
df_a549['Training data R score'] = handle_negative_corr(df_a549['Training data␣
↪R^2 score'])
df_a549['Test data R score'] = handle_negative_corr(df_a549['Test data R^2␣
↪score'])
df_a549
```

[24]:
```
     Unnamed: 0  Correlation threshold  Training data R^2 score  \
323         323                   0.50                 0.852952
324         324                   0.50                 0.860201
325         325                   0.50                 0.865749
326         326                   0.50                 0.887848
327         327                   0.50                 0.888773
328         328                   0.50                 0.892504
329         329                   0.50                 0.908859
330         330                   0.50                 0.909170
331         331                   0.50                 0.913283
332         332                   0.50                 0.918630
333         333                   0.50                 0.917600
334         334                   0.50                 0.915991
335         335                   0.50                 0.921486
336         336                   0.50                 0.924538
337         337                   0.50                 0.921465
338         338                   0.50                 0.922737
339         339                   0.50                 0.924854
340         340                   0.50                 0.927070
341         341                   0.50                 0.920711
342         342                   0.51                 0.852952
343         343                   0.51                 0.860201
344         344                   0.51                 0.865749
345         345                   0.51                 0.887848
346         346                   0.51                 0.888773
347         347                   0.51                 0.892504
348         348                   0.51                 0.908859
349         349                   0.51                 0.909170
350         350                   0.51                 0.913283
351         351                   0.51                 0.918630
352         352                   0.51                 0.917600
353         353                   0.51                 0.915991
354         354                   0.51                 0.921486
```

355	355	0.51	0.924538
356	356	0.51	0.921465
357	357	0.51	0.922737
358	358	0.51	0.924854
359	359	0.51	0.927070
360	360	0.51	0.920711

	Test data R^2 score	Training RMSE	Test RMSE	Number of features	\
323	0.693940	0.357987	0.573479	5	
324	0.699356	0.349052	0.568382	5	
325	0.668966	0.342057	0.596418	5	
326	0.623941	0.312638	0.635685	5	
327	0.648644	0.311347	0.614452	5	
328	0.672695	0.306080	0.593049	5	
329	0.683983	0.281835	0.582732	5	
330	0.668412	0.281354	0.596917	5	
331	0.710288	0.274910	0.557952	5	
332	0.703868	0.266300	0.564101	5	
333	0.707403	0.267980	0.560723	5	
334	0.732924	0.270583	0.535712	5	
335	0.736283	0.261585	0.532333	5	
336	0.734897	0.256449	0.533730	5	
337	0.729516	0.261619	0.539119	5	
338	0.743151	0.259492	0.525356	5	
339	0.739241	0.255913	0.529339	5	
340	0.734946	0.252110	0.533681	5	
341	0.737105	0.262872	0.531502	5	
342	0.693940	0.357987	0.573479	5	
343	0.699356	0.349052	0.568382	5	
344	0.668966	0.342057	0.596418	5	
345	0.623941	0.312638	0.635685	5	
346	0.648644	0.311347	0.614452	5	
347	0.672695	0.306080	0.593049	5	
348	0.683983	0.281835	0.582732	5	
349	0.668412	0.281354	0.596917	5	
350	0.710288	0.274910	0.557952	5	
351	0.703868	0.266300	0.564101	5	
352	0.707403	0.267980	0.560723	5	
353	0.732924	0.270583	0.535712	5	
354	0.736283	0.261585	0.532333	5	
355	0.734897	0.256449	0.533730	5	
356	0.729516	0.261619	0.539119	5	
357	0.743151	0.259492	0.525356	5	
358	0.739241	0.255913	0.529339	5	
359	0.734946	0.252110	0.533681	5	
360	0.737105	0.262872	0.531502	5	

	Number of estimators	Training data R score	Test data R score
323	2	0.923554	0.833031
324	3	0.927470	0.836275
325	4	0.930456	0.817903
326	5	0.942257	0.789899
327	6	0.942747	0.805384
328	7	0.944724	0.820180
329	8	0.953341	0.827033
330	9	0.953504	0.817564
331	10	0.955658	0.842786
332	11	0.958452	0.838968
333	12	0.957914	0.841073
334	13	0.957074	0.856110
335	14	0.959941	0.858069
336	15	0.961529	0.857261
337	16	0.959930	0.854117
338	17	0.960592	0.862062
339	18	0.961693	0.859791
340	19	0.962845	0.857290
341	20	0.959537	0.858548
342	2	0.923554	0.833031
343	3	0.927470	0.836275
344	4	0.930456	0.817903
345	5	0.942257	0.789899
346	6	0.942747	0.805384
347	7	0.944724	0.820180
348	8	0.953341	0.827033
349	9	0.953504	0.817564
350	10	0.955658	0.842786
351	11	0.958452	0.838968
352	12	0.957914	0.841073
353	13	0.957074	0.856110
354	14	0.959941	0.858069
355	15	0.961529	0.857261
356	16	0.959930	0.854117
357	17	0.960592	0.862062
358	18	0.961693	0.859791
359	19	0.962845	0.857290
360	20	0.959537	0.858548

[25]:
```
df_ = pd.read_excel('../Data/Quality_BALB_3T3_42_.xlsx', sheet_name='RF')
df_ = df_[df_['Number of features'] == 6]
df_['Training data R score'] = handle_negative_corr(df_['Training data R^2␣
↪score'])
df_['Test data R score'] = handle_negative_corr(df_['Test data R^2 score'])
df_
```

[25]:

	Unnamed: 0	Correlation threshold	Training data R^2 score
342	342	0.51	0.869233
343	343	0.51	0.923683
344	344	0.51	0.934524
345	345	0.51	0.937457
346	346	0.51	0.929701
347	347	0.51	0.940844
348	348	0.51	0.945992
349	349	0.51	0.943862
350	350	0.51	0.940881
351	351	0.51	0.940214
352	352	0.51	0.939011
353	353	0.51	0.939990
354	354	0.51	0.941761
355	355	0.51	0.945607
356	356	0.51	0.946995
357	357	0.51	0.948830
358	358	0.51	0.946417
359	359	0.51	0.944971
360	360	0.51	0.943980

	Test data R^2 score	Training RMSE	Test RMSE	Number of features
342	0.716186	0.318598	0.424152	6
343	0.597886	0.243391	0.504870	6
344	0.681066	0.225443	0.449630	6
345	0.701507	0.220334	0.434983	6
346	0.675674	0.233598	0.453414	6
347	0.604822	0.214286	0.500497	6
348	0.648896	0.204749	0.471762	6
349	0.649432	0.208749	0.471401	6
350	0.665910	0.214219	0.460189	6
351	0.664213	0.215424	0.461356	6
352	0.683600	0.217581	0.447840	6
353	0.657975	0.215827	0.465622	6
354	0.616112	0.212619	0.493295	6
355	0.597845	0.205478	0.504895	6
356	0.570470	0.202840	0.521797	6
357	0.577984	0.199298	0.517213	6
358	0.591313	0.203942	0.508979	6
359	0.588724	0.206677	0.510589	6
360	0.591825	0.208528	0.508661	6

	Number of estimators	Training data R score	Test data R score
342	2	0.932327	0.846278
343	3	0.961084	0.773231
344	4	0.966708	0.825268
345	5	0.968224	0.837560

346	6	0.964210	0.821994
347	7	0.969971	0.777703
348	8	0.972621	0.805541
349	9	0.971525	0.805873
350	10	0.969990	0.816033
351	11	0.969646	0.814993
352	12	0.969026	0.826801
353	13	0.969531	0.811157
354	14	0.970444	0.784928
355	15	0.972423	0.773204
356	16	0.973136	0.755294
357	17	0.974079	0.760253
358	18	0.972840	0.768969
359	19	0.972096	0.767284
360	20	0.971586	0.769301

[ ]:

[26]:
```
df_ = pd.read_excel('../Data/Quality_LoVo_28_.xlsx', sheet_name='RF')
df_ = df_[df_['Number of features'] == 2]
df_['Training data R score'] = handle_negative_corr(df_['Training data R^2␣
↪score'])
df_['Test data R score'] = handle_negative_corr(df_['Test data R^2 score'])
df_
```

[26]:

	Unnamed: 0	Correlation threshold	Training data R^2 score	\
380	380	0.53	0.726452	
381	381	0.53	0.730699	
382	382	0.53	0.729826	
383	383	0.53	0.755753	
384	384	0.53	0.772016	
385	385	0.53	0.771714	
386	386	0.53	0.773504	
387	387	0.53	0.772194	
388	388	0.53	0.770797	
389	389	0.53	0.777861	
390	390	0.53	0.780498	
391	391	0.53	0.779444	
392	392	0.53	0.783158	
393	393	0.53	0.778982	
394	394	0.53	0.780691	
395	395	0.53	0.781139	
396	396	0.53	0.776384	
397	397	0.53	0.775154	
398	398	0.53	0.775951	
399	399	0.54	0.726452	
400	400	0.54	0.730699	

401	401	0.54	0.729826
402	402	0.54	0.755753
403	403	0.54	0.772016
404	404	0.54	0.771714
405	405	0.54	0.773504
406	406	0.54	0.772194
407	407	0.54	0.770797
408	408	0.54	0.777861
409	409	0.54	0.780498
410	410	0.54	0.779444
411	411	0.54	0.783158
412	412	0.54	0.778982
413	413	0.54	0.780691
414	414	0.54	0.781139
415	415	0.54	0.776384
416	416	0.54	0.775154
417	417	0.54	0.775951

	Test data R^2 score	Training RMSE	Test RMSE	Number of features \
380	0.267403	0.490898	0.573263	2
381	0.199666	0.487072	0.599180	2
382	0.237774	0.487861	0.584741	2
383	0.311228	0.463862	0.555852	2
384	0.352617	0.448153	0.538893	2
385	0.376864	0.448449	0.528704	2
386	0.352702	0.446688	0.538857	2
387	0.319981	0.447978	0.552309	2
388	0.336020	0.449349	0.545757	2
389	0.334133	0.442371	0.546531	2
390	0.299672	0.439737	0.560496	2
391	0.317294	0.440791	0.553399	2
392	0.339342	0.437064	0.544390	2
393	0.395078	0.441253	0.520920	2
394	0.403927	0.439544	0.517096	2
395	0.403071	0.439095	0.517467	2
396	0.431995	0.443838	0.504775	2
397	0.404526	0.445058	0.516836	2
398	0.380702	0.444269	0.527074	2
399	0.267403	0.490898	0.573263	2
400	0.199666	0.487072	0.599180	2
401	0.237774	0.487861	0.584741	2
402	0.311228	0.463862	0.555852	2
403	0.352617	0.448153	0.538893	2
404	0.376864	0.448449	0.528704	2
405	0.352702	0.446688	0.538857	2
406	0.319981	0.447978	0.552309	2
407	0.336020	0.449349	0.545757	2

408	0.334133	0.442371	0.546531	2
409	0.299672	0.439737	0.560496	2
410	0.317294	0.440791	0.553399	2
411	0.339342	0.437064	0.544390	2
412	0.395078	0.441253	0.520920	2
413	0.403927	0.439544	0.517096	2
414	0.403071	0.439095	0.517467	2
415	0.431995	0.443838	0.504775	2
416	0.404526	0.445058	0.516836	2
417	0.380702	0.444269	0.527074	2

	Number of estimators	Training data R score	Test data R score
380	2	0.852321	0.517110
381	3	0.854809	0.446839
382	4	0.854298	0.487620
383	5	0.869341	0.557879
384	6	0.878644	0.593815
385	7	0.878473	0.613892
386	8	0.879491	0.593887
387	9	0.878746	0.565669
388	10	0.877951	0.579672
389	11	0.881964	0.578042
390	12	0.883458	0.547423
391	13	0.882861	0.563289
392	14	0.884962	0.582531
393	15	0.882600	0.628552
394	16	0.883567	0.635553
395	17	0.883821	0.634879
396	18	0.881127	0.657263
397	19	0.880428	0.636024
398	20	0.880881	0.617010
399	2	0.852321	0.517110
400	3	0.854809	0.446839
401	4	0.854298	0.487620
402	5	0.869341	0.557879
403	6	0.878644	0.593815
404	7	0.878473	0.613892
405	8	0.879491	0.593887
406	9	0.878746	0.565669
407	10	0.877951	0.579672
408	11	0.881964	0.578042
409	12	0.883458	0.547423
410	13	0.882861	0.563289
411	14	0.884962	0.582531
412	15	0.882600	0.628552
413	16	0.883567	0.635553
414	17	0.883821	0.634879

```
415                   18           0.881127           0.657263
416                   19           0.880428           0.636024
417                   20           0.880881           0.617010
```

[ ]:

[27]:
```
df_ = pd.read_excel('../Data/Quality_LoVo_DX_42_.xlsx', sheet_name='RF')
df_ = df_[df_['Number of features'] == 5]
df_['Training data R score'] = handle_negative_corr(df_['Training data R^2␣
 ↪score'])
df_['Test data R score'] = handle_negative_corr(df_['Test data R^2 score'])
df_
```

[27]:
```
     Unnamed: 0  Correlation threshold  Training data R^2 score  \
570         570                   0.63                 0.849392
571         571                   0.63                 0.877695
572         572                   0.63                 0.886607
573         573                   0.63                 0.894969
574         574                   0.63                 0.904921
575         575                   0.63                 0.918333
576         576                   0.63                 0.916942
577         577                   0.63                 0.923954
578         578                   0.63                 0.918480
579         579                   0.63                 0.920255
580         580                   0.63                 0.916592
581         581                   0.63                 0.916344
582         582                   0.63                 0.923535
583         583                   0.63                 0.927928
584         584                   0.63                 0.931174
585         585                   0.63                 0.934467
586         586                   0.63                 0.935167
587         587                   0.63                 0.937938
588         588                   0.63                 0.937747

     Test data R^2 score  Training RMSE  Test RMSE  Number of features  \
570             0.290146       0.373186   0.608242                   5
571             0.320146       0.336297   0.595251                   5
572             0.387461       0.323813   0.565014                   5
573             0.467702       0.311645   0.526707                   5
574             0.516674       0.296513   0.501895                   5
575             0.530785       0.274806   0.494514                   5
576             0.617333       0.277136   0.446583                   5
577             0.602381       0.265179   0.455225                   5
578             0.586091       0.274558   0.464456                   5
579             0.563115       0.271552   0.477173                   5
580             0.592591       0.277719   0.460795                   5
581             0.612007       0.278132   0.449681                   5
```

582	0.624988	0.265908	0.442094	5
583	0.607313	0.258158	0.452393	5
584	0.616693	0.252278	0.446957	5
585	0.605643	0.246168	0.453354	5
586	0.608711	0.244850	0.451587	5
587	0.602155	0.239559	0.455354	5
588	0.598890	0.239929	0.457218	5

	Number of estimators	Training data R score	Test data R score
570	2	0.921625	0.538652
571	3	0.936854	0.565815
572	4	0.941598	0.622463
573	5	0.946028	0.683888
574	6	0.951273	0.718800
575	7	0.958297	0.728550
576	8	0.957571	0.785706
577	9	0.961225	0.776132
578	10	0.958374	0.765566
579	11	0.959299	0.750410
580	12	0.957388	0.769800
581	13	0.957258	0.782309
582	14	0.961007	0.790562
583	15	0.963290	0.779303
584	16	0.964973	0.785298
585	17	0.966678	0.778231
586	18	0.967040	0.780199
587	19	0.968472	0.775987
588	20	0.968373	0.773880

[ ]:

[28]:
```
df_ = pd.read_excel('../Data/Quality_MCF-7_15_.xlsx', sheet_name='RF')
df_ = df_[df_['Number of features'] == 4]
df_['Training data R score'] = handle_negative_corr(df_['Training data R^2␣
↪score'])
df_['Test data R score'] = handle_negative_corr(df_['Test data R^2 score'])
df_
```

[28]:

	Unnamed: 0	Correlation threshold	Training data R^2 score	\
323	323	0.50	0.823725	
324	324	0.50	0.819379	
325	325	0.50	0.861725	
326	326	0.50	0.861772	
327	327	0.50	0.876076	
328	328	0.50	0.887302	
329	329	0.50	0.891312	
330	330	0.50	0.885151	

331	331	0.50	0.897883
332	332	0.50	0.900694
333	333	0.50	0.898891
334	334	0.50	0.897355
335	335	0.50	0.903932
336	336	0.50	0.902161
337	337	0.50	0.897602
338	338	0.50	0.901345
339	339	0.50	0.903325
340	340	0.50	0.909007
341	341	0.50	0.910494
342	342	0.51	0.823725
343	343	0.51	0.819379
344	344	0.51	0.861725
345	345	0.51	0.861772
346	346	0.51	0.876076
347	347	0.51	0.887302
348	348	0.51	0.891312
349	349	0.51	0.885151
350	350	0.51	0.897883
351	351	0.51	0.900694
352	352	0.51	0.898891
353	353	0.51	0.897355
354	354	0.51	0.903932
355	355	0.51	0.902161
356	356	0.51	0.897602
357	357	0.51	0.901345
358	358	0.51	0.903325
359	359	0.51	0.909007
360	360	0.51	0.910494

	Test data R^2 score	Training RMSE	Test RMSE	Number of features	\
323	0.492083	0.390625	0.767939	4	
324	0.614284	0.395412	0.669213	4	
325	0.495651	0.345969	0.765237	4	
326	0.472072	0.345911	0.782920	4	
327	0.532895	0.327523	0.736440	4	
328	0.499692	0.312337	0.762165	4	
329	0.494367	0.306729	0.766211	4	
330	0.462619	0.315304	0.789899	4	
331	0.422784	0.297314	0.818652	4	
332	0.424585	0.293192	0.817374	4	
333	0.451898	0.295842	0.797739	4	
334	0.480492	0.298081	0.776652	4	
335	0.482197	0.288373	0.775377	4	
336	0.522720	0.291019	0.744418	4	
337	0.531393	0.297722	0.737623	4	

338	0.536501	0.292231	0.733593	4
339	0.532348	0.289282	0.736871	4
340	0.534079	0.280653	0.735506	4
341	0.539028	0.278349	0.731589	4
342	0.492083	0.390625	0.767939	4
343	0.614284	0.395412	0.669213	4
344	0.495651	0.345969	0.765237	4
345	0.472072	0.345911	0.782920	4
346	0.532895	0.327523	0.736440	4
347	0.499692	0.312337	0.762165	4
348	0.494367	0.306729	0.766211	4
349	0.462619	0.315304	0.789899	4
350	0.422784	0.297314	0.818652	4
351	0.424585	0.293192	0.817374	4
352	0.451898	0.295842	0.797739	4
353	0.480492	0.298081	0.776652	4
354	0.482197	0.288373	0.775377	4
355	0.522720	0.291019	0.744418	4
356	0.531393	0.297722	0.737623	4
357	0.536501	0.292231	0.733593	4
358	0.532348	0.289282	0.736871	4
359	0.534079	0.280653	0.735506	4
360	0.539028	0.278349	0.731589	4

	Number of estimators	Training data R score	Test data R score
323	2	0.907593	0.701486
324	3	0.905196	0.783763
325	4	0.928291	0.704025
326	5	0.928317	0.687075
327	6	0.935989	0.729997
328	7	0.941967	0.706889
329	8	0.944093	0.703112
330	9	0.940824	0.680161
331	10	0.947567	0.650219
332	11	0.949049	0.651602
333	12	0.948099	0.672234
334	13	0.947288	0.693176
335	14	0.950753	0.694404
336	15	0.949821	0.722994
337	16	0.947419	0.728967
338	17	0.949392	0.732462
339	18	0.950434	0.729622
340	19	0.953419	0.730807
341	20	0.954198	0.734186
342	2	0.907593	0.701486
343	3	0.905196	0.783763
344	4	0.928291	0.704025

345	5	0.928317	0.687075
346	6	0.935989	0.729997
347	7	0.941967	0.706889
348	8	0.944093	0.703112
349	9	0.940824	0.680161
350	10	0.947567	0.650219
351	11	0.949049	0.651602
352	12	0.948099	0.672234
353	13	0.947288	0.693176
354	14	0.950753	0.694404
355	15	0.949821	0.722994
356	16	0.947419	0.728967
357	17	0.949392	0.732462
358	18	0.950434	0.729622
359	19	0.953419	0.730807
360	20	0.954198	0.734186

[ ]:

```
```

Notebook

January 18, 2024

1 File 31

[1]:
```
import sys
from pathlib import Path
prediction_mode_path = Path("../module")
sys.path.append(prediction_mode_path.as_posix())
import models_creation as pred_model

import pandas as pd
import numpy as np

from sklearn.metrics import r2_score, mean_squared_error, mean_absolute_error
import math

import matplotlib.pyplot as plt
from sklearn.tree import plot_tree
#from sklearn import tree

from matplotlib import pyplot as plt

import joblib

from mordred import Calculator, descriptors
import mordred
from rdkit import Chem

import warnings
warnings.filterwarnings('ignore')
```

[2]:
```
load_prepared_data = pd.read_excel('../Data/Kolchicyna_machine_learning.xlsx')
load_prepared_data = load_prepared_data.drop(columns=['Unnamed: 0'])
```

[3]:
```
def is_morder_missing(x):
    return np.nan if type(x) == mordred.error.Missing or type(x) == mordred.
↪error.Error else x
```

[4]:
```
to_prediction = pd.read_excel('../Data/
↪Proposed_structures_with_AI_colchicyne_tanimoto_similarity_.xlsx')
mol_objs = [Chem.MolFromSmiles(smi) for smi in␣
↪to_prediction['AI_generated_SMILES']]

calculate_descriptors = False
if calculate_descriptors == True:
    calc = Calculator(descriptors, ignore_3D=True)
    molecular_descriptors = calc.pandas(mol_objs)
    molecular_descriptors['SMILES'] = to_prediction['AI_generated_SMILES']
    molecular_descriptors = molecular_descriptors.applymap(is_morder_missing)
    molecular_descriptors = molecular_descriptors.dropna(subset=['MDEO-12'])
    molecular_descriptors.to_excel('../Data/
↪New_structures_molecular_descriptors.xlsx')
else:
    molecular_descriptors = pd.read_excel('../Data/
↪New_structures_molecular_descriptors.xlsx')
```

[5]:
```
molecular_descriptors.head()
```

[5]:
```
   Unnamed: 0        ABC      ABCGG  nAcid  nBase    SpAbs_A   SpMax_A  \
0           0  23.120393  18.971182      0      1  39.868485  2.525781
1           1  23.120393  18.971182      0      1  39.868485  2.525781
2           2  23.827499  19.325995      0      1  41.234073  2.525784
3           3  25.908380  21.011762      0      1  44.265079  2.539061
4           4  23.948820  19.806455      0      1  40.972555  2.454223

   SpDiam_A     SpAD_A   SpMAD_A  …     TSRW10          MW       AMW  WPath  \
0  4.995962  39.868485  1.286080  …  76.110947  429.226371  6.923006   2466
1  4.995962  39.868485  1.286080  …  76.110947  426.251858  6.557721   2466
2  4.995966  41.234073  1.288565  …  77.202427  440.267508  6.474522   2760
3  5.010823  44.265079  1.301914  …  84.705631  465.237604  7.157502   3124
4  4.851643  40.972555  1.280392  …  81.511277  439.221954  7.200360   2754

   WPol  Zagreb1  Zagreb2   mZagreb1  mZagreb2  \
0    61      156      187  10.861111  7.388889
1    61      156      187  10.861111  7.388889
2    62      160      191  11.111111  7.638889
3    67      178      216  11.333333  7.916667
4    57      160      188  11.111111  7.555556

                                              SMILES
0  COC1=C2C3=CC=C(NC)C(=O)C=C3[C@H1](CCC2=CC(OC)=…
1  COC1=C2C3=CC=C(NC)C(=O)C=C3[C@H1](CCC2=CC(OC)=…
2  COC1=C2C3=CC=C(NC)C(=O)C=C3[C@H1](CCC2=CC(OC)=…
3  COC1=C2C3=CC=C(NC)C(=O)C=C3[C@H1](CCC2=CC(OC)=…
4  COC1=CC2=CC=C(NC)C(C=C2[C@@H1](N3C=C(N=N3)CN)C…
```

```
[5 rows x 1615 columns]
```

[6]:
```
molecular_descriptors.shape
```

[6]:
```
(1289, 1615)
```

2 A549

[7]:
```
target = 'A549'
data = load_prepared_data[list(load_prepared_data.columns)[0:-10]]
data[target] = load_prepared_data[load_prepared_data.columns[-5]]
data.head()
```

[7]:
```
      AATS0Z  AATS0are    AATS0d   AATS0dv      AATS0i     AATS0m    AATS0p  \
0  25.090909  6.046518  3.109091  6.662626  160.775045  99.075564  1.555512
1  24.448276  6.008422  3.051724  6.386973  161.021674  96.473315  1.538471
2  23.868852  5.974074  3.000000  6.138434  161.244045  94.127025  1.523105
3  23.868852  5.974074  3.032787  6.171220  161.244045  94.127025  1.523105
4  23.343750  5.942945  2.953125  5.913194  161.445569  92.000700  1.509180

    AATS0pe   AATS0s  AATS0se  …    piPC10     piPC2     piPC3     piPC4  \
0  6.126680  3.435416  7.629859  …  7.999655  4.266195  4.915408  5.516700
1  6.088791  3.330998  7.596891  …  8.018726  4.280132  4.922714  5.520711
2  6.054630  3.236850  7.567166  …  8.028799  4.293878  4.929967  5.524706
3  6.054630  3.257798  7.567166  …  8.037440  4.307438  4.929967  5.524706
4  6.023670  3.151529  7.540228  …  8.034892  4.307438  4.937168  5.528685

      piPC5     piPC6     piPC7     piPC8     piPC9      A549
0  6.098566  6.595759  6.979116  7.337313  7.681445  7.966576
1  6.103048  6.601209  6.985613  7.349442  7.695531  7.935542
2  6.105281  6.603923  6.989306  7.353932  7.704023  7.962574
3  6.107510  6.606629  6.992068  7.361425  7.709420  7.978811
4  6.107510  6.605277  6.991148  7.356489  7.707175  7.048177

[5 rows x 1212 columns]
```

[8]:
```
## create best model and make predictions to selected structures with␣
↪retransformation
```

[9]:
```
## The best model is: Random Forest (correlation_threshold = 0.51, 5 features,␣
↪17 estimators, random_state=15)
```

[11]:
```
model, train_r2_, test_r2_, hist1, hist2, target_column_name,␣
↪training_data_RMSE, test_data_RMSE  = pred_model.
↪prepare_data_and_create_model(molecular_descriptors_df=data,
```

```
                    correlation_threshold=0.51,
                    standardization=False,
                    model_type='RandomForestRegressor',
                    n_estimators_=17,
                    target_column_name = target,
                    random_state=15,
                    train_test_split_=True,
                    verbose=True)
print("R^2 score: " + str(r2_score(data[target], model.
 ↪predict(data[hist2['molecular descriptor name']]))))
print("Mean squared error: "+str(mean_squared_error(data[target], model.
 ↪predict(data[hist2['molecular descriptor name']]))))
print('Mean absolute error: ' + str(mean_absolute_error(data[target], model.
 ↪predict(data[hist2['molecular descriptor name']]))))
print("Root Mean Square Error: "+ str(math.
 ↪sqrt(mean_squared_error(data[target], model.predict(data[hist2['molecular␣
 ↪descriptor name']])))))
```

```
I am not doing standardization…
  molecular descriptor name
0                    AATS0Z
1                  AATS0are
2                    AATS0d
3                   AATS0dv
4                    AATS0i
  molecular descriptor name  corr_value
0                    AATS0Z   -0.022797
1                  AATS0are   -0.139064
2                    AATS0d    0.027592
3                   AATS0dv   -0.136049
4                    AATS0i    0.168958
  molecular descriptor name  corr_value  absolute correlation value
0                    AATS0Z   -0.022797                    0.022797
1                  AATS0are   -0.139064                    0.139064
2                    AATS0d    0.027592                    0.027592
3                   AATS0dv   -0.136049                    0.136049
4                    AATS0i    0.168958                    0.168958
    molecular descriptor name  corr_value  absolute correlation value
226                    AMID_O   -0.557717                    0.557717
```

```
505                EState_VSA5   -0.534748                    0.534748
791                    MDEO-12   -0.555724                    0.555724
921                      SaasC    0.513071                    0.513071
1091               VSA_EState5    0.526082                    0.526082
     molecular descriptor name  corr_value  absolute correlation value
226                     AMID_O   -0.557717                    0.557717
505                EState_VSA5   -0.534748                    0.534748
791                    MDEO-12   -0.555724                    0.555724
921                      SaasC    0.513071                    0.513071
1091               VSA_EState5    0.526082                    0.526082
The model used is: RandomForest…
Return the coefficient of determination of the prediction:
0.7431506499986544
R^2 score: 0.9227368657388829
Correlation coefficient: 0.960591935079034
Test data - unseen during training:
R^2 score: 0.7431506499986544
Correlation coefficient: 0.8620618597285548
[7.97245978 5.83683289 7.16157908 7.97048418 8.00084813 7.68188043
 8.04390248 7.33278952 8.03891061 7.50094682 8.25677164 8.11146208
 8.17909706 7.8550048  7.77872021 7.96616182 5.9492495  5.60331737]
102    7.920819
38     6.019997
8      5.996841
109    7.886057
11     7.962574
51     7.886057
88     8.075721
21     7.325139
82     7.978811
98     7.481486
58     7.677781
64     8.537602
74     8.142668
103    8.397940
91     8.958607
43     7.823909
25     4.886525
30     6.009661
Name: A549, dtype: float64
Training Root Mean Square Error: 0.2594924364766403
Testing Root Mean Square Error: 0.5253556041537444
R^2 score: 0.8906447986609566
Mean squared error: 0.09863565252265759
Mean absolute error: 0.20534614429271014
Root Mean Square Error: 0.31406313461254504
```

[12]:
```
# save
joblib.dump(model, "Random_forest/random_forest_model_17_estimators_A549.
 ↪joblib")
```

[12]: ['Random_forest/random_forest_model_17_estimators_A549.joblib']

[13]:
```
for x, decision_tree in enumerate(model.estimators_):
    plt.figure(figsize=(10,8), dpi=150)
    plot_tree(decision_tree, feature_names=list(hist2['molecular descriptor␣
 ↪name']),
              filled=True)
    plt.savefig('Random_forest/random_forest_17_'+str(x)+'_model_A549.
 ↪pdf',bbox_inches = "tight")
```

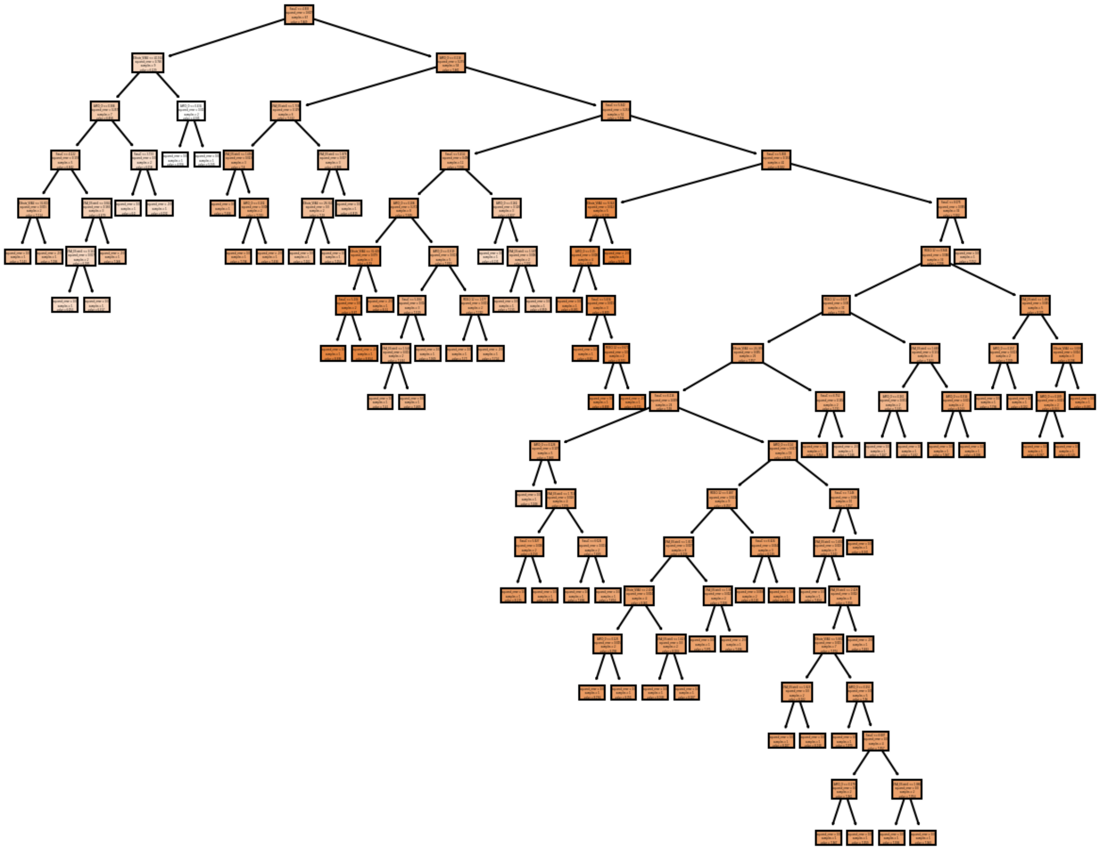

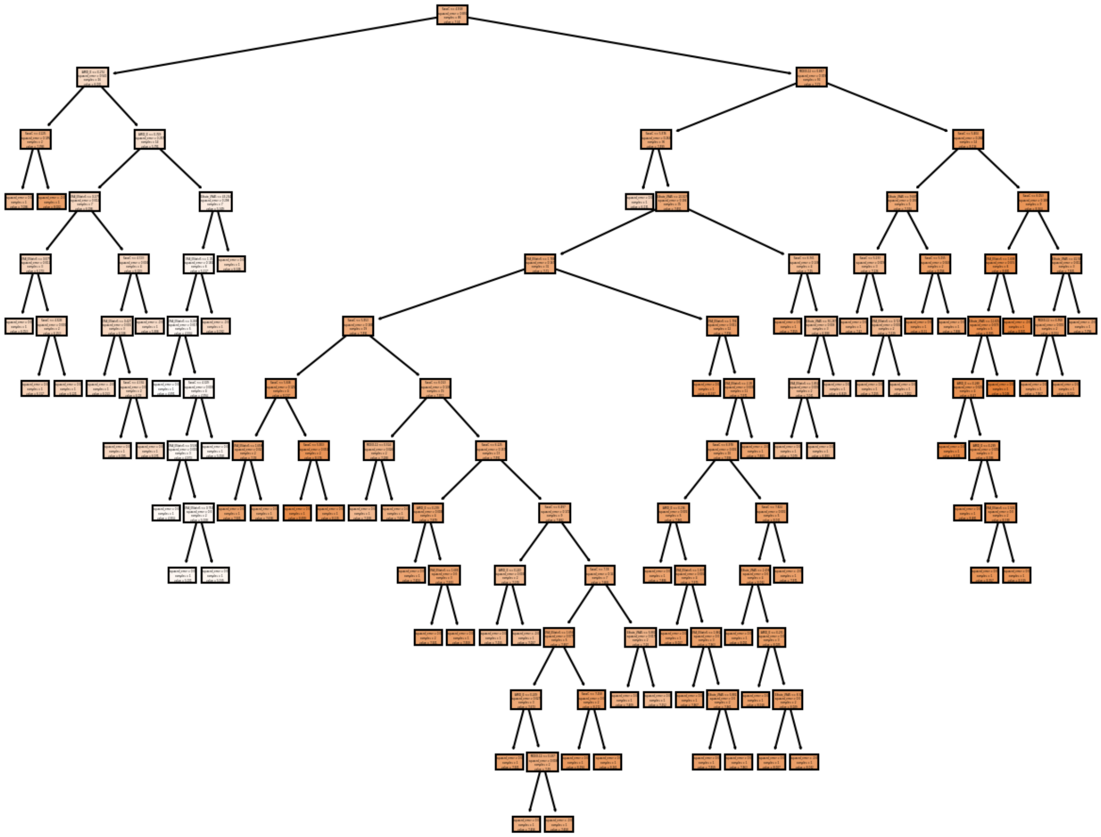

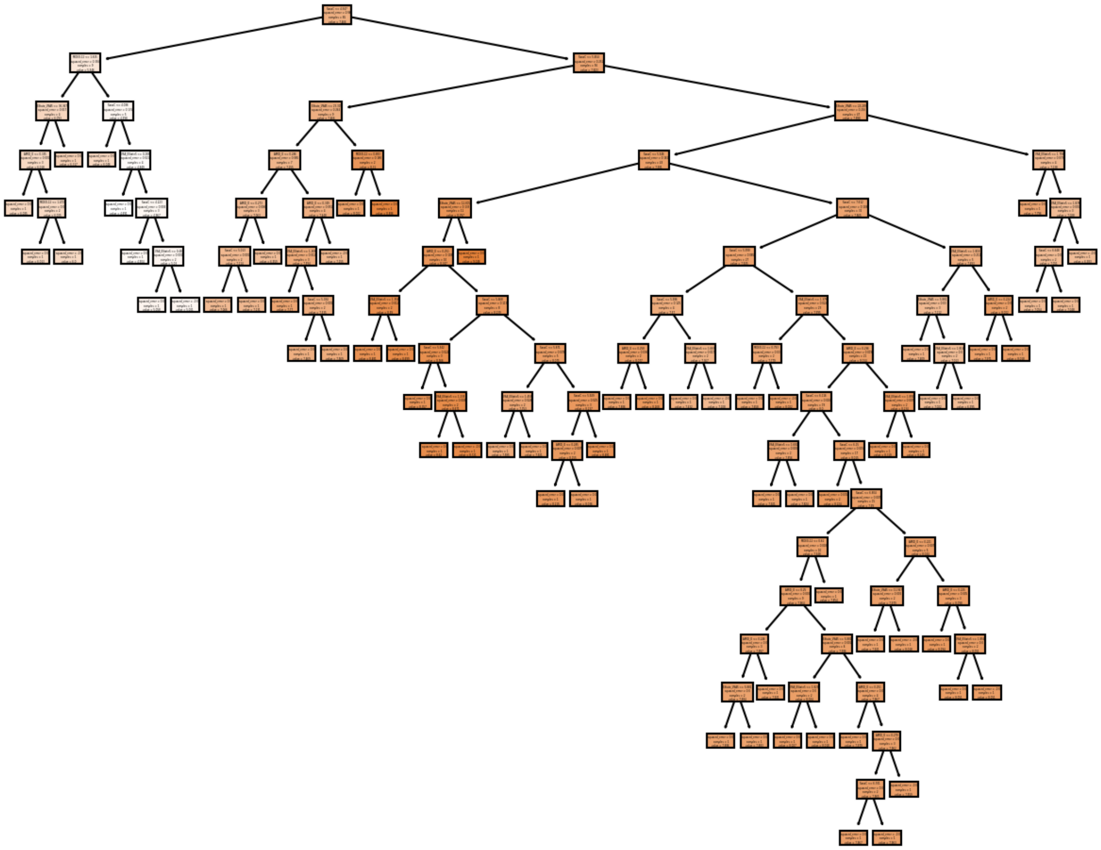

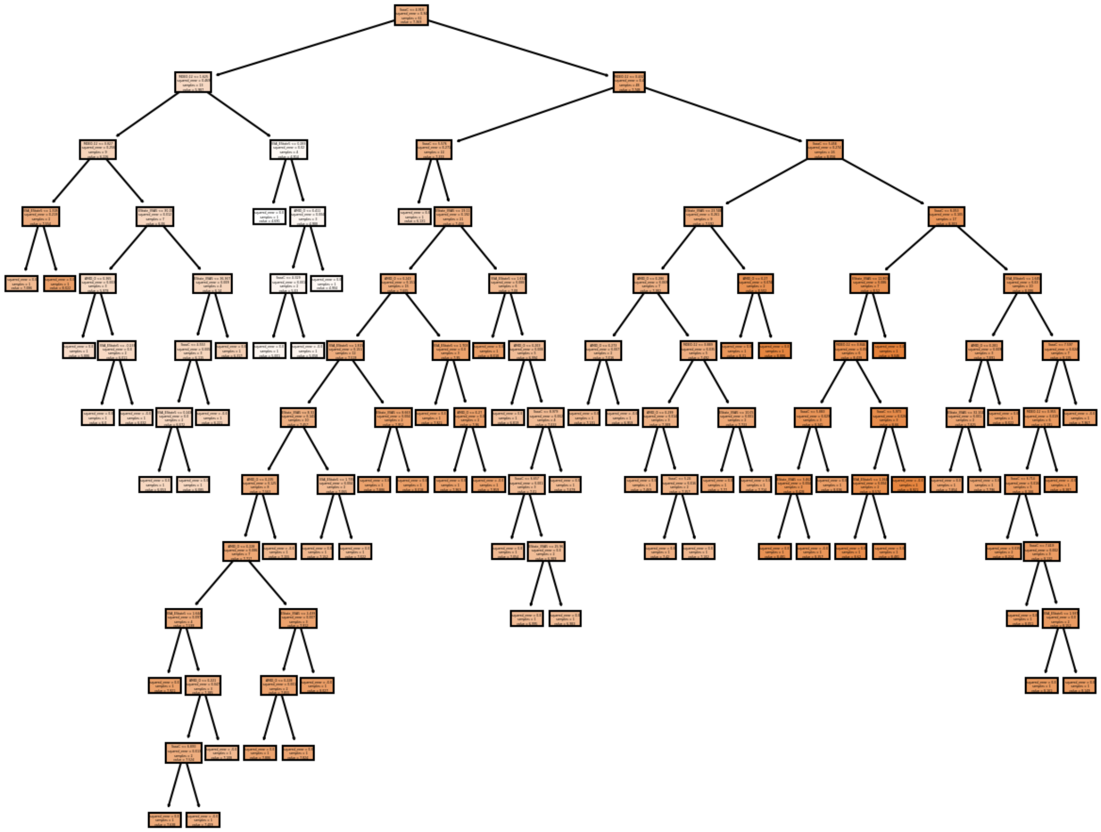

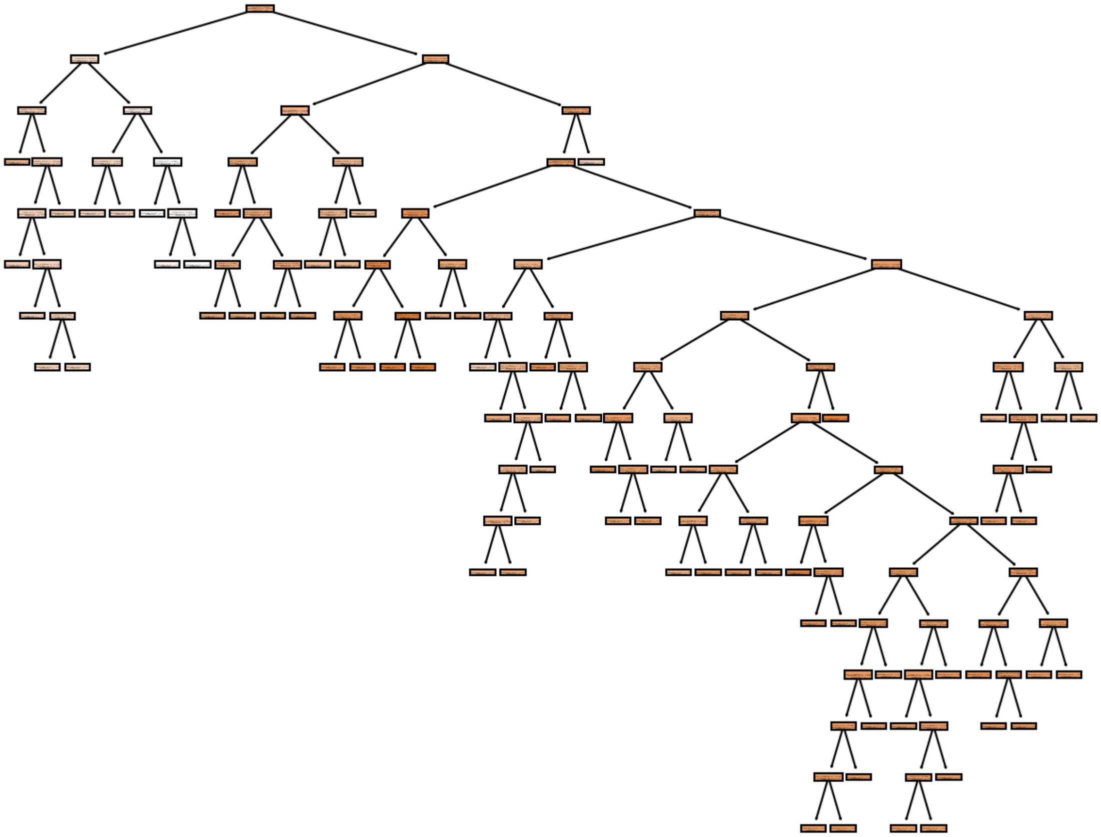

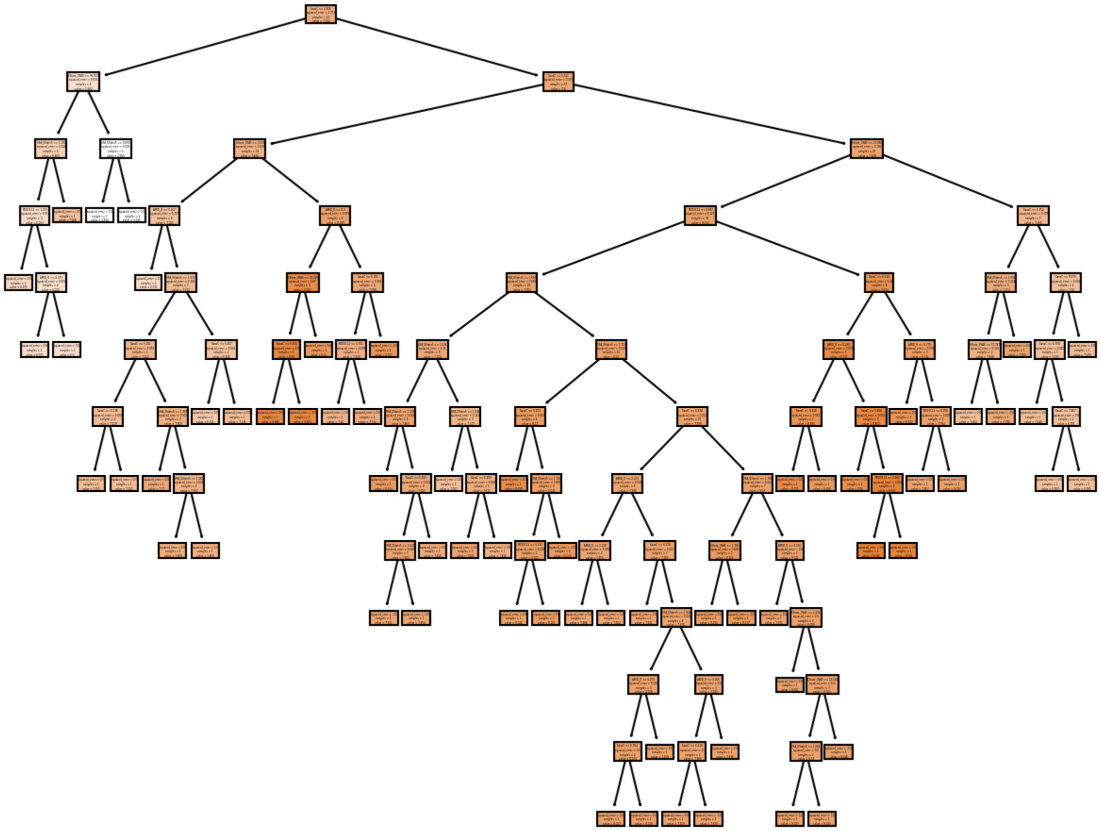

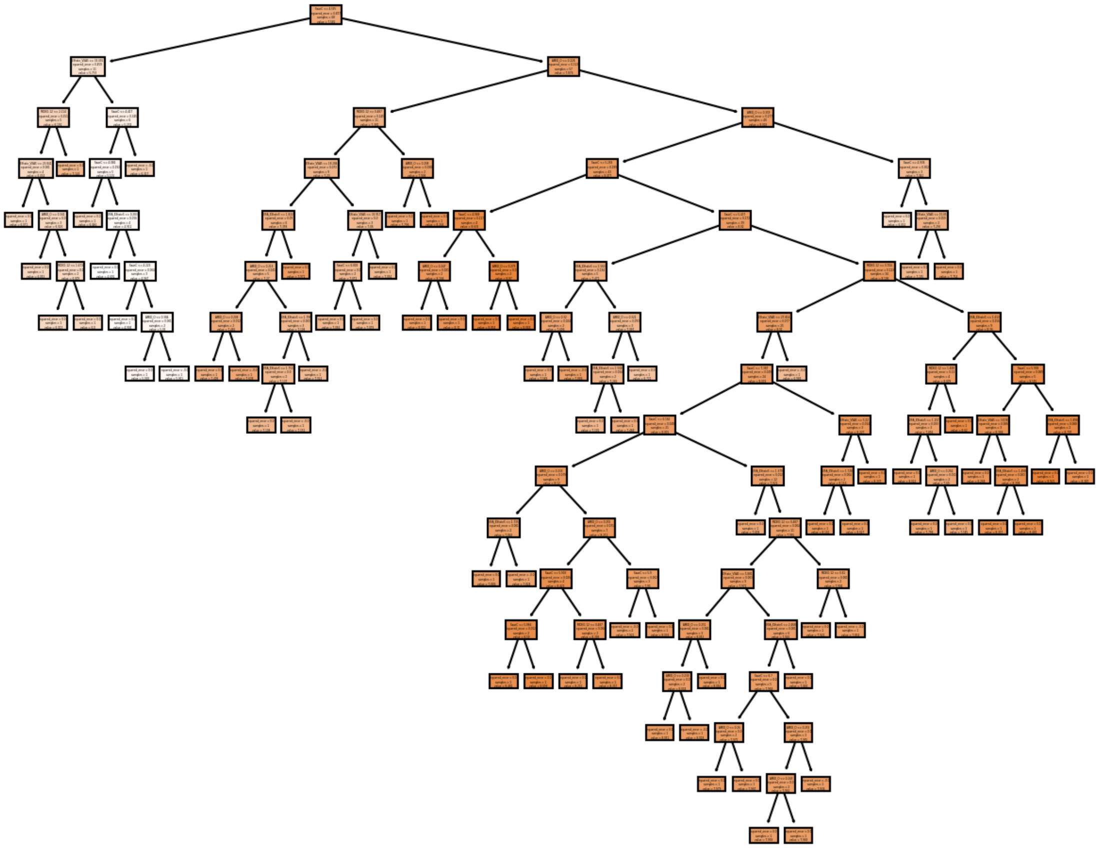

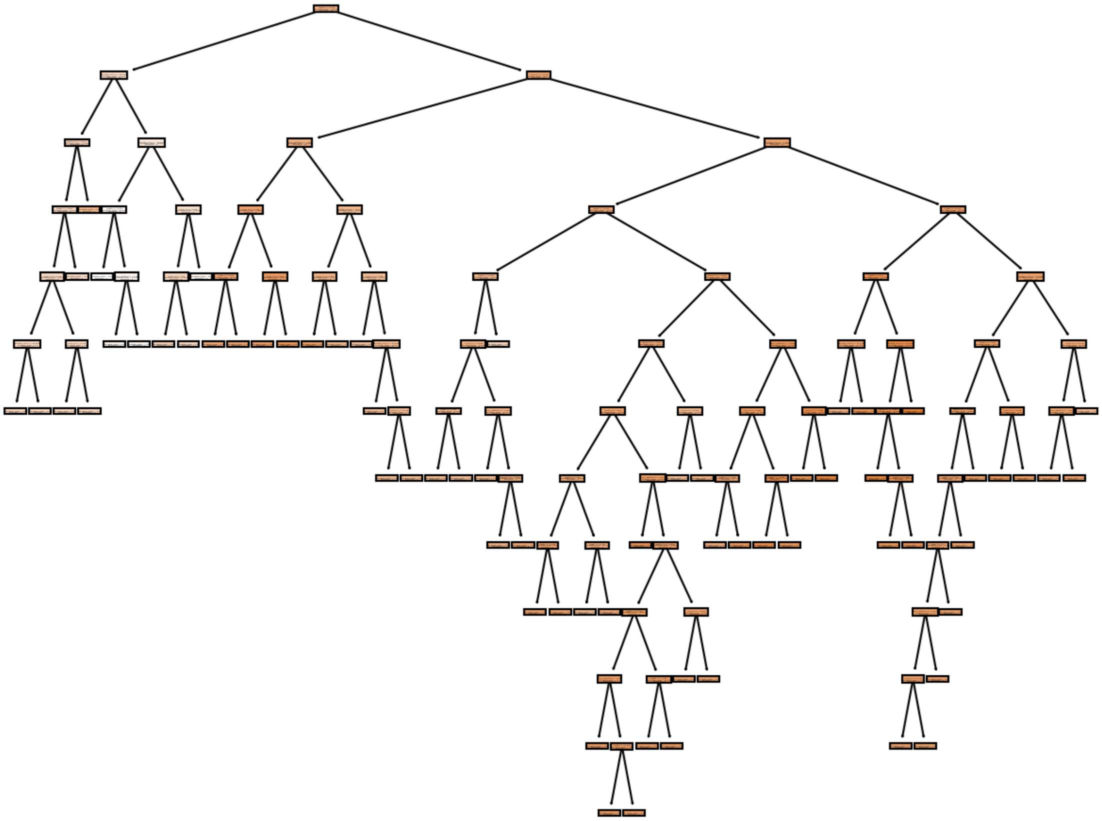

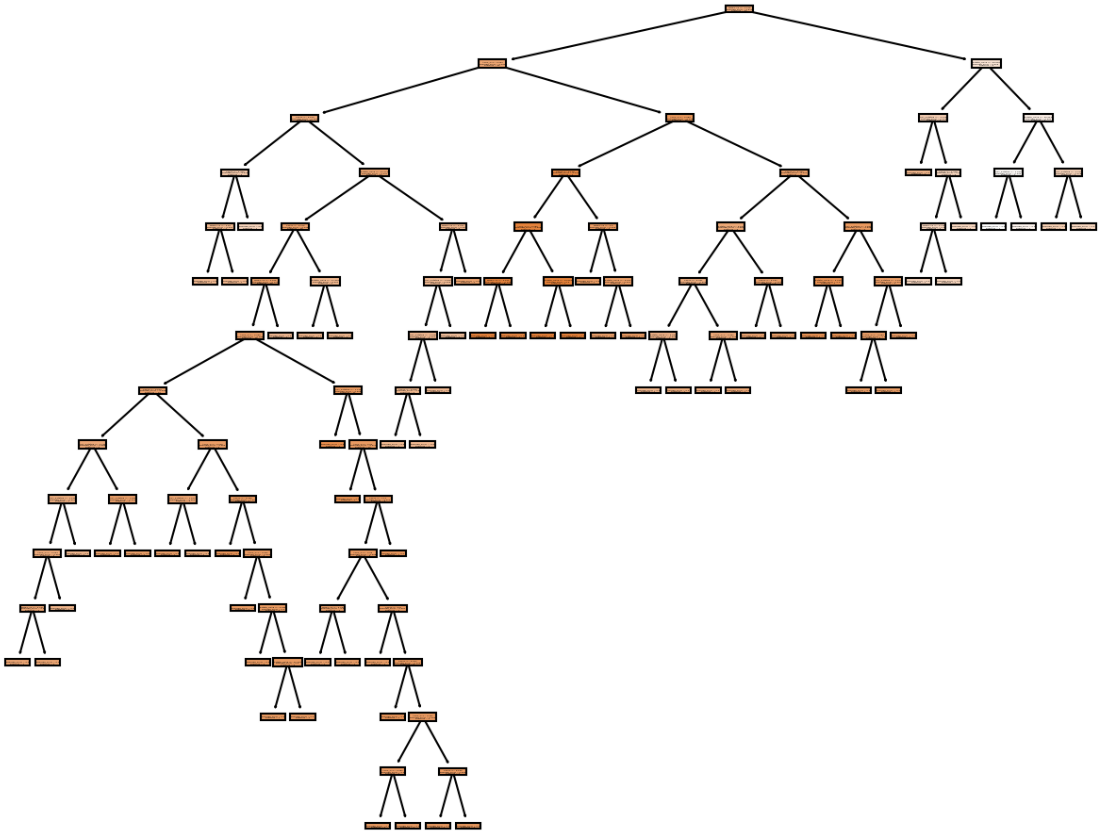

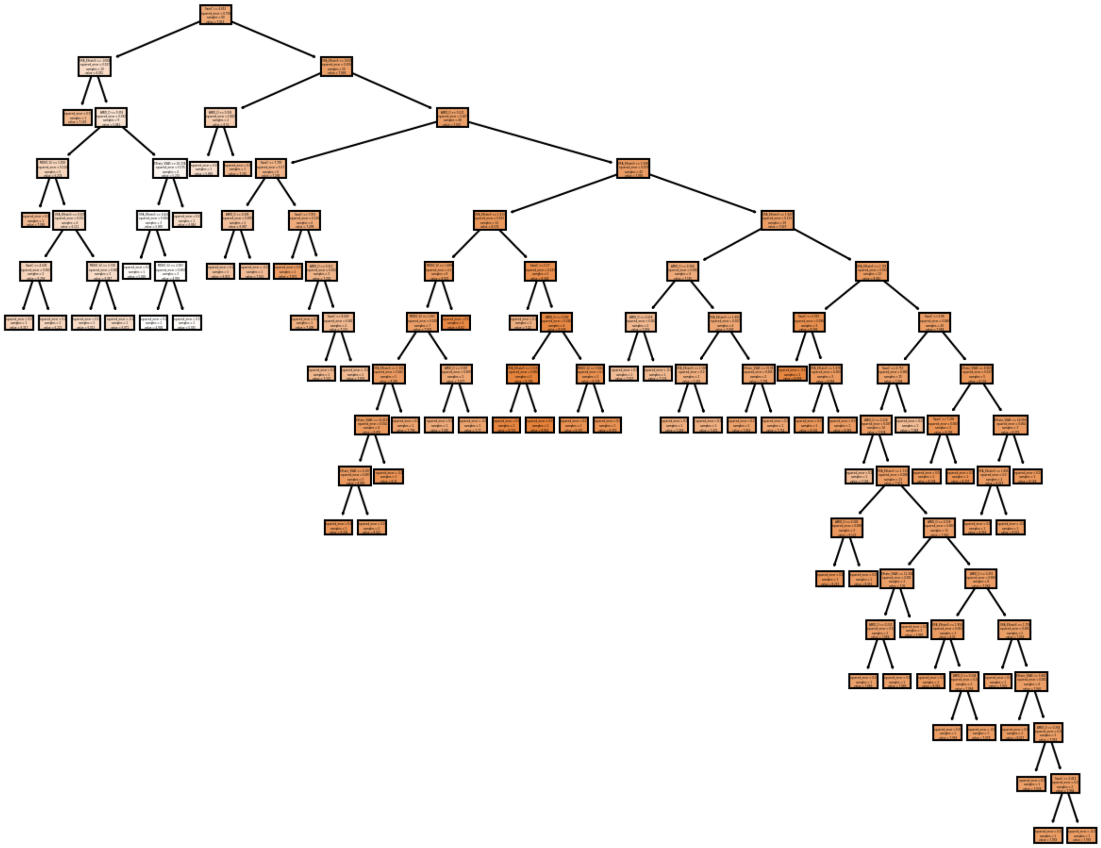

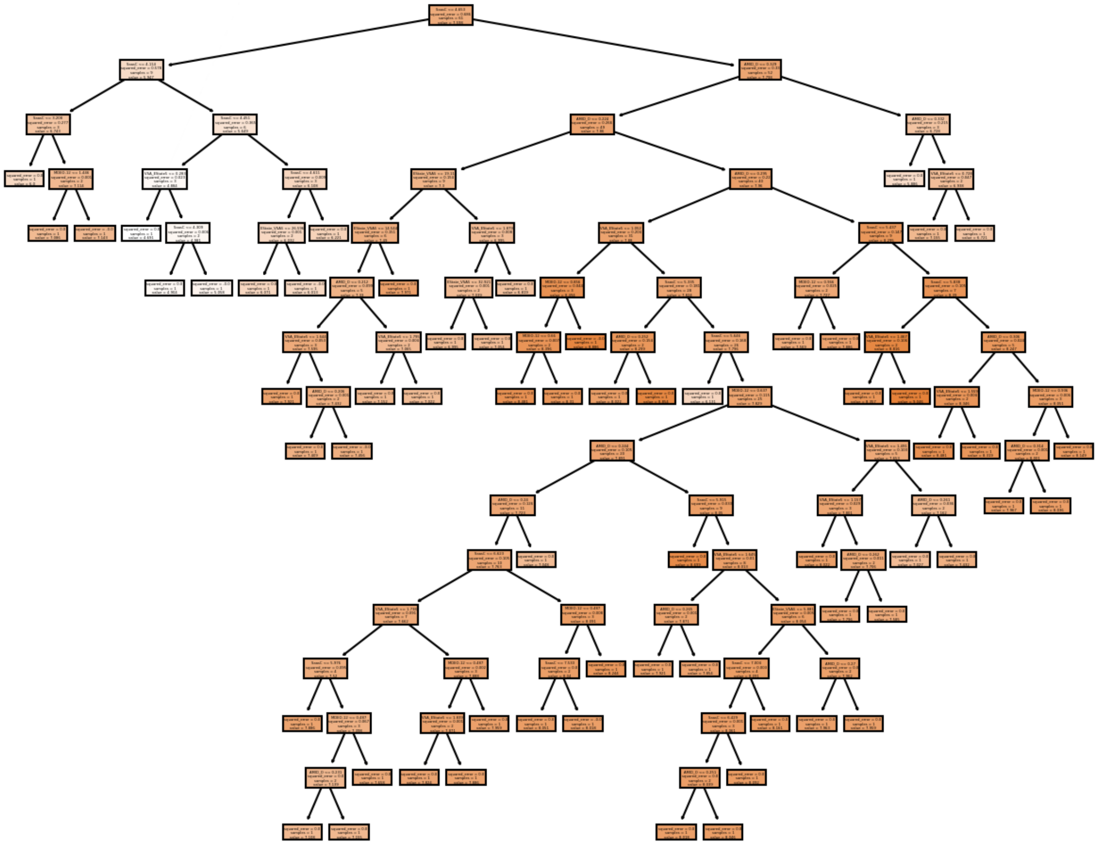

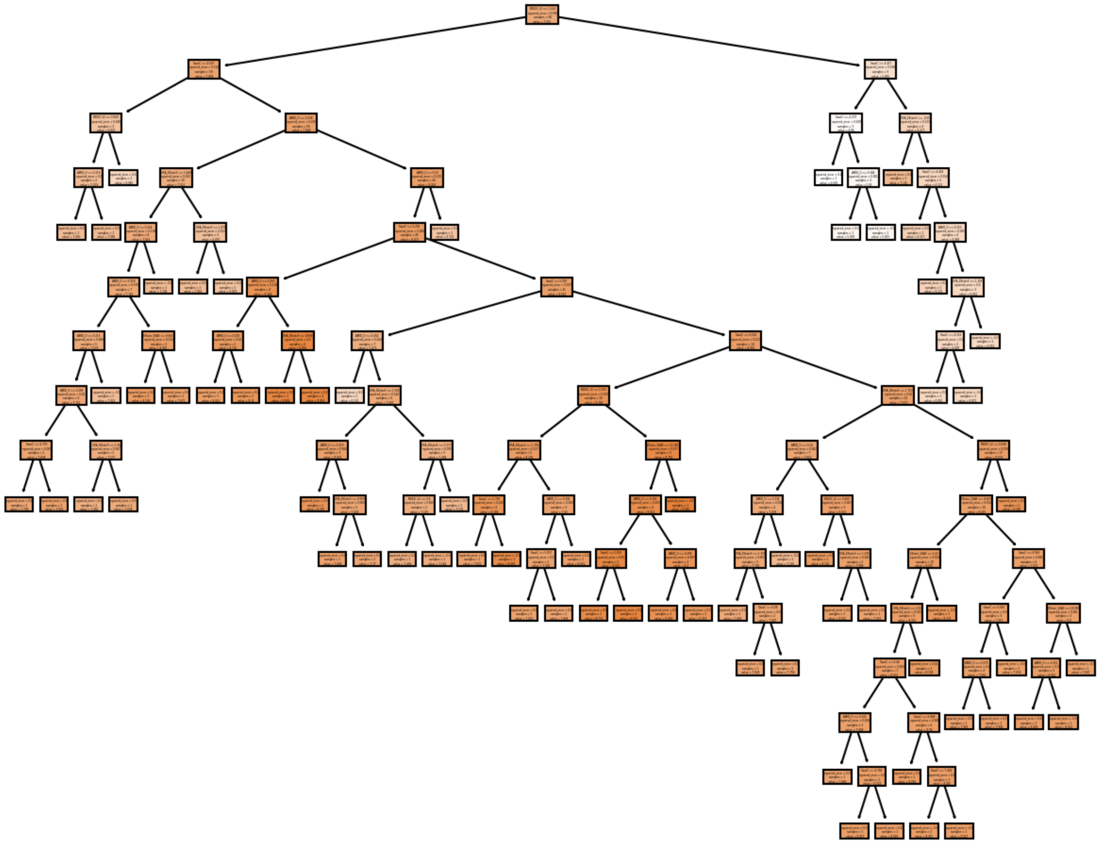

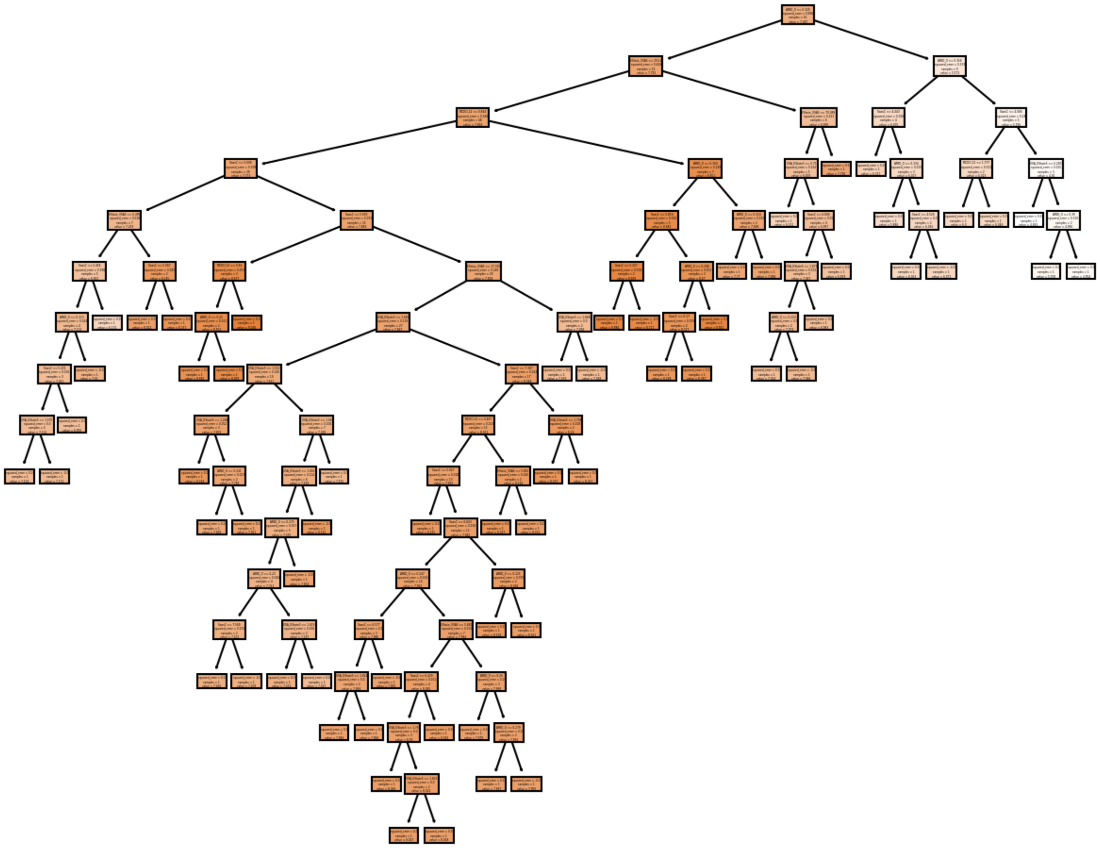

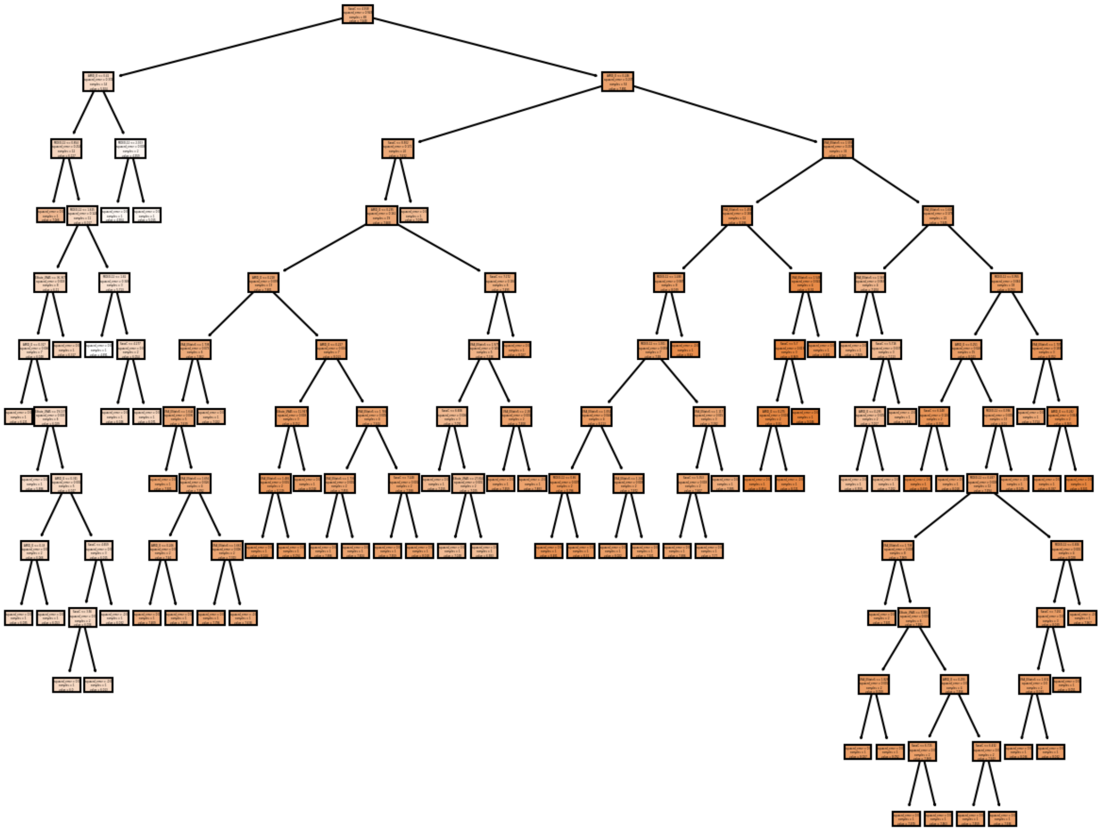

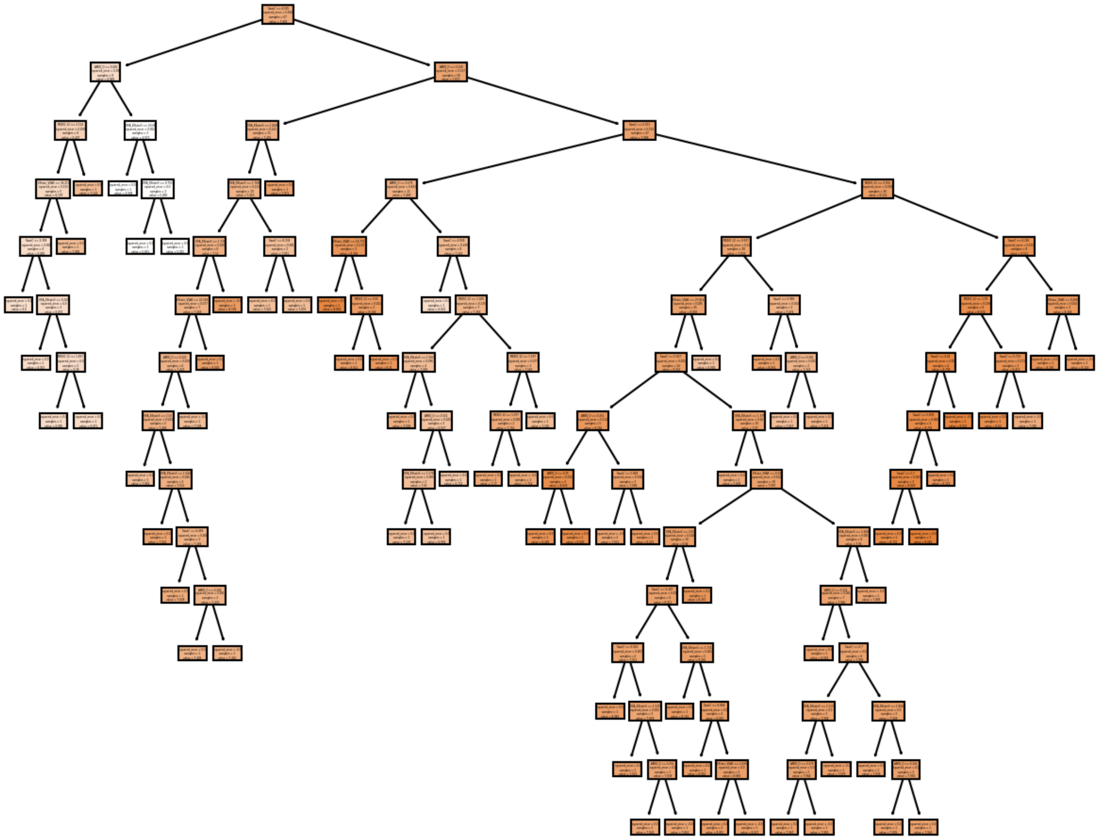

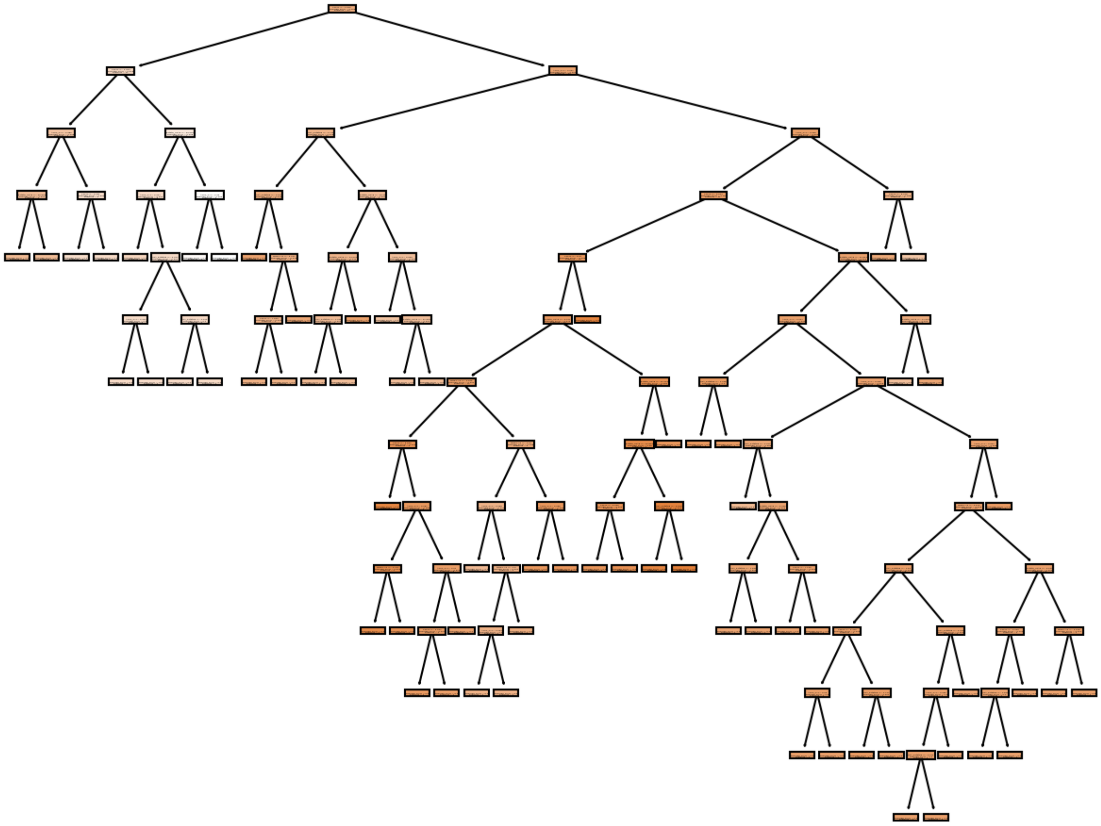

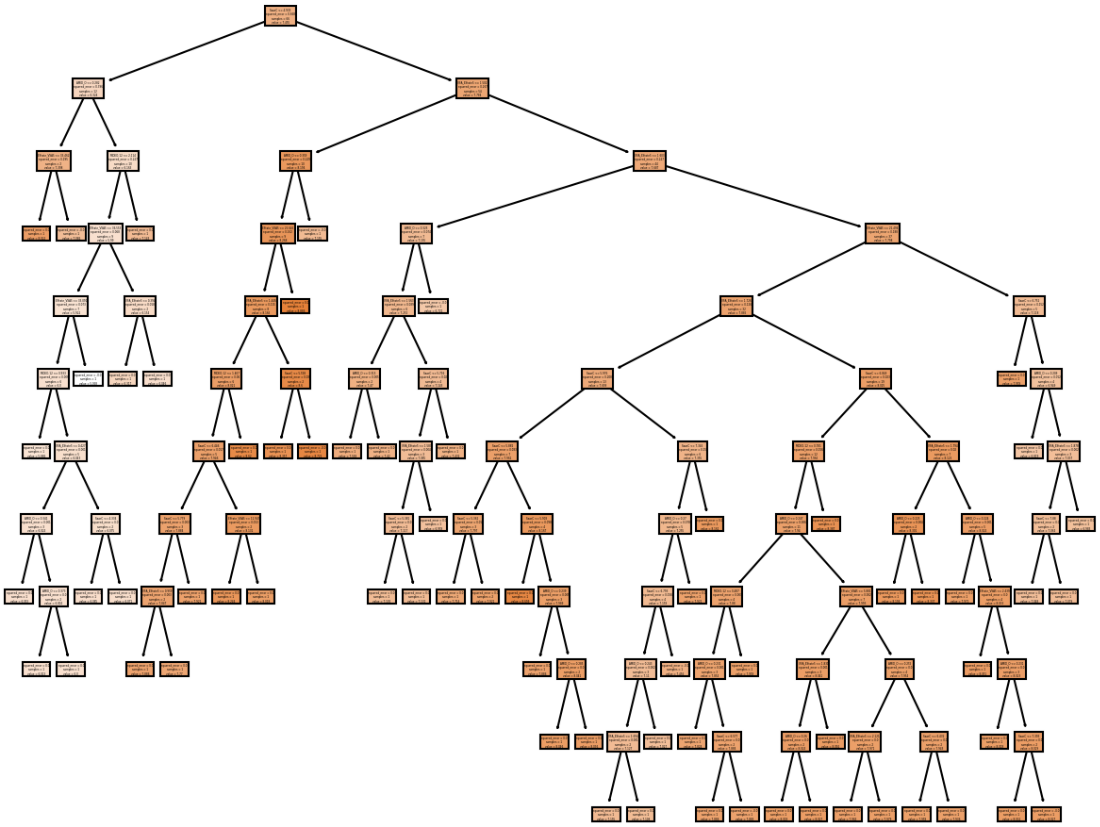

```
[14]: hist2
```

```
[14]:      molecular descriptor name  corr_value  absolute correlation value
226                       AMID_O   -0.557717                    0.557717
505                   EState_VSA5   -0.534748                    0.534748
791                      MDEO-12   -0.555724                    0.555724
921                        SaasC    0.513071                    0.513071
1091                  VSA_EState5    0.526082                    0.526082
```

```
[15]: predicted_activity = model.predict(molecular_descriptors[hist2['molecular␣
       ↪descriptor name']])
```

```
[16]: len(predicted_activity)
```

```
[16]: 1289
```

```
[17]: smiles = molecular_descriptors['SMILES'].to_list()
      save_to_df = pd.DataFrame(data=smiles, columns=['SMILES'])
```

[18]:
```
save_to_df['Predicted A549'] = predicted_activity
```

[19]:
```
save_to_df.head()
```

[19]:
```
                                              SMILES  Predicted A549
0  COC1=C2C3=CC=C(NC)C(=O)C=C3[C@H1](CCC2=CC(OC)=…        8.078406
1  COC1=C2C3=CC=C(NC)C(=O)C=C3[C@H1](CCC2=CC(OC)=…        7.867157
2  COC1=C2C3=CC=C(NC)C(=O)C=C3[C@H1](CCC2=CC(OC)=…        7.785028
3  COC1=C2C3=CC=C(NC)C(=O)C=C3[C@H1](CCC2=CC(OC)=…        7.742135
4  COC1=CC2=CC=C(NC)C(C=C2[C@@H1](N3C=C(N=N3)CN)C…        6.789342
```

3 BALB_3T3

[20]:
```
target = 'BALB/3T3'
data = load_prepared_data[list(load_prepared_data.columns)[0:-10]]
data[target] = load_prepared_data[load_prepared_data.columns[-1]]
data.head()
```

[20]:
```
     AATS0Z  AATS0are    AATS0d   AATS0dv      AATS0i     AATS0m    AATS0p  \
0  25.090909  6.046518  3.109091  6.662626  160.775045  99.075564  1.555512
1  24.448276  6.008422  3.051724  6.386973  161.021674  96.473315  1.538471
2  23.868852  5.974074  3.000000  6.138434  161.244045  94.127025  1.523105
3  23.868852  5.974074  3.032787  6.171220  161.244045  94.127025  1.523105
4  23.343750  5.942945  2.953125  5.913194  161.445569  92.000700  1.509180

   AATS0pe   AATS0s  AATS0se  …   piPC10    piPC2    piPC3    piPC4  \
0  6.126680  3.435416  7.629859  …  7.999655  4.266195  4.915408  5.516700
1  6.088791  3.330998  7.596891  …  8.018726  4.280132  4.922714  5.520711
2  6.054630  3.236850  7.567166  …  8.028799  4.293878  4.929967  5.524706
3  6.054630  3.257798  7.567166  …  8.037440  4.307438  4.929967  5.524706
4  6.023670  3.151529  7.540228  …  8.034892  4.307438  4.937168  5.528685

     piPC5    piPC6    piPC7    piPC8    piPC9  BALB/3T3
0  6.098566  6.595759  6.979116  7.337313  7.681445  7.991400
1  6.103048  6.601209  6.985613  7.349442  7.695531  7.844664
2  6.105281  6.603923  6.989306  7.353932  7.704023  7.931814
3  6.107510  6.606629  6.992068  7.361425  7.709420  7.958607
4  6.107510  6.605277  6.991148  7.356489  7.707175  7.002614

[5 rows x 1212 columns]
```

[21]:
```
## The best model is: Random Forest (correlation_threshold = 0.51, 6 features,␣
↪19 estimators, random_state=15)
```

[22]:
```
model, train_r2_, test_r2_, hist1, hist2, target_column_name,␣
↪training_data_RMSE, test_data_RMSE  = pred_model.
↪prepare_data_and_create_model(molecular_descriptors_df=data,
```

```
                    correlation_threshold=0.51,
                    standardization=False,
                    model_type='RandomForestRegressor',
                    n_estimators_=19,
                    target_column_name = target,
                    random_state=15,
                    train_test_split_=True,
                    verbose=True)
print("R^2 score: " + str(r2_score(data[target], model.
 ↪predict(data[hist2['molecular descriptor name']]))))
print("Mean squared error: "+str(mean_squared_error(data[target], model.
 ↪predict(data[hist2['molecular descriptor name']]))))
print('Mean absolute error: ' + str(mean_absolute_error(data[target], model.
 ↪predict(data[hist2['molecular descriptor name']]))))
print("Root Mean Square Error: "+ str(math.
 ↪sqrt(mean_squared_error(data[target], model.predict(data[hist2['molecular
 ↪descriptor name']])))))
```

```
I am not doing standardization…
  molecular descriptor name
0                    AATS0Z
1                  AATS0are
2                    AATS0d
3                   AATS0dv
4                    AATS0i
  molecular descriptor name  corr_value
0                    AATS0Z   -0.020817
1                  AATS0are   -0.148257
2                    AATS0d    0.022999
3                   AATS0dv   -0.137980
4                    AATS0i    0.133257
  molecular descriptor name  corr_value  absolute correlation value
0                    AATS0Z   -0.020817                    0.020817
1                  AATS0are   -0.148257                    0.148257
2                    AATS0d    0.022999                    0.022999
3                   AATS0dv   -0.137980                    0.137980
4                    AATS0i    0.133257                    0.133257
    molecular descriptor name  corr_value  absolute correlation value
226                    AMID_O   -0.546843                    0.546843
```

```
505              EState_VSA5   -0.588780                    0.588780
556                   GATS2c    0.511671                    0.511671
791                  MDEO-12   -0.582747                    0.582747
851                    NdssC   -0.526783                    0.526783
    molecular descriptor name  corr_value  absolute correlation value
226                   AMID_O   -0.546843                    0.546843
505              EState_VSA5   -0.588780                    0.588780
556                   GATS2c    0.511671                    0.511671
791                  MDEO-12   -0.582747                    0.582747
851                    NdssC   -0.526783                    0.526783
The model used is: RandomForest…
Return the coefficient of determination of the prediction:
0.7470132420088307
R^2 score: 0.9324873059163808
Correlation coefficient: 0.9656538230216772
Test data - unseen during training:
R^2 score: 0.7470132420088307
Correlation coefficient: 0.8642992780332693
[7.8032879  5.96743537 7.05720098 7.99770833 8.01718256 7.37593994
 7.99139143 7.21584713 8.01638597 7.67277552 7.18454636 7.91178639
 7.95499438 7.90669765 7.43673091 7.92913317 5.9970933  5.56800923]
102    7.698970
38     5.986741
8      5.984515
109    7.886057
11     7.962574
51     7.698970
88     8.229148
21     7.298432
82     8.065502
98     7.376751
58     7.602060
64     8.060481
74     7.853872
103    7.853872
91     8.346787
43     7.619789
25     4.989276
30     6.085657
Name: BALB/3T3, dtype: float64
Training Root Mean Square Error: 0.22547718186980922
Testing Root Mean Square Error: 0.46260702873007903
R^2 score: 0.9018620203956247
Mean squared error: 0.07531475506692918
Mean absolute error: 0.18296175995878303
Root Mean Square Error: 0.2744353385898565
```

[23]:
```
# save
joblib.dump(model, "Random_forest/random_forest_model_19_estimators_BALB_3T3.
↪joblib")
```

[23]: ['Random_forest/random_forest_model_19_estimators_BALB_3T3.joblib']

[24]:
```
for x, decision_tree in enumerate(model.estimators_):
    plt.figure(figsize=(10,8), dpi=150)
    plot_tree(decision_tree, feature_names=list(hist2['molecular descriptor␣
↪name']),
              filled=True)
    plt.savefig('Random_forest/random_forest_19_'+str(x)+'_model_BALB_3T3.
↪pdf',bbox_inches = "tight")
```

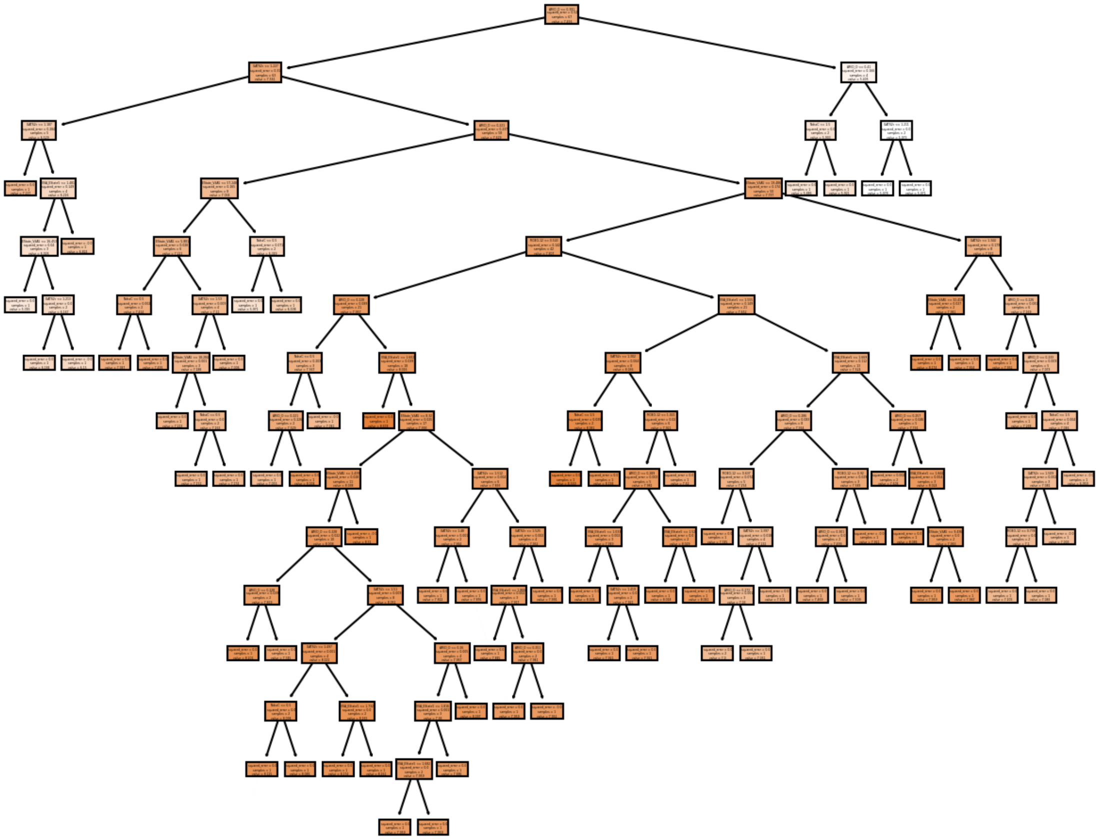

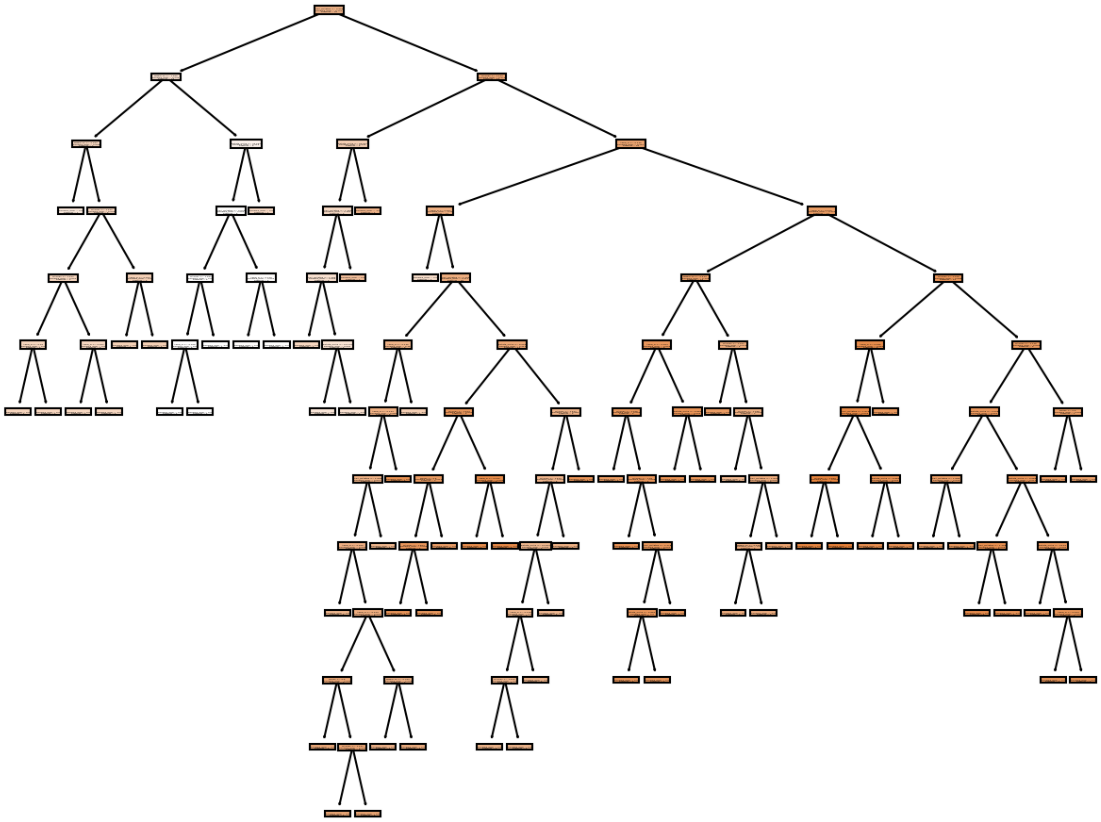

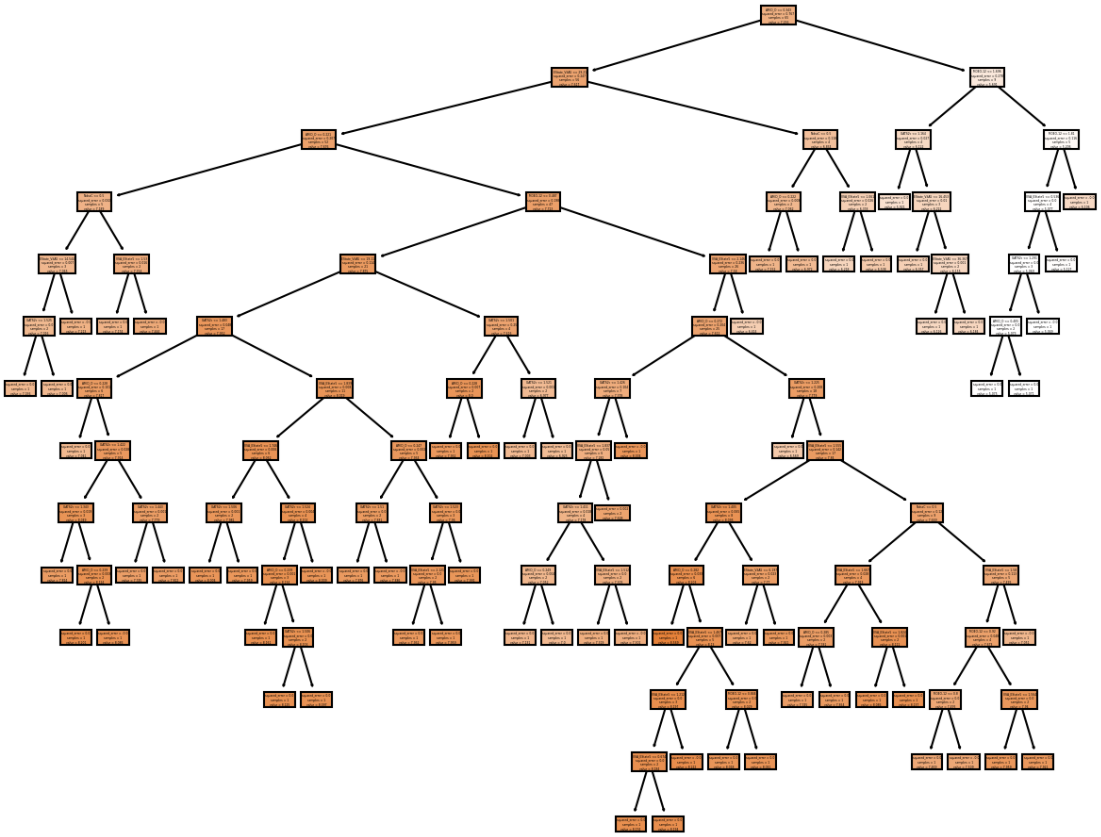

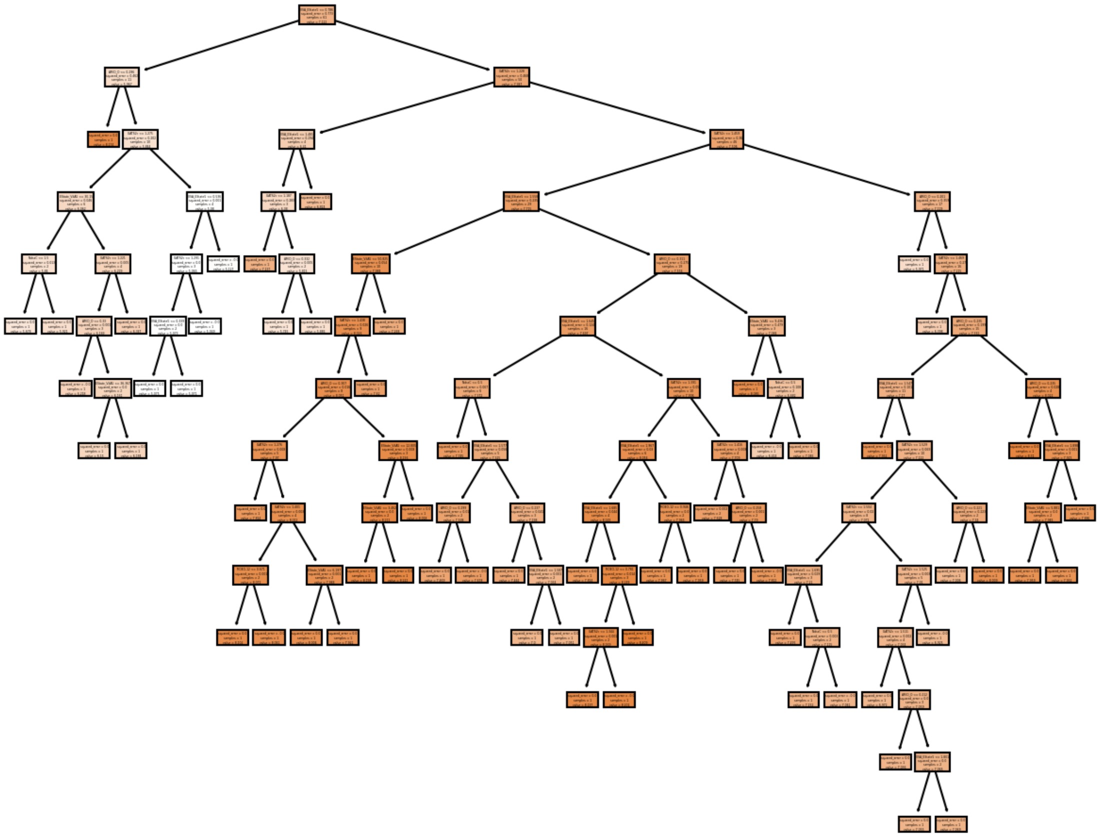

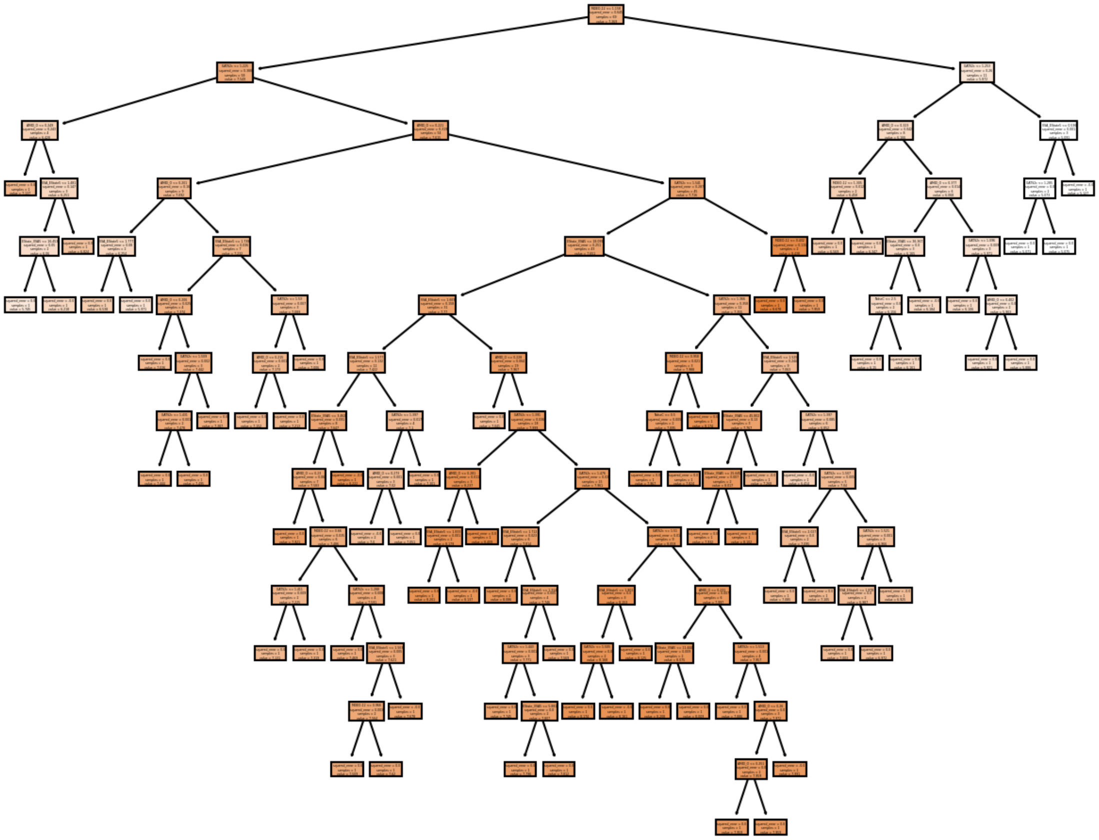

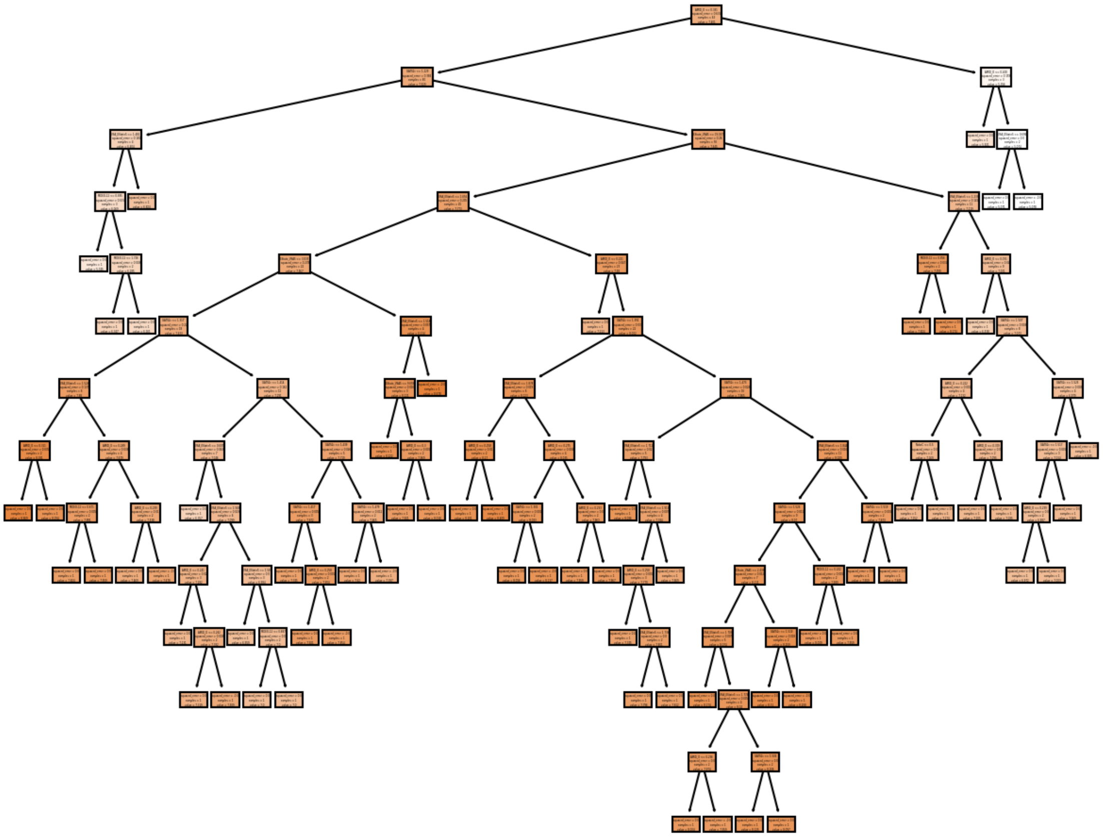

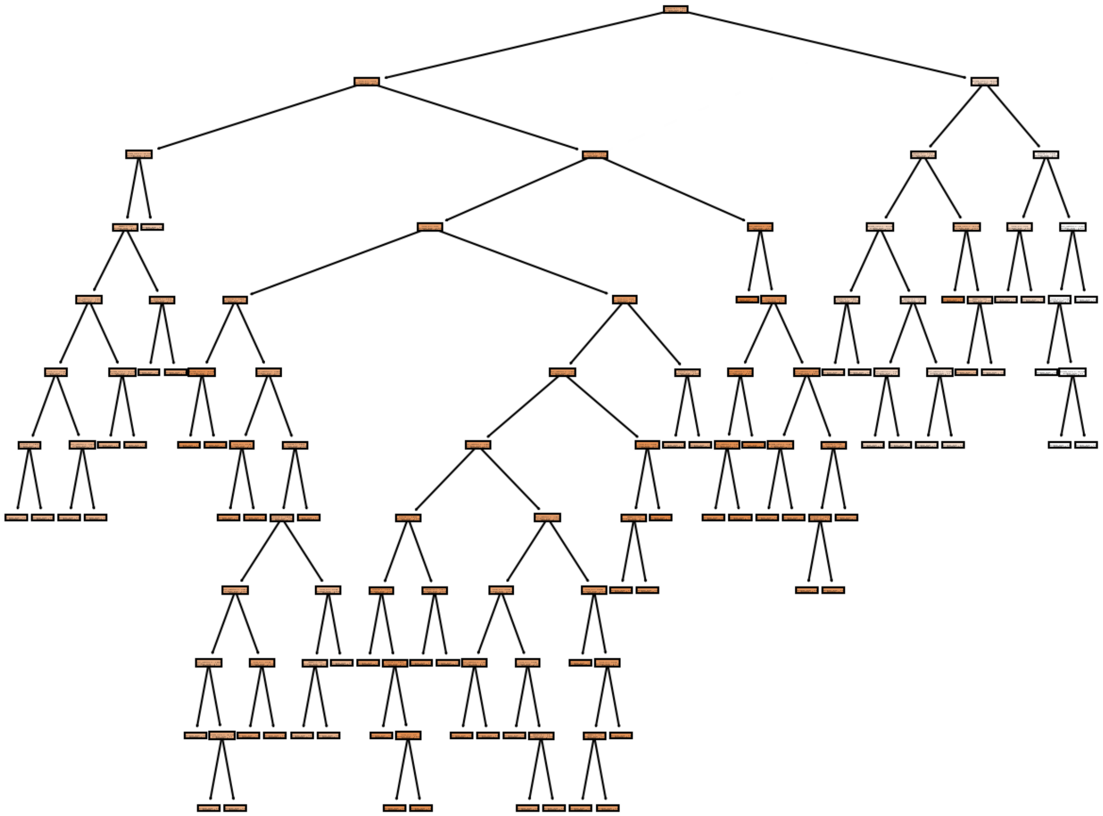

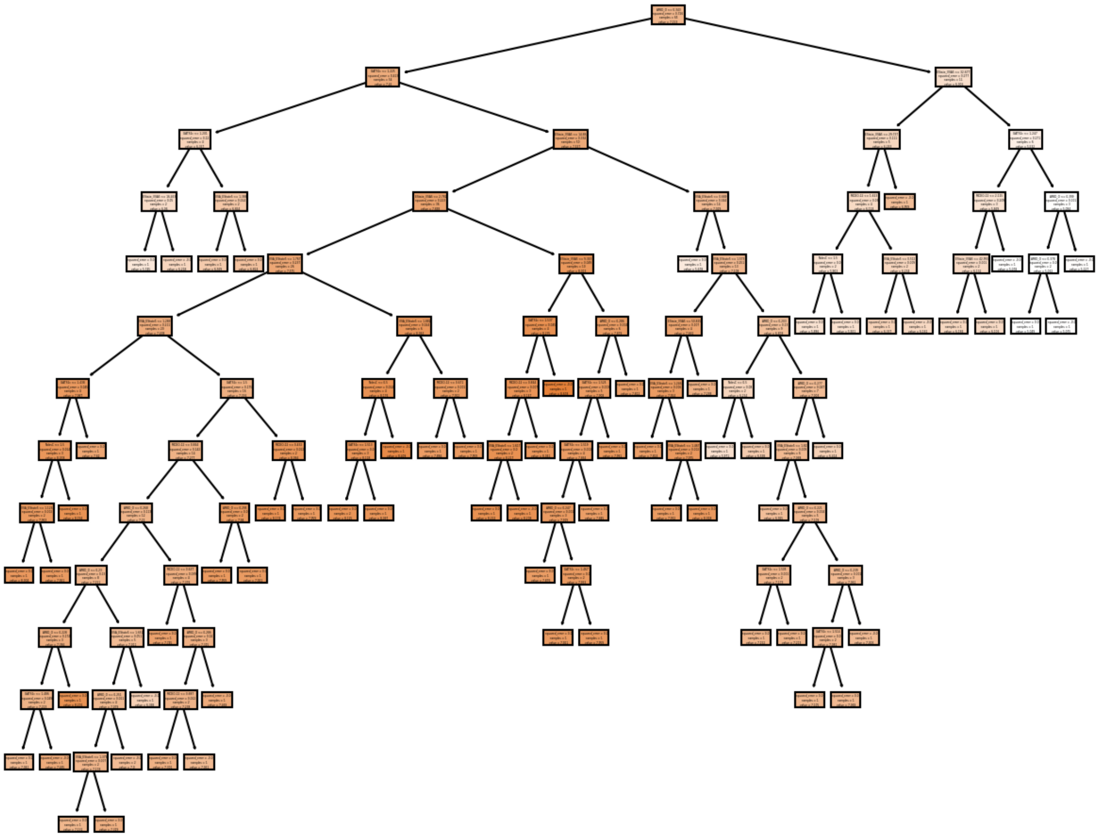

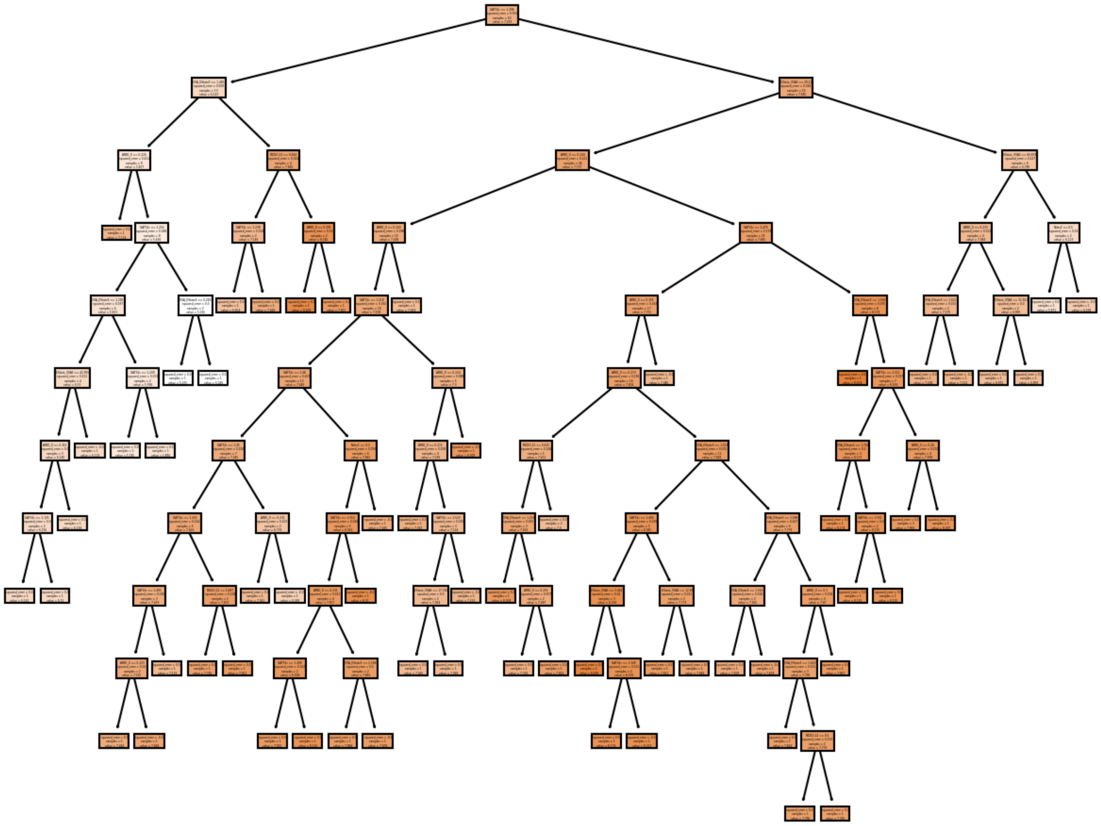

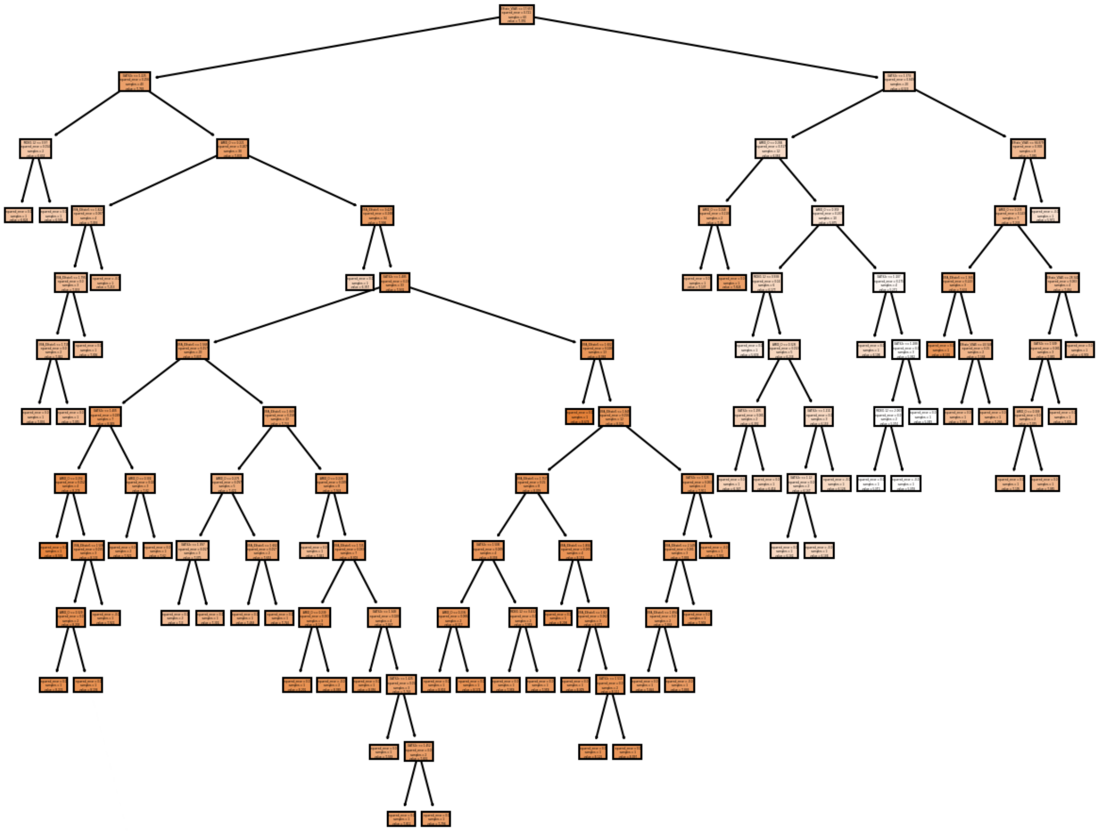

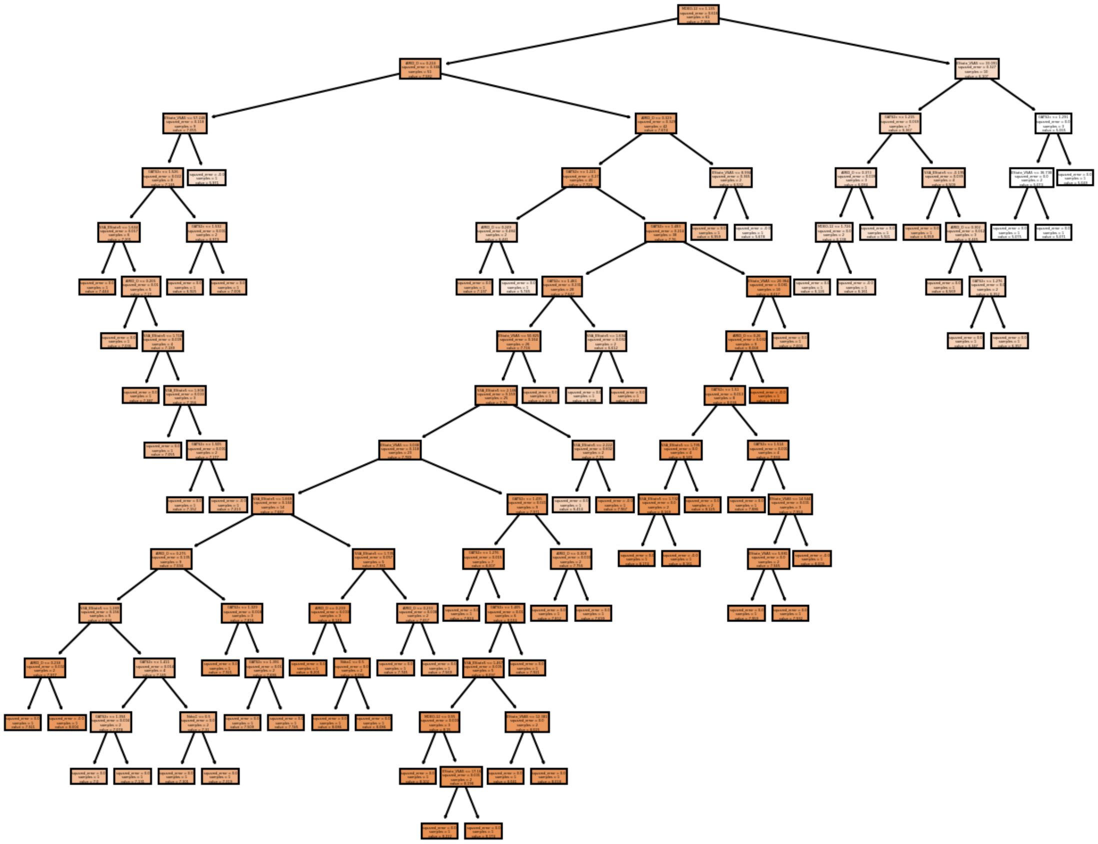

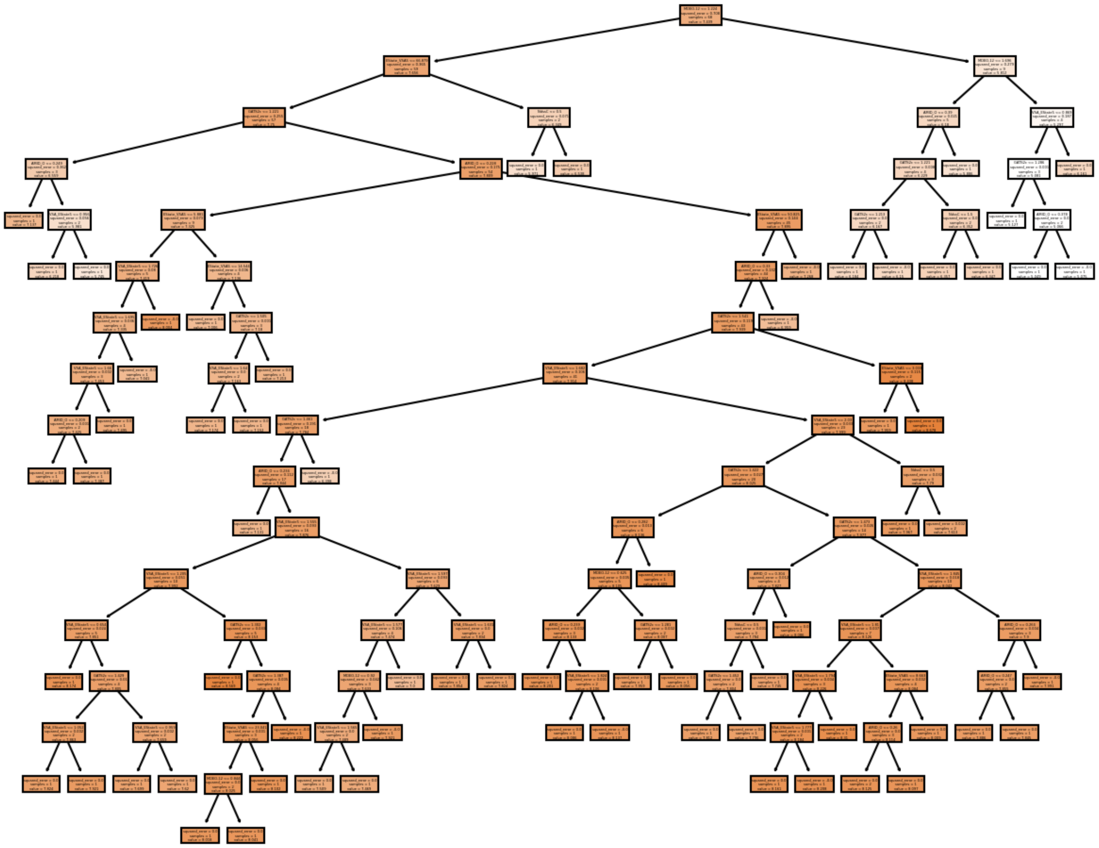

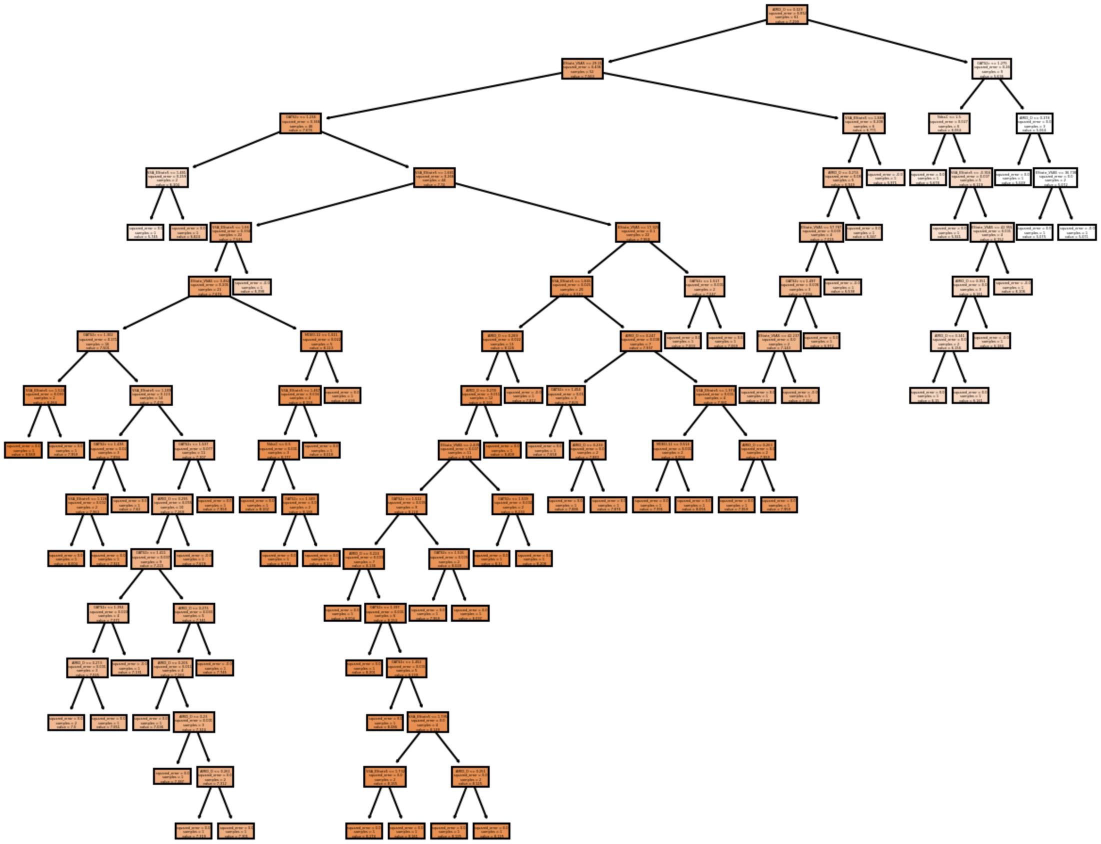

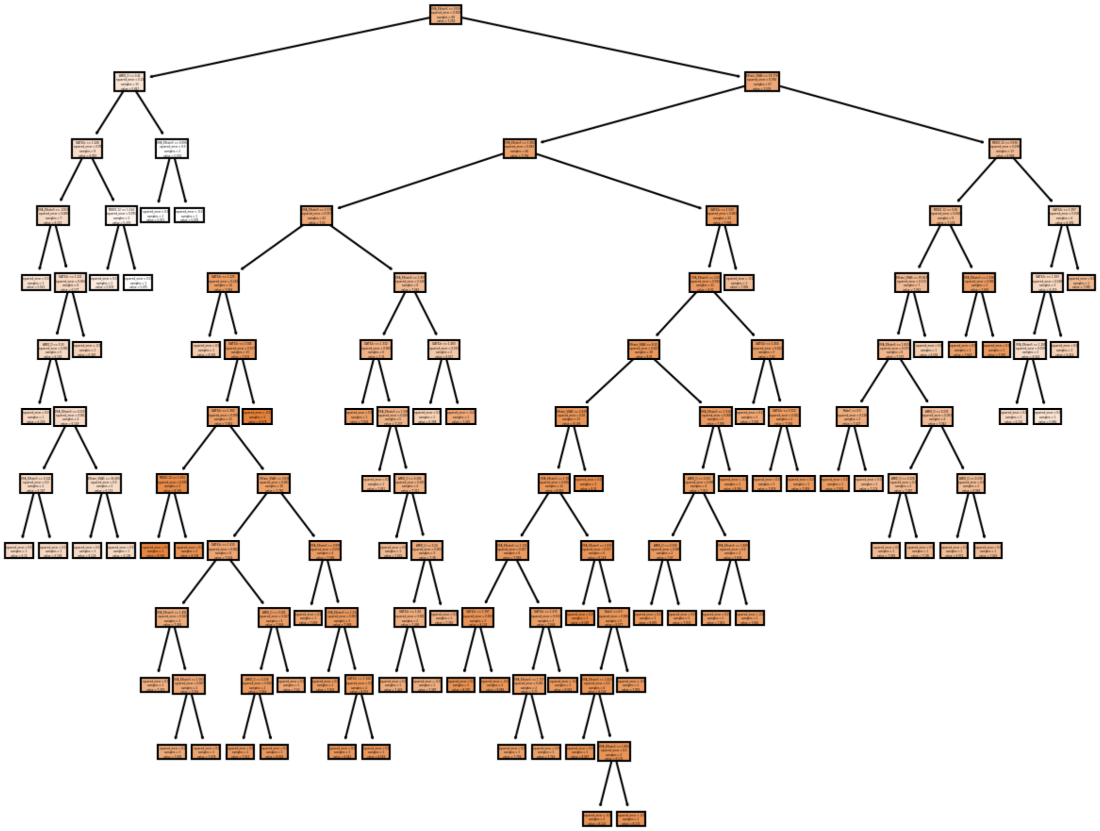

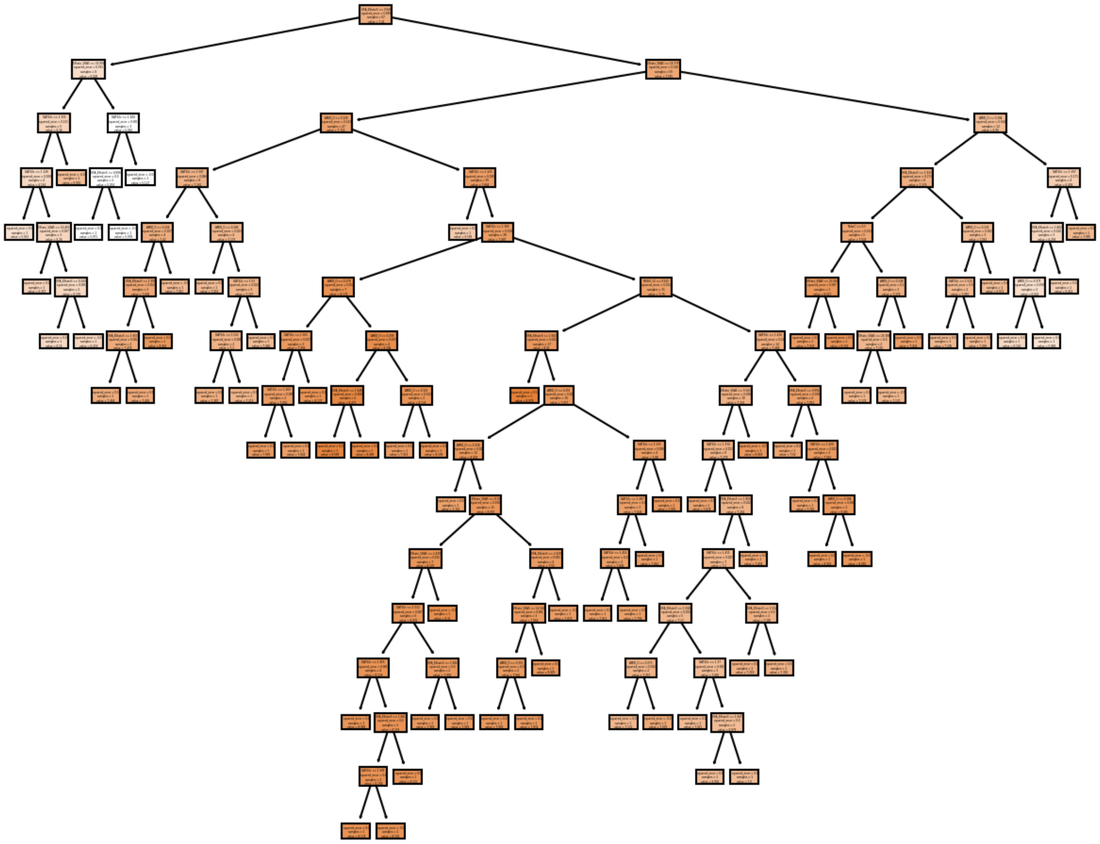

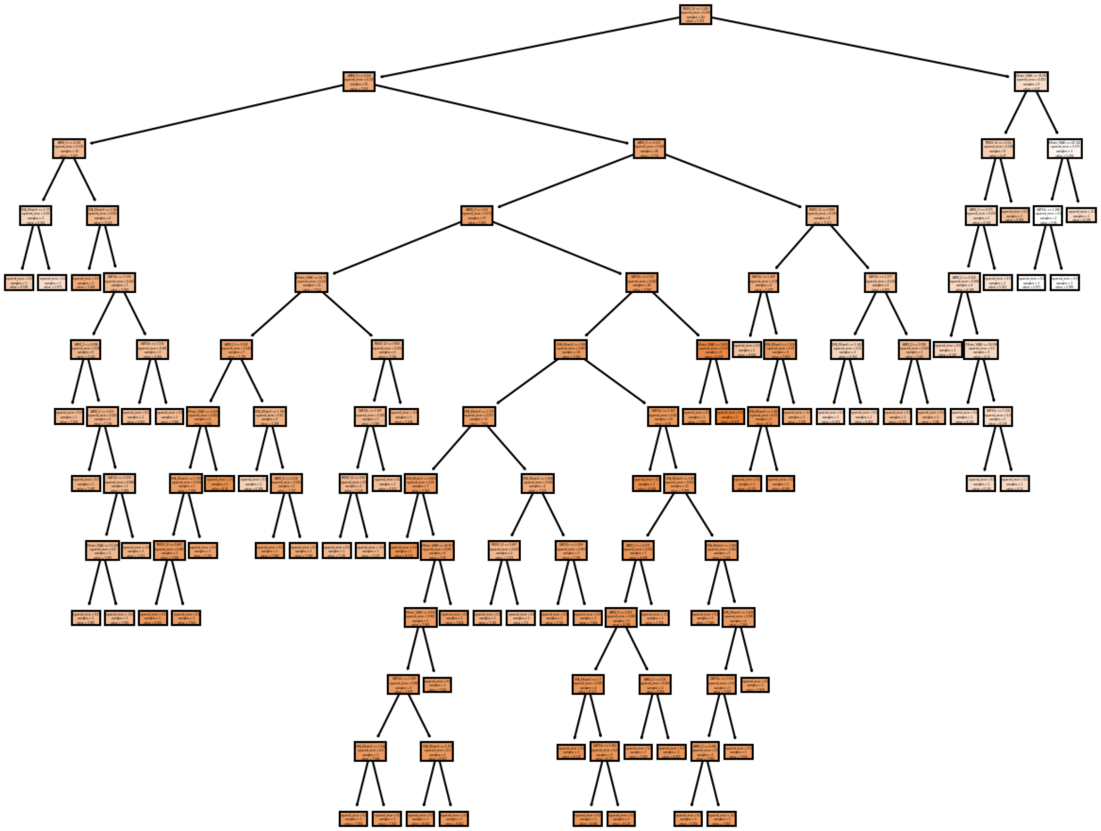

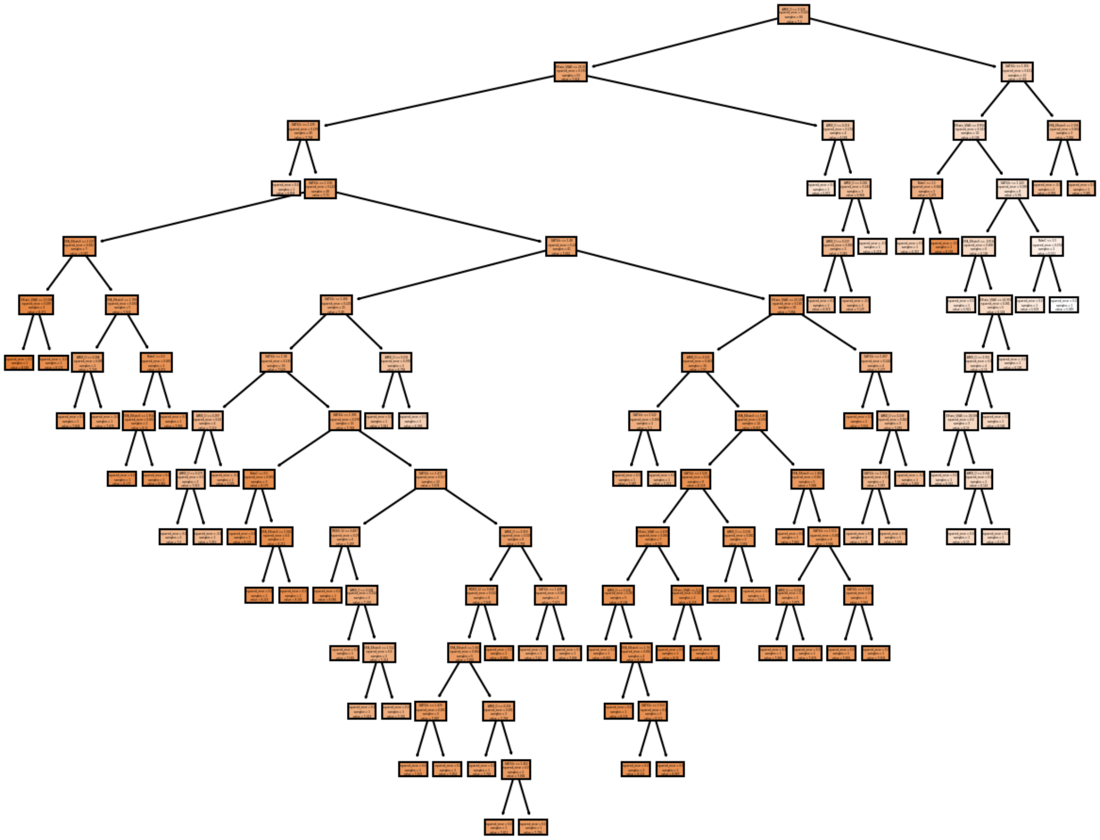

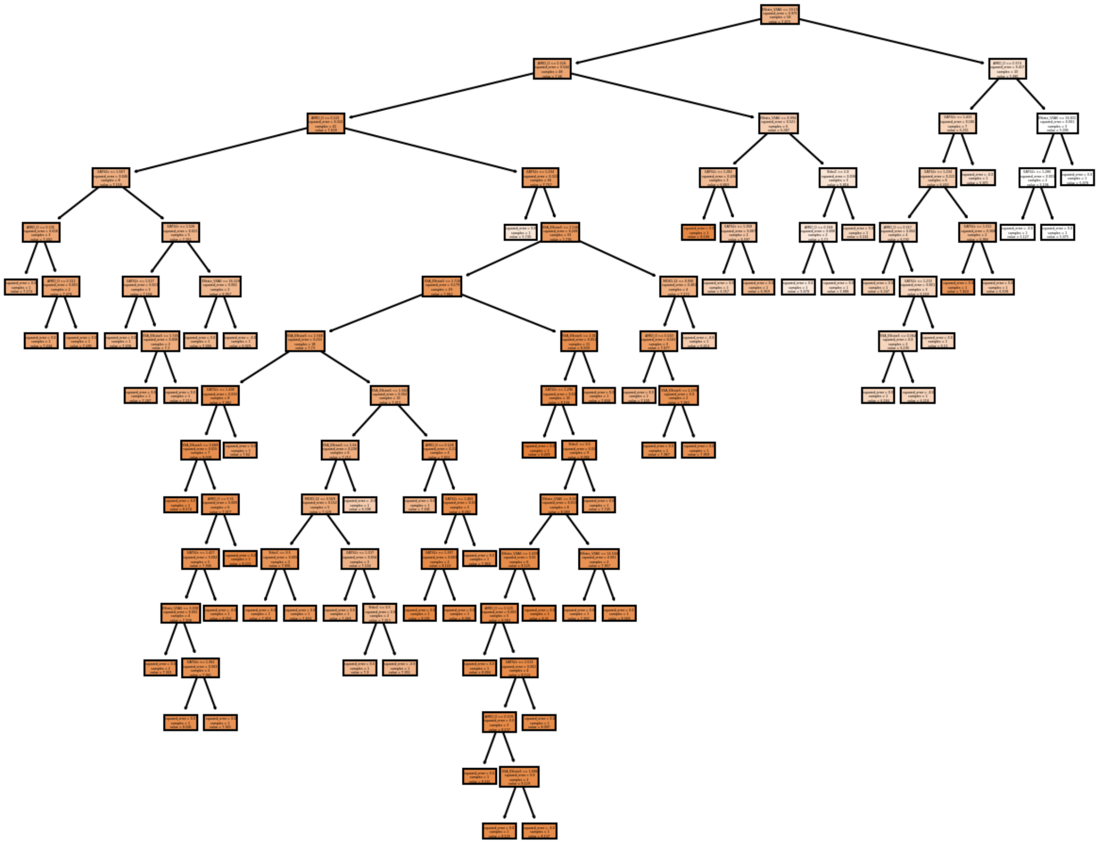

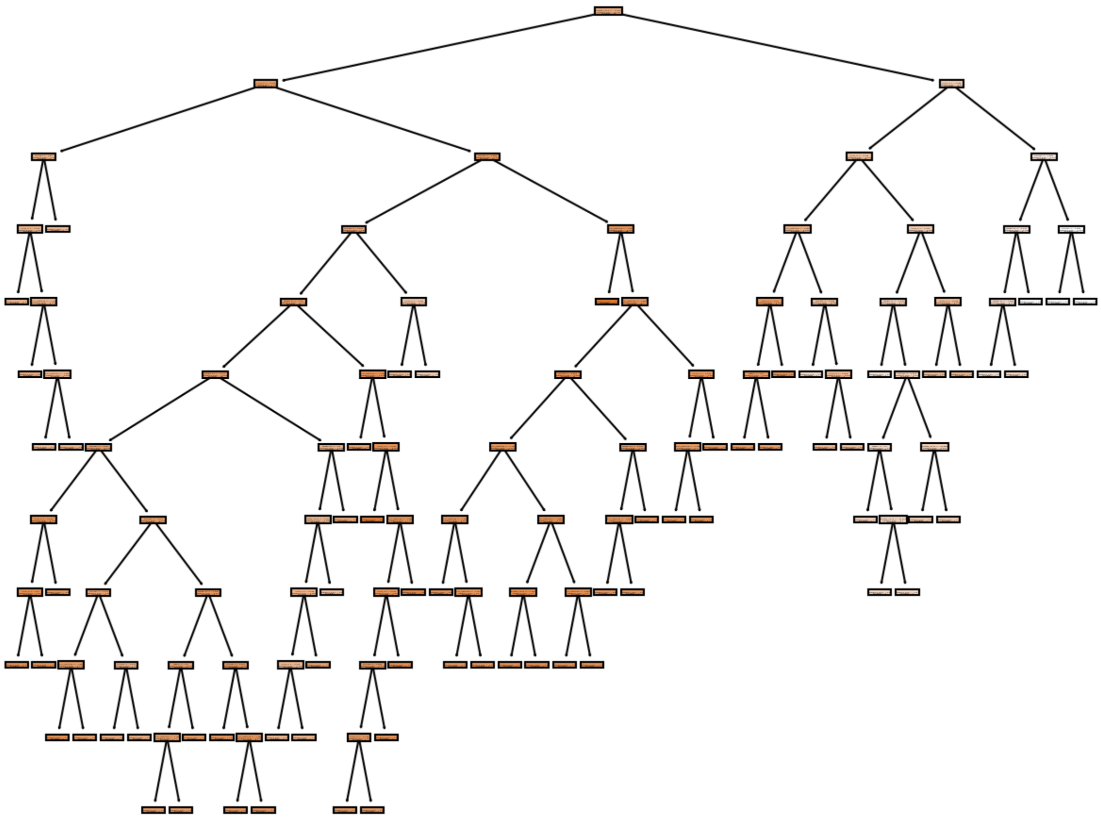

[25]:
```
hist2
```

[25]:
```
     molecular descriptor name  corr_value  absolute correlation value
226                     AMID_O   -0.546843                    0.546843
505                 EState_VSA5  -0.588780                    0.588780
556                     GATS2c    0.511671                    0.511671
791                   MDEO-12    -0.582747                    0.582747
851                      NdssC   -0.526783                    0.526783
1091                VSA_EState5   0.529677                    0.529677
```

[26]:
```
predicted_activity = model.predict(molecular_descriptors[hist2['molecular␣
↪descriptor name']])
```

[27]:
```
save_to_df['Predicted BALB/3T3'] = predicted_activity
```

[28]:
```
save_to_df.head()
```

[28]:
```
                                              SMILES  Predicted A549  \
0  COC1=C2C3=CC=C(NC)C(=O)C=C3[C@H1](CCC2=CC(OC)=…        8.078406
```

```
1  COC1=C2C3=CC=C(NC)C(=O)C=C3[C@H1](CCC2=CC(OC)=…        7.867157
2  COC1=C2C3=CC=C(NC)C(=O)C=C3[C@H1](CCC2=CC(OC)=…        7.785028
3  COC1=C2C3=CC=C(NC)C(=O)C=C3[C@H1](CCC2=CC(OC)=…        7.742135
4  COC1=CC2=CC=C(NC)C(C=C2[C@@H1](N3C=C(N=N3)CN)C…        6.789342

   Predicted BALB/3T3
0            7.961224
1            7.934181
2            7.407610
3            7.568620
4            7.974253
```

4 LoVo

[29]:
```
## The best model is: Random Forest (correlation_threshold = 0.54, 2 features,
↪18 estimators, random_state=28)
```

[30]:
```
target = 'LoVo'
data = load_prepared_data[list(load_prepared_data.columns)[0:-10]]
data[target] = load_prepared_data[load_prepared_data.columns[-3]]
data.head()
```

[30]:
```
      AATS0Z  AATS0are    AATS0d   AATS0dv      AATS0i     AATS0m    AATS0p  \
0  25.090909  6.046518  3.109091  6.662626  160.775045  99.075564  1.555512
1  24.448276  6.008422  3.051724  6.386973  161.021674  96.473315  1.538471
2  23.868852  5.974074  3.000000  6.138434  161.244045  94.127025  1.523105
3  23.868852  5.974074  3.032787  6.171220  161.244045  94.127025  1.523105
4  23.343750  5.942945  2.953125  5.913194  161.445569  92.000700  1.509180

    AATS0pe   AATS0s  AATS0se  …    piPC10     piPC2     piPC3     piPC4  \
0  6.126680  3.435416  7.629859  …  7.999655  4.266195  4.915408  5.516700
1  6.088791  3.330998  7.596891  …  8.018726  4.280132  4.922714  5.520711
2  6.054630  3.236850  7.567166  …  8.028799  4.293878  4.929967  5.524706
3  6.054630  3.257798  7.567166  …  8.037440  4.307438  4.929967  5.524706
4  6.023670  3.151529  7.540228  …  8.034892  4.307438  4.937168  5.528685

      piPC5     piPC6     piPC7     piPC8     piPC9      LoVo
0  6.098566  6.595759  6.979116  7.337313  7.681445  8.187087
1  6.103048  6.601209  6.985613  7.349442  7.695531  8.070581
2  6.105281  6.603923  6.989306  7.353932  7.704023  8.055517
3  6.107510  6.606629  6.992068  7.361425  7.709420  8.070581
4  6.107510  6.605277  6.991148  7.356489  7.707175  7.277366

[5 rows x 1212 columns]
```

[31]:
```
model, train_r2_, test_r2_, hist1, hist2, target_column_name,␣
↪training_data_RMSE, test_data_RMSE  = pred_model.
↪prepare_data_and_create_model(molecular_descriptors_df=data,
                                                                              ␣
↪                   correlation_threshold=0.54,
                                                                              ␣
↪                   standardization=False,
                                                                              ␣
↪                   model_type='RandomForestRegressor',
                                                                              ␣
↪                   n_estimators_=18,
                                                                              ␣
↪                   target_column_name = target,
                                                                              ␣
↪                   random_state=28,
                                                                              ␣
↪                   train_test_split_=True,
                                                                              ␣
↪                   verbose=True)
print("R^2 score: " + str(r2_score(data[target], model.
↪predict(data[hist2['molecular descriptor name']]))))
print("Mean squared error: "+str(mean_squared_error(data[target], model.
↪predict(data[hist2['molecular descriptor name']]))))
print('Mean absolute error: ' + str(mean_absolute_error(data[target], model.
↪predict(data[hist2['molecular descriptor name']]))))
print("Root Mean Square Error: "+ str(math.
↪sqrt(mean_squared_error(data[target], model.predict(data[hist2['molecular␣
↪descriptor name']])))))
```

```
I am not doing standardization…
  molecular descriptor name
0                    AATS0Z
1                  AATS0are
2                    AATS0d
3                   AATS0dv
4                    AATS0i
  molecular descriptor name  corr_value
0                    AATS0Z   -0.027037
1                  AATS0are   -0.129823
2                    AATS0d    0.042740
3                   AATS0dv   -0.120173
4                    AATS0i    0.132395
  molecular descriptor name  corr_value  absolute correlation value
0                    AATS0Z   -0.027037                    0.027037
1                  AATS0are   -0.129823                    0.129823
2                    AATS0d    0.042740                    0.042740
3                   AATS0dv   -0.120173                    0.120173
```

```
4                   AATS0i    0.132395                   0.132395
     molecular descriptor name  corr_value  absolute correlation value
505               EState_VSA5   -0.579664                    0.579664
791                   MDEO-12   -0.558727                    0.558727
1211                     LoVo    1.000000                    1.000000
    molecular descriptor name  corr_value  absolute correlation value
505               EState_VSA5   -0.579664                    0.579664
791                   MDEO-12   -0.558727                    0.558727
The model used is: RandomForest…
Return the coefficient of determination of the prediction:
0.43199473372238906
R^2 score: 0.7763844047145243
Correlation coefficient: 0.8811267812945673
Test data - unseen during training:
R^2 score: 0.43199473372238906
Correlation coefficient: 0.6572630628008767
[8.01614039 6.10216465 8.29736491 6.10216465 7.84848772 8.06296524
 7.84018633 8.01614039 7.54269918 7.84848772 8.01614039 7.84848772
 8.13682138 8.01615479 8.06296524 6.58534596 7.84018633 6.97001882]
113    7.795880
35     6.254925
101    7.275724
36     7.017729
100    7.337242
13     8.055517
0      8.187087
114    7.638272
104    8.318759
96     7.769551
40     8.050610
103    8.136677
48     7.853872
39     8.267606
14     7.273273
117    6.167491
21     7.554396
9      6.343902
Name: LoVo, dtype: float64
Training Root Mean Square Error: 0.4438384819944741
Testing Root Mean Square Error: 0.5047746604903675
R^2 score: 0.7487186984561868
Mean squared error: 0.20566332706526017
Mean absolute error: 0.319905742678649
Root Mean Square Error: 0.45350118750148843
```

[32]:
```
# save
```

```
joblib.dump(model, "Random_forest/random_forest_model_18_estimators_LoVo.
 ↪joblib")
```

[32]: ['Random_forest/random_forest_model_18_estimators_LoVo.joblib']

[33]:
```
for x, decision_tree in enumerate(model.estimators_):
    plt.figure(figsize=(10,8), dpi=150)
    plot_tree(decision_tree, feature_names=list(hist2['molecular descriptor␣
 ↪name']),
              filled=True)
    plt.savefig('Random_forest/random_forest_18_'+str(x)+'_model_LoVo.
 ↪pdf',bbox_inches = "tight")
```

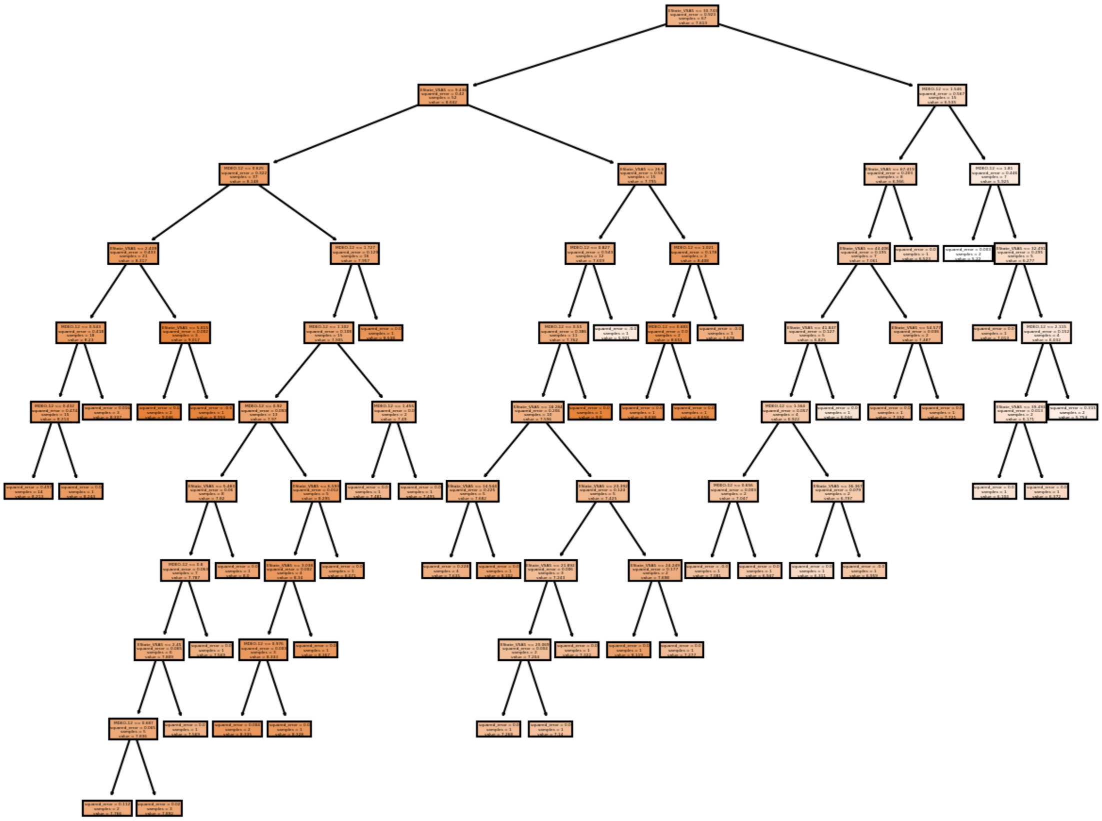

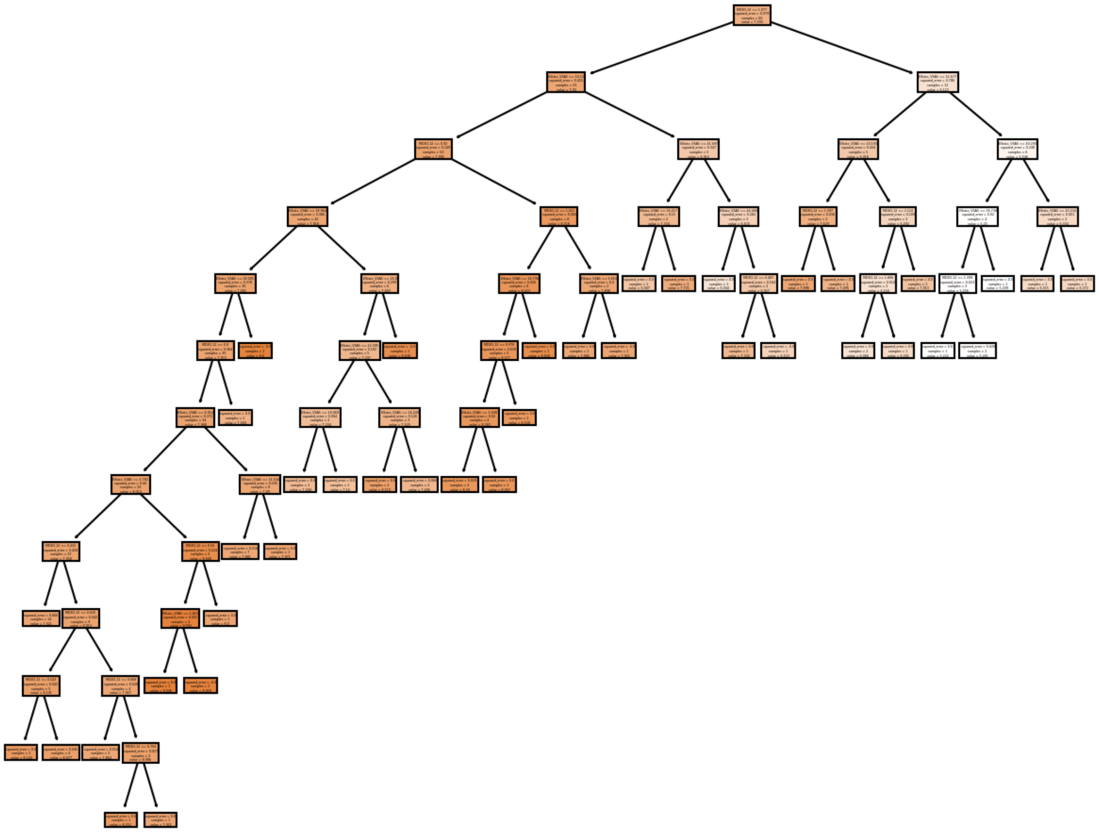

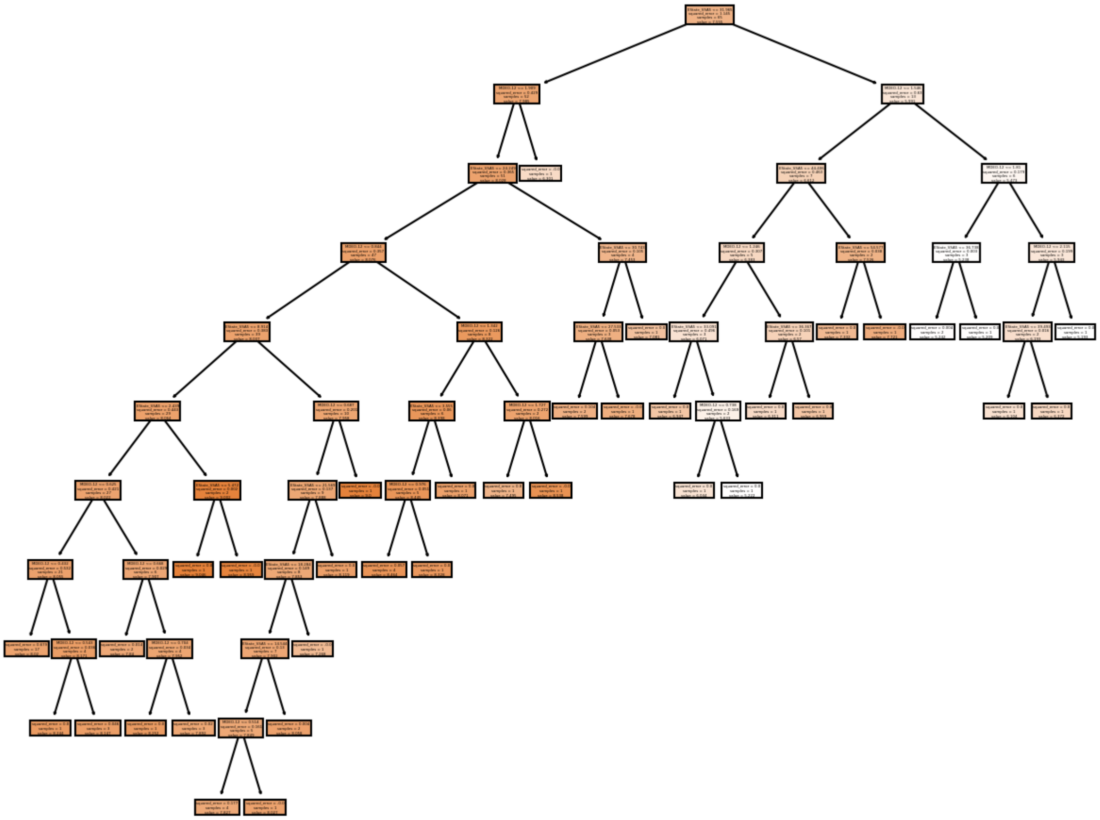

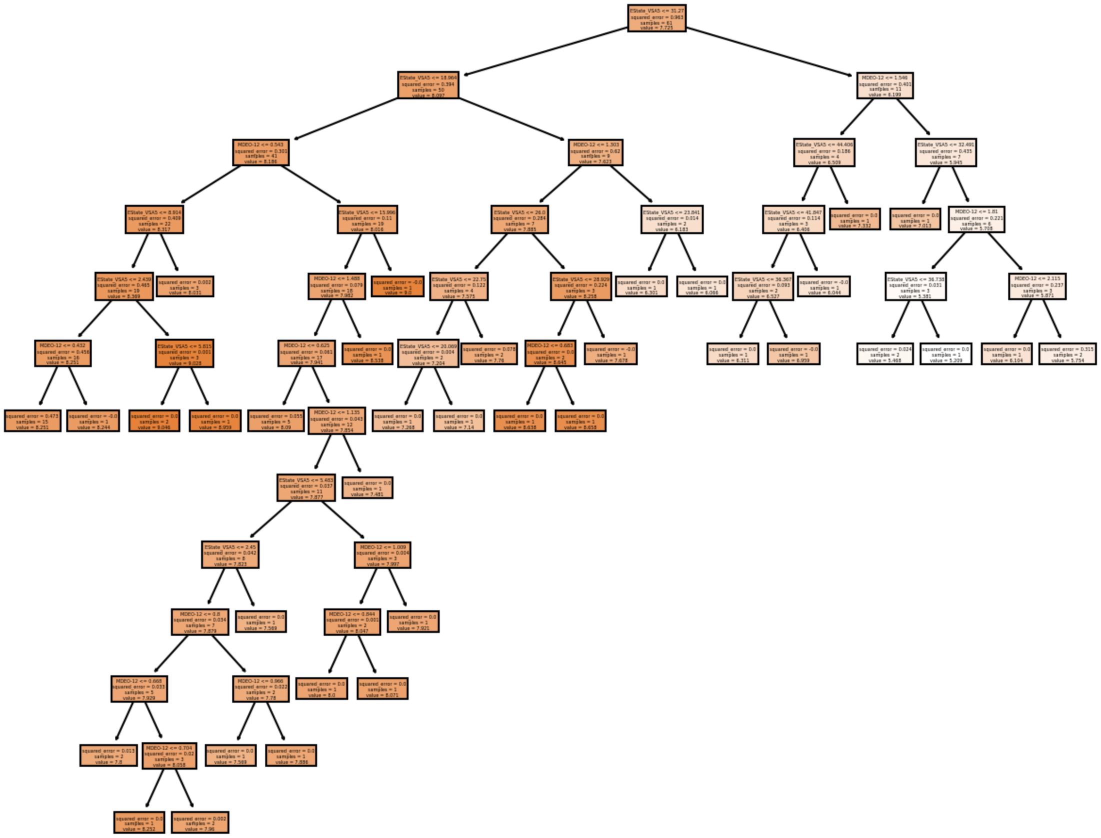

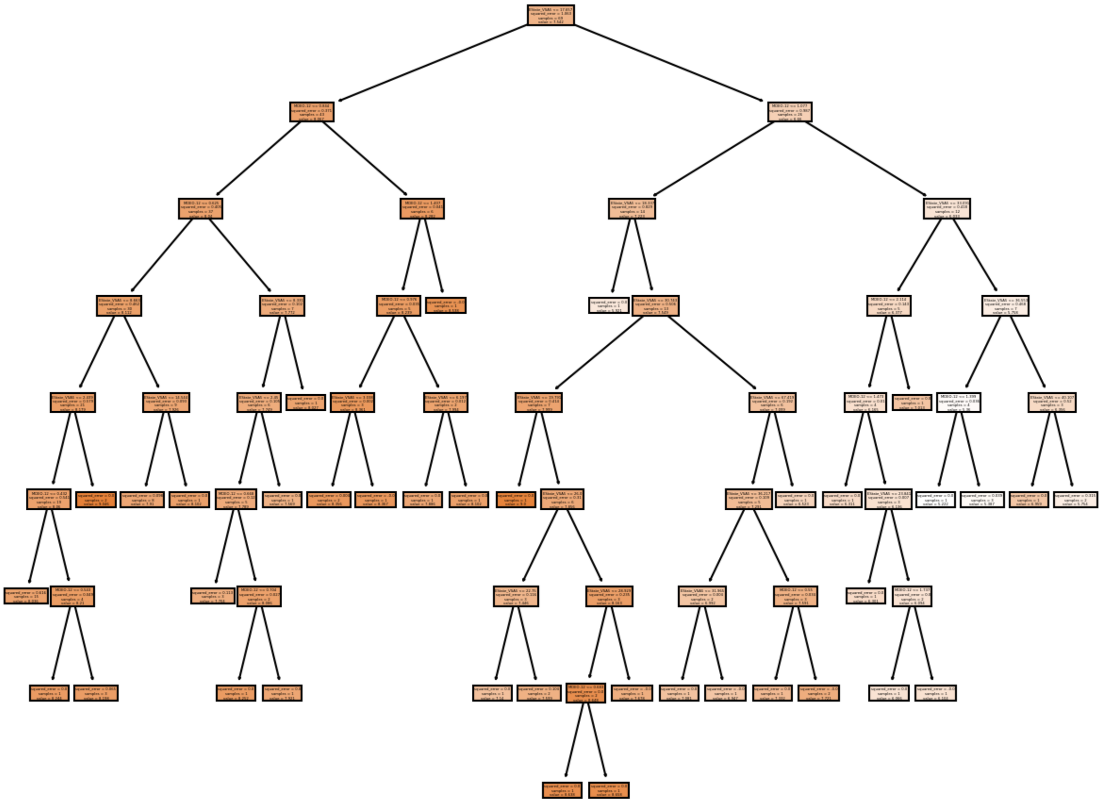

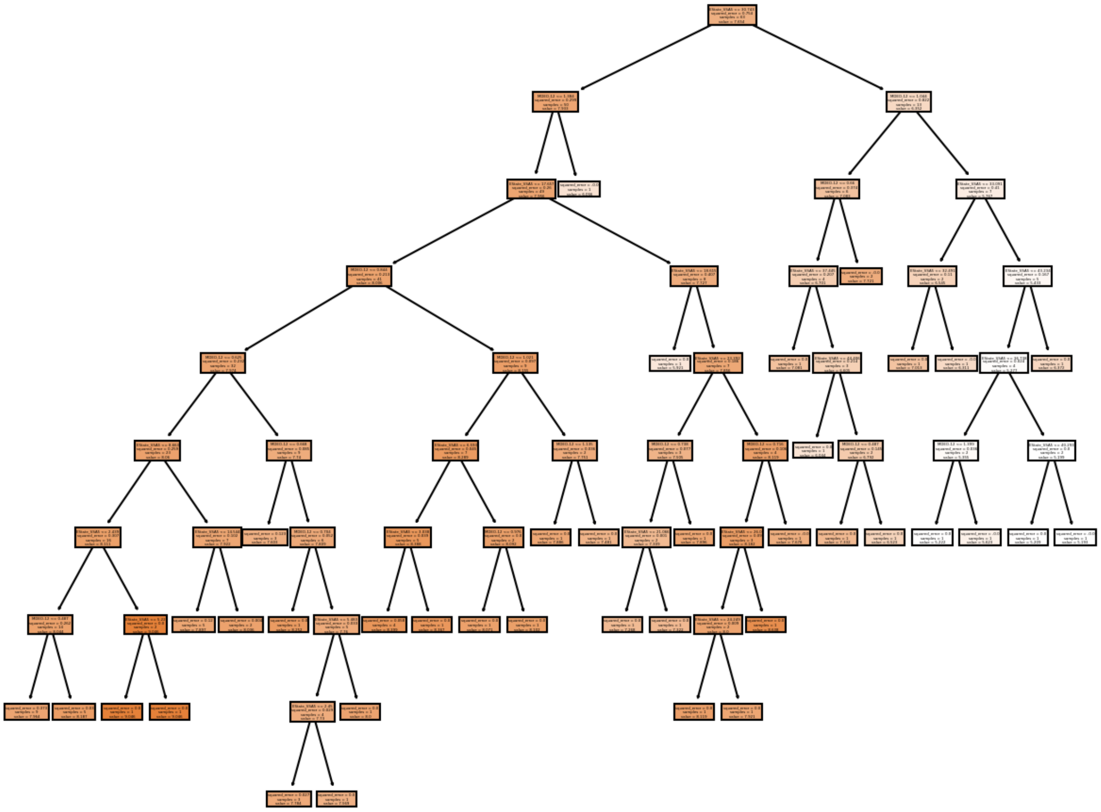

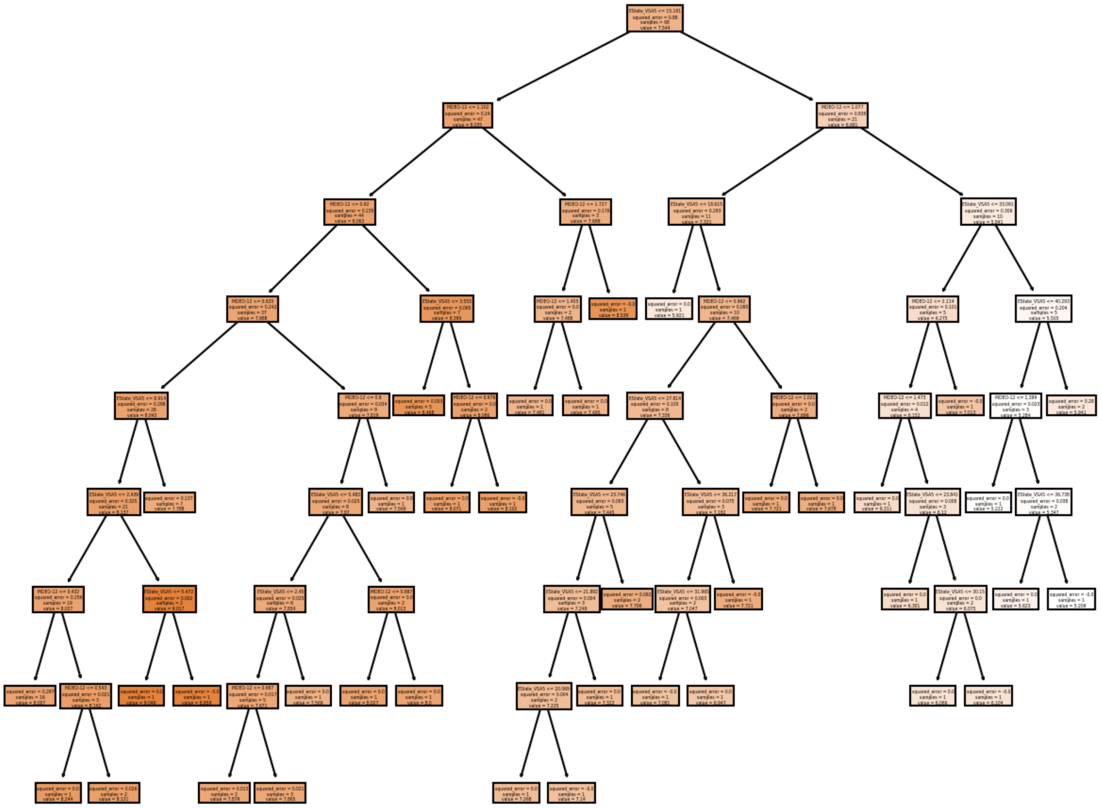

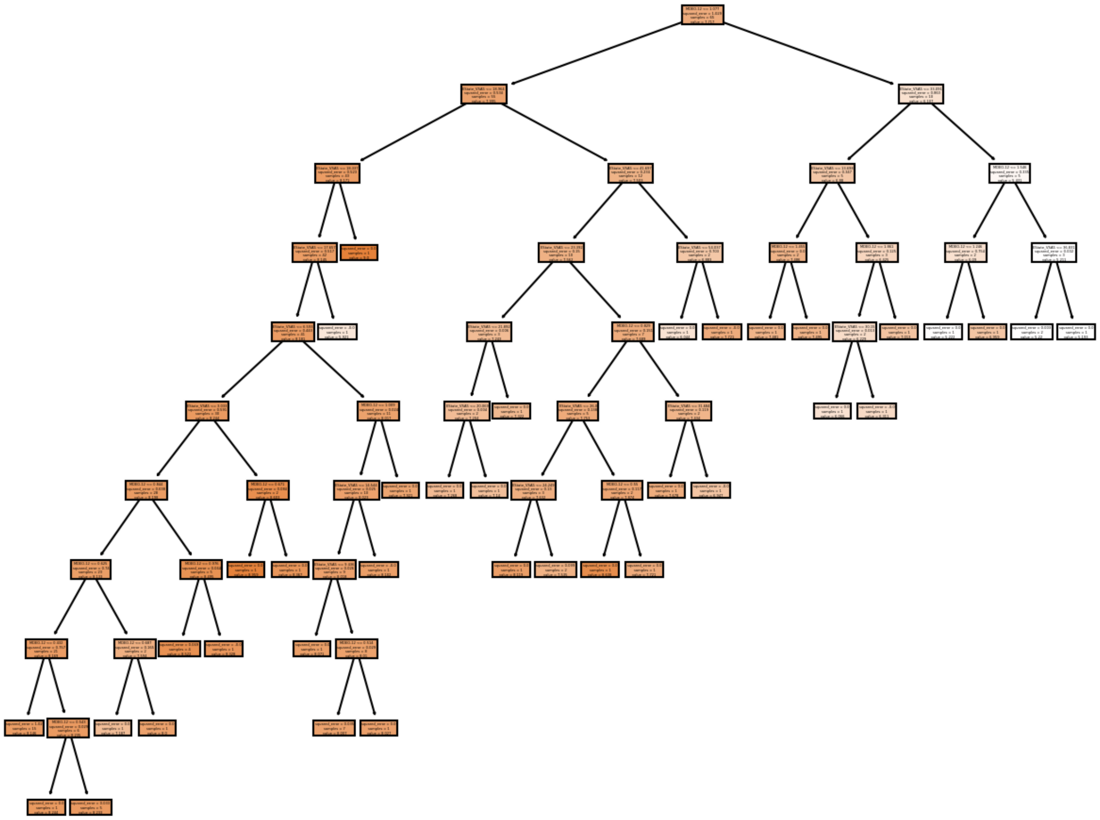

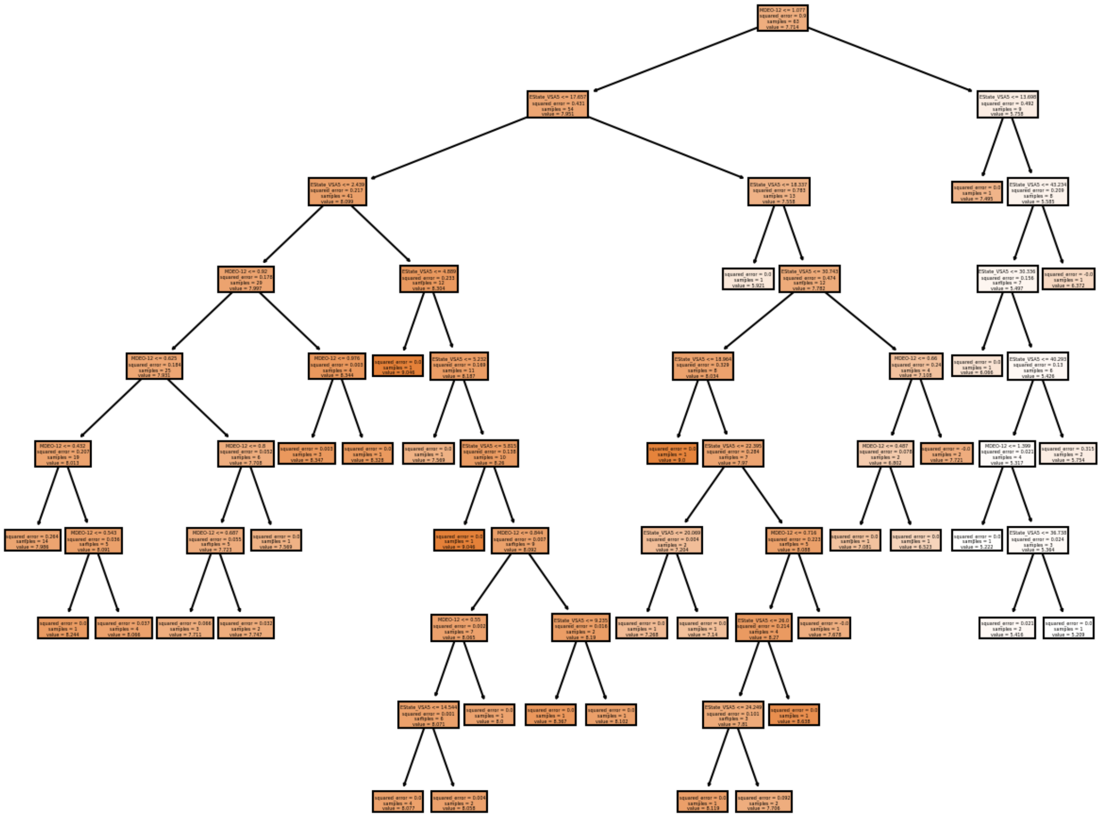

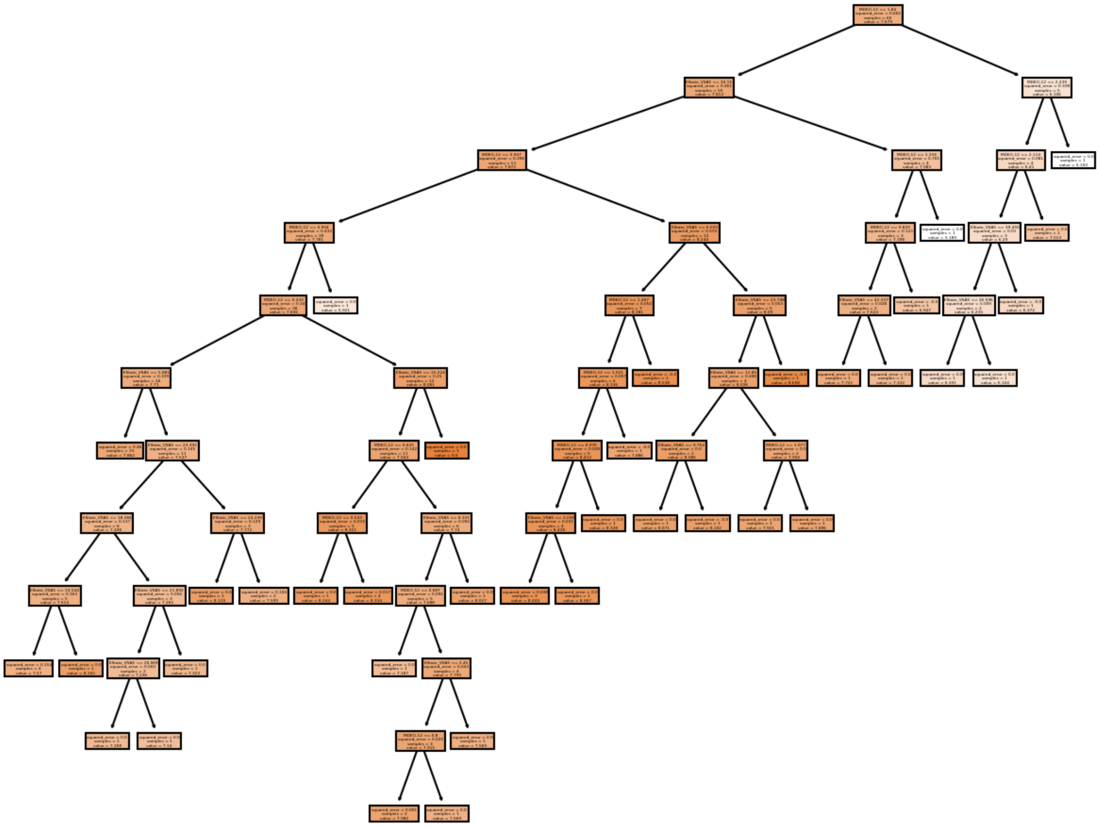

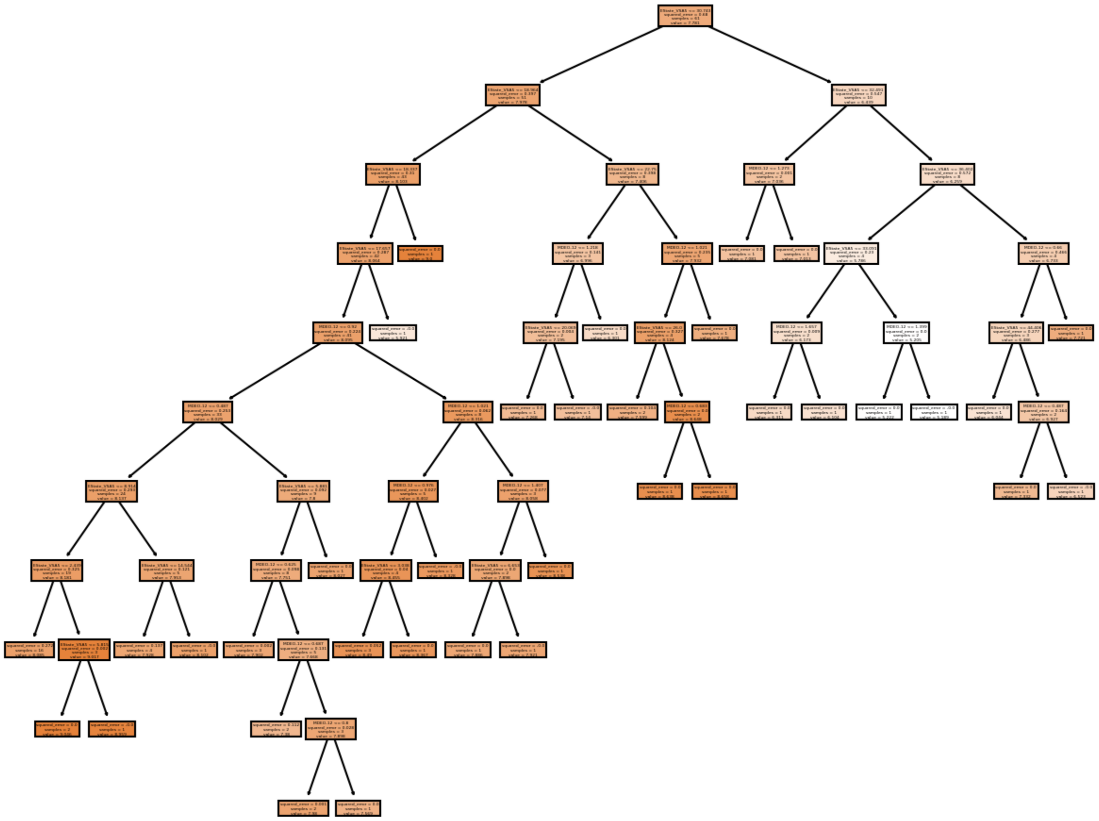

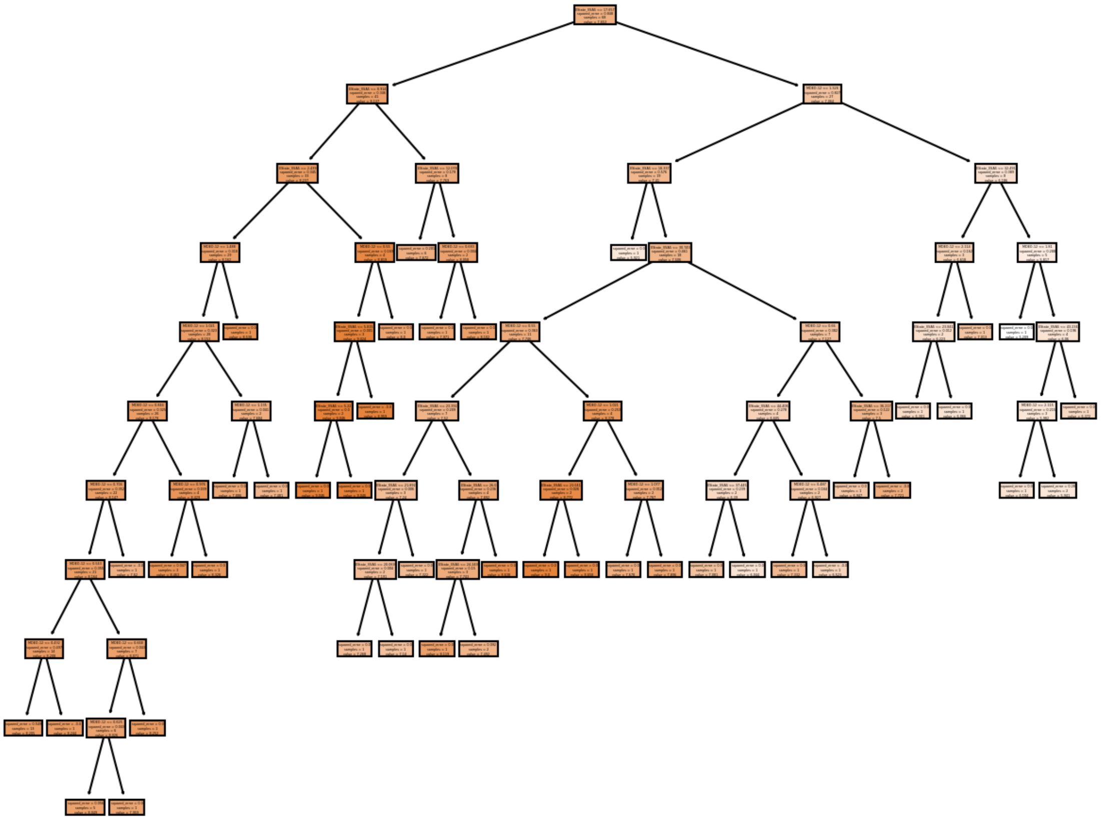

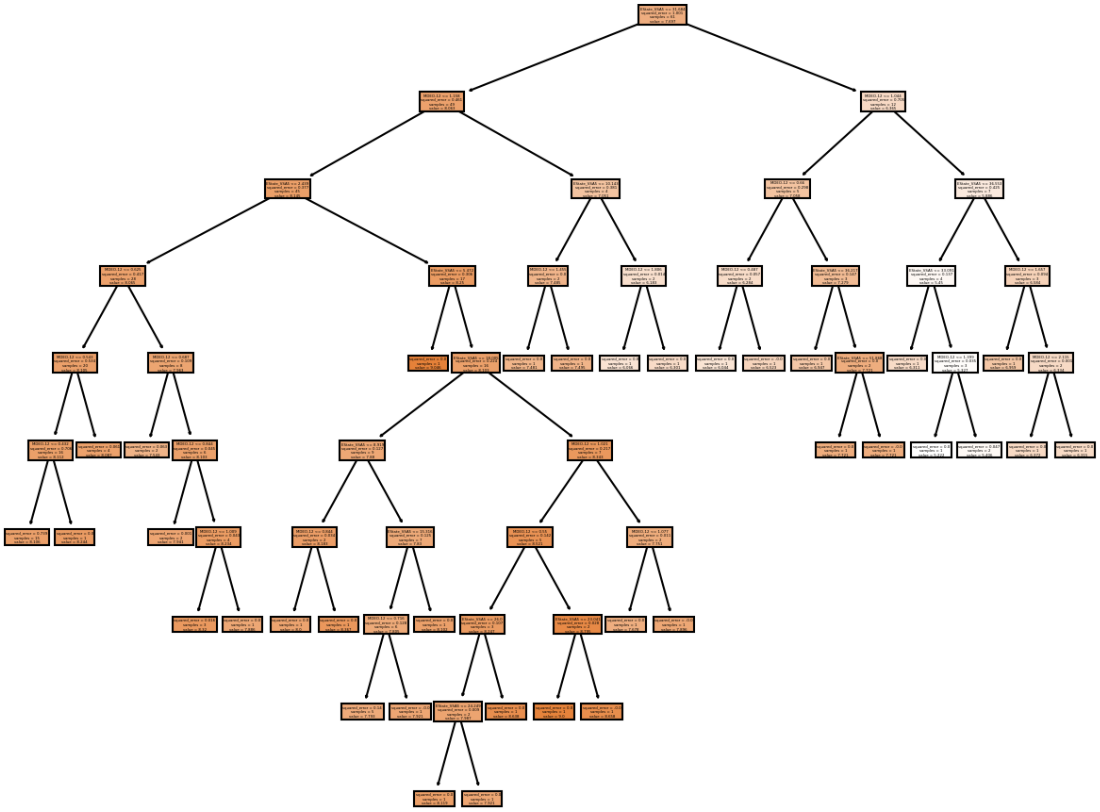

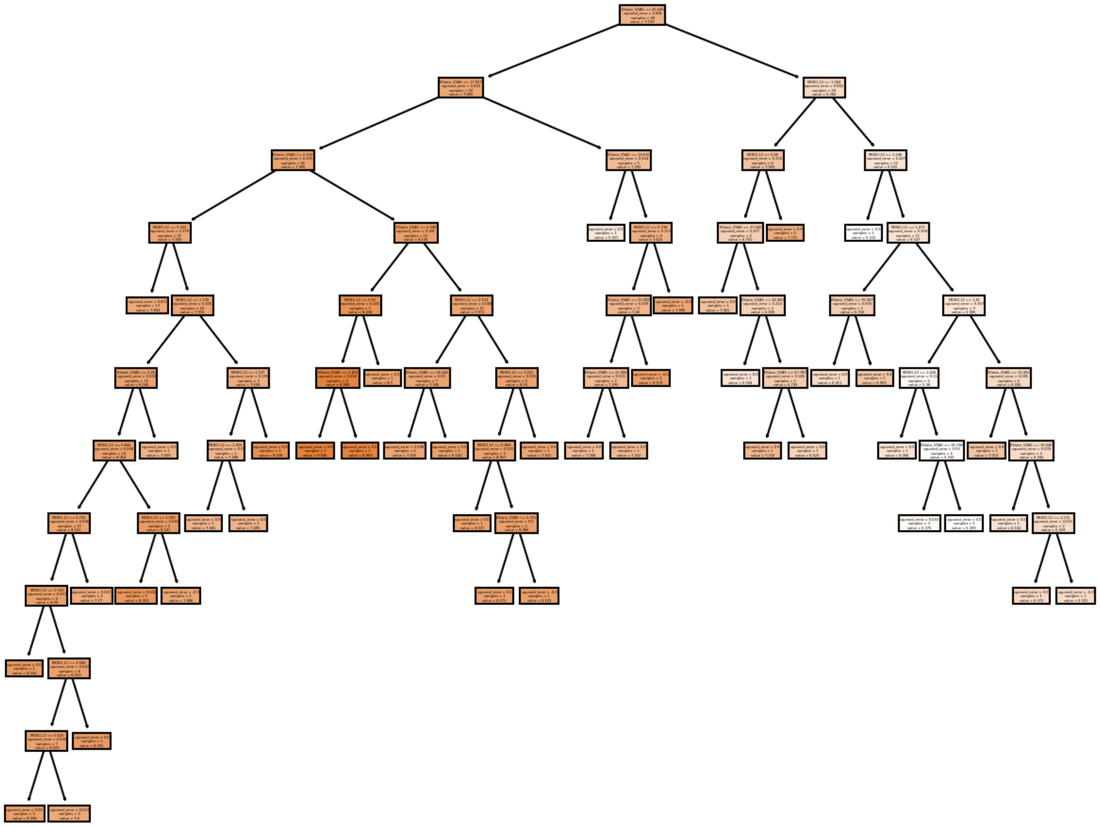

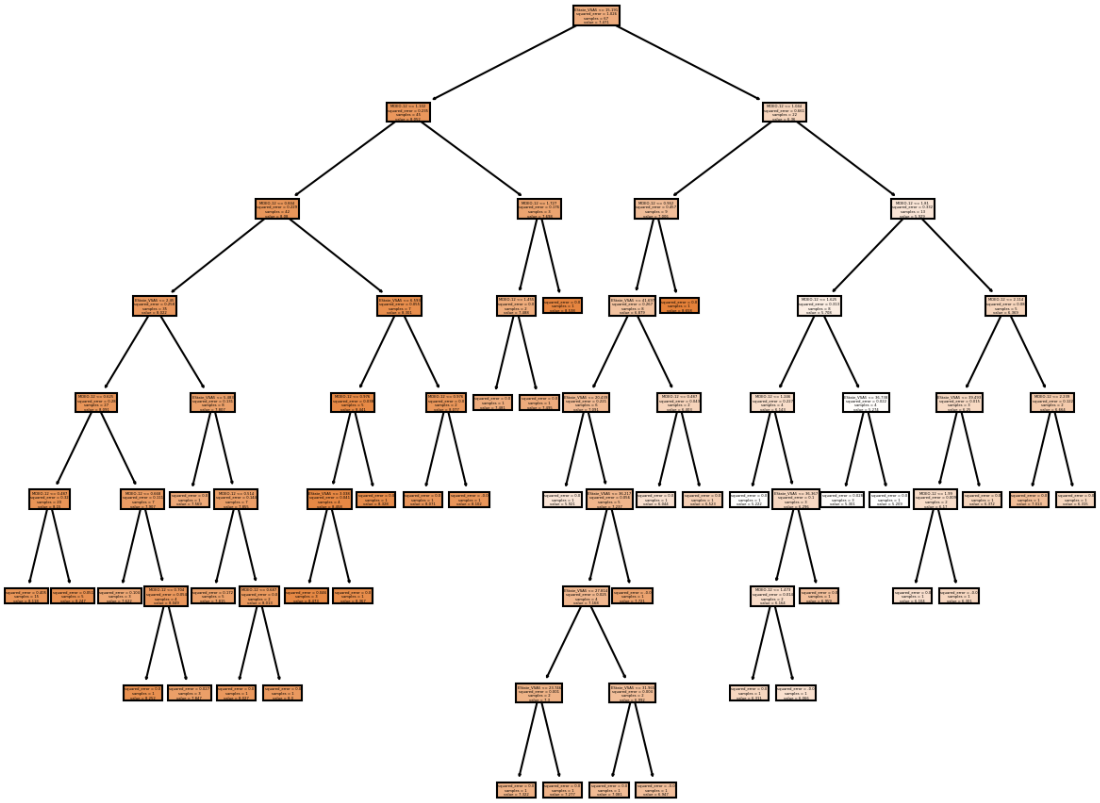

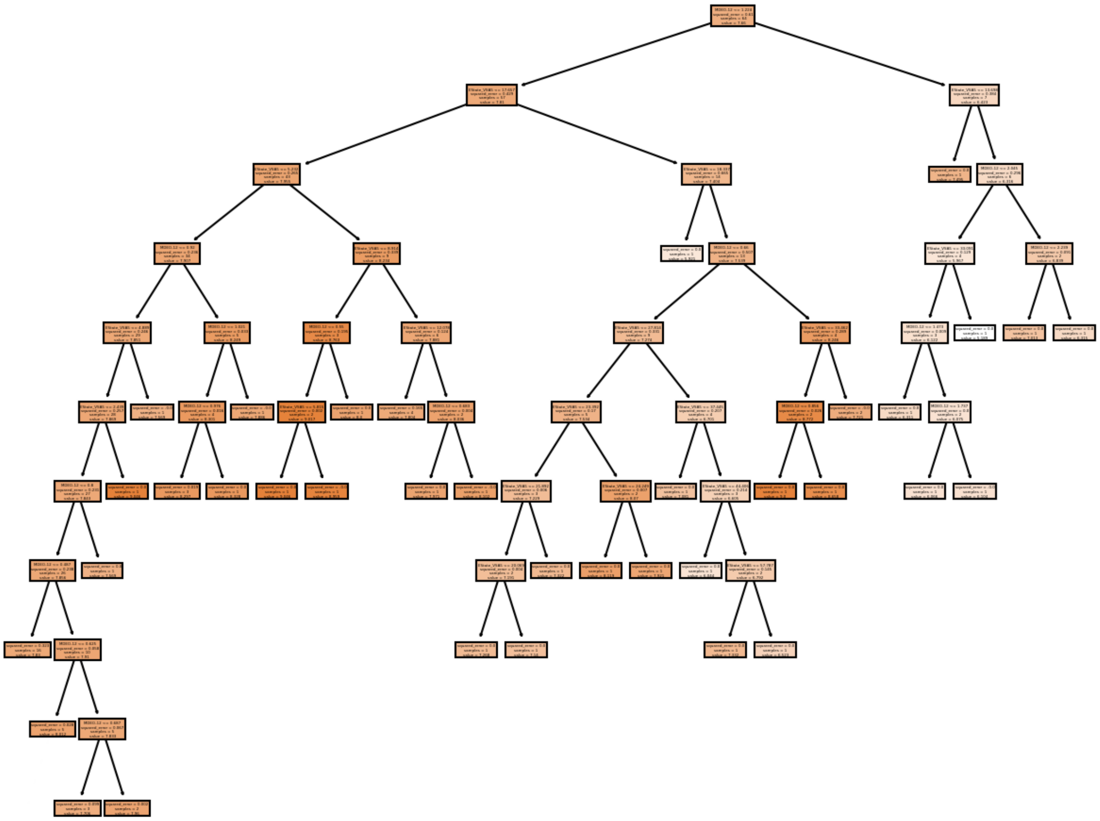

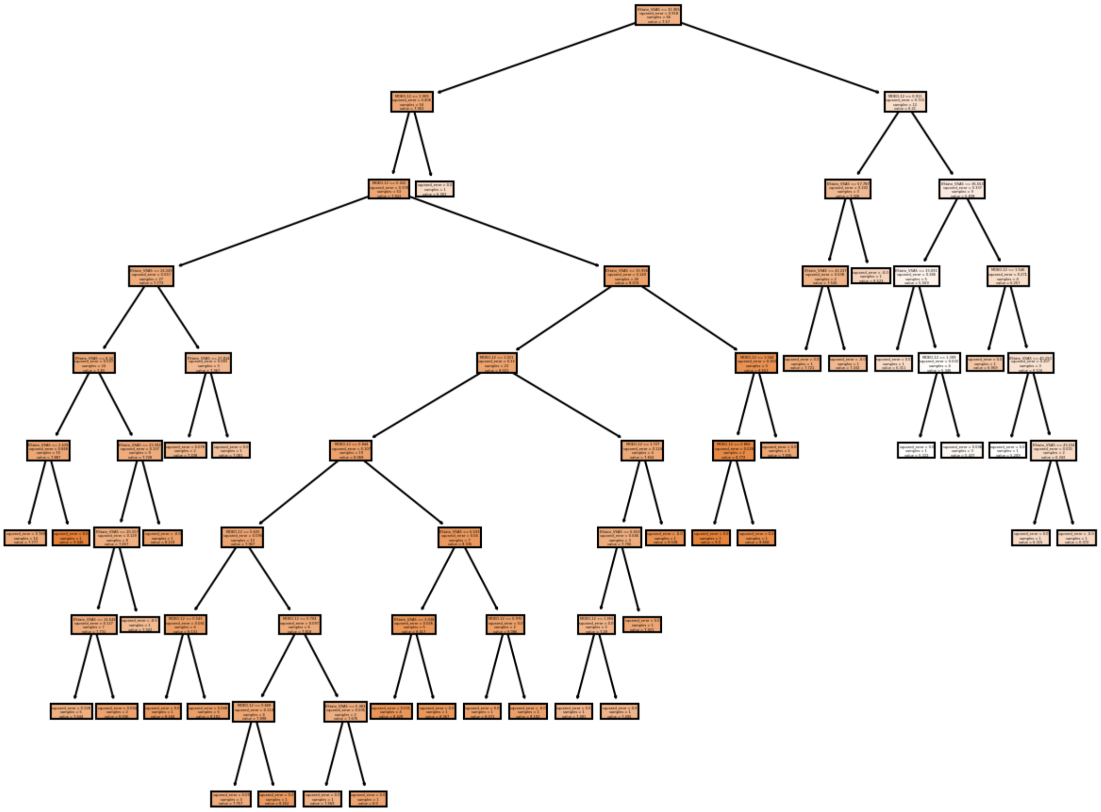

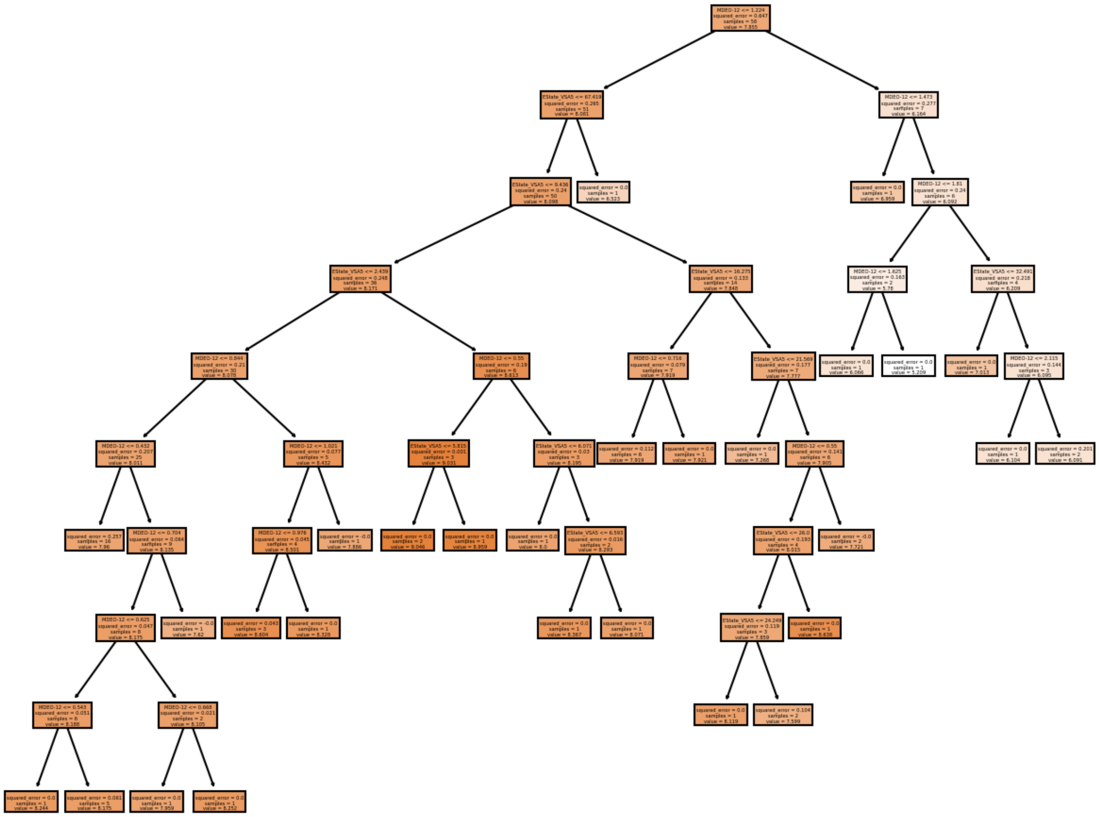

[34]:
```
hist2
```

[34]:

	molecular descriptor name	corr_value	absolute correlation value
505	EState_VSA5	-0.579664	0.579664
791	MDEO-12	-0.558727	0.558727

[35]:
```
predicted_activity = model.predict(molecular_descriptors[hist2['molecular␣
↪descriptor name']])
```

[36]:
```
save_to_df['Predicted LoVo'] = predicted_activity
```

[37]:
```
save_to_df.head()
```

[37]:

	SMILES	Predicted A549 \
0	COC1=C2C3=CC=C(NC)C(=O)C=C3[C@H1](CCC2=CC(OC)=…	8.078406
1	COC1=C2C3=CC=C(NC)C(=O)C=C3[C@H1](CCC2=CC(OC)=…	7.867157
2	COC1=C2C3=CC=C(NC)C(=O)C=C3[C@H1](CCC2=CC(OC)=…	7.785028
3	COC1=C2C3=CC=C(NC)C(=O)C=C3[C@H1](CCC2=CC(OC)=…	7.742135
4	COC1=CC2=CC=C(NC)C(C=C2[C@@H1](N3C=C(N=N3)CN)C…	6.789342

	Predicted BALB/3T3	Predicted LoVo
0	7.961224	8.209224
1	7.934181	7.866056
2	7.407610	7.379055
3	7.568620	8.016140
4	7.974253	8.730218

5 LoVo_DX

[38]:
```
## The best model is: Random Forest (correlation_threshold = 0.63, 5 features,␣
↪14 estimators, random_state=42)
```

[39]:
```
target = 'LoVo/DX'
data = load_prepared_data[list(load_prepared_data.columns)[0:-10]]
data[target] = load_prepared_data[load_prepared_data.columns[-2]]
data.head()
```

[39]:

	AATS0Z	AATS0are	AATS0d	AATS0dv	AATS0i	AATS0m	AATS0p	\
0	25.090909	6.046518	3.109091	6.662626	160.775045	99.075564	1.555512	
1	24.448276	6.008422	3.051724	6.386973	161.021674	96.473315	1.538471	
2	23.868852	5.974074	3.000000	6.138434	161.244045	94.127025	1.523105	
3	23.868852	5.974074	3.032787	6.171220	161.244045	94.127025	1.523105	
4	23.343750	5.942945	2.953125	5.913194	161.445569	92.000700	1.509180	

	AATS0pe	AATS0s	AATS0se	…	piPC10	piPC2	piPC3	piPC4	\
0	6.126680	3.435416	7.629859	…	7.999655	4.266195	4.915408	5.516700	
1	6.088791	3.330998	7.596891	…	8.018726	4.280132	4.922714	5.520711	
2	6.054630	3.236850	7.567166	…	8.028799	4.293878	4.929967	5.524706	
3	6.054630	3.257798	7.567166	…	8.037440	4.307438	4.929967	5.524706	
4	6.023670	3.151529	7.540228	…	8.034892	4.307438	4.937168	5.528685	

	piPC5	piPC6	piPC7	piPC8	piPC9	LoVo/DX
0	6.098566	6.595759	6.979116	7.337313	7.681445	7.260428
1	6.103048	6.601209	6.985613	7.349442	7.695531	7.507240
2	6.105281	6.603923	6.989306	7.353932	7.704023	7.747147
3	6.107510	6.606629	6.992068	7.361425	7.709420	7.991400
4	6.107510	6.605277	6.991148	7.356489	7.707175	7.109020

[5 rows x 1212 columns]

[40]:
```
print(data.shape)
data = data.dropna()
```

(120, 1212)

```
[41]: model, train_r2_, test_r2_, hist1, hist2, target_column_name,␣
        ↪training_data_RMSE, test_data_RMSE  = pred_model.
        ↪prepare_data_and_create_model(molecular_descriptors_df=data,
                                                                            ␣
        ↪                    correlation_threshold=0.63,
                                                                            ␣
        ↪                    standardization=False,
                                                                            ␣
        ↪                    model_type='RandomForestRegressor',
                                                                            ␣
        ↪                    n_estimators_=14,
                                                                            ␣
        ↪                    target_column_name = target,
                                                                            ␣
        ↪                    random_state=42,
                                                                            ␣
        ↪                    train_test_split_=True,
                                                                            ␣
        ↪                    verbose=True)
      print("R^2 score: " + str(r2_score(data[target], model.
        ↪predict(data[hist2['molecular descriptor name']]))))
      print("Mean squared error: "+str(mean_squared_error(data[target], model.
        ↪predict(data[hist2['molecular descriptor name']]))))
      print('Mean absolute error: ' + str(mean_absolute_error(data[target], model.
        ↪predict(data[hist2['molecular descriptor name']]))))
      print("Root Mean Square Error: "+ str(math.
        ↪sqrt(mean_squared_error(data[target], model.predict(data[hist2['molecular␣
        ↪descriptor name']])))))
```

```
I am not doing standardization…
  molecular descriptor name
0                    AATS0Z
1                  AATS0are
2                    AATS0d
3                   AATS0dv
4                    AATS0i
  molecular descriptor name  corr_value
0                    AATS0Z    0.018677
1                  AATS0are   -0.341313
2                    AATS0d   -0.123443
3                   AATS0dv   -0.265670
4                    AATS0i   -0.428929
  molecular descriptor name  corr_value  absolute correlation value
0                    AATS0Z    0.018677                    0.018677
1                  AATS0are   -0.341313                    0.341313
2                    AATS0d   -0.123443                    0.123443
3                   AATS0dv   -0.265670                    0.265670
```

```
4                    AATS0i   -0.428929                   0.428929
     molecular descriptor name  corr_value  absolute correlation value
556                     GATS2c    0.652245                    0.652245
696                     MATS2c   -0.638624                    0.638624
851                      NdssC   -0.647960                    0.647960
881                       RNCG    0.663041                    0.663041
1022                TopoPSA(NO)   -0.635724                    0.635724
     molecular descriptor name  corr_value  absolute correlation value
556                     GATS2c    0.652245                    0.652245
696                     MATS2c   -0.638624                    0.638624
851                      NdssC   -0.647960                    0.647960
881                       RNCG    0.663041                    0.663041
1022                TopoPSA(NO)   -0.635724                    0.635724
The model used is: RandomForest…
Return the coefficient of determination of the prediction:
0.6249884711849997
R^2 score: 0.923535411990395
Correlation coefficient: 0.9610074984048745
Test data - unseen during training:
R^2 score: 0.6249884711849997
Correlation coefficient: 0.790562123545645
[6.1547412  7.13053465 7.59814903 4.84627594 6.51359131 6.38096941
 7.25966313 7.13539448 6.42648883 7.85741914 5.76764772 6.07409969
 7.60026782 6.41520183 7.16325348 6.0335248 ]
114    6.026872
10     7.856985
4      7.109020
95     5.200659
111    6.853872
79     6.327902
44     7.638272
47     7.022276
107    6.000000
11     7.250264
61     5.481486
56     6.657577
0      7.260428
92     7.141463
18     6.630970
78     6.244125
Name: LoVo/DX, dtype: float64
Training Root Mean Square Error: 0.26590821262128245
Testing Root Mean Square Error: 0.4420939409154765
R^2 score: 0.8967153598641877
Mean squared error: 0.08953583792506502
Mean absolute error: 0.21690719242962295
Root Mean Square Error: 0.2992253965242005
```

[42]:
```python
# save
joblib.dump(model, "Random_forest/random_forest_model_14_estimators_LoVo_DX.
↪joblib")
```

[42]: ['Random_forest/random_forest_model_14_estimators_LoVo_DX.joblib']

[43]:
```python
for x, decision_tree in enumerate(model.estimators_):
    plt.figure(figsize=(10,8), dpi=150)
    plot_tree(decision_tree, feature_names=list(hist2['molecular descriptor␣
↪name']),
              filled=True)
    plt.savefig('Random_forest/random_forest_14_'+str(x)+'_model_LoVo_DX.
↪pdf',bbox_inches = "tight")
```

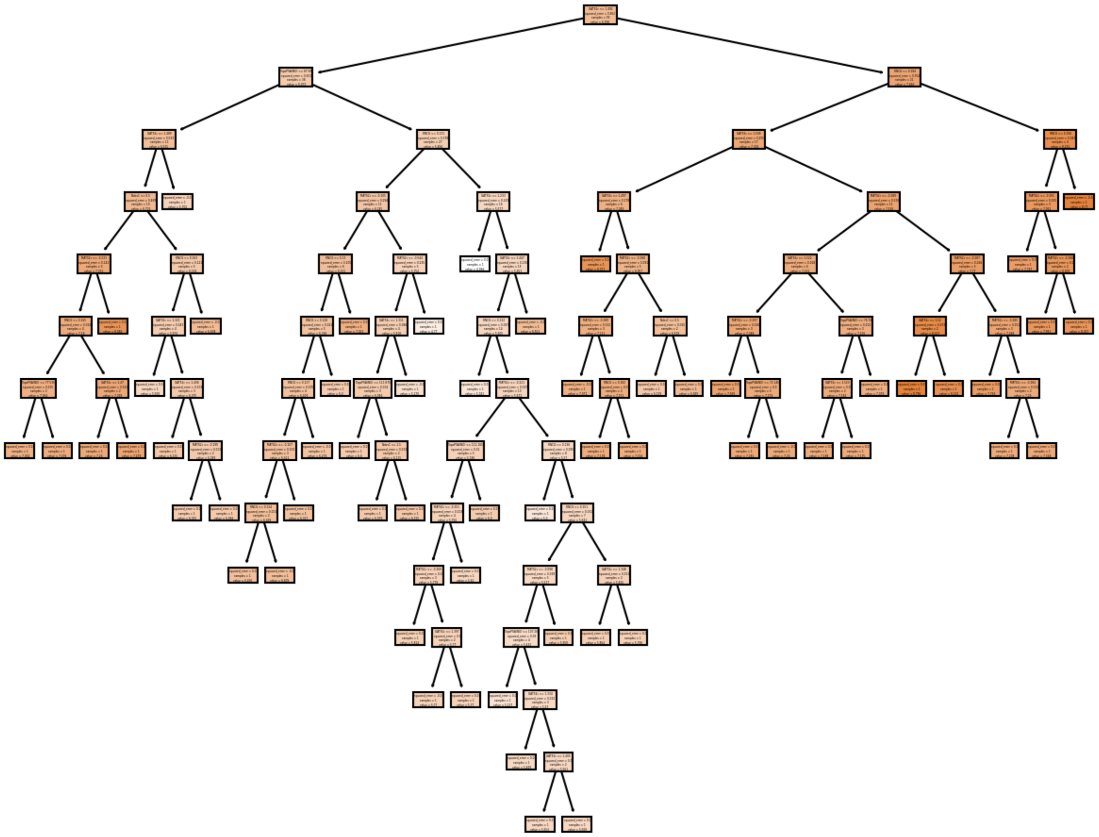

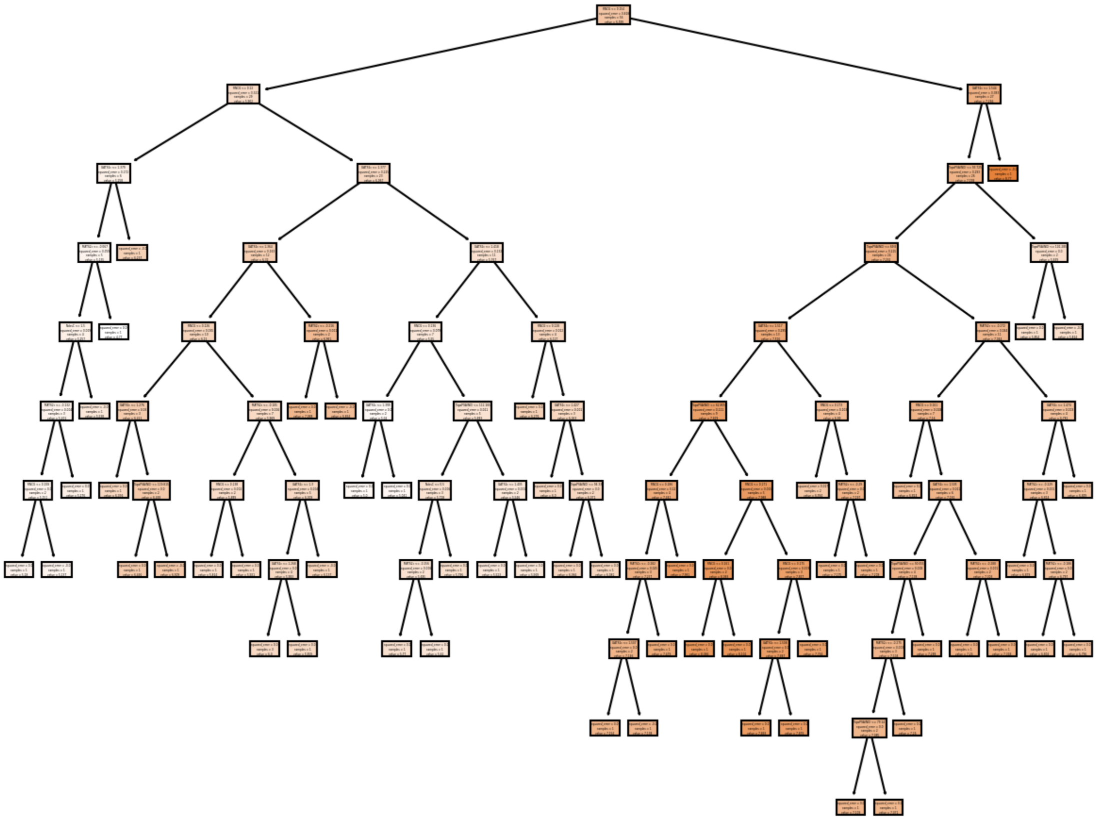

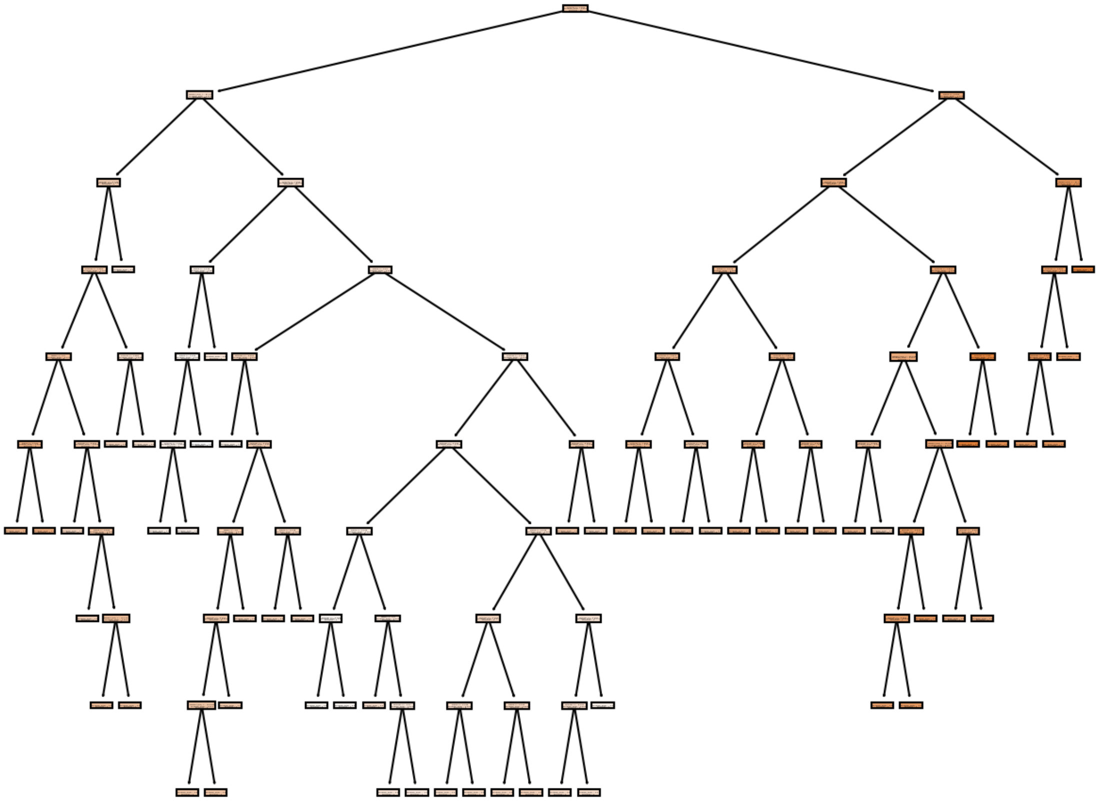

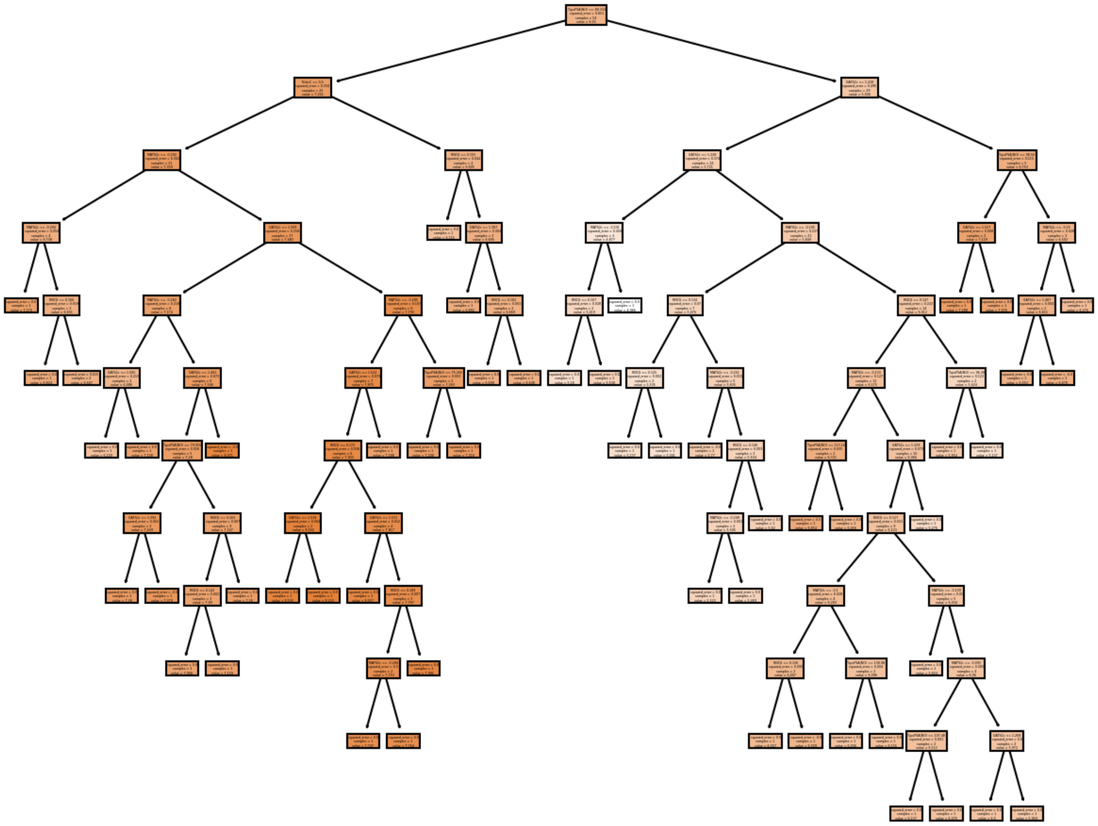

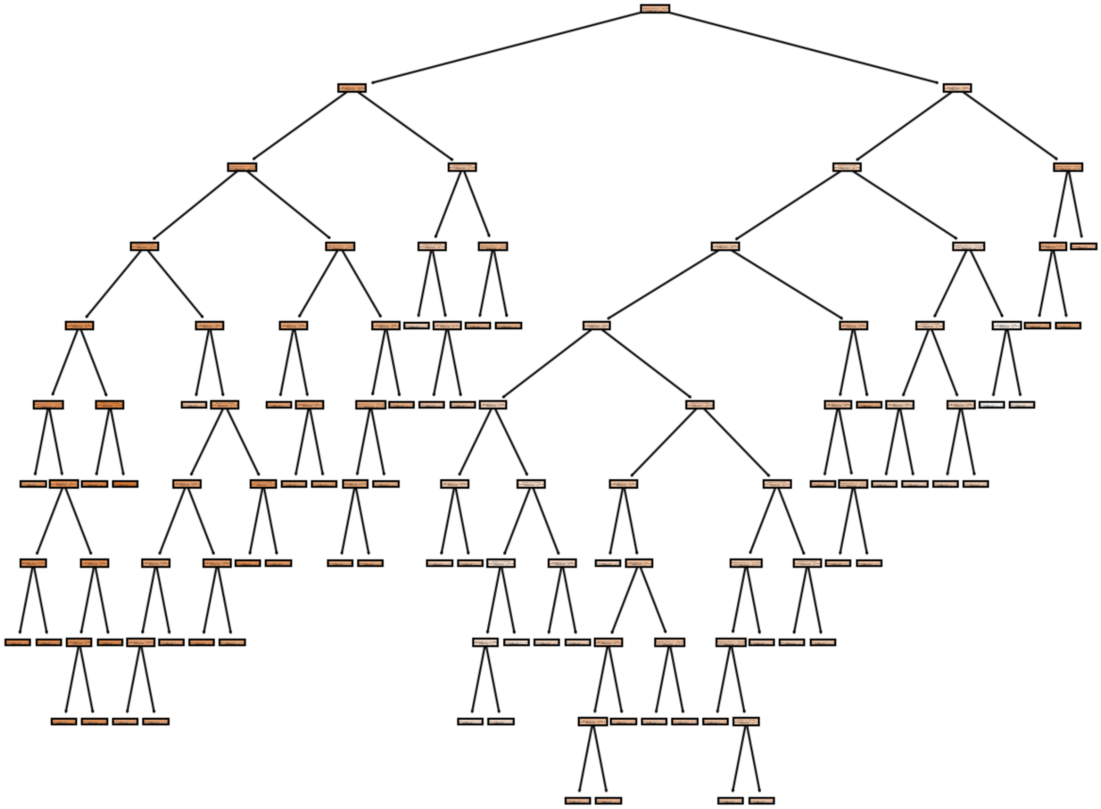

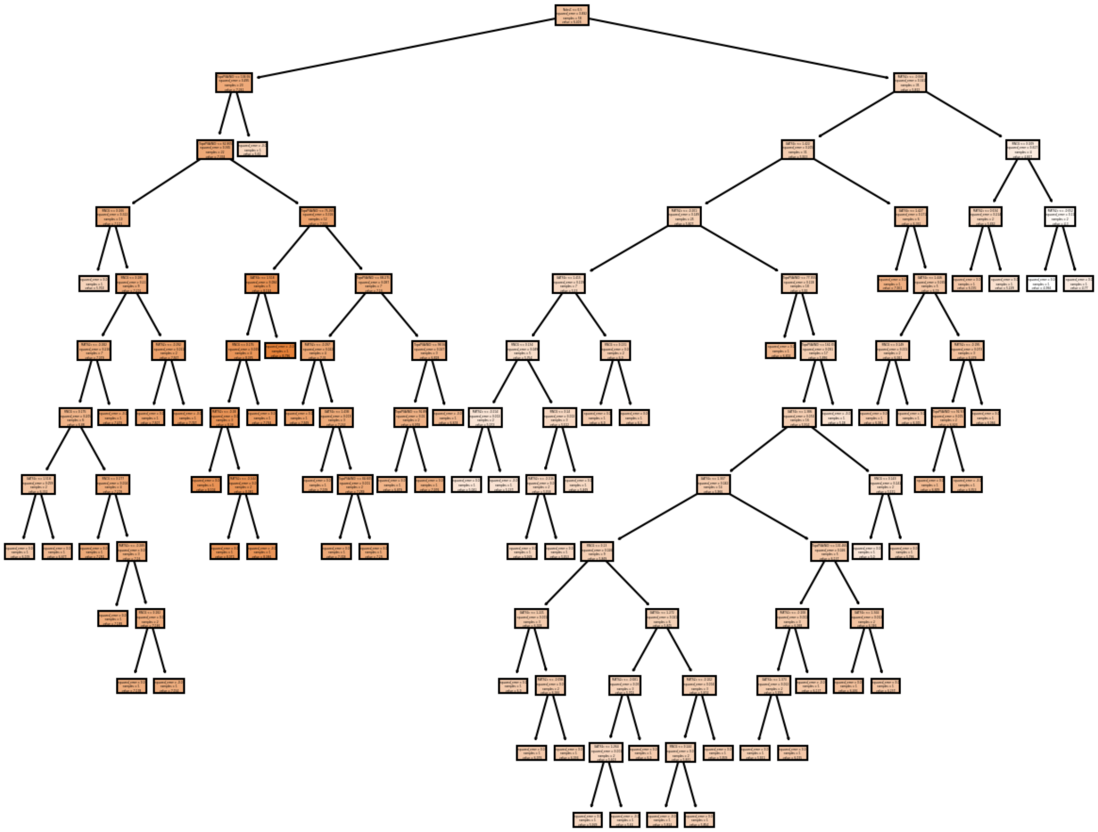

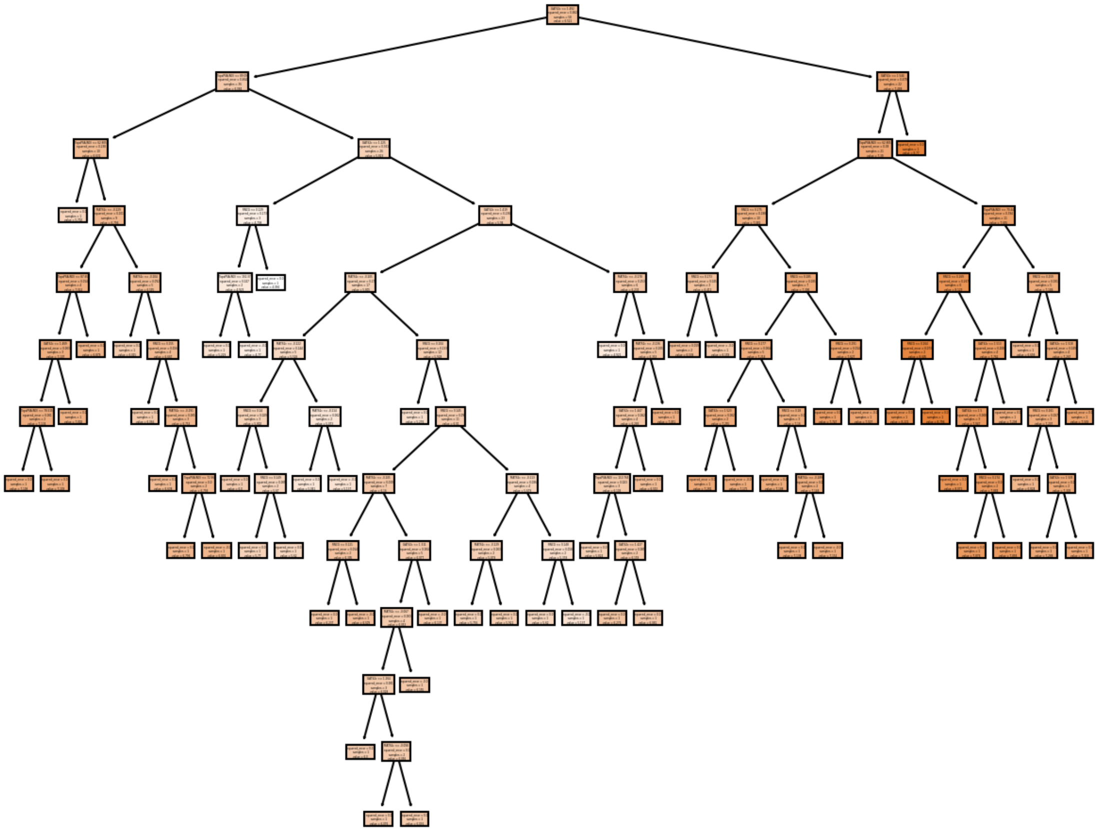

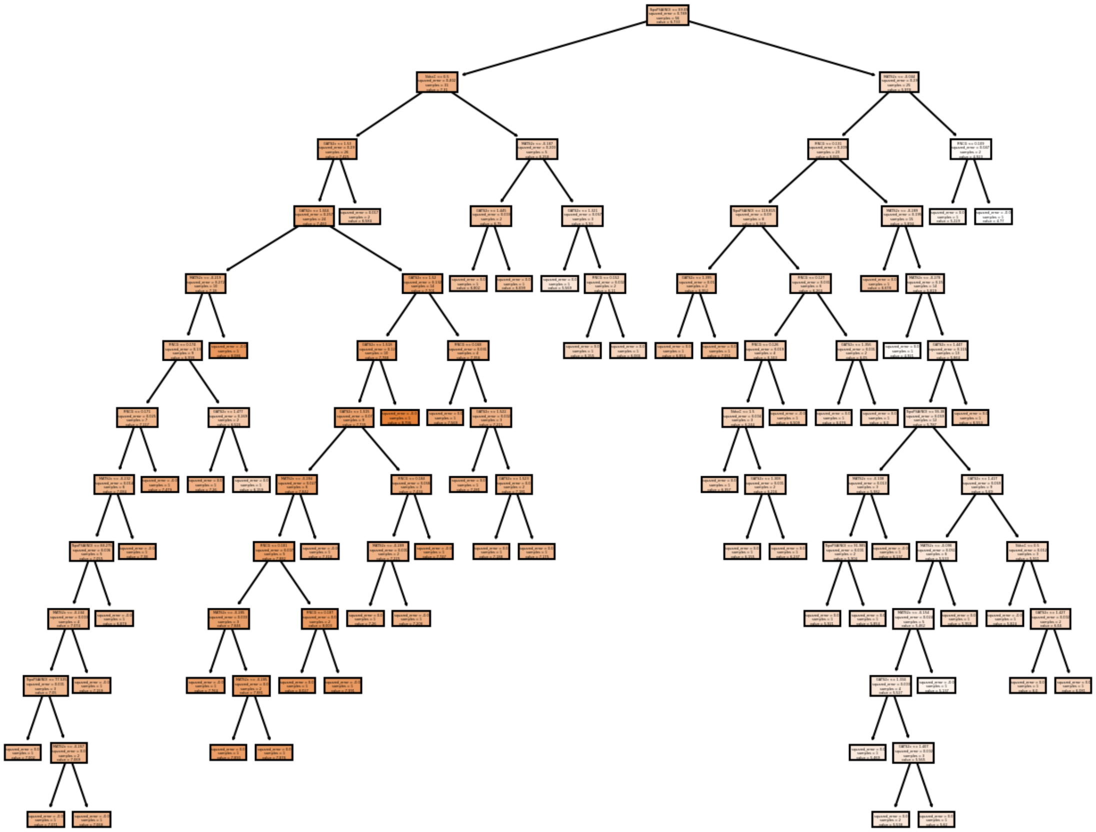

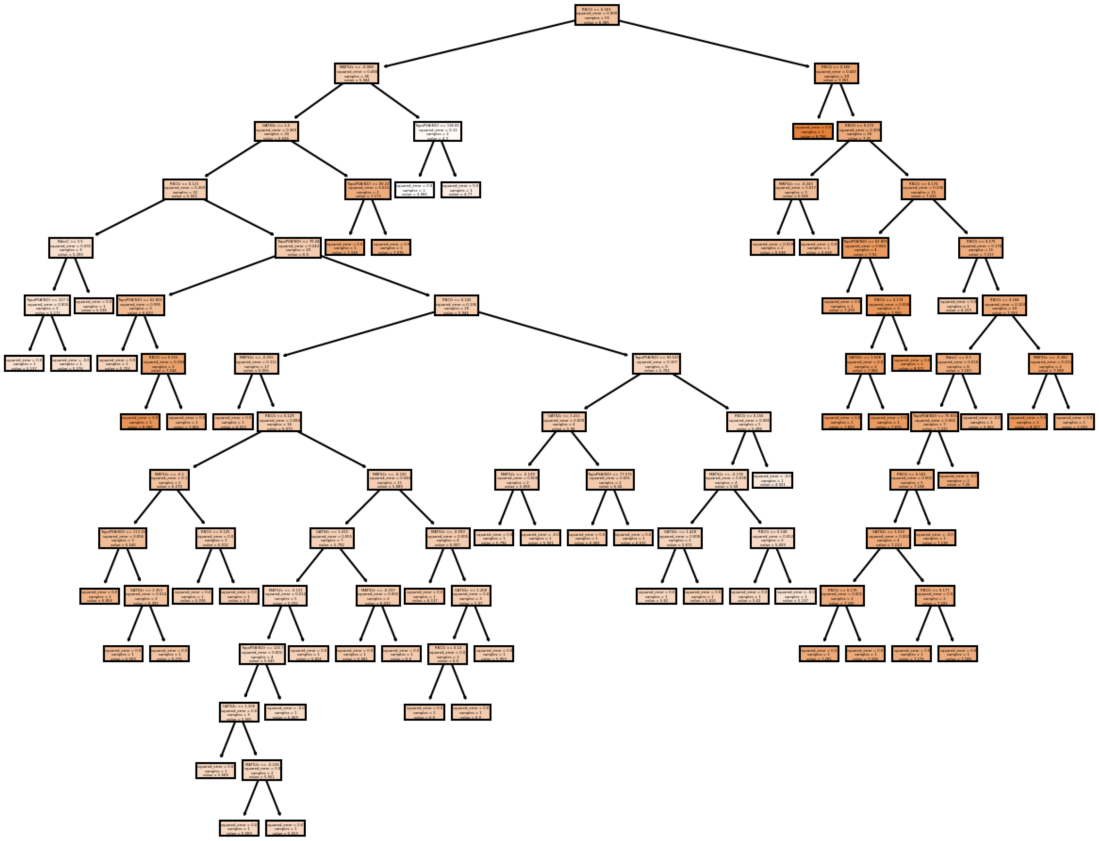

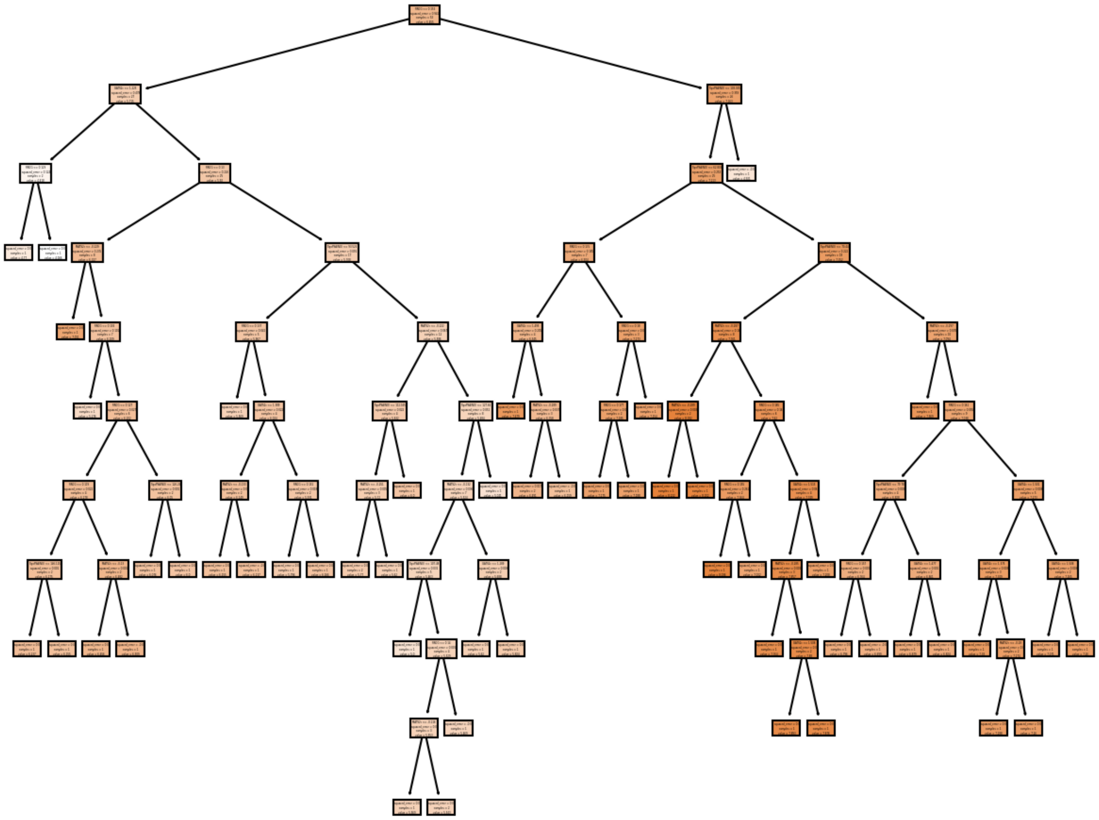

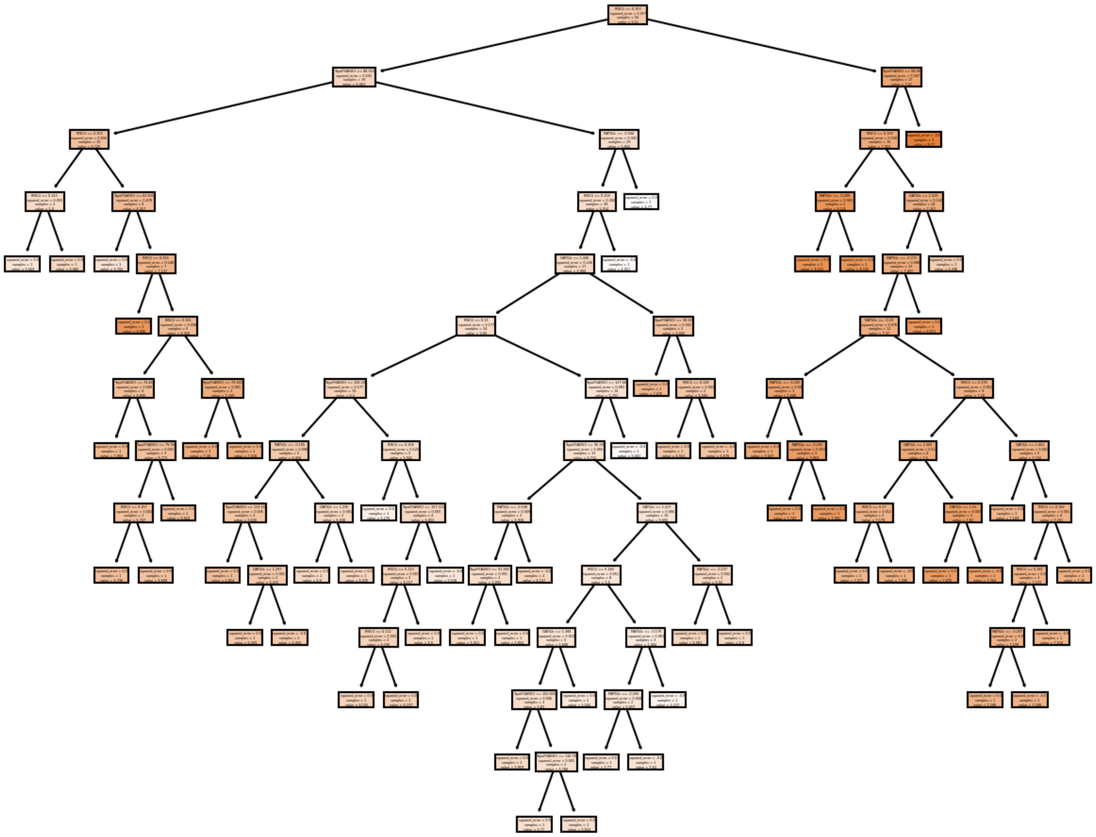

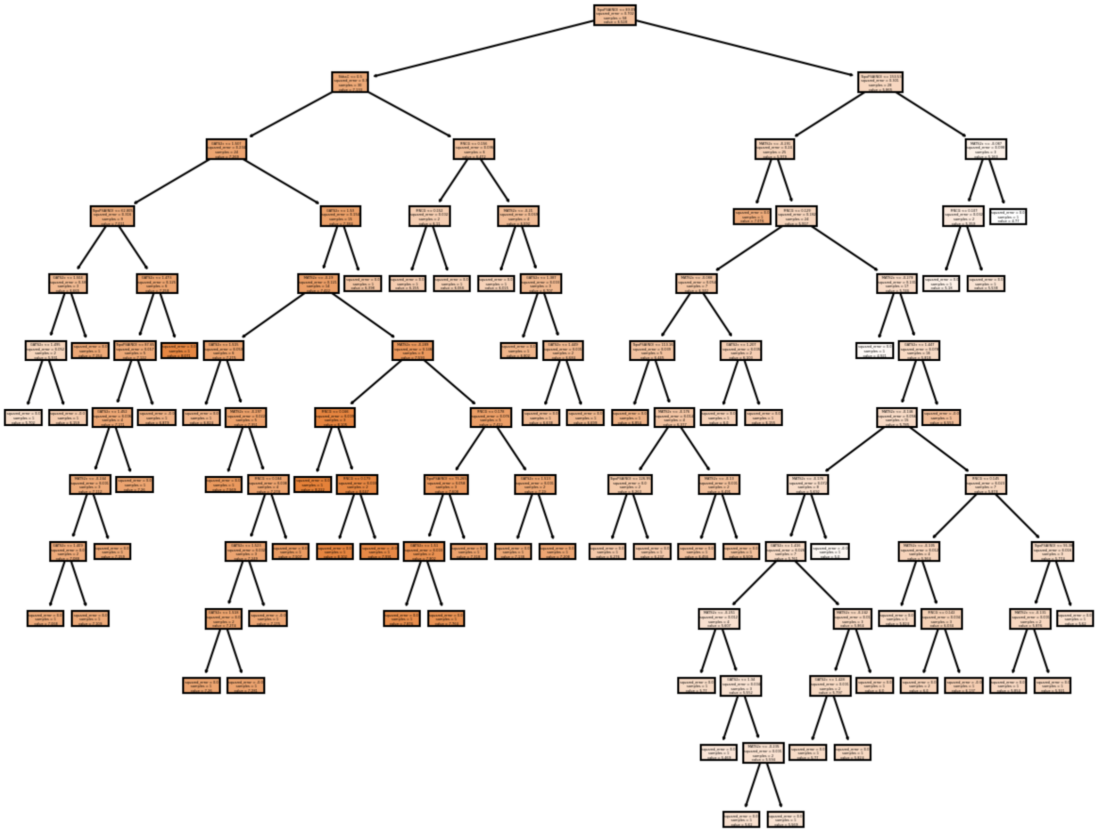

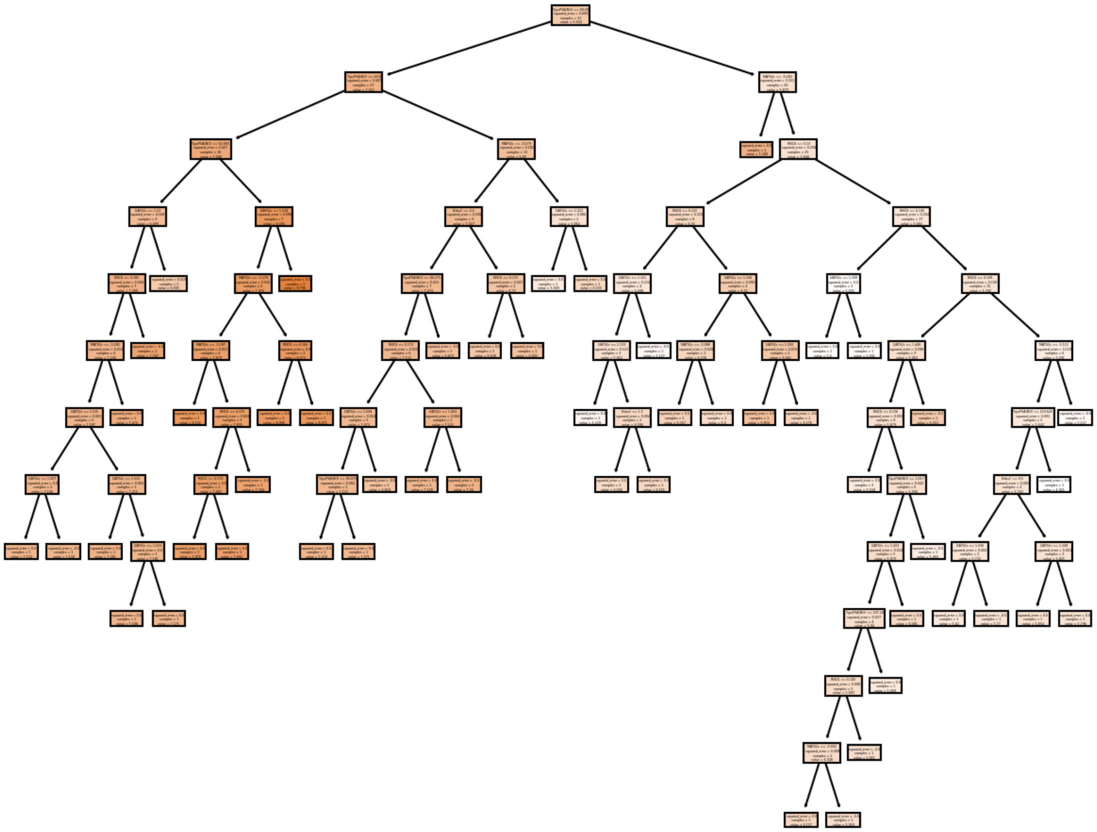

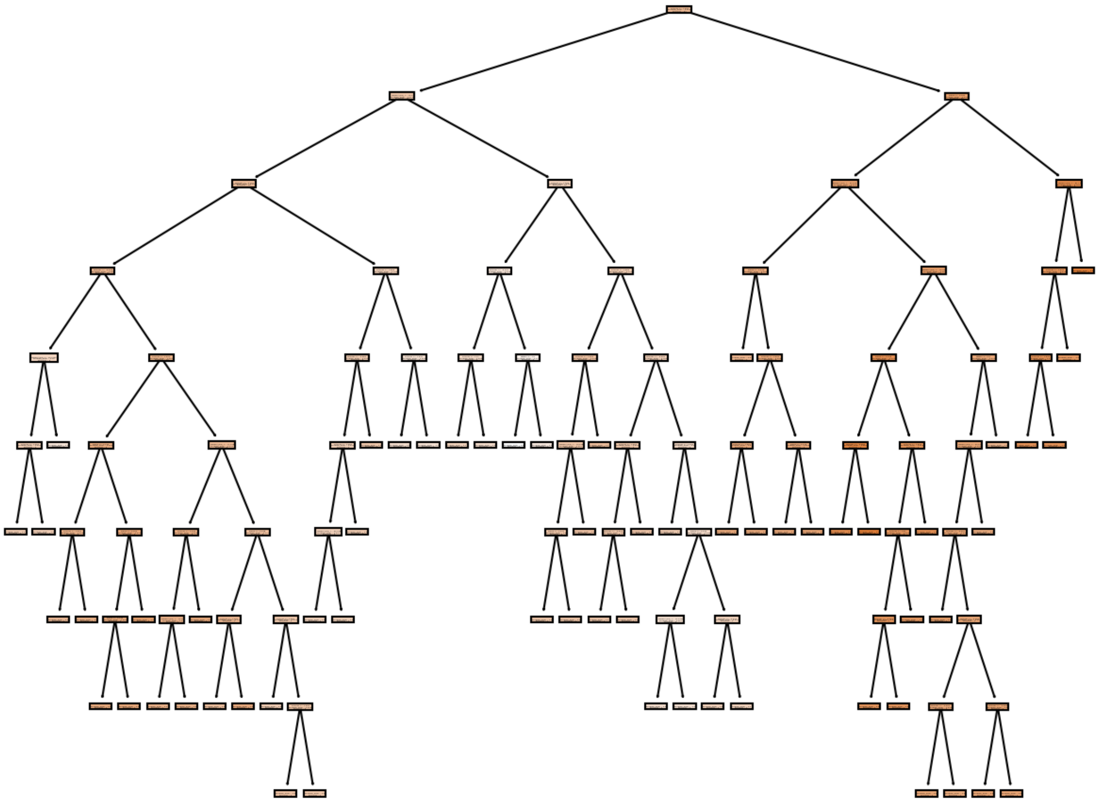

[44]:
```
hist2
```

[44]:

	molecular descriptor name	corr_value	absolute correlation value
556	GATS2c	0.652245	0.652245
696	MATS2c	-0.638624	0.638624
851	NdssC	-0.647960	0.647960
881	RNCG	0.663041	0.663041
1022	TopoPSA(NO)	-0.635724	0.635724

[45]:
```
predicted_activity = model.predict(molecular_descriptors[hist2['molecular␣
↪descriptor name']])
```

[46]:
```
save_to_df['Predicted LoVo/DX'] = predicted_activity
```

[47]:
```
save_to_df.head()
```

[47]:

	SMILES	Predicted A549 \
0	COC1=C2C3=CC=C(NC)C(=O)C=C3[C@H1](CCC2=CC(OC)=…	8.078406
1	COC1=C2C3=CC=C(NC)C(=O)C=C3[C@H1](CCC2=CC(OC)=…	7.867157

```
2  COC1=C2C3=CC=C(NC)C(=O)C=C3[C@H1](CCC2=CC(OC)=…        7.785028
3  COC1=C2C3=CC=C(NC)C(=O)C=C3[C@H1](CCC2=CC(OC)=…        7.742135
4  COC1=CC2=CC=C(NC)C(C=C2[C@@H1](N3C=C(N=N3)CN)C…        6.789342

   Predicted BALB/3T3  Predicted LoVo  Predicted LoVo/DX
0            7.961224        8.209224           7.025589
1            7.934181        7.866056           7.480642
2            7.407610        7.379055           7.617192
3            7.568620        8.016140           6.634258
4            7.974253        8.730218           5.695813
```

6 MCF-7

[48]:
```
## The best model is: Random Forest (correlation_threshold = 0.51, 4 features,␣
↪3 estimators, random_state=15)
```

[49]:
```
target = 'MCF-7'
data = load_prepared_data[list(load_prepared_data.columns)[0:-10]]
data[target] = load_prepared_data[load_prepared_data.columns[-4]]
data.head()
```

[49]:
```
      AATS0Z  AATS0are    AATS0d   AATS0dv      AATS0i     AATS0m    AATS0p  \
0  25.090909  6.046518  3.109091  6.662626  160.775045  99.075564  1.555512
1  24.448276  6.008422  3.051724  6.386973  161.021674  96.473315  1.538471
2  23.868852  5.974074  3.000000  6.138434  161.244045  94.127025  1.523105
3  23.868852  5.974074  3.032787  6.171220  161.244045  94.127025  1.523105
4  23.343750  5.942945  2.953125  5.913194  161.445569  92.000700  1.509180

    AATS0pe    AATS0s   AATS0se  …    piPC10     piPC2     piPC3     piPC4  \
0  6.126680  3.435416  7.629859  …  7.999655  4.266195  4.915408  5.516700
1  6.088791  3.330998  7.596891  …  8.018726  4.280132  4.922714  5.520711
2  6.054630  3.236850  7.567166  …  8.028799  4.293878  4.929967  5.524706
3  6.054630  3.257798  7.567166  …  8.037440  4.307438  4.929967  5.524706
4  6.023670  3.151529  7.540228  …  8.034892  4.307438  4.937168  5.528685

      piPC5     piPC6     piPC7     piPC8     piPC9     MCF-7
0  6.098566  6.595759  6.979116  7.337313  7.681445  7.987163
1  6.103048  6.601209  6.985613  7.349442  7.695531  7.920819
2  6.105281  6.603923  6.989306  7.353932  7.704023  7.913640
3  6.107510  6.606629  6.992068  7.361425  7.709420  7.946922
4  6.107510  6.605277  6.991148  7.356489  7.707175  7.032920

[5 rows x 1212 columns]
```

[50]:
```
model, train_r2_, test_r2_, hist1, hist2, target_column_name,␣
↪training_data_RMSE, test_data_RMSE  = pred_model.
↪prepare_data_and_create_model(molecular_descriptors_df=data,
```

```
                                                                                ␣
↪                    correlation_threshold=0.51,
                                                                                ␣
↪                    standardization=False,
                                                                                ␣
↪                    model_type='RandomForestRegressor',
                                                                                ␣
↪                    n_estimators_=3,
                                                                                ␣
↪                    target_column_name = target,
                                                                                ␣
↪                    random_state=15,
                                                                                ␣
↪                    train_test_split_=True,
                                                                                ␣
↪                    verbose=True)
print("R^2 score: " + str(r2_score(data[target], model.
 ↪predict(data[hist2['molecular descriptor name']]))))
print("Mean squared error: "+str(mean_squared_error(data[target], model.
 ↪predict(data[hist2['molecular descriptor name']]))))
print('Mean absolute error: ' + str(mean_absolute_error(data[target], model.
 ↪predict(data[hist2['molecular descriptor name']]))))
print("Root Mean Square Error: "+ str(math.
 ↪sqrt(mean_squared_error(data[target], model.predict(data[hist2['molecular␣
 ↪descriptor name']])))))
```

```
I am not doing standardization…
  molecular descriptor name
0                    AATS0Z
1                  AATS0are
2                    AATS0d
3                   AATS0dv
4                    AATS0i
  molecular descriptor name  corr_value
0                    AATS0Z   -0.038502
1                  AATS0are   -0.120847
2                    AATS0d    0.041648
3                   AATS0dv   -0.115899
4                    AATS0i    0.191765
  molecular descriptor name  corr_value  absolute correlation value
0                    AATS0Z   -0.038502                    0.038502
1                  AATS0are   -0.120847                    0.120847
2                    AATS0d    0.041648                    0.041648
3                   AATS0dv   -0.115899                    0.115899
4                    AATS0i    0.191765                    0.191765
    molecular descriptor name  corr_value  absolute correlation value
226                    AMID_O   -0.520578                    0.520578
```

```
505               EState_VSA5   -0.578904                     0.578904
506               EState_VSA6    0.519794                     0.519794
791                   MDEO-12   -0.525441                     0.525441
1211                    MCF-7    1.000000                     1.000000
    molecular descriptor name  corr_value  absolute correlation value
226                    AMID_O   -0.520578                     0.520578
505               EState_VSA5   -0.578904                     0.578904
506               EState_VSA6    0.519794                     0.519794
791                   MDEO-12   -0.525441                     0.525441
The model used is: RandomForest…
Return the coefficient of determination of the prediction:
0.6142840305325452
R^2 score: 0.819378937516738
Correlation coefficient: 0.9051955244679118
Test data - unseen during training:
R^2 score: 0.6142840305325452
Correlation coefficient: 0.7837627386732194
[7.51646048 5.63977394 6.68863667 8.06699682 8.02507489 7.52053863
 8.11761239 7.30778129 7.59968192 8.0589289  7.82677649 8.48554246
 8.51560134 7.36599904 7.51207137 8.08489178 6.30627483 5.35904447]
102    7.958607
38     6.022276
8      5.949079
109    8.086186
11     8.013228
51     8.000000
88     8.119186
21     8.113509
82     7.982967
98     7.795880
58     7.677781
64     8.619789
74     8.327902
103    8.376751
91     9.154902
43     7.958607
25     4.949659
30     6.010550
Name: MCF-7, dtype: float64
Training Root Mean Square Error: 0.39541164003880247
Testing Root Mean Square Error: 0.6692126081310851
R^2 score: 0.7801557253334048
Mean squared error: 0.20007463754869056
Mean absolute error: 0.2585070583354094
Root Mean Square Error: 0.4472970350323044
```

[51]:
```
# save
joblib.dump(model, "Random_forest/random_forest_model_3_estimators_MCF-7.
↪joblib")
```

[51]: ['Random_forest/random_forest_model_3_estimators_MCF-7.joblib']

[52]:
```
for x, decision_tree in enumerate(model.estimators_):
    plt.figure(figsize=(10,8), dpi=150)
    plot_tree(decision_tree, feature_names=list(hist2['molecular descriptor␣
↪name']),
              filled=True)
    plt.savefig('Random_forest/random_forest_3_'+str(x)+'_model_MCF-7.
↪pdf',bbox_inches = "tight")
```

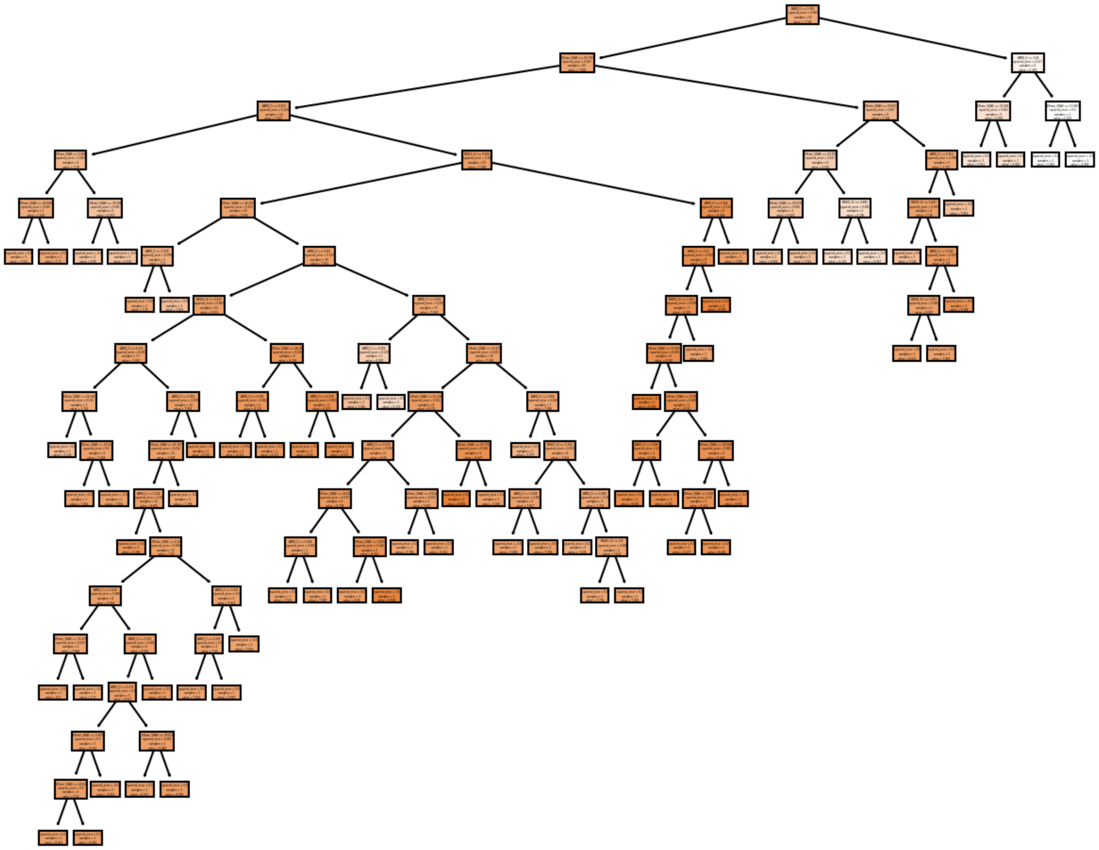

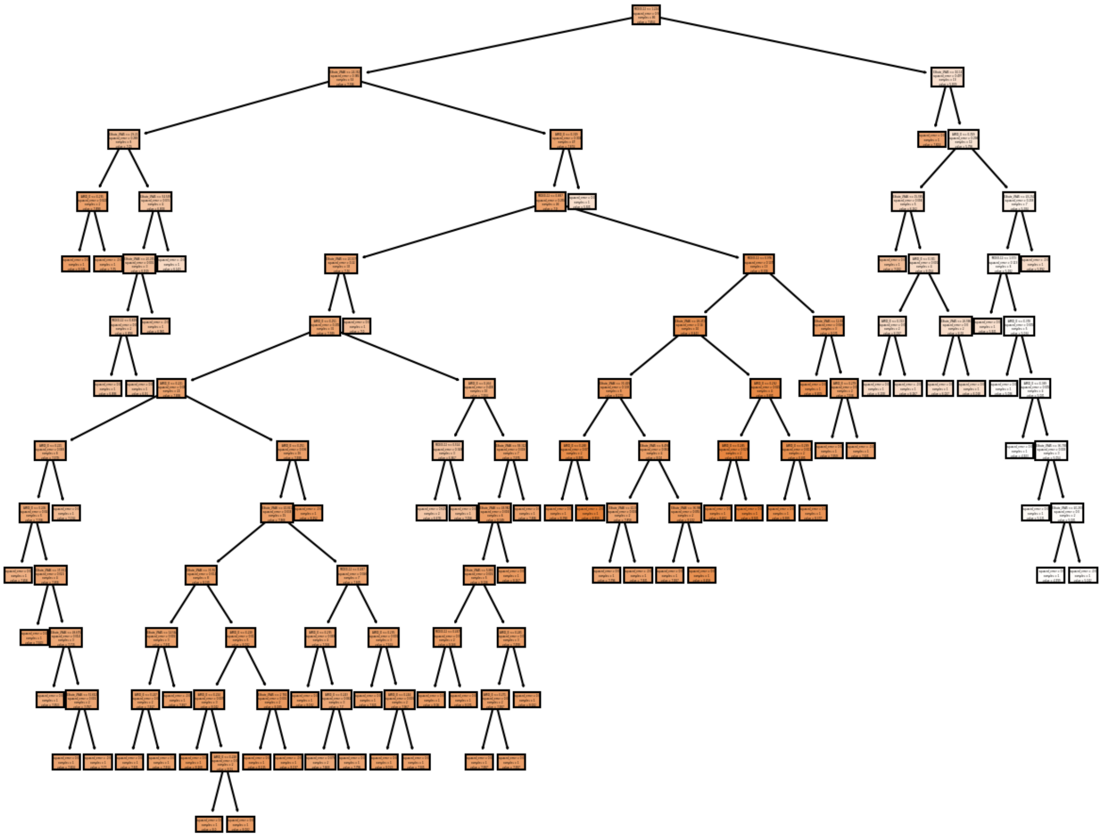

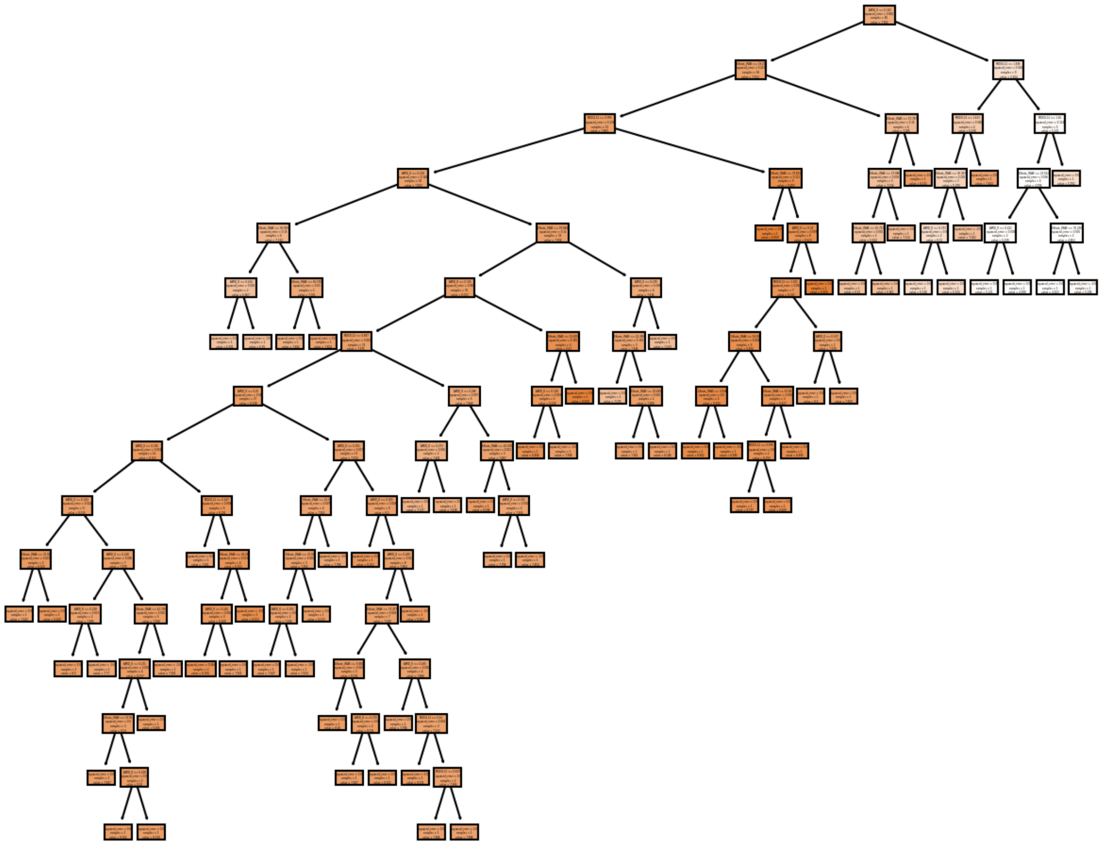

[53]:
```
hist2
```

[53]:

	molecular descriptor name	corr_value	absolute correlation value
226	AMID_O	-0.520578	0.520578
505	EState_VSA5	-0.578904	0.578904
506	EState_VSA6	0.519794	0.519794
791	MDEO-12	-0.525441	0.525441

[54]:
```
predicted_activity = model.predict(molecular_descriptors[hist2['molecular␣
↪descriptor name']])
```

[55]:
```
save_to_df['Predicted MCF-7'] = predicted_activity
```

[56]:
```
save_to_df.head()
```

[56]:

	SMILES	Predicted A549 \
0	COC1=C2C3=CC=C(NC)C(=O)C=C3[C@H1](CCC2=CC(OC)=…	8.078406
1	COC1=C2C3=CC=C(NC)C(=O)C=C3[C@H1](CCC2=CC(OC)=…	7.867157
2	COC1=C2C3=CC=C(NC)C(=O)C=C3[C@H1](CCC2=CC(OC)=…	7.785028

```
3  COC1=C2C3=CC=C(NC)C(=O)C=C3[C@H1](CCC2=CC(OC)=…        7.742135
4  COC1=CC2=CC=C(NC)C(C=C2[C@@H1](N3C=C(N=N3)CN)C…        6.789342

   Predicted BALB/3T3  Predicted LoVo  Predicted LoVo/DX  Predicted MCF-7
0            7.961224        8.209224           7.025589         8.077917
1            7.934181        7.866056           7.480642         7.992772
2            7.407610        7.379055           7.617192         8.040999
3            7.568620        8.016140           6.634258         8.267606
4            7.974253        8.730218           5.695813         8.042845
```

[57]:
```python
save_to_df['A549 [nM]'] = pred_model.inverse_transform(save_to_df['Predicted␣
↪A549'])
save_to_df['BALB/3T3 [nM]'] = pred_model.
↪inverse_transform(save_to_df['Predicted BALB/3T3'])
save_to_df['LoVo [nM]'] = pred_model.inverse_transform(save_to_df['Predicted␣
↪LoVo'])
save_to_df['LoVo/DX [nM]'] = pred_model.inverse_transform(save_to_df['Predicted␣
↪LoVo/DX'])
save_to_df['MCF-7 [nM]'] = pred_model.inverse_transform(save_to_df['Predicted␣
↪MCF-7'])
save_to_df.head()
```

[57]:
```
                                              SMILES  Predicted A549  \
0  COC1=C2C3=CC=C(NC)C(=O)C=C3[C@H1](CCC2=CC(OC)=…        8.078406
1  COC1=C2C3=CC=C(NC)C(=O)C=C3[C@H1](CCC2=CC(OC)=…        7.867157
2  COC1=C2C3=CC=C(NC)C(=O)C=C3[C@H1](CCC2=CC(OC)=…        7.785028
3  COC1=C2C3=CC=C(NC)C(=O)C=C3[C@H1](CCC2=CC(OC)=…        7.742135
4  COC1=CC2=CC=C(NC)C(C=C2[C@@H1](N3C=C(N=N3)CN)C…        6.789342

   Predicted BALB/3T3  Predicted LoVo  Predicted LoVo/DX  Predicted MCF-7  \
0            7.961224        8.209224           7.025589         8.077917
1            7.934181        7.866056           7.480642         7.992772
2            7.407610        7.379055           7.617192         8.040999
3            7.568620        8.016140           6.634258         8.267606
4            7.974253        8.730218           5.695813         8.042845

    A549 [nM]  BALB/3T3 [nM]  LoVo [nM]  LoVo/DX [nM]  MCF-7 [nM]
0    8.348220      10.933929   6.176982     94.278083    8.357629
1   13.578222      11.636406  13.612706     33.064228   10.167834
2   16.404839      39.119200  41.777761     24.143942    9.099151
3   18.107777      27.000988   9.635175    232.135864    5.400000
4  162.427093      10.610769   1.861153   2014.589795    9.060555
```

[58]:
```python
save_to_df.to_excel('../Data/Predicted_activities.xlsx')
```

File 37

```
import pandas as pd
from rdkit import Chem
from rdkit.Chem import Draw

file = pd.read_excel(r'../Data/Kolchicyna_prepared_data.xlsx')

file.head()
```

```
   Unnamed: 0                              Publikacja DOI  \
0           0  https://doi.org/10.1016/j.bmcl.2021.128382
1           1  https://doi.org/10.1016/j.bmcl.2021.128382
2           2  https://doi.org/10.1016/j.bmcl.2021.128382
3           3  https://doi.org/10.1016/j.bmcl.2021.128382
4           4  https://doi.org/10.1016/j.bmcl.2021.128382

  Numer związku w publikacji  \
0                          1
1                          2
2                          3
3                          4
4                          5

                                              SMILES  Atywność [nM]
A549  \
0  COc2c3C1=CC=C(SC)C(=O)C=C1[C@H](CCc3cc(OC)c2OC...  Atywność [nM]
10,8
1  COc2c3C1=CC=C(SC)C(=O)C=C1[C@H](CCc3cc(OC)c2OC...  Atywność [nM]
11,6
2  COc2c3C1=CC=C(SC)C(=O)C=C1[C@H](CCc3cc(OC)c2OC...  Atywność [nM]
10,9
3  CC(C)CN[C@H]2CCc3cc(OC)c(OC)c(OC)c3C1=CC=C(SC)...  Atywność [nM]
10,5
4  COc2c3C1=CC=C(SC)C(=O)C=C1[C@H](CCc3cc(OC)c2OC...  Atywność [nM]
89,5

   MCF-7    LoVo  LoVo/DX  BALB/3T3  A549_float  MCF-7_float  LoVo_float
\
0  10,3    6,5     54,9     10,2          10.8         10.3         6.5

1  12,0    8,5      31.1     14.3         11.6         12.0         8.5

2  12,2    8,8     17,9     11,7          10.9         12.2         8.8

3  11,3    8,5     10,2     11,0          10.5         11.3         8.5

4  92,7   52,8     77,8     99,4          89.5         92.7        52.8
```

```
   LoVo/DX_float  BALB/3T3_float
0           54.9            10.2
1           31.1            14.3
2           17.9            11.7
3           10.2            11.0
4           77.8            99.4
```

```
from rdkit import Chem
from rdkit.Chem import Draw
from rdkit.Chem.Draw import IPythonConsole
from rdkit.Chem import rdFMCS
from rdkit.Chem.Draw import rdDepictor
rdDepictor.SetPreferCoordGen(True)
#IPythonConsole.drawOptions.minFontSize=20
def view_difference(mol1, mol2):
    mcs = rdFMCS.FindMCS([mol1,mol2])
    mcs_mol = Chem.MolFromSmarts(mcs.smartsString)
    match1 = mol1.GetSubstructMatch(mcs_mol)
    target_atm1 = []
    for atom in mol1.GetAtoms():
        if atom.GetIdx() not in match1:
            target_atm1.append(atom.GetIdx())
    match2 = mol2.GetSubstructMatch(mcs_mol)
    target_atm2 = []
    for atom in mol2.GetAtoms():
        if atom.GetIdx() not in match2:
            target_atm2.append(atom.GetIdx())
    return Draw.MolsToGridImage([mol1,
mol2],highlightAtomLists=[target_atm1, target_atm2]) #, useSVG=True


mol1 = Chem.MolFromSmiles(file['SMILES'][0])
mol2 = Chem.MolFromSmiles('C1Cc2ccccc2C2=CC=CCC=C2C1')

view_difference(mol1,mol2)
```

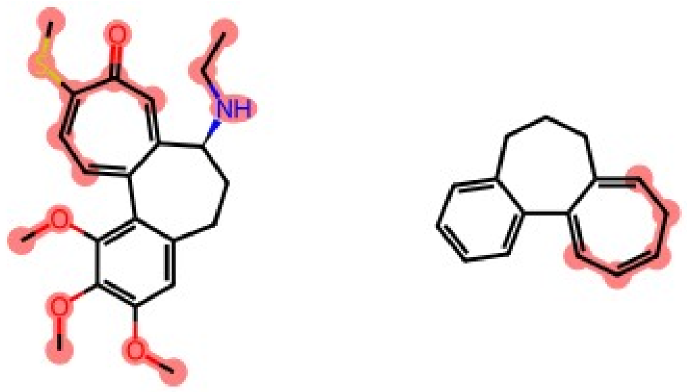

```
l_mn = list(range(len(file['SMILES'])))
mol1 = Chem.MolFromSmiles('C1Cc2ccccc2C2=CC=CCC=C2C1')
for i in range(len(file['SMILES'])):
    mol = Chem.MolFromSmiles(file['SMILES'][i])
    l_mn[i] = view_difference(mol1, mol)

l_mn[0]
```

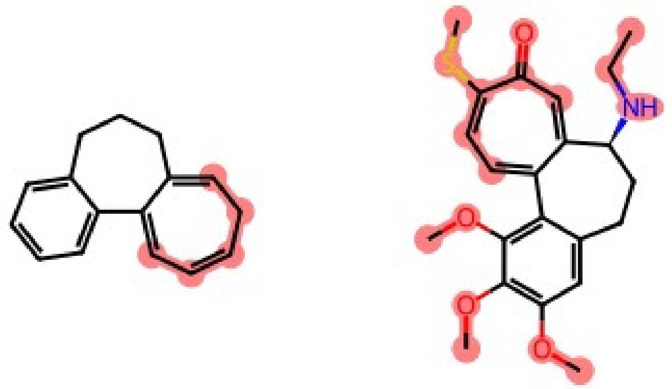

```
l_mn[1]
```

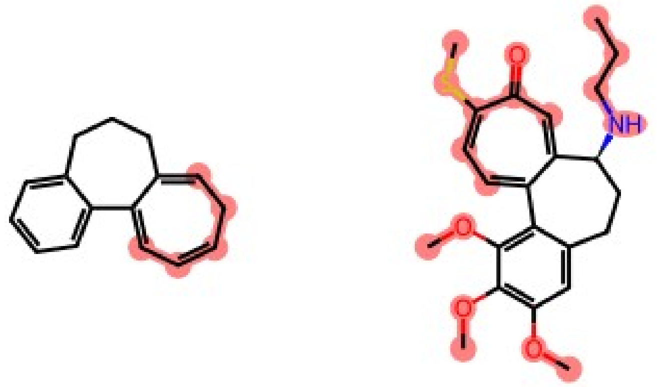

```
l_mn[7]
```

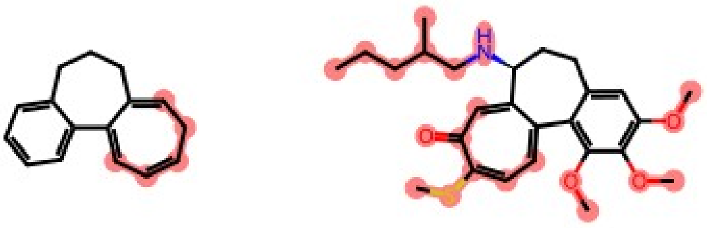


```
m = Chem.MolFromSmiles(file['SMILES'][0])
substructure =
Chem.MolFromSmarts('CC(=O)NC1CCC2=CC(=C(C(=C2C3=CC=C(C(=O)C=C13)OC)OC)
OC)OC')
print(m.GetSubstructMatch(substructure)) #GetSubstructMatch
#GetSubstructMatches
```

```
()
```

```
m
```

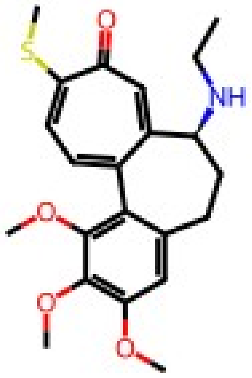


```
file['SMILES'][0]
```

```
'COc2c3C1=CC=C(SC)C(=O)C=C1[C@H](CCc3cc(OC)c2OC)NCC'
```

```
Chem.MolFromSmiles('CC(=O)NC1CCC2=CC(=C(C(=C2C3=CC=C(C(=O)C=C13)OC)OC)
OC)OC')
```

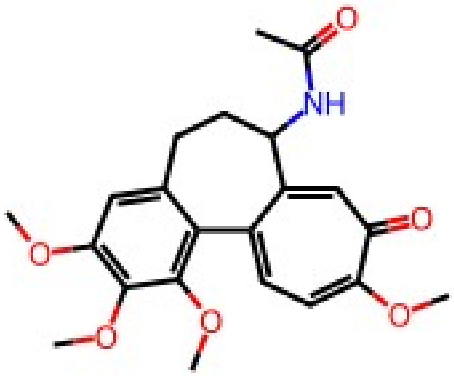

```
# draw structure and get SMILES -> only of the core
interesting_smiles = 'C1Cc2ccccc2C2=CC=CCC=C2C1'
#'C1CC2=CCC=CC=C2C3=CC=CC=C3C1' # OLD'C1CC2=C(C=CC=C2)C2=CC=CCC=C2C1'
#C1CC2=CCC=CC=C2C3=CC=CC=C3C1

Chem.MolFromSmiles(interesting_smiles)
```

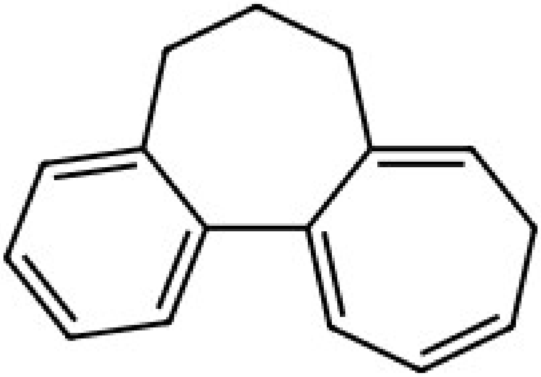

```
m = Chem.MolFromSmiles(file['SMILES'][0])
substructure = Chem.MolFromSmarts(interesting_smiles)
print(m.GetSubstructMatch(substructure))

()

m
```

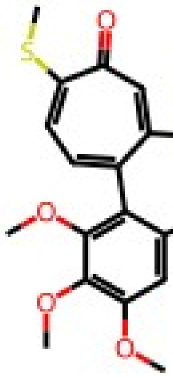


```
ne_att = 'CC\C(C)=C/C(=C)C=C'

Chem.MolFromSmiles(ne_att)
```

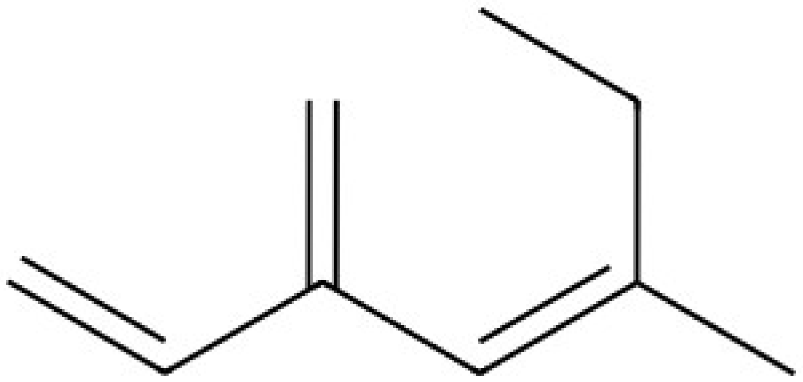

```
bnm = Chem.MolToSmiles(Chem.MolFromSmiles(file['SMILES'][0]),
canonical=True, isomericSmiles=False)
m = Chem.MolFromSmiles(bnm)
substructure = Chem.MolFromSmarts('c1ccccc1')
substructure2 = Chem.MolFromSmarts('CCC')
print(m.GetSubstructMatches(substructure))
#print(m)
#print(m.GetSubstructMatches(substructure2))

((6, 7, 8, 11, 14, 17),)

m
```

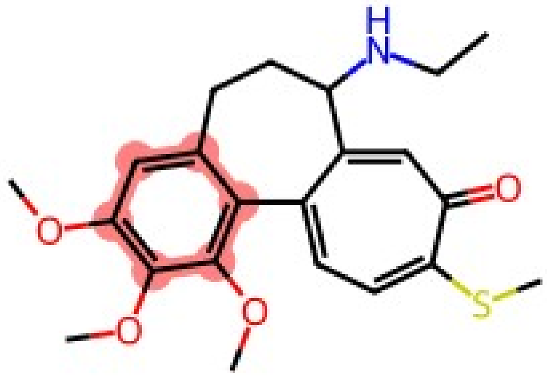

```
bnm
```

```
'CCNC1CCc2cc(OC)c(OC)c(OC)c2-c2ccc(SC)c(=O)cc21'
```

```
bnmo = 'C1Cc2ccccc2C2=CC=CCC=C2C1'
bnmo = Chem.MolToSmiles(Chem.MolFromSmiles(bnmo), canonical=True,
isomericSmiles=False)
```

```
m = Chem.MolFromSmiles(bnm)
substructure = Chem.MolFromSmarts(bnmo)
print(m.GetSubstructMatch(substructure))
```

```
()
```

```
m
```

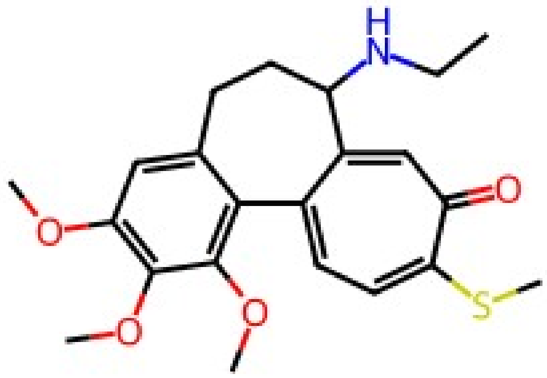

```
all_mols = [Chem.MolFromSmiles(smi) for smi in file['SMILES']]
```

```
my_svg = Draw.MolsToGridImage(all_mols,legends=[str("Molecule number:
"+str(x+1)) for x in range(len(all_mols))],
                              maxMols=int(len(all_mols)),
molsPerRow=5, useSVG = True, subImgSize=(300,300))
my_svg
```

```
#with open('initial_colchicine.svg','wb+') as outfile:
   # outfile.write(my_svg.data.encode('utf-8'))

## create the charset and other stuff.... check this and try to train the model...
```

Tanimoto similarity between...

```
from rdkit.DataStructs.cDataStructs import TanimotoSimilarity
import matplotlib.pyplot as plt

def prepare_fingerprints(smi_1, smi_list2):
    mol_l1 = Chem.MolFromSmiles(smi_1)
    mol_l2 = [Chem.MolFromSmiles(smi) for smi in smi_list2]

    fingerprint_list1 = Chem.RDKFingerprint(mol_l1)

    fingerprint_list2 = [Chem.RDKFingerprint(mol) for mol in mol_l2]

    return fingerprint_list1, fingerprint_list2

def calculate_similarity(finger, finger_list):

    similarity = []
    for sim in finger_list:
        similarity.append(TanimotoSimilarity(sim, finger))

    return similarity

nea = prepare_fingerprints(interesting_smiles, file['SMILES'])

simi = calculate_similarity(nea[0], nea[1])

final_df = pd.DataFrame(columns=['test'])
final_df['Similarity'] = 0
final_df['Similarity'] = simi

fig = plt.figure(figsize=(20,20))
ax = fig.add_subplot(111)

plt.hist(final_df['Similarity'], bins=10)
plt.title('Tanimoto similarity ...', fontweight='bold', fontsize=28)
plt.xlabel('Tanimoto similarity', fontweight='bold', fontsize=24)
plt.ylabel('Counts', fontweight='bold', fontsize=24)
plt.rc('grid', linestyle="-.", color='black')
plt.grid(True)
plt.show()
```

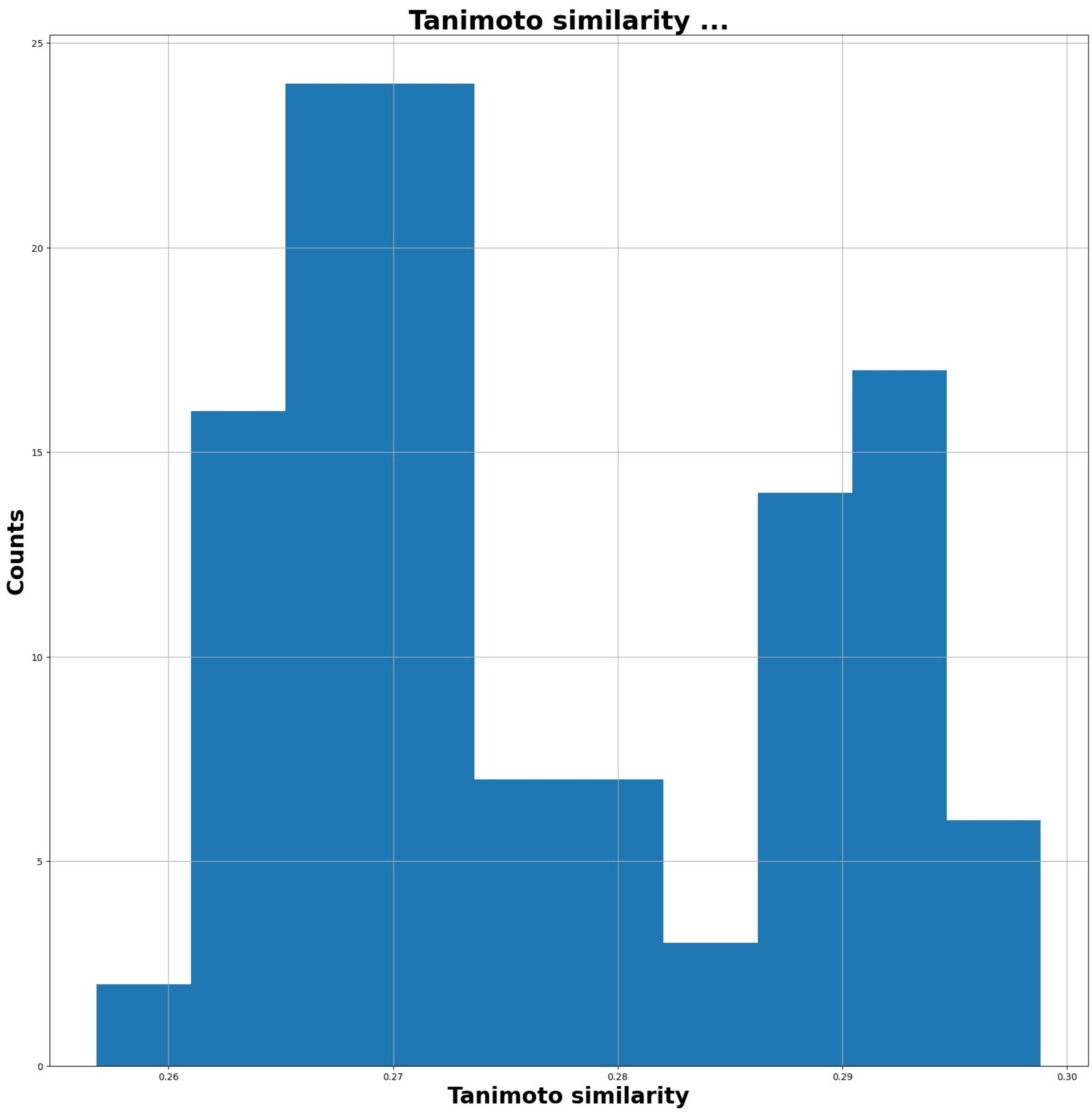


```
min(final_df['Similarity'])
```

```
0.2568064753495217
```

Notebook

December 19, 2023

1 File 38

[1]:
```
import pandas as pd
from rdkit import Chem
from rdkit.Chem import Draw
from rdkit.DataStructs.cDataStructs import TanimotoSimilarity
import matplotlib.pyplot as plt

import warnings
warnings.filterwarnings(action='ignore')
```

[2]:
```
dat_1 = pd.read_excel(r'../Data/AI_generated_Molecules_0_1_.xlsx')
dat_2 = pd.read_excel(r'../Data/AI_generated_Molecules_0_2_.xlsx')
```

[3]:
```
dat_1.head()
```

[3]:
```
   Unnamed: 0                                 AI_generated_SMILES
0           0  COC1=C2C3=CC=C(NC)C(=O)C=C3[C@H1](CCC2=CC(OC)=…
1           1  COC1=C2C3=CC=C(NC)C(=O)C=C3[C@H1](CCC2=CC(OC)=…
2           2  COC1=C2C3=CC=C(NC)C(=O)C=C3[C@H1](CCC2=CC(OC)=…
3           3  COC1=C2C3=CC=C(NC)C(=O)C=C3[C@H1](CCC2=CC(OC)=…
4           4  COC1=C2C3=CC=C(NC)C(=O)C=C3[C@H1](CCC2=CC(OC)=…
```

[4]:
```
dat_2.head()
```

[4]:
```
   Unnamed: 0                                 AI_generated_SMILES
0           0  COC1=C2C3=CC=C(NC)C(=O)C=C3[C@H1](CCC2=CC(OC)=…
1           1  COC1=C2C3=CC=C(NC)C(=O)C=C3[C@H1](CCC2=CC(OC)=…
2           2  CSCC(=O)C=C1C(C2=C(OC)C(OC)=C(C=C2CC[C@@H1]1NC…
3           3  CNCCC=C1C(C2=C(OC)C(=C(C=C2CC[C@@H1]1NCN)C)OO)COC
4           4  COC1=CC2=C(C3=C(OC)C(OC)=CC=C3CC[C@@H1]2NOC)CC…
```

[5]:
```
all_frames = [dat_1, dat_2]
all_dat = pd.concat(all_frames)
```

[6]:
```
all_dat.shape
```

[6]:
```
(2175, 2)
```

[7]:
```
all_dat = all_dat.drop_duplicates(subset=['AI_generated_SMILES'])
all_dat.shape
```

[7]: (1786, 2)

[8]:
```
all_dat.head()
```

[8]:

	Unnamed: 0	AI_generated_SMILES
0	0	COC1=C2C3=CC=C(NC)C(=O)C=C3[C@H1](CCC2=CC(OC)=…
1	1	COC1=C2C3=CC=C(NC)C(=O)C=C3[C@H1](CCC2=CC(OC)=…
2	2	COC1=C2C3=CC=C(NC)C(=O)C=C3[C@H1](CCC2=CC(OC)=…
3	3	COC1=C2C3=CC=C(NC)C(=O)C=C3[C@H1](CCC2=CC(OC)=…
4	4	COC1=C2C3=CC=C(NC)C(=O)C=C3[C@H1](CCC2=CC(OC)=…

[9]:
```
## Similar structures
Colchicine_core = 'C1Cc2ccccc2C2=CC=CCC=C2C1'
```

[10]:
```
def prepare_fingerprints(smi_1, smi_list2):
    mol_l1 = Chem.MolFromSmiles(smi_1)
    mol_l2 = [Chem.MolFromSmiles(smi) for smi in smi_list2]

    fingerprint_list1 = Chem.RDKFingerprint(mol_l1)

    fingerprint_list2 = [Chem.RDKFingerprint(mol) for mol in mol_l2]

    return fingerprint_list1, fingerprint_list2
```

[11]:
```
def calculate_similarity(finger, finger_list):

    similarity = []
    for sim in finger_list:
        similarity.append(TanimotoSimilarity(sim, finger))

    return similarity
```

[12]:
```
nea = prepare_fingerprints(Colchicine_core, all_dat['AI_generated_SMILES'])
```

[13]:
```
simi = calculate_similarity(nea[0], nea[1])
```

[14]:
```
final_df = pd.DataFrame(columns=['test'])
final_df['Similarity'] = 0
final_df['Similarity'] = simi
all_dat['Similarity to colchicine core'] = simi
```

[15]:
```
fig = plt.figure(figsize=(20,20))
ax = fig.add_subplot(111)

plt.hist(final_df['Similarity'], bins=10)
```

```
plt.title('Tanimoto similarity of newly generated structures to colchicine␣
 ↪core', fontweight='bold', fontsize=28)
plt.xlabel('Tanimoto similarity', fontweight='bold', fontsize=24)
plt.ylabel('Counts', fontweight='bold', fontsize=24)
plt.rc('grid', linestyle="-.", color='black')
plt.axvline(x = 0.2568064753495217, color = 'r', label = 'left-side cut off')
plt.grid(True)
plt.show()
```

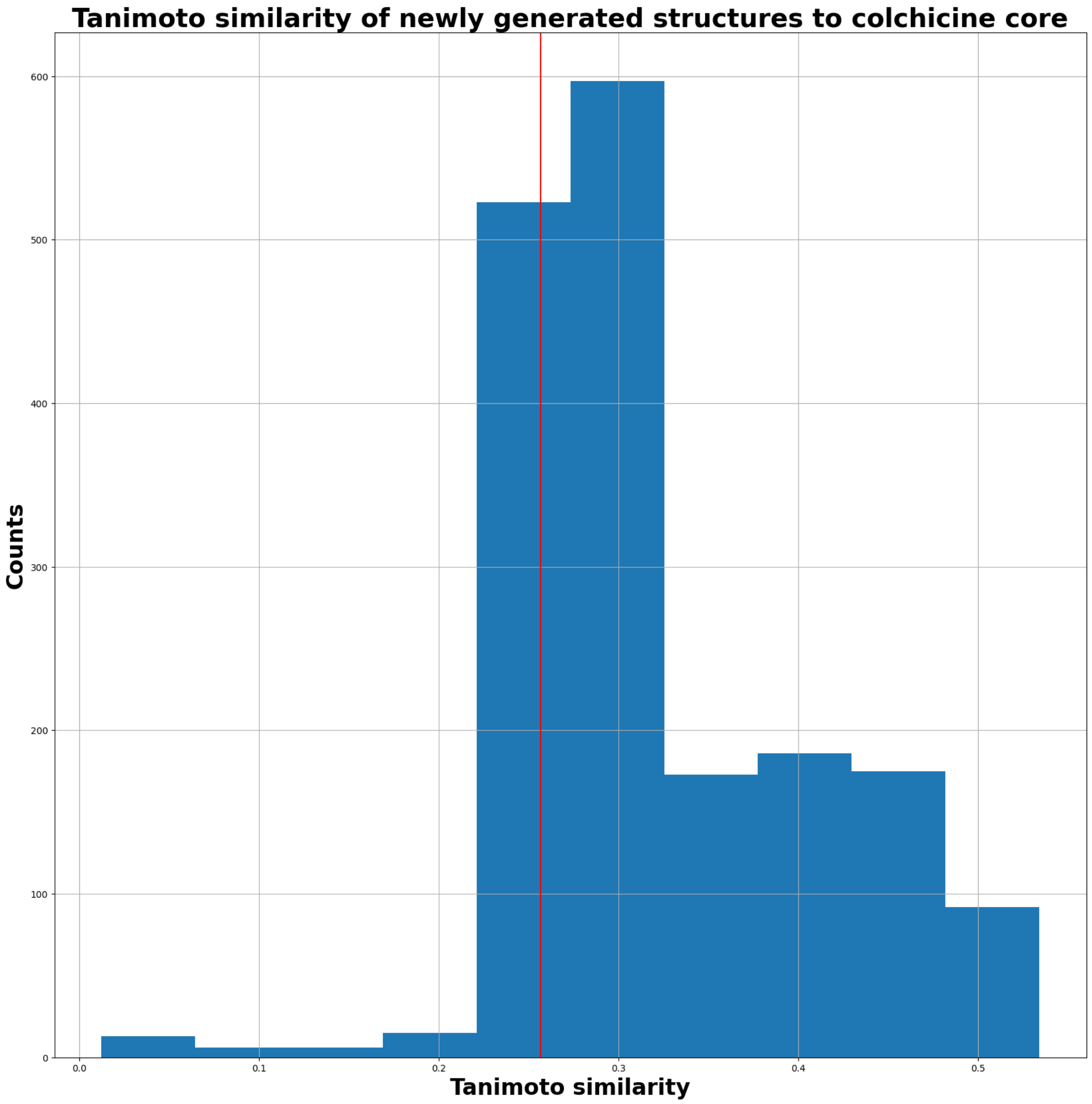

[16]:
```
final_df['Similarity'].min()
```

[16]: 0.01228878648233487

[ ]:

```python

```

[17]:

```python
Colchicine = 'CC(=O)NC1CCC2=CC(=C(C(=C2C3=CC=C(C(=O)C=C13)OC)OC)OC)OC'
```

[18]:

```python
nea = prepare_fingerprints(Colchicine, all_dat['AI_generated_SMILES'])
```

[19]:

```python
simi = calculate_similarity(nea[0], nea[1])
```

[20]:

```python
final_df = pd.DataFrame(columns=['test'])
final_df['Similarity'] = 0
final_df['Similarity'] = simi
all_dat['Similarity to colchicine'] = simi
```

[21]:

```python
fig = plt.figure(figsize=(20,20))
ax = fig.add_subplot(111)

plt.hist(final_df['Similarity'], bins=10)
plt.title('Tanimoto similarity of newly generated structures to colchicine',␣
 ↪fontweight='bold', fontsize=28)
plt.xlabel('Tanimoto similarity', fontweight='bold', fontsize=24)
plt.ylabel('Counts', fontweight='bold', fontsize=24)
plt.rc('grid', linestyle="-.", color='black')
plt.axvline(x = 0.2568064753495217, color = 'r', label = 'left-side cut off')
plt.grid(True)
plt.show()
```

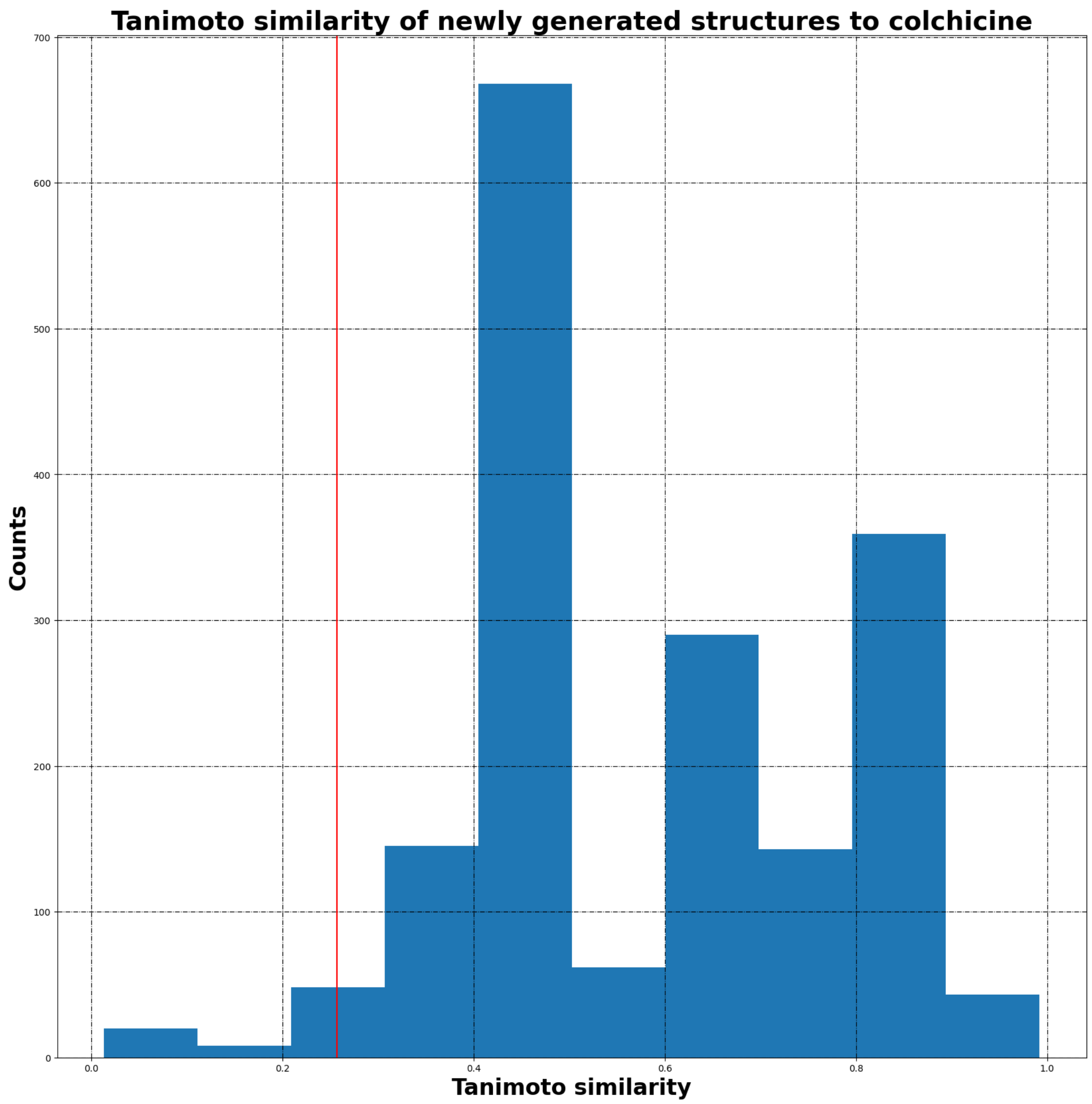


```
[22]: final_df['Similarity'].min()
```

[22]: 0.013404825737265416

```
[23]: all_dat.head()
```

```
[23]:    Unnamed: 0                                    AI_generated_SMILES  \
0             0  COC1=C2C3=CC=C(NC)C(=O)C=C3[C@H1](CCC2=CC(OC)=…
1             1  COC1=C2C3=CC=C(NC)C(=O)C=C3[C@H1](CCC2=CC(OC)=…
2             2  COC1=C2C3=CC=C(NC)C(=O)C=C3[C@H1](CCC2=CC(OC)=…
3             3  COC1=C2C3=CC=C(NC)C(=O)C=C3[C@H1](CCC2=CC(OC)=…
4             4  COC1=C2C3=CC=C(NC)C(=O)C=C3[C@H1](CCC2=CC(OC)=…
```

```
   Similarity to colchicine core  Similarity to colchicine
0                       0.266997                  0.871074
1                       0.268667                  0.844730
2                       0.268310                  0.862520
3                       0.266807                  0.862969
4                       0.270042                  0.860522
```

[24]:
```
to_colchicyne_core = all_dat[all_dat['Similarity to colchicine core'] > 0.
 ↪2568064753495217]
to_colchicyne_core.shape
```

[24]:
```
(1678, 4)
```

[25]:
```
to_colchicyne = all_dat[all_dat['Similarity to colchicine'] > 0.
 ↪2568064753495217]
to_colchicyne.shape
```

[25]:
```
(1740, 4)
```

[ ]:
```
## Delete structures that are recreated => 100% similar (the same as initial)
```

[26]:
```
initial_data = pd.read_excel(r'../Data/Kolchicyna_prepared_data.xlsx')
```

[27]:
```
new_df = pd.DataFrame(data=to_colchicyne_core, columns=['AI_generated_SMILES'])
for element in initial_data['SMILES']:
    new_df[element] = 0
```

[28]:
```
new_df = new_df.reset_index()
new_df = new_df.drop(columns=['index'])
new_df.head()
```

[28]:
```
                                 AI_generated_SMILES  \
0  COC1=C2C3=CC=C(NC)C(=O)C=C3[C@H1](CCC2=CC(OC)=…
1  COC1=C2C3=CC=C(NC)C(=O)C=C3[C@H1](CCC2=CC(OC)=…
2  COC1=C2C3=CC=C(NC)C(=O)C=C3[C@H1](CCC2=CC(OC)=…
3  COC1=C2C3=CC=C(NC)C(=O)C=C3[C@H1](CCC2=CC(OC)=…
4  COC1=C2C3=CC=C(NC)C(=O)C=C3[C@H1](CCC2=CC(OC)=…

   COc2c3C1=CC=C(SC)C(=O)C=C1[C@H](CCc3cc(OC)c2OC)NCC  \
0                                                  0
1                                                  0
2                                                  0
3                                                  0
4                                                  0

   COc2c3C1=CC=C(SC)C(=O)C=C1[C@H](CCc3cc(OC)c2OC)NCCC  \
0                                                   0
```

```
1                                                  0
2                                                  0
3                                                  0
4                                                  0

   COc2c3C1=CC=C(SC)C(=O)C=C1[C@H](CCc3cc(OC)c2OC)NCCCC  \
0                                                  0
1                                                  0
2                                                  0
3                                                  0
4                                                  0

   CC(C)CN[C@H]2CCc3cc(OC)c(OC)c(OC)c3C1=CC=C(SC)C(=O)C=C12  \
0                                                  0
1                                                  0
2                                                  0
3                                                  0
4                                                  0

   COc2c3C1=CC=C(SC)C(=O)C=C1[C@H](CCc3cc(OC)c2OC)NCCCCC  \
0                                                  0
1                                                  0
2                                                  0
3                                                  0
4                                                  0

   CC(C)CCN[C@H]2CCc3cc(OC)c(OC)c(OC)c3C1=CC=C(SC)C(=O)C=C12  \
0                                                  0
1                                                  0
2                                                  0
3                                                  0
4                                                  0

   COc2c3C1=CC=C(SC)C(=O)C=C1[C@H](CCc3cc(OC)c2OC)NCCCCCC  \
0                                                  0
1                                                  0
2                                                  0
3                                                  0
4                                                  0

   CC(CCC)CN[C@H]2CCc3cc(OC)c(OC)c(OC)c3C1=CC=C(SC)C(=O)C=C12  \
0                                                  0
1                                                  0
2                                                  0
3                                                  0
4                                                  0
```

```
   COc2c3C1=CC=C(SC)C(=O)C=C1[C@H](CCc3cc(OC)c2OC)NCCCCCCCC  …  \
0                                                          0          …
1                                                          0          …
2                                                          0          …
3                                                          0          …
4                                                          0          …

   CCN(CC)C(=S)N[C@H]2CCc3cc(OC)c(OC)c(OC)c3C1=CC=C(NC)C(=O)C=C12  \
0                                                          0
1                                                          0
2                                                          0
3                                                          0
4                                                          0

   C=CCN(CC=C)C(=S)N[C@H]2CCc3cc(OC)c(OC)c(OC)c3C1=CC=C(NC)C(=O)C=C12  \
0                                                          0
1                                                          0
2                                                          0
3                                                          0
4                                                          0

   OCCN(CCO)C(=S)N[C@H]2CCc3cc(OC)c(OC)c(OC)c3C1=CC=C(NC)C(=O)C=C12  \
0                                                          0
1                                                          0
2                                                          0
3                                                          0
4                                                          0

   S=C(N[C@H]1CCc3cc(OC)c(OC)c(OC)c3C2=CC=C(NC)C(=O)C=C12)N4CCCC4  \
0                                                          0
1                                                          0
2                                                          0
3                                                          0
4                                                          0

   Clc1ccc(cc1)NC(=S)N[C@H]3CCc4cc(OC)c(OC)c(OC)c4C2=CC=C(NC)C(=O)C=C23  \
0                                                          0
1                                                          0
2                                                          0
3                                                          0
4                                                          0

   FC(F)(F)c1ccc(cc1)NC(=S)N[C@H]3CCc4cc(OC)c(OC)c(OC)c4C2=CC=C(NC)C(=O)C=C23  \
0                                                          0
1                                                          0
2                                                          0
3                                                          0
```

```
4                                                     0

   O[C@@H](CNC(=S)N[C@H]2CCc3cc(OC)c(OC)c(OC)c3C1=CC=C(NC)C(=O)C=C12)[C@H]4OC(C)
(C)O[C@@H]4[C@H]5COC(C)(C)O5  \
0                                                     0
1                                                     0
2                                                     0
3                                                     0
4                                                     0

   OC[C@@H](O)[C@@H](O)[C@H](O)[C@@H](O)CNC(=S)N[C@H]2CCc3cc(OC)c(OC)c(OC)c3C1=C
C=C(NC)C(=O)C=C12  \
0                                                     0
1                                                     0
2                                                     0
3                                                     0
4                                                     0

   CC(C)(C)OC(=O)\N=C(/NC(=O)OC(C)(C)C)N[C@H]2CCc3cc(OC)c(OC)c(OC)c3C1=CC=C(NC)C
(=O)C=C12  \
0                                                     0
1                                                     0
2                                                     0
3                                                     0
4                                                     0

   N=C(N)N[C@H]2CCc3cc(OC)c(OC)c(OC)c3C1=CC=C(NC)C(=O)C=C12
0                                                     0
1                                                     0
2                                                     0
3                                                     0
4                                                     0

[5 rows x 119 columns]
```

[29]:
```
new_df.tail(n=5)
```

[29]:
```
                                     AI_generated_SMILES  \
1673  N([C@H1]1CCC2=CC(OC)=C(OC)C(OC)=C2C3=CC=C(NC)C…
1674  O=C(CNC=C1C(C2=C(OC)C(OC)=C(OC)C=C2CC[C@@H1]1N…
1675  CNC(CNC(=O)OCCN[C@H1]1)CCC2=CC(OC)=C(OC)C(OC)=…
1676  O=C(N[C@H1]1CCC2=CC(OC)=C(OC)C(OC)=C2C3=CC=C(N…
1677  C12=C3C(C4=C(OC)C(OC)=C(OC)C=C4CC[C@@H1]3NC(=O…

      COc2c3C1=CC=C(SC)C(=O)C=C1[C@H](CCc3cc(OC)c2OC)NCC  \
1673                                                     0
1674                                                     0
```

```
1675                                                    0
1676                                                    0
1677                                                    0

     COc2c3C1=CC=C(SC)C(=O)C=C1[C@H](CCc3cc(OC)c2OC)NCCC  \
1673                                                    0
1674                                                    0
1675                                                    0
1676                                                    0
1677                                                    0

     COc2c3C1=CC=C(SC)C(=O)C=C1[C@H](CCc3cc(OC)c2OC)NCCCC  \
1673                                                    0
1674                                                    0
1675                                                    0
1676                                                    0
1677                                                    0

     CC(C)CN[C@H]2CCc3cc(OC)c(OC)c(OC)c3C1=CC=C(SC)C(=O)C=C12  \
1673                                                    0
1674                                                    0
1675                                                    0
1676                                                    0
1677                                                    0

     COc2c3C1=CC=C(SC)C(=O)C=C1[C@H](CCc3cc(OC)c2OC)NCCCCC  \
1673                                                    0
1674                                                    0
1675                                                    0
1676                                                    0
1677                                                    0

     CC(C)CCN[C@H]2CCc3cc(OC)c(OC)c(OC)c3C1=CC=C(SC)C(=O)C=C12  \
1673                                                    0
1674                                                    0
1675                                                    0
1676                                                    0
1677                                                    0

     COc2c3C1=CC=C(SC)C(=O)C=C1[C@H](CCc3cc(OC)c2OC)NCCCCCC  \
1673                                                    0
1674                                                    0
1675                                                    0
1676                                                    0
1677                                                    0

     CC(CCC)CN[C@H]2CCc3cc(OC)c(OC)c(OC)c3C1=CC=C(SC)C(=O)C=C12  \
```

```
1673                                             0
1674                                             0
1675                                             0
1676                                             0
1677                                             0

     COc2c3C1=CC=C(SC)C(=O)C=C1[C@H](CCc3cc(OC)c2OC)NCCCCCCCC  …  \
1673                                             0          …
1674                                             0          …
1675                                             0          …
1676                                             0          …
1677                                             0          …

     CCN(CC)C(=S)N[C@H]2CCc3cc(OC)c(OC)c(OC)c3C1=CC=C(NC)C(=O)C=C12  \
1673                                             0
1674                                             0
1675                                             0
1676                                             0
1677                                             0

     C=CCN(CC=C)C(=S)N[C@H]2CCc3cc(OC)c(OC)c(OC)c3C1=CC=C(NC)C(=O)C=C12  \
1673                                             0
1674                                             0
1675                                             0
1676                                             0
1677                                             0

     OCCN(CCO)C(=S)N[C@H]2CCc3cc(OC)c(OC)c(OC)c3C1=CC=C(NC)C(=O)C=C12  \
1673                                             0
1674                                             0
1675                                             0
1676                                             0
1677                                             0

     S=C(N[C@H]1CCc3cc(OC)c(OC)c(OC)c3C2=CC=C(NC)C(=O)C=C12)N4CCCC4  \
1673                                             0
1674                                             0
1675                                             0
1676                                             0
1677                                             0

     Clc1ccc(cc1)NC(=S)N[C@H]3CCc4cc(OC)c(OC)c(OC)c4C2=CC=C(NC)C(=O)C=C23  \
1673                                             0
1674                                             0
1675                                             0
1676                                             0
1677                                             0
```

```
      FC(F)(F)c1ccc(cc1)NC(=S)N[C@H]3CCc4cc(OC)c(OC)c(OC)c4C2=CC=C(NC)C(=O)C=C23
\
1673                                                      0
1674                                                      0
1675                                                      0
1676                                                      0
1677                                                      0

      O[C@@H](CNC(=S)N[C@H]2CCc3cc(OC)c(OC)c(OC)c3C1=CC=C(NC)C(=O)C=C12)[C@H]4OC
(C)(C)O[C@@H]4[C@H]5COC(C)(C)O5  \
1673                                                      0
1674                                                      0
1675                                                      0
1676                                                      0
1677                                                      0

      OC[C@@H](O)[C@@H](O)[C@H](O)[C@@H](O)CNC(=S)N[C@H]2CCc3cc(OC)c(OC)c(OC)c3C
1=CC=C(NC)C(=O)C=C12  \
1673                                                      0
1674                                                      0
1675                                                      0
1676                                                      0
1677                                                      0

      CC(C)(C)OC(=O)\N=C(/NC(=O)OC(C)(C)C)N[C@H]2CCc3cc(OC)c(OC)c(OC)c3C1=CC=C(N
C)C(=O)C=C12  \
1673                                                      0
1674                                                      0
1675                                                      0
1676                                                      0
1677                                                      0

      N=C(N)N[C@H]2CCc3cc(OC)c(OC)c(OC)c3C1=CC=C(NC)C(=O)C=C12
1673                                                      0
1674                                                      0
1675                                                      0
1676                                                      0
1677                                                      0

[5 rows x 119 columns]
```

[30]:

```
for element in initial_data['SMILES']:
    finger_1 = Chem.RDKFingerprint(Chem.MolFromSmiles(element))
    for i, smi in enumerate(new_df['AI_generated_SMILES']):
        finger_2 = Chem.RDKFingerprint(Chem.MolFromSmiles(smi))
        ta_sim = TanimotoSimilarity(finger_1, finger_2)
```

```
        new_df[element].loc[i] = ta_sim
        if ta_sim == 1:
            new_df[element][i] = 100
```

[31]:
```
new_df.head()
```

[31]:
```
                                   AI_generated_SMILES  \
0  COC1=C2C3=CC=C(NC)C(=O)C=C3[C@H1](CCC2=CC(OC)=…
1  COC1=C2C3=CC=C(NC)C(=O)C=C3[C@H1](CCC2=CC(OC)=…
2  COC1=C2C3=CC=C(NC)C(=O)C=C3[C@H1](CCC2=CC(OC)=…
3  COC1=C2C3=CC=C(NC)C(=O)C=C3[C@H1](CCC2=CC(OC)=…
4  COC1=C2C3=CC=C(NC)C(=O)C=C3[C@H1](CCC2=CC(OC)=…

   COc2c3C1=CC=C(SC)C(=O)C=C1[C@H](CCc3cc(OC)c2OC)NCC  \
0                                           0.854604
1                                           0.802326
2                                           0.846400
3                                           0.843949
4                                           0.844498

   COc2c3C1=CC=C(SC)C(=O)C=C1[C@H](CCc3cc(OC)c2OC)NCCC  \
0                                           0.846400
1                                           0.794931
2                                           0.856000
3                                           0.837411
4                                           0.854067

   COc2c3C1=CC=C(SC)C(=O)C=C1[C@H](CCc3cc(OC)c2OC)NCCCC  \
0                                           0.843700
1                                           0.792496
2                                           0.853270
3                                           0.834776
4                                           0.857257

   CC(C)CN[C@H]2CCc3cc(OC)c(OC)c(OC)c3C1=CC=C(SC)C(=O)C=C12  \
0                                           0.842482
1                                           0.791444
2                                           0.852029
3                                           0.833595
4                                           0.850119

   COc2c3C1=CC=C(SC)C(=O)C=C1[C@H](CCc3cc(OC)c2OC)NCCCCC  \
0                                           0.841687
1                                           0.790680
2                                           0.851233
3                                           0.832810
4                                           0.855211
```

```
   CC(C)CCN[C@H]2CCc3cc(OC)c(OC)c(OC)c3C1=CC=C(SC)C(=O)C=C12  \
0                                           0.841144
1                                           0.790236
2                                           0.850675
3                                           0.832288
4                                           0.854647

   COc2c3C1=CC=C(SC)C(=O)C=C1[C@H](CCc3cc(OC)c2OC)NCCCCCC  \
0                                           0.841270
1                                           0.790396
2                                           0.850794
3                                           0.832420
4                                           0.854762

   CC(CCC)CN[C@H]2CCc3cc(OC)c(OC)c(OC)c3C1=CC=C(SC)C(=O)C=C12  \
0                                           0.833989
1                                           0.783825
2                                           0.843430
3                                           0.825311
4                                           0.847364

   COc2c3C1=CC=C(SC)C(=O)C=C1[C@H](CCc3cc(OC)c2OC)NCCCCCCCC  …  \
0                                           0.840603         …
1                                           0.789794         …
2                                           0.850119         …
3                                           0.831768         …
4                                           0.854084         …

   CCN(CC)C(=S)N[C@H]2CCc3cc(OC)c(OC)c(OC)c3C1=CC=C(NC)C(=O)C=C12  \
0                                           0.901929
1                                           0.917534
2                                           0.893312
3                                           0.893651
4                                           0.889770

   C=CCN(CC=C)C(=S)N[C@H]2CCc3cc(OC)c(OC)c(OC)c3C1=CC=C(NC)C(=O)C=C12  \
0                                           0.891270
1                                           0.905213
2                                           0.882862
3                                           0.883229
4                                           0.879405

   OCCN(CCO)C(=S)N[C@H]2CCc3cc(OC)c(OC)c(OC)c3C1=CC=C(NC)C(=O)C=C12  \
0                                           0.891892
1                                           0.908875
2                                           0.884949
```

```
3                                             0.886792
4                                             0.881476

   S=C(N[C@H]1CCc3cc(OC)c(OC)c(OC)c3C2=CC=C(NC)C(=O)C=C12)N4CCCC4  \
0                                             0.893566
1                                             0.906003
2                                             0.885130
3                                             0.885490
4                                             0.887579

   Clc1ccc(cc1)NC(=S)N[C@H]3CCc4cc(OC)c(OC)c(OC)c4C2=CC=C(NC)C(=O)C=C23  \
0                                             0.877743
1                                             0.901961
2                                             0.871018
3                                             0.869969
4                                             0.870543

   FC(F)(F)c1ccc(cc1)NC(=S)N[C@H]3CCc4cc(OC)c(OC)c(OC)c4C2=CC=C(NC)C(=O)C=C23  \
0                                             0.877839
1                                             0.899061
2                                             0.872572
3                                             0.870070
4                                             0.870643

   O[C@@H](CNC(=S)N[C@H]2CCc3cc(OC)c(OC)c(OC)c3C1=CC=C(NC)C(=O)C=C12)[C@H]4OC(C)
(C)O[C@@H]4[C@H]5COC(C)(C)O5  \
0                                             0.801847
1                                             0.823738
2                                             0.797602
3                                             0.800704
4                                             0.801128

   OC[C@@H](O)[C@@H](O)[C@H](O)[C@@H](O)CNC(=S)N[C@H]2CCc3cc(OC)c(OC)c(OC)c3C1=C
C=C(NC)C(=O)C=C12  \
0                                             0.862519
1                                             0.883346
2                                             0.856055
3                                             0.857903
4                                             0.858447

   CC(C)(C)OC(=O)\N=C(/NC(=O)OC(C)(C)C)N[C@H]2CCc3cc(OC)c(OC)c(OC)c3C1=CC=C(NC)C
(=O)C=C12  \
0                                             0.824652
1                                             0.848641
2                                             0.820102
3                                             0.816667
4                                             0.817126
```

```
   N=C(N)N[C@H]2CCc3cc(OC)c(OC)c(OC)c3C1=CC=C(NC)C(=O)C=C12
0                                            0.926325
1                                            0.945500
2                                            0.917213
3                                            0.912795
4                                            0.913469

[5 rows x 119 columns]
```

[32]:
```
new_df.columns[3:][0]
```

[32]:
```
'COc2c3C1=CC=C(SC)C(=O)C=C1[C@H](CCc3cc(OC)c2OC)NCCCC'
```

[33]:
```
new_df.columns[1:][0]
```

[33]:
```
'COc2c3C1=CC=C(SC)C(=O)C=C1[C@H](CCc3cc(OC)c2OC)NCC'
```

[34]:
```
new_df.tail(n=5)
```

[34]:
```
                                    AI_generated_SMILES  \
1673  N([C@H1]1CCC2=CC(OC)=C(OC)C(OC)=C2C3=CC=C(NC)C…
1674  O=C(CNC=C1C(C2=C(OC)C(OC)=C(OC)C=C2CC[C@@H1]1N…
1675  CNC(CNC(=O)OCCN[C@H1]1)CCC2=CC(OC)=C(OC)C(OC)=…
1676  O=C(N[C@H1]1CCC2=CC(OC)=C(OC)C(OC)=C2C3=CC=C(N…
1677  C12=C3C(C4=C(OC)C(OC)=C(OC)C=C4CC[C@@H1]3NC(=O…

      COc2c3C1=CC=C(SC)C(=O)C=C1[C@H](CCc3cc(OC)c2OC)NCC  \
1673                                           0.441733
1674                                           0.468137
1675                                           0.729483
1676                                           0.836622
1677                                           0.702219

      COc2c3C1=CC=C(SC)C(=O)C=C1[C@H](CCc3cc(OC)c2OC)NCCC  \
1673                                           0.444647
1674                                           0.472783
1675                                           0.725490
1676                                           0.828772
1677                                           0.697443

      COc2c3C1=CC=C(SC)C(=O)C=C1[C@H](CCc3cc(OC)c2OC)NCCCC  \
1673                                           0.445322
1674                                           0.474328
1675                                           0.727204
1676                                           0.826189
1677                                           0.697872
```

```
     CC(C)CN[C@H]2CCc3cc(OC)c(OC)c(OC)c3C1=CC=C(SC)C(=O)C=C12  \
1673                                         0.446873
1674                                         0.474070
1675                                         0.722431
1676                                         0.825039
1677                                         0.697098

     COc2c3C1=CC=C(SC)C(=O)C=C1[C@H](CCc3cc(OC)c2OC)NCCCCC  \
1673                                         0.445388
1674                                         0.475260
1675                                         0.725564
1676                                         0.824261
1677                                         0.696391

     CC(C)CCN[C@H]2CCc3cc(OC)c(OC)c(OC)c3C1=CC=C(SC)C(=O)C=C12  \
1673                                         0.447209
1674                                         0.475290
1675                                         0.726521
1676                                         0.823758
1677                                         0.697313

     COc2c3C1=CC=C(SC)C(=O)C=C1[C@H](CCc3cc(OC)c2OC)NCCCCCC  \
1673                                         0.446667
1674                                         0.477411
1675                                         0.726727
1676                                         0.823894
1677                                         0.695131

     CC(CCC)CN[C@H]2CCc3cc(OC)c(OC)c(OC)c3C1=CC=C(SC)C(=O)C=C12  \
1673                                         0.449819
1674                                         0.476914
1675                                         0.720774
1676                                         0.818322
1677                                         0.692146

     COc2c3C1=CC=C(SC)C(=O)C=C1[C@H](CCc3cc(OC)c2OC)NCCCCCCCC  …  \
1673                                         0.447273        …
1674                                         0.478022        …
1675                                         0.726182        …
1676                                         0.823256        …
1677                                         0.694640        …

     CCN(CC)C(=S)N[C@H]2CCc3cc(OC)c(OC)c(OC)c3C1=CC=C(NC)C(=O)C=C12  \
1673                                         0.473652
1674                                         0.476647
1675                                         0.785388
```

```
1676                                            0.885917
1677                                            0.704735

      C=CCN(CC=C)C(=S)N[C@H]2CCc3cc(OC)c(OC)c(OC)c3C1=CC=C(NC)C(=O)C=C12  \
1673                                            0.475904
1674                                            0.481504
1675                                            0.779367
1676                                            0.875680
1677                                            0.701173

      OCCN(CCO)C(=S)N[C@H]2CCc3cc(OC)c(OC)c(OC)c3C1=CC=C(NC)C(=O)C=C12  \
1673                                            0.475875
1674                                            0.479714
1675                                            0.783825
1676                                            0.876265
1677                                            0.701451

      S=C(N[C@H]1CCc3cc(OC)c(OC)c(OC)c3C2=CC=C(NC)C(=O)C=C12)N4CCCC4  \
1673                                            0.476506
1674                                            0.482100
1675                                            0.782805
1676                                            0.879377
1677                                            0.701863

      Clc1ccc(cc1)NC(=S)N[C@H]3CCc4cc(OC)c(OC)c(OC)c4C2=CC=C(NC)C(=O)C=C23  \
1673                                            0.481949
1674                                            0.485714
1675                                            0.767857
1676                                            0.866923
1677                                            0.708966

      FC(F)(F)c1ccc(cc1)NC(=S)N[C@H]3CCc4cc(OC)c(OC)c(OC)c4C2=CC=C(NC)C(=O)C=C23
\
1673                                            0.484940
1674                                            0.484254
1675                                            0.765405
1676                                            0.865591
1677                                            0.710345

      O[C@@H](CNC(=S)N[C@H]2CCc3cc(OC)c(OC)c(OC)c3C1=CC=C(NC)C(=O)C=C12)[C@H]4OC
(C)(C)O[C@@H]4[C@H]5COC(C)(C)O5  \
1673                                            0.493976
1674                                            0.506857
1675                                            0.728709
1676                                            0.802105
1677                                            0.682081
```

```
      OC[C@@H](O)[C@@H](O)[C@H](O)[C@@H](O)CNC(=S)N[C@H]2CCc3cc(OC)c(OC)c(OC)c3C
1=CC=C(NC)C(=O)C=C12  \
1673                                              0.481570
1674                                              0.492317
1675                                              0.773599
1676                                              0.853585
1677                                              0.699661

      CC(C)(C)OC(=O)\N=C(/NC(=O)OC(C)(C)C)N[C@H]2CCc3cc(OC)c(OC)c(OC)c3C1=CC=C(N
C)C(=O)C=C12  \
1673                                              0.483159
1674                                              0.496241
1675                                              0.741002
1676                                              0.816739
1677                                              0.691244

      N=C(N)N[C@H]2CCc3cc(OC)c(OC)c(OC)c3C1=CC=C(NC)C(=O)C=C12
1673                                              0.468712
1674                                              0.469128
1675                                              0.802344
1676                                              0.907767
1677                                              0.712154

[5 rows x 119 columns]
```

[35]:
```
for element in new_df.columns[1:]:

    new_df = new_df.drop(new_df[new_df[element] > 99].index)
```

[36]:
```
new_df.shape
```

[36]: (1356, 119)

[37]:
```
new_df.to_excel('../Data/
 ↪Proposed_structures_with_AI_colchicyne_tanimoto_similarity_.xlsx')
```

Notebook

January 18, 2024

[ ]:
```
##########
# FILE 40
##########

#Libraries import
import pandas as pd
import numpy as np
from mordred import Calculator, descriptors
import mordred
from rdkit import Chem
from syba.syba import SybaClassifier
from rdkit.Chem import PandasTools
import joblib

import sys
from pathlib import Path
prediction_mode_path = Path("../module")
sys.path.append(prediction_mode_path.as_posix())
import models_creation as pred_model

def is_morder_missing(x):
    return np.nan if type(x) == mordred.error.Missing or type(x) == mordred.
 ↪error.Error else x

if __name__ == "__main__":
    #load initial structures
    initial_structures = pd.read_excel('../Data/Kolchicyna_prepared_data.xlsx')
    initial_structures = list(initial_structures['SMILES'])
    #load generated structures
    generated_structures = pd.read_excel('../Data/
 ↪Proposed_structures_with_AI_colchicyne_tanimoto_similarity_.xlsx')
    generated_structures = list(generated_structures['AI_generated_SMILES'])

    #SYBA application
    #SYBA classifier compilation
    mols_ini = [Chem.MolFromSmiles(smi) for smi in initial_structures]
    mols_gen = [Chem.MolFromSmiles(smi) for smi in generated_structures]
```

```python
    syba = SybaClassifier()
    syba.fitDefaultScore()
    SYBA_score_to_initial_structures = [syba.predict(mol=mol) for mol in␣
↪mols_ini]
    SYBA_score_to_generated_structures = [syba.predict(mol=mol) for mol in␣
↪mols_gen]

    threshold = min(SYBA_score_to_initial_structures)
    print('The minimal SYBA score is: '+str(threshold))
    df_gen = pd.DataFrame(data=generated_structures, columns=['SMILES'])
    df_gen['SYBA score'] = SYBA_score_to_generated_structures

    df_gen_fin = df_gen[df_gen['SYBA score'] > threshold]
    df_gen_fin = df_gen_fin.round({'SYBA score': 2})

    #calculate molecular descriptors
    mol_objs = [Chem.MolFromSmiles(smi) for smi in df_gen_fin['SMILES']]

    calculate_descriptors = True
    if calculate_descriptors == True:
        calc = Calculator(descriptors, ignore_3D=True)
        molecular_descriptors = calc.pandas(mol_objs)
        molecular_descriptors = molecular_descriptors.
↪applymap(is_morder_missing)
        molecular_descriptors['SMILES'] = df_gen_fin['SMILES']
        molecular_descriptors.to_excel('../Data/Final_selection.xlsx')
    else:
        molecular_descriptors = pd.read_excel('../Data/Final_selection.xlsx')

    #assign predicted value
    df_gen_fin['A549 [nM]'] = 0
    df_gen_fin['BALB/3T3 [nM]'] = 0
    df_gen_fin['LoVo [nM]'] = 0
    df_gen_fin['LoVo/DX [nM]'] = 0
    df_gen_fin['MCF-7 [nM]'] = 0

    try:
       #A549
        model = joblib.load('../Activity/Random_forest/
↪random_forest_model_17_estimators_A549.joblib')
        mob = model.predict(molecular_descriptors[list(model.
↪feature_names_in_)])
        converted = pred_model.inverse_transform(mob)
        df_gen_fin['A549 [nM]'] = converted

        #BALB/3T3
```

```
        model = joblib.load('../Activity/Random_forest/
↪random_forest_model_19_estimators_BALB_3T3.joblib')
        mob = model.predict(molecular_descriptors[list(model.
↪feature_names_in_)])
        converted = pred_model.inverse_transform(mob)
        df_gen_fin['BALB/3T3 [nM]'] = converted

        #LoVo
        model = joblib.load('../Activity/Random_forest/
↪random_forest_model_18_estimators_LoVo.joblib')
        mob = model.predict(molecular_descriptors[list(model.
↪feature_names_in_)])
        converted = pred_model.inverse_transform(mob)
        df_gen_fin['LoVo [nM]'] = converted

        #LoVo/DX
        model = joblib.load('../Activity/Random_forest/
↪random_forest_model_14_estimators_LoVo_DX.joblib')
        mob = model.predict(molecular_descriptors[list(model.
↪feature_names_in_)])
        converted = pred_model.inverse_transform(mob)
        df_gen_fin['LoVo/DX [nM]'] = converted

        #MCF-7
        model = joblib.load('../Activity/Random_forest/
↪random_forest_model_3_estimators_MCF-7.joblib')
        mob = model.predict(molecular_descriptors[list(model.
↪feature_names_in_)])
        converted = pred_model.inverse_transform(mob)
        df_gen_fin['MCF-7 [nM]'] = converted

    except:
        print("Error with predicted value...")


    try:

        df_gen_fin = df_gen_fin.drop_duplicates(subset=['SYBA score', 'A549␣
↪[nM]', 'BALB/3T3 [nM]', 'LoVo [nM]', 'LoVo/DX [nM]', 'MCF-7 [nM]'],␣
↪keep='first')

        df_gen_fin['Mol Image'] = [Chem.MolFromSmiles(smi) for smi in␣
↪df_gen_fin['SMILES']]

        PandasTools.SaveXlsxFromFrame(df_gen_fin, '../Data/Whole_report.xlsx',␣
↪molCol='Mol Image')
```

```
    except:
        print("Error when inserting images...")
```

Notebook

December 19, 2023

1 File 42

[1]:
```
import pandas as pd
from rdkit import Chem
import matplotlib.pyplot as plt
%matplotlib inline
import numpy as np

from rdkit.Chem import PandasTools

from rdkit import Chem
from rdkit.Chem.Draw import IPythonConsole

def show_atom_number(mol, label):
    for atom in mol.GetAtoms():
        atom.SetProp(label, str(atom.GetIdx()+1))
    return mol
```

[2]:
```
## Starting structures stereochemistry
start = pd.read_excel('../Data/Kolchicyna_prepared_data.xlsx')
```

[3]:
```
start.head()
```

[3]:
```
   Unnamed: 0                              Publikacja DOI  \
0           0  https://doi.org/10.1016/j.bmcl.2021.128382
1           1  https://doi.org/10.1016/j.bmcl.2021.128382
2           2  https://doi.org/10.1016/j.bmcl.2021.128382
3           3  https://doi.org/10.1016/j.bmcl.2021.128382
4           4  https://doi.org/10.1016/j.bmcl.2021.128382

  Numer związku w publikacji  \
0                          1
1                          2
2                          3
3                          4
4                          5

                                              SMILES  Atywność [nM]   A549  \
```

```
0  COc2c3C1=CC=C(SC)C(=O)C=C1[C@H](CCc3cc(OC)c2OC...  Atywność [nM]  10,8
1  COc2c3C1=CC=C(SC)C(=O)C=C1[C@H](CCc3cc(OC)c2OC...  Atywność [nM]  11,6
2  COc2c3C1=CC=C(SC)C(=O)C=C1[C@H](CCc3cc(OC)c2OC...  Atywność [nM]  10,9
3  CC(C)CN[C@H]2CCc3cc(OC)c(OC)c(OC)c3C1=CC=C(SC)...  Atywność [nM]  10,5
4  COc2c3C1=CC=C(SC)C(=O)C=C1[C@H](CCc3cc(OC)c2OC...  Atywność [nM]  89,5

   MCF-7   LoVo  LoVo/DX  BALB/3T3  A549_float  MCF-7_float  LoVo_float  \
0  10,3    6,5     54,9     10,2         10.8         10.3         6.5
1  12,0    8,5      31.1     14.3        11.6         12.0         8.5
2  12,2    8,8     17,9     11,7         10.9         12.2         8.8
3  11,3    8,5     10,2     11,0         10.5         11.3         8.5
4  92,7   52,8     77,8     99,4         89.5         92.7        52.8

   LoVo/DX_float  BALB/3T3_float
0           54.9            10.2
1           31.1            14.3
2           17.9            11.7
3           10.2            11.0
4           77.8            99.4
```

[4]:
```
## https://www.rdkit.org/docs/RDKit_Book.html
```

[5]:
```
Chem.MolFromSmiles(start['SMILES'][0])
```

[5]:

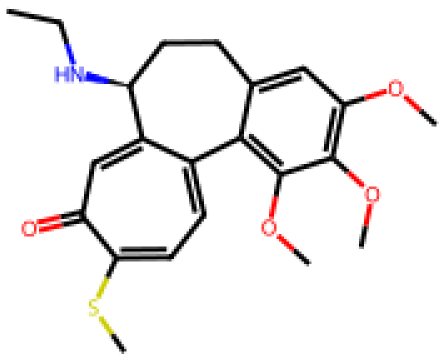

[6]:
```
x = Chem.MolFromSmiles(start['SMILES'][0])
Chem.AssignStereochemistry(x)
x
```

[6]:

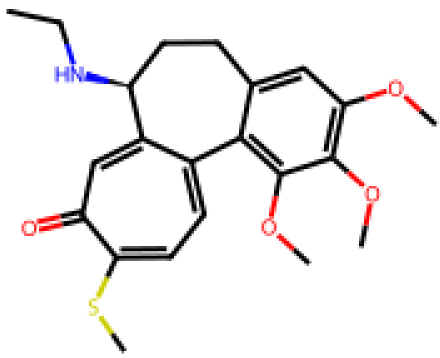

[7]:
```
xx = Chem.FindMolChiralCenters(x)
xx
```

[7]: [(14, 'S')]

[8]:
```
xx[0][1]
```

[8]: 'S'

[9]:
```
atom_num = []
chirality = []

for smiles in start['SMILES']:
    mol = Chem.MolFromSmiles(smiles)
    chiral = Chem.FindMolChiralCenters(mol)
    atom_num.append(chiral[0][0])
    chirality.append(chiral[0][1])
print("Job finished...")
```

Job finished…

[10]:
```
start['atom_num'] = atom_num
start['chirality'] = chirality
start.head()
```

[10]:
```
   Unnamed: 0                              Publikacja DOI  \
0           0  https://doi.org/10.1016/j.bmcl.2021.128382
1           1  https://doi.org/10.1016/j.bmcl.2021.128382
2           2  https://doi.org/10.1016/j.bmcl.2021.128382
3           3  https://doi.org/10.1016/j.bmcl.2021.128382
4           4  https://doi.org/10.1016/j.bmcl.2021.128382

  Numer związku w publikacji  \
0                          1
```

```
1                          2
2                          3
3                          4
4                          5

                                                  SMILES  Atywność [nM]   A549  \
0  COc2c3C1=CC=C(SC)C(=O)C=C1[C@H](CCc3cc(OC)c2OC…  Atywność [nM]  10,8
1  COc2c3C1=CC=C(SC)C(=O)C=C1[C@H](CCc3cc(OC)c2OC…  Atywność [nM]  11,6
2  COc2c3C1=CC=C(SC)C(=O)C=C1[C@H](CCc3cc(OC)c2OC…  Atywność [nM]  10,9
3  CC(C)CN[C@H]2CCc3cc(OC)c(OC)c(OC)c3C1=CC=C(SC)…  Atywność [nM]  10,5
4  COc2c3C1=CC=C(SC)C(=O)C=C1[C@H](CCc3cc(OC)c2OC…  Atywność [nM]  89,5

  MCF-7   LoVo LoVo/DX BALB/3T3  A549_float  MCF-7_float  LoVo_float  \
0  10,3    6,5    54,9     10,2        10.8         10.3         6.5
1  12,0    8,5     31.1     14.3        11.6         12.0         8.5
2  12,2    8,8    17,9     11,7        10.9         12.2         8.8
3  11,3    8,5    10,2     11,0        10.5         11.3         8.5
4  92,7   52,8    77,8     99,4        89.5         92.7        52.8

  LoVo/DX_float  BALB/3T3_float  atom_num chirality
0          54.9            10.2        14         S
1          31.1            14.3        14         S
2          17.9            11.7        14         S
3          10.2            11.0         5         S
4          77.8            99.4        14         S
```

[11]:
```python
r_struct = start[start['chirality'] == 'R']
```

[12]:
```python
r_struct
```

[12]:
```
     Unnamed: 0                              Publikacja DOI  \
22           22  https://doi.org/10.1016/j.bmcl.2021.128382
117         117  https://doi.org/10.1016/j.bmcl.2021.128197

    Numer związku w publikacji  \
22                       COLCH
117                         26

                                                  SMILES  Atywność [nM]   A549  \
22   COc3cc2CC[C@@H](NC(C)=O)c1cc(=O)c(OC)ccc1c2c(O…  Atywność [nM]  19,3
117  OC[C@@H](O)[C@@H](O)[C@H](O)[C@@H](O)CNC(=S)N[…  Atywność [nM]    930

     MCF-7   LoVo LoVo/DX BALB/3T3  A549_float  MCF-7_float  LoVo_float  \
22    10,1   12,7   966,8     82,3        19.3         10.1        12.7
117   1200    680    6600     1300       930.0       1200.0       680.0

     LoVo/DX_float  BALB/3T3_float  atom_num chirality
```

```
22             966.8            82.3         7          R
117           6600.0          1300.0         2          R
```

[13]:
```
r_struct_0 = Chem.MolFromSmiles(r_struct['SMILES'][22]) # structure no. 23 in␣
↪Kolchicyna_prepared_data.xlsx
```

[14]:
```
r_struct_0 ## correct SMILES is ->␣
↪COc1cc2CC[C@H](NC(C)=O)c3cc(=O)c(OC)ccc3-c2c(OC)c1OC (Marvin Sketch␣
↪Generated)
```

[14]:

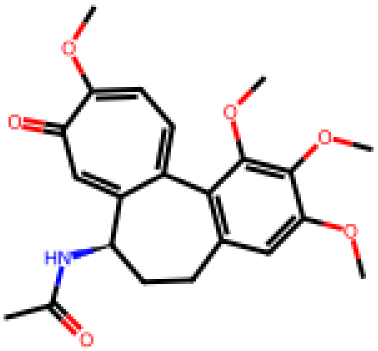

[15]:
```
xx = Chem.FindMolChiralCenters(r_struct_0)
xx
```

[15]: [(7, 'R')]

[16]:
```
r_struct['SMILES'][22] # -> This SMILES is for R absolute configuration␣
↪colchicine, and the experiment was conducted for S absolute configuration␣
↪colchicine
```

[16]: 'COc3cc2CC[C@@H](NC(C)=O)c1cc(=O)c(OC)ccc1c2c(OC)c3OC'

[17]:
```
Chem.FindMolChiralCenters(Chem.
↪MolFromSmiles('COc1cc2CC[C@H](NC(C)=O)c3cc(=O)c(OC)ccc3-c2c(OC)c1OC'))
```

[17]: [(7, 'S')]

[18]:
```
Chem.MolFromSmiles('COc1cc2CC[C@H](NC(C)=O)c3cc(=O)c(OC)ccc3-c2c(OC)c1OC')
```

[18]:

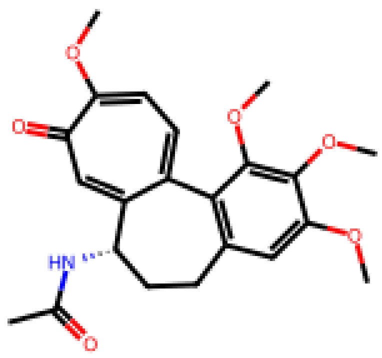

[19]:
```
start['chirality'][22] = 'S'
```

```
C:\Users\aleks\AppData\Local\Temp\ipykernel_26592\131833170.py:1:
SettingWithCopyWarning:
A value is trying to be set on a copy of a slice from a DataFrame

See the caveats in the documentation: https://pandas.pydata.org/pandas-
docs/stable/user_guide/indexing.html#returning-a-view-versus-a-copy
  start['chirality'][22] = 'S'
```

[20]:
```
r_struct_1 = Chem.MolFromSmiles(r_struct['SMILES'][117])
r_struct_1
```

[20]:

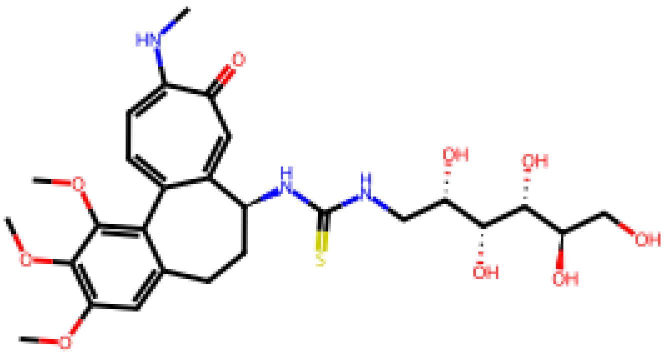

[21]:
```
xx = Chem.FindMolChiralCenters(r_struct_1)
xx
```

[21]: [(2, 'R'), (4, 'R'), (6, 'R'), (8, 'S'), (15, 'S')]

[22]:
```
show_atom_number(r_struct_1, 'atomLabel')
```

[22]:

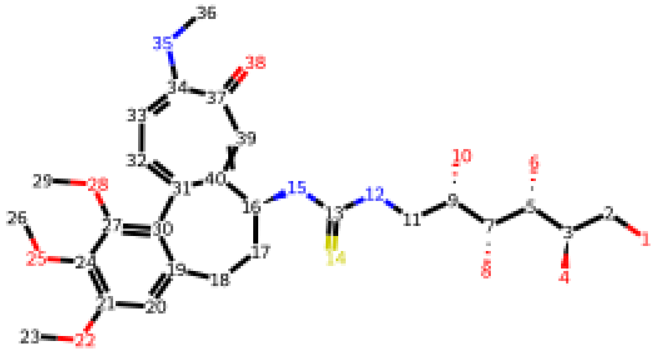


```
[23]: start['chirality'][117] = 'S'
```

```
C:\Users\aleks\AppData\Local\Temp\ipykernel_26592\2686388605.py:1:
SettingWithCopyWarning:
A value is trying to be set on a copy of a slice from a DataFrame

See the caveats in the documentation: https://pandas.pydata.org/pandas-
docs/stable/user_guide/indexing.html#returning-a-view-versus-a-copy
  start['chirality'][117] = 'S'
```

```
[24]: fig, ax = plt.subplots()
bar_container = ax.bar(['S', 'R', 'No chiral center'],␣
  ↪[len(start[start['chirality'] == 'S']), len(start[start['chirality'] ==␣
  ↪'R']), len(start[start['chirality'] == 'No chiral center'])])
ax.set(xlabel = 'Absolute configuration of starting structures', ylabel='Number␣
  ↪of compounds')
plt.rc('grid', linestyle="-.", color='black')
plt.grid(False)
ax.bar_label(bar_container, padding=2, label_type='edge')
plt.savefig('chirality_starting.pdf', bbox_inches='tight')
```

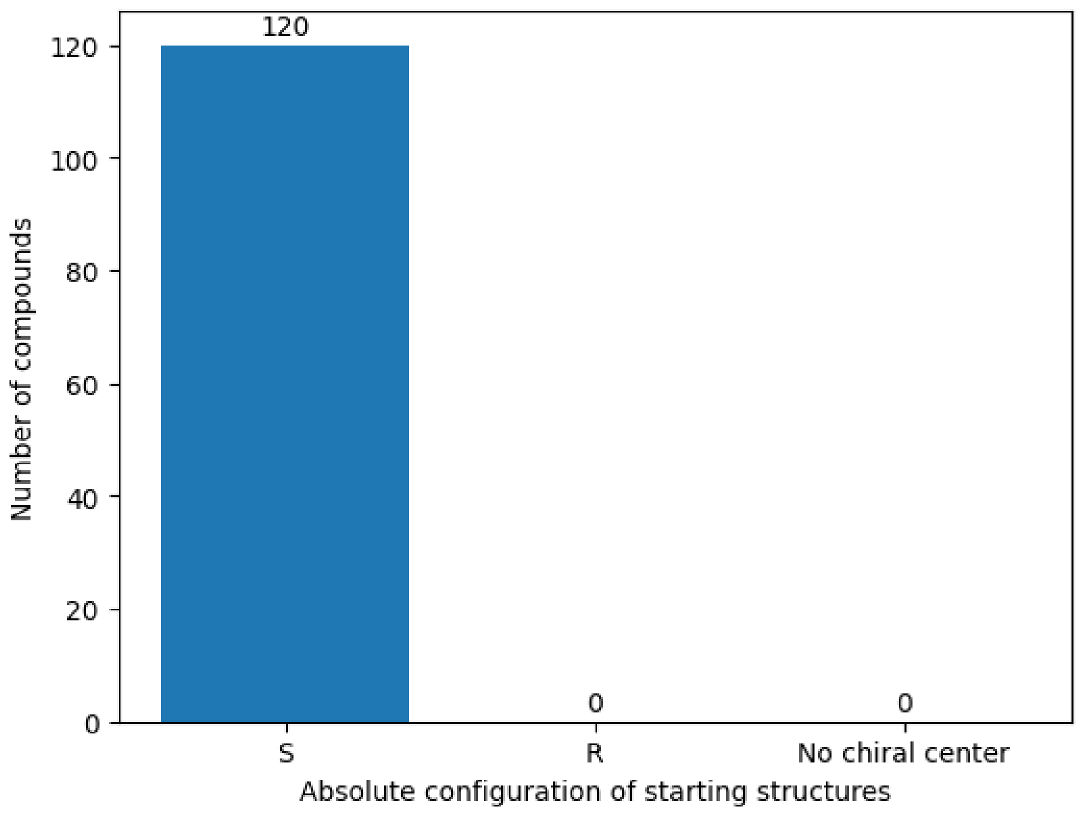

[25]:
```
len(start[start['chirality'] == 'No chiral centre'])
```

[25]: 0

[ ]:

[ ]:

[26]:
```
for i, smiles in enumerate(start['SMILES']):
    mol = Chem.MolFromSmiles(smiles)
    chiral = Chem.FindMolChiralCenters(mol)
    print(i, chiral)
```

```
0 [(14, 'S')]
1 [(14, 'S')]
2 [(14, 'S')]
3 [(5, 'S')]
4 [(14, 'S')]
5 [(6, 'S')]
6 [(14, 'S')]
7 [(7, 'S')]
8 [(14, 'S')]
```

```
9 [(14, 'S')]
10 [(14, 'S')]
11 [(13, 'S')]
12 [(9, 'S')]
13 [(11, 'S')]
14 [(11, 'S')]
15 [(9, 'S')]
16 [(9, 'S')]
17 [(9, 'S')]
18 [(9, 'S')]
19 [(14, 'S')]
20 [(14, 'S')]
21 [(14, 'S')]
22 [(7, 'R')]
23 [(7, 'S')]
24 [(7, 'S')]
25 [(4, 'S')]
26 [(12, 'S')]
27 [(13, 'S')]
28 [(14, 'S')]
29 [(4, 'S')]
30 [(13, 'S')]
31 [(14, 'S')]
32 [(4, 'S')]
33 [(16, 'S')]
34 [(17, 'S')]
35 [(18, 'S')]
36 [(4, 'S')]
37 [(17, 'S')]
38 [(18, 'S')]
39 [(4, 'S')]
40 [(14, 'S')]
41 [(14, 'S')]
42 [(13, 'S')]
43 [(13, 'S')]
44 [(13, 'S')]
45 [(13, 'S')]
46 [(13, 'S')]
47 [(13, 'S')]
48 [(13, 'S')]
49 [(9, 'S')]
50 [(12, 'S')]
51 [(13, 'S')]
52 [(12, 'S')]
53 [(13, 'S')]
54 [(13, 'S')]
55 [(9, 'S')]
56 [(10, 'S')]
```

```
57 [(8, 'S')]
58 [(16, 'S')]
59 [(10, 'S')]
60 [(8, 'S')]
61 [(24, 'S')]
62 [(15, 'S')]
63 [(19, 'S')]
64 [(16, 'S')]
65 [(13, 'S')]
66 [(12, 'S')]
67 [(24, 'S')]
68 [(12, 'S')]
69 [(14, 'S')]
70 [(14, 'S')]
71 [(19, 'S')]
72 [(16, 'S')]
73 [(13, 'S')]
74 [(11, 'S')]
75 [(12, 'S')]
76 [(13, 'S')]
77 [(14, 'S')]
78 [(14, 'S')]
79 [(15, 'S')]
80 [(16, 'S')]
81 [(14, 'S')]
82 [(14, 'S')]
83 [(14, 'S')]
84 [(14, 'S')]
85 [(5, 'S')]
86 [(14, 'S')]
87 [(14, 'S')]
88 [(14, 'S')]
89 [(7, 'S')]
90 [(14, 'S')]
91 [(9, 'S')]
92 [(9, 'S')]
93 [(13, 'S')]
94 [(13, 'S')]
95 [(4, 'S')]
96 [(5, 'S')]
97 [(7, 'S')]
98 [(6, 'S')]
99 [(8, 'S')]
100 [(10, 'S')]
101 [(3, 'S')]
102 [(14, 'S')]
103 [(11, 'S')]
104 [(11, 'S')]
```

```
105 [(14, 'S')]
106 [(4, 'S')]
107 [(5, 'S')]
108 [(8, 'S')]
109 [(6, 'S')]
110 [(8, 'S')]
111 [(10, 'S')]
112 [(10, 'S')]
113 [(3, 'S')]
114 [(11, 'S')]
115 [(14, 'S')]
116 [(1, 'S'), (7, 'S'), (32, 'R'), (38, 'R'), (39, 'R')]
117 [(2, 'R'), (4, 'R'), (6, 'R'), (8, 'S'), (15, 'S')]
118 [(18, 'S')]
119 [(4, 'S')]
```

[ ]:

[27]:
```
to_be_printed = pd.DataFrame(data=start['SMILES'], columns=['SMILES'])
to_be_printed['Chirality'] = [Chem.FindMolChiralCenters(Chem.
 ↪MolFromSmiles(smiles)) for smiles in to_be_printed['SMILES']]
to_be_printed['Mol Image'] = [Chem.MolFromSmiles(smi) for smi in␣
 ↪to_be_printed['SMILES']]
PandasTools.SaveXlsxFromFrame(to_be_printed, '../Data/
 ↪start_structures_chirality.xlsx', molCol='Mol Image')
```

[28]:
```
to_be_printed.head()
```

[28]:
```
                                               SMILES  Chirality  \
0  COc2c3C1=CC=C(SC)C(=O)C=C1[C@H](CCc3cc(OC)c2OC…  [(14, S)]
1  COc2c3C1=CC=C(SC)C(=O)C=C1[C@H](CCc3cc(OC)c2OC…  [(14, S)]
2  COc2c3C1=CC=C(SC)C(=O)C=C1[C@H](CCc3cc(OC)c2OC…  [(14, S)]
3  CC(C)CN[C@H]2CCc3cc(OC)c(OC)c(OC)c3C1=CC=C(SC)…   [(5, S)]
4  COc2c3C1=CC=C(SC)C(=O)C=C1[C@H](CCc3cc(OC)c2OC…  [(14, S)]

                                        Mol Image
0  <rdkit.Chem.rdchem.Mol object at 0x00000218896…
1  <rdkit.Chem.rdchem.Mol object at 0x00000218896…
2  <rdkit.Chem.rdchem.Mol object at 0x00000218896…
3  <rdkit.Chem.rdchem.Mol object at 0x00000218896…
4  <rdkit.Chem.rdchem.Mol object at 0x00000218896…
```

[29]:
```
mol = Chem.
 ↪MolFromSmiles('OC[C@@H](O)[C@@H](O)[C@H](O)[C@@H](O)CNC(=S)N[C@H]2CCc3cc(OC)c(OC)c(OC)c3C1=
show_atom_number(mol, 'atomLabel')
```

[29]:

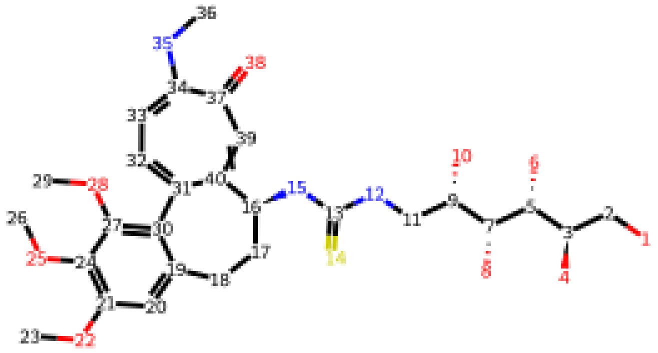

```
[30]: mol = Chem.MolFromSmiles(to_be_printed.iloc[-2]['SMILES'])
      show_atom_number(mol, 'atomLabel')
```

[30]:

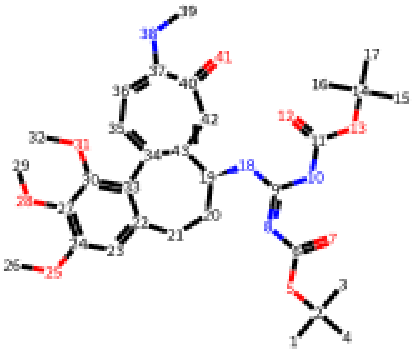

```
[31]: to_be_printed.iloc[-2]['SMILES']
```

[31]: 'CC(C)(C)OC(=O)\\N=C(/NC(=O)OC(C)(C)C)N[C@H]2CCc3cc(OC)c(OC)c(OC)c3C1=CC=C(NC)C(=O)C=C12'

```
[ ]:
```

```
[32]: ## new structures stereochemistry
      new = pd.read_excel('../Data/Whole_report.xlsx')
```

```
[33]: new.head()
```

[33]:

```
   Unnamed: 0                                             SMILES  SYBA score  \
0         NaN  COC1=C2C3=CC=C(NC)C(=O)C=C3[C@H1](CCC2=CC(OC)=...      45.88
1         NaN  COC1=C2C3=CC=C(NC)C(=O)C=C3[C@H1](CCC2=CC(OC)=...      68.25
2         NaN  COC1=C2C3=CC=C(NC)C(=O)C=C3[C@H1](CCC2=CC(OC)=...      74.22
3         NaN  COC1=C2C3=CC=C(NC)C(=O)C=C3[C@H1](CCC2=CC(OC)=...      40.43
```

```
4          NaN   O=CC1=CN=NN1[C@H1]2CCC3=CC(OC)=C(OC)C(OC)=C3C4…       41.84

   A549 [nM]  BALB/3T3 [nM]  LoVo [nM]  LoVo/DX [nM]  MCF-7 [nM]
0  10.168579       6.200000   5.700000    136.586050    8.357629
1  10.648944       9.439809  17.460496     49.797629   10.167834
2  11.794066      30.305940  54.000000     37.606395    9.099151
3   6.400000      23.752895   8.570105    210.367783    5.400000
4   4.774935      11.772850  10.000000   1806.439871   12.029326
```

[34]:

```python
atom_num = []
chirality = []
issue = []
for smiles in new['SMILES']:
    try:
        mol = Chem.MolFromSmiles(smiles)
        chiral = Chem.FindMolChiralCenters(mol)
        atom_num.append(chiral[0][0])
        chirality.append(chiral[0][1])
    except:
        issue.append(smiles)
print("Job finished...")
```

```
Job finished…
```

[35]:

```python
issue
```

[35]:

```
['CC(=O)N[C@@H1]C1=C2C(C(NC)=CC=C1C3=C(OC)C(OC)=C(OC)C=C3CC2)=O',
 'CNC(NCCCC)=S[C@@H1]C1=CC(=O)C(NC)=CC=C1C2=C(C=C(OC)C(=C2OC)OC)CCF',
 'C1=2[C@H1]CCC3=CC(OC)=C(OC)C(OC(CC)=O)=C3C1=CC=C(C(C=2)=O)SCNC(=O)CSC',
 'O=C(C1=C(C(OCC)=O)N(N=N1)[C@@H1]C2=C3C(C(NC)=CC=C2C4=C(C(OC)=C(OC)C=C4CC3)OC)=
O)O',
 'C=12N(N=NC=1C(OCC3=CC=C(C=C3)F)=O)C(OC)=C(OC)C(OC)=CC4=CC=C(C(=O)C=C24)NC',
 'C=1N(N=NC=1C(=O)OCC2=CC=C(C=C2)F)CC(OC)=C(OC)C(OC)=CC3=CC=C(NC)C(=O)C=C3',
 'O=CC1=C(C(OCC)=O)N(N=N1)[C@@H1]C2=C3C(C(NC)=CC=C2C4=C(C(OC)=C(OC)C=C4CC3)OC)=O
',
 'O=C(O)C1=C(C(OCC)=O)N=NN1[C@H1]CCC2=CC(OC)=C(OC)C(OC)=C2C3=CC=C(C(C=C3)=O)NC',
 'CCCCCCCCCCCCCCCC(OCC1=CN(N=N1)[C@H1]C2CC3=CC(OC)=C(OC)C(OC)=C3C4=CC=C(C(=O)C=C
24)NC)=O',
 'OC(=O)C1=C(C(OCC)=O)N(N=N1)[C@H1]C2CC3=CC(OC)=C(OC)C(OC)=C3C4=CC=C(NC)C(=O)C=C
42',
 'N(C)C(=O)NCCC(OCC1=CN(N=N1)[C@H1]CCC2=CC(OC)=C(OC)C(OC)=C2C3=CC=C(C(=O)C=C3)N)
C=O',
 'CC(C)(C)OC(=O)NCCC(NCC=1N=NN(C=1)[C@H1]C2CC3=CC(OC)=C(OC)C(OC)=C3C4=CC=C(C(=O)
C=C24)N)C=O',
 'CC(C)(C)OC(=O)NCCC(OCC=1N=NN(C=1)[C@H1]C2CC3=CC(OC)=C(OC)C(OC)=C3C4=CC=C(C(=O)
C=C24)NC)=O',
 'N(C(C)C)CC(=O)OCC1=C2N(N=N1)[C@H1]CCC3=CC(OC)=C(OC)C(OC)=C3C4=CC=C(NC)C(C=C24)
=O',
```

```
 'CC(CC)N[C@@H1]C1=C2C(C(NC)=CC=C1C3=C(OC)C(OC)=C(OC)C=C3CC2)=O',
 'NC(=O)N[C@@H1]C1=C2C(C(NC)=CC=C1C3=C(OC)C(OC)=C(OC)C=C3CC2)=O',
 'CC(C)NC(=O)N1[C@@H1]C2=CC(C(NC)=CC=C2C3=C(OC)C(OC)=C(OC)C=C3CC1)=O',
 'CC(C)N(C(C)C)C(=O)N[C@@H1]C1=CC(C(NC)=CC=C1C2=C(OC)C(OC)=C(OC)C=C2CC)=O',
 'C1=2[C@H1]CCC3=CC(OC)=C(OC)C(OC)=C3C1=CC=C(C(C=2)=O)SCNC(C)=O',
 'N(C(C)C)(C(C)C)C(=O)N1[C@H1]CCC2=C(C(OC)=C(OC)C(OC)=C2)C3=CC=C(NC)C(C=C31)=O',
 'NC(=S)N1[C@@H1]C2=CC(C(NC)=CC=C2C3=C(OC)C(OC)=C(OC)C=C3CC1)=O',
 'OCCN(CCO)C(=S)N1[C@@H1]C2=CC(C(NC)=CC=C2C3=C(OC)C(OC)=C(OC)C=C3CC1)=O',
 'CC(C)CCN[C@H1]C1CC2=CC(OC)=C(OC)C(OC)=C2C3=CC=C(SC)C(C=C31)=O',
 'OC1=C(C=CC=C1)CN2[C@H1]CCC3=CC(OC)=C(OC)C(OC)=C3C4=CC=C(SC)C(C=C24)=O',
 'CC(=O)N1[C@H1]CCC2=CC(OC)=C(OC)C(OC)=C2C3=CC=C(NC)C(C=C31)=O',
 'CC(C)(C)OC1=CN(N=N1)[C@H1]C2CC3=CC(OC)=C(OC)C(OC)=C3C4=CC=C(NC)C(=O)C=C42',
 'OC(CCC(C)C)OCC1=CN(N=N1)[C@H1]CCC2=CC(OC)=C(OC)C(OC)=C2C3=CC=C(C(=O)C=C3)NC',
 'OC1=C(C=CC=C1)C=2N=NN(C=2)[C@H1]C3CC4=CC(OC)=C(OC)C(OC)=C4C5=CC=C(NC)C(=O)C=C5
3',
'OC1=C(C=CC=C1)C=2N=NN(C=2)[C@H1]CCC3=CC(OC)=C(OC)C(OC)=C3C4=CC=C(NC)C(=O)C=C4',
 'O=C(OCC)CN(CCC)C[C@H1]C1CC2=CC(OC)=C(OC)C(OC)=C2C3=CC=C(SC)C(=O)C=C31',
 'CC(C)(C)OC(=O)NCCC(NCC=1N=NN(C=1)[C@H1]CCC2=CC(OC)=C(OC)C=C2C3=CC=C(NC)C(C=C3)
=O)OC=O',
 'CC(C)(C)OCNCCC(=O)NCC1=CN(N=N1)[C@H1]C2CC3=CC(OC)=C(OC)C(OC)=C3C4=CC=C(C(=O)C=
C24)NC=O',
 'CC(C)(C)OC(=O)NCCC(OCC=1N=NN(C=1)[C@H1]CCC2=CC(OC)=C(OC)C(=C2C3=CC=C(NC)C(=O)C
=C3)O)C=O',
 'O=C(OC(C)(C)C)NCC=1N=NN(C=1)[C@H1]C2CC3=CC(OC)=C(OC)C(OC)=C3C4=CC=C(NC)C(=O)C=
C42',
 'CC(C)COC(NCC1=CN(N=N1)[C@H1]C2CC3=CC(OC)=C(OC)C(OC)=C3C4=CC=C(C(=O)C=C24)NC)=O
',
 'O=C(OCC)C1=C(C(OCC)=O)N=NN1[C@H1]CCC2=CC(OC)=C(OC)C(OC)=C2C3=CC=C(NC)C(C=C3)=O
',
 'CC(CC)N[C@H1]C1CC2=CC(OC)=C(OC)C(OC)=C2C3=CC=C(C(C=C31)=O)NCC',
 'CC(CC)N[C@H1]C1CC2=CC(OC)=C(OC)C(OC)=C2C3=CC=C(NC)C(=O)C=C31',
 'CCN(CC)C(=O)N[C@H1]C1CC2=CC(OC)=C(OC)C(OC)=C2C3=CC=C(C(C=C31)=O)NC',
 'CCN(CC)C(=O)N1[C@H1]CCC2=CC(OC)=C(OC)C(OC)=C2C3=CC=C(NC)C(C=C31)=O',
 'NC(=O)N1[C@@H1]C2=CC(C(NC)=CC=C2C3=C(OC)C(OC)=C(OC)C=C3CC1)=O',
 'C1=2[C@H1]CCC3=CC(OC)=C(OC)C(OC)=C3C1=CC=C(C(=O)C=2)NCNC(C)=O']
```

[36]:

```
mol = Chem.MolFromSmiles(issue[0])
chiral = Chem.FindMolChiralCenters(mol)
chiral
```

[36]:

```
[]
```

[37]:

```
mol
```

[37]:

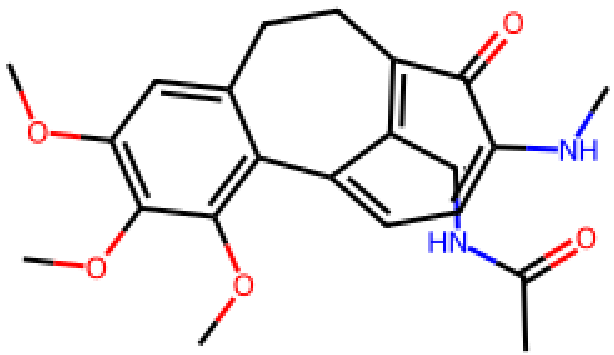

[38]:
```
mol = Chem.MolFromSmiles(issue[1])
chiral = Chem.FindMolChiralCenters(mol)
print(chiral)
mol
```

```
[]
```

[38]:

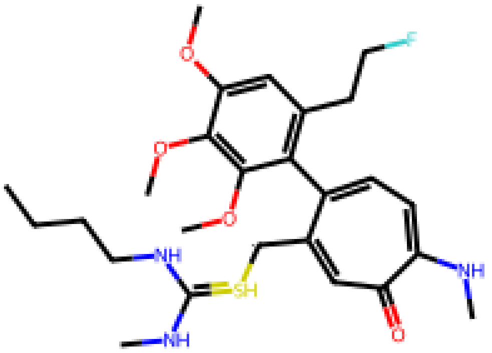

[39]:
```
mol = Chem.MolFromSmiles(issue[2])
chiral = Chem.FindMolChiralCenters(mol)
print(chiral)
mol
```

```
[]
```

[39]:

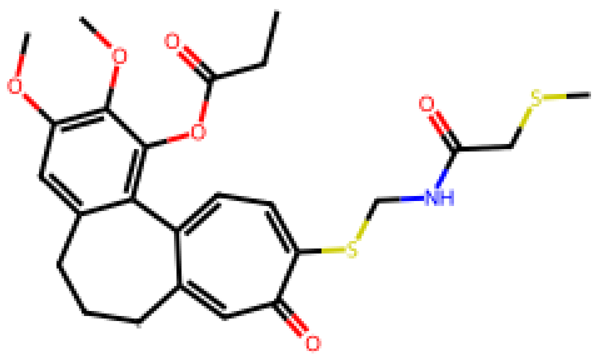

[40]:
```
len(issue)
```

[40]: 42

[41]:
```
atom_num = []
chirality = []
issue = []
for smiles in new['SMILES']:
    mol = Chem.MolFromSmiles(smiles)
    chiral = Chem.FindMolChiralCenters(mol)
    try:
        atom_num.append(chiral[0][0])
        chirality.append(chiral[0][1])
    except:
        atom_num.append(0)
        chirality.append('No chiral center')
print("Job finished...")
```

```
Job finished…
```

[42]:
```
new['atom_num'] = atom_num
new['chirality'] = chirality
new.head()
```

[42]:
```
   Unnamed: 0                                             SMILES  SYBA score  \
0         NaN  COC1=C2C3=CC=C(NC)C(=O)C=C3[C@H1](CCC2=CC(OC)=…       45.88
1         NaN  COC1=C2C3=CC=C(NC)C(=O)C=C3[C@H1](CCC2=CC(OC)=…       68.25
2         NaN  COC1=C2C3=CC=C(NC)C(=O)C=C3[C@H1](CCC2=CC(OC)=…       74.22
3         NaN  COC1=C2C3=CC=C(NC)C(=O)C=C3[C@H1](CCC2=CC(OC)=…       40.43
4         NaN  O=CC1=CN=NN1[C@H1]2CCC3=CC(OC)=C(OC)C(OC)=C3C4…       41.84

   A549 [nM]  BALB/3T3 [nM]  LoVo [nM]  LoVo/DX [nM]  MCF-7 [nM]  atom_num  \
0  10.168579       6.200000   5.700000    136.586050    8.357629        14
1  10.648944       9.439809  17.460496     49.797629   10.167834        14
```

```
2  11.794066       30.305940  54.000000     37.606395    9.099151          14
3   6.400000       23.752895   8.570105    210.367783    5.400000          14
4   4.774935       11.772850  10.000000   1806.439871   12.029326           7

  chirality
0         S
1         S
2         S
3         S
4         S
```

[ ]:

```

```

[43]:

```
to_be_printed = pd.DataFrame(data=new['SMILES'], columns=['SMILES'])
chirality = []
for i, smiles in enumerate(new['SMILES']):
    mol = Chem.MolFromSmiles(smiles)

    chiral = Chem.FindMolChiralCenters(mol)
    if len(chiral) > 0:

        chirality.append(chiral)
    else:
        chirality.append('No chiral center')

to_be_printed['Chirality'] = chirality
to_be_printed['Mol Image'] = [Chem.MolFromSmiles(smi) for smi in␣
 ↪to_be_printed['SMILES']]
PandasTools.SaveXlsxFromFrame(to_be_printed, '../Data/new_structures_chirality.
 ↪xlsx', molCol='Mol Image')
```

[ ]:

```

```

[44]:

```
mol = Chem.
 ↪MolFromSmiles('CC(=O)N[C@@H1]C1=C2C(C(NC)=CC=C1C3=C(OC)C(OC)=C(OC)C=C3CC2)=O')
show_atom_number(mol, 'atomLabel')
```

[44]:

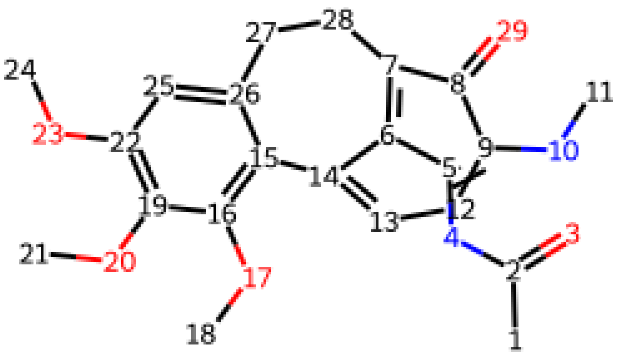

[45]:
```
mol = Chem.
  ↪MolFromSmiles('CN(C(=S)N[C@@H1]1C2=CC(=O)C(NC)=CC=C2C3=C(C=C(OC)C(OC)=C3OC)CC1C)C#N')
show_atom_number(mol, 'atomLabel')
```

[45]:

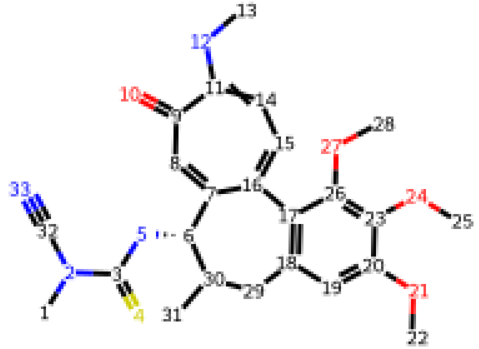

[46]:
```
mol = Chem.
  ↪MolFromSmiles('C1=2[C@H1](CCC3=CC(OC)=C(OC)C(OC)=C3C1=CC=C(C(=O)C=2)NCO)C(C(OC)=O)NC(=O)CNC
show_atom_number(mol, 'atomLabel')
```

[46]:

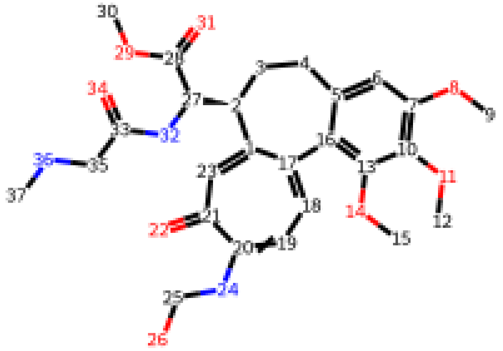

[47]:
```
mol = Chem.
  ↪MolFromSmiles('C[C@H1](NCC)CC1=CC(C(NC)=CC=C1C2=C(C=C(OC)C(OC)=C2OCC3=CC=CC=C3)OSCC)=O')
show_atom_number(mol, 'atomLabel')
```

[47]:

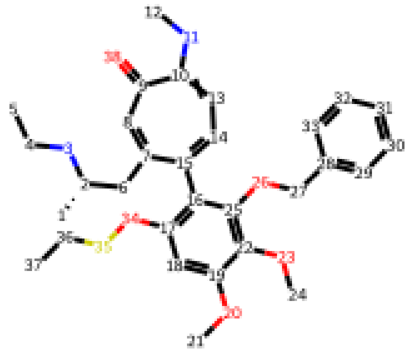

[48]:
```
mol = Chem.
  ↪MolFromSmiles('N1(CCCC1)C(=O)OC2=C(OC)C(OC3)=CC=C2C4=CC=C(SC)C(C=C4[C@@H1](NC(=O)C)CC3)=O')
show_atom_number(mol, 'atomLabel')
```

[48]:

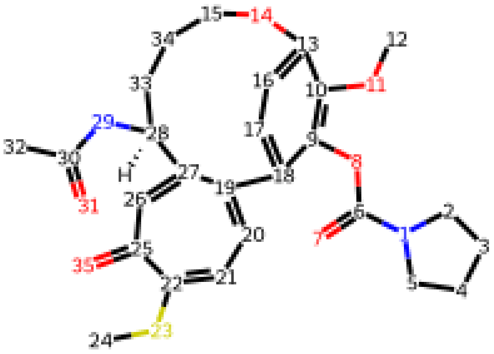

[49]:
```
mol = Chem.
  ↪MolFromSmiles('C1[C@H1](C2=CC(C(SC)=CC=C2C3=C(OC)C(=C(OC)C=C3C1)OC)=O)NCC')
show_atom_number(mol, 'atomLabel')
```

[49]:

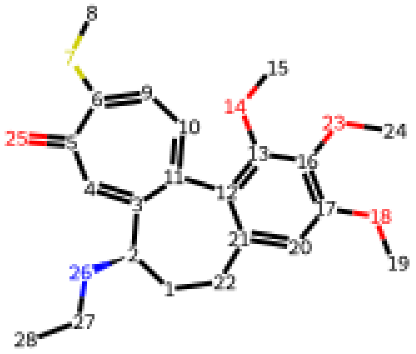

[50]:
```
mol = Chem.
 ↪MolFromSmiles('O=C(OCC)C=1N(N=NC=1C(=O)O2)[C@H1]2NC(=S)N[C@H1]3CCC4=CC(OC)=C(OC)C(OC)=C4C=5
show_atom_number(mol, 'atomLabel')
```

[50]:

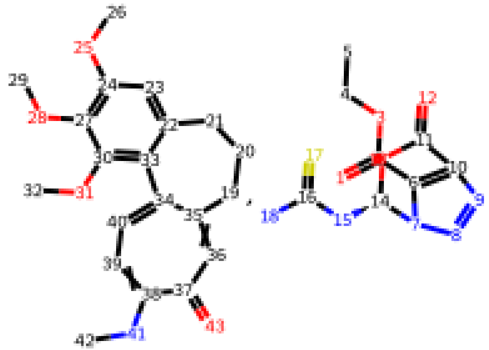

[ ]:

[ ]:

[ ]:

[51]:
```
fig, ax = plt.subplots()
bar_container = ax.bar(['S', 'R', 'No chiral center'],␣
 ↪[len(new[new['chirality'] == 'S']), len(new[new['chirality'] == 'R']),␣
 ↪len(new[new['chirality'] == 'No chiral center'])])
ax.set(xlabel = 'Absolute configuration of new structures', ylabel='Number of␣
 ↪compounds')
plt.rc('grid', linestyle="-.", color='black')
plt.grid(False)
ax.bar_label(bar_container, padding=2, label_type='edge')
plt.savefig('chirality_new.pdf', bbox_inches='tight')
```

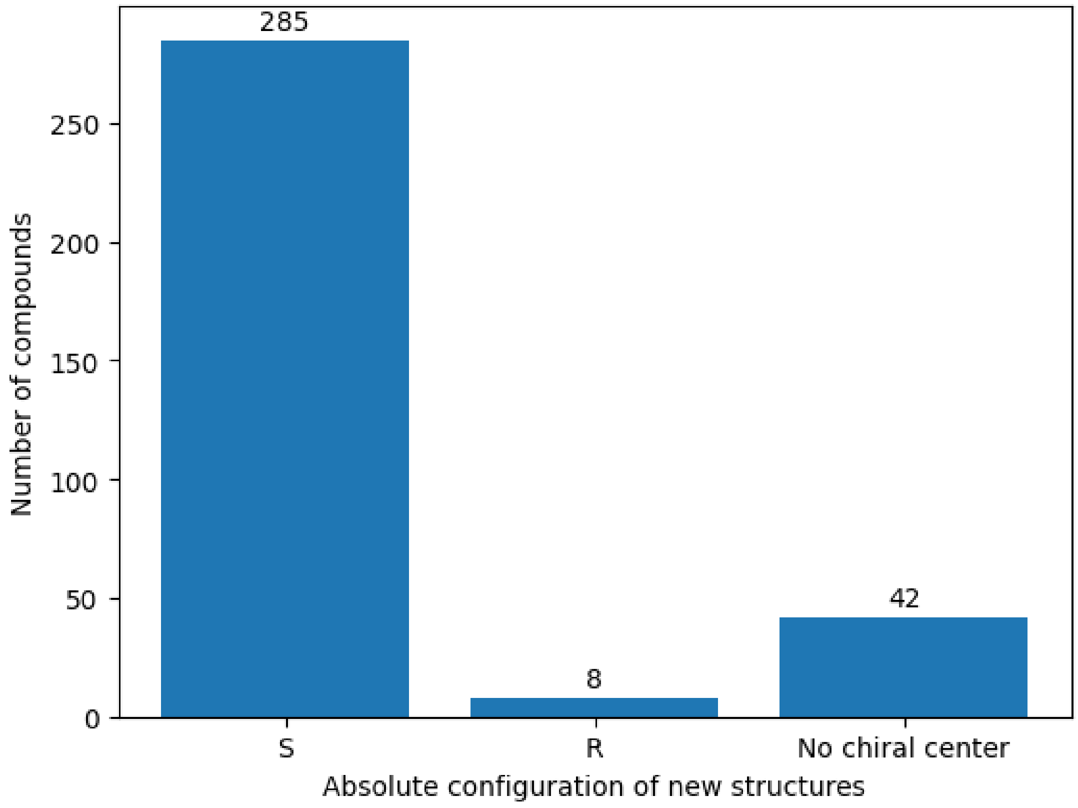

[52]:
```
len(new[new['chirality'] =='S'])
```

[52]: 285

[53]:
```
len(new[new['chirality'] =='R'])
```

[53]: 8

[54]:
```
len(new[new['chirality'] =='No chiral center'])
```

[54]: 42

[55]:
```
to_save = new[new['chirality'] =='S']
to_save.to_excel('../Data/new_structures_S_chirality.xlsx')
```

[ ]:
```

```

[56]:
```
for i, smiles in enumerate(new['SMILES']):
    mol = Chem.MolFromSmiles(smiles)

    chiral = Chem.FindMolChiralCenters(mol)
```

```python
    if len(chiral) > 0:

        print(i, chiral)
    else:
        print(i, 'No chiral center')
```

```
0 [(14, 'S')]
1 [(14, 'S')]
2 [(14, 'S')]
3 [(14, 'S')]
4 [(7, 'S')]
5 [(9, 'S')]
6 [(9, 'S')]
7 [(4, 'S')]
8 [(1, 'S')]
9 [(5, 'S')]
10 [(1, 'S')]
11 [(1, 'R')]
12 [(14, 'S')]
13 [(14, 'S')]
14 [(4, 'S')]
15 [(11, 'S')]
16 [(18, 'S')]
17 [(18, 'S')]
18 [(18, 'S')]
19 [(13, 'S')]
20 [(4, 'S')]
21 [(19, 'S')]
22 [(4, 'S')]
23 [(10, 'S')]
24 [(10, 'S')]
25 [(4, 'S')]
26 No chiral center
27 [(14, 'S')]
28 [(14, 'S')]
29 [(14, 'S')]
30 [(14, 'S')]
31 [(13, 'S')]
32 No chiral center
33 [(5, 'S')]
34 [(7, 'S')]
35 [(13, 'S')]
36 [(13, 'S')]
37 [(24, 'S')]
38 No chiral center
39 [(14, 'S')]
40 [(12, 'S')]
41 [(6, 'S')]
```

```
42 [(11, 'S')]
43 No chiral center
44 [(1, 'S')]
45 No chiral center
46 No chiral center
47 No chiral center
48 [(9, 'S')]
49 [(12, 'S')]
50 [(9, 'S')]
51 [(13, 'S')]
52 [(13, 'S')]
53 [(10, 'S')]
54 [(8, 'S')]
55 No chiral center
56 No chiral center
57 No chiral center
58 [(12, 'R')]
59 No chiral center
60 [(16, 'S')]
61 [(1, 'S')]
62 [(10, 'S')]
63 [(3, 'S')]
64 [(14, 'S')]
65 [(3, 'S')]
66 [(12, 'S')]
67 [(9, 'S')]
68 [(7, 'S')]
69 [(27, 'S')]
70 [(7, 'S')]
71 No chiral center
72 [(9, 'S')]
73 [(18, 'S')]
74 [(9, 'S')]
75 No chiral center
76 [(18, 'S')]
77 [(14, 'S')]
78 [(14, 'S')]
79 [(11, 'S')]
80 [(9, 'S')]
81 [(12, 'S')]
82 [(13, 'S')]
83 [(11, 'S')]
84 [(11, 'S')]
85 [(10, 'S')]
86 [(12, 'S')]
87 [(20, 'S')]
88 [(11, 'S')]
89 [(15, 'S')]
```

90 [(13, 'S')]
91 [(20, 'S')]
92 [(20, 'S')]
93 No chiral center
94 [(5, 'S')]
95 No chiral center
96 [(14, 'S')]
97 [(7, 'R')]
98 [(6, 'S')]
99 [(2, 'S')]
100 [(10, 'S')]
101 [(10, 'S')]
102 [(8, 'S')]
103 [(8, 'S')]
104 [(1, 'S')]
105 [(1, 'S')]
106 No chiral center
107 No chiral center
108 [(6, 'S')]
109 [(6, 'S')]
110 [(1, 'S')]
111 [(1, 'S')]
112 [(3, 'S')]
113 No chiral center
114 [(6, 'S')]
115 [(3, 'S')]
116 [(3, 'S')]
117 [(14, 'S')]
118 [(14, 'S')]
119 [(5, 'S')]
120 No chiral center
121 [(1, 'S')]
122 [(1, 'S')]
123 [(8, 'S')]
124 No chiral center
125 [(11, 'S')]
126 No chiral center
127 [(4, 'S')]
128 [(5, 'S')]
129 [(8, 'S')]
130 [(1, 'R')]
131 [(6, 'S')]
132 [(6, 'S')]
133 No chiral center
134 [(8, 'S')]
135 [(1, 'S')]
136 [(6, 'S')]
137 [(1, 'S')]

```
138 [(1, 'S')]
139 [(1, 'S')]
140 [(7, 'S')]
141 [(13, 'S')]
142 [(7, 'S')]
143 [(3, 'S')]
144 [(14, 'S')]
145 [(6, 'S')]
146 [(14, 'S')]
147 [(10, 'S')]
148 [(18, 'S')]
149 [(14, 'S')]
150 [(6, 'S')]
151 [(5, 'S')]
152 [(6, 'S')]
153 No chiral center
154 [(3, 'S')]
155 [(14, 'S')]
156 [(13, 'S')]
157 [(23, 'S')]
158 [(7, 'S')]
159 [(8, 'S')]
160 [(8, 'S')]
161 [(9, 'S')]
162 [(9, 'S')]
163 [(7, 'S')]
164 [(23, 'S')]
165 [(8, 'S')]
166 [(7, 'S')]
167 [(8, 'S')]
168 [(7, 'S')]
169 No chiral center
170 [(14, 'S')]
171 [(7, 'S')]
172 [(10, 'S')]
173 [(2, 'S')]
174 [(18, 'S')]
175 [(24, 'S')]
176 [(18, 'S')]
177 [(19, 'S')]
178 [(5, 'S')]
179 [(13, 'S')]
180 [(13, 'S')]
181 [(20, 'S')]
182 [(23, 'S')]
183 [(10, 'S')]
184 [(17, 'S')]
185 [(4, 'S')]
```

```
186 [(4, 'S')]
187 [(8, 'S')]
188 [(5, 'S')]
189 [(5, 'S')]
190 [(11, 'S')]
191 [(8, 'S')]
192 [(6, 'S')]
193 [(6, 'S')]
194 [(12, 'S')]
195 [(4, 'S')]
196 [(22, 'S')]
197 [(4, 'S')]
198 [(4, 'S')]
199 No chiral center
200 [(14, 'S')]
201 [(1, 'S')]
202 [(7, 'S')]
203 [(7, 'S')]
204 [(5, 'S')]
205 [(1, 'S')]
206 [(23, 'S')]
207 [(13, 'S')]
208 [(13, 'S')]
209 [(5, 'S')]
210 No chiral center
211 [(10, 'S')]
212 [(7, 'S')]
213 No chiral center
214 [(12, 'S')]
215 [(1, 'S')]
216 [(11, 'S')]
217 No chiral center
218 No chiral center
219 [(1, 'S')]
220 [(8, 'S')]
221 [(11, 'S')]
222 [(9, 'S')]
223 No chiral center
224 [(9, 'S')]
225 [(9, 'S')]
226 [(13, 'S')]
227 [(13, 'S')]
228 [(13, 'S')]
229 [(12, 'S')]
230 [(12, 'S')]
231 [(4, 'S')]
232 [(9, 'S')]
233 [(10, 'S')]
```

```
234 [(11, 'S')]
235 [(12, 'S')]
236 [(14, 'S')]
237 [(9, 'S')]
238 [(11, 'S')]
239 [(13, 'S')]
240 [(11, 'S')]
241 [(7, 'S')]
242 [(10, 'R')]
243 [(10, 'S')]
244 [(10, 'S')]
245 [(12, 'S')]
246 [(10, 'S')]
247 [(7, 'S')]
248 [(15, 'S')]
249 [(7, 'S')]
250 No chiral center
251 No chiral center
252 [(9, 'S')]
253 [(18, 'S')]
254 No chiral center
255 [(9, 'S')]
256 [(10, 'S')]
257 [(7, 'S')]
258 [(14, 'S')]
259 [(3, 'S')]
260 [(3, 'S')]
261 [(3, 'S')]
262 [(11, 'S')]
263 No chiral center
264 [(8, 'S')]
265 [(13, 'R'), (18, 'S')]
266 [(14, 'S')]
267 No chiral center
268 [(10, 'S')]
269 [(13, 'S')]
270 [(20, 'S')]
271 No chiral center
272 [(1, 'S')]
273 No chiral center
274 No chiral center
275 [(14, 'S')]
276 [(14, 'S')]
277 [(14, 'S')]
278 [(15, 'S')]
279 [(8, 'S')]
280 [(7, 'S')]
281 [(5, 'S')]
```

282 [(24, 'S')]
283 [(16, 'S')]
284 [(6, 'S')]
285 [(7, 'S')]
286 [(10, 'S')]
287 [(10, 'S')]
288 No chiral center
289 No chiral center
290 [(8, 'S')]
291 [(16, 'S')]
292 [(11, 'S')]
293 [(14, 'S')]
294 [(1, 'S')]
295 [(3, 'S')]
296 [(3, 'S')]
297 [(1, 'S')]
298 [(3, 'S')]
299 [(4, 'S')]
300 [(11, 'S')]
301 [(1, 'S')]
302 [(1, 'S')]
303 [(10, 'S')]
304 [(13, 'S')]
305 [(1, 'R')]
306 No chiral center
307 [(5, 'S')]
308 [(1, 'S')]
309 [(10, 'S')]
310 [(16, 'S')]
311 [(4, 'S')]
312 [(1, 'S')]
313 [(9, 'S')]
314 No chiral center
315 [(21, 'S')]
316 [(4, 'S')]
317 [(4, 'S')]
318 [(4, 'S')]
319 [(6, 'S')]
320 [(7, 'S')]
321 [(1, 'S')]
322 [(1, 'R')]
323 [(1, 'S')]
324 [(25, 'S')]
325 [(20, 'S')]
326 [(5, 'S')]
327 [(10, 'S')]
328 [(7, 'S')]
329 [(16, 'S')]

```
330 [(1, 'S')]
331 [(8, 'S')]
332 [(10, 'S')]
333 [(10, 'S')]
334 [(17, 'S')]
```

```
[ ]:
```

```
[ ]:
```

Notebook

December 19, 2023

1 File 43

[1]:
```
# libraries import
import pandas as pd
import numpy as np
from sklearn.decomposition import PCA
from sklearn.manifold import TSNE

%matplotlib inline
import matplotlib.pyplot as plt
from mpl_toolkits.mplot3d import Axes3D

from rdkit import Chem
from rdkit import DataStructs
from rdkit.Chem import AllChem
import seaborn as sns
```

[46]:
```
#initial structures load
initials = pd.read_excel('../Data/Kolchicyna_prepared_data.xlsx')
initials['Initial/new'] = 'Initial'
```

[47]:
```
new_structures = pd.read_excel('../Data/
↪Proposed_structures_with_AI_colchicyne_tanimoto_similarity_.xlsx')
new_structures['Initial/new'] = 'New'
new_structures = new_structures.rename(columns={"AI_generated_SMILES" :␣
↪"SMILES"})
```

[48]:
```
whole_df = initials[['SMILES', 'Initial/new']]
whole_df = whole_df.append(new_structures[['SMILES', 'Initial/new']])
```

[49]:
```
whole_df.head()
```

[49]:
```
                                              SMILES Initial/new
0  COc2c3C1=CC=C(SC)C(=O)C=C1[C@H](CCc3cc(OC)c2OC…     Initial
1  COc2c3C1=CC=C(SC)C(=O)C=C1[C@H](CCc3cc(OC)c2OC…     Initial
2  COc2c3C1=CC=C(SC)C(=O)C=C1[C@H](CCc3cc(OC)c2OC…     Initial
3  CC(C)CN[C@H]2CCc3cc(OC)c(OC)c(OC)c3C1=CC=C(SC)…     Initial
4  COc2c3C1=CC=C(SC)C(=O)C=C1[C@H](CCc3cc(OC)c2OC…     Initial
```

[50]:
```
# Molecular fingerprints calculations
class FP:
    """
    Molecular fingerprint class, useful to pack features in pandas df

    Parameters
    ----------
    fp : np.array
        Features stored in numpy array
    names : list, np.array
        Names of the features
    """
    def __init__(self, fp, names):
        self.fp = fp
        self.names = names
    def __str__(self):
        return "%d bit FP" % len(self.fp)
    def __len__(self):
        return len(self.fp)
```

[51]:
```
def fingerprint(mol, radius=2, nBits=1024, useFeatures=False, counts=False,␣
 ↪dtype=np.float32):
    arr = np.zeros((1,), dtype)
    DataStructs.ConvertToNumpyArray(AllChem.GetMorganFingerprintAsBitVect(mol,␣
 ↪radius, nBits=nBits, useFeatures=useFeatures), arr)
    return FP(arr, range(nBits))
```

[52]:
```
# Molecular fingerprints calculations
whole_df['FP'] = [fingerprint(Chem.MolFromSmiles(smi)) for smi in␣
 ↪whole_df['SMILES']]
```

[53]:
```
data_ = np.array([x.fp for x in whole_df['FP']])
```

[54]:
```
# t-SNE analysis
model = TSNE(n_components=2, random_state=0, perplexity=30, n_iter=5000)
tsne_whole_set = model.fit_transform(data_)
```

[55]:
```
whole_df['TSNE_C1'] = tsne_whole_set.T[0]
whole_df['TSNE_C2'] = tsne_whole_set.T[1]
```

[56]:
```
# t-SNE plot
sns.pairplot(whole_df, hue='Initial/new', vars=['TSNE_C1', 'TSNE_C2'],␣
 ↪palette='viridis')
```

[56]:
```
<seaborn.axisgrid.PairGrid at 0x165bcb0db20>
```

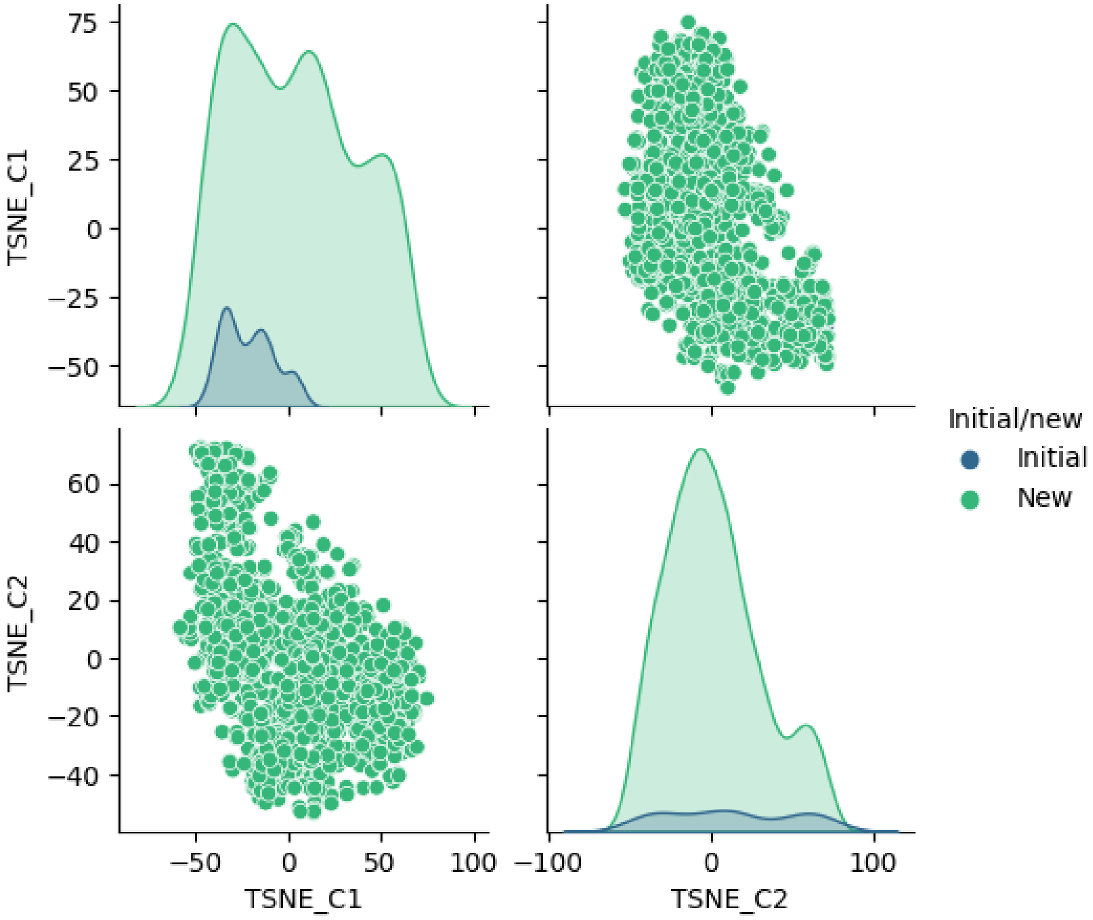

[57]:
```
x_t = whole_df.loc[whole_df['Initial/new'] == 'Initial']
len(x_t)
```

[57]: 120

[58]:
```
x_r = whole_df.loc[whole_df['Initial/new'] == 'New']
len(x_r)
```

[58]: 1356

[59]:
```
fig = plt.figure(figsize=(20,20))
ax = fig.add_subplot(111)

ax.scatter(x_r['TSNE_C1'], x_r['TSNE_C2'], c='r', marker="o", label='New') #,
  ↪alpha=1
ax.scatter(x_t['TSNE_C1'], x_t['TSNE_C2'], c='b', marker="s",
  ↪label='Initial')#, alpha=0.1
plt.legend(loc='lower right', fontsize=24)
```

```
plt.xlabel('TSNE_C1', fontweight='bold', fontsize=24)
plt.ylabel('TSNE_C2', fontweight='bold', fontsize=24)
plt.rc('grid', linestyle="-.", color='black')
plt.grid(True)
plt.savefig('t-SNE_analysis_all_generated_no_PCA.pdf', bbox_inches='tight')
plt.show()
```

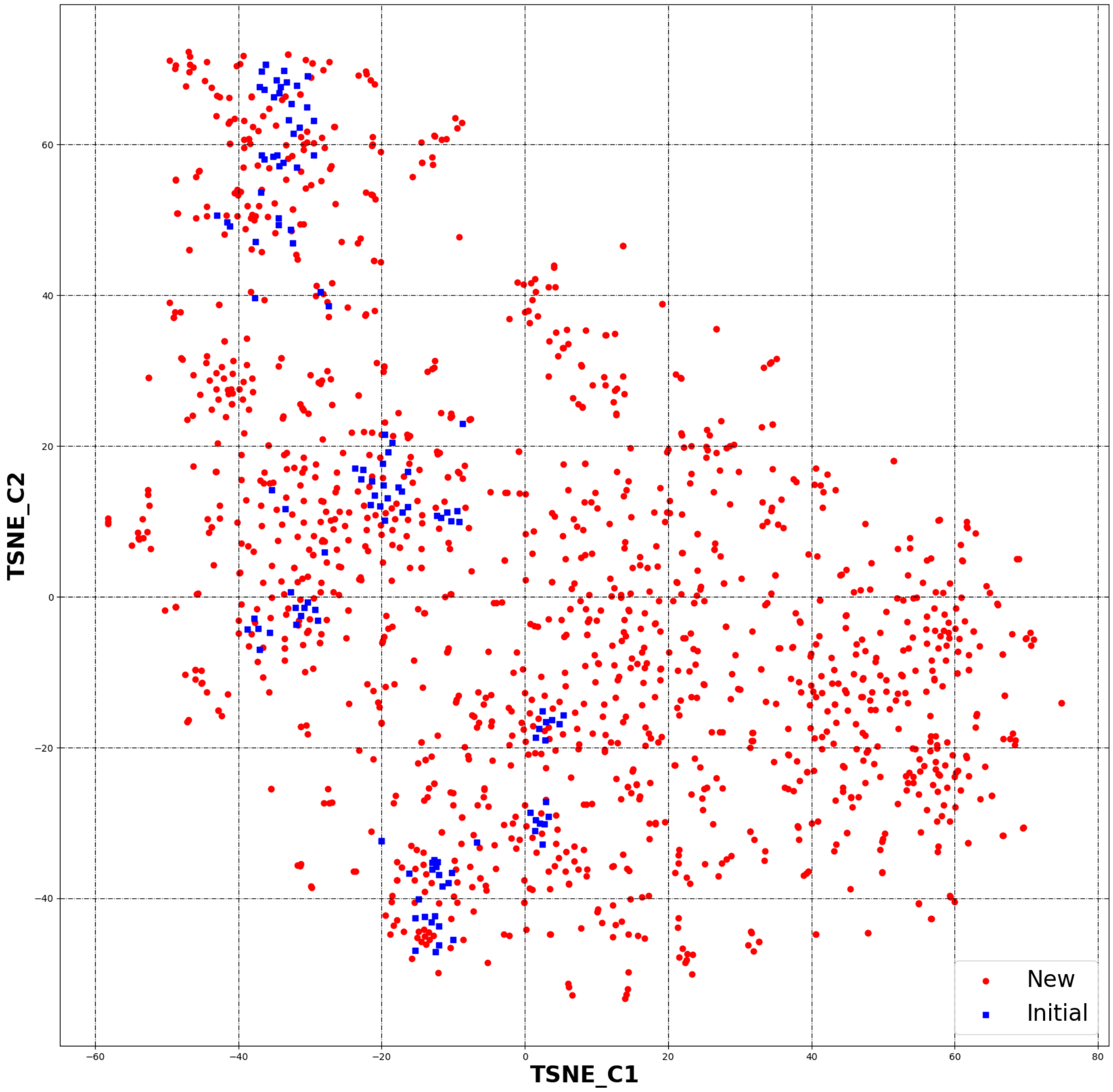

[16]:

```
# PCA analysis
pca = PCA(n_components=5, random_state=42) #n_components=20 in the case of␣
↪activity_pred_app
pca_training_whole = pca.fit_transform(data_)
```

[17]:
```
list_pca = []
for i in range(5):
    list_pca.append('PC'+str(i+1))
```

[18]:
```
for pca_ in range(5):
    whole_df[list_pca[pca_]] = pca_training_whole.T[pca_]
```

[19]:
```
sns.pairplot(whole_df, hue='Initial/new', vars=list_pca, palette='viridis')
```

[19]:
```
<seaborn.axisgrid.PairGrid at 0x165b06e2940>
```

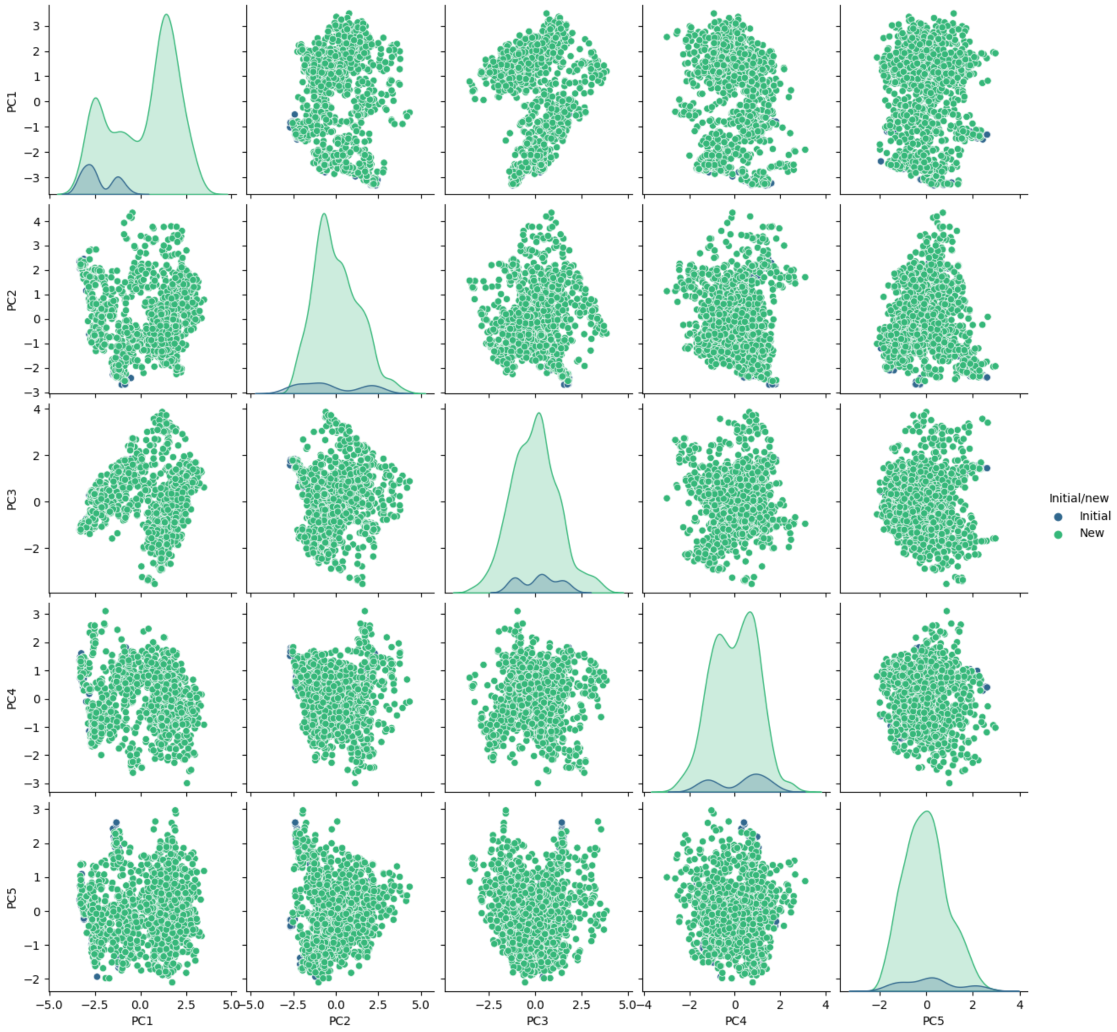

[20]:
```
# t-SNE analysis
model = TSNE(n_components=2, random_state=0, perplexity=30, n_iter=5000)
tsne_whole_set = model.fit_transform(pca_training_whole)
```

[21]:
```
whole_df['TSNE_C1'] = tsne_whole_set.T[0]
whole_df['TSNE_C2'] = tsne_whole_set.T[1]
```

[22]:
```
# t-SNE plot
sns.pairplot(whole_df, hue='Initial/new', vars=['TSNE_C1', 'TSNE_C2'],␣
 ↪palette='viridis')
```

[22]: <seaborn.axisgrid.PairGrid at 0x165b53903a0>

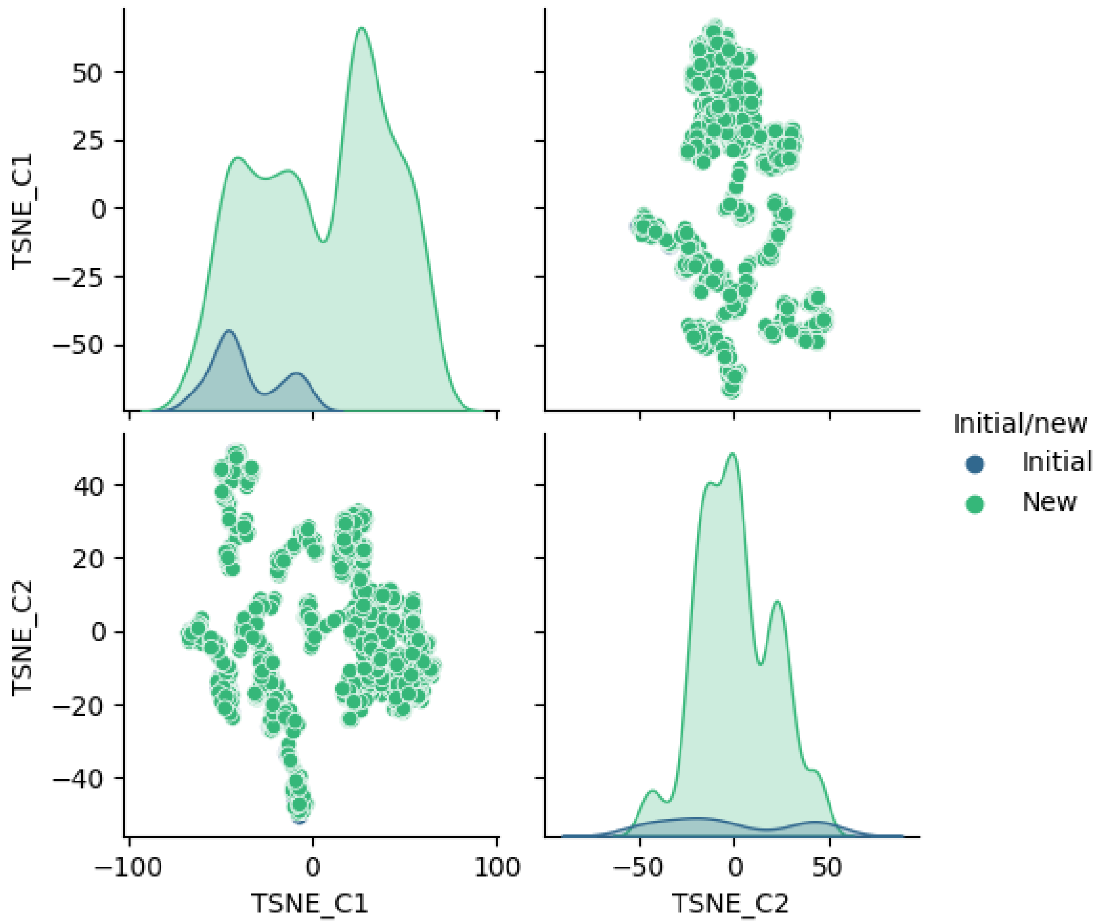

[23]:
```
x_t = whole_df.loc[whole_df['Initial/new'] == 'Initial']
print(len(x_t))
x_r = whole_df.loc[whole_df['Initial/new'] == 'New']
len(x_r)
```

120

[23]: 1356

[24]:
```
fig = plt.figure(figsize=(20,20))
ax = fig.add_subplot(111)

ax.scatter(x_r['TSNE_C1'], x_r['TSNE_C2'], c='r', marker="o", label='New') #,␣
 ↪alpha=1
ax.scatter(x_t['TSNE_C1'], x_t['TSNE_C2'], c='b', marker="s",␣
 ↪label='Initial')#, alpha=0.1
plt.legend(loc='lower right', fontsize=24)
plt.xlabel('TSNE_C1', fontweight='bold', fontsize=24)
plt.ylabel('TSNE_C2', fontweight='bold', fontsize=24)
plt.rc('grid', linestyle="-.", color='black')
plt.grid(True)
plt.savefig('t-SNE_analysis_all_generated.pdf', bbox_inches='tight')
plt.show()
```

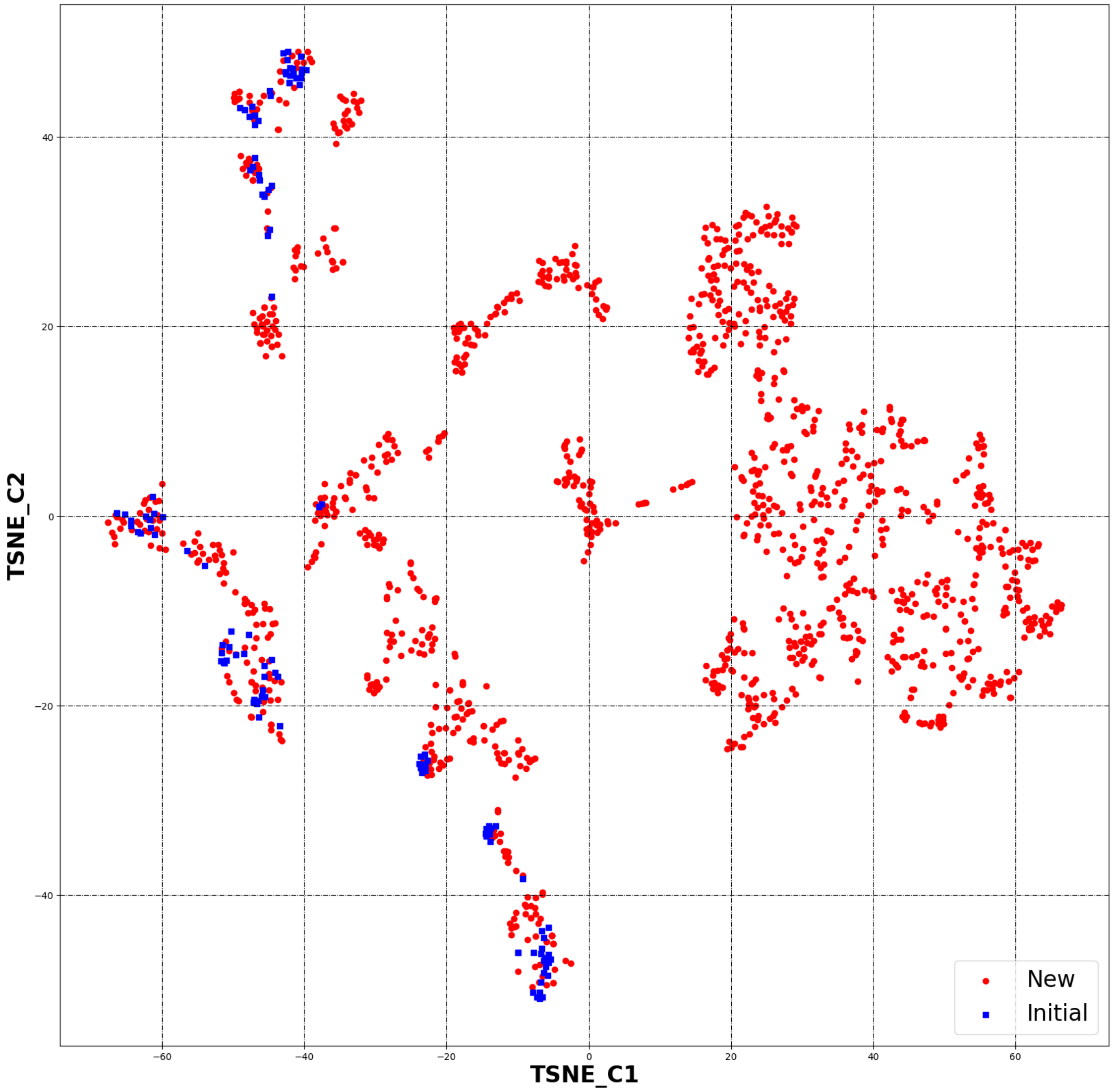

1.1 Only structures that are selected

[25]:
```
new_structures = pd.read_excel('../Data/Whole_report.xlsx')
new_structures['Initial/new'] = 'New'
```

[26]:
```
whole_df_ = initials[['SMILES', 'Initial/new']]
whole_df_ = whole_df_.append(new_structures[['SMILES', 'Initial/new']])
```

[27]:
```
# Molecular fingerprints calculations
whole_df_['FP'] = [fingerprint(Chem.MolFromSmiles(smi)) for smi in␣
↪whole_df_['SMILES']]
```

[28]:
```
data_ = np.array([x.fp for x in whole_df_['FP']])
```

[29]:
```
# t-SNE analysis
model = TSNE(n_components=2, random_state=0, perplexity=30, n_iter=5000)
tsne_whole_set = model.fit_transform(data_)
```

[30]:
```
whole_df_['TSNE_C1'] = tsne_whole_set.T[0]
whole_df_['TSNE_C2'] = tsne_whole_set.T[1]
```

[31]:
```
# t-SNE plot
sns.pairplot(whole_df_, hue='Initial/new', vars=['TSNE_C1', 'TSNE_C2'],␣
↪palette='viridis')
```

[31]:
```
<seaborn.axisgrid.PairGrid at 0x165a8391d30>
```

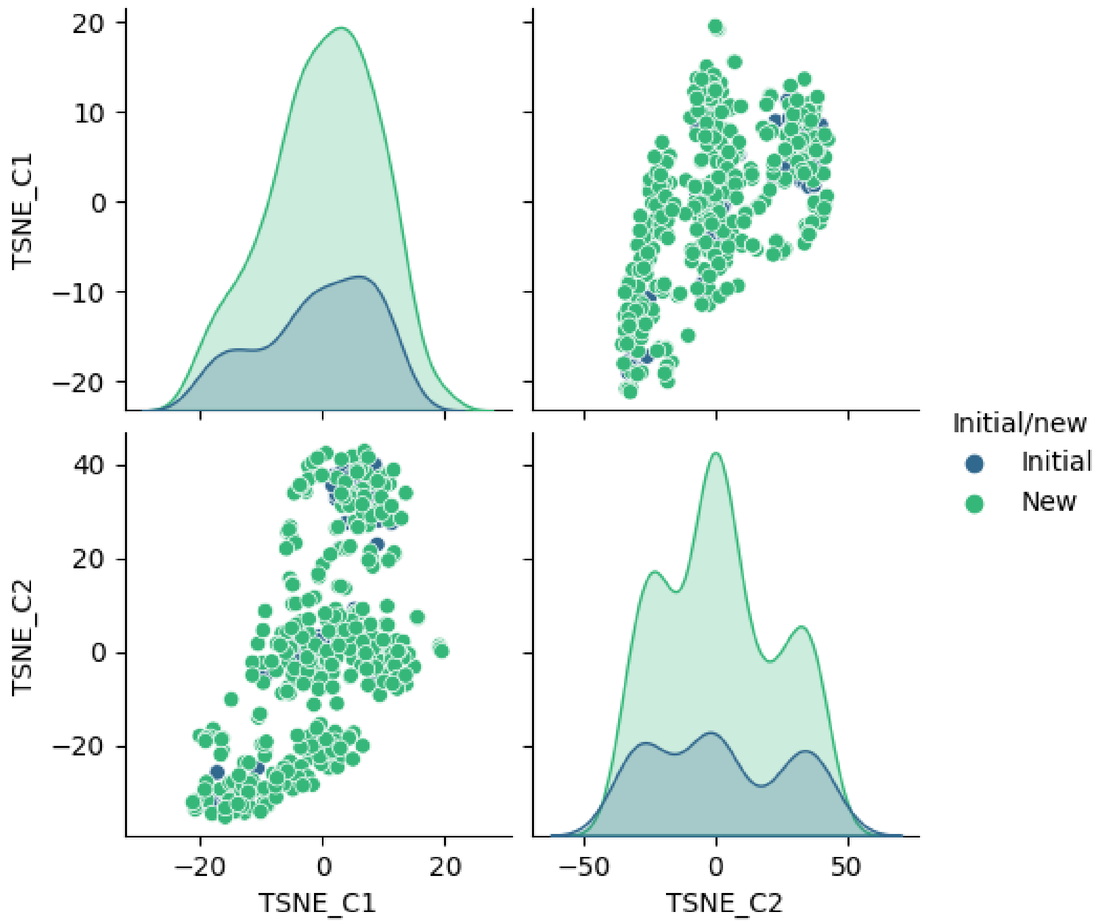

[32]:
```
x_t = whole_df_.loc[whole_df_['Initial/new'] == 'Initial']
len(x_t)
```

[32]: 120

[33]:
```
x_r = whole_df_.loc[whole_df_['Initial/new'] == 'New']
len(x_r)
```

[33]: 335

[34]:
```
fig = plt.figure(figsize=(20,20))
ax = fig.add_subplot(111)

ax.scatter(x_r['TSNE_C1'], x_r['TSNE_C2'], c='r', marker="o", label='New') #,␣
 ↪alpha=1
ax.scatter(x_t['TSNE_C1'], x_t['TSNE_C2'], c='b', marker="s",␣
 ↪label='Initial')#, alpha=0.1
plt.legend(loc='lower right', fontsize=24)
```

```
plt.xlabel('TSNE_C1', fontweight='bold', fontsize=24)
plt.ylabel('TSNE_C2', fontweight='bold', fontsize=24)
plt.rc('grid', linestyle="-.", color='black')
plt.grid(True)
plt.savefig('t-SNE_analysis_selected_generated_PCA_2.pdf', bbox_inches='tight')
plt.show()
```

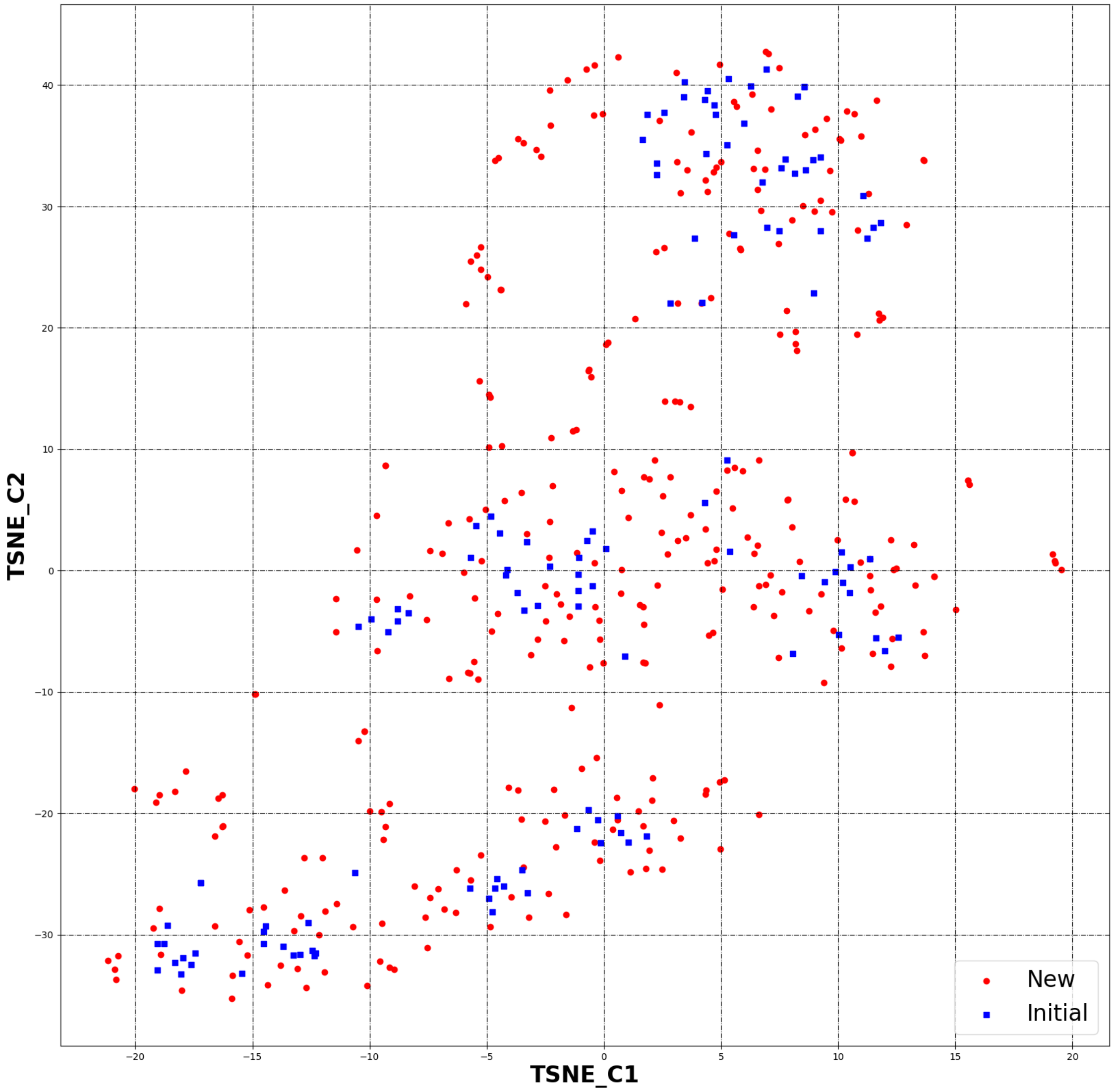

[ ]:

```

```

[35]:

```
# PCA analysis
pca = PCA(n_components=5, random_state=42) #n_components=20 in the case of␣
↪activity_pred_app
pca_training_whole = pca.fit_transform(data_)
```

[36]:
```
list_pca = []
for i in range(5):
    list_pca.append('PC'+str(i+1))
```

[37]:
```
for pca_ in range(5):
    whole_df_[list_pca[pca_]] = pca_training_whole.T[pca_]
```

[38]:
```
sns.pairplot(whole_df_, hue='Initial/new', vars=list_pca, palette='viridis')
```

[38]: <seaborn.axisgrid.PairGrid at 0x165b08eef70>

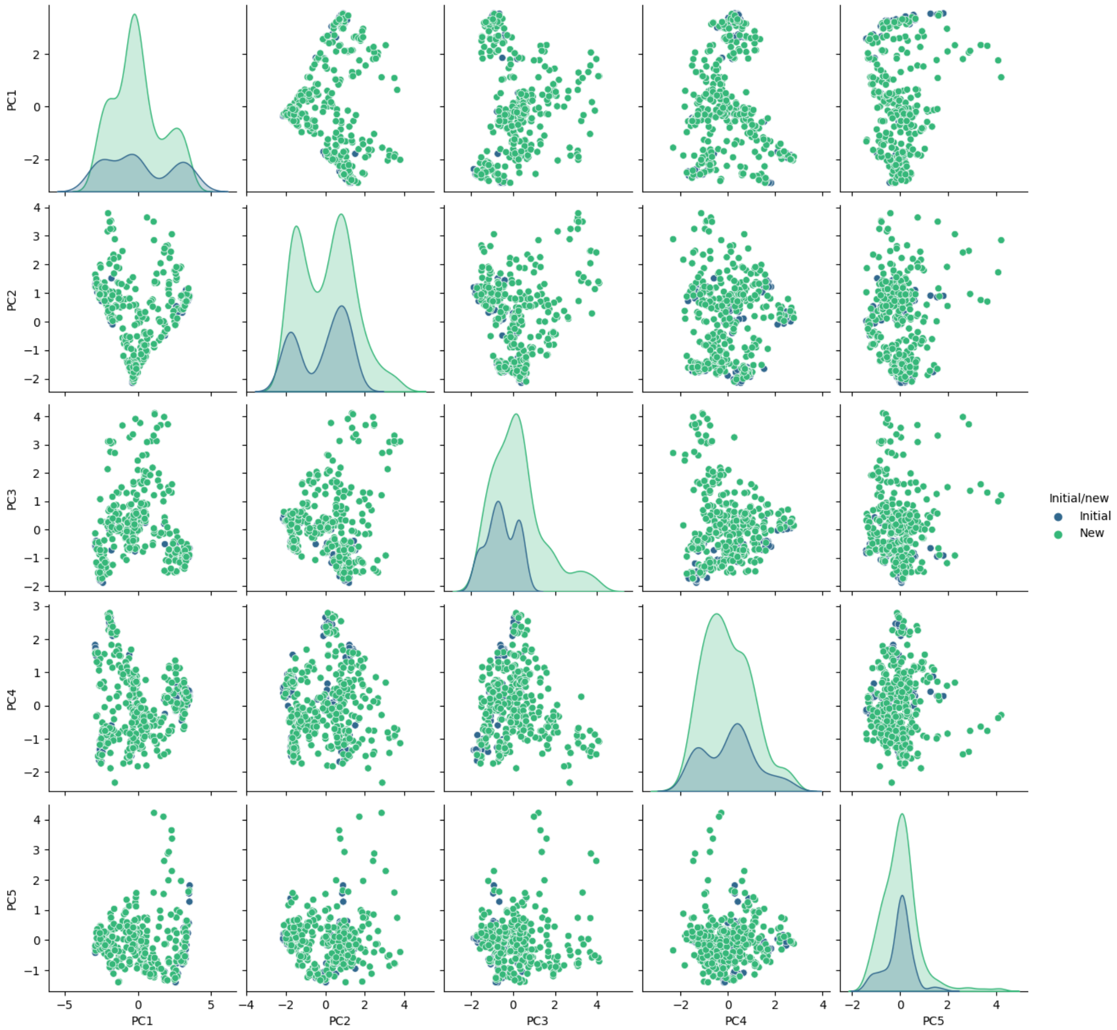

[39]:
```
# t-SNE analysis
model = TSNE(n_components=2, random_state=0, perplexity=30, n_iter=5000)
tsne_whole_set = model.fit_transform(pca_training_whole)
```

[40]:
```
whole_df_['TSNE_C1'] = tsne_whole_set.T[0]
whole_df_['TSNE_C2'] = tsne_whole_set.T[1]
```

[41]:
```
# t-SNE plot
sns.pairplot(whole_df_, hue='Initial/new', vars=['TSNE_C1', 'TSNE_C2'],␣
↪palette='viridis')
```

[41]: <seaborn.axisgrid.PairGrid at 0x165ba9243a0>

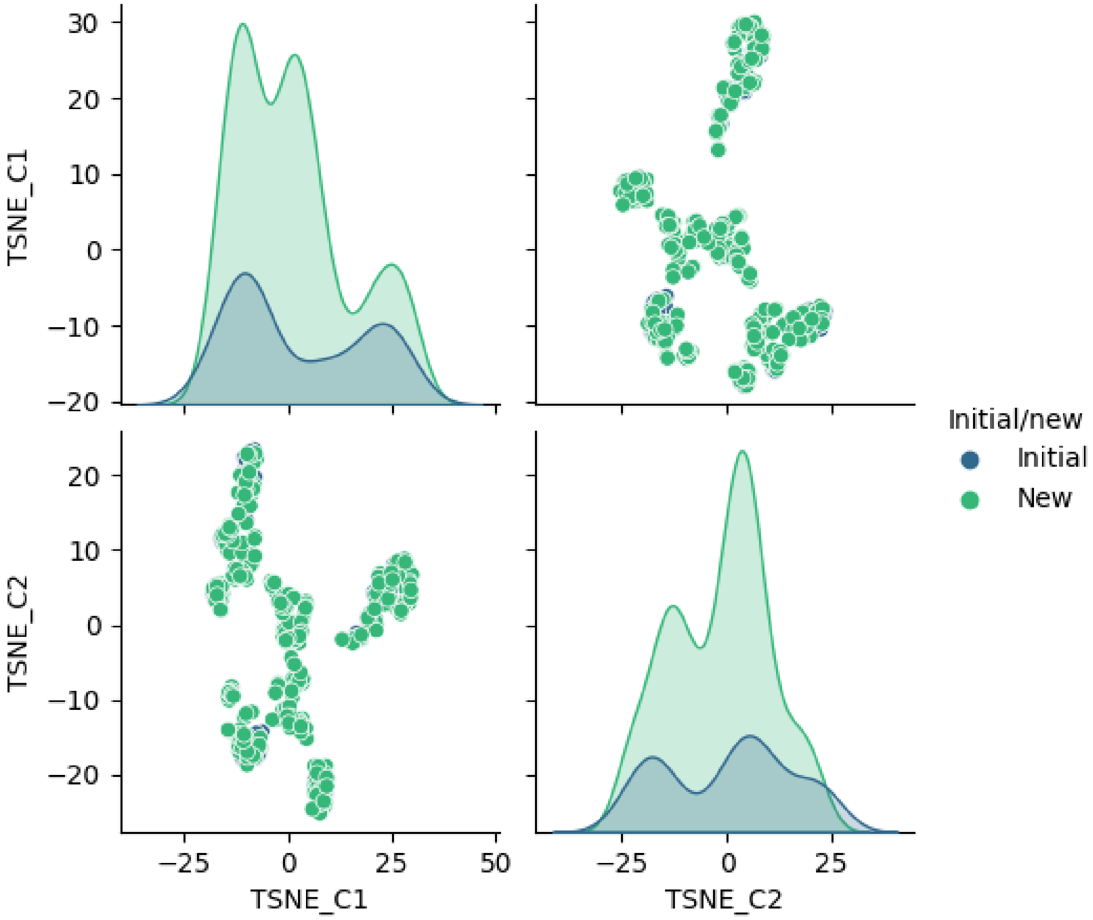

[42]:
```
x_t = whole_df_.loc[whole_df_['Initial/new'] == 'Initial']
print(len(x_t))
x_r = whole_df_.loc[whole_df_['Initial/new'] == 'New']
len(x_r)
```

120

[42]: 335

[43]:
```
fig = plt.figure(figsize=(20,20))
ax = fig.add_subplot(111)

ax.scatter(x_r['TSNE_C1'], x_r['TSNE_C2'], c='r', marker="o", label='New') #,␣
 ↪alpha=1
ax.scatter(x_t['TSNE_C1'], x_t['TSNE_C2'], c='b', marker="s",␣
 ↪label='Initial')#, alpha=0.1
plt.legend(loc='lower right', fontsize=24)
plt.xlabel('TSNE_C1', fontweight='bold', fontsize=24)
plt.ylabel('TSNE_C2', fontweight='bold', fontsize=24)
plt.rc('grid', linestyle="-.", color='black')
plt.grid(True)
plt.savefig('t-SNE_analysis_all_generated_PCA_5.pdf', bbox_inches='tight')
plt.show()
```

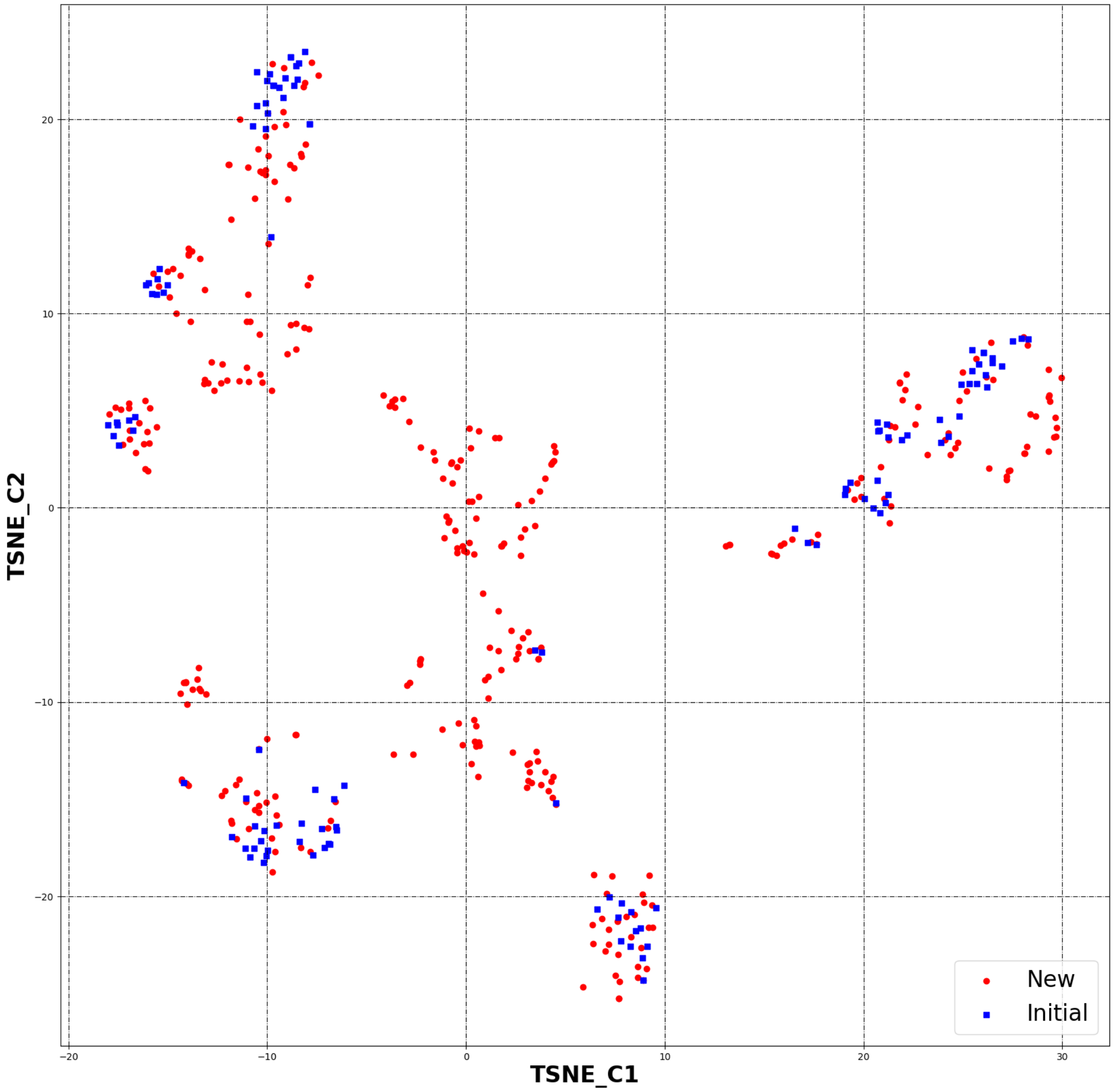

[44]:
```
whole_df_.head()
```

[44]:
```
                                              SMILES Initial/new           FP  \
0  COc2c3C1=CC=C(SC)C(=O)C=C1[C@H](CCc3cc(OC)c2OC...     Initial  1024 bit FP
1  COc2c3C1=CC=C(SC)C(=O)C=C1[C@H](CCc3cc(OC)c2OC...     Initial  1024 bit FP
2  COc2c3C1=CC=C(SC)C(=O)C=C1[C@H](CCc3cc(OC)c2OC...     Initial  1024 bit FP
3  CC(C)CN[C@H]2CCc3cc(OC)c(OC)c(OC)c3C1=CC=C(SC)...     Initial  1024 bit FP
4  COc2c3C1=CC=C(SC)C(=O)C=C1[C@H](CCc3cc(OC)c2OC...     Initial  1024 bit FP

     TSNE_C1    TSNE_C2       PC1       PC2       PC3       PC4       PC5
0 -10.516629  20.702110 -2.562503  0.952497 -1.505651 -1.099564 -0.178360
1  -9.104795  22.109497 -2.609674  0.952670 -1.650509 -1.440522  0.029450
2  -8.091981  23.488331 -2.470824  0.717305 -1.650362 -1.689266  0.085596
3 -10.093107  20.833193 -2.575260  1.009453 -1.548183 -1.154107 -0.071839
4  -8.543242  22.754614 -2.625635  0.947688 -1.711178 -1.624480  0.108450
```

[45]:
```
len(whole_df_)
```

[45]: 455

Notebook

December 19, 2023

1 File 44

[1]:
```
import pubchempy as pcp
import pandas as pd
import warnings
warnings.filterwarnings("ignore")
```

[2]:
```
df_search = pd.read_excel('../Data/
↪Proposed_structures_with_AI_colchicyne_tanimoto_similarity_.xlsx')
```

[3]:
```
data_ = pd.DataFrame(data=df_search['AI_generated_SMILES'])
zeros = [0 for i in range(len(data_))]
data_['CID'] = zeros
data_['PUBCHEM_SMILES'] = zeros
data_.head()
```

[3]:

	AI_generated_SMILES	CID	PUBCHEM_SMILES
0	COC1=C2C3=CC=C(NC)C(=O)C=C3[C@H1](CCC2=CC(OC)=…	0	0
1	COC1=C2C3=CC=C(NC)C(=O)C=C3[C@H1](CCC2=CC(OC)=…	0	0
2	COC1=C2C3=CC=C(NC)C(=O)C=C3[C@H1](CCC2=CC(OC)=…	0	0
3	COC1=C2C3=CC=C(NC)C(=O)C=C3[C@H1](CCC2=CC(OC)=…	0	0
4	COC1=CC2=CC=C(NC)C(C=C2[C@@H1](N3C=C(N=N3)CN)C…	0	0

[4]:
```
len(data_)
```

[4]: 1356

[5]:
```
knowon_l = []
for i, smiles in enumerate(data_['AI_generated_SMILES']):
    try:
        compound = pcp.get_compounds(smiles, 'smiles')
        data_['CID'][i] = compound[0].cid
        data_['PUBCHEM_SMILES'][i] = compound[0].canonical_smiles
        if math.isnan(data_['CID'][i]):
            data_['CID'][i] = 0
            data_['PUBCHEM_SMILES'][i] = 0
        else:
            pass
```

```
        knowon_l.append(compound)
    except:
        print("PUGREST.BadRequest: error: ", smiles)
```

```
PUGREST.BadRequest: error:
COC1=C2C3=CC=C(NC)C(=O)C=C3[C@H1](CCC2=CC(OC)=C1OC)NCCONC
PUGREST.BadRequest: error:
COC1=C2C3=CC=C(NC)C(=O)C=C3[C@H1](CCC2=CC(OC)=C1OC)NCCCCC
PUGREST.BadRequest: error:
COC1=C2C3=CC=C(NC)C(=O)C=C3[C@H1](CCC2=CC(OC)=C1OC)NCCCCCC
PUGREST.BadRequest: error:
COC1=C2C3=CC=C(NC)C(=O)C=C3[C@H1](CCC2=CC(OC)=C1OC)N4C=C(N=N4)CNCC
PUGREST.BadRequest: error:
COC1=CC2=CC=C(NC)C(C=C2[C@@H1](N3C=C(N=N3)CN)CCC1=CC(=COC)OC)=O
PUGREST.BadRequest: error:
C1=C2C(C3=C(OC)C(=C(C=C3CC4[C@@H1]2NCC4CCC)OC)OC)=CC=C(NC)C1=O
PUGREST.BadRequest: error:
C12=CC(C(NC)=CC=C1C=C(CC[C@@H1]2NCCC(F)(F)F)C=C(OC)C(OC)=COC)=O
PUGREST.BadRequest: error:
C1=C2C([C@H1](CCC3=CC(OC)=C(C(=C32)OC)OC)NCCCCl)(C(NC)=C1)O
PUGREST.BadRequest: error:
C=1=C2C([C@H1](CCC3=CC(OC)=C(C(=C32)OC)OC)NCCCCl)(C(NC)=CC=1)O
PUGREST.BadRequest: error:
C1=C2C([C@H1](CCC3=CC(OC)=C(C(=C32)OC)OC)NCCCCl)(C(=C1)NC)O
PUGREST.BadRequest: error:  C12=CC(C(NC)=CC=C1C=C(CC[C@H1]2NCCC(F)F)F)OC=O
PUGREST.BadRequest: error:  C12=NCCC1[C@H1]CNC32C4CC=CC=C3C4=CC
PUGREST.BadRequest: error:
O=CC1=CN=NN1[C@H1]2CCC3=CC(OC)=C(OC)C(OC)=C3C4=CC=C(C(=O)C=C42)NCC
PUGREST.BadRequest: error:
C1=NCCC1NC[C@@H1]2C3=CC(C(SC)=CC=C3C4=C(OC)C(OC)=C(C=C4CC2)OC=O)NC(=O)C
PUGREST.BadRequest: error:
C1=NCC(F)=CC=C1CN[C@H1]2CCC3=CC(OC)=C(OC)C(OC)=C3C4=CC=C(C(=O)C=C24)SCC
PUGREST.BadRequest: error:
OC1=CC=C(C=C1)CN[C@H1]2CCC3=C(C(OC)=C(OC)C(OC)=C3)C4=CC=C(SC)C(=O)C=C24
PUGREST.BadRequest: error:
OC1=CC=C(C=C1)CN[C@H1]2CCC3=CC(OC)=C(OC)C(OC)=C3C4=CC=C(C(C=C42)=O)SCC
PUGREST.BadRequest: error:
O=CC1=C[C@H1](CCC=C2C(OC)=C(OC)C(OC(C)=O)=C2C1=CC=C(SC)C=O)CC=O
PUGREST.BadRequest: error:
OC1=CC=C(C=C1)CN[C@H1]2CCC3=CC(OC)=C(OC)C(OC)=C3C4=CC=C(SC)C(=O)C=C42
PUGREST.BadRequest: error:
OC1=CC=C(C=C1)CN[C@H1]2CCC3=C(C(OC)=C(OC)C(OC)=C3)C4=CC=C(SC)C(C=C24)=O
PUGREST.BadRequest: error:
OC1=CC=C(C=C1)CN[C@H1]2CCC3=C(C(OC)=C(OC)C(OC)=C3)C4=CC=C(SC)C(=O)C=C42
PUGREST.BadRequest: error:
OC1=CC=C(C=C1)CN[C@H1]2CCC3=CC(OC)=C(OC)C(OC)=C3C4=CC=C(SC)C(=O)C=C24
PUGREST.BadRequest: error:
O=CC1=C2[C@H1](CCC3=CC(OC)=C(OC)C(OC(=O)C)=C3C1=CC=C(SC)C2=O)CC=O
```

```
PUGREST.BadRequest: error:
O=CC1=C2[C@H1](CCC3=CC(OC)=C(OC)C(=C3C2=CC=C(SC)C1=O)OCC4=CC=CC=C4)ONC
PUGREST.BadRequest: error:
COC1=CC=C(C(OC)=C1OC)C2=CC=C(SC)C(C=C2[C@@H1]NC(N(COC)C)=C)=CNC=O
PUGREST.BadRequest: error:
C1=CCC2[C@H1](N3C(C(OCC)=O)=C(N=N3)C=CC(=O)C4NC)CC=CC=C4C2=C(OC)C(OC)=C1OCNC=O
PUGREST.BadRequest: error:
C1OC2=CC=C(C(OC)=C2OC)C3=CC=C(SC)C1(C=C3[C@@H1](NC(N(CC)CC)=O)CC=C)NC=O
PUGREST.BadRequest: error:
CC1=CC=C(SC)C(C=C1[C@@H1](NC(=O)C)CCC=CC(OC)=C(OC)C(OC=CCOC)=C)=O
PUGREST.BadRequest: error:
C1C2=CC=C(SC)C(C=C2[C@@H1](NC(C)=O)CCC=CC(OC)=C(OC)C(OC(=O)C=C)C1)SC=O
PUGREST.BadRequest: error:
CC1=C2C=C(SC)C(C=C1[C@@H1](NC(=O)C)CCC=CC(OC)=C(OC)C(OC=CCOC)=C2)=O
PUGREST.BadRequest: error:
CCOC(OC1=C2CC([C@H1](CCC2=CC(OC)=C1OC)NC(C)=O)=CC(C(SC)=CC)=O)SC
PUGREST.BadRequest: error:
CCOC(OC1=CC=CC2=C(OC)C(OC)=C(OC)C=C2CC[C@@H1](C=CC1=O)NC(C)=O)SSC=O
PUGREST.BadRequest: error:
OC(NC(=O)OC)C=CC=C1C2=C(OC)C(OC)=C(OC)C=C2CC[C@@H1](C1=CC=O)NC(C)=O
PUGREST.BadRequest: error:
O1C(OC(=O)OC)=CC=C2C([C@@H1](NC(C)=O)CCC3=CC(OC)=C(OC)C(OC)=C32)=CC1NC
PUGREST.BadRequest: error:
OC(NCCOCOC1)[C@@H1]C2=CC(C(NC)=CC=C2C3=C(OC)C(OC)=C(OC)C=C3CC1)=O
PUGREST.BadRequest: error:
C1[C@H1](CCC2=CC(OC)=C(OC)C(OC)=C2C1=CC=C(OC(OC)=O)C=O)CSCNC(=O)C
PUGREST.BadRequest: error:
C=1=C2C(C3=C(OC)C(OC)=C(OC)C=C3CC[C@@H1]2NC(C)=O)C=C(OC(CC)=O)C(C=1)=O
PUGREST.BadRequest: error:
N([C@H1]1CCC=C(C(OC)=C(OC)C(OC)=C1C2=CC=C(SC)CC=C2)OC3=CC)=NC=C3
PUGREST.BadRequest: error:
OC(N1CCOC12)[C@@H1]C3=CC(C(NC)=CC=C3C4=C(OC)C(OC)=C(OC)C=C4CC2)=O
PUGREST.BadRequest: error:
C1[C@H1](CCC2=CC(OC)=C(OC)C(OC)=C2C1=CC=C(SC)CC)ONC(C)=O
PUGREST.BadRequest: error:
C1=2[C@H1](CCC3=CC(OC)=C(OC)C(OC)=C3C1=CC=C(SC)C(=O)C=2)NC(C)CC=CSC
PUGREST.BadRequest: error:
C1[C@@H1](C2=CCC(SC)=CC=C2C3=C(OC)C(OC)=C(OC)C=C3C1)ONC(C)=O
PUGREST.BadRequest: error:
N([C@H1]1CC2C=C(C(OC)=C(OC)C(OC)=C1C3=CC=C(SC)CC=C23)OC=CC)=NC=C
PUGREST.BadRequest: error:
C1N(C(=S)N[C@@H1]2C3=CC(C(NC)=CC=C3C4=C(OC)C(OC)=C(OC)C=C4CC2)=O)C=C1
PUGREST.BadRequest: error:
C=1=CC(C(C=C2[C@H1](CCC3=C(C(OC)=C(OC)C(OC)=C3)C2=1)SC(C)=O)C)=O
PUGREST.BadRequest: error:
C1=CC=C(S1C)C2=CN(NC2)[C@H1]C3C4C5=CC(OC)=C(OC)C(OC)=C5C=CC=C(NC)C(=O)C=C34
PUGREST.BadRequest: error:
OC=C(OC)C=C1C(C2=CC=C(SC)C(C=C2[C@H1](CC1)NC(C)=O)OCC(=O)OCS)C=O
```

```
PUGREST.BadRequest: error:
C1=2[C@H1](CCC3=CC(OC)=C(OC)C(OC)=C3C1=CC=C(C(C=2)=O)SCNC(C)=O)C
PUGREST.BadRequest: error:
C1[C@H1](C2=CCC(SC)=CC=C2C3=C(OC)C(OC)=C(OC)C=C3C1=O)C=O
PUGREST.BadRequest: error:
C1=CC2=C(S1C)C(=O)C3=C[C@H1](CCC4=C(C(OC)=C(OC)C(OC)=C4)C23)NCSC
PUGREST.BadRequest: error:
C1[C@H1](CCC2=CC(OC)=C(OC)C(OC(=O)CC)=C2C1=CC=C(SC)C(=O)CNC)SC
PUGREST.BadRequest: error:
C1[C@@H1](C2=CC(C(SC)=CC=C2C3=C(OC)C(OC)=C(OC)C=C3CCNCC)=O)C1SC=O
PUGREST.BadRequest: error:
C[C@H1](CCC1=CC(OC)=C(OC)C(OC(OCC)=O)=C1C2=CC=C(SC)C(=O)C=C2)NC
PUGREST.BadRequest: error:
C1[C@H1](CCC2=CC(OC)=C(OC)C(OC)=C2C3=CC=C(C(C=C3)=O)SCNCC)OC1
PUGREST.BadRequest: error:
C[C@H1]1CCC2=CC(OC)=C(OC)C(OC)=C2C3=CC=C(C(=O)C=C31)SCNC(C)=O
PUGREST.BadRequest: error:
C=CC=1C(=CCC(OC(C(C)C=2)=O)=CC=1OC(NCC=2)C)CC=C(OC3)C(OC)=CC(C)=C3NCNC
PUGREST.BadRequest: error:
C12=CC=3C(=CCC(OC(C(C)C)=O)=CC=3OC([C@@H1]N)C=C)C=CC=C1SC=CC(OC)=C2OC
PUGREST.BadRequest: error:
C1[C@H1](CCC2=CC(OC)=C(OC)C(OC(CC)=O)=C2C1=CC=C(SC)C(=O)CNC)SC
PUGREST.BadRequest: error:
C1=CC=2C(=CCC(OC(C3=CC=CC4=C3)=O)=CC=2O[C@H1]CCC1)NC=C(OC)C4OC
PUGREST.BadRequest: error:
COC1=C2C3=CC=C(NC)C(=O)C=C3[C@H1](CCC2=CC(OC)=C1OC)NCCC4=CC=CC=C4
PUGREST.BadRequest: error:
C1=C2CC[C@H1](C3=CCC(NC)=CC=C3C2=C(OC)C(=C1OC)OC)ONCCCCCCC
PUGREST.BadRequest: error:
COC1=C2C3=CC=C(NC)C(=O)C=C3[C@H1](CCC2=CC(OC)=C1OC)NC/C=C/C4=CC=CC=C4
PUGREST.BadRequest: error:
CSC=1C(C=C2C(=CC=1)C3=C(OC)C(=C(OC)C=C3CC[C@@H1]2NCC)OO)C=O
PUGREST.BadRequest: error:
CSC=1C(C=C2C(=CC=1)C3=C(OC)C(=C(OC)C=C3CC[C@@H1]2NCC)O)OC=O
PUGREST.BadRequest: error:
CSC=1C(C=C2C(=CC=1)C3=C(OC)C=CC=C3CC[C@@H1]2NCN)COCOC=O
PUGREST.BadRequest: error:  CSCCC=C1C(C2=C(C=C(OC)C(OC)=C2OC)CC[C@@H1]1N=O)CC=O
PUGREST.BadRequest: error:
CSC=1C(C=C2C(=CC=1)C3=C(OC)C(OC)=CC=C3CC[C@@H1]2NCC)=O
PUGREST.BadRequest: error:
CSC=1C(C=C2C(=CC=1)C3=C(OC)C(OC)=C(C=C3CC[C@@H1]2NCC)OO)C=O
PUGREST.BadRequest: error:
COC=CC=1CC[C@H1](NC(C)=O)C2=CC(=O)C(SC)=CC=C2C=1C(O)=COC
PUGREST.BadRequest: error:  C=1OC=CC2CC[C@H1](N)CCC3=CC(=C(OC)C=C3C2=CC=1OCS)C
PUGREST.BadRequest: error:  C1OC2=CCCC[C@H1](N)C3=CC(=O)C(SC)=CC=C3C2C(OC)=C1OC
PUGREST.BadRequest: error:
C1N2CC1(C=C3C(=O)C(NC)=CC=C3C=C(OC)C(OC)=C(OC)C=C)CC[C@@H1]2N=O
PUGREST.BadRequest: error:
```

```
COC=C1CC=2C[C@H1](N)C3=CC(C(=CC=C3C=2C(OC)=C1O)CN)C=O
PUGREST.BadRequest: error:  COC1=CC2CC[C@H1](N)C3=CC(=O)C(SC)=CC=C3C1C(OC)=C2OC
PUGREST.BadRequest: error:
C=1SCC(C=C2C=1C3=C(CC[C@@H1]2NC(N)=O)C=C(OC)C(=C3OC)OC)SC=O
PUGREST.BadRequest: error:
CSC=1C2(C=CC(=CC=1)C3=C(OC)C(OC)=CC=C3CC[C@@H1]2NOC)NC=O
PUGREST.BadRequest: error:
CSC=1C(C2=CC(=CC=1)C3=C(OC)C(OC)=CC=C3CC[C@@H1]2NOC)NC=O
PUGREST.BadRequest: error:
CC(=O)C(S1C2)CC(OC)=C(OC)C(O)=C2C=3C1=CC(C(SC)=CC=3)=O
PUGREST.BadRequest: error:  CC(=O)C=C1C(C2=C(OC)C(OC)=C(OC)C=C2CC[C@@H1]1NCC)=O
PUGREST.BadRequest: error:
CC(=O)N[C@H1]1CCC2=CC(OC)=C(OC)C(OC)=C2C=3C1=CC(=O)C(SC)=CC=3N
PUGREST.BadRequest: error:
C1=2C(=O)C(=C3C=C1C4=C(CC[C@@H1]3NC(C)=O)C=C(OC)C(OC)=C4OC(OC)=O)C=2
PUGREST.BadRequest: error:
C1C(=O)C(=C2C=C1C3=C(CC[C@@H1]2NC(C)=O)C=C(OC)C(OC)=C3OC(OC)=O)C
PUGREST.BadRequest: error:
CC(=O)OC1=CC=C2C3=C(C(OC)=C(C=C3CC[C@@H1](C2=CC1=O)OC(=O)CO)CO)C
PUGREST.BadRequest: error:
CC(=O)OC1=CC=C2C3=C(C(OC)=C(C=C3CC[C@@H1](C2=CC1=O)OC(=O)CO)CN)C(=O)COC
PUGREST.BadRequest: error:
CC(=O)OC1=CC=C2C3=C(C(OC)=C(C=C3CC[C@@H1](C2=CC1=O)OC(=O)C)OC)NC(=O)C
PUGREST.BadRequest: error:
CC(=O)OC1=CC=C2C3=C(C(OC)=C(C=C3CC[C@@H1](C2=CC1=O)OC(=O)C)OC)NC(=O)COC
PUGREST.BadRequest: error:
CC(=O)OC1=CC=C2C3=C(C(OC)=C(C=C3CC[C@@H1](C2=CC1=O)OCNCC)O)OC
PUGREST.BadRequest: error:
CC(=O)OC1=CC=C2C3=C(C(OC)=C(C=C3CC[C@@H1](C2=CC1=O)OC(=O)C)OC)OC
PUGREST.BadRequest: error:
CCC(=O)OC1=CC=C2C=C(C3C[C@H1](NC(=O)C)C2=CC1=O)C=C(OC)C(=C3OC)OC(=O)OC
PUGREST.BadRequest: error:
CCC(=O)OC1=CC=C2C3=C(C(OC)=C(C=C3CC[C@@H1](C2=CC1=O)OC(=O)C)NC)C=O
PUGREST.BadRequest: error:
CC(C)C(=O)OC1=C2CC([C@@H1](NC(=O)C)CCC2=CC(=C1OC)OC)=CC(C(SC)=CC)=O
PUGREST.BadRequest: error:
CC(C)C(=O)OC1=C2C3=CC=C(C(C=C3[C@H1](CCC2=CC(OC)=C1OC)NC=O)C)OOC
PUGREST.BadRequest: error:
CC(C)C(OC1=CC=C2C3=C(CC[C@@H1](C2=CC1=O)NC(C)=O)C=C(OC)C(OC)=C3OC)(O)C
PUGREST.BadRequest: error:
CC(C1)C(=O)OC=CC2=CC([C@@H1](NC(C)=O)CCC3=CC(=C(OC)C(O)=C3C2)O)OC=CC1=O
PUGREST.BadRequest: error:
CC(=O)N[C@@H1]1C2=CC(C(SC)=CC=C2C3=C(OC)C(=C(OC)C=C3CC1)OC(=O)C4=CC=CC=C4)=O
PUGREST.BadRequest: error:
CC(=O)N[C@@H1]1C2=CC(C(SC)=CC=C2C3=C(C(OC)=C(OC)C=C3CC1)OC(=O)C4=CC=CC)=C4O
PUGREST.BadRequest: error:
O=C(OC)OC1=CC=C2C3=C(C(OC)=C(C=C3CC[C@@H1](C2=CC1=O)NC(=O)C)OC)OC(=O)C
PUGREST.BadRequest: error:
```

```
O=C(OC1)OC=C2C3=CC=C(C(C=C3[C@H1](CCC2=CC(=C1OC)OC)NCC)=O)OOC(=O)C
PUGREST.BadRequest: error:
O=C(OC)OC1=CC=C2C3=C(C(OC)=C(C=C3CC[C@@H1](C2=CC1=O)OC(=O)C)NC)C=O
PUGREST.BadRequest: error:
O=C(OC1)OC=C2C3=CC=C(C(C=C3[C@H1](CCC2=CC(=C1OC)OC)NCC)=O)OOCO
PUGREST.BadRequest: error:
O=C(OC)OCC(C=C1C(C2=C(C=C(OC)C(OC)=C2OC)CC[C@@H1]1NC(=O)C)=C)=O
PUGREST.BadRequest: error:
O=C(OC)OC1=C2C3=CC=C(C(C=C3[C@H1](CCC2=CC(OC)=C1OC)NC=O)C)OOC
PUGREST.BadRequest: error:
O=C(C)N[C@@H1]1C=2C(C3=C(C(=C(OC)C=C3CC1)OC)OC)C=C(OC)C(C=2)=O
PUGREST.BadRequest: error:
O=C(O1)C=C(C(OC)=CC=2CC[C@@H1](C3=CC(=O)C(SC)=CC=C3C1=2)NC(C)=O)COC
PUGREST.BadRequest: error:
O=C(C)N[C@@H1]1CC(C2=C(C(=C(OC)C=C2CC1)OC)OC)C=C(OC)C(OCC=C)O
PUGREST.BadRequest: error:
O=C(C)N[C@H1]1CCC2=CC(=C(OC)C(=C2C=3C1=CC(C(SC)=CC=3)=O)OC(=O)CC)COC
PUGREST.BadRequest: error:
O=C(C)N[C@H1]1CCC2=CC(=C(OC)C(=C2C=3C1=CC(C(=CC=3)OC)CC)OOCOC)C=O
PUGREST.BadRequest: error:
C1C(=O)C=C(C2=CC=C(SC)C(C=C2[C@H1](NC(C)=O)C)C)OC(OC)=C1O
PUGREST.BadRequest: error:
CC(=O)OC=C1C2=CC=C(SC)C(=O)C=C2[C@H1](CCC1=C(OC)C(OC)=C)OC(=O)OC
PUGREST.BadRequest: error:
CC(=O)OC=C1C2=CC=C(SC)C(=O)C=C2[C@H1](CCC1=C(OC)C(OC)=C)OCNC(=O)C
PUGREST.BadRequest: error:  C1=2C=CCC[C@H1](NC(=O)C)C1=CC(C(=CC=2)SC)=O
PUGREST.BadRequest: error:
C1=2C=C(CC[C@H1](NC(=O)C)C1=CC(C(=CC=2)SC)=O)C(OC)C(=COC)OC
PUGREST.BadRequest: error:
C12C=C(CC[C@H1](NC(=O)C)C1=CC(C(=CC)SC)=O)C(OC)C(OC)=C2OC
PUGREST.BadRequest: error:
C123C=C(CC[C@H1](NC(=O)C)C1=CC(C(SC)=C2)=O)C(C(=C3OC)OC)OC
PUGREST.BadRequest: error:
C12C=C(CC[C@H1](NC(=O)C)C1=CC(C(=CC)SC)=O)C(OC)C(=C2OC)OC(=O)C
PUGREST.BadRequest: error:
C1=2C=C(CC[C@H1](NC(=O)C)C1=CC(C(=CC=2)SC)=O)C(OC)C(OC)=COC(=O)C
PUGREST.BadRequest: error:
CC(=O)OCC1=CN(N=N1)[C@H1]2CCC3=CC(OC)=C(OC)C(OC)=C3C=4C2=CC(=O)C(NC)=CC=4C
PUGREST.BadRequest: error:
CC(=O)OCC1=CN(N=N1)[C@H1]2CCC3=CC(OC)=C(OC)C(=C3C=4C2=CC(C(NC)=CC=4)=O)OC
PUGREST.BadRequest: error:
O=C(OC1)OC=CC=C2C3=C(OC)C(=C(OC)C=C3CC[C@H1](NC(=O)C)C2=CC1)OOC
PUGREST.BadRequest: error:
O=C(OC1)OC=CC=C2C3=C(OC)C(=C(OC)C=C3CC[C@@H1](NC(C)=O)C2=CC1)OOC
PUGREST.BadRequest: error:
O=C(NC)NCC=CN(COCC)C1C(C2=C(OC)C=C(OC)C=C2CC[C@@H1]1NC=O)C=O
PUGREST.BadRequest: error:
O=C(C)N[C@H1]CC1C2=CC(OC)=C(C(OC)=C2C3=CC=C(SC)C(=O)C=C13)OOCC
```

```
PUGREST.BadRequest: error:
O=C(NC)N1CC=CC=C2C([C@H1](CCC3=CC(OC)=C(C(OC)=C32)OC)NC=O)C=C1O
PUGREST.BadRequest: error:
O=C(CC)OC1=C2CC([C@@H1](NC(C)=O)CCC2=CC(OC)=C1OC)=CC(C(=CC)SC)=O
PUGREST.BadRequest: error:
O=C(C)N[C@H1]1CCC2=CC(OC)=C(C(OC)=C2CC1=CC(C(OC(CC)=O)=CC)=O)OCNC
PUGREST.BadRequest: error:
O=C(NC)NCC=CN(COC1C)CC(C2=C(OC)C(=C(OC)C=C2CC[C@@H1]1)NC(=O)C)OC=O
PUGREST.BadRequest: error:
O=C(C)N[C@H1]1CCC2=CC(OC)=C(C(OC)=C2CC1=CC(C(OC(CC)=O)=CC)=O)OC
PUGREST.BadRequest: error:
O=C(C)N[C@H1]CC1C2=CC(OC)=C(C(OC)=C2C3=CC=C(SC)C(=O)C=C13)OCC
PUGREST.BadRequest: error:
O=C(C)N[C@H1]1CCC2=CC(OC)=C(C(OC)=C2C=CC=C(OC(C)=O)C(C=C1)=O)OC
PUGREST.BadRequest: error:
CC(=O)N[C@@H1]C1=C2C(C(NC)=CC=C1C3=C(OC)C(OC)=C(OC)C=C3CC2)NC=O
PUGREST.BadRequest: error:
CC(=O)N[C@@H1]1C2=CC(C(NC)=CC=C2C3=C(OC)C(OC)=C(OC)C=C3C1)=O
PUGREST.BadRequest: error:
CC(=O)N[C@@H1]C1=CC(C(NC)=CC=C1C2=C(OC)C(OC)=C(OC)C=C2CC)N=O
PUGREST.BadRequest: error:
CC(=O)N[C@@H1]C1=C2C(C(NC)=CC=C1C3=C(OC)C(OC)=C(OC)C=C3CC2)N=O
PUGREST.BadRequest: error:
CC(=O)N[C@@H1]1C2=CC(C(NC)=CC=C2C3=C(OC)C(OC)=C(OC)C=C3C1)NC=O
PUGREST.BadRequest: error:
CC(=O)N[C@@H1]C1=C2C(C(NC)=CC=C1C3=C(OC)C(OC)=C(OC)C=C3CC2)=O
PUGREST.BadRequest: error:
CC(=O)N1[C@@H1]C2=CC(C(NC)=CC=C2C3=C(OC)C(OC)=C(OC)C=C3CC1)N=O
PUGREST.BadRequest: error:
C1OC=CC2C([C@@H1](N)CCC1=CCOC(=O)OCC=C)(OC)C(=C2)OCSC
PUGREST.BadRequest: error:  COC=C1C2=CC=C(NC)C(=O)C=C2[C@H1](CCC1C=COC)NC(=O)CNC
PUGREST.BadRequest: error:  C1OC=C2C=3C([C@H1](CCC2=C1)N=CCC(=CC=3)SC)=O
PUGREST.BadRequest: error:  COC=C1C2=CC=C(NC)C(=O)C=C2[C@H1](CCC1C=COC)NC(=O)NNC
PUGREST.BadRequest: error:  COC=C1C2=CC=C(NC)C(=O)C=C2[C@H1](CCC1C=COC)NC(=O)NC
PUGREST.BadRequest: error:  COC=C1C2=CC=C(NC)C(=O)C=C2[C@H1](CCC1C=COC)NC(=O)N
PUGREST.BadRequest: error:
COC=CC=C(C1=C2C=C(NC)CC=C1[C@H1](C)N=[N+1])[N-1]CC(OC)=C2OC
PUGREST.BadRequest: error:
COC1=C2C3=CC=C(NC)C(=O)C=C3[C@@H1](NCCC)CCC2=CC(=C1OC)NC
PUGREST.BadRequest: error:
C=1OC=CC2=C(C3=C(OC)C(OC)=CC=C3CC[C@@H1]2N=[N+1]=[N-1])NC=CC=1
PUGREST.BadRequest: error:
COC1=C2C3=CC=C(NC)C(=O)C=C3[C@H1](CCC2=CC(OC)=C1OC)NN=NC=C
PUGREST.BadRequest: error:
COC=CC1=C(C2=CC=C(NC)CC=C2[C@H1](C)N=[N+1])[N-1]CC(OC)=C1OC
PUGREST.BadRequest: error:
COC1=C2C3=CC=C(NC)C(=O)C=C3[C@@H1](NCCC)CCC2=CC(=C1OC)OSC
PUGREST.BadRequest: error:
```

```
COC=C1C2=CC=C(NC)C(=O)C=C2[C@@H1](N3N=NC=C3)CCC1=CC(=COC)OC
PUGREST.BadRequest: error:
COC1=C2C3=CC=C(NC)C(=O)C=C3[C@H1](CCC2=CC(=C1OC)OC)NCCCS
PUGREST.BadRequest: error:
COC1=CC=C(C2=CC=C(NC)CC=C2[C@H1](C)N=[N+1])[N-1]CC(OC)=C1OC
PUGREST.BadRequest: error:
C=C1C([C@H1](CCC2=CC(=C(OC)C(=C21)OC)OC)NC=CNCOCC(C)C)=O
PUGREST.BadRequest: error:
C12=CC(C(SC)=CC=C1C3=C(C=C(C(OC)=C3OC)OC)CC[C@@H1]2NCC(C)CN)C=O
PUGREST.BadRequest: error:
COC=C(OC)C(OC)=C1CCC[C@H1](N2C=CN=N2)C3=CC(=O)C(=CC=C3C1)NC
PUGREST.BadRequest: error:
COC=C(OC)C(OC)=CC=1CC[C@H1](NC(=N)N)C2=CC(=O)C(=CC=C2C=1)CNC
PUGREST.BadRequest: error:
COC1=C(OC)C(OC)=CC=2CC[C@H1](NC(=N)N)C3=CC(=O)C(=CC=C3C1=2)CNC
PUGREST.BadRequest: error:
CNC(NCCCC)=S[C@@H1]C1=CC(=O)C(NC)=CC=C1C2=C(C=C(OC)C(=C2OC)OC)CCF
PUGREST.BadRequest: error:
C12=CC(C(NC)=CC=C1C3=C(C=C(C(OC)=C3OC)OC)CC[C@@H1]2NCCCCCCCCCC)CSC=O
PUGREST.BadRequest: error:
CN(C(=S)N[C@@H1]1C2=CC(=O)C(NC)=CC=C2C3=C(C=C(OC)C(OC)=C3OC)CC1C)C#N
PUGREST.BadRequest: error:
C1=C2C(C3=C(C=C(OC)C(=C3OC)OC)CC[C@@H1]2NCCCCCCCCCCC=C)C=C(SC)C1=O
PUGREST.BadRequest: error:
C12=CC3(C(NC)=CC=C1C4=C(C=C(C(OC)=C4OC)OC)CC[C@@H1]2NCCCCCCCCCC)C=C3
PUGREST.BadRequest: error:
C12=CC(C(NC)=CC=C1C3=C(C=C(C(OC)=C3OC)OC)CC[C@@H1]2N4C=C(N=N4)CCC)C=CNC=O
PUGREST.BadRequest: error:
C=C1C([C@H1](CCC2=CC(=C(OC)C(=C21)OC)OC)NCCCCCC)CC=CC(C(SC)=C)=O
PUGREST.BadRequest: error:
C=C1C(C2=C(C=C(OC)C(=C2OC)OC)CC[C@@H1]1NC/C=C/C3=CC=CC=C3CC)=C(SC)C=O
PUGREST.BadRequest: error:
C12=CCC(NC)=CC=C1C3=C(C=C(C(OC)=C3OC)OC)CC[C@@H1]2NCCCCCCCCCC=O
PUGREST.BadRequest: error:
CNC(C(C)C)N[C@@H1]1C=2C(C3=C(C=C(OC)C(OC)=C3OC)CC1)=CC=C(C(=O)C=2)NC
PUGREST.BadRequest: error:
C=C1C([C@H1](C2CC3=CC(=C(OC)C(=C31)OC)OC)NCCCCCC)CCC=CC(C(=C2)SC)=O
PUGREST.BadRequest: error:
COC1=C(OC)C(OC)=CC=2CC[C@@H1](C3=CC(=O)C(NC)=CC=C3C=21)N4C=C(N=N4)CON
PUGREST.BadRequest: error:
C12=CC(C(NC)=CC=C1C3=C(C=C(C(OC)=C3OC)OC)CC[C@@H1]2NCCCCCCCCCC=O)SC
PUGREST.BadRequest: error:
C12=CCC(NC)=CC=C1C3=C(C=C(C(OC)=C3OC)OC)CC[C@@H1]2NCCC4CCCCC4=O
PUGREST.BadRequest: error:
COC1=C(OC)C(OC)=CC=2CC[C@@H1](C3=CC(=O)C(NC)=CC=C3C=21)NCCC4CCCCC4
PUGREST.BadRequest: error:
C12=CC(C(NC)=CC=C1C3=C(C=C(C(OC)=C3OC)OC)CC[C@@H1]2NCCC4=CC=CC=C4C)SC
PUGREST.BadRequest: error:
```

```
COC1=C(OC)C(OC)=CC=2CC[C@@H1](C3=CC(=O)C(NC)=CC=C3C=21)NCCC4=CC=CC=C4
PUGREST.BadRequest: error:
CNC(C(C)O)N[C@@H1]1CC(C2=C(C=C(OC)C(OC)=C2OC)CC1)=CC=C(C(=O)C)NC
PUGREST.BadRequest: error:
C12=CC(C(NC)=CC=C1C3=C(C=C(C(OC)=C3OC)OC)CC[C@@H1]2NCCC4=CC=CC=C4C)NC=O
PUGREST.BadRequest: error:
C12=CCC(NC)=CC=C1C3=C(C=C(C(OC)=C3OC)OC)CC[C@@H1]2NCCC4=CC=CC=C4C
PUGREST.BadRequest: error:
C=C1C2([C@@H1](NCCCCCCCCC)CCC3=CC=C(OC)C(=C31)OC)OC=CC(C(=C2)NC)=O
PUGREST.BadRequest: error:
C=C1C([C@H1](CCC2=CC(=C(OC)C(=C21)OC)OC)NCCCCC)=CC(C(SC)=C)=O
PUGREST.BadRequest: error:
C1=C2C([C@H1](CCC3=CC(=C(OC)C(=C32)OC)OC)NCCCCC)C=CC(C(=C1)SC)=O
PUGREST.BadRequest: error:
CC(C)(C)C(OC1=CC=CC2=C(OC)C(OC)=C(OC)C=C2CC[C@H1](NC(=O)C)C1=CC=O)=O
PUGREST.BadRequest: error:
CC(C)C(=O)OCCC=C1[C@H1](CCC2=CC(OC)=C(OC)C(OC)=C2C1=CCN)SC=O
PUGREST.BadRequest: error:
C=1C(C)(C)C(OC2=CC=CC3=C(OC)C(OC)=C(OC)C=C3CC[C@H1](NC(=O)C)C2=CC=1O)SC
PUGREST.BadRequest: error:
CC(C)(C)COC1=CC=C2C3=C(OC)C(OC)=C(OC)C=C3CC[C@@H1](C2=CC1=O)SC=O
PUGREST.BadRequest: error:
O=C(C=CNCC(=O)OCCN[C@H1]1)CCC2=C(C(OC)=C(OC)C(OC)=C2)C3=CC=C(NC)C(=O)C=C13
PUGREST.BadRequest: error:
C1=2[C@H1]CCC3=CC(OC)=C(OC)C(OC(CC)=O)=C3C1=CC=C(C(C=2)=O)SCNC(=O)CSC
PUGREST.BadRequest: error:
OC(CCC(C)C)OCC1=CN(N=N1)[C@H1]2CCC3=CC(OC)=C(OC)C(OC)=C3C4=CC=C(NC)C(=O)C=C24
PUGREST.BadRequest: error:
O=CCN(CC=C)N(OCC)O[C@H1]1CCC2=CC(OC)=C(OC)C(OC)=C2C=CC=C(NC)C(=O)C=C1
PUGREST.BadRequest: error:
N([C@@H1]1C=2CC3=C(C(OC)=C(OC)C=C3CC1)OC=CC=C(NC)CC=2)=O
PUGREST.BadRequest: error:
OC(CCC(C)C)OCC1=CN(N=N1)[C@H1]CCC2=CC(OC)=C(OC)C(OC)=C2C=CC=C(C(C)N)C
PUGREST.BadRequest: error:
C=C1C(C2=C(C=C(OC)C(=C2OC)OC)CC[C@@H1]1N3C=C(N=N3)C4=CC=CC=C4)=CC=C(NC)C=O
PUGREST.BadRequest: error:
C=C1C([C@H1](CCC2=CC(=C(OC)C(=C21)OC)OC)NCCCCCCCCCC)C=CC(CNC)=O
PUGREST.BadRequest: error:
C=C1C(C2=CC=C(C(=O)C=C2[C@H1](CC1)N3C=C(N=N3)C=CC=CC=N)C=C)OCNC(OC)=O
PUGREST.BadRequest: error:
OC1=C(C=CC=C1)C=2N=NN(C=2)[C@H1]3CCC4=CC(OC)=C(OC)C(OC)=C4C5=CC=C(NC)C(=O)C=C35
PUGREST.BadRequest: error:
NC(=C(N=NCC(=O)CCC=C1N=CC(C(OC(C)=O)=CC=C1C2=C(OC)C)(OC)COC)C)C2CCOF
PUGREST.BadRequest: error:
N1=CC=C(C2=C1)COC3=CC=C4C5=C(OC)C(OC)=C(OC)C=C5CC[C@@H1](C4=CC3=O)N2
PUGREST.BadRequest: error:
NC1=CN(N=N1)[C@H1]2CCC3=C(C(OC)=C(OC)C(OC)=C3)C4=CC=C(NC)C(C=C24)=O
PUGREST.BadRequest: error:
```

```
NC1=C(N=NC1C(=O)CCC=CC=C2C3=C(OC)C(OC)=C(OC)C=C3CC[C@H1](CC2N)CN)C=O
PUGREST.BadRequest: error:
C[C@@H1](C1=CCC(SC)=CC=C1C2=C(OC)C(OC)=C(C=C2CC)OCOCC=CCSC=O)O
PUGREST.BadRequest: error:
N=C=C(N=NC(C(C)OC)[C@H1]CCC1=CC(OC)=C(OC)C(OC)=C1C2=CC=C(NC)C=O)C=C2
PUGREST.BadRequest: error:
C=C1C(C2=C(C(=C(OC)C=C2CC[C@@H1]1N3C=C(N=N3)C4=CC)CC)N4O)COC=CC=C(CON)C
PUGREST.BadRequest: error:
C=C1C(C2=CC=CC(=O)C=C2[C@@H1](N3C=C(N=N3)C4=CC=CC=N4)CC1)NC=C(OC)C(OC)SCOC
PUGREST.BadRequest: error:
C1=C2C(C3=C(C=C(OC)C=C3CC[C@@H1]2N4C=C(N=N4)C=CC)OCOC)OC=CC=C(C1=O)C
PUGREST.BadRequest: error:
C=C1C([C@H1](CCC2=CC(=C(OC)C(=C21)OC)OC)NCCCCCCCCCC)C=CCC(NC)=O
PUGREST.BadRequest: error:
C=1=C2C(C3=C(C=C(OC)C=C3CC[C@@H1]2N4C=C(N=N4)C=CC)OCOC)OC=CC=CC=1O
PUGREST.BadRequest: error:
C1=C2C(C3=CC=CC(=O)C=C3[C@H1](CC2)N4C=C(N=N4)C=CC=CC=N)(OC)C(OC)=C1OCNC
PUGREST.BadRequest: error:
C=C1C2(C3=C(C=C(OC)C=C3CC[C@@H1]1N4C=C(N=N4)C=CC)OCOC)OC=CC=C(NC)C2=O
PUGREST.BadRequest: error:
C1=C2C(C3=C(C=C(OC)C=C3CC[C@@H1]2N4C=C(N=N4)C=CC)OCOC)OC=CC=C(C1=O)NC
PUGREST.BadRequest: error:
C=C1C(C2=C(C=C(OC)C=C2CC[C@@H1]1N3C=C(N=N3)CCC)COCO)C=CC=C(NC)C=O
PUGREST.BadRequest: error:
C=C1C(C2=CC=C(C(=O)C=C2[C@@H1](N3C=C(N=N3)C4=NC=CC=C4)CC1)NC)=C(OC)C(OC)=COC
PUGREST.BadRequest: error:
C1=C2C(C3=CC=C(C(C=C3[C@H1](CC2)N4C=C(N=N4)C5=NC=CC=C5O)N)C=C)(C(=C1OC)OC)OC
PUGREST.BadRequest: error:  C12=CC([C@@H1]1NCC3=CC=C(C)C=C3CCCC)C(SC)=CC2O
PUGREST.BadRequest: error:
O=C(OCC)CN(CCO)N[C@H1]1CCC2=CC(OC)=C(OC)C(OC)=C2C3=CC=C(NC)C(=O)C=C13
PUGREST.BadRequest: error:
O=C(O)C=C(OC)CC=C1C(C2=C(OC)C(OC)=C(OC)C=C2C=3C[C@@H1]1N=CC=3S)C
PUGREST.BadRequest: error:
O=C(O)C=C(OC)CC=C1C(C2=C(OC)C(OC)=C(OC)C=C2C=3C[C@@H1]1N=CC=3SC)=O
PUGREST.BadRequest: error:
O=C(C)N[C@@H1]1CC(C2=C(C(OC)=C(OC)C=C2CC1)OC(CC)=O)=CC=C(SC)C(=O)C
PUGREST.BadRequest: error:
O=C(O)C=C(OC)CC=C1C(C2=C(OC)C(OC)=C(OC)C=C2C=3C[C@@H1]1N=CC=3S)C=O
PUGREST.BadRequest: error:
O=C(C)N[C@@H1]1C2=CCC(SC)=CC=C2C3=C(C(OC)=C(OC)C=C3CC1)OCOO
PUGREST.BadRequest: error:
O=C(C1)N[C@@H1]2CC(C3=C(C(OC)=C(OC)C=C3CC2)OC(CC)=O)=CC=C(SC)C1=O
PUGREST.BadRequest: error:
O=C(C)N[C@@H1]1C2=CCC(SC)=CC=C2C3=C(C(OC)=C(OC)C=C3CC1)OC=O
PUGREST.BadRequest: error:  OC(OC(C)CC)CC(NCC1=CN(N=N1)[C@H1]C2CC3=CC(OC)=C(OC)C
(OC)=C3C4=CC=CC(=O)C=C24)NC=O
PUGREST.BadRequest: error:
O=CC(OOC1=C)C=CC=C1OC(=O)N[C@H1]2CCC3=CC(OC)=C(OC)C(OC)=C3C4=CC=C(NC)C(=O)C=C24
```

```
PUGREST.BadRequest: error:
OC(OC(C)CC)CC=1OCCC=C2[C@H1](CCC3=C(C(OC)=C(OC)C(OC)=C3)C2=CC=1)NSC=O
PUGREST.BadRequest: error:  O=C(C1=C(C(OCC)=O)N(N=N1)[C@@H1]C2=C3C(C(NC)=CC=C2C4
=C(C(OC)=C(OC)C=C4CC3)OC)=O)O
PUGREST.BadRequest: error:
C1[C@@H1](C2=CC(C(NC)=CC=C2C3=C(OC)C(OC)=C(C=C3C1)O)C)ONC(C(=O)O)=CNC
PUGREST.BadRequest: error:
C1=2[C@H1](CCC3=CC(OC)=C(OC)C(OC)=C3C1=CC=C(C(=O)C=2)NCO)C(C(OC)=O)NC(=O)CNC
PUGREST.BadRequest: error:
C1[C@@H1](C2=CC(C(NC)=CC=C2C3=C(OC)C(OC)=C(C=C3C1)O)C)NC(C(O)=O)=CC(=O)ONC=O
PUGREST.BadRequest: error:
C1[C@@H1](C2=CC(C(SC)=CC=C2C3=C(OC)C(OC)=C(C=C3C1)O)C)NC(C(O)=O)=C(COCC)OOSC=O
PUGREST.BadRequest: error:
CN([C@H1]1CC2=CC(OC)=C(OC)C(OC)=C2C=C3C=C)NC(=O)C=C13
PUGREST.BadRequest: error:
C=12N(N=NC=1C(OCC3=CC=C(C=C3)F)=O)C(OC)=C(OC)C(OC)=CC4=CC=C(C(=O)C=C24)NC
PUGREST.BadRequest: error:
C1=CC1(C=CN(COC(OC)=O)N=NN)[C@H1]C2CC3=CC(OC)=C(OC)C(OC)=C3C=CC=C(NC)C(=O)C=C2
PUGREST.BadRequest: error:
O=C(C1=C(C(OCC)=O)N(N=N1)[C@@H1]C2=C3CC(NC)=CC=C2C4=C(C(OC)=C(OC)C=C4CC3)OC)OO
PUGREST.BadRequest: error:
C=1N(N=NC=1C(=O)OCC2=CC=C(C=C2)F)CC(OC)=C(OC)C(OC)=CC3=CC=C(NC)C(=O)C=C3
PUGREST.BadRequest: error:
O=CC1=C(C(OCC)=O)N(N=N1)[C@@H1]C2=C3C(C(NC)=CC=C2C4=C(C(OC)=C(OC)C=C4CC3)OC)=O
PUGREST.BadRequest: error:
C1[C@@H1](C2=CC3(C(SC)=CC=C2C4=C(OC)C(OC)=C(C=C4C1)O)C)NC(C(O)=O)=C(C3)ONC
PUGREST.BadRequest: error:  N([C@@H1]1CCC2=CC(OC)=C(OC)C=C2CC1)=CC=C(C=C)NC
PUGREST.BadRequest: error:
O=C(C(Cl)Cl)NC(=O)N[C@H1]1CCC2=C(C(OC)=C(OC)C(OC)=C2)C3=CC=C(NC)C(=O)C=C13
PUGREST.BadRequest: error:
O=C(C(Cl)Cl)NCC1=CN(N=N1)[C@H1]2CCC3=CC(OC)=C(OC)C(OC)=C3C4=CC=C(C(=O)C=C42)NCCO
PUGREST.BadRequest: error:  O=C(C(Cl)Cl)NC(=O)N[C@H1]1CCC2=C(C(OC)=C(OC)C(OC)=C2
)C3=CC=C(C(C(C=C31)=O)NCC(=O)OC
PUGREST.BadRequest: error:
O=C(C(Cl)Cl)NC(=O)N[C@H1]1CCC2=C(C(OC)=C(OC)C(OC)=C2)C3=CC=C(NC)C(=O)C=C31
PUGREST.BadRequest: error:
O=C(O)C=C(C(OCC)=O)N=NN[C@H1]1CCC2=CC(OC)=C(OC)C(OC)=C2C3=CC=C(NC)C(=O)C=C13
PUGREST.BadRequest: error:
O=C(O)C1=C(C(OCC)=O)N=NN1[C@H1]2CCC3=CC(OC)=C(OC)C(OC)=C3C4=CC=C(NC)C(=O)C=C24
PUGREST.BadRequest: error:
O=C(C)N[C@@H1]C1=C2C(C(SC)=CC=C1C3=C(C(OC)=C(OC)C=C3CC2)OC(C(C)C)=O)C=O
PUGREST.BadRequest: error:
O=C(OCC)C1=CN(N=N1)[C@H1]2CCC3=CC(OC)=C(OC)C(=C3C4=CC=C(NC)C(C=C42)=O)OCC
PUGREST.BadRequest: error:
O=C(C)N[C@@H1]C1=C2C(C(SC)=CC=C1C3=C(C(OC)=C(OC)C=C3CC2)OC(C4=CC=CC=C4)=O)SC=O
PUGREST.BadRequest: error:
O=C(C(C)C)OC=1C(C=C2[C@H1](CCC3=CC(OC)=C(OC)C(OC)=C3C2=CC=1N)C(C)=O)SC=O
PUGREST.BadRequest: error:
```

```
O=C(O)C1=CN(N=N1)[C@H1]2CCC3=C(C(OC)=C(OC)C(OC)=C3)C4=CC=C(C(=O)C=C42)NCC
PUGREST.BadRequest: error:
O=C(O)C1=C(C(OCC)=O)N=NN1[C@H1]CCC2=CC(OC)=C(OC)C(OC)=C2C3=CC=C(C(C=C3)=O)NC
PUGREST.BadRequest: error:
O=C(C(C)C)OCC(C=C1[C@H1](CCC2=CC(OC)=C(OC)C(O)=C2C1=CC=O)C(C)=O)C=O
PUGREST.BadRequest: error:
O=C(C(C)C)OC=1C(C=C2[C@H1](CCC3=CC(OC)=C(OC)C(OC)=C3C2=CC=1S)C(C)=O)OC
PUGREST.BadRequest: error:
O=C(O)C=C(OC)C(C(NC)=CC=C1C2=C(OC)C(OC)=C(OC)C=C2CC[C@@H1](C1)NCC)=O
PUGREST.BadRequest: error:
O=C(O)C1=CN(N=N1)[C@H1]2CCC3=C(C(OC)=C(OC)C(OC)=C3)C4=CC=C(C(C=C42)=O)NCC
PUGREST.BadRequest: error:  CCCCCCCCCCCCCCCC(OCC1=CN(N=N1)[C@H1]C2CC3=CC(OC)=C(O
C)C(OC)=C3C4=CC=C(C(=O)C=C24)NC)=O
PUGREST.BadRequest: error:
CCCCCCCCCCCCCCCC=1OCCC=C2[C@H1](CCC3=C(C(OC)=C(OC)C(OC)=C3)C2=CC=1)NSC=O
PUGREST.BadRequest: error:
OC(=O)C1=C(C(OCC)=O)N=NN1[C@H1]CCC2=CC(OC)=C(OC)C(OC)=C2C3=CC=C(NC)C(C=C3)=O
PUGREST.BadRequest: error:
C[C@H1](NCC)CC1=CCC(NC)=CC=C1C2=C(C=C(OC)C(OC)=C2OCC3=CC=CC=C3)OSC
PUGREST.BadRequest: error:  C1[C@H1](OC1)O[C@@H1]([C@H1]O)COOCNC2(N=NC(=C2)C=CC3
=C(C=CC4=C3)Cl)CC(OC)=C(OC)C=C4CCNC=O
PUGREST.BadRequest: error:
OC(=O)C1=C(C(OCC)=O)N(N=N1)[C@H1]C2CC3=CC(OC)=C(OC)C(OC)=C3C4=CC=C(NC)C(=O)C=C42
PUGREST.BadRequest: error:
C1[C@H1](OCCC)CC2=CC(C(NC)=CC=C2C3=C(C(OC)=C(OC)C=C3CC1)OC(OCC)=O)NC=O
PUGREST.BadRequest: error:
C1=C(OC)C=C(OC)C=C1CC[C@H1](C=CC(C(OC(C2=CC=CC=C2)=O)=CC=CC)=O)C
PUGREST.BadRequest: error:  N(C)C(=O)NCCC(OCC1=CN(N=N1)[C@H1]CCC2=CC(OC)=C(OC)C(
OC)=C2C3=CC=C(C(=O)C=C3)N)C=O
PUGREST.BadRequest: error:
C1[C@H1](OC1)OCOC(=S)N[C@H1]C2CC=C3C(OC)=C(OC)C(OC)C=C3C=4C2=CC(=O)C(=CC=4)NCC=C
PUGREST.BadRequest: error:
C1[C@H1](NCC)C2C=CC(OC)=C(OC)C=C2CC[C@H1]C3=CCC(OC(OCC)=O)=CC=C31
PUGREST.BadRequest: error:
C12=C(OC)C=C(OC)C=C1CC[C@H1](NC(C(O)=O)(C)C2=CC=C(NC)C)CON=COC
PUGREST.BadRequest: error:
N(C)CNCC1=CN(N=N1)[C@H1]2CCC3=CC(OC)=C(OC)C(OC)=C3C4=CC=C(C(C=C24)=O)NC=O
PUGREST.BadRequest: error:
OC(=O)C=C(C(OCC)=O)N(NCCC)N[C@H1]1CCC2=CC(OC)=C(OC)C(OC)=C2C3=CC=C(NC)C(=O)C=C31
PUGREST.BadRequest: error:
C[C@H1](NCC)CC1=CC(C(NC)=CC=C1C2=C(C=C(OC)C(OC)=C2OCC3=CC=CC=C3)OSCC)=O
PUGREST.BadRequest: error:  C1[C@H1](OC1)OOC(O)(C)CN=CN([C@@H1]2C3=CC(C(SC)=CC=C
3C4=C(OC)C(OC)=C(C=C4CC2)O)O)C=O
PUGREST.BadRequest: error:
C12=C(OC)C=C(OC)C=C1CC[C@H1](NC(C(O)=O)(C)C2=CC=C(NC)C)COCNCOC
PUGREST.BadRequest: error:  C=1C(C)(C)OC(=O)/N=C(/N[C@H1]2CCC3=C(C(OC)=C(OC)C(OC
)=C3)C4=CC=C(NC)C(=O)C=C24)N=1
PUGREST.BadRequest: error:  C=1C(C)(C)OC(=O)/N=C(/N[C@H1]2CCC3=C(C(OC)=C(OC)C(OC
```

```
)=C3)C4=CC=C(C(C=C42)=O)NC)N=1
PUGREST.BadRequest: error:
N1=NN1[C@@H1]2C3=CC(C(NC)=CC=C3C4=C(C(OC)=C(OC)C=C4CC2)OC)=O
PUGREST.BadRequest: error:
CNCC1C=CC=C(C=C1)NC(=O)N[C@@H1]2C3=CC(C(NC)=CC=C3C4=C(OC)C(OC)=C(C=C4CC2)OC)=O
PUGREST.BadRequest: error:
CNC=CC=C1C[C@H1](CCC2=CC(OC)=C(OC)C(=C21)OC)N3C(C(O)=O)=C(N=N3)C(=O)OOSC
PUGREST.BadRequest: error:
CNC=1CC=C2[C@H1](CCC3=C(C(OC)=C(OC)C(=C3)OC)C2=CC=1)NC(C(=O)O)=CC=CNC=O
PUGREST.BadRequest: error:
CNC=1CC2=C3[C@H1](CCC4=C(C(OC)=C(OC)C(=C4)OC)C3=C5C=1)NC(C(=O)O)=C5ON2
PUGREST.BadRequest: error:
N=NN([C@H1]1CCC2=C(C(OC)=C(OC)C(OC)=C2)C3=CC=C(C(=O)C=C13)NC)COOCCOC=O
PUGREST.BadRequest: error:
CNCC1C=C2[C@H1](CCC3=C(C(OC)=C(OC)C(=C3)OC)C2=CC=C1)NCOC(C4=CC=NC=C4)=O
PUGREST.BadRequest: error:
O=CC=CC=CC(NCC1=CN(N=N1)[C@H1]2CCC3=CC(OC)=C(OC)C(OC)=C3C4=CC=C(C(C=C24)=O)NC)=O
PUGREST.BadRequest: error:
O=CC=CC=CC(NCC1=CN(N=N1)[C@H1]2CCC3=CC(OC)=C(OC)C(OC)=C3C4=CC=C(NC)C(=O)C=C24)O
PUGREST.BadRequest: error:
CNC=1CC=C2[C@H1](CCC3=C(C(OC)=C(OC)C(OC)=C3)C2=CC=1)NC(C(=O)O)=CC=CNC=O
PUGREST.BadRequest: error:
N=NN([C@H1]1CCC2=C(C(OC)=C(OC)C(OC)=C2)C3=CC=C(C(=O)C=C13)NC)COCC=COCOCC
PUGREST.BadRequest: error:
C12=C3C(C=4C([C@@H1](NCC5=CC=C(C)C=C5CCC1)C2)=CC(=O)C(=CC=4)SC=C)(OC)C(=C3OC)OC
PUGREST.BadRequest: error:
C1=C2C(C3=C(CC[C@@H1]2N4N=NC(=C4)C5(O)CCCCC5)C=C(C(OC)=C3OC)OC=C)C=C(C1=O)O
PUGREST.BadRequest: error:
C1=C2C(C=C(CC[C@@H1]2N3C=C(N=N3)COCC4=CC=CC=C4)C=C(C(=COC)OC)OC)=CC=C(NC)C1=O
PUGREST.BadRequest: error:  C1=C2C(C3=CC=C(C(=O)C=C3[C@H1](CC2)N4C=C(N=N4)COC(=O
)C5=CC=NC=C5)COC)C(OC)=C1OCNC=O
PUGREST.BadRequest: error:
C12=C3C(CC([C@@H1](NCC4=CC=C(C)C=C4CCC1)C2)=CC(=O)C(=CC)SC=C)(OC)C(=C3OC)OC
PUGREST.BadRequest: error:
C1=C2C(CC([C@@H1]1NCCCCCCCCCCCCCCCC)C(=CC2)SC=COC)C(OC)=COC
PUGREST.BadRequest: error:
CN(N([C@@H1]1C2=CC(=O)C(NC)=CC=C2C3=CC(=C(OC)C=C3CC1)OC)O)CNC(=S)CCCCC
PUGREST.BadRequest: error:
C=C1C(C=C(CC[C@@H1]1N2N=NC(=C2)C3=CC=C(Cl)C=C3)C=C(C(OC)=COC)OC)=CC=C(C=O)NC
PUGREST.BadRequest: error:
CC(C)C(=O)OCC1=CN(N=N1)[C@H1]2CCC3=CC(OC)=C(OC)C(OC)=C3C4=CC=C(NC)C(=O)C=C24
PUGREST.BadRequest: error:
CC(C)C=1OCCC=C2[C@H1](CCC3=C(C(OC)=C(OC)C(OC)=C3)C2=CC=1)NSC=O
PUGREST.BadRequest: error:
CC(C)(O)C1N=NN(CN1CC[C@H1]2CC)C3=CC(OC)=C(OC)C(OC)=C3C4=CC=C(C(C=C24)=O)NC
PUGREST.BadRequest: error:
N(C(C1=CC=NC=C12)=O)[C@@H1]3CC(C4=C(C(OC)=C(OC)C=C4CC3)OC(OC)=O)=CC=C(SC)C2=O
PUGREST.BadRequest: error:
```

```
N(C(C1=CC=NC=C1)=O)[C@@H1]2C=3C(C4=C(C(OC)=C(OC)C=C4CC2)OC)=CC=C(SC)C(=O)C=3
PUGREST.BadRequest: error:
C12=C(C(=C(OC)C(OC)=C1)O)CC3=CC=C(NC)CC=C3[C@H1](CC2)NC(C)=O
PUGREST.BadRequest: error:
N1(CCCC1)CN[C@H1]2CCC3=C(C(OC)=C(OC)C(OC)=C3)C4=CC=C(SC)C(=O)C=C24
PUGREST.BadRequest: error:
C1[C@H1](NC(O)=O)C2=CCC=CC=C2C3=C(OC)C(OC)=C(OC)C=C3CC=C1SC=O
PUGREST.BadRequest: error:
N1(CCCC1)C(=O)OC2=C(OC)C(OC3)=CC=C2C4=CC=C(SC)C(C=C4[C@@H1](NC(=O)C)CC3)=O
PUGREST.BadRequest: error:
C=CC(=O)OCCC=C1[C@H1](CCC2=C(C(OC)=C(OC)C(OC)=C2)C1=CC)N=O
PUGREST.BadRequest: error:
N(C(C1=CC=NC=C1)=O)[C@@H1]2C=3C(C4=C(C(OC)=C(OC)C=C4CC2)OC=C)C=C(SC)C(=O)C=3
PUGREST.BadRequest: error:
C12=CC(OC)=C(OC)CCC([C@H1](CCCC=C1OC)NC(C)=O)(OC(=O)OC)C2=O
PUGREST.BadRequest: error:
C1[C@H1](NC(O)=O)C=CCC(SC)=CCC=C(C2=C(OC)C(OC)=C(OC)C=C2CC1)SC=O
PUGREST.BadRequest: error:  C12=C(C(OC)=C(OC)CCC([C@@H1]2CCC(C)C1N=C)=O)OSC
PUGREST.BadRequest: error:
C=CC(OC)=C(OC)CC1=CC=C(NC)CC=C1[C@@H1](NC(N(CCO)CCO)=S)CCCNC=O
PUGREST.BadRequest: error:
CNC=1C(=O)C=C2[C@H1](CCC3=CC(OC)=C(OC)C(OC)=C3C2=CC=1)NC4(C(=O)O)C=CN=N4
PUGREST.BadRequest: error:
CNC=1C(=O)C=C2[C@H1](CCC3=CC(OC)=C(OC)C(OC)=C3C2=CC=1N)C4(C(=O)O)C=CN=N4
PUGREST.BadRequest: error:
N1=CC=CC2=C1C(OC=CC(OC=3)=CCCC[C@H1](NC(C)=O)C4=CCC(SC)=CC=C4C=32)C
PUGREST.BadRequest: error:
N=1N(C=C(CNC(C(C)C)=O)N=1)[C@H1]CCC2=C3C(OC)=C(OC)C(OC)=C2C=CC=C(CC=CC=C3)CC
PUGREST.BadRequest: error:
NN(C=C(CNC(C1CCCC1)=O)N=N)[C@H1]CCC2=CC(OC)=C(OC)C(OC)=C2C3=CC=C(C(=O)C=C13)NC
PUGREST.BadRequest: error:
C=C1[C@H1](CCC2=C(C(OC)=C(OC)C(OC)=C2)C1=CC=CC(C)=O)NCNC(C)=O
PUGREST.BadRequest: error:
N=1N(C=C(CNC(C(C)C)=O)N=1)[C@H1]CCC2=CC(OC)=C(OC)C(OC)=C2C=CC3=CCC=CC=C3NC=O
PUGREST.BadRequest: error:
C=C1[C@H1](CCC2=CC(OC)=C(OC)C(OC)=C2C1=CC=C(C(=O)C)NC)NC(C(=O)O)=CN=CNC
PUGREST.BadRequest: error:  CC(C)(C)OC(=O)NCCC(NCC=1N=NN(C=1)[C@H1]C2CC3=CC(OC)=
C(OC)C(OC)=C3C4=CC=C(C(=O)C=C24)N)C=O
PUGREST.BadRequest: error:
C1C(C)(C)OC/N=C(/N[C@H1]2CCC3=CC(OC)=C(OC)C(=C3C4=CC=C(NC)C(C=C42)=O)OC)N=N1
PUGREST.BadRequest: error:  CC(C)(C)OCNCCC(=O)NCC1=CN(N=N1)[C@H1]2CCC3=CC(OC)=C(
OC)C(OC)=C3C4=CC=C(C(C=C24)=O)NC=O
PUGREST.BadRequest: error:
C1C(C)(C)OC/N=C(/N[C@H1]2CCC3=CC(OC)=C(OC)C(=C3C4=CC=C(NC)C(C=C42)=O)OC)N1C
PUGREST.BadRequest: error:  CC(C)(C)OC(=O)NCCC(OCC=1N=NN(C=1)[C@H1]C2CC3=CC(OC)=
C(OC)C(OC)=C3C4=CC=C(C(=O)C=C24)NC)=O
PUGREST.BadRequest: error:  CC(C)(C)OC(=O)NCCC(NCC=1N=NN(C=1)[C@H1]2CCC3=CC(OC)=
C(OC)C(OC)=C3C4=CC=C(C(C=C24)=O)N)C=O
```

```
PUGREST.BadRequest: error:
CNCCC1=CC=C(C=C1)NC(=O)N[C@H1]2CCC3=CC(OC)=C(OC)C(OC)=C3C4=CC=C(C(C=C24)=O)NC=O
PUGREST.BadRequest: error:
N=NN([C@@H1]1C=2C(C3=C(C(OC)=C(OC)C=C3CC1)OC)=CC=C(NC)CC=2)OC(O)=O
PUGREST.BadRequest: error:
N1=NN[C@@H1]2CCC3=C(C(OC)=C(OC)C=C3CC2=CC(OC)=C(OC)COC)CN1
PUGREST.BadRequest: error:
O=CC=CC=CC(NCC1=CN(N=N1)[C@H1]C2CC3=CC(OC)=C(OC)C(OC)=C3C4=CC=C(C(=O)C=C24)NC)=O
PUGREST.BadRequest: error:  CNCC(C1=CC=C(C=C1)NC(=O)N[C@@H1]2C3=CC(C(NC)=CC=C3C4
=C(C(OC)=C(OC)C=C4CC2)OC)=O)OC
PUGREST.BadRequest: error:
CNCC1C=C2[C@H1](CCC3=C(C(OC)=C(OC)C(=C3)OC)C2=CC=C1)NCOC(C4=CC=CC=C4)=O
PUGREST.BadRequest: error:
N=NN([C@H1]1CCC2=C(C(OC)=C(OC)C(=C2)OC)C3=CC=C(NC)C(C=C13)=O)COC4CC=CC=C4O
PUGREST.BadRequest: error:
C1=C2C(C3=CC=C(CC=C3[C@@H1](N4C=C(N=N4)CNC(=O)C(Cl)Cl)C1)ONC=C)(C(=C2OC)OC)OC
PUGREST.BadRequest: error:
C12=C3C(CC([C@@H1](NCC4=CC=C(C)C=C4CCC1)C2)NN=NC=C(NC)C=O)=C=C(C(=C3)OC)OCOCC
PUGREST.BadRequest: error:
C=C1C(C=C(CC[C@@H1]1N2N=NC(=C2)CNC(=O)OCCC)CC=C(C(OC)=COC)O)C=CC=C(C=O)NC
PUGREST.BadRequest: error:
C=C1C(C=C(CC[C@@H1]1N2C=C(N=N2)CNC(=O)C3=CC=NC=C3)C=CC(OC)=COC)OC=CC=C(C=O)NC
PUGREST.BadRequest: error:
O(C=CC=CC1=C(OC)C(OC)=C(OC)C=C1CC[C@H1](C=CCCOCC2=CC=CC=C2)OCC)ONC
PUGREST.BadRequest: error:
O(C=CC=CC1=C(OC)C(OC)=C(OC)C=C1CC[C@H1](C=CCCOCC2=CC=CC=C2)OSC=O)NC
PUGREST.BadRequest: error:
O=C(OC)OCC1=CN(N=N1)[C@H1]2CCC3=CC(OC)=C(OC)C(OC)=C3C4=CC=C(NC)C(=O)C=C24
PUGREST.BadRequest: error:
O(C=CC=CC1=C(OC)C(OC)=C(OC)C=C1C2C[C@H1](C=CCC(OCC(C)C)=O)N=C2ON)C
PUGREST.BadRequest: error:
O=C(C(C)C)NC(=S)N[C@@H1]1C=2C(C3=C(C(OC)=C(OC)C=C3CC1)OC)=CC=C(NC)C(=O)C=2
PUGREST.BadRequest: error:
O=C(OCC)OCC=1N=NN(C=1)[C@H1]2CCC3=CC(OC)=C(OC)C(OC)=C3C4=CC=C(NC)C(=O)C=C24
PUGREST.BadRequest: error:
O=C(OCC)CN(CCO)C(=S)N[C@H1]1CCC2=CC(OC)=C(OC)C(OC)=C2C3=CC=C(NC)C(=O)C=C13
PUGREST.BadRequest: error:
CC(C)CNCC1=CN(N=N1)[C@H1]2CCC3=CC(OC)=C(OC)C(OC)=C3C4=CC=C(C(C=C24)=O)NC=O
PUGREST.BadRequest: error:
CC(C)CNCC1=CN(N=N1)[C@H1]2CCC3=CC(OC)=C(OC)C(OC)=C3C4=CC=C(NC)C(=O)C=C24
PUGREST.BadRequest: error:
C(=CN(C(=O)OCC)N[C@@H1]1CC=2)(C3=C(C(OC)=C(OC)C=C3CC1)OC)CC=C(NC)C(C=2)=O
PUGREST.BadRequest: error:
COCNCC1=CN(N=N1)[C@H1]2CCC3=CC(OC)=C(OC)C(OC)=C3C4=CC=C(C(C=C42)=O)NC=O
PUGREST.BadRequest: error:
C(=NC=C(NC)CC(=O)N)CC1=CN(N=N1)[C@H1]CCC2=CC(OC)=C(OC)C(OC)=C2C=CC=C(C)CNC=O
PUGREST.BadRequest: error:
C(=C1N(C(=O)OCC)N12)CCC3=C(C(OC)=C(OC)C(OC)=C3)C4=CC=C(NC)C(C=C24)=O
```

```
PUGREST.BadRequest: error:
N(C(C)C)C(NCC1=CN(N=N1)[C@H1]2CCC3=CC(OC)=C(OC)C(OC)=C3C4=CC=C(NC)C(=O)C=C24)=O
PUGREST.BadRequest: error:
N(C)C1=CC=C2C3=C(OC)C(OC)=C(OC)C=C3CC[C@@H1](C2=CC1=O)NC(C(=O)O)CCON=CCF
PUGREST.BadRequest: error:
N(C(C(C(C)C)=O)C1=CN(N=N1)[C@H1]2CCC3=C(C(OC)=C(OC)C(OC)=C3)C4=CC=C(NC)C(=O)C=C24
PUGREST.BadRequest: error:
N(CCCCCCC=O)CC1=CN(N=N1)[C@H1]2CCC3=CC(OC)=C(OC)C(OC)=C3C4=CC=C(NC)C(=O)C=C24
PUGREST.BadRequest: error:  C1=C(C=CC(Cl)=C1)COC(=O)CN[C@@H1]2C=3C(C4=C(C(OC)=C(
OC)C=C4CC2)OC)=CC=C(SC)C(=O)C=3
PUGREST.BadRequest: error:
N(C)C1=CC=C2C3=C(OC)C(OC)=C(OC)C=C3CC[C@@H1](C2=CC1=O)NC(C(=O)O)CCONC(=O)C=C
PUGREST.BadRequest: error:
N(C)C1=CC=C2C3=C(OC)C(OC)=C(OC)C=C3CC[C@@H1](C2=CC1=O)NC(C(=O)O)CCONC(=O)CNC
PUGREST.BadRequest: error:
N(C(C)C)CC(=O)OCC1=C2N(N=N1)[C@H1]CCC3=CC(OC)=C(OC)C(OC)=C3C4=CC=C(NC)C(C=C24)=O
PUGREST.BadRequest: error:
C[C@H1](C1OC=CN[C@@H1]2C=C(C3=CC(OC)=C(OC)C(OC)=C3C1)CC=C(NC)CC2)ONCC
PUGREST.BadRequest: error:  C1=C2CC[C@H1](C3=CC(C(SC)=CC=C3C2C=C1O)CNC=O)CN=O
PUGREST.BadRequest: error:
COC=C1C2=CC=C(NC)C(=O)C=C2[C@@H1](NCC(C)C)CCC1=CC(OC)=COC
PUGREST.BadRequest: error:
C1=C2CC[C@H1](C3=CCC(SC)=CC=C3C2=C(C(OC)=C1OC)OC=O)[N-1]
PUGREST.BadRequest: error:
C=C1C=2C[C@H1](CC(C3=CC(OC)=C(OC)C=C31)C=CC=CSC)C(=O)C=2N
PUGREST.BadRequest: error:
COC=C1C2=CC=C(NC)C(=O)C=C2[C@@H1](NCCC)CCC1=CC(OC)=COC
PUGREST.BadRequest: error:
C1OC=C2C3=CC=C(NC)C(=O)C=C3[C@H1](CCC2C=COC)NCCCCCOC=C1
PUGREST.BadRequest: error:
C1=C2CC[C@H1](C3=CCC(NC)=CC=C3C2=C(C(OC)=C1OC)OC)ONCCC#N
PUGREST.BadRequest: error:
CC(CC)N[C@@H1]1C2=CC(C(SC)=CC=C2C3=C(OC)C(OC)=C(C=C3CC1)OC)=O
PUGREST.BadRequest: error:
CC(CC)N[C@@H1]C1=C2C(C(NC)=CC=C1C3=C(OC)C(OC)=C(OC)C=C3CC2)=O
PUGREST.BadRequest: error:
CC(CC)N[C@@H1]1C2=CC(C(SC)=CC=C2C3=C(CC1)C=C(OC)C(OC)=C3OC)=O
PUGREST.BadRequest: error:
C1=C2CC[C@H1](C3=CCC(SC)=CC=C3C2=C(C(OC)=C1OC)OC=O)N=[N+1]=[N-1]
PUGREST.BadRequest: error:
COC1=C2C3=CC=C(NC)C(=O)C=C3[C@H1](CCC2=CC(OC)=C1OC)NCC4=CC=NS4C
PUGREST.BadRequest: error:
N(CCCC)C(CN1[C@@H1]C2=CCC(O)=CC=C2C3=C(CC1)C=C(OC)C(OC)=C3OC)=O
PUGREST.BadRequest: error:
C1[C@H1](NCC)CC2=CC(C(NC)=CC=C2C3=C(OC)C(OC)=C(OC)C=C3C1N)C=O
PUGREST.BadRequest: error:
N(C)C=1C(=O)C=C2[C@H1](CCC3=CC(OC)=C(OC)C(OC)=C3C2=CC=1N)C(C)CCSC
PUGREST.BadRequest: error:
```

```
C1=C(OC)C(OC)=C(OC=2)CC3=CC=C(SC)C1(C=C3[C@@H1](NC(N(C)C)=O)CCC=2)O
PUGREST.BadRequest: error:
N(C(C)=O)[C@@H1]C1=CC(C(NC)=CC=C1C2=C(OC)C(OC)=C(OC)C=C2CCNC=O)C
PUGREST.BadRequest: error:
C[C@H1](OCCC)[N-1]C1=CC(C(NC)=CC=C1C2=C(C(OC)=C(OC)C=C2C)OC)NC(=O)C
PUGREST.BadRequest: error:
C1=C(OC)C(OC)=C(OC)CC2=CC=C(SC)C1(C=C2[C@@H1](NC(N(C)C)=O)CCC)O
PUGREST.BadRequest: error:
N1=NN([C@@H1]C2=CC(C(O)=CC=C2C3=C(CC)C=C(OC)C(OC)=C3OC)=O)CC=C1F
PUGREST.BadRequest: error:
CNC=1C(C=C2[C@H1](CCC3=C(C(OC)=C(OC)C(OC)=C3)C2=CC=1)NC(C)C=O)SC
PUGREST.BadRequest: error:
CNC=1C(C=C2[C@H1](CCC3=C(C(OC)=C(OC)C(OC)=C3)C2=CC=1)NC(C)C)=O
PUGREST.BadRequest: error:
CNC=1C(C=C2[C@H1](CCC3=C(C(OC)=C(OC)C(OC)=C3)C2=CC=1)NC(C)C)CSCSC=O
PUGREST.BadRequest: error:
CNC=1C(C=C2[C@H1](CCC3=C(C(OC)=C(OC)C(OC)=C3)C2=CC=1)NC(C)C=O)SCSC
PUGREST.BadRequest: error:
C=1NC=2C(C=C3[C@H1](CCC4=C(C(OC)=C(OC)C(OC)=C4)C3=CC=2)NC(C)C)N=CN=1
PUGREST.BadRequest: error:
NN([C@H1]1CCC2=CC(OC)=C(OC)C(OC)=C2C3=CC=C(NC)C(=O)C=C13)C=O
PUGREST.BadRequest: error:
CNC=1C(C=C2[C@H1](CCC3=C(C(OC)=C(OC)C(OC)=C3)C2=CC=1)NC(C)C)C=O
PUGREST.BadRequest: error:
N1=NC(=CN(NC1)C=CC2(CC[C@H1](N)C3=CC(C(NC)=CC=C32)=O)CCOC)COC=C
PUGREST.BadRequest: error:
N1=NC(=C2N(NC1)C=CC3=CC4=C(OC)C(OC)=CC=C4CC[C@H1](NC(N)=O)C3=C2)OC=O
PUGREST.BadRequest: error:
N1=NC(=CN(N=N1)[C@H1]2CCC3=CC(OC)=C(OC)C(OC)=C3C4=CC=C(C(C=C42)=O)N)C
PUGREST.BadRequest: error:
OCCN(CC=C)C(=O)N[C@@H1]1C2=CC(C(NC)=CC=C2C3=C(OC)C(OC)=C(OC)C=C3CC1)=O
PUGREST.BadRequest: error:
N1=NC=CN(NC1)C=CC=C2C3=C(OC)C(OC)=C(OC)C=C3CC[C@@H1](C2=C)NC=CC=C
PUGREST.BadRequest: error:
N1=NC=CN(N=N1)[C@H1]2CCC3=CC(OC)=C(OC)C(OC)=C3C4=CC=C(NC)C(=O)C=C24
PUGREST.BadRequest: error:
N1=NC=CN(NC1)[C@H1]2CCC3=CC(OC)=C(OC)C(OC)=C3C4=CC=C(NC)C(=O)C=C24
PUGREST.BadRequest: error:
N1=NC(=CN(NC12)C=CC3=C(O)C(OC)=C(OC)C=C3CC[C@H1](N)C4=CCC(NC)=CC=C24)OC
PUGREST.BadRequest: error:
N1=NC(=CN(NC1)C=CC2=C(O)C(OC)=C(OC)C=C2CC3[C@H1](N)C=CCC(NC)=CC=C3)OC
PUGREST.BadRequest: error:
OCCN(CC=C)C(=S)N[C@H1]1CCC2=CC(OC)=C(OC)C(OC)=C2C3=CC=C(NC)C(=O)C=C13
PUGREST.BadRequest: error:
CNC(N(C)C)CN[C@H1]1CCC2=CC(OC)=C(C(OC)=C2C=3C1=CC(=O)C(=CC=3)NC)OC
PUGREST.BadRequest: error:
C1=C2C([C@H1](CCC3=CC(OC)=C(C(=C32)OC)OC)NCCC=CC)(C(SC)=C1)O
PUGREST.BadRequest: error:
```

```
C=C1C([C@H1](CCC2=CC(OC)=C(C(OC)=C12)OC)NCC=CC=O)C(=CC)NC
PUGREST.BadRequest: error:
C12=CC(C(SC)=CC=C1C3=C(CC[C@@H1]2NCC4=CC=NC=C4)C=C(OC)C(OC)=C3O)C=O
PUGREST.BadRequest: error:
C12=CC(C(NC)=CC=C1C3=C(OC)C(=C(C=C3CC[C@@H1]2N4CC=CC=C4)O)CO)C=O
PUGREST.BadRequest: error:
C12=CC(C(NC)=CC=C1C3=C(OC)C3=C(CCC[C@@H1]2NCC=CC)CS)OC=O
PUGREST.BadRequest: error:
COC1=C(OC)C(OC)=CCCC[C@H1](NCC2=CC=CS2)C3=CC(=O)C(NC)=CC=C3C1
PUGREST.BadRequest: error:
C12=CC(C(SC)=CC=C1C3=C(OC)C(=C(C=C3CC[C@@H1]2N4CC=CC=C4)O)CO)C=O
PUGREST.BadRequest: error:
COC=C(OC)C(OC)=C1CCC[C@H1](NCC(C)C)C2=CC(=O)C(SC)=CC=C2C1
PUGREST.BadRequest: error:
NC(N(CC)OC)N[C@H1]1CCC2=CC(OC)=C(OC)C(OC)=C2C=3C1=CC(=O)C(=CC=3)NC
PUGREST.BadRequest: error:
N1C(C(CCC)=C)N1[C@H1]2CCC3=CC(OC)=C(OC)C(OC)=C3C4=CC=C(C(=O)C=C24)NC
PUGREST.BadRequest: error:
C12=CC=C(NC)C2C=C1[C@@H1](NC(C)=O)CCC=CC(OC)=C(OC)C(OC)=C
PUGREST.BadRequest: error:
NC(CCN=N[C@H1]1CCC2=CC(OC)=C(OC)C(OC)=C2C3=CC=CC(=O)C=C13)NCC
PUGREST.BadRequest: error:
C1=2[C@H1](CCC3=CC(OC)=C(OC)C(OC)=C3C1=CC=C(NC)C(=O)C=2NC)C=O
PUGREST.BadRequest: error:
C=C1C2=C(OC)C(OC)=C(OC)C=C2CC[C@H1](NC(C=3)=O)C1=CC=C(C(C=3)=O)SC
PUGREST.BadRequest: error:
C=C1C2=C(OC)C(OC)=C(OC)C=C2CC[C@H1](NC(N)=O)C1=CC=C(NC)C(=O)C
PUGREST.BadRequest: error:  C1[C@H1](CC(C2=CC(OC)=C(OC)C=C21)C=CC=C(NC)CC)ON
PUGREST.BadRequest: error:
C12=CC3=C(OC)C(OC)=C(OC)C=C3CC[C@H1](NC(N)=O)C=C1C=C(OC)C(=C2)OO
PUGREST.BadRequest: error:
C=C1C2=C(OC)C(OC)=C(OC)C=C2CC[C@H1](NC(C)=O)C1=CC=C(C(C)=O)SC
PUGREST.BadRequest: error:
C1[C@@H1](C2=CC(C(NC)=CC=C2C3=C(OC)C(OC)=C(OC)C=C3C1=O)N)C(=O)C
PUGREST.BadRequest: error:
C1=2[C@H1](CCC3=CC(OC)=C(OC)C(OC)=C3C1=CC=C(C(C=2)=O)SCNC)C=O
PUGREST.BadRequest: error:
C1=2[C@H1](CCC3=CC(OC)=C(OC)C(OC)=C3C1=CC=C(SC)C(=O)C=2NC)C
PUGREST.BadRequest: error:
C1=2[C@H1](CCC3=CC(OC)=C(OC)C(OC)=C3C1=CC=C(C(C=2)=O)SCN)C(=O)C
PUGREST.BadRequest: error:
NC(=O)N[C@@H1]C1=C2C(C(NC)=CC=C1C3=C(OC)C(OC)=C(OC)C=C3CC2)=O
PUGREST.BadRequest: error:
CC(C)NC(=O)N1[C@@H1]C2=CC(C(NC)=CC=C2C3=C(OC)C(OC)=C(OC)C=C3CC1)=O
PUGREST.BadRequest: error:
CC(N)(CCO)[C@H1]1CCC2=CC(OC)=C(OC)C(OC)=C2C3=CC=C(SC)C(=O)C=C13
PUGREST.BadRequest: error:
CC(N)(CCO)[C@H1]1CCC2=CC(OC)=C(OC)C(OC)=C2C3=CC=C(C(C=C31)=O)NCC
```

```
PUGREST.BadRequest: error:
C1#CC(C=C2[C@H1](CCC3=CC(OC)=C(OC)C(OC)=C3C2=C1N)C(C)=O)=C
PUGREST.BadRequest: error:
C1[C@H1](NC(C)=O)C2=CC(C(NC)=CC=C2C3=C(OC)C(OC)=C(C=C3C1)OCNC)=O
PUGREST.BadRequest: error:
C12=C(C2C=C3[C@H1](CCC4=CC(OC)=C(OC)C(OC)=C4C3=C1N)C(C)=O)OSC
PUGREST.BadRequest: error:
C1[C@H1](NC(C)=O)C2=CC(C(SC)=CC=C2C3=C(OC)C(OC)=C(C=C3C1)OCNC)=O
PUGREST.BadRequest: error:
C12=C(C(C=C3[C@H1](CCC4=CC(OC)=C(OC)C(OC)=C4C3=C1N)CC)=O)C2=O
PUGREST.BadRequest: error:
N1(CC2=CC=CN=C23)C=CC(C4=CN(OC)C=C3CC[C@H1](C4)C=C)CC(OC)=COC=C1
PUGREST.BadRequest: error:
C1[C@H1](NC(C)=O)C2=CCC(NC)=CC=C2C3=C(OC)C(OC)=C(OC)C=C3C1=O
PUGREST.BadRequest: error:
C=C(N[C@H1]1CCC2=C(C(OC)=C(OC)C(OC)=C2)C3=CC=C(SC)C(C=C31)=O)C
PUGREST.BadRequest: error:
C=12C=C(NC=3)CC4=CC=1CC=C2[C@H1](CCC=3C=C(OC)C(OC)=C4O)CNC(=O)CSCSC=O
PUGREST.BadRequest: error:
C1[C@H1](NC(C)=O)C2=CCC(SC)=CC=C2C3=C(OC)C(OC)=C(OC)C=C3C1NC=O
PUGREST.BadRequest: error:
C=1C=C(NC)CC=C2C=1C3=C(OC)C(OC)=C(OC)C=C3CC[C@@H1]2NC(C)=O
PUGREST.BadRequest: error:
C=12C=C(NC=3)CC4=CC=1CC=C2[C@H1](CCC=3C=C(OC)C(OC)=C4O)CNC=O
PUGREST.BadRequest: error:
CC(C)N(C(C)C)C(=O)N[C@@H1]C1=CC(C(NC)=CC=C1C2=C(OC)C(OC)=C(OC)C=C2CC)=O
PUGREST.BadRequest: error:
C1C(C)NC1N[C@H1]2CCC3=CC(OC)=C(OC)C(OC)=C3C4=CC=C(C(=O)C=C42)NC=O
PUGREST.BadRequest: error:
CC(C)COC1=C(OC)C(OC)=CC=2CC[C@H1](NC(=O)C)C3=CCC(SC)=CC=C3C=21
PUGREST.BadRequest: error:
O=C(N[C@H1]1CCC2=CC(OC)=C(OC)C(OC)=C2C3=CC=C(NC)C(=O)C=C31)N(S)CC
PUGREST.BadRequest: error:
O=C(N[C@H1]1CCC2=CC(OC)=C(OC)C(OC)=C2C3=CC=C(NC)C(=O)C=C13)CCOCC
PUGREST.BadRequest: error:
O=C(N[C@H1]1CCC2=CC(OC)=C(OC)C(OC)=C2C3=CC=C(NC)C(=O)C=C31)CCOCC
PUGREST.BadRequest: error:
COC1=CC2=CC=C(NC)C(=O)C=C2[C@@H1](NC(=O)NC3=CC=C(F)C=C3)CCC=CC(OC)=C1OC
PUGREST.BadRequest: error:
COC1=C2C3=CC=C(NC)C(=O)C=C3[C@H1](CCC2=CC(OC)=C1OC)NC(=O)N(CCCCCl)O
PUGREST.BadRequest: error:
COC=C1C2=CC=C(NC)C(=O)C=C2[C@@H1](NC(=O)NC3=CC=C(F)C=C3)CCC1C=C(OC)C=COC
PUGREST.BadRequest: error:
COC=CC1=CC=C(NC)C(=O)C=C1[C@@H1](NC(=S)NC2=CC=CC=C2)Cl
PUGREST.BadRequest: error:
N=1C(=CN(N=1)[C@H1]2CCC3=CC(OC)=C(OC)C(OC)=C3C4=CC=C(NC)C(=O)C=C42)C=O
PUGREST.BadRequest: error:
C[C@H1](CCC1=C(C(OC)=C(OC)C(OC)=C1)C2=CC=C(C(C(=C2)N)C=O)CC=O
```

```
PUGREST.BadRequest: error:
C1=2[C@H1]CCC3=CC(OC)=C(OC)C(OC)=C3C1=CC=C(C(C=2)=O)SCNC(C)=O
PUGREST.BadRequest: error:
N1([C@H1]2CCC3=C(C(OC)=C(OC)C(OC)=C3)C4=CC=C(NC)C(C=C24)=O)C=C1I
PUGREST.BadRequest: error:
C1=CC(C2=CN(N=N2)[C@@H1]3C4=CC(C(NC)=CC=C4C5=C(OC)C(OC)=C(OC)C=C5CC3)=O)=C1
PUGREST.BadRequest: error:
N1([C@H1]2CCC3=C(C(OC)=C(OC)C(OC)=C3)C4=CC=C(NC)C(C=C24)=O)C=C1OCC
PUGREST.BadRequest: error:
C12N([C@@H1]3C4=CCC(NC)=CC=C4C5=C(OC)C(OC)=C(OC)C=C5CC3)OC=C1CC=C2O
PUGREST.BadRequest: error:
N1([C@H1]2CCC3=C(C(OC)=C(OC)C(OC)=C3)C4=CC=C(NC)C(=O)C=C42)COCC=CC=C1
PUGREST.BadRequest: error:
N1([C@H1]2CCC3=C(C(OC)=C(OC)C(OC)=C3)C4=CC=C(NC)C(C=C24)=O)C=C1Cl
PUGREST.BadRequest: error:
C=1N([C@H1]2CCC3=C(C(OC)=C(OC)C(OC)=C3)C4=CC=C(NC)C(=O)C=C24)CCNC(=O)OCCN=1
PUGREST.BadRequest: error:
N1C(OC)=C(C=C1)N[C@H1]2CCC3=C(C(OC)=C(OC)C(OC)=C3)C4=CC=C(SC)C(C=C42)=O
PUGREST.BadRequest: error:
C1[C@@H1](C2=CC(C(SC)=CC=C2C3=C(OC)C(OC)=C(C=C3C1)O)C)ONC(C)=O
PUGREST.BadRequest: error:
C1N([C@H1]2CCC3=CC(OC)=C(OC)C(OC)=C3C4=CC=C(NC)C(=O)C=C24)OC(=CC=C1)OSC
PUGREST.BadRequest: error:
C1N([C@H1]2CCC3=C(C(OC)=C(OC)C(OC)=C3)C4=CC=C(NC)C(=O)C=C42)CC=C1OC
PUGREST.BadRequest: error:
N(C(C)C)(C(C)C)C(=O)N1[C@H1]CCC2=C(C(OC)=C(OC)C(OC)=C2)C3=CC=C(NC)C(C=C31)=O
PUGREST.BadRequest: error:
N(CCCC)CC1=CN(N=N1)[C@H1]2CCC3=CC(OC)=C(OC)C(OC)=C3C4=CC=C(NC)C(=O)C=C24
PUGREST.BadRequest: error:
N(CCCC)CC1=CN(N=N1)[C@H1]2CCC3=C(C(OC)=C(OC)C(OC)=C3)C4=CC=C(NC)C(=O)C=C42
PUGREST.BadRequest: error:
C1[C@H1](NCC)CC2=CC(C(NC)=CC=C2C3=C(C(OC)=C(OC)C=C3CC1)OC(C)=O)CC=O
PUGREST.BadRequest: error:
C1=C(OC)C(=C(OC)C=C1CC2[C@H1](NC(C(O)=O)C(=O)OCC=CC2=CC=C(NC)CC)OOCN)C
PUGREST.BadRequest: error:
C12=C(OC)C(=C(OC)C=C1CC[C@H1](NC(C(O)=O)=CNCC2=CC=C(NC)CC)ONCC)ONC
PUGREST.BadRequest: error:
C1=C(OC)C(=C(OC)C=C1CC[C@H1](N2C(C(OCC)=O)=C(N=N2)C(OCC)=O)CCC(NC)=CC=C)ONCOC
PUGREST.BadRequest: error:
NC(=S)N1[C@@H1]C2=CC(C(NC)=CC=C2C3=C(OC)C(OC)=C(OC)C=C3CC1)=O
PUGREST.BadRequest: error:
C[C@H1](CC1C2=CC(OC)=C(OC)C(OC)=C2C3=CC=C(C(=O)C=C31)SCN)CC
PUGREST.BadRequest: error:
N=NC(CO)C(O)(OCC)O[C@@H1]1C2=CC(C(NC)=CC=C2C3=C(OC)C(OC)=C(C=C3CC1)NC)=O
PUGREST.BadRequest: error:
C12=CC3=C(OC)C(OC)=C(OC)C=C3CC[C@H1](NC=CC=C)C1=CC(=O)C2SC
PUGREST.BadRequest: error:
C1[C@@H1](C2=CCC(NC)=CC=C2C3=C(OC)C(OC)=C(C=C3C1)OC=O)CCO
```

```
PUGREST.BadRequest: error:
COC1=C(OC)C=2C3=CC=C(SC)C(C=C3[C@H1](CCC=2C=C1OC)NCC4=CC=CC)=C4O
PUGREST.BadRequest: error:
C=C1C2=C(OC)C(OC)=C(OC)C=C2CC[C@H1](NCCCCl)C1=CC(=O)C(NC)=CNC
PUGREST.BadRequest: error:
C=C1C2=C(OC)C(OC)=C(OC)C=C2CC[C@H1](NCCCCl)C1=CC(C(=C)NC)=O
PUGREST.BadRequest: error:
C=1=C2C3=C(OC)C(OC)=C(OC)C=C3CC[C@H1](NCC)C2=CC(=O)C(SC)=CC=1NC
PUGREST.BadRequest: error:
C1=2[C@@H1](CCC3=CC(OC)=C(OC)C(OC)=C3C1=CC=C(C(C=2)=O)NCNC)CONC
PUGREST.BadRequest: error:
C1C2=CC(OC)=C(OC)C(OC)=C2C3=CC=C(NC)C(=O)C=C3[C@H1](C1)NC4C=CC=CS4
PUGREST.BadRequest: error:
C[C@@H1](C1=CC(C(NC2)=CC=C1C3=C(OC)C(OC)=C(OC)C=C32)NC)CC
PUGREST.BadRequest: error:
N(C=C(O)C=C[C@@H1]1C2)=CC(C(SC)=CC=C2C3=C(OC)C(OC)=C(OC)C=C3CC1)=O
PUGREST.BadRequest: error:
C=1[C@H1](NC(N)=N)CC(C2=C(C(OC)=C(OC)C=C2C)OC)=CC=C(NC)C(C=1)=O
PUGREST.BadRequest: error:
C1C(C2=C(OC)C(OC)=C(OC)C=C2CC[C@@H1]1NC(N)C)C=CC=C(C(C)=O)NC
PUGREST.BadRequest: error:
C1[C@H1](NC(N)=S)C2=CCC(NC)=CC=C2C3=C(OC)C(OC)=C(OC)C=C3C1=O
PUGREST.BadRequest: error:
C=C(CN[C@H1]1CCC2=CC(OC)=C(OC)C(OC)=C2C3=CC=C(NC)C(C=C13)=O)C=O
PUGREST.BadRequest: error:
C1[C@H1](NCCC)C2=CCC(SC)=CC=C2C3=C(OC)C(OC)=C(OC)C=C3CC1=O
PUGREST.BadRequest: error:
C1=C(N=NN1[C@H1]2CCC3=CC(OC)=C(OC)C(OC)=C3C4=CC=C(NC)C(=O)C=C24)C
PUGREST.BadRequest: error:
C1=C(C(C=C2[C@H1](CCC3=CC(OC)=C(OC)C(OC)=C3C2=C1)NC(C)C)C)OSC
PUGREST.BadRequest: error:
C1[C@H1](NC(N)=S)C2=CC(C(NC)=CC=C2C3=C(OC)C(OC)=C(OC)C=C3C1=O)C
PUGREST.BadRequest: error:
C=1C=C(NC)C(C=C2C=1C3=C(OC)C(OC)=C(OC)C=C3CC[C@@H1]2NC(C(C)C)=O)SNC
PUGREST.BadRequest: error:
C=12C=C(NC)C(C=C3C=1C4=C(OC)C(OC)=C(OC)C=C4CC[C@@H1]3NC(NCCCC=O)N)=C2O
PUGREST.BadRequest: error:
C=1C=C(NC)C(C=C2C=1C3=C(OC)C(OC)=C(OC)C=C3CC[C@@H1]2NC(C(C)C)=O)SNC=O
PUGREST.BadRequest: error:
C1[C@H1](NC(N(CC=C)CC=C)=S)C2=CC(C(NC)=CC=C2C3=C(OC)C(OC)=C(C=C3C1)OC)NC=O
PUGREST.BadRequest: error:
C=CC=C(NC(=O)N[C@H1]1CCC2=CC(OC)=C(OC)C(OC)=C2C3=CC=C(NC)C(=O)C=C13)C
PUGREST.BadRequest: error:
C=CC=C(NC([C@@H1]C1=CCC(SC)=CC=C1C2=C(OC)C(OC)=C(OC)C=C2CC)=O)N=O
PUGREST.BadRequest: error:
C1=2[C@H1](CCC3=CC(OC)=C(OC)C(OC)=C3C1=CC=C(C(=O)C=2)SCNC(C)=O)C=C
PUGREST.BadRequest: error:
C=CC=C(NC(=O)N[C@H1]1CCC2=CC(OC)=C(OC)C(OC)=C2C3=CC=C(NC)C(C=C13)=O)C
```

```
PUGREST.BadRequest: error:
C=1NCC(=O)C=C2[C@H1](CCC3=C(C(OC)=C(OC)C(OC)=C3)C2=CC=1)NC(C(C)C)=O
PUGREST.BadRequest: error:
C=CC=C(NC(=O)N[C@H1]1CCC2=CC(OC)=C(OC)C(OC)=C2C3=CC=C(NC)C(=O)C=C31)C
PUGREST.BadRequest: error:
C1=CC=C(NC(=O)N[C@H1]2CCC3=CC(OC)=C(OC)C(OC)=C3C=4C2=CC(C(NC)=CC=4)=O)C1
PUGREST.BadRequest: error:
C[C@H1](CCC1=CC(OC)=C(OC)C(OC(OCC)=O)=C1C2=CC=C(SC)C(=O)C=C2)NCC
PUGREST.BadRequest: error:  CCN[C@H1]([C@H1])C1OC(C)(C2)O[C@@H1]1[C@H1](CNC(=S)N
[C@H1]CCC3=C(C(OC)=C(OC)C(OC)=C3)C4=CC=C(C(=O)C=C24)NC)O
PUGREST.BadRequest: error:
CNCC(O)C(OC=C1C=C2C3=C(OC)C(OC)=CC=C3CC[C@H1](NC(C)=O)C2=CC1)=O
PUGREST.BadRequest: error:
COCC(CN[C@@H1]1C=2C(C3=C(C(OC)=C(OC)C=C3CC1)OC)=CC=C(NC)C(C=2)=O)F
PUGREST.BadRequest: error:
N=NC(CN1C(=O)OC)=C=C(C=CC=CNCC=CC)C2(NC)C(C(O)=CC2C3=C)(OC)C(OC)=C(OC)C=C3CC1OC
PUGREST.BadRequest: error:
NC1=NN=NN1[C@@H1]2C=3C(C4=C(C(OC)=C(OC)C=C4CC2)OC)=CC=C(NC)C(C=3)=O
PUGREST.BadRequest: error:
N=NC(CNC(=O)OC)=C=C(C=CC=CNCC=CC12)C(NC)C(OC)=C(OC)C=C1CC[C@H1](NC(=O)C)C2F
PUGREST.BadRequest: error:
N=NC(CNC(=O)OC)=C=C(C=CC=CNCC=CC12)C(NC)C(OC)=C(OC)C=C1CC[C@H1](NC(=O)C)C2=CC
PUGREST.BadRequest: error:
N1C=NN=NN1[C@H1]2CCC3=C(C(OC)=C(OC)C(OC)=C3)C4=CC=C(NC)C(=O)C=C24
PUGREST.BadRequest: error:
OCCN(CCO)C(=S)N1[C@@H1]C2=CC(C(NC)=CC=C2C3=C(OC)C(OC)=C(OC)C=C3CC1)=O
PUGREST.BadRequest: error:
N12C=NN=NN1[C@H1]3CCC4=C(C(OC)=C(OC)C=C4C=5C3=CC(C(NC)=CC=5)=O)OCC=C2
PUGREST.BadRequest: error:  N=NC(CNC(=O)OC)=C=C(C=CC=CNCC=CC=1)C2(NC)C(C(O)=CC=1
C3=C)(OC)C(OC)=C(OC)C=C3CC2OOC
PUGREST.BadRequest: error:
N1CC=CN([C@@H1]2C3=CC(C(NC)=CC=C3C4=C(CC2)C=C(OC)C(OC)=C4OC)=O)C1C
PUGREST.BadRequest: error:
N=NC(CNC(=O)OC)=C=C(C=CC=CNCC=CC1)C(NC)C(OC)=C(OC)C=C1CC[C@H1](NC(=O)C)C=CC
PUGREST.BadRequest: error:
NC(C(OCC)=C)N[C@H1]1CCC2=CC(OC)=C(OC)C(OC)=C2C3=CC=C(NC)C(=O)C=C31
PUGREST.BadRequest: error:
NC(C(OCC)=C)N[C@H1]1CCC2=CC(OC)=C(OC)C(OC)=C2C3=CC=C(NC)C(=O)C=C13
PUGREST.BadRequest: error:
N([C@H1]1CCC2=CC(OC)=C(OC)C(OC)=C2C3=CC=C(NC)C(C=C13)=O)=N
PUGREST.BadRequest: error:
NC(C(OCC)=C)N[C@H1]1CCC2=CC(OC)=C(OC)C(OC)=C2C3=CC=C(NC)C(C=C13)=O
PUGREST.BadRequest: error:
NC(CCC)N[C@@H1]1C2=CC(C(NC)=CC=C2C3=C(OC)C(OC)=C(OC)C=C3CC1)=O
PUGREST.BadRequest: error:
N([C@H1]1CCC2=CC(OC)=C(OC)C(OC)=C2C3=CC=C(NC)C(C=C31)=O)=C
PUGREST.BadRequest: error:
N([C@H1]1CCC2=CC(OC)=C(OC)C(OC)=C2C3=CC=C(NC)C(C=C13)=O)C=CC=C
```

```
PUGREST.BadRequest: error:
N([C@H1]1CCC2=CC(OC)=C(OC)C(OC)=C2C3=CC=C(NC)C(C=C13)=O)=CC
PUGREST.BadRequest: error:
C1=2[C@H1](CCC3=C(C(OC)=C(OC)C(OC)=C3)C1=CC=C(C(C=2)=O)NCNCC(C)C)ONC
PUGREST.BadRequest: error:
C1=2[C@H1](CCC3=C(C(OC)=C(OC)C(OC)=C3)C1=CC=C(C(C=2)=O)NCNCC)OCSC
PUGREST.BadRequest: error:
C12[C@@H1](CC(C3=C2C(OC)=C(OC)C=C3C1)OC=CC=C(NC)CC)ONC(C)=O
PUGREST.BadRequest: error:  N(C)C=1C(=O)C=C2[C@H1](CCC3=C(C(OC)=C(OC)C(OC)=C3)C2
=CC=1)N4C(C(=O)O)=C(N=N4)C(=O)N
PUGREST.BadRequest: error:
C1[C@H1](NCC)CC2=CC(C(NC)=CC=C2C3=C(C(OC)=C(OC)C=C3CC1)OC(C)=O)(C)ONC=O
PUGREST.BadRequest: error:
N(CCCC)CNCC1=CN(N=N1)[C@H1]2CCC3=CC(OC)=C(OC)C(OC)=C3C4=CC=C(NC)C(=O)C=C24
PUGREST.BadRequest: error:
C12[C@H1](NCC)CC3=CC2(C(SC)=CC=C3C4=C(C(OC)=C(OC)C=C4CC1)OCNC(C)=O)NC
PUGREST.BadRequest: error:
C1=C(O)C(OC)=C(OC)C=CCC[C@H1](NN=NCC(O)=O)OC2=CC(C(NC)=CC=C12)=O
PUGREST.BadRequest: error:
C12=C(OC)C(=C(OC)C=C1CC[C@H1](NC(C(=O)OCC)=C(COCC)OCCC(NC)=CC=CON)C=C2N)C
PUGREST.BadRequest: error:
N(C)C=1C(=O)C=C2[C@H1](CCC3=C(C(OC)=C(OC)C(OC)=C3)C2=CC=1)NC(C(=O)O)=C(COCC)C=O
PUGREST.BadRequest: error:  N(C)C=1C(=O)C=C2[C@H1](CCC3=CC(OC)=C(OC)C(OC)=C3C2=C
C=1N4)C(C(=O)O)=C(N=N4)C(O)=O
PUGREST.BadRequest: error:
C12=C(OC)C(=C(OC)C=C1CC[C@H1](NC(C(=O)OCC)=C(COCC)OCCC(NC)=CC=CON)C=C2)NC
PUGREST.BadRequest: error:  O=C(C=C(C(OC1C2)=O)N(N=N2)[C@@H1]3C=4C(C5=C(C(OC)=C(
OC)C=C5CC3)OC)=CC=C(NC)CC=4)OC(Cl)=C1C
PUGREST.BadRequest: error:  O=C(C=C(C(OCC1)=O)N(N=N1)[C@@H1]2C=3C(C4=C(C(OC)=C(O
C)C=C4CC2)OC)=CC=C(NC)CC=3)OC(Cl)=CC
PUGREST.BadRequest: error:  O=C(C=C(C(OCC1)=O)N(N=N1)[C@@H1]2C=3C(C4=C(C(OC)=C(O
C)C=C4CC2)OC)=CC=C(NC)CC=3)OC(Cl)=C
PUGREST.BadRequest: error:  O=C(C=C(C(OCC1)=O)N(N=N1)[C@@H1]2C=3C(C4=C(C(OC)=C(O
C)C=C4CC2)OC)=CC=C(NC)CC=3)OC=CCCC
PUGREST.BadRequest: error:
OC(\N[C@H1]1CCC2=CC(OC)=C(OC)C(OC)=C2C3=CC=C(C(C=C31)=O)NCNC(C)C)=O
PUGREST.BadRequest: error:
O1CC(C2)=CC(=C13)C4=C(OC)C(OC)=C(OC)C=C4CC[C@H1](NC(NC5=CC=C(C=C5)Cl)=S)C3=CC2=O
PUGREST.BadRequest: error:  N1=CC=C(C=C1)C(NCC=2N=NN(C=2)[C@H1]3CCC4=CC(OC)=C(OC
)C(OC)=C4C5=CC=C(C(C=C53)=O)N)C=O
PUGREST.BadRequest: error:
OC(NCC1=CC(OC)=C(OC)C=C1CC[C@H1](NCN(CC)CC)C2=CCC(NC)=CC=C2)=O
PUGREST.BadRequest: error:
NC(=CN/C(=NC(O)C=CCI)C1=CN(NC1)C=CC2=CC(NC)=CC(OC)=C2C=CC(OC3)C(OC)=CC=C3)C=CC=O
PUGREST.BadRequest: error:
O=CC(=CC1=CC([C@@H1](NC(C(O)=O)=C(N=C)CCCCC2)CC(OC)=C(OC)C(OC)=C21)NC)O
PUGREST.BadRequest: error:  NC(=CN/C(=NC(O)C=CCI)C1=CN(N2C1)C=CC3=CC(NC)=CC(OC)=
C3C=CC4(OC)C(OC)=CC=C4)CC[C@@H1]2N=O
```

PUGREST.BadRequest: error: O=CC(=CC=C1C([C@@H1](N2C(C(O)=O)=C(N=C2)C(=O)C)CCC3=CC(OC)=C(OC)C(OC)=C31)=C)NC=O
PUGREST.BadRequest: error:
OCC(C)=C1C([C@H1](CCC2=CC(OC)=C(OC)C(OC)=C12)NC(NC3=CC=C(C=C3)F)=O)PNC=O
PUGREST.BadRequest: error: O=CC(=CC=C1C([C@@H1](N2C(C(=O)O)=C(C(=O)O)N=N2)CCC3=CC(OC)=C(OC)C(OC)=C31)=C)NC=O
PUGREST.BadRequest: error:
OC(\N[C@H1]1CCC2=CC(OC)=C(OC)C(OC)=C2C3=CC=C(C(C=C13)=O)NCNC(C)C)=O
PUGREST.BadRequest: error: N1=CC=C(C=C1)C(NCC=2N=NN(C=2)[C@@H1]3C4=CCC(NC)=CC=C4C5=C(OC)C(OC)=C(OC)C=C5CC3)=O
PUGREST.BadRequest: error: N1=CC=C(C=C1)C(NCC=2N=NN(C=2)[C@@H1]3C4=CCC(NC)=CC=C4C5=C(OC)C(OC)=C(C=C5CC3)OC)=O
PUGREST.BadRequest: error:
CC(C)(C)OC(=O)/N=C(/N[C@H1]1CCC2=C(C(OC)=C(OC)C(OC)=C2)C3=CC=C(NC)C(C=C13)=O)N=N
PUGREST.BadRequest: error: CC(C)(C)OC(=O)NCCCNCC1=CN(N=N1)[C@H1]2CCC3=CC(OC)=C(OC)C(OC)=C3C4=CC=C(NC)C(=O)C=C42
PUGREST.BadRequest: error:
CC(C)(C)OC(=O)/N=C(/N[C@H1]1CCC2=C(C(OC)=C(OC)C(OC)=C2)C3=CC=C(NC)C(C=C31)=O)N=N
PUGREST.BadRequest: error:
C(=CNCNC(=O)OCC(Cl)(Cl)Cl)[C@H1]CCC1=C(C(OC)=C(OC)C(OC)=C1)C2=CC=C(NC)C(=O)C=C2
PUGREST.BadRequest: error: CC(C)(C)OC(=O)NCCCNCC1=CN(N=N1)[C@H1]2CCC3=CC(OC)=C(OC)C(OC)=C3C4=CC=C(NC)C(=O)C=C24
PUGREST.BadRequest: error: C1=C(OC)C(OC)=C(OC=2)C=3CC([C@H1](CCC=3)C(C(O)=O)=C(COC(=O)CCC=C1)CCC(=CC=2)NC)=O
PUGREST.BadRequest: error:
CSCC(=O)C=C1C(C2=C(OC)C(OC)=C(C=C2CC[C@@H1]1NCN)N)OCC
PUGREST.BadRequest: error: CNCCC=C1C(C2=C(OC)C(=C(C=C2CC[C@@H1]1NCN)C)OO)COC
PUGREST.BadRequest: error: COC1=CC2=C(C3=C(OC)C(OC)=CC=C3CC[C@@H1]2NOC)CC=C1OC
PUGREST.BadRequest: error: CNCC(C=C1C(C2=C(OC)C(=CC=C2CC[C@@H1]1NCN)C)=O)COC
PUGREST.BadRequest: error: CNCC(C=C1C(C2=C(OC)C=C(OC)C=C2CC[C@@H1]1NC=O)C)OC=O
PUGREST.BadRequest: error:
C=C1C=2C[C@H1](CC(C3=CC(OC)=C(OC)C=C31)C=CC=CSC)C(=O)C=2
PUGREST.BadRequest: error: COC=C1C2=CC=C(NC)C(=O)C=C2[C@H1](CCC1C=COC)NCCCCl
PUGREST.BadRequest: error:
C=1NCC=1C=C2C(C3=C(OC)C(=C(OC)C=C3CC[C@@H1]2N)[N+1]=[N-1])OC=CC
PUGREST.BadRequest: error: C1=C2CC[C@H1](C3=CCC(SC)=CC=C3C2=C(OC)C(=C1OC)OC=O)N
PUGREST.BadRequest: error:
C=C1CC[C@H1](CC(C2=CC(OC)=C(OC)C=C21)C=CC=CSC)C(=O)CN
PUGREST.BadRequest: error:
C=C1CC[C@H1](C=2C(C3=CC(OC)=C(OC)C=C31)C=CC=CSC)C(C=2)=O
PUGREST.BadRequest: error:
COC1=C2C3=CC=C(NC)C(C=C3[C@@H1](NCCCCCC)CCC2=CC(=C1OC)O)C=O
PUGREST.BadRequest: error:
C=C1CC=2[C@H1](CC(C3=CC(OC)=C(OC)C=C31)C=CC=CSC)C(C=2)=O
PUGREST.BadRequest: error:
COC1=C2C3=CC=C(NC)C(=O)C=C3[C@H1](CCC2=CC(OC)=C1OC)NCCCCCCSC
PUGREST.BadRequest: error:
C=C(CO)CN[C@H1]1CCC2=CC(OC)=C(OC)C(OC)=C2C3=CC=C(SC)C(=O)C=C31

```
PUGREST.BadRequest: error:
CC(C)CN[C@H1]1CCC2=CC(OC)=C(OC)C(OC)=C2C3=CC=C(C(C=C31)=O)SCC
PUGREST.BadRequest: error:
CC(C)CCN[C@H1]1CCC2=CC(OC)=C(OC)C(OC)=C2C3=CC=C(C(C=C13)=O)SCC
PUGREST.BadRequest: error:
C=1NCC(=O)C=C2C(C3=C(OC)C(OC)=C(C=C3CC[C@@H1]2NCCC#N)C)=CC=1
PUGREST.BadRequest: error:  C12=CCC[C@H1]2CC1(C3=CC(OC)=C(OC)C(OC)=C3)CC=C
PUGREST.BadRequest: error:
C1=C2CC[C@H1](C3=CCC(SC)=CC=C3C2=C(C(OC)=C1OC)OON)CNCCC
PUGREST.BadRequest: error:
C1=C2CC[C@H1](C3=CCC(SC)=CC=C3C2=C(OC)C(=C1OC)OC=O)NCCCC
PUGREST.BadRequest: error:
CC(C)CCN[C@H1]C1CC2=CC(OC)=C(OC)C(OC)=C2C3=CC=C(SC)C(C=C31)=O
PUGREST.BadRequest: error:
C1=C(CO2)CN1C[C@@H1]C3=CC(C(SC)=CC=C3C4=C(OC)C(OC)=C(OC)C=C4CC2)=O
PUGREST.BadRequest: error:
C=C1CC=2[C@H1](CC(C3=CC(OC)=C(OC)C=C31)C=CC=CSC)C(=O)C=2N=[N+1]=[N-1]
PUGREST.BadRequest: error:
CNCC(C1=C2C(C3=C(OC)C(=C(OC)C=C3CC[C@@H1]2NCCC#N)OC)=CC1)=O
PUGREST.BadRequest: error:
N=1NC=1[C@@H1]2C3=CC(C(NC)=CC=C3C4=C(C=C(OC)C(=C4OC)OC)CC2)=O
PUGREST.BadRequest: error:
C1=C2CC[C@H1](C3=CCC(NC)=CC=C3C2=C(OC)C(=C1OC)OC)ONCCC#N
PUGREST.BadRequest: error:
COC=C1C2=CC=C(NC)C(C=C2[C@@H1](NCCCCCC)CCC1=CC(=COC)OC)=O
PUGREST.BadRequest: error:
COC1=C2CC([C@H1](CCC2=CC(OC)=C1OC)NCCC#N)C(C(=CC)NC)=O
PUGREST.BadRequest: error:
C1=C2CC[C@H1](C3=CCC(NC)=CC=C3C2=C(OC)C(=C1OC)OC=O)NCCCCCCCCCC
PUGREST.BadRequest: error:
COC=C1C2=CC=C(NC)C(C=C2[C@@H1](NCCCCCCCCC)CCC1=CC(=COC)OC)=O
PUGREST.BadRequest: error:
COC=C1C2=CC=C(NC)C(C=C2[C@@H1](NCCCCCCCC)CC=CC(=C1OC)OC=O)CC
PUGREST.BadRequest: error:
COC1=C2CC([C@H1](CCC2=CC(OC)=C1OC)N3N=NC=C3)CN=CC(C(=CC)NC)=O
PUGREST.BadRequest: error:
C1NCC(=O)C=C2C(C3=C(OC)C(OC)=C(OC)C=C3CC[C@@H1]2N4C=C(N=N4)CNN)=CC=C1
PUGREST.BadRequest: error:
COC=C1C2=CC=C(NC)C(C=C2[C@@H1](N3C=C(N=N3)CCCC)CCC=CC(=C1OC)O)C=O
PUGREST.BadRequest: error:
COC1=C2CC([C@H1](CCC2=CC(OC)=C1OC)NC/C=C/C3=CC=CC)=C3CC(C(SC)=CC)=O
PUGREST.BadRequest: error:
COC1=C2CC([C@H1](CCC2=CC(OC)=C1OC)N3N=NC=C3)CN=CC(=O)C(NC)=CC
PUGREST.BadRequest: error:
COC1=C2C3=CC=C(NC)C(=O)C=C3[C@H1](CCC2=CC(OC)=C1OC)NCC(C)C
PUGREST.BadRequest: error:
COC=C1C2=CC=C(NC)C(C=C2[C@@H1](NCC(C)C)CCC1=CC(=COC)OC)=O
PUGREST.BadRequest: error:
```

```
C1=C2CC[C@H1](C3=CCC(SC)=CC=C3C2=C(OC)C(=C1OC)OC=O)NCCC
PUGREST.BadRequest: error:
C1=C2CC[C@H1](C3=CCC(SC)=CC=C3C2=C(C(OC)=C1OC)OON)COCC
PUGREST.BadRequest: error:
COC1=C(OC)C(OC)=CC=2CC[C@@H1](C3=CC(C(=CC=C3C1=2)SC)=O)NCCC(C)CC
PUGREST.BadRequest: error:
C12=CC(C(SC)=CC=C1C=C(CC[C@@H1]2NCCC3=CC=CC=C3)C=C(OC)C(OC)=CO)C=O
PUGREST.BadRequest: error:
C1=C2C(C3=C(C(OC)=C(OC)C=C3CC[C@@H1]2NCCC(F)F)F)OC=CC=C(C1=O)NC
PUGREST.BadRequest: error:
C12=CC(C(NC)=CC=C1C3=C(C=C(OC)C(OC)=C3OC)CC[C@@H1]2NCCC(F)F)F
PUGREST.BadRequest: error:
C1=C2C([C@H1](CCC3=CC(OC)=C(C(OC)=C23)OC)NCCCCl)C(=O)C(=C1)NC
PUGREST.BadRequest: error:
C12=CC(C(NC)=CC=C1C3=C(C=C(OC)C(OC)=C3OC)CC[C@@H1]2NCC=4SC=CC=4OC)=O
PUGREST.BadRequest: error:
C12=CCC(NC)=CC=C1C3=C(OC)C(=C(C=C3CC4[C@@H1]2NCC4CCCOC)OC)O
PUGREST.BadRequest: error:
O=CC1=CN=NN1[C@H1]2CCC3=CC(OC)=C(OC)C(OC)=C3C4=CC=C(C(C=C24)=O)NCCC
PUGREST.BadRequest: error:
C12=NCCC13[C@H1](CNC(CCC=CC=CC3=O)=C(OC)C(OC)=C(OC)C)C2SC=CCNC
PUGREST.BadRequest: error:
C1=CN=CC=C1CN[C@H1]2CCC3=CC(OC)=C(OC)C(OC)=C3C4=CC=C(C(C=C42)=O)SCC(=O)C
PUGREST.BadRequest: error:
O=CC1=CN=NN1[C@H1]2CCC3=CC(OC)=C(OC)C(OC)=C3C4=CC=C(C(C=C42)=O)NCCC
PUGREST.BadRequest: error:
C1=CN=CC=C1CN[C@H1]2CCC3=CC(OC)=C(OC)C(OC)=C3C4=CC=C(C(C=C42)=O)SCC
PUGREST.BadRequest: error:
C1=NCCC12[C@H1](CC3C=CC(OC)=C(OC)C(OC(=O)C)=CC=C23)C(C)=O
PUGREST.BadRequest: error:
C12[C@H1]C=CCC[C@H1](N=C(COCC)CC3=CCC(NC)=CC=C31)C(OC)C(OC)=C2OC=O
PUGREST.BadRequest: error:
OC1=CC=C(C=C1)CN[C@@H1]2C3=CC(C(SC)=CC=C3C4=C(C(OC)=C(OC)C=C4CC2)OC)=O
PUGREST.BadRequest: error:
O=CC1=C2[C@H1](CCC3=CC(OC)=C(OC)C(OC)=C3C2=CC=C(OC(=O)CC)C1=O)CC=O
PUGREST.BadRequest: error:
C=C1N=CC(C)N1CN[C@H1]2CCC3=C(C(OC)=C(OC)C(OC)=C3)C4=CC=C(C(C=C24)=O)NC
PUGREST.BadRequest: error:
OC1=CC=C(C=C1)CN[C@H1]2CCC3=C(C(OC)=C(OC)C(OC)=C3)C4=CC=C(C(C=C42)=O)SC
PUGREST.BadRequest: error:
O=CC1=C2[C@H1](CCC=C3C(OC)=C(OC)C(OC(C)=O)=C3C1=CC=C(SC)C2=O)CC=O
PUGREST.BadRequest: error:
C1=CC2C[C@H1](NC(C)=O)C3=CC(C(SC)=CC=C3C2=C(OC)C(OC)=C1OC(C4=CC=CC=C4)=O)SC=O
PUGREST.BadRequest: error:
OC1=CC=C(C=C1)CN[C@@H1]2C3=CC(C(SC)=CC=C3C4=C(OC)C(OC)=C(OC)C=C4CC2)=O
PUGREST.BadRequest: error:
O=CC1=C[C@H1](CCC2=CC(OC)=C(OC)C(OC(=O)C)=C2C1=CC=C(SC)C=O)CC=O
PUGREST.BadRequest: error:
```

```
CNCC(C)OC(=O)N[C@H1]1CCC2=C(C(OC)=C(OC)C(OC)=C2)C3=CC=C(C(=O)C=C31)NCF
PUGREST.BadRequest: error:
C1OC2=CC=C(C(OC)=C2OC)C3=CC=C(SC)C1(C=C3[C@@H1](NC(N(COC)C)O)C=C)ONC
PUGREST.BadRequest: error:
COC1=C2C=C(C(OC)=C1OC)C3=CC=C(SC)C(C=C3[C@@H1](NCN=CC=C(F)C=COC)C2)NC=O
PUGREST.BadRequest: error:
C1OC2=CC=C(C(OC)=C2OC)C3=CC=C(SC)C(C=C3[C@@H1]1CCC(=O)C(OC)=C)(O)CSCSC
PUGREST.BadRequest: error:
C=C1CC[C@H1](N2C(C(OCC)=O)=C(N=N2)C=CC(=O)C3NC)CC=CC=C3C=C1CNCOCOC
PUGREST.BadRequest: error:
OC=CC=CCCN[C@H1]1CCC2=CC(OC)=C(OC)C(=C2C3=CC=C(SC)C(=O)C=C13)OCC
PUGREST.BadRequest: error:
COC1=CC=C(C(OC)=C1OC)C2=CC=C(SC)C(C=C2[C@@H1]NC(N(COC)C)=C)ONC=O
PUGREST.BadRequest: error:
C=C1CC2[C@H1](NC(C(=O)OCC)=C2)C3=CC(C(NC)=CC=C3C4=C(C(OC)=C(C=C41)OC)OC)=O
PUGREST.BadRequest: error:
C1=CC=2C[C@H1](NCN(C(C)C)C(C)C)OC3=CC(C(NC)=CC=C3C=2C=C(C(=C1)OC)OC)NC=O
PUGREST.BadRequest: error:
COC1=C2C=C(C(OC)=C1OC)C3=CC=C(NC)C(C=C3[C@@H1](NC(N(COC)C)O)CC2)NC=O
PUGREST.BadRequest: error:
C1OC2=CC=C(C(OC)=C2OC)C3=CC=C(SC)C(C=C3[C@@H1]1CCC(=O)C(OC)=C)C=O
PUGREST.BadRequest: error:
COC1=CC2=C(C(OC)=C1OC)C3=CC=C(SC)C2(C=C3[C@@H1]NC(N(COC)C)=C)ONC
PUGREST.BadRequest: error:
CC12[C@H1](CCC3=CC(OC)=C(OC)C(OC)=C3C1=CC=CC(=O)C2)SCNC(C)=O
PUGREST.BadRequest: error:
C=CC=C(CCN[C@H1]1CCC2=CC(OC)=C(OC)C(OC)=C2C3=CC=C(SC)C(C=C31)=O)OC
PUGREST.BadRequest: error:
CCOC(OC1=C2CC([C@H1](CCC2=CC(OC)=C1OC)NC(C)=O)=CC(C(SC)=CC)=O)SC=O
PUGREST.BadRequest: error:
CC12[C@H1](CCC3=CC(OC)=C(OC)C(OC)=C3C1=CC=CC(C2)=O)SCNC(C)=O
PUGREST.BadRequest: error:
C1C=CC=C(SCC1)C=CN(N2C3OC)[C@@H1]2NC(=O)CNC(=O)OCCCC4C=CC(=C)C=C4C=CC=C(NC)CCC3
PUGREST.BadRequest: error:
CC1=C2C=C(SC)C(C=C1[C@@H1](NC(C)=O)CCC=CC(OC)=C(OC)COC=CC=C2SC)=O
PUGREST.BadRequest: error:
CC1=CC=C(S=2C)C(C=C1[C@@H1](NC(=O)C)CCC=CC(OC)=C(OC)C(OC=CC=O)C=2)SC=O
PUGREST.BadRequest: error:
C1COC1(OC2=CC=C3C4=C(OC)C(OC)=C(OC)C=C4CC[C@@H1](C3=CC2=O)SC(C)=O)SC
PUGREST.BadRequest: error:
CC1=CC=C(S=2C)C(C=C1[C@@H1](NC(C)=O)CCC=CC(OC)=C(OC)C(OC=CC=O)C=2)SC=O
PUGREST.BadRequest: error:
C1C=CC=C(SC2C1)C3=C(C(OC)=C(OC)C=C3CC[C@@H1]2NC(C)=O)OC=CC=C(SC)C=O
PUGREST.BadRequest: error:
CC1=C2C=C(SC)CC=C1[C@@H1](NC(=O)C)CCC=CC(OC)=C(OC)C(OC=CC2)=O
PUGREST.BadRequest: error:
C=C1C(C2=C(C(OC)=C(OC)C=C2CC[C@@H1]1NC(C)=O)OC)(C3=CC=CC=C3)OCC=C(SC)C=O
PUGREST.BadRequest: error:
```

```
C1=C2C(C(OC(CC)=O)=CC=C1C3=C(OC)C(OC)=C(OC)C=C3CC[C@@H1]NSC=CC2)=O
PUGREST.BadRequest: error:
OC(NC(=O)OC)C=CC=C1C([C@H1](CCC2=CC(OC)=C(OC)C(OC)=C21)N=C)C=O
PUGREST.BadRequest: error:
O1C(OC(=O)OC)=CC2=CC([C@@H1](NC(C)=O)CCC3=CC(OC)=C(OC)C(OC)=C32)=CC1NC
PUGREST.BadRequest: error:
C=1=CC(C(C=C2[C@H1](CCC3=C(C(OC)=C(OC)C(OC)=C3)C2=1)NC(C)=O)=C)=O
PUGREST.BadRequest: error:
C1[C@@H1](C2=CC(C(OC)=CC=C2C3=C1C=C(OC)COC)=C3OC=O)CC=O
PUGREST.BadRequest: error:
C=CC(C(OC(CC)=O)=CC=CC1=C(OC)C(OC)=C(OC)C=C1CC[C@@H1]NSC(=O)CC)=O
PUGREST.BadRequest: error:
O=C(OC1=C2C(CC[C@H1](NC(=O)C)C3=CC(C(SC)=CC=C32)=O)=CC(OC)=C1OC)SCC
PUGREST.BadRequest: error:
CN(C(=S)N[C@@H1]1C2=CCC(NC)=CC=C2C3=C(C(OC)=C(OC)C=C3CC1)OC)OC
PUGREST.BadRequest: error:
C1=C2C3(C(OC(C)=O)=CC=C1C4=C(OC)C(OC)=C(OC)C=C4CC[C@@H1]3NSC=C)C2=O
PUGREST.BadRequest: error:
O1C(OC(=O)OC2)=CC=CC([C@@H1](NC(C)=O)CCC3=CC(OC)=C(OC)C(OC)=C32)=CC1NC
PUGREST.BadRequest: error:
N1([C@H1]2CCC=C(C(OC)=C(OC)C(OC)=C2C3=CC=C(SC)CC=C3)OC=C1)F
PUGREST.BadRequest: error:
O=C(OC1=C2CC([C@H1](CCC2=CC(OC)=C1OC)NC(C)=O)=CC(C(SC)=CC)=O)C
PUGREST.BadRequest: error:
C1N(C(=O)N[C@H1]2CCC3=CC(OC)=C(OC)C(OC)=C3C4=CC=C(NC)CC=C24)OC=C1
PUGREST.BadRequest: error:
NC(=CN(NCC)CCCCN1[C@@H1]C=C2CC(C)=CC=C2C3=C(CC1)C=C(OC)C(OC)=C3OC)OF
PUGREST.BadRequest: error:
OC=C(OC)C=C1C(C2=CC=C(SC)C(C=C2[C@H1](CC1)NC(C)=O)OCC)=O
PUGREST.BadRequest: error:
O=C1C=CC=C2C([C@H1](CCC3=CC(OC)=C(OC)C(OC)=C32)NC(C)=O)=C1SC
PUGREST.BadRequest: error:
C1=CC=C(C=C1)CN[C@H1]2CCC3=CC(OC)=C(OC)C(OC)=C3C4=CC=C(C(C=C42)=O)SCC
PUGREST.BadRequest: error:
O=CC(=CC=C1C([C@H1](CCC2=CC(OC)=C(OC)C(OC)=C21)NC(C)=O)SC)O
PUGREST.BadRequest: error:
N=1C(=CN(N=1)[C@@H1]2C3=CCC(NC)=CC=C3C4=C(OC)C(OC)=C(OC)C=C4CC2)F
PUGREST.BadRequest: error:
CC=CCCCN[C@H1]1CCC2=CC(OC)=C(OC)C(OC)=C2C3=CC=C(SC)C(C=C13)=O
PUGREST.BadRequest: error:
C1=CC=C(S1C)C2=CN(NC2)[C@H1]3CCC4=CC(OC)=C(OC)C(OC)=C4C5=CC=C(NC)C(=O)C=C35
PUGREST.BadRequest: error:
OC1=C(C=CC=C1)CN2[C@H1]CCC3=CC(OC)=C(OC)C(OC)=C3C4=CC=C(SC)C(C=C24)=O
PUGREST.BadRequest: error:
OC=C(OC)C=C1C(C2=CC=C(SC)C(C=C2[C@@H1](NC(C)=O)CC1)=O)(C)OSC
PUGREST.BadRequest: error:  C=C1CC(=C2C=C(SC)CC=C[C@H1]CCC2=CC(OC)=C1O)C(OCC)=O
PUGREST.BadRequest: error:
OC1=C(C=CC=C1)CN[C@@H1]2C3=CC(C(SC)=CC=C3C4=C(CC2)C=C(OC)C(OC)=C4OC)SC=O
```

```
PUGREST.BadRequest: error:
C1=C2CC[C@H1](C3=CCC(NC)=CC=C3C2=C(C(OC)=C1OC)OC=O)NCCCCC
PUGREST.BadRequest: error:
COC1=C2C3=CC=C(NC)C(C=C3[C@@H1](NCCCCCCC)CCC1=CC=C2OC)OC=O
PUGREST.BadRequest: error:
C=1NCC(=O)C=C2C=1C3=C(OC)C(OC)=C(C=C3CC[C@@H1]2NCCCCCO)C=CC
PUGREST.BadRequest: error:
C=C1CC2[C@H1](CCC3=C(C(OC)=C(OC)C(=C3)OC)C2=CC=C1)SC=O
PUGREST.BadRequest: error:
C1OC2=C3C4=CC=C(NC)C(=O)C=C4[C@H1](CCC3=CC(OC)=C2OC)NCCCCC=C1
PUGREST.BadRequest: error:
COC=C1C2=C3C=C(NC)C(C=C2[C@@H1](NCCCCCCCCC)CCC1=CC(=C3OC)O)OC
PUGREST.BadRequest: error:
COC1=C2C3=CC=C(NC)C(=O)C=C3[C@H1](CCC2=CC(OC)=C1OC)NCCCCCCCCSC
PUGREST.BadRequest: error:
COC1=C2C3=CC=C(NC)C(C=C3[C@@H1](NCCCCCCC)CCC1=CC(=C2OC)OCS)C=O
PUGREST.BadRequest: error:
C1=C2CC[C@H1](C3=CCC(SC)=CC=C3C2=C(C(OC)=C1OC)OC=O)NCCCCCC
PUGREST.BadRequest: error:
COC=CC1=C2C=C(NC)C(C=C1[C@@H1](NCCCCCCCCC)CCC=CC(=C2OC)O)C=O
PUGREST.BadRequest: error:
C1=C2CC[C@H1](C3=CCC(NC)=CC=C3C2=C(OC)C(=C1OC)OC)ONCCCCCC
PUGREST.BadRequest: error:
COC=C1C2=CC=C(NC)C(C=C2[C@@H1](NCCCCCC)CCC1=CC(=COC)OC)SC=O
PUGREST.BadRequest: error:
C1=C2CC[C@H1](C3=CCC(SC)=CC=C3C2=C(OC)C(=C1OC)OC=O)NCCCCCC
PUGREST.BadRequest: error:  CNC=CC=C1C([C@H1](CCC2=CC(=C(OC)C(OC)=C12)OC)NCC)=O
PUGREST.BadRequest: error:
CSC=1C(C=C2C(=CC=1)C3=C(OC)C=CC=C3CC[C@@H1]2NCN)COCOC
PUGREST.BadRequest: error:
C=1SC=2C=1C=C3C(=CC=2)C4=C(OC)C(=CC=C4CC[C@@H1]3NCN)C
PUGREST.BadRequest: error:
COC1=CC=2CC[C@@H1](C=3C(C=2C(=C1OC)OC)=CC=C(NC)C(=O)C=3)NC(=O)CNC
PUGREST.BadRequest: error:
CSC1=CC=C2C(=CC1=O)[C@H1](CCC3=CC(OC)=C(OC)C(=C23)O)CNC(=O)C
PUGREST.BadRequest: error:
COC1=CC=2CC[C@@H1](C3=CC(=O)C(NC)=CC=C3C=2C(OC)=C1OCN)C(=O)C
PUGREST.BadRequest: error:
CSC=1C(C=C2C(=CC=1)C3=C(OC)C(=C(OC)C=C3CC[C@@H1]2NCN)O)OCNC
PUGREST.BadRequest: error:
CNC=CC=C1C[C@H1](CCC2=CC(=C(OC)C(OC)=C12)OC)N=CC(=O)OCOO
PUGREST.BadRequest: error:
C1=C2[C@H1](CCC3=C(C(OC)=C(C(=C3)OC)OC)C2=CC=C(C1=O)NC)NC
PUGREST.BadRequest: error:
C1SCC(C=C2C(C3=C(C=C(OC)C(OC)=C3OC)CC[C@@H1]2N)=C1)=O
PUGREST.BadRequest: error:
CNC=1C(C=C2C(=CC=1)C3=C(OC)C(OC)=C(C=C3CC[C@@H1]2NC(C)=O)O)C=O
PUGREST.BadRequest: error:
```

```
COC=C1C=2CC[C@H1](NC(C)=O)C3=CC(=O)C(SC)=CC=C3C=2C(O)=C1OC
PUGREST.BadRequest: error:
COC=C1CCC2[C@H1](O)CCC3=CC(=C(OC)C(OC)=C3C2=CC1OCN)C(=O)C
PUGREST.BadRequest: error:
COC=C1CCC2[C@H1](O)CCC3=CC(=C(OC)C(OC)=C3C2=CC1OCNC)C=O
PUGREST.BadRequest: error:
COC=C1CCC[C@H1](O)CCC2=CC(=C(OC)C(OC)=C2C=CC1OC)NC(=O)C
PUGREST.BadRequest: error:
CNC=1C(C=C2C(=CC=1)C3=C(OC)C(OC)=CC=C3CC[C@@H1]2NC(C)=O)OC=O
PUGREST.BadRequest: error:
C1OC=CC=2CC[C@H1](NC(N)=O)C3=CC(C(=CC=C3C=2C(OC)=C1OC)NC)=O
PUGREST.BadRequest: error:  COC=C1CCC[C@H1](NC(N)=O)C2=CCC(=CC=C2C1C(O)C=O)NC
PUGREST.BadRequest: error:
CSC=1CC=CC(=C2C=1)C3=C(OC)C(OC)=C(C=C3CC[C@@H1]2NOCNC)O
PUGREST.BadRequest: error:
NN(C[C@H1](OC[C@@H1][C@@H1](CO)O)OS1C2=CCC(NC)=CC=C2C3=C(C=C(C(=C3OC)OC)OC)CC1)O
PUGREST.BadRequest: error:  C=12OC3=CCCC[C@H1](N)CC(C=1C(=C2OC)OC)=CC=C(O)CC3=O
PUGREST.BadRequest: error:
CNC=1C(C=CC(=C2C=1)C3=C(OC)C(OC)=CC=C3CC[C@@H1]2NOCS)C=O
PUGREST.BadRequest: error:  CSCCC=C1C(C2=CC=C(OC)C(OC)=C2OC)CC[C@@H1]1N=O
PUGREST.BadRequest: error:
CNC=1CC=CC(=C2C=1)C3=C(OC)C(OC)=C(C=C3CC[C@@H1]2NOCS)C=O
PUGREST.BadRequest: error:  COC=C1C2CC[C@H1](N)CCC3=CC(=C(OC)C=C3C2=CC1OCS)C
PUGREST.BadRequest: error:
CC(=O)C(S1=2C3)CC(OC)=C(OC)C(C)=C1C3=CC=C(SC)C(C=2)=O
PUGREST.BadRequest: error:
C1C(=O)C=CC2=C(SC)C(OC)=C(OC)C(C)=CCC[C@@H1](C2=C1)NC(=O)C
PUGREST.BadRequest: error:  C12=C(CC1NC(=O)C=CC(C(SC)=CC2)=O)C(OC)=C(C(OC)=C)OC
PUGREST.BadRequest: error:
C1=C(C(OC(=O)C)=C2C(OC)=C1)OCC=3C([C@H1](CC2)NC(=O)C)=CC(=O)C(O)=CC=3
PUGREST.BadRequest: error:
CC(=O)OC1=CC=C2C3=C(C(OC)=C(C=C3CC[C@@H1](C2=CC1=O)NC(=O)CO)CO)C
PUGREST.BadRequest: error:
C12C(=O)C(=C3C=C1C4=C(CC[C@@H1]3NC(C)=O)C=C(OC)C(=C4OC)OC=C2SC)OC(=O)C
PUGREST.BadRequest: error:
C12C(=O)C2=C3C=C1C4=C(CC[C@@H1]3NC(C)=O)C=C(OC)C(OC)=C4OC(OC)=O
PUGREST.BadRequest: error:
C12C(=O)C(=CC=C1C3=C(CC[C@@H1]2NC(C)=O)C=C(OC)C(=C3OC)OC(OC)=O)SC
PUGREST.BadRequest: error:
CC(=O)OC=1C(=O)C=C2C(=CC=1)C3=C(OC)C(OC)=C(C=C3CC[C@@H1]2NC(C)=O)C(=O)C
PUGREST.BadRequest: error:
C12C(=O)C=C3C=C1C4=C(CC[C@@H1]3NC(C)=O)C=C(OC)C(=C4OC(OC)=O)OC=C2
PUGREST.BadRequest: error:
C12C(=O)C(=CC=C1C3=C(CC[C@@H1]2NC(C)=O)C=C(OC)C(OC)=C3OC(OC)=O)SC
PUGREST.BadRequest: error:
CC(=O)OC1=CC=C2C3=C(C(OC)=C(C=C3CC[C@@H1](C2=CC1=O)NC(=O)C)OC)OC(=O)C
PUGREST.BadRequest: error:
C1C(=O)C(=C2C=C1C3=C(CC[C@@H1]2NC(C)=O)C=C(OC)C(OC)=C3OC(OC)=O)SC
```

```
PUGREST.BadRequest: error:
C=1C(=O)C(OC(=O)CC)=CC=CC2=C(C3=C(OC)C(OC)=C2OC)CC[C@H1](NC(=O)C)C3=1
PUGREST.BadRequest: error:
C1C(=O)OC2C=CC=CC(=C2)[C@@H1](NC(=O)C)CCC3=CC(OC)=C(OC)C(=C3)OC=C1
PUGREST.BadRequest: error:
C=12C=3C(=CC(=O)C(SC)=CC=3)[C@H1]CCC=1C=C(OC)C(=C2OC)OCNC(C)=O
PUGREST.BadRequest: error:
CCC(=O)OC1=CC=C2C3=C(C(OC)=C(C=C3CC[C@@H1](C2=CC1=O)OC(=O)C)OC)OC
PUGREST.BadRequest: error:
CCC(=O)N[C@H1]1CCC2=CC(=C(OC)C(OC)=C2C=3C1=CC(=O)C(=CC=3)OC(OC)=O)OC
PUGREST.BadRequest: error:
CCC(=O)OCC(C=C1C(=C)C2=C(OC)C(=C(C=C2CC[C@@H1]1N)CC)OO)COC=CO
PUGREST.BadRequest: error:
CC(C)C1(OC=C2C3=CC=C(C(C=C3[C@H1](CCC2=CC(=C1OC)OC)NCC)=O)O)OC=O
PUGREST.BadRequest: error:
CC(C)C(OC1=CC=C2C3=C(CC[C@@H1](C2=CC1=O)NC(C)=O)C=C(OC)C(OC)=C3OC)(O)C=O
PUGREST.BadRequest: error:
CC(C)C1(OC=CC2=CC=C(C(C=C2[C@H1](CCC=CC(=C1OC)OC)NCC)=O)O)OC=O
PUGREST.BadRequest: error:
CC(C)C(OC1=CC=C2C3=C(CC[C@@H1](C2=CC1=O)NC(C)=O)C=C(OC)C(OC)=C3OC=O)C
PUGREST.BadRequest: error:
CC(C)C(=O)OC1=CC=C2C3=C(C(OC)=C(C=C3CC[C@@H1](C2=CC1=O)NC(C)=O)OC)OC(=O)C
PUGREST.BadRequest: error:
CC(C)C(OC1=CC=C2C3=C(C=C(OC)C(OC)=C3OC)CC[C@@H1](C2=CC1=O)NC(=O)CC)(O)C
PUGREST.BadRequest: error:
CC(=O)N[C@H1]CCC1=CC(OC)=C(OC)C(=C1C2=CC=C(SC)C(=O)C=C2)CC=C
PUGREST.BadRequest: error:
CC(=O)N[C@@H1]1C2=CC(C(SC)=CC=C2C3=C(OC)C(=C(OC)C=C3CC1)OC(=O)C=CC=CC=C)O
PUGREST.BadRequest: error:
C12=C(C(=C(OC)C(OC)=C1)OC(C)=O)CC([C@H1](CC2)NC(C)=O)=CC(C(SC)=CC)=O
PUGREST.BadRequest: error:
CC(=O)OCC1=CN(N=N1)[C@H1]2CCC3=CC(OC)=C(OC)C(OC)=C3C=4C2=CC(=O)C(=CC=4)NCC
PUGREST.BadRequest: error:
C1=C(C2=C(OC)C(OC)=C1OC(=O)CCC)C[C@@H1](C3=CC(=O)C(SC)=CC=C32)NC(C)=O
PUGREST.BadRequest: error:
CC(=O)N[C@H1]1CCC2=CC(OC)=C(OC)C(=C2C3=CC=C(C(C=C31)=O)OCC4=CC=CC=C4)OOC
PUGREST.BadRequest: error:
O=C(OC)OC=C(OC)C(=C1C(CC[C@@H1](C2=CC(C(=CC=C12)SC)=O)N=C)O)C
PUGREST.BadRequest: error:
O=C(OC1)OC=C2C3=CC=C(C(C=C3[C@H1](CCC2=CC(=C1OC)OC)NCC)=O)OOC
PUGREST.BadRequest: error:
O=C(OC)OCC(=O)C=C1C(C2=C(C(OC)=C(C=C2CC[C@@H1]1NC(=O)C)OC)OC)(O)CC
PUGREST.BadRequest: error:
O=C(OC)OC1=C2C3=CC=C(C(C=C3[C@H1](CCC2=CC(OC)=C1O)CNC=O)C)OOC(=O)C
PUGREST.BadRequest: error:
O=C(OC)OC1=CC=C2C3=C(C(OC)=C(C=C3CC[C@@H1](C2=CC1=O)OC(=O)C)NC)CC=O
PUGREST.BadRequest: error:
O=C(OC)OC=CC=C1C([C@@H1](NC(=O)C)CCC2=CC(=C(OC)C(OC)=C21)O)C=CC=O
```

```
PUGREST.BadRequest: error:
O=C(OC)OC=CC=C1C2=C(C(OC)=C(C=C2CC[C@@H1](C1=CC=O)NC(=O)C)OCO)C(=O)C
PUGREST.BadRequest: error:
O=C(OC)OC1=CC=C2C3=C(C(OC)=C(C=C3CC[C@@H1](C2=CC1=O)OC(=O)O)CO)SC
PUGREST.BadRequest: error:
O=C(OC1)OC=C2C3=CC=C(C(C=C3[C@H1](CCC2=CC(=C1OC)OC)NC=O)C)OOC
PUGREST.BadRequest: error:
O=C(OC)OC=1C(C=C2C(=CC=1)C3=C(OC)C(=C(OC)C=C3CC[C@@H1]2NC(=O)C)O)C=O
PUGREST.BadRequest: error:
O=C(OC)OCC(C=C1C(C2=C(C=C(OC)C(OC)=C2OC)CC[C@@H1]1NC(C)=O)=CC)=O
PUGREST.BadRequest: error:
O=C(C)N[C@H1]1CCC2=CC(=C(OC)C(=C2C=3C1=CC(C(=CC=3)OC(=O)OC)=O)CN)C(=O)COCC
PUGREST.BadRequest: error:
O=C(C)N[C@@H1]1CC(C2=C(C(=C(OC)C=C2CC1)OC)OC)C=C(SC)C(C)=O
PUGREST.BadRequest: error:
O=C(C(C)C)OC1=C2C3=CC=C(C(C=C3[C@H1](CCC2=CC(OC)=C1O)CNCC)=O)OOC
PUGREST.BadRequest: error:
O=C(C)N[C@@H1]1CC2(C3=C(C(=C(OC)C=C3CC1)OC)OC)C=C(OC)C(C2)=O
PUGREST.BadRequest: error:
O=C(C)N[C@H1]1CCC2=CC(=C(OC)C(=C2C=3C1=CC(C(=CC=3)OC)O)COC(=O)OC)NC(=O)C=O
PUGREST.BadRequest: error:
O=C(CC)OC1=C2CC([C@@H1](NC(C)=O)CCC2=CC(=C1OC)OC)=CC(C(=CC)SC)=O
PUGREST.BadRequest: error:
O=C(C)N[C@H1]1CCC2=CC(=C(OC)C(=C2C=3C1=CC(C(=CC=3)OC(OC)=O)=O)CC)OOC
PUGREST.BadRequest: error:
O=C(C)N[C@H1]1CCC2=CC(=C(OC)C(=C2C=3C1=CC(C(=CC=3)OC)O)COOCOC)C=O
PUGREST.BadRequest: error:
CC(=O)N[C@H1]C1CC2=CC(OC)=C(OC)C(OC)=C2C3=CC=C(C(=O)C=C31)OC
PUGREST.BadRequest: error:
CC(=O)C1=CC=C(SC)C(C=C1[C@H1]CCC=CC(OC)=COC)NC(=O)CSC
PUGREST.BadRequest: error:
C=1C(=O)C2=C(C3=CC=C(SC)C(C=C3[C@H1](CC2)NC=O)COC)C(OC)C=1
PUGREST.BadRequest: error:
CC(=O)N[C@H1]C1CC2=CC(OC)=C(OC)C(OC)=C2C3=CC=C(C(=O)C=C31)O
PUGREST.BadRequest: error:
C1C(=O)C=C(C2=CC=C(SC)C(C=C2[C@H1](NC(C)=O)C)COC)C(OC)C(=C1)SC
PUGREST.BadRequest: error:  CC(=O)C=C(C1=CC2=C(SC)C(C=C1[C@H1](NC(C)=O)C)C)OC2OC
PUGREST.BadRequest: error:  C12=C(C3=CC=C(SC)C(C=C3[C@@H1](N)CC1)=CCOC)C2OC=C
PUGREST.BadRequest: error:
C=12C(=CC(C(SC)=CC=1)=O)[C@H1](CCC3=CC(OC)=C(OC)C(=C32)N)C
PUGREST.BadRequest: error:
C=1C(=O)C(=CC=C2C3=C(OC)C(OC)=CC=C3CC[C@@H1](C2=1)NC=O)COC
PUGREST.BadRequest: error:
C=1C(=O)C(OC(=O)C)=CC=C2C3=C(OC)C(=C(OC)C=C3CC[C@@H1](C2=1)OCNC=O)C
PUGREST.BadRequest: error:
C=12C(=O)C(=CC=C3C=1[C@H1](CCC4=CC(OC)=C(OC)C(OC)=C34)NCC)OC2
PUGREST.BadRequest: error:
CC(=O)OC1=C2C3=CC=C(SC)C(=O)C=C3[C@H1](CCC2=C(OC)C=C1)OCNC(C)=O
```

```
PUGREST.BadRequest: error:
C=12C(=O)C(=CC=C3C=1[C@H1](CCC4=CC(OC)=C(OC)C(OC)=C34)NC(C)=O)C2
PUGREST.BadRequest: error:
C1=C(C(O)=CC=C1CC([C@@H1](NC(=O)C)CC)=CCC(=CC)SC)OOC(=O)CCOC
PUGREST.BadRequest: error:
CCC(=O)OC=C1C2=CC=C(SC)C(=O)C=C2[C@H1](CCC1=C(OC)C=C)OCOC(=O)OC
PUGREST.BadRequest: error:
C=1C=C(OC(=O)OC)C(C=C2C=1C3=C(OC)C(=C(OC)C=C3CC[C@@H1]2NC)C)C=O
PUGREST.BadRequest: error:
CCC(=O)OC=CC=C1C([C@@H1](NC(C)=O)CCC2=CC(OC)=CC(OC)=C21)OC=CC=O
PUGREST.BadRequest: error:
CCC(=O)OC=C1C2=CC=C(SC)C(=O)C=C2[C@H1](CCC1=C(OC)C=C)OCOCNC(=O)C
PUGREST.BadRequest: error:
C=1C=C(OC(=O)OC)C2(C=CC=1C3=C(OC)C(=C(C(OC)=C3CC[C@@H1]2NC)C)O)COC=O
PUGREST.BadRequest: error:
C12C=C(C3=CC=C(SC)C(=O)C=C3[C@@H1](NC(=O)C)C1)C(=C(OC)C(OC)=C2)OC
PUGREST.BadRequest: error:
CCC(=O)N[C@H1]1CCC2=C(C(OC)=C(C(OC)=C2)OC)C=3C1=CC(C(SC)=CC=3)=O
PUGREST.BadRequest: error:
CCC(=O)OC=C1C2=CC=C(SC)C(=O)C=C2[C@@H1](NC(=O)C)CCC1=CC(OC)=COC
PUGREST.BadRequest: error:  C1C=C1C2=CC=C(SC)C(=O)C=C2[C@H1]
PUGREST.BadRequest: error:
C=1C=C(OC(=O)OC)C(C=C2C=1C3=C(OC)C(=C(C=C3CC[C@@H1]2NC(C)=O)OC)O)C=O
PUGREST.BadRequest: error:
CCC(=O)N[C@H1]1CCC2=CC(OC)=C(C(OC)=C2C=3C1=CC(=O)C(=CC=3)SC)OC(=O)OCC
PUGREST.BadRequest: error:
C12C=C(CC[C@H1](NC(=O)C)C1=CC(C(=CC)SC)=O)C=C(OC)C(OC)=C2OC
PUGREST.BadRequest: error:
CC=1C(=CC(C(=CC=1)SC)=O)[C@H1](CCC2=CC(OC)=C(OC)C(=C2)OC)NC(=O)C
PUGREST.BadRequest: error:
CC(C)COCCN[C@H1]1CCC2=CC(OC)=C(C(=C2C=3C1=CC(C(=CC=3)SC)=O)OC)OC(=O)C
PUGREST.BadRequest: error:
CC(C)CCN[C@H1]1CCC2=C(C3=CC=C(SC)C(C=C31)=O)C(=C(C(OC)=C2)OC)OCCC
PUGREST.BadRequest: error:
CC(C)CCN[C@H1]1CCC2=C(C3=CC=C(SC)C(C=C31)=O)C(=C(C(OC)=C2)OC)OC(=O)C
PUGREST.BadRequest: error:
CC(C)C(OC1=C2C3=CC=C(SC)C(=O)C=C3[C@H1](CCC2=CC(OC)=C1OC)NC=O)C=O
PUGREST.BadRequest: error:
C1=C(OC)C(=C(OC)C2=C1CC[C@H1](NC(=O)C)C=3C2=CC=C(OC(=O)OC)C(C=3)=O)C
PUGREST.BadRequest: error:
CC(C)CCN[C@H1]1CCC2=C(C3=CC=C(SC)C(C=C13)=O)C(=C(C(OC)=C2)OC)OCCC
PUGREST.BadRequest: error:
CC(=O)N1[C@H1]CCC2=CC(OC)=C(OC)C(OC(=O)C)=C2C=3C1=CC(C(=CC=3)SC=O)CC=O
PUGREST.BadRequest: error:
C=1C(=CC(C(SC)=CC=1)=O)[C@H1](CCC=CC(OC(=O)C(C)C=COC)C(=C)OCNC=O)CSC
PUGREST.BadRequest: error:
C12=C(C=C(OC)C(OC)=C1OC(=O)C3=CC)CC=C3C=CC=C(CC2O[C@@H1]NC(C)=O)C
PUGREST.BadRequest: error:
```

```
O=C(OC1)OC=CC=C2C3=C(OC)C(=C(OC)C=C3CC[C@@H1](NC(C)=O)C2=CC1)OO
PUGREST.BadRequest: error:
O=C(OC)OC1=CC=C2C3=C(OC)C(=C(OC)C=C3CC[C@@H1](C2=CC1=O)NC=O)COC
PUGREST.BadRequest: error:
O=C(OC1)OC=CC=C2C3=C(OC)C(=C(OC)C=C3CC[C@H1](NC(=O)C)C2=CC1)OO
PUGREST.BadRequest: error:
O=C(OC)OC1=CC=C2C3=C(OC)C(=C(OC)C=C3CC[C@@H1](C2=CC1=O)NCC)OOC
PUGREST.BadRequest: error:
O=C(CC1)OC=CC=C2C3=C(OC)C(=C(OC)C=C3CC[C@H1](NC(=O)C)C2=CC1)OOC
PUGREST.BadRequest: error:
O=C(C)N[C@H1]1CCC2=CC(OC)=C(C(OC)=C2C3=CC=C(C(=O)C=C13)OCCC)OOC
PUGREST.BadRequest: error:
O=C(C)N[C@H1]C1=CC(C(OC(OCC)=O)=CC=C1C2=C(OC)C=C(OC)C=C2CC)OC=O
PUGREST.BadRequest: error:
O=C(C)N[C@H1]CC1C2=CC(OC)=CC(OC)=C2C=CC=C(OC(=O)OCC)C(=O)C=C1
PUGREST.BadRequest: error:
O=C(NC)NCC=CN(C=1OCC)CN(OCCC)CC=CC2=C(OC)C(=C(OC)C=C2CC[C@H1](NC(=O)C)OC=1)C=O
PUGREST.BadRequest: error:
O=C(N(C)C)NC1=CC=C2C3=C(OC)C(=C(OC)C=C3CC[C@@H1](C2=CC1=O)NCC)OOC
PUGREST.BadRequest: error:
O=C(C)N[C@H1]1CCC=C(C2=CC=C(SC)C(C=C12)=O)C(OC(CC)=O)=C(C(OC)=C)OC
PUGREST.BadRequest: error:
O=C(N)N[C@H1]1CCC2=CC(OC)=C(C(OC)=C2C=3C1=CC(C(OC(=O)C)=CC=3)=O)OC
PUGREST.BadRequest: error:
O=C(C)N[C@H1]1CCC2=CC(OC)=C(C(OC)=C2C3=CC=C(C(=O)C=C31)OC(=O)O)CCOC
PUGREST.BadRequest: error:
O=C(C)N[C@H1]1CCC2=CC(OC)=C(C(OC)=C2C=3C1=CC(C(OC(=O)CC)=CC=3)O)C=O
PUGREST.BadRequest: error:
O=C(N)N[C@H1]1CCC2=CC(OC)=C(C(OC)=C2C3=CC=C(C(C=C31)=O)O)C(=O)OCNC
PUGREST.BadRequest: error:
CC(=O)N1[C@H1]CCC2=CC(OC)=C(OC)C(OC)=C2C3=CC=C(NC)C(C=C31)=O
PUGREST.BadRequest: error:
C=1C(=CC=C(C2=O)OCC[C@H1]CCC3=CC(OC)=C(OC)C(OC)=C3C2)CC=1SC
PUGREST.BadRequest: error:
C1C(=O)OCC2=CC=CC3=C(OC)C(OC)=C(OC)C=C3CC[C@H1](NC(C)=O)C2=CC1=O
PUGREST.BadRequest: error:
CC(=O)OCC=CC=C1C2=C(OC)C(OC)=C(OC)C=C2CC[C@H1](NC(C)=O)C1=CSC
PUGREST.BadRequest: error:
CC(=O)N1[C@@H1]C2=CC(C(NC)=CC=C2C3=C(C(OC)=C(OC)C=C3CC1)OC)NC=O
PUGREST.BadRequest: error:
CC(=O)OC=CC=C1C2=C(OC)C(OC)=C(OC)C=C2CC[C@H1](NC(C)=O)C1=CCSC=O
PUGREST.BadRequest: error:
CC(=O)N[C@H1]C1CC2=CC(OC)=C(OC)C(OC)=C2C3=CC=C(SC)C(C=C31)=O
PUGREST.BadRequest: error:
C12=C(C=3C([C@H1])(C4C1)N=C(Cl)C=C4CCC(SC)=CC=3)OC=C2OCNC
PUGREST.BadRequest: error:  C1OC=C2C3=CC=C(NC)C(=O)C=C3[C@H1](CCC2=CC(OC)=C1OC)N
PUGREST.BadRequest: error:  CNCCC=C1C(C2=C(OC)C(OC)=CC=C2CC[C@@H1]1NOC)=O
PUGREST.BadRequest: error:  C1OC=CC2=C(CC(O)CC1=CC=C3C(OC)=C(OC)C)P=C3C2OCSC
```

```
PUGREST.BadRequest: error:  CSCC=CC=CC1=C(OC)C=C(C=C1CC[C@@H1]NC(N)=NOCOC)O
PUGREST.BadRequest: error:  C1OC=C2CC([C@@H1](N)CCC2=CC(OC)=C1OC=CC)(C(=CC)SC)O
PUGREST.BadRequest: error:
COC1=C2C3=CC=C(NC)C(=O)C=C3[C@H1](CCC2=CC(OC)=C1OC)NO
PUGREST.BadRequest: error:
C=C1CC2[C@H1](CCC3=C(C(OC)=C(OC)C(=C3)OC)C2)CC=C1N=[N+1]=[N-1]
PUGREST.BadRequest: error:
C=1NCC(C=C2C(C3=C(OC)C=C(C=C3CC[C@@H1]2N=[N+1]=[N-1])OC)OC=C)C=1O
PUGREST.BadRequest: error:  COC=C1C2=CC=C(NC)C(=O)C=C2[C@H1](CCC1C=COC)N3N=NC=C3
PUGREST.BadRequest: error:
COC=C1C2=CC=C(SC)C(=O)C=C2[C@@H1](NCCCCCCC)CCC1=CC(=COC)SC
PUGREST.BadRequest: error:
COC=C1C2=CC=C(NC)C(=O)C=C2[C@@H1](N=[N+1]=[N-1])CCC1=CC(=COC)OC
PUGREST.BadRequest: error:
C1OC=CC2=C(C3=C(OC)C(OC)=CC=C3CC[C@@H1]2N=[N+1]=[N-1])CC=C1NC
PUGREST.BadRequest: error:
C=CCC1[C@H1](CC=[N+1]C2=C(C(OC)=C(OC)C=C2C1O)C=O)[N-1]CCC
PUGREST.BadRequest: error:
C=12[C@@H1](C=CC(=O)C(C)=CC=1C3=C(CC2)C=C(OC)C(OC)=C3OCNC)CNC=C
PUGREST.BadRequest: error:
C1[C@@H1](C2=CC(=O)C(SC)=CC=C2C3=C(C=C(OC)C(=C3OC)OC)C1N)CCNC
PUGREST.BadRequest: error:
COC1=C(OC)C(OC)=C2CCC[C@H1](N3C=CN=N3)C4=CC(=O)C(=CC=C4C21)NC
PUGREST.BadRequest: error:
C12=CC(C(=CC=C1C3=C(C=C(OC)C(OC)=C3OC)CC[C@@H1]2NCCO)C)NC
PUGREST.BadRequest: error:
C1[C@@H1](C2=CC(=O)C(NC)=CC=C2C3=C(C=C(OC)C(=C3OC)OC)C1)NCCSC
PUGREST.BadRequest: error:
N(C1=CC=C2C3=C(CC[C@@H1](C2=CC1=O)N4C=CN=N4)C=C(OC)C(OC)=C3O)CC
PUGREST.BadRequest: error:
C=1=C2C([C@H1](CCC3=CC(=C(OC)C(=C32)OC)OC)NCCCCCC)CC=CC(C=1SC)=O
PUGREST.BadRequest: error:
N1C(COC(=O)OC=CN1[C@@H1]2)C3=CC(=O)C(NC)=CC=C3C4=C(CC2)C=C(C(OC)=C4OC)OC
PUGREST.BadRequest: error:
N1C(COC(=O)OC=CN1[C@@H1]2)C3=CC(=O)C(NC)=CC=C3C4=C(CC2)C=C(C(=C4OC)OC)OC
PUGREST.BadRequest: error:
CNC(C(C)O)N[C@@H1]1CC(C2=C(C(=C(OC)C=C2CC1)OC)OC)=CC=C(NC)C(C)=O
PUGREST.BadRequest: error:
C=C1C([C@H1](CCC2=CC(=C(OC)C(=C21)OC)OC)NCCCCCC)CC=CC(C(=C)SC)=O
PUGREST.BadRequest: error:
COC1=C(OC)C(=CC=2CC[C@@H1](C3=CC(C(=CC=C3C=21)NC)=O)N4C=C(N=N4)CCC)NCOC
PUGREST.BadRequest: error:
CNC(C(C)O)ON(C=1C(C=C2C(=CC=1)C3=C(OC)C=C(C=C3CC[C@@H1]2NCC)CC)O)COC=O
PUGREST.BadRequest: error:
C1=C2C(C3=C(C=C(OC)C(=C3OC)OC)CC[C@@H1]2N4C=C(N=N4)CCCC=C)C=C(NC)C1=O
PUGREST.BadRequest: error:
CNC(C(C)O)N([C@H1]1CCC2=CC(=C(OC)C(OC)=C2C=3C1=CC(=O)C(=CC=3)NC)OCC)C
PUGREST.BadRequest: error:
```

```
CNC(C(C)O)N[C@@H1]1C=2C(C3=C(C=C(OC)C(OC)=C3OC)CC1)=CC=C(C(=O)C=2)NC
PUGREST.BadRequest: error:
C=C1C(C2=C(C=C(OC)C(=C2OC)OC)CC[C@@H1]1NCCCCCCCCCCCC)=CC=C(SC)C=O
PUGREST.BadRequest: error:
CNC(=S)N[C@@H1]1C2=CC(=O)C(NC)=CC=C2C3=C(OC)C(=C(C=C3CC1)OC)OCO
PUGREST.BadRequest: error:
C1=2C3=C(C(=C(C=C3CC[C@H1](NCCCCCCCC)C1=CCC(=CC=2)NC)OO)CO)COC
PUGREST.BadRequest: error:
C12=CC(C(NC)=CC=C1C3=C(C=C(C(OC)=C3OC)OC)CC[C@@H1]2N4C=C(N=N4)COO)NC
PUGREST.BadRequest: error:
N([C@H1]1CCC2=C(C3=CC=C(C(C=C13)=O)NC)C(OC)=C(OC)C(OC)=C2)N=N
PUGREST.BadRequest: error:
COC=C(OC)C(OC)=C1CCC[C@@H1](C2=CC(=O)C(NC)=CC=C12)NCCCCCC
PUGREST.BadRequest: error:
C=C1C([C@H1](CCC2=CC(=C(OC)C(=C21)OC)OC)N3N=NC=C3)CCl
PUGREST.BadRequest: error:
COC=C(OC)C(OC)=C1CCC[C@@H1](C2=CC(=O)C(SC)=CC=C12)NCCCCCCCC
PUGREST.BadRequest: error:
COC=C(OC)C(OC)=C1CCC[C@@H1](C2=CC(=O)C(NC)=CC=C12)NCCCCCCCC
PUGREST.BadRequest: error:
C12C3=CC(OC)=C(OC)C=C3CC([C@H1]1NCCCCCCCCC)=CC(=C2OC)OC
PUGREST.BadRequest: error:
C12=CC(C(NC)=CC=C1C3=C(C=C(C(OC)=C3OC)OC)CC[C@@H1]2NCCCCCCCCCCF)=O
PUGREST.BadRequest: error:
C12=CC(C(NC)=CC=C1C3=C(C=C(C(OC)=C3OC)OC)CC[C@@H1]2N4C=C(N=N4)CCl)NC
PUGREST.BadRequest: error:
C1=C2C(C3=CC=C(C(=O)C=C3[C@H1](CC2)NCCC4=CC=CC=C4)OS)CC(OC)=C(OC)C(=C1)OC
PUGREST.BadRequest: error:
COC1=C(OC)C(OC)=CC=2CC[C@@H1](C3=CC(=O)C(NC)=CC=C3C=21)NCCC4=CC=CC=C4NC
PUGREST.BadRequest: error:
C1[C@@H1](C2=CC(=O)C(SC)=CC=C2C3=C(C(=C(OC)C=C3C1)OC)OC)NCCC=CC=CC
PUGREST.BadRequest: error:
CNC(C(C)C)N[C@@H1]1C2=CC(=O)C(NC)=CC=C2C3=C(C(=C(OC)C=C3CC1)OC)OC
PUGREST.BadRequest: error:
C=C1C([C@H1](CCC2=CC(=C(OC)C(=C21)OC)OC)NCCCCC=C)C(C(SC)=C)=O
PUGREST.BadRequest: error:
C=C1C([C@H1](CCC2=CC(=C(OC)C(=C21)OC)OC)NCCCCCC)C=CC(C(SC)=C)=O
PUGREST.BadRequest: error:
COC1=C(OC)C(OC)=CC=2CC[C@@H1](C3=CC(=O)C(NC)=CC=C3C=21)N4C=C(N=N4)CCONC
PUGREST.BadRequest: error:
C12=CC(C(NC)=CC=C1C3=C(C=C(C(OC)=C3OC)OC)CC[C@@H1]2N4C=C(N=N4)CCC)Cl
PUGREST.BadRequest: error:
CN(C(=S)N[C@@H1]1C2=CC(=O)C(NC)=CC=C2C3=C(C(=C(OC)C=C3CC1)OC)OC)CC#N
PUGREST.BadRequest: error:
C1OC=C(OC)C(OC)=C2C1CC[C@@H1](C3=CC(=O)C(NC)=CC=C23)NCCCCCCCC=C
PUGREST.BadRequest: error:
C[C@H1](OCCC)CCC1=C(OC)C(OC)=C(OC)C=C1CC=CC=CC(=O)CSCN
PUGREST.BadRequest: error:
```

```
CC(C)(C)OC1=CN(N=N1)[C@H1]C2CC3=CC(OC)=C(OC)C(OC)=C3C4=CC=C(NC)C(=O)C=C42
PUGREST.BadRequest: error:  C=C(OC)C1=C(OC)C=2C3=CC=C(SC)CC=C3[C@H1](CCC1=2)C=O
PUGREST.BadRequest: error:
CC(C)CCCCCCN[C@@H1]1C2=CC(C(SC)=CC=C2C3=C(C(OC)=C(OC)C=C3CC1)OC)=O
PUGREST.BadRequest: error:
CC(C)(C)C(=O)N[C@@H1]1C=2C(C3=C(C(OC)=C(OC)C=C3CC1)OC)=CC=C(NC)C(=O)C=2
PUGREST.BadRequest: error:
C1=C(OC)C(OC=2)=C(OC)C3CC([C@H1](CCC3=C1)NC(C)=O)SC=CC=2OSC
PUGREST.BadRequest: error:
C=CCCOCC(C=C1[C@H1](CCC2=CC(OC)=C(OC)C(OC)=C2C1=CCN)C(=O)C=O)SC=O
PUGREST.BadRequest: error:
OC(CCC(C)C)OCC1=CN(N=N1)[C@H1]CCC2=CC(OC)=C(OC)C(OC)=C2C3=CC=C(C(=O)C=C3)NC
PUGREST.BadRequest: error:
O=C(NCC1=CC=CC=C1Cl)N[C@H1]2CCC3=C(C(OC)=C(OC)C(OC)=C3)C4=CC=C(NC)C(=O)C=C24
PUGREST.BadRequest: error:
N([C@H1]1CCC2=C(C(OC)=C(OC)C(OC)=C2)C3=CC=C(NC)C(C=C13)=O)C(O)=O
PUGREST.BadRequest: error:
O=CC=CCC=CNNC(N[C@H1]1CCC2=CC(OC)=C(OC)C(OC)=C2C3=CC=C(NC)C(=O)C=C13)=O
PUGREST.BadRequest: error:
C12[C@H1]CC(C3=C(OC)C(OC)=C(OC)C=C3C1=CC=COC(OCC)=O)=C2SC=CC=O
PUGREST.BadRequest: error:
OC(CCC1(C)C)OC=C(C(OC)=C2CCC[C@H1](NC(C)=O)C3=CCC(SC)=CC=C3C21)O
PUGREST.BadRequest: error:
OC(CCC1(C)C)OC=C(C(OC=2)=CCCC[C@H1](NC(C)=O)C3=CCC(SC)=CC=C3C=21)O
PUGREST.BadRequest: error:
O=C(C(=O)OCC)C1OC=CC=CC2=C(OC)C(OC)=C(OC)C=C2CC[C@H1](NC(=O)C)C=CC1=O
PUGREST.BadRequest: error:
C=CCC(=O)OCCC=C1[C@H1](CCC2=C(C(OC)=C(OC)C(OC)=C2)C1=C)N=O
PUGREST.BadRequest: error:
C12[C@H1]CC(C3=C(OC)C(OC)=C(OC)C=C3C1=CC=C(SC)C)(C=C2)ONC(=O)C
PUGREST.BadRequest: error:
C=C1C(C2=CC=C(C(=O)C=C2[C@@H1](NCCCCCCCCCCC)CC1)COC)C(OC)=COCOC
PUGREST.BadRequest: error:
C=1N([C@H1]2CCC3=C(C4=CC=C(C(C=C24)=O)NC)C(OC)=C(OC)C(=C3)OC)N=NC=1C
PUGREST.BadRequest: error:
C1=C2C(C3=CC=C(C(=O)C=C3[C@H1](CC2)N4C=C(N=N4)CC(Cl)Cl)ONC)C(OC)=C(OC)C(=C1)OC
PUGREST.BadRequest: error:
C1=C2C(C3=CC=C(C(=O)C=C3[C@H1](CC2)N4C=C(N=N4)C=CC=CC=N)C=C)OCNC(OC)=C1OC
PUGREST.BadRequest: error:
C=1N([C@H1]2CCC3=C(C(=C(OC)C(OC)=C3)OC)C=4C2=CC(C(=CC=4)NC)=O)N=NC=1C
PUGREST.BadRequest: error:
C=C1C(C2=CC=C(C(=O)C=C2[C@H1](CC1)N3C=C(N=N3)C=CC=CC=N)C=C)OCNCOCOCC
PUGREST.BadRequest: error:
C1C2=CC(OC)=C(OC)C(=C2CC([C@H1](C1)NC/C=C/C3=CC=CC=C3)=CC(=O)C(=CC)SCO)C
PUGREST.BadRequest: error:
C1=C2C(C3=CC=CC(=O)C=C3[C@H1](CC2)N4C=C(N=N4)C=CC=CC=C)(OC)C(OC)=C1OCNC
PUGREST.BadRequest: error:
C=CC=CC(=O)OCCC=C1[C@H1](CCC2=CC(OC)=C(OC)C(OC)=C2C1=CN)OSCC
```

```
PUGREST.BadRequest: error:
N1=CC=C(C=C1)C(OC=CC=C2C3=C(OC)C(OC)=C(OC)C=C3CC[C@@H1](C2=CC=O)NC(=O)CSC)=O
PUGREST.BadRequest: error:
C=[C@H1]C1=CCC(OC(C2=CC=CC=C2)=O)=CC=C1C3=C(OC)C(OC)=C(OC)C=C3CC
PUGREST.BadRequest: error:
OC1=C(C=CC=C1)C=2N=NN(C=2)[C@H1]C3CC4=CC(OC)=C(OC)C(OC)=C4C5=CC=C(NC)C(=O)C=C53
PUGREST.BadRequest: error:
C12=CC=C(S1=C3)C(=O)C=C2[C@H1](CCC=CC(OC)=C(OC)C(OC)=C3)NC(C)=O
PUGREST.BadRequest: error:
OC=C(SC)C(C(OC=1)=CCC2([C@H1](NC(C)C)C=CCC2=CC=C=1)NC=O)NCOC
PUGREST.BadRequest: error:
OC1=C(C=CC=C1)C=2N=NN(C=2)[C@H1]CCC3=CC(OC)=C(OC)C(OC)=C3C4=CC=C(NC)C(=O)C=C4
PUGREST.BadRequest: error:
CNCC(O)(C1OC=CC=C1CN[C@@H1]2C3=C)C(C(NC)=CC=C3C4=C(C(OC)=C(OC)C=C4CC2)OC)=O
PUGREST.BadRequest: error:
C=CC1=C(COC2=CC(CC[C@H1](NC(C)=O)C3=CC(C(SC)=CC=C32)=C)C(OC)=C1OOC)OC
PUGREST.BadRequest: error:
C=CC=CCOC=CC=C1C([C@H1](CCC2=CC(OC)=C(OC)C(OC)=C21)NC(=O)C)=O
PUGREST.BadRequest: error:
C1[C@@H1](C2=CC(=O)C(NC)=CC=C2C3=C(C(=C(OC)C=C3C1)OC)OC)N4C=C(N=N4)COC
PUGREST.BadRequest: error:
C=1N([C@H1]2CCC3=C(C(=C(OC)C(OC)=C3)OC)C=4C2=CC(=O)C(=CC=4)NC)N=NC=1C
PUGREST.BadRequest: error:
NC(C1OC(=O)OC=CN1[C@@H1]2)C3=CC(C(=CC=C3C4=C(CC2)C=C(C(OC)=C4OC)OC)NC)=O
PUGREST.BadRequest: error:
C=C1C(C2=CC=C(C(=O)C=C2[C@H1](CC1)N3C=C(N=N3)COCC4=CC=CC=C4)COC)C(OC)=COCNC
PUGREST.BadRequest: error:
C1=CC(C2=CC=C(C(=O)C=C2[C@@H1](N3C=C(N=N3)C4=NC=CC=C4)C1N)C=C)(OC)C(OC)=COC
PUGREST.BadRequest: error:
C1=C2C(C3=CC=C(C(C=C3[C@H1](CC2)N4C=C(N=N4)C5=CC=CC=N5)N)C=C)(C(=C1OC)OC)OC
PUGREST.BadRequest: error:
C=1N([C@H1]2CCC=C(C3=CC=C(C(C=C23)=O)NC)C(OC)=C(OC)C(=C)OC)N=NC=1C(=O)OC
PUGREST.BadRequest: error:
O1CC=CC=C1CN[C@H1]2CCC3=CC(OC)=C(OC)C(OC)=C3C4=CC=C(NC)C(C=C24)=O
PUGREST.BadRequest: error:
O=C(OCC)CN(OCC)C(CN[C@@H1]1C2=CCC(NC)=CC3=C2C=C(CC1)C=C(OC)C(=C3OC)N)C=O
PUGREST.BadRequest: error:
O1CC=C2C=C1C3=C(OC)C(OC)=C(OC)C=C3CC[C@H1](CCCC=C2O)CN=CCSC
PUGREST.BadRequest: error:  OC=CC=CC1=C(C=C(OC)C(OC)=C1OC(OCC)=O)NC(C)=O
PUGREST.BadRequest: error:
O=C(C(C)C=1)OCCC=C2[C@H1](CCC3=CC(OC)=C(OC)C(OC)=C3C2=CC=1N)ONC
PUGREST.BadRequest: error:
O=C(OC(C)(C)C)N[C@H1]1CCC2=C(C(OC)=C(OC)C(OC)=C2)C3=CC=C(NC)C(=O)C=C13
PUGREST.BadRequest: error:
O=C(C(C)C=1)OCCC=C2[C@H1]CCC3=CC(OC)=C(OC)C(OC)=C3C2=CC=1N
PUGREST.BadRequest: error:
O=C(OCC)CN(OCC)C[C@@H1]1C2=CC(C(NC)=CC=C2C3=C(C(OC)=C(OC)C=C3CC1)OC)=O
PUGREST.BadRequest: error:
```

```
O=C(C)OC1=CN(N=N1)[C@H1]2CCC3=CC(OC)=C(OC)C(OC)=C3C4=CC=C(NC)C(=O)C=C24
PUGREST.BadRequest: error:
O=C(O)C=C(OC)CC=C1C(C2=C(OC)C(OC)=C(OC)C=C2CC[C@@H1]1N=CCS)C=O
PUGREST.BadRequest: error:
O=C(C)N[C@@H1]1C2=CCC(SC)=CC=C2C3=C(C(OC)=C(OC)C=C3CC1)OCSC=O
PUGREST.BadRequest: error:
O=C(C)N[C@@H1]1C2=CCC(SC)=CC=C2C3=C(C(OC)=C(OC)C=C3CC1)OCSCOC=O
PUGREST.BadRequest: error:
O=C(O)C=C(C(OC1C)=O)[C@H1]CCC2=CC(OC)=C(OC)C(OC)=C2C3=CC=C(SC)C(=O)C=C31
PUGREST.BadRequest: error:
O=C(C)N[C@@H1]1C2=CCC(SC)=CC=C2C3=C(C(OC)=C(OC)C=C3CC1)OCOC=O
PUGREST.BadRequest: error:
O=C(OCC)CN(CCC)C[C@H1]C1CC2=CC(OC)=C(OC)C(OC)=C2C3=CC=C(SC)C(=O)C=C31
PUGREST.BadRequest: error:
O=C(C(C)C=1)OCCC=C2[C@H1](CCC3=CC(OC)=C(OC)C(OC)=C3C2=CC=1)NSC=O
PUGREST.BadRequest: error:
C1=C2CC(OC(C3=CC=CC=C3)=O)=CC=C1C4=C(OC)C(OC)=C(OC)C=C4CC[C@H1](NC(=O)C)C2=O
PUGREST.BadRequest: error:
C1=CCC(OC(C2=CC=CC=C2)=O)=CC=C1C3=C(OC)C(OC)=C(OC)C=C3CC[C@H1](NC(=O)C)C=O
PUGREST.BadRequest: error:
C1N([C@@H1]2C3=CCC(SC)=CC=C3C4=C(OC)C(OC)=C(C=C4CC2)OCN)C(C(O)=O)=C1O
PUGREST.BadRequest: error:
C1=CC(C=C(C1(OC(C)=C=2)OC(=O)CCC=C3N=C4CNC)CC=C3C=2C(OC)=COC)C=C4NC=O
PUGREST.BadRequest: error:
C1N([C@@H1]2C3=CCC(SC)=CC=C3C4=C(OC)C(OC)=C(C=C4CC2)OC=O)N(C(C)C)OC1
PUGREST.BadRequest: error:
C1[C@@H1](C2=CCC(SC)=CC=C2C3=C(OC)C(OC)=C(C=C3C1)OC=O)NC(C(=O)O)=CNC
PUGREST.BadRequest: error:
N1([C@@H1]CCC1=C2C=C(CNC)CC=C2C3=C(OC)C(OC)=C(OC)C=C3C)NC(Cl)=O
PUGREST.BadRequest: error:  OC(OC(C)CC12)=C(C(OCC)=O)N=NN1[C@H1]3CCC4=CC(OC)=C(O
C)C(OC)=C4C=CC=C(NC)C(=O)C=C32
PUGREST.BadRequest: error:
C=C1C(C2=C(OC)C(OC)=C(OC)C=C2CC[C@@H1]1NC(C(O)=O)=C(NCO)OC=CC=CC(=O)C)SCNC
PUGREST.BadRequest: error:  O=C(C(Cl)Cl)NC(=O)N[C@H1]1CCC2=C(C(OC)=C(OC)C(OC)=C2
)C3=CC=C(C(C=C31)=O)NCN(O)CCC=O
PUGREST.BadRequest: error:  O=C(C(Cl)Cl)NC(=O)N[C@H1]1CCC2=C(C(OC)=C(OC)C(OC)=C2
)C3=CC=C(C(C=C31)=O)NCC4=CC=CC=C4
PUGREST.BadRequest: error:
O=C(C(Cl)Cl)NCC1=CN(N=N1)[C@H1]2CCC3=CC(OC)=C(OC)C(OC)=C3C4=CC=C(C(=O)C=C24)NCCO
PUGREST.BadRequest: error:
O=C(OCCC)OCC1=CN(N=N1)[C@H1]2CCC3=CC(OC)=C(OC)C(OC)=C3C4=CC=C(C(=O)C=C24)NCCO
PUGREST.BadRequest: error:
O(C=CC=CC1=C2C(OC)=C(OC)C=C1CC[C@H1](NC(N(CCO)CCO)=S)C2=CCCNC)ONCC
PUGREST.BadRequest: error:  O=C(OCCC)OCC1=CN(N=N1)[C@H1]2CCC3=CC(OC)=C(OC)C(OC)=
C3C4=CC=C(C(=O)C=C42)NCC(=O)C
PUGREST.BadRequest: error:  O=C(OCC=C)NCC1=CN(N=N1)[C@H1]2CCC3=CC(OC)=C(OC)C(OC)
=C3C4=CC=C(C(=O)C=C24)NCC(=O)C
PUGREST.BadRequest: error:  O=C(C(Cl)Cl)NCC1=CN(N=N1)[C@H1]2CCC3=CC(OC)=C(OC)C(O
```

```
C)=C3C4=CC=C(C(=O)C=C24)NCC(=O)C
PUGREST.BadRequest: error:  O=C(C(Cl)Cl)N1C(=O)N[C@H1]2CCC3=C(C(OC)=C(OC)C(OC)=C
3)C4=CC=C(C(C=C42)=O)NCC=CC=C1
PUGREST.BadRequest: error:  O=C(C(Cl)Cl)NCC1=CN(N=N1)[C@H1]2CCC3=CC(OC)=C(OC)C(=
C3C=4C2=CC(C(NC)=CC=4)=O)OCCCl
PUGREST.BadRequest: error:  O=C(OCC=C)NCC1=CN(N=N1)[C@H1]2CCC3=CC(OC)=C(OC)C(OC)
=C3C4=CC=C(C(C=C24)=O)NCC(=O)C
PUGREST.BadRequest: error:
O=C(C)N[C@@H1]1CC(C2=C(C(OC)=C(OC)C=C2CC1)OCC3=CC=CC=C3)=O
PUGREST.BadRequest: error:
O=C(C)N[C@@H1]1C=2C(C3=C(C(OC)=C(OC)C=C3CC1)OC(C)=O)C=C(SC)C(C=2)=O
PUGREST.BadRequest: error:
O=C(C(Cl)Cl)NC(=O)N[C@H1]1CCC2=CC(OC)=C(OC)C(OC)=C2C3=CC=C(C(C=C31)=O)NCC(=O)O
PUGREST.BadRequest: error:
O=C(C(C)C)OCC(C=C1[C@H1](CCC2=CC(OC)=C(OC)C(O)=C2C1=CC=CO)CSCC)=O
PUGREST.BadRequest: error:
O=C(OCC)C1=CN(N=N1)[C@H1]2CCC3=CC(OC)=C(OC)C(OC)=C3C4=CC=C(C(C=C24)=O)NCC(=O)C
PUGREST.BadRequest: error:
O=C(C)N[C@@H1]1C=2C(C3=C(C(OC)=C(OC)C=C3CC1)CC=C(SC)C)(C=2)ONC(C)=O
PUGREST.BadRequest: error:
O=C(O)CN(CCO)C(=O)N[C@H1]1CCC2=CC(OC)=C(OC)C(OC)=C2C3=CC=C(C(C=C13)=O)NCC
PUGREST.BadRequest: error:
O=C(OCC)C=C(C(OCC)=O)N[C@H1]1CCC2=CC(OC)=C(OC)C(OC)=C2C3=CC=C(C(C=C31)S)CC
PUGREST.BadRequest: error:
O=C(O)C=C(OC)C(C(NC)=CC=CC1=C(OC)C(OC)=C(OC)C=C1CC[C@@H1](C)NCC)=O
PUGREST.BadRequest: error:
O=C(OCC(C)COC1=C)N(N=N1)[C@H1]2CCC3=CC(OC)=C(OC)C(OC)=C3C4=CC=C(C(=O)C=C24)NC
PUGREST.BadRequest: error:
O(C=CC=CC1=C(OC)C(OC)=CC=C1CC[C@H1](CC=COC(C2=CC=CC=C2)=O)C(OC)=C)OSC=O
PUGREST.BadRequest: error:
CCCCCCCCCCCCC=1CCCOCC(C=C2[C@H1](CCC3=CC(OC)=C(OC)C(OC)=C3C2=CC=1N)C(=O)C=O)SC
PUGREST.BadRequest: error:
CCCCCCCCCCCCCCCC=1OCC(C=C2[C@H1](CCC3=CC(OC)=C(OC)C(OC)=C3C2=CC=1N)C(=O)C=O)SC
PUGREST.BadRequest: error:
CC(C)OC(NCC1=CN(N=N1)[C@H1]2CCC3=CC(OC)=C(OC)C(OC)=C3C4=CC=CC(C=C24)=O)NC=O
PUGREST.BadRequest: error:
C1=C(OC)C(OC)=C(OC)C=C1CC[C@@H1](C2=CC(C(OC(C3=CC=CC=C3)=O)=CC=C2)N)C=O
PUGREST.BadRequest: error:
OC1=C(OC)C(OC)=CC=C1C2=CC=C(NC)CC=C2[C@@H1](NC(C(C)C)=O)NCC
PUGREST.BadRequest: error:
C1=C(OC2)C=C(OC)C=C1CC[C@H1](NC(C(OCC)=O)=C(NCO)OOC3=CCC(NC)=CC=C23)OOC
PUGREST.BadRequest: error:
C12=C(OC)C(OC)=C(OC)C=C1CC[C@H1](NC2(C(O)=O)CC=CCC(NC)=C)OSC
PUGREST.BadRequest: error:
C12=C(OC)C(OC)=C(OC)C=C1CC[C@H1](NC(C(O)=O)(O)CC3=CC(OC)=C(C=C3C2)N)C=O
PUGREST.BadRequest: error:
C1=C(OC)C(=CC=C1C2=C(C(OC)=C(OC)C=C2CC[C@H1](C=CC(C(O)=CC)O)C(C(C)C)=O)S)C
PUGREST.BadRequest: error:
```

```
OC1=C(OC)C(OC)=CC=C1C2=CC=C(NC)CC=C2[C@@H1](N3C(C(OCC)=O)=C(N=N3)C=O)OC
PUGREST.BadRequest: error:
COC(NCCCOCC1=CN(N=N1)[C@H1]2CCC3=CC(OC)=C(OC)C(=C3C4=CC=C(NC)C(C=C24)=O)OC=O)=O
PUGREST.BadRequest: error:  C(=CC=CN(COCC)OCC1)=CN(N=N1)[C@H1]2CCC3=C(C(OC)=C(OC
)C(OC)=C3)C4=CC=C(C(C=C24)=O)NC
PUGREST.BadRequest: error:  C=1C(C)(C)OC(=O)/N=C(/N[C@H1]2CCC3=C(C(OC)=C(OC)C(OC
)=C3)C4=CC=C(NC)C(=O)C=C42)N=1
PUGREST.BadRequest: error:  C=1C(C)(C)OC(=O)/N=C(/N[C@H1]2CCC3=C(C(OC)=C(OC)C(OC
)=C3)C4=CC=C(C(C=C24)=O)NC)N=1
PUGREST.BadRequest: error:
C=1C(C)OC(=O)/N=C(/N[C@H1]2CCC3=C(C(OC)=C(OC)C(OC)=C3)C4=CC=C(NC)C(=O)C=C42)N=1
PUGREST.BadRequest: error:
C=1NC=2CC=C3[C@H1](CCC4=C(C(OC)=C(OC)C(=C4)OC)C3=CC=2)NC(C(=O)O)C=CN=1
PUGREST.BadRequest: error:
O=CC=CC=CC(NCC1=CN(N=N1)[C@H1]CCC2=CC(OC)=C(OC)C(OC)=C2C3=CC=C(C(=O)C=C3)N)C=O
PUGREST.BadRequest: error:
O=CC=CC1=CC2=C(SC)CC=C1[C@H1](CC3C=CC(OC)=C(C(=C32)C=O)OCN)NOCNCO
PUGREST.BadRequest: error:
CNCC1(C2=CC=C(C=C2)NC(=O)N[C@H1]C3CC4=CC(OC)=C(OC)C(OC)=C4C=CC=C(NC)C(=O)C=C31)C
PUGREST.BadRequest: error:
C1=CN=CC=C1C2=CN(N=N2)[C@H1]3CCC4=C(C(OC)=C(OC)C(OC)=C4)C5=CC=C(C(=O)C=C35)NCO
PUGREST.BadRequest: error:
COC=CC1CC[C@H1](NC(=O)NC=C[C@@H1](OC(C=CC=C)=O)C2=CC(OC)=C(OC)C(OC)=C2)C1NC=O
PUGREST.BadRequest: error:
O=CC=C1C=CC(NCC2=CN(N=N2)[C@H1]C3CC4=CC(OC)=C(OC)C(OC)=C4C=CC=C(NC)C(=O)C=C31)NC
PUGREST.BadRequest: error:
CNC=CC=C1C[C@H1](CCC2=CC(OC)=C(OC)C(=C12)OC)N3C(C(O)=O)=C(N=N3)C(=O)OONC
PUGREST.BadRequest: error:
CNC=C1C=C2C[C@H1](CCC3=CC(OC)=C(OC)C(=C23)OCC(=O)OC=C)C1=O
PUGREST.BadRequest: error:
COC=CC1C=2C[C@H1]NC(=O)NC3=CC=C(Cl)C=C3C4=CCC(NC)=CC=C4C=2C(OC)=C1OCNC=O
PUGREST.BadRequest: error:
C=NC=CCC1OCCC=C2[C@H1](CCC3=C(C(OC)=C(OC)C(OC)=C3)C2=C1)N=O
PUGREST.BadRequest: error:
COC=C(OC)C(OC)=CCCC[C@H1](N1C=C(N=N1)COC(=O)C2=CC=NC=C2)C3=CC(=O)C(=CC=C3CNCN)C
PUGREST.BadRequest: error:
C12=CC(C=3C([C@@H1](NCC4=CC=C(C)C=C4CCC1)C2)=CC(=O)C(=CC=3)SC=C)(OC)C(OC)=COC
PUGREST.BadRequest: error:
C12=C3C(C4=CC=C(C(=O)C=C4[C@H1](CC3)N5C=C(N=N5)COC(=O)C6=CC=NC=C6)C1)NC=C2OCOCOC
PUGREST.BadRequest: error:
COC1=C(OC)C=CC=2CC[C@@H1](C3=CC(=O)C(NC)=CC=C3C1=2)N4C=C(N=N4)COCC5=CC=CC=C5OC
PUGREST.BadRequest: error:
C1=C2C(CC([C@H1](CC2)N3N=NC(=C3)C4(O)CCCCC4)=O)CC(OC)=C(C(=C1)OC)OC
PUGREST.BadRequest: error:
C=C1C(C=C(CC[C@@H1]1N2N=NC(=C2)C3=CC=C(Cl)C=C3)C=C(C(=COC)OC)OC)=CC(C(NC)=C)=O
PUGREST.BadRequest: error:
C=C1C(C2=C(CC[C@@H1]1N3C=C(N=N3)COCC4=CC=CC=C4)C=C(C(OC)=C2OC)OC)=CC=C(CN)C=O
PUGREST.BadRequest: error:
```

```
CC(C)COC=1CC=C2[C@H1](CCC3=C(C(OC)=C(OC)C(OC)=C3)C2=CC=1)NSC=O
PUGREST.BadRequest: error:
CC(C)(O)C=CN([C@H1]1CCC2=CC(OC)=C(OC)C(OC)=C2C3=CC=C(C(C=C13)=O)NC)O
PUGREST.BadRequest: error:  CCCCCCCCCCCCCCCCOCC1=CN(N=N1)[C@H1]2CCC3=CC(OC)=C(OC
)C(OC)=C3C=CC=C(NC)C(=O)C=C2O
PUGREST.BadRequest: error:  CCCCCCCCCCCCCCCCOCC1=CN(N=N1)[C@H1]2CCC3=CC(OC)=C(OC
)C(OC)=C3C=CC=C(NC)C(=O)C=C2OC
PUGREST.BadRequest: error:
C[C@H1](NC(C)=O)C1=CCC(SC)=CC=C1C2=C(C=C(OC)C(OC)=C2OCC3=CC=CC=C3)OSC
PUGREST.BadRequest: error:
C=12C(=CC(C(NC)=CC=1)=O)[C@H1](CCC3=CC(OC)=C(OC)C(=C32)OC)C(C)C=O
PUGREST.BadRequest: error:
NC(=O)NCC1=CN(N=N1)[C@H1]2CCC3=CC(OC)=C(OC)C(OC)=C3C4=CC=C(NC)C(=O)C=C24
PUGREST.BadRequest: error:
N(C(C1=CC=NC=C12)=O)[C@@H1]3CC(C4=C(C(OC)=C(OC)C=C4CC3)OC(CC)=O)=CC=C(SC)C2=O
PUGREST.BadRequest: error:
C=12C(=CC(C(NC)=CC=1)=O)[C@H1](CCC3=CC(OC)=C(OC)C(=C3C2)NC(C)C)CSCSC
PUGREST.BadRequest: error:
C12[C@H1](NC(O)=O)C3=CCC(SC)=CC=C3C4=C(C(OC)=C(OC)C=C4CC1)OC=CC2=O
PUGREST.BadRequest: error:
C1=CC(OC)=CC=C1CN[C@H1]2CCC3=C(C(OC)=C(OC)C(OC)=C3)C=4C2=CC(C(=CC=4)NC)=O
PUGREST.BadRequest: error:
C=CC(OC)=C(OC)C(OC)=CC1(C2=CC=C(NC)CC=C2[C@H1](CCC1)NC(=O)C=O)SC
PUGREST.BadRequest: error:
O(C1=CC(=CC(OC)=C1OC)CC[C@H1](NC(N(C(C)C)C(C2)C)=O)C=CCC(NC)=CC=C2ONC)C
PUGREST.BadRequest: error:
N(C(C1=CC=CC=C1)=O)CCN[C@H1]2CCC3=CC(OC)=C(OC)C(OC)=C3C4=CC=C(C(C=C24)=O)SC
PUGREST.BadRequest: error:
C1[C@H1](NC(O)=O)C2=CCC=CC=C2C3=C(OC)C(OC)=C(OC)C=C3CCNC=C1O
PUGREST.BadRequest: error:
C1=C(C(OC)=C(OC=2)CC3=CC=C(NC)C(C=C3[C@@H1](NC(N(COC)C)O)SCCC=21)NC)OC
PUGREST.BadRequest: error:
N1=NC1=C(C(=O)O)NC(=O)N[C@H1]2CCC3=CC(OC)=C(OC)C(OC)=C3C=CC=C(NC)C(=O)C=C2
PUGREST.BadRequest: error:
N=CC=C(NCN[C@H1]1CCC2=CC(OC)=C(OC)C(OC)=C2C3=CC=C(C(C=C13)=O)N)C=O
PUGREST.BadRequest: error:
N=CC=C(NCN[C@H1]1CCC2=CC(OC)=C(OC)C(OC)=C2C3=CC=C(NC)C(=O)C=C13)O
PUGREST.BadRequest: error:
N=NC1=CN(N=N1)[C@H1]C2CC3=CC(OC)=C(OC)C(OC)=C3C4=CC=C(C(=O)C=C42)NCOC
PUGREST.BadRequest: error:
N=NC(=C(C(OCC)=O)N[C@H1]1CCC2=CC(OC)=C(OC)C(OC)=C2C3=CC=C(C(C=C13)=O)N)C
PUGREST.BadRequest: error:
CNC=1C(=O)C=C2[C@H1](CCC3=C(C(OC)=C(OC)C(OC)=C3)C2=CC=1)NC(C(=O)O)=CN=CNC
PUGREST.BadRequest: error:
C=CC1C[C@H1](NC(C2)=O)C=CC=3C(OC)=C2C1=CC=C(SC)C(C=3)=O
PUGREST.BadRequest: error:
N1=CC=CC2=C1C(OC=CC(OC=3)=CCCC[C@H1](NC(C)=O)C4=CCC(NC)=CC=C4C=32)=O
PUGREST.BadRequest: error:
```

```
CNC=1C(=O)C=C2[C@H1](CCC3=CC(OC)=C(OC)C(OC)=C3C2=CC=1)NC4(C(=O)O)C=CN=C4NC
PUGREST.BadRequest: error:
N1=CC=CC=C1COC=C(C(OC=2)=CCCC[C@H1](NC(C)=O)C3=CC(C(SC)=CC=C3C=2OCO)=O)O
PUGREST.BadRequest: error:
C=C1[C@H1](CCC2=C(C(OC)=C(OC)C(OC)=C2)C1=CC=CC(C)=O)NCNC(=O)CSC
PUGREST.BadRequest: error:
CN1CC(=O)C=C2C(C3=C(OC)C(OC)=C(OC)C=C3CC[C@@H1]2N4C(C(O)=O)=C(N=N4)OCNC)=CC1
PUGREST.BadRequest: error:
NN(C=C(CNC(CCCCCl)=O)N=N)[C@H1]1CCC2=CC(OC)=C(OC)C(OC)=C2C3=CC=C(NC)C(=O)C=C31
PUGREST.BadRequest: error:
C=C1[C@H1](C=2CC=CC(OC)=C(OC)C(OC(C3=CC=CC=C3)=O)=CC1=CC=C)C(C=2)=O
PUGREST.BadRequest: error:
N=NC1=CN(N=N1)[C@H1]2CCC3=CC(OC)=C(OC)C(=C3C4=CC=C(NC)C(C=C42)=O)OC
PUGREST.BadRequest: error:
N=NC=C(C(=O)O)NC(=O)N[C@H1]CCC1=CC(OC)=C(OC)C(OC)=C1C2=CC=C(NC)C(=O)C=C2
PUGREST.BadRequest: error:
COC1=CC(=CC(OC)=C1OC)CC[C@H1](NC(N(C(C)C)C(C2)C)=O)C=CC(C(NC)=CC=C2NC=O)NC
PUGREST.BadRequest: error:
C=C1[C@H1](CCC2=CC(OC)=C(OC)C(OC)=C2C1=CC=C(C(C)=O)N3C)NC(C(=O)O)=C3NC
PUGREST.BadRequest: error:
CNC=1C(=O)C=C2[C@H1](CCC3=CC(OC)=C(OC)C(OC)=C3C2=CC=1)NC(C(=O)O)C=CN=CNC
PUGREST.BadRequest: error:
N=NC(=C(C(=O)O)NC(=O)N[C@H1]C1CC2=CC(OC)=C(OC)C(OC)=C2C=CC=C(NC)C(=O)C=C1)CC
PUGREST.BadRequest: error:  CC(C)(C)OC(=O)NCCC(NCC=1N=NN(C=1)[C@H1]CCC2=CC(OC)=C
(OC)C=C2C3=CC=C(NC)C(C=C3)=O)OC=O
PUGREST.BadRequest: error:  CC(C)(C)OCNCCC(=O)NCC1=CN(N=N1)[C@H1]C2CC3=CC(OC)=C(
OC)C(OC)=C3C4=CC=C(C(=O)C=C24)NC=O
PUGREST.BadRequest: error:
COC(=O)C1=C(N=NN(CNCC)OCCCCC(=O)C2=CC(C3=C(OC)C(OC)=C(OC)C=C3CC[C@@H1]2N1)C)O
PUGREST.BadRequest: error:  CC(C)(C)OCNCCC(=O)NCC1=CN(N=N1)[C@@H1]2C3=CC(C(NC)=C
C=C3C=C(CC2)C=C(OC)C(OC)NC)=O
PUGREST.BadRequest: error:
C1(=CN(C(=O)OCC)N[C@@H1]2C=3C(C4=C(OC)C(OC)=C(OC)C=C4CC2)=CC=C(C(=O)C=3)NC)N=C1
PUGREST.BadRequest: error:  CC(C)(C)OCNCCC(=O)NCC1=CN(N=N1)[C@@H1]2C3=CC(C(NC)=C
C=C3C4=C(CC2)C=C(OC)C(=C4OC)NC)=O
PUGREST.BadRequest: error:  C1C(C)(C)OC(=O)NCCC1NCC=2N=NN(C=2)[C@H1]3CCC4=CC(OC)
=C(OC)C(OC)=C4C=CC=C(NC)C(=O)C=C3O
PUGREST.BadRequest: error:  CC(C)(C)OC(=O)NCCC(OCC=1N=NN(C=1)[C@H1]CCC2=CC(OC)=C
(OC)C(=C2C3=CC=C(NC)C(=O)C=C3)O)C=O
PUGREST.BadRequest: error:
CC(C)OC(=O)/N=C(/N[C@H1]1CCC2=C(C(OC)=C(OC)C(OC)=C2)C3=CC=C(NC)C(=O)C=C13)N=N
PUGREST.BadRequest: error:
C=1N(N=NC=1C(OCC)=O)[C@H1]2CCC3=C(C(OC)=C(OC)C(OC)=C3)C4=CC=C(C(=O)C=C42)NCOCC
PUGREST.BadRequest: error:
O=CC=CN=NN[C@H1]1CCC2=C(C(OC)=C(OC)C(=C2)OC)C3=CC=C(NC)C(=O)C=C13
PUGREST.BadRequest: error:
CNC=CC=C1C[C@H1](CCC2=CC(OC)=C(OC)C(=C12)OC)NC3(C(=O)O)C=CN=N3
PUGREST.BadRequest: error:
```

```
CNC=CC=C1C([C@H1](CCC2=CC(OC)=C(OC)C(=C12)OC)NC(C(=O)OCC)=C(C(OCO)=O)ONC)SC
PUGREST.BadRequest: error:
C=1NC=2CC=C3[C@H1](CCC4=C(C(OC)=C(OC)C(OC)=C4)C3=CC=2)NC(C(=O)O)C=CN=1
PUGREST.BadRequest: error:  C1=CN=CC=C1C(NCC2=CN(N=N2)[C@H1]3CCC4=CC(OC)=C(OC)C(
=C4C5=CC=C(C(C=C53)=O)OC)OO)C
PUGREST.BadRequest: error:
N=NN([C@H1]1CCC2=C(C(OC)=C(OC)C(OC)=C2)C3=CC=C(C(=O)C=C13)NC)COC4CC=CC=C4O
PUGREST.BadRequest: error:
N=NN([C@H1]1CCC2=C(C(OC)=C(OC)C(OC)=C2)C3=CC=C(C(=O)C=C13)NC)C(=O)OCC
PUGREST.BadRequest: error:
N=NN([C@@H1]1C2=CC(C(NC)=CC=C2C3=C(C(OC)=C(OC)C=C3CC1)OC)=O)OCC=C=O
PUGREST.BadRequest: error:
C=C1C(C=C(CC[C@@H1]1N2N=NC(=C2)CNC(=O)OCC(C)C)C=C(C(OC)=COC)O)C=CC=C(C=O)NC
PUGREST.BadRequest: error:
C=C1C(C2=C(CC[C@@H1]1N3N=NC(=C3)CNC(=O)OCC(C)C)C=C(C(OC)=C2OC)O)C=CC=C(C=O)NC
PUGREST.BadRequest: error:
C1=C2C(CC([C@H1](CC2)N3N=NC(=C3)CNC(=O)C(Cl)Cl)=CC(=O)C(=CC)NC)=C(OC)C(OC)=C1OC
PUGREST.BadRequest: error:
CN(N([C@@H1]1C2=CC(=O)C(NC)=CC=C2C3=CC(=C(OC)C=C3CC1)OC)O)CNC(=S)NCCCCC
PUGREST.BadRequest: error:
N([C@H1]1CCC2=CC(=C(OC)C(=C2C3=CC=C(C(C=C31)=O)NC)OC4)OC[C@H1])(OC4)NC(=S)O
PUGREST.BadRequest: error:
CN(N([C@@H1]1C2=CC(=O)C(NC)=CC=C2C3=CC(=C(OC)C=C3CC1)OC)O)CNC(=S)CCCCCC
PUGREST.BadRequest: error:  CN(N([C@@H1]1C2=CC(=O)C(NC)=CC=C2C3=CC(=C(OC)C=C3CC1
)OC)O)CNC(=S)N4C[C@@H1](O)[C@@H1]OC4=CC
PUGREST.BadRequest: error:
OC=CC=C1C2=C(OC)C(OC)=C(OC)C=C2CC[C@H1](C3=CCC(=CC=C31)NC)ON
PUGREST.BadRequest: error:  O1C=CC=CC2=C(OC)C(OC)=C(OC)C=C2CC[C@@H1]1C=CCC(F)F
PUGREST.BadRequest: error:
O(C=CC=CC1=C(OC)C(OC)=C(OC)C=C1CC[C@H1](C=C2CC(OCC(C)C)=O)N=C2ON)C
PUGREST.BadRequest: error:
O(C=CC=CC1=C(OC)C(OC)=C(OC)C=C1CC[C@H1]C=CCC(OC(=O)OCC)=CC)C
PUGREST.BadRequest: error:
O=C(CC)OCC1=CN(N=N1)[C@H1]2CCC3=CC(OC)=C(OC)C(OC)=C3C4=CC=C(NC)C(=O)C=C24
PUGREST.BadRequest: error:
O=C(OC)CCOC=CC=C1C[C@H1](CCC2=CC(OC)=C(OC)C(OC)=C21)NC=O
PUGREST.BadRequest: error:
O(C=CC=CC1=C(OC)C(OC)=C(OC)C=C1CC[C@H1](C=CCCOCC2=CC=CC=C2)OONC)ONCC
PUGREST.BadRequest: error:
OC=CC=CC1=C(OC)C(OC)=C(OC)C=C1C2C[C@H1](C=CCC(OCC(C)C)=O)N=C2ONC
PUGREST.BadRequest: error:
O=C(OCC=C)N1C(=O)N[C@H1]CCC2=C(C(OC)=C(OC)C(OC)=C2)C3=CC=C(NC)C(C=C13)=O
PUGREST.BadRequest: error:  O=C(OC(C)(C)C)NCC=1N=NN(C=1)[C@H1]C2CC3=CC(OC)=C(OC)
C(OC)=C3C4=CC=C(NC)C(=O)C=C42
PUGREST.BadRequest: error:
O=C(C(C)C)NCN[C@H1]1CCC2=CC(OC)=C(OC)C(OC)=C2C3=CC=C(NC)C(=O)C=C13
PUGREST.BadRequest: error:
O=C(OCC(C)COCC=1)N=NN(C=1)[C@@H1]2C3=CC(C(NC)=CC=C3C4=C(OC)C(OC)=C(OC)C=C4CC2)=O
```

```
PUGREST.BadRequest: error:  O=C(OCC)C=1N(N=NC=1C(=O)O2)[C@H1]2NC(=S)N[C@H1]3CCC4
=CC(OC)=C(OC)C(OC)=C4C=5C3=CC(C(=CC=5)NC)=O
PUGREST.BadRequest: error:
OC1=C2C3=CC=C(NC)CC=C3[C@H1](CCC2=CC(OC)=C1OC)NC(C(O)=O)=O
PUGREST.BadRequest: error:
CC(O)(OC(=O)CC1)CN(N=N1)[C@H1]2CCC3=CC(OC)=C(OC)C(OC)=C3C4=CC=C(NC)C(=O)C=C24
PUGREST.BadRequest: error:
C1=2C(O)CN(CC3O)N1[C@H1]CCSC(C4=C(C=C(OC)C(OC)=C4OC(O)CCC3=O)CC=2)C
PUGREST.BadRequest: error:  CC(O)(C)CC(=O)OCC1=CN(N=N1)[C@H1]2CCC3=CC(OC)=C(OC)C
(OC)=C3C=4C2=CC(C(=CC=4)NC)=O
PUGREST.BadRequest: error:
CC(C)COC(NCC1=CN(N=N1)[C@H1]C2CC3=CC(OC)=C(OC)C(OC)=C3C4=CC=C(C(=O)C=C24)NC)=O
PUGREST.BadRequest: error:
C=C(NC)CC=C1CC2=C(OC)C(OC)=C(OC)C=C2CC[C@@H1]1N3C(C=CC=C3OC(=O)OC)=O
PUGREST.BadRequest: error:
COCNCC1=CN(N=N1)[C@H1]2CCC3=CC(OC)=C(OC)C(OC)=C3C4=CC=C(NC)C(=O)C=C24
PUGREST.BadRequest: error:  CC(C)(C)OC(NCC=1N=NN(C=1)[C@H1]2CCC3=CC(OC)=C(OC)C(O
C)=C3C4=CC=C(C(C=C24)=O)N)C=O
PUGREST.BadRequest: error:
OC1=C(C(OC)=CC2=C1C3=CC=C(NC)CC=C3[C@H1](CC2)N4C(C(O)=O)=C(N=N4)OC=O)O
PUGREST.BadRequest: error:
C[C@H1](COC=CN=NN[C@H1]1CCC2=CC(OC)=C(OC)C(OC)=C2C=3C1=CCC(=CC=3)NC)OCCC
PUGREST.BadRequest: error:
N(C)C1=CC=C2C3=C(OC)C(OC)=C(OC)C=C3CC[C@@H1](C2=CC1=O)NC(C(=O)O)CCONCOCNCOC
PUGREST.BadRequest: error:
N(CCCCC)CC1C2C=C[C@H1](CCC3=CC(OC)=C(OC)C(OC(C)=O)=C3C2=CC=C1SCSC=O)C
PUGREST.BadRequest: error:
O(C1=C(C(OC)=CC2=C1C3=CC=C(NC)CC=C3[C@H1](CC2)N4C(C(O)=O)=C(N=N4)OC=O)O)OCC
PUGREST.BadRequest: error:
N(C(C)C)C(NCC1=CN(N=N1)[C@H1]2CCC3=CC(OC)=C(OC)C(OC)=C3C4=CC=C(C(C=C24)=O)NC)=O
PUGREST.BadRequest: error:
N(CCCCCCC=O)CC1=CN(N=N1)[C@H1]2CCC3=CC(OC)=C(OC)C(OC)=C3C4=CC=C(C(C=C24)=O)NC
PUGREST.BadRequest: error:
N(C(C)=O)[C@@H1]1C2=CC(C(SC)=CC=C2C3=C(C(OC)=C(OC)C=C3CC1)OC(C4=CCCC=C4)=O)C
PUGREST.BadRequest: error:
N(CCCCC)CC1CC2=C[C@H1](CCC3=CC(OC)=C(OC)C(OC(C)=O)=C3C2=CC=C1SCNC=O)C
PUGREST.BadRequest: error:
O=C(OCC)C1=C(C(OCC)=O)N=NN1[C@H1]CCC2=CC(OC)=C(OC)C(OC)=C2C3=CC=C(NC)C(C=C3)=O
PUGREST.BadRequest: error:
N([C@H1]1CCC2=CC(OC)=C(OC)C(OC)=C2C3=CC=C(NC)C(C=C13)=O)C=CCOC(=O)CCCC=O
PUGREST.BadRequest: error:
O=C(OCC1=CC2=C(C=C1)F)N[C@@H1]CC(C3=C(C=C(OC)C(OC)=C3OC(OCC)=O)NCCC2)=CC=C(O)C=O
PUGREST.BadRequest: error:
C1OC=CC2=C(C3=CC=C(NC)CC=C3[C@@H1](N)CC2)ONC(OC)=C1OC
PUGREST.BadRequest: error:  C1=C2CC[C@H1](C3=CCC(SC)=CC=C3C2=C(C(OC)=C1OC)OC=O)N
PUGREST.BadRequest: error:
C=CCC1[C@H1](CC=[N+1]C2=C1CC(C(OC)=C2OCOC=C)C=CSC)C=O
PUGREST.BadRequest: error:
```

```
C1OC=C2C3=CC=C(NC)C(=O)C=C3[C@H1](CCC2C=COC)NCCCCC=C1
PUGREST.BadRequest: error:
C1OC=C2C3=CC=C(NC)C(=O)C=C3[C@H1](CCC2C=COC)NCCCCC=C1OC
PUGREST.BadRequest: error:
C1=C2CC[C@H1](CC(C2=C(OC)C(=C1OC)OC)=CC=C(NC)C=O)CN=[N+1]=[N-1]
PUGREST.BadRequest: error:
COC=C1C2=C3C=C(NC)C(C=C2[C@@H1](NCCCCCCCCC)CCC1=CC(=C3OC)O)C=O
PUGREST.BadRequest: error:
CC(CC)N[C@H1]C1CC2=CC(OC)=C(OC)C(OC)=C2C3=CC=C(C(C=C31)=O)NCC
PUGREST.BadRequest: error:
CC(CC)N[C@H1]C1CC2=CC(OC)=C(OC)C(OC)=C2C3=CC=C(NC)C(=O)C=C31
PUGREST.BadRequest: error:
C(CCCCN1[C@H1]CCC2=CC(OC)=C(OC)C(OC)=C2C3=CC=C(SC)CC=C13)=O
PUGREST.BadRequest: error:
CC(CC)N[C@H1]C1CC2=CC(OC)=C(OC)C(OC)=C2C3=CC=C(C(C=C31)=O)NC
PUGREST.BadRequest: error:
C1(=NC=C(O)C(C)=CC1)C2=C(C(OC)=C(OC)C=C2CC[C@H1](O)C=CN)CSC
PUGREST.BadRequest: error:
C1(=CC=C(O)C(CCC)C=O)C2=C(OC)C(OC)=C(OC)C=C2CC[C@H1](NC(=O)C)C1SC
PUGREST.BadRequest: error:
COC=C1C2=CC=C(NC)C(=O)C=C2[C@@H1](NCC(C)CCC)CCC1=CC(=COC)OC
PUGREST.BadRequest: error:
C1OC=C2C3=CC=C(NC)C(=O)C=C3[C@@H1](NC(=N)N)CCC2=CC(OC)=C1OC
PUGREST.BadRequest: error:
COC1=C2C3=CC=C(NC)C(=O)C=C3[C@@H1](NCC(CCC)C)CCC2=CC(OC)=C1OC
PUGREST.BadRequest: error:
NC1=CC=CC=C1C2=CC3=C(OC)C(=C(OC)C=C3CC[C@H1](NCCC)C2NCOC)O
PUGREST.BadRequest: error:
COC=C1C2=CC=C(NC)CC=C2[C@@H1](NCCC#N)CCC1=CC(=COC)OC=O
PUGREST.BadRequest: error:
C1=C2CC[C@H1](C3=CCC(SC)=CC=C3C2=C(C(OC)=C1OC)OC=O)NCCC#N
PUGREST.BadRequest: error:
C1=C2CC[C@H1](C3=CCC(SC)=CC=C3C2=C(C(OC)=C1OC)OC=O)NCCC
PUGREST.BadRequest: error:
COC1=C2C3=CC=C(NC)C(=O)C=C3[C@H1](CCC2=CC(OC)=C1OC)NCC4=CC=NS4
PUGREST.BadRequest: error:
COC1=C2C3=CC=C(SC)C(=O)C=C3[C@H1](CCC2=CC(OC)=C1OC)NCCCCl
PUGREST.BadRequest: error:
COC1=C2C3=CC=C(NC)C(=O)C=C3[C@H1](CCC2=CC(OC)=C1OC)NCC=CC=NCN
PUGREST.BadRequest: error:
COC1=C2CC([C@H1](CCC2=CC(OC)=C1OC)NCCCCl)C(C(=CC)NC)=O
PUGREST.BadRequest: error:
C=1NCC(=O)C=C2C=1C3=C(OC)C(OC)=C(C=C3CC[C@@H1]2NCCCCl)C=CC
PUGREST.BadRequest: error:
O(C1=C(OC)C(OC)=CC23C([C@@H1](NC(N)=S)CCC2=C1)=CC(C(NC)=CC3)=O)NCC
PUGREST.BadRequest: error:
O(C1=C(OC)C=2C3=CC=C(SC)CC=C3[C@H1](CCC=2C=C1OC)NC(C)CC=C)OC
PUGREST.BadRequest: error:
```

```
C1=C(OC)C(OC)=C(OC)CC2=CC=C(SC)C(C=C2[C@@H1](NC(N(C)C)=O)CCC1)=O
PUGREST.BadRequest: error:
C12=C(OC)C(OC)=C(OC)C=C1CC[C@H1](NC(C3)=O)C(C(NC)=CC=C32)=O
PUGREST.BadRequest: error:
C1[C@H1](OCCC2)CC=C(C(OC)=C(OC)C=C2CC1N3C=CC=C(NC)CC3)ONC(C)=O
PUGREST.BadRequest: error:
N(C)C=1C(=O)C=C2[C@H1](CCC3=CC(OC)=C(OC)C(OC)=C3C2=CC=1N)C(C)CCSCSC
PUGREST.BadRequest: error:
O(C1=C(OC)C=2C3=CC=C(SC)C(C=C3[C@H1](CCC=2C=C1OC)NC(C)CC=C)=O)C
PUGREST.BadRequest: error:
C12=C(OC)C(=C(OC)C=C1CC[C@H1](NCC(O)=O)C3=CCC(NC)=CC=C32)OOC
PUGREST.BadRequest: error:
C1[C@H1](NCC)CC2=CC(C(NC)=CC=C2C3=C(OC)C(OC)=C(OC)C=C3C1N)C(=O)C
PUGREST.BadRequest: error:
C1=C(OC)C(OC)=C(OC=2)CC3=CC=C(SC)C(C=C3[C@@H1](NC(N(C)C)=O)CCC=21)=O
PUGREST.BadRequest: error:
N(C)C=1C(=O)C=C2[C@H1](CCC3=CC(OC)=C(OC)C(OC)=C3C2=CC=1N)C(C)CCNC
PUGREST.BadRequest: error:
C1=C2CC[C@H1](C3=CCC(SC)=CC=C3C2=C(C(OC)=C1OC=4)OC=O)NCC=CC=4
PUGREST.BadRequest: error:
COC1=CC2=CC=C(SC)C(=O)C=C2[C@@H1](NCC3=CC=CC=C3)CCC1=CC(OC)=COC
PUGREST.BadRequest: error:
N1=NN([C@@H1]C2=CC(C(O)=CC=C2C3=C(CC)C=C(OC)C(OC)=C3OC)=O)CC(O)=C1F
PUGREST.BadRequest: error:
N1=NN([C@@H1]C2=C3C(C(C)=CC=C3C4=C(CC2)C=C(OC)C(OC)=C4OC)=O)N=CC=N1
PUGREST.BadRequest: error:
CNC=1C(C=C2[C@H1](CCC3=C(C(OC)=C(OC)C(OC)=C3)C2=CC=1)NC(C)=O)SC=O
PUGREST.BadRequest: error:
N1=NC=CN=C2C1(C3=C[C@H1](CCC=C2C(OC)=C(OC)C(OC)=C3N)C(C)C=C)OF
PUGREST.BadRequest: error:
O=CC=C1N=NN(C1)[C@@H1]2C3=CC(C(NC)=CC=C3C4=C(OC)C(OC)=C(C=C4CC2)OC)NC
PUGREST.BadRequest: error:
N1=NN([C@@H1]C2=CC(C(O)=CC=C2C3=C(CC)C=C(OC)C(OC)=C3OC)=O)CC=C1
PUGREST.BadRequest: error:
O=CC1=CN=NN1[C@H1]2CCC3=C(C(OC)=C(OC)C(OC)=C3)C4=CC=C(C(C=C42)N)COC
PUGREST.BadRequest: error:
C1=CN=CC=C1CN[C@H1]2CCC3=CC(OC)=C(OC)C(OC)=C3C4=CC=C(C(C=C24)=O)NCC
PUGREST.BadRequest: error:
CSC=1C(C=C2[C@H1](CCC3=C(C(OC)=C(OC)C(OC)=C3)C2=CC=1)NC(C)=O)NC=O
PUGREST.BadRequest: error:
C1NC=CC=C2C([C@@H1](NC(C(C)C)=O)CCC3=CC(OC)=C(OC)C(OC)=C23)C1=O
PUGREST.BadRequest: error:
O=CC1=CN=NN1[C@H1]2CCC3=CC(OC)=C(OC)C(OC)=C3C4=CC=C(C(C=C42)=O)NC
PUGREST.BadRequest: error:
OCCN(CC=C)C(=O)N[C@H1]1CCC2=CC(OC)=C(OC)C(OC)=C2C3=CC=C(NC)C(=O)C=C31
PUGREST.BadRequest: error:
O=C1C2=C[C@H1](CCC=C3C(OC)=C(OC)C(OC(C)=O)=C3C2=CC=C1NC(C)=O)NC
PUGREST.BadRequest: error:
```

```
OCCN(CC=C)C(=O)N[C@H1]1CCC2=CC(OC)=C(OC)C(OC)=C2C3=CC=C(NC)C(=O)C=C13
PUGREST.BadRequest: error:
C=CN=C1C(C)NC(=O)OC=CC=C2C3=C(OC)C(OC)=C(OC)C=C3CC[C@@H1](C2=C1)NCCC
PUGREST.BadRequest: error:
O=C1C=C2[C@H1]CCC3=CC(OC)=C(OC)C(OC)=C3C2=CC=C1SCNC(C)=O
PUGREST.BadRequest: error:
N1=NC(=CN(N=N1)[C@H1]2CCC3=CC(OC)=C(OC)C(OC)=C3C4=CC=C(C(C=C24)=O)N)C
PUGREST.BadRequest: error:
C12=CC(C(NC)=CC=C1C3=C(C=C(OC)C(OC)=C3OC)CC[C@@H1]2NCCC(F)(F)F)=CO
PUGREST.BadRequest: error:
C1=C2C([C@H1](CCC3=CC(OC)=C(C(=C32)OC)OC)NCCCCl)C(C(=C1)NC)=O
PUGREST.BadRequest: error:
CNC(N(C)C)CN[C@H1]1CCC2=CC(OC)=C(C(OC)=C2C=3C1=CC(=O)C(NC)=CC=3)OC
PUGREST.BadRequest: error:
N1C(NC)=CC=C2C([C@H1](CCC3=CC(OC)=C(C(OC)=C23)OC)NCCC(C)C)=CC1=O
PUGREST.BadRequest: error:
C12=CCC(NC)=CC=C1C3=C(C=C(OC)C(OC)=C3OC)CC[C@@H1]2NCC=4SC=CC=4O
PUGREST.BadRequest: error:
C12=CC(C(NC)=CC=C1C3=C(OC)C(=C(C=C3CC[C@@H1]2N4C=C(CO)N=N4)O)CO)C=O
PUGREST.BadRequest: error:
C=1=C2C([C@H1](CCC3=CC(OC)=C(C(OC)=C23)OC)NCC=CC=O)C(=CC=1)SC
PUGREST.BadRequest: error:
COC=C(OC)C(OC)=CCCC=1[C@H1](NCC(C)C)C2=CC(=O)C(SC)=CC=C2C=1
PUGREST.BadRequest: error:
C1=C2C(C3=C(OC)C(=C(C=C3CC[C@@H1]2NCC4=CC=CS4)O)CO)C=CC=C(NC)C1=O
PUGREST.BadRequest: error:
COC1=C(OC)C(OC)=CCCC=2[C@H1](NCC(C)C)C3=CC(=O)C(SC)=CC=C3C1=2
PUGREST.BadRequest: error:
C1=CC(C(=CC=C1C2=C(OC)C(OC)=C(C=C2CC[C@@H1]NCCOO)C)OC)(CC)O
PUGREST.BadRequest: error:
NC(N(CC)OC)N[C@H1]1CCC2=CC(OC)=C(OC)C(OC)=C2C=3C1=CC(=O)C(NC)=CC=3F
PUGREST.BadRequest: error:
O=CC(SC)=CC=C1C([C@H1](CCC2=CC(OC)=C(OC)C(OC)=C12)N)=CCNC
PUGREST.BadRequest: error:
O=C1C(SC)=CC=C2C([C@H1](CCC3=CC(OC)=C(OC)C(OC)=C23)NCC)C=C1
PUGREST.BadRequest: error:
C12=CC=C(NC)C(C=C1[C@@H1](NC(C)=O)CCC3=CC(OC)=C(OC)C(OC)=C23)SC=O
PUGREST.BadRequest: error:
OC(OC1=C2C3=CC=C(SC)C(C=C3[C@H1]CCC2=CC(=C1OC)O)CNC(C)=O)=O
PUGREST.BadRequest: error:
O1C(OC2=C3C4=CC=C(SC)C(C=C4[C@H1]CCC3=CCOC)=C2OCNC=CC=C1)C
PUGREST.BadRequest: error:
C12=CC=C(NC)C(C=C1[C@@H1](NC(N)=O)CCC=CC(OC)=C(OC)C(OC)=C2)O
PUGREST.BadRequest: error:
NC(CCN[C@H1]1CCC2=CC(OC)=C(OC)C(OC)=C2C=3C1=CC(=O)C=CC=3)NCF
PUGREST.BadRequest: error:
O=C1C(NC)=CC=C2C3=C(OC)C(OC)=C(C=C3CC[C@@H1](C2=C1)NCC)OOCSC
PUGREST.BadRequest: error:
```

```
C1[C@H1](C2=CC(C(SC)=CC=C2C3=C(OC)C(OC)=C(C=C3C1)O)C)ONC
PUGREST.BadRequest: error:
C12=CC=C(NC)C2(C=C1[C@@H1](NC(C)=O)CCC3=CC(OC)=C(OC)C(OC)=C3)SC
PUGREST.BadRequest: error:
C1=C2C=C(NC)C(C=C1[C@H1]CCC3=CC(OC)=C(OC)C(OC)=C23)NC(N)=O
PUGREST.BadRequest: error:
C1=C2C=C(NC)C(C=C1[C@H1]CCC3=C2C(OC)=C(OC)C(OC)=C3)NC(C)=O
PUGREST.BadRequest: error:
COCC(C)=CC1=CC([C@@H1](NC(C)=O)CCC2=CC(OC)=C(OC)C(OC)=C21)=CSC
PUGREST.BadRequest: error:
C1[C@H1](C2=CCC(SC)=CC=C2C3=C(OC)C(OC)=C(OC)C=C3C1)ONCC
PUGREST.BadRequest: error:
C=C1C2=C(OC)C(OC)=C(OC)C=C2CC[C@H1](NC(C)=O)C1=CC=C(NC)C(=O)CNC
PUGREST.BadRequest: error:
C=1C2=CC(=CC(C=1OC)=O)[C@H1](CCC3=CC(OC)=C(OC)C(OC)=C32)NC(C)=O
PUGREST.BadRequest: error:
NC(N=C1C=CC2)=CC=C1C3=C(OC)C(OC)=C(OC)C=C3CC[C@@H1]2NC(N)=O
PUGREST.BadRequest: error:
C=CC1=C(NC)C(OC)=C(OC)C=C1CC[C@H1](NC(C)=O)C2=CC(C(=CC=C2)NC)=O
PUGREST.BadRequest: error:
C[C@H1](CCC1=CC(OC)=C(OC)C(OC)=C1C2=CC=C(C(C=C2)N)C=O)NCC
PUGREST.BadRequest: error:
C=1=C2C3=C(OC)C(OC)=C(OC)C=C3CC[C@H1](NC(N)=O)C2=CC=C(SC)C(=O)C=1NC
PUGREST.BadRequest: error:
C[C@@H1](C1CC2=CC(OC)=C(OC)C(OC)=C2C3=CC=C(C(C=C31)=O)SCN)C(=O)C
PUGREST.BadRequest: error:
C1=2[C@H1](CC(C3=CC(OC)=C(OC)C=C31)C=CC=C(NC)CC=2)OSC
PUGREST.BadRequest: error:
C=C(C)C=C1C(C(OC(C)=O)=CC=CC2=C(OC)C(OC)=C(OC)C=C2CC[C@@H1]1N=O)C
PUGREST.BadRequest: error:
CC(N)(CCO)[C@H1]1CCC2=CC(OC)=C(OC)C(OC)=C2C3=CC=C(NC)C(=O)C=C31
PUGREST.BadRequest: error:
CC(N)(CCO)[C@H1]1CCC2=CC(OC)=C(OC)C(OC)=C2C3=CC=C(C(=O)C=C31)NCC
PUGREST.BadRequest: error:
C=C1[C@@H1](NC(C)=O)CCC2=CC(OC)=C(OC)C(OC)=C2C1=CC=C(NC)C=O
PUGREST.BadRequest: error:
C12[C@H1](NC(C)=O)C3=CC(C(SC)=CC=C3C4=C(OC)C(OC)=C(C=C4C1)OC=C2)NC=O
PUGREST.BadRequest: error:
N(NCCCCC1)[C@H1]1CCC2=CC(OC)=C(OC)C(OC)=C2C3=CC=C(NC)C(=O)C=C31
PUGREST.BadRequest: error:
N(C1=CC=C(C=C1)C)CN[C@H1]2CCC3=CC(OC)=C(OC)C(OC)=C3C=4C2=CC(C(NC)=CC=4)=O
PUGREST.BadRequest: error:
C12[C@H1](NC(C)=O)C3=CC(C(NC)=CC=C3C4=C(OC)C(OC)=C(OC)C=C4C1=C2)NC=O
PUGREST.BadRequest: error:
N1(C(=O)OC2=CC=CC3=C(OC)C(OC)=C(OC)C=C3CC[C@H1](NC(C)=O)C2=CC1)SC
PUGREST.BadRequest: error:
C1[C@H1](NC(N)=O)C2=CC(C(NC)=CC=C2C3=C(C(OC)=C(OC)C=C3CC1)OC)NC=O
PUGREST.BadRequest: error:
```

```
N(C1=CC=C(C=C1)C)CN[C@H1]2CCC3=CC(OC)=C(OC)C(OC)=C3C=4C2=CC(C(=CC=4)SC)=O
PUGREST.BadRequest: error:
N(C1=CC=C(C=C1)Cl)C=CN(OC(CC)=O)[C@H1]CCC2=CC(OC)=C(OC)C(OC)=C2C=CC=C(C)CNC=O
PUGREST.BadRequest: error:
C12=C(CC=C3[C@H1](CCC4=CC(OC)=C(OC)C(OC)=C4C3=C1)NCC)OC2=O
PUGREST.BadRequest: error:
C=C(CN[C@H1]1CCC2=CC(OC)=C(OC)C(OC)=C2C3=CC=C(NC)C(C=C31)=O)C=O
PUGREST.BadRequest: error:
C1#CC(C=C2[C@H1](CCC3=CC(OC)=C(OC)C(OC)=C3C2=C1)NC(C)=O)=C
PUGREST.BadRequest: error:
C=C1[C@@H1](NC(N)=O)CCC2=CC(OC)=C(OC)C(OC)=C2C1=CC=C(NC)C=O
PUGREST.BadRequest: error:
CCN(CC)C(=O)N[C@H1]C1CC2=CC(OC)=C(OC)C(OC)=C2C3=CC=C(C(C=C31)=O)NC
PUGREST.BadRequest: error:
C1[C@H1](NC(C)=O)C2=CCC(SC)=CC=C2C3=C(OC)C(OC)=C(C=C3C1)OCNC(=O)C
PUGREST.BadRequest: error:
C=1C=C(NC)C(C=C2[C@H1](CCC3=CC(OC)=C(OC)C(OC)=C3C=12)CC=O)NSC=O
PUGREST.BadRequest: error:
C1=CC(=CC=C1CCCN[C@H1]2CCC3)CC(OC)=C(OC)C(OC)=C3C4=CC=C(SC)C(C=C42)=O
PUGREST.BadRequest: error:
N1([C@@H1]2C3=CC(C(SC)=CC=C3C4=C(OC)C(OC)=C(C=C4CC2)OC=CC=C1)O)CS
PUGREST.BadRequest: error:
CCN(CC)C(=O)N1[C@H1]CCC2=CC(OC)=C(OC)C(OC)=C2C3=CC=C(NC)C(C=C31)=O
PUGREST.BadRequest: error:
C1=CC(=CC=C1CCN[C@H1]2CCC3=C)C(OC)=C(OC)C(OC)=C3C4=CC=C(SC)C(C=C42)=O
PUGREST.BadRequest: error:
C1=CC(=CC=C1O)CN[C@H1]2CCC3=CC(OC)=C(OC)C(OC)=C3C4=CC=C(SC)C(C=C42)=O
PUGREST.BadRequest: error:
CCN(CC)C(=O)N[C@H1]1CCC2=CC(OC)=C(OC)C(OC)=C2C3=CC=C(C(C=C31)=O)NCC
PUGREST.BadRequest: error:
CCN[C@@H1]1C2=CC(C(NC)=CC=C2C3=C(OC)C(OC)=C(OC)C=C3CC1=O)=O
PUGREST.BadRequest: error:
CC(C)COC1=C(OC)C(OC)=CC=2CC[C@H1](NC(=O)C)C3=CC(C(SC)=CC=C3C1=2)=O
PUGREST.BadRequest: error:
CC(C)NC(N[C@H1]1CCC2=CC(OC)=C(OC)C(=C2C3=CC=C(NC)C(C=C31)=O)OC=O)C
PUGREST.BadRequest: error:
C=C(C)CN(CCO)C(=S)N[C@H1]1CCC2=C(C(OC)=C(OC)C(OC)=C2)C3=CC=C(NC)C(C=C31)=O
PUGREST.BadRequest: error:
CC(C)C(OC=C(C(OC)=CC=1CC[C@H1](NC(=O)C)C2=CC(C(SC)=CC=C2C=1)S)C=O)=O
PUGREST.BadRequest: error:
C=C(O)C(OC)=CC(=C)CN[C@H1]1CCC2=CC(OC)=C(OC)C(OC)=C2C3=CC=C(SC)C(C=C31)=O
PUGREST.BadRequest: error:
CC(C)C(OC=C(C(OC)=CCCC[C@H1](NC(=O)C)C1=CC(C(SC)=CC=C1C)=O)C)=O
PUGREST.BadRequest: error:
CC(CO1)(C(C)CO[C@H1]CCC2=C)C(OC)=C(OC)C(OC(=O)C)=C2C3=CC=C(NC)C(C=C31)=O
PUGREST.BadRequest: error:
O=C(N[C@@H1]1C2=CCC(NC)=CC=C2C3=C(CC1)C=C(OC)C(OC)=C3OC)OCC=C
PUGREST.BadRequest: error:
```

```
N([C@H1]1CCC2=CC(OC)=C(OC)C(OC)=C2C3=CC=C(NC)C(=O)C=C31)=C(Cl)C=C
PUGREST.BadRequest: error:
O=C(N[C@H1]1CCC2=CC(OC)=C(OC)C(OC)=C2C3=CC=C(C(C=C31)=O)NCN)C4=CC=CC=C4
PUGREST.BadRequest: error:
N([C@H1]1CCC2=CC(OC)=C(OC)C(OC)=C2C3=CC=C(NC)C(C=C13)=O)C(C)C
PUGREST.BadRequest: error:
O=C(N[C@@H1]1C2=CC(C(NC)=CC=C2C3=C(CC1)C=C(OC)C(OC)=C3OC)=O)N=N
PUGREST.BadRequest: error:
N([C@H1]1CCC2=CC(OC)=C(OC)C(OC)=C2C3=CC=C(NC)C(C=C13)=O)=CC=C
PUGREST.BadRequest: error:
O=C(N[C@@H1]1C2=CCC(NC)=CC=C2C3=C(CC1)C=C(OC)C(OC)=C3OC)ON=N
PUGREST.BadRequest: error:
N([C@H1]1CCC2=CC(OC)=C(OC)C(OC)=C2C3=CC=C(NC)C(C=C13)=O)CNC(=O)OCCONC
PUGREST.BadRequest: error:
O=C(N[C@H1]1CCC2=C3C(OC)=C(OC)C(OC)=C2C4=CC=C(NC)C(C=C14)=O)CCOC3
PUGREST.BadRequest: error:
O=C(N[C@H1]1CCC2=CC(OC)=C(OC)C(OC)=C2C3=CC=C(NC)C(=O)C=C31)CCOC(=O)O
PUGREST.BadRequest: error:
C=C1CC[C@@H1](C2=CCC(OC(C3=CC=CC=C3)=O)=CC=C2C=C1C=COC)C(OC)=COC=O
PUGREST.BadRequest: error:
C1=CCC[C@H1](NC(=S)NC2=CC=C(C=C2)C(F)(F)F)CC(C1)=CC=C(NC)C(=O)COCOC
PUGREST.BadRequest: error:
COC1=CC2=CC=C(NC)C(C=C2[C@@H1](NC(=O)NC3=CC=CC=C3)CCC1=CC(=COC)O)C=O
PUGREST.BadRequest: error:
C1=C2CC[C@@H1](C3=CCC(SC)=CC=C3C2=C(OC)C(=C1OC)OC=O)NCC4=C(C=CC=C4)O
PUGREST.BadRequest: error:
COC1=CC2=C3C=C(NC)C(=O)C=C2[C@@H1](NC(=S)N(CCO)CCCCC)C3=CC(OC)=C1OC
PUGREST.BadRequest: error:
COC=CC1=C(C2=CC=C(SC)CC=C2[C@H1](CC1)NC(=O)NC3=CC=C(F)C)C3=O
PUGREST.BadRequest: error:
C1=C2CC[C@@H1](C3=CCC(SC)=CC=C3C2=C(OC)C(=C1OC)OC=O)NC(=O)NC4=CC=CC=C4
PUGREST.BadRequest: error:
C=C1C2C[C@H1](N3C=C(COC(=O)C4=CC=CC=C4Cl)N=N3)C5=CC(C(NC)=CC=C5C2=CC1O)C=O
PUGREST.BadRequest: error:
C=CC1=C(C2=C(OC)C(OC)=C1OC(OCC)=O)CC[C@H1](NC(C)=O)C3=CC(C(SC)=CC=C23)=O
PUGREST.BadRequest: error:
O=CC(=CC1=CC[C@H1](CCC2=CC(OC)=C(OC)C(OC(=O)C)=C21)CC=O)OSC
PUGREST.BadRequest: error:
C1[C@@H1](C2=CC(C(SC)=CC=C2C3=C(OC)C(OC)=C(C=C3CC)OCNCC)=O)C1NC=O
PUGREST.BadRequest: error:
C[C@@H1](C1=CCC(OC(OC)=O)=CC=C1C2=C(OC)C(OC)=C(OC)C=C2CN)C(C)=O
PUGREST.BadRequest: error:
OCC(C(C1)=O)N(N=N1)[C@H1]2CCC3=CC(OC)=C(OC)C(OC)=C3C4=CC=C(NC)C(=O)C=C24
PUGREST.BadRequest: error:
CNCC(O)(C)C1=CN(N=N1)[C@H1]2CCC3=CC(OC)=C(OC)C(OC)=C3C4=CC=C(C(=O)C=C24)NC
PUGREST.BadRequest: error:
O1C=C(NC)CC=C1CN[C@H1]2CCC3=CC(OC)=C(OC)C(OC)=C3C4=CC=C(SC)C(C=C24)=O
PUGREST.BadRequest: error:
```

```
N=1C(=CN(N=1)[C@H1]2CCC3=CC(OC)=C(OC)C(OC)=C3C4=CC=C(NC)C(C=C24)=O)C=O
PUGREST.BadRequest: error:
C=CC=C(C(=O)OC=CC1=CC([C@@H1](NC(C)=O)CCC2=CC(OC)=C(OC)C(OC)=C21)SC=C)C
PUGREST.BadRequest: error:
N1([C@H1]2CCC3=C(C(OC)=C(OC)C(OC)=C3)C4=CC=C(NC)C(C=C24)=O)C(Cl)=C1
PUGREST.BadRequest: error:
N1([C@H1]2CCC3=C(C(OC)=C(OC)C(OC)=C3)C4=CC=C(NC)C(C=C42)=O)C=C1Cl
PUGREST.BadRequest: error:
N1([C@H1]2CCC3=C(C(OC)=C(OC)C(OC)=C3)C4=CC=C(NC)C(C=C42)=O)C(Cl)=C1
PUGREST.BadRequest: error:
O=CC1=CN(N=N1)[C@H1]2CCC3=CC(OC)=C(OC)C(OC)=C3C4=CC=C(NC)C(=O)C=C24
PUGREST.BadRequest: error:
C1N([C@H1]2CCC3=CC(OC)=C(OC)C(OC)=C3C4=CC=C(NC)C(=O)C=C24)CC(OC)=C1
PUGREST.BadRequest: error:
N1([C@H1]2CCC3=C(C(OC)=C(OC)C(OC)=C3)C4=CC=C(NC)C(C=C24)=O)C(O)=C1C
PUGREST.BadRequest: error:
N1([C@H1]2CCC3=C(C(OC)=C(OC)C(OC)=C3)C4=CC=C(NC)C(C=C24)=O)C(O)=C1
PUGREST.BadRequest: error:
O=CC1=CN(N=N1)[C@H1]2CCC3=CC(OC)=C(OC)C(OC)=C3C4=CC=C(NC)C(=O)C=C42
PUGREST.BadRequest: error:
C1N([C@H1]2CCC3=CC(OC)=C(OC)C(OC)=C3C4=CC=C(C(C=C42)=O)NCC=C)C(OC)=C1OC
PUGREST.BadRequest: error:
N([C@H1]1CCC2=C(C(OC)=C(OC)C(OC)=C2)C3=CC=C(NC)C(C=C13)=O)NC=CC=CCl
PUGREST.BadRequest: error:
N1([C@H1]2CCC3=C(C(OC)=C(OC)C(OC)=C3)C4=CC=C(NC)C(C=C42)=O)C=C1I
PUGREST.BadRequest: error:
N(CCCC)CC1=CN(N=N1)[C@H1]2CCC3=C(C(OC)=C(OC)C(OC)=C3)C4=CC=C(NC)C(=O)C=C24
PUGREST.BadRequest: error:
C1=C(O)C(OC)=CC(CN[C@H1]2CCC3=C(C(OC)=C(OC)C(OC)=C3)C4=CC=C(SC)C(=O)C=C42)=C1C
PUGREST.BadRequest: error:
N(C(C)C)(C(C)C)C(=O)N[C@@H1]1CC(C2=C(C(OC)=C(OC)C=C2CC1)OC)(CC)OCC=C(NC)C(C)=O
PUGREST.BadRequest: error:
N(C)C[C@H1](C1=CC(C(NC)=CC=C1C2=C(OC)C(OC)=C(C=C2CC)OCNCC)=O)C=C
PUGREST.BadRequest: error:
C1=2[C@H1](OC1)CCCC=C(C3=C(OC)C(OC)=C(OC)C=C3CC=CC=CNC)C(C=2)=O
PUGREST.BadRequest: error:
N(CCCC)CC1=CN(N=N1)[C@H1]2CCC3=C(C(OC)=C(OC)C(OC)=C3)C4=CC=C(NC)C(C=C24)=O
PUGREST.BadRequest: error:
C1=C(OC)C(=C(OC)C=C1CC[C@H1](NC(C(O)=O)C(=O)OCC=CC(OC)=C(OC)C=C)OCNC)O
PUGREST.BadRequest: error:  C12=C(NC)CC([C@@H1](C3N=NCO3)C=C1OC=CCC2SC)=O
PUGREST.BadRequest: error:
C1=C(OC)C(=C(OC)C=C1CC[C@H1](NC(C(OCC)=O)=C(NCO)OOC2=CCC(NC)=CC=C2)ON)C
PUGREST.BadRequest: error:
N1(CCCC)CC2=CN(N=N2)[C@H1]3CCC4=CC(OC)=C(OC)C(OC)=C4C5=CC=C(C(C=C53)=O)NCC=C1
PUGREST.BadRequest: error:
N(C)C(=O)NCCC(OCC(C=C1[C@H1](CCC2=CC(OC)=C(OC)C(OC)=C2C1=CCN)C(C)=O)S)C=O
PUGREST.BadRequest: error:
C1=C(OC2)C(=CC=C1C3=CC(OC)=C(OC)C=C3CC4[C@H1](CCC=CC=C4COC(C)=O)C2OCOCC)ONC
```

```
PUGREST.BadRequest: error:
C1[C@H1](C2=CCC(SC)=CC=C2C3=C(OC)C(OC)=C(C=C3C1)OC)ONC
PUGREST.BadRequest: error:
C12=CC=C(NC)C(C=C1[C@@H1](NCC)CCCC=CC(OC)=C(OC)C(OC)=C2)SC=O
PUGREST.BadRequest: error:
C1[C@@H1](C2=CC(C(NC)=CC=C2C3=C(OC)C(OC)=C(C=C3C1)O)C)ONCC
PUGREST.BadRequest: error:
N=CC(C(OC(OCC)=O)=CC=CC1=C(OC)C(OC)=COC)C=C1CC[C@H1](NCCC)C=O
PUGREST.BadRequest: error:
COCC(C)=C(C(OC)=CCCC[C@H1](NCC)C1=CC(C(SC)=CC=C1)=O)OC
PUGREST.BadRequest: error:
C=CC(N[C@H1]C1CC2=C(C(OC)=C(OC)C(OC)=C2)C3=CC=C(NC)C(=O)C=C31)OO
PUGREST.BadRequest: error:
COC1C(C)=C(C=2C3=CC=C(SC)CC=C3[C@H1]CCC=2C=C1)NCNCCOOOC
PUGREST.BadRequest: error:
C1[C@@H1](C2=CC(C(NC)=CC=C2C3=C(OC)C(OC)=C(C=C3C1)OC=O)N)CCO
PUGREST.BadRequest: error:
CC1=C2C(=C(OC)C(OC)=C1C3=CC=C(NC)C(C=C3[C@H1](C2)NC)C)OONC
PUGREST.BadRequest: error:
COC1=C(OC)C=2C3=CC=C(SC)CC=C3[C@H1](CCC=2C=C1OC)NCC4=CC=CC=C4O
PUGREST.BadRequest: error:
C[C@H1](CCC1=CC(OC)=C(OC)C(OC)=C1C2=CC=C(C(=O)C=C2)NCN)CCO
PUGREST.BadRequest: error:
NC(=O)N1[C@@H1]C2=CC(C(NC)=CC=C2C3=C(OC)C(OC)=C(OC)C=C3CC1)=O
PUGREST.BadRequest: error:
NN(CCO)[C@@H1]1C2=CC(C(NC)=CC=C2C3=C(OC)C(OC)=C(C=C3CC1)OC)=O
PUGREST.BadRequest: error:
C1=2[C@H1](CCC3=CC(OC)=C(OC)C(OC)=C3C1=CC=C(NC)C(=O)C=2NC)CO
PUGREST.BadRequest: error:
C12=CC(C=CC=C1C3=C(OC)C(OC)=C(OC)C=C3CC[C@@H1]2NCC=CC=CC=C)SCC
PUGREST.BadRequest: error:
N=C1C(C(OC(C)=O)CCC2=C(OC)C(OC)=C(OC)C=C2CC[C@H1]1)N=O
PUGREST.BadRequest: error:
NC1(N=CC=C1S)[C@H1]2CCC3=CC(OC)=C(OC)C(OC)=C3C4=CC=C(C(C=C24)N)C=O
PUGREST.BadRequest: error:
NC(N=NCOCCC)C[C@H1]1C2=CC(C(NC)=CC=C2C3=C(CC1)C=C(OC)C(OC)=C3OC)=O
PUGREST.BadRequest: error:
CCCCCC=1N2[C@@H1]CC(C3=C(C(OC)=C(OC)C=C3C2=C)C=C)(SC)C(C=1)=O
PUGREST.BadRequest: error:
C1C2(C3=C(OC)C(OC)=C(OC)C=C3CC[C@@H1]1NC(N)C)C=CC=C(C(C2)=O)NC
PUGREST.BadRequest: error:
C1C2(C3=C(OC)C(OC)=C(OC)C=C3CC[C@@H1]1NC(N)=NC)C=C(C(=O)C2)NC
PUGREST.BadRequest: error:
C1=2C(C3=C(OC)C(OC)=C(OC)C=C3CC[C@@H1]1NC(C)CC)=CC=C(NC)C(=O)C=2NC
PUGREST.BadRequest: error:
C12=C(C=CN(NC1)OC3=CC=C(NC)CC=C3[C@@H1](NC(C)=O)CC)CC(OC)=C2OC=O
PUGREST.BadRequest: error:
C1=C(CN[C@H1]2CCC3=CC(OC)=C(OC)C(OC)=C3C4=CC=C(NC)C(=O)C=C42)C=NC=C1
```

```
PUGREST.BadRequest: error:
N(C1=CC=C(C=C1)Cl)C=CN(OC=CC=CC=C)C=CC(N2C)=C(C(OC)=CC=CC=CC2C)[C@H1]SOC
PUGREST.BadRequest: error:
C1[C@H1](NC(N=2)=N)CC(C3=C(C(OC)=C(OC)C=C3C1)OC)=CC=C(NC)C(C=2)=O
PUGREST.BadRequest: error:
C1=C(C(C=C2[C@H1](CCC3=CC(OC)=C(OC)C(OC)=C3C2=C1)NC(C)C)=O)NC
PUGREST.BadRequest: error:
N(C1=CC=C(C=C1)Cl)C=CN(OC=CC=CC=C)C=CC(N2C)=C(C(OC)=CC=CC=CC2C)[C@H1]NOC=O
PUGREST.BadRequest: error:
C12=C[C@@H1](NC(N(C)C)=O)CCC3=CC(OC)=C(OC)C(OC)=C3C1=CC=C(NC)C2=O
PUGREST.BadRequest: error:
C1=C(CN[C@H1]2CCC3=CC(OC)=C(OC)C(OC)=C3C4=CC=C(NC)C(=O)C=C42)C=NC=CC1
PUGREST.BadRequest: error:
C=1C=C(NC)C(C=C2C=1C3=C(OC)C(OC)=C(OC)C=C3CC[C@@H1]2NC(N(C)C)=O)SNC
PUGREST.BadRequest: error:
C1CN[C@@H1]2C3=CC(C(NC)=CC=C3C4=C(C(OC)=C(OC)C=C4CC2)OC=O)C=C1
PUGREST.BadRequest: error:
N=1[C@@H1]2C3=CCC(SC)=CC=C3C4=C(OC)C(OC)=C(C=C4CC2)OC=CC=1C
PUGREST.BadRequest: error:
C1[C@H1](NC(NC2=CC=C(C=C2)F)=O)C3=CC(C(NC)=CC=C3C4=C(OC)C(OC)=CC=C4OCC1)=O
PUGREST.BadRequest: error:
C=1C=C(NC)C(C=C2[C@H1](CCC3=CC(OC)=C(OC)C(OC)=C3C2=1)CCC=O)C
PUGREST.BadRequest: error:
C=12C=C(NC)C2(C=C3C=1C4=C(OC)C(OC)=C(OC)C=C4CC[C@@H1]3NC(N(CC)CC)=O)NC
PUGREST.BadRequest: error:
C1=CC(=CC=N1)CCN[C@@H1]2C3=CC(C(SC)=CC=C3C4=C(CC2)C=C(OC)C(OC)=C4OC)=O
PUGREST.BadRequest: error:
C=CC=C(NC(=O)C=CC=C1C2=C(OC)C(OC)=C(OC)C=C2CC[C@H1](NC(C)=O)C1=C)SC=O
PUGREST.BadRequest: error:
N=NC(CNC(=O)OC)=C=C(CCO[C@H1]CCC1=CC(OC)=C(OC)C(OC)=C1C2=CC=CNC)C(=O)C=C2
PUGREST.BadRequest: error:
C1=2[C@H1]CCC3=CC(OC)=C(OC)C(OC)=C3C1=CC=C(C(=O)C=2)NCNC(C)=O
PUGREST.BadRequest: error:
CC=C1C2=C(C=C(OC)C(OC)=C2OC(CC)=O)C[C@H1](NC(C)=O)C=CC(C(SC)=CC=C1)=O
PUGREST.BadRequest: error:
C1=2[C@H1]CCC3=CC(OC)=C(OC)C(OC)=C3C1=CC=C(C(C=2)=O)NCNC(C)=O
PUGREST.BadRequest: error:
C1[C@@H1](CC(C2=C(C(OC)=C(OC)C=C2C1)OC)=CC=C(SC)C(C)=O)NC(C)=O
PUGREST.BadRequest: error:
CC1[C@H1](NC(=O)NC2=CC=C(C(F)(F)F)C=C2C3=CCC(NC)=CC=C3C=C(OC)C(OC)=C(C=C1)OO)C
PUGREST.BadRequest: error:
C12C=CC3=C(C=C(OC)C(OC)=C3OC(C)=O)[C@H1](NC(C)=O)C1=CC(C(SC)=C2)=O
PUGREST.BadRequest: error:
CC=CC1=C(C=C(OC)C(OC)=C1OC(OCC)=O)CC[C@H1](NC(C)=O)C2=CC(C(SC)=CC=C2)=O
PUGREST.BadRequest: error:
C=CC=C(NC1CCC=CN=C(CC)CC=C1[C@H1]CCC2=CC(OC)=C(OC)C(OC)=C2)ONC
PUGREST.BadRequest: error:
C=CC=C(NC([C@@H1]C1=CCC(SC)=CC=C1C2=C(OC)C(OC)=C(OC)C=C2CC)=O)NC=O
```

```
PUGREST.BadRequest: error:
O=C1C=C2[C@H1](CCC3=C(C(OC)=C(OC)C(OC)=C3)C2=CC=C1SC)NC(N(C(C)C)C(Cl)=O)NC
PUGREST.BadRequest: error:
O=C1C=C2[C@H1](CCC3=C(C(OC)=C(OC)C(OC)=C3)C2=CC=C1SC)NC(C(=O)OCC)=CNC
PUGREST.BadRequest: error:
N=NC(CNC(=O)OC)=C=C(C=CC=CNCC=CC1)C(OC)C(OC)=C(OC)C=C1CC[C@H1](NC(=O)C)C=CC
PUGREST.BadRequest: error:
C1=C2CC[C@H1](NC(N(C)C)=O)C3=CC(C(NC)=CC=C3C2=C(OC)C(OC)=C1OC)NC(C)=O
PUGREST.BadRequest: error:
O=C1C=C2[C@H1](CCC3=C(C(OC)=C(OC)C(OC)=C3)C2=CC=C1SC)NC(C(C)C)=O
PUGREST.BadRequest: error:
N1=NC(COC(=O)C2=CC=NC=C2)=CN1NCC=CC3C4=CC=C(NC)CC=C4[C@H1]CCC3=CCNCOCC
PUGREST.BadRequest: error:
N1C=NN=NN1[C@H1]2CCC3=C(C(OC)=C(OC)C(OC)=C3)C4=CC=C(C(C=C42)=O)NCCC
PUGREST.BadRequest: error:
CNCC(OC1=2)=C(OC)C=3CC([C@H1](CCC=3C=C1OC)NC(N(C)C)=O)OC(=CC=2)NC=O
PUGREST.BadRequest: error:
CNC=1C(=O)C=C2[C@H1](CCC3=CC(OC)=C(OC)C(OC)=C3C2=CC=1N)C(C(C)C)=O
PUGREST.BadRequest: error:
N=NC(CNC(=O)OC)=C=C(C=CC=CNCC=C1C)C2(NC)C(C(O)=CC2[C@H1]NC(C)=O)CCC1SCOCC
PUGREST.BadRequest: error:
O=CC(NC)=CC=C1C([C@H1](CCC2=CC(OC)=C(OC)C(OC)=C21)N=[N+1])=[N-1]
PUGREST.BadRequest: error:
N([C@H1]1CCC2=CC(OC)=C(OC)C(OC)=C2C3=CC=C(NC)C(C=C31)=O)CCCCF
PUGREST.BadRequest: error:
C[C@H1](CCC1=CC(OC)=C(OC)C(OC)=C1C2=CC=C(C(C=C2)=O)SC)NC/C=C/C3=CC=CC=C3
PUGREST.BadRequest: error:
O=CC(NC)=C1C2=CC([C@@H1](N=[N+1]=[N-1])CCC3=CC(OC)=C(OC)C(OC)=C32)NC=CC1
PUGREST.BadRequest: error:
N([C@H1]1CCC2=C(C(OC)=C(OC)C(OC)=C2)C3=CC=C(SC)C(C=C31)=O)CCCO
PUGREST.BadRequest: error:
O=CC(NC)=CC=CC1([C@H1](CCC2=CC(OC)=C(OC)C(OC)=C21)N=[N+1]=[N-1])O
PUGREST.BadRequest: error:
C1NC(CNC(=O)OCCN=N1)CC2=CC(OC)=C(OC)C(OC)=C2C3=CC=C(NC)C(=O)C=C3
PUGREST.BadRequest: error:
O=CC(NC)=CC=C1C([C@H1](CCC2=CC(OC)=C(OC)C(OC)=C21)N)=[N+1]=[N-1]
PUGREST.BadRequest: error:
C=CC=C(C(=O)OCC1=CN(N=N1)[C@H1]2CCC3=CC(OC)=C(OC)C(OC)=C3C4=CC=C(NC)CC=C24)O
PUGREST.BadRequest: error:
C=CC1=C(C(=O)OC=CC=C2C3=C(OC)C(OC)=C(OC)C=C3CC[C@H1](NC(C)=O)C2=C1SC)C
PUGREST.BadRequest: error:
C=C1C2=C(C=C(OC)C(OC)=C2OC(OCC)=O)CC[C@H1](NC(C)=O)C3=CC(C(NC)=CC=C13)=O
PUGREST.BadRequest: error:
C=CC=CC(=O)OC1=C(C(OC)=CCCC[C@H1](NC(C)=O)C2=CC(C(SC)=CC=C21)=O)C
PUGREST.BadRequest: error:
OCC(C1(C)OCN)=NN(C1)[C@H1]2C3=CC(C(NC)=CC=C3C4=C(CC2)C=C(OC)C(OC)=C4OC)=O
PUGREST.BadRequest: error:
OCC(C1(C)OCN)=NN(C1)[C@H1]2C3=CC(C(NC)=CC=C3C4=C(OC)C(OC)=C(C=C4CC2)OC)=O
```

```
PUGREST.BadRequest: error:
C1=2[C@H1](CCC3=C(C(OC)=C(OC)C(OC)=C3)C1=CC=C(C(C=2)=O)SCNCC)OC=C
PUGREST.BadRequest: error:
CC=CC(N[C@H1]1CCC2=C(C(OC)=C(OC)C(OC)=C2)C3=CC=C(SC)C(C=C13)=O)CCOC
PUGREST.BadRequest: error:
OC(=O)NCC1=CN(N=N1)[C@@H1]2C=3C(C4=C(C(OC)=C(OC)C=C4CC2)OC)=CC=C(NC)C(C=3)=O
PUGREST.BadRequest: error:
O(C=C(OC=1)CC2=CC=C(NC)CC=C2[C@@H1](N3C(C(O)=O)=C(N=N3)C(O)=O)CCC=1C=COC=O)C
PUGREST.BadRequest: error:
N(C)C=1C(=O)C=C2[C@H1](CCC3=C(C(OC)=C(OC)C(OC)=C3)C2=CC=1)N4C(C(=O)O)C=CN=C4NC
PUGREST.BadRequest: error:
C1=C(OC)C(=C(OC)C=C1CC[C@H1](NC(C(=O)OCC)=C(COCC)OCCC(NC)=CC=CON)C=C)NC
PUGREST.BadRequest: error:  N(C)C=1C(=O)C=C2[C@H1](CCC3=C(C(OC)=C(OC)C(OC)=C3)C2
=CC=1)N4C(C(=O)O)=C(N=N4)C(=O)NNC
PUGREST.BadRequest: error:  N1(C)C=2C(=O)C=C3[C@H1](CCC4=C(C(OC)=C(OC)C(OC)=C4)C
3=CC=2)NC(C(=O)O5)C=CN=C5C=C1NC
PUGREST.BadRequest: error:
C1[C@H1](NCC)CC2=CC(C(SC)=CC=C2C3=C(C(OC)=C(OC)C=C3CC1)OC(C)=O)CC=O
PUGREST.BadRequest: error:
C12[C@H1](NCC)CC3=CC(C(SC)=CC=C3C4=C(C(OC)=C(OC)C=C4CC1)OC=C2NCC)=O
PUGREST.BadRequest: error:
C=C(NC)C1C([C@H1](CCC2=CC(OC)=C(OC)C(OC)=C21)NC(NC3=CC=C(C=C3)F)=O)PNC=O
PUGREST.BadRequest: error:
O(C1=C(OC=2)C=3CC([C@@H1](N4N=NC(C(O)=O)=C4CO)OCCC=2C=C1)OC=CCC(NC)=CC=3)OC
PUGREST.BadRequest: error:  O=C(NC=C1C=C(C(F)(F)F)CC=C1)N[C@@H1]2C=3C(C4=C(C(OC)
=C(OC)C=C4CC2)OC)=CC=C(NC)C(C=3)=O
PUGREST.BadRequest: error:  O=C(C=C(C(OCC1)=O)N(N=N1)[C@@H1]2C=3C(C4=C(C(OC)=C(O
C)C=C4CC2)OC)=CC=C(NC)CC=3)OCOCC=CC=CO
PUGREST.BadRequest: error:  O=C(C=C(C(OCC1)=O)N(N=N1)[C@@H1]2C=3C(C4=C(C(OC)=C(O
C)C=C4CC2)OC)=CC=C(NC)CC=3)OC5OCCCC5=O
PUGREST.BadRequest: error:
O=C(C=C(C(O)CCCCCC)[C@H1]1CC)C2=C(C(OC)=C(OC)C(OC)=C2)C3=CC=C(NC)C(C=C31)=O
PUGREST.BadRequest: error:  O=C(C=C(C(O1)CCCCCC1)CN=N)N[C@H1]2CCC3=CC(OC)=C(OC)C
(OC)=C3C=4C2=CC(C(NC)=CC=4)=O
PUGREST.BadRequest: error:
N([C@H1]1CCC2=C(C(OC)=C(OC)C(OC)=C2)C3=CC=C(NC)C(=O)C=C13)CNCC4=CC=C(C=C4)C=O
PUGREST.BadRequest: error:  O=C(C=C(Cl)C1=CN(N=N1)C(=S)N[C@@H1]2C=3C(C4=C(C(OC)=
C(OC)C=C4CC2)OC)=CC=C(NC)CC=3)OC(O)=O
PUGREST.BadRequest: error:  O=C(C=C(Cl)C=CN([C@H1]1CCC2=C(C(OC)=C(OC)C(OC)=C2)C3
=CC=C(NC)C(C=C13)=O)OC(=O)CC)NC(OC4=CC=CC=C4)=O
PUGREST.BadRequest: error:  O=CC(=CC=C1C([C@@H1](N2C(C(O)=O)=C(N=N2)C(O)=O)CCC3=
CC(OC)=C(OC)C(OC)=C31)=C)NC=O
PUGREST.BadRequest: error:
O=CC(=C1C=C2C[C@H1](CCC3=CC(OC)=C(OC)C(OC)=C32)NC(C(=O)OCC)=C(NCC)OON=C1)NC
PUGREST.BadRequest: error:
N=1C(=C(C(=O)O)NCCN=NN(C=1)[C@H1]2CCC3=CC(OC)=C(OC)C(OC)=C3C4=CC=C(NC)CC=C42)OC
PUGREST.BadRequest: error:  NC(=CN/C(=NC(O)C=CCI)C1=CN(NC12)C=CC3=CC(NC)=CC(OC)=
C3C=CC(OC4)C(OC)=CC5=C4)C=CC=CC5S[C@@H1]2NC
```

```
PUGREST.BadRequest: error:  OC(\N[C@H1]1CCC2=CC(OC)=C(OC)C(OC)=C2C3=CC=C(C(=O)C=
C31)OC(=O)N)(C[C@@H1])C4=CCC(OC)=C4CCO
PUGREST.BadRequest: error:
CNCC(O)(C1OC=CC=C1N=NNC)[C@@H1]2C3=CC(C(NC)=CC=C3C4=C(OC)C(OC)=C(C=C4CC2)OC)=O
PUGREST.BadRequest: error:
O=C1C(=CC=C2C([C@H1](CCC3=CC(OC)=C(OC)C(OC)=C32)N4C(C(=O)OCC)=C(N=N4)CC1=O)N)C
PUGREST.BadRequest: error:  O=C1C(=CC=C2C([C@@H1](N3C(C(=O)O)=C(C(O)=O)N=N3)CCC4
=CC(OC)=C(OC)C(OC)=C42)=C1)NC=O
PUGREST.BadRequest: error:  CNCC(O)(C1OC=CC=C1C=2N=NNC=2)[C@H1]3CCC4=CC(OC)=C(OC
)C(OC)=C4C5=CC=C(C(=O)C=C53)NC
PUGREST.BadRequest: error:
N=1C(=C(C(=O)O)NCCN=NN(C=1)[C@H1]2CCC3=CC(OC)=C(OC)C(OC)=C3C4=CC=C(NC)CC=C42)O
PUGREST.BadRequest: error:  CC(C)(C)OC(=O)/N=C(/N[C@H1]1CCC2=C(C(OC)=C(OC)C(OC)=
C2)C3=CC=C(NC)C(C=C31)=O)NCOC(C)=O
PUGREST.BadRequest: error:
C1(=CC=C1NCOCC)C=2N=NN(C=2)[C@H1]3CCC4=CC(OC)=C(OC)C(OC)=C4C5=CC=C(NC)C(C=C53)=O
PUGREST.BadRequest: error:  CC(C)(C)OC(=O)/N=C(/N[C@H1]1CCC2=C(C(OC)=C(OC)C(OC)=
C2)C3=CC=C(NC)C(C=C13)=O)NCOC(C)(F)F
PUGREST.BadRequest: error:
C1(OC(C=C(N1[C@H1]2CCC3=C(C(OC)=C(OC)C(OC)=C3)C4=CC=C(NC)C(C=C24)=O)CC)CC)=CO
PUGREST.BadRequest: error:
CC(C)(C)OC(=O)/N=C(/N[C@H1]1CCC2=C(C(OC)=C(OC)C(OC)=C2)C3=CC=C(NC)C(=O)C=C31)N=N
PUGREST.BadRequest: error:
N([C@H1]1CCC2=CC(OC)=C(OC)C(OC)=C2C3=CC=C(NC)CC=C13)=O
PUGREST.BadRequest: error:
O=C(CNC=C1C(C2=C(OC)C(OC)=C(OC)C=C2CC[C@@H1]1NC=CC=C)OC)C(=O)CSC
PUGREST.BadRequest: error:
CNC(CNC(=O)OCCN[C@H1]1)CCC2=CC(OC)=C(OC)C(OC)=C2C=3C1=CC(C(=CC=3)NC)=O
PUGREST.BadRequest: error:
C12=C3C(C4=C(OC)C(OC)=C(OC)C=C4CC[C@@H1]3NC(=O)N)=CC=C1NC=C2
PUGREST.BadRequest: error:
CC(C)C(=O)OCCC=C1[C@H1](CCC2=CC(OC)=C(OC)C(OC)=C2C1=CCN)SC=O
PUGREST.BadRequest: error:
C=1C(C)(C)C(OC2=CC=CC3=C(OC)C(OC)=C(OC)C=C3CC[C@H1](NC(=O)C)C2=CC=1O)SC
PUGREST.BadRequest: error:
CC(C)(C)COC1=CC=C2C3=C(OC)C(OC)=C(OC)C=C3CC[C@@H1](C2=CC1=O)SC=O
PUGREST.BadRequest: error:
O=C(C=CNCC(=O)OCCN[C@H1]1)CCC2=C(C(OC)=C(OC)C(OC)=C2)C3=CC=C(NC)C(=O)C=C13
PUGREST.BadRequest: error:
C1=2[C@H1]CCC3=CC(OC)=C(OC)C(OC(CC)=O)=C3C1=CC=C(C(C=2)=O)SCNC(=O)CSC
PUGREST.BadRequest: error:
OC(CCC(C)C)OCC1=CN(N=N1)[C@H1]2CCC3=CC(OC)=C(OC)C(OC)=C3C4=CC=C(NC)C(=O)C=C24
PUGREST.BadRequest: error:
O=CCN(CC=C)N(OCC)O[C@H1]1CCC2=CC(OC)=C(OC)C(OC)=C2C=CC=C(NC)C(=O)C=C1
PUGREST.BadRequest: error:
N([C@@H1]1C=2CC3=C(C(OC)=C(OC)C=C3CC1)OC=CC=C(NC)CC=2)=O
PUGREST.BadRequest: error:
OC(CCC(C)C)OCC1=CN(N=N1)[C@H1]CCC2=CC(OC)=C(OC)C(OC)=C2C=CC=C(C(C)N)C
```

```
PUGREST.BadRequest: error:
C=C1C(C2=C(C=C(OC)C(=C2OC)OC)CC[C@@H1]1N3C=C(N=N3)C4=CC=CC=C4)=CC=C(NC)C=O
PUGREST.BadRequest: error:
C=C1C([C@H1](CCC2=CC(=C(OC)C(=C21)OC)OC)NCCCCCCCCCC)C=CC(CNC)=O
PUGREST.BadRequest: error:
C=C1C(C2=CC=C(C(=O)C=C2[C@H1](CC1)N3C=C(N=N3)C=CC=CC=N)C=C)OCNC(OC)=O
PUGREST.BadRequest: error:
OC1=C(C=CC=C1)C=2N=NN(C=2)[C@H1]3CCC4=CC(OC)=C(OC)C(OC)=C4C5=CC=C(NC)C(=O)C=C35
PUGREST.BadRequest: error:
NC(=C(N=NCC(=O)CCC=C1N=CC(C(OC(C)=O)=CC=C1C2=C(OC)C)(OC)COC)C)C2CCOF
PUGREST.BadRequest: error:
N1=CC=C(C2=C1)COC3=CC=C4C5=C(OC)C(OC)=C(OC)C=C5CC[C@@H1](C4=CC3=O)N2
PUGREST.BadRequest: error:
NC1=CN(N=N1)[C@H1]2CCC3=C(C(OC)=C(OC)C(OC)=C3)C4=CC=C(NC)C(C=C24)=O
PUGREST.BadRequest: error:
NC1=C(N=NC1C(=O)CCC=CC=C2C3=C(OC)C(OC)=C(OC)C=C3CC[C@H1](CC2N)CN)C=O
PUGREST.BadRequest: error:
C[C@@H1](C1=CCC(SC)=CC=C1C2=C(OC)C(OC)=C(C=C2CC)OCOCC=CCSC=O)O
PUGREST.BadRequest: error:
N=C=C(N=NC(C(C)OC)[C@H1]CCC1=CC(OC)=C(OC)C(OC)=C1C2=CC=C(NC)C=O)C=C2
PUGREST.BadRequest: error:
C=C1C(C2=C(C(=C(OC)C=C2CC[C@@H1]1N3C=C(N=N3)C4=CC)CC)N4O)COC=CC=C(CON)C
PUGREST.BadRequest: error:
C=C1C(C2=CC=CC(=O)C=C2[C@@H1](N3C=C(N=N3)C4=CC=CC=N4)CC1)NC=C(OC)C(OC)SCOC
PUGREST.BadRequest: error:
C1=C2C(C3=C(C=C(OC)C=C3CC[C@@H1]2N4C=C(N=N4)C=CC)OCOC)OC=CC=C(C1=O)C
PUGREST.BadRequest: error:
C=C1C([C@H1](CCC2=CC(=C(OC)C(=C21)OC)OC)NCCCCCCCCCC)C=CCC(NC)=O
PUGREST.BadRequest: error:
C=1=C2C(C3=C(C=C(OC)C=C3CC[C@@H1]2N4C=C(N=N4)C=CC)OCOC)OC=CC=CC=1O
PUGREST.BadRequest: error:
C1=C2C(C3=CC=CC(=O)C=C3[C@H1](CC2)N4C=C(N=N4)C=CC=CC=N)(OC)C(OC)=C1OCNC
PUGREST.BadRequest: error:
C=C1C2(C3=C(C=C(OC)C=C3CC[C@@H1]1N4C=C(N=N4)C=CC)OCOC)OC=CC=C(NC)C2=O
PUGREST.BadRequest: error:
C1=C2C(C3=C(C=C(OC)C=C3CC[C@@H1]2N4C=C(N=N4)C=CC)OCOC)OC=CC=C(C1=O)NC
PUGREST.BadRequest: error:
C=C1C(C2=C(C=C(OC)C=C2CC[C@@H1]1N3C=C(N=N3)CCC)COCO)C=CC=C(NC)C=O
PUGREST.BadRequest: error:
C=C1C(C2=CC=C(C(=O)C=C2[C@@H1](N3C=C(N=N3)C4=NC=CC=C4)CC1)NC)=C(OC)C(OC)=COC
PUGREST.BadRequest: error:
C1=C2C(C3=CC=C(C(C=C3[C@H1](CC2)N4C=C(N=N4)C5=NC=CC=C5O)N)C=C)(C(=C1OC)OC)OC
PUGREST.BadRequest: error:  C12=CC([C@@H1]1NCC3=CC=C(C)C=C3CCCC)C(SC)=CC2O
PUGREST.BadRequest: error:
O=C(OCC)CN(CCO)N[C@H1]1CCC2=CC(OC)=C(OC)C(OC)=C2C3=CC=C(NC)C(=O)C=C13
PUGREST.BadRequest: error:
O=C(O)C=C(OC)CC=C1C(C2=C(OC)C(OC)=C(OC)C=C2C=3C[C@@H1]1N=CC=3S)C
PUGREST.BadRequest: error:
```

```
O=C(O)C=C(OC)CC=C1C(C2=C(OC)C(OC)=C(OC)C=C2C=3C[C@@H1]1N=CC=3SC)=O
PUGREST.BadRequest: error:
O=C(C)N[C@@H1]1CC(C2=C(C(OC)=C(OC)C=C2CC1)OC(CC)=O)=CC=C(SC)C(=O)C
PUGREST.BadRequest: error:
O=C(O)C=C(OC)CC=C1C(C2=C(OC)C(OC)=C(OC)C=C2C=3C[C@@H1]1N=CC=3S)C=O
PUGREST.BadRequest: error:
O=C(C)N[C@@H1]1C2=CCC(SC)=CC=C2C3=C(C(OC)=C(OC)C=C3CC1)OCOO
PUGREST.BadRequest: error:
O=C(C1)N[C@@H1]2CC(C3=C(C(OC)=C(OC)C=C3CC2)OC(CC)=O)=CC=C(SC)C1=O
PUGREST.BadRequest: error:
O=C(C)N[C@@H1]1C2=CCC(SC)=CC=C2C3=C(C(OC)=C(OC)C=C3CC1)OC=O
PUGREST.BadRequest: error:  OC(OC(C)CC)CC(NCC1=CN(N=N1)[C@H1]C2CC3=CC(OC)=C(OC)C
(OC)=C3C4=CC=CC(=O)C=C24)NC=O
PUGREST.BadRequest: error:
O=CC(OOC1=C)C=CC=C1OC(=O)N[C@H1]2CCC3=CC(OC)=C(OC)C(OC)=C3C4=CC=C(NC)C(=O)C=C24
PUGREST.BadRequest: error:
OC(OC(C)CC)CC=1OCCC=C2[C@H1](CCC3=C(C(OC)=C(OC)C(OC)=C3)C2=CC=1)NSC=O
PUGREST.BadRequest: error:  O=C(C1=C(C(OCC)=O)N(N=N1)[C@@H1]C2=C3C(C(NC)=CC=C2C4
=C(C(OC)=C(OC)C=C4CC3)OC)=O)O
PUGREST.BadRequest: error:
C1[C@@H1](C2=CC(C(NC)=CC=C2C3=C(OC)C(OC)=C(C=C3C1)O)C)ONC(C(=O)O)=CNC
PUGREST.BadRequest: error:
C1=2[C@H1](CCC3=CC(OC)=C(OC)C(OC)=C3C1=CC=C(C(=O)C=2)NCO)C(C(OC)=O)NC(=O)CNC
PUGREST.BadRequest: error:
C1[C@@H1](C2=CC(C(NC)=CC=C2C3=C(OC)C(OC)=C(C=C3C1)O)C)NC(C(O)=O)=CC(=O)ONC=O
PUGREST.BadRequest: error:
C1[C@@H1](C2=CC(C(SC)=CC=C2C3=C(OC)C(OC)=C(C=C3C1)O)C)NC(C(O)=O)=C(COCC)OOSC=O
PUGREST.BadRequest: error:
CN([C@H1]1CC2=CC(OC)=C(OC)C(OC)=C2C=C3C=C)NC(=O)C=C13
PUGREST.BadRequest: error:
C=12N(N=NC=1C(OCC3=CC=C(C=C3)F)=O)C(OC)=C(OC)C(OC)=CC4=CC=C(C(=O)C=C24)NC
PUGREST.BadRequest: error:
C1=CC1(C=CN(COC(OC)=O)N=NN)[C@H1]C2CC3=CC(OC)=C(OC)C(OC)=C3C=CC=C(NC)C(=O)C=C2
PUGREST.BadRequest: error:
O=C(C1=C(C(OCC)=O)N(N=N1)[C@@H1]C2=C3CC(NC)=CC=C2C4=C(C(OC)=C(OC)C=C4CC3)OC)OO
PUGREST.BadRequest: error:
C=1N(N=NC=1C(=O)OCC2=CC=C(C=C2)F)CC(OC)=C(OC)C(OC)=CC3=CC=C(NC)C(=O)C=C3
PUGREST.BadRequest: error:
O=CC1=C(C(OCC)=O)N(N=N1)[C@@H1]C2=C3C(C(NC)=CC=C2C4=C(C(OC)=C(OC)C=C4CC3)OC)=O
PUGREST.BadRequest: error:
C1[C@@H1](C2=CC3(C(SC)=CC=C2C4=C(OC)C(OC)=C(C=C4C1)O)C)NC(C(O)=O)=C(C3)ONC
PUGREST.BadRequest: error:  N([C@@H1]1CCC2=CC(OC)=C(OC)C=C2CC1)=CC=C(C=C)NC
PUGREST.BadRequest: error:
O=C(C(Cl)Cl)NC(=O)N[C@H1]1CCC2=C(C(OC)=C(OC)C(OC)=C2)C3=CC=C(NC)C(=O)C=C13
PUGREST.BadRequest: error:
O=C(C(Cl)Cl)NCC1=CN(N=N1)[C@H1]2CCC3=CC(OC)=C(OC)C(OC)=C3C4=CC=C(C(=O)C=C42)NCCO
PUGREST.BadRequest: error:  O=C(C(Cl)Cl)NC(=O)N[C@H1]1CCC2=C(C(OC)=C(OC)C(OC)=C2
)C3=CC=C(C(C(=C31)=O)NCC(=O)OC
```

```
PUGREST.BadRequest: error:
O=C(C(Cl)Cl)NC(=O)N[C@H1]1CCC2=C(C(OC)=C(OC)C(OC)=C2)C3=CC=C(NC)C(=O)C=C31
PUGREST.BadRequest: error:
O=C(O)C=C(C(OCC)=O)N=NN[C@H1]1CCC2=CC(OC)=C(OC)C(OC)=C2C3=CC=C(NC)C(=O)C=C13
PUGREST.BadRequest: error:
O=C(O)C1=C(C(OCC)=O)N=NN1[C@H1]2CCC3=CC(OC)=C(OC)C(OC)=C3C4=CC=C(NC)C(=O)C=C24
PUGREST.BadRequest: error:
O=C(C)N[C@@H1]C1=C2C(C(SC)=CC=C1C3=C(C(OC)=C(OC)C=C3CC2)OC(C(C)C)=O)C=O
PUGREST.BadRequest: error:
O=C(OCC)C1=CN(N=N1)[C@H1]2CCC3=CC(OC)=C(OC)C(=C3C4=CC=C(NC)C(C=C42)=O)OCC
PUGREST.BadRequest: error:
O=C(C)N[C@@H1]C1=C2C(C(SC)=CC=C1C3=C(C(OC)=C(OC)C=C3CC2)OC(C4=CC=CC=C4)=O)SC=O
PUGREST.BadRequest: error:
O=C(C(C)C)OC=1C(C=C2[C@H1](CCC3=CC(OC)=C(OC)C(OC)=C3C2=CC=1N)C(C)=O)SC=O
PUGREST.BadRequest: error:
O=C(O)C1=CN(N=N1)[C@H1]2CCC3=C(C(OC)=C(OC)C(OC)=C3)C4=CC=C(C(=O)C=C42)NCC
PUGREST.BadRequest: error:
O=C(O)C1=C(C(OCC)=O)N=NN1[C@H1]CCC2=CC(OC)=C(OC)C(OC)=C2C3=CC=C(C(C=C3)=O)NC
PUGREST.BadRequest: error:
O=C(C(C)C)OCC(C=C1[C@H1](CCC2=CC(OC)=C(OC)C(O)=C2C1=CC=O)C(C)=O)C=O
PUGREST.BadRequest: error:
O=C(C(C)C)OC=1C(C=C2[C@H1](CCC3=CC(OC)=C(OC)C(OC)=C3C2=CC=1S)C(C)=O)OC
PUGREST.BadRequest: error:
O=C(O)C=C(OC)C(C(NC)=CC=C1C2=C(OC)C(OC)=C(OC)C=C2CC[C@@H1](C1)NCC)=O
PUGREST.BadRequest: error:
O=C(O)C1=CN(N=N1)[C@H1]2CCC3=C(C(OC)=C(OC)C(OC)=C3)C4=CC=C(C(C=C42)=O)NCC
PUGREST.BadRequest: error:  CCCCCCCCCCCCCCCC(OCC1=CN(N=N1)[C@H1]C2CC3=CC(OC)=C(O
C)C(OC)=C3C4=CC=C(C(=O)C=C24)NC)=O
PUGREST.BadRequest: error:
CCCCCCCCCCCCCCCC=1OCCC=C2[C@H1](CCC3=C(C(OC)=C(OC)C(OC)=C3)C2=CC=1)NSC=O
PUGREST.BadRequest: error:
OC(=O)C1=C(C(OCC)=O)N=NN1[C@H1]CCC2=CC(OC)=C(OC)C(OC)=C2C3=CC=C(NC)C(C=C3)=O
PUGREST.BadRequest: error:
C[C@H1](NCC)CC1=CCC(NC)=CC=C1C2=C(C=C(OC)C(OC)=C2OCC3=CC=CC=C3)OSC
PUGREST.BadRequest: error:  C1[C@H1](OC1)O[C@@H1]([C@H1]O)COOCNC2(N=NC(=C2)C=CC3
=C(C=CC4=C3)Cl)CC(OC)=C(OC)C=C4CCNC=O
PUGREST.BadRequest: error:
OC(=O)C1=C(C(OCC)=O)N(N=N1)[C@H1]C2CC3=CC(OC)=C(OC)C(OC)=C3C4=CC=C(NC)C(=O)C=C42
PUGREST.BadRequest: error:
C1[C@H1](OCCC)CC2=CC(C(NC)=CC=C2C3=C(C(OC)=C(OC)C=C3CC1)OC(OCC)=O)NC=O
PUGREST.BadRequest: error:
C1=C(OC)C=C(OC)C=C1CC[C@H1](C=CC(C(OC(C2=CC=CC=C2)=O)=CC=CC)=O)C
PUGREST.BadRequest: error:  N(C)C(=O)NCCC(OCC1=CN(N=N1)[C@H1]CCC2=CC(OC)=C(OC)C(
OC)=C2C3=CC=C(C(=O)C=C3)N)C=O
PUGREST.BadRequest: error:
C1[C@H1](OC1)OCOC(=S)N[C@H1]C2CC=C3C(OC)=C(OC)C(OC)C=C3C=4C2=CC(=O)C(=CC=4)NCC=C
PUGREST.BadRequest: error:
C1[C@H1](NCC)C2C=CC(OC)=C(OC)C=C2CC[C@H1]C3=CCC(OC(OCC)=O)=CC=C31
```

```
PUGREST.BadRequest: error:
C12=C(OC)C=C(OC)C=C1CC[C@H1](NC(C(O)=O)(C)C2=CC=C(NC)C)CON=COC
PUGREST.BadRequest: error:
N(C)CNCC1=CN(N=N1)[C@H1]2CCC3=CC(OC)=C(OC)C(OC)=C3C4=CC=C(C(C=C24)=O)NC=O
PUGREST.BadRequest: error:
OC(=O)C=C(C(OCC)=O)N(NCCC)N[C@H1]1CCC2=CC(OC)=C(OC)C(OC)=C2C3=CC=C(NC)C(=O)C=C31
PUGREST.BadRequest: error:
C[C@H1](NCC)CC1=CC(C(NC)=CC=C1C2=C(C=C(OC)C(OC)=C2OCC3=CC=CC=C3)OSCC)=O
PUGREST.BadRequest: error:  C1[C@H1](OC1)OOC(O)(C)CN=CN([C@@H1]2C3=CC(C(SC)=CC=C
3C4=C(OC)C(OC)=C(C=C4CC2)O)O)C=O
PUGREST.BadRequest: error:
C12=C(OC)C=C(OC)C=C1CC[C@H1](NC(C(O)=O)(C)C2=CC=C(NC)C)COCNCOC
PUGREST.BadRequest: error:  C=1C(C)(C)OC(=O)/N=C(/N[C@H1]2CCC3=C(C(OC)=C(OC)C(OC
)=C3)C4=CC=C(NC)C(=O)C=C24)N=1
PUGREST.BadRequest: error:  C=1C(C)(C)OC(=O)/N=C(/N[C@H1]2CCC3=C(C(OC)=C(OC)C(OC
)=C3)C4=CC=C(C(C=C42)=O)NC)N=1
PUGREST.BadRequest: error:
N1=NN1[C@@H1]2C3=CC(C(NC)=CC=C3C4=C(C(OC)=C(OC)C=C4CC2)OC)=O
PUGREST.BadRequest: error:
CNCC1C=CC=C(C=C1)NC(=O)N[C@@H1]2C3=CC(C(NC)=CC=C3C4=C(OC)C(OC)=C(C=C4CC2)OC)=O
PUGREST.BadRequest: error:
CNC=CC=C1C[C@H1](CCC2=CC(OC)=C(OC)C(=C21)OC)N3C(C(O)=O)=C(N=N3)C(=O)OOSC
PUGREST.BadRequest: error:
CNC=1CC=C2[C@H1](CCC3=C(C(OC)=C(OC)C(=C3)OC)C2=CC=1)NC(C(=O)O)=CC=CNC=O
PUGREST.BadRequest: error:
CNC=1CC2=C3[C@H1](CCC4=C(C(OC)=C(OC)C(=C4)OC)C3=C5C=1)NC(C(=O)O)=C5ON2
PUGREST.BadRequest: error:
N=NN([C@H1]1CCC2=C(C(OC)=C(OC)C(OC)=C2)C3=CC=C(C(=O)C=C13)NC)COOCCOC=O
PUGREST.BadRequest: error:
CNCC1C=C2[C@H1](CCC3=C(C(OC)=C(OC)C(=C3)OC)C2=CC=C1)NCOC(C4=CC=NC=C4)=O
PUGREST.BadRequest: error:
O=CC=CC=CC(NCC1=CN(N=N1)[C@H1]2CCC3=CC(OC)=C(OC)C(OC)=C3C4=CC=C(C(C=C24)=O)NC)=O
PUGREST.BadRequest: error:
O=CC=CC=CC(NCC1=CN(N=N1)[C@H1]2CCC3=CC(OC)=C(OC)C(OC)=C3C4=CC=C(NC)C(=O)C=C24)O
PUGREST.BadRequest: error:
CNC=1CC=C2[C@H1](CCC3=C(C(OC)=C(OC)C(OC)=C3)C2=CC=1)NC(C(=O)O)=CC=CNC=O
PUGREST.BadRequest: error:
N=NN([C@H1]1CCC2=C(C(OC)=C(OC)C(OC)=C2)C3=CC=C(C(=O)C=C13)NC)COCC=COCOCC
PUGREST.BadRequest: error:
C12=C3C(C=4C([C@@H1](NCC5=CC=C(C)C=C5CCC1)C2)=CC(=O)C(=CC=4)SC=C)(OC)C(=C3OC)OC
PUGREST.BadRequest: error:
C1=C2C(C3=C(CC[C@@H1]2N4N=NC(=C4)C5(O)CCCCC5)C=C(C(OC)=C3OC)OC=C)C=C(C1=O)O
PUGREST.BadRequest: error:
C1=C2C(C=C(CC[C@@H1]2N3C=C(N=N3)COCC4=CC=CC=C4)C=C(C(=COC)OC)OC)=CC=C(NC)C1=O
PUGREST.BadRequest: error:  C1=C2C(C3=CC=C(C(=O)C=C3[C@H1](CC2)N4C=C(N=N4)COC(=O
)C5=CC=NC=C5)COC)C(OC)=C1OCNC=O
PUGREST.BadRequest: error:
C12=C3C(CC([C@@H1](NCC4=CC=C(C)C=C4CCC1)C2)=CC(=O)C(=CC)SC=C)(OC)C(=C3OC)OC
```

```
PUGREST.BadRequest: error:
C1=C2C(CC([C@@H1]1NCCCCCCCCCCCCCCCC)C(=CC2)SC=COC)C(OC)=COC
PUGREST.BadRequest: error:
CN(N([C@@H1]1C2=CC(=O)C(NC)=CC=C2C3=CC(=C(OC)C=C3CC1)OC)O)CNC(=S)CCCCC
PUGREST.BadRequest: error:
C=C1C(C=C(CC[C@@H1]1N2N=NC(=C2)C3=CC=C(Cl)C=C3)C=C(C(OC)=COC)OC)=CC=C(C=O)NC
PUGREST.BadRequest: error:
CC(C)C(=O)OCC1=CN(N=N1)[C@H1]2CCC3=CC(OC)=C(OC)C(OC)=C3C4=CC=C(NC)C(=O)C=C24
PUGREST.BadRequest: error:
CC(C)C=1OCCC=C2[C@H1](CCC3=C(C(OC)=C(OC)C(OC)=C3)C2=CC=1)NSC=O
PUGREST.BadRequest: error:
CC(C)(O)C1N=NN(CN1CC[C@H1]2CC)C3=CC(OC)=C(OC)C(OC)=C3C4=CC=C(C(C=C24)=O)NC
PUGREST.BadRequest: error:
N(C(C1=CC=NC=C12)=O)[C@@H1]3CC(C4=C(C(OC)=C(OC)C=C4CC3)OC(OC)=O)=CC=C(SC)C2=O
PUGREST.BadRequest: error:
N(C(C1=CC=NC=C1)=O)[C@@H1]2C=3C(C4=C(C(OC)=C(OC)C=C4CC2)OC)=CC=C(SC)C(=O)C=3
PUGREST.BadRequest: error:
C12=C(C(=C(OC)C(OC)=C1)O)CC3=CC=C(NC)CC=C3[C@H1](CC2)NC(C)=O
PUGREST.BadRequest: error:
N1(CCCC1)CN[C@H1]2CCC3=C(C(OC)=C(OC)C(OC)=C3)C4=CC=C(SC)C(=O)C=C24
PUGREST.BadRequest: error:
C1[C@H1](NC(O)=O)C2=CCC=CC=C2C3=C(OC)C(OC)=C(OC)C=C3CC=C1SC=O
PUGREST.BadRequest: error:
N1(CCCC1)C(=O)OC2=C(OC)C(OC3)=CC=C2C4=CC=C(SC)C(C=C4[C@@H1](NC(=O)C)CC3)=O
PUGREST.BadRequest: error:
C=CC(=O)OCCC=C1[C@H1](CCC2=C(C(OC)=C(OC)C(OC)=C2)C1=CC)N=O
PUGREST.BadRequest: error:
N(C(C1=CC=NC=C1)=O)[C@@H1]2C=3C(C4=C(C(OC)=C(OC)C=C4CC2)OC=C)C=C(SC)C(=O)C=3
PUGREST.BadRequest: error:
C12=CC(OC)=C(OC)CCC([C@H1](CCCC=C1OC)NC(C)=O)(OC(=O)OC)C2=O
PUGREST.BadRequest: error:
C1[C@H1](NC(O)=O)C=CCC(SC)=CCC=C(C2=C(OC)C(OC)=C(OC)C=C2CC1)SC=O
PUGREST.BadRequest: error:  C12=C(C(OC)=C(OC)CCC([C@@H1]2CCC(C)C1N=C)=O)OSC
PUGREST.BadRequest: error:
C=CC(OC)=C(OC)CC1=CC=C(NC)CC=C1[C@@H1](NC(N(CCO)CCO)=S)CCCNC=O
PUGREST.BadRequest: error:
CNC=1C(=O)C=C2[C@H1](CCC3=CC(OC)=C(OC)C(OC)=C3C2=CC=1)NC4(C(=O)O)C=CN=N4
PUGREST.BadRequest: error:
CNC=1C(=O)C=C2[C@H1](CCC3=CC(OC)=C(OC)C(OC)=C3C2=CC=1N)C4(C(=O)O)C=CN=N4
PUGREST.BadRequest: error:
N1=CC=CC2=C1C(OC=CC(OC=3)=CCCC[C@H1](NC(C)=O)C4=CCC(SC)=CC=C4C=32)C
PUGREST.BadRequest: error:
N=1N(C=C(CNC(C(C)C)=O)N=1)[C@H1]CCC2=C3C(OC)=C(OC)C(OC)=C2C=CC=C(CC=CC=C3)CC
PUGREST.BadRequest: error:
NN(C=C(CNC(C1CCCC1)=O)N=N)[C@H1]CCC2=CC(OC)=C(OC)C(OC)=C2C3=CC=C(C(=O)C=C13)NC
PUGREST.BadRequest: error:
C=C1[C@H1](CCC2=C(C(OC)=C(OC)C(OC)=C2)C1=CC=CC(C)=O)NCNC(C)=O
PUGREST.BadRequest: error:
```

```
N=1N(C=C(CNC(C(C)C)=O)N=1)[C@H1]CCC2=CC(OC)=C(OC)C(OC)=C2C=CC3=CCC=CC=C3NC=O
PUGREST.BadRequest: error:
C=C1[C@H1](CCC2=CC(OC)=C(OC)C(OC)=C2C1=CC=C(C(=O)C)NC)NC(C(=O)O)=CN=CNC
PUGREST.BadRequest: error:  CC(C)(C)OC(=O)NCCC(NCC=1N=NN(C=1)[C@H1]C2CC3=CC(OC)=
C(OC)C(OC)=C3C4=CC=C(C(=O)C=C24)N)C=O
PUGREST.BadRequest: error:
C1C(C)(C)OC/N=C(/N[C@H1]2CCC3=CC(OC)=C(OC)C(=C3C4=CC=C(NC)C(C=C42)=O)OC)N=N1
PUGREST.BadRequest: error:  CC(C)(C)OCNCCC(=O)NCC1=CN(N=N1)[C@H1]2CCC3=CC(OC)=C(
OC)C(OC)=C3C4=CC=C(C(C=C24)=O)NC=O
PUGREST.BadRequest: error:
C1C(C)(C)OC/N=C(/N[C@H1]2CCC3=CC(OC)=C(OC)C(=C3C4=CC=C(NC)C(C=C42)=O)OC)N1C
PUGREST.BadRequest: error:  CC(C)(C)OC(=O)NCCC(OCC=1N=NN(C=1)[C@H1]C2CC3=CC(OC)=
C(OC)C(OC)=C3C4=CC=C(C(=O)C=C24)NC)=O
PUGREST.BadRequest: error:  CC(C)(C)OC(=O)NCCC(NCC=1N=NN(C=1)[C@H1]2CCC3=CC(OC)=
C(OC)C(OC)=C3C4=CC=C(C(C=C24)=O)N)C=O
PUGREST.BadRequest: error:
CNCCC1=CC=C(C=C1)NC(=O)N[C@H1]2CCC3=CC(OC)=C(OC)C(OC)=C3C4=CC=C(C(C=C24)=O)NC=O
PUGREST.BadRequest: error:
N=NN([C@@H1]1C=2C(C3=C(C(OC)=C(OC)C=C3CC1)OC)=CC=C(NC)CC=2)OC(O)=O
PUGREST.BadRequest: error:
N1=NN[C@@H1]2CCC3=C(C(OC)=C(OC)C=C3CC2=CC(OC)=C(OC)COC)CN1
PUGREST.BadRequest: error:
O=CC=CC=CC(NCC1=CN(N=N1)[C@H1]C2CC3=CC(OC)=C(OC)C(OC)=C3C4=CC=C(C(=O)C=C24)NC)=O
PUGREST.BadRequest: error:  CNCC(C1=CC=C(C=C1)NC(=O)N[C@@H1]2C3=CC(C(NC)=CC=C3C4
=C(C(OC)=C(OC)C=C4CC2)OC)=O)OC
PUGREST.BadRequest: error:
CNCC1C=C2[C@H1](CCC3=C(C(OC)=C(OC)C(=C3)OC)C2=CC=C1)NCOC(C4=CC=CC=C4)=O
PUGREST.BadRequest: error:
N=NN([C@H1]1CCC2=C(C(OC)=C(OC)C(=C2)OC)C3=CC=C(NC)C(C=C13)=O)COC4CC=CC=C4O
PUGREST.BadRequest: error:
C1=C2C(C3=CC=C(CC=C3[C@@H1](N4C=C(N=N4)CNC(=O)C(Cl)Cl)C1)ONC=C)(C(=C2OC)OC)OC
PUGREST.BadRequest: error:
C12=C3C(CC([C@@H1](NCC4=CC=C(C)C=C4CCC1)C2)NN=NC=C(NC)C=O)=C=C(C(=C3)OC)OCOCC
PUGREST.BadRequest: error:
C=C1C(C=C(CC[C@@H1]1N2N=NC(=C2)CNC(=O)OCCC)CC=C(C(OC)=COC)O)C=CC=C(C=O)NC
PUGREST.BadRequest: error:
C=C1C(C=C(CC[C@@H1]1N2C=C(N=N2)CNC(=O)C3=CC=NC=C3)C=CC(OC)=COC)OC=CC=C(C=O)NC
PUGREST.BadRequest: error:
O(C=CC=CC1=C(OC)C(OC)=C(OC)C=C1CC[C@H1](C=CCCOCC2=CC=CC=C2)OCC)ONC
PUGREST.BadRequest: error:
O(C=CC=CC1=C(OC)C(OC)=C(OC)C=C1CC[C@H1](C=CCCOCC2=CC=CC=C2)OSC=O)NC
PUGREST.BadRequest: error:
O=C(OC)OCC1=CN(N=N1)[C@H1]2CCC3=CC(OC)=C(OC)C(OC)=C3C4=CC=C(NC)C(=O)C=C24
PUGREST.BadRequest: error:
O(C=CC=CC1=C(OC)C(OC)=C(OC)C=C1C2C[C@H1](C=CCC(OCC(C)C)=O)N=C2ON)C
PUGREST.BadRequest: error:
O=C(C(C)C)NC(=S)N[C@@H1]1C=2C(C3=C(C(OC)=C(OC)C=C3CC1)OC)=CC=C(NC)C(=O)C=2
PUGREST.BadRequest: error:
```

```
O=C(OCC)OCC=1N=NN(C=1)[C@H1]2CCC3=CC(OC)=C(OC)C(OC)=C3C4=CC=C(NC)C(=O)C=C24
PUGREST.BadRequest: error:
O=C(OCC)CN(CCO)C(=S)N[C@H1]1CCC2=CC(OC)=C(OC)C(OC)=C2C3=CC=C(NC)C(=O)C=C13
PUGREST.BadRequest: error:
CC(C)CNCC1=CN(N=N1)[C@H1]2CCC3=CC(OC)=C(OC)C(OC)=C3C4=CC=C(C(C=C24)=O)NC=O
PUGREST.BadRequest: error:
CC(C)CNCC1=CN(N=N1)[C@H1]2CCC3=CC(OC)=C(OC)C(OC)=C3C4=CC=C(NC)C(=O)C=C24
PUGREST.BadRequest: error:
C(=CN(C(=O)OCC)N[C@@H1]1CC=2)(C3=C(C(OC)=C(OC)C=C3CC1)OC)CC=C(NC)C(C=2)=O
PUGREST.BadRequest: error:
COCNCC1=CN(N=N1)[C@H1]2CCC3=CC(OC)=C(OC)C(OC)=C3C4=CC=C(C(C=C42)=O)NC=O
PUGREST.BadRequest: error:
C(=NC=C(NC)CC(=O)N)CC1=CN(N=N1)[C@H1]CCC2=CC(OC)=C(OC)C(OC)=C2C=CC=C(C)CNC=O
PUGREST.BadRequest: error:
C(=C1N(C(=O)OCC)N12)CCC3=C(C(OC)=C(OC)C(OC)=C3)C4=CC=C(NC)C(C=C24)=O
PUGREST.BadRequest: error:
N(C(C)C)C(NCC1=CN(N=N1)[C@H1]2CCC3=CC(OC)=C(OC)C(OC)=C3C4=CC=C(NC)C(=O)C=C24)=O
PUGREST.BadRequest: error:
N(C)C1=CC=C2C3=C(OC)C(OC)=C(OC)C=C3CC[C@@H1](C2=CC1=O)NC(C(=O)O)CCON=CCF
PUGREST.BadRequest: error:
N(C(C(C(C)C)=O)C1=CN(N=N1)[C@H1]2CCC3=C(C(OC)=C(OC)C(OC)=C3)C4=CC=C(NC)C(=O)C=C24
PUGREST.BadRequest: error:
N(CCCCCCC=O)CC1=CN(N=N1)[C@H1]2CCC3=CC(OC)=C(OC)C(OC)=C3C4=CC=C(NC)C(=O)C=C24
PUGREST.BadRequest: error:  C1=C(C=CC(Cl)=C1)COC(=O)CN[C@@H1]2C=3C(C4=C(C(OC)=C(
OC)C=C4CC2)OC)=CC=C(SC)C(=O)C=3
PUGREST.BadRequest: error:
N(C)C1=CC=C2C3=C(OC)C(OC)=C(OC)C=C3CC[C@@H1](C2=CC1=O)NC(C(=O)O)CCONC(=O)C=C
PUGREST.BadRequest: error:
N(C)C1=CC=C2C3=C(OC)C(OC)=C(OC)C=C3CC[C@@H1](C2=CC1=O)NC(C(=O)O)CCONC(=O)CNC
PUGREST.BadRequest: error:
N(C(C)C)CC(=O)OCC1=C2N(N=N1)[C@H1]CCC3=CC(OC)=C(OC)C(OC)=C3C4=CC=C(NC)C(C=C24)=O
PUGREST.BadRequest: error:
C[C@H1](C1OC=CN[C@@H1]2C=C(C3=CC(OC)=C(OC)C(OC)=C3C1)CC=C(NC)CC2)ONCC
PUGREST.BadRequest: error:  C1=C2CC[C@H1](C3=CC(C(SC)=CC=C3C2C=C1O)CNC=O)CN=O
PUGREST.BadRequest: error:
COC=C1C2=CC=C(NC)C(=O)C=C2[C@@H1](NCC(C)C)CCC1=CC(OC)=COC
PUGREST.BadRequest: error:
C1=C2CC[C@H1](C3=CCC(SC)=CC=C3C2=C(C(OC)=C1OC)OC=O)[N-1]
PUGREST.BadRequest: error:
C=C1C=2C[C@H1](CC(C3=CC(OC)=C(OC)C=C31)C=CC=CSC)C(=O)C=2N
PUGREST.BadRequest: error:
COC=C1C2=CC=C(NC)C(=O)C=C2[C@@H1](NCCC)CCC1=CC(OC)=COC
PUGREST.BadRequest: error:
C1OC=C2C3=CC=C(NC)C(=O)C=C3[C@H1](CCC2C=COC)NCCCCCOC=C1
PUGREST.BadRequest: error:
C1=C2CC[C@H1](C3=CCC(NC)=CC=C3C2=C(C(OC)=C1OC)OC)ONCCC#N
PUGREST.BadRequest: error:
CC(CC)N[C@@H1]1C2=CC(C(SC)=CC=C2C3=C(OC)C(OC)=C(C=C3CC1)OC)=O
```

```
PUGREST.BadRequest: error:
CC(CC)N[C@@H1]C1=C2C(C(NC)=CC=C1C3=C(OC)C(OC)=C(OC)C=C3CC2)=O
PUGREST.BadRequest: error:
CC(CC)N[C@@H1]1C2=CC(C(SC)=CC=C2C3=C(CC1)C=C(OC)C(OC)=C3OC)=O
PUGREST.BadRequest: error:
C1=C2CC[C@H1](C3=CCC(SC)=CC=C3C2=C(C(OC)=C1OC)OC=O)N=[N+1]=[N-1]
PUGREST.BadRequest: error:
COC1=C2C3=CC=C(NC)C(=O)C=C3[C@H1](CCC2=CC(OC)=C1OC)NCC4=CC=NS4C
PUGREST.BadRequest: error:
N(CCCC)C(CN1[C@@H1]C2=CCC(O)=CC=C2C3=C(CC1)C=C(OC)C(OC)=C3OC)=O
PUGREST.BadRequest: error:
C1[C@H1](NCC)CC2=CC(C(NC)=CC=C2C3=C(OC)C(OC)=C(OC)C=C3C1N)C=O
PUGREST.BadRequest: error:
N(C)C=1C(=O)C=C2[C@H1](CCC3=CC(OC)=C(OC)C(OC)=C3C2=CC=1N)C(C)CCSC
PUGREST.BadRequest: error:
C1=C(OC)C(OC)=C(OC=2)CC3=CC=C(SC)C1(C=C3[C@@H1](NC(N(C)C)=O)CCC=2)O
PUGREST.BadRequest: error:
N(C(C)=O)[C@@H1]C1=CC(C(NC)=CC=C1C2=C(OC)C(OC)=C(OC)C=C2CCNC=O)C
PUGREST.BadRequest: error:
C[C@H1](OCCC)[N-1]C1=CC(C(NC)=CC=C1C2=C(C(OC)=C(OC)C=C2C)OC)NC(=O)C
PUGREST.BadRequest: error:
C1=C(OC)C(OC)=C(OC)CC2=CC=C(SC)C1(C=C2[C@@H1](NC(N(C)C)=O)CCC)O
PUGREST.BadRequest: error:
N1=NN([C@@H1]C2=CC(C(O)=CC=C2C3=C(CC)C=C(OC)C(OC)=C3OC)=O)CC=C1F
PUGREST.BadRequest: error:
CNC=1C(C=C2[C@H1](CCC3=C(C(OC)=C(OC)C(OC)=C3)C2=CC=1)NC(C)C=O)SC
PUGREST.BadRequest: error:
CNC=1C(C=C2[C@H1](CCC3=C(C(OC)=C(OC)C(OC)=C3)C2=CC=1)NC(C)C)=O
PUGREST.BadRequest: error:
CNC=1C(C=C2[C@H1](CCC3=C(C(OC)=C(OC)C(OC)=C3)C2=CC=1)NC(C)C)CSCSC=O
PUGREST.BadRequest: error:
CNC=1C(C=C2[C@H1](CCC3=C(C(OC)=C(OC)C(OC)=C3)C2=CC=1)NC(C)C=O)SCSC
PUGREST.BadRequest: error:
C=1NC=2C(C=C3[C@H1](CCC4=C(C(OC)=C(OC)C(OC)=C4)C3=CC=2)NC(C)C)N=CN=1
PUGREST.BadRequest: error:
NN([C@H1]1CCC2=CC(OC)=C(OC)C(OC)=C2C3=CC=C(NC)C(=O)C=C13)C=O
PUGREST.BadRequest: error:
CNC=1C(C=C2[C@H1](CCC3=C(C(OC)=C(OC)C(OC)=C3)C2=CC=1)NC(C)C)C=O
PUGREST.BadRequest: error:
N1=NC(=CN(NC1)C=CC2(CC[C@H1](N)C3=CC(C(NC)=CC=C32)=O)CCOC)COC=C
PUGREST.BadRequest: error:
N1=NC(=C2N(NC1)C=CC3=CC4=C(OC)C(OC)=CC=C4CC[C@H1](NC(N)=O)C3=C2)OC=O
PUGREST.BadRequest: error:
N1=NC(=CN(N=N1)[C@H1]2CCC3=CC(OC)=C(OC)C(OC)=C3C4=CC=C(C(C=C42)=O)N)C
PUGREST.BadRequest: error:
OCCN(CC=C)C(=O)N[C@@H1]1C2=CC(C(NC)=CC=C2C3=C(OC)C(OC)=C(OC)C=C3CC1)=O
PUGREST.BadRequest: error:
N1=NC=CN(NC1)C=CC=C2C3=C(OC)C(OC)=C(OC)C=C3CC[C@@H1](C2=C)NC=CC=C
```

```
PUGREST.BadRequest: error:
N1=NC=CN(N=N1)[C@H1]2CCC3=CC(OC)=C(OC)C(OC)=C3C4=CC=C(NC)C(=O)C=C24
PUGREST.BadRequest: error:
N1=NC=CN(NC1)[C@H1]2CCC3=CC(OC)=C(OC)C(OC)=C3C4=CC=C(NC)C(=O)C=C24
PUGREST.BadRequest: error:
N1=NC(=CN(NC12)C=CC3=C(O)C(OC)=C(OC)C=C3CC[C@H1](N)C4=CCC(NC)=CC=C24)OC
PUGREST.BadRequest: error:
N1=NC(=CN(NC1)C=CC2=C(O)C(OC)=C(OC)C=C2CC3[C@H1](N)C=CCC(NC)=CC=C3)OC
PUGREST.BadRequest: error:
OCCN(CC=C)C(=S)N[C@H1]1CCC2=CC(OC)=C(OC)C(OC)=C2C3=CC=C(NC)C(=O)C=C13
PUGREST.BadRequest: error:
CNC(N(C)C)CN[C@H1]1CCC2=CC(OC)=C(C(OC)=C2C=3C1=CC(=O)C(=CC=3)NC)OC
PUGREST.BadRequest: error:
C1=C2C([C@H1](CCC3=CC(OC)=C(C(=C32)OC)OC)NCCC=CC)(C(SC)=C1)O
PUGREST.BadRequest: error:
C=C1C([C@H1](CCC2=CC(OC)=C(C(OC)=C12)OC)NCC=CC=O)C(=CC)NC
PUGREST.BadRequest: error:
C12=CC(C(SC)=CC=C1C3=C(CC[C@@H1]2NCC4=CC=NC=C4)C=C(OC)C(OC)=C3O)C=O
PUGREST.BadRequest: error:
C12=CC(C(NC)=CC=C1C3=C(OC)C(=C(C=C3CC[C@@H1]2N4CC=CC=C4)O)CO)C=O
PUGREST.BadRequest: error:
C12=CC(C(NC)=CC=C1C3=C(OC)C3=C(CCC[C@@H1]2NCC=CC)CS)OC=O
PUGREST.BadRequest: error:
COC1=C(OC)C(OC)=CCCC[C@H1](NCC2=CC=CS2)C3=CC(=O)C(NC)=CC=C3C1
PUGREST.BadRequest: error:
C12=CC(C(SC)=CC=C1C3=C(OC)C(=C(C=C3CC[C@@H1]2N4CC=CC=C4)O)CO)C=O
PUGREST.BadRequest: error:
COC=C(OC)C(OC)=C1CCC[C@H1](NCC(C)C)C2=CC(=O)C(SC)=CC=C2C1
PUGREST.BadRequest: error:
NC(N(CC)OC)N[C@H1]1CCC2=CC(OC)=C(OC)C(OC)=C2C=3C1=CC(=O)C(=CC=3)NC
PUGREST.BadRequest: error:
N1C(C(CCC)=C)N1[C@H1]2CCC3=CC(OC)=C(OC)C(OC)=C3C4=CC=C(C(=O)C=C24)NC
PUGREST.BadRequest: error:
C12=CC=C(NC)C2C=C1[C@@H1](NC(C)=O)CCC=CC(OC)=C(OC)C(OC)=C
PUGREST.BadRequest: error:
C1[C@H1](C2=CC(C(SC)=CC=C2C3=C(OC)C(=C(OC)C=C3C1)OC)=O)NCC
PUGREST.BadRequest: error:
NC(CCN=N[C@H1]1CCC2=CC(OC)=C(OC)C(OC)=C2C3=CC=CC(=O)C=C13)NCC
PUGREST.BadRequest: error:
C1=2[C@H1](CCC3=CC(OC)=C(OC)C(OC)=C3C1=CC=C(NC)C(=O)C=2NC)C=O
PUGREST.BadRequest: error:
C=C1C2=C(OC)C(OC)=C(OC)C=C2CC[C@H1](NC(C=3)=O)C1=CC=C(C(C=3)=O)SC
PUGREST.BadRequest: error:
C=C1C2=C(OC)C(OC)=C(OC)C=C2CC[C@H1](NC(N)=O)C1=CC=C(NC)C(=O)C
PUGREST.BadRequest: error:  C1[C@H1](CC(C2=CC(OC)=C(OC)C=C21)C=CC=C(NC)CC)ON
PUGREST.BadRequest: error:
C12=CC3=C(OC)C(OC)=C(OC)C=C3CC[C@H1](NC(N)=O)C=C1C=C(OC)C(=C2)OO
PUGREST.BadRequest: error:
```

```
C=C1C2=C(OC)C(OC)=C(OC)C=C2CC[C@H1](NC(C)=O)C1=CC=C(C(C)=O)SC
PUGREST.BadRequest: error:
C1[C@@H1](C2=CC(C(NC)=CC=C2C3=C(OC)C(OC)=C(OC)C=C3C1=O)N)C(=O)C
PUGREST.BadRequest: error:
C1=2[C@H1](CCC3=CC(OC)=C(OC)C(OC)=C3C1=CC=C(C(C=2)=O)SCNC)C=O
PUGREST.BadRequest: error:
C1=2[C@H1](CCC3=CC(OC)=C(OC)C(OC)=C3C1=CC=C(SC)C(=O)C=2NC)C
PUGREST.BadRequest: error:
C1=2[C@H1](CCC3=CC(OC)=C(OC)C(OC)=C3C1=CC=C(C(C=2)=O)SCN)C(=O)C
PUGREST.BadRequest: error:
NC(=O)N[C@@H1]C1=C2C(C(NC)=CC=C1C3=C(OC)C(OC)=C(OC)C=C3CC2)=O
PUGREST.BadRequest: error:
CC(C)NC(=O)N1[C@@H1]C2=CC(C(NC)=CC=C2C3=C(OC)C(OC)=C(OC)C=C3CC1)=O
PUGREST.BadRequest: error:
CC(N)(CCO)[C@H1]1CCC2=CC(OC)=C(OC)C(OC)=C2C3=CC=C(SC)C(=O)C=C13
PUGREST.BadRequest: error:
CC(N)(CCO)[C@H1]1CCC2=CC(OC)=C(OC)C(OC)=C2C3=CC=C(C(C=C31)=O)NCC
PUGREST.BadRequest: error:
C1#CC(C=C2[C@H1](CCC3=CC(OC)=C(OC)C(OC)=C3C2=C1N)C(C)=O)=C
PUGREST.BadRequest: error:
C1[C@H1](NC(C)=O)C2=CC(C(NC)=CC=C2C3=C(OC)C(OC)=C(C=C3C1)OCNC)=O
PUGREST.BadRequest: error:
C12=C(C2C=C3[C@H1](CCC4=CC(OC)=C(OC)C(OC)=C4C3=C1N)C(C)=O)OSC
PUGREST.BadRequest: error:
C1[C@H1](NC(C)=O)C2=CC(C(SC)=CC=C2C3=C(OC)C(OC)=C(C=C3C1)OCNC)=O
PUGREST.BadRequest: error:
C12=C(C(C=C3[C@H1](CCC4=CC(OC)=C(OC)C(OC)=C4C3=C1N)CC)=O)C2=O
PUGREST.BadRequest: error:
N1(CC2=CC=CN=C23)C=CC(C4=CN(OC)C=C3CC[C@H1](C4)C=C)CC(OC)=COC=C1
PUGREST.BadRequest: error:
C1[C@H1](NC(C)=O)C2=CCC(NC)=CC=C2C3=C(OC)C(OC)=C(OC)C=C3C1=O
PUGREST.BadRequest: error:
C=C(N[C@H1]1CCC2=C(C(OC)=C(OC)C(OC)=C2)C3=CC=C(SC)C(C=C31)=O)C
PUGREST.BadRequest: error:
C=12C=C(NC=3)CC4=CC=1CC=C2[C@H1](CCC=3C=C(OC)C(OC)=C4O)CNC(=O)CSCSC=O
PUGREST.BadRequest: error:
C1[C@H1](NC(C)=O)C2=CCC(SC)=CC=C2C3=C(OC)C(OC)=C(OC)C=C3C1NC=O
PUGREST.BadRequest: error:
C=1C=C(NC)CC=C2C=1C3=C(OC)C(OC)=C(OC)C=C3CC[C@@H1]2NC(C)=O
PUGREST.BadRequest: error:
C=12C=C(NC=3)CC4=CC=1CC=C2[C@H1](CCC=3C=C(OC)C(OC)=C4O)CNC=O
PUGREST.BadRequest: error:
CC(C)N(C(C)C)C(=O)N[C@@H1]C1=CC(C(NC)=CC=C1C2=C(OC)C(OC)=C(OC)C=C2CC)=O
PUGREST.BadRequest: error:
C1C(C)NC1N[C@H1]2CCC3=CC(OC)=C(OC)C(OC)=C3C4=CC=C(C(=O)C=C42)NC=O
PUGREST.BadRequest: error:
CC(C)COC1=C(OC)C(OC)=CC=2CC[C@H1](NC(=O)C)C3=CCC(SC)=CC=C3C=21
PUGREST.BadRequest: error:
```

```
O=C(N[C@H1]1CCC2=CC(OC)=C(OC)C(OC)=C2C3=CC=C(NC)C(=O)C=C31)N(S)CC
PUGREST.BadRequest: error:
O=C(N[C@H1]1CCC2=CC(OC)=C(OC)C(OC)=C2C3=CC=C(NC)C(=O)C=C13)CCOCC
PUGREST.BadRequest: error:
O=C(N[C@H1]1CCC2=CC(OC)=C(OC)C(OC)=C2C3=CC=C(NC)C(=O)C=C31)CCOCC
PUGREST.BadRequest: error:
COC1=CC2=CC=C(NC)C(=O)C=C2[C@@H1](NC(=O)NC3=CC=C(F)C=C3)CCC=CC(OC)=C1OC
PUGREST.BadRequest: error:
COC1=C2C3=CC=C(NC)C(=O)C=C3[C@H1](CCC2=CC(OC)=C1OC)NC(=O)N(CCCCCl)O
PUGREST.BadRequest: error:
COC=C1C2=CC=C(NC)C(=O)C=C2[C@@H1](NC(=O)NC3=CC=C(F)C=C3)CCC1C=C(OC)C=COC
PUGREST.BadRequest: error:
COC=CC1=CC=C(NC)C(=O)C=C1[C@@H1](NC(=S)NC2=CC=CC=C2)Cl
PUGREST.BadRequest: error:
N=1C(=CN(N=1)[C@H1]2CCC3=CC(OC)=C(OC)C(OC)=C3C4=CC=C(NC)C(=O)C=C42)C=O
PUGREST.BadRequest: error:
C[C@H1](CCC1=C(C(OC)=C(OC)C(OC)=C1)C2=CC=C(C(C=C2)N)C=O)CC=O
PUGREST.BadRequest: error:
C1=2[C@H1]CCC3=CC(OC)=C(OC)C(OC)=C3C1=CC=C(C(C=2)=O)SCNC(C)=O
PUGREST.BadRequest: error:
N1([C@H1]2CCC3=C(C(OC)=C(OC)C(OC)=C3)C4=CC=C(NC)C(C=C24)=O)C=C1I
PUGREST.BadRequest: error:
C1=CC(C2=CN(N=N2)[C@@H1]3C4=CC(C(NC)=CC=C4C5=C(OC)C(OC)=C(OC)C=C5CC3)=O)=C1
PUGREST.BadRequest: error:
N1([C@H1]2CCC3=C(C(OC)=C(OC)C(OC)=C3)C4=CC=C(NC)C(C=C24)=O)C=C1OCC
PUGREST.BadRequest: error:
C12N([C@@H1]3C4=CCC(NC)=CC=C4C5=C(OC)C(OC)=C(OC)C=C5CC3)OC=C1CC=C2O
PUGREST.BadRequest: error:
N1([C@H1]2CCC3=C(C(OC)=C(OC)C(OC)=C3)C4=CC=C(NC)C(=O)C=C42)COCC=CC=C1
PUGREST.BadRequest: error:
N1([C@H1]2CCC3=C(C(OC)=C(OC)C(OC)=C3)C4=CC=C(NC)C(C=C24)=O)C=C1Cl
PUGREST.BadRequest: error:
C=1N([C@H1]2CCC3=C(C(OC)=C(OC)C(OC)=C3)C4=CC=C(NC)C(=O)C=C24)CCNC(=O)OCCN=1
PUGREST.BadRequest: error:
N1C(OC)=C(C=C1)N[C@H1]2CCC3=C(C(OC)=C(OC)C(OC)=C3)C4=CC=C(SC)C(C=C42)=O
PUGREST.BadRequest: error:
C1[C@@H1](C2=CC(C(SC)=CC=C2C3=C(OC)C(OC)=C(C=C3C1)O)C)ONC(C)=O
PUGREST.BadRequest: error:
C1N([C@H1]2CCC3=CC(OC)=C(OC)C(OC)=C3C4=CC=C(NC)C(=O)C=C24)OC(=CC=C1)OSC
PUGREST.BadRequest: error:
C1N([C@H1]2CCC3=C(C(OC)=C(OC)C(OC)=C3)C4=CC=C(NC)C(=O)C=C42)CC=C1OC
PUGREST.BadRequest: error:
N(C(C)C)(C(C)C)C(=O)N1[C@H1]CCC2=C(C(OC)=C(OC)C(OC)=C2)C3=CC=C(NC)C(C=C31)=O
PUGREST.BadRequest: error:
N(CCCC)CC1=CN(N=N1)[C@H1]2CCC3=CC(OC)=C(OC)C(OC)=C3C4=CC=C(NC)C(=O)C=C24
PUGREST.BadRequest: error:
N(CCCC)CC1=CN(N=N1)[C@H1]2CCC3=C(C(OC)=C(OC)C(OC)=C3)C4=CC=C(NC)C(=O)C=C42
PUGREST.BadRequest: error:
```

```
C1[C@H1](NCC)CC2=CC(C(NC)=CC=C2C3=C(C(OC)=C(OC)C=C3CC1)OC(C)=O)CC=O
PUGREST.BadRequest: error:
C1=C(OC)C(=C(OC)C=C1CC2[C@H1](NC(C(O)=O)C(=O)OCC=CC2=CC=C(NC)CC)OOCN)C
PUGREST.BadRequest: error:
C12=C(OC)C(=C(OC)C=C1CC[C@H1](NC(C(O)=O)=CNCC2=CC=C(NC)CC)ONCC)ONC
PUGREST.BadRequest: error:
C1=C(OC)C(=C(OC)C=C1CC[C@H1](N2C(C(OCC)=O)=C(N=N2)C(OCC)=O)CCC(NC)=CC=C)ONCOC
PUGREST.BadRequest: error:
NC(=S)N1[C@@H1]C2=CC(C(NC)=CC=C2C3=C(OC)C(OC)=C(OC)C=C3CC1)=O
PUGREST.BadRequest: error:
C[C@H1](CC1C2=CC(OC)=C(OC)C(OC)=C2C3=CC=C(C(=O)C=C31)SCN)CC
PUGREST.BadRequest: error:
N=NC(CO)C(O)(OCC)O[C@@H1]1C2=CC(C(NC)=CC=C2C3=C(OC)C(OC)=C(C=C3CC1)NC)=O
PUGREST.BadRequest: error:
C12=CC3=C(OC)C(OC)=C(OC)C=C3CC[C@H1](NC=CC=C)C1=CC(=O)C2SC
PUGREST.BadRequest: error:
C1[C@@H1](C2=CCC(NC)=CC=C2C3=C(OC)C(OC)=C(C=C3C1)OC=O)CCO
PUGREST.BadRequest: error:
COC1=C(OC)C=2C3=CC=C(SC)C(C=C3[C@H1](CCC=2C=C1OC)NCC4=CC=CC)=C4O
PUGREST.BadRequest: error:
C=C1C2=C(OC)C(OC)=C(OC)C=C2CC[C@H1](NCCCCl)C1=CC(=O)C(NC)=CNC
PUGREST.BadRequest: error:
C=C1C2=C(OC)C(OC)=C(OC)C=C2CC[C@H1](NCCCCl)C1=CC(C(=C)NC)=O
PUGREST.BadRequest: error:
C=1=C2C3=C(OC)C(OC)=C(OC)C=C3CC[C@H1](NCC)C2=CC(=O)C(SC)=CC=1NC
PUGREST.BadRequest: error:
C1=2[C@@H1](CCC3=CC(OC)=C(OC)C(OC)=C3C1=CC=C(C(C=2)=O)NCNC)CONC
PUGREST.BadRequest: error:
C1C2=CC(OC)=C(OC)C(OC)=C2C3=CC=C(NC)C(=O)C=C3[C@H1](C1)NC4C=CC=CS4
PUGREST.BadRequest: error:
C[C@@H1](C1=CC(C(NC2)=CC=C1C3=C(OC)C(OC)=C(OC)C=C32)NC)CC
PUGREST.BadRequest: error:
N(C=C(O)C=C[C@@H1]1C2)=CC(C(SC)=CC=C2C3=C(OC)C(OC)=C(OC)C=C3CC1)=O
PUGREST.BadRequest: error:
C=1[C@H1](NC(N)=N)CC(C2=C(C(OC)=C(OC)C=C2C)OC)=CC=C(NC)C(C=1)=O
PUGREST.BadRequest: error:
C1C(C2=C(OC)C(OC)=C(OC)C=C2CC[C@@H1]1NC(N)C)C=CC=C(C(C)=O)NC
PUGREST.BadRequest: error:
C1[C@H1](NC(N)=S)C2=CCC(NC)=CC=C2C3=C(OC)C(OC)=C(OC)C=C3C1=O
PUGREST.BadRequest: error:
C=C(CN[C@H1]1CCC2=CC(OC)=C(OC)C(OC)=C2C3=CC=C(NC)C(C=C13)=O)C=O
PUGREST.BadRequest: error:
C1[C@H1](NCCC)C2=CCC(SC)=CC=C2C3=C(OC)C(OC)=C(OC)C=C3CC1=O
PUGREST.BadRequest: error:
C1=C(N=NN1[C@H1]2CCC3=CC(OC)=C(OC)C(OC)=C3C4=CC=C(NC)C(=O)C=C24)C
PUGREST.BadRequest: error:
C1=C(C(C=C2[C@H1](CCC3=CC(OC)=C(OC)C(OC)=C3C2=C1)NC(C)C)C)OSC
PUGREST.BadRequest: error:
```

```
C1[C@H1](NC(N)=S)C2=CC(C(NC)=CC=C2C3=C(OC)C(OC)=C(OC)C=C3C1=O)C
PUGREST.BadRequest: error:
C=1C=C(NC)C(C=C2C=1C3=C(OC)C(OC)=C(OC)C=C3CC[C@@H1]2NC(C(C)C)=O)SNC
PUGREST.BadRequest: error:
C=12C=C(NC)C(C=C3C=1C4=C(OC)C(OC)=C(OC)C=C4CC[C@@H1]3NC(NCCCC=O)N)=C2O
PUGREST.BadRequest: error:
C=1C=C(NC)C(C=C2C=1C3=C(OC)C(OC)=C(OC)C=C3CC[C@@H1]2NC(C(C)C)=O)SNC=O
PUGREST.BadRequest: error:
C1[C@H1](NC(N(CC=C)CC=C)=S)C2=CC(C(NC)=CC=C2C3=C(OC)C(OC)=C(C=C3C1)OC)NC=O
PUGREST.BadRequest: error:
C=CC=C(NC(=O)N[C@H1]1CCC2=CC(OC)=C(OC)C(OC)=C2C3=CC=C(NC)C(=O)C=C13)C
PUGREST.BadRequest: error:
C=CC=C(NC([C@@H1]C1=CCC(SC)=CC=C1C2=C(OC)C(OC)=C(OC)C=C2CC)=O)N=O
PUGREST.BadRequest: error:
C1=2[C@H1](CCC3=CC(OC)=C(OC)C(OC)=C3C1=CC=C(C(=O)C=2)SCNC(C)=O)C=C
PUGREST.BadRequest: error:
C=CC=C(NC(=O)N[C@H1]1CCC2=CC(OC)=C(OC)C(OC)=C2C3=CC=C(NC)C(C=C13)=O)C
PUGREST.BadRequest: error:
C=1NCC(=O)C=C2[C@H1](CCC3=C(C(OC)=C(OC)C(OC)=C3)C2=CC=1)NC(C(C)C)=O
PUGREST.BadRequest: error:
C=CC=C(NC(=O)N[C@H1]1CCC2=CC(OC)=C(OC)C(OC)=C2C3=CC=C(NC)C(=O)C=C31)C
PUGREST.BadRequest: error:
C1=CC=C(NC(=O)N[C@H1]2CCC3=CC(OC)=C(OC)C(OC)=C3C=4C2=CC(C(NC)=CC=4)=O)C1
PUGREST.BadRequest: error:
C[C@H1](CCC1=CC(OC)=C(OC)C(OC(OCC)=O)=C1C2=CC=C(SC)C(=O)C=C2)NCC
PUGREST.BadRequest: error:  CCN[C@H1]([C@H1])C1OC(C)(C2)O[C@@H1]1[C@H1](CNC(=S)N
[C@H1]CCC3=C(C(OC)=C(OC)C(OC)=C3)C4=CC=C(C(=O)C=C24)NC)O
PUGREST.BadRequest: error:
CNCC(O)C(OC=C1C=C2C3=C(OC)C(OC)=CC=C3CC[C@H1](NC(C)=O)C2=CC1)=O
PUGREST.BadRequest: error:
COCC(CN[C@@H1]1C=2C(C3=C(C(OC)=C(OC)C=C3CC1)OC)=CC=C(NC)C(C=2)=O)F
PUGREST.BadRequest: error:
N=NC(CN1C(=O)OC)=C=C(C=CC=CNCC=CC)C2(NC)C(C(O)=CC2C3=C)(OC)C(OC)=C(OC)C=C3CC1OC
PUGREST.BadRequest: error:
NC1=NN=NN1[C@@H1]2C=3C(C4=C(C(OC)=C(OC)C=C4CC2)OC)=CC=C(NC)C(C=3)=O
PUGREST.BadRequest: error:
N=NC(CNC(=O)OC)=C=C(C=CC=CNCC=CC12)C(NC)C(OC)=C(OC)C=C1CC[C@H1](NC(=O)C)C2F
PUGREST.BadRequest: error:
N=NC(CNC(=O)OC)=C=C(C=CC=CNCC=CC12)C(NC)C(OC)=C(OC)C=C1CC[C@H1](NC(=O)C)C2=CC
PUGREST.BadRequest: error:
N1C=NN=NN1[C@H1]2CCC3=C(C(OC)=C(OC)C(OC)=C3)C4=CC=C(NC)C(=O)C=C24
PUGREST.BadRequest: error:
OCCN(CCO)C(=S)N1[C@@H1]C2=CC(C(NC)=CC=C2C3=C(OC)C(OC)=C(OC)C=C3CC1)=O
PUGREST.BadRequest: error:
N12C=NN=NN1[C@H1]3CCC4=C(C(OC)=C(OC)C=C4C=5C3=CC(C(NC)=CC=5)=O)OCC=C2
PUGREST.BadRequest: error:  N=NC(CNC(=O)OC)=C=C(C=CC=CNCC=CC=1)C2(NC)C(C(O)=CC=1
C3=C)(OC)C(OC)=C(OC)C=C3CC2OOC
PUGREST.BadRequest: error:
```

```
N1CC=CN([C@@H1]2C3=CC(C(NC)=CC=C3C4=C(CC2)C=C(OC)C(OC)=C4OC)=O)C1C
PUGREST.BadRequest: error:
N=NC(CNC(=O)OC)=C=C(C=CC=CNCC=CC1)C(NC)C(OC)=C(OC)C=C1CC[C@H1](NC(=O)C)C=CC
PUGREST.BadRequest: error:
NC(C(OCC)=C)N[C@H1]1CCC2=CC(OC)=C(OC)C(OC)=C2C3=CC=C(NC)C(=O)C=C31
PUGREST.BadRequest: error:
NC(C(OCC)=C)N[C@H1]1CCC2=CC(OC)=C(OC)C(OC)=C2C3=CC=C(NC)C(=O)C=C13
PUGREST.BadRequest: error:
N([C@H1]1CCC2=CC(OC)=C(OC)C(OC)=C2C3=CC=C(NC)C(C=C13)=O)=N
PUGREST.BadRequest: error:
NC(C(OCC)=C)N[C@H1]1CCC2=CC(OC)=C(OC)C(OC)=C2C3=CC=C(NC)C(C=C13)=O
PUGREST.BadRequest: error:
NC(CCC)N[C@@H1]1C2=CC(C(NC)=CC=C2C3=C(OC)C(OC)=C(OC)C=C3CC1)=O
PUGREST.BadRequest: error:
N([C@H1]1CCC2=CC(OC)=C(OC)C(OC)=C2C3=CC=C(NC)C(C=C31)=O)=C
PUGREST.BadRequest: error:
N([C@H1]1CCC2=CC(OC)=C(OC)C(OC)=C2C3=CC=C(NC)C(C=C13)=O)C=CC=C
PUGREST.BadRequest: error:
N([C@H1]1CCC2=CC(OC)=C(OC)C(OC)=C2C3=CC=C(NC)C(C=C13)=O)=CC
PUGREST.BadRequest: error:
C1=2[C@H1](CCC3=C(C(OC)=C(OC)C(OC)=C3)C1=CC=C(C(C=2)=O)NCNCC(C)C)ONC
PUGREST.BadRequest: error:
C1=2[C@H1](CCC3=C(C(OC)=C(OC)C(OC)=C3)C1=CC=C(C(C=2)=O)NCNCC)OCSC
PUGREST.BadRequest: error:
C12[C@@H1](CC(C3=C2C(OC)=C(OC)C=C3C1)OC=CC=C(NC)CC)ONC(C)=O
PUGREST.BadRequest: error:  N(C)C=1C(=O)C=C2[C@H1](CCC3=C(C(OC)=C(OC)C(OC)=C3)C2
=CC=1)N4C(C(=O)O)=C(N=N4)C(=O)N
PUGREST.BadRequest: error:
C1[C@H1](NCC)CC2=CC(C(NC)=CC=C2C3=C(C(OC)=C(OC)C=C3CC1)OC(C)=O)(C)ONC=O
PUGREST.BadRequest: error:
N(CCCC)CNCC1=CN(N=N1)[C@H1]2CCC3=CC(OC)=C(OC)C(OC)=C3C4=CC=C(NC)C(=O)C=C24
PUGREST.BadRequest: error:
C12[C@H1](NCC)CC3=CC2(C(SC)=CC=C3C4=C(C(OC)=C(OC)C=C4CC1)OCNC(C)=O)NC
PUGREST.BadRequest: error:
C1=C(O)C(OC)=C(OC)C=CCC[C@H1](NN=NCC(O)=O)OC2=CC(C(NC)=CC=C12)=O
PUGREST.BadRequest: error:
C12=C(OC)C(=C(OC)C=C1CC[C@H1](NC(C(=O)OCC)=C(COCC)OCCC(NC)=CC=CON)C=C2N)C
PUGREST.BadRequest: error:
N(C)C=1C(=O)C=C2[C@H1](CCC3=C(C(OC)=C(OC)C(OC)=C3)C2=CC=1)NC(C(=O)O)=C(COCC)C=O
PUGREST.BadRequest: error:  N(C)C=1C(=O)C=C2[C@H1](CCC3=CC(OC)=C(OC)C(OC)=C3C2=C
C=1N4)C(C(=O)O)=C(N=N4)C(O)=O
PUGREST.BadRequest: error:
C12=C(OC)C(=C(OC)C=C1CC[C@H1](NC(C(=O)OCC)=C(COCC)OCCC(NC)=CC=CON)C=C2)NC
PUGREST.BadRequest: error:  O=C(C=C(C(OC1C2)=O)N(N=N2)[C@@H1]3C=4C(C5=C(C(OC)=C(
OC)C=C5CC3)OC)=CC=C(NC)CC=4)OC(Cl)=C1C
PUGREST.BadRequest: error:  O=C(C=C(C(OCC1)=O)N(N=N1)[C@@H1]2C=3C(C4=C(C(OC)=C(O
C)C=C4CC2)OC)=CC=C(NC)CC=3)OC(Cl)=CC
PUGREST.BadRequest: error:  O=C(C=C(C(OCC1)=O)N(N=N1)[C@@H1]2C=3C(C4=C(C(OC)=C(O
```

```
C)C=C4CC2)OC)=CC=C(NC)CC=3)OC(Cl)=C
PUGREST.BadRequest: error:  O=C(C=C(C(OCC1)=O)N(N=N1)[C@@H1]2C=3C(C4=C(C(OC)=C(O
C)C=C4CC2)OC)=CC=C(NC)CC=3)OC=CCCC
PUGREST.BadRequest: error:
OC(\N[C@H1]1CCC2=CC(OC)=C(OC)C(OC)=C2C3=CC=C(C(C=C31)=O)NCNC(C)C)=O
PUGREST.BadRequest: error:
O1CC(C2)=CC(=C13)C4=C(OC)C(OC)=C(OC)C=C4CC[C@H1](NC(NC5=CC=C(C=C5)Cl)=S)C3=CC2=O
PUGREST.BadRequest: error:  N1=CC=C(C=C1)C(NCC=2N=NN(C=2)[C@H1]3CCC4=CC(OC)=C(OC
)C(OC)=C4C5=CC=C(C(C=C53)=O)N)C=O
PUGREST.BadRequest: error:
OC(NCC1=CC(OC)=C(OC)C=C1CC[C@H1](NCN(CC)CC)C2=CCC(NC)=CC=C2)=O
PUGREST.BadRequest: error:
NC(=CN/C(=NC(O)C=CCI)C1=CN(NC1)C=CC2=CC(NC)=CC(OC)=C2C=CC(OC3)C(OC)=CC=C3)C=CC=O
PUGREST.BadRequest: error:
O=CC(=CC1=CC([C@@H1](NC(C(O)=O)=C(N=C)CCCCC2)CC(OC)=C(OC)C(OC)=C21)NC)O
PUGREST.BadRequest: error:  NC(=CN/C(=NC(O)C=CCI)C1=CN(N2C1)C=CC3=CC(NC)=CC(OC)=
C3C=CC4(OC)C(OC)=CC=C4)CC[C@@H1]2N=O
PUGREST.BadRequest: error:  O=CC(=CC=C1C([C@@H1](N2C(C(O)=O)=C(N=C2)C(=O)C)CCC3=
CC(OC)=C(OC)C(OC)=C31)=C)NC=O
PUGREST.BadRequest: error:
OCC(C)=C1C([C@H1](CCC2=CC(OC)=C(OC)C(OC)=C12)NC(NC3=CC=C(C=C3)F)=O)PNC=O
PUGREST.BadRequest: error:  O=CC(=CC=C1C([C@@H1](N2C(C(=O)O)=C(C(=O)O)N=N2)CCC3=
CC(OC)=C(OC)C(OC)=C31)=C)NC=O
PUGREST.BadRequest: error:
OC(\N[C@H1]1CCC2=CC(OC)=C(OC)C(OC)=C2C3=CC=C(C(C=C13)=O)NCNC(C)C)=O
PUGREST.BadRequest: error:  N1=CC=C(C=C1)C(NCC=2N=NN(C=2)[C@@H1]3C4=CCC(NC)=CC=C
4C5=C(OC)C(OC)=C(OC)C=C5CC3)=O
PUGREST.BadRequest: error:  N1=CC=C(C=C1)C(NCC=2N=NN(C=2)[C@@H1]3C4=CCC(NC)=CC=C
4C5=C(OC)C(OC)=C(C=C5CC3)OC)=O
PUGREST.BadRequest: error:
CC(C)(C)OC(=O)/N=C(/N[C@H1]1CCC2=C(C(OC)=C(OC)C(OC)=C2)C3=CC=C(NC)C(C=C13)=O)N=N
PUGREST.BadRequest: error:  CC(C)(C)OC(=O)NCCCNCC1=CN(N=N1)[C@H1]2CCC3=CC(OC)=C(
OC)C(OC)=C3C4=CC=C(NC)C(=O)C=C42
PUGREST.BadRequest: error:
CC(C)(C)OC(=O)/N=C(/N[C@H1]1CCC2=C(C(OC)=C(OC)C(OC)=C2)C3=CC=C(NC)C(C=C31)=O)N=N
PUGREST.BadRequest: error:
C(=CNCNC(=O)OCC(Cl)(Cl)Cl)[C@H1]CCC1=C(C(OC)=C(OC)C(OC)=C1)C2=CC=C(NC)C(=O)C=C2
PUGREST.BadRequest: error:  CC(C)(C)OC(=O)NCCCNCC1=CN(N=N1)[C@H1]2CCC3=CC(OC)=C(
OC)C(OC)=C3C4=CC=C(NC)C(=O)C=C24
PUGREST.BadRequest: error:  C1=C(OC)C(OC)=C(OC=2)C=3CC([C@H1](CCC=3)C(C(O)=O)=C(
COC(=O)CCC=C1)CCC(=CC=2)NC)=O
PUGREST.BadRequest: error:
CSCC(=O)C=C1C(C2=C(OC)C(OC)=C(C=C2CC[C@@H1]1NCN)N)OCC
PUGREST.BadRequest: error:  CNCCC=C1C(C2=C(OC)C(=C(C=C2CC[C@@H1]1NCN)C)OO)COC
PUGREST.BadRequest: error:  COC1=CC2=C(C3=C(OC)C(OC)=CC=C3CC[C@@H1]2NOC)CC=C1OC
PUGREST.BadRequest: error:  CNCC(C=C1C(C2=C(OC)C(=CC=C2CC[C@@H1]1NCN)C)=O)COC
PUGREST.BadRequest: error:  CNCC(C=C1C(C2=C(OC)C=C(OC)C=C2CC[C@@H1]1NC=O)C)OC=O
PUGREST.BadRequest: error:
```

```
COC1=C2C3=CC=C(SC)C(=O)C=C3[C@H1](CCC2=CC(OC)=C1OC)NCCC
PUGREST.BadRequest: error:
C=C1C=2C[C@H1](CC(C3=CC(OC)=C(OC)C=C31)C=CC=CSC)C(=O)C=2
PUGREST.BadRequest: error:  COC=C1C2=CC=C(NC)C(=O)C=C2[C@H1](CCC1C=COC)NCCCCl
PUGREST.BadRequest: error:
C=1NCC=1C=C2C(C3=C(OC)C(=C(OC)C=C3CC[C@@H1]2N)[N+1]=[N-1])OC=CC
PUGREST.BadRequest: error:  C1=C2CC[C@H1](C3=CCC(SC)=CC=C3C2=C(OC)C(=C1OC)OC=O)N
PUGREST.BadRequest: error:
C=C1CC[C@H1](CC(C2=CC(OC)=C(OC)C=C21)C=CC=CSC)C(=O)CN
PUGREST.BadRequest: error:
C=C1CC[C@H1](C=2C(C3=CC(OC)=C(OC)C=C31)C=CC=CSC)C(C=2)=O
PUGREST.BadRequest: error:
COC1=C2C3=CC=C(NC)C(C=C3[C@@H1](NCCCCCC)CCC2=CC(=C1OC)O)C=O
PUGREST.BadRequest: error:
C=C1CC=2[C@H1](CC(C3=CC(OC)=C(OC)C=C31)C=CC=CSC)C(C=2)=O
PUGREST.BadRequest: error:
COC1=C2C3=CC=C(NC)C(=O)C=C3[C@H1](CCC2=CC(OC)=C1OC)NCCCCCCSC
PUGREST.BadRequest: error:
C=C(CO)CN[C@H1]1CCC2=CC(OC)=C(OC)C(OC)=C2C3=CC=C(SC)C(=O)C=C31
PUGREST.BadRequest: error:
CC(C)CN[C@H1]1CCC2=CC(OC)=C(OC)C(OC)=C2C3=CC=C(C(C=C31)=O)SCC
PUGREST.BadRequest: error:
CC(C)CCN[C@H1]1CCC2=CC(OC)=C(OC)C(OC)=C2C3=CC=C(C(C=C13)=O)SCC
PUGREST.BadRequest: error:
C=1NCC(=O)C=C2C(C3=C(OC)C(OC)=C(C=C3CC[C@@H1]2NCCC#N)C)=CC=1
PUGREST.BadRequest: error:  C12=CCC[C@H1]2CC1(C3=CC(OC)=C(OC)C(OC)=C3)CC=C
PUGREST.BadRequest: error:
C1=C2CC[C@H1](C3=CCC(SC)=CC=C3C2=C(C(OC)=C1OC)OON)CNCCC
PUGREST.BadRequest: error:
C1=C2CC[C@H1](C3=CCC(SC)=CC=C3C2=C(OC)C(=C1OC)OC=O)NCCCC
PUGREST.BadRequest: error:
CC(C)CCN[C@H1]C1CC2=CC(OC)=C(OC)C(OC)=C2C3=CC=C(SC)C(C=C31)=O
PUGREST.BadRequest: error:
C1=C(CO2)CN1C[C@@H1]C3=CC(C(SC)=CC=C3C4=C(OC)C(OC)=C(OC)C=C4CC2)=O
PUGREST.BadRequest: error:
C=C1CC=2[C@H1](CC(C3=CC(OC)=C(OC)C=C31)C=CC=CSC)C(=O)C=2N=[N+1]=[N-1]
PUGREST.BadRequest: error:
CNCC(C1=C2C(C3=C(OC)C(=C(OC)C=C3CC[C@@H1]2NCCC#N)OC)=CC1)=O
PUGREST.BadRequest: error:
N=1NC=1[C@@H1]2C3=CC(C(NC)=CC=C3C4=C(C=C(OC)C(=C4OC)OC)CC2)=O
PUGREST.BadRequest: error:
C1=C2CC[C@H1](C3=CCC(NC)=CC=C3C2=C(OC)C(=C1OC)OC)ONCCC#N
PUGREST.BadRequest: error:
COC=C1C2=CC=C(NC)C(C=C2[C@@H1](NCCCCCC)CCC1=CC(=COC)OC)=O
PUGREST.BadRequest: error:
COC1=C2CC([C@H1](CCC2=CC(OC)=C1OC)NCCC#N)C(C(=CC)NC)=O
PUGREST.BadRequest: error:
C1=C2CC[C@H1](C3=CCC(NC)=CC=C3C2=C(OC)C(=C1OC)OC=O)NCCCCCCCCCCC
```

```
PUGREST.BadRequest: error:
COC=C1C2=CC=C(NC)C(C=C2[C@@H1](NCCCCCCCCCC)CCC1=CC(=COC)OC)=O
PUGREST.BadRequest: error:
COC=C1C2=CC=C(NC)C(C=C2[C@@H1](NCCCCCCCCC)CC=CC(=C1OC)OC=O)CC
PUGREST.BadRequest: error:
COC1=C2CC([C@H1](CCC2=CC(OC)=C1OC)N3N=NC=C3)CN=CC(C(=CC)NC)=O
PUGREST.BadRequest: error:
C1NCC(=O)C=C2C(C3=C(OC)C(OC)=C(OC)C=C3CC[C@@H1]2N4C=C(N=N4)CNN)=CC=C1
PUGREST.BadRequest: error:
COC=C1C2=CC=C(NC)C(C=C2[C@@H1](N3C=C(N=N3)CCCC)CCC=CC(=C1OC)O)C=O
PUGREST.BadRequest: error:
COC1=C2CC([C@H1](CCC2=CC(OC)=C1OC)NC/C=C/C3=CC=CC)=C3CC(C(SC)=CC)=O
PUGREST.BadRequest: error:
COC1=C2CC([C@H1](CCC2=CC(OC)=C1OC)N3N=NC=C3)CN=CC(=O)C(NC)=CC
PUGREST.BadRequest: error:
COC1=C2C3=CC=C(NC)C(=O)C=C3[C@H1](CCC2=CC(OC)=C1OC)NCC(C)C
PUGREST.BadRequest: error:
COC=C1C2=CC=C(NC)C(C=C2[C@@H1](NCC(C)C)CCC1=CC(=COC)OC)=O
PUGREST.BadRequest: error:
C1=C2CC[C@H1](C3=CCC(SC)=CC=C3C2=C(OC)C(=C1OC)OC=O)NCCC
PUGREST.BadRequest: error:
C1=C2CC[C@H1](C3=CCC(SC)=CC=C3C2=C(C(OC)=C1OC)OON)COCC
PUGREST.BadRequest: error:
COC1=C(OC)C(OC)=CC=2CC[C@@H1](C3=CC(C(=CC=C3C1=2)SC)=O)NCCC(C)CC
PUGREST.BadRequest: error:
C12=CC(C(SC)=CC=C1C=C(CC[C@@H1]2NCCC3=CC=CC=C3)C=C(OC)C(OC)=CO)C=O
PUGREST.BadRequest: error:
C1=C2C(C3=C(C(OC)=C(OC)C=C3CC[C@@H1]2NCCC(F)F)F)OC=CC=C(C1=O)NC
PUGREST.BadRequest: error:
C12=CC(C(NC)=CC=C1C3=C(C=C(OC)C(OC)=C3OC)CC[C@@H1]2NCCC(F)F)F
PUGREST.BadRequest: error:
C1=C2C([C@H1](CCC3=CC(OC)=C(C(OC)=C23)OC)NCCCCl)C(=O)C(=C1)NC
PUGREST.BadRequest: error:
C12=CC(C(NC)=CC=C1C3=C(C=C(OC)C(OC)=C3OC)CC[C@@H1]2NCC=4SC=CC=4OC)=O
PUGREST.BadRequest: error:
C12=CCC(NC)=CC=C1C3=C(OC)C(=C(C=C3CC4[C@@H1]2NCC4CCCOC)OC)O
PUGREST.BadRequest: error:
O=CC1=CN=NN1[C@H1]2CCC3=CC(OC)=C(OC)C(OC)=C3C4=CC=C(C(C(=C24)=O)NCCC
PUGREST.BadRequest: error:
C12=NCCC13[C@H1](CNC(CCC=CC=CC3=O)=C(OC)C(OC)=C(OC)C)C2SC=CCNC
PUGREST.BadRequest: error:
C1=CN=CC=C1CN[C@H1]2CCC3=CC(OC)=C(OC)C(OC)=C3C4=CC=C(C(C(=C42)=O)SCC(=O)C
PUGREST.BadRequest: error:
O=CC1=CN=NN1[C@H1]2CCC3=CC(OC)=C(OC)C(OC)=C3C4=CC=C(C(C(=C42)=O)NCCC
PUGREST.BadRequest: error:
C1=CN=CC=C1CN[C@H1]2CCC3=CC(OC)=C(OC)C(OC)=C3C4=CC=C(C(C(=C42)=O)SCC
PUGREST.BadRequest: error:
C1=NCCC12[C@H1](CC3C=CC(OC)=C(OC)C(OC(=O)C)=CC=C23)C(C)=O
```

```
PUGREST.BadRequest: error:
C12[C@H1]C=CCC[C@H1](N=C(COCC)CC3=CCC(NC)=CC=C31)C(OC)C(OC)=C2OC=O
PUGREST.BadRequest: error:
OC1=CC=C(C=C1)CN[C@@H1]2C3=CC(C(SC)=CC=C3C4=C(C(OC)=C(OC)C=C4CC2)OC)=O
PUGREST.BadRequest: error:
O=CC1=C2[C@H1](CCC3=CC(OC)=C(OC)C(OC)=C3C2=CC=C(OC(=O)CC)C1=O)CC=O
PUGREST.BadRequest: error:
C=C1N=CC(C)N1CN[C@H1]2CCC3=C(C(OC)=C(OC)C(OC)=C3)C4=CC=C(C(C=C24)=O)NC
PUGREST.BadRequest: error:
OC1=CC=C(C=C1)CN[C@H1]2CCC3=C(C(OC)=C(OC)C(OC)=C3)C4=CC=C(C(C=C42)=O)SC
PUGREST.BadRequest: error:
O=CC1=C2[C@H1](CCC=C3C(OC)=C(OC)C(OC(C)=O)=C3C1=CC=C(SC)C2=O)CC=O
PUGREST.BadRequest: error:
C1=CC2C[C@H1](NC(C)=O)C3=CC(C(SC)=CC=C3C2=C(OC)C(OC)=C1OC(C4=CC=CC=C4)=O)SC=O
PUGREST.BadRequest: error:
OC1=CC=C(C=C1)CN[C@@H1]2C3=CC(C(SC)=CC=C3C4=C(OC)C(OC)=C(OC)C=C4CC2)=O
PUGREST.BadRequest: error:
O=CC1=C[C@H1](CCC2=CC(OC)=C(OC)C(OC(=O)C)=C2C1=CC=C(SC)C=O)CC=O
PUGREST.BadRequest: error:
CNCC(C)OC(=O)N[C@H1]1CCC2=C(C(OC)=C(OC)C(OC)=C2)C3=CC=C(C(=O)C=C31)NCF
PUGREST.BadRequest: error:
C1OC2=CC=C(C(OC)=C2OC)C3=CC=C(SC)C1(C=C3[C@@H1](NC(N(COC)C)O)C=C)ONC
PUGREST.BadRequest: error:
COC1=C2C=C(C(OC)=C1OC)C3=CC=C(SC)C(C=C3[C@@H1](NCN=CC=C(F)C=COC)C2)NC=O
PUGREST.BadRequest: error:
C1OC2=CC=C(C(OC)=C2OC)C3=CC=C(SC)C(C=C3[C@@H1]1CCC(=O)C(OC)=C)(O)CSCSC
PUGREST.BadRequest: error:
C=C1CC[C@H1](N2C(C(OCC)=O)=C(N=N2)C=CC(=O)C3NC)CC=CC=C3C=C1CNCOCOC
PUGREST.BadRequest: error:
OC=CC=CCCN[C@H1]1CCC2=CC(OC)=C(OC)C(=C2C3=CC=C(SC)C(=O)C=C13)OCC
PUGREST.BadRequest: error:
COC1=CC=C(C(OC)=C1OC)C2=CC=C(SC)C(C=C2[C@@H1]NC(N(COC)C)=C)ONC=O
PUGREST.BadRequest: error:
C=C1CC2[C@H1](NC(C(=O)OCC)=C2)C3=CC(C(NC)=CC=C3C4=C(C(OC)=C(C=C41)OC)OC)=O
PUGREST.BadRequest: error:
C1=CC=2C[C@H1](NCN(C(C)C)C(C)C)OC3=CC(C(NC)=CC=C3C=2C=C(C(=C1)OC)OC)NC=O
PUGREST.BadRequest: error:
COC1=C2C=C(C(OC)=C1OC)C3=CC=C(NC)C(C=C3[C@@H1](NC(N(COC)C)O)CC2)NC=O
PUGREST.BadRequest: error:
C1OC2=CC=C(C(OC)=C2OC)C3=CC=C(SC)C(C=C3[C@@H1]1CCC(=O)C(OC)=C)C=O
PUGREST.BadRequest: error:
COC1=CC2=C(C(OC)=C1OC)C3=CC=C(SC)C2(C=C3[C@@H1]NC(N(COC)C)=C)ONC
PUGREST.BadRequest: error:
CC12[C@H1](CCC3=CC(OC)=C(OC)C(OC)=C3C1=CC=CC(=O)C2)SCNC(C)=O
PUGREST.BadRequest: error:
C=CC=C(CCN[C@H1]1CCC2=CC(OC)=C(OC)C(OC)=C2C3=CC=C(SC)C(C=C31)=O)OC
PUGREST.BadRequest: error:
CCOC(OC1=C2CC([C@H1](CCC2=CC(OC)=C1OC)NC(C)=O)=CC(C(SC)=CC)=O)SC=O
```

```
PUGREST.BadRequest: error:
CC12[C@H1](CCC3=CC(OC)=C(OC)C(OC)=C3C1=CC=CC(C2)=O)SCNC(C)=O
PUGREST.BadRequest: error:
C1C=CC=C(SCC1)C=CN(N2C3OC)[C@@H1]2NC(=O)CNC(=O)OCCCC4C=CC(=C)C=C4C=CC=C(NC)CCC3
PUGREST.BadRequest: error:
CC1=C2C=C(SC)C(C=C1[C@@H1](NC(C)=O)CCC=CC(OC)=C(OC)COC=CC=C2SC)=O
PUGREST.BadRequest: error:
CC1=CC=C(S=2C)C(C=C1[C@@H1](NC(=O)C)CCC=CC(OC)=C(OC)C(OC=CC=O)C=2)SC=O
PUGREST.BadRequest: error:
C1COC1(OC2=CC=C3C4=C(OC)C(OC)=C(OC)C=C4CC[C@@H1](C3=CC2=O)SC(C)=O)SC
PUGREST.BadRequest: error:
CC1=CC=C(S=2C)C(C=C1[C@@H1](NC(C)=O)CCC=CC(OC)=C(OC)C(OC=CC=O)C=2)SC=O
PUGREST.BadRequest: error:
C1C=CC=C(SC2C1)C3=C(C(OC)=C(OC)C=C3CC[C@@H1]2NC(C)=O)OC=CC=C(SC)C=O
PUGREST.BadRequest: error:
CC1=C2C=C(SC)CC=C1[C@@H1](NC(=O)C)CCC=CC(OC)=C(OC)C(OC=CC2)=O
PUGREST.BadRequest: error:
C=C1C(C2=C(C(OC)=C(OC)C=C2CC[C@@H1]1NC(C)=O)OC)(C3=CC=CC=C3)OCC=C(SC)C=O
PUGREST.BadRequest: error:
C1=C2C(C(OC(CC)=O)=CC=C1C3=C(OC)C(OC)=C(OC)C=C3CC[C@@H1]NSC=CC2)=O
PUGREST.BadRequest: error:
OC(NC(=O)OC)C=CC=C1C([C@H1](CCC2=CC(OC)=C(OC)C(OC)=C21)N=C)C=O
PUGREST.BadRequest: error:
O1C(OC(=O)OC)=CC2=CC([C@@H1](NC(C)=O)CCC3=CC(OC)=C(OC)C(OC)=C32)=CC1NC
PUGREST.BadRequest: error:
C=1=CC(C(C=C2[C@H1](CCC3=C(C(OC)=C(OC)C(OC)=C3)C2=1)NC(C)=O)=C)=O
PUGREST.BadRequest: error:
C1[C@@H1](C2=CC(C(OC)=CC=C2C3=C1C=C(OC)COC)=C3OC=O)CC=O
PUGREST.BadRequest: error:
C=CC(C(OC(CC)=O)=CC=CC1=C(OC)C(OC)=C(OC)C=C1CC[C@@H1]NSC(=O)CC)=O
PUGREST.BadRequest: error:
O=C(OC1=C2C(CC[C@H1](NC(=O)C)C3=CC(C(SC)=CC=C32)=O)=CC(OC)=C1OC)SCC
PUGREST.BadRequest: error:
CN(C(=S)N[C@@H1]1C2=CCC(NC)=CC=C2C3=C(C(OC)=C(OC)C=C3CC1)OC)OC
PUGREST.BadRequest: error:
C1=C2C3(C(OC(C)=O)=CC=C1C4=C(OC)C(OC)=C(OC)C=C4CC[C@@H1]3NSC=C)C2=O
PUGREST.BadRequest: error:
O1C(OC(=O)OC2)=CC=CC([C@@H1](NC(C)=O)CCC3=CC(OC)=C(OC)C(OC)=C32)=CC1NC
PUGREST.BadRequest: error:
N1([C@H1]2CCC=C(C(OC)=C(OC)C(OC)=C2C3=CC=C(SC)CC=C3)OC=C1)F
PUGREST.BadRequest: error:
O=C(OC1=C2CC([C@H1](CCC2=CC(OC)=C1OC)NC(C)=O)=CC(C(SC)=CC)=O)C
PUGREST.BadRequest: error:
C1N(C(=O)N[C@H1]2CCC3=CC(OC)=C(OC)C(OC)=C3C4=CC=C(NC)CC=C24)OC=C1
PUGREST.BadRequest: error:
NC(=CN(NCC)CCCCN1[C@@H1]C=C2CC(C)=CC=C2C3=C(CC1)C=C(OC)C(OC)=C3OC)OF
PUGREST.BadRequest: error:
OC=C(OC)C=C1C(C2=CC=C(SC)C(C=C2[C@H1](CC1)NC(C)=O)OCC)=O
```

PUGREST.BadRequest: error:
O=C1C=CC=C2C([C@H1](CCC3=CC(OC)=C(OC)C(OC)=C32)NC(C)=O)=C1SC
PUGREST.BadRequest: error:
C1=CC=C(C=C1)CN[C@H1]2CCC3=CC(OC)=C(OC)C(OC)=C3C4=CC=C(C(C=C42)=O)SCC
PUGREST.BadRequest: error:
O=CC(=CC=C1C([C@H1](CCC2=CC(OC)=C(OC)C(OC)=C21)NC(C)=O)SC)O
PUGREST.BadRequest: error:
N=1C(=CN(N=1)[C@@H1]2C3=CCC(NC)=CC=C3C4=C(OC)C(OC)=C(OC)C=C4CC2)F
PUGREST.BadRequest: error:
CC=CCCCN[C@H1]1CCC2=CC(OC)=C(OC)C(OC)=C2C3=CC=C(SC)C(C=C13)=O
PUGREST.BadRequest: error:
C1=CC=C(S1C)C2=CN(NC2)[C@H1]3CCC4=CC(OC)=C(OC)C(OC)=C4C5=CC=C(NC)C(=O)C=C35
PUGREST.BadRequest: error:
OC1=C(C=CC=C1)CN2[C@H1]CCC3=CC(OC)=C(OC)C(OC)=C3C4=CC=C(SC)C(C=C24)=O
PUGREST.BadRequest: error:
OC=C(OC)C=C1C(C2=CC=C(SC)C(C=C2[C@@H1](NC(C)=O)CC1)=O)(C)OSC
PUGREST.BadRequest: error: C=C1CC(=C2C=C(SC)CC=C[C@H1]CCC2=CC(OC)=C1O)C(OCC)=O
PUGREST.BadRequest: error:
OC1=C(C=CC=C1)CN[C@@H1]2C3=CC(C(SC)=CC=C3C4=C(CC2)C=C(OC)C(OC)=C4OC)SC=O
PUGREST.BadRequest: error:
C1=C2CC[C@H1](C3=CCC(NC)=CC=C3C2=C(C(OC)=C1OC)OC=O)NCCCCC
PUGREST.BadRequest: error:
COC1=C2C3=CC=C(NC)C(C=C3[C@@H1](NCCCCCCC)CCC1=CC=C2OC)OC=O
PUGREST.BadRequest: error:
C=1NCC(=O)C=C2C=1C3=C(OC)C(OC)=C(C=C3CC[C@@H1]2NCCCCCO)C=CC
PUGREST.BadRequest: error:
C=C1CC2[C@H1](CCC3=C(C(OC)=C(OC)C(=C3)OC)C2=CC=C1)SC=O
PUGREST.BadRequest: error:
C1OC2=C3C4=CC=C(NC)C(=O)C=C4[C@H1](CCC3=CC(OC)=C2OC)NCCCCC=C1
PUGREST.BadRequest: error:
COC=C1C2=C3C=C(NC)C(C=C2[C@@H1](NCCCCCCCCC)CCC1=CC(=C3OC)O)OC
PUGREST.BadRequest: error:
COC1=C2C3=CC=C(NC)C(=O)C=C3[C@H1](CCC2=CC(OC)=C1OC)NCCCCCCCCSC
PUGREST.BadRequest: error:
COC1=C2C3=CC=C(NC)C(C=C3[C@@H1](NCCCCCCC)CCC1=CC(=C2OC)OCS)C=O
PUGREST.BadRequest: error:
C1=C2CC[C@H1](C3=CCC(SC)=CC=C3C2=C(C(OC)=C1OC)OC=O)NCCCCCC
PUGREST.BadRequest: error:
COC=CC1=C2C=C(NC)C(C=C1[C@@H1](NCCCCCCCCC)CCC=CC(=C2OC)O)C=O
PUGREST.BadRequest: error:
C1=C2CC[C@H1](C3=CCC(NC)=CC=C3C2=C(OC)C(=C1OC)OC)ONCCCCCC
PUGREST.BadRequest: error:
COC=C1C2=CC=C(NC)C(C=C2[C@@H1](NCCCCCC)CCC1=CC(=COC)OC)SC=O
PUGREST.BadRequest: error:
C1=C2CC[C@H1](C3=CCC(SC)=CC=C3C2=C(OC)C(=C1OC)OC=O)NCCCCCC
PUGREST.BadRequest: error: CNC=CC=C1C([C@H1](CCC2=CC(=C(OC)C(OC)=C12)OC)NCC)=O
PUGREST.BadRequest: error:
CSC=1C(C=C2C(=CC=1)C3=C(OC)C=CC=C3CC[C@@H1]2NCN)COCOC

```
PUGREST.BadRequest: error:
C=1SC=2C=1C=C3C(=CC=2)C4=C(OC)C(=CC=C4CC[C@@H1]3NCN)C
PUGREST.BadRequest: error:
COC1=CC=2CC[C@@H1](C=3C(C=2C(=C1OC)OC)=CC=C(NC)C(=O)C=3)NC(=O)CNC
PUGREST.BadRequest: error:
CSC1=CC=C2C(=CC1=O)[C@H1](CCC3=CC(OC)=C(OC)C(=C23)O)CNC(=O)C
PUGREST.BadRequest: error:
COC1=CC=2CC[C@@H1](C3=CC(=O)C(NC)=CC=C3C=2C(OC)=C1OCN)C(=O)C
PUGREST.BadRequest: error:
CSC=1C(C=C2C(=CC=1)C3=C(OC)C(=C(OC)C=C3CC[C@@H1]2NCN)O)OCNC
PUGREST.BadRequest: error:
CNC=CC=C1C[C@H1](CCC2=CC(=C(OC)C(OC)=C12)OC)N=CC(=O)OCOO
PUGREST.BadRequest: error:
C1=C2[C@H1](CCC3=C(C(OC)=C(C(=C3)OC)OC)C2=CC=C(C1=O)NC)NC
PUGREST.BadRequest: error:
C1SCC(C=C2C(C3=C(C=C(OC)C(OC)=C3OC)CC[C@@H1]2N)=C1)=O
PUGREST.BadRequest: error:
CNC=1C(C=C2C(=CC=1)C3=C(OC)C(OC)=C(C=C3CC[C@@H1]2NC(C)=O)O)C=O
PUGREST.BadRequest: error:
CSC1=CC=C2C(=CC1=O)[C@H1](CCC3=CC(OC)=C(OC)C(OC)=C23)NCC
PUGREST.BadRequest: error:
COC=C1C=2CC[C@H1](NC(C)=O)C3=CC(=O)C(SC)=CC=C3C=2C(O)=C1OC
PUGREST.BadRequest: error:
COC=C1CCC2[C@H1](O)CCC3=CC(=C(OC)C(OC)=C3C2=CC1OCN)C(=O)C
PUGREST.BadRequest: error:
COC=C1CCC2[C@H1](O)CCC3=CC(=C(OC)C(OC)=C3C2=CC1OCNC)C=O
PUGREST.BadRequest: error:
COC=C1CCC[C@H1](O)CCC2=CC(=C(OC)C(OC)=C2C=CC1OC)NC(=O)C
PUGREST.BadRequest: error:
CNC=1C(C=C2C(=CC=1)C3=C(OC)C(OC)=CC=C3CC[C@@H1]2NC(C)=O)OC=O
PUGREST.BadRequest: error:
C1OC=CC=2CC[C@H1](NC(N)=O)C3=CC(C(=CC=C3C=2C(OC)=C1OC)NC)=O
PUGREST.BadRequest: error:  COC=C1CCC[C@H1](NC(N)=O)C2=CCC(=CC=C2C1C(O)C=O)NC
PUGREST.BadRequest: error:
CSC=1CC=CC(=C2C=1)C3=C(OC)C(OC)=C(C=C3CC[C@@H1]2NOCNC)O
PUGREST.BadRequest: error:
NN(C[C@H1](OC[C@@H1][C@@H1](CO)O)OS1C2=CCC(NC)=CC=C2C3=C(C=C(C(=C3OC)OC)OC)CC1)O
PUGREST.BadRequest: error:  C=12OC3=CCCC[C@H1](N)CC(C=1C(=C2OC)OC)=CC=C(O)CC3=O
PUGREST.BadRequest: error:
CNC=1C(C=CC(=C2C=1)C3=C(OC)C(OC)=CC=C3CC[C@@H1]2NOCS)C=O
PUGREST.BadRequest: error:  CSCCC=C1C(C2=CC=C(OC)C(OC)=C2OC)CC[C@@H1]1N=O
PUGREST.BadRequest: error:
CNC=1CC=CC(=C2C=1)C3=C(OC)C(OC)=C(C=C3CC[C@@H1]2NOCS)C=O
PUGREST.BadRequest: error:  COC=C1C2CC[C@H1](N)CCC3=CC(=C(OC)C=C3C2=CC1OCS)C
PUGREST.BadRequest: error:
CC(=O)C(S1=2C3)CC(OC)=C(OC)C(C)=C1C3=CC=C(SC)C(C=2)=O
PUGREST.BadRequest: error:
C1C(=O)C=CC2=C(SC)C(OC)=C(OC)C(C)=CCC[C@@H1](C2=C1)NC(=O)C
```

```
PUGREST.BadRequest: error:  C12=C(CC1NC(=O)C=CC(C(SC)=CC2)=O)C(OC)=C(C(OC)=C)OC
PUGREST.BadRequest: error:
C1=C(C(OC(=O)C)=C2C(OC)=C1)OCC=3C([C@H1](CC2)NC(=O)C)=CC(=O)C(O)=CC=3
PUGREST.BadRequest: error:
CC(=O)OC1=CC=C2C3=C(C(OC)=C(C=C3CC[C@@H1](C2=CC1=O)NC(=O)CO)CO)C
PUGREST.BadRequest: error:
C12C(=O)C(=C3C=C1C4=C(CC[C@@H1]3NC(C)=O)C=C(OC)C(=C4OC)OC=C2SC)OC(=O)C
PUGREST.BadRequest: error:
C12C(=O)C2=C3C=C1C4=C(CC[C@@H1]3NC(C)=O)C=C(OC)C(OC)=C4OC(OC)=O
PUGREST.BadRequest: error:
C12C(=O)C(=CC=C1C3=C(CC[C@@H1]2NC(C)=O)C=C(OC)C(=C3OC)OC(OC)=O)SC
PUGREST.BadRequest: error:
CC(=O)OC=1C(=O)C=C2C(=CC=1)C3=C(OC)C(OC)=C(C=C3CC[C@@H1]2NC(C)=O)C(=O)C
PUGREST.BadRequest: error:
C12C(=O)C=C3C=C1C4=C(CC[C@@H1]3NC(C)=O)C=C(OC)C(=C4OC(OC)=O)OC=C2
PUGREST.BadRequest: error:
C12C(=O)C(=CC=C1C3=C(CC[C@@H1]2NC(C)=O)C=C(OC)C(OC)=C3OC(OC)=O)SC
PUGREST.BadRequest: error:
CC(=O)OC1=CC=C2C3=C(C(OC)=C(C=C3CC[C@@H1](C2=CC1=O)NC(=O)C)OC)OC(=O)C
PUGREST.BadRequest: error:
C1C(=O)C(=C2C=C1C3=C(CC[C@@H1]2NC(C)=O)C=C(OC)C(OC)=C3OC(OC)=O)SC
PUGREST.BadRequest: error:
C=1C(=O)C(OC(=O)CC)=CC=CC2=C(C3=C(OC)C(OC)=C2OC)CC[C@H1](NC(=O)C)C3=1
PUGREST.BadRequest: error:
C1C(=O)OC2C=CC=CC(=C2)[C@@H1](NC(=O)C)CCC3=CC(OC)=C(OC)C(=C3)OC=C1
PUGREST.BadRequest: error:
C=12C=3C(=CC(=O)C(SC)=CC=3)[C@H1]CCC=1C=C(OC)C(=C2OC)OCNC(C)=O
PUGREST.BadRequest: error:
CCC(=O)OC1=CC=C2C3=C(C(OC)=C(C=C3CC[C@@H1](C2=CC1=O)OC(=O)C)OC)OC
PUGREST.BadRequest: error:
CCC(=O)N[C@H1]1CCC2=CC(=C(OC)C(OC)=C2C=3C1=CC(=O)C(=CC=3)OC(OC)=O)OC
PUGREST.BadRequest: error:
CCC(=O)OCC(C=C1C(=C)C2=C(OC)C(=C(C=C2CC[C@@H1]1N)CC)OO)COC=CO
PUGREST.BadRequest: error:
CC(C)C1(OC=C2C3=CC=C(C(C=C3[C@H1](CCC2=CC(=C1OC)OC)NCC)=O)O)OC=O
PUGREST.BadRequest: error:
CC(C)C(OC1=CC=C2C3=C(CC[C@@H1](C2=CC1=O)NC(C)=O)C=C(OC)C(OC)=C3OC)(O)C=O
PUGREST.BadRequest: error:
CC(C)C1(OC=CC2=CC=C(C(C=C2[C@H1](CCC=CC(=C1OC)OC)NCC)=O)O)OC=O
PUGREST.BadRequest: error:
CC(C)C(OC1=CC=C2C3=C(CC[C@@H1](C2=CC1=O)NC(C)=O)C=C(OC)C(OC)=C3OC=O)C
PUGREST.BadRequest: error:
CC(C)C(=O)OC1=CC=C2C3=C(C(OC)=C(C=C3CC[C@@H1](C2=CC1=O)NC(C)=O)OC)OC(=O)C
PUGREST.BadRequest: error:
CC(C)C(OC1=CC=C2C3=C(C=C(OC)C(OC)=C3OC)CC[C@@H1](C2=CC1=O)NC(=O)CC)(O)C
PUGREST.BadRequest: error:
CC(=O)N[C@H1]CCC1=CC(OC)=C(OC)C(=C1C2=CC=C(SC)C(=O)C=C2)CC=C
PUGREST.BadRequest: error:
```

```
CC(=O)N[C@@H1]1C2=CC(C(SC)=CC=C2C3=C(OC)C(=C(OC)C=C3CC1)OC(=O)C=CC=CC=C)O
PUGREST.BadRequest: error:
C12=C(C(=C(OC)C(OC)=C1)OC(C)=O)CC([C@H1](CC2)NC(C)=O)=CC(C(SC)=CC)=O
PUGREST.BadRequest: error:
CC(=O)OCC1=CN(N=N1)[C@H1]2CCC3=CC(OC)=C(OC)C(OC)=C3C=4C2=CC(=O)C(=CC=4)NCC
PUGREST.BadRequest: error:
C1=C(C2=C(OC)C(OC)=C1OC(=O)CCC)C[C@@H1](C3=CC(=O)C(SC)=CC=C32)NC(C)=O
PUGREST.BadRequest: error:
CC(=O)N[C@H1]1CCC2=CC(OC)=C(OC)C(=C2C3=CC=C(C(C=C31)=O)OCC4=CC=CC=C4)OOC
PUGREST.BadRequest: error:
O=C(OC)OC=C(OC)C(=C1C(CC[C@@H1](C2=CC(C(=CC=C12)SC)=O)N=C)O)C
PUGREST.BadRequest: error:
O=C(OC1)OC=C2C3=CC=C(C(C=C3[C@H1](CCC2=CC(=C1OC)OC)NCC)=O)OOC
PUGREST.BadRequest: error:
O=C(OC)OCC(=O)C=C1C(C2=C(C(OC)=C(C=C2CC[C@@H1]1NC(=O)C)OC)OC)(O)CC
PUGREST.BadRequest: error:
O=C(OC)OC1=C2C3=CC=C(C(C=C3[C@H1](CCC2=CC(OC)=C1O)CNC=O)C)OOC(=O)C
PUGREST.BadRequest: error:
O=C(OC)OC1=CC=C2C3=C(C(OC)=C(C=C3CC[C@@H1](C2=CC1=O)OC(=O)C)NC)CC=O
PUGREST.BadRequest: error:
O=C(OC)OC=CC=C1C([C@@H1](NC(=O)C)CCC2=CC(=C(OC)C(OC)=C21)O)C=CC=O
PUGREST.BadRequest: error:
O=C(OC)OC=CC=C1C2=C(C(OC)=C(C=C2CC[C@@H1](C1=CC=O)NC(=O)C)OCO)C(=O)C
PUGREST.BadRequest: error:
O=C(OC)OC1=CC=C2C3=C(C(OC)=C(C=C3CC[C@@H1](C2=CC1=O)OC(=O)O)CO)SC
PUGREST.BadRequest: error:
O=C(OC1)OC=C2C3=CC=C(C(C=C3[C@H1](CCC2=CC(=C1OC)OC)NC=O)C)OOC
PUGREST.BadRequest: error:
O=C(OC)OC=1C(C=C2C(=CC=1)C3=C(OC)C(=C(OC)C=C3CC[C@@H1]2NC(=O)C)O)C=O
PUGREST.BadRequest: error:
O=C(OC)OCC(C=C1C(C2=C(C=C(OC)C(OC)=C2OC)CC[C@@H1]1NC(C)=O)=CC)=O
PUGREST.BadRequest: error:
O=C(C)N[C@H1]1CCC2=CC(=C(OC)C(=C2C=3C1=CC(C(=CC=3)OC(=O)OC)=O)CN)C(=O)COCC
PUGREST.BadRequest: error:
O=C(C)N[C@@H1]1CC(C2=C(C(=C(OC)C=C2CC1)OC)OC)C=C(SC)C(C)=O
PUGREST.BadRequest: error:
O=C(C(C)C)OC1=C2C3=CC=C(C(C=C3[C@H1](CCC2=CC(OC)=C1O)CNCC)=O)OOC
PUGREST.BadRequest: error:
O=C(C)N[C@@H1]1CC2(C3=C(C(=C(OC)C=C3CC1)OC)OC)C=C(OC)C(C2)=O
PUGREST.BadRequest: error:
O=C(C)N[C@H1]1CCC2=CC(=C(OC)C(=C2C=3C1=CC(C(=CC=3)OC)O)COC(=O)OC)NC(=O)C=O
PUGREST.BadRequest: error:
O=C(CC)OC1=C2CC([C@@H1](NC(C)=O)CCC2=CC(=C1OC)OC)=CC(C(=CC)SC)=O
PUGREST.BadRequest: error:
O=C(C)N[C@H1]1CCC2=CC(=C(OC)C(=C2C=3C1=CC(C(=CC=3)OC(OC)=O)=O)CC)OOC
PUGREST.BadRequest: error:
O=C(C)N[C@H1]1CCC2=CC(=C(OC)C(=C2C=3C1=CC(C(=CC=3)OC)O)COOCOC)C=O
PUGREST.BadRequest: error:
```

```
CC(=O)N[C@H1]C1CC2=CC(OC)=C(OC)C(OC)=C2C3=CC=C(C(=O)C=C31)OC
PUGREST.BadRequest: error:
CC(=O)C1=CC=C(SC)C(C=C1[C@H1]CCC=CC(OC)=COC)NC(=O)CSC
PUGREST.BadRequest: error:
C=1C(=O)C2=C(C3=CC=C(SC)C(C=C3[C@H1](CC2)NC=O)COC)C(OC)C=1
PUGREST.BadRequest: error:
CC(=O)N[C@H1]C1CC2=CC(OC)=C(OC)C(OC)=C2C3=CC=C(C(=O)C=C31)O
PUGREST.BadRequest: error:
C1C(=O)C=C(C2=CC=C(SC)C(C=C2[C@H1](NC(C)=O)C)COC)C(OC)C(=C1)SC
PUGREST.BadRequest: error:  CC(=O)C=C(C1=CC2=C(SC)C(C=C1[C@H1](NC(C)=O)C)C)OC2OC
PUGREST.BadRequest: error:  C12=C(C3=CC=C(SC)C(C=C3[C@@H1](N)CC1)=CCOC)C2OC=C
PUGREST.BadRequest: error:
C=12C(=CC(C(SC)=CC=1)=O)[C@H1](CCC3=CC(OC)=C(OC)C(=C32)N)C
PUGREST.BadRequest: error:
C=1C(=O)C(=CC=C2C3=C(OC)C(OC)=CC=C3CC[C@@H1](C2=1)NC=O)COC
PUGREST.BadRequest: error:
C=1C(=O)C(OC(=O)C)=CC=C2C3=C(OC)C(=C(OC)C=C3CC[C@@H1](C2=1)OCNC=O)C
PUGREST.BadRequest: error:
C=12C(=O)C(=CC=C3C=1[C@H1](CCC4=CC(OC)=C(OC)C(OC)=C34)NCC)OC2
PUGREST.BadRequest: error:
CC(=O)OC1=C2C3=CC=C(SC)C(=O)C=C3[C@H1](CCC2=C(OC)C=C1)OCNC(C)=O
PUGREST.BadRequest: error:
C=12C(=O)C(=CC=C3C=1[C@H1](CCC4=CC(OC)=C(OC)C(OC)=C34)NC(C)=O)C2
PUGREST.BadRequest: error:
C1=C(C(O)=CC=C1CC([C@@H1](NC(=O)C)CC)=CCC(=CC)SC)OOC(=O)CCOC
PUGREST.BadRequest: error:
CCC(=O)OC=C1C2=CC=C(SC)C(=O)C=C2[C@H1](CCC1=C(OC)C=C)OCOC(=O)OC
PUGREST.BadRequest: error:
C=1C=C(OC(=O)OC)C(C=C2C=1C3=C(OC)C(=C(OC)C=C3CC[C@@H1]2NC)C)C=O
PUGREST.BadRequest: error:
CCC(=O)OC=CC=C1C([C@@H1](NC(C)=O)CCC2=CC(OC)=CC(OC)=C21)OC=CC=O
PUGREST.BadRequest: error:
CCC(=O)OC=C1C2=CC=C(SC)C(=O)C=C2[C@H1](CCC1=C(OC)C=C)OCOCNC(=O)C
PUGREST.BadRequest: error:
C=1C=C(OC(=O)OC)C2(C=CC=1C3=C(OC)C(=C(C(OC)=C3CC[C@@H1]2NC)C)O)COC=O
PUGREST.BadRequest: error:
C12C=C(C3=CC=C(SC)C(=O)C=C3[C@@H1](NC(=O)C)C1)C(=C(OC)C(OC)=C2)OC
PUGREST.BadRequest: error:
CCC(=O)N[C@H1]1CCC2=C(C(OC)=C(C(OC)=C2)OC)C=3C1=CC(C(SC)=CC=3)=O
PUGREST.BadRequest: error:
CCC(=O)OC=C1C2=CC=C(SC)C(=O)C=C2[C@@H1](NC(=O)C)CCC1=CC(OC)=COC
PUGREST.BadRequest: error:  C1C=C1C2=CC=C(SC)C(=O)C=C2[C@H1]
PUGREST.BadRequest: error:
C=1C=C(OC(=O)OC)C(C=C2C=1C3=C(OC)C(=C(C=C3CC[C@@H1]2NC(C)=O)OC)O)C=O
PUGREST.BadRequest: error:
CCC(=O)N[C@H1]1CCC2=CC(OC)=C(C(OC)=C2C=3C1=CC(=O)C(=CC=3)SC)OC(=O)OCC
PUGREST.BadRequest: error:
C12C=C(CC[C@H1](NC(=O)C)C1=CC(C(=CC)SC)=O)C=C(OC)C(OC)=C2OC
```

```
PUGREST.BadRequest: error:
CC=1C(=CC(C(=CC=1)SC)=O)[C@H1](CCC2=CC(OC)=C(OC)C(=C2)OC)NC(=O)C
PUGREST.BadRequest: error:
CC(C)COCCN[C@H1]1CCC2=CC(OC)=C(C(=C2C=3C1=CC(C(=CC=3)SC)=O)OC)OC(=O)C
PUGREST.BadRequest: error:
CC(C)CCN[C@H1]1CCC2=C(C3=CC=C(SC)C(C=C31)=O)C(=C(C(OC)=C2)OC)OCCC
PUGREST.BadRequest: error:
CC(C)CCN[C@H1]1CCC2=C(C3=CC=C(SC)C(C=C31)=O)C(=C(C(OC)=C2)OC)OC(=O)C
PUGREST.BadRequest: error:
CC(C)C(OC1=C2C3=CC=C(SC)C(=O)C=C3[C@H1](CCC2=CC(OC)=C1OC)NC=O)C=O
PUGREST.BadRequest: error:
C1=C(OC)C(=C(OC)C2=C1CC[C@H1](NC(=O)C)C=3C2=CC=C(OC(=O)OC)C(C=3)=O)C
PUGREST.BadRequest: error:
CC(C)CCN[C@H1]1CCC2=C(C3=CC=C(SC)C(C=C13)=O)C(=C(C(OC)=C2)OC)OCCC
PUGREST.BadRequest: error:
CC(=O)N1[C@H1]CCC2=CC(OC)=C(OC)C(OC(=O)C)=C2C=3C1=CC(C(=CC=3)SC=O)CC=O
PUGREST.BadRequest: error:
C=1C(=CC(C(SC)=CC=1)=O)[C@H1](CCC=CC(OC(=O)C(C)C=COC)C(=C)OCNC=O)CSC
PUGREST.BadRequest: error:
C12=C(C=C(OC)C(OC)=C1OC(=O)C3=CC)CC=C3C=CC=C(CC2O[C@@H1]NC(C)=O)C
PUGREST.BadRequest: error:
O=C(OC1)OC=CC=C2C3=C(OC)C(=C(OC)C=C3CC[C@@H1](NC(C)=O)C2=CC1)OO
PUGREST.BadRequest: error:
O=C(OC)OC1=CC=C2C3=C(OC)C(=C(OC)C=C3CC[C@@H1](C2=CC1=O)NC=O)COC
PUGREST.BadRequest: error:
O=C(OC1)OC=CC=C2C3=C(OC)C(=C(OC)C=C3CC[C@H1](NC(=O)C)C2=CC1)OO
PUGREST.BadRequest: error:
O=C(OC)OC1=CC=C2C3=C(OC)C(=C(OC)C=C3CC[C@@H1](C2=CC1=O)NCC)OOC
PUGREST.BadRequest: error:
O=C(CC1)OC=CC=C2C3=C(OC)C(=C(OC)C=C3CC[C@H1](NC(=O)C)C2=CC1)OOC
PUGREST.BadRequest: error:
O=C(C)N[C@H1]1CCC2=CC(OC)=C(C(OC)=C2C3=CC=C(C(=O)C=C13)OCCC)OOC
PUGREST.BadRequest: error:
O=C(C)N[C@H1]C1=CC(C(OC(OCC)=O)=CC=C1C2=C(OC)C=C(OC)C=C2CC)OC=O
PUGREST.BadRequest: error:
O=C(C)N[C@H1]CC1C2=CC(OC)=CC(OC)=C2C=CC=C(OC(=O)OCC)C(=O)C=C1
PUGREST.BadRequest: error:
O=C(NC)NCC=CN(C=1OCC)CN(OCCC)CC=CC2=C(OC)C(=C(OC)C=C2CC[C@H1](NC(=O)C)OC=1)C=O
PUGREST.BadRequest: error:
O=C(N(C)C)NC1=CC=C2C3=C(OC)C(=C(OC)C=C3CC[C@@H1](C2=CC1=O)NCC)OOC
PUGREST.BadRequest: error:
O=C(C)N[C@H1]1CCC=C(C2=CC=C(SC)C(C=C12)=O)C(OC(CC)=O)=C(C(OC)=C)OC
PUGREST.BadRequest: error:
O=C(N)N[C@H1]1CCC2=CC(OC)=C(C(OC)=C2C=3C1=CC(C(OC(=O)C)=CC=3)=O)OC
PUGREST.BadRequest: error:
O=C(C)N[C@H1]1CCC2=CC(OC)=C(C(OC)=C2C3=CC=C(C(=O)C=C31)OC(=O)O)CCOC
PUGREST.BadRequest: error:
O=C(C)N[C@H1]1CCC2=CC(OC)=C(C(OC)=C2C=3C1=CC(C(OC(=O)CC)=CC=3)O)C=O
```

```
PUGREST.BadRequest: error:
O=C(N)N[C@H1]1CCC2=CC(OC)=C(C(OC)=C2C3=CC=C(C(C=C31)=O)O)C(=O)OCNC
PUGREST.BadRequest: error:
CC(=O)N1[C@H1]CCC2=CC(OC)=C(OC)C(OC)=C2C3=CC=C(NC)C(C=C31)=O
PUGREST.BadRequest: error:
C=1C(=CC=C(C2=O)OCC[C@H1]CCC3=CC(OC)=C(OC)C(OC)=C3C2)CC=1SC
PUGREST.BadRequest: error:
C1C(=O)OCC2=CC=CC3=C(OC)C(OC)=C(OC)C=C3CC[C@H1](NC(C)=O)C2=CC1=O
PUGREST.BadRequest: error:
CC(=O)OCC=CC=C1C2=C(OC)C(OC)=C(OC)C=C2CC[C@H1](NC(C)=O)C1=CSC
PUGREST.BadRequest: error:
CC(=O)N1[C@@H1]C2=CC(C(NC)=CC=C2C3=C(C(OC)=C(OC)C=C3CC1)OC)NC=O
PUGREST.BadRequest: error:
CC(=O)OC=CC=C1C2=C(OC)C(OC)=C(OC)C=C2CC[C@H1](NC(C)=O)C1=CCSC=O
PUGREST.BadRequest: error:
CC(=O)N[C@H1]C1CC2=CC(OC)=C(OC)C(OC)=C2C3=CC=C(SC)C(C=C31)=O
PUGREST.BadRequest: error:
C12=C(C=3C([C@H1])(C4C1)N=C(Cl)C=C4CCC(SC)=CC=3)OC=C2OCNC
PUGREST.BadRequest: error:  C1OC=C2C3=CC=C(NC)C(=O)C=C3[C@H1](CCC2=CC(OC)=C1OC)N
PUGREST.BadRequest: error:  CNCCC=C1C(C2=C(OC)C(OC)=CC=C2CC[C@@H1]1NOC)=O
PUGREST.BadRequest: error:  C1OC=CC2=C(CC(O)CC1=CC=C3C(OC)=C(OC)C)P=C3C2OCSC
PUGREST.BadRequest: error:  CSCC=CC=CC1=C(OC)C=C(C=C1CC[C@@H1]NC(N)=NOCOC)O
PUGREST.BadRequest: error:  C1OC=C2CC([C@@H1](N)CCC2=CC(OC)=C1OC=CC)(C(=CC)SC)O
PUGREST.BadRequest: error:
COC1=C2C3=CC=C(NC)C(=O)C=C3[C@H1](CCC2=CC(OC)=C1OC)NO
PUGREST.BadRequest: error:
C=C1CC2[C@H1](CCC3=C(C(OC)=C(OC)C(=C3)OC)C2)CC=C1N=[N+1]=[N-1]
PUGREST.BadRequest: error:
C=1NCC(C=C2C(C3=C(OC)C=C(C=C3CC[C@@H1]2N=[N+1]=[N-1])OC)OC=C)C=1O
PUGREST.BadRequest: error:  COC=C1C2=CC=C(NC)C(=O)C=C2[C@H1](CCC1C=COC)N3N=NC=C3
PUGREST.BadRequest: error:
COC=C1C2=CC=C(SC)C(=O)C=C2[C@@H1](NCCCCCCC)CCC1=CC(=COC)SC
PUGREST.BadRequest: error:
COC=C1C2=CC=C(NC)C(=O)C=C2[C@@H1](N=[N+1]=[N-1])CCC1=CC(=COC)OC
PUGREST.BadRequest: error:
C1OC=CC2=C(C3=C(OC)C(OC)=CC=C3CC[C@@H1]2N=[N+1]=[N-1])CC=C1NC
PUGREST.BadRequest: error:
C=CCC1[C@H1](CC=[N+1]C2=C(C(OC)=C(OC)C=C2C1O)C=O)[N-1]CCC
PUGREST.BadRequest: error:
C=12[C@@H1](C=CC(=O)C(C)=CC=1C3=C(CC2)C=C(OC)C(OC)=C3OCNC)CNC=C
PUGREST.BadRequest: error:
C1[C@@H1](C2=CC(=O)C(SC)=CC=C2C3=C(C=C(OC)C(=C3OC)OC)C1N)CCNC
PUGREST.BadRequest: error:
COC1=C(OC)C(OC)=C2CCC[C@H1](N3C=CN=N3)C4=CC(=O)C(=CC=C4C21)NC
PUGREST.BadRequest: error:
C12=CC(C(=CC=C1C3=C(C=C(OC)C(OC)=C3OC)CC[C@@H1]2NCCO)C)NC
PUGREST.BadRequest: error:
C1[C@@H1](C2=CC(=O)C(NC)=CC=C2C3=C(C=C(OC)C(=C3OC)OC)C1)NCCSC
```

```
PUGREST.BadRequest: error:
N(C1=CC=C2C3=C(CC[C@@H1](C2=CC1=O)N4C=CN=N4)C=C(OC)C(OC)=C3O)CC
PUGREST.BadRequest: error:
C=1=C2C([C@H1](CCC3=CC(=C(OC)C(=C32)OC)OC)NCCCCCC)CC=CC(C=1SC)=O
PUGREST.BadRequest: error:
N1C(COC(=O)OC=CN1[C@@H1]2)C3=CC(=O)C(NC)=CC=C3C4=C(CC2)C=C(C(OC)=C4OC)OC
PUGREST.BadRequest: error:
N1C(COC(=O)OC=CN1[C@@H1]2)C3=CC(=O)C(NC)=CC=C3C4=C(CC2)C=C(C(=C4OC)OC)OC
PUGREST.BadRequest: error:
CNC(C(C)O)N[C@@H1]1CC(C2=C(C(=C(OC)C=C2CC1)OC)OC)=CC=C(NC)C(C)=O
PUGREST.BadRequest: error:
C=C1C([C@H1](CCC2=CC(=C(OC)C(=C21)OC)OC)NCCCCCC)CC=CC(C(=C)SC)=O
PUGREST.BadRequest: error:
COC1=C(OC)C(=CC=2CC[C@@H1](C3=CC(C(=CC=C3C=21)NC)=O)N4C=C(N=N4)CCC)NCOC
PUGREST.BadRequest: error:
CNC(C(C)O)ON(C=1C(C=C2C(=CC=1)C3=C(OC)C=C(C=C3CC[C@@H1]2NCC)CC)O)COC=O
PUGREST.BadRequest: error:
C1=C2C(C3=C(C=C(OC)C(=C3OC)OC)CC[C@@H1]2N4C=C(N=N4)CCCC=C)C=C(NC)C1=O
PUGREST.BadRequest: error:
CNC(C(C)O)N([C@H1]1CCC2=CC(=C(OC)C(OC)=C2C=3C1=CC(=O)C(=CC=3)NC)OCC)C
PUGREST.BadRequest: error:
CNC(C(C)O)N[C@@H1]1C=2C(C3=C(C=C(OC)C(OC)=C3OC)CC1)=CC=C(C(=O)C=2)NC
PUGREST.BadRequest: error:
C=C1C(C2=C(C=C(OC)C(=C2OC)OC)CC[C@@H1]1NCCCCCCCCCCC)=CC=C(SC)C=O
PUGREST.BadRequest: error:
CNC(=S)N[C@@H1]1C2=CC(=O)C(NC)=CC=C2C3=C(OC)C(=C(C=C3CC1)OC)OCO
PUGREST.BadRequest: error:
C1=2C3=C(C(=C(C=C3CC[C@H1](NCCCCCCC)C1=CCC(=CC=2)NC)OO)CO)COC
PUGREST.BadRequest: error:
C12=CC(C(NC)=CC=C1C3=C(C=C(C(OC)=C3OC)OC)CC[C@@H1]2N4C=C(N=N4)COO)NC
PUGREST.BadRequest: error:
N([C@H1]1CCC2=C(C3=CC=C(C(C=C13)=O)NC)C(OC)=C(OC)C(OC)=C2)N=N
PUGREST.BadRequest: error:
COC=C(OC)C(OC)=C1CCC[C@@H1](C2=CC(=O)C(NC)=CC=C12)NCCCCCC
PUGREST.BadRequest: error:
C=C1C([C@H1](CCC2=CC(=C(OC)C(=C21)OC)OC)N3N=NC=C3)CCl
PUGREST.BadRequest: error:
COC=C(OC)C(OC)=C1CCC[C@@H1](C2=CC(=O)C(SC)=CC=C12)NCCCCCCCC
PUGREST.BadRequest: error:
COC=C(OC)C(OC)=C1CCC[C@@H1](C2=CC(=O)C(NC)=CC=C12)NCCCCCCCC
PUGREST.BadRequest: error:
C12C3=CC(OC)=C(OC)C=C3CC([C@H1]1NCCCCCCCCC)=CC(=C2OC)OC
PUGREST.BadRequest: error:
C12=CC(C(NC)=CC=C1C3=C(C=C(C(OC)=C3OC)OC)CC[C@@H1]2NCCCCCCCCCCF)=O
PUGREST.BadRequest: error:
C12=CC(C(NC)=CC=C1C3=C(C=C(C(OC)=C3OC)OC)CC[C@@H1]2N4C=C(N=N4)CCl)NC
PUGREST.BadRequest: error:
C1=C2C(C3=CC=C(C(=O)C=C3[C@H1](CC2)NCCC4=CC=CC=C4)OS)CC(OC)=C(OC)C(=C1)OC
```

```
PUGREST.BadRequest: error:
COC1=C(OC)C(OC)=CC=2CC[C@@H1](C3=CC(=O)C(NC)=CC=C3C=21)NCCC4=CC=CC=C4NC
PUGREST.BadRequest: error:
C1[C@@H1](C2=CC(=O)C(SC)=CC=C2C3=C(C(=C(OC)C=C3C1)OC)OC)NCCC=CC=CC
PUGREST.BadRequest: error:
CNC(C(C)C)N[C@@H1]1C2=CC(=O)C(NC)=CC=C2C3=C(C(=C(OC)C=C3CC1)OC)OC
PUGREST.BadRequest: error:
C=C1C([C@H1](CCC2=CC(=C(OC)C(=C21)OC)OC)NCCCCC=C)C(C(SC)=C)=O
PUGREST.BadRequest: error:
C=C1C([C@H1](CCC2=CC(=C(OC)C(=C21)OC)OC)NCCCCCC)C=CC(C(SC)=C)=O
PUGREST.BadRequest: error:
COC1=C(OC)C(OC)=CC=2CC[C@@H1](C3=CC(=O)C(NC)=CC=C3C=21)N4C=C(N=N4)CCONC
PUGREST.BadRequest: error:
C12=CC(C(NC)=CC=C1C3=C(C=C(C(OC)=C3OC)OC)CC[C@@H1]2N4C=C(N=N4)CCC)Cl
PUGREST.BadRequest: error:
CN(C(=S)N[C@@H1]1C2=CC(=O)C(NC)=CC=C2C3=C(C(=C(OC)C=C3CC1)OC)OC)CC#N
PUGREST.BadRequest: error:
C1OC=C(OC)C(OC)=C2C1CC[C@@H1](C3=CC(=O)C(NC)=CC=C23)NCCCCCCC=C
PUGREST.BadRequest: error:
C[C@H1](OCCC)CCC1=C(OC)C(OC)=C(OC)C=C1CC=CC=CC(=O)CSCN
PUGREST.BadRequest: error:
CC(C)(C)OC1=CN(N=N1)[C@H1]C2CC3=CC(OC)=C(OC)C(OC)=C3C4=CC=C(NC)C(=O)C=C42
PUGREST.BadRequest: error:  C=C(OC)C1=C(OC)C=2C3=CC=C(SC)CC=C3[C@H1](CCC1=2)C=O
PUGREST.BadRequest: error:
CC(C)CCCCCCN[C@@H1]1C2=CC(C(SC)=CC=C2C3=C(C(OC)=C(OC)C=C3CC1)OC)=O
PUGREST.BadRequest: error:
CC(C)(C)C(=O)N[C@@H1]1C=2C(C3=C(C(OC)=C(OC)C=C3CC1)OC)=CC=C(NC)C(=O)C=2
PUGREST.BadRequest: error:
C1=C(OC)C(OC=2)=C(OC)C3CC([C@H1](CCC3=C1)NC(C)=O)SC=CC=2OSC
PUGREST.BadRequest: error:
C=CCCOCC(C=C1[C@H1](CCC2=CC(OC)=C(OC)C(OC)=C2C1=CCN)C(=O)C=O)SC=O
PUGREST.BadRequest: error:
OC(CCC(C)C)OCC1=CN(N=N1)[C@H1]CCC2=CC(OC)=C(OC)C(OC)=C2C3=CC=C(C(=O)C=C3)NC
PUGREST.BadRequest: error:
O=C(NCC1=CC=CC=C1Cl)N[C@H1]2CCC3=C(C(OC)=C(OC)C(OC)=C3)C4=CC=C(NC)C(=O)C=C24
PUGREST.BadRequest: error:
N([C@H1]1CCC2=C(C(OC)=C(OC)C(OC)=C2)C3=CC=C(NC)C(C=C13)=O)C(O)=O
PUGREST.BadRequest: error:
O=CC=CCC=CNNC(N[C@H1]1CCC2=CC(OC)=C(OC)C(OC)=C2C3=CC=C(NC)C(=O)C=C13)=O
PUGREST.BadRequest: error:
C12[C@H1]CC(C3=C(OC)C(OC)=C(OC)C=C3C1=CC=COC(OCC)=O)=C2SC=CC=O
PUGREST.BadRequest: error:
OC(CCC1(C)C)OC=C(C(OC)=C2CCC[C@H1](NC(C)=O)C3=CCC(SC)=CC=C3C21)O
PUGREST.BadRequest: error:
OC(CCC1(C)C)OC=C(C(OC=2)=CCCC[C@H1](NC(C)=O)C3=CCC(SC)=CC=C3C=21)O
PUGREST.BadRequest: error:
O=C(C(=O)OCC)C1OC=CC=CC2=C(OC)C(OC)=C(OC)C=C2CC[C@H1](NC(=O)C)C=CC1=O
PUGREST.BadRequest: error:
```

```
C=CCC(=O)OCCC=C1[C@H1](CCC2=C(C(OC)=C(OC)C(OC)=C2)C1=C)N=O
PUGREST.BadRequest: error:
C12[C@H1]CC(C3=C(OC)C(OC)=C(OC)C=C3C1=CC=C(SC)C)(C=C2)ONC(=O)C
PUGREST.BadRequest: error:
C=C1C(C2=CC=C(C(=O)C=C2[C@@H1](NCCCCCCCCCCC)CC1)COC)C(OC)=COCOC
PUGREST.BadRequest: error:
C=1N([C@H1]2CCC3=C(C4=CC=C(C(C=C24)=O)NC)C(OC)=C(OC)C(=C3)OC)N=NC=1C
PUGREST.BadRequest: error:
C1=C2C(C3=CC=C(C(=O)C=C3[C@H1](CC2)N4C=C(N=N4)CC(Cl)Cl)ONC)C(OC)=C(OC)C(=C1)OC
PUGREST.BadRequest: error:
C1=C2C(C3=CC=C(C(=O)C=C3[C@H1](CC2)N4C=C(N=N4)C=CC=CC=N)C=C)OCNC(OC)=C1OC
PUGREST.BadRequest: error:
C=1N([C@H1]2CCC3=C(C(=C(OC)C(OC)=C3)OC)C=4C2=CC(C(=CC=4)NC)=O)N=NC=1C
PUGREST.BadRequest: error:
C=C1C(C2=CC=C(C(=O)C=C2[C@H1](CC1)N3C=C(N=N3)C=CC=CC=N)C=C)OCNCOCOCC
PUGREST.BadRequest: error:
C1C2=CC(OC)=C(OC)C(=C2CC([C@H1](C1)NC/C=C/C3=CC=CC=C3)=CC(=O)C(=CC)SCO)C
PUGREST.BadRequest: error:
C1=C2C(C3=CC=CC(=O)C=C3[C@H1](CC2)N4C=C(N=N4)C=CC=CC=C)(OC)C(OC)=C1OCNC
PUGREST.BadRequest: error:
C=CC=CC(=O)OCCC=C1[C@H1](CCC2=CC(OC)=C(OC)C(OC)=C2C1=CN)OSCC
PUGREST.BadRequest: error:
N1=CC=C(C=C1)C(OC=CC=C2C3=C(OC)C(OC)=C(OC)C=C3CC[C@@H1](C2=CC=O)NC(=O)CSC)=O
PUGREST.BadRequest: error:
C=[C@H1]C1=CCC(OC(C2=CC=CC=C2)=O)=CC=C1C3=C(OC)C(OC)=C(OC)C=C3CC
PUGREST.BadRequest: error:
OC1=C(C=CC=C1)C=2N=NN(C=2)[C@H1]C3CC4=CC(OC)=C(OC)C(OC)=C4C5=CC=C(NC)C(=O)C=C53
PUGREST.BadRequest: error:
C12=CC=C(S1=C3)C(=O)C=C2[C@H1](CCC=CC(OC)=C(OC)C(OC)=C3)NC(C)=O
PUGREST.BadRequest: error:
OC=C(SC)C(C(OC=1)=CCC2([C@H1](NC(C)C)C=CCC2=CC=C=1)NC=O)NCOC
PUGREST.BadRequest: error:
OC1=C(C=CC=C1)C=2N=NN(C=2)[C@H1]CCC3=CC(OC)=C(OC)C(OC)=C3C4=CC=C(NC)C(=O)C=C4
PUGREST.BadRequest: error:
CNCC(O)(C1OC=CC=C1CN[C@@H1]2C3=C)C(C(NC)=CC=C3C4=C(C(OC)=C(OC)C=C4CC2)OC)=O
PUGREST.BadRequest: error:
C=CC1=C(COC2=CC(CC[C@H1](NC(C)=O)C3=CC(C(SC)=CC=C32)=C)C(OC)=C1OOC)OC
PUGREST.BadRequest: error:
C=CC=CCOC=CC=C1C([C@H1](CCC2=CC(OC)=C(OC)C(OC)=C21)NC(=O)C)=O
PUGREST.BadRequest: error:
C1[C@@H1](C2=CC(=O)C(NC)=CC=C2C3=C(C(=C(OC)C=C3C1)OC)OC)N4C=C(N=N4)COC
PUGREST.BadRequest: error:
C=1N([C@H1]2CCC3=C(C(=C(OC)C(OC)=C3)OC)C=4C2=CC(=O)C(=CC=4)NC)N=NC=1C
PUGREST.BadRequest: error:
NC(C1OC(=O)OC=CN1[C@@H1]2)C3=CC(C(=CC=C3C4=C(CC2)C=C(C(OC)=C4OC)OC)NC)=O
PUGREST.BadRequest: error:
C=C1C(C2=CC=C(C(=O)C=C2[C@H1](CC1)N3C=C(N=N3)COCC4=CC=CC=C4)COC)C(OC)=COCNC
PUGREST.BadRequest: error:
```

```
C1=CC(C2=CC=C(C(=O)C=C2[C@@H1](N3C=C(N=N3)C4=NC=CC=C4)C1N)C=C)(OC)C(OC)=COC
PUGREST.BadRequest: error:
C1=C2C(C3=CC=C(C(C=C3[C@H1](CC2)N4C=C(N=N4)C5=CC=CC=N5)N)C=C)(C(=C1OC)OC)OC
PUGREST.BadRequest: error:
C=1N([C@H1]2CCC=C(C3=CC=C(C(C=C23)=O)NC)C(OC)=C(OC)C(=C)OC)N=NC=1C(=O)OC
PUGREST.BadRequest: error:
O1CC=CC=C1CN[C@H1]2CCC3=CC(OC)=C(OC)C(OC)=C3C4=CC=C(NC)C(C=C24)=O
PUGREST.BadRequest: error:
O=C(OCC)CN(OCC)C(CN[C@@H1]1C2=CCC(NC)=CC3=C2C=C(CC1)C=C(OC)C(=C3OC)N)C=O
PUGREST.BadRequest: error:
O1CC=C2C=C1C3=C(OC)C(OC)=C(OC)C=C3CC[C@H1](CCCC=C2O)CN=CCSC
PUGREST.BadRequest: error:  OC=CC=CC1=C(C=C(OC)C(OC)=C1OC(OCC)=O)NC(C)=O
PUGREST.BadRequest: error:
O=C(C(C)C=1)OCCC=C2[C@H1](CCC3=CC(OC)=C(OC)C(OC)=C3C2=CC=1N)ONC
PUGREST.BadRequest: error:
O=C(OC(C)(C)C)N[C@H1]1CCC2=C(C(OC)=C(OC)C(OC)=C2)C3=CC=C(NC)C(=O)C=C13
PUGREST.BadRequest: error:
O=C(C(C)C=1)OCCC=C2[C@H1]CCC3=CC(OC)=C(OC)C(OC)=C3C2=CC=1N
PUGREST.BadRequest: error:
O=C(OCC)CN(OCC)C[C@@H1]1C2=CC(C(NC)=CC=C2C3=C(C(OC)=C(OC)C=C3CC1)OC)=O
PUGREST.BadRequest: error:
O=C(C)OC1=CN(N=N1)[C@H1]2CCC3=CC(OC)=C(OC)C(OC)=C3C4=CC=C(NC)C(=O)C=C24
PUGREST.BadRequest: error:
O=C(O)C=C(OC)CC=C1C(C2=C(OC)C(OC)=C(OC)C=C2CC[C@@H1]1N=CCS)C=O
PUGREST.BadRequest: error:
O=C(C)N[C@@H1]1C2=CCC(SC)=CC=C2C3=C(C(OC)=C(OC)C=C3CC1)OCSC=O
PUGREST.BadRequest: error:
O=C(C)N[C@@H1]1C2=CCC(SC)=CC=C2C3=C(C(OC)=C(OC)C=C3CC1)OCSCOC=O
PUGREST.BadRequest: error:
O=C(O)C=C(C(OC1C)=O)[C@H1]CCC2=CC(OC)=C(OC)C(OC)=C2C3=CC=C(SC)C(=O)C=C31
PUGREST.BadRequest: error:
O=C(C)N[C@@H1]1C2=CCC(SC)=CC=C2C3=C(C(OC)=C(OC)C=C3CC1)OCOC=O
PUGREST.BadRequest: error:
O=C(OCC)CN(CCC)C[C@H1]C1CC2=CC(OC)=C(OC)C(OC)=C2C3=CC=C(SC)C(=O)C=C31
PUGREST.BadRequest: error:
O=C(C(C)C=1)OCCC=C2[C@H1](CCC3=CC(OC)=C(OC)C(OC)=C3C2=CC=1)NSC=O
PUGREST.BadRequest: error:
C1=C2CC(OC(C3=CC=CC=C3)=O)=CC=C1C4=C(OC)C(OC)=C(OC)C=C4CC[C@H1](NC(=O)C)C2=O
PUGREST.BadRequest: error:
C1=CCC(OC(C2=CC=CC=C2)=O)=CC=C1C3=C(OC)C(OC)=C(OC)C=C3CC[C@H1](NC(=O)C)C=O
PUGREST.BadRequest: error:
C1N([C@@H1]2C3=CCC(SC)=CC=C3C4=C(OC)C(OC)=C(C=C4CC2)OCN)C(C(O)=O)=C1O
PUGREST.BadRequest: error:
C1=CC(C=C(C1(OC(C)=C=2)OC(=O)CCC=C3N=C4CNC)CC=C3C=2C(OC)=COC)C=C4NC=O
PUGREST.BadRequest: error:
C1N([C@@H1]2C3=CCC(SC)=CC=C3C4=C(OC)C(OC)=C(C=C4CC2)OC=O)N(C(C)C)OC1
PUGREST.BadRequest: error:
C1[C@@H1](C2=CCC(SC)=CC=C2C3=C(OC)C(OC)=C(C=C3C1)OC=O)NC(C(=O)O)=CNC
```

```
PUGREST.BadRequest: error:
N1([C@@H1]CCC1=C2C=C(CNC)CC=C2C3=C(OC)C(OC)=C(OC)C=C3C)NC(Cl)=O
PUGREST.BadRequest: error:  OC(OC(C)CC12)=C(C(OCC)=O)N=NN1[C@H1]3CCC4=CC(OC)=C(O
C)C(OC)=C4C=CC=C(NC)C(=O)C=C32
PUGREST.BadRequest: error:
C=C1C(C2=C(OC)C(OC)=C(OC)C=C2CC[C@@H1]1NC(C(O)=O)=C(NCO)OC=CC=CC(=O)C)SCNC
PUGREST.BadRequest: error:  O=C(C(Cl)Cl)NC(=O)N[C@H1]1CCC2=C(C(OC)=C(OC)C(OC)=C2
)C3=CC=C(C(C=C31)=O)NCN(O)CCC=O
PUGREST.BadRequest: error:  O=C(C(Cl)Cl)NC(=O)N[C@H1]1CCC2=C(C(OC)=C(OC)C(OC)=C2
)C3=CC=C(C(C=C31)=O)NCC4=CC=CC=C4
PUGREST.BadRequest: error:
O=C(C(Cl)Cl)NCC1=CN(N=N1)[C@H1]2CCC3=CC(OC)=C(OC)C(OC)=C3C4=CC=C(C(=O)C=C24)NCCO
PUGREST.BadRequest: error:
O=C(OCCC)OCC1=CN(N=N1)[C@H1]2CCC3=CC(OC)=C(OC)C(OC)=C3C4=CC=C(C(=O)C=C24)NCCO
PUGREST.BadRequest: error:
O(C=CC=CC1=C2C(OC)=C(OC)C=C1CC[C@H1](NC(N(CCO)CCO)=S)C2=CCCNC)ONCC
PUGREST.BadRequest: error:  O=C(OCCC)OCC1=CN(N=N1)[C@H1]2CCC3=CC(OC)=C(OC)C(OC)=
C3C4=CC=C(C(=O)C=C42)NCC(=O)C
PUGREST.BadRequest: error:  O=C(OCC=C)NCC1=CN(N=N1)[C@H1]2CCC3=CC(OC)=C(OC)C(OC)
=C3C4=CC=C(C(=O)C=C24)NCC(=O)C
PUGREST.BadRequest: error:  O=C(C(Cl)Cl)NCC1=CN(N=N1)[C@H1]2CCC3=CC(OC)=C(OC)C(O
C)=C3C4=CC=C(C(=O)C=C24)NCC(=O)C
PUGREST.BadRequest: error:  O=C(C(Cl)Cl)N1C(=O)N[C@H1]2CCC3=C(C(OC)=C(OC)C(OC)=C
3)C4=CC=C(C(C=C42)=O)NCC=CC=C1
PUGREST.BadRequest: error:  O=C(C(Cl)Cl)NCC1=CN(N=N1)[C@H1]2CCC3=CC(OC)=C(OC)C(=
C3C=4C2=CC(C(NC)=CC=4)=O)OCCCl
PUGREST.BadRequest: error:  O=C(OCC=C)NCC1=CN(N=N1)[C@H1]2CCC3=CC(OC)=C(OC)C(OC)
=C3C4=CC=C(C(C=C24)=O)NCC(=O)C
PUGREST.BadRequest: error:
O=C(C)N[C@@H1]1CC(C2=C(C(OC)=C(OC)C=C2CC1)OCC3=CC=CC=C3)=O
PUGREST.BadRequest: error:
O=C(C)N[C@@H1]1C=2C(C3=C(C(OC)=C(OC)C=C3CC1)OC(C)=O)C=C(SC)C(C=2)=O
PUGREST.BadRequest: error:
O=C(C(Cl)Cl)NC(=O)N[C@H1]1CCC2=CC(OC)=C(OC)C(OC)=C2C3=CC=C(C(C=C31)=O)NCC(=O)O
PUGREST.BadRequest: error:
O=C(C(C)C)OCC(C=C1[C@H1](CCC2=CC(OC)=C(OC)C(O)=C2C1=CC=CO)CSCC)=O
PUGREST.BadRequest: error:
O=C(OCC)C1=CN(N=N1)[C@H1]2CCC3=CC(OC)=C(OC)C(OC)=C3C4=CC=C(C(C=C24)=O)NCC(=O)C
PUGREST.BadRequest: error:
O=C(C)N[C@@H1]1C=2C(C3=C(C(OC)=C(OC)C=C3CC1)CC=C(SC)C)(C=2)ONC(C)=O
PUGREST.BadRequest: error:
O=C(O)CN(CCO)C(=O)N[C@H1]1CCC2=CC(OC)=C(OC)C(OC)=C2C3=CC=C(C(C=C13)=O)NCC
PUGREST.BadRequest: error:
O=C(OCC)C=C(C(OCC)=O)N[C@H1]1CCC2=CC(OC)=C(OC)C(OC)=C2C3=CC=C(C(C=C31)S)CC
PUGREST.BadRequest: error:
O=C(O)C=C(OC)C(C(NC)=CC=CC1=C(OC)C(OC)=C(OC)C=C1CC[C@@H1](C)NCC)=O
PUGREST.BadRequest: error:
O=C(OCC(C)COC1=C)N(N=N1)[C@H1]2CCC3=CC(OC)=C(OC)C(OC)=C3C4=CC=C(C(=O)C=C24)NC
```

```
PUGREST.BadRequest: error:
O(C=CC=CC1=C(OC)C(OC)=CC=C1CC[C@H1](CC=COC(C2=CC=CC=C2)=O)C(OC)=C)OSC=O
PUGREST.BadRequest: error:
CCCCCCCCCCCCC=1CCCOCC(C=C2[C@H1](CCC3=CC(OC)=C(OC)C(OC)=C3C2=CC=1N)C(=O)C=O)SC
PUGREST.BadRequest: error:
CCCCCCCCCCCCCCCC=1OCC(C=C2[C@H1](CCC3=CC(OC)=C(OC)C(OC)=C3C2=CC=1N)C(=O)C=O)SC
PUGREST.BadRequest: error:
CC(C)OC(NCC1=CN(N=N1)[C@H1]2CCC3=CC(OC)=C(OC)C(OC)=C3C4=CC=CC(C=C24)=O)NC=O
PUGREST.BadRequest: error:
C1=C(OC)C(OC)=C(OC)C=C1CC[C@@H1](C2=CC(C(OC(C3=CC=CC=C3)=O)=CC=C2)N)C=O
PUGREST.BadRequest: error:
OC1=C(OC)C(OC)=CC=C1C2=CC=C(NC)CC=C2[C@@H1](NC(C(C)C)=O)NCC
PUGREST.BadRequest: error:
C1=C(OC2)C=C(OC)C=C1CC[C@H1](NC(C(OCC)=O)=C(NCO)OOC3=CCC(NC)=CC=C23)OOC
PUGREST.BadRequest: error:
C12=C(OC)C(OC)=C(OC)C=C1CC[C@H1](NC2(C(O)=O)CC=CCC(NC)=C)OSC
PUGREST.BadRequest: error:
C12=C(OC)C(OC)=C(OC)C=C1CC[C@H1](NC(C(O)=O)(O)CC3=CC(OC)=C(C=C3C2)N)C=O
PUGREST.BadRequest: error:
C1=C(OC)C(=CC=C1C2=C(C(OC)=C(OC)C=C2CC[C@H1](C=CC(C(O)=CC)O)C(C(C)C)=O)S)C
PUGREST.BadRequest: error:
OC1=C(OC)C(OC)=CC=C1C2=CC=C(NC)CC=C2[C@@H1](N3C(C(OCC)=O)=C(N=N3)C=O)OC
PUGREST.BadRequest: error:
COC(NCCCOCC1=CN(N=N1)[C@H1]2CCC3=CC(OC)=C(OC)C(=C3C4=CC=C(NC)C(C=C24)=O)OC=O)=O
PUGREST.BadRequest: error:  C(=CC=CN(COCC)OCC1)=CN(N=N1)[C@H1]2CCC3=C(C(OC)=C(OC
)C(OC)=C3)C4=CC=C(C(C=C24)=O)NC
PUGREST.BadRequest: error:  C=1C(C)(C)OC(=O)/N=C(/N[C@H1]2CCC3=C(C(OC)=C(OC)C(OC
)=C3)C4=CC=C(NC)C(=O)C=C42)N=1
PUGREST.BadRequest: error:  C=1C(C)(C)OC(=O)/N=C(/N[C@H1]2CCC3=C(C(OC)=C(OC)C(OC
)=C3)C4=CC=C(C(C=C24)=O)NC)N=1
PUGREST.BadRequest: error:
C=1C(C)OC(=O)/N=C(/N[C@H1]2CCC3=C(C(OC)=C(OC)C(OC)=C3)C4=CC=C(NC)C(=O)C=C42)N=1
PUGREST.BadRequest: error:
C=1NC=2CC=C3[C@H1](CCC4=C(C(OC)=C(OC)C(=C4)OC)C3=CC=2)NC(C(=O)O)C=CN=1
PUGREST.BadRequest: error:
O=CC=CC=CC(NCC1=CN(N=N1)[C@H1]CCC2=CC(OC)=C(OC)C(OC)=C2C3=CC=C(C(=O)C=C3)N)C=O
PUGREST.BadRequest: error:
O=CC=CC1=CC2=C(SC)CC=C1[C@H1](CC3C=CC(OC)=C(C(=C32)C=O)OCN)NOCNCO
PUGREST.BadRequest: error:
CNCC1(C2=CC=C(C=C2)NC(=O)N[C@H1]C3CC4=CC(OC)=C(OC)C(OC)=C4C=CC=C(NC)C(=O)C=C31)C
PUGREST.BadRequest: error:
C1=CN=CC=C1C2=CN(N=N2)[C@H1]3CCC4=C(C(OC)=C(OC)C(OC)=C4)C5=CC=C(C(=O)C=C35)NCO
PUGREST.BadRequest: error:
COC=CC1CC[C@H1](NC(=O)NC=C[C@@H1](OC(C=CC=C)=O)C2=CC(OC)=C(OC)C(OC)=C2)C1NC=O
PUGREST.BadRequest: error:
O=CC=C1C=CC(NCC2=CN(N=N2)[C@H1]C3CC4=CC(OC)=C(OC)C(OC)=C4C=CC=C(NC)C(=O)C=C31)NC
PUGREST.BadRequest: error:
CNC=CC=C1C[C@H1](CCC2=CC(OC)=C(OC)C(=C12)OC)N3C(C(O)=O)=C(N=N3)C(=O)OONC
```

```
PUGREST.BadRequest: error:
CNC=C1C=C2C[C@H1](CCC3=CC(OC)=C(OC)C(=C23)OCC(=O)OC=C)C1=O
PUGREST.BadRequest: error:
COC=CC1C=2C[C@H1]NC(=O)NC3=CC=C(Cl)C=C3C4=CCC(NC)=CC=C4C=2C(OC)=C1OCNC=O
PUGREST.BadRequest: error:
C=NC=CCC1OCCC=C2[C@H1](CCC3=C(C(OC)=C(OC)C(OC)=C3)C2=C1)N=O
PUGREST.BadRequest: error:
COC=C(OC)C(OC)=CCCC[C@H1](N1C=C(N=N1)COC(=O)C2=CC=NC=C2)C3=CC(=O)C(=CC=C3CNCN)C
PUGREST.BadRequest: error:
C12=CC(C=3C([C@@H1](NCC4=CC=C(C)C=C4CCC1)C2)=CC(=O)C(=CC=3)SC=C)(OC)C(OC)=COC
PUGREST.BadRequest: error:
C12=C3C(C4=CC=C(C(=O)C=C4[C@H1](CC3)N5C=C(N=N5)COC(=O)C6=CC=NC=C6)C1)NC=C2OCOCOC
PUGREST.BadRequest: error:
COC1=C(OC)C=CC=2CC[C@@H1](C3=CC(=O)C(NC)=CC=C3C1=2)N4C=C(N=N4)COCC5=CC=CC=C5OC
PUGREST.BadRequest: error:
C1=C2C(CC([C@H1](CC2)N3N=NC(=C3)C4(O)CCCCC4)=O)CC(OC)=C(C(=C1)OC)OC
PUGREST.BadRequest: error:
C=C1C(C=C(CC[C@@H1]1N2N=NC(=C2)C3=CC=C(Cl)C=C3)C=C(C(=COC)OC)OC)=CC(C(NC)=C)=O
PUGREST.BadRequest: error:
C=C1C(C2=C(CC[C@@H1]1N3C=C(N=N3)COCC4=CC=CC=C4)C=C(C(OC)=C2OC)OC)=CC=C(CN)C=O
PUGREST.BadRequest: error:
CC(C)COC=1CC=C2[C@H1](CCC3=C(C(OC)=C(OC)C(OC)=C3)C2=CC=1)NSC=O
PUGREST.BadRequest: error:
CC(C)(O)C=CN([C@H1]1CCC2=CC(OC)=C(OC)C(OC)=C2C3=CC=C(C(C=C13)=O)NC)O
PUGREST.BadRequest: error:  CCCCCCCCCCCCCCCCOCC1=CN(N=N1)[C@H1]2CCC3=CC(OC)=C(OC
)C(OC)=C3C=CC=C(NC)C(=O)C=C2O
PUGREST.BadRequest: error:  CCCCCCCCCCCCCCCCOCC1=CN(N=N1)[C@H1]2CCC3=CC(OC)=C(OC
)C(OC)=C3C=CC=C(NC)C(=O)C=C2OC
PUGREST.BadRequest: error:
C[C@H1](NC(C)=O)C1=CCC(SC)=CC=C1C2=C(C=C(OC)C(OC)=C2OCC3=CC=CC=C3)OSC
PUGREST.BadRequest: error:
C=12C(=CC(C(NC)=CC=1)=O)[C@H1](CCC3=CC(OC)=C(OC)C(=C32)OC)C(C)C=O
PUGREST.BadRequest: error:
NC(=O)NCC1=CN(N=N1)[C@H1]2CCC3=CC(OC)=C(OC)C(OC)=C3C4=CC=C(NC)C(=O)C=C24
PUGREST.BadRequest: error:
N(C(C1=CC=NC=C12)=O)[C@@H1]3CC(C4=C(C(OC)=C(OC)C=C4CC3)OC(CC)=O)=CC=C(SC)C2=O
PUGREST.BadRequest: error:
C=12C(=CC(C(NC)=CC=1)=O)[C@H1](CCC3=CC(OC)=C(OC)C(=C3C2)NC(C)C)CSCSC
PUGREST.BadRequest: error:
C12[C@H1](NC(O)=O)C3=CCC(SC)=CC=C3C4=C(C(OC)=C(OC)C=C4CC1)OC=CC2=O
PUGREST.BadRequest: error:
C1=CC(OC)=CC=C1CN[C@H1]2CCC3=C(C(OC)=C(OC)C(OC)=C3)C=4C2=CC(C(=CC=4)NC)=O
PUGREST.BadRequest: error:
C=CC(OC)=C(OC)C(OC)=CC1(C2=CC=C(NC)CC=C2[C@H1](CCC1)NC(=O)C=O)SC
PUGREST.BadRequest: error:
O(C1=CC(=CC(OC)=C1OC)CC[C@H1](NC(N(C(C)C)C(C2)C)=O)C=CCC(NC)=CC=C2ONC)C
PUGREST.BadRequest: error:
N(C(C1=CC=CC=C1)=O)CCN[C@H1]2CCC3=CC(OC)=C(OC)C(OC)=C3C4=CC=C(C(C=C24)=O)SC
```

```
PUGREST.BadRequest: error:
C1[C@H1](NC(O)=O)C2=CCC=CC=C2C3=C(OC)C(OC)=C(OC)C=C3CCNC=C1O
PUGREST.BadRequest: error:
C1=C(C(OC)=C(OC=2)CC3=CC=C(NC)C(C=C3[C@@H1](NC(N(COC)C)O)SCCC=21)NC)OC
PUGREST.BadRequest: error:
N1=NC1=C(C(=O)O)NC(=O)N[C@H1]2CCC3=CC(OC)=C(OC)C(OC)=C3C=CC=C(NC)C(=O)C=C2
PUGREST.BadRequest: error:
N=CC=C(NCN[C@H1]1CCC2=CC(OC)=C(OC)C(OC)=C2C3=CC=C(C(C=C13)=O)N)C=O
PUGREST.BadRequest: error:
N=CC=C(NCN[C@H1]1CCC2=CC(OC)=C(OC)C(OC)=C2C3=CC=C(NC)C(=O)C=C13)O
PUGREST.BadRequest: error:
N=NC1=CN(N=N1)[C@H1]C2CC3=CC(OC)=C(OC)C(OC)=C3C4=CC=C(C(=O)C=C42)NCOC
PUGREST.BadRequest: error:
N=NC(=C(C(OCC)=O)N[C@H1]1CCC2=CC(OC)=C(OC)C(OC)=C2C3=CC=C(C(C=C13)=O)N)C
PUGREST.BadRequest: error:
CNC=1C(=O)C=C2[C@H1](CCC3=C(C(OC)=C(OC)C(OC)=C3)C2=CC=1)NC(C(=O)O)=CN=CNC
PUGREST.BadRequest: error:
C=CC1C[C@H1](NC(C2)=O)C=CC=3C(OC)=C2C1=CC=C(SC)C(C=3)=O
PUGREST.BadRequest: error:
N1=CC=CC2=C1C(OC=CC(OC=3)=CCCC[C@H1](NC(C)=O)C4=CCC(NC)=CC=C4C=32)=O
PUGREST.BadRequest: error:
CNC=1C(=O)C=C2[C@H1](CCC3=CC(OC)=C(OC)C(OC)=C3C2=CC=1)NC4(C(=O)O)C=CN=C4NC
PUGREST.BadRequest: error:
N1=CC=CC=C1COC=C(C(OC=2)=CCCC[C@H1](NC(C)=O)C3=CC(C(SC)=CC=C3C=2OCO)=O)O
PUGREST.BadRequest: error:
C=C1[C@H1](CCC2=C(C(OC)=C(OC)C(OC)=C2)C1=CC=CC(C)=O)NCNC(=O)CSC
PUGREST.BadRequest: error:
CN1CC(=O)C=C2C(C3=C(OC)C(OC)=C(OC)C=C3CC[C@@H1]2N4C(C(O)=O)=C(N=N4)OCNC)=CC1
PUGREST.BadRequest: error:
NN(C=C(CNC(CCCCCl)=O)N=N)[C@H1]1CCC2=CC(OC)=C(OC)C(OC)=C2C3=CC=C(NC)C(=O)C=C31
PUGREST.BadRequest: error:
C=C1[C@H1](C=2CC=CC(OC)=C(OC)C(OC(C3=CC=CC=C3)=O)=CC1=CC=C)C(C=2)=O
PUGREST.BadRequest: error:
N=NC1=CN(N=N1)[C@H1]2CCC3=CC(OC)=C(OC)C(=C3C4=CC=C(NC)C(C=C42)=O)OC
PUGREST.BadRequest: error:
N=NC=C(C(=O)O)NC(=O)N[C@H1]CCC1=CC(OC)=C(OC)C(OC)=C1C2=CC=C(NC)C(=O)C=C2
PUGREST.BadRequest: error:
COC1=CC(=CC(OC)=C1OC)CC[C@H1](NC(N(C(C)C)C(C2)C)=O)C=CC(C(NC)=CC=C2NC=O)NC
PUGREST.BadRequest: error:
C=C1[C@H1](CCC2=CC(OC)=C(OC)C(OC)=C2C1=CC=C(C(C)=O)N3C)NC(C(=O)O)=C3NC
PUGREST.BadRequest: error:
CNC=1C(=O)C=C2[C@H1](CCC3=CC(OC)=C(OC)C(OC)=C3C2=CC=1)NC(C(=O)O)C=CN=CNC
PUGREST.BadRequest: error:
N=NC(=C(C(=O)O)NC(=O)N[C@H1]C1CC2=CC(OC)=C(OC)C(OC)=C2C=CC=C(NC)C(=O)C=C1)CC
PUGREST.BadRequest: error:  CC(C)(C)OC(=O)NCCC(NCC=1N=NN(C=1)[C@H1]CCC2=CC(OC)=C
(OC)C=C2C3=CC=C(NC)C(C=C3)=O)OC=O
PUGREST.BadRequest: error:  CC(C)(C)OCNCCC(=O)NCC1=CN(N=N1)[C@H1]C2CC3=CC(OC)=C(
OC)C(OC)=C3C4=CC=C(C(=O)C=C24)NC=O
```

PUGREST.BadRequest: error:
COC(=O)C1=C(N=NN(CNCC)OCCCCC(=O)C2=CC(C3=C(OC)C(OC)=C(OC)C=C3CC[C@@H1]2N1)C)O
PUGREST.BadRequest: error: CC(C)(C)OCNCCC(=O)NCC1=CN(N=N1)[C@@H1]2C3=CC(C(NC)=C
C=C3C=C(CC2)C=C(OC)C(OC)NC)=O
PUGREST.BadRequest: error:
C1(=CN(C(=O)OCC)N[C@@H1]2C=3C(C4=C(OC)C(OC)=C(OC)C=C4CC2)=CC=C(C(=O)C=3)NC)N=C1
PUGREST.BadRequest: error: CC(C)(C)OCNCCC(=O)NCC1=CN(N=N1)[C@@H1]2C3=CC(C(NC)=C
C=C3C4=C(CC2)C=C(OC)C(=C4OC)NC)=O
PUGREST.BadRequest: error: C1C(C)(C)OC(=O)NCCC1NCC=2N=NN(C=2)[C@H1]3CCC4=CC(OC)
=C(OC)C(OC)=C4C=CC=C(NC)C(=O)C=C3O
PUGREST.BadRequest: error: CC(C)(C)OC(=O)NCCC(OCC=1N=NN(C=1)[C@H1]CCC2=CC(OC)=C
(OC)C(=C2C3=CC=C(NC)C(=O)C=C3)O)C=O
PUGREST.BadRequest: error:
CC(C)OC(=O)/N=C(/N[C@H1]1CCC2=C(C(OC)=C(OC)C(OC)=C2)C3=CC=C(NC)C(=O)C=C13)N=N
PUGREST.BadRequest: error:
C=1N(N=NC=1C(OCC)=O)[C@H1]2CCC3=C(C(OC)=C(OC)C(OC)=C3)C4=CC=C(C(=O)C=C42)NCOCC
PUGREST.BadRequest: error:
O=CC=CN=NN[C@H1]1CCC2=C(C(OC)=C(OC)C(=C2)OC)C3=CC=C(NC)C(=O)C=C13
PUGREST.BadRequest: error:
CNC=CC=C1C[C@H1](CCC2=CC(OC)=C(OC)C(=C12)OC)NC3(C(=O)O)C=CN=N3
PUGREST.BadRequest: error:
CNC=CC=C1C([C@H1](CCC2=CC(OC)=C(OC)C(=C12)OC)NC(C(=O)OCC)=C(C(OCO)=O)ONC)SC
PUGREST.BadRequest: error:
C=1NC=2CC=C3[C@H1](CCC4=C(C(OC)=C(OC)C(OC)=C4)C3=CC=2)NC(C(=O)O)C=CN=1
PUGREST.BadRequest: error: C1=CN=CC=C1C(NCC2=CN(N=N2)[C@H1]3CCC4=CC(OC)=C(OC)C(
=C4C5=CC=C(C(C(C=C53)=O)OC)OO)C
PUGREST.BadRequest: error:
N=NN([C@H1]1CCC2=C(C(OC)=C(OC)C(OC)=C2)C3=CC=C(C(=O)C=C13)NC)COC4CC=CC=C4O
PUGREST.BadRequest: error:
N=NN([C@H1]1CCC2=C(C(OC)=C(OC)C(OC)=C2)C3=CC=C(C(=O)C=C13)NC)C(=O)OCC
PUGREST.BadRequest: error:
N=NN([C@@H1]1C2=CC(C(NC)=CC=C2C3=C(C(OC)=C(OC)C=C3CC1)OC)=O)OCC=C=O
PUGREST.BadRequest: error:
C=C1C(C=C(CC[C@@H1]1N2N=NC(=C2)CNC(=O)OCC(C)C)C=C(C(OC)=COC)O)C=CC=C(C=O)NC
PUGREST.BadRequest: error:
C=C1C(C2=C(CC[C@@H1]1N3N=NC(=C3)CNC(=O)OCC(C)C)C=C(C(OC)=C2OC)O)C=CC=C(C=O)NC
PUGREST.BadRequest: error:
C1=C2C(CC([C@H1](CC2)N3N=NC(=C3)CNC(=O)C(Cl)Cl)=CC(=O)C(=CC)NC)=C(OC)C(OC)=C1OC
PUGREST.BadRequest: error:
CN(N([C@@H1]1C2=CC(=O)C(NC)=CC=C2C3=CC(=C(OC)C=C3CC1)OC)O)CNC(=S)NCCCCC
PUGREST.BadRequest: error:
N([C@H1]1CCC2=CC(=C(OC)C(=C2C3=CC=C(C(C=C31)=O)NC)OC4)OC[C@H1])(OC4)NC(=S)O
PUGREST.BadRequest: error:
CN(N([C@@H1]1C2=CC(=O)C(NC)=CC=C2C3=CC(=C(OC)C=C3CC1)OC)O)CNC(=S)CCCCCC
PUGREST.BadRequest: error: CN(N([C@@H1]1C2=CC(=O)C(NC)=CC=C2C3=CC(=C(OC)C=C3CC1
)OC)O)CNC(=S)N4C[C@@H1](O)[C@@H1]OC4=CC
PUGREST.BadRequest: error:
OC=CC=C1C2=C(OC)C(OC)=C(OC)C=C2CC[C@H1](C3=CCC(=CC=C31)NC)ON

PUGREST.BadRequest: error: O1C=CC=CC2=C(OC)C(OC)=C(OC)C=C2CC[C@@H1]1C=CCC(F)F
PUGREST.BadRequest: error:
O(C=CC=CC1=C(OC)C(OC)=C(OC)C=C1CC[C@H1](C=C2CC(OCC(C)C)=O)N=C2ON)C
PUGREST.BadRequest: error:
O(C=CC=CC1=C(OC)C(OC)=C(OC)C=C1CC[C@H1]C=CCC(OC(=O)OCC)=CC)C
PUGREST.BadRequest: error:
O=C(CC)OCC1=CN(N=N1)[C@H1]2CCC3=CC(OC)=C(OC)C(OC)=C3C4=CC=C(NC)C(=O)C=C24
PUGREST.BadRequest: error:
O=C(OC)CCOC=CC=C1C[C@H1](CCC2=CC(OC)=C(OC)C(OC)=C21)NC=O
PUGREST.BadRequest: error:
O(C=CC=CC1=C(OC)C(OC)=C(OC)C=C1CC[C@H1](C=CCCOCC2=CC=CC=C2)OONC)ONCC
PUGREST.BadRequest: error:
OC=CC=CC1=C(OC)C(OC)=C(OC)C=C1C2C[C@H1](C=CCC(OCC(C)C)=O)N=C2ONC
PUGREST.BadRequest: error:
O=C(OCC=C)N1C(=O)N[C@H1]CCC2=C(C(OC)=C(OC)C(OC)=C2)C3=CC=C(NC)C(C=C13)=O
PUGREST.BadRequest: error: O=C(OC(C)(C)C)NCC=1N=NN(C=1)[C@H1]C2CC3=CC(OC)=C(OC)
C(OC)=C3C4=CC=C(NC)C(=O)C=C42
PUGREST.BadRequest: error:
O=C(C(C)C)NCN[C@H1]1CCC2=CC(OC)=C(OC)C(OC)=C2C3=CC=C(NC)C(=O)C=C13
PUGREST.BadRequest: error:
O=C(OCC(C)COCC=1)N=NN(C=1)[C@@H1]2C3=CC(C(NC)=CC=C3C4=C(OC)C(OC)=C(OC)C=C4CC2)=O
PUGREST.BadRequest: error: O=C(OCC)C=1N(N=NC=1C(=O)O2)[C@H1]2NC(=S)N[C@H1]3CCC4
=CC(OC)=C(OC)C(OC)=C4C=5C3=CC(C(=CC=5)NC)=O
PUGREST.BadRequest: error:
OC1=C2C3=CC=C(NC)CC=C3[C@H1](CCC2=CC(OC)=C1OC)NC(C(O)=O)=O
PUGREST.BadRequest: error:
CC(O)(OC(=O)CC1)CN(N=N1)[C@H1]2CCC3=CC(OC)=C(OC)C(OC)=C3C4=CC=C(NC)C(=O)C=C24
PUGREST.BadRequest: error:
C1=2C(O)CN(CC3O)N1[C@H1]CCSC(C4=C(C=C(OC)C(OC)=C4OC(O)CCC3=O)CC=2)C
PUGREST.BadRequest: error: CC(O)(C)CC(=O)OCC1=CN(N=N1)[C@H1]2CCC3=CC(OC)=C(OC)C
(OC)=C3C=4C2=CC(C(=CC=4)NC)=O
PUGREST.BadRequest: error:
CC(C)COC(NCC1=CN(N=N1)[C@H1]C2CC3=CC(OC)=C(OC)C(OC)=C3C4=CC=C(C(=O)C=C24)NC)=O
PUGREST.BadRequest: error:
C=C(NC)CC=C1CC2=C(OC)C(OC)=C(OC)C=C2CC[C@@H1]1N3C(C=CC=C3OC(=O)OC)=O
PUGREST.BadRequest: error:
COCNCC1=CN(N=N1)[C@H1]2CCC3=CC(OC)=C(OC)C(OC)=C3C4=CC=C(NC)C(=O)C=C24
PUGREST.BadRequest: error: CC(C)(C)OC(NCC=1N=NN(C=1)[C@H1]2CCC3=CC(OC)=C(OC)C(O
C)=C3C4=CC=C(C(C=C24)=O)N)C=O
PUGREST.BadRequest: error:
OC1=C(C(OC)=CC2=C1C3=CC=C(NC)CC=C3[C@H1](CC2)N4C(C(O)=O)=C(N=N4)OC=O)O
PUGREST.BadRequest: error:
C[C@H1](COC=CN=NN[C@H1]1CCC2=CC(OC)=C(OC)C(OC)=C2C=3C1=CCC(=CC=3)NC)OCCC
PUGREST.BadRequest: error:
N(C)C1=CC=C2C3=C(OC)C(OC)=C(OC)C=C3CC[C@@H1](C2=CC1=O)NC(C(=O)O)CCONCOCNCOC
PUGREST.BadRequest: error:
N(CCCCC)CC1C2C=C[C@H1](CCC3=CC(OC)=C(OC)C(OC(C)=O)=C3C2=CC=C1SCSC=O)C
PUGREST.BadRequest: error:

```
O(C1=C(C(OC)=CC2=C1C3=CC=C(NC)CC=C3[C@H1](CC2)N4C(C(O)=O)=C(N=N4)OC=O)O)OCC
PUGREST.BadRequest: error:
N(C(C)C)C(NCC1=CN(N=N1)[C@H1]2CCC3=CC(OC)=C(OC)C(OC)=C3C4=CC=C(C(C=C24)=O)NC)=O
PUGREST.BadRequest: error:
N(CCCCCCC=O)CC1=CN(N=N1)[C@H1]2CCC3=CC(OC)=C(OC)C(OC)=C3C4=CC=C(C(C=C24)=O)NC
PUGREST.BadRequest: error:
N(C(C)=O)[C@@H1]1C2=CC(C(SC)=CC=C2C3=C(C(OC)=C(OC)C=C3CC1)OC(C4=CCCC=C4)=O)C
PUGREST.BadRequest: error:
N(CCCCC)CC1CC2=C[C@H1](CCC3=CC(OC)=C(OC)C(OC(C)=O)=C3C2=CC=C1SCNC=O)C
PUGREST.BadRequest: error:
O=C(OCC)C1=C(C(OCC)=O)N=NN1[C@H1]CCC2=CC(OC)=C(OC)C(OC)=C2C3=CC=C(NC)C(C=C3)=O
PUGREST.BadRequest: error:
N([C@H1]1CCC2=CC(OC)=C(OC)C(OC)=C2C3=CC=C(NC)C(C=C13)=O)C=CCOC(=O)CCCC=O
PUGREST.BadRequest: error:
O=C(OCC1=CC2=C(C=C1)F)N[C@@H1]CC(C3=C(C=C(OC)C(OC)=C3OC(OCC)=O)NCCC2)=CC=C(O)C=O
PUGREST.BadRequest: error:
C1OC=CC2=C(C3=CC=C(NC)CC=C3[C@@H1](N)CC2)ONC(OC)=C1OC
PUGREST.BadRequest: error:  C1=C2CC[C@H1](C3=CCC(SC)=CC=C3C2=C(C(OC)=C1OC)OC=O)N
PUGREST.BadRequest: error:
C=CCC1[C@H1](CC=[N+1]C2=C1CC(C(OC)=C2OCOC=C)C=CSC)C=O
PUGREST.BadRequest: error:
C1OC=C2C3=CC=C(NC)C(=O)C=C3[C@H1](CCC2C=COC)NCCCCC=C1
PUGREST.BadRequest: error:
C1OC=C2C3=CC=C(NC)C(=O)C=C3[C@H1](CCC2C=COC)NCCCCC=C1OC
PUGREST.BadRequest: error:
C1=C2CC[C@H1](CC(C2=C(OC)C(=C1OC)OC)=CC=C(NC)C=O)CN=[N+1]=[N-1]
PUGREST.BadRequest: error:
COC=C1C2=C3C=C(NC)C(C=C2[C@@H1](NCCCCCCCCC)CCC1=CC(=C3OC)O)C=O
PUGREST.BadRequest: error:
CC(CC)N[C@H1]C1CC2=CC(OC)=C(OC)C(OC)=C2C3=CC=C(C(C=C31)=O)NCC
PUGREST.BadRequest: error:
CC(CC)N[C@H1]C1CC2=CC(OC)=C(OC)C(OC)=C2C3=CC=C(NC)C(=O)C=C31
PUGREST.BadRequest: error:
C(CCCCN1[C@H1]CCC2=CC(OC)=C(OC)C(OC)=C2C3=CC=C(SC)CC=C13)=O
PUGREST.BadRequest: error:
CC(CC)N[C@H1]C1CC2=CC(OC)=C(OC)C(OC)=C2C3=CC=C(C(C=C31)=O)NC
PUGREST.BadRequest: error:
C1(=NC=C(O)C(C)=CC1)C2=C(C(OC)=C(OC)C=C2CC[C@H1](O)C=CN)CSC
PUGREST.BadRequest: error:
C1(=CC=C(O)C(CCC)C=O)C2=C(OC)C(OC)=C(OC)C=C2CC[C@H1](NC(=O)C)C1SC
PUGREST.BadRequest: error:
COC=C1C2=CC=C(NC)C(=O)C=C2[C@@H1](NCC(C)CCC)CCC1=CC(=COC)OC
PUGREST.BadRequest: error:
C1OC=C2C3=CC=C(NC)C(=O)C=C3[C@@H1](NC(=N)N)CCC2=CC(OC)=C1OC
PUGREST.BadRequest: error:
COC1=C2C3=CC=C(NC)C(=O)C=C3[C@@H1](NCC(CCC)C)CCC2=CC(OC)=C1OC
PUGREST.BadRequest: error:
NC1=CC=CC=C1C2=CC3=C(OC)C(=C(OC)C=C3CC[C@H1](NCCC)C2NCOC)O
```

```
PUGREST.BadRequest: error:
COC=C1C2=CC=C(NC)CC=C2[C@@H1](NCCC#N)CCC1=CC(=COC)OC=O
PUGREST.BadRequest: error:
C1=C2CC[C@H1](C3=CCC(SC)=CC=C3C2=C(C(OC)=C1OC)OC=O)NCCC#N
PUGREST.BadRequest: error:
C1=C2CC[C@H1](C3=CCC(SC)=CC=C3C2=C(C(OC)=C1OC)OC=O)NCCC
PUGREST.BadRequest: error:
COC1=C2C3=CC=C(NC)C(=O)C=C3[C@H1](CCC2=CC(OC)=C1OC)NCC4=CC=NS4
PUGREST.BadRequest: error:
COC1=C2C3=CC=C(SC)C(=O)C=C3[C@H1](CCC2=CC(OC)=C1OC)NCCCCl
PUGREST.BadRequest: error:
COC1=C2C3=CC=C(NC)C(=O)C=C3[C@H1](CCC2=CC(OC)=C1OC)NCC=CC=NCN
PUGREST.BadRequest: error:
COC1=C2CC([C@H1](CCC2=CC(OC)=C1OC)NCCCCl)C(C(=CC)NC)=O
PUGREST.BadRequest: error:
C=1NCC(=O)C=C2C=1C3=C(OC)C(OC)=C(C=C3CC[C@@H1]2NCCCCl)C=CC
PUGREST.BadRequest: error:
O(C1=C(OC)C(OC)=CC23C([C@@H1](NC(N)=S)CCC2=C1)=CC(C(NC)=CC3)=O)NCC
PUGREST.BadRequest: error:
O(C1=C(OC)C=2C3=CC=C(SC)CC=C3[C@H1](CCC=2C=C1OC)NC(C)CC=C)OC
PUGREST.BadRequest: error:
C1=C(OC)C(OC)=C(OC)CC2=CC=C(SC)C(C=C2[C@@H1](NC(N(C)C)=O)CCC1)=O
PUGREST.BadRequest: error:
C12=C(OC)C(OC)=C(OC)C=C1CC[C@H1](NC(C3)=O)C(C(NC)=CC=C32)=O
PUGREST.BadRequest: error:
C1[C@H1](OCCC2)CC=C(C(OC)=C(OC)C=C2CC1N3C=CC=C(NC)CC3)ONC(C)=O
PUGREST.BadRequest: error:
N(C)C=1C(=O)C=C2[C@H1](CCC3=CC(OC)=C(OC)C(OC)=C3C2=CC=1N)C(C)CCSCSC
PUGREST.BadRequest: error:
O(C1=C(OC)C=2C3=CC=C(SC)C(C=C3[C@H1](CCC=2C=C1OC)NC(C)CC=C)=O)C
PUGREST.BadRequest: error:
C12=C(OC)C(=C(OC)C=C1CC[C@H1](NCC(O)=O)C3=CCC(NC)=CC=C32)OOC
PUGREST.BadRequest: error:
C1[C@H1](NCC)CC2=CC(C(NC)=CC=C2C3=C(OC)C(OC)=C(OC)C=C3C1N)C(=O)C
PUGREST.BadRequest: error:
C1=C(OC)C(OC)=C(OC=2)CC3=CC=C(SC)C(C=C3[C@@H1](NC(N(C)C)=O)CCC=21)=O
PUGREST.BadRequest: error:
N(C)C=1C(=O)C=C2[C@H1](CCC3=CC(OC)=C(OC)C(OC)=C3C2=CC=1N)C(C)CCNC
PUGREST.BadRequest: error:
C1=C2CC[C@H1](C3=CCC(SC)=CC=C3C2=C(C(OC)=C1OC=4)OC=O)NCC=CC=4
PUGREST.BadRequest: error:
COC1=CC2=CC=C(SC)C(=O)C=C2[C@@H1](NCC3=CC=CC=C3)CCC1=CC(OC)=COC
PUGREST.BadRequest: error:
N1=NN([C@@H1]C2=CC(C(O)=CC=C2C3=C(CC)C=C(OC)C(OC)=C3OC)=O)CC(O)=C1F
PUGREST.BadRequest: error:
N1=NN([C@@H1]C2=C3C(C(C)=CC=C3C4=C(CC2)C=C(OC)C(OC)=C4OC)=O)N=CC=N1
PUGREST.BadRequest: error:
CNC=1C(C=C2[C@H1](CCC3=C(C(OC)=C(OC)C(OC)=C3)C2=CC=1)NC(C)=O)SC=O
```

```
PUGREST.BadRequest: error:
N1=NC=CN=C2C1(C3=C[C@H1](CCC=C2C(OC)=C(OC)C(OC)=C3N)C(C)C=C)OF
PUGREST.BadRequest: error:
O=CC=C1N=NN(C1)[C@@H1]2C3=CC(C(NC)=CC=C3C4=C(OC)C(OC)=C(C=C4CC2)OC)NC
PUGREST.BadRequest: error:
N1=NN([C@@H1]C2=CC(C(O)=CC=C2C3=C(CC)C=C(OC)C(OC)=C3OC)=O)CC=C1
PUGREST.BadRequest: error:
O=CC1=CN=NN1[C@H1]2CCC3=C(C(OC)=C(OC)C(OC)=C3)C4=CC=C(C(C=C42)N)COC
PUGREST.BadRequest: error:
C1=CN=CC=C1CN[C@H1]2CCC3=CC(OC)=C(OC)C(OC)=C3C4=CC=C(C(C=C24)=O)NCC
PUGREST.BadRequest: error:
CSC=1C(C=C2[C@H1](CCC3=C(C(OC)=C(OC)C(OC)=C3)C2=CC=1)NC(C)=O)NC=O
PUGREST.BadRequest: error:
C1NC=CC=C2C([C@@H1](NC(C(C)C)=O)CCC3=CC(OC)=C(OC)C(OC)=C23)C1=O
PUGREST.BadRequest: error:
O=CC1=CN=NN1[C@H1]2CCC3=CC(OC)=C(OC)C(OC)=C3C4=CC=C(C(C=C42)=O)NC
PUGREST.BadRequest: error:
OCCN(CC=C)C(=O)N[C@H1]1CCC2=CC(OC)=C(OC)C(OC)=C2C3=CC=C(NC)C(=O)C=C31
PUGREST.BadRequest: error:
O=C1C2=C[C@H1](CCC=C3C(OC)=C(OC)C(OC(C)=O)=C3C2=CC=C1NC(C)=O)NC
PUGREST.BadRequest: error:
OCCN(CC=C)C(=O)N[C@H1]1CCC2=CC(OC)=C(OC)C(OC)=C2C3=CC=C(NC)C(=O)C=C13
PUGREST.BadRequest: error:
C=CN=C1C(C)NC(=O)OC=CC=C2C3=C(OC)C(OC)=C(OC)C=C3CC[C@@H1](C2=C1)NCCC
PUGREST.BadRequest: error:
O=C1C=C2[C@H1]CCC3=CC(OC)=C(OC)C(OC)=C3C2=CC=C1SCNC(C)=O
PUGREST.BadRequest: error:
N1=NC(=CN(N=N1)[C@H1]2CCC3=CC(OC)=C(OC)C(OC)=C3C4=CC=C(C(C=C24)=O)N)C
PUGREST.BadRequest: error:
C12=CC(C(NC)=CC=C1C3=C(C=C(OC)C(OC)=C3OC)CC[C@@H1]2NCCC(F)(F)F)=CO
PUGREST.BadRequest: error:
C1=C2C([C@H1](CCC3=CC(OC)=C(C(=C32)OC)OC)NCCCCl)C(C(=C1)NC)=O
PUGREST.BadRequest: error:
CNC(N(C)C)CN[C@H1]1CCC2=CC(OC)=C(C(OC)=C2C=3C1=CC(=O)C(NC)=CC=3)OC
PUGREST.BadRequest: error:
N1C(NC)=CC=C2C([C@H1](CCC3=CC(OC)=C(C(OC)=C23)OC)NCCC(C)C)=CC1=O
PUGREST.BadRequest: error:
C12=CCC(NC)=CC=C1C3=C(C=C(OC)C(OC)=C3OC)CC[C@@H1]2NCC=4SC=CC=4O
PUGREST.BadRequest: error:
C12=CC(C(NC)=CC=C1C3=C(OC)C(=C(C=C3CC[C@@H1]2N4C=C(CO)N=N4)O)CO)C=O
PUGREST.BadRequest: error:
C=1=C2C([C@H1](CCC3=CC(OC)=C(C(OC)=C23)OC)NCC=CC=O)C(=CC=1)SC
PUGREST.BadRequest: error:
COC=C(OC)C(OC)=CCCC=1[C@H1](NCC(C)C)C2=CC(=O)C(SC)=CC=C2C=1
PUGREST.BadRequest: error:
C1=C2C(C3=C(OC)C(=C(C=C3CC[C@@H1]2NCC4=CC=CS4)O)CO)C=CC=C(NC)C1=O
PUGREST.BadRequest: error:
COC1=C(OC)C(OC)=CCCC=2[C@H1](NCC(C)C)C3=CC(=O)C(SC)=CC=C3C1=2
```

```
PUGREST.BadRequest: error:
C1=CC(C(=CC=C1C2=C(OC)C(OC)=C(C=C2CC[C@@H1]NCCOO)C)OC)(CC)O
PUGREST.BadRequest: error:
NC(N(CC)OC)N[C@H1]1CCC2=CC(OC)=C(OC)C(OC)=C2C=3C1=CC(=O)C(NC)=CC=3F
PUGREST.BadRequest: error:
O=CC(SC)=CC=C1C([C@H1](CCC2=CC(OC)=C(OC)C(OC)=C12)N)=CCNC
PUGREST.BadRequest: error:
O=C1C(SC)=CC=C2C([C@H1](CCC3=CC(OC)=C(OC)C(OC)=C23)NCC)C=C1
PUGREST.BadRequest: error:
C12=CC=C(NC)C(C=C1[C@@H1](NC(C)=O)CCC3=CC(OC)=C(OC)C(OC)=C23)SC=O
PUGREST.BadRequest: error:
OC(OC1=C2C3=CC=C(SC)C(C=C3[C@H1]CCC2=CC(=C1OC)O)CNC(C)=O)=O
PUGREST.BadRequest: error:
O1C(OC2=C3C4=CC=C(SC)C(C=C4[C@H1]CCC3=CCOC)=C2OCNC=CC=C1)C
PUGREST.BadRequest: error:
C12=CC=C(NC)C(C=C1[C@@H1](NC(N)=O)CCC=CC(OC)=C(OC)C(OC)=C2)O
PUGREST.BadRequest: error:
NC(CCN[C@H1]1CCC2=CC(OC)=C(OC)C(OC)=C2C=3C1=CC(=O)C=CC=3)NCF
PUGREST.BadRequest: error:
O=C1C(NC)=CC=C2C3=C(OC)C(OC)=C(C=C3CC[C@@H1](C2=C1)NCC)OOCSC
PUGREST.BadRequest: error:
C1[C@H1](C2=CC(C(SC)=CC=C2C3=C(OC)C(OC)=C(C=C3C1)O)C)ONC
PUGREST.BadRequest: error:
C12=CC=C(NC)C2(C=C1[C@@H1](NC(C)=O)CCC3=CC(OC)=C(OC)C(OC)=C3)SC
PUGREST.BadRequest: error:
C1=C2C=C(NC)C(C=C1[C@H1]CCC3=CC(OC)=C(OC)C(OC)=C23)NC(N)=O
PUGREST.BadRequest: error:
C1=C2C=C(NC)C(C=C1[C@H1]CCC3=C2C(OC)=C(OC)C(OC)=C3)NC(C)=O
PUGREST.BadRequest: error:
COCC(C)=CC1=CC([C@@H1](NC(C)=O)CCC2=CC(OC)=C(OC)C(OC)=C21)=CSC
PUGREST.BadRequest: error:
C1[C@H1](C2=CCC(SC)=CC=C2C3=C(OC)C(OC)=C(OC)C=C3C1)ONCC
PUGREST.BadRequest: error:
C=C1C2=C(OC)C(OC)=C(OC)C=C2CC[C@H1](NC(C)=O)C1=CC=C(NC)C(=O)CNC
PUGREST.BadRequest: error:
C=1C2=CC(=CC(C=1OC)=O)[C@H1](CCC3=CC(OC)=C(OC)C(OC)=C32)NC(C)=O
PUGREST.BadRequest: error:
NC(N=C1C=CC2)=CC=C1C3=C(OC)C(OC)=C(OC)C=C3CC[C@@H1]2NC(N)=O
PUGREST.BadRequest: error:
C=CC1=C(NC)C(OC)=C(OC)C=C1CC[C@H1](NC(C)=O)C2=CC(C(=CC=C2)NC)=O
PUGREST.BadRequest: error:
C[C@H1](CCC1=CC(OC)=C(OC)C(OC)=C1C2=CC=C(C(C=C2)N)C=O)NCC
PUGREST.BadRequest: error:
C=1=C2C3=C(OC)C(OC)=C(OC)C=C3CC[C@H1](NC(N)=O)C2=CC=C(SC)C(=O)C=1NC
PUGREST.BadRequest: error:
C[C@@H1](C1CC2=CC(OC)=C(OC)C(OC)=C2C3=CC=C(C(C=C31)=O)SCN)C(=O)C
PUGREST.BadRequest: error:
C1=2[C@H1](CC(C3=CC(OC)=C(OC)C=C31)C=CC=C(NC)CC=2)OSC
```

```
PUGREST.BadRequest: error:
C=C(C)C=C1C(C(OC(C)=O)=CC=CC2=C(OC)C(OC)=C(OC)C=C2CC[C@@H1]1N=O)C
PUGREST.BadRequest: error:
CC(N)(CCO)[C@H1]1CCC2=CC(OC)=C(OC)C(OC)=C2C3=CC=C(NC)C(=O)C=C31
PUGREST.BadRequest: error:
CC(N)(CCO)[C@H1]1CCC2=CC(OC)=C(OC)C(OC)=C2C3=CC=C(C(=O)C=C31)NCC
PUGREST.BadRequest: error:
C=C1[C@@H1](NC(C)=O)CCC2=CC(OC)=C(OC)C(OC)=C2C1=CC=C(NC)C=O
PUGREST.BadRequest: error:
C12[C@H1](NC(C)=O)C3=CC(C(SC)=CC=C3C4=C(OC)C(OC)=C(C=C4C1)OC=C2)NC=O
PUGREST.BadRequest: error:
N(NCCCCC1)[C@H1]1CCC2=CC(OC)=C(OC)C(OC)=C2C3=CC=C(NC)C(=O)C=C31
PUGREST.BadRequest: error:
N(C1=CC=C(C=C1)C)CN[C@H1]2CCC3=CC(OC)=C(OC)C(OC)=C3C=4C2=CC(C(NC)=CC=4)=O
PUGREST.BadRequest: error:
C12[C@H1](NC(C)=O)C3=CC(C(NC)=CC=C3C4=C(OC)C(OC)=C(OC)C=C4C1=C2)NC=O
PUGREST.BadRequest: error:
N1(C(=O)OC2=CC=CC3=C(OC)C(OC)=C(OC)C=C3CC[C@H1](NC(C)=O)C2=CC1)SC
PUGREST.BadRequest: error:
C1[C@H1](NC(N)=O)C2=CC(C(NC)=CC=C2C3=C(C(OC)=C(OC)C=C3CC1)OC)NC=O
PUGREST.BadRequest: error:
N(C1=CC=C(C=C1)C)CN[C@H1]2CCC3=CC(OC)=C(OC)C(OC)=C3C=4C2=CC(C(=CC=4)SC)=O
PUGREST.BadRequest: error:
N(C1=CC=C(C=C1)Cl)C=CN(OC(CC)=O)[C@H1]CCC2=CC(OC)=C(OC)C(OC)=C2C=CC=C(C)CNC=O
PUGREST.BadRequest: error:
C12=C(CC=C3[C@H1](CCC4=CC(OC)=C(OC)C(OC)=C4C3=C1)NCC)OC2=O
PUGREST.BadRequest: error:
C=C(CN[C@H1]1CCC2=CC(OC)=C(OC)C(OC)=C2C3=CC=C(NC)C(C=C31)=O)C=O
PUGREST.BadRequest: error:
C1#CC(C=C2[C@H1](CCC3=CC(OC)=C(OC)C(OC)=C3C2=C1)NC(C)=O)=C
PUGREST.BadRequest: error:
C=C1[C@@H1](NC(N)=O)CCC2=CC(OC)=C(OC)C(OC)=C2C1=CC=C(NC)C=O
PUGREST.BadRequest: error:
CCN(CC)C(=O)N[C@H1]C1CC2=CC(OC)=C(OC)C(OC)=C2C3=CC=C(C(C=C31)=O)NC
PUGREST.BadRequest: error:
C1[C@H1](NC(C)=O)C2=CCC(SC)=CC=C2C3=C(OC)C(OC)=C(C=C3C1)OCNC(=O)C
PUGREST.BadRequest: error:
C=1C=C(NC)C(C=C2[C@H1](CCC3=CC(OC)=C(OC)C(OC)=C3C=12)CC=O)NSC=O
PUGREST.BadRequest: error:
C1=CC(=CC=C1CCCN[C@H1]2CCC3)CC(OC)=C(OC)C(OC)=C3C4=CC=C(SC)C(C=C42)=O
PUGREST.BadRequest: error:
N1([C@@H1]2C3=CC(C(SC)=CC=C3C4=C(OC)C(OC)=C(C=C4CC2)OC=CC=C1)O)CS
PUGREST.BadRequest: error:
CCN(CC)C(=O)N1[C@H1]CCC2=CC(OC)=C(OC)C(OC)=C2C3=CC=C(NC)C(C=C31)=O
PUGREST.BadRequest: error:
C1=CC(=CC=C1CCN[C@H1]2CCC3=C)C(OC)=C(OC)C(OC)=C3C4=CC=C(SC)C(C=C42)=O
PUGREST.BadRequest: error:
C1=CC(=CC=C1O)CN[C@H1]2CCC3=CC(OC)=C(OC)C(OC)=C3C4=CC=C(SC)C(C=C42)=O
```

```
PUGREST.BadRequest: error:
CCN(CC)C(=O)N[C@H1]1CCC2=CC(OC)=C(OC)C(OC)=C2C3=CC=C(C(C=C31)=O)NCC
PUGREST.BadRequest: error:
CCN[C@@H1]1C2=CC(C(NC)=CC=C2C3=C(OC)C(OC)=C(OC)C=C3CC1=O)=O
PUGREST.BadRequest: error:
CC(C)COC1=C(OC)C(OC)=CC=2CC[C@H1](NC(=O)C)C3=CC(C(SC)=CC=C3C1=2)=O
PUGREST.BadRequest: error:
CC(C)NC(N[C@H1]1CCC2=CC(OC)=C(OC)C(=C2C3=CC=C(NC)C(C=C31)=O)OC=O)C
PUGREST.BadRequest: error:
C=C(C)CN(CCO)C(=S)N[C@H1]1CCC2=C(C(OC)=C(OC)C(OC)=C2)C3=CC=C(NC)C(C=C31)=O
PUGREST.BadRequest: error:
CC(C)C(OC=C(C(OC)=CC=1CC[C@H1](NC(=O)C)C2=CC(C(SC)=CC=C2C=1)S)C=O)=O
PUGREST.BadRequest: error:
C=C(O)C(OC)=CC(=C)CN[C@H1]1CCC2=CC(OC)=C(OC)C(OC)=C2C3=CC=C(SC)C(C=C31)=O
PUGREST.BadRequest: error:
CC(C)C(OC=C(C(OC)=CCCC[C@H1](NC(=O)C)C1=CC(C(SC)=CC=C1C)=O)C)=O
PUGREST.BadRequest: error:
CC(CO1)(C(C)CO[C@H1]CCC2=C)C(OC)=C(OC)C(OC(=O)C)=C2C3=CC=C(NC)C(C=C31)=O
PUGREST.BadRequest: error:
O=C(N[C@@H1]1C2=CCC(NC)=CC=C2C3=C(CC1)C=C(OC)C(OC)=C3OC)OCC=C
PUGREST.BadRequest: error:
N([C@H1]1CCC2=CC(OC)=C(OC)C(OC)=C2C3=CC=C(NC)C(=O)C=C31)=C(Cl)C=C
PUGREST.BadRequest: error:
O=C(N[C@H1]1CCC2=CC(OC)=C(OC)C(OC)=C2C3=CC=C(C(C=C31)=O)NCN)C4=CC=CC=C4
PUGREST.BadRequest: error:
N([C@H1]1CCC2=CC(OC)=C(OC)C(OC)=C2C3=CC=C(NC)C(C=C13)=O)C(C)C
PUGREST.BadRequest: error:
O=C(N[C@@H1]1C2=CC(C(NC)=CC=C2C3=C(CC1)C=C(OC)C(OC)=C3OC)=O)N=N
PUGREST.BadRequest: error:
N([C@H1]1CCC2=CC(OC)=C(OC)C(OC)=C2C3=CC=C(NC)C(C=C13)=O)=CC=C
PUGREST.BadRequest: error:
O=C(N[C@@H1]1C2=CCC(NC)=CC=C2C3=C(CC1)C=C(OC)C(OC)=C3OC)ON=N
PUGREST.BadRequest: error:
N([C@H1]1CCC2=CC(OC)=C(OC)C(OC)=C2C3=CC=C(NC)C(C=C13)=O)CNC(=O)OCCONC
PUGREST.BadRequest: error:
O=C(N[C@H1]1CCC2=C3C(OC)=C(OC)C(OC)=C2C4=CC=C(NC)C(C=C14)=O)CCOC3
PUGREST.BadRequest: error:
O=C(N[C@H1]1CCC2=CC(OC)=C(OC)C(OC)=C2C3=CC=C(NC)C(=O)C=C31)CCOC(=O)O
PUGREST.BadRequest: error:
C=C1CC[C@@H1](C2=CCC(OC(C3=CC=CC=C3)=O)=CC=C2C=C1C=COC)C(OC)=COC=O
PUGREST.BadRequest: error:
C1=CCC[C@H1](NC(=S)NC2=CC=C(C=C2)C(F)(F)F)CC(C1)=CC=C(NC)C(=O)COCOC
PUGREST.BadRequest: error:
COC1=CC2=CC=C(NC)C(C=C2[C@@H1](NC(=O)NC3=CC=CC=C3)CCC1=CC(=COC)O)C=O
PUGREST.BadRequest: error:
C1=C2CC[C@@H1](C3=CCC(SC)=CC=C3C2=C(OC)C(=C1OC)OC=O)NCC4=C(C=CC=C4)O
PUGREST.BadRequest: error:
COC1=CC2=C3C=C(NC)C(=O)C=C2[C@@H1](NC(=S)N(CCO)CCCCC)C3=CC(OC)=C1OC
```

```
PUGREST.BadRequest: error:
COC=CC1=C(C2=CC=C(SC)CC=C2[C@H1](CC1)NC(=O)NC3=CC=C(F)C)C3=O
PUGREST.BadRequest: error:
C1=C2CC[C@@H1](C3=CCC(SC)=CC=C3C2=C(OC)C(=C1OC)OC=O)NC(=O)NC4=CC=CC=C4
PUGREST.BadRequest: error:
C=C1C2C[C@H1](N3C=C(COC(=O)C4=CC=CC=C4Cl)N=N3)C5=CC(C(NC)=CC=C5C2=CC1O)C=O
PUGREST.BadRequest: error:
C=CC1=C(C2=C(OC)C(OC)=C1OC(OCC)=O)CC[C@H1](NC(C)=O)C3=CC(C(SC)=CC=C23)=O
PUGREST.BadRequest: error:
O=CC(=CC1=CC[C@H1](CCC2=CC(OC)=C(OC)C(OC(=O)C)=C21)CC=O)OSC
PUGREST.BadRequest: error:
C1[C@@H1](C2=CC(C(SC)=CC=C2C3=C(OC)C(OC)=C(C=C3CC)OCNCC)=O)C1NC=O
PUGREST.BadRequest: error:
C[C@@H1](C1=CCC(OC(OC)=O)=CC=C1C2=C(OC)C(OC)=C(OC)C=C2CN)C(C)=O
PUGREST.BadRequest: error:
OCC(C(C1)=O)N(N=N1)[C@H1]2CCC3=CC(OC)=C(OC)C(OC)=C3C4=CC=C(NC)C(=O)C=C24
PUGREST.BadRequest: error:
CNCC(O)(C)C1=CN(N=N1)[C@H1]2CCC3=CC(OC)=C(OC)C(OC)=C3C4=CC=C(C(=O)C=C24)NC
PUGREST.BadRequest: error:
O1C=C(NC)CC=C1CN[C@H1]2CCC3=CC(OC)=C(OC)C(OC)=C3C4=CC=C(SC)C(C=C24)=O
PUGREST.BadRequest: error:
N=1C(=CN(N=1)[C@H1]2CCC3=CC(OC)=C(OC)C(OC)=C3C4=CC=C(NC)C(C=C24)=O)C=O
PUGREST.BadRequest: error:
C=CC=C(C(=O)OC=CC1=CC([C@@H1](NC(C)=O)CCC2=CC(OC)=C(OC)C(OC)=C21)SC=C)C
PUGREST.BadRequest: error:
N1([C@H1]2CCC3=C(C(OC)=C(OC)C(OC)=C3)C4=CC=C(NC)C(C=C24)=O)C(Cl)=C1
PUGREST.BadRequest: error:
N1([C@H1]2CCC3=C(C(OC)=C(OC)C(OC)=C3)C4=CC=C(NC)C(C=C42)=O)C=C1Cl
PUGREST.BadRequest: error:
N1([C@H1]2CCC3=C(C(OC)=C(OC)C(OC)=C3)C4=CC=C(NC)C(C=C42)=O)C(Cl)=C1
PUGREST.BadRequest: error:
O=CC1=CN(N=N1)[C@H1]2CCC3=CC(OC)=C(OC)C(OC)=C3C4=CC=C(NC)C(=O)C=C24
PUGREST.BadRequest: error:
C1N([C@H1]2CCC3=CC(OC)=C(OC)C(OC)=C3C4=CC=C(NC)C(=O)C=C24)CC(OC)=C1
PUGREST.BadRequest: error:
N1([C@H1]2CCC3=C(C(OC)=C(OC)C(OC)=C3)C4=CC=C(NC)C(C=C24)=O)C(O)=C1C
PUGREST.BadRequest: error:
N1([C@H1]2CCC3=C(C(OC)=C(OC)C(OC)=C3)C4=CC=C(NC)C(C=C24)=O)C(O)=C1
PUGREST.BadRequest: error:
O=CC1=CN(N=N1)[C@H1]2CCC3=CC(OC)=C(OC)C(OC)=C3C4=CC=C(NC)C(=O)C=C42
PUGREST.BadRequest: error:
C1N([C@H1]2CCC3=CC(OC)=C(OC)C(OC)=C3C4=CC=C(C(C=C42)=O)NCC=C)C(OC)=C1OC
PUGREST.BadRequest: error:
N([C@H1]1CCC2=C(C(OC)=C(OC)C(OC)=C2)C3=CC=C(NC)C(C=C13)=O)NC=CC=CCl
PUGREST.BadRequest: error:
N1([C@H1]2CCC3=C(C(OC)=C(OC)C(OC)=C3)C4=CC=C(NC)C(C=C42)=O)C=C1I
PUGREST.BadRequest: error:
N(CCCC)CC1=CN(N=N1)[C@H1]2CCC3=C(C(OC)=C(OC)C(OC)=C3)C4=CC=C(NC)C(=O)C=C24
```

```
PUGREST.BadRequest: error:
C1=C(O)C(OC)=CC(CN[C@H1]2CCC3=C(C(OC)=C(OC)C(OC)=C3)C4=CC=C(SC)C(=O)C=C42)=C1C
PUGREST.BadRequest: error:
N(C(C)C)(C(C)C)C(=O)N[C@@H1]1CC(C2=C(C(OC)=C(OC)C=C2CC1)OC)(CC)OCC=C(NC)C(C)=O
PUGREST.BadRequest: error:
N(C)C[C@H1](C1=CC(C(NC)=CC=C1C2=C(OC)C(OC)=C(C=C2CC)OCNCC)=O)C=C
PUGREST.BadRequest: error:
C1=2[C@H1](OC1)CCCC=C(C3=C(OC)C(OC)=C(OC)C=C3CC=CC=CNC)C(C=2)=O
PUGREST.BadRequest: error:
N(CCCC)CC1=CN(N=N1)[C@H1]2CCC3=C(C(OC)=C(OC)C(OC)=C3)C4=CC=C(NC)C(C=C24)=O
PUGREST.BadRequest: error:
C1=C(OC)C(=C(OC)C=C1CC[C@H1](NC(C(O)=O)C(=O)OCC=CC(OC)=C(OC)C=C)OCNC)O
PUGREST.BadRequest: error:  C12=C(NC)CC([C@@H1](C3N=NCO3)C=C1OC=CCC2SC)=O
PUGREST.BadRequest: error:
C1=C(OC)C(=C(OC)C=C1CC[C@H1](NC(C(OCC)=O)=C(NCO)OOC2=CCC(NC)=CC=C2)ON)C
PUGREST.BadRequest: error:
N1(CCCC)CC2=CN(N=N2)[C@H1]3CCC4=CC(OC)=C(OC)C(OC)=C4C5=CC=C(C(C=C53)=O)NCC=C1
PUGREST.BadRequest: error:
N(C)C(=O)NCCC(OCC(C=C1[C@H1](CCC2=CC(OC)=C(OC)C(OC)=C2C1=CCN)C(C)=O)S)C=O
PUGREST.BadRequest: error:
C1=C(OC2)C(=CC=C1C3=CC(OC)=C(OC)C=C3CC4[C@H1](CCC=CC=C4COC(C)=O)C2OCOCC)ONC
PUGREST.BadRequest: error:
C1[C@H1](C2=CCC(SC)=CC=C2C3=C(OC)C(OC)=C(C=C3C1)OC)ONC
PUGREST.BadRequest: error:
C12=CC=C(NC)C(C=C1[C@@H1](NCC)CCCC=CC(OC)=C(OC)C(OC)=C2)SC=O
PUGREST.BadRequest: error:
C1[C@@H1](C2=CC(C(NC)=CC=C2C3=C(OC)C(OC)=C(C=C3C1)O)C)ONCC
PUGREST.BadRequest: error:
N=CC(C(OC(OCC)=O)=CC=CC1=C(OC)C(OC)=COC)C=C1CC[C@H1](NCCC)C=O
PUGREST.BadRequest: error:
COCC(C)=C(C(OC)=CCCC[C@H1](NCC)C1=CC(C(SC)=CC=C1)=O)OC
PUGREST.BadRequest: error:
C=CC(N[C@H1]C1CC2=C(C(OC)=C(OC)C(OC)=C2)C3=CC=C(NC)C(=O)C=C31)OO
PUGREST.BadRequest: error:
COC1C(C)=C(C=2C3=CC=C(SC)CC=C3[C@H1]CCC=2C=C1)NCNCCOOOC
PUGREST.BadRequest: error:
C1[C@@H1](C2=CC(C(NC)=CC=C2C3=C(OC)C(OC)=C(C=C3C1)OC=O)N)CCO
PUGREST.BadRequest: error:
CC1=C2C(=C(OC)C(OC)=C1C3=CC=C(NC)C(C=C3[C@H1](C2)NC)C)OONC
PUGREST.BadRequest: error:
COC1=C(OC)C=2C3=CC=C(SC)CC=C3[C@H1](CCC=2C=C1OC)NCC4=CC=CC=C4O
PUGREST.BadRequest: error:
C[C@H1](CCC1=CC(OC)=C(OC)C(OC)=C1C2=CC=C(C(=O)C=C2)NCN)CCO
PUGREST.BadRequest: error:
NC(=O)N1[C@@H1]C2=CC(C(NC)=CC=C2C3=C(OC)C(OC)=C(OC)C=C3CC1)=O
PUGREST.BadRequest: error:
NN(CCO)[C@@H1]1C2=CC(C(NC)=CC=C2C3=C(OC)C(OC)=C(C=C3CC1)OC)=O
PUGREST.BadRequest: error:
```

```
C1=2[C@H1](CCC3=CC(OC)=C(OC)C(OC)=C3C1=CC=C(NC)C(=O)C=2NC)CO
PUGREST.BadRequest: error:
C12=CC(C=CC=C1C3=C(OC)C(OC)=C(OC)C=C3CC[C@@H1]2NCC=CC=CC=C)SCC
PUGREST.BadRequest: error:
N=C1C(C(OC(C)=O)CCC2=C(OC)C(OC)=C(OC)C=C2CC[C@H1]1)N=O
PUGREST.BadRequest: error:
NC1(N=CC=C1S)[C@H1]2CCC3=CC(OC)=C(OC)C(OC)=C3C4=CC=C(C(C=C24)N)C=O
PUGREST.BadRequest: error:
NC(N=NCOCCC)C[C@H1]1C2=CC(C(NC)=CC=C2C3=C(CC1)C=C(OC)C(OC)=C3OC)=O
PUGREST.BadRequest: error:
CCCCCC=1N2[C@@H1]CC(C3=C(C(OC)=C(OC)C=C3C2=C)C=C)(SC)C(C=1)=O
PUGREST.BadRequest: error:
C1C2(C3=C(OC)C(OC)=C(OC)C=C3CC[C@@H1]1NC(N)C)C=CC=C(C(C2)=O)NC
PUGREST.BadRequest: error:
C1C2(C3=C(OC)C(OC)=C(OC)C=C3CC[C@@H1]1NC(N)=NC)C=C(C(=O)C2)NC
PUGREST.BadRequest: error:
C1=2C(C3=C(OC)C(OC)=C(OC)C=C3CC[C@@H1]1NC(C)CC)=CC=C(NC)C(=O)C=2NC
PUGREST.BadRequest: error:
C12=C(C=CN(NC1)OC3=CC=C(NC)CC=C3[C@@H1](NC(C)=O)CC)CC(OC)=C2OC=O
PUGREST.BadRequest: error:
C1=C(CN[C@H1]2CCC3=CC(OC)=C(OC)C(OC)=C3C4=CC=C(NC)C(=O)C=C42)C=NC=C1
PUGREST.BadRequest: error:
N(C1=CC=C(C=C1)Cl)C=CN(OC=CC=CC=C)C=CC(N2C)=C(C(OC)=CC=CC=CC2C)[C@H1]SOC
PUGREST.BadRequest: error:
C1[C@H1](NC(N=2)=N)CC(C3=C(C(OC)=C(OC)C=C3C1)OC)=CC=C(NC)C(C=2)=O
PUGREST.BadRequest: error:
C1=C(C(C=C2[C@H1](CCC3=CC(OC)=C(OC)C(OC)=C3C2=C1)NC(C)C)=O)NC
PUGREST.BadRequest: error:
N(C1=CC=C(C=C1)Cl)C=CN(OC=CC=CC=C)C=CC(N2C)=C(C(OC)=CC=CC=CC2C)[C@H1]NOC=O
PUGREST.BadRequest: error:
C12=C[C@@H1](NC(N(C)C)=O)CCC3=CC(OC)=C(OC)C(OC)=C3C1=CC=C(NC)C2=O
PUGREST.BadRequest: error:
C1=C(CN[C@H1]2CCC3=CC(OC)=C(OC)C(OC)=C3C4=CC=C(NC)C(=O)C=C42)C=NC=CC1
PUGREST.BadRequest: error:
C=1C=C(NC)C(C=C2C=1C3=C(OC)C(OC)=C(OC)C=C3CC[C@@H1]2NC(N(C)C)=O)SNC
PUGREST.BadRequest: error:
C1CN[C@@H1]2C3=CC(C(NC)=CC=C3C4=C(C(OC)=C(OC)C=C4CC2)OC=O)C=C1
PUGREST.BadRequest: error:
N=1[C@@H1]2C3=CCC(SC)=CC=C3C4=C(OC)C(OC)=C(C=C4CC2)OC=CC=1C
PUGREST.BadRequest: error:
C1[C@H1](NC(NC2=CC=C(C=C2)F)=O)C3=CC(C(NC)=CC=C3C4=C(OC)C(OC)=CC=C4OCC1)=O
PUGREST.BadRequest: error:
C=1C=C(NC)C(C=C2[C@H1](CCC3=CC(OC)=C(OC)C(OC)=C3C2=1)CCC=O)C
PUGREST.BadRequest: error:
C=12C=C(NC)C2(C=C3C=1C4=C(OC)C(OC)=C(OC)C=C4CC[C@@H1]3NC(N(CC)CC)=O)NC
PUGREST.BadRequest: error:
C1=CC(=CC=N1)CCN[C@@H1]2C3=CC(C(SC)=CC=C3C4=C(CC2)C=C(OC)C(OC)=C4OC)=O
PUGREST.BadRequest: error:
```

```
C=CC=C(NC(=O)C=CC=C1C2=C(OC)C(OC)=C(OC)C=C2CC[C@H1](NC(C)=O)C1=C)SC=O
PUGREST.BadRequest: error:
N=NC(CNC(=O)OC)=C=C(CCO[C@H1]CCC1=CC(OC)=C(OC)C(OC)=C1C2=CC=CNC)C(=O)C=C2
PUGREST.BadRequest: error:
C1=2[C@H1]CCC3=CC(OC)=C(OC)C(OC)=C3C1=CC=C(C(=O)C=2)NCNC(C)=O
PUGREST.BadRequest: error:
CC=C1C2=C(C=C(OC)C(OC)=C2OC(CC)=O)C[C@H1](NC(C)=O)C=CC(C(SC)=CC=C1)=O
PUGREST.BadRequest: error:
C1=2[C@H1]CCC3=CC(OC)=C(OC)C(OC)=C3C1=CC=C(C(C=2)=O)NCNC(C)=O
PUGREST.BadRequest: error:
C1[C@@H1](CC(C2=C(C(OC)=C(OC)C=C2C1)OC)=CC=C(SC)C(C)=O)NC(C)=O
PUGREST.BadRequest: error:
CC1[C@H1](NC(=O)NC2=CC=C(C(F)(F)F)C=C2C3=CCC(NC)=CC=C3C=C(OC)C(OC)=C(C=C1)OO)C
PUGREST.BadRequest: error:
C12C=CC3=C(C=C(OC)C(OC)=C3OC(C)=O)[C@H1](NC(C)=O)C1=CC(C(SC)=C2)=O
PUGREST.BadRequest: error:
CC=CC1=C(C=C(OC)C(OC)=C1OC(OCC)=O)CC[C@H1](NC(C)=O)C2=CC(C(SC)=CC=C2)=O
PUGREST.BadRequest: error:
C=CC=C(NC1CCC=CN=C(CC)CC=C1[C@H1]CCC2=CC(OC)=C(OC)C(OC)=C2)ONC
PUGREST.BadRequest: error:
C=CC=C(NC([C@@H1]C1=CCC(SC)=CC=C1C2=C(OC)C(OC)=C(OC)C=C2CC)=O)NC=O
PUGREST.BadRequest: error:
O=C1C=C2[C@H1](CCC3=C(C(OC)=C(OC)C(OC)=C3)C2=CC=C1SC)NC(N(C(C)C)C(Cl)=O)NC
PUGREST.BadRequest: error:
O=C1C=C2[C@H1](CCC3=C(C(OC)=C(OC)C(OC)=C3)C2=CC=C1SC)NC(C(=O)OCC)=CNC
PUGREST.BadRequest: error:
N=NC(CNC(=O)OC)=C=C(C=CC=CNCC=CC1)C(OC)C(OC)=C(OC)C=C1CC[C@H1](NC(=O)C)C=CC
PUGREST.BadRequest: error:
C1=C2CC[C@H1](NC(N(C)C)=O)C3=CC(C(NC)=CC=C3C2=C(OC)C(OC)=C1OC)NC(C)=O
PUGREST.BadRequest: error:
O=C1C=C2[C@H1](CCC3=C(C(OC)=C(OC)C(OC)=C3)C2=CC=C1SC)NC(C(C)C)=O
PUGREST.BadRequest: error:
N1=NC(COC(=O)C2=CC=NC=C2)=CN1NCC=CC3C4=CC=C(NC)CC=C4[C@H1]CCC3=CCNCOCC
PUGREST.BadRequest: error:
N1C=NN=NN1[C@H1]2CCC3=C(C(OC)=C(OC)C(OC)=C3)C4=CC=C(C(C=C42)=O)NCCC
PUGREST.BadRequest: error:
CNCC(OC1=2)=C(OC)C=3CC([C@H1](CCC=3C=C1OC)NC(N(C)C)=O)OC(=CC=2)NC=O
PUGREST.BadRequest: error:
CNC=1C(=O)C=C2[C@H1](CCC3=CC(OC)=C(OC)C(OC)=C3C2=CC=1N)C(C(C)C)=O
PUGREST.BadRequest: error:
N=NC(CNC(=O)OC)=C=C(C=CC=CNCC=C1C)C2(NC)C(C(O)=CC2[C@H1]NC(C)=O)CCC1SCOCC
PUGREST.BadRequest: error:
O=CC(NC)=CC=C1C([C@H1](CCC2=CC(OC)=C(OC)C(OC)=C21)N=[N+1])=[N-1]
PUGREST.BadRequest: error:
N([C@H1]1CCC2=CC(OC)=C(OC)C(OC)=C2C3=CC=C(NC)C(C=C31)=O)CCCCF
PUGREST.BadRequest: error:
C[C@H1](CCC1=CC(OC)=C(OC)C(OC)=C1C2=CC=C(C(C=C2)=O)SC)NC/C=C/C3=CC=CC=C3
PUGREST.BadRequest: error:
```

```
O=CC(NC)=C1C2=CC([C@@H1](N=[N+1]=[N-1])CCC3=CC(OC)=C(OC)C(OC)=C32)NC=CC1
PUGREST.BadRequest: error:
N([C@H1]1CCC2=C(C(OC)=C(OC)C(OC)=C2)C3=CC=C(SC)C(C=C31)=O)CCCO
PUGREST.BadRequest: error:
O=CC(NC)=CC=CC1([C@H1](CCC2=CC(OC)=C(OC)C(OC)=C21)N=[N+1]=[N-1])O
PUGREST.BadRequest: error:
C1NC(CNC(=O)OCCN=N1)CC2=CC(OC)=C(OC)C(OC)=C2C3=CC=C(NC)C(=O)C=C3
PUGREST.BadRequest: error:
O=CC(NC)=CC=C1C([C@H1](CCC2=CC(OC)=C(OC)C(OC)=C21)N)=[N+1]=[N-1]
PUGREST.BadRequest: error:
C=CC=C(C(=O)OCC1=CN(N=N1)[C@H1]2CCC3=CC(OC)=C(OC)C(OC)=C3C4=CC=C(NC)CC=C24)O
PUGREST.BadRequest: error:
C=CC1=C(C(=O)OC=CC=C2C3=C(OC)C(OC)=C(OC)C=C3CC[C@H1](NC(C)=O)C2=C1SC)C
PUGREST.BadRequest: error:
C=C1C2=C(C=C(OC)C(OC)=C2OC(OCC)=O)CC[C@H1](NC(C)=O)C3=CC(C(NC)=CC=C13)=O
PUGREST.BadRequest: error:
C=CC=CC(=O)OC1=C(C(OC)=CCCC[C@H1](NC(C)=O)C2=CC(C(SC)=CC=C21)=O)C
PUGREST.BadRequest: error:
OCC(C1(C)OCN)=NN(C1)[C@H1]2C3=CC(C(NC)=CC=C3C4=C(CC2)C=C(OC)C(OC)=C4OC)=O
PUGREST.BadRequest: error:
OCC(C1(C)OCN)=NN(C1)[C@H1]2C3=CC(C(NC)=CC=C3C4=C(OC)C(OC)=C(C=C4CC2)OC)=O
PUGREST.BadRequest: error:
C1=2[C@H1](CCC3=C(C(OC)=C(OC)C(OC)=C3)C1=CC=C(C(C=2)=O)SCNCC)OC=C
PUGREST.BadRequest: error:
CC=CC(N[C@H1]1CCC2=C(C(OC)=C(OC)C(OC)=C2)C3=CC=C(SC)C(C=C13)=O)CCOC
PUGREST.BadRequest: error:
OC(=O)NCC1=CN(N=N1)[C@@H1]2C=3C(C4=C(C(OC)=C(OC)C=C4CC2)OC)=CC=C(NC)C(C=3)=O
PUGREST.BadRequest: error:
O(C=C(OC=1)CC2=CC=C(NC)CC=C2[C@@H1](N3C(C(O)=O)=C(N=N3)C(O)=O)CCC=1C=COC=O)C
PUGREST.BadRequest: error:
N(C)C=1C(=O)C=C2[C@H1](CCC3=C(C(OC)=C(OC)C(OC)=C3)C2=CC=1)N4C(C(=O)O)C=CN=C4NC
PUGREST.BadRequest: error:
C1=C(OC)C(=C(OC)C=C1CC[C@H1](NC(C(=O)OCC)=C(COCC)OCCC(NC)=CC=CON)C=C)NC
PUGREST.BadRequest: error:  N(C)C=1C(=O)C=C2[C@H1](CCC3=C(C(OC)=C(OC)C(OC)=C3)C2
=CC=1)N4C(C(=O)O)=C(N=N4)C(=O)NNC
PUGREST.BadRequest: error:  N1(C)C=2C(=O)C=C3[C@H1](CCC4=C(C(OC)=C(OC)C(OC)=C4)C
3=CC=2)NC(C(=O)O5)C=CN=C5C=C1NC
PUGREST.BadRequest: error:
C1[C@H1](NCC)CC2=CC(C(SC)=CC=C2C3=C(C(OC)=C(OC)C=C3CC1)OC(C)=O)CC=O
PUGREST.BadRequest: error:
C12[C@H1](NCC)CC3=CC(C(SC)=CC=C3C4=C(C(OC)=C(OC)C=C4CC1)OC=C2NCC)=O
PUGREST.BadRequest: error:
C=C(NC)C1C([C@H1](CCC2=CC(OC)=C(OC)C(OC)=C21)NC(NC3=CC=C(C=C3)F)=O)PNC=O
PUGREST.BadRequest: error:
O(C1=C(OC=2)C=3CC([C@@H1](N4N=NC(C(O)=O)=C4CO)OCCC=2C=C1)OC=CCC(NC)=CC=3)OC
PUGREST.BadRequest: error:  O=C(NC=C1C=C(C(F)(F)F)CC=C1)N[C@@H1]2C=3C(C4=C(C(OC)
=C(OC)C=C4CC2)OC)=CC=C(NC)C(C=3)=O
PUGREST.BadRequest: error:  O=C(C=C(C(OCC1)=O)N(N=N1)[C@@H1]2C=3C(C4=C(C(OC)=C(O
```

```
C)C=C4CC2)OC)=CC=C(NC)CC=3)OCOCC=CC=CO
PUGREST.BadRequest: error:  O=C(C=C(C(OCC1)=O)N(N=N1)[C@@H1]2C=3C(C4=C(C(OC)=C(O
C)C=C4CC2)OC)=CC=C(NC)CC=3)OC5OCCCC5=O
PUGREST.BadRequest: error:
O=C(C=C(C(O)CCCCCC)[C@H1]1CC)C2=C(C(OC)=C(OC)C(OC)=C2)C3=CC=C(NC)C(C=C31)=O
PUGREST.BadRequest: error:  O=C(C=C(C(O1)CCCCCC1)CN=N)N[C@H1]2CCC3=CC(OC)=C(OC)C
(OC)=C3C=4C2=CC(C(NC)=CC=4)=O
PUGREST.BadRequest: error:
N([C@H1]1CCC2=C(C(OC)=C(OC)C(OC)=C2)C3=CC=C(NC)C(=O)C=C13)CNCC4=CC=C(C=C4)C=O
PUGREST.BadRequest: error:  O=C(C=C(Cl)C1=CN(N=N1)C(=S)N[C@@H1]2C=3C(C4=C(C(OC)=
C(OC)C=C4CC2)OC)=CC=C(NC)CC=3)OC(O)=O
PUGREST.BadRequest: error:  O=C(C=C(Cl)C=CN([C@H1]1CCC2=C(C(OC)=C(OC)C(OC)=C2)C3
=CC=C(NC)C(C=C13)=O)OC(=O)CC)NC(OC4=CC=CC=C4)=O
PUGREST.BadRequest: error:  O=CC(=CC=C1C([C@@H1](N2C(C(O)=O)=C(N=N2)C(O)=O)CCC3=
CC(OC)=C(OC)C(OC)=C31)=C)NC=O
PUGREST.BadRequest: error:
O=CC(=C1C=C2C[C@H1](CCC3=CC(OC)=C(OC)C(OC)=C32)NC(C(=O)OCC)=C(NCC)OON=C1)NC
PUGREST.BadRequest: error:
N=1C(=C(C(=O)O)NCCN=NN(C=1)[C@H1]2CCC3=CC(OC)=C(OC)C(OC)=C3C4=CC=C(NC)CC=C42)OC
PUGREST.BadRequest: error:  NC(=CN/C(=NC(O)C=CCI)C1=CN(NC12)C=CC3=CC(NC)=CC(OC)=
C3C=CC(OC4)C(OC)=CC5=C4)C=CC=CC5S[C@@H1]2NC
PUGREST.BadRequest: error:  OC(\N[C@H1]1CCC2=CC(OC)=C(OC)C(OC)=C2C3=CC=C(C(=O)C=
C31)OC(=O)N)(C[C@@H1])C4=CCC(OC)=C4CCO
PUGREST.BadRequest: error:
CNCC(O)(C1OC=CC=C1N=NNC)[C@@H1]2C3=CC(C(NC)=CC=C3C4=C(OC)C(OC)=C(C=C4CC2)OC)=O
PUGREST.BadRequest: error:
O=C1C(=CC=C2C([C@H1](CCC3=CC(OC)=C(OC)C(OC)=C32)N4C(C(=O)OCC)=C(N=N4)CC1=O)N)C
PUGREST.BadRequest: error:  O=C1C(=CC=C2C([C@@H1](N3C(C(=O)O)=C(C(O)=O)N=N3)CCC4
=CC(OC)=C(OC)C(OC)=C42)=C1)NC=O
PUGREST.BadRequest: error:  CNCC(O)(C1OC=CC=C1C=2N=NNC=2)[C@H1]3CCC4=CC(OC)=C(OC
)C(OC)=C4C5=CC=C(C(=O)C=C53)NC
PUGREST.BadRequest: error:
N=1C(=C(C(=O)O)NCCN=NN(C=1)[C@H1]2CCC3=CC(OC)=C(OC)C(OC)=C3C4=CC=C(NC)CC=C42)O
PUGREST.BadRequest: error:  CC(C)(C)OC(=O)/N=C(/N[C@H1]1CCC2=C(C(OC)=C(OC)C(OC)=
C2)C3=CC=C(NC)C(C=C31)=O)NCOC(C)=O
PUGREST.BadRequest: error:
C1(=CC=C1NCOCC)C=2N=NN(C=2)[C@H1]3CCC4=CC(OC)=C(OC)C(OC)=C4C5=CC=C(NC)C(C=C53)=O
PUGREST.BadRequest: error:  CC(C)(C)OC(=O)/N=C(/N[C@H1]1CCC2=C(C(OC)=C(OC)C(OC)=
C2)C3=CC=C(NC)C(C=C13)=O)NCOC(C)(F)F
PUGREST.BadRequest: error:
C1(OC(C=C(N1[C@H1]2CCC3=C(C(OC)=C(OC)C(OC)=C3)C4=CC=C(NC)C(C=C24)=O)CC)CC)=CO
PUGREST.BadRequest: error:
CC(C)(C)OC(=O)/N=C(/N[C@H1]1CCC2=C(C(OC)=C(OC)C(OC)=C2)C3=CC=C(NC)C(=O)C=C31)N=N
PUGREST.BadRequest: error:
N([C@H1]1CCC2=CC(OC)=C(OC)C(OC)=C2C3=CC=C(NC)CC=C13)=O
PUGREST.BadRequest: error:
O=C(CNC=C1C(C2=C(OC)C(OC)=C(OC)C=C2CC[C@@H1]1NC=CC=C)OC)C(=O)CSC
PUGREST.BadRequest: error:
```

```
CNC(CNC(=O)OCCN[C@H1]1)CCC2=CC(OC)=C(OC)C(OC)=C2C=3C1=CC(C(=CC=3)NC)=O
PUGREST.BadRequest: error:
C12=C3C(C4=C(OC)C(OC)=C(OC)C=C4CC[C@@H1]3NC(=O)N)=CC=C1NC=C2
```

[6]:
```
compound = pcp.get_compounds(data_['AI_generated_SMILES'][2], 'smiles')
```

[7]:
```
compound
```

[7]:
```
[Compound()]
```

[8]:
```
compound[0].cid
```

[9]:
```
compound[0].canonical_smiles
```

[10]:
```
data_.CID.unique()
```

[10]:
```
array([           nan, 0.00000000e+00, 6.35135500e+06, 1.62648725e+08,
       1.64628185e+08, 1.62672356e+08])
```

[ ]:
```

```

[11]:
```
len(data_.CID.unique())
```

[11]:
```
6
```

[12]:
```
data_.to_excel('../Data/PubChemSearch_colchicyne.xlsx')
```

[ ]:
```

```

Notebook

December 19, 2023

1 File 46

[1]:
```
import pubchempy as pcp
import pandas as pd
import warnings
warnings.filterwarnings("ignore")
```

[2]:
```
df_search = pd.read_excel('../Data/Whole_report.xlsx')
```

[3]:
```
data_ = pd.DataFrame(data=df_search['SMILES'])
zeros = [0 for i in range(len(data_))]
data_['CID'] = zeros
data_['PUBCHEM_SMILES'] = zeros
data_.head()
```

[3]:

	SMILES	CID	PUBCHEM_SMILES
0	COC1=C2C3=CC=C(NC)C(=O)C=C3[C@H1](CCC2=CC(OC)=…	0	0
1	COC1=C2C3=CC=C(NC)C(=O)C=C3[C@H1](CCC2=CC(OC)=…	0	0
2	COC1=C2C3=CC=C(NC)C(=O)C=C3[C@H1](CCC2=CC(OC)=…	0	0
3	COC1=C2C3=CC=C(NC)C(=O)C=C3[C@H1](CCC2=CC(OC)=…	0	0
4	O=CC1=CN=NN1[C@H1]2CCC3=CC(OC)=C(OC)C(OC)=C3C4…	0	0

[4]:
```
len(data_)
```

[4]: 335

[5]:
```
knowon_l = []
for i, smiles in enumerate(data_['SMILES']):
    try:
        compound = pcp.get_compounds(smiles, 'smiles')
        data_['CID'][i] = compound[0].cid
        data_['PUBCHEM_SMILES'][i] = compound[0].canonical_smiles
        if math.isnan(data_['CID'][i]):
            data_['CID'][i] = 0
            data_['PUBCHEM_SMILES'][i] = 0
        else:
            pass
        knowon_l.append(compound)
```

```
    except:
        print("PUGREST.BadRequest: error: ", smiles)
```

```
PUGREST.BadRequest: error:
COC1=C2C3=CC=C(NC)C(=O)C=C3[C@H1](CCC2=CC(OC)=C1OC)NCCONC
PUGREST.BadRequest: error:
COC1=C2C3=CC=C(NC)C(=O)C=C3[C@H1](CCC2=CC(OC)=C1OC)NCCCCC
PUGREST.BadRequest: error:
COC1=C2C3=CC=C(NC)C(=O)C=C3[C@H1](CCC2=CC(OC)=C1OC)NCCCCCC
PUGREST.BadRequest: error:
COC1=C2C3=CC=C(NC)C(=O)C=C3[C@H1](CCC2=CC(OC)=C1OC)N4C=C(N=N4)CNCC
PUGREST.BadRequest: error:
O=CC1=CN=NN1[C@H1]2CCC3=CC(OC)=C(OC)C(OC)=C3C4=CC=C(C(=O)C=C42)NCC
PUGREST.BadRequest: error:
OC1=CC=C(C=C1)CN[C@H1]2CCC3=C(C(OC)=C(OC)C(OC)=C3)C4=CC=C(SC)C(=O)C=C24
PUGREST.BadRequest: error:
OC1=CC=C(C=C1)CN[C@H1]2CCC3=CC(OC)=C(OC)C(OC)=C3C4=CC=C(C(C=C42)=O)SCC
PUGREST.BadRequest: error:
O=CC1=C2[C@H1](CCC3=CC(OC)=C(OC)C(=C3C2=CC=C(SC)C1=O)OCC4=CC=CC=C4)ONC
PUGREST.BadRequest: error:
C1=2[C@H1](CCC3=CC(OC)=C(OC)C(OC)=C3C1=CC=C(SC)C(=O)C=2)NC(C)CC=CSC
PUGREST.BadRequest: error:
C1N(C(=S)N[C@@H1]2C3=CC(C(NC)=CC=C3C4=C(OC)C(OC)=C(OC)C=C4CC2)=O)C=C1
PUGREST.BadRequest: error:
C1=2[C@H1](CCC3=CC(OC)=C(OC)C(OC)=C3C1=CC=C(C(C=2)=O)SCNC(C)=O)C
PUGREST.BadRequest: error:
C[C@H1](CCC1=CC(OC)=C(OC)C(OC(OCC)=O)=C1C2=CC=C(SC)C(=O)C=C2)NC
PUGREST.BadRequest: error:
COC1=C2C3=CC=C(NC)C(=O)C=C3[C@H1](CCC2=CC(OC)=C1OC)NCCC4=CC=CC=C4
PUGREST.BadRequest: error:
COC1=C2C3=CC=C(NC)C(=O)C=C3[C@H1](CCC2=CC(OC)=C1OC)NC/C=C/C4=CC=CC=C4
PUGREST.BadRequest: error:
CC(=O)N[C@H1]1CCC2=CC(OC)=C(OC)C(OC)=C2C=3C1=CC(=O)C(SC)=CC=3N
PUGREST.BadRequest: error:
C1=2C(=O)C(=C3C=C1C4=C(CC[C@@H1]3NC(C)=O)C=C(OC)C(OC)=C4OC(OC)=O)C=2
PUGREST.BadRequest: error:
CC(=O)OC1=CC=C2C3=C(C(OC)=C(C=C3CC[C@@H1](C2=CC1=O)OC(=O)C)OC)NC(=O)C
PUGREST.BadRequest: error:
CC(=O)OC1=CC=C2C3=C(C(OC)=C(C=C3CC[C@@H1](C2=CC1=O)OC(=O)C)OC)NC(=O)COC
PUGREST.BadRequest: error:
CC(=O)OC1=CC=C2C3=C(C(OC)=C(C=C3CC[C@@H1](C2=CC1=O)OC(=O)C)OC)OC
PUGREST.BadRequest: error:
CC(C)C(OC1=CC=C2C3=C(CC[C@@H1](C2=CC1=O)NC(C)=O)C=C(OC)C(OC)=C3OC)(O)C
PUGREST.BadRequest: error:
CC(=O)N[C@@H1]1C2=CC(C(SC)=CC=C2C3=C(OC)C(=C(OC)C=C3CC1)OC(=O)C4=CC=CC=C4)=O
PUGREST.BadRequest: error:
O=C(OC)OC1=CC=C2C3=C(C(OC)=C(C=C3CC[C@@H1](C2=CC1=O)NC(=O)C)OC)OC(=O)C
PUGREST.BadRequest: error:
```

```
O=C(C)N[C@H1]1CCC2=CC(=C(OC)C(=C2C=3C1=CC(C(SC)=CC=3)=O)OC(=O)CC)COC
PUGREST.BadRequest: error:
CC(=O)OCC1=CN(N=N1)[C@H1]2CCC3=CC(OC)=C(OC)C(OC)=C3C=4C2=CC(=O)C(NC)=CC=4C
PUGREST.BadRequest: error:
CC(=O)OCC1=CN(N=N1)[C@H1]2CCC3=CC(OC)=C(OC)C(=C3C=4C2=CC(C(NC)=CC=4)=O)OC
PUGREST.BadRequest: error:
CC(=O)N[C@@H1]1C2=CC(C(NC)=CC=C2C3=C(OC)C(OC)=C(OC)C=C3C1)=O
PUGREST.BadRequest: error:
CC(=O)N[C@@H1]C1=C2C(C(NC)=CC=C1C3=C(OC)C(OC)=C(OC)C=C3CC2)=O
PUGREST.BadRequest: error:
COC1=C2C3=CC=C(NC)C(=O)C=C3[C@@H1](NCCC)CCC2=CC(=C1OC)NC
PUGREST.BadRequest: error:
COC1=C2C3=CC=C(NC)C(=O)C=C3[C@H1](CCC2=CC(OC)=C1OC)NN=NC=C
PUGREST.BadRequest: error:
COC1=C2C3=CC=C(NC)C(=O)C=C3[C@@H1](NCCC)CCC2=CC(=C1OC)OSC
PUGREST.BadRequest: error:
COC1=C2C3=CC=C(NC)C(=O)C=C3[C@H1](CCC2=CC(=C1OC)OC)NCCCS
PUGREST.BadRequest: error:
COC1=C(OC)C(OC)=CC=2CC[C@H1](NC(=N)N)C3=CC(=O)C(=CC=C3C1=2)CNC
PUGREST.BadRequest: error:
CNC(NCCCC)=S[C@@H1]C1=CC(=O)C(NC)=CC=C1C2=C(C=C(OC)C(=C2OC)OC)CCF
PUGREST.BadRequest: error:
CN(C(=S)N[C@@H1]1C2=CC(=O)C(NC)=CC=C2C3=C(C=C(OC)C(OC)=C3OC)CC1C)C#N
PUGREST.BadRequest: error:
CNC(C(C)C)N[C@@H1]1C=2C(C3=C(C=C(OC)C(OC)=C3OC)CC1)=CC=C(C(=O)C=2)NC
PUGREST.BadRequest: error:
COC1=C(OC)C(OC)=CC=2CC[C@@H1](C3=CC(=O)C(NC)=CC=C3C=21)N4C=C(N=N4)CON
PUGREST.BadRequest: error:
COC1=C(OC)C(OC)=CC=2CC[C@@H1](C3=CC(=O)C(NC)=CC=C3C=21)NCCC4CCCCC4
PUGREST.BadRequest: error:
CC(C)(C)COC1=CC=C2C3=C(OC)C(OC)=C(OC)C=C3CC[C@@H1](C2=CC1=O)SC=O
PUGREST.BadRequest: error:
C1=2[C@H1]CCC3=CC(OC)=C(OC)C(OC(CC)=O)=C3C1=CC=C(C(C=2)=O)SCNC(=O)CSC
PUGREST.BadRequest: error:
OC(CCC(C)C)OCC1=CN(N=N1)[C@H1]2CCC3=CC(OC)=C(OC)C(OC)=C3C4=CC=C(NC)C(=O)C=C24
PUGREST.BadRequest: error:
OC1=C(C=CC=C1)C=2N=NN(C=2)[C@H1]3CCC4=CC(OC)=C(OC)C(OC)=C4C5=CC=C(NC)C(=O)C=C35
PUGREST.BadRequest: error:
NC1=CN(N=N1)[C@H1]2CCC3=C(C(OC)=C(OC)C(OC)=C3)C4=CC=C(NC)C(C=C24)=O
PUGREST.BadRequest: error:
O=C(OCC)CN(CCO)N[C@H1]1CCC2=CC(OC)=C(OC)C(OC)=C2C3=CC=C(NC)C(=O)C=C13
PUGREST.BadRequest: error:  O=C(C1=C(C(OCC)=O)N(N=N1)[C@@H1]C2=C3C(C(NC)=CC=C2C4
=C(C(OC)=C(OC)C=C4CC3)OC)=O)O
PUGREST.BadRequest: error:
C1=2[C@H1](CCC3=CC(OC)=C(OC)C(OC)=C3C1=CC=C(C(=O)C=2)NCO)C(C(OC)=O)NC(=O)CNC
PUGREST.BadRequest: error:
C=12N(N=NC=1C(OCC3=CC=C(C=C3)F)=O)C(OC)=C(OC)C(OC)=CC4=CC=C(C(=O)C=C24)NC
PUGREST.BadRequest: error:
```

```
C=1N(N=NC=1C(=O)OCC2=CC=C(C=C2)F)CC(OC)=C(OC)C(OC)=CC3=CC=C(NC)C(=O)C=C3
PUGREST.BadRequest: error:
O=CC1=C(C(OCC)=O)N(N=N1)[C@@H1]C2=C3C(C(NC)=CC=C2C4=C(C(OC)=C(OC)C=C4CC3)OC)=O
PUGREST.BadRequest: error:
O=C(C(Cl)Cl)NC(=O)N[C@H1]1CCC2=C(C(OC)=C(OC)C(OC)=C2)C3=CC=C(NC)C(=O)C=C13
PUGREST.BadRequest: error:
O=C(C(Cl)Cl)NCC1=CN(N=N1)[C@H1]2CCC3=CC(OC)=C(OC)C(OC)=C3C4=CC=C(C(=O)C=C42)NCCO
PUGREST.BadRequest: error:  O=C(C(Cl)Cl)NC(=O)N[C@H1]1CCC2=C(C(OC)=C(OC)C(OC)=C2
)C3=CC=C(C(C=C31)=O)NCC(=O)OC
PUGREST.BadRequest: error:
O=C(O)C=C(C(OCC)=O)N=NN[C@H1]1CCC2=CC(OC)=C(OC)C(OC)=C2C3=CC=C(NC)C(=O)C=C13
PUGREST.BadRequest: error:
O=C(O)C1=C(C(OCC)=O)N=NN1[C@H1]2CCC3=CC(OC)=C(OC)C(OC)=C3C4=CC=C(NC)C(=O)C=C24
PUGREST.BadRequest: error:
O=C(OCC)C1=CN(N=N1)[C@H1]2CCC3=CC(OC)=C(OC)C(=C3C4=CC=C(NC)C(C=C42)=O)OCC
PUGREST.BadRequest: error:
O=C(O)C1=CN(N=N1)[C@H1]2CCC3=C(C(OC)=C(OC)C(OC)=C3)C4=CC=C(C(=O)C=C42)NCC
PUGREST.BadRequest: error:
O=C(O)C1=C(C(OCC)=O)N=NN1[C@H1]CCC2=CC(OC)=C(OC)C(OC)=C2C3=CC=C(C(C=C3)=O)NC
PUGREST.BadRequest: error:  CCCCCCCCCCCCCCCC(OCC1=CN(N=N1)[C@H1]C2CC3=CC(OC)=C(O
C)C(OC)=C3C4=CC=C(C(=O)C=C24)NC)=O
PUGREST.BadRequest: error:
OC(=O)C1=C(C(OCC)=O)N(N=N1)[C@H1]C2CC3=CC(OC)=C(OC)C(OC)=C3C4=CC=C(NC)C(=O)C=C42
PUGREST.BadRequest: error:
C1=C(OC)C=C(OC)C=C1CC[C@H1](C=CC(C(OC(C2=CC=CC=C2)=O)=CC=CC)=O)C
PUGREST.BadRequest: error:  N(C)C(=O)NCCC(OCC1=CN(N=N1)[C@H1]CCC2=CC(OC)=C(OC)C(
OC)=C2C3=CC=C(C(=O)C=C3)N)C=O
PUGREST.BadRequest: error:
OC(=O)C=C(C(OCC)=O)N(NCCC)N[C@H1]1CCC2=CC(OC)=C(OC)C(OC)=C2C3=CC=C(NC)C(=O)C=C31
PUGREST.BadRequest: error:
C[C@H1](NCC)CC1=CC(C(NC)=CC=C1C2=C(C=C(OC)C(OC)=C2OCC3=CC=CC=C3)OSCC)=O
PUGREST.BadRequest: error:  C=1C(C)(C)OC(=O)/N=C(/N[C@H1]2CCC3=C(C(OC)=C(OC)C(OC
)=C3)C4=CC=C(NC)C(=O)C=C24)N=1
PUGREST.BadRequest: error:
N1=NN1[C@@H1]2C3=CC(C(NC)=CC=C3C4=C(C(OC)=C(OC)C=C4CC2)OC)=O
PUGREST.BadRequest: error:
O=CC=CC=CC(NCC1=CN(N=N1)[C@H1]2CCC3=CC(OC)=C(OC)C(OC)=C3C4=CC=C(C(C=C24)=O)NC)=O
PUGREST.BadRequest: error:
CN(N([C@@H1]1C2=CC(=O)C(NC)=CC=C2C3=CC(=C(OC)C=C3CC1)OC)O)CNC(=S)CCCCC
PUGREST.BadRequest: error:
CC(C)C(=O)OCC1=CN(N=N1)[C@H1]2CCC3=CC(OC)=C(OC)C(OC)=C3C4=CC=C(NC)C(=O)C=C24
PUGREST.BadRequest: error:
N(C(C1=CC=NC=C1)=O)[C@@H1]2C=3C(C4=C(C(OC)=C(OC)C=C4CC2)OC)=CC=C(SC)C(=O)C=3
PUGREST.BadRequest: error:
N1(CCCC1)CN[C@H1]2CCC3=C(C(OC)=C(OC)C(OC)=C3)C4=CC=C(SC)C(=O)C=C24
PUGREST.BadRequest: error:
N1(CCCC1)C(=O)OC2=C(OC)C(OC3)=CC=C2C4=CC=C(SC)C(C=C4[C@@H1](NC(=O)C)CC3)=O
PUGREST.BadRequest: error:
```

CNC=1C(=O)C=C2[C@H1](CCC3=CC(OC)=C(OC)C(OC)=C3C2=CC=1)NC4(C(=O)O)C=CN=N4
PUGREST.BadRequest: error: CC(C)(C)OC(=O)NCCC(NCC=1N=NN(C=1)[C@H1]C2CC3=CC(OC)=C(OC)C(OC)=C3C4=CC=C(C(=O)C=C24)N)C=O
PUGREST.BadRequest: error:
C1C(C)(C)OC/N=C(/N[C@H1]2CCC3=CC(OC)=C(OC)C(=C3C4=CC=C(NC)C(C=C42)=O)OC)N=N1
PUGREST.BadRequest: error: CC(C)(C)OCNCCC(=O)NCC1=CN(N=N1)[C@H1]2CCC3=CC(OC)=C(OC)C(OC)=C3C4=CC=C(C(C=C24)=O)NC=O
PUGREST.BadRequest: error:
C1C(C)(C)OC/N=C(/N[C@H1]2CCC3=CC(OC)=C(OC)C(=C3C4=CC=C(NC)C(C=C42)=O)OC)N1C
PUGREST.BadRequest: error: CC(C)(C)OC(=O)NCCC(OCC=1N=NN(C=1)[C@H1]2CCC3=CC(OC)=C(OC)C(OC)=C3C4=CC=C(C(=O)C=C24)NC)=O
PUGREST.BadRequest: error: CC(C)(C)OC(=O)NCCC(NCC=1N=NN(C=1)[C@H1]2CCC3=CC(OC)=C(OC)C(OC)=C3C4=CC=C(C(C=C24)=O)N)C=O
PUGREST.BadRequest: error:
CNCCC1=CC=C(C=C1)NC(=O)N[C@H1]2CCC3=CC(OC)=C(OC)C(OC)=C3C4=CC=C(C(C=C24)=O)NC=O
PUGREST.BadRequest: error: CNCC(C1=CC=C(C=C1)NC(=O)N[C@@H1]2C3=CC(C(NC)=CC=C3C4=C(C(OC)=C(OC)C=C4CC2)OC)=O)OC
PUGREST.BadRequest: error:
O=C(OC)OCC1=CN(N=N1)[C@H1]2CCC3=CC(OC)=C(OC)C(OC)=C3C4=CC=C(NC)C(=O)C=C24
PUGREST.BadRequest: error:
O=C(C(C)C)NC(=S)N[C@@H1]1C=2C(C3=C(C(OC)=C(OC)C=C3CC1)OC)=CC=C(NC)C(=O)C=2
PUGREST.BadRequest: error:
O=C(OCC)OCC=1N=NN(C=1)[C@H1]2CCC3=CC(OC)=C(OC)C(OC)=C3C4=CC=C(NC)C(=O)C=C24
PUGREST.BadRequest: error:
O=C(OCC)CN(CCO)C(=S)N[C@H1]1CCC2=CC(OC)=C(OC)C(OC)=C2C3=CC=C(NC)C(=O)C=C13
PUGREST.BadRequest: error:
CC(C)CNCC1=CN(N=N1)[C@H1]2CCC3=CC(OC)=C(OC)C(OC)=C3C4=CC=C(C(C=C24)=O)NC=O
PUGREST.BadRequest: error:
CC(C)CNCC1=CN(N=N1)[C@H1]2CCC3=CC(OC)=C(OC)C(OC)=C3C4=CC=C(NC)C(=O)C=C24
PUGREST.BadRequest: error:
COCNCC1=CN(N=N1)[C@H1]2CCC3=CC(OC)=C(OC)C(OC)=C3C4=CC=C(C(C=C42)=O)NC=O
PUGREST.BadRequest: error:
N(C(C)C)C(NCC1=CN(N=N1)[C@H1]2CCC3=CC(OC)=C(OC)C(OC)=C3C4=CC=C(NC)C(=O)C=C24)=O
PUGREST.BadRequest: error:
N(C)C1=CC=C2C3=C(OC)C(OC)=C(OC)C=C3CC[C@@H1](C2=CC1=O)NC(C(=O)O)CCON=CCF
PUGREST.BadRequest: error:
N(C(C(C)C)=O)C1=CN(N=N1)[C@H1]2CCC3=C(C(OC)=C(OC)C(OC)=C3)C4=CC=C(NC)C(=O)C=C24
PUGREST.BadRequest: error:
N(CCCCCCC=O)CC1=CN(N=N1)[C@H1]2CCC3=CC(OC)=C(OC)C(OC)=C3C4=CC=C(NC)C(=O)C=C24
PUGREST.BadRequest: error: C1=C(C=CC(Cl)=C1)COC(=O)CN[C@@H1]2C=3C(C4=C(C(OC)=C(OC)C=C4CC2)OC)=CC=C(SC)C(=O)C=3
PUGREST.BadRequest: error:
N(C)C1=CC=C2C3=C(OC)C(OC)=C(OC)C=C3CC[C@@H1](C2=CC1=O)NC(C(=O)O)CCONC(=O)C=C
PUGREST.BadRequest: error:
N(C)C1=CC=C2C3=C(OC)C(OC)=C(OC)C=C3CC[C@@H1](C2=CC1=O)NC(C(=O)O)CCONC(=O)CNC
PUGREST.BadRequest: error:
N(C(C)C)CC(=O)OCC1=C2N(N=N1)[C@H1]CCC3=CC(OC)=C(OC)C(OC)=C3C4=CC=C(NC)C(C=C24)=O
PUGREST.BadRequest: error:

```
CC(CC)N[C@@H1]1C2=CC(C(SC)=CC=C2C3=C(OC)C(OC)=C(C=C3CC1)OC)=O
PUGREST.BadRequest: error:
CC(CC)N[C@@H1]C1=C2C(C(NC)=CC=C1C3=C(OC)C(OC)=C(OC)C=C3CC2)=O
PUGREST.BadRequest: error:
COC1=C2C3=CC=C(NC)C(=O)C=C3[C@H1](CCC2=CC(OC)=C1OC)NCC4=CC=NS4C
PUGREST.BadRequest: error:
N(C)C=1C(=O)C=C2[C@H1](CCC3=CC(OC)=C(OC)C(OC)=C3C2=CC=1N)C(C)CCSC
PUGREST.BadRequest: error:
CNC=1C(C=C2[C@H1](CCC3=C(C(OC)=C(OC)C(OC)=C3)C2=CC=1)NC(C)C)=O
PUGREST.BadRequest: error:
NN([C@H1]1CCC2=CC(OC)=C(OC)C(OC)=C2C3=CC=C(NC)C(=O)C=C13)C=O
PUGREST.BadRequest: error:
OCCN(CC=C)C(=O)N[C@@H1]1C2=CC(C(NC)=CC=C2C3=C(OC)C(OC)=C(OC)C=C3CC1)=O
PUGREST.BadRequest: error:
OCCN(CC=C)C(=S)N[C@H1]1CCC2=CC(OC)=C(OC)C(OC)=C2C3=CC=C(NC)C(=O)C=C13
PUGREST.BadRequest: error:
CNC(N(C)C)CN[C@H1]1CCC2=CC(OC)=C(C(OC)=C2C=3C1=CC(=O)C(=CC=3)NC)OC
PUGREST.BadRequest: error:
NC(N(CC)OC)N[C@H1]1CCC2=CC(OC)=C(OC)C(OC)=C2C=3C1=CC(=O)C(=CC=3)NC
PUGREST.BadRequest: error:
C1=2[C@H1](CCC3=CC(OC)=C(OC)C(OC)=C3C1=CC=C(SC)C(=O)C=2NC)C
PUGREST.BadRequest: error:
C1=2[C@H1](CCC3=CC(OC)=C(OC)C(OC)=C3C1=CC=C(C(C=2)=O)SCN)C(=O)C
PUGREST.BadRequest: error:
NC(=O)N[C@@H1]C1=C2C(C(NC)=CC=C1C3=C(OC)C(OC)=C(OC)C=C3CC2)=O
PUGREST.BadRequest: error:
CC(C)NC(=O)N1[C@@H1]C2=CC(C(NC)=CC=C2C3=C(OC)C(OC)=C(OC)C=C3CC1)=O
PUGREST.BadRequest: error:
CC(N)(CCO)[C@H1]1CCC2=CC(OC)=C(OC)C(OC)=C2C3=CC=C(SC)C(=O)C=C13
PUGREST.BadRequest: error:
CC(N)(CCO)[C@H1]1CCC2=CC(OC)=C(OC)C(OC)=C2C3=CC=C(C(C=C31)=O)NCC
PUGREST.BadRequest: error:
C1[C@H1](NC(C)=O)C2=CC(C(NC)=CC=C2C3=C(OC)C(OC)=C(C=C3C1)OCNC)=O
PUGREST.BadRequest: error:
C1[C@H1](NC(C)=O)C2=CC(C(SC)=CC=C2C3=C(OC)C(OC)=C(C=C3C1)OCNC)=O
PUGREST.BadRequest: error:
C=C(N[C@H1]1CCC2=C(C(OC)=C(OC)C(OC)=C2)C3=CC=C(SC)C(C=C31)=O)C
PUGREST.BadRequest: error:
CC(C)N(C(C)C)C(=O)N[C@@H1]C1=CC(C(NC)=CC=C1C2=C(OC)C(OC)=C(OC)C=C2CC)=O
PUGREST.BadRequest: error:
C1C(C)NC1N[C@H1]2CCC3=CC(OC)=C(OC)C(OC)=C3C4=CC=C(C(=O)C=C42)NC=O
PUGREST.BadRequest: error:
O=C(N[C@H1]1CCC2=CC(OC)=C(OC)C(OC)=C2C3=CC=C(NC)C(=O)C=C31)N(S)CC
PUGREST.BadRequest: error:
O=C(N[C@H1]1CCC2=CC(OC)=C(OC)C(OC)=C2C3=CC=C(NC)C(=O)C=C13)CCOCC
PUGREST.BadRequest: error:
COC1=C2C3=CC=C(NC)C(=O)C=C3[C@H1](CCC2=CC(OC)=C1OC)NC(=O)N(CCCCCl)O
PUGREST.BadRequest: error:
```

```
COC=CC1=CC=C(NC)C(=O)C=C1[C@@H1](NC(=S)NC2=CC=CC=C2)Cl
PUGREST.BadRequest: error:
N=1C(=CN(N=1)[C@H1]2CCC3=CC(OC)=C(OC)C(OC)=C3C4=CC=C(NC)C(=O)C=C42)C=O
PUGREST.BadRequest: error:
C1=2[C@H1]CCC3=CC(OC)=C(OC)C(OC)=C3C1=CC=C(C(C=2)=O)SCNC(C)=O
PUGREST.BadRequest: error:
N1([C@H1]2CCC3=C(C(OC)=C(OC)C(OC)=C3)C4=CC=C(NC)C(C=C24)=O)C=C1I
PUGREST.BadRequest: error:
N1([C@H1]2CCC3=C(C(OC)=C(OC)C(OC)=C3)C4=CC=C(NC)C(C=C24)=O)C=C1OCC
PUGREST.BadRequest: error:
N1C(OC)=C(C=C1)N[C@H1]2CCC3=C(C(OC)=C(OC)C(OC)=C3)C4=CC=C(SC)C(C=C42)=O
PUGREST.BadRequest: error:
N(C(C)C)(C(C)C)C(=O)N1[C@H1]CCC2=C(C(OC)=C(OC)C(OC)=C2)C3=CC=C(NC)C(C=C31)=O
PUGREST.BadRequest: error:
N(CCCC)CC1=CN(N=N1)[C@H1]2CCC3=CC(OC)=C(OC)C(OC)=C3C4=CC=C(NC)C(=O)C=C24
PUGREST.BadRequest: error:
NC(=S)N1[C@@H1]C2=CC(C(NC)=CC=C2C3=C(OC)C(OC)=C(OC)C=C3CC1)=O
PUGREST.BadRequest: error:
C=C(CN[C@H1]1CCC2=CC(OC)=C(OC)C(OC)=C2C3=CC=C(NC)C(C=C13)=O)C=O
PUGREST.BadRequest: error:
C1=C(N=NN1[C@H1]2CCC3=CC(OC)=C(OC)C(OC)=C3C4=CC=C(NC)C(=O)C=C24)C
PUGREST.BadRequest: error:
C=CC=C(NC(=O)N[C@H1]1CCC2=CC(OC)=C(OC)C(OC)=C2C3=CC=C(NC)C(=O)C=C13)C
PUGREST.BadRequest: error:
C[C@H1](CCC1=CC(OC)=C(OC)C(OC(OCC)=O)=C1C2=CC=C(SC)C(=O)C=C2)NCC
PUGREST.BadRequest: error:
COCC(CN[C@@H1]1C=2C(C3=C(C(OC)=C(OC)C=C3CC1)OC)=CC=C(NC)C(C=2)=O)F
PUGREST.BadRequest: error:
NC1=NN=NN1[C@@H1]2C=3C(C4=C(C(OC)=C(OC)C=C4CC2)OC)=CC=C(NC)C(C=3)=O
PUGREST.BadRequest: error:
OCCN(CCO)C(=S)N1[C@@H1]C2=CC(C(NC)=CC=C2C3=C(OC)C(OC)=C(OC)C=C3CC1)=O
PUGREST.BadRequest: error:
NC(C(OCC)=C)N[C@H1]1CCC2=CC(OC)=C(OC)C(OC)=C2C3=CC=C(NC)C(=O)C=C31
PUGREST.BadRequest: error:
N([C@H1]1CCC2=CC(OC)=C(OC)C(OC)=C2C3=CC=C(NC)C(C=C13)=O)=N
PUGREST.BadRequest: error:
NC(CCC)N[C@@H1]1C2=CC(C(NC)=CC=C2C3=C(OC)C(OC)=C(OC)C=C3CC1)=O
PUGREST.BadRequest: error:
N([C@H1]1CCC2=CC(OC)=C(OC)C(OC)=C2C3=CC=C(NC)C(C=C31)=O)=C
PUGREST.BadRequest: error:
N([C@H1]1CCC2=CC(OC)=C(OC)C(OC)=C2C3=CC=C(NC)C(C=C13)=O)=CC
PUGREST.BadRequest: error:
C1=2[C@H1](CCC3=C(C(OC)=C(OC)C(OC)=C3)C1=CC=C(C(C=2)=O)NCNCC(C)C)ONC
PUGREST.BadRequest: error:  N(C)C=1C(=O)C=C2[C@H1](CCC3=C(C(OC)=C(OC)C(OC)=C3)C2
=CC=1)N4C(C(=O)O)=C(N=N4)C(=O)N
PUGREST.BadRequest: error:
N(CCCC)CNCC1=CN(N=N1)[C@H1]2CCC3=CC(OC)=C(OC)C(OC)=C3C4=CC=C(NC)C(=O)C=C24
PUGREST.BadRequest: error:
```

```
N(C)C=1C(=O)C=C2[C@H1](CCC3=C(C(OC)=C(OC)C(OC)=C3)C2=CC=1)NC(C(=O)O)=C(COCC)C=O
PUGREST.BadRequest: error:
OC(\N[C@H1]1CCC2=CC(OC)=C(OC)C(OC)=C2C3=CC=C(C(C=C31)=O)NCNC(C)C)=O
PUGREST.BadRequest: error:  N1=CC=C(C=C1)C(NCC=2N=NN(C=2)[C@H1]3CCC4=CC(OC)=C(OC
)C(OC)=C4C5=CC=C(C(C=C53)=O)N)C=O
PUGREST.BadRequest: error:
OCC(C)=C1C([C@H1](CCC2=CC(OC)=C(OC)C(OC)=C12)NC(NC3=CC=C(C=C3)F)=O)PNC=O
PUGREST.BadRequest: error:  N1=CC=C(C=C1)C(NCC=2N=NN(C=2)[C@@H1]3C4=CCC(NC)=CC=C
4C5=C(OC)C(OC)=C(OC)C=C5CC3)=O
PUGREST.BadRequest: error:
CC(C)(C)OC(=O)/N=C(/N[C@H1]1CCC2=C(C(OC)=C(OC)C(OC)=C2)C3=CC=C(NC)C(C=C13)=O)N=N
PUGREST.BadRequest: error:  CC(C)(C)OC(=O)NCCCNCC1=CN(N=N1)[C@H1]2CCC3=CC(OC)=C(
OC)C(OC)=C3C4=CC=C(NC)C(=O)C=C42
PUGREST.BadRequest: error:
COC1=C2C3=CC=C(NC)C(=O)C=C3[C@H1](CCC2=CC(OC)=C1OC)NCCCCCCSC
PUGREST.BadRequest: error:
C=C(CO)CN[C@H1]1CCC2=CC(OC)=C(OC)C(OC)=C2C3=CC=C(SC)C(=O)C=C31
PUGREST.BadRequest: error:
CC(C)CN[C@H1]1CCC2=CC(OC)=C(OC)C(OC)=C2C3=CC=C(C(C=C31)=O)SCC
PUGREST.BadRequest: error:
CC(C)CCN[C@H1]1CCC2=CC(OC)=C(OC)C(OC)=C2C3=CC=C(C(C=C13)=O)SCC
PUGREST.BadRequest: error:
CC(C)CCN[C@H1]C1CC2=CC(OC)=C(OC)C(OC)=C2C3=CC=C(SC)C(C=C31)=O
PUGREST.BadRequest: error:
N=1NC=1[C@@H1]2C3=CC(C(NC)=CC=C3C4=C(C=C(OC)C(=C4OC)OC)CC2)=O
PUGREST.BadRequest: error:
COC1=C2C3=CC=C(NC)C(=O)C=C3[C@H1](CCC2=CC(OC)=C1OC)NCC(C)C
PUGREST.BadRequest: error:
COC1=C(OC)C(OC)=CC=2CC[C@@H1](C3=CC(C(=CC=C3C1=2)SC)=O)NCCC(C)CC
PUGREST.BadRequest: error:
C12=CC(C(NC)=CC=C1C3=C(C=C(OC)C(OC)=C3OC)CC[C@@H1]2NCC=4SC=CC=4OC)=O
PUGREST.BadRequest: error:
O=CC1=CN=NN1[C@H1]2CCC3=CC(OC)=C(OC)C(OC)=C3C4=CC=C(C(C=C24)=O)NCCC
PUGREST.BadRequest: error:
C1=CN=CC=C1CN[C@H1]2CCC3=CC(OC)=C(OC)C(OC)=C3C4=CC=C(C(C=C42)=O)SCC(=O)C
PUGREST.BadRequest: error:
C1=CN=CC=C1CN[C@H1]2CCC3=CC(OC)=C(OC)C(OC)=C3C4=CC=C(C(C=C42)=O)SCC
PUGREST.BadRequest: error:
C=C1N=CC(C)N1CN[C@H1]2CCC3=C(C(OC)=C(OC)C(OC)=C3)C4=CC=C(C(C=C24)=O)NC
PUGREST.BadRequest: error:
CNCC(C)OC(=O)N[C@H1]1CCC2=C(C(OC)=C(OC)C(OC)=C2)C3=CC=C(C(=O)C=C31)NCF
PUGREST.BadRequest: error:
C=CC=C(CCN[C@H1]1CCC2=CC(OC)=C(OC)C(OC)=C2C3=CC=C(SC)C(C=C31)=O)OC
PUGREST.BadRequest: error:
C1COC1(OC2=CC=C3C4=C(OC)C(OC)=C(OC)C=C4CC[C@@H1](C3=CC2=O)SC(C)=O)SC
PUGREST.BadRequest: error:
O=C(OC1=C2C(CC[C@H1](NC(=O)C)C3=CC(C(SC)=CC=C32)=O)=CC(OC)=C1OC)SCC
PUGREST.BadRequest: error:
```

```
O=C1C=CC=C2C([C@H1](CCC3=CC(OC)=C(OC)C(OC)=C32)NC(C)=O)=C1SC
PUGREST.BadRequest: error:
C1=CC=C(C=C1)CN[C@H1]2CCC3=CC(OC)=C(OC)C(OC)=C3C4=CC=C(C(C=C42)=O)SCC
PUGREST.BadRequest: error:
CC=CCCCN[C@H1]1CCC2=CC(OC)=C(OC)C(OC)=C2C3=CC=C(SC)C(C=C13)=O
PUGREST.BadRequest: error:
OC1=C(C=CC=C1)CN2[C@H1]CCC3=CC(OC)=C(OC)C(OC)=C3C4=CC=C(SC)C(C=C24)=O
PUGREST.BadRequest: error:
COC1=C2C3=CC=C(NC)C(=O)C=C3[C@H1](CCC2=CC(OC)=C1OC)NCCCCCCCSC
PUGREST.BadRequest: error:
COC1=CC=2CC[C@@H1](C=3C(C=2C(=C1OC)OC)=CC=C(NC)C(=O)C=3)NC(=O)CNC
PUGREST.BadRequest: error:
CSC1=CC=C2C(=CC1=O)[C@H1](CCC3=CC(OC)=C(OC)C(=C23)O)CNC(=O)C
PUGREST.BadRequest: error:
C1=C2[C@H1](CCC3=C(C(OC)=C(C(=C3)OC)OC)C2=CC=C(C1=O)NC)NC
PUGREST.BadRequest: error:
CC(=O)OC1=CC=C2C3=C(C(OC)=C(C=C3CC[C@@H1](C2=CC1=O)NC(=O)CO)CO)C
PUGREST.BadRequest: error:
CC(=O)OC=1C(=O)C=C2C(=CC=1)C3=C(OC)C(OC)=C(C=C3CC[C@@H1]2NC(C)=O)C(=O)C
PUGREST.BadRequest: error:
CC(=O)OC1=CC=C2C3=C(C(OC)=C(C=C3CC[C@@H1](C2=CC1=O)NC(=O)C)OC)OC(=O)C
PUGREST.BadRequest: error:
CCC(=O)OC1=CC=C2C3=C(C(OC)=C(C=C3CC[C@@H1](C2=CC1=O)OC(=O)C)OC)OC
PUGREST.BadRequest: error:
CCC(=O)N[C@H1]1CCC2=CC(=C(OC)C(OC)=C2C=3C1=CC(=O)C(=CC=3)OC(OC)=O)OC
PUGREST.BadRequest: error:
CC(C)C(OC1=CC=C2C3=C(CC[C@@H1](C2=CC1=O)NC(C)=O)C=C(OC)C(OC)=C3OC)(O)C=O
PUGREST.BadRequest: error:
CC(C)C(OC1=CC=C2C3=C(CC[C@@H1](C2=CC1=O)NC(C)=O)C=C(OC)C(OC)=C3OC=O)C
PUGREST.BadRequest: error:
CC(C)C(=O)OC1=CC=C2C3=C(C(OC)=C(C=C3CC[C@@H1](C2=CC1=O)NC(C)=O)OC)OC(=O)C
PUGREST.BadRequest: error:
CC(C)C(OC1=CC=C2C3=C(C=C(OC)C(OC)=C3OC)CC[C@@H1](C2=CC1=O)NC(=O)CC)(O)C
PUGREST.BadRequest: error:
CC(=O)OCC1=CN(N=N1)[C@H1]2CCC3=CC(OC)=C(OC)C(OC)=C3C=4C2=CC(=O)C(=CC=4)NCC
PUGREST.BadRequest: error:
C1=C(C2=C(OC)C(OC)=C1OC(=O)CCC)C[C@@H1](C3=CC(=O)C(SC)=CC=C32)NC(C)=O
PUGREST.BadRequest: error:
CC(=O)N[C@H1]1CCC2=CC(OC)=C(OC)C(=C2C3=CC=C(C(C=C31)=O)OCC4=CC=CC=C4)OOC
PUGREST.BadRequest: error:
O=C(C)N[C@H1]1CCC2=CC(=C(OC)C(=C2C=3C1=CC(C(=CC=3)OC(=O)OC)=O)CN)C(=O)COCC
PUGREST.BadRequest: error:
C=12C(=O)C(=CC=C3C=1[C@H1](CCC4=CC(OC)=C(OC)C(OC)=C34)NC(C)=O)C2
PUGREST.BadRequest: error:
CCC(=O)N[C@H1]1CCC2=C(C(OC)=C(C(OC)=C2)OC)C=3C1=CC(C(SC)=CC=3)=O
PUGREST.BadRequest: error:
CCC(=O)N[C@H1]1CCC2=CC(OC)=C(C(OC)=C2C=3C1=CC(=O)C(=CC=3)SC)OC(=O)OCC
PUGREST.BadRequest: error:
```

```
CC=1C(=CC(C(=CC=1)SC)=O)[C@H1](CCC2=CC(OC)=C(OC)C(=C2)OC)NC(=O)C
PUGREST.BadRequest: error:
CC(C)COCCN[C@H1]1CCC2=CC(OC)=C(C(=C2C=3C1=CC(C(=CC=3)SC)=O)OC)OC(=O)C
PUGREST.BadRequest: error:
CC(C)CCN[C@H1]1CCC2=C(C3=CC=C(SC)C(C=C31)=O)C(=C(C(OC)=C2)OC)OCCC
PUGREST.BadRequest: error:
CC(C)CCN[C@H1]1CCC2=C(C3=CC=C(SC)C(C=C31)=O)C(=C(C(OC)=C2)OC)OC(=O)C
PUGREST.BadRequest: error:
C1=C(OC)C(=C(OC)C2=C1CC[C@H1](NC(=O)C)C=3C2=CC=C(OC(=O)OC)C(C=3)=O)C
PUGREST.BadRequest: error:
O=C(C)N[C@H1]1CCC2=CC(OC)=C(C(OC)=C2C3=CC=C(C(=O)C=C13)OCCC)OOC
PUGREST.BadRequest: error:
O=C(N(C)C)NC1=CC=C2C3=C(OC)C(=C(OC)C=C3CC[C@@H1](C2=CC1=O)NCC)OOC
PUGREST.BadRequest: error:
O=C(N)N[C@H1]1CCC2=CC(OC)=C(C(OC)=C2C=3C1=CC(C(OC(=O)C)=CC=3)=O)OC
PUGREST.BadRequest: error:
O=C(C)N[C@H1]1CCC2=CC(OC)=C(C(OC)=C2C3=CC=C(C(=O)C=C31)OC(=O)O)CCOC
PUGREST.BadRequest: error:
CC(=O)N1[C@H1]CCC2=CC(OC)=C(OC)C(OC)=C2C3=CC=C(NC)C(C=C31)=O
PUGREST.BadRequest: error:
COC1=C2C3=CC=C(NC)C(=O)C=C3[C@H1](CCC2=CC(OC)=C1OC)NO
PUGREST.BadRequest: error:
C1[C@@H1](C2=CC(=O)C(NC)=CC=C2C3=C(C=C(OC)C(=C3OC)OC)C1)NCCSC
PUGREST.BadRequest: error:
CNC(C(C)O)N([C@H1]1CCC2=CC(=C(OC)C(OC)=C2C=3C1=CC(=O)C(=CC=3)NC)OCC)C
PUGREST.BadRequest: error:
CNC(C(C)O)N[C@@H1]1C=2C(C3=C(C=C(OC)C(OC)=C3OC)CC1)=CC=C(C(=O)C=2)NC
PUGREST.BadRequest: error:
CNC(=S)N[C@@H1]1C2=CC(=O)C(NC)=CC=C2C3=C(OC)C(=C(C=C3CC1)OC)OCO
PUGREST.BadRequest: error:
N([C@H1]1CCC2=C(C3=CC=C(C(C=C13)=O)NC)C(OC)=C(OC)C(OC)=C2)N=N
PUGREST.BadRequest: error:
C12=CC(C(NC)=CC=C1C3=C(C=C(C(OC)=C3OC)OC)CC[C@@H1]2NCCCCCCCCCF)=O
PUGREST.BadRequest: error:
COC1=C(OC)C(OC)=CC=2CC[C@@H1](C3=CC(=O)C(NC)=CC=C3C=21)NCCC4=CC=CC=C4NC
PUGREST.BadRequest: error:
COC1=C(OC)C(OC)=CC=2CC[C@@H1](C3=CC(=O)C(NC)=CC=C3C=21)N4C=C(N=N4)CCONC
PUGREST.BadRequest: error:
CN(C(=S)N[C@@H1]1C2=CC(=O)C(NC)=CC=C2C3=C(C(=C(OC)C=C3CC1)OC)OC)CC#N
PUGREST.BadRequest: error:
CC(C)(C)OC1=CN(N=N1)[C@H1]C2CC3=CC(OC)=C(OC)C(OC)=C3C4=CC=C(NC)C(=O)C=C42
PUGREST.BadRequest: error:
CC(C)CCCCCCN[C@@H1]1C2=CC(C(SC)=CC=C2C3=C(C(OC)=C(OC)C=C3CC1)OC)=O
PUGREST.BadRequest: error:
CC(C)(C)C(=O)N[C@@H1]1C=2C(C3=C(C(OC)=C(OC)C=C3CC1)OC)=CC=C(NC)C(=O)C=2
PUGREST.BadRequest: error:
OC(CCC(C)C)OCC1=CN(N=N1)[C@H1]CCC2=CC(OC)=C(OC)C(OC)=C2C3=CC=C(C(=O)C=C3)NC
PUGREST.BadRequest: error:
```

```
O=C(NCC1=CC=CC=C1Cl)N[C@H1]2CCC3=C(C(OC)=C(OC)C(OC)=C3)C4=CC=C(NC)C(=O)C=C24
PUGREST.BadRequest: error:
N([C@H1]1CCC2=C(C(OC)=C(OC)C(OC)=C2)C3=CC=C(NC)C(C=C13)=O)C(O)=O
PUGREST.BadRequest: error:
O=CC=CCC=CNNC(N[C@H1]1CCC2=CC(OC)=C(OC)C(OC)=C2C3=CC=C(NC)C(=O)C=C13)=O
PUGREST.BadRequest: error:
OC1=C(C=CC=C1)C=2N=NN(C=2)[C@H1]C3CC4=CC(OC)=C(OC)C(OC)=C4C5=CC=C(NC)C(=O)C=C53
PUGREST.BadRequest: error:
OC1=C(C=CC=C1)C=2N=NN(C=2)[C@H1]CCC3=CC(OC)=C(OC)C(OC)=C3C4=CC=C(NC)C(=O)C=C4
PUGREST.BadRequest: error:
C1[C@@H1](C2=CC(=O)C(NC)=CC=C2C3=C(C(=C(OC)C=C3C1)OC)OC)N4C=C(N=N4)COC
PUGREST.BadRequest: error:
O=C(OC(C)(C)C)N[C@H1]1CCC2=C(C(OC)=C(OC)C(OC)=C2)C3=CC=C(NC)C(=O)C=C13
PUGREST.BadRequest: error:
O=C(OCC)CN(OCC)C[C@@H1]1C2=CC(C(NC)=CC=C2C3=C(C(OC)=C(OC)C=C3CC1)OC)=O
PUGREST.BadRequest: error:
O=C(C)OC1=CN(N=N1)[C@H1]2CCC3=CC(OC)=C(OC)C(OC)=C3C4=CC=C(NC)C(=O)C=C24
PUGREST.BadRequest: error:
O=C(OCC)CN(CCC)C[C@H1]C1CC2=CC(OC)=C(OC)C(OC)=C2C3=CC=C(SC)C(=O)C=C31
PUGREST.BadRequest: error:  O=C(C(Cl)Cl)NC(=O)N[C@H1]1CCC2=C(C(OC)=C(OC)C(OC)=C2
)C3=CC=C(C(C=C31)=O)NCN(O)CCC=O
PUGREST.BadRequest: error:  O=C(C(Cl)Cl)NC(=O)N[C@H1]1CCC2=C(C(OC)=C(OC)C(OC)=C2
)C3=CC=C(C(C=C31)=O)NCC4=CC=CC=C4
PUGREST.BadRequest: error:
O=C(OCCC)OCC1=CN(N=N1)[C@H1]2CCC3=CC(OC)=C(OC)C(OC)=C3C4=CC=C(C(=O)C=C24)NCCO
PUGREST.BadRequest: error:  O=C(OCCC)OCC1=CN(N=N1)[C@H1]2CCC3=CC(OC)=C(OC)C(OC)=
C3C4=CC=C(C(=O)C=C42)NCC(=O)C
PUGREST.BadRequest: error:  O=C(OCC=C)NCC1=CN(N=N1)[C@H1]2CCC3=CC(OC)=C(OC)C(OC)
=C3C4=CC=C(C(=O)C=C24)NCC(=O)C
PUGREST.BadRequest: error:  O=C(C(Cl)Cl)NCC1=CN(N=N1)[C@H1]2CCC3=CC(OC)=C(OC)C(O
C)=C3C4=CC=C(C(=O)C=C24)NCC(=O)C
PUGREST.BadRequest: error:  O=C(C(Cl)Cl)NCC1=CN(N=N1)[C@H1]2CCC3=CC(OC)=C(OC)C(=
C3C=4C2=CC(C(NC)=CC=4)=O)OCCCl
PUGREST.BadRequest: error:
O=C(C)N[C@@H1]1CC(C2=C(C(OC)=C(OC)C=C2CC1)OCC3=CC=CC=C3)=O
PUGREST.BadRequest: error:
O=C(C(Cl)Cl)NC(=O)N[C@H1]1CCC2=CC(OC)=C(OC)C(OC)=C2C3=CC=C(C(C=C31)=O)NCC(=O)O
PUGREST.BadRequest: error:
O=C(OCC)C1=CN(N=N1)[C@H1]2CCC3=CC(OC)=C(OC)C(OC)=C3C4=CC=C(C(C=C24)=O)NCC(=O)C
PUGREST.BadRequest: error:
O=C(O)CN(CCO)C(=O)N[C@H1]1CCC2=CC(OC)=C(OC)C(OC)=C2C3=CC=C(C(C=C13)=O)NCC
PUGREST.BadRequest: error:
CC(C)OC(NCC1=CN(N=N1)[C@H1]2CCC3=CC(OC)=C(OC)C(OC)=C3C4=CC=CC(C=C24)=O)NC=O
PUGREST.BadRequest: error:
COC(NCCCOCC1=CN(N=N1)[C@H1]2CCC3=CC(OC)=C(OC)C(=C3C4=CC=C(NC)C(C=C24)=O)OC=O)=O
PUGREST.BadRequest: error:
C=1C(C)OC(=O)/N=C(/N[C@H1]2CCC3=C(C(OC)=C(OC)C(OC)=C3)C4=CC=C(NC)C(=O)C=C42)N=1
PUGREST.BadRequest: error:
```

```
C1=CN=CC=C1C2=CN(N=N2)[C@H1]3CCC4=C(C(OC)=C(OC)C(OC)=C4)C5=CC=C(C(=O)C=C35)NCO
PUGREST.BadRequest: error:
COC=C(OC)C(OC)=CCCC[C@H1](N1C=C(N=N1)COC(=O)C2=CC=NC=C2)C3=CC(=O)C(=CC=C3CNCN)C
PUGREST.BadRequest: error:
COC1=C(OC)C=CC=2CC[C@@H1](C3=CC(=O)C(NC)=CC=C3C1=2)N4C=C(N=N4)COCC5=CC=CC=C5OC
PUGREST.BadRequest: error:
CC(C)(O)C=CN([C@H1]1CCC2=CC(OC)=C(OC)C(OC)=C2C3=CC=C(C(C=C13)=O)NC)O
PUGREST.BadRequest: error:
C=12C(=CC(C(NC)=CC=1)=O)[C@H1](CCC3=CC(OC)=C(OC)C(=C32)OC)C(C)C=O
PUGREST.BadRequest: error:
NC(=O)NCC1=CN(N=N1)[C@H1]2CCC3=CC(OC)=C(OC)C(OC)=C3C4=CC=C(NC)C(=O)C=C24
PUGREST.BadRequest: error:
C1=CC(OC)=CC=C1CN[C@H1]2CCC3=C(C(OC)=C(OC)C(OC)=C3)C=4C2=CC(C(=CC=4)NC)=O
PUGREST.BadRequest: error:
N(C(C1=CC=CC=C1)=O)CCN[C@H1]2CCC3=CC(OC)=C(OC)C(OC)=C3C4=CC=C(C(C=C24)=O)SC
PUGREST.BadRequest: error:
N=NC(=C(C(OCC)=O)N[C@H1]1CCC2=CC(OC)=C(OC)C(OC)=C2C3=CC=C(C(C=C13)=O)N)C
PUGREST.BadRequest: error:
CNC=1C(=O)C=C2[C@H1](CCC3=C(C(OC)=C(OC)C(OC)=C3)C2=CC=1)NC(C(=O)O)=CN=CNC
PUGREST.BadRequest: error:
NN(C=C(CNC(CCCCCl)=O)N=N)[C@H1]1CCC2=CC(OC)=C(OC)C(OC)=C2C3=CC=C(NC)C(=O)C=C31
PUGREST.BadRequest: error:
N=NC1=CN(N=N1)[C@H1]2CCC3=CC(OC)=C(OC)C(=C3C4=CC=C(NC)C(C=C42)=O)OC
PUGREST.BadRequest: error:  CC(C)(C)OC(=O)NCCC(NCC=1N=NN(C=1)[C@H1]CCC2=CC(OC)=C
(OC)C=C2C3=CC=C(NC)C(C=C3)=O)OC=O
PUGREST.BadRequest: error:  CC(C)(C)OCNCCC(=O)NCC1=CN(N=N1)[C@H1]C2CC3=CC(OC)=C(
OC)C(OC)=C3C4=CC=C(C(=O)C=C24)NC=O
PUGREST.BadRequest: error:
C1(=CN(C(=O)OCC)N[C@@H1]2C=3C(C4=C(OC)C(OC)=C(OC)C=C4CC2)=CC=C(C(=O)C=3)NC)N=C1
PUGREST.BadRequest: error:  CC(C)(C)OCNCCC(=O)NCC1=CN(N=N1)[C@@H1]2C3=CC(C(NC)=C
C=C3C4=C(CC2)C=C(OC)C(=C4OC)NC)=O
PUGREST.BadRequest: error:  CC(C)(C)OC(=O)NCCC(OCC=1N=NN(C=1)[C@H1]CCC2=CC(OC)=C
(OC)C(=C2C3=CC=C(NC)C(=O)C=C3)O)C=O
PUGREST.BadRequest: error:
CC(C)OC(=O)/N=C(/N[C@H1]1CCC2=C(C(OC)=C(OC)C(OC)=C2)C3=CC=C(NC)C(=O)C=C13)N=N
PUGREST.BadRequest: error:
C=1N(N=NC=1C(OCC)=O)[C@H1]2CCC3=C(C(OC)=C(OC)C(OC)=C3)C4=CC=C(C(=O)C=C42)NCOCC
PUGREST.BadRequest: error:
O=CC=CN=NN[C@H1]1CCC2=C(C(OC)=C(OC)C(=C2)OC)C3=CC=C(NC)C(=O)C=C13
PUGREST.BadRequest: error:  C1=CN=CC=C1C(NCC2=CN(N=N2)[C@H1]3CCC4=CC(OC)=C(OC)C(
=C4C5=CC=C(C(C=C53)=O)OC)OO)C
PUGREST.BadRequest: error:
N=NN([C@H1]1CCC2=C(C(OC)=C(OC)C(OC)=C2)C3=CC=C(C(=O)C=C13)NC)C(=O)OCC
PUGREST.BadRequest: error:
CN(N([C@@H1]1C2=CC(=O)C(NC)=CC=C2C3=CC(=C(OC)C=C3CC1)OC)O)CNC(=S)NCCCCC
PUGREST.BadRequest: error:
CN(N([C@@H1]1C2=CC(=O)C(NC)=CC=C2C3=CC(=C(OC)C=C3CC1)OC)O)CNC(=S)CCCCCC
PUGREST.BadRequest: error:
```

O=C(CC)OCC1=CN(N=N1)[C@H1]2CCC3=CC(OC)=C(OC)C(OC)=C3C4=CC=C(NC)C(=O)C=C24
PUGREST.BadRequest: error: O=C(OC(C)(C)C)NCC=1N=NN(C=1)[C@H1]C2CC3=CC(OC)=C(OC)C(OC)=C3C4=CC=C(NC)C(=O)C=C42
PUGREST.BadRequest: error:
O=C(C(C)C)NCN[C@H1]1CCC2=CC(OC)=C(OC)C(OC)=C2C3=CC=C(NC)C(=O)C=C13
PUGREST.BadRequest: error: O=C(OCC)C=1N(N=NC=1C(=O)O2)[C@H1]2NC(=S)N[C@H1]3CCC4=CC(OC)=C(OC)C(OC)=C4C=5C3=CC(C(=CC=5)NC)=O
PUGREST.BadRequest: error: CC(O)(C)CC(=O)OCC1=CN(N=N1)[C@H1]2CCC3=CC(OC)=C(OC)C(OC)=C3C=4C2=CC(C(=CC=4)NC)=O
PUGREST.BadRequest: error:
CC(C)COC(NCC1=CN(N=N1)[C@H1]C2CC3=CC(OC)=C(OC)C(OC)=C3C4=CC=C(C(=O)C=C24)NC)=O
PUGREST.BadRequest: error:
COCNCC1=CN(N=N1)[C@H1]2CCC3=CC(OC)=C(OC)C(OC)=C3C4=CC=C(NC)C(=O)C=C24
PUGREST.BadRequest: error: CC(C)(C)OC(NCC=1N=NN(C=1)[C@H1]2CCC3=CC(OC)=C(OC)C(OC)=C3C4=CC=C(C(C=C24)=O)N)C=O
PUGREST.BadRequest: error:
N(C)C1=CC=C2C3=C(OC)C(OC)=C(OC)C=C3CC[C@@H1](C2=CC1=O)NC(C(=O)O)CCONCOCNCOC
PUGREST.BadRequest: error:
O=C(OCC)C1=C(C(OCC)=O)N=NN1[C@H1]CCC2=CC(OC)=C(OC)C(OC)=C2C3=CC=C(NC)C(C=C3)=O
PUGREST.BadRequest: error:
N([C@H1]1CCC2=CC(OC)=C(OC)C(OC)=C2C3=CC=C(NC)C(C=C13)=O)C=CCOC(=O)CCCC=O
PUGREST.BadRequest: error:
CC(CC)N[C@H1]C1CC2=CC(OC)=C(OC)C(OC)=C2C3=CC=C(C(C=C31)=O)NCC
PUGREST.BadRequest: error:
CC(CC)N[C@H1]C1CC2=CC(OC)=C(OC)C(OC)=C2C3=CC=C(NC)C(=O)C=C31
PUGREST.BadRequest: error:
COC1=C2C3=CC=C(NC)C(=O)C=C3[C@@H1](NCC(CCC)C)CCC2=CC(OC)=C1OC
PUGREST.BadRequest: error:
COC1=C2C3=CC=C(NC)C(=O)C=C3[C@H1](CCC2=CC(OC)=C1OC)NCC4=CC=NS4
PUGREST.BadRequest: error:
COC1=C2C3=CC=C(SC)C(=O)C=C3[C@H1](CCC2=CC(OC)=C1OC)NCCCCl
PUGREST.BadRequest: error:
O(C1=C(OC)C=2C3=CC=C(SC)C(C=C3[C@H1](CCC=2C=C1OC)NC(C)CC=C)=O)C
PUGREST.BadRequest: error:
C1=CN=CC=C1CN[C@H1]2CCC3=CC(OC)=C(OC)C(OC)=C3C4=CC=C(C(C=C24)=O)NCC
PUGREST.BadRequest: error:
O=CC1=CN=NN1[C@H1]2CCC3=CC(OC)=C(OC)C(OC)=C3C4=CC=C(C(C=C42)=O)NC
PUGREST.BadRequest: error:
NC(CCN[C@H1]1CCC2=CC(OC)=C(OC)C(OC)=C2C=3C1=CC(=O)C=CC=3)NCF
PUGREST.BadRequest: error:
NC(N=C1C=CC2)=CC=C1C3=C(OC)C(OC)=C(OC)C=C3CC[C@@H1]2NC(N)=O
PUGREST.BadRequest: error:
C=CC1=C(NC)C(OC)=C(OC)C=C1CC[C@H1](NC(C)=O)C2=CC(C(=CC=C2)NC)=O
PUGREST.BadRequest: error:
CC(N)(CCO)[C@H1]1CCC2=CC(OC)=C(OC)C(OC)=C2C3=CC=C(NC)C(=O)C=C31
PUGREST.BadRequest: error:
N(NCCCCCl)[C@H1]1CCC2=CC(OC)=C(OC)C(OC)=C2C3=CC=C(NC)C(=O)C=C31
PUGREST.BadRequest: error:

```
N(C1=CC=C(C=C1)C)CN[C@H1]2CCC3=CC(OC)=C(OC)C(OC)=C3C=4C2=CC(C(NC)=CC=4)=O
PUGREST.BadRequest: error:
N(C1=CC=C(C=C1)C)CN[C@H1]2CCC3=CC(OC)=C(OC)C(OC)=C3C=4C2=CC(C(=CC=4)SC)=O
PUGREST.BadRequest: error:
CCN(CC)C(=O)N[C@H1]C1CC2=CC(OC)=C(OC)C(OC)=C2C3=CC=C(C(C=C31)=O)NC
PUGREST.BadRequest: error:
CCN(CC)C(=O)N1[C@H1]CCC2=CC(OC)=C(OC)C(OC)=C2C3=CC=C(NC)C(C=C31)=O
PUGREST.BadRequest: error:
CCN(CC)C(=O)N[C@H1]1CCC2=CC(OC)=C(OC)C(OC)=C2C3=CC=C(C(C=C31)=O)NCC
PUGREST.BadRequest: error:
CC(C)COC1=C(OC)C(OC)=CC=2CC[C@H1](NC(=O)C)C3=CC(C(SC)=CC=C3C1=2)=O
PUGREST.BadRequest: error:
C=C(C)CN(CCO)C(=S)N[C@H1]1CCC2=C(C(OC)=C(OC)C(OC)=C2)C3=CC=C(NC)C(C=C31)=O
PUGREST.BadRequest: error:
CC(C)C(OC=C(C(OC)=CCCC[C@H1](NC(=O)C)C1=CC(C(SC)=CC=C1C)=O)C)=O
PUGREST.BadRequest: error:
N([C@H1]1CCC2=CC(OC)=C(OC)C(OC)=C2C3=CC=C(NC)C(=O)C=C31)=C(Cl)C=C
PUGREST.BadRequest: error:
O=C(N[C@H1]1CCC2=CC(OC)=C(OC)C(OC)=C2C3=CC=C(C(C=C31)=O)NCN)C4=CC=CC=C4
PUGREST.BadRequest: error:
O=C(N[C@@H1]1C2=CC(C(NC)=CC=C2C3=C(CC1)C=C(OC)C(OC)=C3OC)=O)N=N
PUGREST.BadRequest: error:
N([C@H1]1CCC2=CC(OC)=C(OC)C(OC)=C2C3=CC=C(NC)C(C=C13)=O)CNC(=O)OCCONC
PUGREST.BadRequest: error:
O=C(N[C@H1]1CCC2=CC(OC)=C(OC)C(OC)=C2C3=CC=C(NC)C(=O)C=C31)CCOC(=O)O
PUGREST.BadRequest: error:
C1=CCC[C@H1](NC(=S)NC2=CC=C(C=C2)C(F)(F)F)CC(C1)=CC=C(NC)C(=O)COCOC
PUGREST.BadRequest: error:
CNCC(O)(C)C1=CN(N=N1)[C@H1]2CCC3=CC(OC)=C(OC)C(OC)=C3C4=CC=C(C(=O)C=C24)NC
PUGREST.BadRequest: error:
N1([C@H1]2CCC3=C(C(OC)=C(OC)C(OC)=C3)C4=CC=C(NC)C(C=C24)=O)C(O)=C1C
PUGREST.BadRequest: error:
N([C@H1]1CCC2=C(C(OC)=C(OC)C(OC)=C2)C3=CC=C(NC)C(C=C13)=O)NC=CC=CCl
PUGREST.BadRequest: error:
C1=C(O)C(OC)=CC(CN[C@H1]2CCC3=C(C(OC)=C(OC)C(OC)=C3)C4=CC=C(SC)C(=O)C=C42)=C1C
PUGREST.BadRequest: error:
COCC(C)=C(C(OC)=CCCC[C@H1](NCC)C1=CC(C(SC)=CC=C1)=O)OC
PUGREST.BadRequest: error:
C[C@H1](CCC1=CC(OC)=C(OC)C(OC)=C1C2=CC=C(C(=O)C=C2)NCN)CCO
PUGREST.BadRequest: error:
NC(=O)N1[C@@H1]C2=CC(C(NC)=CC=C2C3=C(OC)C(OC)=C(OC)C=C3CC1)=O
PUGREST.BadRequest: error:
NN(CCO)[C@@H1]1C2=CC(C(NC)=CC=C2C3=C(OC)C(OC)=C(C=C3CC1)OC)=O
PUGREST.BadRequest: error:
C1=2[C@H1](CCC3=CC(OC)=C(OC)C(OC)=C3C1=CC=C(NC)C(=O)C=2NC)CO
PUGREST.BadRequest: error:
NC(N=NCOCCC)C[C@H1]1C2=CC(C(NC)=CC=C2C3=C(CC1)C=C(OC)C(OC)=C3OC)=O
PUGREST.BadRequest: error:
```

```
C1=2C(C3=C(OC)C(OC)=C(OC)C=C3CC[C@@H1]1NC(C)CC)=CC=C(NC)C(=O)C=2NC
PUGREST.BadRequest: error:
C1=C(CN[C@H1]2CCC3=CC(OC)=C(OC)C(OC)=C3C4=CC=C(NC)C(=O)C=C42)C=NC=C1
PUGREST.BadRequest: error:
C1[C@H1](NC(NC2=CC=C(C=C2)F)=O)C3=CC(C(NC)=CC=C3C4=C(OC)C(OC)=CC=C4OCC1)=O
PUGREST.BadRequest: error:
C1=CC(=CC=N1)CCN[C@@H1]2C3=CC(C(SC)=CC=C3C4=C(CC2)C=C(OC)C(OC)=C4OC)=O
PUGREST.BadRequest: error:
C1=2[C@H1]CCC3=CC(OC)=C(OC)C(OC)=C3C1=CC=C(C(=O)C=2)NCNC(C)=O
PUGREST.BadRequest: error:
CC=CC1=C(C=C(OC)C(OC)=C1OC(OCC)=O)CC[C@H1](NC(C)=O)C2=CC(C(SC)=CC=C2)=O
PUGREST.BadRequest: error:
O=C1C=C2[C@H1](CCC3=C(C(OC)=C(OC)C(OC)=C3)C2=CC=C1SC)NC(N(C(C)C)C(Cl)=O)NC
PUGREST.BadRequest: error:
O=C1C=C2[C@H1](CCC3=C(C(OC)=C(OC)C(OC)=C3)C2=CC=C1SC)NC(C(=O)OCC)=CNC
PUGREST.BadRequest: error:
O=C1C=C2[C@H1](CCC3=C(C(OC)=C(OC)C(OC)=C3)C2=CC=C1SC)NC(C(C)C)=O
PUGREST.BadRequest: error:
N1C=NN=NN1[C@H1]2CCC3=C(C(OC)=C(OC)C(OC)=C3)C4=CC=C(C(C=C42)=O)NCCC
PUGREST.BadRequest: error:
CNC=1C(=O)C=C2[C@H1](CCC3=CC(OC)=C(OC)C(OC)=C3C2=CC=1N)C(C(C)C)=O
PUGREST.BadRequest: error:
N([C@H1]1CCC2=CC(OC)=C(OC)C(OC)=C2C3=CC=C(NC)C(C=C31)=O)CCCCF
PUGREST.BadRequest: error:
C[C@H1](CCC1=CC(OC)=C(OC)C(OC)=C1C2=CC=C(C(C=C2)=O)SC)NC/C=C/C3=CC=CC=C3
PUGREST.BadRequest: error:
N([C@H1]1CCC2=C(C(OC)=C(OC)C(OC)=C2)C3=CC=C(SC)C(C=C31)=O)CCCO
PUGREST.BadRequest: error:
C=CC1=C(C(=O)OC=CC=C2C3=C(OC)C(OC)=C(OC)C=C3CC[C@H1](NC(C)=O)C2=C1SC)C
PUGREST.BadRequest: error:
C=C1C2=C(C=C(OC)C(OC)=C2OC(OCC)=O)CC[C@H1](NC(C)=O)C3=CC(C(NC)=CC=C13)=O
PUGREST.BadRequest: error:
CC=CC(N[C@H1]1CCC2=C(C(OC)=C(OC)C(OC)=C2)C3=CC=C(SC)C(C=C13)=O)CCOC
PUGREST.BadRequest: error:
OC(=O)NCC1=CN(N=N1)[C@@H1]2C=3C(C4=C(C(OC)=C(OC)C=C4CC2)OC)=CC=C(NC)C(C=3)=O
PUGREST.BadRequest: error:  N(C)C=1C(=O)C=C2[C@H1](CCC3=C(C(OC)=C(OC)C(OC)=C3)C2
=CC=1)N4C(C(=O)O)=C(N=N4)C(=O)NNC
PUGREST.BadRequest: error:  O=C(C=C(C(O1)CCCCCC1)CN=N)N[C@H1]2CCC3=CC(OC)=C(OC)C
(OC)=C3C=4C2=CC(C(NC)=CC=4)=O
PUGREST.BadRequest: error:
N([C@H1]1CCC2=C(C(OC)=C(OC)C(OC)=C2)C3=CC=C(NC)C(=O)C=C13)CNCC4=CC=C(C=C4)C=O
PUGREST.BadRequest: error:  O=C(C=C(Cl)C=CN([C@H1]1CCC2=C(C(OC)=C(OC)C(OC)=C2)C3
=CC=C(NC)C(C=C13)=O)OC(=O)CC)NC(OC4=CC=CC=C4)=O
PUGREST.BadRequest: error:  CC(C)(C)OC(=O)/N=C(/N[C@H1]1CCC2=C(C(OC)=C(OC)C(OC)=
C2)C3=CC=C(NC)C(C=C31)=O)NCOC(C)=O
PUGREST.BadRequest: error:  CC(C)(C)OC(=O)/N=C(/N[C@H1]1CCC2=C(C(OC)=C(OC)C(OC)=
C2)C3=CC=C(NC)C(C=C13)=O)NCOC(C)(F)F
PUGREST.BadRequest: error:
```

```
C12=C3C(C4=C(OC)C(OC)=C(OC)C=C4CC[C@@H1]3NC(=O)N)=CC=C1NC=C2
PUGREST.BadRequest: error:
O=C(OC1=C2C(CC[C@H1](NC(=O)C)C3=CC(C(SC)=CC=C32)=O)=CC(OC)=C1OC)SCC
PUGREST.BadRequest: error:
O=C1C=CC=C2C([C@H1](CCC3=CC(OC)=C(OC)C(OC)=C32)NC(C)=O)=C1SC
PUGREST.BadRequest: error:
C1=CC=C(C=C1)CN[C@H1]2CCC3=CC(OC)=C(OC)C(OC)=C3C4=CC=C(C(C=C42)=O)SCC
PUGREST.BadRequest: error:
CC=CCCCN[C@H1]1CCC2=CC(OC)=C(OC)C(OC)=C2C3=CC=C(SC)C(C=C13)=O
PUGREST.BadRequest: error:
OC1=C(C=CC=C1)CN2[C@H1]CCC3=CC(OC)=C(OC)C(OC)=C3C4=CC=C(SC)C(C=C24)=O
PUGREST.BadRequest: error:
COC1=C2C3=CC=C(NC)C(=O)C=C3[C@H1](CCC2=CC(OC)=C1OC)NCCCCCCCCSC
PUGREST.BadRequest: error:
COC1=CC=2CC[C@@H1](C=3C(C=2C(=C1OC)OC)=CC=C(NC)C(=O)C=3)NC(=O)CNC
PUGREST.BadRequest: error:
CSC1=CC=C2C(=CC1=O)[C@H1](CCC3=CC(OC)=C(OC)C(=C23)O)CNC(=O)C
PUGREST.BadRequest: error:
C1=C2[C@H1](CCC3=C(C(OC)=C(C(=C3)OC)OC)C2=CC=C(C1=O)NC)NC
PUGREST.BadRequest: error:
CC(=O)OC1=CC=C2C3=C(C(OC)=C(C=C3CC[C@@H1](C2=CC1=O)NC(=O)CO)CO)C
PUGREST.BadRequest: error:
CC(=O)OC=1C(=O)C=C2C(=CC=1)C3=C(OC)C(OC)=C(C=C3CC[C@@H1]2NC(C)=O)C(=O)C
PUGREST.BadRequest: error:
CC(=O)OC1=CC=C2C3=C(C(OC)=C(C=C3CC[C@@H1](C2=CC1=O)NC(=O)C)OC)OC(=O)C
PUGREST.BadRequest: error:
CCC(=O)OC1=CC=C2C3=C(C(OC)=C(C=C3CC[C@@H1](C2=CC1=O)OC(=O)C)OC)OC
PUGREST.BadRequest: error:
CCC(=O)N[C@H1]1CCC2=CC(=C(OC)C(OC)=C2C=3C1=CC(=O)C(=CC=3)OC(OC)=O)OC
PUGREST.BadRequest: error:
CC(C)C(OC1=CC=C2C3=C(CC[C@@H1](C2=CC1=O)NC(C)=O)C=C(OC)C(OC)=C3OC)(O)C=O
PUGREST.BadRequest: error:
CC(C)C(OC1=CC=C2C3=C(CC[C@@H1](C2=CC1=O)NC(C)=O)C=C(OC)C(OC)=C3OC=O)C
PUGREST.BadRequest: error:
CC(C)C(=O)OC1=CC=C2C3=C(C(OC)=C(C=C3CC[C@@H1](C2=CC1=O)NC(C)=O)OC)OC(=O)C
PUGREST.BadRequest: error:
CC(C)C(OC1=CC=C2C3=C(C=C(OC)C(OC)=C3OC)CC[C@@H1](C2=CC1=O)NC(=O)CC)(O)C
PUGREST.BadRequest: error:
CC(=O)OCC1=CN(N=N1)[C@H1]2CCC3=CC(OC)=C(OC)C(OC)=C3C=4C2=CC(=O)C(=CC=4)NCC
PUGREST.BadRequest: error:
C1=C(C2=C(OC)C(OC)=C1OC(=O)CCC)C[C@@H1](C3=CC(=O)C(SC)=CC=C32)NC(C)=O
PUGREST.BadRequest: error:
CC(=O)N[C@H1]1CCC2=CC(OC)=C(OC)C(=C2C3=CC=C(C(C=C31)=O)OCC4=CC=CC=C4)OOC
PUGREST.BadRequest: error:
O=C(C)N[C@H1]1CCC2=CC(=C(OC)C(=C2C=3C1=CC(C(=CC=3)OC(=O)OC)=O)CN)C(=O)COCC
PUGREST.BadRequest: error:
C=12C(=O)C(=CC=C3C=1[C@H1](CCC4=CC(OC)=C(OC)C(OC)=C34)NC(C)=O)C2
PUGREST.BadRequest: error:
```

```
CCC(=O)N[C@H1]1CCC2=C(C(OC)=C(C(OC)=C2)OC)C=3C1=CC(C(SC)=CC=3)=O
PUGREST.BadRequest: error:
CCC(=O)N[C@H1]1CCC2=CC(OC)=C(C(OC)=C2C=3C1=CC(=O)C(=CC=3)SC)OC(=O)OCC
PUGREST.BadRequest: error:
CC=1C(=CC(C(=CC=1)SC)=O)[C@H1](CCC2=CC(OC)=C(OC)C(=C2)OC)NC(=O)C
PUGREST.BadRequest: error:
CC(C)COCCN[C@H1]1CCC2=CC(OC)=C(C(=C2C=3C1=CC(C(=CC=3)SC)=O)OC)OC(=O)C
PUGREST.BadRequest: error:
CC(C)CCN[C@H1]1CCC2=C(C3=CC=C(SC)C(C=C31)=O)C(=C(C(OC)=C2)OC)OCCC
PUGREST.BadRequest: error:
CC(C)CCN[C@H1]1CCC2=C(C3=CC=C(SC)C(C=C31)=O)C(=C(C(OC)=C2)OC)OC(=O)C
PUGREST.BadRequest: error:
C1=C(OC)C(=C(OC)C2=C1CC[C@H1](NC(=O)C)C=3C2=CC=C(OC(=O)OC)C(C=3)=O)C
PUGREST.BadRequest: error:
O=C(C)N[C@H1]1CCC2=CC(OC)=C(C(OC)=C2C3=CC=C(C(=O)C=C13)OCCC)OOC
PUGREST.BadRequest: error:
O=C(N(C)C)NC1=CC=C2C3=C(OC)C(=C(OC)C=C3CC[C@@H1](C2=CC1=O)NCC)OOC
PUGREST.BadRequest: error:
O=C(N)N[C@H1]1CCC2=CC(OC)=C(C(OC)=C2C=3C1=CC(C(OC(=O)C)=CC=3)=O)OC
PUGREST.BadRequest: error:
O=C(C)N[C@H1]1CCC2=CC(OC)=C(C(OC)=C2C3=CC=C(C(=O)C=C31)OC(=O)O)CCOC
PUGREST.BadRequest: error:
CC(=O)N1[C@H1]CCC2=CC(OC)=C(OC)C(OC)=C2C3=CC=C(NC)C(C=C31)=O
PUGREST.BadRequest: error:
COC1=C2C3=CC=C(NC)C(=O)C=C3[C@H1](CCC2=CC(OC)=C1OC)NO
PUGREST.BadRequest: error:
C1[C@@H1](C2=CC(=O)C(NC)=CC=C2C3=C(C=C(OC)C(=C3OC)OC)C1)NCCSC
PUGREST.BadRequest: error:
CNC(C(C)O)N([C@H1]1CCC2=CC(=C(OC)C(OC)=C2C=3C1=CC(=O)C(=CC=3)NC)OCC)C
PUGREST.BadRequest: error:
CNC(C(C)O)N[C@@H1]1C=2C(C3=C(C=C(OC)C(OC)=C3OC)CC1)=CC=C(C(=O)C=2)NC
PUGREST.BadRequest: error:
CNC(=S)N[C@@H1]1C2=CC(=O)C(NC)=CC=C2C3=C(OC)C(=C(C=C3CC1)OC)OCO
PUGREST.BadRequest: error:
N([C@H1]1CCC2=C(C3=CC=C(C(C=C13)=O)NC)C(OC)=C(OC)C(OC)=C2)N=N
PUGREST.BadRequest: error:
C12=CC(C(NC)=CC=C1C3=C(C=C(C(OC)=C3OC)OC)CC[C@@H1]2NCCCCCCCCCF)=O
PUGREST.BadRequest: error:
COC1=C(OC)C(OC)=CC=2CC[C@@H1](C3=CC(=O)C(NC)=CC=C3C=21)NCCC4=CC=CC=C4NC
PUGREST.BadRequest: error:
COC1=C(OC)C(OC)=CC=2CC[C@@H1](C3=CC(=O)C(NC)=CC=C3C=21)N4C=C(N=N4)CCONC
PUGREST.BadRequest: error:
CN(C(=S)N[C@@H1]1C2=CC(=O)C(NC)=CC=C2C3=C(C(=C(OC)C=C3CC1)OC)OC)CC#N
PUGREST.BadRequest: error:
CC(C)(C)OC1=CN(N=N1)[C@H1]C2CC3=CC(OC)=C(OC)C(OC)=C3C4=CC=C(NC)C(=O)C=C42
PUGREST.BadRequest: error:
CC(C)CCCCCCN[C@@H1]1C2=CC(C(SC)=CC=C2C3=C(C(OC)=C(OC)C=C3CC1)OC)=O
PUGREST.BadRequest: error:
```

```
CC(C)(C)C(=O)N[C@@H1]1C=2C(C3=C(C(OC)=C(OC)C=C3CC1)OC)=CC=C(NC)C(=O)C=2
PUGREST.BadRequest: error:
OC(CCC(C)C)OCC1=CN(N=N1)[C@H1]CCC2=CC(OC)=C(OC)C(OC)=C2C3=CC=C(C(=O)C=C3)NC
PUGREST.BadRequest: error:
O=C(NCC1=CC=CC=C1Cl)N[C@H1]2CCC3=C(C(OC)=C(OC)C(OC)=C3)C4=CC=C(NC)C(=O)C=C24
PUGREST.BadRequest: error:
N([C@H1]1CCC2=C(C(OC)=C(OC)C(OC)=C2)C3=CC=C(NC)C(C=C13)=O)C(O)=O
PUGREST.BadRequest: error:
O=CC=CCC=CNNC(N[C@H1]1CCC2=CC(OC)=C(OC)C(OC)=C2C3=CC=C(NC)C(=O)C=C13)=O
PUGREST.BadRequest: error:
OC1=C(C=CC=C1)C=2N=NN(C=2)[C@H1]C3CC4=CC(OC)=C(OC)C(OC)=C4C5=CC=C(NC)C(=O)C=C53
PUGREST.BadRequest: error:
OC1=C(C=CC=C1)C=2N=NN(C=2)[C@H1]CCC3=CC(OC)=C(OC)C(OC)=C3C4=CC=C(NC)C(=O)C=C4
PUGREST.BadRequest: error:
C1[C@@H1](C2=CC(=O)C(NC)=CC=C2C3=C(C(=C(OC)C=C3C1)OC)OC)N4C=C(N=N4)COC
PUGREST.BadRequest: error:
O=C(OC(C)(C)C)N[C@H1]1CCC2=C(C(OC)=C(OC)C(OC)=C2)C3=CC=C(NC)C(=O)C=C13
PUGREST.BadRequest: error:
O=C(OCC)CN(OCC)C[C@@H1]1C2=CC(C(NC)=CC=C2C3=C(C(OC)=C(OC)C=C3CC1)OC)=O
PUGREST.BadRequest: error:
O=C(C)OC1=CN(N=N1)[C@H1]2CCC3=CC(OC)=C(OC)C(OC)=C3C4=CC=C(NC)C(=O)C=C24
PUGREST.BadRequest: error:
O=C(OCC)CN(CCC)C[C@H1]C1CC2=CC(OC)=C(OC)C(OC)=C2C3=CC=C(SC)C(=O)C=C31
PUGREST.BadRequest: error:  O=C(C(Cl)Cl)NC(=O)N[C@H1]1CCC2=C(C(OC)=C(OC)C(OC)=C2
)C3=CC=C(C(C=C31)=O)NCN(O)CCC=O
PUGREST.BadRequest: error:  O=C(C(Cl)Cl)NC(=O)N[C@H1]1CCC2=C(C(OC)=C(OC)C(OC)=C2
)C3=CC=C(C(C=C31)=O)NCC4=CC=CC=C4
PUGREST.BadRequest: error:
O=C(OCCC)OCC1=CN(N=N1)[C@H1]2CCC3=CC(OC)=C(OC)C(OC)=C3C4=CC=C(C(=O)C=C24)NCCO
PUGREST.BadRequest: error:  O=C(OCCC)OCC1=CN(N=N1)[C@H1]2CCC3=CC(OC)=C(OC)C(OC)=
C3C4=CC=C(C(=O)C=C42)NCC(=O)C
PUGREST.BadRequest: error:  O=C(OCC=C)NCC1=CN(N=N1)[C@H1]2CCC3=CC(OC)=C(OC)C(OC)
=C3C4=CC=C(C(=O)C=C24)NCC(=O)C
PUGREST.BadRequest: error:  O=C(C(Cl)Cl)NCC1=CN(N=N1)[C@H1]2CCC3=CC(OC)=C(OC)C(O
C)=C3C4=CC=C(C(=O)C=C24)NCC(=O)C
PUGREST.BadRequest: error:  O=C(C(Cl)Cl)NCC1=CN(N=N1)[C@H1]2CCC3=CC(OC)=C(OC)C(=
C3C=4C2=CC(C(NC)=CC=4)=O)OCCCl
PUGREST.BadRequest: error:
O=C(C)N[C@@H1]1CC(C2=C(C(OC)=C(OC)C=C2CC1)OCC3=CC=CC=C3)=O
PUGREST.BadRequest: error:
O=C(C(Cl)Cl)NC(=O)N[C@H1]1CCC2=CC(OC)=C(OC)C(OC)=C2C3=CC=C(C(C=C31)=O)NCC(=O)O
PUGREST.BadRequest: error:
O=C(OCC)C1=CN(N=N1)[C@H1]2CCC3=CC(OC)=C(OC)C(OC)=C3C4=CC=C(C(C=C24)=O)NCC(=O)C
PUGREST.BadRequest: error:
O=C(O)CN(CCO)C(=O)N[C@H1]1CCC2=CC(OC)=C(OC)C(OC)=C2C3=CC=C(C(C=C13)=O)NCC
PUGREST.BadRequest: error:
CC(C)OC(NCC1=CN(N=N1)[C@H1]2CCC3=CC(OC)=C(OC)C(OC)=C3C4=CC=CC(C=C24)=O)NC=O
PUGREST.BadRequest: error:
```

COC(NCCCOCC1=CN(N=N1)[C@H1]2CCC3=CC(OC)=C(OC)C(=C3C4=CC=C(NC)C(C=C24)=O)OC=O)=O
PUGREST.BadRequest: error:
C=1C(C)OC(=O)/N=C(/N[C@H1]2CCC3=C(C(OC)=C(OC)C(OC)=C3)C4=CC=C(NC)C(=O)C=C42)N=1
PUGREST.BadRequest: error:
C1=CN=CC=C1C2=CN(N=N2)[C@H1]3CCC4=C(C(OC)=C(OC)C(OC)=C4)C5=CC=C(C(=O)C=C35)NCO
PUGREST.BadRequest: error:
COC=C(OC)C(OC)=CCCC[C@H1](N1C=C(N=N1)COC(=O)C2=CC=NC=C2)C3=CC(=O)C(=CC=C3CNCN)C
PUGREST.BadRequest: error:
COC1=C(OC)C=CC=2CC[C@@H1](C3=CC(=O)C(NC)=CC=C3C1=2)N4C=C(N=N4)COCC5=CC=CC=C5OC
PUGREST.BadRequest: error:
CC(C)(O)C=CN([C@H1]1CCC2=CC(OC)=C(OC)C(OC)=C2C3=CC=C(C(C=C13)=O)NC)O
PUGREST.BadRequest: error:
C=12C(=CC(C(NC)=CC=1)=O)[C@H1](CCC3=CC(OC)=C(OC)C(=C32)OC)C(C)C=O
PUGREST.BadRequest: error:
NC(=O)NCC1=CN(N=N1)[C@H1]2CCC3=CC(OC)=C(OC)C(OC)=C3C4=CC=C(NC)C(=O)C=C24
PUGREST.BadRequest: error:
C1=CC(OC)=CC=C1CN[C@H1]2CCC3=C(C(OC)=C(OC)C(OC)=C3)C=4C2=CC(C(=CC=4)NC)=O
PUGREST.BadRequest: error:
N(C(C1=CC=CC=C1)=O)CCN[C@H1]2CCC3=CC(OC)=C(OC)C(OC)=C3C4=CC=C(C(C=C24)=O)SC
PUGREST.BadRequest: error:
N=NC(=C(C(OCC)=O)N[C@H1]1CCC2=CC(OC)=C(OC)C(OC)=C2C3=CC=C(C(C=C13)=O)N)C
PUGREST.BadRequest: error:
CNC=1C(=O)C=C2[C@H1](CCC3=C(C(OC)=C(OC)C(OC)=C3)C2=CC=1)NC(C(=O)O)=CN=CNC
PUGREST.BadRequest: error:
NN(C=C(CNC(CCCCCl)=O)N=N)[C@H1]1CCC2=CC(OC)=C(OC)C(OC)=C2C3=CC=C(NC)C(=O)C=C31
PUGREST.BadRequest: error:
N=NC1=CN(N=N1)[C@H1]2CCC3=CC(OC)=C(OC)C(=C3C4=CC=C(NC)C(C=C42)=O)OC
PUGREST.BadRequest: error: CC(C)(C)OC(=O)NCCC(NCC=1N=NN(C=1)[C@H1]CCC2=CC(OC)=C(OC)C=C2C3=CC=C(NC)C(C=C3)=O)OC=O
PUGREST.BadRequest: error: CC(C)(C)OCNCCC(=O)NCC1=CN(N=N1)[C@H1]C2CC3=CC(OC)=C(OC)C(OC)=C3C4=CC=C(C(=O)C=C24)NC=O
PUGREST.BadRequest: error:
C1(=CN(C(=O)OCC)N[C@@H1]2C=3C(C4=C(OC)C(OC)=C(OC)C=C4CC2)=CC=C(C(=O)C=3)NC)N=C1
PUGREST.BadRequest: error: CC(C)(C)OCNCCC(=O)NCC1=CN(N=N1)[C@@H1]2C3=CC(C(NC)=CC=C3C4=C(CC2)C=C(OC)C(=C4OC)NC)=O
PUGREST.BadRequest: error: CC(C)(C)OC(=O)NCCC(OCC=1N=NN(C=1)[C@H1]CCC2=CC(OC)=C(OC)C(=C2C3=CC=C(NC)C(=O)C=C3)O)C=O
PUGREST.BadRequest: error:
CC(C)OC(=O)/N=C(/N[C@H1]1CCC2=C(C(OC)=C(OC)C(OC)=C2)C3=CC=C(NC)C(=O)C=C13)N=N
PUGREST.BadRequest: error:
C=1N(N=NC=1C(OCC)=O)[C@H1]2CCC3=C(C(OC)=C(OC)C(OC)=C3)C4=CC=C(C(=O)C=C42)NCOCC
PUGREST.BadRequest: error:
O=CC=CN=NN[C@H1]1CCC2=C(C(OC)=C(OC)C(=C2)OC)C3=CC=C(NC)C(=O)C=C13
PUGREST.BadRequest: error: C1=CN=CC=C1C(NCC2=CN(N=N2)[C@H1]3CCC4=CC(OC)=C(OC)C(=C4C5=CC=C(C(C=C53)=O)OC)OO)C
PUGREST.BadRequest: error:
N=NN([C@H1]1CCC2=C(C(OC)=C(OC)C(OC)=C2)C3=CC=C(C(=O)C=C13)NC)C(=O)OCC
PUGREST.BadRequest: error:

```
CN(N([C@@H1]1C2=CC(=O)C(NC)=CC=C2C3=CC(=C(OC)C=C3CC1)OC)O)CNC(=S)NCCCCC
PUGREST.BadRequest: error:
CN(N([C@@H1]1C2=CC(=O)C(NC)=CC=C2C3=CC(=C(OC)C=C3CC1)OC)O)CNC(=S)CCCCCC
PUGREST.BadRequest: error:
O=C(CC)OCC1=CN(N=N1)[C@H1]2CCC3=CC(OC)=C(OC)C(OC)=C3C4=CC=C(NC)C(=O)C=C24
PUGREST.BadRequest: error:  O=C(OC(C)(C)C)NCC=1N=NN(C=1)[C@H1]C2CC3=CC(OC)=C(OC)
C(OC)=C3C4=CC=C(NC)C(=O)C=C42
PUGREST.BadRequest: error:
O=C(C(C)C)NCN[C@H1]1CCC2=CC(OC)=C(OC)C(OC)=C2C3=CC=C(NC)C(=O)C=C13
PUGREST.BadRequest: error:  O=C(OCC)C=1N(N=NC=1C(=O)O2)[C@H1]2NC(=S)N[C@H1]3CCC4
=CC(OC)=C(OC)C(OC)=C4C=5C3=CC(C(=CC=5)NC)=O
PUGREST.BadRequest: error:  CC(O)(C)CC(=O)OCC1=CN(N=N1)[C@H1]2CCC3=CC(OC)=C(OC)C
(OC)=C3C=4C2=CC(C(=CC=4)NC)=O
PUGREST.BadRequest: error:
CC(C)COC(NCC1=CN(N=N1)[C@H1]C2CC3=CC(OC)=C(OC)C(OC)=C3C4=CC=C(C(=O)C=C24)NC)=O
PUGREST.BadRequest: error:
COCNCC1=CN(N=N1)[C@H1]2CCC3=CC(OC)=C(OC)C(OC)=C3C4=CC=C(NC)C(=O)C=C24
PUGREST.BadRequest: error:  CC(C)(C)OC(NCC=1N=NN(C=1)[C@H1]2CCC3=CC(OC)=C(OC)C(O
C)=C3C4=CC=C(C(C=C24)=O)N)C=O
PUGREST.BadRequest: error:
N(C)C1=CC=C2C3=C(OC)C(OC)=C(OC)C=C3CC[C@@H1](C2=CC1=O)NC(C(=O)O)CCONCOCNCOC
PUGREST.BadRequest: error:
O=C(OCC)C1=C(C(OCC)=O)N=NN1[C@H1]CCC2=CC(OC)=C(OC)C(OC)=C2C3=CC=C(NC)C(C=C3)=O
PUGREST.BadRequest: error:
N([C@H1]1CCC2=CC(OC)=C(OC)C(OC)=C2C3=CC=C(NC)C(C=C13)=O)C=CCOC(=O)CCCC=O
PUGREST.BadRequest: error:
CC(CC)N[C@H1]C1CC2=CC(OC)=C(OC)C(OC)=C2C3=CC=C(C(C=C31)=O)NCC
PUGREST.BadRequest: error:
CC(CC)N[C@H1]C1CC2=CC(OC)=C(OC)C(OC)=C2C3=CC=C(NC)C(=O)C=C31
PUGREST.BadRequest: error:
COC1=C2C3=CC=C(NC)C(=O)C=C3[C@@H1](NCC(CCC)C)CCC2=CC(OC)=C1OC
PUGREST.BadRequest: error:
COC1=C2C3=CC=C(NC)C(=O)C=C3[C@H1](CCC2=CC(OC)=C1OC)NCC4=CC=NS4
PUGREST.BadRequest: error:
COC1=C2C3=CC=C(SC)C(=O)C=C3[C@H1](CCC2=CC(OC)=C1OC)NCCCCl
PUGREST.BadRequest: error:
O(C1=C(OC)C=2C3=CC=C(SC)C(C=C3[C@H1](CCC=2C=C1OC)NC(C)CC=C)=O)C
PUGREST.BadRequest: error:
C1=CN=CC=C1CN[C@H1]2CCC3=CC(OC)=C(OC)C(OC)=C3C4=CC=C(C(C=C24)=O)NCC
PUGREST.BadRequest: error:
O=CC1=CN=NN1[C@H1]2CCC3=CC(OC)=C(OC)C(OC)=C3C4=CC=C(C(C=C42)=O)NC
PUGREST.BadRequest: error:
NC(CCN[C@H1]1CCC2=CC(OC)=C(OC)C(OC)=C2C=3C1=CC(=O)C=CC=3)NCF
PUGREST.BadRequest: error:
NC(N=C1C=CC2)=CC=C1C3=C(OC)C(OC)=C(OC)C=C3CC[C@@H1]2NC(N)=O
PUGREST.BadRequest: error:
C=CC1=C(NC)C(OC)=C(OC)C=C1CC[C@H1](NC(C)=O)C2=CC(C(=CC=C2)NC)=O
PUGREST.BadRequest: error:
```

```
CC(N)(CCO)[C@H1]1CCC2=CC(OC)=C(OC)C(OC)=C2C3=CC=C(NC)C(=O)C=C31
PUGREST.BadRequest: error:
N(NCCCCCl)[C@H1]1CCC2=CC(OC)=C(OC)C(OC)=C2C3=CC=C(NC)C(=O)C=C31
PUGREST.BadRequest: error:
N(C1=CC=C(C=C1)C)CN[C@H1]2CCC3=CC(OC)=C(OC)C(OC)=C3C=4C2=CC(C(NC)=CC=4)=O
PUGREST.BadRequest: error:
N(C1=CC=C(C=C1)C)CN[C@H1]2CCC3=CC(OC)=C(OC)C(OC)=C3C=4C2=CC(C(=CC=4)SC)=O
PUGREST.BadRequest: error:
CCN(CC)C(=O)N[C@H1]C1CC2=CC(OC)=C(OC)C(OC)=C2C3=CC=C(C(C=C31)=O)NC
PUGREST.BadRequest: error:
CCN(CC)C(=O)N1[C@H1]CCC2=CC(OC)=C(OC)C(OC)=C2C3=CC=C(NC)C(C=C31)=O
PUGREST.BadRequest: error:
CCN(CC)C(=O)N[C@H1]1CCC2=CC(OC)=C(OC)C(OC)=C2C3=CC=C(C(C=C31)=O)NCC
PUGREST.BadRequest: error:
CC(C)COC1=C(OC)C(OC)=CC=2CC[C@H1](NC(=O)C)C3=CC(C(SC)=CC=C3C1=2)=O
PUGREST.BadRequest: error:
C=C(C)CN(CCO)C(=S)N[C@H1]1CCC2=C(C(OC)=C(OC)C(OC)=C2)C3=CC=C(NC)C(C=C31)=O
PUGREST.BadRequest: error:
CC(C)C(OC=C(C(OC)=CCCC[C@H1](NC(=O)C)C1=CC(C(SC)=CC=C1C)=O)C)=O
PUGREST.BadRequest: error:
N([C@H1]1CCC2=CC(OC)=C(OC)C(OC)=C2C3=CC=C(NC)C(=O)C=C31)=C(Cl)C=C
PUGREST.BadRequest: error:
O=C(N[C@H1]1CCC2=CC(OC)=C(OC)C(OC)=C2C3=CC=C(C(C=C31)=O)NCN)C4=CC=CC=C4
PUGREST.BadRequest: error:
O=C(N[C@@H1]1C2=CC(C(NC)=CC=C2C3=C(CC1)C=C(OC)C(OC)=C3OC)=O)N=N
PUGREST.BadRequest: error:
N([C@H1]1CCC2=CC(OC)=C(OC)C(OC)=C2C3=CC=C(NC)C(C=C13)=O)CNC(=O)OCCONC
PUGREST.BadRequest: error:
O=C(N[C@H1]1CCC2=CC(OC)=C(OC)C(OC)=C2C3=CC=C(NC)C(=O)C=C31)CCOC(=O)O
PUGREST.BadRequest: error:
C1=CCC[C@H1](NC(=S)NC2=CC=C(C=C2)C(F)(F)F)CC(C1)=CC=C(NC)C(=O)COCOC
PUGREST.BadRequest: error:
CNCC(O)(C)C1=CN(N=N1)[C@H1]2CCC3=CC(OC)=C(OC)C(OC)=C3C4=CC=C(C(=O)C=C24)NC
PUGREST.BadRequest: error:
N1([C@H1]2CCC3=C(C(OC)=C(OC)C(OC)=C3)C4=CC=C(NC)C(C=C24)=O)C(O)=C1C
PUGREST.BadRequest: error:
N([C@H1]1CCC2=C(C(OC)=C(OC)C(OC)=C2)C3=CC=C(NC)C(C=C13)=O)NC=CC=CCl
PUGREST.BadRequest: error:
C1=C(O)C(OC)=CC(CN[C@H1]2CCC3=C(C(OC)=C(OC)C(OC)=C3)C4=CC=C(SC)C(=O)C=C42)=C1C
PUGREST.BadRequest: error:
COCC(C)=C(C(OC)=CCCC[C@H1](NCC)C1=CC(C(SC)=CC=C1)=O)OC
PUGREST.BadRequest: error:
C[C@H1](CCC1=CC(OC)=C(OC)C(OC)=C1C2=CC=C(C(=O)C=C2)NCN)CCO
PUGREST.BadRequest: error:
NC(=O)N1[C@@H1]C2=CC(C(NC)=CC=C2C3=C(OC)C(OC)=C(OC)C=C3CC1)=O
PUGREST.BadRequest: error:
NN(CCO)[C@@H1]1C2=CC(C(NC)=CC=C2C3=C(OC)C(OC)=C(C=C3CC1)OC)=O
PUGREST.BadRequest: error:
```

```
C1=2[C@H1](CCC3=CC(OC)=C(OC)C(OC)=C3C1=CC=C(NC)C(=O)C=2NC)CO
PUGREST.BadRequest: error:
NC(N=NCOCCC)C[C@H1]1C2=CC(C(NC)=CC=C2C3=C(CC1)C=C(OC)C(OC)=C3OC)=O
PUGREST.BadRequest: error:
C1=2C(C3=C(OC)C(OC)=C(OC)C=C3CC[C@@H1]1NC(C)CC)=CC=C(NC)C(=O)C=2NC
PUGREST.BadRequest: error:
C1=C(CN[C@H1]2CCC3=CC(OC)=C(OC)C(OC)=C3C4=CC=C(NC)C(=O)C=C42)C=NC=C1
PUGREST.BadRequest: error:
C1[C@H1](NC(NC2=CC=C(C=C2)F)=O)C3=CC(C(NC)=CC=C3C4=C(OC)C(OC)=CC=C4OCC1)=O
PUGREST.BadRequest: error:
C1=CC(=CC=N1)CCN[C@@H1]2C3=CC(C(SC)=CC=C3C4=C(CC2)C=C(OC)C(OC)=C4OC)=O
PUGREST.BadRequest: error:
C1=2[C@H1]CCC3=CC(OC)=C(OC)C(OC)=C3C1=CC=C(C(=O)C=2)NCNC(C)=O
PUGREST.BadRequest: error:
CC=CC1=C(C=C(OC)C(OC)=C1OC(OCC)=O)CC[C@H1](NC(C)=O)C2=CC(C(SC)=CC=C2)=O
PUGREST.BadRequest: error:
O=C1C=C2[C@H1](CCC3=C(C(OC)=C(OC)C(OC)=C3)C2=CC=C1SC)NC(N(C(C)C)C(Cl)=O)NC
PUGREST.BadRequest: error:
O=C1C=C2[C@H1](CCC3=C(C(OC)=C(OC)C(OC)=C3)C2=CC=C1SC)NC(C(=O)OCC)=CNC
PUGREST.BadRequest: error:
O=C1C=C2[C@H1](CCC3=C(C(OC)=C(OC)C(OC)=C3)C2=CC=C1SC)NC(C(C)C)=O
PUGREST.BadRequest: error:
N1C=NN=NN1[C@H1]2CCC3=C(C(OC)=C(OC)C(OC)=C3)C4=CC=C(C(C=C42)=O)NCCC
PUGREST.BadRequest: error:
CNC=1C(=O)C=C2[C@H1](CCC3=CC(OC)=C(OC)C(OC)=C3C2=CC=1N)C(C(C)C)=O
PUGREST.BadRequest: error:
N([C@H1]1CCC2=CC(OC)=C(OC)C(OC)=C2C3=CC=C(NC)C(C=C31)=O)CCCCF
PUGREST.BadRequest: error:
C[C@H1](CCC1=CC(OC)=C(OC)C(OC)=C1C2=CC=C(C(C=C2)=O)SC)NC/C=C/C3=CC=CC=C3
PUGREST.BadRequest: error:
N([C@H1]1CCC2=C(C(OC)=C(OC)C(OC)=C2)C3=CC=C(SC)C(C=C31)=O)CCCO
PUGREST.BadRequest: error:
C=CC1=C(C(=O)OC=CC=C2C3=C(OC)C(OC)=C(OC)C=C3CC[C@H1](NC(C)=O)C2=C1SC)C
PUGREST.BadRequest: error:
C=C1C2=C(C=C(OC)C(OC)=C2OC(OCC)=O)CC[C@H1](NC(C)=O)C3=CC(C(NC)=CC=C13)=O
PUGREST.BadRequest: error:
CC=CC(N[C@H1]1CCC2=C(C(OC)=C(OC)C(OC)=C2)C3=CC=C(SC)C(C=C13)=O)CCOC
PUGREST.BadRequest: error:
OC(=O)NCC1=CN(N=N1)[C@@H1]2C=3C(C4=C(C(OC)=C(OC)C=C4CC2)OC)=CC=C(NC)C(C=3)=O
PUGREST.BadRequest: error:  N(C)C=1C(=O)C=C2[C@H1](CCC3=C(C(OC)=C(OC)C(OC)=C3)C2
=CC=1)N4C(C(=O)O)=C(N=N4)C(=O)NNC
PUGREST.BadRequest: error:  O=C(C=C(C(O1)CCCCCC1)CN=N)N[C@H1]2CCC3=CC(OC)=C(OC)C
(OC)=C3C=4C2=CC(C(NC)=CC=4)=O
PUGREST.BadRequest: error:
N([C@H1]1CCC2=C(C(OC)=C(OC)C(OC)=C2)C3=CC=C(NC)C(=O)C=C13)CNCC4=CC=C(C=C4)C=O
PUGREST.BadRequest: error:  O=C(C=C(Cl)C=CN([C@H1]1CCC2=C(C(OC)=C(OC)C(OC)=C2)C3
=CC=C(NC)C(C=C13)=O)OC(=O)CC)NC(OC4=CC=CC=C4)=O
PUGREST.BadRequest: error:  CC(C)(C)OC(=O)/N=C(/N[C@H1]1CCC2=C(C(OC)=C(OC)C(OC)=
```

```
C2)C3=CC=C(NC)C(C=C31)=O)NCOC(C)=O
PUGREST.BadRequest: error:  CC(C)(C)OC(=O)/N=C(/N[C@H1]1CCC2=C(C(OC)=C(OC)C(OC)=
C2)C3=CC=C(NC)C(C=C13)=O)NCOC(C)(F)F
PUGREST.BadRequest: error:
C12=C3C(C4=C(OC)C(OC)=C(OC)C=C4CC[C@@H1]3NC(=O)N)=CC=C1NC=C2
```

[6]:
```
compound = pcp.get_compounds(data_['SMILES'][2], 'smiles')
```

[7]:
```
compound
```

[7]: [Compound()]

[8]:
```
compound[0].cid
```

[9]:
```
compound[0].canonical_smiles
```

[10]:
```
data_.CID.unique()
```

[10]:
```
array([          nan, 0.00000000e+00, 6.35135500e+06, 1.62648725e+08,
       1.64628185e+08, 1.62672356e+08])
```

[ ]:
```

```

[11]:
```
len(data_.CID.unique())
```

[11]: 6

[12]:
```
data_.to_excel('../Data/PubChemSearch_colchicyne_selected.xlsx')
```

Notebook

December 19, 2023

1 File 48

[1]:
```
import pandas as pd

import os
import shutil

from rdkit import Chem
from rdkit.Chem import Draw
from rdkit.Chem import AllChem
```

[2]:
```
colchicine = 'CC(=O)NC1CCC2=CC(=C(C(=C2C3=CC=C(C(=O)C=C13)OC)OC)OC)OC'
```

[3]:
```
def create_PDB_from_SMILES(smiles, name):
    try:
        mol = Chem.MolFromSmiles(smiles)
    except:
        print("There is an error when preparing molecule: "+str(smiles))

    try:
        new_mol = Chem.AddHs(mol)
        AllChem.EmbedMolecule(new_mol, randomSeed=0xf00d)
    except:
        print("Error:"+str(smiles))

    try:
        Chem.rdmolfiles.MolToPDBFile(new_mol,␣
↪"3D_structure"+'_'+str(name)+'_'+str(smiles[0:30])+'.pdb')
    except:
        print("Something went wrong while creating PDB file...")

    return new_mol
```

[4]:
```
col = create_PDB_from_SMILES(colchicine, 'raw_colchicine')
```

[5]:
```
col
```

[5]:

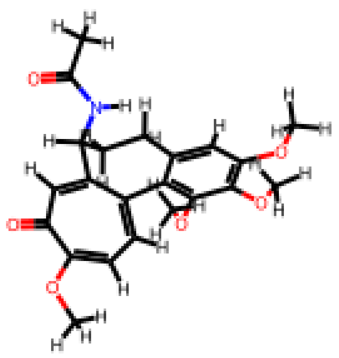

Notebook

December 19, 2023

1 File 49

[1]:
```
import pandas as pd

import os
import shutil

from rdkit import Chem
from rdkit.Chem import Draw
from rdkit.Chem import AllChem

import glob
```

[2]:
```
excel_sheet = glob.glob('*.xlsx')
```

[3]:
```
excel_sheet
```

[3]:
```
['Whole_report.xlsx']
```

[4]:
```
dat_list = pd.read_excel(excel_sheet[0])
```

[5]:
```
dat_list.head()
```

[5]:
```
   Structure                                             SMILES  SYBA score  \
0        NaN  COC1=C2C3=CC=C(NC)C(=O)C=C3[C@H1](CCC2=CC(OC)=...       45.88
1        NaN  COC1=C2C3=CC=C(NC)C(=O)C=C3[C@H1](CCC2=CC(OC)=...       68.25
2        NaN  COC1=C2C3=CC=C(NC)C(=O)C=C3[C@H1](CCC2=CC(OC)=...       74.22
3        NaN  COC1=C2C3=CC=C(NC)C(=O)C=C3[C@H1](CCC2=CC(OC)=...       40.43
4        NaN  O=CC1=CN=NN1[C@H1]2CCC3=CC(OC)=C(OC)C(OC)=C3C4...       41.84

   A549 [nM]  BALB/3T3 [nM]  LoVo [nM]  LoVo/DX [nM]  MCF-7 [nM]
0         12              6          7           145           8
1         12              9         15            39          10
2         12             30         17            29           9
3         12             23          6           235           5
4         12             11          3          1425          12
```

1.1 Create directories

[6]:
```
#to get the current working directory
directory = os.getcwd()
directory
```

[6]:
```
'C:\\Users\\aleks\\Documents\\GitHub\\Kolchicyna\\Molecular docking'
```

[7]:
```
list_dir = []

dat_list['destenation'] = 0

for i, structure in enumerate(dat_list['SMILES']):
    os.mkdir(os.path.join(directory, str(str(i+1)+'_lig'+str(i+1)+'_')))
    dat_list['destenation'][i] = os.path.join(directory,␣
↪str(str(i+1)+'_lig'+str(i+1)+'_'))
    list_dir.append(os.path.join(directory, str(str(i+1)+'_lig'+str(i+1)+'_')))
```

```
C:\Users\aleks\AppData\Local\Temp\ipykernel_31364\2301388344.py:7:
SettingWithCopyWarning:
A value is trying to be set on a copy of a slice from a DataFrame

See the caveats in the documentation: https://pandas.pydata.org/pandas-
docs/stable/user_guide/indexing.html#returning-a-view-versus-a-copy
  dat_list['destenation'][i] = os.path.join(directory,
str(str(i+1)+'_lig'+str(i+1)+'_'))
C:\Users\aleks\anaconda3\envs\cheminf_gpu\lib\site-
packages\pandas\core\indexing.py:1637: SettingWithCopyWarning:
A value is trying to be set on a copy of a slice from a DataFrame

See the caveats in the documentation: https://pandas.pydata.org/pandas-
docs/stable/user_guide/indexing.html#returning-a-view-versus-a-copy
  self._setitem_single_block(indexer, value, name)
```

[8]:
```
tmp_ = pd.DataFrame(data=list_dir, columns=['Name'])
tmp_.to_excel('to_be_moved_to.xlsx')
list_dir
```

[8]:
```
['C:\\Users\\aleks\\Documents\\GitHub\\Kolchicyna\\Molecular docking\\1_lig1_',
 'C:\\Users\\aleks\\Documents\\GitHub\\Kolchicyna\\Molecular docking\\2_lig2_',
 'C:\\Users\\aleks\\Documents\\GitHub\\Kolchicyna\\Molecular docking\\3_lig3_',
 'C:\\Users\\aleks\\Documents\\GitHub\\Kolchicyna\\Molecular docking\\4_lig4_',
 'C:\\Users\\aleks\\Documents\\GitHub\\Kolchicyna\\Molecular docking\\5_lig5_',
 'C:\\Users\\aleks\\Documents\\GitHub\\Kolchicyna\\Molecular docking\\6_lig6_',
 'C:\\Users\\aleks\\Documents\\GitHub\\Kolchicyna\\Molecular docking\\7_lig7_',
 'C:\\Users\\aleks\\Documents\\GitHub\\Kolchicyna\\Molecular docking\\8_lig8_',
 'C:\\Users\\aleks\\Documents\\GitHub\\Kolchicyna\\Molecular docking\\9_lig9_',
 'C:\\Users\\aleks\\Documents\\GitHub\\Kolchicyna\\Molecular
```

```
docking\\10_lig10_',
 'C:\\Users\\aleks\\Documents\\GitHub\\Kolchicyna\\Molecular
docking\\11_lig11_',
 'C:\\Users\\aleks\\Documents\\GitHub\\Kolchicyna\\Molecular
docking\\12_lig12_',
 'C:\\Users\\aleks\\Documents\\GitHub\\Kolchicyna\\Molecular
docking\\13_lig13_',
 'C:\\Users\\aleks\\Documents\\GitHub\\Kolchicyna\\Molecular
docking\\14_lig14_',
 'C:\\Users\\aleks\\Documents\\GitHub\\Kolchicyna\\Molecular
docking\\15_lig15_',
 'C:\\Users\\aleks\\Documents\\GitHub\\Kolchicyna\\Molecular
docking\\16_lig16_',
 'C:\\Users\\aleks\\Documents\\GitHub\\Kolchicyna\\Molecular
docking\\17_lig17_',
 'C:\\Users\\aleks\\Documents\\GitHub\\Kolchicyna\\Molecular
docking\\18_lig18_',
 'C:\\Users\\aleks\\Documents\\GitHub\\Kolchicyna\\Molecular
docking\\19_lig19_',
 'C:\\Users\\aleks\\Documents\\GitHub\\Kolchicyna\\Molecular
docking\\20_lig20_',
 'C:\\Users\\aleks\\Documents\\GitHub\\Kolchicyna\\Molecular
docking\\21_lig21_',
 'C:\\Users\\aleks\\Documents\\GitHub\\Kolchicyna\\Molecular
docking\\22_lig22_',
 'C:\\Users\\aleks\\Documents\\GitHub\\Kolchicyna\\Molecular
docking\\23_lig23_',
 'C:\\Users\\aleks\\Documents\\GitHub\\Kolchicyna\\Molecular
docking\\24_lig24_',
 'C:\\Users\\aleks\\Documents\\GitHub\\Kolchicyna\\Molecular
docking\\25_lig25_',
 'C:\\Users\\aleks\\Documents\\GitHub\\Kolchicyna\\Molecular
docking\\26_lig26_',
 'C:\\Users\\aleks\\Documents\\GitHub\\Kolchicyna\\Molecular
docking\\27_lig27_',
 'C:\\Users\\aleks\\Documents\\GitHub\\Kolchicyna\\Molecular
docking\\28_lig28_',
 'C:\\Users\\aleks\\Documents\\GitHub\\Kolchicyna\\Molecular
docking\\29_lig29_',
 'C:\\Users\\aleks\\Documents\\GitHub\\Kolchicyna\\Molecular
docking\\30_lig30_',
 'C:\\Users\\aleks\\Documents\\GitHub\\Kolchicyna\\Molecular
docking\\31_lig31_',
 'C:\\Users\\aleks\\Documents\\GitHub\\Kolchicyna\\Molecular
docking\\32_lig32_',
 'C:\\Users\\aleks\\Documents\\GitHub\\Kolchicyna\\Molecular
docking\\33_lig33_',
```

```
 'C:\\Users\\aleks\\Documents\\GitHub\\Kolchicyna\\Molecular
docking\\34_lig34_',
 'C:\\Users\\aleks\\Documents\\GitHub\\Kolchicyna\\Molecular
docking\\35_lig35_',
 'C:\\Users\\aleks\\Documents\\GitHub\\Kolchicyna\\Molecular
docking\\36_lig36_',
 'C:\\Users\\aleks\\Documents\\GitHub\\Kolchicyna\\Molecular
docking\\37_lig37_',
 'C:\\Users\\aleks\\Documents\\GitHub\\Kolchicyna\\Molecular
docking\\38_lig38_',
 'C:\\Users\\aleks\\Documents\\GitHub\\Kolchicyna\\Molecular
docking\\39_lig39_',
 'C:\\Users\\aleks\\Documents\\GitHub\\Kolchicyna\\Molecular
docking\\40_lig40_',
 'C:\\Users\\aleks\\Documents\\GitHub\\Kolchicyna\\Molecular
docking\\41_lig41_',
 'C:\\Users\\aleks\\Documents\\GitHub\\Kolchicyna\\Molecular
docking\\42_lig42_',
 'C:\\Users\\aleks\\Documents\\GitHub\\Kolchicyna\\Molecular
docking\\43_lig43_',
 'C:\\Users\\aleks\\Documents\\GitHub\\Kolchicyna\\Molecular
docking\\44_lig44_',
 'C:\\Users\\aleks\\Documents\\GitHub\\Kolchicyna\\Molecular
docking\\45_lig45_',
 'C:\\Users\\aleks\\Documents\\GitHub\\Kolchicyna\\Molecular
docking\\46_lig46_',
 'C:\\Users\\aleks\\Documents\\GitHub\\Kolchicyna\\Molecular
docking\\47_lig47_',
 'C:\\Users\\aleks\\Documents\\GitHub\\Kolchicyna\\Molecular
docking\\48_lig48_',
 'C:\\Users\\aleks\\Documents\\GitHub\\Kolchicyna\\Molecular
docking\\49_lig49_',
 'C:\\Users\\aleks\\Documents\\GitHub\\Kolchicyna\\Molecular
docking\\50_lig50_',
 'C:\\Users\\aleks\\Documents\\GitHub\\Kolchicyna\\Molecular
docking\\51_lig51_',
 'C:\\Users\\aleks\\Documents\\GitHub\\Kolchicyna\\Molecular
docking\\52_lig52_',
 'C:\\Users\\aleks\\Documents\\GitHub\\Kolchicyna\\Molecular
docking\\53_lig53_',
 'C:\\Users\\aleks\\Documents\\GitHub\\Kolchicyna\\Molecular
docking\\54_lig54_',
 'C:\\Users\\aleks\\Documents\\GitHub\\Kolchicyna\\Molecular
docking\\55_lig55_',
 'C:\\Users\\aleks\\Documents\\GitHub\\Kolchicyna\\Molecular
docking\\56_lig56_',
 'C:\\Users\\aleks\\Documents\\GitHub\\Kolchicyna\\Molecular
```

```
docking\\57_lig57_',
 'C:\\Users\\aleks\\Documents\\GitHub\\Kolchicyna\\Molecular
docking\\58_lig58_',
 'C:\\Users\\aleks\\Documents\\GitHub\\Kolchicyna\\Molecular
docking\\59_lig59_',
 'C:\\Users\\aleks\\Documents\\GitHub\\Kolchicyna\\Molecular
docking\\60_lig60_',
 'C:\\Users\\aleks\\Documents\\GitHub\\Kolchicyna\\Molecular
docking\\61_lig61_',
 'C:\\Users\\aleks\\Documents\\GitHub\\Kolchicyna\\Molecular
docking\\62_lig62_',
 'C:\\Users\\aleks\\Documents\\GitHub\\Kolchicyna\\Molecular
docking\\63_lig63_',
 'C:\\Users\\aleks\\Documents\\GitHub\\Kolchicyna\\Molecular
docking\\64_lig64_',
 'C:\\Users\\aleks\\Documents\\GitHub\\Kolchicyna\\Molecular
docking\\65_lig65_',
 'C:\\Users\\aleks\\Documents\\GitHub\\Kolchicyna\\Molecular
docking\\66_lig66_',
 'C:\\Users\\aleks\\Documents\\GitHub\\Kolchicyna\\Molecular
docking\\67_lig67_',
 'C:\\Users\\aleks\\Documents\\GitHub\\Kolchicyna\\Molecular
docking\\68_lig68_',
 'C:\\Users\\aleks\\Documents\\GitHub\\Kolchicyna\\Molecular
docking\\69_lig69_',
 'C:\\Users\\aleks\\Documents\\GitHub\\Kolchicyna\\Molecular
docking\\70_lig70_',
 'C:\\Users\\aleks\\Documents\\GitHub\\Kolchicyna\\Molecular
docking\\71_lig71_',
 'C:\\Users\\aleks\\Documents\\GitHub\\Kolchicyna\\Molecular
docking\\72_lig72_',
 'C:\\Users\\aleks\\Documents\\GitHub\\Kolchicyna\\Molecular
docking\\73_lig73_',
 'C:\\Users\\aleks\\Documents\\GitHub\\Kolchicyna\\Molecular
docking\\74_lig74_',
 'C:\\Users\\aleks\\Documents\\GitHub\\Kolchicyna\\Molecular
docking\\75_lig75_',
 'C:\\Users\\aleks\\Documents\\GitHub\\Kolchicyna\\Molecular
docking\\76_lig76_',
 'C:\\Users\\aleks\\Documents\\GitHub\\Kolchicyna\\Molecular
docking\\77_lig77_',
 'C:\\Users\\aleks\\Documents\\GitHub\\Kolchicyna\\Molecular
docking\\78_lig78_',
 'C:\\Users\\aleks\\Documents\\GitHub\\Kolchicyna\\Molecular
docking\\79_lig79_',
 'C:\\Users\\aleks\\Documents\\GitHub\\Kolchicyna\\Molecular
docking\\80_lig80_',
```

```
 'C:\\Users\\aleks\\Documents\\GitHub\\Kolchicyna\\Molecular
docking\\81_lig81_',
 'C:\\Users\\aleks\\Documents\\GitHub\\Kolchicyna\\Molecular
docking\\82_lig82_',
 'C:\\Users\\aleks\\Documents\\GitHub\\Kolchicyna\\Molecular
docking\\83_lig83_',
 'C:\\Users\\aleks\\Documents\\GitHub\\Kolchicyna\\Molecular
docking\\84_lig84_',
 'C:\\Users\\aleks\\Documents\\GitHub\\Kolchicyna\\Molecular
docking\\85_lig85_',
 'C:\\Users\\aleks\\Documents\\GitHub\\Kolchicyna\\Molecular
docking\\86_lig86_',
 'C:\\Users\\aleks\\Documents\\GitHub\\Kolchicyna\\Molecular
docking\\87_lig87_',
 'C:\\Users\\aleks\\Documents\\GitHub\\Kolchicyna\\Molecular
docking\\88_lig88_',
 'C:\\Users\\aleks\\Documents\\GitHub\\Kolchicyna\\Molecular
docking\\89_lig89_',
 'C:\\Users\\aleks\\Documents\\GitHub\\Kolchicyna\\Molecular
docking\\90_lig90_',
 'C:\\Users\\aleks\\Documents\\GitHub\\Kolchicyna\\Molecular
docking\\91_lig91_',
 'C:\\Users\\aleks\\Documents\\GitHub\\Kolchicyna\\Molecular
docking\\92_lig92_',
 'C:\\Users\\aleks\\Documents\\GitHub\\Kolchicyna\\Molecular
docking\\93_lig93_',
 'C:\\Users\\aleks\\Documents\\GitHub\\Kolchicyna\\Molecular
docking\\94_lig94_',
 'C:\\Users\\aleks\\Documents\\GitHub\\Kolchicyna\\Molecular
docking\\95_lig95_',
 'C:\\Users\\aleks\\Documents\\GitHub\\Kolchicyna\\Molecular
docking\\96_lig96_',
 'C:\\Users\\aleks\\Documents\\GitHub\\Kolchicyna\\Molecular
docking\\97_lig97_',
 'C:\\Users\\aleks\\Documents\\GitHub\\Kolchicyna\\Molecular
docking\\98_lig98_',
 'C:\\Users\\aleks\\Documents\\GitHub\\Kolchicyna\\Molecular
docking\\99_lig99_',
 'C:\\Users\\aleks\\Documents\\GitHub\\Kolchicyna\\Molecular
docking\\100_lig100_',
 'C:\\Users\\aleks\\Documents\\GitHub\\Kolchicyna\\Molecular
docking\\101_lig101_',
 'C:\\Users\\aleks\\Documents\\GitHub\\Kolchicyna\\Molecular
docking\\102_lig102_',
 'C:\\Users\\aleks\\Documents\\GitHub\\Kolchicyna\\Molecular
docking\\103_lig103_',
 'C:\\Users\\aleks\\Documents\\GitHub\\Kolchicyna\\Molecular
```

```
docking\\104_lig104_',
 'C:\\Users\\aleks\\Documents\\GitHub\\Kolchicyna\\Molecular
docking\\105_lig105_',
 'C:\\Users\\aleks\\Documents\\GitHub\\Kolchicyna\\Molecular
docking\\106_lig106_',
 'C:\\Users\\aleks\\Documents\\GitHub\\Kolchicyna\\Molecular
docking\\107_lig107_',
 'C:\\Users\\aleks\\Documents\\GitHub\\Kolchicyna\\Molecular
docking\\108_lig108_',
 'C:\\Users\\aleks\\Documents\\GitHub\\Kolchicyna\\Molecular
docking\\109_lig109_',
 'C:\\Users\\aleks\\Documents\\GitHub\\Kolchicyna\\Molecular
docking\\110_lig110_',
 'C:\\Users\\aleks\\Documents\\GitHub\\Kolchicyna\\Molecular
docking\\111_lig111_',
 'C:\\Users\\aleks\\Documents\\GitHub\\Kolchicyna\\Molecular
docking\\112_lig112_',
 'C:\\Users\\aleks\\Documents\\GitHub\\Kolchicyna\\Molecular
docking\\113_lig113_',
 'C:\\Users\\aleks\\Documents\\GitHub\\Kolchicyna\\Molecular
docking\\114_lig114_',
 'C:\\Users\\aleks\\Documents\\GitHub\\Kolchicyna\\Molecular
docking\\115_lig115_',
 'C:\\Users\\aleks\\Documents\\GitHub\\Kolchicyna\\Molecular
docking\\116_lig116_',
 'C:\\Users\\aleks\\Documents\\GitHub\\Kolchicyna\\Molecular
docking\\117_lig117_',
 'C:\\Users\\aleks\\Documents\\GitHub\\Kolchicyna\\Molecular
docking\\118_lig118_',
 'C:\\Users\\aleks\\Documents\\GitHub\\Kolchicyna\\Molecular
docking\\119_lig119_',
 'C:\\Users\\aleks\\Documents\\GitHub\\Kolchicyna\\Molecular
docking\\120_lig120_',
 'C:\\Users\\aleks\\Documents\\GitHub\\Kolchicyna\\Molecular
docking\\121_lig121_',
 'C:\\Users\\aleks\\Documents\\GitHub\\Kolchicyna\\Molecular
docking\\122_lig122_',
 'C:\\Users\\aleks\\Documents\\GitHub\\Kolchicyna\\Molecular
docking\\123_lig123_',
 'C:\\Users\\aleks\\Documents\\GitHub\\Kolchicyna\\Molecular
docking\\124_lig124_',
 'C:\\Users\\aleks\\Documents\\GitHub\\Kolchicyna\\Molecular
docking\\125_lig125_',
 'C:\\Users\\aleks\\Documents\\GitHub\\Kolchicyna\\Molecular
docking\\126_lig126_',
 'C:\\Users\\aleks\\Documents\\GitHub\\Kolchicyna\\Molecular
docking\\127_lig127_',
```

```
 'C:\\Users\\aleks\\Documents\\GitHub\\Kolchicyna\\Molecular
docking\\128_lig128_',
 'C:\\Users\\aleks\\Documents\\GitHub\\Kolchicyna\\Molecular
docking\\129_lig129_',
 'C:\\Users\\aleks\\Documents\\GitHub\\Kolchicyna\\Molecular
docking\\130_lig130_',
 'C:\\Users\\aleks\\Documents\\GitHub\\Kolchicyna\\Molecular
docking\\131_lig131_',
 'C:\\Users\\aleks\\Documents\\GitHub\\Kolchicyna\\Molecular
docking\\132_lig132_',
 'C:\\Users\\aleks\\Documents\\GitHub\\Kolchicyna\\Molecular
docking\\133_lig133_',
 'C:\\Users\\aleks\\Documents\\GitHub\\Kolchicyna\\Molecular
docking\\134_lig134_',
 'C:\\Users\\aleks\\Documents\\GitHub\\Kolchicyna\\Molecular
docking\\135_lig135_',
 'C:\\Users\\aleks\\Documents\\GitHub\\Kolchicyna\\Molecular
docking\\136_lig136_',
 'C:\\Users\\aleks\\Documents\\GitHub\\Kolchicyna\\Molecular
docking\\137_lig137_',
 'C:\\Users\\aleks\\Documents\\GitHub\\Kolchicyna\\Molecular
docking\\138_lig138_',
 'C:\\Users\\aleks\\Documents\\GitHub\\Kolchicyna\\Molecular
docking\\139_lig139_',
 'C:\\Users\\aleks\\Documents\\GitHub\\Kolchicyna\\Molecular
docking\\140_lig140_',
 'C:\\Users\\aleks\\Documents\\GitHub\\Kolchicyna\\Molecular
docking\\141_lig141_',
 'C:\\Users\\aleks\\Documents\\GitHub\\Kolchicyna\\Molecular
docking\\142_lig142_',
 'C:\\Users\\aleks\\Documents\\GitHub\\Kolchicyna\\Molecular
docking\\143_lig143_',
 'C:\\Users\\aleks\\Documents\\GitHub\\Kolchicyna\\Molecular
docking\\144_lig144_',
 'C:\\Users\\aleks\\Documents\\GitHub\\Kolchicyna\\Molecular
docking\\145_lig145_',
 'C:\\Users\\aleks\\Documents\\GitHub\\Kolchicyna\\Molecular
docking\\146_lig146_',
 'C:\\Users\\aleks\\Documents\\GitHub\\Kolchicyna\\Molecular
docking\\147_lig147_',
 'C:\\Users\\aleks\\Documents\\GitHub\\Kolchicyna\\Molecular
docking\\148_lig148_',
 'C:\\Users\\aleks\\Documents\\GitHub\\Kolchicyna\\Molecular
docking\\149_lig149_',
 'C:\\Users\\aleks\\Documents\\GitHub\\Kolchicyna\\Molecular
docking\\150_lig150_',
 'C:\\Users\\aleks\\Documents\\GitHub\\Kolchicyna\\Molecular
```

```
docking\\151_lig151_',
 'C:\\Users\\aleks\\Documents\\GitHub\\Kolchicyna\\Molecular
docking\\152_lig152_',
 'C:\\Users\\aleks\\Documents\\GitHub\\Kolchicyna\\Molecular
docking\\153_lig153_',
 'C:\\Users\\aleks\\Documents\\GitHub\\Kolchicyna\\Molecular
docking\\154_lig154_',
 'C:\\Users\\aleks\\Documents\\GitHub\\Kolchicyna\\Molecular
docking\\155_lig155_',
 'C:\\Users\\aleks\\Documents\\GitHub\\Kolchicyna\\Molecular
docking\\156_lig156_',
 'C:\\Users\\aleks\\Documents\\GitHub\\Kolchicyna\\Molecular
docking\\157_lig157_',
 'C:\\Users\\aleks\\Documents\\GitHub\\Kolchicyna\\Molecular
docking\\158_lig158_',
 'C:\\Users\\aleks\\Documents\\GitHub\\Kolchicyna\\Molecular
docking\\159_lig159_',
 'C:\\Users\\aleks\\Documents\\GitHub\\Kolchicyna\\Molecular
docking\\160_lig160_',
 'C:\\Users\\aleks\\Documents\\GitHub\\Kolchicyna\\Molecular
docking\\161_lig161_',
 'C:\\Users\\aleks\\Documents\\GitHub\\Kolchicyna\\Molecular
docking\\162_lig162_',
 'C:\\Users\\aleks\\Documents\\GitHub\\Kolchicyna\\Molecular
docking\\163_lig163_',
 'C:\\Users\\aleks\\Documents\\GitHub\\Kolchicyna\\Molecular
docking\\164_lig164_',
 'C:\\Users\\aleks\\Documents\\GitHub\\Kolchicyna\\Molecular
docking\\165_lig165_',
 'C:\\Users\\aleks\\Documents\\GitHub\\Kolchicyna\\Molecular
docking\\166_lig166_',
 'C:\\Users\\aleks\\Documents\\GitHub\\Kolchicyna\\Molecular
docking\\167_lig167_',
 'C:\\Users\\aleks\\Documents\\GitHub\\Kolchicyna\\Molecular
docking\\168_lig168_',
 'C:\\Users\\aleks\\Documents\\GitHub\\Kolchicyna\\Molecular
docking\\169_lig169_',
 'C:\\Users\\aleks\\Documents\\GitHub\\Kolchicyna\\Molecular
docking\\170_lig170_',
 'C:\\Users\\aleks\\Documents\\GitHub\\Kolchicyna\\Molecular
docking\\171_lig171_',
 'C:\\Users\\aleks\\Documents\\GitHub\\Kolchicyna\\Molecular
docking\\172_lig172_',
 'C:\\Users\\aleks\\Documents\\GitHub\\Kolchicyna\\Molecular
docking\\173_lig173_',
 'C:\\Users\\aleks\\Documents\\GitHub\\Kolchicyna\\Molecular
docking\\174_lig174_',
```

```
 'C:\\Users\\aleks\\Documents\\GitHub\\Kolchicyna\\Molecular
docking\\175_lig175_',
 'C:\\Users\\aleks\\Documents\\GitHub\\Kolchicyna\\Molecular
docking\\176_lig176_',
 'C:\\Users\\aleks\\Documents\\GitHub\\Kolchicyna\\Molecular
docking\\177_lig177_',
 'C:\\Users\\aleks\\Documents\\GitHub\\Kolchicyna\\Molecular
docking\\178_lig178_',
 'C:\\Users\\aleks\\Documents\\GitHub\\Kolchicyna\\Molecular
docking\\179_lig179_',
 'C:\\Users\\aleks\\Documents\\GitHub\\Kolchicyna\\Molecular
docking\\180_lig180_',
 'C:\\Users\\aleks\\Documents\\GitHub\\Kolchicyna\\Molecular
docking\\181_lig181_',
 'C:\\Users\\aleks\\Documents\\GitHub\\Kolchicyna\\Molecular
docking\\182_lig182_',
 'C:\\Users\\aleks\\Documents\\GitHub\\Kolchicyna\\Molecular
docking\\183_lig183_',
 'C:\\Users\\aleks\\Documents\\GitHub\\Kolchicyna\\Molecular
docking\\184_lig184_',
 'C:\\Users\\aleks\\Documents\\GitHub\\Kolchicyna\\Molecular
docking\\185_lig185_',
 'C:\\Users\\aleks\\Documents\\GitHub\\Kolchicyna\\Molecular
docking\\186_lig186_',
 'C:\\Users\\aleks\\Documents\\GitHub\\Kolchicyna\\Molecular
docking\\187_lig187_',
 'C:\\Users\\aleks\\Documents\\GitHub\\Kolchicyna\\Molecular
docking\\188_lig188_',
 'C:\\Users\\aleks\\Documents\\GitHub\\Kolchicyna\\Molecular
docking\\189_lig189_',
 'C:\\Users\\aleks\\Documents\\GitHub\\Kolchicyna\\Molecular
docking\\190_lig190_',
 'C:\\Users\\aleks\\Documents\\GitHub\\Kolchicyna\\Molecular
docking\\191_lig191_',
 'C:\\Users\\aleks\\Documents\\GitHub\\Kolchicyna\\Molecular
docking\\192_lig192_',
 'C:\\Users\\aleks\\Documents\\GitHub\\Kolchicyna\\Molecular
docking\\193_lig193_',
 'C:\\Users\\aleks\\Documents\\GitHub\\Kolchicyna\\Molecular
docking\\194_lig194_',
 'C:\\Users\\aleks\\Documents\\GitHub\\Kolchicyna\\Molecular
docking\\195_lig195_',
 'C:\\Users\\aleks\\Documents\\GitHub\\Kolchicyna\\Molecular
docking\\196_lig196_',
 'C:\\Users\\aleks\\Documents\\GitHub\\Kolchicyna\\Molecular
docking\\197_lig197_',
 'C:\\Users\\aleks\\Documents\\GitHub\\Kolchicyna\\Molecular
```

```
docking\\198_lig198_',
 'C:\\Users\\aleks\\Documents\\GitHub\\Kolchicyna\\Molecular
docking\\199_lig199_',
 'C:\\Users\\aleks\\Documents\\GitHub\\Kolchicyna\\Molecular
docking\\200_lig200_',
 'C:\\Users\\aleks\\Documents\\GitHub\\Kolchicyna\\Molecular
docking\\201_lig201_',
 'C:\\Users\\aleks\\Documents\\GitHub\\Kolchicyna\\Molecular
docking\\202_lig202_',
 'C:\\Users\\aleks\\Documents\\GitHub\\Kolchicyna\\Molecular
docking\\203_lig203_',
 'C:\\Users\\aleks\\Documents\\GitHub\\Kolchicyna\\Molecular
docking\\204_lig204_',
 'C:\\Users\\aleks\\Documents\\GitHub\\Kolchicyna\\Molecular
docking\\205_lig205_',
 'C:\\Users\\aleks\\Documents\\GitHub\\Kolchicyna\\Molecular
docking\\206_lig206_',
 'C:\\Users\\aleks\\Documents\\GitHub\\Kolchicyna\\Molecular
docking\\207_lig207_',
 'C:\\Users\\aleks\\Documents\\GitHub\\Kolchicyna\\Molecular
docking\\208_lig208_',
 'C:\\Users\\aleks\\Documents\\GitHub\\Kolchicyna\\Molecular
docking\\209_lig209_',
 'C:\\Users\\aleks\\Documents\\GitHub\\Kolchicyna\\Molecular
docking\\210_lig210_',
 'C:\\Users\\aleks\\Documents\\GitHub\\Kolchicyna\\Molecular
docking\\211_lig211_',
 'C:\\Users\\aleks\\Documents\\GitHub\\Kolchicyna\\Molecular
docking\\212_lig212_',
 'C:\\Users\\aleks\\Documents\\GitHub\\Kolchicyna\\Molecular
docking\\213_lig213_',
 'C:\\Users\\aleks\\Documents\\GitHub\\Kolchicyna\\Molecular
docking\\214_lig214_',
 'C:\\Users\\aleks\\Documents\\GitHub\\Kolchicyna\\Molecular
docking\\215_lig215_',
 'C:\\Users\\aleks\\Documents\\GitHub\\Kolchicyna\\Molecular
docking\\216_lig216_',
 'C:\\Users\\aleks\\Documents\\GitHub\\Kolchicyna\\Molecular
docking\\217_lig217_',
 'C:\\Users\\aleks\\Documents\\GitHub\\Kolchicyna\\Molecular
docking\\218_lig218_',
 'C:\\Users\\aleks\\Documents\\GitHub\\Kolchicyna\\Molecular
docking\\219_lig219_',
 'C:\\Users\\aleks\\Documents\\GitHub\\Kolchicyna\\Molecular
docking\\220_lig220_',
 'C:\\Users\\aleks\\Documents\\GitHub\\Kolchicyna\\Molecular
docking\\221_lig221_',
```

```
 'C:\\Users\\aleks\\Documents\\GitHub\\Kolchicyna\\Molecular
docking\\222_lig222_',
 'C:\\Users\\aleks\\Documents\\GitHub\\Kolchicyna\\Molecular
docking\\223_lig223_',
 'C:\\Users\\aleks\\Documents\\GitHub\\Kolchicyna\\Molecular
docking\\224_lig224_',
 'C:\\Users\\aleks\\Documents\\GitHub\\Kolchicyna\\Molecular
docking\\225_lig225_',
 'C:\\Users\\aleks\\Documents\\GitHub\\Kolchicyna\\Molecular
docking\\226_lig226_',
 'C:\\Users\\aleks\\Documents\\GitHub\\Kolchicyna\\Molecular
docking\\227_lig227_',
 'C:\\Users\\aleks\\Documents\\GitHub\\Kolchicyna\\Molecular
docking\\228_lig228_',
 'C:\\Users\\aleks\\Documents\\GitHub\\Kolchicyna\\Molecular
docking\\229_lig229_',
 'C:\\Users\\aleks\\Documents\\GitHub\\Kolchicyna\\Molecular
docking\\230_lig230_',
 'C:\\Users\\aleks\\Documents\\GitHub\\Kolchicyna\\Molecular
docking\\231_lig231_',
 'C:\\Users\\aleks\\Documents\\GitHub\\Kolchicyna\\Molecular
docking\\232_lig232_',
 'C:\\Users\\aleks\\Documents\\GitHub\\Kolchicyna\\Molecular
docking\\233_lig233_',
 'C:\\Users\\aleks\\Documents\\GitHub\\Kolchicyna\\Molecular
docking\\234_lig234_',
 'C:\\Users\\aleks\\Documents\\GitHub\\Kolchicyna\\Molecular
docking\\235_lig235_',
 'C:\\Users\\aleks\\Documents\\GitHub\\Kolchicyna\\Molecular
docking\\236_lig236_',
 'C:\\Users\\aleks\\Documents\\GitHub\\Kolchicyna\\Molecular
docking\\237_lig237_',
 'C:\\Users\\aleks\\Documents\\GitHub\\Kolchicyna\\Molecular
docking\\238_lig238_',
 'C:\\Users\\aleks\\Documents\\GitHub\\Kolchicyna\\Molecular
docking\\239_lig239_',
 'C:\\Users\\aleks\\Documents\\GitHub\\Kolchicyna\\Molecular
docking\\240_lig240_',
 'C:\\Users\\aleks\\Documents\\GitHub\\Kolchicyna\\Molecular
docking\\241_lig241_',
 'C:\\Users\\aleks\\Documents\\GitHub\\Kolchicyna\\Molecular
docking\\242_lig242_',
 'C:\\Users\\aleks\\Documents\\GitHub\\Kolchicyna\\Molecular
docking\\243_lig243_',
 'C:\\Users\\aleks\\Documents\\GitHub\\Kolchicyna\\Molecular
docking\\244_lig244_',
 'C:\\Users\\aleks\\Documents\\GitHub\\Kolchicyna\\Molecular
```

```
docking\\245_lig245_',
 'C:\\Users\\aleks\\Documents\\GitHub\\Kolchicyna\\Molecular
docking\\246_lig246_',
 'C:\\Users\\aleks\\Documents\\GitHub\\Kolchicyna\\Molecular
docking\\247_lig247_',
 'C:\\Users\\aleks\\Documents\\GitHub\\Kolchicyna\\Molecular
docking\\248_lig248_',
 'C:\\Users\\aleks\\Documents\\GitHub\\Kolchicyna\\Molecular
docking\\249_lig249_',
 'C:\\Users\\aleks\\Documents\\GitHub\\Kolchicyna\\Molecular
docking\\250_lig250_',
 'C:\\Users\\aleks\\Documents\\GitHub\\Kolchicyna\\Molecular
docking\\251_lig251_',
 'C:\\Users\\aleks\\Documents\\GitHub\\Kolchicyna\\Molecular
docking\\252_lig252_',
 'C:\\Users\\aleks\\Documents\\GitHub\\Kolchicyna\\Molecular
docking\\253_lig253_',
 'C:\\Users\\aleks\\Documents\\GitHub\\Kolchicyna\\Molecular
docking\\254_lig254_',
 'C:\\Users\\aleks\\Documents\\GitHub\\Kolchicyna\\Molecular
docking\\255_lig255_',
 'C:\\Users\\aleks\\Documents\\GitHub\\Kolchicyna\\Molecular
docking\\256_lig256_',
 'C:\\Users\\aleks\\Documents\\GitHub\\Kolchicyna\\Molecular
docking\\257_lig257_',
 'C:\\Users\\aleks\\Documents\\GitHub\\Kolchicyna\\Molecular
docking\\258_lig258_',
 'C:\\Users\\aleks\\Documents\\GitHub\\Kolchicyna\\Molecular
docking\\259_lig259_',
 'C:\\Users\\aleks\\Documents\\GitHub\\Kolchicyna\\Molecular
docking\\260_lig260_',
 'C:\\Users\\aleks\\Documents\\GitHub\\Kolchicyna\\Molecular
docking\\261_lig261_',
 'C:\\Users\\aleks\\Documents\\GitHub\\Kolchicyna\\Molecular
docking\\262_lig262_',
 'C:\\Users\\aleks\\Documents\\GitHub\\Kolchicyna\\Molecular
docking\\263_lig263_',
 'C:\\Users\\aleks\\Documents\\GitHub\\Kolchicyna\\Molecular
docking\\264_lig264_',
 'C:\\Users\\aleks\\Documents\\GitHub\\Kolchicyna\\Molecular
docking\\265_lig265_',
 'C:\\Users\\aleks\\Documents\\GitHub\\Kolchicyna\\Molecular
docking\\266_lig266_',
 'C:\\Users\\aleks\\Documents\\GitHub\\Kolchicyna\\Molecular
docking\\267_lig267_',
 'C:\\Users\\aleks\\Documents\\GitHub\\Kolchicyna\\Molecular
docking\\268_lig268_',
```

```
 'C:\\Users\\aleks\\Documents\\GitHub\\Kolchicyna\\Molecular
docking\\269_lig269_',
 'C:\\Users\\aleks\\Documents\\GitHub\\Kolchicyna\\Molecular
docking\\270_lig270_',
 'C:\\Users\\aleks\\Documents\\GitHub\\Kolchicyna\\Molecular
docking\\271_lig271_',
 'C:\\Users\\aleks\\Documents\\GitHub\\Kolchicyna\\Molecular
docking\\272_lig272_',
 'C:\\Users\\aleks\\Documents\\GitHub\\Kolchicyna\\Molecular
docking\\273_lig273_',
 'C:\\Users\\aleks\\Documents\\GitHub\\Kolchicyna\\Molecular
docking\\274_lig274_',
 'C:\\Users\\aleks\\Documents\\GitHub\\Kolchicyna\\Molecular
docking\\275_lig275_',
 'C:\\Users\\aleks\\Documents\\GitHub\\Kolchicyna\\Molecular
docking\\276_lig276_',
 'C:\\Users\\aleks\\Documents\\GitHub\\Kolchicyna\\Molecular
docking\\277_lig277_',
 'C:\\Users\\aleks\\Documents\\GitHub\\Kolchicyna\\Molecular
docking\\278_lig278_',
 'C:\\Users\\aleks\\Documents\\GitHub\\Kolchicyna\\Molecular
docking\\279_lig279_',
 'C:\\Users\\aleks\\Documents\\GitHub\\Kolchicyna\\Molecular
docking\\280_lig280_',
 'C:\\Users\\aleks\\Documents\\GitHub\\Kolchicyna\\Molecular
docking\\281_lig281_',
 'C:\\Users\\aleks\\Documents\\GitHub\\Kolchicyna\\Molecular
docking\\282_lig282_',
 'C:\\Users\\aleks\\Documents\\GitHub\\Kolchicyna\\Molecular
docking\\283_lig283_',
 'C:\\Users\\aleks\\Documents\\GitHub\\Kolchicyna\\Molecular
docking\\284_lig284_',
 'C:\\Users\\aleks\\Documents\\GitHub\\Kolchicyna\\Molecular
docking\\285_lig285_',
 'C:\\Users\\aleks\\Documents\\GitHub\\Kolchicyna\\Molecular
docking\\286_lig286_',
 'C:\\Users\\aleks\\Documents\\GitHub\\Kolchicyna\\Molecular
docking\\287_lig287_',
 'C:\\Users\\aleks\\Documents\\GitHub\\Kolchicyna\\Molecular
docking\\288_lig288_',
 'C:\\Users\\aleks\\Documents\\GitHub\\Kolchicyna\\Molecular
docking\\289_lig289_',
 'C:\\Users\\aleks\\Documents\\GitHub\\Kolchicyna\\Molecular
docking\\290_lig290_',
 'C:\\Users\\aleks\\Documents\\GitHub\\Kolchicyna\\Molecular
docking\\291_lig291_',
 'C:\\Users\\aleks\\Documents\\GitHub\\Kolchicyna\\Molecular
```

```
docking\\292_lig292_',
 'C:\\Users\\aleks\\Documents\\GitHub\\Kolchicyna\\Molecular
docking\\293_lig293_',
 'C:\\Users\\aleks\\Documents\\GitHub\\Kolchicyna\\Molecular
docking\\294_lig294_',
 'C:\\Users\\aleks\\Documents\\GitHub\\Kolchicyna\\Molecular
docking\\295_lig295_',
 'C:\\Users\\aleks\\Documents\\GitHub\\Kolchicyna\\Molecular
docking\\296_lig296_',
 'C:\\Users\\aleks\\Documents\\GitHub\\Kolchicyna\\Molecular
docking\\297_lig297_',
 'C:\\Users\\aleks\\Documents\\GitHub\\Kolchicyna\\Molecular
docking\\298_lig298_',
 'C:\\Users\\aleks\\Documents\\GitHub\\Kolchicyna\\Molecular
docking\\299_lig299_',
 'C:\\Users\\aleks\\Documents\\GitHub\\Kolchicyna\\Molecular
docking\\300_lig300_',
 'C:\\Users\\aleks\\Documents\\GitHub\\Kolchicyna\\Molecular
docking\\301_lig301_',
 'C:\\Users\\aleks\\Documents\\GitHub\\Kolchicyna\\Molecular
docking\\302_lig302_',
 'C:\\Users\\aleks\\Documents\\GitHub\\Kolchicyna\\Molecular
docking\\303_lig303_',
 'C:\\Users\\aleks\\Documents\\GitHub\\Kolchicyna\\Molecular
docking\\304_lig304_',
 'C:\\Users\\aleks\\Documents\\GitHub\\Kolchicyna\\Molecular
docking\\305_lig305_',
 'C:\\Users\\aleks\\Documents\\GitHub\\Kolchicyna\\Molecular
docking\\306_lig306_',
 'C:\\Users\\aleks\\Documents\\GitHub\\Kolchicyna\\Molecular
docking\\307_lig307_',
 'C:\\Users\\aleks\\Documents\\GitHub\\Kolchicyna\\Molecular
docking\\308_lig308_',
 'C:\\Users\\aleks\\Documents\\GitHub\\Kolchicyna\\Molecular
docking\\309_lig309_',
 'C:\\Users\\aleks\\Documents\\GitHub\\Kolchicyna\\Molecular
docking\\310_lig310_',
 'C:\\Users\\aleks\\Documents\\GitHub\\Kolchicyna\\Molecular
docking\\311_lig311_',
 'C:\\Users\\aleks\\Documents\\GitHub\\Kolchicyna\\Molecular
docking\\312_lig312_',
 'C:\\Users\\aleks\\Documents\\GitHub\\Kolchicyna\\Molecular
docking\\313_lig313_',
 'C:\\Users\\aleks\\Documents\\GitHub\\Kolchicyna\\Molecular
docking\\314_lig314_',
 'C:\\Users\\aleks\\Documents\\GitHub\\Kolchicyna\\Molecular
docking\\315_lig315_',
```

```
 'C:\\Users\\aleks\\Documents\\GitHub\\Kolchicyna\\Molecular
docking\\316_lig316_',
 'C:\\Users\\aleks\\Documents\\GitHub\\Kolchicyna\\Molecular
docking\\317_lig317_',
 'C:\\Users\\aleks\\Documents\\GitHub\\Kolchicyna\\Molecular
docking\\318_lig318_',
 'C:\\Users\\aleks\\Documents\\GitHub\\Kolchicyna\\Molecular
docking\\319_lig319_',
 'C:\\Users\\aleks\\Documents\\GitHub\\Kolchicyna\\Molecular
docking\\320_lig320_',
 'C:\\Users\\aleks\\Documents\\GitHub\\Kolchicyna\\Molecular
docking\\321_lig321_',
 'C:\\Users\\aleks\\Documents\\GitHub\\Kolchicyna\\Molecular
docking\\322_lig322_',
 'C:\\Users\\aleks\\Documents\\GitHub\\Kolchicyna\\Molecular
docking\\323_lig323_',
 'C:\\Users\\aleks\\Documents\\GitHub\\Kolchicyna\\Molecular
docking\\324_lig324_',
 'C:\\Users\\aleks\\Documents\\GitHub\\Kolchicyna\\Molecular
docking\\325_lig325_',
 'C:\\Users\\aleks\\Documents\\GitHub\\Kolchicyna\\Molecular
docking\\326_lig326_',
 'C:\\Users\\aleks\\Documents\\GitHub\\Kolchicyna\\Molecular
docking\\327_lig327_',
 'C:\\Users\\aleks\\Documents\\GitHub\\Kolchicyna\\Molecular
docking\\328_lig328_',
 'C:\\Users\\aleks\\Documents\\GitHub\\Kolchicyna\\Molecular
docking\\329_lig329_',
 'C:\\Users\\aleks\\Documents\\GitHub\\Kolchicyna\\Molecular
docking\\330_lig330_',
 'C:\\Users\\aleks\\Documents\\GitHub\\Kolchicyna\\Molecular
docking\\331_lig331_',
 'C:\\Users\\aleks\\Documents\\GitHub\\Kolchicyna\\Molecular
docking\\332_lig332_',
 'C:\\Users\\aleks\\Documents\\GitHub\\Kolchicyna\\Molecular
docking\\333_lig333_',
 'C:\\Users\\aleks\\Documents\\GitHub\\Kolchicyna\\Molecular
docking\\334_lig334_',
 'C:\\Users\\aleks\\Documents\\GitHub\\Kolchicyna\\Molecular
docking\\335_lig335_',
 'C:\\Users\\aleks\\Documents\\GitHub\\Kolchicyna\\Molecular
docking\\336_lig336_',
 'C:\\Users\\aleks\\Documents\\GitHub\\Kolchicyna\\Molecular
docking\\337_lig337_']
```

1.2 Create mol objects instead of SMILES

[9]:
```
def create_PDB_from_SMILES(smiles, initial_dir, destenation_dir, name):
    try:
        mol = Chem.MolFromSmiles(smiles)
    except:
        print("There is an error when preparing molecule: "+str(smiles))

    try:
        new_mol = Chem.AddHs(mol)
        AllChem.EmbedMolecule(new_mol, randomSeed=0xf00d)
    except:
        print("Error:"+str(smiles))

    try:
        Chem.rdmolfiles.MolToPDBFile(new_mol,␣
↪"3D_structure"+'_'+str(name)+'_'+str(smiles[0:30])+'.pdb')
        move_generated_structure(initial_dir,␣
↪"3D_structure"+'_'+str(name)+'_'+str(smiles[0:30])+'.pdb', destenation_dir)
    except:
        print("Something went wrong while creating PDB file...")

    return new_mol
```

[10]:
```
def move_generated_structure(initial_dir, name_of_file, destenation_dir):
    ini = initial_dir+'\\'+name_of_file
    des = destenation_dir+'\\'+name_of_file
    shutil.move(ini, des)
    return print("Correctly moved...")
```

[11]:
```
try:
    for i, smile in enumerate(dat_list['SMILES']):
        create_PDB_from_SMILES(smile, directory, dat_list['destenation'][i],␣
↪'new_colchicine'+str(i))
        print(str(i)+' '+str(smile))
except:
    print("Error when creating PDB for "+str(smile)+' '+str(i))
```

```
Correctly moved…
0 COC1=C2C3=CC=C(NC)C(=O)C=C3[C@H1](CCC2=CC(OC)=C1OC)NCCONC
Correctly moved…
1 COC1=C2C3=CC=C(NC)C(=O)C=C3[C@H1](CCC2=CC(OC)=C1OC)NCCCCC
Correctly moved…
2 COC1=C2C3=CC=C(NC)C(=O)C=C3[C@H1](CCC2=CC(OC)=C1OC)NCCCCCC
Correctly moved…
3 COC1=C2C3=CC=C(NC)C(=O)C=C3[C@H1](CCC2=CC(OC)=C1OC)N4C=C(N=N4)CNCC
Correctly moved…
```

```
4 O=CC1=CN=NN1[C@H1]2CCC3=CC(OC)=C(OC)C(OC)=C3C4=CC=C(C(=O)C=C42)NCC
Correctly moved…
5 OC1=CC=C(C=C1)CN[C@H1]2CCC3=C(C(OC)=C(OC)C(OC)=C3)C4=CC=C(SC)C(=O)C=C24
Correctly moved…
6 OC1=CC=C(C=C1)CN[C@H1]2CCC3=CC(OC)=C(OC)C(OC)=C3C4=CC=C(C(C=C42)=O)SCC
Correctly moved…
7 O=CC1=C2[C@H1](CCC3=CC(OC)=C(OC)C(=C3C2=CC=C(SC)C1=O)OCC4=CC=CC=C4)ONC
Correctly moved…
8 C1=2[C@H1](CCC3=CC(OC)=C(OC)C(OC)=C3C1=CC=C(SC)C(=O)C=2)NC(C)CC=CSC
Correctly moved…
9 C1N(C(=S)N[C@@H1]2C3=CC(C(NC)=CC=C3C4=C(OC)C(OC)=C(OC)C=C4CC2)=O)C=C1
Correctly moved…
10 C1=2[C@H1](CCC3=CC(OC)=C(OC)C(OC)=C3C1=CC=C(C(C=2)=O)SCNC(C)=O)C
Correctly moved…
11 C[C@H1](CCC1=CC(OC)=C(OC)C(OC(OCC)=O)=C1C2=CC=C(SC)C(=O)C=C2)NC
Correctly moved…
12 COC1=C2C3=CC=C(NC)C(=O)C=C3[C@H1](CCC2=CC(OC)=C1OC)NCCC4=CC=CC=C4
Correctly moved…
13 COC1=C2C3=CC=C(NC)C(=O)C=C3[C@H1](CCC2=CC(OC)=C1OC)NC/C=C/C4=CC=CC=C4
Correctly moved…
14 CC(=O)N[C@H1]1CCC2=CC(OC)=C(OC)C(OC)=C2C=3C1=CC(=O)C(SC)=CC=3N
Correctly moved…
15 C1=2C(=O)C(=C3C=C1C4=C(CC[C@@H1]3NC(C)=O)C=C(OC)C(OC)=C4OC(OC)=O)C=2
Correctly moved…
16 CC(=O)OC1=CC=C2C3=C(C(OC)=C(C=C3CC[C@@H1](C2=CC1=O)OC(=O)C)OC)NC(=O)C
Correctly moved…
17 CC(=O)OC1=CC=C2C3=C(C(OC)=C(C=C3CC[C@@H1](C2=CC1=O)OC(=O)C)OC)NC(=O)COC
Correctly moved…
18 CC(=O)OC1=CC=C2C3=C(C(OC)=C(C=C3CC[C@@H1](C2=CC1=O)OC(=O)C)OC)OC
Correctly moved…
19 CC(C)C(OC1=CC=C2C3=C(CC[C@@H1](C2=CC1=O)NC(C)=O)C=C(OC)C(OC)=C3OC)(O)C
Correctly moved…
20 CC(=O)N[C@@H1]1C2=CC(C(SC)=CC=C2C3=C(OC)C(=C(OC)C=C3CC1)OC(=O)C4=CC=CC=C4)=O
Correctly moved…
21 O=C(OC)OC1=CC=C2C3=C(C(OC)=C(C=C3CC[C@@H1](C2=CC1=O)NC(=O)C)OC)OC(=O)C
Correctly moved…
22 O=C(C)N[C@H1]1CCC2=CC(=C(OC)C(=C2C=3C1=CC(C(SC)=CC=3)=O)OC(=O)CC)COC
Correctly moved…
23 CC(=O)OCC1=CN(N=N1)[C@H1]2CCC3=CC(OC)=C(OC)C(OC)=C3C=4C2=CC(=O)C(NC)=CC=4C
Correctly moved…
24 CC(=O)OCC1=CN(N=N1)[C@H1]2CCC3=CC(OC)=C(OC)C(=C3C=4C2=CC(C(NC)=CC=4)=O)OC
Correctly moved…
25 CC(=O)N[C@@H1]1C2=CC(C(NC)=CC=C2C3=C(OC)C(OC)=C(OC)C=C3C1)=O
Correctly moved…
26 CC(=O)N[C@@H1]C1=C2C(C(NC)=CC=C1C3=C(OC)C(OC)=C(OC)C=C3CC2)=O
Correctly moved…
27 COC1=C2C3=CC=C(NC)C(=O)C=C3[C@@H1](NCCC)CCC2=CC(=C1OC)NC
Correctly moved…
```

28 COC1=C2C3=CC=C(NC)C(=O)C=C3[C@H1](CCC2=CC(OC)=C1OC)NN=NC=C
Correctly moved…
29 COC1=C2C3=CC=C(NC)C(=O)C=C3[C@@H1](NCCC)CCC2=CC(=C1OC)OSC
Correctly moved…
30 COC1=C2C3=CC=C(NC)C(=O)C=C3[C@H1](CCC2=CC(=C1OC)OC)NCCCS
Correctly moved…
31 COC1=C(OC)C(OC)=CC=2CC[C@H1](NC(=N)N)C3=CC(=O)C(=CC=C3C1=2)CNC
Correctly moved…
32 CNC(NCCCC)=S[C@@H1]C1=CC(=O)C(NC)=CC=C1C2=C(C=C(OC)C(=C2OC)OC)CCF
Correctly moved…
33 CN(C(=S)N[C@@H1]1C2=CC(=O)C(NC)=CC=C2C3=C(C=C(OC)C(OC)=C3OC)CC1C)C#N
Correctly moved…
34 CNC(C(C)C)N[C@@H1]1C=2C(C3=C(C=C(OC)C(OC)=C3OC)CC1)=CC=C(C(=O)C=2)NC
Correctly moved…
35 COC1=C(OC)C(OC)=CC=2CC[C@@H1](C3=CC(=O)C(NC)=CC=C3C=21)N4C=C(N=N4)CON
Correctly moved…
36 COC1=C(OC)C(OC)=CC=2CC[C@@H1](C3=CC(=O)C(NC)=CC=C3C=21)NCCC4CCCCC4
Correctly moved…
37 CC(C)(C)COC1=CC=C2C3=C(OC)C(OC)=C(OC)C=C3CC[C@@H1](C2=CC1=O)SC=O
Correctly moved…
38 C1=2[C@H1]CCC3=CC(OC)=C(OC)C(OC(CC)=O)=C3C1=CC=C(C(C=2)=O)SCNC(=O)CSC
Correctly moved…
39 OC(CCC(C)C)OCC1=CN(N=N1)[C@H1]2CCC3=CC(OC)=C(OC)C(OC)=C3C4=CC=C(NC)C(=O)C=C24
Correctly moved…
40
OC1=C(C=CC=C1)C=2N=NN(C=2)[C@H1]3CCC4=CC(OC)=C(OC)C(OC)=C4C5=CC=C(NC)C(=O)C=C35
Correctly moved…
41 NC1=CN(N=N1)[C@H1]2CCC3=C(C(OC)=C(OC)C(OC)=C3)C4=CC=C(NC)C(C=C24)=O
Correctly moved…
42 O=C(OCC)CN(CCO)N[C@H1]1CCC2=CC(OC)=C(OC)C(OC)=C2C3=CC=C(NC)C(=O)C=C13
Correctly moved…
43 O=C(C1=C(C(OCC)=O)N(N=N1)[C@@H1]C2=C3C(C(NC)=CC=C2C4=C(C(OC)=C(OC)C=C4CC3)OC)
=O)O
Correctly moved…
44 C1=2[C@H1](CCC3=CC(OC)=C(OC)C(OC)=C3C1=CC=C(C(=O)C=2)NCO)C(C(OC)=O)NC(=O)CNC
Correctly moved…
45 C=12N(N=NC=1C(OCC3=CC=C(C=C3)F)=O)C(OC)=C(OC)C(OC)=CC4=CC=C(C(=O)C=C24)NC
Correctly moved…
46 C=1N(N=NC=1C(=O)OCC2=CC=C(C=C2)F)CC(OC)=C(OC)C(OC)=CC3=CC=C(NC)C(=O)C=C3
Correctly moved…
47
O=CC1=C(C(OCC)=O)N(N=N1)[C@@H1]C2=C3C(C(NC)=CC=C2C4=C(C(OC)=C(OC)C=C4CC3)OC)=O
Correctly moved…
48 O=C(C(Cl)Cl)NC(=O)N[C@H1]1CCC2=C(C(OC)=C(OC)C(OC)=C2)C3=CC=C(NC)C(=O)C=C13
Correctly moved…
49
O=C(C(Cl)Cl)NCC1=CN(N=N1)[C@H1]2CCC3=CC(OC)=C(OC)C(OC)=C3C4=CC=C(C(=O)C=C42)NCCO
Correctly moved…

```
50 O=C(C(Cl)Cl)NC(=O)N[C@H1]1CCC2=C(C(OC)=C(OC)C(OC)=C2)C3=CC=C(C(C=C31)=O)NCC(=
O)OC
Correctly moved…
51 O=C(O)C=C(C(OCC)=O)N=NN[C@H1]1CCC2=CC(OC)=C(OC)C(OC)=C2C3=CC=C(NC)C(=O)C=C13
Correctly moved…
52
O=C(O)C1=C(C(OCC)=O)N=NN1[C@H1]2CCC3=CC(OC)=C(OC)C(OC)=C3C4=CC=C(NC)C(=O)C=C24
Correctly moved…
53 O=C(OCC)C1=CN(N=N1)[C@H1]2CCC3=CC(OC)=C(OC)C(=C3C4=CC=C(NC)C(C=C42)=O)OCC
Correctly moved…
54 O=C(O)C1=CN(N=N1)[C@H1]2CCC3=C(C(OC)=C(OC)C(OC)=C3)C4=CC=C(C(=O)C=C42)NCC
Correctly moved…
55 O=C(O)C1=C(C(OCC)=O)N=NN1[C@H1]CCC2=CC(OC)=C(OC)C(OC)=C2C3=CC=C(C(C=C3)=O)NC
Correctly moved…
56 CCCCCCCCCCCCCCCC(OCC1=CN(N=N1)[C@H1]C2CC3=CC(OC)=C(OC)C(OC)=C3C4=CC=C(C(=O)C=
C24)NC)=O
Correctly moved…
57
OC(=O)C1=C(C(OCC)=O)N(N=N1)[C@H1]C2CC3=CC(OC)=C(OC)C(OC)=C3C4=CC=C(NC)C(=O)C=C42
Correctly moved…
58 C1=C(OC)C=C(OC)C=C1CC[C@H1](C=CC(C(OC(C2=CC=CC=C2)=O)=CC=CC)=O)C
Correctly moved…
59 N(C)C(=O)NCCC(OCC1=CN(N=N1)[C@H1]CCC2=CC(OC)=C(OC)C(OC)=C2C3=CC=C(C(=O)C=C3)N
)C=O
Correctly moved…
60
OC(=O)C=C(C(OCC)=O)N(NCCC)N[C@H1]1CCC2=CC(OC)=C(OC)C(OC)=C2C3=CC=C(NC)C(=O)C=C31
Correctly moved…
61 C[C@H1](NCC)CC1=CC(C(NC)=CC=C1C2=C(C=C(OC)C(OC)=C2OCC3=CC=CC=C3)OSCC)=O
Something went wrong while creating PDB file…
62 C=1C(C)(C)OC(=O)/N=C(/N[C@H1]2CCC3=C(C(OC)=C(OC)C(OC)=C3)C4=CC=C(NC)C(=O)C=C2
4)N=1
Correctly moved…
63 N1=NN1[C@@H1]2C3=CC(C(NC)=CC=C3C4=C(C(OC)=C(OC)C=C4CC2)OC)=O
Correctly moved…
64
O=CC=CC=CC(NCC1=CN(N=N1)[C@H1]2CCC3=CC(OC)=C(OC)C(OC)=C3C4=CC=C(C(C=C24)=O)NC)=O
Correctly moved…
65 CN(N([C@@H1]1C2=CC(=O)C(NC)=CC=C2C3=CC(=C(OC)C=C3CC1)OC)O)CNC(=S)CCCCC
Correctly moved…
66 CC(C)C(=O)OCC1=CN(N=N1)[C@H1]2CCC3=CC(OC)=C(OC)C(OC)=C3C4=CC=C(NC)C(=O)C=C24
Correctly moved…
67 N(C(C1=CC=NC=C1)=O)[C@@H1]2C=3C(C4=C(C(OC)=C(OC)C=C4CC2)OC)=CC=C(SC)C(=O)C=3
Correctly moved…
68 N1(CCCC1)CN[C@H1]2CCC3=C(C(OC)=C(OC)C(OC)=C3)C4=CC=C(SC)C(=O)C=C24
Correctly moved…
69 N1(CCCC1)C(=O)OC2=C(OC)C(OC3)=CC=C2C4=CC=C(SC)C(C=C4[C@@H1](NC(=O)C)CC3)=O
Correctly moved…
```

```
70 CNC=1C(=O)C=C2[C@H1](CCC3=CC(OC)=C(OC)C(OC)=C3C2=CC=1)NC4(C(=O)O)C=CN=N4
Correctly moved…
71 CC(C)(C)OC(=O)NCCC(NCC=1N=NN(C=1)[C@H1]C2CC3=CC(OC)=C(OC)C(OC)=C3C4=CC=C(C(=O
)C=C24)N)C=O
Something went wrong while creating PDB file…
72 C1C(C)(C)OC/N=C(/N[C@H1]2CCC3=CC(OC)=C(OC)C(=C3C4=CC=C(NC)C(C=C42)=O)OC)N=N1
Correctly moved…
73 CC(C)(C)OCNCCC(=O)NCC1=CN(N=N1)[C@H1]2CCC3=CC(OC)=C(OC)C(OC)=C3C4=CC=C(C(C=C2
4)=O)NC=O
Something went wrong while creating PDB file…
74 C1C(C)(C)OC/N=C(/N[C@H1]2CCC3=CC(OC)=C(OC)C(=C3C4=CC=C(NC)C(C=C42)=O)OC)N1C
Correctly moved…
75 CC(C)(C)OC(=O)NCCC(OCC=1N=NN(C=1)[C@H1]C2CC3=CC(OC)=C(OC)C(OC)=C3C4=CC=C(C(=O
)C=C24)NC)=O
Correctly moved…
76 CC(C)(C)OC(=O)NCCC(NCC=1N=NN(C=1)[C@H1]2CCC3=CC(OC)=C(OC)C(OC)=C3C4=CC=C(C(C=
C24)=O)N)C=O
Correctly moved…
77
CNCCC1=CC=C(C=C1)NC(=O)N[C@H1]2CCC3=CC(OC)=C(OC)C(OC)=C3C4=CC=C(C(C=C24)=O)NC=O
Correctly moved…
78 CNCC(C1=CC=C(C=C1)NC(=O)N[C@@H1]2C3=CC(C(NC)=CC=C3C4=C(C(OC)=C(OC)C=C4CC2)OC)
=O)OC
Correctly moved…
79 O=C(OC)OCC1=CN(N=N1)[C@H1]2CCC3=CC(OC)=C(OC)C(OC)=C3C4=CC=C(NC)C(=O)C=C24
Correctly moved…
80 O=C(C(C)C)NC(=S)N[C@@H1]1C=2C(C3=C(C(OC)=C(OC)C=C3CC1)OC)=CC=C(NC)C(=O)C=2
Correctly moved…
81 O=C(OCC)OCC=1N=NN(C=1)[C@H1]2CCC3=CC(OC)=C(OC)C(OC)=C3C4=CC=C(NC)C(=O)C=C24
Correctly moved…
82 O=C(OCC)CN(CCO)C(=S)N[C@H1]1CCC2=CC(OC)=C(OC)C(OC)=C2C3=CC=C(NC)C(=O)C=C13
Correctly moved…
83 CC(C)CNCC1=CN(N=N1)[C@H1]2CCC3=CC(OC)=C(OC)C(OC)=C3C4=CC=C(C(C=C24)=O)NC=O
Correctly moved…
84 CC(C)CNCC1=CN(N=N1)[C@H1]2CCC3=CC(OC)=C(OC)C(OC)=C3C4=CC=C(NC)C(=O)C=C24
Correctly moved…
85 COCNCC1=CN(N=N1)[C@H1]2CCC3=CC(OC)=C(OC)C(OC)=C3C4=CC=C(C(C=C42)=O)NC=O
Correctly moved…
86
N(C(C)C)C(NCC1=CN(N=N1)[C@H1]2CCC3=CC(OC)=C(OC)C(OC)=C3C4=CC=C(NC)C(=O)C=C24)=O
Correctly moved…
87 N(C)C1=CC=C2C3=C(OC)C(OC)=C(OC)C=C3CC[C@@H1](C2=CC1=O)NC(C(=O)O)CCON=CCF
Correctly moved…
88
N(C(C(C)C)=O)C1=CN(N=N1)[C@H1]2CCC3=C(C(OC)=C(OC)C(OC)=C3)C4=CC=C(NC)C(=O)C=C24
Correctly moved…
89 N(CCCCCCC=O)CC1=CN(N=N1)[C@H1]2CCC3=CC(OC)=C(OC)C(OC)=C3C4=CC=C(NC)C(=O)C=C24
Correctly moved…
```

```
90 C1=C(C=CC(Cl)=C1)COC(=O)CN[C@@H1]2C=3C(C4=C(C(OC)=C(OC)C=C4CC2)OC)=CC=C(SC)C(
=O)C=3
Correctly moved…
91 N(C)C1=CC=C2C3=C(OC)C(OC)=C(OC)C=C3CC[C@@H1](C2=CC1=O)NC(C(=O)O)CCONC(=O)C=C
Correctly moved…
92 N(C)C1=CC=C2C3=C(OC)C(OC)=C(OC)C=C3CC[C@@H1](C2=CC1=O)NC(C(=O)O)CCONC(=O)CNC
Correctly moved…
93
N(C(C)C)CC(=O)OCC1=C2N(N=N1)[C@H1]CCC3=CC(OC)=C(OC)C(OC)=C3C4=CC=C(NC)C(C=C24)=O
Correctly moved…
94 CC(CC)N[C@@H1]1C2=CC(C(SC)=CC=C2C3=C(OC)C(OC)=C(C=C3CC1)OC)=O
Correctly moved…
95 CC(CC)N[C@@H1]C1=C2C(C(NC)=CC=C1C3=C(OC)C(OC)=C(OC)C=C3CC2)=O
Correctly moved…
96 COC1=C2C3=CC=C(NC)C(=O)C=C3[C@H1](CCC2=CC(OC)=C1OC)NCC4=CC=NS4C

[13:01:05] UFFTYPER: Unrecognized atom type: S_5+4 (31)

Correctly moved…
97 N(C)C=1C(=O)C=C2[C@H1](CCC3=CC(OC)=C(OC)C(OC)=C3C2=CC=1N)C(C)CCSC
Correctly moved…
98 CNC=1C(C=C2[C@H1](CCC3=C(C(OC)=C(OC)C(OC)=C3)C2=CC=1)NC(C)C)=O
Correctly moved…
99 NN([C@H1]1CCC2=CC(OC)=C(OC)C(OC)=C2C3=CC=C(NC)C(=O)C=C13)C=O
Correctly moved…
100 OCCN(CC=C)C(=O)N[C@@H1]1C2=CC(C(NC)=CC=C2C3=C(OC)C(OC)=C(OC)C=C3CC1)=O
Correctly moved…
101 OCCN(CC=C)C(=S)N[C@H1]1CCC2=CC(OC)=C(OC)C(OC)=C2C3=CC=C(NC)C(=O)C=C13
Correctly moved…
102 CNC(N(C)C)CN[C@H1]1CCC2=CC(OC)=C(C(OC)=C2C=3C1=CC(=O)C(=CC=3)NC)OC
Correctly moved…
103 NC(N(CC)OC)N[C@H1]1CCC2=CC(OC)=C(OC)C(OC)=C2C=3C1=CC(=O)C(=CC=3)NC
Correctly moved…
104 C1[C@H1](C2=CC(C(SC)=CC=C2C3=C(OC)C(=C(OC)C=C3C1)OC)=O)NCC
Correctly moved…
105 C1=2[C@H1](CCC3=CC(OC)=C(OC)C(OC)=C3C1=CC=C(SC)C(=O)C=2NC)C
Correctly moved…
106 C1=2[C@H1](CCC3=CC(OC)=C(OC)C(OC)=C3C1=CC=C(C(C(C=2)=O)SCN)C(=O)C
Correctly moved…
107 NC(=O)N[C@@H1]C1=C2C(C(NC)=CC=C1C3=C(OC)C(OC)=C(OC)C=C3CC2)=O
Correctly moved…
108 CC(C)NC(=O)N1[C@@H1]C2=CC(C(NC)=CC=C2C3=C(OC)C(OC)=C(OC)C=C3CC1)=O
Correctly moved…
109 CC(N)(CCO)[C@H1]1CCC2=CC(OC)=C(OC)C(OC)=C2C3=CC=C(SC)C(=O)C=C13
Correctly moved…
110 CC(N)(CCO)[C@H1]1CCC2=CC(OC)=C(OC)C(OC)=C2C3=CC=C(C(C(C=C31)=O)NCC
Correctly moved…
111 C1[C@H1](NC(C)=O)C2=CC(C(NC)=CC=C2C3=C(OC)C(OC)=C(C=C3C1)OCNC)=O
Correctly moved…
```

112 C1[C@H1](NC(C)=O)C2=CC(C(SC)=CC=C2C3=C(OC)C(OC)=C(C=C3C1)OCNC)=O
Correctly moved…
113 C=C(N[C@H1]1CCC2=C(C(OC)=C(OC)C(OC)=C2)C3=CC=C(SC)C(C=C31)=O)C
Correctly moved…
114 CC(C)N(C(C)C)C(=O)N[C@@H1]C1=CC(C(NC)=CC=C1C2=C(OC)C(OC)=C(OC)C=C2CC)=O
Correctly moved…
115 C1C(C)NC1N[C@H1]2CCC3=CC(OC)=C(OC)C(OC)=C3C4=CC=C(C(=O)C=C42)NC=O
Correctly moved…
116 O=C(N[C@H1]1CCC2=CC(OC)=C(OC)C(OC)=C2C3=CC=C(NC)C(=O)C=C31)N(S)CC
Correctly moved…
117 O=C(N[C@H1]1CCC2=CC(OC)=C(OC)C(OC)=C2C3=CC=C(NC)C(=O)C=C13)CCOCC
Correctly moved…
118 COC1=C2C3=CC=C(NC)C(=O)C=C3[C@H1](CCC2=CC(OC)=C1OC)NC(=O)N(CCCCCl)O
Correctly moved…
119 COC=CC1=CC=C(NC)C(=O)C=C1[C@@H1](NC(=S)NC2=CC=CC=C2)Cl
Correctly moved…
120 N=1C(=CN(N=1)[C@H1]2CCC3=CC(OC)=C(OC)C(OC)=C3C4=CC=C(NC)C(=O)C=C42)C=O
Correctly moved…
121 C1=2[C@H1]CCC3=CC(OC)=C(OC)C(OC)=C3C1=CC=C(C(C=2)=O)SCNC(C)=O
Correctly moved…
122 N1([C@H1]2CCC3=C(C(OC)=C(OC)C(OC)=C3)C4=CC=C(NC)C(C=C24)=O)C=C1I
Correctly moved…
123 N1([C@H1]2CCC3=C(C(OC)=C(OC)C(OC)=C3)C4=CC=C(NC)C(C=C24)=O)C=C1OCC
Correctly moved…
124 N1C(OC)=C(C=C1)N[C@H1]2CCC3=C(C(OC)=C(OC)C(OC)=C3)C4=CC=C(SC)C(C=C42)=O
Correctly moved…
125 N(C(C)C)(C(C)C)C(=O)N1[C@H1]CCC2=C(C(OC)=C(OC)C(OC)=C2)C3=CC=C(NC)C(C=C31)=O
Correctly moved…
126 N(CCCC)CC1=CN(N=N1)[C@H1]2CCC3=CC(OC)=C(OC)C(OC)=C3C4=CC=C(NC)C(=O)C=C24
Correctly moved…
127 NC(=S)N1[C@@H1]C2=CC(C(NC)=CC=C2C3=C(OC)C(OC)=C(OC)C=C3CC1)=O
Correctly moved…
128 C=C(CN[C@H1]1CCC2=CC(OC)=C(OC)C(OC)=C2C3=CC=C(NC)C(C=C13)=O)C=O
Correctly moved…
129 C1=C(N=NN1[C@H1]2CCC3=CC(OC)=C(OC)C(OC)=C3C4=CC=C(NC)C(=O)C=C24)C
Correctly moved…
130 C=CC=C(NC(=O)N[C@H1]1CCC2=CC(OC)=C(OC)C(OC)=C2C3=CC=C(NC)C(=O)C=C13)C
Correctly moved…
131 C[C@H1](CCC1=CC(OC)=C(OC)C(OC(OCC)=O)=C1C2=CC=C(SC)C(=O)C=C2)NCC
Correctly moved…
132 COCC(CN[C@@H1]1C=2C(C3=C(C(OC)=C(OC)C=C3CC1)OC)=CC=C(NC)C(C=2)=O)F
Correctly moved…
133 NC1=NN=NN1[C@@H1]2C=3C(C4=C(C(OC)=C(OC)C=C4CC2)OC)=CC=C(NC)C(C=3)=O
Correctly moved…
134 OCCN(CCO)C(=S)N1[C@@H1]C2=CC(C(NC)=CC=C2C3=C(OC)C(OC)=C(OC)C=C3CC1)=O
Correctly moved…
135 NC(C(OCC)=C)N[C@H1]1CCC2=CC(OC)=C(OC)C(OC)=C2C3=CC=C(NC)C(=O)C=C31
Correctly moved…

```
136 N([C@H1]1CCC2=CC(OC)=C(OC)C(OC)=C2C3=CC=C(NC)C(C=C13)=O)=N
Correctly moved…
137 NC(CCC)N[C@@H1]1C2=CC(C(NC)=CC=C2C3=C(OC)C(OC)=C(OC)C=C3CC1)=O
Correctly moved…
138 N([C@H1]1CCC2=CC(OC)=C(OC)C(OC)=C2C3=CC=C(NC)C(C=C31)=O)=C
Correctly moved…
139 N([C@H1]1CCC2=CC(OC)=C(OC)C(OC)=C2C3=CC=C(NC)C(C=C13)=O)=CC
Correctly moved…
140 C1=2[C@H1](CCC3=C(C(OC)=C(OC)C(OC)=C3)C1=CC=C(C(C=2)=O)NCNCC(C)C)ONC
Correctly moved…
141 N(C)C=1C(=O)C=C2[C@H1](CCC3=C(C(OC)=C(OC)C(OC)=C3)C2=CC=1)N4C(C(=O)O)=C(N=N4
)C(=O)N
Correctly moved…
142 N(CCCC)CNCC1=CN(N=N1)[C@H1]2CCC3=CC(OC)=C(OC)C(OC)=C3C4=CC=C(NC)C(=O)C=C24
Correctly moved…
143
N(C)C=1C(=O)C=C2[C@H1](CCC3=C(C(OC)=C(OC)C(OC)=C3)C2=CC=1)NC(C(=O)O)=C(COCC)C=O
Something went wrong while creating PDB file…
144 OC(\N[C@H1]1CCC2=CC(OC)=C(OC)C(OC)=C2C3=CC=C(C(C=C31)=O)NCNC(C)C)=O
Correctly moved…
145 N1=CC=C(C=C1)C(NCC=2N=NN(C=2)[C@H1]3CCC4=CC(OC)=C(OC)C(OC)=C4C5=CC=C(C(C=C53
)=O)N)C=O
Correctly moved…
146 OCC(C)=C1C([C@H1](CCC2=CC(OC)=C(OC)C(OC)=C12)NC(NC3=CC=C(C=C3)F)=O)PNC=O
Correctly moved…
147 N1=CC=C(C=C1)C(NCC=2N=NN(C=2)[C@@H1]3C4=CCC(NC)=CC=C4C5=C(OC)C(OC)=C(OC)C=C5
CC3)=O
Something went wrong while creating PDB file…
148
CC(C)(C)OC(=O)/N=C(/N[C@H1]1CCC2=C(C(OC)=C(OC)C(OC)=C2)C3=CC=C(NC)C(C=C13)=O)N=N
Correctly moved…
149 CC(C)(C)OC(=O)NCCCNCC1=CN(N=N1)[C@H1]2CCC3=CC(OC)=C(OC)C(OC)=C3C4=CC=C(NC)C(
=O)C=C42
Correctly moved…
150 COC1=C2C3=CC=C(SC)C(=O)C=C3[C@H1](CCC2=CC(OC)=C1OC)NCCC
Correctly moved…
151 COC1=C2C3=CC=C(NC)C(=O)C=C3[C@H1](CCC2=CC(OC)=C1OC)NCCCCCCSC
Correctly moved…
152 C=C(CO)CN[C@H1]1CCC2=CC(OC)=C(OC)C(OC)=C2C3=CC=C(SC)C(=O)C=C31
Correctly moved…
153 CC(C)CN[C@H1]1CCC2=CC(OC)=C(OC)C(OC)=C2C3=CC=C(C(C=C31)=O)SCC
Correctly moved…
154 CC(C)CCN[C@H1]1CCC2=CC(OC)=C(OC)C(OC)=C2C3=CC=C(C(C=C13)=O)SCC
Correctly moved…
155 CC(C)CCN[C@H1]C1CC2=CC(OC)=C(OC)C(OC)=C2C3=CC=C(SC)C(C=C31)=O
Correctly moved…
156 N=1NC=1[C@@H1]2C3=CC(C(NC)=CC=C3C4=C(C=C(OC)C(=C4OC)OC)CC2)=O
Correctly moved…
```

157 COC1=C2C3=CC=C(NC)C(=O)C=C3[C@H1](CCC2=CC(OC)=C1OC)NCC(C)C
Correctly moved…
158 COC1=C(OC)C(OC)=CC=2CC[C@@H1](C3=CC(C(=CC=C3C1=2)SC)=O)NCCC(C)CC
Correctly moved…
159 C12=CC(C(NC)=CC=C1C3=C(C=C(OC)C(OC)=C3OC)CC[C@@H1]2NCC=4SC=CC=4OC)=O
Correctly moved…
160 O=CC1=CN=NN1[C@H1]2CCC3=CC(OC)=C(OC)C(OC)=C3C4=CC=C(C(C=C24)=O)NCCC
Correctly moved…
161 C1=CN=CC=C1CN[C@H1]2CCC3=CC(OC)=C(OC)C(OC)=C3C4=CC=C(C(C=C42)=O)SCC(=O)C
Correctly moved…
162 C1=CN=CC=C1CN[C@H1]2CCC3=CC(OC)=C(OC)C(OC)=C3C4=CC=C(C(C=C42)=O)SCC
Correctly moved…
163 C=C1N=CC(C)N1CN[C@H1]2CCC3=C(C(OC)=C(OC)C(OC)=C3)C4=CC=C(C(C=C24)=O)NC
Correctly moved…
164 CNCC(C)OC(=O)N[C@H1]1CCC2=C(C(OC)=C(OC)C(OC)=C2)C3=CC=C(C(=O)C=C31)NCF
Correctly moved…
165 C=CC=C(CCN[C@H1]1CCC2=CC(OC)=C(OC)C(OC)=C2C3=CC=C(SC)C(C=C31)=O)OC
Correctly moved…
166 C1COC1(OC2=CC=C3C4=C(OC)C(OC)=C(OC)C=C4CC[C@@H1](C3=CC2=O)SC(C)=O)SC
Correctly moved…
167 O=C(OC1=C2C(CC[C@H1](NC(=O)C)C3=CC(C(SC)=CC=C32)=O)=CC(OC)=C1OC)SCC
Correctly moved…
168 O=C1C=CC=C2C([C@H1](CCC3=CC(OC)=C(OC)C(OC)=C32)NC(C)=O)=C1SC
Correctly moved…
169 C1=CC=C(C=C1)CN[C@H1]2CCC3=CC(OC)=C(OC)C(OC)=C3C4=CC=C(C(C=C42)=O)SCC
Correctly moved…
170 CC=CCCCN[C@H1]1CCC2=CC(OC)=C(OC)C(OC)=C2C3=CC=C(SC)C(C=C13)=O
Correctly moved…
171 OC1=C(C=CC=C1)CN2[C@H1]CCC3=CC(OC)=C(OC)C(OC)=C3C4=CC=C(SC)C(C=C24)=O
Correctly moved…
172 COC1=C2C3=CC=C(NC)C(=O)C=C3[C@H1](CCC2=CC(OC)=C1OC)NCCCCCCCCSC
Correctly moved…
173 COC1=CC=2CC[C@@H1](C=3C(C=2C(=C1OC)OC)=CC=C(NC)C(=O)C=3)NC(=O)CNC
Correctly moved…
174 CSC1=CC=C2C(=CC1=O)[C@H1](CCC3=CC(OC)=C(OC)C(=C23)O)CNC(=O)C
Correctly moved…
175 C1=C2[C@H1](CCC3=C(C(OC)=C(C(=C3)OC)OC)C2=CC=C(C1=O)NC)NC
Correctly moved…
176 CC(=O)OC1=CC=C2C3=C(C(OC)=C(C=C3CC[C@@H1](C2=CC1=O)NC(=O)CO)CO)C
Correctly moved…
177 CC(=O)OC=1C(=O)C=C2C(=CC=1)C3=C(OC)C(OC)=C(C=C3CC[C@@H1]2NC(C)=O)C(=O)C
Correctly moved…
178 CC(=O)OC1=CC=C2C3=C(C(OC)=C(C=C3CC[C@@H1](C2=CC1=O)NC(=O)C)OC)OC(=O)C
Correctly moved…
179 CCC(=O)OC1=CC=C2C3=C(C(OC)=C(C=C3CC[C@@H1](C2=CC1=O)OC(=O)C)OC)OC
Correctly moved…
180 CCC(=O)N[C@H1]1CCC2=CC(=C(OC)C(OC)=C2C=3C1=CC(=O)C(=CC=3)OC(OC)=O)OC
Correctly moved…

```
181 CC(C)C(OC1=CC=C2C3=C(CC[C@@H1](C2=CC1=O)NC(C)=O)C=C(OC)C(OC)=C3OC)(O)C=O
Correctly moved…
182 CC(C)C(OC1=CC=C2C3=C(CC[C@@H1](C2=CC1=O)NC(C)=O)C=C(OC)C(OC)=C3OC=O)C
Correctly moved…
183 CC(C)C(=O)OC1=CC=C2C3=C(C(OC)=C(C=C3CC[C@@H1](C2=CC1=O)NC(C)=O)OC)OC(=O)C
Correctly moved…
184 CC(C)C(OC1=CC=C2C3=C(C=C(OC)C(OC)=C3OC)CC[C@@H1](C2=CC1=O)NC(=O)CC)(O)C
Correctly moved…
185 CC(=O)OCC1=CN(N=N1)[C@H1]2CCC3=CC(OC)=C(OC)C(OC)=C3C=4C2=CC(=O)C(=CC=4)NCC
Correctly moved…
186 C1=C(C2=C(OC)C(OC)=C1OC(=O)CCC)C[C@@H1](C3=CC(=O)C(SC)=CC=C32)NC(C)=O
Correctly moved…
187 CC(=O)N[C@H1]1CCC2=CC(OC)=C(OC)C(=C2C3=CC=C(C(C=C31)=O)OCC4=CC=CC=C4)OOC
Correctly moved…
188 O=C(C)N[C@H1]1CCC2=CC(=C(OC)C(=C2C=3C1=CC(C(=CC=3)OC(=O)OC)=O)CN)C(=O)COCC
Correctly moved…
189 C=12C(=O)C(=CC=C3C=1[C@H1](CCC4=CC(OC)=C(OC)C(OC)=C34)NC(C)=O)C2
Correctly moved…
190 CCC(=O)N[C@H1]1CCC2=C(C(OC)=C(C(OC)=C2)OC)C=3C1=CC(C(SC)=CC=3)=O
Correctly moved…
191 CCC(=O)N[C@H1]1CCC2=CC(OC)=C(C(OC)=C2C=3C1=CC(=O)C(=CC=3)SC)OC(=O)OCC
Correctly moved…
192 CC=1C(=CC(C(=CC=1)SC)=O)[C@H1](CCC2=CC(OC)=C(OC)C(=C2)OC)NC(=O)C
Correctly moved…
193 CC(C)COCCN[C@H1]1CCC2=CC(OC)=C(C(=C2C=3C1=CC(C(=CC=3)SC)=O)OC)OC(=O)C
Correctly moved…
194 CC(C)CCN[C@H1]1CCC2=C(C3=CC=C(SC)C(C=C31)=O)C(=C(C(OC)=C2)OC)OCCC
Correctly moved…
195 CC(C)CCN[C@H1]1CCC2=C(C3=CC=C(SC)C(C=C31)=O)C(=C(C(OC)=C2)OC)OC(=O)C
Correctly moved…
196 C1=C(OC)C(=C(OC)C2=C1CC[C@H1](NC(=O)C)C=3C2=CC=C(OC(=O)OC)C(C=3)=O)C
Correctly moved…
197 O=C(C)N[C@H1]1CCC2=CC(OC)=C(C(OC)=C2C3=CC=C(C(=O)C=C13)OCCC)OOC
Correctly moved…
198 O=C(N(C)C)NC1=CC=C2C3=C(OC)C(=C(OC)C=C3CC[C@@H1](C2=CC1=O)NCC)OOC
Correctly moved…
199 O=C(N)N[C@H1]1CCC2=CC(OC)=C(C(OC)=C2C=3C1=CC(C(OC(=O)C)=CC=3)=O)OC
Correctly moved…
200 O=C(C)N[C@H1]1CCC2=CC(OC)=C(C(OC)=C2C3=CC=C(C(=O)C=C31)OC(=O)O)CCOC
Correctly moved…
201 CC(=O)N1[C@H1]CCC2=CC(OC)=C(OC)C(OC)=C2C3=CC=C(NC)C(C=C31)=O
Correctly moved…
202 COC1=C2C3=CC=C(NC)C(=O)C=C3[C@H1](CCC2=CC(OC)=C1OC)NO
Correctly moved…
203 C1[C@@H1](C2=CC(=O)C(NC)=CC=C2C3=C(C=C(OC)C(=C3OC)OC)C1)NCCSC
Correctly moved…
204 CNC(C(C)O)N([C@H1]1CCC2=CC(=C(OC)C(OC)=C2C=3C1=CC(=O)C(=CC=3)NC)OCC)C
Correctly moved…
```

205 CNC(C(C)O)N[C@@H1]1C=2C(C3=C(C=C(OC)C(OC)=C3OC)CC1)=CC=C(C(=O)C=2)NC
Correctly moved…
206 CNC(=S)N[C@@H1]1C2=CC(=O)C(NC)=CC=C2C3=C(OC)C(=C(C=C3CC1)OC)OCO
Correctly moved…
207 N([C@H1]1CCC2=C(C3=CC=C(C(C=C13)=O)NC)C(OC)=C(OC)C(OC)=C2)N=N
Correctly moved…
208 C12=CC(C(NC)=CC=C1C3=C(C=C(C(OC)=C3OC)OC)CC[C@@H1]2NCCCCCCCCCCF)=O
Correctly moved…
209 COC1=C(OC)C(OC)=CC=2CC[C@@H1](C3=CC(=O)C(NC)=CC=C3C=21)NCCC4=CC=CC=C4NC
Correctly moved…
210 COC1=C(OC)C(OC)=CC=2CC[C@@H1](C3=CC(=O)C(NC)=CC=C3C=21)N4C=C(N=N4)CCONC
Correctly moved…
211 CN(C(=S)N[C@@H1]1C2=CC(=O)C(NC)=CC=C2C3=C(C(=C(OC)C=C3CC1)OC)OC)CC#N
Correctly moved…
212 CC(C)(C)OC1=CN(N=N1)[C@H1]C2CC3=CC(OC)=C(OC)C(OC)=C3C4=CC=C(NC)C(=O)C=C42
Correctly moved…
213 CC(C)CCCCCCN[C@@H1]1C2=CC(C(SC)=CC=C2C3=C(C(OC)=C(OC)C=C3CC1)OC)=O
Correctly moved…
214 CC(C)(C)C(=O)N[C@@H1]1C=2C(C3=C(C(OC)=C(OC)C=C3CC1)OC)=CC=C(NC)C(=O)C=2
Correctly moved…
215 OC(CCC(C)C)OCC1=CN(N=N1)[C@H1]CCC2=CC(OC)=C(OC)C(OC)=C2C3=CC=C(C(=O)C=C3)NC
Correctly moved…
216 O=C(NCC1=CC=CC=C1Cl)N[C@H1]2CCC3=C(C(OC)=C(OC)C(OC)=C3)C4=CC=C(NC)C(=O)C=C24
Correctly moved…
217 N([C@H1]1CCC2=C(C(OC)=C(OC)C(OC)=C2)C3=CC=C(NC)C(C=C13)=O)C(O)=O
Correctly moved…
218 O=CC=CCC=CNNC(N[C@H1]1CCC2=CC(OC)=C(OC)C(OC)=C2C3=CC=C(NC)C(=O)C=C13)=O
Correctly moved…
219
OC1=C(C=CC=C1)C=2N=NN(C=2)[C@H1]C3CC4=CC(OC)=C(OC)C(OC)=C4C5=CC=C(NC)C(=O)C=C53
Correctly moved…
220
OC1=C(C=CC=C1)C=2N=NN(C=2)[C@H1]CCC3=CC(OC)=C(OC)C(OC)=C3C4=CC=C(NC)C(=O)C=C4
Correctly moved…
221 C1[C@@H1](C2=CC(=O)C(NC)=CC=C2C3=C(C(=C(OC)C=C3C1)OC)OC)N4C=C(N=N4)COC
Correctly moved…
222 O=C(OC(C)(C)C)N[C@H1]1CCC2=C(C(OC)=C(OC)C(OC)=C2)C3=CC=C(NC)C(=O)C=C13
Correctly moved…
223 O=C(OCC)CN(OCC)C[C@@H1]1C2=CC(C(NC)=CC=C2C3=C(C(OC)=C(OC)C=C3CC1)OC)=O
Correctly moved…
224 O=C(C)OC1=CN(N=N1)[C@H1]2CCC3=CC(OC)=C(OC)C(OC)=C3C4=CC=C(NC)C(=O)C=C24
Correctly moved…
225 O=C(OCC)CN(CCC)C[C@H1]C1CC2=CC(OC)=C(OC)C(OC)=C2C3=CC=C(SC)C(=O)C=C31
Correctly moved…
226 O=C(C(Cl)Cl)NC(=O)N[C@H1]1CCC2=C(C(OC)=C(OC)C(OC)=C2)C3=CC=C(C(C=C31)=O)NCN(
O)CCC=O
Correctly moved…
227 O=C(C(Cl)Cl)NC(=O)N[C@H1]1CCC2=C(C(OC)=C(OC)C(OC)=C2)C3=CC=C(C(C=C31)=O)NCC4

```
=CC=CC=C4
Correctly moved…
228
O=C(OCCC)OCC1=CN(N=N1)[C@H1]2CCC3=CC(OC)=C(OC)C(OC)=C3C4=CC=C(C(=O)C=C24)NCCO
Correctly moved…
229 O=C(OCCC)OCC1=CN(N=N1)[C@H1]2CCC3=CC(OC)=C(OC)C(OC)=C3C4=CC=C(C(=O)C=C42)NCC
(=O)C
Correctly moved…
230 O=C(OCC=C)NCC1=CN(N=N1)[C@H1]2CCC3=CC(OC)=C(OC)C(OC)=C3C4=CC=C(C(=O)C=C24)NC
C(=O)C
Correctly moved…
231 O=C(C(Cl)Cl)NCC1=CN(N=N1)[C@H1]2CCC3=CC(OC)=C(OC)C(OC)=C3C4=CC=C(C(=O)C=C24)
NCC(=O)C
Correctly moved…
232 O=C(C(Cl)Cl)NCC1=CN(N=N1)[C@H1]2CCC3=CC(OC)=C(OC)C(=C3C=4C2=CC(C(NC)=CC=4)=O
)OCCCl
Correctly moved…
233 O=C(C)N[C@@H1]1CC(C2=C(C(OC)=C(OC)C=C2CC1)OCC3=CC=CC=C3)=O
Correctly moved…
234
O=C(C(Cl)Cl)NC(=O)N[C@H1]1CCC2=CC(OC)=C(OC)C(OC)=C2C3=CC=C(C(C=C31)=O)NCC(=O)O
Correctly moved…
235
O=C(OCC)C1=CN(N=N1)[C@H1]2CCC3=CC(OC)=C(OC)C(OC)=C3C4=CC=C(C(C=C24)=O)NCC(=O)C
Correctly moved…
236 O=C(O)CN(CCO)C(=O)N[C@H1]1CCC2=CC(OC)=C(OC)C(OC)=C2C3=CC=C(C(C=C13)=O)NCC
Correctly moved…
237 CC(C)OC(NCC1=CN(N=N1)[C@H1]2CCC3=CC(OC)=C(OC)C(OC)=C3C4=CC=CC(C=C24)=O)NC=O
Correctly moved…
238
COC(NCCCOCC1=CN(N=N1)[C@H1]2CCC3=CC(OC)=C(OC)C(=C3C4=CC=C(NC)C(C=C24)=O)OC=O)=O
Something went wrong while creating PDB file…
239
C=1C(C)OC(=O)/N=C(/N[C@H1]2CCC3=C(C(OC)=C(OC)C(OC)=C3)C4=CC=C(NC)C(=O)C=C42)N=1
Correctly moved…
240
C1=CN=CC=C1C2=CN(N=N2)[C@H1]3CCC4=C(C(OC)=C(OC)C(OC)=C4)C5=CC=C(C(=O)C=C35)NCO
Correctly moved…
241
COC=C(OC)C(OC)=CCCC[C@H1](N1C=C(N=N1)COC(=O)C2=CC=NC=C2)C3=CC(=O)C(=CC=C3CNCN)C
Correctly moved…
242
COC1=C(OC)C=CC=2CC[C@@H1](C3=CC(=O)C(NC)=CC=C3C1=2)N4C=C(N=N4)COCC5=CC=CC=C5OC
Correctly moved…
243 CC(C)(O)C=CN([C@H1]1CCC2=CC(OC)=C(OC)C(OC)=C2C3=CC=C(C(C=C13)=O)NC)O
Correctly moved…
244 C=12C(=CC(C(NC)=CC=1)=O)[C@H1](CCC3=CC(OC)=C(OC)C(=C32)OC)C(C)C=O
Correctly moved…
```

245 NC(=O)NCC1=CN(N=N1)[C@H1]2CCC3=CC(OC)=C(OC)C(OC)=C3C4=CC=C(NC)C(=O)C=C24
Correctly moved…
246 C1=CC(OC)=CC=C1CN[C@H1]2CCC3=C(C(OC)=C(OC)C(OC)=C3)C=4C2=CC(C(=CC=4)NC)=O
Correctly moved…
247 N(C(C1=CC=CC=C1)=O)CCN[C@H1]2CCC3=CC(OC)=C(OC)C(OC)=C3C4=CC=C(C(C=C24)=O)SC
Correctly moved…
248 N=NC(=C(C(OCC)=O)N[C@H1]1CCC2=CC(OC)=C(OC)C(OC)=C2C3=CC=C(C(C=C13)=O)N)C
Correctly moved…
249 CNC=1C(=O)C=C2[C@H1](CCC3=C(C(OC)=C(OC)C(OC)=C3)C2=CC=1)NC(C(=O)O)=CN=CNC
Correctly moved…
250
NN(C=C(CNC(CCCCCl)=O)N=N)[C@H1]1CCC2=CC(OC)=C(OC)C(OC)=C2C3=CC=C(NC)C(=O)C=C31
Correctly moved…
251 N=NC1=CN(N=N1)[C@H1]2CCC3=CC(OC)=C(OC)C(=C3C4=CC=C(NC)C(C=C42)=O)OC
Correctly moved…
252 CC(C)(C)OC(=O)NCCC(NCC=1N=NN(C=1)[C@H1]CCC2=CC(OC)=C(OC)C=C2C3=CC=C(NC)C(C=C
3)=O)OC=O
Correctly moved…
253 CC(C)(C)OCNCCC(=O)NCC1=CN(N=N1)[C@H1]C2CC3=CC(OC)=C(OC)C(OC)=C3C4=CC=C(C(=O)
C=C24)NC=O
Correctly moved…
254
C1(=CN(C(=O)OCC)N[C@@H1]2C=3C(C4=C(OC)C(OC)=C(OC)C=C4CC2)=CC=C(C(=O)C=3)NC)N=C1
Correctly moved…
255 CC(C)(C)OCNCCC(=O)NCC1=CN(N=N1)[C@@H1]2C3=CC(C(NC)=CC=C3C4=C(CC2)C=C(OC)C(=C
4OC)NC)=O
Correctly moved…
256 CC(C)(C)OC(=O)NCCC(OCC=1N=NN(C=1)[C@H1]CCC2=CC(OC)=C(OC)C(=C2C3=CC=C(NC)C(=O
)C=C3)O)C=O
Something went wrong while creating PDB file…
257
CC(C)OC(=O)/N=C(/N[C@H1]1CCC2=C(C(OC)=C(OC)C(OC)=C2)C3=CC=C(NC)C(=O)C=C13)N=N
Correctly moved…
258
C=1N(N=NC=1C(OCC)=O)[C@H1]2CCC3=C(C(OC)=C(OC)C(OC)=C3)C4=CC=C(C(=O)C=C42)NCOCC
Correctly moved…
259 O=CC=CN=NN[C@H1]1CCC2=C(C(OC)=C(OC)C(=C2)OC)C3=CC=C(NC)C(=O)C=C13
Correctly moved…
260 C1=CN=CC=C1C(NCC2=CN(N=N2)[C@H1]3CCC4=CC(OC)=C(OC)C(=C4C5=CC=C(C(C=C53)=O)OC
)OO)C
Correctly moved…
261 N=NN([C@H1]1CCC2=C(C(OC)=C(OC)C(OC)=C2)C3=CC=C(C(=O)C=C13)NC)C(=O)OCC
Correctly moved…
262 CN(N([C@@H1]1C2=CC(=O)C(NC)=CC=C2C3=CC(=C(OC)C=C3CC1)OC)O)CNC(=S)NCCCCC
Correctly moved…
263 CN(N([C@@H1]1C2=CC(=O)C(NC)=CC=C2C3=CC(=C(OC)C=C3CC1)OC)O)CNC(=S)CCCCCC
Correctly moved…
264 O=C(CC)OCC1=CN(N=N1)[C@H1]2CCC3=CC(OC)=C(OC)C(OC)=C3C4=CC=C(NC)C(=O)C=C24

Correctly moved…
265 O=C(OC(C)(C)C)NCC=1N=NN(C=1)[C@H1]C2CC3=CC(OC)=C(OC)C(OC)=C3C4=CC=C(NC)C(=O)
C=C42
Correctly moved…
266 O=C(C(C)C)NCN[C@H1]1CCC2=CC(OC)=C(OC)C(OC)=C2C3=CC=C(NC)C(=O)C=C13
Correctly moved…
267 O=C(OCC)C=1N(N=NC=1C(=O)O2)[C@H1]2NC(=S)N[C@H1]3CCC4=CC(OC)=C(OC)C(OC)=C4C=5
C3=CC(C(=CC=5)NC)=O
Correctly moved…
268 CC(O)(C)CC(=O)OCC1=CN(N=N1)[C@H1]2CCC3=CC(OC)=C(OC)C(OC)=C3C=4C2=CC(C(=CC=4)
NC)=O
Correctly moved…
269
CC(C)COC(NCC1=CN(N=N1)[C@H1]C2CC3=CC(OC)=C(OC)C(OC)=C3C4=CC=C(C(=O)C=C24)NC)=O
Correctly moved…
270 COCNCC1=CN(N=N1)[C@H1]2CCC3=CC(OC)=C(OC)C(OC)=C3C4=CC=C(NC)C(=O)C=C24
Correctly moved…
271 CC(C)(C)OC(NCC=1N=NN(C=1)[C@H1]2CCC3=CC(OC)=C(OC)C(OC)=C3C4=CC=C(C(C=C24)=O)
N)C=O
Correctly moved…
272 N(C)C1=CC=C2C3=C(OC)C(OC)=C(OC)C=C3CC[C@@H1](C2=CC1=O)NC(C(=O)O)CCONCOCNCOC
Correctly moved…
273
O=C(OCC)C1=C(C(OCC)=O)N=NN1[C@H1]CCC2=CC(OC)=C(OC)C(OC)=C2C3=CC=C(NC)C(C=C3)=O
Correctly moved…
274 N([C@H1]1CCC2=CC(OC)=C(OC)C(OC)=C2C3=CC=C(NC)C(C=C13)=O)C=CCOC(=O)CCCC=O
Correctly moved…
275 CC(CC)N[C@H1]C1CC2=CC(OC)=C(OC)C(OC)=C2C3=CC=C(C(C=C31)=O)NCC
Correctly moved…
276 CC(CC)N[C@H1]C1CC2=CC(OC)=C(OC)C(OC)=C2C3=CC=C(NC)C(=O)C=C31
Correctly moved…
277 COC1=C2C3=CC=C(NC)C(=O)C=C3[C@@H1](NCC(CCC)C)CCC2=CC(OC)=C1OC
Correctly moved…
278 COC1=C2C3=CC=C(NC)C(=O)C=C3[C@H1](CCC2=CC(OC)=C1OC)NCC4=CC=NS4
Correctly moved…
279 COC1=C2C3=CC=C(SC)C(=O)C=C3[C@H1](CCC2=CC(OC)=C1OC)NCCCCl
Correctly moved…
280 O(C1=C(OC)C=2C3=CC=C(SC)C(C=C3[C@H1](CCC=2C=C1OC)NC(C)CC=C)=O)C
Correctly moved…
281 C1=CN=CC=C1CN[C@H1]2CCC3=CC(OC)=C(OC)C(OC)=C3C4=CC=C(C(C=C24)=O)NCC
Correctly moved…
282 O=CC1=CN=NN1[C@H1]2CCC3=CC(OC)=C(OC)C(OC)=C3C4=CC=C(C(C=C42)=O)NC
Correctly moved…
283 NC(CCN[C@H1]1CCC2=CC(OC)=C(OC)C(OC)=C2C=3C1=CC(=O)C=CC=3)NCF
Correctly moved…
284 NC(N=C1C=CC2)=CC=C1C3=C(OC)C(OC)=C(OC)C=C3CC[C@@H1]2NC(N)=O
Correctly moved…
285 C=CC1=C(NC)C(OC)=C(OC)C=C1CC[C@H1](NC(C)=O)C2=CC(C(=CC=C2)NC)=O

```
Correctly moved…
286 CC(N)(CCO)[C@H1]1CCC2=CC(OC)=C(OC)C(OC)=C2C3=CC=C(NC)C(=O)C=C31
Correctly moved…
287 N(NCCCCCl)[C@H1]1CCC2=CC(OC)=C(OC)C(OC)=C2C3=CC=C(NC)C(=O)C=C31
Correctly moved…
288 N(C1=CC=C(C=C1)C)CN[C@H1]2CCC3=CC(OC)=C(OC)C(OC)=C3C=4C2=CC(C(NC)=CC=4)=O
Correctly moved…
289 N(C1=CC=C(C=C1)C)CN[C@H1]2CCC3=CC(OC)=C(OC)C(OC)=C3C=4C2=CC(C(=CC=4)SC)=O
Correctly moved…
290 CCN(CC)C(=O)N[C@H1]C1CC2=CC(OC)=C(OC)C(OC)=C2C3=CC=C(C(C=C31)=O)NC
Correctly moved…
291 CCN(CC)C(=O)N1[C@H1]CCC2=CC(OC)=C(OC)C(OC)=C2C3=CC=C(NC)C(C=C31)=O
Correctly moved…
292 CCN(CC)C(=O)N[C@H1]1CCC2=CC(OC)=C(OC)C(OC)=C2C3=CC=C(C(C=C31)=O)NCC
Correctly moved…
293 CC(C)COC1=C(OC)C(OC)=CC=2CC[C@H1](NC(=O)C)C3=CC(C(SC)=CC=C3C1=2)=O
Correctly moved…
294 C=C(C)CN(CCO)C(=S)N[C@H1]1CCC2=C(C(OC)=C(OC)C(OC)=C2)C3=CC=C(NC)C(C=C31)=O
Correctly moved…
295 CC(C)C(OC=C(C(OC)=CCCC[C@H1](NC(=O)C)C1=CC(C(SC)=CC=C1C)=O)C)=O
Correctly moved…
296 N([C@H1]1CCC2=CC(OC)=C(OC)C(OC)=C2C3=CC=C(NC)C(=O)C=C31)=C(Cl)C=C
Correctly moved…
297 O=C(N[C@H1]1CCC2=CC(OC)=C(OC)C(OC)=C2C3=CC=C(C(C=C31)=O)NCN)C4=CC=CC=C4
Correctly moved…
298 O=C(N[C@@H1]1C2=CC(C(NC)=CC=C2C3=C(CC1)C=C(OC)C(OC)=C3OC)=O)N=N
Correctly moved…
299 N([C@H1]1CCC2=CC(OC)=C(OC)C(OC)=C2C3=CC=C(NC)C(C=C13)=O)CNC(=O)OCCONC
Correctly moved…
300 O=C(N[C@H1]1CCC2=CC(OC)=C(OC)C(OC)=C2C3=CC=C(NC)C(=O)C=C31)CCOC(=O)O
Correctly moved…
301 C1=CCC[C@H1](NC(=S)NC2=CC=C(C=C2)C(F)(F)F)CC(C1)=CC=C(NC)C(=O)COCOC
Correctly moved…
302 CNCC(O)(C)C1=CN(N=N1)[C@H1]2CCC3=CC(OC)=C(OC)C(OC)=C3C4=CC=C(C(=O)C=C24)NC
Correctly moved…
303 N1([C@H1]2CCC3=C(C(OC)=C(OC)C(OC)=C3)C4=CC=C(NC)C(C=C24)=O)C(O)=C1C
Correctly moved…
304 N([C@H1]1CCC2=C(C(OC)=C(OC)C(OC)=C2)C3=CC=C(NC)C(C=C13)=O)NC=CC=CCl
Correctly moved…
305
C1=C(O)C(OC)=CC(CN[C@H1]2CCC3=C(C(OC)=C(OC)C(OC)=C3)C4=CC=C(SC)C(=O)C=C42)=C1C
Correctly moved…
306 COCC(C)=C(C(OC)=CCCC[C@H1](NCC)C1=CC(C(SC)=CC=C1)=O)OC
Correctly moved…
307 C[C@H1](CCC1=CC(OC)=C(OC)C(OC)=C1C2=CC=C(C(=O)C=C2)NCN)CCO
Correctly moved…
308 NC(=O)N1[C@@H1]C2=CC(C(NC)=CC=C2C3=C(OC)C(OC)=C(OC)C=C3CC1)=O
Correctly moved…
```

309 NN(CCO)[C@@H1]1C2=CC(C(NC)=CC=C2C3=C(OC)C(OC)=C(C=C3CC1)OC)=O
Correctly moved…
310 C1=2[C@H1](CCC3=CC(OC)=C(OC)C(OC)=C3C1=CC=C(NC)C(=O)C=2NC)CO
Correctly moved…
311 NC(N=NCOCCC)C[C@H1]1C2=CC(C(NC)=CC=C2C3=C(CC1)C=C(OC)C(OC)=C3OC)=O
Correctly moved…
312 C1=2C(C3=C(OC)C(OC)=C(OC)C=C3CC[C@@H1]1NC(C)CC)=CC=C(NC)C(=O)C=2NC
Correctly moved…
313 C1=C(CN[C@H1]2CCC3=CC(OC)=C(OC)C(OC)=C3C4=CC=C(NC)C(=O)C=C42)C=NC=C1
Correctly moved…
314 C1[C@H1](NC(NC2=CC=C(C=C2)F)=O)C3=CC(C(NC)=CC=C3C4=C(OC)C(OC)=CC=C4OCC1)=O
Correctly moved…
315 C1=CC(=CC=N1)CCN[C@@H1]2C3=CC(C(SC)=CC=C3C4=C(CC2)C=C(OC)C(OC)=C4OC)=O
Correctly moved…
316 C1=2[C@H1]CCC3=CC(OC)=C(OC)C(OC)=C3C1=CC=C(C(=O)C=2)NCNC(C)=O
Correctly moved…
317 CC=CC1=C(C=C(OC)C(OC)=C1OC(OCC)=O)CC[C@H1](NC(C)=O)C2=CC(C(SC)=CC=C2)=O
Correctly moved…
318 O=C1C=C2[C@H1](CCC3=C(C(OC)=C(OC)C(OC)=C3)C2=CC=C1SC)NC(N(C(C)C)C(Cl)=O)NC
Correctly moved…
319 O=C1C=C2[C@H1](CCC3=C(C(OC)=C(OC)C(OC)=C3)C2=CC=C1SC)NC(C(=O)OCC)=CNC
Correctly moved…
320 O=C1C=C2[C@H1](CCC3=C(C(OC)=C(OC)C(OC)=C3)C2=CC=C1SC)NC(C(C)C)=O
Correctly moved…
321 N1C=NN=NN1[C@H1]2CCC3=C(C(OC)=C(OC)C(OC)=C3)C4=CC=C(C(C=C42)=O)NCCC
Correctly moved…
322 CNC=1C(=O)C=C2[C@H1](CCC3=CC(OC)=C(OC)C(OC)=C3C2=CC=1N)C(C(C)C)=O
Correctly moved…
323 N([C@H1]1CCC2=CC(OC)=C(OC)C(OC)=C2C3=CC=C(NC)C(C=C31)=O)CCCCF
Correctly moved…
324 C[C@H1](CCC1=CC(OC)=C(OC)C(OC)=C1C2=CC=C(C(C=C2)=O)SC)NC/C=C/C3=CC=CC=C3
Correctly moved…
325 N([C@H1]1CCC2=C(C(OC)=C(OC)C(OC)=C2)C3=CC=C(SC)C(C=C31)=O)CCCO
Correctly moved…
326 C=CC1=C(C(=O)OC=CC=C2C3=C(OC)C(OC)=C(OC)C=C3CC[C@H1](NC(C)=O)C2=C1SC)C
Correctly moved…
327 C=C1C2=C(C=C(OC)C(OC)=C2OC(OCC)=O)CC[C@H1](NC(C)=O)C3=CC(C(NC)=CC=C13)=O
Correctly moved…
328 CC=CC(N[C@H1]1CCC2=C(C(OC)=C(OC)C(OC)=C2)C3=CC=C(SC)C(C=C13)=O)CCOC
Correctly moved…
329 OC(=O)NCC1=CN(N=N1)[C@@H1]2C=3C(C4=C(C(OC)=C(OC)C=C4CC2)OC)=CC=C(NC)C(C=3)=O
Correctly moved…
330 N(C)C=1C(=O)C=C2[C@H1](CCC3=C(C(OC)=C(OC)C(OC)=C3)C2=CC=1)N4C(C(=O)O)=C(N=N4
)C(=O)NNC
Correctly moved…
331 O=C(C=C(C(O1)CCCCCC1)CN=N)N[C@H1]2CCC3=CC(OC)=C(OC)C(OC)=C3C=4C2=CC(C(NC)=CC
=4)=O
Correctly moved…

```
332
N([C@H1]1CCC2=C(C(OC)=C(OC)C(OC)=C2)C3=CC=C(NC)C(=O)C=C13)CNCC4=CC=C(C=C4)C=O
Correctly moved…
333 O=C(C=C(Cl)C=CN([C@H1]1CCC2=C(C(OC)=C(OC)C(OC)=C2)C3=CC=C(NC)C(C=C13)=O)OC(=
O)CC)NC(OC4=CC=CC=C4)=O
Something went wrong while creating PDB file…
334 CC(C)(C)OC(=O)/N=C(/N[C@H1]1CCC2=C(C(OC)=C(OC)C(OC)=C2)C3=CC=C(NC)C(C=C31)=O
)NCOC(C)=O
Something went wrong while creating PDB file…
335 CC(C)(C)OC(=O)/N=C(/N[C@H1]1CCC2=C(C(OC)=C(OC)C(OC)=C2)C3=CC=C(NC)C(C=C13)=O
)NCOC(C)(F)F
Correctly moved…
336 C12=C3C(C4=C(OC)C(OC)=C(OC)C=C4CC[C@@H1]3NC(=O)N)=CC=C1NC=C2
```

1.3 load all the ligands

[12]:
```python
files = glob.glob("*lig*")
print(files)
```

```
['100_lig100_', '101_lig101_', '102_lig102_', '103_lig103_', '104_lig104_',
'105_lig105_', '106_lig106_', '107_lig107_', '108_lig108_', '109_lig109_',
'10_lig10_', '110_lig110_', '111_lig111_', '112_lig112_', '113_lig113_',
'114_lig114_', '115_lig115_', '116_lig116_', '117_lig117_', '118_lig118_',
'119_lig119_', '11_lig11_', '120_lig120_', '121_lig121_', '122_lig122_',
'123_lig123_', '124_lig124_', '125_lig125_', '126_lig126_', '127_lig127_',
'128_lig128_', '129_lig129_', '12_lig12_', '130_lig130_', '131_lig131_',
'132_lig132_', '133_lig133_', '134_lig134_', '135_lig135_', '136_lig136_',
'137_lig137_', '138_lig138_', '139_lig139_', '13_lig13_', '140_lig140_',
'141_lig141_', '142_lig142_', '143_lig143_', '144_lig144_', '145_lig145_',
'146_lig146_', '147_lig147_', '148_lig148_', '149_lig149_', '14_lig14_',
'150_lig150_', '151_lig151_', '152_lig152_', '153_lig153_', '154_lig154_',
'155_lig155_', '156_lig156_', '157_lig157_', '158_lig158_', '159_lig159_',
'15_lig15_', '160_lig160_', '161_lig161_', '162_lig162_', '163_lig163_',
'164_lig164_', '165_lig165_', '166_lig166_', '167_lig167_', '168_lig168_',
'169_lig169_', '16_lig16_', '170_lig170_', '171_lig171_', '172_lig172_',
'173_lig173_', '174_lig174_', '175_lig175_', '176_lig176_', '177_lig177_',
'178_lig178_', '179_lig179_', '17_lig17_', '180_lig180_', '181_lig181_',
'182_lig182_', '183_lig183_', '184_lig184_', '185_lig185_', '186_lig186_',
'187_lig187_', '188_lig188_', '189_lig189_', '18_lig18_', '190_lig190_',
'191_lig191_', '192_lig192_', '193_lig193_', '194_lig194_', '195_lig195_',
'196_lig196_', '197_lig197_', '198_lig198_', '199_lig199_', '19_lig19_',
'1_lig1_', '200_lig200_', '201_lig201_', '202_lig202_', '203_lig203_',
'204_lig204_', '205_lig205_', '206_lig206_', '207_lig207_', '208_lig208_',
'209_lig209_', '20_lig20_', '210_lig210_', '211_lig211_', '212_lig212_',
'213_lig213_', '214_lig214_', '215_lig215_', '216_lig216_', '217_lig217_',
'218_lig218_', '219_lig219_', '21_lig21_', '220_lig220_', '221_lig221_',
'222_lig222_', '223_lig223_', '224_lig224_', '225_lig225_', '226_lig226_',
'227_lig227_', '228_lig228_', '229_lig229_', '22_lig22_', '230_lig230_',
```

```
'231_lig231_', '232_lig232_', '233_lig233_', '234_lig234_', '235_lig235_',
'236_lig236_', '237_lig237_', '238_lig238_', '239_lig239_', '23_lig23_',
'240_lig240_', '241_lig241_', '242_lig242_', '243_lig243_', '244_lig244_',
'245_lig245_', '246_lig246_', '247_lig247_', '248_lig248_', '249_lig249_',
'24_lig24_', '250_lig250_', '251_lig251_', '252_lig252_', '253_lig253_',
'254_lig254_', '255_lig255_', '256_lig256_', '257_lig257_', '258_lig258_',
'259_lig259_', '25_lig25_', '260_lig260_', '261_lig261_', '262_lig262_',
'263_lig263_', '264_lig264_', '265_lig265_', '266_lig266_', '267_lig267_',
'268_lig268_', '269_lig269_', '26_lig26_', '270_lig270_', '271_lig271_',
'272_lig272_', '273_lig273_', '274_lig274_', '275_lig275_', '276_lig276_',
'277_lig277_', '278_lig278_', '279_lig279_', '27_lig27_', '280_lig280_',
'281_lig281_', '282_lig282_', '283_lig283_', '284_lig284_', '285_lig285_',
'286_lig286_', '287_lig287_', '288_lig288_', '289_lig289_', '28_lig28_',
'290_lig290_', '291_lig291_', '292_lig292_', '293_lig293_', '294_lig294_',
'295_lig295_', '296_lig296_', '297_lig297_', '298_lig298_', '299_lig299_',
'29_lig29_', '2_lig2_', '300_lig300_', '301_lig301_', '302_lig302_',
'303_lig303_', '304_lig304_', '305_lig305_', '306_lig306_', '307_lig307_',
'308_lig308_', '309_lig309_', '30_lig30_', '310_lig310_', '311_lig311_',
'312_lig312_', '313_lig313_', '314_lig314_', '315_lig315_', '316_lig316_',
'317_lig317_', '318_lig318_', '319_lig319_', '31_lig31_', '320_lig320_',
'321_lig321_', '322_lig322_', '323_lig323_', '324_lig324_', '325_lig325_',
'326_lig326_', '327_lig327_', '328_lig328_', '329_lig329_', '32_lig32_',
'330_lig330_', '331_lig331_', '332_lig332_', '333_lig333_', '334_lig334_',
'335_lig335_', '336_lig336_', '337_lig337_', '33_lig33_', '34_lig34_',
'35_lig35_', '36_lig36_', '37_lig37_', '38_lig38_', '39_lig39_', '3_lig3_',
'40_lig40_', '41_lig41_', '42_lig42_', '43_lig43_', '44_lig44_', '45_lig45_',
'46_lig46_', '47_lig47_', '48_lig48_', '49_lig49_', '4_lig4_', '50_lig50_',
'51_lig51_', '52_lig52_', '53_lig53_', '54_lig54_', '55_lig55_', '56_lig56_',
'57_lig57_', '58_lig58_', '59_lig59_', '5_lig5_', '60_lig60_', '61_lig61_',
'62_lig62_', '63_lig63_', '64_lig64_', '65_lig65_', '66_lig66_', '67_lig67_',
'68_lig68_', '69_lig69_', '6_lig6_', '70_lig70_', '71_lig71_', '72_lig72_',
'73_lig73_', '74_lig74_', '75_lig75_', '76_lig76_', '77_lig77_', '78_lig78_',
'79_lig79_', '7_lig7_', '80_lig80_', '81_lig81_', '82_lig82_', '83_lig83_',
'84_lig84_', '85_lig85_', '86_lig86_', '87_lig87_', '88_lig88_', '89_lig89_',
'8_lig8_', '90_lig90_', '91_lig91_', '92_lig92_', '93_lig93_', '94_lig94_',
'95_lig95_', '96_lig96_', '97_lig97_', '98_lig98_', '99_lig99_', '9_lig9_']
```

[17]:
```
pdbs = []
for pdb in files:
    pdb = glob.glob(pdb+str('\\3D*'))
    pdbs.append(pdb)
```

[18]:
```
pdbs
```

[18]:
```
[['100_lig100_\\3D_structure_new_colchicine99_NN([C@H1]1CCC2=CC(OC)=C(OC)C(O.pdb
'],
 ['101_lig101_\\3D_structure_new_colchicine100_OCCN(CC=C)C(=O)N[C@@H1]1C2=CC(.pd
```

```
b'],
 ['102_lig102_\\3D_structure_new_colchicine101_OCCN(CC=C)C(=S)N[C@H1]1CCC2=CC.pd
b'],
 ['103_lig103_\\3D_structure_new_colchicine102_CNC(N(C)C)CN[C@H1]1CCC2=CC(OC).pd
b'],
 ['104_lig104_\\3D_structure_new_colchicine103_NC(N(CC)OC)N[C@H1]1CCC2=CC(OC).pd
b'],
 ['105_lig105_\\3D_structure_new_colchicine104_C1[C@H1](C2=CC(C(SC)=CC=C2C3=C.pd
b'],
 ['106_lig106_\\3D_structure_new_colchicine105_C1=2[C@H1](CCC3=CC(OC)=C(OC)C(.pd
b'],
 ['107_lig107_\\3D_structure_new_colchicine106_C1=2[C@H1](CCC3=CC(OC)=C(OC)C(.pd
b'],
 ['108_lig108_\\3D_structure_new_colchicine107_NC(=O)N[C@@H1]C1=C2C(C(NC)=CC=.pd
b'],
 ['109_lig109_\\3D_structure_new_colchicine108_CC(C)NC(=O)N1[C@@H1]C2=CC(C(NC.pd
b'],
 ['10_lig10_\\3D_structure_new_colchicine9_C1N(C(=S)N[C@@H1]2C3=CC(C(NC)=.pdb'],
 ['110_lig110_\\3D_structure_new_colchicine109_CC(N)(CCO)[C@H1]1CCC2=CC(OC)=C.pd
b'],
 ['111_lig111_\\3D_structure_new_colchicine110_CC(N)(CCO)[C@H1]1CCC2=CC(OC)=C.pd
b'],
 ['112_lig112_\\3D_structure_new_colchicine111_C1[C@H1](NC(C)=O)C2=CC(C(NC)=C.pd
b'],
 ['113_lig113_\\3D_structure_new_colchicine112_C1[C@H1](NC(C)=O)C2=CC(C(SC)=C.pd
b'],
 ['114_lig114_\\3D_structure_new_colchicine113_C=C(N[C@H1]1CCC2=C(C(OC)=C(OC).pd
b'],
 ['115_lig115_\\3D_structure_new_colchicine114_CC(C)N(C(C)C)C(=O)N[C@@H1]C1=C.pd
b'],
 ['116_lig116_\\3D_structure_new_colchicine115_C1C(C)NC1N[C@H1]2CCC3=CC(OC)=C.pd
b'],
 ['117_lig117_\\3D_structure_new_colchicine116_O=C(N[C@H1]1CCC2=CC(OC)=C(OC)C.pd
b'],
 ['118_lig118_\\3D_structure_new_colchicine117_O=C(N[C@H1]1CCC2=CC(OC)=C(OC)C.pd
b'],
 ['119_lig119_\\3D_structure_new_colchicine118_COC1=C2C3=CC=C(NC)C(=O)C=C3[C@.pd
b'],
['11_lig11_\\3D_structure_new_colchicine10_C1=2[C@H1](CCC3=CC(OC)=C(OC)C(.pdb'],
 ['120_lig120_\\3D_structure_new_colchicine119_COC=CC1=CC=C(NC)C(=O)C=C1[C@@H.pd
b'],
 ['121_lig121_\\3D_structure_new_colchicine120_N=1C(=CN(N=1)[C@H1]2CCC3=CC(OC.pd
b'],
 ['122_lig122_\\3D_structure_new_colchicine121_C1=2[C@H1]CCC3=CC(OC)=C(OC)C(O.pd
b'],
 ['123_lig123_\\3D_structure_new_colchicine122_N1([C@H1]2CCC3=C(C(OC)=C(OC)C(.pd
b'],
```

```
 ['124_lig124_\\3D_structure_new_colchicine123_N1([C@H1]2CCC3=C(C(OC)=C(OC)C(.pd
b'],
 ['125_lig125_\\3D_structure_new_colchicine124_N1C(OC)=C(C=C1)N[C@H1]2CCC3=C(.pd
b'],
 ['126_lig126_\\3D_structure_new_colchicine125_N(C(C)C)(C(C)C)C(=O)N1[C@H1]CC.pd
b'],
 ['127_lig127_\\3D_structure_new_colchicine126_N(CCCC)CC1=CN(N=N1)[C@H1]2CCC3.pd
b'],
 ['128_lig128_\\3D_structure_new_colchicine127_NC(=S)N1[C@@H1]C2=CC(C(NC)=CC=.pd
b'],
 ['129_lig129_\\3D_structure_new_colchicine128_C=C(CN[C@H1]1CCC2=CC(OC)=C(OC).pd
b'],
['12_lig12_\\3D_structure_new_colchicine11_C[C@H1](CCC1=CC(OC)=C(OC)C(OC(.pdb'],
 ['130_lig130_\\3D_structure_new_colchicine129_C1=C(N=NN1[C@H1]2CCC3=CC(OC)=C.pd
b'],
 ['131_lig131_\\3D_structure_new_colchicine130_C=CC=C(NC(=O)N[C@H1]1CCC2=CC(O.pd
b'],
 ['132_lig132_\\3D_structure_new_colchicine131_C[C@H1](CCC1=CC(OC)=C(OC)C(OC(.pd
b'],
 ['133_lig133_\\3D_structure_new_colchicine132_COCC(CN[C@@H1]1C=2C(C3=C(C(OC).pd
b'],
 ['134_lig134_\\3D_structure_new_colchicine133_NC1=NN=NN1[C@@H1]2C=3C(C4=C(C(.pd
b'],
 ['135_lig135_\\3D_structure_new_colchicine134_OCCN(CCO)C(=S)N1[C@@H1]C2=CC(C.pd
b'],
 ['136_lig136_\\3D_structure_new_colchicine135_NC(C(OCC)=C)N[C@H1]1CCC2=CC(OC.pd
b'],
 ['137_lig137_\\3D_structure_new_colchicine136_N([C@H1]1CCC2=CC(OC)=C(OC)C(OC.pd
b'],
 ['138_lig138_\\3D_structure_new_colchicine137_NC(CCC)N[C@@H1]1C2=CC(C(NC)=CC.pd
b'],
 ['139_lig139_\\3D_structure_new_colchicine138_N([C@H1]1CCC2=CC(OC)=C(OC)C(OC.pd
b'],
['13_lig13_\\3D_structure_new_colchicine12_COC1=C2C3=CC=C(NC)C(=O)C=C3[C@.pdb'],
 ['140_lig140_\\3D_structure_new_colchicine139_N([C@H1]1CCC2=CC(OC)=C(OC)C(OC.pd
b'],
 ['141_lig141_\\3D_structure_new_colchicine140_C1=2[C@H1](CCC3=C(C(OC)=C(OC)C.pd
b'],
 ['142_lig142_\\3D_structure_new_colchicine141_N(C)C=1C(=O)C=C2[C@H1](CCC3=C(.pd
b'],
 ['143_lig143_\\3D_structure_new_colchicine142_N(CCCC)CNCC1=CN(N=N1)[C@H1]2CC.pd
b'],
 ['144_lig144_\\3D_structure_new_colchicine143_N(C)C=1C(=O)C=C2[C@H1](CCC3=C(.pd
b'],
 ['145_lig145_\\3D_LtvuU3.pdb'],
 ['146_lig146_\\3D_structure_new_colchicine145_N1=CC=C(C=C1)C(NCC=2N=NN(C=2)[.pd
b'],
```

```
 ['147_lig147_\\3D_structure_new_colchicine146_OCC(C)=C1C([C@H1](CCC2=CC(OC)=.pd
b'],
 ['148_lig148_\\3D_structure_new_colchicine147_N1=CC=C(C=C1)C(NCC=2N=NN(C=2)[.pd
b'],
 ['149_lig149_\\3D_bJov87.pdb'],
['14_lig14_\\3D_structure_new_colchicine13_COC1=C2C3=CC=C(NC)C(=O)C=C3[C@.pdb'],
 ['150_lig150_\\3D_structure_new_colchicine149_CC(C)(C)OC(=O)NCCCNCC1=CN(N=N1.pd
b'],
 ['151_lig151_\\3D_structure_new_colchicine150_COC1=C2C3=CC=C(SC)C(=O)C=C3[C@.pd
b'],
 ['152_lig152_\\3D_structure_new_colchicine151_COC1=C2C3=CC=C(NC)C(=O)C=C3[C@.pd
b'],
 ['153_lig153_\\3D_structure_new_colchicine152_C=C(CO)CN[C@H1]1CCC2=CC(OC)=C(.pd
b'],
 ['154_lig154_\\3D_structure_new_colchicine153_CC(C)CN[C@H1]1CCC2=CC(OC)=C(OC.pd
b'],
 ['155_lig155_\\3D_structure_new_colchicine154_CC(C)CCN[C@H1]1CCC2=CC(OC)=C(O.pd
b'],
 ['156_lig156_\\3D_structure_new_colchicine155_CC(C)CCN[C@H1]C1CC2=CC(OC)=C(O.pd
b'],
 ['157_lig157_\\3D_structure_new_colchicine156_N=1NC=1[C@@H1]2C3=CC(C(NC)=CC=.pd
b'],
 ['158_lig158_\\3D_structure_new_colchicine157_COC1=C2C3=CC=C(NC)C(=O)C=C3[C@.pd
b'],
 ['159_lig159_\\3D_structure_new_colchicine158_COC1=C(OC)C(OC)=CC=2CC[C@@H1](.pd
b'],
['15_lig15_\\3D_structure_new_colchicine14_CC(=O)N[C@H1]1CCC2=CC(OC)=C(OC.pdb'],
 ['160_lig160_\\3D_structure_new_colchicine159_C12=CC(C(NC)=CC=C1C3=C(C=C(OC).pd
b'],
 ['161_lig161_\\3D_structure_new_colchicine160_O=CC1=CN=NN1[C@H1]2CCC3=CC(OC).pd
b'],
 ['162_lig162_\\3D_structure_new_colchicine161_C1=CN=CC=C1CN[C@H1]2CCC3=CC(OC.pd
b'],
 ['163_lig163_\\3D_structure_new_colchicine162_C1=CN=CC=C1CN[C@H1]2CCC3=CC(OC.pd
b'],
 ['164_lig164_\\3D_structure_new_colchicine163_C=C1N=CC(C)N1CN[C@H1]2CCC3=C(C.pd
b'],
 ['165_lig165_\\3D_structure_new_colchicine164_CNCC(C)OC(=O)N[C@H1]1CCC2=C(C(.pd
b'],
 ['166_lig166_\\3D_structure_new_colchicine165_C=CC=C(CCN[C@H1]1CCC2=CC(OC)=C.pd
b'],
 ['167_lig167_\\3D_structure_new_colchicine166_C1COC1(OC2=CC=C3C4=C(OC)C(OC)=.pd
b'],
 ['168_lig168_\\3D_structure_new_colchicine167_O=C(OC1=C2C(CC[C@H1](NC(=O)C)C.pd
b'],
 ['169_lig169_\\3D_structure_new_colchicine168_O=C1C=CC=C2C([C@H1](CCC3=CC(OC.pd
b'],
```

['16_lig16_\\3D_structure_new_colchicine15_C1=2C(=O)C(=C3C=C1C4=C(CC[C@@H.pdb'],
['170_lig170_\\3D_structure_new_colchicine169_C1=CC=C(C=C1)CN[C@H1]2CCC3=CC(.pdb'],
['171_lig171_\\3D_structure_new_colchicine170_CC=CCCCN[C@H1]1CCC2=CC(OC)=C(O.pdb'],
['172_lig172_\\3D_structure_new_colchicine171_OC1=C(C=CC=C1)CN2[C@H1]CCC3=CC.pdb'],
['173_lig173_\\3D_structure_new_colchicine172_COC1=C2C3=CC=C(NC)C(=O)C=C3[C@.pdb'],
['174_lig174_\\3D_structure_new_colchicine173_COC1=CC=2CC[C@@H1](C=3C(C=2C(=.pdb'],
['175_lig175_\\3D_structure_new_colchicine174_CSC1=CC=C2C(=CC1=O)[C@H1](CCC3.pdb'],
['176_lig176_\\3D_structure_new_colchicine175_C1=C2[C@H1](CCC3=C(C(OC)=C(C(=.pdb'],
['177_lig177_\\3D_structure_new_colchicine176_CC(=O)OC1=CC=C2C3=C(C(OC)=C(C=.pdb'],
['178_lig178_\\3D_structure_new_colchicine177_CC(=O)OC=1C(=O)C=C2C(=CC=1)C3=.pdb'],
['179_lig179_\\3D_structure_new_colchicine178_CC(=O)OC1=CC=C2C3=C(C(OC)=C(C=.pdb'],
['17_lig17_\\3D_structure_new_colchicine16_CC(=O)OC1=CC=C2C3=C(C(OC)=C(C=.pdb'],
['180_lig180_\\3D_structure_new_colchicine179_CCC(=O)OC1=CC=C2C3=C(C(OC)=C(C.pdb'],
['181_lig181_\\3D_structure_new_colchicine180_CCC(=O)N[C@H1]1CCC2=CC(=C(OC)C.pdb'],
['182_lig182_\\3D_structure_new_colchicine181_CC(C)C(OC1=CC=C2C3=C(CC[C@@H1].pdb'],
['183_lig183_\\3D_structure_new_colchicine182_CC(C)C(OC1=CC=C2C3=C(CC[C@@H1].pdb'],
['184_lig184_\\3D_structure_new_colchicine183_CC(C)C(=O)OC1=CC=C2C3=C(C(OC)=.pdb'],
['185_lig185_\\3D_structure_new_colchicine184_CC(C)C(OC1=CC=C2C3=C(C=C(OC)C(.pdb'],
['186_lig186_\\3D_structure_new_colchicine185_CC(=O)OCC1=CN(N=N1)[C@H1]2CCC3.pdb'],
['187_lig187_\\3D_structure_new_colchicine186_C1=C(C2=C(OC)C(OC)=C1OC(=O)CCC.pdb'],
['188_lig188_\\3D_structure_new_colchicine187_CC(=O)N[C@H1]1CCC2=CC(OC)=C(OC.pdb'],
['189_lig189_\\3D_structure_new_colchicine188_O=C(C)N[C@H1]1CCC2=CC(=C(OC)C(.pdb'],
['18_lig18_\\3D_structure_new_colchicine17_CC(=O)OC1=CC=C2C3=C(C(OC)=C(C=.pdb'],
['190_lig190_\\3D_structure_new_colchicine189_C=12C(=O)C(=CC=C3C=1[C@H1](CCC.pdb'],
['191_lig191_\\3D_structure_new_colchicine190_CCC(=O)N[C@H1]1CCC2=C(C(OC)=C(.pdb'],

```
 ['192_lig192_\\3D_structure_new_colchicine191_CCC(=O)N[C@H1]1CCC2=CC(OC)=C(C.pd
b'],
 ['193_lig193_\\3D_structure_new_colchicine192_CC=1C(=CC(C(=CC=1)SC)=O)[C@H1].pd
b'],
 ['194_lig194_\\3D_structure_new_colchicine193_CC(C)COCCN[C@H1]1CCC2=CC(OC)=C.pd
b'],
 ['195_lig195_\\3D_structure_new_colchicine194_CC(C)CCN[C@H1]1CCC2=C(C3=CC=C(.pd
b'],
 ['196_lig196_\\3D_structure_new_colchicine195_CC(C)CCN[C@H1]1CCC2=C(C3=CC=C(.pd
b'],
 ['197_lig197_\\3D_structure_new_colchicine196_C1=C(OC)C(=C(OC)C2=C1CC[C@H1](.pd
b'],
 ['198_lig198_\\3D_structure_new_colchicine197_O=C(C)N[C@H1]1CCC2=CC(OC)=C(C(.pd
b'],
 ['199_lig199_\\3D_structure_new_colchicine198_O=C(N(C)C)NC1=CC=C2C3=C(OC)C(=.pd
b'],
['19_lig19_\\3D_structure_new_colchicine18_CC(=O)OC1=CC=C2C3=C(C(OC)=C(C=.pdb'],
 ['1_lig1_\\3D_structure_new_colchicine0_COC1=C2C3=CC=C(NC)C(=O)C=C3[C@.pdb'],
 ['200_lig200_\\3D_structure_new_colchicine199_O=C(N)N[C@H1]1CCC2=CC(OC)=C(C(.pd
b'],
 ['201_lig201_\\3D_structure_new_colchicine200_O=C(C)N[C@H1]1CCC2=CC(OC)=C(C(.pd
b'],
 ['202_lig202_\\3D_structure_new_colchicine201_CC(=O)N1[C@H1]CCC2=CC(OC)=C(OC.pd
b'],
 ['203_lig203_\\3D_structure_new_colchicine202_COC1=C2C3=CC=C(NC)C(=O)C=C3[C@.pd
b'],
 ['204_lig204_\\3D_structure_new_colchicine203_C1[C@@H1](C2=CC(=O)C(NC)=CC=C2.pd
b'],
 ['205_lig205_\\3D_structure_new_colchicine204_CNC(C(C)O)N([C@H1]1CCC2=CC(=C(.pd
b'],
 ['206_lig206_\\3D_structure_new_colchicine205_CNC(C(C)O)N[C@@H1]1C=2C(C3=C(C.pd
b'],
 ['207_lig207_\\3D_structure_new_colchicine206_CNC(=S)N[C@@H1]1C2=CC(=O)C(NC).pd
b'],
 ['208_lig208_\\3D_structure_new_colchicine207_N([C@H1]1CCC2=C(C3=CC=C(C(C=C1.pd
b'],
 ['209_lig209_\\3D_structure_new_colchicine208_C12=CC(C(NC)=CC=C1C3=C(C=C(C(O.pd
b'],
['20_lig20_\\3D_structure_new_colchicine19_CC(C)C(OC1=CC=C2C3=C(CC[C@@H1].pdb'],
 ['210_lig210_\\3D_structure_new_colchicine209_COC1=C(OC)C(OC)=CC=2CC[C@@H1](.pd
b'],
 ['211_lig211_\\3D_structure_new_colchicine210_COC1=C(OC)C(OC)=CC=2CC[C@@H1](.pd
b'],
 ['212_lig212_\\3D_structure_new_colchicine211_CN(C(=S)N[C@@H1]1C2=CC(=O)C(NC.pd
b'],
 ['213_lig213_\\3D_structure_new_colchicine212_CC(C)(C)OC1=CN(N=N1)[C@H1]C2CC.pd
b'],
```

['214_lig214_\\3D_structure_new_colchicine213_CC(C)CCCCCCN[C@@H1]1C2=CC(C(SC.pd
b'],
['215_lig215_\\3D_structure_new_colchicine214_CC(C)(C)C(=O)N[C@@H1]1C=2C(C3=.pd
b'],
['216_lig216_\\3D_structure_new_colchicine215_OC(CCC(C)C)OCC1=CN(N=N1)[C@H1].pd
b'],
['217_lig217_\\3D_structure_new_colchicine216_O=C(NCC1=CC=CC=C1Cl)N[C@H1]2CC.pd
b'],
['218_lig218_\\3D_structure_new_colchicine217_N([C@H1]1CCC2=C(C(OC)=C(OC)C(O.pd
b'],
['219_lig219_\\3D_structure_new_colchicine218_O=CC=CCC=CNNC(N[C@H1]1CCC2=CC(.pd
b'],
['21_lig21_\\3D_structure_new_colchicine20_CC(=O)N[C@@H1]1C2=CC(C(SC)=CC=.pdb'],
['220_lig220_\\3D_structure_new_colchicine219_OC1=C(C=CC=C1)C=2N=NN(C=2)[C@H.pd
b'],
['221_lig221_\\3D_structure_new_colchicine220_OC1=C(C=CC=C1)C=2N=NN(C=2)[C@H.pd
b'],
['222_lig222_\\3D_structure_new_colchicine221_C1[C@@H1](C2=CC(=O)C(NC)=CC=C2.pd
b'],
['223_lig223_\\3D_structure_new_colchicine222_O=C(OC(C)(C)C)N[C@H1]1CCC2=C(C.pd
b'],
['224_lig224_\\3D_structure_new_colchicine223_O=C(OCC)CN(OCC)C[C@@H1]1C2=CC(.pd
b'],
['225_lig225_\\3D_structure_new_colchicine224_O=C(C)OC1=CN(N=N1)[C@H1]2CCC3=.pd
b'],
['226_lig226_\\3D_structure_new_colchicine225_O=C(OCC)CN(CCC)C[C@H1]C1CC2=CC.pd
b'],
['227_lig227_\\3D_structure_new_colchicine226_O=C(C(Cl)Cl)NC(=O)N[C@H1]1CCC2.pd
b'],
['228_lig228_\\3D_structure_new_colchicine227_O=C(C(Cl)Cl)NC(=O)N[C@H1]1CCC2.pd
b'],
['229_lig229_\\3D_structure_new_colchicine228_O=C(OCCC)OCC1=CN(N=N1)[C@H1]2C.pd
b'],
['22_lig22_\\3D_structure_new_colchicine21_O=C(OC)OC1=CC=C2C3=C(C(OC)=C(C.pdb'],
['230_lig230_\\3D_structure_new_colchicine229_O=C(OCCC)OCC1=CN(N=N1)[C@H1]2C.pd
b'],
['231_lig231_\\3D_structure_new_colchicine230_O=C(OCC=C)NCC1=CN(N=N1)[C@H1]2.pd
b'],
['232_lig232_\\3D_structure_new_colchicine231_O=C(C(Cl)Cl)NCC1=CN(N=N1)[C@H1.pd
b'],
['233_lig233_\\3D_structure_new_colchicine232_O=C(C(Cl)Cl)NCC1=CN(N=N1)[C@H1.pd
b'],
['234_lig234_\\3D_structure_new_colchicine233_O=C(C)N[C@@H1]1CC(C2=C(C(OC)=C.pd
b'],
['235_lig235_\\3D_structure_new_colchicine234_O=C(C(Cl)Cl)NC(=O)N[C@H1]1CCC2.pd
b'],
['236_lig236_\\3D_structure_new_colchicine235_O=C(OCC)C1=CN(N=N1)[C@H1]2CCC3.pd

```
b'],
 ['237_lig237_\\3D_structure_new_colchicine236_O=C(O)CN(CCO)C(=O)N[C@H1]1CCC2.pd
b'],
 ['238_lig238_\\3D_structure_new_colchicine237_CC(C)OC(NCC1=CN(N=N1)[C@H1]2CC.pd
b'],
 ['239_lig239_\\3D_structure_new_colchicine238_COC(NCCCOCC1=CN(N=N1)[C@H1]2CC.pd
b'],
['23_lig23_\\3D_structure_new_colchicine22_O=C(C)N[C@H1]1CCC2=CC(=C(OC)C(.pdb'],
 ['240_lig240_\\3D_zEatBw.pdb'],
 ['241_lig241_\\3D_structure_new_colchicine240_C1=CN=CC=C1C2=CN(N=N2)[C@H1]3C.pd
b'],
 ['242_lig242_\\3D_structure_new_colchicine241_COC=C(OC)C(OC)=CCCC[C@H1](N1C=.pd
b'],
 ['243_lig243_\\3D_structure_new_colchicine242_COC1=C(OC)C=CC=2CC[C@@H1](C3=C.pd
b'],
 ['244_lig244_\\3D_structure_new_colchicine243_CC(C)(O)C=CN([C@H1]1CCC2=CC(OC.pd
b'],
 ['245_lig245_\\3D_structure_new_colchicine244_C=12C(=CC(C(NC)=CC=1)=O)[C@H1].pd
b'],
 ['246_lig246_\\3D_structure_new_colchicine245_NC(=O)NCC1=CN(N=N1)[C@H1]2CCC3.pd
b'],
 ['247_lig247_\\3D_structure_new_colchicine246_C1=CC(OC)=CC=C1CN[C@H1]2CCC3=C.pd
b'],
 ['248_lig248_\\3D_structure_new_colchicine247_N(C(C1=CC=CC=C1)=O)CCN[C@H1]2C.pd
b'],
 ['249_lig249_\\3D_structure_new_colchicine248_N=NC(=C(C(OCC)=O)N[C@H1]1CCC2=.pd
b'],
['24_lig24_\\3D_structure_new_colchicine23_CC(=O)OCC1=CN(N=N1)[C@H1]2CCC3.pdb'],
 ['250_lig250_\\3D_structure_new_colchicine249_CNC=1C(=O)C=C2[C@H1](CCC3=C(C(.pd
b'],
 ['251_lig251_\\3D_structure_new_colchicine250_NN(C=C(CNC(CCCCCl)=O)N=N)[C@H1.pd
b'],
 ['252_lig252_\\3D_structure_new_colchicine251_N=NC1=CN(N=N1)[C@H1]2CCC3=CC(O.pd
b'],
 ['253_lig253_\\3D_structure_new_colchicine252_CC(C)(C)OC(=O)NCCC(NCC=1N=NN(C.pd
b'],
 ['254_lig254_\\3D_structure_new_colchicine253_CC(C)(C)OCNCCC(=O)NCC1=CN(N=N1.pd
b'],
 ['255_lig255_\\3D_structure_new_colchicine254_C1(=CN(C(=O)OCC)N[C@@H1]2C=3C(.pd
b'],
 ['256_lig256_\\3D_structure_new_colchicine255_CC(C)(C)OCNCCC(=O)NCC1=CN(N=N1.pd
b'],
 ['257_lig257_\\3D_structure_new_colchicine256_CC(C)(C)OC(=O)NCCC(OCC=1N=NN(C.pd
b'],
 ['258_lig258_\\3D_QxX7hq.pdb'],
 ['259_lig259_\\3D_structure_new_colchicine258_C=1N(N=NC=1C(OCC)=O)[C@H1]2CCC.pd
b'],
```

```
['25_lig25_\\3D_structure_new_colchicine24_CC(=O)OCC1=CN(N=N1)[C@H1]2CCC3.pdb'],
 ['260_lig260_\\3D_structure_new_colchicine259_O=CC=CN=NN[C@H1]1CCC2=C(C(OC)=.pd
b'],
 ['261_lig261_\\3D_structure_new_colchicine260_C1=CN=CC=C1C(NCC2=CN(N=N2)[C@H.pd
b'],
 ['262_lig262_\\3D_structure_new_colchicine261_N=NN([C@H1]1CCC2=C(C(OC)=C(OC).pd
b'],
 ['263_lig263_\\3D_structure_new_colchicine262_CN(N([C@@H1]1C2=CC(=O)C(NC)=CC.pd
b'],
 ['264_lig264_\\3D_structure_new_colchicine263_CN(N([C@@H1]1C2=CC(=O)C(NC)=CC.pd
b'],
 ['265_lig265_\\3D_structure_new_colchicine264_O=C(CC)OCC1=CN(N=N1)[C@H1]2CCC.pd
b'],
 ['266_lig266_\\3D_structure_new_colchicine265_O=C(OC(C)(C)C)NCC=1N=NN(C=1)[C.pd
b'],
 ['267_lig267_\\3D_structure_new_colchicine266_O=C(C(C)C)NCN[C@H1]1CCC2=CC(OC.pd
b'],
 ['268_lig268_\\3D_structure_new_colchicine267_O=C(OCC)C=1N(N=NC=1C(=O)O2)[C@.pd
b'],
 ['269_lig269_\\3D_structure_new_colchicine268_CC(O)(C)CC(=O)OCC1=CN(N=N1)[C@.pd
b'],
['26_lig26_\\3D_structure_new_colchicine25_CC(=O)N[C@@H1]1C2=CC(C(NC)=CC=.pdb'],
 ['270_lig270_\\3D_structure_new_colchicine269_CC(C)COC(NCC1=CN(N=N1)[C@H1]C2.pd
b'],
 ['271_lig271_\\3D_structure_new_colchicine270_COCNCC1=CN(N=N1)[C@H1]2CCC3=CC.pd
b'],
 ['272_lig272_\\3D_structure_new_colchicine271_CC(C)(C)OC(NCC=1N=NN(C=1)[C@H1.pd
b'],
 ['273_lig273_\\3D_structure_new_colchicine272_N(C)C1=CC=C2C3=C(OC)C(OC)=C(OC.pd
b'],
 ['274_lig274_\\3D_structure_new_colchicine273_O=C(OCC)C1=C(C(OCC)=O)N=NN1[C@.pd
b'],
 ['275_lig275_\\3D_structure_new_colchicine274_N([C@H1]1CCC2=CC(OC)=C(OC)C(OC.pd
b'],
 ['276_lig276_\\3D_structure_new_colchicine275_CC(CC)N[C@H1]C1CC2=CC(OC)=C(OC.pd
b'],
 ['277_lig277_\\3D_structure_new_colchicine276_CC(CC)N[C@H1]C1CC2=CC(OC)=C(OC.pd
b'],
 ['278_lig278_\\3D_structure_new_colchicine277_COC1=C2C3=CC=C(NC)C(=O)C=C3[C@.pd
b'],
 ['279_lig279_\\3D_structure_new_colchicine278_COC1=C2C3=CC=C(NC)C(=O)C=C3[C@.pd
b'],
['27_lig27_\\3D_structure_new_colchicine26_CC(=O)N[C@@H1]C1=C2C(C(NC)=CC=.pdb'],
 ['280_lig280_\\3D_structure_new_colchicine279_COC1=C2C3=CC=C(SC)C(=O)C=C3[C@.pd
b'],
 ['281_lig281_\\3D_structure_new_colchicine280_O(C1=C(OC)C=2C3=CC=C(SC)C(C=C3.pd
b'],
```

```
 ['282_lig282_\\3D_structure_new_colchicine281_C1=CN=CC=C1CN[C@H1]2CCC3=CC(OC.pd
b'],
 ['283_lig283_\\3D_structure_new_colchicine282_O=CC1=CN=NN1[C@H1]2CCC3=CC(OC).pd
b'],
 ['284_lig284_\\3D_structure_new_colchicine283_NC(CCN[C@H1]1CCC2=CC(OC)=C(OC).pd
b'],
 ['285_lig285_\\3D_structure_new_colchicine284_NC(N=C1C=CC2)=CC=C1C3=C(OC)C(O.pd
b'],
 ['286_lig286_\\3D_structure_new_colchicine285_C=CC1=C(NC)C(OC)=C(OC)C=C1CC[C.pd
b'],
 ['287_lig287_\\3D_structure_new_colchicine286_CC(N)(CCO)[C@H1]1CCC2=CC(OC)=C.pd
b'],
 ['288_lig288_\\3D_structure_new_colchicine287_N(NCCCCCl)[C@H1]1CCC2=CC(OC)=C.pd
b'],
 ['289_lig289_\\3D_structure_new_colchicine288_N(C1=CC=C(C=C1)C)CN[C@H1]2CCC3.pd
b'],
['28_lig28_\\3D_structure_new_colchicine27_COC1=C2C3=CC=C(NC)C(=O)C=C3[C@.pdb'],
 ['290_lig290_\\3D_structure_new_colchicine289_N(C1=CC=C(C=C1)C)CN[C@H1]2CCC3.pd
b'],
 ['291_lig291_\\3D_structure_new_colchicine290_CCN(CC)C(=O)N[C@H1]C1CC2=CC(OC.pd
b'],
 ['292_lig292_\\3D_structure_new_colchicine291_CCN(CC)C(=O)N1[C@H1]CCC2=CC(OC.pd
b'],
 ['293_lig293_\\3D_structure_new_colchicine292_CCN(CC)C(=O)N[C@H1]1CCC2=CC(OC.pd
b'],
 ['294_lig294_\\3D_structure_new_colchicine293_CC(C)COC1=C(OC)C(OC)=CC=2CC[C@.pd
b'],
 ['295_lig295_\\3D_structure_new_colchicine294_C=C(C)CN(CCO)C(=S)N[C@H1]1CCC2.pd
b'],
 ['296_lig296_\\3D_structure_new_colchicine295_CC(C)C(OC=C(C(OC)=CCCC[C@H1](N.pd
b'],
 ['297_lig297_\\3D_structure_new_colchicine296_N([C@H1]1CCC2=CC(OC)=C(OC)C(OC.pd
b'],
 ['298_lig298_\\3D_structure_new_colchicine297_O=C(N[C@H1]1CCC2=CC(OC)=C(OC)C.pd
b'],
 ['299_lig299_\\3D_structure_new_colchicine298_O=C(N[C@@H1]1C2=CC(C(NC)=CC=C2.pd
b'],
['29_lig29_\\3D_structure_new_colchicine28_COC1=C2C3=CC=C(NC)C(=O)C=C3[C@.pdb'],
 ['2_lig2_\\3D_structure_new_colchicine1_COC1=C2C3=CC=C(NC)C(=O)C=C3[C@.pdb'],
 ['300_lig300_\\3D_structure_new_colchicine299_N([C@H1]1CCC2=CC(OC)=C(OC)C(OC.pd
b'],
 ['301_lig301_\\3D_structure_new_colchicine300_O=C(N[C@H1]1CCC2=CC(OC)=C(OC)C.pd
b'],
 ['302_lig302_\\3D_structure_new_colchicine301_C1=CCC[C@H1](NC(=S)NC2=CC=C(C=.pd
b'],
 ['303_lig303_\\3D_structure_new_colchicine302_CNCC(O)(C)C1=CN(N=N1)[C@H1]2CC.pd
b'],
```

```
 ['304_lig304_\\3D_structure_new_colchicine303_N1([C@H1]2CCC3=C(C(OC)=C(OC)C(.pd
b'],
 ['305_lig305_\\3D_structure_new_colchicine304_N([C@H1]1CCC2=C(C(OC)=C(OC)C(O.pd
b'],
 ['306_lig306_\\3D_structure_new_colchicine305_C1=C(O)C(OC)=CC(CN[C@H1]2CCC3=.pd
b'],
 ['307_lig307_\\3D_structure_new_colchicine306_COCC(C)=C(C(OC)=CCCC[C@H1](NCC.pd
b'],
 ['308_lig308_\\3D_structure_new_colchicine307_C[C@H1](CCC1=CC(OC)=C(OC)C(OC).pd
b'],
 ['309_lig309_\\3D_structure_new_colchicine308_NC(=O)N1[C@@H1]C2=CC(C(NC)=CC=.pd
b'],
['30_lig30_\\3D_structure_new_colchicine29_COC1=C2C3=CC=C(NC)C(=O)C=C3[C@.pdb'],
 ['310_lig310_\\3D_structure_new_colchicine309_NN(CCO)[C@@H1]1C2=CC(C(NC)=CC=.pd
b'],
 ['311_lig311_\\3D_structure_new_colchicine310_C1=2[C@H1](CCC3=CC(OC)=C(OC)C(.pd
b'],
 ['312_lig312_\\3D_structure_new_colchicine311_NC(N=NCOCCC)C[C@H1]1C2=CC(C(NC.pd
b'],
 ['313_lig313_\\3D_structure_new_colchicine312_C1=2C(C3=C(OC)C(OC)=C(OC)C=C3C.pd
b'],
 ['314_lig314_\\3D_structure_new_colchicine313_C1=C(CN[C@H1]2CCC3=CC(OC)=C(OC.pd
b'],
 ['315_lig315_\\3D_structure_new_colchicine314_C1[C@H1](NC(NC2=CC=C(C=C2)F)=O.pd
b'],
 ['316_lig316_\\3D_structure_new_colchicine315_C1=CC(=CC=N1)CCN[C@@H1]2C3=CC(.pd
b'],
 ['317_lig317_\\3D_structure_new_colchicine316_C1=2[C@H1]CCC3=CC(OC)=C(OC)C(O.pd
b'],
 ['318_lig318_\\3D_structure_new_colchicine317_CC=CC1=C(C=C(OC)C(OC)=C1OC(OCC.pd
b'],
 ['319_lig319_\\3D_structure_new_colchicine318_O=C1C=C2[C@H1](CCC3=C(C(OC)=C(.pd
b'],
['31_lig31_\\3D_structure_new_colchicine30_COC1=C2C3=CC=C(NC)C(=O)C=C3[C@.pdb'],
 ['320_lig320_\\3D_structure_new_colchicine319_O=C1C=C2[C@H1](CCC3=C(C(OC)=C(.pd
b'],
 ['321_lig321_\\3D_structure_new_colchicine320_O=C1C=C2[C@H1](CCC3=C(C(OC)=C(.pd
b'],
 ['322_lig322_\\3D_structure_new_colchicine321_N1C=NN=NN1[C@H1]2CCC3=C(C(OC)=.pd
b'],
 ['323_lig323_\\3D_structure_new_colchicine322_CNC=1C(=O)C=C2[C@H1](CCC3=CC(O.pd
b'],
 ['324_lig324_\\3D_structure_new_colchicine323_N([C@H1]1CCC2=CC(OC)=C(OC)C(OC.pd
b'],
 ['325_lig325_\\3D_structure_new_colchicine324_C[C@H1](CCC1=CC(OC)=C(OC)C(OC).pd
b'],
 ['326_lig326_\\3D_structure_new_colchicine325_N([C@H1]1CCC2=C(C(OC)=C(OC)C(O.pd
```

```
b'],
 ['327_lig327_\\3D_structure_new_colchicine326_C=CC1=C(C(=O)OC=CC=C2C3=C(OC)C.pd
b'],
 ['328_lig328_\\3D_structure_new_colchicine327_C=C1C2=C(C=C(OC)C(OC)=C2OC(OCC.pd
b'],
 ['329_lig329_\\3D_structure_new_colchicine328_CC=CC(N[C@H1]1CCC2=C(C(OC)=C(O.pd
b'],
['32_lig32_\\3D_structure_new_colchicine31_COC1=C(OC)C(OC)=CC=2CC[C@H1](N.pdb'],
 ['330_lig330_\\3D_structure_new_colchicine329_OC(=O)NCC1=CN(N=N1)[C@@H1]2C=3.pd
b'],
 ['331_lig331_\\3D_structure_new_colchicine330_N(C)C=1C(=O)C=C2[C@H1](CCC3=C(.pd
b'],
 ['332_lig332_\\3D_structure_new_colchicine331_O=C(C=C(C(O1)CCCCC1)CN=N)N[C@.pd
b'],
 ['333_lig333_\\3D_structure_new_colchicine332_N([C@H1]1CCC2=C(C(OC)=C(OC)C(O.pd
b'],
 ['334_lig334_\\3D_structure_new_colchicine333_O=C(C=C(Cl)C=CN([C@H1]1CCC2=C(.pd
b'],
 ['335_lig335_\\3D_O4ZgW2.pdb'],
 ['336_lig336_\\3D_L9ZpgB.pdb'],
 ['337_lig337_\\3D_structure_new_colchicine336_C12=C3C(C4=C(OC)C(OC)=C(OC)C=C.pd
b'],
['33_lig33_\\3D_structure_new_colchicine32_CNC(NCCCC)=S[C@@H1]C1=CC(=O)C(.pdb'],
['34_lig34_\\3D_structure_new_colchicine33_CN(C(=S)N[C@@H1]1C2=CC(=O)C(NC.pdb'],
['35_lig35_\\3D_structure_new_colchicine34_CNC(C(C)C)N[C@@H1]1C=2C(C3=C(C.pdb'],
['36_lig36_\\3D_structure_new_colchicine35_COC1=C(OC)C(OC)=CC=2CC[C@@H1](.pdb'],
['37_lig37_\\3D_structure_new_colchicine36_COC1=C(OC)C(OC)=CC=2CC[C@@H1](.pdb'],
['38_lig38_\\3D_structure_new_colchicine37_CC(C)(C)COC1=CC=C2C3=C(OC)C(OC.pdb'],
['39_lig39_\\3D_structure_new_colchicine38_C1=2[C@H1]CCC3=CC(OC)=C(OC)C(O.pdb'],
 ['3_lig3_\\3D_structure_new_colchicine2_COC1=C2C3=CC=C(NC)C(=O)C=C3[C@.pdb'],
['40_lig40_\\3D_structure_new_colchicine39_OC(CCC(C)C)OCC1=CN(N=N1)[C@H1].pdb'],
['41_lig41_\\3D_structure_new_colchicine40_OC1=C(C=CC=C1)C=2N=NN(C=2)[C@H.pdb'],
['42_lig42_\\3D_structure_new_colchicine41_NC1=CN(N=N1)[C@H1]2CCC3=C(C(OC.pdb'],
['43_lig43_\\3D_structure_new_colchicine42_O=C(OCC)CN(CCO)N[C@H1]1CCC2=CC.pdb'],
['44_lig44_\\3D_structure_new_colchicine43_O=C(C1=C(C(OCC)=O)N(N=N1)[C@@H.pdb'],
['45_lig45_\\3D_structure_new_colchicine44_C1=2[C@H1](CCC3=CC(OC)=C(OC)C(.pdb'],
['46_lig46_\\3D_structure_new_colchicine45_C=12N(N=NC=1C(OCC3=CC=C(C=C3)F.pdb'],
['47_lig47_\\3D_structure_new_colchicine46_C=1N(N=NC=1C(=O)OCC2=CC=C(C=C2.pdb'],
['48_lig48_\\3D_structure_new_colchicine47_O=CC1=C(C(OCC)=O)N(N=N1)[C@@H1.pdb'],
['49_lig49_\\3D_structure_new_colchicine48_O=C(C(Cl)Cl)NC(=O)N[C@H1]1CCC2.pdb'],
 ['4_lig4_\\3D_structure_new_colchicine3_COC1=C2C3=CC=C(NC)C(=O)C=C3[C@.pdb'],
['50_lig50_\\3D_structure_new_colchicine49_O=C(C(Cl)Cl)NCC1=CN(N=N1)[C@H1.pdb'],
['51_lig51_\\3D_structure_new_colchicine50_O=C(C(Cl)Cl)NC(=O)N[C@H1]1CCC2.pdb'],
['52_lig52_\\3D_structure_new_colchicine51_O=C(O)C=C(C(OCC)=O)N=NN[C@H1]1.pdb'],
['53_lig53_\\3D_structure_new_colchicine52_O=C(O)C1=C(C(OCC)=O)N=NN1[C@H1.pdb'],
['54_lig54_\\3D_structure_new_colchicine53_O=C(OCC)C1=CN(N=N1)[C@H1]2CCC3.pdb'],
['55_lig55_\\3D_structure_new_colchicine54_O=C(O)C1=CN(N=N1)[C@H1]2CCC3=C.pdb'],
```

['56_lig56_\\3D_structure_new_colchicine55_O=C(O)C1=C(C(OCC)=O)N=NN1[C@H1.pdb'],
['57_lig57_\\3D_structure_new_colchicine56_CCCCCCCCCCCCCCC(OCC1=CN(N=N1).pdb'],
['58_lig58_\\3D_structure_new_colchicine57_OC(=O)C1=C(C(OCC)=O)N(N=N1)[C@.pdb'],
['59_lig59_\\3D_structure_new_colchicine58_C1=C(OC)C=C(OC)C=C1CC[C@H1](C=.pdb'],
['5_lig5_\\3D_structure_new_colchicine4_O=CC1=CN=NN1[C@H1]2CCC3=CC(OC).pdb'],
['60_lig60_\\3D_structure_new_colchicine59_N(C)C(=O)NCCC(OCC1=CN(N=N1)[C@.pdb'],
['61_lig61_\\3D_structure_new_colchicine60_OC(=O)C=C(C(OCC)=O)N(NCCC)N[C@.pdb'],
['62_lig62_\\3D_structure_new_colchicine61_C[C@H1](NCC)CC1=CC(C(NC)=CC=C1.pdb'],
['63_lig63_\\3D_NsTQ4B.pdb'],
['64_lig64_\\3D_structure_new_colchicine63_N1=NN1[C@@H1]2C3=CC(C(NC)=CC=C.pdb'],
['65_lig65_\\3D_structure_new_colchicine64_O=CC=CC=CC(NCC1=CN(N=N1)[C@H1].pdb'],
['66_lig66_\\3D_structure_new_colchicine65_CN(N([C@@H1]1C2=CC(=O)C(NC)=CC.pdb'],
['67_lig67_\\3D_structure_new_colchicine66_CC(C)C(=O)OCC1=CN(N=N1)[C@H1]2.pdb'],
['68_lig68_\\3D_structure_new_colchicine67_N(C(C1=CC=NC=C1)=O)[C@@H1]2C=3.pdb'],
['69_lig69_\\3D_structure_new_colchicine68_N1(CCCC1)CN[C@H1]2CCC3=C(C(OC).pdb'],
['6_lig6_\\3D_structure_new_colchicine5_OC1=CC=C(C=C1)CN[C@H1]2CCC3=C(.pdb'],
['70_lig70_\\3D_structure_new_colchicine69_N1(CCCC1)C(=O)OC2=C(OC)C(OC3)=.pdb'],
['71_lig71_\\3D_structure_new_colchicine70_CNC=1C(=O)C=C2[C@H1](CCC3=CC(O.pdb'],
['72_lig72_\\3D_structure_new_colchicine71_CC(C)(C)OC(=O)NCCC(NCC=1N=NN(C.pdb'],
['73_lig73_\\3D_8godGN.pdb'],
['74_lig74_\\3D_structure_new_colchicine73_CC(C)(C)OCNCCC(=O)NCC1=CN(N=N1.pdb'],
['75_lig75_\\3D_YDrpzN.pdb'],
['76_lig76_\\3D_structure_new_colchicine75_CC(C)(C)OC(=O)NCCC(OCC=1N=NN(C.pdb'],
['77_lig77_\\3D_structure_new_colchicine76_CC(C)(C)OC(=O)NCCC(NCC=1N=NN(C.pdb'],
['78_lig78_\\3D_structure_new_colchicine77_CNCCC1=CC=C(C=C1)NC(=O)N[C@H1].pdb'],
['79_lig79_\\3D_structure_new_colchicine78_CNCC(C1=CC=C(C=C1)NC(=O)N[C@@H.pdb'],
['7_lig7_\\3D_structure_new_colchicine6_OC1=CC=C(C=C1)CN[C@H1]2CCC3=CC.pdb'],
['80_lig80_\\3D_structure_new_colchicine79_O=C(OC)OCC1=CN(N=N1)[C@H1]2CCC.pdb'],
['81_lig81_\\3D_structure_new_colchicine80_O=C(C(C)C)NC(=S)N[C@@H1]1C=2C(.pdb'],
['82_lig82_\\3D_structure_new_colchicine81_O=C(OCC)OCC=1N=NN(C=1)[C@H1]2C.pdb'],
['83_lig83_\\3D_structure_new_colchicine82_O=C(OCC)CN(CCO)C(=S)N[C@H1]1CC.pdb'],
['84_lig84_\\3D_structure_new_colchicine83_CC(C)CNCC1=CN(N=N1)[C@H1]2CCC3.pdb'],
['85_lig85_\\3D_structure_new_colchicine84_CC(C)CNCC1=CN(N=N1)[C@H1]2CCC3.pdb'],
['86_lig86_\\3D_structure_new_colchicine85_COCNCC1=CN(N=N1)[C@H1]2CCC3=CC.pdb'],
['87_lig87_\\3D_structure_new_colchicine86_N(C(C)C)C(NCC1=CN(N=N1)[C@H1]2.pdb'],
['88_lig88_\\3D_structure_new_colchicine87_N(C)C1=CC=C2C3=C(OC)C(OC)=C(OC.pdb'],
['89_lig89_\\3D_structure_new_colchicine88_N(C(C(C)C)=O)C1=CN(N=N1)[C@H1].pdb'],
['8_lig8_\\3D_structure_new_colchicine7_O=CC1=C2[C@H1](CCC3=CC(OC)=C(O.pdb'],
['90_lig90_\\3D_structure_new_colchicine89_N(CCCCCCC=O)CC1=CN(N=N1)[C@H1].pdb'],
['91_lig91_\\3D_structure_new_colchicine90_C1=C(C=CC(Cl)=C1)COC(=O)CN[C@@.pdb'],
['92_lig92_\\3D_structure_new_colchicine91_N(C)C1=CC=C2C3=C(OC)C(OC)=C(OC.pdb'],
['93_lig93_\\3D_structure_new_colchicine92_N(C)C1=CC=C2C3=C(OC)C(OC)=C(OC.pdb'],
['94_lig94_\\3D_structure_new_colchicine93_N(C(C)C)CC(=O)OCC1=C2N(N=N1)[C.pdb'],
['95_lig95_\\3D_structure_new_colchicine94_CC(CC)N[C@@H1]1C2=CC(C(SC)=CC=.pdb'],
['96_lig96_\\3D_structure_new_colchicine95_CC(CC)N[C@@H1]C1=C2C(C(NC)=CC=.pdb'],
['97_lig97_\\3D_structure_new_colchicine96_COC1=C2C3=CC=C(NC)C(=O)C=C3[C@.pdb'],
['98_lig98_\\3D_structure_new_colchicine97_N(C)C=1C(=O)C=C2[C@H1](CCC3=CC.pdb'],

```
['99_lig99_\\3D_structure_new_colchicine98_CNC=1C(C=C2[C@H1](CCC3=C(C(OC).pdb'],
 ['9_lig9_\\3D_structure_new_colchicine8_C1=2[C@H1](CCC3=CC(OC)=C(OC)C(.pdb']]
```

[19]:
```
pdbs[1][0]
```

[19]:
```
'101_lig101_\\3D_structure_new_colchicine100_OCCN(CC=C)C(=O)N[C@@H1]1C2=CC(.pdb'
```

[20]:
```
with open('Prepare_pdbqt_from_pdb.txt', 'w') as f:
    for line in pdbs:
        f.write('obabel ')
        f.write(str('"')+line[0]+str('"'))
        f.write(' -O ')
        f.write(line[0].split('\\')[0]+str('\\')+line[0].split('\\')[0]+str('.
 ↪pdbqt'))
        f.write('\n')
```

1.4 Run generated code in the command prompt in the direcotry where the file is created

1.5 distribute protein - the receptor should be prepared before running below code in the *.pdbqt file (with polar H)

1.6 1SA0_AB_chains_prot_polar_H.pdbqt

[21]:
```
name_of_prepared_receptor = str(input('Please use the name given for your␣
 ↪protein domain after preparation... (For example 5v5z_polar_H.pdbqt) : '))
```

```
Please use the name given for your protein domain after preparation… (For
example 5v5z_polar_H.pdbqt) : 1SA0_AB_chains_prot_polar_H.pdbqt
```

[22]:
```
to_be_moved_to = list_dir
```

[23]:
```
to_be_moved_to[0].split("\\")[-1]
```

[23]:
```
'1_lig1_'
```

[24]:
```
to_be_moved_to[0]
```

[24]:
```
'C:\\Users\\aleks\\Documents\\GitHub\\Kolchicyna\\Molecular docking\\1_lig1_'
```

1.7 The initial config file should be prepared before running this code, exemplary one is in the repo

[25]:
```
with open('config.txt') as f:
    lines = f.readlines()
```

[26]:
```
lines
```

[26]:
```
['receptor = 1SA0_AB_chains_prot_polar_H.pdbqt\n',
 'ligand = 1SA0_AB_chains_colchicine_only.pdbqt\n',
 '\n',
 'center_x = 119.743\n',
 'center_y = 92.779\n',
 'center_z = 10.765\n',
 '\n',
 'size_x = 44\n',
 'size_y = 44\n',
 'size_z = 60\n',
 '\n',
 'out = blind_try_lig_1_pub_1.pdbqt\n',
 '\n',
 'exhaustiveness = 32\n']
```

[27]:
```
run_multiple_mol_doc = True #it changes the config.txt file in order to conduct␣
↪more MDs
if run_multiple_mol_doc == True:

    for folder in to_be_moved_to:
        shutil.copy(name_of_prepared_receptor, folder)
        shutil.copy('config.txt', folder)
        with open(str(folder+str('\\config.txt'))) as f:
            lines = f.readlines()
            lines[0] = str('receptor = '+folder.
↪split("\\")[-1]+'\\'+'1SA0_AB_chains_prot_polar_H.pdbqt')
            lines[1] = str('ligand = '+folder.split("\\")[-1]+'\\'+folder.
↪split("\\")[-1]+'.pdbqt')
            lines[11] = str('out = '+folder.
↪split("\\")[-1]+'\\docking_result_'+folder.split("\\")[-1]+'.pdbqt')
        with open(str(folder+str('\\config.txt')), 'w') as f:

            for line in lines:
                f.write(line)
                f.write('\n')
else:
    for folder in to_be_moved_to:
        shutil.copy(name_of_prepared_receptor, folder)
        shutil.copy('config.txt', folder)
        with open(str(folder+str('\\config.txt'))) as f:
            lines = f.readlines()
            lines[1] = str('ligand = '+folder.split("\\")[-1]+'.pdbqt')
            lines[11] = str('out = docking_result_'+folder.split("\\")[-1]+'.
↪pdbqt')
        with open(str(folder+str('\\config.txt')), 'w') as f:

            for line in lines:
```

```
                f.write(line)
                f.write('\n')



## Copy ligands to one directory
try:
    os.mkdir("Prepared_ligands")
except:
    pass
ligands_in_one_dir = False
if ligands_in_one_dir is not True:
    ligands_names = []
    for folder_name in to_be_moved_to:
        ligands_names.append(folder_name.split("\\")[-1]+str('\\')+folder_name.
↪split("\\")[-1]+'.pdbqt')
    for directory, file in enumerate(ligands_names):
        shutil.copy(file, 'Prepared_ligands\\')


## Distribute ligands if prepared
pdbqt_lingands_prepared = True
if pdbqt_lingands_prepared == True:
    ligands_names = []
    for folder_name in to_be_moved_to:
        ligands_names.append(folder_name.split("\\")[-1]+'.pdbqt')
    for directory, ligand in enumerate(ligands_names):
        shutil.copy('Prepared_ligands\\'+str(ligand), to_be_moved_to[directory])
```

1.8 prepare one file to be executed via prompt

[30]:
```
with open('to_be_executed.txt', 'w') as f:
    for element in to_be_moved_to:
        f.write('vina --config ')
        f.write(str(element.split("\\")[-1]))
        f.write('\config.txt --log ')
        f.write(str(element.split("\\")[-1]))
        f.write('\MD_log_.txt &')
        f.write('\n')
```

2 Run after molecular docking procedure is completed - after execution of “to_be_executed.txt”

2.1 load molecular docking results into table

[1]:
```
import pandas as pd
```

[2]:
```
to_be_moved_to = pd.read_excel('to_be_moved_to.xlsx')
to_be_moved_to = list(to_be_moved_to['Name'])
```

[3]:
```
to_be_moved_to[0]
```

[3]:
```
'C:\\Users\\aleks\\Documents\\GitHub\\Kolchicyna\\Molecular docking\\1_lig1_'
```

[4]:
```
with open(str(to_be_moved_to[0]+str('\\MD_log_.txt'))) as f:
    lines = f.readlines()
```

[5]:
```
lines
```

[5]:
```
['#################################################################\n',
 '# If you used AutoDock Vina in your work, please cite:          #\n',
 '#                                                               #\n',
 '# O. Trott, A. J. Olson,                                        #\n',
 '# AutoDock Vina: improving the speed and accuracy of docking    #\n',
 '# with a new scoring function, efficient optimization and       #\n',
 '# multithreading, Journal of Computational Chemistry 31 (2010)  #\n',
 '# 455-461                                                       #\n',
 '#                                                               #\n',
 '# DOI 10.1002/jcc.21334                                         #\n',
 '#                                                               #\n',
 '# Please see http://vina.scripps.edu for more information.      #\n',
 '#################################################################\n',
 '\n',
 'WARNING: The search space volume > 27000 Angstrom^3 (See FAQ)\n',
 'Detected 16 CPUs\n',
 'Reading input … done.\n',
 'Setting up the scoring function … done.\n',
 'Analyzing the binding site … done.\n',
 'Using random seed: 1838683264\n',
 'Performing search … done.\n',
 'Refining results … done.\n',
 '\n',
 'mode |   affinity | dist from best mode\n',
 '     | (kcal/mol) | rmsd l.b.| rmsd u.b.\n',
 '-----+------------+----------+----------\n',
 '   1         -7.8      0.000      0.000\n',
 '   2         -7.1     12.902     16.240\n',
 '   3         -7.0      4.737      7.990\n',
```

```
 '   4          -7.0       6.668      11.525\n',
 '   5          -6.8      14.405      17.577\n',
 '   6          -6.8       3.849       6.534\n',
 '   7          -6.8       2.870       6.691\n',
 '   8          -6.7       5.120       8.342\n',
 '   9          -6.7      11.843      15.491\n',
 'Writing output … done.\n']
```

[6]:
```
lines[-10]
```

[6]:
```
'   1          -7.8      0.000      0.000\n'
```

[7]:
```
lines[-10][12:18]
```

[7]:
```
' -7.8 '
```

[8]:
```
affinities = []
for element in lines[-10:-1]:
    affinities.append(element[12:18])
```

[9]:
```
affinities
```

[9]:
```
[' -7.8 ',
 ' -7.1 ',
 ' -7.0 ',
 ' -7.0 ',
 ' -6.8 ',
 ' -6.8 ',
 ' -6.8 ',
 ' -6.7 ',
 ' -6.7 ']
```

[10]:
```
float(affinities[0])
```

[10]:
```
-7.8
```

[11]:
```
floats = [float(x) for x in affinities]
```

2.2 construct dataframe

[12]:
```
df = pd.DataFrame(data=floats, columns=[str(to_be_moved_to[0].split("\\")[-1])])
```

[13]:
```
df
```

[13]:

	1_lig1_
0	-7.8
1	-7.1
2	-7.0

```
3     -7.0
4     -6.8
5     -6.8
6     -6.8
7     -6.7
8     -6.7
```

[14]:
```
to_be_moved_to__ = to_be_moved_to.copy()
try:
    for i, directory in enumerate(to_be_moved_to):

        with open(str(directory+str('\\MD_log_.txt'))) as f:
            lines = f.readlines()

        affinities = []
        for element in lines[-10:-1]:
            affinities.append(element[12:18])

        floats = [float(x) for x in affinities]

        to_be_moved_to__[i] = pd.DataFrame(data=floats, columns=[str(directory.
 ↪split("\\")[-1])])
except:
    print("Error: "+str(i)+' '+str(directory))
```

[15]:
```
to_be_moved_to__[0]
```

[15]:
```
   1_lig1_
0     -7.8
1     -7.1
2     -7.0
3     -7.0
4     -6.8
5     -6.8
6     -6.8
7     -6.7
8     -6.7
```

[16]:
```
concat = pd.concat(to_be_moved_to__, axis=1)
```

[17]:
```
concat
```

[17]:
```
   1_lig1_  2_lig2_  3_lig3_  4_lig4_  5_lig5_  6_lig6_  7_lig7_  8_lig8_  \
0     -7.8     -7.6     -8.4     -8.2     -8.1     -9.4     -9.0     -7.4
1     -7.1     -7.4     -8.4     -8.1     -7.6     -8.7     -8.6     -7.4
2     -7.0     -7.3     -8.3     -8.1     -7.5     -8.4     -8.5     -7.1
3     -7.0     -7.2     -8.3     -8.1     -7.2     -8.0     -8.4     -7.1
```

```
4     -6.8     -7.2     -8.2     -8.0     -7.2     -7.9     -8.2     -6.9
5     -6.8     -7.0     -8.2     -7.9     -7.1     -7.9     -8.1     -6.8
6     -6.8     -6.9     -7.8     -7.9     -7.1     -7.9     -7.9     -6.8
7     -6.7     -6.8     -7.7     -7.7     -7.1     -7.7     -7.9     -6.7
8     -6.7     -6.8     -7.7     -7.7     -7.0     -7.7     -7.9     -6.7

   9_lig9_  10_lig10_  …  328_lig328_  329_lig329_  330_lig330_  \
0     -8.0       -8.3  …         -7.8         -7.7         -9.0
1     -8.0       -8.0  …         -7.8         -7.5         -8.9
2     -7.3       -7.7  …         -7.7         -7.4         -8.9
3     -7.1       -7.4  …         -7.5         -7.3         -8.4
4     -7.1       -7.4  …         -7.5         -7.2         -8.1
5     -6.9       -7.3  …         -7.3         -7.2         -8.0
6     -6.8       -7.1  …         -7.3         -7.0         -8.0
7     -6.8       -7.0  …         -7.3         -6.9         -8.0
8     -6.8       -7.0  …         -7.2         -6.9         -7.9

   331_lig331_  332_lig332_  333_lig333_  334_lig334_  335_lig335_  \
0         -8.9         -8.8         -8.7         -9.9         -7.2
1         -8.8         -8.5         -8.2         -9.0         -7.2
2         -8.7         -8.4         -8.1         -9.0         -7.0
3         -8.5         -8.4         -7.9         -8.9         -6.8
4         -8.4         -8.3         -7.9         -8.5         -6.6
5         -7.9         -8.3         -7.6         -8.4         -6.6
6         -7.9         -8.2         -7.6         -8.4         -6.5
7         -7.9         -8.1         -7.6         -8.3         -6.5
8         -7.9         -8.0         -7.5         -8.1         -6.5

   336_lig336_  337_lig337_
0         -8.5         -9.4
1         -7.8         -9.4
2         -7.8         -8.8
3         -7.6         -8.8
4         -7.6         -8.7
5         -7.3         -8.5
6         -7.3         -8.2
7         -7.2         -8.2
8         -7.1         -8.2

[9 rows x 337 columns]
```

[18]:
```
concat.to_excel('docking_results_'+str(input("Name of protein domain...
↪"))+str('.xlsx'))
```

```
Name of protein domain…1SA0_chain_AB
```

```
##########
# FILE 50
##########
obabel
'100_lig100_\3D_structure_new_colchicine99_NN([C@H1]1CCC2=CC(OC)=C(OC)C(O
.pdb' -O 100_lig100_\100_lig100_.pdbqt
obabel
'101_lig101_\3D_structure_new_colchicine100_OCCN(CC=C)C(=O)N[C@@H1]1C2=CC
(.pdb' -O 101_lig101_\101_lig101_.pdbqt
obabel
'102_lig102_\3D_structure_new_colchicine101_OCCN(CC=C)C(=S)N[C@H1]1CCC2=C
C.pdb' -O 102_lig102_\102_lig102_.pdbqt
obabel
'103_lig103_\3D_structure_new_colchicine102_CNC(N(C)C)CN[C@H1]1CCC2=CC(OC
).pdb' -O 103_lig103_\103_lig103_.pdbqt
obabel
'104_lig104_\3D_structure_new_colchicine103_NC(N(CC)OC)N[C@H1]1CCC2=CC(OC
).pdb' -O 104_lig104_\104_lig104_.pdbqt
obabel
'105_lig105_\3D_structure_new_colchicine104_C1[C@H1](C2=CC(C(SC)=CC=C2C3=
C.pdb' -O 105_lig105_\105_lig105_.pdbqt
obabel
'106_lig106_\3D_structure_new_colchicine105_C1=2[C@H1](CCC3=CC(OC)=C(OC)C
(.pdb' -O 106_lig106_\106_lig106_.pdbqt
obabel
'107_lig107_\3D_structure_new_colchicine106_C1=2[C@H1](CCC3=CC(OC)=C(OC)C
(.pdb' -O 107_lig107_\107_lig107_.pdbqt
obabel
'108_lig108_\3D_structure_new_colchicine107_NC(=O)N[C@@H1]C1=C2C(C(NC)=CC
=.pdb' -O 108_lig108_\108_lig108_.pdbqt
obabel
'109_lig109_\3D_structure_new_colchicine108_CC(C)NC(=O)N1[C@@H1]C2=CC(C(N
C.pdb' -O 109_lig109_\109_lig109_.pdbqt
obabel
'10_lig10_\3D_structure_new_colchicine9_C1N(C(=S)N[C@@H1]2C3=CC(C(NC)=.pd
b' -O 10_lig10_\10_lig10_.pdbqt
obabel
'110_lig110_\3D_structure_new_colchicine109_CC(N)(CCO)[C@H1]1CCC2=CC(OC)=
C.pdb' -O 110_lig110_\110_lig110_.pdbqt
obabel
'111_lig111_\3D_structure_new_colchicine110_CC(N)(CCO)[C@H1]1CCC2=CC(OC)=
C.pdb' -O 111_lig111_\111_lig111_.pdbqt
obabel
'112_lig112_\3D_structure_new_colchicine111_C1[C@H1](NC(C)=O)C2=CC(C(NC)=
C.pdb' -O 112_lig112_\112_lig112_.pdbqt
obabel
'113_lig113_\3D_structure_new_colchicine112_C1[C@H1](NC(C)=O)C2=CC(C(SC)=
C.pdb' -O 113_lig113_\113_lig113_.pdbqt
obabel
'114_lig114_\3D_structure_new_colchicine113_C=C(N[C@H1]1CCC2=C(C(OC)=C(OC
).pdb' -O 114_lig114_\114_lig114_.pdbqt
obabel
'115_lig115_\3D_structure_new_colchicine114_CC(C)N(C(C)C)C(=O)N[C@@H1]C1=
C.pdb' -O 115_lig115_\115_lig115_.pdbqt
```

```
obabel
'116_lig116_\3D_structure_new_colchicine115_C1C(C)NC1N[C@H1]2CCC3=CC(OC)=
C.pdb' -O 116_lig116_\116_lig116_.pdbqt
obabel
'117_lig117_\3D_structure_new_colchicine116_O=C(N[C@H1]1CCC2=CC(OC)=C(OC)
C.pdb' -O 117_lig117_\117_lig117_.pdbqt
obabel
'118_lig118_\3D_structure_new_colchicine117_O=C(N[C@H1]1CCC2=CC(OC)=C(OC)
C.pdb' -O 118_lig118_\118_lig118_.pdbqt
obabel
'119_lig119_\3D_structure_new_colchicine118_COC1=C2C3=CC=C(NC)C(=O)C=C3[C
@.pdb' -O 119_lig119_\119_lig119_.pdbqt
obabel
'11_lig11_\3D_structure_new_colchicine10_C1=2[C@H1](CCC3=CC(OC)=C(OC)C(.p
db' -O 11_lig11_\11_lig11_.pdbqt
obabel
'120_lig120_\3D_structure_new_colchicine119_COC=CC1=CC=C(NC)C(=O)C=C1[C@@
H.pdb' -O 120_lig120_\120_lig120_.pdbqt
obabel
'121_lig121_\3D_structure_new_colchicine120_N=1C(=CN(N=1)[C@H1]2CCC3=CC(O
C.pdb' -O 121_lig121_\121_lig121_.pdbqt
obabel
'122_lig122_\3D_structure_new_colchicine121_C1=2[C@H1]CCC3=CC(OC)=C(OC)C(
O.pdb' -O 122_lig122_\122_lig122_.pdbqt
obabel
'123_lig123_\3D_structure_new_colchicine122_N1([C@H1]2CCC3=C(C(OC)=C(OC)C
(.pdb' -O 123_lig123_\123_lig123_.pdbqt
obabel
'124_lig124_\3D_structure_new_colchicine123_N1([C@H1]2CCC3=C(C(OC)=C(OC)C
(.pdb' -O 124_lig124_\124_lig124_.pdbqt
obabel
'125_lig125_\3D_structure_new_colchicine124_N1C(OC)=C(C=C1)N[C@H1]2CCC3=C
(.pdb' -O 125_lig125_\125_lig125_.pdbqt
obabel
'126_lig126_\3D_structure_new_colchicine125_N(C(C)C)(C(C)C)C(=O)N1[C@H1]C
C.pdb' -O 126_lig126_\126_lig126_.pdbqt
obabel
'127_lig127_\3D_structure_new_colchicine126_N(CCCC)CC1=CN(N=N1)[C@H1]2CCC
3.pdb' -O 127_lig127_\127_lig127_.pdbqt
obabel
'128_lig128_\3D_structure_new_colchicine127_NC(=S)N1[C@@H1]C2=CC(C(NC)=CC
=.pdb' -O 128_lig128_\128_lig128_.pdbqt
obabel
'129_lig129_\3D_structure_new_colchicine128_C=C(CN[C@H1]1CCC2=CC(OC)=C(OC
).pdb' -O 129_lig129_\129_lig129_.pdbqt
obabel
'12_lig12_\3D_structure_new_colchicine11_C[C@H1](CCC1=CC(OC)=C(OC)C(OC(.p
db' -O 12_lig12_\12_lig12_.pdbqt
obabel
'130_lig130_\3D_structure_new_colchicine129_C1=C(N=NN1[C@H1]2CCC3=CC(OC)=
C.pdb' -O 130_lig130_\130_lig130_.pdbqt
obabel
'131_lig131_\3D_structure_new_colchicine130_C=CC=C(NC(=O)N[C@H1]1CCC2=CC(
O.pdb' -O 131_lig131_\131_lig131_.pdbqt
```

```
obabel
'132_lig132_\3D_structure_new_colchicine131_C[C@H1](CCC1=CC(OC)=C(OC)C(OC
(.pdb' -O 132_lig132_\132_lig132_.pdbqt
obabel
'133_lig133_\3D_structure_new_colchicine132_COCC(CN[C@@H1]1C=2C(C3=C(C(OC
).pdb' -O 133_lig133_\133_lig133_.pdbqt
obabel
'134_lig134_\3D_structure_new_colchicine133_NC1=NN=NN1[C@@H1]2C=3C(C4=C(C
(.pdb' -O 134_lig134_\134_lig134_.pdbqt
obabel
'135_lig135_\3D_structure_new_colchicine134_OCCN(CCO)C(=S)N1[C@@H1]C2=CC(
C.pdb' -O 135_lig135_\135_lig135_.pdbqt
obabel
'136_lig136_\3D_structure_new_colchicine135_NC(C(OCC)=C)N[C@H1]1CCC2=CC(O
C.pdb' -O 136_lig136_\136_lig136_.pdbqt
obabel
'137_lig137_\3D_structure_new_colchicine136_N([C@H1]1CCC2=CC(OC)=C(OC)C(O
C.pdb' -O 137_lig137_\137_lig137_.pdbqt
obabel
'138_lig138_\3D_structure_new_colchicine137_NC(CCC)N[C@@H1]1C2=CC(C(NC)=C
C.pdb' -O 138_lig138_\138_lig138_.pdbqt
obabel
'139_lig139_\3D_structure_new_colchicine138_N([C@H1]1CCC2=CC(OC)=C(OC)C(O
C.pdb' -O 139_lig139_\139_lig139_.pdbqt
obabel
'13_lig13_\3D_structure_new_colchicine12_COC1=C2C3=CC=C(NC)C(=O)C=C3[C@.p
db' -O 13_lig13_\13_lig13_.pdbqt
obabel
'140_lig140_\3D_structure_new_colchicine139_N([C@H1]1CCC2=CC(OC)=C(OC)C(O
C.pdb' -O 140_lig140_\140_lig140_.pdbqt
obabel
'141_lig141_\3D_structure_new_colchicine140_C1=2[C@H1](CCC3=C(C(OC)=C(OC)
C.pdb' -O 141_lig141_\141_lig141_.pdbqt
obabel
'142_lig142_\3D_structure_new_colchicine141_N(C)C=1C(=O)C=C2[C@H1](CCC3=C
(.pdb' -O 142_lig142_\142_lig142_.pdbqt
obabel
'143_lig143_\3D_structure_new_colchicine142_N(CCCC)CNCC1=CN(N=N1)[C@H1]2C
C.pdb' -O 143_lig143_\143_lig143_.pdbqt
obabel
'144_lig144_\3D_structure_new_colchicine143_N(C)C=1C(=O)C=C2[C@H1](CCC3=C
(.pdb' -O 144_lig144_\144_lig144_.pdbqt
obabel '145_lig145_\3D_LtvuU3.pdb' -O 145_lig145_\145_lig145_.pdbqt
obabel
'146_lig146_\3D_structure_new_colchicine145_N1=CC=C(C=C1)C(NCC=2N=NN(C=2)
[.pdb' -O 146_lig146_\146_lig146_.pdbqt
obabel
'147_lig147_\3D_structure_new_colchicine146_OCC(C)=C1C([C@H1](CCC2=CC(OC)
=.pdb' -O 147_lig147_\147_lig147_.pdbqt
obabel
'148_lig148_\3D_structure_new_colchicine147_N1=CC=C(C=C1)C(NCC=2N=NN(C=2)
[.pdb' -O 148_lig148_\148_lig148_.pdbqt
obabel '149_lig149_\3D_bJov87.pdb' -O 149_lig149_\149_lig149_.pdbqt
```

```
obabel
'14_lig14_\3D_structure_new_colchicine13_COC1=C2C3=CC=C(NC)C(=O)C=C3[C@.p
db' -O 14_lig14_\14_lig14_.pdbqt
obabel
'150_lig150_\3D_structure_new_colchicine149_CC(C)(C)OC(=O)NCCCNCC1=CN(N=N
1.pdb' -O 150_lig150_\150_lig150_.pdbqt
obabel
'151_lig151_\3D_structure_new_colchicine150_COC1=C2C3=CC=C(SC)C(=O)C=C3[C
@.pdb' -O 151_lig151_\151_lig151_.pdbqt
obabel
'152_lig152_\3D_structure_new_colchicine151_COC1=C2C3=CC=C(NC)C(=O)C=C3[C
@.pdb' -O 152_lig152_\152_lig152_.pdbqt
obabel
'153_lig153_\3D_structure_new_colchicine152_C=C(CO)CN[C@H1]1CCC2=CC(OC)=C
(.pdb' -O 153_lig153_\153_lig153_.pdbqt
obabel
'154_lig154_\3D_structure_new_colchicine153_CC(C)CN[C@H1]1CCC2=CC(OC)=C(O
C.pdb' -O 154_lig154_\154_lig154_.pdbqt
obabel
'155_lig155_\3D_structure_new_colchicine154_CC(C)CCN[C@H1]1CCC2=CC(OC)=C(
O.pdb' -O 155_lig155_\155_lig155_.pdbqt
obabel
'156_lig156_\3D_structure_new_colchicine155_CC(C)CCN[C@H1]C1CC2=CC(OC)=C(
O.pdb' -O 156_lig156_\156_lig156_.pdbqt
obabel
'157_lig157_\3D_structure_new_colchicine156_N=1NC=1[C@@H1]2C3=CC(C(NC)=CC
=.pdb' -O 157_lig157_\157_lig157_.pdbqt
obabel
'158_lig158_\3D_structure_new_colchicine157_COC1=C2C3=CC=C(NC)C(=O)C=C3[C
@.pdb' -O 158_lig158_\158_lig158_.pdbqt
obabel
'159_lig159_\3D_structure_new_colchicine158_COC1=C(OC)C(OC)=CC=2CC[C@@H1]
(.pdb' -O 159_lig159_\159_lig159_.pdbqt
obabel
'15_lig15_\3D_structure_new_colchicine14_CC(=O)N[C@H1]1CCC2=CC(OC)=C(OC.p
db' -O 15_lig15_\15_lig15_.pdbqt
obabel
'160_lig160_\3D_structure_new_colchicine159_C12=CC(C(NC)=CC=C1C3=C(C=C(OC
).pdb' -O 160_lig160_\160_lig160_.pdbqt
obabel
'161_lig161_\3D_structure_new_colchicine160_O=CC1=CN=NN1[C@H1]2CCC3=CC(OC
).pdb' -O 161_lig161_\161_lig161_.pdbqt
obabel
'162_lig162_\3D_structure_new_colchicine161_C1=CN=CC=C1CN[C@H1]2CCC3=CC(O
C.pdb' -O 162_lig162_\162_lig162_.pdbqt
obabel
'163_lig163_\3D_structure_new_colchicine162_C1=CN=CC=C1CN[C@H1]2CCC3=CC(O
C.pdb' -O 163_lig163_\163_lig163_.pdbqt
obabel
'164_lig164_\3D_structure_new_colchicine163_C=C1N=CC(C)N1CN[C@H1]2CCC3=C(
C.pdb' -O 164_lig164_\164_lig164_.pdbqt
obabel
'165_lig165_\3D_structure_new_colchicine164_CNCC(C)OC(=O)N[C@H1]1CCC2=C(C
(.pdb' -O 165_lig165_\165_lig165_.pdbqt
```

```
obabel
'166_lig166_\3D_structure_new_colchicine165_C=CC=C(CCN[C@H1]1CCC2=CC(OC)=
C.pdb' -O 166_lig166_\166_lig166_.pdbqt
obabel
'167_lig167_\3D_structure_new_colchicine166_C1COC1(OC2=CC=C3C4=C(OC)C(OC)
=.pdb' -O 167_lig167_\167_lig167_.pdbqt
obabel
'168_lig168_\3D_structure_new_colchicine167_O=C(OC1=C2C(CC[C@H1](NC(=O)C)
C.pdb' -O 168_lig168_\168_lig168_.pdbqt
obabel
'169_lig169_\3D_structure_new_colchicine168_O=C1C=CC=C2C([C@H1](CCC3=CC(O
C.pdb' -O 169_lig169_\169_lig169_.pdbqt
obabel
'16_lig16_\3D_structure_new_colchicine15_C1=2C(=O)C(=C3C=C1C4=C(CC[C@@H.p
db' -O 16_lig16_\16_lig16_.pdbqt
obabel
'170_lig170_\3D_structure_new_colchicine169_C1=CC=C(C=C1)CN[C@H1]2CCC3=CC
(.pdb' -O 170_lig170_\170_lig170_.pdbqt
obabel
'171_lig171_\3D_structure_new_colchicine170_CC=CCCCN[C@H1]1CCC2=CC(OC)=C(
O.pdb' -O 171_lig171_\171_lig171_.pdbqt
obabel
'172_lig172_\3D_structure_new_colchicine171_OC1=C(C=CC=C1)CN2[C@H1]CCC3=C
C.pdb' -O 172_lig172_\172_lig172_.pdbqt
obabel
'173_lig173_\3D_structure_new_colchicine172_COC1=C2C3=CC=C(NC)C(=O)C=C3[C
@.pdb' -O 173_lig173_\173_lig173_.pdbqt
obabel
'174_lig174_\3D_structure_new_colchicine173_COC1=CC=2CC[C@@H1](C=3C(C=2C(
=.pdb' -O 174_lig174_\174_lig174_.pdbqt
obabel
'175_lig175_\3D_structure_new_colchicine174_CSC1=CC=C2C(=CC1=O)[C@H1](CCC
3.pdb' -O 175_lig175_\175_lig175_.pdbqt
obabel
'176_lig176_\3D_structure_new_colchicine175_C1=C2[C@H1](CCC3=C(C(OC)=C(C(
=.pdb' -O 176_lig176_\176_lig176_.pdbqt
obabel
'177_lig177_\3D_structure_new_colchicine176_CC(=O)OC1=CC=C2C3=C(C(OC)=C(C
=.pdb' -O 177_lig177_\177_lig177_.pdbqt
obabel
'178_lig178_\3D_structure_new_colchicine177_CC(=O)OC=1C(=O)C=C2C(=CC=1)C3
=.pdb' -O 178_lig178_\178_lig178_.pdbqt
obabel
'179_lig179_\3D_structure_new_colchicine178_CC(=O)OC1=CC=C2C3=C(C(OC)=C(C
=.pdb' -O 179_lig179_\179_lig179_.pdbqt
obabel
'17_lig17_\3D_structure_new_colchicine16_CC(=O)OC1=CC=C2C3=C(C(OC)=C(C=.p
db' -O 17_lig17_\17_lig17_.pdbqt
obabel
'180_lig180_\3D_structure_new_colchicine179_CCC(=O)OC1=CC=C2C3=C(C(OC)=C(
C.pdb' -O 180_lig180_\180_lig180_.pdbqt
obabel
'181_lig181_\3D_structure_new_colchicine180_CCC(=O)N[C@H1]1CCC2=CC(=C(OC)
C.pdb' -O 181_lig181_\181_lig181_.pdbqt
```

```
obabel
'182_lig182_\3D_structure_new_colchicine181_CC(C)C(OC1=CC=C2C3=C(CC[C@@H1
].pdb' -O 182_lig182_\182_lig182_.pdbqt
obabel
'183_lig183_\3D_structure_new_colchicine182_CC(C)C(OC1=CC=C2C3=C(CC[C@@H1
].pdb' -O 183_lig183_\183_lig183_.pdbqt
obabel
'184_lig184_\3D_structure_new_colchicine183_CC(C)C(=O)OC1=CC=C2C3=C(C(OC)
=.pdb' -O 184_lig184_\184_lig184_.pdbqt
obabel
'185_lig185_\3D_structure_new_colchicine184_CC(C)C(OC1=CC=C2C3=C(C=C(OC)C
(.pdb' -O 185_lig185_\185_lig185_.pdbqt
obabel
'186_lig186_\3D_structure_new_colchicine185_CC(=O)OCC1=CN(N=N1)[C@H1]2CCC
3.pdb' -O 186_lig186_\186_lig186_.pdbqt
obabel
'187_lig187_\3D_structure_new_colchicine186_C1=C(C2=C(OC)C(OC)=C1OC(=O)CC
C.pdb' -O 187_lig187_\187_lig187_.pdbqt
obabel
'188_lig188_\3D_structure_new_colchicine187_CC(=O)N[C@H1]1CCC2=CC(OC)=C(O
C.pdb' -O 188_lig188_\188_lig188_.pdbqt
obabel
'189_lig189_\3D_structure_new_colchicine188_O=C(C)N[C@H1]1CCC2=CC(=C(OC)C
(.pdb' -O 189_lig189_\189_lig189_.pdbqt
obabel
'18_lig18_\3D_structure_new_colchicine17_CC(=O)OC1=CC=C2C3=C(C(OC)=C(C=.p
db' -O 18_lig18_\18_lig18_.pdbqt
obabel
'190_lig190_\3D_structure_new_colchicine189_C=12C(=O)C(=CC=C3C=1[C@H1](CC
C.pdb' -O 190_lig190_\190_lig190_.pdbqt
obabel
'191_lig191_\3D_structure_new_colchicine190_CCC(=O)N[C@H1]1CCC2=C(C(OC)=C
(.pdb' -O 191_lig191_\191_lig191_.pdbqt
obabel
'192_lig192_\3D_structure_new_colchicine191_CCC(=O)N[C@H1]1CCC2=CC(OC)=C(
C.pdb' -O 192_lig192_\192_lig192_.pdbqt
obabel
'193_lig193_\3D_structure_new_colchicine192_CC=1C(=CC(C(=CC=1)SC)=O)[C@H1
].pdb' -O 193_lig193_\193_lig193_.pdbqt
obabel
'194_lig194_\3D_structure_new_colchicine193_CC(C)COCCN[C@H1]1CCC2=CC(OC)=
C.pdb' -O 194_lig194_\194_lig194_.pdbqt
obabel
'195_lig195_\3D_structure_new_colchicine194_CC(C)CCN[C@H1]1CCC2=C(C3=CC=C
(.pdb' -O 195_lig195_\195_lig195_.pdbqt
obabel
'196_lig196_\3D_structure_new_colchicine195_CC(C)CCN[C@H1]1CCC2=C(C3=CC=C
(.pdb' -O 196_lig196_\196_lig196_.pdbqt
obabel
'197_lig197_\3D_structure_new_colchicine196_C1=C(OC)C(=C(OC)C2=C1CC[C@H1]
(.pdb' -O 197_lig197_\197_lig197_.pdbqt
obabel
'198_lig198_\3D_structure_new_colchicine197_O=C(C)N[C@H1]1CCC2=CC(OC)=C(C
(.pdb' -O 198_lig198_\198_lig198_.pdbqt
```

```
obabel
'199_lig199_\3D_structure_new_colchicine198_O=C(N(C)C)NC1=CC=C2C3=C(OC)C(
=.pdb' -O 199_lig199_\199_lig199_.pdbqt
obabel
'19_lig19_\3D_structure_new_colchicine18_CC(=O)OC1=CC=C2C3=C(C(OC)=C(C=.p
db' -O 19_lig19_\19_lig19_.pdbqt
obabel
'1_lig1_\3D_structure_new_colchicine0_COC1=C2C3=CC=C(NC)C(=O)C=C3[C@.pdb'
-O 1_lig1_\1_lig1_.pdbqt
obabel
'200_lig200_\3D_structure_new_colchicine199_O=C(N)N[C@H1]1CCC2=CC(OC)=C(C
(.pdb' -O 200_lig200_\200_lig200_.pdbqt
obabel
'201_lig201_\3D_structure_new_colchicine200_O=C(C)N[C@H1]1CCC2=CC(OC)=C(C
(.pdb' -O 201_lig201_\201_lig201_.pdbqt
obabel
'202_lig202_\3D_structure_new_colchicine201_CC(=O)N1[C@H1]CCC2=CC(OC)=C(O
C.pdb' -O 202_lig202_\202_lig202_.pdbqt
obabel
'203_lig203_\3D_structure_new_colchicine202_COC1=C2C3=CC=C(NC)C(=O)C=C3[C
@.pdb' -O 203_lig203_\203_lig203_.pdbqt
obabel
'204_lig204_\3D_structure_new_colchicine203_C1[C@@H1](C2=CC(=O)C(NC)=CC=C
2.pdb' -O 204_lig204_\204_lig204_.pdbqt
obabel
'205_lig205_\3D_structure_new_colchicine204_CNC(C(C)O)N([C@H1]1CCC2=CC(=C
(.pdb' -O 205_lig205_\205_lig205_.pdbqt
obabel
'206_lig206_\3D_structure_new_colchicine205_CNC(C(C)O)N[C@@H1]1C=2C(C3=C(
C.pdb' -O 206_lig206_\206_lig206_.pdbqt
obabel
'207_lig207_\3D_structure_new_colchicine206_CNC(=S)N[C@@H1]1C2=CC(=O)C(NC
).pdb' -O 207_lig207_\207_lig207_.pdbqt
obabel
'208_lig208_\3D_structure_new_colchicine207_N([C@H1]1CCC2=C(C3=CC=C(C(C=C
1.pdb' -O 208_lig208_\208_lig208_.pdbqt
obabel
'209_lig209_\3D_structure_new_colchicine208_C12=CC(C(NC)=CC=C1C3=C(C=C(C(
O.pdb' -O 209_lig209_\209_lig209_.pdbqt
obabel
'20_lig20_\3D_structure_new_colchicine19_CC(C)C(OC1=CC=C2C3=C(CC[C@@H1].p
db' -O 20_lig20_\20_lig20_.pdbqt
obabel
'210_lig210_\3D_structure_new_colchicine209_COC1=C(OC)C(OC)=CC=2CC[C@@H1]
(.pdb' -O 210_lig210_\210_lig210_.pdbqt
obabel
'211_lig211_\3D_structure_new_colchicine210_COC1=C(OC)C(OC)=CC=2CC[C@@H1]
(.pdb' -O 211_lig211_\211_lig211_.pdbqt
obabel
'212_lig212_\3D_structure_new_colchicine211_CN(C(=S)N[C@@H1]1C2=CC(=O)C(N
C.pdb' -O 212_lig212_\212_lig212_.pdbqt
obabel
'213_lig213_\3D_structure_new_colchicine212_CC(C)(C)OC1=CN(N=N1)[C@H1]C2C
C.pdb' -O 213_lig213_\213_lig213_.pdbqt
```

```
obabel
'214_lig214_\3D_structure_new_colchicine213_CC(C)CCCCCCN[C@@H1]1C2=CC(C(S
C.pdb' -O 214_lig214_\214_lig214_.pdbqt
obabel
'215_lig215_\3D_structure_new_colchicine214_CC(C)(C)C(=O)N[C@@H1]1C=2C(C3
=.pdb' -O 215_lig215_\215_lig215_.pdbqt
obabel
'216_lig216_\3D_structure_new_colchicine215_OC(CCC(C)C)OCC1=CN(N=N1)[C@H1
].pdb' -O 216_lig216_\216_lig216_.pdbqt
obabel
'217_lig217_\3D_structure_new_colchicine216_O=C(NCC1=CC=CC=C1Cl)N[C@H1]2C
C.pdb' -O 217_lig217_\217_lig217_.pdbqt
obabel
'218_lig218_\3D_structure_new_colchicine217_N([C@H1]1CCC2=C(C(OC)=C(OC)C(
O.pdb' -O 218_lig218_\218_lig218_.pdbqt
obabel
'219_lig219_\3D_structure_new_colchicine218_O=CC=CCC=CNNC(N[C@H1]1CCC2=CC
(.pdb' -O 219_lig219_\219_lig219_.pdbqt
obabel
'21_lig21_\3D_structure_new_colchicine20_CC(=O)N[C@@H1]1C2=CC(C(SC)=CC=.p
db' -O 21_lig21_\21_lig21_.pdbqt
obabel
'220_lig220_\3D_structure_new_colchicine219_OC1=C(C=CC=C1)C=2N=NN(C=2)[C@
H.pdb' -O 220_lig220_\220_lig220_.pdbqt
obabel
'221_lig221_\3D_structure_new_colchicine220_OC1=C(C=CC=C1)C=2N=NN(C=2)[C@
H.pdb' -O 221_lig221_\221_lig221_.pdbqt
obabel
'222_lig222_\3D_structure_new_colchicine221_C1[C@@H1](C2=CC(=O)C(NC)=CC=C
2.pdb' -O 222_lig222_\222_lig222_.pdbqt
obabel
'223_lig223_\3D_structure_new_colchicine222_O=C(OC(C)(C)C)N[C@H1]1CCC2=C(
C.pdb' -O 223_lig223_\223_lig223_.pdbqt
obabel
'224_lig224_\3D_structure_new_colchicine223_O=C(OCC)CN(OCC)C[C@@H1]1C2=CC
(.pdb' -O 224_lig224_\224_lig224_.pdbqt
obabel
'225_lig225_\3D_structure_new_colchicine224_O=C(C)OC1=CN(N=N1)[C@H1]2CCC3
=.pdb' -O 225_lig225_\225_lig225_.pdbqt
obabel
'226_lig226_\3D_structure_new_colchicine225_O=C(OCC)CN(CCC)C[C@H1]C1CC2=C
C.pdb' -O 226_lig226_\226_lig226_.pdbqt
obabel
'227_lig227_\3D_structure_new_colchicine226_O=C(C(Cl)Cl)NC(=O)N[C@H1]1CCC
2.pdb' -O 227_lig227_\227_lig227_.pdbqt
obabel
'228_lig228_\3D_structure_new_colchicine227_O=C(C(Cl)Cl)NC(=O)N[C@H1]1CCC
2.pdb' -O 228_lig228_\228_lig228_.pdbqt
obabel
'229_lig229_\3D_structure_new_colchicine228_O=C(OCCC)OCC1=CN(N=N1)[C@H1]2
C.pdb' -O 229_lig229_\229_lig229_.pdbqt
obabel
'22_lig22_\3D_structure_new_colchicine21_O=C(OC)OC1=CC=C2C3=C(C(OC)=C(C.p
db' -O 22_lig22_\22_lig22_.pdbqt
```

```
obabel
'230_lig230_\3D_structure_new_colchicine229_O=C(OCCC)OCC1=CN(N=N1)[C@H1]2
C.pdb' -O 230_lig230_\230_lig230_.pdbqt
obabel
'231_lig231_\3D_structure_new_colchicine230_O=C(OCC=C)NCC1=CN(N=N1)[C@H1]
2.pdb' -O 231_lig231_\231_lig231_.pdbqt
obabel
'232_lig232_\3D_structure_new_colchicine231_O=C(C(Cl)Cl)NCC1=CN(N=N1)[C@H
1.pdb' -O 232_lig232_\232_lig232_.pdbqt
obabel
'233_lig233_\3D_structure_new_colchicine232_O=C(C(Cl)Cl)NCC1=CN(N=N1)[C@H
1.pdb' -O 233_lig233_\233_lig233_.pdbqt
obabel
'234_lig234_\3D_structure_new_colchicine233_O=C(C)N[C@@H1]1CC(C2=C(C(OC)=
C.pdb' -O 234_lig234_\234_lig234_.pdbqt
obabel
'235_lig235_\3D_structure_new_colchicine234_O=C(C(Cl)Cl)NC(=O)N[C@H1]1CCC
2.pdb' -O 235_lig235_\235_lig235_.pdbqt
obabel
'236_lig236_\3D_structure_new_colchicine235_O=C(OCC)C1=CN(N=N1)[C@H1]2CCC
3.pdb' -O 236_lig236_\236_lig236_.pdbqt
obabel
'237_lig237_\3D_structure_new_colchicine236_O=C(O)CN(CCO)C(=O)N[C@H1]1CCC
2.pdb' -O 237_lig237_\237_lig237_.pdbqt
obabel
'238_lig238_\3D_structure_new_colchicine237_CC(C)OC(NCC1=CN(N=N1)[C@H1]2C
C.pdb' -O 238_lig238_\238_lig238_.pdbqt
obabel
'239_lig239_\3D_structure_new_colchicine238_COC(NCCCOCC1=CN(N=N1)[C@H1]2C
C.pdb' -O 239_lig239_\239_lig239_.pdbqt
obabel
'23_lig23_\3D_structure_new_colchicine22_O=C(C)N[C@H1]1CCC2=CC(=C(OC)C(.p
db' -O 23_lig23_\23_lig23_.pdbqt
obabel '240_lig240_\3D_zEatBw.pdb' -O 240_lig240_\240_lig240_.pdbqt
obabel
'241_lig241_\3D_structure_new_colchicine240_C1=CN=CC=C1C2=CN(N=N2)[C@H1]3
C.pdb' -O 241_lig241_\241_lig241_.pdbqt
obabel
'242_lig242_\3D_structure_new_colchicine241_COC=C(OC)C(OC)=CCCC[C@H1](N1C
=.pdb' -O 242_lig242_\242_lig242_.pdbqt
obabel
'243_lig243_\3D_structure_new_colchicine242_COC1=C(OC)C=CC=2CC[C@@H1](C3=
C.pdb' -O 243_lig243_\243_lig243_.pdbqt
obabel
'244_lig244_\3D_structure_new_colchicine243_CC(C)(O)C=CN([C@H1]1CCC2=CC(O
C.pdb' -O 244_lig244_\244_lig244_.pdbqt
obabel
'245_lig245_\3D_structure_new_colchicine244_C=12C(=CC(C(NC)=CC=1)=O)[C@H1
].pdb' -O 245_lig245_\245_lig245_.pdbqt
obabel
'246_lig246_\3D_structure_new_colchicine245_NC(=O)NCC1=CN(N=N1)[C@H1]2CCC
3.pdb' -O 246_lig246_\246_lig246_.pdbqt
```

```
obabel
'247_lig247_\3D_structure_new_colchicine246_C1=CC(OC)=CC=C1CN[C@H1]2CCC3=
C.pdb' -O 247_lig247_\247_lig247_.pdbqt
obabel
'248_lig248_\3D_structure_new_colchicine247_N(C(C1=CC=CC=C1)=O)CCN[C@H1]2
C.pdb' -O 248_lig248_\248_lig248_.pdbqt
obabel
'249_lig249_\3D_structure_new_colchicine248_N=NC(=C(C(OCC)=O)N[C@H1]1CCC2
=.pdb' -O 249_lig249_\249_lig249_.pdbqt
obabel
'24_lig24_\3D_structure_new_colchicine23_CC(=O)OCC1=CN(N=N1)[C@H1]2CCC3.p
db' -O 24_lig24_\24_lig24_.pdbqt
obabel
'250_lig250_\3D_structure_new_colchicine249_CNC=1C(=O)C=C2[C@H1](CCC3=C(C
(.pdb' -O 250_lig250_\250_lig250_.pdbqt
obabel
'251_lig251_\3D_structure_new_colchicine250_NN(C=C(CNC(CCCCCl)=O)N=N)[C@H
1.pdb' -O 251_lig251_\251_lig251_.pdbqt
obabel
'252_lig252_\3D_structure_new_colchicine251_N=NC1=CN(N=N1)[C@H1]2CCC3=CC(
O.pdb' -O 252_lig252_\252_lig252_.pdbqt
obabel
'253_lig253_\3D_structure_new_colchicine252_CC(C)(C)OC(=O)NCCC(NCC=1N=NN(
C.pdb' -O 253_lig253_\253_lig253_.pdbqt
obabel
'254_lig254_\3D_structure_new_colchicine253_CC(C)(C)OCNCCC(=O)NCC1=CN(N=N
1.pdb' -O 254_lig254_\254_lig254_.pdbqt
obabel
'255_lig255_\3D_structure_new_colchicine254_C1(=CN(C(=O)OCC)N[C@@H1]2C=3C
(.pdb' -O 255_lig255_\255_lig255_.pdbqt
obabel
'256_lig256_\3D_structure_new_colchicine255_CC(C)(C)OCNCCC(=O)NCC1=CN(N=N
1.pdb' -O 256_lig256_\256_lig256_.pdbqt
obabel
'257_lig257_\3D_structure_new_colchicine256_CC(C)(C)OC(=O)NCCC(OCC=1N=NN(
C.pdb' -O 257_lig257_\257_lig257_.pdbqt
obabel '258_lig258_\3D_QxX7hq.pdb' -O 258_lig258_\258_lig258_.pdbqt
obabel
'259_lig259_\3D_structure_new_colchicine258_C=1N(N=NC=1C(OCC)=O)[C@H1]2CC
C.pdb' -O 259_lig259_\259_lig259_.pdbqt
obabel
'25_lig25_\3D_structure_new_colchicine24_CC(=O)OCC1=CN(N=N1)[C@H1]2CCC3.p
db' -O 25_lig25_\25_lig25_.pdbqt
obabel
'260_lig260_\3D_structure_new_colchicine259_O=CC=CN=NN[C@H1]1CCC2=C(C(OC)
=.pdb' -O 260_lig260_\260_lig260_.pdbqt
obabel
'261_lig261_\3D_structure_new_colchicine260_C1=CN=CC=C1C(NCC2=CN(N=N2)[C@
H.pdb' -O 261_lig261_\261_lig261_.pdbqt
obabel
'262_lig262_\3D_structure_new_colchicine261_N=NN([C@H1]1CCC2=C(C(OC)=C(OC
).pdb' -O 262_lig262_\262_lig262_.pdbqt
```

obabel
'263_lig263_\3D_structure_new_colchicine262_CN(N([C@@H1]1C2=CC(=O)C(NC)=C
C.pdb' -O 263_lig263_\263_lig263_.pdbqt
obabel
'264_lig264_\3D_structure_new_colchicine263_CN(N([C@@H1]1C2=CC(=O)C(NC)=C
C.pdb' -O 264_lig264_\264_lig264_.pdbqt
obabel
'265_lig265_\3D_structure_new_colchicine264_O=C(CC)OCC1=CN(N=N1)[C@H1]2CC
C.pdb' -O 265_lig265_\265_lig265_.pdbqt
obabel
'266_lig266_\3D_structure_new_colchicine265_O=C(OC(C)(C)C)NCC=1N=NN(C=1)[
C.pdb' -O 266_lig266_\266_lig266_.pdbqt
obabel
'267_lig267_\3D_structure_new_colchicine266_O=C(C(C)C)NCN[C@H1]1CCC2=CC(O
C.pdb' -O 267_lig267_\267_lig267_.pdbqt
obabel
'268_lig268_\3D_structure_new_colchicine267_O=C(OCC)C=1N(N=NC=1C(=O)O2)[C
@.pdb' -O 268_lig268_\268_lig268_.pdbqt
obabel
'269_lig269_\3D_structure_new_colchicine268_CC(O)(C)CC(=O)OCC1=CN(N=N1)[C
@.pdb' -O 269_lig269_\269_lig269_.pdbqt
obabel
'26_lig26_\3D_structure_new_colchicine25_CC(=O)N[C@@H1]1C2=CC(C(NC)=CC=.p
db' -O 26_lig26_\26_lig26_.pdbqt
obabel
'270_lig270_\3D_structure_new_colchicine269_CC(C)COC(NCC1=CN(N=N1)[C@H1]C
2.pdb' -O 270_lig270_\270_lig270_.pdbqt
obabel
'271_lig271_\3D_structure_new_colchicine270_COCNCC1=CN(N=N1)[C@H1]2CCC3=C
C.pdb' -O 271_lig271_\271_lig271_.pdbqt
obabel
'272_lig272_\3D_structure_new_colchicine271_CC(C)(C)OC(NCC=1N=NN(C=1)[C@H
1.pdb' -O 272_lig272_\272_lig272_.pdbqt
obabel
'273_lig273_\3D_structure_new_colchicine272_N(C)C1=CC=C2C3=C(OC)C(OC)=C(O
C.pdb' -O 273_lig273_\273_lig273_.pdbqt
obabel
'274_lig274_\3D_structure_new_colchicine273_O=C(OCC)C1=C(C(OCC)=O)N=NN1[C
@.pdb' -O 274_lig274_\274_lig274_.pdbqt
obabel
'275_lig275_\3D_structure_new_colchicine274_N([C@H1]1CCC2=CC(OC)=C(OC)C(O
C.pdb' -O 275_lig275_\275_lig275_.pdbqt
obabel
'276_lig276_\3D_structure_new_colchicine275_CC(CC)N[C@H1]C1CC2=CC(OC)=C(O
C.pdb' -O 276_lig276_\276_lig276_.pdbqt
obabel
'277_lig277_\3D_structure_new_colchicine276_CC(CC)N[C@H1]C1CC2=CC(OC)=C(O
C.pdb' -O 277_lig277_\277_lig277_.pdbqt
obabel
'278_lig278_\3D_structure_new_colchicine277_COC1=C2C3=CC=C(NC)C(=O)C=C3[C
@.pdb' -O 278_lig278_\278_lig278_.pdbqt
obabel
'279_lig279_\3D_structure_new_colchicine278_COC1=C2C3=CC=C(NC)C(=O)C=C3[C
@.pdb' -O 279_lig279_\279_lig279_.pdbqt

```
obabel
'27_lig27_\3D_structure_new_colchicine26_CC(=O)N[C@@H1]C1=C2C(C(NC)=CC=.p
db' -O 27_lig27_\27_lig27_.pdbqt
obabel
'280_lig280_\3D_structure_new_colchicine279_COC1=C2C3=CC=C(SC)C(=O)C=C3[C
@.pdb' -O 280_lig280_\280_lig280_.pdbqt
obabel
'281_lig281_\3D_structure_new_colchicine280_O(C1=C(OC)C=2C3=CC=C(SC)C(C=C
3.pdb' -O 281_lig281_\281_lig281_.pdbqt
obabel
'282_lig282_\3D_structure_new_colchicine281_C1=CN=CC=C1CN[C@H1]2CCC3=CC(O
C.pdb' -O 282_lig282_\282_lig282_.pdbqt
obabel
'283_lig283_\3D_structure_new_colchicine282_O=CC1=CN=NN1[C@H1]2CCC3=CC(OC
).pdb' -O 283_lig283_\283_lig283_.pdbqt
obabel
'284_lig284_\3D_structure_new_colchicine283_NC(CCN[C@H1]1CCC2=CC(OC)=C(OC
).pdb' -O 284_lig284_\284_lig284_.pdbqt
obabel
'285_lig285_\3D_structure_new_colchicine284_NC(N=C1C=CC2)=CC=C1C3=C(OC)C(
O.pdb' -O 285_lig285_\285_lig285_.pdbqt
obabel
'286_lig286_\3D_structure_new_colchicine285_C=CC1=C(NC)C(OC)=C(OC)C=C1CC[
C.pdb' -O 286_lig286_\286_lig286_.pdbqt
obabel
'287_lig287_\3D_structure_new_colchicine286_CC(N)(CCO)[C@H1]1CCC2=CC(OC)=
C.pdb' -O 287_lig287_\287_lig287_.pdbqt
obabel
'288_lig288_\3D_structure_new_colchicine287_N(NCCCCCl)[C@H1]1CCC2=CC(OC)=
C.pdb' -O 288_lig288_\288_lig288_.pdbqt
obabel
'289_lig289_\3D_structure_new_colchicine288_N(C1=CC=C(C=C1)C)CN[C@H1]2CCC
3.pdb' -O 289_lig289_\289_lig289_.pdbqt
obabel
'28_lig28_\3D_structure_new_colchicine27_COC1=C2C3=CC=C(NC)C(=O)C=C3[C@.p
db' -O 28_lig28_\28_lig28_.pdbqt
obabel
'290_lig290_\3D_structure_new_colchicine289_N(C1=CC=C(C=C1)C)CN[C@H1]2CCC
3.pdb' -O 290_lig290_\290_lig290_.pdbqt
obabel
'291_lig291_\3D_structure_new_colchicine290_CCN(CC)C(=O)N[C@H1]C1CC2=CC(O
C.pdb' -O 291_lig291_\291_lig291_.pdbqt
obabel
'292_lig292_\3D_structure_new_colchicine291_CCN(CC)C(=O)N1[C@H1]CCC2=CC(O
C.pdb' -O 292_lig292_\292_lig292_.pdbqt
obabel
'293_lig293_\3D_structure_new_colchicine292_CCN(CC)C(=O)N[C@H1]1CCC2=CC(O
C.pdb' -O 293_lig293_\293_lig293_.pdbqt
obabel
'294_lig294_\3D_structure_new_colchicine293_CC(C)COC1=C(OC)C(OC)=CC=2CC[C
@.pdb' -O 294_lig294_\294_lig294_.pdbqt
obabel
'295_lig295_\3D_structure_new_colchicine294_C=C(C)CN(CCO)C(=S)N[C@H1]1CCC
2.pdb' -O 295_lig295_\295_lig295_.pdbqt
```

```
obabel
'296_lig296_\3D_structure_new_colchicine295_CC(C)C(OC=C(C(OC)=CCCC[C@H1](
N.pdb' -O 296_lig296_\296_lig296_.pdbqt
obabel
'297_lig297_\3D_structure_new_colchicine296_N([C@H1]1CCC2=CC(OC)=C(OC)C(O
C.pdb' -O 297_lig297_\297_lig297_.pdbqt
obabel
'298_lig298_\3D_structure_new_colchicine297_O=C(N[C@H1]1CCC2=CC(OC)=C(OC)
C.pdb' -O 298_lig298_\298_lig298_.pdbqt
obabel
'299_lig299_\3D_structure_new_colchicine298_O=C(N[C@@H1]1C2=CC(C(NC)=CC=C
2.pdb' -O 299_lig299_\299_lig299_.pdbqt
obabel
'29_lig29_\3D_structure_new_colchicine28_COC1=C2C3=CC=C(NC)C(=O)C=C3[C@.p
db' -O 29_lig29_\29_lig29_.pdbqt
obabel
'2_lig2_\3D_structure_new_colchicine1_COC1=C2C3=CC=C(NC)C(=O)C=C3[C@.pdb'
-O 2_lig2_\2_lig2_.pdbqt
obabel
'300_lig300_\3D_structure_new_colchicine299_N([C@H1]1CCC2=CC(OC)=C(OC)C(O
C.pdb' -O 300_lig300_\300_lig300_.pdbqt
obabel
'301_lig301_\3D_structure_new_colchicine300_O=C(N[C@H1]1CCC2=CC(OC)=C(OC)
C.pdb' -O 301_lig301_\301_lig301_.pdbqt
obabel
'302_lig302_\3D_structure_new_colchicine301_C1=CCC[C@H1](NC(=S)NC2=CC=C(C
=.pdb' -O 302_lig302_\302_lig302_.pdbqt
obabel
'303_lig303_\3D_structure_new_colchicine302_CNCC(O)(C)C1=CN(N=N1)[C@H1]2C
C.pdb' -O 303_lig303_\303_lig303_.pdbqt
obabel
'304_lig304_\3D_structure_new_colchicine303_N1([C@H1]2CCC3=C(C(OC)=C(OC)C
(.pdb' -O 304_lig304_\304_lig304_.pdbqt
obabel
'305_lig305_\3D_structure_new_colchicine304_N([C@H1]1CCC2=C(C(OC)=C(OC)C(
O.pdb' -O 305_lig305_\305_lig305_.pdbqt
obabel
'306_lig306_\3D_structure_new_colchicine305_C1=C(O)C(OC)=CC(CN[C@H1]2CCC3
=.pdb' -O 306_lig306_\306_lig306_.pdbqt
obabel
'307_lig307_\3D_structure_new_colchicine306_COCC(C)=C(C(OC)=CCCC[C@H1](NC
C.pdb' -O 307_lig307_\307_lig307_.pdbqt
obabel
'308_lig308_\3D_structure_new_colchicine307_C[C@H1](CCC1=CC(OC)=C(OC)C(OC
).pdb' -O 308_lig308_\308_lig308_.pdbqt
obabel
'309_lig309_\3D_structure_new_colchicine308_NC(=O)N1[C@@H1]C2=CC(C(NC)=CC
=.pdb' -O 309_lig309_\309_lig309_.pdbqt
obabel
'30_lig30_\3D_structure_new_colchicine29_COC1=C2C3=CC=C(NC)C(=O)C=C3[C@.p
db' -O 30_lig30_\30_lig30_.pdbqt
obabel
'310_lig310_\3D_structure_new_colchicine309_NN(CCO)[C@@H1]1C2=CC(C(NC)=CC
=.pdb' -O 310_lig310_\310_lig310_.pdbqt
```

```
obabel
'311_lig311_\3D_structure_new_colchicine310_C1=2[C@H1](CCC3=CC(OC)=C(OC)C
(.pdb' -O 311_lig311_\311_lig311_.pdbqt
obabel
'312_lig312_\3D_structure_new_colchicine311_NC(N=NCOCCC)C[C@H1]1C2=CC(C(N
C.pdb' -O 312_lig312_\312_lig312_.pdbqt
obabel
'313_lig313_\3D_structure_new_colchicine312_C1=2C(C3=C(OC)C(OC)=C(OC)C=C3
C.pdb' -O 313_lig313_\313_lig313_.pdbqt
obabel
'314_lig314_\3D_structure_new_colchicine313_C1=C(CN[C@H1]2CCC3=CC(OC)=C(O
C.pdb' -O 314_lig314_\314_lig314_.pdbqt
obabel
'315_lig315_\3D_structure_new_colchicine314_C1[C@H1](NC(NC2=CC=C(C=C2)F)=
O.pdb' -O 315_lig315_\315_lig315_.pdbqt
obabel
'316_lig316_\3D_structure_new_colchicine315_C1=CC(=CC=N1)CCN[C@@H1]2C3=CC
(.pdb' -O 316_lig316_\316_lig316_.pdbqt
obabel
'317_lig317_\3D_structure_new_colchicine316_C1=2[C@H1]CCC3=CC(OC)=C(OC)C(
O.pdb' -O 317_lig317_\317_lig317_.pdbqt
obabel
'318_lig318_\3D_structure_new_colchicine317_CC=CC1=C(C=C(OC)C(OC)=C1OC(OC
C.pdb' -O 318_lig318_\318_lig318_.pdbqt
obabel
'319_lig319_\3D_structure_new_colchicine318_O=C1C=C2[C@H1](CCC3=C(C(OC)=C
(.pdb' -O 319_lig319_\319_lig319_.pdbqt
obabel
'31_lig31_\3D_structure_new_colchicine30_COC1=C2C3=CC=C(NC)C(=O)C=C3[C@.p
db' -O 31_lig31_\31_lig31_.pdbqt
obabel
'320_lig320_\3D_structure_new_colchicine319_O=C1C=C2[C@H1](CCC3=C(C(OC)=C
(.pdb' -O 320_lig320_\320_lig320_.pdbqt
obabel
'321_lig321_\3D_structure_new_colchicine320_O=C1C=C2[C@H1](CCC3=C(C(OC)=C
(.pdb' -O 321_lig321_\321_lig321_.pdbqt
obabel
'322_lig322_\3D_structure_new_colchicine321_N1C=NN=NN1[C@H1]2CCC3=C(C(OC)
=.pdb' -O 322_lig322_\322_lig322_.pdbqt
obabel
'323_lig323_\3D_structure_new_colchicine322_CNC=1C(=O)C=C2[C@H1](CCC3=CC(
O.pdb' -O 323_lig323_\323_lig323_.pdbqt
obabel
'324_lig324_\3D_structure_new_colchicine323_N([C@H1]1CCC2=CC(OC)=C(OC)C(O
C.pdb' -O 324_lig324_\324_lig324_.pdbqt
obabel
'325_lig325_\3D_structure_new_colchicine324_C[C@H1](CCC1=CC(OC)=C(OC)C(OC
).pdb' -O 325_lig325_\325_lig325_.pdbqt
obabel
'326_lig326_\3D_structure_new_colchicine325_N([C@H1]1CCC2=C(C(OC)=C(OC)C(
O.pdb' -O 326_lig326_\326_lig326_.pdbqt
obabel
'327_lig327_\3D_structure_new_colchicine326_C=CC1=C(C(=O)OC=CC=C2C3=C(OC)
C.pdb' -O 327_lig327_\327_lig327_.pdbqt
```

```
obabel
'328_lig328_\3D_structure_new_colchicine327_C=C1C2=C(C=C(OC)C(OC)=C2OC(OC
C.pdb' -O 328_lig328_\328_lig328_.pdbqt
obabel
'329_lig329_\3D_structure_new_colchicine328_CC=CC(N[C@H1]1CCC2=C(C(OC)=C(
O.pdb' -O 329_lig329_\329_lig329_.pdbqt
obabel
'32_lig32_\3D_structure_new_colchicine31_COC1=C(OC)C(OC)=CC=2CC[C@H1](N.p
db' -O 32_lig32_\32_lig32_.pdbqt
obabel
'330_lig330_\3D_structure_new_colchicine329_OC(=O)NCC1=CN(N=N1)[C@@H1]2C=
3.pdb' -O 330_lig330_\330_lig330_.pdbqt
obabel
'331_lig331_\3D_structure_new_colchicine330_N(C)C=1C(=O)C=C2[C@H1](CCC3=C
(.pdb' -O 331_lig331_\331_lig331_.pdbqt
obabel
'332_lig332_\3D_structure_new_colchicine331_O=C(C=C(C(O1)CCCCC1)CN=N)N[C
@.pdb' -O 332_lig332_\332_lig332_.pdbqt
obabel
'333_lig333_\3D_structure_new_colchicine332_N([C@H1]1CCC2=C(C(OC)=C(OC)C(
O.pdb' -O 333_lig333_\333_lig333_.pdbqt
obabel
'334_lig334_\3D_structure_new_colchicine333_O=C(C=C(Cl)C=CN([C@H1]1CCC2=C
(.pdb' -O 334_lig334_\334_lig334_.pdbqt
obabel '335_lig335_\3D_O4ZgW2.pdb' -O 335_lig335_\335_lig335_.pdbqt
obabel '336_lig336_\3D_L9ZpgB.pdb' -O 336_lig336_\336_lig336_.pdbqt
obabel
'337_lig337_\3D_structure_new_colchicine336_C12=C3C(C4=C(OC)C(OC)=C(OC)C=
C.pdb' -O 337_lig337_\337_lig337_.pdbqt
obabel
'33_lig33_\3D_structure_new_colchicine32_CNC(NCCCC)=S[C@@H1]C1=CC(=O)C(.p
db' -O 33_lig33_\33_lig33_.pdbqt
obabel
'34_lig34_\3D_structure_new_colchicine33_CN(C(=S)N[C@@H1]1C2=CC(=O)C(NC.p
db' -O 34_lig34_\34_lig34_.pdbqt
obabel
'35_lig35_\3D_structure_new_colchicine34_CNC(C(C)C)N[C@@H1]1C=2C(C3=C(C.p
db' -O 35_lig35_\35_lig35_.pdbqt
obabel
'36_lig36_\3D_structure_new_colchicine35_COC1=C(OC)C(OC)=CC=2CC[C@@H1](.p
db' -O 36_lig36_\36_lig36_.pdbqt
obabel
'37_lig37_\3D_structure_new_colchicine36_COC1=C(OC)C(OC)=CC=2CC[C@@H1](.p
db' -O 37_lig37_\37_lig37_.pdbqt
obabel
'38_lig38_\3D_structure_new_colchicine37_CC(C)(C)COC1=CC=C2C3=C(OC)C(OC.p
db' -O 38_lig38_\38_lig38_.pdbqt
obabel
'39_lig39_\3D_structure_new_colchicine38_C1=2[C@H1]CCC3=CC(OC)=C(OC)C(O.p
db' -O 39_lig39_\39_lig39_.pdbqt
obabel
'3_lig3_\3D_structure_new_colchicine2_COC1=C2C3=CC=C(NC)C(=O)C=C3[C@.pdb'
-O 3_lig3_\3_lig3_.pdbqt
```

```
obabel
'40_lig40_\3D_structure_new_colchicine39_OC(CCC(C)C)OCC1=CN(N=N1)[C@H1].p
db' -O 40_lig40_\40_lig40_.pdbqt
obabel
'41_lig41_\3D_structure_new_colchicine40_OC1=C(C=CC=C1)C=2N=NN(C=2)[C@H.p
db' -O 41_lig41_\41_lig41_.pdbqt
obabel
'42_lig42_\3D_structure_new_colchicine41_NC1=CN(N=N1)[C@H1]2CCC3=C(C(OC.p
db' -O 42_lig42_\42_lig42_.pdbqt
obabel
'43_lig43_\3D_structure_new_colchicine42_O=C(OCC)CN(CCO)N[C@H1]1CCC2=CC.p
db' -O 43_lig43_\43_lig43_.pdbqt
obabel
'44_lig44_\3D_structure_new_colchicine43_O=C(C1=C(C(OCC)=O)N(N=N1)[C@@H.p
db' -O 44_lig44_\44_lig44_.pdbqt
obabel
'45_lig45_\3D_structure_new_colchicine44_C1=2[C@H1](CCC3=CC(OC)=C(OC)C(.p
db' -O 45_lig45_\45_lig45_.pdbqt
obabel
'46_lig46_\3D_structure_new_colchicine45_C=12N(N=NC=1C(OCC3=CC=C(C=C3)F.p
db' -O 46_lig46_\46_lig46_.pdbqt
obabel
'47_lig47_\3D_structure_new_colchicine46_C=1N(N=NC=1C(=O)OCC2=CC=C(C=C2.p
db' -O 47_lig47_\47_lig47_.pdbqt
obabel
'48_lig48_\3D_structure_new_colchicine47_O=CC1=C(C(OCC)=O)N(N=N1)[C@@H1.p
db' -O 48_lig48_\48_lig48_.pdbqt
obabel
'49_lig49_\3D_structure_new_colchicine48_O=C(C(Cl)Cl)NC(=O)N[C@H1]1CCC2.p
db' -O 49_lig49_\49_lig49_.pdbqt
obabel
'4_lig4_\3D_structure_new_colchicine3_COC1=C2C3=CC=C(NC)C(=O)C=C3[C@.pdb'
-O 4_lig4_\4_lig4_.pdbqt
obabel
'50_lig50_\3D_structure_new_colchicine49_O=C(C(Cl)Cl)NCC1=CN(N=N1)[C@H1.p
db' -O 50_lig50_\50_lig50_.pdbqt
obabel
'51_lig51_\3D_structure_new_colchicine50_O=C(C(Cl)Cl)NC(=O)N[C@H1]1CCC2.p
db' -O 51_lig51_\51_lig51_.pdbqt
obabel
'52_lig52_\3D_structure_new_colchicine51_O=C(O)C=C(C(OCC)=O)N=NN[C@H1]1.p
db' -O 52_lig52_\52_lig52_.pdbqt
obabel
'53_lig53_\3D_structure_new_colchicine52_O=C(O)C1=C(C(OCC)=O)N=NN1[C@H1.p
db' -O 53_lig53_\53_lig53_.pdbqt
obabel
'54_lig54_\3D_structure_new_colchicine53_O=C(OCC)C1=CN(N=N1)[C@H1]2CCC3.p
db' -O 54_lig54_\54_lig54_.pdbqt
obabel
'55_lig55_\3D_structure_new_colchicine54_O=C(O)C1=CN(N=N1)[C@H1]2CCC3=C.p
db' -O 55_lig55_\55_lig55_.pdbqt
obabel
'56_lig56_\3D_structure_new_colchicine55_O=C(O)C1=C(C(OCC)=O)N=NN1[C@H1.p
db' -O 56_lig56_\56_lig56_.pdbqt
```

```
obabel
'57_lig57_\3D_structure_new_colchicine56_CCCCCCCCCCCCCCCC(OCC1=CN(N=N1).p
db' -O 57_lig57_\57_lig57_.pdbqt
obabel
'58_lig58_\3D_structure_new_colchicine57_OC(=O)C1=C(C(OCC)=O)N(N=N1)[C@.p
db' -O 58_lig58_\58_lig58_.pdbqt
obabel
'59_lig59_\3D_structure_new_colchicine58_C1=C(OC)C=C(OC)C=C1CC[C@H1](C=.p
db' -O 59_lig59_\59_lig59_.pdbqt
obabel
'5_lig5_\3D_structure_new_colchicine4_O=CC1=CN=NN1[C@H1]2CCC3=CC(OC).pdb'
-O 5_lig5_\5_lig5_.pdbqt
obabel
'60_lig60_\3D_structure_new_colchicine59_N(C)C(=O)NCCC(OCC1=CN(N=N1)[C@.p
db' -O 60_lig60_\60_lig60_.pdbqt
obabel
'61_lig61_\3D_structure_new_colchicine60_OC(=O)C=C(C(OCC)=O)N(NCCC)N[C@.p
db' -O 61_lig61_\61_lig61_.pdbqt
obabel
'62_lig62_\3D_structure_new_colchicine61_C[C@H1](NCC)CC1=CC(C(NC)=CC=C1.p
db' -O 62_lig62_\62_lig62_.pdbqt
obabel '63_lig63_\3D_NsTQ4B.pdb' -O 63_lig63_\63_lig63_.pdbqt
obabel
'64_lig64_\3D_structure_new_colchicine63_N1=NN1[C@@H1]2C3=CC(C(NC)=CC=C.p
db' -O 64_lig64_\64_lig64_.pdbqt
obabel
'65_lig65_\3D_structure_new_colchicine64_O=CC=CC=CC(NCC1=CN(N=N1)[C@H1].p
db' -O 65_lig65_\65_lig65_.pdbqt
obabel
'66_lig66_\3D_structure_new_colchicine65_CN(N([C@@H1]1C2=CC(=O)C(NC)=CC.p
db' -O 66_lig66_\66_lig66_.pdbqt
obabel
'67_lig67_\3D_structure_new_colchicine66_CC(C)C(=O)OCC1=CN(N=N1)[C@H1]2.p
db' -O 67_lig67_\67_lig67_.pdbqt
obabel
'68_lig68_\3D_structure_new_colchicine67_N(C(C1=CC=NC=C1)=O)[C@@H1]2C=3.p
db' -O 68_lig68_\68_lig68_.pdbqt
obabel
'69_lig69_\3D_structure_new_colchicine68_N1(CCCC1)CN[C@H1]2CCC3=C(C(OC).p
db' -O 69_lig69_\69_lig69_.pdbqt
obabel
'6_lig6_\3D_structure_new_colchicine5_OC1=CC=C(C=C1)CN[C@H1]2CCC3=C(.pdb'
-O 6_lig6_\6_lig6_.pdbqt
obabel
'70_lig70_\3D_structure_new_colchicine69_N1(CCCC1)C(=O)OC2=C(OC)C(OC3)=.p
db' -O 70_lig70_\70_lig70_.pdbqt
obabel
'71_lig71_\3D_structure_new_colchicine70_CNC=1C(=O)C=C2[C@H1](CCC3=CC(O.p
db' -O 71_lig71_\71_lig71_.pdbqt
obabel
'72_lig72_\3D_structure_new_colchicine71_CC(C)(C)OC(=O)NCCC(NCC=1N=NN(C.p
db' -O 72_lig72_\72_lig72_.pdbqt
obabel '73_lig73_\3D_8godGN.pdb' -O 73_lig73_\73_lig73_.pdbqt
```

```
obabel
'74_lig74_\3D_structure_new_colchicine73_CC(C)(C)OCNCCC(=O)NCC1=CN(N=N1.p
db' -O 74_lig74_\74_lig74_.pdbqt
obabel '75_lig75_\3D_YDrpzN.pdb' -O 75_lig75_\75_lig75_.pdbqt
obabel
'76_lig76_\3D_structure_new_colchicine75_CC(C)(C)OC(=O)NCCC(OCC=1N=NN(C.p
db' -O 76_lig76_\76_lig76_.pdbqt
obabel
'77_lig77_\3D_structure_new_colchicine76_CC(C)(C)OC(=O)NCCC(NCC=1N=NN(C.p
db' -O 77_lig77_\77_lig77_.pdbqt
obabel
'78_lig78_\3D_structure_new_colchicine77_CNCCC1=CC=C(C=C1)NC(=O)N[C@H1].p
db' -O 78_lig78_\78_lig78_.pdbqt
obabel
'79_lig79_\3D_structure_new_colchicine78_CNCC(C1=CC=C(C=C1)NC(=O)N[C@@H.p
db' -O 79_lig79_\79_lig79_.pdbqt
obabel
'7_lig7_\3D_structure_new_colchicine6_OC1=CC=C(C=C1)CN[C@H1]2CCC3=CC.pdb'
-O 7_lig7_\7_lig7_.pdbqt
obabel
'80_lig80_\3D_structure_new_colchicine79_O=C(OC)OCC1=CN(N=N1)[C@H1]2CCC.p
db' -O 80_lig80_\80_lig80_.pdbqt
obabel
'81_lig81_\3D_structure_new_colchicine80_O=C(C(C)C)NC(=S)N[C@@H1]1C=2C(.p
db' -O 81_lig81_\81_lig81_.pdbqt
obabel
'82_lig82_\3D_structure_new_colchicine81_O=C(OCC)OCC=1N=NN(C=1)[C@H1]2C.p
db' -O 82_lig82_\82_lig82_.pdbqt
obabel
'83_lig83_\3D_structure_new_colchicine82_O=C(OCC)CN(CCO)C(=S)N[C@H1]1CC.p
db' -O 83_lig83_\83_lig83_.pdbqt
obabel
'84_lig84_\3D_structure_new_colchicine83_CC(C)CNCC1=CN(N=N1)[C@H1]2CCC3.p
db' -O 84_lig84_\84_lig84_.pdbqt
obabel
'85_lig85_\3D_structure_new_colchicine84_CC(C)CNCC1=CN(N=N1)[C@H1]2CCC3.p
db' -O 85_lig85_\85_lig85_.pdbqt
obabel
'86_lig86_\3D_structure_new_colchicine85_COCNCC1=CN(N=N1)[C@H1]2CCC3=CC.p
db' -O 86_lig86_\86_lig86_.pdbqt
obabel
'87_lig87_\3D_structure_new_colchicine86_N(C(C)C)C(NCC1=CN(N=N1)[C@H1]2.p
db' -O 87_lig87_\87_lig87_.pdbqt
obabel
'88_lig88_\3D_structure_new_colchicine87_N(C)C1=CC=C2C3=C(OC)C(OC)=C(OC.p
db' -O 88_lig88_\88_lig88_.pdbqt
obabel
'89_lig89_\3D_structure_new_colchicine88_N(C(C(C)C)=O)C1=CN(N=N1)[C@H1].p
db' -O 89_lig89_\89_lig89_.pdbqt
obabel
'8_lig8_\3D_structure_new_colchicine7_O=CC1=C2[C@H1](CCC3=CC(OC)=C(O.pdb'
-O 8_lig8_\8_lig8_.pdbqt
```

```
obabel
'90_lig90_\3D_structure_new_colchicine89_N(CCCCCCC=O)CC1=CN(N=N1)[C@H1].p
db' -O 90_lig90_\90_lig90_.pdbqt
obabel
'91_lig91_\3D_structure_new_colchicine90_C1=C(C=CC(Cl)=C1)COC(=O)CN[C@@.p
db' -O 91_lig91_\91_lig91_.pdbqt
obabel
'92_lig92_\3D_structure_new_colchicine91_N(C)C1=CC=C2C3=C(OC)C(OC)=C(OC.p
db' -O 92_lig92_\92_lig92_.pdbqt
obabel
'93_lig93_\3D_structure_new_colchicine92_N(C)C1=CC=C2C3=C(OC)C(OC)=C(OC.p
db' -O 93_lig93_\93_lig93_.pdbqt
obabel
'94_lig94_\3D_structure_new_colchicine93_N(C(C)C)CC(=O)OCC1=C2N(N=N1)[C.p
db' -O 94_lig94_\94_lig94_.pdbqt
obabel
'95_lig95_\3D_structure_new_colchicine94_CC(CC)N[C@@H1]1C2=CC(C(SC)=CC=.p
db' -O 95_lig95_\95_lig95_.pdbqt
obabel
'96_lig96_\3D_structure_new_colchicine95_CC(CC)N[C@@H1]C1=C2C(C(NC)=CC=.p
db' -O 96_lig96_\96_lig96_.pdbqt
obabel
'97_lig97_\3D_structure_new_colchicine96_COC1=C2C3=CC=C(NC)C(=O)C=C3[C@.p
db' -O 97_lig97_\97_lig97_.pdbqt
obabel
'98_lig98_\3D_structure_new_colchicine97_N(C)C=1C(=O)C=C2[C@H1](CCC3=CC.p
db' -O 98_lig98_\98_lig98_.pdbqt
obabel
'99_lig99_\3D_structure_new_colchicine98_CNC=1C(C=C2[C@H1](CCC3=C(C(OC).p
db' -O 99_lig99_\99_lig99_.pdbqt
obabel
'9_lig9_\3D_structure_new_colchicine8_C1=2[C@H1](CCC3=CC(OC)=C(OC)C(.pdb'
-O 9_lig9_\9_lig9_.pdbqt
```

```
##########
# FILE 51
##########
vina --config 1_lig1_\config.txt --log 1_lig1_\MD_log_.txt &
vina --config 2_lig2_\config.txt --log 2_lig2_\MD_log_.txt &
vina --config 3_lig3_\config.txt --log 3_lig3_\MD_log_.txt &
vina --config 4_lig4_\config.txt --log 4_lig4_\MD_log_.txt &
vina --config 5_lig5_\config.txt --log 5_lig5_\MD_log_.txt &
vina --config 6_lig6_\config.txt --log 6_lig6_\MD_log_.txt &
vina --config 7_lig7_\config.txt --log 7_lig7_\MD_log_.txt &
vina --config 8_lig8_\config.txt --log 8_lig8_\MD_log_.txt &
vina --config 9_lig9_\config.txt --log 9_lig9_\MD_log_.txt &
vina --config 10_lig10_\config.txt --log 10_lig10_\MD_log_.txt &
vina --config 11_lig11_\config.txt --log 11_lig11_\MD_log_.txt &
vina --config 12_lig12_\config.txt --log 12_lig12_\MD_log_.txt &
vina --config 13_lig13_\config.txt --log 13_lig13_\MD_log_.txt &
vina --config 14_lig14_\config.txt --log 14_lig14_\MD_log_.txt &
vina --config 15_lig15_\config.txt --log 15_lig15_\MD_log_.txt &
vina --config 16_lig16_\config.txt --log 16_lig16_\MD_log_.txt &
vina --config 17_lig17_\config.txt --log 17_lig17_\MD_log_.txt &
vina --config 18_lig18_\config.txt --log 18_lig18_\MD_log_.txt &
vina --config 19_lig19_\config.txt --log 19_lig19_\MD_log_.txt &
vina --config 20_lig20_\config.txt --log 20_lig20_\MD_log_.txt &
vina --config 21_lig21_\config.txt --log 21_lig21_\MD_log_.txt &
vina --config 22_lig22_\config.txt --log 22_lig22_\MD_log_.txt &
vina --config 23_lig23_\config.txt --log 23_lig23_\MD_log_.txt &
vina --config 24_lig24_\config.txt --log 24_lig24_\MD_log_.txt &
vina --config 25_lig25_\config.txt --log 25_lig25_\MD_log_.txt &
vina --config 26_lig26_\config.txt --log 26_lig26_\MD_log_.txt &
vina --config 27_lig27_\config.txt --log 27_lig27_\MD_log_.txt &
vina --config 28_lig28_\config.txt --log 28_lig28_\MD_log_.txt &
vina --config 29_lig29_\config.txt --log 29_lig29_\MD_log_.txt &
vina --config 30_lig30_\config.txt --log 30_lig30_\MD_log_.txt &
vina --config 31_lig31_\config.txt --log 31_lig31_\MD_log_.txt &
vina --config 32_lig32_\config.txt --log 32_lig32_\MD_log_.txt &
vina --config 33_lig33_\config.txt --log 33_lig33_\MD_log_.txt &
vina --config 34_lig34_\config.txt --log 34_lig34_\MD_log_.txt &
vina --config 35_lig35_\config.txt --log 35_lig35_\MD_log_.txt &
vina --config 36_lig36_\config.txt --log 36_lig36_\MD_log_.txt &
vina --config 37_lig37_\config.txt --log 37_lig37_\MD_log_.txt &
vina --config 38_lig38_\config.txt --log 38_lig38_\MD_log_.txt &
vina --config 39_lig39_\config.txt --log 39_lig39_\MD_log_.txt &
vina --config 40_lig40_\config.txt --log 40_lig40_\MD_log_.txt &
vina --config 41_lig41_\config.txt --log 41_lig41_\MD_log_.txt &
vina --config 42_lig42_\config.txt --log 42_lig42_\MD_log_.txt &
vina --config 43_lig43_\config.txt --log 43_lig43_\MD_log_.txt &
vina --config 44_lig44_\config.txt --log 44_lig44_\MD_log_.txt &
vina --config 45_lig45_\config.txt --log 45_lig45_\MD_log_.txt &
vina --config 46_lig46_\config.txt --log 46_lig46_\MD_log_.txt &
vina --config 47_lig47_\config.txt --log 47_lig47_\MD_log_.txt &
vina --config 48_lig48_\config.txt --log 48_lig48_\MD_log_.txt &
vina --config 49_lig49_\config.txt --log 49_lig49_\MD_log_.txt &
vina --config 50_lig50_\config.txt --log 50_lig50_\MD_log_.txt &
vina --config 51_lig51_\config.txt --log 51_lig51_\MD_log_.txt &
```

```
vina --config 52_lig52_\config.txt --log 52_lig52_\MD_log_.txt &
vina --config 53_lig53_\config.txt --log 53_lig53_\MD_log_.txt &
vina --config 54_lig54_\config.txt --log 54_lig54_\MD_log_.txt &
vina --config 55_lig55_\config.txt --log 55_lig55_\MD_log_.txt &
vina --config 56_lig56_\config.txt --log 56_lig56_\MD_log_.txt &
vina --config 57_lig57_\config.txt --log 57_lig57_\MD_log_.txt &
vina --config 58_lig58_\config.txt --log 58_lig58_\MD_log_.txt &
vina --config 59_lig59_\config.txt --log 59_lig59_\MD_log_.txt &
vina --config 60_lig60_\config.txt --log 60_lig60_\MD_log_.txt &
vina --config 61_lig61_\config.txt --log 61_lig61_\MD_log_.txt &
vina --config 62_lig62_\config.txt --log 62_lig62_\MD_log_.txt &
vina --config 63_lig63_\config.txt --log 63_lig63_\MD_log_.txt &
vina --config 64_lig64_\config.txt --log 64_lig64_\MD_log_.txt &
vina --config 65_lig65_\config.txt --log 65_lig65_\MD_log_.txt &
vina --config 66_lig66_\config.txt --log 66_lig66_\MD_log_.txt &
vina --config 67_lig67_\config.txt --log 67_lig67_\MD_log_.txt &
vina --config 68_lig68_\config.txt --log 68_lig68_\MD_log_.txt &
vina --config 69_lig69_\config.txt --log 69_lig69_\MD_log_.txt &
vina --config 70_lig70_\config.txt --log 70_lig70_\MD_log_.txt &
vina --config 71_lig71_\config.txt --log 71_lig71_\MD_log_.txt &
vina --config 72_lig72_\config.txt --log 72_lig72_\MD_log_.txt &
vina --config 73_lig73_\config.txt --log 73_lig73_\MD_log_.txt &
vina --config 74_lig74_\config.txt --log 74_lig74_\MD_log_.txt &
vina --config 75_lig75_\config.txt --log 75_lig75_\MD_log_.txt &
vina --config 76_lig76_\config.txt --log 76_lig76_\MD_log_.txt &
vina --config 77_lig77_\config.txt --log 77_lig77_\MD_log_.txt &
vina --config 78_lig78_\config.txt --log 78_lig78_\MD_log_.txt &
vina --config 79_lig79_\config.txt --log 79_lig79_\MD_log_.txt &
vina --config 80_lig80_\config.txt --log 80_lig80_\MD_log_.txt &
vina --config 81_lig81_\config.txt --log 81_lig81_\MD_log_.txt &
vina --config 82_lig82_\config.txt --log 82_lig82_\MD_log_.txt &
vina --config 83_lig83_\config.txt --log 83_lig83_\MD_log_.txt &
vina --config 84_lig84_\config.txt --log 84_lig84_\MD_log_.txt &
vina --config 85_lig85_\config.txt --log 85_lig85_\MD_log_.txt &
vina --config 86_lig86_\config.txt --log 86_lig86_\MD_log_.txt &
vina --config 87_lig87_\config.txt --log 87_lig87_\MD_log_.txt &
vina --config 88_lig88_\config.txt --log 88_lig88_\MD_log_.txt &
vina --config 89_lig89_\config.txt --log 89_lig89_\MD_log_.txt &
vina --config 90_lig90_\config.txt --log 90_lig90_\MD_log_.txt &
vina --config 91_lig91_\config.txt --log 91_lig91_\MD_log_.txt &
vina --config 92_lig92_\config.txt --log 92_lig92_\MD_log_.txt &
vina --config 93_lig93_\config.txt --log 93_lig93_\MD_log_.txt &
vina --config 94_lig94_\config.txt --log 94_lig94_\MD_log_.txt &
vina --config 95_lig95_\config.txt --log 95_lig95_\MD_log_.txt &
vina --config 96_lig96_\config.txt --log 96_lig96_\MD_log_.txt &
vina --config 97_lig97_\config.txt --log 97_lig97_\MD_log_.txt &
vina --config 98_lig98_\config.txt --log 98_lig98_\MD_log_.txt &
vina --config 99_lig99_\config.txt --log 99_lig99_\MD_log_.txt &
vina --config 100_lig100_\config.txt --log 100_lig100_\MD_log_.txt &
vina --config 101_lig101_\config.txt --log 101_lig101_\MD_log_.txt &
vina --config 102_lig102_\config.txt --log 102_lig102_\MD_log_.txt &
vina --config 103_lig103_\config.txt --log 103_lig103_\MD_log_.txt &
vina --config 104_lig104_\config.txt --log 104_lig104_\MD_log_.txt &
vina --config 105_lig105_\config.txt --log 105_lig105_\MD_log_.txt &
```

```
vina --config 106_lig106_\config.txt --log 106_lig106_\MD_log_.txt &
vina --config 107_lig107_\config.txt --log 107_lig107_\MD_log_.txt &
vina --config 108_lig108_\config.txt --log 108_lig108_\MD_log_.txt &
vina --config 109_lig109_\config.txt --log 109_lig109_\MD_log_.txt &
vina --config 110_lig110_\config.txt --log 110_lig110_\MD_log_.txt &
vina --config 111_lig111_\config.txt --log 111_lig111_\MD_log_.txt &
vina --config 112_lig112_\config.txt --log 112_lig112_\MD_log_.txt &
vina --config 113_lig113_\config.txt --log 113_lig113_\MD_log_.txt &
vina --config 114_lig114_\config.txt --log 114_lig114_\MD_log_.txt &
vina --config 115_lig115_\config.txt --log 115_lig115_\MD_log_.txt &
vina --config 116_lig116_\config.txt --log 116_lig116_\MD_log_.txt &
vina --config 117_lig117_\config.txt --log 117_lig117_\MD_log_.txt &
vina --config 118_lig118_\config.txt --log 118_lig118_\MD_log_.txt &
vina --config 119_lig119_\config.txt --log 119_lig119_\MD_log_.txt &
vina --config 120_lig120_\config.txt --log 120_lig120_\MD_log_.txt &
vina --config 121_lig121_\config.txt --log 121_lig121_\MD_log_.txt &
vina --config 122_lig122_\config.txt --log 122_lig122_\MD_log_.txt &
vina --config 123_lig123_\config.txt --log 123_lig123_\MD_log_.txt &
vina --config 124_lig124_\config.txt --log 124_lig124_\MD_log_.txt &
vina --config 125_lig125_\config.txt --log 125_lig125_\MD_log_.txt &
vina --config 126_lig126_\config.txt --log 126_lig126_\MD_log_.txt &
vina --config 127_lig127_\config.txt --log 127_lig127_\MD_log_.txt &
vina --config 128_lig128_\config.txt --log 128_lig128_\MD_log_.txt &
vina --config 129_lig129_\config.txt --log 129_lig129_\MD_log_.txt &
vina --config 130_lig130_\config.txt --log 130_lig130_\MD_log_.txt &
vina --config 131_lig131_\config.txt --log 131_lig131_\MD_log_.txt &
vina --config 132_lig132_\config.txt --log 132_lig132_\MD_log_.txt &
vina --config 133_lig133_\config.txt --log 133_lig133_\MD_log_.txt &
vina --config 134_lig134_\config.txt --log 134_lig134_\MD_log_.txt &
vina --config 135_lig135_\config.txt --log 135_lig135_\MD_log_.txt &
vina --config 136_lig136_\config.txt --log 136_lig136_\MD_log_.txt &
vina --config 137_lig137_\config.txt --log 137_lig137_\MD_log_.txt &
vina --config 138_lig138_\config.txt --log 138_lig138_\MD_log_.txt &
vina --config 139_lig139_\config.txt --log 139_lig139_\MD_log_.txt &
vina --config 140_lig140_\config.txt --log 140_lig140_\MD_log_.txt &
vina --config 141_lig141_\config.txt --log 141_lig141_\MD_log_.txt &
vina --config 142_lig142_\config.txt --log 142_lig142_\MD_log_.txt &
vina --config 143_lig143_\config.txt --log 143_lig143_\MD_log_.txt &
vina --config 144_lig144_\config.txt --log 144_lig144_\MD_log_.txt &
vina --config 145_lig145_\config.txt --log 145_lig145_\MD_log_.txt &
vina --config 146_lig146_\config.txt --log 146_lig146_\MD_log_.txt &
vina --config 147_lig147_\config.txt --log 147_lig147_\MD_log_.txt &
vina --config 148_lig148_\config.txt --log 148_lig148_\MD_log_.txt &
vina --config 149_lig149_\config.txt --log 149_lig149_\MD_log_.txt &
vina --config 150_lig150_\config.txt --log 150_lig150_\MD_log_.txt &
vina --config 151_lig151_\config.txt --log 151_lig151_\MD_log_.txt &
vina --config 152_lig152_\config.txt --log 152_lig152_\MD_log_.txt &
vina --config 153_lig153_\config.txt --log 153_lig153_\MD_log_.txt &
vina --config 154_lig154_\config.txt --log 154_lig154_\MD_log_.txt &
vina --config 155_lig155_\config.txt --log 155_lig155_\MD_log_.txt &
vina --config 156_lig156_\config.txt --log 156_lig156_\MD_log_.txt &
vina --config 157_lig157_\config.txt --log 157_lig157_\MD_log_.txt &
vina --config 158_lig158_\config.txt --log 158_lig158_\MD_log_.txt &
vina --config 159_lig159_\config.txt --log 159_lig159_\MD_log_.txt &
```

```
vina --config 160_lig160_\config.txt --log 160_lig160_\MD_log_.txt &
vina --config 161_lig161_\config.txt --log 161_lig161_\MD_log_.txt &
vina --config 162_lig162_\config.txt --log 162_lig162_\MD_log_.txt &
vina --config 163_lig163_\config.txt --log 163_lig163_\MD_log_.txt &
vina --config 164_lig164_\config.txt --log 164_lig164_\MD_log_.txt &
vina --config 165_lig165_\config.txt --log 165_lig165_\MD_log_.txt &
vina --config 166_lig166_\config.txt --log 166_lig166_\MD_log_.txt &
vina --config 167_lig167_\config.txt --log 167_lig167_\MD_log_.txt &
vina --config 168_lig168_\config.txt --log 168_lig168_\MD_log_.txt &
vina --config 169_lig169_\config.txt --log 169_lig169_\MD_log_.txt &
vina --config 170_lig170_\config.txt --log 170_lig170_\MD_log_.txt &
vina --config 171_lig171_\config.txt --log 171_lig171_\MD_log_.txt &
vina --config 172_lig172_\config.txt --log 172_lig172_\MD_log_.txt &
vina --config 173_lig173_\config.txt --log 173_lig173_\MD_log_.txt &
vina --config 174_lig174_\config.txt --log 174_lig174_\MD_log_.txt &
vina --config 175_lig175_\config.txt --log 175_lig175_\MD_log_.txt &
vina --config 176_lig176_\config.txt --log 176_lig176_\MD_log_.txt &
vina --config 177_lig177_\config.txt --log 177_lig177_\MD_log_.txt &
vina --config 178_lig178_\config.txt --log 178_lig178_\MD_log_.txt &
vina --config 179_lig179_\config.txt --log 179_lig179_\MD_log_.txt &
vina --config 180_lig180_\config.txt --log 180_lig180_\MD_log_.txt &
vina --config 181_lig181_\config.txt --log 181_lig181_\MD_log_.txt &
vina --config 182_lig182_\config.txt --log 182_lig182_\MD_log_.txt &
vina --config 183_lig183_\config.txt --log 183_lig183_\MD_log_.txt &
vina --config 184_lig184_\config.txt --log 184_lig184_\MD_log_.txt &
vina --config 185_lig185_\config.txt --log 185_lig185_\MD_log_.txt &
vina --config 186_lig186_\config.txt --log 186_lig186_\MD_log_.txt &
vina --config 187_lig187_\config.txt --log 187_lig187_\MD_log_.txt &
vina --config 188_lig188_\config.txt --log 188_lig188_\MD_log_.txt &
vina --config 189_lig189_\config.txt --log 189_lig189_\MD_log_.txt &
vina --config 190_lig190_\config.txt --log 190_lig190_\MD_log_.txt &
vina --config 191_lig191_\config.txt --log 191_lig191_\MD_log_.txt &
vina --config 192_lig192_\config.txt --log 192_lig192_\MD_log_.txt &
vina --config 193_lig193_\config.txt --log 193_lig193_\MD_log_.txt &
vina --config 194_lig194_\config.txt --log 194_lig194_\MD_log_.txt &
vina --config 195_lig195_\config.txt --log 195_lig195_\MD_log_.txt &
vina --config 196_lig196_\config.txt --log 196_lig196_\MD_log_.txt &
vina --config 197_lig197_\config.txt --log 197_lig197_\MD_log_.txt &
vina --config 198_lig198_\config.txt --log 198_lig198_\MD_log_.txt &
vina --config 199_lig199_\config.txt --log 199_lig199_\MD_log_.txt &
vina --config 200_lig200_\config.txt --log 200_lig200_\MD_log_.txt &
vina --config 201_lig201_\config.txt --log 201_lig201_\MD_log_.txt &
vina --config 202_lig202_\config.txt --log 202_lig202_\MD_log_.txt &
vina --config 203_lig203_\config.txt --log 203_lig203_\MD_log_.txt &
vina --config 204_lig204_\config.txt --log 204_lig204_\MD_log_.txt &
vina --config 205_lig205_\config.txt --log 205_lig205_\MD_log_.txt &
vina --config 206_lig206_\config.txt --log 206_lig206_\MD_log_.txt &
vina --config 207_lig207_\config.txt --log 207_lig207_\MD_log_.txt &
vina --config 208_lig208_\config.txt --log 208_lig208_\MD_log_.txt &
vina --config 209_lig209_\config.txt --log 209_lig209_\MD_log_.txt &
vina --config 210_lig210_\config.txt --log 210_lig210_\MD_log_.txt &
vina --config 211_lig211_\config.txt --log 211_lig211_\MD_log_.txt &
vina --config 212_lig212_\config.txt --log 212_lig212_\MD_log_.txt &
vina --config 213_lig213_\config.txt --log 213_lig213_\MD_log_.txt &
```

```
vina --config 214_lig214_\config.txt --log 214_lig214_\MD_log_.txt &
vina --config 215_lig215_\config.txt --log 215_lig215_\MD_log_.txt &
vina --config 216_lig216_\config.txt --log 216_lig216_\MD_log_.txt &
vina --config 217_lig217_\config.txt --log 217_lig217_\MD_log_.txt &
vina --config 218_lig218_\config.txt --log 218_lig218_\MD_log_.txt &
vina --config 219_lig219_\config.txt --log 219_lig219_\MD_log_.txt &
vina --config 220_lig220_\config.txt --log 220_lig220_\MD_log_.txt &
vina --config 221_lig221_\config.txt --log 221_lig221_\MD_log_.txt &
vina --config 222_lig222_\config.txt --log 222_lig222_\MD_log_.txt &
vina --config 223_lig223_\config.txt --log 223_lig223_\MD_log_.txt &
vina --config 224_lig224_\config.txt --log 224_lig224_\MD_log_.txt &
vina --config 225_lig225_\config.txt --log 225_lig225_\MD_log_.txt &
vina --config 226_lig226_\config.txt --log 226_lig226_\MD_log_.txt &
vina --config 227_lig227_\config.txt --log 227_lig227_\MD_log_.txt &
vina --config 228_lig228_\config.txt --log 228_lig228_\MD_log_.txt &
vina --config 229_lig229_\config.txt --log 229_lig229_\MD_log_.txt &
vina --config 230_lig230_\config.txt --log 230_lig230_\MD_log_.txt &
vina --config 231_lig231_\config.txt --log 231_lig231_\MD_log_.txt &
vina --config 232_lig232_\config.txt --log 232_lig232_\MD_log_.txt &
vina --config 233_lig233_\config.txt --log 233_lig233_\MD_log_.txt &
vina --config 234_lig234_\config.txt --log 234_lig234_\MD_log_.txt &
vina --config 235_lig235_\config.txt --log 235_lig235_\MD_log_.txt &
vina --config 236_lig236_\config.txt --log 236_lig236_\MD_log_.txt &
vina --config 237_lig237_\config.txt --log 237_lig237_\MD_log_.txt &
vina --config 238_lig238_\config.txt --log 238_lig238_\MD_log_.txt &
vina --config 239_lig239_\config.txt --log 239_lig239_\MD_log_.txt &
vina --config 240_lig240_\config.txt --log 240_lig240_\MD_log_.txt &
vina --config 241_lig241_\config.txt --log 241_lig241_\MD_log_.txt &
vina --config 242_lig242_\config.txt --log 242_lig242_\MD_log_.txt &
vina --config 243_lig243_\config.txt --log 243_lig243_\MD_log_.txt &
vina --config 244_lig244_\config.txt --log 244_lig244_\MD_log_.txt &
vina --config 245_lig245_\config.txt --log 245_lig245_\MD_log_.txt &
vina --config 246_lig246_\config.txt --log 246_lig246_\MD_log_.txt &
vina --config 247_lig247_\config.txt --log 247_lig247_\MD_log_.txt &
vina --config 248_lig248_\config.txt --log 248_lig248_\MD_log_.txt &
vina --config 249_lig249_\config.txt --log 249_lig249_\MD_log_.txt &
vina --config 250_lig250_\config.txt --log 250_lig250_\MD_log_.txt &
vina --config 251_lig251_\config.txt --log 251_lig251_\MD_log_.txt &
vina --config 252_lig252_\config.txt --log 252_lig252_\MD_log_.txt &
vina --config 253_lig253_\config.txt --log 253_lig253_\MD_log_.txt &
vina --config 254_lig254_\config.txt --log 254_lig254_\MD_log_.txt &
vina --config 255_lig255_\config.txt --log 255_lig255_\MD_log_.txt &
vina --config 256_lig256_\config.txt --log 256_lig256_\MD_log_.txt &
vina --config 257_lig257_\config.txt --log 257_lig257_\MD_log_.txt &
vina --config 258_lig258_\config.txt --log 258_lig258_\MD_log_.txt &
vina --config 259_lig259_\config.txt --log 259_lig259_\MD_log_.txt &
vina --config 260_lig260_\config.txt --log 260_lig260_\MD_log_.txt &
vina --config 261_lig261_\config.txt --log 261_lig261_\MD_log_.txt &
vina --config 262_lig262_\config.txt --log 262_lig262_\MD_log_.txt &
vina --config 263_lig263_\config.txt --log 263_lig263_\MD_log_.txt &
vina --config 264_lig264_\config.txt --log 264_lig264_\MD_log_.txt &
vina --config 265_lig265_\config.txt --log 265_lig265_\MD_log_.txt &
vina --config 266_lig266_\config.txt --log 266_lig266_\MD_log_.txt &
vina --config 267_lig267_\config.txt --log 267_lig267_\MD_log_.txt &
```

```
vina --config 268_lig268_\config.txt --log 268_lig268_\MD_log_.txt &
vina --config 269_lig269_\config.txt --log 269_lig269_\MD_log_.txt &
vina --config 270_lig270_\config.txt --log 270_lig270_\MD_log_.txt &
vina --config 271_lig271_\config.txt --log 271_lig271_\MD_log_.txt &
vina --config 272_lig272_\config.txt --log 272_lig272_\MD_log_.txt &
vina --config 273_lig273_\config.txt --log 273_lig273_\MD_log_.txt &
vina --config 274_lig274_\config.txt --log 274_lig274_\MD_log_.txt &
vina --config 275_lig275_\config.txt --log 275_lig275_\MD_log_.txt &
vina --config 276_lig276_\config.txt --log 276_lig276_\MD_log_.txt &
vina --config 277_lig277_\config.txt --log 277_lig277_\MD_log_.txt &
vina --config 278_lig278_\config.txt --log 278_lig278_\MD_log_.txt &
vina --config 279_lig279_\config.txt --log 279_lig279_\MD_log_.txt &
vina --config 280_lig280_\config.txt --log 280_lig280_\MD_log_.txt &
vina --config 281_lig281_\config.txt --log 281_lig281_\MD_log_.txt &
vina --config 282_lig282_\config.txt --log 282_lig282_\MD_log_.txt &
vina --config 283_lig283_\config.txt --log 283_lig283_\MD_log_.txt &
vina --config 284_lig284_\config.txt --log 284_lig284_\MD_log_.txt &
vina --config 285_lig285_\config.txt --log 285_lig285_\MD_log_.txt &
vina --config 286_lig286_\config.txt --log 286_lig286_\MD_log_.txt &
vina --config 287_lig287_\config.txt --log 287_lig287_\MD_log_.txt &
vina --config 288_lig288_\config.txt --log 288_lig288_\MD_log_.txt &
vina --config 289_lig289_\config.txt --log 289_lig289_\MD_log_.txt &
vina --config 290_lig290_\config.txt --log 290_lig290_\MD_log_.txt &
vina --config 291_lig291_\config.txt --log 291_lig291_\MD_log_.txt &
vina --config 292_lig292_\config.txt --log 292_lig292_\MD_log_.txt &
vina --config 293_lig293_\config.txt --log 293_lig293_\MD_log_.txt &
vina --config 294_lig294_\config.txt --log 294_lig294_\MD_log_.txt &
vina --config 295_lig295_\config.txt --log 295_lig295_\MD_log_.txt &
vina --config 296_lig296_\config.txt --log 296_lig296_\MD_log_.txt &
vina --config 297_lig297_\config.txt --log 297_lig297_\MD_log_.txt &
vina --config 298_lig298_\config.txt --log 298_lig298_\MD_log_.txt &
vina --config 299_lig299_\config.txt --log 299_lig299_\MD_log_.txt &
vina --config 300_lig300_\config.txt --log 300_lig300_\MD_log_.txt &
vina --config 301_lig301_\config.txt --log 301_lig301_\MD_log_.txt &
vina --config 302_lig302_\config.txt --log 302_lig302_\MD_log_.txt &
vina --config 303_lig303_\config.txt --log 303_lig303_\MD_log_.txt &
vina --config 304_lig304_\config.txt --log 304_lig304_\MD_log_.txt &
vina --config 305_lig305_\config.txt --log 305_lig305_\MD_log_.txt &
vina --config 306_lig306_\config.txt --log 306_lig306_\MD_log_.txt &
vina --config 307_lig307_\config.txt --log 307_lig307_\MD_log_.txt &
vina --config 308_lig308_\config.txt --log 308_lig308_\MD_log_.txt &
vina --config 309_lig309_\config.txt --log 309_lig309_\MD_log_.txt &
vina --config 310_lig310_\config.txt --log 310_lig310_\MD_log_.txt &
vina --config 311_lig311_\config.txt --log 311_lig311_\MD_log_.txt &
vina --config 312_lig312_\config.txt --log 312_lig312_\MD_log_.txt &
vina --config 313_lig313_\config.txt --log 313_lig313_\MD_log_.txt &
vina --config 314_lig314_\config.txt --log 314_lig314_\MD_log_.txt &
vina --config 315_lig315_\config.txt --log 315_lig315_\MD_log_.txt &
vina --config 316_lig316_\config.txt --log 316_lig316_\MD_log_.txt &
vina --config 317_lig317_\config.txt --log 317_lig317_\MD_log_.txt &
vina --config 318_lig318_\config.txt --log 318_lig318_\MD_log_.txt &
vina --config 319_lig319_\config.txt --log 319_lig319_\MD_log_.txt &
vina --config 320_lig320_\config.txt --log 320_lig320_\MD_log_.txt &
vina --config 321_lig321_\config.txt --log 321_lig321_\MD_log_.txt &
```

```
vina --config 322_lig322_\config.txt --log 322_lig322_\MD_log_.txt &
vina --config 323_lig323_\config.txt --log 323_lig323_\MD_log_.txt &
vina --config 324_lig324_\config.txt --log 324_lig324_\MD_log_.txt &
vina --config 325_lig325_\config.txt --log 325_lig325_\MD_log_.txt &
vina --config 326_lig326_\config.txt --log 326_lig326_\MD_log_.txt &
vina --config 327_lig327_\config.txt --log 327_lig327_\MD_log_.txt &
vina --config 328_lig328_\config.txt --log 328_lig328_\MD_log_.txt &
vina --config 329_lig329_\config.txt --log 329_lig329_\MD_log_.txt &
vina --config 330_lig330_\config.txt --log 330_lig330_\MD_log_.txt &
vina --config 331_lig331_\config.txt --log 331_lig331_\MD_log_.txt &
vina --config 332_lig332_\config.txt --log 332_lig332_\MD_log_.txt &
vina --config 333_lig333_\config.txt --log 333_lig333_\MD_log_.txt &
vina --config 334_lig334_\config.txt --log 334_lig334_\MD_log_.txt &
vina --config 335_lig335_\config.txt --log 335_lig335_\MD_log_.txt &
vina --config 336_lig336_\config.txt --log 336_lig336_\MD_log_.txt &
vina --config 337_lig337_\config.txt --log 337_lig337_\MD_log_.txt &
```

Notebook

January 18, 2024

1 File 53

[1]:
```
import pandas as pd
import matplotlib.pyplot as plt
%matplotlib inline
from rdkit import Chem
from rdkit.Chem import PandasTools
```

[2]:
```
all_docked = pd.read_excel('../Data/Colchicine - AI, MD.xlsx')
```

[3]:
```
all_docked.head()
```

[3]:
```
   Index  Structure                                             SMILES  \
0      1        NaN  COC1=C2C3=CC=C(NC)C(=O)C=C3[C@H1](CCC2=CC(OC)=…
1      2        NaN  COC1=C2C3=CC=C(NC)C(=O)C=C3[C@H1](CCC2=CC(OC)=…
2      3        NaN  COC1=C2C3=CC=C(NC)C(=O)C=C3[C@H1](CCC2=CC(OC)=…
3      4        NaN  COC1=C2C3=CC=C(NC)C(=O)C=C3[C@H1](CCC2=CC(OC)=…
4      5        NaN  O=CC1=CN=NN1[C@H1]2CCC3=CC(OC)=C(OC)C(OC)=C3C4…

   SYBA score  A549 [nM]  BALB/3T3 [nM]  LoVo [nM]  LoVo/DX [nM]  MCF-7 [nM]  \
0       45.88   8.348220      10.933929   6.176982     94.278083    8.357629
1       68.25  13.578222      11.636406  13.612706     33.064228   10.167834
2       74.22  16.404839      39.119200  41.777761     24.143942    9.099151
3       40.43  18.107777      27.000988   9.635175    232.135864    5.400000
4       41.84   6.445819      10.817093   8.763690   1553.892060   12.029326

   RI LoVo  SI BALB3/T3 (A549)  SI BALB3/T3 (LoVo)  SI BALB3/T3 (LoVo/DX)  \
0    15.26                1.31                1.77                   0.12
1     2.43                0.86                0.85                   0.35
2     0.58                2.38                0.94                   1.62
3    24.09                1.49                2.80                   0.12
4   177.31                1.68                1.23                   0.01

   SI BALB3/T3 (MCF-7)  Affinity to 1SA0 [kcal/mol]
0                 1.31                         -7.8
1                 1.14                         -7.6
2                 4.30                         -8.4
3                 5.00                         -8.2
```

```
4                 0.90                         -8.1
```

[4]:
```python
all_docked.rename(columns={'Index': 'ligand number'}, inplace=True)
all_docked.head()
```

[4]:
```
   ligand number  Structure  \
0              1        NaN
1              2        NaN
2              3        NaN
3              4        NaN
4              5        NaN

                                             SMILES  SYBA score  A549 [nM]  \
0  COC1=C2C3=CC=C(NC)C(=O)C=C3[C@H1](CCC2=CC(OC)=…       45.88   8.348220
1  COC1=C2C3=CC=C(NC)C(=O)C=C3[C@H1](CCC2=CC(OC)=…       68.25  13.578222
2  COC1=C2C3=CC=C(NC)C(=O)C=C3[C@H1](CCC2=CC(OC)=…       74.22  16.404839
3  COC1=C2C3=CC=C(NC)C(=O)C=C3[C@H1](CCC2=CC(OC)=…       40.43  18.107777
4  O=CC1=CN=NN1[C@H1]2CCC3=CC(OC)=C(OC)C(OC)=C3C4…       41.84   6.445819

   BALB/3T3 [nM]  LoVo [nM]  LoVo/DX [nM]  MCF-7 [nM]  RI LoVo  \
0      10.933929   6.176982     94.278083    8.357629    15.26
1      11.636406  13.612706     33.064228   10.167834     2.43
2      39.119200  41.777761     24.143942    9.099151     0.58
3      27.000988   9.635175    232.135864    5.400000    24.09
4      10.817093   8.763690   1553.892060   12.029326   177.31

   SI BALB3/T3 (A549)  SI BALB3/T3 (LoVo)  SI BALB3/T3 (LoVo/DX)  \
0                1.31                1.77                   0.12
1                0.86                0.85                   0.35
2                2.38                0.94                   1.62
3                1.49                2.80                   0.12
4                1.68                1.23                   0.01

   SI BALB3/T3 (MCF-7)  Affinity to 1SA0 [kcal/mol]
0                 1.31                         -7.8
1                 1.14                         -7.6
2                 4.30                         -8.4
3                 5.00                         -8.2
4                 0.90                         -8.1
```

[5]:
```python
chirality_selection = pd.read_excel('../Data/new_structures_S_chirality.xlsx')
```

[6]:
```python
chirality_selection.head()
```

[6]:
```
   Unnamed: 0  Unnamed: 0.1  \
0           0           NaN
1           1           NaN
```

```
2           2           NaN
3           3           NaN
4           4           NaN

                                                  SMILES  SYBA score  A549 [nM]  \
0  COC1=C2C3=CC=C(NC)C(=O)C=C3[C@H1](CCC2=CC(OC)=…       45.88  10.168579
1  COC1=C2C3=CC=C(NC)C(=O)C=C3[C@H1](CCC2=CC(OC)=…       68.25  10.648944
2  COC1=C2C3=CC=C(NC)C(=O)C=C3[C@H1](CCC2=CC(OC)=…       74.22  11.794066
3  COC1=C2C3=CC=C(NC)C(=O)C=C3[C@H1](CCC2=CC(OC)=…       40.43   6.400000
4  O=CC1=CN=NN1[C@H1]2CCC3=CC(OC)=C(OC)C(OC)=C3C4…       41.84   4.774935

   BALB/3T3 [nM]  LoVo [nM]  LoVo/DX [nM]  MCF-7 [nM]  atom_num chirality
0       6.200000   5.700000    136.586050    8.357629        14         S
1       9.439809  17.460496     49.797629   10.167834        14         S
2      30.305940  54.000000     37.606395    9.099151        14         S
3      23.752895   8.570105    210.367783    5.400000        14         S
4      11.772850  10.000000   1806.439871   12.029326         7         S
```

[7]:
```python
chirality_selection.rename(columns={'Unnamed: 0': 'ligand number'},␣
 ↪inplace=True)
chirality_selection.head()
```

[7]:
```
   ligand number  Unnamed: 0.1  \
0              0           NaN
1              1           NaN
2              2           NaN
3              3           NaN
4              4           NaN

                                                  SMILES  SYBA score  A549 [nM]  \
0  COC1=C2C3=CC=C(NC)C(=O)C=C3[C@H1](CCC2=CC(OC)=…       45.88  10.168579
1  COC1=C2C3=CC=C(NC)C(=O)C=C3[C@H1](CCC2=CC(OC)=…       68.25  10.648944
2  COC1=C2C3=CC=C(NC)C(=O)C=C3[C@H1](CCC2=CC(OC)=…       74.22  11.794066
3  COC1=C2C3=CC=C(NC)C(=O)C=C3[C@H1](CCC2=CC(OC)=…       40.43   6.400000
4  O=CC1=CN=NN1[C@H1]2CCC3=CC(OC)=C(OC)C(OC)=C3C4…       41.84   4.774935

   BALB/3T3 [nM]  LoVo [nM]  LoVo/DX [nM]  MCF-7 [nM]  atom_num chirality
0       6.200000   5.700000    136.586050    8.357629        14         S
1       9.439809  17.460496     49.797629   10.167834        14         S
2      30.305940  54.000000     37.606395    9.099151        14         S
3      23.752895   8.570105    210.367783    5.400000        14         S
4      11.772850  10.000000   1806.439871   12.029326         7         S
```

[8]:
```python
dock_res = all_docked[all_docked['SMILES'].
 ↪isin(list(chirality_selection['SMILES']))]
```

[9]:
```python
dock_res
```

```
[9]:      ligand number  Structure  \
     0                1        NaN
     1                2        NaN
     2                3        NaN
     3                4        NaN
     4                5        NaN
     ..             ...        ...
     332            333        NaN
     333            334        NaN
     334            335        NaN
     335            336        NaN
     336            337        NaN

                                                     SMILES  SYBA score  \
     0    COC1=C2C3=CC=C(NC)C(=O)C=C3[C@H1](CCC2=CC(OC)=…       45.88
     1    COC1=C2C3=CC=C(NC)C(=O)C=C3[C@H1](CCC2=CC(OC)=…       68.25
     2    COC1=C2C3=CC=C(NC)C(=O)C=C3[C@H1](CCC2=CC(OC)=…       74.22
     3    COC1=C2C3=CC=C(NC)C(=O)C=C3[C@H1](CCC2=CC(OC)=…       40.43
     4    O=CC1=CN=NN1[C@H1]2CCC3=CC(OC)=C(OC)C(OC)=C3C4…       41.84
     ..                                                 …          …
     332  N([C@H1]1CCC2=C(C(OC)=C(OC)C(OC)=C2)C3=CC=C(NC…       50.61
     333  O=C(C=C(Cl)C=CN([C@H1]1CCC2=C(C(OC)=C(OC)C(OC)…       55.61
     334  CC(C)(C)OC(=O)/N=C(/N[C@H1]1CCC2=C(C(OC)=C(OC)…       77.77
     335  CC(C)(C)OC(=O)/N=C(/N[C@H1]1CCC2=C(C(OC)=C(OC)…       74.50
     336  C12=C3C(C4=C(OC)C(OC)=C(OC)C=C4CC[C@@H1]3NC(=O…       44.20

           A549 [nM]  BALB/3T3 [nM]   LoVo [nM]  LoVo/DX [nM]  MCF-7 [nM]  RI LoVo  \
     0      8.348220      10.933929    6.176982     94.278083    8.357629    15.26
     1     13.578222      11.636406   13.612706     33.064228   10.167834     2.43
     2     16.404839      39.119200   41.777761     24.143942    9.099151     0.58
     3     18.107777      27.000988    9.635175    232.135864    5.400000    24.09
     4      6.445819      10.817093    8.763690   1553.892060   12.029326   177.31
     ..          …              …           …            …           …        …
     332    7.718822      14.078016    6.176982    342.962430    9.577028    55.52
     333  697.197228    1057.618868  803.006371   6999.610248  449.451787     8.72
     334  640.864950     503.426708   63.156498   9415.565981  438.651686   149.08
     335  738.759051     483.152174  164.873587  10571.477341   45.510081    64.12
     336   10.449190     122.159988    9.635175   1952.664035   24.303734   202.66

          SI BALB3/T3 (A549)  SI BALB3/T3 (LoVo)  SI BALB3/T3 (LoVo/DX)  \
     0                  1.31                1.77                   0.12
     1                  0.86                0.85                   0.35
     2                  2.38                0.94                   1.62
     3                  1.49                2.80                   0.12
     4                  1.68                1.23                   0.01
     ..                    …                   …                      …
     332                1.82                2.28                   0.04
```

```
333                1.52              1.32                0.15
334                0.79              7.97                0.05
335                0.65              2.93                0.05
336               11.69             12.68                0.06

     SI BALB3/T3 (MCF-7)  Affinity to 1SA0 [kcal/mol]
0                   1.31                         -7.8
1                   1.14                         -7.6
2                   4.30                         -8.4
3                   5.00                         -8.2
4                   0.90                         -8.1
..                   …                          …
332                 1.47                         -8.7
333                 2.35                         -9.9
334                 1.15                         -7.2
335                10.62                         -8.5
336                 5.03                         -9.4

[285 rows x 15 columns]
```

[10]:
```
dock_res['Structure'] = [Chem.MolFromSmiles(smi) for smi in dock_res['SMILES']]
PandasTools.SaveXlsxFromFrame(dock_res, '../Data/molecular_docking_chirality.
 ↪xlsx', molCol='Structure')
#dock_res.to_excel('../Data/molecular_docking_chirality.xlsx')
```

```
C:\Users\aleks\AppData\Local\Temp\ipykernel_19496\1886125816.py:1:
SettingWithCopyWarning:
A value is trying to be set on a copy of a slice from a DataFrame.
Try using .loc[row_indexer,col_indexer] = value instead

See the caveats in the documentation: https://pandas.pydata.org/pandas-
docs/stable/user_guide/indexing.html#returning-a-view-versus-a-copy
  dock_res['Structure'] = [Chem.MolFromSmiles(smi) for smi in
dock_res['SMILES']]
```

[11]:
```
dock_res['Affinity to 1SA0 [kcal/mol]'].hist(bins=10)
#plt.title("Distribution of binarized activity")
#plt.xlim(xmin=-0.5, xmax = 1.5)
x = [-10.5, -10, -9, -8.6, -8.2, -8, -7, -6.5]
labels = [-11, -10, -9, -8.6, -8.2, -8, -7, -6]
plt.xlabel('Binding energy [kcal/mol]')
plt.ylabel('Number of compounds')
plt.axvline(x=-8.6, color='r') #native docked
plt.axvline(x=-8.2, color='r') #colchicine
plt.rc('grid', linestyle="-.", color='black')
plt.grid(True)
plt.xticks(x, labels)
```

```
#plt.savefig('distribution_activity.pdf', bbox_inches='tight')
plt.savefig('chirality_binding_energy.pdf', bbox_inches='tight')
```

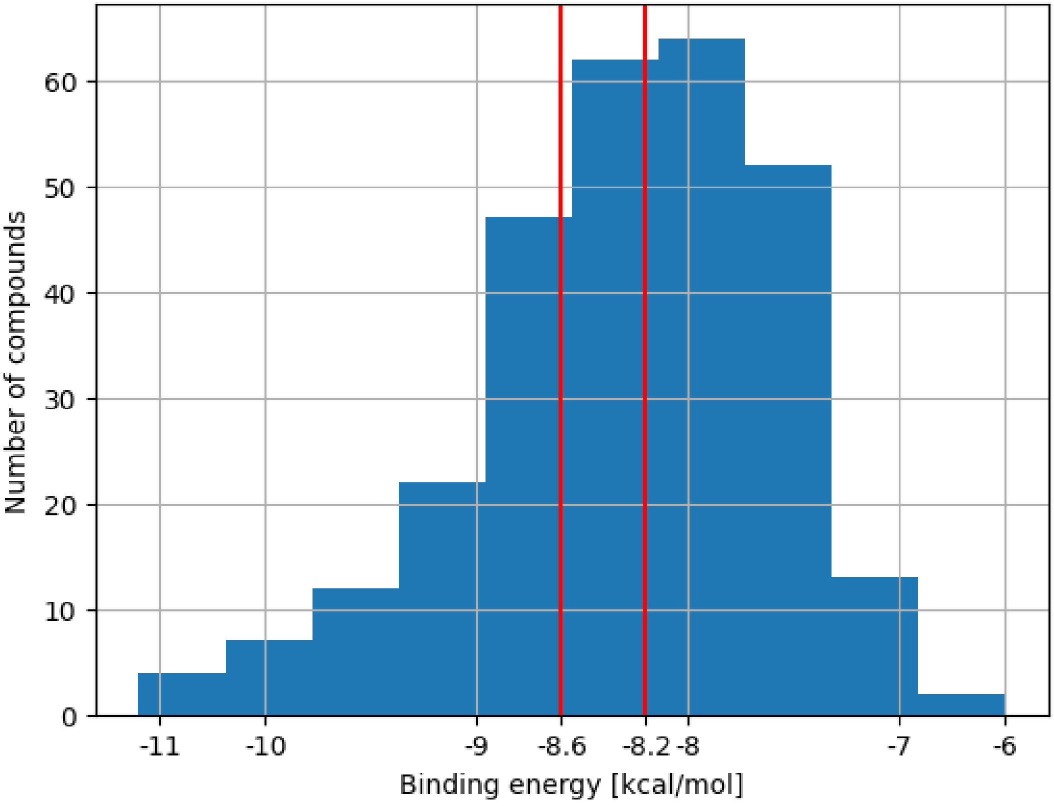

[12]:
```
in_range = dock_res[dock_res['Affinity to 1SA0 [kcal/mol]'] > -8.6]
in_range = in_range[in_range['Affinity to 1SA0 [kcal/mol]'] < -8.2]
```

[13]:
```
the_same_0 = dock_res[dock_res['Affinity to 1SA0 [kcal/mol]'] == -8.6]
len(the_same_0)
```

[13]: 14

[14]:
```
the_same_1 = dock_res[dock_res['Affinity to 1SA0 [kcal/mol]'] == -8.2]
len(the_same_1)
```

[14]: 15

[15]:
```
len(in_range)
```

[15]: 47

[16]:
```
in_range
```

[16]:
```
     ligand number                                         Structure  \
2                3  <rdkit.Chem.rdchem.Mol object at 0x0000021E330…
9               10  <rdkit.Chem.rdchem.Mol object at 0x0000021E330…
16              17  <rdkit.Chem.rdchem.Mol object at 0x0000021E330…
25              26  <rdkit.Chem.rdchem.Mol object at 0x0000021E330…
28              29  <rdkit.Chem.rdchem.Mol object at 0x0000021E330…
31              32  <rdkit.Chem.rdchem.Mol object at 0x0000021E331…
51              52  <rdkit.Chem.rdchem.Mol object at 0x0000021E330…
60              61  <rdkit.Chem.rdchem.Mol object at 0x0000021E330…
69              70  <rdkit.Chem.rdchem.Mol object at 0x0000021E330…
80              81  <rdkit.Chem.rdchem.Mol object at 0x0000021E330…
126            127  <rdkit.Chem.rdchem.Mol object at 0x0000021E330…
130            131  <rdkit.Chem.rdchem.Mol object at 0x0000021E330…
136            137  <rdkit.Chem.rdchem.Mol object at 0x0000021E330…
137            138  <rdkit.Chem.rdchem.Mol object at 0x0000021E330…
146            147  <rdkit.Chem.rdchem.Mol object at 0x0000021E330…
152            153  <rdkit.Chem.rdchem.Mol object at 0x0000021E330…
164            165  <rdkit.Chem.rdchem.Mol object at 0x0000021E330…
170            171  <rdkit.Chem.rdchem.Mol object at 0x0000021E330…
173            174  <rdkit.Chem.rdchem.Mol object at 0x0000021E330…
174            175  <rdkit.Chem.rdchem.Mol object at 0x0000021E330…
176            177  <rdkit.Chem.rdchem.Mol object at 0x0000021E330…
178            179  <rdkit.Chem.rdchem.Mol object at 0x0000021E330…
181            182  <rdkit.Chem.rdchem.Mol object at 0x0000021E330…
182            183  <rdkit.Chem.rdchem.Mol object at 0x0000021E330…
185            186  <rdkit.Chem.rdchem.Mol object at 0x0000021E330…
189            190  <rdkit.Chem.rdchem.Mol object at 0x0000021E330…
195            196  <rdkit.Chem.rdchem.Mol object at 0x0000021E330…
200            201  <rdkit.Chem.rdchem.Mol object at 0x0000021E330…
206            207  <rdkit.Chem.rdchem.Mol object at 0x0000021E330…
224            225  <rdkit.Chem.rdchem.Mol object at 0x0000021E330…
226            227  <rdkit.Chem.rdchem.Mol object at 0x0000021E330…
228            229  <rdkit.Chem.rdchem.Mol object at 0x0000021E330…
232            233  <rdkit.Chem.rdchem.Mol object at 0x0000021E330…
233            234  <rdkit.Chem.rdchem.Mol object at 0x0000021E330…
241            242  <rdkit.Chem.rdchem.Mol object at 0x0000021E330…
255            256  <rdkit.Chem.rdchem.Mol object at 0x0000021E330…
258            259  <rdkit.Chem.rdchem.Mol object at 0x0000021E330…
261            262  <rdkit.Chem.rdchem.Mol object at 0x0000021E330…
270            271  <rdkit.Chem.rdchem.Mol object at 0x0000021E330…
271            272  <rdkit.Chem.rdchem.Mol object at 0x0000021E330…
281            282  <rdkit.Chem.rdchem.Mol object at 0x0000021E330…
284            285  <rdkit.Chem.rdchem.Mol object at 0x0000021E330…
296            297  <rdkit.Chem.rdchem.Mol object at 0x0000021E330…
309            310  <rdkit.Chem.rdchem.Mol object at 0x0000021E330…
311            312  <rdkit.Chem.rdchem.Mol object at 0x0000021E330…
325            326  <rdkit.Chem.rdchem.Mol object at 0x0000021E330…
```

```
335           336  <rdkit.Chem.rdchem.Mol object at 0x0000021E330…

                                                  SMILES  SYBA score  \
2    COC1=C2C3=CC=C(NC)C(=O)C=C3[C@H1](CCC2=CC(OC)=…       74.22
9    C1N(C(=S)N[C@@H1]2C3=CC(C(NC)=CC=C3C4=C(OC)C(O…       49.88
16   CC(=O)OC1=CC=C2C3=C(C(OC)=C(C=C3CC[C@@H1](C2=C…       34.35
25   CC(=O)N[C@@H1]1C2=CC(C(NC)=CC=C2C3=C(OC)C(OC)=…       25.63
28   COC1=C2C3=CC=C(NC)C(=O)C=C3[C@H1](CCC2=CC(OC)=…       27.84
31   COC1=C(OC)C(OC)=CC=2CC[C@H1](NC(=N)N)C3=CC(=O)…       40.64
51   O=C(O)C=C(C(OCC)=O)N=NN[C@H1]1CCC2=CC(OC)=C(OC…       38.99
60   OC(=O)C=C(C(OCC)=O)N(NCCC)N[C@H1]1CCC2=CC(OC)=…       45.62
69   N1(CCCC1)C(=O)OC2=C(OC)C(OC3)=CC=C2C4=CC=C(SC)…       37.14
80   O=C(C(C)C)NC(=S)N[C@@H1]1C=2C(C3=C(C(OC)=C(OC)…       92.22
126  N(CCCC)CC1=CN(N=N1)[C@H1]2CCC3=CC(OC)=C(OC)C(O…       52.78
130  C=CC=C(NC(=O)N[C@H1]1CCC2=CC(OC)=C(OC)C(OC)=C2…       42.50
136  N([C@H1]1CCC2=CC(OC)=C(OC)C(OC)=C2C3=CC=C(NC)C…       24.37
137  NC(CCC)N[C@@H1]1C2=CC(C(NC)=CC=C2C3=C(OC)C(OC)…       55.10
146  OCC(C)=C1C([C@H1](CCC2=CC(OC)=C(OC)C(OC)=C12)N…       21.41
152  C=C(CO)CN[C@H1]1CCC2=CC(OC)=C(OC)C(OC)=C2C3=CC…       46.58
164  CNCC(C)OC(=O)N[C@H1]1CCC2=C(C(OC)=C(OC)C(OC)=C…       59.33
170  CC=CCCCN[C@H1]1CCC2=CC(OC)=C(OC)C(OC)=C2C3=CC=…       39.12
173  COC1=CC=2CC[C@@H1](C=3C(C=2C(=C1OC)OC)=CC=C(NC…       67.57
174  CSC1=CC=C2C(=CC1=O)[C@H1](CCC3=CC(OC)=C(OC)C(=…       22.94
176  CC(=O)OC1=CC=C2C3=C(C(OC)=C(C=C3CC[C@@H1](C2=C…       32.22
178  CC(=O)OC1=CC=C2C3=C(C(OC)=C(C=C3CC[C@@H1](C2=C…       64.15
181  CC(C)C(OC1=CC=C2C3=C(CC[C@@H1](C2=CC1=O)NC(C)=…       71.31
182  CC(C)C(OC1=CC=C2C3=C(CC[C@@H1](C2=CC1=O)NC(C)=…       39.74
185  CC(=O)OCC1=CN(N=N1)[C@H1]2CCC3=CC(OC)=C(OC)C(O…       67.53
189  C=12C(=O)C(=CC=C3C=1[C@H1](CCC4=CC(OC)=C(OC)C(…       25.47
195  CC(C)CCN[C@H1]1CCC2=C(C3=CC=C(SC)C(C=C31)=O)C(…       56.33
200  O=C(C)N[C@H1]1CCC2=CC(OC)=C(C(OC)=C2C3=CC=C(C(…       50.84
206  CNC(=S)N[C@@H1]1C2=CC(=O)C(NC)=CC=C2C3=C(OC)C(…       41.73
224  O=C(C)OC1=CN(N=N1)[C@H1]2CCC3=CC(OC)=C(OC)C(OC…       45.43
226  O=C(C(Cl)Cl)NC(=O)N[C@H1]1CCC2=C(C(OC)=C(OC)C(…       63.92
228  O=C(OCCC)OCC1=CN(N=N1)[C@H1]2CCC3=CC(OC)=C(OC)…       77.90
232  O=C(C(Cl)Cl)NCC1=CN(N=N1)[C@H1]2CCC3=CC(OC)=C(…       41.81
233  O=C(C)N[C@@H1]1CC(C2=C(C(OC)=C(OC)C=C2CC1)OCC3…       66.26
241  COC=C(OC)C(OC)=CCCC[C@H1](N1C=C(N=N1)COC(=O)C2…       26.59
255  CC(C)(C)OCNCCC(=O)NCC1=CN(N=N1)[C@@H1]2C3=CC(C…       42.74
258  C=1N(N=NC=1C(OCC)=O)[C@H1]2CCC3=C(C(OC)=C(OC)C…       61.54
261  N=NN([C@H1]1CCC2=C(C(OC)=C(OC)C(OC)=C2)C3=CC=C…       36.59
270  COCNCC1=CN(N=N1)[C@H1]2CCC3=CC(OC)=C(OC)C(OC)=…       32.69
271  CC(C)(C)OC(NCC=1N=NN(C=1)[C@H1]2CCC3=CC(OC)=C(…       29.68
281  C1=CN=CC=C1CN[C@H1]2CCC3=CC(OC)=C(OC)C(OC)=C3C…      102.60
284  NC(N=C1C=CC2)=CC=C1C3=C(OC)C(OC)=C(OC)C=C3CC[C…       21.44
296  N([C@H1]1CCC2=CC(OC)=C(OC)C(OC)=C2C3=CC=C(NC)C…       20.12
309  NN(CCO)[C@@H1]1C2=CC(C(NC)=CC=C2C3=C(OC)C(OC)=…       40.49
```

311	NC(N=NCOCCC)C[C@H1]1C2=CC(C(NC)=CC=C2C3=C(CC1)…	25.56
325	N([C@H1]1CCC2=C(C(OC)=C(OC)C(OC)=C2)C3=CC=C(SC…	59.06
335	CC(C)(C)OC(=O)/N=C(/N[C@H1]1CCC2=C(C(OC)=C(OC)…	74.50

	A549 [nM]	BALB/3T3 [nM]	LoVo [nM]	LoVo/DX [nM]	MCF-7 [nM]	\
2	16.404839	39.119200	41.777761	24.143942	9.099151	
9	13.217962	14.260864	9.635175	229.598512	7.928371	
16	1681.944585	2890.205397	803.006371	12855.961370	2110.336219	
25	33.218544	18.995642	9.634856	187.241318	57.881645	
28	5.536264	11.530736	1.861153	103.052597	8.098140	
31	6.748411	279.996735	9.635175	16246.471197	5.600000	
51	982.609443	1238.334197	353.700943	2195.134387	1129.719436	
60	296.687449	475.939825	259.046049	693.847187	101.723846	
69	529.630535	505.849181	253.706239	11735.188746	21.123908	
80	22.340738	7.469565	20.818933	1471.601346	10.474466	
126	22.386835	41.374984	9.635175	200.432032	31.962900	
130	7.992238	12.093598	14.174648	1114.393739	13.416494	
136	61.044735	26.926048	9.635175	175.441092	9.869470	
137	14.908827	15.993569	9.635175	1680.576300	8.921289	
146	391.053934	300.023346	443.206857	3859.227656	125.290418	
152	10.207992	14.512168	10.467981	171.783838	8.498039	
164	494.148508	90.560531	83.116794	750.970712	5.256002	
170	12.382034	11.641270	14.448198	102.472568	10.738748	
173	20.868913	16.060210	14.174648	464.533245	14.536965	
174	531.338692	513.912071	65.109453	330.193355	125.290418	
176	316.540118	994.480486	265.734143	17945.838427	1055.562319	
178	1963.484234	2890.205397	803.006371	12855.961370	2110.336219	
181	1772.816249	5522.772718	717.774803	1553.425204	4524.591036	
182	1077.713531	368.507201	237.556250	2051.494925	497.112620	
185	7.018461	12.196628	7.361318	2427.856798	2.976257	
189	26.301301	36.613507	9.634856	130.118997	25.543106	
195	5.413134	12.419313	36.306653	136.685884	5.994254	
200	1363.359377	1421.011999	188.272301	13148.227551	4259.023504	
206	10.421510	12.028252	25.783470	1581.577231	5.256002	
224	31.601559	23.170112	9.104401	1130.556772	52.560022	
226	955.969641	787.317900	202.638588	4289.948579	226.520273	
228	77.838408	397.581054	11.866527	1732.985256	107.539405	
232	8.381523	15.400336	7.297576	1207.120828	6.155925	
233	391.022971	56.890864	16.674135	136.860581	12.198248	
241	215.275841	36.603252	13.491703	1064.762116	21.351236	
255	17.986795	37.001121	7.297576	809.375787	30.162086	
258	39.978429	128.326379	25.620457	662.578905	25.105421	
261	540.390454	180.550219	102.812412	1491.681105	11.323713	
270	10.622728	18.973084	6.460893	212.139231	14.590242	
271	11.815905	16.030302	8.869165	1783.604598	13.904107	
281	13.819017	14.748669	9.635175	167.127833	5.400000	
284	93.417816	124.794930	9.635175	21333.691258	111.536289	

296	16.155076	13.007130	1.861153	52.778252	8.476131
309	15.072236	14.600783	11.542807	1436.190429	8.357629
311	7.715502	28.126916	6.460893	969.121368	9.869470
325	12.260896	12.654742	10.467981	49.329453	8.498039
335	738.759051	483.152174	164.873587	10571.477341	45.510081

	RI LoVo	SI BALB3/T3 (A549)	SI BALB3/T3 (LoVo)	SI BALB3/T3 (LoVo/DX) \
2	0.58	2.38	0.94	1.62
9	23.83	1.08	1.48	0.06
16	16.01	1.72	3.60	0.22
25	19.43	0.57	1.97	0.10
28	55.37	2.08	6.20	0.11
31	1686.16	41.49	29.06	0.02
51	6.21	1.26	3.50	0.56
60	2.68	1.60	1.84	0.69
69	46.26	0.96	1.99	0.04
80	70.69	0.33	0.36	0.01
126	20.80	1.85	4.29	0.21
130	78.62	1.51	0.85	0.01
136	18.21	0.44	2.79	0.15
137	174.42	1.07	1.66	0.01
146	8.71	0.77	0.68	0.08
152	16.41	1.42	1.39	0.08
164	9.04	0.18	1.09	0.12
170	7.09	0.94	0.81	0.11
173	32.77	0.77	1.13	0.03
174	5.07	0.97	7.89	1.56
176	67.53	3.14	3.74	0.06
178	16.01	1.47	3.60	0.22
181	2.16	3.12	7.69	3.56
182	8.64	0.34	1.55	0.18
185	329.81	1.74	1.66	0.01
189	13.51	1.39	3.80	0.28
195	3.76	2.29	0.34	0.09
200	69.84	1.04	7.55	0.11
206	61.34	1.15	0.47	0.01
224	124.18	0.73	2.54	0.02
226	21.17	0.82	3.89	0.18
228	146.04	5.11	33.50	0.23
232	165.41	1.84	2.11	0.01
233	8.21	0.15	3.41	0.42
241	78.92	0.17	2.71	0.03
255	110.91	2.06	5.07	0.05
258	25.86	3.21	5.01	0.19
261	14.51	0.33	1.76	0.12
270	32.83	1.79	2.94	0.09
271	201.10	1.36	1.81	0.01

281	17.35	1.07	1.53	0.09
284	2214.15	1.34	12.95	0.01
296	28.36	0.81	6.99	0.25
309	124.42	0.97	1.26	0.01
311	150.00	3.65	4.35	0.03
325	4.71	1.03	1.21	0.26
335	64.12	0.65	2.93	0.05

	SI BALB3/T3 (MCF-7)	Affinity to 1SA0 [kcal/mol]
2	4.30	-8.4
9	1.80	-8.3
16	1.37	-8.4
25	0.33	-8.3
28	1.42	-8.5
31	50.00	-8.3
51	1.10	-8.5
60	4.68	-8.5
69	23.95	-8.5
80	0.71	-8.5
126	1.29	-8.4
130	0.90	-8.3
136	2.73	-8.4
137	1.79	-8.3
146	2.39	-8.3
152	1.71	-8.3
164	17.23	-8.4
170	1.08	-8.5
173	1.10	-8.3
174	4.10	-8.5
176	0.94	-8.5
178	1.37	-8.5
181	1.22	-8.5
182	0.74	-8.4
185	4.10	-8.3
189	1.43	-8.5
195	2.07	-8.3
200	0.33	-8.4
206	2.29	-8.4
224	0.44	-8.4
226	3.48	-8.5
228	3.70	-8.3
232	2.50	-8.5
233	4.66	-8.3
241	1.71	-8.5
255	1.23	-8.5
258	5.11	-8.3
261	15.94	-8.3

270	1.30	-8.5
271	1.15	-8.3
281	2.73	-8.4
284	1.12	-8.5
296	1.53	-8.3
309	1.75	-8.3
311	2.85	-8.3
325	1.49	-8.3
335	10.62	-8.5

[ ]:

```

```

[17]:

```
higher_aff = dock_res[dock_res['Affinity to 1SA0 [kcal/mol]'] < -8.6]
len(higher_aff)
```

[17]: 78

[18]:

```
lower_aff = dock_res[dock_res['Affinity to 1SA0 [kcal/mol]'] > -8.2]
len(lower_aff)
```

[18]: 131

[ ]:

```

```

Notebook

January 18, 2024

1 File 55

[1]:
```
import pandas as pd

from sklearn.model_selection import train_test_split

import joblib

from sklearn.metrics import r2_score, mean_squared_error
import math

import numpy as np
```

[2]:
```
def transform(target_val):
    transformed = []
    for element in target_val:
        transformed.append(-1.0 * np.log10(element/1000000000))
    return transformed

def inverse_transform(transformed_values):
    inversed = []
    for element in transformed_values:
        inversed.append(np.power(10,-element)*1000000000)
    return inversed
```

[3]:
```
smiles_df = pd.read_excel('../Data/Kolchicyna_prepared_data.xlsx')
```

[4]:
```
smiles_df.head()
```

[4]:
```
   Unnamed: 0                             Publication DOI  \
0           0  https://doi.org/10.1016/j.bmcl.2021.128382
1           1  https://doi.org/10.1016/j.bmcl.2021.128382
2           2  https://doi.org/10.1016/j.bmcl.2021.128382
3           3  https://doi.org/10.1016/j.bmcl.2021.128382
4           4  https://doi.org/10.1016/j.bmcl.2021.128382

  Number of compound in publication  \
0                                 1
```

```
1                                  2
2                                  3
3                                  4
4                                  5

                                              SMILES  Activity [nM]   A549  \
0  COc2c3C1=CC=C(SC)C(=O)C=C1[C@H](CCc3cc(OC)c2OC…  Activity [nM]   10,8
1  COc2c3C1=CC=C(SC)C(=O)C=C1[C@H](CCc3cc(OC)c2OC…  Activity [nM]   11,6
2  COc2c3C1=CC=C(SC)C(=O)C=C1[C@H](CCc3cc(OC)c2OC…  Activity [nM]   10,9
3  CC(C)CN[C@H]2CCc3cc(OC)c(OC)c(OC)c3C1=CC=C(SC)…  Activity [nM]   10,5
4  COc2c3C1=CC=C(SC)C(=O)C=C1[C@H](CCc3cc(OC)c2OC…  Activity [nM]   89,5

   MCF-7   LoVo  LoVo/DX  BALB/3T3  A549_float  MCF-7_float  LoVo_float  \
0  10,3    6,5    54,9     10,2          10.8         10.3         6.5
1  12,0    8,5     31.1     14.3         11.6         12.0         8.5
2  12,2    8,8    17,9     11,7          10.9         12.2         8.8
3  11,3    8,5    10,2     11,0          10.5         11.3         8.5
4  92,7   52,8    77,8     99,4          89.5         92.7        52.8

   LoVo/DX_float  BALB/3T3_float
0           54.9            10.2
1           31.1            14.3
2           17.9            11.7
3           10.2            11.0
4           77.8            99.4
```

[5]:
```python
molecular_descriptors = pd.read_excel('../Data/Kolchicyna_machine_learning.
  ↪xlsx')
```

[6]:
```python
molecular_descriptors.head()
```

[6]:
```
   Unnamed: 0     AATS0Z  AATS0are    AATS0d   AATS0dv      AATS0i     AATS0m  \
0           0  25.090909  6.046518  3.109091  6.662626  160.775045  99.075564
1           1  24.448276  6.008422  3.051724  6.386973  161.021674  96.473315
2           2  23.868852  5.974074  3.000000  6.138434  161.244045  94.127025
3           3  23.868852  5.974074  3.032787  6.171220  161.244045  94.127025
4           4  23.343750  5.942945  2.953125  5.913194  161.445569  92.000700

     AATS0p   AATS0pe    AATS0s  …  A549_float  MCF-7_float  LoVo_float  \
0  1.555512  6.126680  3.435416  …        10.8         10.3         6.5
1  1.538471  6.088791  3.330998  …        11.6         12.0         8.5
2  1.523105  6.054630  3.236850  …        10.9         12.2         8.8
3  1.523105  6.054630  3.257798  …        10.5         11.3         8.5
4  1.509180  6.023670  3.151529  …        89.5         92.7        52.8

   LoVo/DX_float  BALB/3T3_float  A549_float_transformed  \
0           54.9            10.2                7.966576
```

```
1          31.1          14.3               7.935542
2          17.9          11.7               7.962574
3          10.2          11.0               7.978811
4          77.8          99.4               7.048177

   MCF-7_float_transformed  LoVo_float_transformed  LoVo/DX_float_transformed  \
0                 7.987163                8.187087                   7.260428
1                 7.920819                8.070581                   7.507240
2                 7.913640                8.055517                   7.747147
3                 7.946922                8.070581                   7.991400
4                 7.032920                7.277366                   7.109020

   BALB/3T3_float_transformed
0                    7.991400
1                    7.844664
2                    7.931814
3                    7.958607
4                    7.002614

[5 rows x 1222 columns]
```

[7]:
```
frames = [smiles_df['SMILES'], molecular_descriptors]
res = pd.concat(frames, axis=1)
res = res.drop(['Unnamed: 0'], axis=1)
```

[8]:
```
res
```

[8]:
```
                                                SMILES     AATS0Z  AATS0are  \
0    COc2c3C1=CC=C(SC)C(=O)C=C1[C@H](CCc3cc(OC)c2OC…  25.090909  6.046518
1    COc2c3C1=CC=C(SC)C(=O)C=C1[C@H](CCc3cc(OC)c2OC…  24.448276  6.008422
2    COc2c3C1=CC=C(SC)C(=O)C=C1[C@H](CCc3cc(OC)c2OC…  23.868852  5.974074
3    CC(C)CN[C@H]2CCc3cc(OC)c(OC)c(OC)c3C1=CC=C(SC)…  23.868852  5.974074
4    COc2c3C1=CC=C(SC)C(=O)C=C1[C@H](CCc3cc(OC)c2OC…  23.343750  5.942945
..                                                 …        …        …
115  FC(F)(F)c1ccc(cc1)NC(=S)N[C@H]3CCc4cc(OC)c(OC)…  28.925373  6.629527
116  O[C@@H](CNC(=S)N[C@H]2CCc3cc(OC)c(OC)c(OC)c3C1…  24.307692  6.247564
117  OC[C@@H](O)[C@@H](O)[C@H](O)[C@@H](O)CNC(=S)N[…  25.818182  6.393614
118  CC(C)(C)OC(=O)\N=C(/NC(=O)OC(C)(C)C)N[C@H]2CCc…  21.952941  6.267407
119  N=C(N)N[C@H]2CCc3cc(OC)c(OC)c(OC)c3C1=CC=C(NC)…  22.436364  6.250720

       AATS0d   AATS0dv     AATS0i     AATS0m    AATS0p   AATS0pe    AATS0s  \
0    3.109091  6.662626  160.775045  99.075564  1.555512  6.126680  3.435416
1    3.051724  6.386973  161.021674  96.473315  1.538471  6.088791  3.330998
2    3.000000  6.138434  161.244045  94.127025  1.523105  6.054630  3.236850
3    3.032787  6.171220  161.244045  94.127025  1.523105  6.054630  3.257798
4    2.953125  5.913194  161.445569  92.000700  1.509180  6.023670  3.151529
..        …         …          …          …         …         …         …
```

```
115  3.492537  9.558872  165.991839  116.283088  1.583312  6.669057  6.199313
116  3.241758  7.235653  163.907908   95.896512  1.427144  6.299627  3.659034
117  3.103896  7.512266  164.617043  101.985317  1.423114  6.435469  5.205806
118  3.105882  7.623529  165.008670   86.439890  1.354297  6.311679  4.432190
119  3.200000  7.600000  164.678288   88.447644  1.409812  6.303515  4.205556

     …  A549_float  MCF-7_float  LoVo_float  LoVo/DX_float  BALB/3T3_float  \
0    …        10.8         10.3         6.5           54.9            10.2
1    …        11.6         12.0         8.5           31.1            14.3
2    …        10.9         12.2         8.8           17.9            11.7
3    …        10.5         11.3         8.5           10.2            11.0
4    …        89.5         92.7        52.8           77.8            99.4
..   …         …            …          …              …               …
115  …        86.0        120.0        54.0         2700.0            67.0
116  …        70.0         15.0        32.0         5300.0           440.0
117  …       930.0       1200.0       680.0         6600.0          1300.0
118  …       850.0        940.0       500.0         5900.0           690.0
119  …       740.0        760.0       920.0        86000.0          1800.0

     A549_float_transformed  MCF-7_float_transformed  LoVo_float_transformed  \
0                  7.966576                 7.987163                8.187087
1                  7.935542                 7.920819                8.070581
2                  7.962574                 7.913640                8.055517
3                  7.978811                 7.946922                8.070581
4                  7.048177                 7.032920                7.277366
..                     …                        …                       …
115                7.065502                 6.920819                7.267606
116                7.154902                 7.823909                7.494850
117                6.031517                 5.920819                6.167491
118                6.070581                 6.026872                6.301030
119                6.130768                 6.119186                6.036212

     LoVo/DX_float_transformed  BALB/3T3_float_transformed
0                     7.260428                    7.991400
1                     7.507240                    7.844664
2                     7.747147                    7.931814
3                     7.991400                    7.958607
4                     7.109020                    7.002614
..                        …                           …
115                   5.568636                    7.173925
116                   5.275724                    6.356547
117                   5.180456                    5.886057
118                   5.229148                    6.161151
119                   4.065502                    5.744727

[120 rows x 1222 columns]
```

[9]:
```
## load models
model_A549 = joblib.load('Random_forest/random_forest_model_17_estimators_A549.
↪joblib') #Random state 15
model_BALB3_t3 = joblib.load('Random_forest/
↪random_forest_model_19_estimators_BALB_3T3.joblib') #Random state 42
model_LoVo_DX = joblib.load('Random_forest/
↪random_forest_model_14_estimators_LoVo_DX.joblib') #Random state 28
model_LoVo = joblib.load('Random_forest/random_forest_model_18_estimators_LoVo.
↪joblib') #Random state 42
model_MCF_7 = joblib.load('Random_forest/random_forest_model_3_estimators_MCF-7.
↪joblib') #Random state 15
```

2 Construct a dataframe for each Random Seed and predict values, additionally calculate R score and RMSE…

2.1 A549

[10]:
```
list(model_A549.feature_names_in_)
```

[10]:
```
['AMID_O', 'EState_VSA5', 'MDEO-12', 'SaasC', 'VSA_EState5']
```

[11]:
```
res.columns[0:-9]
```

[11]:
```
Index(['SMILES', 'AATS0Z', 'AATS0are', 'AATS0d', 'AATS0dv', 'AATS0i', 'AATS0m',
       'AATS0p', 'AATS0pe', 'AATS0s',
       …
       'piPC10', 'piPC2', 'piPC3', 'piPC4', 'piPC5', 'piPC6', 'piPC7', 'piPC8',
       'piPC9', 'A549_float'],
      dtype='object', length=1213)
```

[12]:
```
X_train, X_test, y_train, y_test = train_test_split(res[res.columns[0:-9]],
                                                                          ␣
↪res['A549_float_transformed'],
                                                                   test_size=0.15,␣
↪random_state=15)

X_train['predicted A549 transformed IC50'] = model_A549.
↪predict(X_train[list(model_A549.feature_names_in_)])
X_train['observed A549 transformed IC50'] = y_train
X_train['predicted A549 IC50'] = inverse_transform(X_train['predicted A549␣
↪transformed IC50'])
X_test['predicted A549 transformed IC50'] = model_A549.
↪predict(X_test[list(model_A549.feature_names_in_)])
X_test['observed A549 transformed IC50'] = y_test
X_test['predicted A549 IC50'] = inverse_transform(X_test['predicted A549␣
↪transformed IC50'])
```

```
print('Train R score: '+str(math.sqrt(r2_score(X_train['observed A549␣
↪transformed IC50'], X_train['predicted A549 transformed IC50']))))
print('Test R score: '+str(math.sqrt(r2_score(X_test['observed A549 transformed␣
↪IC50'], X_test['predicted A549 transformed IC50']))))
print('Train RMSE score: '+str(math.sqrt(mean_squared_error(X_train['observed␣
↪A549 transformed IC50'], X_train['predicted A549 transformed IC50']))))
print('Test RMSE score: '+str(math.sqrt(mean_squared_error(X_test['observed␣
↪A549 transformed IC50'], X_test['predicted A549 transformed IC50']))))
```

```
Train R score: 0.960591935079034
Test R score: 0.8620618597285548
Train RMSE score: 0.2594924364766403
Test RMSE score: 0.5253556041537444
```

[13]:
```
with pd.ExcelWriter("../Data/A549_random_state_15.xlsx") as writer:
    X_train.to_excel(writer, sheet_name="Train", index=True)
    X_test.to_excel(writer, sheet_name="Test", index=True)
```

[14]:
```
X_train, X_test, y_train, y_test = train_test_split(res[res.columns[0:-9]],
                                                                          ␣
↪res['A549_float_transformed'],
                                                                           test_size=0.15,␣
↪random_state=28)

X_train['predicted A549 transformed IC50'] = model_A549.
↪predict(X_train[list(model_A549.feature_names_in_)])
X_train['observed A549 transformed IC50'] = y_train
X_train['predicted A549 IC50'] = inverse_transform(X_train['predicted A549␣
↪transformed IC50'])
X_test['predicted A549 transformed IC50'] = model_A549.
↪predict(X_test[list(model_A549.feature_names_in_)])
X_test['observed A549 transformed IC50'] = y_test
X_test['predicted A549 IC50'] = inverse_transform(X_test['predicted A549␣
↪transformed IC50'])

#print('Train R score: '+str(math.sqrt(r2_score(X_train['observed A549␣
↪transformed IC50'], X_train['predicted A549 transformed IC50']))))
#print('Test R score: '+str(math.sqrt(r2_score(X_test['observed A549␣
↪transformed IC50'], X_test['predicted A549 transformed IC50']))))
#print('Train RMSE score: '+str(math.sqrt(mean_squared_error(X_train['observed␣
↪A549 transformed IC50'], X_train['predicted A549 transformed IC50']))))
#print('Test RMSE score: '+str(math.sqrt(mean_squared_error(X_test['observed␣
↪A549 transformed IC50'], X_test['predicted A549 transformed IC50']))))
```

[15]:
```
with pd.ExcelWriter("../Data/A549_random_state_28.xlsx") as writer:
    X_train.to_excel(writer, sheet_name="Train", index=True)
    X_test.to_excel(writer, sheet_name="Test", index=True)
```

[16]:
```
X_train, X_test, y_train, y_test = train_test_split(res[res.columns[0:-9]],␣
 ↪res['A549_float_transformed'],
                                                     test_size=0.15,␣
 ↪random_state=42)

X_train['predicted A549 transformed IC50'] = model_A549.
 ↪predict(X_train[list(model_A549.feature_names_in_)])
X_train['observed A549 transformed IC50'] = y_train
X_train['predicted A549 IC50'] = inverse_transform(X_train['predicted A549␣
 ↪transformed IC50'])
X_test['predicted A549 transformed IC50'] = model_A549.
 ↪predict(X_test[list(model_A549.feature_names_in_)])
X_test['observed A549 transformed IC50'] = y_test
X_test['predicted A549 IC50'] = inverse_transform(X_test['predicted A549␣
 ↪transformed IC50'])

#print('Train R score: '+str(math.sqrt(r2_score(X_train['observed A549␣
 ↪transformed IC50'], X_train['predicted A549 transformed IC50']))))
#print('Test R score: '+str(math.sqrt(r2_score(X_test['observed A549␣
 ↪transformed IC50'], X_test['predicted A549 transformed IC50']))))
#print('Train RMSE score: '+str(math.sqrt(mean_squared_error(X_train['observed␣
 ↪A549 transformed IC50'], X_train['predicted A549 transformed IC50']))))
#print('Test RMSE score: '+str(math.sqrt(mean_squared_error(X_test['observed␣
 ↪A549 transformed IC50'], X_test['predicted A549 transformed IC50']))))
```

[17]:
```
with pd.ExcelWriter("../Data/A549_random_state_42.xlsx") as writer:
    X_train.to_excel(writer, sheet_name="Train", index=True)
    X_test.to_excel(writer, sheet_name="Test", index=True)
```

2.2 BALB 3T3

[18]:
```
list(model_BALB3_t3.feature_names_in_)
```

[18]:
```
['AMID_O', 'EState_VSA5', 'GATS2c', 'MDEO-12', 'NdssC', 'VSA_EState5']
```

[19]:
```
res.columns[0:-5]
```

[19]:
```
Index(['SMILES', 'AATS0Z', 'AATS0are', 'AATS0d', 'AATS0dv', 'AATS0i', 'AATS0m',
       'AATS0p', 'AATS0pe', 'AATS0s',
       …
       'piPC5', 'piPC6', 'piPC7', 'piPC8', 'piPC9', 'A549_float',
       'MCF-7_float', 'LoVo_float', 'LoVo/DX_float', 'BALB/3T3_float'],
```

```
    dtype='object', length=1217)
```

[20]:
```
X_train, X_test, y_train, y_test = train_test_split(res[res.columns[0:-5]],
                                                    res['BALB/
↪3T3_float_transformed'],
                                                    test_size=0.15,␣
↪random_state=15)

X_train['predicted BALB 3T3 transformed IC50'] = model_BALB3_t3.
↪predict(X_train[list(model_BALB3_t3.feature_names_in_)])
X_train['observed BALB 3T3 transformed IC50'] = y_train
X_train['predicted BALB 3T3 IC50'] = inverse_transform(X_train['predicted BALB␣
↪3T3 transformed IC50'])
X_test['predicted BALB 3T3 transformed IC50'] = model_BALB3_t3.
↪predict(X_test[list(model_BALB3_t3.feature_names_in_)])
X_test['observed BALB 3T3 transformed IC50'] = y_test
X_test['predicted BALB 3T3 IC50'] = inverse_transform(X_test['predicted BALB␣
↪3T3 transformed IC50'])

print('Train R score: '+str(math.sqrt(r2_score(X_train['observed BALB 3T3␣
↪transformed IC50'], X_train['predicted BALB 3T3 transformed IC50']))))
print('Test R score: '+str(math.sqrt(r2_score(X_test['observed BALB 3T3␣
↪transformed IC50'], X_test['predicted BALB 3T3 transformed IC50']))))
print('Train RMSE score: '+str(math.sqrt(mean_squared_error(X_train['observed␣
↪BALB 3T3 transformed IC50'], X_train['predicted BALB 3T3 transformed␣
↪IC50']))))
print('Test RMSE score: '+str(math.sqrt(mean_squared_error(X_test['observed␣
↪BALB 3T3 transformed IC50'], X_test['predicted BALB 3T3 transformed␣
↪IC50']))))
```

```
Train R score: 0.9656538230216772
Test R score: 0.8642992780332693
Train RMSE score: 0.22547718186980922
Test RMSE score: 0.46260702873007903
```

[21]:
```
with pd.ExcelWriter("../Data/BALB_3T3_random_state_15.xlsx") as writer:
    X_train.to_excel(writer, sheet_name="Train", index=True)
    X_test.to_excel(writer, sheet_name="Test", index=True)
```

[22]:
```
X_train, X_test, y_train, y_test = train_test_split(res[res.columns[0:-5]],
                                                    res['BALB/
↪3T3_float_transformed'],
                                                    test_size=0.15,␣
↪random_state=28)

X_train['predicted BALB 3T3 transformed IC50'] = model_BALB3_t3.
↪predict(X_train[list(model_BALB3_t3.feature_names_in_)])
```

```
X_train['observed BALB 3T3 transformed IC50'] = y_train
X_train['predicted BALB 3T3 IC50'] = inverse_transform(X_train['predicted BALB␣
↪3T3 transformed IC50'])
X_test['predicted BALB 3T3 transformed IC50'] = model_BALB3_t3.
↪predict(X_test[list(model_BALB3_t3.feature_names_in_)])
X_test['observed BALB 3T3 transformed IC50'] = y_test
X_test['predicted BALB 3T3 IC50'] = inverse_transform(X_test['predicted BALB␣
↪3T3 transformed IC50'])

#print('Train R score: '+str(math.sqrt(r2_score(X_train['observed BALB 3T3␣
↪transformed IC50'], X_train['predicted BALB 3T3 transformed IC50']))))
#print('Test R score: '+str(math.sqrt(r2_score(X_test['observed BALB 3T3␣
↪transformed IC50'], X_test['predicted BALB 3T3 transformed IC50']))))
#print('Train RMSE score: '+str(math.sqrt(mean_squared_error(X_train['observed␣
↪BALB 3T3 transformed IC50'], X_train['predicted BALB 3T3 transformed␣
↪IC50']))))
#print('Test RMSE score: '+str(math.sqrt(mean_squared_error(X_test['observed␣
↪BALB 3T3 transformed IC50'], X_test['predicted BALB 3T3 transformed␣
↪IC50']))))
```

[23]:
```
with pd.ExcelWriter("../Data/BALB_3T3_random_state_28.xlsx") as writer:
    X_train.to_excel(writer, sheet_name="Train", index=True)
    X_test.to_excel(writer, sheet_name="Test", index=True)
```

[24]:
```
X_train, X_test, y_train, y_test = train_test_split(res[res.columns[0:-5]],
                                                        res['BALB/
↪3T3_float_transformed'],
                                                        test_size=0.15,␣
↪random_state=42)

X_train['predicted BALB 3T3 transformed IC50'] = model_BALB3_t3.
↪predict(X_train[list(model_BALB3_t3.feature_names_in_)])
X_train['observed BALB 3T3 transformed IC50'] = y_train
X_train['predicted BALB 3T3 IC50'] = inverse_transform(X_train['predicted BALB␣
↪3T3 transformed IC50'])
X_test['predicted BALB 3T3 transformed IC50'] = model_BALB3_t3.
↪predict(X_test[list(model_BALB3_t3.feature_names_in_)])
X_test['observed BALB 3T3 transformed IC50'] = y_test
X_test['predicted BALB 3T3 IC50'] = inverse_transform(X_test['predicted BALB␣
↪3T3 transformed IC50'])

#print('Train R score: '+str(math.sqrt(r2_score(X_train['observed BALB 3T3␣
↪transformed IC50'], X_train['predicted BALB 3T3 transformed IC50']))))
#print('Test R score: '+str(math.sqrt(r2_score(X_test['observed BALB 3T3␣
↪transformed IC50'], X_test['predicted BALB 3T3 transformed IC50']))))
```

```
#print('Train RMSE score: '+str(math.sqrt(mean_squared_error(X_train['observed␣
↪BALB 3T3 transformed IC50'], X_train['predicted BALB 3T3 transformed␣
↪IC50']))))
#print('Test RMSE score: '+str(math.sqrt(mean_squared_error(X_test['observed␣
↪BALB 3T3 transformed IC50'], X_test['predicted BALB 3T3 transformed␣
↪IC50']))))
```

[25]:
```
with pd.ExcelWriter("../Data/BALB_3T3_random_state_42.xlsx") as writer:
    X_train.to_excel(writer, sheet_name="Train", index=True)
    X_test.to_excel(writer, sheet_name="Test", index=True)
```

2.3 LoVo/dx

[26]:
```
list(model_LoVo_DX.feature_names_in_)
```

[26]:
```
['GATS2c', 'MATS2c', 'NdssC', 'RNCG', 'TopoPSA(NO)']
```

[27]:
```
res.columns[0:-6]
```

[27]:
```
Index(['SMILES', 'AATS0Z', 'AATS0are', 'AATS0d', 'AATS0dv', 'AATS0i', 'AATS0m',
       'AATS0p', 'AATS0pe', 'AATS0s',
       …
       'piPC4', 'piPC5', 'piPC6', 'piPC7', 'piPC8', 'piPC9', 'A549_float',
       'MCF-7_float', 'LoVo_float', 'LoVo/DX_float'],
      dtype='object', length=1216)
```

[28]:
```
print(res.shape)
res_ = res.dropna()
res_.shape
```

```
(120, 1222)
```

[28]:
```
(106, 1222)
```

[29]:
```
X_train, X_test, y_train, y_test = train_test_split(res_[res_.columns[0:-6]],
                                                    res_['LoVo/
 ↪DX_float_transformed'],
                                                    test_size=0.15,␣
 ↪random_state=15)

X_train['predicted LoVo/DX transformed IC50'] = model_LoVo_DX.
 ↪predict(X_train[list(model_LoVo_DX.feature_names_in_)])
X_train['observed LoVo/DX transformed IC50'] = y_train
X_train['predicted LoVo/DX IC50'] = inverse_transform(X_train['predicted LoVo/
 ↪DX transformed IC50'])
X_test['predicted LoVo/DX transformed IC50'] = model_LoVo_DX.
 ↪predict(X_test[list(model_LoVo_DX.feature_names_in_)])
```

```
X_test['observed LoVo/DX transformed IC50'] = y_test
X_test['predicted LoVo/DX IC50'] = inverse_transform(X_test['predicted LoVo/DX␣
↪transformed IC50'])

#print('Train R score: '+str(math.sqrt(r2_score(X_train['observed LoVo/DX␣
↪transformed IC50'], X_train['predicted LoVo/DX transformed IC50']))))
#print('Test R score: '+str(math.sqrt(r2_score(X_test['observed LoVo/DX␣
↪transformed IC50'], X_test['predicted LoVo/DX transformed IC50']))))
#print('Train RMSE score: '+str(math.sqrt(mean_squared_error(X_train['observed␣
↪LoVo/DX transformed IC50'], X_train['predicted LoVo/DX transformed IC50']))))
#print('Test RMSE score: '+str(math.sqrt(mean_squared_error(X_test['observed␣
↪LoVo/DX transformed IC50'], X_test['predicted LoVo/DX transformed IC50']))))
```

[30]:
```
with pd.ExcelWriter("../Data/LoVo_DX_random_state_15.xlsx") as writer:
    X_train.to_excel(writer, sheet_name="Train", index=True)
    X_test.to_excel(writer, sheet_name="Test", index=True)
```

[31]:
```
X_train, X_test, y_train, y_test = train_test_split(res_[res_.columns[0:-6]],
                                                        res_['LoVo/
↪DX_float_transformed'],
                                                        test_size=0.15,␣
↪random_state=28)

X_train['predicted LoVo/DX transformed IC50'] = model_LoVo_DX.
↪predict(X_train[list(model_LoVo_DX.feature_names_in_)])
X_train['observed LoVo/DX transformed IC50'] = y_train
X_train['predicted LoVo/DX IC50'] = inverse_transform(X_train['predicted LoVo/
↪DX transformed IC50'])
X_test['predicted LoVo/DX transformed IC50'] = model_LoVo_DX.
↪predict(X_test[list(model_LoVo_DX.feature_names_in_)])
X_test['observed LoVo/DX transformed IC50'] = y_test
X_test['predicted LoVo/DX IC50'] = inverse_transform(X_test['predicted LoVo/DX␣
↪transformed IC50'])

#print('Train R score: '+str(math.sqrt(r2_score(X_train['observed LoVo/DX␣
↪transformed IC50'], X_train['predicted LoVo/DX transformed IC50']))))
#print('Test R score: '+str(math.sqrt(r2_score(X_test['observed LoVo/DX␣
↪transformed IC50'], X_test['predicted LoVo/DX transformed IC50']))))
#print('Train RMSE score: '+str(math.sqrt(mean_squared_error(X_train['observed␣
↪LoVo/DX transformed IC50'], X_train['predicted LoVo/DX transformed IC50']))))
#print('Test RMSE score: '+str(math.sqrt(mean_squared_error(X_test['observed␣
↪LoVo/DX transformed IC50'], X_test['predicted LoVo/DX transformed IC50']))))
```

[32]:
```
with pd.ExcelWriter("../Data/LoVo_DX_random_state_28.xlsx") as writer:
    X_train.to_excel(writer, sheet_name="Train", index=True)
    X_test.to_excel(writer, sheet_name="Test", index=True)
```

[33]:
```
X_train, X_test, y_train, y_test = train_test_split(res_[res_.columns[0:-6]],
                                                     res_['LoVo/
 ↪DX_float_transformed'],
                                                     test_size=0.15,␣
 ↪random_state=42)

X_train['predicted LoVo/DX transformed IC50'] = model_LoVo_DX.
 ↪predict(X_train[list(model_LoVo_DX.feature_names_in_)])
X_train['observed LoVo/DX transformed IC50'] = y_train
X_train['predicted LoVo/DX IC50'] = inverse_transform(X_train['predicted LoVo/
 ↪DX transformed IC50'])
X_test['predicted LoVo/DX transformed IC50'] = model_LoVo_DX.
 ↪predict(X_test[list(model_LoVo_DX.feature_names_in_)])
X_test['observed LoVo/DX transformed IC50'] = y_test
X_test['predicted LoVo/DX IC50'] = inverse_transform(X_test['predicted LoVo/DX␣
 ↪transformed IC50'])

print('Train R score: '+str(math.sqrt(r2_score(X_train['observed LoVo/DX␣
 ↪transformed IC50'], X_train['predicted LoVo/DX transformed IC50']))))
print('Test R score: '+str(math.sqrt(r2_score(X_test['observed LoVo/DX␣
 ↪transformed IC50'], X_test['predicted LoVo/DX transformed IC50']))))
print('Train RMSE score: '+str(math.sqrt(mean_squared_error(X_train['observed␣
 ↪LoVo/DX transformed IC50'], X_train['predicted LoVo/DX transformed IC50']))))
print('Test RMSE score: '+str(math.sqrt(mean_squared_error(X_test['observed␣
 ↪LoVo/DX transformed IC50'], X_test['predicted LoVo/DX transformed IC50']))))
```

```
Train R score: 0.9610074984048745
Test R score: 0.790562123545645
Train RMSE score: 0.26590821262128245
Test RMSE score: 0.4420939409154765
```

[34]:
```
with pd.ExcelWriter("../Data/LoVo_DX_random_state_42.xlsx") as writer:
    X_train.to_excel(writer, sheet_name="Train", index=True)
    X_test.to_excel(writer, sheet_name="Test", index=True)
```

2.4 LoVo

[35]:
```
list(model_LoVo.feature_names_in_)
```

[35]:
```
['EState_VSA5', 'MDEO-12']
```

[36]:
```
res.columns[0:-7]
```

[36]:
```
Index(['SMILES', 'AATS0Z', 'AATS0are', 'AATS0d', 'AATS0dv', 'AATS0i', 'AATS0m',
       'AATS0p', 'AATS0pe', 'AATS0s',
       …
       'piPC3', 'piPC4', 'piPC5', 'piPC6', 'piPC7', 'piPC8', 'piPC9',
```

```
'A549_float', 'MCF-7_float', 'LoVo_float'],
dtype='object', length=1215)
```

[37]:
```
X_train, X_test, y_train, y_test = train_test_split(res[res.columns[0:-7]],␣
↪res['LoVo_float_transformed'],
                                                   test_size=0.15,␣
↪random_state=15)

X_train['predicted LoVo transformed IC50'] = model_LoVo.
↪predict(X_train[list(model_LoVo.feature_names_in_)])
X_train['observed LoVo transformed IC50'] = y_train
X_train['predicted LoVo IC50'] = inverse_transform(X_train['predicted LoVo␣
↪transformed IC50'])
X_test['predicted LoVo transformed IC50'] = model_LoVo.
↪predict(X_test[list(model_LoVo.feature_names_in_)])
X_test['observed LoVo transformed IC50'] = y_test
X_test['predicted LoVo IC50'] = inverse_transform(X_test['predicted LoVo␣
↪transformed IC50'])

#print('Train R score: '+str(math.sqrt(r2_score(X_train['observed LoVo␣
↪transformed IC50'], X_train['predicted LoVo transformed IC50']))))
#print('Test R score: '+str(math.sqrt(r2_score(X_test['observed LoVo␣
↪transformed IC50'], X_test['predicted LoVo transformed IC50']))))
#print('Train RMSE score: '+str(math.sqrt(mean_squared_error(X_train['observed␣
↪LoVo transformed IC50'], X_train['predicted LoVo transformed IC50']))))
#print('Test RMSE score: '+str(math.sqrt(mean_squared_error(X_test['observed␣
↪LoVo transformed IC50'], X_test['predicted LoVo transformed IC50']))))
```

[38]:
```
with pd.ExcelWriter("../Data/LoVo_random_state_15.xlsx") as writer:
    X_train.to_excel(writer, sheet_name="Train", index=True)
    X_test.to_excel(writer, sheet_name="Test", index=True)
```

[39]:
```
X_train, X_test, y_train, y_test = train_test_split(res[res.columns[0:-7]],␣
↪res['LoVo_float_transformed'],
                                                   test_size=0.15,␣
↪random_state=28)

X_train['predicted LoVo transformed IC50'] = model_LoVo.
↪predict(X_train[list(model_LoVo.feature_names_in_)])
X_train['observed LoVo transformed IC50'] = y_train
X_train['predicted LoVo IC50'] = inverse_transform(X_train['predicted LoVo␣
↪transformed IC50'])
X_test['predicted LoVo transformed IC50'] = model_LoVo.
↪predict(X_test[list(model_LoVo.feature_names_in_)])
```

```
X_test['observed LoVo transformed IC50'] = y_test
X_test['predicted LoVo IC50'] = inverse_transform(X_test['predicted LoVo␣
↪transformed IC50'])

print('Train R score: '+str(math.sqrt(r2_score(X_train['observed LoVo␣
↪transformed IC50'], X_train['predicted LoVo transformed IC50']))))
print('Test R score: '+str(math.sqrt(r2_score(X_test['observed LoVo transformed␣
↪IC50'], X_test['predicted LoVo transformed IC50']))))
print('Train RMSE score: '+str(math.sqrt(mean_squared_error(X_train['observed␣
↪LoVo transformed IC50'], X_train['predicted LoVo transformed IC50']))))
print('Test RMSE score: '+str(math.sqrt(mean_squared_error(X_test['observed␣
↪LoVo transformed IC50'], X_test['predicted LoVo transformed IC50']))))
```

```
Train R score: 0.8811267812945673
Test R score: 0.6572630628008767
Train RMSE score: 0.4438384819944741
Test RMSE score: 0.5047746604903675
```

[40]:
```
with pd.ExcelWriter("../Data/LoVo_random_state_28.xlsx") as writer:
    X_train.to_excel(writer, sheet_name="Train", index=True)
    X_test.to_excel(writer, sheet_name="Test", index=True)
```

[41]:
```
X_train, X_test, y_train, y_test = train_test_split(res[res.columns[0:-7]],
                                                                      ␣
↪res['LoVo_float_transformed'],
                                                                test_size=0.15,␣
↪random_state=42)

X_train['predicted LoVo transformed IC50'] = model_LoVo.
↪predict(X_train[list(model_LoVo.feature_names_in_)])
X_train['observed LoVo transformed IC50'] = y_train
X_train['predicted LoVo IC50'] = inverse_transform(X_train['predicted LoVo␣
↪transformed IC50'])
X_test['predicted LoVo transformed IC50'] = model_LoVo.
↪predict(X_test[list(model_LoVo.feature_names_in_)])
X_test['observed LoVo transformed IC50'] = y_test
X_test['predicted LoVo IC50'] = inverse_transform(X_test['predicted LoVo␣
↪transformed IC50'])

#print('Train R score: '+str(math.sqrt(r2_score(X_train['observed LoVo␣
↪transformed IC50'], X_train['predicted LoVo transformed IC50']))))
#print('Test R score: '+str(math.sqrt(r2_score(X_test['observed LoVo␣
↪transformed IC50'], X_test['predicted LoVo transformed IC50']))))
#print('Train RMSE score: '+str(math.sqrt(mean_squared_error(X_train['observed␣
↪LoVo transformed IC50'], X_train['predicted LoVo transformed IC50']))))
#print('Test RMSE score: '+str(math.sqrt(mean_squared_error(X_test['observed␣
↪LoVo transformed IC50'], X_test['predicted LoVo transformed IC50']))))
```

```
[42]: with pd.ExcelWriter("../Data/LoVo_random_state_42.xlsx") as writer:
          X_train.to_excel(writer, sheet_name="Train", index=True)
          X_test.to_excel(writer, sheet_name="Test", index=True)
```

2.5 MCF-7

```
[43]: list(model_MCF_7.feature_names_in_)
```

```
[43]: ['AMID_O', 'EState_VSA5', 'EState_VSA6', 'MDEO-12']
```

```
[44]: res.columns[0:-8]
```

```
[44]: Index(['SMILES', 'AATS0Z', 'AATS0are', 'AATS0d', 'AATS0dv', 'AATS0i', 'AATS0m',
             'AATS0p', 'AATS0pe', 'AATS0s',
             …
             'piPC2', 'piPC3', 'piPC4', 'piPC5', 'piPC6', 'piPC7', 'piPC8', 'piPC9',
             'A549_float', 'MCF-7_float'],
            dtype='object', length=1214)
```

```
[45]: X_train, X_test, y_train, y_test = train_test_split(res[res.columns[0:-8]],
      ␣
        ↪res['LoVo_float_transformed'],
                                                             test_size=0.15,␣
        ↪random_state=15)

      X_train['predicted MCF-7 transformed IC50'] = model_MCF_7.
        ↪predict(X_train[list(model_MCF_7.feature_names_in_)])
      X_train['observed MCF-7 transformed IC50'] = y_train
      X_train['predicted MCF-7 IC50'] = inverse_transform(X_train['predicted MCF-7␣
        ↪transformed IC50'])
      X_test['predicted MCF-7 transformed IC50'] = model_MCF_7.
        ↪predict(X_test[list(model_MCF_7.feature_names_in_)])
      X_test['observed MCF-7 transformed IC50'] = y_test
      X_test['predicted MCF-7 IC50'] = inverse_transform(X_test['predicted MCF-7␣
        ↪transformed IC50'])

      print('Train R score: '+str(math.sqrt(r2_score(X_train['observed MCF-7␣
        ↪transformed IC50'], X_train['predicted MCF-7 transformed IC50']))))
      print('Test R score: '+str(math.sqrt(r2_score(X_test['observed MCF-7␣
        ↪transformed IC50'], X_test['predicted MCF-7 transformed IC50']))))
      print('Train RMSE score: '+str(math.sqrt(mean_squared_error(X_train['observed␣
        ↪MCF-7 transformed IC50'], X_train['predicted MCF-7 transformed IC50']))))
      print('Test RMSE score: '+str(math.sqrt(mean_squared_error(X_test['observed␣
        ↪MCF-7 transformed IC50'], X_test['predicted MCF-7 transformed IC50']))))
```

```
Train R score: 0.85288466325432
Test R score: 0.677226040545383
```

```
Train RMSE score: 0.4524890646893892
Test RMSE score: 0.8054094630462401
```

[46]:
```
with pd.ExcelWriter("../Data/MCF-7_random_state_15.xlsx") as writer:
    X_train.to_excel(writer, sheet_name="Train", index=True)
    X_test.to_excel(writer, sheet_name="Test", index=True)
```

[47]:
```
X_train, X_test, y_train, y_test = train_test_split(res[res.columns[0:-8]], 
 ↪res['LoVo_float_transformed'],
                                                        test_size=0.15, 
 ↪random_state=28)

X_train['predicted MCF-7 transformed IC50'] = model_MCF_7.
 ↪predict(X_train[list(model_MCF_7.feature_names_in_)])
X_train['observed MCF-7 transformed IC50'] = y_train
X_train['predicted MCF-7 IC50'] = inverse_transform(X_train['predicted MCF-7 
 ↪transformed IC50'])
X_test['predicted MCF-7 transformed IC50'] = model_MCF_7.
 ↪predict(X_test[list(model_MCF_7.feature_names_in_)])
X_test['observed MCF-7 transformed IC50'] = y_test
X_test['predicted MCF-7 IC50'] = inverse_transform(X_test['predicted MCF-7 
 ↪transformed IC50'])

#print('Train R score: '+str(math.sqrt(r2_score(X_train['observed MCF-7 
 ↪transformed IC50'], X_train['predicted MCF-7 transformed IC50']))))
#print('Test R score: '+str(math.sqrt(r2_score(X_test['observed MCF-7 
 ↪transformed IC50'], X_test['predicted MCF-7 transformed IC50']))))
#print('Train RMSE score: '+str(math.sqrt(mean_squared_error(X_train['observed 
 ↪MCF-7 transformed IC50'], X_train['predicted MCF-7 transformed IC50']))))
#print('Test RMSE score: '+str(math.sqrt(mean_squared_error(X_test['observed 
 ↪MCF-7 transformed IC50'], X_test['predicted MCF-7 transformed IC50']))))
```

[48]:
```
with pd.ExcelWriter("../Data/MCF-7_random_state_28.xlsx") as writer:
    X_train.to_excel(writer, sheet_name="Train", index=True)
    X_test.to_excel(writer, sheet_name="Test", index=True)
```

[49]:
```
X_train, X_test, y_train, y_test = train_test_split(res[res.columns[0:-8]], 
 ↪res['LoVo_float_transformed'],
                                                        test_size=0.15, 
 ↪random_state=42)

X_train['predicted MCF-7 transformed IC50'] = model_MCF_7.
 ↪predict(X_train[list(model_MCF_7.feature_names_in_)])
X_train['observed MCF-7 transformed IC50'] = y_train
```

```
X_train['predicted MCF-7 IC50'] = inverse_transform(X_train['predicted MCF-7␣
↪transformed IC50'])
X_test['predicted MCF-7 transformed IC50'] = model_MCF_7.
↪predict(X_test[list(model_MCF_7.feature_names_in_)])
X_test['observed MCF-7 transformed IC50'] = y_test
X_test['predicted MCF-7 IC50'] = inverse_transform(X_test['predicted MCF-7␣
↪transformed IC50'])

#print('Train R score: '+str(math.sqrt(r2_score(X_train['observed MCF-7␣
↪transformed IC50'], X_train['predicted MCF-7 transformed IC50']))))
#print('Test R score: '+str(math.sqrt(r2_score(X_test['observed MCF-7␣
↪transformed IC50'], X_test['predicted MCF-7 transformed IC50']))))
#print('Train RMSE score: '+str(math.sqrt(mean_squared_error(X_train['observed␣
↪MCF-7 transformed IC50'], X_train['predicted MCF-7 transformed IC50']))))
#print('Test RMSE score: '+str(math.sqrt(mean_squared_error(X_test['observed␣
↪MCF-7 transformed IC50'], X_test['predicted MCF-7 transformed IC50']))))
```

[50]:
```
with pd.ExcelWriter("../Data/MCF-7_random_state_42.xlsx") as writer:
    X_train.to_excel(writer, sheet_name="Train", index=True)
    X_test.to_excel(writer, sheet_name="Test", index=True)
```

```
######
#Molecular docking config
######
receptor = 1SA0_AB_chains_prot_polar_H.pdbqt
ligand = 1SA0_AB_chains_colchicine_only.pdbqt

center_x = 119.743
center_y = 92.779
center_z = 10.765

size_x = 44
size_y = 44
size_z = 60

out = blind_try_lig_1_pub_1.pdbqt

exhaustiveness = 32
```

Notebook

January 18, 2024

[ ]:
```
#########################

# THE SOURCE CODE FOR MACHINE LEARNING AND FINAL MODELS CREATION

#########################




## Load libraries
#Libraries import
import pandas as pd
from mordred import Calculator, descriptors
import mordred
import numpy as np
from rdkit import Chem

from sklearn.ensemble import RandomForestRegressor
from sklearn.tree import DecisionTreeRegressor
from sklearn.neighbors import KNeighborsRegressor
from sklearn.svm import SVR
from sklearn import linear_model


from sklearn.model_selection import train_test_split
from sklearn.metrics import r2_score, mean_squared_error
import math

def prepare_data(file):

    df = pd.read_excel(file)
    targets = list(df.columns)[10:]

    try:
        mol_objs = [Chem.MolFromSmiles(smi) for smi in df['SMILES']]
    except:
```

```
        mol_objs = [Chem.MolFromSmiles(smi) for smi in␣
↪df['AI_generated_SMILES']]

    calculate_descriptors = True

    if calculate_descriptors:
        calc = Calculator(descriptors, ignore_3D=True)
        molecular_descriptors = calc.pandas(mol_objs)
        molecular_descriptors = molecular_descriptors.
↪applymap(is_morder_missing)
        molecular_descriptors =␣
↪molecular_descriptors[sorted(molecular_descriptors.columns)]
    else:
        pass
    print("Data size (rows, columns): "+ str(molecular_descriptors.shape))

    simple_preprocessing = True
    if simple_preprocessing:
        molecular_descriptors_cleaned = molecular_descriptors.dropna(axis=1,␣
↪how='any')
        molecular_descriptors_cleaned
    print("Data size after first reduction (rows, columns): "+␣
↪str(molecular_descriptors_cleaned.shape))
    molecular_descriptors_cleaned = molecular_descriptors_cleaned.loc[:,␣
↪(molecular_descriptors_cleaned != 0).any(axis=0)]
    print("Data size after second reduction (rows, columns): "+␣
↪str(molecular_descriptors_cleaned.shape))

    try:
        molecular_descriptors_cleaned[targets] = df[targets]
    except:
        print('There is an issue with the target values...')


    return molecular_descriptors_cleaned

def is_morder_missing(x):
    return np.nan if type(x) == mordred.error.Missing or type(x) == mordred.
↪error.Error else x


def correlation_dataframe(molecular_descriptors_cleaned, correlation_threshold,␣
↪target_column_name, verbose = False):

    if verbose:
```

```
        correlation_table = pd.DataFrame(data=molecular_descriptors_cleaned.
↪columns.to_list(),
                                        columns=["molecular descriptor name"])
        print(correlation_table.head())
        correlation_to_target = []
        for mol_desc in correlation_table['molecular descriptor name']:
            x = np.corrcoef(np.array(molecular_descriptors_cleaned[mol_desc]),
                            np.
↪array(molecular_descriptors_cleaned[target_column_name]))
            x = x.tolist()[0][1]
            correlation_to_target.append(x)
        correlation_table['corr_value'] = correlation_to_target
        print(correlation_table.head())
        correlation_table['absolute correlation value'] = [abs(x) for x in␣
↪correlation_table['corr_value']]
        print(correlation_table[:-1].head())

        mol_desc_best_corr = correlation_table[correlation_table['absolute␣
↪correlation value'] > correlation_threshold]

        print(mol_desc_best_corr.head())
        table_with_descriptors_to_be_used = mol_desc_best_corr[:-1]
        print(table_with_descriptors_to_be_used.head())
    else:
        correlation_table = pd.DataFrame(data=molecular_descriptors_cleaned.
↪columns.to_list(),
                                        columns=["molecular descriptor name"])

        correlation_to_target = []
        for mol_desc in correlation_table['molecular descriptor name']:
            x = np.corrcoef(np.array(molecular_descriptors_cleaned[mol_desc]),
                            np.
↪array(molecular_descriptors_cleaned[target_column_name]))
            x = x.tolist()[0][1]
            correlation_to_target.append(x)
        correlation_table['corr_value'] = correlation_to_target

        correlation_table['absolute correlation value'] = [abs(x) for x in␣
↪correlation_table['corr_value']]


        mol_desc_best_corr = correlation_table[correlation_table['absolute␣
↪correlation value'] > correlation_threshold]


        table_with_descriptors_to_be_used = mol_desc_best_corr[:-1]
```

```
    return table_with_descriptors_to_be_used



def transform(target_val):
    transformed = []
    for element in target_val:
        transformed.append(-1.0 * np.log10(element/1000000000))
    return transformed

def inverse_transform(transformed_values):
    inversed = []
    for element in transformed_values:
        inversed.append(np.power(10,-element)*1000000000)
    return inversed



def prepare_model(data, features, model_type, test_data, target_column_name,␣
 ↪random_state = 15, n_estimators_ = 2, max_depth = 2, kernel_ = 'linear',␣
 ↪gamma_ = 'auto', train_test_split_ = False, verbose = False):


    if verbose:
        if model_type == 'RandomForestRegressor':
            model = RandomForestRegressor(random_state=15,␣
 ↪n_estimators=n_estimators_)
            print("The model used is: RandomForest...")
        elif model_type == 'DecisionTreeRegressor':
            model = DecisionTreeRegressor(random_state=15, max_depth=max_depth)
            print("The model used is: DecisionTree...")
        elif model_type == 'KNeighborsRegressor':
            model = KNeighborsRegressor()
            print("The model used is: KNeighbors...")
        elif model_type == 'SVR':
            model = SVR(gamma = gamma_, kernel=kernel_)
            print("The model used is: SVR...")
        elif model_type == 'linear_model':
            model = linear_model.LinearRegression()
            print("The model used is: LinearReg...")
        else:
            model = linear_model.LinearRegression()
            print("The model used is: Linear...")
    else:
        if model_type == 'RandomForestRegressor':
```

```
            model = RandomForestRegressor(random_state=15,␣
↪n_estimators=n_estimators_)

        elif model_type == 'DecisionTreeRegressor':
            model = DecisionTreeRegressor(random_state=15, max_depth=max_depth)

        elif model_type == 'KNeighborsRegressor':
            model = KNeighborsRegressor()

        elif model_type == 'SVR':
            model = SVR(gamma=gamma_, kernel=kernel_)

        elif model_type == 'linear_model':
            model = linear_model.LinearRegression()

        else:
            model = linear_model.LinearRegression()



    if verbose:
        if train_test_split_:
            X_train, X_test, y_train, y_test =␣
↪train_test_split(data[features['molecular descriptor name']],
                                                                    ␣
↪data[target_column_name],
                                                                     test_size=0.15,␣
↪random_state=random_state)

            model.fit(X_train, y_train)
            try:
                print("Return the coefficient of determination of the␣
↪prediction: ")
                print(model.score(X_test, y_test))
            except:
                pass

            pred = model.predict(X_train)
            print("R^2 score: "+ str(r2_score(y_train, pred)))
            sqrt_r2 = np.sqrt(r2_score(y_train, pred))
            training_data_r2 = r2_score(y_train, pred)
            print('Correlation coefficient: '+ str(sqrt_r2))
            print("Test data - unseen during training:")
            pred = model.predict(X_test)
            print("R^2 score: "+ str(r2_score(y_test, pred)))
            sqrt_r2 = np.sqrt(r2_score(y_test, pred))
            print('Correlation coefficient: '+ str(sqrt_r2))
```

```
            print(pred)
            print(y_test)
            test_data_r2 = r2_score(y_test, pred)
            pred = model.predict(X_train)
            training_data_RMSE = math.sqrt(mean_squared_error(y_train, pred))
            print('Training Root Mean Square Error: '+str(training_data_RMSE))
            pred = model.predict(X_test)
            test_data_RMSE = math.sqrt(mean_squared_error(y_test, pred))
            print('Testing Root Mean Square Error: '+str(test_data_RMSE))

        else:
            X = data[features['molecular descriptor name']]

            y = data[target_column_name]


            model.fit(X, y)
            print("Return the coefficient of determination of the prediction: ")
            print(model.score(test_data[features['molecular descriptor name']],␣
↪test_data[target_column_name]))

            pred = model.predict(X)
            print("R^2 score: "+ str(r2_score(y, pred)))
            sqrt_r2 = np.sqrt(r2_score(y, pred))
            training_data_r2 = r2_score(y, pred)
            print('Correlation coefficient: '+ str(sqrt_r2))
            print("Test data - unseen during training:")
            pred = model.predict(test_data[features['molecular descriptor␣
↪name']])
            print("R^2 score: "+ str(r2_score(test_data[target_column_name],␣
↪pred)))
            sqrt_r2 = np.sqrt(r2_score(test_data[target_column_name], pred))
            print('Correlation coefficient: '+ str(sqrt_r2))
            print(pred)
            print(test_data[target_column_name])
            test_data_r2 = r2_score(test_data[target_column_name], pred)
            pred = model.predict(X_train)
            training_data_RMSE = math.
↪sqrt(mean_squared_error(test_data[target_column_name], pred))
            print('Training Root Mean Square Error: '+str(training_data_RMSE))
            pred = model.predict(X_test)
            test_data_RMSE = math.
↪sqrt(mean_squared_error(test_data[target_column_name], pred))
            print('Testing Root Mean Square Error: '+str(test_data_RMSE))

    else:
        if train_test_split_:
```

```
            X_train, X_test, y_train, y_test =␣
↪train_test_split(data[features['molecular descriptor name']],
                                                                    ␣
↪data[target_column_name],
                                                                     test_size=0.15,␣
↪random_state=random_state)

            model.fit(X_train, y_train)

            pred = model.predict(X_train)
            sqrt_r2 = np.sqrt(r2_score(y_train, pred))
            training_data_r2 = r2_score(y_train, pred)
            pred = model.predict(X_test)
            sqrt_r2 = np.sqrt(r2_score(y_test, pred))

            test_data_r2 = r2_score(y_test, pred)

            pred = model.predict(X_train)
            training_data_RMSE = math.sqrt(mean_squared_error(y_train, pred))

            pred = model.predict(X_test)
            test_data_RMSE = math.sqrt(mean_squared_error(y_test, pred))


        else:
            X = data[features['molecular descriptor name']]

            y = data[target_column_name]


            model.fit(X, y)

            pred = model.predict(X)

            sqrt_r2 = np.sqrt(r2_score(y, pred))
            training_data_r2 = r2_score(y, pred)
            pred = model.predict(test_data[features['molecular descriptor␣
↪name']])
            sqrt_r2 = np.sqrt(r2_score(test_data[target_column_name], pred))
            test_data_r2 = r2_score(test_data[target_column_name], pred)
            pred = model.predict(X_train)
            training_data_RMSE = math.
↪sqrt(mean_squared_error(test_data[target_column_name], pred))

            pred = model.predict(X_test)
            test_data_RMSE = math.
↪sqrt(mean_squared_error(test_data[target_column_name], pred))
```

```
    return model, training_data_r2, test_data_r2, training_data_RMSE,␣
↪test_data_RMSE


def data_standardization(dataframe, target_column_name):

    dataframe_ = dataframe.drop([target_column_name], axis=1)

    to_be_returned = (dataframe_ - dataframe_.mean()) / dataframe_.std()
    to_be_returned[target_column_name] = dataframe[target_column_name]

    return to_be_returned


def prepare_data_and_create_model(molecular_descriptors_df,␣
↪correlation_threshold, standardization, model_type, target_column_name,␣
↪random_state = 15, n_estimators_ = 12, max_depth = 2, kernel_ = 'linear',␣
↪gamma_ = 'auto', train_test_split_ = True, verbose = False):

    if standardization == True:

        if verbose:
            print("I am doing standardization...")
        else:
            pass

        data_to_be_prepared = molecular_descriptors_df

        stand = data_standardization(data_to_be_prepared, target_column_name)

        corr = correlation_dataframe(stand, correlation_threshold,␣
↪target_column_name, verbose)

        if train_test_split_:
            test_data_ = 'None'
            model, train_r2, test_r2, training_data_RMSE, test_data_RMSE =␣
↪prepare_model(data_to_be_prepared, corr, model_type, test_data_,␣
↪target_column_name, random_state, n_estimators_, max_depth, kernel_, gamma_,␣
↪train_test_split_, verbose)
        else:
            print("There is no hardcoded validation data...")

    elif standardization == False:
```

```
        if verbose:
            print("I am not doing standardization...")
        else:
            pass

        data_to_be_prepared = molecular_descriptors_df

        corr = correlation_dataframe(data_to_be_prepared,␣
↪correlation_threshold, target_column_name, verbose)

        if train_test_split_:
            test_data_ = 'None'
            model, train_r2, test_r2, training_data_RMSE, test_data_RMSE =␣
↪prepare_model(data_to_be_prepared, corr, model_type, test_data_,␣
↪target_column_name, random_state, n_estimators_, max_depth, kernel_, gamma_,␣
↪train_test_split_, verbose)
        else:
            print("There is no hardcoded validation data...")
    else:
        print("Error...")

    return model, train_r2, test_r2, data_to_be_prepared, corr,␣
↪target_column_name, training_data_RMSE, test_data_RMSE
```
