# Supplementary material for: Machine Learning Application for Medicinal Chemistry: Colchicine Case, New Structures, and Anticancer Activity Prediction
Source: Pharmaceuticals (Basel). 2024 Jan 29;17(2):173. doi: 10.3390/ph17020173 (PMC10892630; doi:10.3390/ph17020173)
Supplement: Supplementary file 1 [file pharmaceuticals-17-00173-s001.zip › Additional Supplementary Figures.pdf]

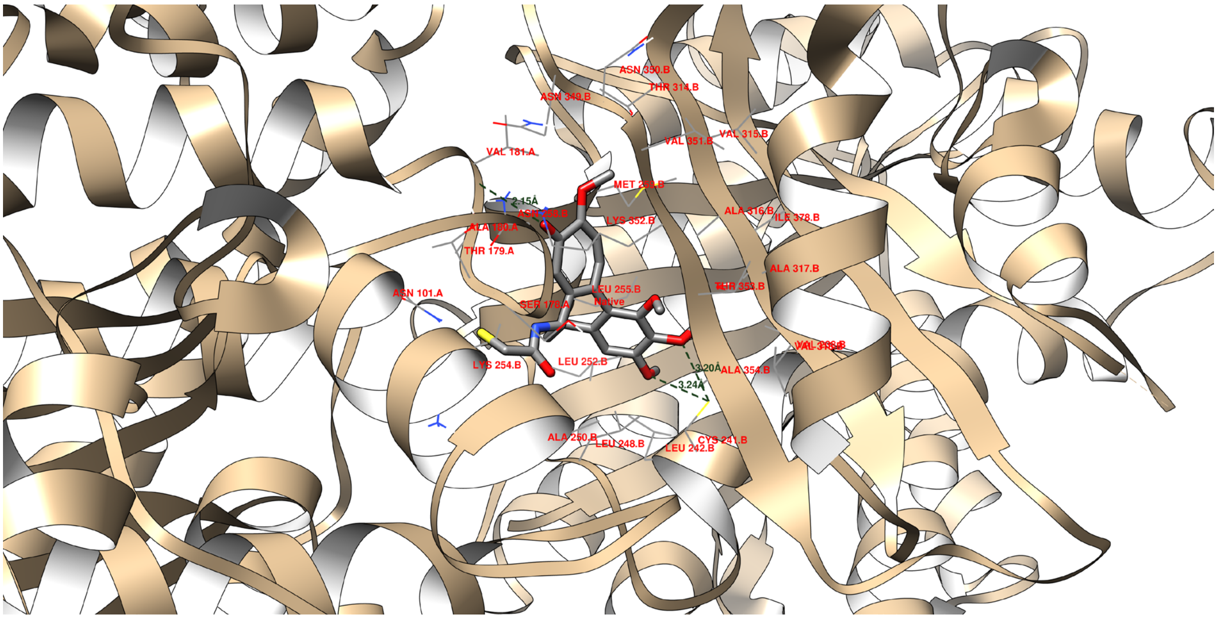

Figure S1. A native ligand from 1SA0 protein domain redocked to the 1SA0 protein domain – potential interactions visualization.

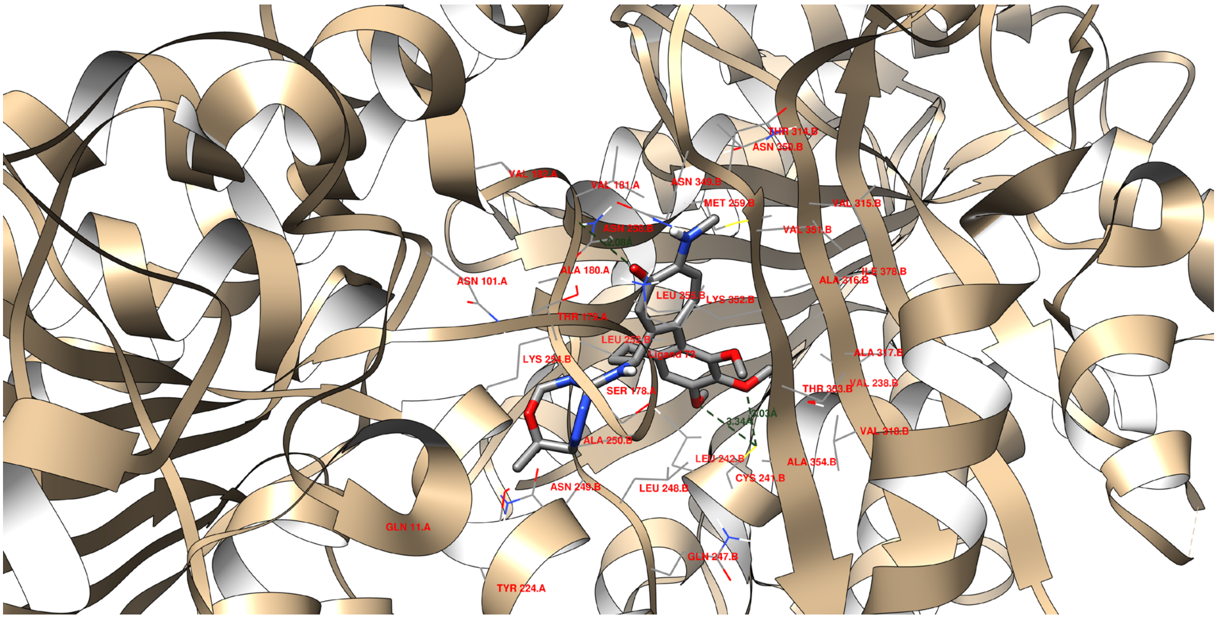

Figure S2. AI-proposed new colchicine-based ligand number 73 in the binding site of 1SA0 protein domain.

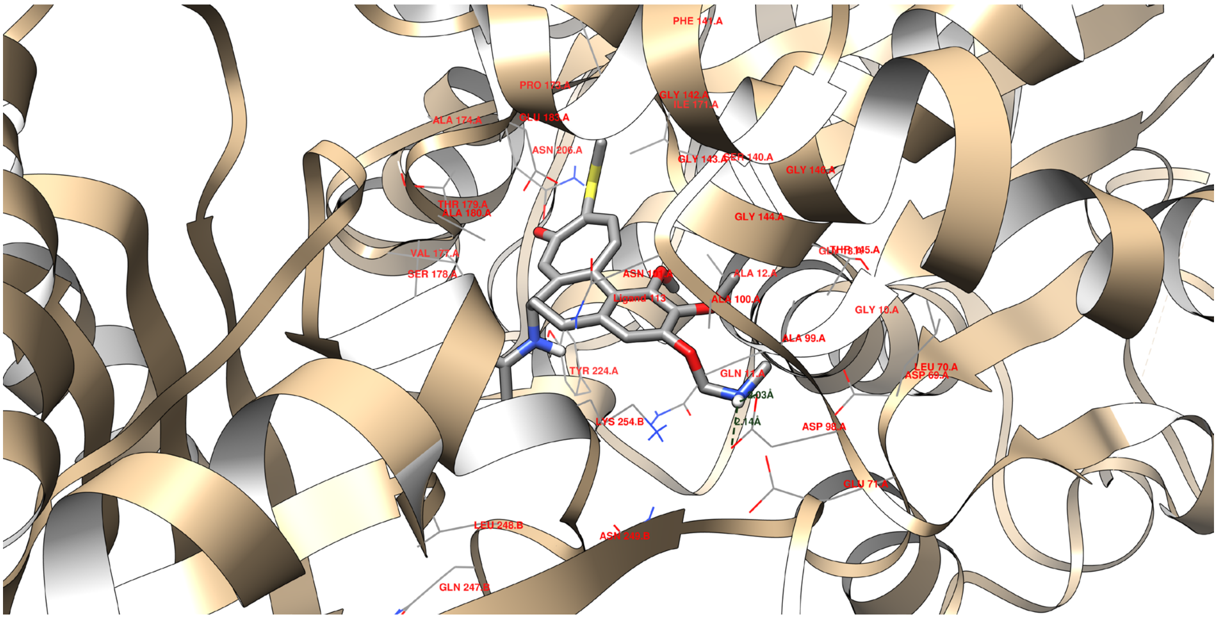

Figure S3. AI-proposed new colchicine-based ligand number 113 in the binding site of 1SA0 protein domain.

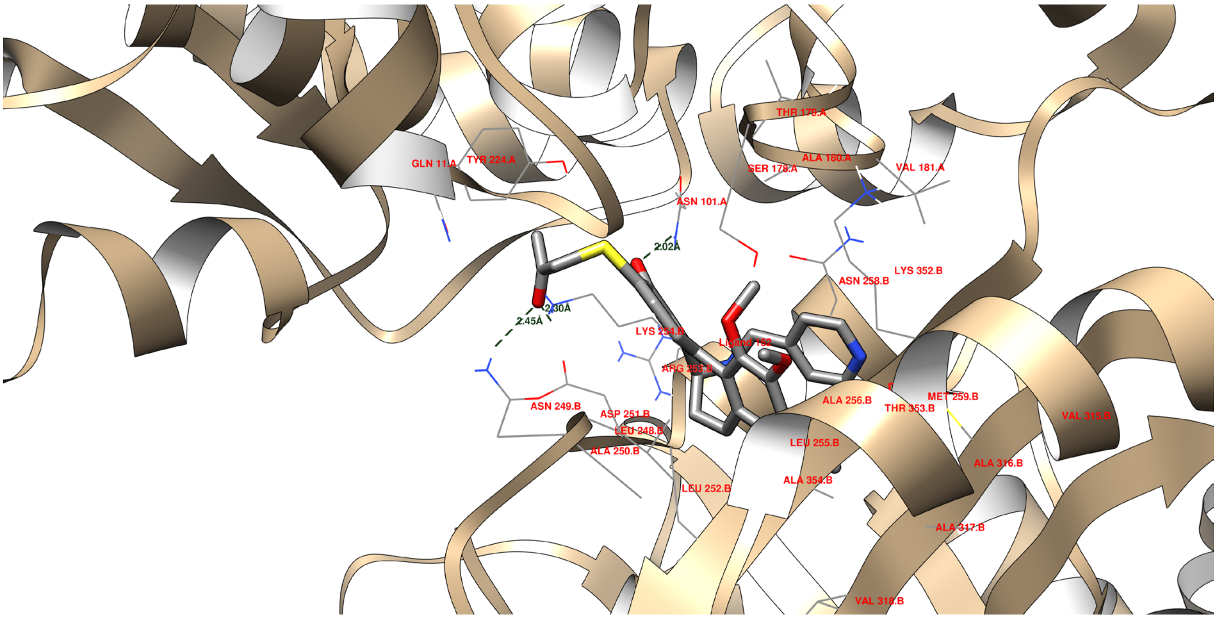

Figure S4. AI-proposed new colchicine-based ligand number 162 in the binding site of 1SA0 protein domain.

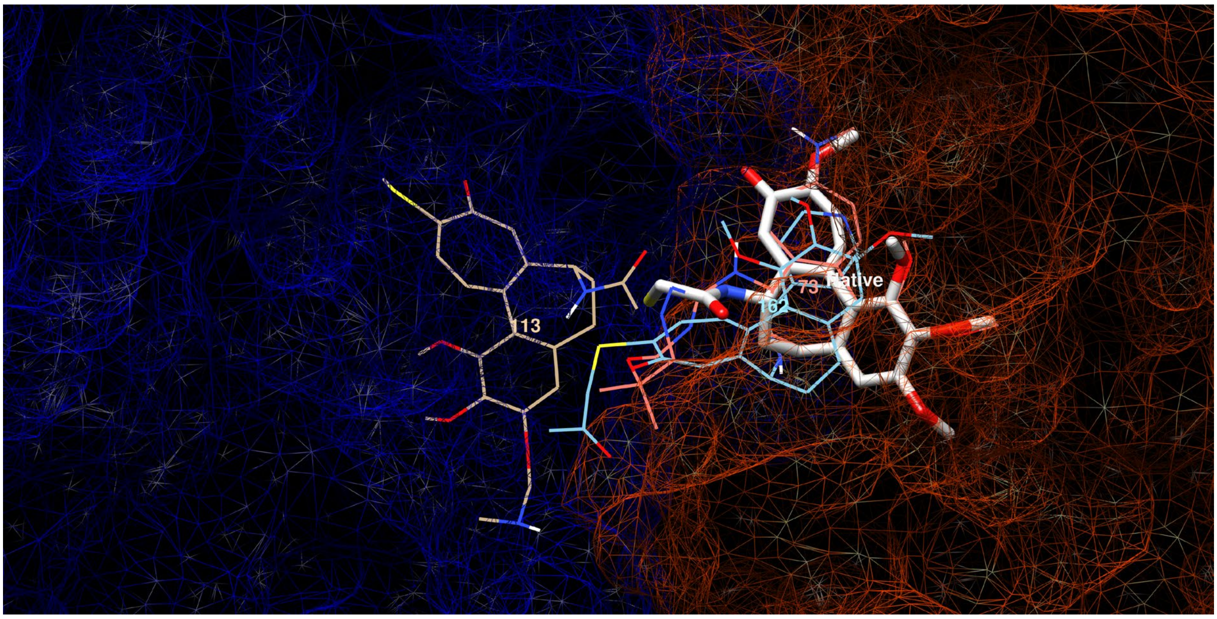

Figure S5. All previously visualized structures into a mesh-like 1SA0 protein domain.

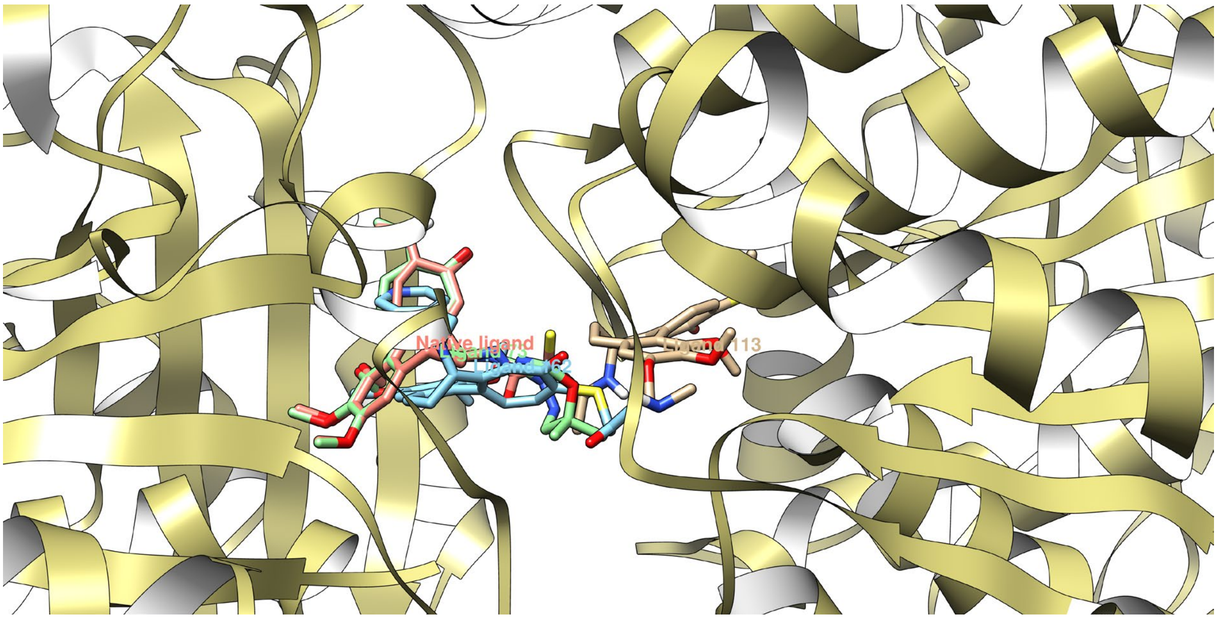

Figure S6. All the ligands (native, 73, 113, 162) visualized at once.
