# Supplementary material for: Machine Learning Application for Medicinal Chemistry: Colchicine Case, New Structures, and Anticancer Activity Prediction
Source: Pharmaceuticals (Basel). 2024 Jan 29;17(2):173. doi: 10.3390/ph17020173 (PMC10892630; doi:10.3390/ph17020173)
Supplement: Supplementary file 1 [file pharmaceuticals-17-00173-s001.zip › Files - colchicine AI.pdf]

Files

The files pertaining to the conducted study have been consolidated on GitHub's platform and are accessible through this link: https://github.com/XDamianX-coder/Colchicine_ML

File 1.	Kolchicyna_prepared_data.xlsx
	https://github.com/XDamianX-coder/Colchicine_ML/blob/main/Data/Kolchicyna_prepared_data.xlsx
File 2.	training_data_preparation.py
	https://github.com/XDamianX-coder/Colchicine_ML/blob/main/New_structures/training_data_preparation.py
File 3.	training_smiles.parquet
	https://github.com/XDamianX-coder/Colchicine_ML/blob/main/Data/training_smiles.parquet
File 4.	neural_network.py
	https://github.com/XDamianX-coder/Colchicine_ML/blob/main/New_structures/neural_network.py
File 5.	mol_seq2lat.h5
	https://github.com/XDamianX-coder/Colchicine_ML/blob/main/New_structures/mol_seq2lat.h5
File 6.	lat2state.h5
	https://github.com/XDamianX-coder/Colchicine_ML/blob/main/New_structures/lat2state.h5
File 7.	samplemodel.h5
	https://github.com/XDamianX-coder/Colchicine_ML/blob/main/New_structures/samplemodel.h5
File 8.	Neural_network_log.ipynb
	https://github.com/XDamianX-coder/Colchicine_ML/blob/main/New_structures/Neural_network_log.ipynb
File 9.	do_predictions.py
	https://github.com/XDamianX-coder/Colchicine_ML/blob/main/New_structures/do_predictions.py
File 10.	AI_generated_Molecules_0_1_.xlsx
	https://github.com/XDamianX-coder/Colchicine_ML/blob/main/Data/AI_generated_Molecules_0_1_.xlsx
File 11.	AI_generated_Molecules_0_2_.xlsx
	https://github.com/XDamianX-coder/Colchicine_ML/blob/main/Data/AI_generated_Molecules_0_2_.xlsx
File 12.	Data_transformation.ipynb

https://github.com/XDamianX-coder/Colchicine_ML/blob/main/Activity/Data_transformation.ipynb

File 13. Kolchicyna_machine_learning.xlsx

https://github.com/XDamianX-coder/Colchicine_ML/blob/main/Data/Kolchicyna_machine_learning.xlsx

File 14. Correlation_to_targets.ipynb

https://github.com/XDamianX-coder/Colchicine_ML/blob/main/Activity/Correlation_to_targets.ipynb

File 15. Models_A549_rs_15.ipynb

https://github.com/XDamianX-coder/Colchicine_ML/blob/main/Activity/Models_A549_rs_15.ipynb

File 16. Models_A549_rs_28.ipynb

https://github.com/XDamianX-coder/Colchicine_ML/blob/main/Activity/Models_A549_rs_28.ipynb

File 17. Models_A549_rs_42.ipynb

https://github.com/XDamianX-coder/Colchicine_ML/blob/main/Activity/Models_A549_rs_42.ipynb

File 18. Models_BALB_3T3_rs_15.ipynb

https://github.com/XDamianX-coder/Colchicine_ML/blob/main/Activity/Models_BALB_3T3_rs_15.ipynb

File 19. Models_BALB_3T3_rs_28.ipynb

https://github.com/XDamianX-coder/Colchicine_ML/blob/main/Activity/Models_BALB_3T3_rs_28.ipynb

File 20. Models_BALB_3T3_rs_42.ipynb

https://github.com/XDamianX-coder/Colchicine_ML/blob/main/Activity/Models_BALB_3T3_rs_42.ipynb

File 21. Models_LoVo_DX_rs_15.ipynb

https://github.com/XDamianX-coder/Colchicine_ML/blob/main/Activity/Models_LoVo_DX_rs_15.ipynb

File 22. Models_LoVo_DX_rs_28.ipynb

https://github.com/XDamianX-coder/Colchicine_ML/blob/main/Activity/Models_LoVo_DX_rs_28.ipynb

File 23. Models_LoVo_DX_rs_42.ipynb

https://github.com/XDamianX-coder/Colchicine_ML/blob/main/Activity/Models_LoVo_DX_rs_42.ipynb

File 24. Models_LoVo_rs_15.ipynb

https://github.com/XDamianX-coder/Colchicine_ML/blob/main/Activity/Models_LoVo_rs_15.ipynb

File 25. Models_LoVo_rs_28.ipynb

https://github.com/XDamianX-coder/Colchicine_ML/blob/main/Activity/Models_LoVo_rs_28.ipynb

File 26. Models_LoVo_rs_42.ipynb

https://github.com/XDamianX-coder/Colchicine_ML/blob/main/Activity/Models_LoVo_rs_42.ipynb

File 27. Models_MCF-7_rs_15.ipynb

https://github.com/XDamianX-coder/Colchicine_ML/blob/main/Activity/Models_MCF-7_rs_15.ipynb

File 28. Models_MCF-7_rs_28.ipynb

https://github.com/XDamianX-coder/Colchicine_ML/blob/main/Activity/Models_MCF-7_rs_28.ipynb

File 29. Models_MCF-7_rs_42.ipynb

https://github.com/XDamianX-coder/Colchicine_ML/blob/main/Activity/Models_MCF-7_rs_42.ipynb

File 30. Load data and analyze results.ipynb

https://github.com/XDamianX-coder/Colchicine_ML/blob/main/Activity/Load%20data%20and%20analyze%20results.ipynb

a. Load data and analyze results-RMSE.ipynb

https://github.com/XDamianX-coder/Colchicine_ML/blob/main/Activity/Load%20data%20and%20analyze%20results-RMSE.ipynb

File 31. Activity_prediction.ipynb

https://github.com/XDamianX-coder/Colchicine_ML/blob/main/Activity/Activity_prediction.ipynb

File 32. random_forest_model_17_estimators_A549.joblib

https://github.com/XDamianX-coder/Colchicine_ML/blob/main/Activity/Random_forest/random_forest_model_17_estimators_A549.joblib

File 33. random_forest_model_19_estimators_BALB_3T3.joblib

https://github.com/XDamianX-coder/Colchicine_ML/blob/main/Activity/Random_forest/random_forest_model_19_estimators_BALB_3T3.joblib

File 34. random_forest_model_14_estimators_LoVo_DX.joblib

https://github.com/XDamianX-coder/Colchicine_ML/blob/main/Activity/Random_forest/random_forest_model_14_estimators_LoVo_DX.joblib

File 35. random_forest_model_18_estimators_LoVo.joblib

	https://github.com/XDamianX-coder/Colchicine_ML/blob/main/Activity/Random_forest/random_forest_model_18_estimators_LoVo.joblib
File 36.	random_forest_model_3_estimators_MCF-7.joblib
	https://github.com/XDamianX-coder/Colchicine_ML/blob/main/Activity/Random_forest/random_forest_model_3_estimators_MCF-7.joblib
File 37.	Structures_comparision.ipynb
	https://github.com/XDamianX-coder/Colchicine_ML/blob/main/New_structures/Structures_comparision.ipynb
File 38.	Preserve_colchicine_similar_structures.ipynb
	https://github.com/XDamianX-coder/Colchicine_ML/blob/main/New_structures/Preserve_colchicine_similar_structures.ipynb
File 39.	Proposed_structures_with_AI_colchicyne_tanimoto_similarity_.xlsx
	https://github.com/XDamianX-coder/Colchicine_ML/blob/main/Data/Proposed_structures_with_AI_colchicyne_tanimoto_similarity_.xlsx
File 40.	SYBA_selection.py
	https://github.com/XDamianX-coder/Colchicine_ML/blob/main/New_structures/SYBA_selection.py
File 41.	Whole_report.xlsx
	https://github.com/XDamianX-coder/Colchicine_ML/blob/main/Data/Whole_report.xlsx
File 42.	Stereochemistry_selection.ipynb
	https://github.com/XDamianX-coder/Colchicine_ML/blob/main/New_structures/Sterochemistry_selection.ipynb
File 43.	t-SNE.ipynb
	https://github.com/XDamianX-coder/Colchicine_ML/blob/main/New_structures/t-SNE.ipynb
File 44.	PubChemPy_search.ipynb
	https://github.com/XDamianX-coder/Colchicine_ML/blob/main/New_structures/PubChemPy_search.ipynb
File 45.	PubChemSearch_colchicyne.xlsx
	https://github.com/XDamianX-coder/Colchicine_ML/blob/main/Data/PubChemSearch_colchicyne.xlsx
File 46.	PubChemPy_search-selected_structures.ipynb
	https://github.com/XDamianX-coder/Colchicine_ML/blob/main/New_structures/PubChemPy_search-selected_structures.ipynb
File 47.	PubChemSearch_colchicyne_selected.xlsx

https://github.com/XDamianX-coder/Colchicine_ML/blob/main/Data/PubChemSearch_colchicyne_selected.xlsx

File 48. Colchicine PDB.ipynb

https://github.com/XDamianX-coder/Colchicine_ML/blob/main/Molecular_docking/Colchicine%20PDB.ipynb

File 49. Create folders for structures, 3D structures, distribute files and read results.ipynb

https://github.com/XDamianX-coder/Colchicine_ML/blob/main/Molecular_docking/Create%20folders%20for%20structures%2C%203D%20structures%2C%20distribute%20files%20and%20read%20results.ipynb

File 50. Prepare_pdbqt_from_pdb.txt

https://github.com/XDamianX-coder/Colchicine_ML/blob/main/Molecular_docking/Prepare_pdbqt_from_pdb.txt

File 51. to_be_executed.txt

https://github.com/XDamianX-coder/Colchicine_ML/blob/main/Molecular_docking/to_be_executed.txt

File 52. docking_results_1SA0_chain_AB.xlsx

https://github.com/XDamianX-coder/Colchicine_ML/blob/main/Molecular_docking/docking_results_1SA0_chain_AB.xlsx

File 53. Save_docking_results.ipynb

https://github.com/XDamianX-coder/Colchicine_ML/blob/main/Molecular_docking/Save_docking_results.ipynb

File 54. molecular_docking_chirality.xlsx

https://github.com/XDamianX-coder/Colchicine_ML/blob/main/Data/molecular_docking_chirality.xlsx

File 55. Random_state_explanation.ipynb

https://github.com/XDamianX-coder/Colchicine_ML/blob/main/Activity/Random_state_explanation.ipynb

File 56. A549_random_state_15.xlsx

https://github.com/XDamianX-coder/Colchicine_ML/blob/main/Data/A549_random_state_15.xlsx

File 57. A549_random_state_28.xlsx

https://github.com/XDamianX-coder/Colchicine_ML/blob/main/Data/A549_random_state_28.xlsx

File 58. A549_random_state_42.xlsx

https://github.com/XDamianX-coder/Colchicine_ML/blob/main/Data/A549_random_state_42.xlsx

File 59. BALB_3T3_random_state_15.xlsx

https://github.com/XDamianX-coder/Colchicine_ML/blob/main/Data/BALB_3T3_random_state_15.xlsx

File 60.	BALB_3T3_random_state_28.xlsx
	https://github.com/XDamianX-coder/Colchicine_ML/blob/main/Data/BALB_3T3_random_state_28.xlsx
File 61.	BALB_3T3_random_state_42.xlsx
	https://github.com/XDamianX-coder/Colchicine_ML/blob/main/Data/BALB_3T3_random_state_42.xlsx
File 62.	LoVo_random_state_15.xlsx
	https://github.com/XDamianX-coder/Colchicine_ML/blob/main/Data/LoVo_random_state_15.xlsx
File 63.	LoVo_random_state_28.xlsx
	https://github.com/XDamianX-coder/Colchicine_ML/blob/main/Data/LoVo_random_state_28.xlsx
File 64.	LoVo_random_state_42.xlsx
	https://github.com/XDamianX-coder/Colchicine_ML/blob/main/Data/LoVo_random_state_42.xlsx
File 65.	LoVo_DX_random_state_15.xlsx
	https://github.com/XDamianX-coder/Colchicine_ML/blob/main/Data/LoVo_DX_random_state_15.xlsx
File 66.	LoVo_DX_random_state_28.xlsx
	https://github.com/XDamianX-coder/Colchicine_ML/blob/main/Data/LoVo_DX_random_state_28.xlsx
File 67.	LoVo_DX_random_state_42.xlsx
	https://github.com/XDamianX-coder/Colchicine_ML/blob/main/Data/LoVo_DX_random_state_42.xlsx
File 68.	MCF-7_random_state_15.xlsx
	https://github.com/XDamianX-coder/Colchicine_ML/blob/main/Data/MCF-7_random_state_15.xlsx
File 69.	MCF-7_random_state_28.xlsx
	https://github.com/XDamianX-coder/Colchicine_ML/blob/main/Data/MCF-7_random_state_28.xlsx
File 70.	MCF-7_random_state_42.xlsx
	https://github.com/XDamianX-coder/Colchicine_ML/blob/main/Data/MCF-7_random_state_42.xlsx
